# Precipitation-first synthesis and deep profiling of a 1200-member acrylamide library for covalent drug discovery

**DOI:** 10.1039/d5gc04440e

**Published:** 2025-10-23

**Authors:** Pravin Patil, Emis Ingenito, Riccardo Fusco, Imma Capriello, Zhirui Guan, Anuja Bhalkikar, John Gibbons, Tomas Korba, Stanislav Bashkyrtsev, Oleh Tkachenko, Alexander Dömling

**Affiliations:** a Institute of Molecular and Translational Medicine (IMTM), Faculty of Medicine and Dentistry and Czech Advanced Technology and Research Institute, Palacký University in, Olomouc Krížkovského 511/8 779 00 Olomouc Czech Republic alexander.domling@upol.cz; b SCIEX, Echo® MS Center of Excellence Marlborough Massachusetts 01752 USA; c SCIEX 71 Four Valley Drive Concord Ontario Canada; d AMEDIS Bobkova 786/4 198 00 Praha Czech Republic; e Elsci LLC Arabkir district Komitas ave 1st lane b. 20 0051 Yerevan Armenia

## Abstract

Screening libraries of low molecular weight (LMW) compounds are foundational in early-stage drug discovery. However, the parallel synthesis of milligram-scale large libraries – especially for covalent inhibitors – remains constrained by resource-intensive purification protocols, high solvent consumption, and limited scalability. Electrophilic fragments such as acrylamides are widely used to target nucleophilic cysteines in disease-relevant proteins, but practical workflows to synthesize diverse covalent libraries in sufficient quantities and quality are lacking. Here, we report a high-throughput, chromatography-free workflow to synthesize and analyze 1235 acrylamides using a modified Ugi four-component reaction (U-4CR) featuring ammonia and acrylic acid. This strategy exploits direct product precipitation to replace chromatographic purification steps with simple filtration, enabling synthesis at scale with common parallel lab instrumentation. Our protocol provides a sustainable and modular solution to library generation, especially suitable for covalent drug discovery. Using complementary analytical methods – ^1^H NMR, UPLC-MS-UV, and AEMS – we systematically evaluated compound purity, identity, and long-term stability. The large dataset was systematically processed using PeakSel® (live dashboard at https://peaksel.elsci.io/a/domling_group/post/8gY7CqUMVtb), providing complementary information on compound quality and long-term stability. This platform delivers the largest fully profiled covalent electrophile library to date and offers a generalizable framework for sustainable library production and high-content analytical workflows. It complements emerging analytics-only approaches by offering a complete synthesis-to-analysis pipeline accessible with standard laboratory instrumentation.

## Abstract 2

We developed an efficient Ugi-based route to 1200 acrylamides, delivering millimolar quantities of pure precipitated products. Library quality was verified by 1H NMR, UPLC-UV-MS, and AEMS, yielding a sustainable drug-discovery-ready set.

## Graphical abstract

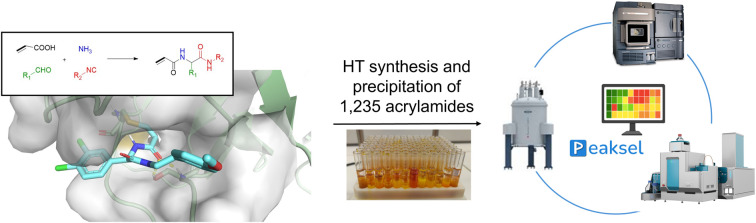

**Green foundation**

1. We establish a precipitation-first workflow for library synthesis that eliminates chromatographic purification, significantly reducing solvent and energy consumption. This enables sustainable, scalable production of high-quality covalent libraries.

2. Our protocol synthesized 1235 acrylamides with >80% purity while consuming ∼10 L of solvent in total – an order of magnitude less than chromatography-based approaches. The workflow is accessible with standard lab equipment and achieves milligram-scale yields suitable for screening.

3. Future efforts could substitute trifluoroethanol and ether with greener solvents, automate solid handling to minimize losses, and integrate solvent recycling. Extending precipitation-first workflows to other electrophile classes would further broaden sustainable library design.

## Introduction

**Fig. 1:**
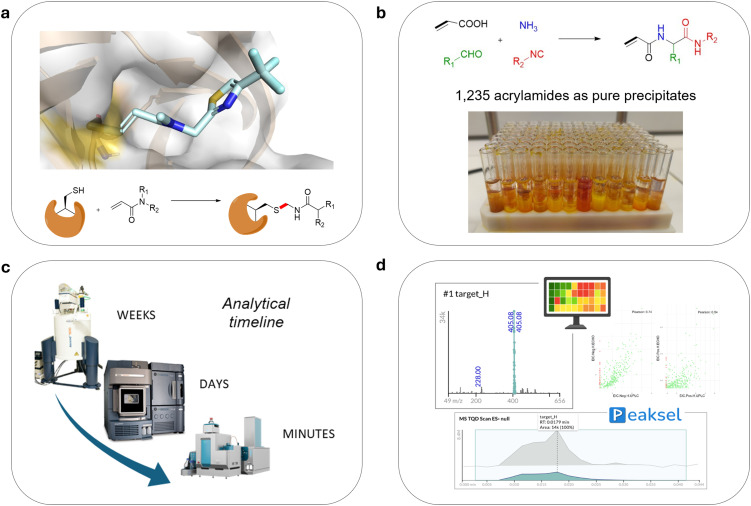
Background and study design. (a) Acrylamides as validated nucleophiles for covalent bond formation to reactive cysteines in protein targets, exemplified by the crystal structure of a compound targeting the active site cysteine of the 3C protease of Enterovirus 71 (PDB 7WYL). (b) The Ugi-4CR ammonia/acrylic acid variation. (c) High throughput analysis of the library in different time regiments, using three different methods. (d) Performed data analysis by Peaksel®.

Screening libraries of low molecular weight (LMW) compounds remain a cornerstone in early drug discovery.^1,2^ Recently, covalent inhibitor libraries based on acrylamides became popular in covalent modification of proteins and several drugs are clinically in use. Examples include the RAS G12C inhibitor Sotorasib or the Bruton kinase inhibitor Ibrutinib.^3,4^ Typically consisting of hundreds of thousands to millions of compounds, screening libraries are essential assets in the identification of bioactive hits during the initial phases of drug development projects.^5^ In pharmaceutical settings, screening libraries are predominantly composed of legacy compounds derived from historical project pipelines, and expanded through targeted synthesis or commercial acquisitions.^6,7^ While many clinically approved drugs have originated from high-throughput screening (HTS) campaigns using such collections, these libraries frequently lag behind contemporary developments in synthetic chemistry, particularly in incorporating novel bond-forming reactions and underexplored chemical space such as acrylamides. Despite a trend toward miniaturizing parallel reactions, the amount of material required for screening assays remains in the milligram range (sub-millimolar scale).^8,9^ Unlike proprietary pharmaceutical libraries, academic libraries are generally composed of commercially available compounds with publicly known structures. Such libraries can include both general, untargeted compounds and targeted compounds designed for specific proteins or applications – for instance, electrophiles targeting nucleophilic side chains in proteins (Fig. 1a). Although high compound purity generally improves the quality of biological readouts, purities >85% are commonly accepted for screening libraries. Achieving such purity, however, often requires extensive workup procedures involving chromatographic purification, which is time-consuming, solvent-intensive, and resource-demanding, making it unsustainable and expensive for producing LMW libraries. To overcome some of those limitations, pharmaceutical companies employ high-throughput purification systems (auto-purifiers) integrated into automated workflows.

An illustrative large scale library synthesis effort is the European Lead Factory (ELF), a pan-European public-private partnership project, involving major pharmaceutical companies, contract research organizations (CROs), and academic partners. Over several years, the ELF partners synthesized over 300 000 novel LMW compounds, collectively known as the Joint European Compound Library (JECL), accessible to small and medium-sized enterprises (SMEs), academic labs, and patient-driven initiatives.^10^ Despite the many methods described for producing compound libraries, no protocols enable the synthesis of hundreds to thousands of acrylamide compounds at high purity, on a milligram scale, using sustainable practices and simple instrumentation, without sacrificing structural diversity. Moreover, the library management poses additional challenges, particularly in their high-throughput analysis and rigorous quality control.

To address these needs, here we present an integrated approach from synthetic generation to analytical resolution, supported by machine learning analyses that retrospectively uncover correlations between molecular descriptors and the experimental outcomes of precipitation and stability. Building on our previous proof-of-concept MCR-based electrophile libraries synthesis,^11^ this work advances the approach toward a practical, chromatography-free workflow that yields multi-milligram quantities of high-quality acrylamides directly compatible with high-throughput biological screening, which is also offered free of charge to interested academical collaborators in a stock solution plate format (Fig. 1a).

## Results and discussion

### Reaction selection, optimization and exploration

The library was designed as a modified Ugi four-component reaction (U-4CR) involving acrylic acid, aldehydes, isocyanides, and ammonia as alternative to primary amines (Fig. 1b).

The U-4CR is known to be an established approach for library production due to its ability to explore vast chemical space, thanks to its high variability of building blocks, excellent functional group compatibility, and reliable synthesis.^12^ Replacing primary amines with ammonia provided several advantages:

1. Increased rotational flexibility: the use of ammonia provides enhanced flexibility around the bond adjacent to the amide group (Fig. S1).

2. Optimized molecular weight: ammonia reduces the molecular weight of the products, yielding compounds with superior physicochemical properties and serving as an ideal starting point for hit-to-lead optimization.

3. Precipitation-driven workflow: the presence of two secondary amides in the core structure promotes the formation of intermolecular hydrogen bonds, facilitating product precipitation directly from the reaction solution.

**Fig. 2:**
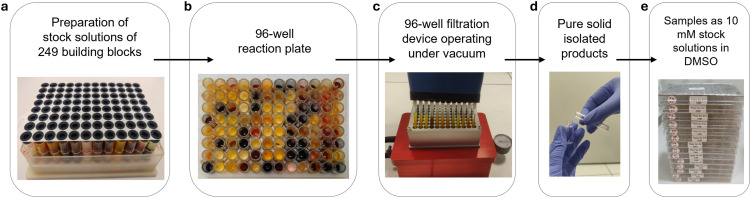
Optimized synthetic process. (a) Stock solutions of building blocks prepared in trifluoroethanol at 2.5 M or 5 M. (b) Top view of a 96-well reaction plate. (c) Transferring of precipitated products to metal 96-well filtration plate using 12-channel pipettes. (d) Single glass vial with pure solid product. (e) Final 10 mM stock solutions in DMSO within 96-well plates for storage and biological evaluation.

These last two features are central to our sustainable high-throughput synthetic workflow (Fig. 2), which avoids solvent intensive chromatographic purification, one of the major contributors to the unsustainability of multi-milligram-scale library production. Recognizing the critical impact of solvent polarity, concentration, and reagent combinations, we conducted a detailed optimization campaign (SI – section 1). Starting from a model Ugi reaction, we screened different solvents (ethanol, methanol, trifluoroethanol, H_2_O and mixtures), concentrations (0.1–1.0 M) and reactant ratios to allow for the direct precipitation of pure products. Notably, this was accomplished using methanolic or aqueous ammonia, with TFE and aqueous ammonia giving the best results in terms of yields and purity.

Challenges in filtration and solid transfer were addressed by developing a custom aluminium filtration plate and modifying pipette tips to facilitate the efficient transfer (Fig. S10). Together, these advancements enabled the high-throughput synthesis and isolation (excluding analysis) at an unprecedented speed of up to four 96-well reaction plates per day.

A total of 2551 reactions were performed, using stock solutions of 249 building blocks (SI – section 4) prepared in trifluoroethanol (TFE) at 2.5 or 5.0 M, depending on solubility (Fig. 2a and Fig. S12). In a general multi-well reaction plate, starting materials were combined in 2 mL glass vials with magnetic stirring (Fig. 2b) and let at room temperature for 18 hours, resulting in significant precipitation, enhanced upon the addition of diethyl ether and cooling to 0 °C. Crude products were then transferred manually using 8- or 12-channel pipettes into the custom vacuum filtration device (Fig. 2c) and washed sequentially with diethyl ether and a diethyl ether/petroleum ether mixture. The total solvent consumption for synthesis and purification was ∼10 L, yielding approximately 100 mg compounds per reaction. Out of 2551 reactions performed, 1612 produced a solid precipitate (63%).

### Analytical evaluation

To evaluate the quality, identity, and stability of the resulting library, we applied three analytical techniques (Fig. 1c): proton nuclear magnetic resonance (^1^H NMR), ultra-performance liquid chromatography coupled to UV detection and mass spectrometry (UPLC-UV-MS), and acoustic ejection mass spectrometry (AEMS).

Following the precipitation-based isolation, the first analytical step involved individual ^1^H NMR analysis of each product, which took approximately 7 minutes to acquire, resulting in the analysis of an entire 96-well plate within 7–8 hours (SI, Fig. S14). Based on the NMR analysis, 1235 pure solid isolated products (Fig. 2d) with a purity exceeding 80% were successfully identified and retained, while impure samples were discarded.

**Fig. 3:**
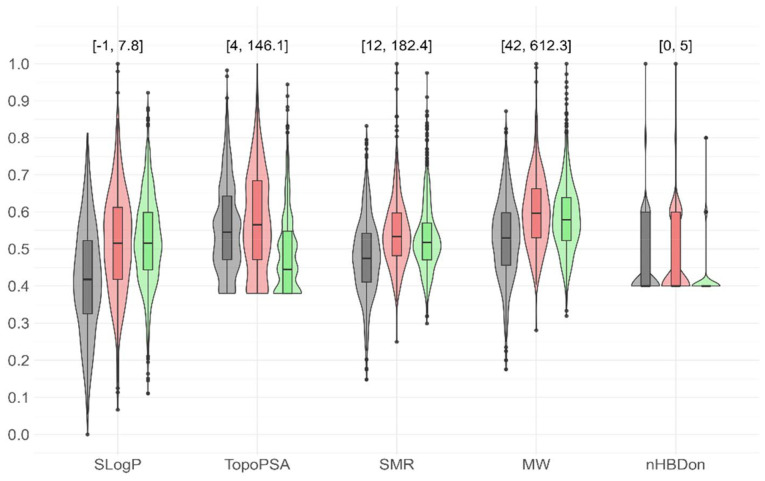
Distribution of five key molecular descriptors in the 2551 compound library. The violin plots show the distribution of the key molecular descriptors – SLogP, TopoPSA, SMR (Simple Molar Refractivity), MW (Molecular Weight), and nHBDon (Number of Hydrogen Bond Donors) – which were identified as the most influential features for the precipitation success through machine learning analysis. These plots are arranged by reaction outcomes: “Green” for successful products, “Red” for incorrect precipitates, and “Black” for no precipitation. All descriptor values have been normalized from 0 to 1.

According to precipitation behaviour and NMR results, compounds were classified into three categories (Fig. 3 and Fig. S16):

• Black compounds: not precipitated. These had significantly lower Simulated Log Partition Coefficient (SLogP) values and lower polarity, indicating retention in the TFE phase and absence of solid formation.

• Green compounds: precipitated successfully and pure by NMR check (>80%). These compounds had higher a-polarity and lower Topological Polar Surface Area (TopoPSA), correlating with favorable precipitation (SI, section 7.2).

• Red compounds: precipitated but showed inconsistent NMR spectra. No clear physicochemical pattern emerged from machine learning or statistical analysis, although elevated TopoPSA values in the aldehyde building blocks suggested that unreacted starting materials may have co-precipitated.

After compound purity was initially confirmed by ^1^H NMR analysis of the precipitated products, the products were stored both as solid powders and as 10 mM stock solutions in DMSO within 96-well plates (Fig. 2d, e and Fig. S15).

Long-term stability was evaluated using both AEMS and UPLC-MS-UV following two years of storage at room temperature. While ^1^H NMR analysis required several days to complete, the second analytical technique applied, AEMS, enabled rapid assessment of the entire library in a matter of minutes. This last unites acoustic droplet ejection, an open-port interface, and ESI-MS (SCIEX Triple Quad 6500+ or ZenoTOF 7600) into a non-liquid chromatography-based, high-throughput platform capable of analysing up to one sample every 2.5 seconds. In addition, it allows for direct analysis in DMSO, the same solvent used for compound storage and biological screening, simplifying sample preparation and making it particularly suited for high-throughput library profiling.^13,14^

**Fig. 4:**
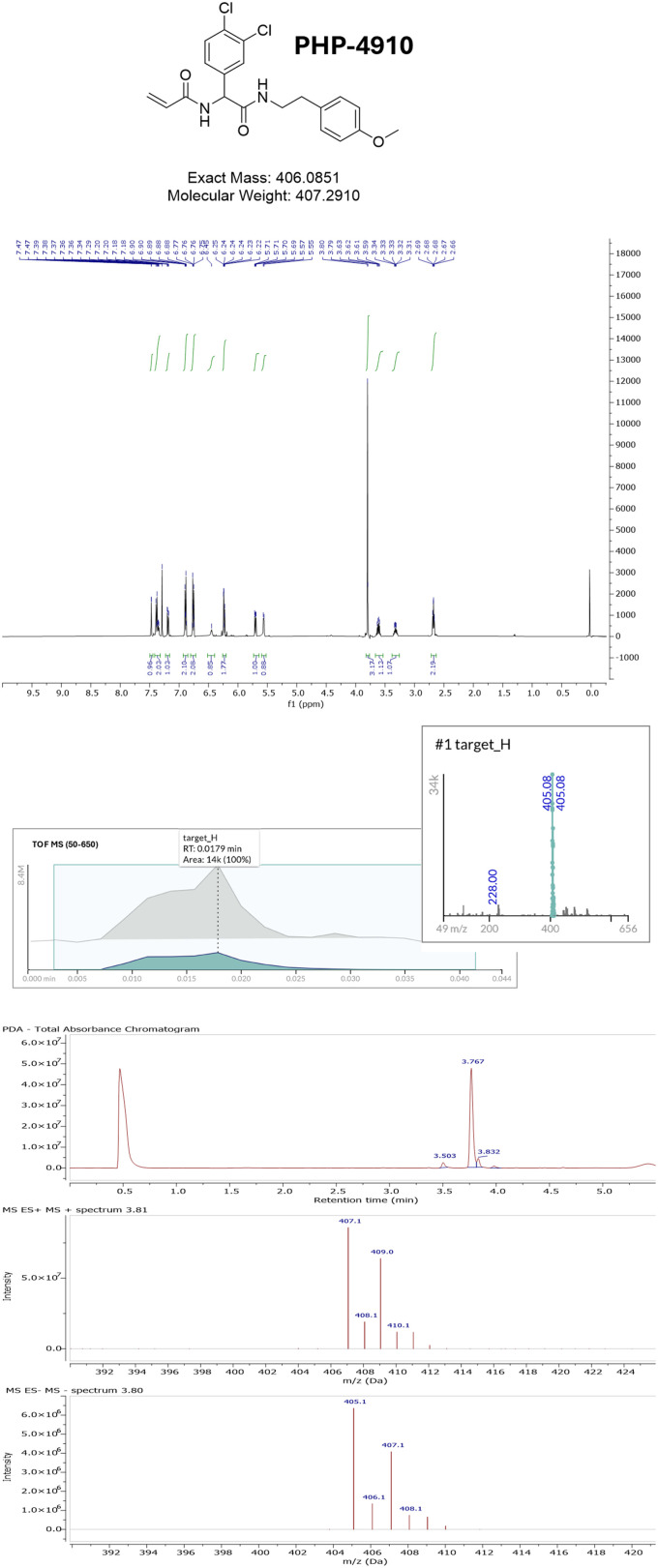
Example of the comprehensive analytical profile of compound PHP-4910. Reported there are structure and characteristics of the compound, ^1^H NMR spectra of the compound in CDCl_3_, AEMS profile in negative mode, as visualized in PeakSel® and UPLC-MS profile in positive and negative modes.

**Fig. 5:**
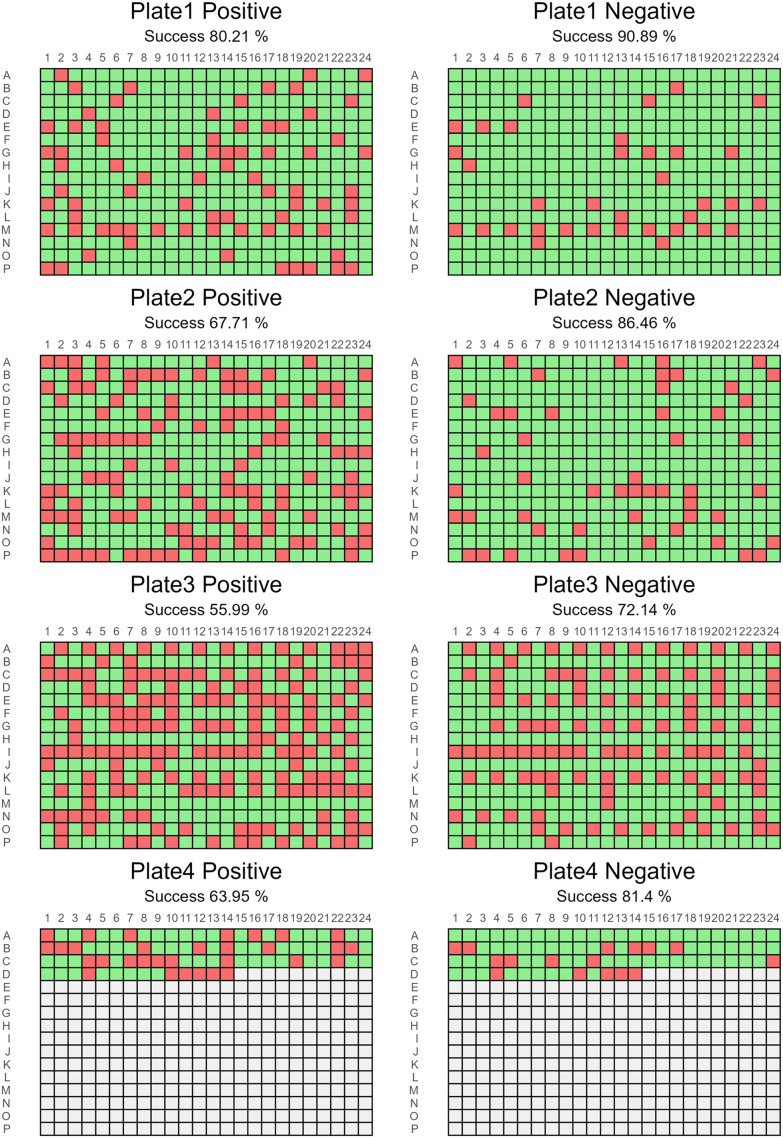
AEMS positive and negative mode success heatmaps. Green cells correspond to detected compounds for all the possible adducts, red correspond to the absence of ionized forms.

**Fig. 6:**
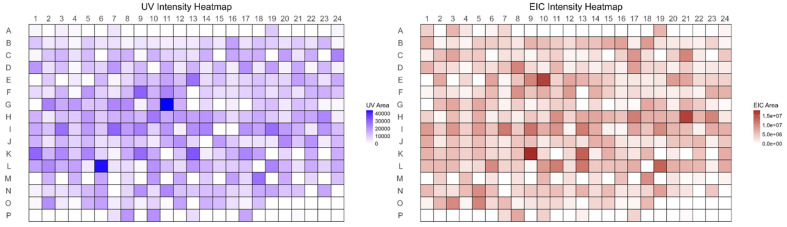
Heatmaps illustrating compound stability in the representative 384-member subset. (Left) UV Intensity Heatmap showing relative absorbance (AUC values) from UPLC-UV analysis. (Right) EIC Intensity Heatmap showing relative signal intensity from UPLC-MS extracted ion chromatograms.

Exploiting this throughput, the 1235 samples were reformatted into a 384-well plate format, diluted to 100 μM in DMSO and analysed through the Echo® MS+ system with a ZenoTOF 7600 system, enabling a comprehensive mass detection from 50 to 650 Da, in both positive and negative ion modes, achieving full plate analysis within just 17 minutes per plate (Fig. 5). As the third analytical technique, UPLC-UV-MS was applied to a representative subset of 384 compounds from the 1235 purified products, diluted from the original stock solutions to 0.7 mg mL^−1^ in a 50 : 50 (v/v) acetonitrile/water mixture to ensure compatibility with the chromatographic method. This final method, operating in both ionization modes, provided a comprehensive analytical profile for each of the 384 compounds (Fig. 6), combining retention time, UV absorbance, and mass spectrometric data, as exemplified in Fig. 4, and it concluded the analytical acquisition stage before data interpretation.

### Data analysis and interpretation

As first analytical output, the library analysed by AEMS after two years of storage in DMSO resulted in 88% of the compounds (1092 out of 1235) remaining stable and detectable (Fig. 5).

**Fig. 7:**
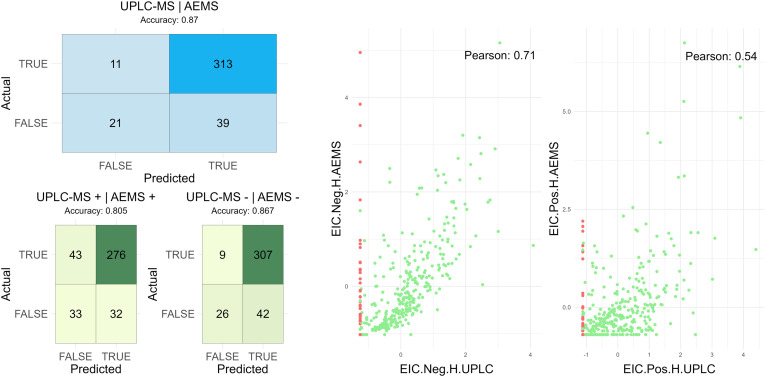
Correlation diagrams between AEMS and UPLC-MS for the 384-compounds subset. (a) Heatmaps comparing the detection sensitivity of AEMS to UPLC-MS, established as ground truth. The blue heatmap illustrates the overall agreement in detecting adducts (*e.g.*, H^+^, Na^+^, H^−^) across all modes, with an accuracy of 0.88. (b) The green heatmaps evaluate performance in specific configurations: both methods in positive mode (accuracy 0.82) and in negative mode (accuracy 0.86). (c) Correlation plot in negative ([M − H]^−^) mode (left) and in positive mode ([M + H]^+^) (right) between the UPLC (*x*-axis) *versus* the AEMS (*y*-axis) considering the AUC (Area Under the Curve). Green dots represent compounds that were present in the methods of interest. Red dots represent compounds that were not present (in any method: ([M + H]^+^), ([M + Na]^+^), ([M − H]^−^)), which have been stripped off from the Pearson Correlation.

This high retention of analysable material is particularly notable given that acrylamides are inherently reactive electrophiles prone to degradation processes such as hydrolysis and polymerization, especially under long-term storage conditions. Additional validation on the representative subset of 384 compounds using both AEMS and UPLC-MS provided two stability estimates: a conservative value of 81%, requiring detection by both methods, and a more permissive estimate of 94%, based on detection by either platform. Together, these findings demonstrate the robust stability of the synthesized library over time and highlight some systematic differences in analytical performance between the two complementary detection platforms. Taking as input the .raw files, our further analysis was focused on the detection of the differences in sensitivity and specificity between AEMS and UPLC-MS-UV. To quantitatively assess these parameters for each method, we generated confusion matrix heatmaps, reported in Fig. 7a and b, with UPLC-MS serving as the analytical reference (ground truth), rather than UV detection, due to the presence of non-UV-absorbing compounds in the library. Compound detection *via* AEMS and UPLC-MS was based on both ionization modes—positive mode (protonated [M + H]+) and sodium adducts ([M + Na]+) and negative mode (deprotonated [M − H]−).

Based on the obtained results, AEMS demonstrated high sensitivity for compound verification and degradation assessment, despite lacking chromatographic separation. When UPLC-MS was employed as the reference method for the comparison presented in Fig. 7a, AEMS failed to detect only a small number of compounds that were identified by UPLC-MS (11 – False Negative). Conversely, this direct comparison indicated lower specificity for AEMS, as it identified a notable number of additional compounds not detected by UPLC-MS (39 – False Positive). While these additional detections are categorized as false positives within the framework of Fig. 7a where UPLC-MS serves as the reference, subsequent manual inspection of the data revealed that AEMS exclusively detected several compounds confirmed to be genuinely present. This observation reveals that UPLC-MS, due to the specific nature of its detection system, did underestimate the presence of some analytes (SI, section 8.4).

Performance differences between the two platforms were influenced by several factors. AEMS showed variability in signal-to-noise (S/N) ratios, particularly in positive mode, where elevated background noise increased the Total Ion Current (TIC) and impaired the precision of Extracted Ion Current (EIC) measurements. This led to reduced specificity and lower correlation with UPLC-MS data in positive mode.^15^ Conversely, AEMS data in negative mode ([M − H]−) exhibited stronger correlation with UPLC-MS, as illustrated in Fig. 7c, where green dots represent detected compounds and red dots indicate undetected compounds, excluded from correlation analysis. The observed discrepancy in analytical performance between AEMS and UPLC-MS-UV can be attributed to two main factors: (1) the use of a standardized chromatographic method in UPLC-MS across all compounds, regardless of their individual physicochemical properties; and (2) the inherently higher sensitivity of the Time-of-Flight (TOF) detector used in AEMS compared to the triple quadrupole system employed in UPLC-MS.

## Conclusions

This work demonstrates a high-throughput, resource-efficient method for synthesizing a high quality ∼1200-member library of LMW acrylamides targeting protein cysteines. Using a modified Ugi-4CR with ammonia, we implemented a precipitation-driven workflow that eliminates chromatographic purification, reducing solvent use and enhancing sustainability. The protocol is scalable to multi-milligram synthesis in 96-well plates, yielding compounds suitable for screening.

To ensure quality, we employed ^1^H NMR, UPLC-MS-UV, and AEMS, each offering unique insights into purity, identity, and stability. Together, these complementary techniques enabled a robust and comprehensive evaluation of the compound library. Our head-to-head comparison of AEMS and UPLC-MS revealed complementary strengths and limitations, under-scoring the importance of systematic method evaluation for robust library assessment. Moreover, the comprehensive dataset of 1235 rigorously characterized acrylamides is, to our knowledge, the largest such collection ever published, offering an invaluable resource for machine-learning and data-driven drug-discovery efforts. As covalent therapeutics gain traction and the demand for green, cost-effective screening tools grows, this workflow addresses a critical gap between innovation and implementation. Taken together, this work outlines a sustainable and scalable strategy for the generation of screening libraries with minimal resource expenditure. By integrating efficient multicomponent synthesis with high-throughput analytical workflows, we provide a practical framework that balances purity, scalability, and environmental impact. We anticipate its utility across academic screening centres, PROTAC warhead discovery, and early-phase hit expansion campaigns. The resulting library is currently being screened in multiple research projects, with results either already published or forthcoming.^16,17^

## Author contributions

A. D. conceived and supervised the overall project and secured funding. P. P. designed and optimized the synthetic process and carried out the NMR experiments. E. I. and I. C. analysed and processed NMR data and prepared samples for the different analytical methods. E. I. and Z. G. carried out the UPLC-UV-MS experiments and interpreted the resulting spectra. A. B., T. K., and J. G. performed the AEMS experiments and critically reviewed the corresponding sections. R. F. developed the code. R. F and E. I. performed data analysis and data interpretation. S. B. and O. T. furnished the Peaksel® system and reviewed the software-related portions of the manuscript. A. D., P. P., E. I. and R. F. contributed to figure preparation and drafted the original manuscript. All authors critically reviewed and edited the manuscript and approved the final version.

## Conflicts of interest

There are no conflicts to declare.

## Supplementary Material

GC-027-D5GC04440E-s001

## Data Availability

Data for this article, including details about method optimization, chemical synthesis, prototypes, materials, NMR spectroscopy, UPLC-MS and AEMS analysis, data analysis and code structure are available in the Zenodo repository at the https://doi.org/10.5281/zenodo.15496516. Supplementary information (SI) is available. See DOI: https://doi.org/10.1039/d5gc04440e.
