# Supplementary material for: Precipitation-first synthesis and deep profiling of a 1200-member acrylamide library for covalent drug discovery
Source: Green Chem. 2025 Oct 23;27(45):14640–7. doi: 10.1039/d5gc04440e (PMC12577537; doi:10.1039/d5gc04440e)
Supplement: GC-027-D5GC04440E-s001 [file GC-027-D5GC04440E-s001.pdf]

Supporting Information

Precipitation-first Synthesis and Deep Profiling of a 1,200-Member Acrylamide Library for Covalent Drug Discovery

Pravin Patil+,[a] Emis Ingenito+,[a] Riccardo Fusco+,[a] Imma Capriello,[a] Zhirui Guan,[a] Anuja Bhalkikar,[b] , John Gibbons [b], Tomas Korba,[c] Stanislav Bashkyrtsev,[d] Oleh Tkachenko,[d] and Alexander Dömling*[a]

[a] Institute of Molecular and Translational Medicine (IMTM), Faculty of Medicine and Dentistry and Czech Advanced Technology and Research Institute, Palacký University in Olomouc, Krížkovského 511/8, 779 00 Olomouc, Czech Republic.

[b] SCIEX, Echo® MS Center of Excellence, Marlborough, Massachusetts, 01752, United States; SCIEX, 71 Four Valley Drive, Concord, Ontario, Canada.

[c] AMEDIS, Bobkova 786/4, 198 00 Praha, Czech Republic.

[d] Elsci LLC, Yerevan, Armenia Arabkir district, Komitas ave 1st lane, b. 20, 0051

[+] These authors contributed equally.

Correspondence to alexander.domling@upol.cz

Table of contents

1 General information 3

2 Optimization of the multi-well conditions for synthesis of acrylamides 4

2.1 Optimization of reaction conditions and solvents testing 4

2.2 Optimization of the purification process 9

3 New prototypes of metal filtration plates 12

4 General synthetic procedure for the Ugi reaction 13

4.1 List of building blocks used in the study 14

5 Stock solution preparation of starting compounds 19

6 96-well plates synthesis 20

7 Analytical techniques 21

7.1 NMR analysis of isolated solid product and storage 21

7.2 Structures of the isolated products 23

7.3 Reformatting of stored compounds 33

7.4 AEMS 33

7.4.1 Dilution of compounds stock solutions for the AEMS analysis 33

7.4.2 AEMS (acoustic droplet ejection mass spectrometry) analysis 33

7.5 UPLC-MS 33

7.5.1 Dilution of compounds stock solutions for the UPLC-MS analysis 33

7.5.2 UPLC-MS analysis 34

8 Data analysis 35

8.1 Choice of the relevant descriptors 35

8.2 Statistical analysis on synthetic outcome 35

8.3 Automated Analysis of AEMS and UPLC-MS data 39

8.4 In-depth analysis of confusion heatmaps 42

8.5 Code-structure overview 44

8.6 Chemical space insights 45

9 Analytical data 47

9.1 ^{1}H spectra of the 1235 pure products 48

9.2 UPLC-MS spectra of the 384-compounds subset 666

1 General information

Reagents and solvents were purchased from commercial suppliers (Sigma Aldrich, ABCR, BLD and Fluorochem) and were used without further purification. All isocyanides were prepared according to literature. Nuclear magnetic resonance spectra were recorded on a Bruker Avance 500 spectrometer ^{1}H NMR (500 MHz).

2 Optimization of the multi-well conditions for synthesis of acrylamides

2.1 Optimization of reaction conditions and solvents testing

The Ugi reaction was initially performed using a model reaction between 4-chlorobenzaldehyde, benzylamine, acrylic acid, and 2-(isocyanomethyl)thiophene under a range of solvent conditions. These included methanol, ethanol, trifluoroethanol and binary mixtures of water: methanol (1:1) and water: ethanol (1:1) at concentrations of 0.1 M, 0.5 M, and 1.0 M. Despite the variation in solvents and concentrations, none of the reactions resulted in the desired product precipitation, indicating that the tested conditions were not conducive to successful product formation (**Scheme S1**). To further explore its applicability, the same reactions were performed with isocyanoglycine methyl ester and morpholinoethylisocyanide, as well as cyclohexanone, resulting in no precipitation observed.

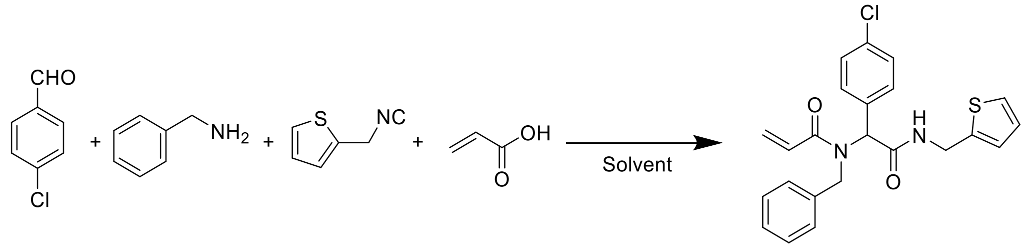

Scheme S1: Schematic reaction of 4-chlorobenzaldehyde, benzylamine, acrylic acid, and 2-(isocyanomethyl)thiophene.

Replacement of the primary amine with aqueous ammonia or methanolic ammonia under the same conditions resulted in the product precipitation (**Scheme S2**).

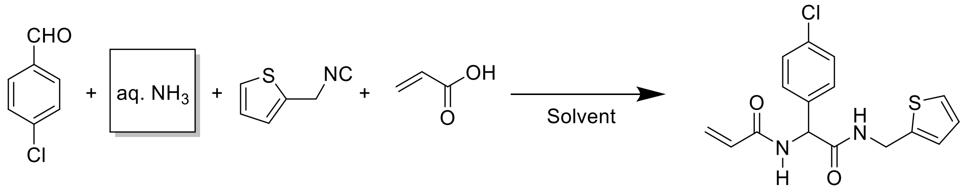

Scheme S2: To test Ugi reaction of 4-chlorobenzldehyde, aqueous ammonia, acrylic acid with 2-(isocyanomethyl)thiophene in various solvents to obtained precipitated product.

The conformational analyses and their corresponding dihedral driver charts presented in **Figures S1A-D** provide strong evidence for the observed differences in precipitation behavior, demonstrating how nitrogen substitution fundamentally alters the conformational energy landscapes of our compounds.

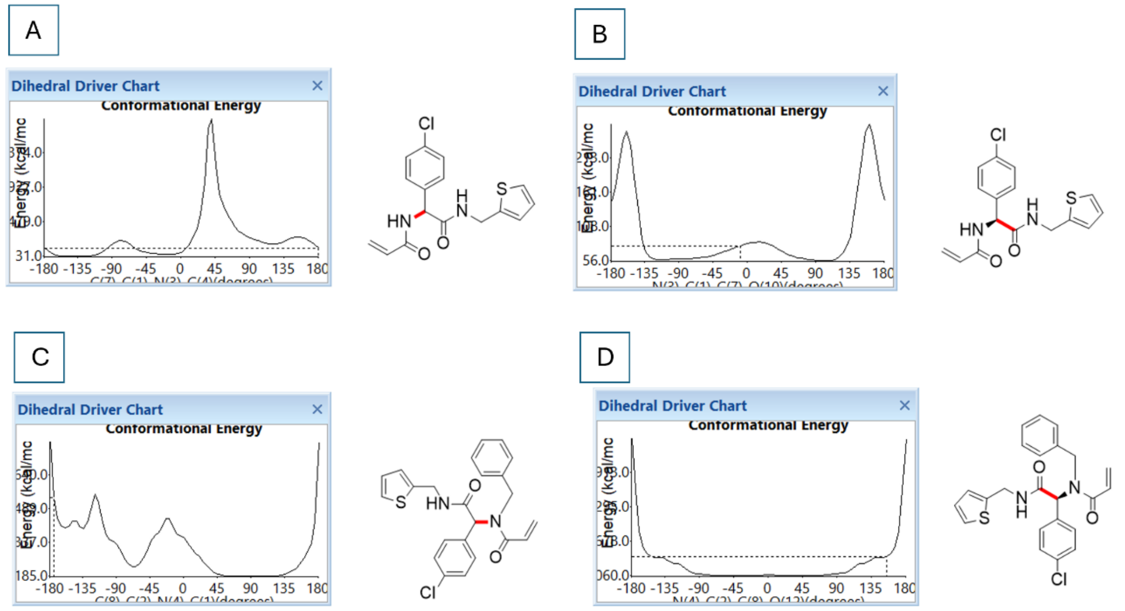

Figure S1. Conformational energy profiles for the rotational bonds (shown in red) of ammonia-derived and N-benzyl substituted Ugi products. **(a)** Conformational energy profile for the CONH-C rotational bond of the ammonia derivative. **(b)** Conformational energy profile for the CONHC-C rotational bond of the ammonia derivative. **(c)** Conformational energy profile for the CONH-C rotational bond of the N-benzyl substituted Ugi product. **(d)** Conformational energy profile for the CONHC-C rotational bond of the N-benzyl substituted Ugi product.

The ammonia derivatives (**S1A and S1B**) exhibit broader energy minima, enabling the molecules to adopt conformations favorable for crystal packing through enhanced intermolecular hydrogen bonding via their two secondary amide groups. This conformational flexibility promotes precipitation from solution, streamlining purification and improving synthetic efficiency.

In contrast, the N-benzyl substituted derivatives (**S1C and S1D**) display more restricted conformational landscapes with pronounced energy barriers. The bulky benzyl group introduces significant spatial constraints around the nitrogen atom. The substantial volume occupied by this substituent creates unfavorable steric interactions with neighboring groups during rotation, particularly evident in the energy maxima observed in **Figure S1C**. These steric constraints limit the accessible conformations, hindering the compounds' ability to form efficient packing arrangements necessary for crystallization.

To expand the study, three different aldehydes (4-chlorobenzaldehyde, 2-chloro-4-fluorobenzaldehyde, and 1H-indole-3-carbaldehyde) combined with four different isocyanides(2-(isocyanomethyl)thiophene, 1-isocyano-4-isopropylbenzene, 5-(isocyanomethyl)benzo[d][1,3]dioxole, and (2-isocyanoethyl)benzene) were employed

using aqueous ammonia and methanolic ammonia in methanol and trifluoroethanol (TFE) at a 1 M concentration. After stirring for 18 hours at room temperature, reactions conducted in TFE resulted in an increased precipitation. The addition of 1 mL diethyl ether facilitated the formation of free-flowing precipitates (**Figure S2**).

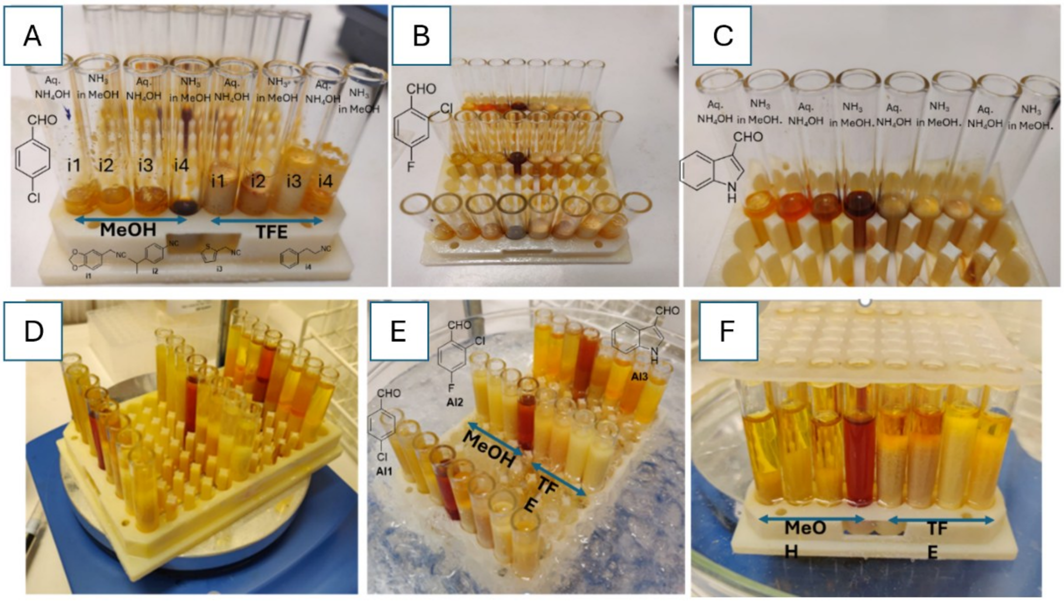

Figure S2: Ugi reaction in 2 mL glass wells exploring the effect of methanol and trifluoroethanol as solvents on product precipitation with different ammonia sources (methanolic ammonia and aqueous ammonia) in combination with various aldehydes and isocyanides. **(a)** 4-Chlorobenzaldehyde after 18 hours at room temperature. **(b)** 4-Fluoro-2-chlorobenzaldehyde after 18 hours at room temperature. **(c)** Indole-3-carboxaldehyde after 18 hours at room temperature. **(d)** Reaction wells after dilution with diethyl ether. **(e)** Reaction wells after dilution with diethyl ether and cooling in an ice bath to enhance precipitation. **(f)** Comparison of precipitate formation after 15 minutes of stirring in an ice bath for 4-chlorobenzaldehyde shows that TFE leads to more precipitate than methanol.

Notably, most reactions led to product precipitation at room temperature, and cooling to 0 °C further increased solid formation. Reactions with aqueous ammonia showed greater precipitation compared to those with methanolic ammonia.

To isolate the solid products, we filtered the reactions using a 96-well polyvinylidene fluoride (PVDF) filtration plate with 0.45 μm filters. The glass vials were manually transferred to the filtration plate, and the precipitates were washed sequentially with 1 mL of diethyl ether, followed by 1 mL of 50% diethyl ether in petroleum ether (see **Figure S3**).

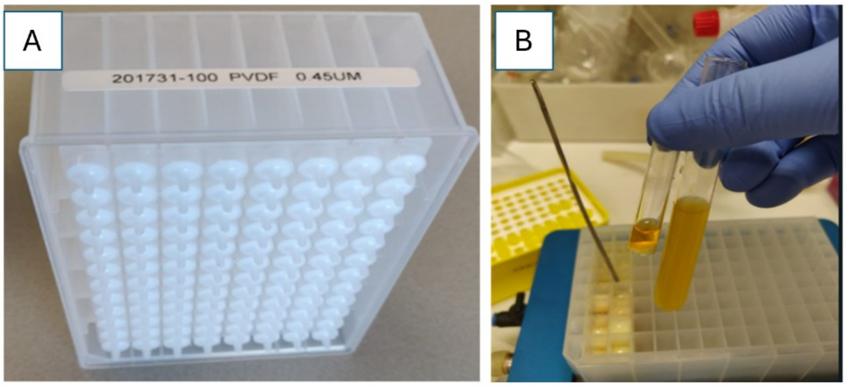

Figure S3: Initial attempt to use a PVDF 96-well filtration plate with fixed filters: (**a**) PVDF 96-well filtration plate with an inbuilt 0.45 μm filter. **(b)** PVDF filtration plate during precipitate transfer and filtration.

NMR analysis of the solid products (**Figure S4**) showed that reactions with aqueous ammonia yielded pure precipitates (**Figure S4b**), whereas those with methanolic ammonia resulted in impure precipitates (**Figure S4a**).

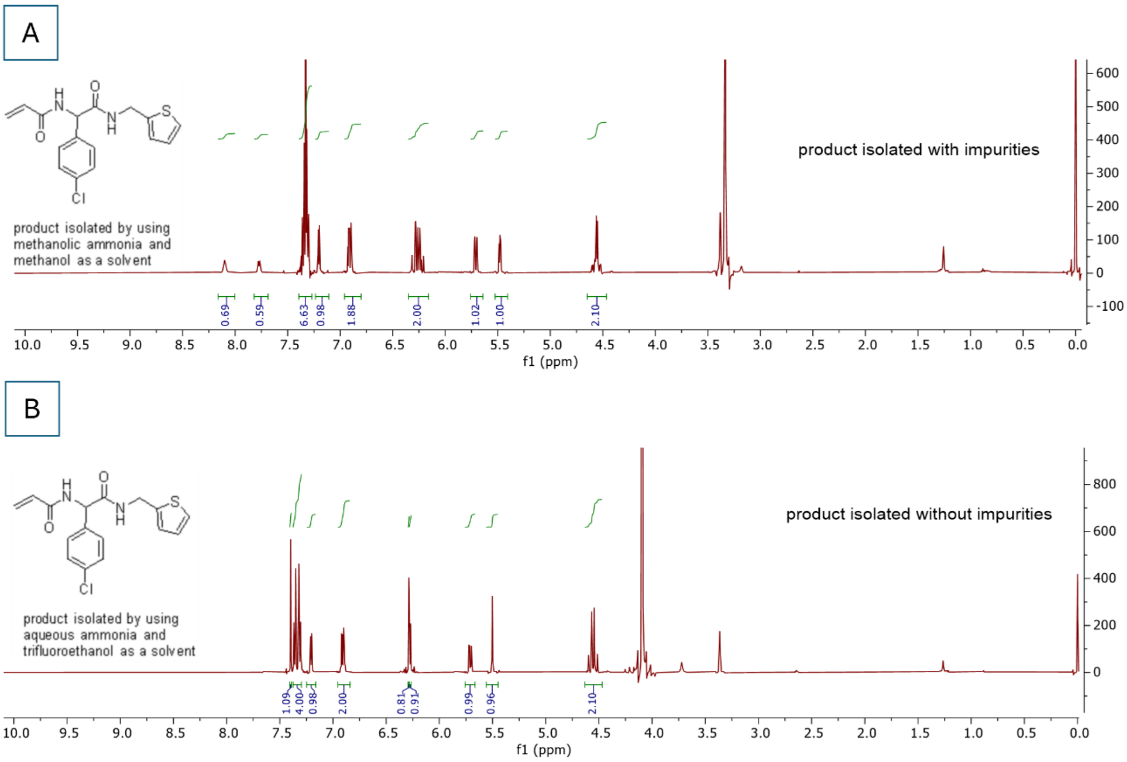

Figure S4: a) Proton NMR of precipitate obtained from the reaction using methanolic ammonia and methanol as solvent; **b**) Proton NMR of precipitate obtained from the reaction using aqueous ammonia and TFE as solvent.

Encouraged by the successful precipitation observed with aqueous ammonia in TFE, we attempted the reaction with different ketones (cyclohexanone, 1-methylpiperidin-4-one, and pentan-2-one). However, no precipitates could be isolated from these reactions.

With these findings, reactions involving a single isocyanide and a diverse range of aldehydes were performed, to explore the precipitation rate. 96 different aldehydes (**Figure S5B**) were tested at a 0.5 mmol scale in a 96-well plate using aqueous ammonia, benzyl isocyanide, and acrylic acid in TFE at a 1 M concentration. After 18 hours of stirring at room temperature, more than 78 reactions showed solid formation. (**Figure S5**).

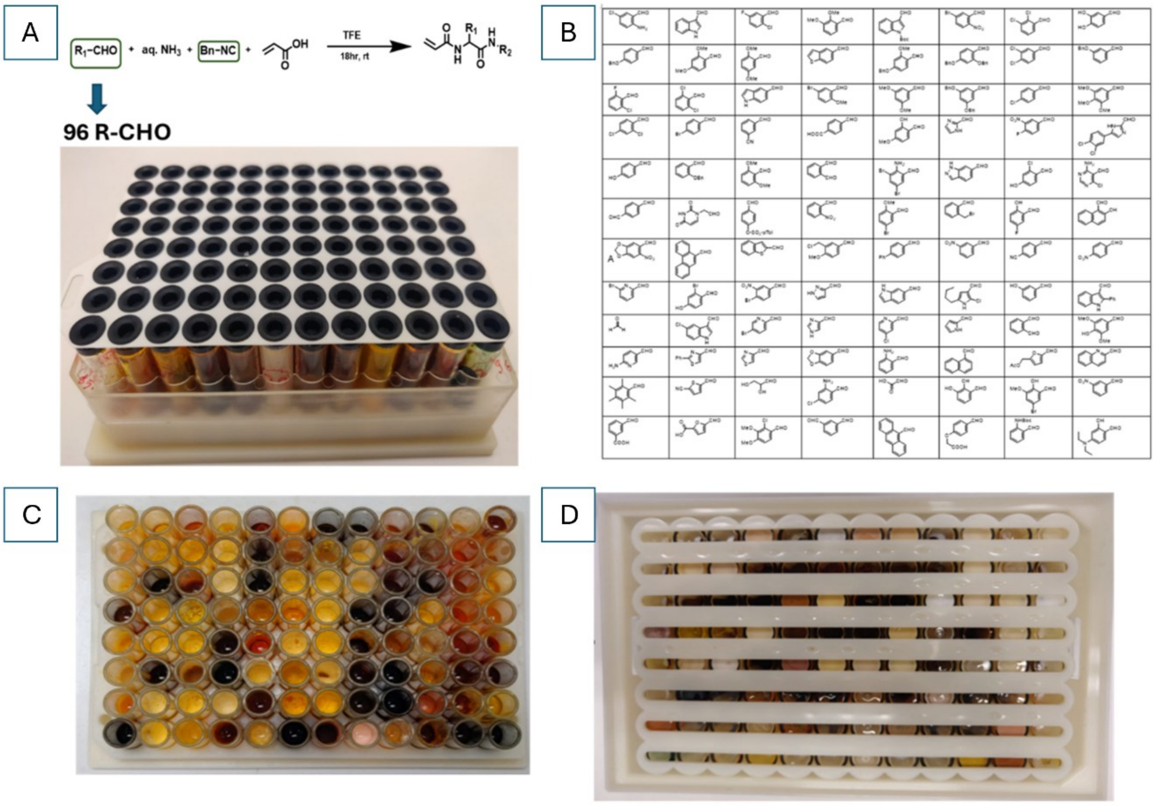

Figure S5: Overview of the 96-well plate setup for screening aldehydes. The figure displays (**a**) the synthetic scheme of the reaction (**b**) the list and well positions of the 96 aldehydes tested, (**c**) a top view of the reaction plate after 18 hours of stirring at room temperature, and (**d**) a bottom view of the plate highlighting wells where solid formation was observed.

Following dilution with diethyl ether and petroleum ether and subsequent filtration, 68 products were successfully obtained as solid powders.

2.2 Optimization of the purification process

The filtration process by manual transferring proved challenging, as many products became cross-contaminated during transferring to the storage vials (**Figure S6a).** To address this issue, a Whatman filter plate with detachable bottom filters was initially tested, but this method failed due to insufficient pressure retention (Figure **S6b**) and for its high cost and the inability to reuse the filters after detachment. To improve filtration, the plate was equipped with four additional screws (Figure **S6c**), but also this resulted in insufficient due to the plate bending at the center, leading to contamination of many vials. For this reason, more screws and filter layers (Figure **S6d**) were added leading to further enhancement of the solid isolation. In fact, after removing the filters, the solid products were easily transferred to another 96-well glass plate by gently pushing the solid product with a spatula. The products were then collected in the corresponding wells of the storage 96-well glass vials (Figure **S6e**). Anyway, the plate was prone to breaking after the second or third use due to its material.

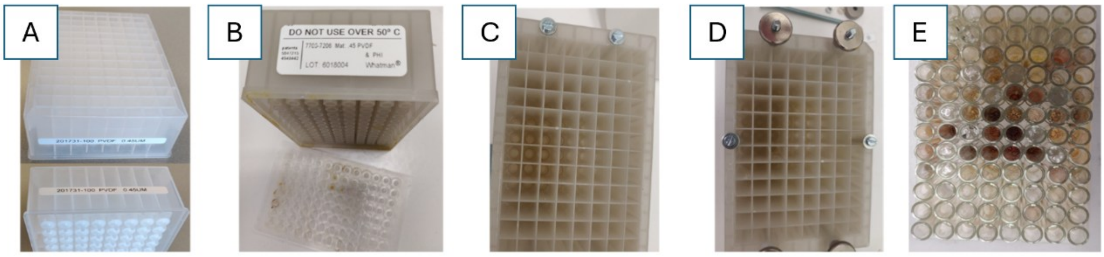

Figure S6: Designing of filtration well plate **a**) Initial attempt using a PVDF 96-well filtration plate with fixed filters. **b**) Whatman PVDF 96-well filtration plate with detachable bottom filters. **c**) Modified Whatman PVDF 96-well filtration plate with filter paper, secured with four screws. **d**) Modified Whatman PVDF 96-well filtration plate with filter paper, secured with six screws and stronger support for multilayer filter papers. **e**) Wells for storage.

As a result, a prototype aluminum filtration plate (**Figure S7**) was designed and built. The plate consists of an upper cover cap to close and protect the solids while removing the filter paper, allowing the entire plate to be inverted. It features a central metal well block with a 4 cm height to accommodate a 2.5 mL solvent volume, and a bottom plate designed to hold filter paper for the 96 wells. The bottom has broad-mouth openings that allow the plate to be securely placed on another 96-well plate, enabling the transfer of solids after the removal of the filter paper.

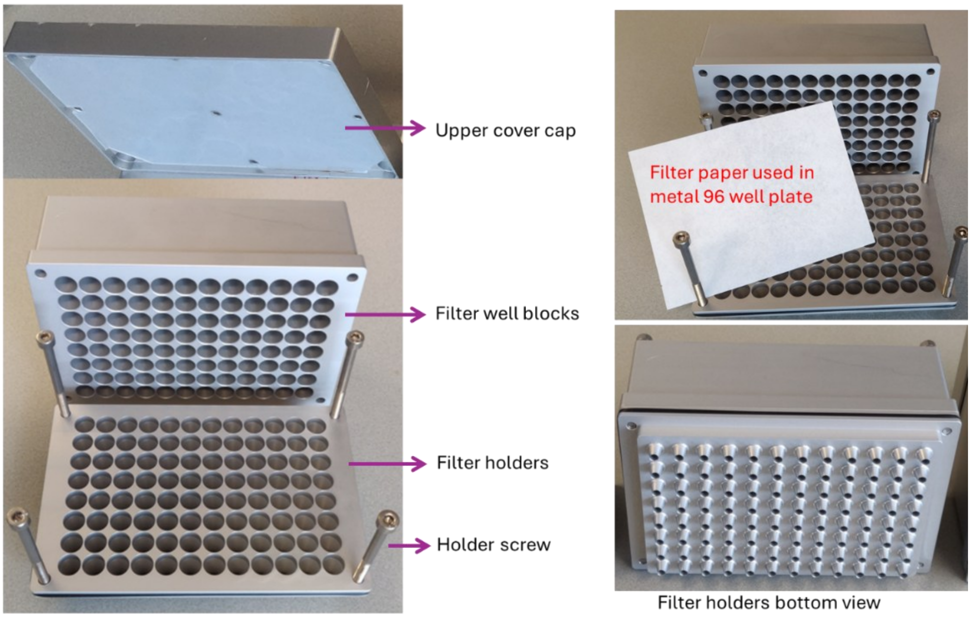

Figure S7: Components of an in-house made aluminum 96-well filter plate.

As the newly designed and constructed metal plate demonstrated strength, no leakage at the bottom of the filters, easy transfer of the product to storage vials without contamination, reusability, and easy cleaning, we replace the expensive one-time use Whatman® filtration plate with it in our purification process (**Figure S8**).

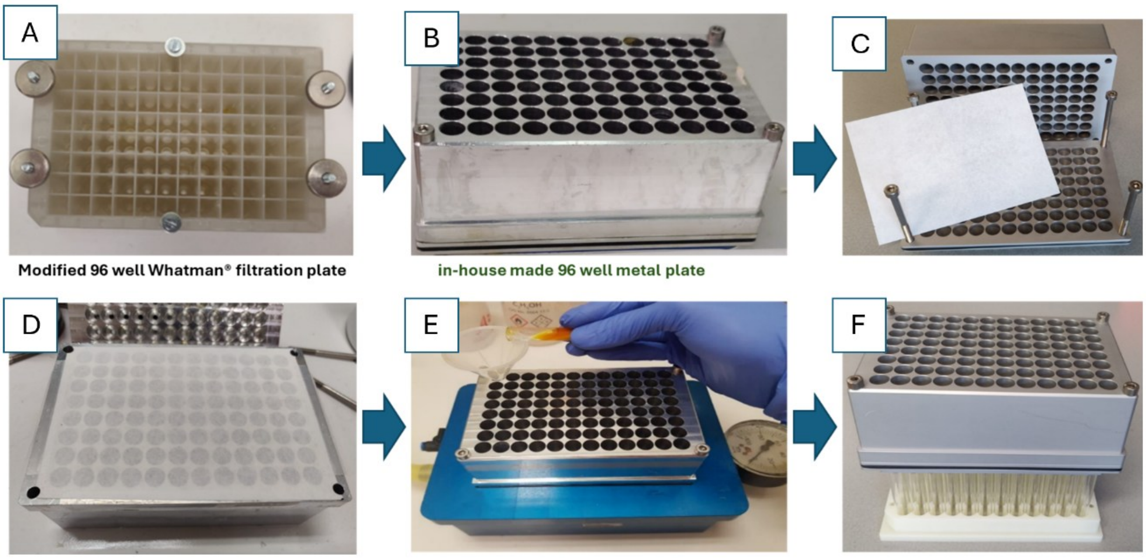

Figure S8: Steps for using the in-house made 96-well metal filtration plate: **(a-b)** Whatman® filtration plate and in-house 96 well metal plate. **(c)** Unscrew to add filter paper, which is cut to the exact size of the filtration plate. (**d**) Position the filter paper between the metal well holder block and the lower filter holder. **(e)** Manually add each reaction well to the metal filtration plate. **(f)** After filtration and drying under vacuum, unscrew the metal well plate, protecting the upper opening. Carefully remove the screws without disturbing the solids. After removing the filter paper, clean the base plate and reassemble by closing the screws. The filter plate is then placed on another 96-well glass plate, and the solid material is pushed down using a spatula.

One additional challenge was represented by the time-consuming transfer of precipitated products from reaction plates to filtration plates, which required picking up each glass well and transferring it to the metal filtration plate (**Figure S8e**). To increase efficiency, we initially used 8-channel and 12-channel pipettes. However, this method proved ineffective as the small tip openings of the pipettes made it difficult to transfer the precipitate. To address this, we modified the pipette tips by cutting off a few millimeters from the tips to create a broader opening. This modification allowed for easier mixing of solvents and smoother transfer of precipitates to the filtration plate (**Figure S9b**), leading to the processing of four reaction plates per day.

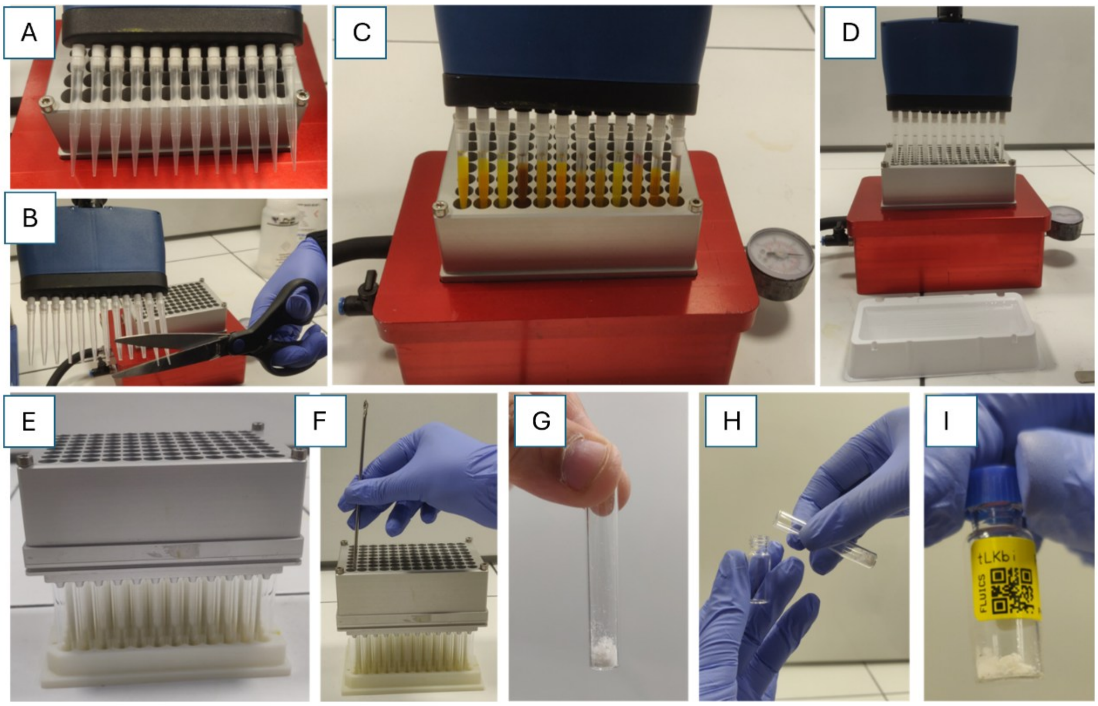

Figure S9: Trial workflow for efficient transfer, isolation and storage of precipitated products. (**a**) 12-channel pipette used for dilution of 96-well reaction plate; (**b**) Pipette tips modified by cutting the ends to widen the opening, ensuring complete transfer of precipitates using a multi-channel pipette; (**c**) Transfer of precipitated products to the metal 96-well filtration plate using a 12-channel pipette; (**d**) Washing of precipitates with diethyl ether; (**e**) Transferring the solid products to a 96-well glass collection plate; (**f**) A typical single glass vial containing the isolated product; (**g**) Transferring the product to a 1.5 mL storage vial; (**h**) Final storage vial labeled with a QR code for tracking and long-term storage.

Once resolved the filtration and isolation challenges, some initial reactions in a 96-well glass plate following a trial protocol (**Figure S9**) and then transferred them to the newly modified metal filtration plate for the isolation of pure precipitated products were conducted.

3 New prototypes of metal filtration plates

Encouraged by these promising results, we developed in-house prototypes of metal filtration plates for various reaction scales: a 384-well metal filtration plate for 0.1 mmol reactions, a 96-well metal filtration plate for 0.5–1 mmol reactions, and a 24-well metal filtration plate for 10 mmol scale reactions (**Figure S10 and S11**). All metal filtration plates, along with their corresponding vacuum manifold setups, are shown in the following figure.

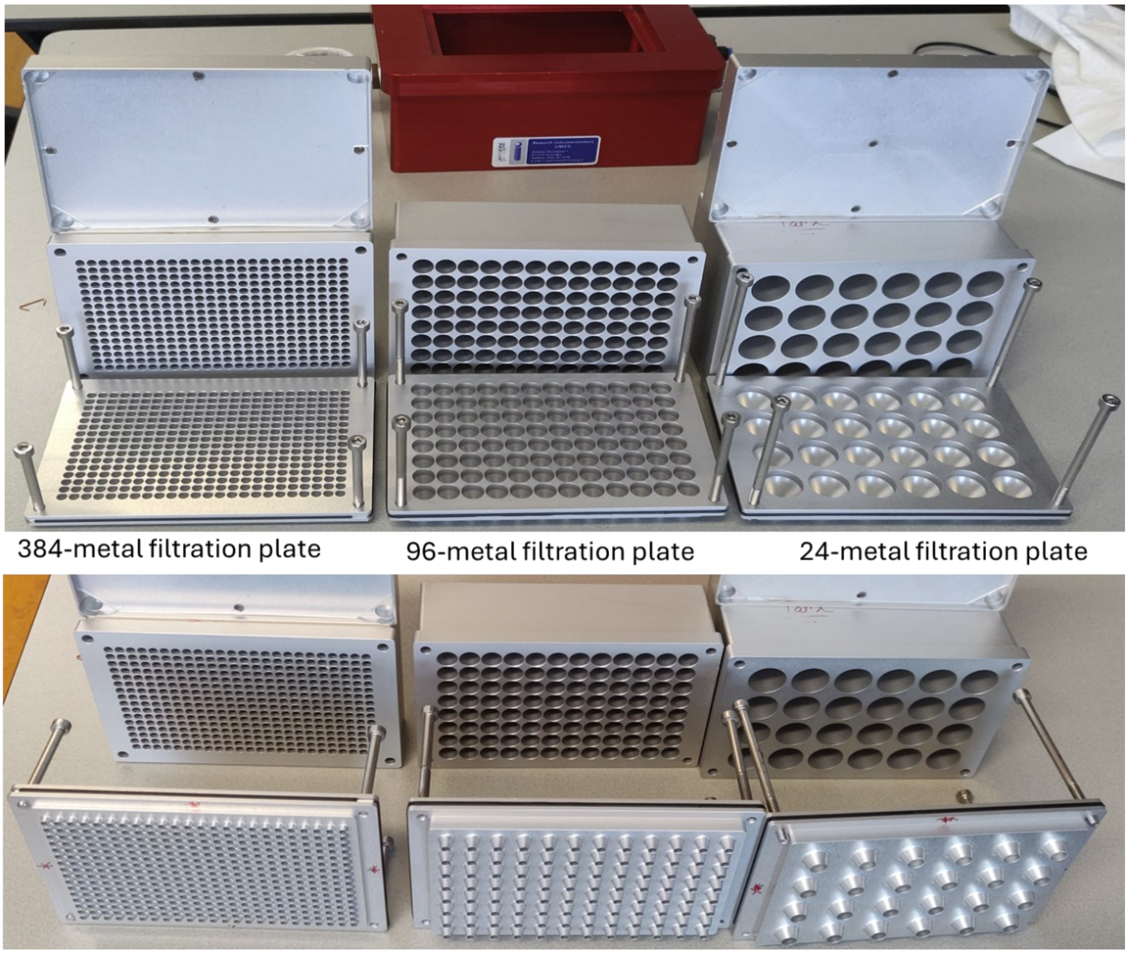

Figure S10: In-house constructed metal filtration plates designed for different reaction scales. The setups include a 384-well metal filtration plate for 0.1 mmol reactions, a 96-well metal filtration plate for 0.5–1 mmol reactions, and a 24-well metal filtration plate for 10 mmol scale reactions.

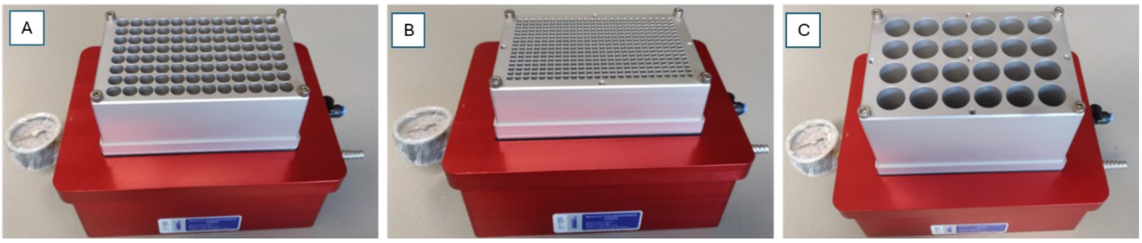

Figure S11: Vacuum manifold setups for metal filtration plates. (**a**) Typical 96-well filtration plate with vacuum manifold. (**b**) 384-well filtration plate with vacuum manifold. (**c**) 24-well filtration plate with vacuum manifold.

4 General synthetic procedure for the Ugi reaction

To a 96-well glass plate equipped with a stirring bar, 0.3 mL of trifluoroethanol (TFE) was added, followed by the addition of stock solutions of aldehydes in TFE (0.5 mmol, 1.0 equiv., 5 M solution, 0.1 mL or 2.5 M solution, 0.2 mL). Next, an aqueous ammonia solution (1 mmol, 2.0 equiv., 57 µL of 30% ammonia in water) was added using multichannel pipettes, and the reaction was stirred at room temperature for 5 minutes. Then, stock solutions of isocyanides in TFE (0.5 mmol, 1.0 equiv., 0.1 mL of 5 M or 0.2 mL of 2.5 M) and acrylic acid (35 µL, 1.0 equiv., 0.5 mmol) were added using multichannel pipettes. The reaction was stirred for 18 hours at room temperature. After 18 hours, the reaction wells were diluted with 1 mL of diethyl ether and stirred at 0°C for 15 minutes. Wells that did not show free-flowing solids were stirred with a small spatula to encourage precipitation. For wells where no precipitation occurred, 0.5 mL of petroleum ether was added, and the mixture was stirred for an additional 15 minutes at 0°C. The reactions were then filtered through a metal filtration plate using multichannel pipettes with modified tips. The residue obtained on the filtration plate was washed with 1 mL of diethyl ether, followed by washing with 50% petroleum ether in diethyl ether to remove unreacted acrylic acid. The reaction plates were dried under vacuum on the filtration assembly for 30 minutes to 1 hour. After drying, the filter was carefully removed by unscrewing the filtration plate and inverting it while closing the top of the plate. A clean base plate was then attached, and the assembly was placed on a new 96-well glass plate. After gently pushing the solids from the top, all products were collected in the collection 96-well plates. Wells where no precipitation occurred were left empty.

4.1 List of building blocks used in the study

List of Isocyanides used in the parallel synthesis (part 1)

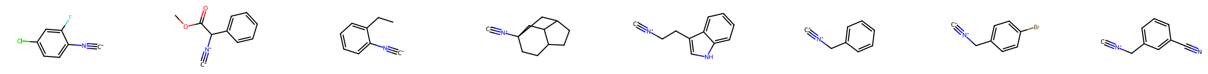

Iso_0 Iso_1 Iso_2 Iso_3 Iso_4 Iso_5 Iso_6 Iso_7

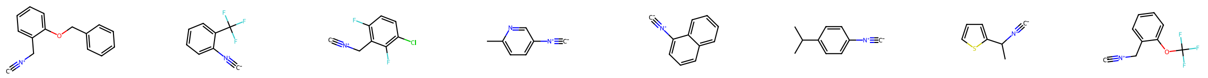

Iso_8 Iso_9 Iso_10 Iso_11 Iso_12 Iso_13 Iso_14 Iso_15

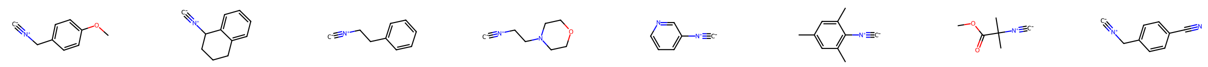

Iso_16 Iso_17 Iso_18 Iso_19 Iso_20 Iso_21 Iso_22 Iso_23

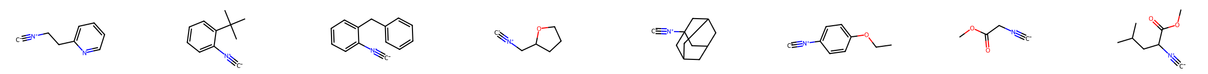

Iso_24 Iso_25 Iso_26 Iso_27 Iso_28 Iso_29 Iso_30 Iso_31

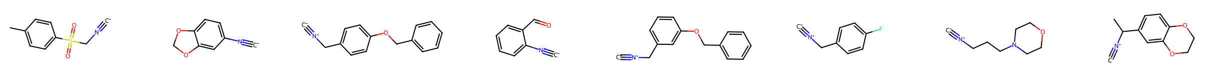

Iso_32 Iso_33 Iso_34 Iso_35 Iso_36 Iso_37 Iso_38 Iso_39

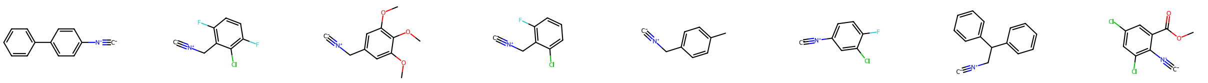

Iso_40 Iso_41 Iso_42 Iso_43 Iso_44 Iso_45 Iso_46 Iso_47

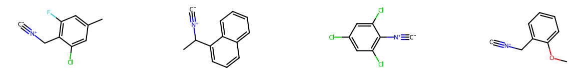

Iso_48 Iso_49 Iso_50 Iso_51

List of Isocyanides used in the parallel synthesis (part 2)

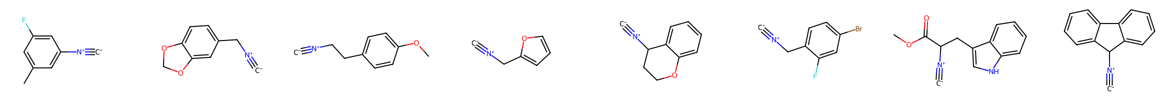

Iso_52 Iso_53 Iso_54 Iso_55 Iso_56 Iso_57 Iso_58 Iso_59

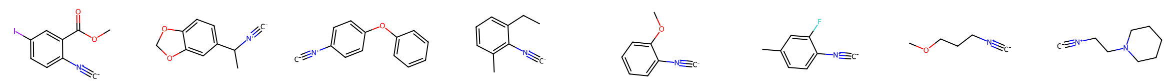

Iso_60 Iso_61 Iso_62 Iso_63 Iso_64 Iso_65 Iso_66 Iso_67

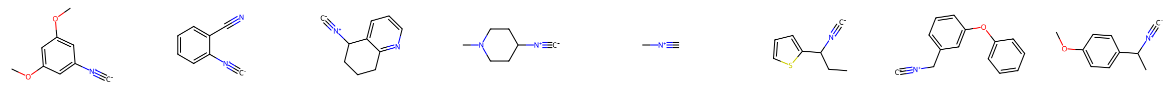

Iso_68 Iso_69 Iso_70 Iso_71 Iso_72 Iso_73 Iso_74 Iso_75

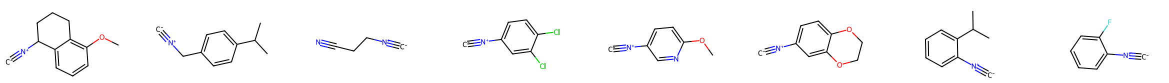

Iso_76 Iso_77 Iso_78 Iso_79 Iso_80 Iso_81 Iso_82 Iso_83

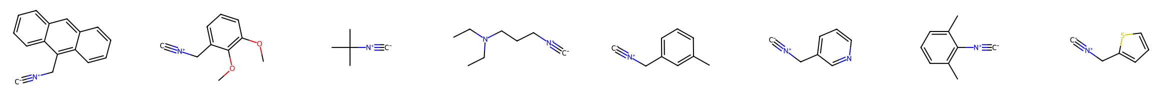

Iso_84 Iso_85 Iso_86 Iso_87 Iso_88 Iso_89 Iso_90 Iso_91

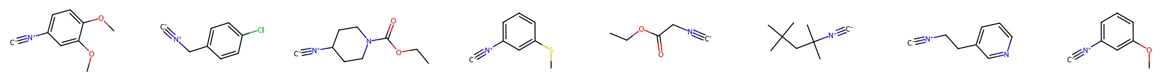

Iso_92 Iso_93 Iso_94 Iso_95 Iso_96 Iso_97 Iso_98 Iso_99

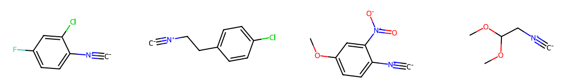

Iso_100 Iso_101 Iso_102 Iso_103

List of aldehydes used in the parallel synthesis (part 1)

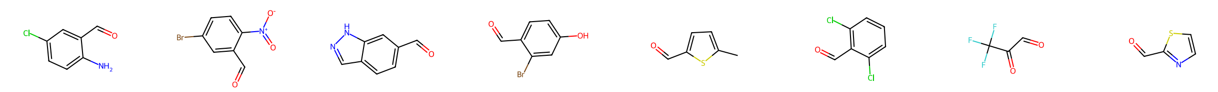
Ald_0 Ald_1 Ald_2 Ald_3 Ald_4 Ald_5 Ald_6 Ald_7

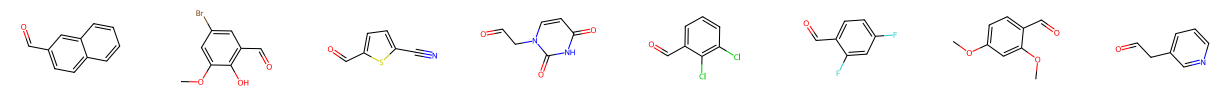
Ald_8 Ald_9 Ald_10 Ald_11 Ald_12 Ald_13 Ald_14 Ald_15

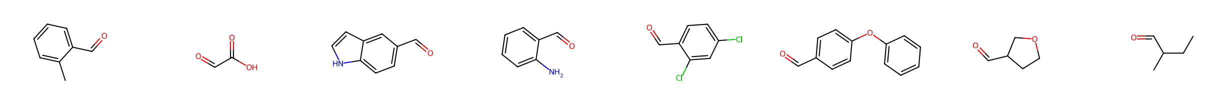
Ald_16 Ald_17 Ald_18 Ald_19 Ald_20 Ald_21 Ald_22 Ald_23

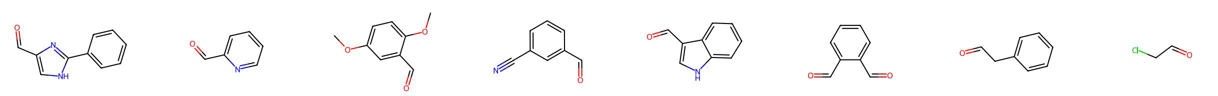
Ald_24 Ald_25 Ald_26 Ald_27 Ald_28 Ald_29 Ald_30 Ald_31

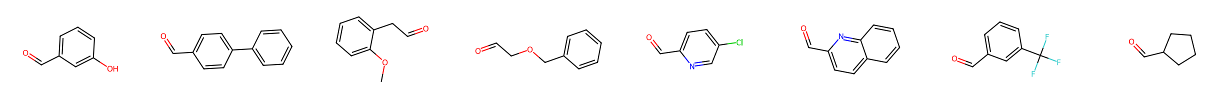
Ald_32 Ald_33 Ald_34 Ald_35 Ald_36 Ald_37 Ald_38 Ald_39

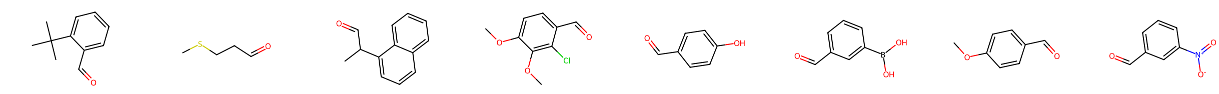
Ald_40 Ald_41 Ald_42 Ald_43 Ald_44 Ald_45 Ald_46 Ald_47

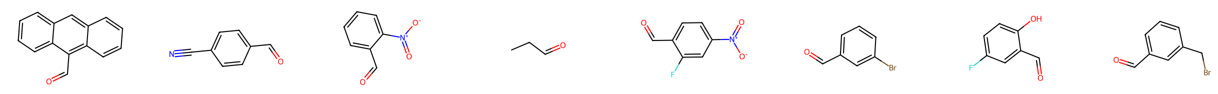
Ald_48 Ald_49 Ald_50 Ald_51 Ald_52 Ald_53 Ald_54 Ald_55

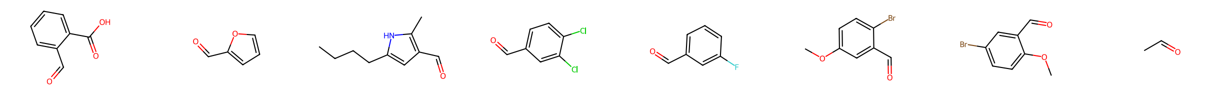
Ald_56 Ald_57 Ald_58 Ald_59 Ald_60 Ald_61 Ald_62 Ald_63

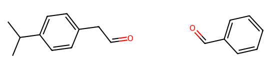
Ald_64 Ald_65

List of aldehydes used in the parallel synthesis (part 2)

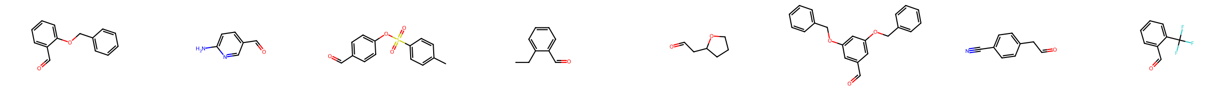

Ald_66 Ald_67 Ald_68 Ald_69 Ald_70 Ald_71 Ald_72 Ald_73

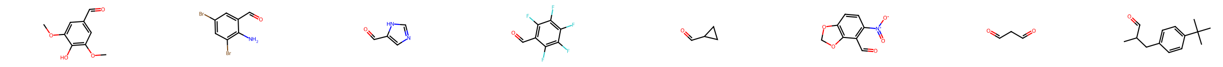

Ald_74 Ald_75 Ald_76 Ald_77 Ald_78 Ald_79 Ald_80 Ald_81

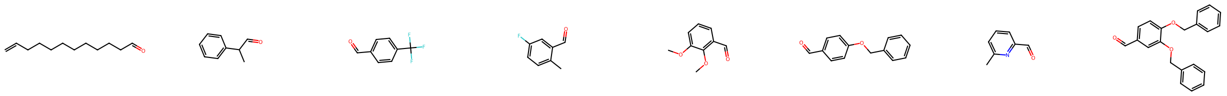

Ald_82 Ald_83 Ald_84 Ald_85 Ald_86 Ald_87 Ald_88 Ald_89

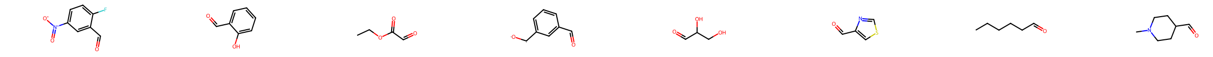

Ald_90 Ald_91 Ald_92 Ald_93 Ald_94 Ald_95 Ald_96 Ald_97

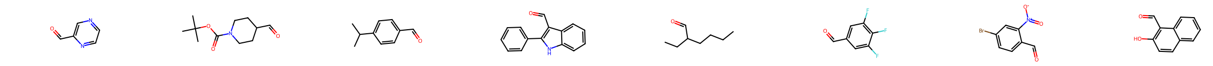

Ald_98 Ald_99 Ald_100 Ald_101 Ald_102 Ald_103 Ald_104 Ald_105

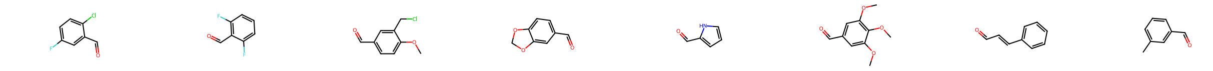

Ald_106 Ald_107 Ald_108 Ald_109 Ald_110 Ald_111 Ald_112 Ald_113

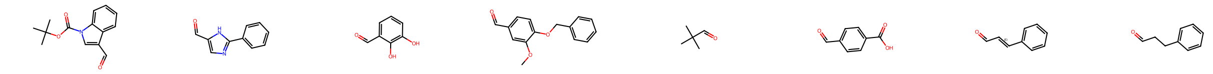

Ald_114 Ald_115 Ald_116 Ald_117 Ald_118 Ald_119 Ald_120 Ald_121

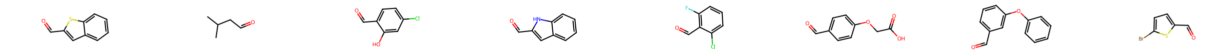

Ald_122 Ald_123 Ald_124 Ald_125 Ald_126 Ald_127 Ald_128 Ald_129

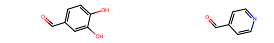

Ald_130 Ald_131

List of aldehydes used in the parallel synthesis (part 3)

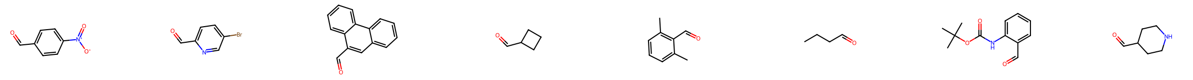

Ald_132 Ald_133 Ald_134 Ald_135 Ald_136 Ald_137 Ald_138 Ald_139

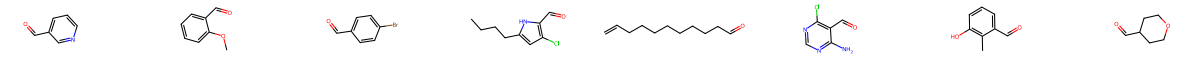

Ald_140 Ald_141 Ald_142 Ald_143 Ald_144 Ald_145 Ald_146 Ald_147

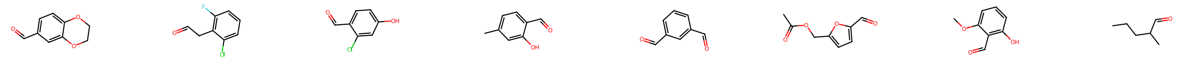

Ald_148 Ald_149 Ald_150 Ald_151 Ald_152 Ald_153 Ald_154 Ald_155

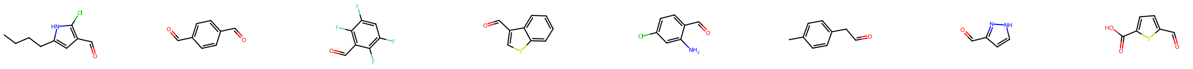

Ald_156 Ald_157 Ald_158 Ald_159 Ald_160 Ald_161 Ald_162 Ald_163

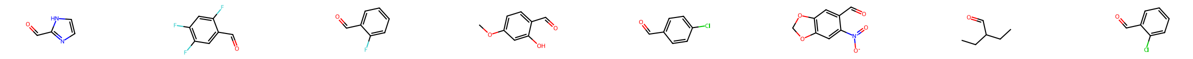

Ald_164 Ald_165 Ald_166 Ald_167 Ald_168 Ald_169 Ald_170 Ald_171

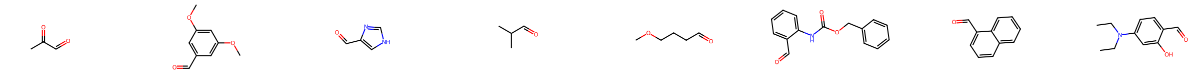

Ald_172 Ald_173 Ald_174 Ald_175 Ald_176 Ald_177 Ald_178 Ald_179

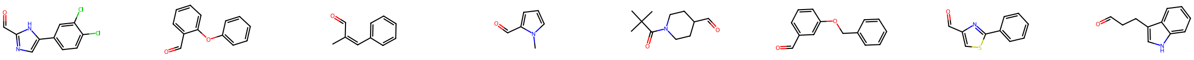

Ald_180 Ald_181 Ald_182 Ald_183 Ald_184 Ald_185 Ald_186 Ald_187

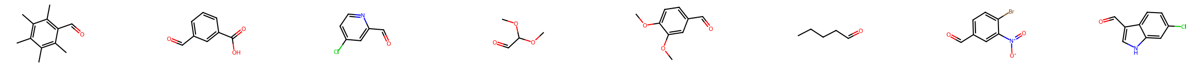

Ald_188 Ald_189 Ald_190 Ald_191 Ald_192 Ald_193 Ald_194 Ald_195

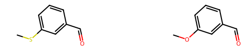

Ald_196 Ald_197

5 Stock solution preparation of starting compounds

96 solid aldehydes were dissolved in trifluoroethanol (TFE) to a concentration of 2.5 M (**Figure S12C**), while 68 liquid aldehydes were prepared as 5 M solutions in TFE (**Figure S12B**) and stored in a refrigerator to maintain stability. Similarly, 83 isocyanide stock solutions were prepared at 5 M and in TFE and stored at 0 °C (**Figure S12A**). For isocyanides that were poorly soluble at this concentration, 2. 5 M stock solutions were prepared.

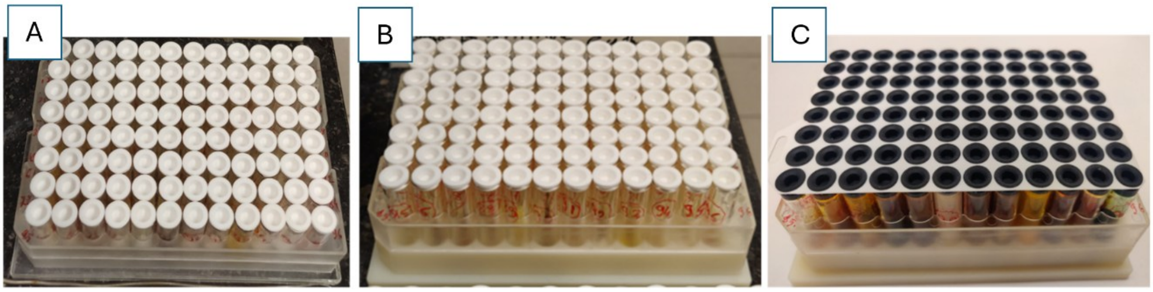

Figure S12: Preparation of aldehyde and isocyanide stock solutions. **(a)** Isocyanide stock solutions in TFE. **(b)** 5 M solutions of liquid aldehydes in TFE. **(c)** 2.5 M solutions of solid aldehydes in TFE.

6 96-well plates synthesis

Starting materials were randomly selected and mixed to perform a total of twenty-nine 96-well plates, at a 0.5 mmol scale. All synthesis took a three-weeks period, with less than an hour of set up for each 96-well plate and a more time-consuming work-up process.

The optimized standardized purification protocol included dilution of reaction mixture using diethyl ether, mixing, brief scratching of solidified products in wells (when needed), cooling at 0–5 °C for 20 minutes under stirring, transferring the reaction plate to the filtration plate, washing with 1 mL of 50% diethyl ether in petroleum ether, followed by 1 mL of petroleum ether alone, drying, and final transfer. The entire workup process took approximately 5–6 hours. Therefore, under optimized conditions, a chemist could conduct 2–3 reaction plates per day and successfully isolate products from one reaction plate within the same timeframe (**Figure S13**).

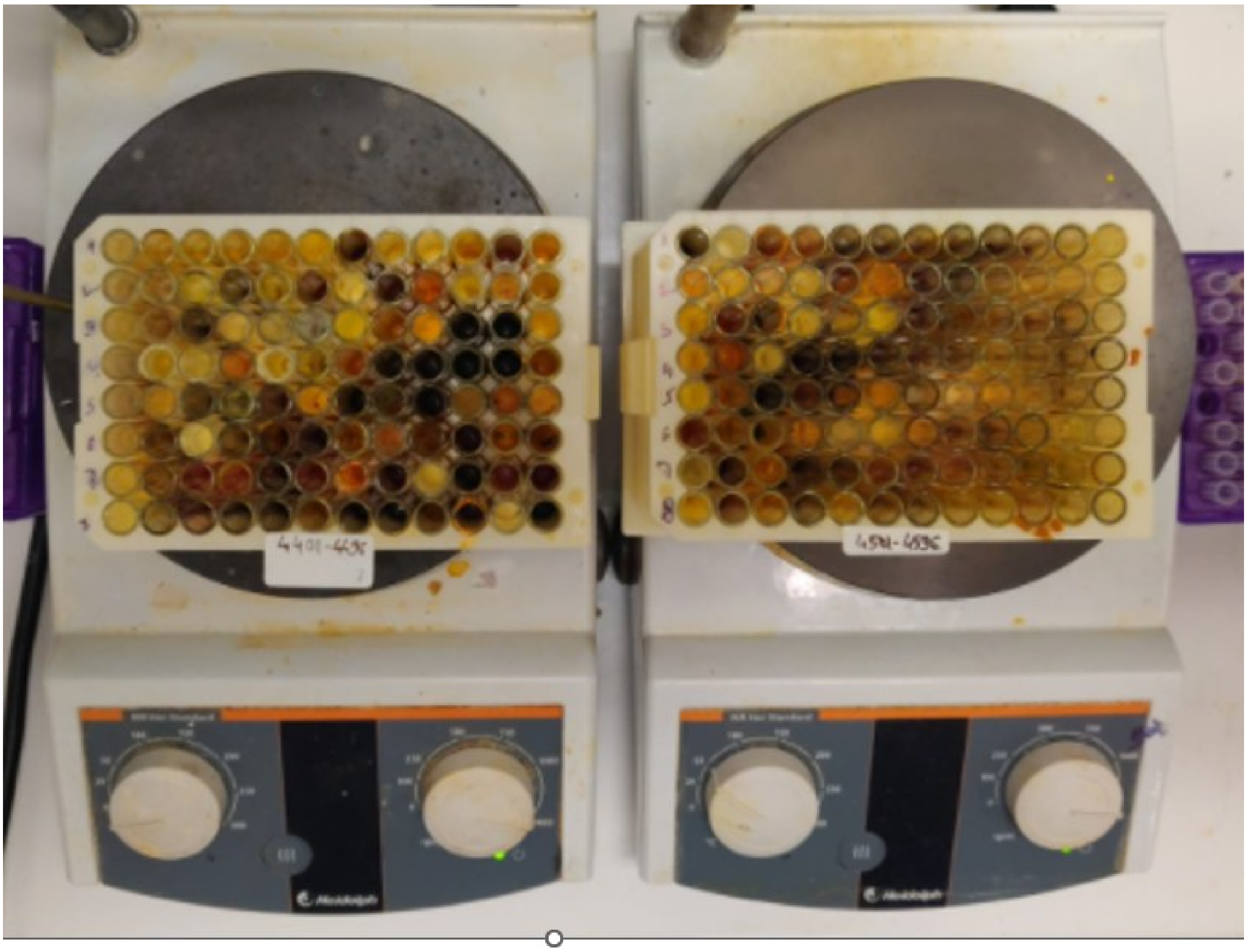

Figure S13: Two parallel 96-well plate reactions.

7 Analytical techniques

7.1 NMR analysis of isolated solid product and storage

After transferring the products to collection plates, each solid was analyzed by NMR spectroscopy (**Figure S14**), equipped with an autosampler.

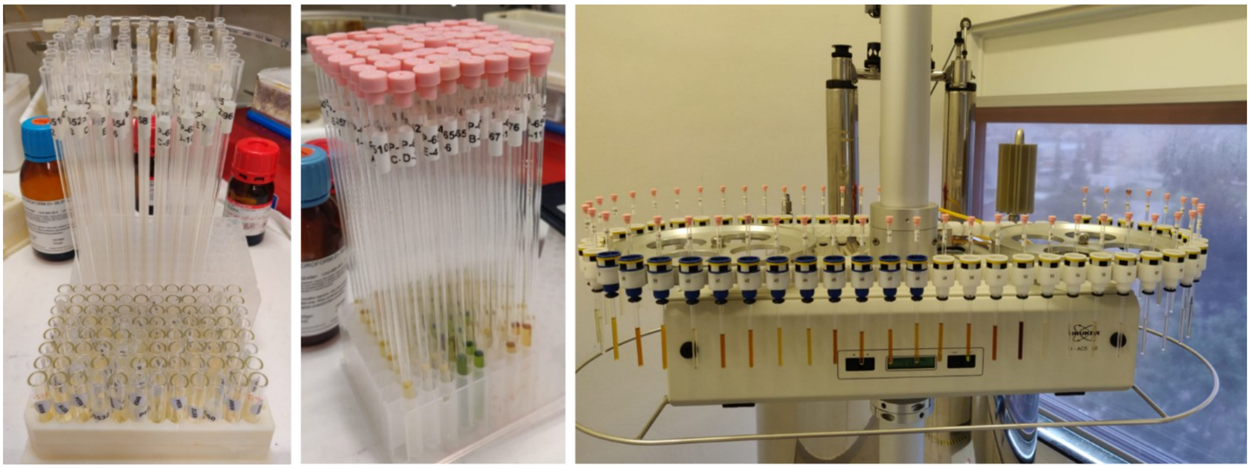

Figure S14: NMR analysis of isolated solid products after transfer to collection plates.

After the NMR analysis, 1235 products exhibited more than 80% purity by NMR. These were selected as successful and stored as solids and stock solutions in DMSO at 10 mM concentration in thirteen plates of 96-well plate format to use for further analysis, while the not pure ones were discarded (**Figure S15**).

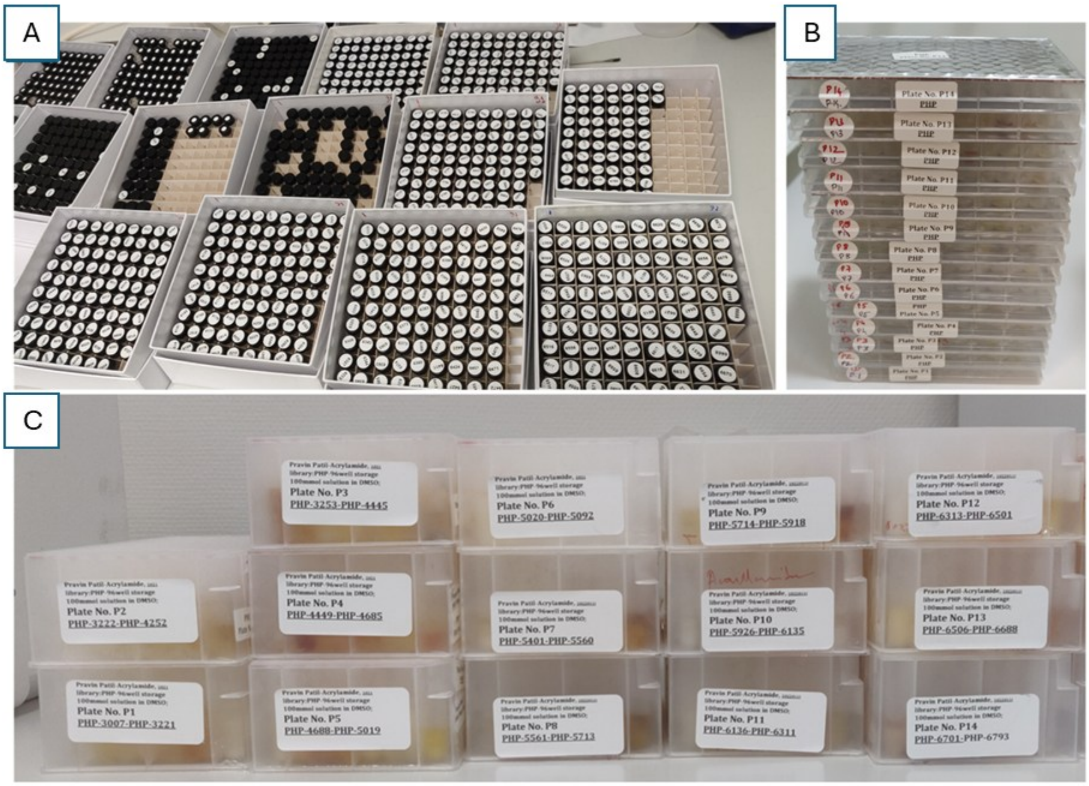

Figure S15: Storage and preparation of acrylamide compound library**. (a**) Library of acrylamide compounds organized in storage boxes as solids; (**b**) Stock solutions prepared at 10 mM concentration and stored in 96-well plates, ready for dispatch for biological studies; (**c**) Stock solutions of each product prepared at 100 mM concentration (1 mL per well) and stored in 96-well plates at room temperature for further biological testing.

7.2 Structures of the isolated products

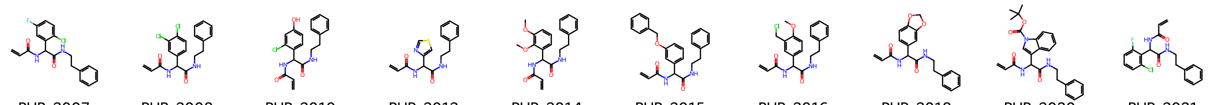

PHP-3007 PHP-3008 PHP-3010 PHP-3013 PHP-3014 PHP-3015 PHP-3016 PHP-3018 PHP-3020 PHP-3021

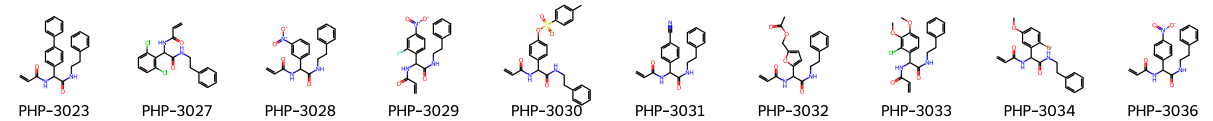

PHP-3023 PHP-3027 PHP-3028 PHP-3029 PHP-3030 PHP-3031 PHP-3032 PHP-3033 PHP-3034 PHP-3036

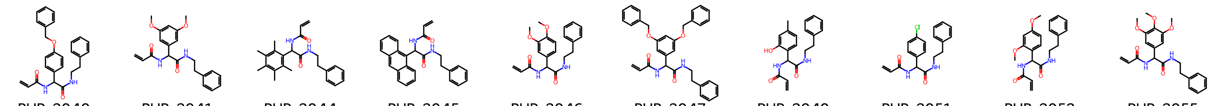

PHP-3040 PHP-3041 PHP-3044 PHP-3045 PHP-3046 PHP-3047 PHP-3049 PHP-3051 PHP-3052 PHP-3055

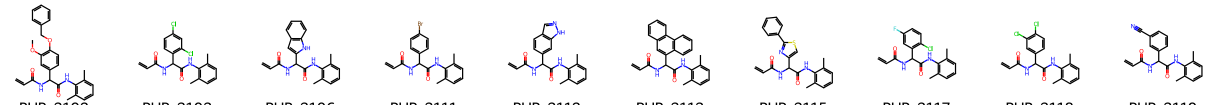

PHP-3102 PHP-3103 PHP-3106 PHP-3111 PHP-3112 PHP-3113 PHP-3115 PHP-3117 PHP-3118 PHP-3119

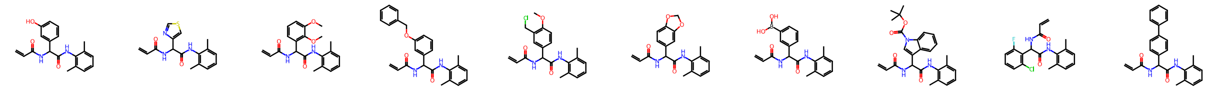

PHP-3122 PHP-3123 PHP-3125 PHP-3126 PHP-3129 PHP-3131 PHP-3132 PHP-3133 PHP-3134 PHP-3137

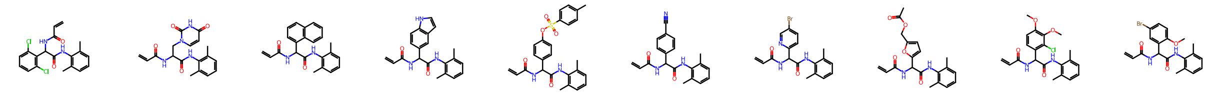

PHP-3142 PHP-3144 PHP-3147 PHP-3150 PHP-3152 PHP-3153 PHP-3154 PHP-3155 PHP-3156 PHP-3158

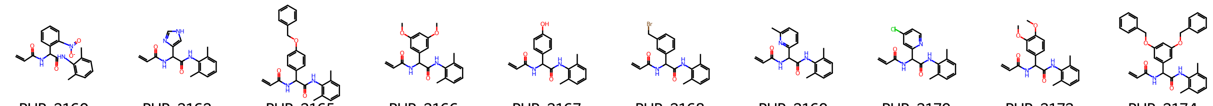

PHP-3160 PHP-3162 PHP-3165 PHP-3166 PHP-3167 PHP-3168 PHP-3169 PHP-3170 PHP-3173 PHP-3174

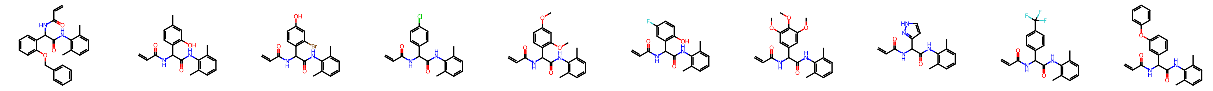

PHP-3175 PHP-3176 PHP-3177 PHP-3182 PHP-3183 PHP-3184 PHP-3190 PHP-3193 PHP-3201 PHP-3202

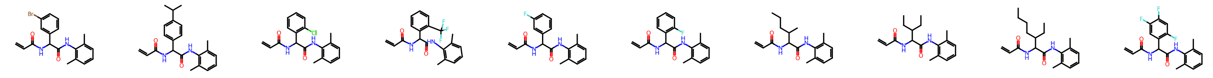

PHP-3203 PHP-3204 PHP-3205 PHP-3206 PHP-3207 PHP-3208 PHP-3209 PHP-3210 PHP-3212 PHP-3213

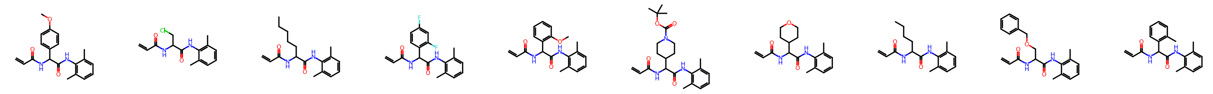

PHP-3214 PHP-3216 PHP-3217 PHP-3218 PHP-3220 PHP-3221 PHP-3222 PHP-3224 PHP-3225 PHP-3226

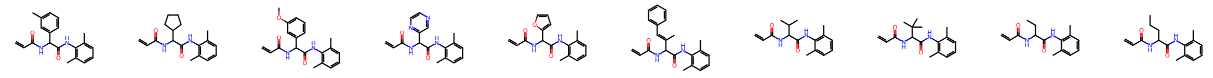

PHP-3227 PHP-3229 PHP-3230 PHP-3231 PHP-3232 PHP-3234 PHP-3236 PHP-3238 PHP-3239 PHP-3240

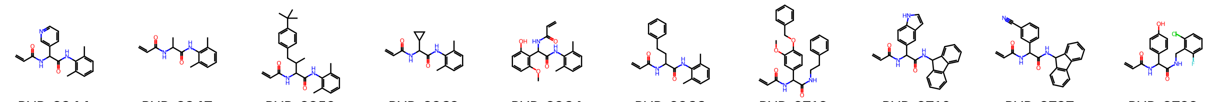

PHP-3244 PHP-3247 PHP-3259 PHP-3262 PHP-3264 PHP-3266 PHP-3713 PHP-3719 PHP-3727 PHP-3733

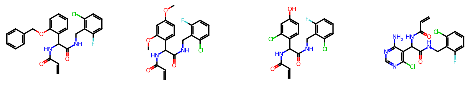

PHP-3734 PHP-3735 PHP-3739 PHP-3740

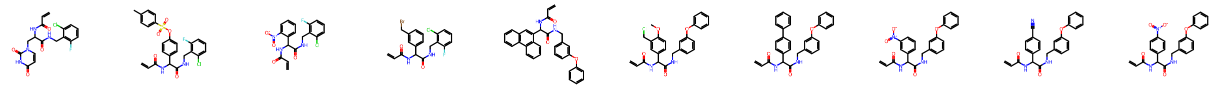

PHP-3742 PHP-3743 PHP-3744 PHP-3745 PHP-3750 PHP-3752 PHP-3753 PHP-3754 PHP-3755 PHP-3756

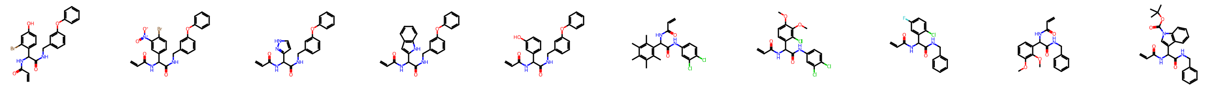

PHP-3758 PHP-3759 PHP-3760 PHP-3761 PHP-3763 PHP-3781 PHP-3791 PHP-3803 PHP-3804 PHP-3805

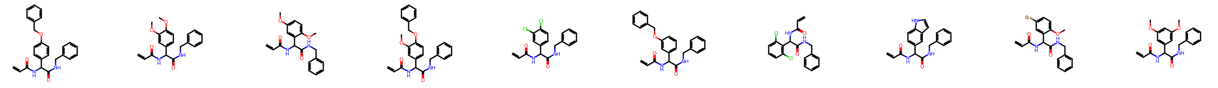

PHP-3809 PHP-3810 PHP-3811 PHP-3813 PHP-3815 PHP-3816 PHP-3818 PHP-3819 PHP-3820 PHP-3821

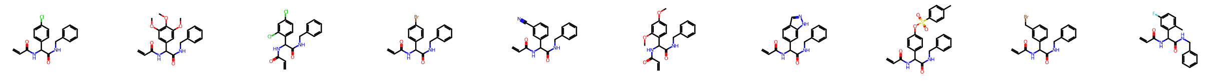

PHP-3823 PHP-3824 PHP-3825 PHP-3826 PHP-3827 PHP-3834 PHP-3838 PHP-3843 PHP-3845 PHP-3847

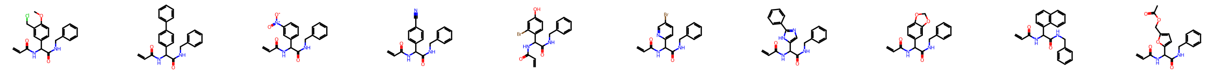

PHP-3852 PHP-3853 PHP-3854 PHP-3855 PHP-3858 PHP-3867 PHP-3874 PHP-3876 PHP-3878 PHP-3879

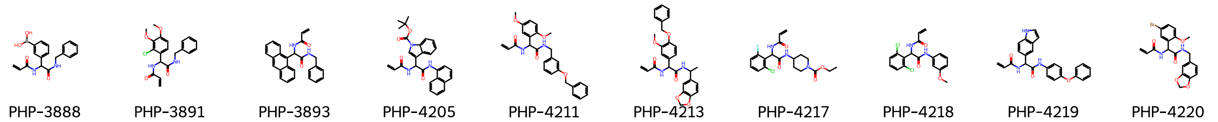

PHP-3888 PHP-3891 PHP-3893 PHP-4205 PHP-4211 PHP-4213 PHP-4217 PHP-4218 PHP-4219 PHP-4220

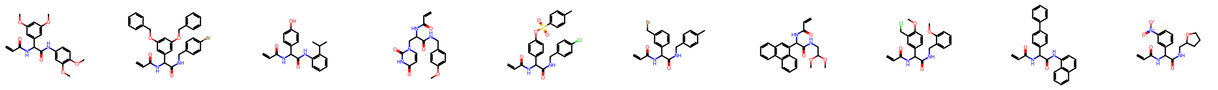

PHP-4221 PHP-4222 PHP-4233 PHP-4242 PHP-4243 PHP-4245 PHP-4250 PHP-4252 PHP-4253 PHP-4254

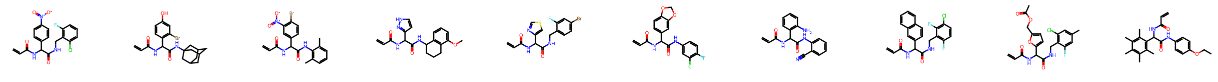

PHP-4256 PHP-4258 PHP-4259 PHP-4260 PHP-4275 PHP-4276 PHP-4277 PHP-4278 PHP-4279 PHP-4281

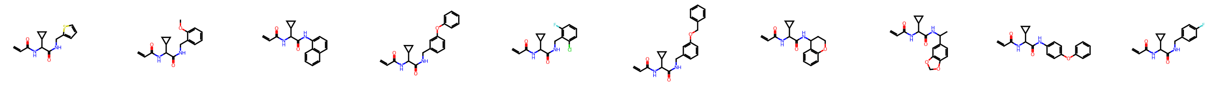

PHP-4301 PHP-4304 PHP-4305 PHP-4307 PHP-4308 PHP-4311 PHP-4312 PHP-4313 PHP-4314 PHP-4315

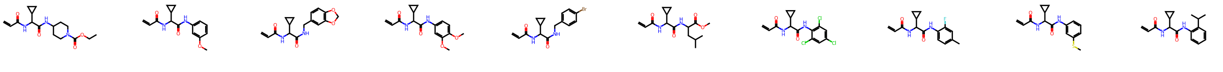

PHP-4317 PHP-4318 PHP-4320 PHP-4321 PHP-4322 PHP-4325 PHP-4327 PHP-4330 PHP-4332 PHP-4333

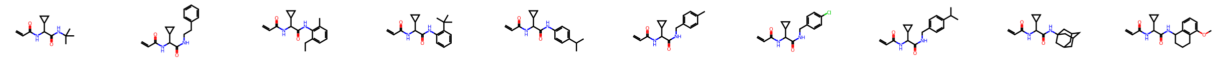

PHP-4334 PHP-4335 PHP-4336 PHP-4337 PHP-4340 PHP-4341 PHP-4343 PHP-4348 PHP-4349 PHP-4352

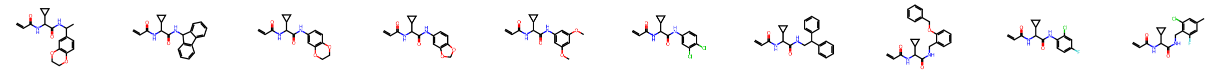

PHP-4354 PHP-4355 PHP-4357 PHP-4358 PHP-4359 PHP-4362 PHP-4363 PHP-4364 PHP-4365 PHP-4367

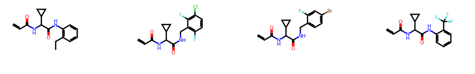

PHP-4368 PHP-4370 PHP-4372 PHP-4373

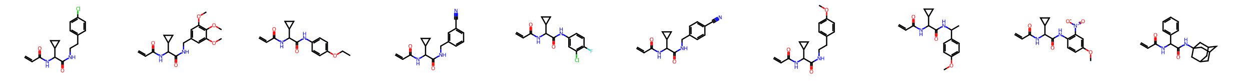

PHP-4374 PHP-4375 PHP-4376 PHP-4378 PHP-4380 PHP-4381 PHP-4383 PHP-4384 PHP-4388 PHP-4389

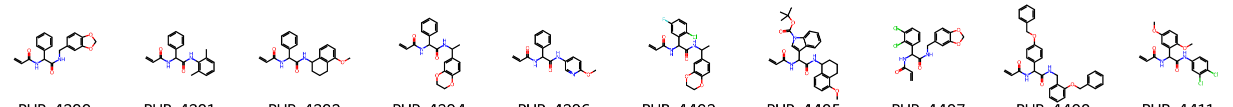

PHP-4390 PHP-4391 PHP-4392 PHP-4394 PHP-4396 PHP-4403 PHP-4405 PHP-4407 PHP-4409 PHP-4411

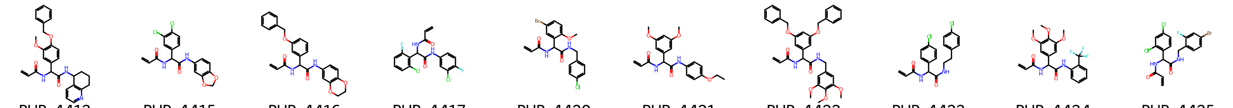

PHP-4413 PHP-4415 PHP-4416 PHP-4417 PHP-4420 PHP-4421 PHP-4422 PHP-4423 PHP-4424 PHP-4425

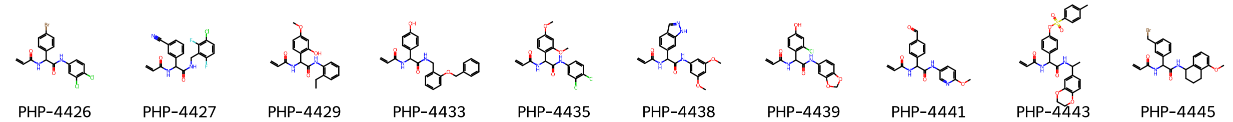

PHP-4426 PHP-4427 PHP-4429 PHP-4433 PHP-4435 PHP-4438 PHP-4439 PHP-4441 PHP-4443 PHP-4445

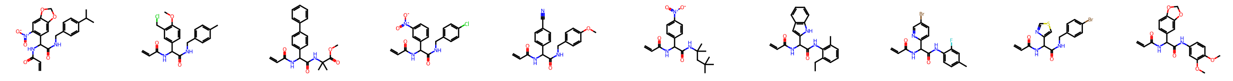

PHP-4449 PHP-4452 PHP-4453 PHP-4454 PHP-4455 PHP-4456 PHP-4461 PHP-4467 PHP-4475 PHP-4476

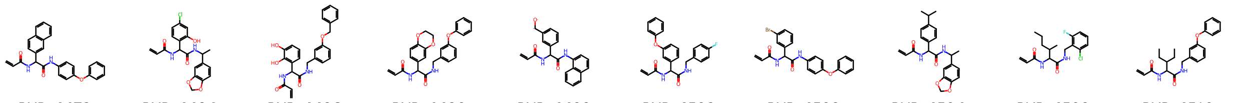

PHP-4478 PHP-4484 PHP-4486 PHP-4490 PHP-4492 PHP-4502 PHP-4503 PHP-4504 PHP-4509 PHP-4510

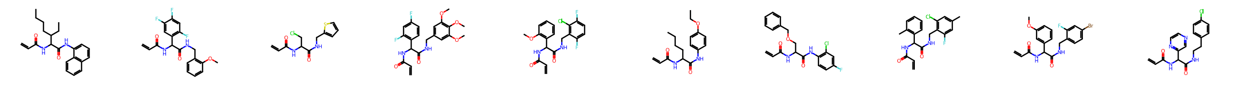

PHP-4512 PHP-4513 PHP-4516 PHP-4518 PHP-4520 PHP-4524 PHP-4525 PHP-4526 PHP-4530 PHP-4531

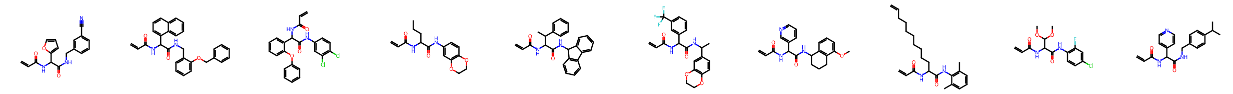

PHP-4532 PHP-4533 PHP-4535 PHP-4540 PHP-4542 PHP-4543 PHP-4545 PHP-4546 PHP-4548 PHP-4549

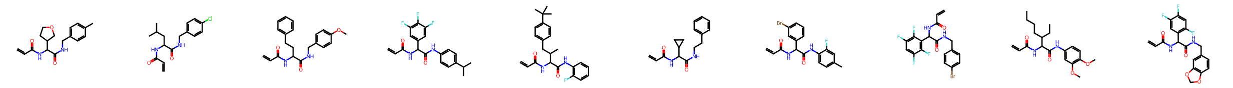

PHP-4552 PHP-4554 PHP-4555 PHP-4557 PHP-4559 PHP-4562 PHP-4567 PHP-4575 PHP-4576 PHP-4577

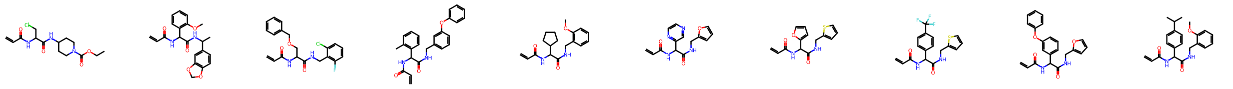

PHP-4580 PHP-4584 PHP-4589 PHP-4590 PHP-4593 PHP-4595 PHP-4596 PHP-4601 PHP-4602 PHP-4604

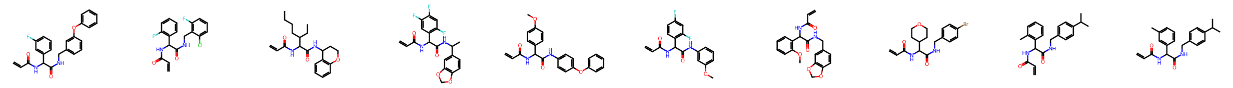

PHP-4607 PHP-4608 PHP-4612 PHP-4613 PHP-4614 PHP-4618 PHP-4620 PHP-4622 PHP-4626 PHP-4627

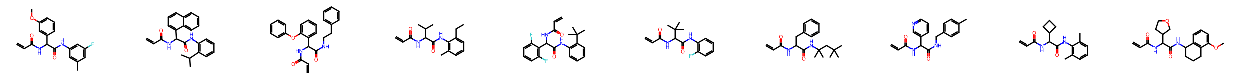

PHP-4630 PHP-4633 PHP-4635 PHP-4636 PHP-4637 PHP-4638 PHP-4641 PHP-4645 PHP-4651 PHP-4652

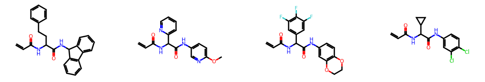

PHP-4655 PHP-4656 PHP-4657 PHP-4662

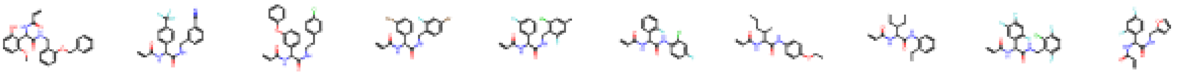

PHP-4664 PHP-4665 PHP-4666 PHP-4667 PHP-4671 PHP-4672 PHP-4673 PHP-4674 PHP-4677 PHP-4682

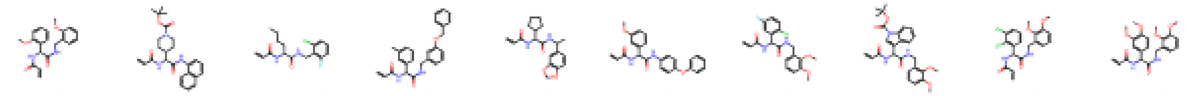

PHP-4684 PHP-4685 PHP-4688 PHP-4691 PHP-4693 PHP-4694 PHP-4801 PHP-4803 PHP-4805 PHP-4807

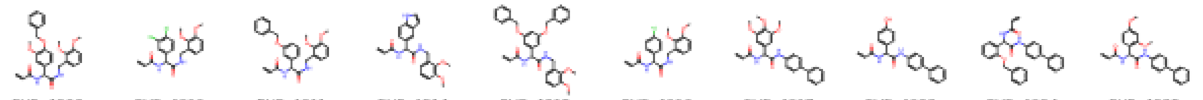

PHP-4809 PHP-4810 PHP-4811 PHP-4814 PHP-4815 PHP-4816 PHP-4817 PHP-4823 PHP-4824 PHP-4825

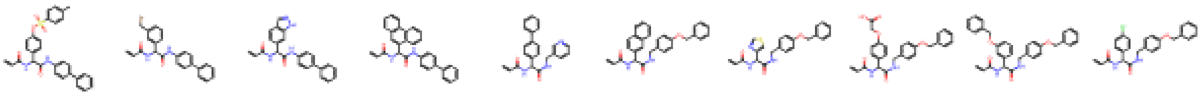

PHP-4827 PHP-4828 PHP-4831 PHP-4832 PHP-4834 PHP-4849 PHP-4851 PHP-4854 PHP-4859 PHP-4864

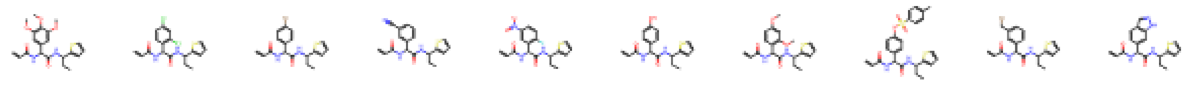

PHP-4865 PHP-4866 PHP-4867 PHP-4868 PHP-4869 PHP-4871 PHP-4873 PHP-4875 PHP-4876 PHP-4879

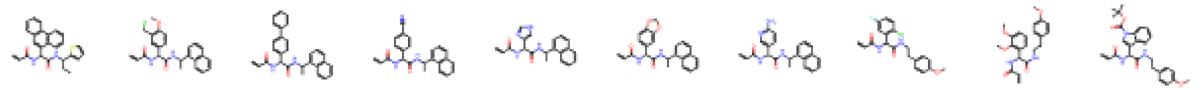

PHP-4880 PHP-4881 PHP-4882 PHP-4884 PHP-4894 PHP-4895 PHP-4896 PHP-4901 PHP-4902 PHP-4903

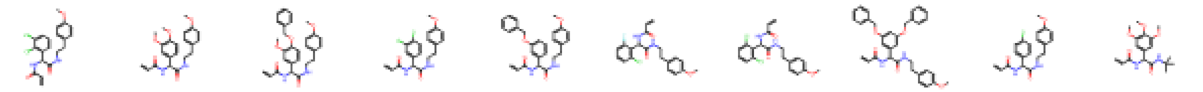

PHP-4905 PHP-4907 PHP-4909 PHP-4910 PHP-4911 PHP-4912 PHP-4913 PHP-4915 PHP-4916 PHP-4917

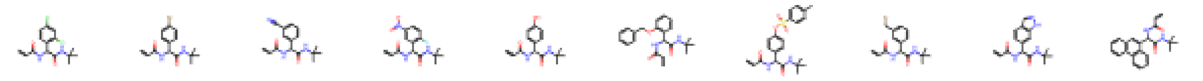

PHP-4918 PHP-4919 PHP-4920 PHP-4921 PHP-4923 PHP-4924 PHP-4927 PHP-4928 PHP-4931 PHP-4932

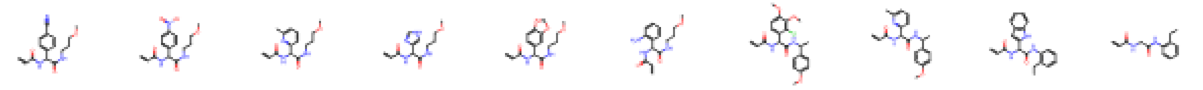

PHP-4936 PHP-4937 PHP-4938 PHP-4946 PHP-4947 PHP-4948 PHP-4953 PHP-4962 PHP-4965 PHP-4968

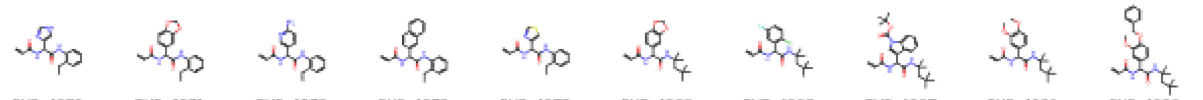

PHP-4970 PHP-4971 PHP-4972 PHP-4973 PHP-4975 PHP-4983 PHP-4985 PHP-4987 PHP-4991 PHP-4993

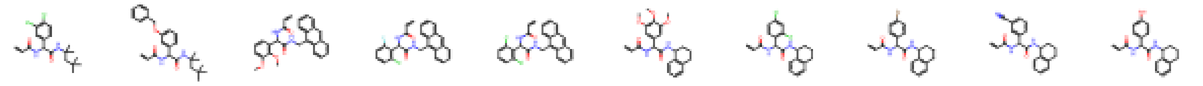

PHP-4994 PHP-4995 PHP-5002 PHP-5012 PHP-5013 PHP-5017 PHP-5018 PHP-5019 PHP-5020 PHP-5023

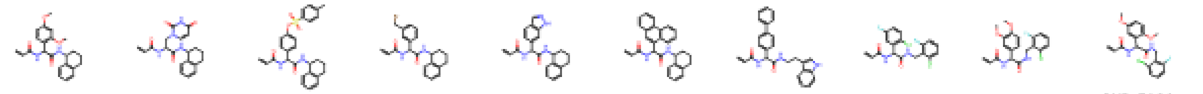

PHP-5025 PHP-5026 PHP-5027 PHP-5028 PHP-5031 PHP-5032 PHP-5034 PHP-5073 PHP-5079 PHP-5080

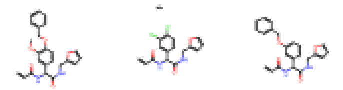

PHP-5081 PHP-5082 PHP-5083

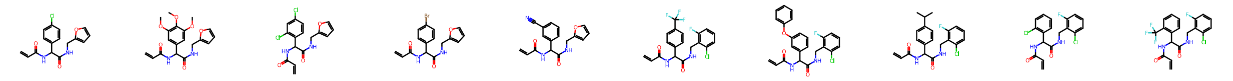

PHP-5088 PHP-5089 PHP-5090 PHP-5091 PHP-5092 PHP-5401 PHP-5402 PHP-5404 PHP-5405 PHP-5406

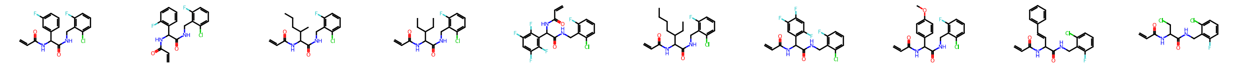

PHP-5407 PHP-5408 PHP-5409 PHP-5410 PHP-5411 PHP-5412 PHP-5413 PHP-5414 PHP-5415 PHP-5416

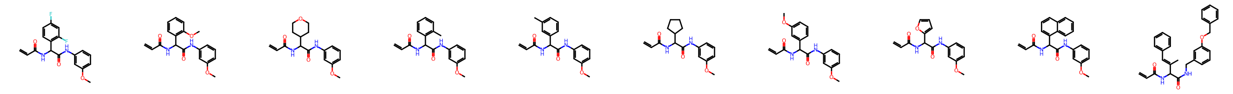

PHP-5418 PHP-5420 PHP-5422 PHP-5426 PHP-5427 PHP-5429 PHP-5430 PHP-5431 PHP-5432 PHP-5433

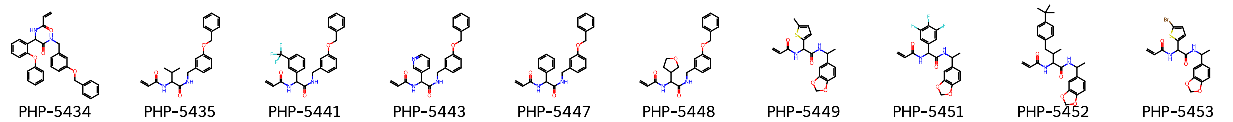

PHP-5434 PHP-5435 PHP-5441 PHP-5443 PHP-5447 PHP-5448 PHP-5449 PHP-5451 PHP-5452 PHP-5453

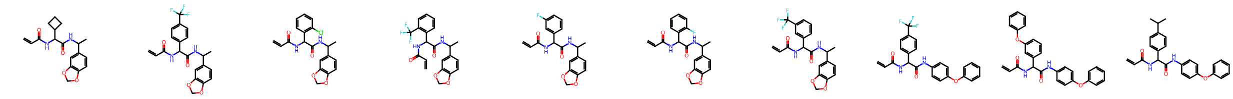

PHP-5457 PHP-5459 PHP-5460 PHP-5461 PHP-5462 PHP-5463 PHP-5464 PHP-5465 PHP-5466 PHP-5468

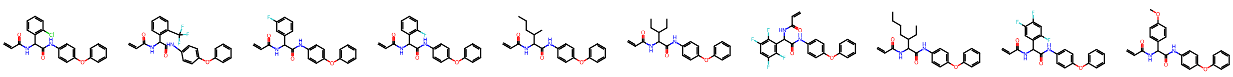

PHP-5469 PHP-5470 PHP-5471 PHP-5472 PHP-5473 PHP-5474 PHP-5475 PHP-5476 PHP-5477 PHP-5478

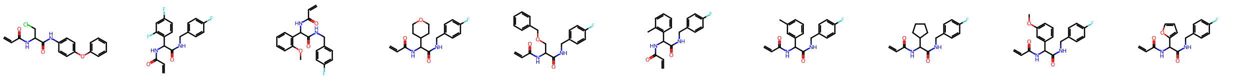

PHP-5480 PHP-5482 PHP-5484 PHP-5486 PHP-5489 PHP-5490 PHP-5491 PHP-5493 PHP-5494 PHP-5495

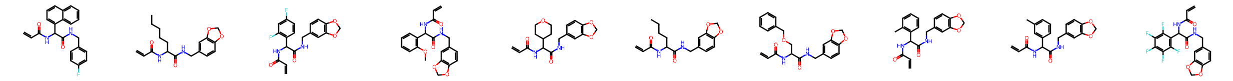

PHP-5496 PHP-5517 PHP-5518 PHP-5520 PHP-5522 PHP-5524 PHP-5525 PHP-5526 PHP-5527 PHP-5528

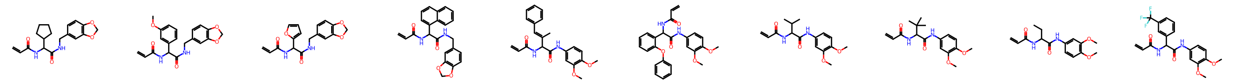

PHP-5529 PHP-5530 PHP-5531 PHP-5532 PHP-5533 PHP-5534 PHP-5535 PHP-5536 PHP-5537 PHP-5541

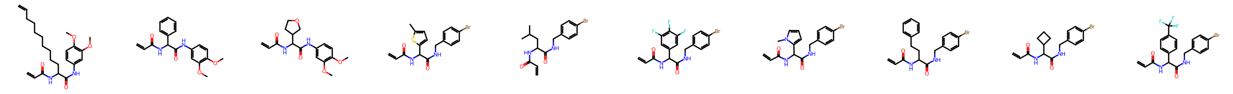

PHP-5544 PHP-5547 PHP-5548 PHP-5549 PHP-5550 PHP-5551 PHP-5554 PHP-5556 PHP-5557 PHP-5559

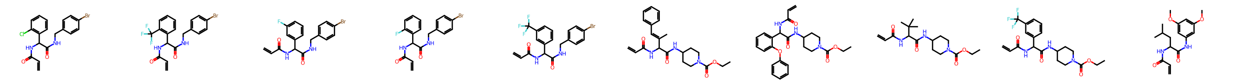

PHP-5560 PHP-5561 PHP-5562 PHP-5563 PHP-5564 PHP-5565 PHP-5566 PHP-5568 PHP-5573 PHP-5582

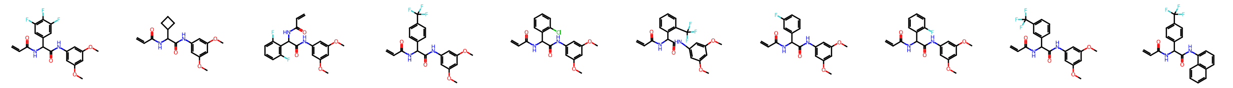

PHP-5583 PHP-5589 PHP-5590 PHP-5591 PHP-5592 PHP-5593 PHP-5594 PHP-5595 PHP-5596 PHP-5601

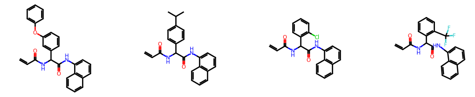

PHP-5602 PHP-5604 PHP-5605 PHP-5606

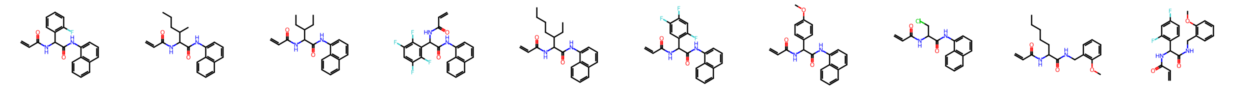

PHP-5608 PHP-5609 PHP-5610 PHP-5611 PHP-5612 PHP-5613 PHP-5614 PHP-5616 PHP-5617 PHP-5618

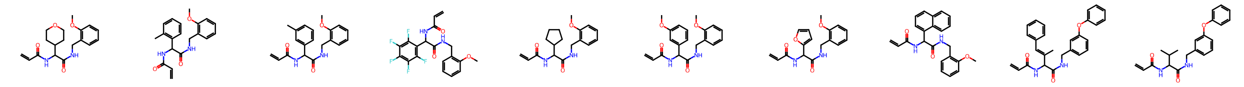

PHP-5622 PHP-5626 PHP-5627 PHP-5628 PHP-5629 PHP-5630 PHP-5631 PHP-5632 PHP-5633 PHP-5635

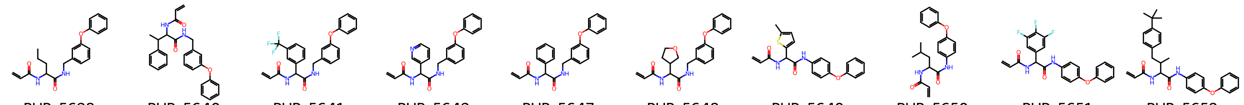

PHP-5638 PHP-5640 PHP-5641 PHP-5643 PHP-5647 PHP-5648 PHP-5649 PHP-5650 PHP-5651 PHP-5652

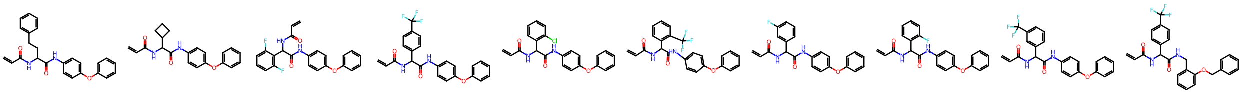

PHP-5656 PHP-5657 PHP-5658 PHP-5659 PHP-5660 PHP-5661 PHP-5662 PHP-5663 PHP-5664 PHP-5665

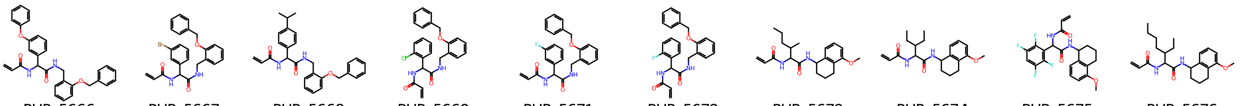

PHP-5666 PHP-5667 PHP-5668 PHP-5669 PHP-5671 PHP-5672 PHP-5673 PHP-5674 PHP-5675 PHP-5676

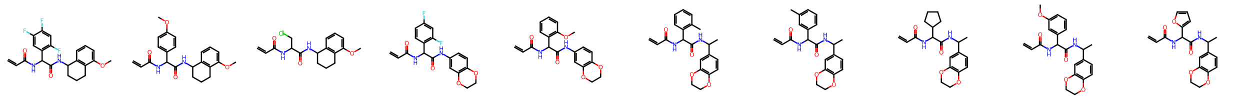

PHP-5677 PHP-5678 PHP-5680 PHP-5682 PHP-5684 PHP-5690 PHP-5691 PHP-5693 PHP-5694 PHP-5695

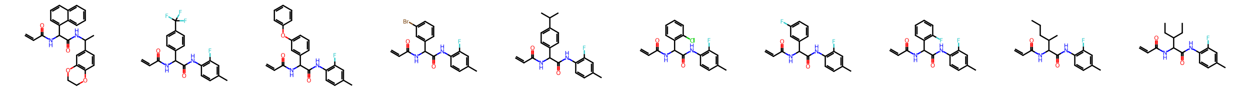

PHP-5696 PHP-5701 PHP-5702 PHP-5703 PHP-5704 PHP-5705 PHP-5707 PHP-5708 PHP-5709 PHP-5710

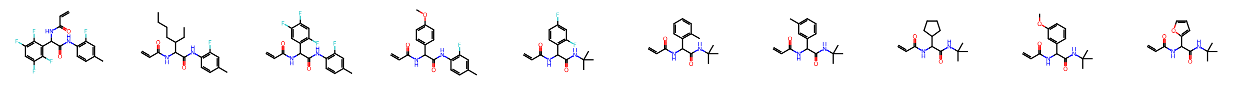

PHP-5711 PHP-5712 PHP-5713 PHP-5714 PHP-5718 PHP-5726 PHP-5727 PHP-5729 PHP-5730 PHP-5731

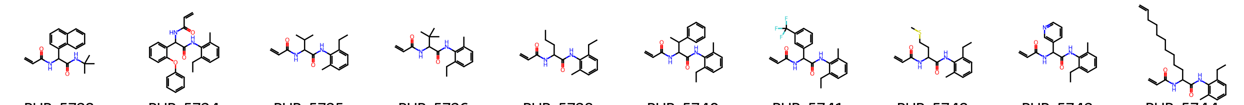

PHP-5732 PHP-5734 PHP-5735 PHP-5736 PHP-5738 PHP-5740 PHP-5741 PHP-5742 PHP-5743 PHP-5744

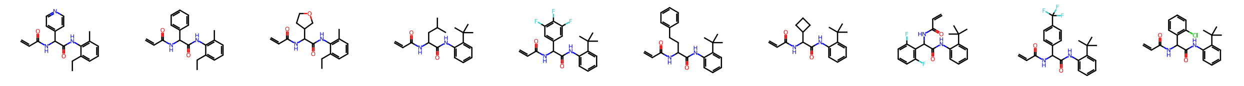

PHP-5746 PHP-5747 PHP-5748 PHP-5750 PHP-5751 PHP-5756 PHP-5757 PHP-5758 PHP-5759 PHP-5760

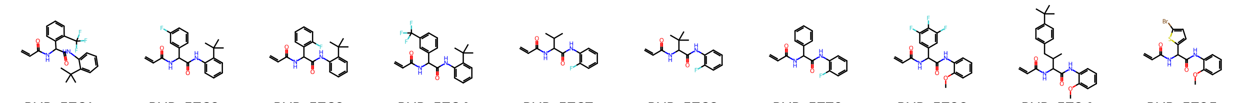

PHP-5761 PHP-5762 PHP-5763 PHP-5764 PHP-5767 PHP-5768 PHP-5779 PHP-5783 PHP-5784 PHP-5785

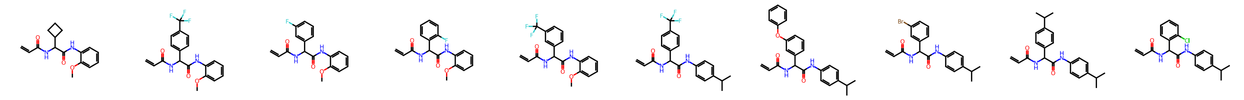

PHP-5789 PHP-5791 PHP-5794 PHP-5795 PHP-5796 PHP-5801 PHP-5802 PHP-5803 PHP-5804 PHP-5805

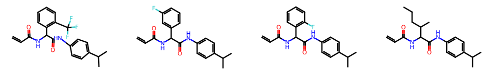

PHP-5806 PHP-5807 PHP-5808 PHP-5809

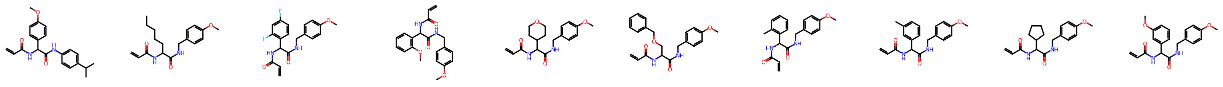

PHP-5814 PHP-5817 PHP-5818 PHP-5820 PHP-5822 PHP-5825 PHP-5826 PHP-5827 PHP-5829 PHP-5830

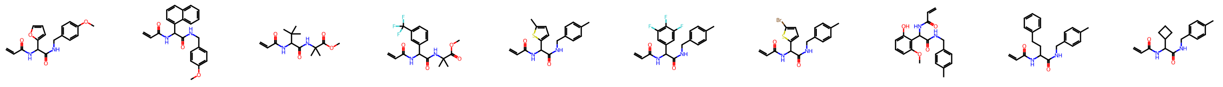

PHP-5831 PHP-5832 PHP-5836 PHP-5841 PHP-5849 PHP-5851 PHP-5853 PHP-5855 PHP-5856 PHP-5857

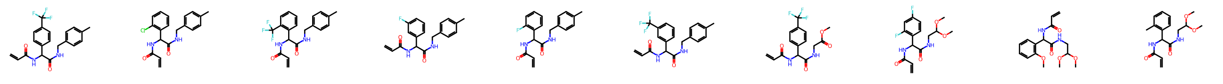

PHP-5859 PHP-5860 PHP-5861 PHP-5862 PHP-5863 PHP-5864 PHP-5865 PHP-5882 PHP-5884 PHP-5890

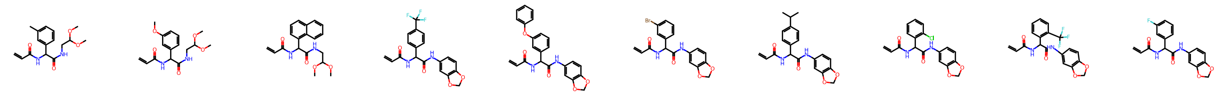

PHP-5891 PHP-5894 PHP-5896 PHP-5901 PHP-5902 PHP-5903 PHP-5904 PHP-5905 PHP-5906 PHP-5907

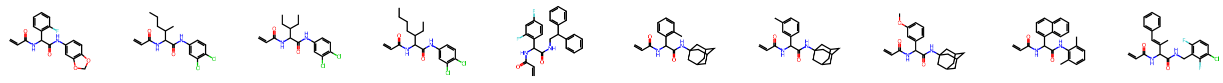

PHP-5908 PHP-5909 PHP-5910 PHP-5912 PHP-5918 PHP-5926 PHP-5927 PHP-5930 PHP-5932 PHP-5933

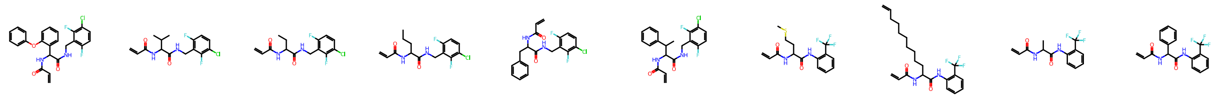

PHP-5934 PHP-5935 PHP-5937 PHP-5938 PHP-5939 PHP-5940 PHP-5942 PHP-5944 PHP-5945 PHP-5947

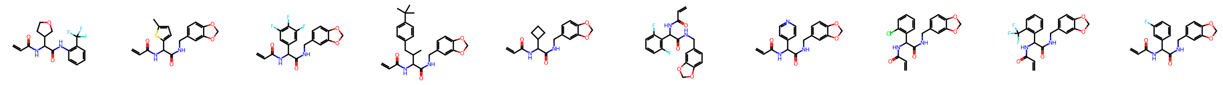

PHP-5948 PHP-5949 PHP-5951 PHP-5952 PHP-5957 PHP-5958 PHP-5959 PHP-5960 PHP-5961 PHP-5962

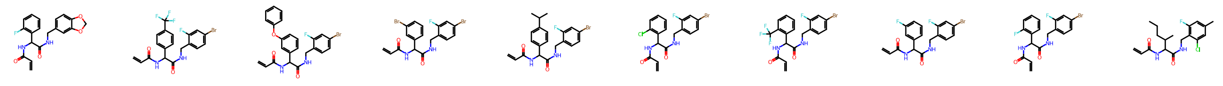

PHP-5963 PHP-5965 PHP-5966 PHP-5967 PHP-5968 PHP-5969 PHP-5970 PHP-5971 PHP-5972 PHP-5973

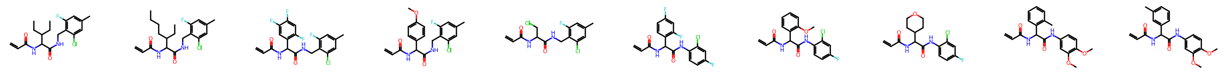

PHP-5974 PHP-5976 PHP-5977 PHP-5978 PHP-5980 PHP-5982 PHP-5984 PHP-5986 PHP-5990 PHP-5991

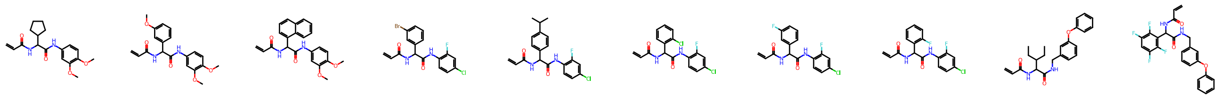

PHP-5993 PHP-5994 PHP-5996 PHP-6003 PHP-6004 PHP-6005 PHP-6007 PHP-6008 PHP-6010 PHP-6011

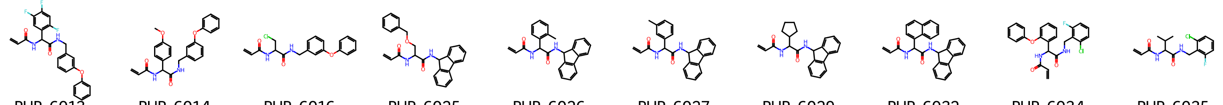

PHP-6013 PHP-6014 PHP-6016 PHP-6025 PHP-6026 PHP-6027 PHP-6029 PHP-6032 PHP-6034 PHP-6035

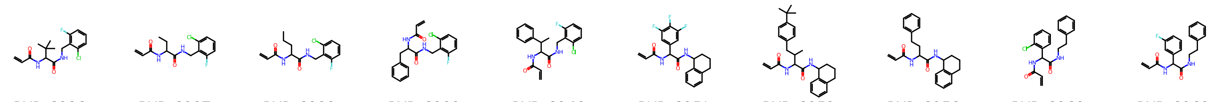

PHP-6036 PHP-6037 PHP-6038 PHP-6039 PHP-6040 PHP-6051 PHP-6052 PHP-6056 PHP-6060 PHP-6062

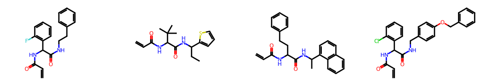

PHP-6063 PHP-6068 PHP-6088 PHP-6092

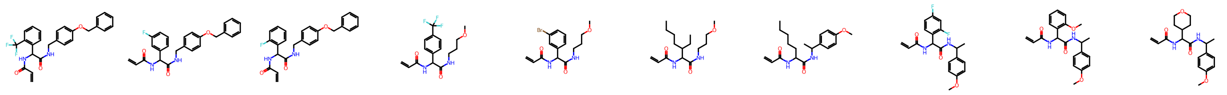

PHP-6093 PHP-6094 PHP-6095 PHP-6101 PHP-6103 PHP-6112 PHP-6117 PHP-6118 PHP-6120 PHP-6122

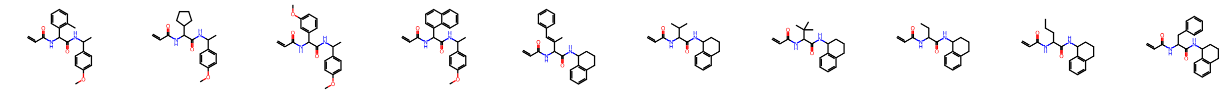

PHP-6126 PHP-6129 PHP-6130 PHP-6132 PHP-6133 PHP-6135 PHP-6136 PHP-6137 PHP-6138 PHP-6139

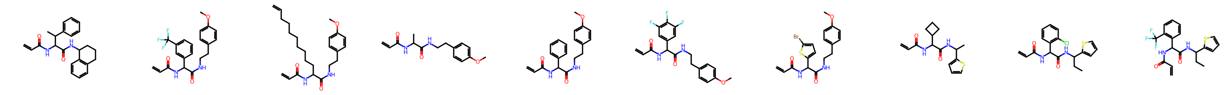

PHP-6140 PHP-6141 PHP-6144 PHP-6145 PHP-6147 PHP-6151 PHP-6153 PHP-6157 PHP-6160 PHP-6161

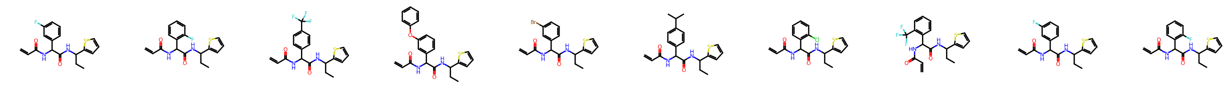

PHP-6162 PHP-6163 PHP-6165 PHP-6166 PHP-6167 PHP-6168 PHP-6169 PHP-6170 PHP-6171 PHP-6172

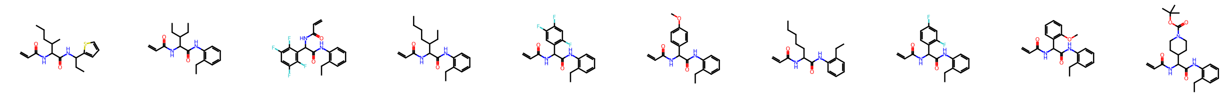

PHP-6173 PHP-6174 PHP-6175 PHP-6176 PHP-6177 PHP-6178 PHP-6181 PHP-6182 PHP-6184 PHP-6185

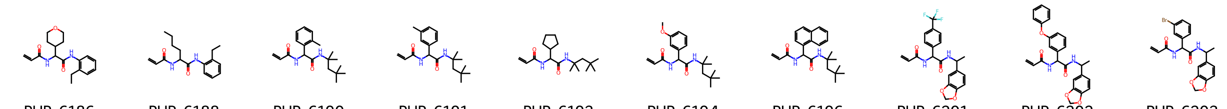

PHP-6186 PHP-6188 PHP-6190 PHP-6191 PHP-6193 PHP-6194 PHP-6196 PHP-6201 PHP-6202 PHP-6203

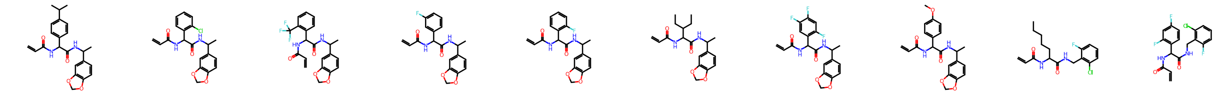

PHP-6204 PHP-6205 PHP-6206 PHP-6207 PHP-6208 PHP-6210 PHP-6213 PHP-6214 PHP-6217 PHP-6218

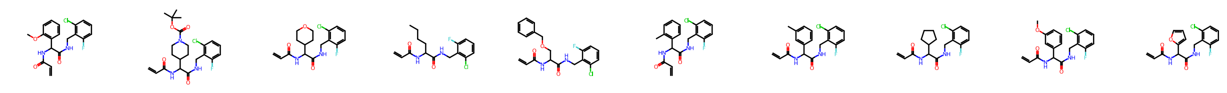

PHP-6220 PHP-6221 PHP-6222 PHP-6224 PHP-6225 PHP-6226 PHP-6227 PHP-6228 PHP-6230 PHP-6231

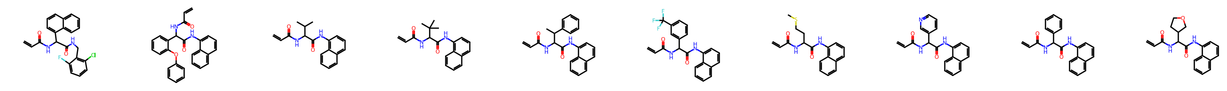

PHP-6232 PHP-6234 PHP-6235 PHP-6236 PHP-6240 PHP-6241 PHP-6242 PHP-6243 PHP-6247 PHP-6248

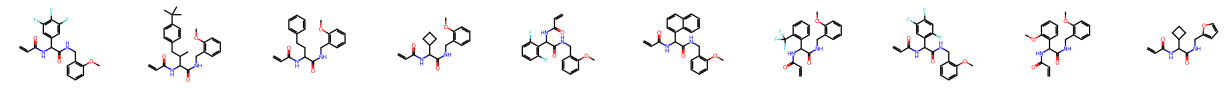

PHP-6251 PHP-6252 PHP-6256 PHP-6257 PHP-6258 PHP-6260 PHP-6261 PHP-6262 PHP-6263 PHP-6289

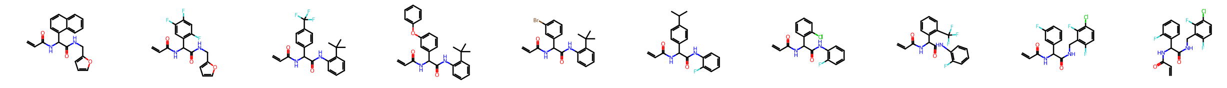

PHP-6292 PHP-6294 PHP-6301 PHP-6302 PHP-6303 PHP-6304 PHP-6305 PHP-6306 PHP-6307 PHP-6308

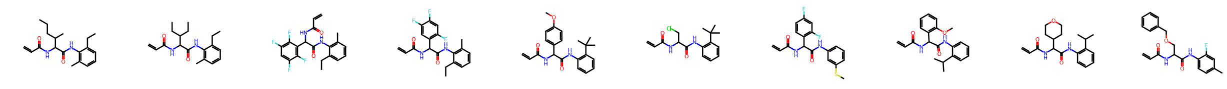

PHP-6309 PHP-6310 PHP-6311 PHP-6313 PHP-6314 PHP-6316 PHP-6318 PHP-6320 PHP-6322 PHP-6325

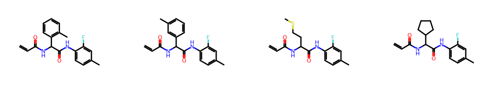

PHP-6326 PHP-6327 PHP-6328 PHP-6329

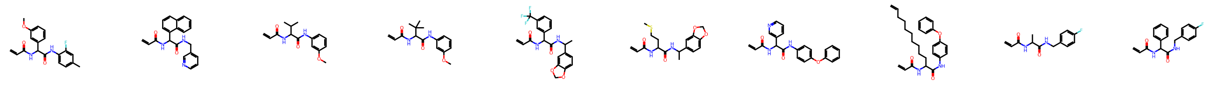

PHP-6330 PHP-6332 PHP-6335 PHP-6336 PHP-6341 PHP-6342 PHP-6343 PHP-6344 PHP-6345 PHP-6347

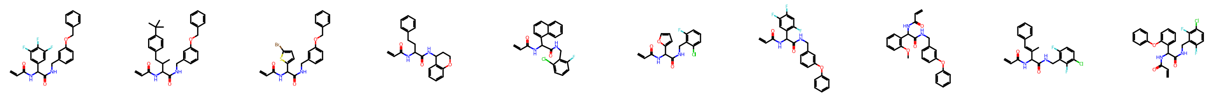

PHP-6351 PHP-6352 PHP-6353 PHP-6356 PHP-6360 PHP-6361 PHP-6362 PHP-6363 PHP-6365 PHP-6366

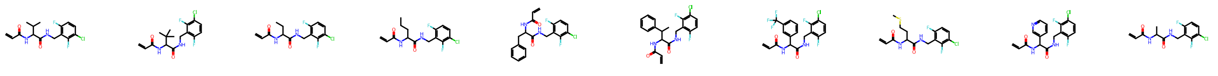

PHP-6367 PHP-6368 PHP-6369 PHP-6370 PHP-6371 PHP-6372 PHP-6373 PHP-6374 PHP-6375 PHP-6377

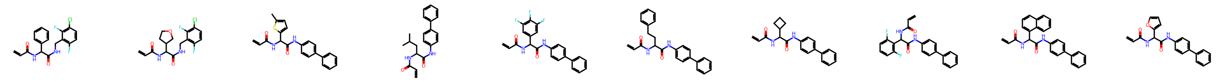

PHP-6379 PHP-6380 PHP-6381 PHP-6382 PHP-6383 PHP-6388 PHP-6389 PHP-6390 PHP-6392 PHP-6393

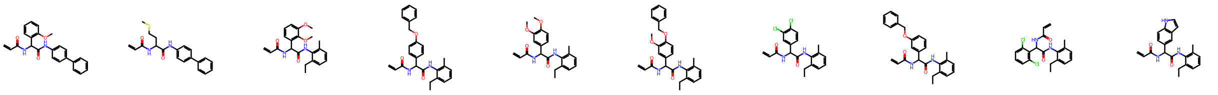

PHP-6395 PHP-6396 PHP-6402 PHP-6406 PHP-6407 PHP-6409 PHP-6410 PHP-6411 PHP-6413 PHP-6414

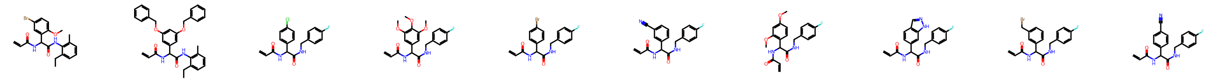

PHP-6415 PHP-6416 PHP-6417 PHP-6418 PHP-6420 PHP-6422 PHP-6423 PHP-6424 PHP-6427 PHP-6430

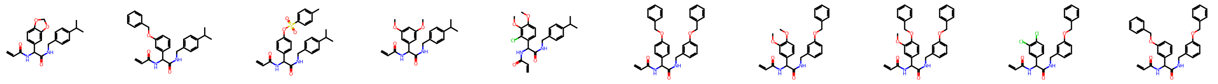

PHP-6436 PHP-6440 PHP-6441 PHP-6447 PHP-6448 PHP-6454 PHP-6455 PHP-6457 PHP-6458 PHP-6459

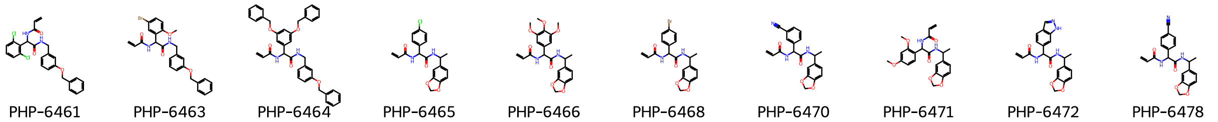

PHP-6461 PHP-6463 PHP-6464 PHP-6465 PHP-6466 PHP-6468 PHP-6470 PHP-6471 PHP-6472 PHP-6478

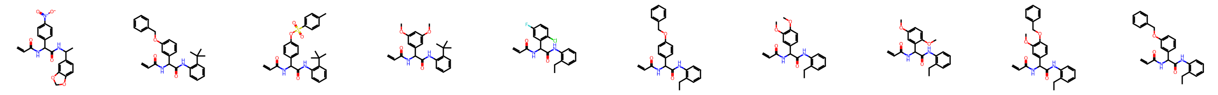

PHP-6479 PHP-6488 PHP-6489 PHP-6495 PHP-6501 PHP-6506 PHP-6507 PHP-6508 PHP-6509 PHP-6511

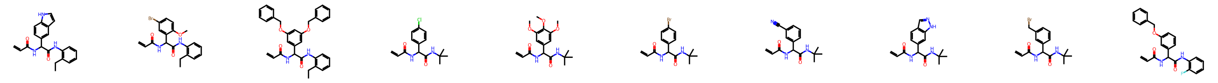

PHP-6514 PHP-6515 PHP-6516 PHP-6517 PHP-6518 PHP-6520 PHP-6522 PHP-6524 PHP-6527 PHP-6540

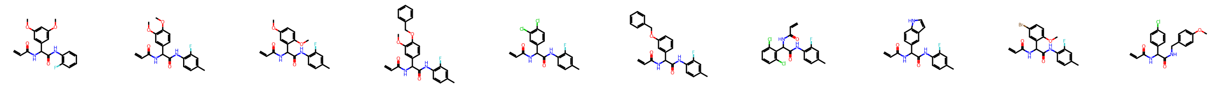

PHP-6547 PHP-6555 PHP-6556 PHP-6557 PHP-6558 PHP-6559 PHP-6561 PHP-6562 PHP-6563 PHP-6565

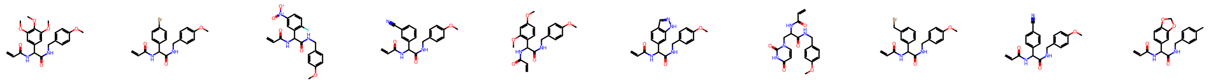

PHP-6566 PHP-6568 PHP-6569 PHP-6570 PHP-6571 PHP-6572 PHP-6573 PHP-6575 PHP-6578 PHP-6584

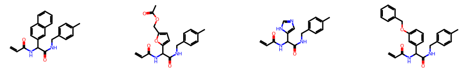

PHP-6585 PHP-6586 PHP-6587 PHP-6588

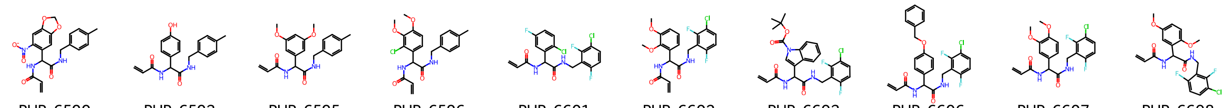

PHP-6590 PHP-6593 PHP-6595 PHP-6596 PHP-6601 PHP-6602 PHP-6603 PHP-6606 PHP-6607 PHP-6608

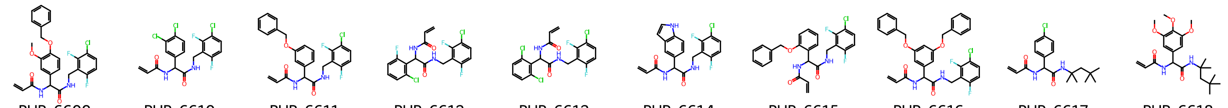

PHP-6609 PHP-6610 PHP-6611 PHP-6612 PHP-6613 PHP-6614 PHP-6615 PHP-6616 PHP-6617 PHP-6618

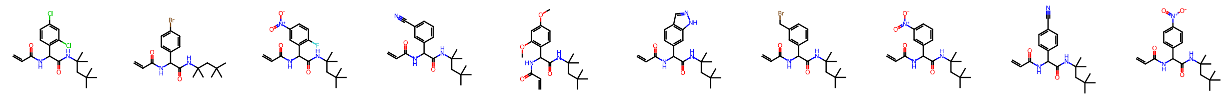

PHP-6619 PHP-6620 PHP-6621 PHP-6622 PHP-6623 PHP-6624 PHP-6627 PHP-6629 PHP-6630 PHP-6631

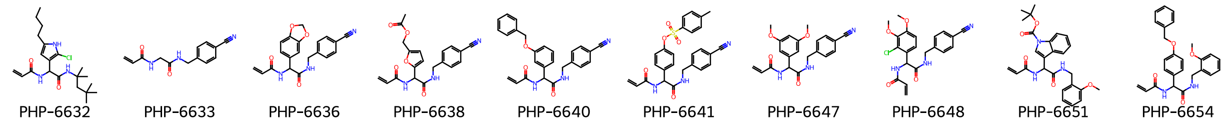

PHP-6632 PHP-6633 PHP-6636 PHP-6638 PHP-6640 PHP-6641 PHP-6647 PHP-6648 PHP-6651 PHP-6654

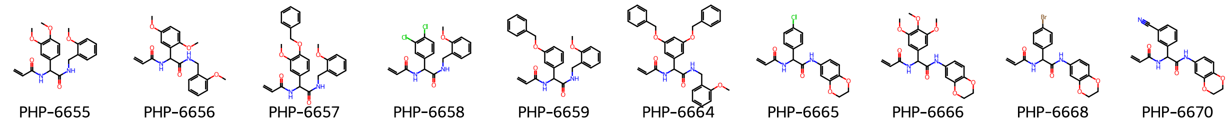

PHP-6655 PHP-6656 PHP-6657 PHP-6658 PHP-6659 PHP-6664 PHP-6665 PHP-6666 PHP-6668 PHP-6670

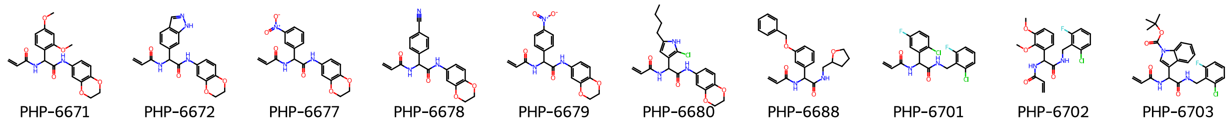

PHP-6671 PHP-6672 PHP-6677 PHP-6678 PHP-6679 PHP-6680 PHP-6688 PHP-6701 PHP-6702 PHP-6703

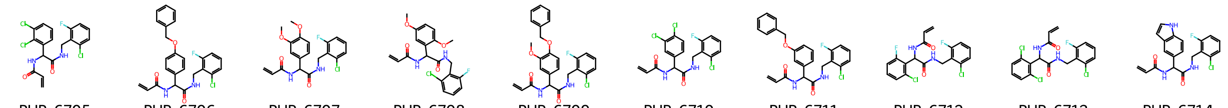

PHP-6705 PHP-6706 PHP-6707 PHP-6708 PHP-6709 PHP-6710 PHP-6711 PHP-6712 PHP-6713 PHP-6714

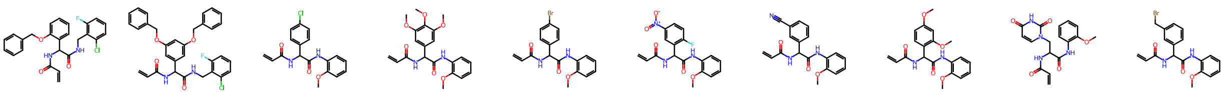

PHP-6715 PHP-6716 PHP-6717 PHP-6718 PHP-6720 PHP-6721 PHP-6722 PHP-6723 PHP-6725 PHP-6727

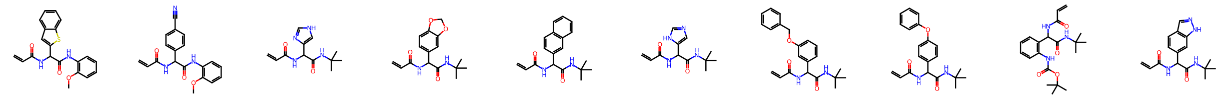

PHP-6728 PHP-6730 PHP-6734 PHP-6736 PHP-6737 PHP-6739 PHP-6740 PHP-6741 PHP-6747 PHP-6748

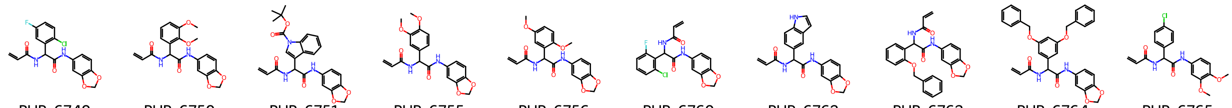

PHP-6749 PHP-6750 PHP-6751 PHP-6755 PHP-6756 PHP-6760 PHP-6762 PHP-6763 PHP-6764 PHP-6765

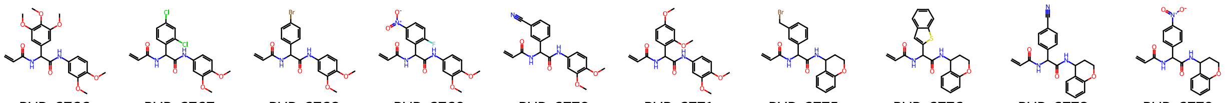

PHP-6766 PHP-6767 PHP-6768 PHP-6769 PHP-6770 PHP-6771 PHP-6775 PHP-6776 PHP-6778 PHP-6779

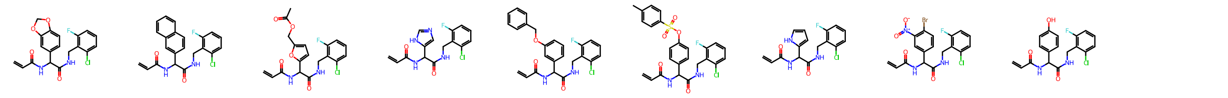

PHP-6784 PHP-6785 PHP-6786 PHP-6787 PHP-6788 PHP-6789 PHP-6791 PHP-6792 PHP-6793

7.3 Reformatting of stored compounds

The samples within 96-well plates over a 2-year time period were reformatted into a 384-well plate format using the Tecan Freedom EVO® robotic liquid handler to enhance compatibility with the AEMS system. Four 96-well plates were mixed to form a single 384-well plate, for a total of three and half plates of the 384-well format containing the 1235 samples.

7.4 AEMS (acoustic ejection mass spectrometry)

7.4.1 Dilution of compounds stock solutions for the AEMS analysis

The Echo ® 550 liquid handler was used to transfer 0.5 microliters (200 drops) of the initial stock solutions from the 384-well source plate to the 384-well destination plate. To these 49.5 microliters of DMSO (HPLC-MS grade) were added to reach the 100 µM concentration required for the AEMS analysis.

7.4.2 AEMS analysis.

The mass analysis was performed on the Echo® MS+ system with ZenoTOF 7600 system.

Prior to full-scale analysis, method optimization was performed to establish optimal analytical conditions. This optimization process evaluated several carrier solvent systems, including 0.1% formic acid in methanol, 0.1% formic acid in acetonitrile:water (70:30), and 5 mM ammonium fluoride in acetonitrile:water (70:30). Through systematic evaluation of these parameters, along with variations in ejection volume and flow rate, optimal conditions were determined. The final method utilized 0.1% formic acid in methanol as the carrier solvent, operating at a flow rate of 300 µL/min with an ejection volume of 60 nL and an ejection interval of 2500 ms.

A TOF MS experiment was conducted with positive and negative ionization modes. In positive mode, the spray voltage was set to 5000 V with a declustering potential of 80 V, while negative mode employed a spray voltage of -4500 V and a declustering potential of -80 V. The ion source parameters were optimized with ion source gas 1 (nebulizing gas, air) at 90 psi, ion source gas 2 (spray drying gas, air) at 60 psi, and a curtain gas (nitrogen) setting of 35. The ion source temperature was maintained at 400°C throughout the analysis. The TOF MS scan range was set from 50 to 650 Da with an accumulation time of 0.1 seconds. To ensure data quality and reliability, automated mass calibration of the mass spectrometer was performed throughout the batch acquisition, maintaining consistent mass accuracy across all analyses.

7.5 UPLC-MS

7.5.1 Dilution of compounds stock solutions for the UPLC-MS analysis

Samples for the UPLC-MS analysis were prepared by diluting the stock solutions (10 mM in DMSO) to a working concentration of 0.7 mg/mL in a solvent mixture of 50%

acetonitrile in water. Approximately 200 µL of the diluted sample was prepared for the analysis into a 384-well PP plate, ensuring the absence of air bubbles.

7.5.2 UPLC-MS analysis

The diluted samples in the 384-weel plate format at the 0.7 mg/mL concentration in a 50% acetonitrile/water mixture were analyzed using a Waters ACQUITY UPLC® system, coupled with a PDA detector and a Xevo TQ-S Cronos Triple Quadrupole Mass Spectrometer. A reversed-phase UPLC column (C18, 1.7 µm particle size, 2.1 mm × 50 mm) was used for the chromatographic separation. The mobile phases consisted of water with 0.1% formic acid (Mobile Phase A) and acetonitrile with 0.1% formic acid (MobilePhase B). The gradient was initiated at 10% B (90% A) at 0 minutes and increased linearly to 100% B (0% A) over the course of 6 minutes. Following the gradient, the column was washed by holding it at 100% B for an additional 1 minute. The total runtime for each analysis was 7 minutes, comprising a 6-minute gradient phase and a 1-minute column wash phase. The flow rate was maintained at 0.6 mL/min throughout the run, and the column temperature was set at 40 °C to ensure reproducibility and optimal peak shape. Injections were performed with a volume of 2 µL per sample. 3D absorbance data were collected by PDA detector, with a range from 200 nm to 400 nm. Detection was carried out contextually using electrospray ionization mass spectrometry (ESI-MS) in positive and negative ion mode, with a mass range of m/z 100–1000. The ionization source was operated at a temperature of 150–250 °C, with nitrogen as the desolvation gas at a flow rate of approximately 500 L/h.

8 Data analysis

8.1 Choice of the relevant descriptors

The selection of molecular descriptors followed a systematic machine learning approach based on Random Forest Classification with optimized hyperparameters through grid search. The analysis incorporated all 202 Mordred descriptors calculated across the library to predict synthesis outcomes, which were classified into three categories: "Red", "Green", or "Black". **Tables S1** and **S2** present the model's performance metrics and feature importance rankings, respectively, with the latter directly informing the choice of descriptors for distribution analysis.

Notably, the low F1-Score (0.23) for the "Red" category, representing compounds that precipitated but were not the desired products, indicates that physicochemical properties (Mordred descriptors) have limited predictive power for these cases. This reduced predictive accuracy aligns with the experimental observations where the target products were not successfully formed.

Class	Precision	Recall	F1-Score	Support
Black	0.62	0.62	0.62	188
Green	0.67	0.80	0.73	247
Red	0.44	0.16	0.23	76
/////	/////	/////	/////	/////
Macro Avg	0.58	0.53	0.53	511
Weighted Avg	0.62	0.64	0.62	511

Table S1: Performance metrics of the Random Forest Classification model for predicting synthesis outcomes classified as 'Black,' 'Green,' or 'Red' using 202 Mordred descriptors. Metrics include precision, recall, F1-score, and support for each class, as well as macro and weighted averages.

Descriptor	Value
SLogP	0.063087
TopoPSA	0.030602
SMR	0.028843
MW	0.026984
nHBDon	0.025379

Table S2: Feature importance values of molecular descriptors from the Random Forest Classification model used to predict synthesis outcomes.

8.2 Statistical analysis on synthetic outcome

Statistical validation of physicochemical property differences across reaction outcome categories (Black, Red, and Green) was performed through one-way ANOVA analysis for five key molecular descriptors: SLogP, TopoPSA, SMR, MW, and nHBDon. Data scaling preceded the analysis to normalize descriptor values, ensuring fair comparability across the dataset and preventing magnitude-based bias. The ANOVA tests evaluated the statistical significance of mean descriptor variations across the "Label" categories. The analysis revealed highly significant differences for all five descriptors, conclusively rejecting the null hypothesis of equal means across

categories. "Black" compounds exhibited no precipitation in either the reaction solvent or diethyl ether. This classification refers only to the isolation method's outcome - whether the compound could be separated by precipitation - and does not indicate whether the chemical reaction itself was successful or not. "Red" compounds, while successfully precipitating, revealed NMR spectral profiles inconsistent with the expected target structures. "Green" compounds demonstrated both successful precipitation and NMR spectral characteristics aligned with the desired products. Analysis of physicochemical properties, with particular emphasis on SLogP, TopoPSA, revealed distinct patterns. Precipitation represents a complex physicochemical process governed by multiple parameters, including temperature, solvent composition, and concentration.

The analysis framework establishes a simplified model to elucidate the observed trends. "Black" compounds exhibited distinctive characteristics through a combination of higher median polarity coupled with lower median molecular weights (**Figure S16**). This specific physicochemical profile directly influenced the precipitation behavior: the lower molecular weight inhibited precipitation, while the elevated polarity facilitated retention within the TFA phase under the experimental conditions, where diethyl ether served as washing solvent and petroleum ether as precipitation inducer. Statistical analysis revealed a significant difference in average SLogP between "Black" and "Green" compounds (estimate = 0.96, $p < 0.001$. **Table S3**), suggesting a dual mechanism for precipitation failure. First, polar compounds remained dissolved in the TFA phase, confirming the synthesis strategy limitation. Second, the 75th quantile of apolar compounds demonstrated no precipitation, indicating potential reaction failure. Conversely, "Green" compounds demonstrated optimal physicochemical properties under the experimental conditions, characterized by higher apolarity and notably low TopoPSA values. These specific molecular features directly correlated with successful precipitation outcomes. The analysis of "Red" compounds revealed no consistent patterns in their physicochemical properties, a finding supported by both machine learning analysis and statistical testing. The machine learning approach demonstrated no correlation between structural features and this classification, while statistical analysis showed that comparisons involving "Red" compounds consistently yielded lower significance levels (**Table S3**). Distribution analysis of the aldehyde starting material (R1) provided additional insights: while "Red" compounds maintained LogP values similar to other outcomes, they distinctively exhibited elevated TopoPSA values (**Figure S16**). These observations led to a mechanistic hypothesis: the unsuccessful product precipitation in TFE, followed by diethyl ether/petroleum ether addition, likely resulted in the precipitation of aldehyde starting materials rather than desired products, thereby explaining the inconsistent NMR profiles observed.

Metric	Effect	DFn	DFd	F	p	p<.05	ges	p.adj
SLogP	Label	2	2548	176.33	1.89e-72	*	0.122	1.89e-72
TopoPSA	Label	2	2548	123.286	7.8e-52	*	0.088	7.8e-52

SMR	Label	2	2548	100.579	9.07e-43	*	0.073	9.07e-43
MW	Label	2	2548	108.412	6.51e-46	*	0.078	6.51e-46
nHBDon	Label	2	2548	108.504	5.98e-46	*	0.078	5.98e-46

Table S3: Anova analysis of key cheminformatic descriptors.

Metric	group 1	group 2	null.value	estimate	conf.low	conf.high	p.adj	p.adj.signif	p.adj.corrected
SLogP	Black	Red	0	0.92	0.75	1.1	0	****	0
SLogP	Black	Green	0	0.96	0.84	1.09	0	****	0
SLogP	Red	Green	0	0.04	-0.13	0.21	0.86	ns	1
TopoPSA	Black	Red	0	2.48	0.14	4.81	0.04	*	0.1
TopoPSA	Black	Green	0	-9.34	-11	-7.68	0	****	0
TopoPSA	Red	Green	0	-11.81	-14.07	-9.55	0	****	0
SMR	Black	Red	0	11.47	9.12	13.83	0	****	0
SMR	Black	Green	0	8.77	7.1	10.44	0	****	0
SMR	Red	Green	0	-2.71	-4.98	-0.43	0.01	*	0.04
MW	Black	Red	0	40.69	32.51	48.87	0	****	0
MW	Black	Green	0	32	26.2	37.81	0	****	0
MW	Red	Green	0	-8.69	-16.58	-0.8	0.03	*	0.08

nHBD on	Black	Red	0	0.07	0.01	0.13	0.02	*	0.07
nHBD on	Black	Green	0	-0.22	-0.26	-0.18	0	****	0
nHBD on	Red	Green	0	-0.29	-0.35	-0.23	0	****	0

Table S4: Posthoc analysis of key cheminformatic descriptors.

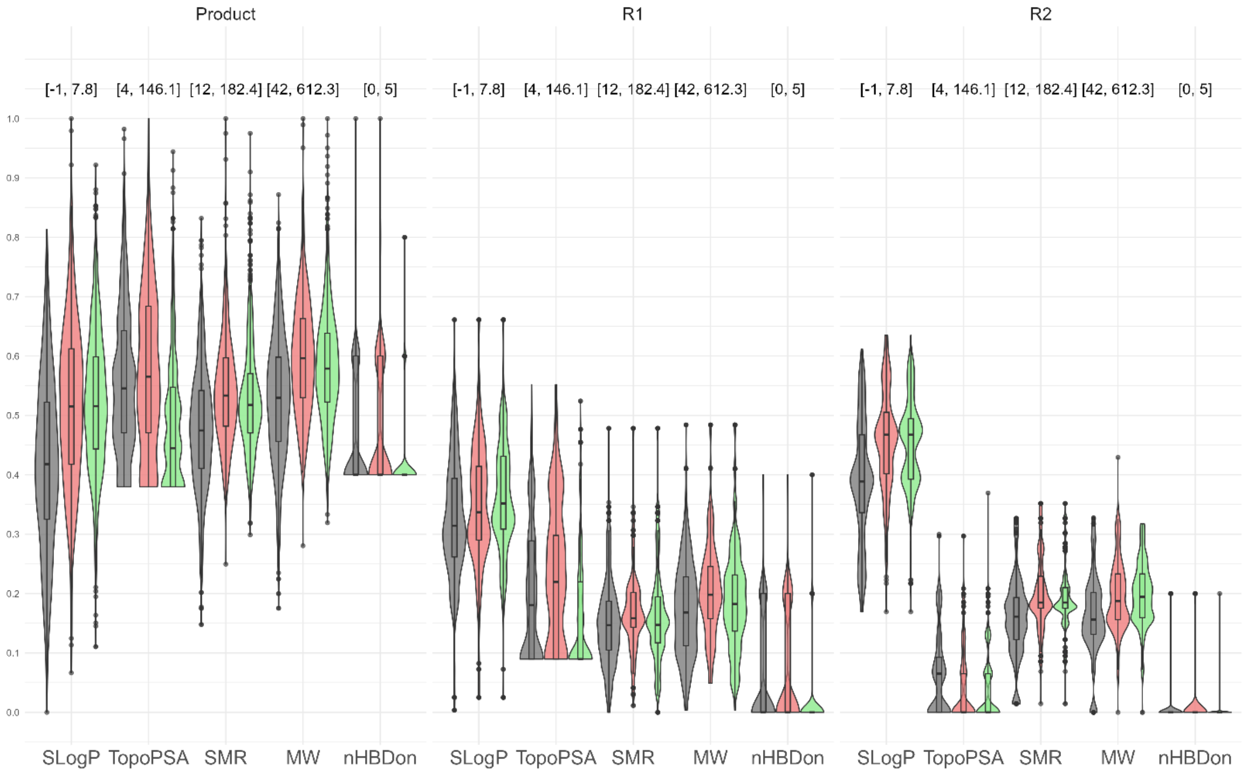

Figure S16: Distribution of five key molecular descriptors among products and starting materials. The violin plots show the distribution of SLogP, TopoPSA, SMR, MW, and nHBDon - which were identified as the most influential features through machine learning analysis. These plots are arranged by reaction outcomes: "Green" for successful products, "Red" for incorrect precipitates, and "Black" for no precipitation. All descriptor values have been normalized from 0 to 1.

8.3 Automated Analysis of AEMS and UPLC-MS data

msconvert.exe --noindex --zlib --ignoreUnknownInstrumentError --filter 'titleMaker <RunId>.<ScanNumber>.<ScanNumber>.<ChargeState> File:"""^<SourcePath^>""", NativeID:"""^<Id^>"""' "Path/to/file.wiff2"

Snippet 1: Script for converting Wiff file into multiple mzML files. The filter flag is responsible for the fragmentation into multiple files, each file will have the name of the fields in brackets. This exact script was run on a docker image through a Python subprocess meaning that the bracket type may differ from a command-line execution.

The complete analytical workflow, implemented through a combination of Python and R scripts, is designed to process data originating from two distinct mass spectrometry instrumentation platforms: a Waters UPLC-MS-UV system and a SCIEX AEMS. Each platform necessitates a tailored data preprocessing pipeline before converging on a common analysis strategy using the Peaksel ® software.

Data Acquisition and Preprocessing

- **Waters UPLC-MS-UV System:** This system generates data in MassLynx RAW format. These files can be directly integrated into the Peaksel® analysis pipeline following minimal preprocessing steps.
- **Sciex AEMS System:** produces .wiff2 files that aren't yet supported by Peaksel®, and therefore had to be converted into mzML format first. The conversion is accomplished using the ProteoWizard software. To ensure a reproducible and streamlined environment, ProteoWizard is configured with specific parameters (as exemplified in Snippet 1 of the original methodology) and integrated within a Docker container. Additionally, AEMS produces a file per plate, thus an extra step is required to split the spectra into an mzML file for each individual well. While Peaksel® has an experimental feature for splitting, doing this in Python script made the workflow more autonomous and systematic.

Peaksel Analysis Platform

All mass spectrometry data have been analyzed using Peaksel ®. 2 peak detection capabilities were used during the analysis:

- **Chromatographic peak detection**. For which the peak needs to be smoothed and sharp enough. For this, the respective parameters (level of smoothing and curvature threshold) were configured using trial and error.
- **Isotopic pattern matching** for peak confirmation. The chromatographic peak is already being detected on an Extracted Ion Chromatogram (EIC), so if the analyte's m/z weren't present in the measured spectra at high enough intensities, there wouldn't be a peak to detect. However, if an isobar were to be present, it could potentially get identified as a peak too. To lower the probability of isobar being detected, additionally the isotope pattern of the compound was

compared to the measured spectra. Peaksel® was configured to match isotopes with ±0.02m/z resolution in AEMS and ±0.5m/z in UPLC, while the intensity ratio was set to 40% to both the methods.

The high ratio threshold was chosen because when measuring some samples the m/z of the analytes were heavily shifting between multiple measurements, especially in the case of UPLC. In these cases, the resulting mean peak spectrum had intensities smeared across multiple m/z values and therefore the isotope pattern didn't match. However, because we were interested only in the reaction products, the analytes of interest had the largest MW across the sample, and therefore we didn't expect any isobars.

The comprehensive details of the algorithm employed by Peaksel ® are described at https://elsci.io/docs/peaksel/mass-spec-ions.html

The optimal values for these parameters are inherently dependent on the specific chemical properties of the substances being analyzed and the characteristics of the dataset. A key strength of Peaksel ® is that it does not rely on rigidly coded settings for these parameters. Instead, it offers the capability to adjust these parameters flexibly, not only for individual substances but also for entire batches. This batch-wise adjustment, which can be tailored based on clusters of substances with similar characteristics, allows for a more sensible and effective hyperparameter optimization across diverse datasets. The specific parameters utilized for the analyses described herein can be found on the project's dashboard (accessible at https://peaksel.elsci.io/a/domling_group/post/8gY7CqUMVtb).

Data Visualization Strategies

The advanced data visualization capabilities of Peaksel® are integral to high-throughput analysis. While an exhaustive discussion of all visualization features is beyond the scope of this document, it is important to highlight that distinct visualization approaches are required for UPLC-MS-UV and AEMS data, primarily due to the differential number of analytical channels available from each platform.

UPLC-MS-UV Data Visualizations

For the UPLC-MS-UV platform, the following visualizations were typically generated:

- The number of peaks detected by the isotopic peak algorithm in negative ionization mode.
- The number of peaks detected by the isotopic peak algorithm in positive ionization mode.
- The total number of compounds detected in the mass spectra, derived by combining detections from both positive mode (including sodium adducts) and negative mode using an OR logic.

- A plot of maximum UV absorbance (y-axis) against each injection (x-axis), providing an overview of chromophore species present.
- Identification of UV-visible versus UV-inactive substances.
- Temporal correlation analysis between UV and MS peak signals to confirm that detected UV peaks correspond to the target substances.

Further elaboration on the UV analysis from the UPLC system is warranted. The system's UV spectra are generated by a Diode Array Detector (DAD), necessitating the extraction of UV chromatograms to assess compound presence. While UV detection can traditionally facilitate precise quantification via the Lambert-Beer law (provided reference standards are available), the diverse compound libraries inherent in high-throughput screening make absolute quantification infeasible. Consequently, UV data were analyzed by plotting the maximum absorbance against the injection number. Specifically, wavelength extraction was performed at 216 ± 20 nm (representing the median wavelength ± one standard deviation across the relevant spectral range). Although not universally optimal for every compound, this standardized approach provides a reliable basis for comparative analysis across the diverse compound library.

AEMS Data Visualizations

Given that the AEMS system provides data primarily from mass spectrometry, resulting in fewer analytical channels compared to the UPLC-MS-UV platform, a more focused subset of visualizations was selected for data interpretation. These visualizations aim to provide analogous insights where possible, while also leveraging the specific strengths of direct MS analysis. The key visualizations for AEMS data include:

- **Substance Presence:** A binary plot indicating the presence or absence of a target substance. In positive ionization mode, this is determined by applying an OR logic to the detection of protonated ($[M+H]^+$) and sodium ($[M+Na]^+$) adducts. In negative ionization mode, presence typically corresponds to the detection of the deprotonated ($[M-H]^-$) adduct. This visualization is conceptually similar to the total compound detection for UPLC data, adapted for MS-only information.
- **Predominant Adduct Identification (Positive Mode):** A strip plot specifically for positive ionization mode data, designed to identify the most abundant adduct (e.g., $[M+H]^+$ versus $[M+Na]^+$) for each detected substance. This helps in understanding the ionization behavior of compounds under the given MS conditions and is unique to the MS-focused analysis of AEMS data.
- **Total Peak Count:** A quantitative measure of the total number of distinct peaks detected by PeakSel®. For positive ionization mode, this count sums the peaks corresponding to both $[M+H]^+$ and $[M+Na]^+$ adducts. For negative ionization mode, it reflects the total count of peaks associated with the $[M-H]^-$ adduct. This

serves a similar purpose to the individual positive and negative mode peak counts in the UPLC analysis, providing a measure of overall detection events.

8.4 In-depth analysis of confusion heatmaps

The heatmap visualization in **Figure S17** provides critical comparative insights between detection methods. For the blue heatmaps (top), detection was considered successful if the substance was observed in either negative or positive ionization mode, applying an OR condition also across Na^+ and H^+ adducts (permissive criterion). For the green heatmaps (bottom), only the positive ionization mode was considered, and as before, an OR condition was applied across Na^+ and H^+ adducts. UPLC-MS was selected as the preferred ground truth reference because it yielded more true positives (313) than the UV detector (UPLC-UV, 304), likely due to its ability to detect substances not visible by UV.

The sensitivity-specificity analysis reveals important detection characteristics. Sensitivity, defined as TP/(TP+FN), measures the true positive rate, while specificity, defined as TN/(TN+TP), quantifies the true negative rate. In comparing UPLC-MS with AEMS, the analysis yielded a sensitivity of 96.6% and a specificity of 35.0%. These metrics indicate that AEMS exhibits excellent capability in detecting compounds present in UPLC-MS analysis but shows limited accuracy in confirming compound absence. The notably low specificity prompted a manual inspection, which revealed that certain compounds demonstrated superior detection in AEMS compared to UPLC-MS. This enhanced detection capability can be attributed to the higher mass accuracy of the Time-of-Flight (TOF) detector in the AEMS system. For manual inspection of these results, we provide a DataWarrior file (datawarrior_confusion_results.dwar) in the Supporting downloads on Zenodo (**10.5281/zenodo.15496516**)

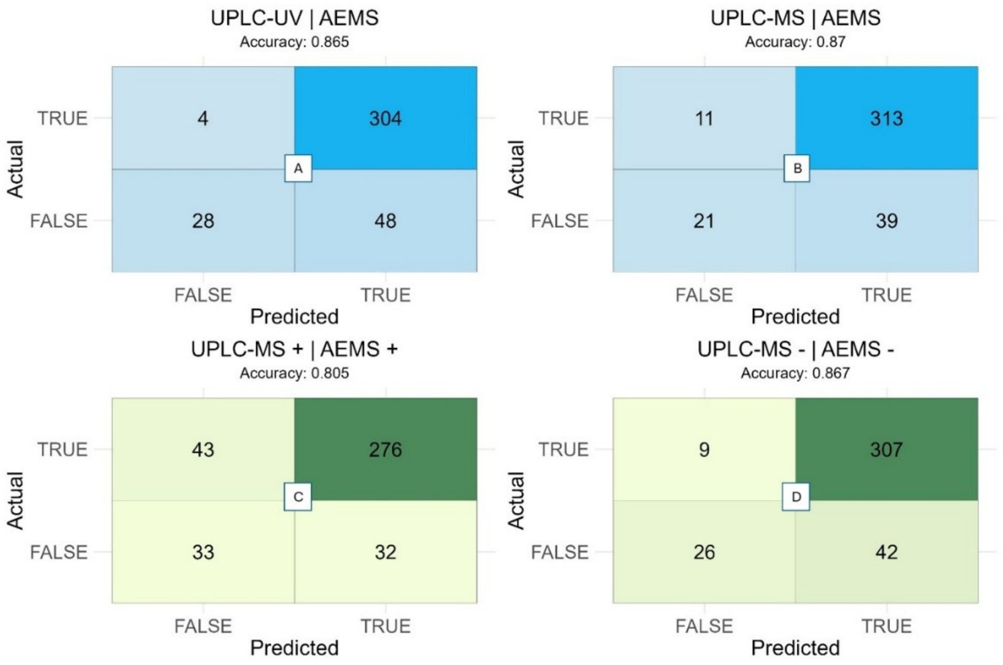

Figure S17. Confusion matrices for binary classification of detected substances: (A) UPLC-UV as ground truth versus AEMS inference; (B) UPLC-MS as ground truth versus AEMS inference. The blue matrices (A, B) include both negative and positive ionization modes under a permissive OR rule across Na^+ and H^+ adducts; (C) shows positive-mode only comparisons (Na^+ and H^+ adducts) and (D) shows negative-mode only comparisons (deprotonated H^- adduct).

8.5 Code-structure overview

All the analysis performed and described in the manuscript have been performed with the attached codes and directories accessible on Zenodo.

The code architecture comprises three integrated components, each with a distinct analytical purpose: *PeakSel_Parser.py* for systematic parsing of .wiff2 files, *Analysis_Notebook.ipynb* for executing core cheminformatic operations, and *Data_Analysis_R.R* for implementing statistical analyses and data visualization protocols.

PeaksSel_Parser.py

A fully functional code that performs the conversion from .wiff2 files to .mzML format. Note that this implementation is specifically tailored for this use case and is not yet generalized.

- Locates the required .wiff2 files based on user input
- Convert the .wiff2 files to .mzML format using a Docker installation of ProteoWizard with a sophisticated combination of flags (Snippet 1)
- Compresses the files in a PeakSel-compatible format
- General cleanup

Analysis_Notebook.ipynb

- Loads SMILES structures of the library, synthetic outcomes, and PeakSel analytical data in both positive and negative modes
- Calculates molecular descriptors and visualizes the chemical space using Mordred
- Implements Random Forest classification with grid search optimization
- Performs R-Group decomposition to obtain reactants components, since SMILES structures only show final products
- Merge the experimental CSVs —containing mass spectrum data— with the structural and synthetic data to create a comprehensive dataframe for the R-based pipeline
- Performs various percentage calculations presented in the main article

Data_Analysis_R.R

The R script orchestrates comprehensive data visualization for both analytical and publication purposes. The script systematically implements statistical analyses, including Analysis of Variance (ANOVA) and corresponding post-hoc tests, alongside their non-parametric alternatives, to ensure robust statistical validation of the findings.

8.6 Chemical space insights

The chemical space analysis utilized two complementary visualization approaches based on different molecular descriptors. The first approach leveraged Mordred molecular descriptors to generate a comprehensive descriptor matrix for the compound library. This matrix underwent systematic refinement through a dual regression strategy: LASSO regression for continuous variables and logistic regression for categorical variables. Such rigorous feature selection effectively eliminated non-informative descriptors, thereby enhancing the signal-to-noise ratio in subsequent analyses. Dimensionality reduction followed a two-step process: initial compression to 50 dimensions via Principal Component Analysis (PCA), followed by t-SNE algorithm application for final two-dimensional visualization. The resulting chemical space representation effectively captures molecular similarity relationships, with spatial proximity indicating structural and property-based similarities between compounds. The Tanimoto-based descriptor analysis employs a fundamentally different visualization approach. The process begins by computing pairwise Tanimoto distances between all compounds using Morgan fingerprints, resulting in an NxN similarity matrix. This matrix undergoes dimensional reduction through a two-step process: initial compression to 50 dimensions via Principal Component Analysis (PCA), followed by t-SNE transformation to achieve the final two-dimensional representation. Unlike the previous chemical space visualization, this approach specifically captures structural relationships between compounds, where spatial proximity in the plot directly corresponds to structural similarity between molecules.

The results are shown in **Figures S18top** and **S18bottom**. The Silhouette scores for both plotting methods indicate that the molecular differences are not well-defined or clearly separated, suggesting that these features failed to fully explain the reaction outcomes. Despite these quantitatively poor results, some qualitative observations can be made. The green dots (successful reactions) appear more clustered together, suggesting that reaction success may be more closely tied to chemical identity rather than molecular similarities. This observation aligns with our methodology, where synthesis outcomes were determined based on precipitation rather than chemical synthetic success.

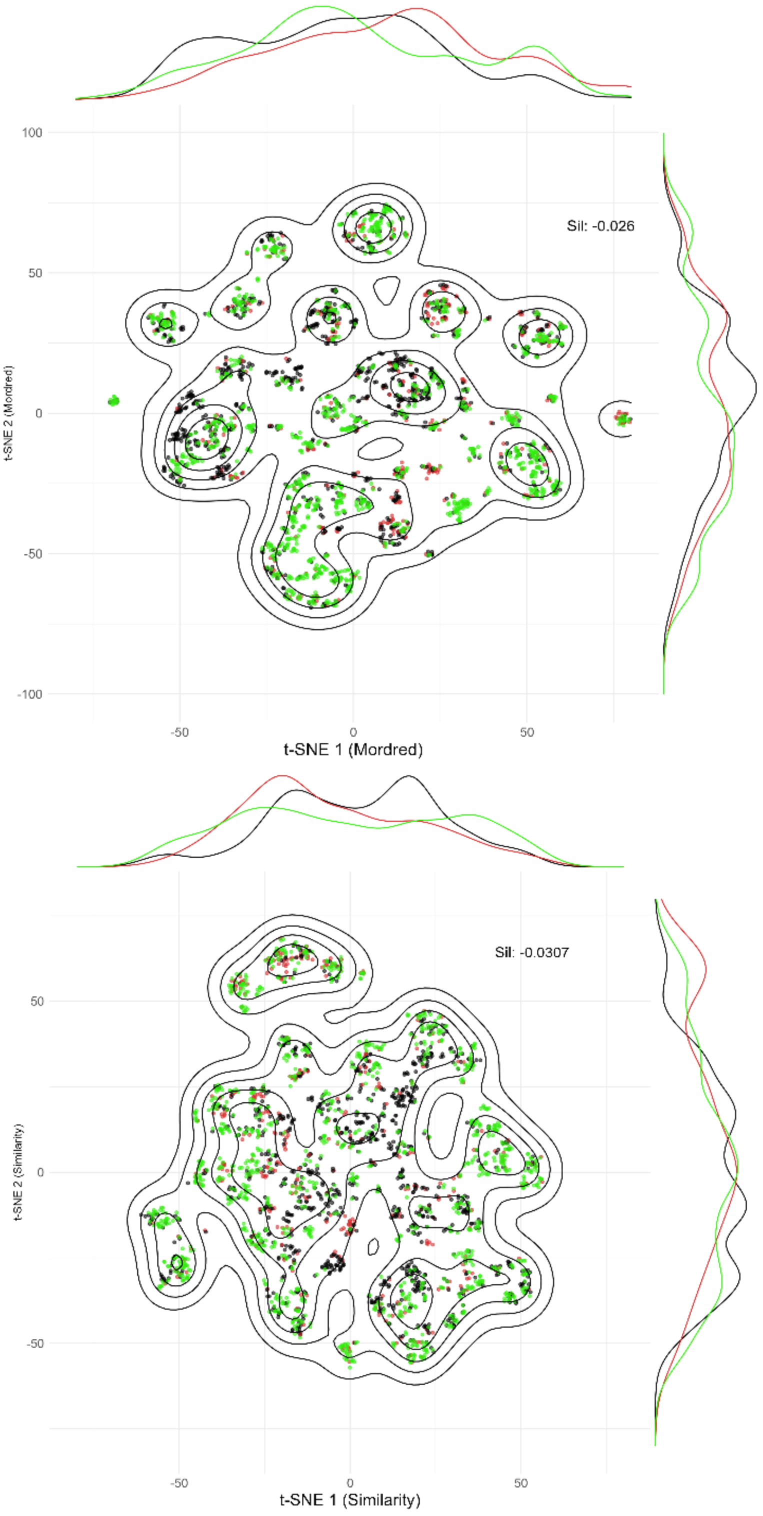

Figures S18: Chemical space visualization of the library. Colors represent the precipitation category. In Green compounds that precipitated and were the correct product based on NMR, in red the compounds that precipitated, but that were not the product, in black the compounds that did not precipitated. In the top diagram, the chemical space is compressed through t-SNE from molecules described using the Mordred descriptors. In the bottom one, the chemical space is compressed from a Tanimoto matrix of similarity through the ECFP fingerprints.

9 Analytical data

9.1 ^{1}H spectra of the 1235 pure products

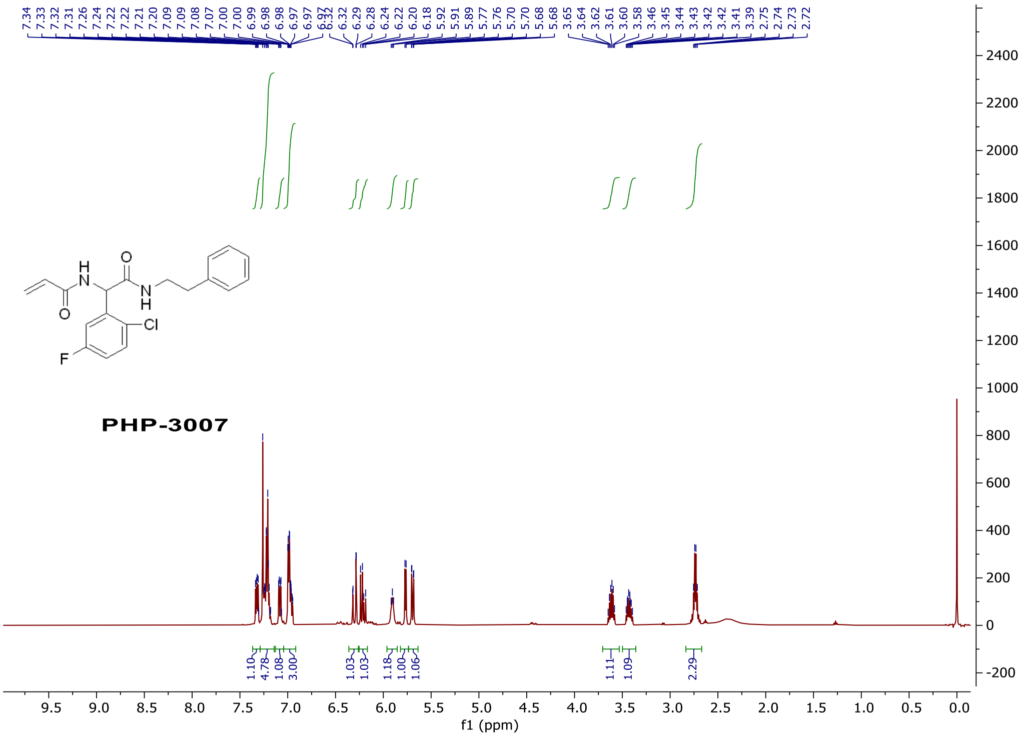

Supplementary Figure 19. ^{1}H NMR (500 MHz, $CDCl_3$) of compound **PHP-3007.**

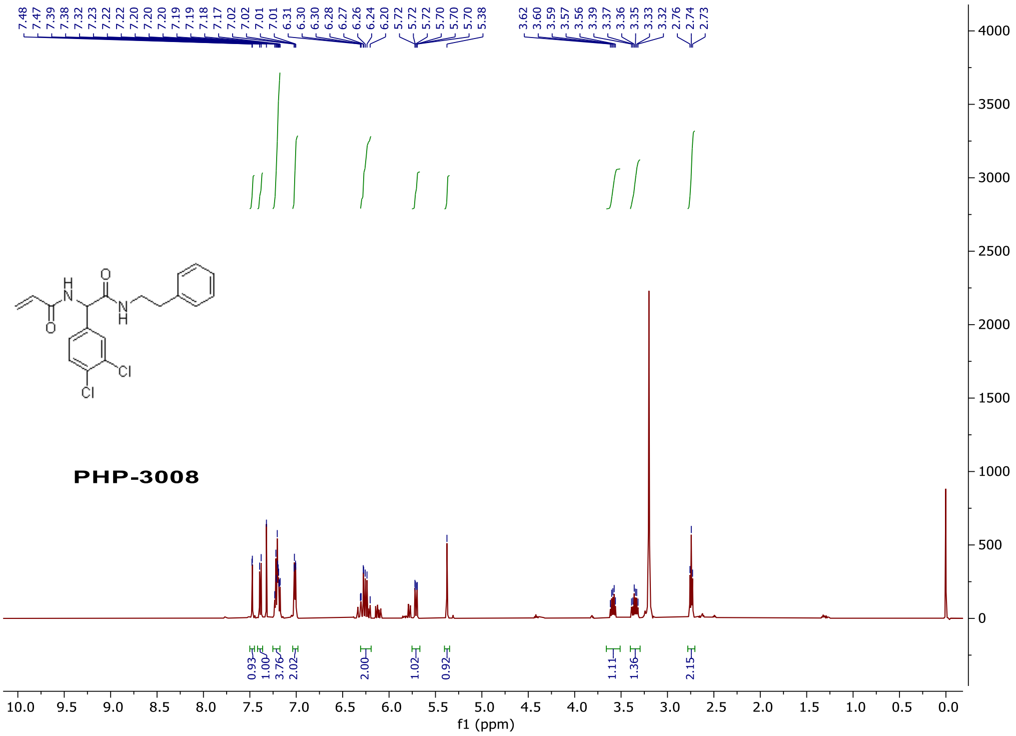

Supplementary Figure 20. ^{1}H NMR (500 MHz, $CDCl_3$+one drop of CD_3OD) of compound **PHP-3008.**

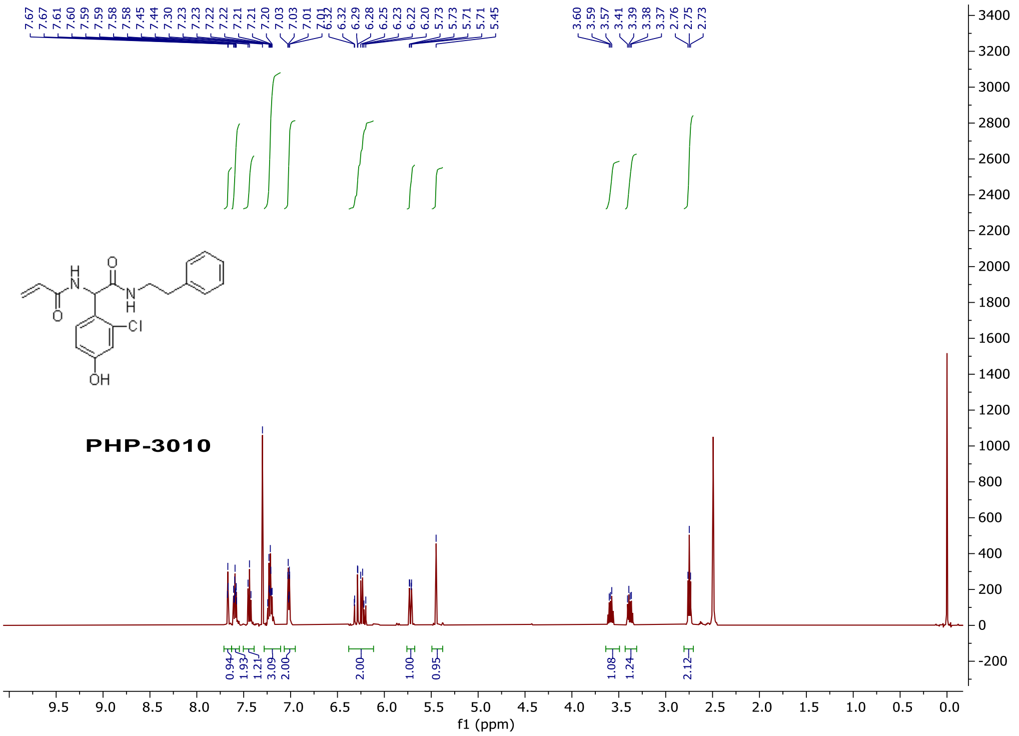

Supplementary Figure 21. ^{1}H NMR (500 MHz, $CDCl_3$+one drop of DMSO-d_6) of compound **PHP-3010.**

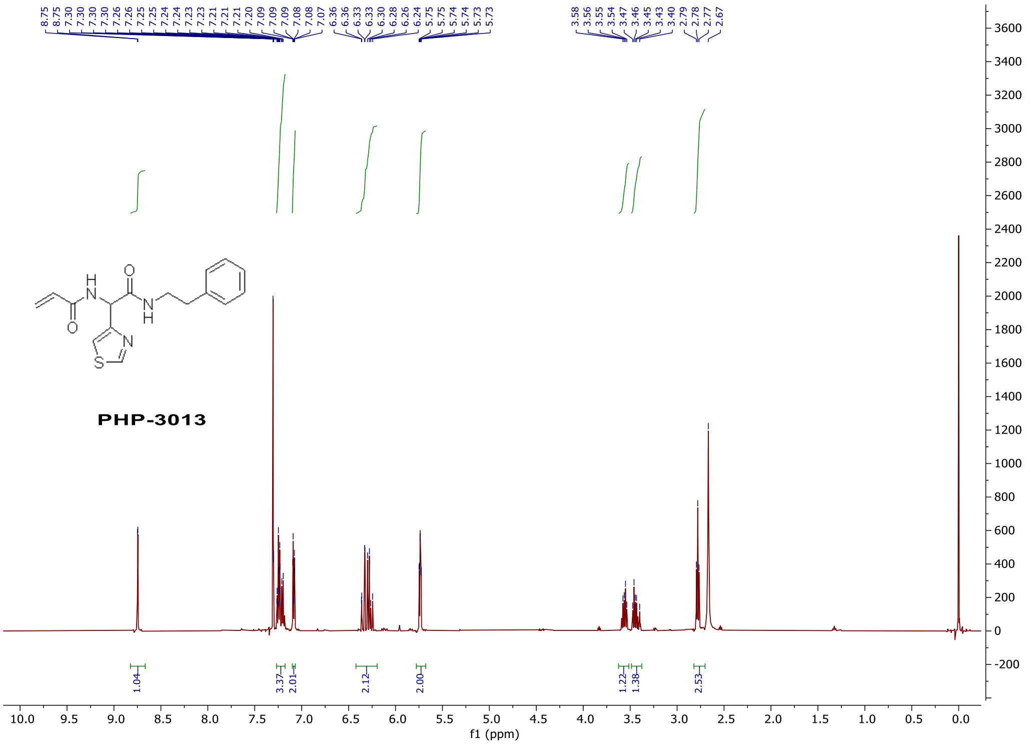

Supplementary Figure 22. ^{1}H NMR (500 MHz, $CDCl_3$+one drop of DMSO-d_6) of compound **PHP-3013.**

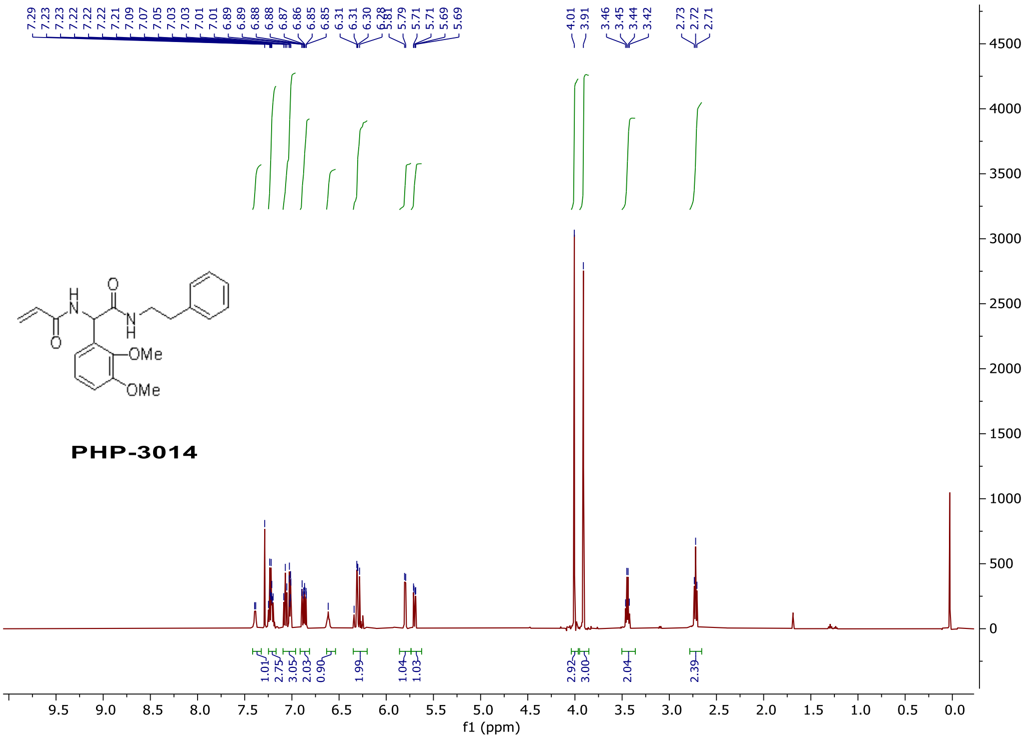

Supplementary Figure 23. ^{1}H NMR (500 MHz, $CDCl_3$) of compound **PHP-3014.**

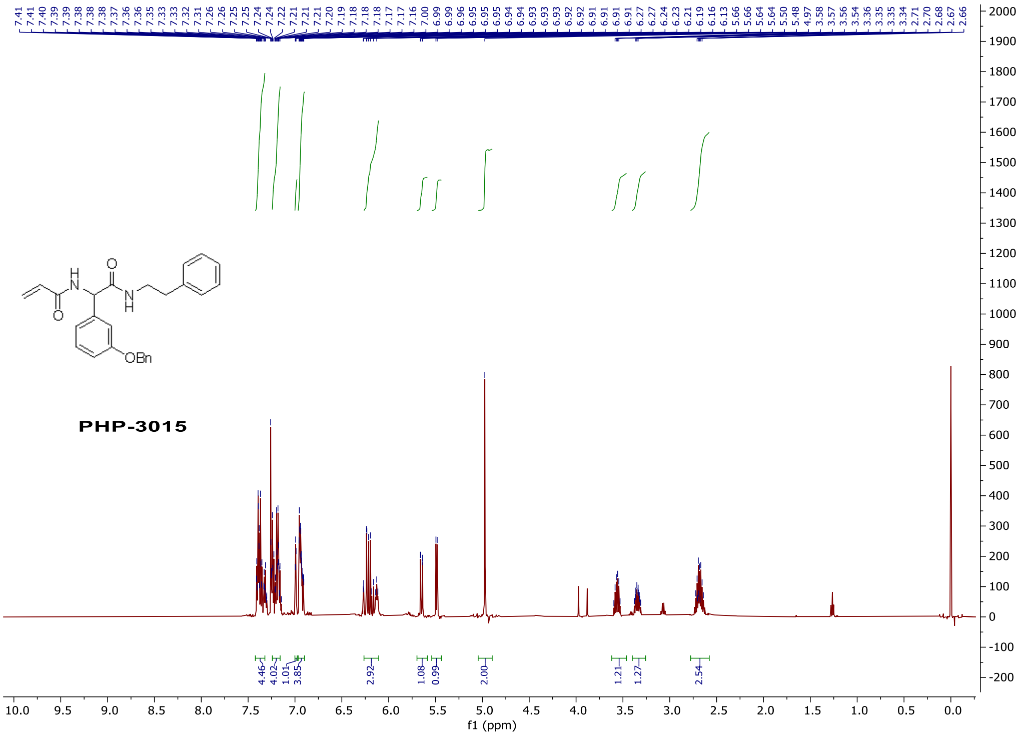

Supplementary Figure 24. ^{1}H NMR (500 MHz, $CDCl_3$) of compound **PHP-3015.**

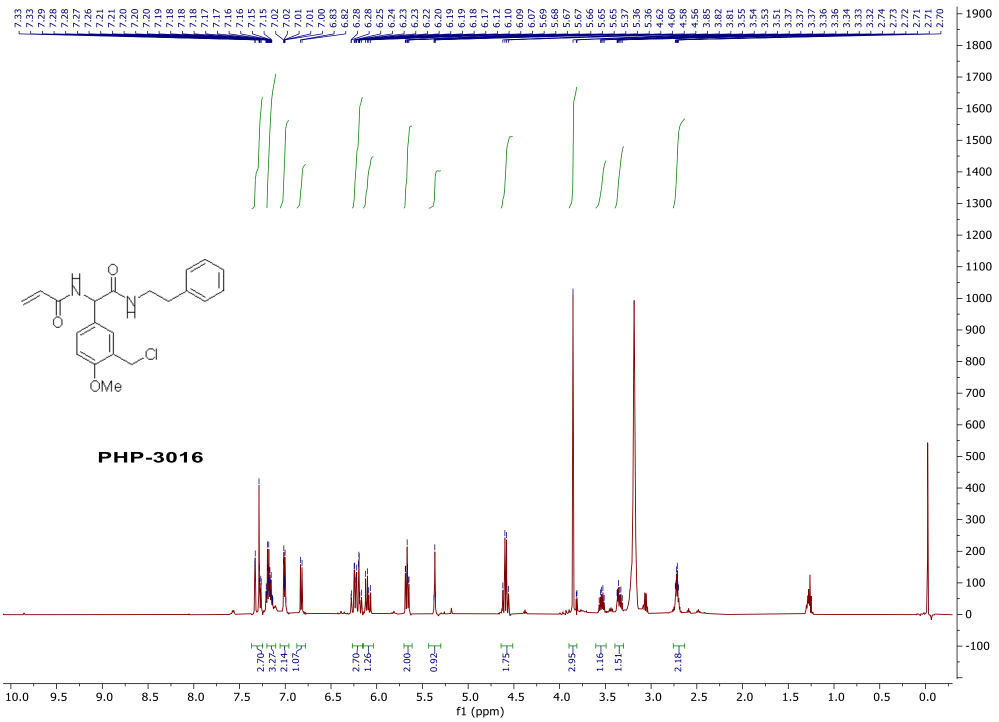

Supplementary Figure 25. ^{1}H NMR (500 MHz, $CDCl_3$+one drop of CD_3OD) of compound **PHP-3016.**

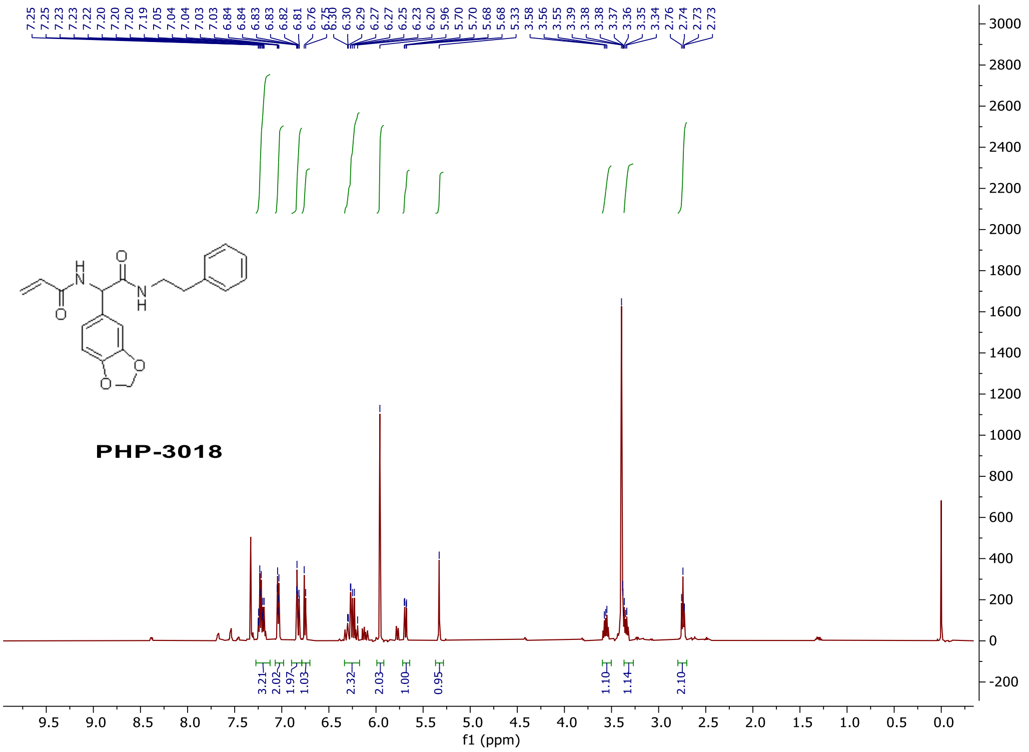

Supplementary Figure 26. ^{1}H NMR (500 MHz, $CDCl_3$+one drop of CD_3OD) of compound **PHP-3018.**

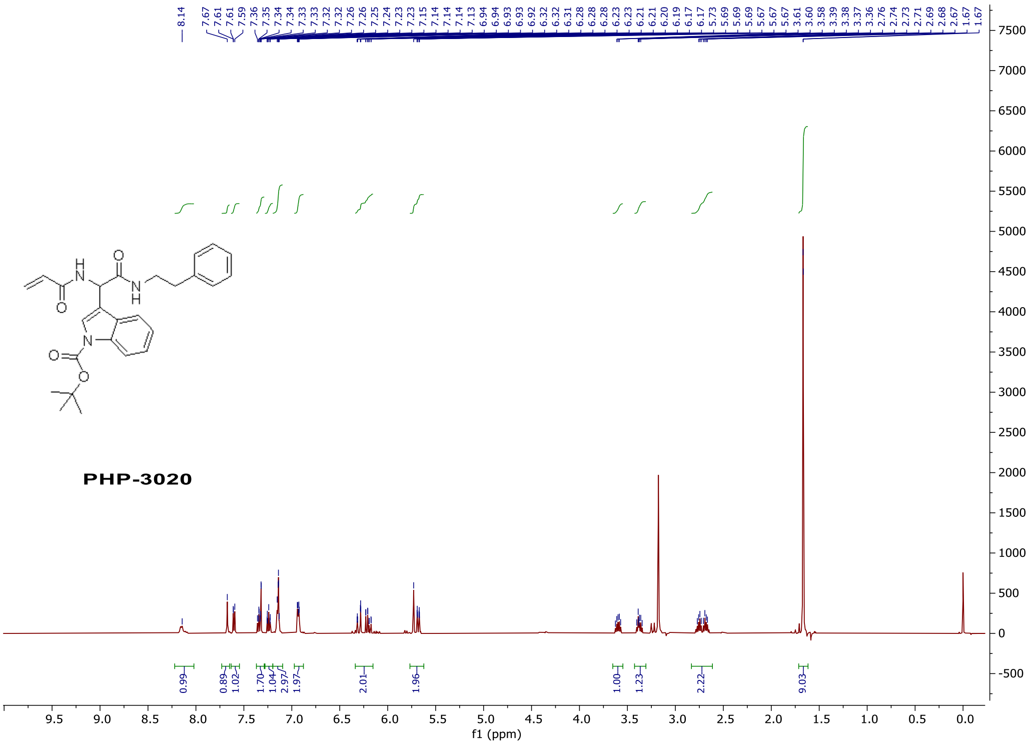

Supplementary Figure 27. ^{1}H NMR (500 MHz, $CDCl_3$) of compound **PHP-3020.**

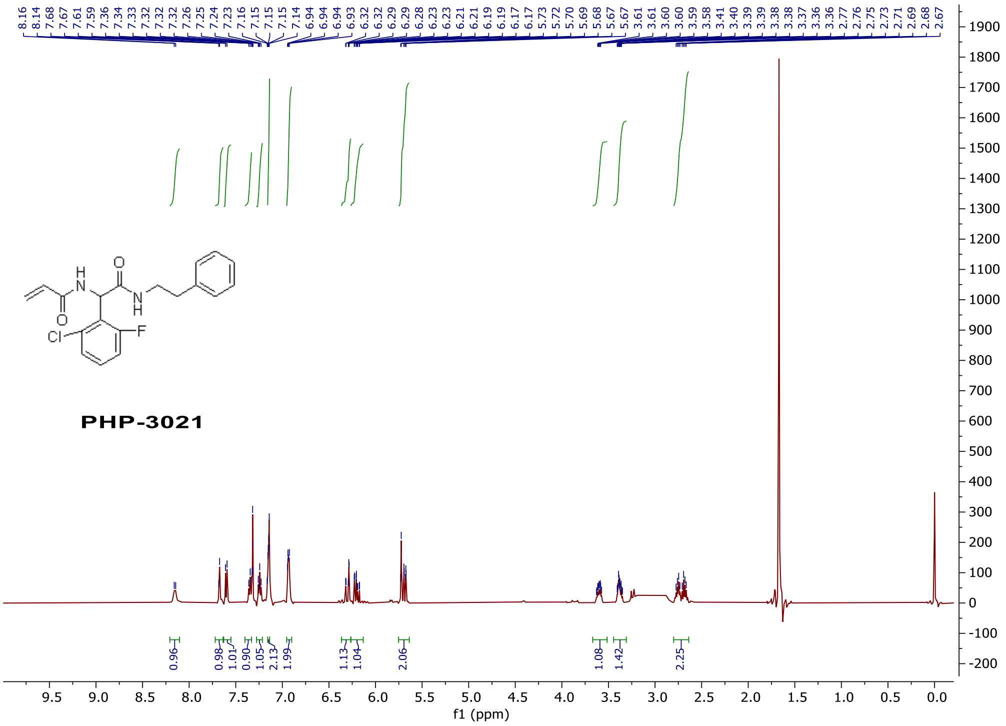

Supplementary Figure 28. ^{1}H NMR (500 MHz, $CDCl_3$) of compound **PHP-3021.**

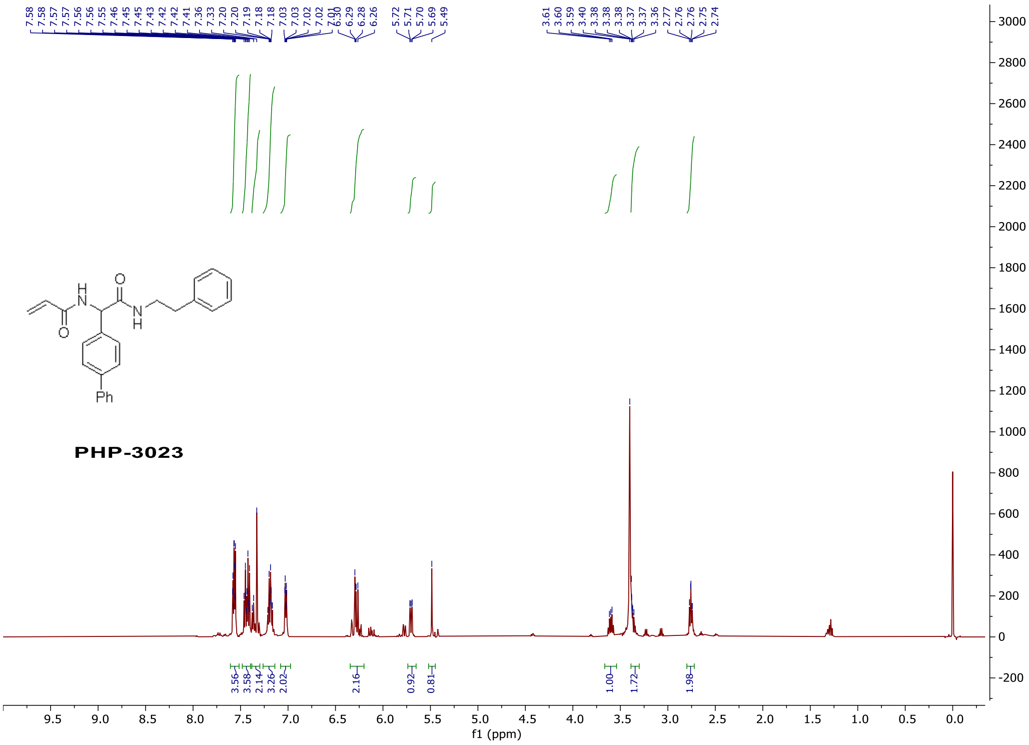

Supplementary Figure 29. ^{1}H NMR (500 MHz, $CDCl_3$) of compound **PHP-3023.**

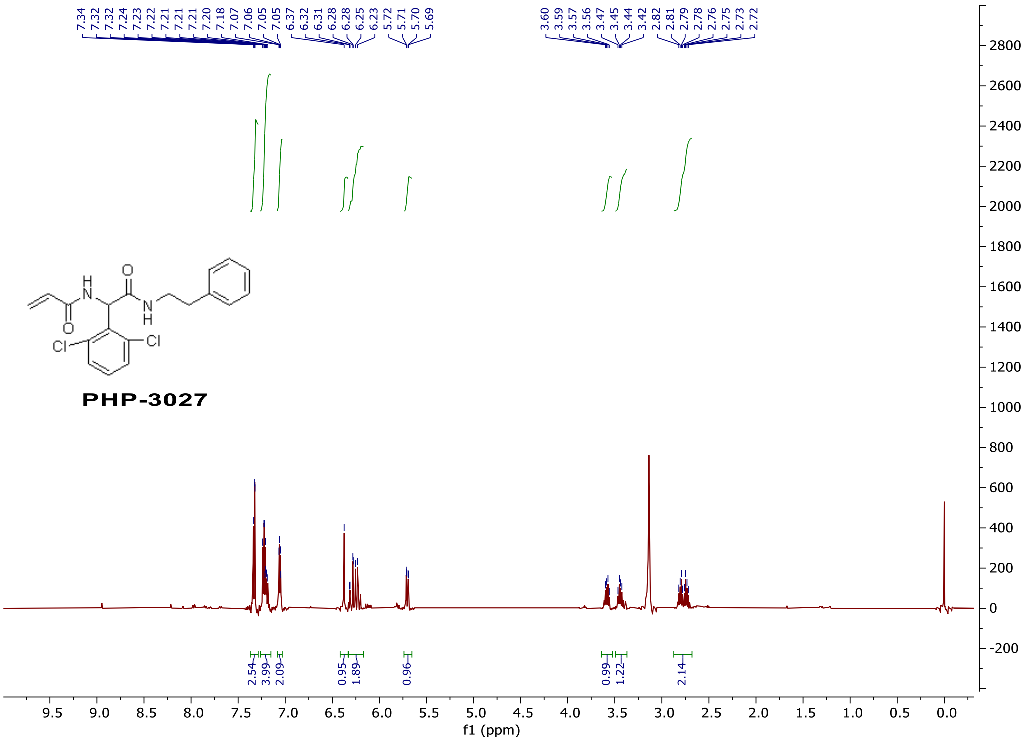

Supplementary Figure 30. ^{1}H NMR (500 MHz, $CDCl_3$+one drop of CD_3OD) of compound **PHP-3027.**

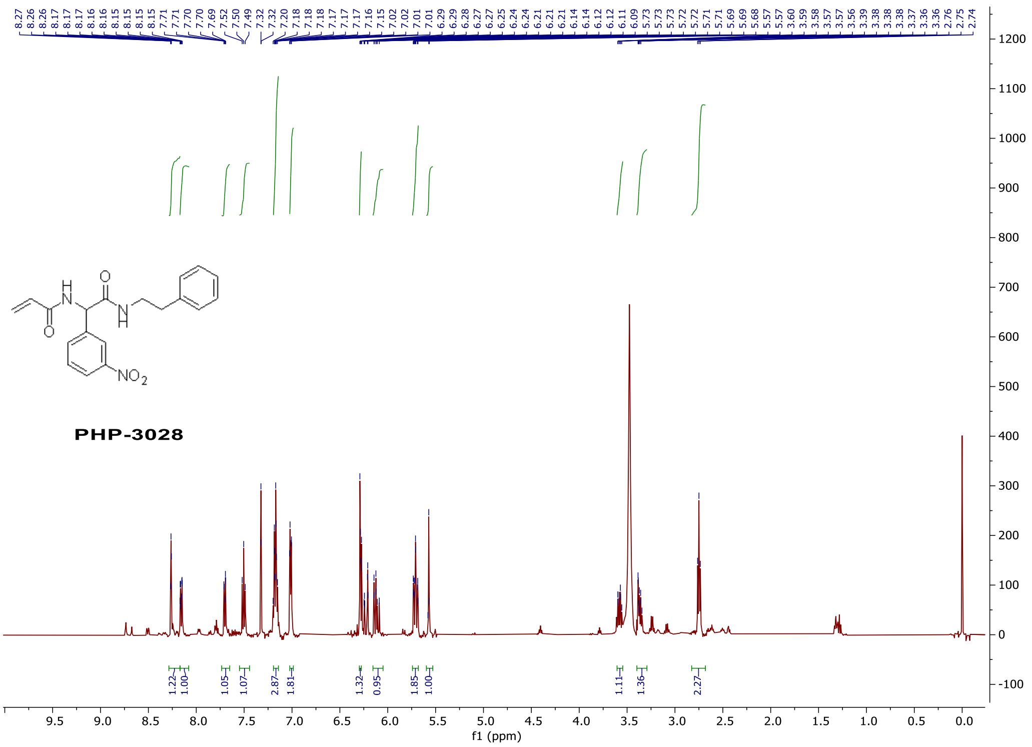

Supplementary Figure 31. ^{1}H NMR (500 MHz, $CDCl_3$+one drop of CD_3OD) of compound **PHP-3028.**

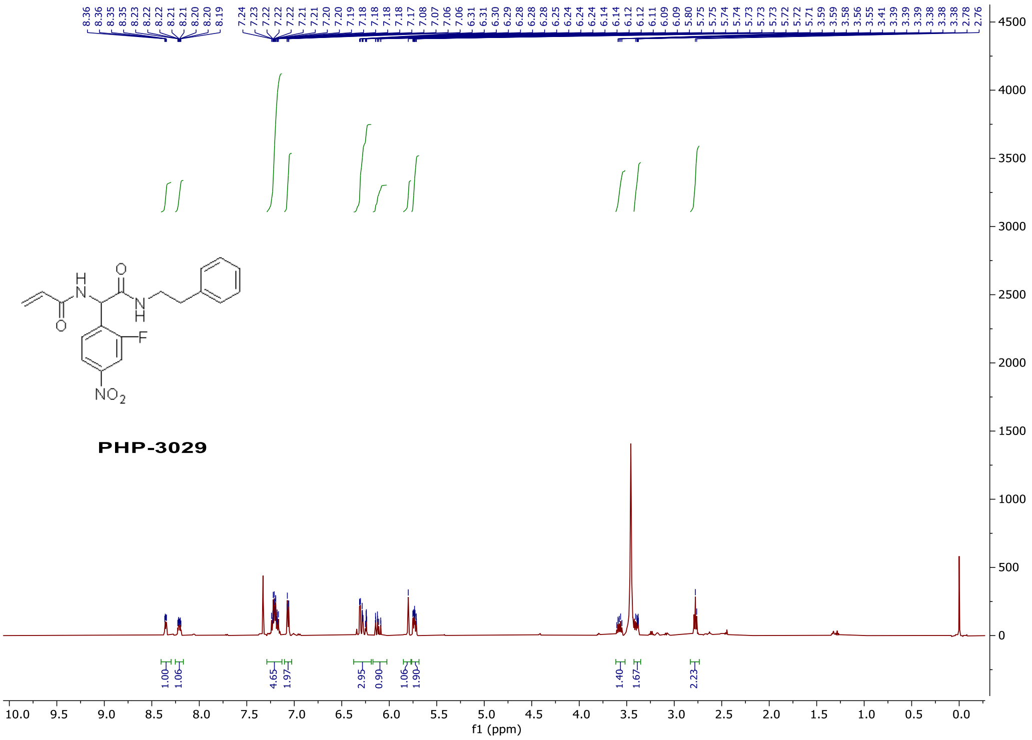

Supplementary Figure 32. ^{1}H NMR (500 MHz, $CDCl_3$+one drop of CD_3OD) of compound **PHP-3029.**

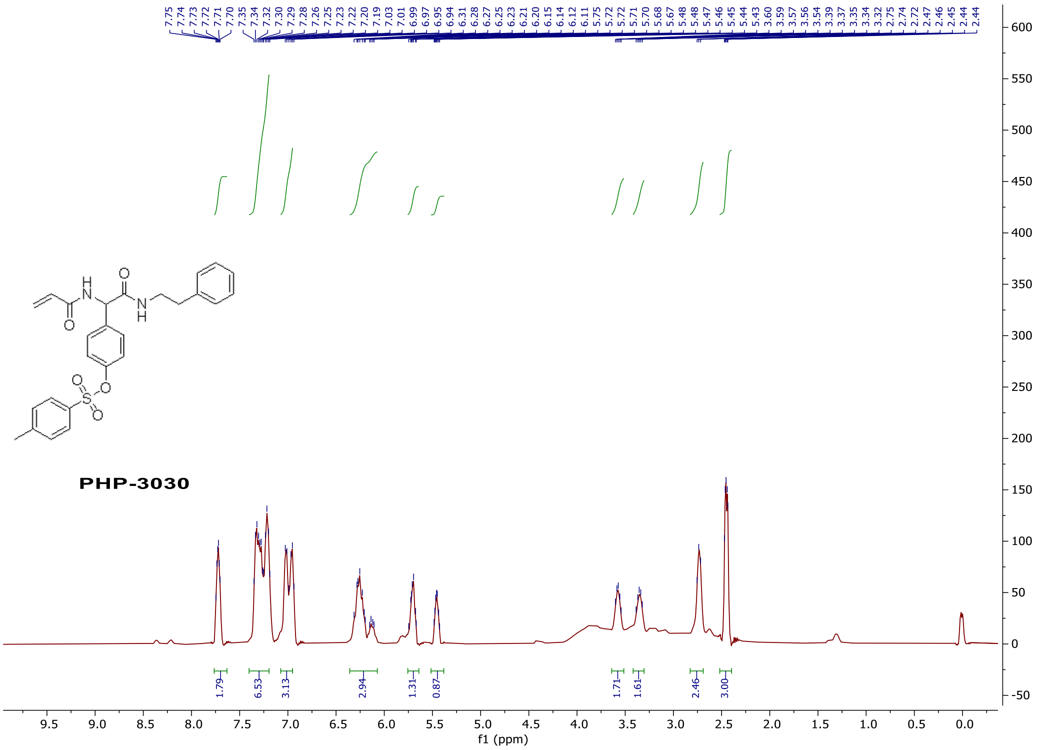

Supplementary Figure 33. ^{1}H NMR (500 MHz, $CDCl_3$) of compound **PHP-3030.**

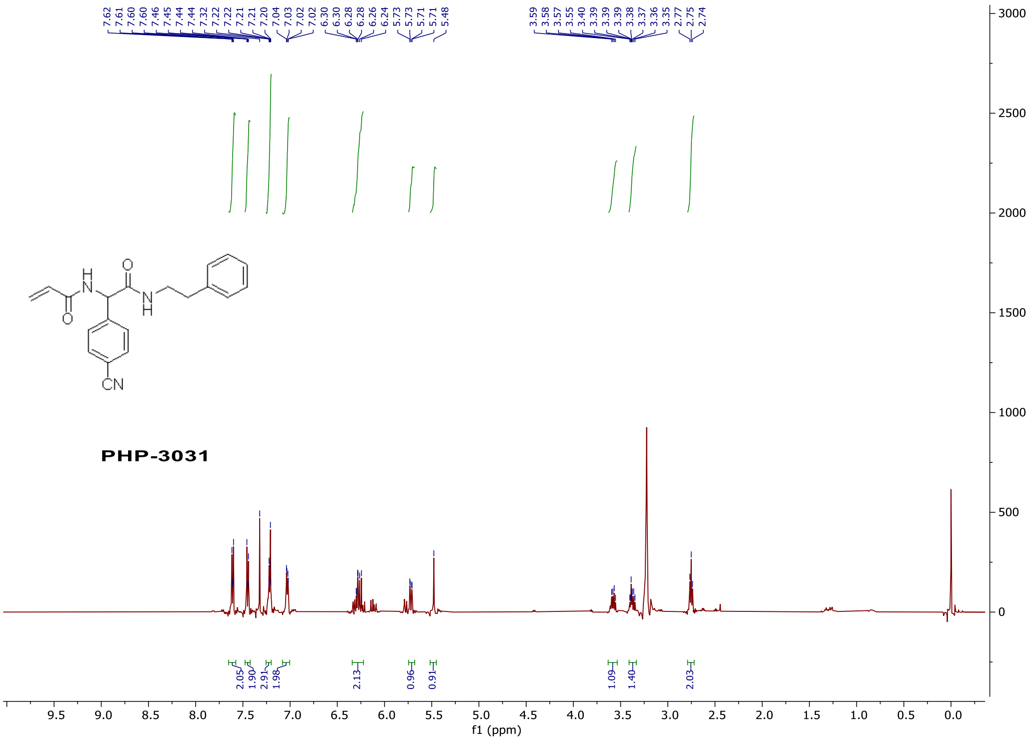

Supplementary Figure 34. ^{1}H NMR (500 MHz, $CDCl_3$+one drop of CD_3OD) of compound **PHP-3031.**

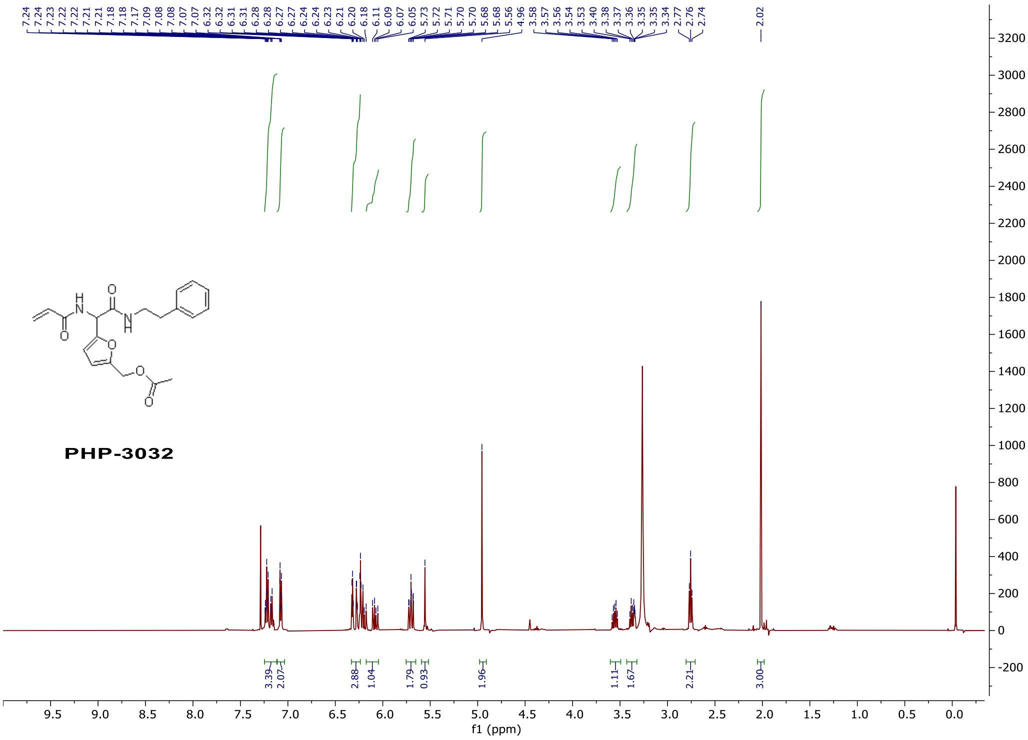

Supplementary Figure 35. ^{1}H NMR (500 MHz, $CDCl_3$+one drop of CD_3OD) of compound **PHP-3032.**

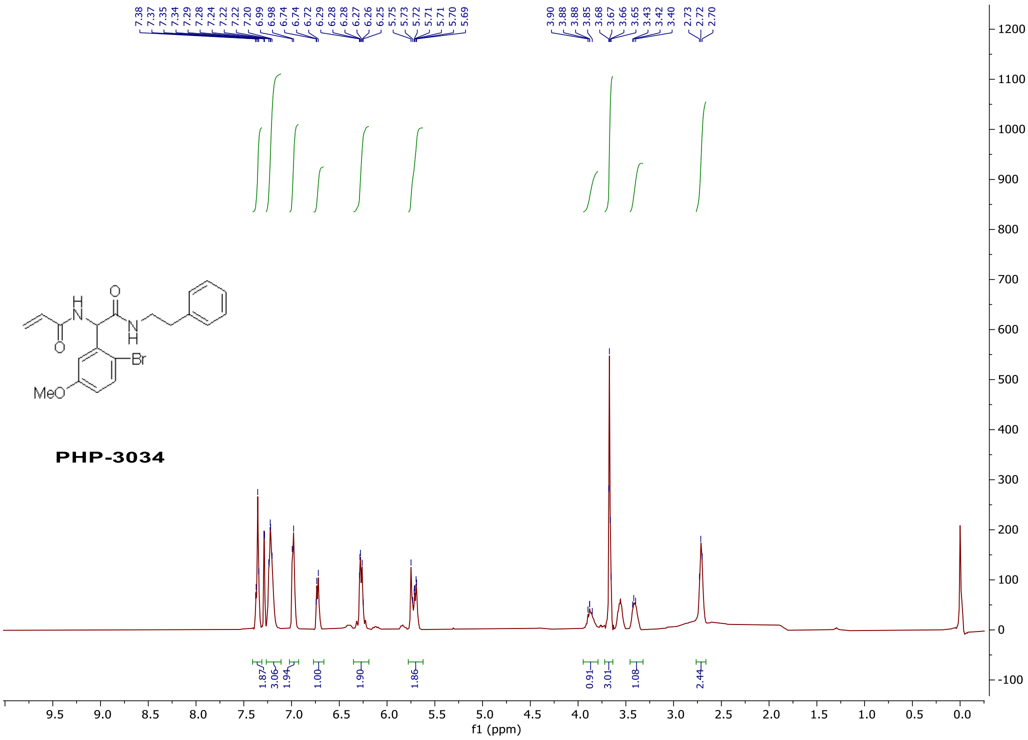

Supplementary Figure 36. ^{1}H NMR (500 MHz, $CDCl_3$+one drop of CD_3OD) of compound **PHP-3034.**

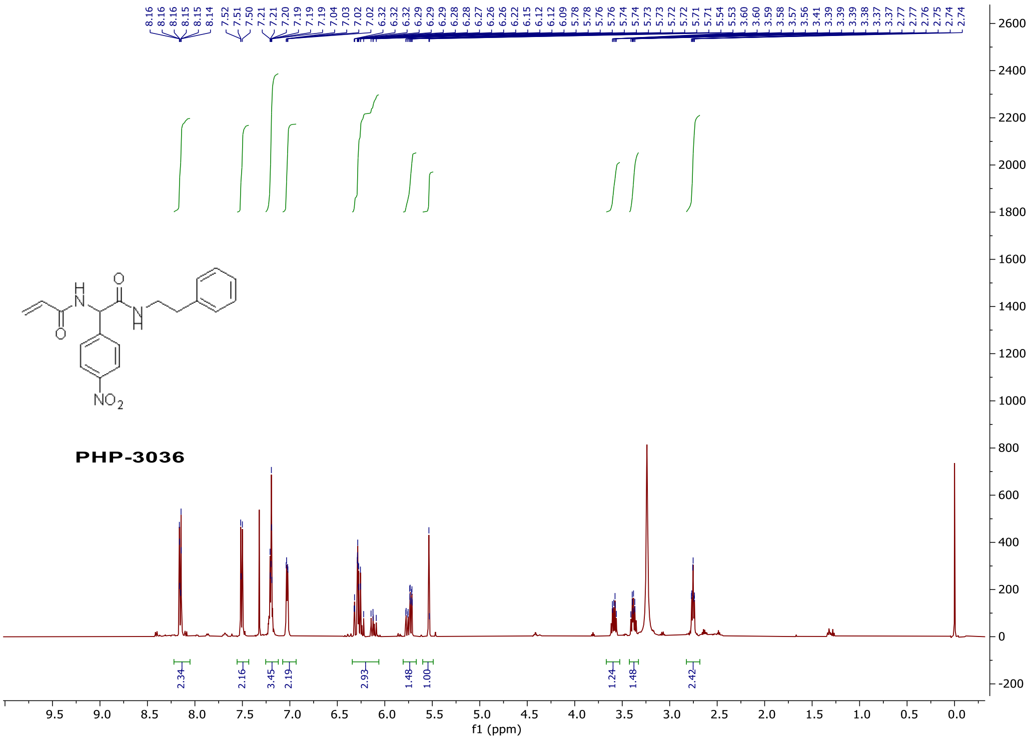

Supplementary Figure 37. ^{1}H NMR (500 MHz, $CDCl_3$+one drop of CD_3OD) of compound **PHP-3036.**

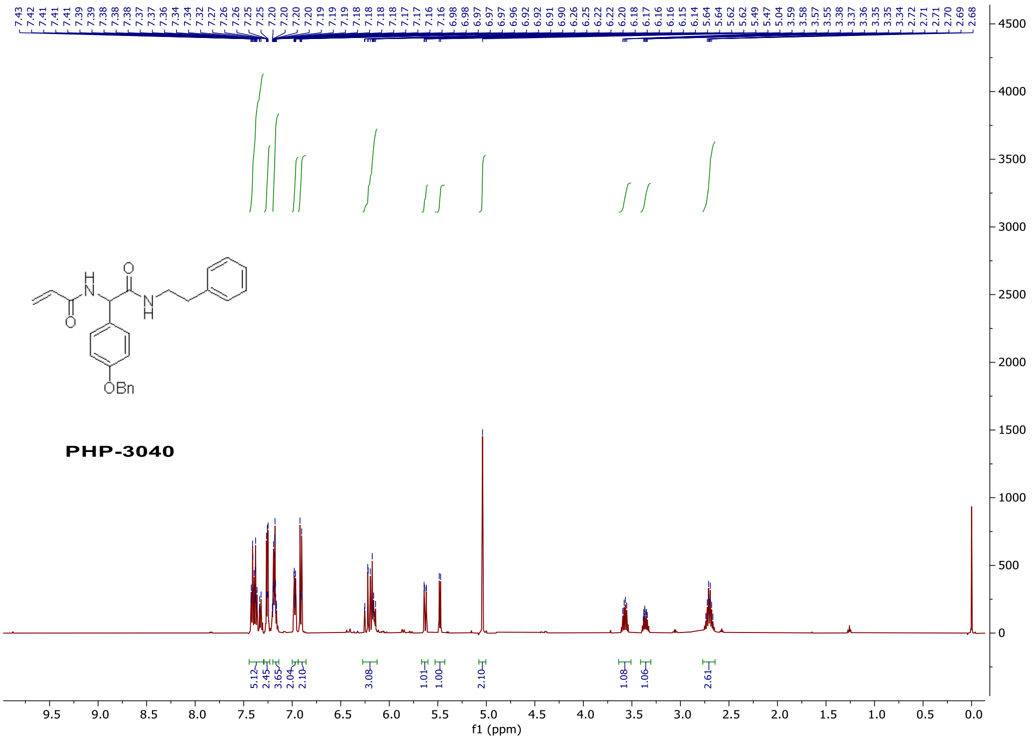

Supplementary Figure 38. ^{1}H NMR (500 MHz, $CDCl_3$) of compound **PHP-3040.**

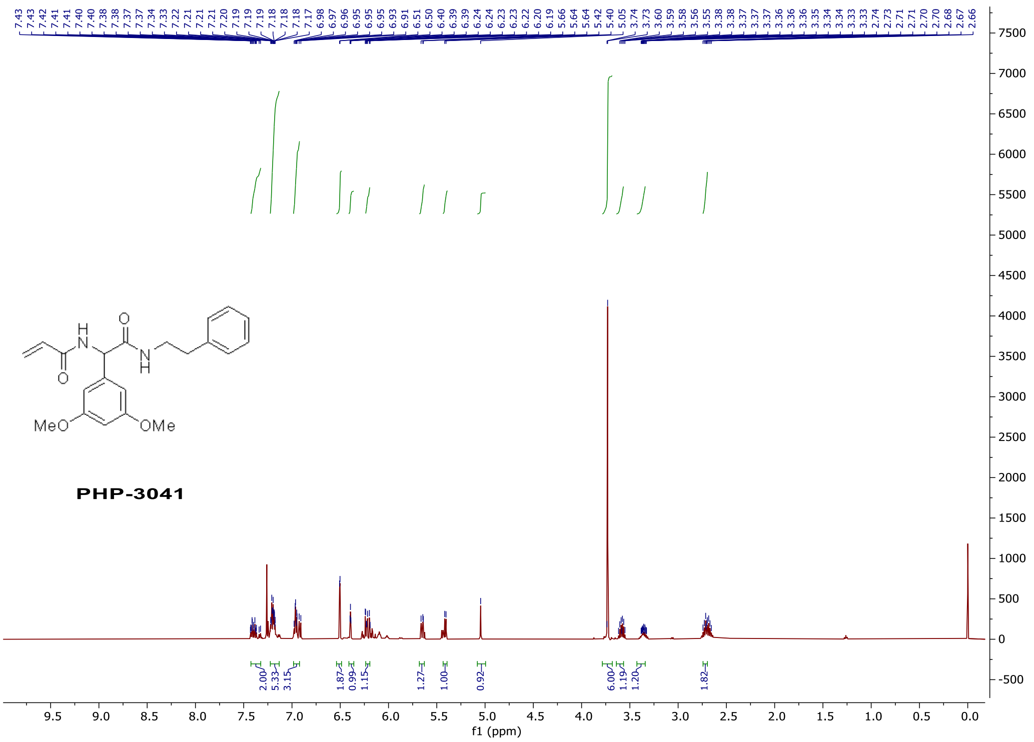

Supplementary Figure 39. ^{1}H NMR (500 MHz, $CDCl_3$) of compound **PHP-3041.**

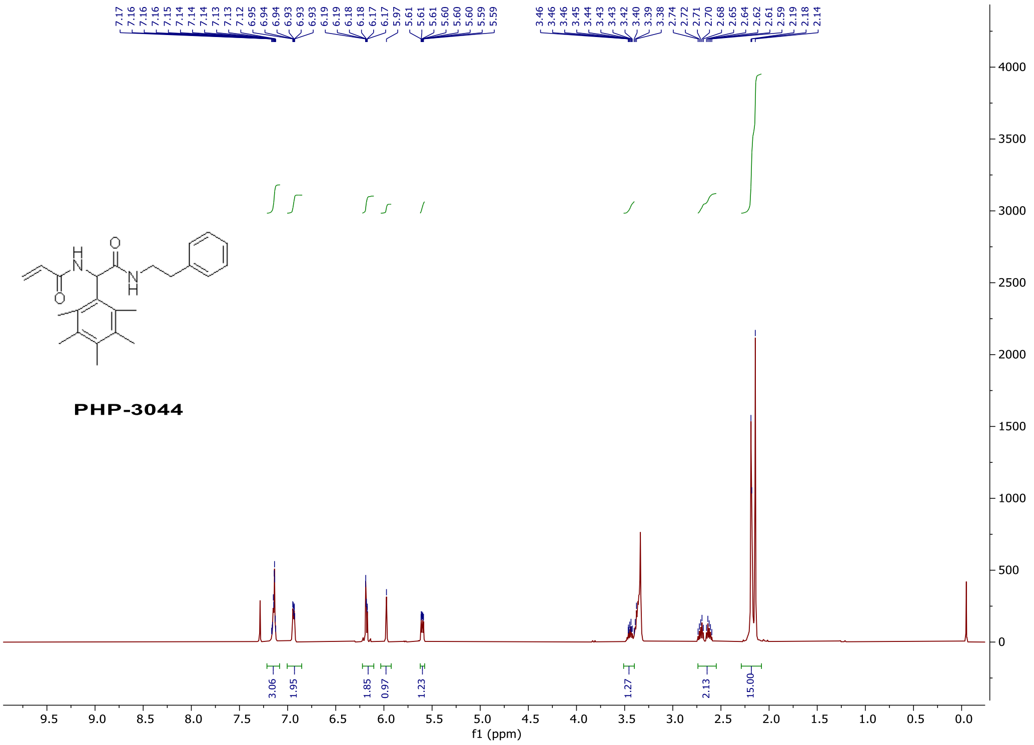

Supplementary Figure 40. ^{1}H NMR (500 MHz, $CDCl_3$+one drop of CD_3OD) of compound **PHP-3044.**

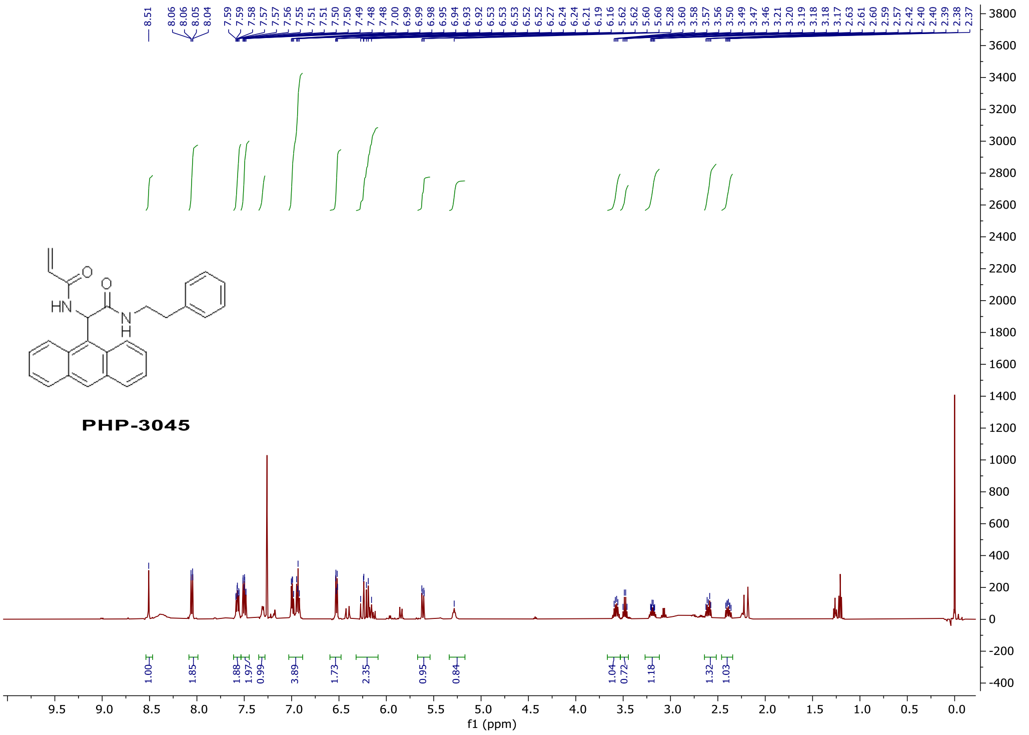

Supplementary Figure 41. ^{1}H NMR (500 MHz, $CDCl_3$) of compound **PHP-3045.**

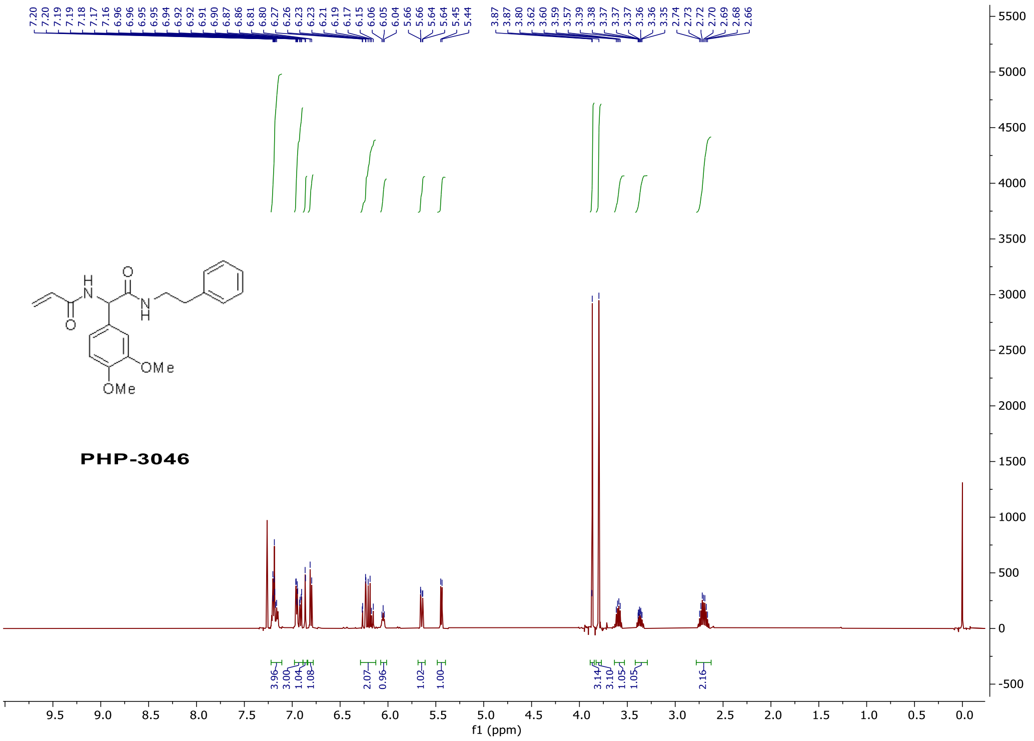

Supplementary Figure 42. ^{1}H NMR (500 MHz, $CDCl_3$) of compound **PHP-3046.**

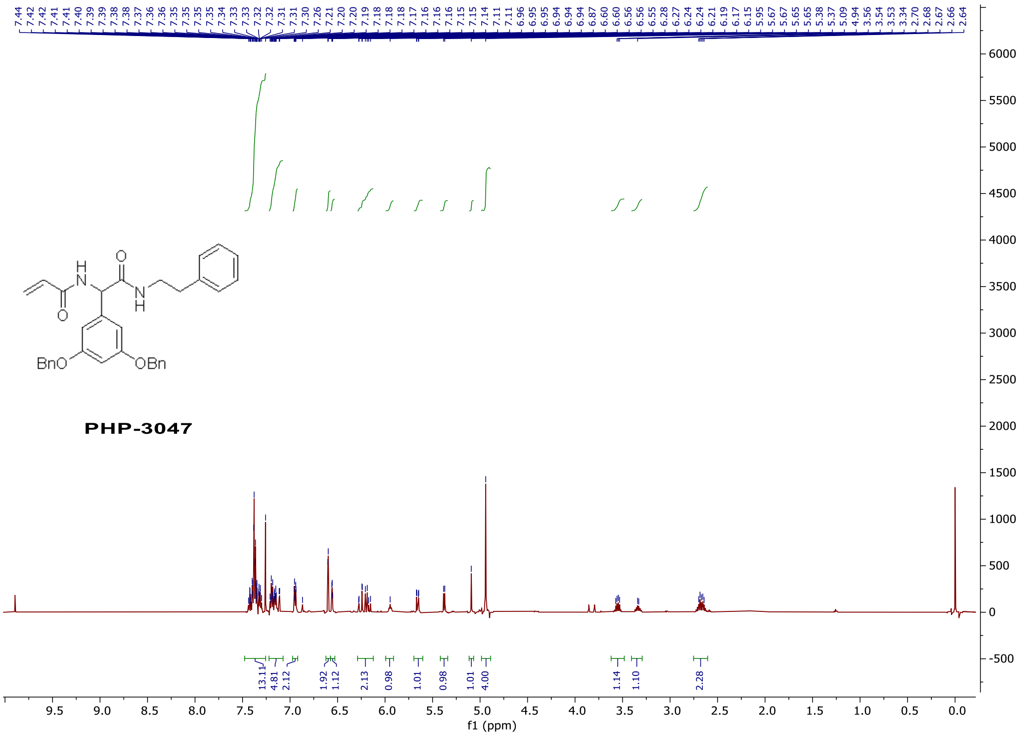

Supplementary Figure 43. ^{1}H NMR (500 MHz, $CDCl_3$) of compound **PHP-3047.**

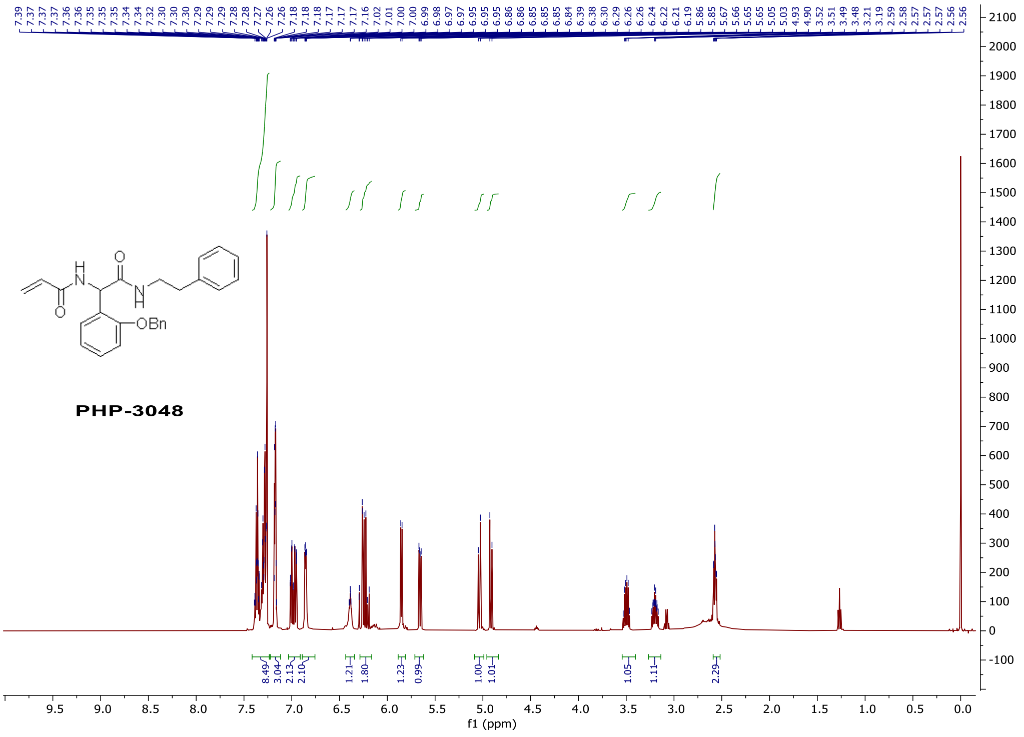

Supplementary Figure 44. ^{1}H NMR (500 MHz, $CDCl_3$) of compound **PHP-3048.**

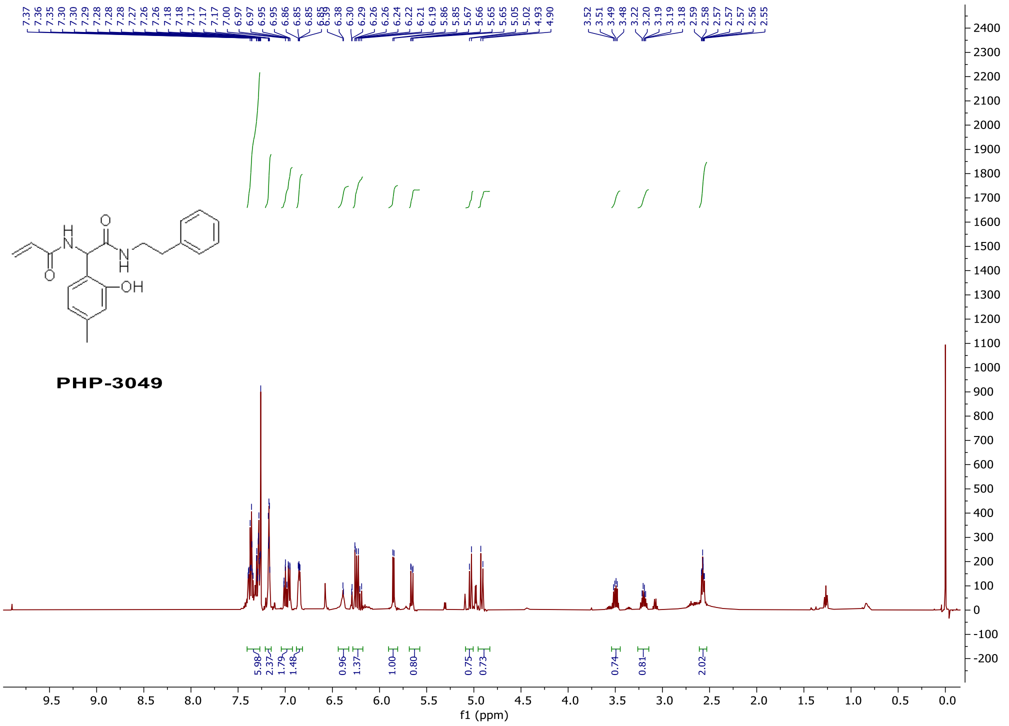

Supplementary Figure 45. ^{1}H NMR (500 MHz, $CDCl_3$) of compound **PHP-3049.**

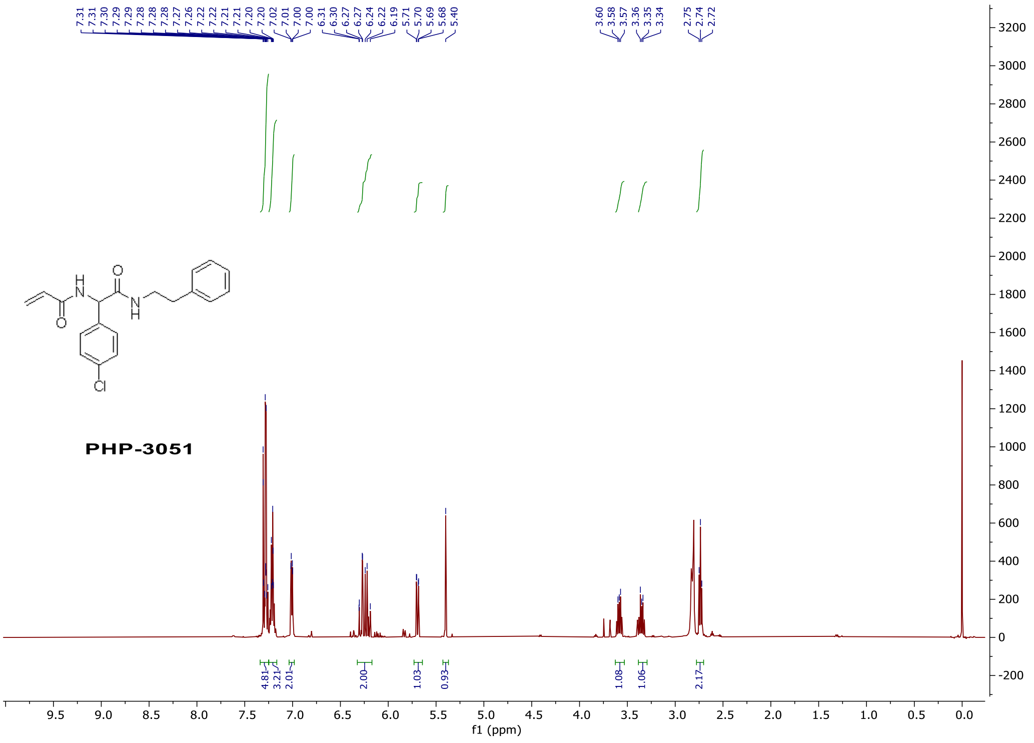

Supplementary Figure 46. ^{1}H NMR (500 MHz, $CDCl_3$+one drop of DMSO-d_6) of compound **PHP-3051.**

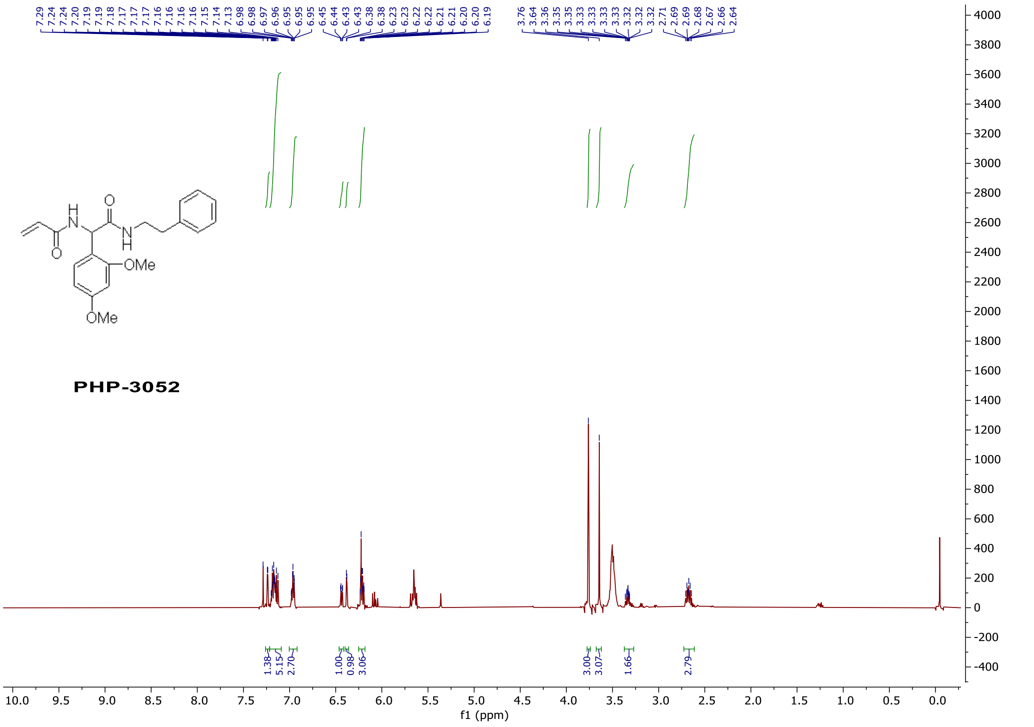

Supplementary Figure 47. ^{1}H NMR (500 MHz, $CDCl_3$+one drop of CD_3OD) of compound **PHP-3052.**

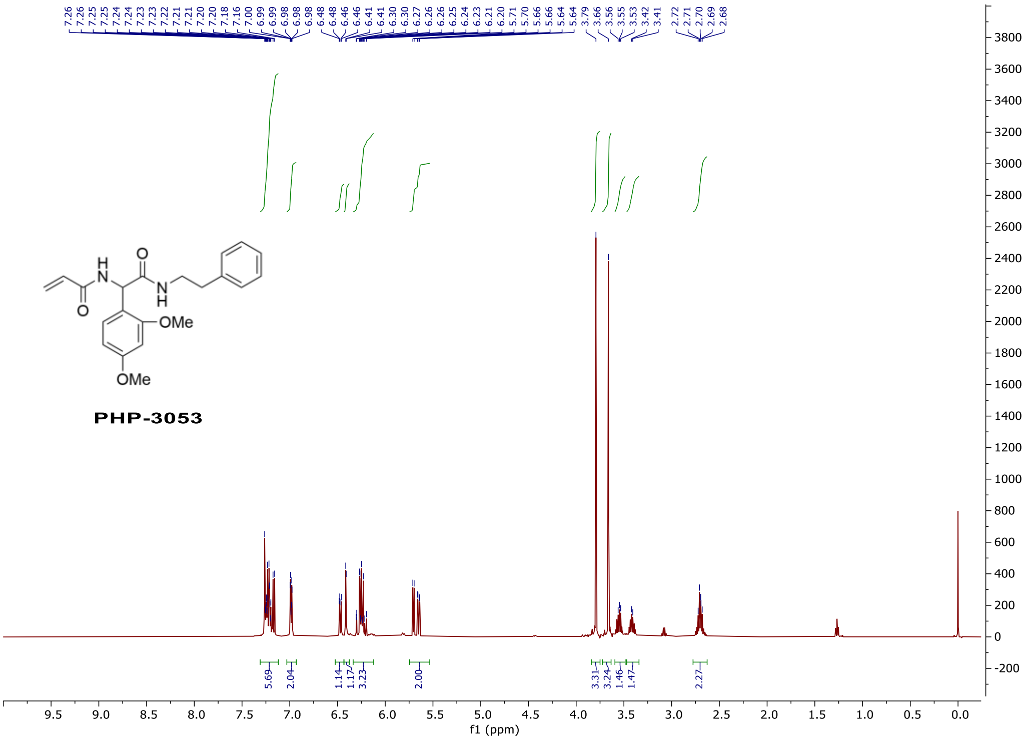

Supplementary Figure 48. ^{1}H NMR (500 MHz, $CDCl_3$) of compound **PHP-3053.**

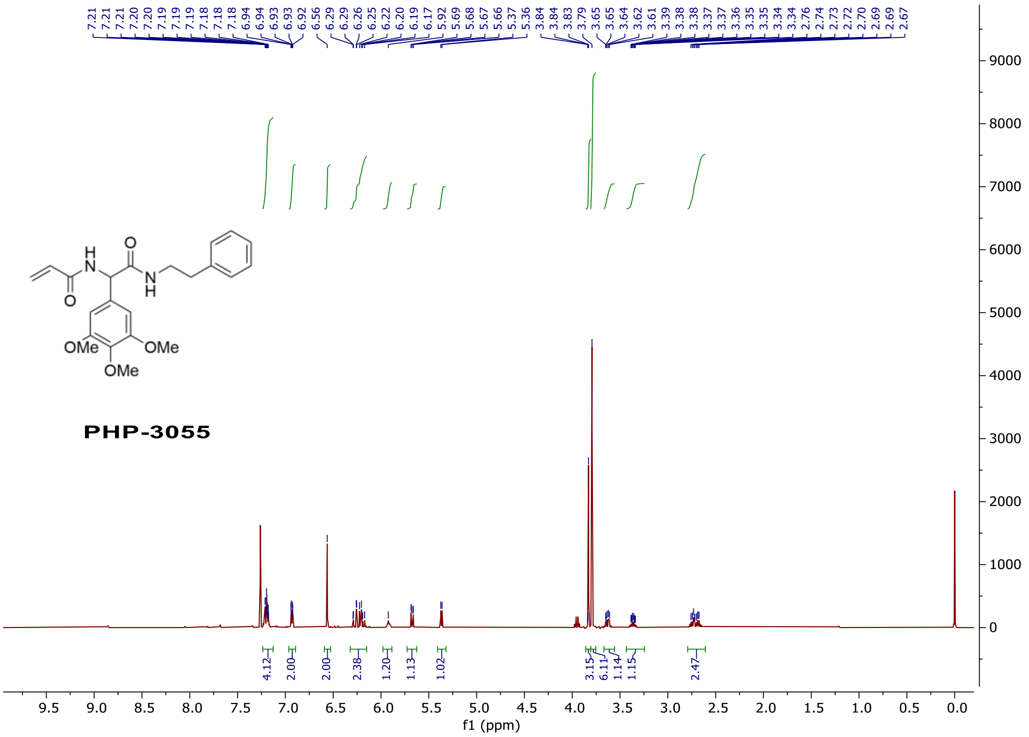

Supplementary Figure 49. ^{1}H NMR (500 MHz, $CDCl_3$) of compound **PHP-3055.**

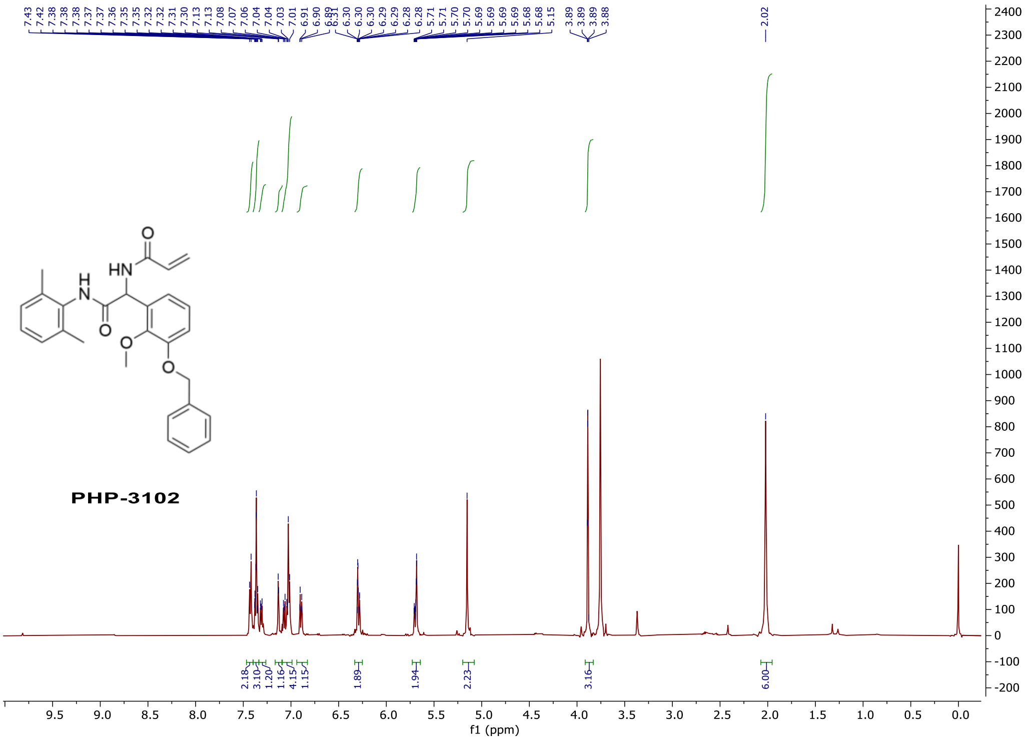

Supplementary Figure 50. ^{1}H NMR (500 MHz, $CDCl_3$) of compound **PHP-3102.**

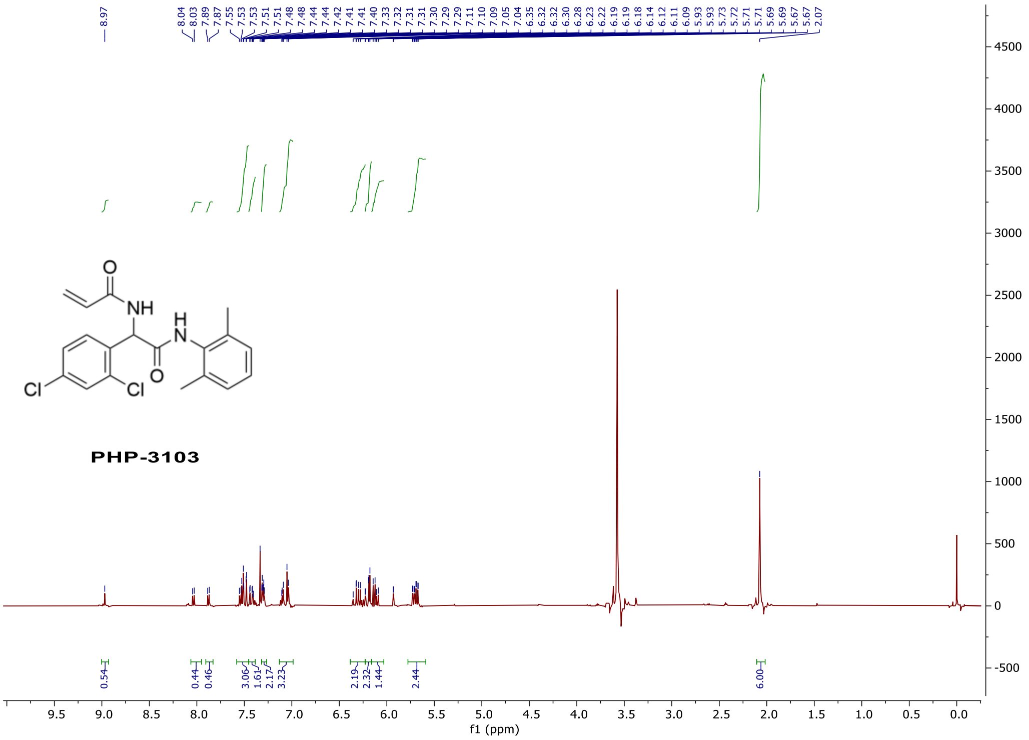

Supplementary Figure 51. ^{1}H NMR (500 MHz, $CDCl_3$+one drop of CD_3OD) of compound **PHP-3103.**

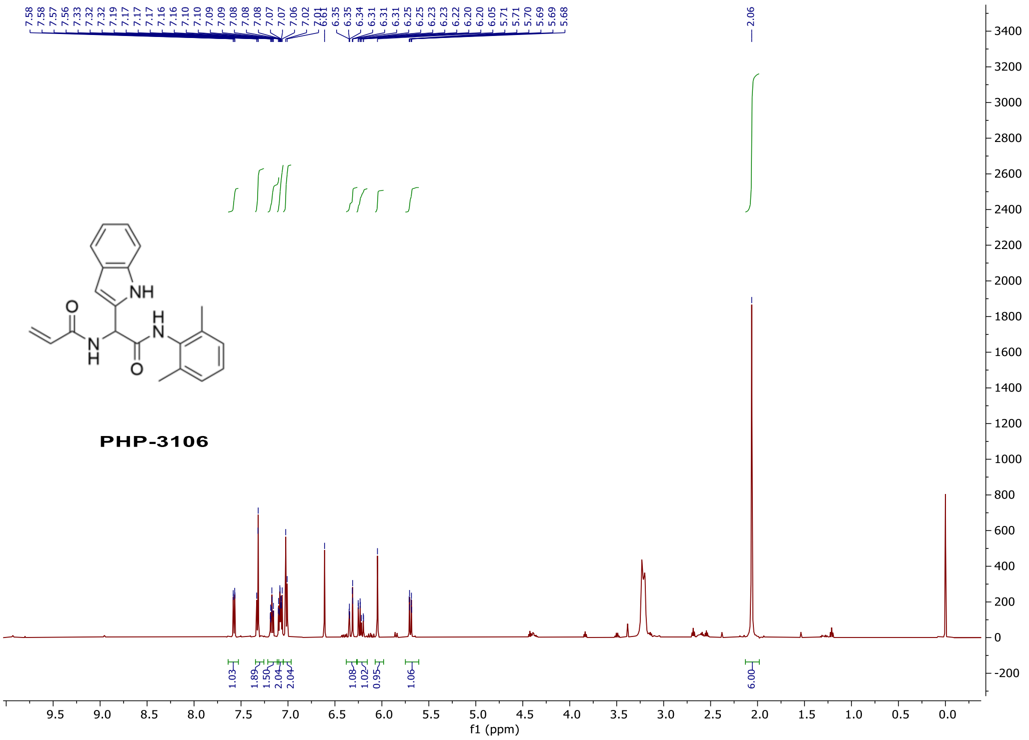

Supplementary Figure 52. ^{1}H NMR (500 MHz, $CDCl_3$+one drop of CD_3OD) of compound **PHP-3106.**

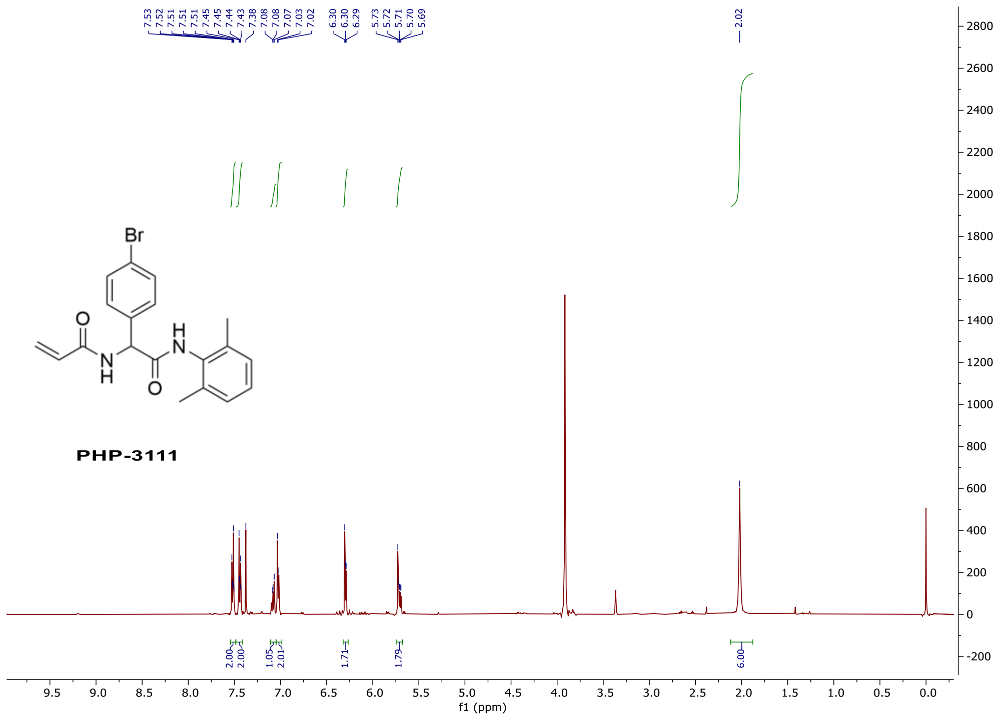

Supplementary Figure 53. ^{1}H NMR (500 MHz, $CDCl_3$) of compound **PHP-3111.**

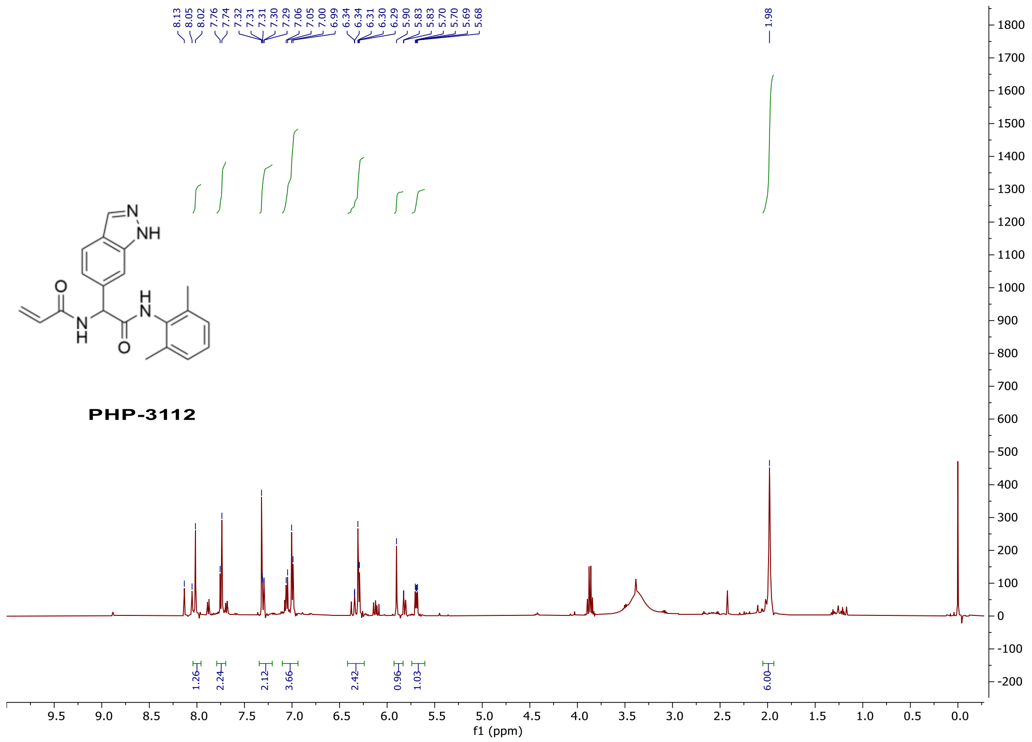

Supplementary Figure 54. ^{1}H NMR (500 MHz, $CDCl_3$+one drop of CD_3OD) of compound **PHP-3112.**

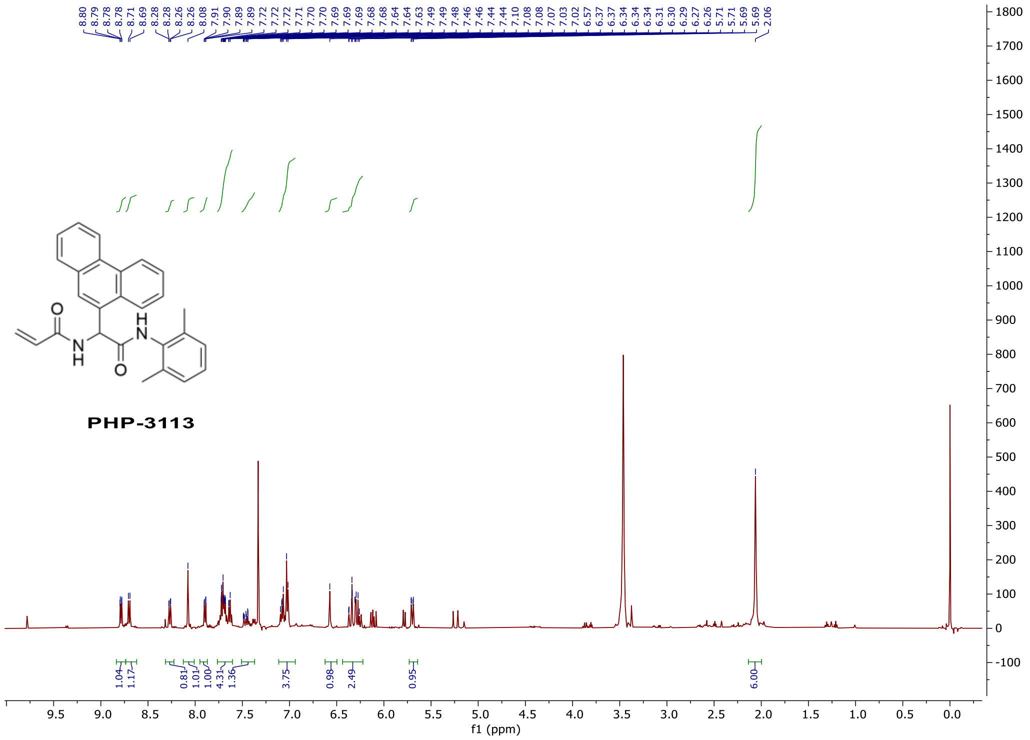

Supplementary Figure 55. ^{1}H NMR (500 MHz, $CDCl_3$+one drop of CD_3OD) of compound **PHP-3113.**

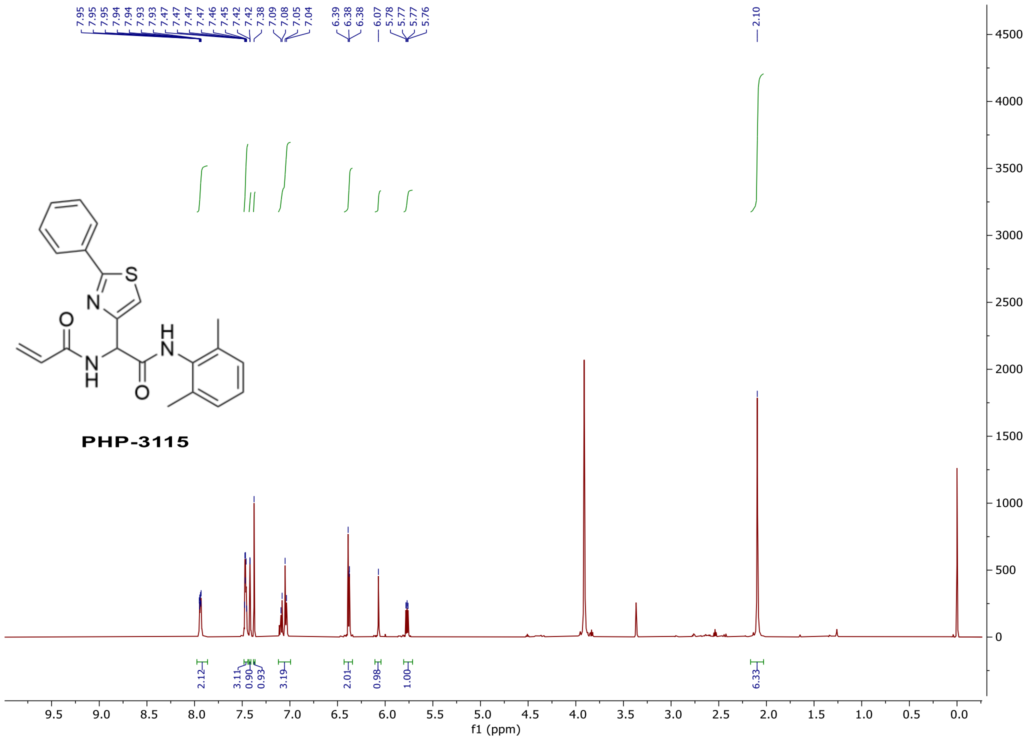

Supplementary Figure 56. ^{1}H NMR (500 MHz, $CDCl_3$+one drop of CD_3OD) of compound **PHP-3115.**

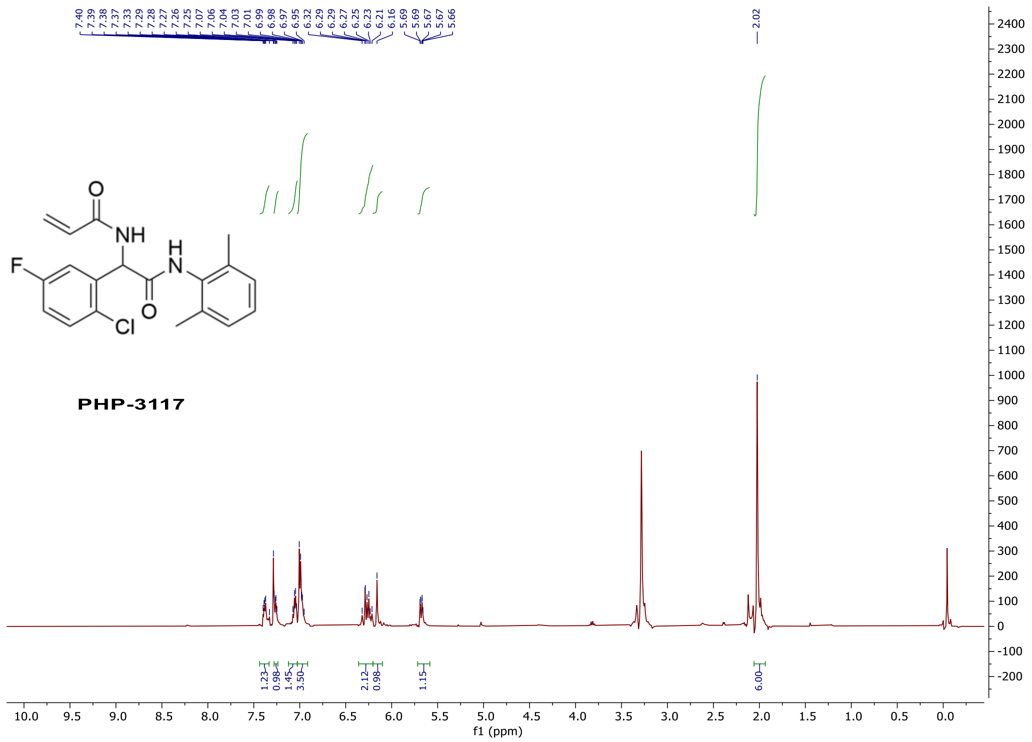

Supplementary Figure 57. ^{1}H NMR (500 MHz, $CDCl_3$+one drop of CD_3OD) of compound **PHP-3117.**

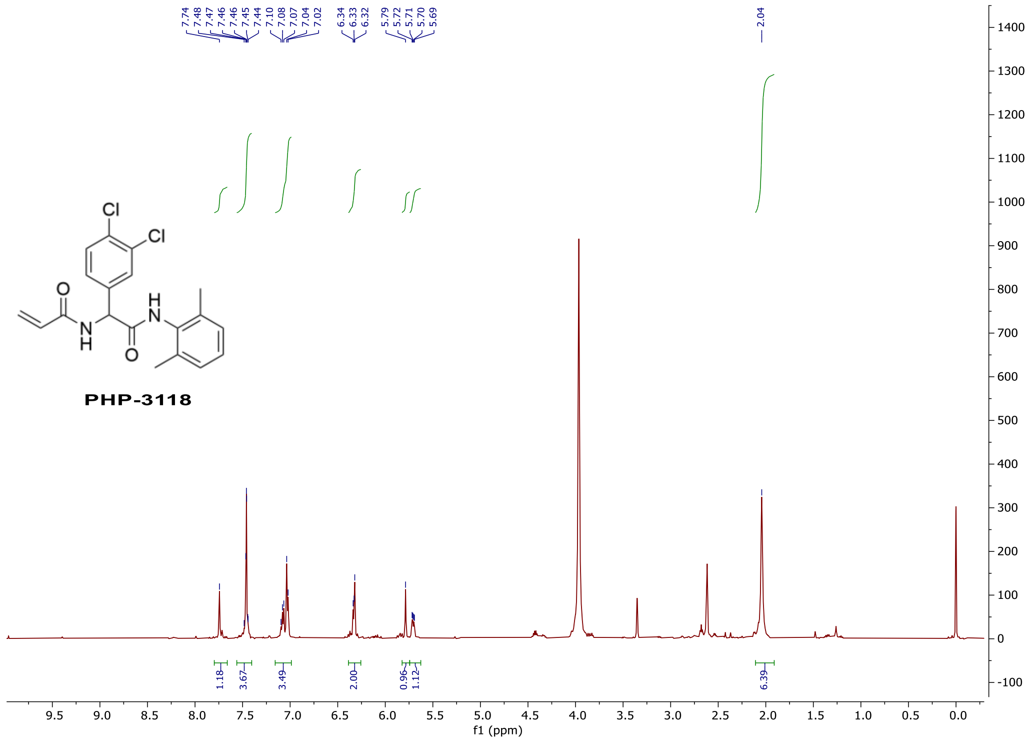

Supplementary Figure 58. ^{1}H NMR (500 MHz, $CDCl_3$+one drop of CD_3OD) of compound **PHP-3118.**

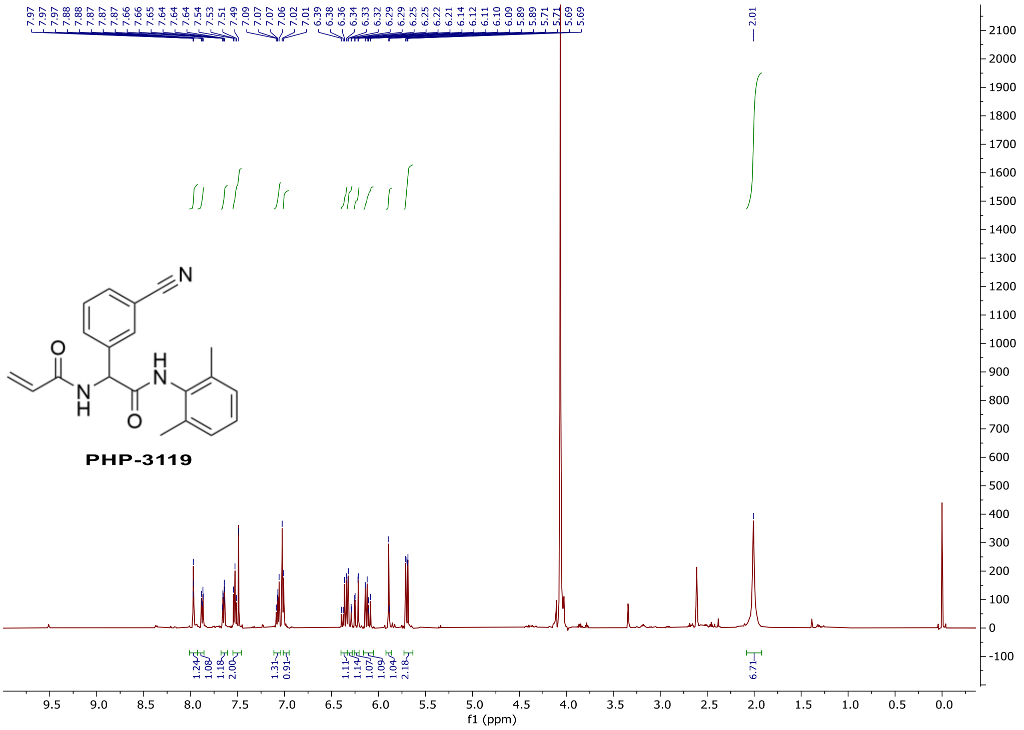

Supplementary Figure 59. ^{1}H NMR (500 MHz, $CDCl_3$+one drop of CD_3OD) of compound **PHP-3119.**

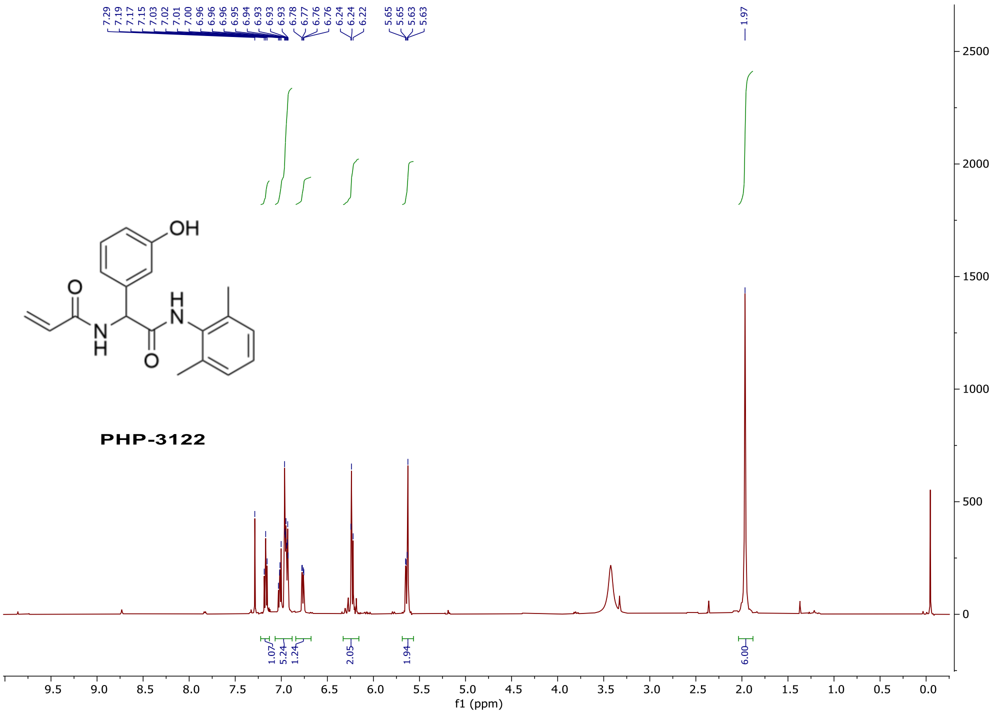

Supplementary Figure 60. ^{1}H NMR (500 MHz, $CDCl_3$+one drop of CD_3OD) of compound **PHP-3122.**

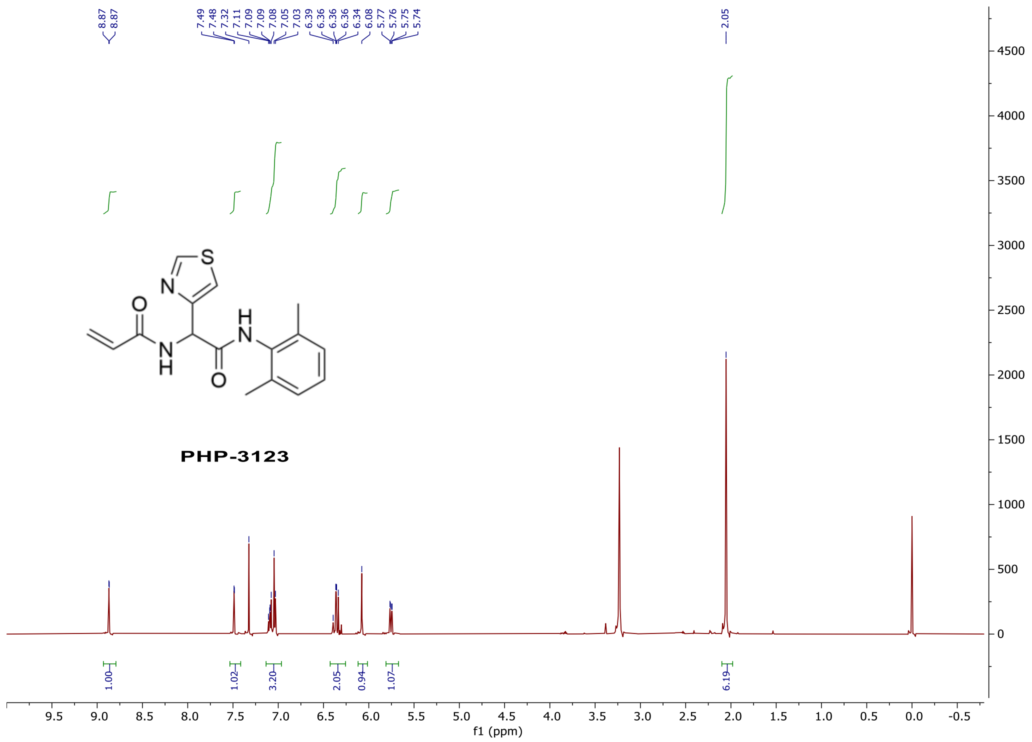

Supplementary Figure 61. ^{1}H NMR (500 MHz, $CDCl_3$+one drop of CD_3OD) of compound **PHP-3123.**

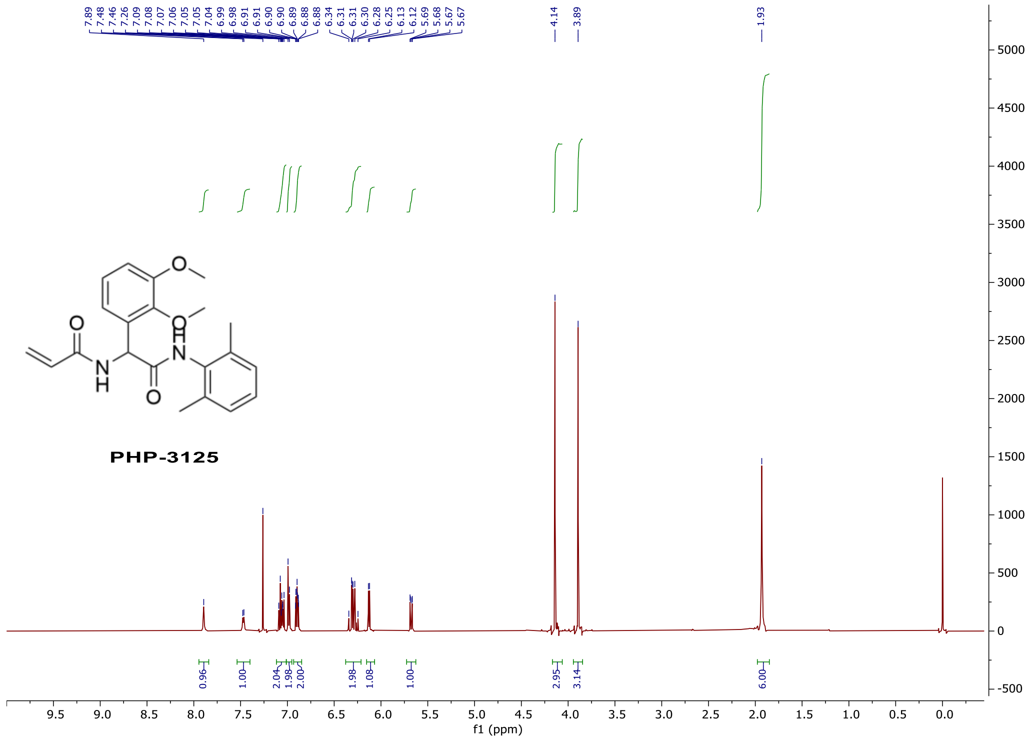

Supplementary Figure 62. ^{1}H NMR (500 MHz, $CDCl_3$) of compound **PHP-3125.**

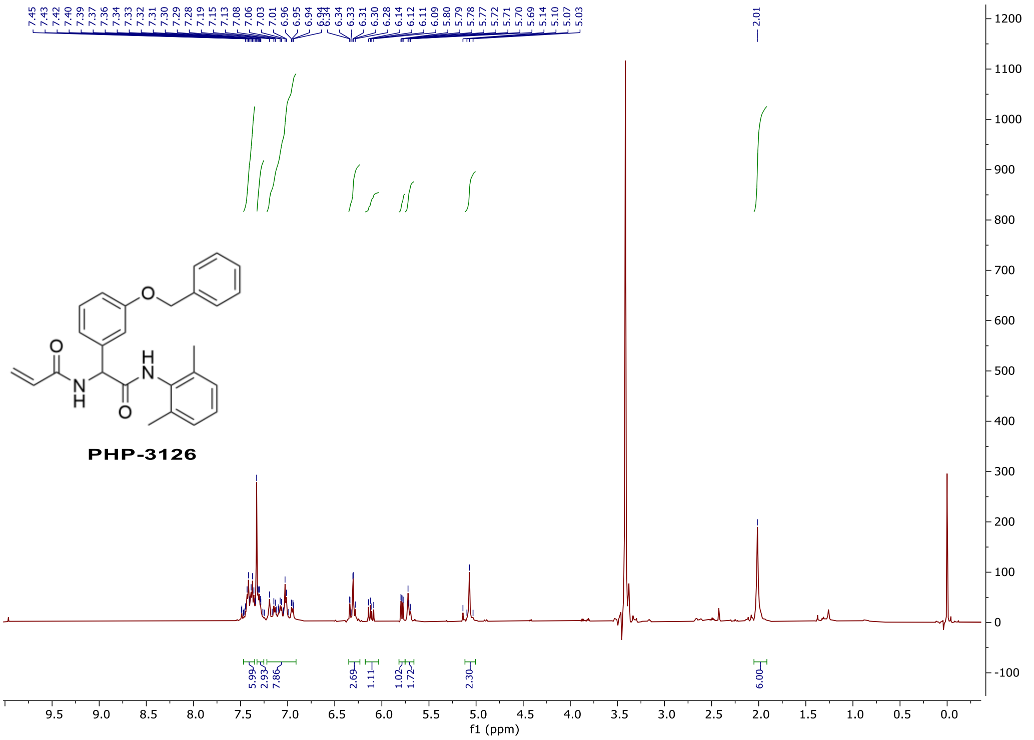

Supplementary Figure 63. ^{1}H NMR (500 MHz, $CDCl_3$+one drop of CD_3OD) of compound **PHP-3126.**

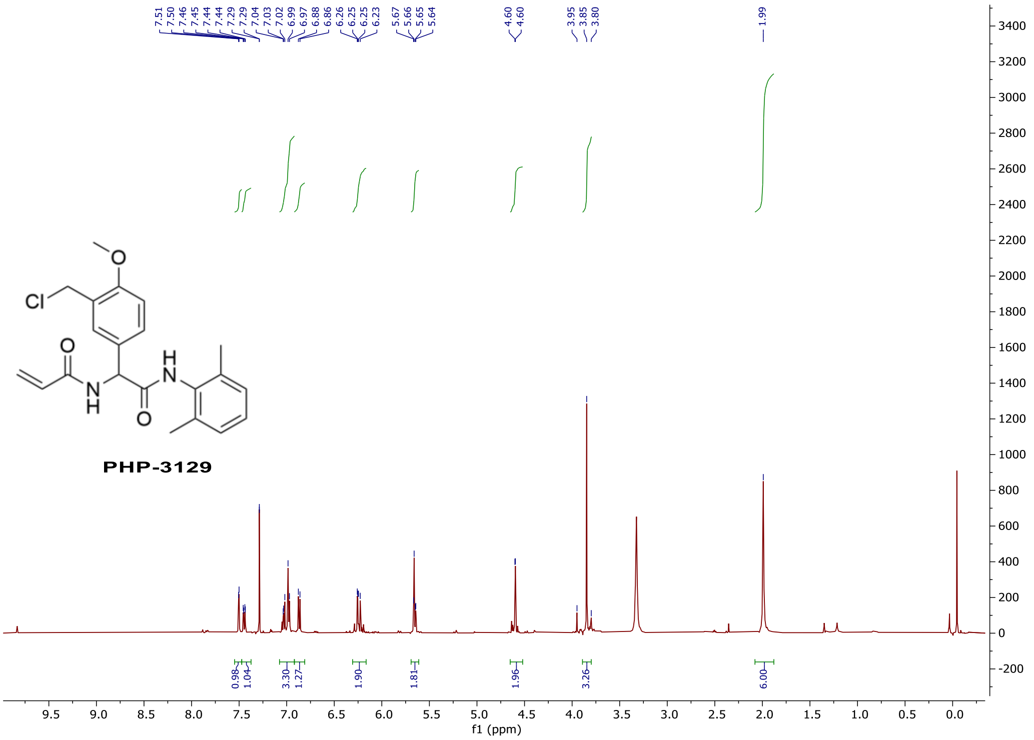

Supplementary Figure 64. ^{1}H NMR (500 MHz, $CDCl_3$+one drop of CD_3OD) of compound **PHP-3129.**

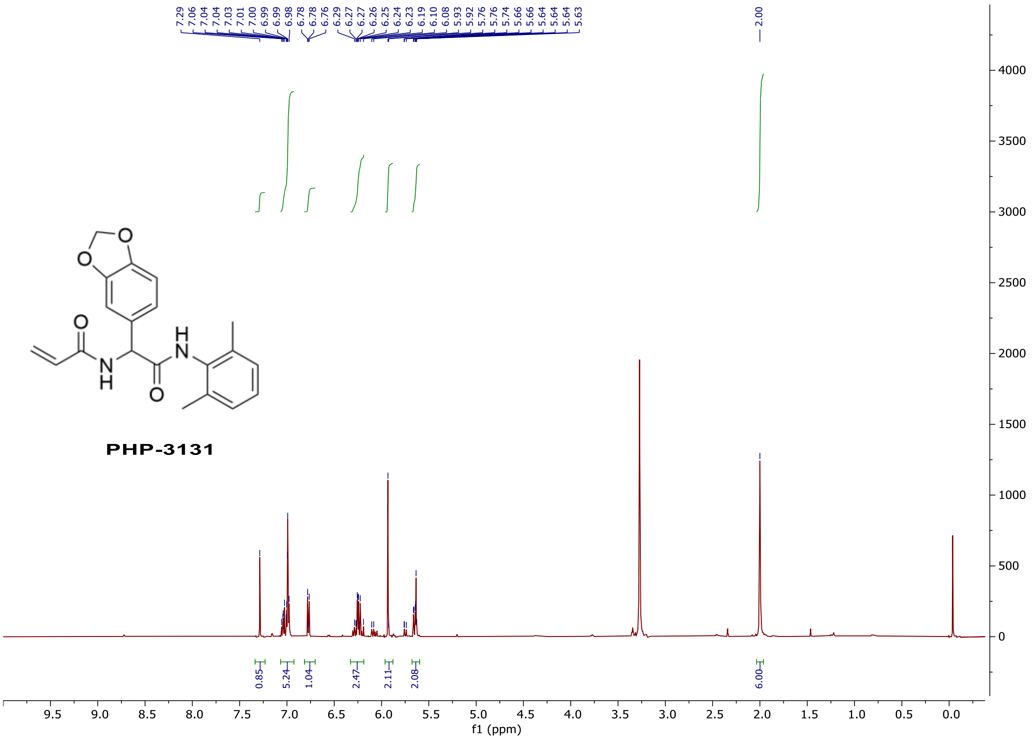

PHP-3131

Supplementary Figure 65. ^{1}H NMR (500 MHz, $CDCl_3$+one drop of CD_3OD) of compound **PHP-3131.**

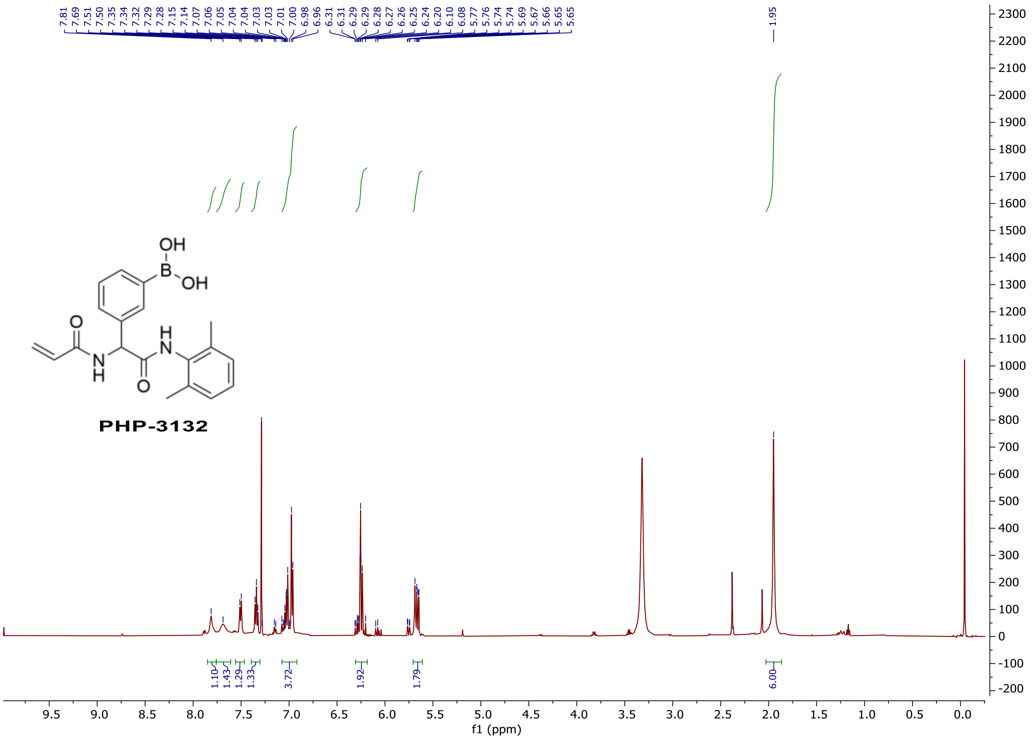

PHP-3132

Supplementary Figure 66. ^{1}H NMR (500 MHz, $CDCl_3$+one drop of CD_3OD) of compound **PHP-3132.**

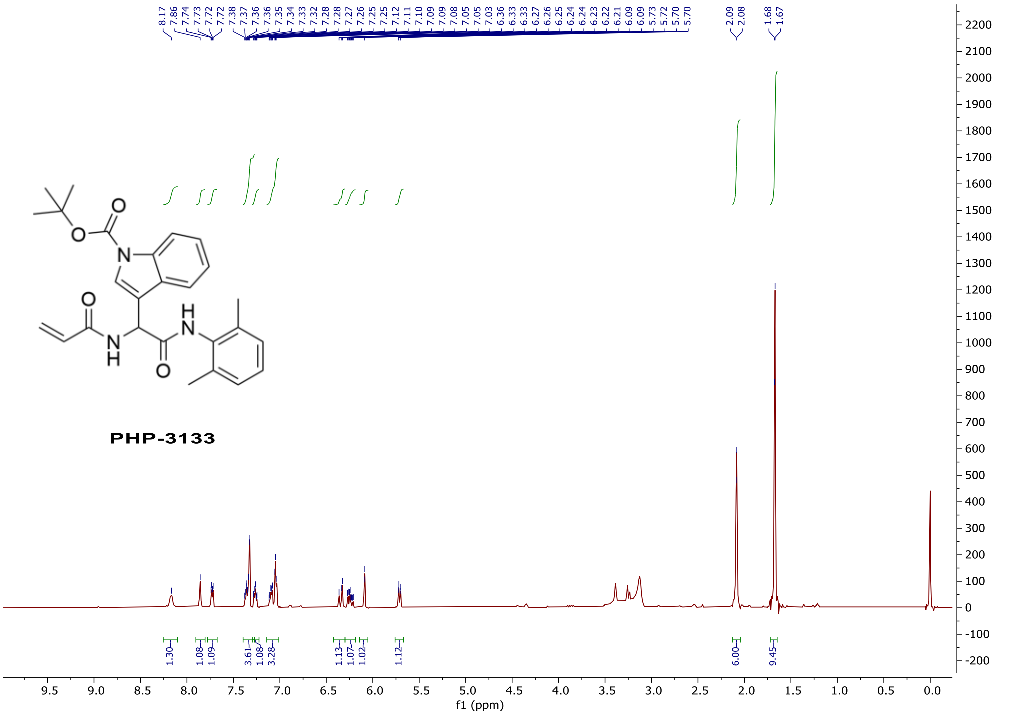

Supplementary Figure 67. ^{1}H NMR (500 MHz, $CDCl_3$+one drop of CD_3OD) of compound **PHP-3133.**

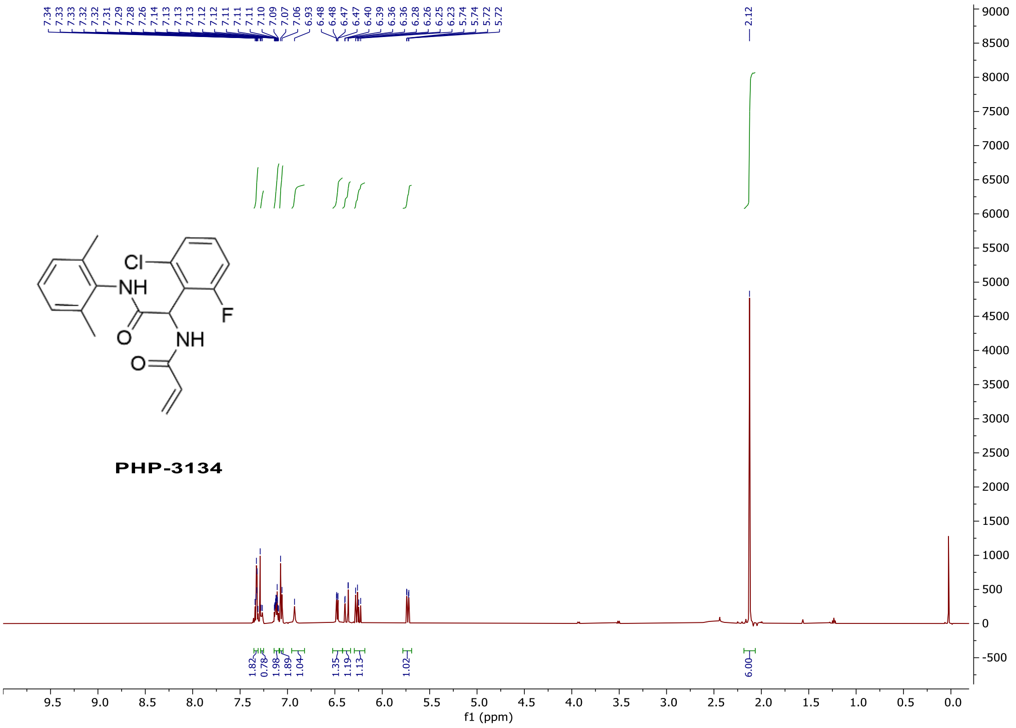

Supplementary Figure 68. ^{1}H NMR (500 MHz, $CDCl_3$) of compound **PHP-3134.**

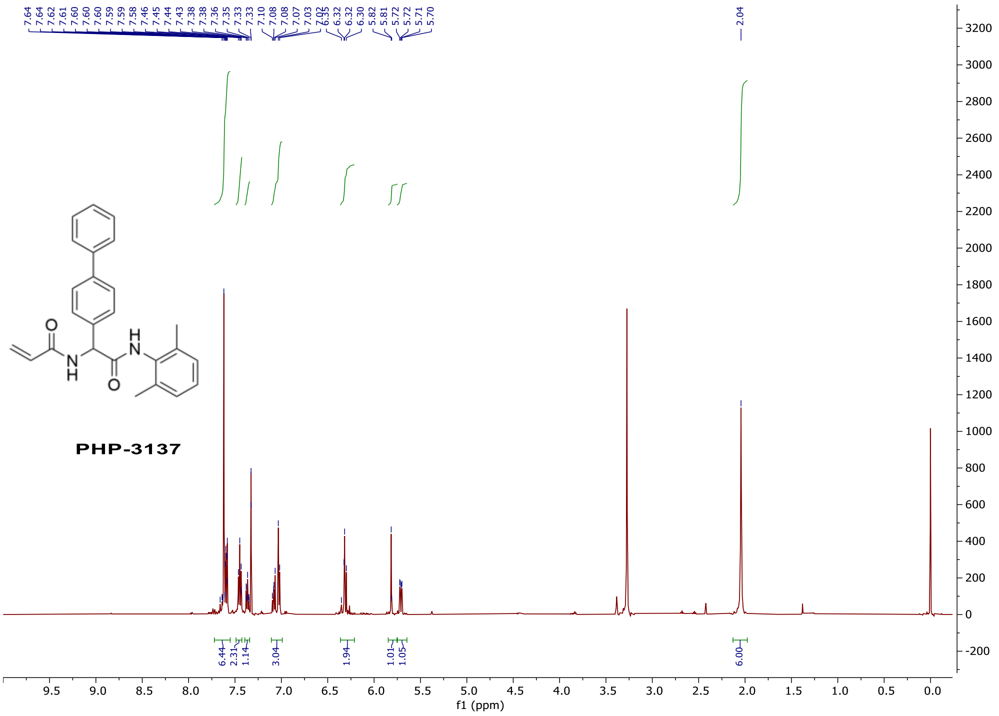

Supplementary Figure 69. ^{1}H NMR (500 MHz, $CDCl_3$+one drop of CD_3OD) of compound **PHP-3137.**

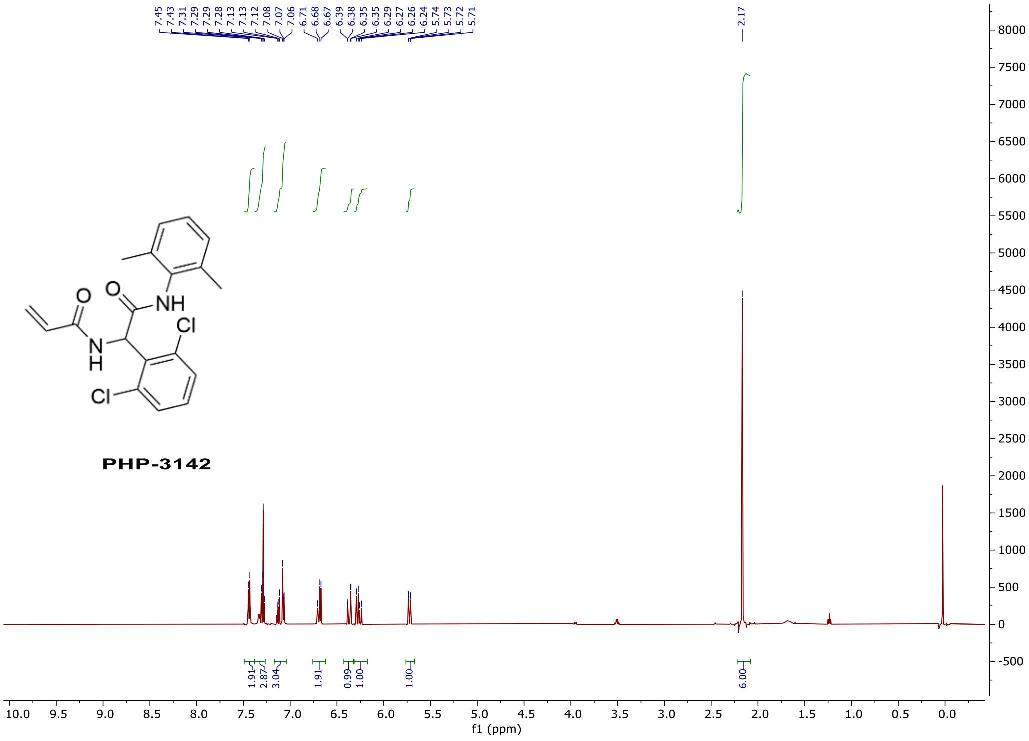

Supplementary Figure 70. ^{1}H NMR (500 MHz, $CDCl_3$) of compound **PHP-3142.**

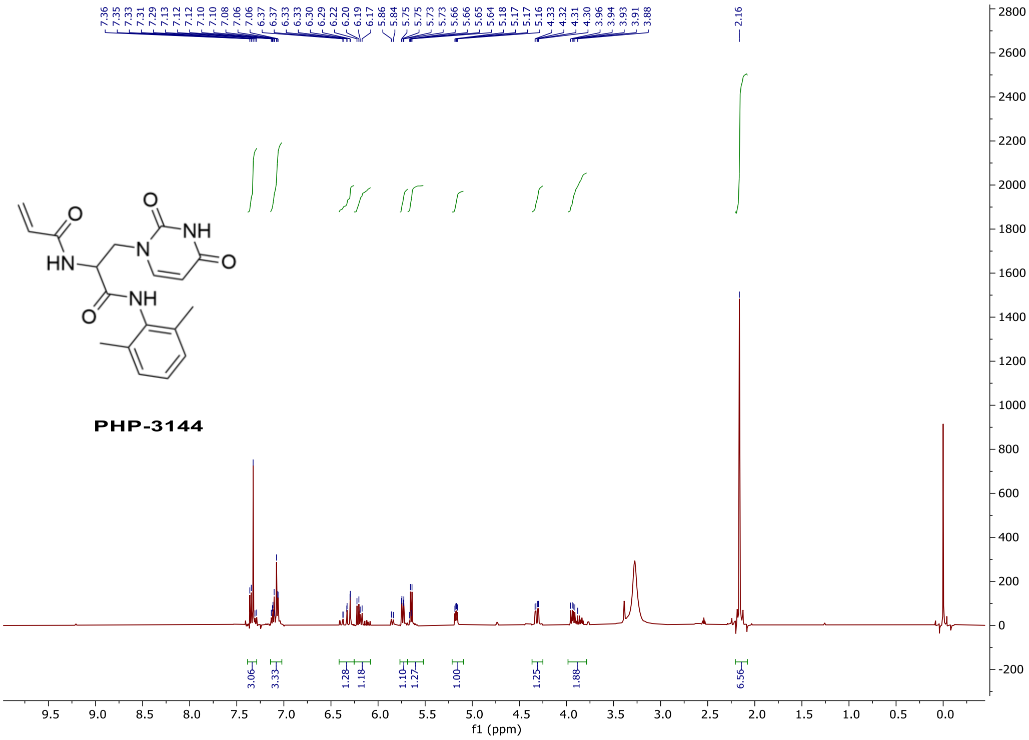

Supplementary Figure 71. ^{1}H NMR (500 MHz, $CDCl_3$+one drop of CD_3OD) of compound **PHP-3144.**

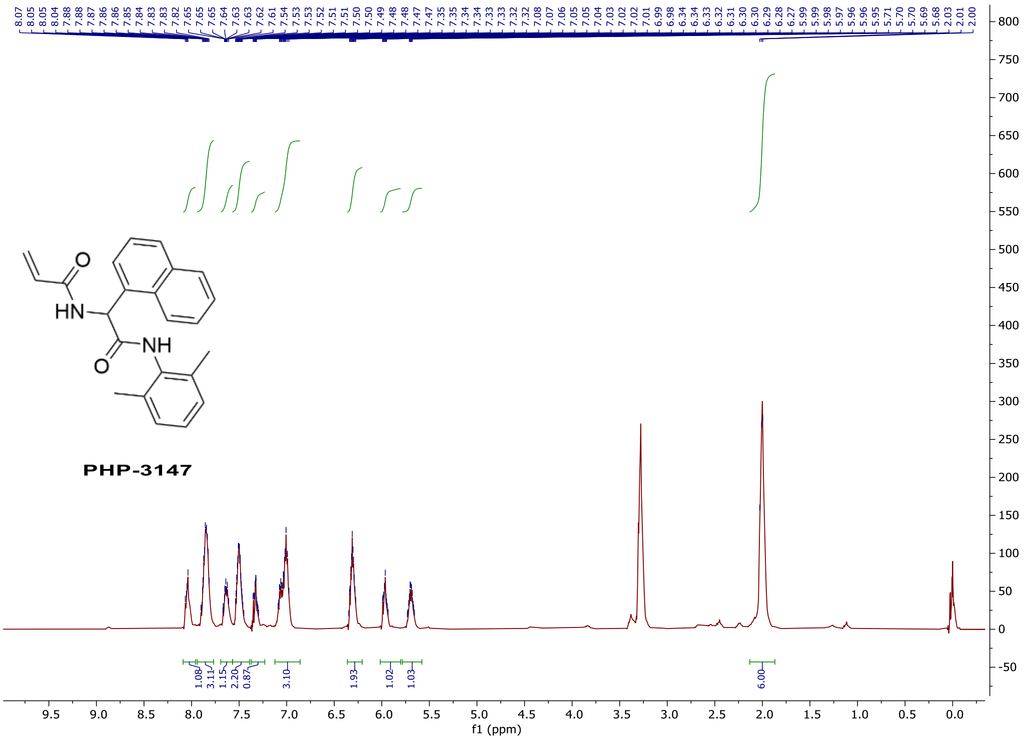

Supplementary Figure 72. ^{1}H NMR (500 MHz, $CDCl_3$+one drop of CD_3OD) of compound **PHP-3147.**

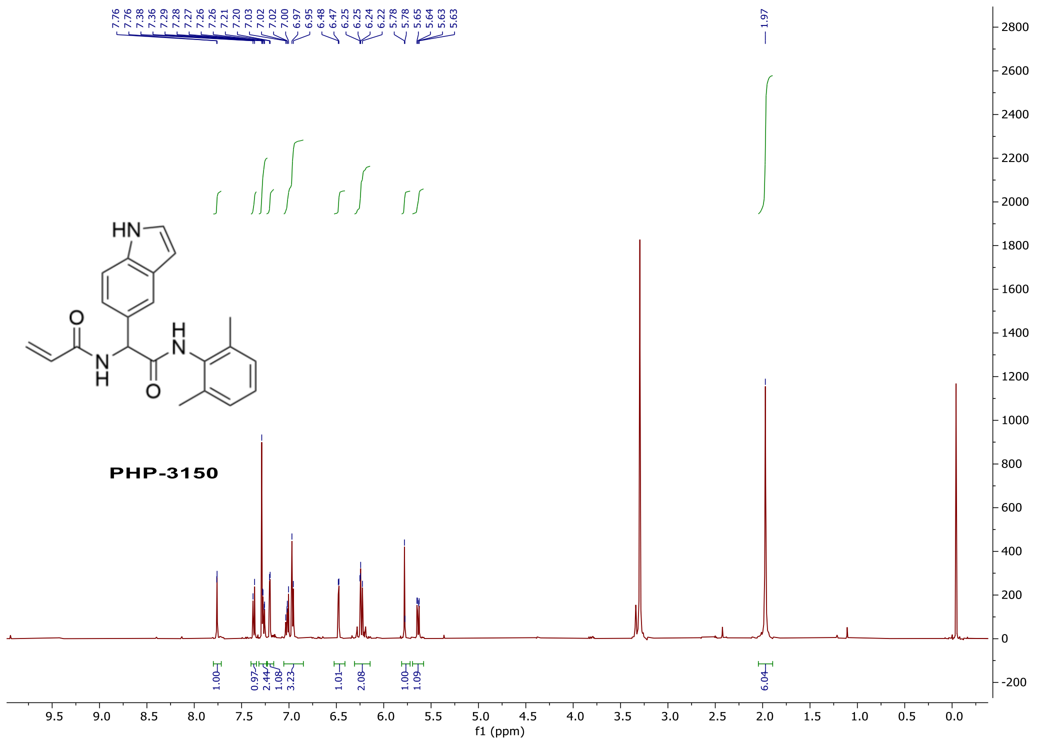

Supplementary Figure 73. ^{1}H NMR (500 MHz, $CDCl_3$+one drop of CD_3OD) of compound **PHP-3150.**

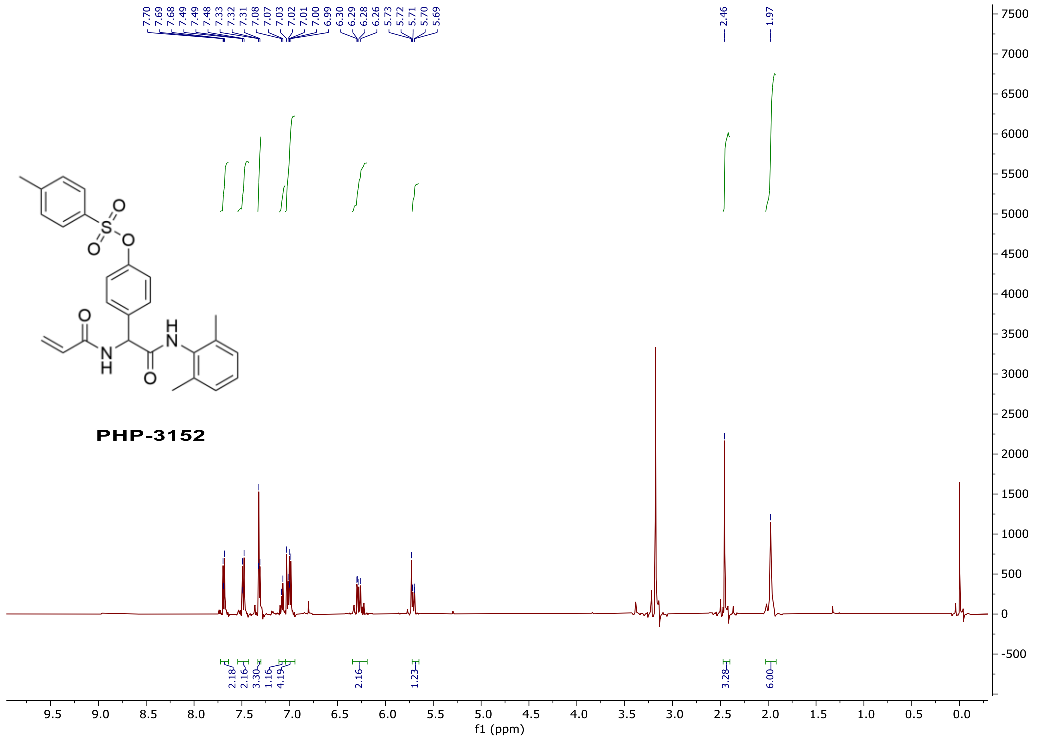

Supplementary Figure 74. ^{1}H NMR (500 MHz, $CDCl_3$+one drop of CD_3OD) of compound **PHP-3152.**

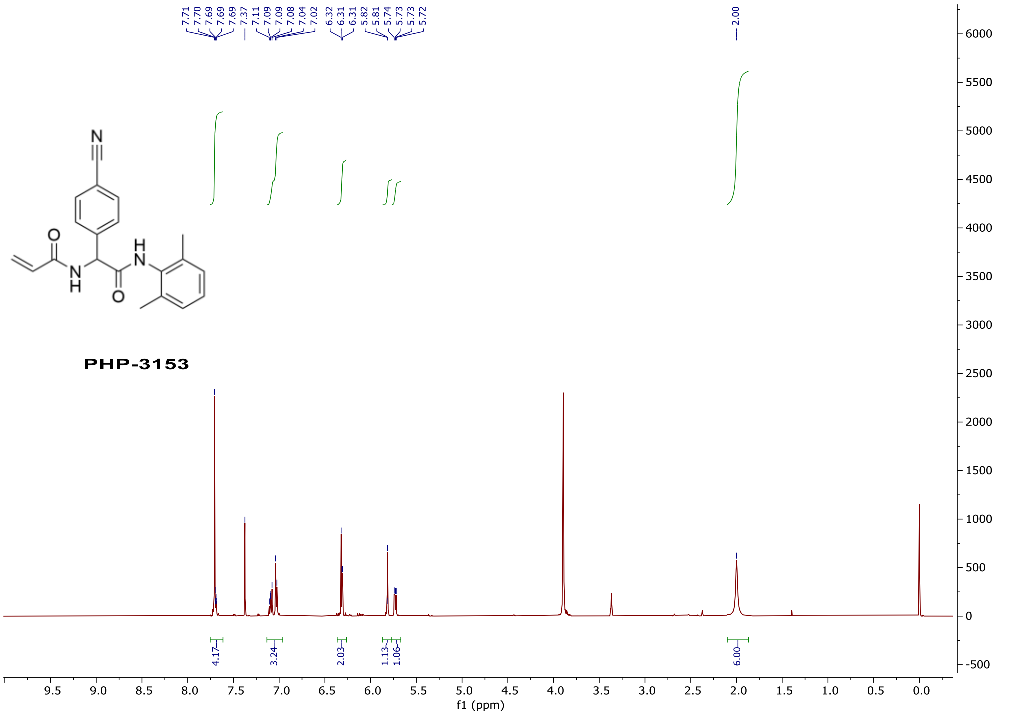

Supplementary Figure 75. ^{1}H NMR (500 MHz, $CDCl_3$+one drop of CD_3OD) of compound **PHP-3153.**

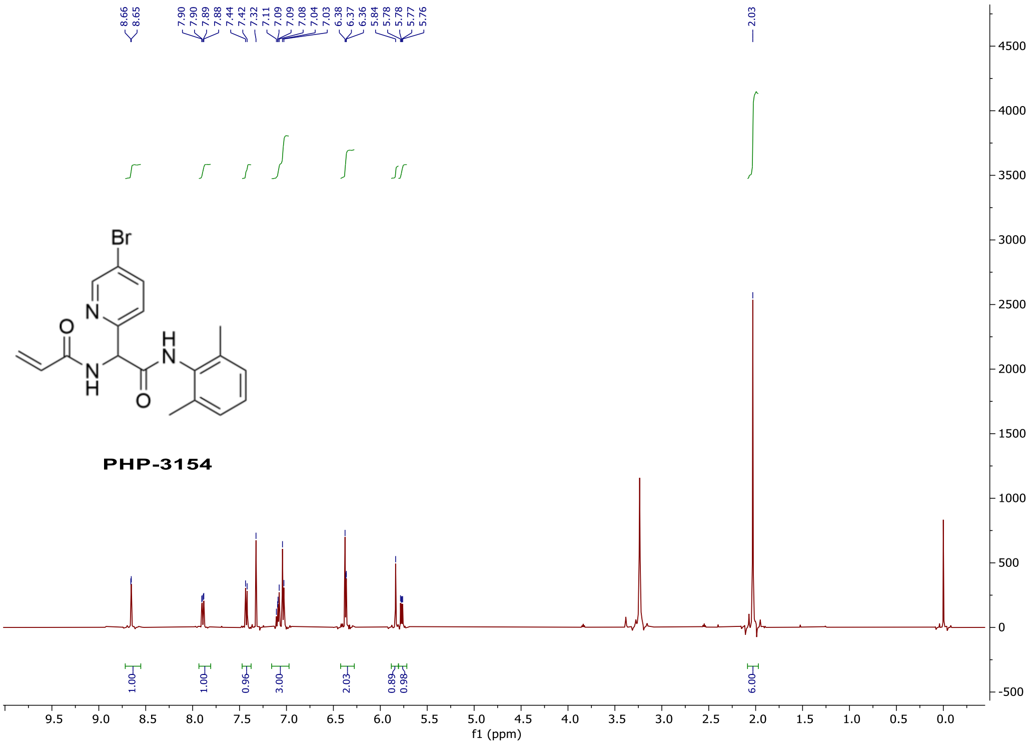

Supplementary Figure 76. ^{1}H NMR (500 MHz, $CDCl_3$+one drop of CD_3OD) of compound **PHP-3154.**

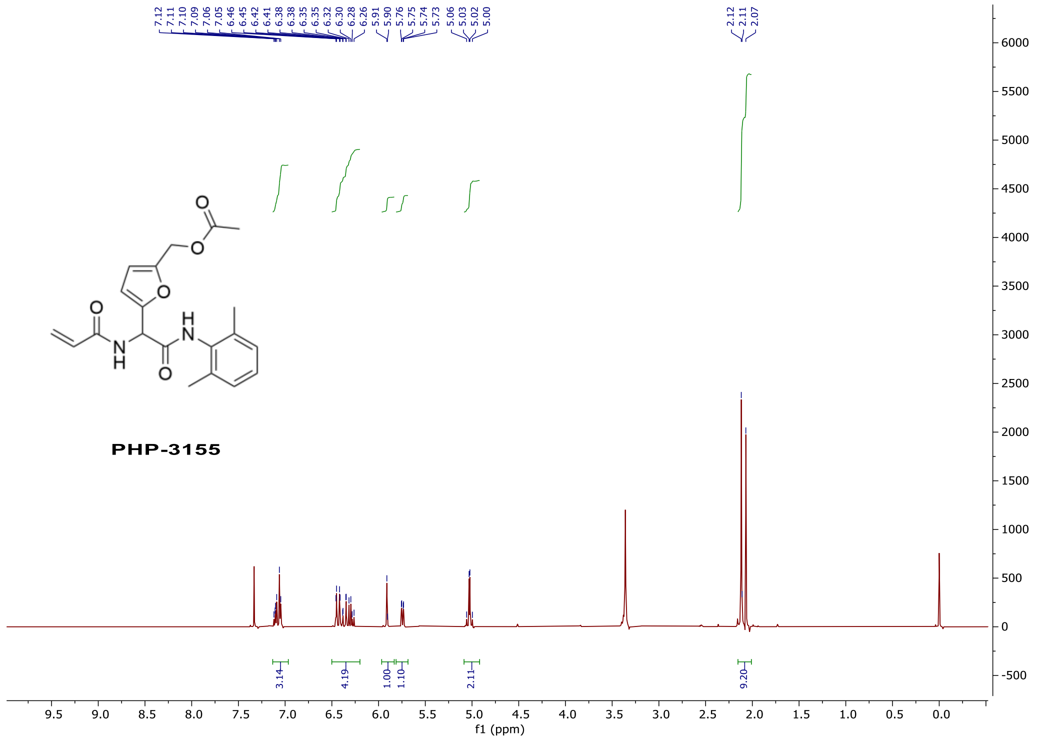

Supplementary Figure 77. ^{1}H NMR (500 MHz, $CDCl_3$+one drop of CD_3OD) of compound **PHP-3155.**

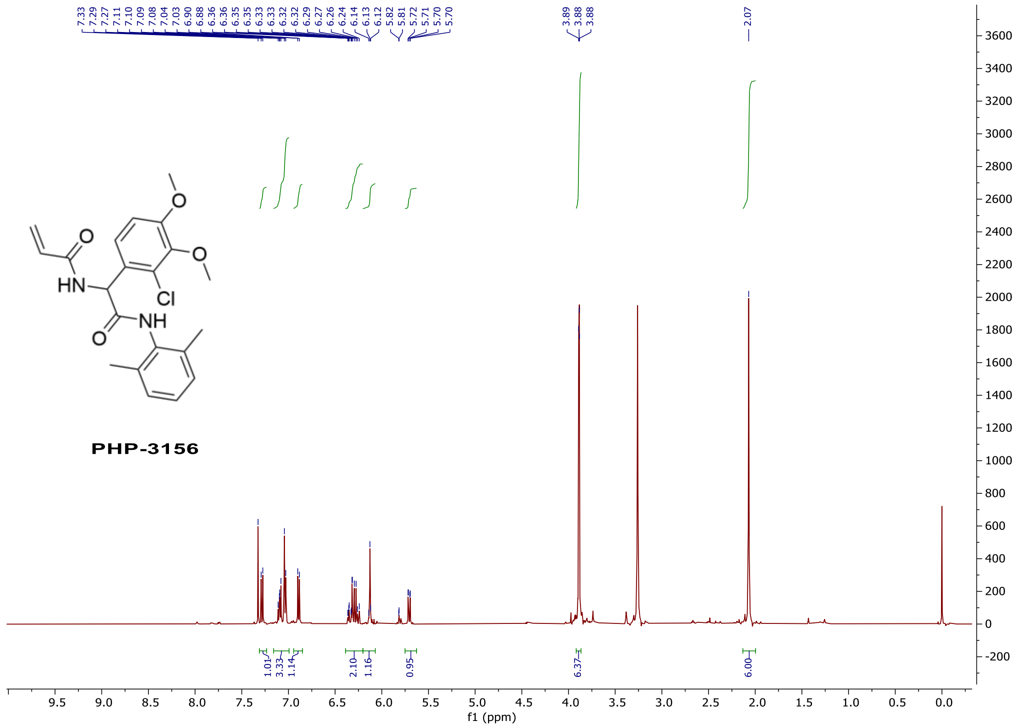

Supplementary Figure 78. ^{1}H NMR (500 MHz, $CDCl_3$+one drop of CD_3OD) of compound **PHP-3156.**

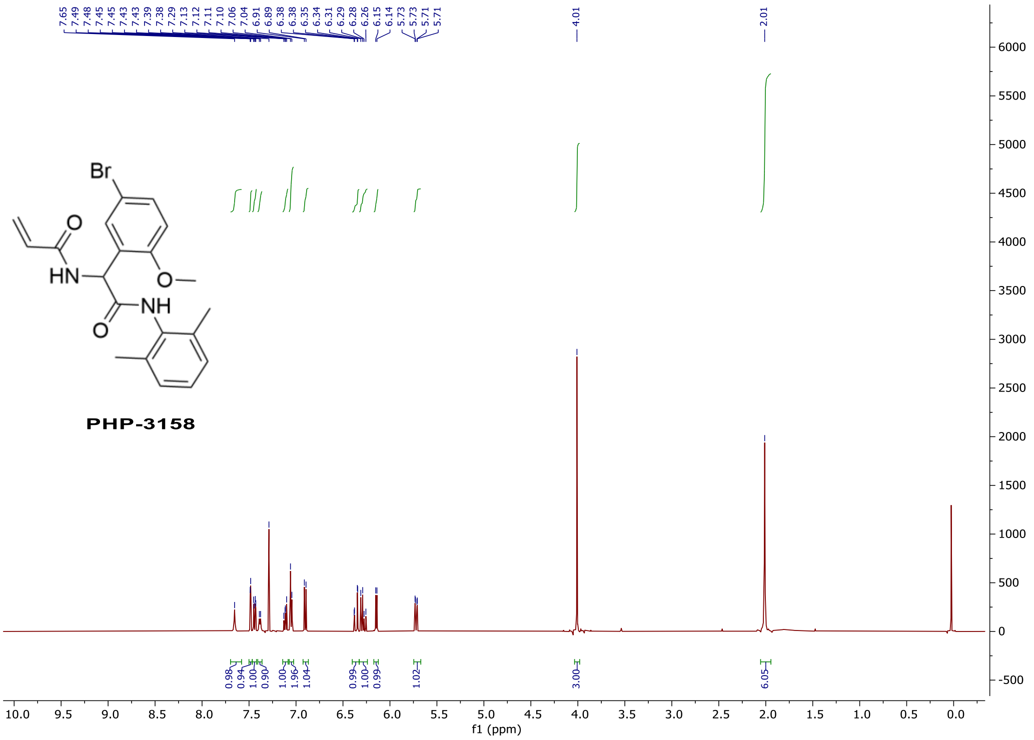

Supplementary Figure 79. ^{1}H NMR (500 MHz, $CDCl_3$) of compound **PHP-3158.**

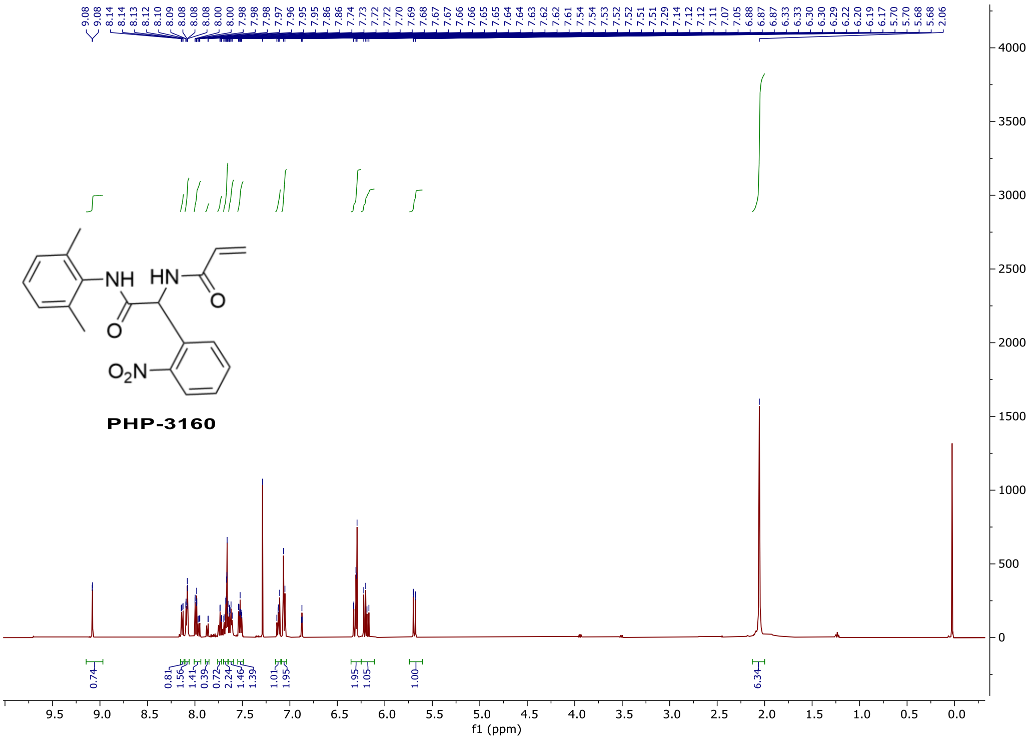

Supplementary Figure 80. ^{1}H NMR (500 MHz, $CDCl_3$) of compound **PHP-3160.**

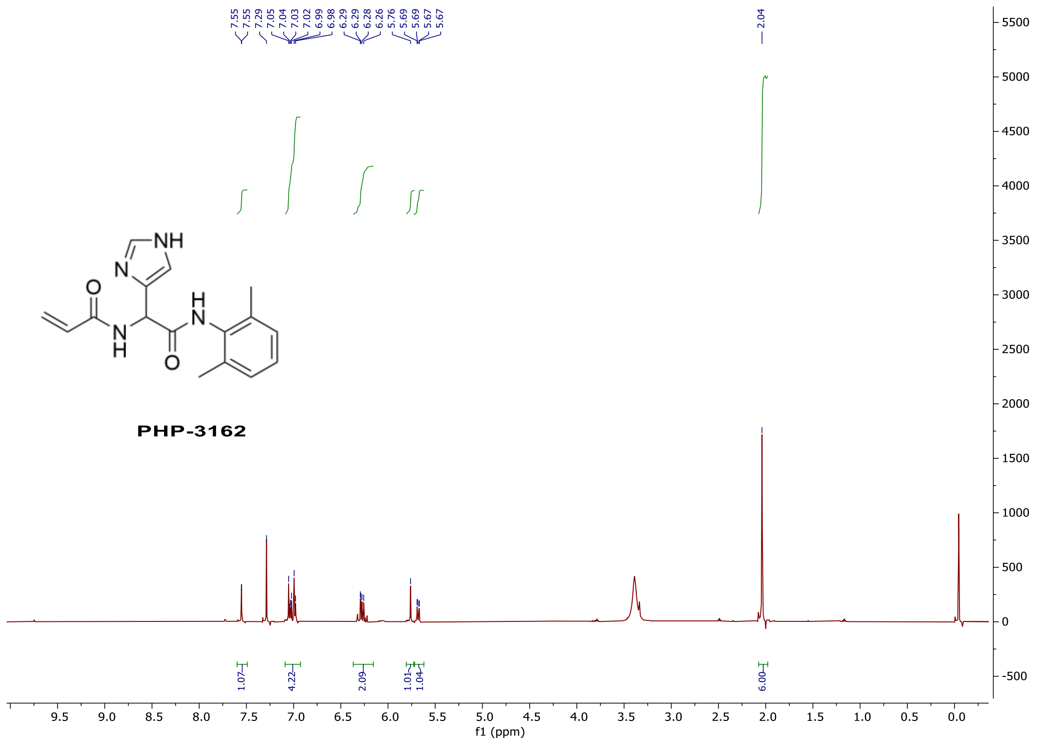

Supplementary Figure 81. ^{1}H NMR (500 MHz, $CDCl_3$+one drop of CD_3OD) of compound **PHP-3162.**

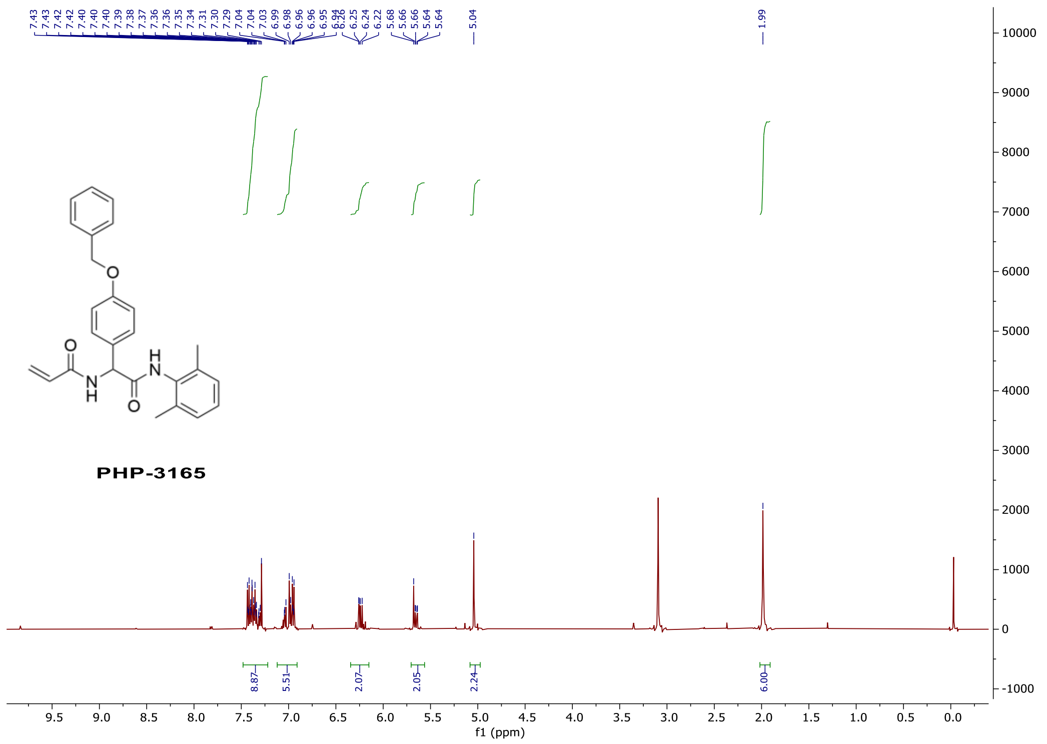

Supplementary Figure 82. ^{1}H NMR (500 MHz, $CDCl_3$+one drop of CD_3OD) of compound **PHP-3165.**

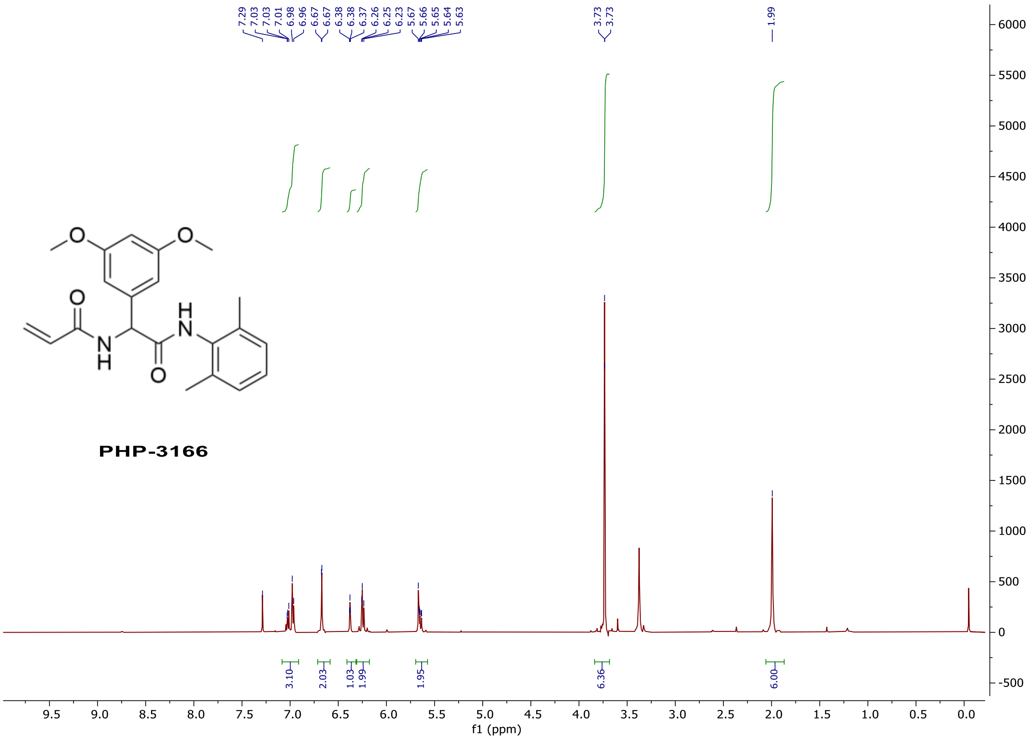

Supplementary Figure 83. ^{1}H NMR (500 MHz, $CDCl_3$+one drop of CD_3OD) of compound **PHP-3166.**

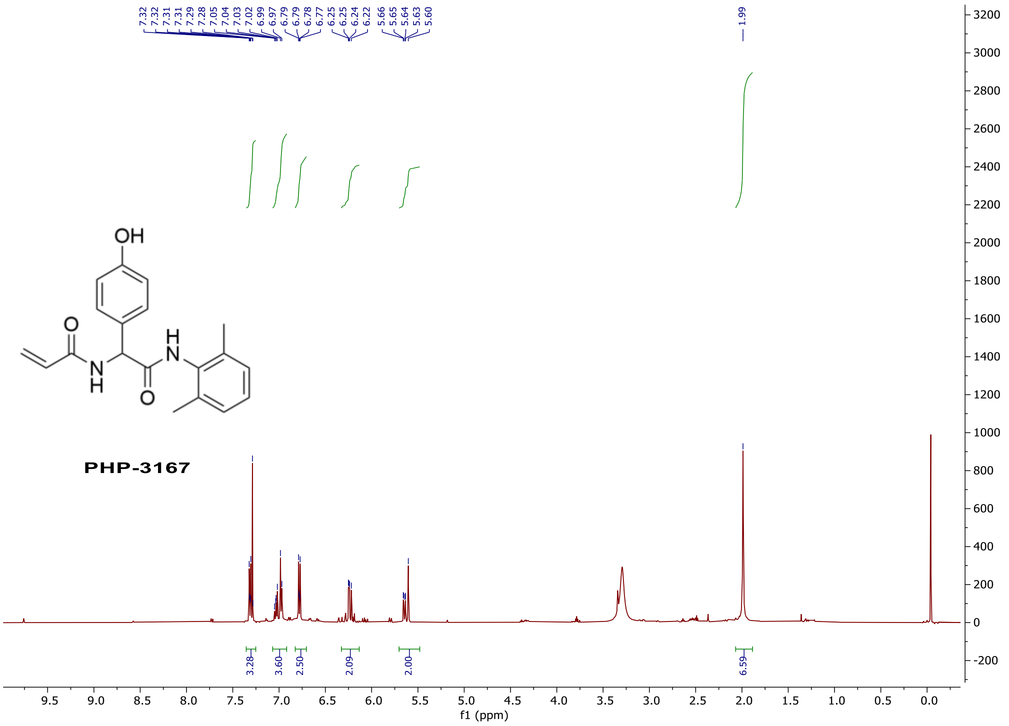

Supplementary Figure 84. ^{1}H NMR (500 MHz, $CDCl_3$+one drop of CD_3OD) of compound **PHP-3167.**

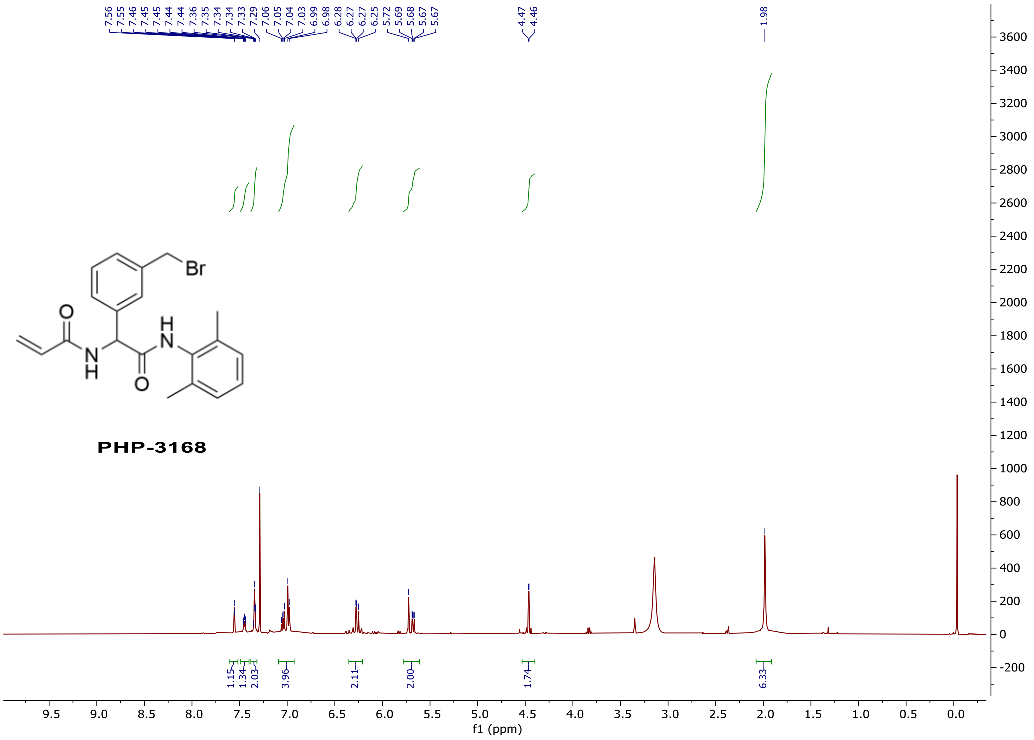

Supplementary Figure 85. ^{1}H NMR (500 MHz, $CDCl_3$+one drop of CD_3OD) of compound **PHP-3168.**

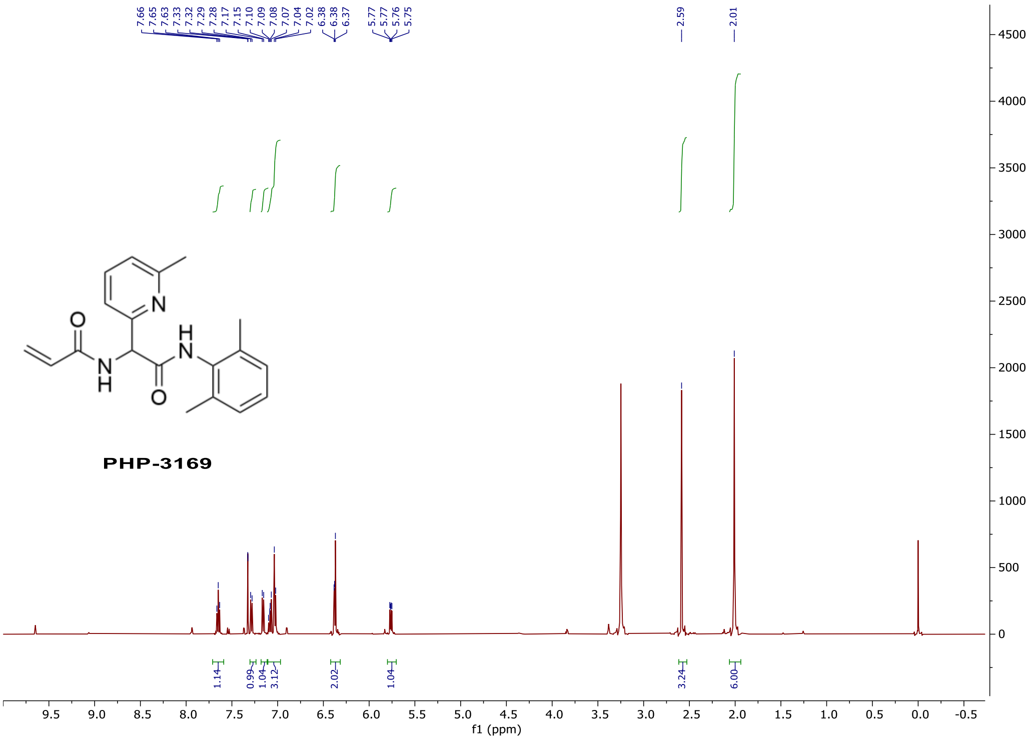

Supplementary Figure 86. ^{1}H NMR (500 MHz, $CDCl_3$+one drop of CD_3OD) of compound **PHP-3169.**

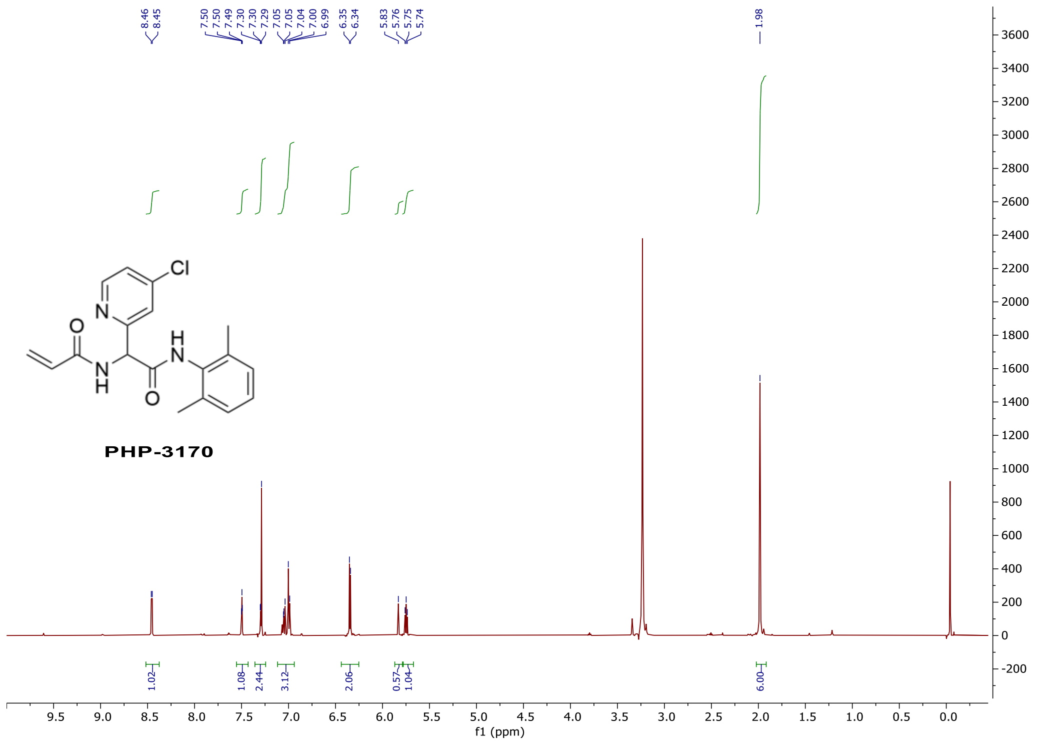

Supplementary Figure 87. ^{1}H NMR (500 MHz, $CDCl_3$+one drop of CD_3OD) of compound **PHP-3170.**

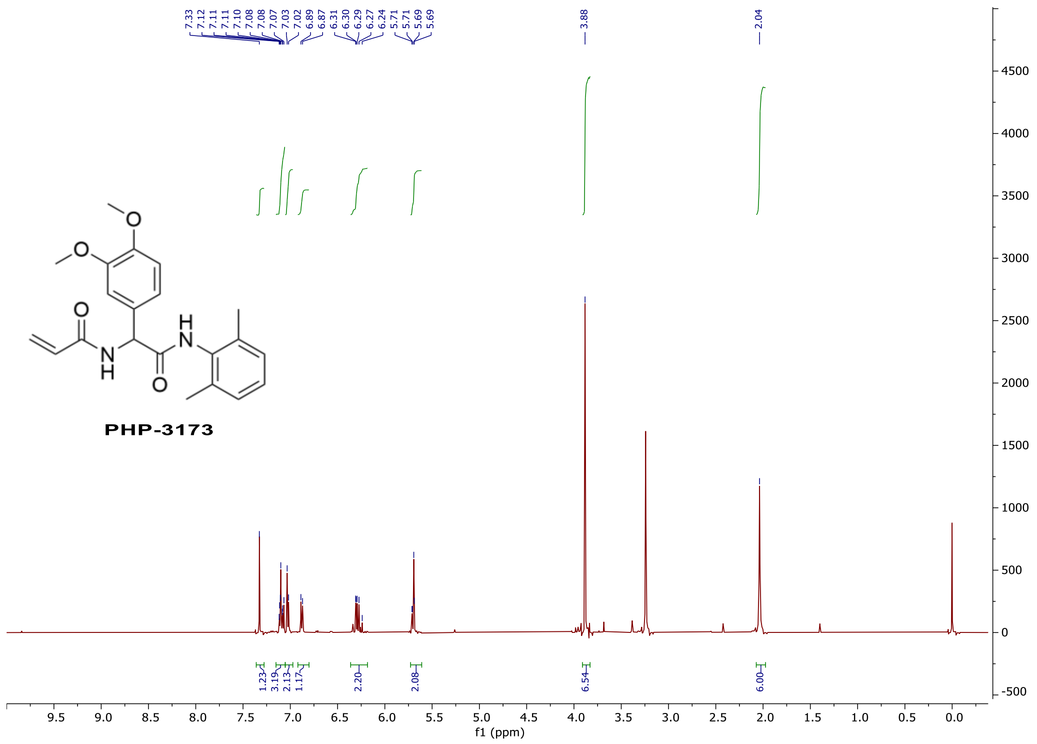

Supplementary Figure 88. ^{1}H NMR (500 MHz, $CDCl_3$+one drop of CD_3OD) of compound **PHP-3173.**

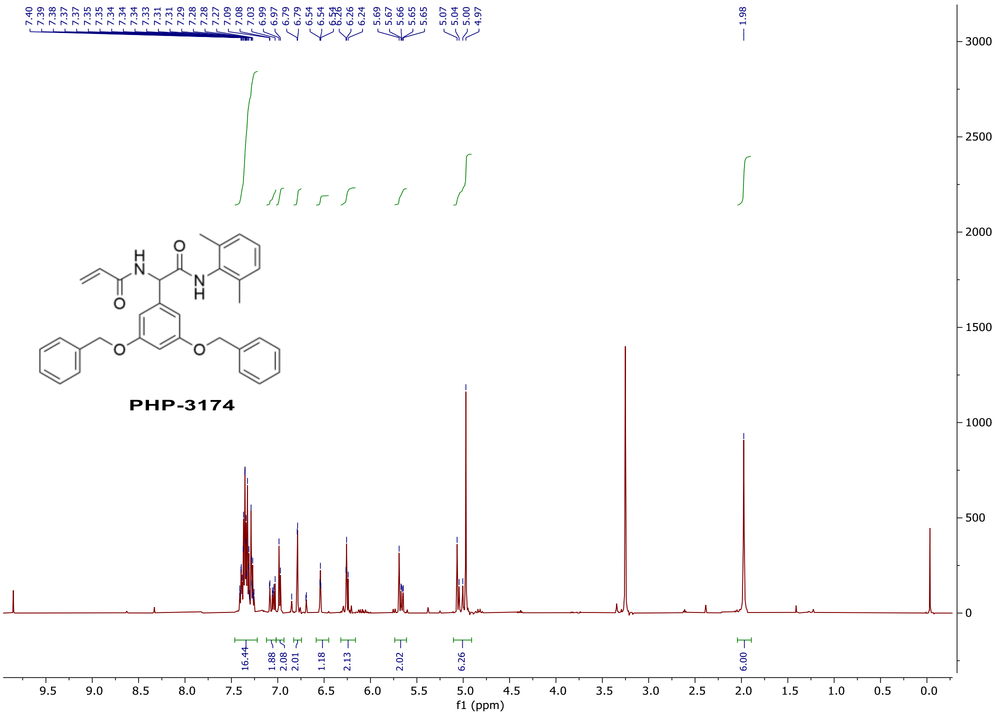

Supplementary Figure 89. ^{1}H NMR (500 MHz, $CDCl_3$+one drop of CD_3OD) of compound **PHP-3174.**

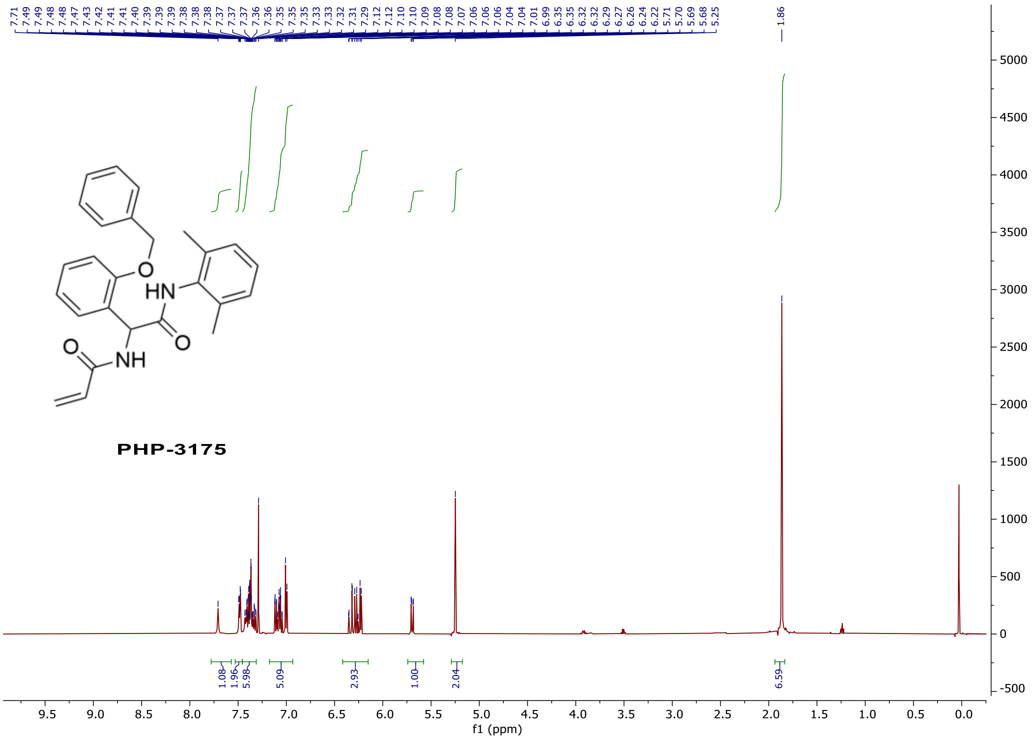

Supplementary Figure 90. ^{1}H NMR (500 MHz, $CDCl_3$) of compound **PHP-3175.**

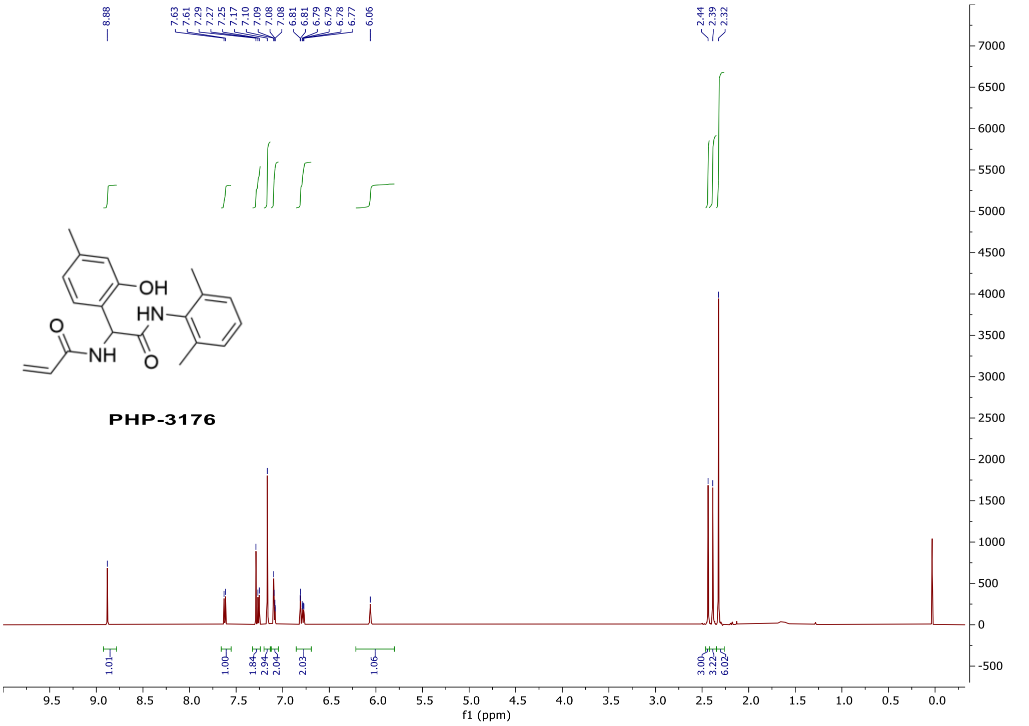

Supplementary Figure 91. ^{1}H NMR (500 MHz, $CDCl_3$) of compound **PHP-3176.**

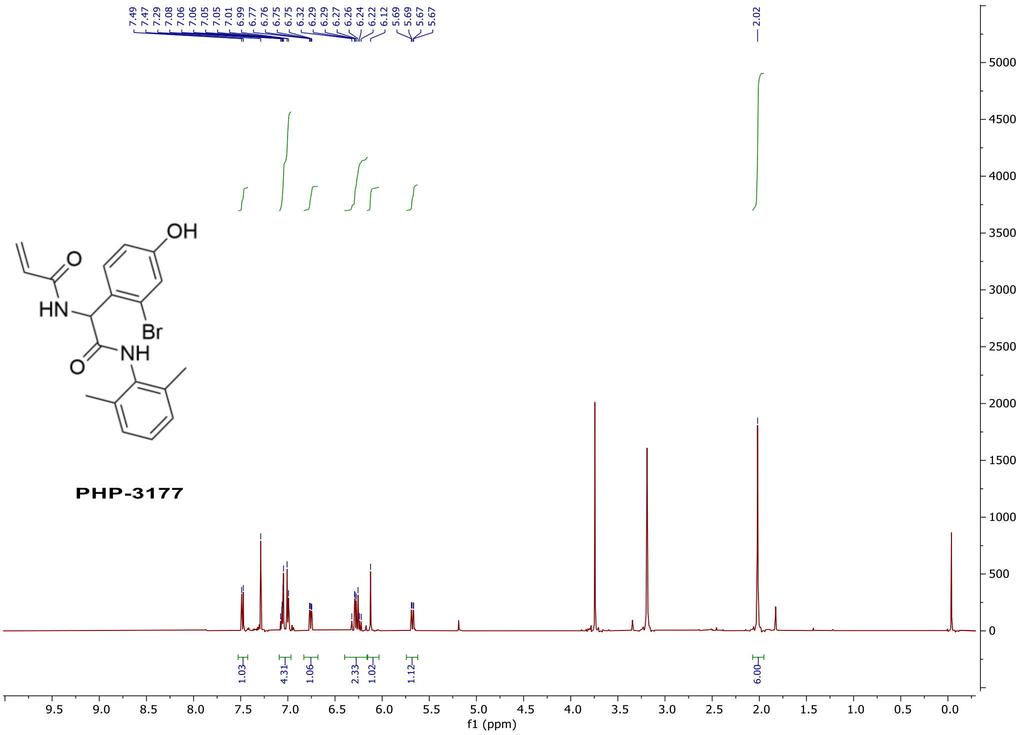

Supplementary Figure 92. ^{1}H NMR (500 MHz, $CDCl_3$+one drop of CD_3OD) of compound **PHP-3177.**

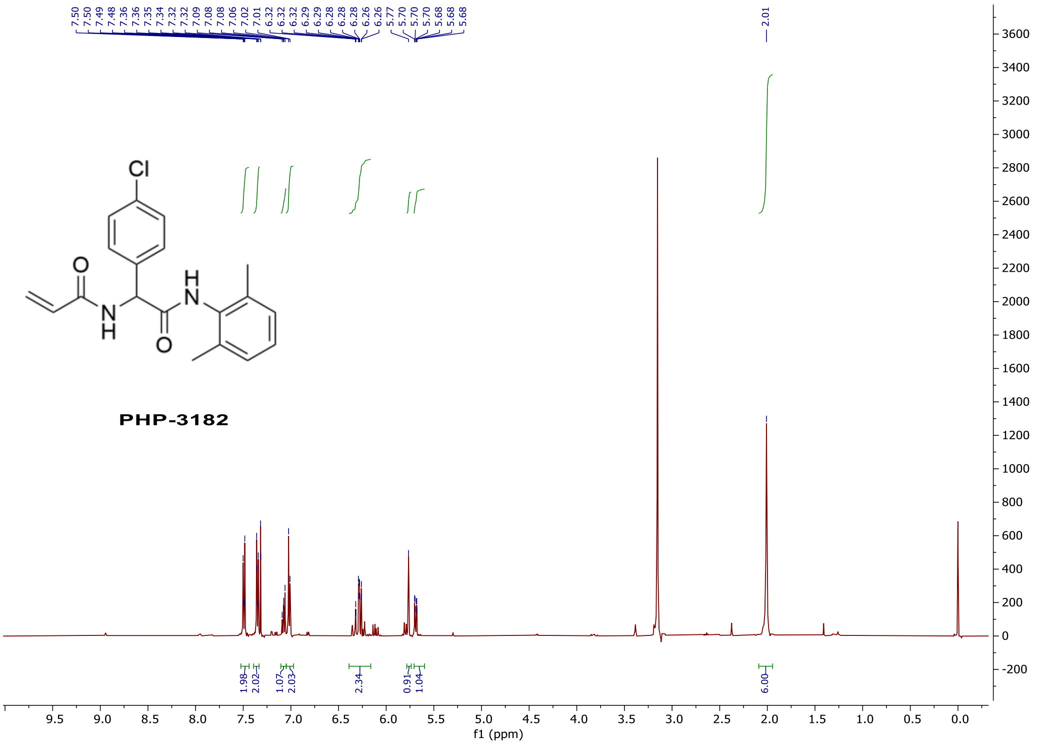

Supplementary Figure 93. ^{1}H NMR (500 MHz, $CDCl_3$+one drop of CD_3OD) of compound **PHP-3182.**

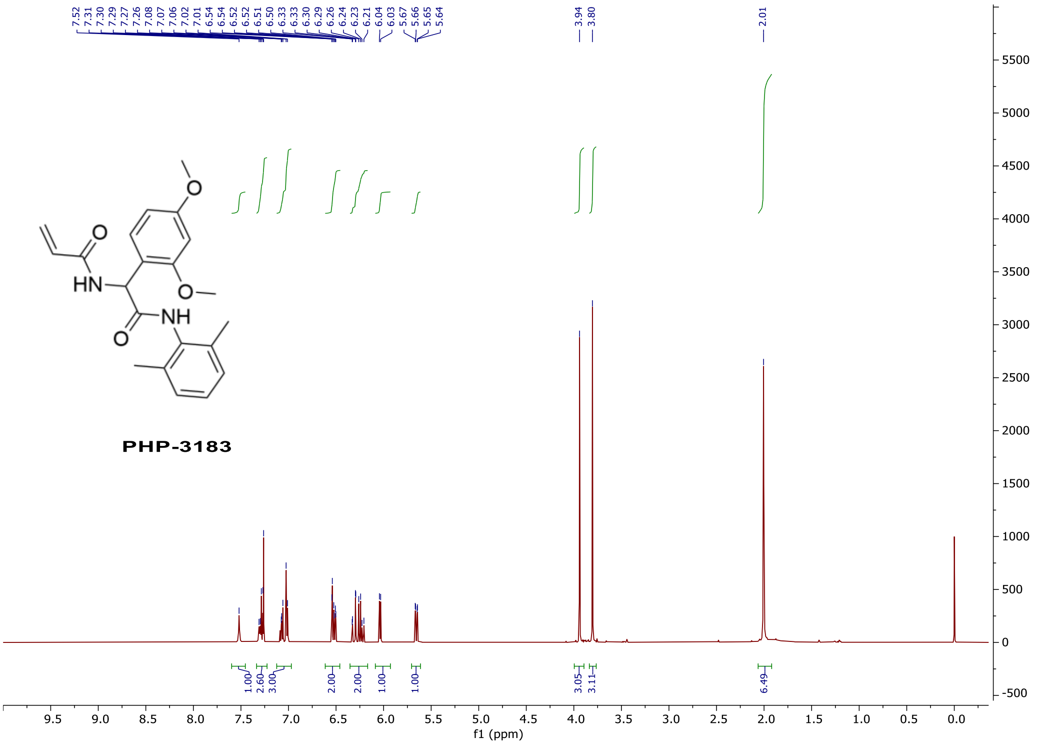

Supplementary Figure 94. ^{1}H NMR (500 MHz, $CDCl_3$) of compound **PHP-3183.**

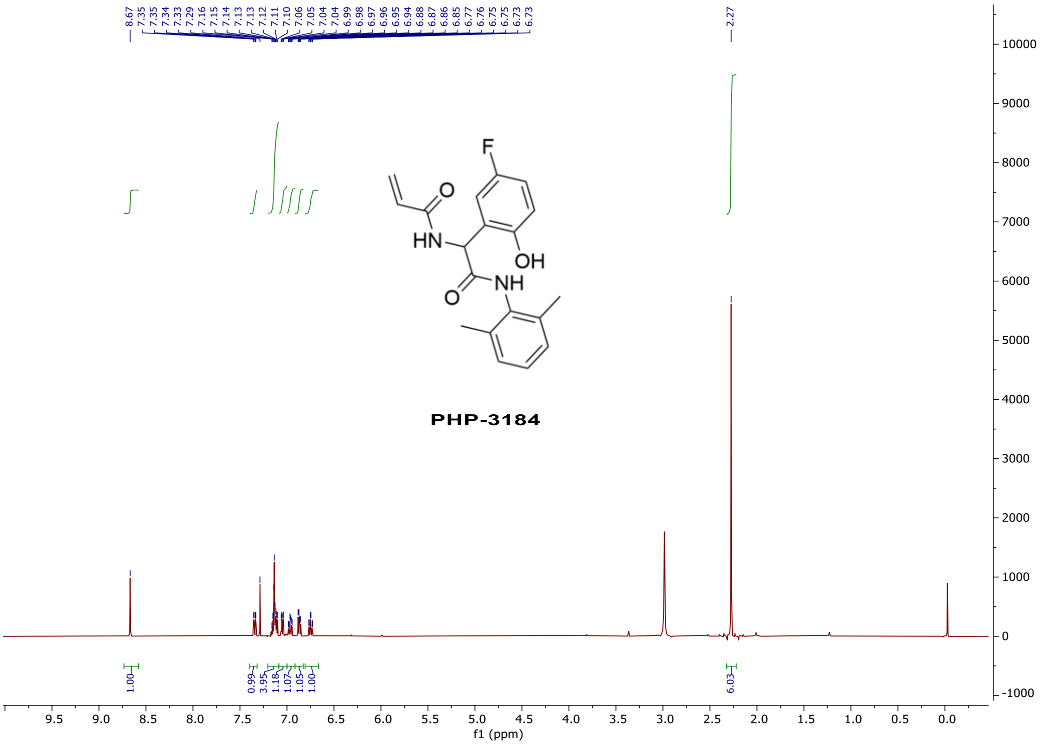

Supplementary Figure 95. ^{1}H NMR (500 MHz, $CDCl_3$) of compound **PHP-3184.**

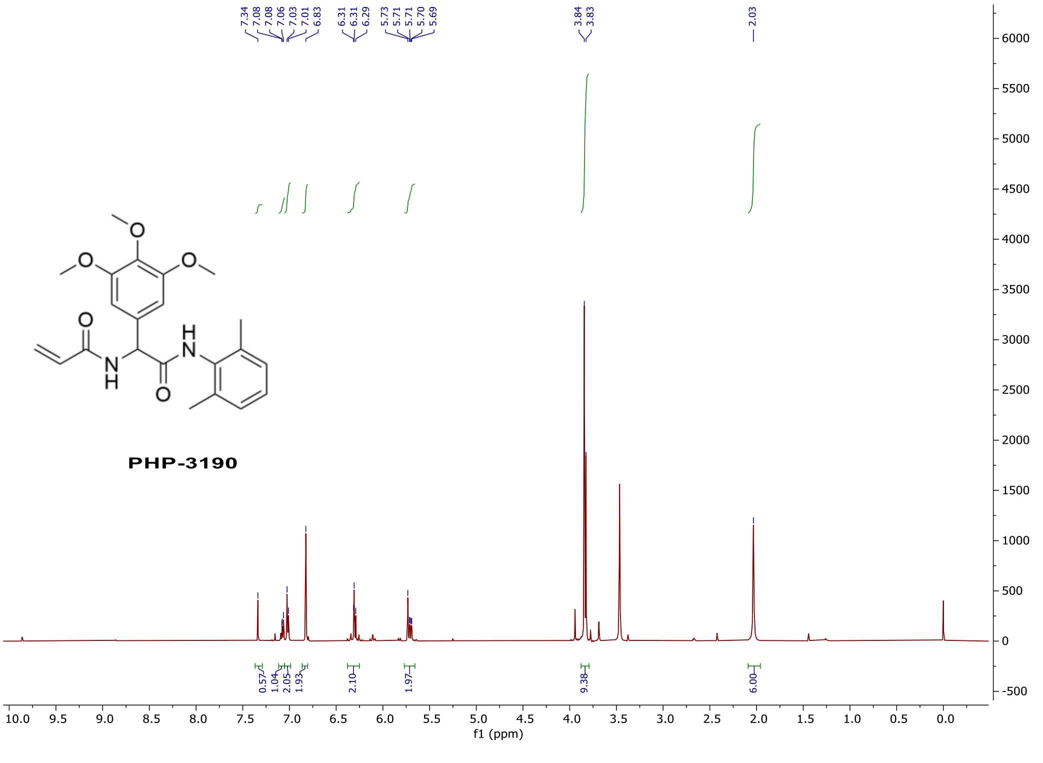

Supplementary Figure 96. ^{1}H NMR (500 MHz, $CDCl_3$+one drop of CD_3OD) of compound **PHP-3190.**

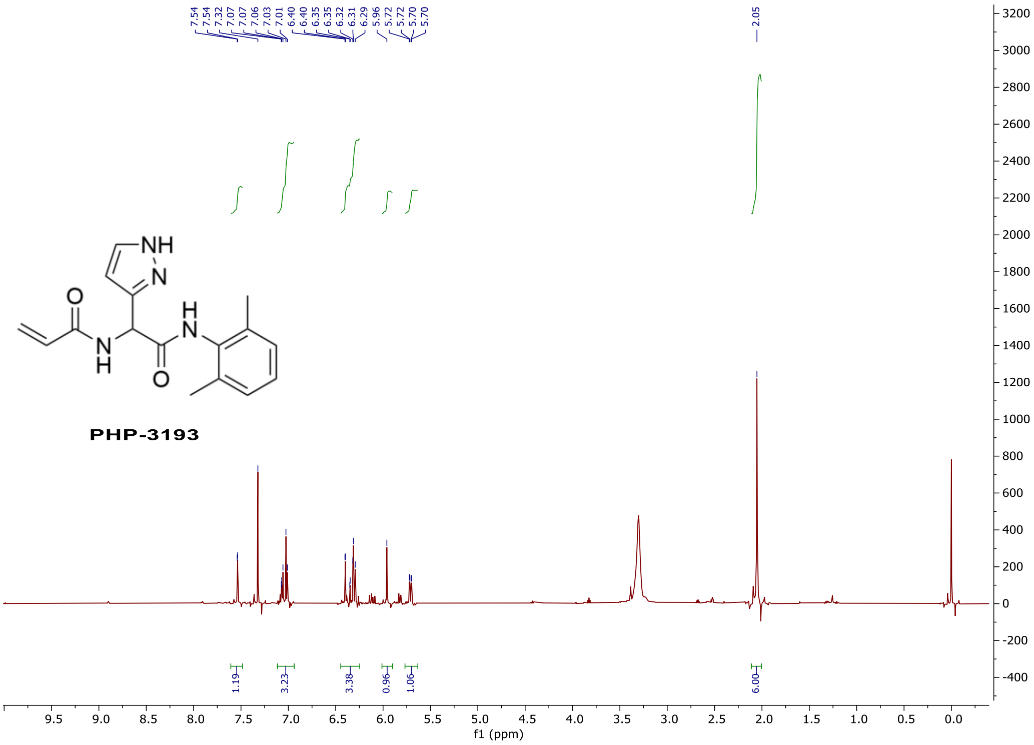

Supplementary Figure 97. ^{1}H NMR (500 MHz, $CDCl_3$+one drop of CD_3OD) of compound **PHP-3193.**

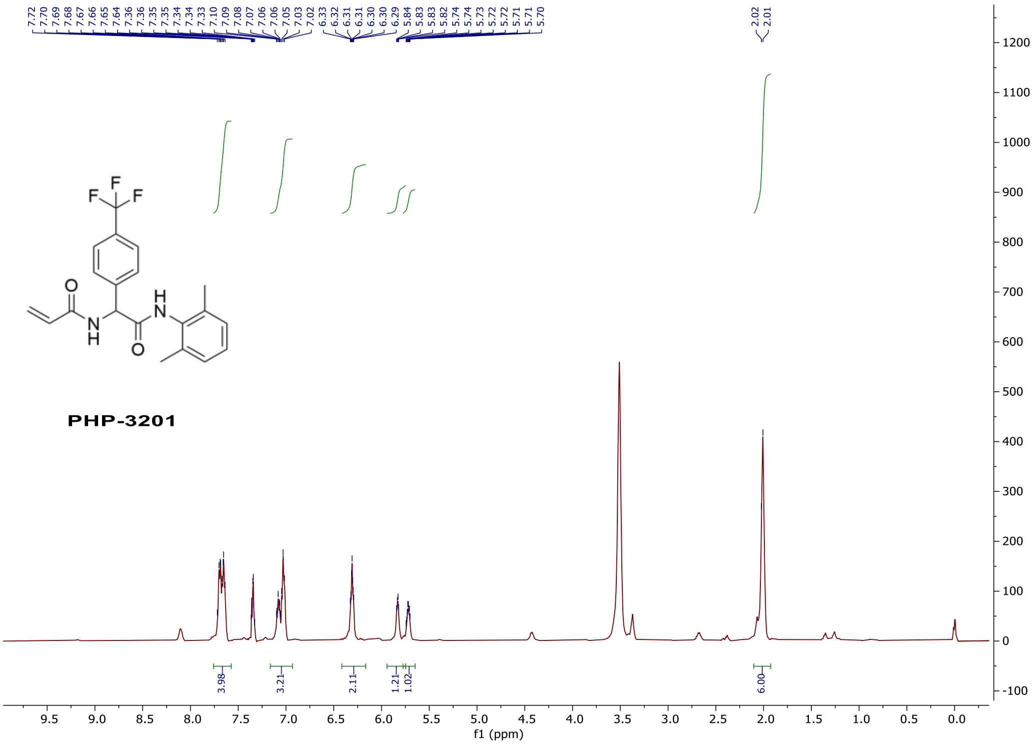

Supplementary Figure 98. ^{1}H NMR (500 MHz, $CDCl_3$+one drop of CD_3OD) of compound **PHP-3201.**

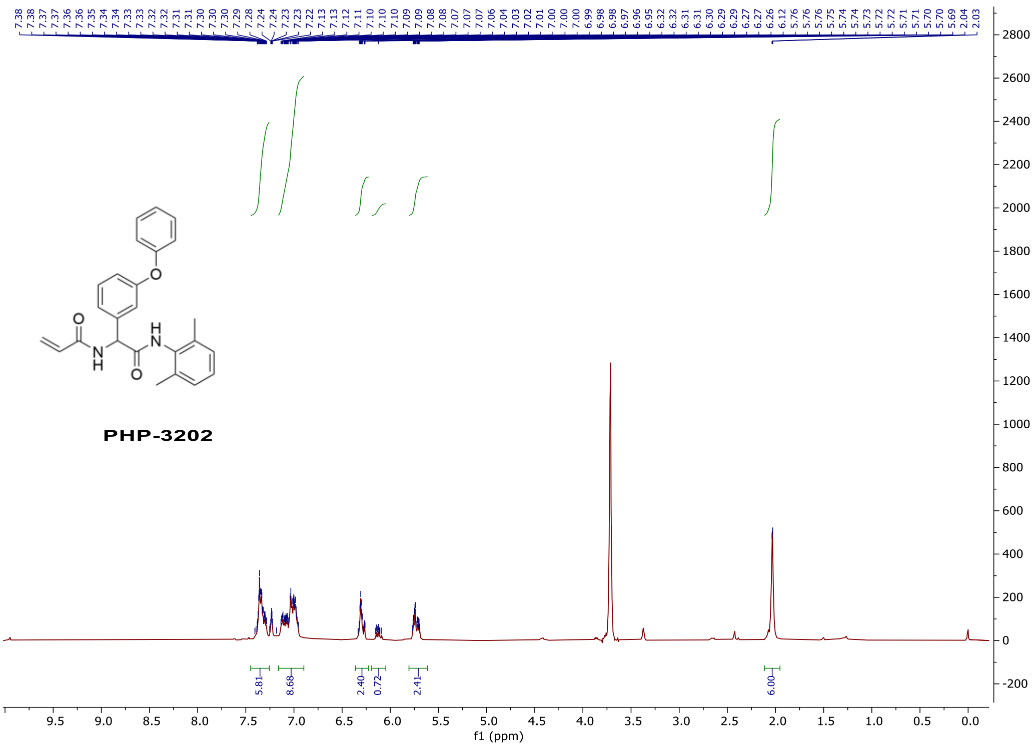

Supplementary Figure 99. ^{1}H NMR (500 MHz, $CDCl_3$+one drop of CD_3OD) of compound **PHP-3202.**

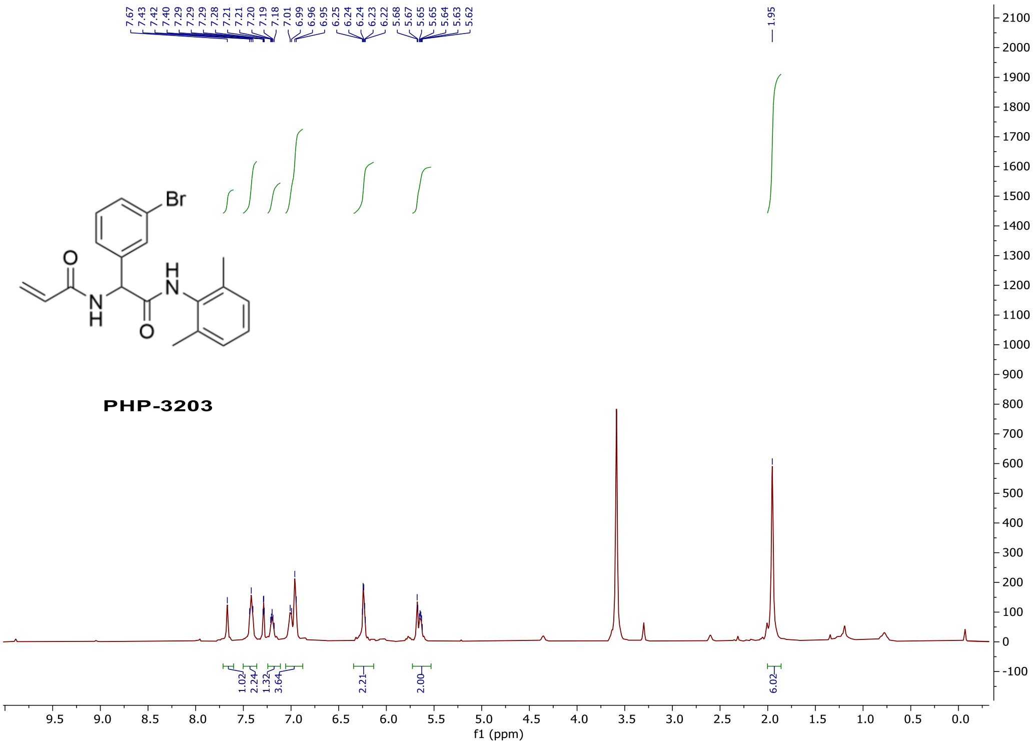

Supplementary Figure 100. ^{1}H NMR (500 MHz, $CDCl_3$+one drop of CD_3OD) of compound **PHP-3203.**

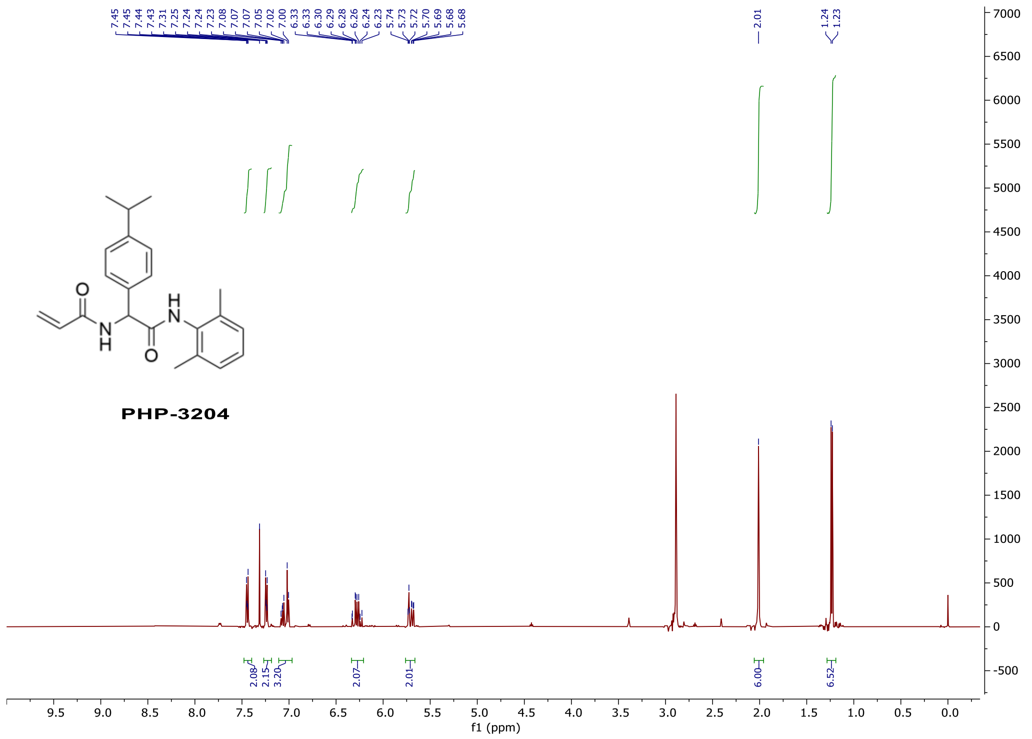

Supplementary Figure 101. ^{1}H NMR (500 MHz, $CDCl_3$+one drop of CD_3OD) of compound **PHP-3204.**

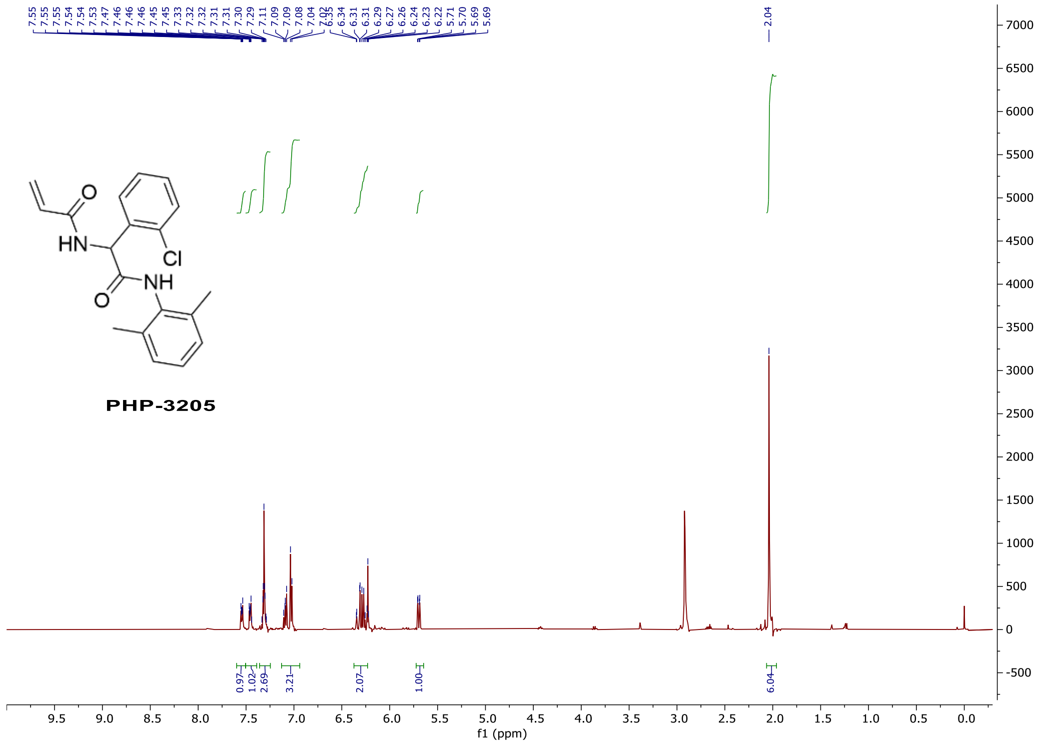

Supplementary Figure 102. ^{1}H NMR (500 MHz, $CDCl_3$) of compound **PHP-3205.**

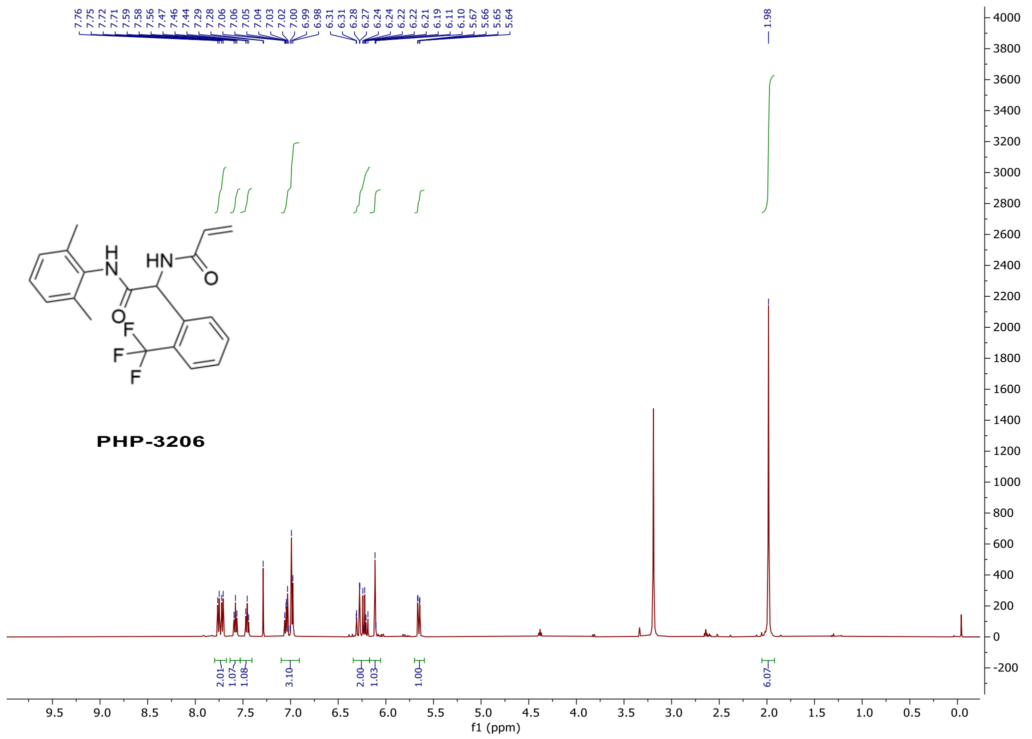

Supplementary Figure 103. ^{1}H NMR (500 MHz, $CDCl_3$+one drop of CD_3OD) of compound **PHP-3206.**

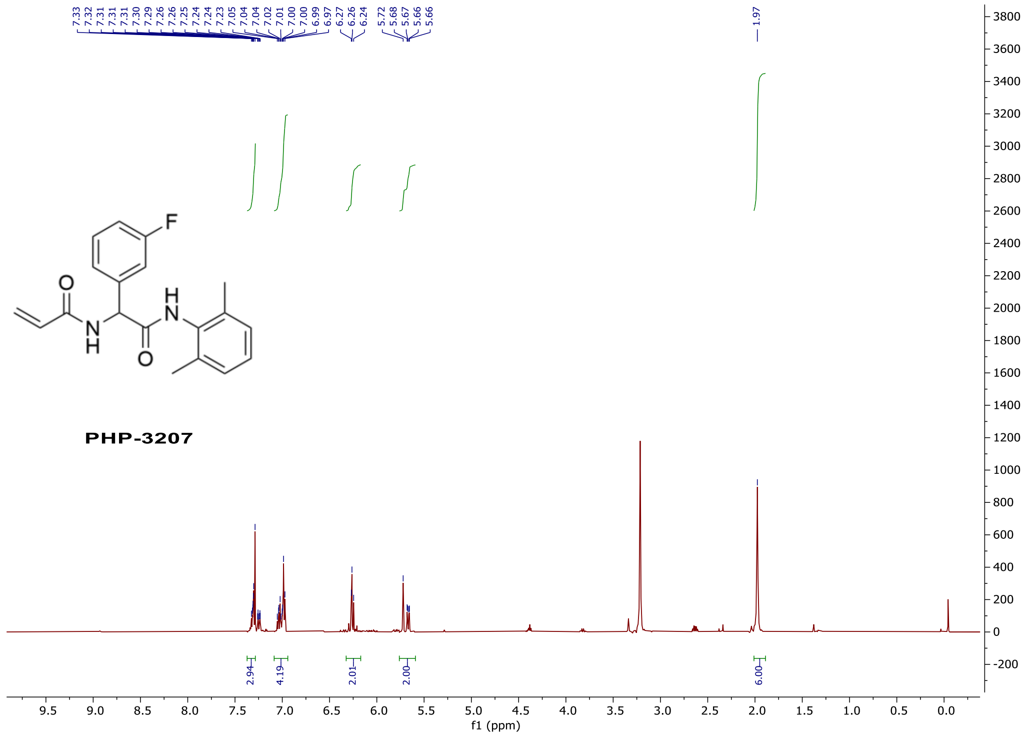

Supplementary Figure 104. ^{1}H NMR (500 MHz, $CDCl_3$+one drop of CD_3OD) of compound **PHP-3207.**

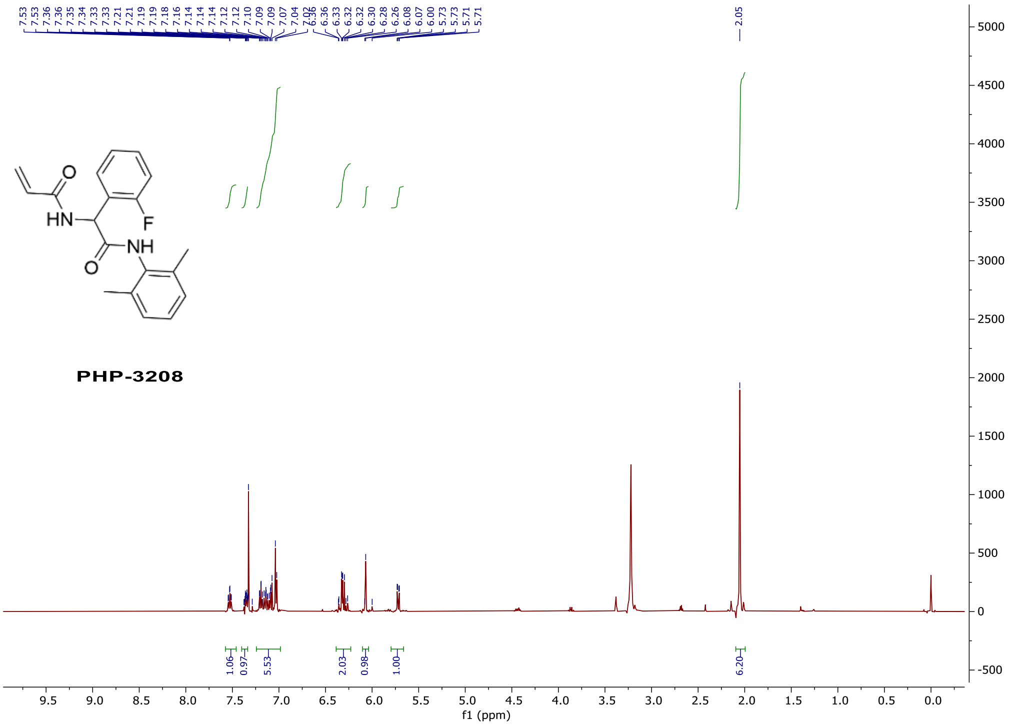

Supplementary Figure 105. ^{1}H NMR (500 MHz, $CDCl_3$+one drop of CD_3OD) of compound **PHP-3208.**

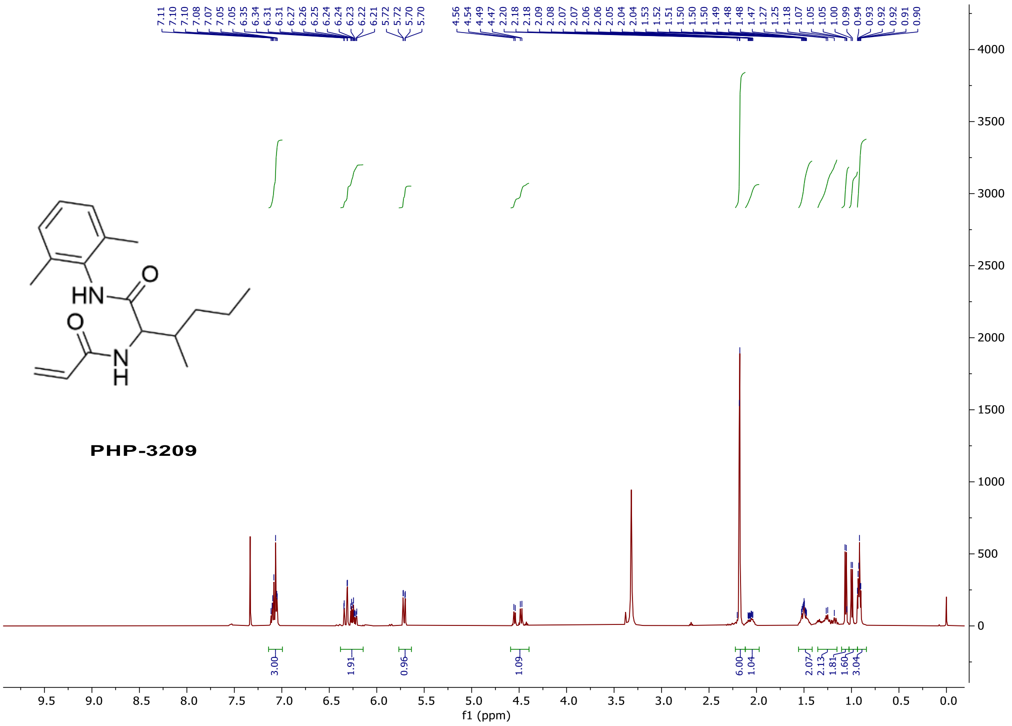

Supplementary Figure 106. ^{1}H NMR (500 MHz, $CDCl_3$+one drop of CD_3OD) of compound **PHP-3209.**

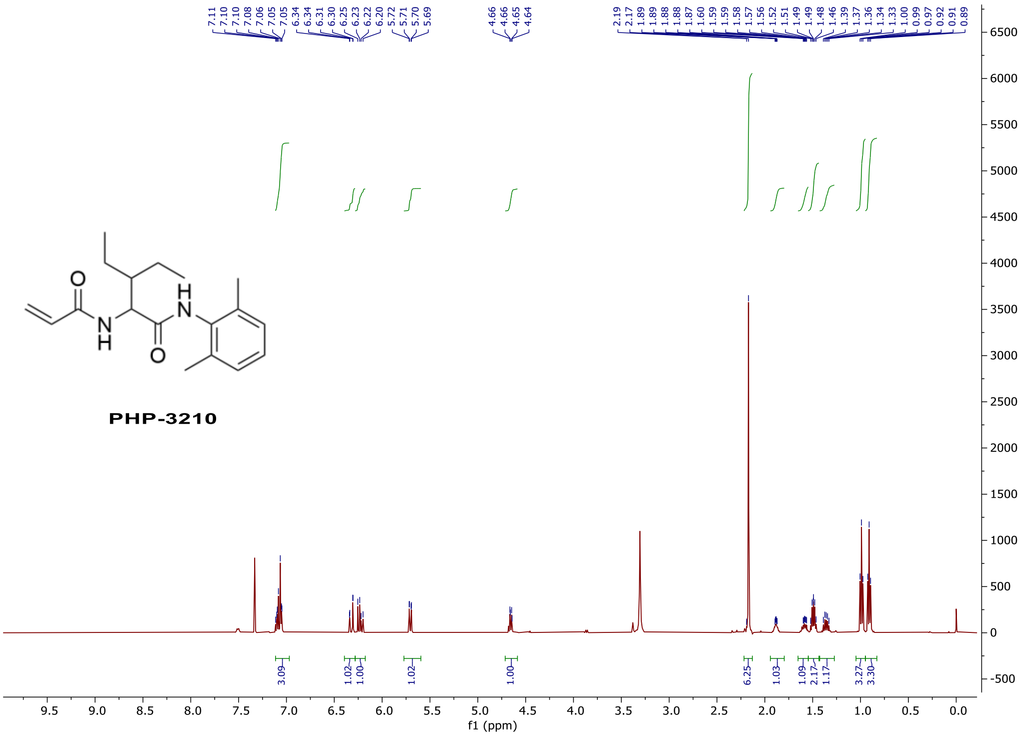

Supplementary Figure 107. ^{1}H NMR (500 MHz, $CDCl_3$+one drop of CD_3OD) of compound **PHP-3210.**

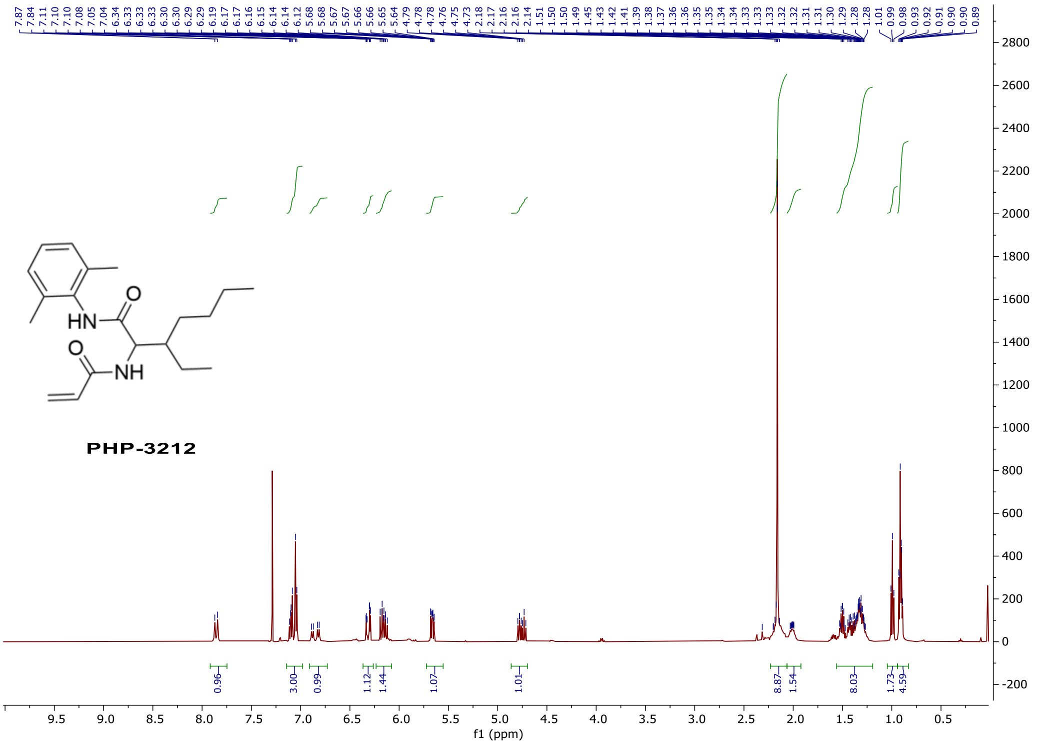

Supplementary Figure 108. ^{1}H NMR (500 MHz, $CDCl_3$) of compound **PHP-3212.**

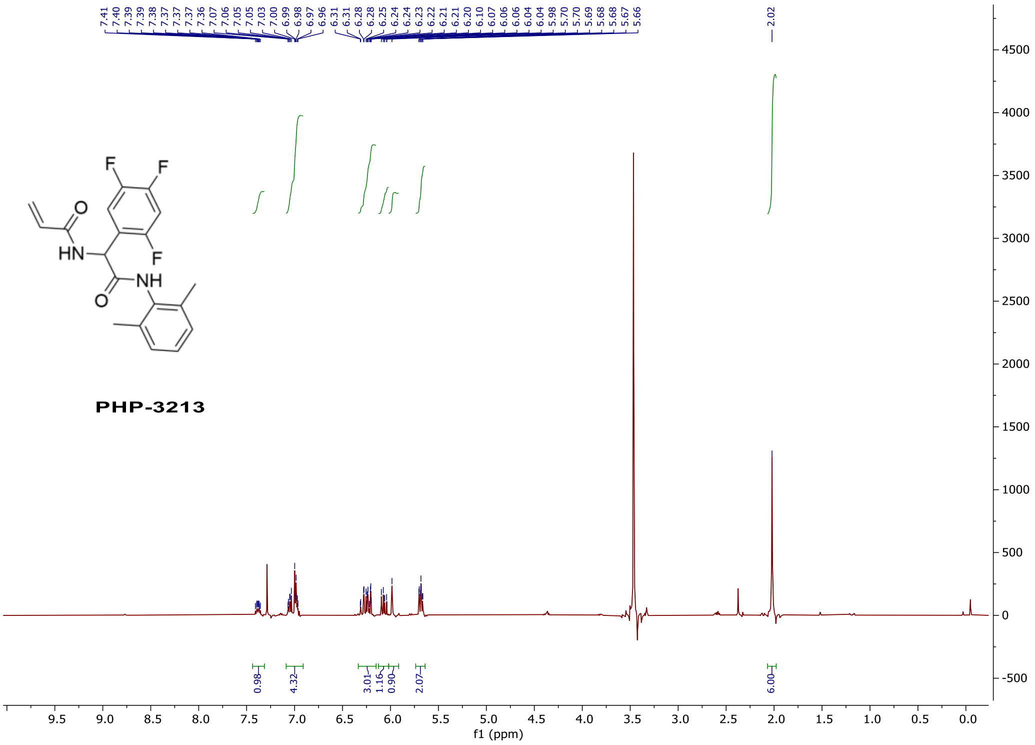

Supplementary Figure 109. ^{1}H NMR (500 MHz, $CDCl_3$+one drop of CD_3OD) of compound **PHP-3213.**

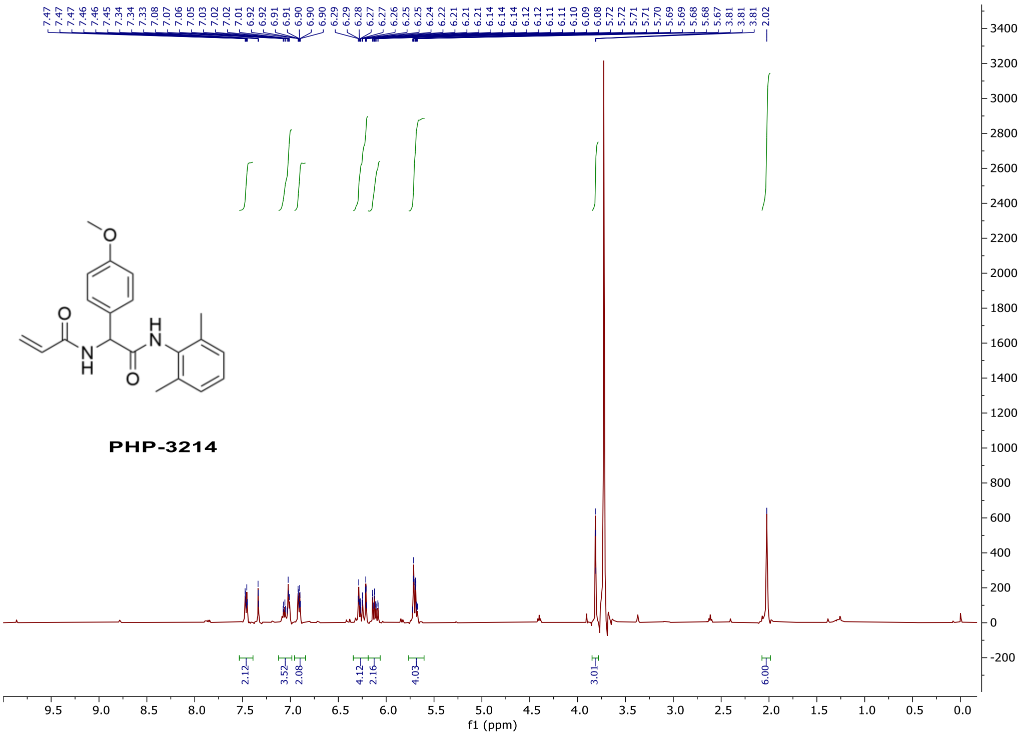

Supplementary Figure 110. ^{1}H NMR (500 MHz, CD_3OD) of compound **PHP-3214.**

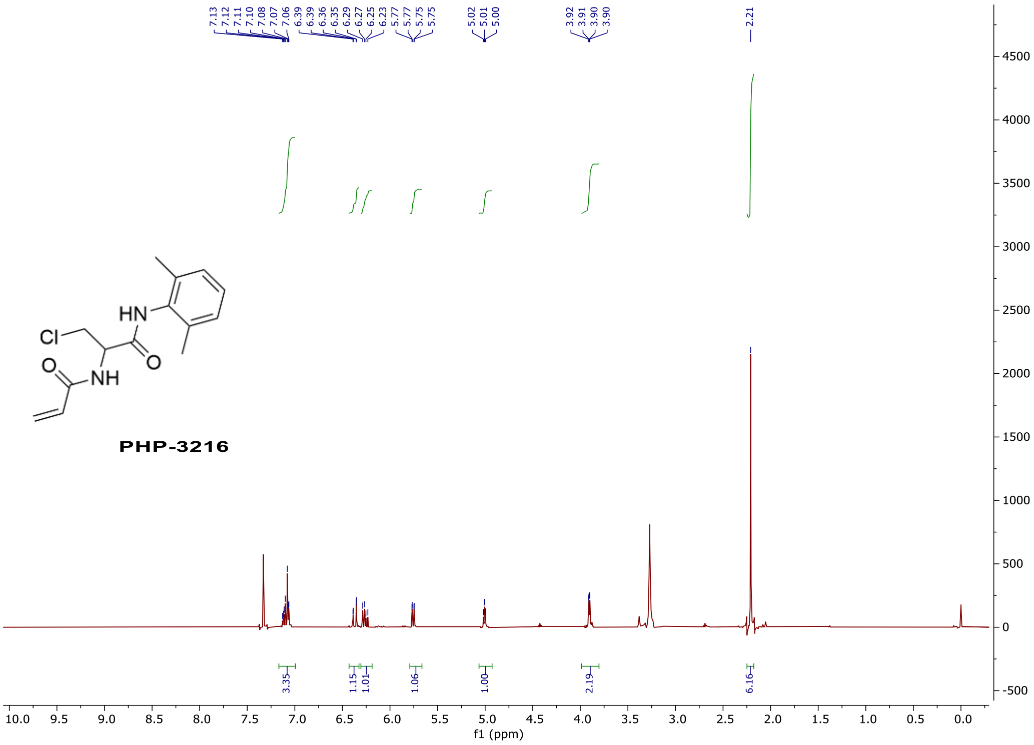

Supplementary Figure 111. ^{1}H NMR (500 MHz, $CDCl_3$+one drop of CD_3OD) of compound **PHP-3216.**

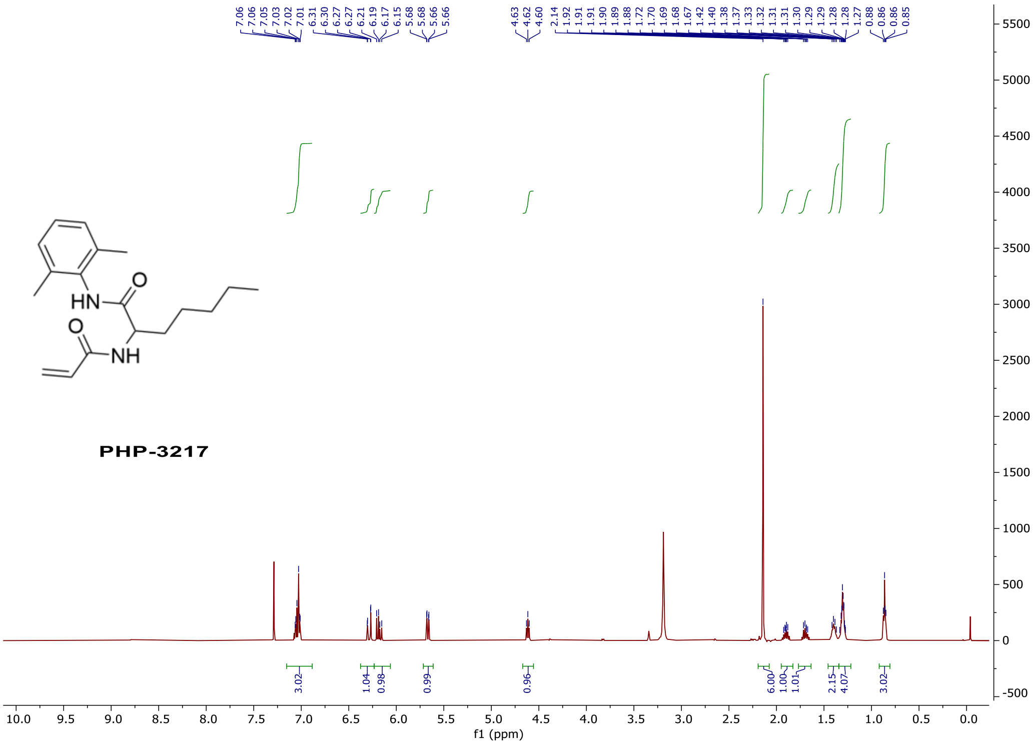

Supplementary Figure 112. ^{1}H NMR (500 MHz, $CDCl_3$+one drop of CD_3OD) of compound **PHP-3217.**

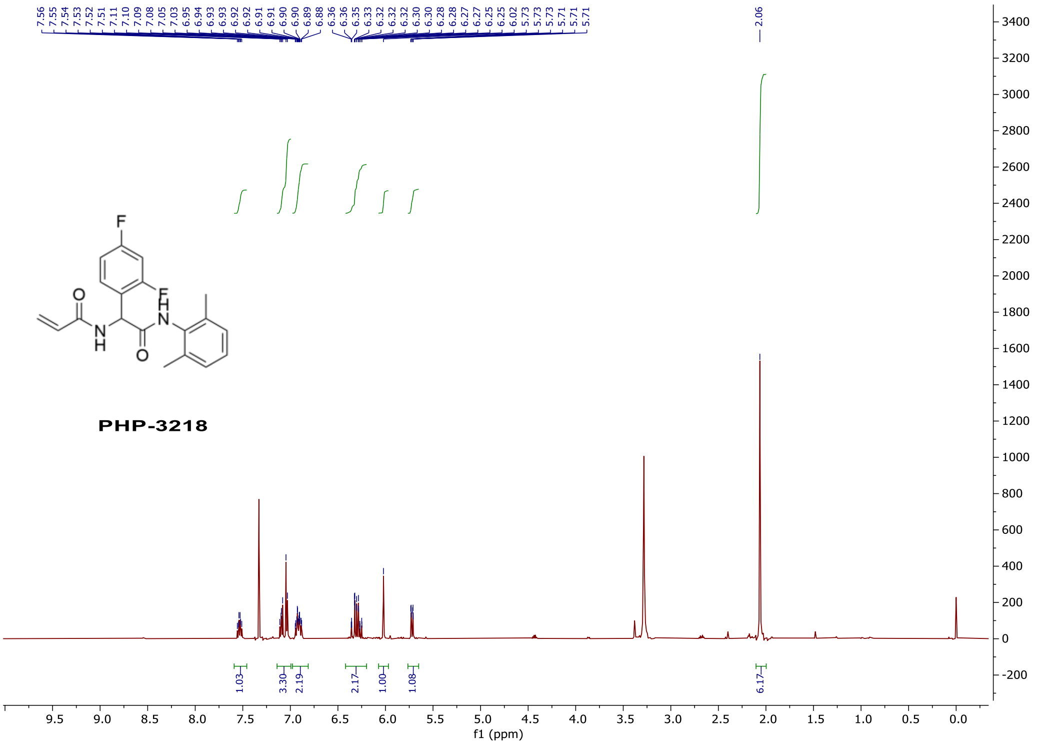

Supplementary Figure 113. ^{1}H NMR (500 MHz, $CDCl_3$+one drop of CD_3OD) of compound **PHP-3218.**

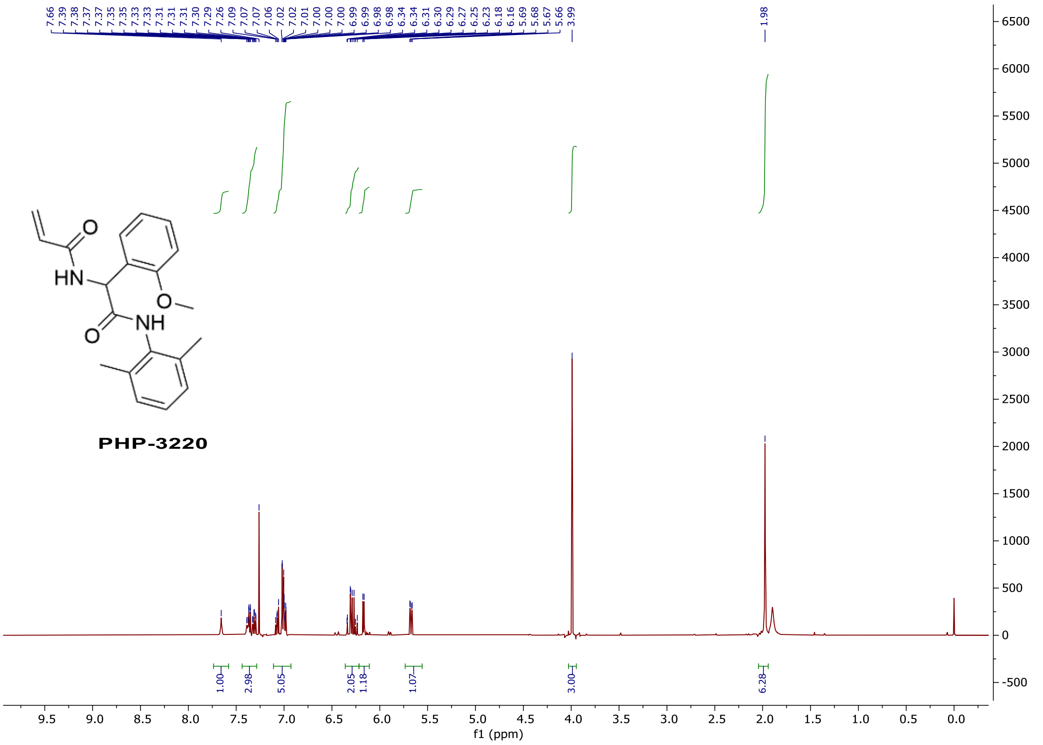

Supplementary Figure 114. ^{1}H NMR (500 MHz, $CDCl_3$) of compound **PHP-3220.**

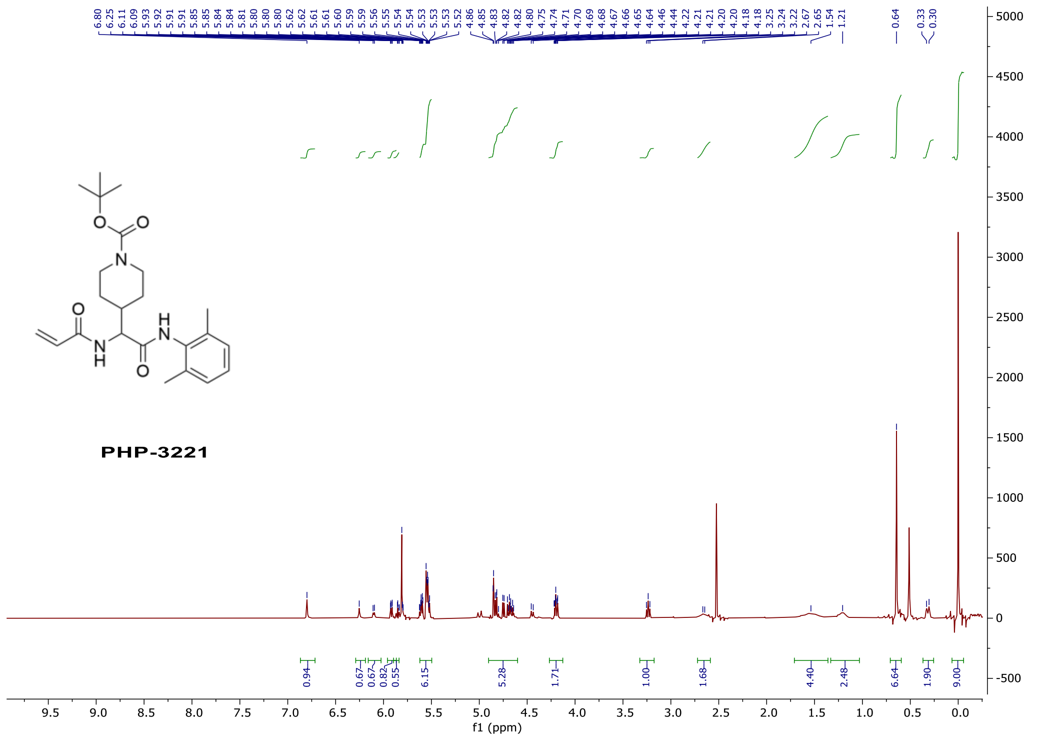

Supplementary Figure 115. ^{1}H NMR (500 MHz, $CDCl_3$) of compound **PHP-3221.**

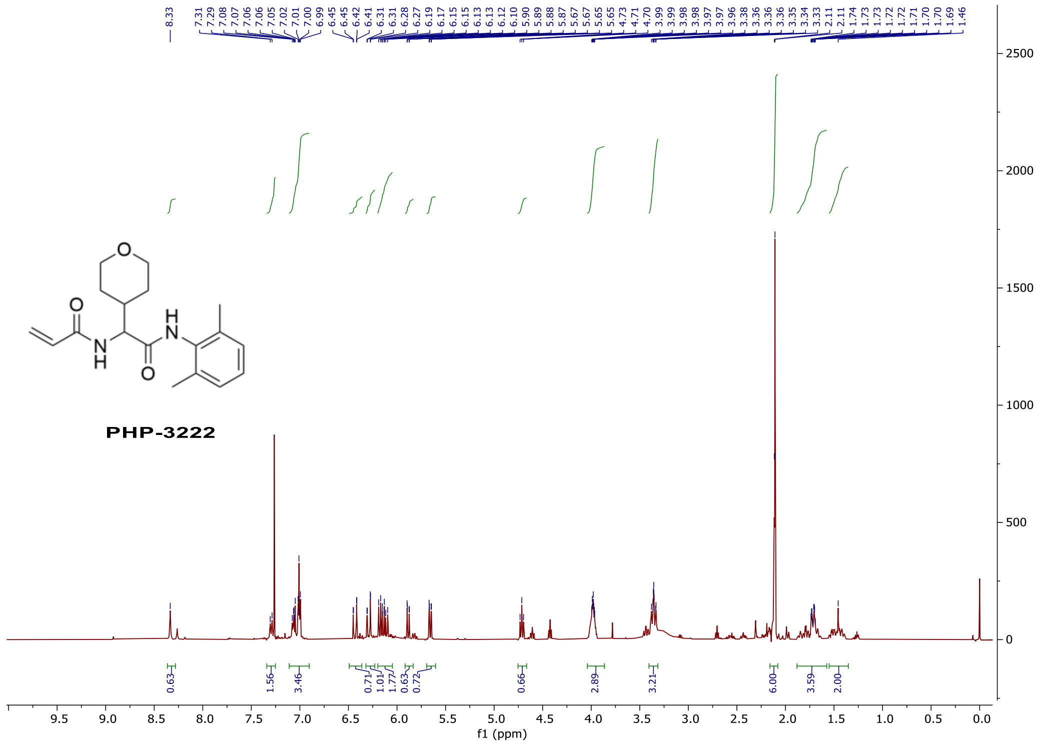

Supplementary Figure 116. ^{1}H NMR (500 MHz, $CDCl_3$) of compound **PHP-3222.**

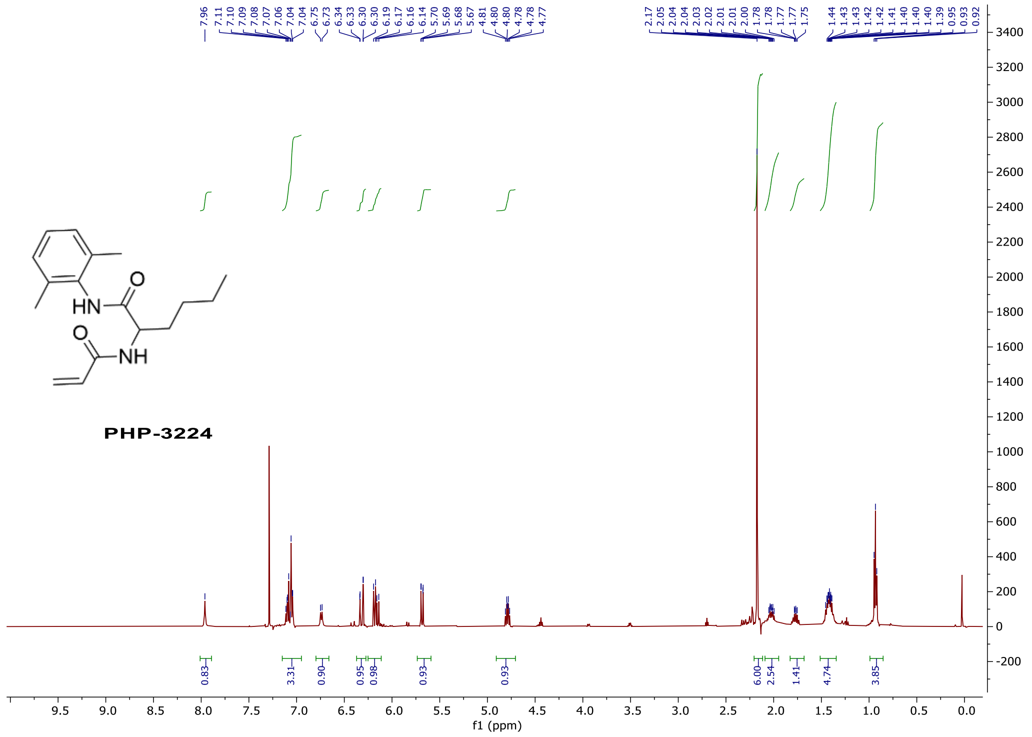

Supplementary Figure 117. ^{1}H NMR (500 MHz, $CDCl_3$) of compound **PHP-3224.**

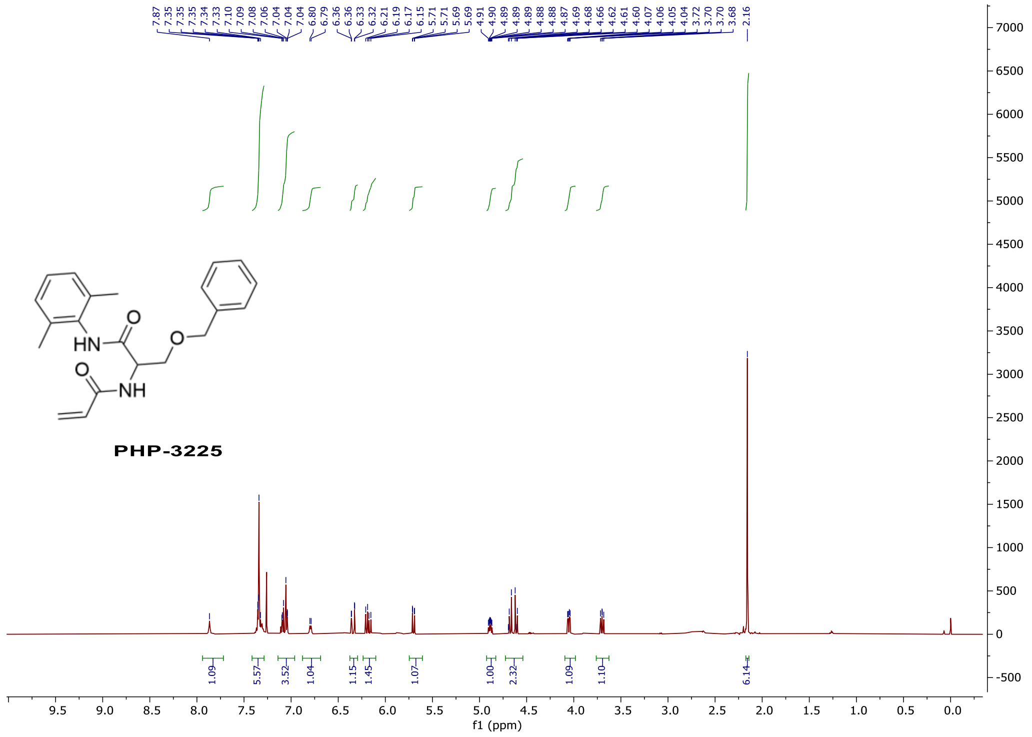

Supplementary Figure 118. ^{1}H NMR (500 MHz, $CDCl_3$) of compound **PHP-3225.**

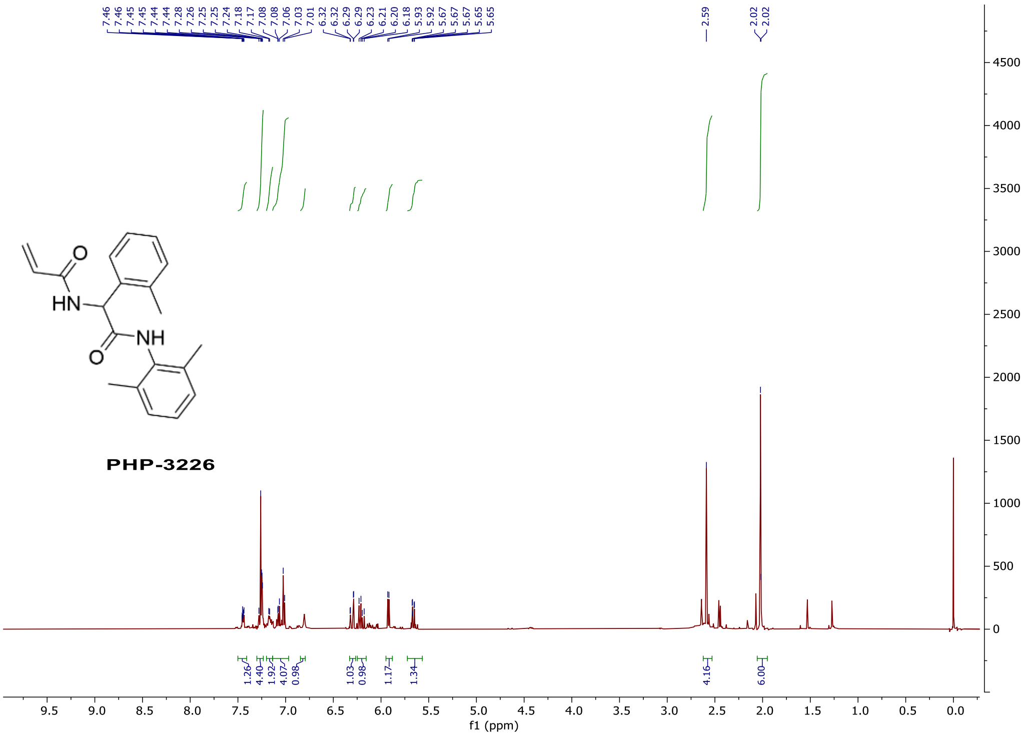

Supplementary Figure 119. ^{1}H NMR (500 MHz, $CDCl_3$) of compound **PHP-3226.**

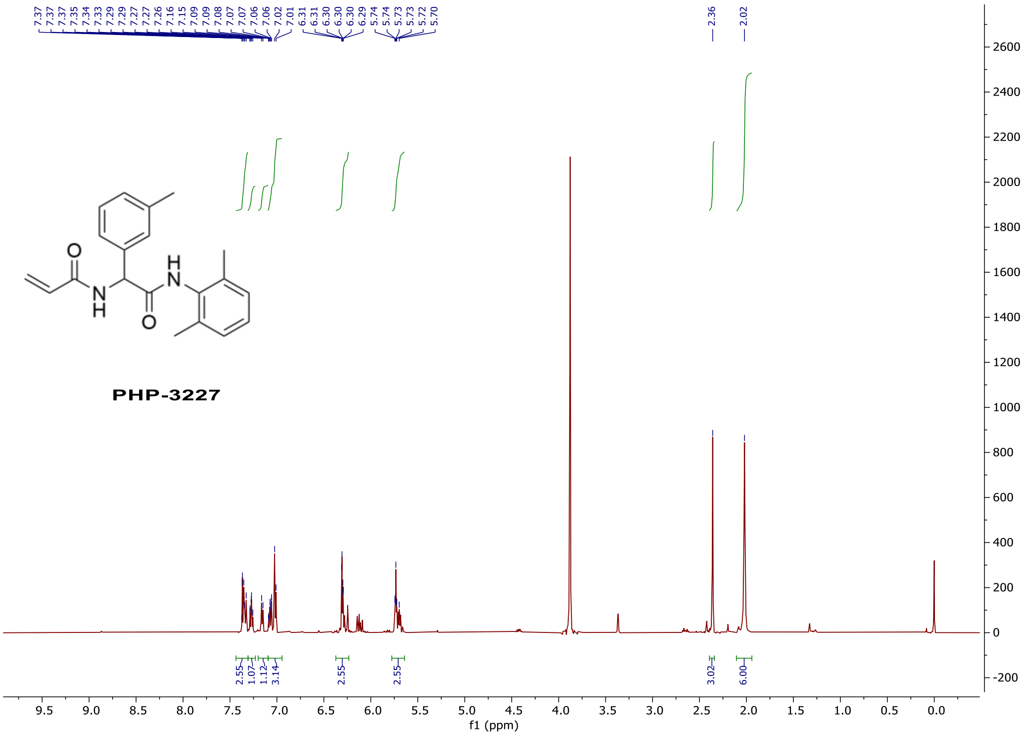

Supplementary Figure 120. ^{1}H NMR (500 MHz, $CDCl_3$+one drop of CD_3OD) of compound **PHP-3227.**

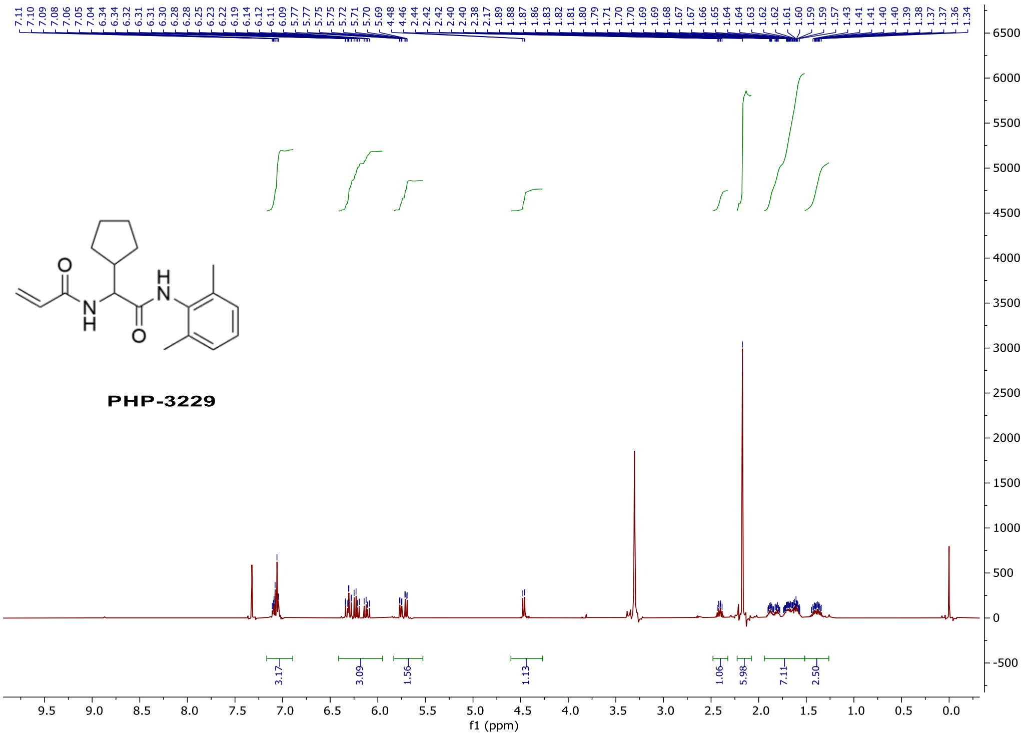

Supplementary Figure 121. ^{1}H NMR (500 MHz, $CDCl_3$+one drop of CD_3OD) of compound **PHP-3229.**

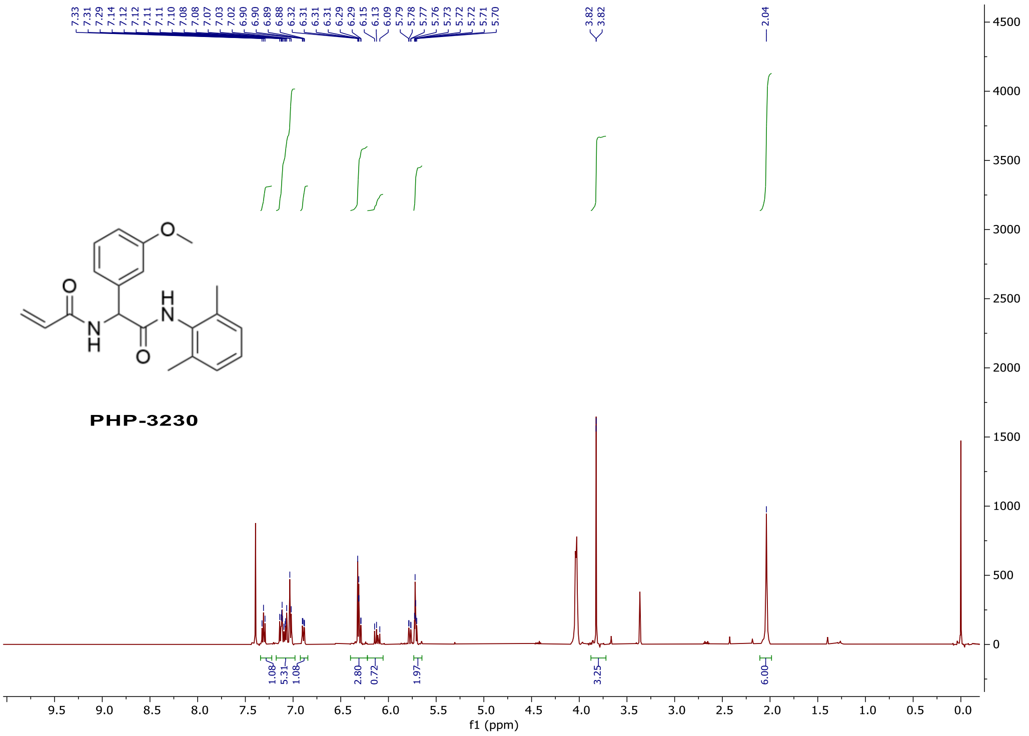

Supplementary Figure 122. ^{1}H NMR (500 MHz, $CDCl_3$+one drop of CD_3OD) of compound **PHP-3230.**

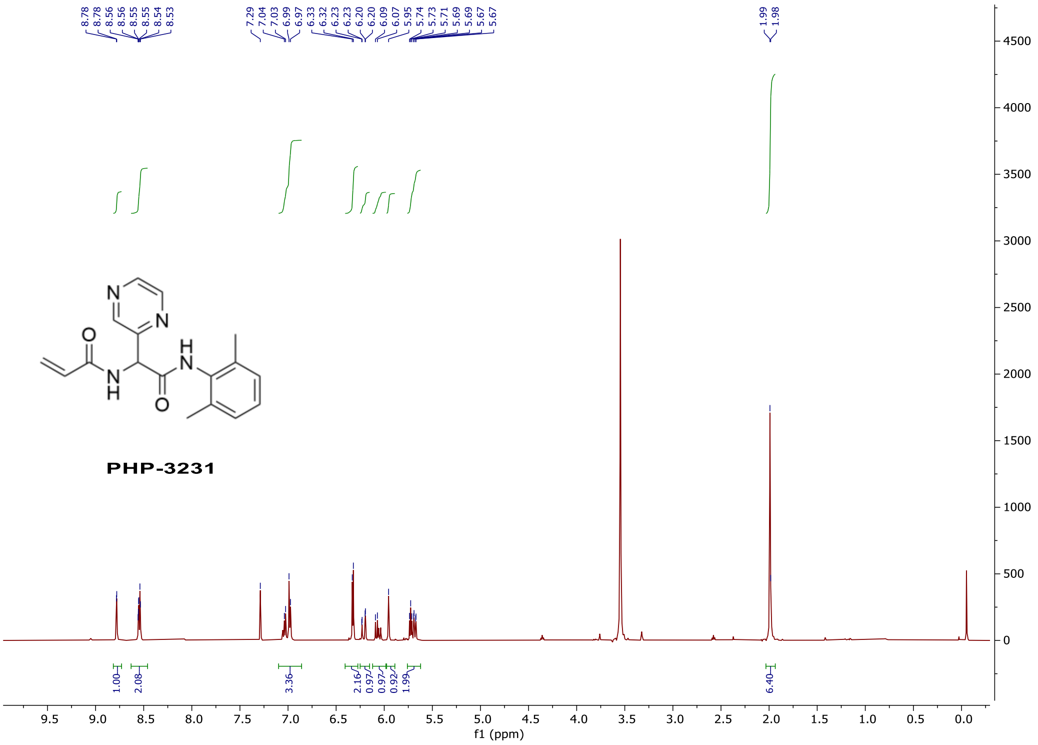

Supplementary Figure 123. ^{1}H NMR (500 MHz, $CDCl_3$+one drop of CD_3OD) of compound **PHP-3231.**

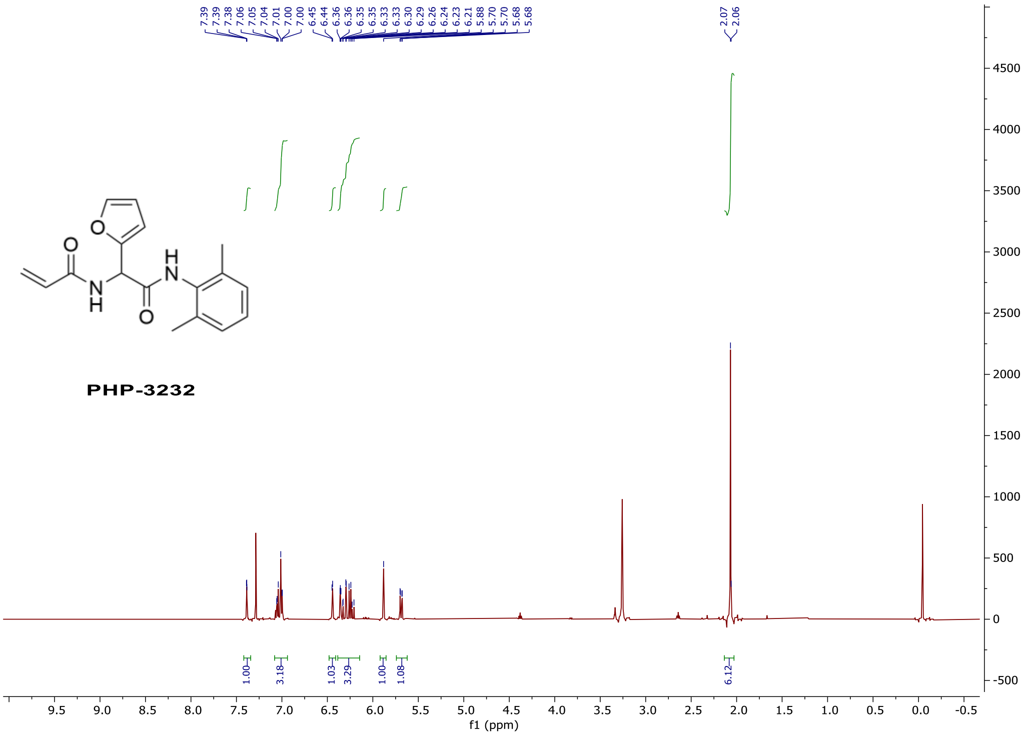

Supplementary Figure 124. ^{1}H NMR (500 MHz, $CDCl_3$+one drop of CD_3OD) of compound **PHP-3232.**

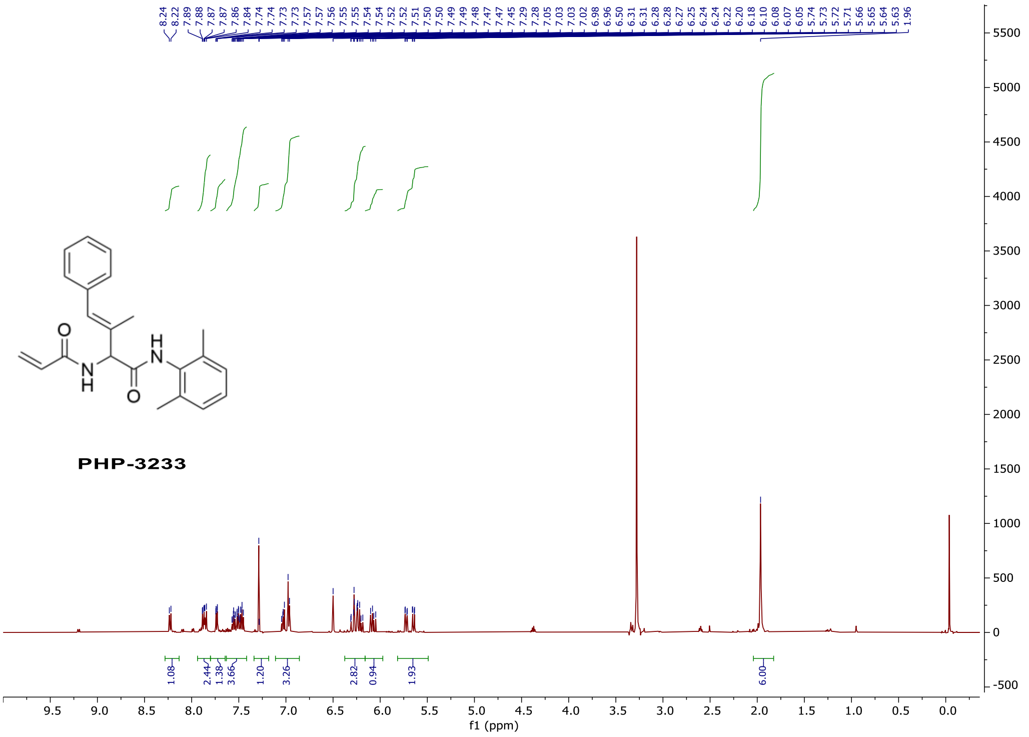

Supplementary Figure 125. ^{1}H NMR (500 MHz, $CDCl_3$+one drop of CD_3OD) of compound **PHP-3233.**

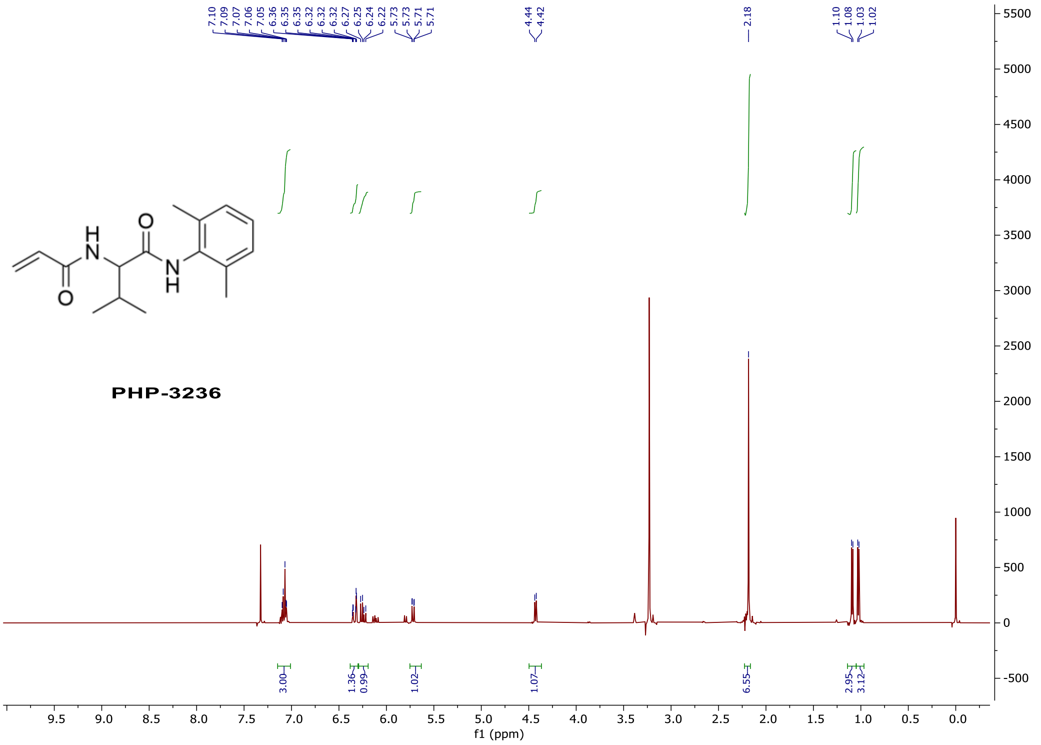

Supplementary Figure 126. ^{1}H NMR (500 MHz, $CDCl_3$+one drop of CD_3OD) of compound **PHP-3236.**

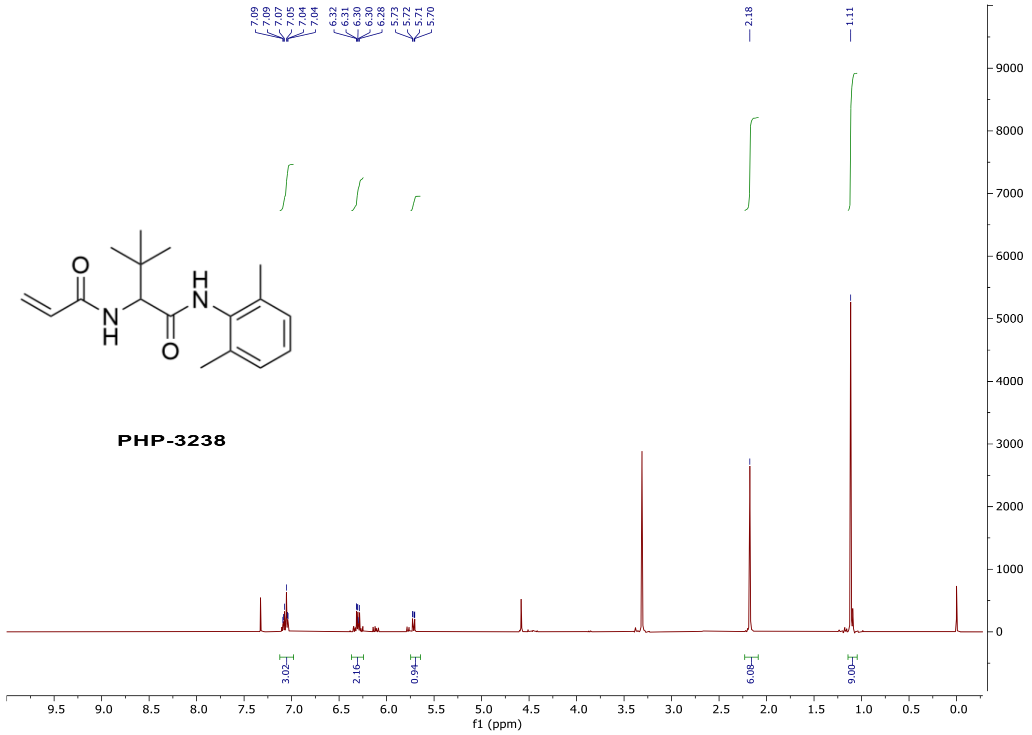

Supplementary Figure 127. ^{1}H NMR (500 MHz, $CDCl_3$+one drop of CD_3OD) of compound **PHP-3238.**

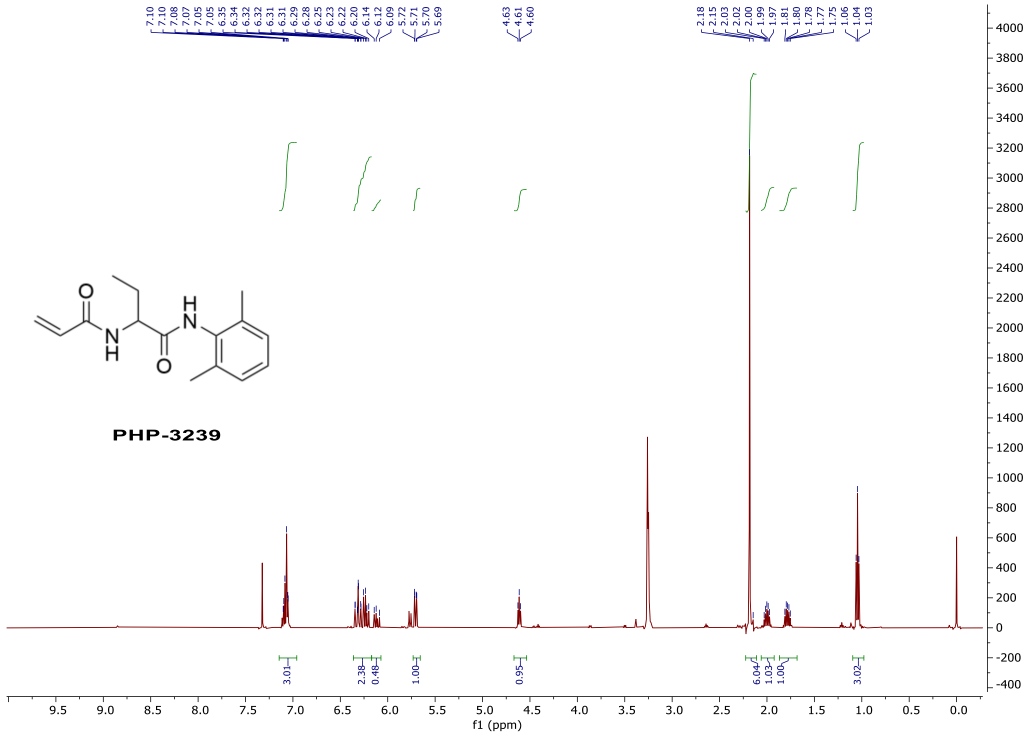

Supplementary Figure 128. ^{1}H NMR (500 MHz, $CDCl_3$+one drop of CD_3OD) of compound **PHP-3239.**

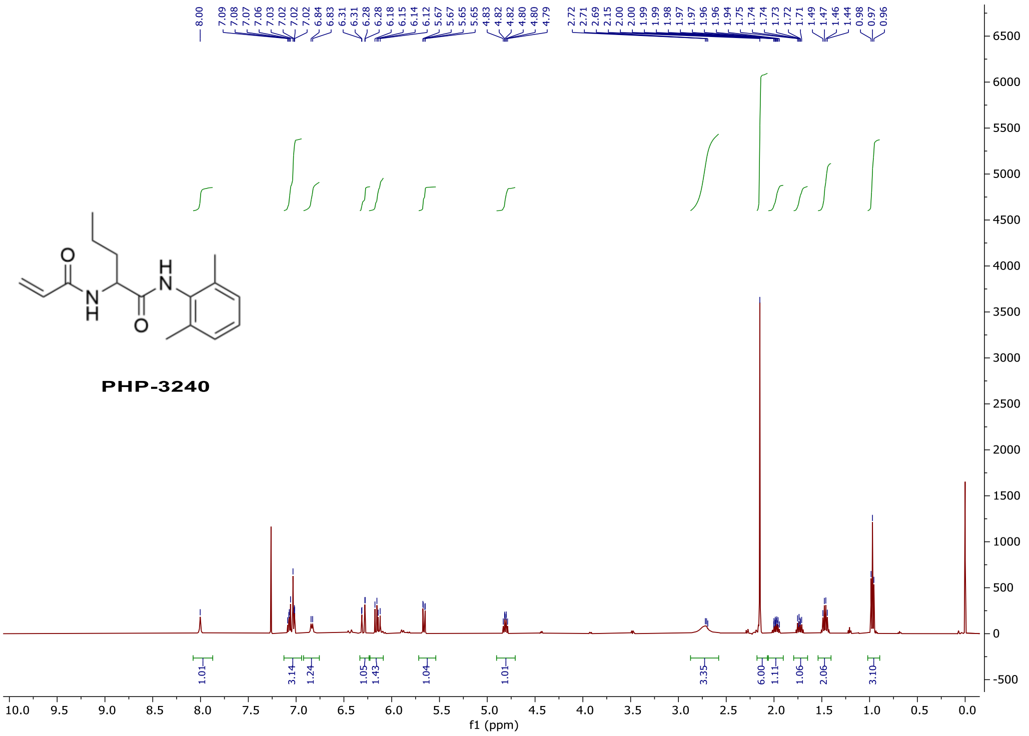

Supplementary Figure 129. ¹H NMR (500 MHz, $CDCl_3$) of compound **PHP-3240.**

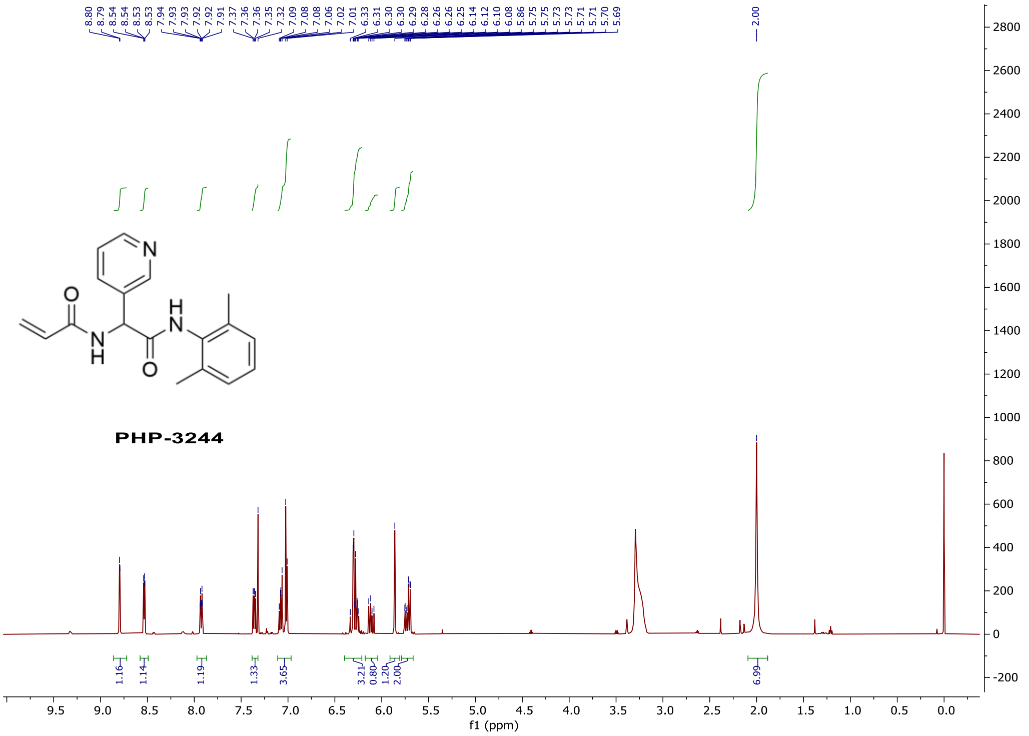

Supplementary Figure 130. ¹H NMR (500 MHz, $CDCl_3$+one drop of CD_3OD) of compound **PHP-3244.**

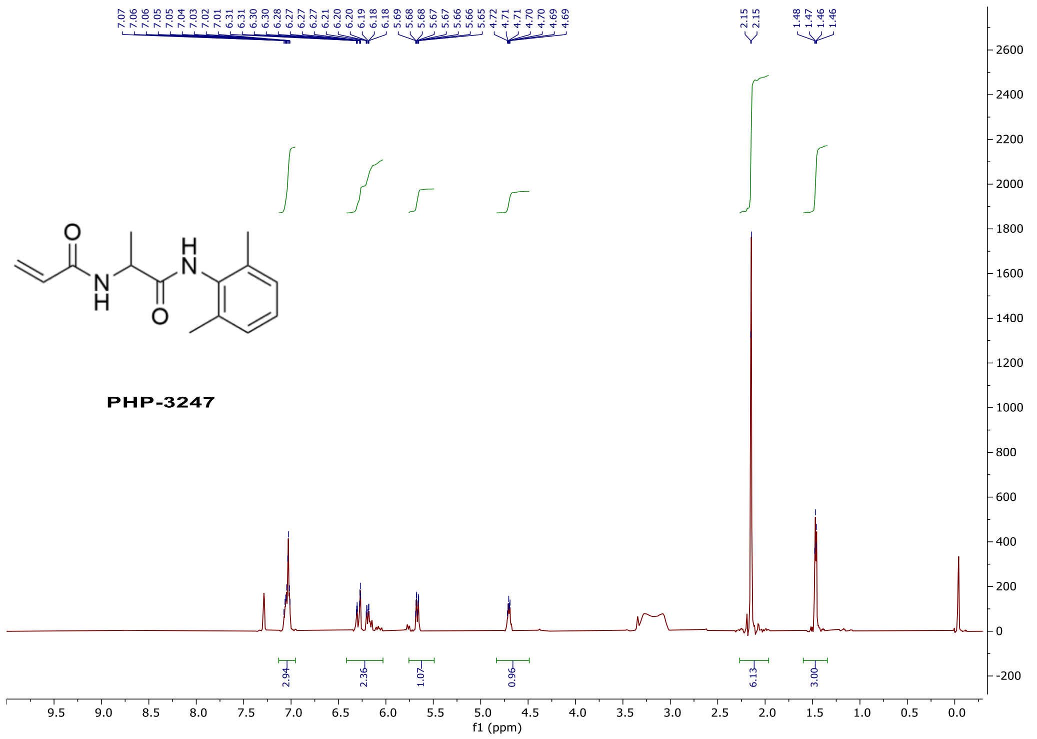

Supplementary Figure 131. ^{1}H NMR (500 MHz, $CDCl_3$+one drop of CD_3OD) of compound **PHP-3247.**

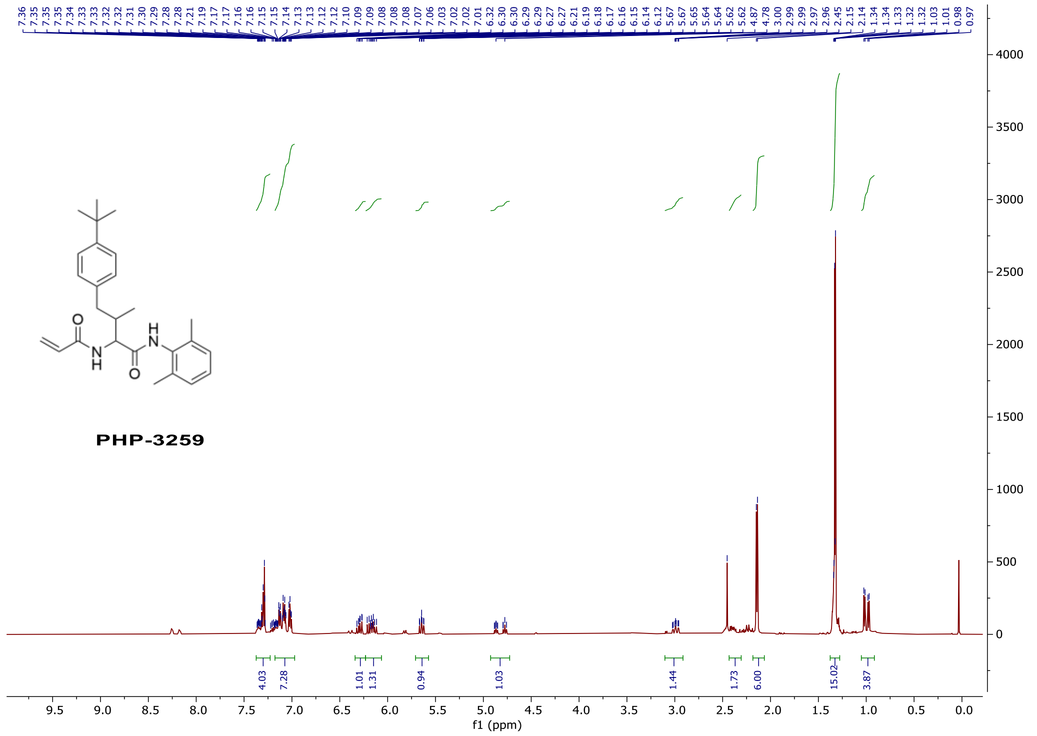

Supplementary Figure 132. ^{1}H NMR (500 MHz, $CDCl_3$) of compound **PHP-3259.**

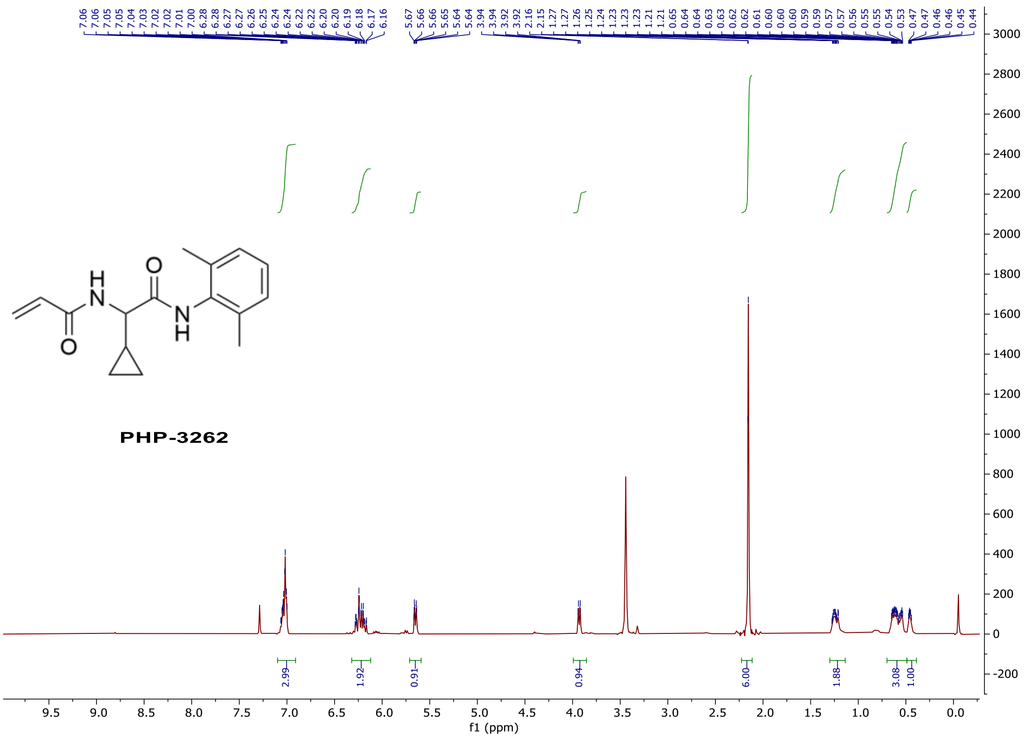

Supplementary Figure 133. ^{1}H NMR (500 MHz, $CDCl_3$+one drop of CD_3OD) of compound **PHP-3262.**

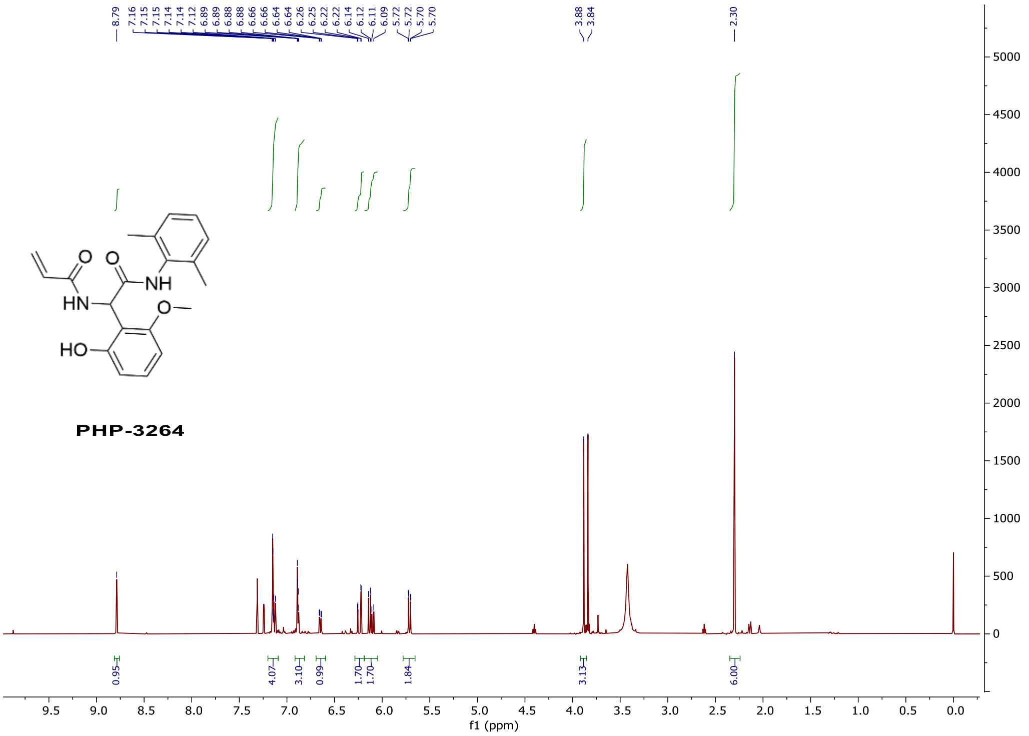

Supplementary Figure 134. ^{1}H NMR (500 MHz, $CDCl_3$+one drop of CD_3OD) of compound **PHP-3264.**

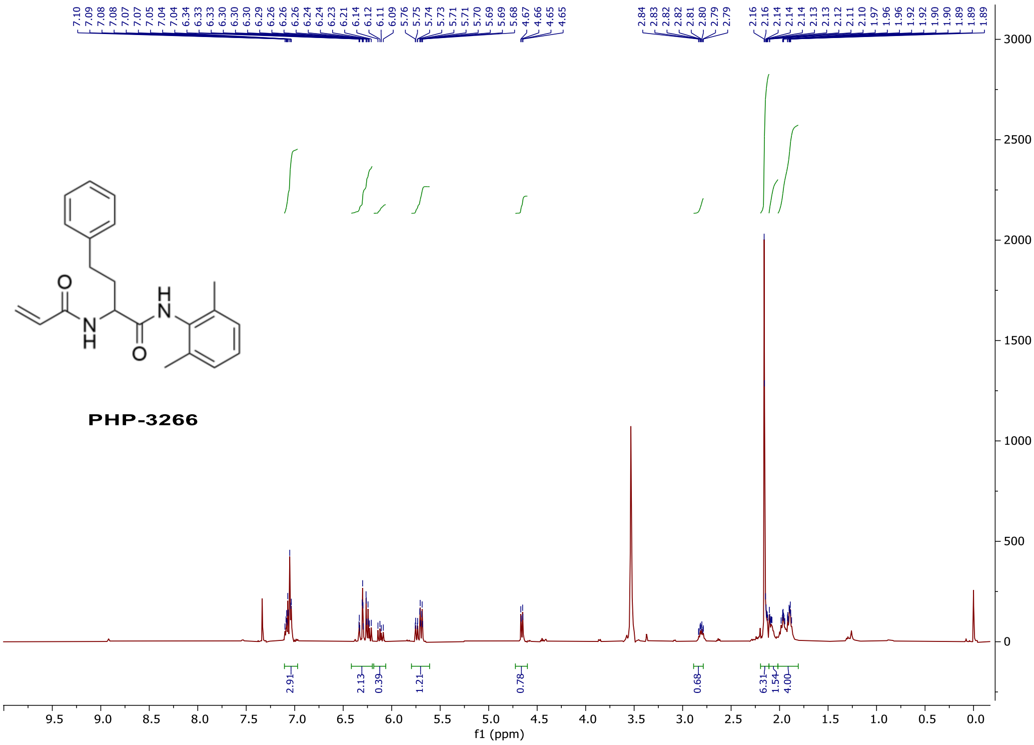

Supplementary Figure 135. ^{1}H NMR (500 MHz, $CDCl_3$+one drop of CD_3OD) of compound **PHP-3266.**

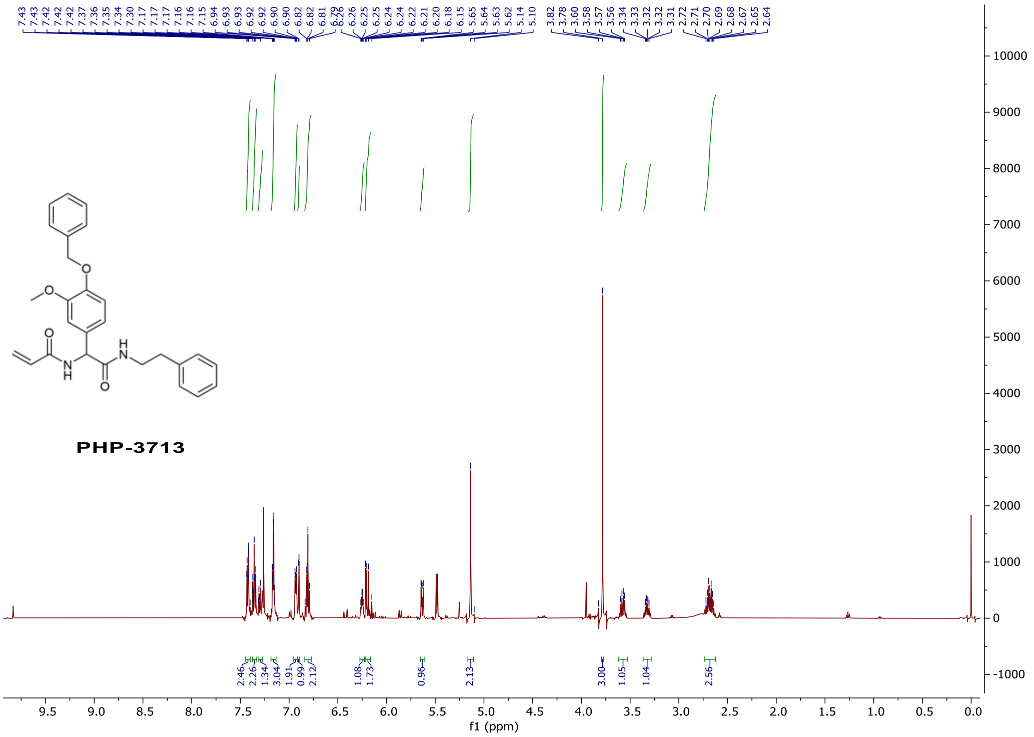

Supplementary Figure 136. ^{1}H NMR (500 MHz, $CDCl_3$) of compound **PHP-3713**.

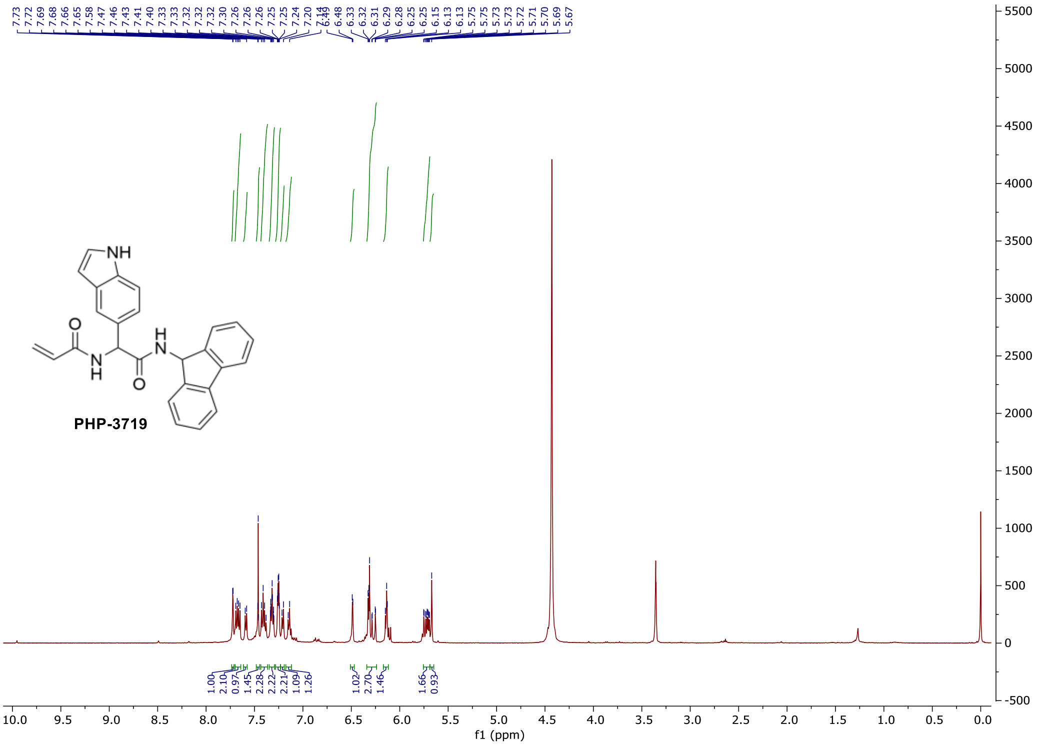

Supplementary Figure 137. ^{1}H NMR (500 MHz, $CDCl_3$) of compound **PHP-3719**.

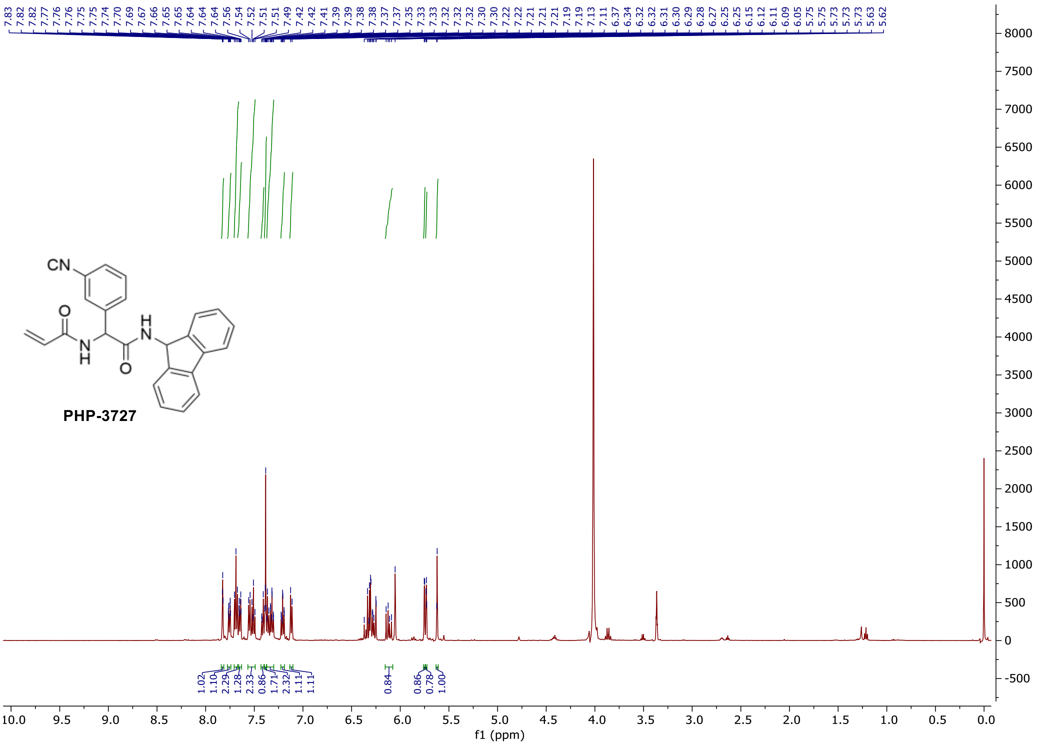

Supplementary Figure 138. ^{1}H NMR (500 MHz, $CDCl_3$ + one drop of CD_3OD) of compound **PHP-3727**.

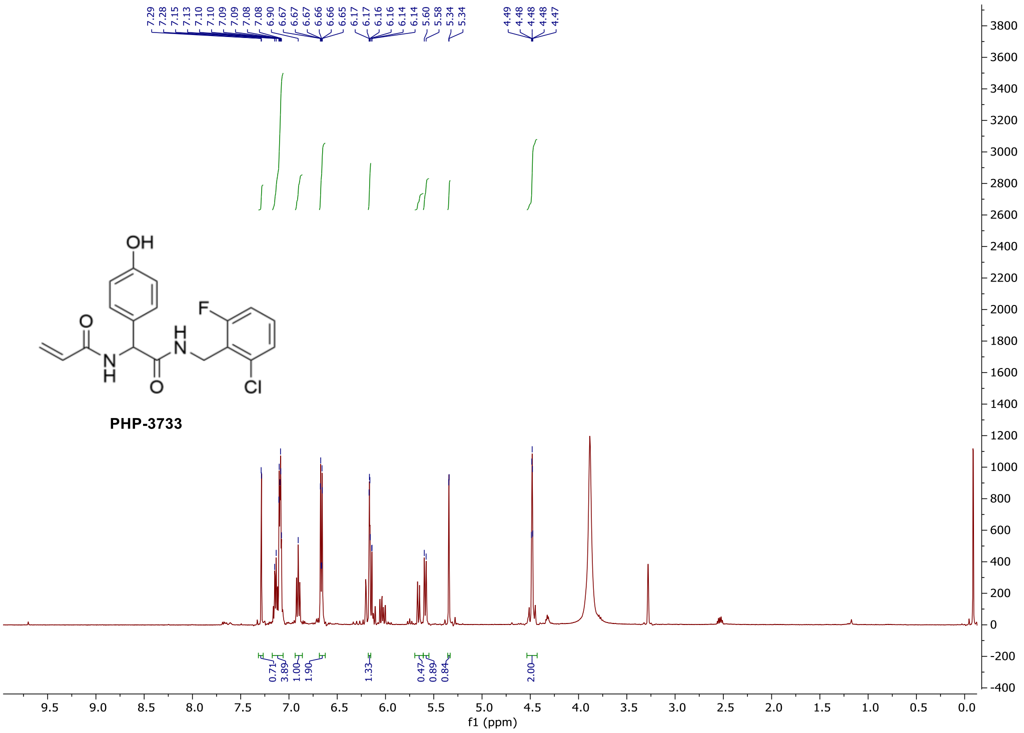

Supplementary Figure 139. ^{1}H NMR (500 MHz, $CDCl_3$ + one drop of CD_3OD) of compound **PHP-3733**.

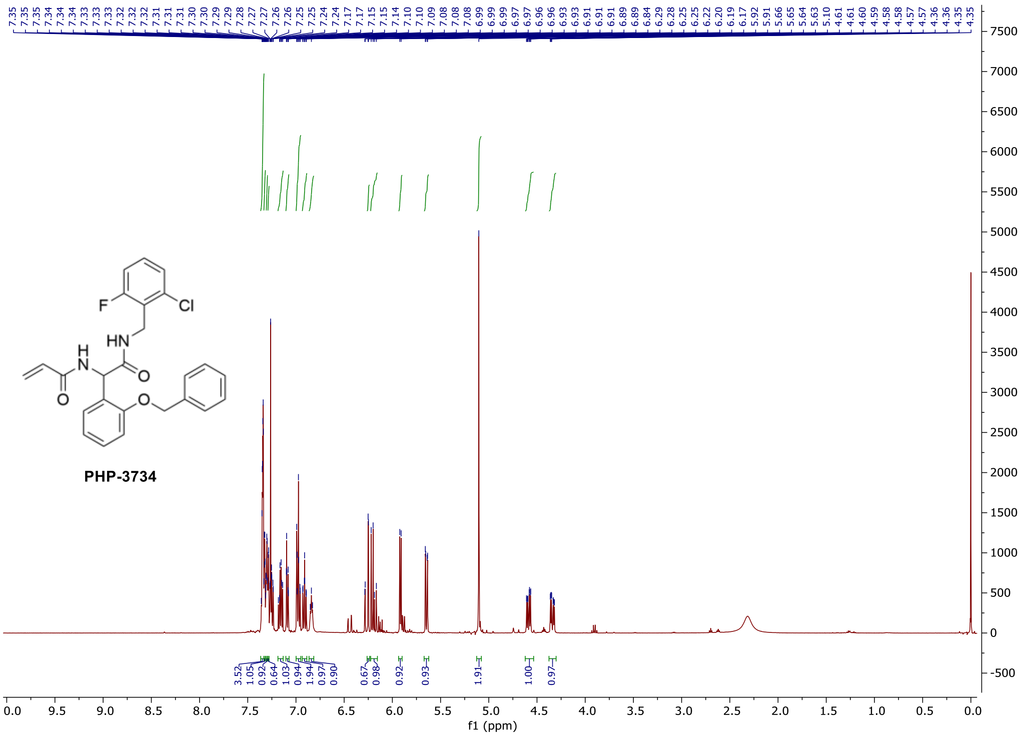

Supplementary Figure 140. ^{1}H NMR (500 MHz, $CDCl_3$) of compound **PHP-3734**.

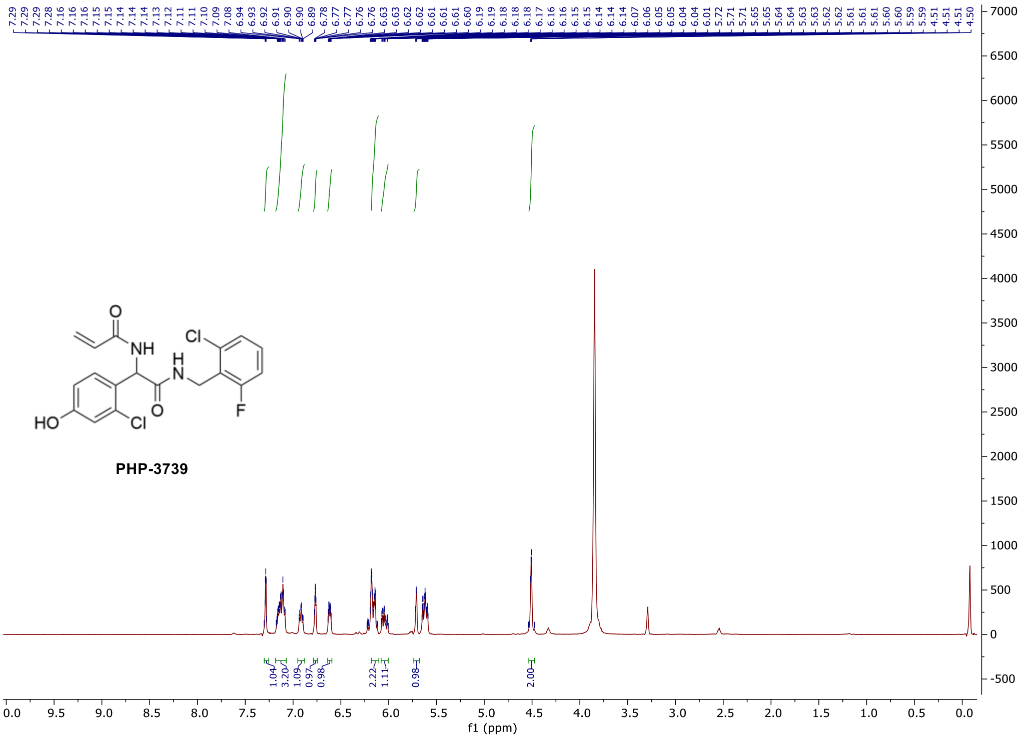

Supplementary Figure 141. ^{1}H NMR (500 MHz, $CDCl_3$ + one drop of CD_3OD) of compound **PHP-3739**.

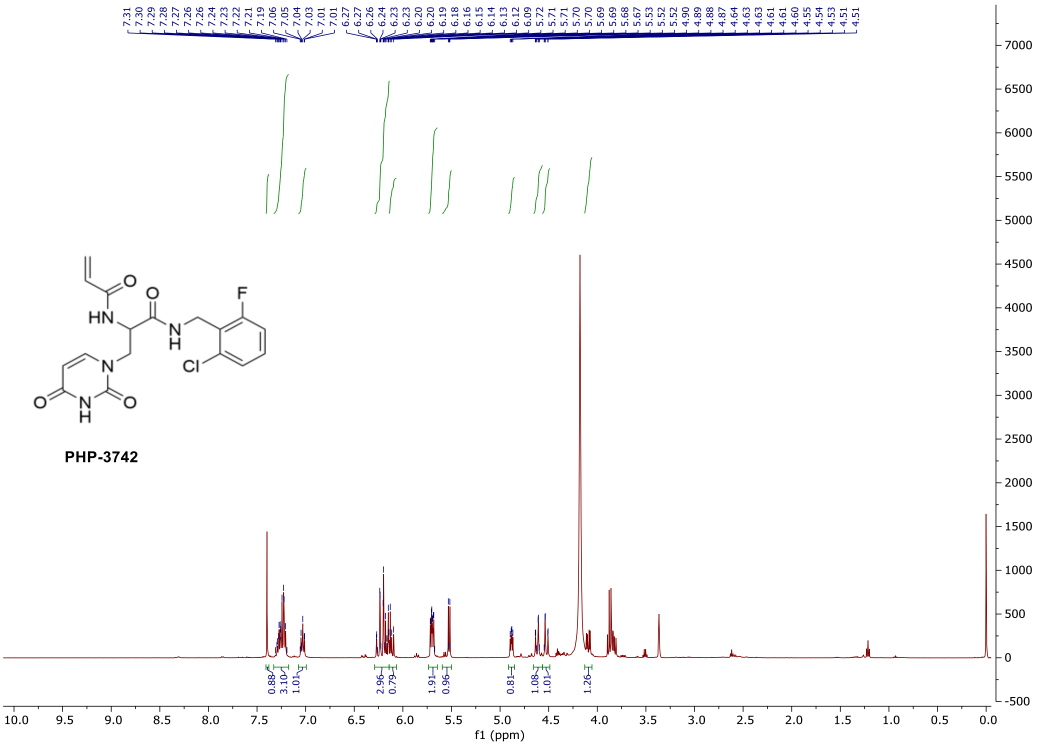

Supplementary Figure 142. ^{1}H NMR (500 MHz, $CDCl_3$ + one drop of CD_3OD) of compound **PHP-3742**.

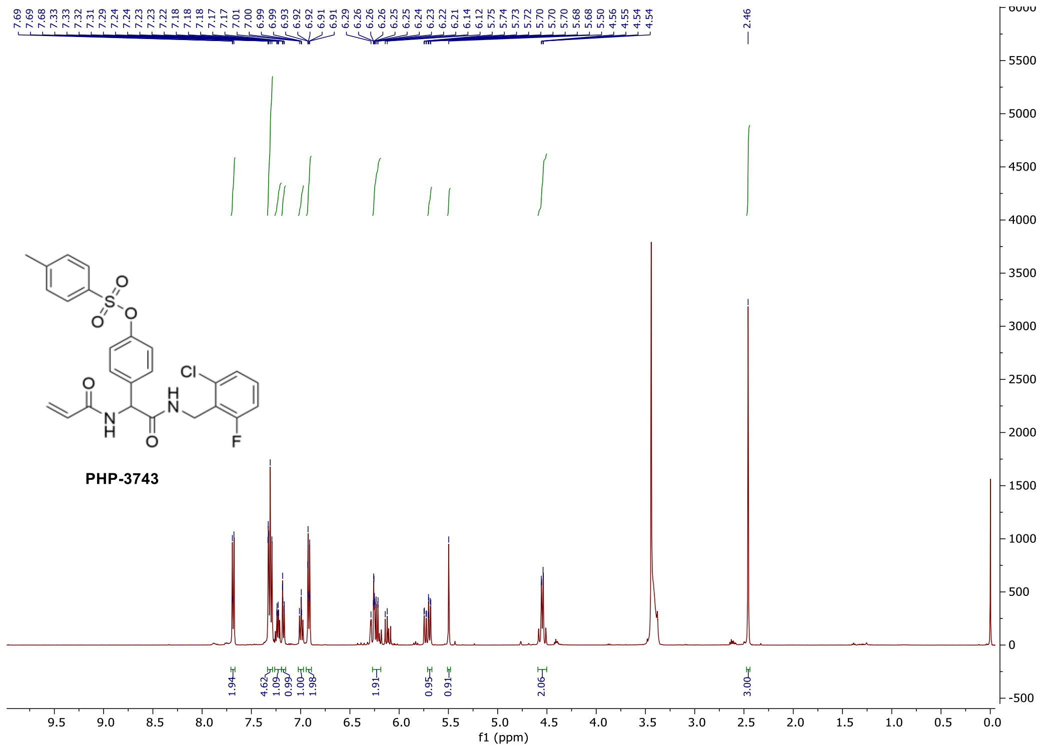

Supplementary Figure 143. ^{1}H NMR (500 MHz, $CDCl_3$ + one drop of CD_3OD) of compound **PHP-3743**.

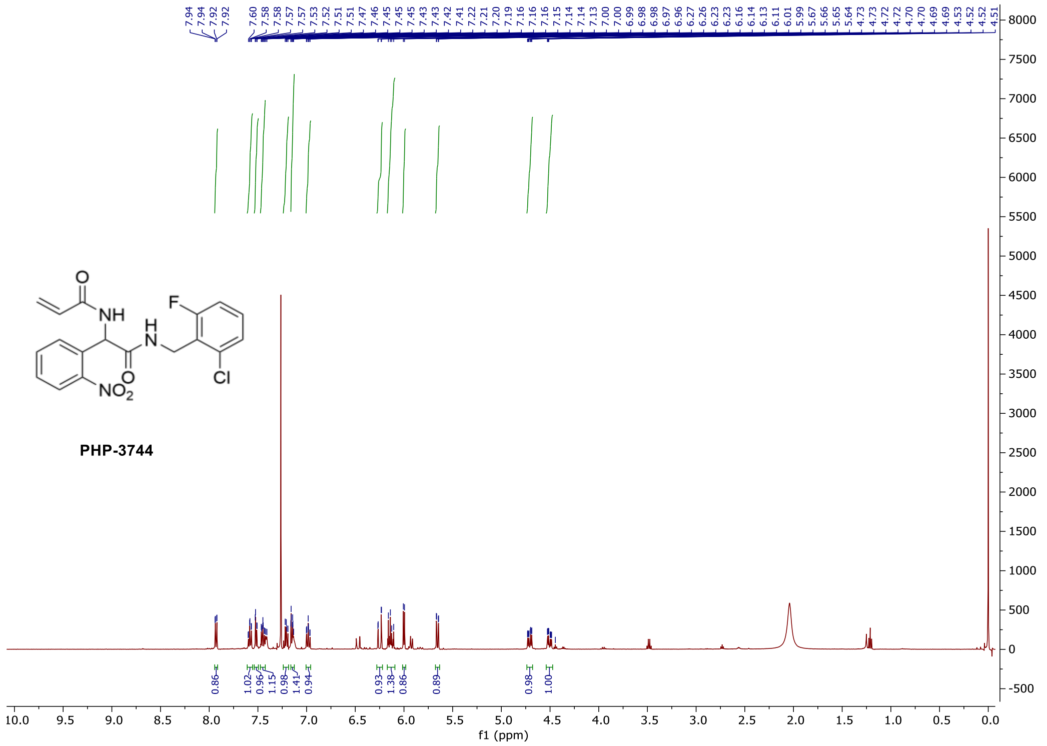

Supplementary Figure 144. ^{1}H NMR (500 MHz, $CDCl_3$) of compound **PHP-3744**.

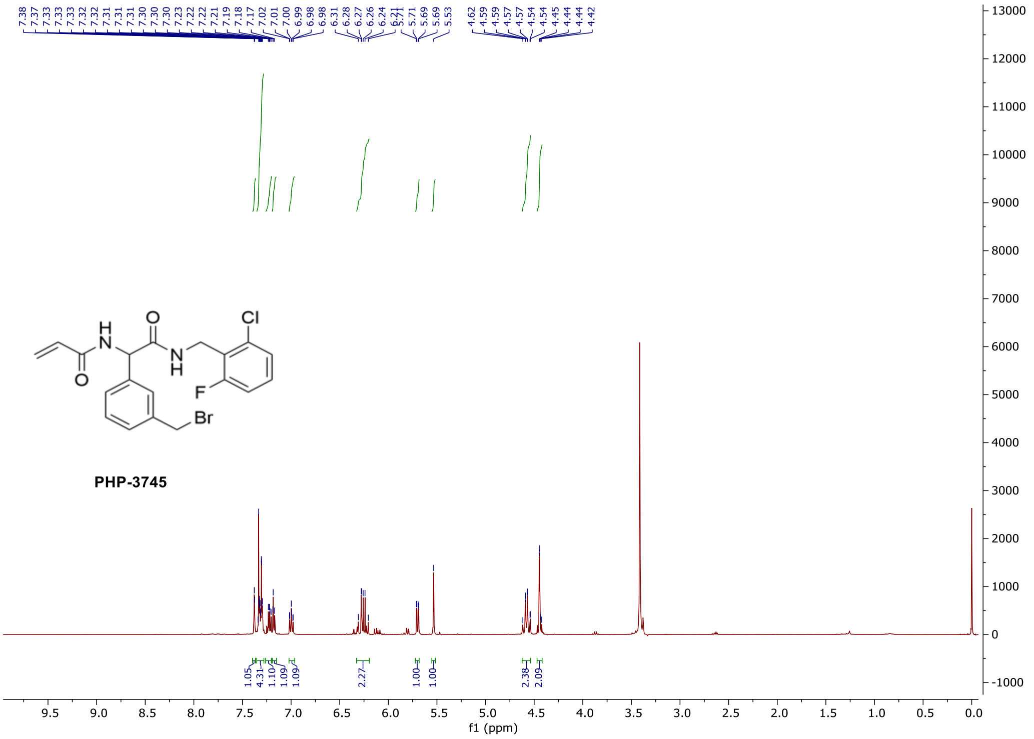

Supplementary Figure 145. ^{1}H NMR (500 MHz, $CDCl_3$ + one drop of CD_3OD) of compound **PHP-3745**.

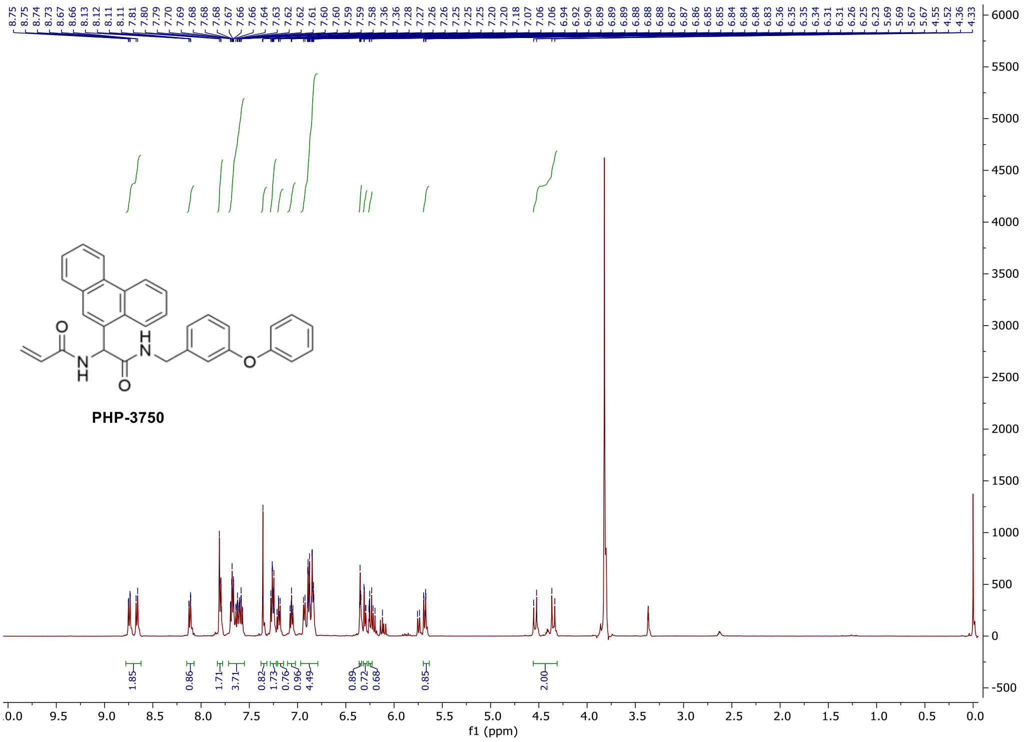

Supplementary Figure 146. ^{1}H NMR (500 MHz, $CDCl_3$ + one drop of CD_3OD) of compound **PHP-3750**.

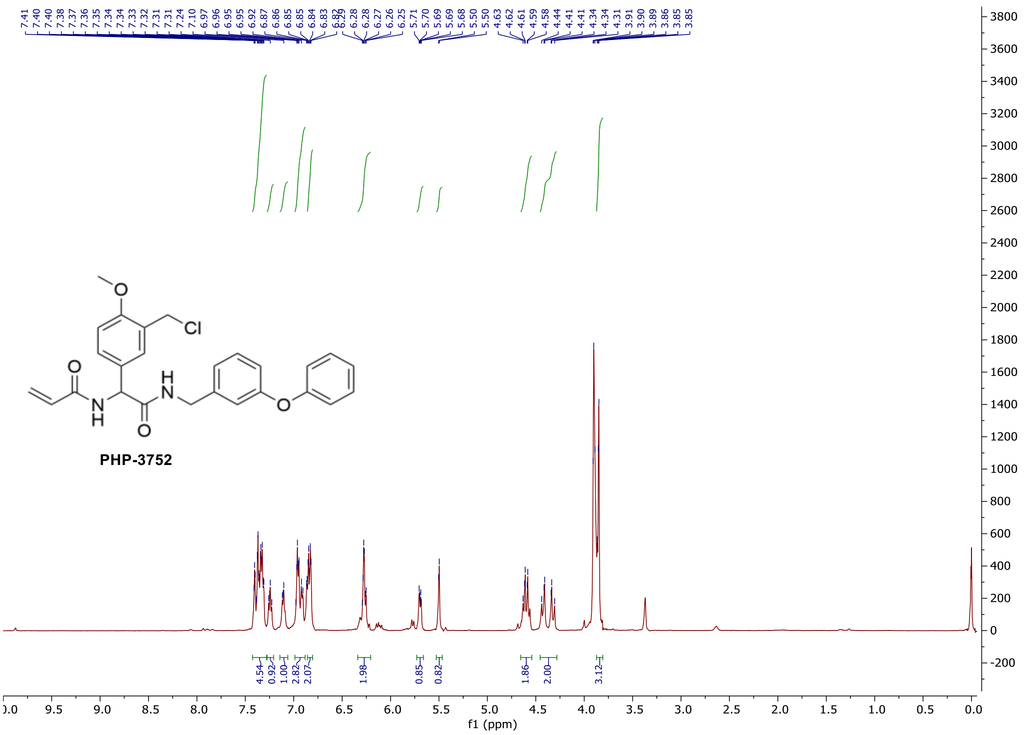

Supplementary Figure 147. ^{1}H NMR (500 MHz, $CDCl_3$ + one drop of CD_3OD) of compound **PHP-3752**.

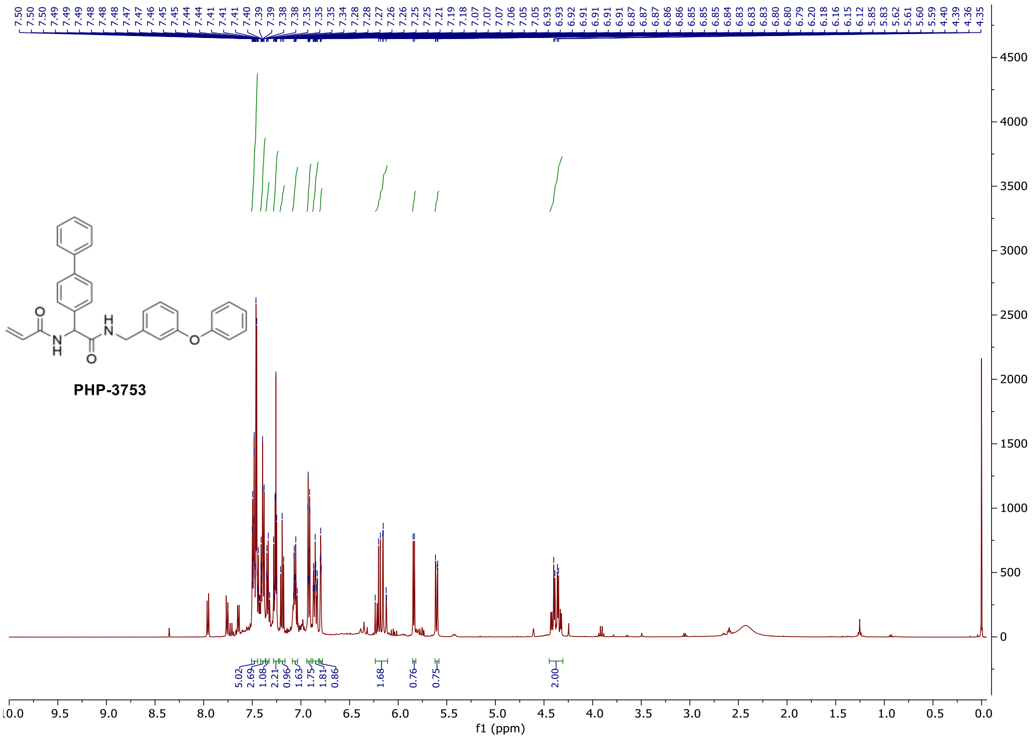

Supplementary Figure 148. ^{1}H NMR (500 MHz, $CDCl_3$ + one drop of CD_3OD) of compound **PHP-3753**.

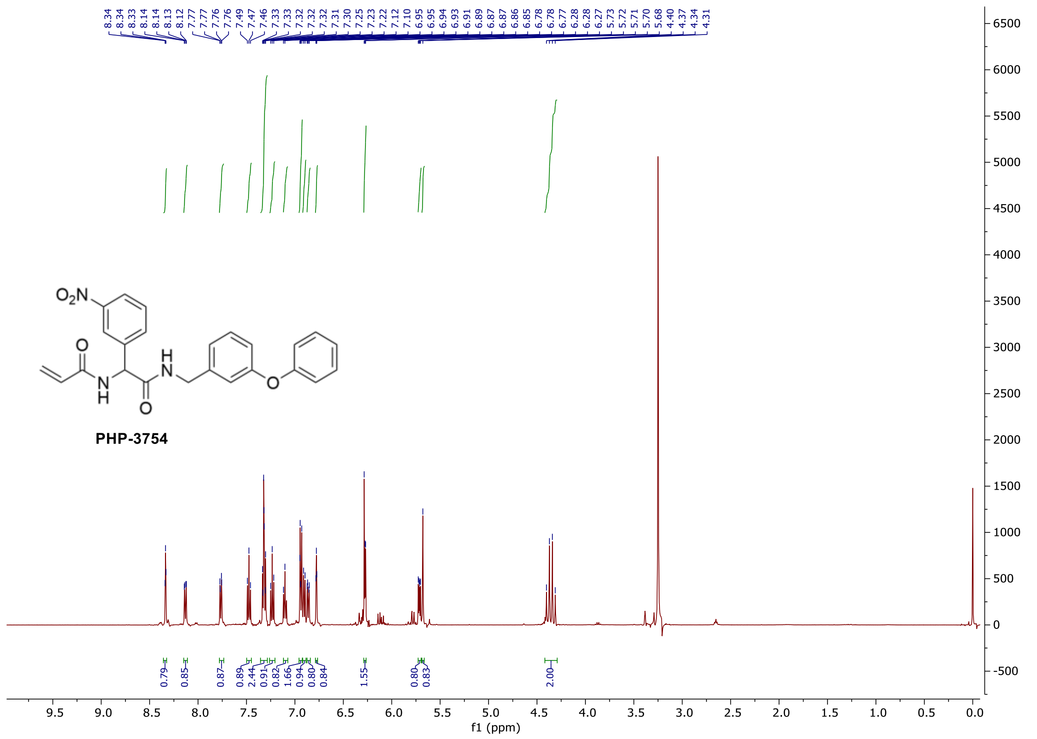

Supplementary Figure 149. ^{1}H NMR (500 MHz, $CDCl_3$ + one drop of CD_3OD) of compound **PHP-3754**.

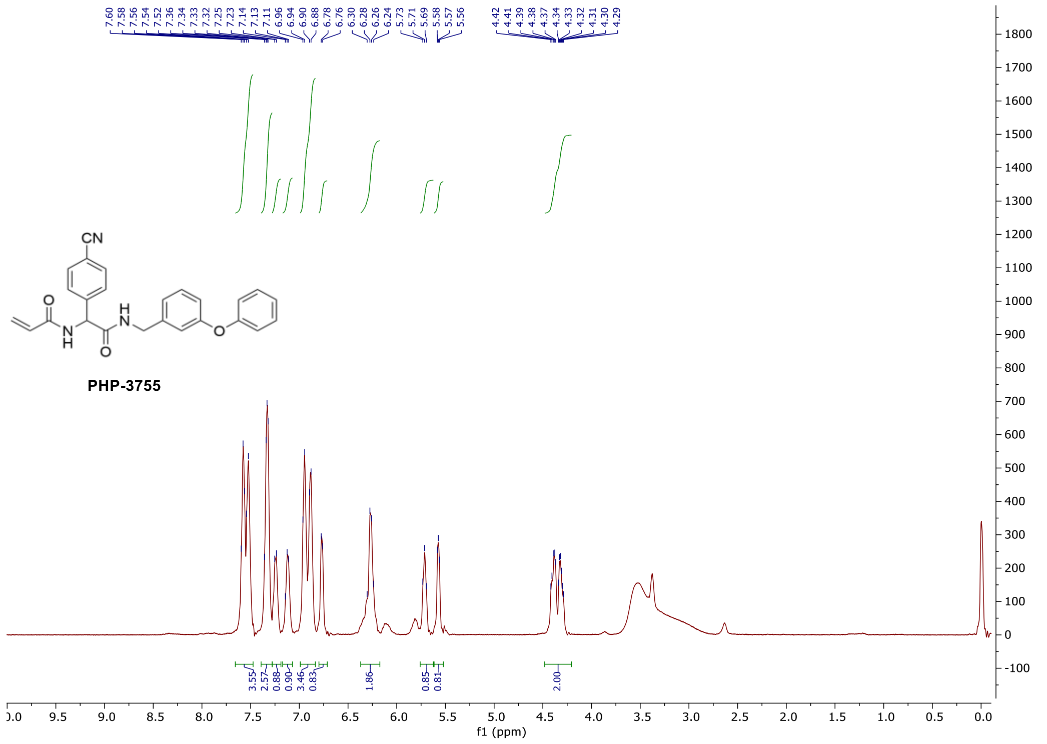

Supplementary Figure 150. ^{1}H NMR (500 MHz, $CDCl_3$ + one drop of CD_3OD) of compound **PHP-3755**.

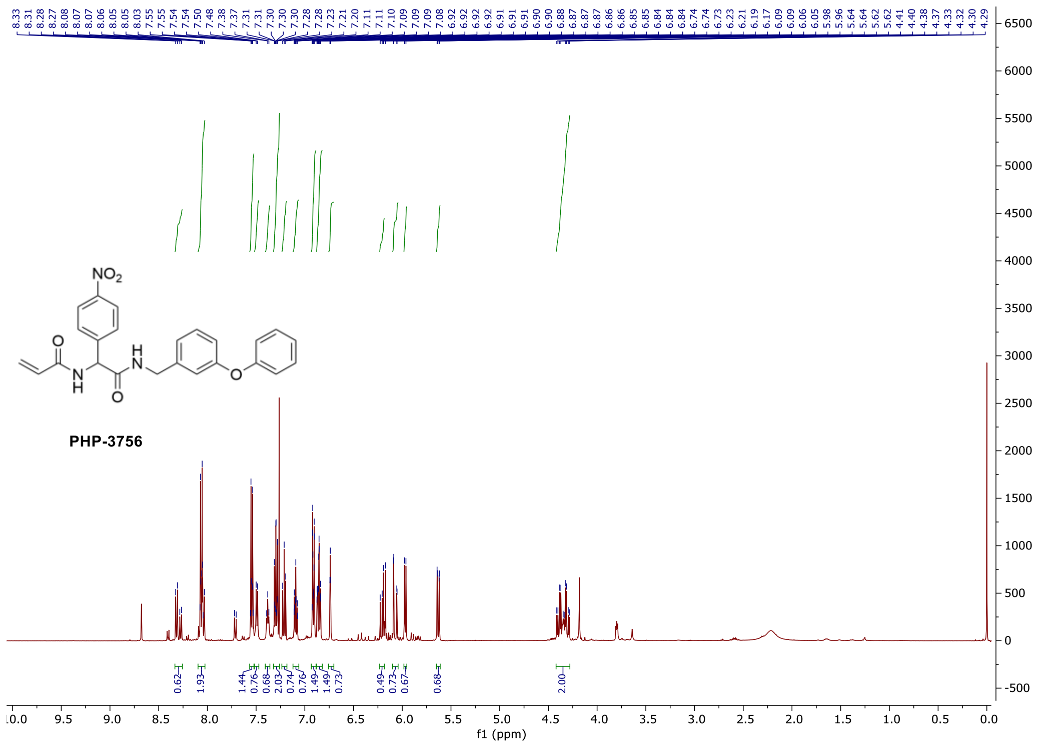

Supplementary Figure 151. ¹H NMR (500 MHz, $CDCl_3$ + one drop of CD_3OD) of compound **PHP-3756**.

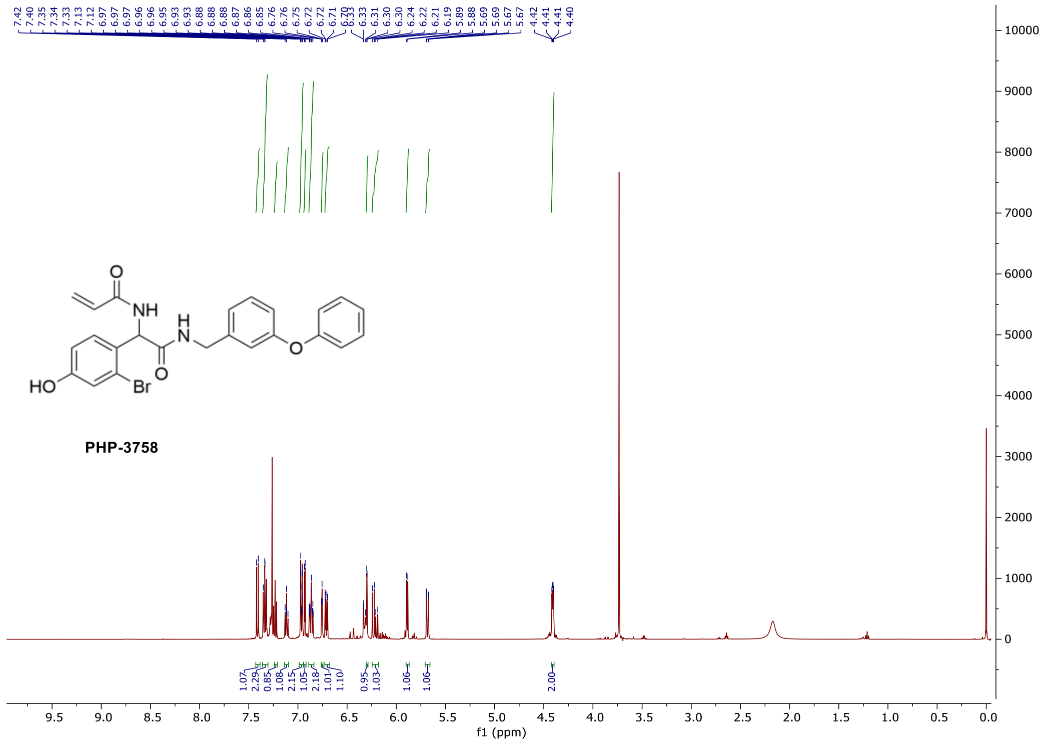

Supplementary Figure 152. ¹H NMR (500 MHz, $CDCl_3$) of compound **PHP-3758**.

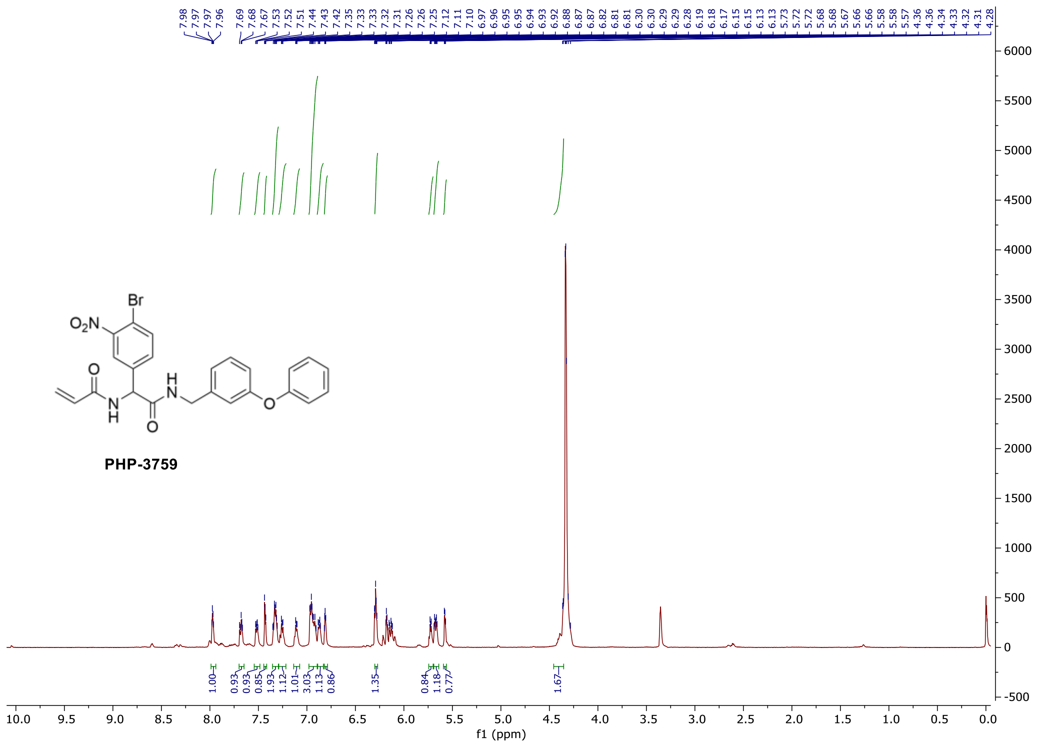

Supplementary Figure 153. ^{1}H NMR (500 MHz, $CDCl_3$ + one drop of CD_3OD) of compound **PHP-3759**.

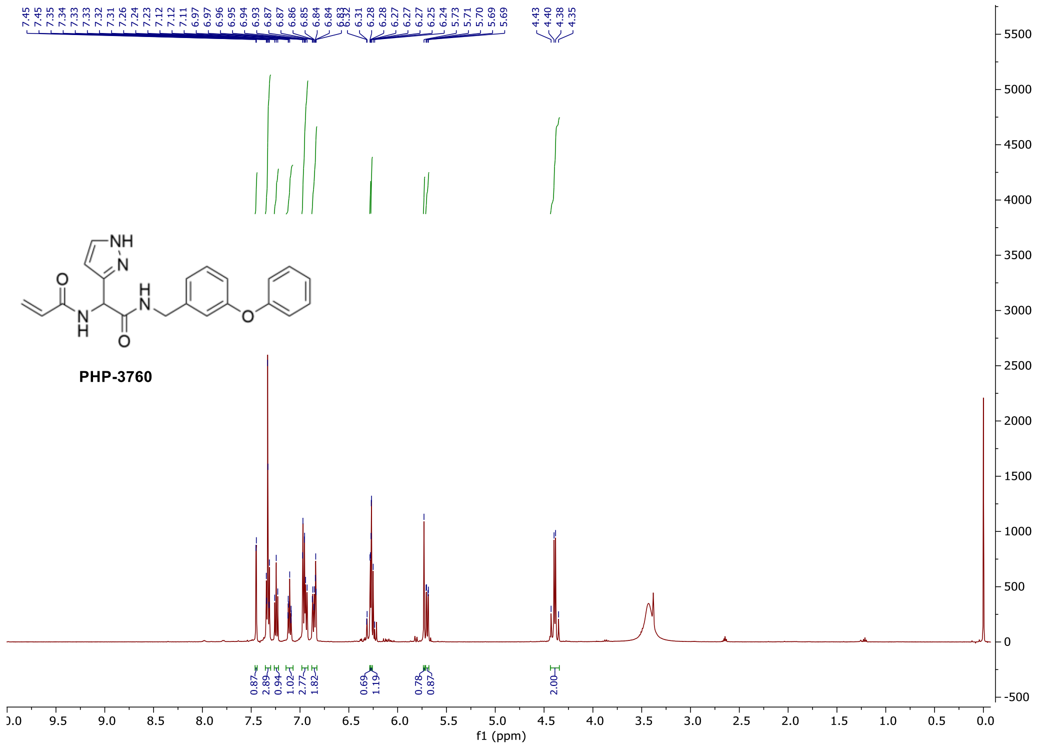

Supplementary Figure 154. ^{1}H NMR (500 MHz, $CDCl_3$ + one drop of CD_3OD) of compound **PHP-3760**.

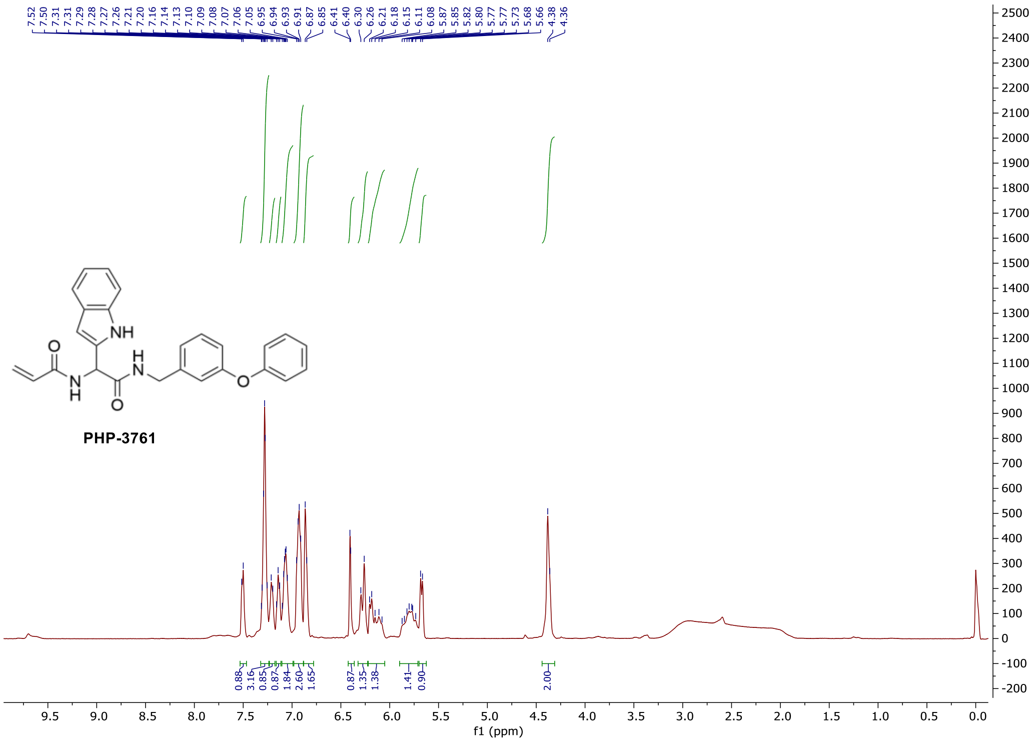

Supplementary Figure 155. ^{1}H NMR (500 MHz, $CDCl_3$ + one drop of CD_3OD) of compound **PHP-3761**.

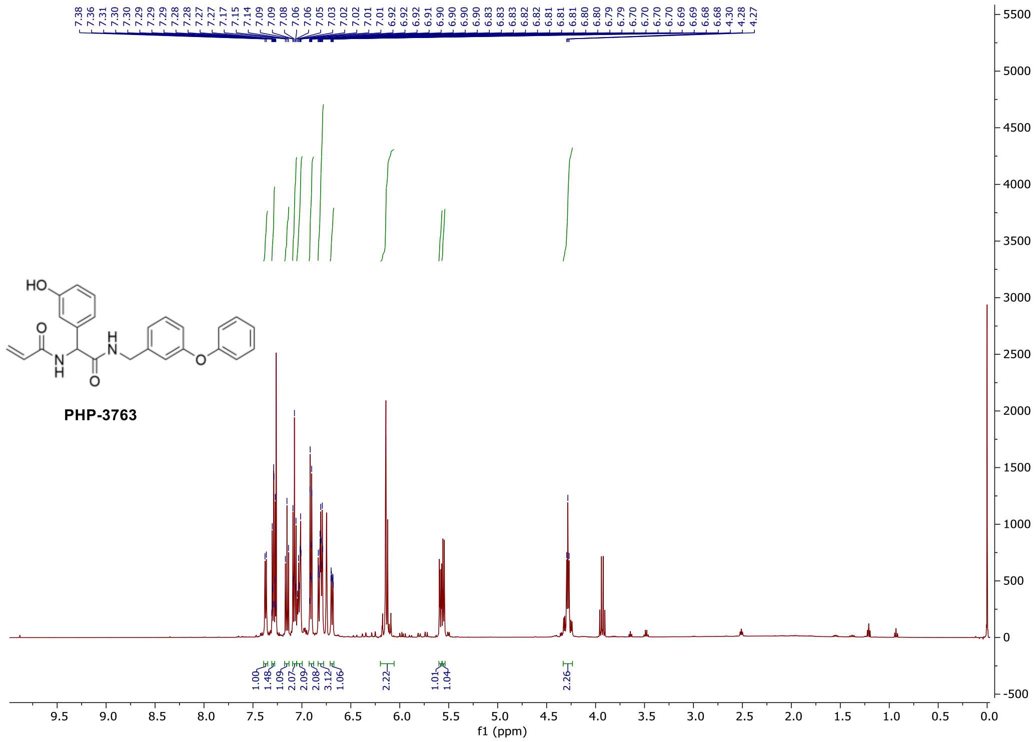

Supplementary Figure 156. ^{1}H NMR (500 MHz, $CDCl_3$) of compound **PHP-3763**.

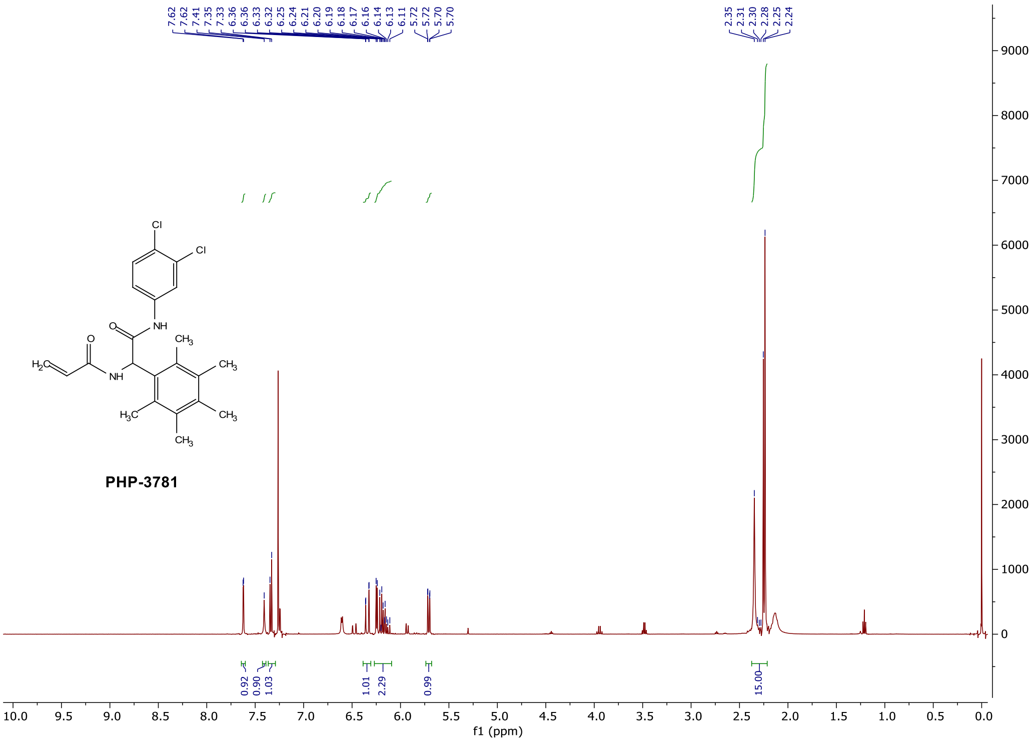

Supplementary Figure 157. ^{1}H NMR (500 MHz, $CDCl_3$) of compound **PHP-3781**.

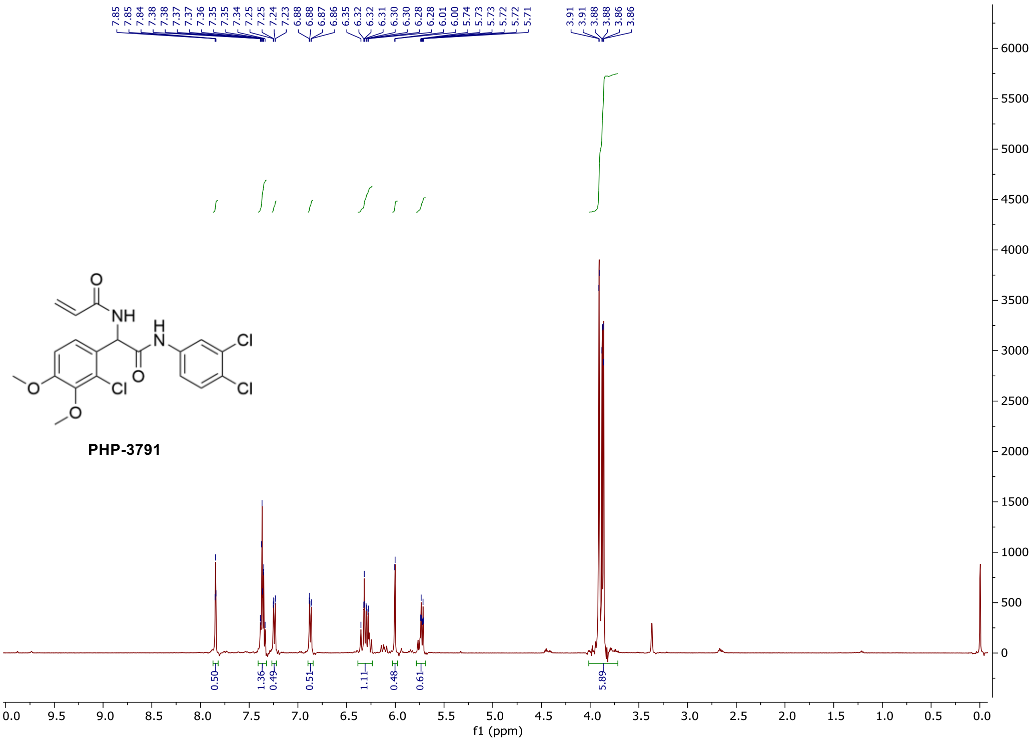

Supplementary Figure 158. ^{1}H NMR (500 MHz, $CDCl_3$ + one drop of CD_3OD) of compound **PHP-3791**.

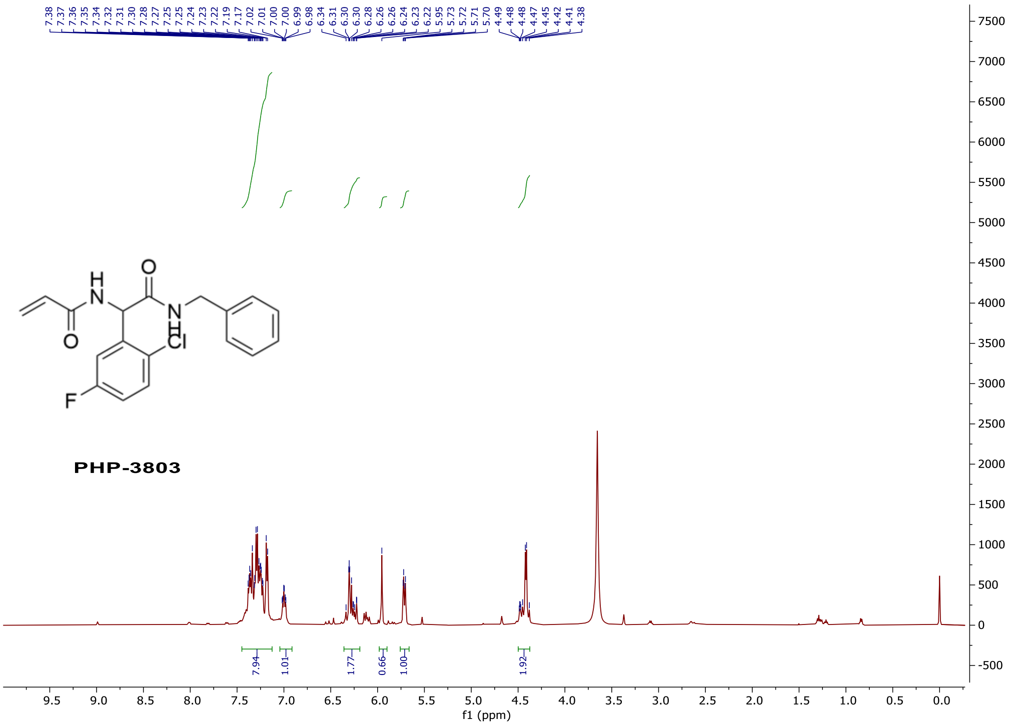

Supplementary Figure 159. ^{1}H NMR (500 MHz, $CDCl_3$+one drop of CD_3OD) of compound **PHP-3803.**

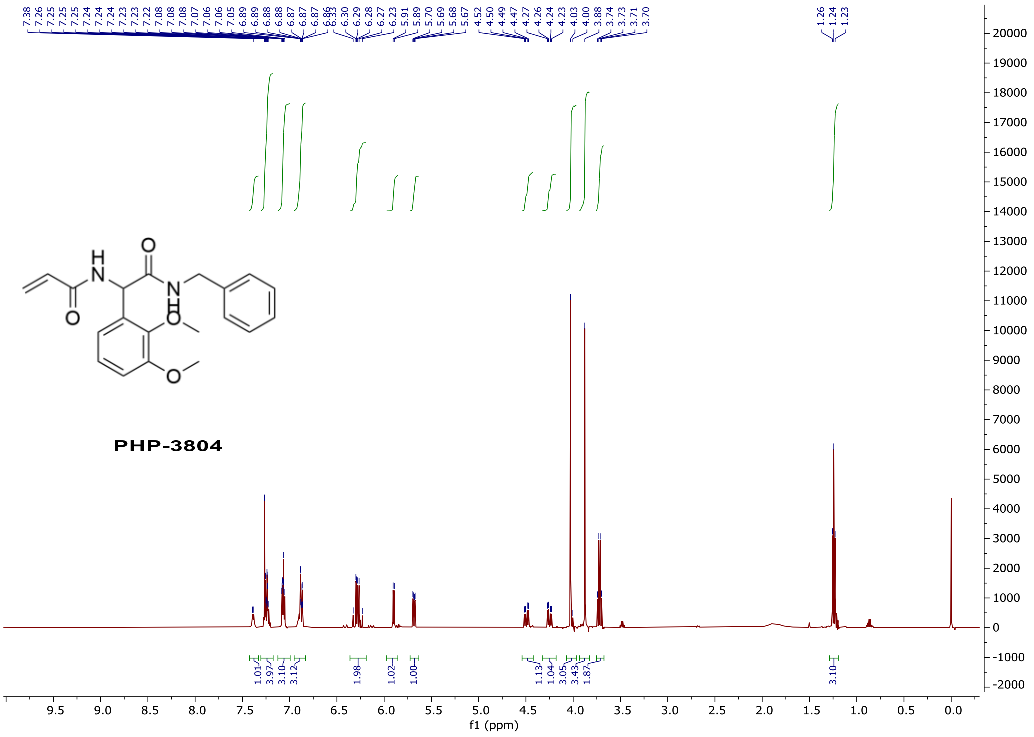

Supplementary Figure 160. ^{1}H NMR (500 MHz, $CDCl_3$) of compound **PHP-3804.**

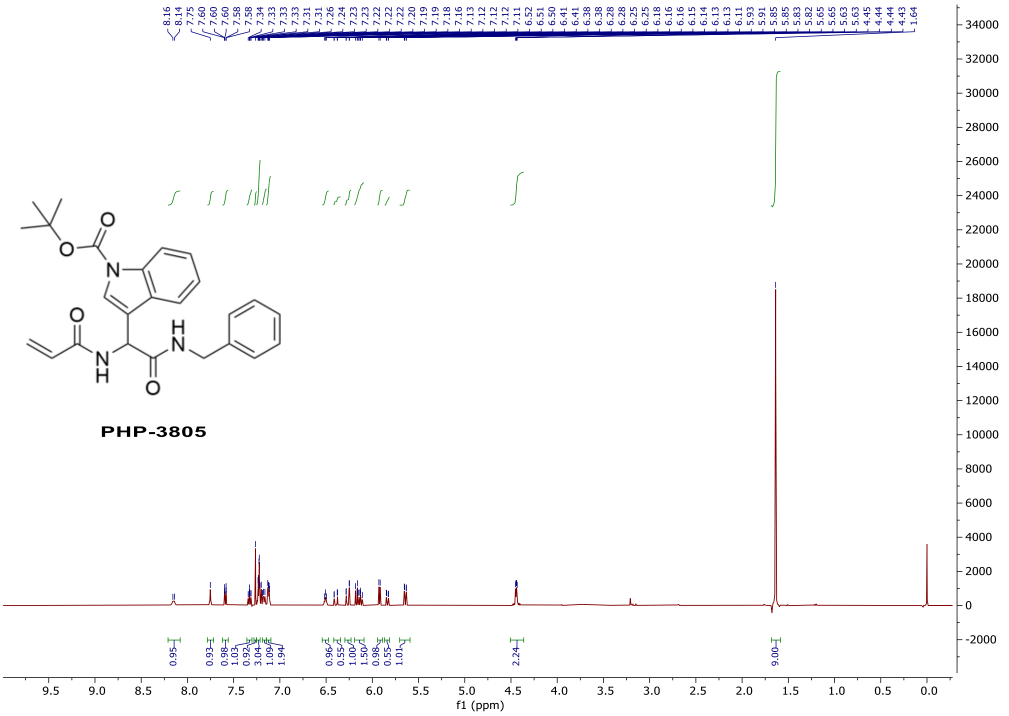

Supplementary Figure 161. ^{1}H NMR (500 MHz, $CDCl_3$+one drop of CD_3OD) of compound **PHP-3805.**

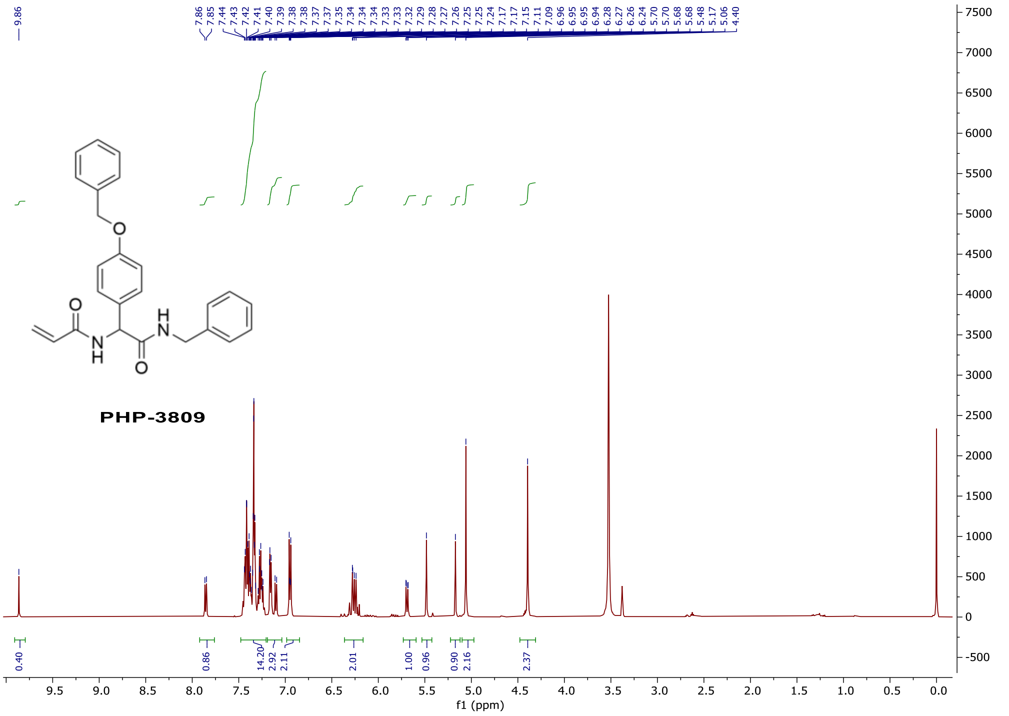

Supplementary Figure 162. ^{1}H NMR (500 MHz, $CDCl_3$+one drop of CD_3OD) of compound **PHP-3809.**

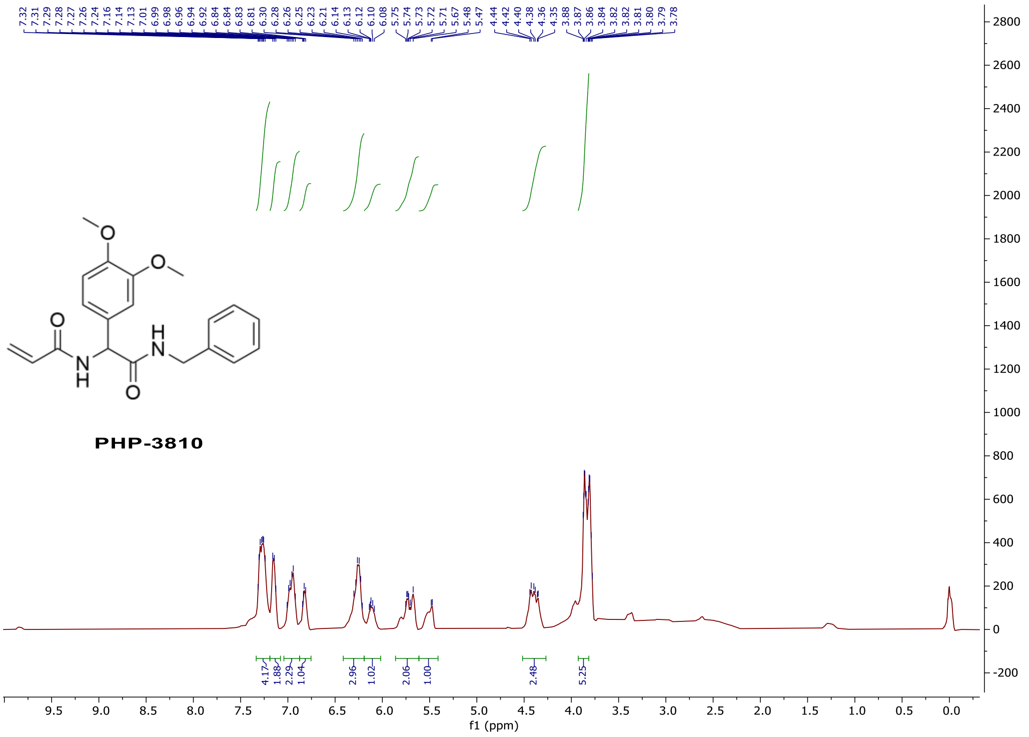

Supplementary Figure 163. ^{1}H NMR (500 MHz, $CDCl_3$) of compound **PHP-3810.**

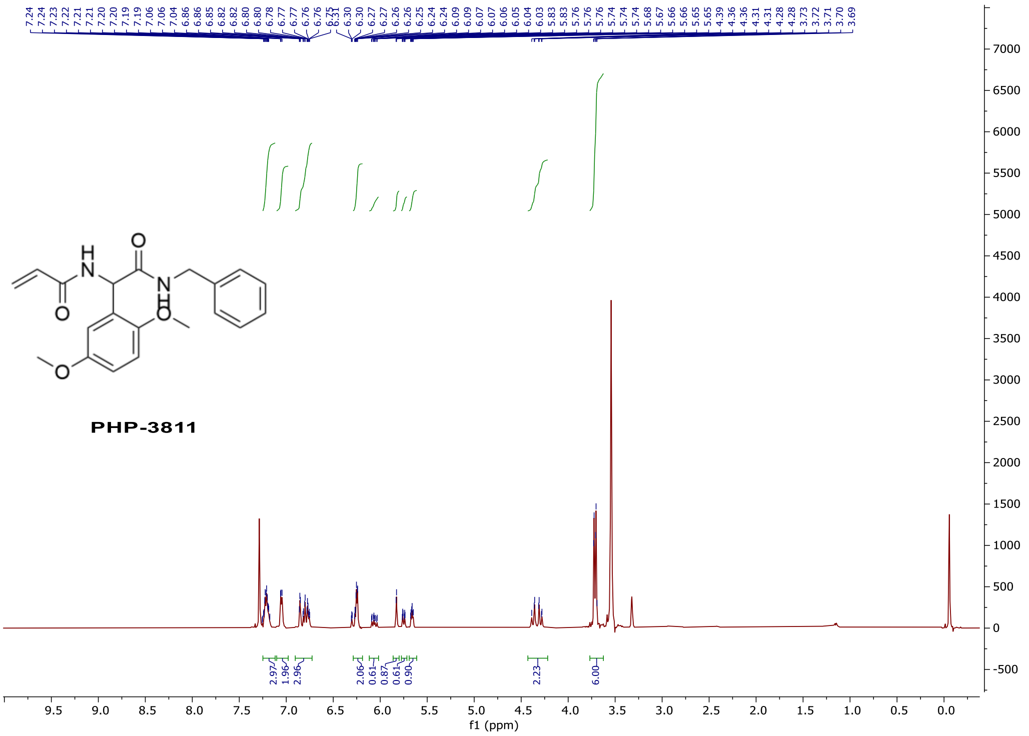

Supplementary Figure 164. ^{1}H NMR (500 MHz, $CDCl_3$+one drop of CD_3OD) of compound **PHP-3811.**

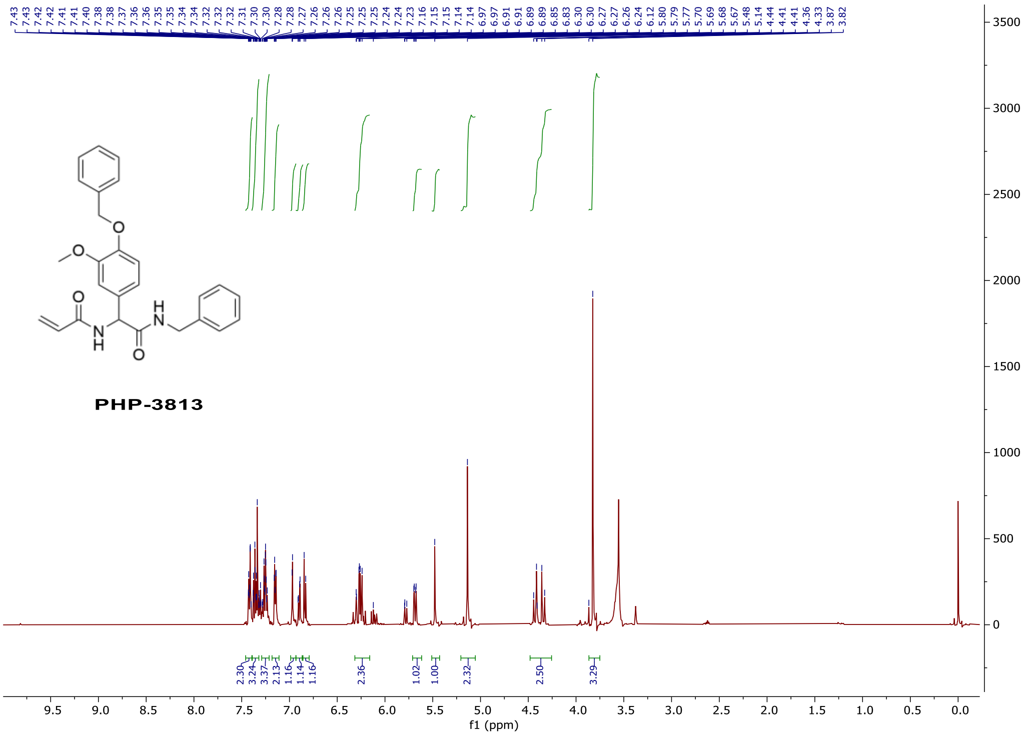

Supplementary Figure 165. ^{1}H NMR (500 MHz, $CDCl_3$+one drop of CD_3OD) of compound **PHP-3813.**

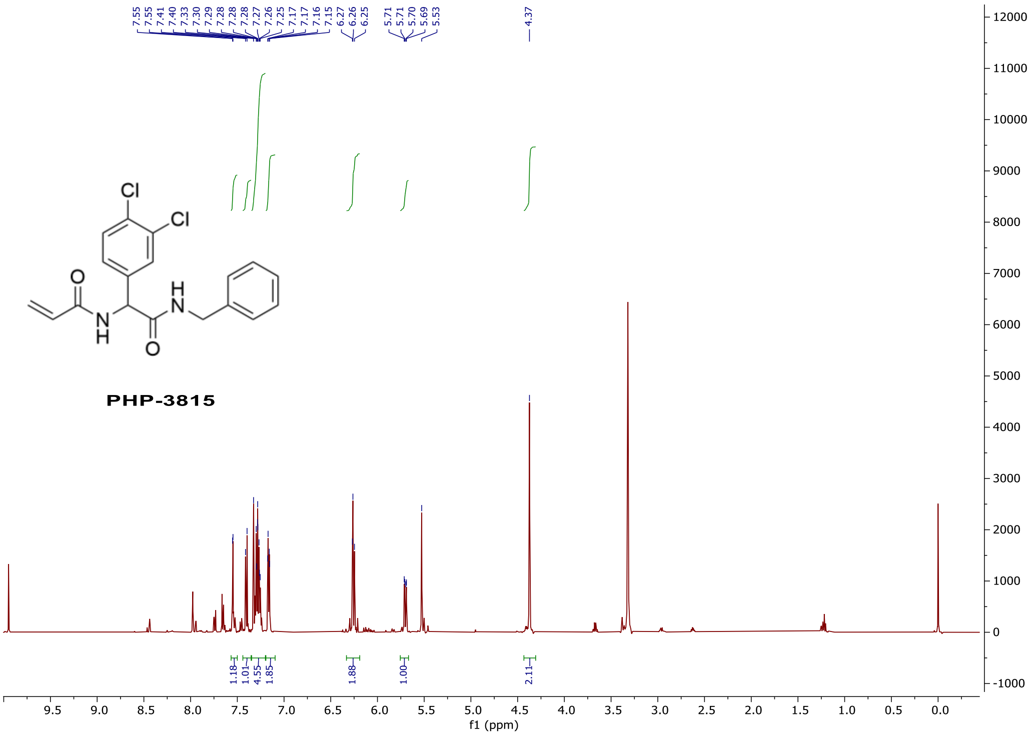

Supplementary Figure 166. ^{1}H NMR (500 MHz, $CDCl_3$+one drop of CD_3OD) of compound **PHP-3815.**

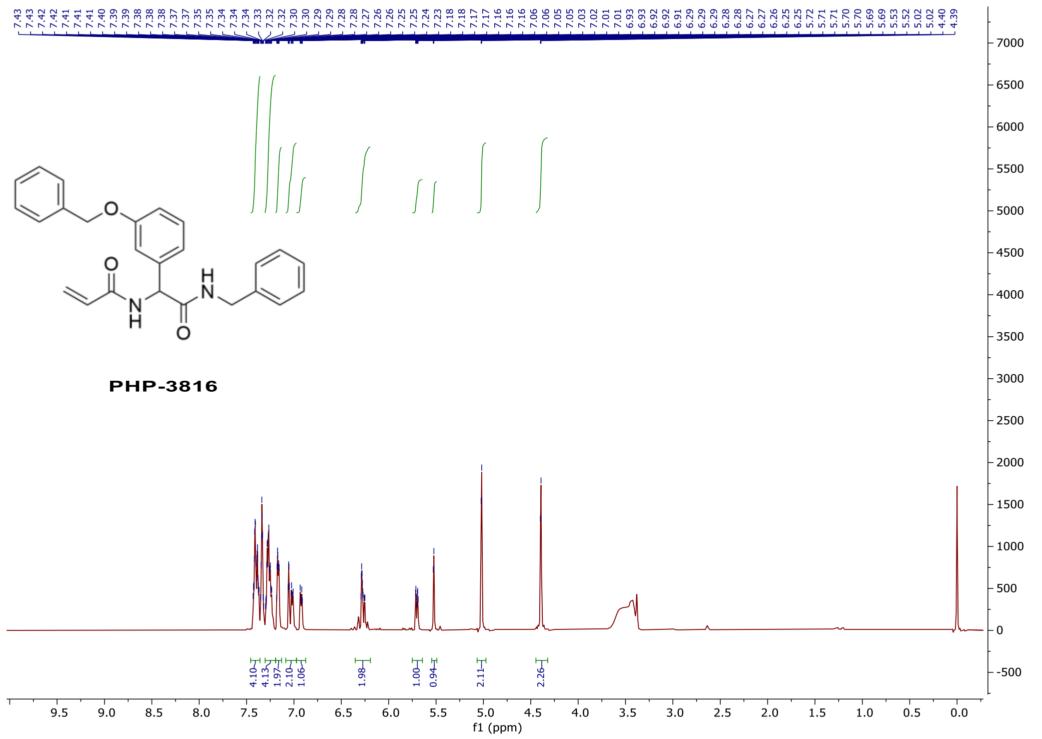

Supplementary Figure 167. ^{1}H NMR (500 MHz, $CDCl_3$+one drop of CD_3OD) of compound **PHP-3816.**

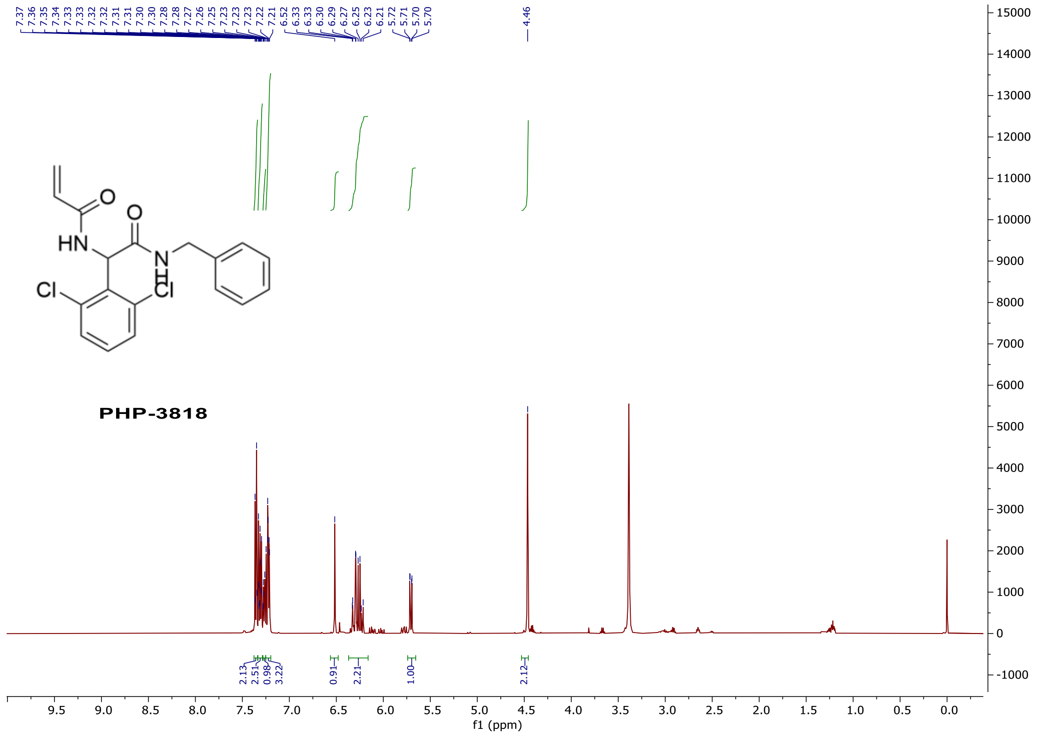

Supplementary Figure 168. ^{1}H NMR (500 MHz, $CDCl_3$+one drop of CD_3OD) of compound **PHP-3818.**

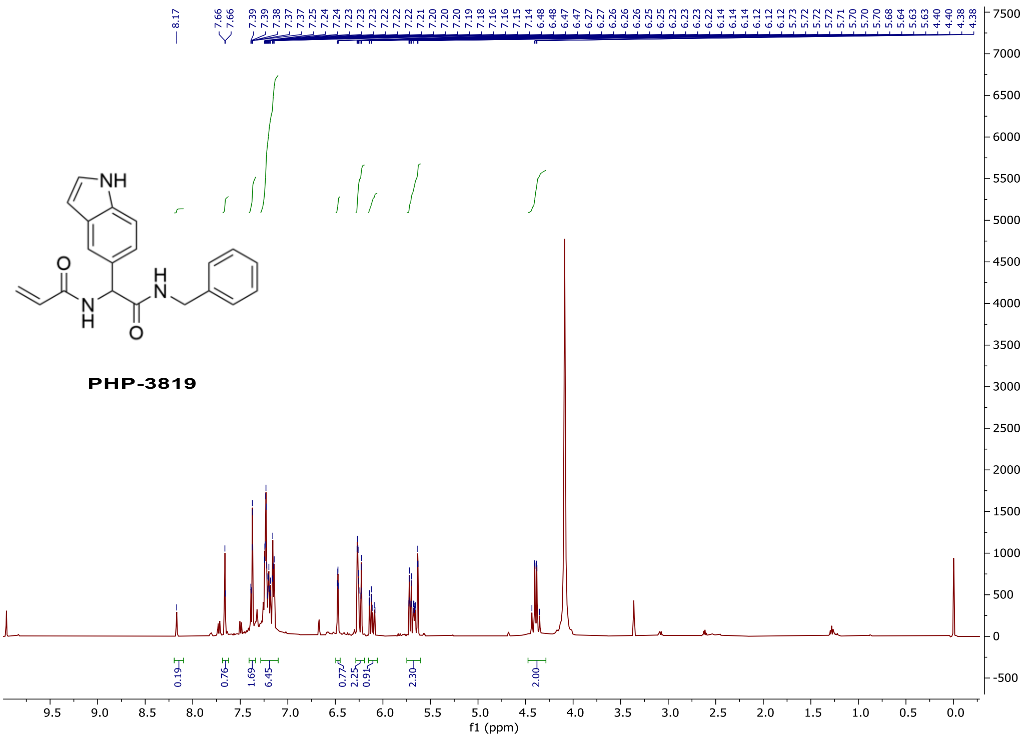

Supplementary Figure 169. ^{1}H NMR (500 MHz, $CDCl_3$+one drop of CD_3OD) of compound **PHP-3819.**

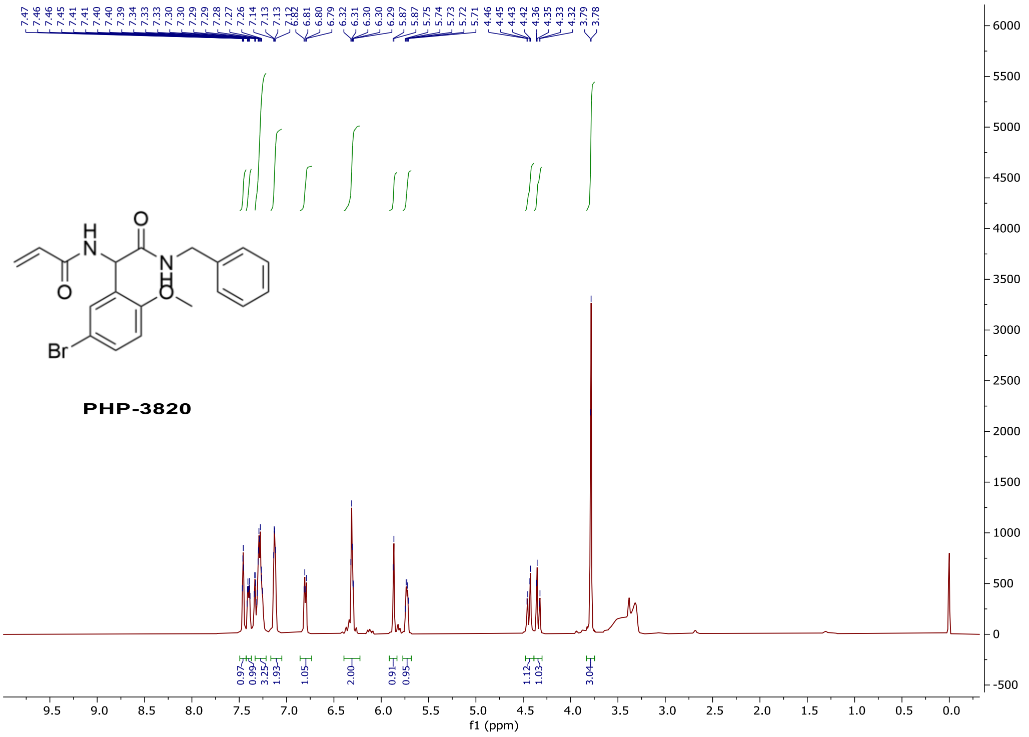

Supplementary Figure 170. ^{1}H NMR (500 MHz, $CDCl_3$+one drop of CD_3OD) of compound **PHP-3820.**

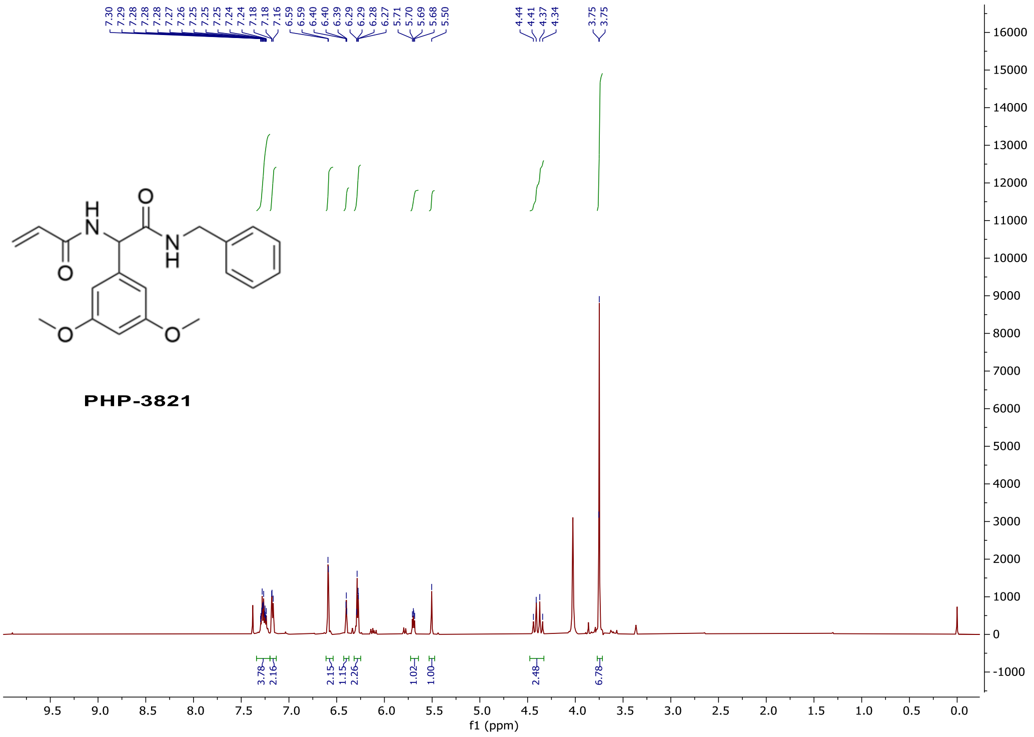

Supplementary Figure 171. ^{1}H NMR (500 MHz, $CDCl_3$) of compound **PHP-3821.**

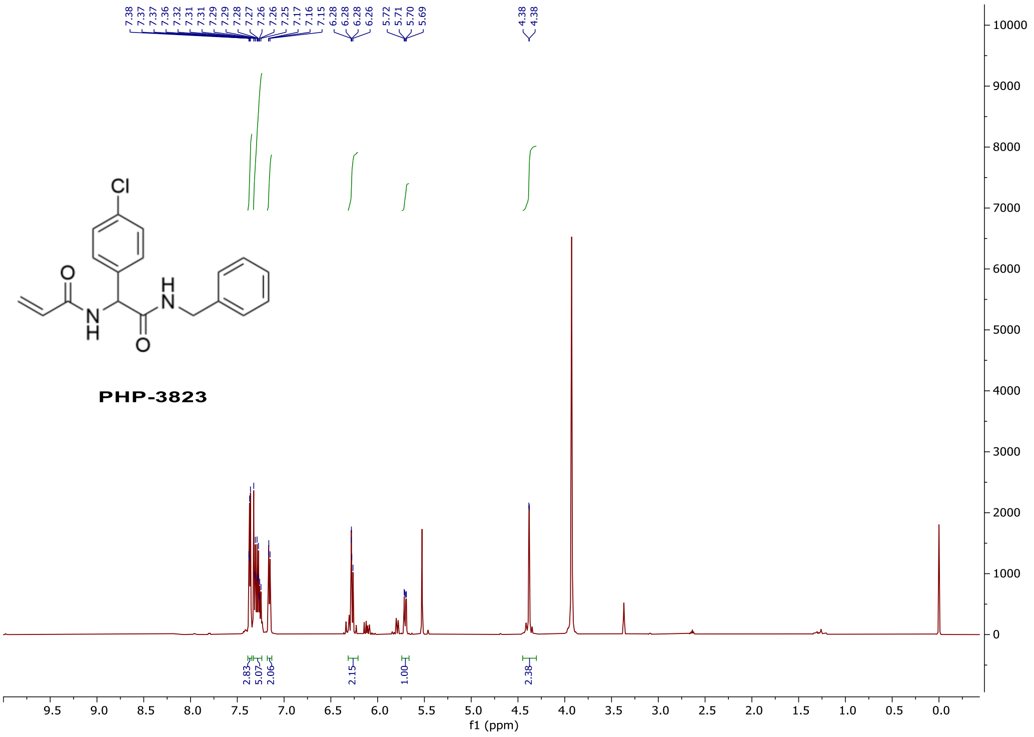

Supplementary Figure 172. ^{1}H NMR (500 MHz, $CDCl_3$+one drop of CD_3OD) of compound **PHP-3823.**

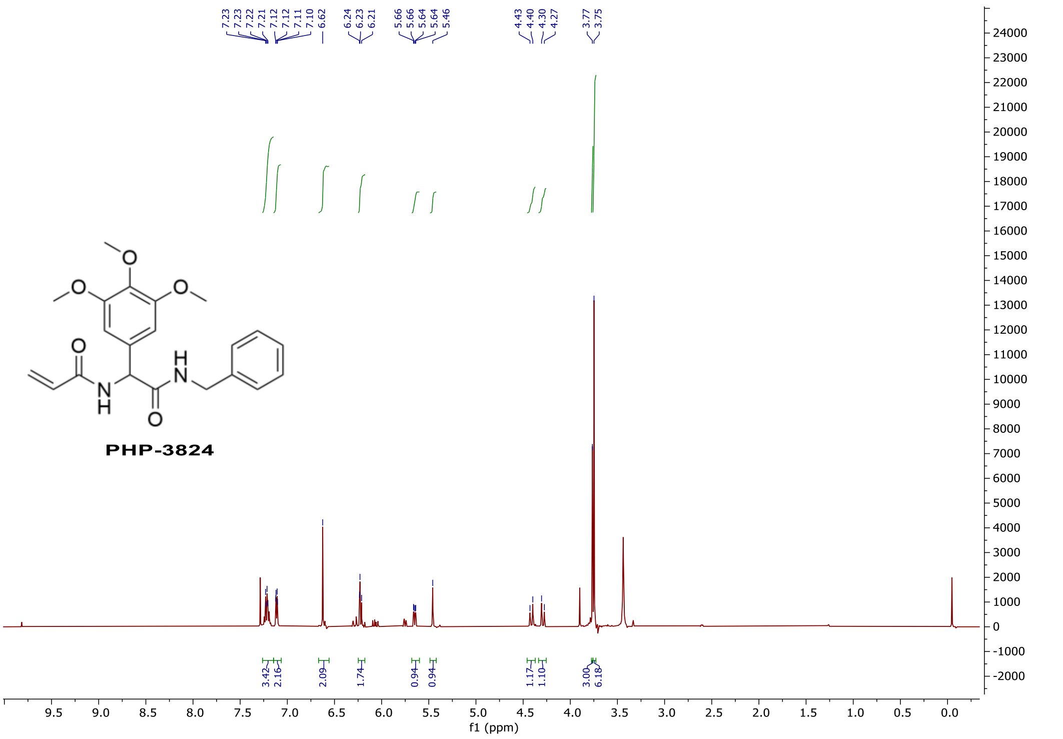

Supplementary Figure 173. ^{1}H NMR (500 MHz, $CDCl_3$+one drop of CD_3OD) of compound **PHP-3824.**

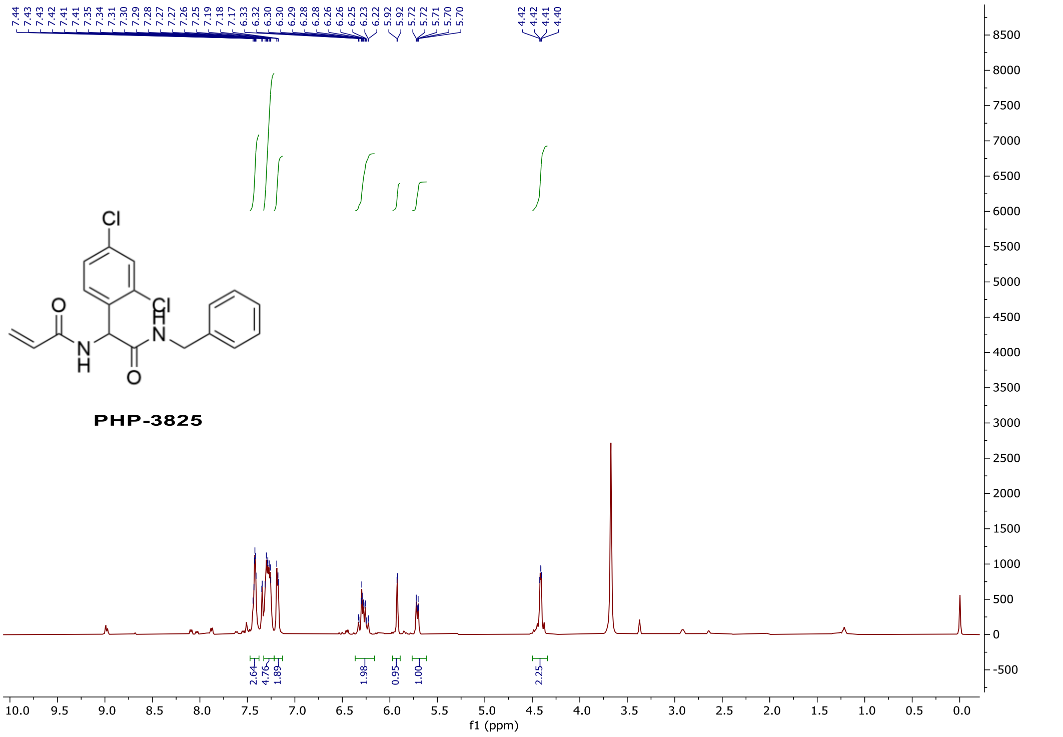

Supplementary Figure 174. ^{1}H NMR (500 MHz, $CDCl_3$+one drop of CD_3OD) of compound **PHP-3825.**

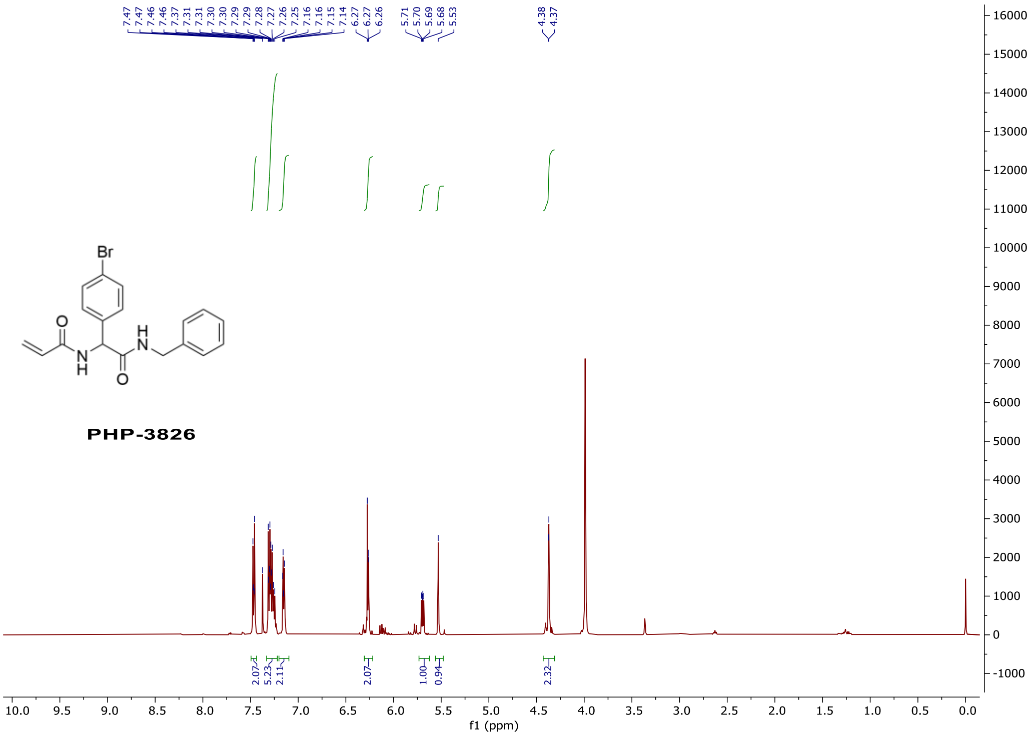

Supplementary Figure 175. ^{1}H NMR (500 MHz, $CDCl_3$+one drop of CD_3OD) of compound **PHP-3826.**

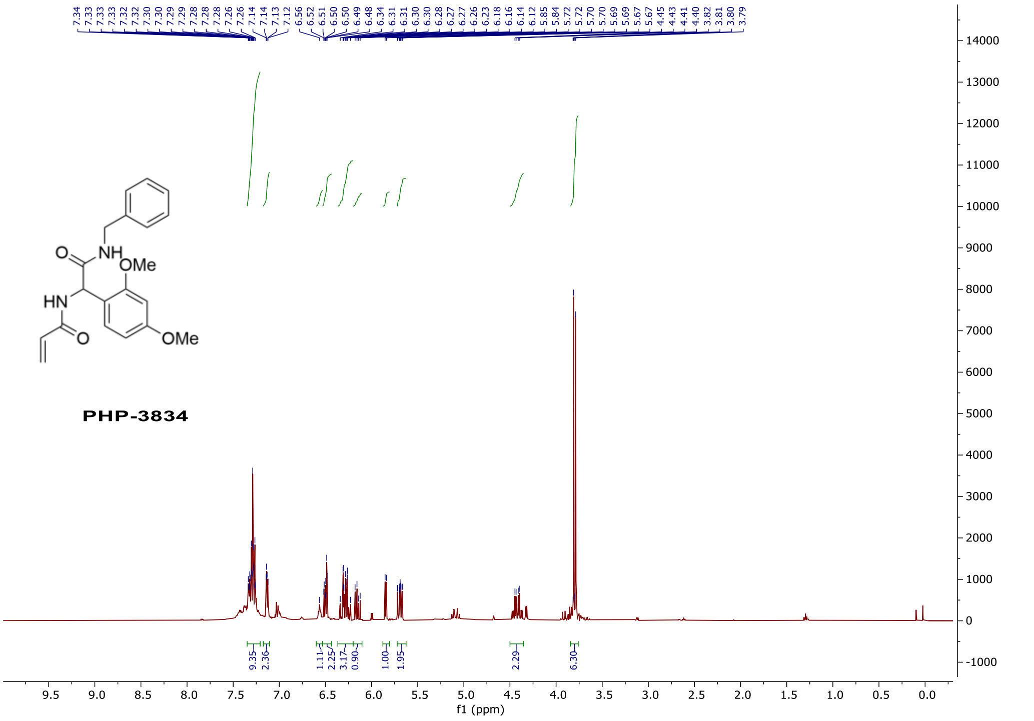

Supplementary Figure 176. ^{1}H NMR (500 MHz, $CDCl_3$) of compound **PHP-3834.**

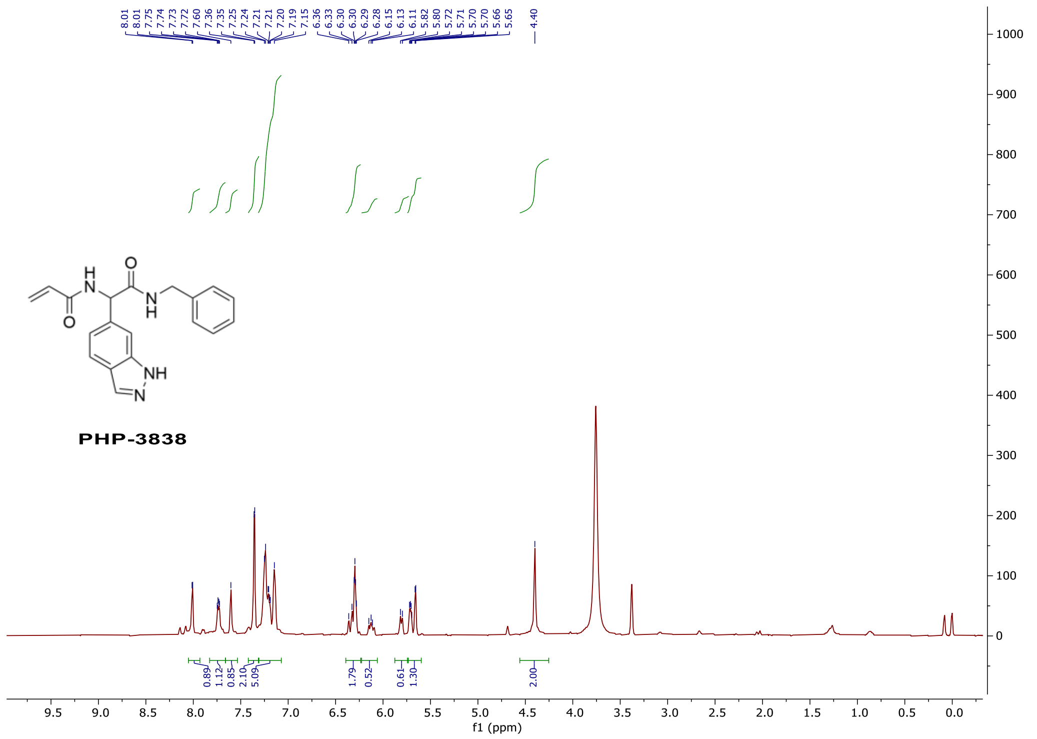

Supplementary Figure 177. ^{1}H NMR (500 MHz, $CDCl_3$+one drop of CD_3OD) of compound **PHP-3838.**

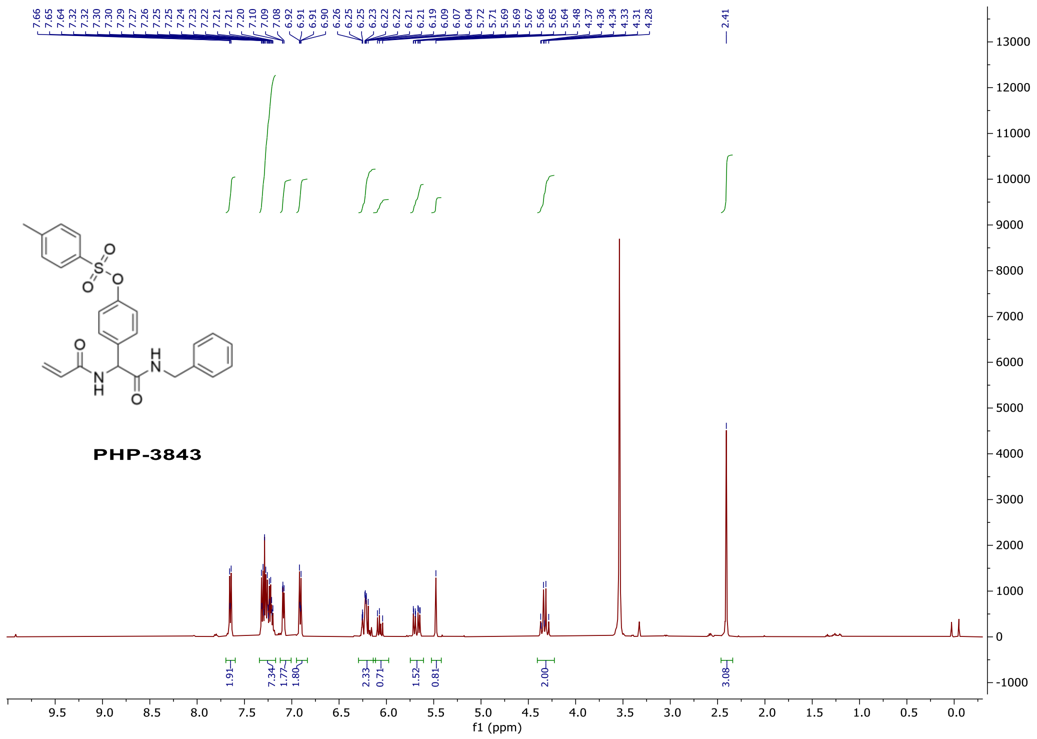

Supplementary Figure 178. ^{1}H NMR (500 MHz, $CDCl_3$+one drop of CD_3OD) of compound **PHP-3843.**

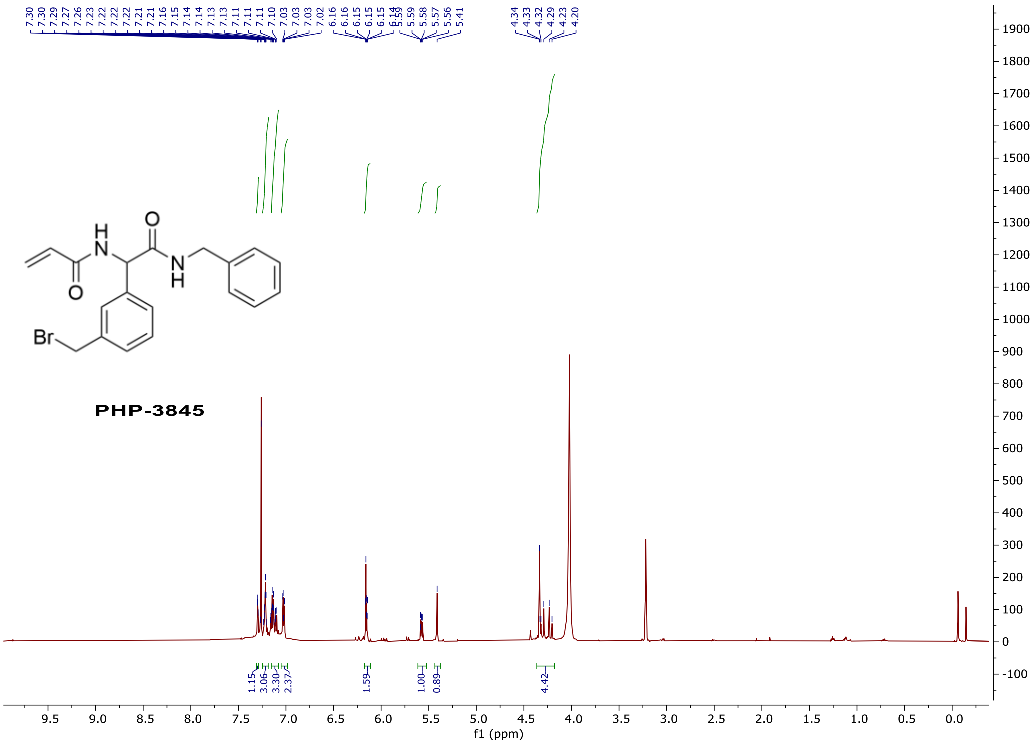

Supplementary Figure 179. ^{1}H NMR (500 MHz, $CDCl_3$+one drop of CD_3OD) of compound **PHP-3845.**

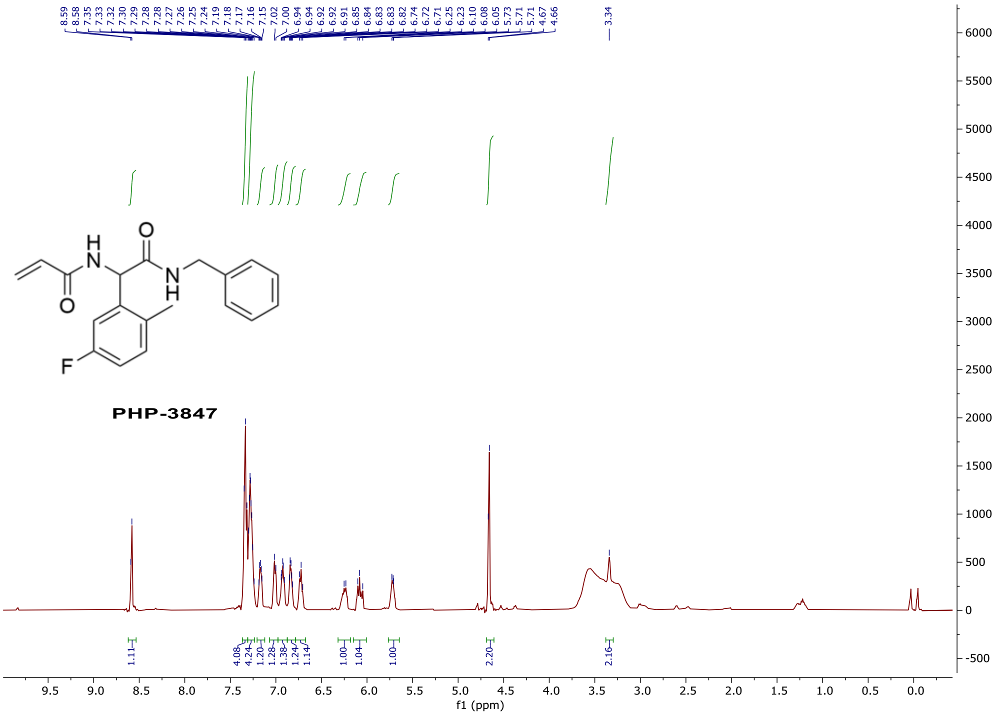

Supplementary Figure 180. ^{1}H NMR (500 MHz, $CDCl_3$+one drop of CD_3OD) of compound **PHP-3847.**

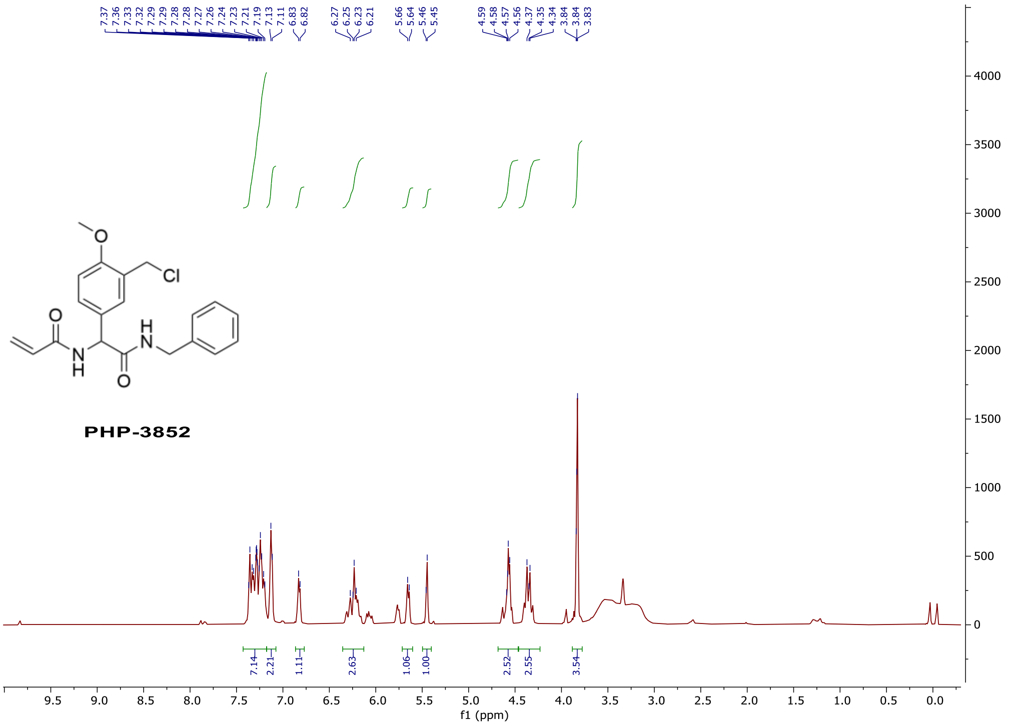

Supplementary Figure 181. ^{1}H NMR (500 MHz, $CDCl_3$+one drop of CD_3OD) of compound **PHP-3852.**

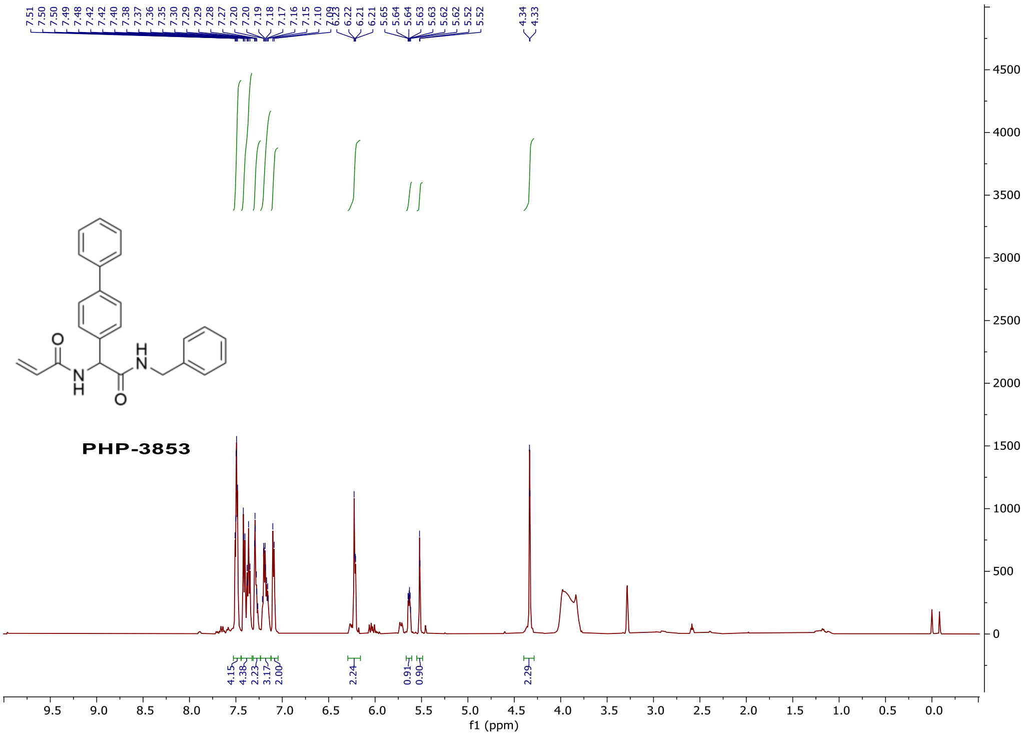

Supplementary Figure 182. ^{1}H NMR (500 MHz, $CDCl_3$+one drop of CD_3OD) of compound **PHP-3853.**

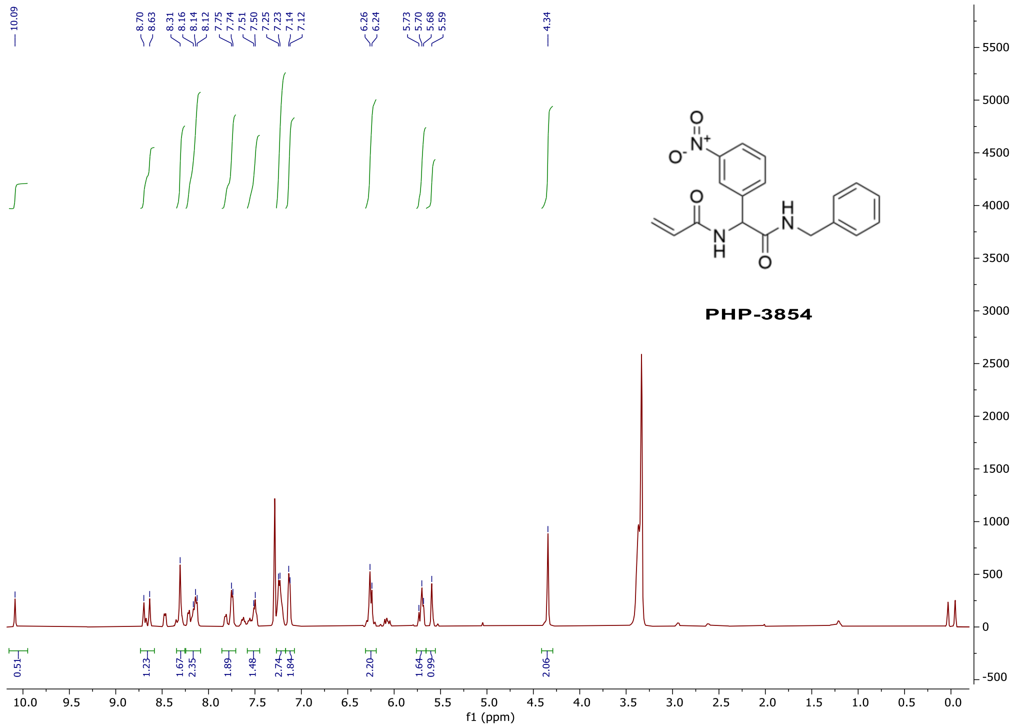

Supplementary Figure 183. ^{1}H NMR (500 MHz, $CDCl_3$+one drop of CD_3OD) of compound **PHP-3854.**

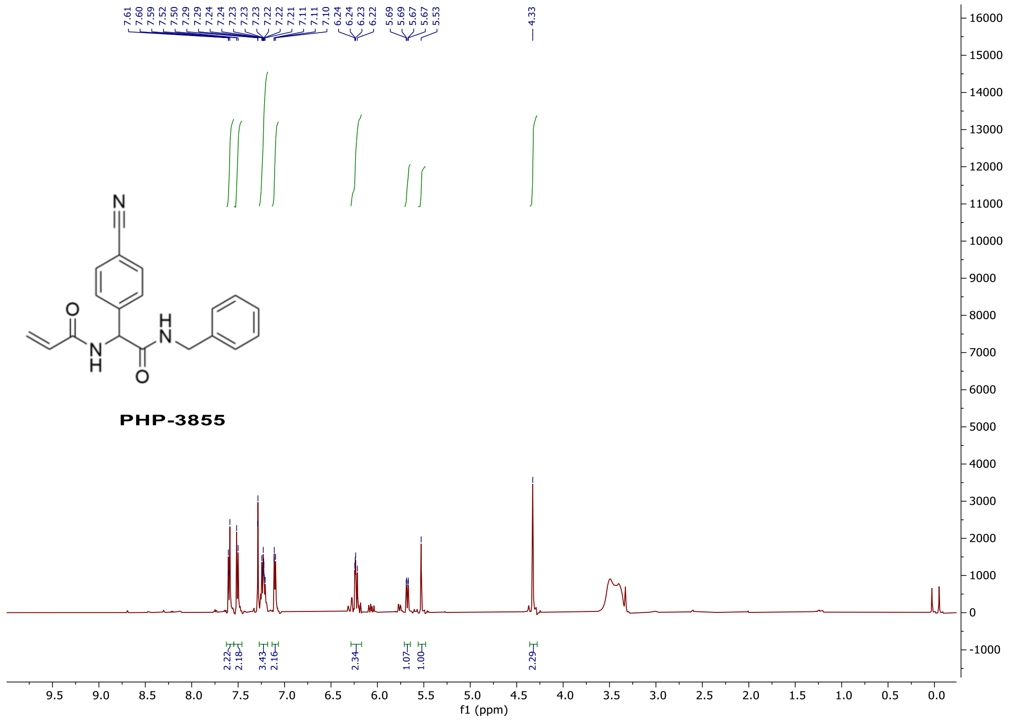

Supplementary Figure 184. ^{1}H NMR (500 MHz, $CDCl_3$+one drop of CD_3OD) of compound **PHP-3855.**

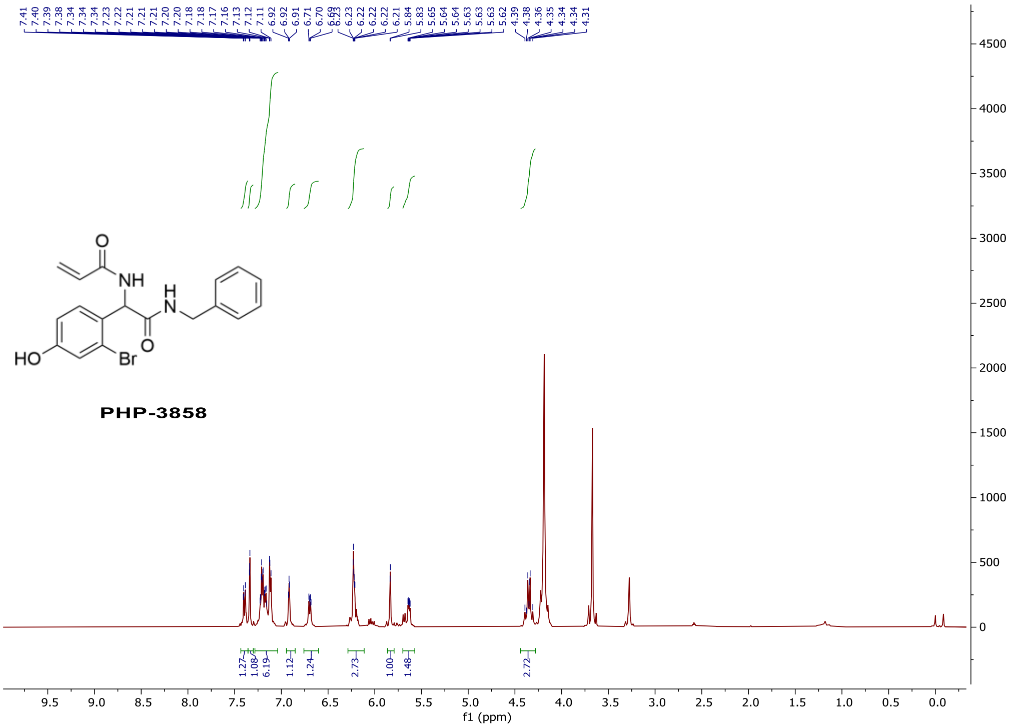

Supplementary Figure 185. ^{1}H NMR (500 MHz, $CDCl_3$+one drop of CD_3OD) of compound **PHP-3858.**

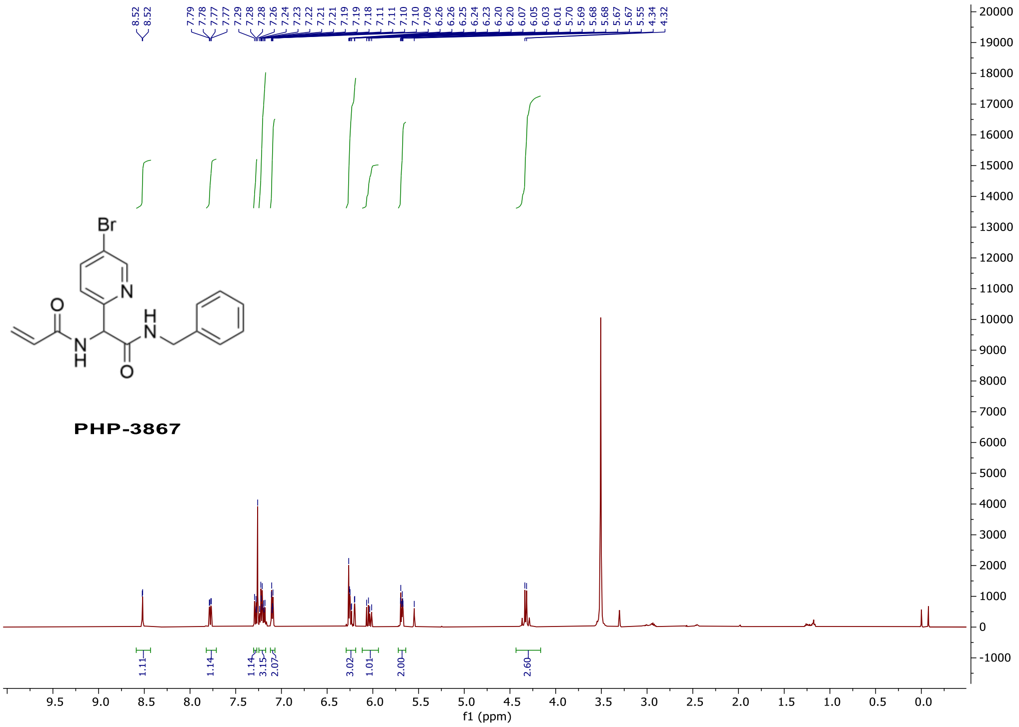

Supplementary Figure 186. ^{1}H NMR (500 MHz, $CDCl_3$+one drop of CD_3OD) of compound **PHP-3867.**

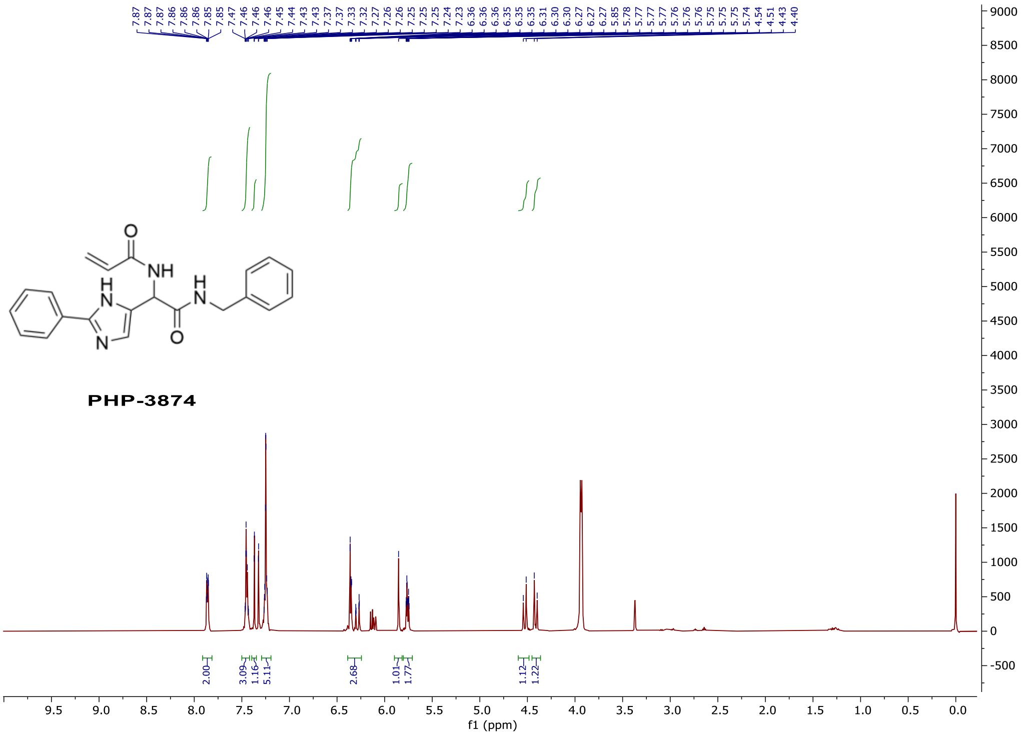

Supplementary Figure 187. ^{1}H NMR (500 MHz, $CDCl_3$+one drop of CD_3OD) of compound **PHP-3874.**

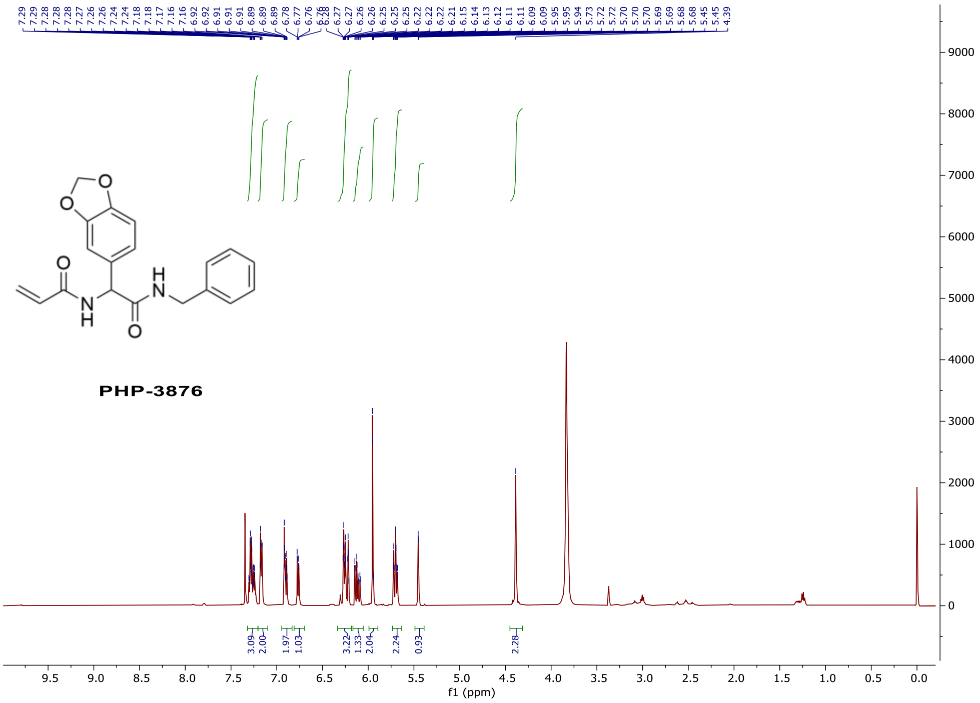

Supplementary Figure 188. ^{1}H NMR (500 MHz, $CDCl_3$+one drop of CD_3OD) of compound **PHP-3876.**

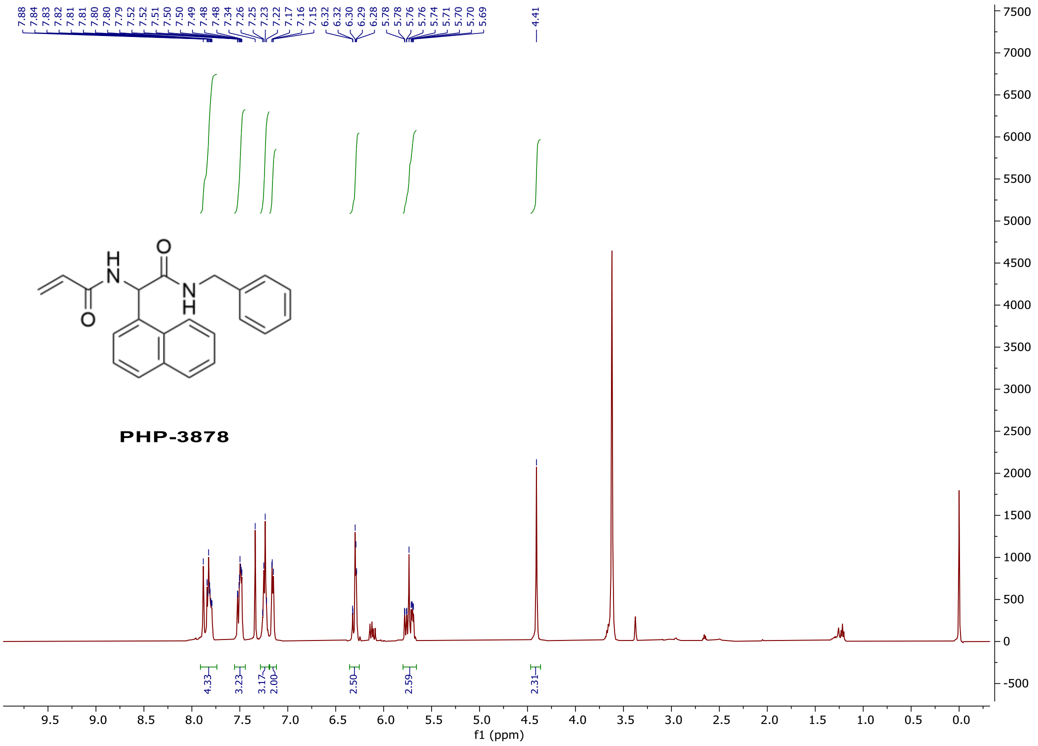

Supplementary Figure 189. ^{1}H NMR (500 MHz, $CDCl_3$+one drop of CD_3OD) of compound **PHP-3878.**

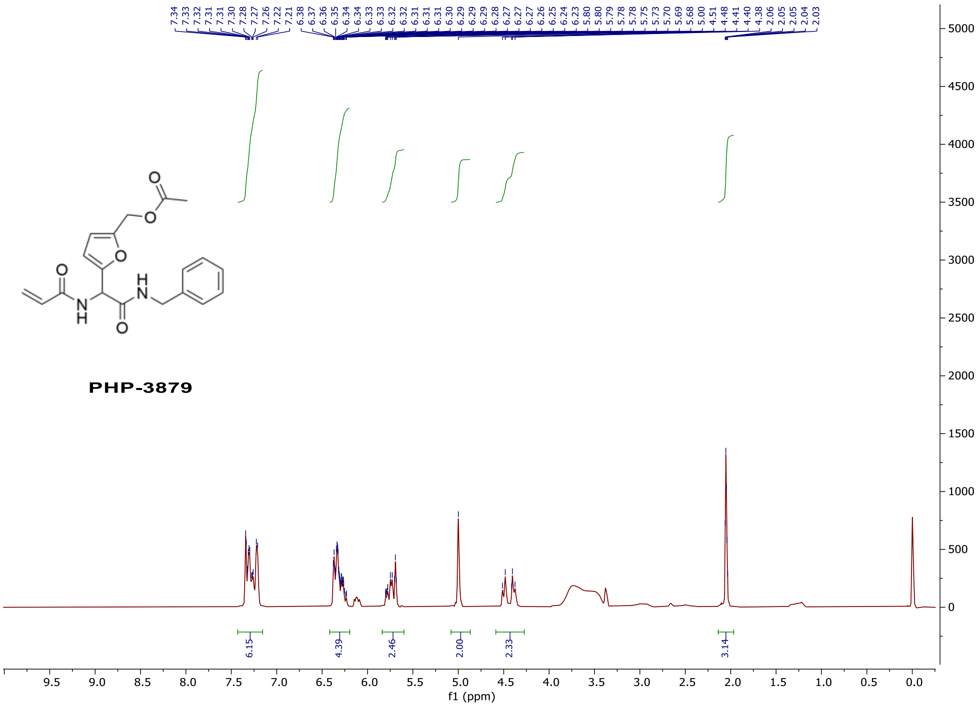

Supplementary Figure 190. ^{1}H NMR (500 MHz, $CDCl_3$+one drop of CD_3OD) of compound **PHP-3879.**

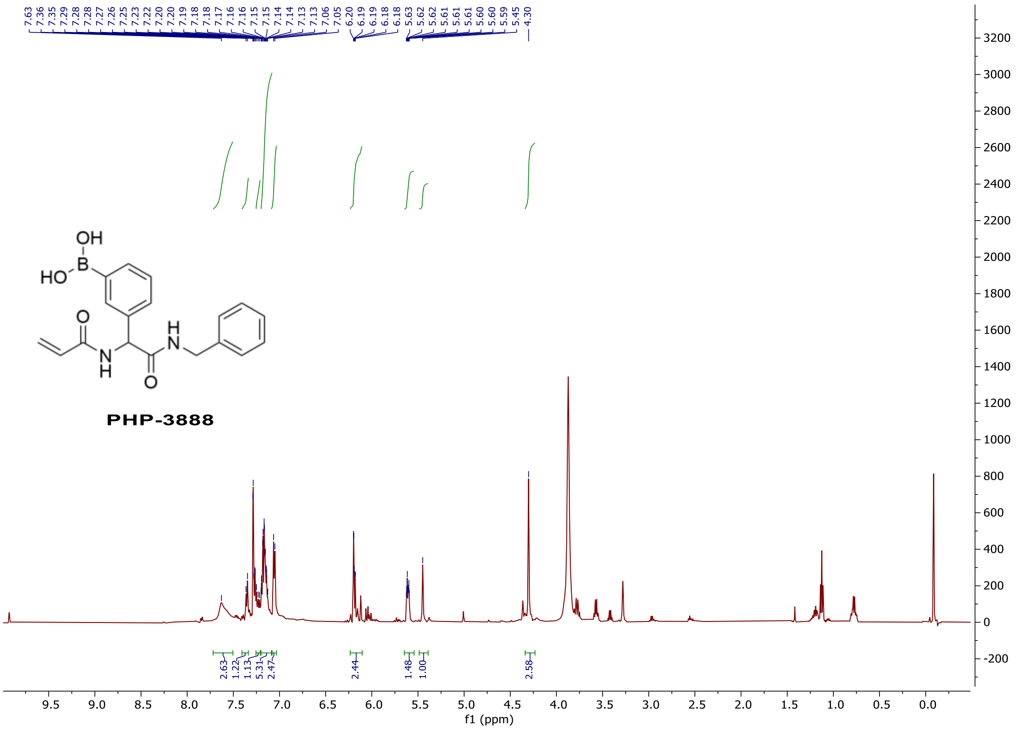

Supplementary Figure 191. ^{1}H NMR (500 MHz, $CDCl_3$+one drop of CD_3OD) of compound **PHP-3888.**

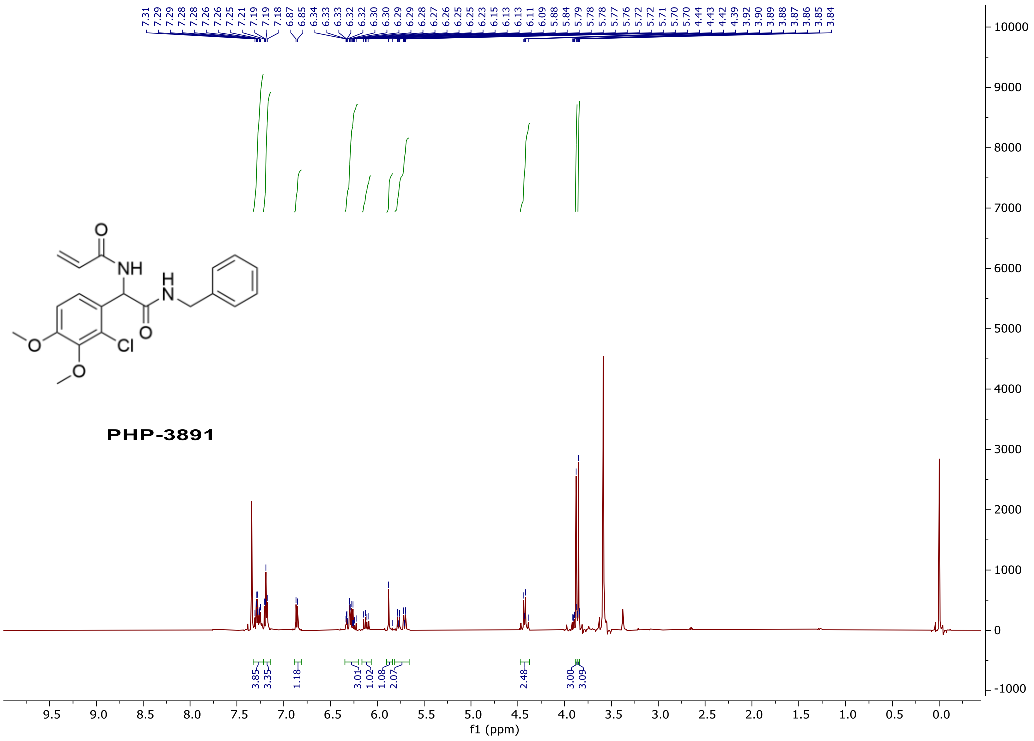

Supplementary Figure 192. ^{1}H NMR (500 MHz, $CDCl_3$+one drop of CD_3OD) of compound **PHP-3891.**

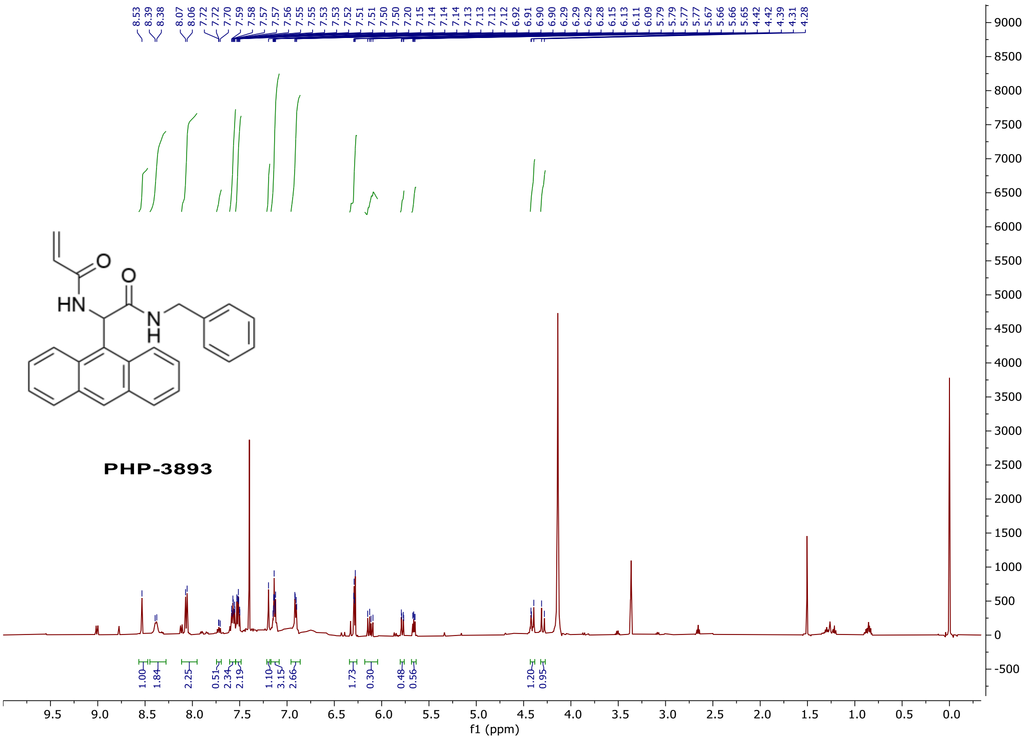

Supplementary Figure 193. ^{1}H NMR (500 MHz, $CDCl_3$+one drop of CD_3OD) of compound **PHP-3893.**

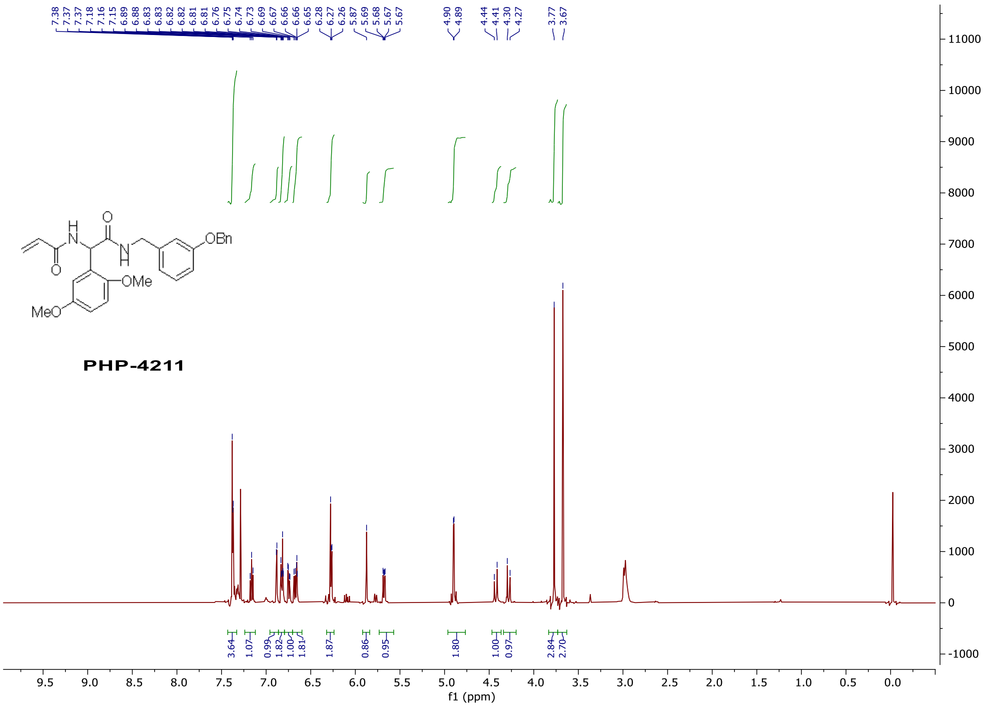

Supplementary Figure 194. ^{1}H NMR (500 MHz, $CDCl_3$+one drop of CD_3OD) of compound **PHP-4211.**

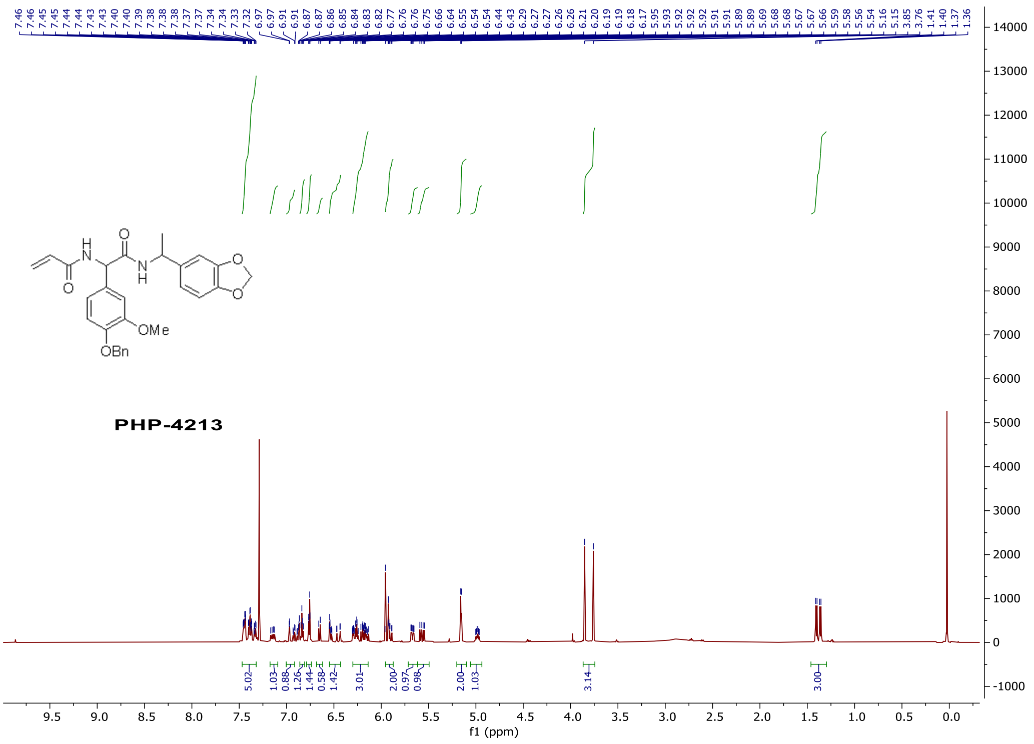

Supplementary Figure 195. ^{1}H NMR (500 MHz, CDCl3) of compound **PHP-4213.**

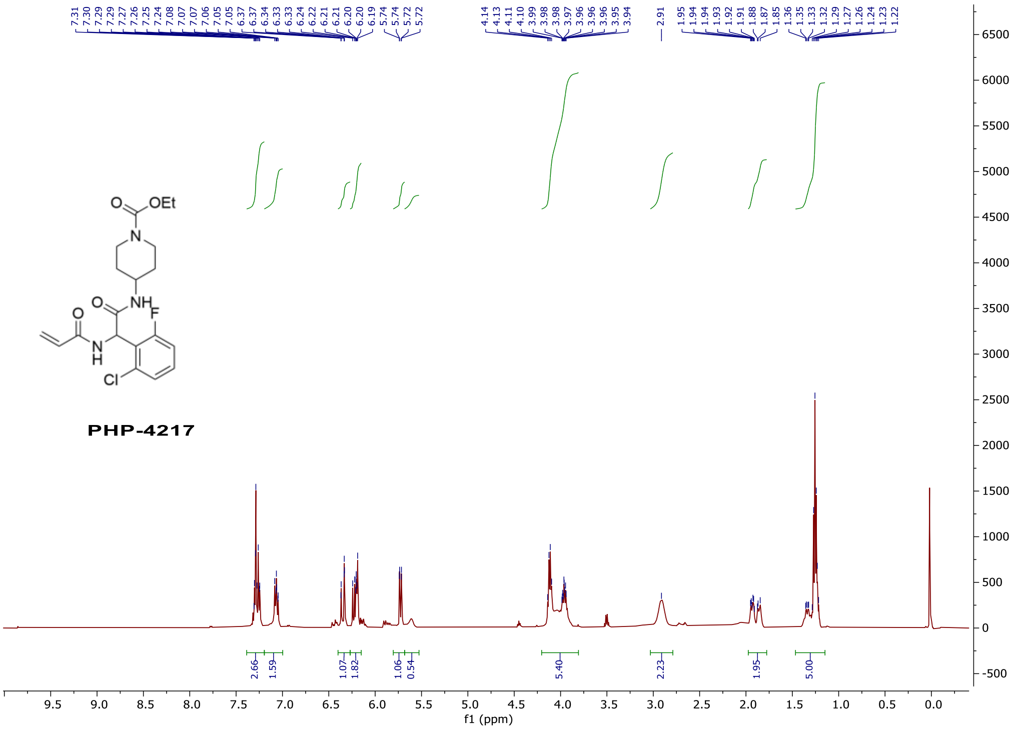

Supplementary Figure 196. ^{1}H NMR (500 MHz, CDCl3) of compound **PHP-4217.**

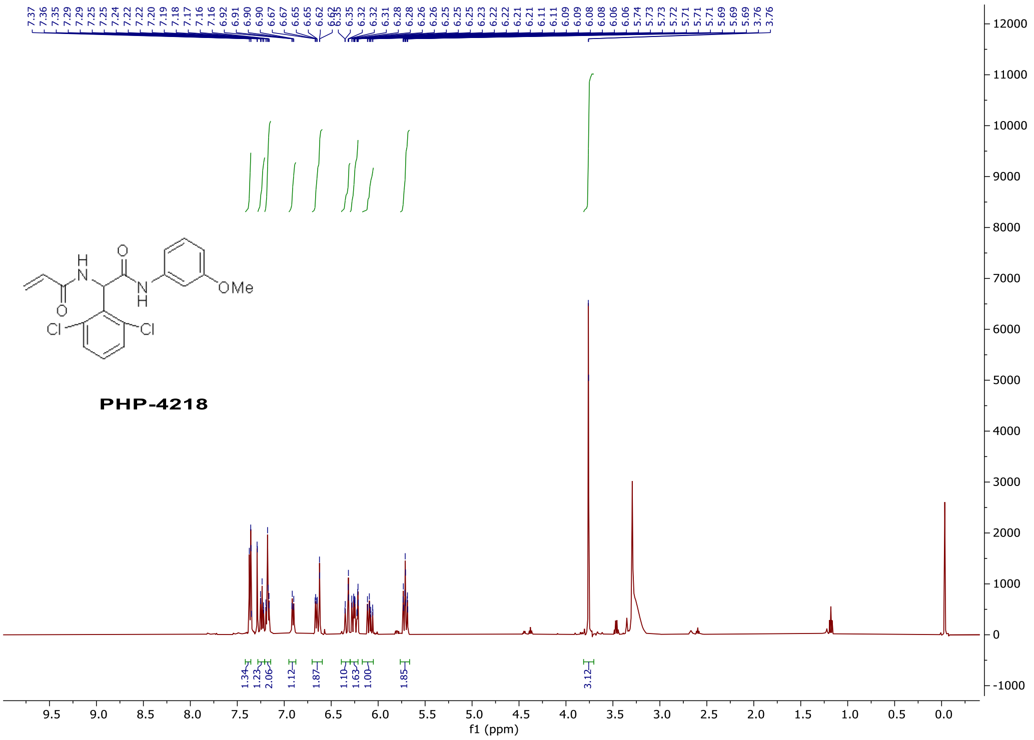

Supplementary Figure 197. ^{1}H NMR (500 MHz, $CDCl_3$+one drop of CD_3OD) of compound **PHP-4218.**

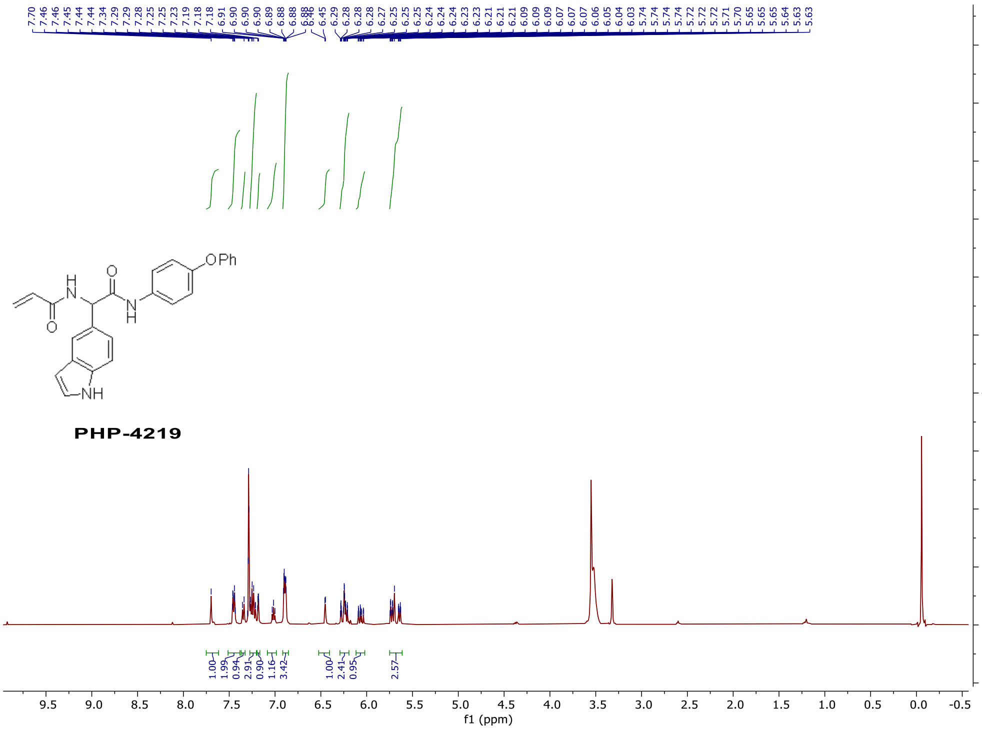

Supplementary Figure 198. ^{1}H NMR (500 MHz, $CDCl_3$+one drop of CD_3OD) of compound **PHP-4219.**

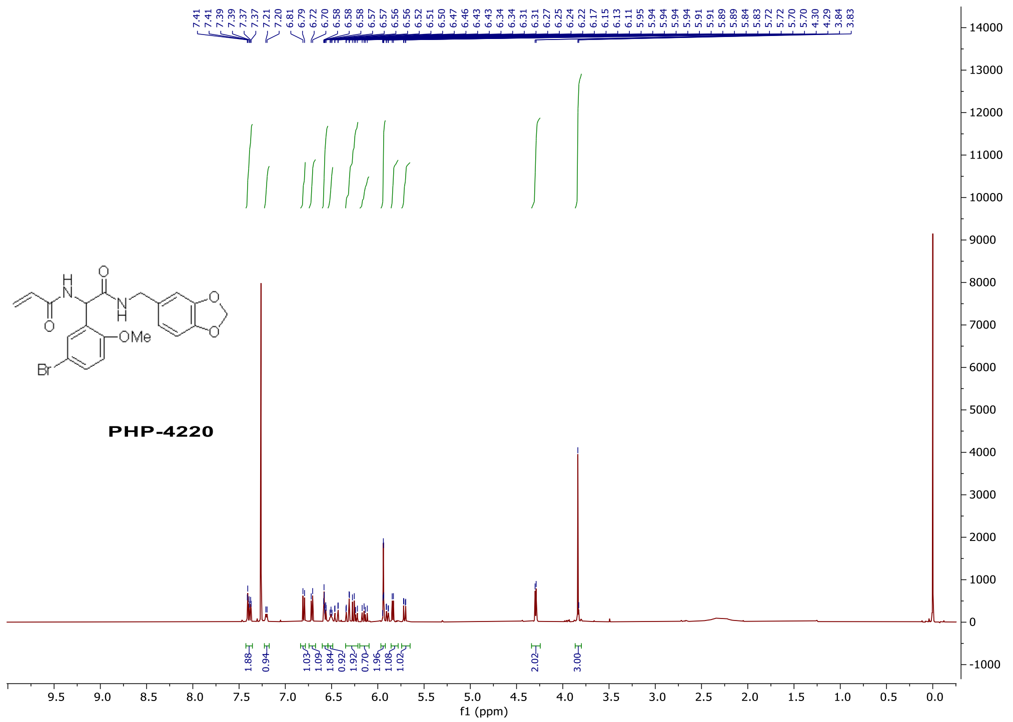

Supplementary Figure 199. ^{1}H NMR (500 MHz, CDCl$_3$) of compound **PHP-4220.**

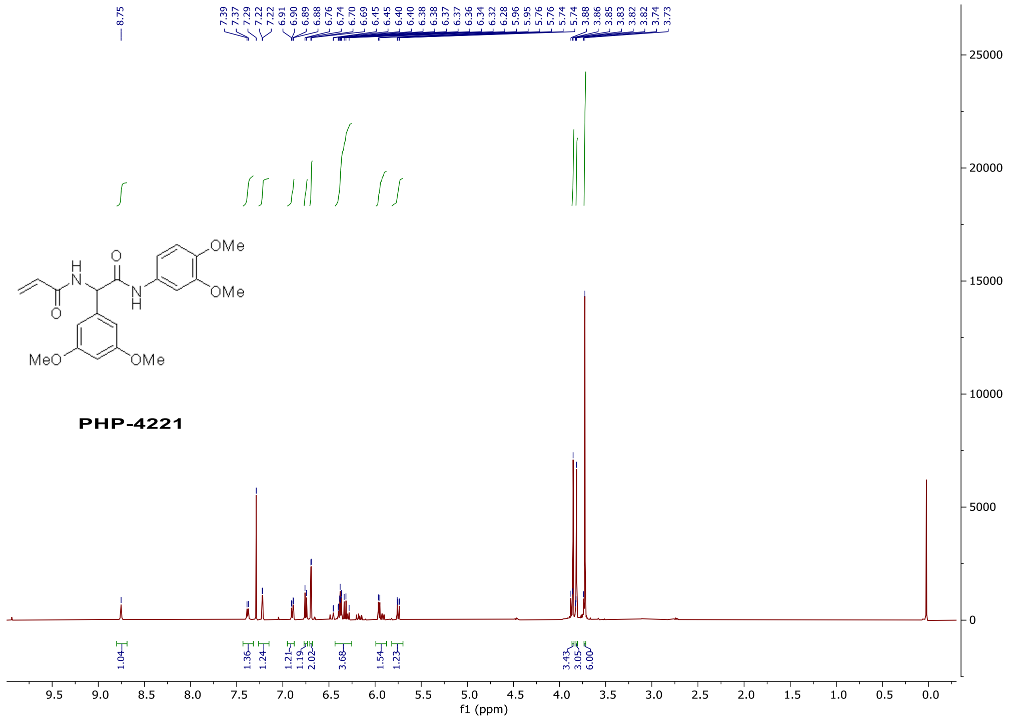

Supplementary Figure 200. ^{1}H NMR (500 MHz, CDCl$_3$) of compound **PHP-4221.**

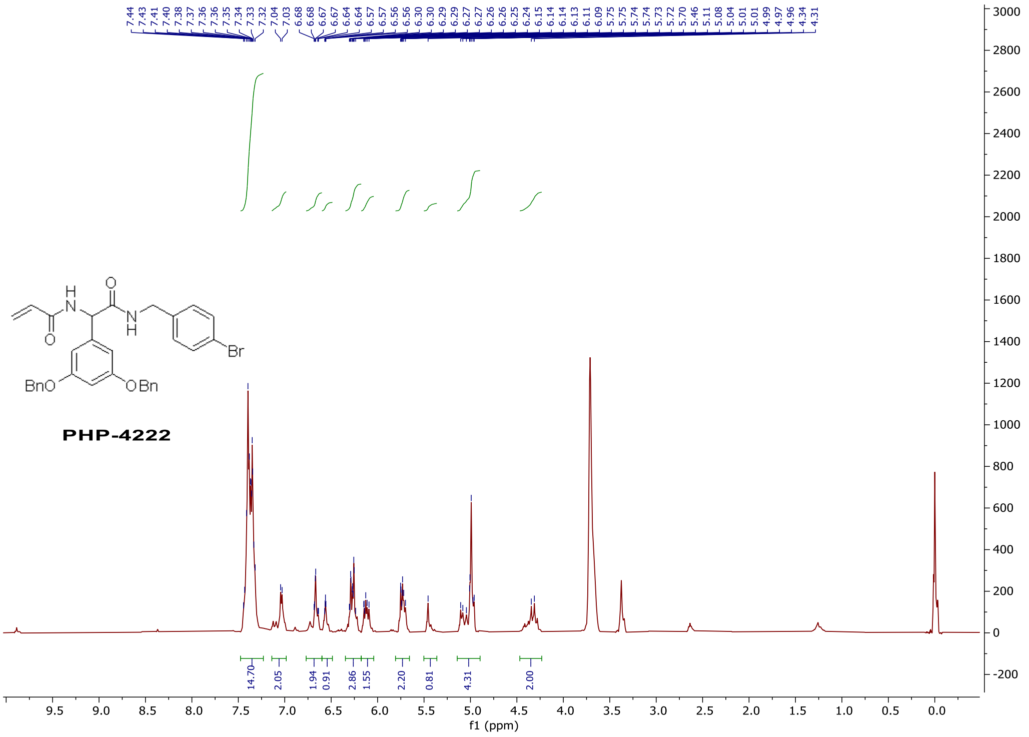

Supplementary Figure 201. ^{1}H NMR (500 MHz, $CDCl_3$+one drop of CD_3OD) of compound **PHP-4222.**

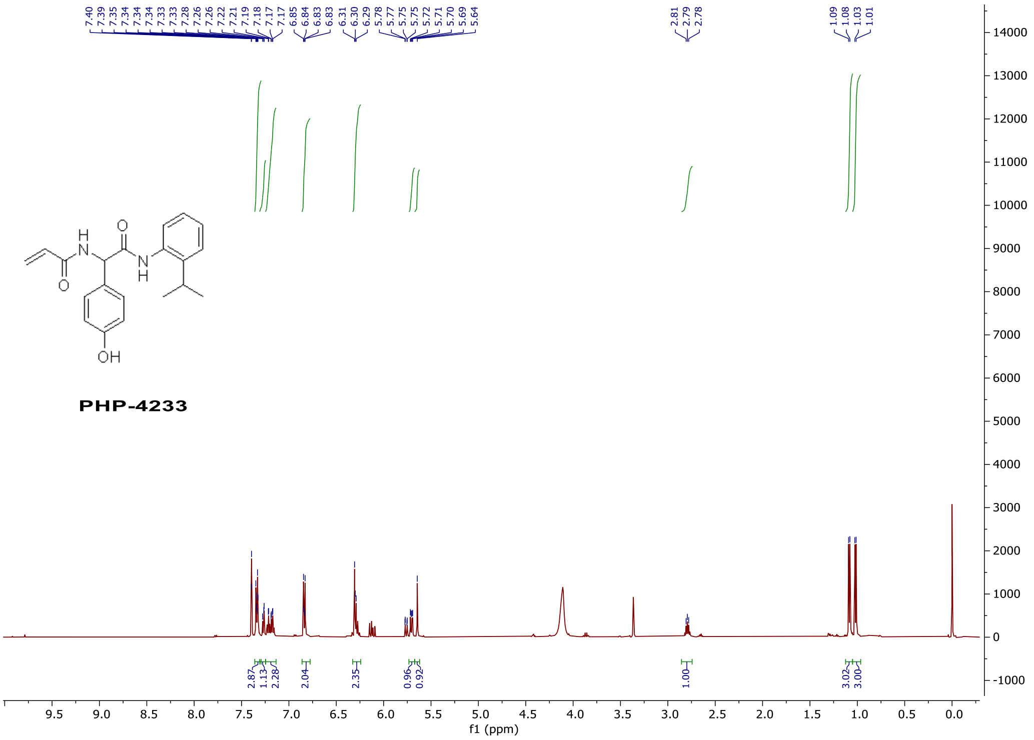

Supplementary Figure 202. ^{1}H NMR (500 MHz, $CDCl_3$+one drop of CD_3OD) of compound **PHP-4233.**

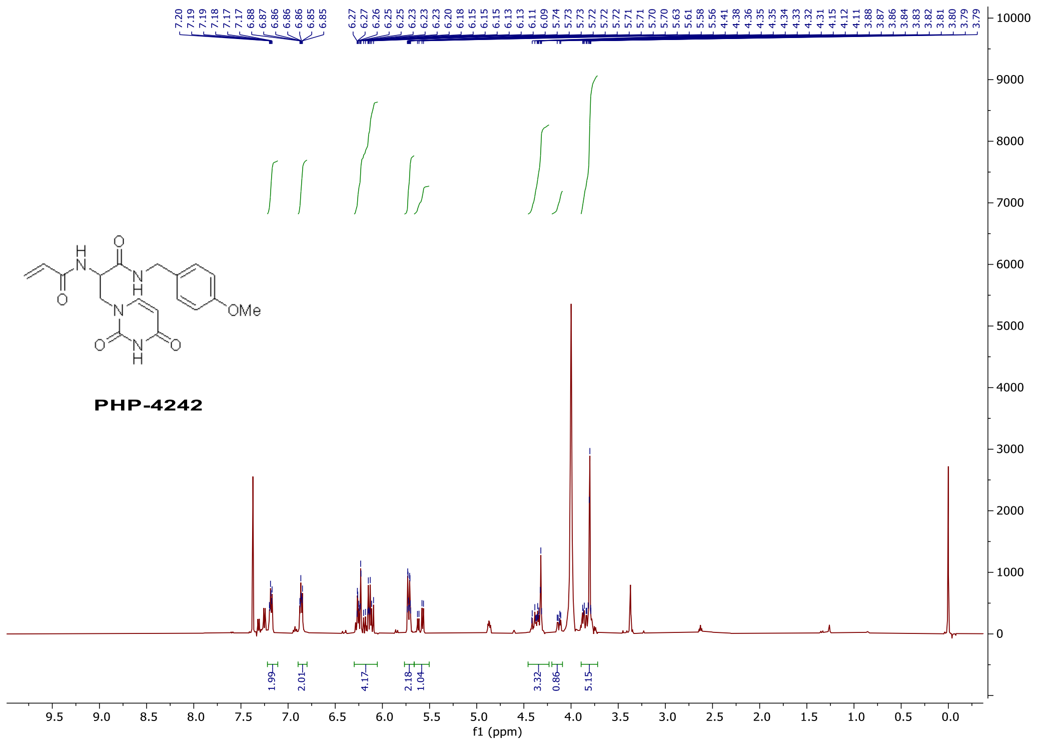

Supplementary Figure 203. ^{1}H NMR (500 MHz, $CDCl_3$+one drop of CD_3OD) of compound **PHP-4242.**

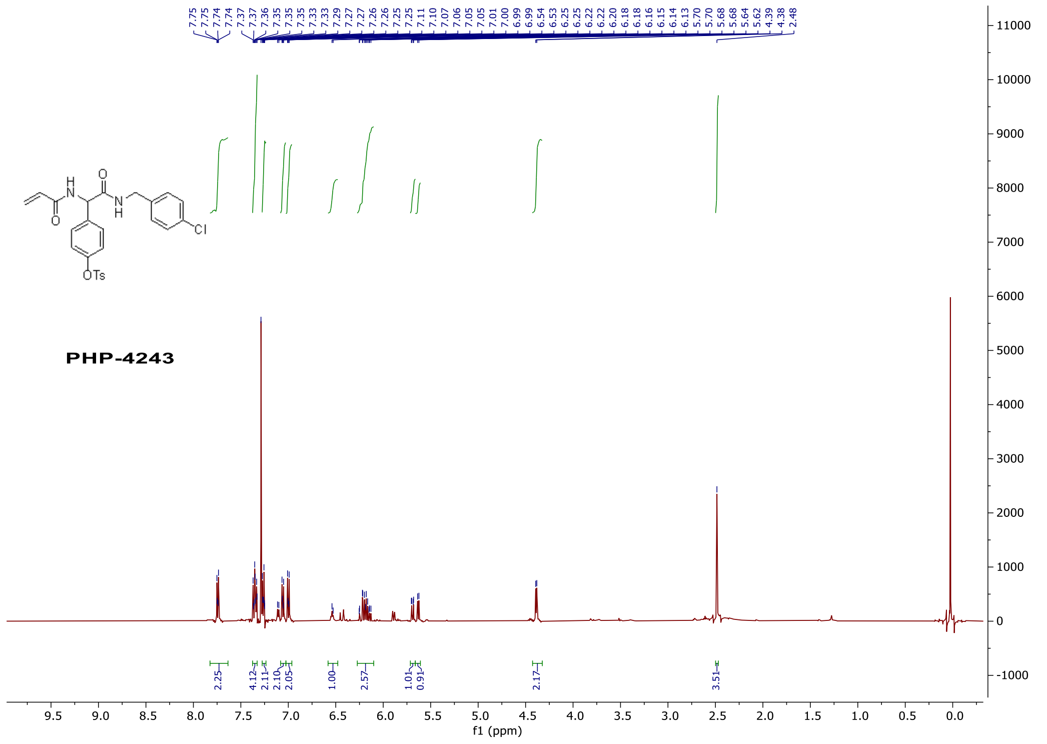

Supplementary Figure 204. ^{1}H NMR (500 MHz, $CDCl_3$) of compound **PHP-4243.**

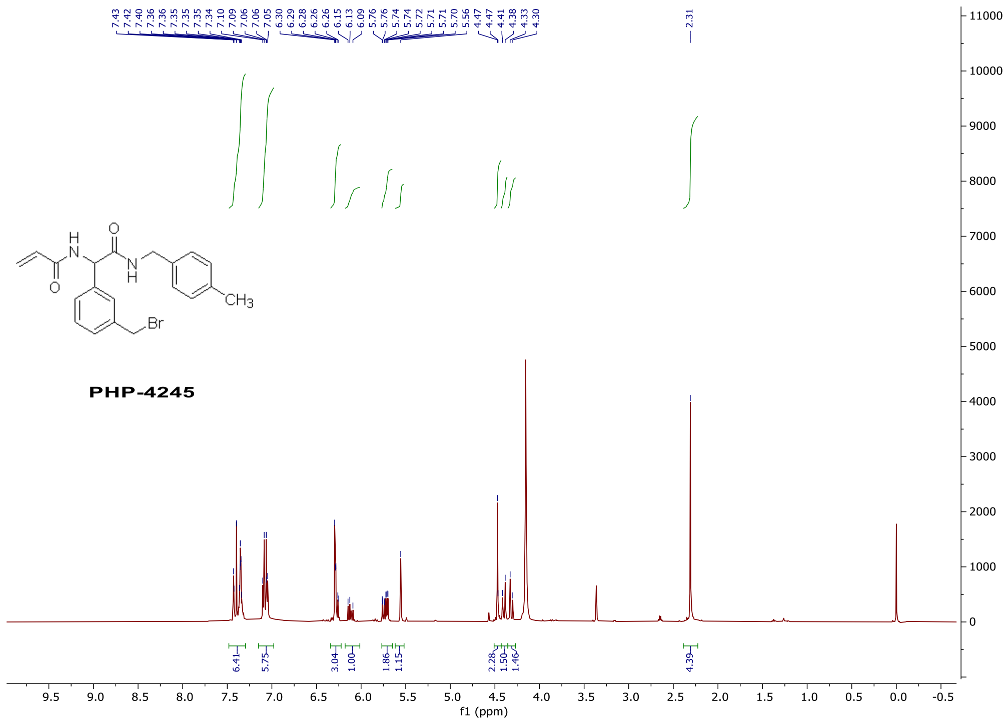

Supplementary Figure 205. ^{1}H NMR (500 MHz, $CDCl_3$+one drop of CD_3OD) of compound **PHP-4245.**

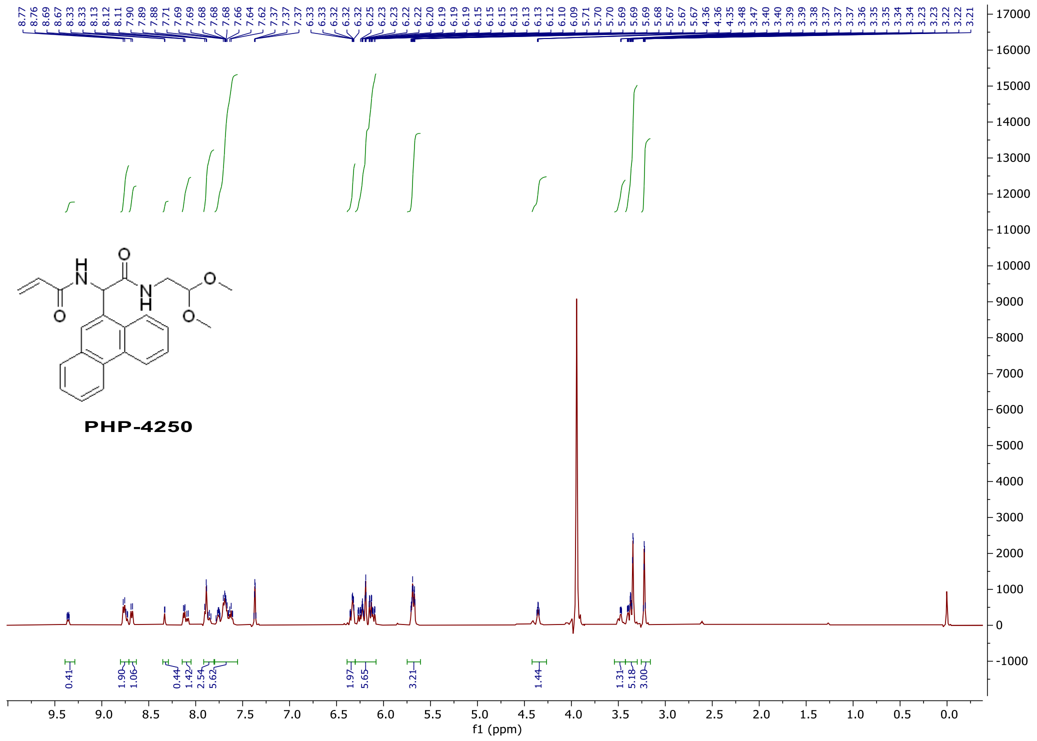

Supplementary Figure 206. ^{1}H NMR (500 MHz, $CDCl_3$+one drop of CD_3OD) of compound **PHP-4250.**

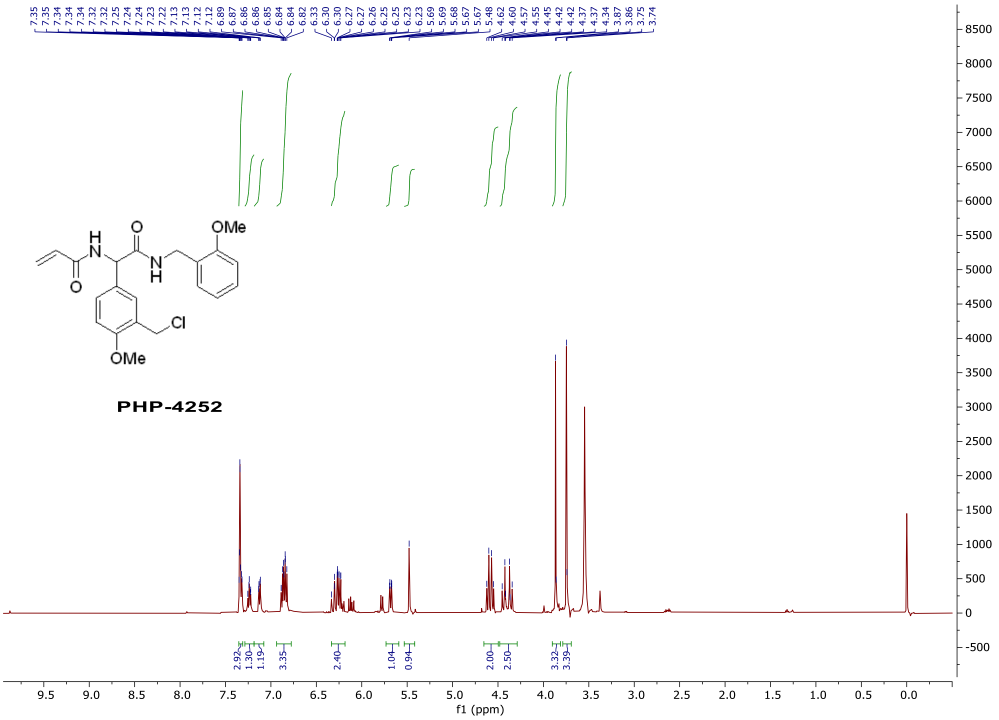

Supplementary Figure 207. ^{1}H NMR (500 MHz, $CDCl_3$+one drop of CD_3OD) of compound **PHP-4252.**

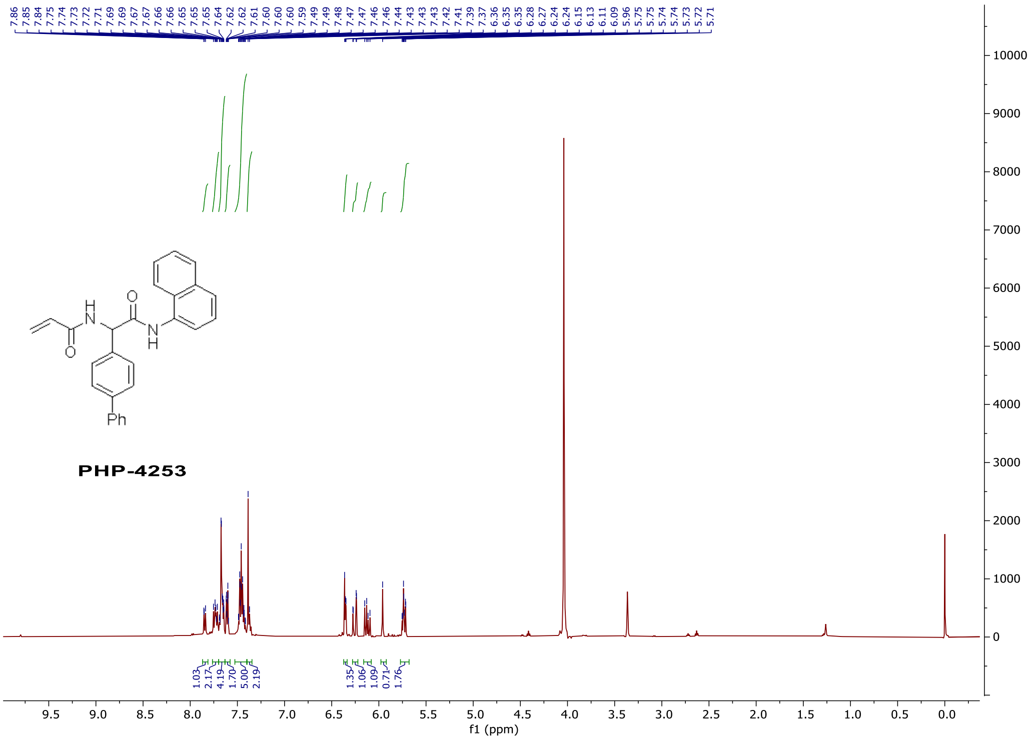

Supplementary Figure 208. ^{1}H NMR (500 MHz, $CDCl_3$+one drop of CD_3OD) of compound **PHP-4253.**

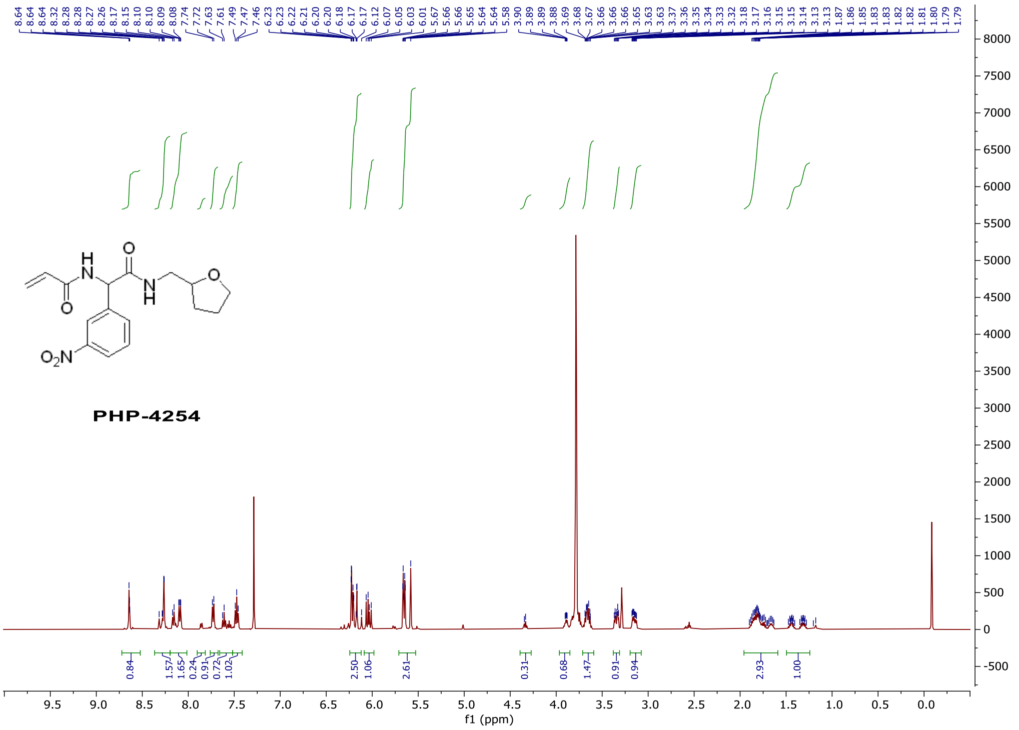

Supplementary Figure 209. ^{1}H NMR (500 MHz, $CDCl_3$+one drop of CD_3OD) of compound **PHP-4254.**

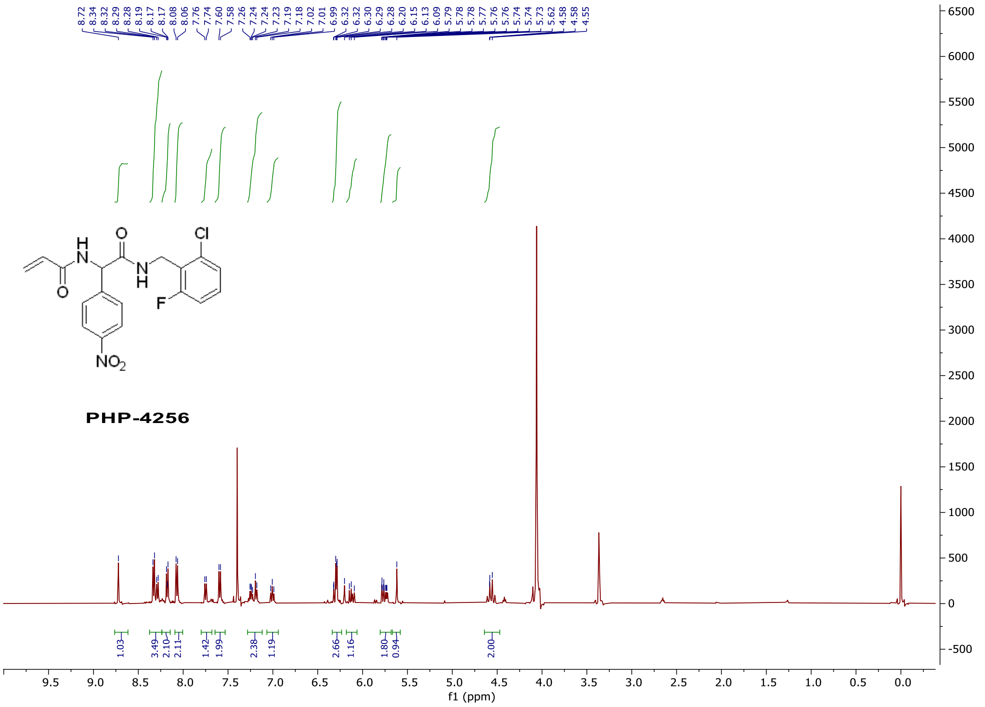

Supplementary Figure 210. ^{1}H NMR (500 MHz, $CDCl_3$) of compound **PHP-4256.**

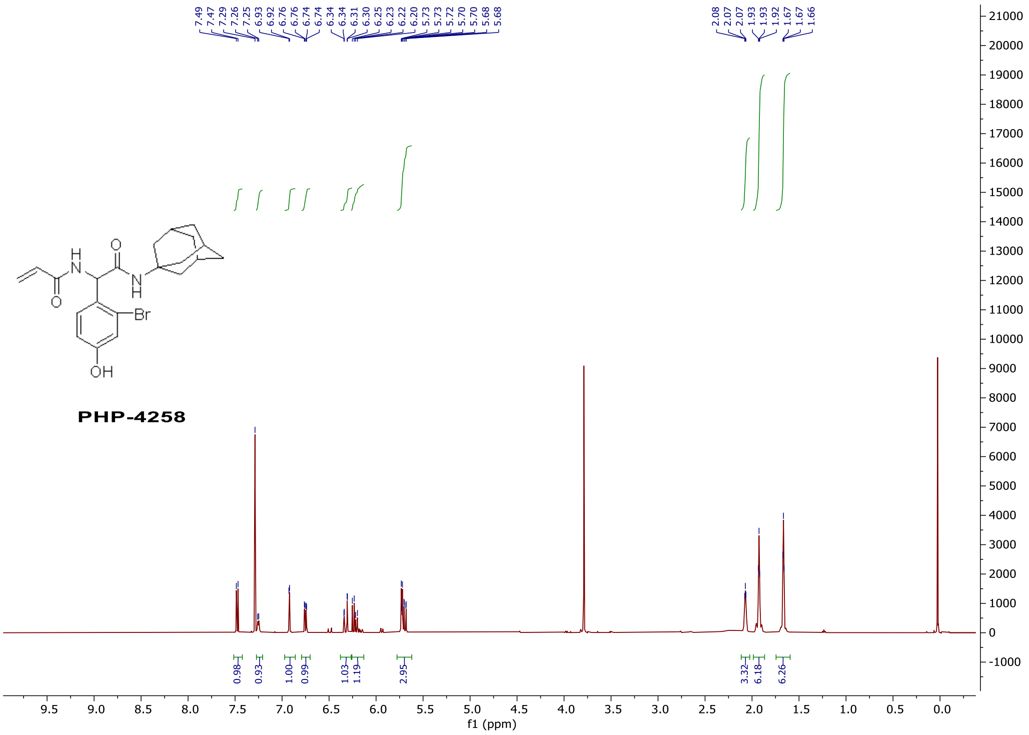

Supplementary Figure 211. ^{1}H NMR (500 MHz, $CDCl_3$+one drop of CD_3OD) of compound **PHP-4258.**

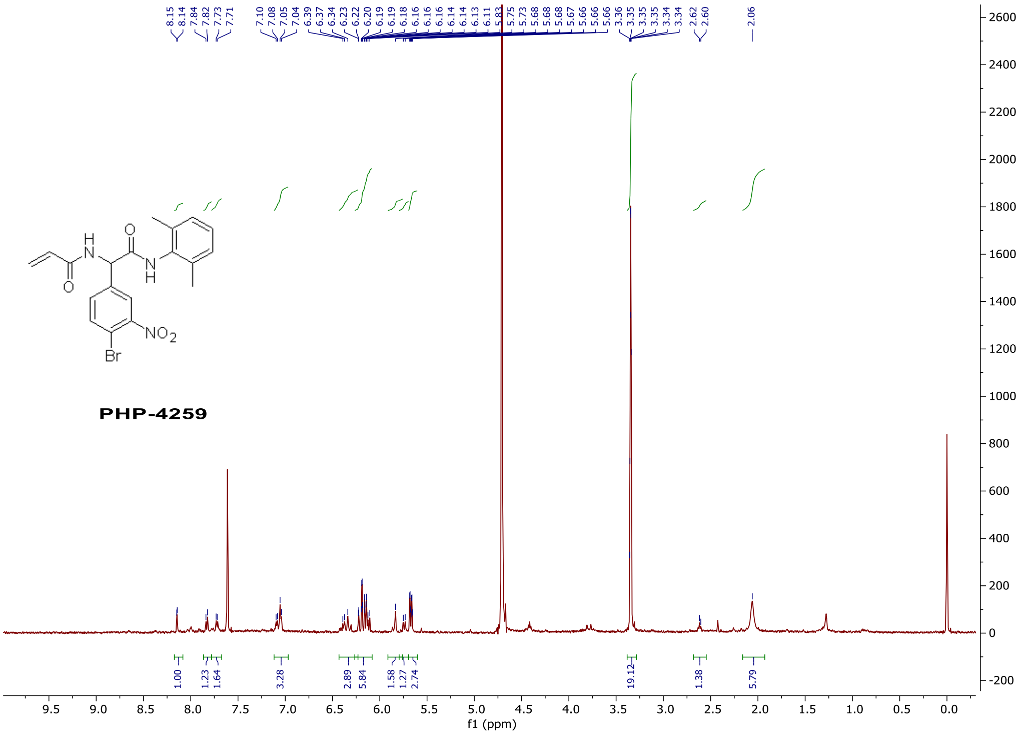

Supplementary Figure 212. ^{1}H NMR (500 MHz, $CDCl_3$+one drop of CD_3OD) of compound **PHP-4259.**

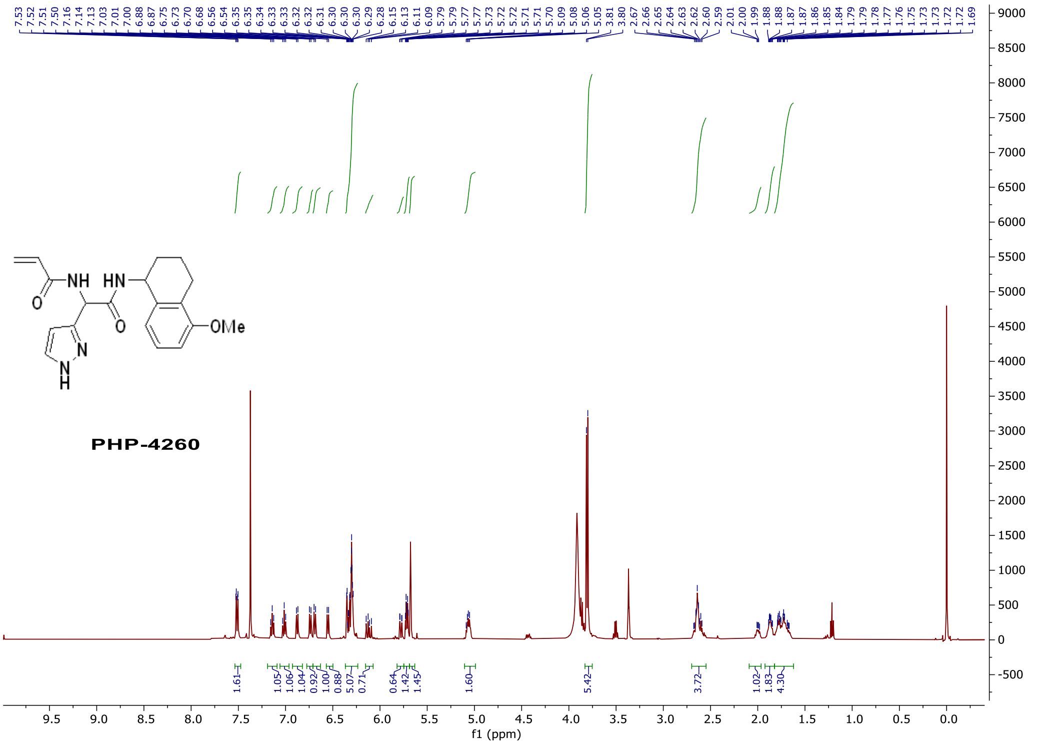

Supplementary Figure 213. ^{1}H NMR (500 MHz, $CDCl_3$) of compound **PHP-4260.**

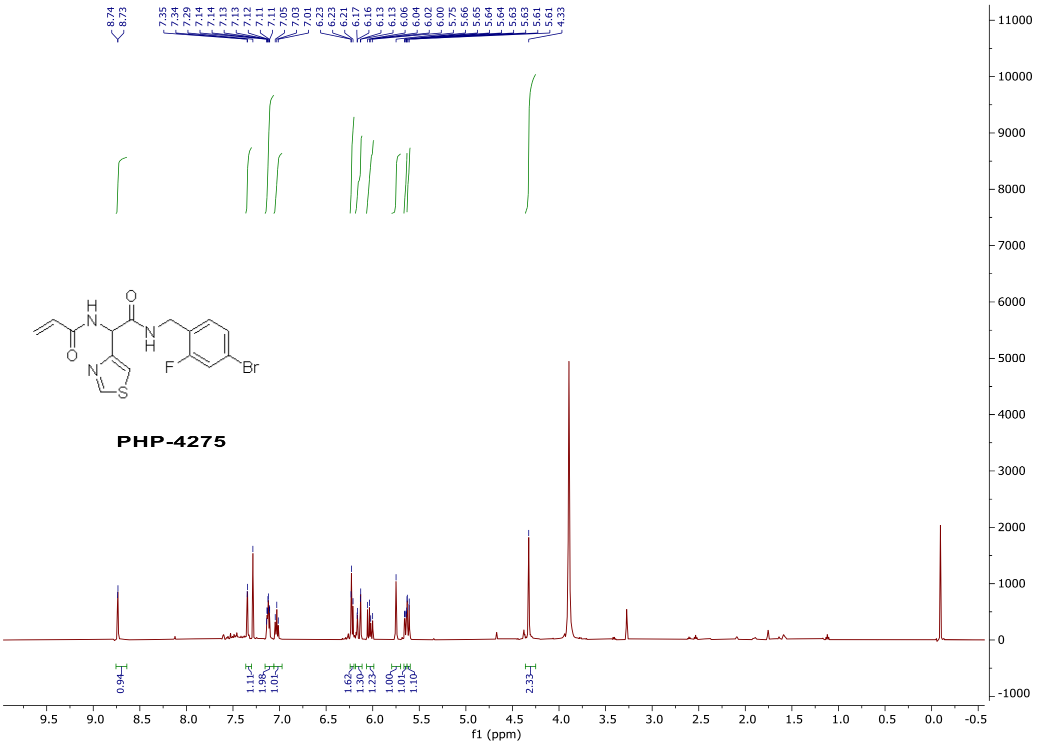

Supplementary Figure 214. ^{1}H NMR (500 MHz, $CDCl_3$+one drop of CD_3OD) of compound **PHP-4275.**

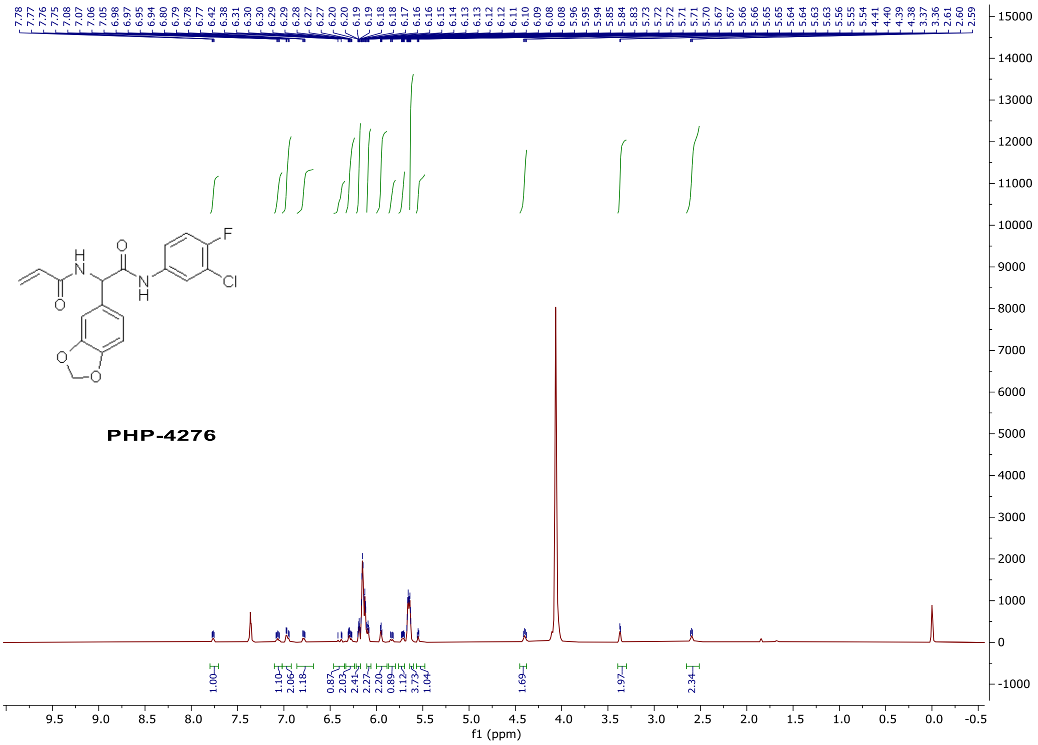

Supplementary Figure 215. ^{1}H NMR (500 MHz, $CDCl_3$+one drop of CD_3OD) of compound **PHP-4276.**

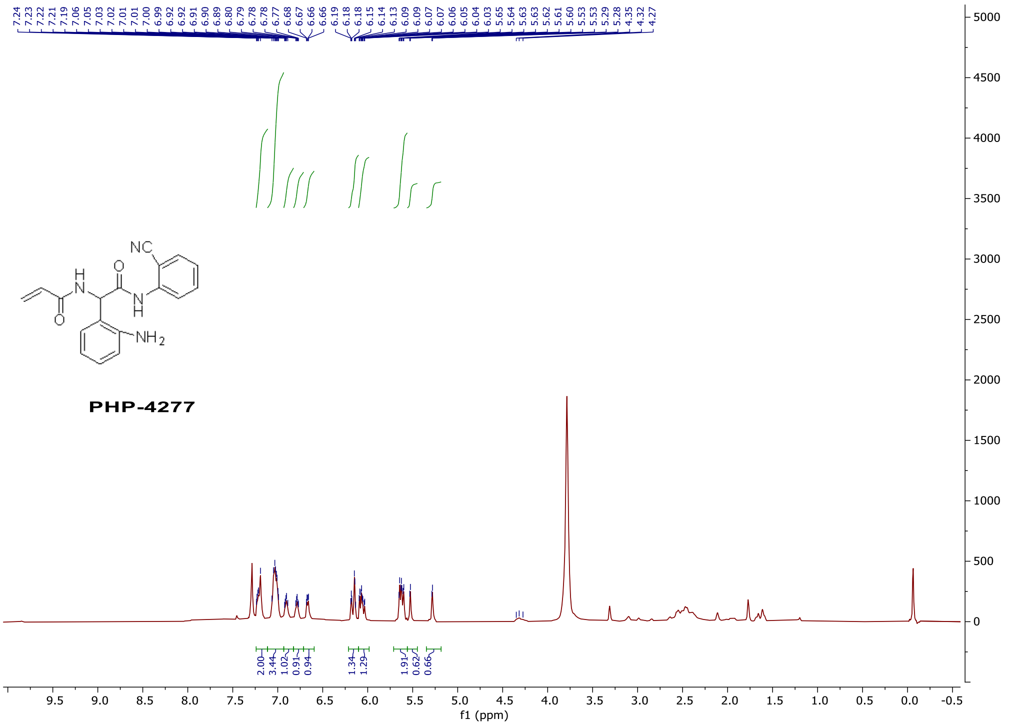

Supplementary Figure 216. ^{1}H NMR (500 MHz, $CDCl_3$+one drop of CD_3OD) of compound **PHP-4277.**

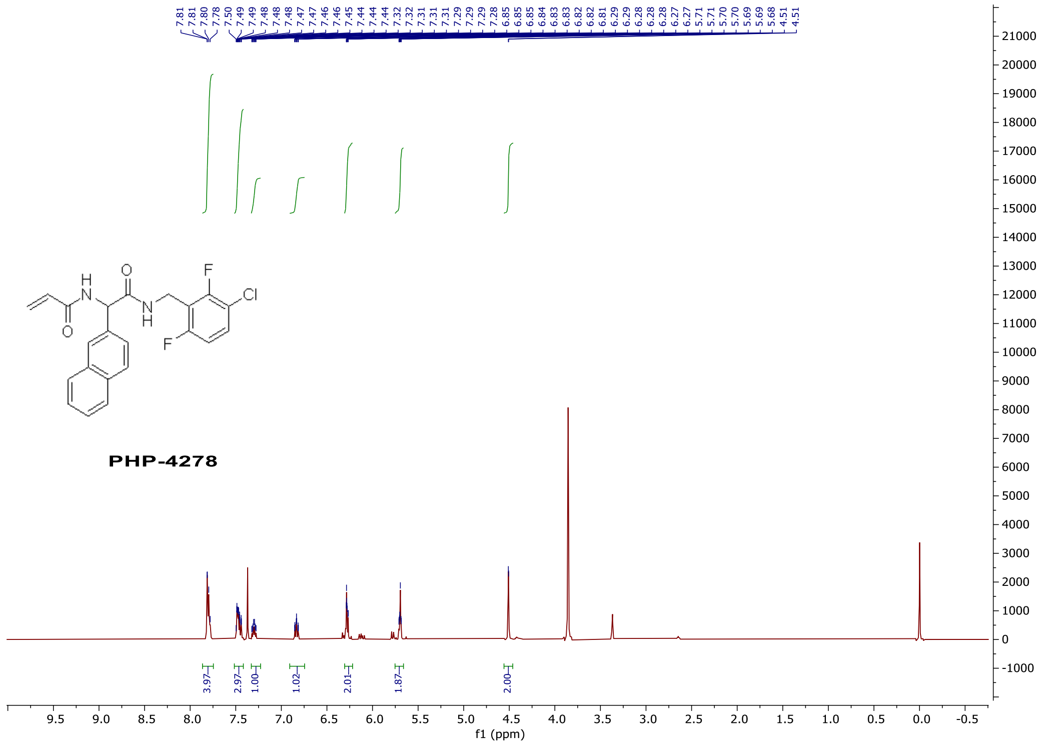

Supplementary Figure 217. ^{1}H NMR (500 MHz, $CDCl_3$+one drop of CD_3OD) of compound **PHP-4278.**

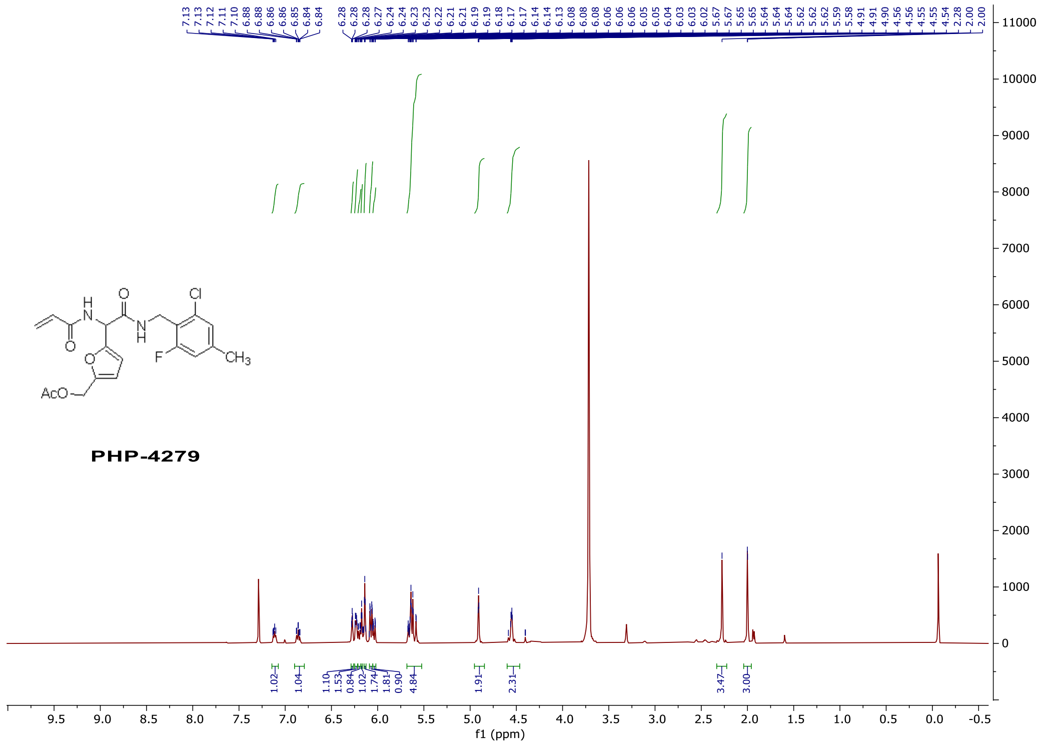

Supplementary Figure 218. ^{1}H NMR (500 MHz, $CDCl_3$+one drop of CD_3OD) of compound **PHP-4279.**

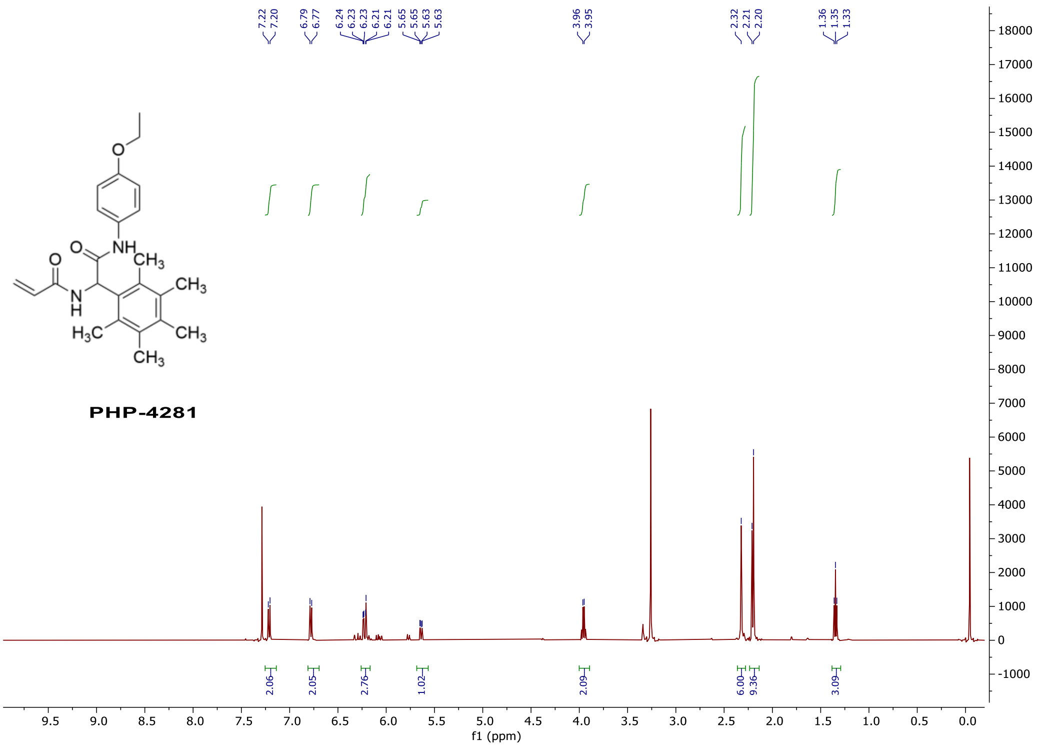

Supplementary Figure 219. ^{1}H NMR (500 MHz, $CDCl_3$+one drop of CD_3OD) of compound **PHP-4281.**

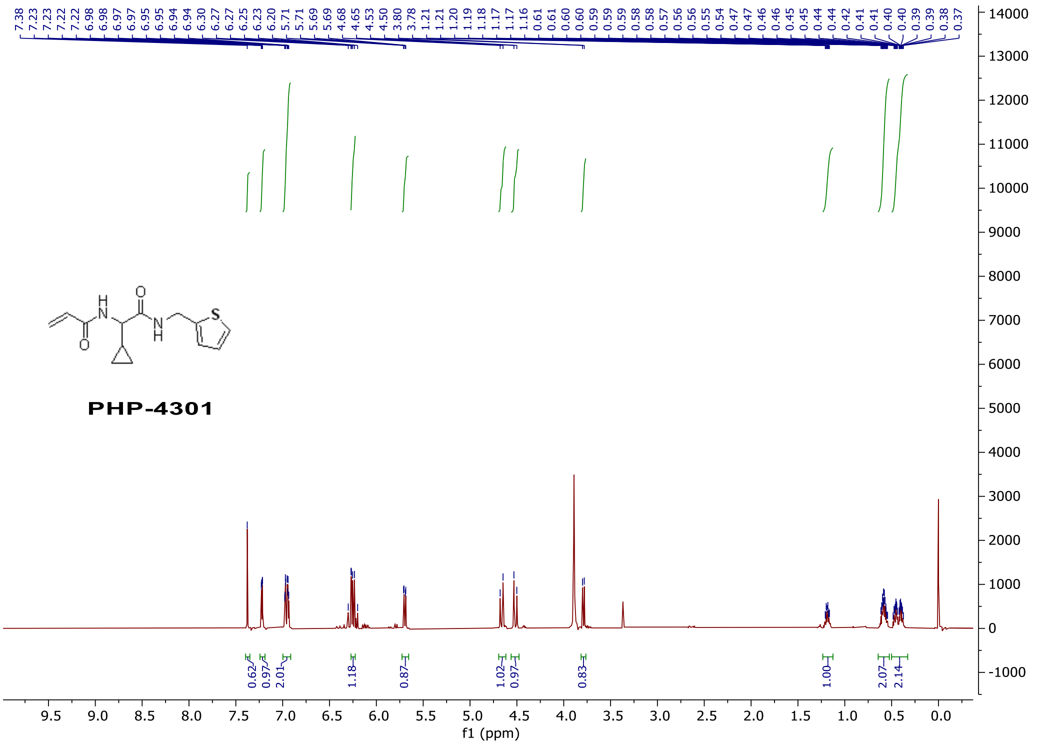

Supplementary Figure 220. ^{1}H NMR (500 MHz, $CDCl_3$+one drop of CD_3OD) of compound **PHP-4301.**

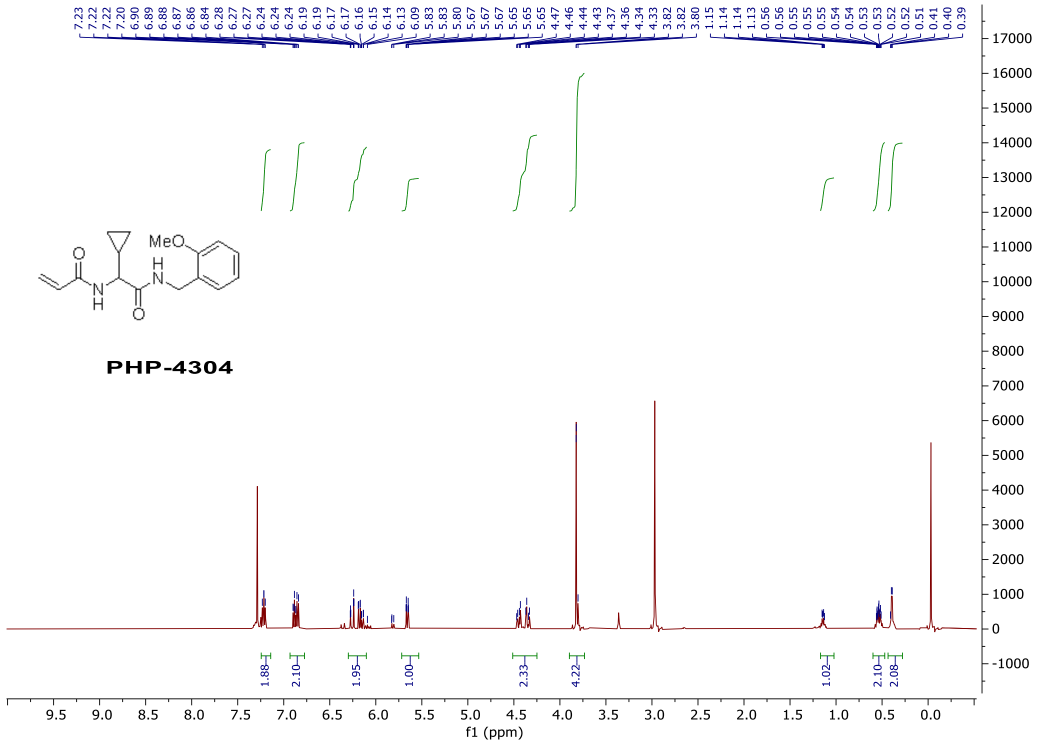

Supplementary Figure 221. ^{1}H NMR (500 MHz, $CDCl_3$+one drop of CD_3OD) of compound **PHP-4304.**

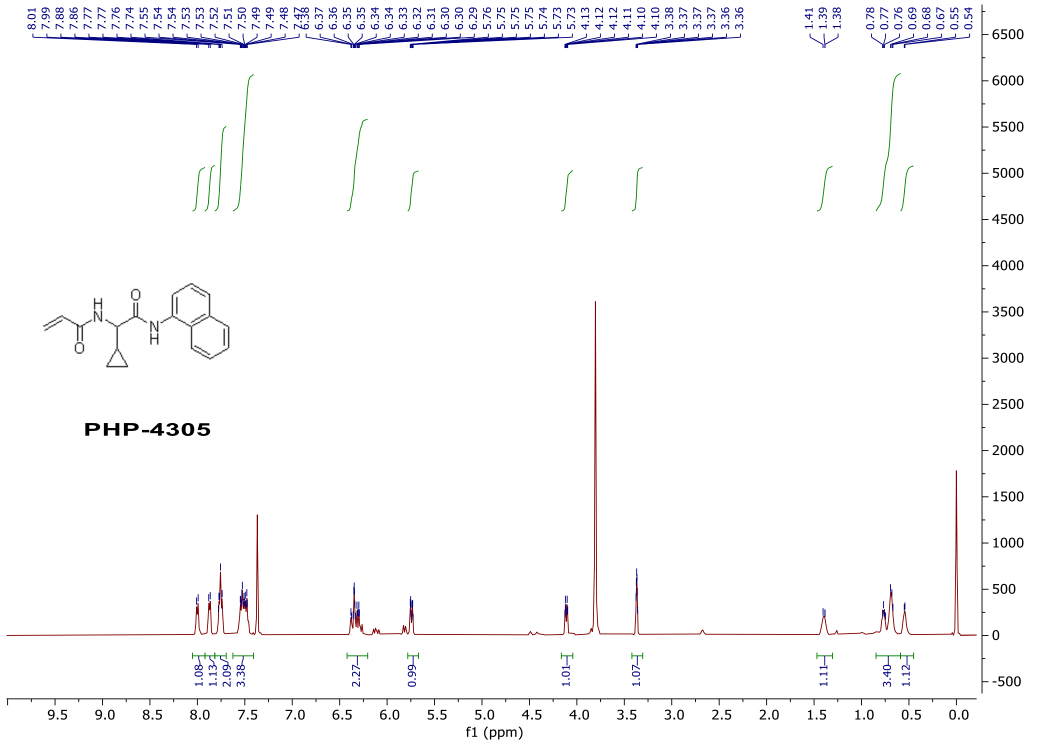

Supplementary Figure 222. ^{1}H NMR (500 MHz, $CDCl_3$+one drop of CD_3OD) of compound **PHP-4305.**

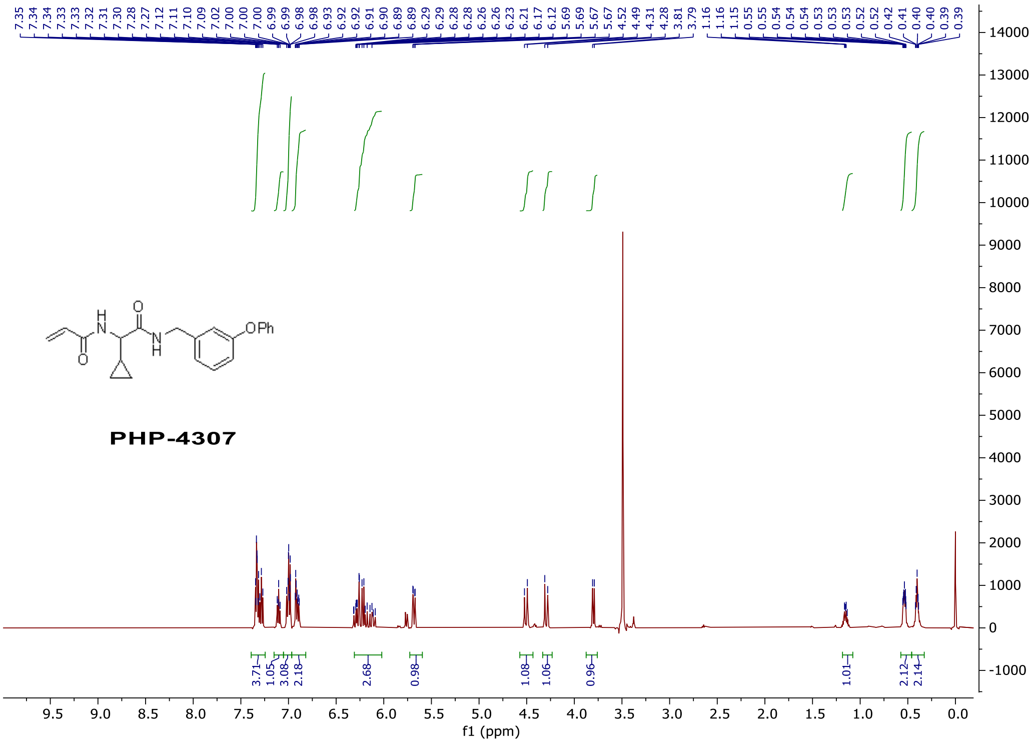

Supplementary Figure 223. ^{1}H NMR (500 MHz, $CDCl_3$+one drop of CD_3OD) of compound **PHP-4307.**

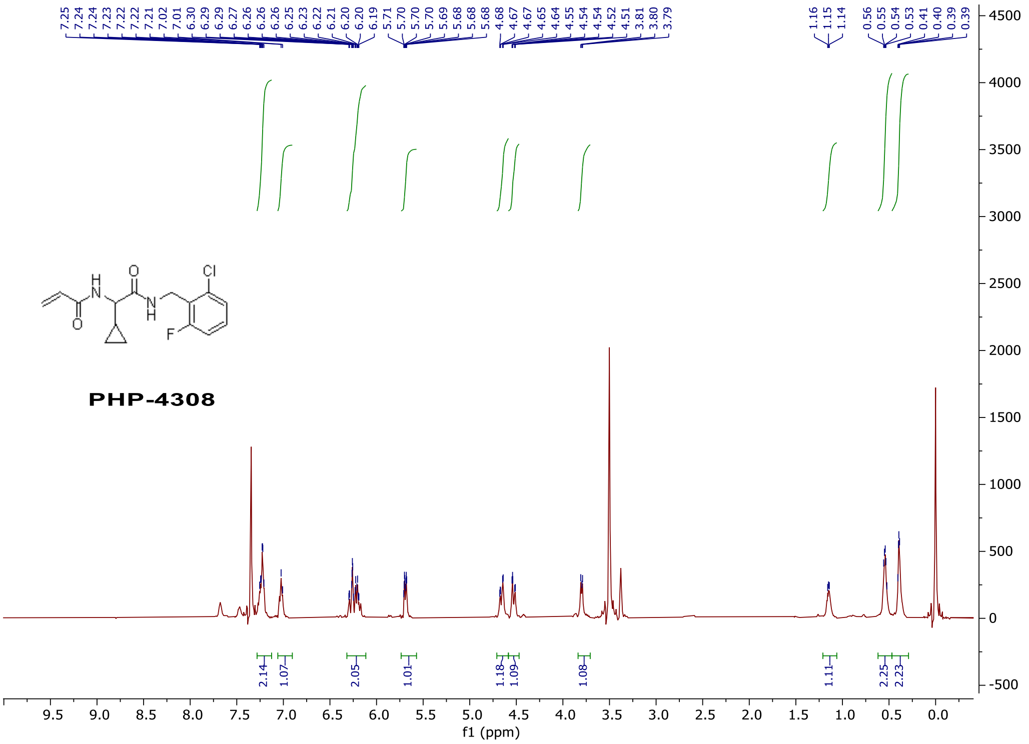

Supplementary Figure 224. ^{1}H NMR (500 MHz, $CDCl_3$+one drop of CD_3OD) of compound **PHP-4308.**

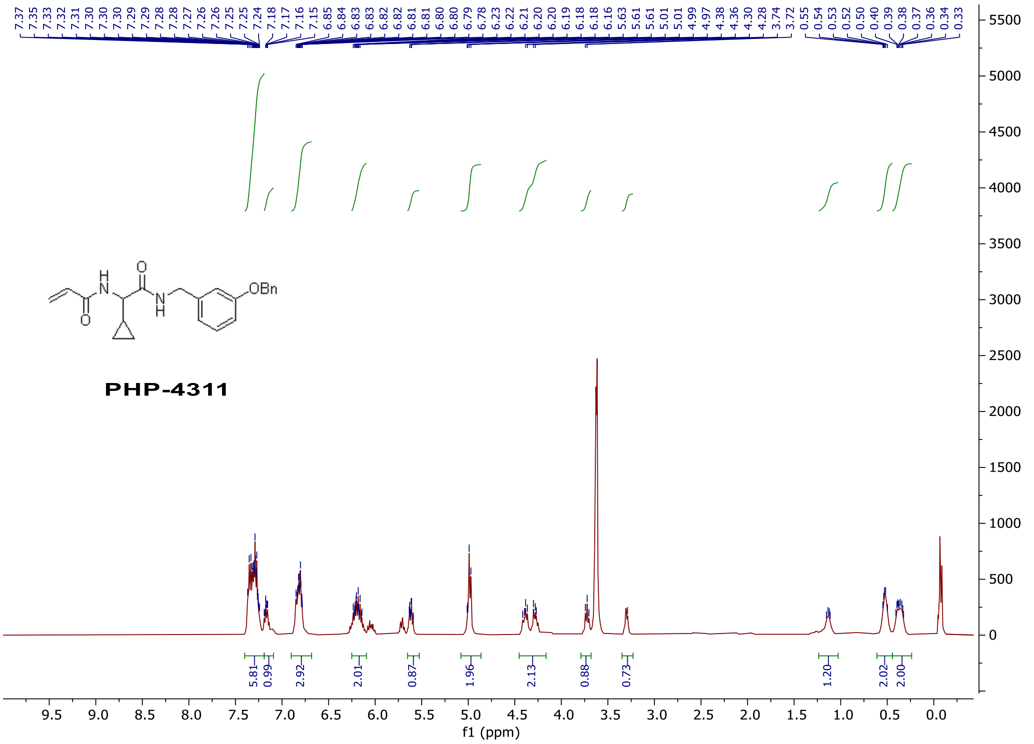

Supplementary Figure 225. ^{1}H NMR (500 MHz, $CDCl_3$+one drop of CD_3OD) of compound **PHP-4311.**

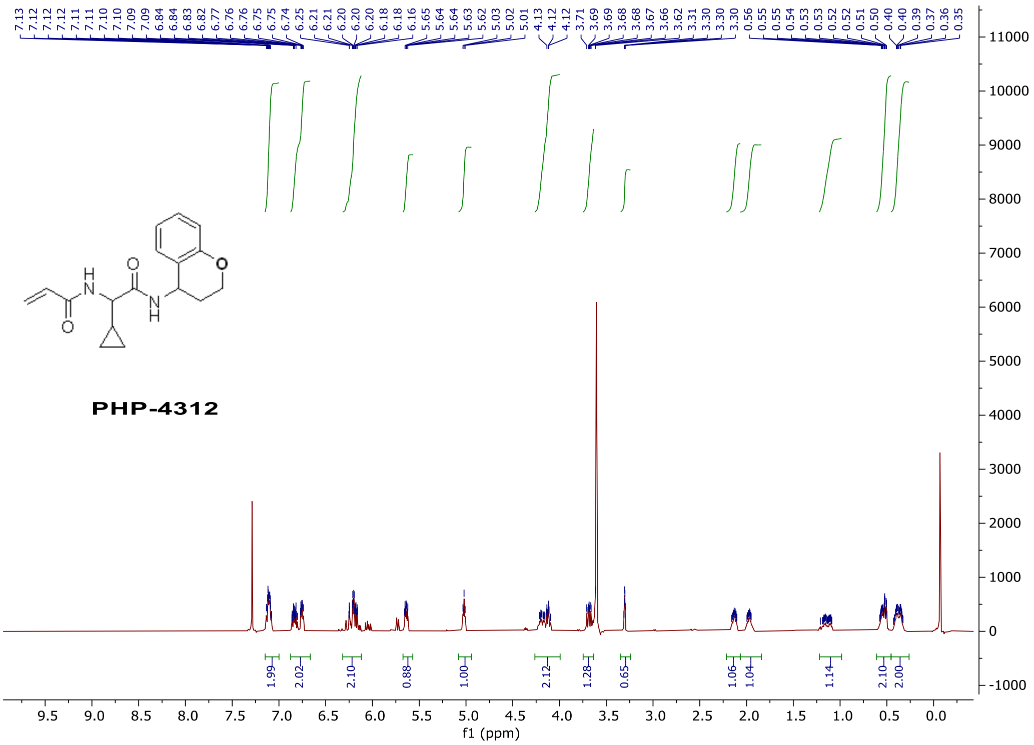

Supplementary Figure 226. ^{1}H NMR (500 MHz, $CDCl_3$+one drop of CD_3OD) of compound **PHP-4312.**

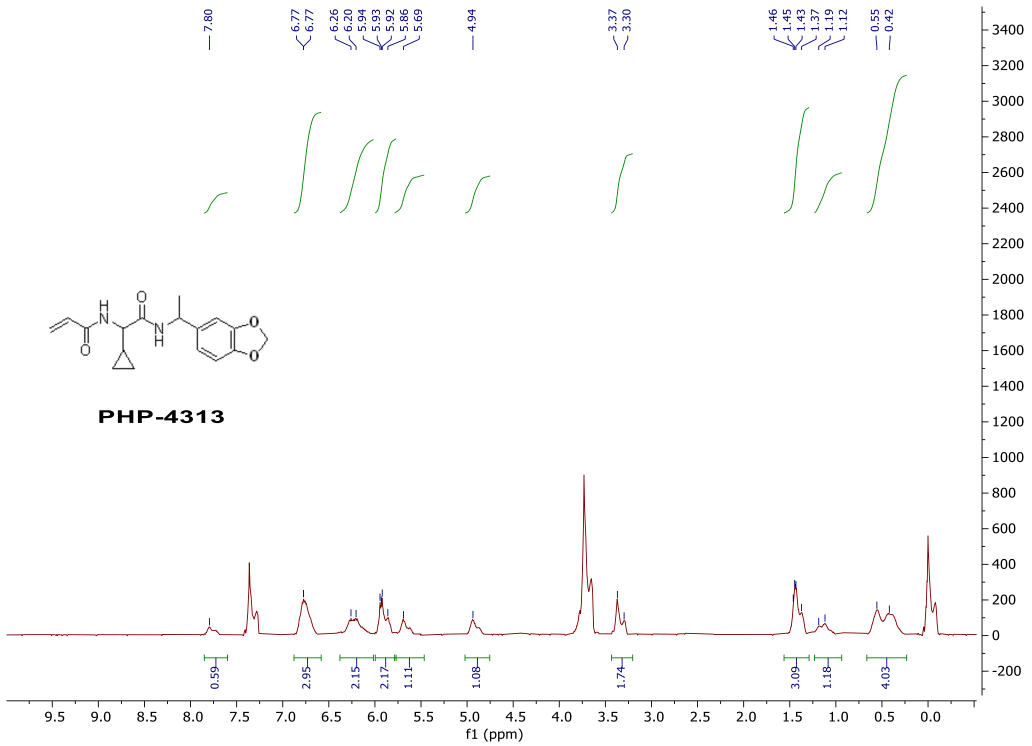

Supplementary Figure 227. ^{1}H NMR (500 MHz, $CDCl_3$+one drop of CD_3OD) of compound **PHP-4313.**

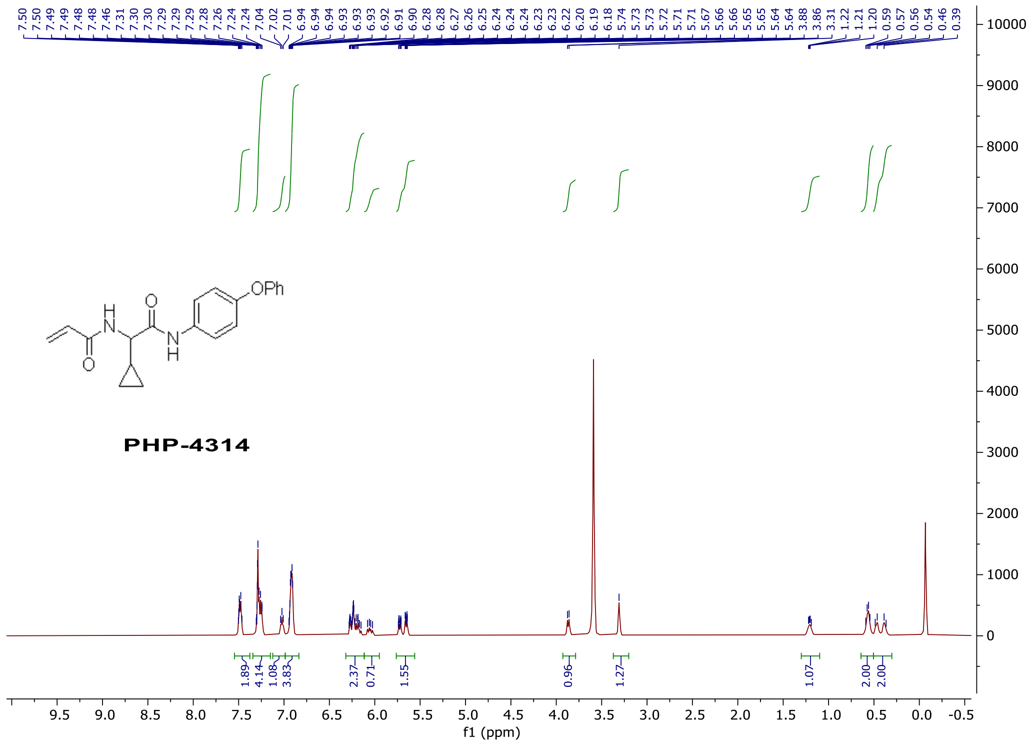

Supplementary Figure 228. ^{1}H NMR (500 MHz, $CDCl_3$+one drop of CD_3OD) of compound **PHP-4314.**

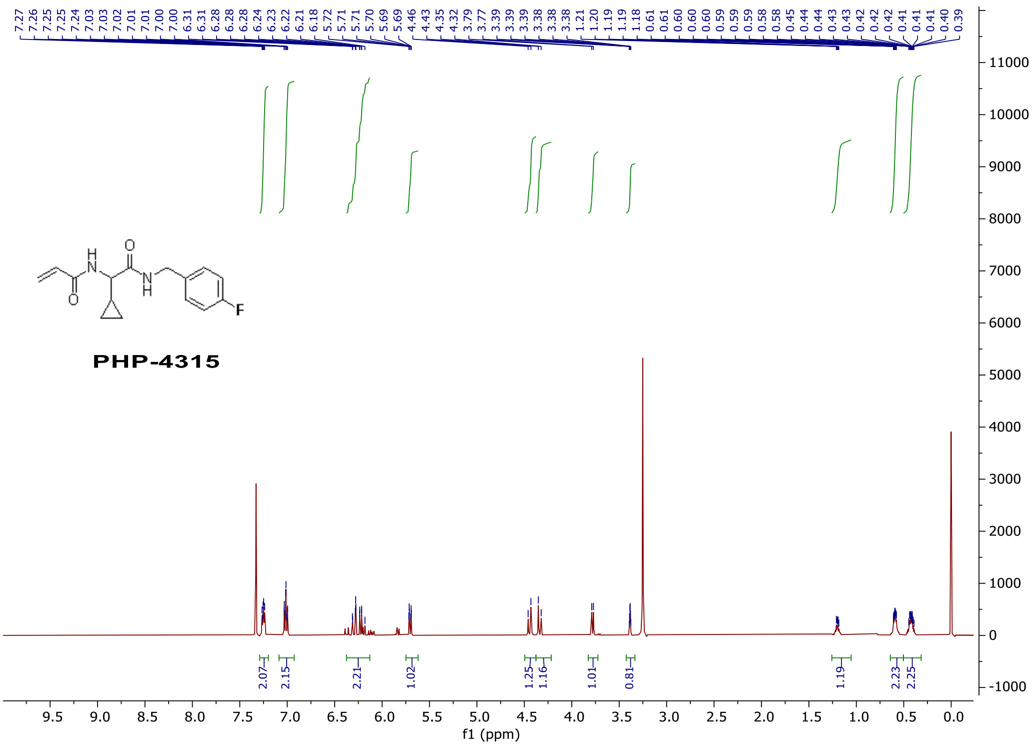

Supplementary Figure 229. ^{1}H NMR (500 MHz, $CDCl_3$+one drop of CD_3OD) of compound **PHP-4315.**

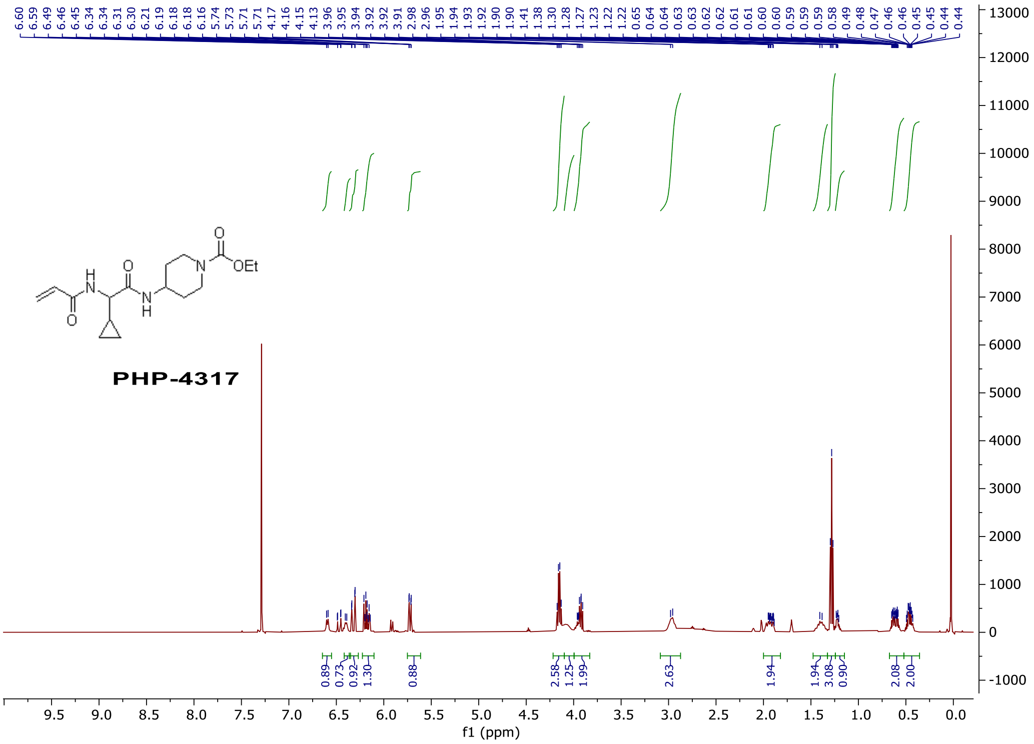

Supplementary Figure 230. ^{1}H NMR (500 MHz, $CDCl_3$) of compound **PHP-4317.**

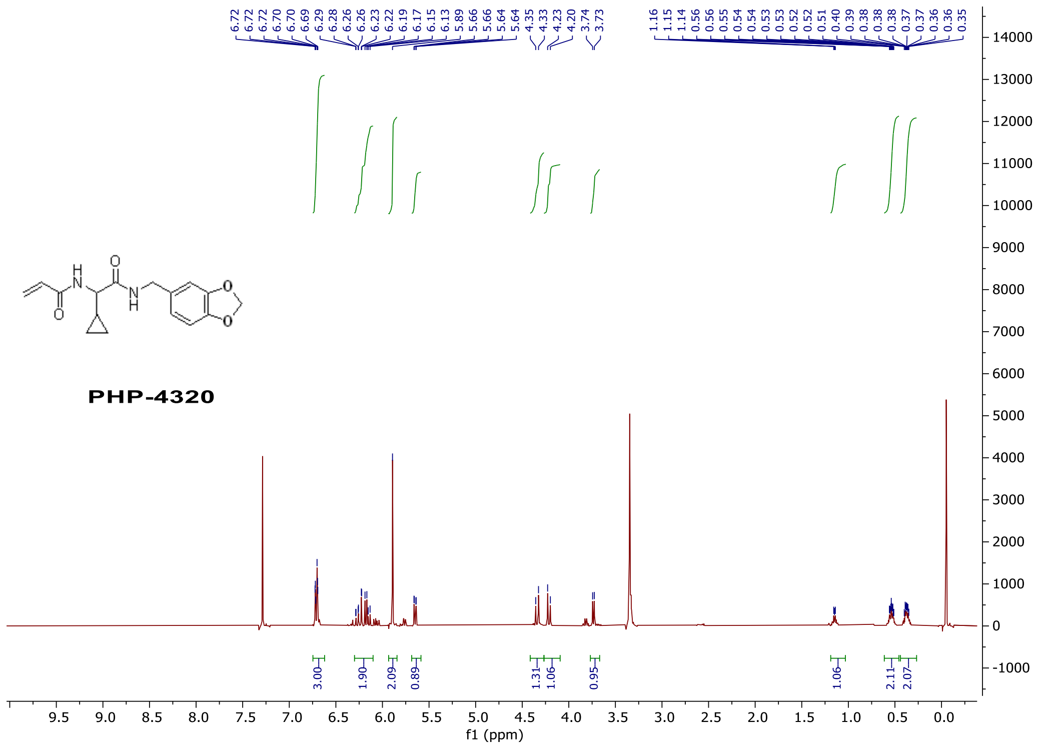

Supplementary Figure 231. ^{1}H NMR (500 MHz, $CDCl_3$+one drop of CD_3OD) of compound **PHP-4320.**

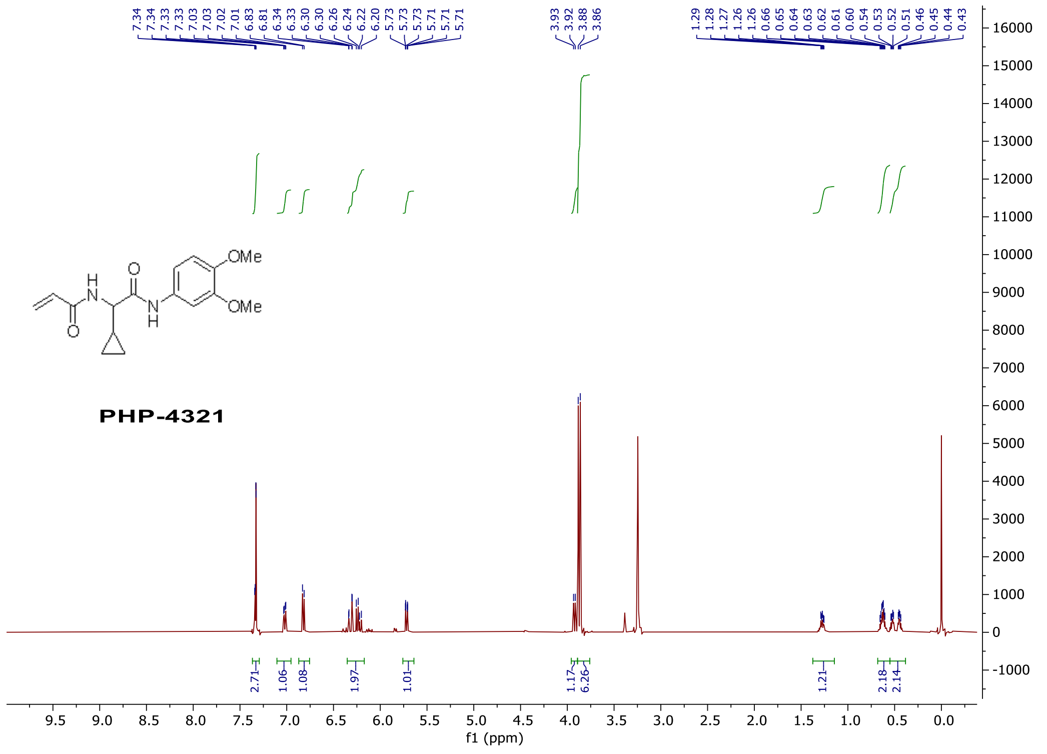

Supplementary Figure 232. ^{1}H NMR (500 MHz, $CDCl_3$+one drop of CD_3OD) of compound **PHP-4321.**

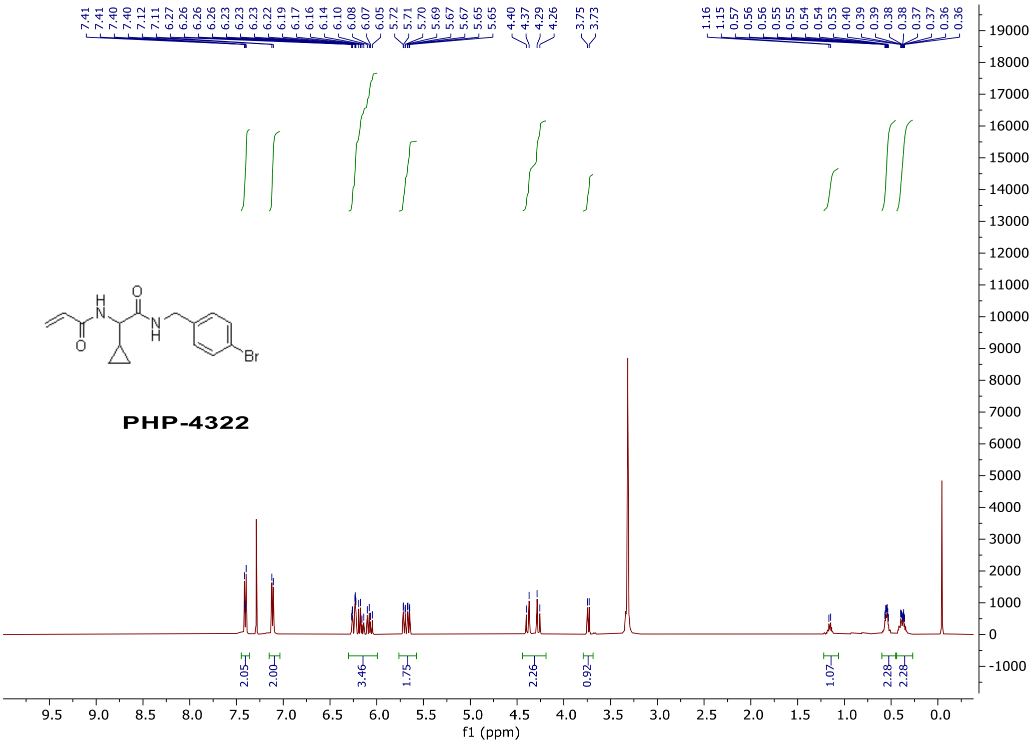

Supplementary Figure 233. ^{1}H NMR (500 MHz, $CDCl_3$+one drop of CD_3OD) of compound **PHP-4322.**

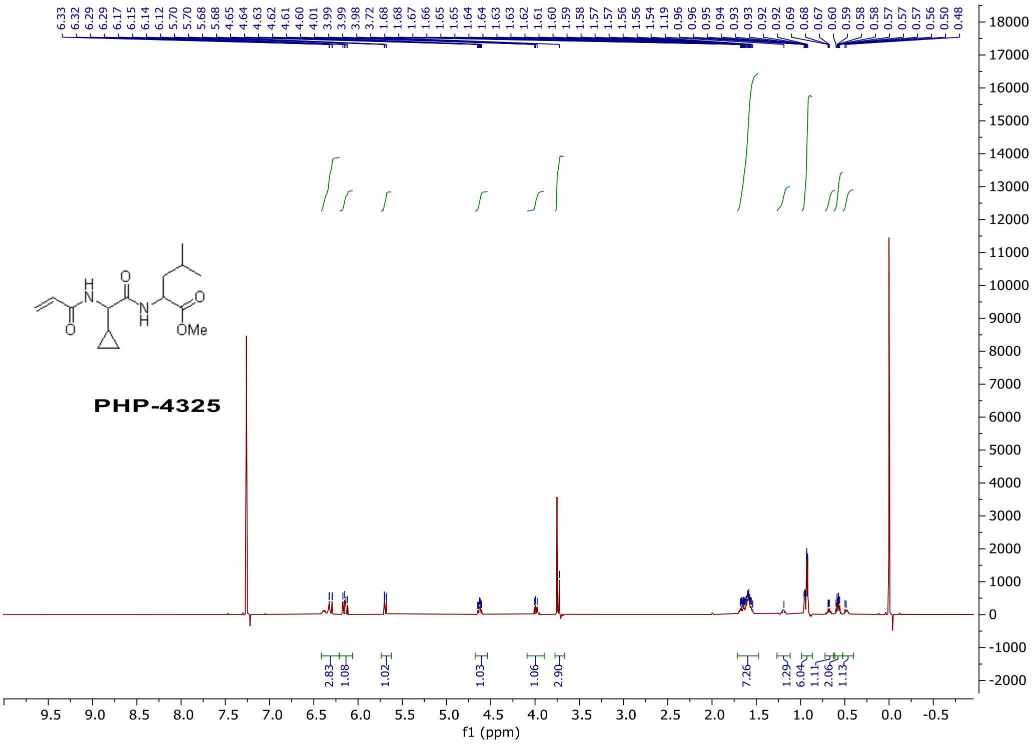

Supplementary Figure 234. ^{1}H NMR (500 MHz, $CDCl_3$+one drop of CD_3OD) of compound **PHP-4325.**

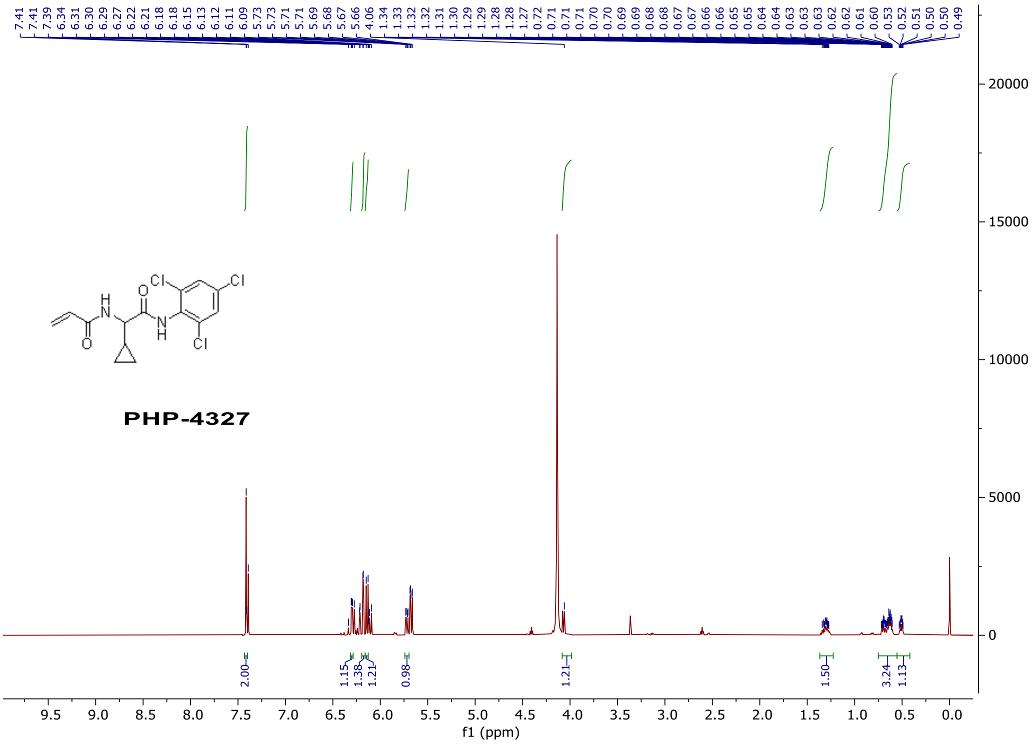

Supplementary Figure 235. ^{1}H NMR (500 MHz, $CDCl_3$+one drop of CD_3OD) of compound **PHP-4327.**

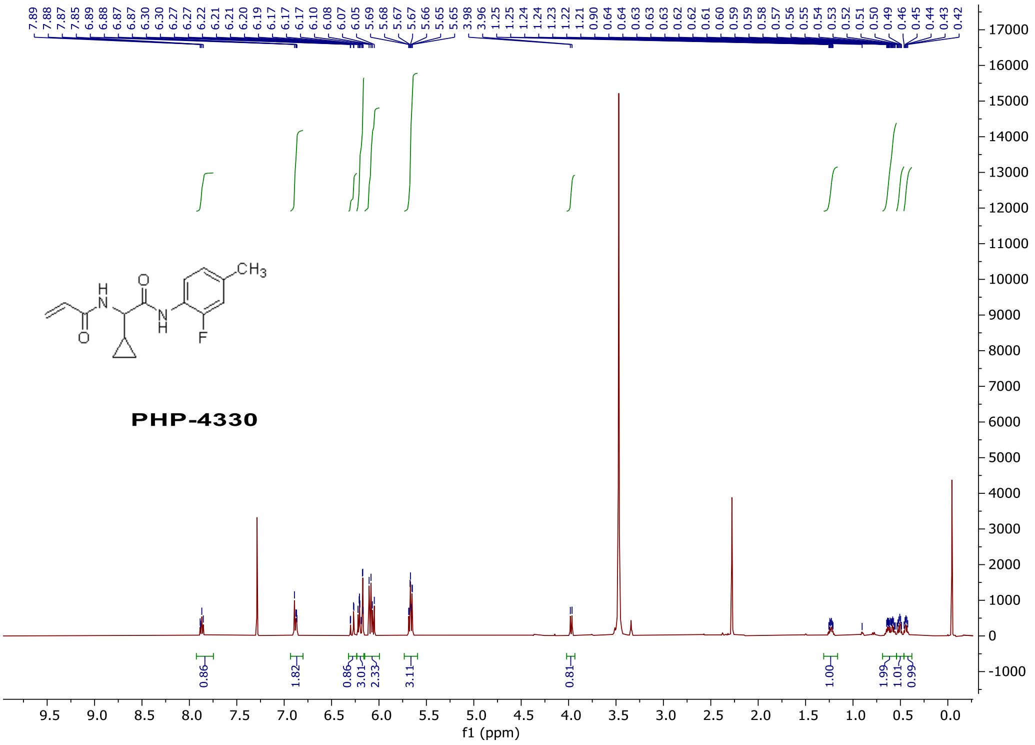

Supplementary Figure 236. ^{1}H NMR (500 MHz, $CDCl_3$+one drop of CD_3OD) of compound **PHP-4330.**

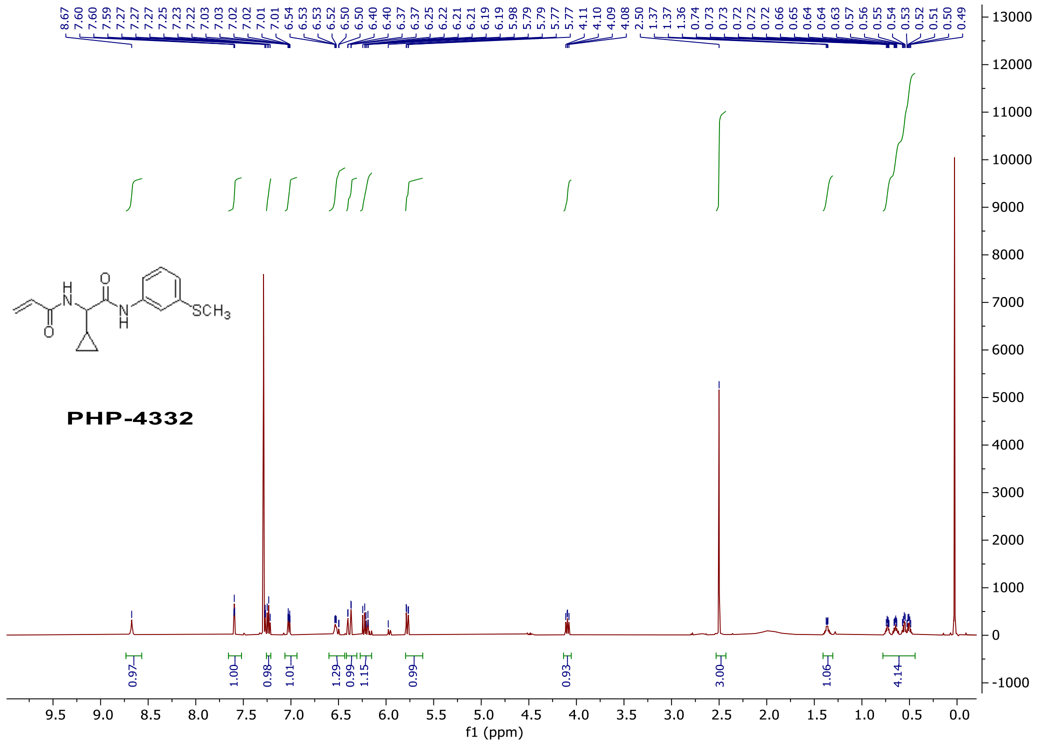

Supplementary Figure 237. ^{1}H NMR (500 MHz, $CDCl_3$) of compound **PHP-4332.**

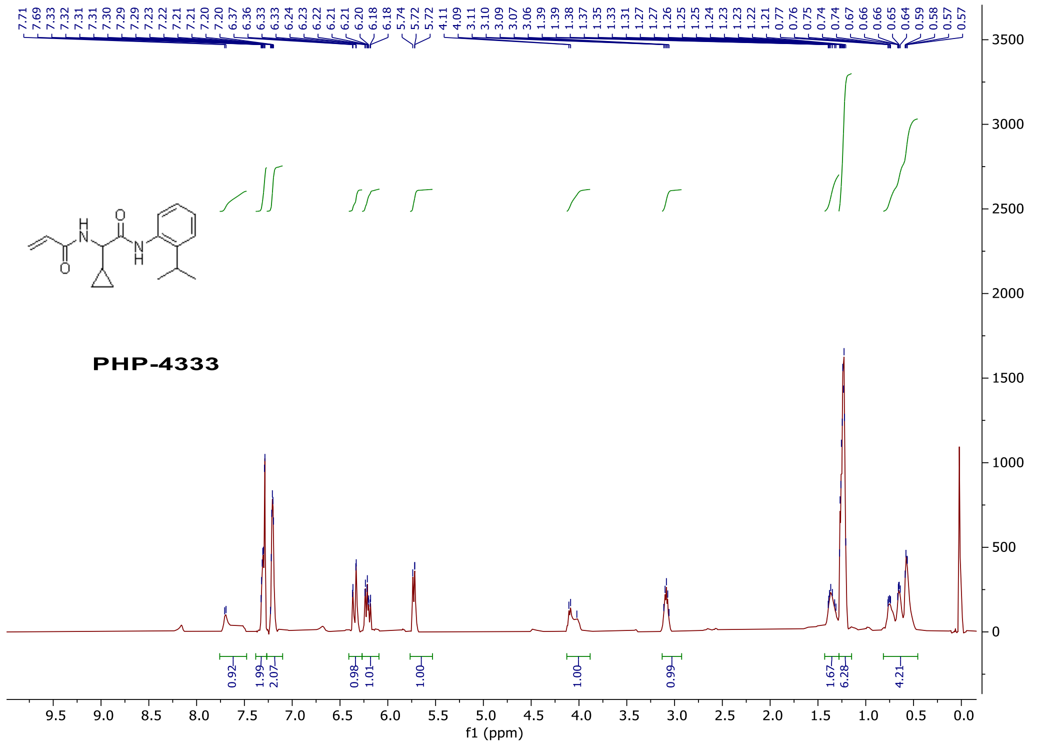

Supplementary Figure 238. ^{1}H NMR (500 MHz, $CDCl_3$) of compound **PHP-4333.**

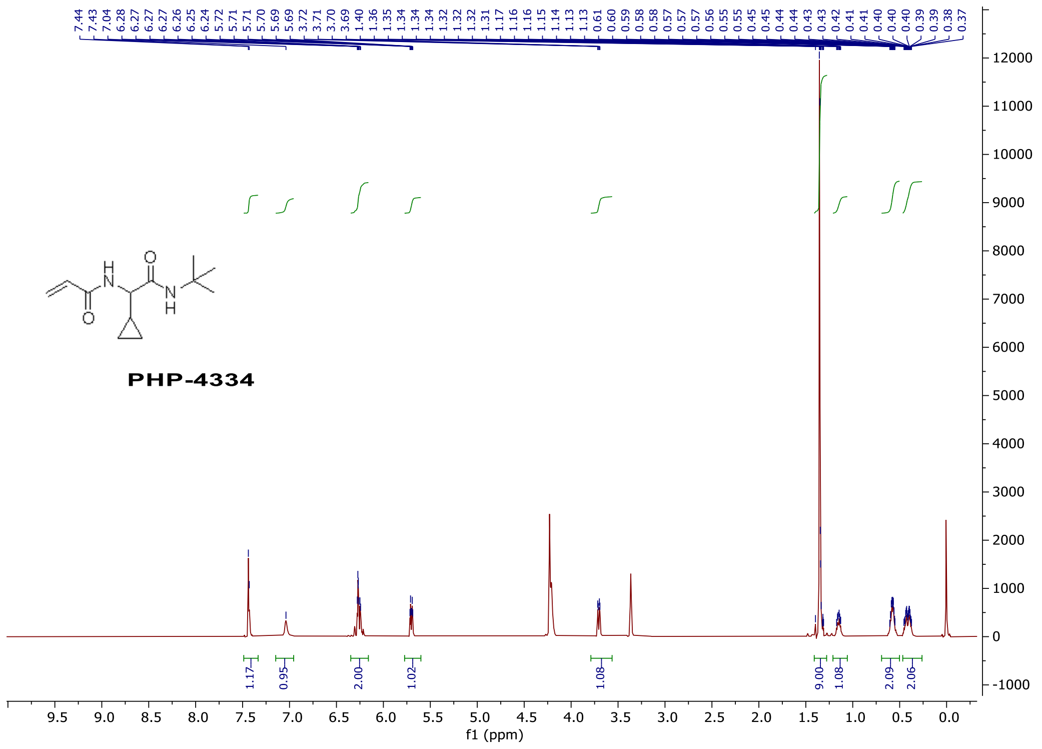

Supplementary Figure 239. ^{1}H NMR (500 MHz, $CDCl_3$+one drop of CD_3OD) of compound **PHP-4334.**

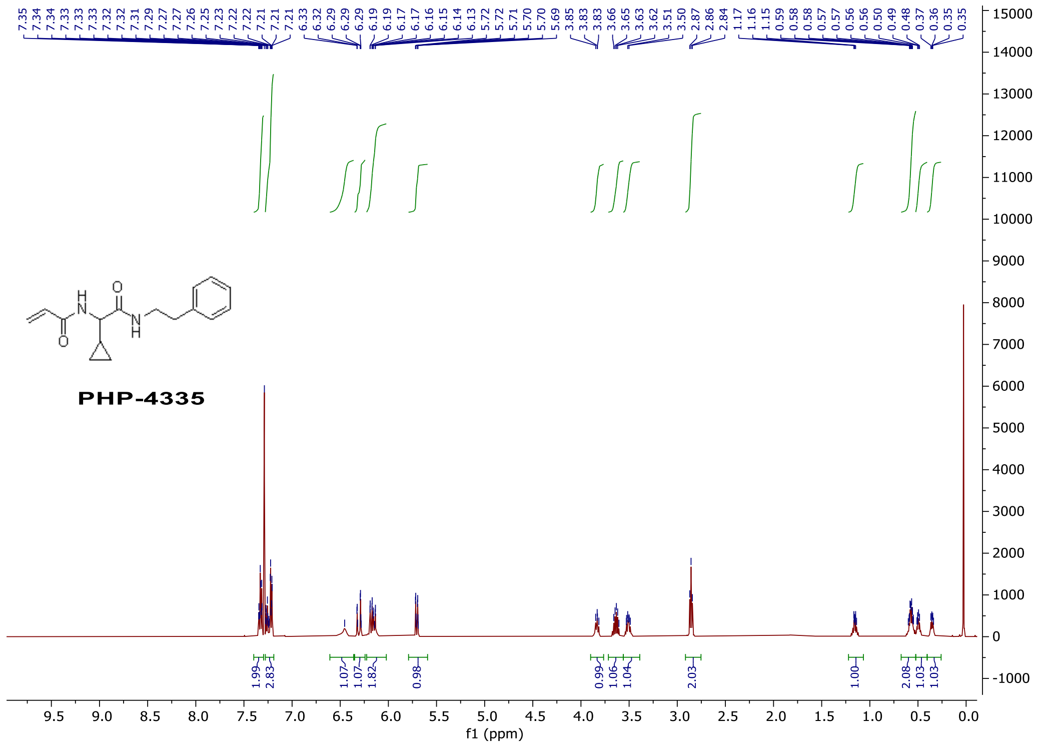

Supplementary Figure 240. ^{1}H NMR (500 MHz, $CDCl_3$) of compound **PHP-4335.**

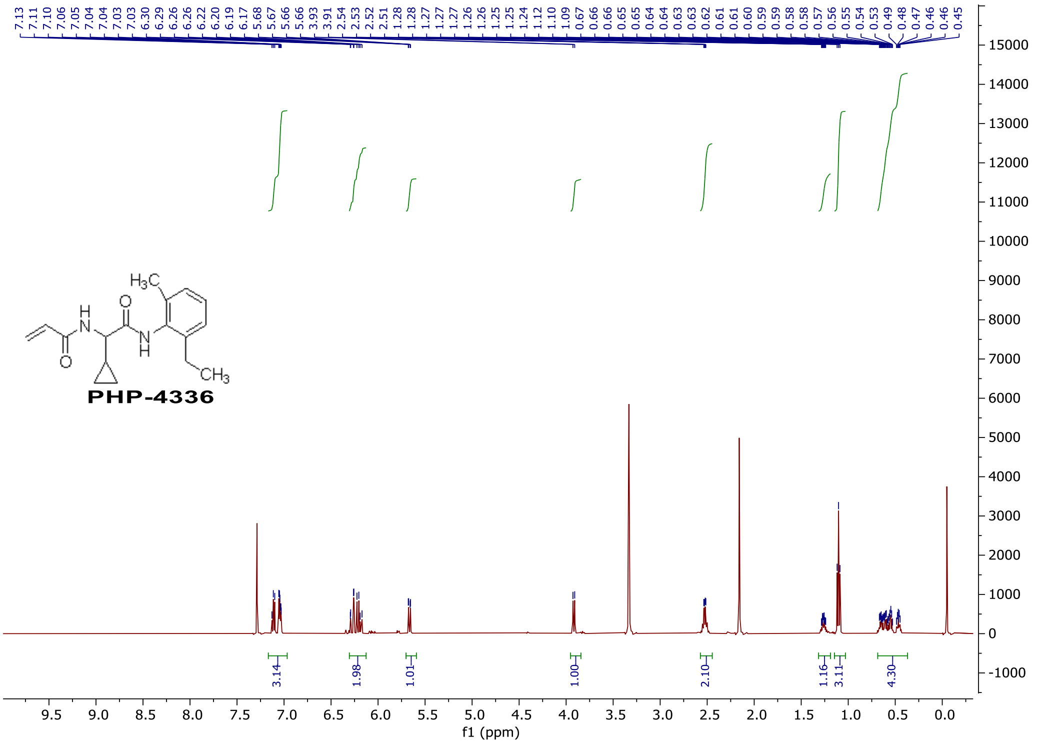

Supplementary Figure 241. ^{1}H NMR (500 MHz, $CDCl_3$+one drop of CD_3OD) of compound **PHP-4336.**

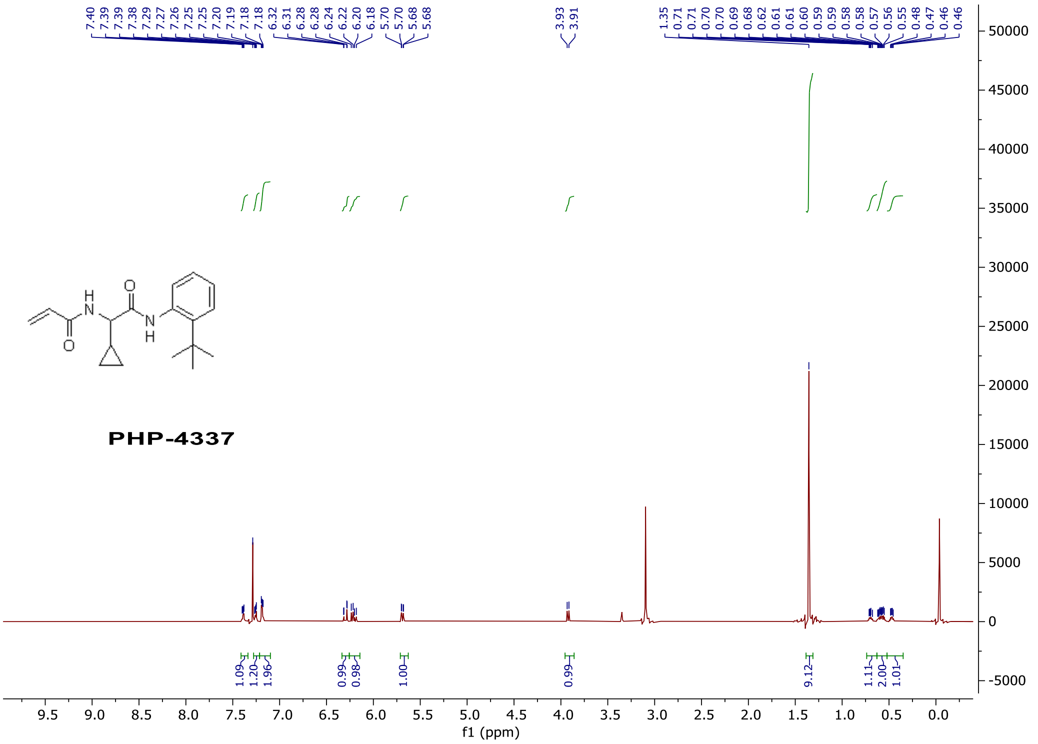

Supplementary Figure 242. ^{1}H NMR (500 MHz, $CDCl_3$+one drop of CD_3OD) of compound **PHP-4337.**

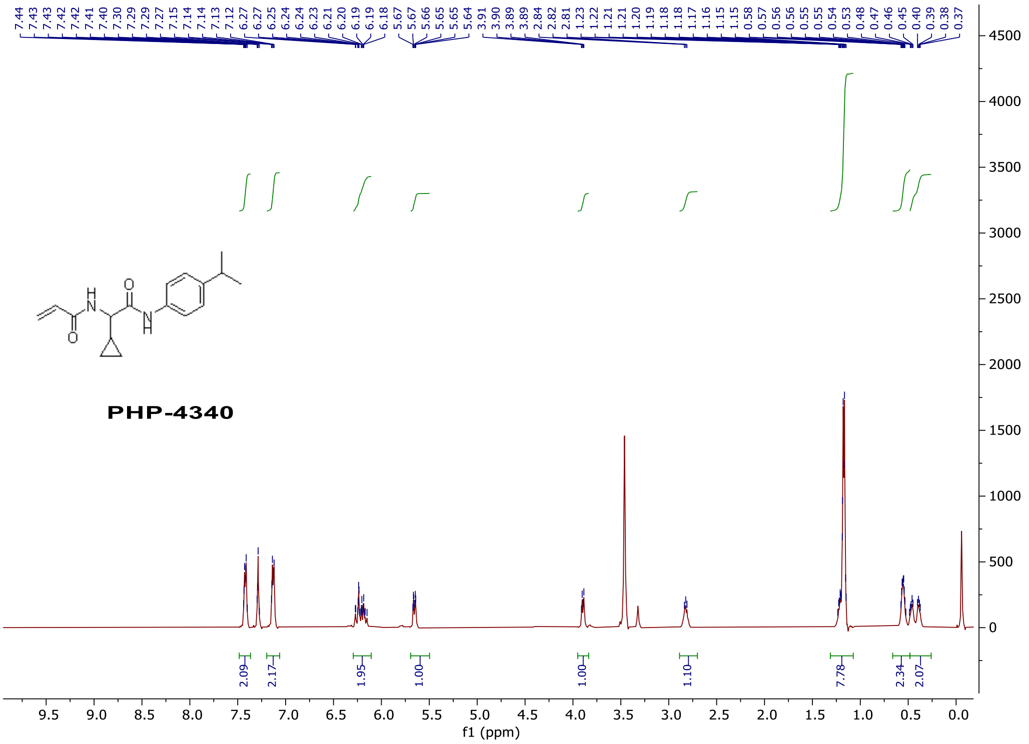

Supplementary Figure 243. ^{1}H NMR (500 MHz, $CDCl_3$+one drop of CD_3OD) of compound **PHP-4340.**

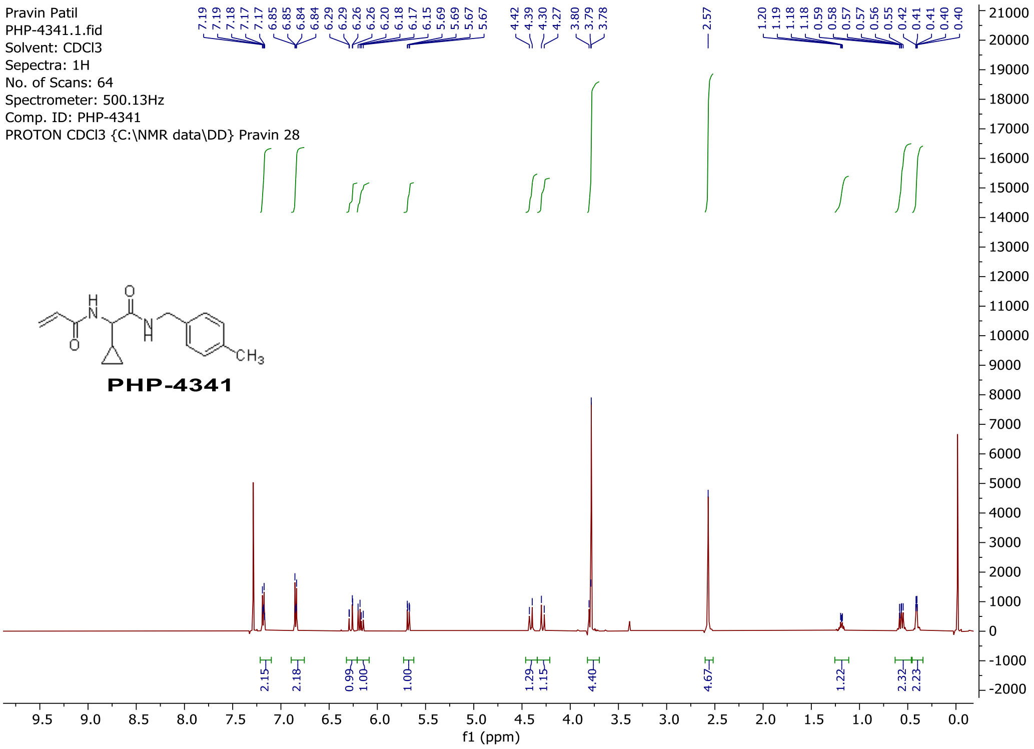

Supplementary Figure 244. ^{1}H NMR (500 MHz, $CDCl_3$) of compound **PHP-4341.**

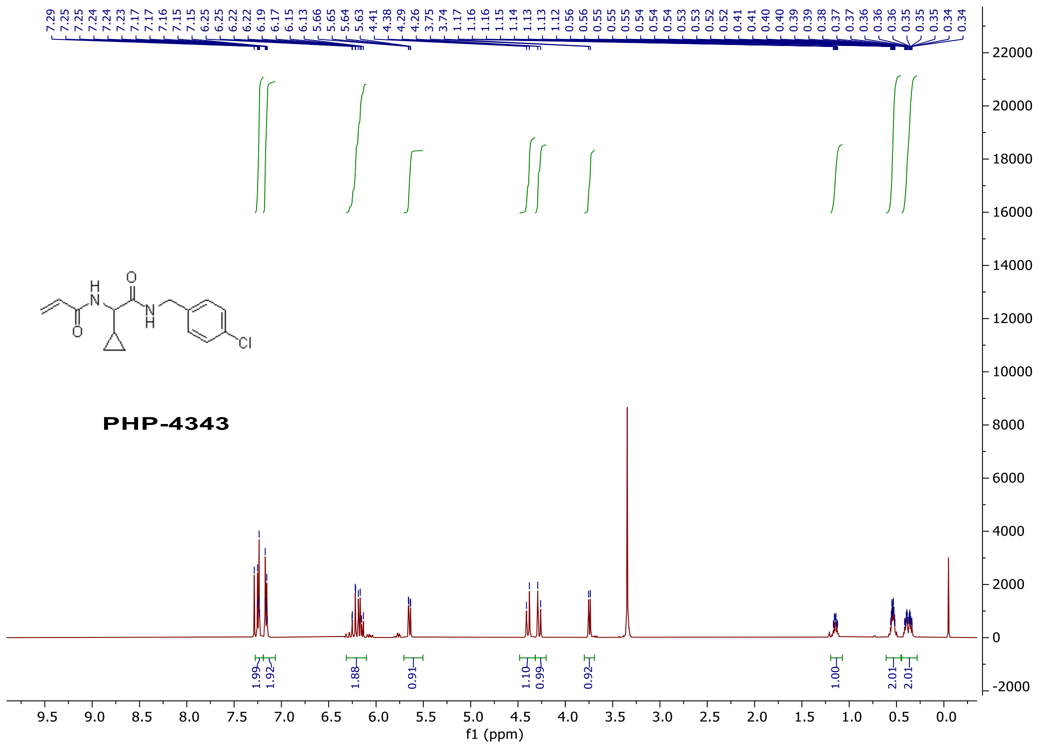

Supplementary Figure 245. ^{1}H NMR (500 MHz, $CDCl_3$+one drop of CD_3OD) of compound **PHP-4343.**

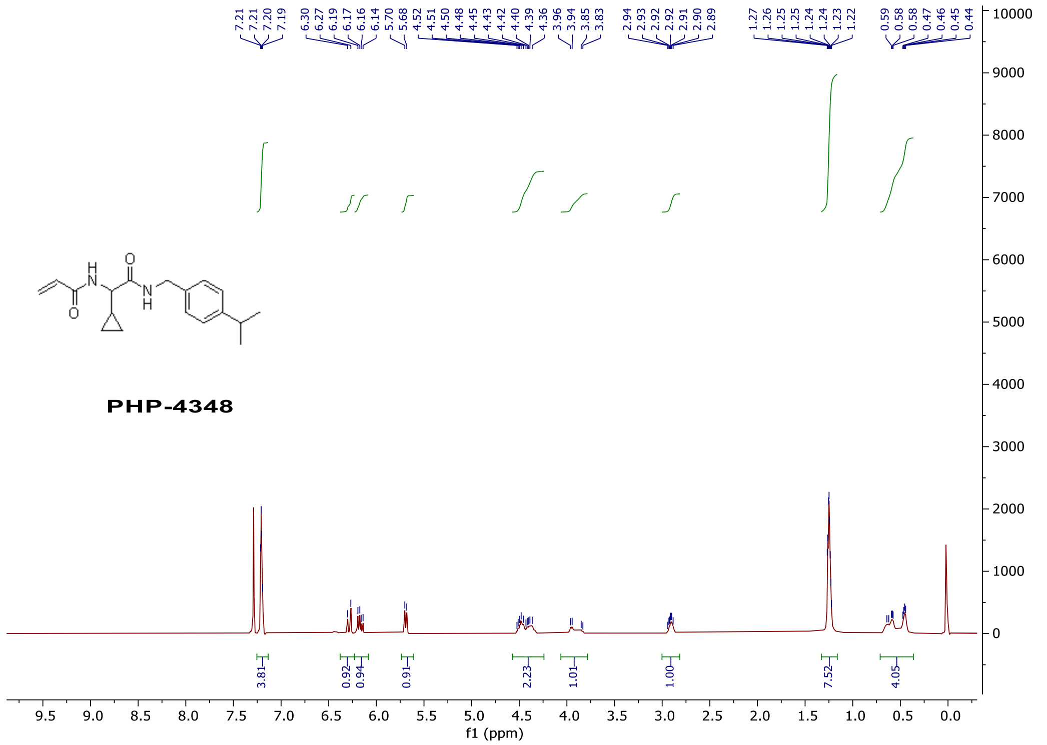

Supplementary Figure 246. ^{1}H NMR (500 MHz, $CDCl_3$) of compound **PHP-4348.**

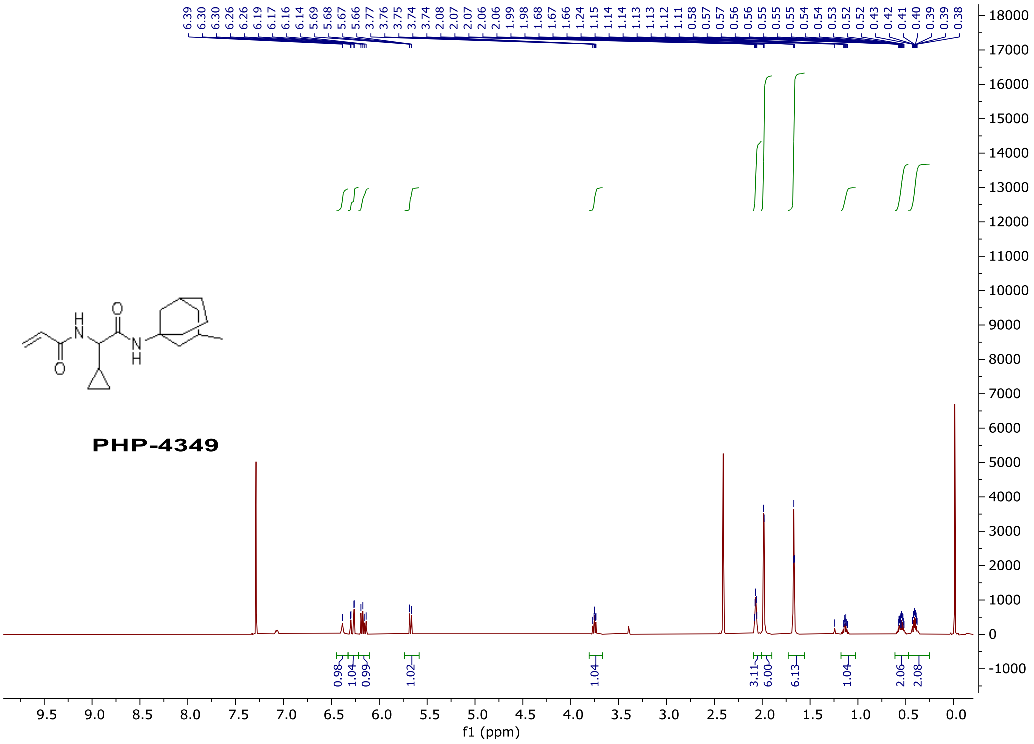

Supplementary Figure 247. ^{1}H NMR (500 MHz, $CDCl_3$) of compound **PHP-4349.**

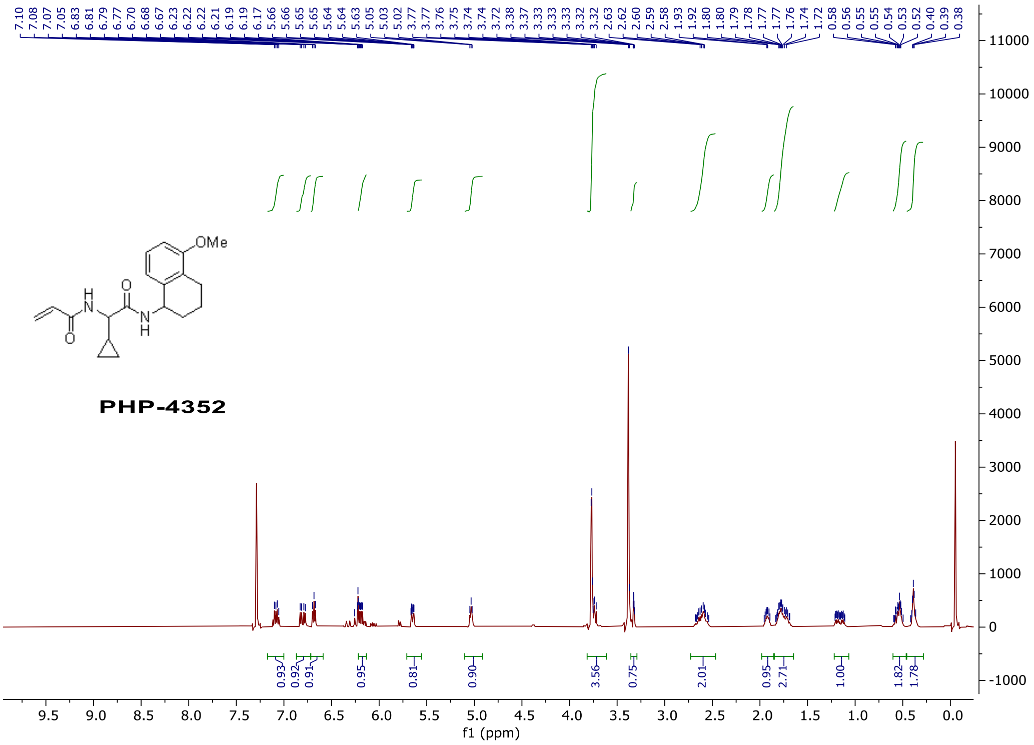

Supplementary Figure 248. ^{1}H NMR (500 MHz, $CDCl_3$+one drop of CD_3OD) of compound **PHP-4352.**

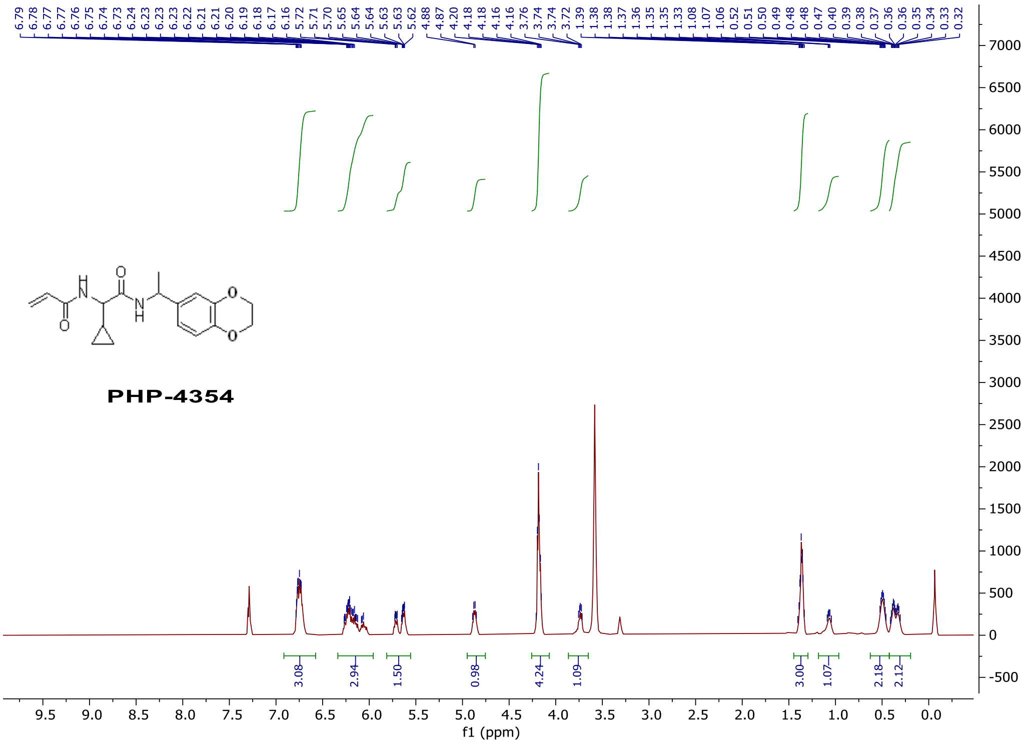

Supplementary Figure 249. ^{1}H NMR (500 MHz, $CDCl_3$+one drop of CD_3OD) of compound **PHP-4354.**

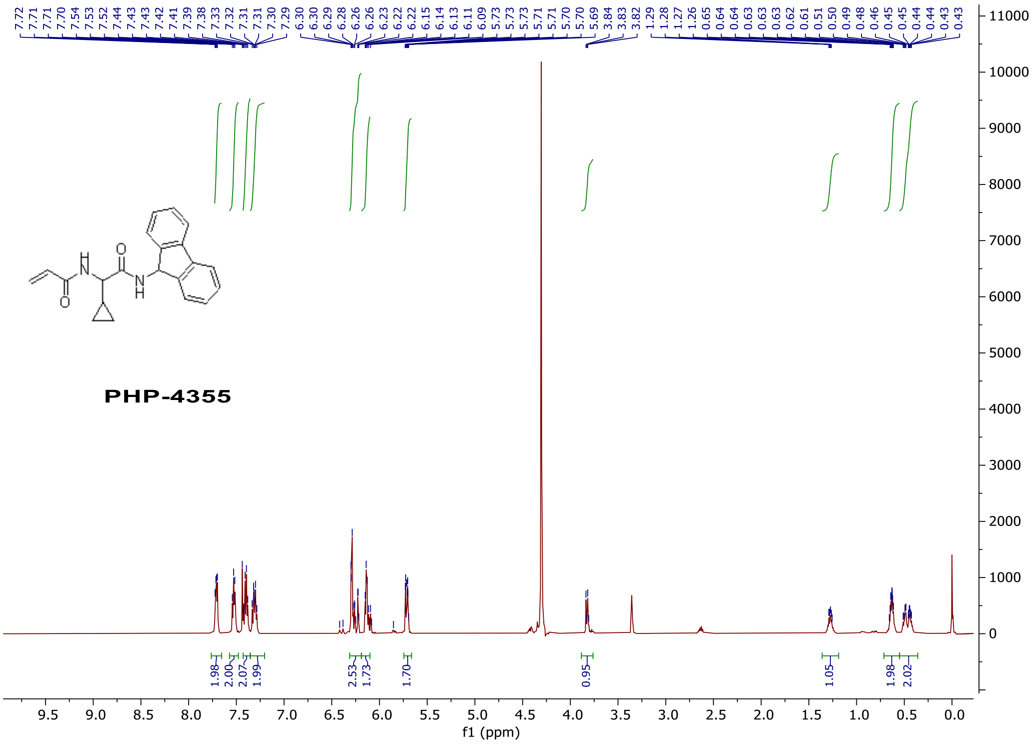

Supplementary Figure 250. ^{1}H NMR (500 MHz, $CDCl_3$+one drop of CD_3OD) of compound **PHP-4355.**

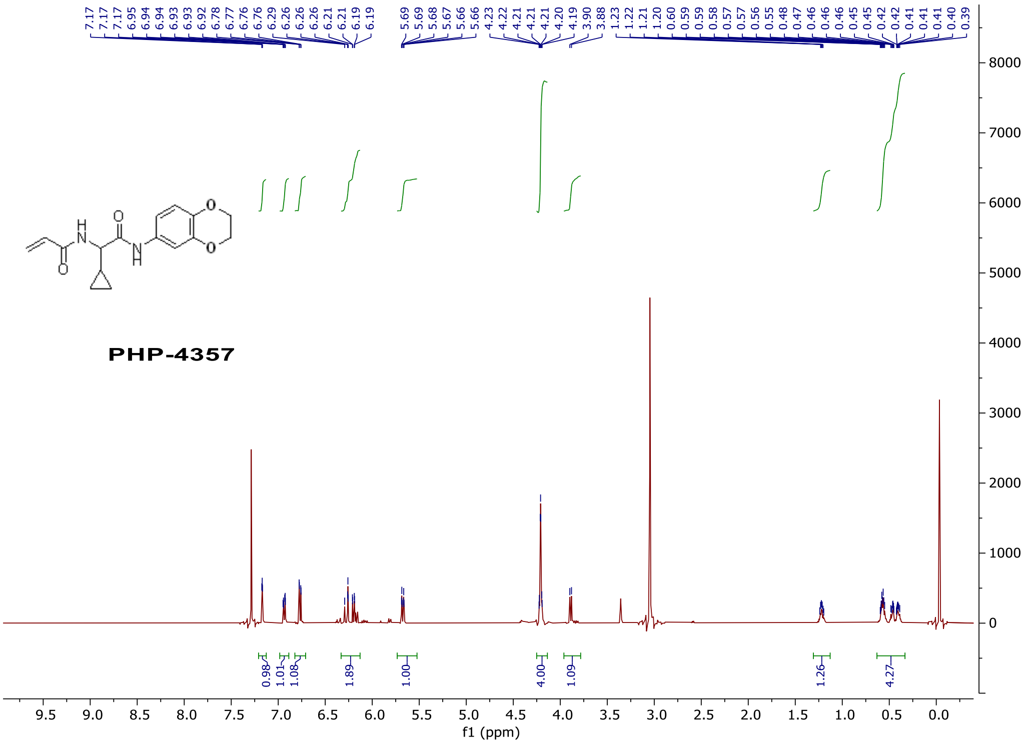

Supplementary Figure 251. ^{1}H NMR (500 MHz, $CDCl_3$+one drop of CD_3OD) of compound **PHP-4357.**

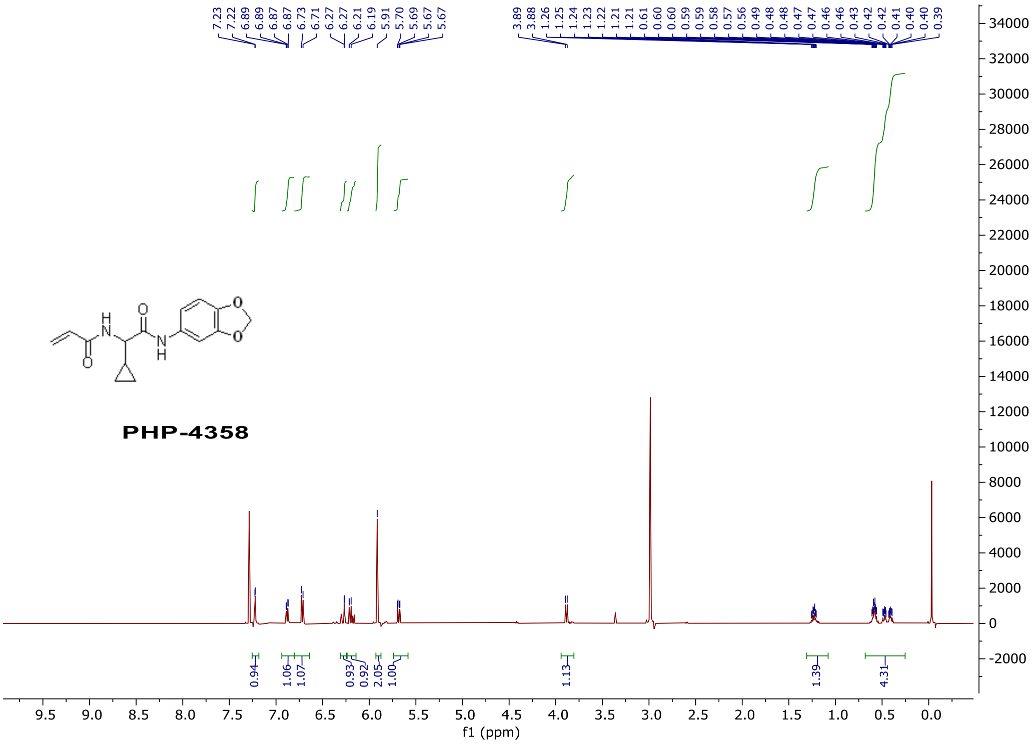

Supplementary Figure 252. ^{1}H NMR (500 MHz, $CDCl_3$+one drop of CD_3OD) of compound **PHP-4358.**

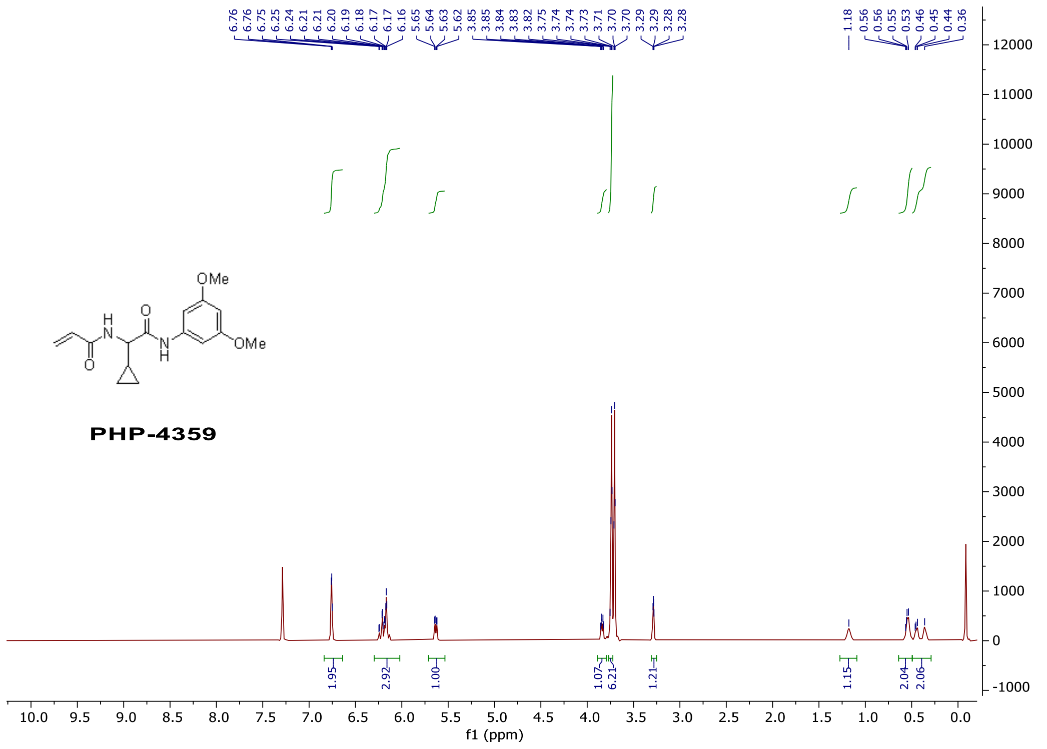

Supplementary Figure 253. ^{1}H NMR (500 MHz, $CDCl_3$) of compound **PHP-4359.**

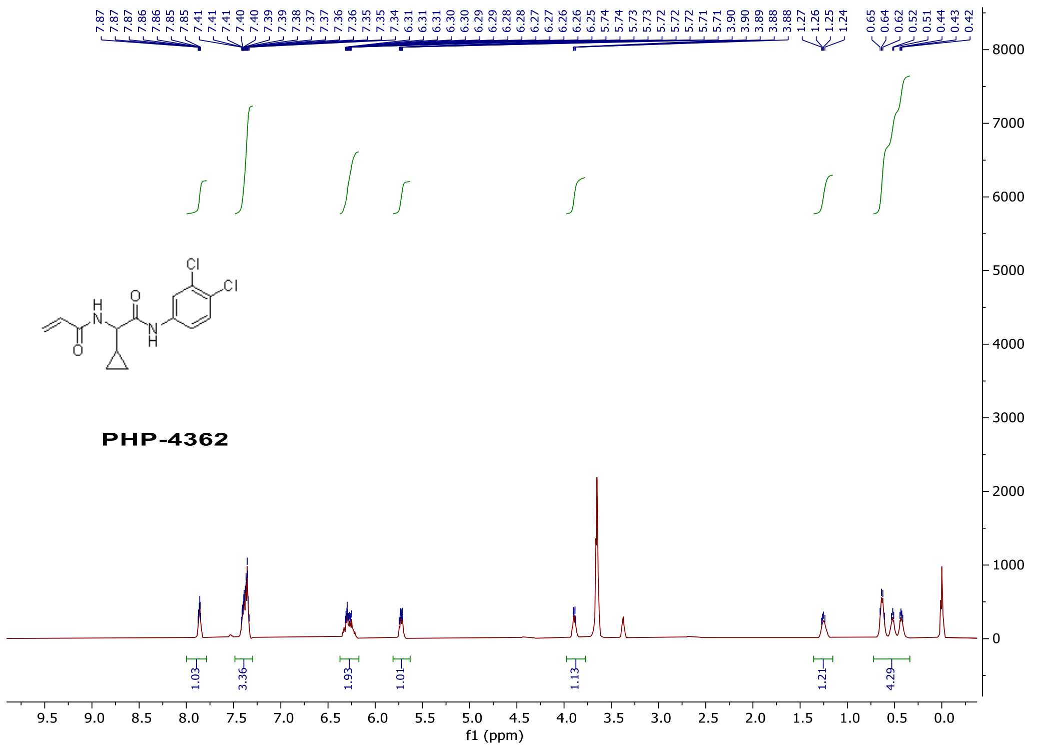

Supplementary Figure 254. ^{1}H NMR (500 MHz, $CDCl_3$+one drop of CD_3OD) of compound **PHP-4362.**

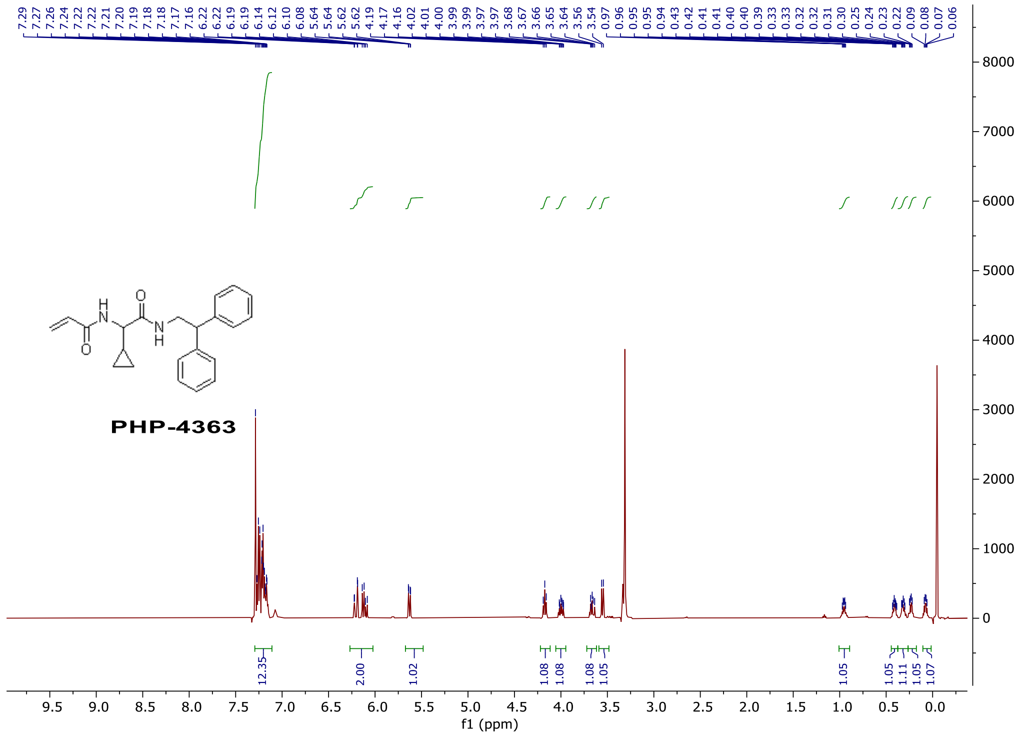

PHP-4363

Supplementary Figure 255. ^{1}H NMR (500 MHz, $CDCl_3$+one drop of CD_3OD) of compound **PHP-4363.**

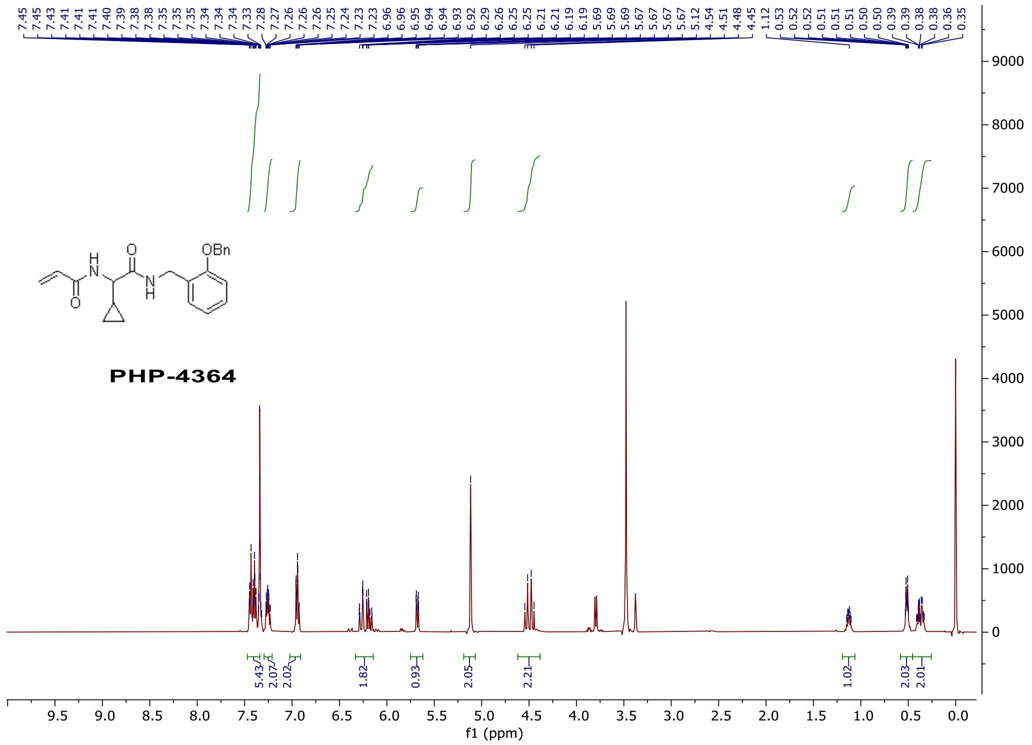

PHP-4364

Supplementary Figure 256. ^{1}H NMR (500 MHz, $CDCl_3$+one drop of CD_3OD) of compound **PHP-4364.**

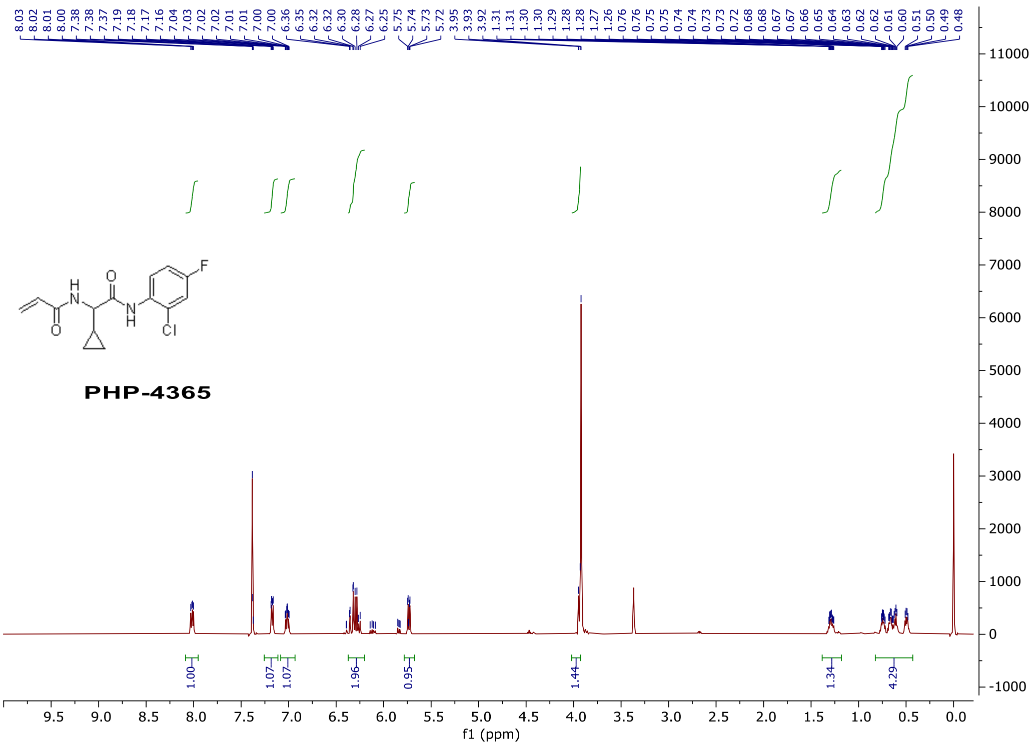

Supplementary Figure 257. ^{1}H NMR (500 MHz, $CDCl_3$+one drop of CD_3OD) of compound **PHP-4365.**

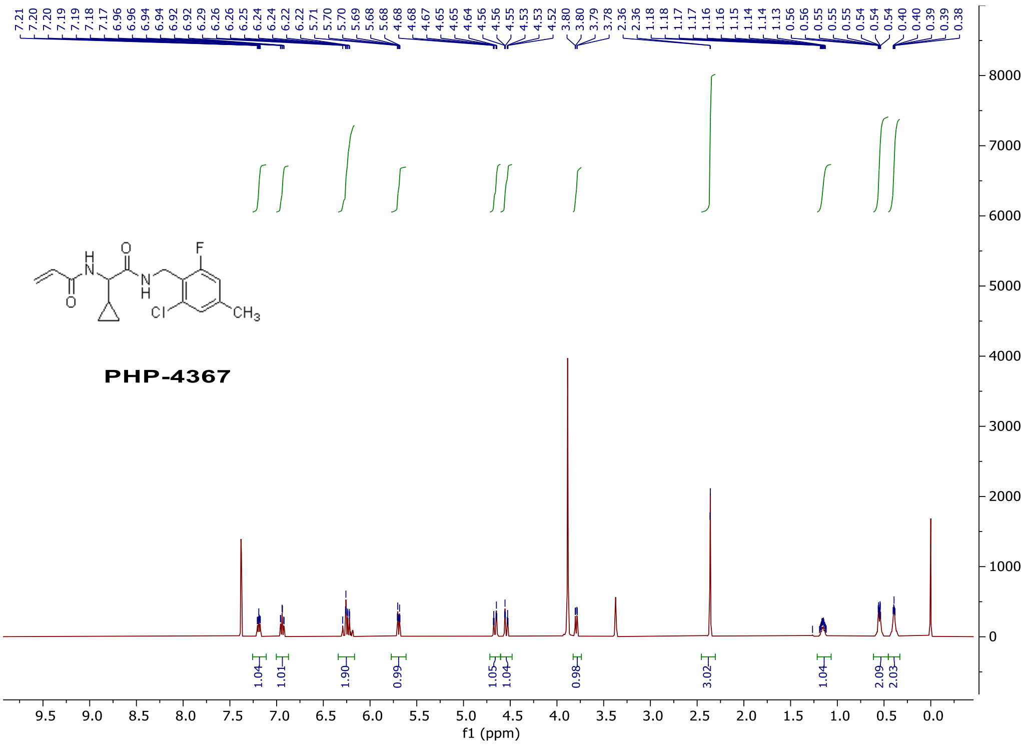

Supplementary Figure 258. ^{1}H NMR (500 MHz, $CDCl_3$+one drop of CD_3OD) of compound **PHP-4367.**

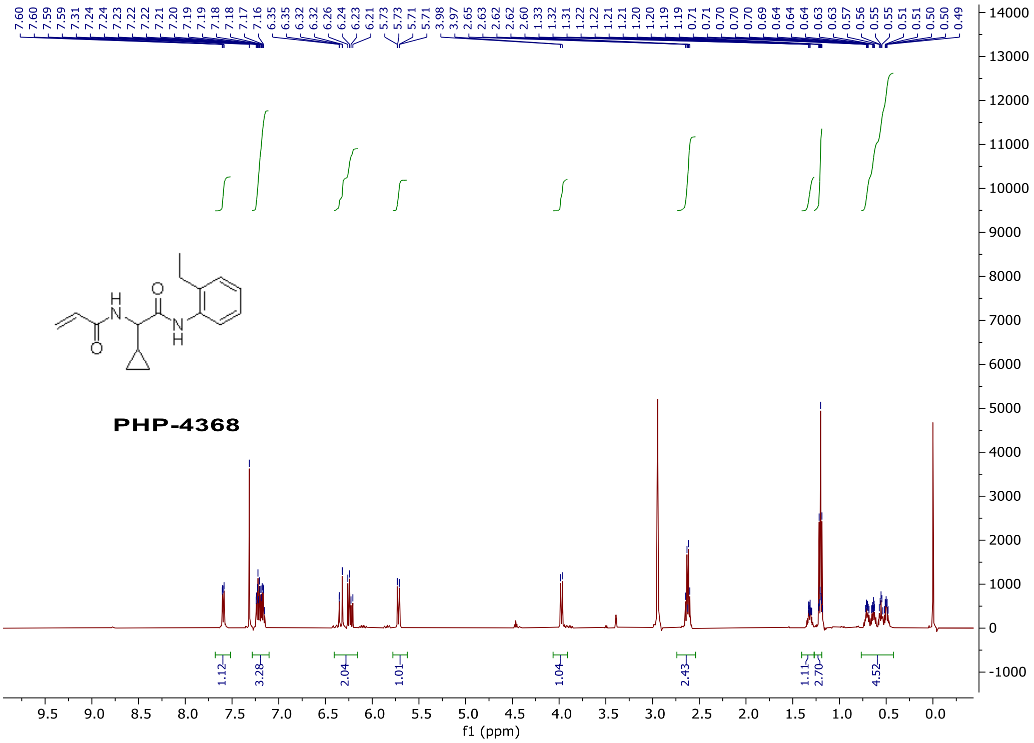

Supplementary Figure 259. ^{1}H NMR (500 MHz, $CDCl_3$+one drop of CD_3OD) of compound **PHP-4368.**

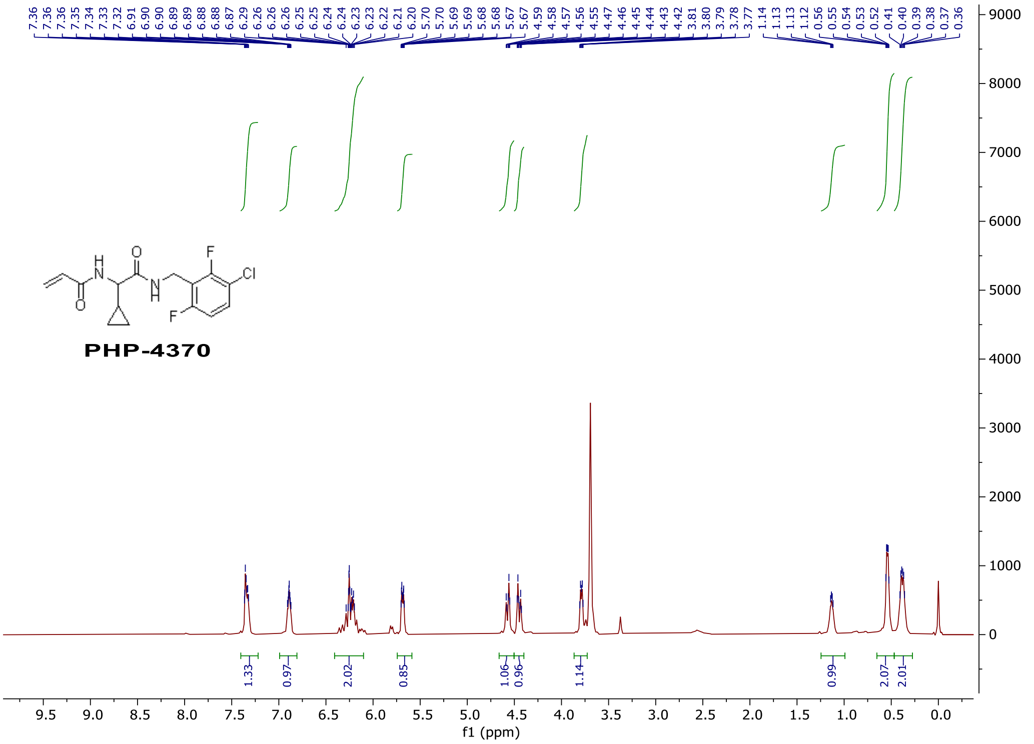

Supplementary Figure 260. ^{1}H NMR (500 MHz, $CDCl_3$+one drop of CD_3OD) of compound **PHP-4370.**

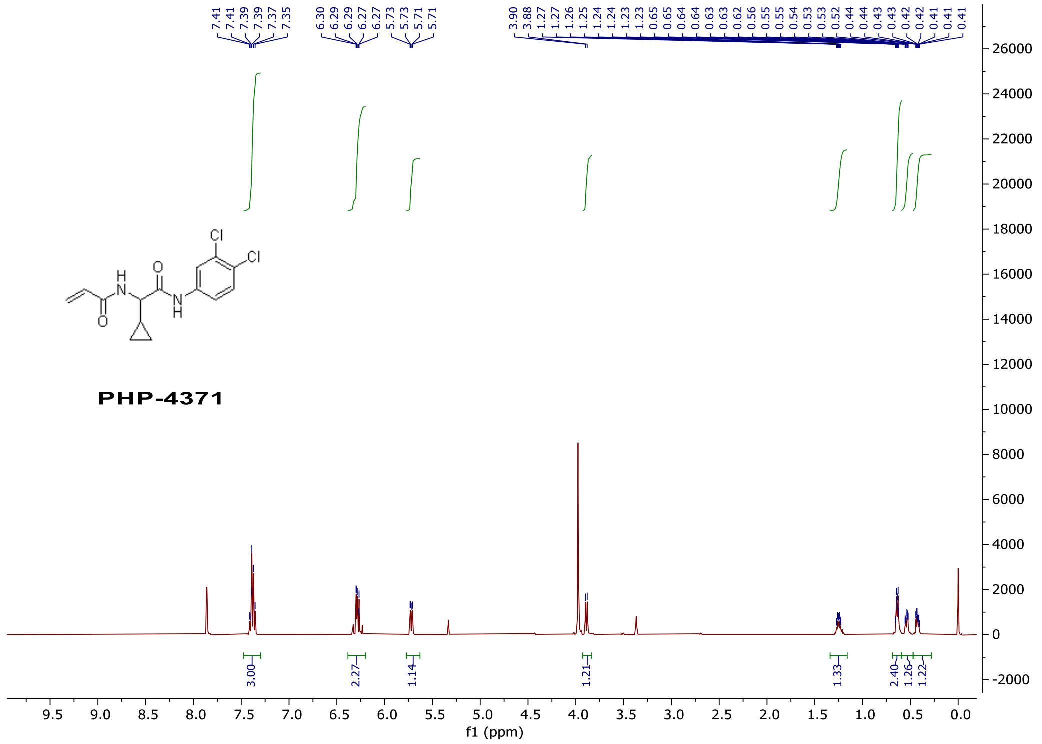

Supplementary Figure 261. ^{1}H NMR (500 MHz, $CDCl_3$+one drop of CD_3OD) of compound **PHP-4371.**

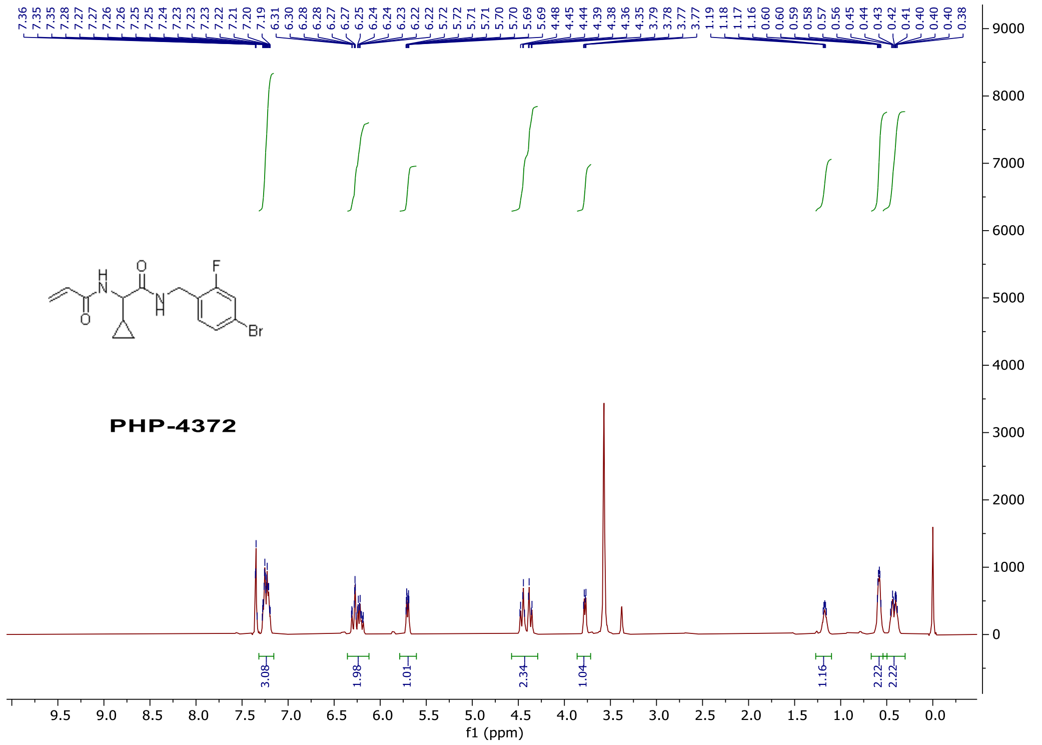

Supplementary Figure 262. ^{1}H NMR (500 MHz, $CDCl_3$+one drop of CD_3OD) of compound **PHP-4372.**

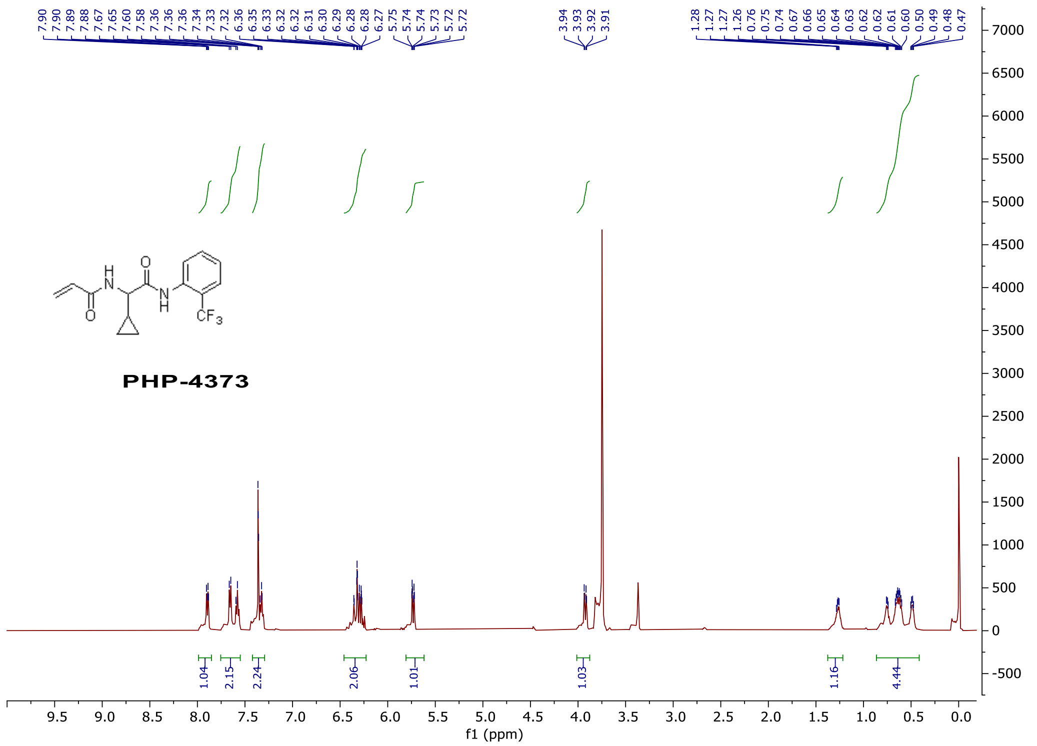

Supplementary Figure 263. ^{1}H NMR (500 MHz, $CDCl_3$+one drop of CD_3OD) of compound **PHP-4373.**

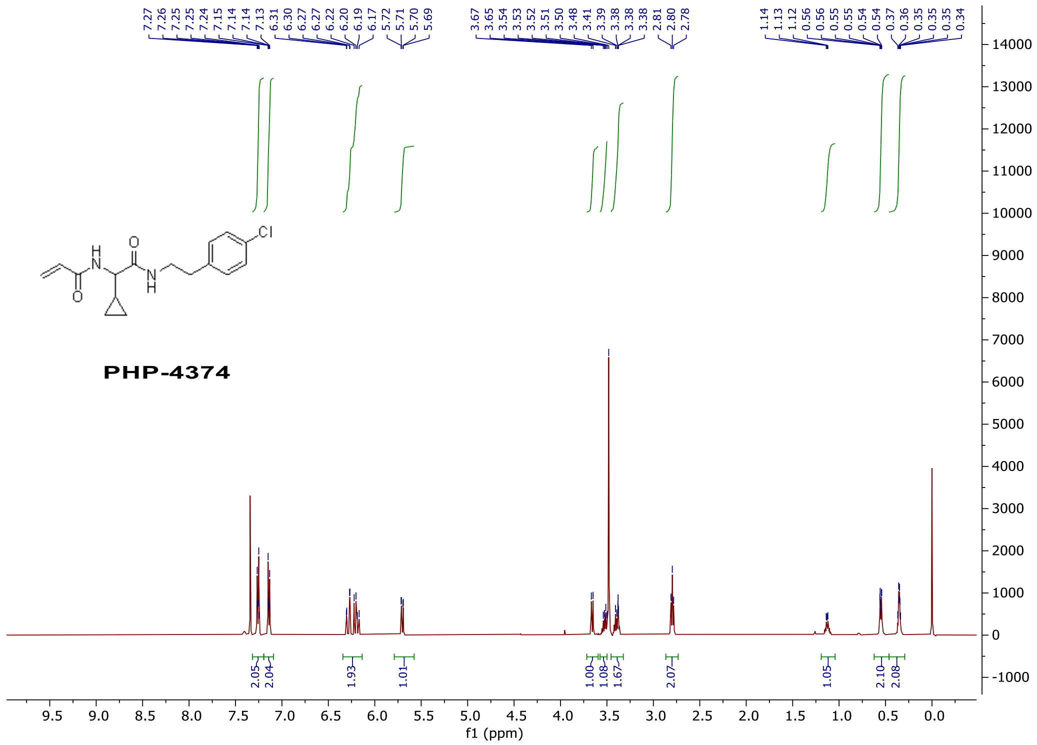

Supplementary Figure 264. ^{1}H NMR (500 MHz, $CDCl_3$+one drop of CD_3OD) of compound **PHP-4374.**

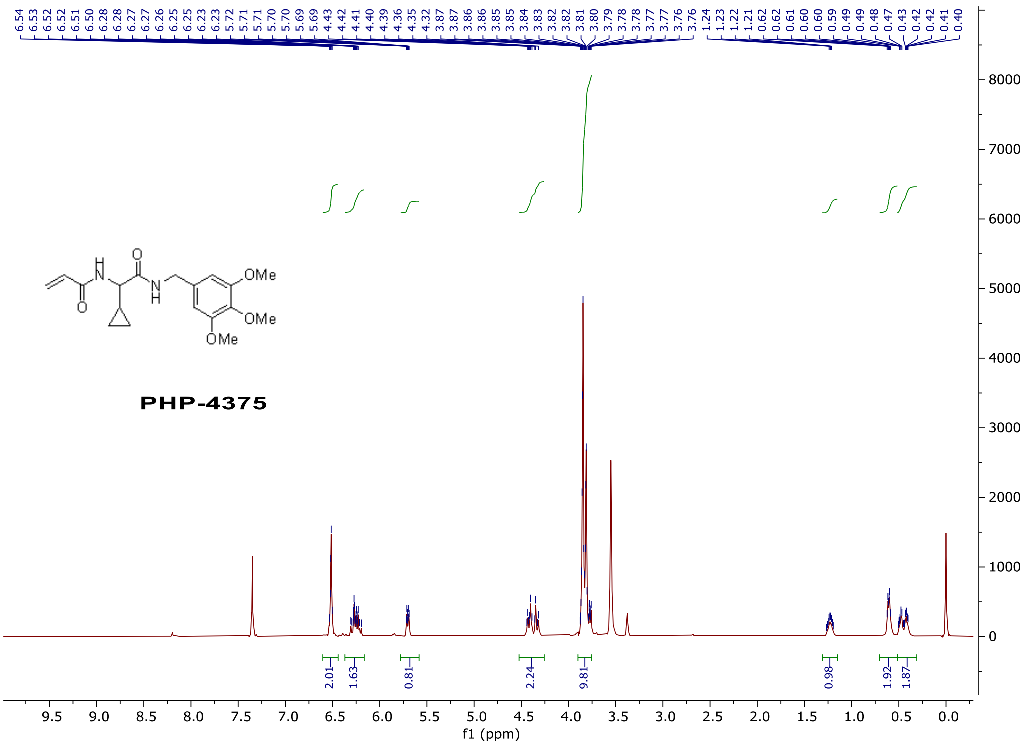

Supplementary Figure 265. ^{1}H NMR (500 MHz, $CDCl_3$+one drop of CD_3OD) of compound **PHP-4375.**

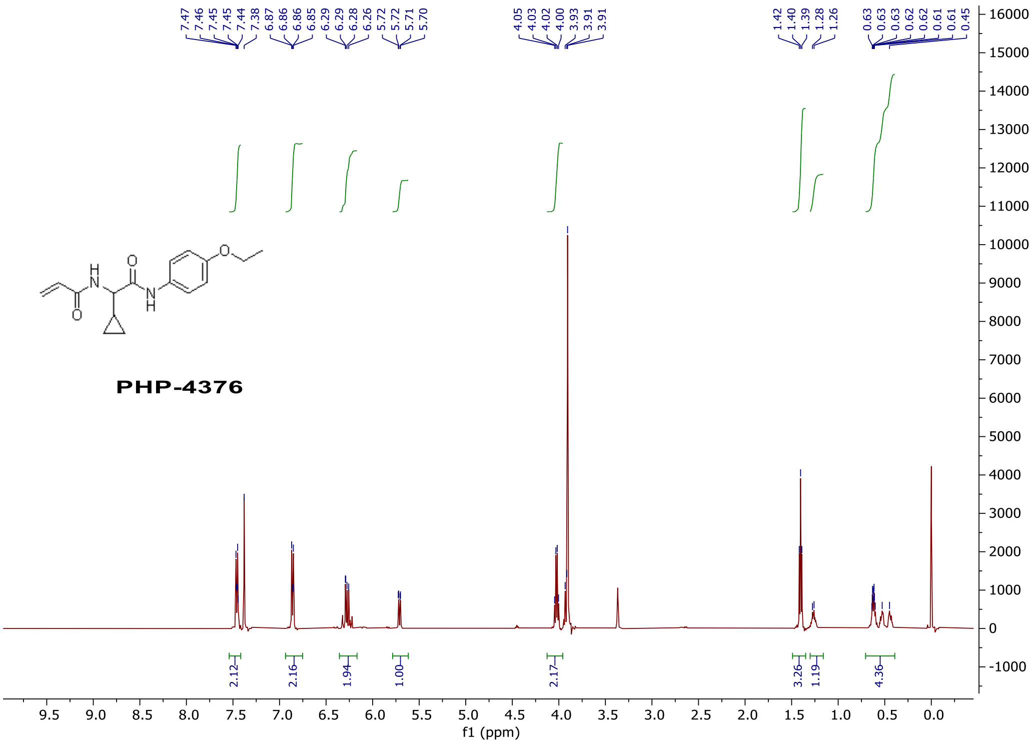

Supplementary Figure 266. ^{1}H NMR (500 MHz, $CDCl_3$+one drop of CD_3OD) of compound **PHP-4376.**

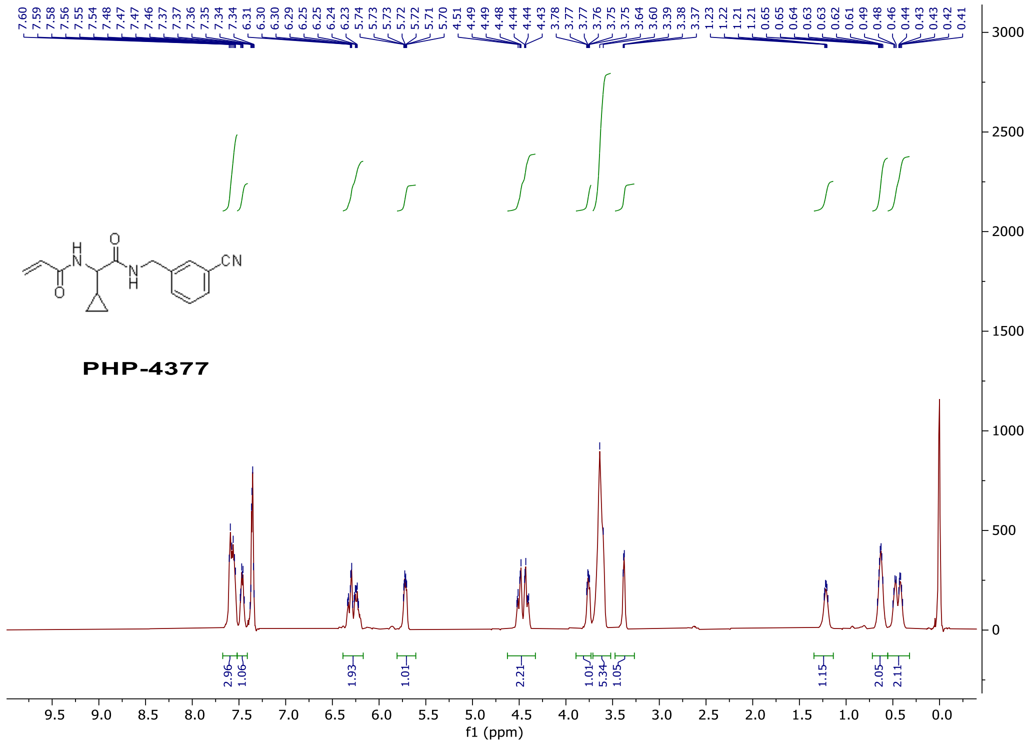

Supplementary Figure 267. ^{1}H NMR (500 MHz, $CDCl_3$+one drop of CD_3OD) of compound **PHP-4377.**

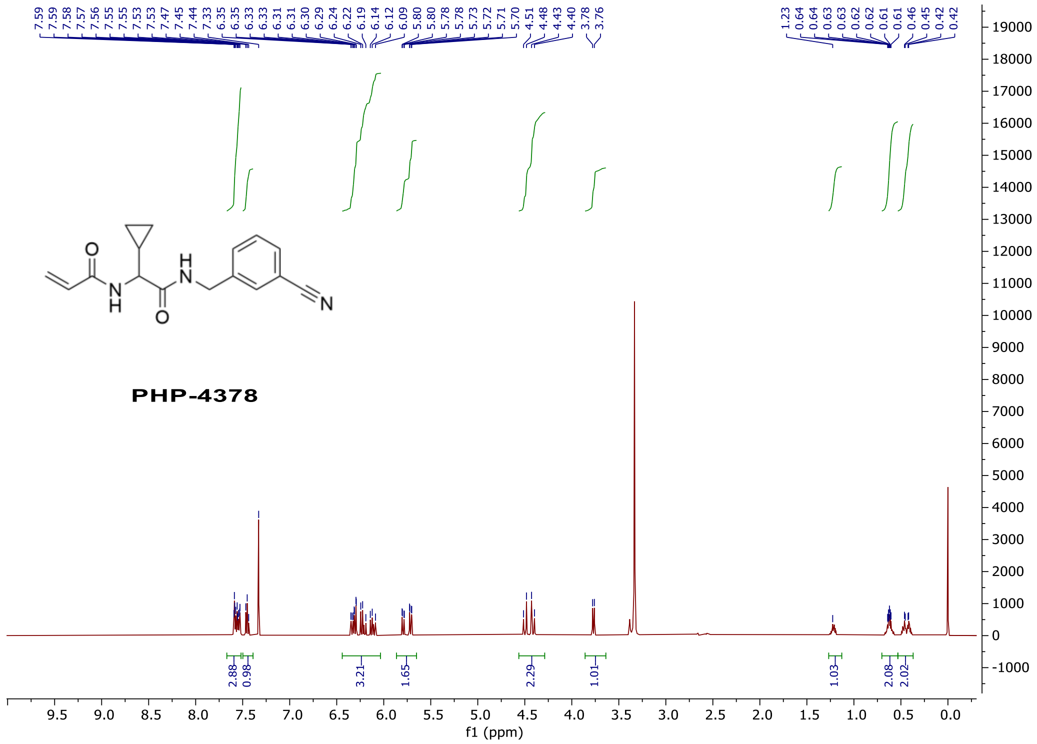

Supplementary Figure 268. ^{1}H NMR (500 MHz, $CDCl_3$+one drop of CD_3OD) of compound **PHP-4378.**

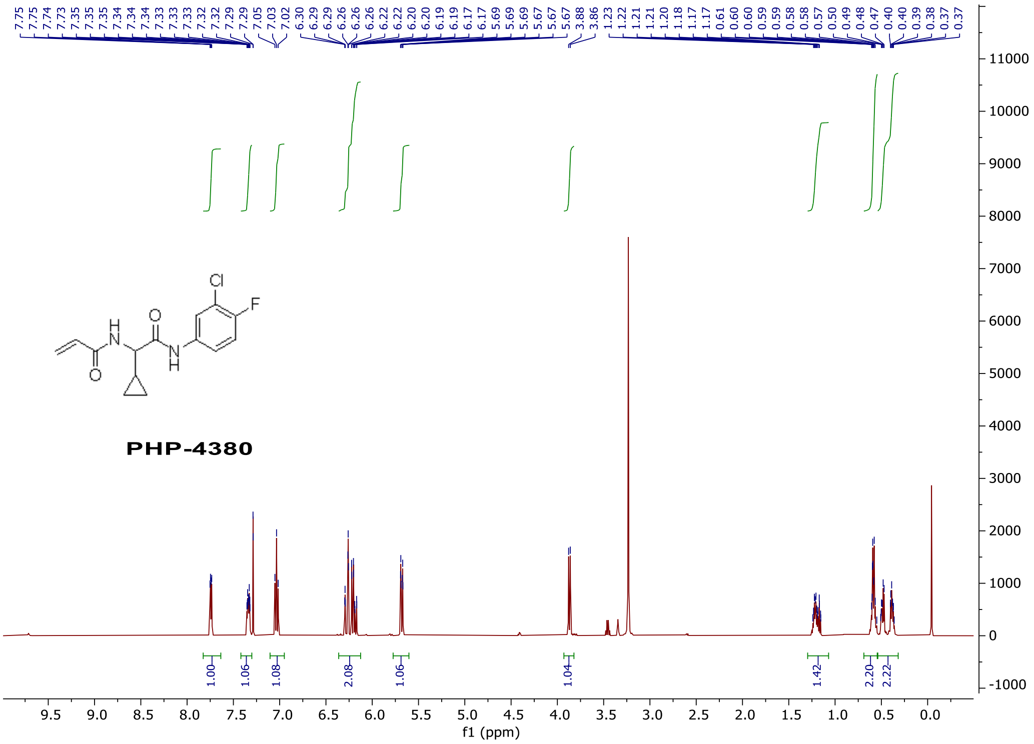

Supplementary Figure 269. ^{1}H NMR (500 MHz, $CDCl_3$+one drop of CD_3OD) of compound **PHP-4380.**

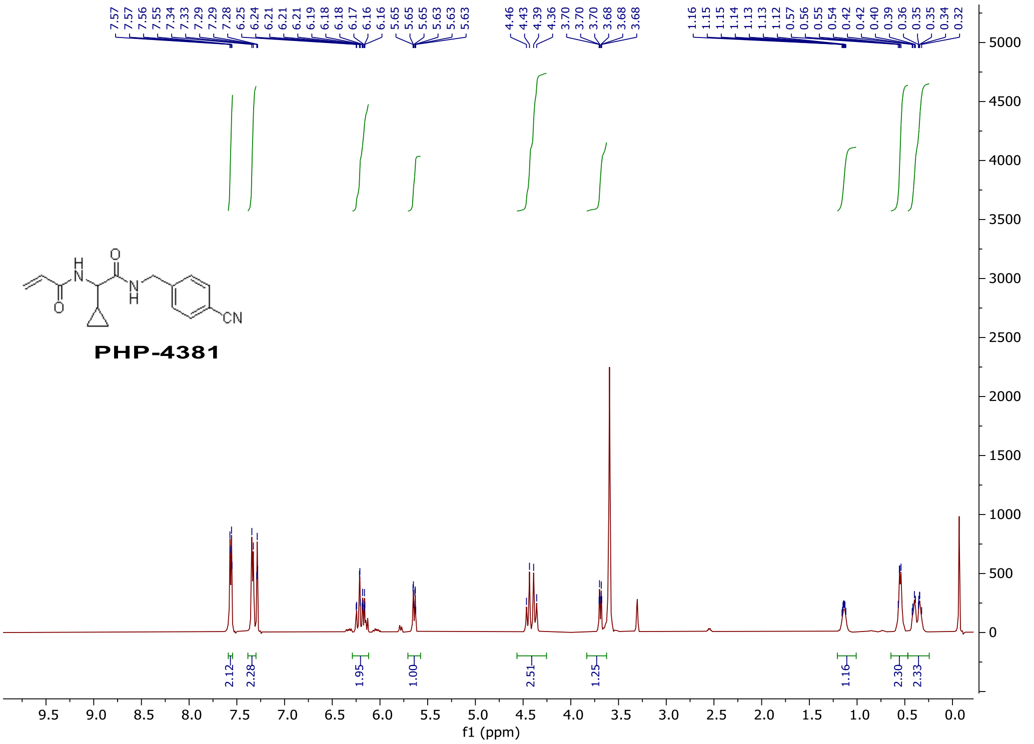

Supplementary Figure 270. ^{1}H NMR (500 MHz, $CDCl_3$+one drop of CD_3OD) of compound **PHP-4381.**

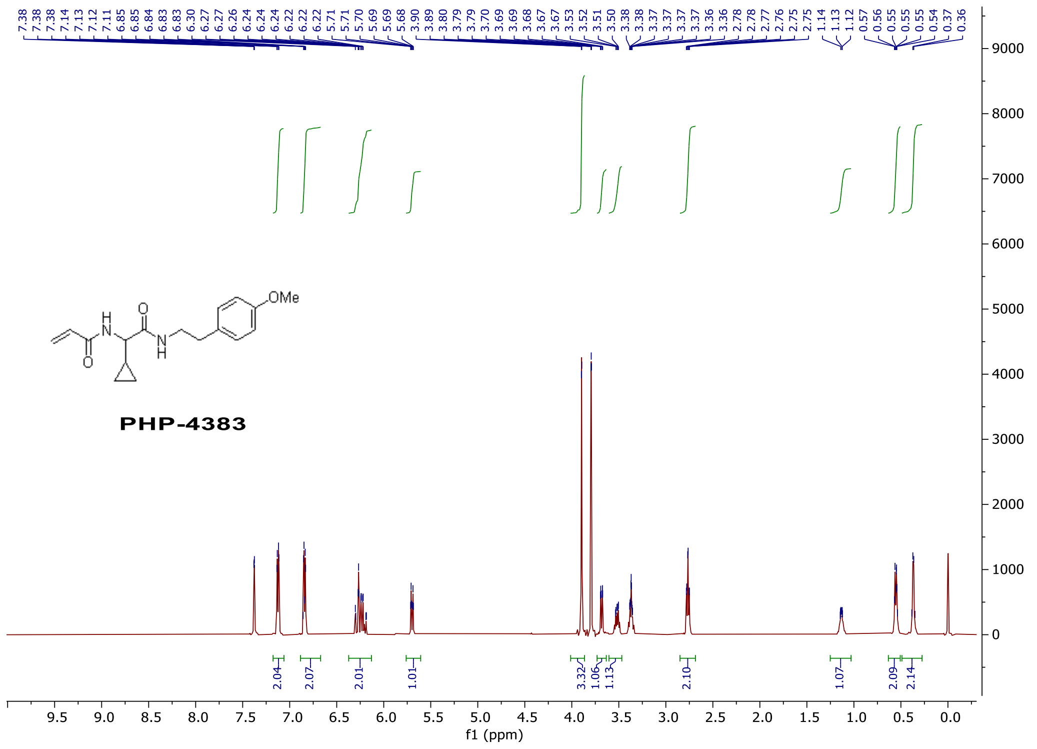

Supplementary Figure 271. ^{1}H NMR (500 MHz, $CDCl_3$+one drop of CD_3OD) of compound **PHP-4383.**

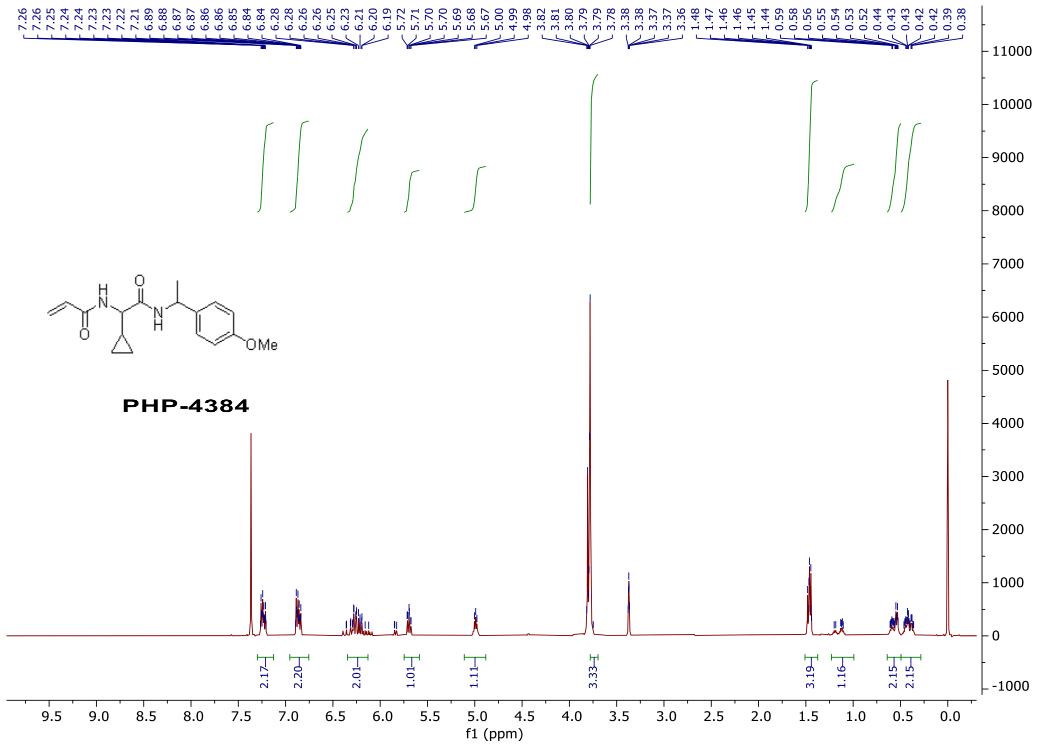

Supplementary Figure 272. ^{1}H NMR (500 MHz, $CDCl_3$) of compound **PHP-4384.**

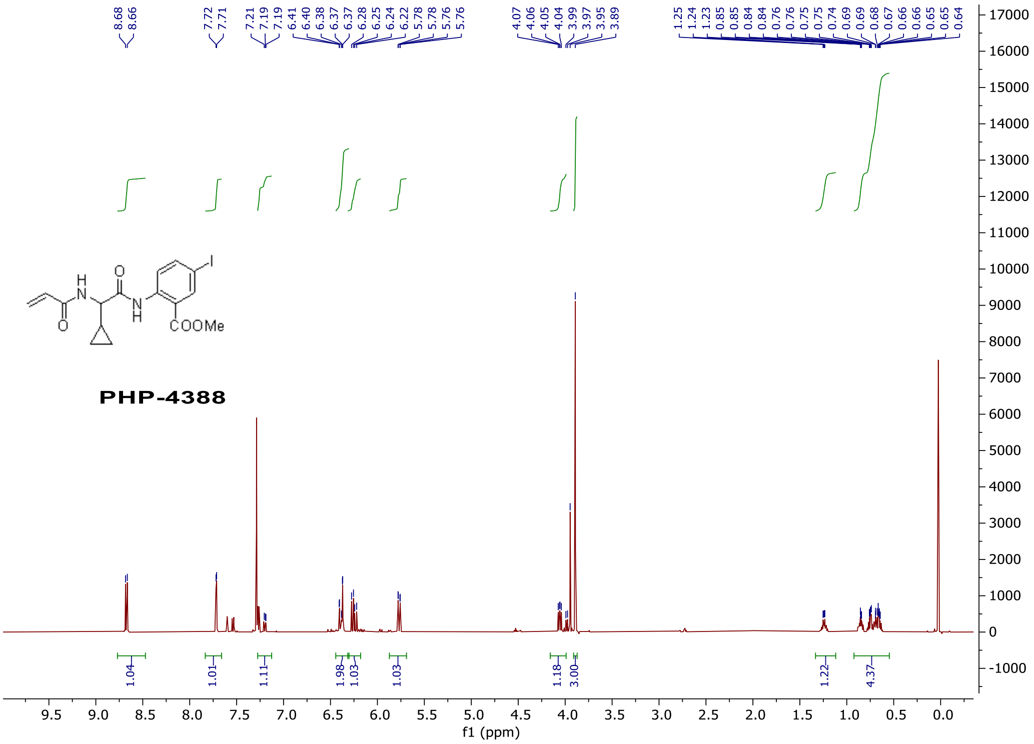

Supplementary Figure 273. ^{1}H NMR (500 MHz, $CDCl_3$) of compound **PHP-4388.**

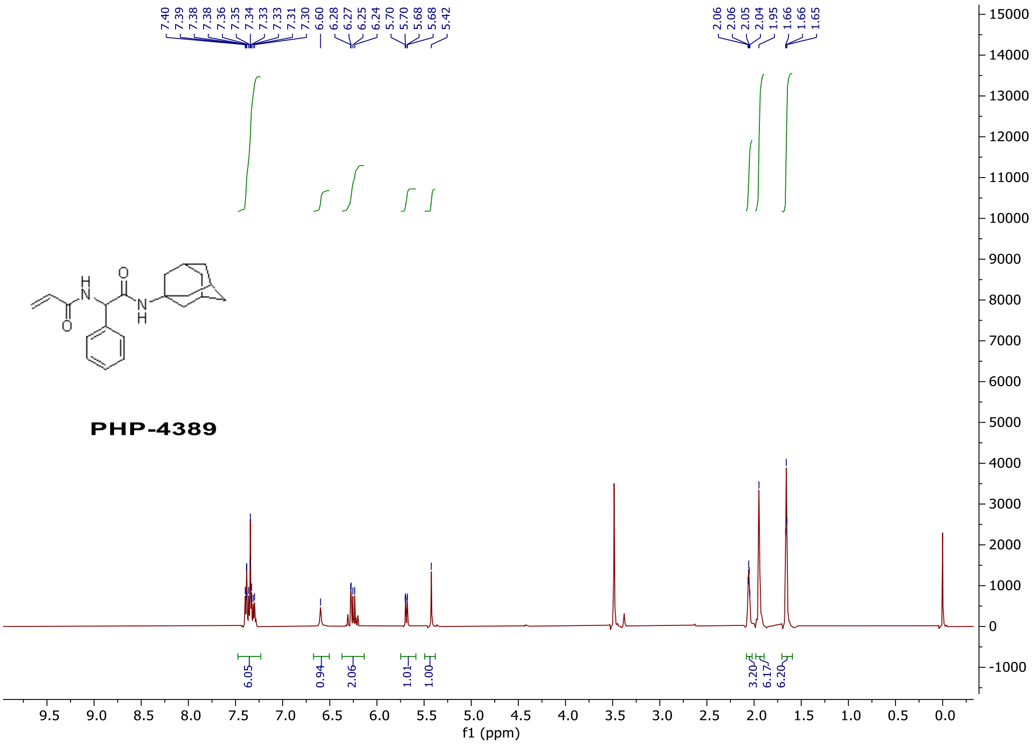

Supplementary Figure 274. ^{1}H NMR (500 MHz, $CDCl_3$+one drop of CD_3OD) of compound **PHP-4389.**

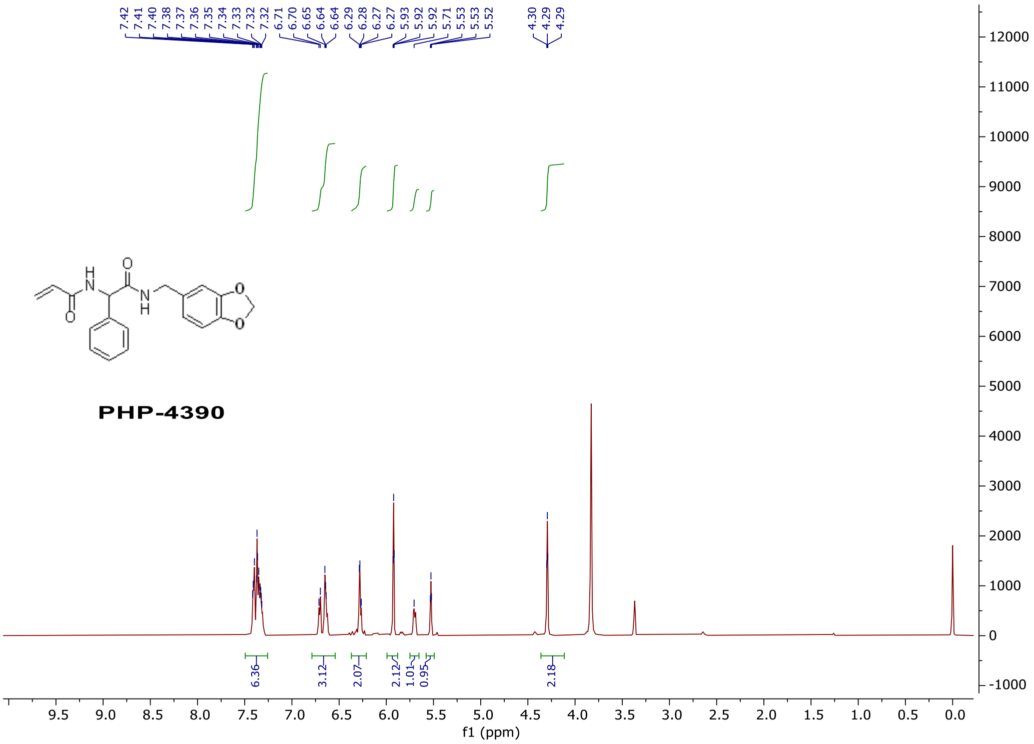

Supplementary Figure 275. ^{1}H NMR (500 MHz, $CDCl_3$+one drop of CD_3OD) of compound **PHP-4390.**

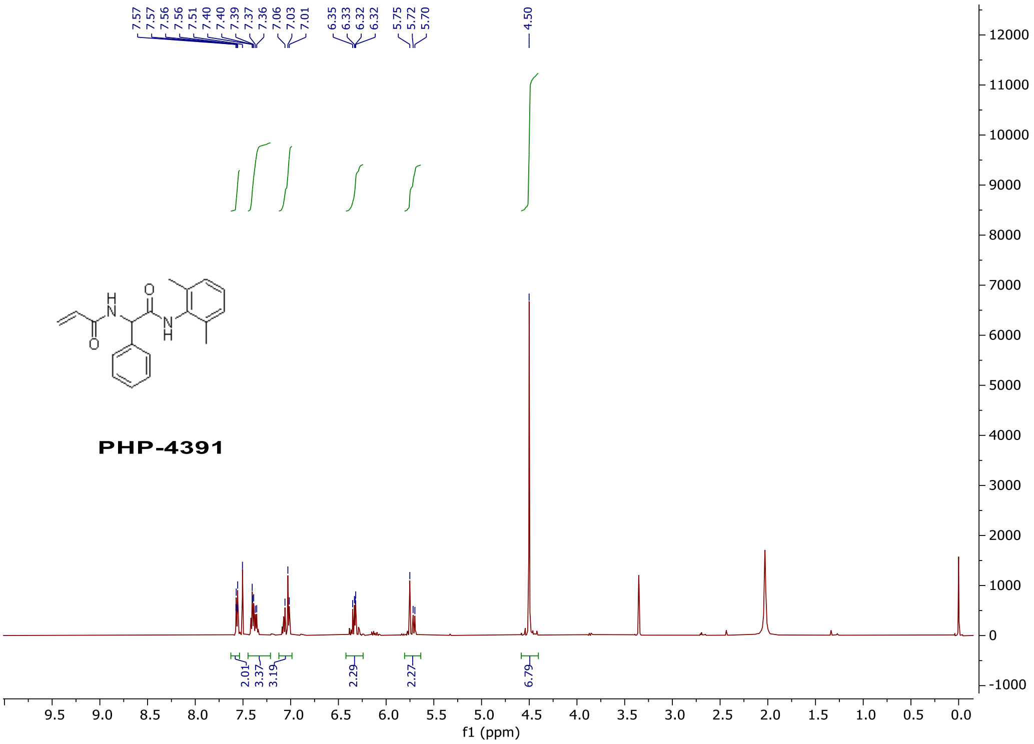

Supplementary Figure 276. ^{1}H NMR (500 MHz, $CDCl_3$+one drop of CD_3OD) of compound **PHP-4391.**

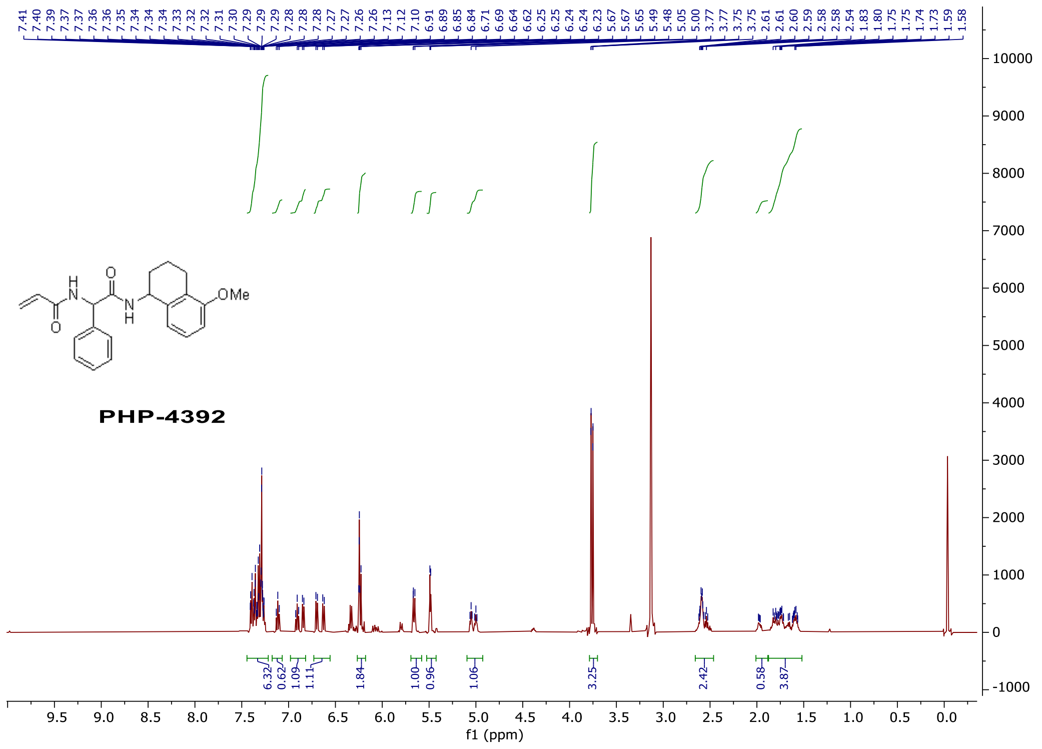

Supplementary Figure 277. ^{1}H NMR (500 MHz, $CDCl_3$+one drop of CD_3OD) of compound **PHP-4392.**

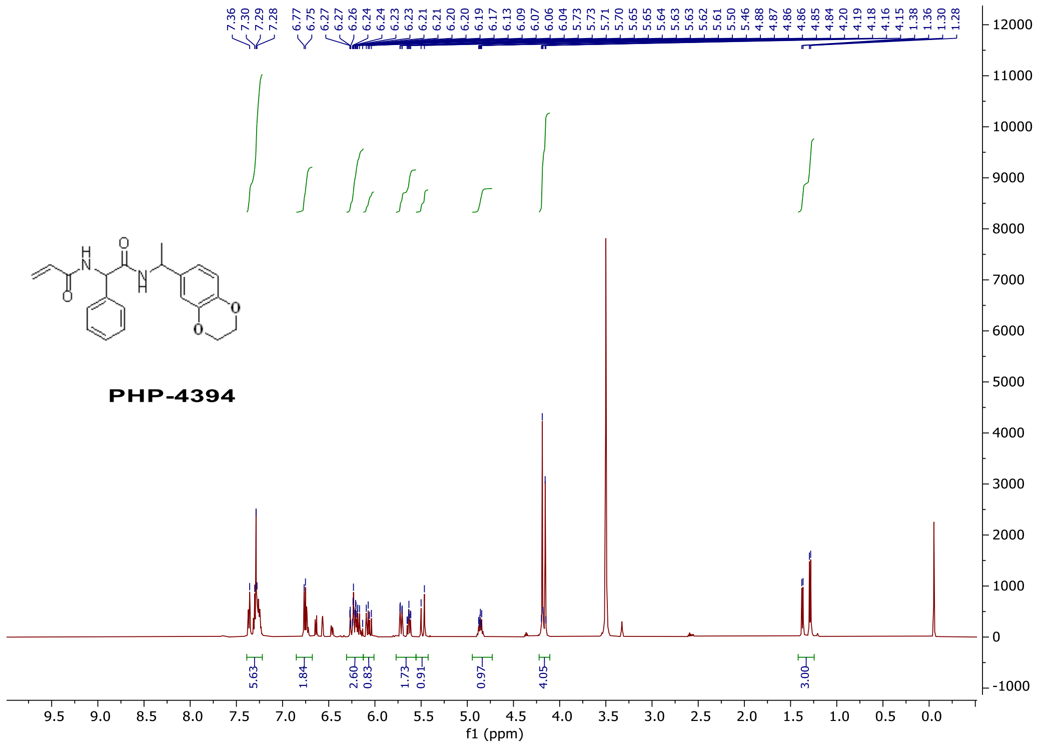

Supplementary Figure 278. ^{1}H NMR (500 MHz, $CDCl_3$+one drop of CD_3OD) of compound **PHP-4394.**

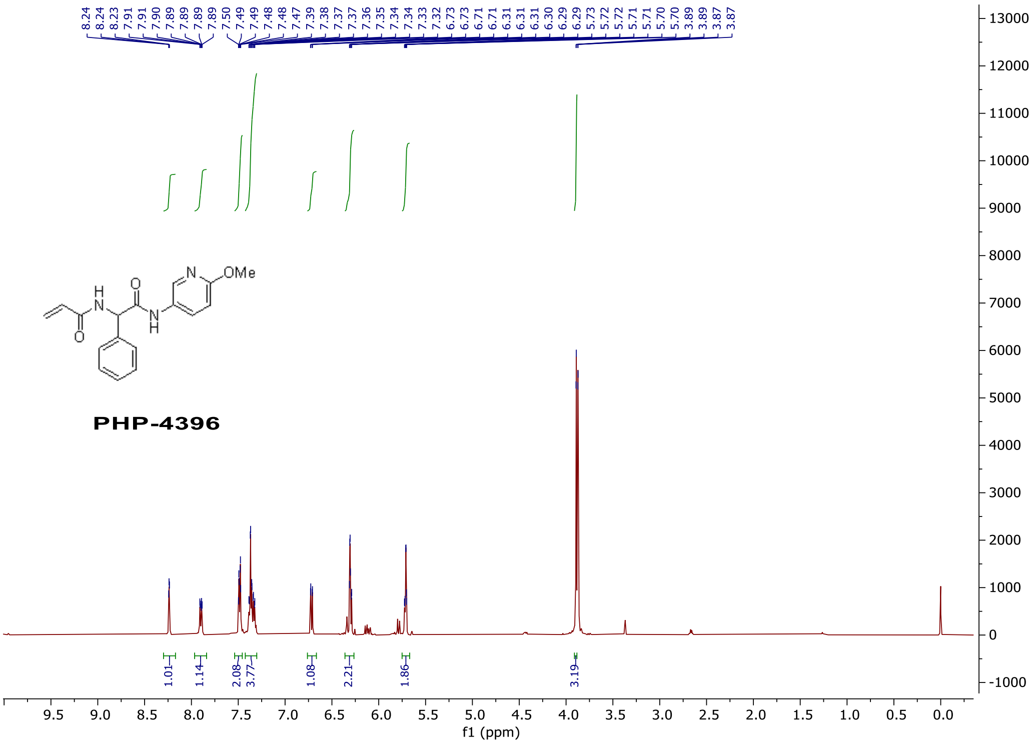

Supplementary Figure 279. ^{1}H NMR (500 MHz, $CDCl_3$) of compound **PHP-4396.**

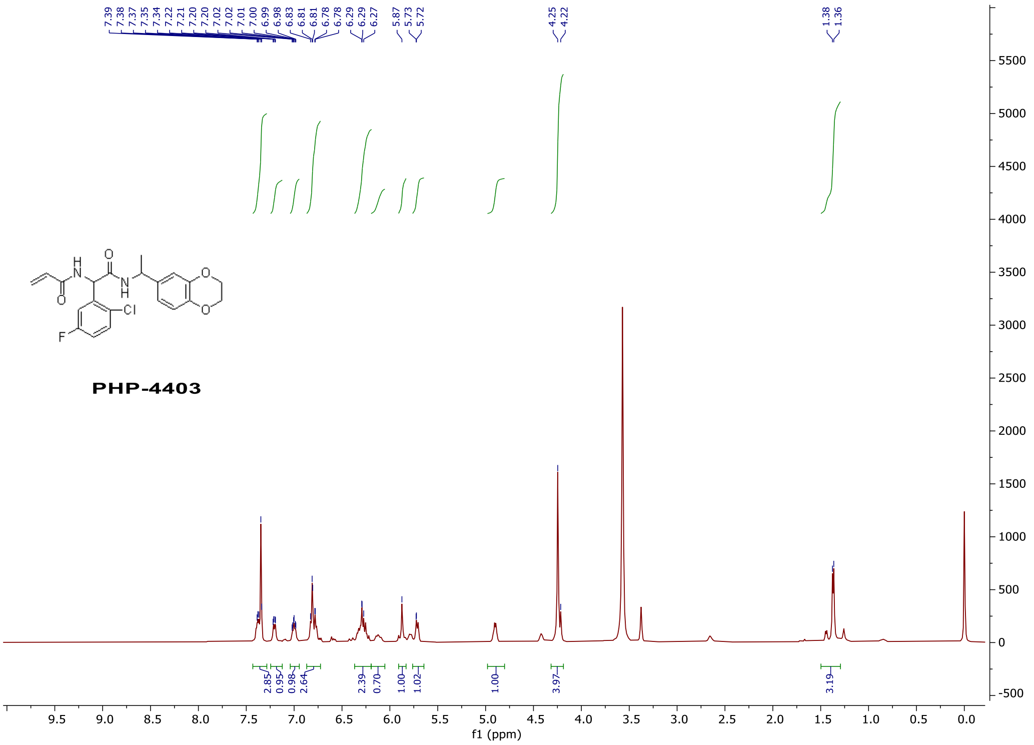

Supplementary Figure 280. ^{1}H NMR (500 MHz, $CDCl_3$+one drop of CD_3OD) of compound **PHP-4403.**

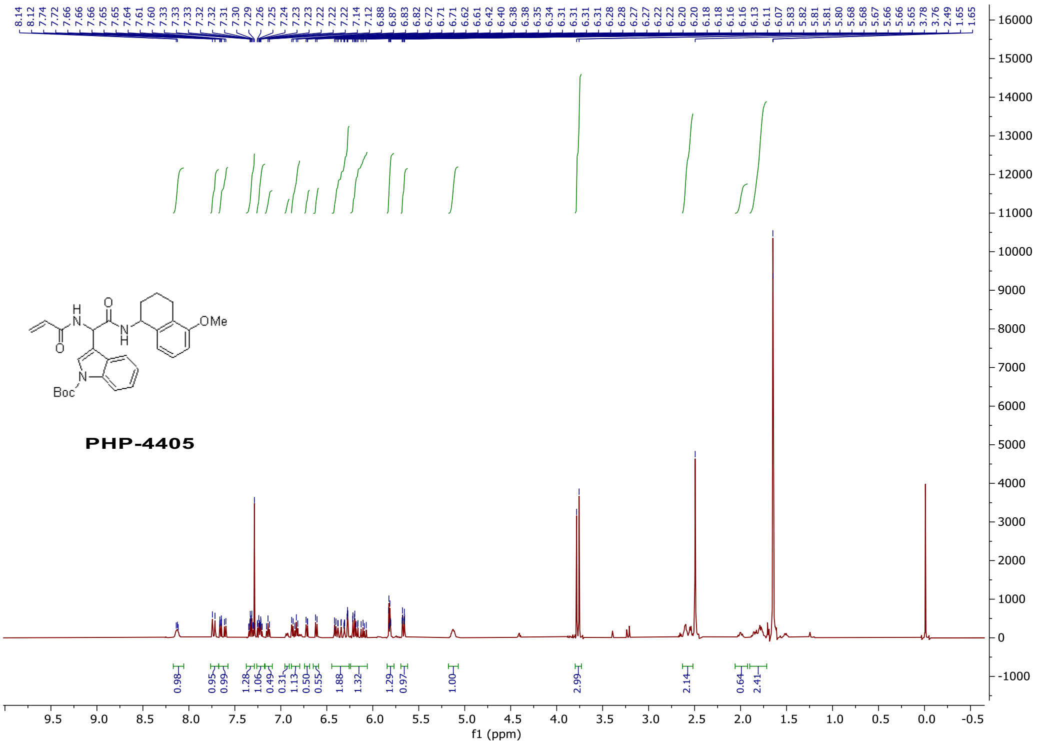

Supplementary Figure 281. ^{1}H NMR (500 MHz, $CDCl_3$) of compound **PHP-4405.**

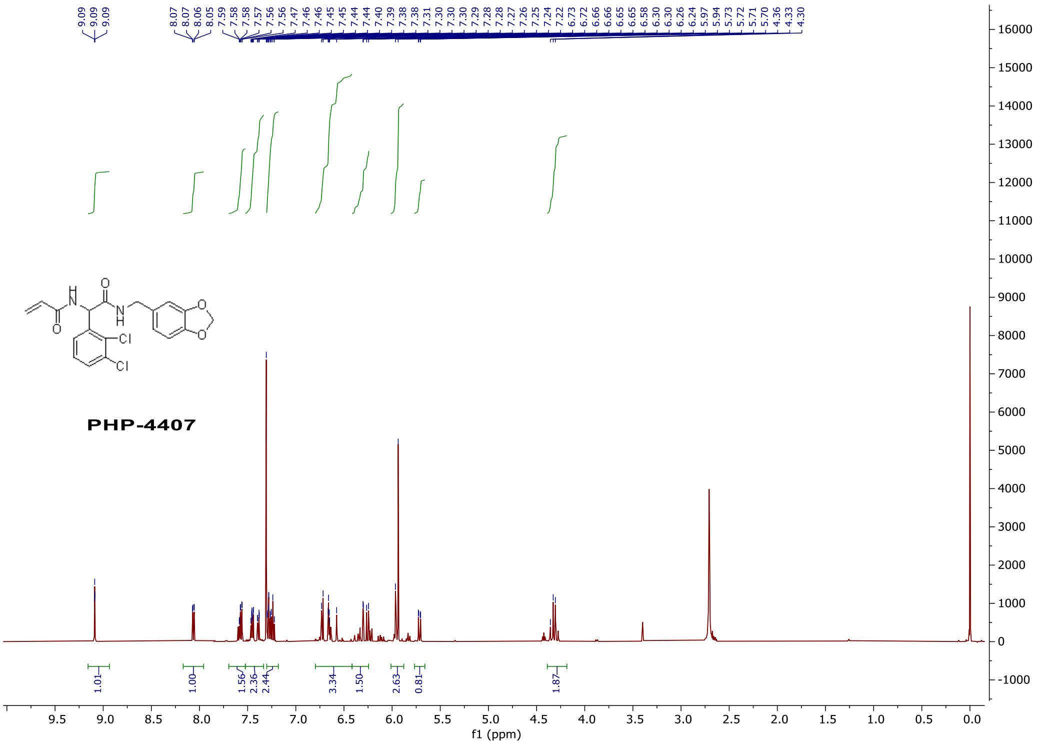

Supplementary Figure 282. ^{1}H NMR (500 MHz, $CDCl_3$) of compound **PHP-4407.**

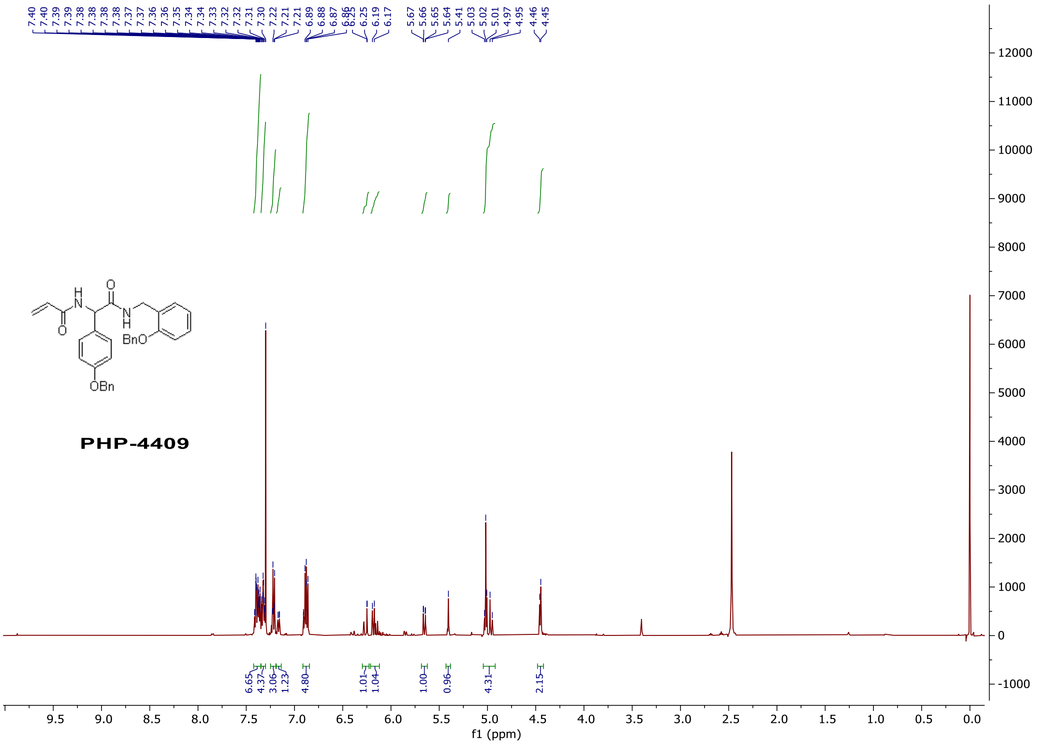

Supplementary Figure 283. ^{1}H NMR (500 MHz, $CDCl_3$) of compound **PHP-4409.**

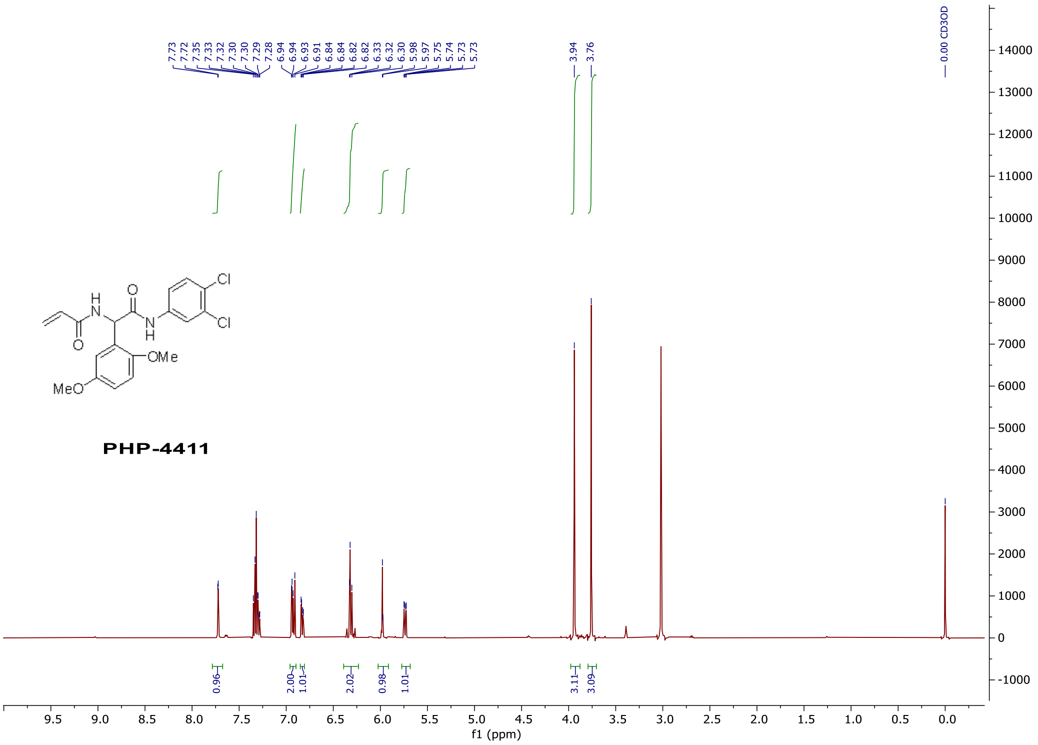

Supplementary Figure 284. ^{1}H NMR (500 MHz, $CDCl_3$) of compound **PHP-4411.**

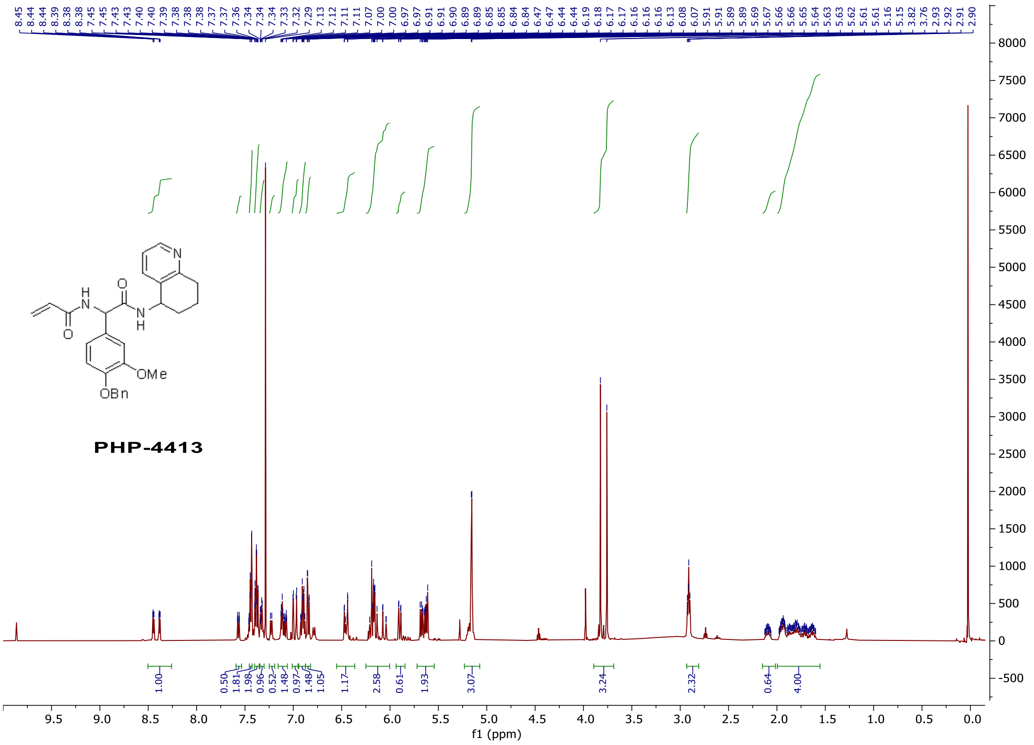

Supplementary Figure 285. ^{1}H NMR (500 MHz, $CDCl_3$) of compound **PHP-4413.**

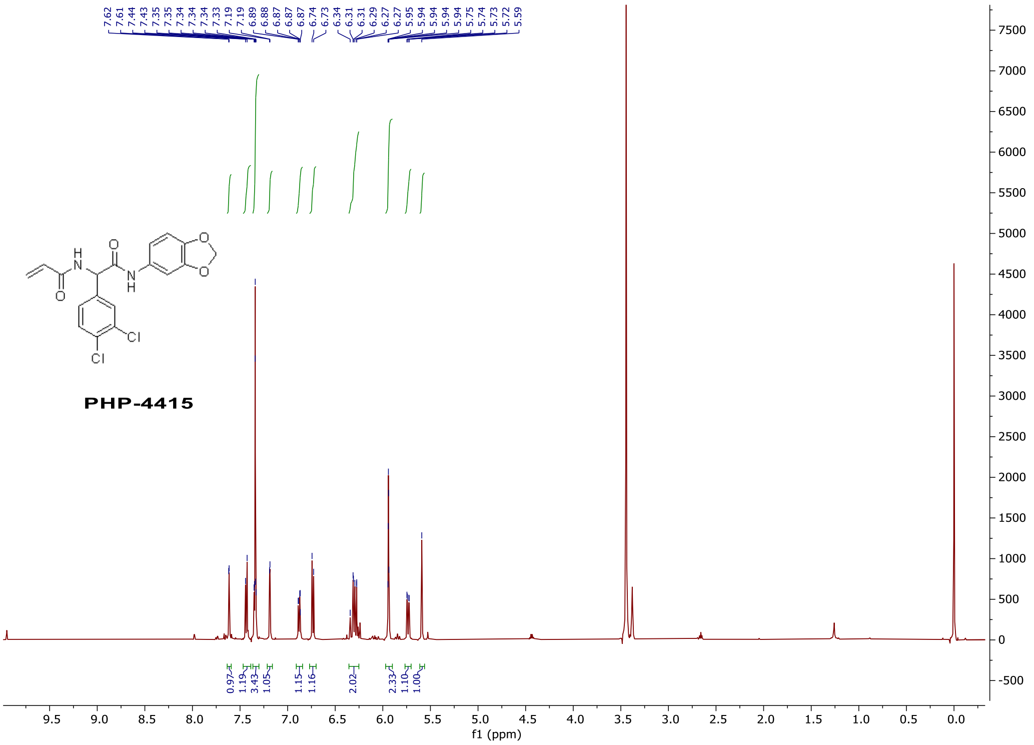

Supplementary Figure 286. ^{1}H NMR (500 MHz, $CDCl_3$+one drop of CD_3OD) of compound **PHP-4415.**

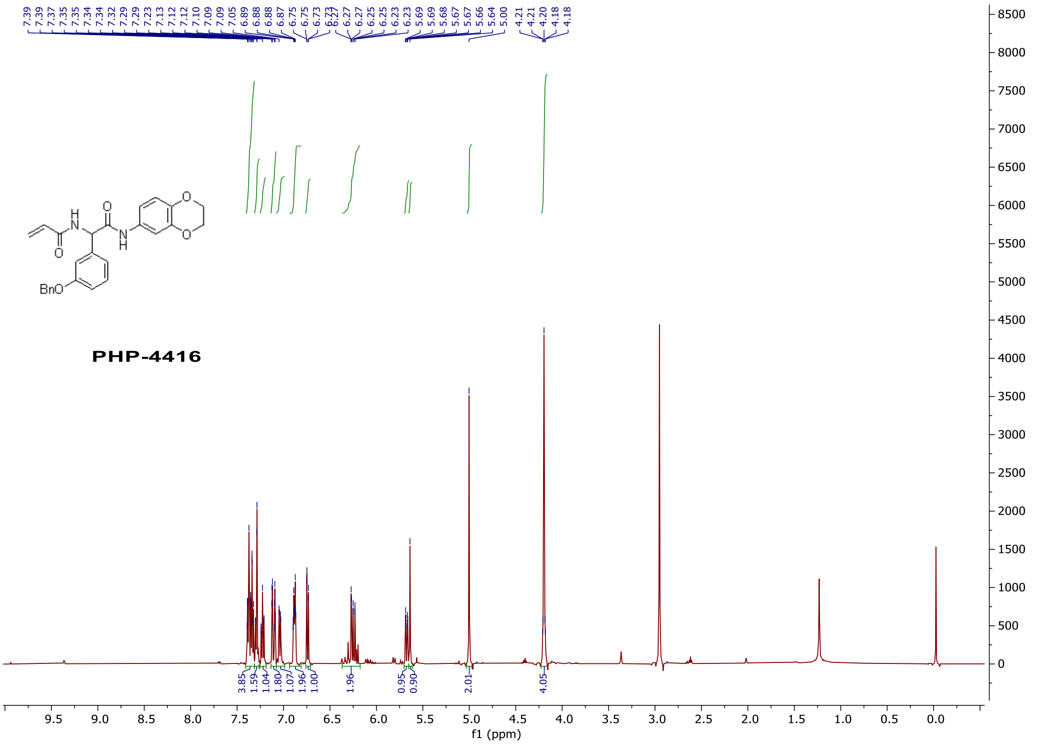

Supplementary Figure 287. ^{1}H NMR (500 MHz, $CDCl_3$) of compound **PHP-4416.**

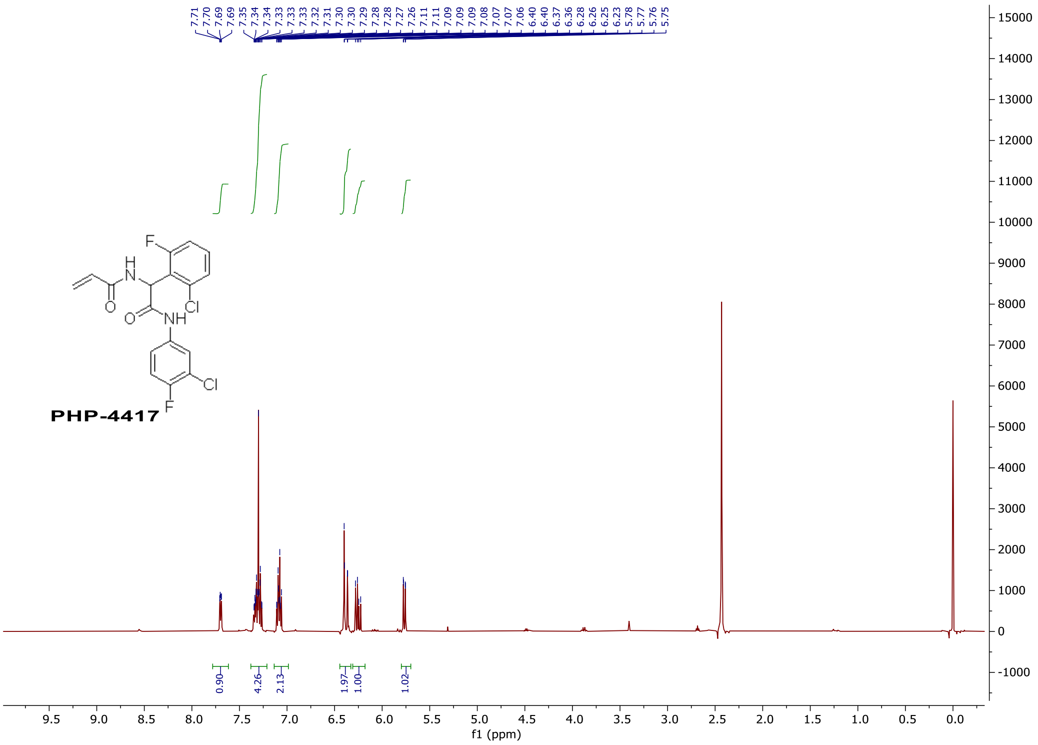

Supplementary Figure 288. ^{1}H NMR (500 MHz, $CDCl_3$+one drop of DMSO-d6) of compound **PHP-4417.**

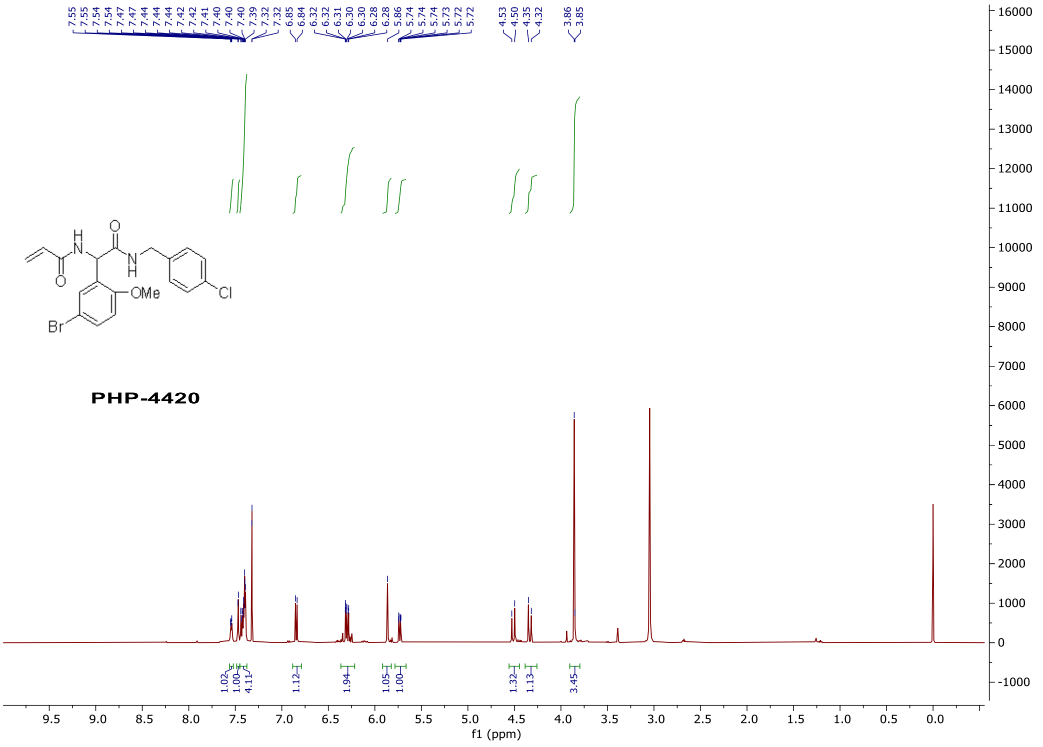

Supplementary Figure 289. ^{1}H NMR (500 MHz, $CDCl_3$) of compound **PHP-4420.**

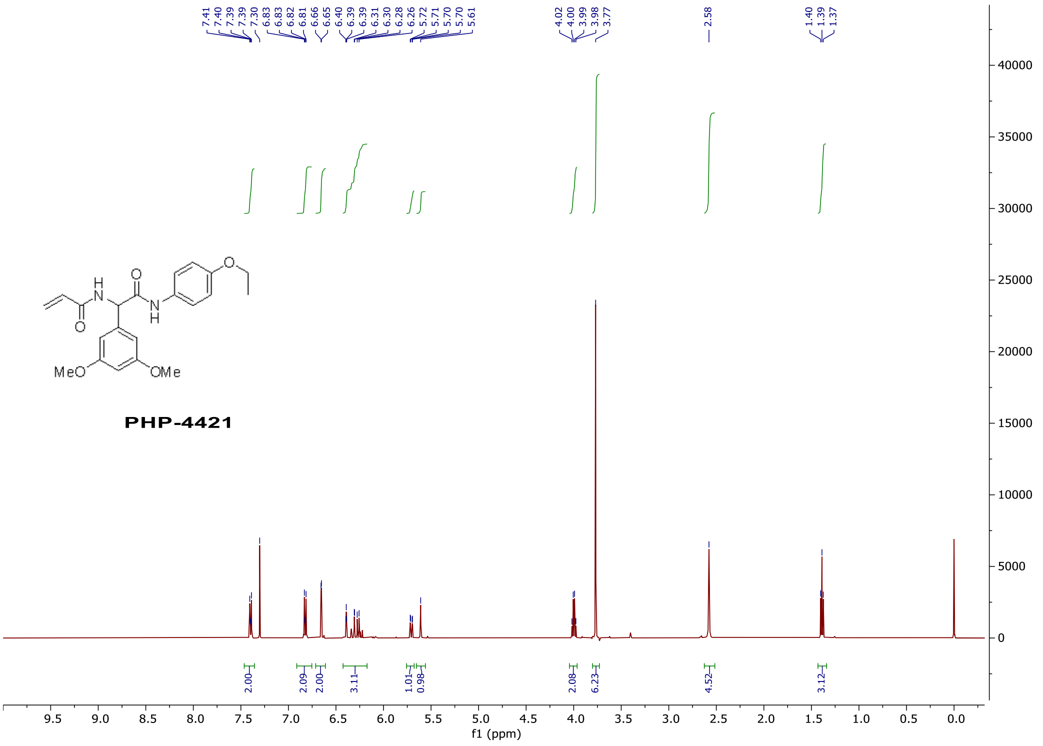

Supplementary Figure 290. ^{1}H NMR (500 MHz, $CDCl_3$) of compound **PHP-4421.**

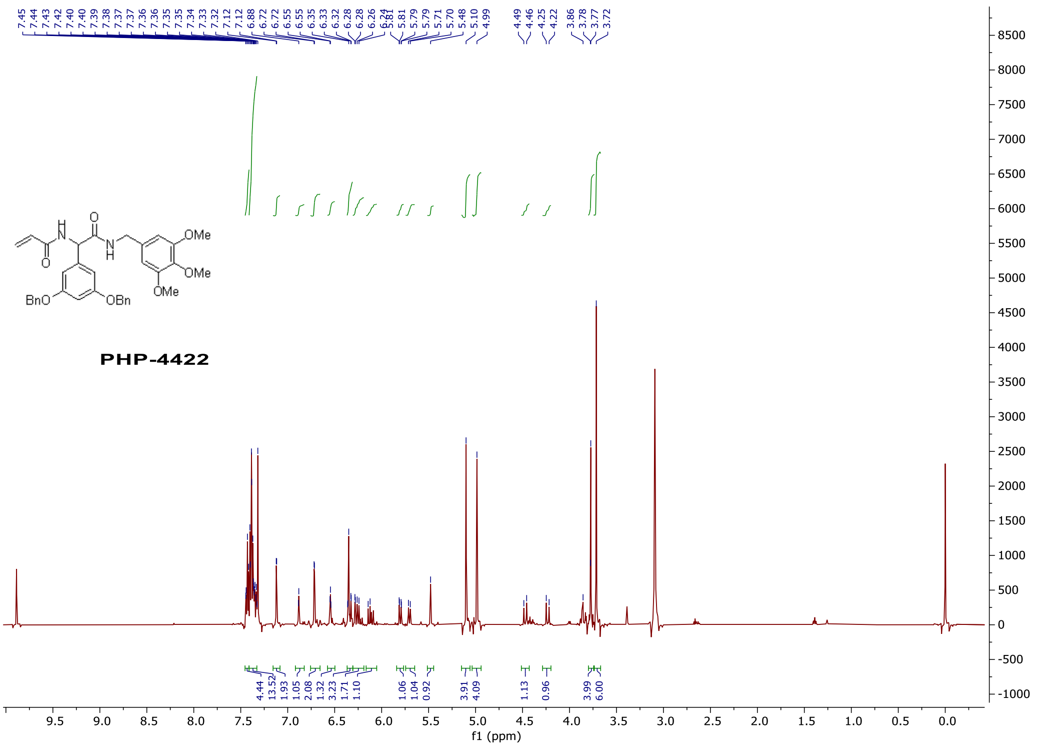

Supplementary Figure 291. ^{1}H NMR (500 MHz, CDCl$_3$) of compound **PHP-4422.**

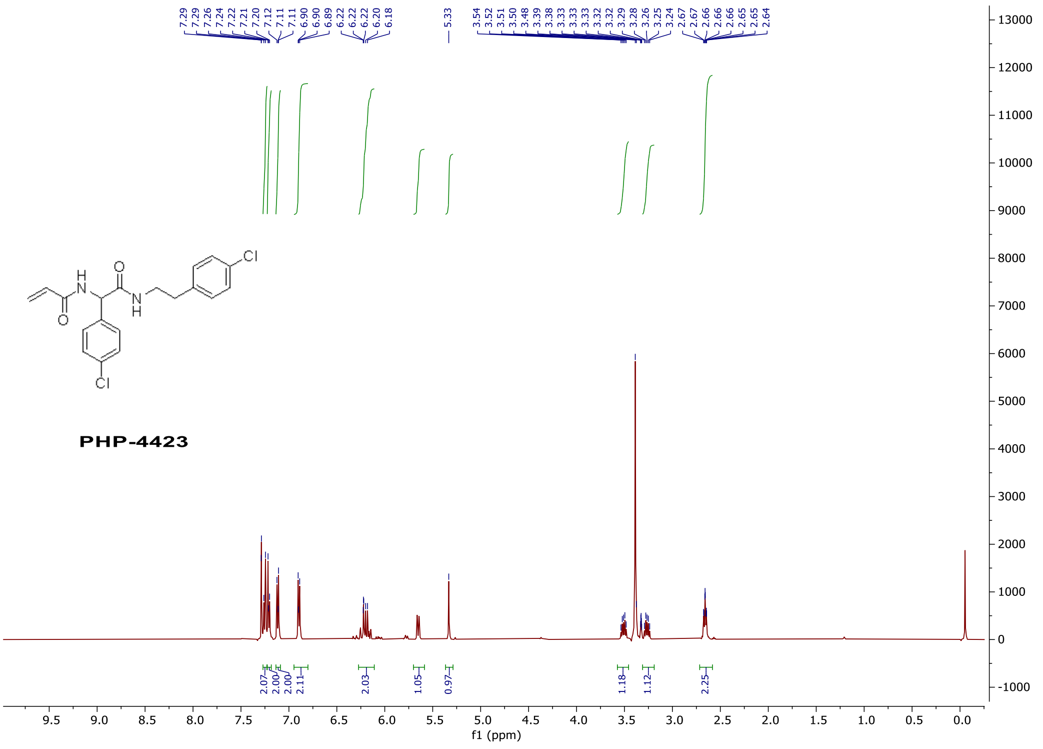

Supplementary Figure 292. ^{1}H NMR (500 MHz, CDCl$_3$) of compound **PHP-4423.**

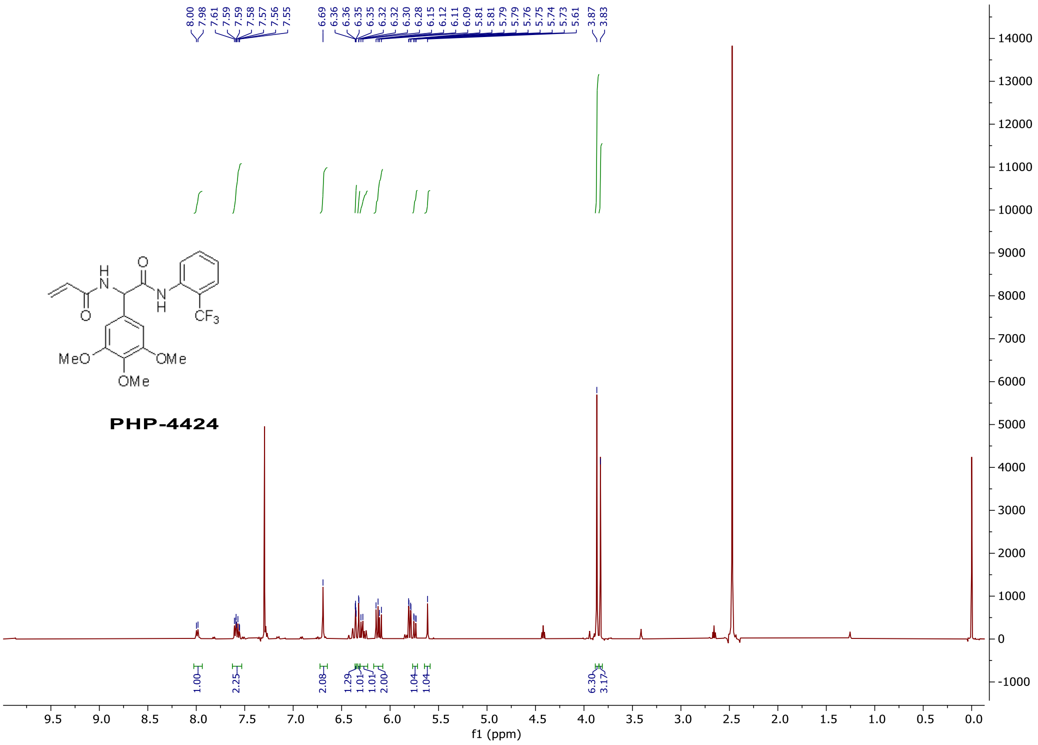

Supplementary Figure 293. ^{1}H NMR (500 MHz, $CDCl_3$+one drop of DMSO-d6) of compound **PHP-4424.**

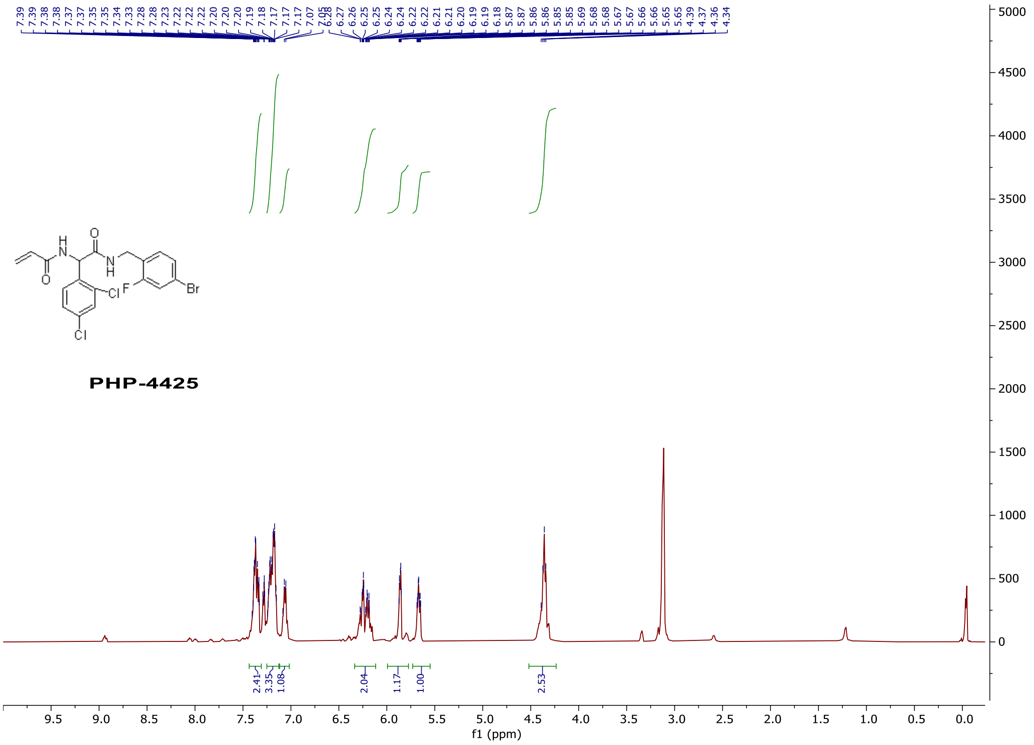

Supplementary Figure 294. ^{1}H NMR (500 MHz, $CDCl_3$+one drop of CD_3OD) of compound **PHP-4425.**

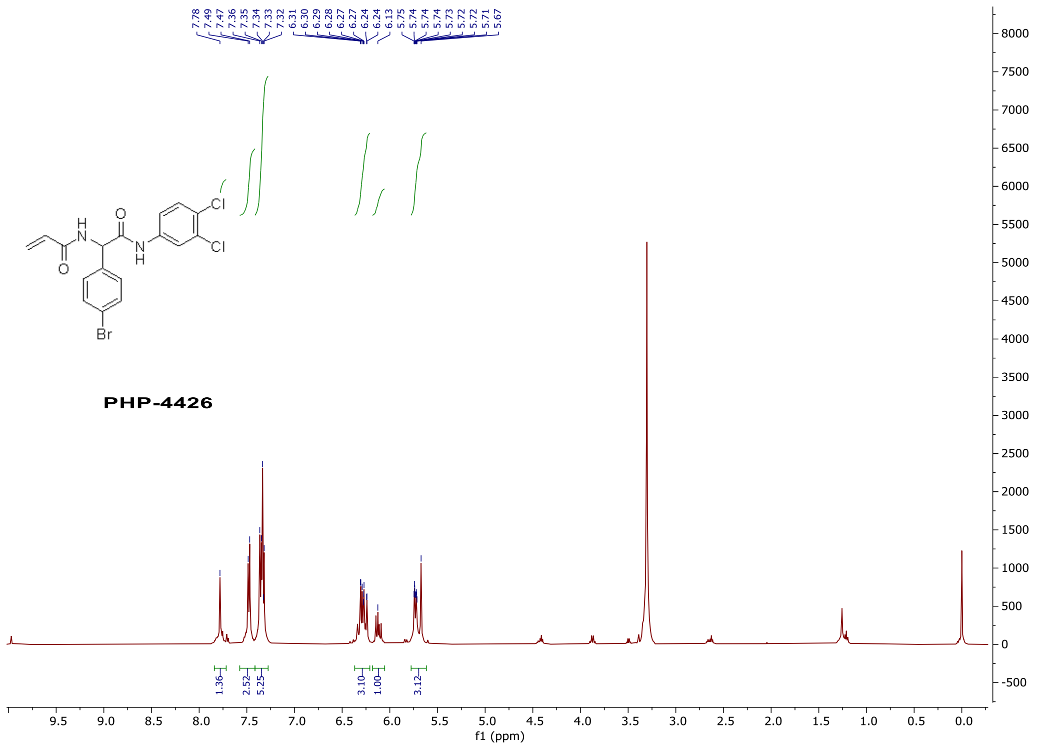

Supplementary Figure 295. ^{1}H NMR (500 MHz, $CDCl_3$+one drop of CD_3OD) of compound **PHP-4426.**

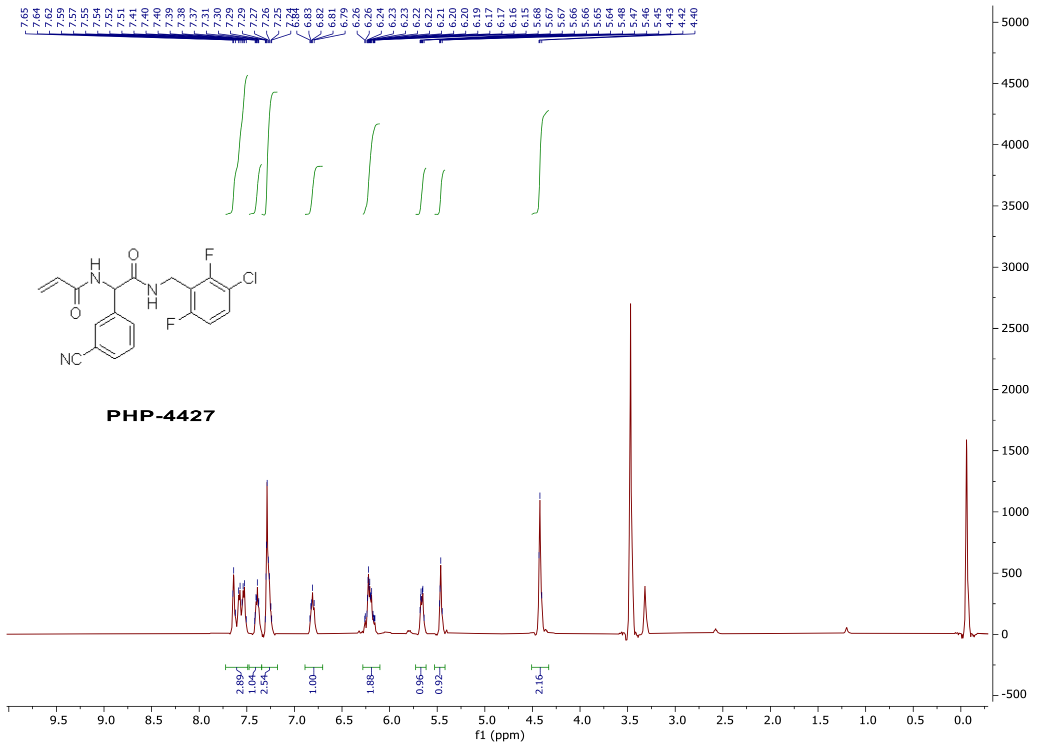

Supplementary Figure 296. ^{1}H NMR (500 MHz, $CDCl_3$+one drop of CD_3OD) of compound **PHP-4427.**

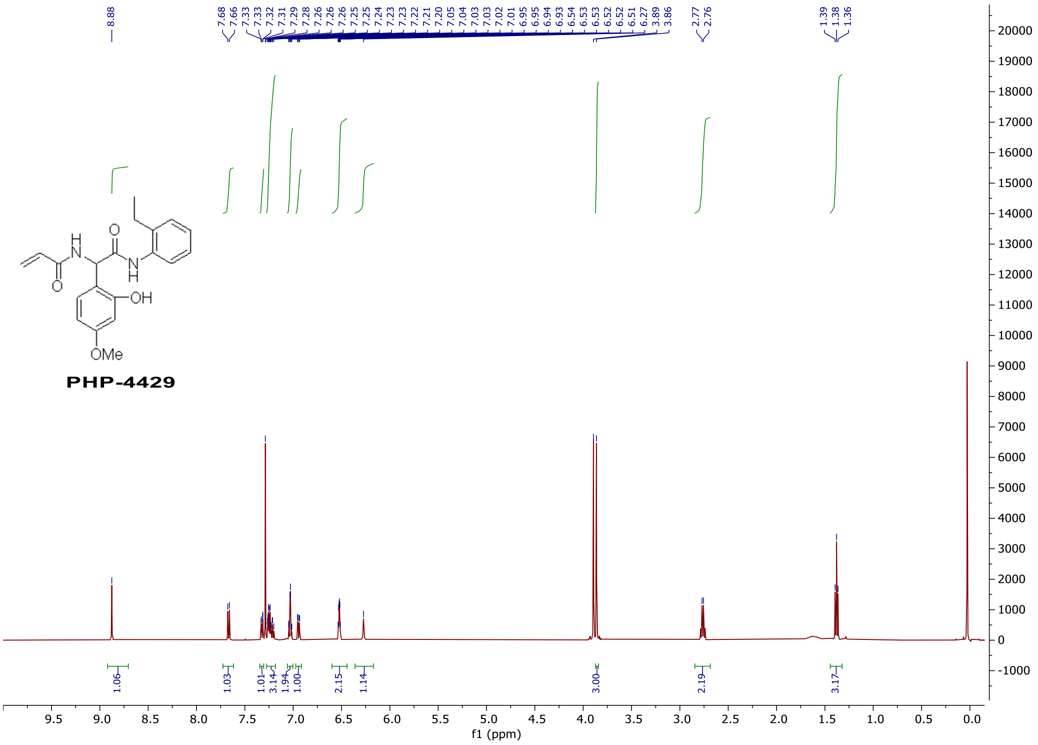

Supplementary Figure 297. ^{1}H NMR (500 MHz, $CDCl_3$) of compound **PHP-4429.**

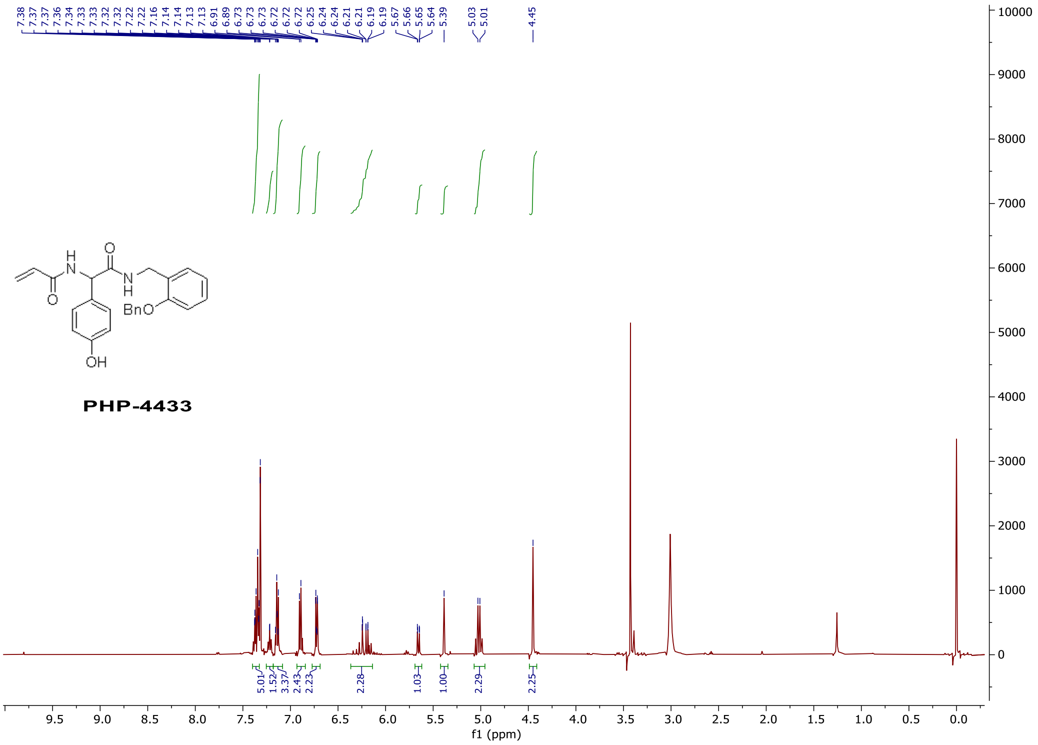

Supplementary Figure 298. ^{1}H NMR (500 MHz, $CDCl_3$+one drop of CD_3OD) of compound **PHP-4433.**

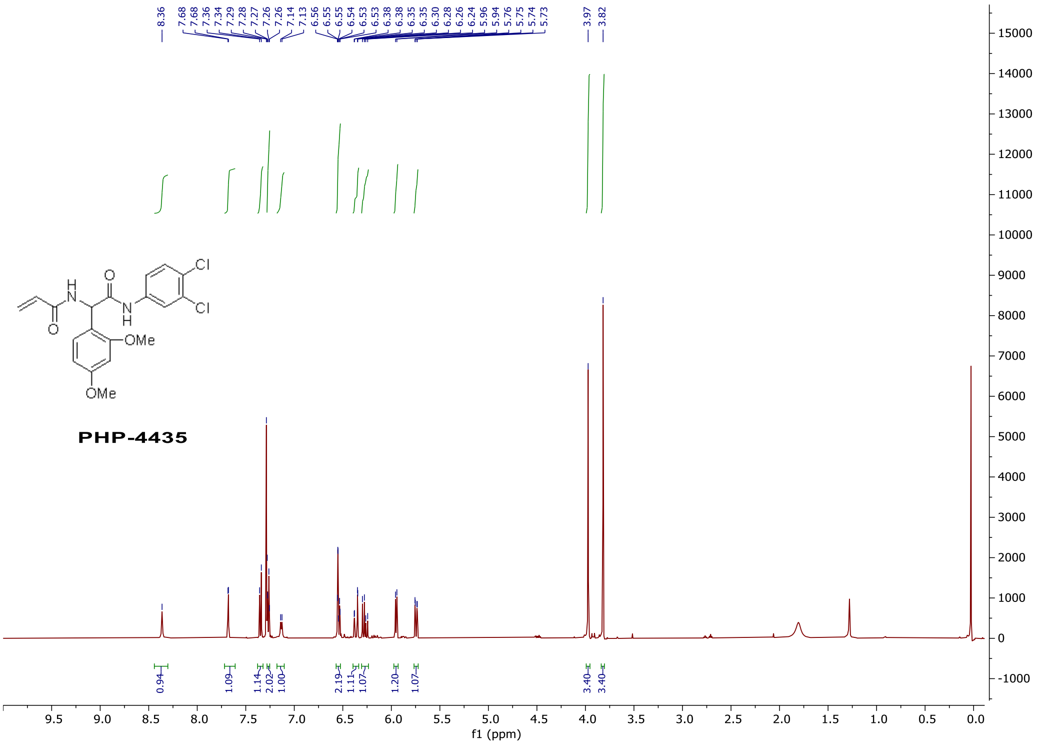

Supplementary Figure 299. ¹H NMR (500 MHz, CDCl₃) of compound **PHP-4435.**

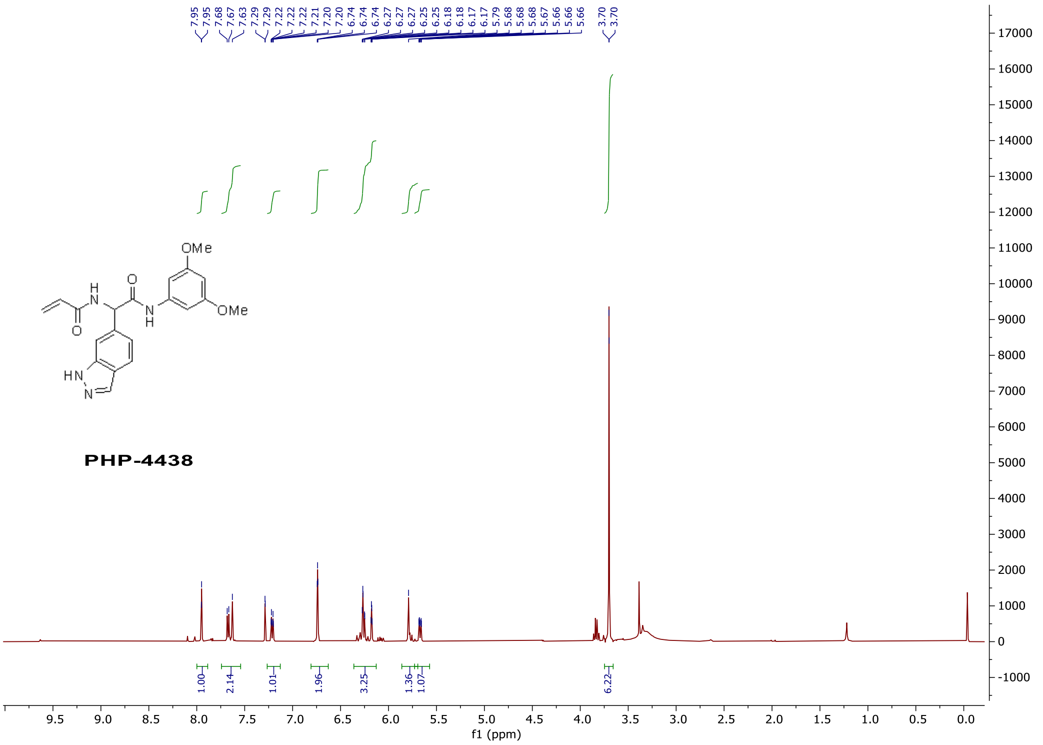

Supplementary Figure 300. ¹H NMR (500 MHz, CDCl₃) of compound **PHP-4438.**

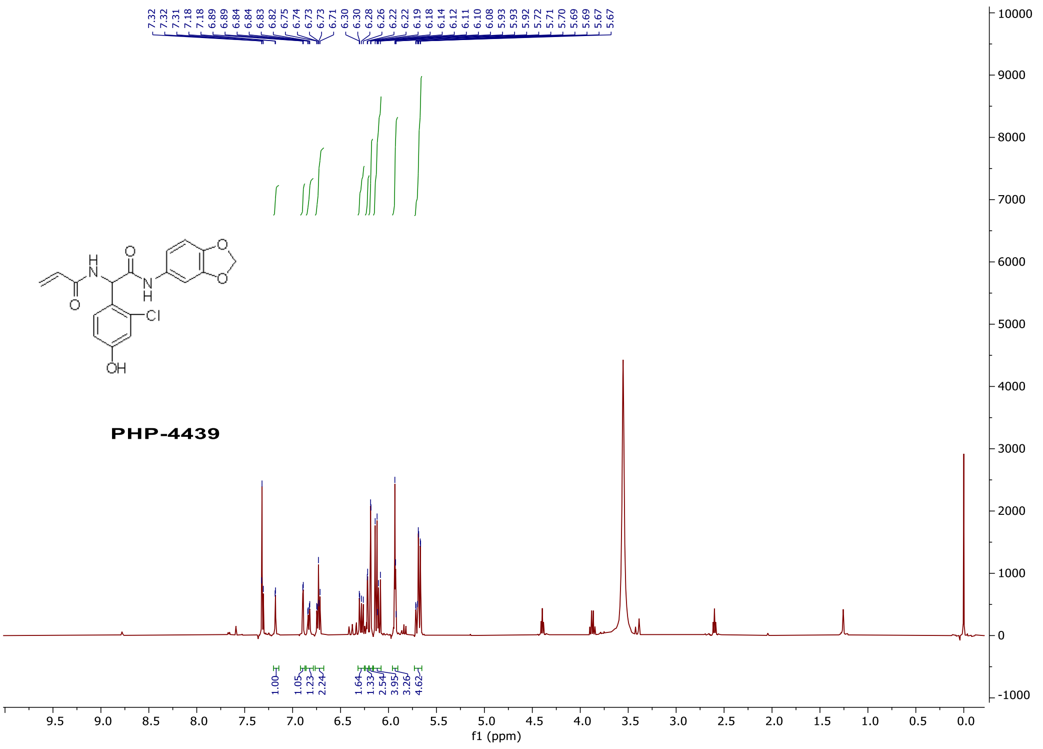

Supplementary Figure 301. ^{1}H NMR (500 MHz, $CDCl_3$+one drop of CD_3OD) of compound **PHP-4439.**

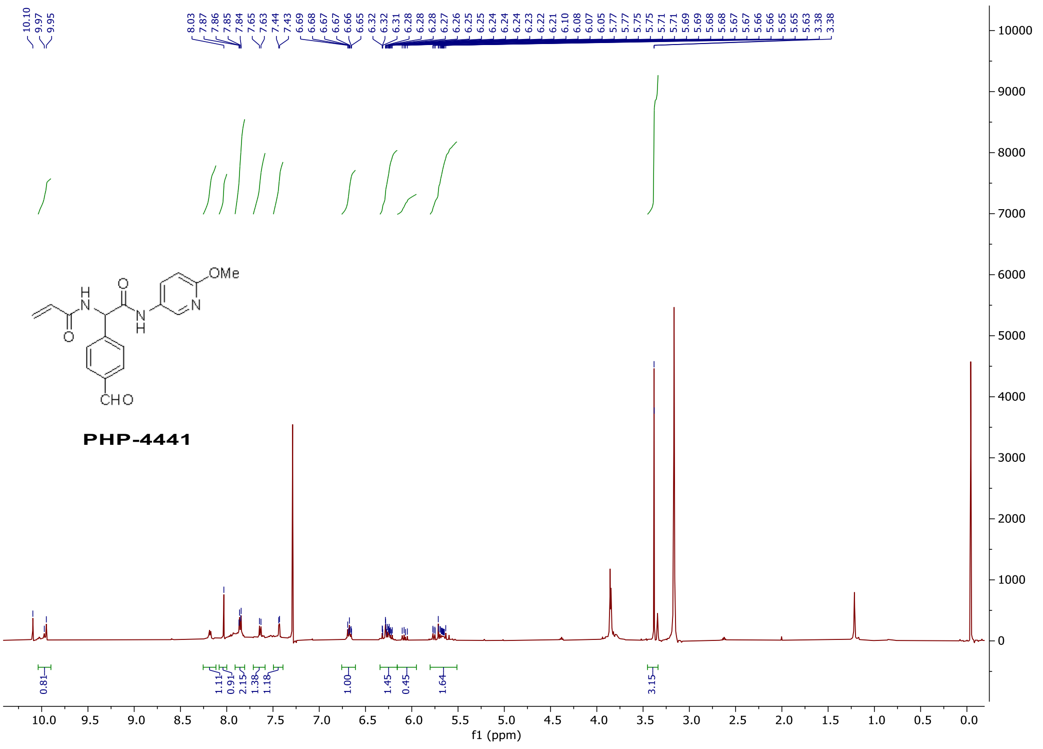

Supplementary Figure 302. ^{1}H NMR (500 MHz, $CDCl_3$+one drop of CD_3OD) of compound **PHP-4441.**

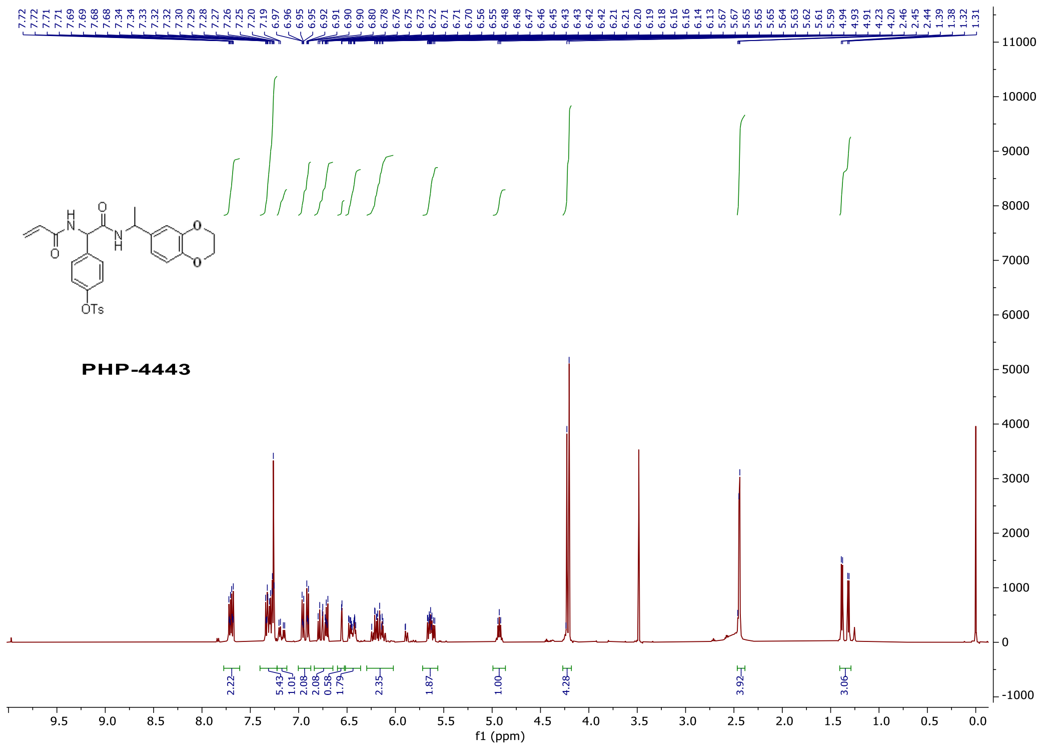

Supplementary Figure 303. ^{1}H NMR (500 MHz, $CDCl_3$+one drop of CD_3OD) of compound **PHP-4443.**

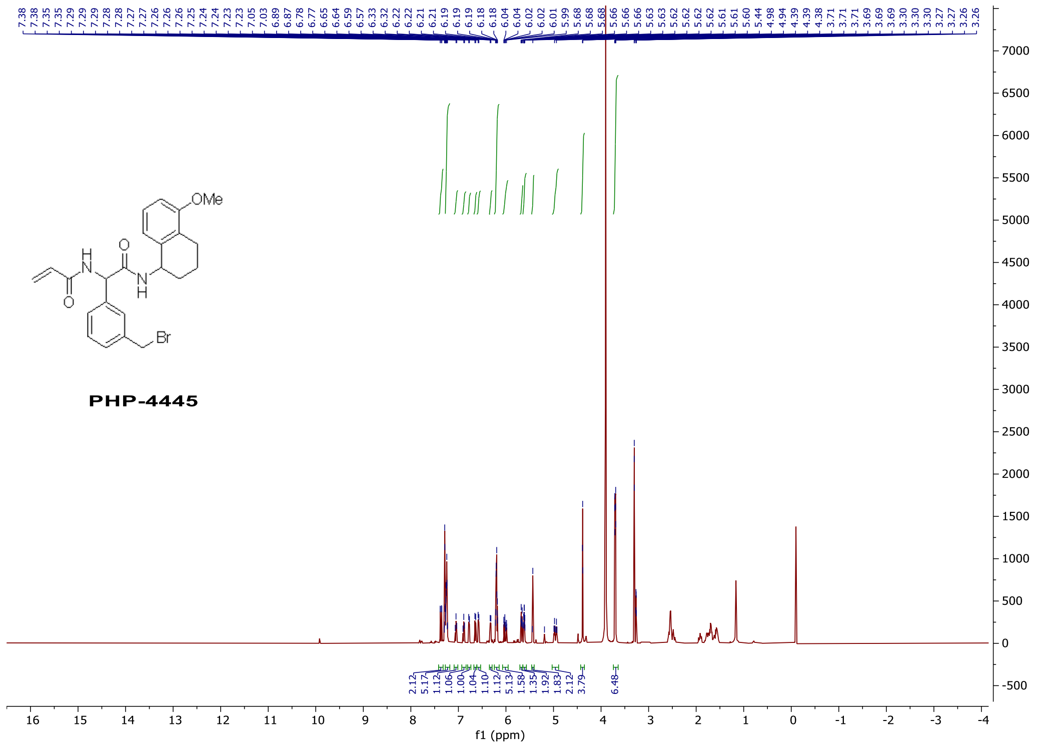

Supplementary Figure 304. ^{1}H NMR (500 MHz, $CDCl_3$+one drop of CD_3OD) of compound **PHP-4445.**

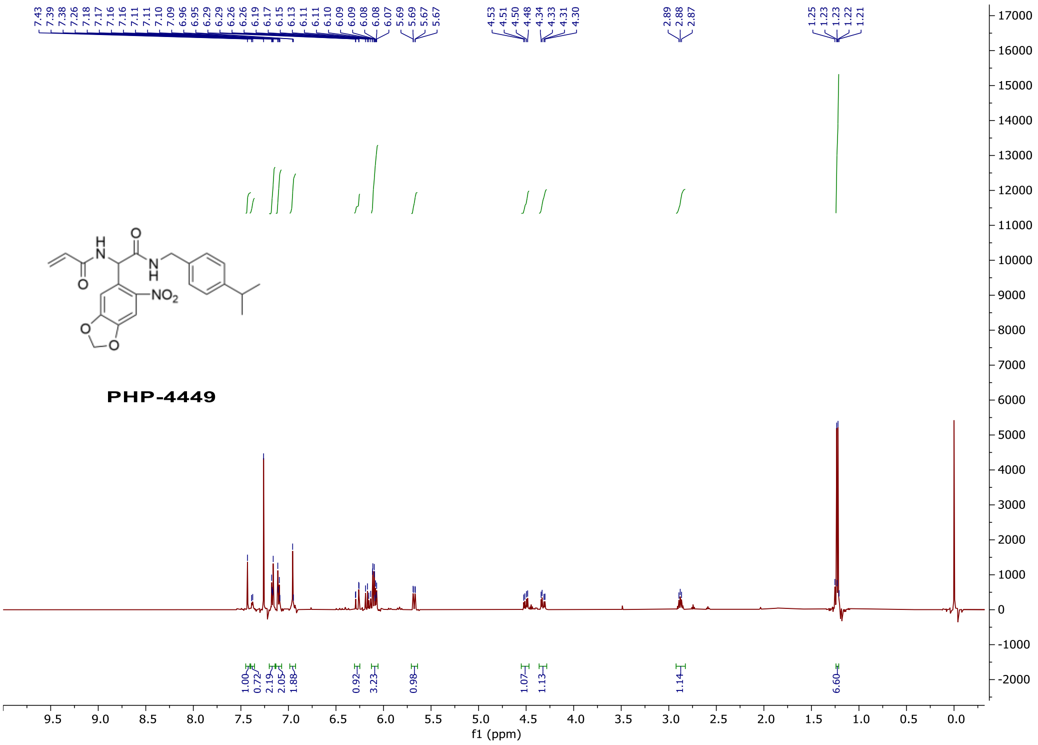

Supplementary Figure 305. ^{1}H NMR (500 MHz, $CDCl_3$) of compound **PHP-4449.**

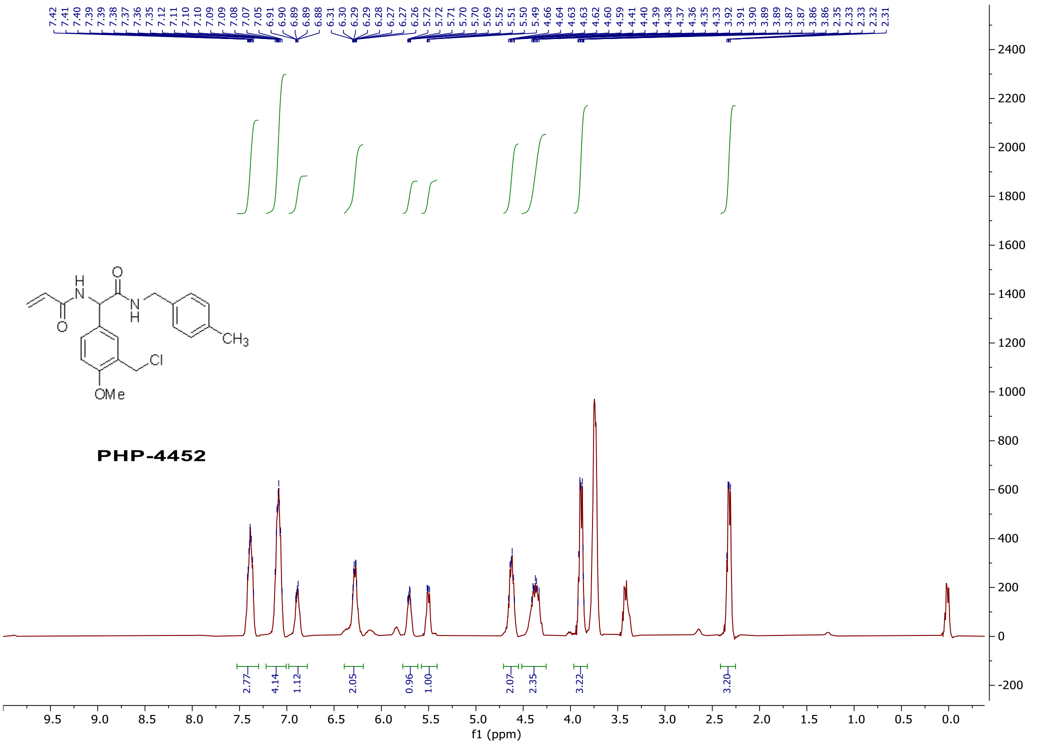

Supplementary Figure 306. ^{1}H NMR (500 MHz, $CDCl_3$+one drop of CD_3OD) of compound **PHP-4452.**

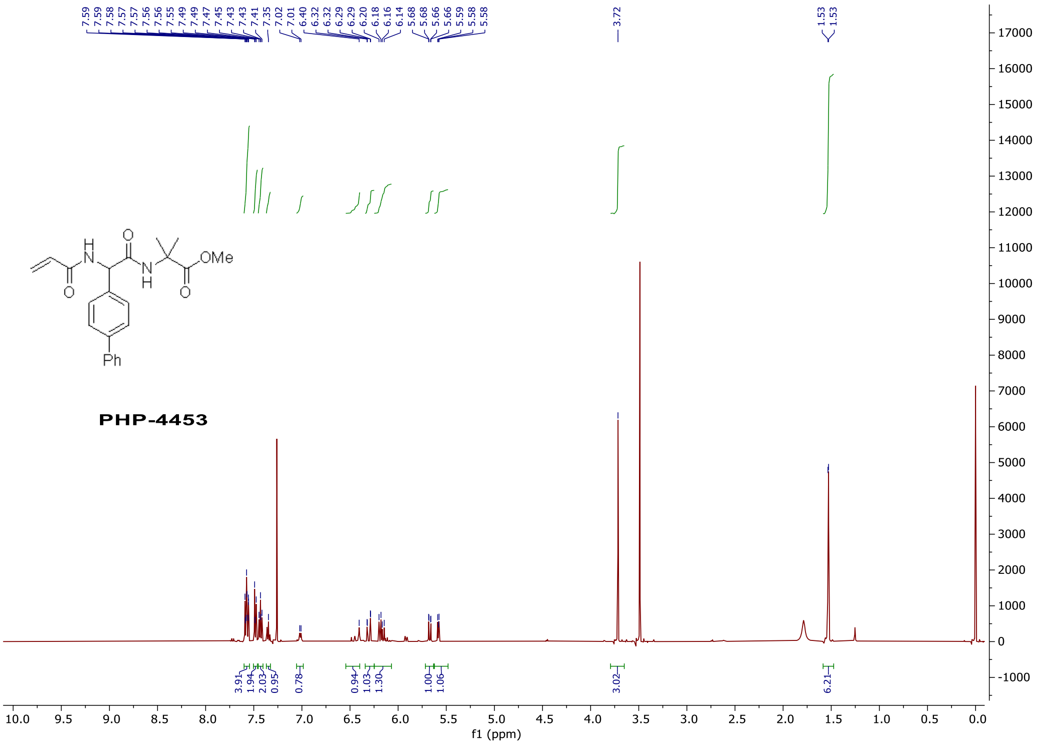

Supplementary Figure 307. ^{1}H NMR (500 MHz, $CDCl_3$+one drop of CD_3OD) of compound **PHP-4453.**

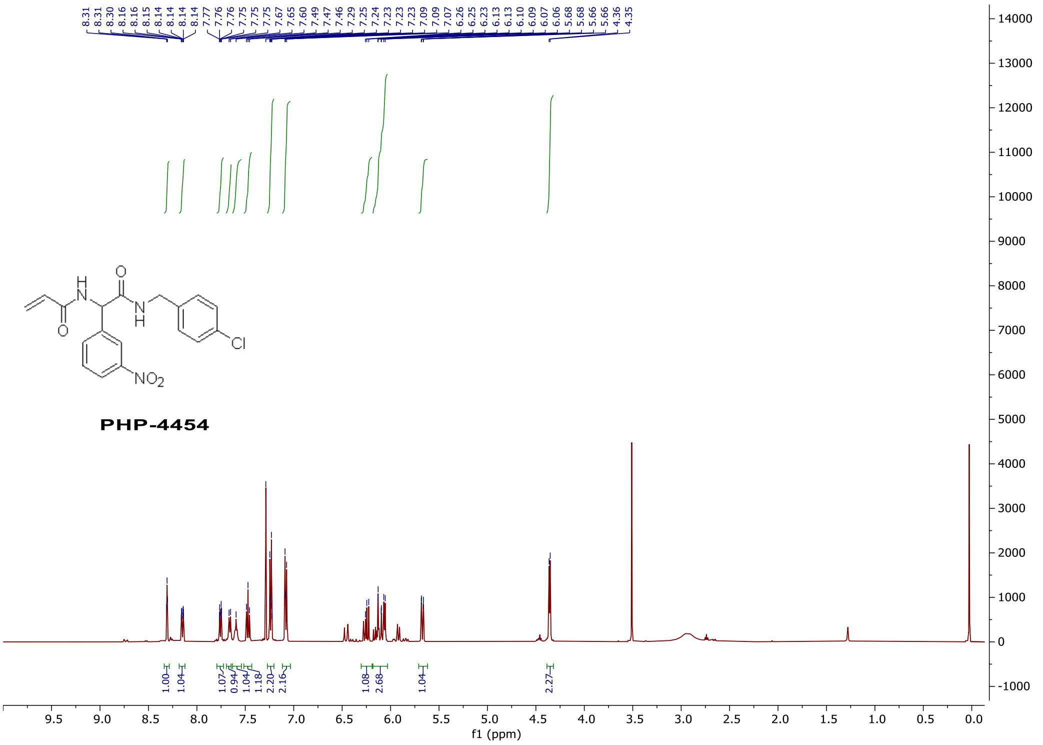

Supplementary Figure 308. ^{1}H NMR (500 MHz, $CDCl_3$+one drop of CD_3OD) of compound **PHP-4454.**

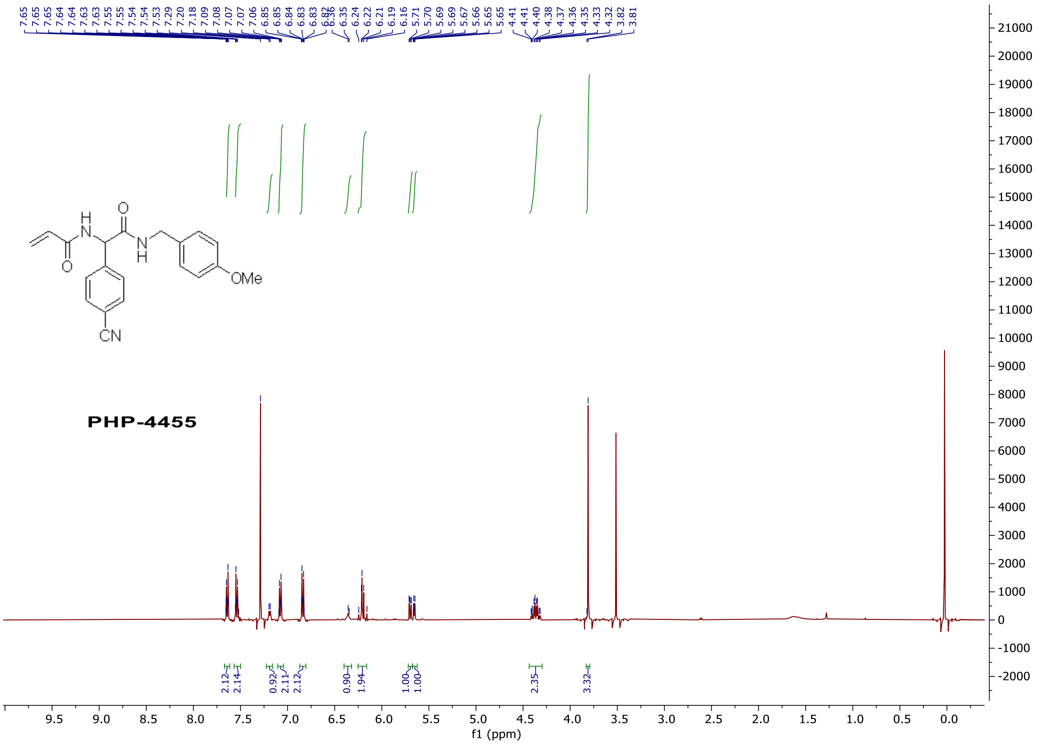

Supplementary Figure 309. ^{1}H NMR (500 MHz, $CDCl_3$+one drop of CD_3OD) of compound **PHP-4455.**

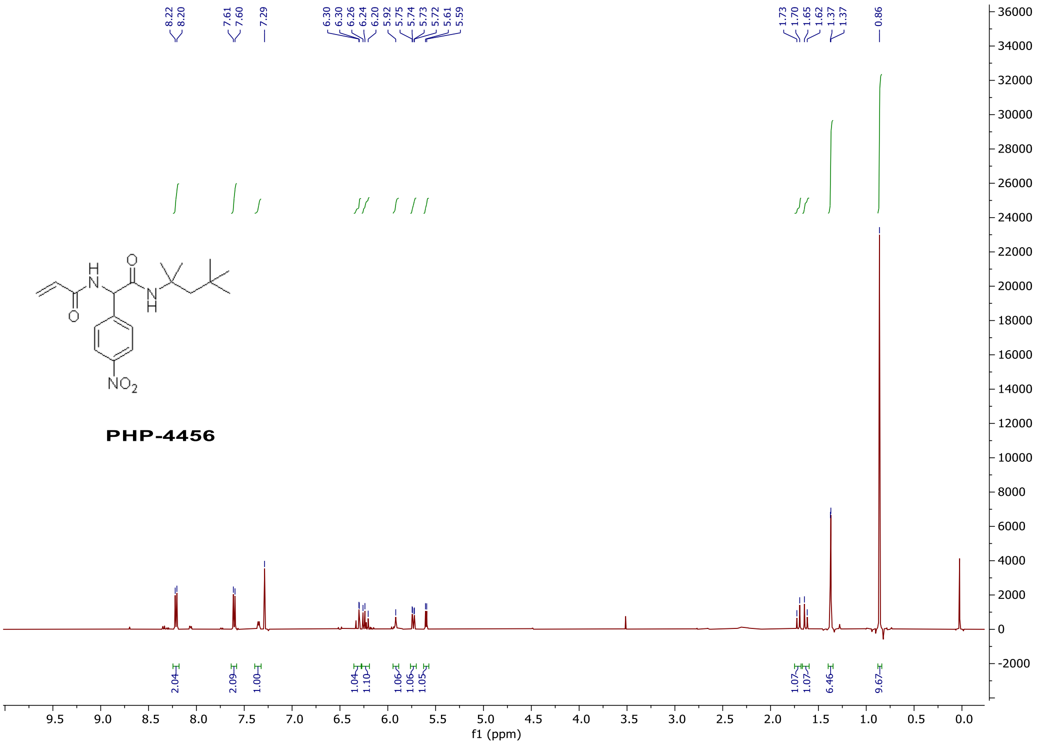

Supplementary Figure 310. ^{1}H NMR (500 MHz, $CDCl_3$) of compound **PHP-4456.**

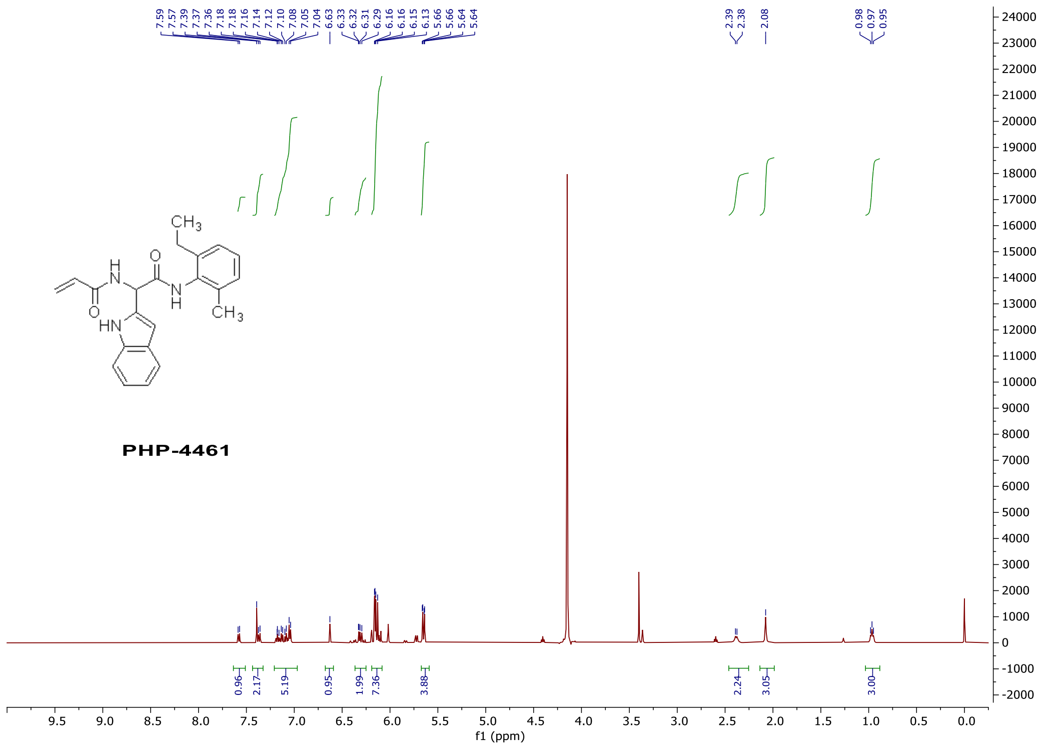

Supplementary Figure 311. ^{1}H NMR (500 MHz, $CDCl_3$+one drop of CD_3OD) of compound **PHP-4461.**

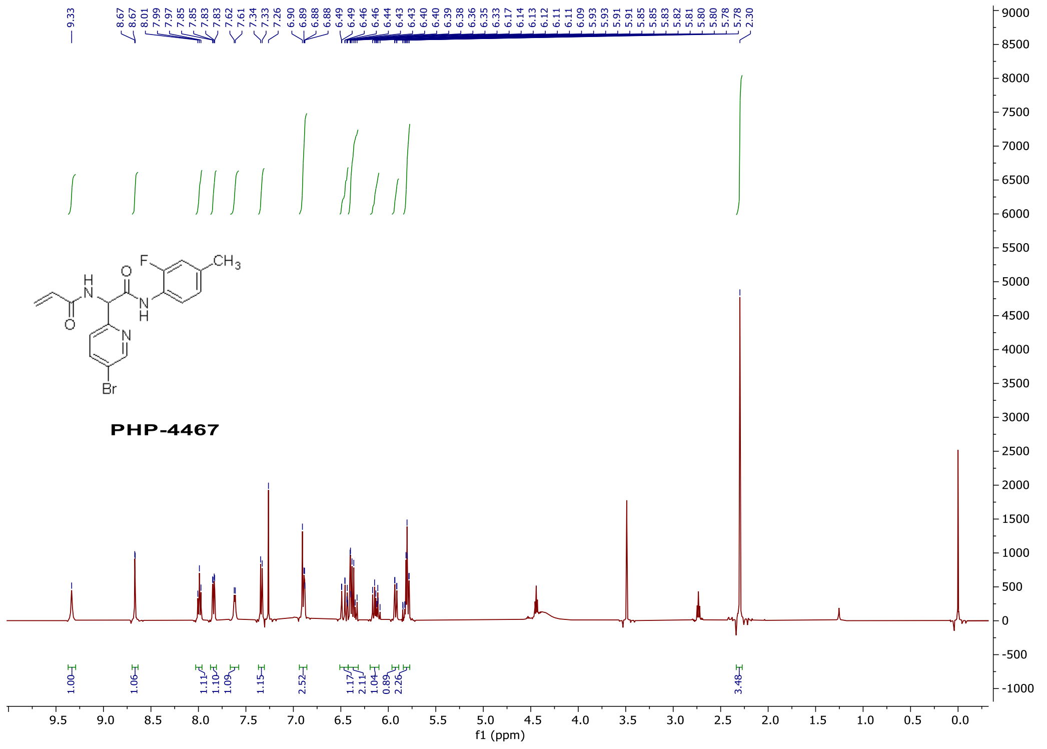

Supplementary Figure 312. ^{1}H NMR (500 MHz, $CDCl_3$+one drop of CD_3OD) of compound **PHP-4467.**

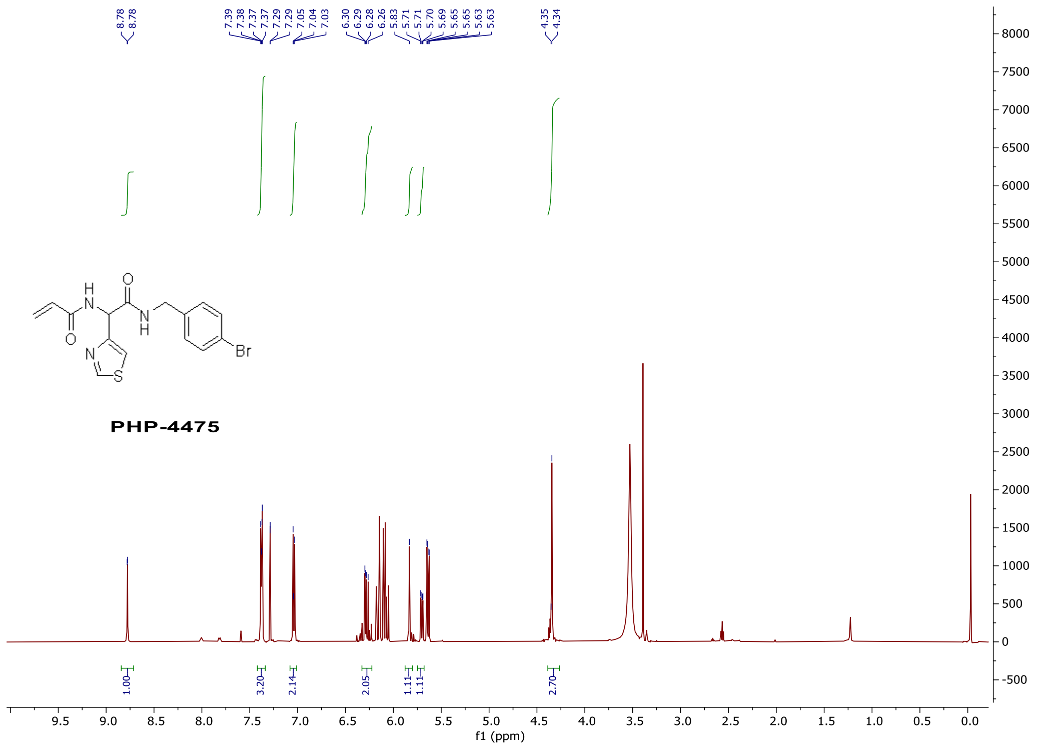

Supplementary Figure 313. ^1H NMR (500 MHz, $CDCl_3$+one drop of CD_3OD) of compound **PHP-4475.**

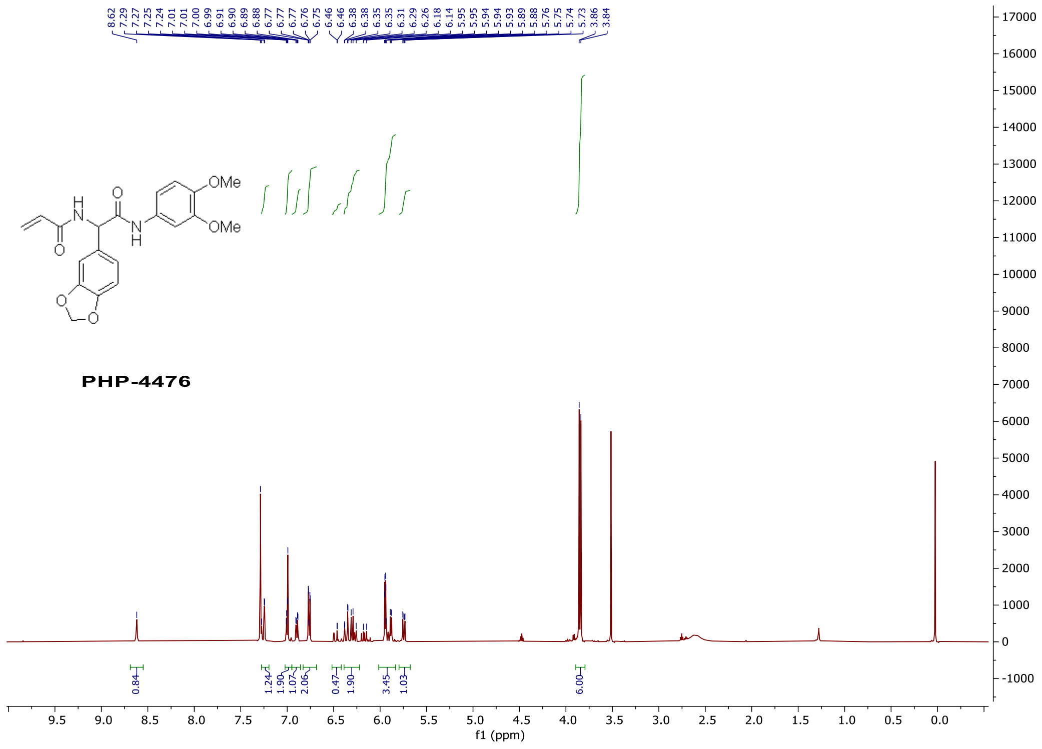

Supplementary Figure 314. ^1H NMR (500 MHz, $CDCl_3$+one drop of CD_3OD) of compound **PHP-4476.**

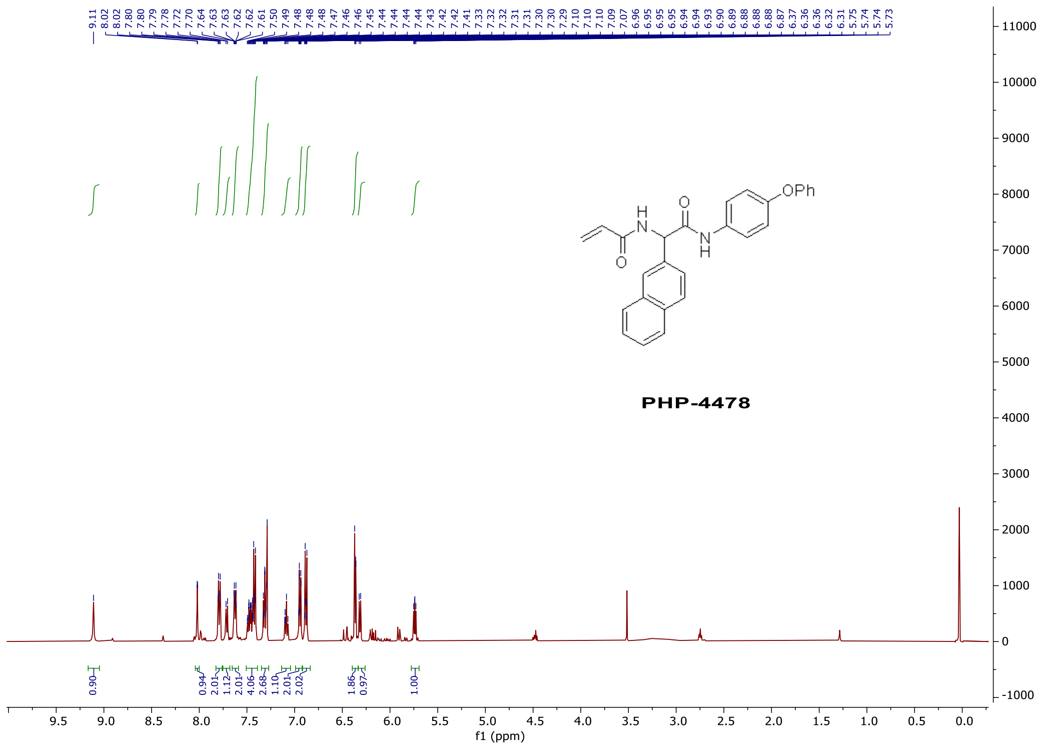

Supplementary Figure 315. ^{1}H NMR (500 MHz, $CDCl_3$+one drop of CD_3OD) of compound **PHP-4478.**

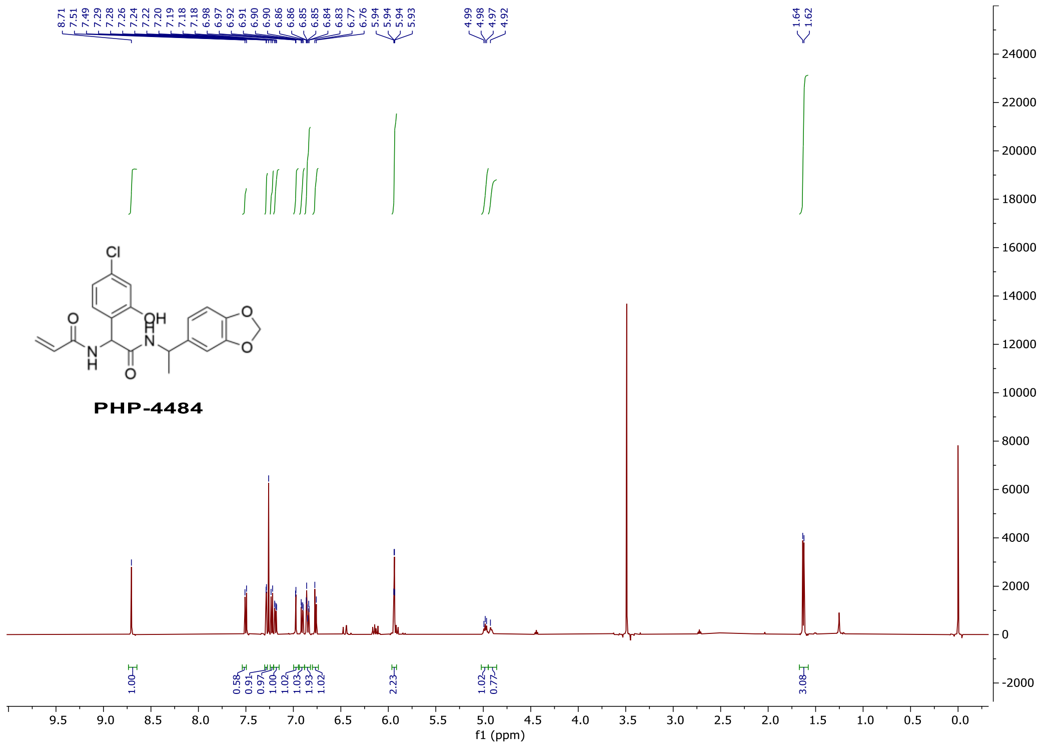

Supplementary Figure 316. ^{1}H NMR (500 MHz, $CDCl_3$+one drop of CD_3OD) of compound **PHP-4484.**

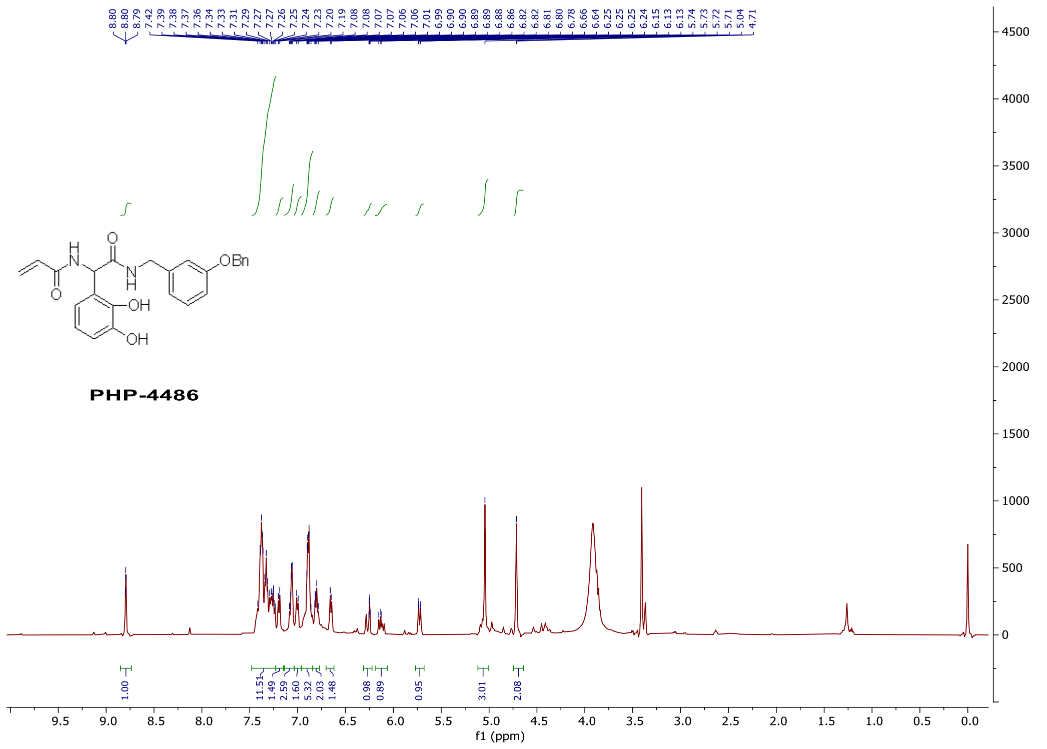

Supplementary Figure 317. ^{1}H NMR (500 MHz, $CDCl_3$+one drop of CD_3OD) of compound **PHP-4486.**

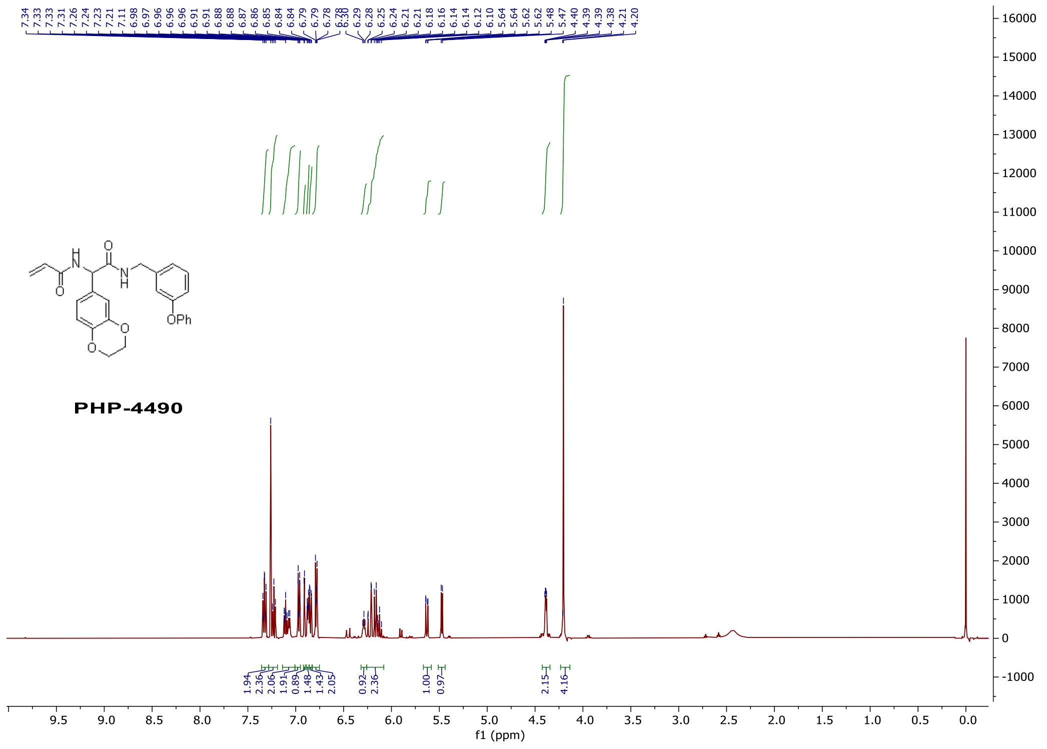

Supplementary Figure 318. ^{1}H NMR (500 MHz, $CDCl_3$) of compound **PHP-4490.**

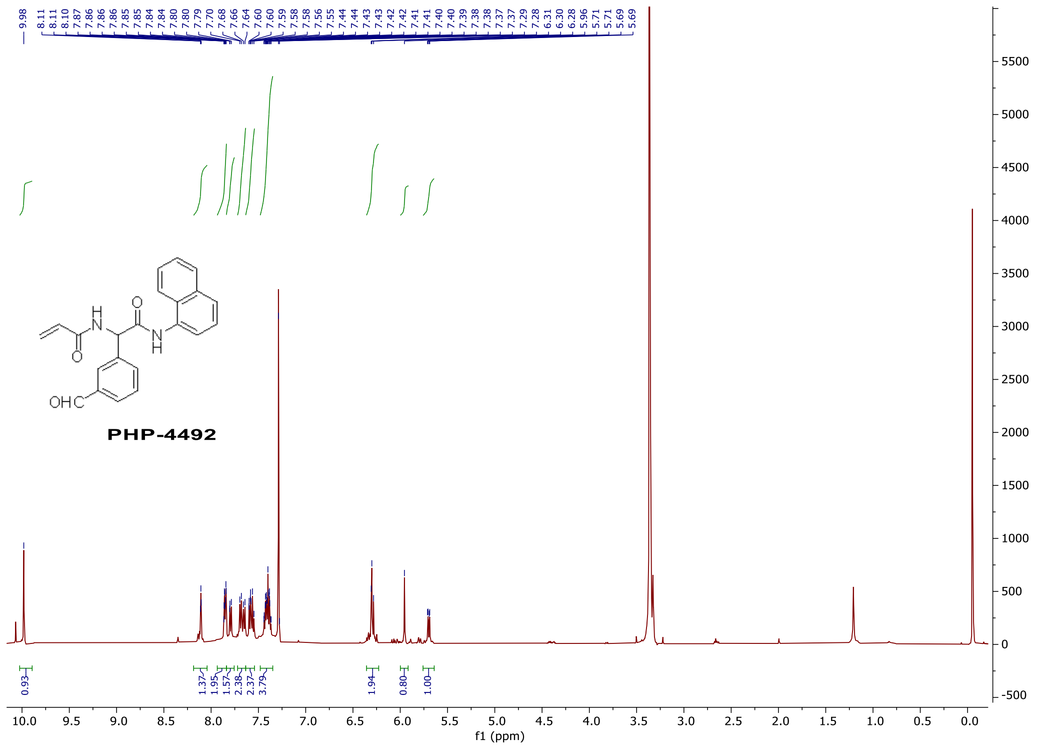

Supplementary Figure 319. ^{1}H NMR (500 MHz, $CDCl_3$+one drop of CD_3OD) of compound **PHP-4492.**

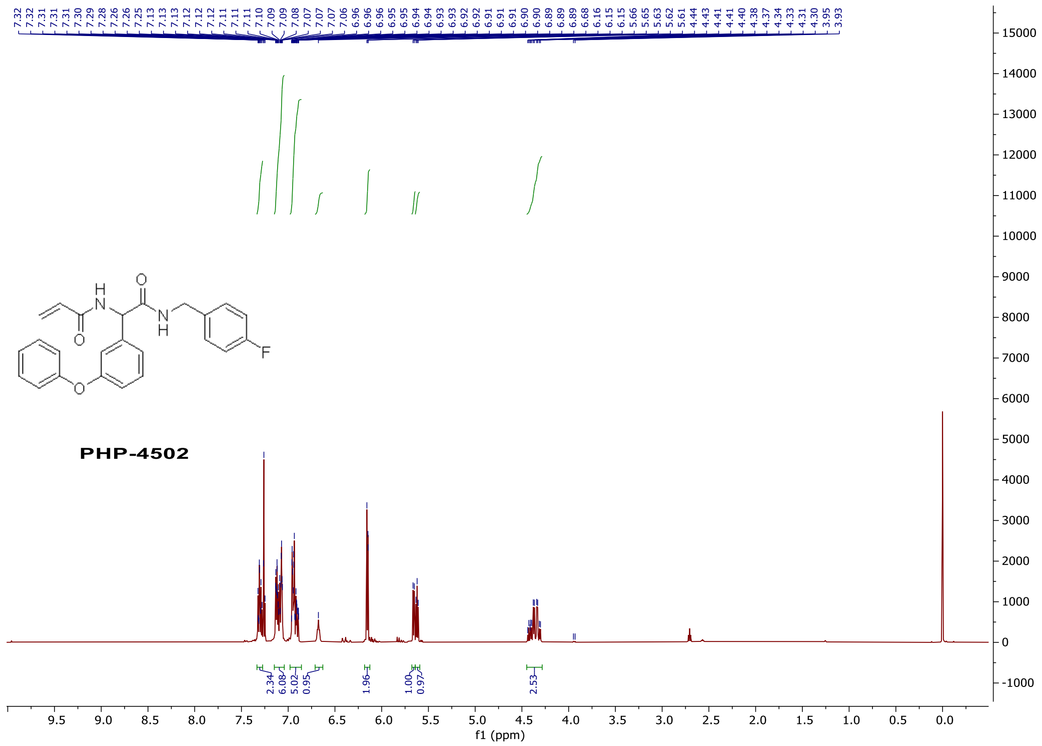

Supplementary Figure 320. ^{1}H NMR (500 MHz, $CDCl_3$) of compound **PHP-4502.**

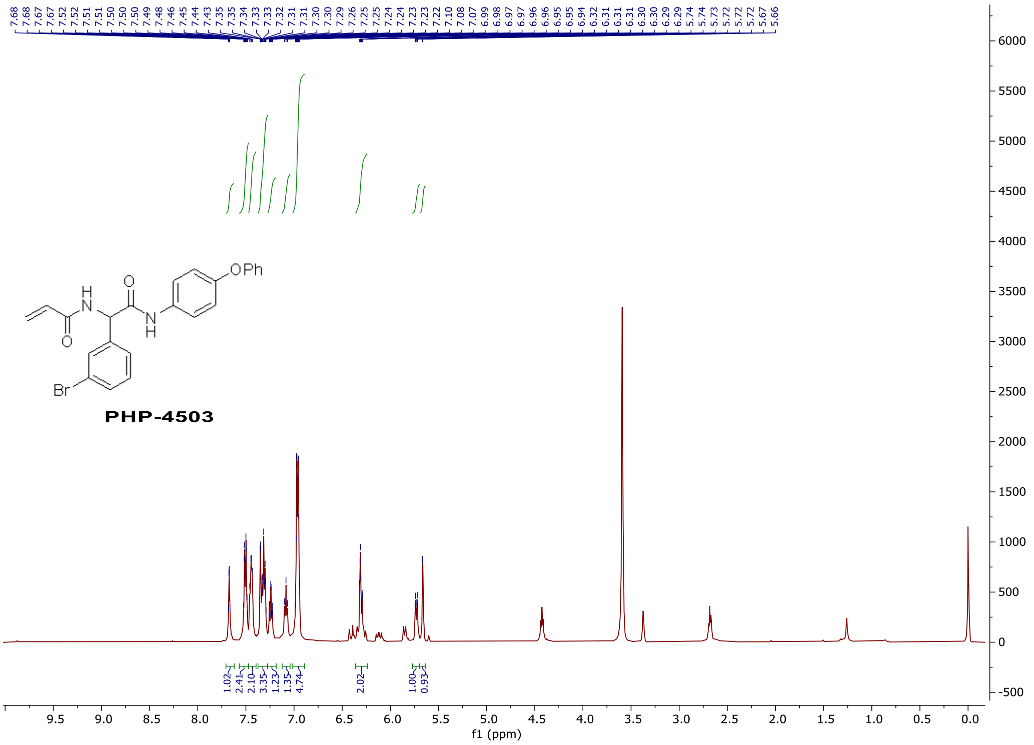

Supplementary Figure 321. ^{1}H NMR (500 MHz, $CDCl_3$+one drop of CD_3OD) of compound **PHP-4503.**

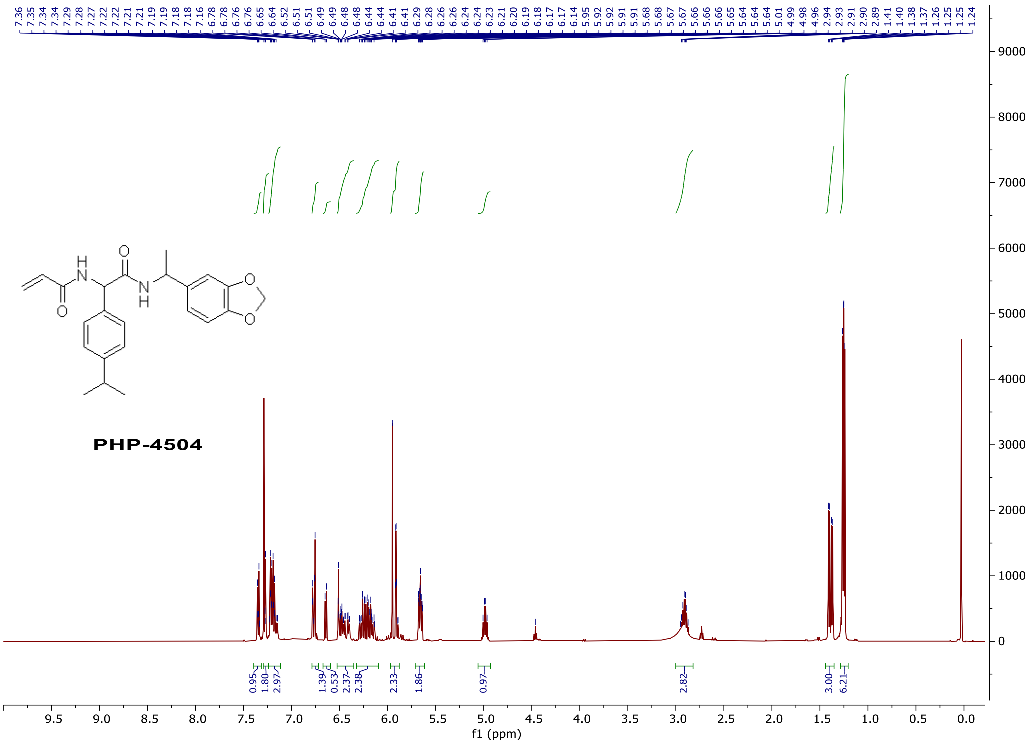

Supplementary Figure 322. ^{1}H NMR (500 MHz, $CDCl_3$) of compound **PHP-4504.**

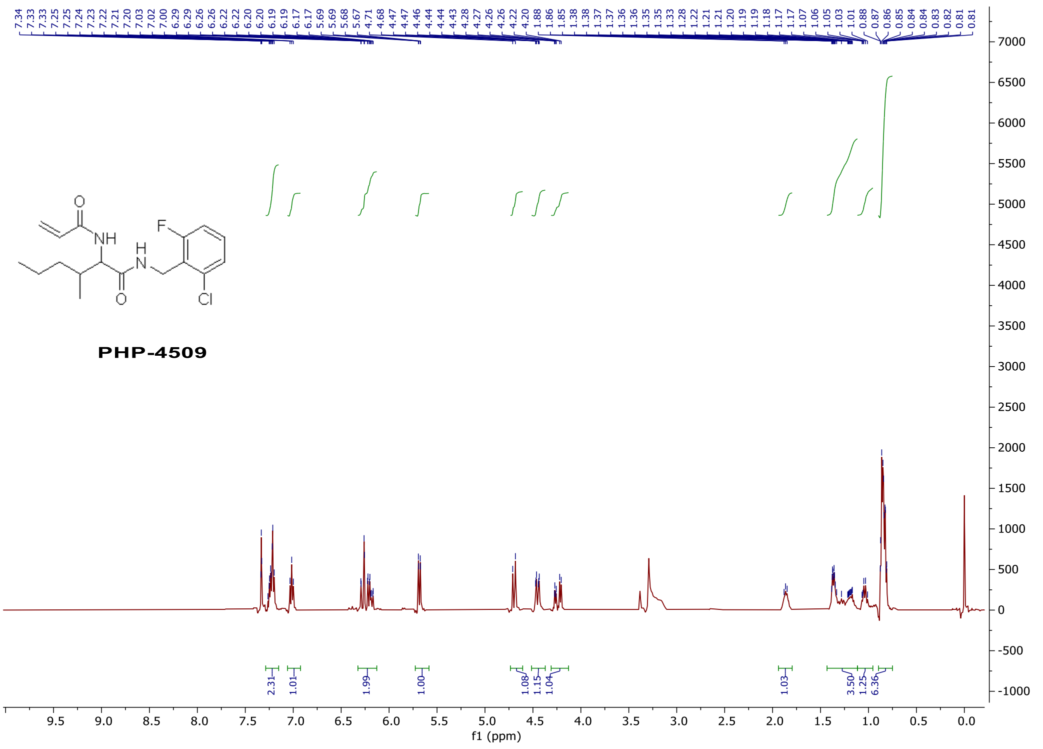

Supplementary Figure 323. ^{1}H NMR (500 MHz, $CDCl_3$+one drop of CD_3OD) of compound **PHP-4509.**

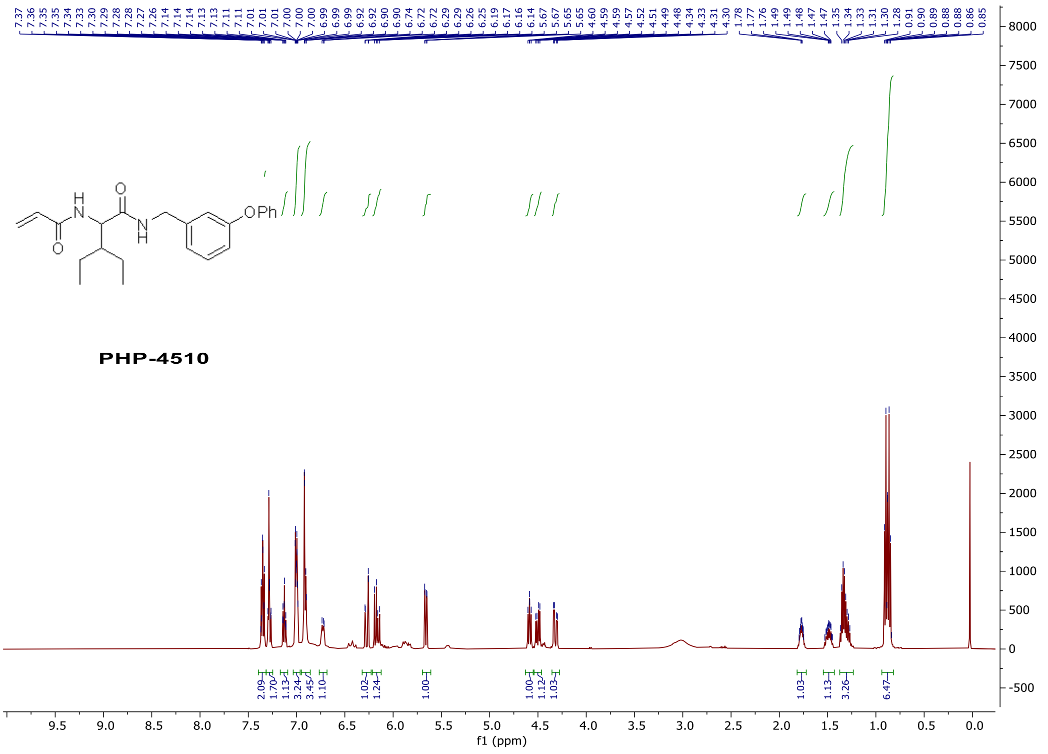

Supplementary Figure 324. ^{1}H NMR (500 MHz, $CDCl_3$) of compound **PHP-4510.**

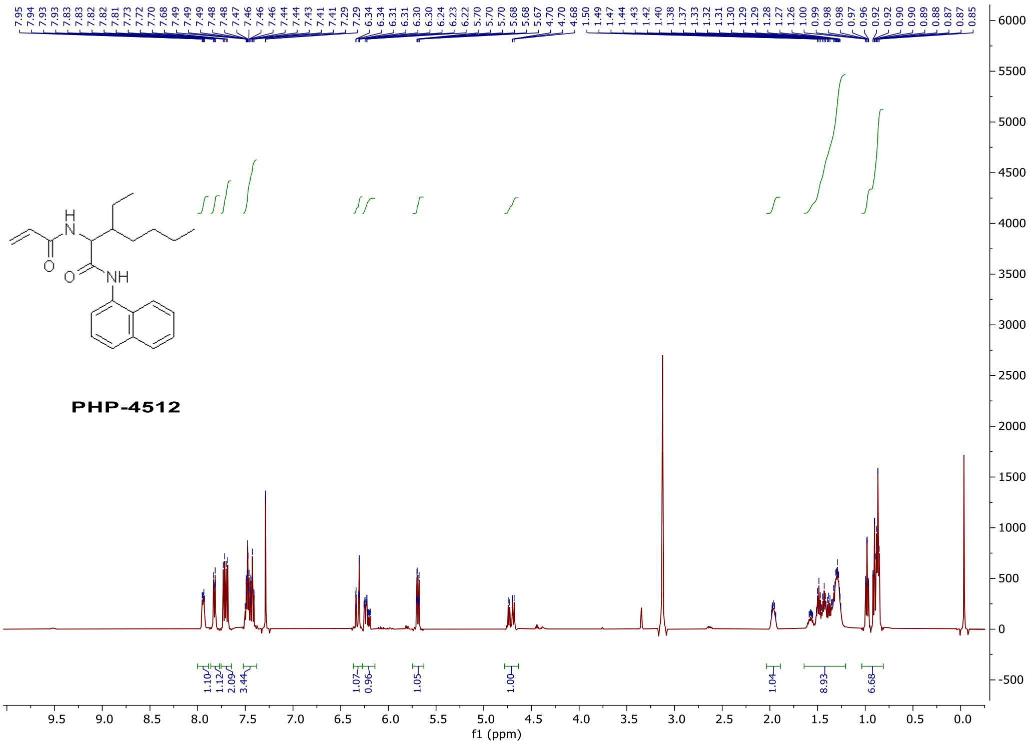

Supplementary Figure 325. ^{1}H NMR (500 MHz, $CDCl_3$+one drop of CD_3OD) of compound **PHP-4512.**

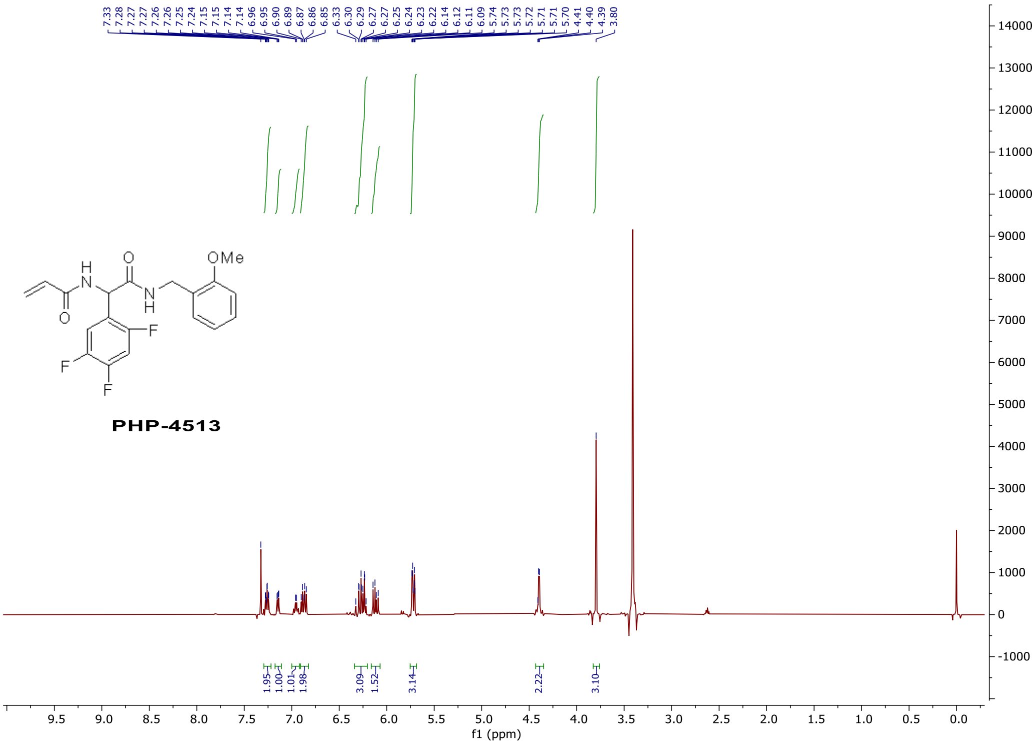

Supplementary Figure 326. ^{1}H NMR (500 MHz, $CDCl_3$+one drop of CD_3OD) of compound **PHP-4513.**

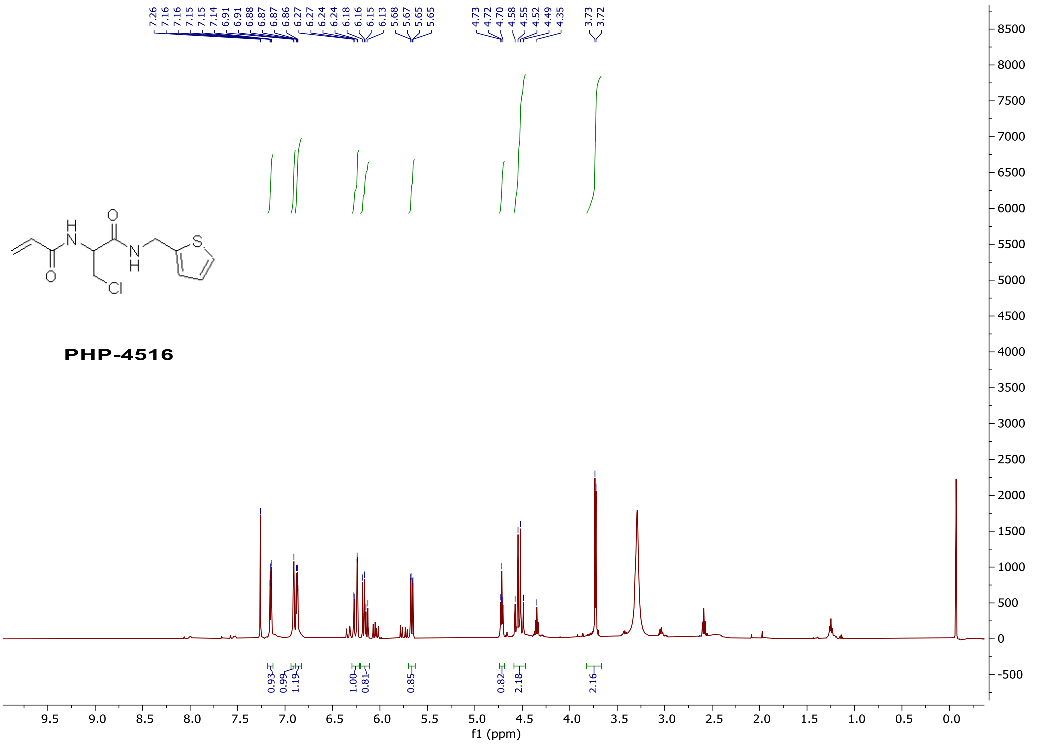

Supplementary Figure 327. ^{1}H NMR (500 MHz, $CDCl_3$+one drop of CD_3OD) of compound **PHP-4516.**

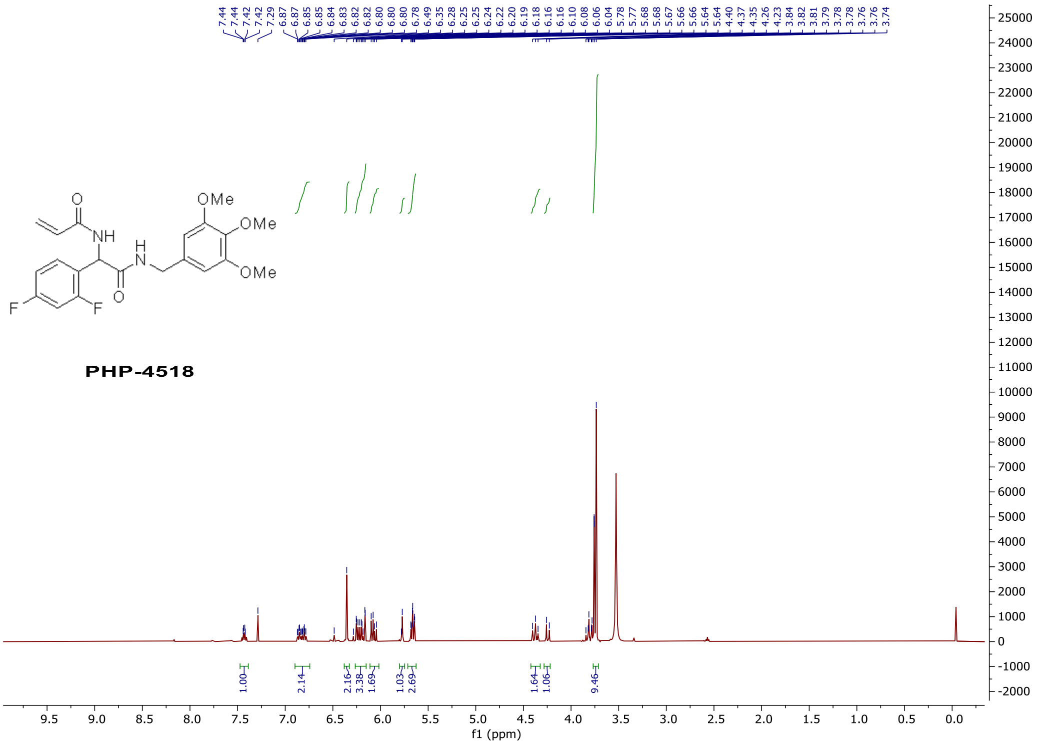

Supplementary Figure 328. ^{1}H NMR (500 MHz, $CDCl_3$+one drop of CD_3OD) of compound **PHP-4518.**

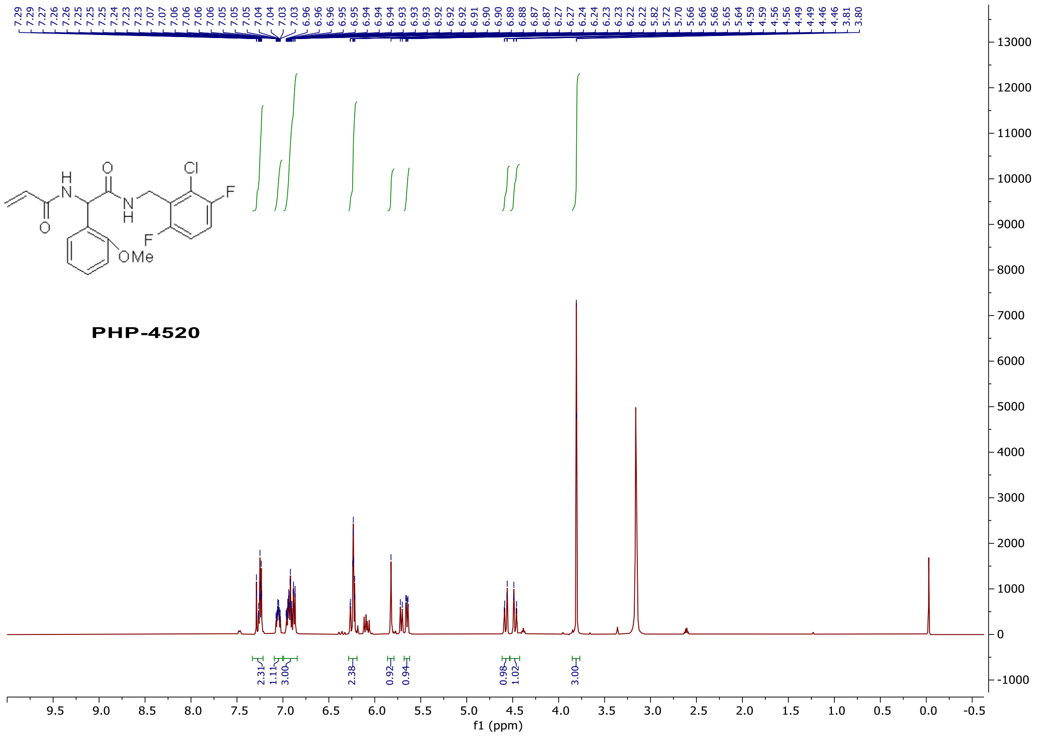

Supplementary Figure 329. ^{1}H NMR (500 MHz, $CDCl_3$+one drop of CD_3OD) of compound **PHP-4520.**

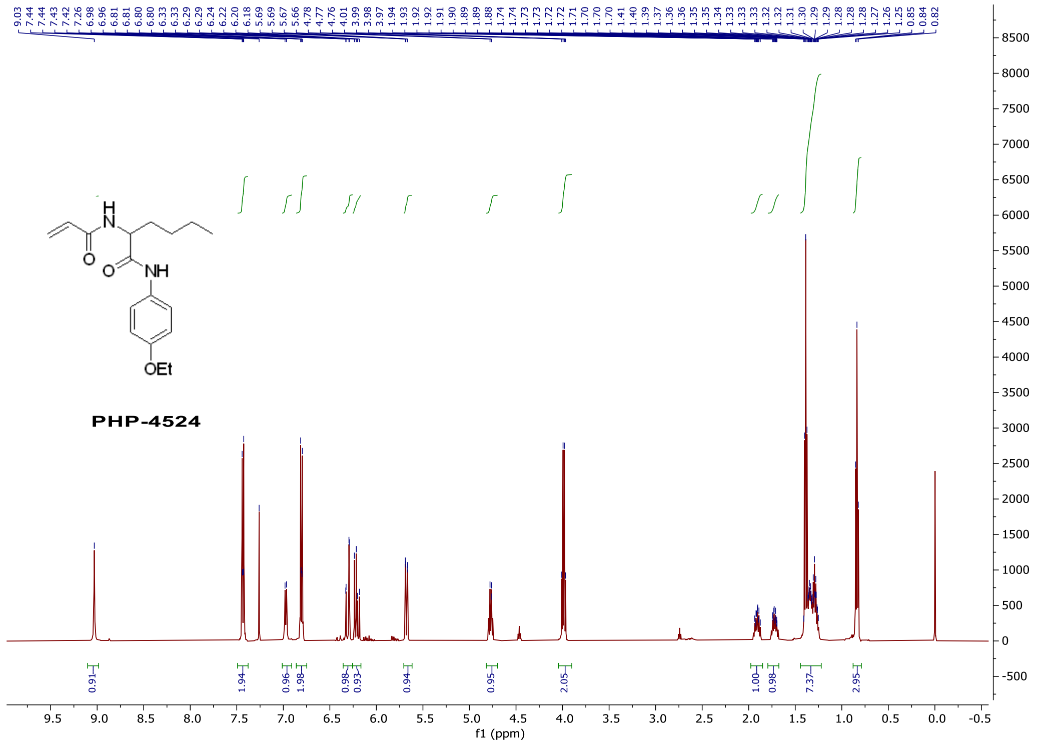

Supplementary Figure 330. ^{1}H NMR (500 MHz, $CDCl_3$) of compound **PHP-4524.**

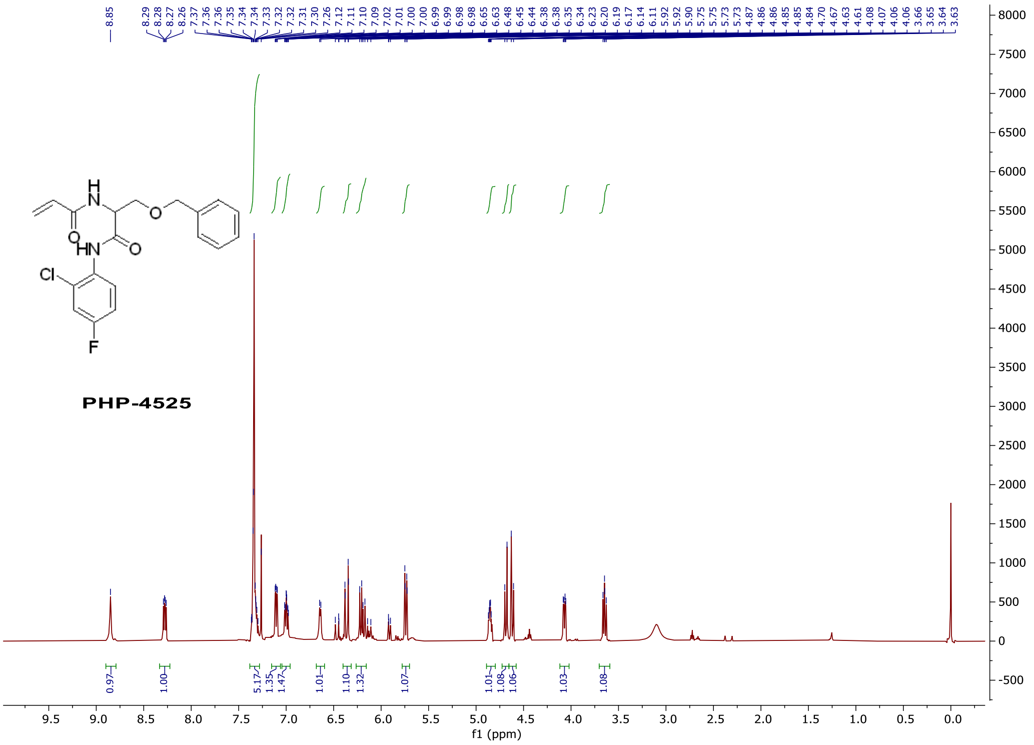

Supplementary Figure 331. ^{1}H NMR (500 MHz, $CDCl_3$) of compound **PHP-4525.**

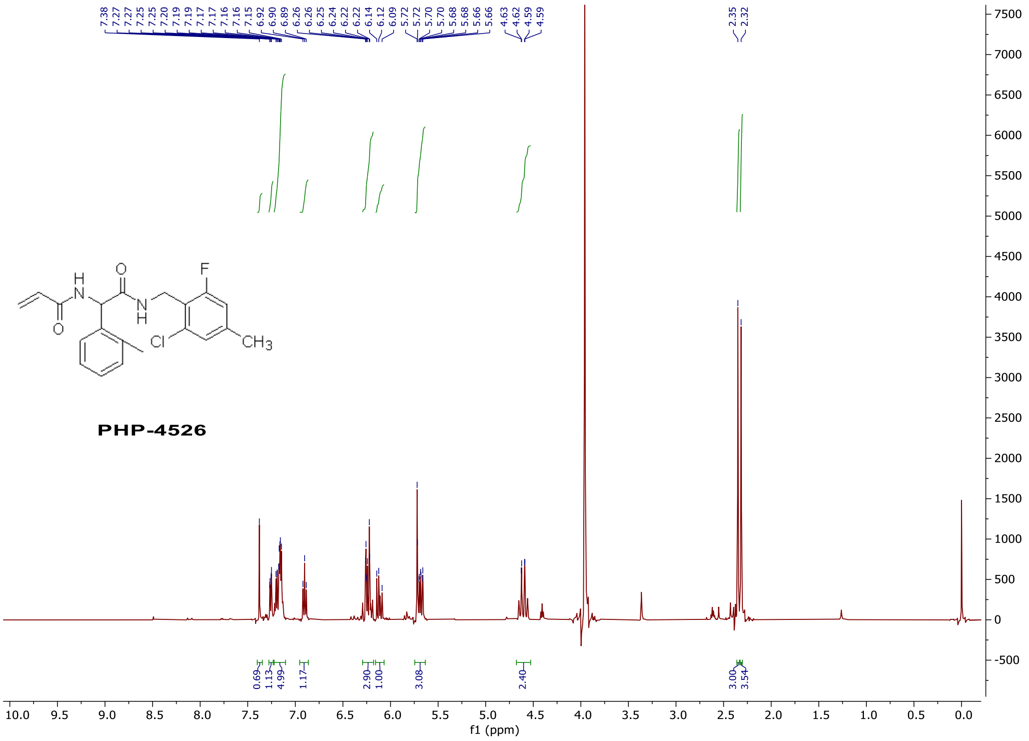

Supplementary Figure 332. ^{1}H NMR (500 MHz, $CDCl_3$+one drop of CD_3OD) of compound **PHP-4526.**

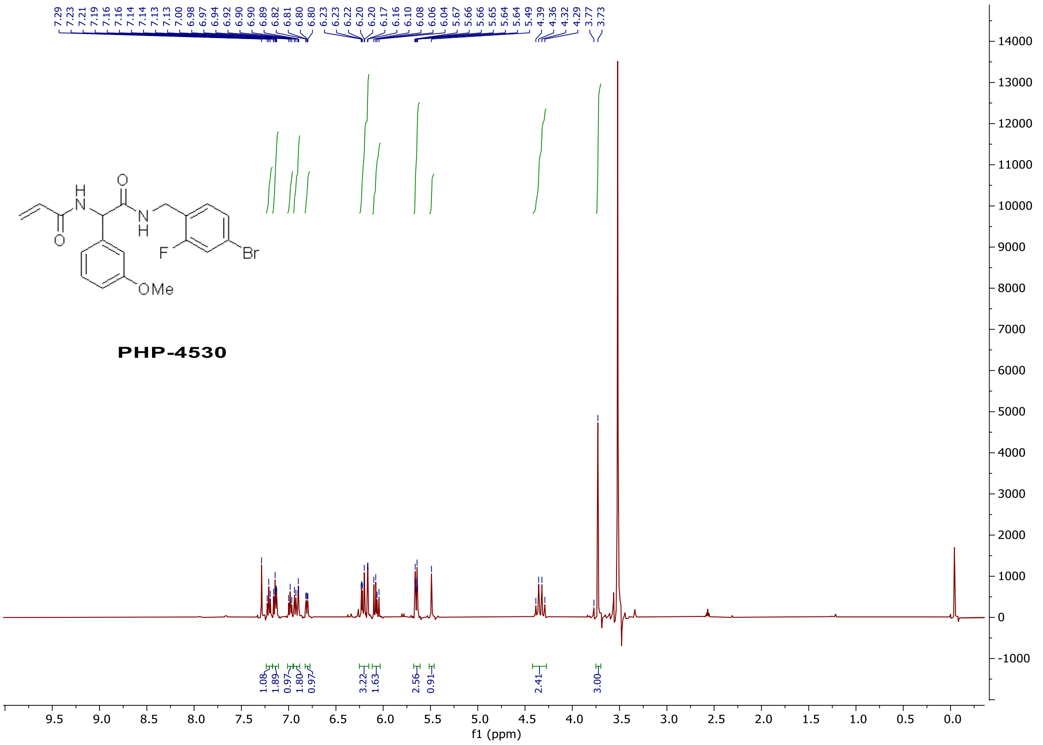

Supplementary Figure 333. ^{1}H NMR (500 MHz, $CDCl_3$+one drop of CD_3OD) of compound **PHP-4530.**

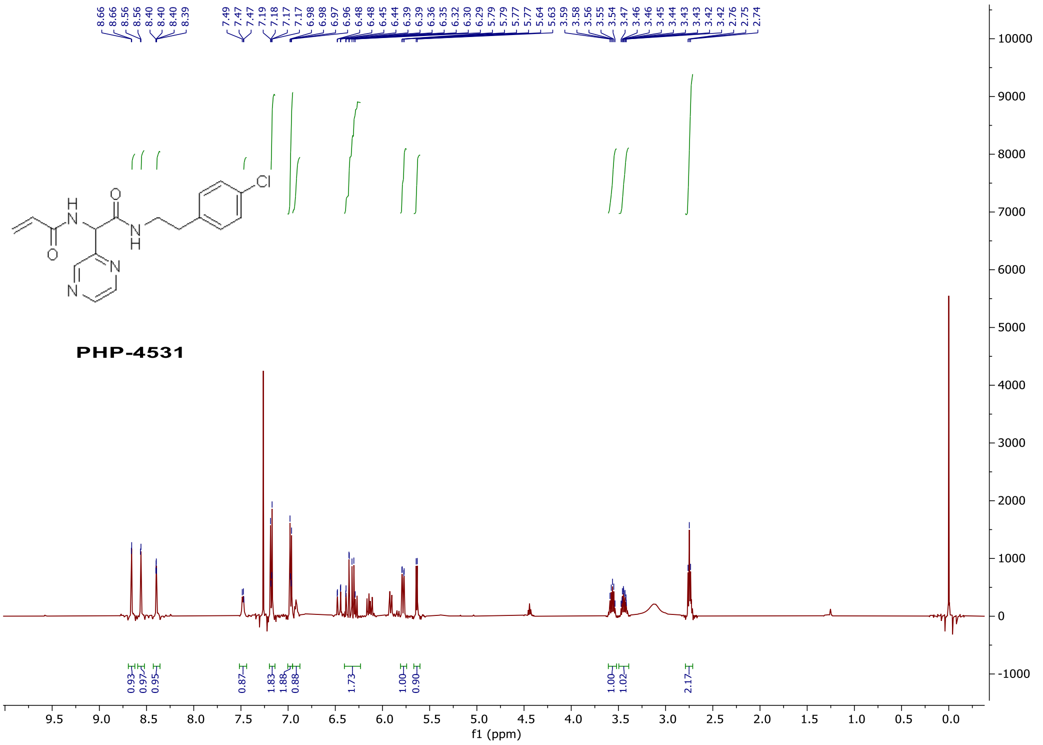

Supplementary Figure 334. ^{1}H NMR (500 MHz, $CDCl_3$) of compound **PHP-4531.**

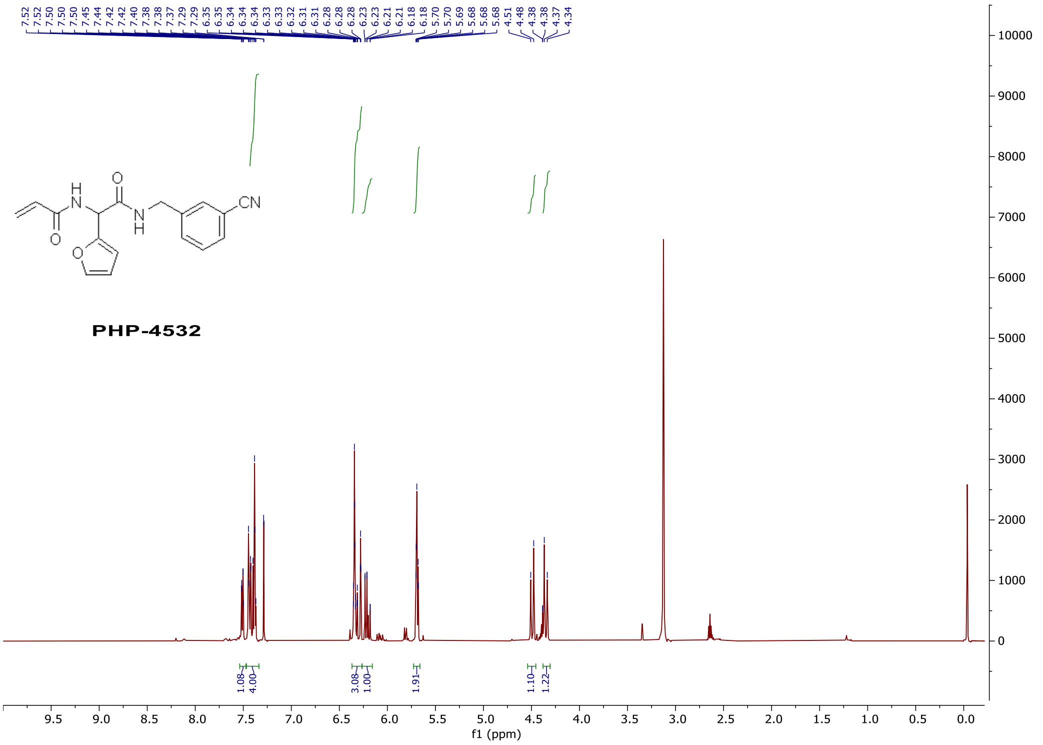

Supplementary Figure 335. ^{1}H NMR (500 MHz, $CDCl_3$+one drop of CD_3OD) of compound **PHP-4532.**

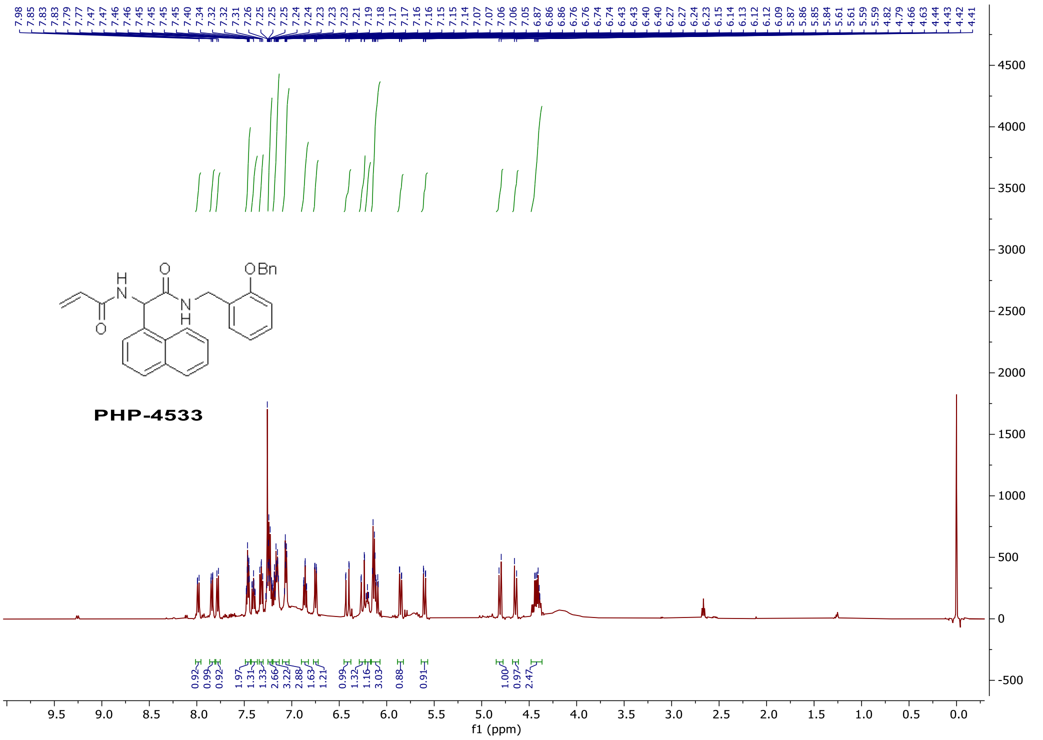

Supplementary Figure 336. ^{1}H NMR (500 MHz, $CDCl_3$) of compound **PHP-4533.**

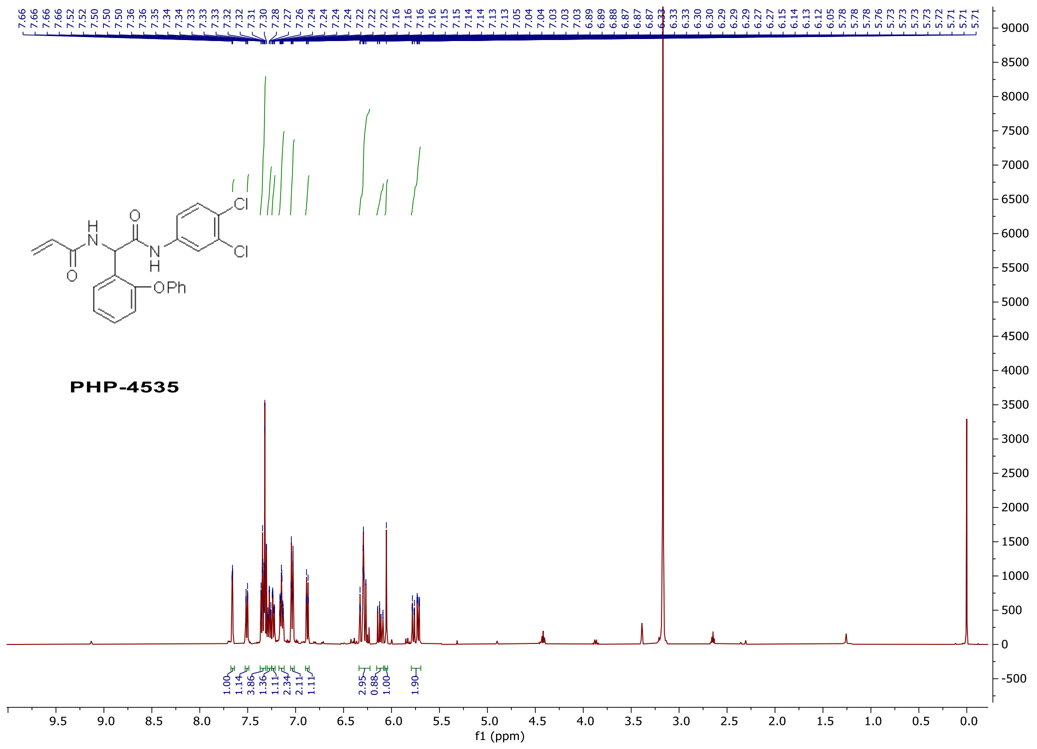

Supplementary Figure 337. ^{1}H NMR (500 MHz, $CDCl_3$+one drop of CD_3OD) of compound **PHP-4535.**

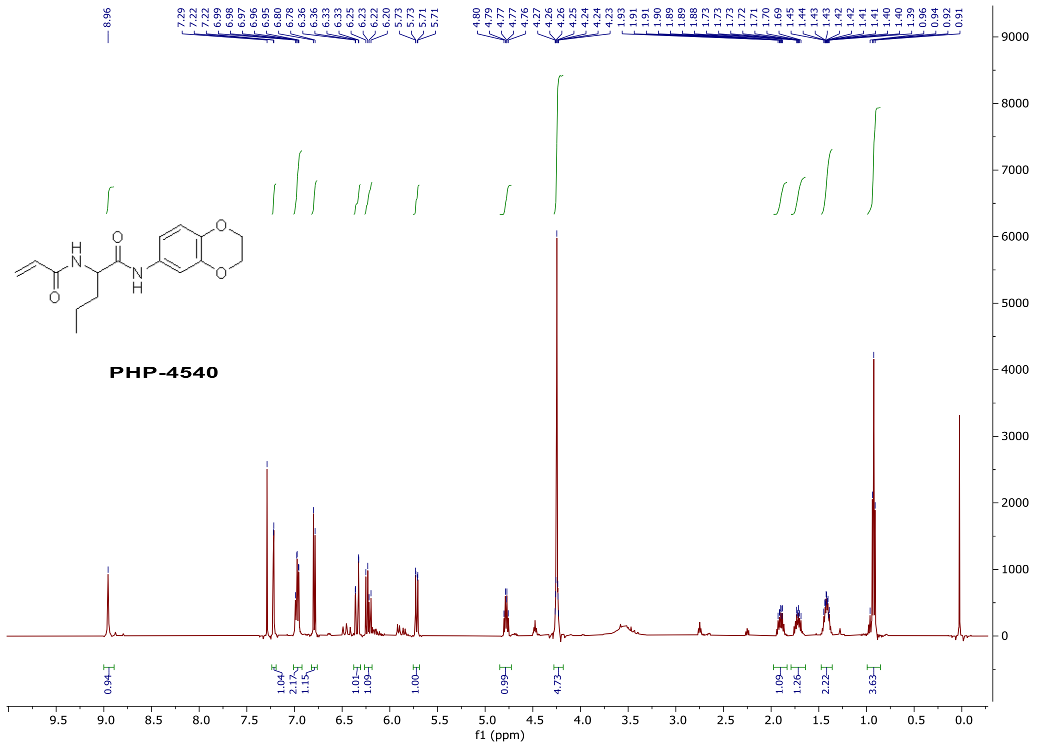

Supplementary Figure 338. ^{1}H NMR (500 MHz, $CDCl_3$+one drop of CD_3OD) of compound **PHP-4540.**

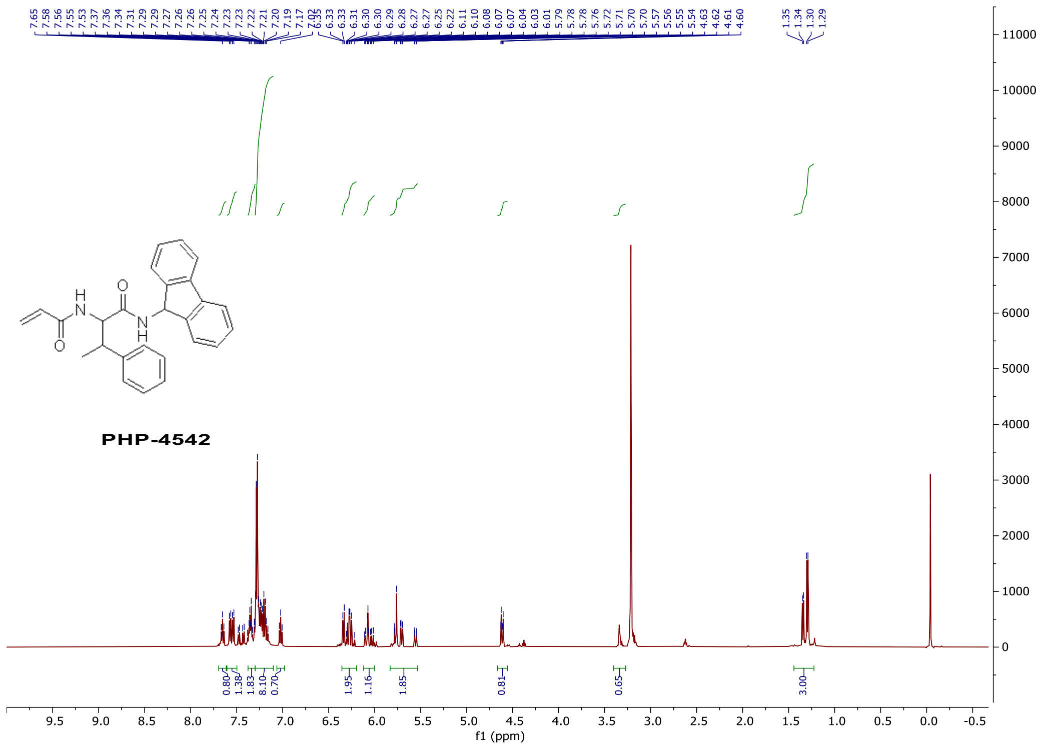

Supplementary Figure 339. ^{1}H NMR (500 MHz, $CDCl_3$+one drop of CD_3OD) of compound **PHP-4542.**

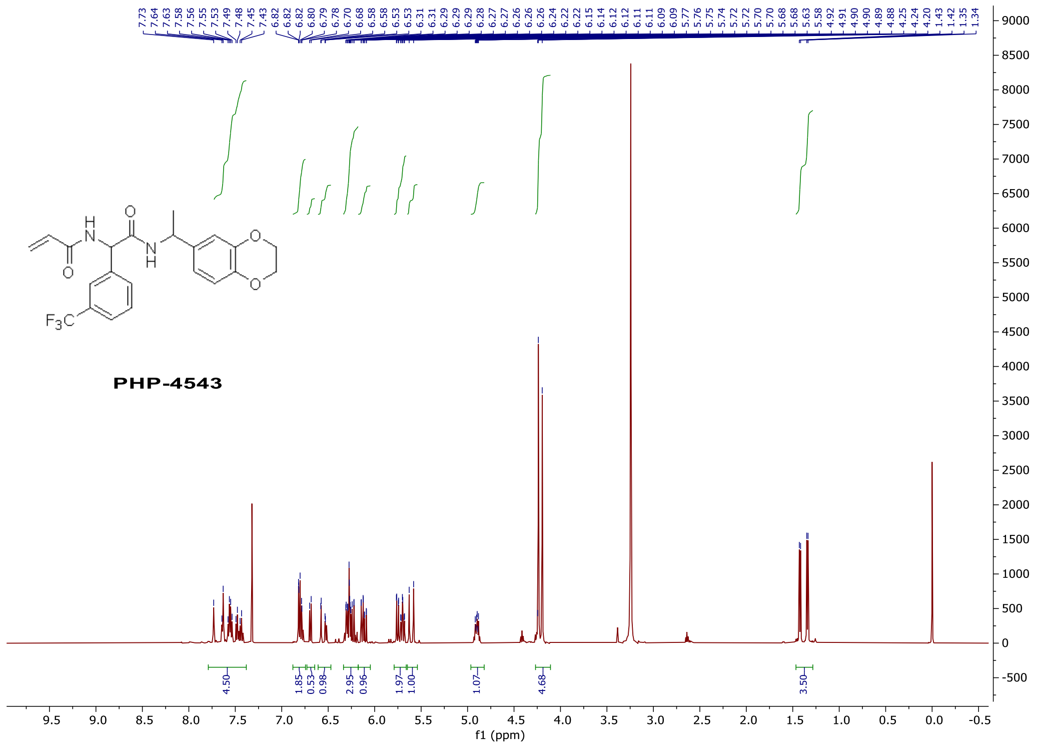

Supplementary Figure 340. ^{1}H NMR (500 MHz, $CDCl_3$+one drop of CD_3OD) of compound **PHP-4543.**

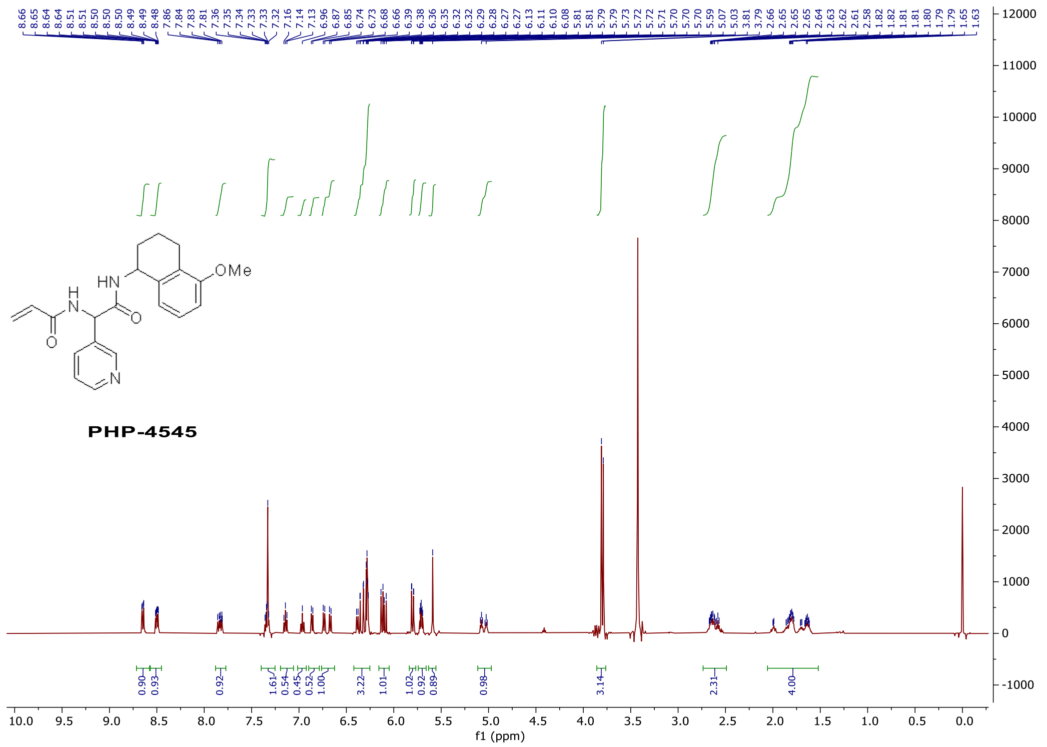

Supplementary Figure 341. ^{1}H NMR (500 MHz, $CDCl_3$+one drop of CD_3OD) of compound **PHP-4545.**

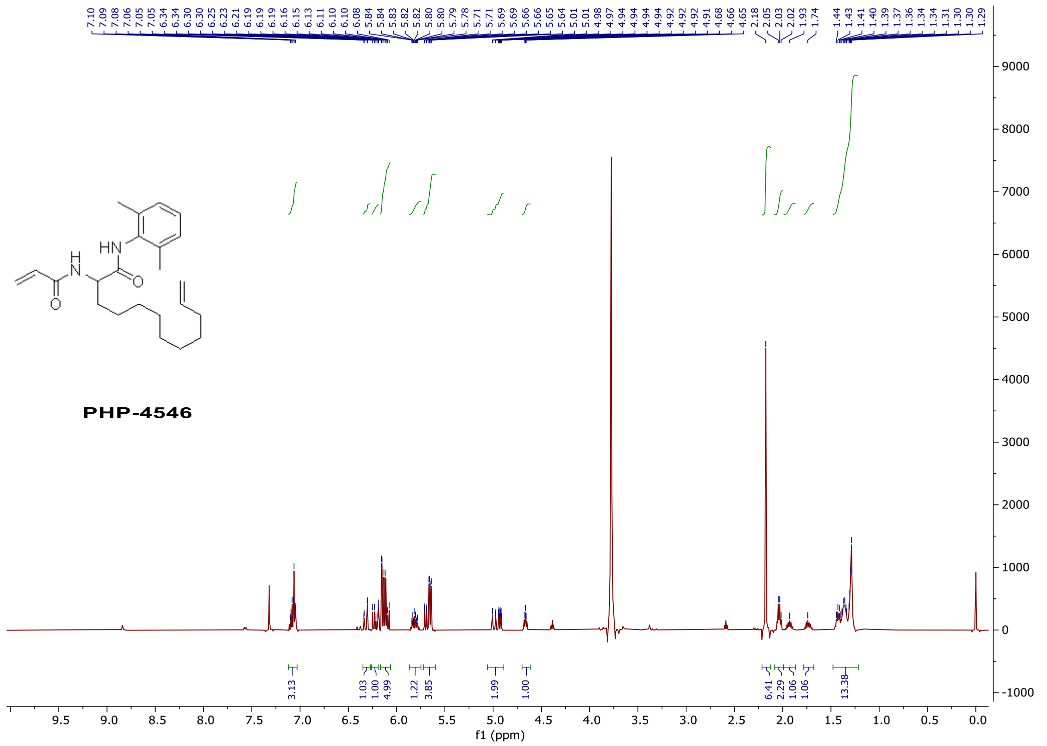

Supplementary Figure 342. ^{1}H NMR (500 MHz, $CDCl_3$+one drop of CD_3OD) of compound **PHP-4546.**

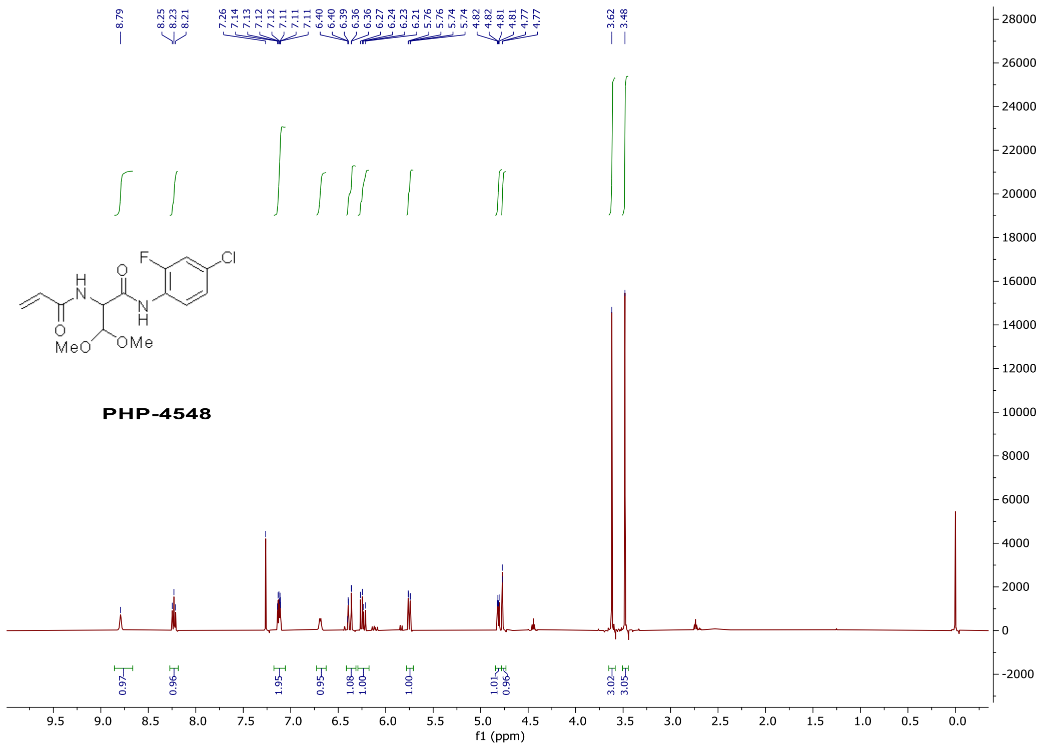

Supplementary Figure 343. ^{1}H NMR (500 MHz, $CDCl_3$) of compound **PHP-4548.**

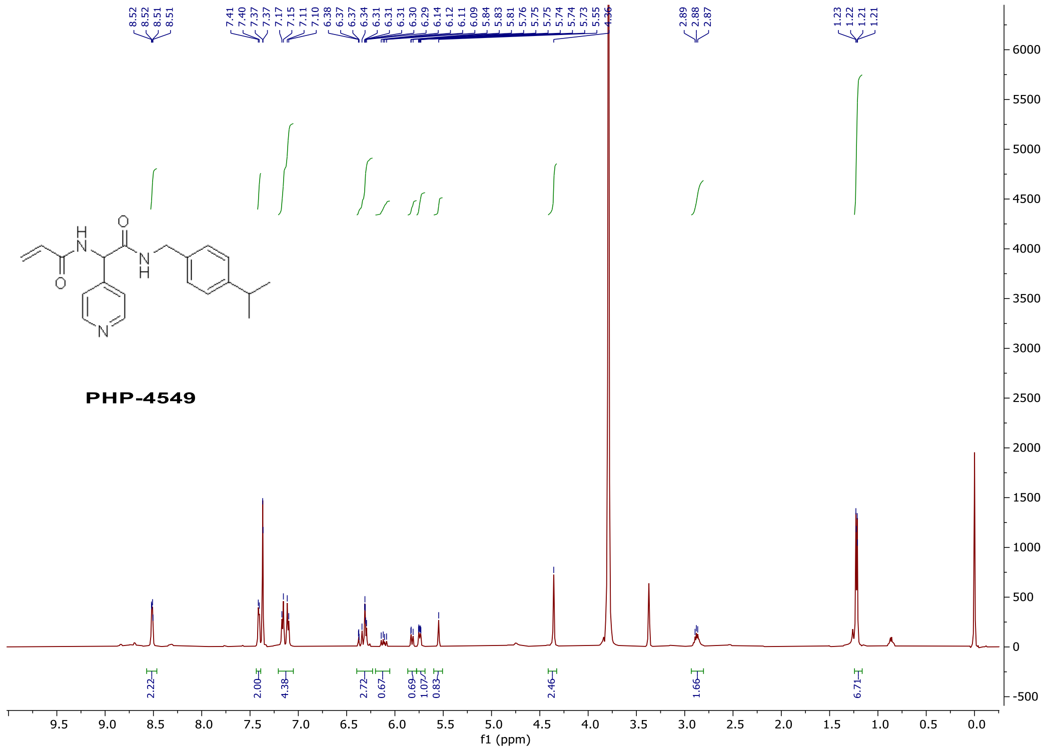

Supplementary Figure 344. ^{1}H NMR (500 MHz, $CDCl_3$+one drop of CD_3OD) of compound **PHP-4549.**

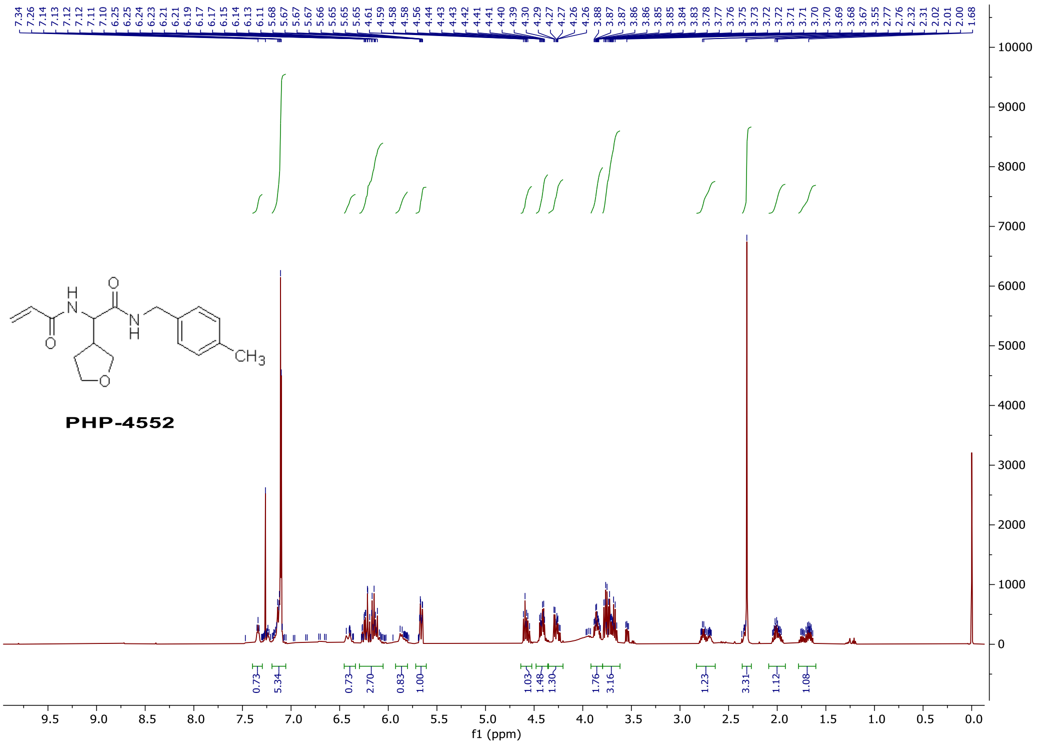

Supplementary Figure 345. ^{1}H NMR (500 MHz, $CDCl_3$) of compound **PHP-4552.**

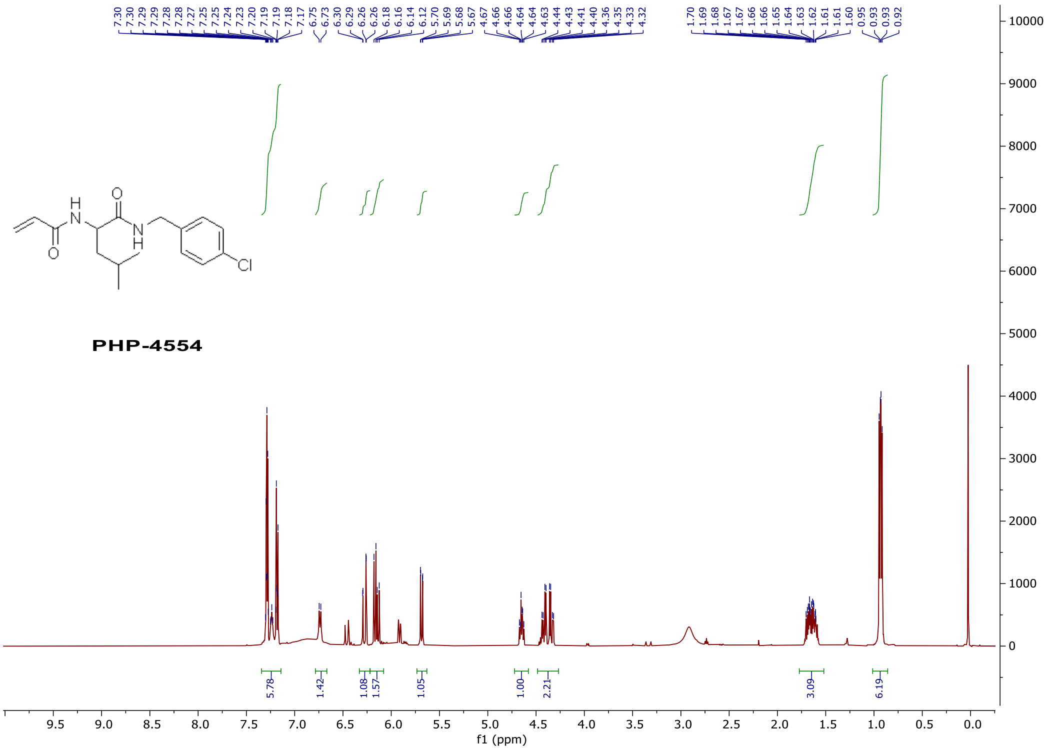

Supplementary Figure 346. ^{1}H NMR (500 MHz, $CDCl_3$) of compound **PHP-4554.**

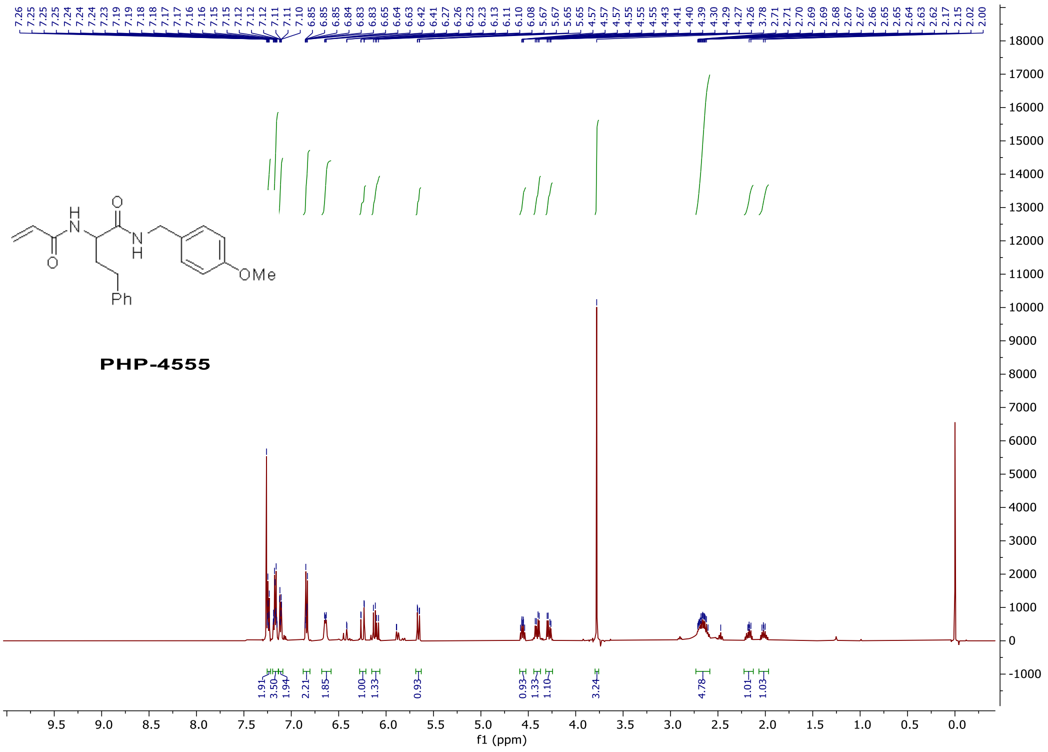

Supplementary Figure 347. ¹H NMR (500 MHz, $CDCl_3$) of compound **PHP-4555.**

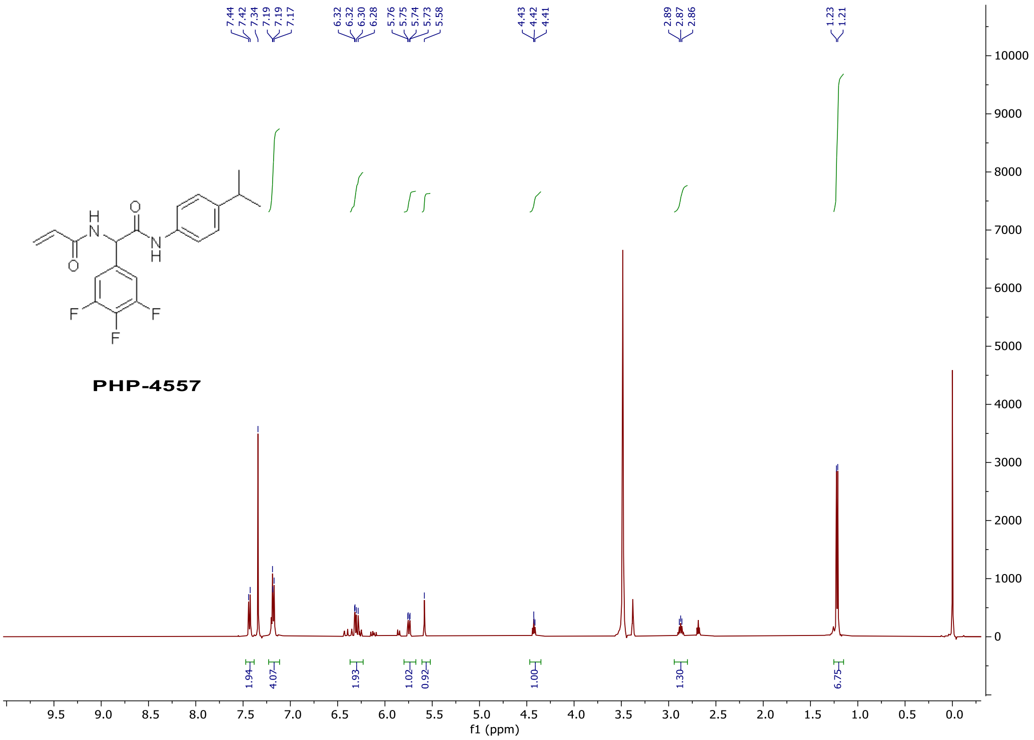

Supplementary Figure 348. ¹H NMR (500 MHz, $CDCl_3$+one drop of CD_3OD) of compound **PHP-4557.**

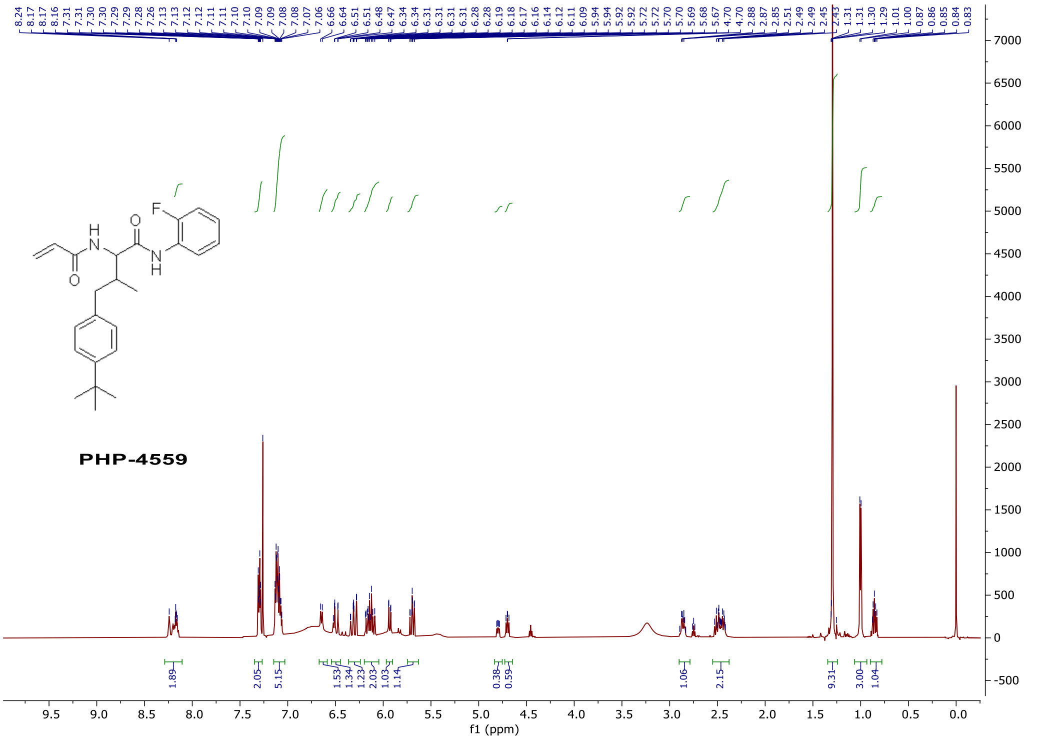

Supplementary Figure 349. ^{1}H NMR (500 MHz, $CDCl_3$) of compound **PHP-4559.**

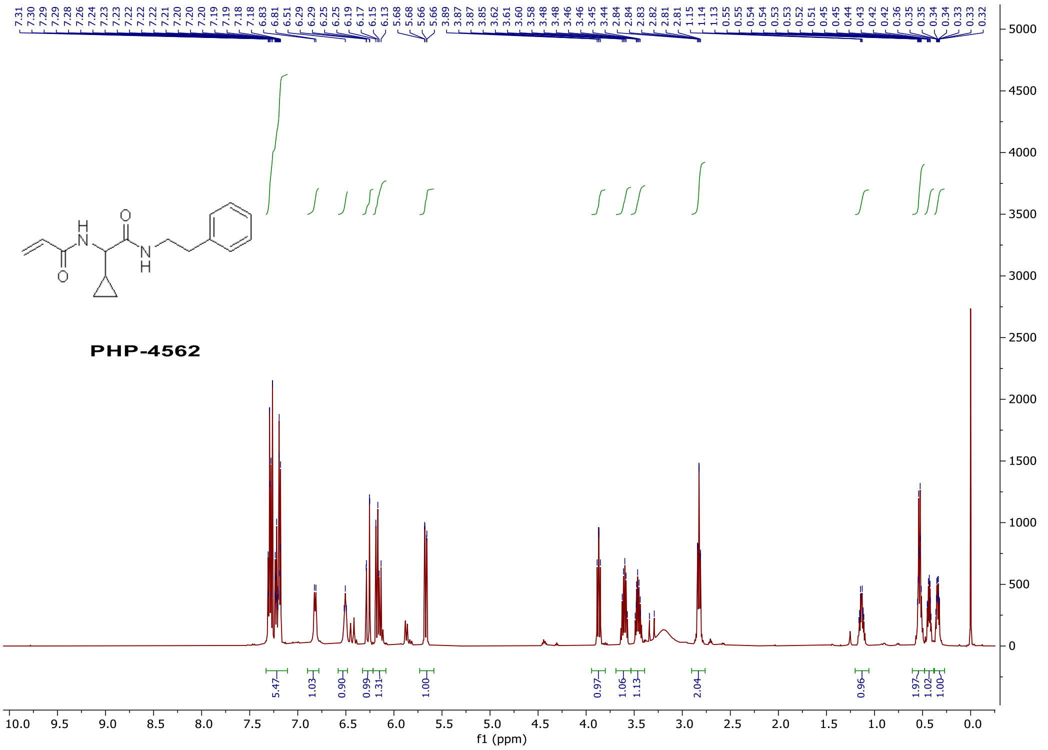

Supplementary Figure 350. ^{1}H NMR (500 MHz, $CDCl_3$) of compound **PHP-4562.**

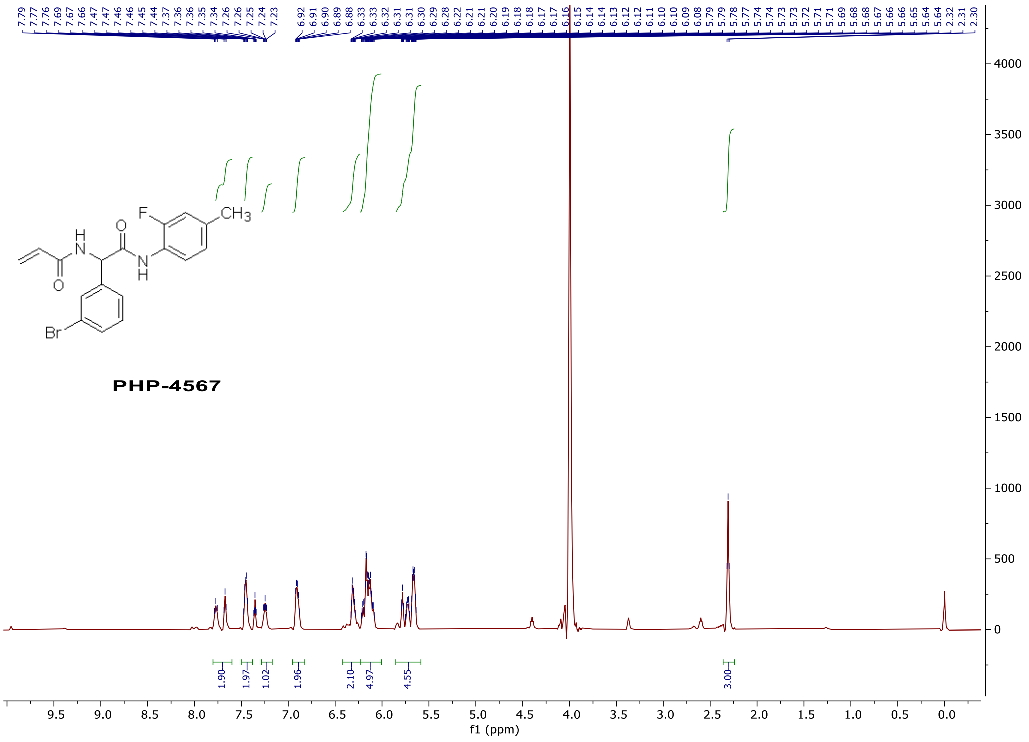

Supplementary Figure 351. ^{1}H NMR (500 MHz, $CDCl_3$+one drop of CD_3OD) of compound **PHP-4567.**

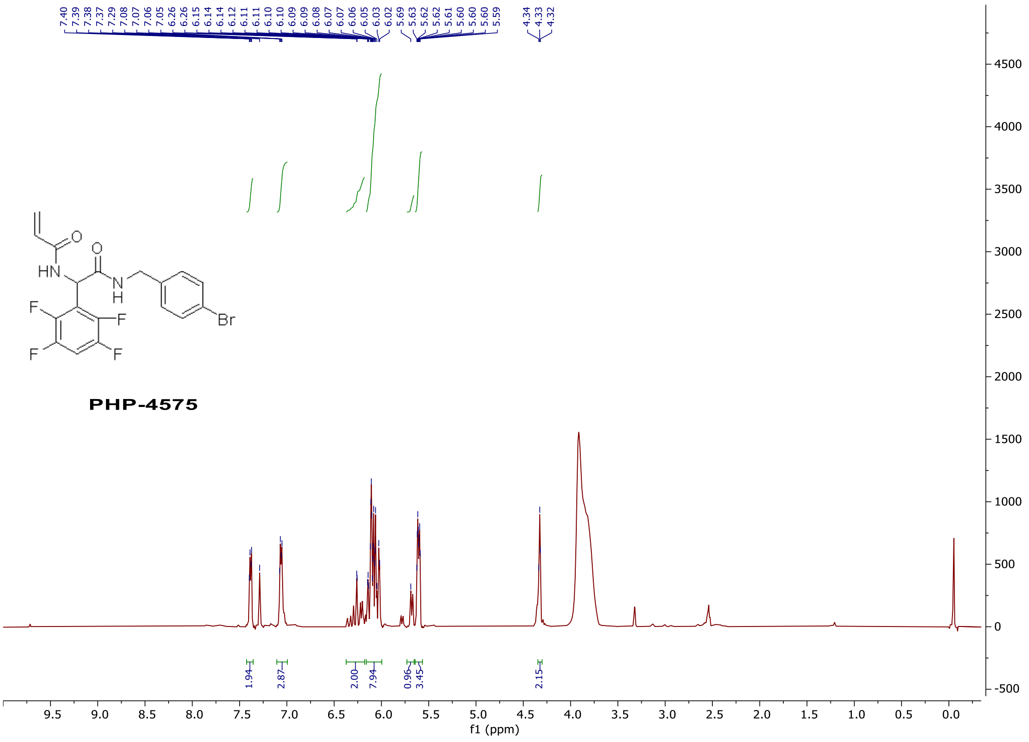

Supplementary Figure 352. ^{1}H NMR (500 MHz, $CDCl_3$+one drop of CD_3OD) of compound **PHP-4575.**

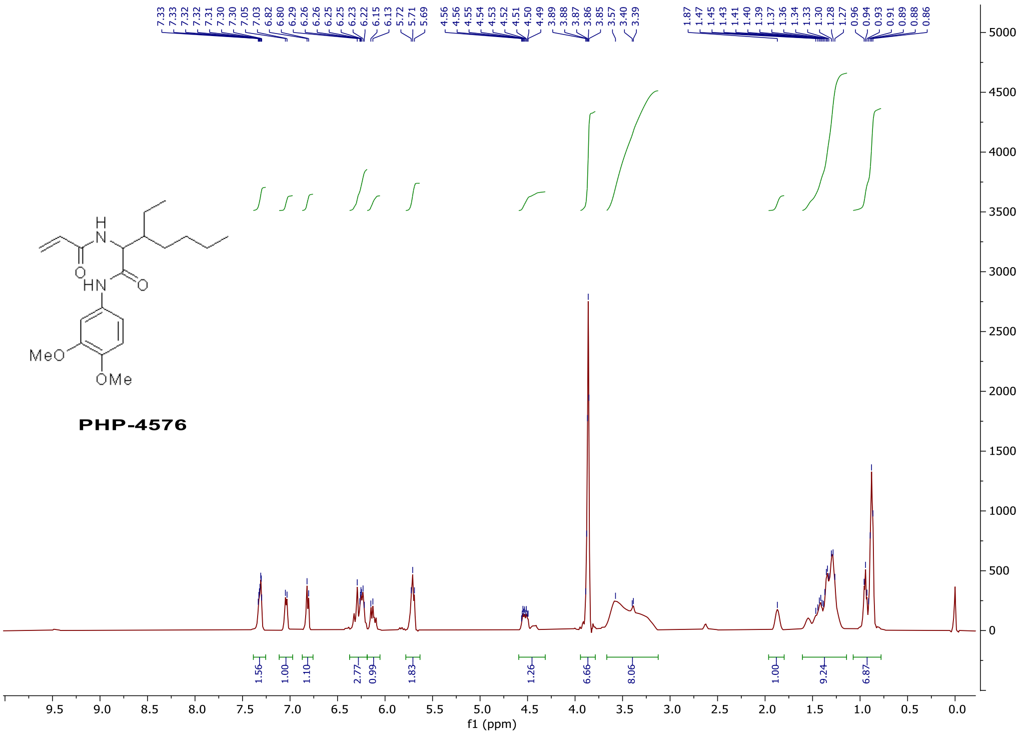

Supplementary Figure 353. ^{1}H NMR (500 MHz, $CDCl_3$+one drop of CD_3OD) of compound **PHP-4576.**

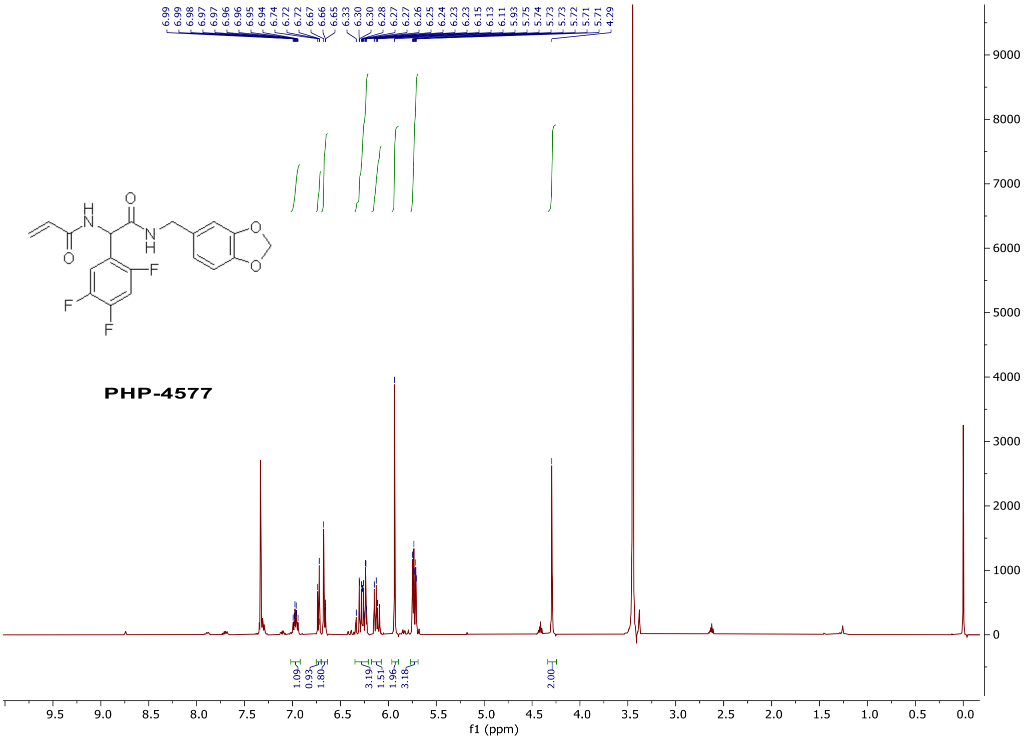

Supplementary Figure 354. ^{1}H NMR (500 MHz, $CDCl_3$+one drop of CD_3OD) of compound **PHP-4577.**

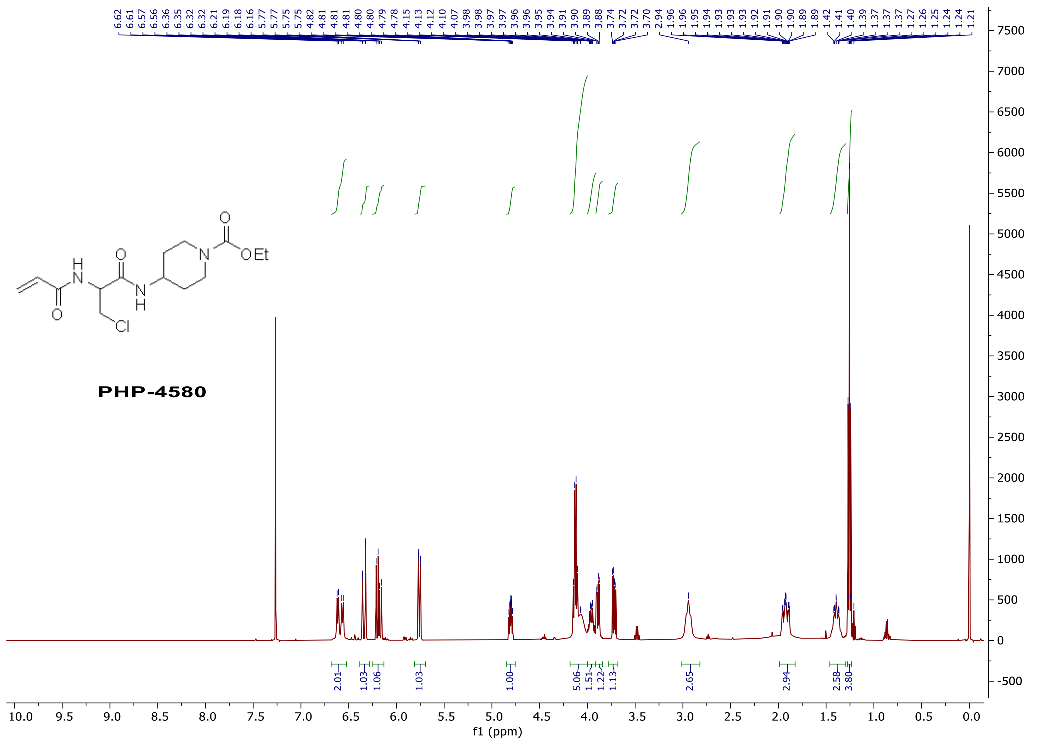

Supplementary Figure 355. ^{1}H NMR (500 MHz, $CDCl_3$) of compound **PHP-4580.**

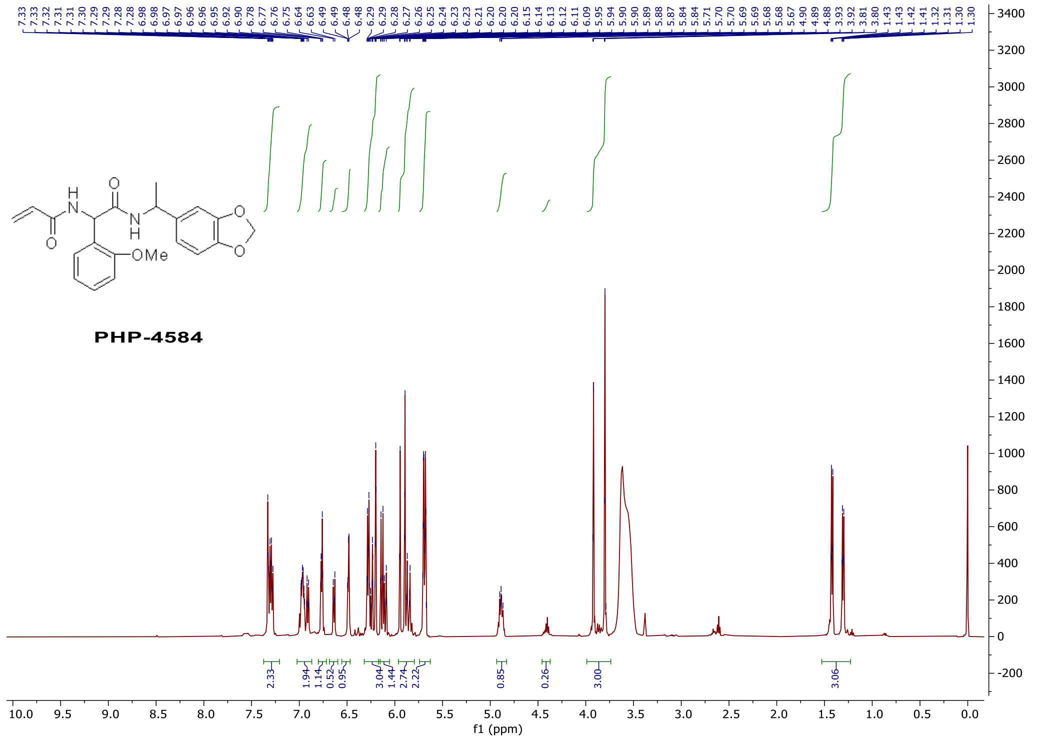

Supplementary Figure 356. ^{1}H NMR (500 MHz, $CDCl_3$+one drop of CD_3OD) of compound **PHP-4584.**

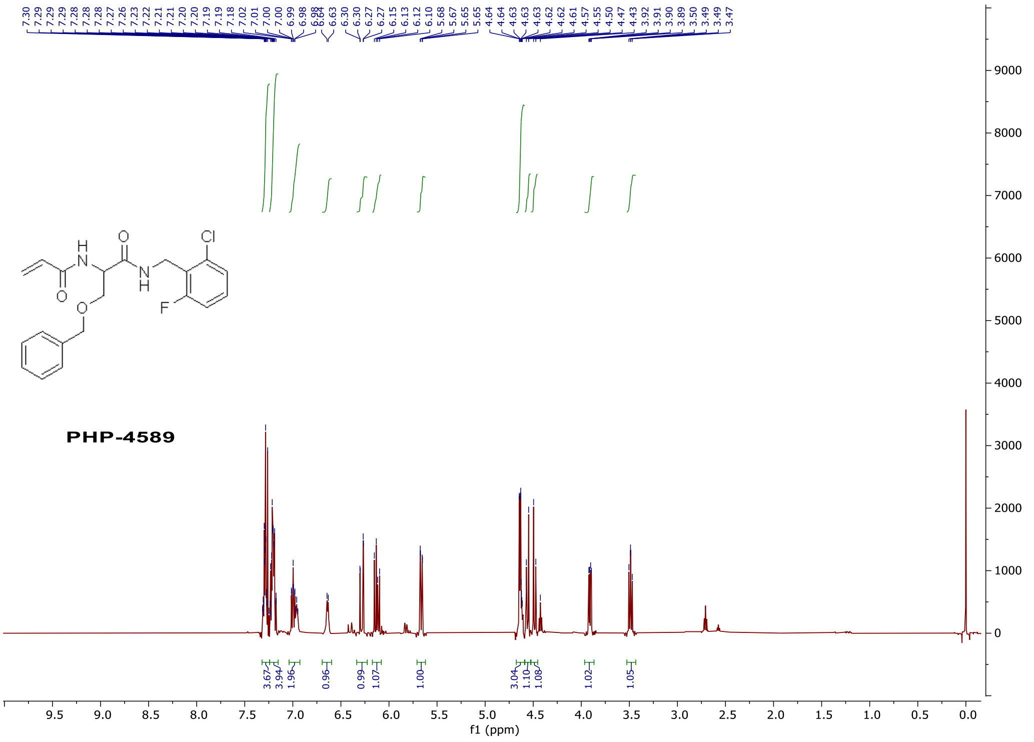

Supplementary Figure 357. ^{1}H NMR (500 MHz, $CDCl_3$) of compound **PHP-4589.**

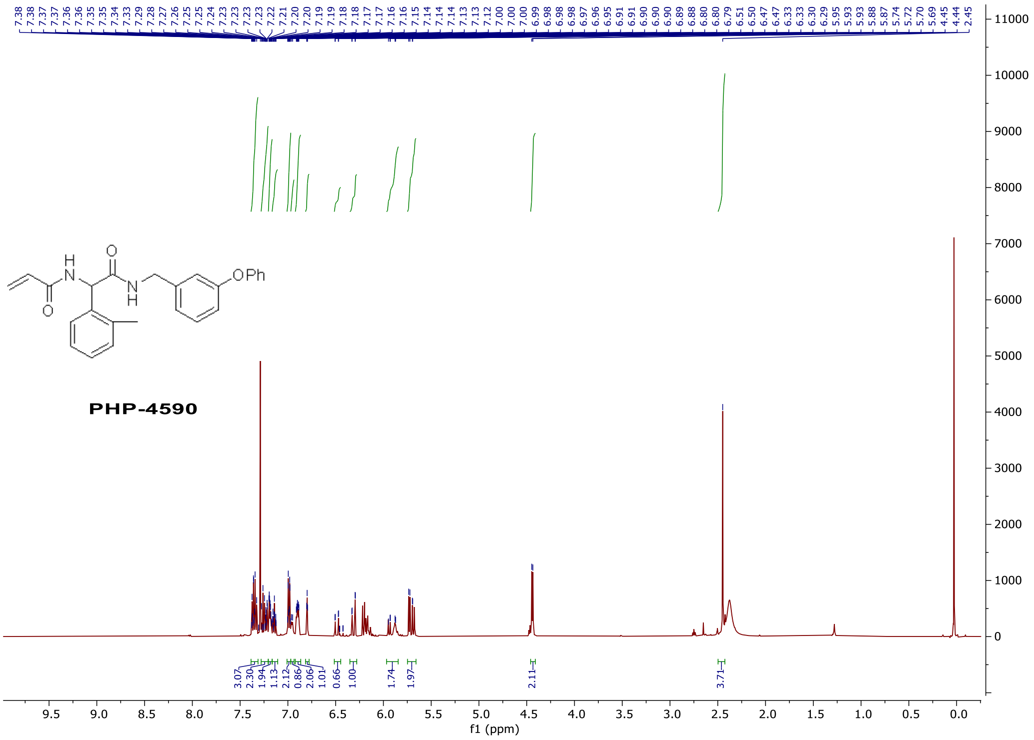

Supplementary Figure 358. ^{1}H NMR (500 MHz, $CDCl_3$) of compound **PHP-4590.**

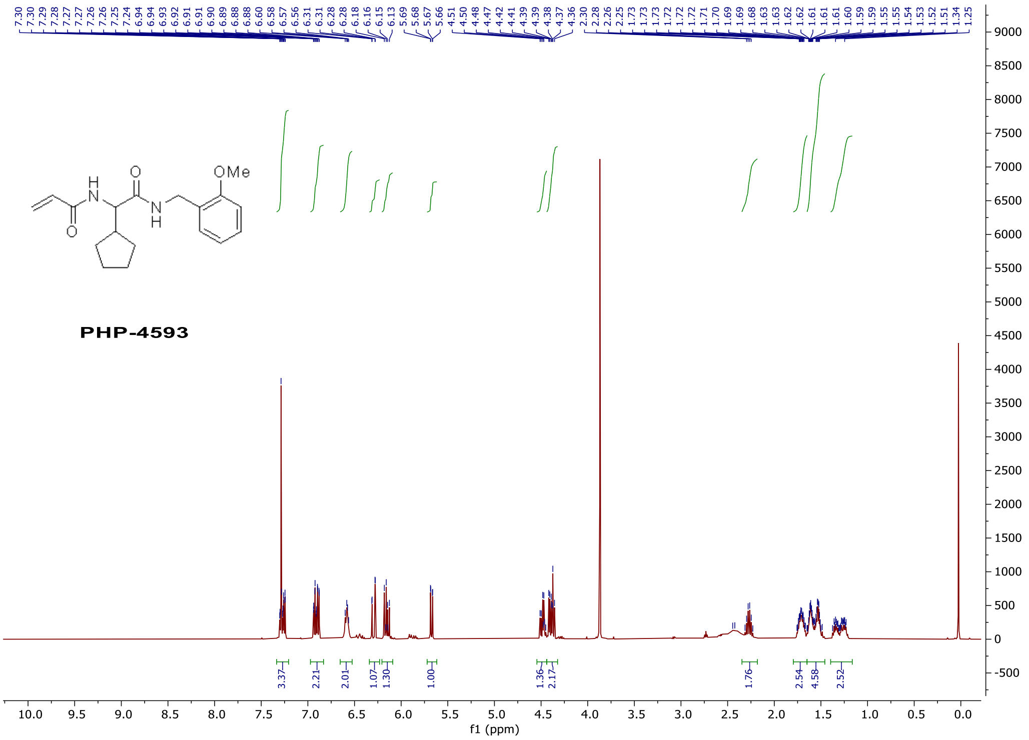

Supplementary Figure 359. ^{1}H NMR (500 MHz, $CDCl_3$+one drop of CD_3OD) of compound **PHP-4593.**

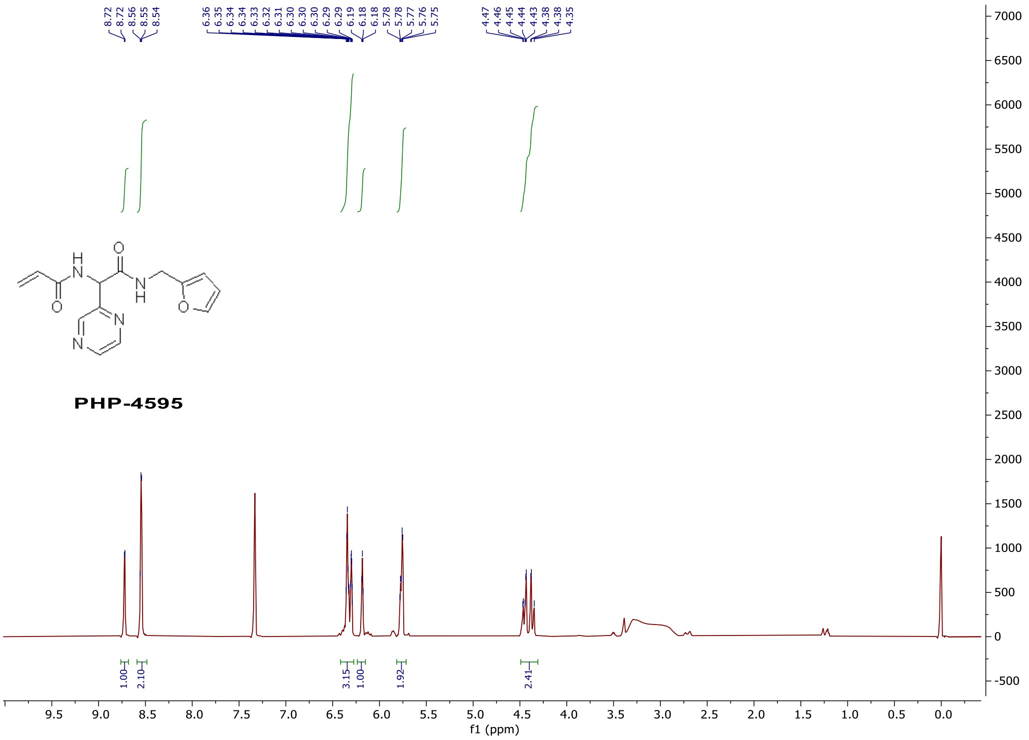

Supplementary Figure 360. ^{1}H NMR (500 MHz, $CDCl_3$+one drop of CD_3OD) of compound **PHP-4595.**

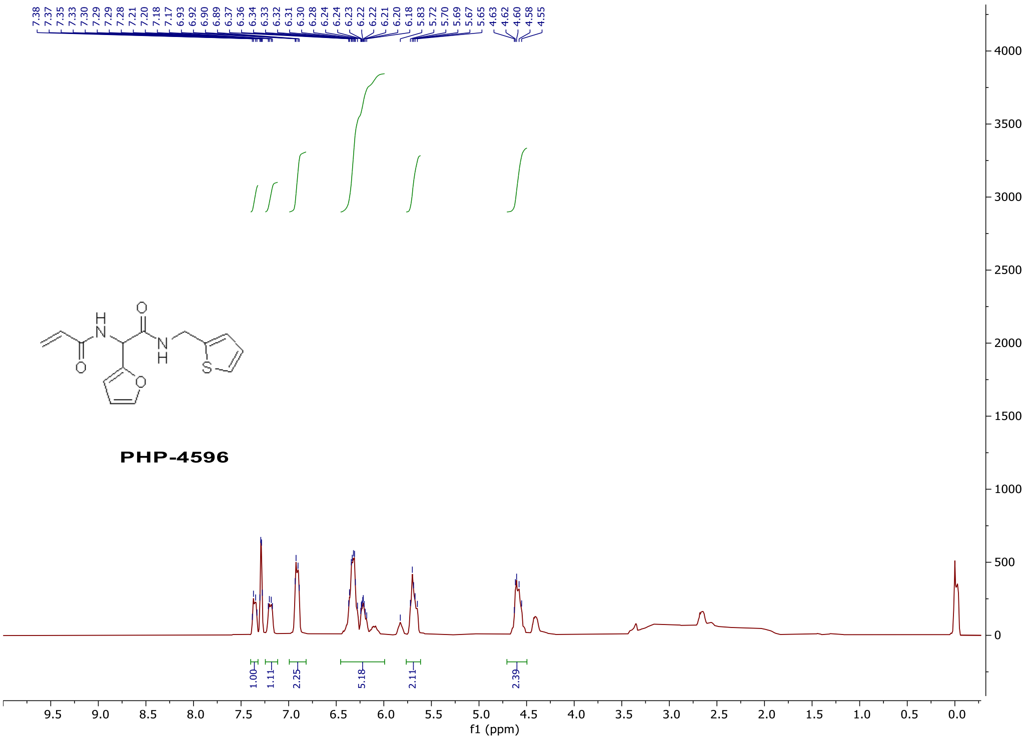

Supplementary Figure 361. ^{1}H NMR (500 MHz, $CDCl_3$+one drop of CD_3OD) of compound **PHP-4596.**

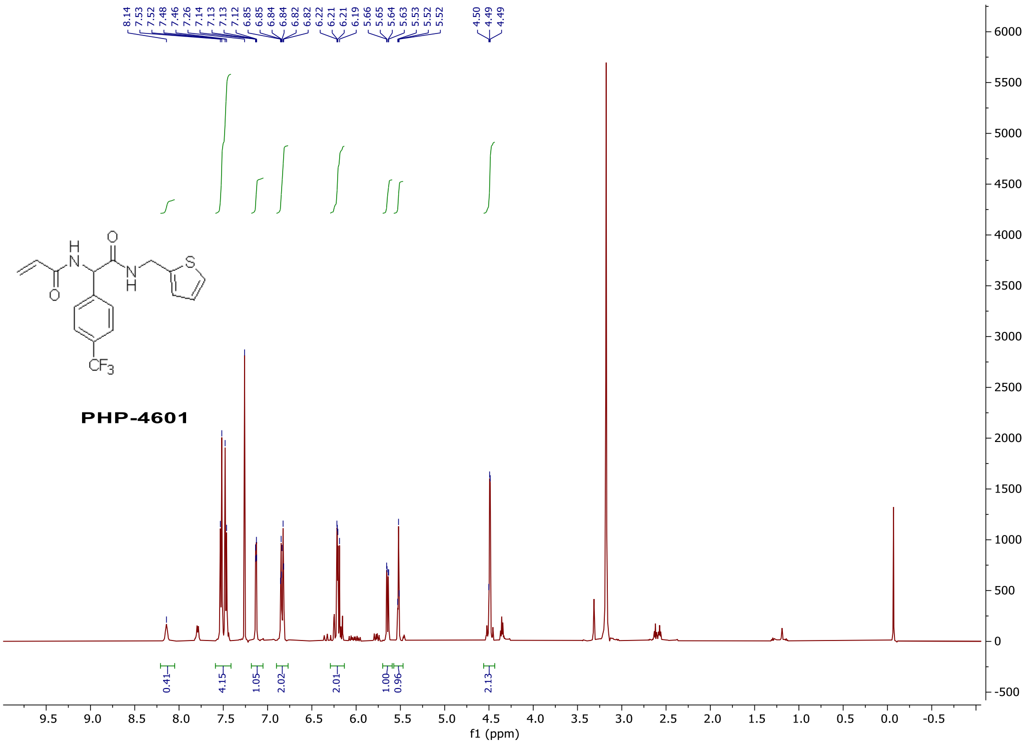

Supplementary Figure 362. ^{1}H NMR (500 MHz, $CDCl_3$+one drop of CD_3OD) of compound **PHP-4601.**

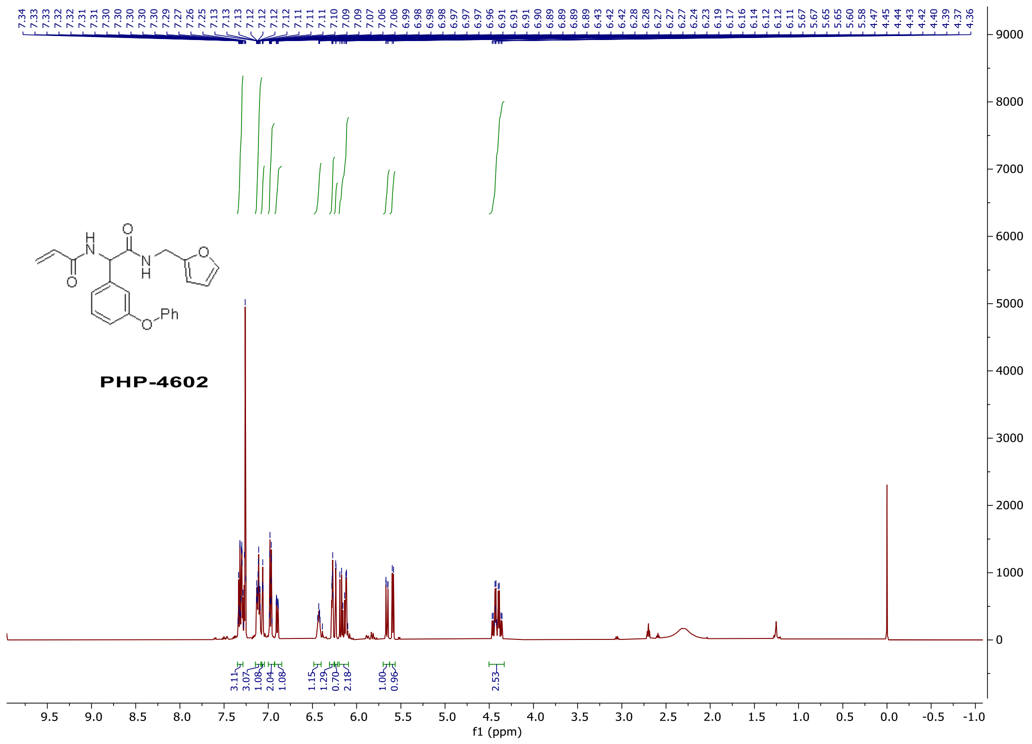

Supplementary Figure 363. ^{1}H NMR (500 MHz, $CDCl_3$) of compound **PHP-4602.**

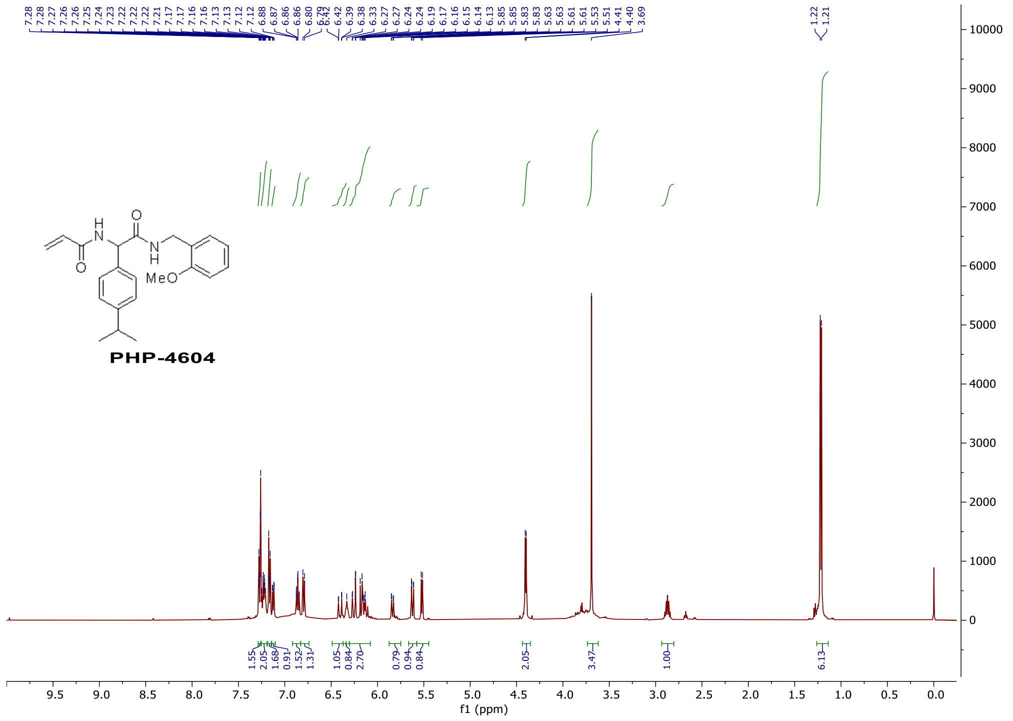

Supplementary Figure 364. ^{1}H NMR (500 MHz, $CDCl_3$) of compound **PHP-4604.**

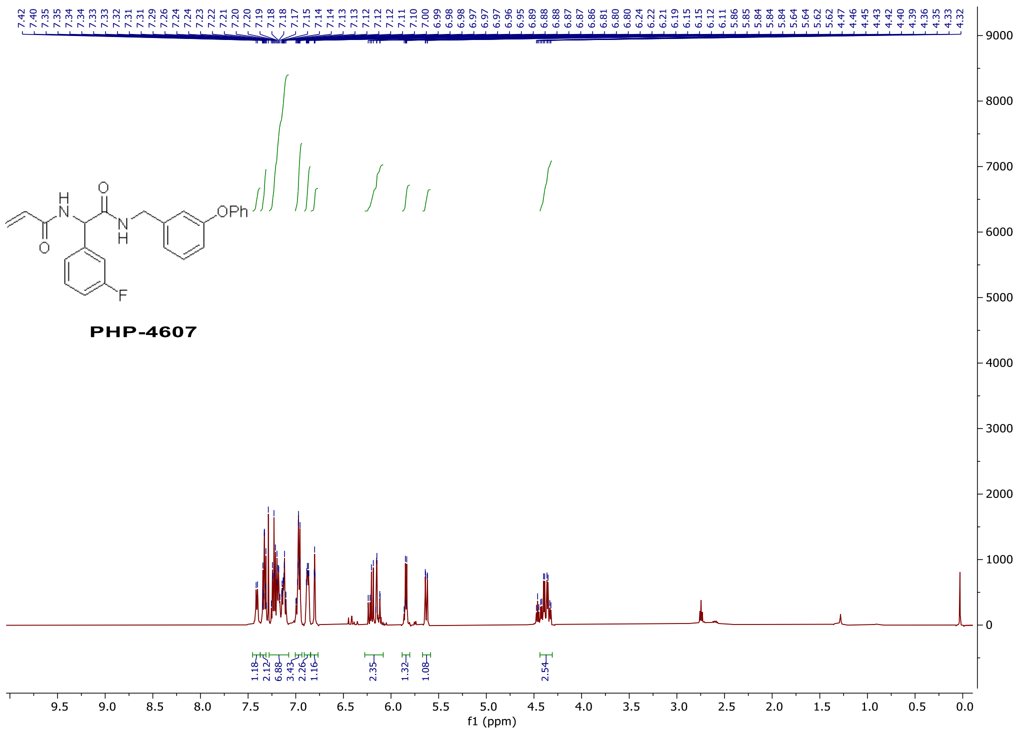

Supplementary Figure 365. ^1H NMR (500 MHz, $CDCl_3$) of compound **PHP-4607.**

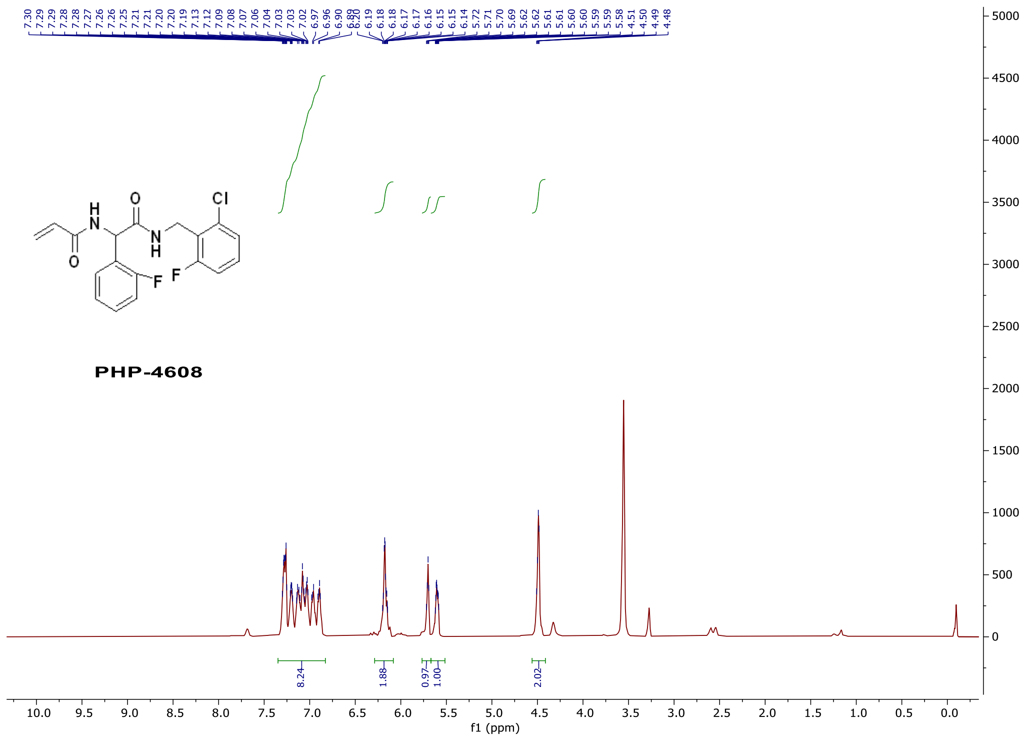

Supplementary Figure 366. ^1H NMR (500 MHz, $CDCl_3$) of compound **PHP-4608.**

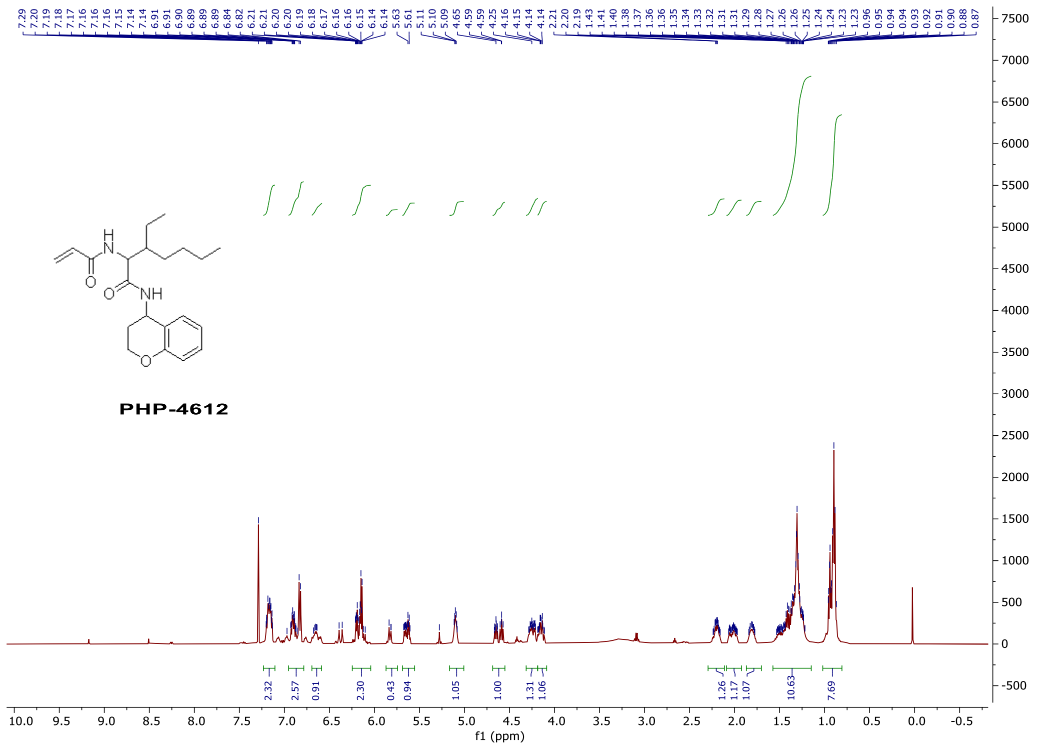

Supplementary Figure 367. ^{1}H NMR (500 MHz, $CDCl_3$) of compound **PHP-4612.**

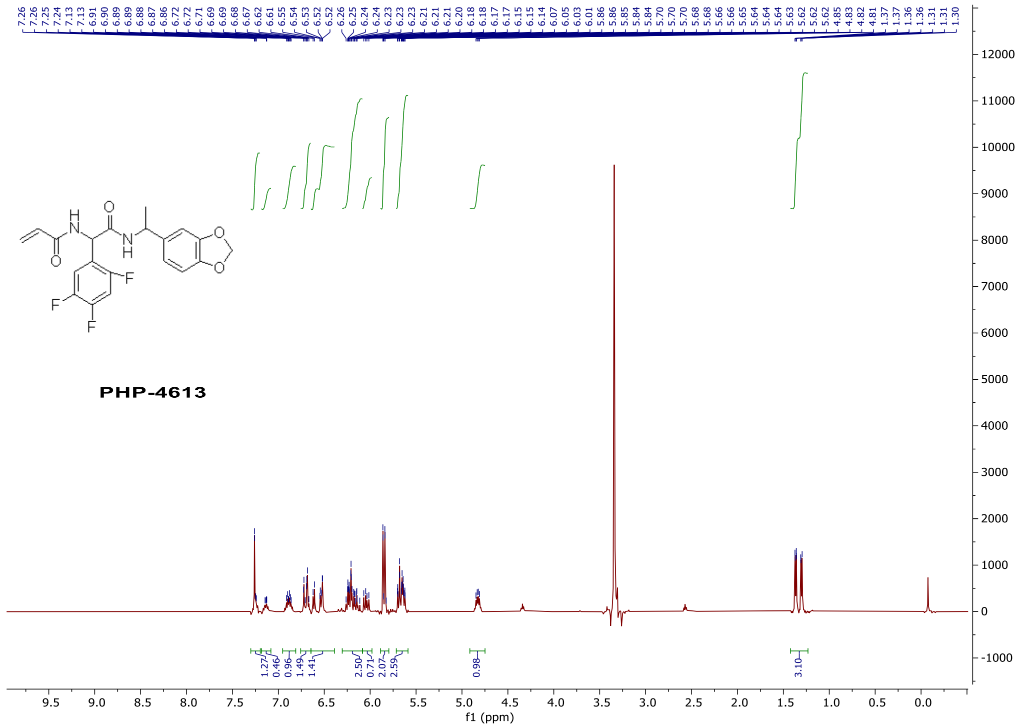

Supplementary Figure 368. ^{1}H NMR (500 MHz, $CDCl_3$) of compound **PHP-4613.**

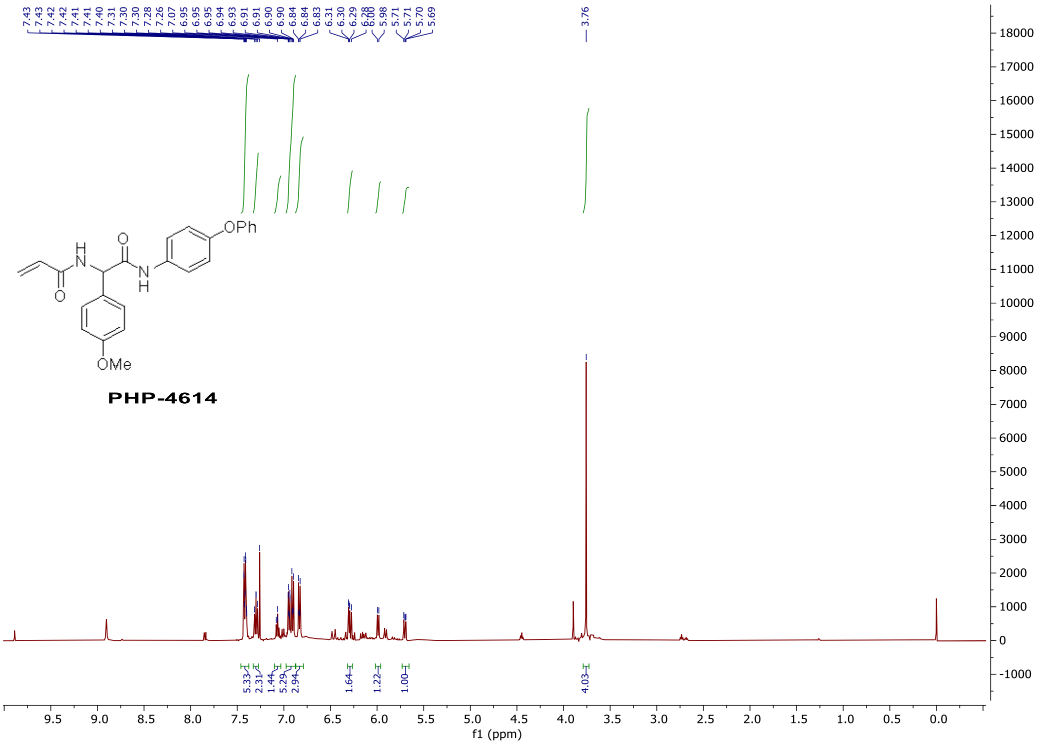

Supplementary Figure 369. ^{1}H NMR (500 MHz, $CDCl_3$) of compound **PHP-4614.**

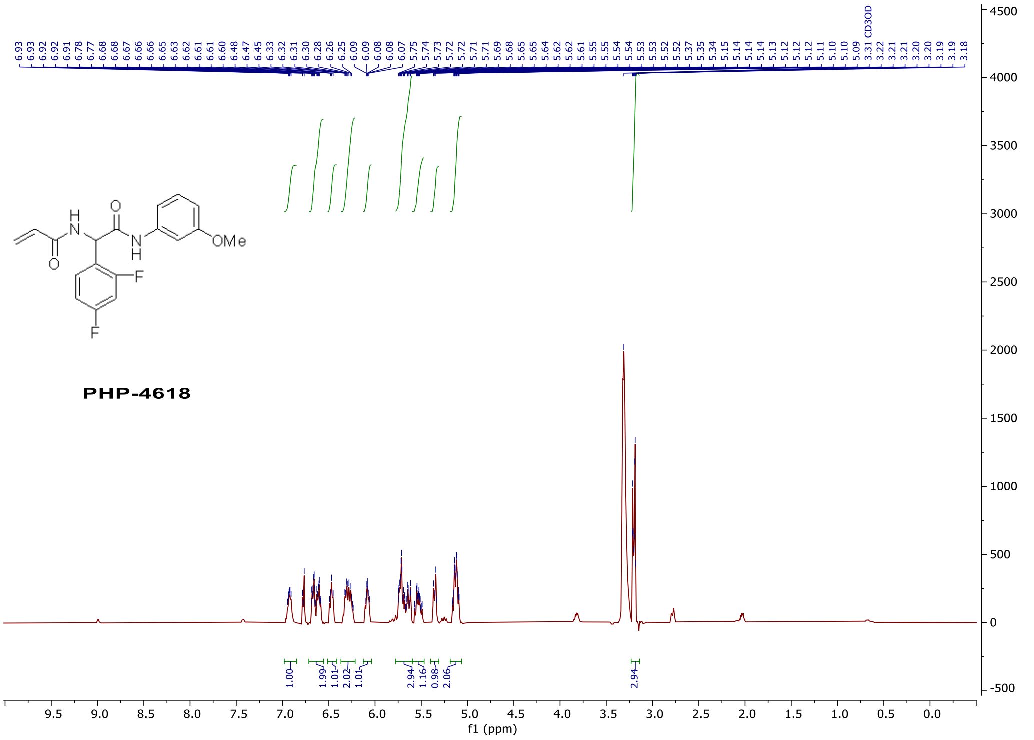

Supplementary Figure 370. ^{1}H NMR (500 MHz, $CDCl_3$+one drop of CD_3OD) of compound **PHP-4618.**

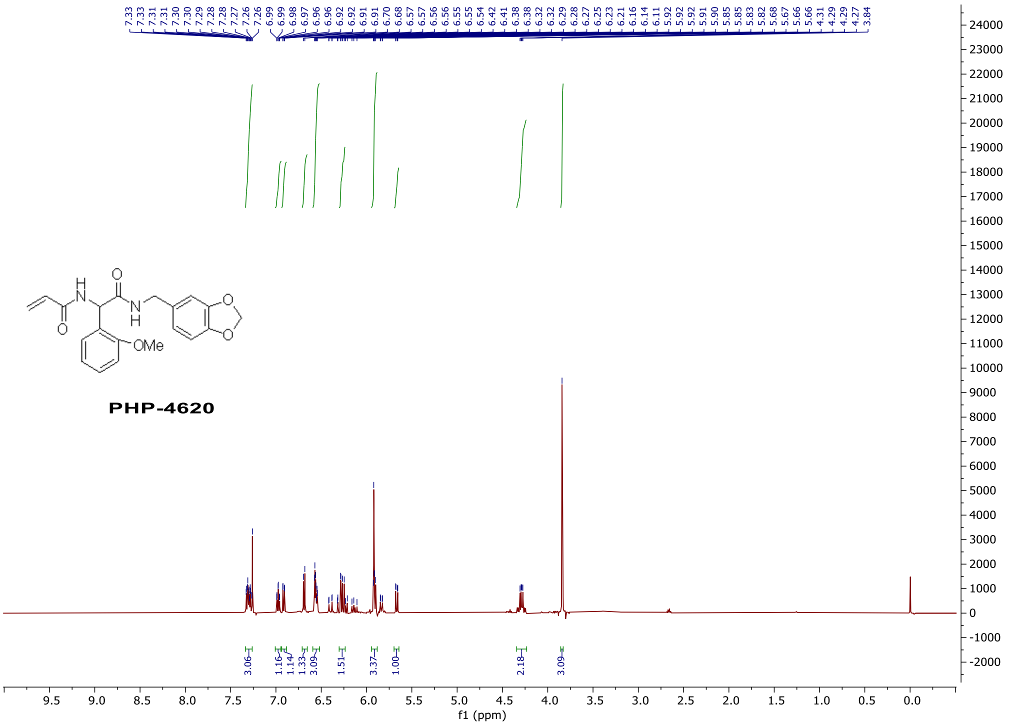

Supplementary Figure 371. ^{1}H NMR (500 MHz, $CDCl_3$) of compound **PHP-4620.**

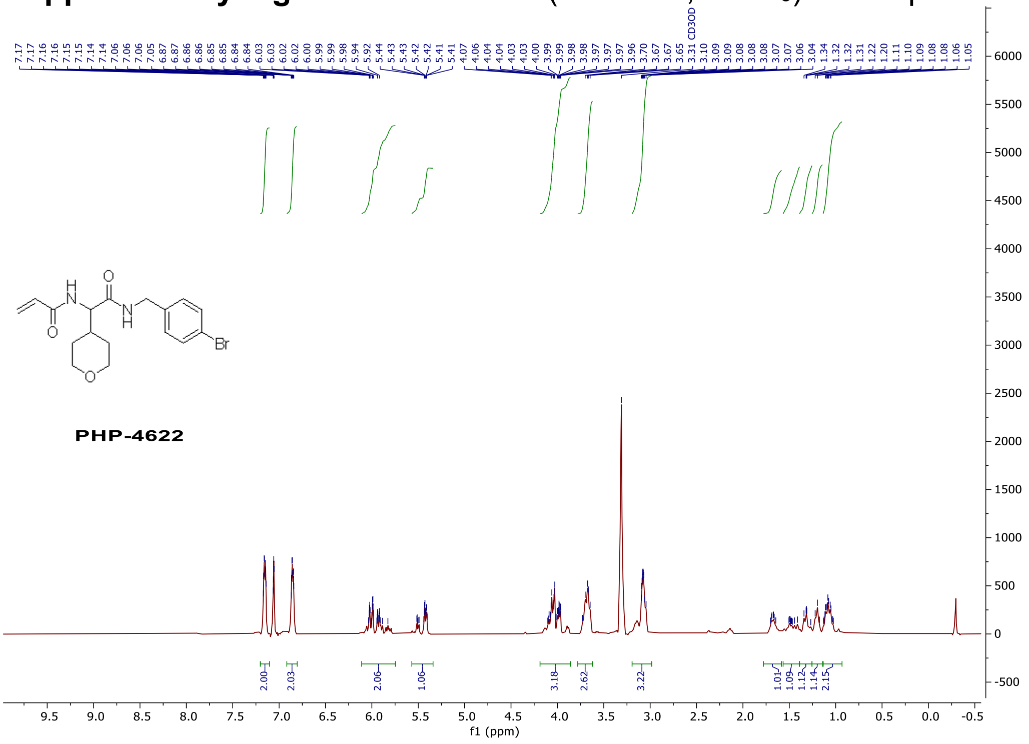

Supplementary Figure 372. ^{1}H NMR (500 MHz, $CDCl_3$+one drop of CD_3OD) of compound **PHP-4622.**

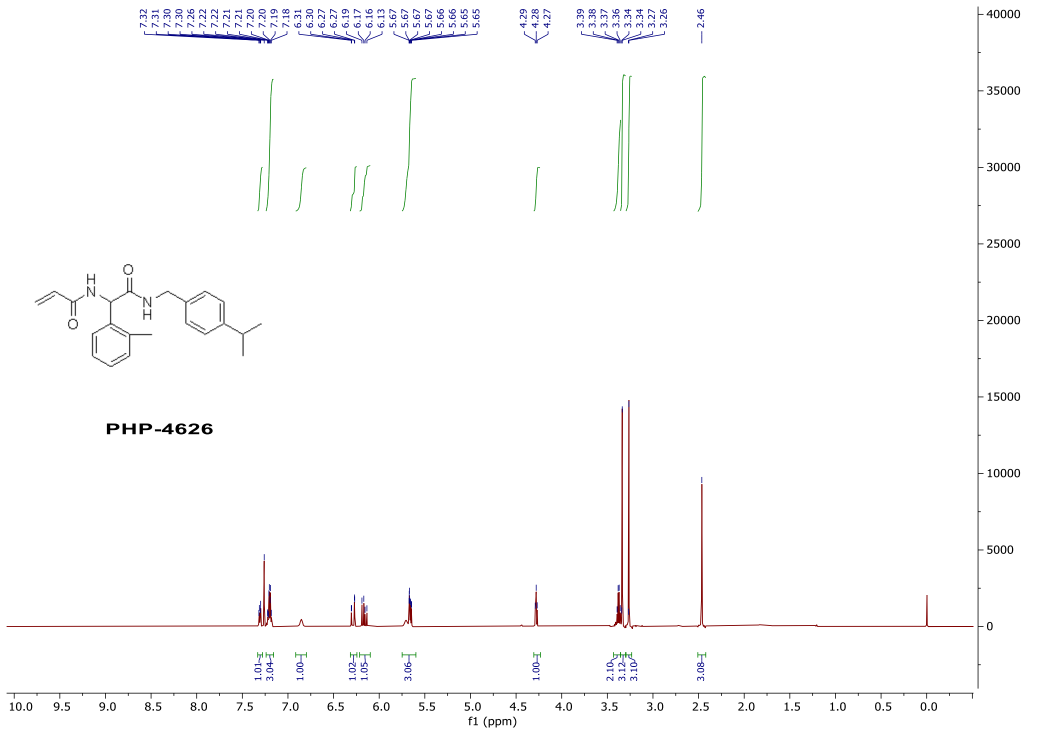

Supplementary Figure 373. ^{1}H NMR (500 MHz, CDCl3) of compound **PHP-4626.**

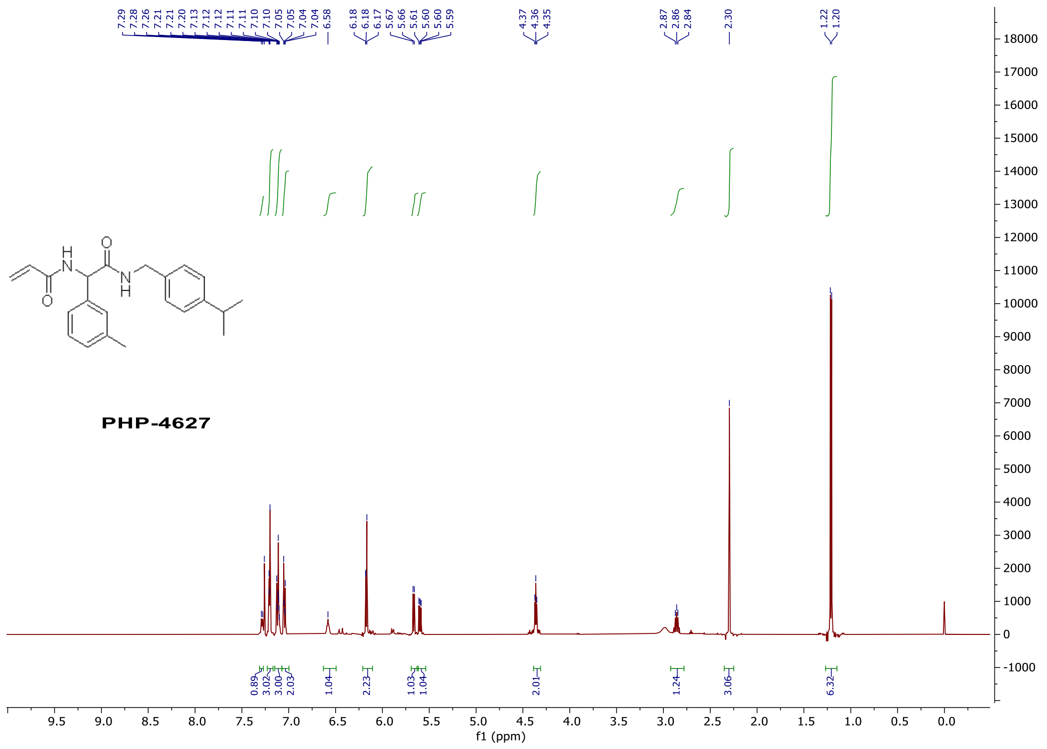

Supplementary Figure 374. ^{1}H NMR (500 MHz, CDCl3) of compound **PHP-4627.**

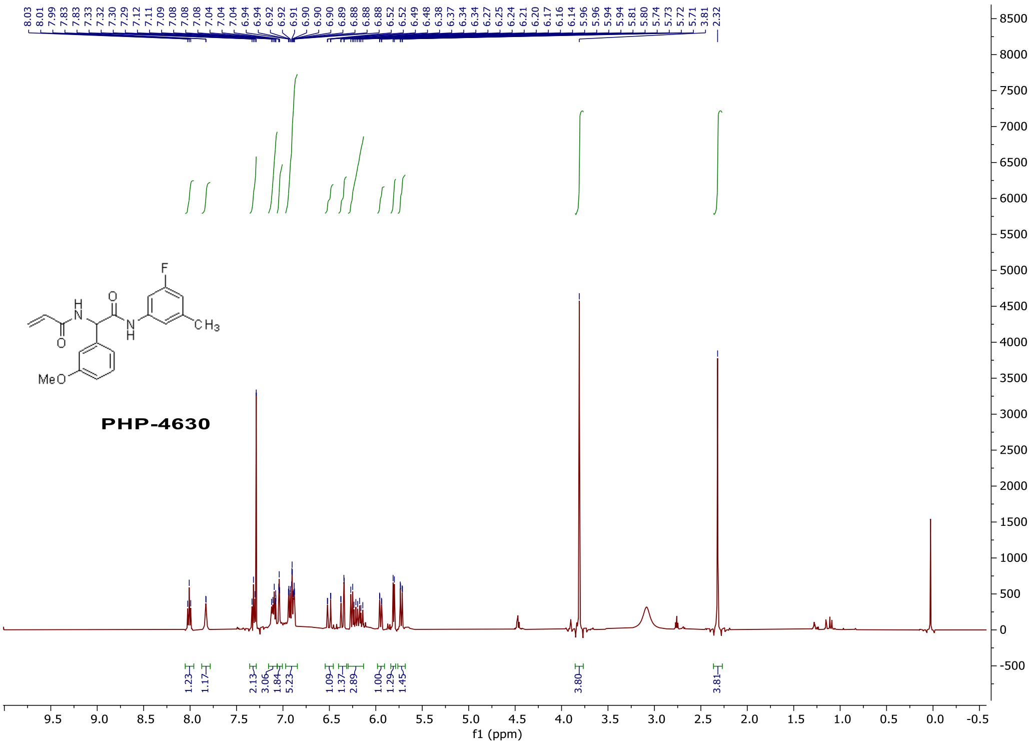

Supplementary Figure 375. ^{1}H NMR (500 MHz, $CDCl_3$) of compound **PHP-4630.**

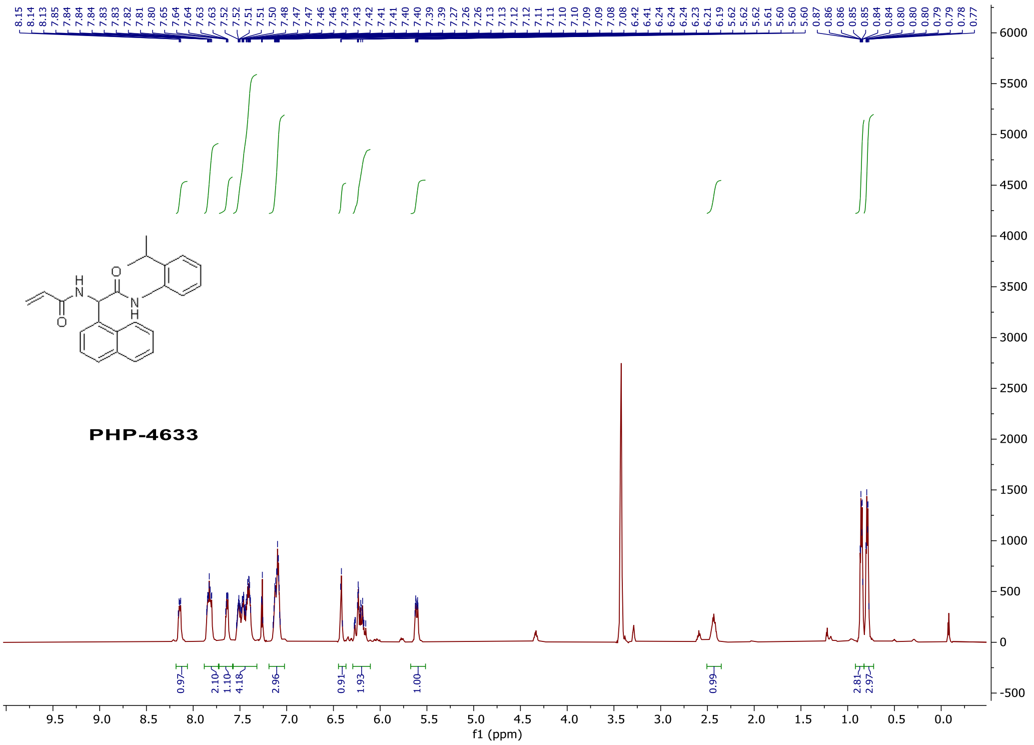

Supplementary Figure 376. ^{1}H NMR (500 MHz, $CDCl_3$+one drop of CD_3OD) of compound **PHP-4633.**

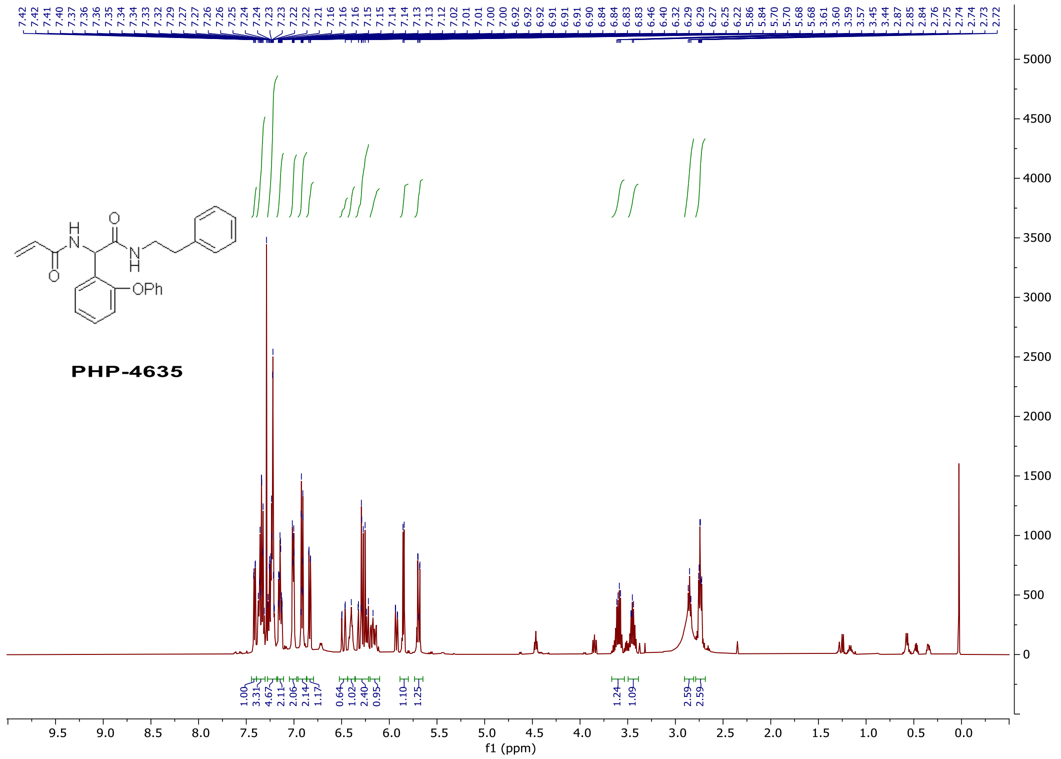

Supplementary Figure 377. ^{1}H NMR (500 MHz, $CDCl_3$) of compound **PHP-4635.**

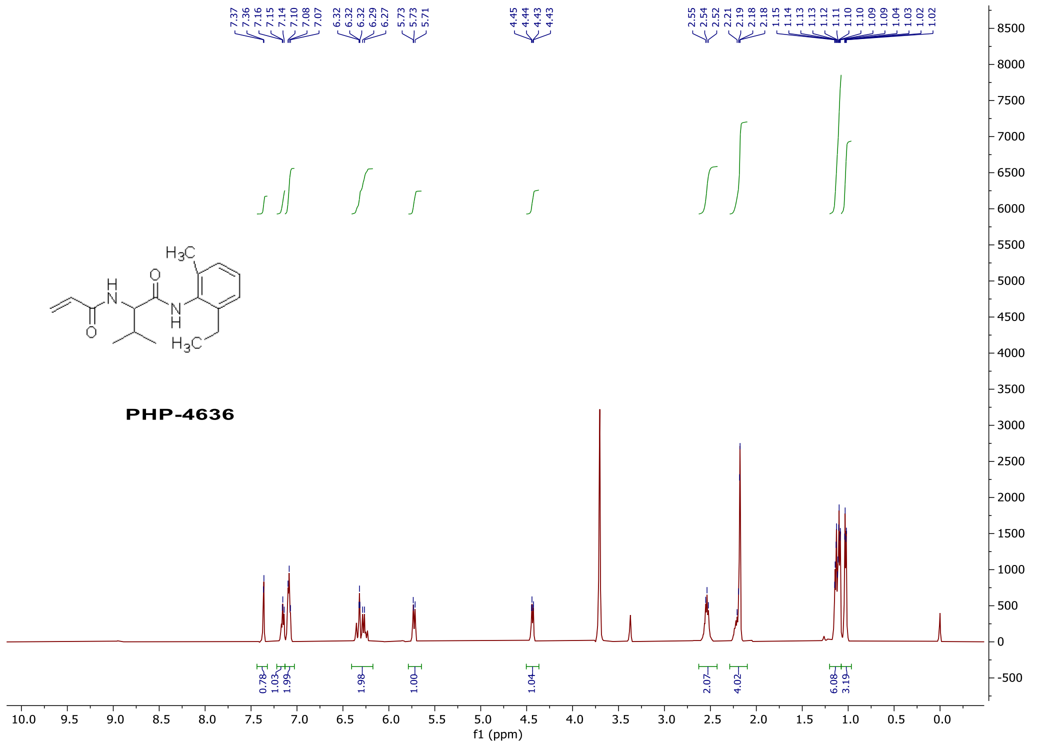

Supplementary Figure 378. ^{1}H NMR (500 MHz, $CDCl_3$+one drop of CD_3OD) of compound **PHP-4636.**

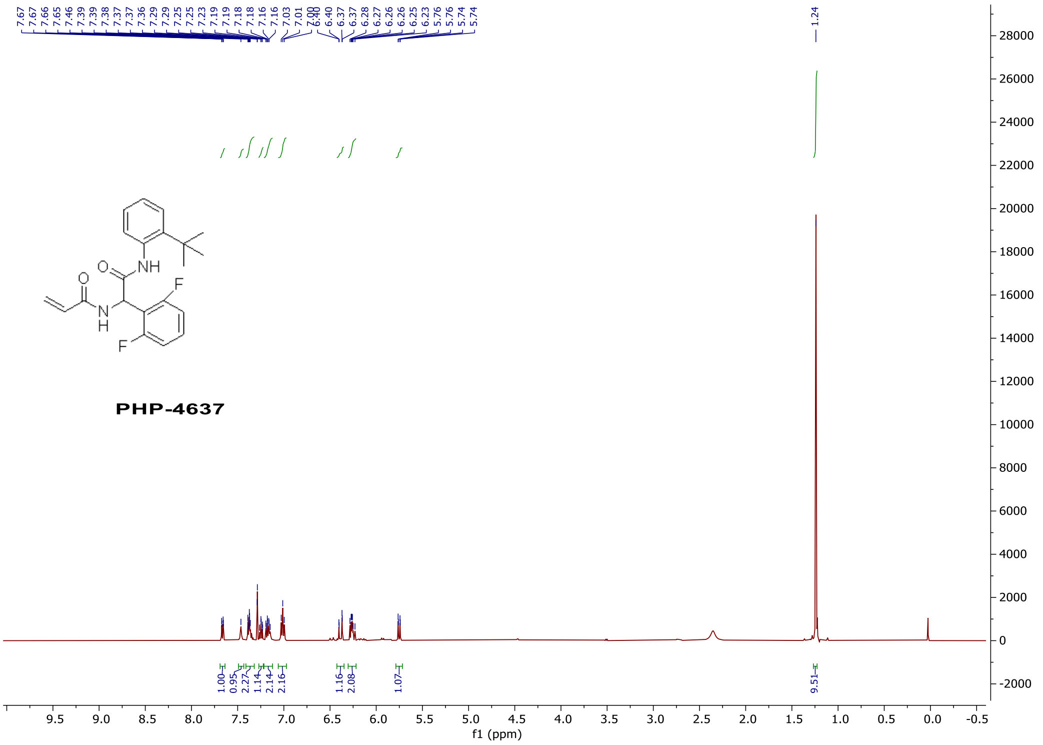

Supplementary Figure 379. ^{1}H NMR (500 MHz, $CDCl_3$) of compound **PHP-4637.**

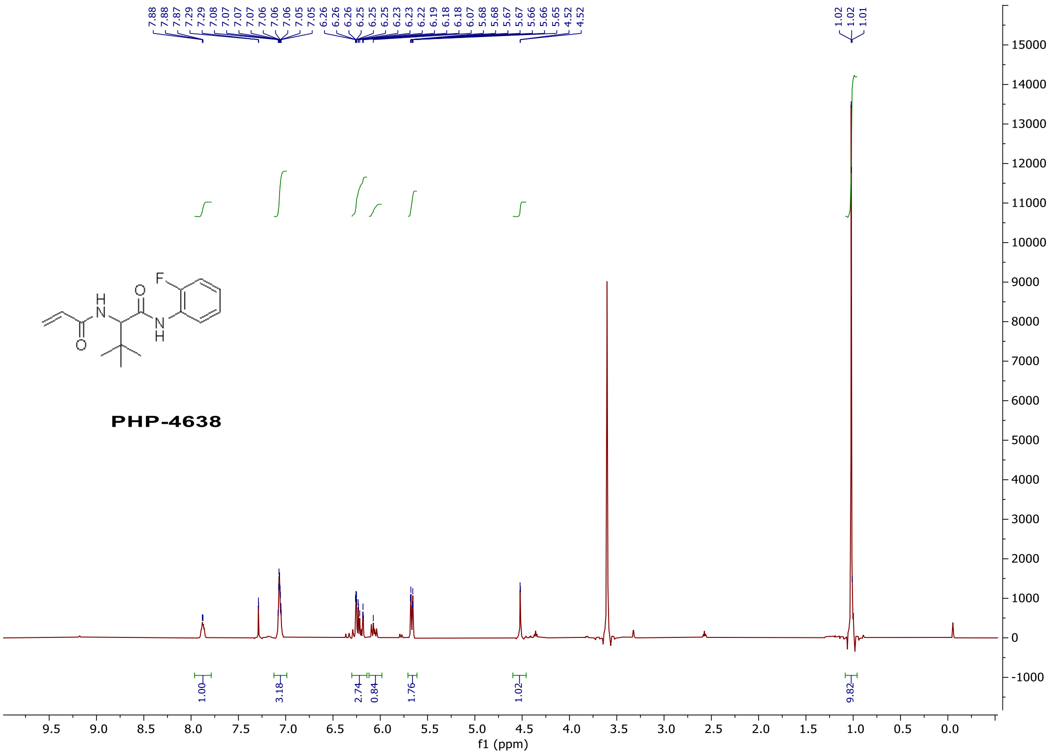

Supplementary Figure 380. ^{1}H NMR (500 MHz, $CDCl_3$+one drop of CD_3OD) of compound **PHP-4638.**

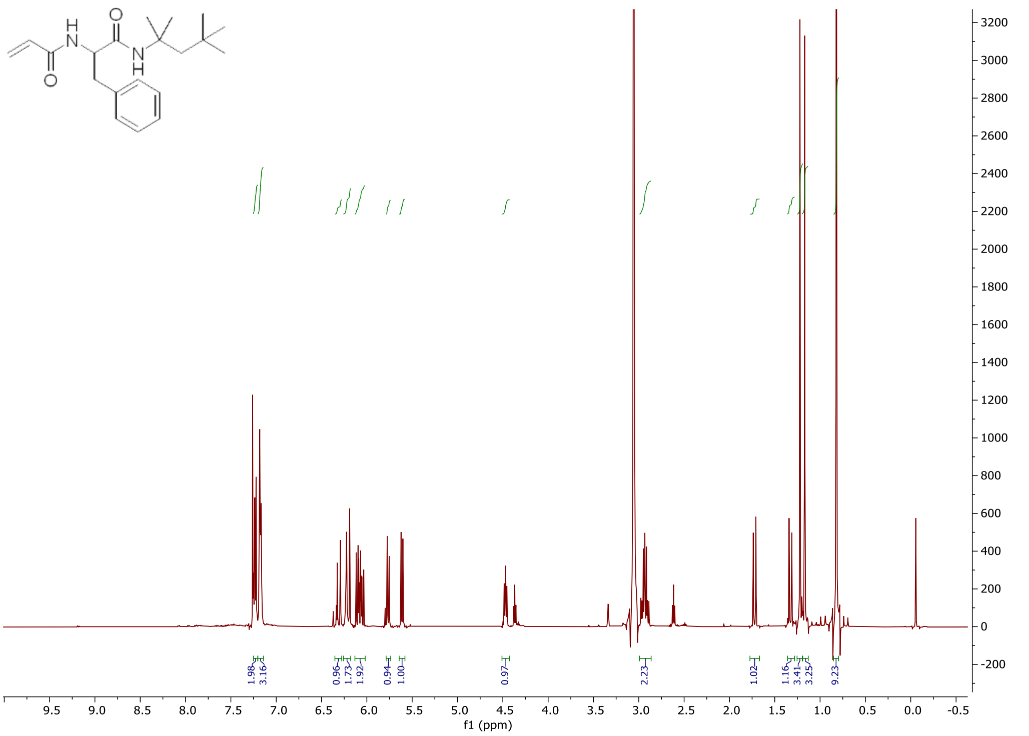

Supplementary Figure 381. ^{1}H NMR (500 MHz, $CDCl_3$+one drop of CD_3OD) of compound **PHP-4641.**

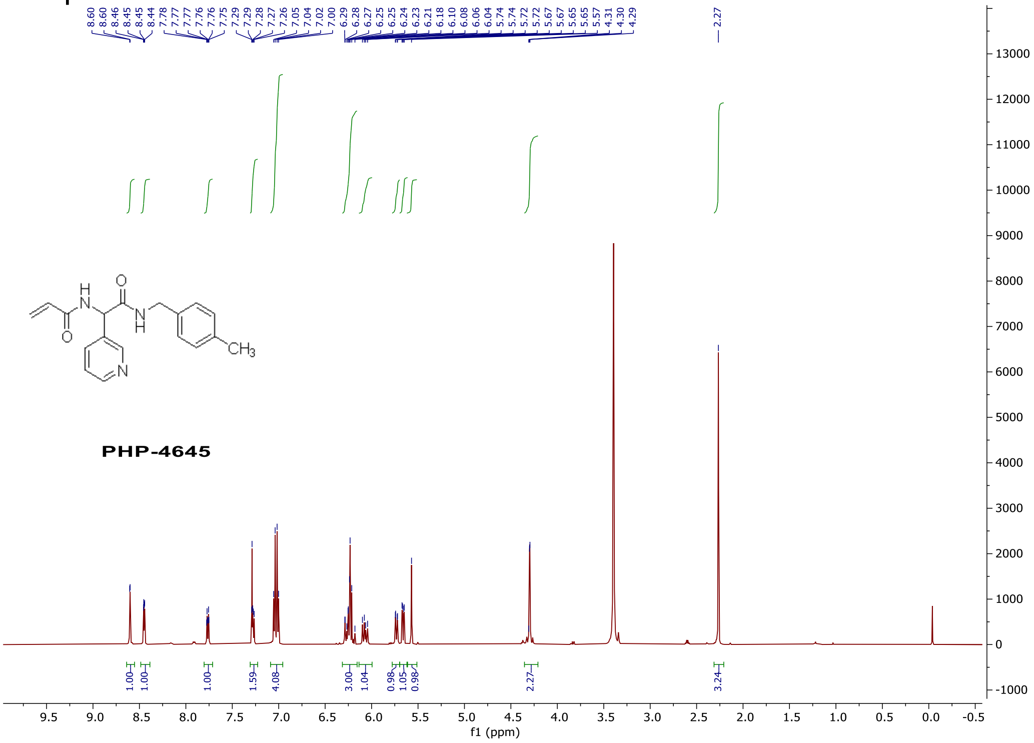

Supplementary Figure 382. ^{1}H NMR (500 MHz, $CDCl_3$+one drop of CD_3OD) of compound **PHP-4645.**

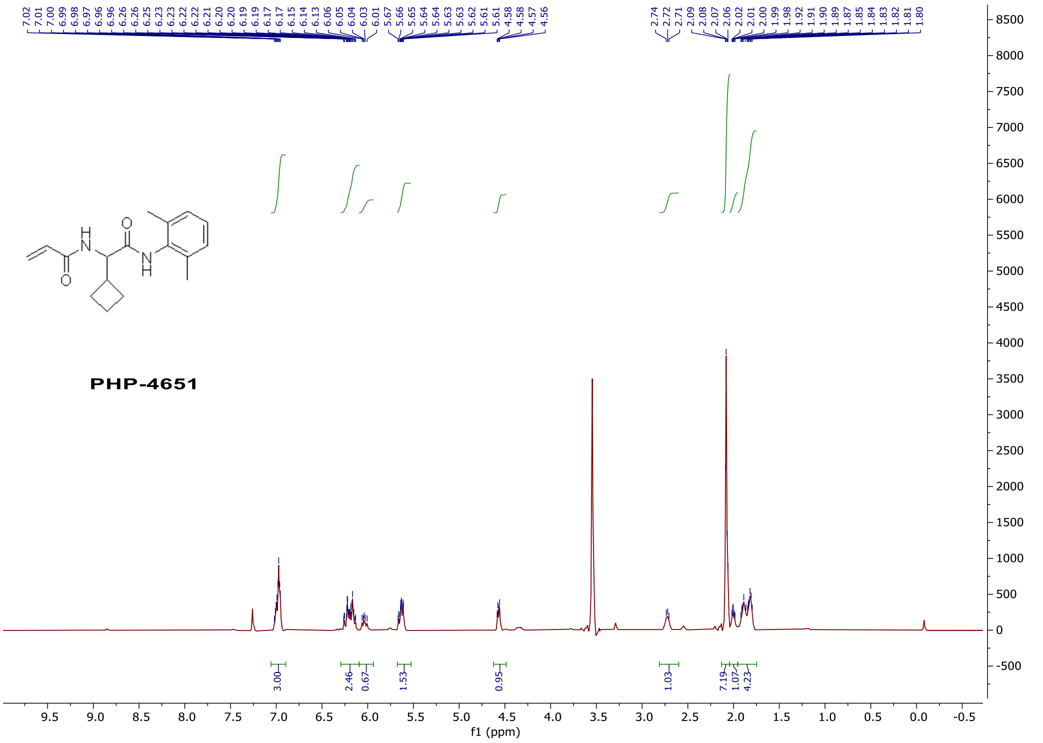

Supplementary Figure 383. ^{1}H NMR (500 MHz, $CDCl_3$+one drop of CD_3OD) of compound **PHP-4651.**

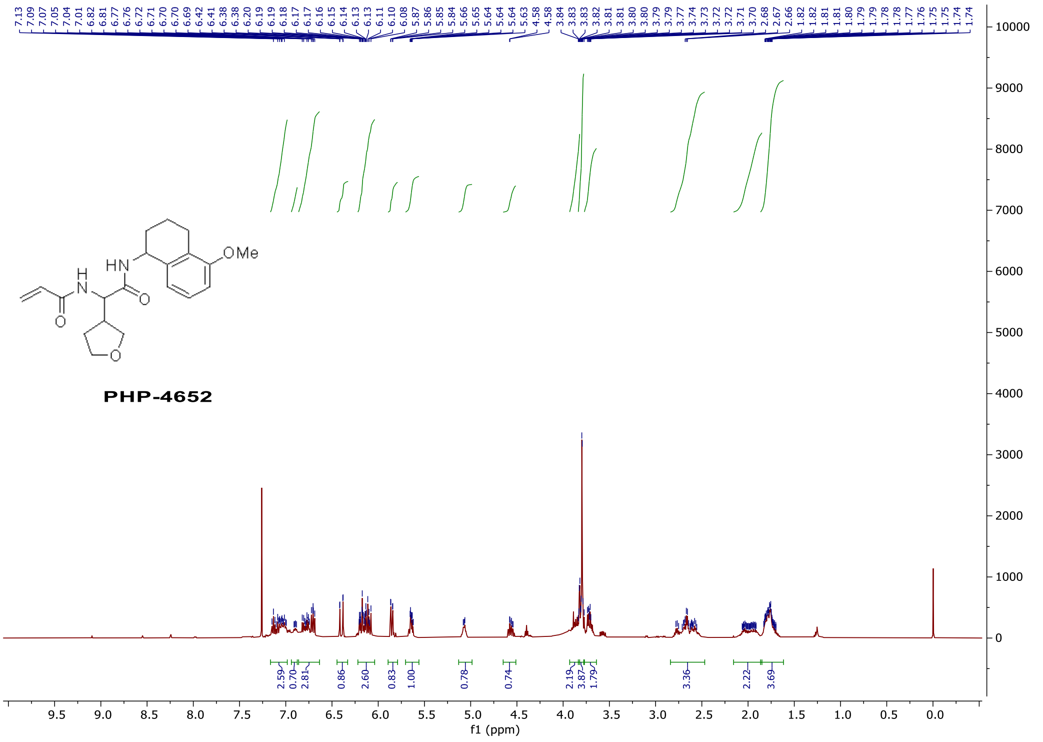

Supplementary Figure 384. ^{1}H NMR (500 MHz, $CDCl_3$) of compound **PHP-4652.**

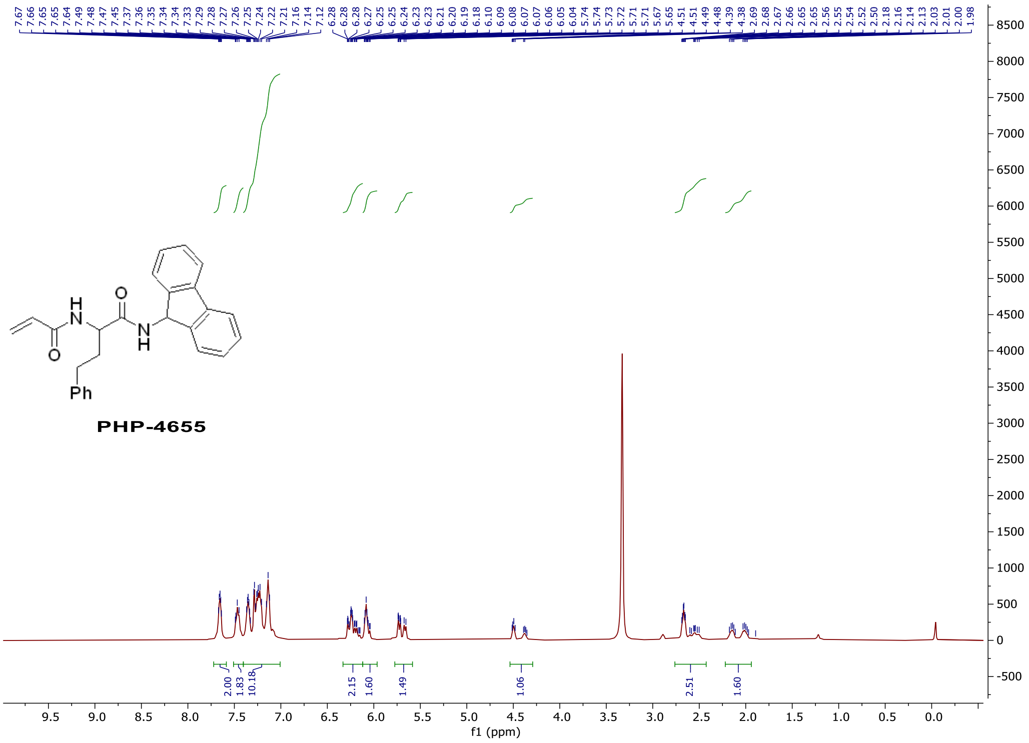

Supplementary Figure 385. ^{1}H NMR (500 MHz, $CDCl_3$+one drop of CD_3OD) of compound **PHP-4655.**

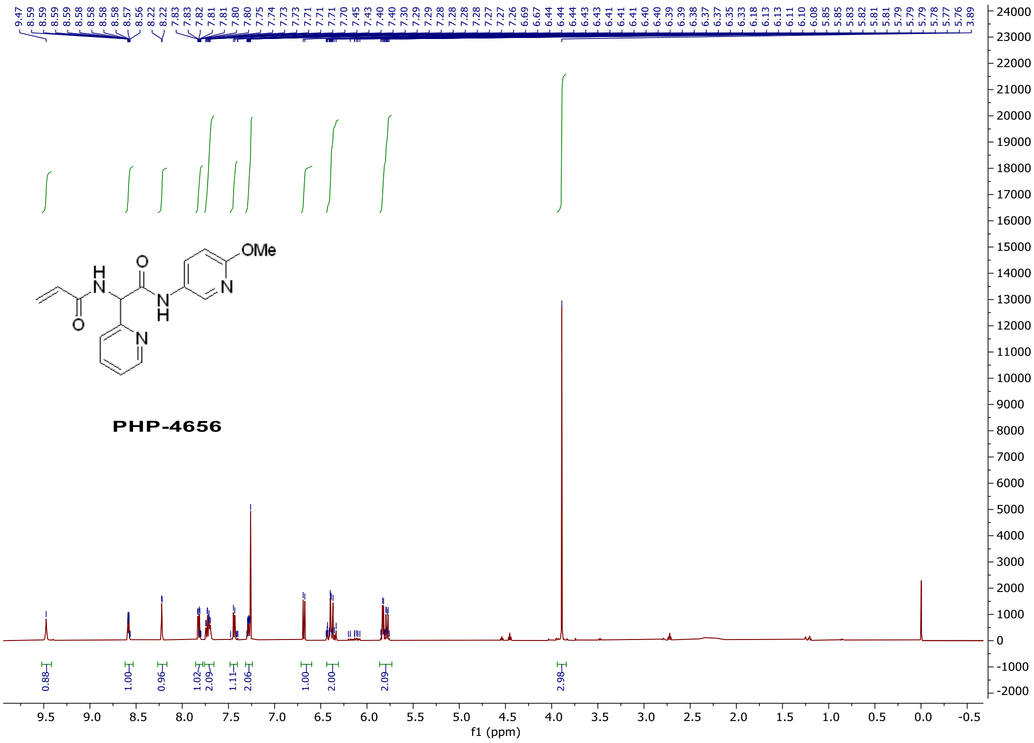

Supplementary Figure 386. ^{1}H NMR (500 MHz, $CDCl_3$) of compound **PHP-4656.**

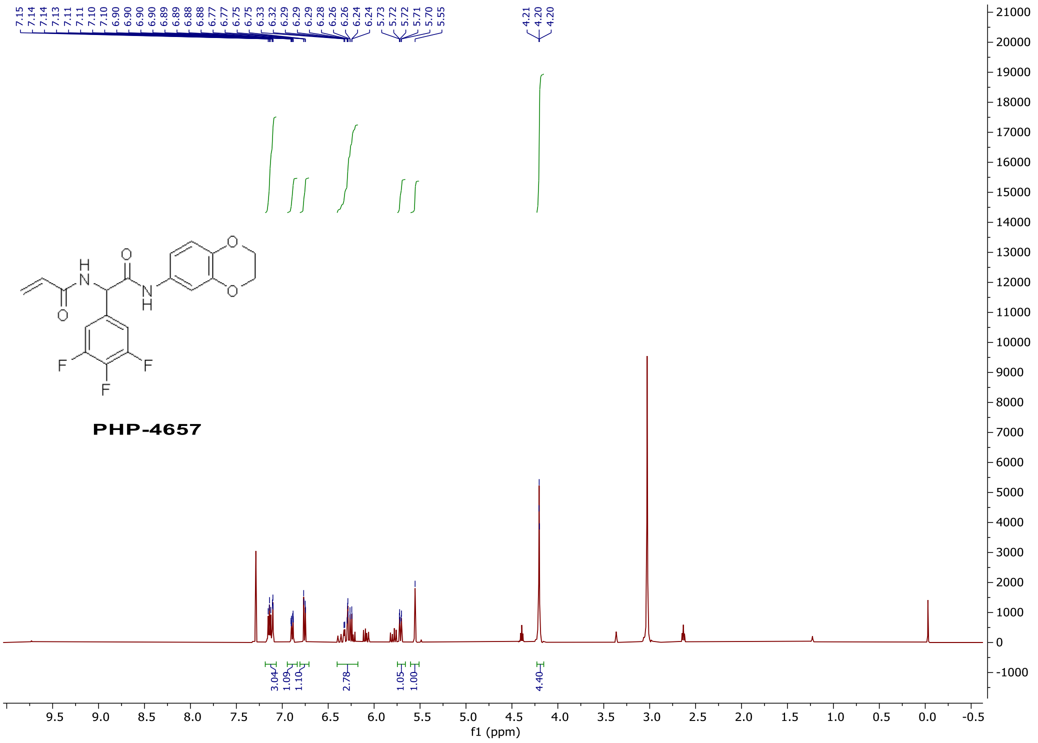

Supplementary Figure 387. ^{1}H NMR (500 MHz, $CDCl_3$+one drop of CD_3OD) of compound **PHP-4657.**

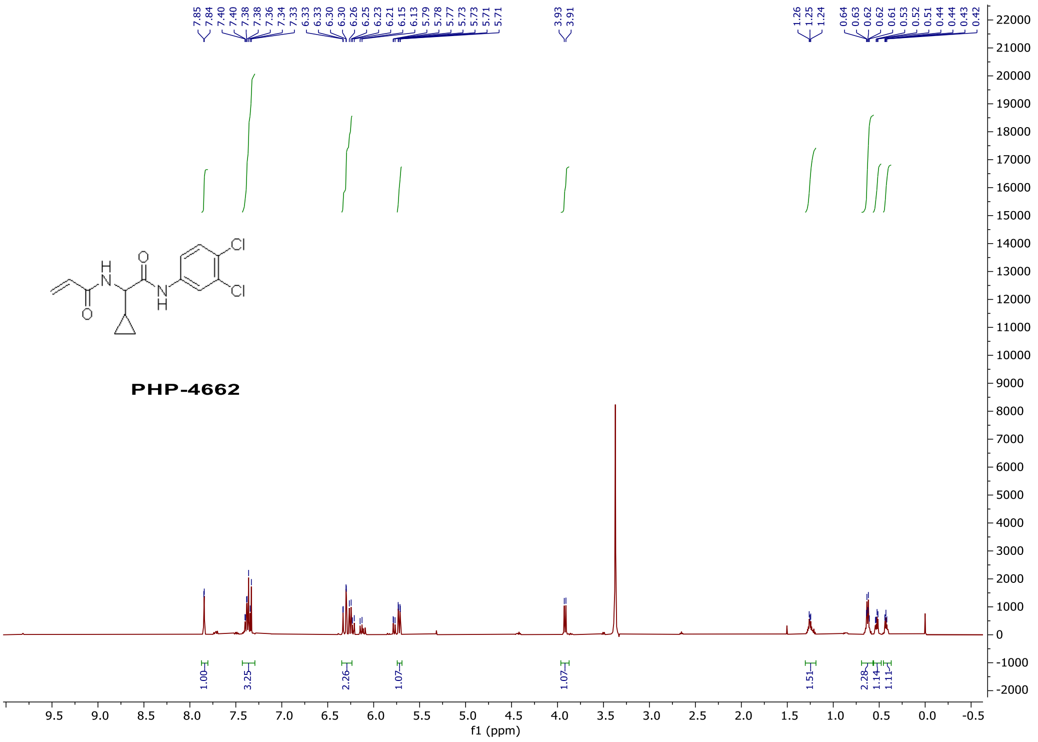

Supplementary Figure 388. ^{1}H NMR (500 MHz, $CDCl_3$+one drop of CD_3OD) of compound **PHP-4662.**

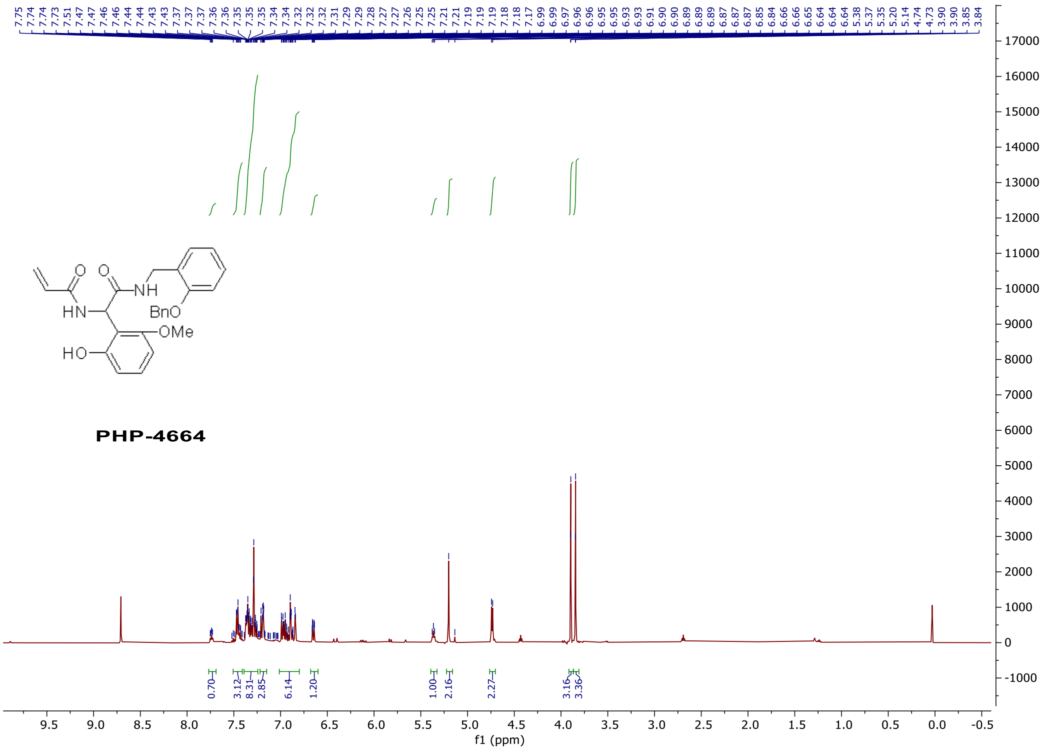

Supplementary Figure 389. ^{1}H NMR (500 MHz, $CDCl_3$) of compound **PHP-4664.**

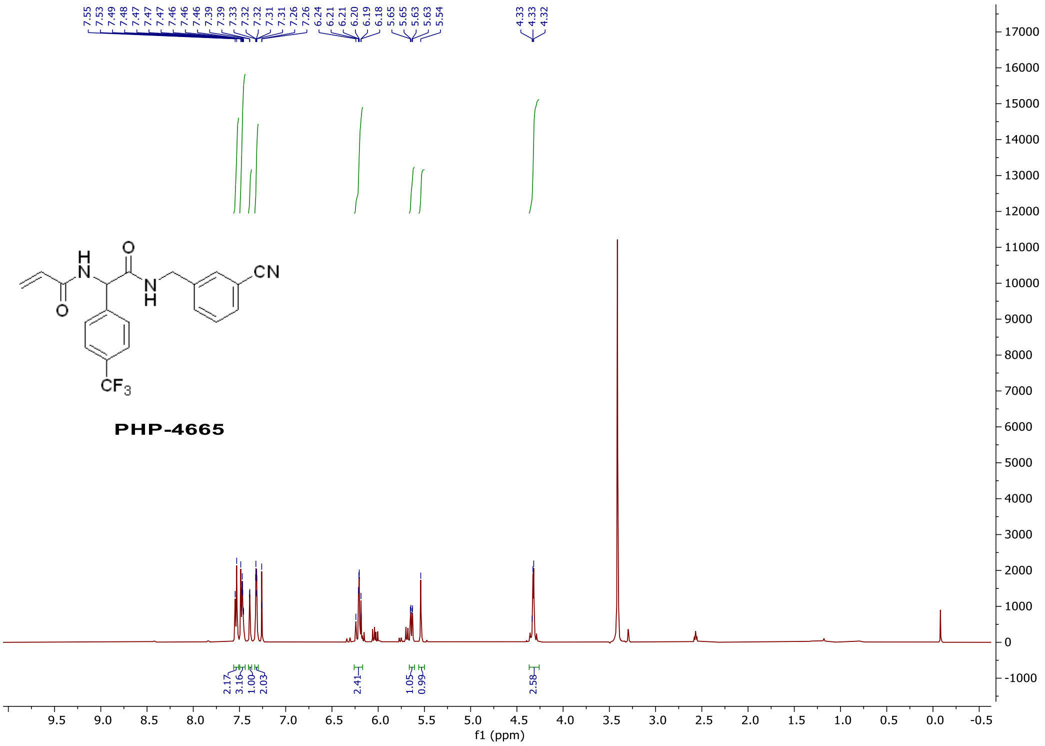

Supplementary Figure 390. ^{1}H NMR (500 MHz, $CDCl_3$+one drop of CD_3OD) of compound **PHP-4665.**

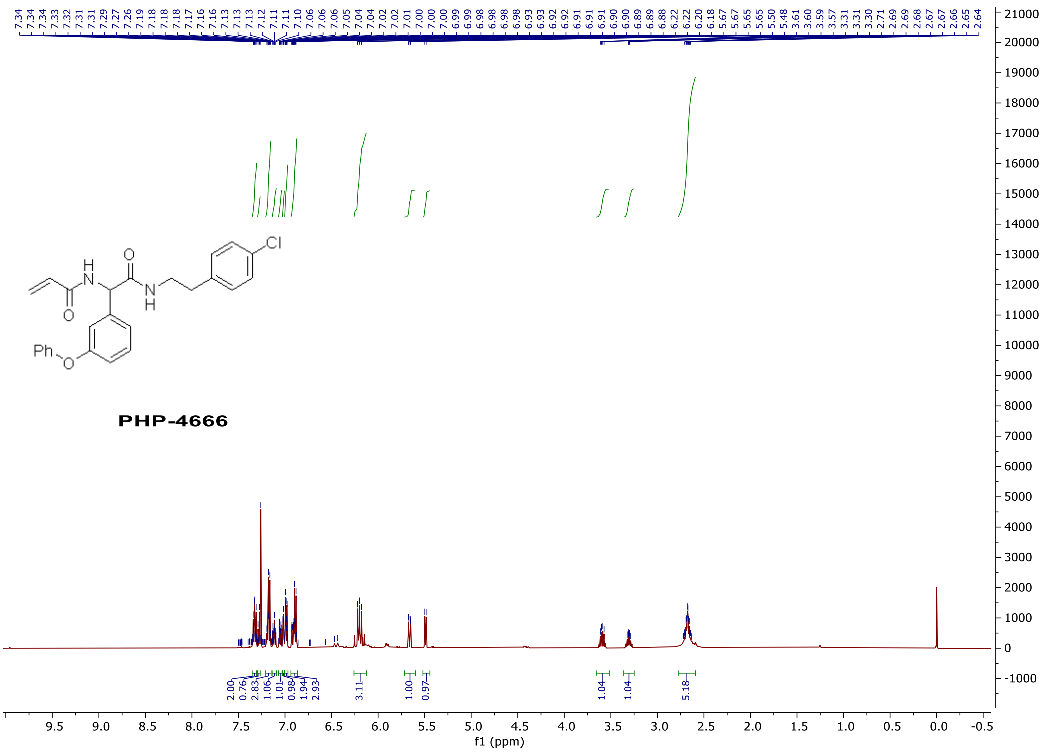

Supplementary Figure 391. ^{1}H NMR (500 MHz, $CDCl_3$) of compound **PHP-4666.**

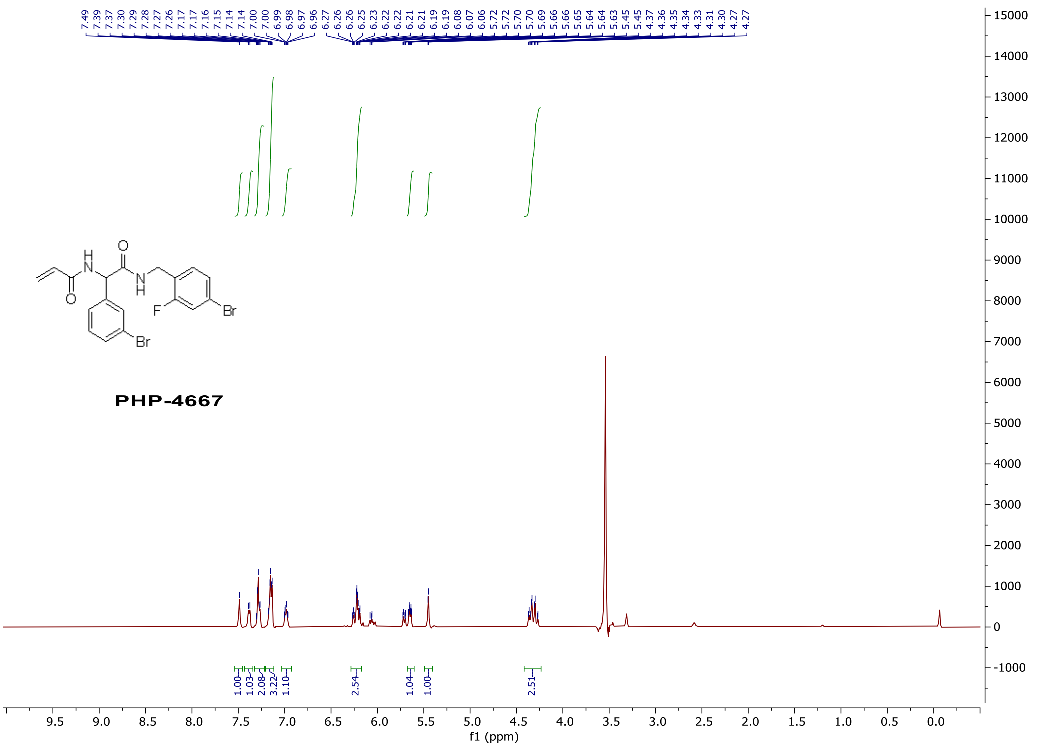

Supplementary Figure 392. ^{1}H NMR (500 MHz, $CDCl_3$+one drop of CD_3OD) of compound **PHP-4667.**

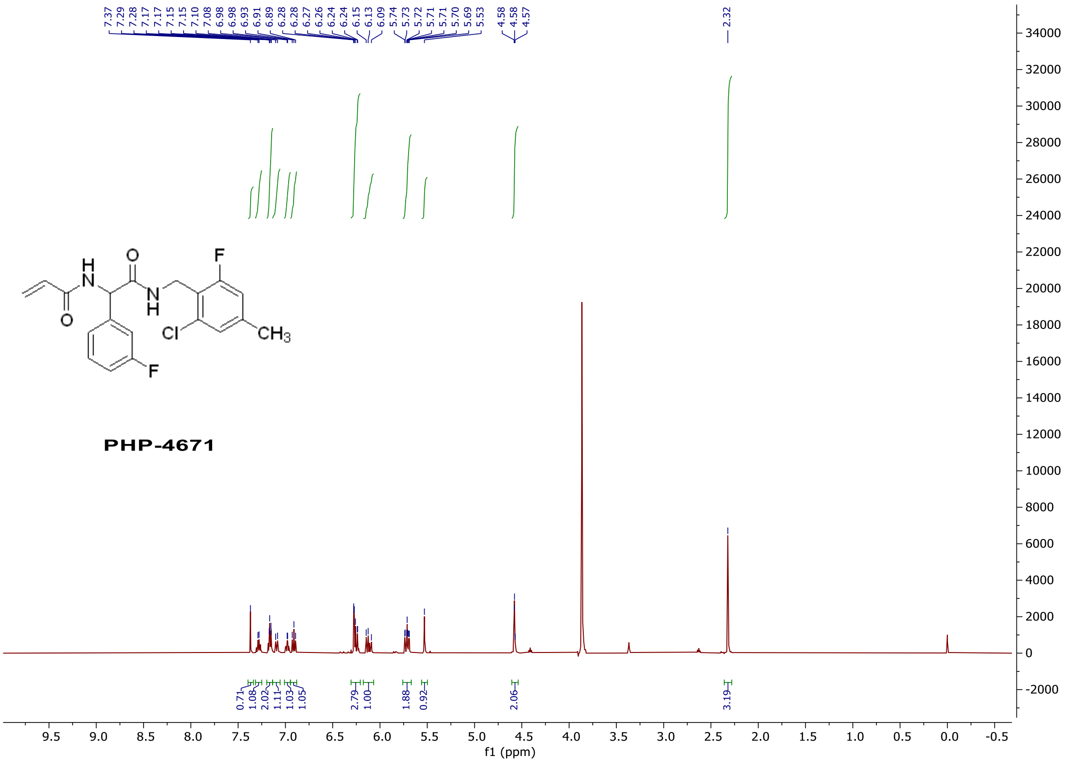

Supplementary Figure 393. ^{1}H NMR (500 MHz, $CDCl_3$) of compound **PHP-4671.**

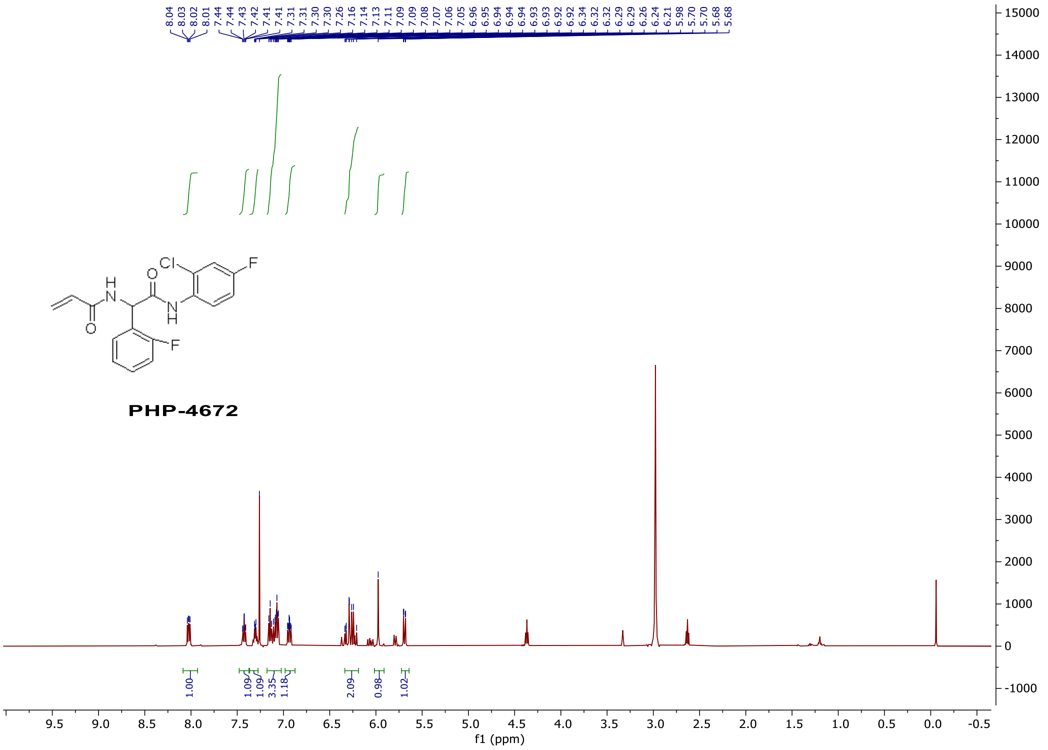

Supplementary Figure 394. ^{1}H NMR (500 MHz, $CDCl_3$+one drop of CD_3OD) of compound **PHP-4672.**

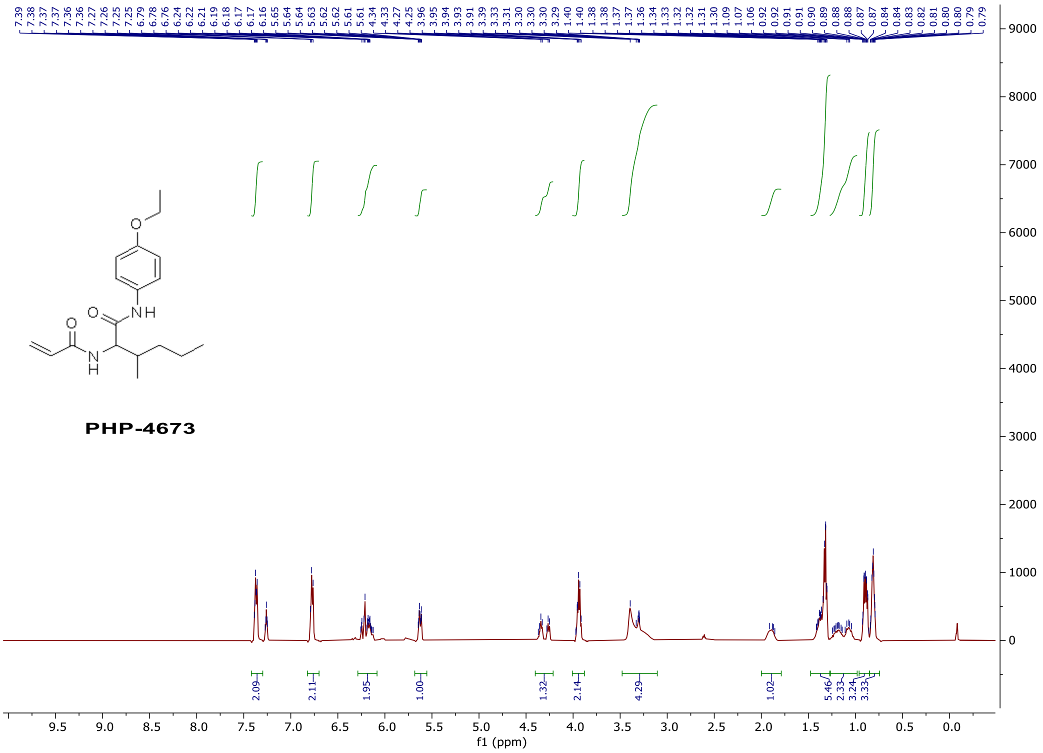

Supplementary Figure 395. ^{1}H NMR (500 MHz, $CDCl_3$+one drop of CD_3OD) of compound **PHP-4673.**

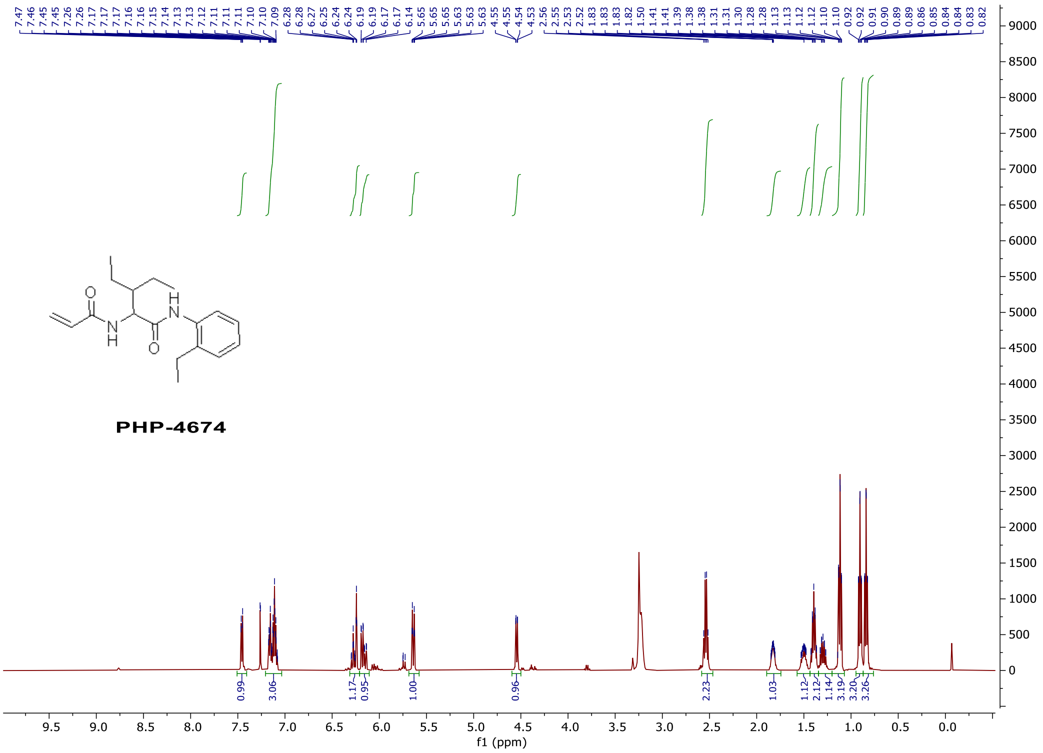

Supplementary Figure 396. ^{1}H NMR (500 MHz, $CDCl_3$+one drop of CD_3OD) of compound **PHP-4674.**

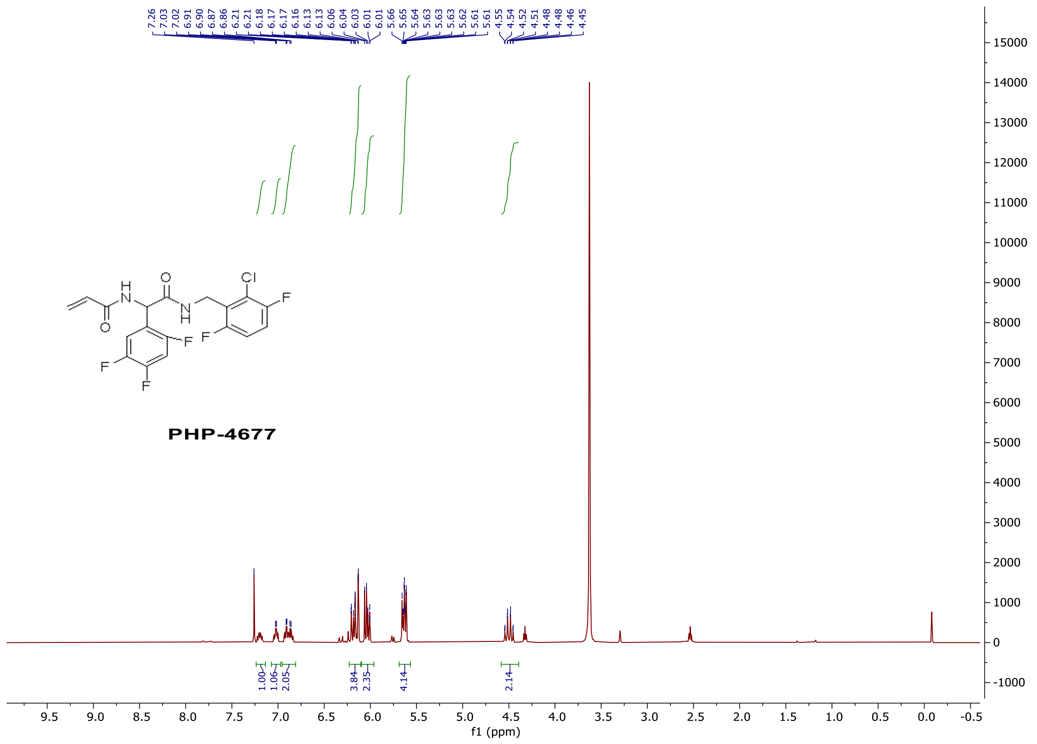

Supplementary Figure 397. ^{1}H NMR (500 MHz, $CDCl_3$+one drop of CD_3OD) of compound **PHP-4677.**

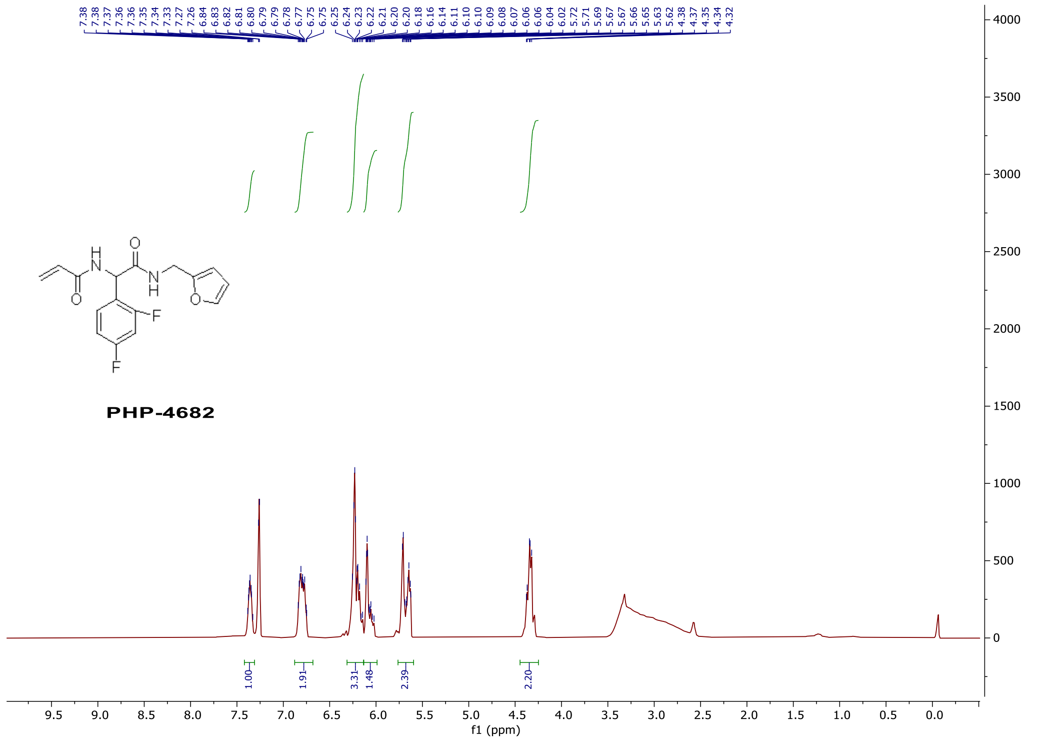

Supplementary Figure 398. ^{1}H NMR (500 MHz, $CDCl_3$+one drop of CD_3OD) of compound **PHP-4682.**

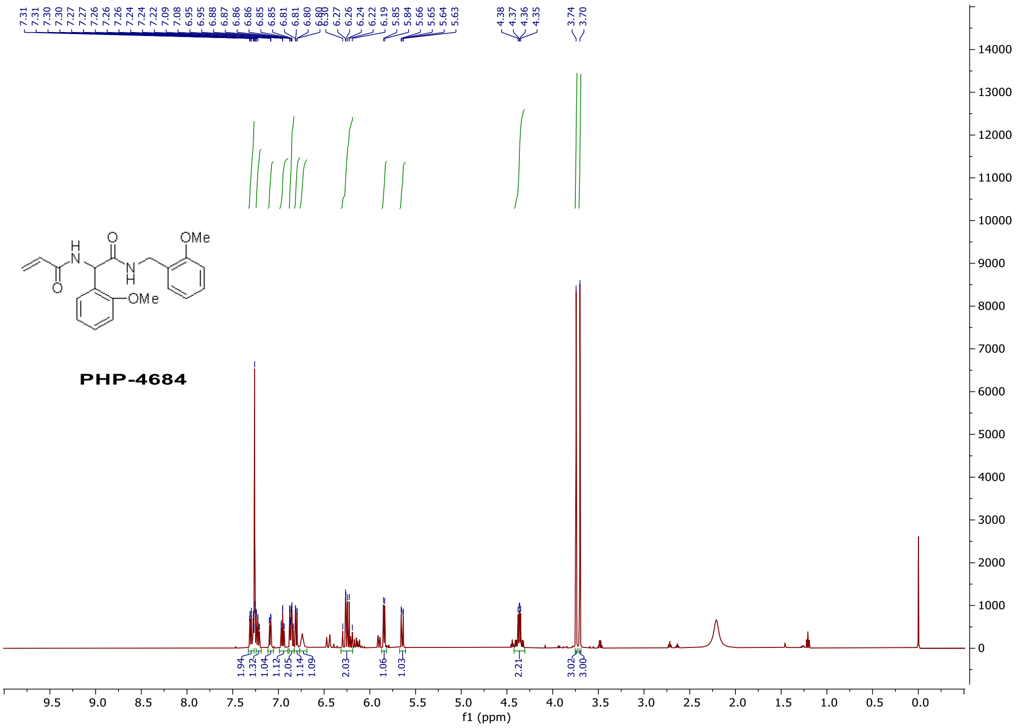

Supplementary Figure 399. ¹H NMR (500 MHz, $CDCl_3$) of compound **PHP-4684.**

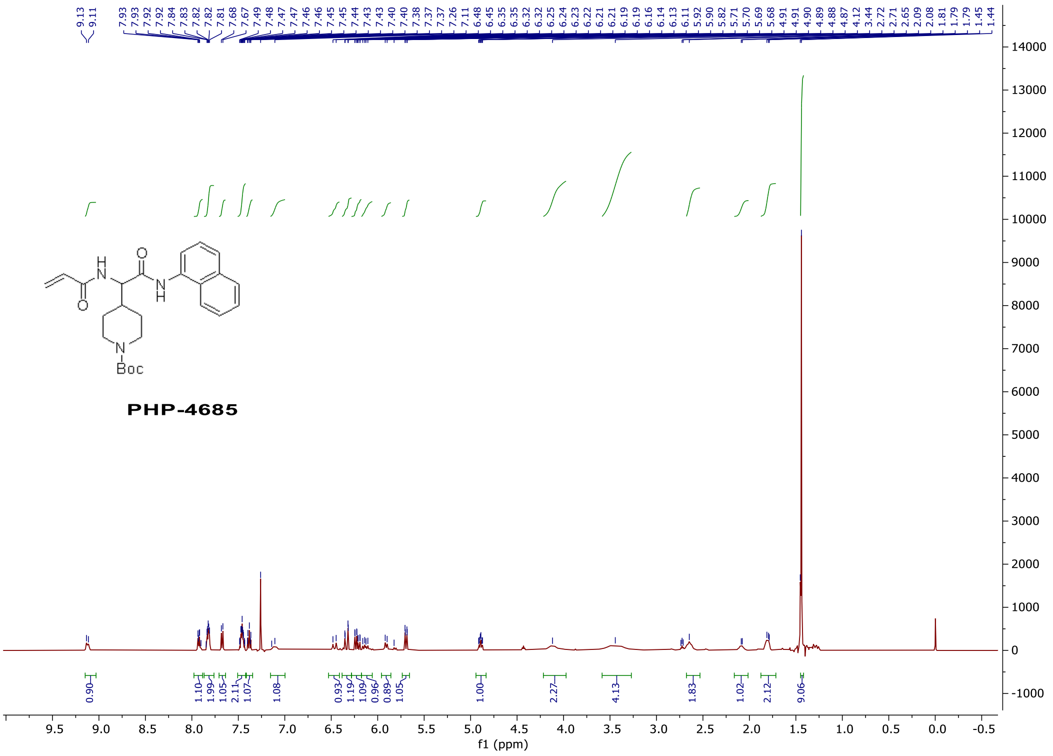

Supplementary Figure 400. ¹H NMR (500 MHz, $CDCl_3$+one drop of CD_3OD) of compound **PHP-4685.**

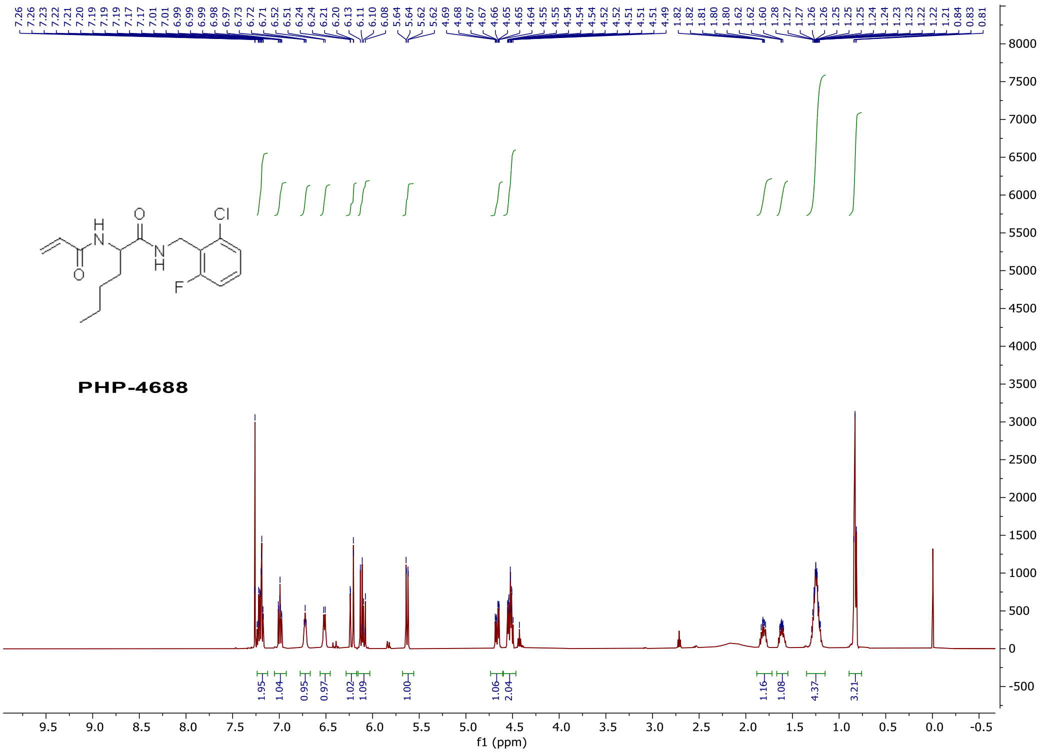

Supplementary Figure 401. ^{1}H NMR (500 MHz, $CDCl_3$) of compound **PHP-4688.**

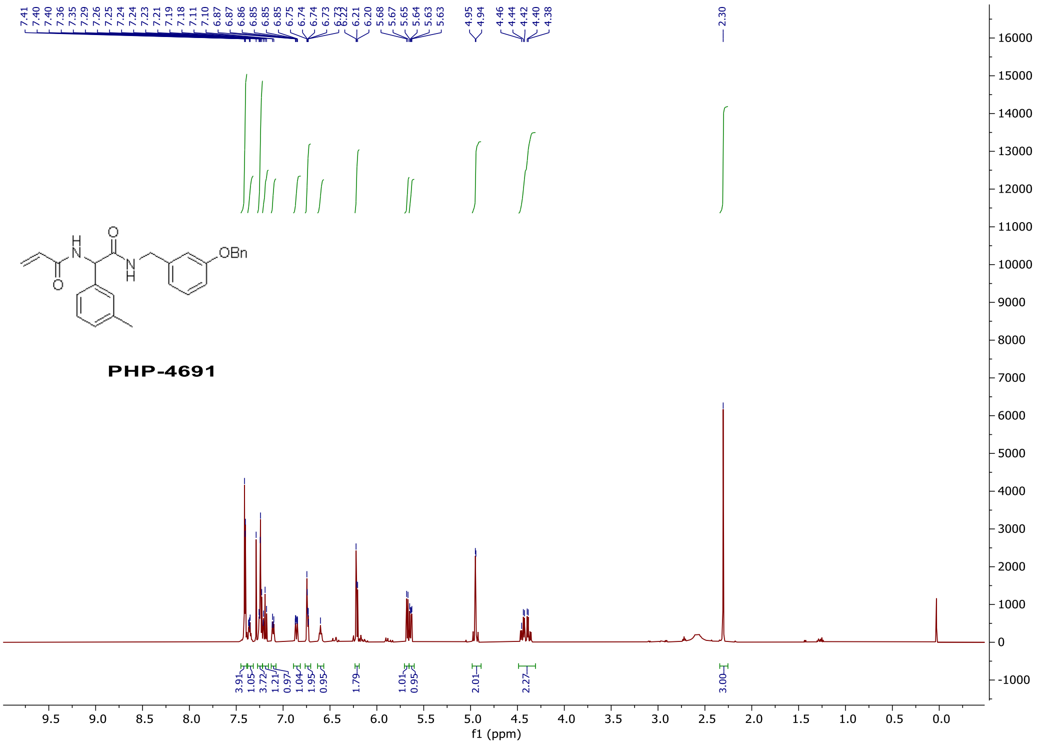

Supplementary Figure 402. ^{1}H NMR (500 MHz, $CDCl_3$) of compound **PHP-4691.**

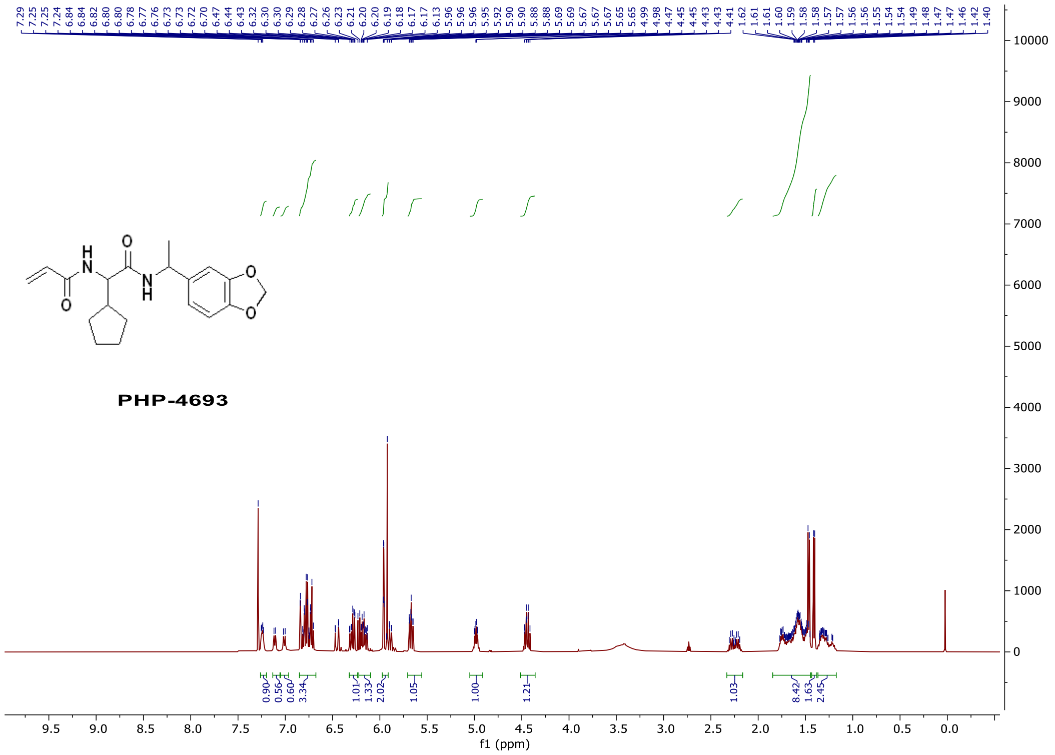

Supplementary Figure 403. ^{1}H NMR (500 MHz, $CDCl_3$+one drop of CD_3OD) of compound **PHP-4693.**

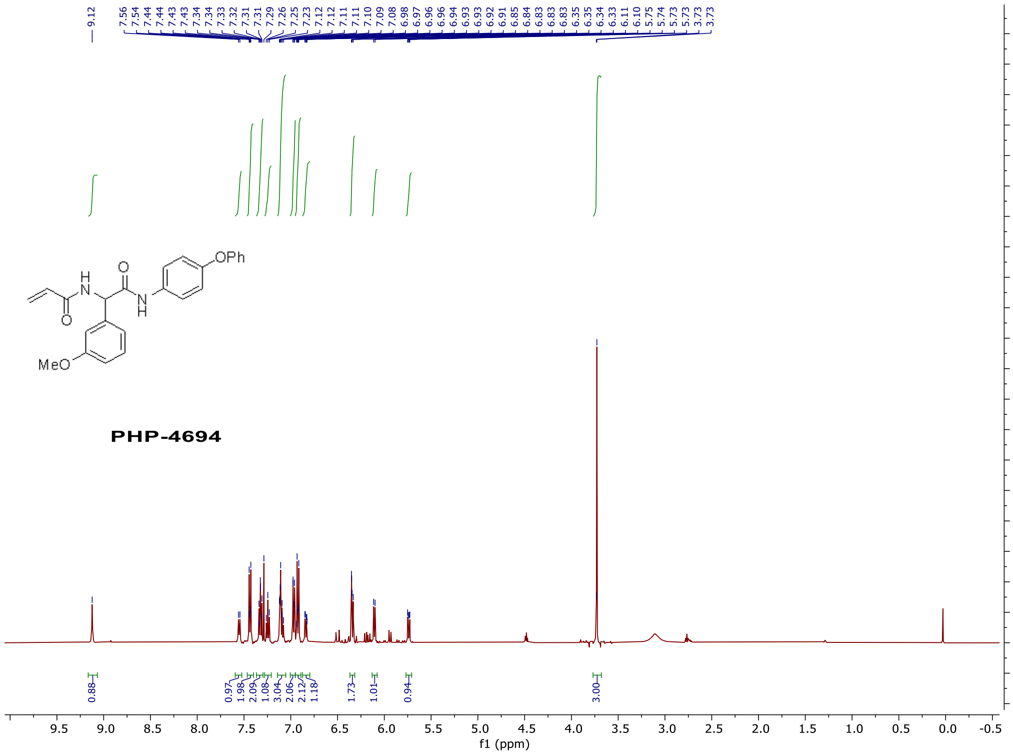

Supplementary Figure 404. ^{1}H NMR (500 MHz, $CDCl_3$+one drop of CD_3OD) of compound **PHP-4694.**

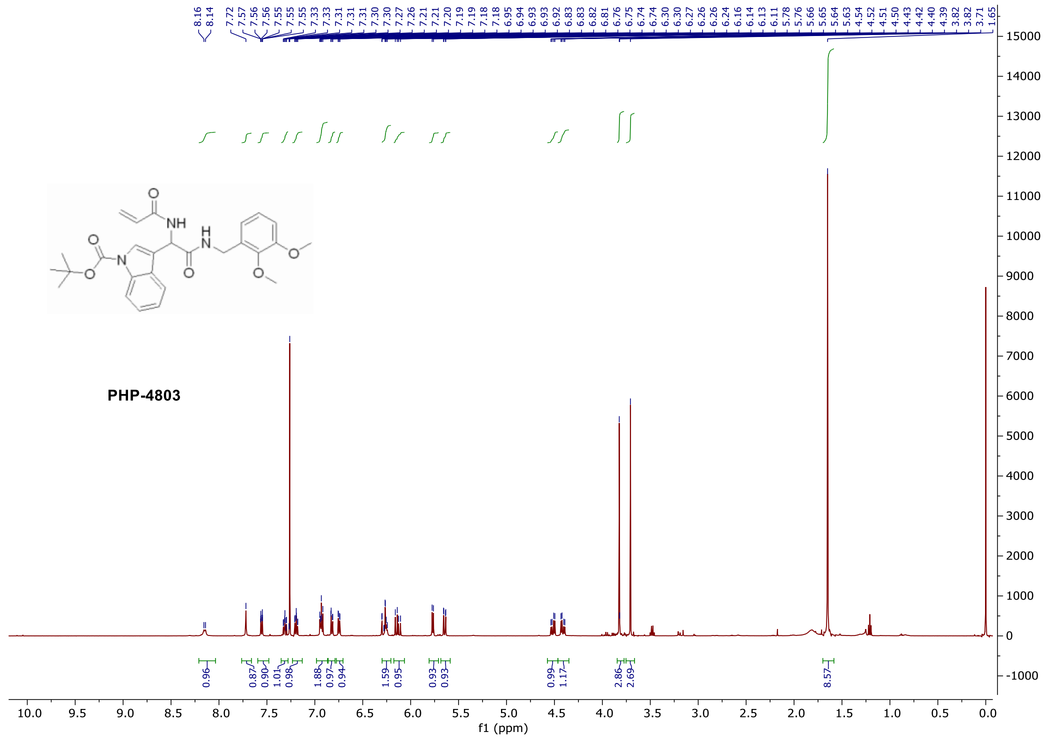

Supplementary Figure 405. ^{1}H NMR (500 MHz, $CDCl_3$) of compound **PHP-4803**.

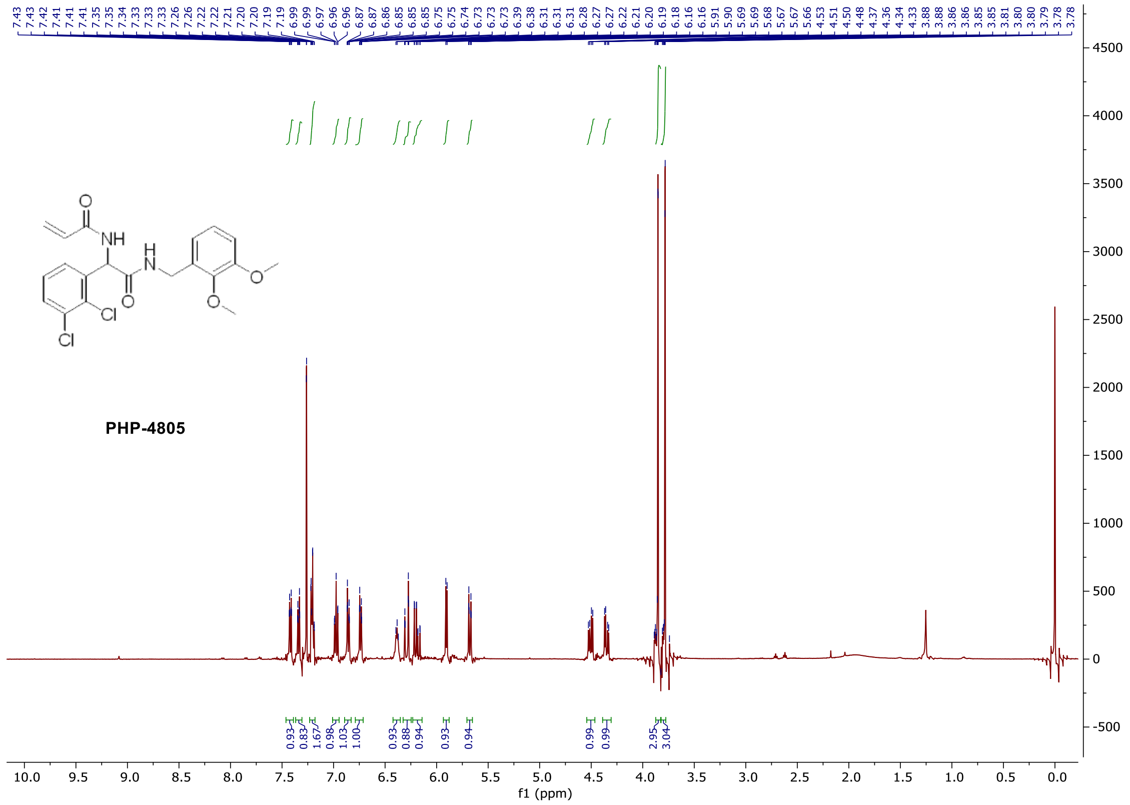

Supplementary Figure 406. ^{1}H NMR (500 MHz, $CDCl_3$) of compound **PHP-4805**.

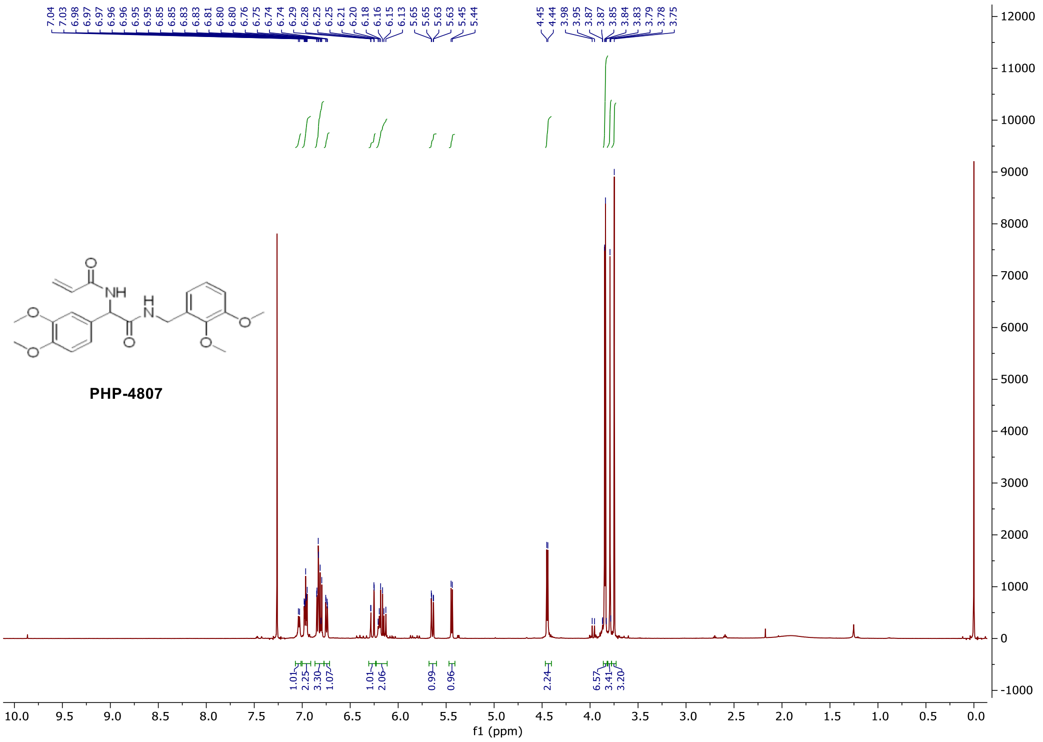

Supplementary Figure 407. ^{1}H NMR (500 MHz, $CDCl_3$) of compound **PHP-4807**.

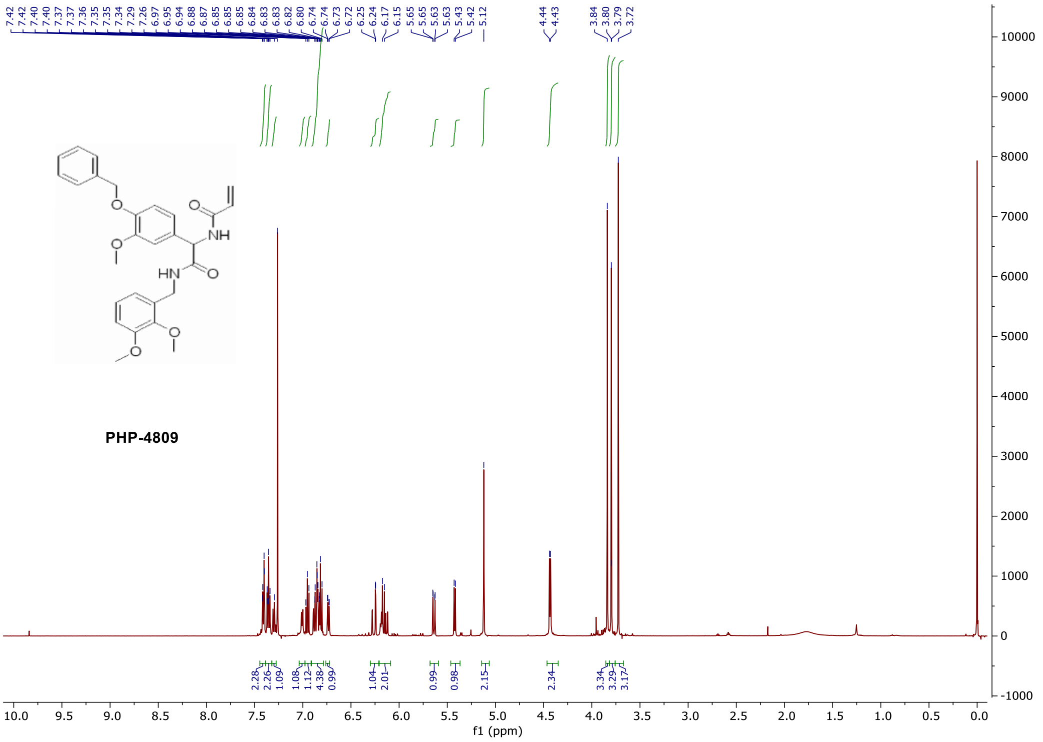

Supplementary Figure 408. ^{1}H NMR (500 MHz, $CDCl_3$) of compound **PHP-4809**.

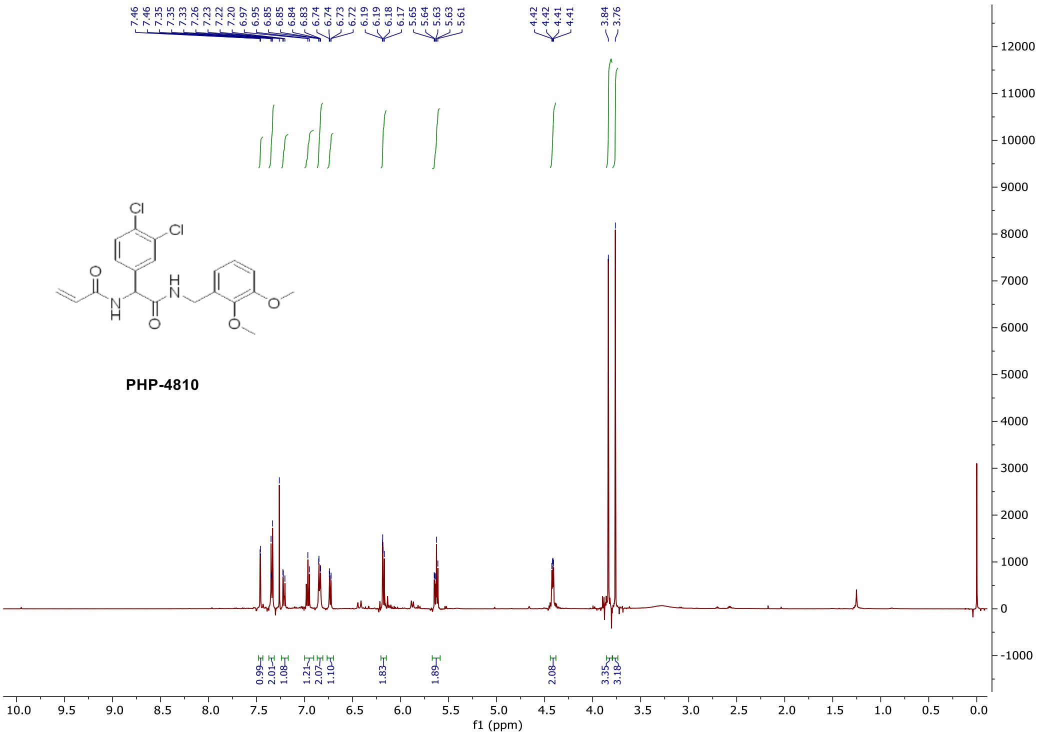

Supplementary Figure 409. ^{1}H NMR (500 MHz, $CDCl_3$) of compound **PHP-4810**.

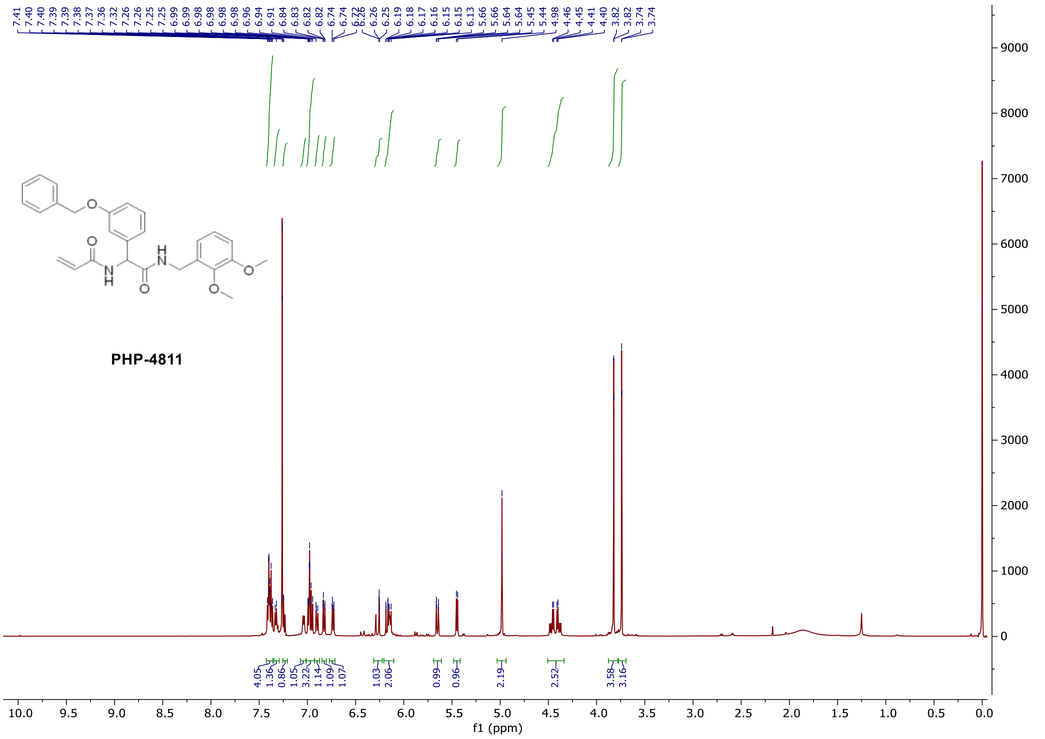

Supplementary Figure 410. ^{1}H NMR (500 MHz, $CDCl_3$) of compound **PHP-4811**.

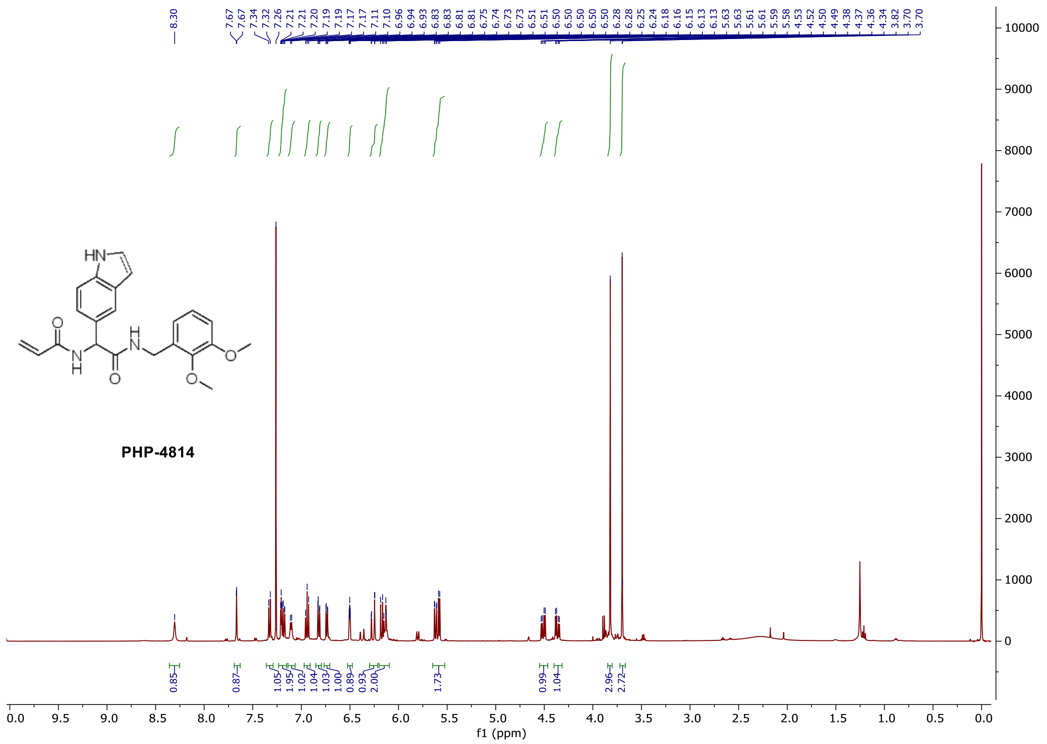

Supplementary Figure 411. ^{1}H NMR (500 MHz, $CDCl_3$) of compound **PHP-4814**.

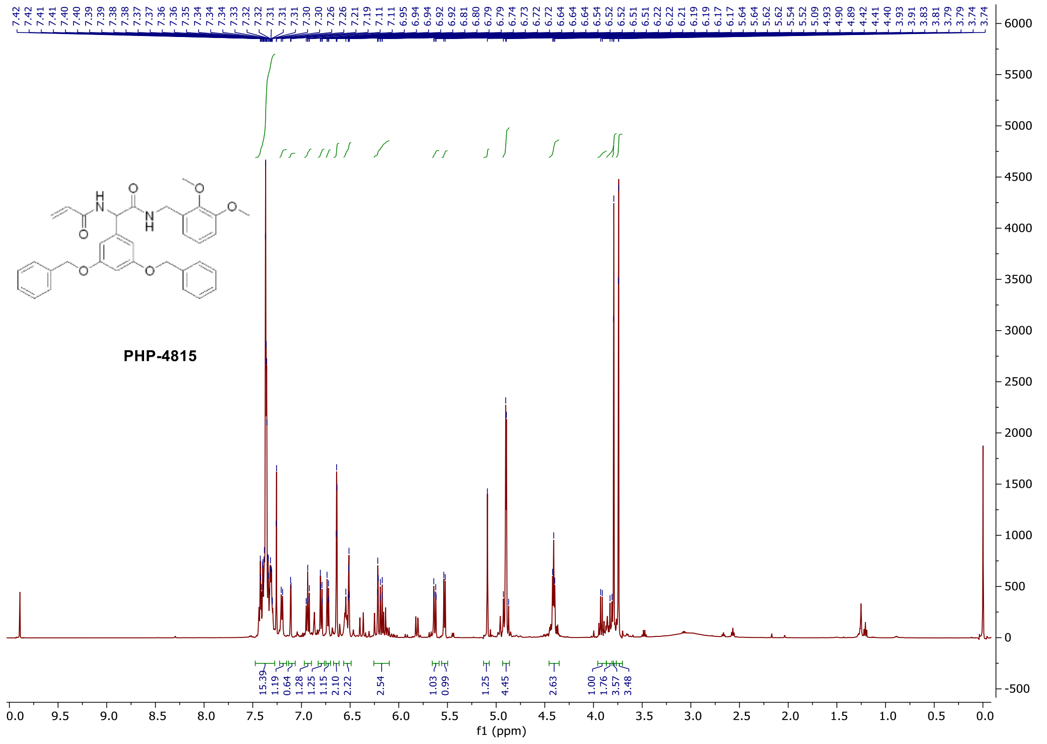

Supplementary Figure 412. ^{1}H NMR (500 MHz, $CDCl_3$) of compound **PHP-4815**.

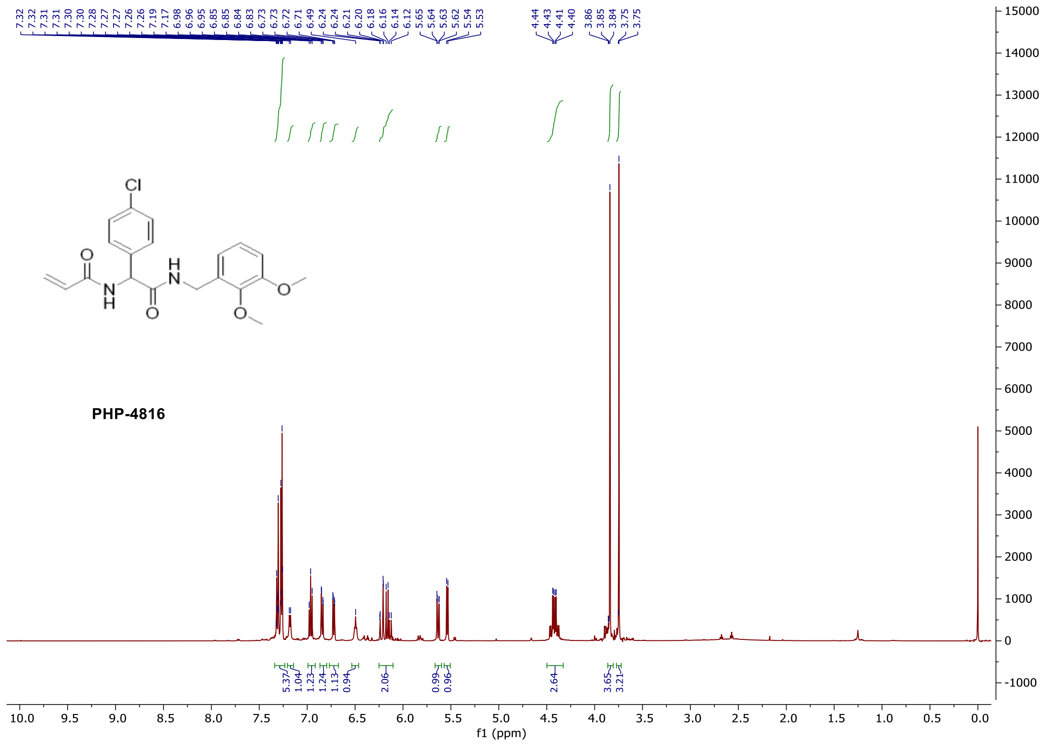

Supplementary Figure 413. ^{1}H NMR (500 MHz, $CDCl_3$) of compound **PHP-4816**.

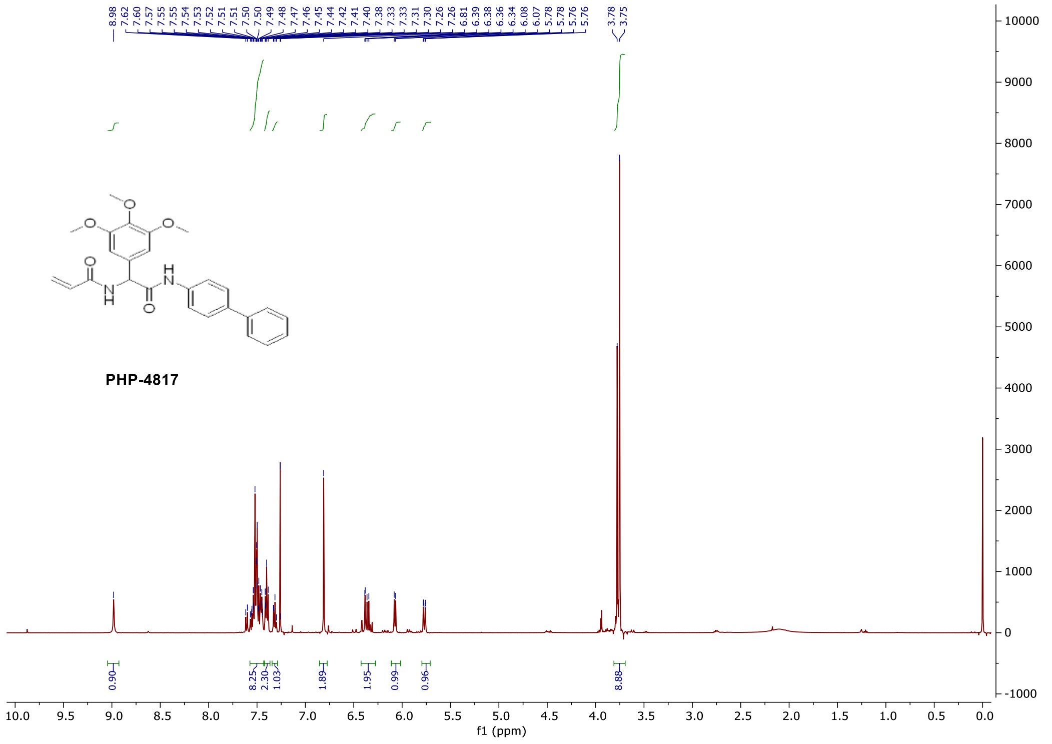

Supplementary Figure 414. ^{1}H NMR (500 MHz, $CDCl_3$) of compound **PHP-4817**.

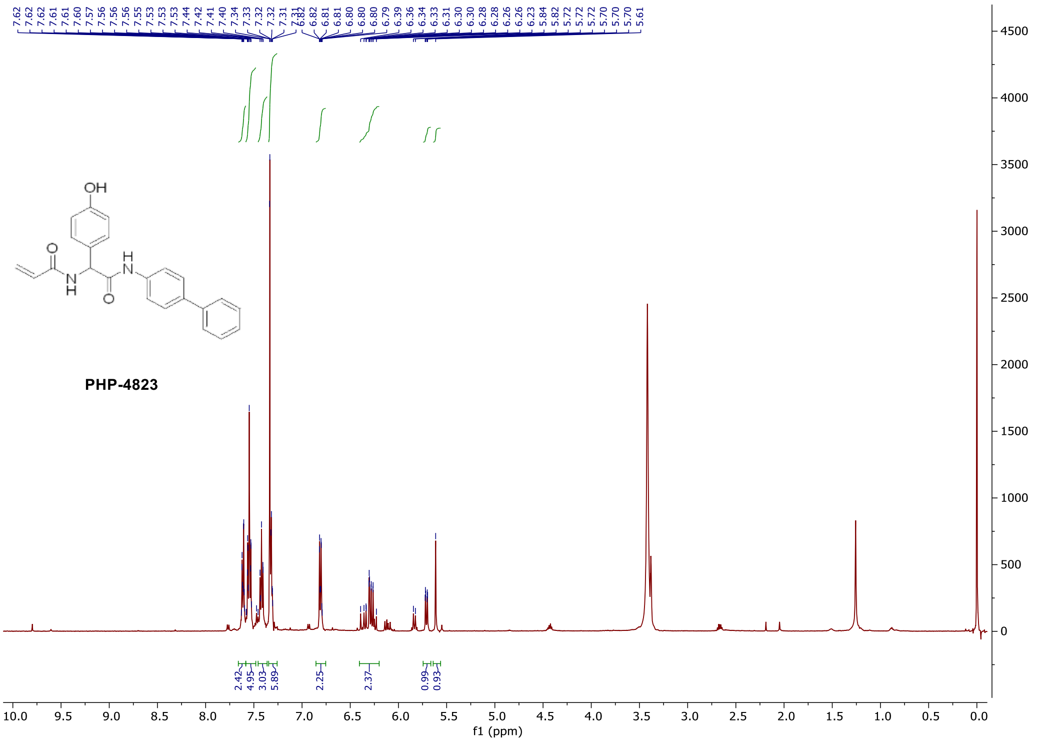

Supplementary Figure 415. ^{1}H NMR (500 MHz, $CDCl_3$ + one drop of CD_3OD) of compound **PHP-4823**.

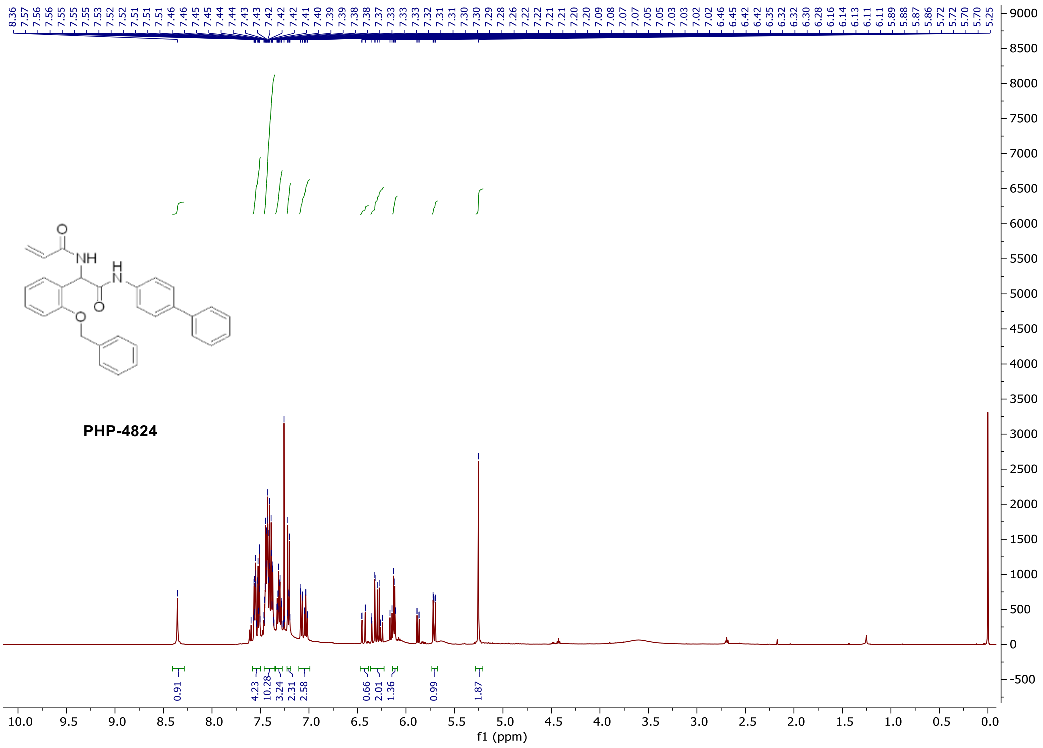

Supplementary Figure 416. ^{1}H NMR (500 MHz, $CDCl_3$) of compound **PHP-4824**.

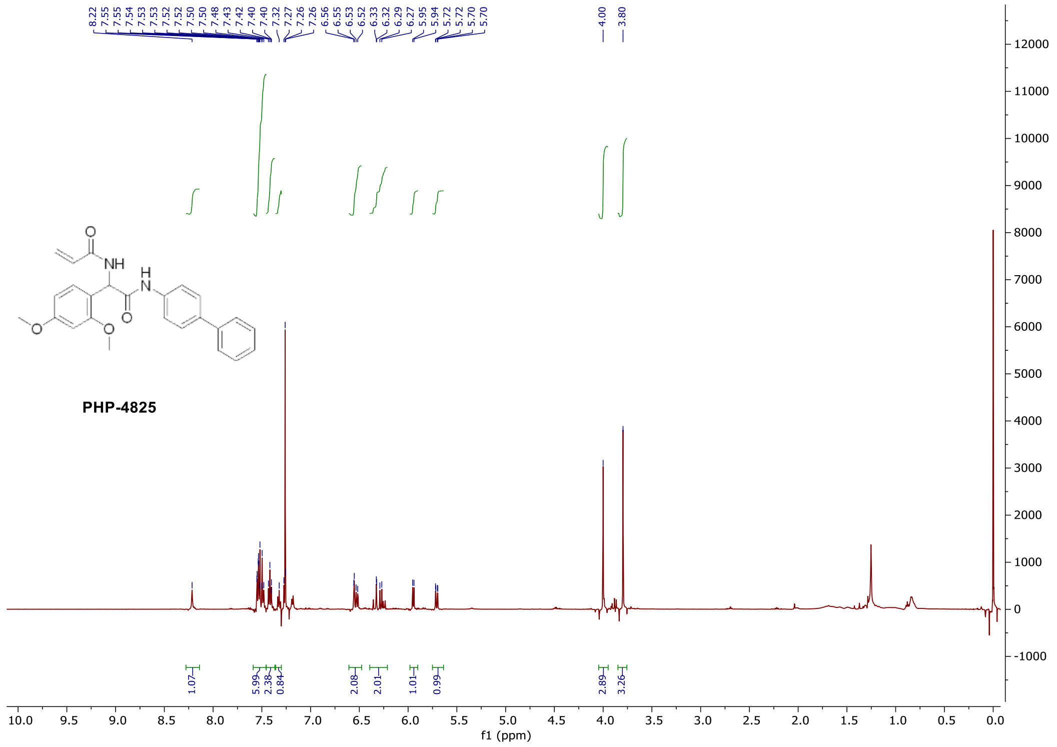

Supplementary Figure 417. ^{1}H NMR (500 MHz, $CDCl_3$) of compound **PHP-4825**.

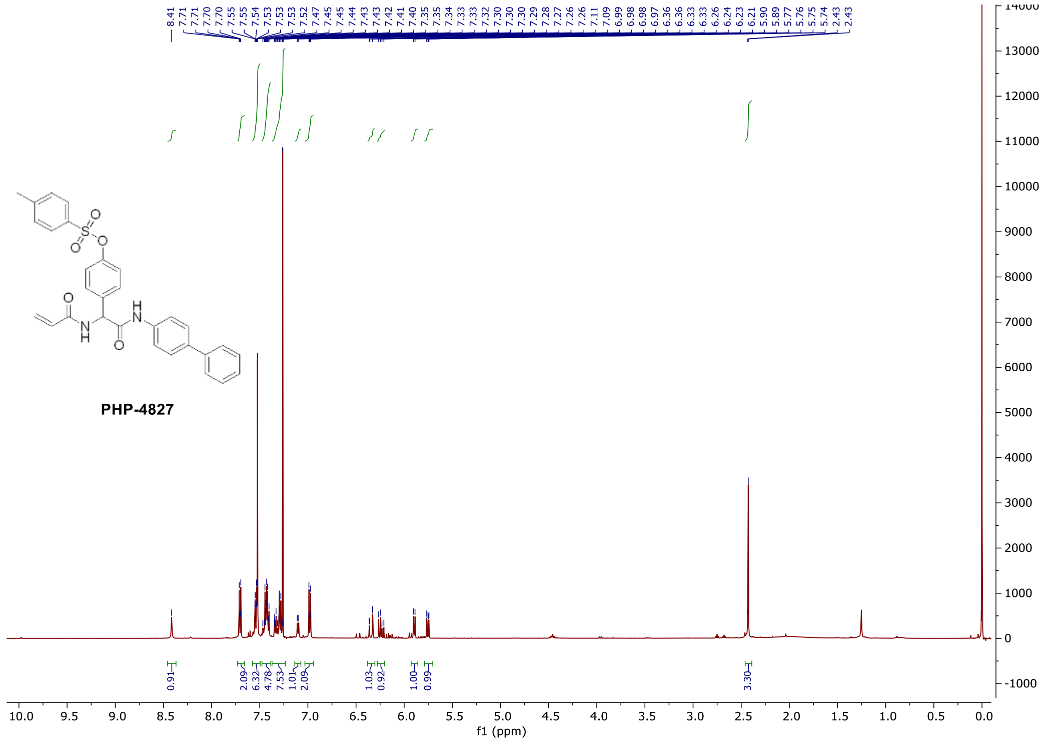

Supplementary Figure 418. ^{1}H NMR (500 MHz, $CDCl_3$) of compound **PHP-4827**.

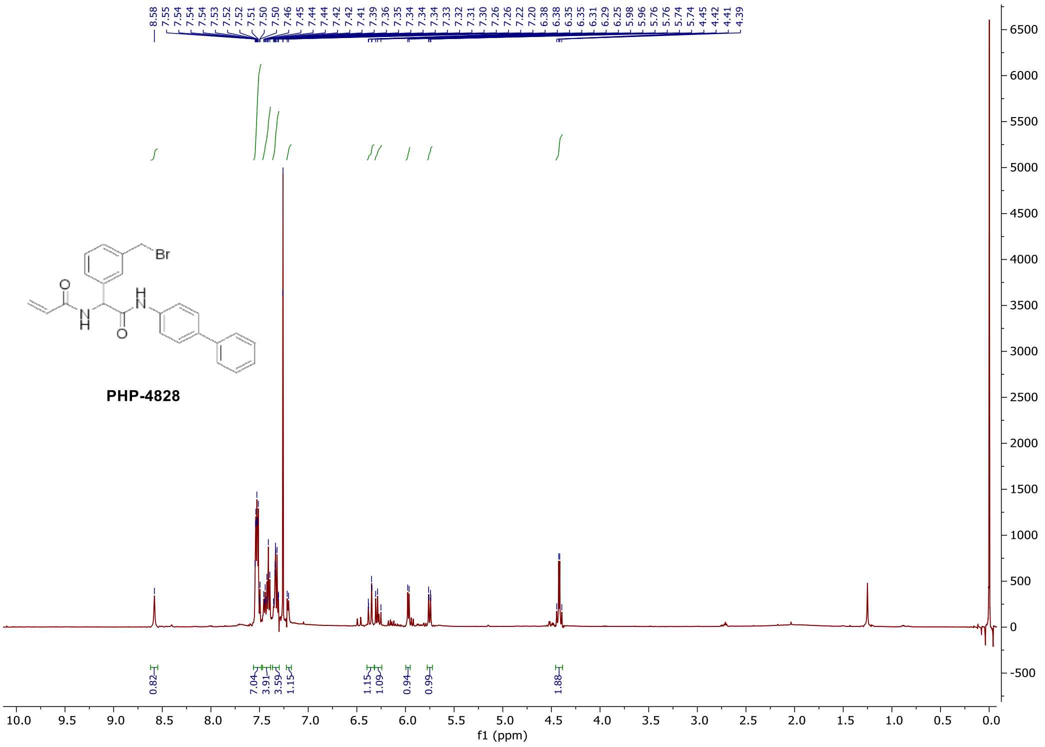

Supplementary Figure 419. ^{1}H NMR (500 MHz, $CDCl_3$) of compound **PHP-4828**.

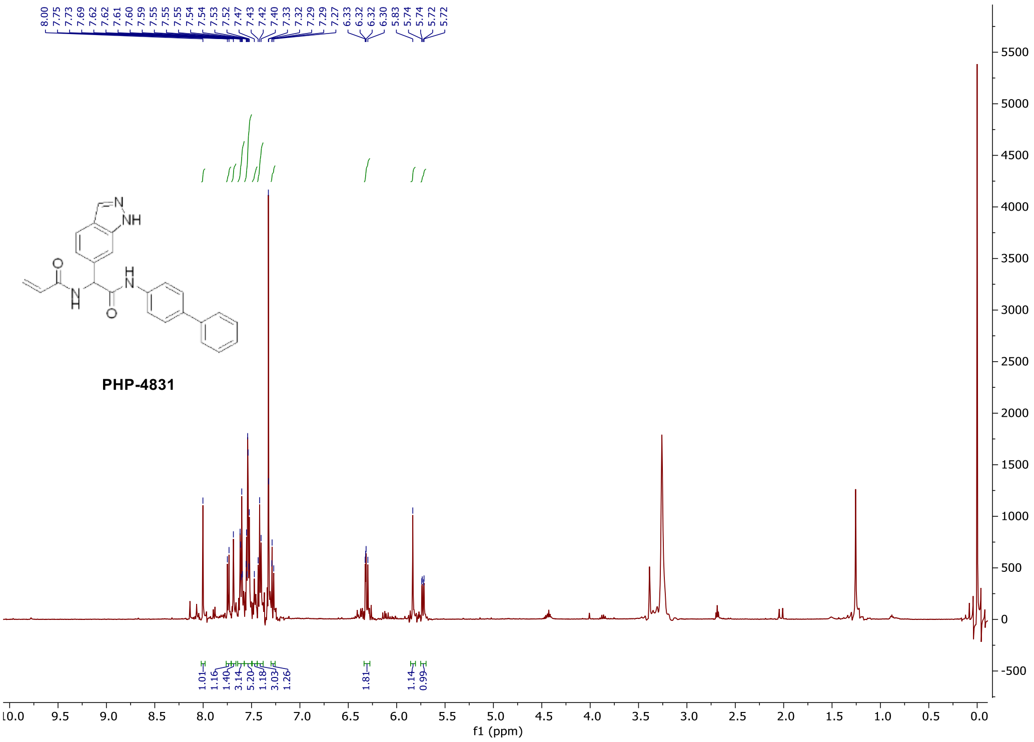

Supplementary Figure 420. ^{1}H NMR (500 MHz, $CDCl_3$ + one drop of CD_3OD) of compound **PHP-4831**.

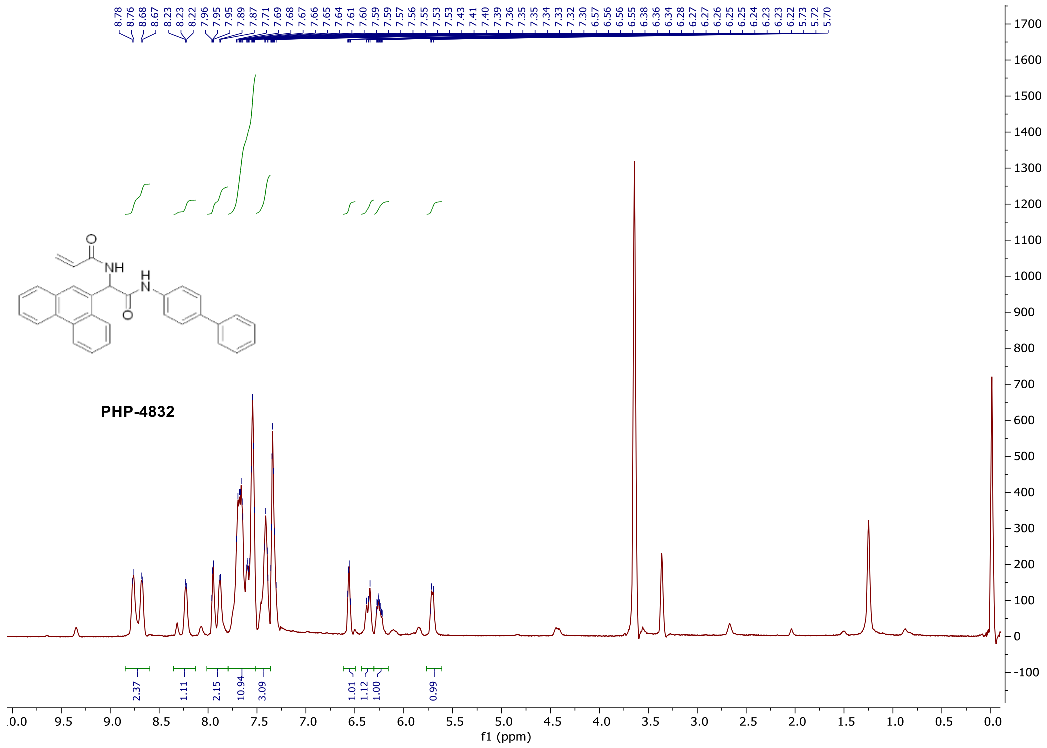

Supplementary Figure 421. ^{1}H NMR (500 MHz, $CDCl_3$ + one drop of CD_3OD) of compound **PHP-4832**.

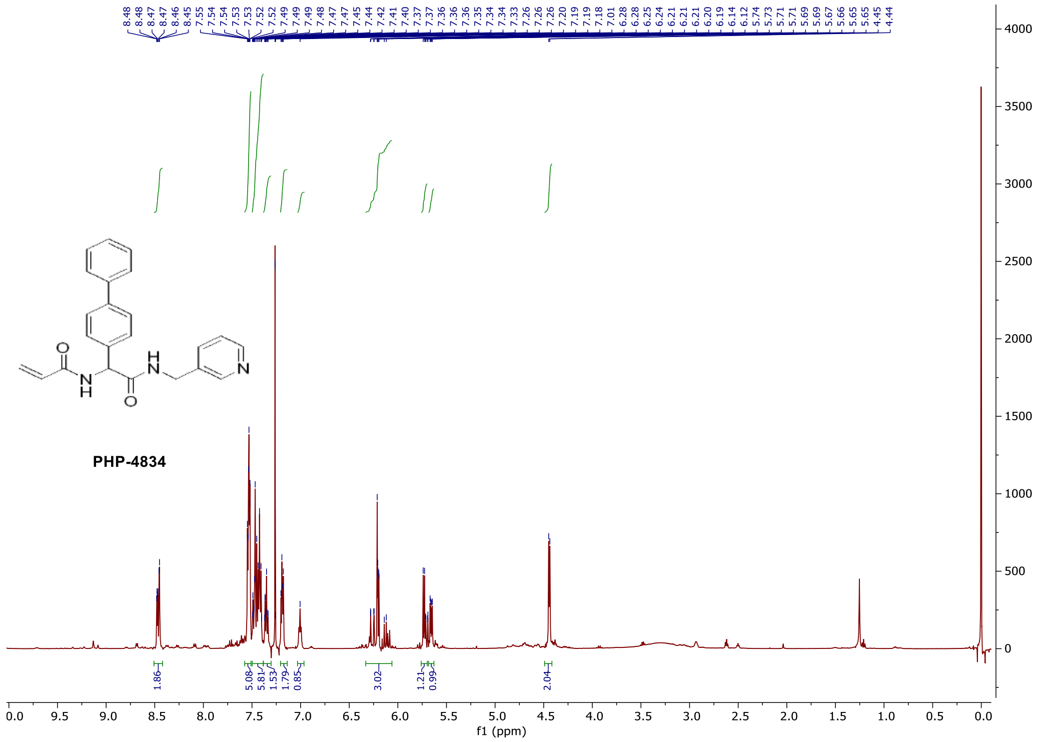

Supplementary Figure 422. ^{1}H NMR (500 MHz, $CDCl_3$) of compound **PHP-4834**.

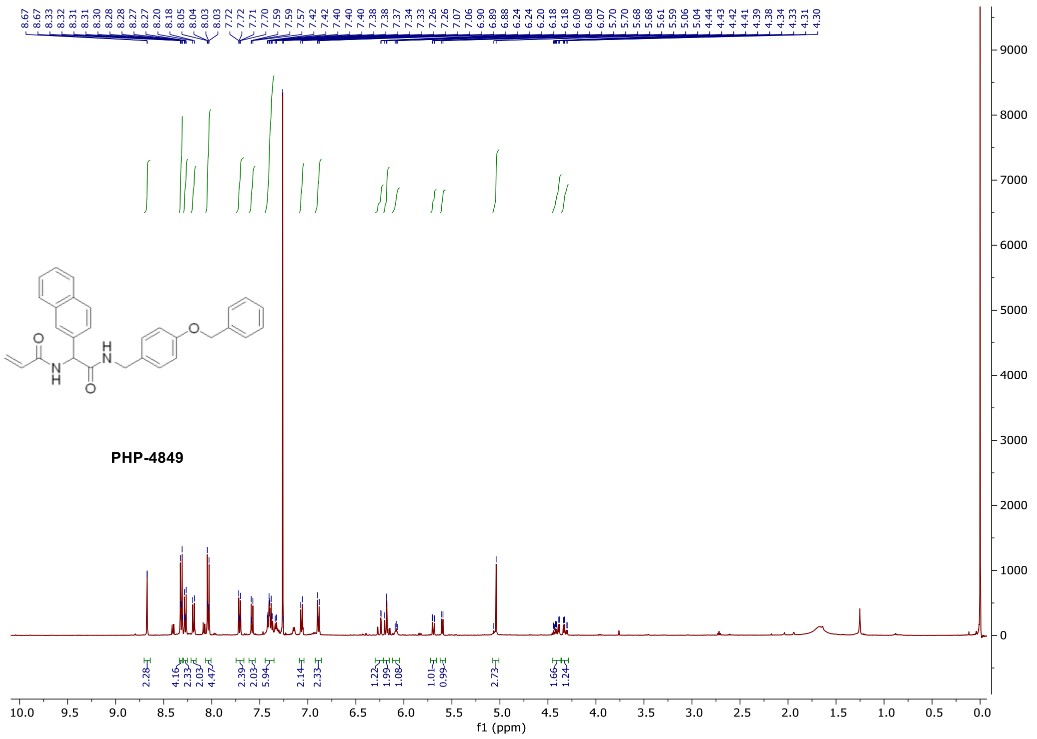

Supplementary Figure 423. ^{1}H NMR (500 MHz, $CDCl_3$) of compound **PHP-4849**.

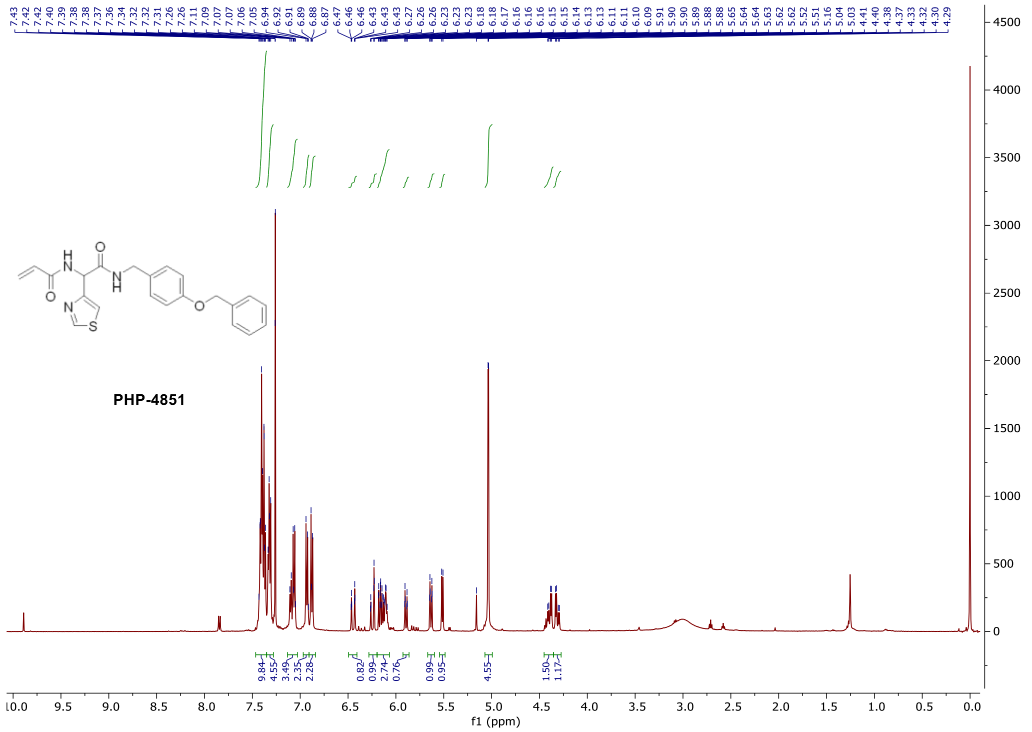

Supplementary Figure 424. ^{1}H NMR (500 MHz, $CDCl_3$ + one drop of CD_3OD) of compound **PHP-4851**.

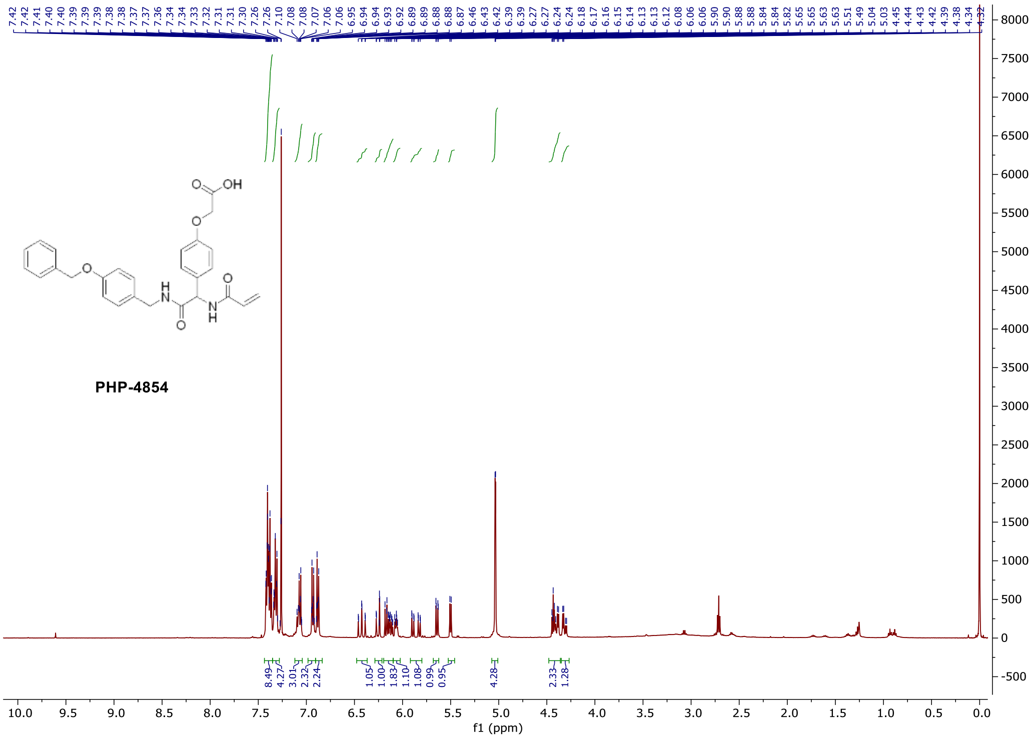

Supplementary Figure 425. ^{1}H NMR (500 MHz, $CDCl_3$) of compound **PHP-4854**.

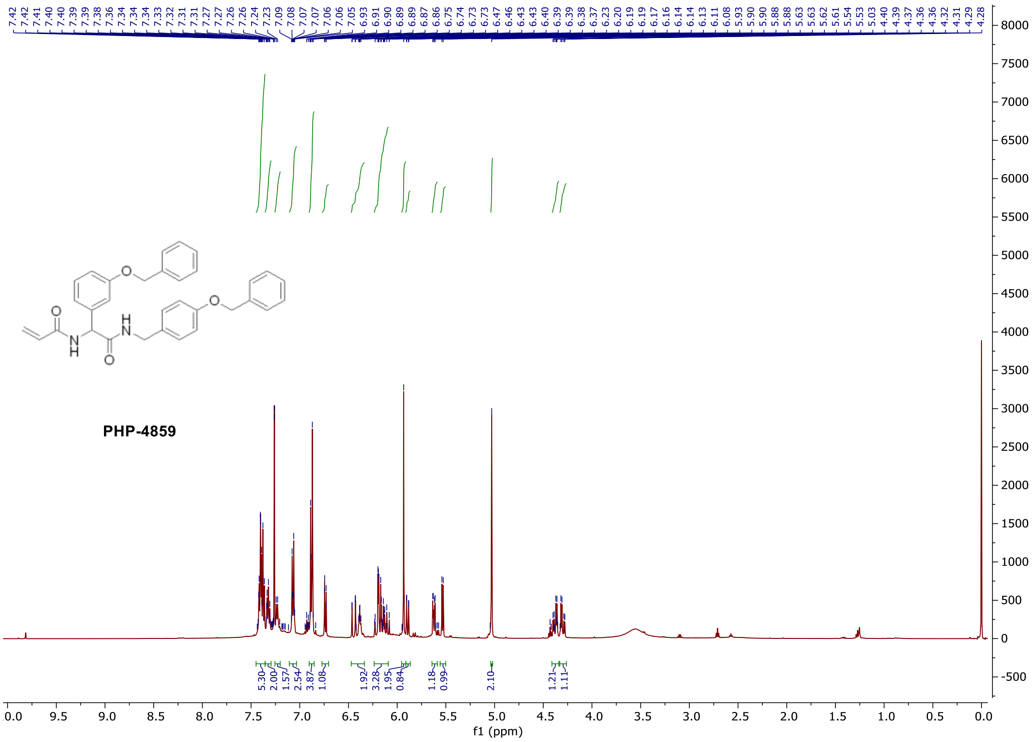

Supplementary Figure 426. ^{1}H NMR (500 MHz, $CDCl_3$ + one drop of CD_3OD) of compound **PHP-4859**.

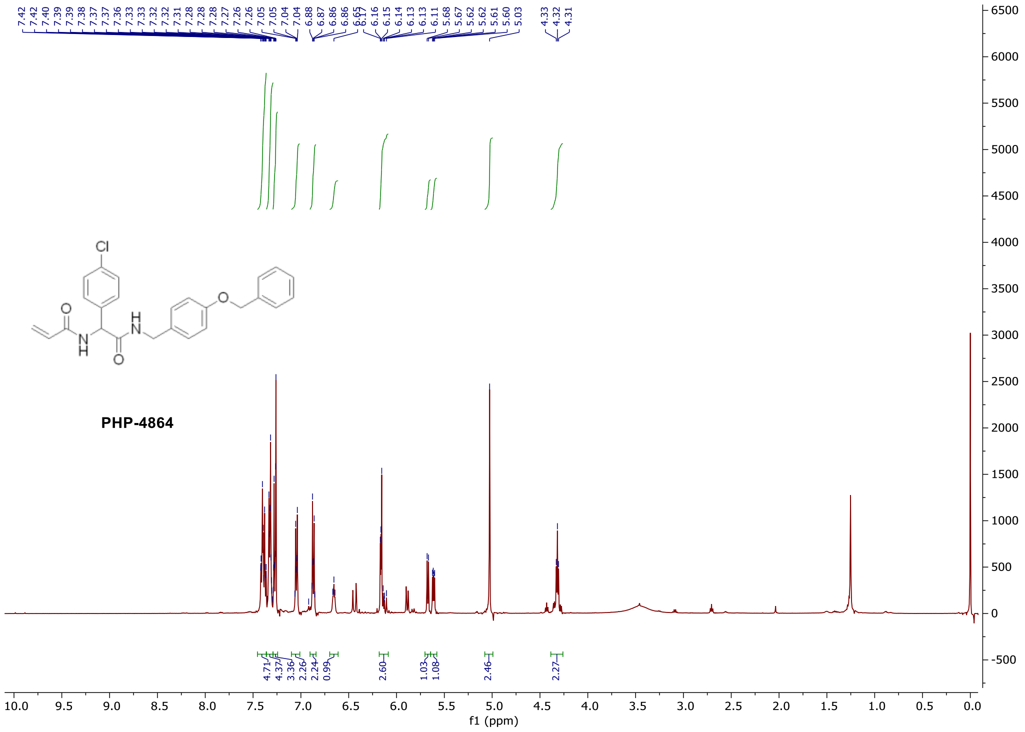

Supplementary Figure 427. ^{1}H NMR (500 MHz, $CDCl_3$ + one drop of CD_3OD) of compound **PHP-4864**.

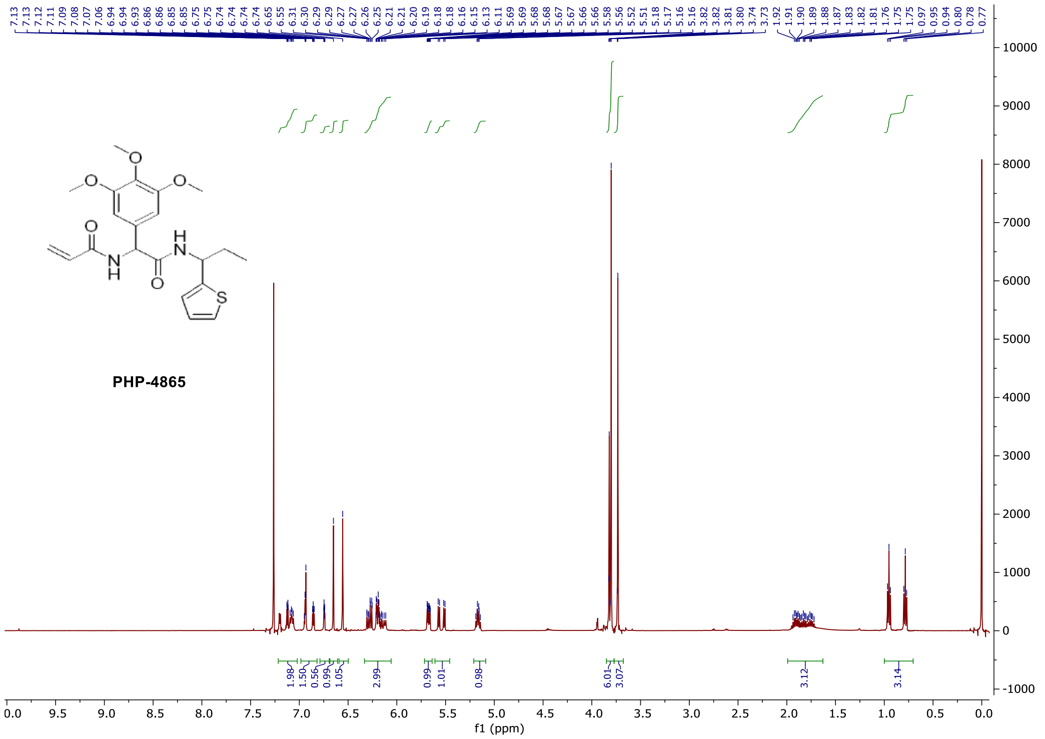

Supplementary Figure 428. ^{1}H NMR (500 MHz, $CDCl_3$) of compound **PHP-4865**.

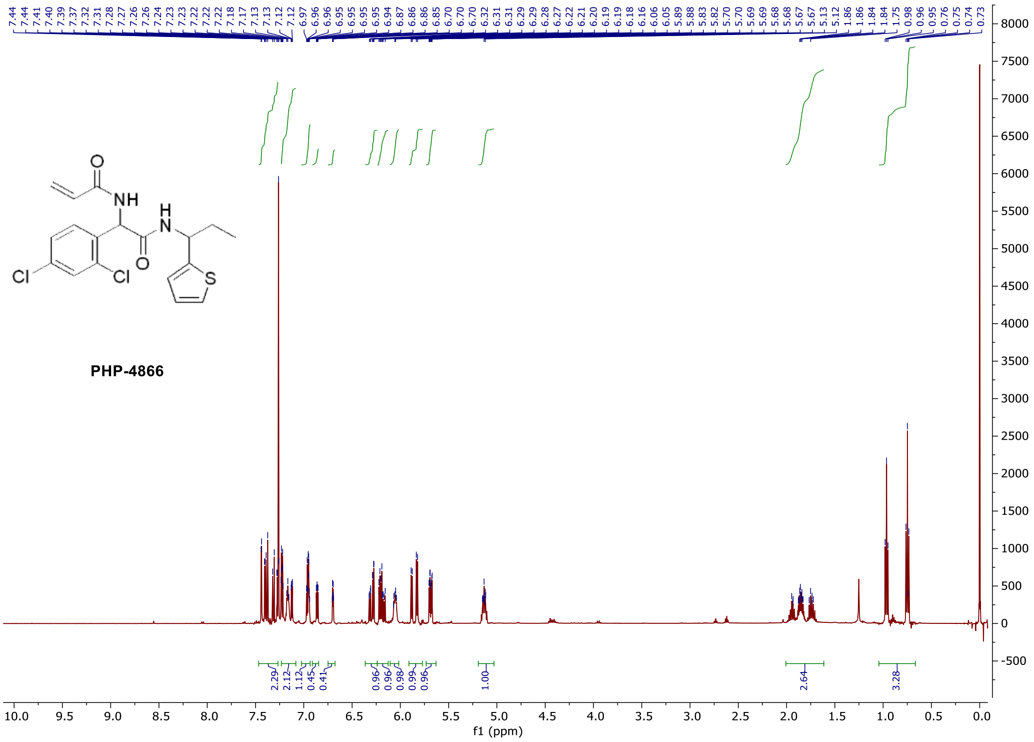

Supplementary Figure 429. ^{1}H NMR (500 MHz, $CDCl_3$) of compound **PHP-4866**.

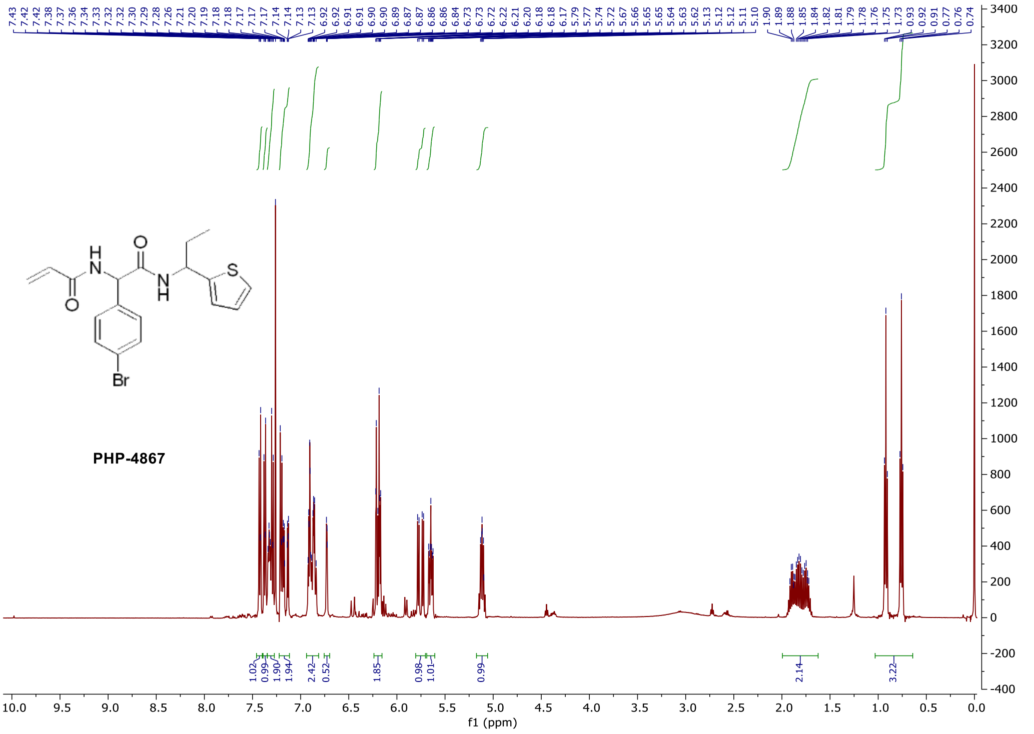

Supplementary Figure 430. ^{1}H NMR (500 MHz, $CDCl_3$ + one drop of CD_3OD) of compound **PHP-4867**.

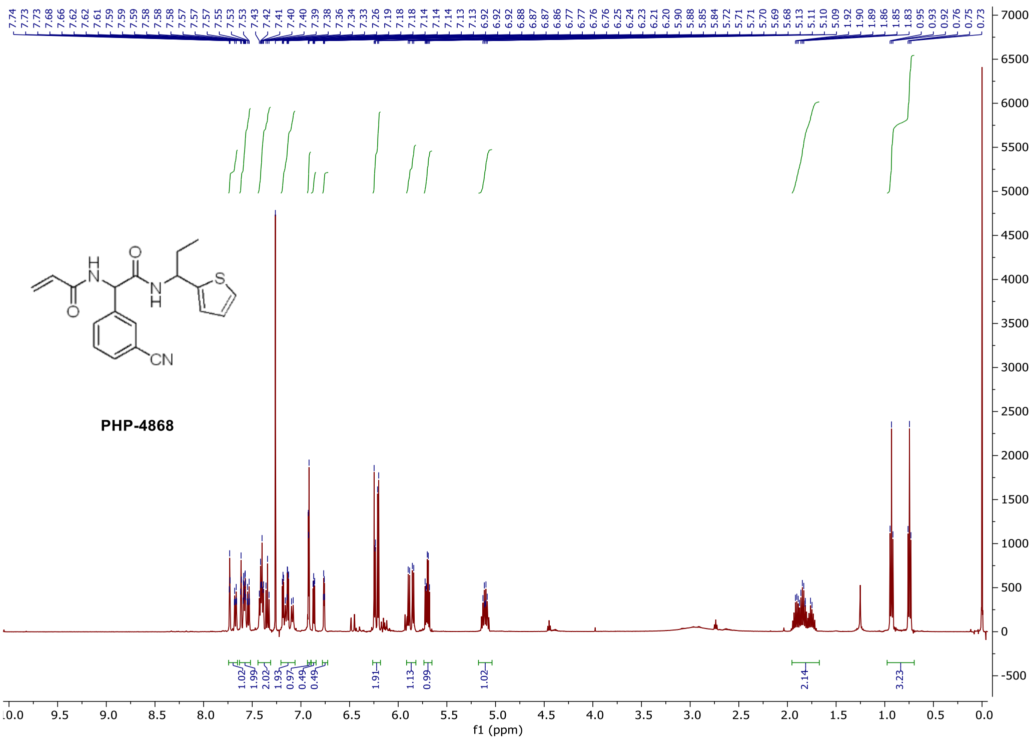

Supplementary Figure 431. ^{1}H NMR (500 MHz, $CDCl_3$ + one drop of CD_3OD) of compound **PHP-4868**.

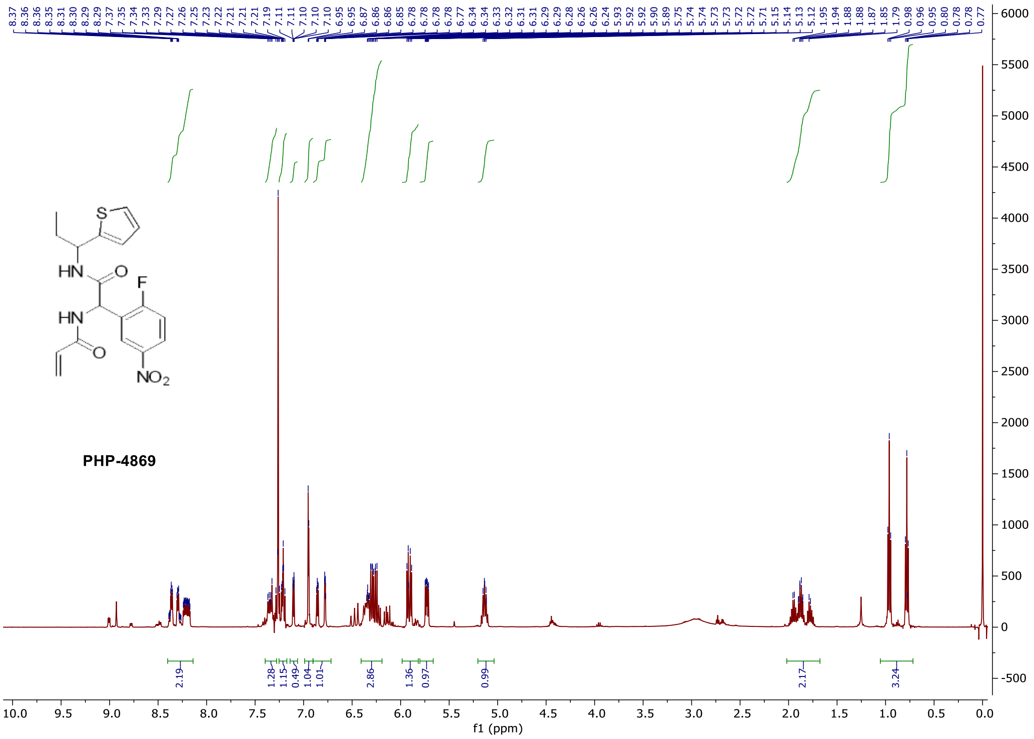

Supplementary Figure 432. ^{1}H NMR (500 MHz, $CDCl_3$ + one drop of CD_3OD) of compound **PHP-4869**.

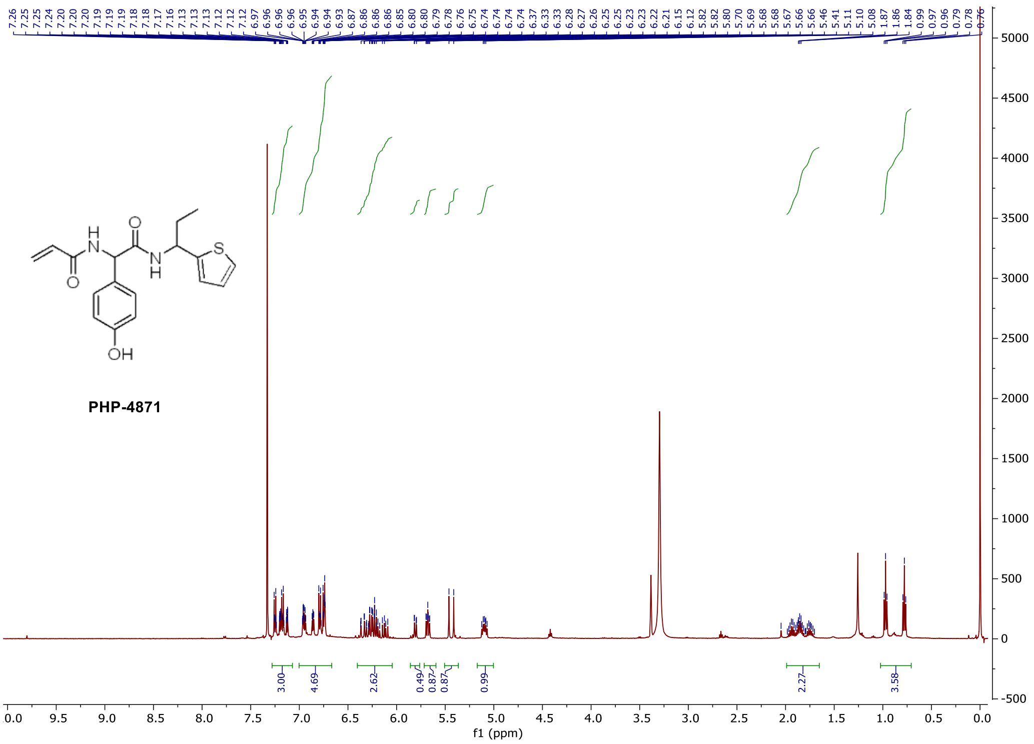

Supplementary Figure 433. ^{1}H NMR (500 MHz, $CDCl_3$ + one drop of CD_3OD) of compound **PHP-4871**.

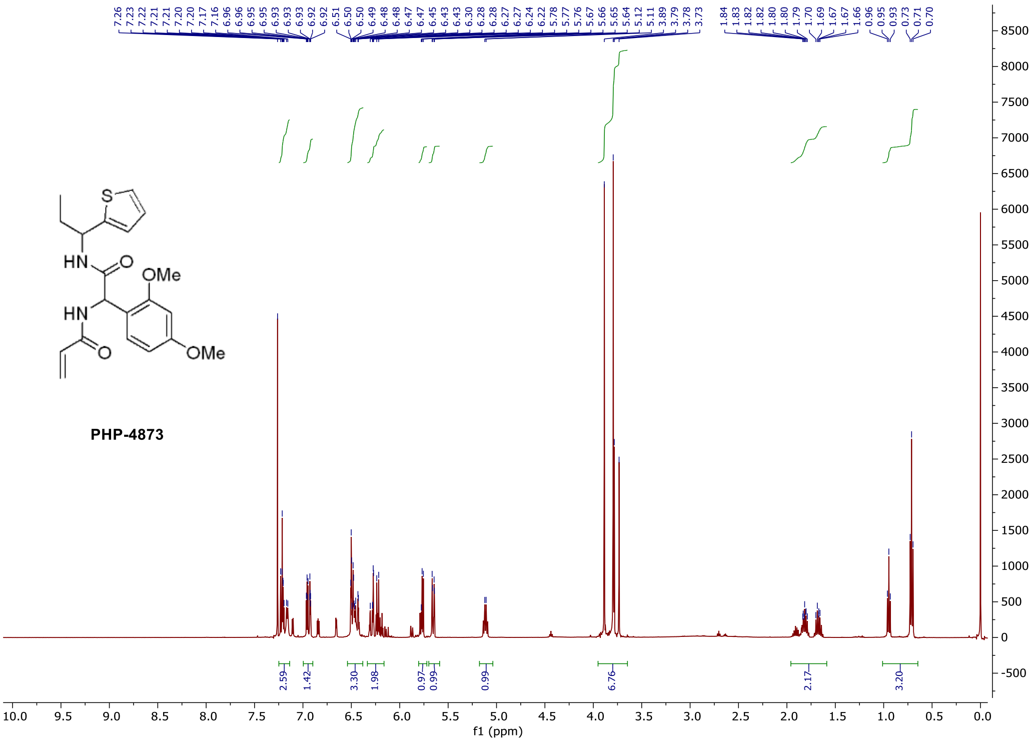

Supplementary Figure 434. ^{1}H NMR (500 MHz, $CDCl_3$) of compound **PHP-4873**.

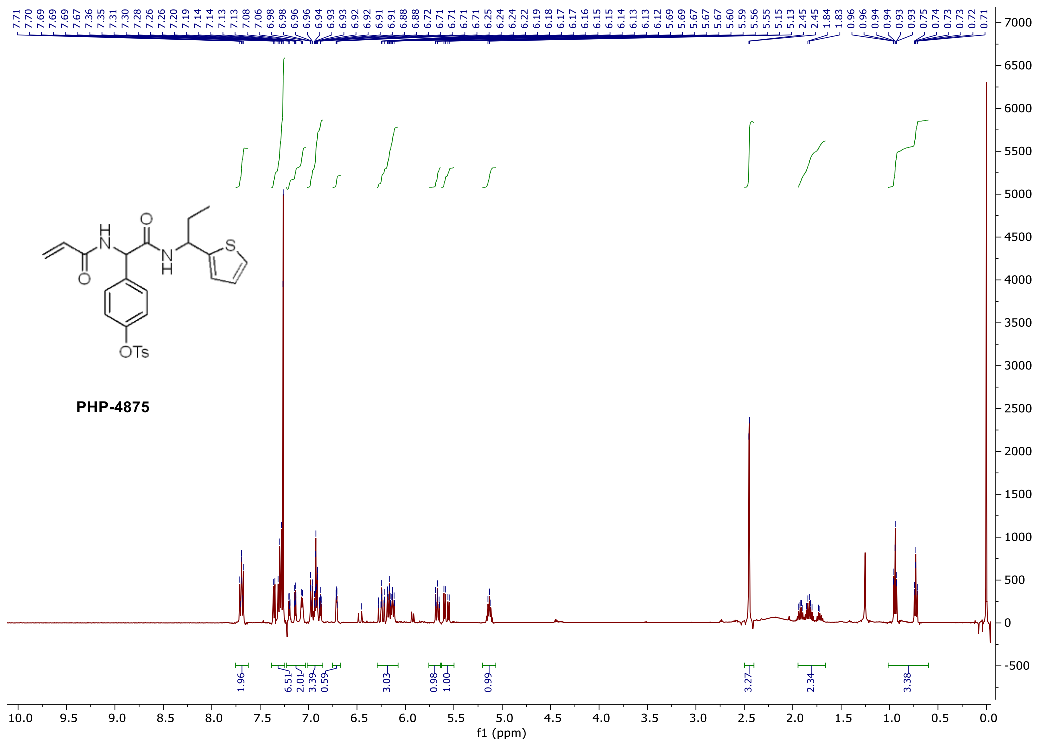

Supplementary Figure 435. ^{1}H NMR (500 MHz, $CDCl_3$) of compound **PHP-4875**.

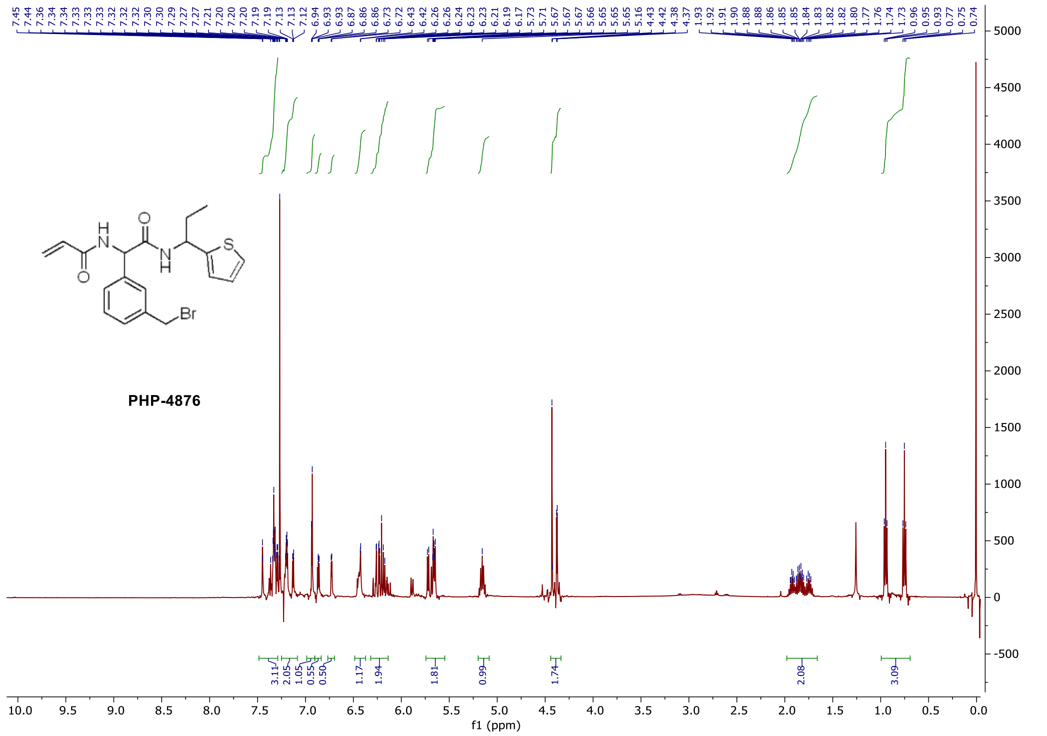

Supplementary Figure 436. ^{1}H NMR (500 MHz, $CDCl_3$) of compound **PHP-4876**.

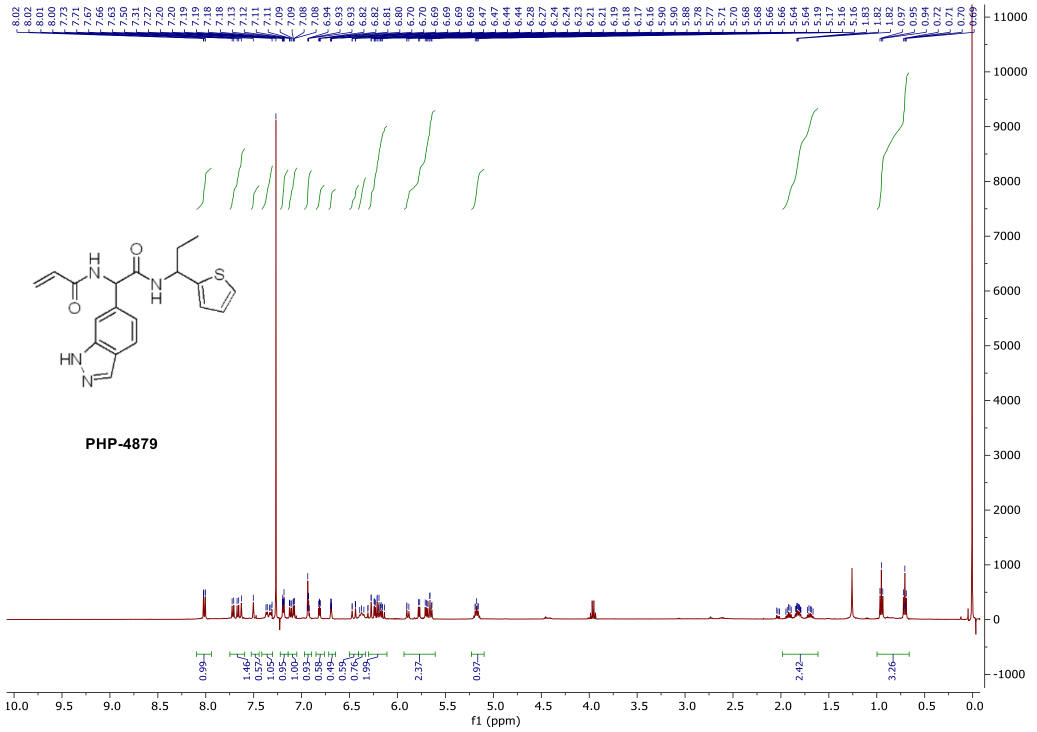

Supplementary Figure 437. ¹H NMR (500 MHz, $CDCl_3$) of compound **PHP-4879**.

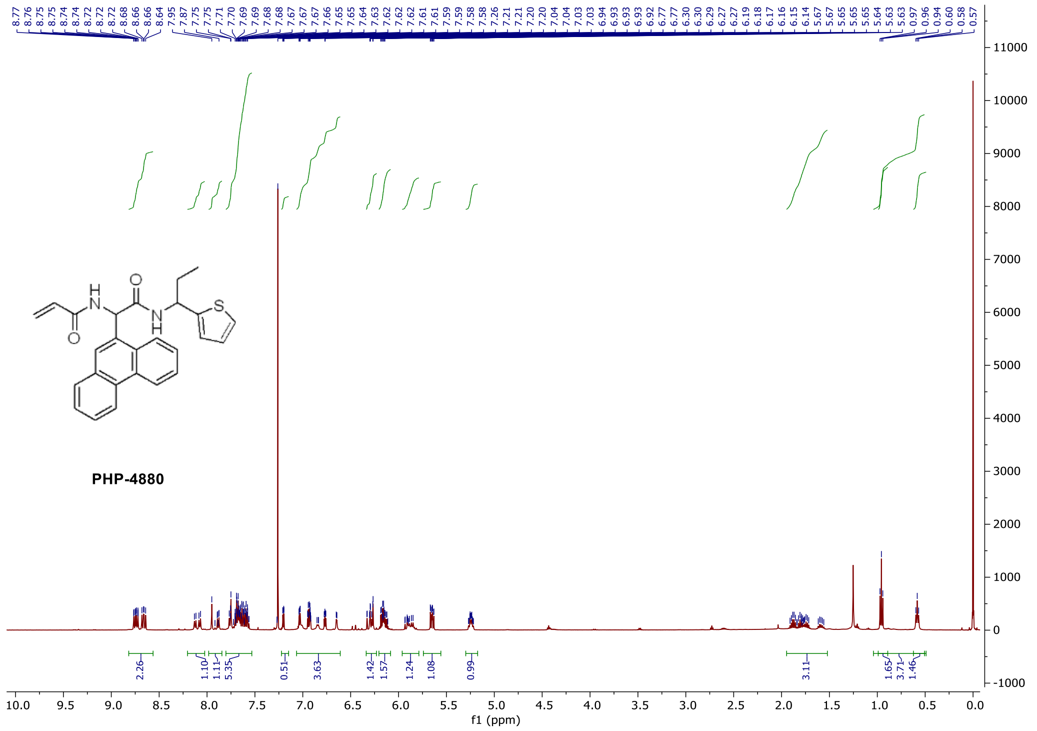

Supplementary Figure 438. ¹H NMR (500 MHz, $CDCl_3$) of compound **PHP-4880**.

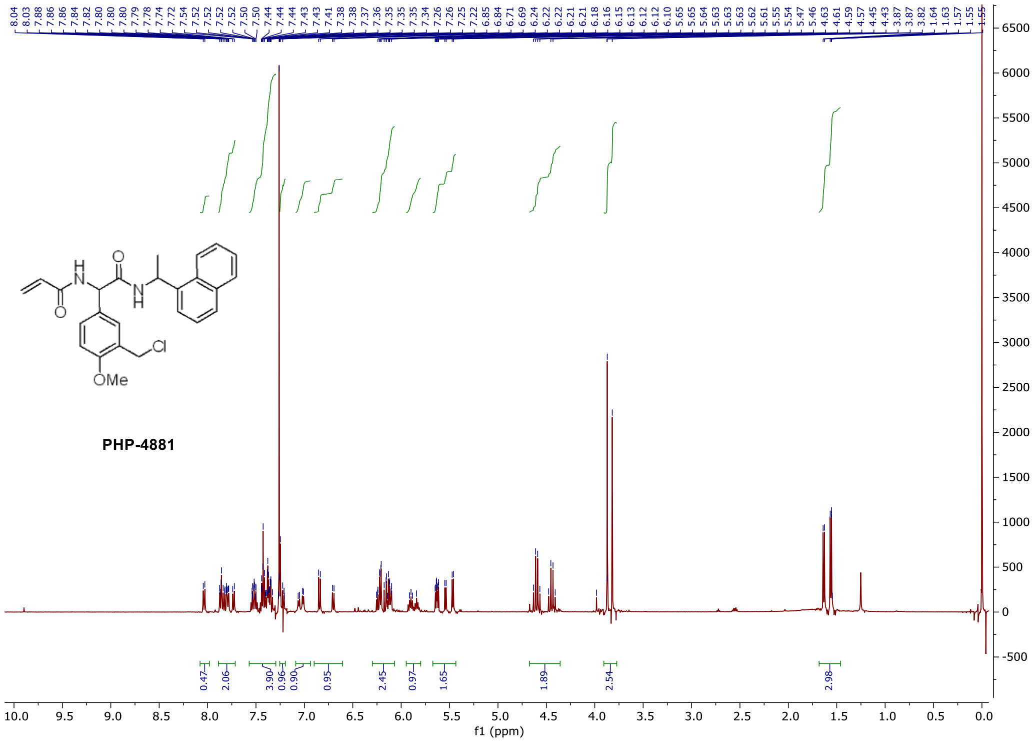

Supplementary Figure 439. ^{1}H NMR (500 MHz, $CDCl_3$) of compound **PHP-4881**.

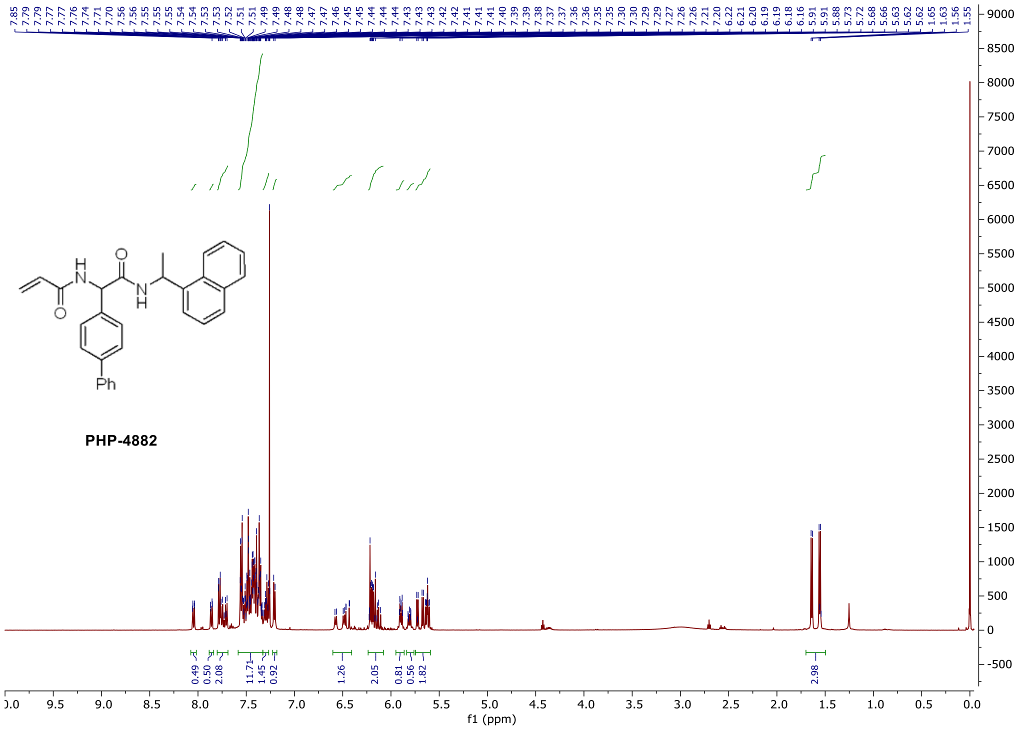

Supplementary Figure 440. ^{1}H NMR (500 MHz, $CDCl_3$) of compound **PHP-4882**.

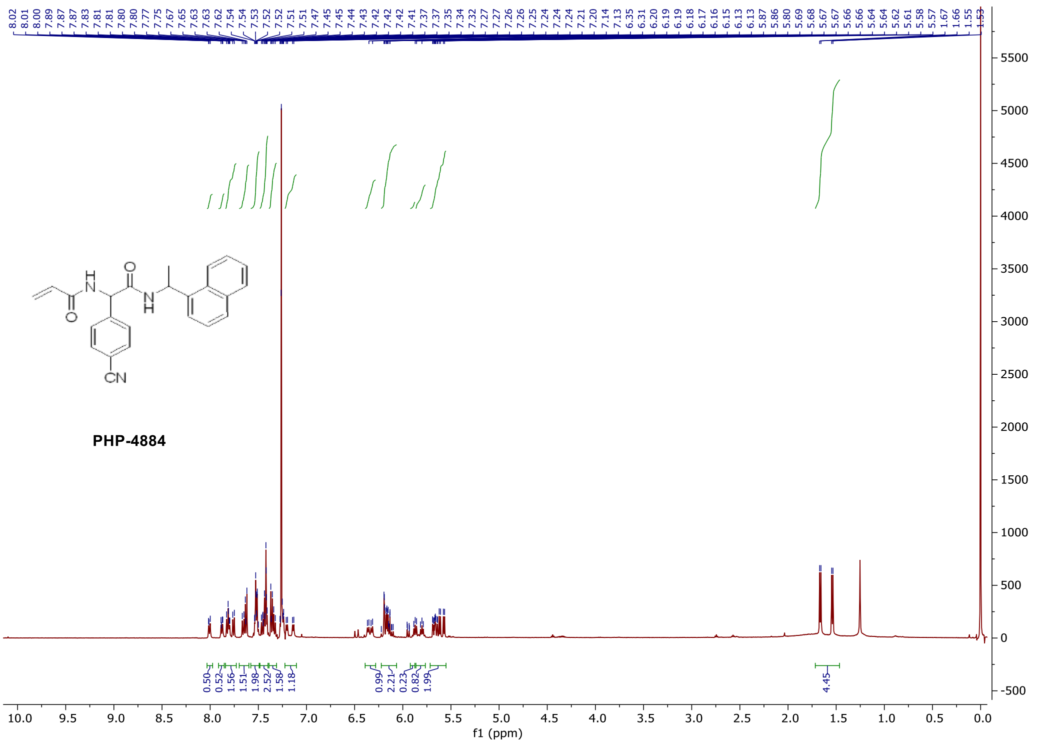

Supplementary Figure 441. ^{1}H NMR (500 MHz, $CDCl_3$) of compound **PHP-4884**.

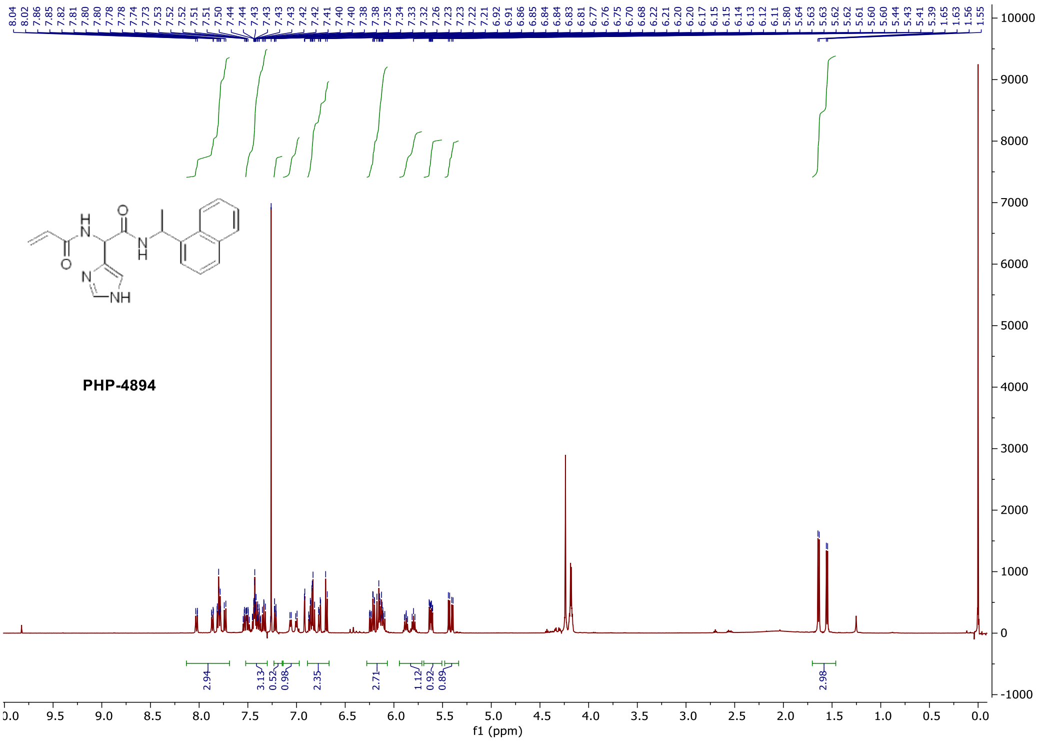

Supplementary Figure 442. ^{1}H NMR (500 MHz, $CDCl_3$) of compound **PHP-4894**.

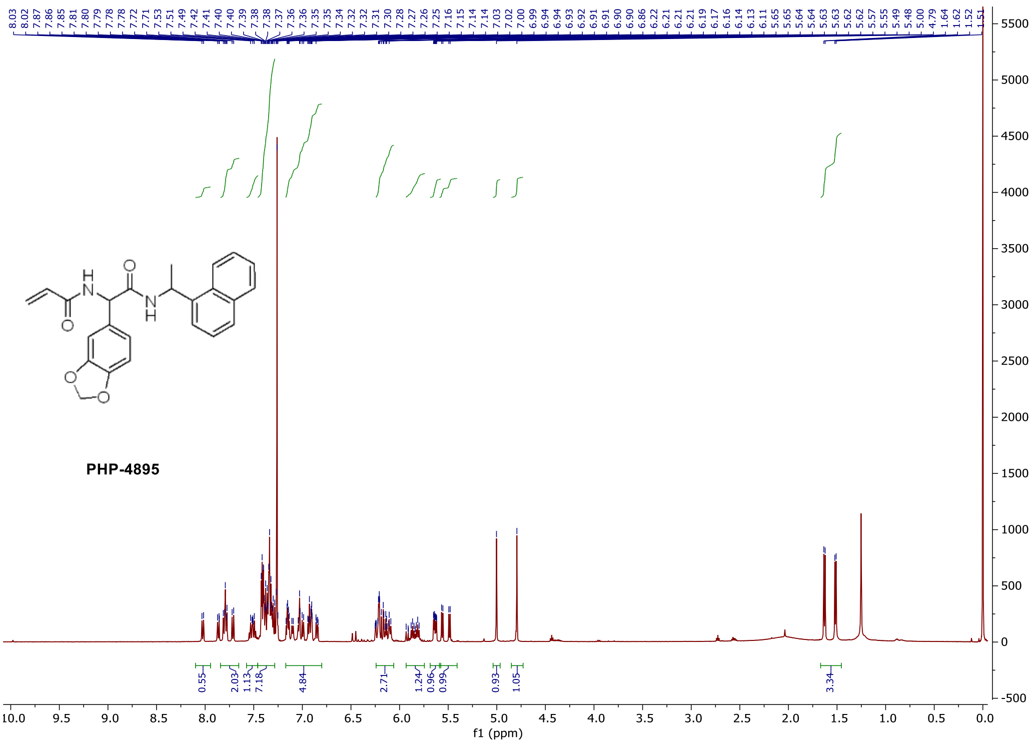

Supplementary Figure 443. ^{1}H NMR (500 MHz, $CDCl_3$) of compound **PHP-4895**.

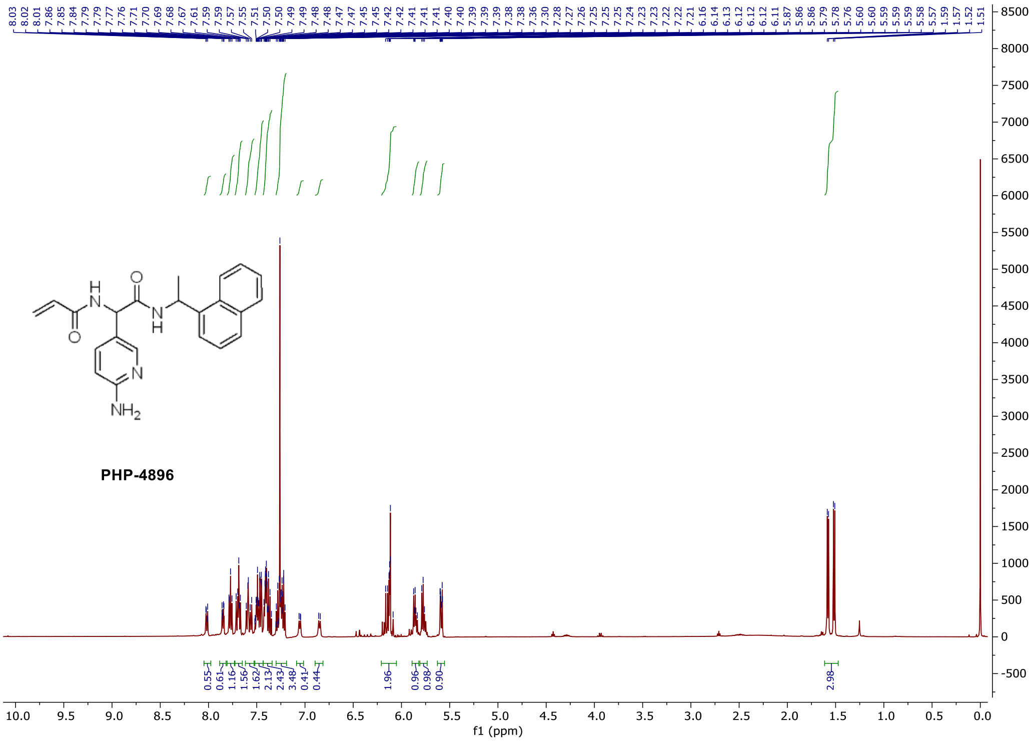

Supplementary Figure 444. ^{1}H NMR (500 MHz, $CDCl_3$) of compound **PHP-4896**.

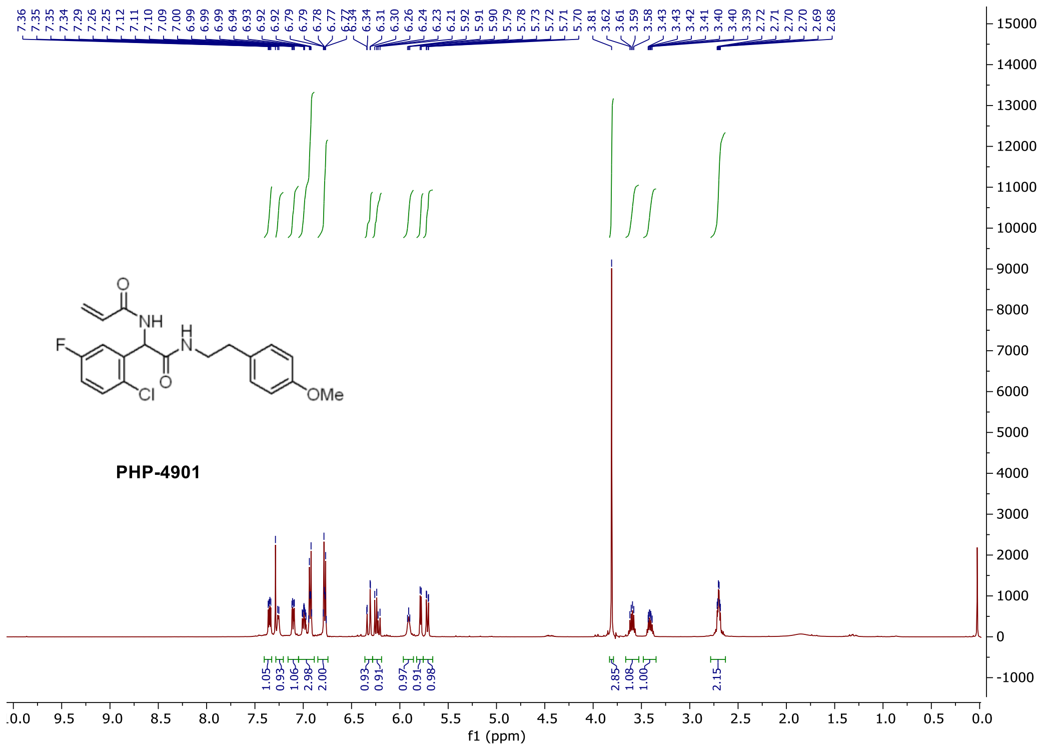

Supplementary Figure 445. ^{1}H NMR (500 MHz, $CDCl_3$) of compound **PHP-4901**.

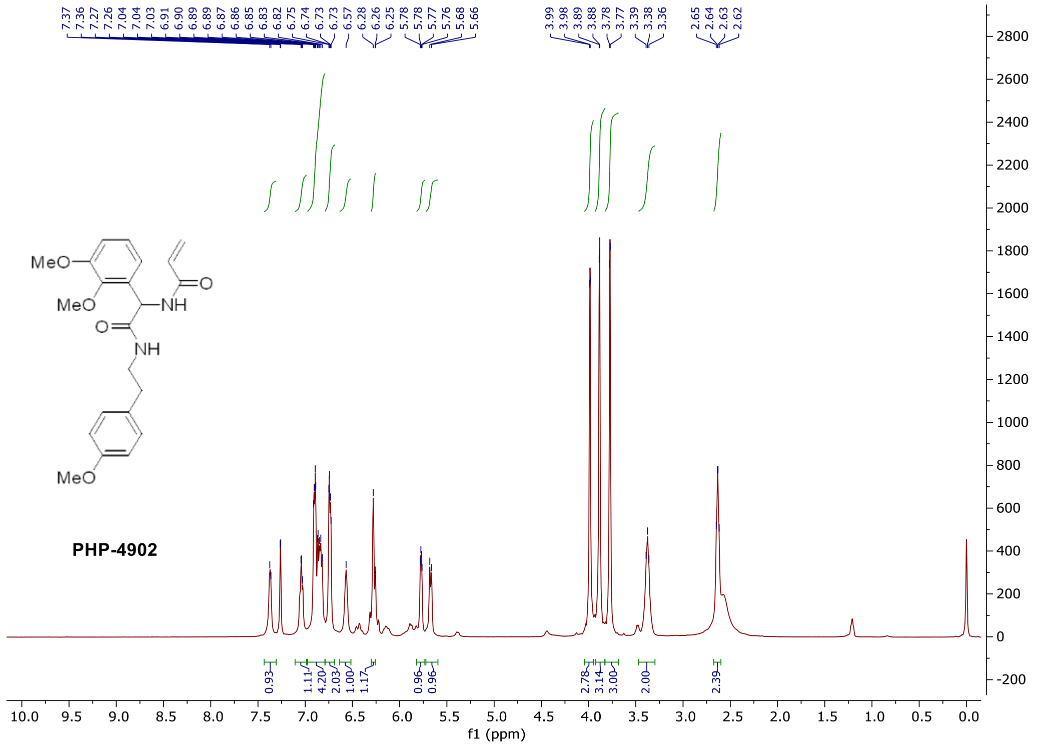

Supplementary Figure 446. ^{1}H NMR (500 MHz, $CDCl_3$) of compound **PHP-4902**.

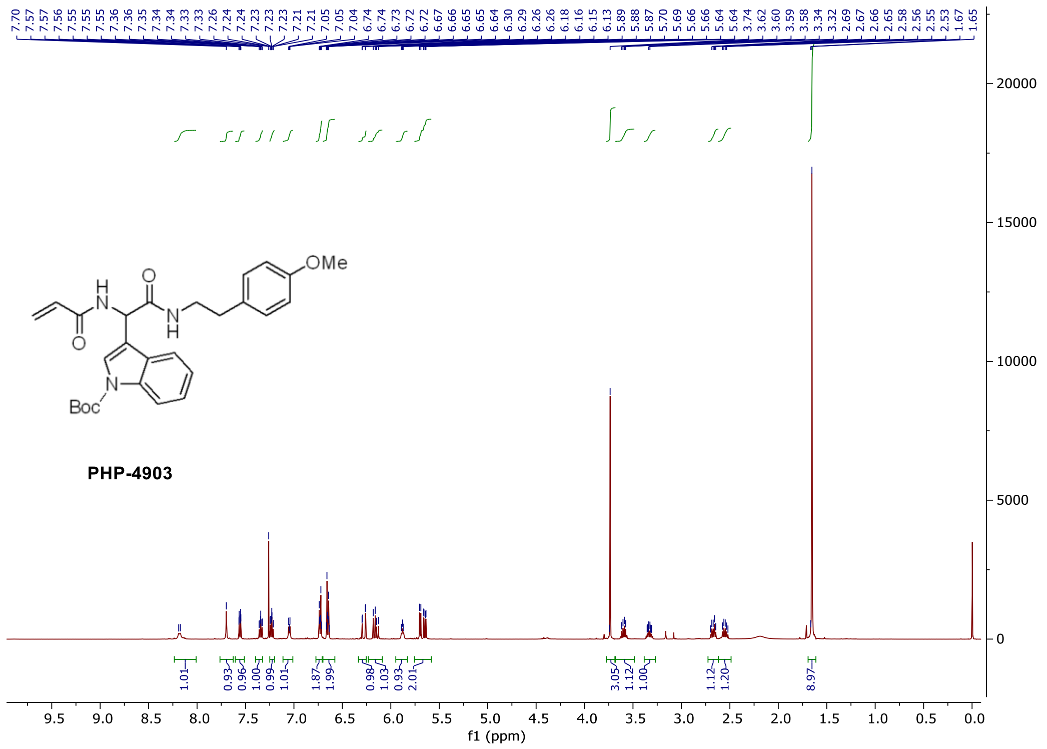

Supplementary Figure 447. ¹H NMR (500 MHz, CDCl₃) of compound **PHP-4903**.

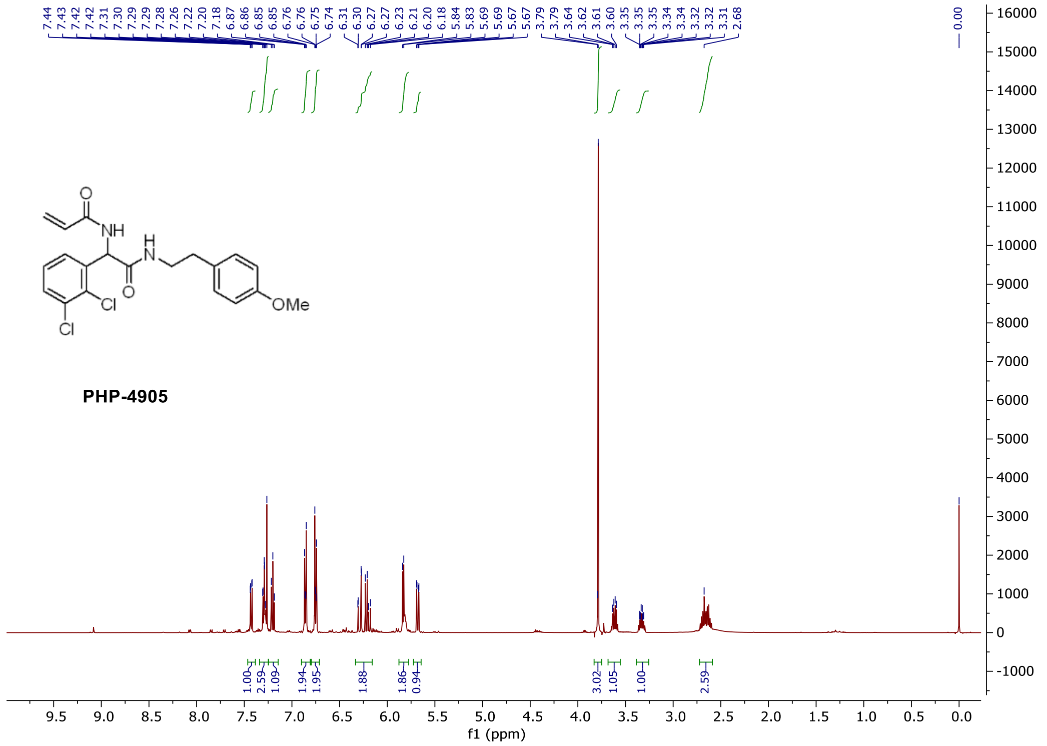

Supplementary Figure 448. ¹H NMR (500 MHz, CDCl₃) of compound **PHP-4905**.

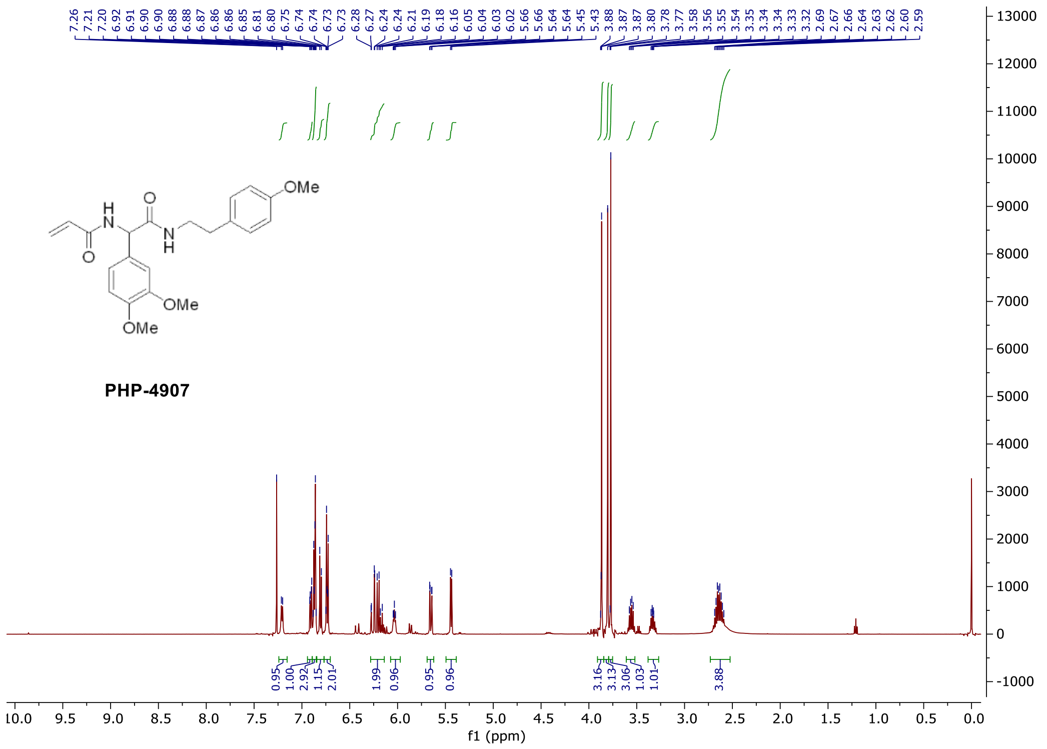

Supplementary Figure 449. ^{1}H NMR (500 MHz, $CDCl_3$) of compound **PHP-4907**.

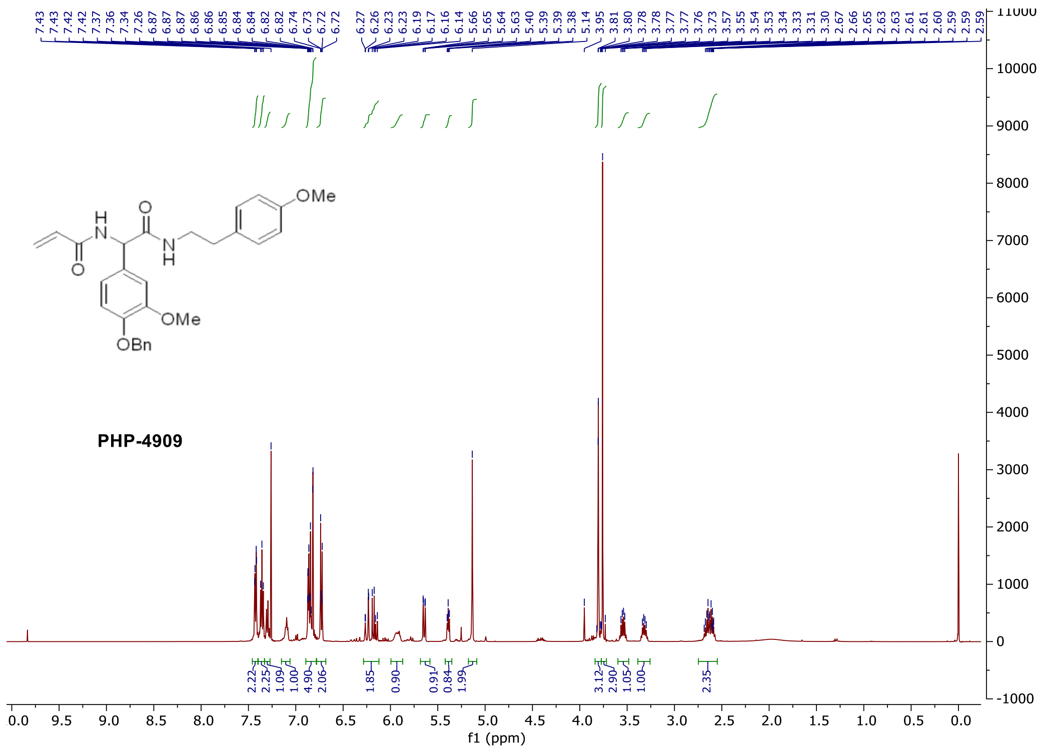

Supplementary Figure 450. ^{1}H NMR (500 MHz, $CDCl_3$) of compound **PHP-4909**.

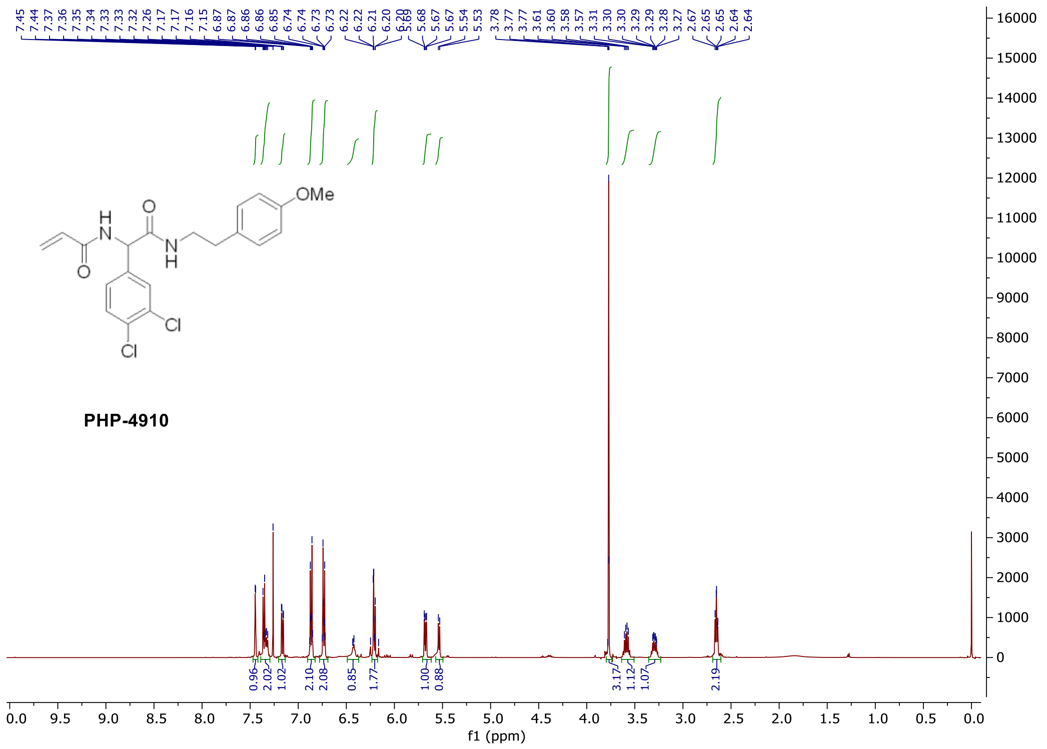

Supplementary Figure 451. ^{1}H NMR (500 MHz, $CDCl_3$) of compound **PHP-4910**.

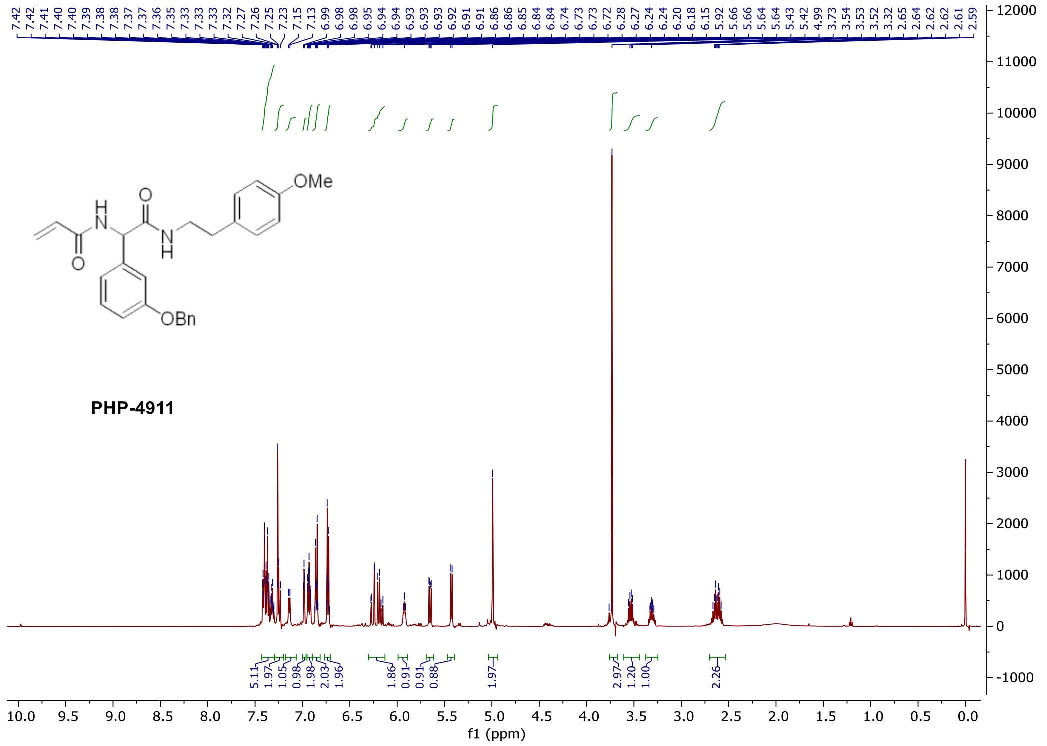

Supplementary Figure 452. ^{1}H NMR (500 MHz, $CDCl_3$) of compound **PHP-4911**.

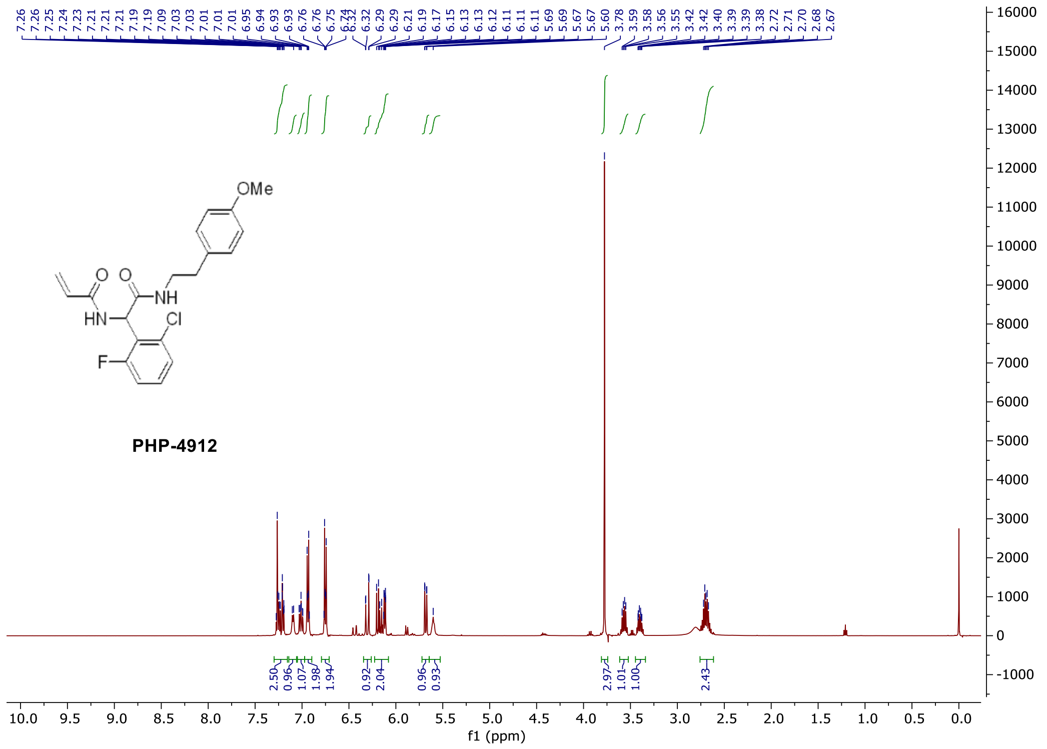

Supplementary Figure 453. ^{1}H NMR (500 MHz, $CDCl_3$) of compound **PHP-4912**.

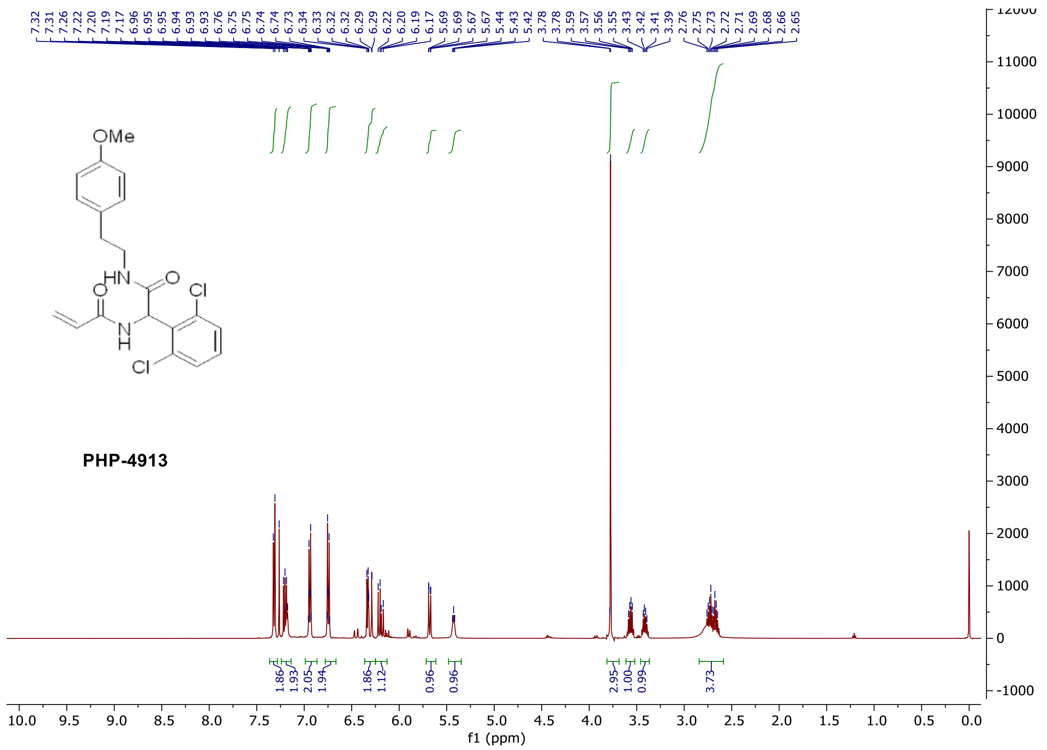

Supplementary Figure 454. ^{1}H NMR (500 MHz, $CDCl_3$) of compound **PHP-4913**.

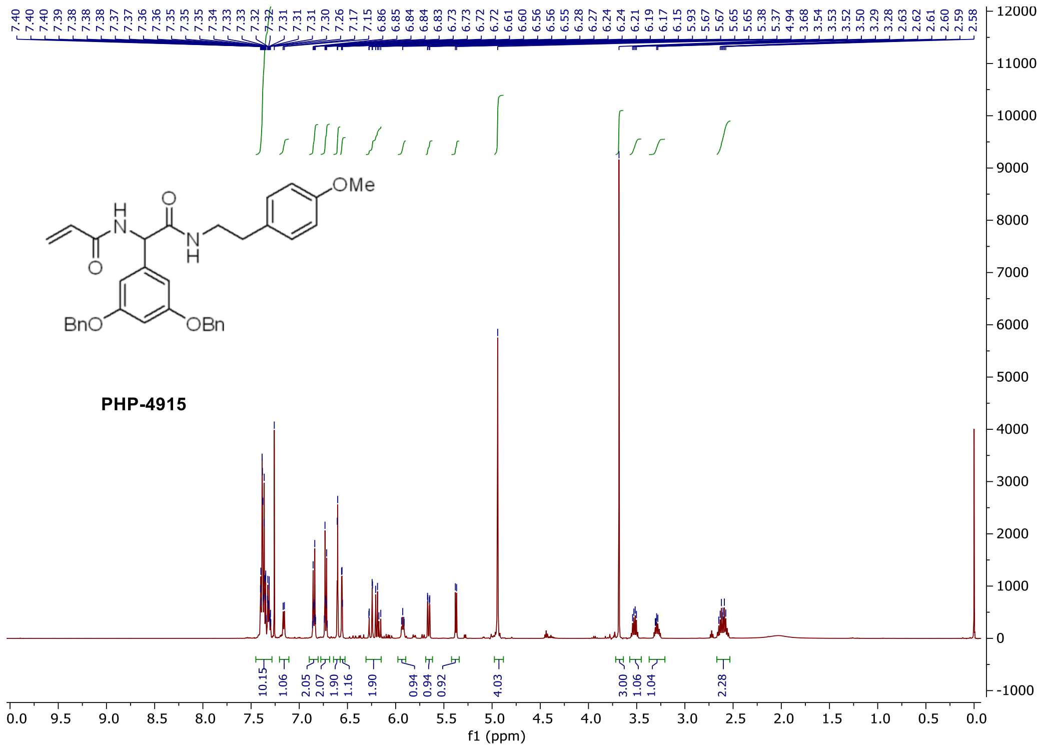

Supplementary Figure 455. ^{1}H NMR (500 MHz, $CDCl_3$) of compound **PHP-4915**.

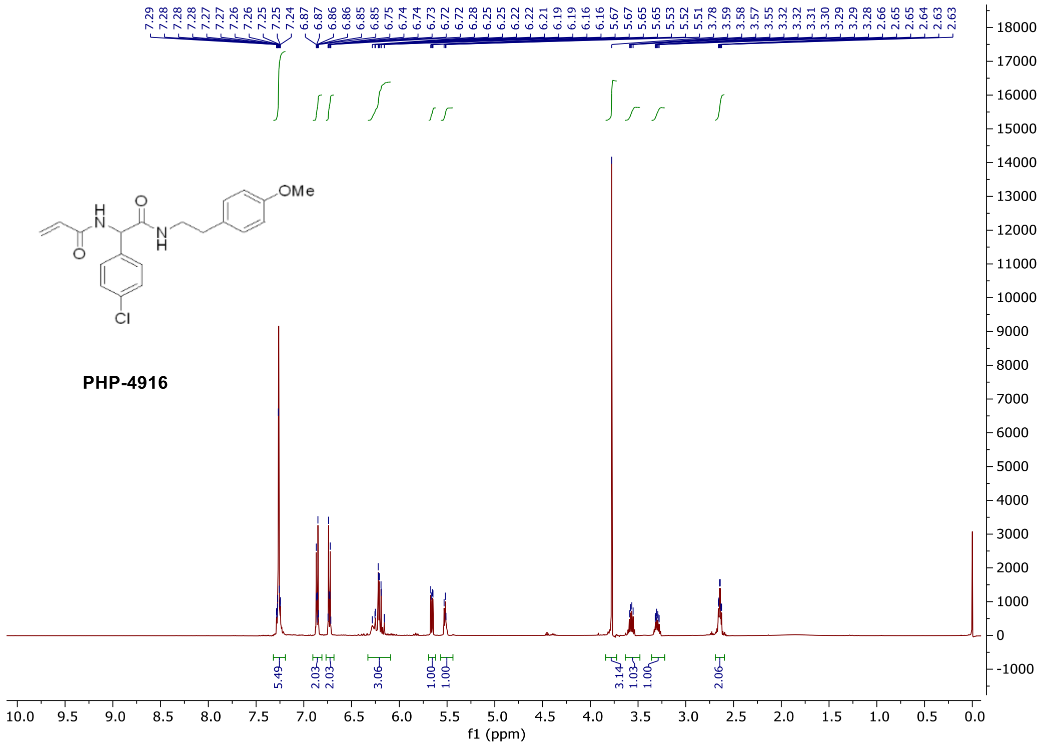

Supplementary Figure 456. ^{1}H NMR (500 MHz, $CDCl_3$) of compound **PHP-4916**.

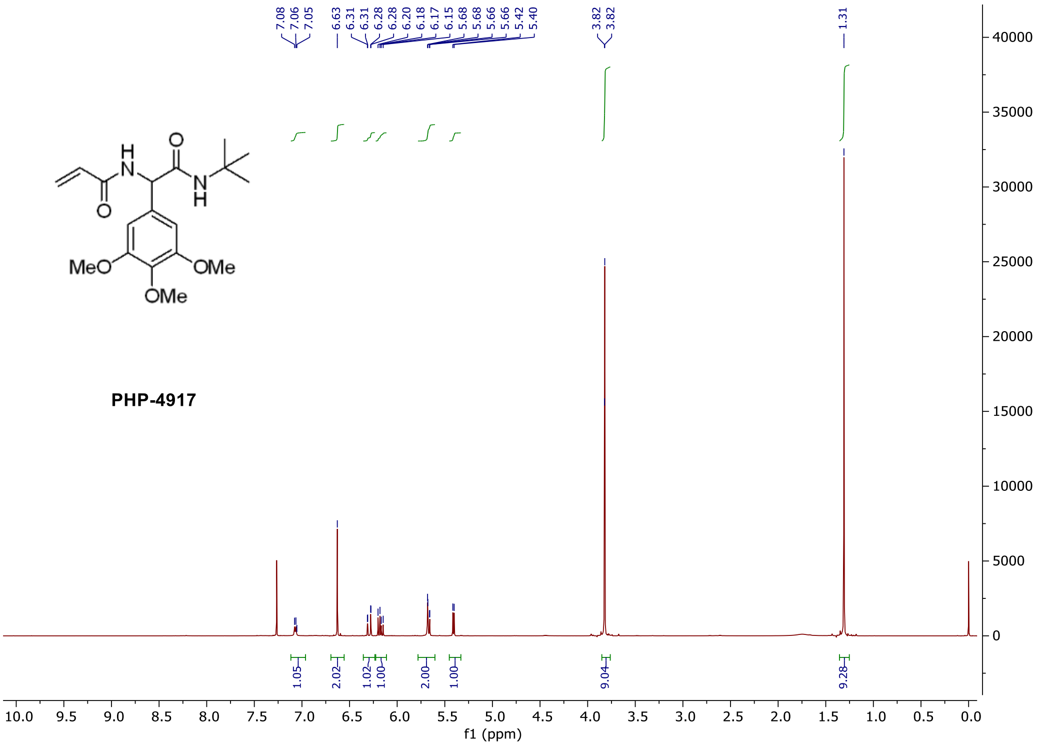

Supplementary Figure 457. ^{1}H NMR (500 MHz, CDCl3) of compound PHP-4917.

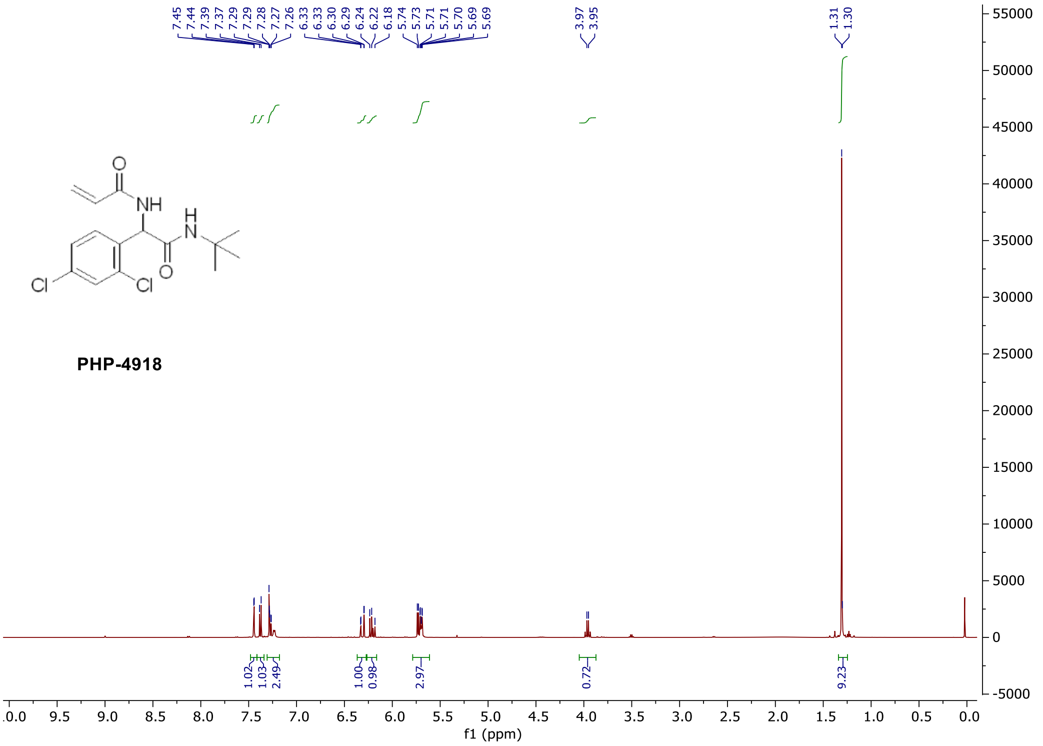

Supplementary Figure 458. ^{1}H NMR (500 MHz, CDCl3) of compound PHP-4918.

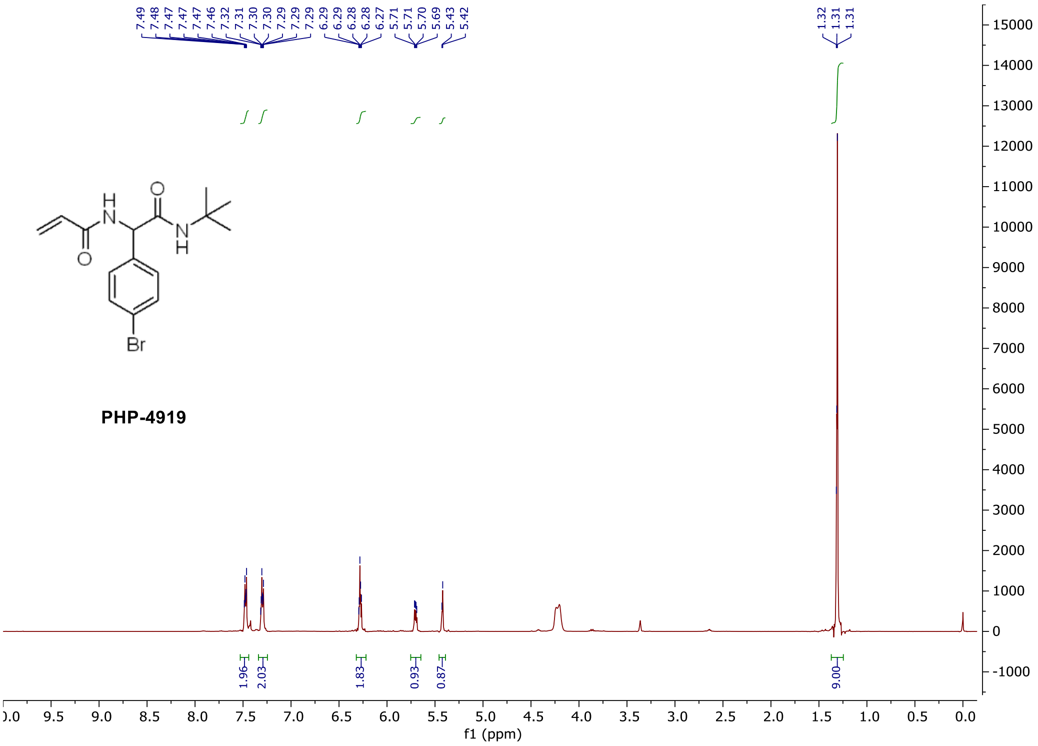

Supplementary Figure 459. ^{1}H NMR (500 MHz, $CDCl_3$ + one drop of CD_3OD) of compound **PHP-4919**.

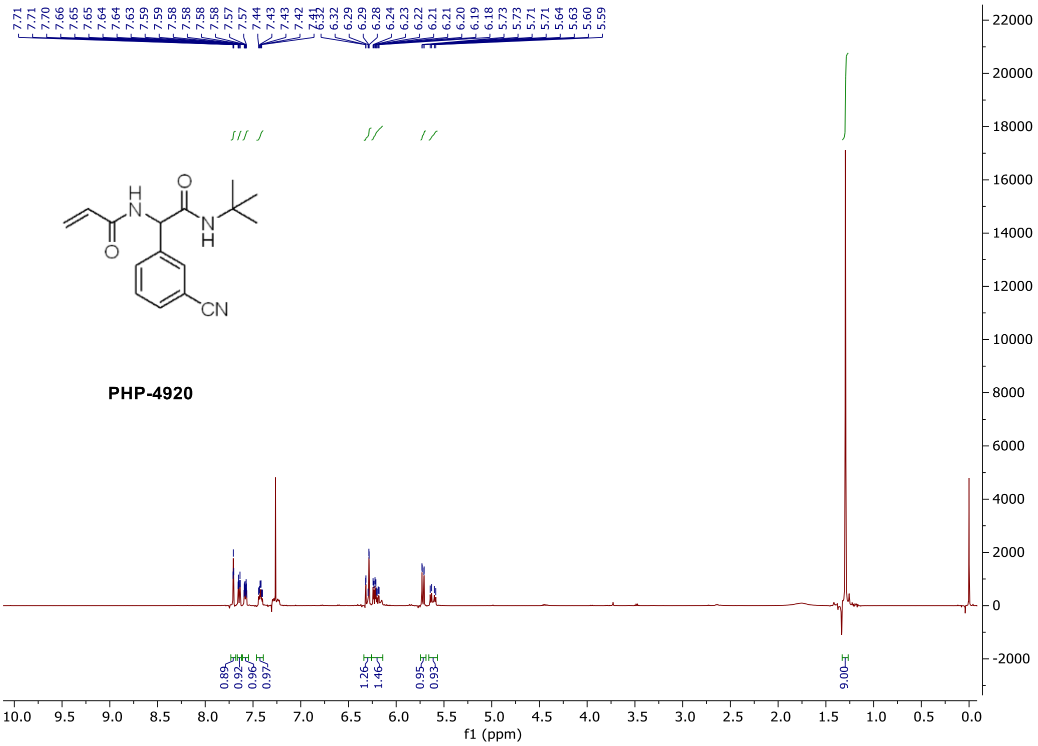

Supplementary Figure 460. ^{1}H NMR (500 MHz, $CDCl_3$) of compound **PHP-4920**.

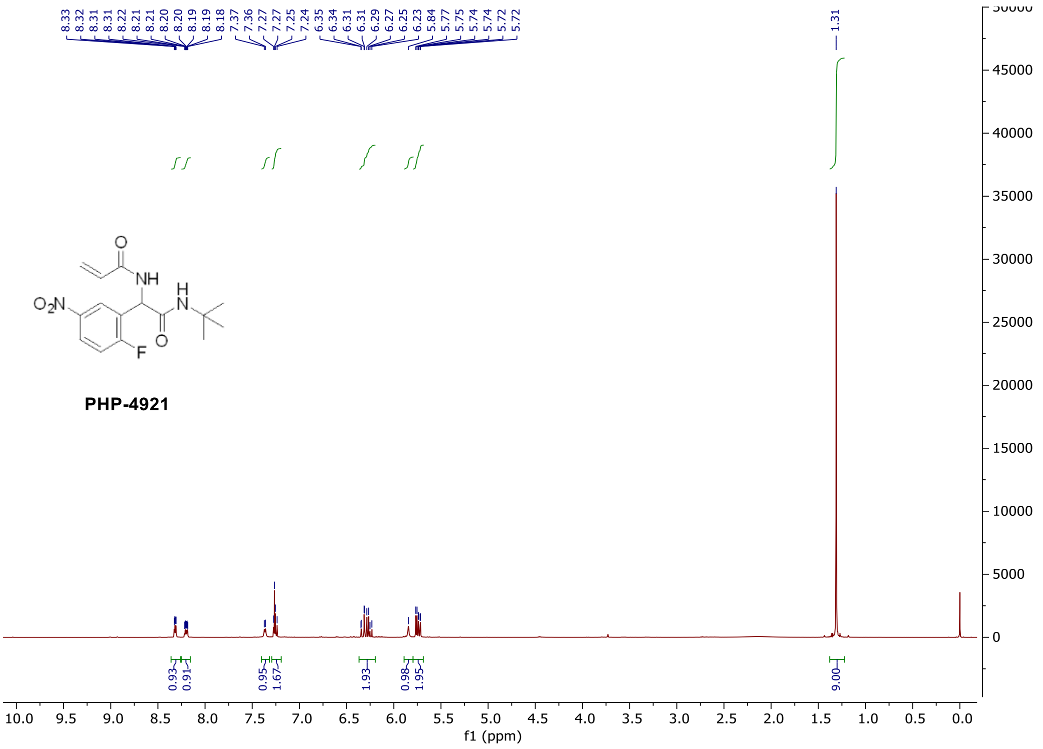

Supplementary Figure 461. ^{1}H NMR (500 MHz, $CDCl_3$) of compound **PHP-4921**.

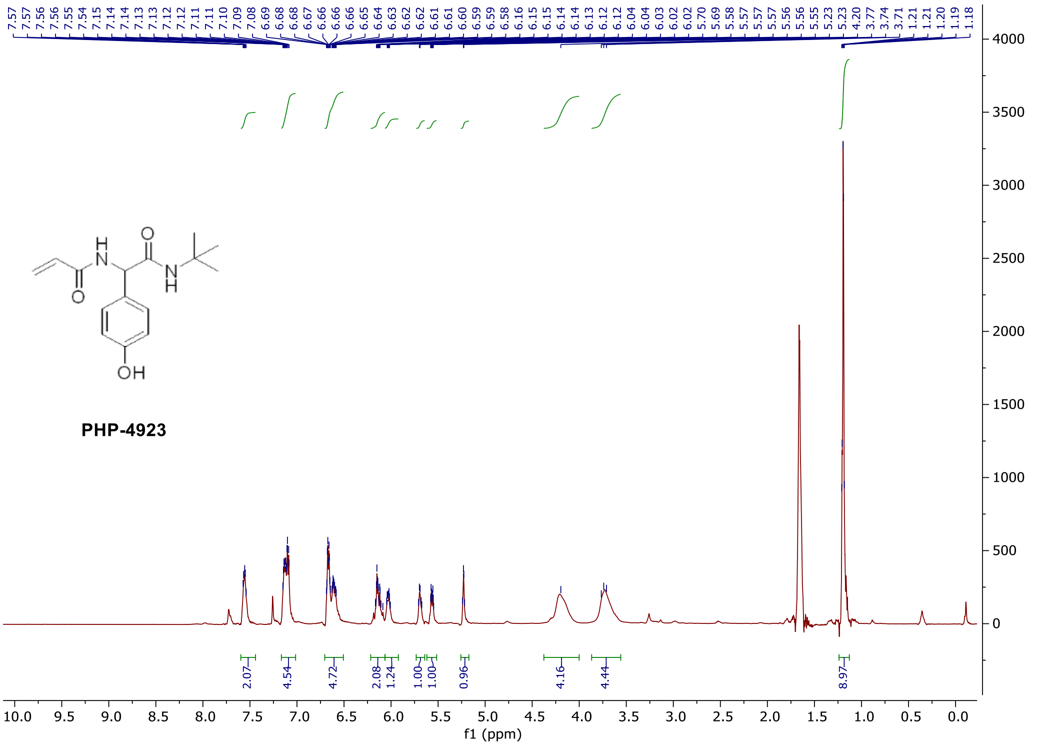

Supplementary Figure 462. ^{1}H NMR (500 MHz, $CDCl_3$ + one drop of CD_3OD) of compound **PHP-4923**.

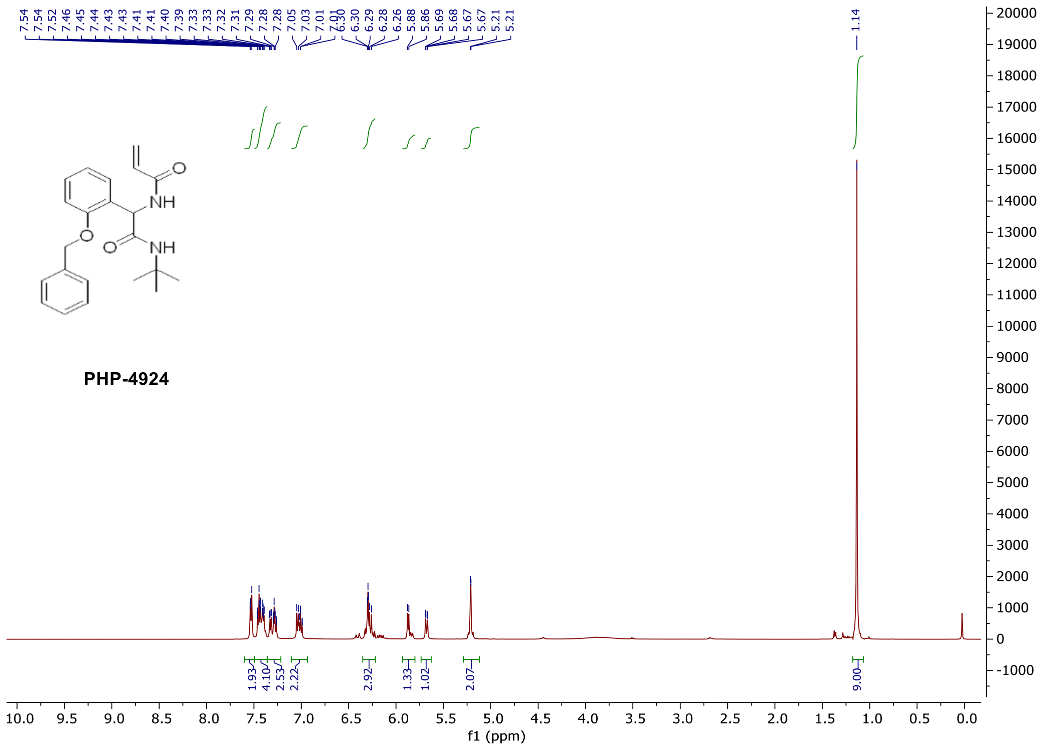

Supplementary Figure 463. ^{1}H NMR (500 MHz, $CDCl_3$) of compound **PHP-4924**.

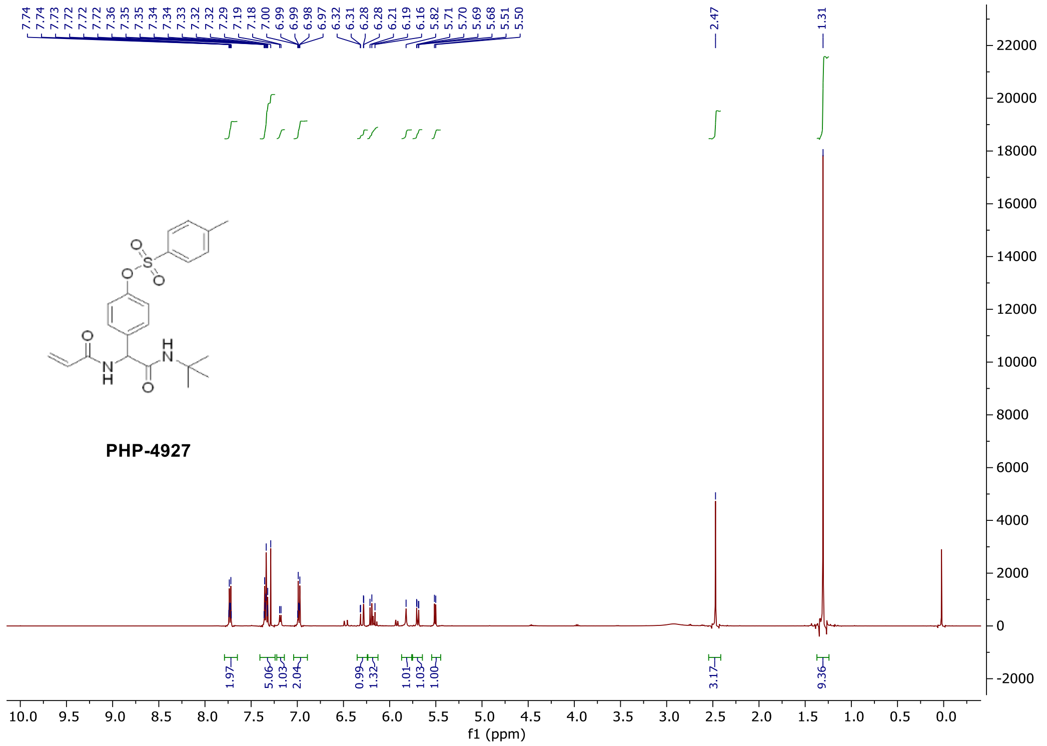

Supplementary Figure 464. ^{1}H NMR (500 MHz, $CDCl_3$ + one drop of CD_3OD) of compound **PHP-4927**.

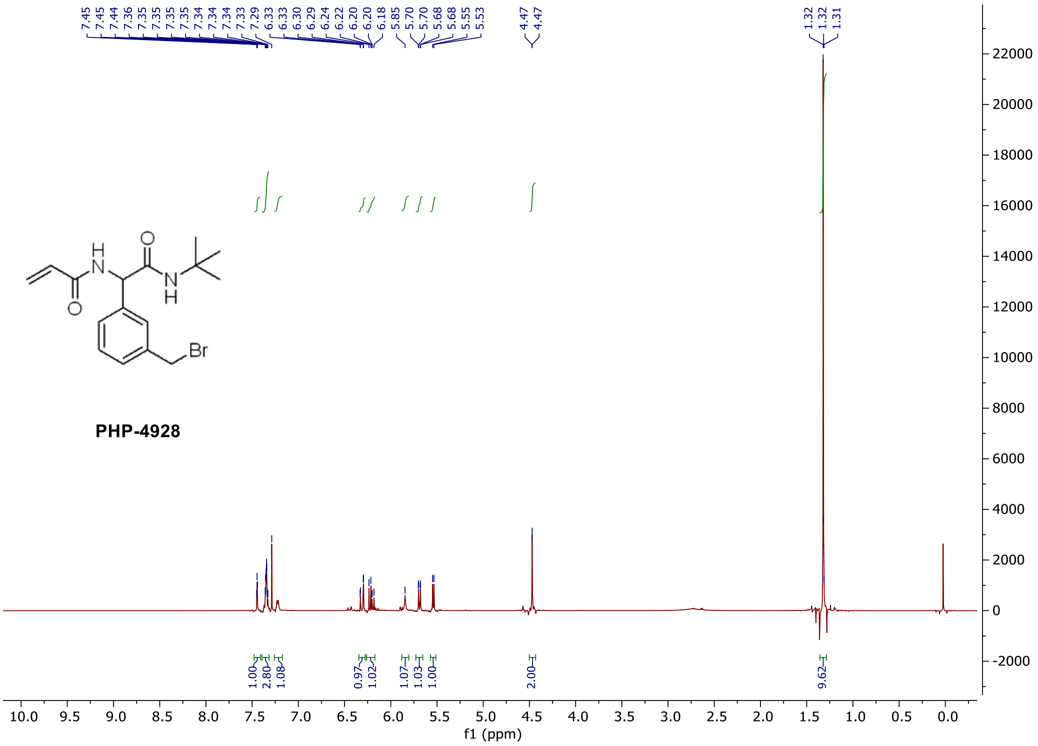

Supplementary Figure 465. ^{1}H NMR (500 MHz, $CDCl_3$ + one drop of CD_3OD) of compound **PHP-4928**.

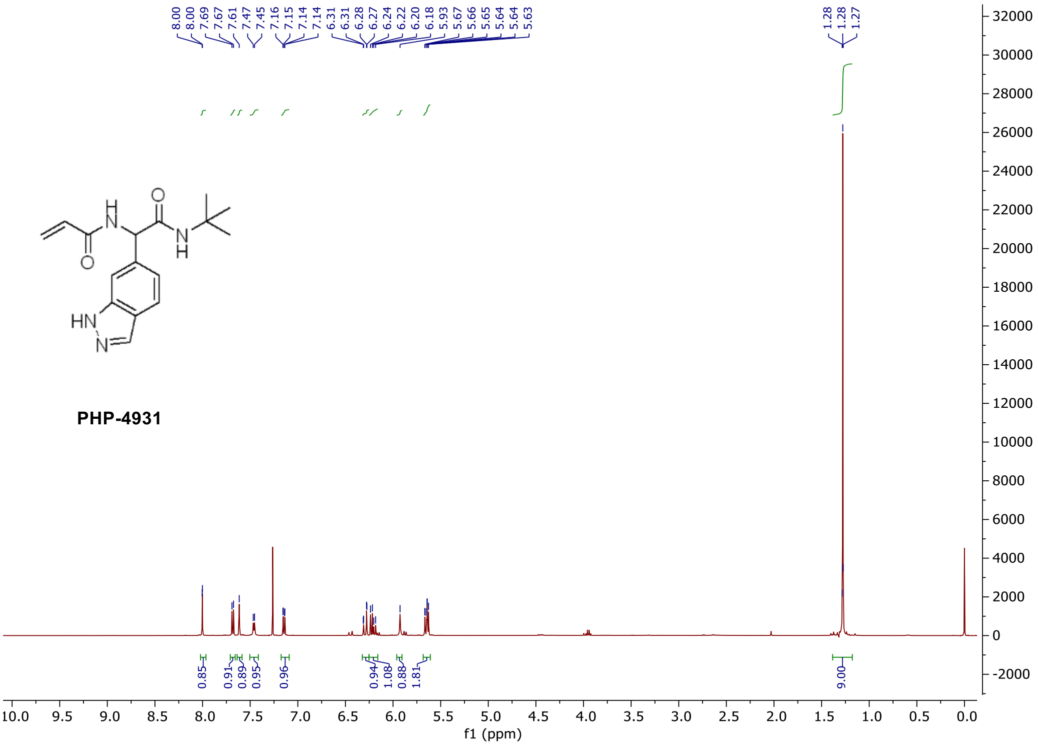

Supplementary Figure 466. ^{1}H NMR (500 MHz, $CDCl_3$) of compound **PHP-4931**.

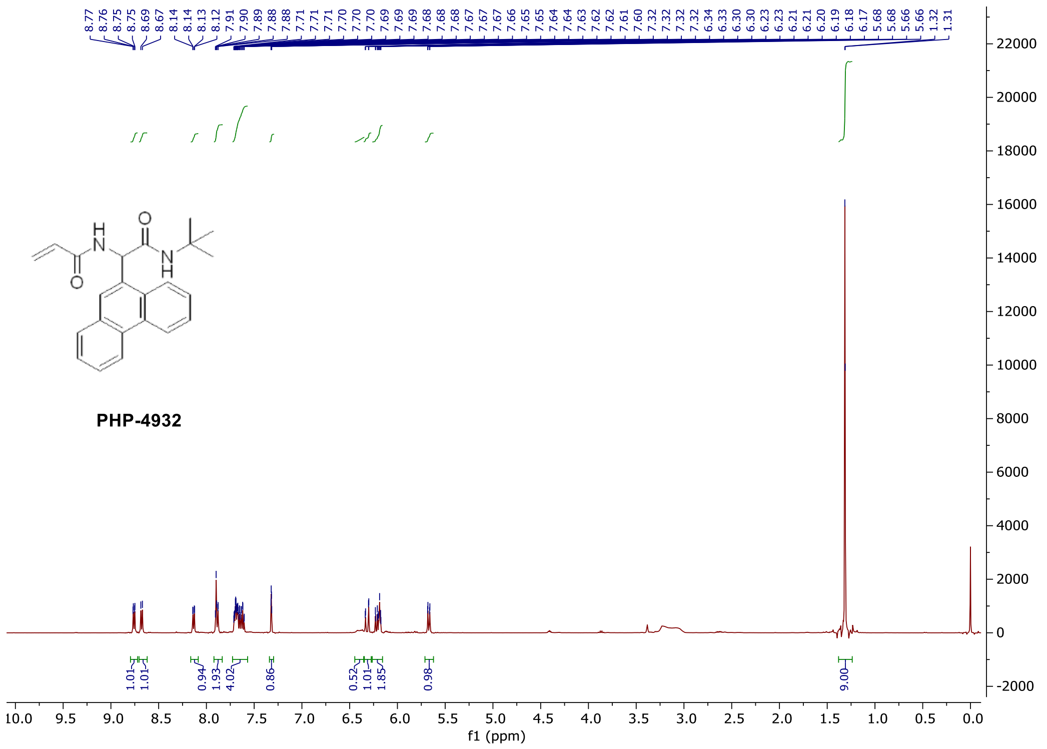

Supplementary Figure 467. ^{1}H NMR (500 MHz, $CDCl_3$ + one drop of CD_3OD) of compound **PHP-4932**.

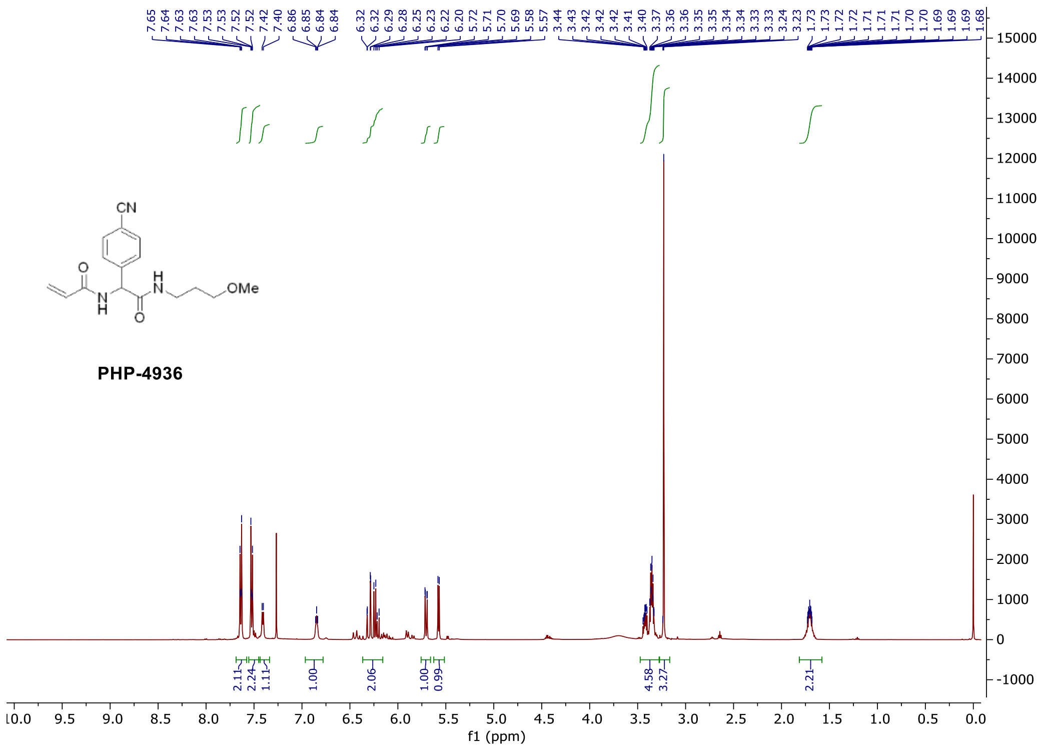

Supplementary Figure 468. ^{1}H NMR (500 MHz, $CDCl_3$) of compound **PHP-4936**.

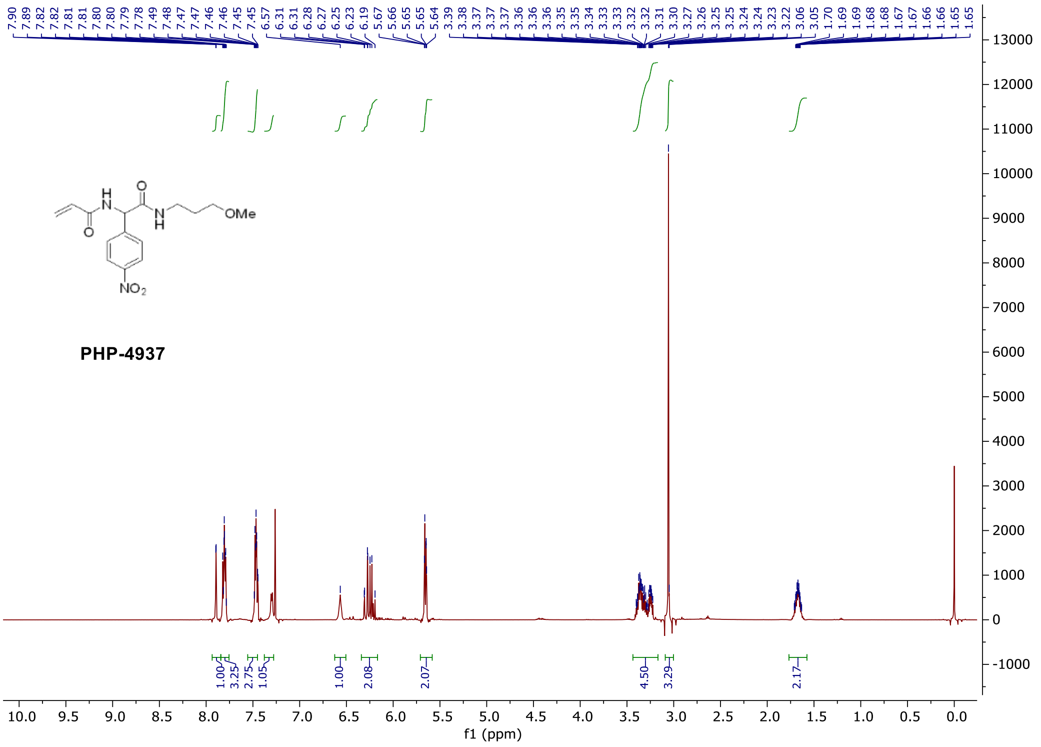

Supplementary Figure 469. ^{1}H NMR (500 MHz, $CDCl_3$) of compound **PHP-4937**.

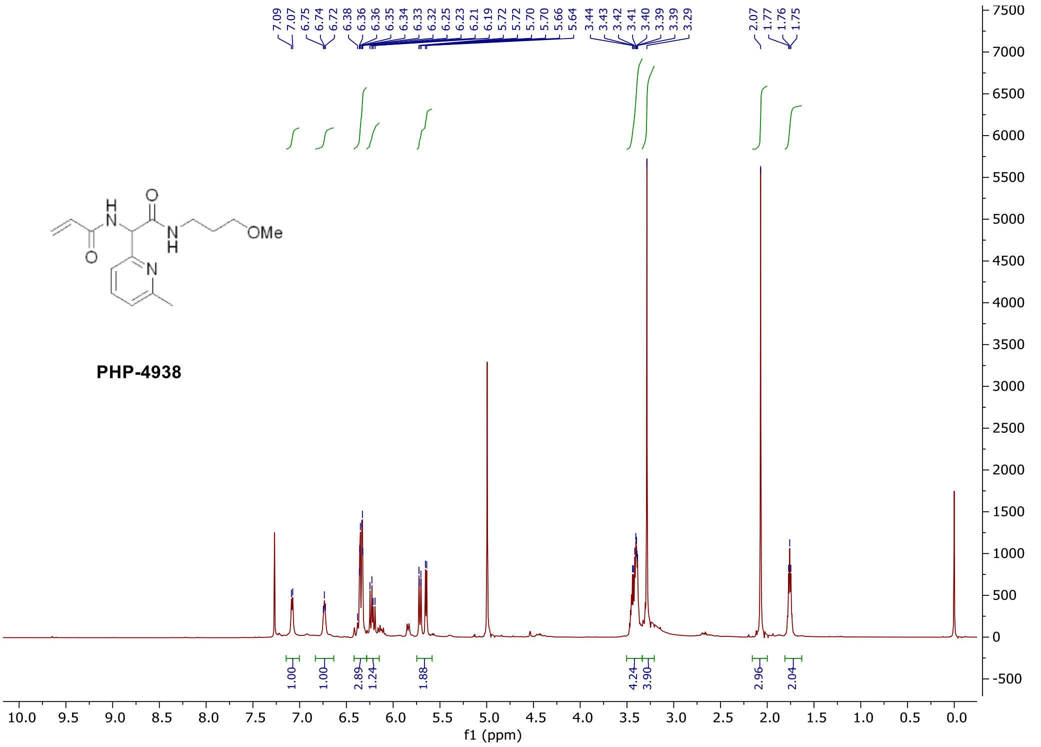

Supplementary Figure 470. ^{1}H NMR (500 MHz, $CDCl_3$ + one drop of CD_3OD) of compound **PHP-4938**.

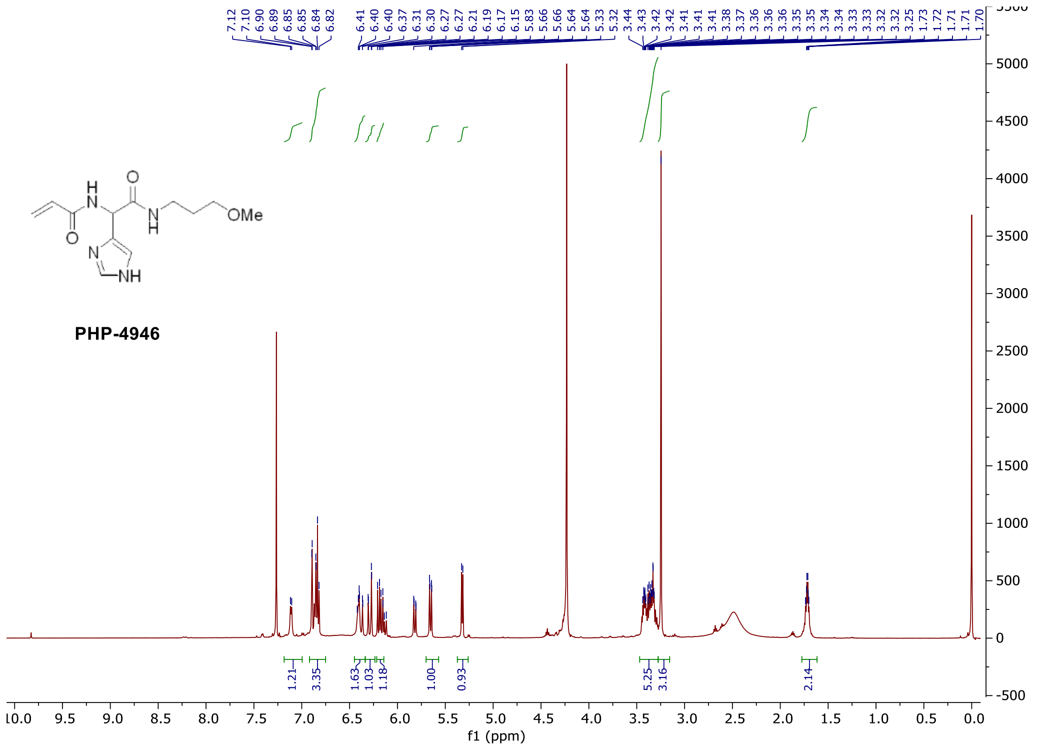

Supplementary Figure 471. ^{1}H NMR (500 MHz, $CDCl_3$) of compound **PHP-4946**.

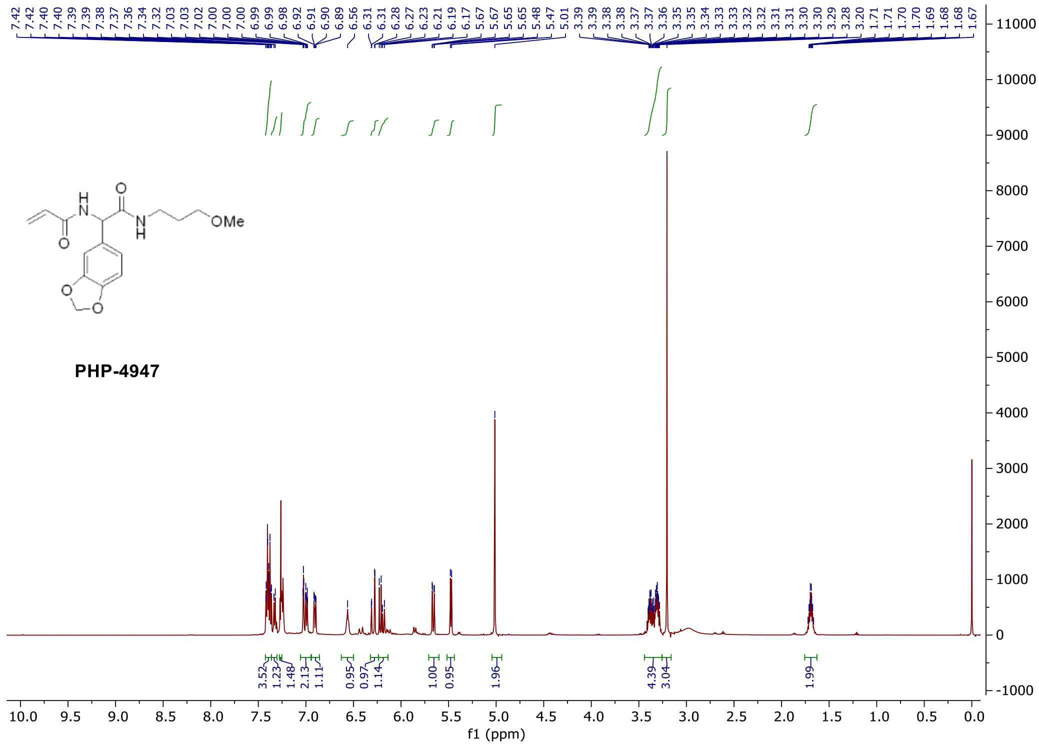

Supplementary Figure 472. ^{1}H NMR (500 MHz, $CDCl_3$ + one drop of CD_3OD) of compound **PHP-4947**.

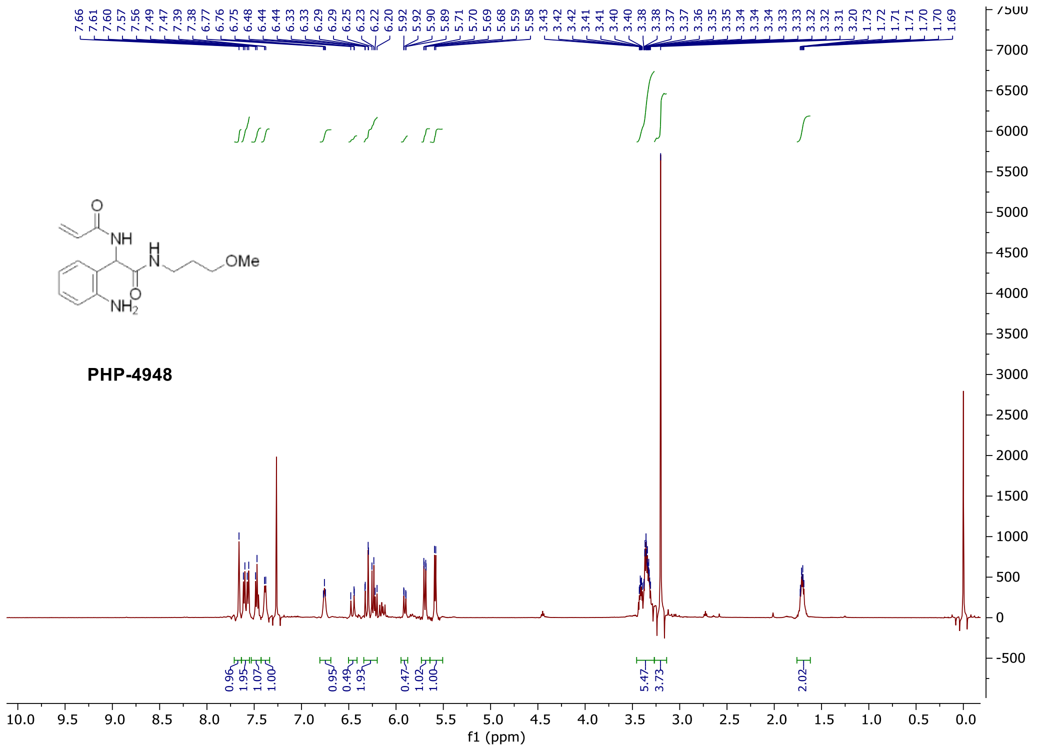

Supplementary Figure 473. ^{1}H NMR (500 MHz, $CDCl_3$) of compound **PHP-4948**.

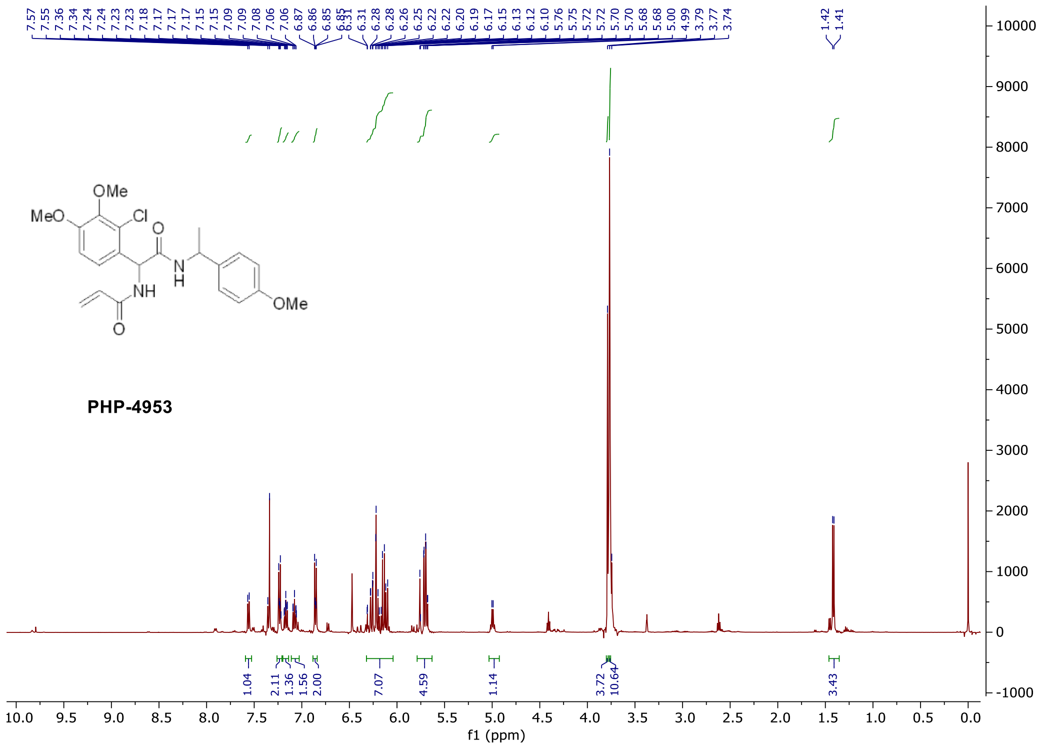

Supplementary Figure 474. ^{1}H NMR (500 MHz, $CDCl_3$) of compound **PHP-4953**.

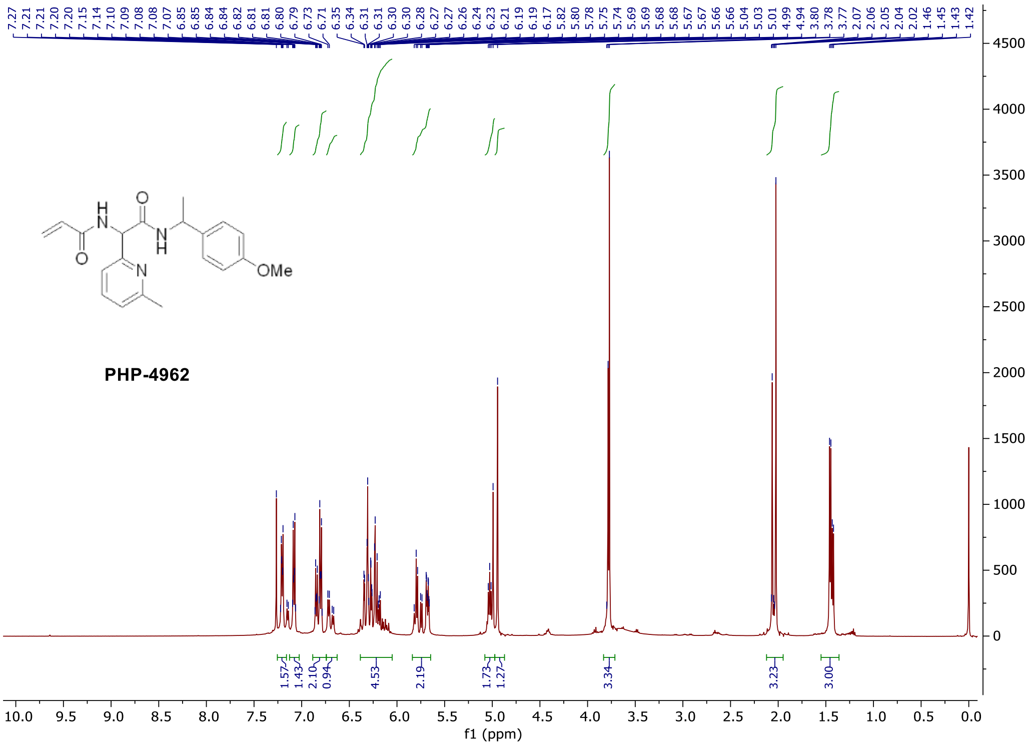

Supplementary Figure 475. ^{1}H NMR (500 MHz, $CDCl_3$) of compound **PHP-4962**.

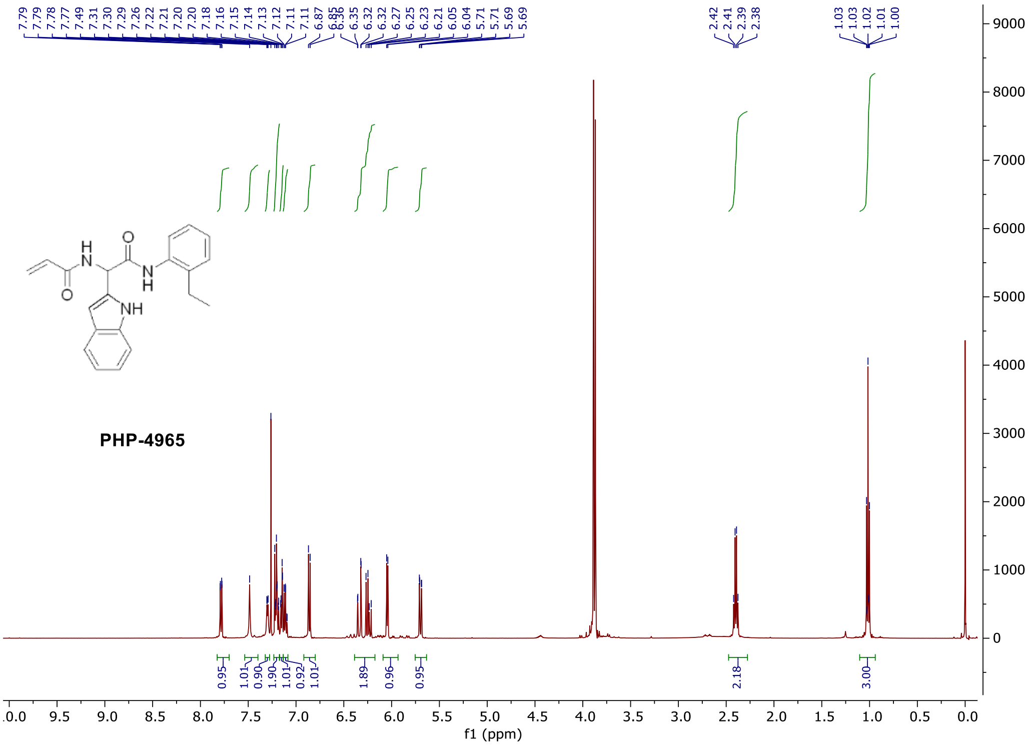

Supplementary Figure 476. ^{1}H NMR (500 MHz, $CDCl_3$) of compound **PHP-4965**.

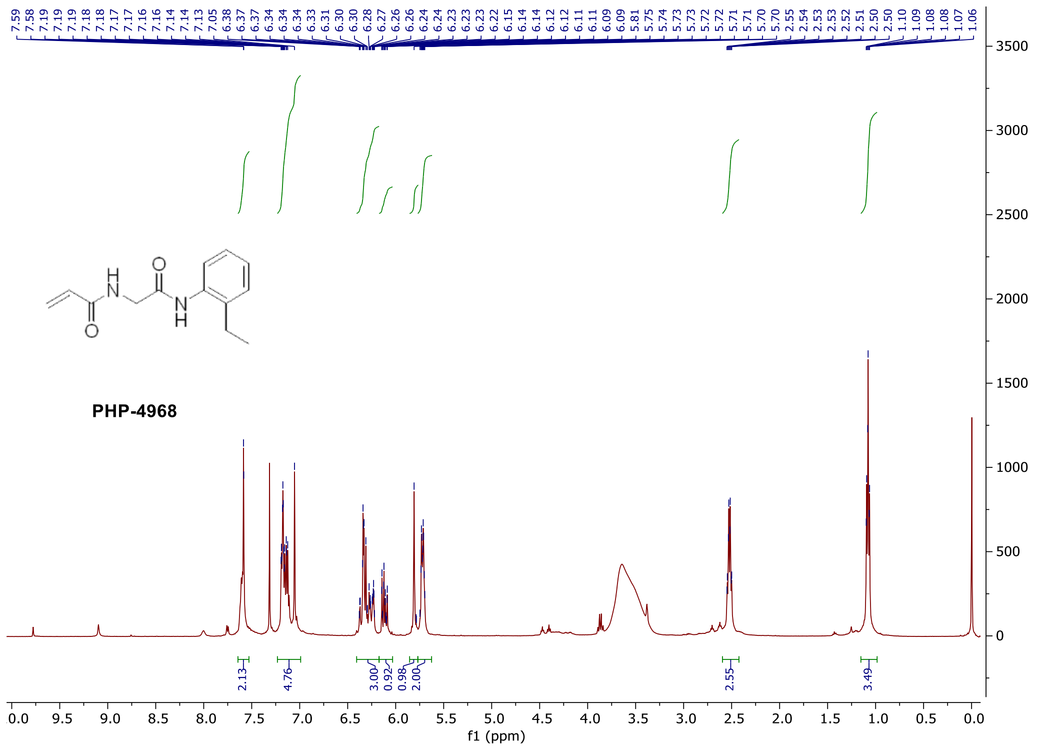

Supplementary Figure 477. ^{1}H NMR (500 MHz, $CDCl_3$ + one drop of CD_3OD) of compound **PHP-4968**.

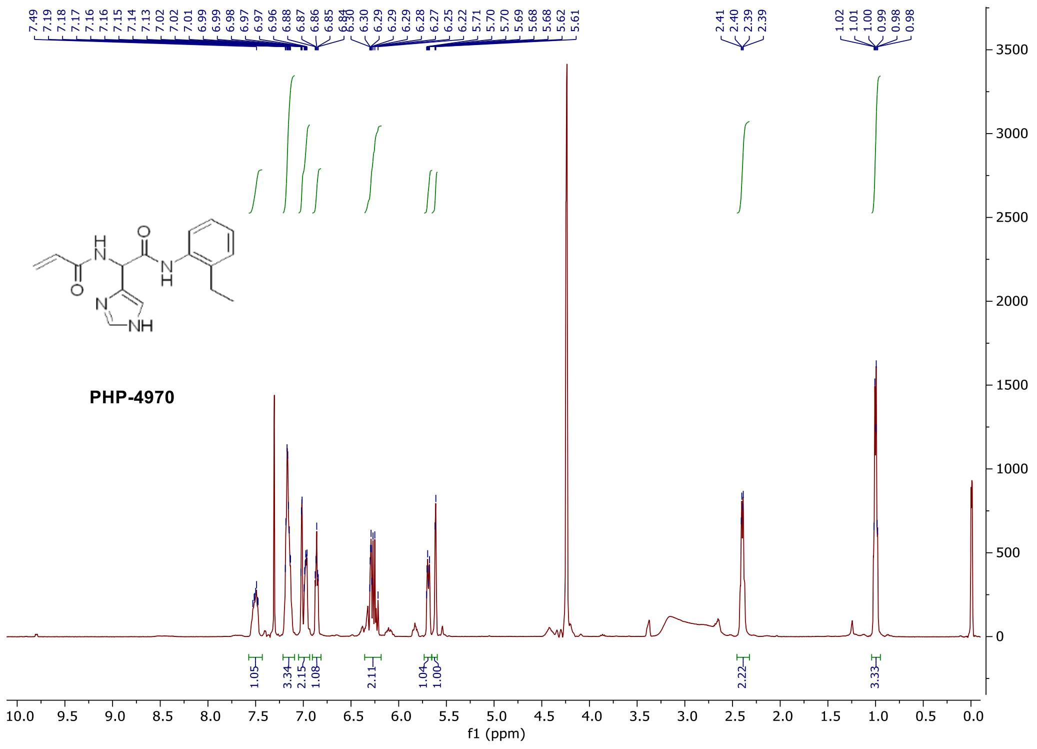

Supplementary Figure 478. ^{1}H NMR (500 MHz, $CDCl_3$ + one drop of CD_3OD) of compound **PHP-4970**.

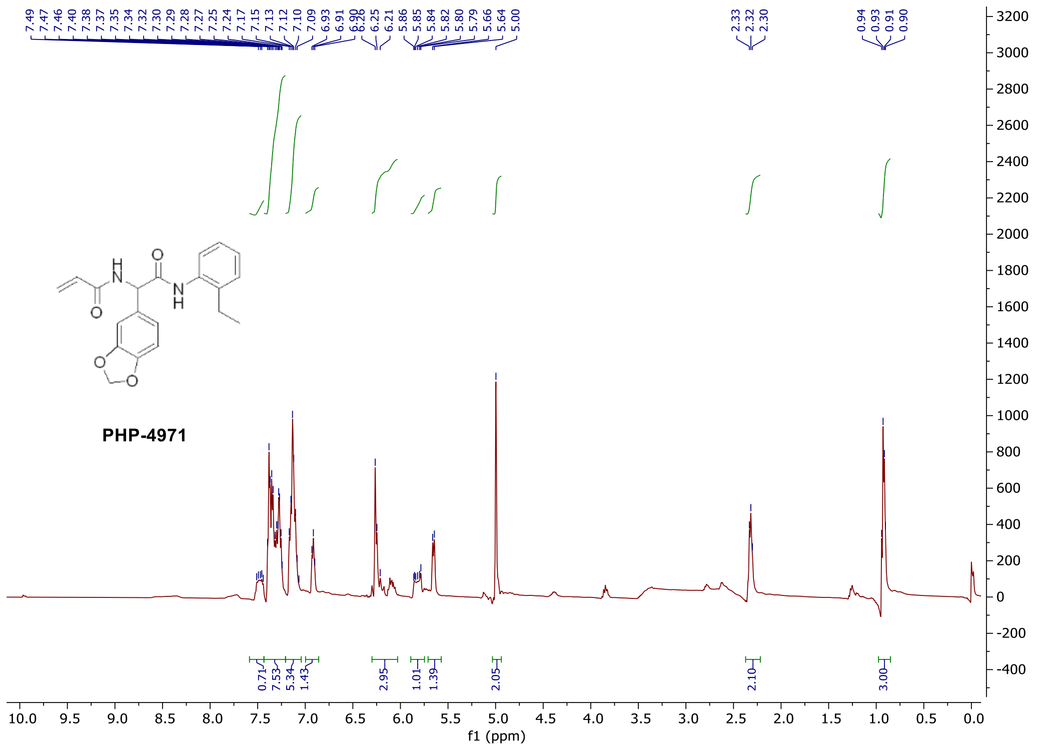

Supplementary Figure 479. ^{1}H NMR (500 MHz, $CDCl_3$ + one drop of CD_3OD) of compound **PHP-4971**.

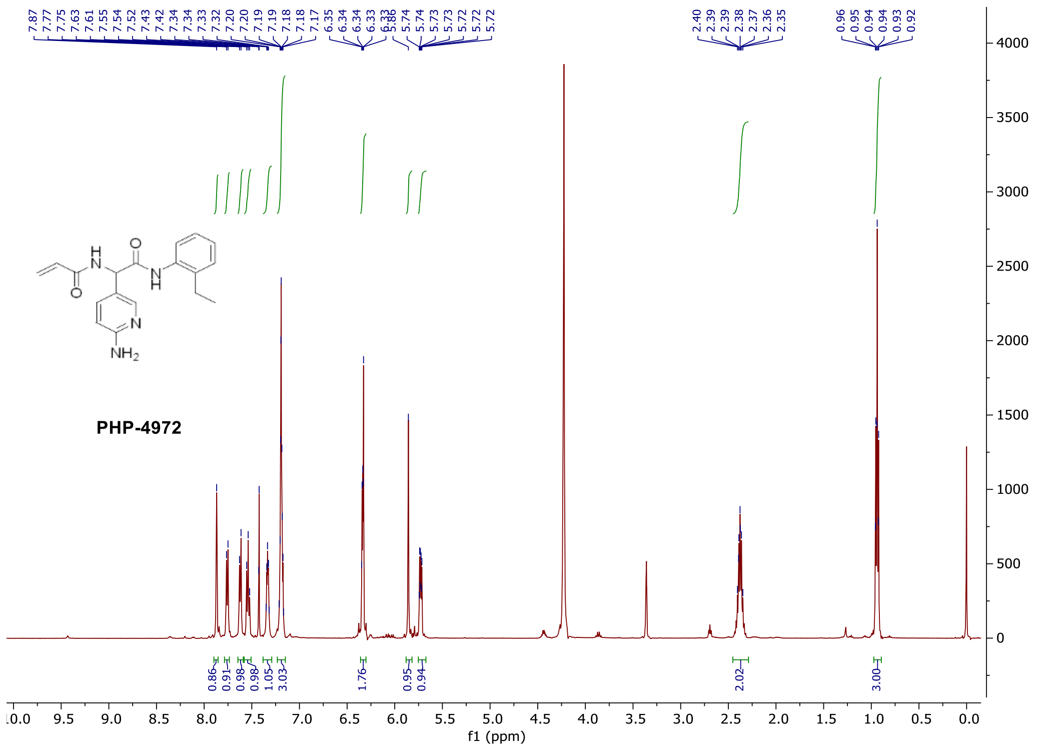

Supplementary Figure 480. ^{1}H NMR (500 MHz, $CDCl_3$) of compound **PHP-4972**.

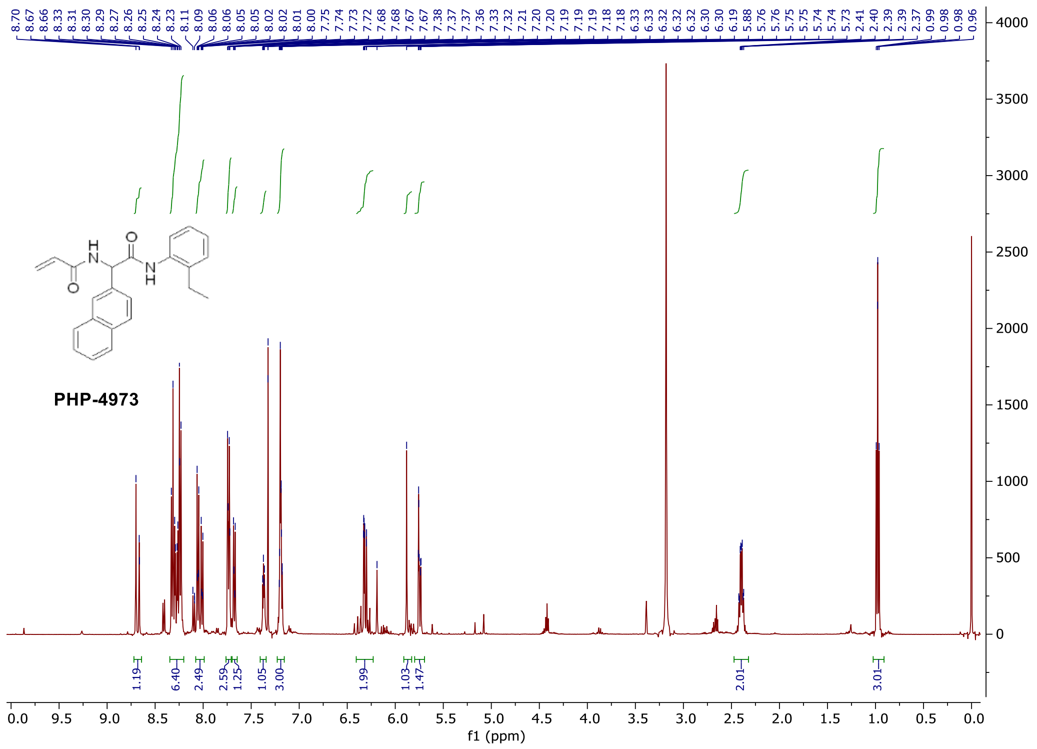

Supplementary Figure 481. ^{1}H NMR (500 MHz, $CDCl_3$ + one drop of CD_3OD) of compound **PHP-4973**.

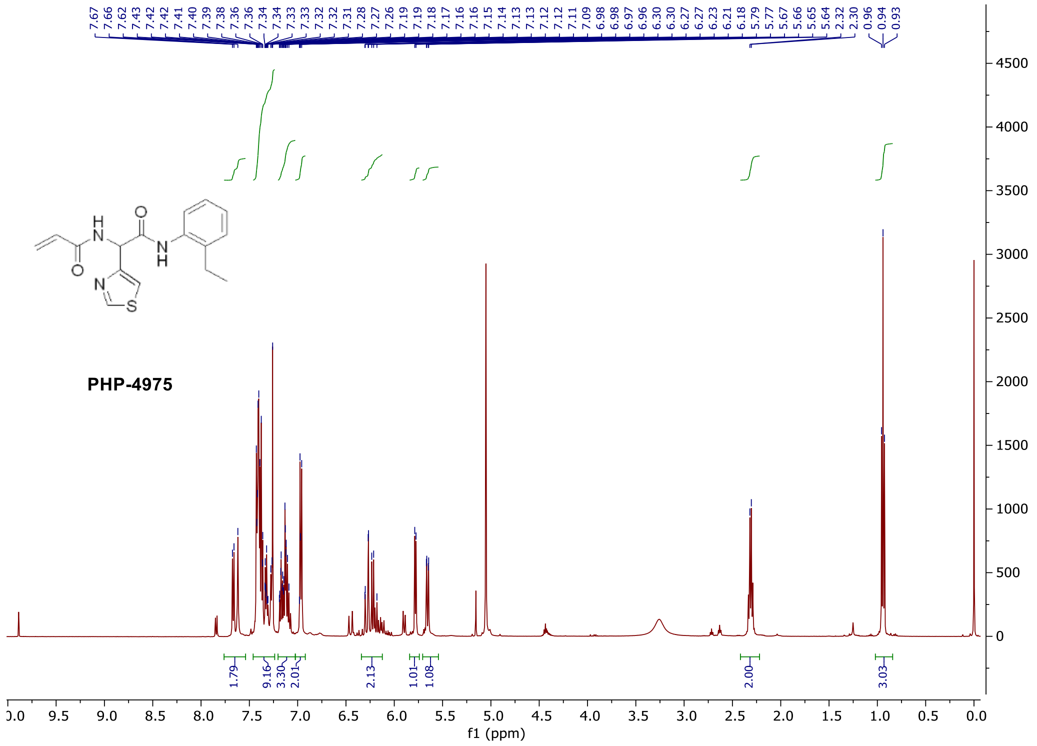

Supplementary Figure 482. ^{1}H NMR (500 MHz, $CDCl_3$ + one drop of CD_3OD) of compound **PHP-4975**.

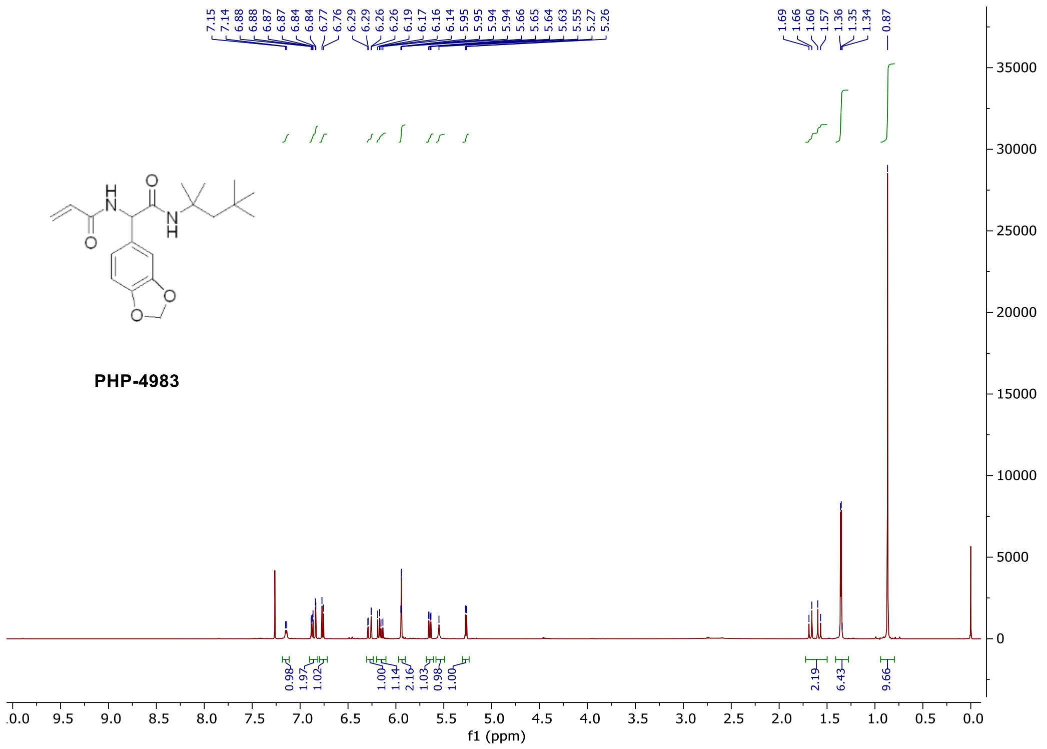

Supplementary Figure 483. ^{1}H NMR (500 MHz, $CDCl_3$) of compound **PHP-4983**.

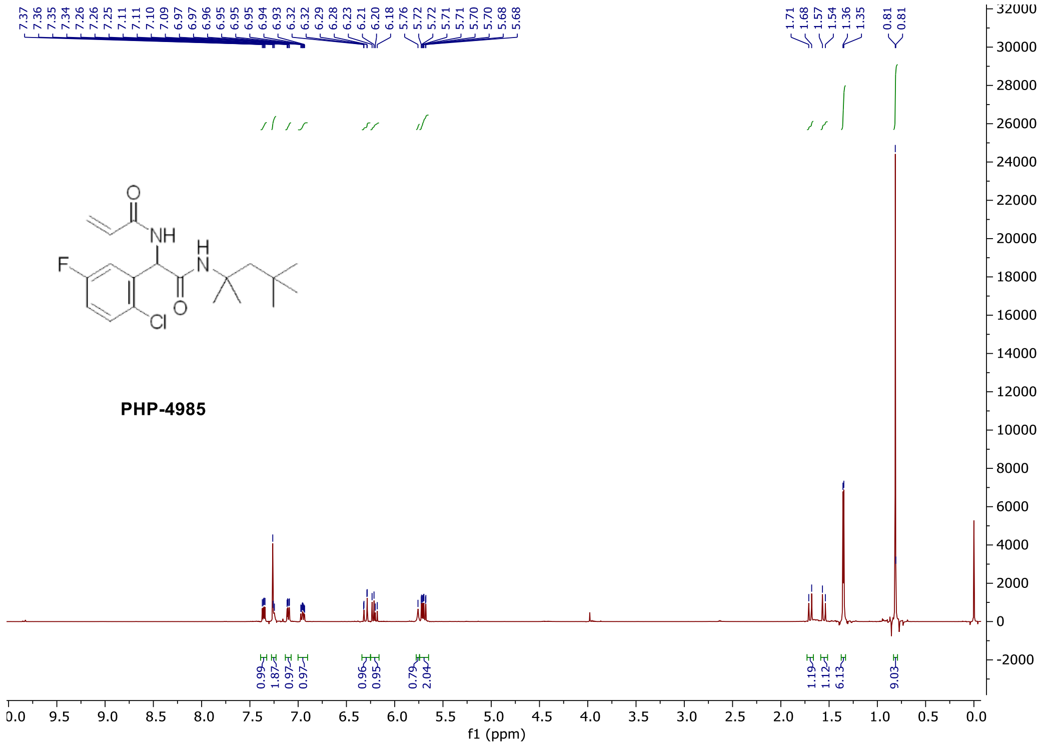

Supplementary Figure 484. ^{1}H NMR (500 MHz, $CDCl_3$) of compound **PHP-4985**.

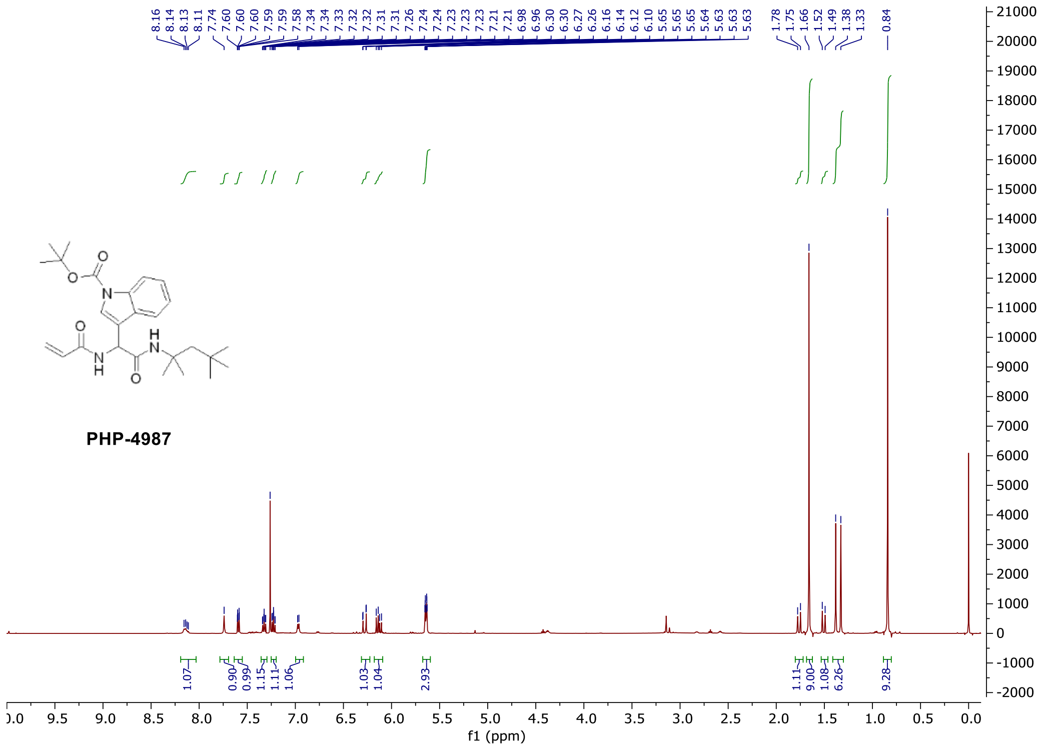

Supplementary Figure 485. ^{1}H NMR (500 MHz, $CDCl_3$) of compound **PHP-4987**.

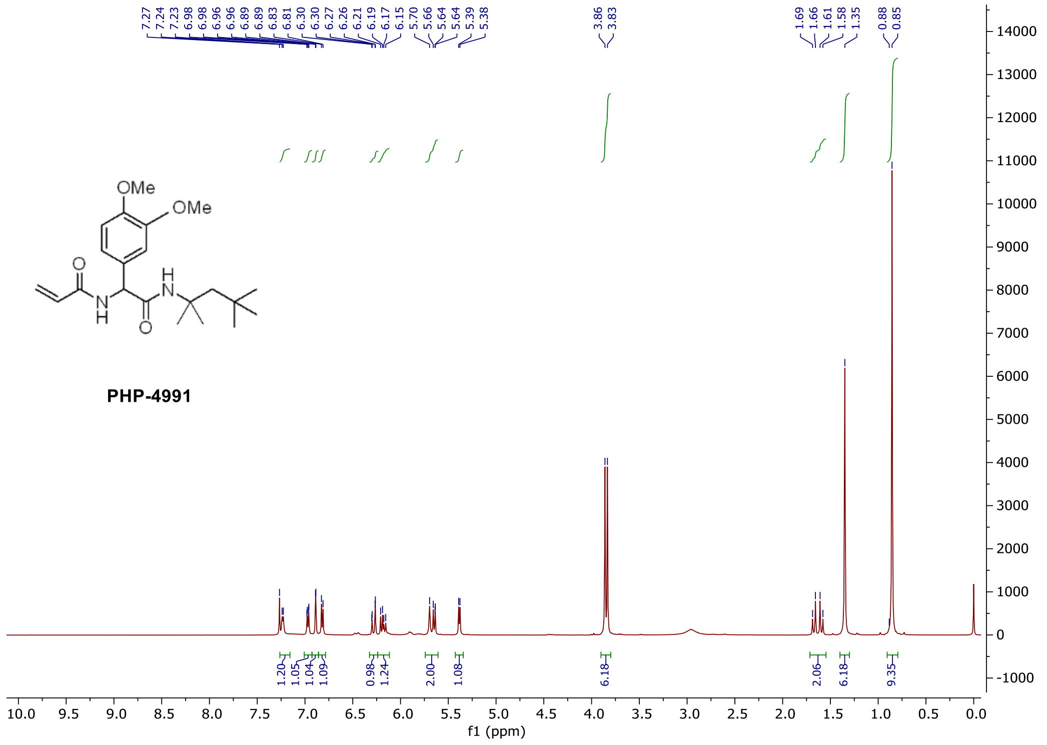

Supplementary Figure 486. ^{1}H NMR (500 MHz, $CDCl_3$ + one drop of CD_3OD) of compound **PHP-4991**.

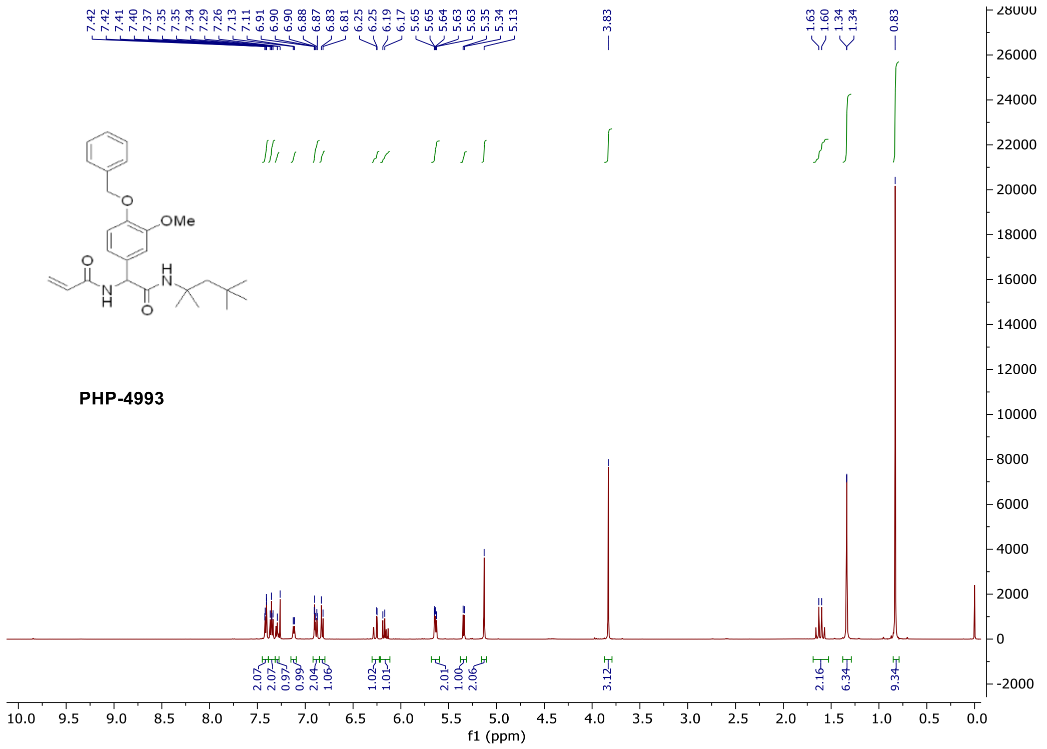

Supplementary Figure 487. ^{1}H NMR (500 MHz, $CDCl_3$) of compound **PHP-4993**.

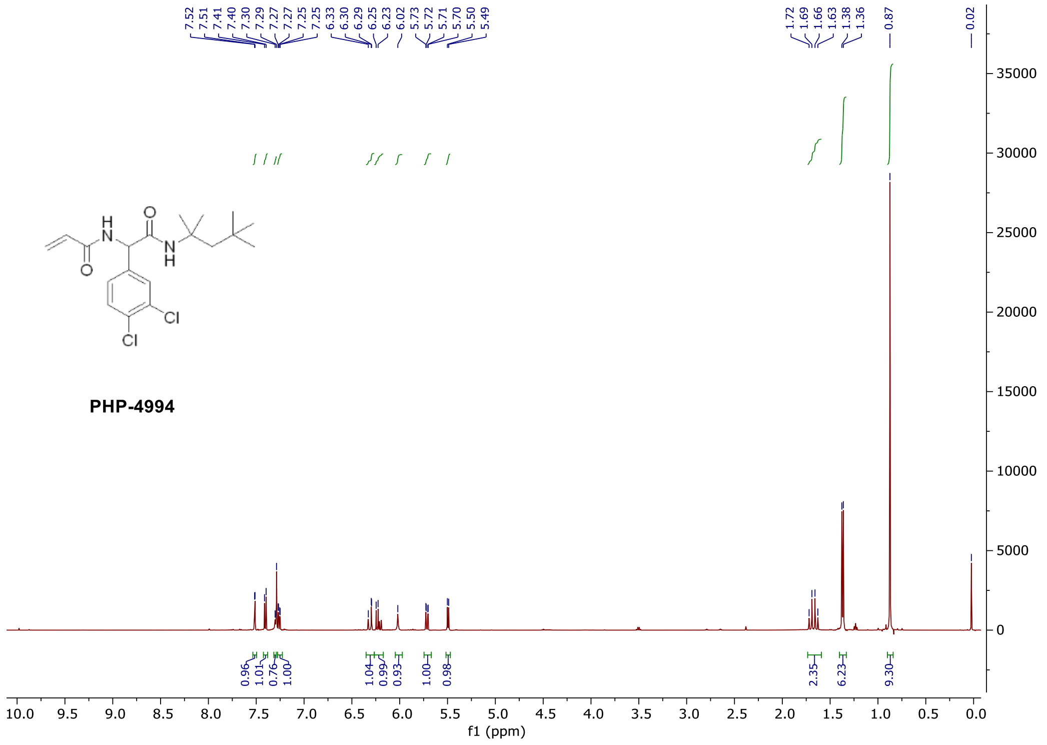

Supplementary Figure 488. ^{1}H NMR (500 MHz, $CDCl_3$) of compound **PHP-4994**.

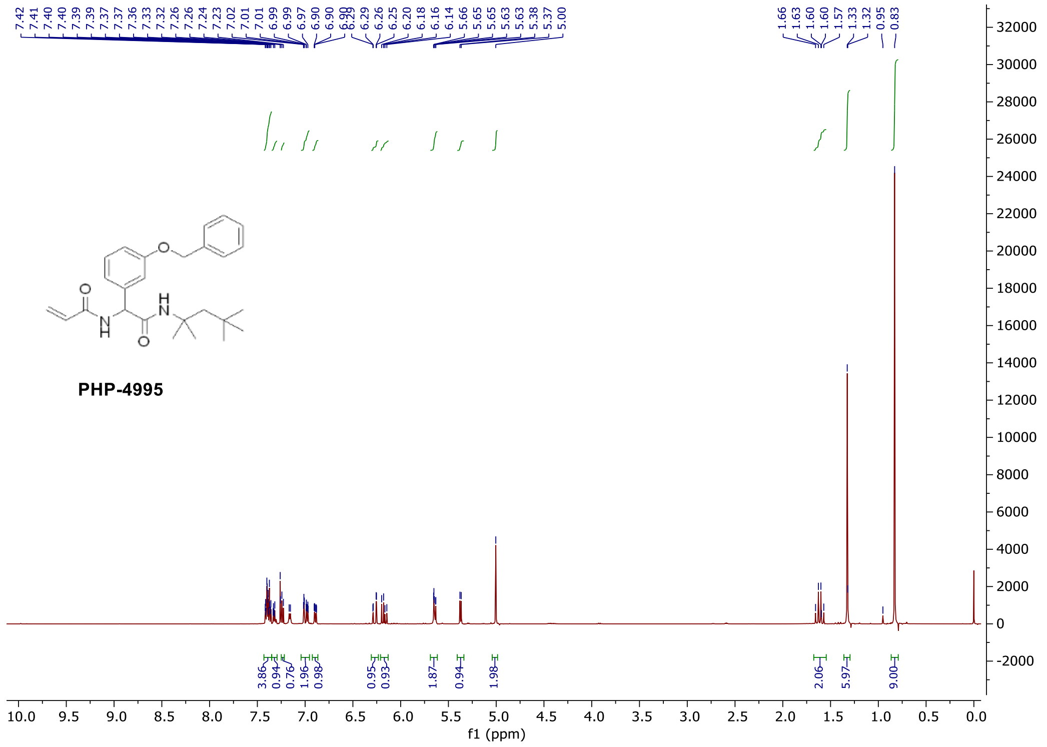

Supplementary Figure 489. ¹H NMR (500 MHz, CDCl3) of compound **PHP-4995**.

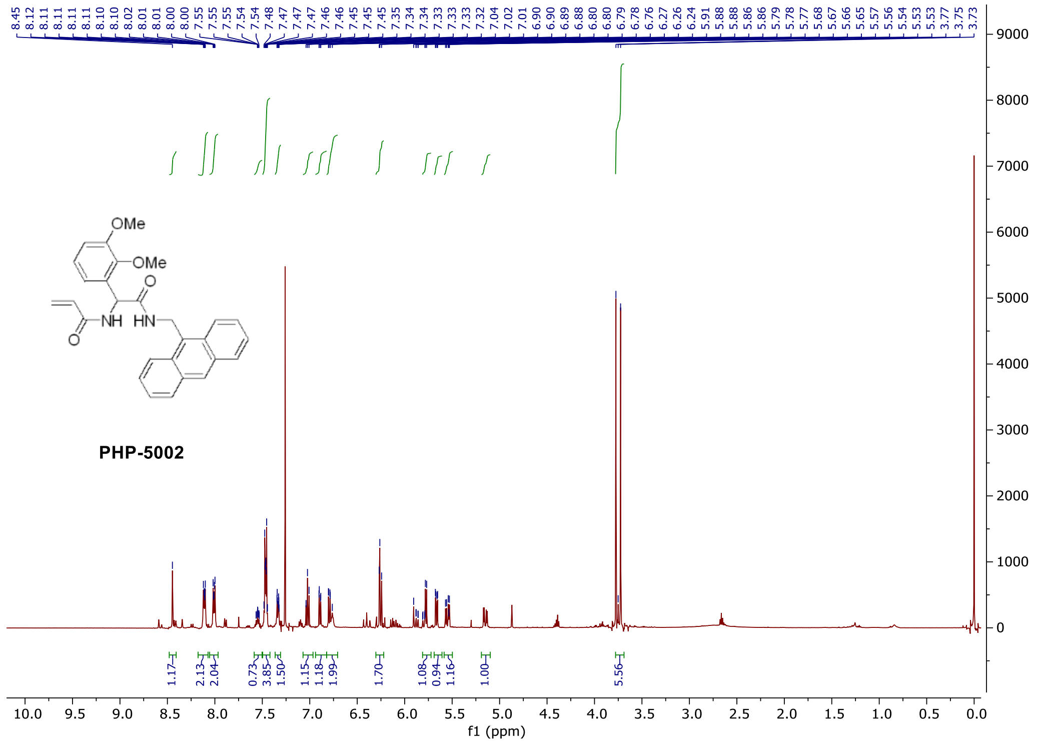

Supplementary Figure 490. ¹H NMR (500 MHz, CDCl3) of compound **PHP-5002**.

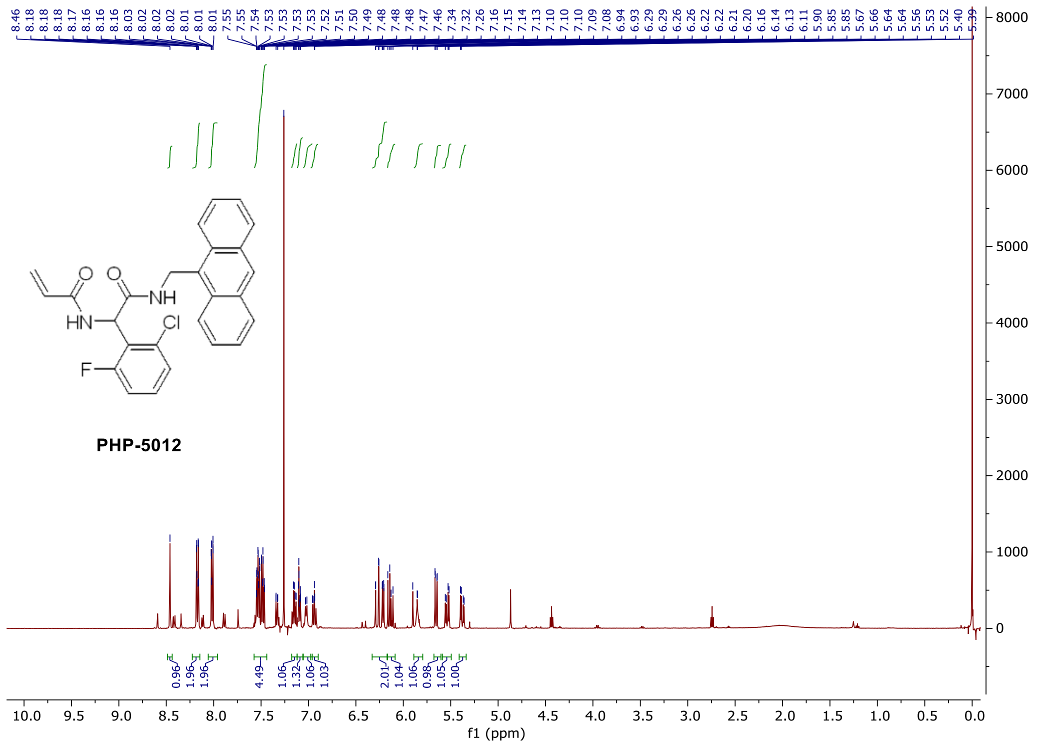

Supplementary Figure 491. ^{1}H NMR (500 MHz, $CDCl_3$) of compound **PHP-5012**.

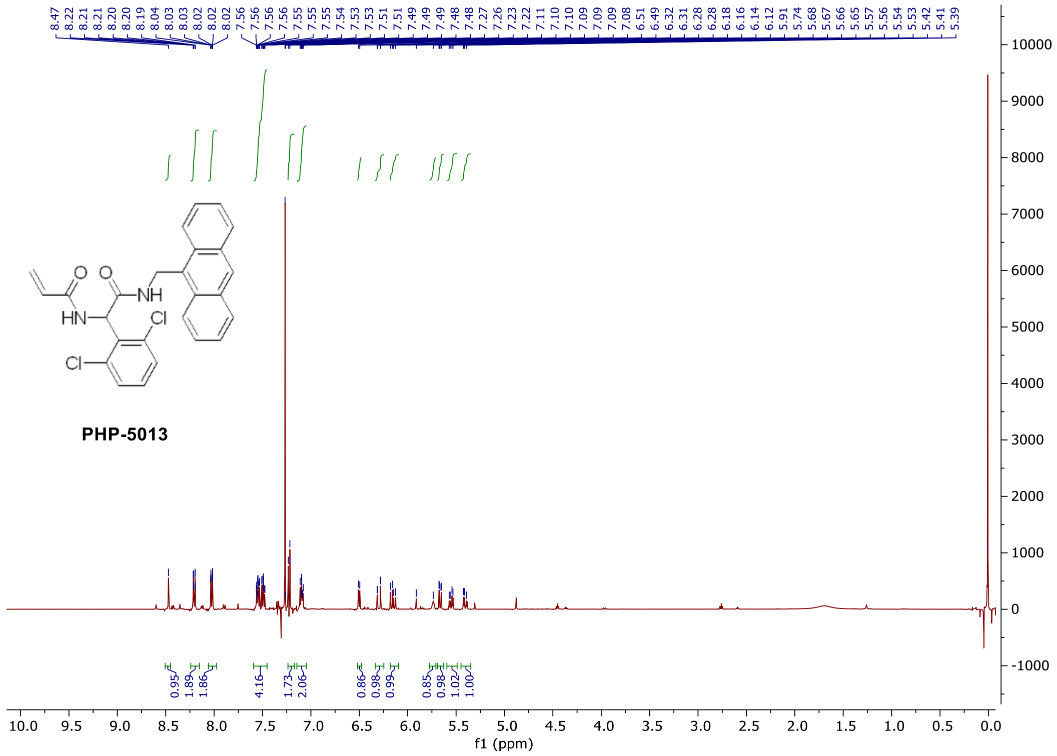

Supplementary Figure 492. ^{1}H NMR (500 MHz, $CDCl_3$) of compound **PHP-5013**.

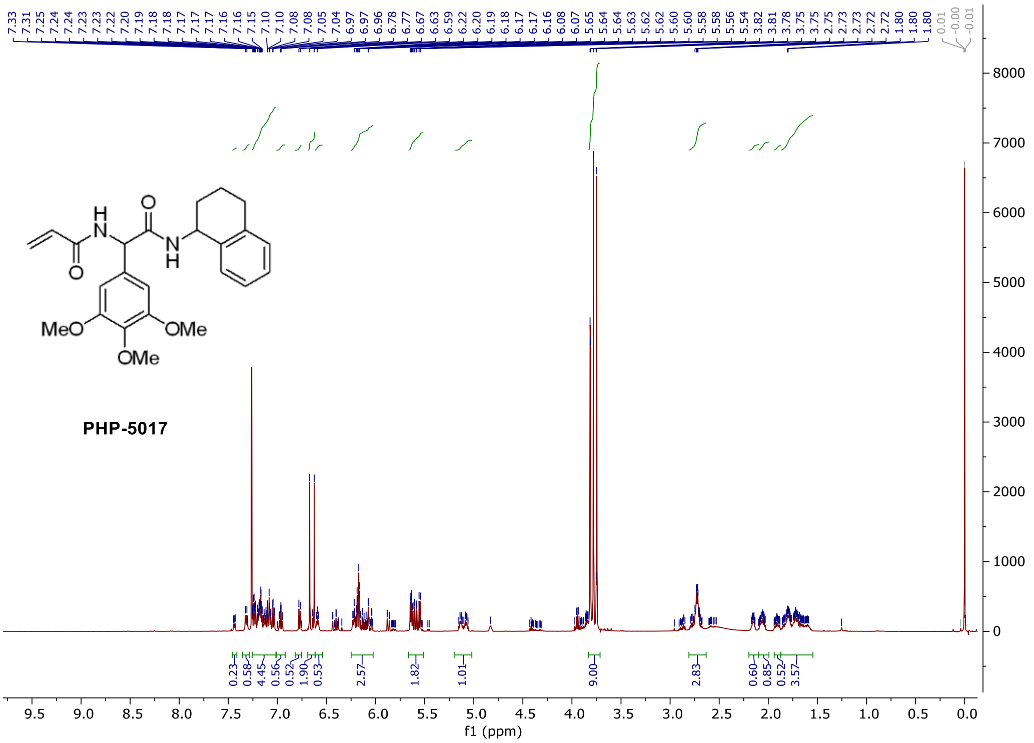

Supplementary Figure 493. ^{1}H NMR (500 MHz, CDCl$_3$) of compound **PHP-5017**.

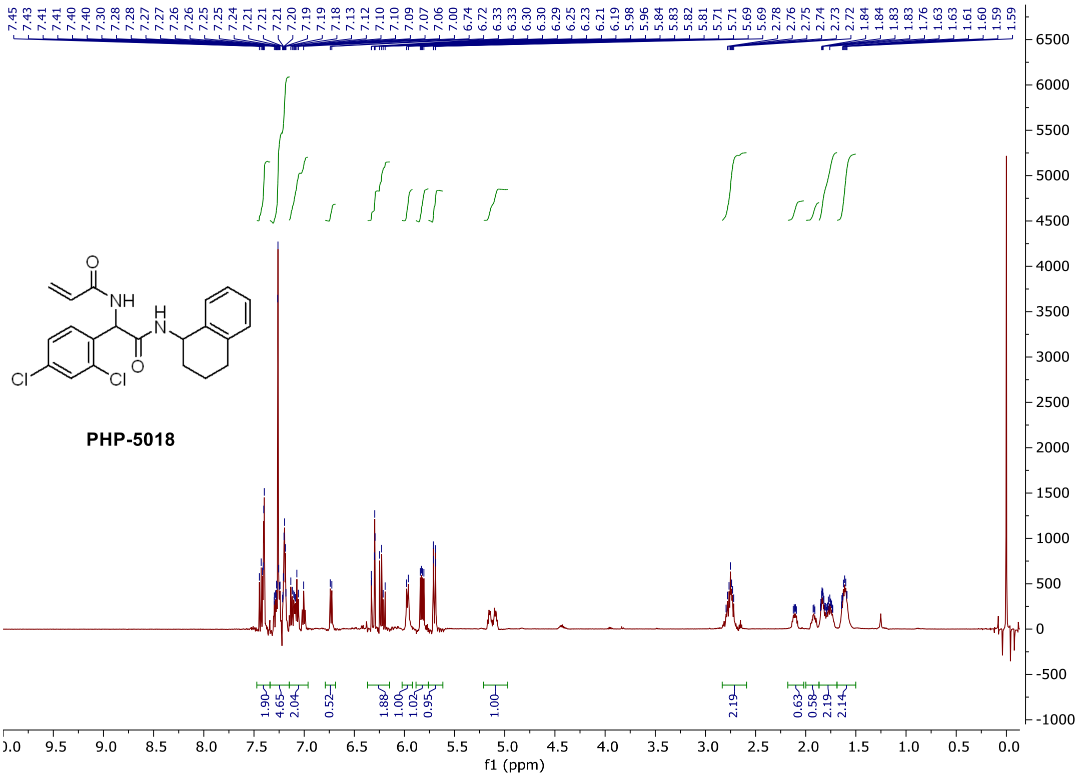

Supplementary Figure 494. ^{1}H NMR (500 MHz, CDCl$_3$) of compound **PHP-5018**.

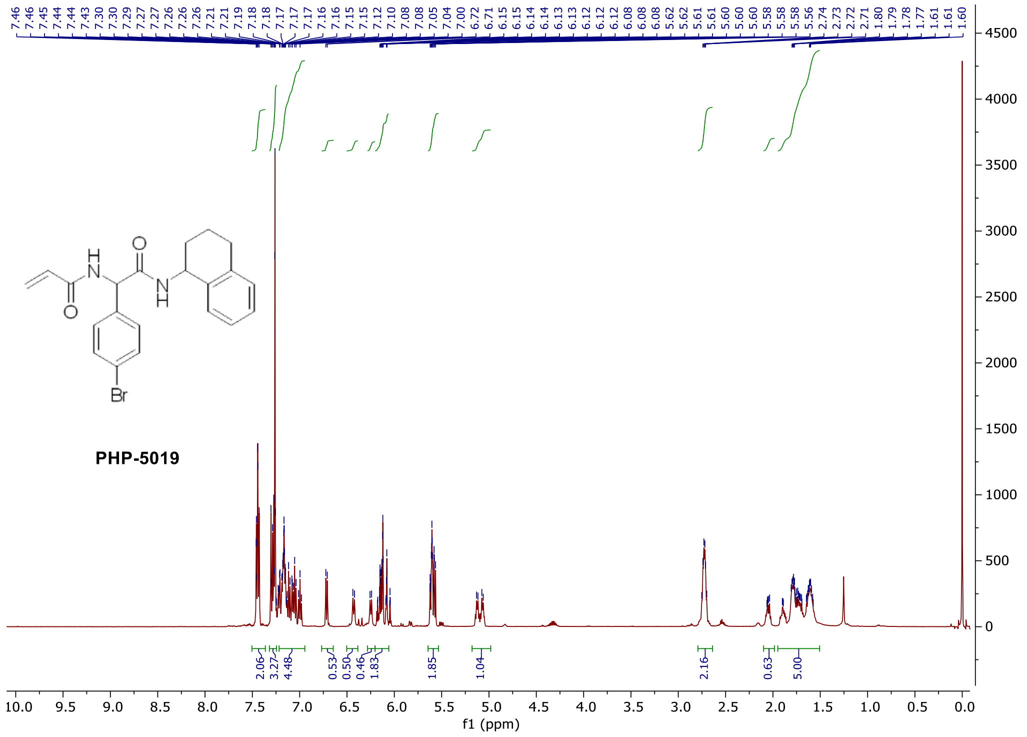

Supplementary Figure 495. ^{1}H NMR (500 MHz, $CDCl_3$) of compound **PHP-5019**.

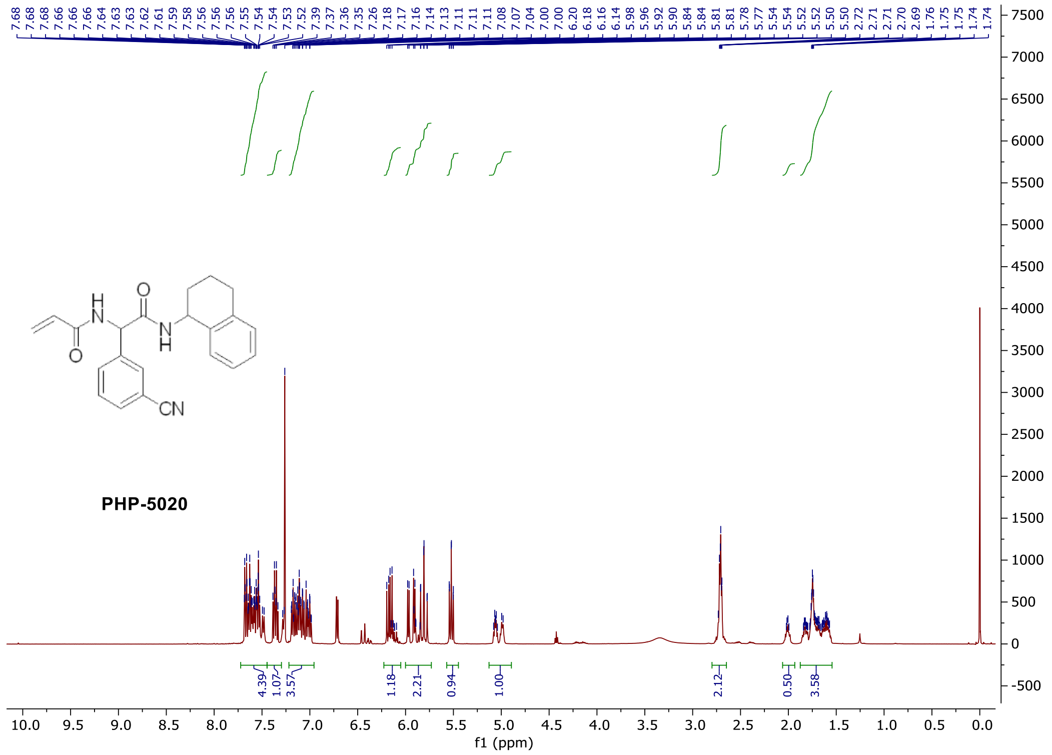

Supplementary Figure 496. ^{1}H NMR (500 MHz, $CDCl_3$ + one drop of CD_3OD) of compound **PHP-5020**.

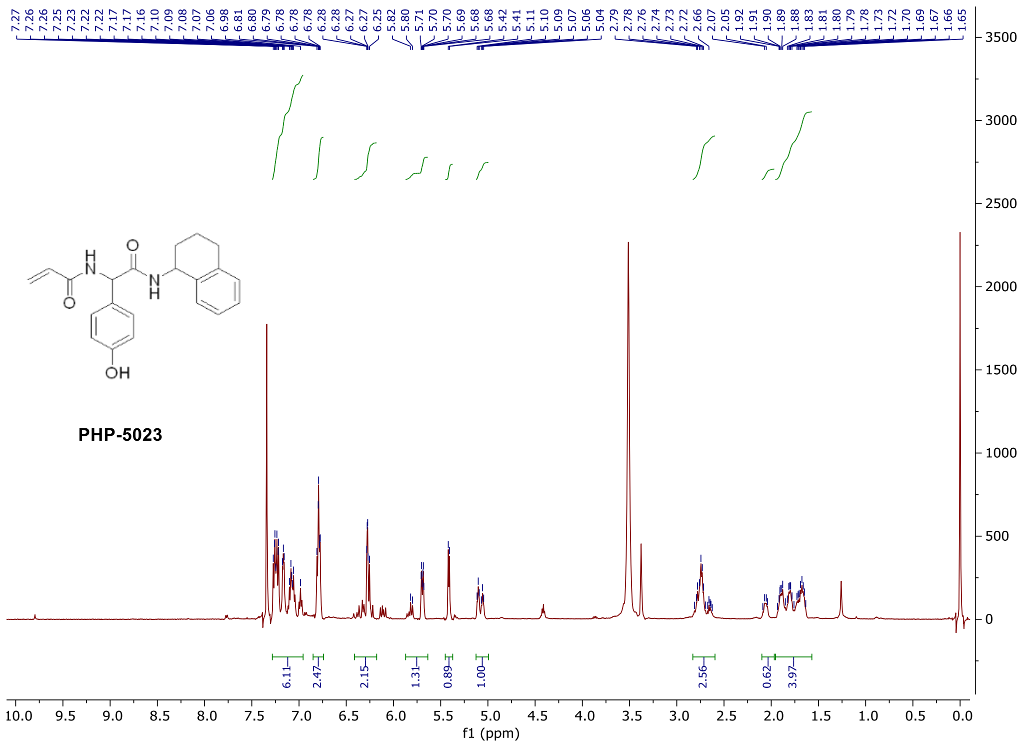

Supplementary Figure 497. ^{1}H NMR (500 MHz, $CDCl_3$ + one drop of CD_3OD) of compound **PHP-5023**.

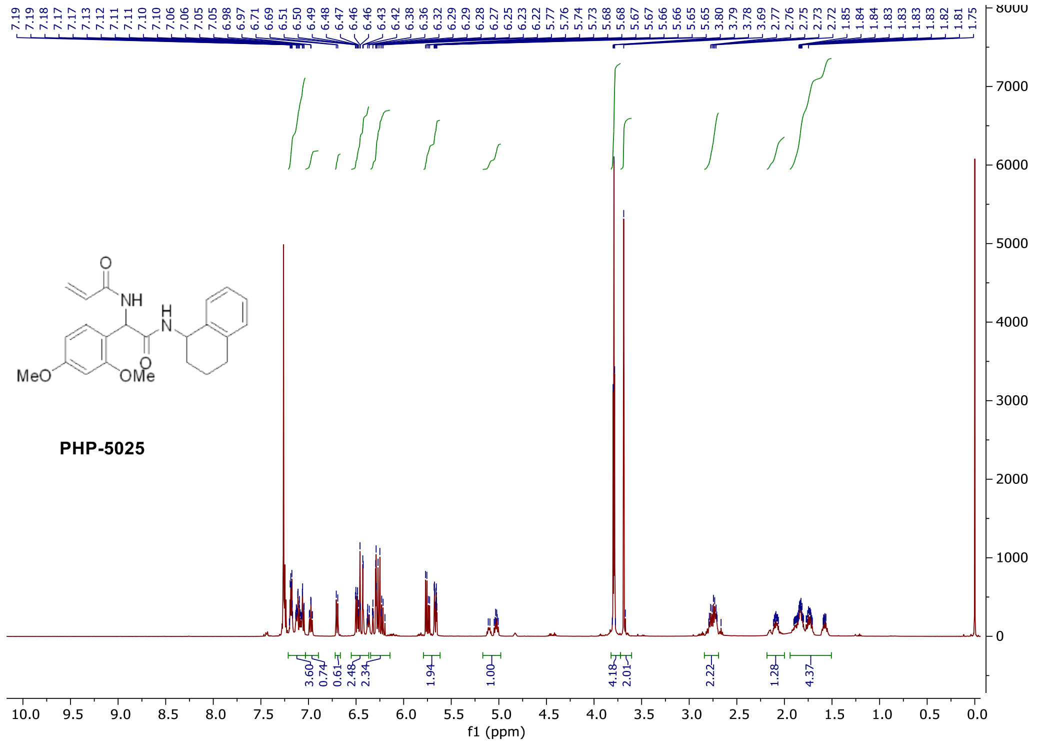

Supplementary Figure 498. ^{1}H NMR (500 MHz, $CDCl_3$) of compound **PHP-5025**.

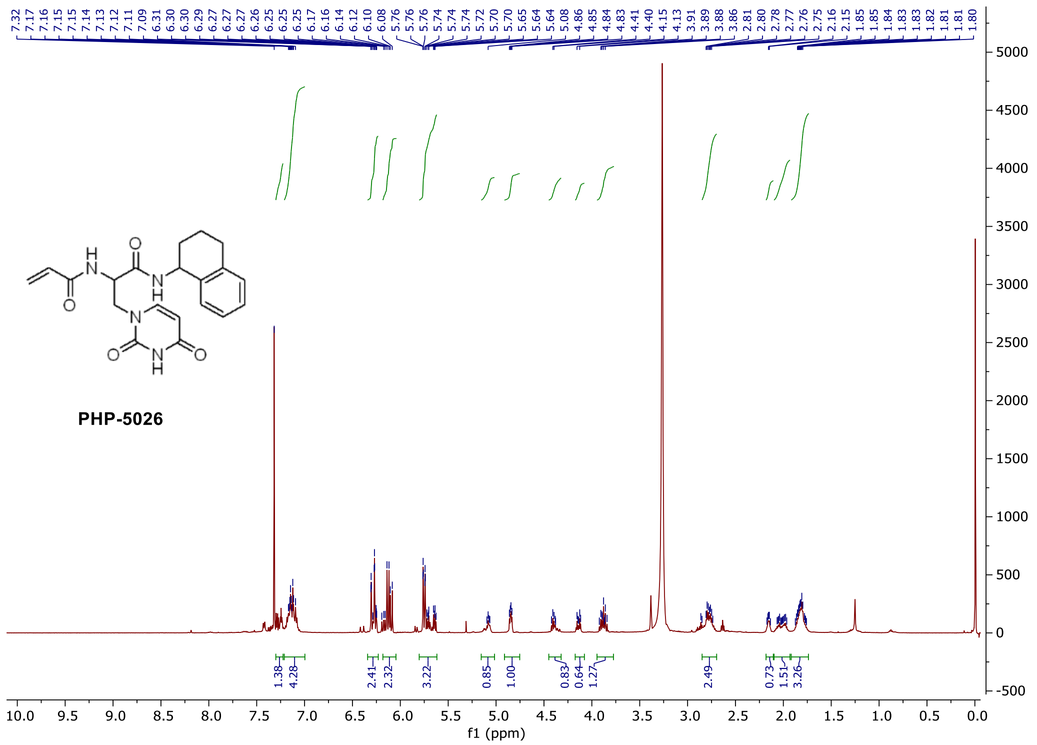

Supplementary Figure 499. ^{1}H NMR (500 MHz, $CDCl_3$ + one drop of CD_3OD) of compound **PHP-5026**.

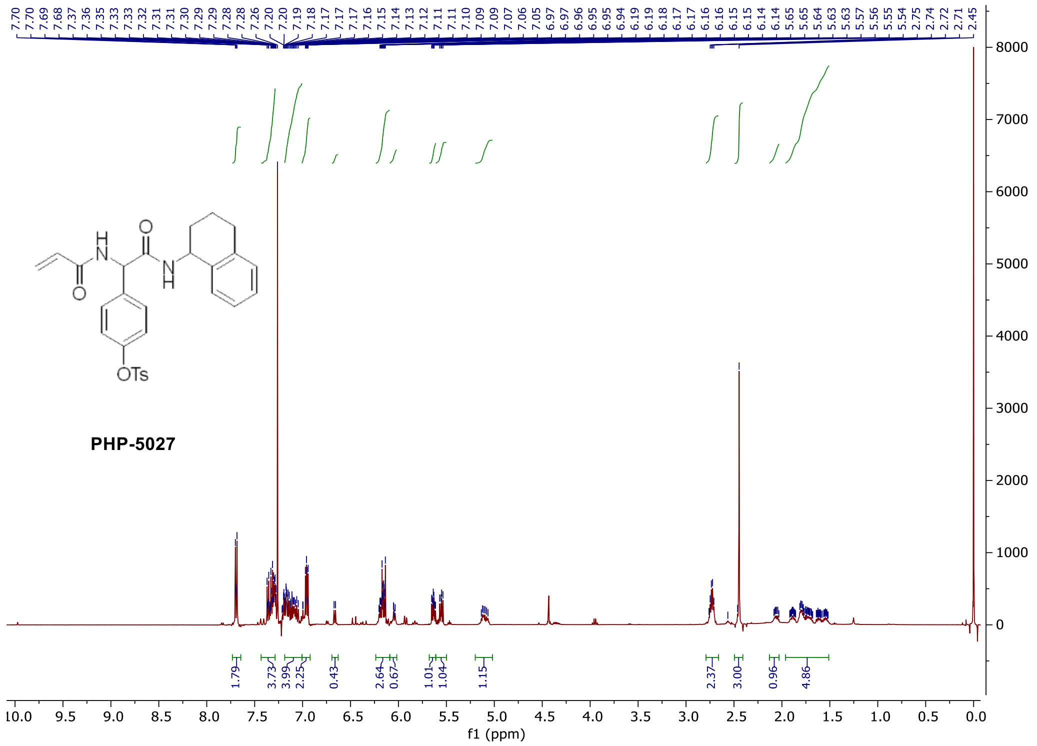

Supplementary Figure 500. ^{1}H NMR (500 MHz, $CDCl_3$) of compound **PHP-5027**.

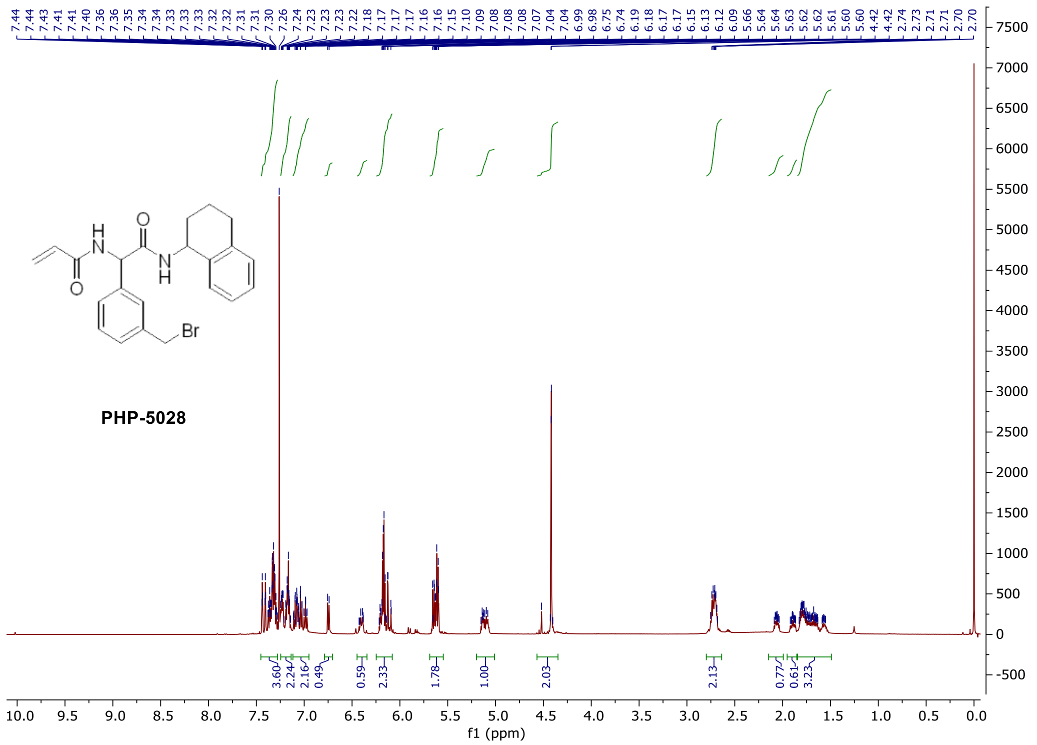

Supplementary Figure 501. ^{1}H NMR (500 MHz, $CDCl_3$) of compound **PHP-5028**.

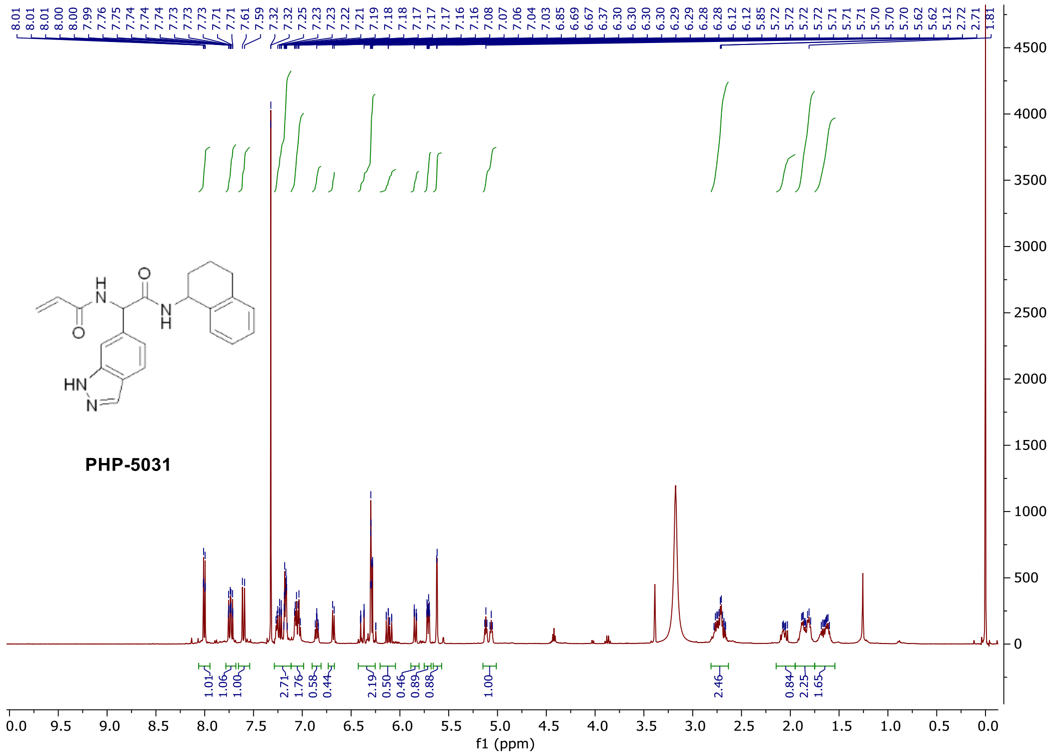

Supplementary Figure 502. ^{1}H NMR (500 MHz, $CDCl_3$ + one drop of CD_3OD) of compound **PHP-5031**.

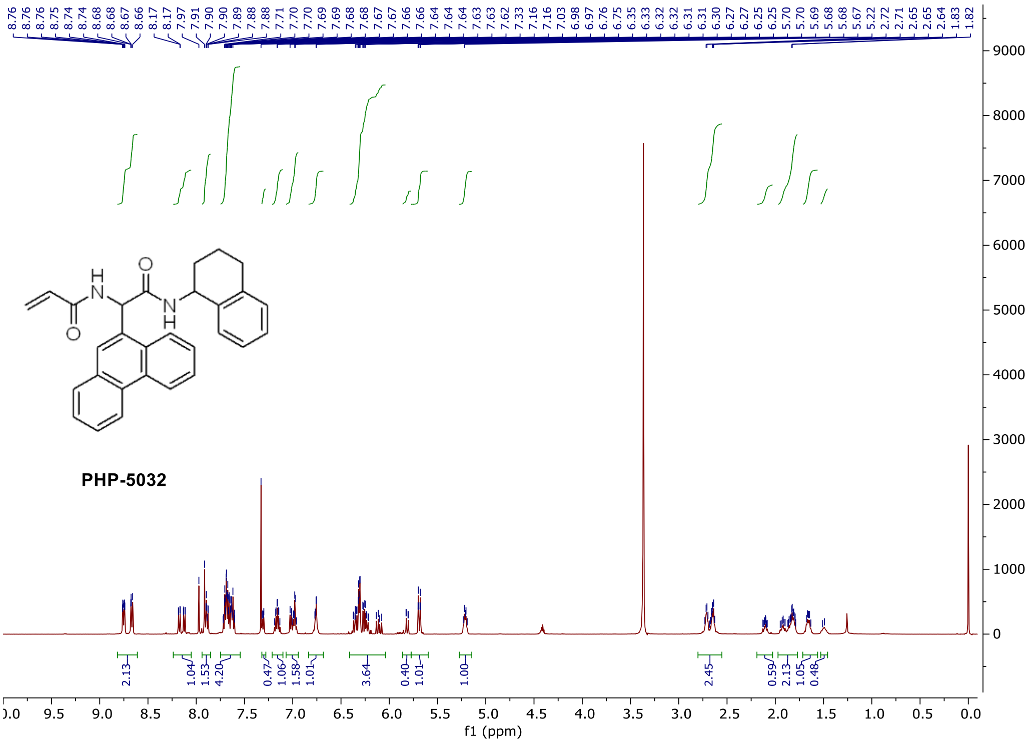

Supplementary Figure 503. ^{1}H NMR (500 MHz, $CDCl_3$) of compound **PHP-5032**.

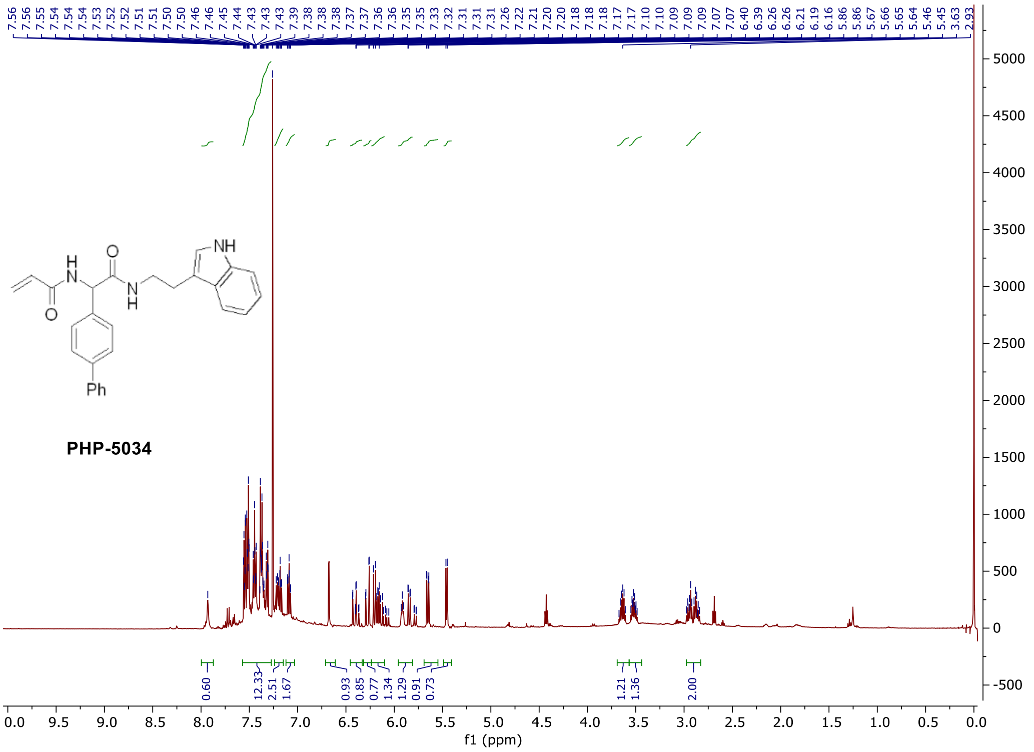

Supplementary Figure 504. ^{1}H NMR (500 MHz, $CDCl_3$) of compound **PHP-5034**.

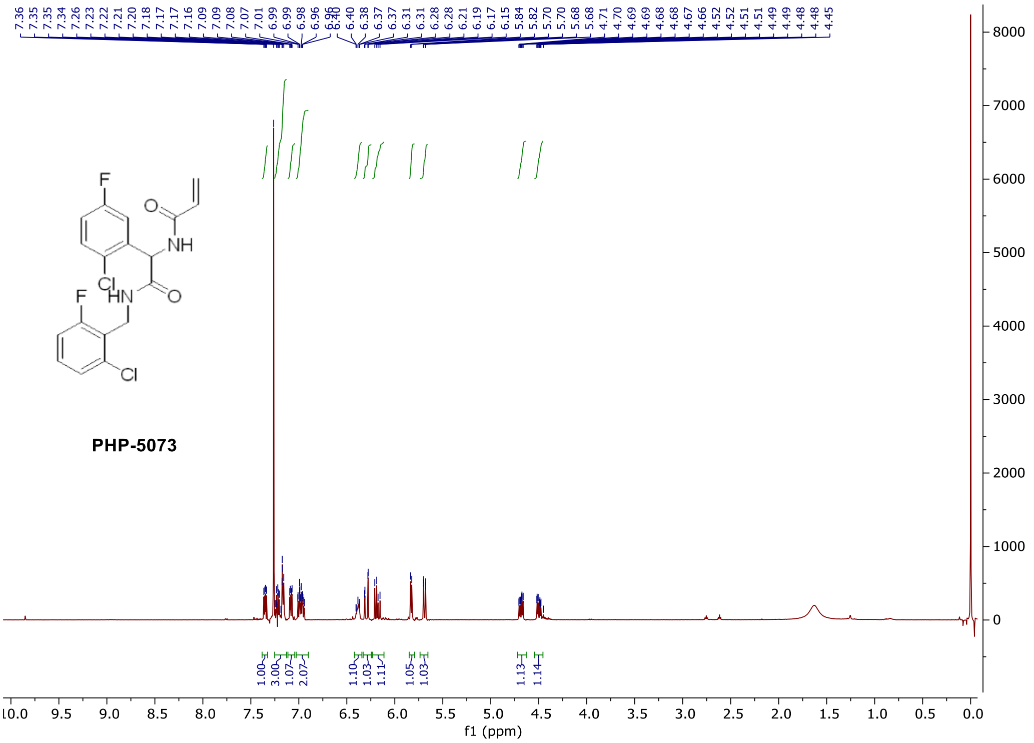

Supplementary Figure 505. ^{1}H NMR (500 MHz, $CDCl_3$) of compound **PHP-5073**.

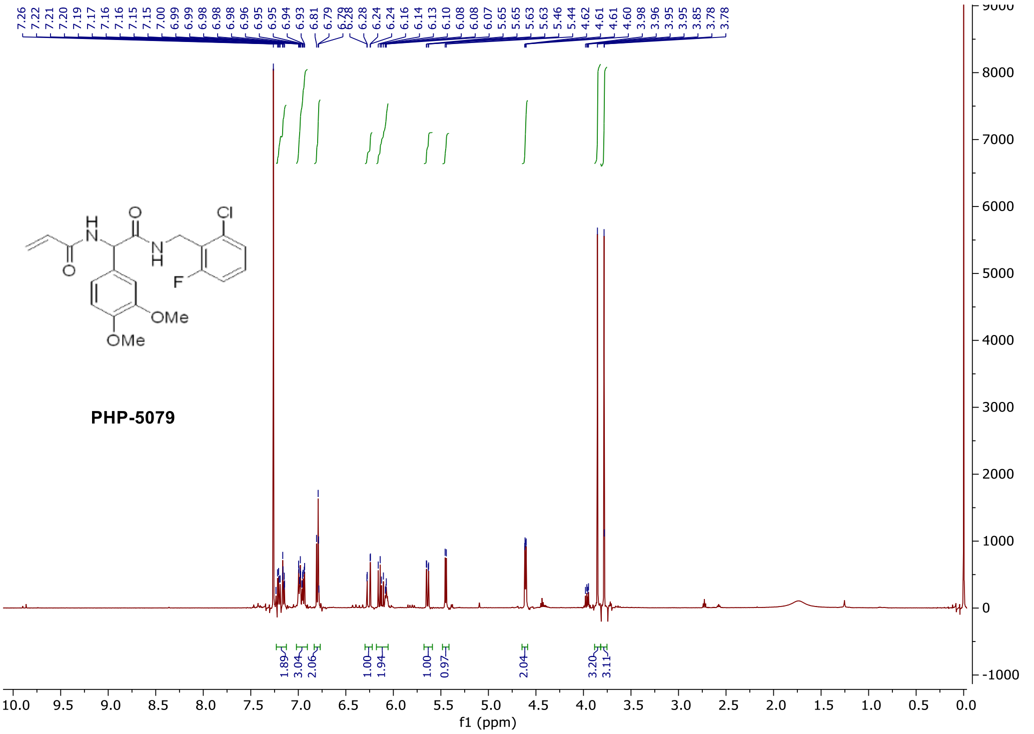

Supplementary Figure 507. ^{1}H NMR (500 MHz, $CDCl_3$) of compound **PHP-5079**.

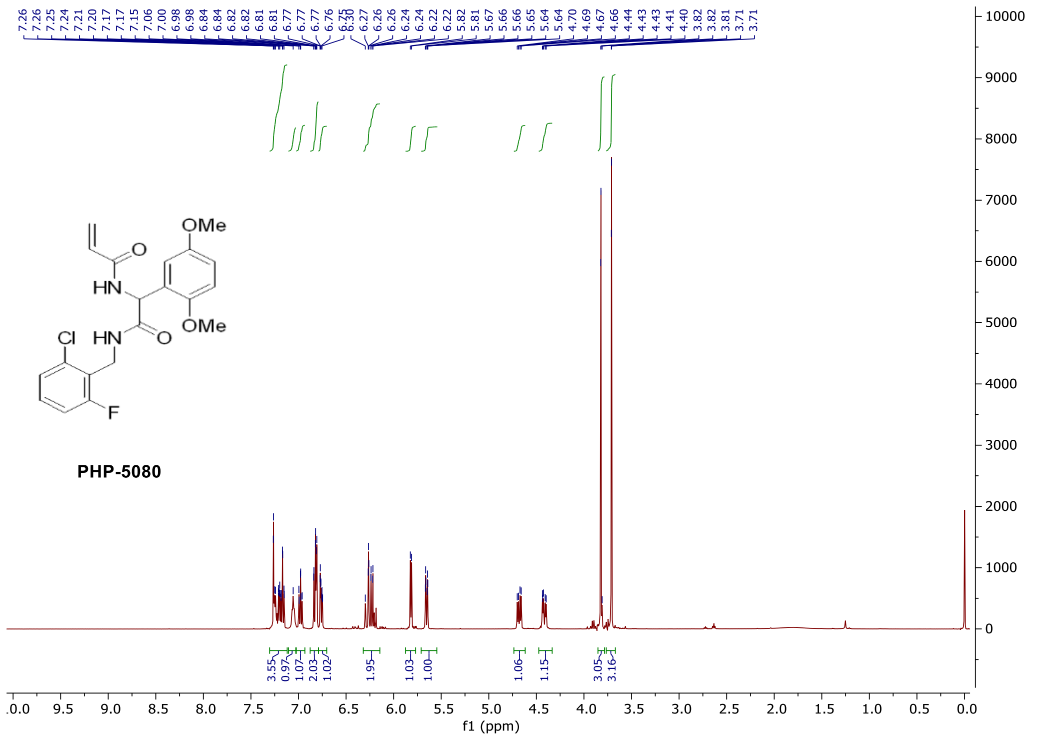

Supplementary Figure 508. ^{1}H NMR (500 MHz, $CDCl_3$) of compound **PHP-5080**.

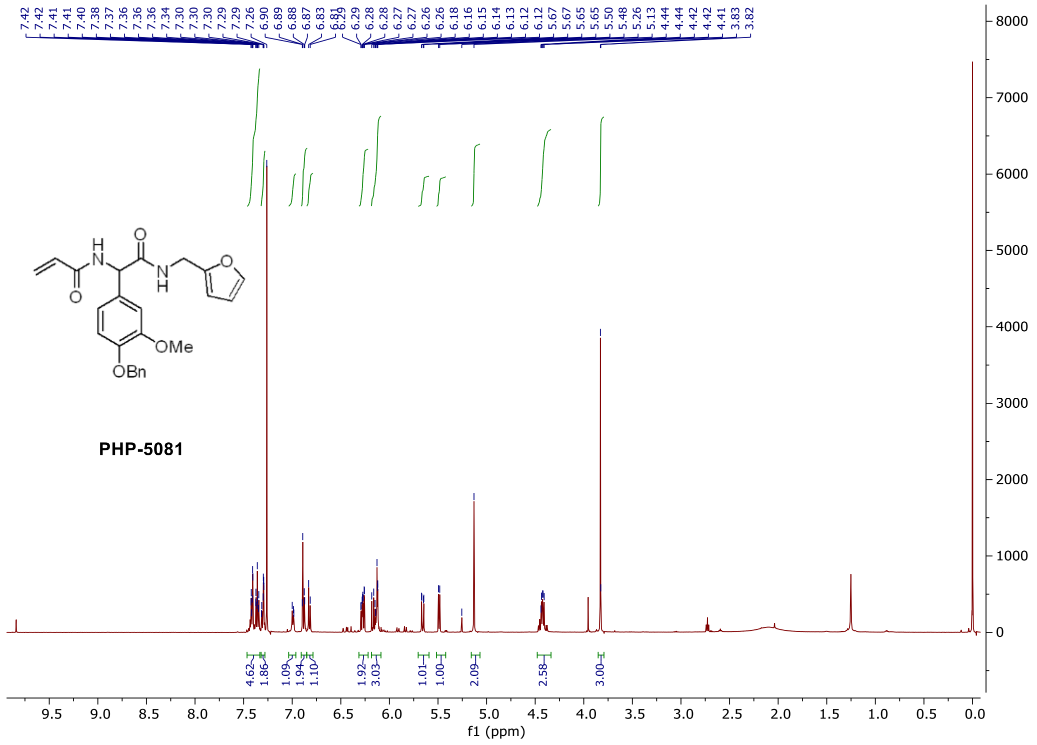

Supplementary Figure 509. ^{1}H NMR (500 MHz, $CDCl_3$) of compound **PHP-5081**.

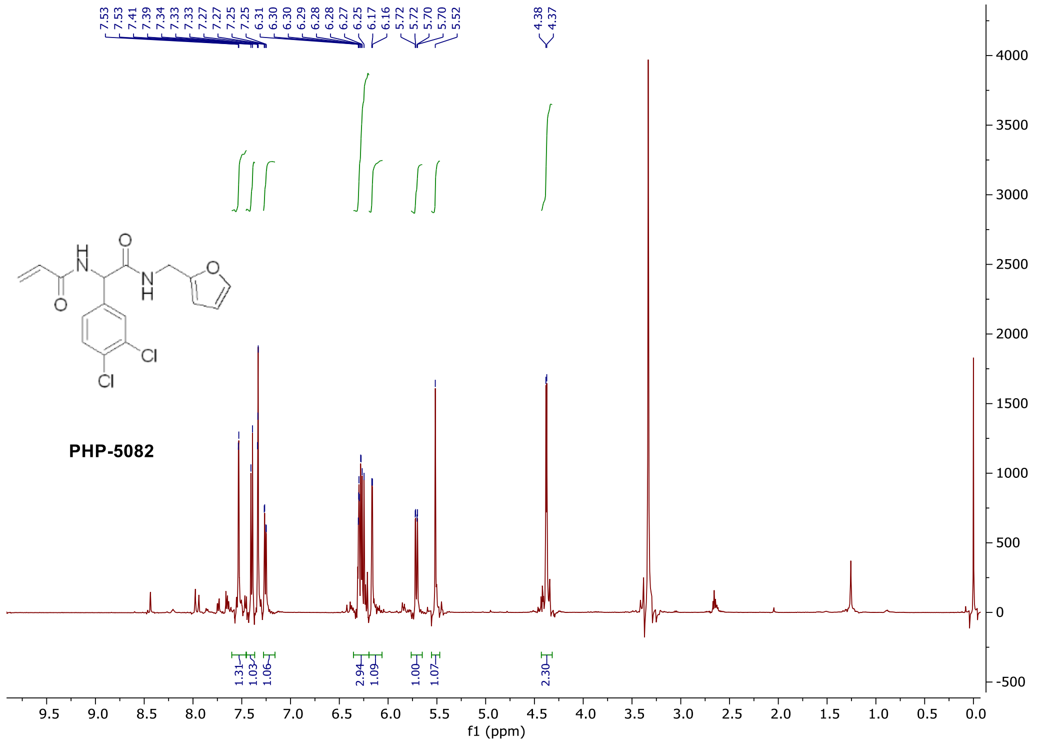

Supplementary Figure 510. ^{1}H NMR (500 MHz, $CDCl_3$ + one drop of CD_3OD) of compound **PHP-5082**.

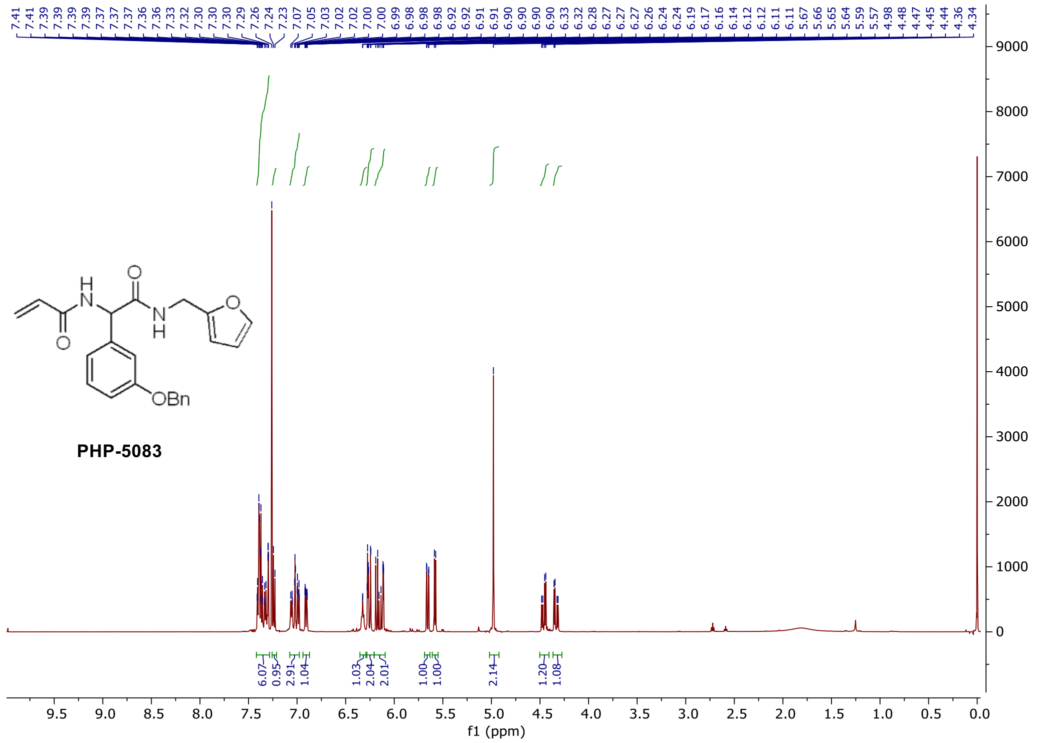

Supplementary Figure 511. ^{1}H NMR (500 MHz, $CDCl_3$) of compound **PHP-5083**.

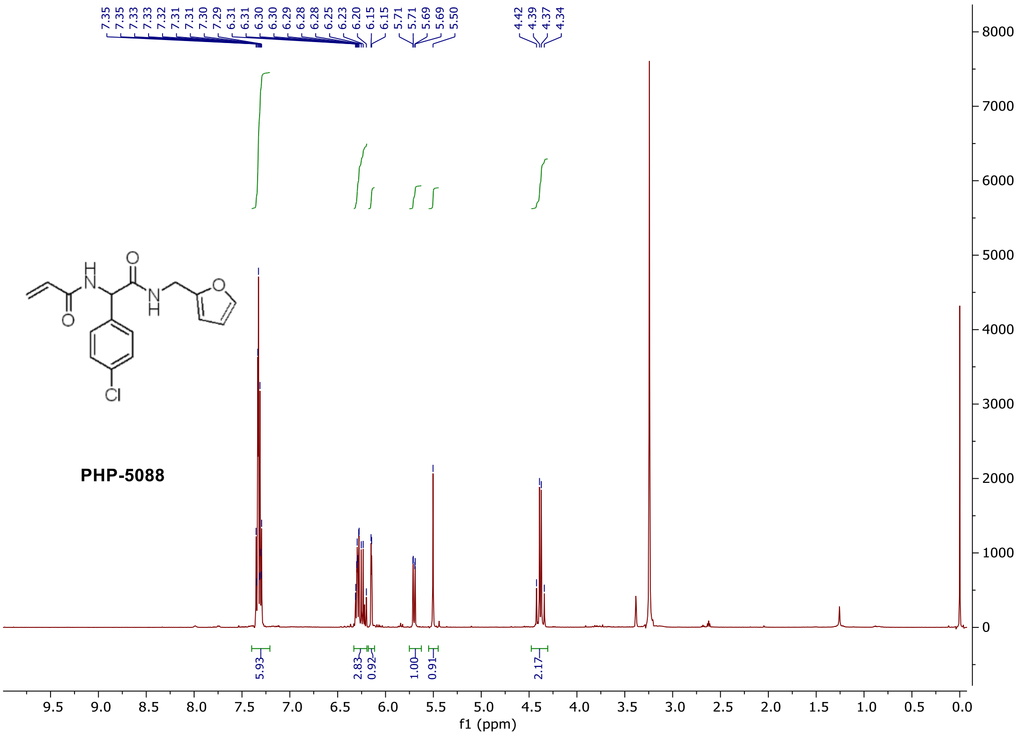

Supplementary Figure 512. ^{1}H NMR (500 MHz, $CDCl_3$ + one drop of CD_3OD) of compound **PHP-5088**.

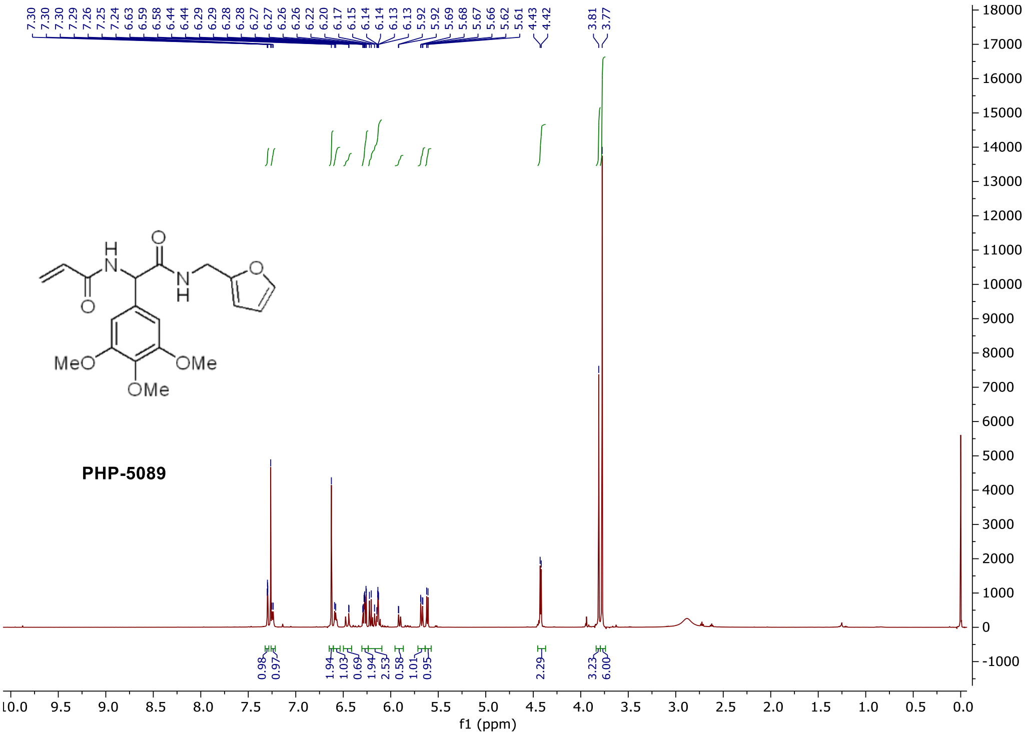

Supplementary Figure 513. ^{1}H NMR (500 MHz, $CDCl_3$) of compound **PHP-5089**.

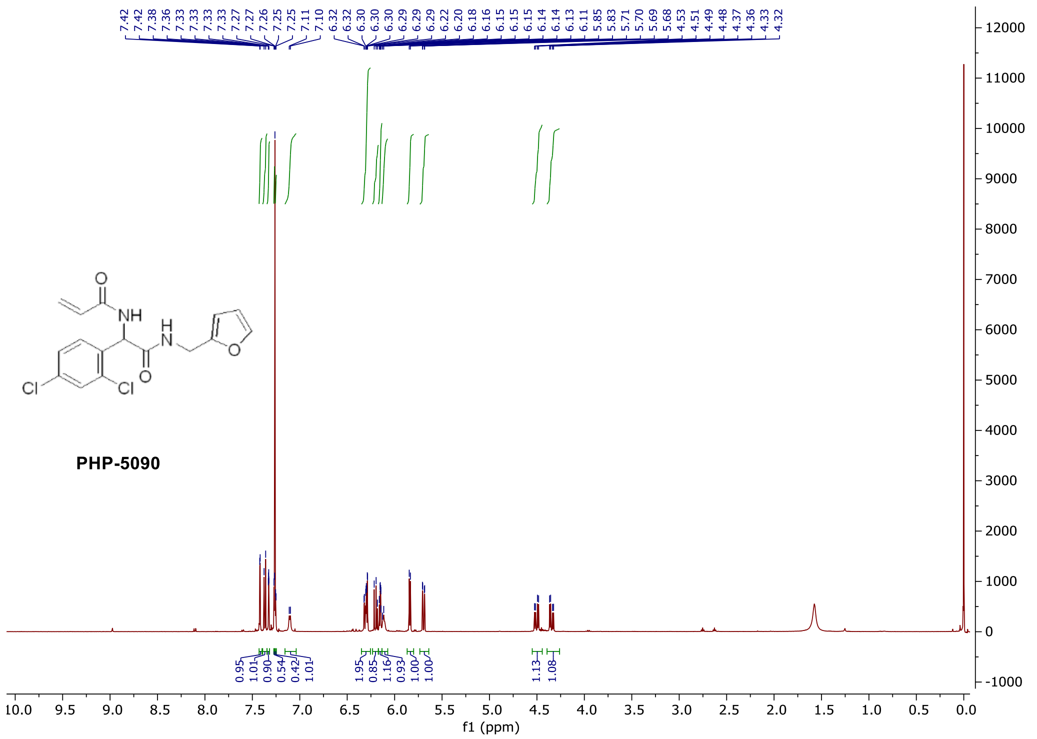

Supplementary Figure 514. ^{1}H NMR (500 MHz, CDCl3) of compound **PHP-5090**.

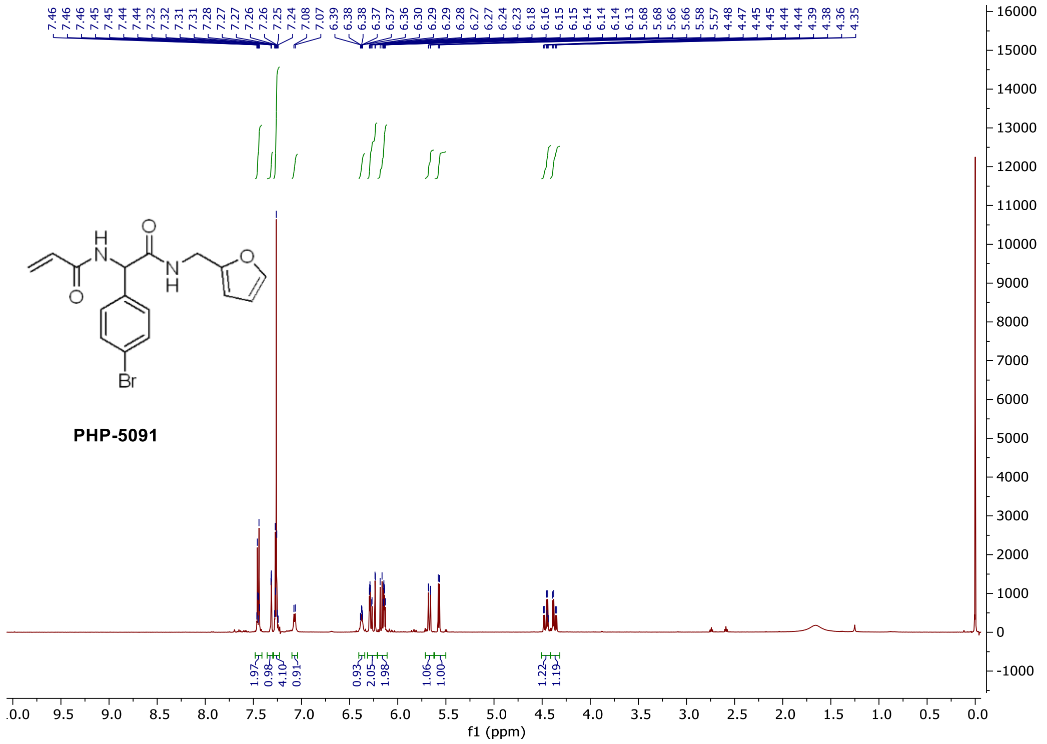

Supplementary Figure 515. ^{1}H NMR (500 MHz, CDCl3) of compound **PHP-5091**.

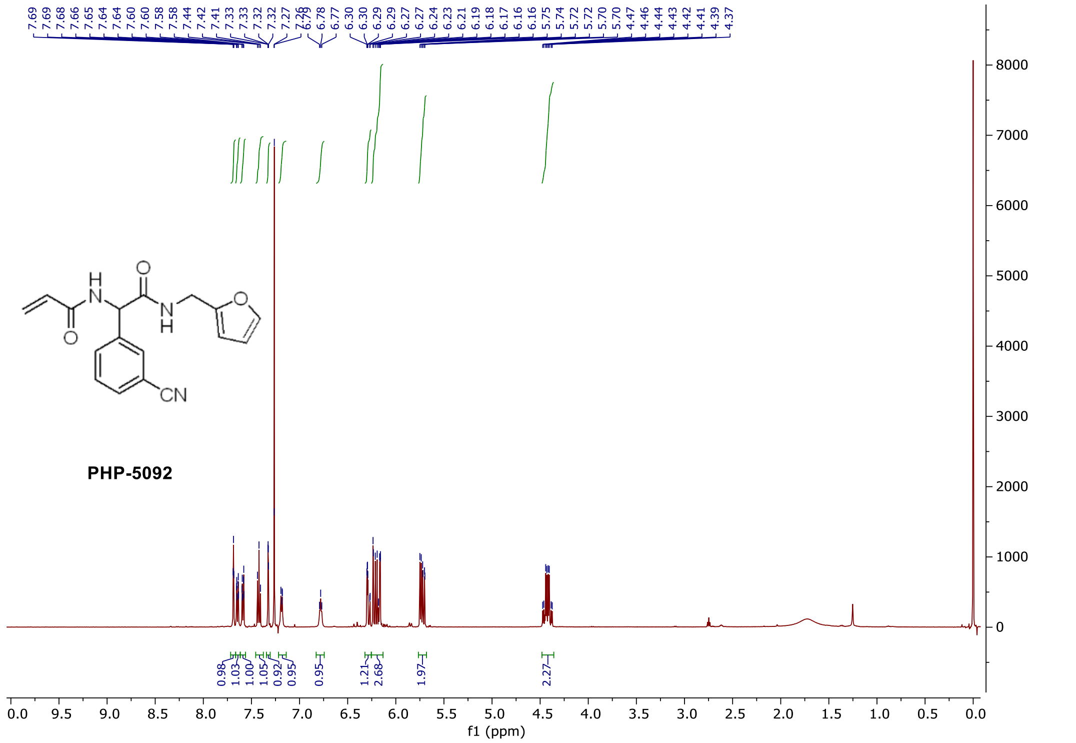

Supplementary Figure 516. ^{1}H NMR (500 MHz, $CDCl_3$) of compound **PHP-5092**.

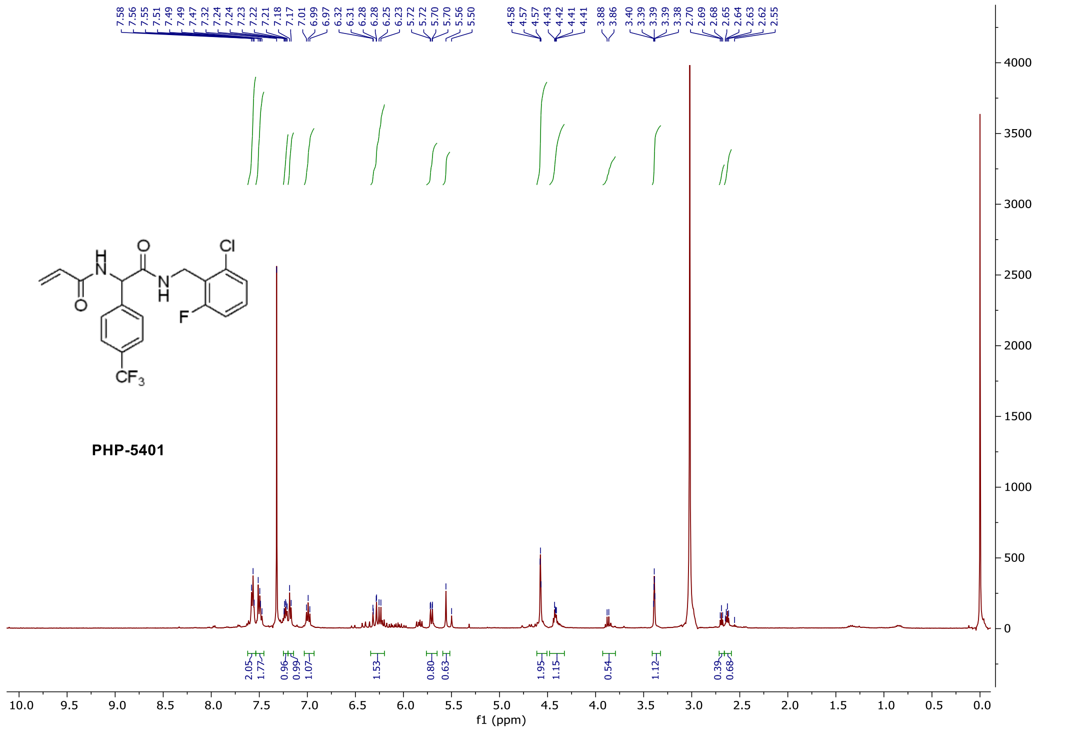

Supplementary Figure 517. ^{1}H NMR (500 MHz, $CDCl_3$ + one drop of CD_3OD) of compound **PHP-5401**.

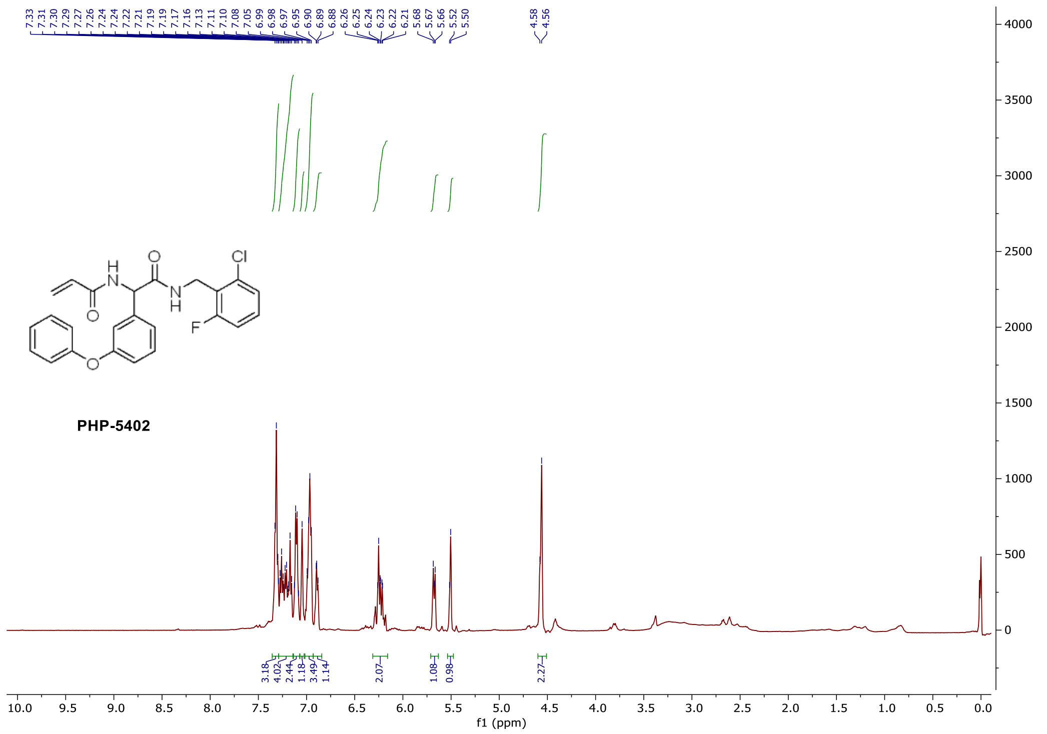

Supplementary Figure 518. ^{1}H NMR (500 MHz, $CDCl_3$ + one drop of CD_3OD) of compound **PHP-5402**.

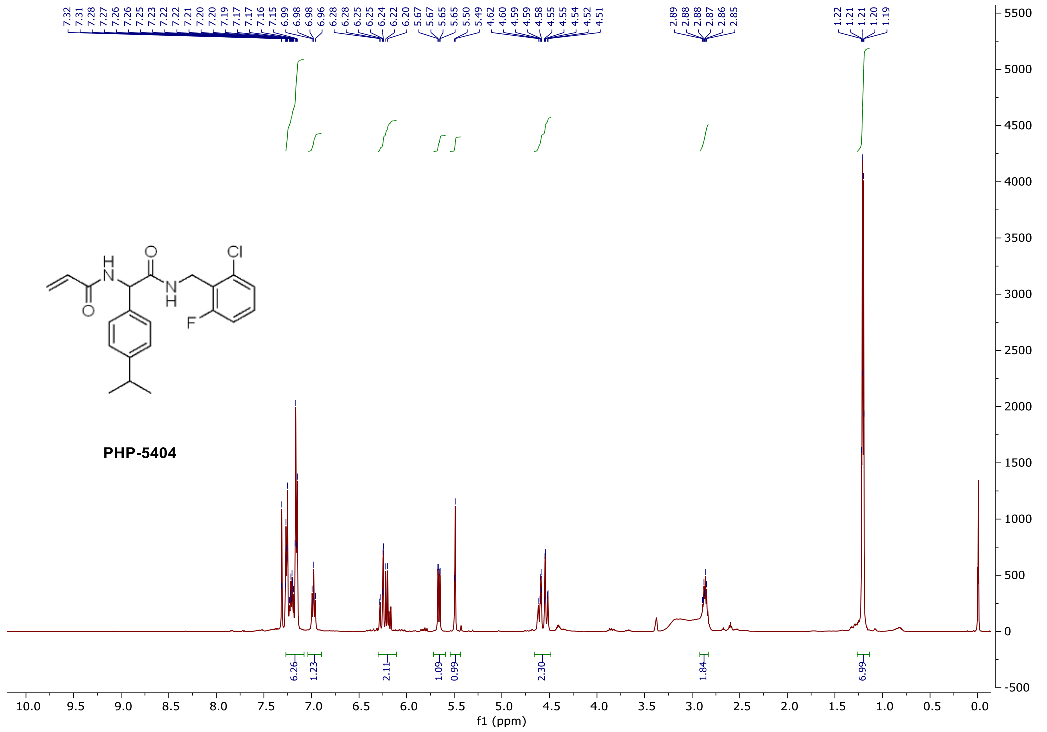

Supplementary Figure 519. ^{1}H NMR (500 MHz, $CDCl_3$ + one drop of CD_3OD) of compound **PHP-5404**.

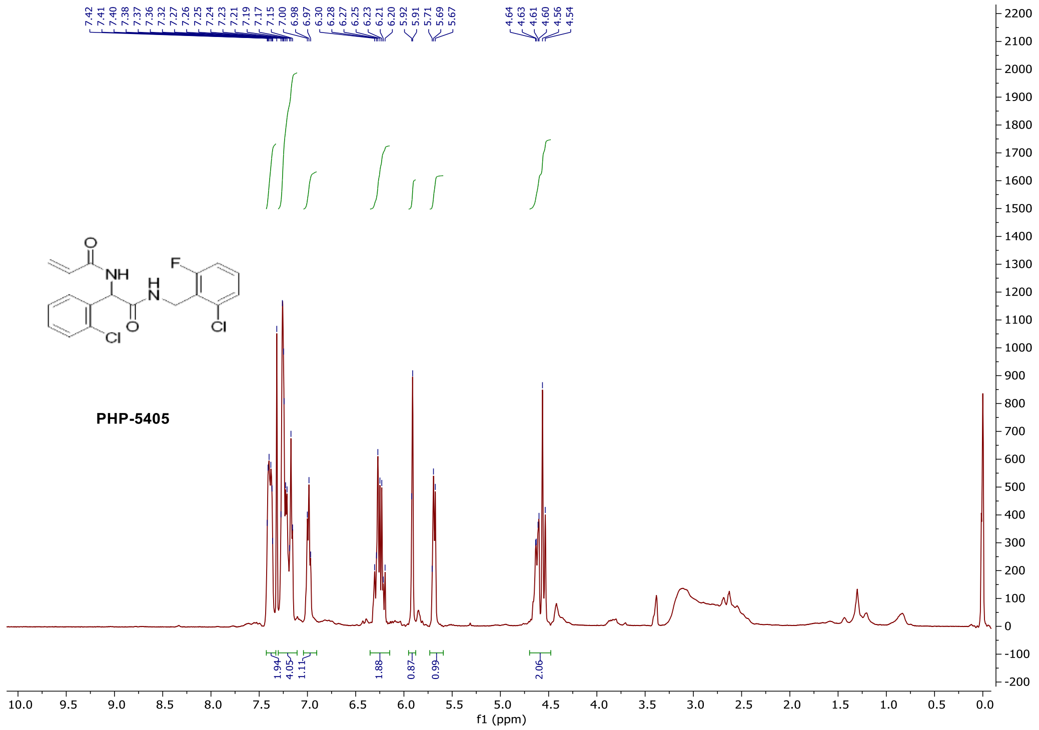

Supplementary Figure 520. ^{1}H NMR (500 MHz, $CDCl_3$ + one drop of CD_3OD) of compound **PHP-5405**.

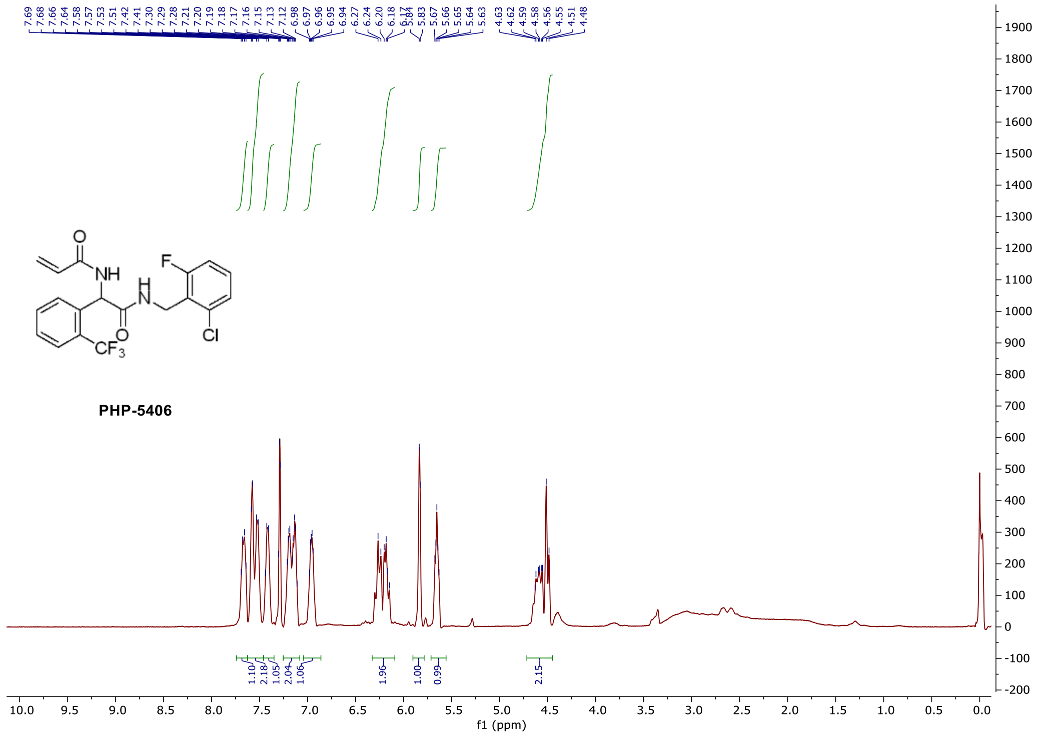

Supplementary Figure 521. ^{1}H NMR (500 MHz, $CDCl_3$ + one drop of CD_3OD) of compound **PHP-5406**.

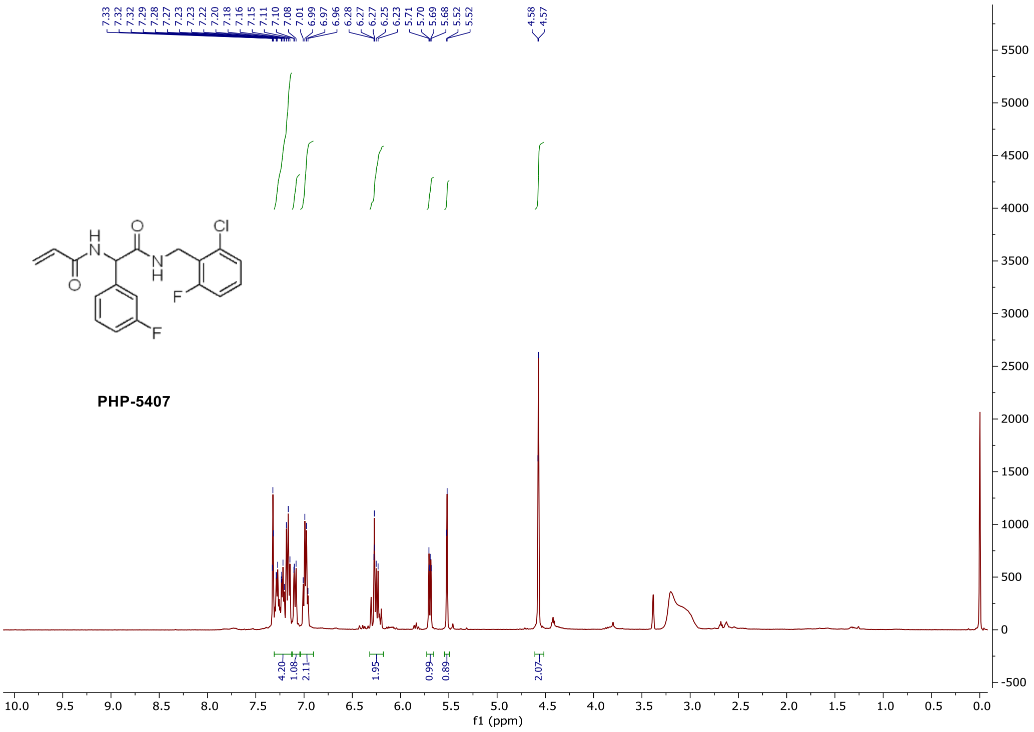

Supplementary Figure 522. ^{1}H NMR (500 MHz, $CDCl_3$ + one drop of CD_3OD) of compound **PHP-5407**.

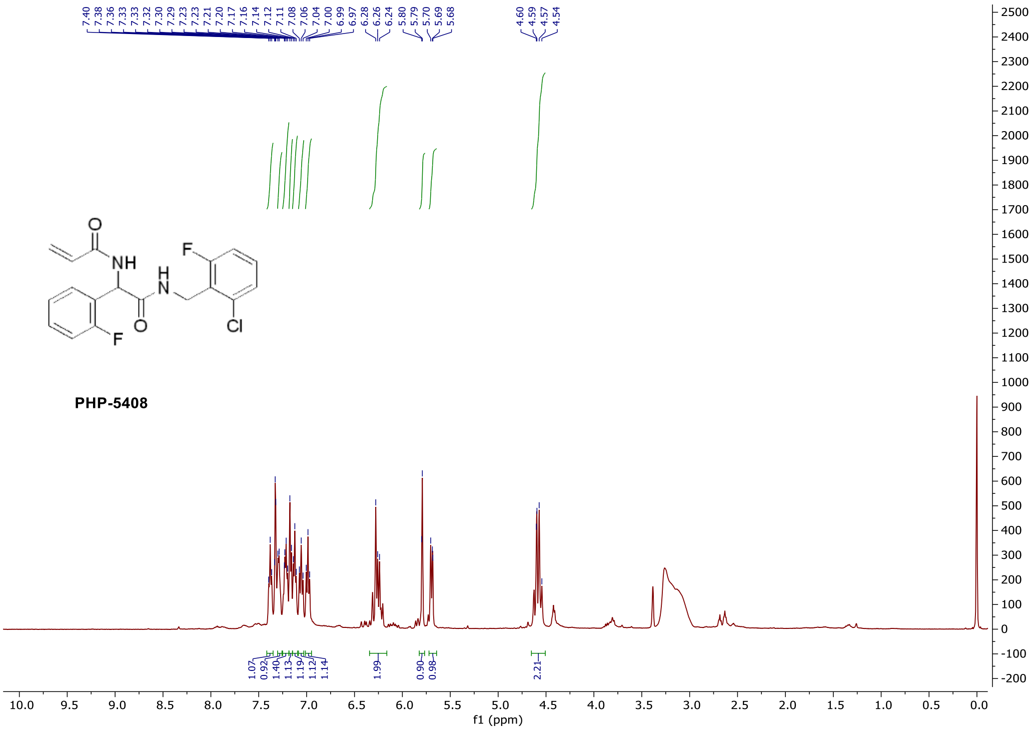

Supplementary Figure 523. ^{1}H NMR (500 MHz, $CDCl_3$ + one drop of CD_3OD) of compound **PHP-5408**.

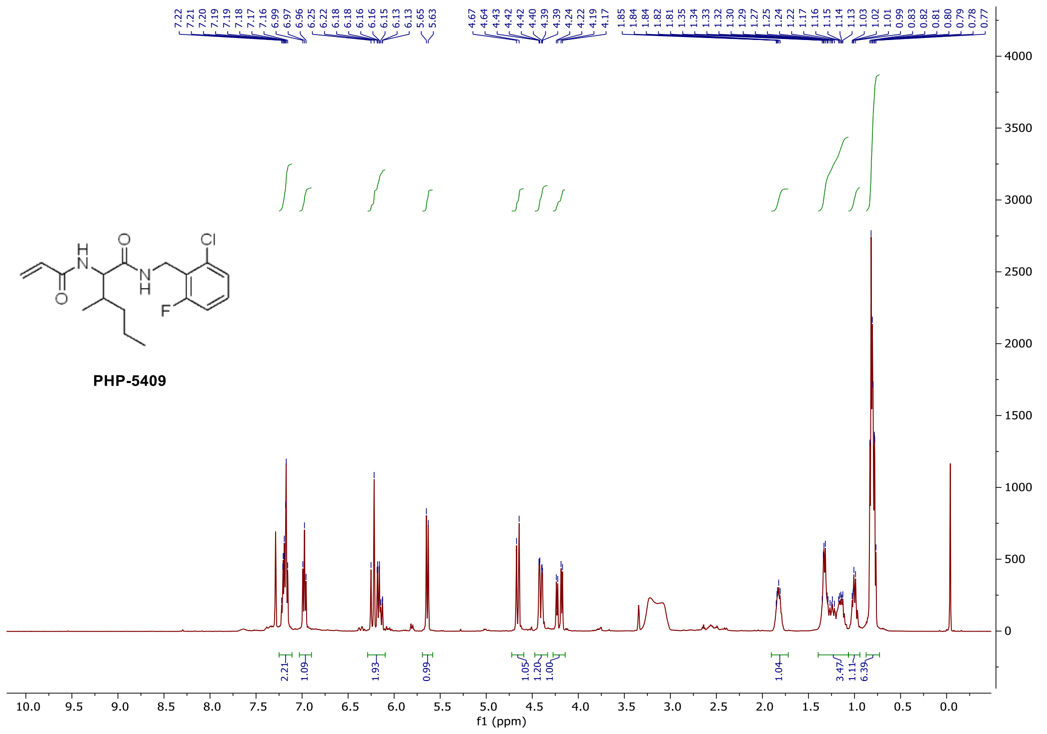

Supplementary Figure 524. ^{1}H NMR (500 MHz, $CDCl_3$ + one drop of CD_3OD) of compound **PHP-5409**.

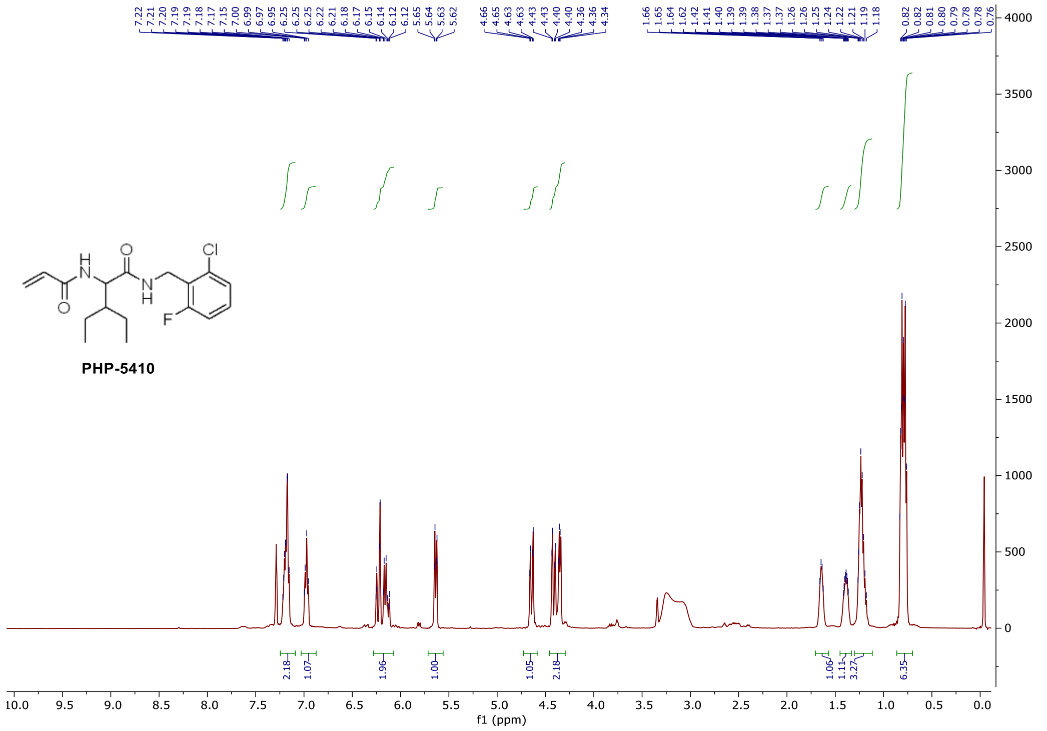

Supplementary Figure 525. ^{1}H NMR (500 MHz, $CDCl_3$ + one drop of CD_3OD) of compound **PHP-5410**.

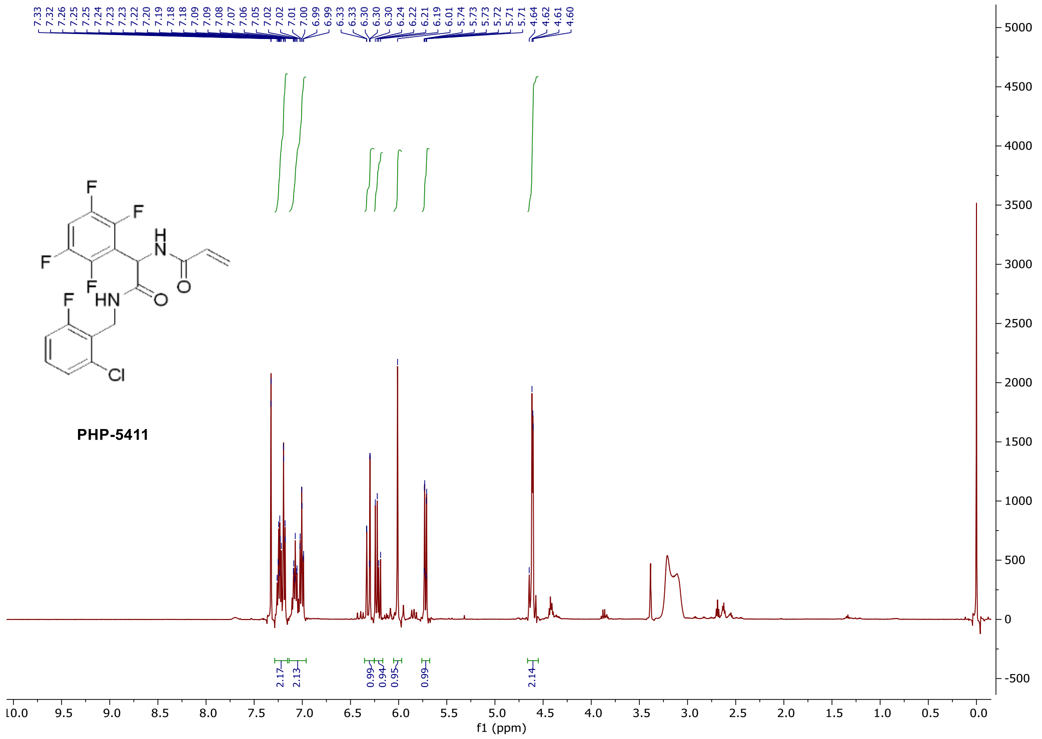

Supplementary Figure 526. ^{1}H NMR (500 MHz, $CDCl_3$ + one drop of CD_3OD) of compound **PHP-5411**.

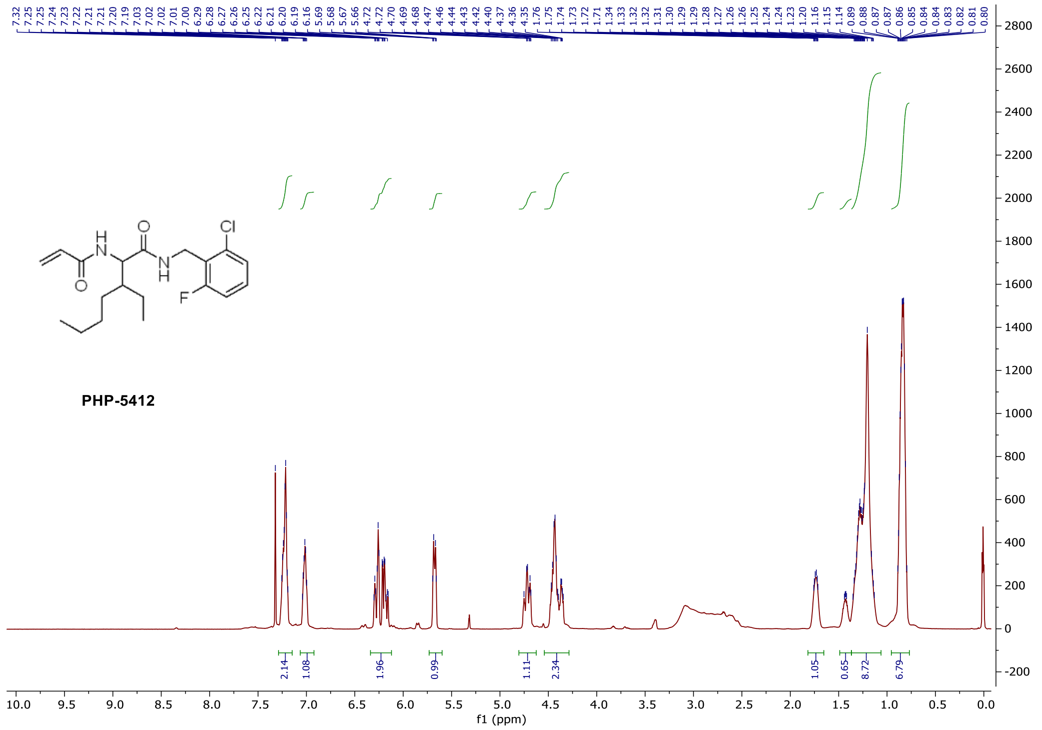

Supplementary Figure 527. ^{1}H NMR (500 MHz, $CDCl_3$ + one drop of CD_3OD) of compound **PHP-5412**.

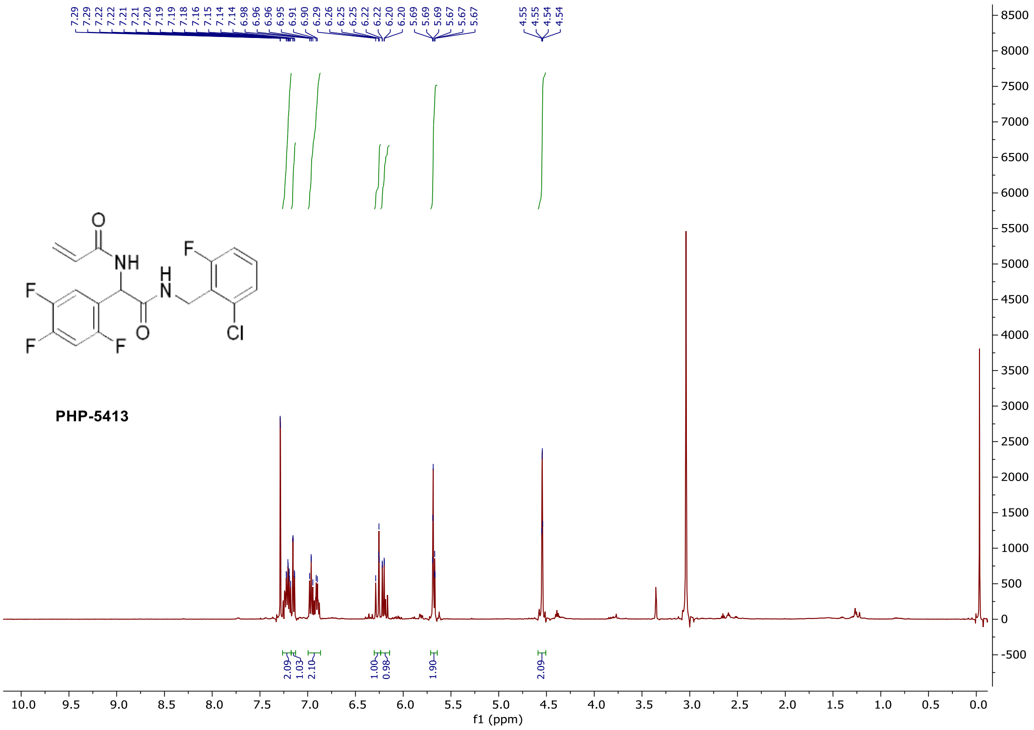

Supplementary Figure 528. ^{1}H NMR (500 MHz, $CDCl_3$ + one drop of CD_3OD) of compound **PHP-5413**.

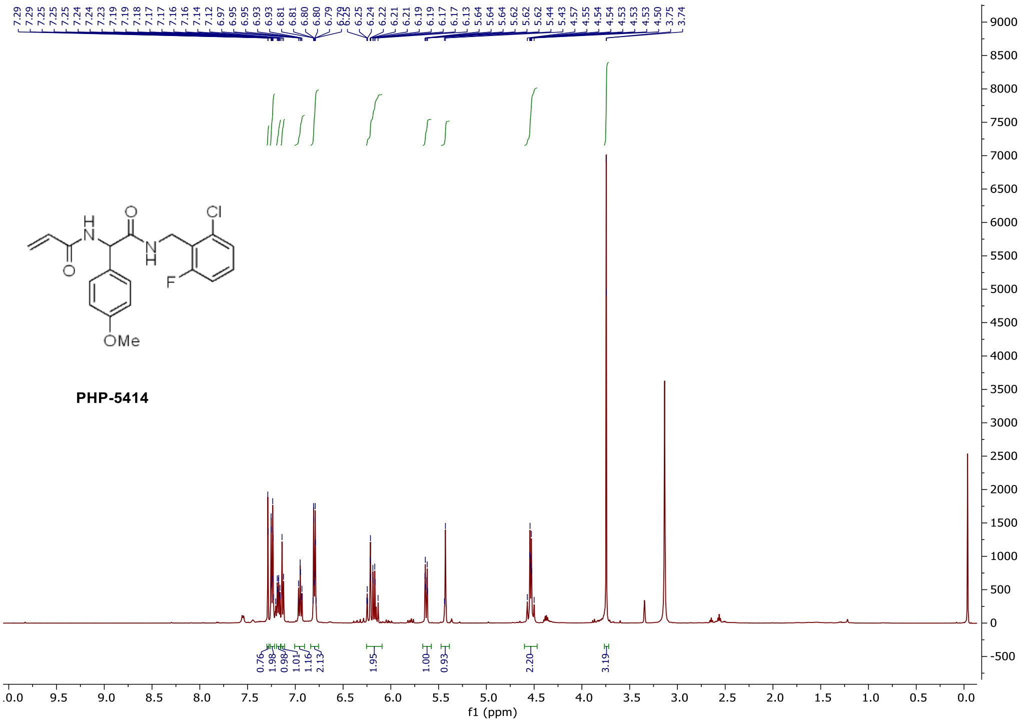

Supplementary Figure 529. ^{1}H NMR (500 MHz, $CDCl_3$ + one drop of CD_3OD) of compound **PHP-5414**.

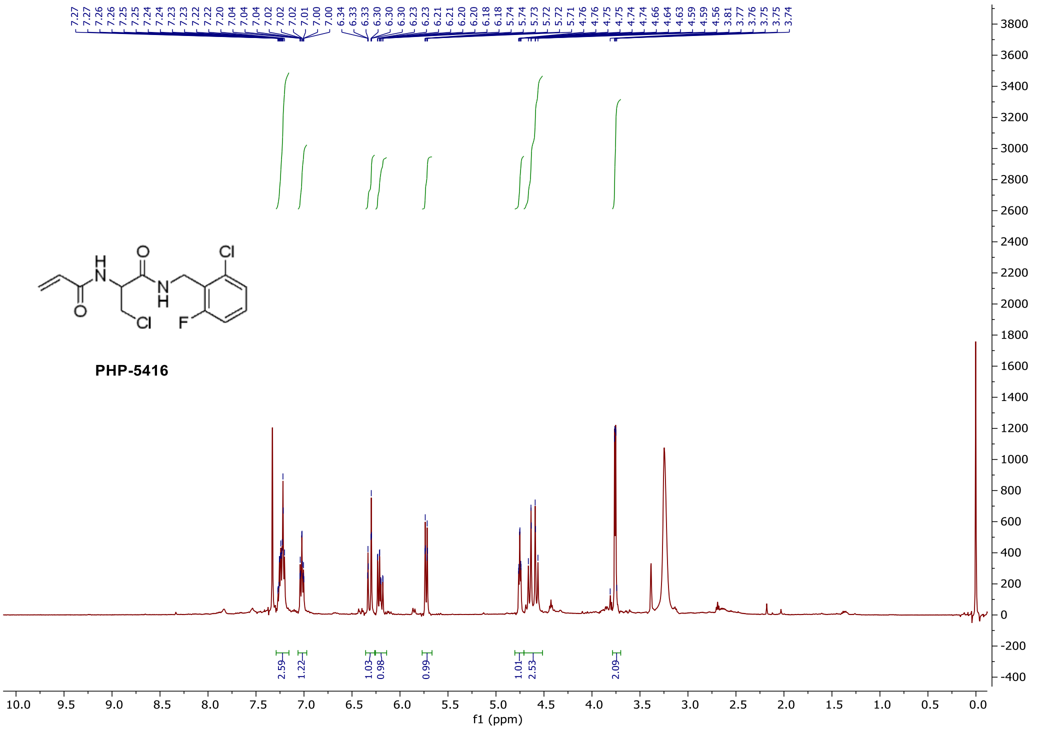

Supplementary Figure 530. ^{1}H NMR (500 MHz, $CDCl_3$ + one drop of CD_3OD) of compound **PHP-5416**.

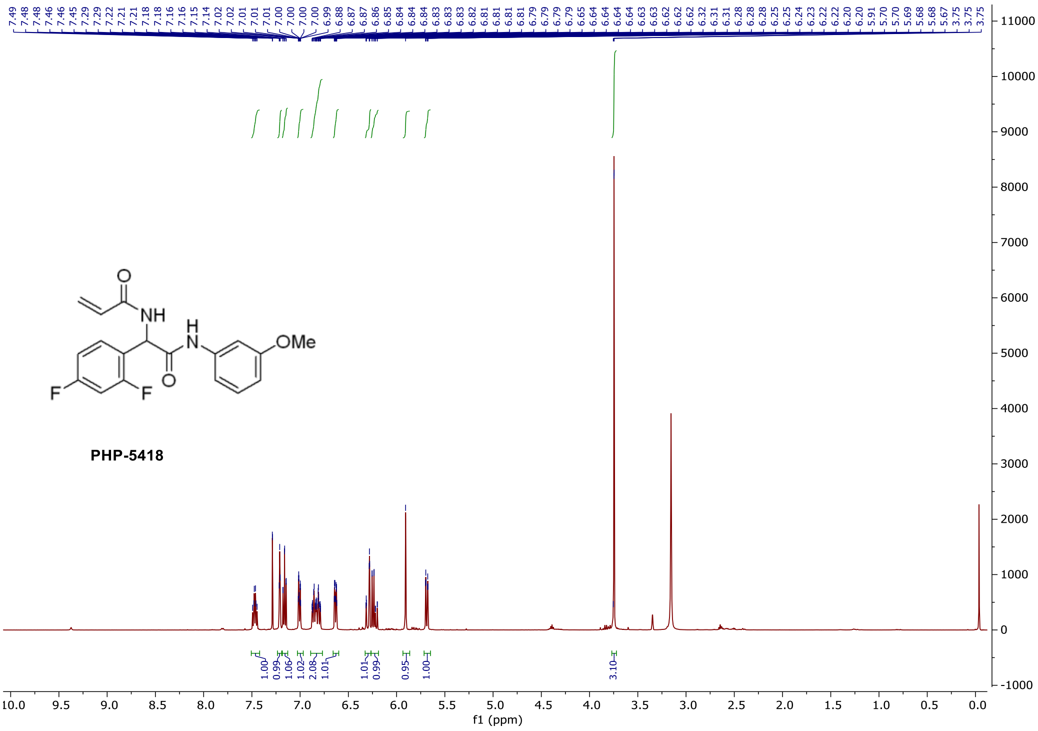

Supplementary Figure 531. ^{1}H NMR (500 MHz, $CDCl_3$ + one drop of CD_3OD) of compound **PHP-5418**.

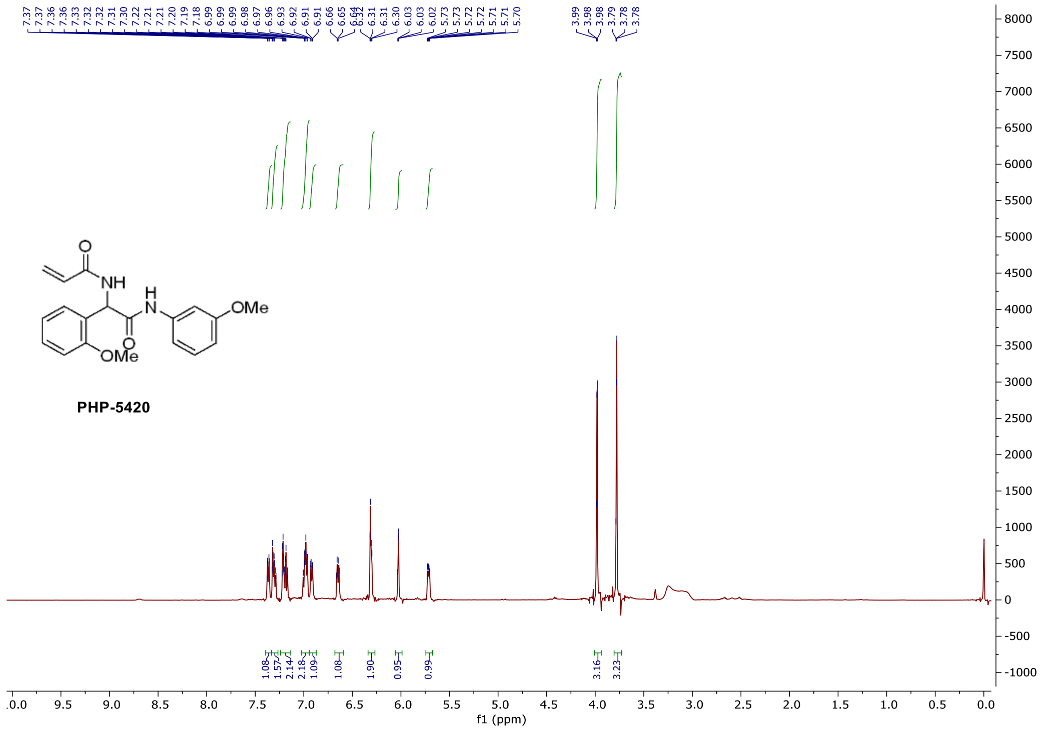

Supplementary Figure 532. ^{1}H NMR (500 MHz, $CDCl_3$ + one drop of CD_3OD) of compound **PHP-5420**.

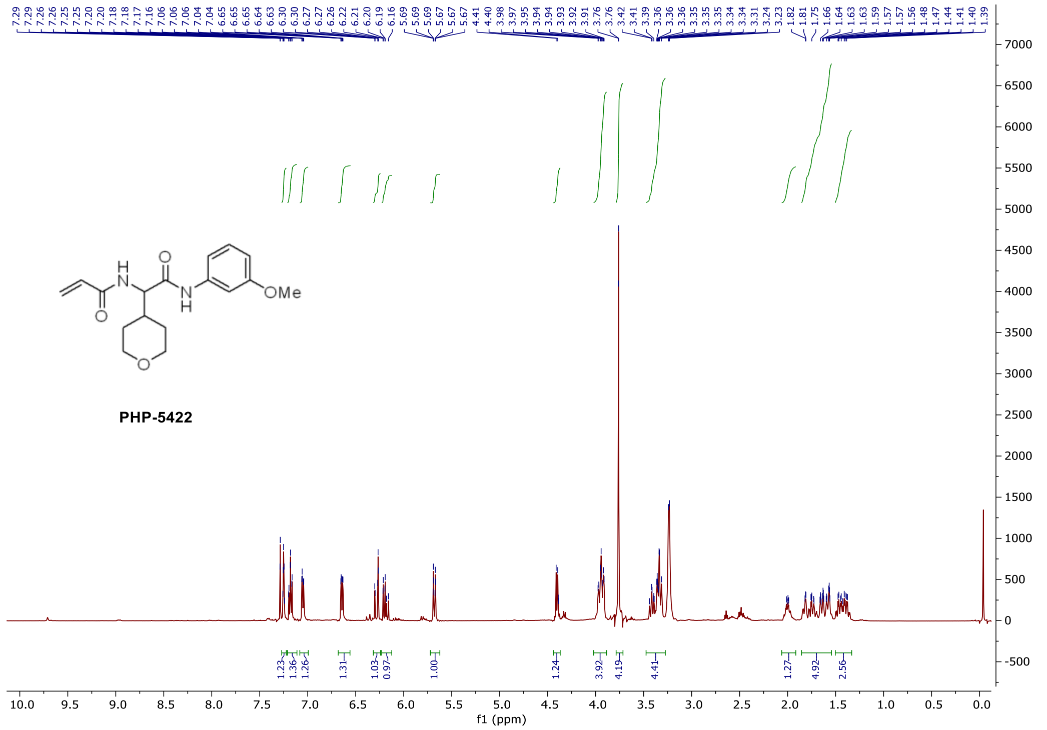

Supplementary Figure 533. ^{1}H NMR (500 MHz, $CDCl_3$ + one drop of CD_3OD) of compound **PHP-5422**.

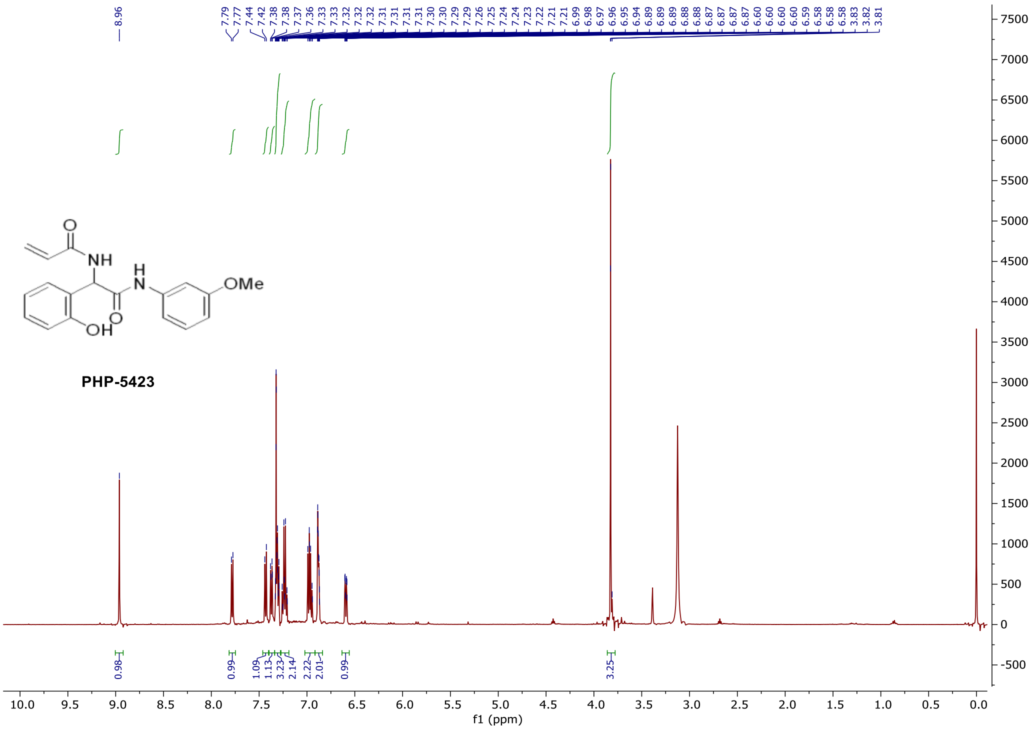

Supplementary Figure 534. ^{1}H NMR (500 MHz, $CDCl_3$ + one drop of CD_3OD) of compound **PHP-5423**.

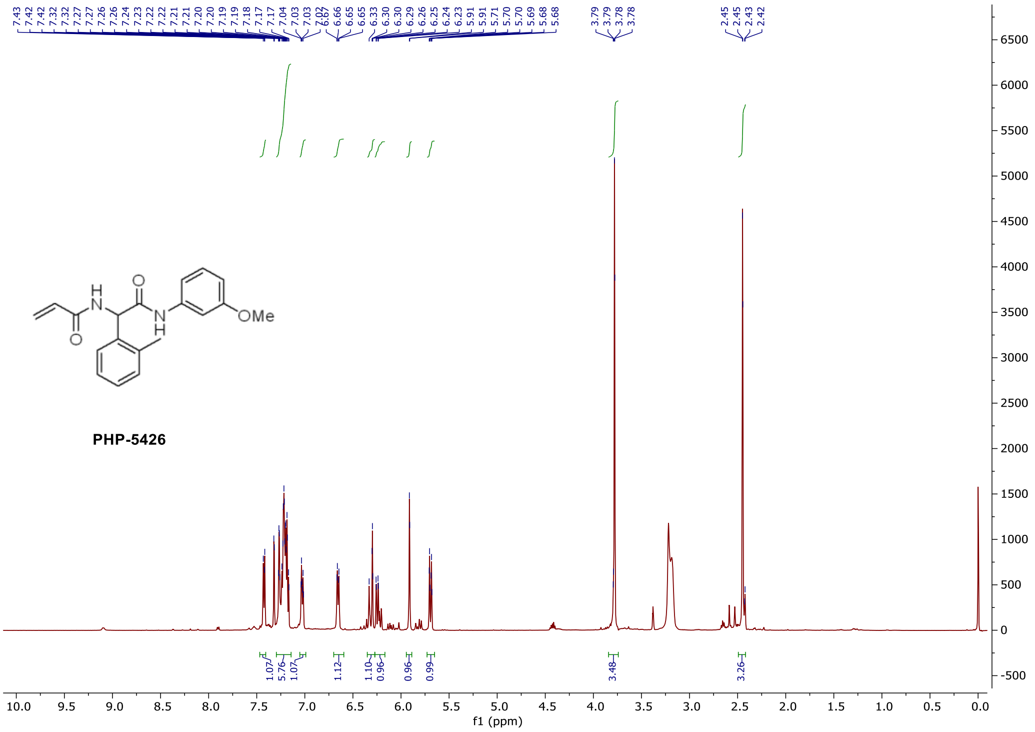

Supplementary Figure 535. ^{1}H NMR (500 MHz, $CDCl_3$ + one drop of CD_3OD) of compound **PHP-5426**.

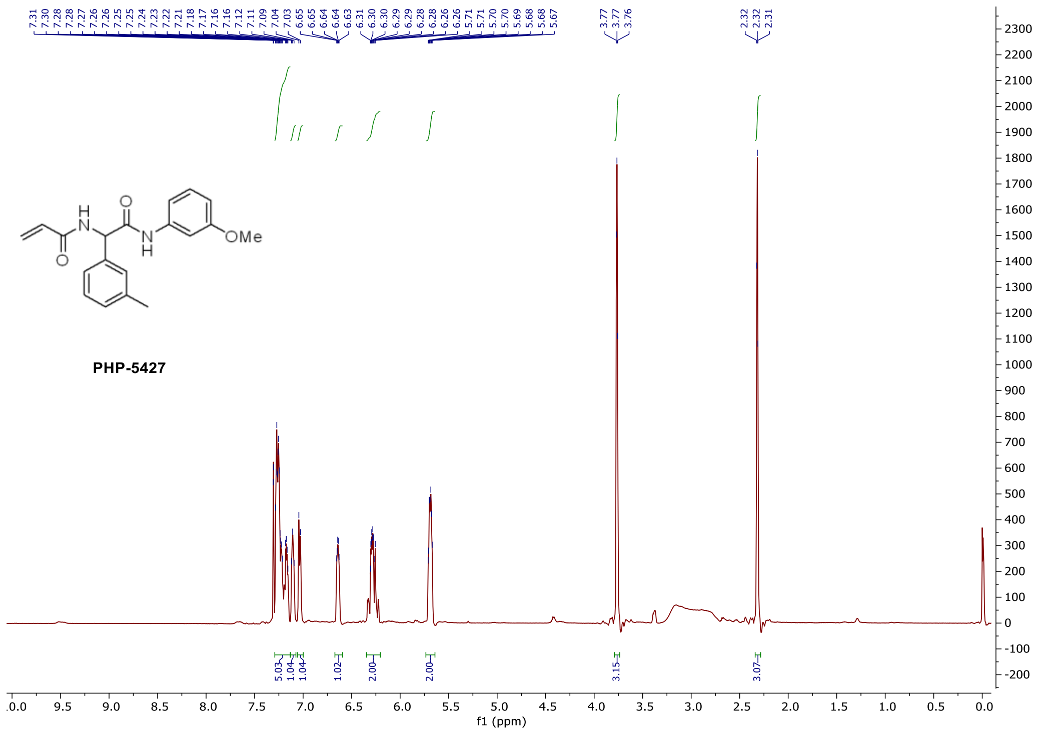

Supplementary Figure 536. ^{1}H NMR (500 MHz, $CDCl_3$ + one drop of CD_3OD) of compound **PHP-5427**.

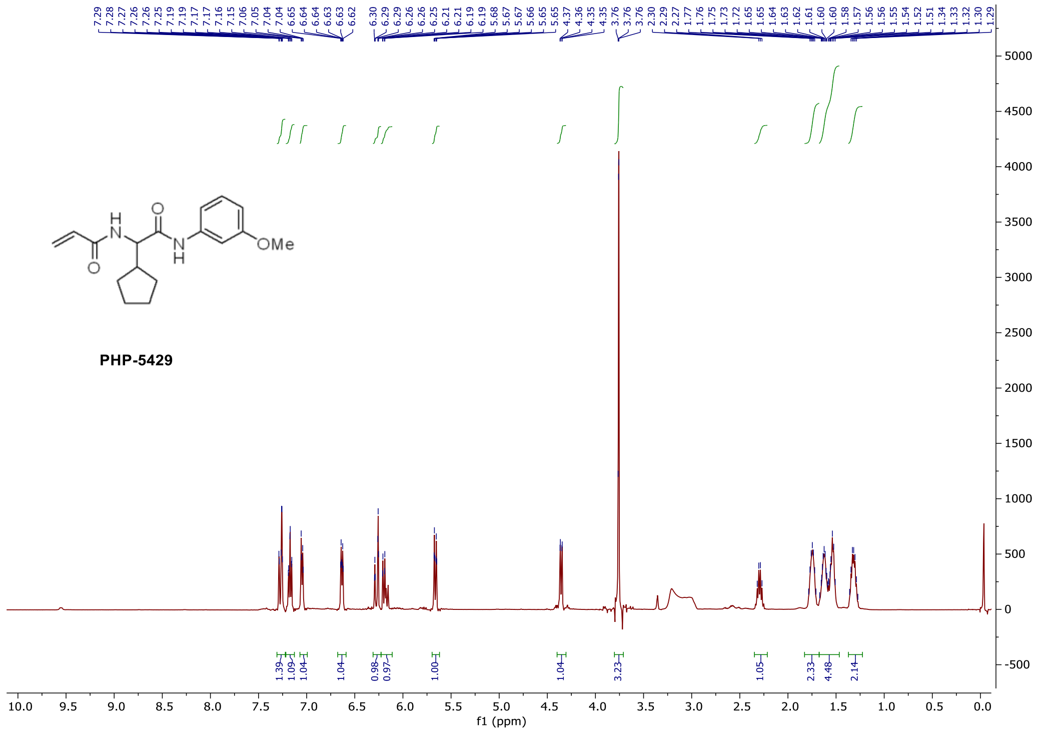

Supplementary Figure 537. ^{1}H NMR (500 MHz, $CDCl_3$ + one drop of CD_3OD) of compound **PHP-5429**.

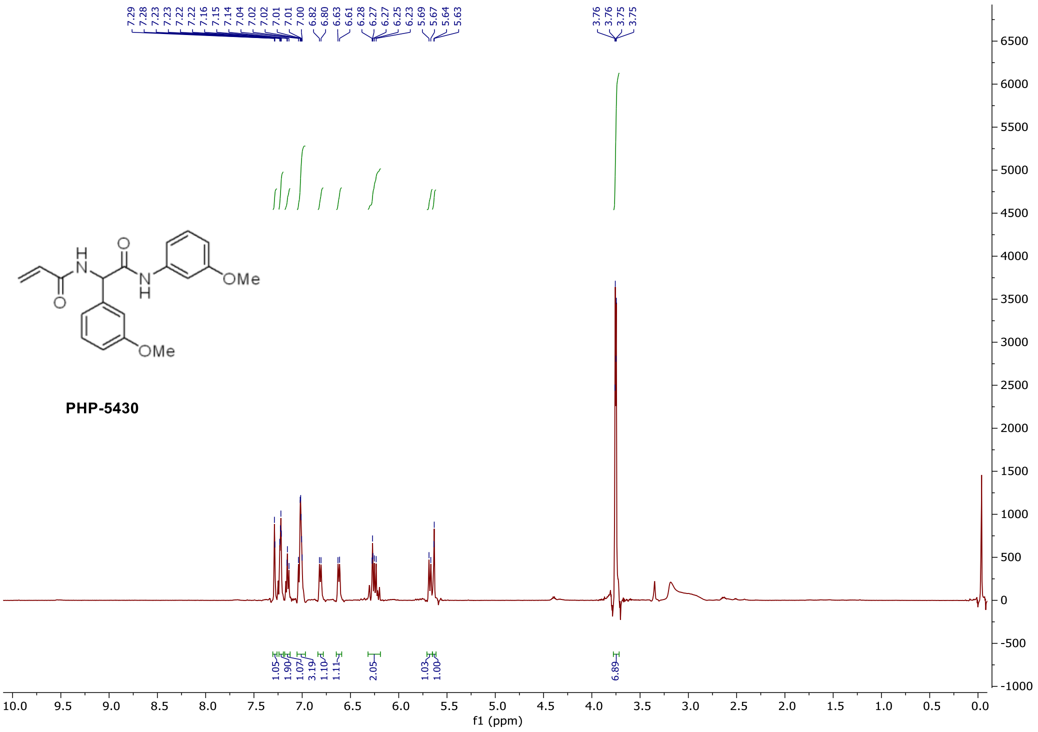

Supplementary Figure 538. ^{1}H NMR (500 MHz, $CDCl_3$ + one drop of CD_3OD) of compound **PHP-5430**.

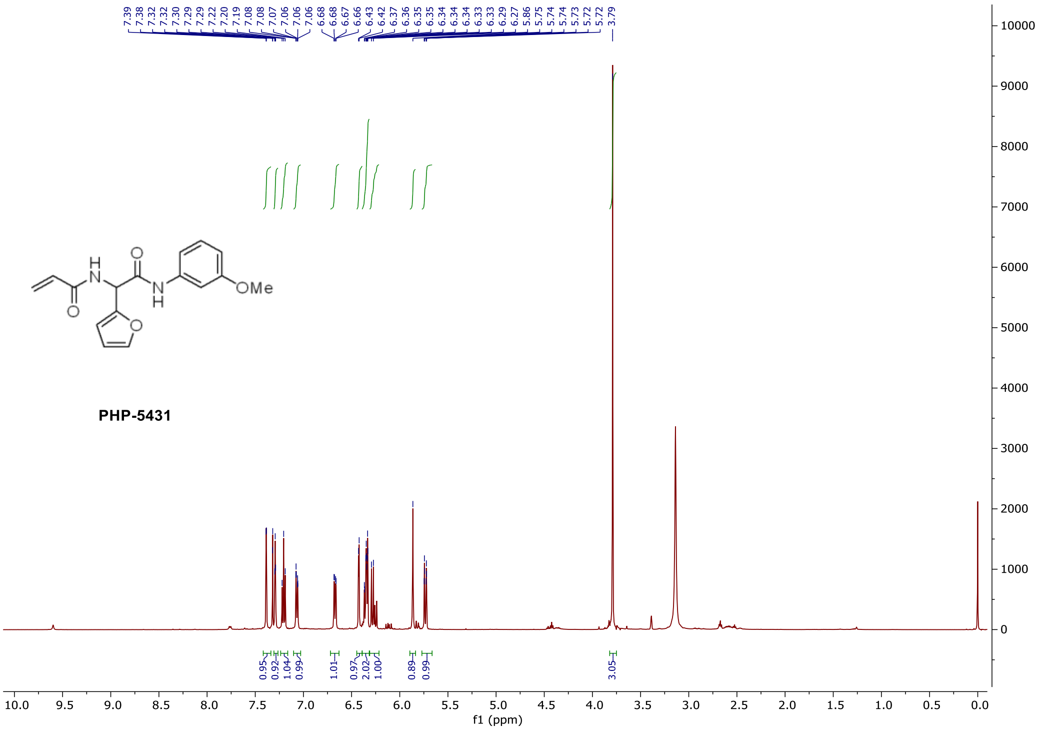

Supplementary Figure 539. ^{1}H NMR (500 MHz, $CDCl_3$ + one drop of CD_3OD) of compound **PHP-5431**.

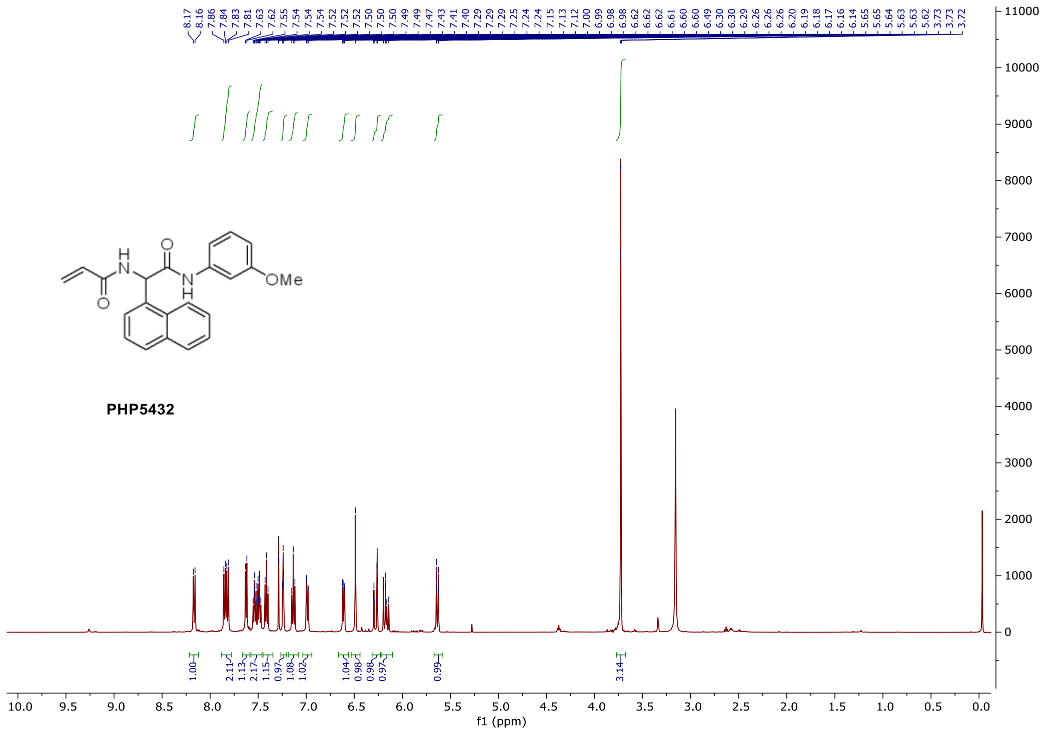

Supplementary Figure 540. ^{1}H NMR (500 MHz, $CDCl_3$ + one drop of CD_3OD) of compound **PHP-5432**.

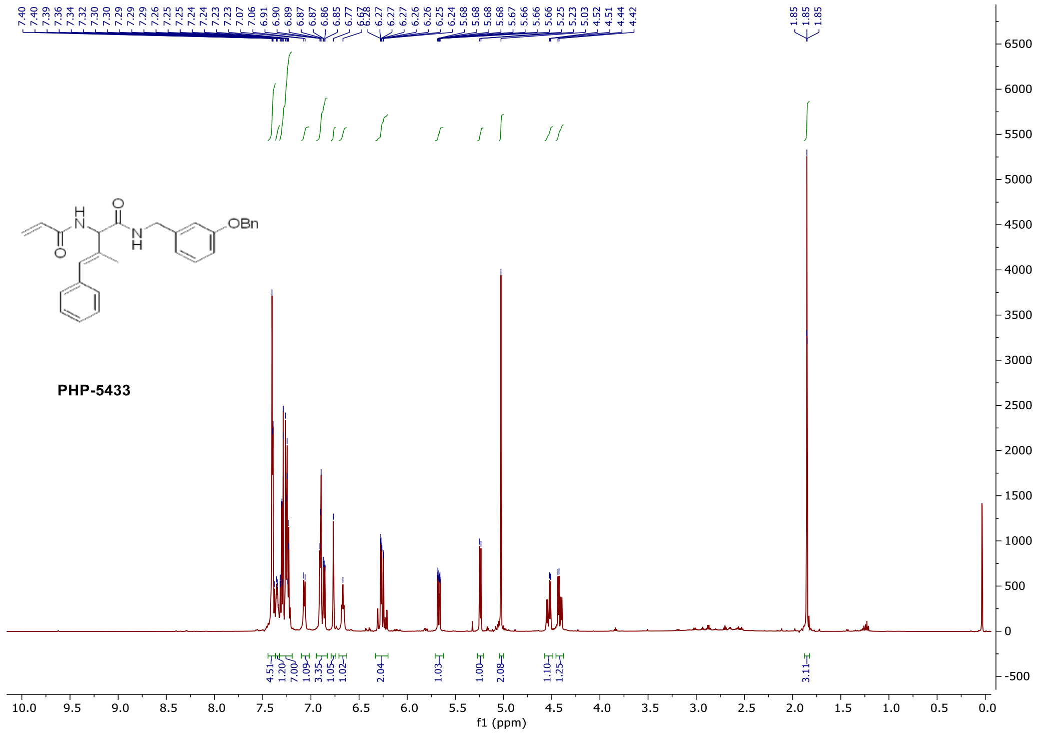

Supplementary Figure 541. ^{1}H NMR (500 MHz, $CDCl_3$) of compound **PHP-5433**.

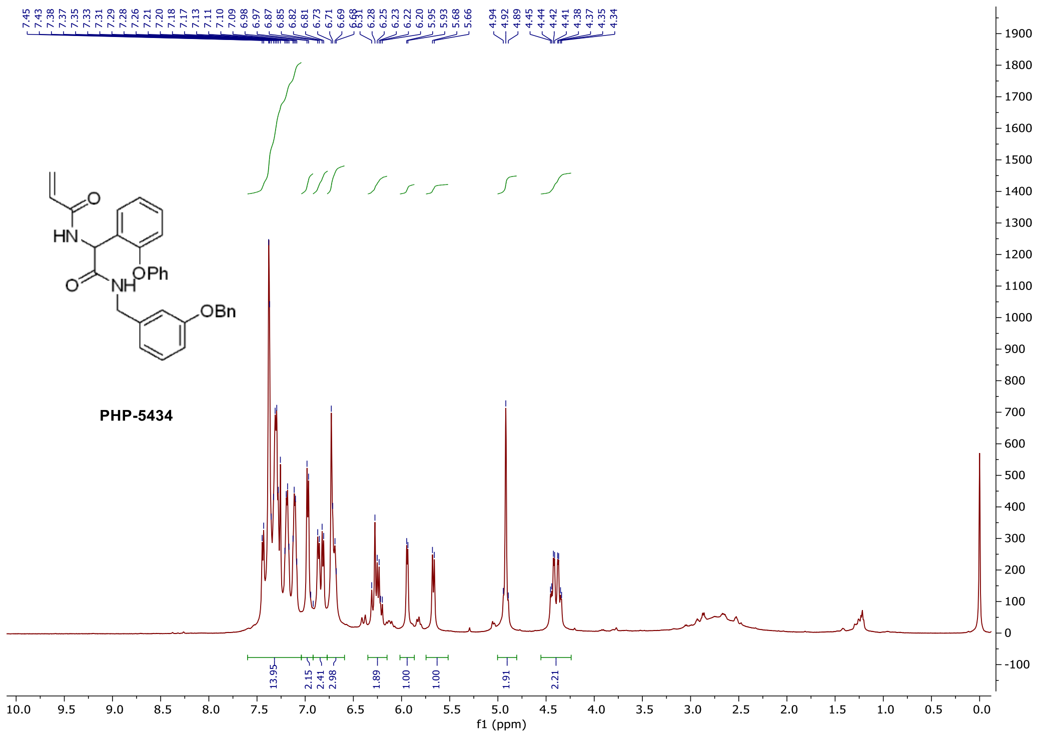

Supplementary Figure 542. ^{1}H NMR (500 MHz, $CDCl_3$ + one drop of CD_3OD) of compound **PHP-5434**.

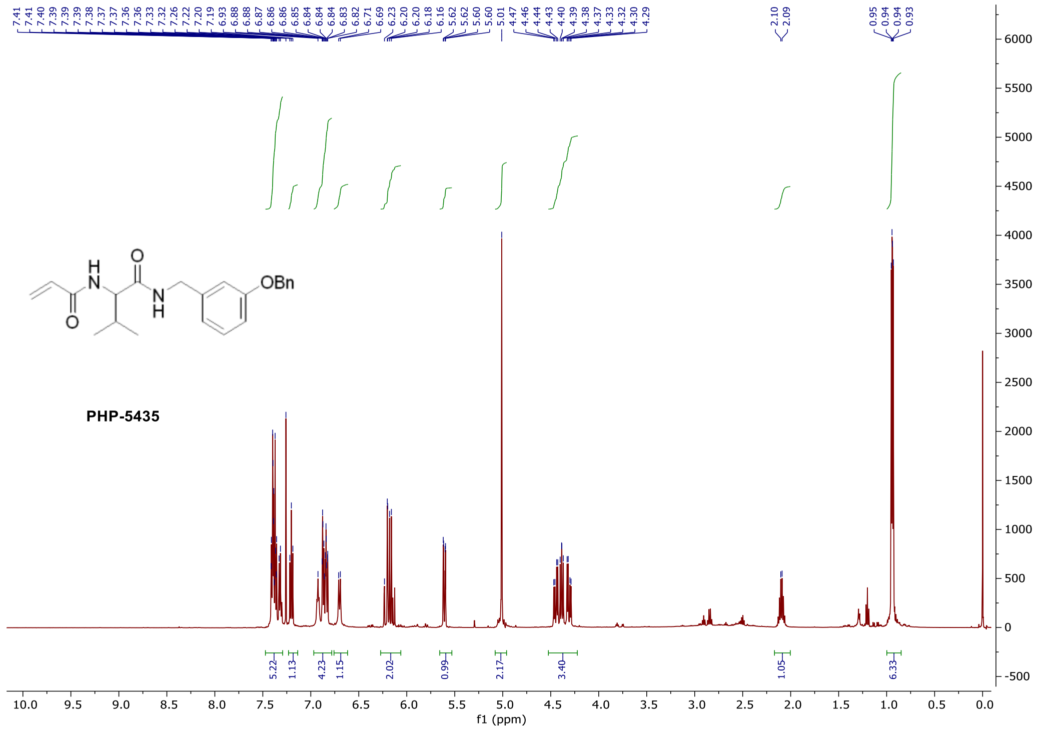

Supplementary Figure 543. ^{1}H NMR (500 MHz, $CDCl_3$) of compound **PHP-5435**.

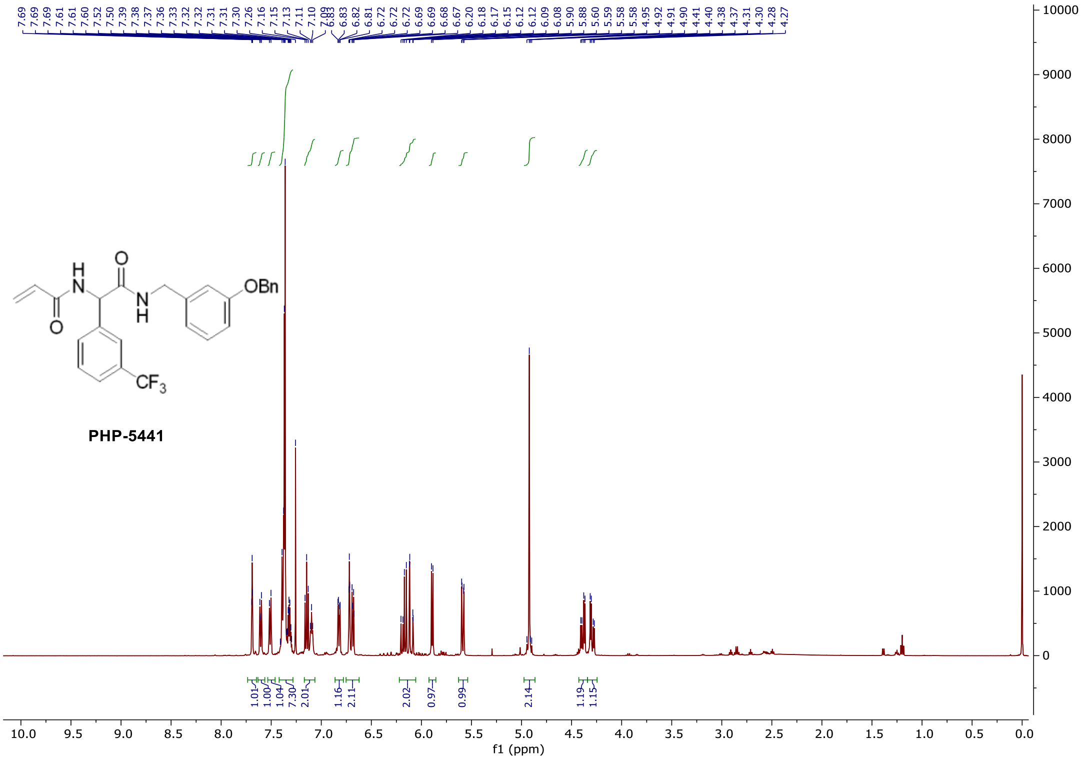

Supplementary Figure 544. ^{1}H NMR (500 MHz, $CDCl_3$) of compound **PHP-5441**.

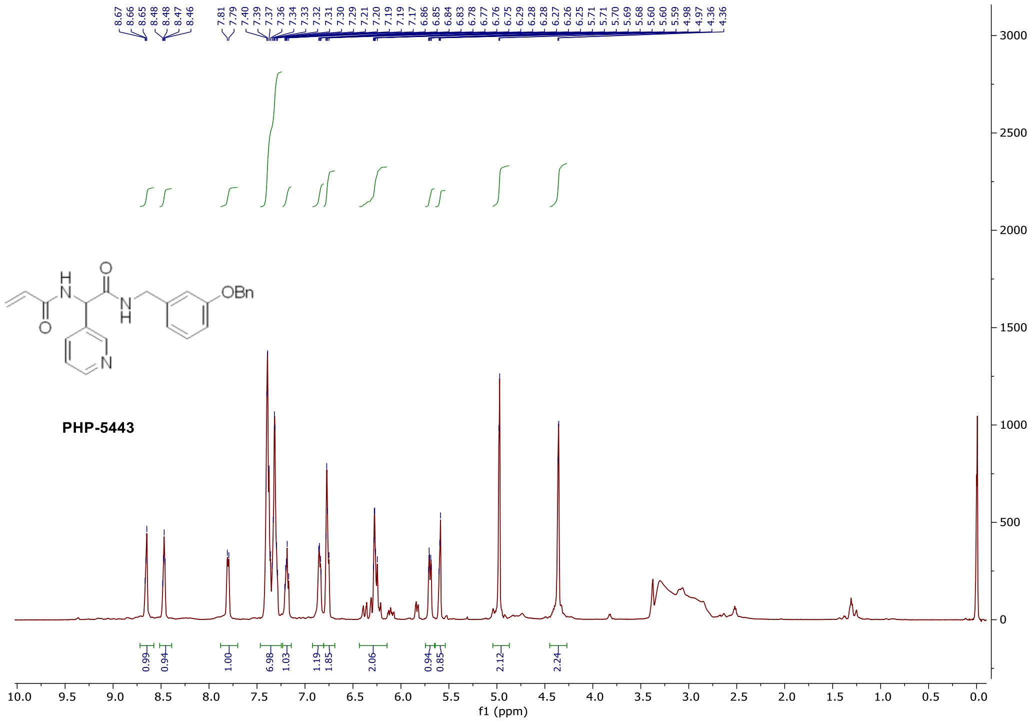

Supplementary Figure 545. ^{1}H NMR (500 MHz, $CDCl_3$ + one drop of CD_3OD) of compound **PHP-5443**.

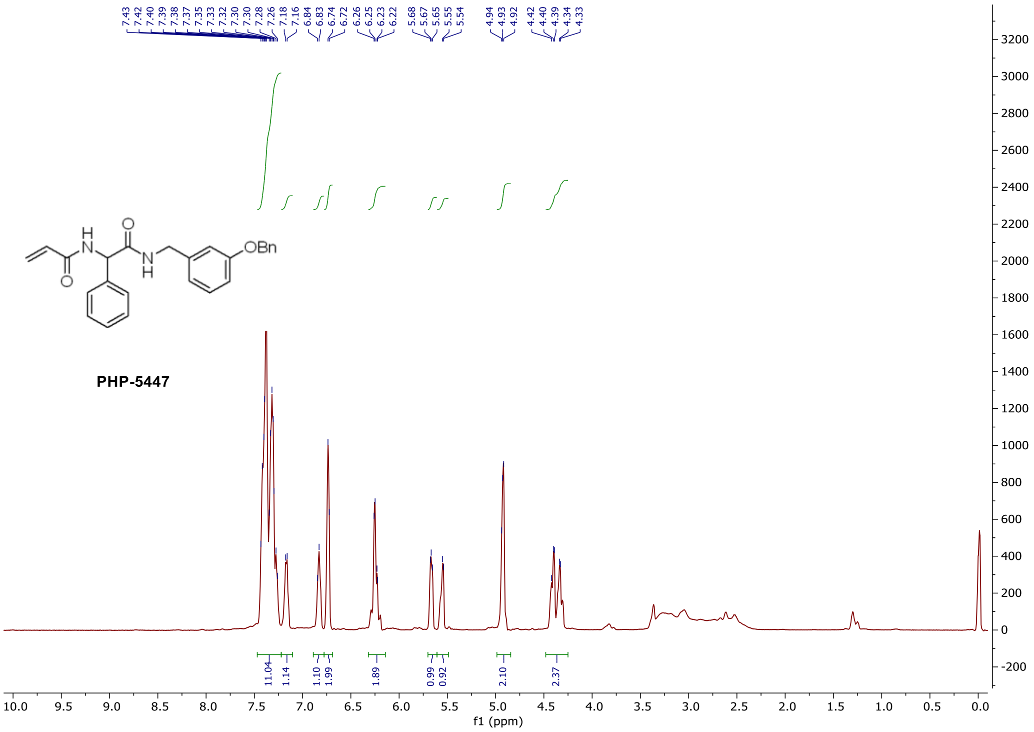

Supplementary Figure 546. ^{1}H NMR (500 MHz, $CDCl_3$ + one drop of CD_3OD) of compound **PHP-5447**.

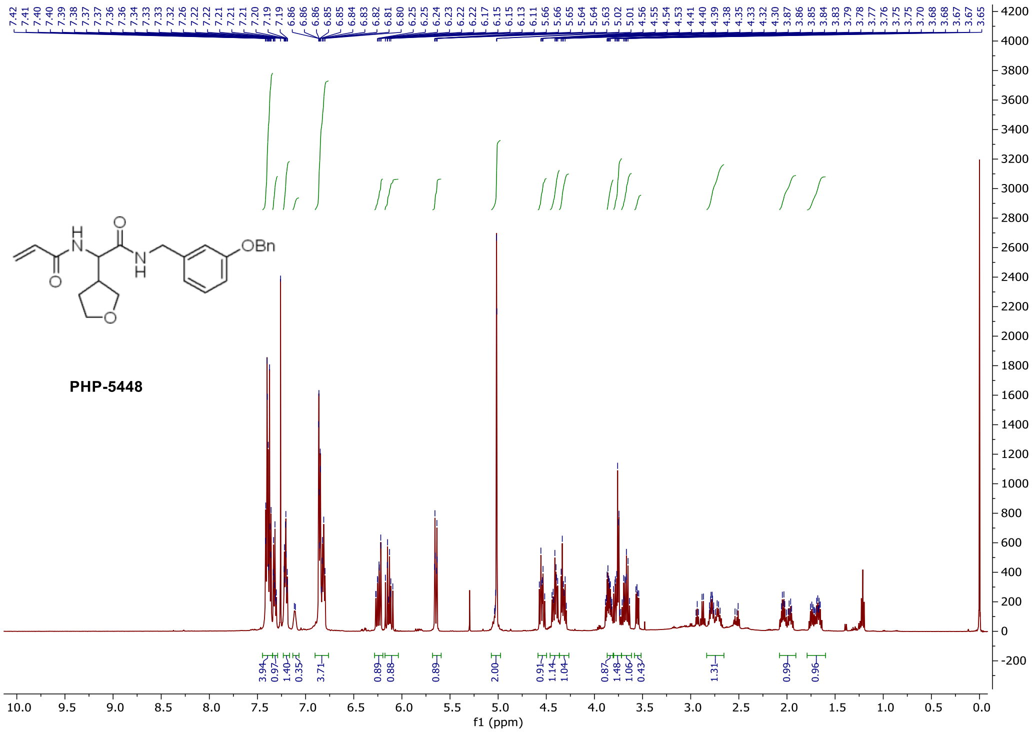

Supplementary Figure 547. ^{1}H NMR (500 MHz, $CDCl_3$) of compound **PHP-5448**.

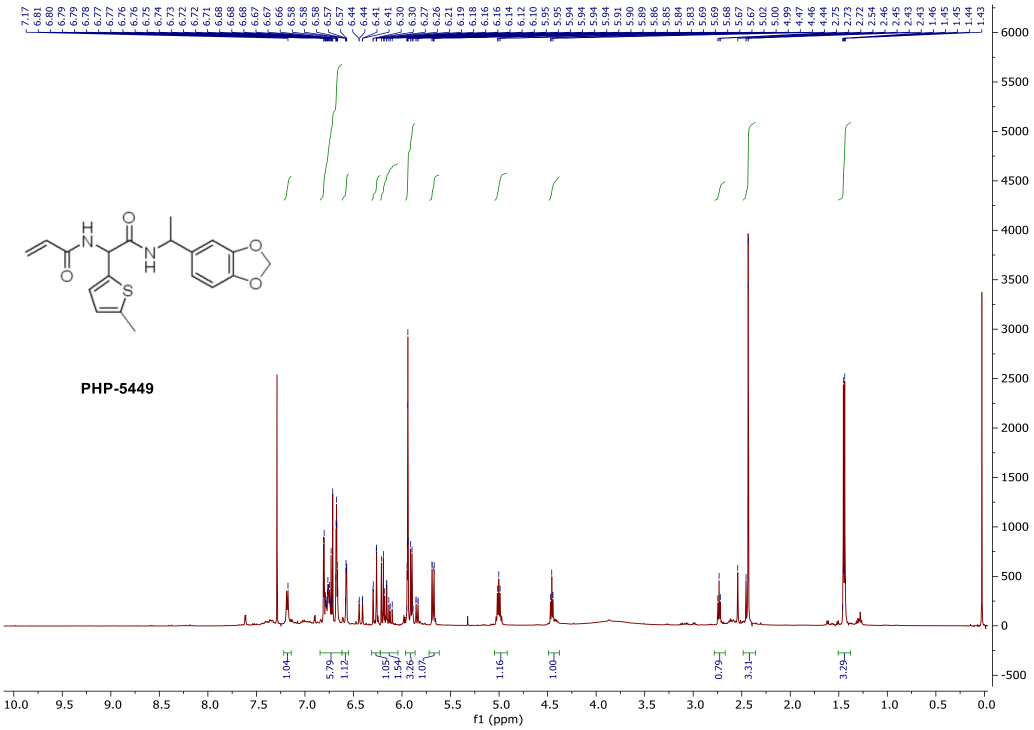

Supplementary Figure 548. ^{1}H NMR (500 MHz, $CDCl_3$) of compound **PHP-5449**.

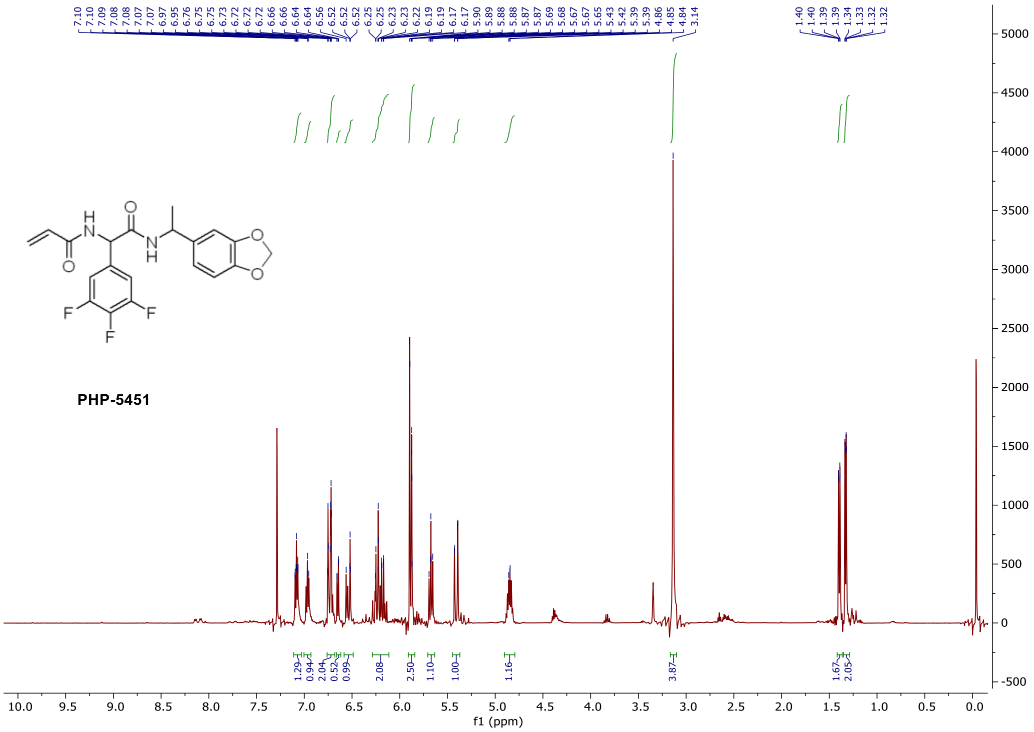

Supplementary Figure 549. ^{1}H NMR (500 MHz, $CDCl_3$) of compound **PHP-5451**.

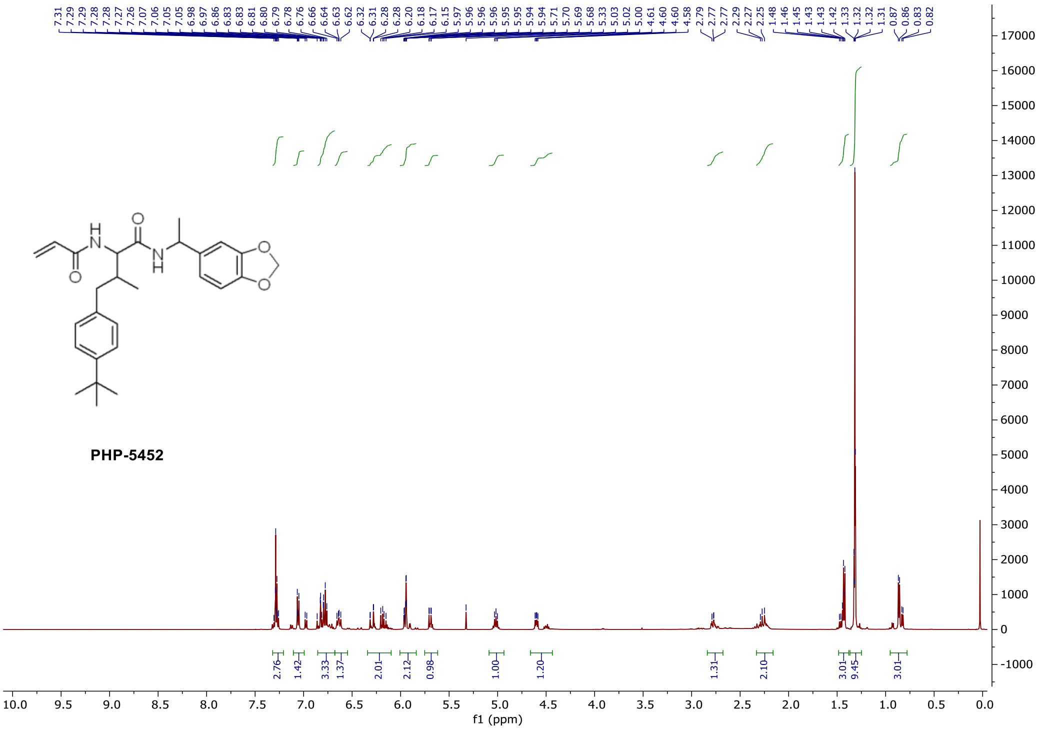

Supplementary Figure 550. ^{1}H NMR (500 MHz, $CDCl_3$) of compound **PHP-5452**.

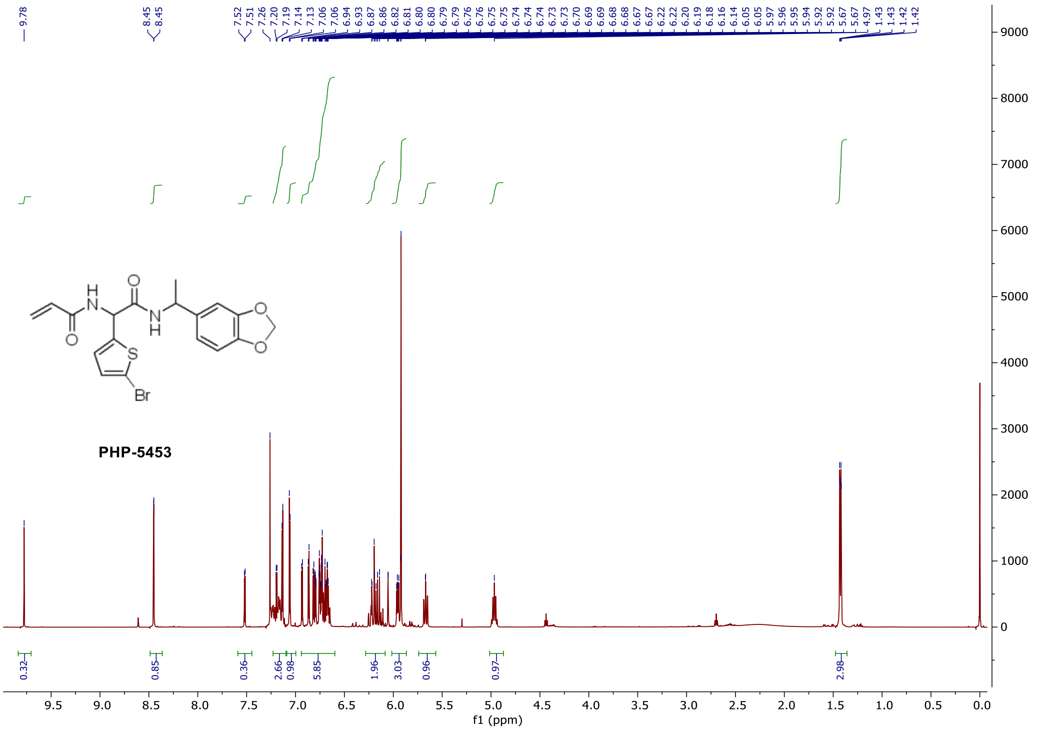

Supplementary Figure 551. ^{1}H NMR (500 MHz, $CDCl_3$) of compound **PHP-5453**.

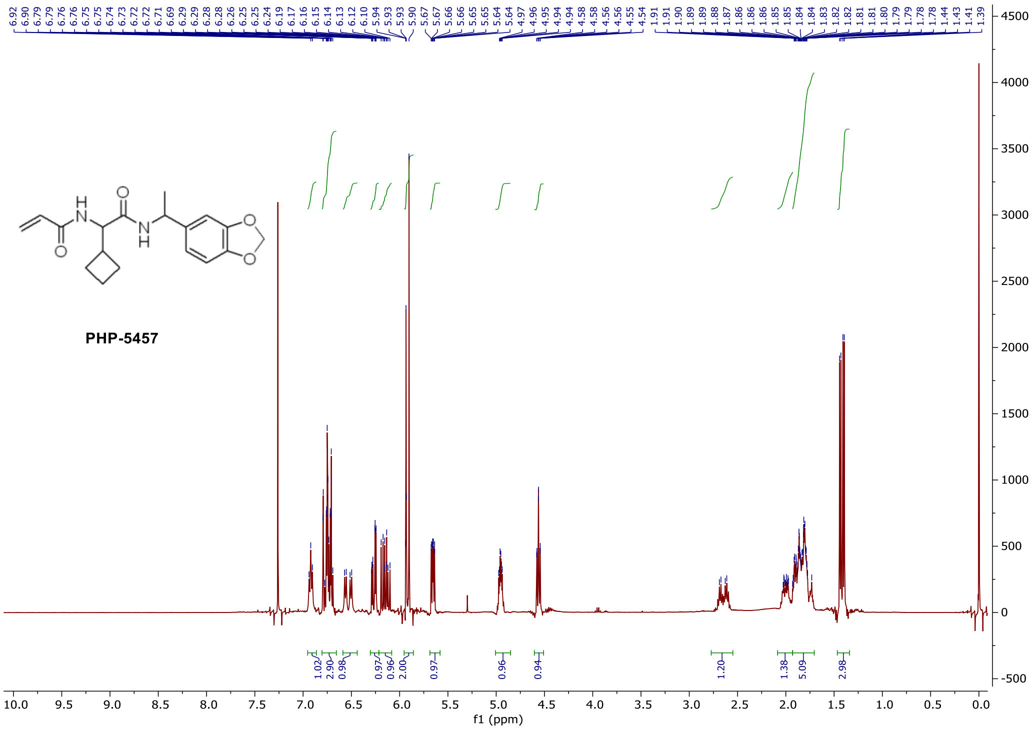

Supplementary Figure 552. ^{1}H NMR (500 MHz, $CDCl_3$) of compound **PHP-5457**.

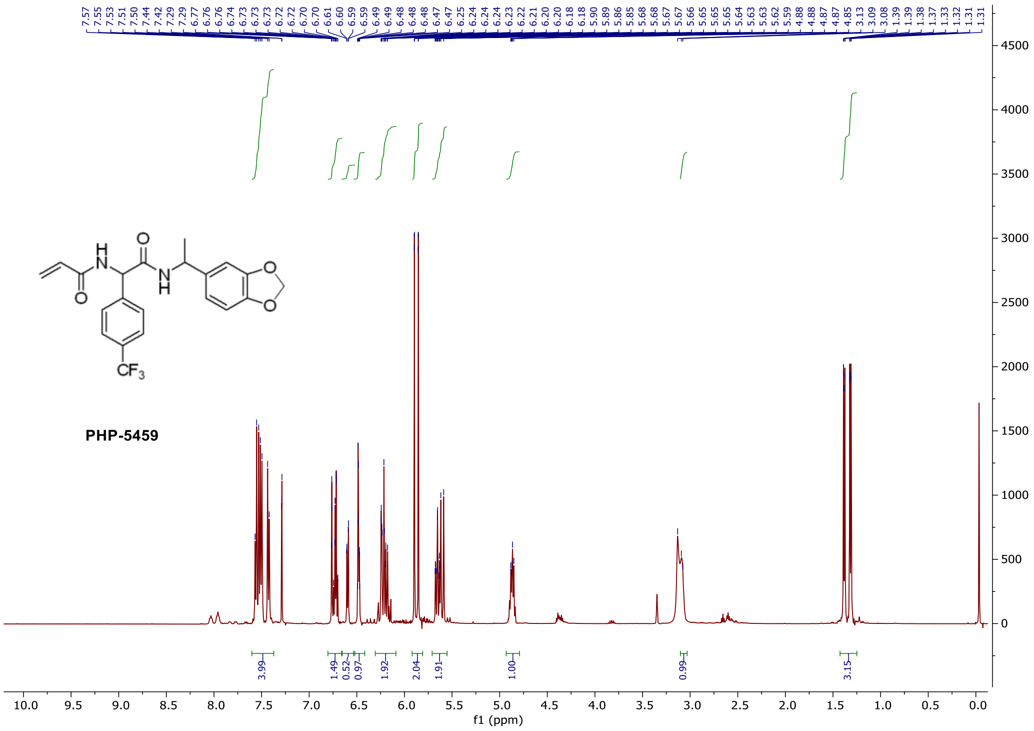

Supplementary Figure 553. ^{1}H NMR (500 MHz, $CDCl_3$ + one drop of CD_3OD) of compound **PHP-5459**.

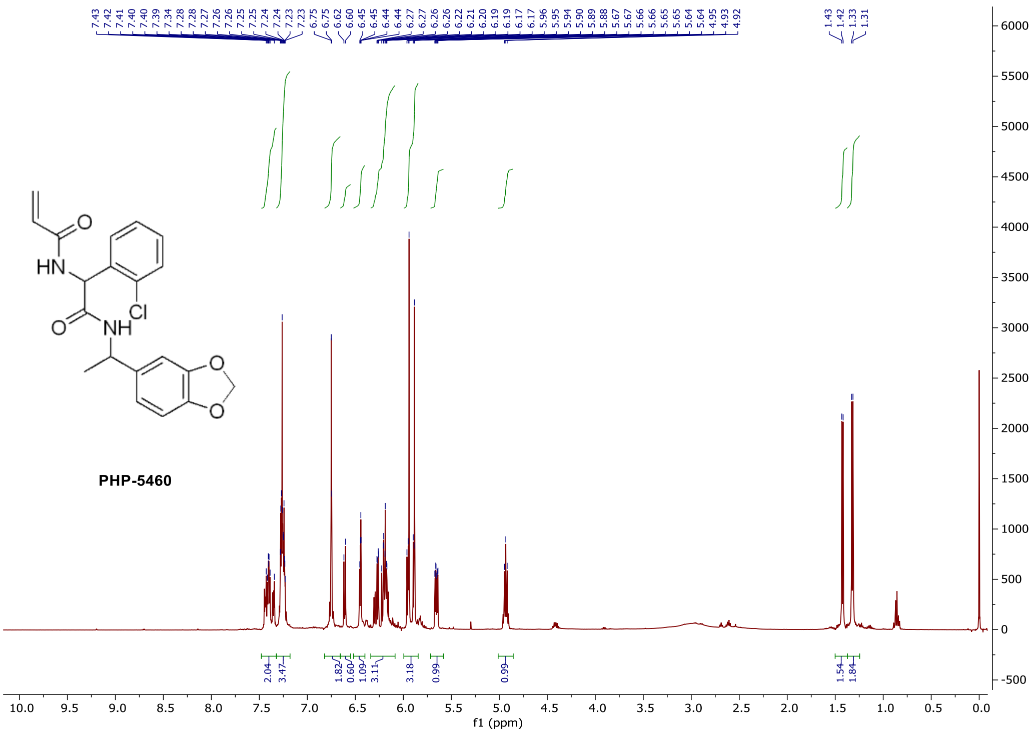

Supplementary Figure 554. ^{1}H NMR (500 MHz, $CDCl_3$) of compound **PHP-5460**.

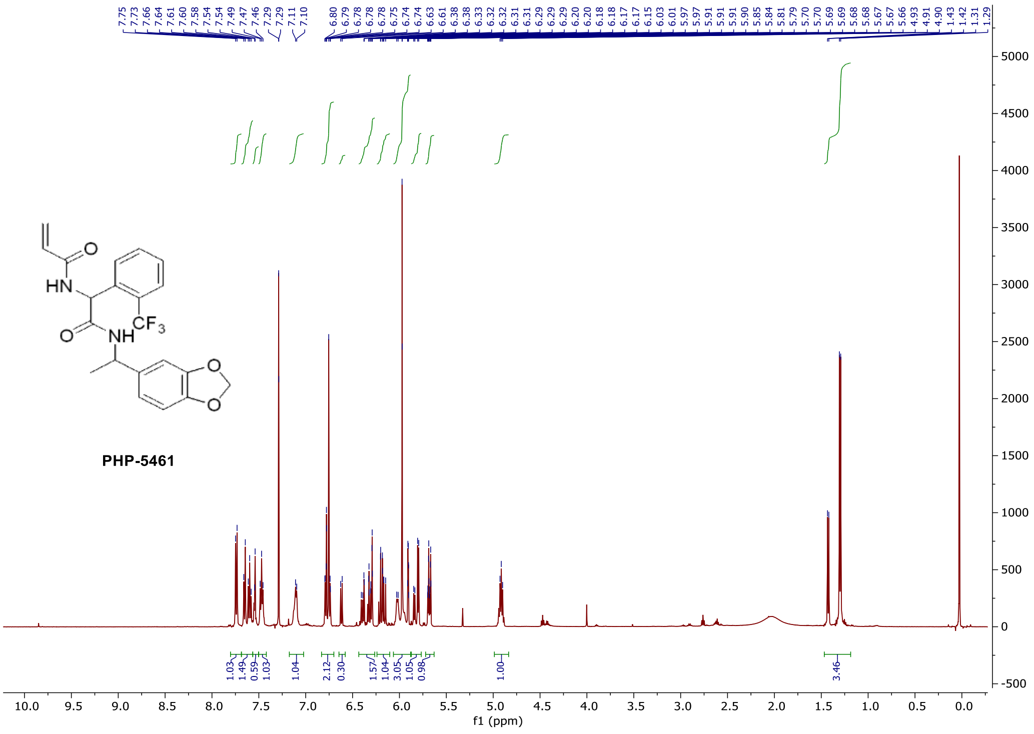

Supplementary Figure 555. ^{1}H NMR (500 MHz, $CDCl_3$) of compound **PHP-5461**.

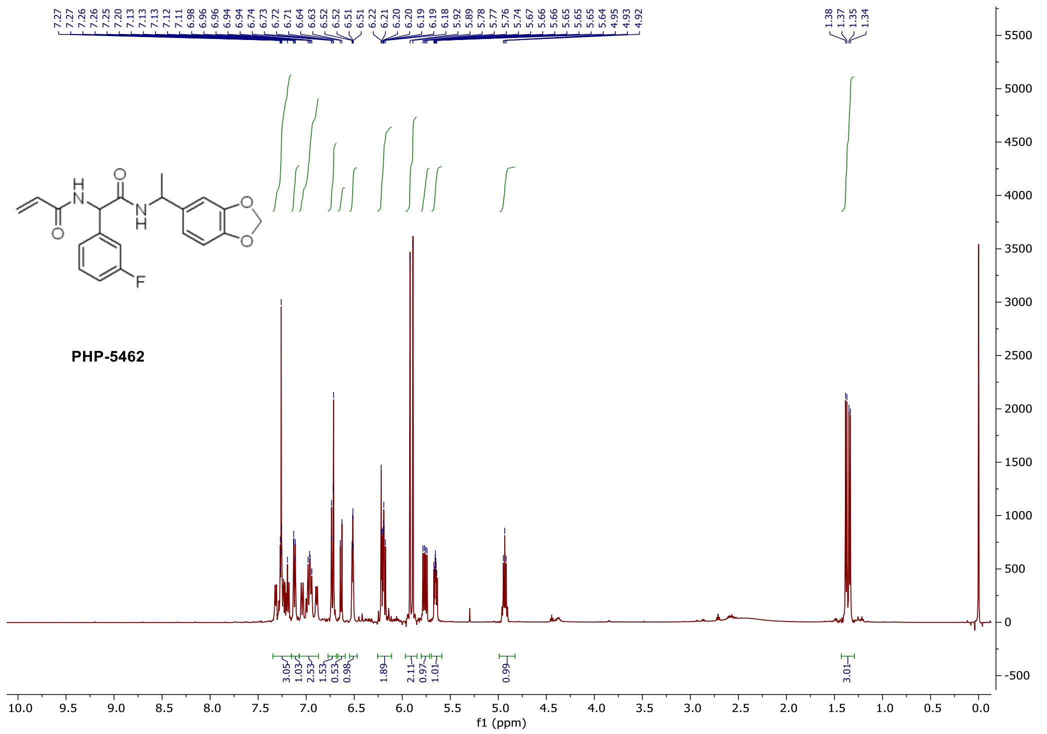

Supplementary Figure 556. ^{1}H NMR (500 MHz, $CDCl_3$) of compound **PHP-5462**.

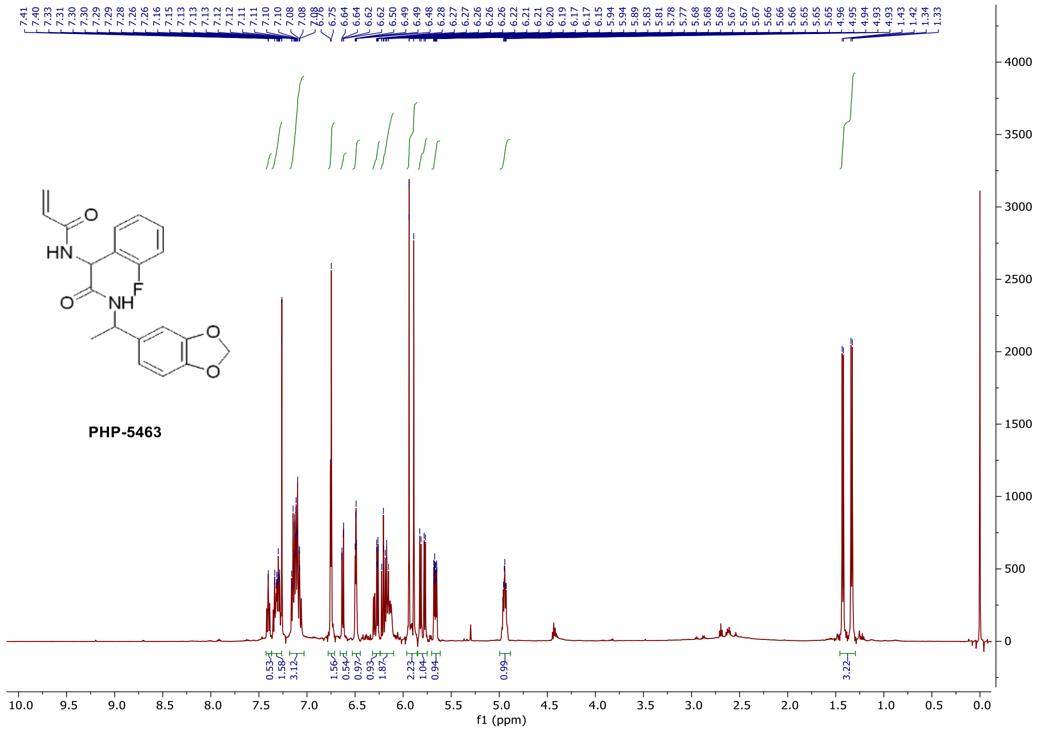

Supplementary Figure 557. ^{1}H NMR (500 MHz, $CDCl_3$) of compound **PHP-5463**.

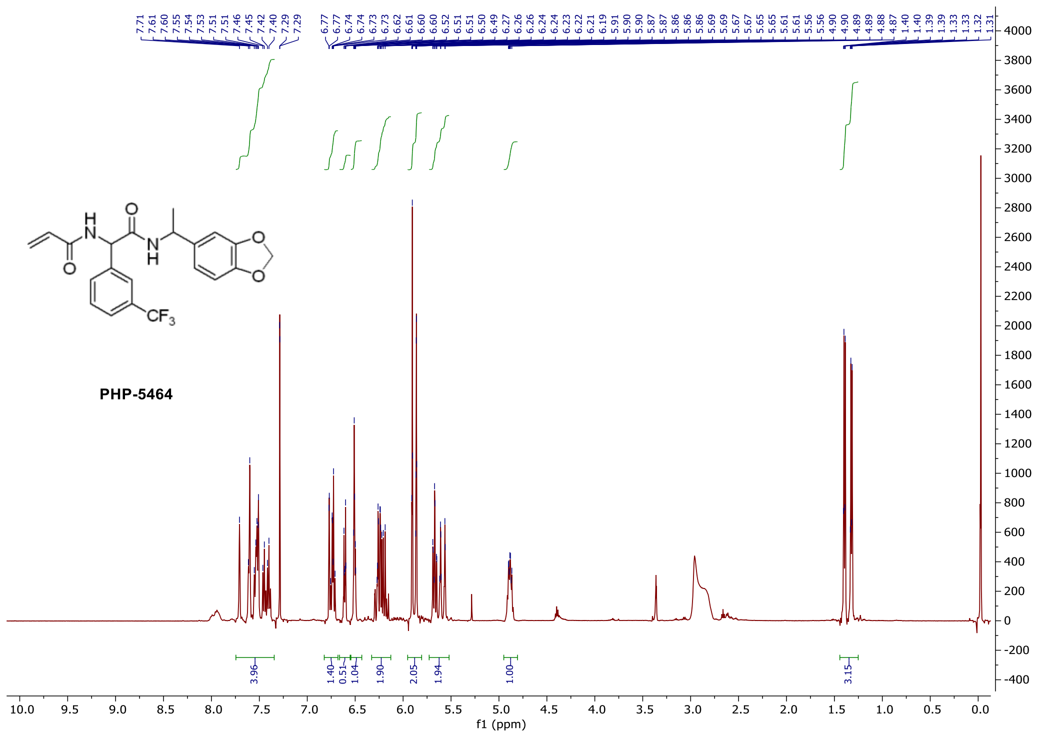

Supplementary Figure 558. ^{1}H NMR (500 MHz, $CDCl_3$ + one drop of CD_3OD) of compound **PHP-5464**.

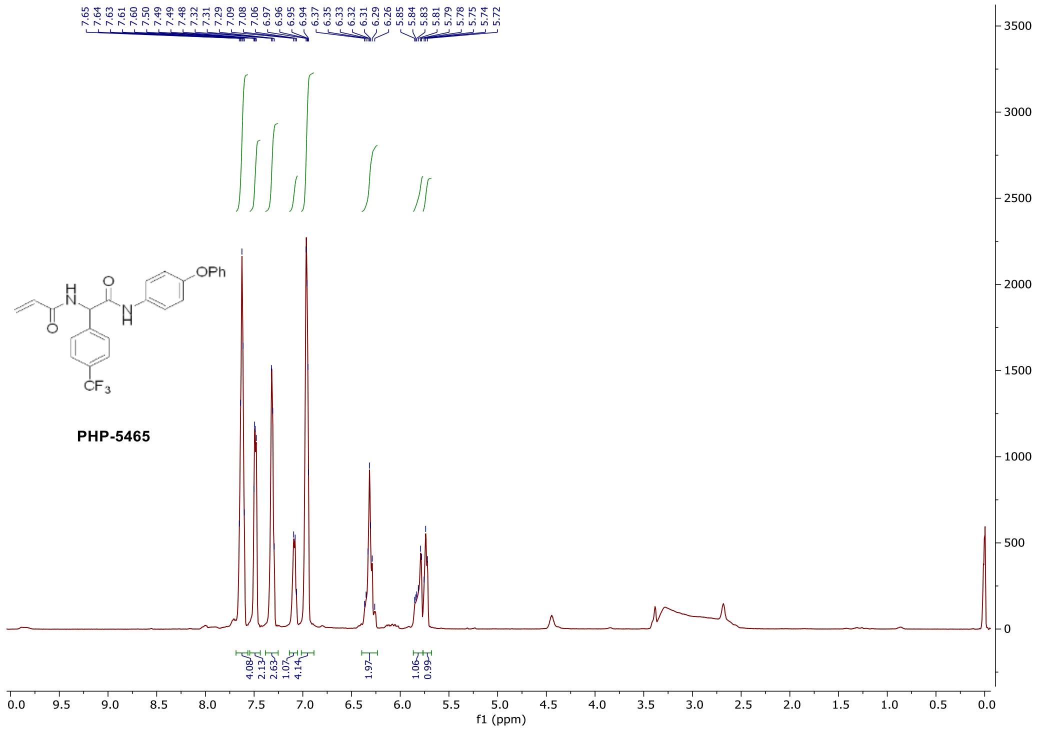

Supplementary Figure 559. ^{1}H NMR (500 MHz, $CDCl_3$) of compound **PHP-5465**.

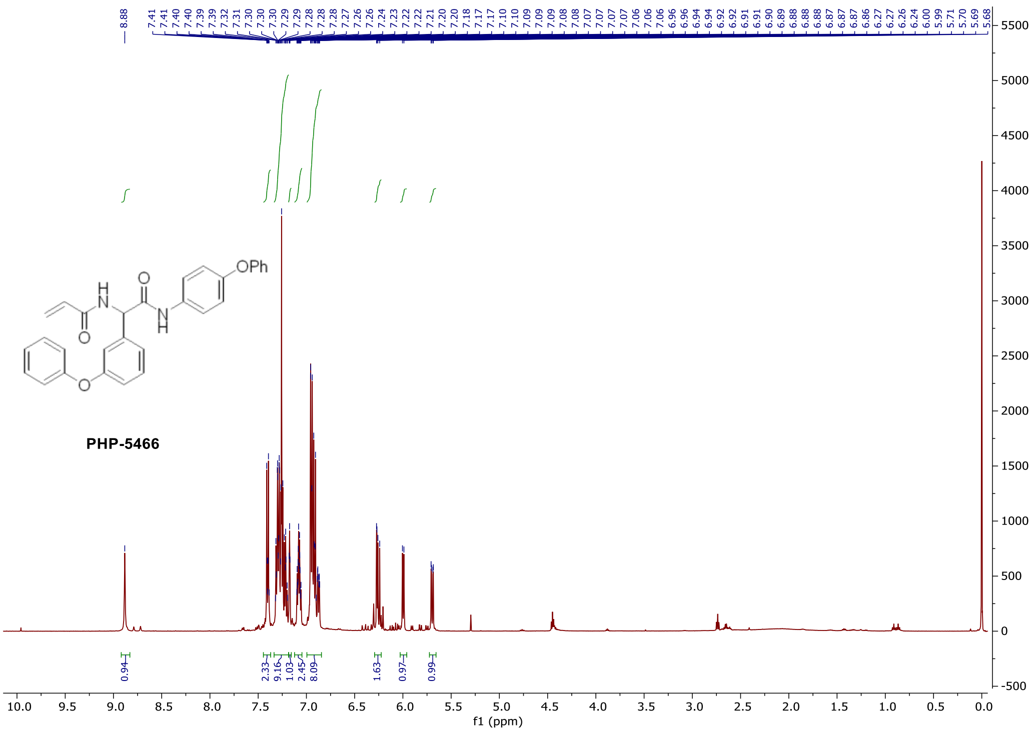

Supplementary Figure 560. ^{1}H NMR (500 MHz, $CDCl_3$) of compound **PHP-5466**.

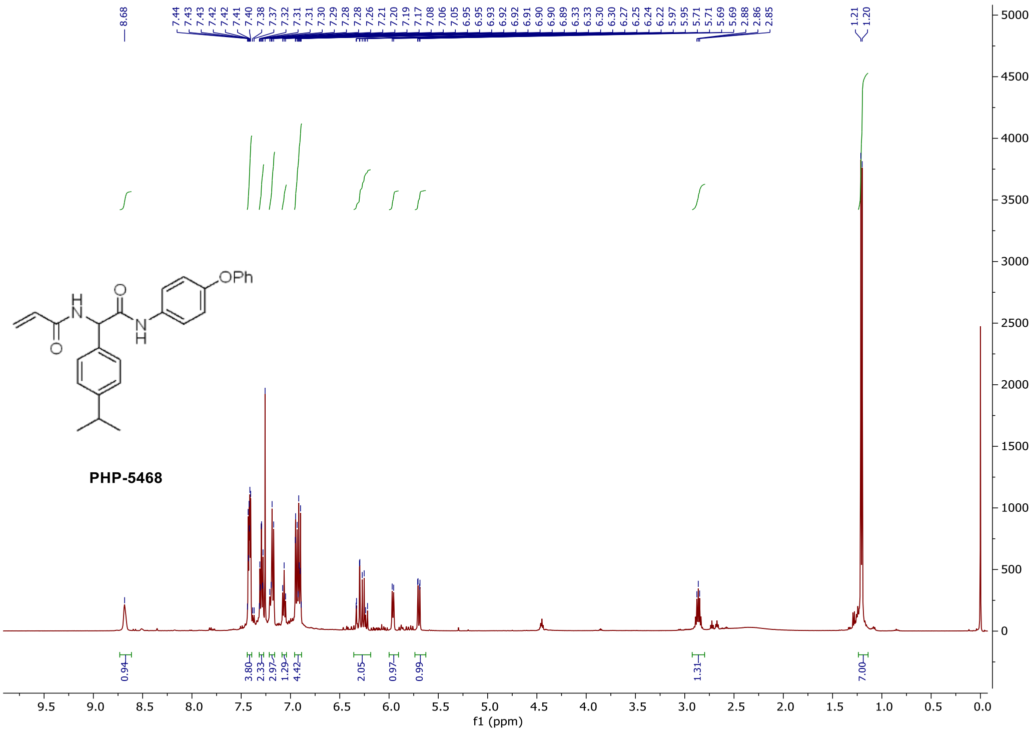

Supplementary Figure 561. ^{1}H NMR (500 MHz, $CDCl_3$) of compound **PHP-5468**.

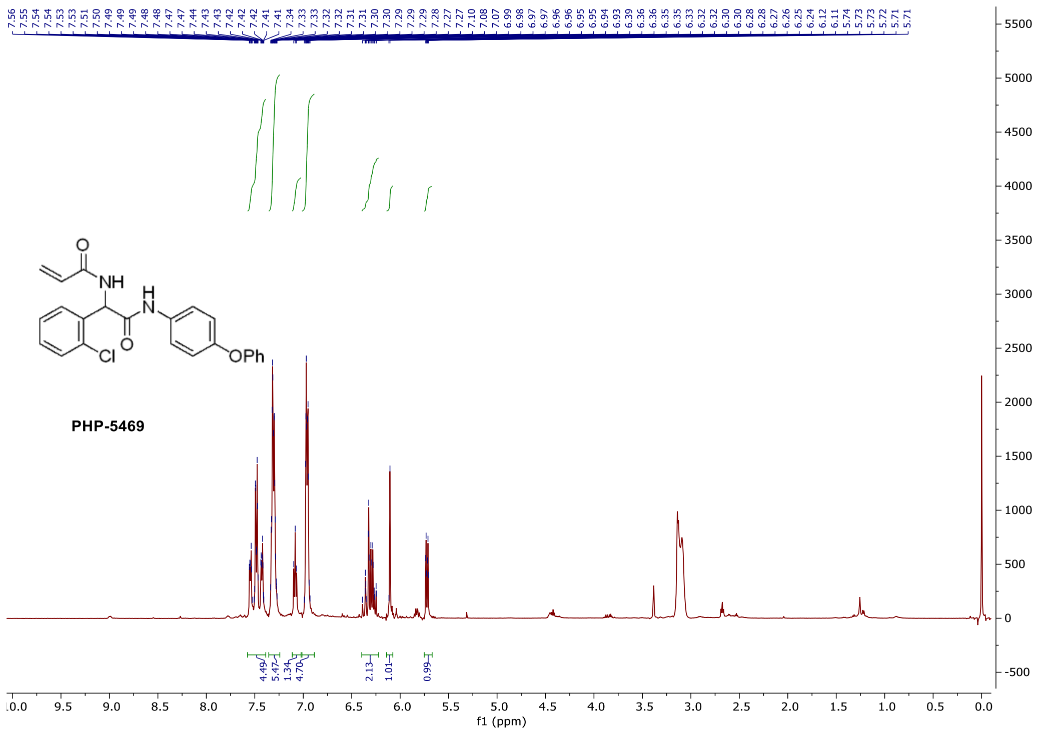

Supplementary Figure 562. ¹H NMR (500 MHz, $CDCl_3$ + one drop of CD_3OD) of compound **PHP-5469**.

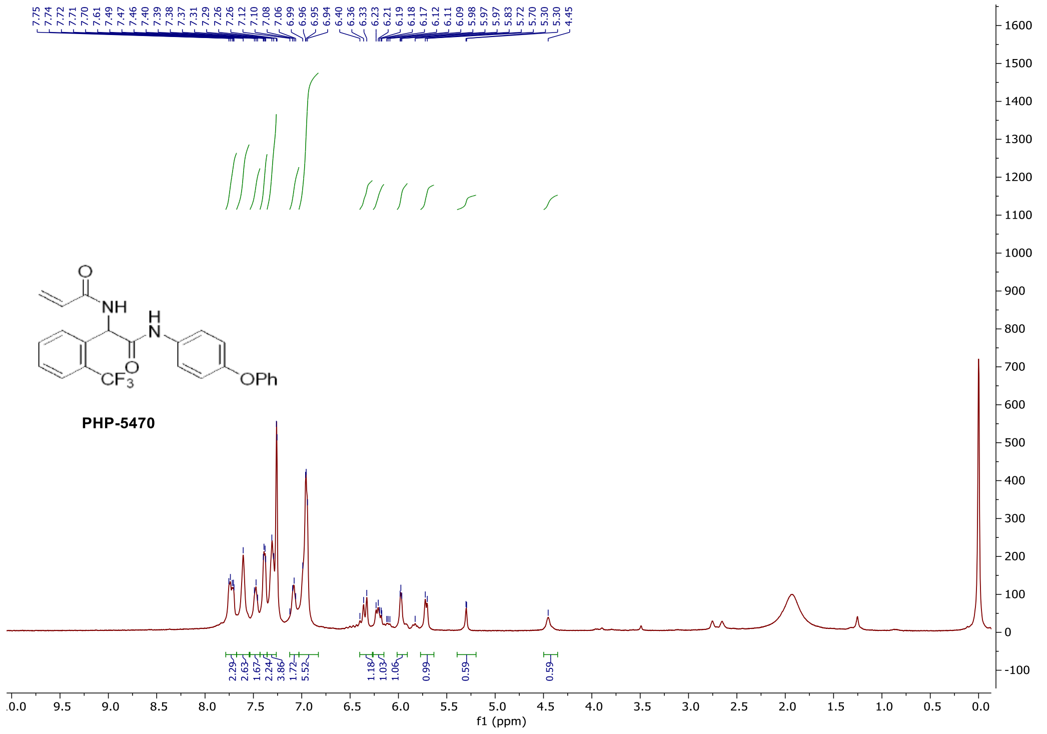

Supplementary Figure 563. ¹H NMR (500 MHz, $CDCl_3$) of compound **PHP-5470**.

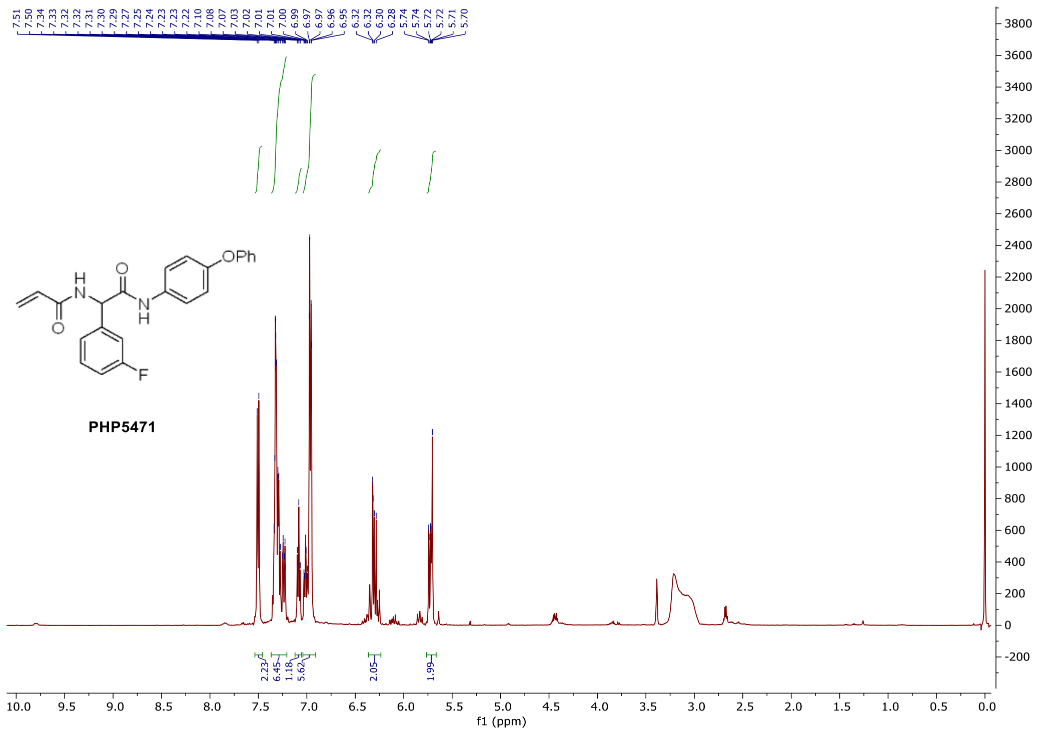

Supplementary Figure 564. ^{1}H NMR (500 MHz, $CDCl_3$ + one drop of CD_3OD) of compound **PHP-5471**.

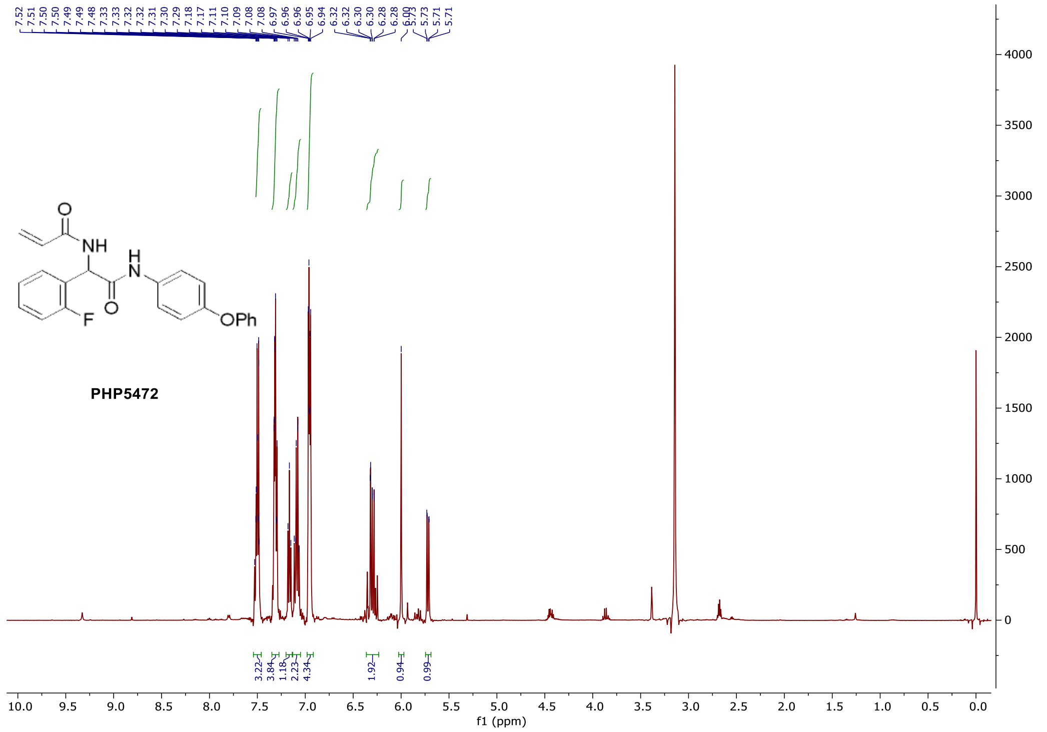

Supplementary Figure 565. ^{1}H NMR (500 MHz, $CDCl_3$ + one drop of CD_3OD) of compound **PHP-5472**.

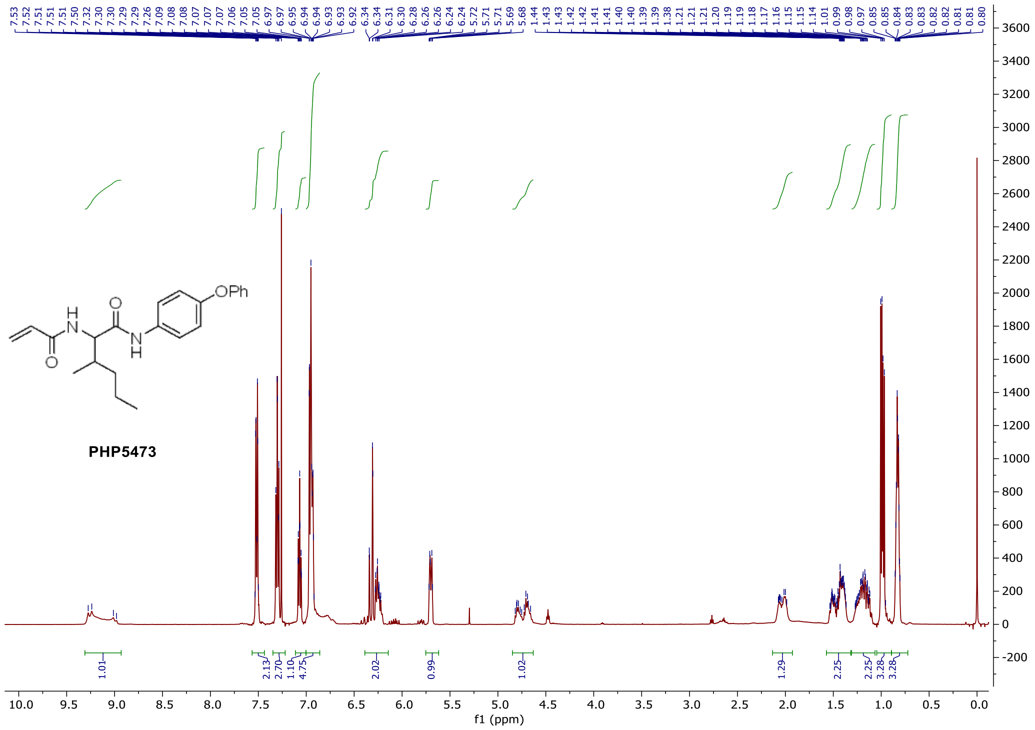

Supplementary Figure 566. ^{1}H NMR (500 MHz, $CDCl_3$) of compound **PHP-5473**.

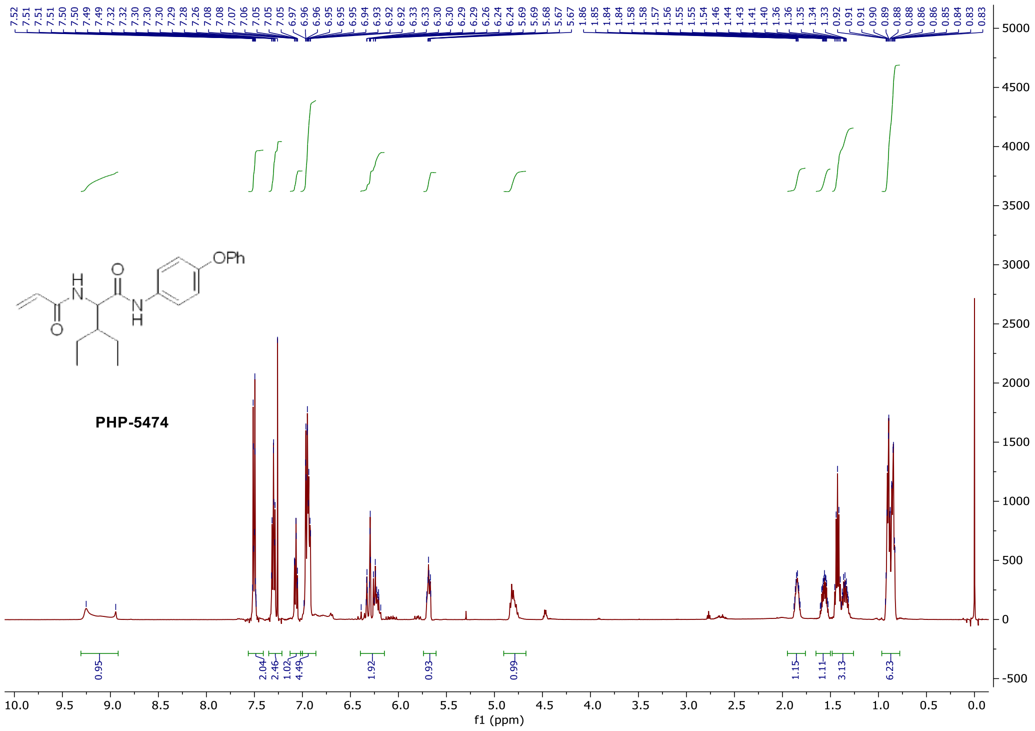

Supplementary Figure 567. ^{1}H NMR (500 MHz, $CDCl_3$) of compound **PHP-5474**.

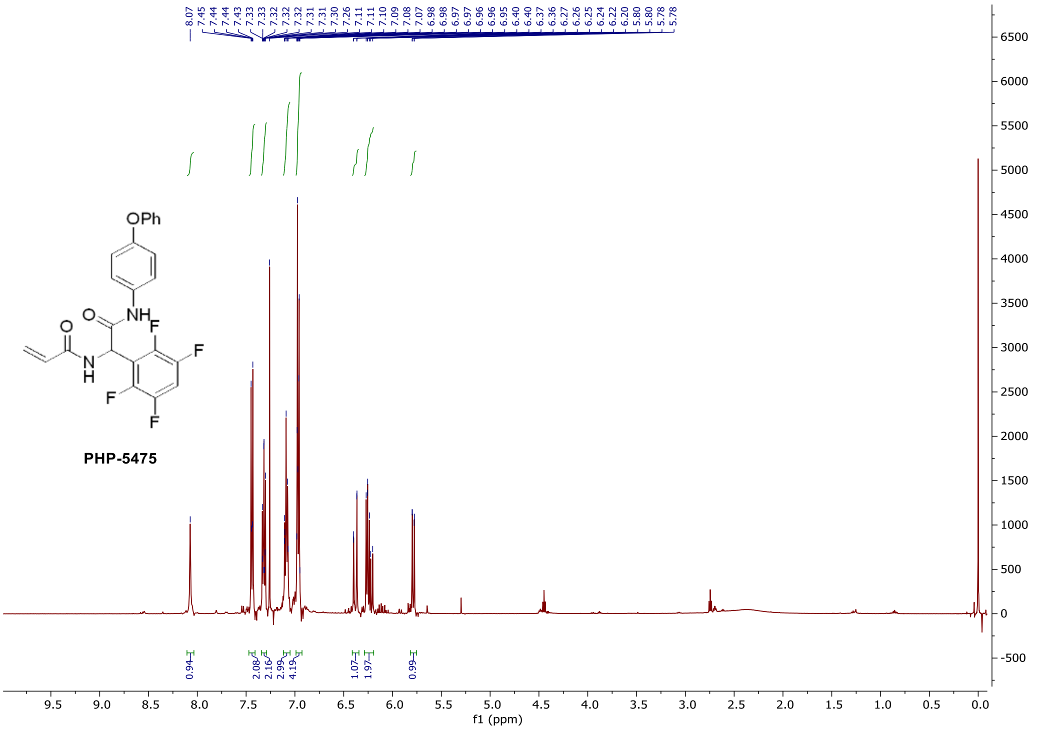

Supplementary Figure 568. ^{1}H NMR (500 MHz, CDCl3) of compound **PHP-5475**.

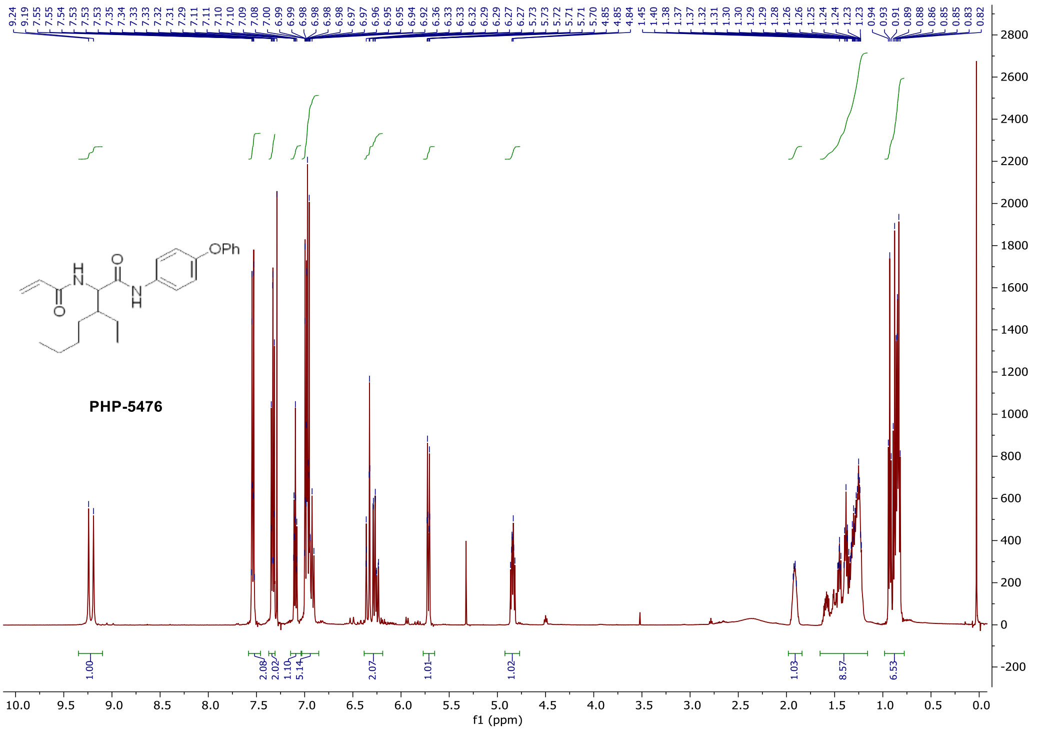

Supplementary Figure 569. ^{1}H NMR (500 MHz, CDCl3) of compound **PHP-5476**.

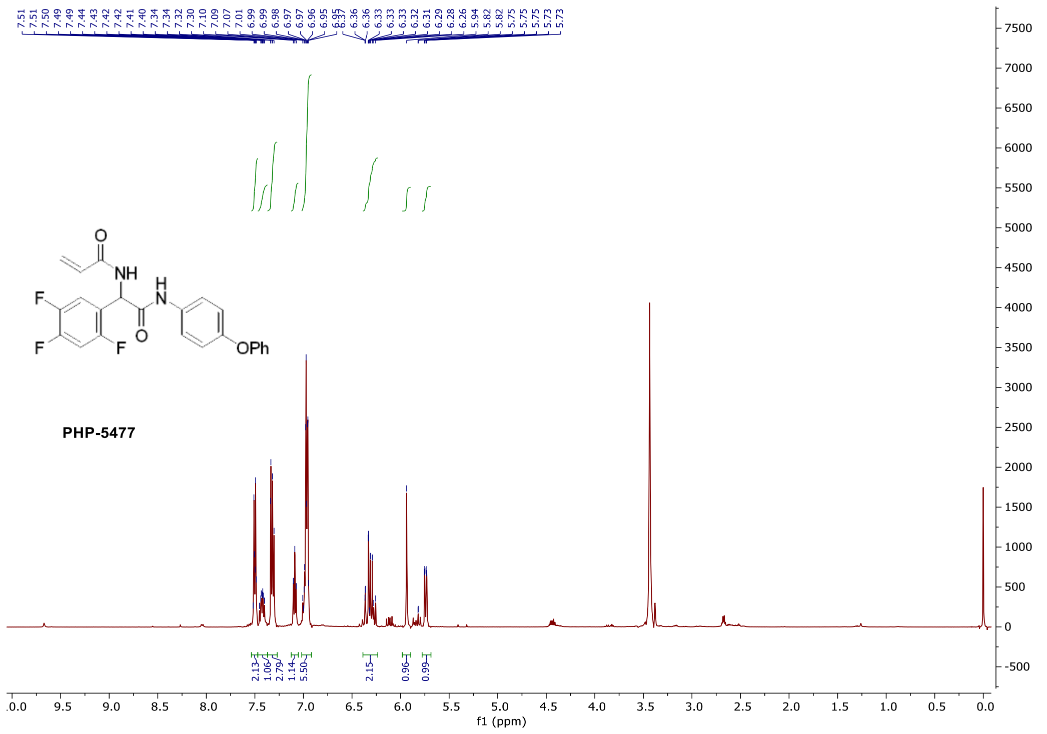

Supplementary Figure 570. ^{1}H NMR (500 MHz, $CDCl_3$) of compound **PHP-5477**.

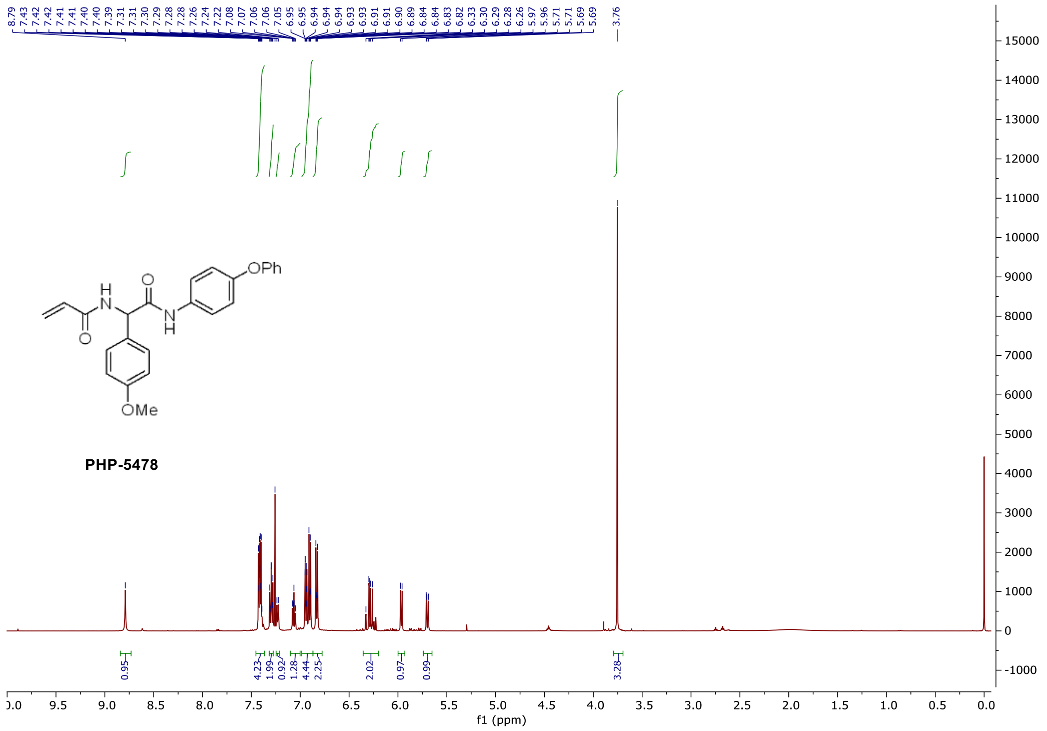

Supplementary Figure 571. ^{1}H NMR (500 MHz, $CDCl_3$) of compound **PHP-5478**.

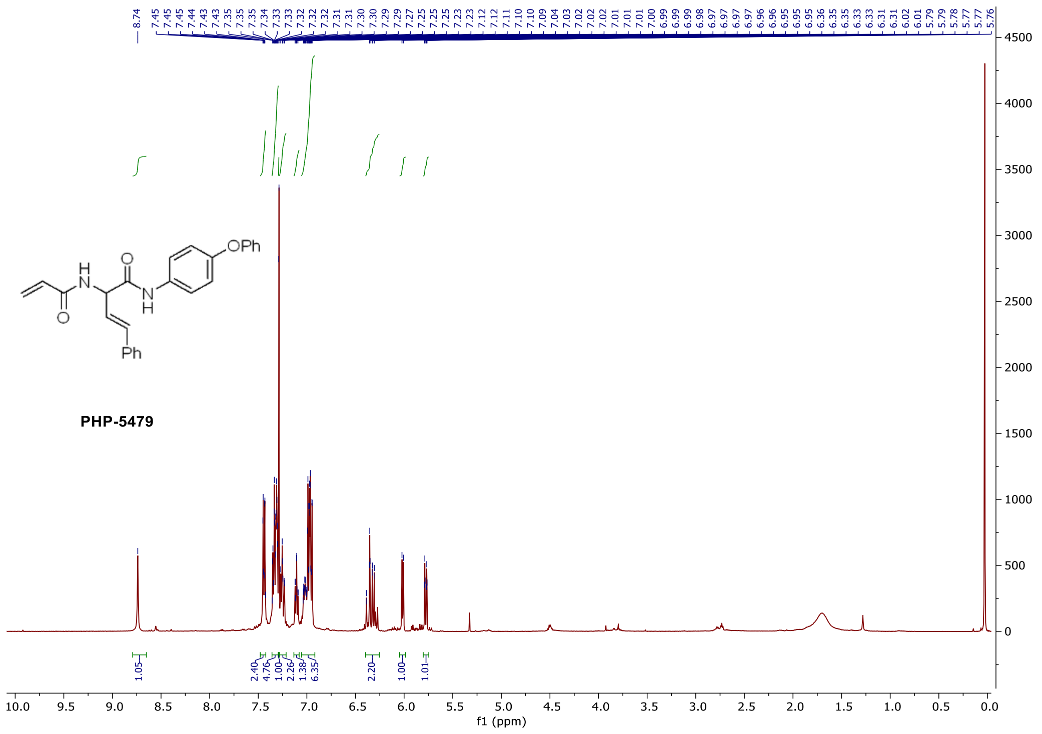

Supplementary Figure 572. ¹H NMR (500 MHz, $CDCl_3$) of compound **PHP-5479**.

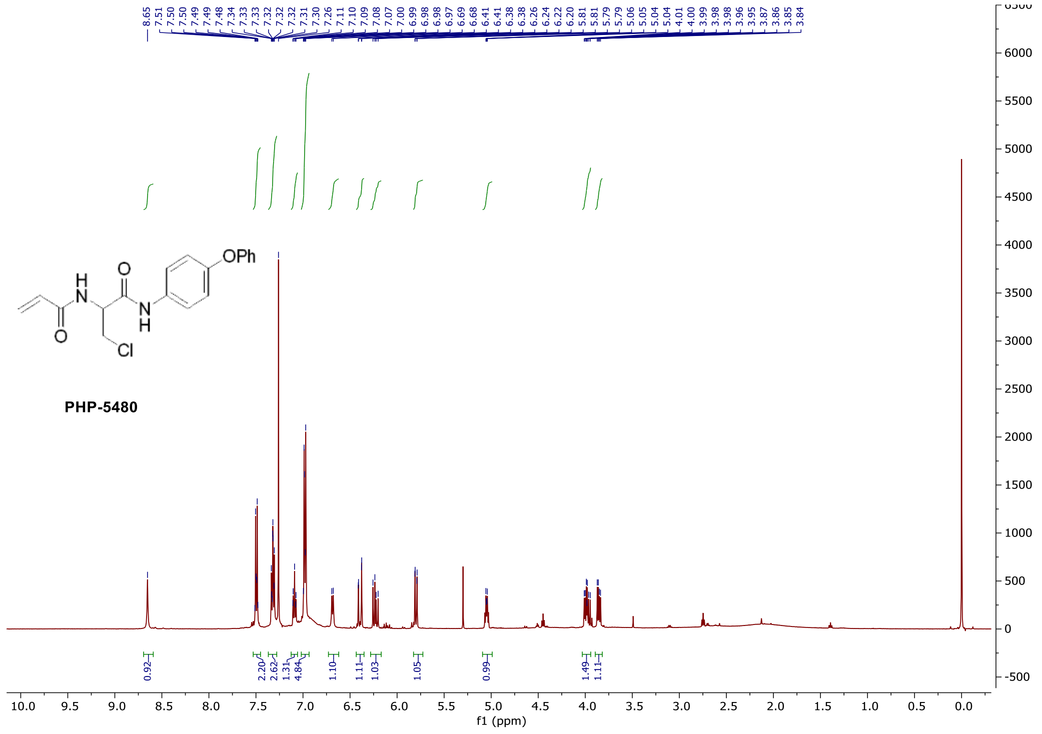

Supplementary Figure 573. ¹H NMR (500 MHz, $CDCl_3$) of compound **PHP-5480**.

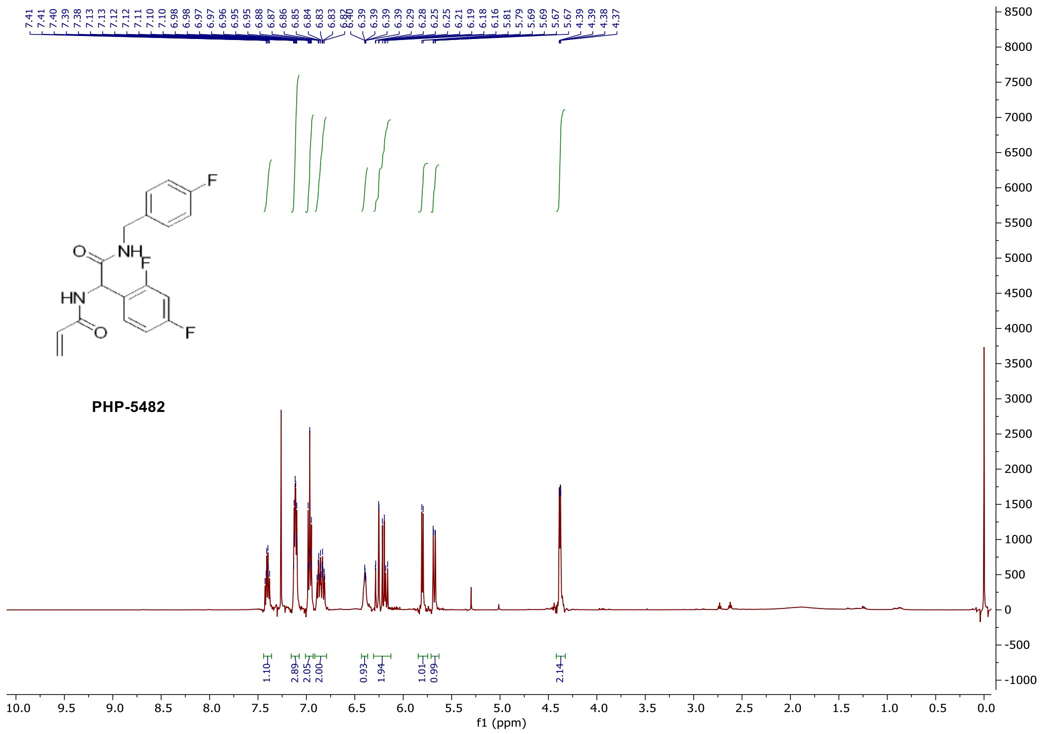

Supplementary Figure 574. ^{1}H NMR (500 MHz, $CDCl_3$) of compound **PHP-5482**.

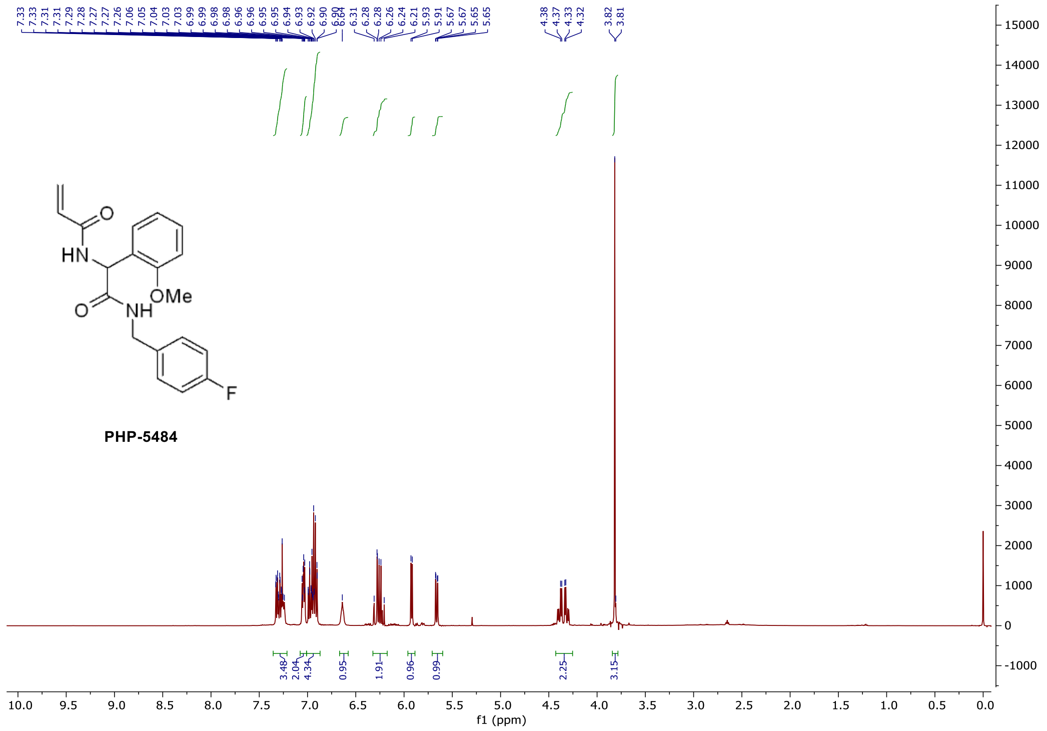

Supplementary Figure 575. ^{1}H NMR (500 MHz, $CDCl_3$) of compound **PHP-5484**.

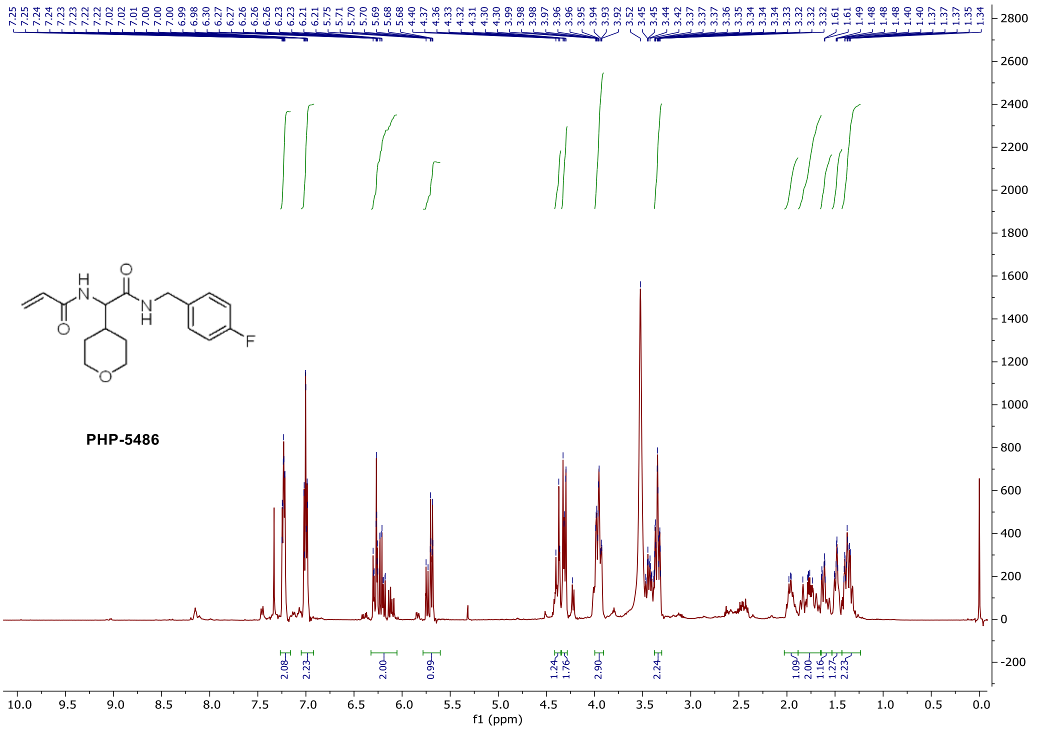

Supplementary Figure 576. ^{1}H NMR (500 MHz, $CDCl_3$) of compound **PHP-5486**.

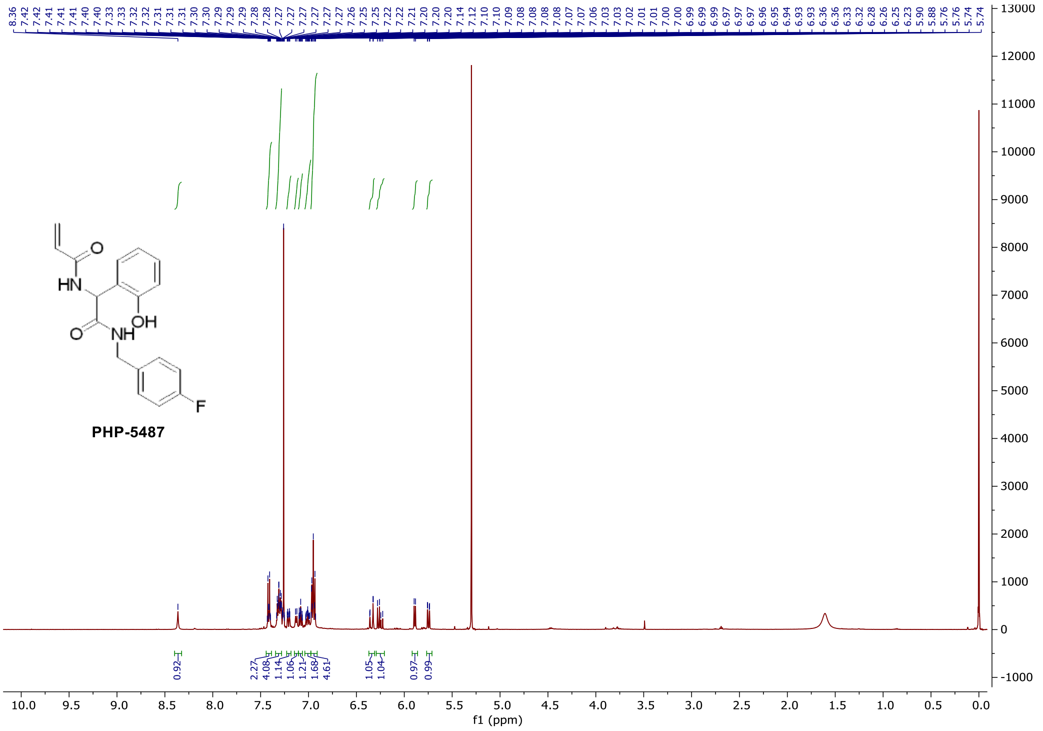

Supplementary Figure 577. ^{1}H NMR (500 MHz, $CDCl_3$) of compound **PHP-5487**.

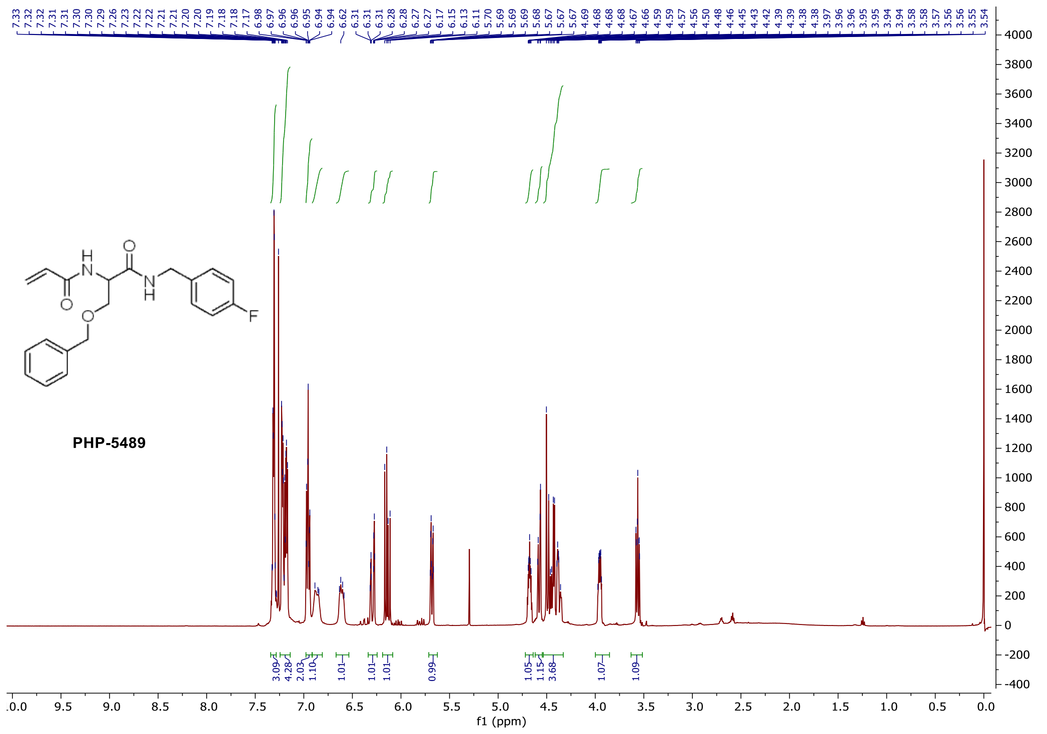

Supplementary Figure 578. ^{1}H NMR (500 MHz, $CDCl_3$) of compound **PHP-5489**.

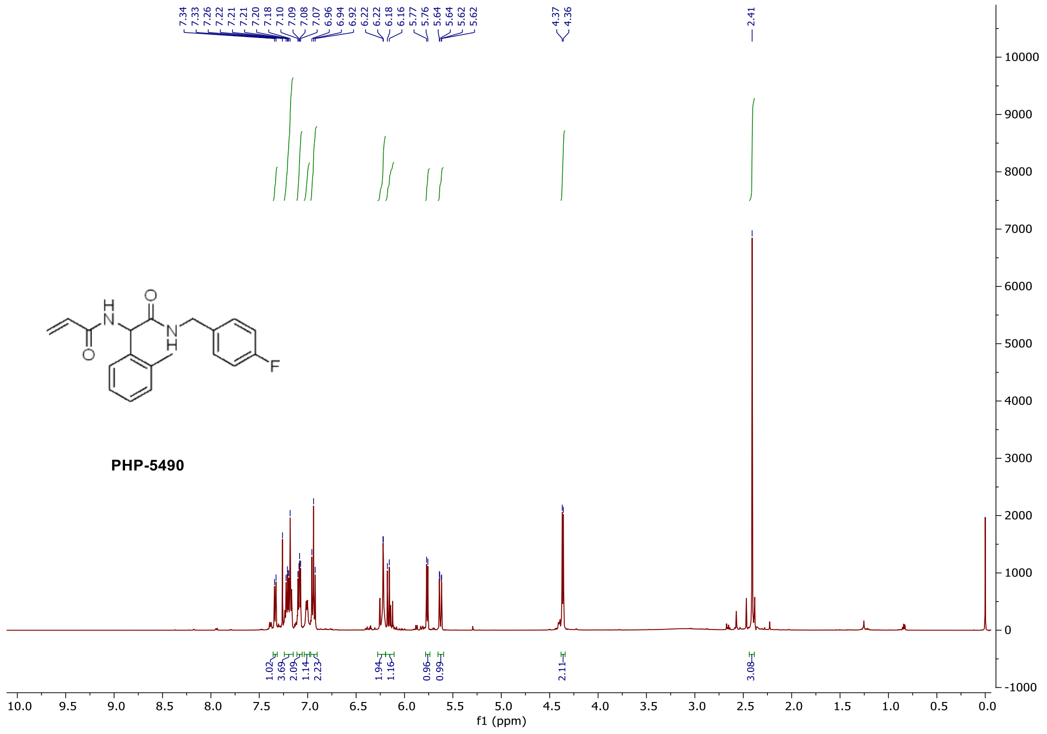

Supplementary Figure 579. ^{1}H NMR (500 MHz, $CDCl_3$) of compound **PHP-5490**.

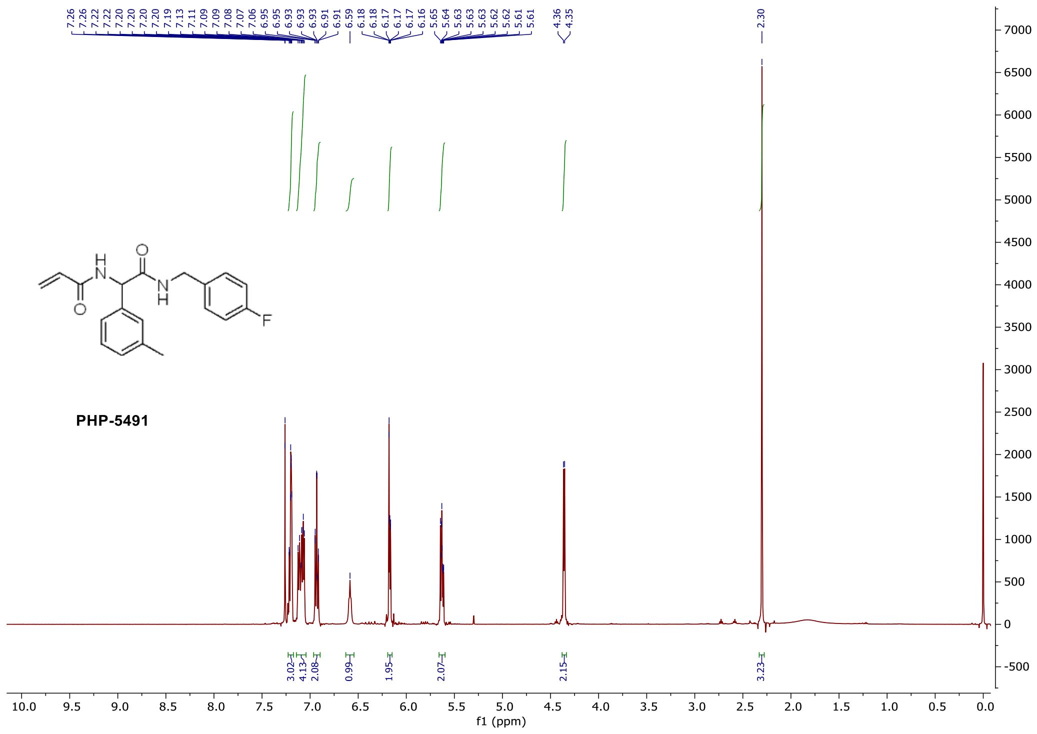

Supplementary Figure 580. ^{1}H NMR (500 MHz, $CDCl_3$) of compound **PHP-5491**.

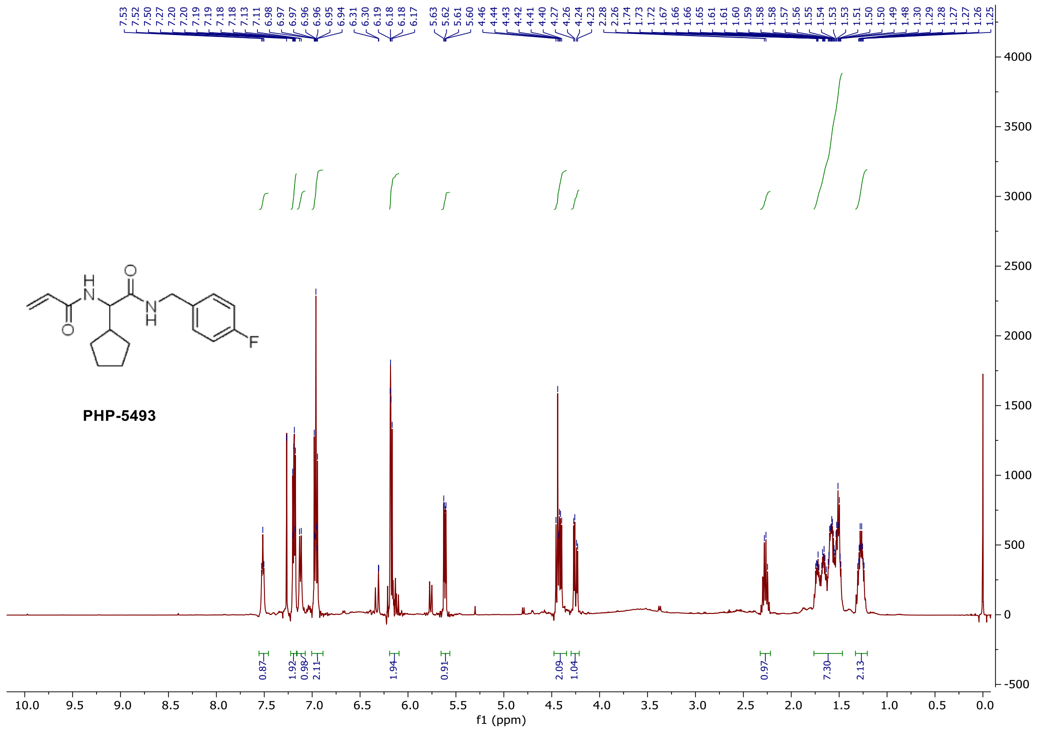

Supplementary Figure 581. ^{1}H NMR (500 MHz, $CDCl_3$) of compound **PHP-5493**.

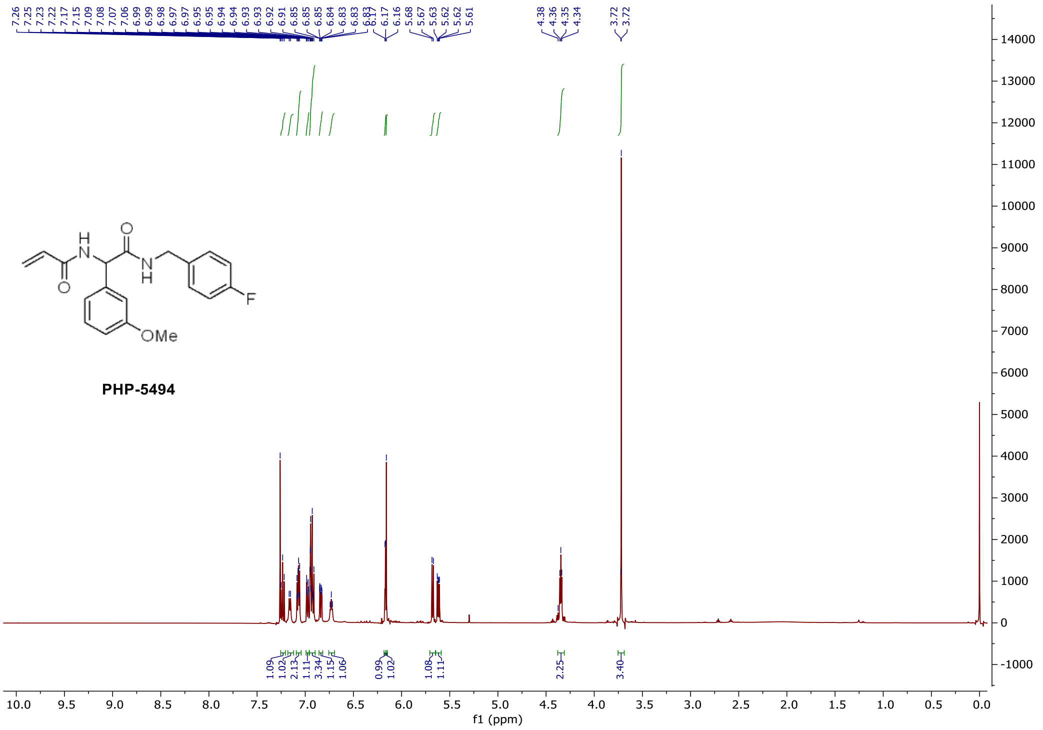

Supplementary Figure 582. ^{1}H NMR (500 MHz, $CDCl_3$) of compound **PHP-5494**.

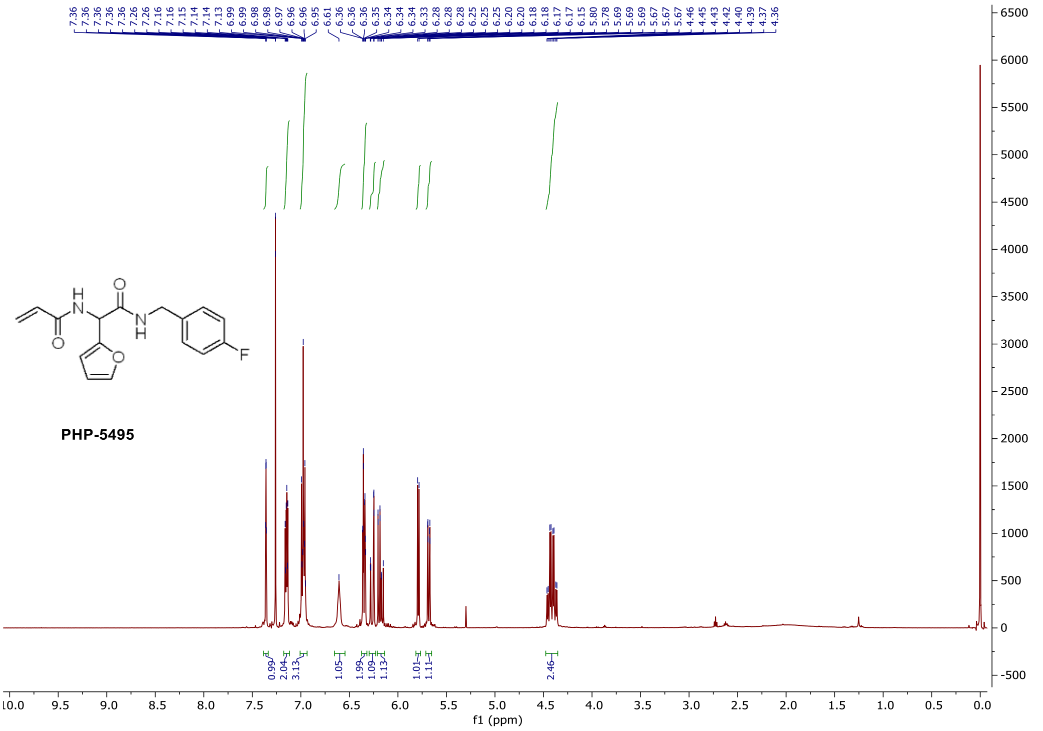

Supplementary Figure 583. ^{1}H NMR (500 MHz, $CDCl_3$) of compound **PHP-5495**.

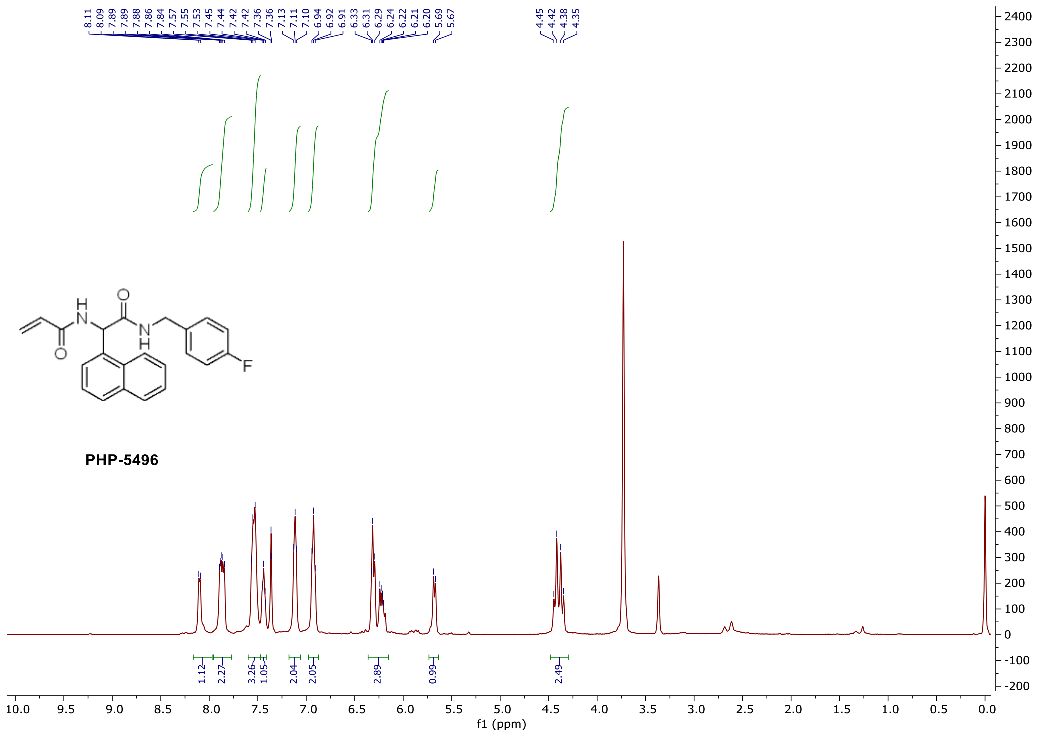

Supplementary Figure 584. ^{1}H NMR (500 MHz, $CDCl_3$ + one drop of CD_3OD) of compound **PHP-5496**.

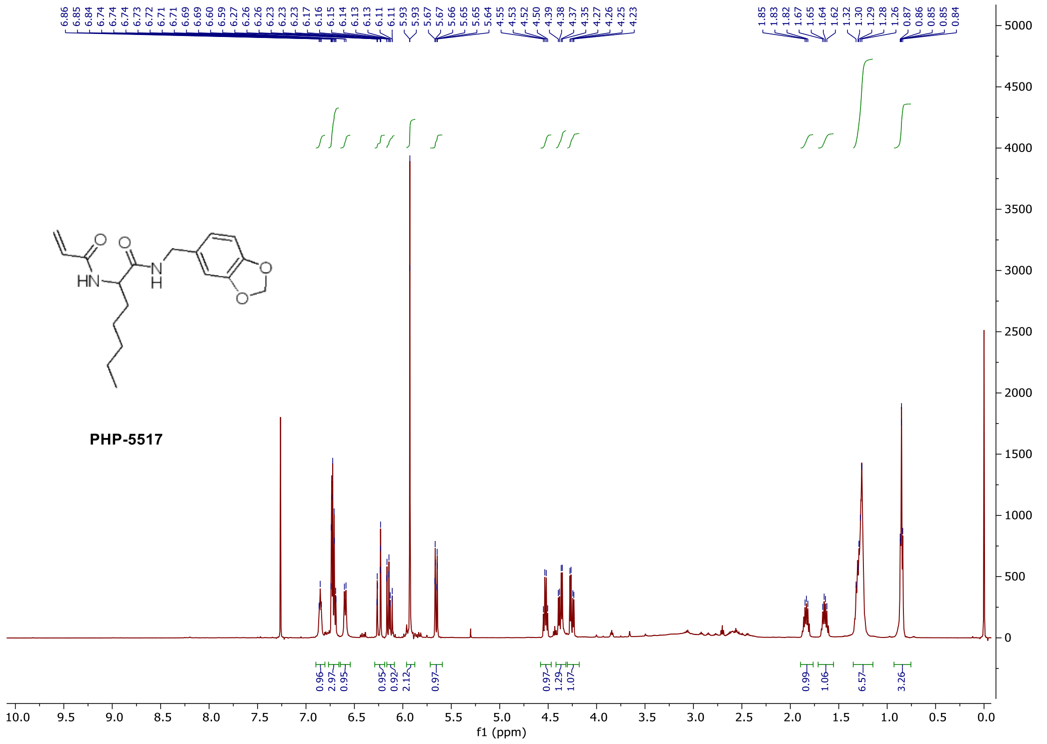

Supplementary Figure 585. ^{1}H NMR (500 MHz, $CDCl_3$) of compound **PHP-5517**.

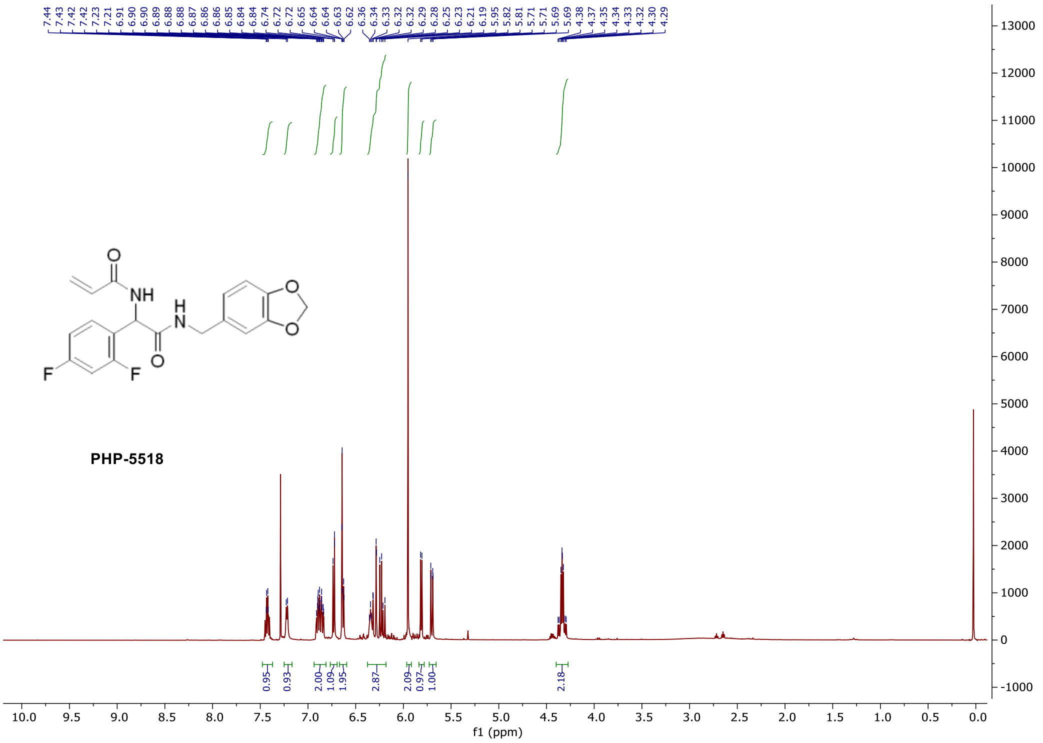

Supplementary Figure 586. ^{1}H NMR (500 MHz, $CDCl_3$) of compound **PHP-5518**.

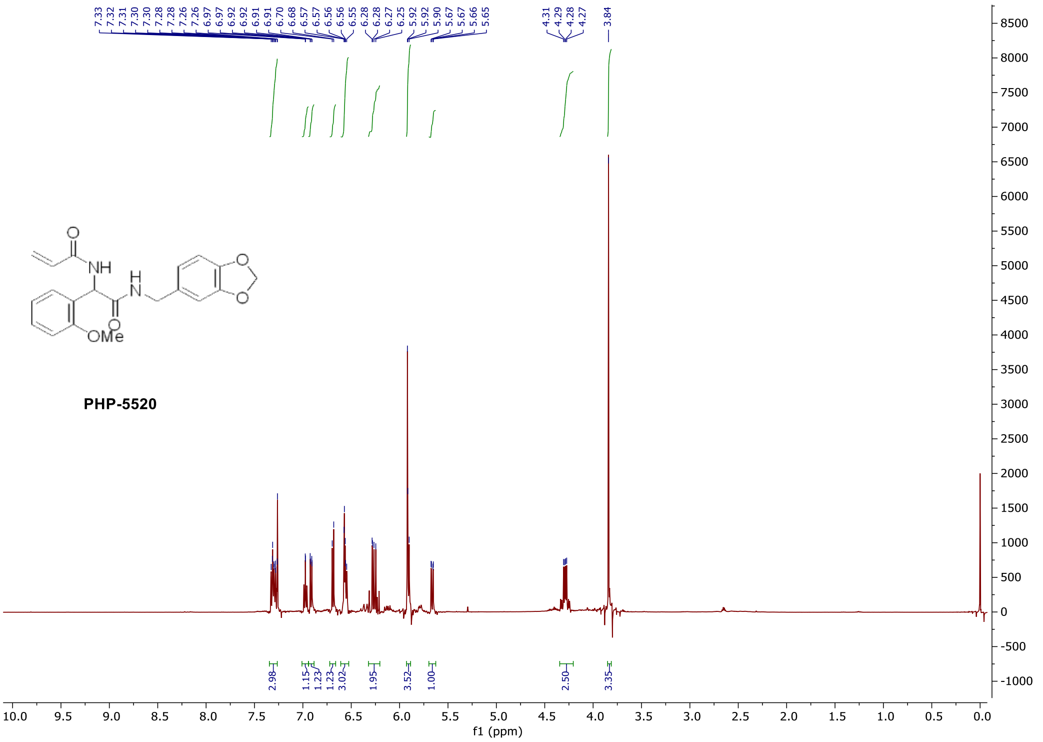

Supplementary Figure 587. ^{1}H NMR (500 MHz, $CDCl_3$) of compound **PHP-5520**.

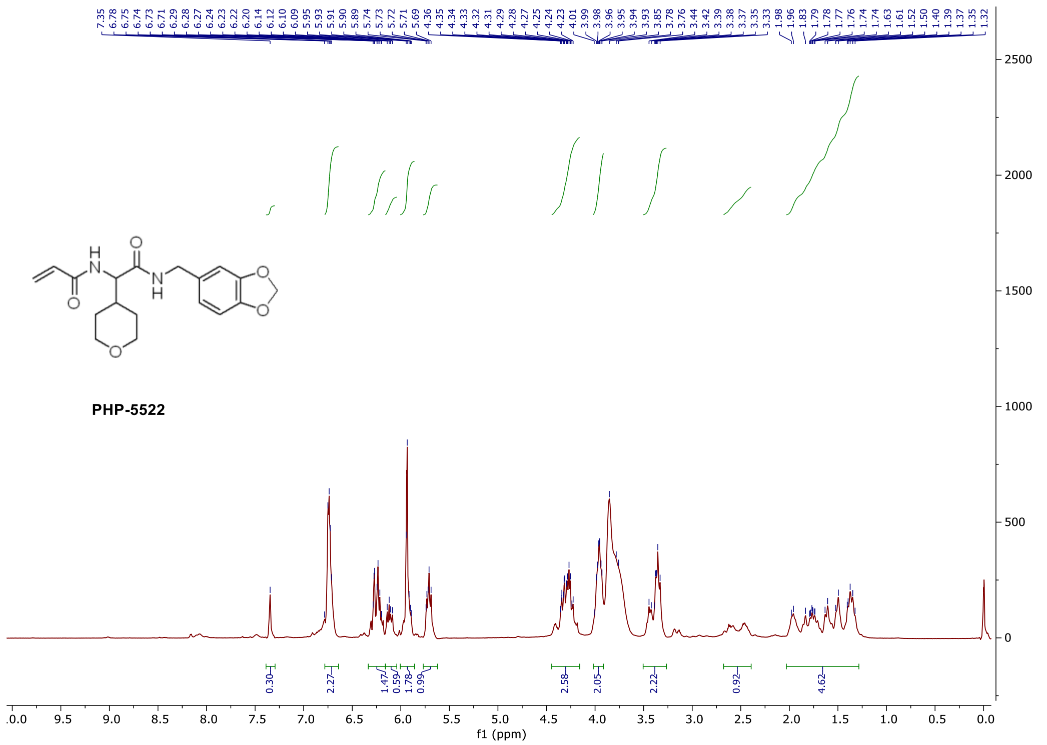

Supplementary Figure 588. ^{1}H NMR (500 MHz, $CDCl_3$) of compound **PHP-5522**.

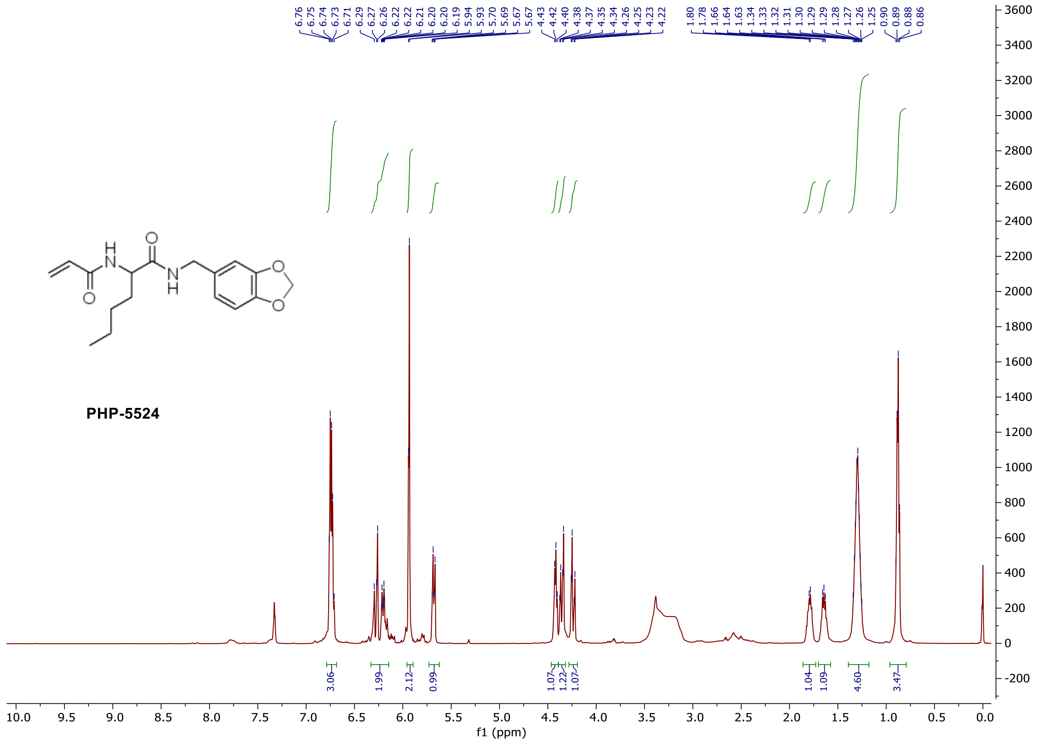

Supplementary Figure 589. ^{1}H NMR (500 MHz, $CDCl_3$ + one drop of CD_3OD) of compound **PHP-5524**.

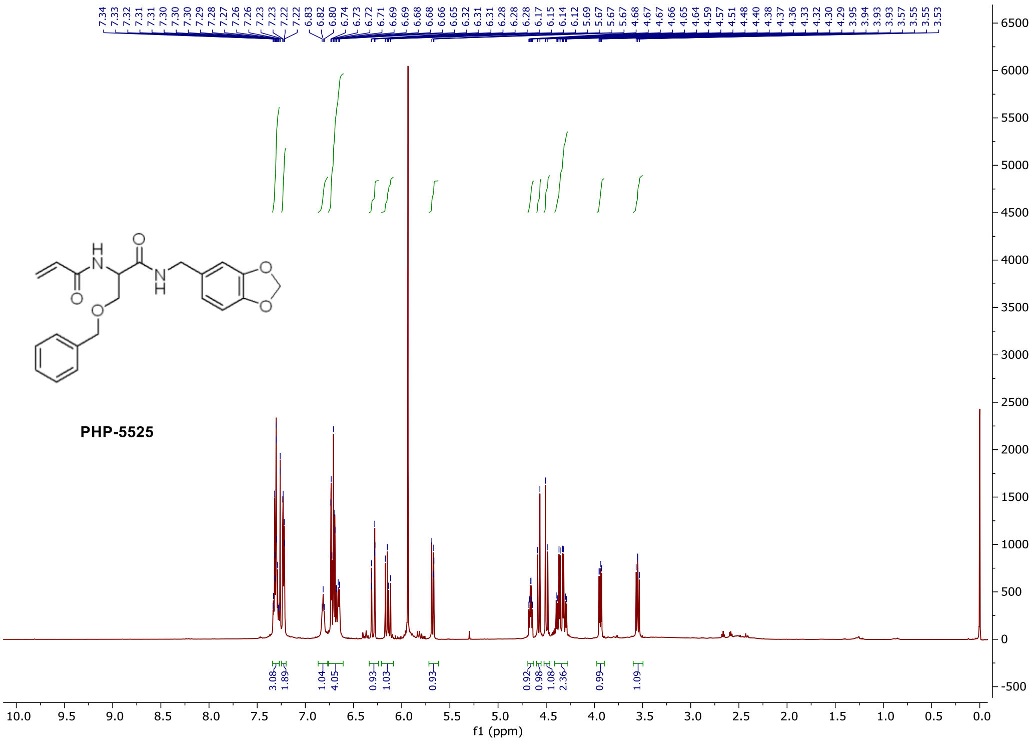

Supplementary Figure 590. ^{1}H NMR (500 MHz, $CDCl_3$) of compound **PHP-5525**.

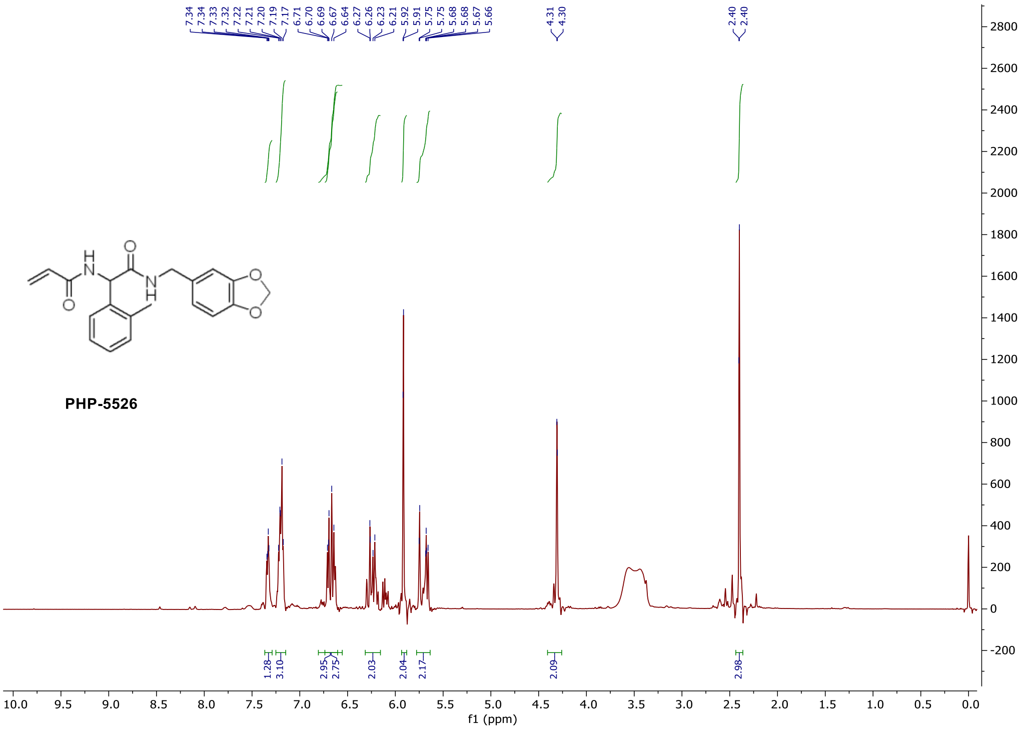

Supplementary Figure 591. ^{1}H NMR (500 MHz, $CDCl_3$ + one drop of CD_3OD) of compound **PHP-5526**.

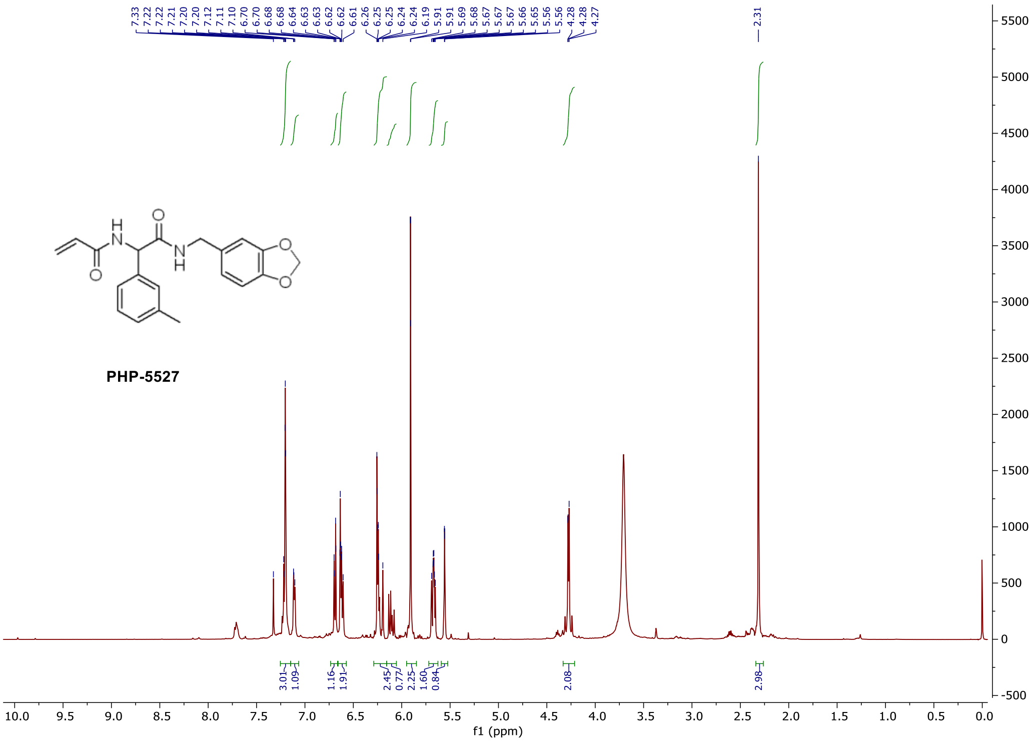

Supplementary Figure 592. ^{1}H NMR (500 MHz, $CDCl_3$ + one drop of CD_3OD) of compound **PHP-5527**.

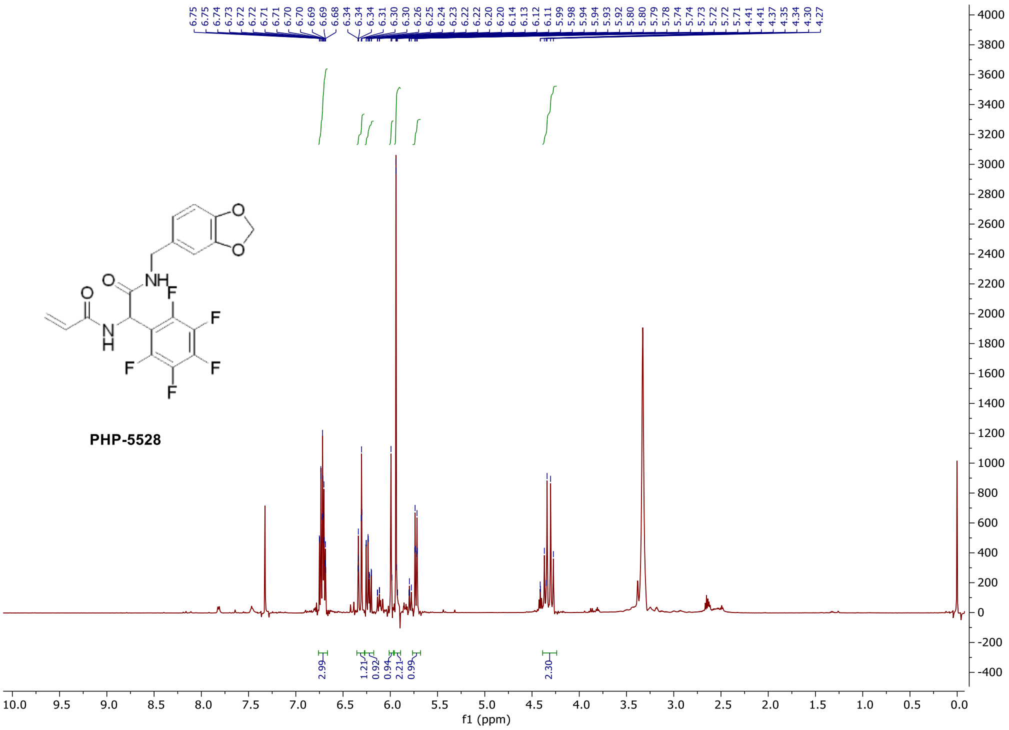

Supplementary Figure 593. ^{1}H NMR (500 MHz, $CDCl_3$ + one drop of CD_3OD) of compound **PHP-5528**.

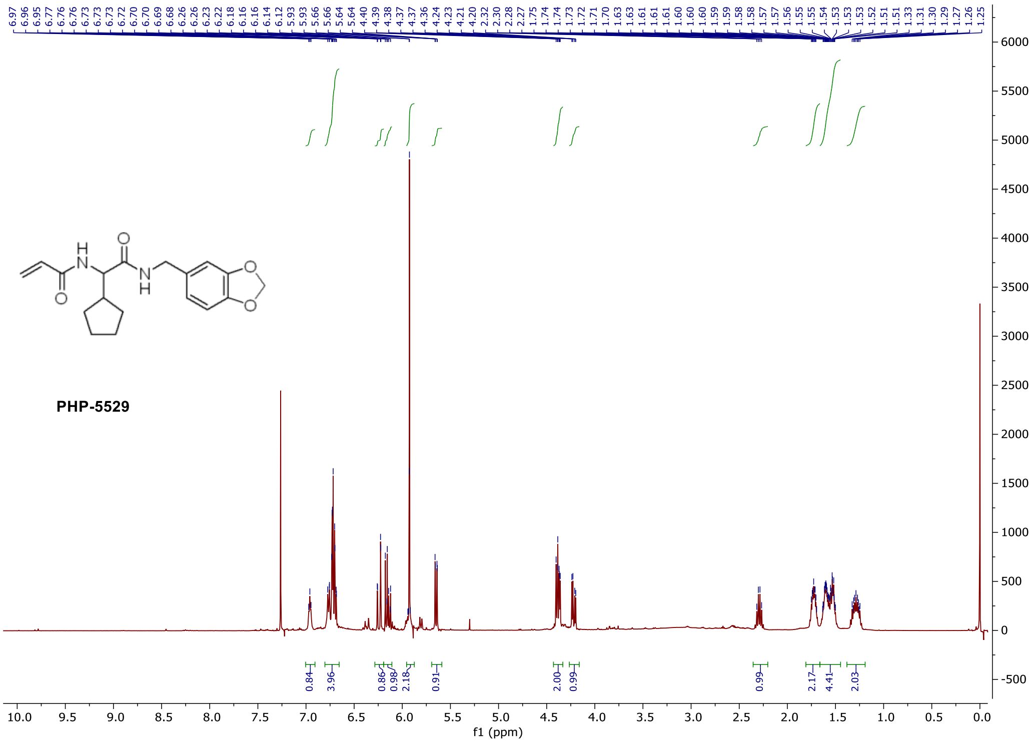

Supplementary Figure x. ^{1}H NMR (500 MHz, $CDCl_3$) of compound **PHP-5529**.

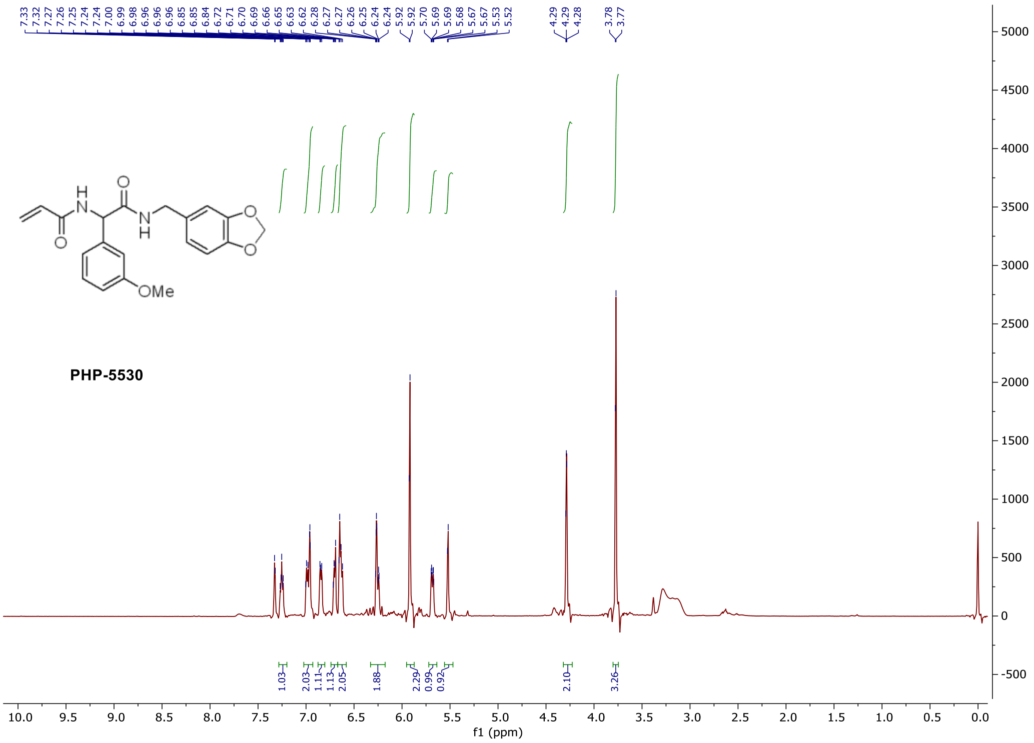

Supplementary Figure 594. ^{1}H NMR (500 MHz, $CDCl_3$ + one drop of CD_3OD) of compound **PHP-5530**.

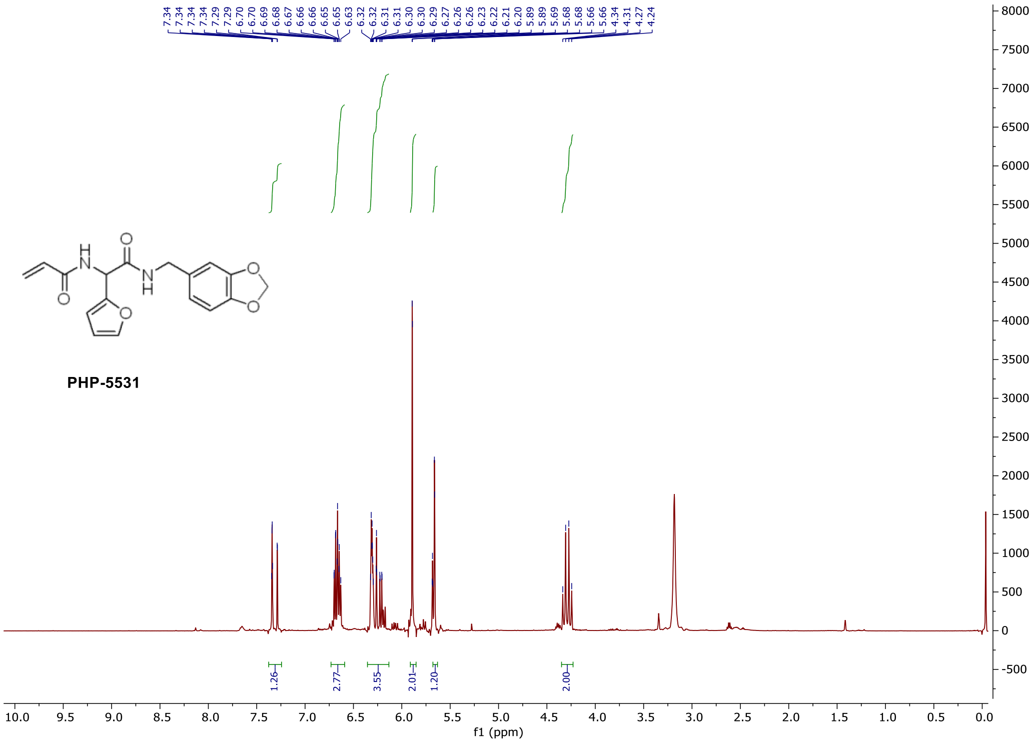

Supplementary Figure 595. ^{1}H NMR (500 MHz, $CDCl_3$ + one drop of CD_3OD) of compound **PHP-5531**.

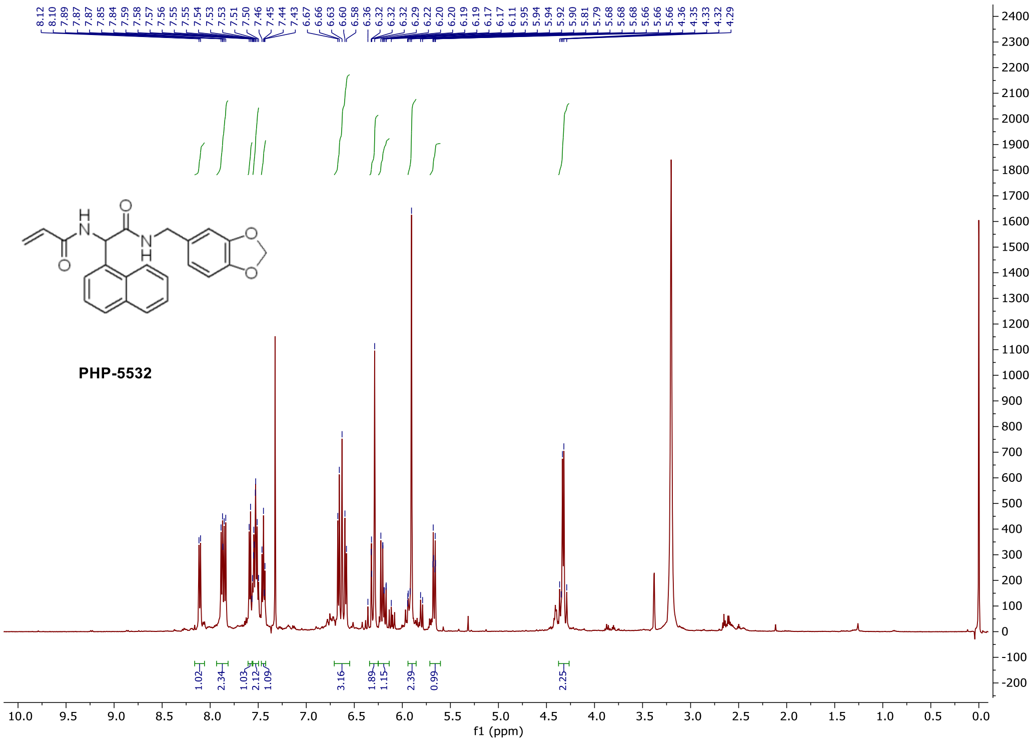

Supplementary Figure 596. ^{1}H NMR (500 MHz, $CDCl_3$ + one drop of CD_3OD) of compound **PHP-5532**.

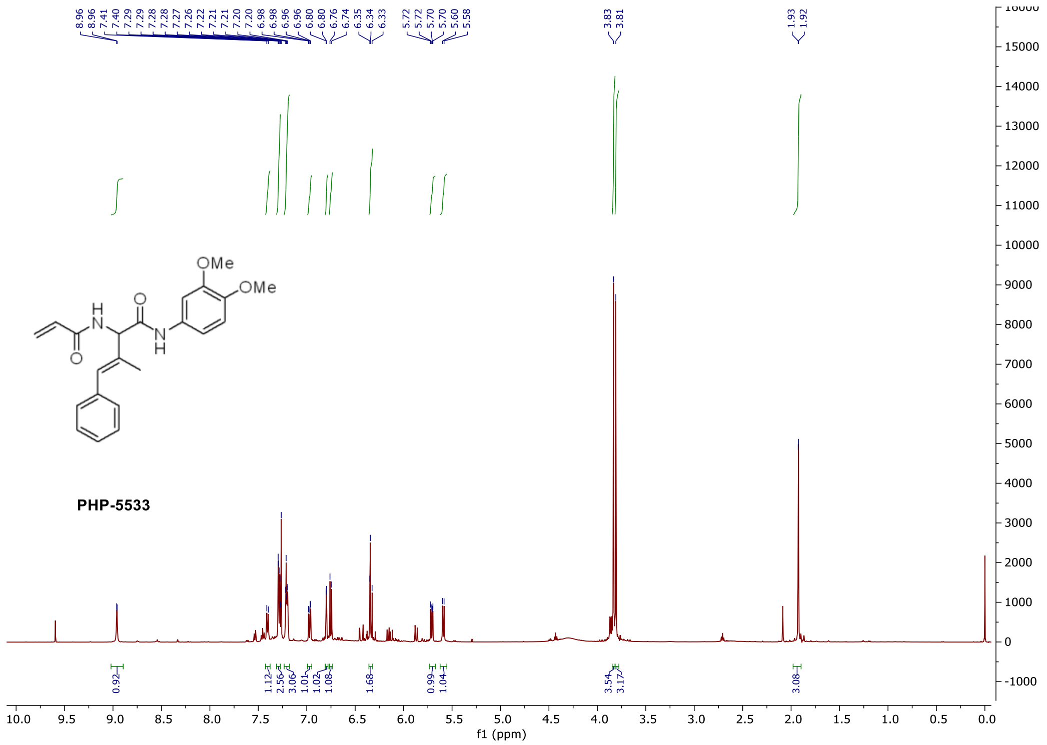

Supplementary Figure 597. ^{1}H NMR (500 MHz, $CDCl_3$) of compound **PHP-5533**.

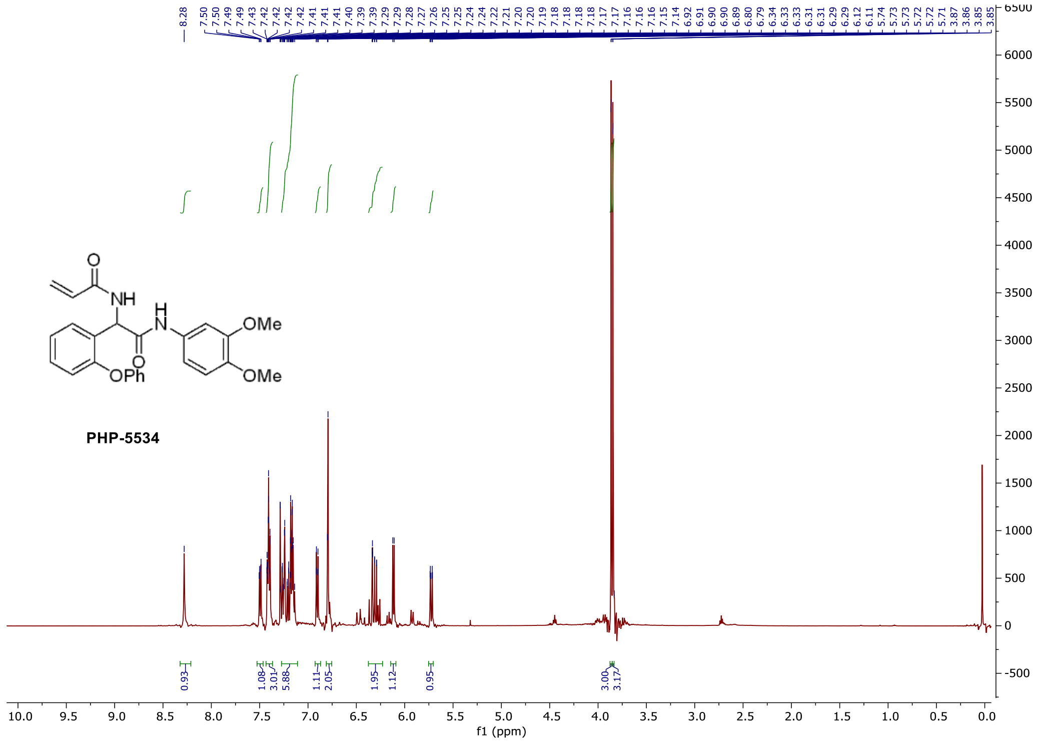

Supplementary Figure 598. ^{1}H NMR (500 MHz, $CDCl_3$) of compound **PHP-5534**.

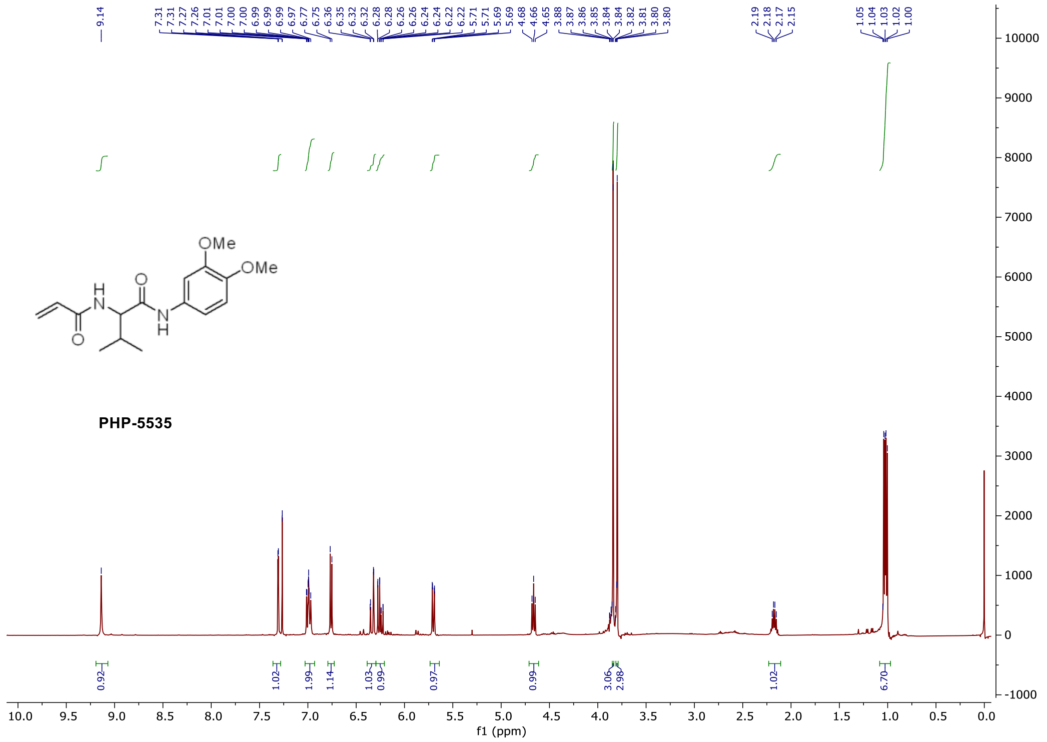

Supplementary Figure 599. ^{1}H NMR (500 MHz, $CDCl_3$) of compound **PHP-5535**.

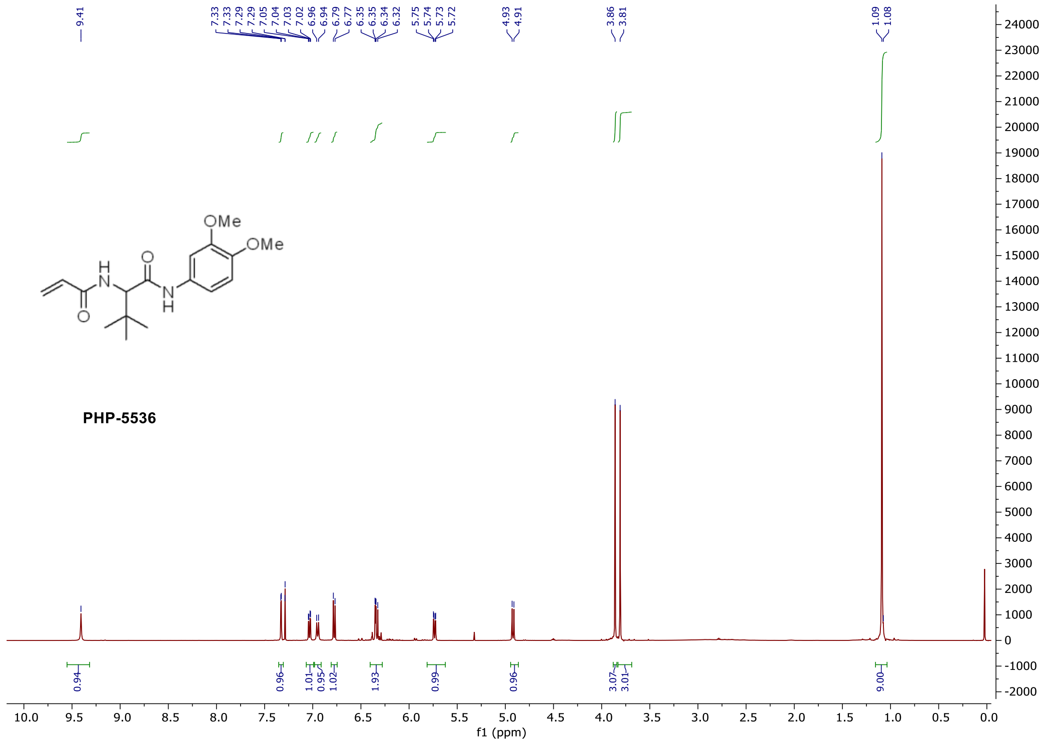

Supplementary Figure 600. ^{1}H NMR (500 MHz, $CDCl_3$) of compound **PHP-5536**.

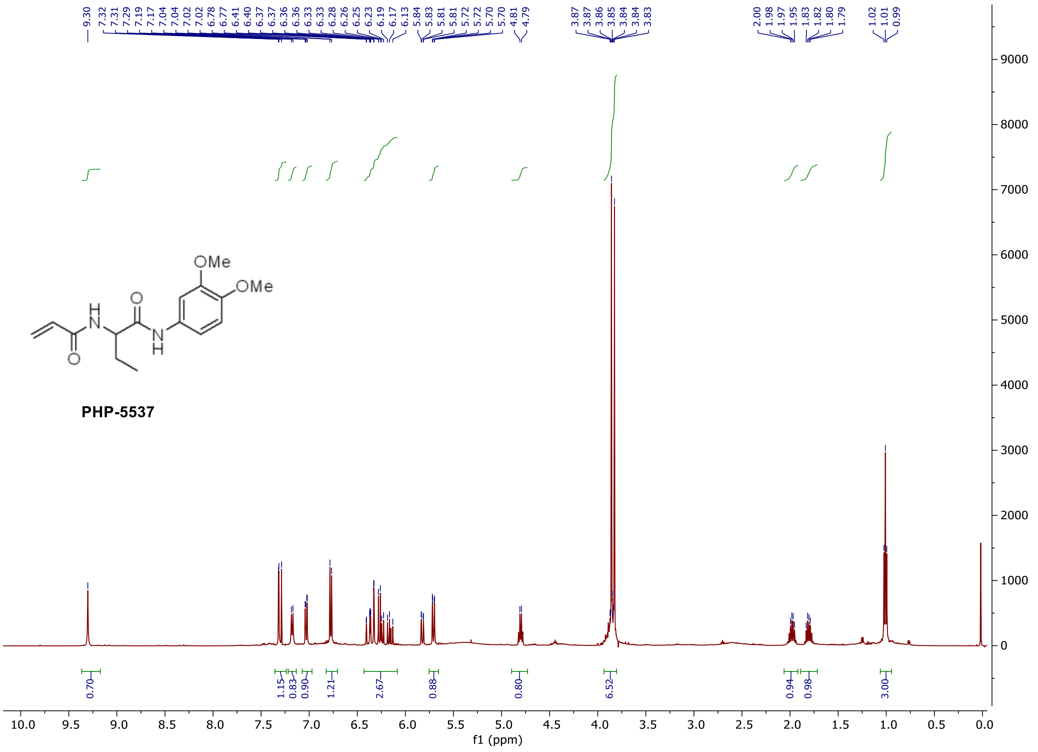

Supplementary Figure 601. ^{1}H NMR (500 MHz, $CDCl_3$) of compound **PHP-5537**.

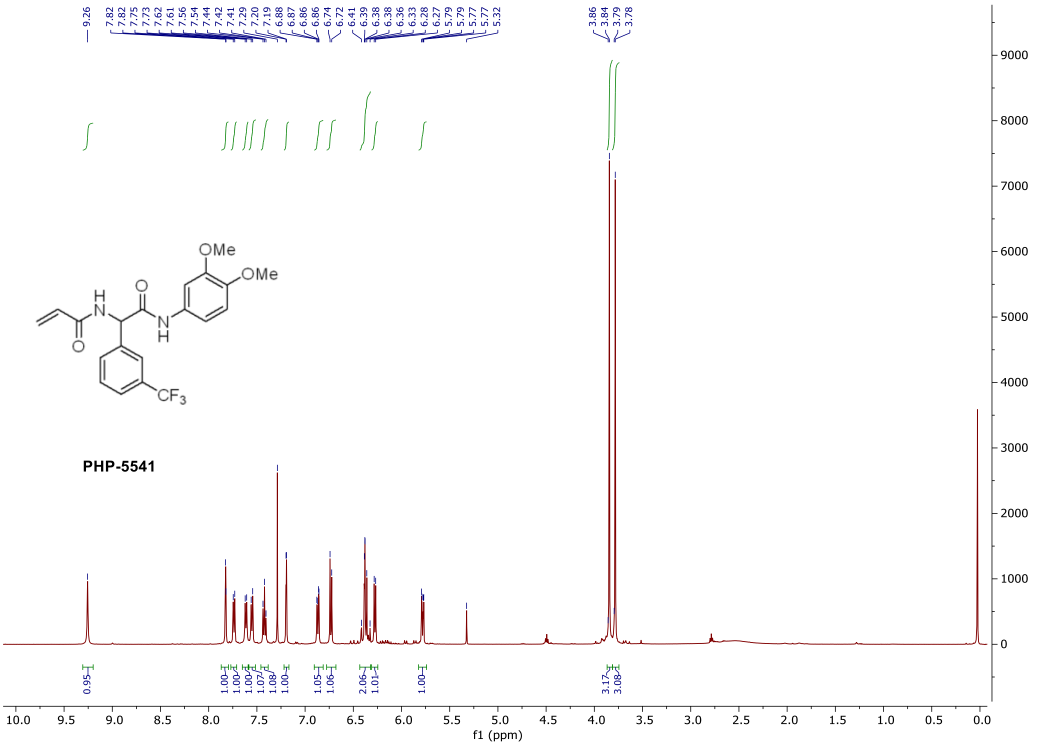

Supplementary Figure 602. ^{1}H NMR (500 MHz, $CDCl_3$) of compound **PHP-5541**.

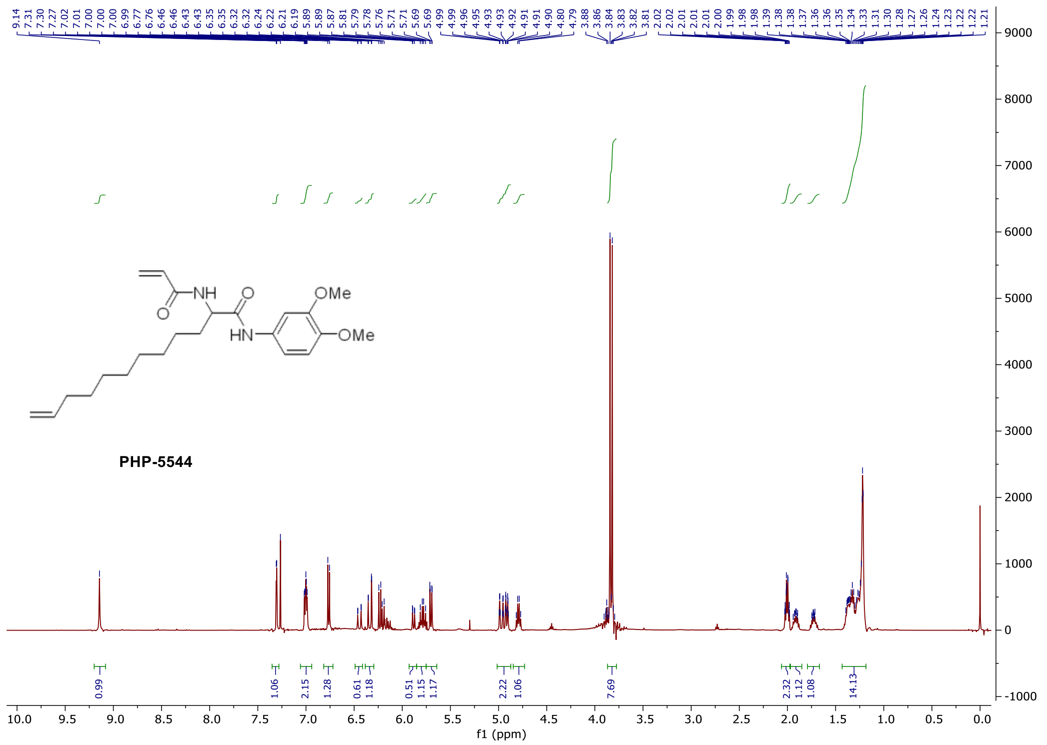

Supplementary Figure 603. ^{1}H NMR (500 MHz, $CDCl_3$) of compound **PHP-5544**.

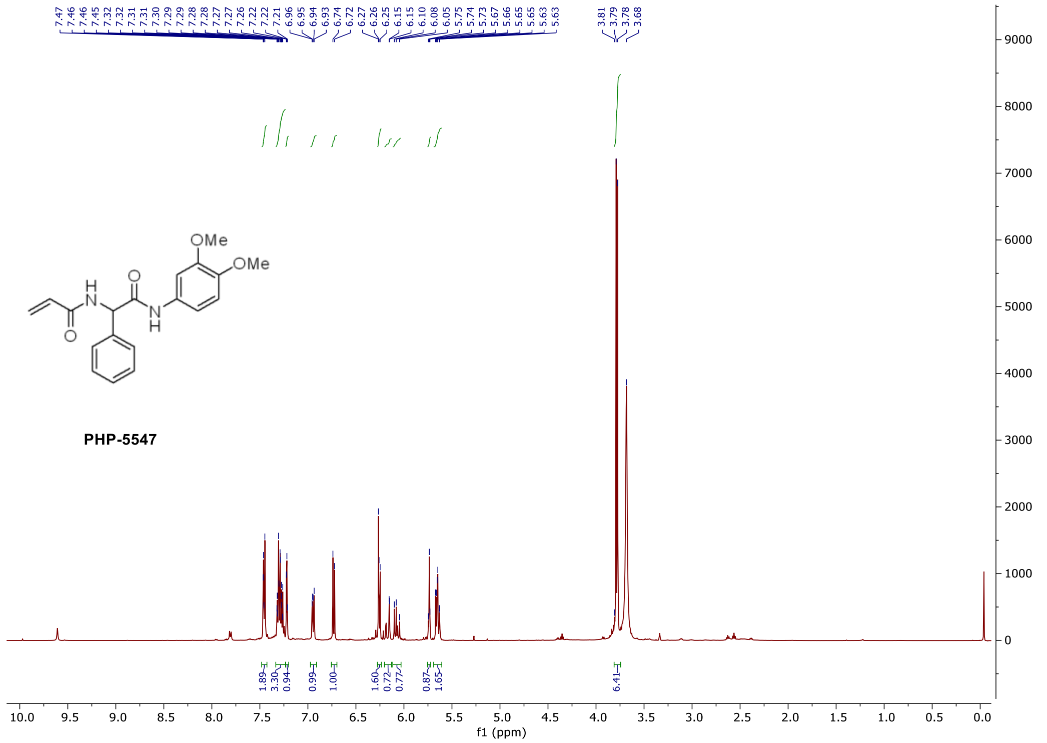

Supplementary Figure 604. ^{1}H NMR (500 MHz, $CDCl_3$) of compound **PHP-5547**.

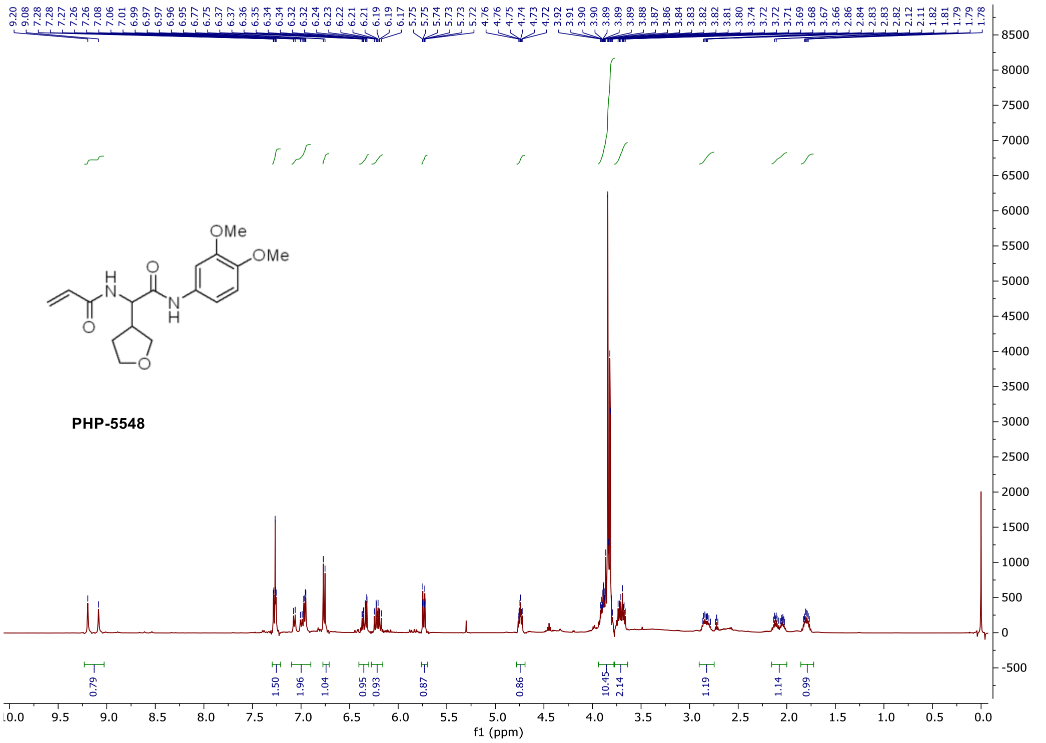

Supplementary Figure 605. ^{1}H NMR (500 MHz, $CDCl_3$) of compound **PHP-5548**.

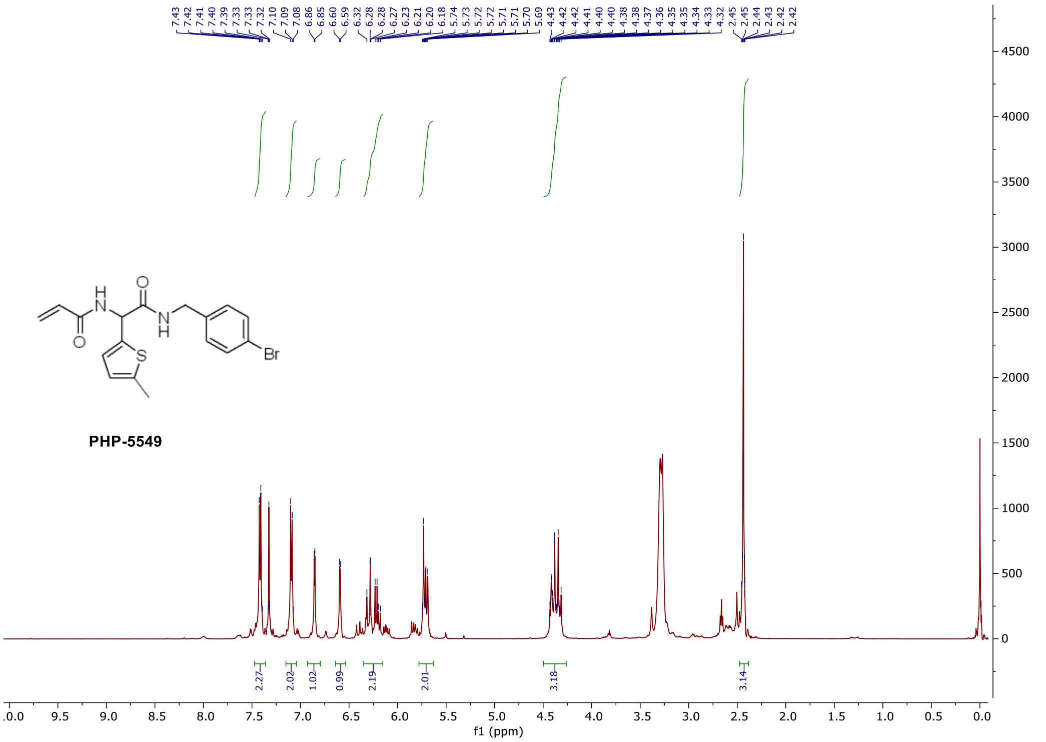

Supplementary Figure 606. ^{1}H NMR (500 MHz, $CDCl_3$ + one drop of CD_3OD) of compound **PHP-5549**.

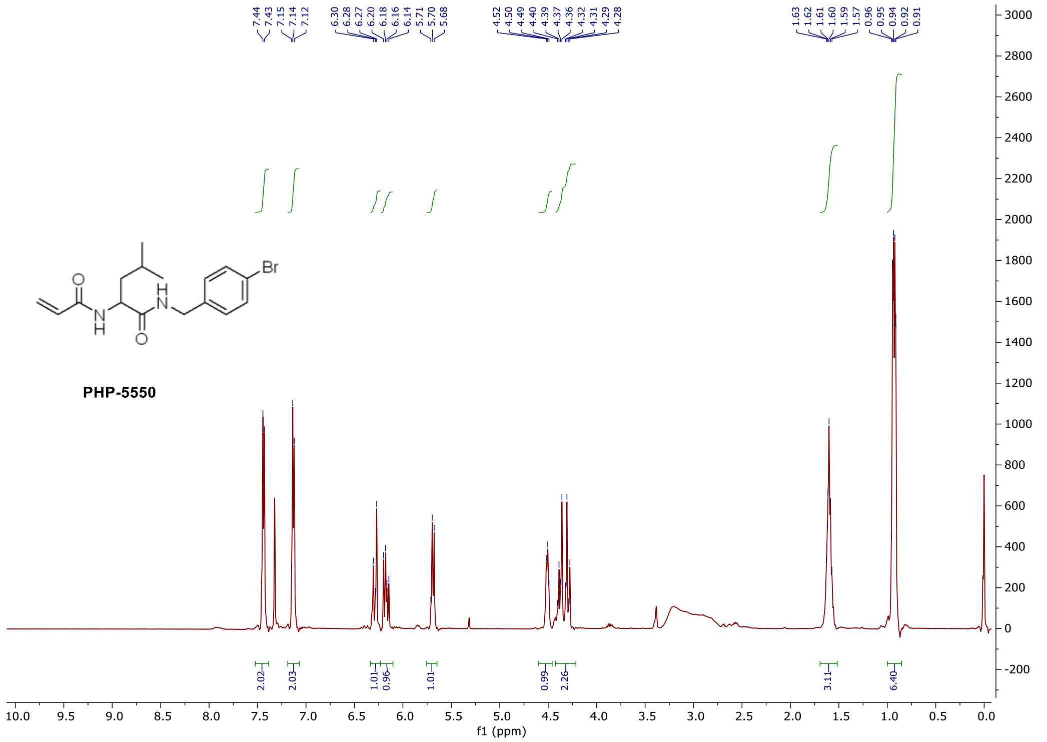

Supplementary Figure 607. ^{1}H NMR (500 MHz, $CDCl_3$ + one drop of CD_3OD) of compound **PHP-5550**.

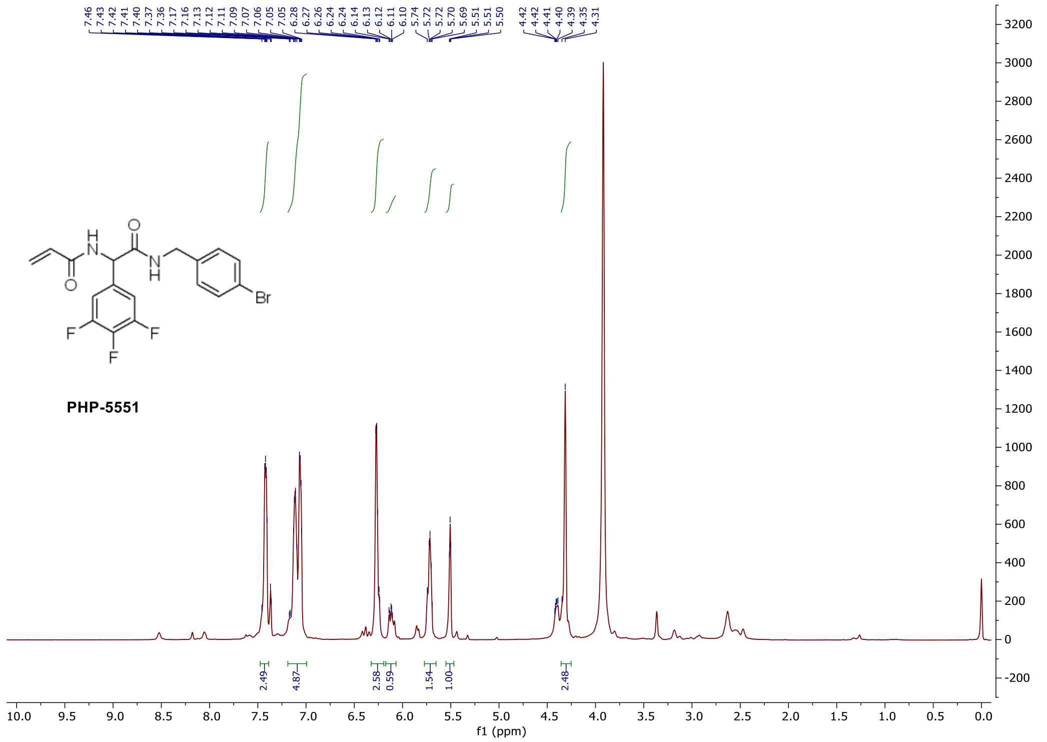

Supplementary Figure 608. ^{1}H NMR (500 MHz, $CDCl_3$ + one drop of CD_3OD) of compound **PHP-5551**.

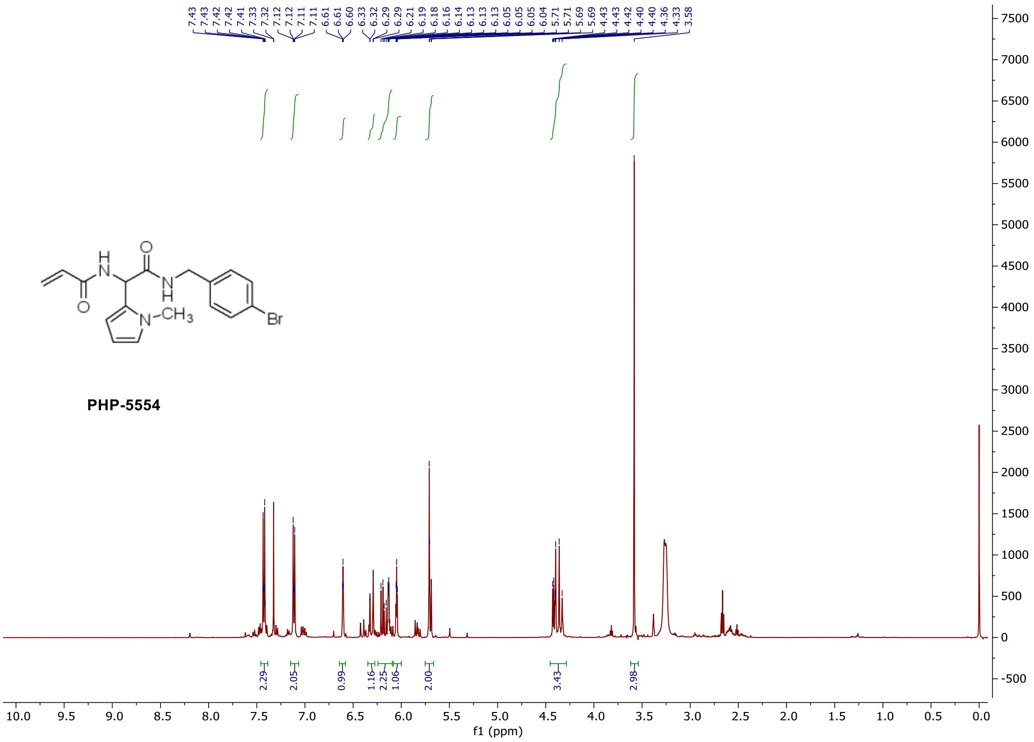

Supplementary Figure 609. ^{1}H NMR (500 MHz, $CDCl_3$ + one drop of CD_3OD) of compound **PHP-5554**.

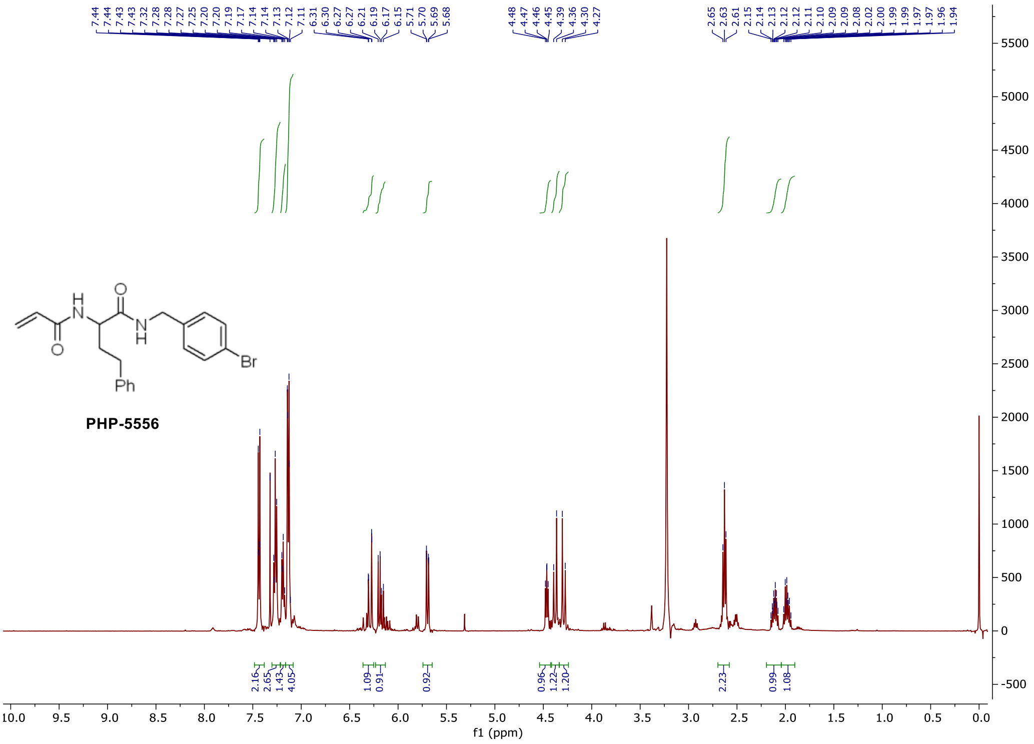

Supplementary Figure 610. ^{1}H NMR (500 MHz, $CDCl_3$ + one drop of CD_3OD) of compound **PHP-5556**.

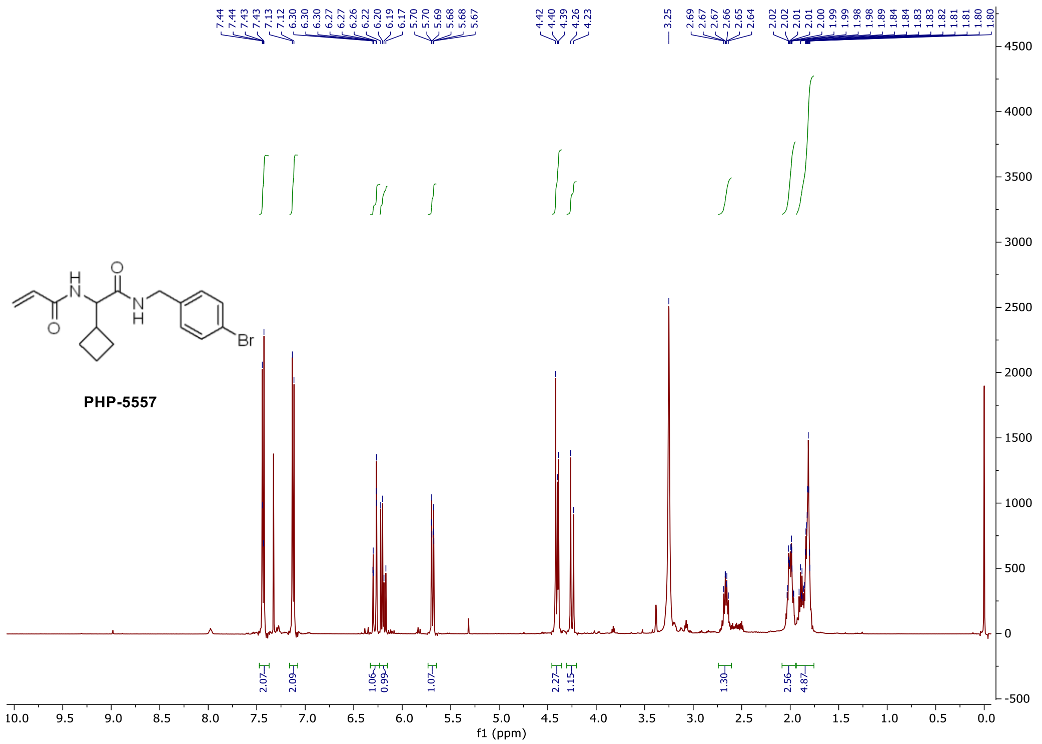

Supplementary Figure 611. ^{1}H NMR (500 MHz, $CDCl_3$ + one drop of CD_3OD) of compound **PHP-5557**.

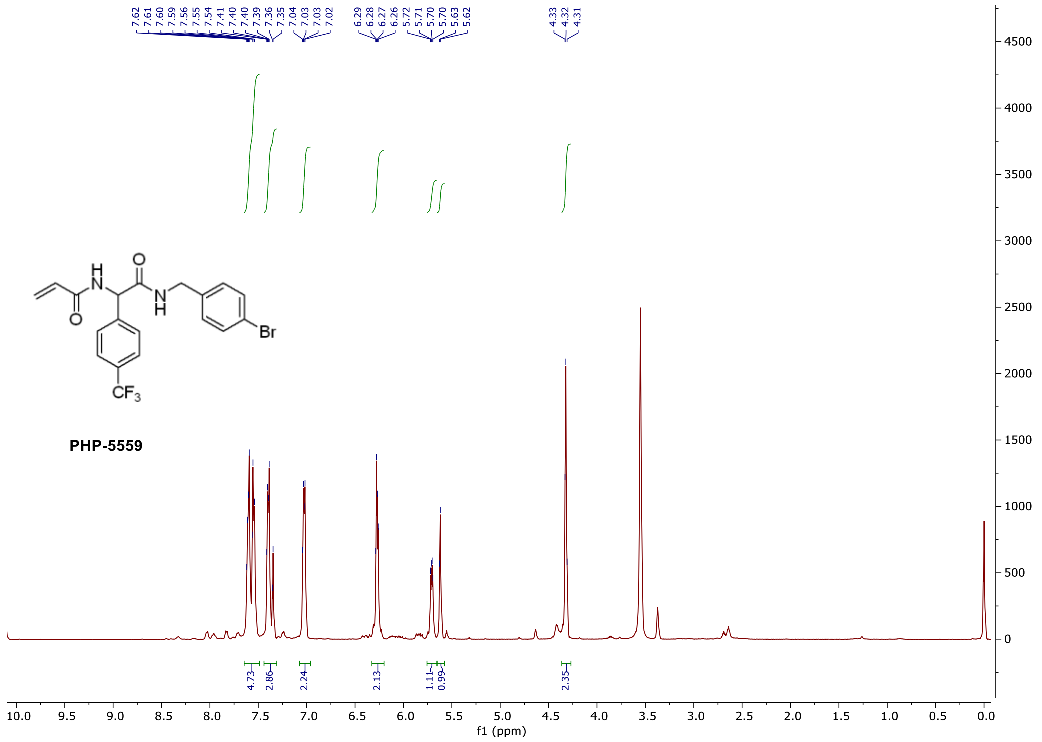

Supplementary Figure 612. ^{1}H NMR (500 MHz, $CDCl_3$ + one drop of CD_3OD) of compound **PHP-5559**.

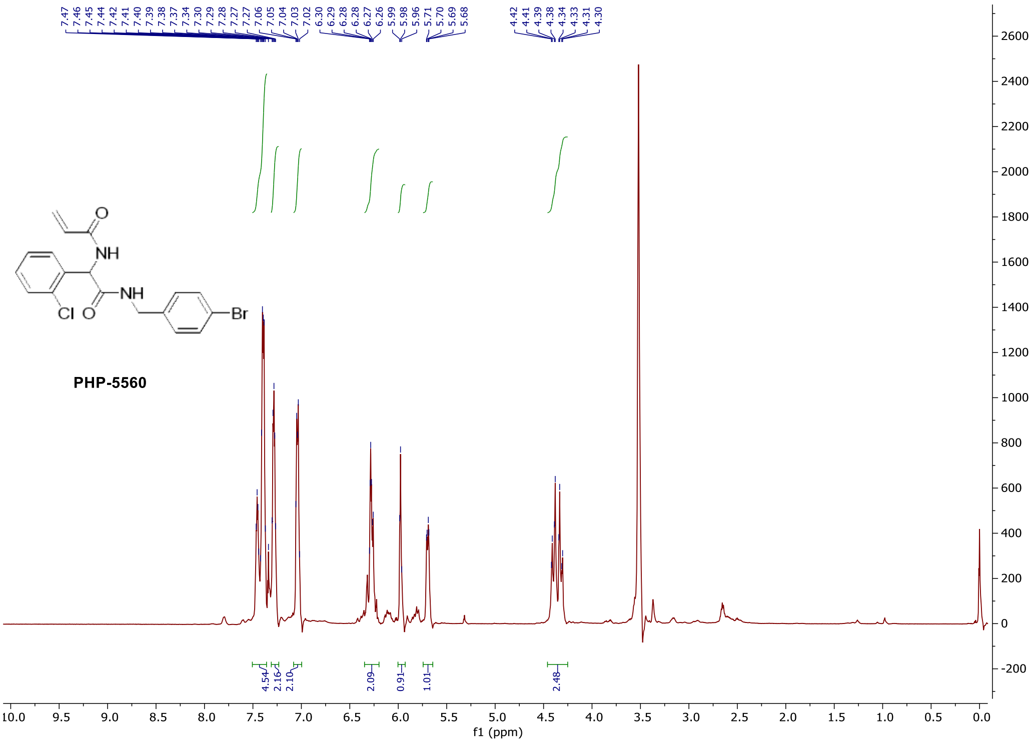

Supplementary Figure 613. ^{1}H NMR (500 MHz, $CDCl_3$ + one drop of CD_3OD) of compound **PHP-5560**.

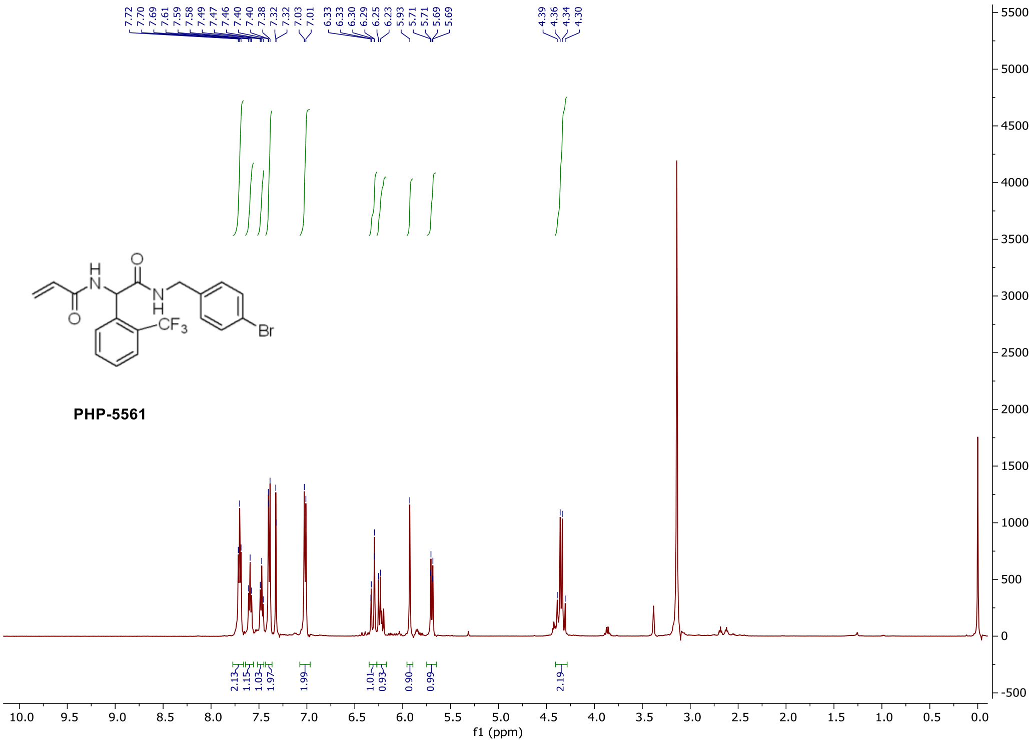

Supplementary Figure 614. ^{1}H NMR (500 MHz, $CDCl_3$ + one drop of CD_3OD) of compound **PHP-5561**.

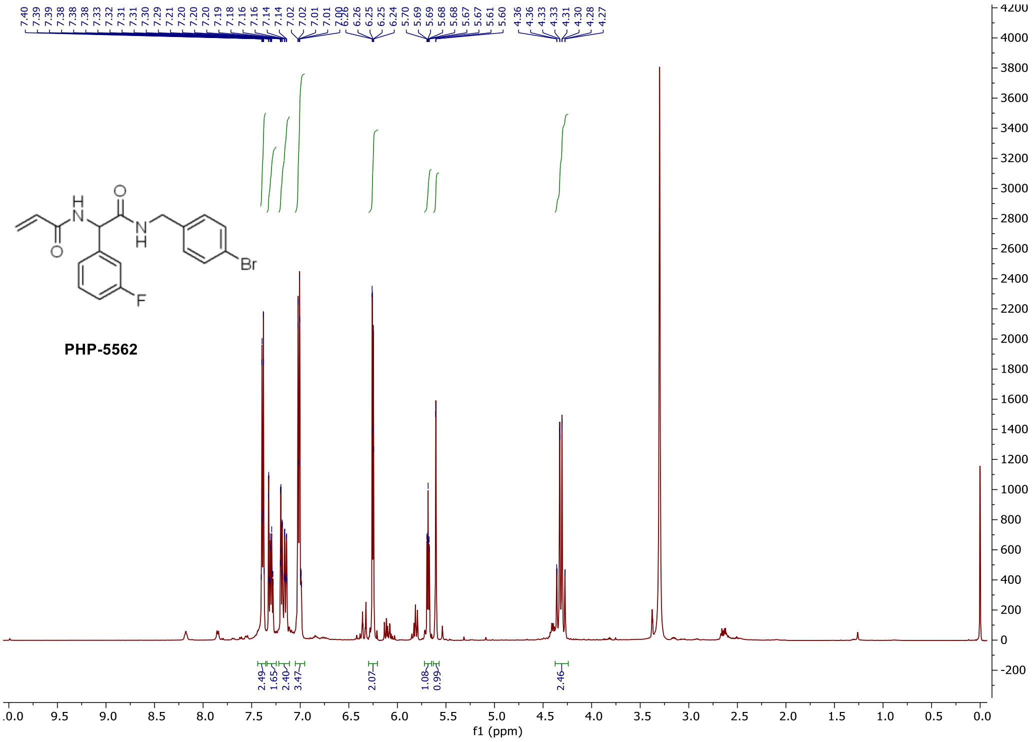

Supplementary Figure 615. ^{1}H NMR (500 MHz, $CDCl_3$ + one drop of CD_3OD) of compound **PHP-5562**.

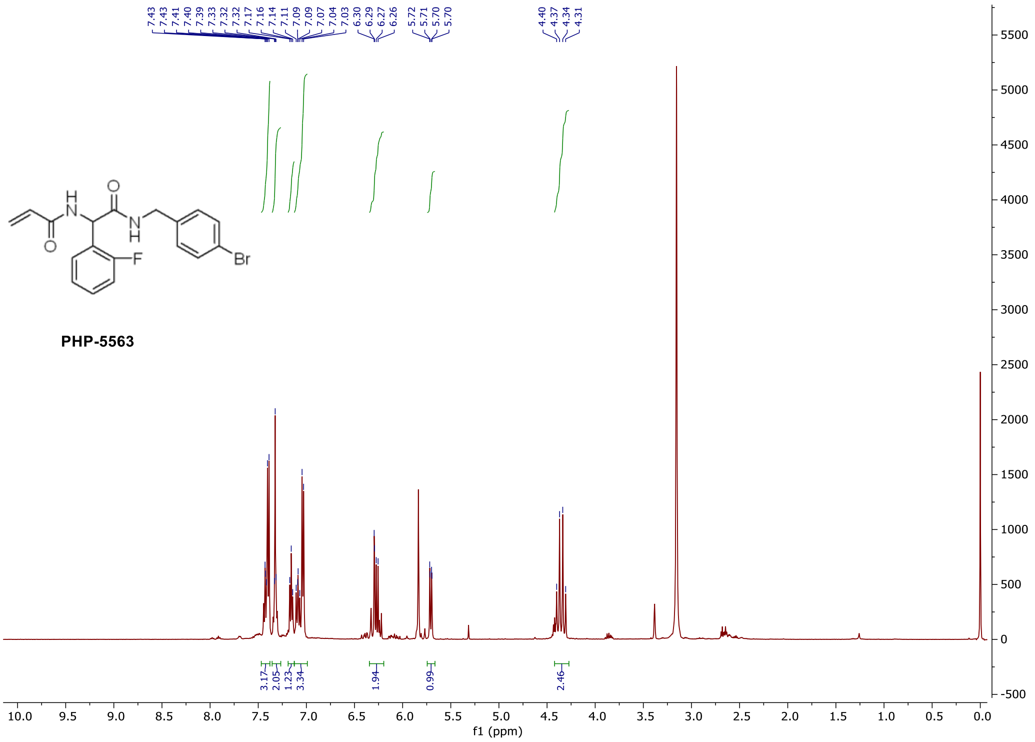

Supplementary Figure 616. ^{1}H NMR (500 MHz, $CDCl_3$ + one drop of CD_3OD) of compound **PHP-5563**.

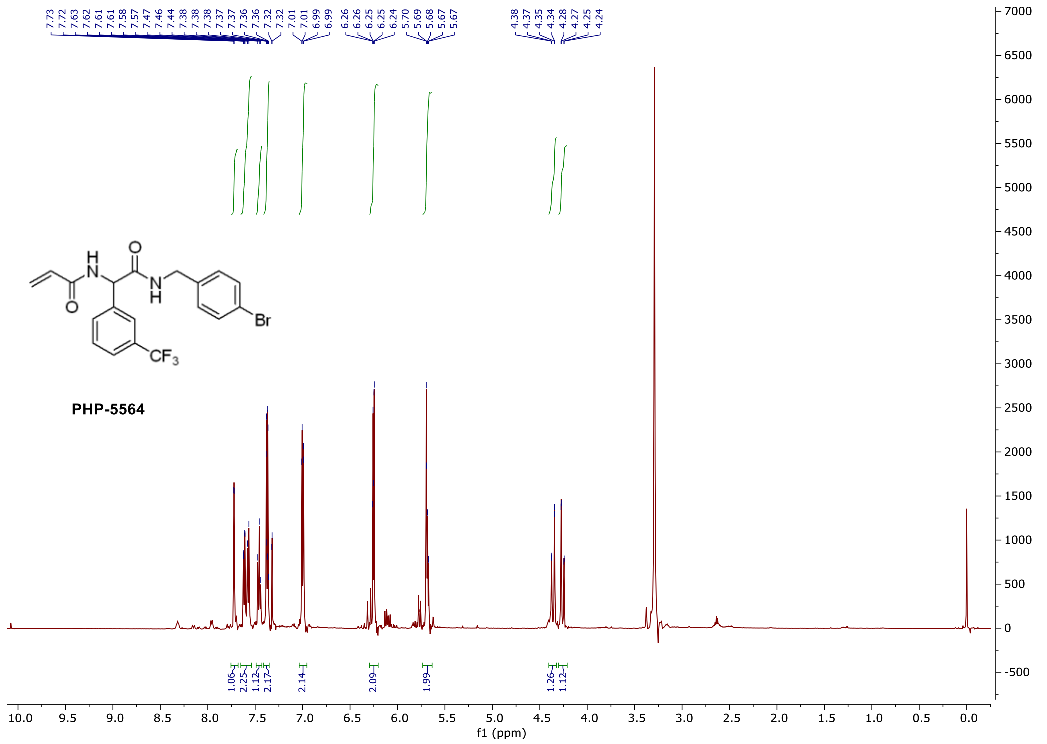

Supplementary Figure 617. ^{1}H NMR (500 MHz, $CDCl_3$ + one drop of CD_3OD) of compound **PHP-5564**.

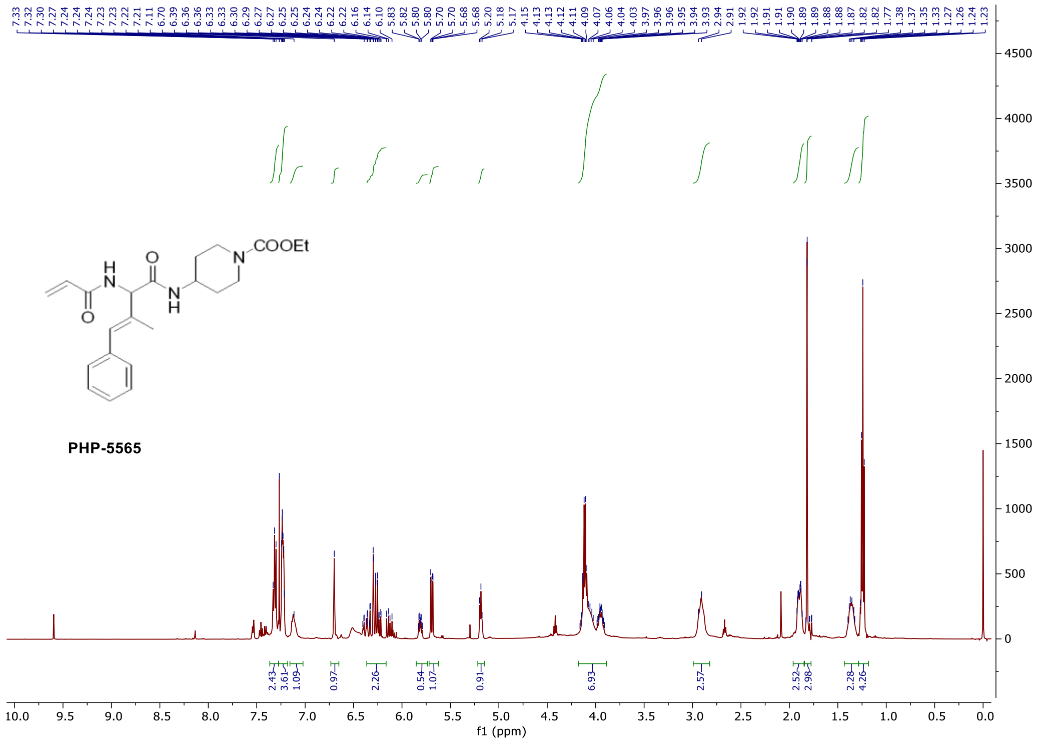

Supplementary Figure 618. ^{1}H NMR (500 MHz, $CDCl_3$) of compound **PHP-5565**.

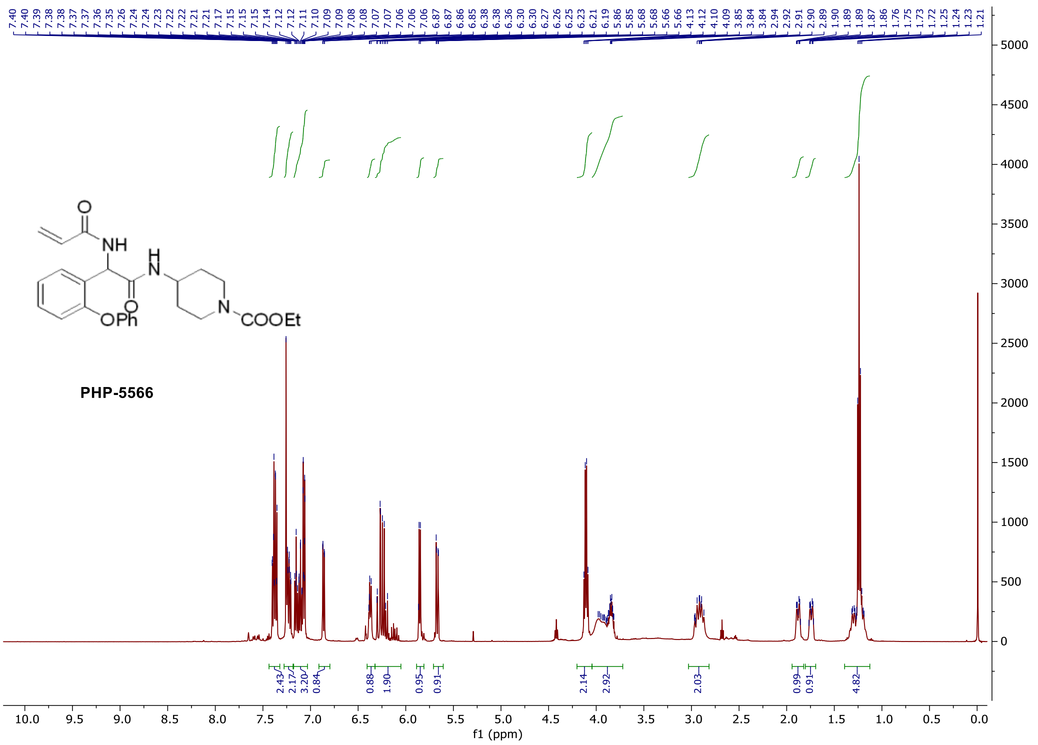

Supplementary Figure 619. ^{1}H NMR (500 MHz, $CDCl_3$) of compound **PHP-5566**.

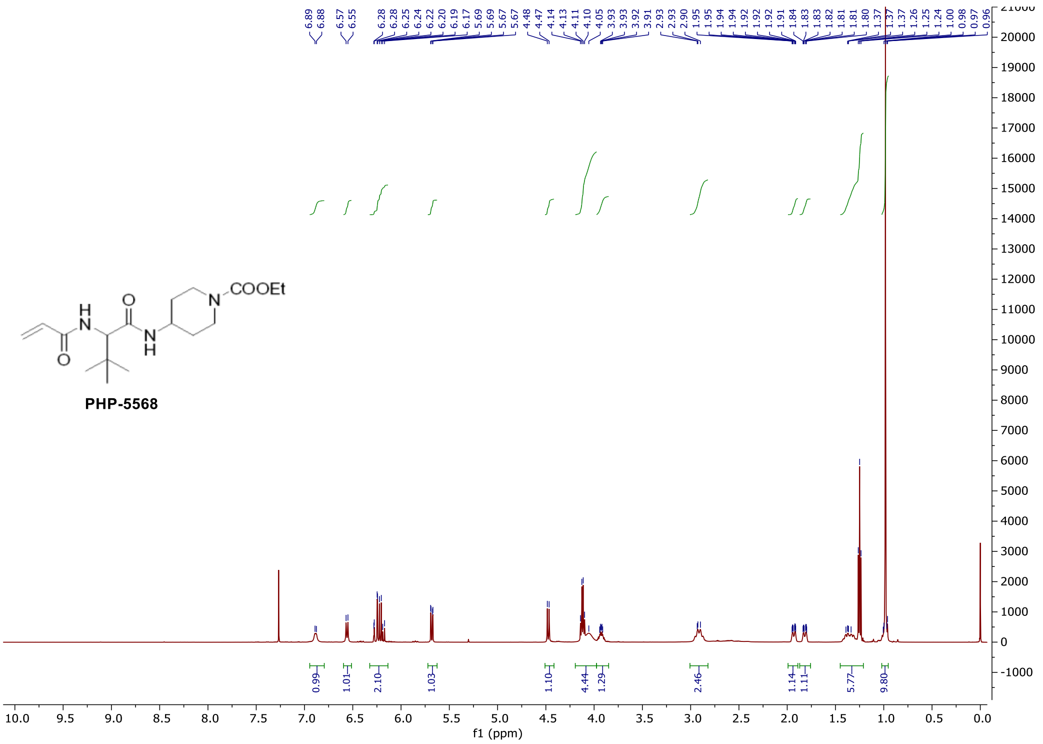

Supplementary Figure 620. ^{1}H NMR (500 MHz, $CDCl_3$) of compound **PHP-5568**.

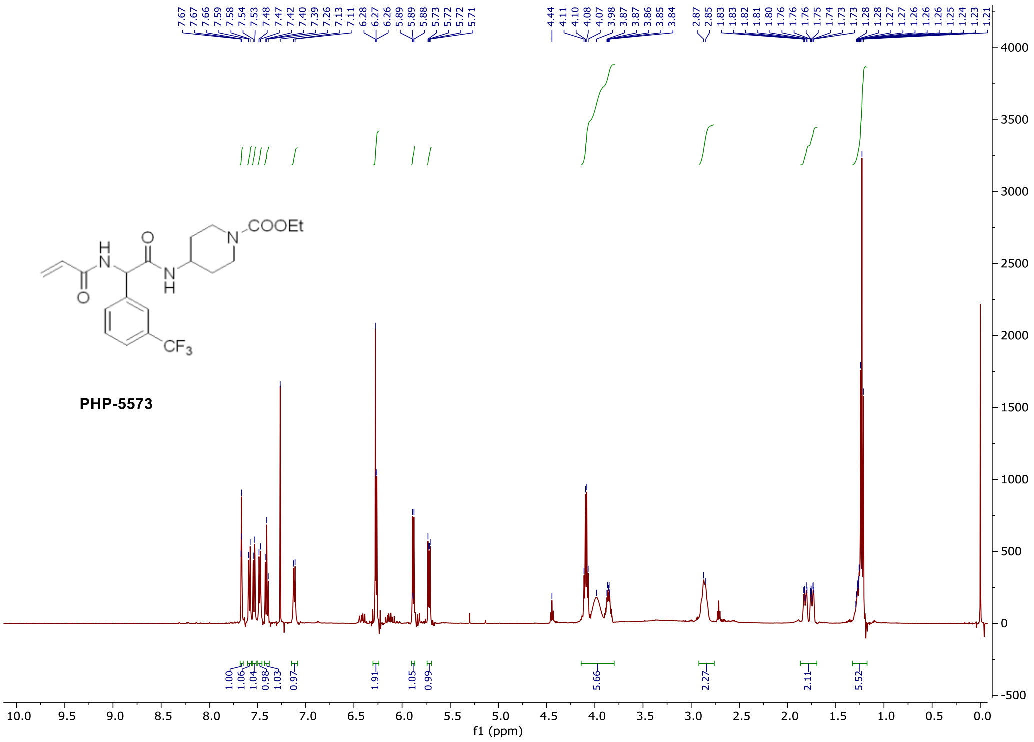

Supplementary Figure 621. ^{1}H NMR (500 MHz, $CDCl_3$) of compound **PHP-5573**.

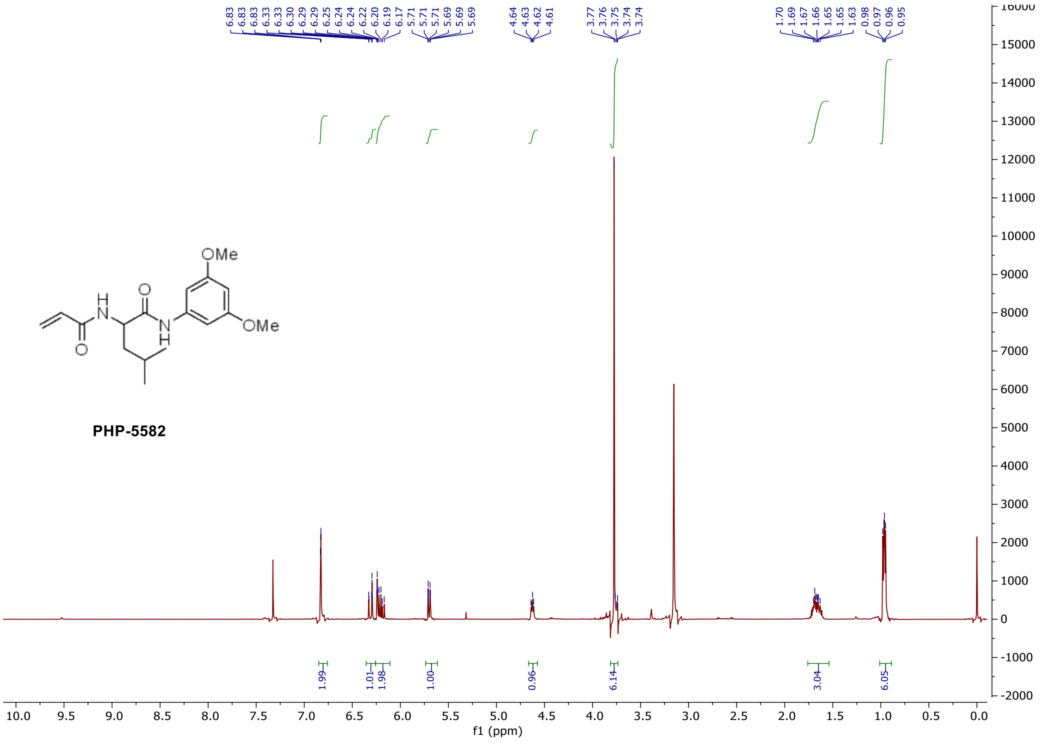

Supplementary Figure 622. ^{1}H NMR (500 MHz, $CDCl_3$ + one drop of CD_3OD) of compound **PHP-5582**.

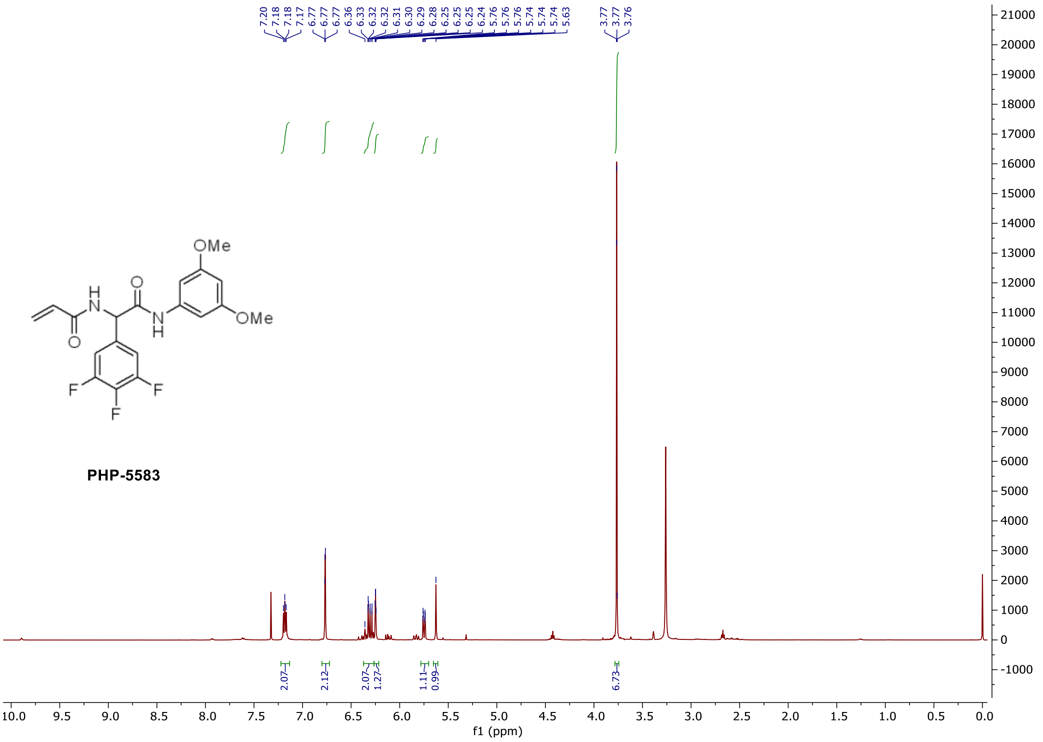

PHP-5583

Supplementary Figure 623. ^{1}H NMR (500 MHz, $CDCl_3$ + one drop of CD_3OD) of compound **PHP-5583**.

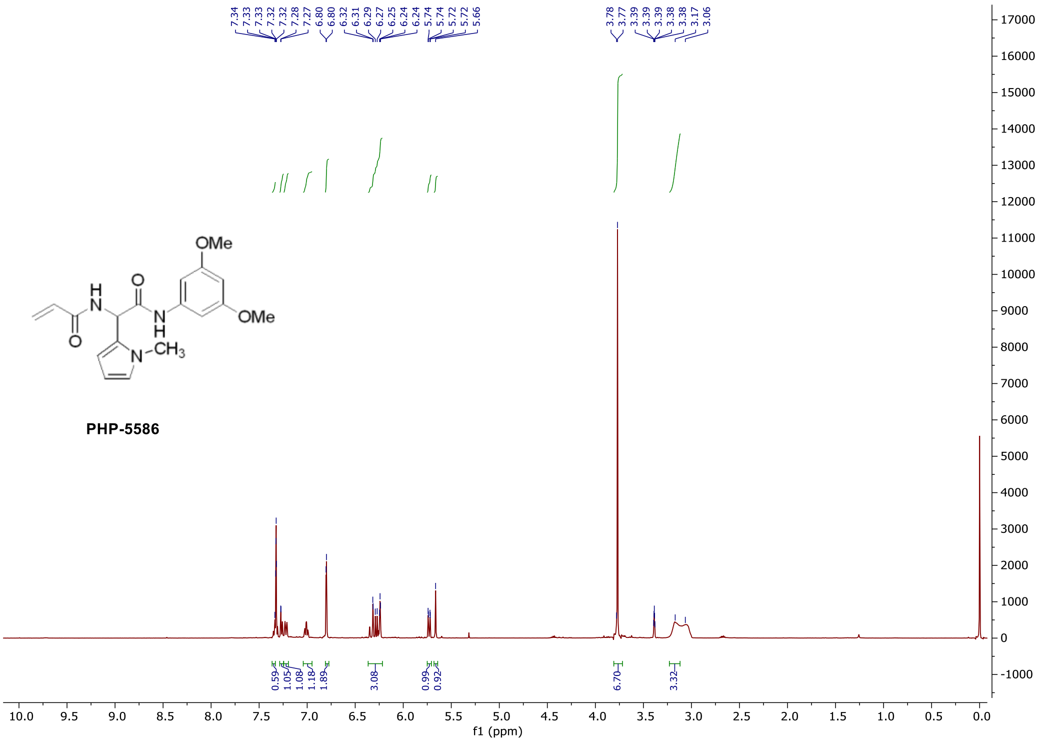

PHP-5586

Supplementary Figure 624. ^{1}H NMR (500 MHz, $CDCl_3$ + one drop of CD_3OD) of compound **PHP-5586**.

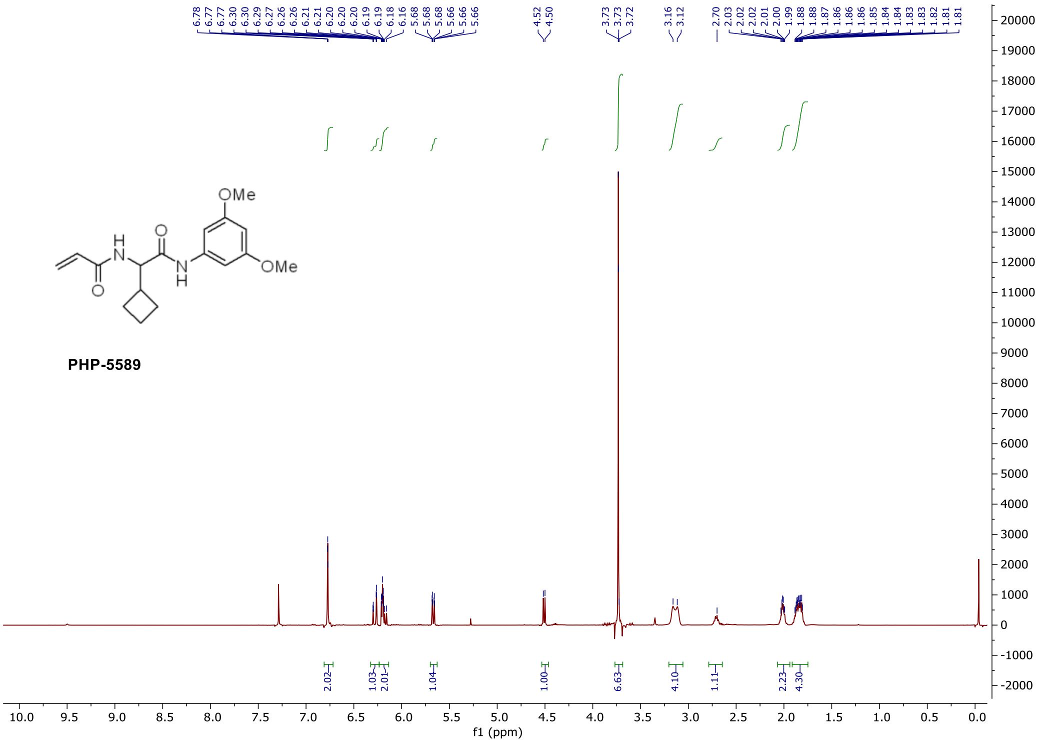

Supplementary Figure 625. ^{1}H NMR (500 MHz, $CDCl_3$) of compound **PHP-5589**.

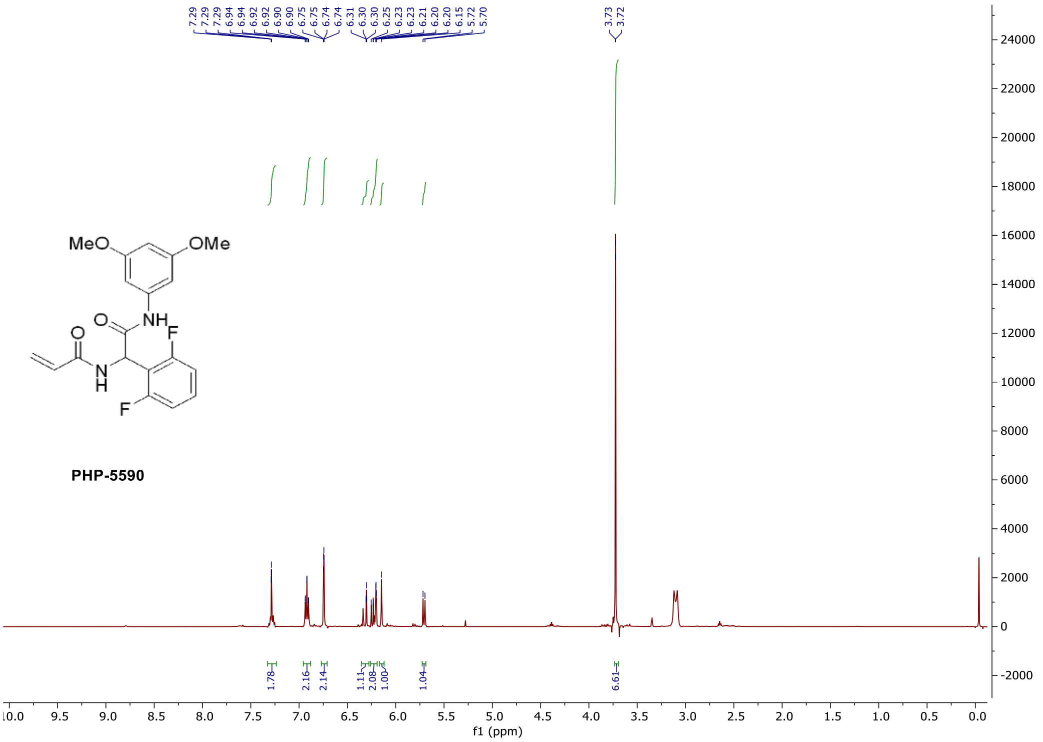

Supplementary Figure 626. ^{1}H NMR (500 MHz, $CDCl_3$ + one drop of CD_3OD) of compound **PHP-5590**.

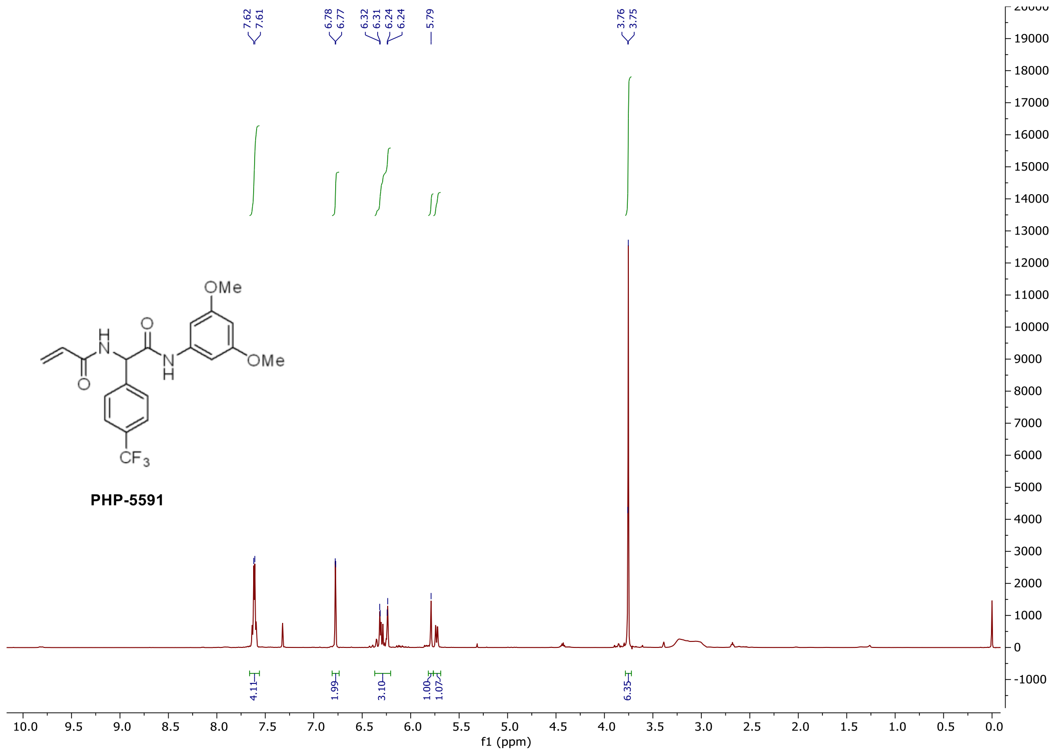

Supplementary Figure 627. ^{1}H NMR (500 MHz, $CDCl_3$ + one drop of CD_3OD) of compound **PHP-5591**.

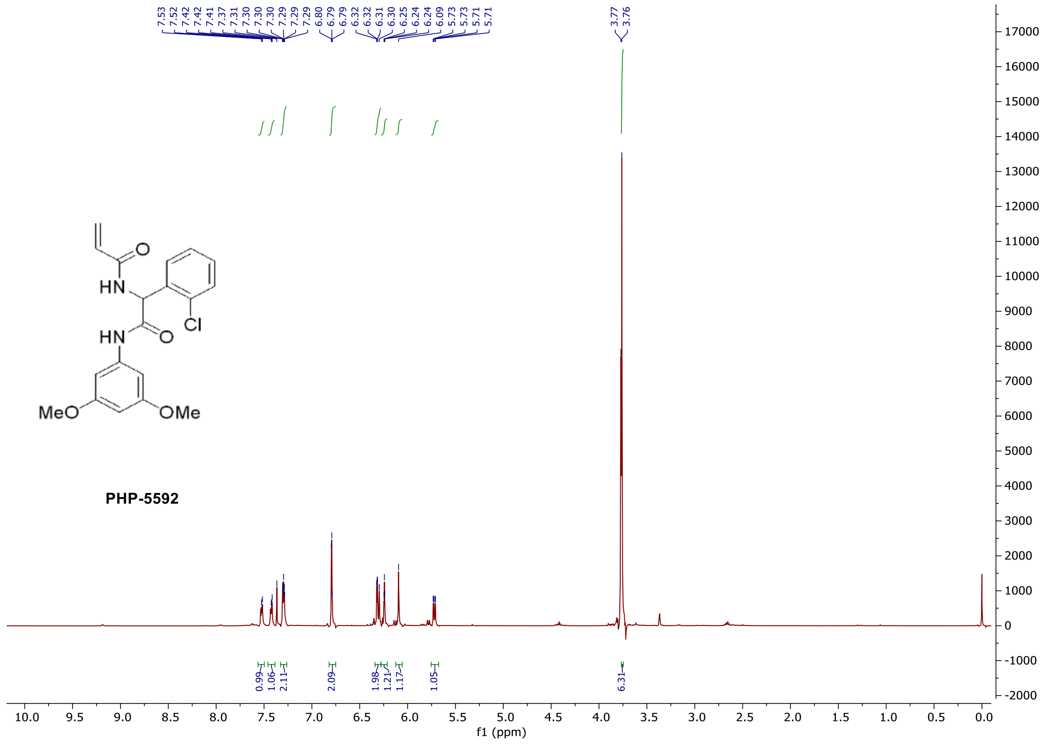

Supplementary Figure 628. ^{1}H NMR (500 MHz, $CDCl_3$) of compound **PHP-5592**.

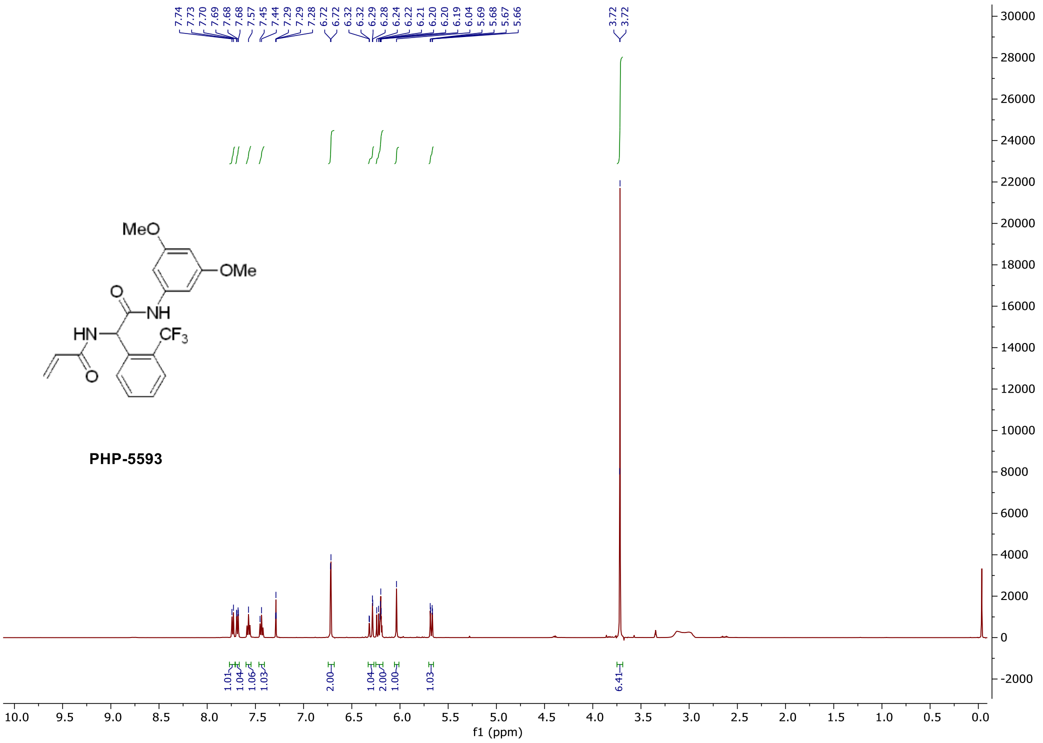

Supplementary Figure 629. ^{1}H NMR (500 MHz, $CDCl_3$) of compound **PHP-5593**.

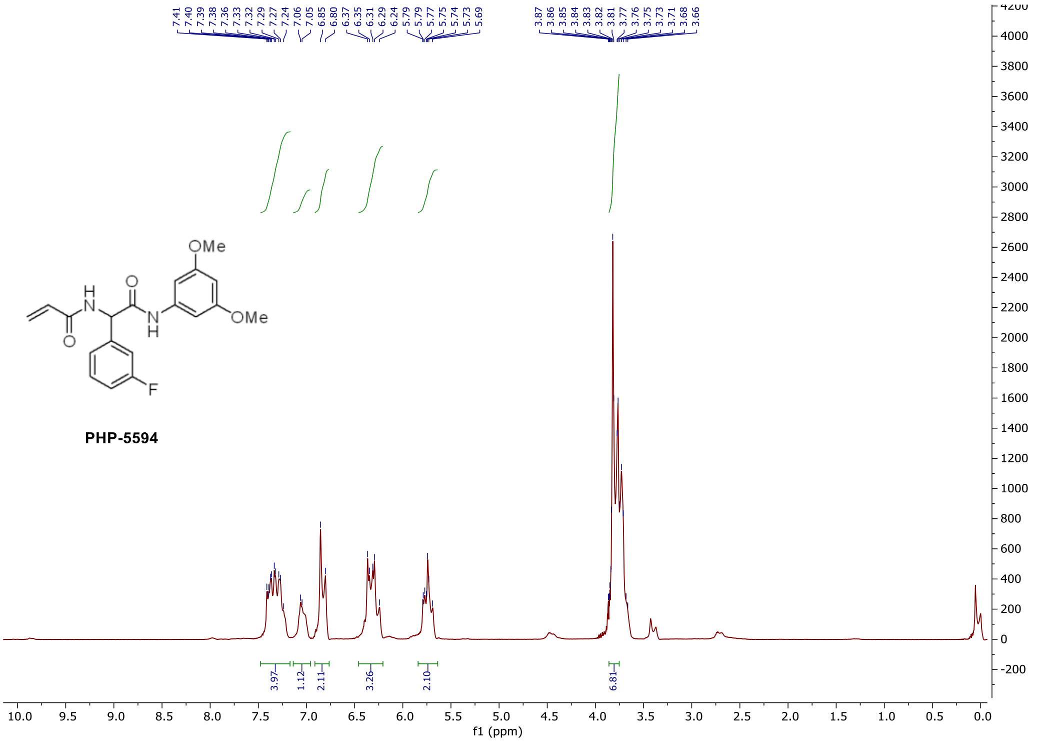

Supplementary Figure 630. ^{1}H NMR (500 MHz, $CDCl_3$) of compound **PHP-5594**.

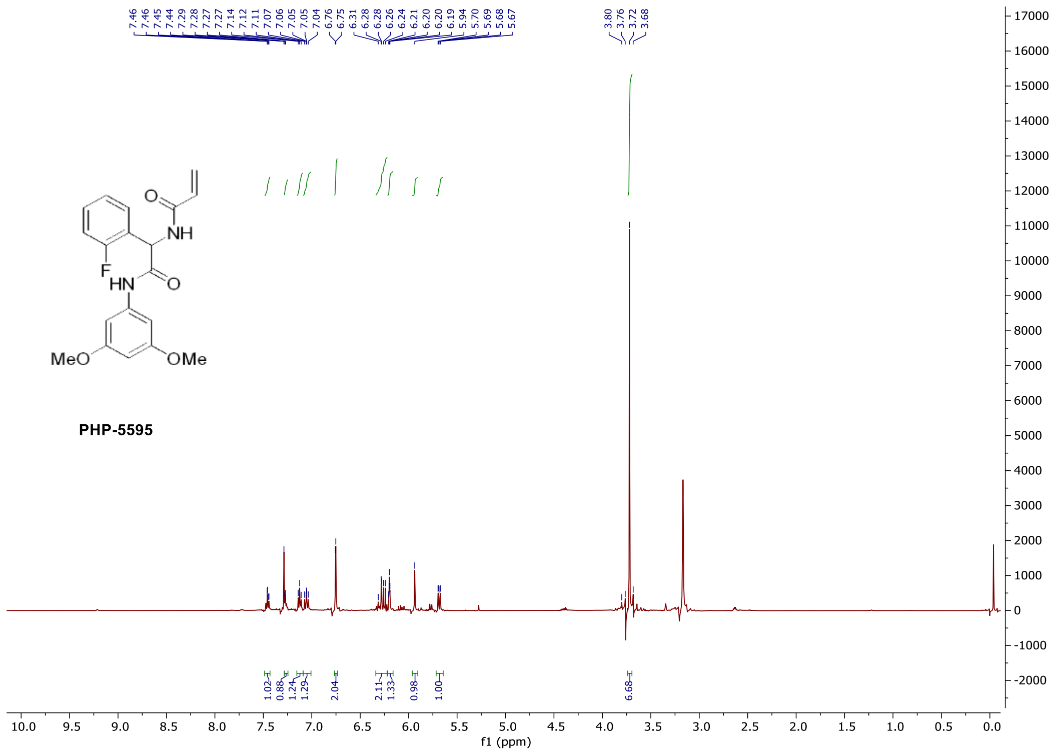

Supplementary Figure 631. ^{1}H NMR (500 MHz, $CDCl_3$ + one drop of CD_3OD) of compound **PHP-5595**.

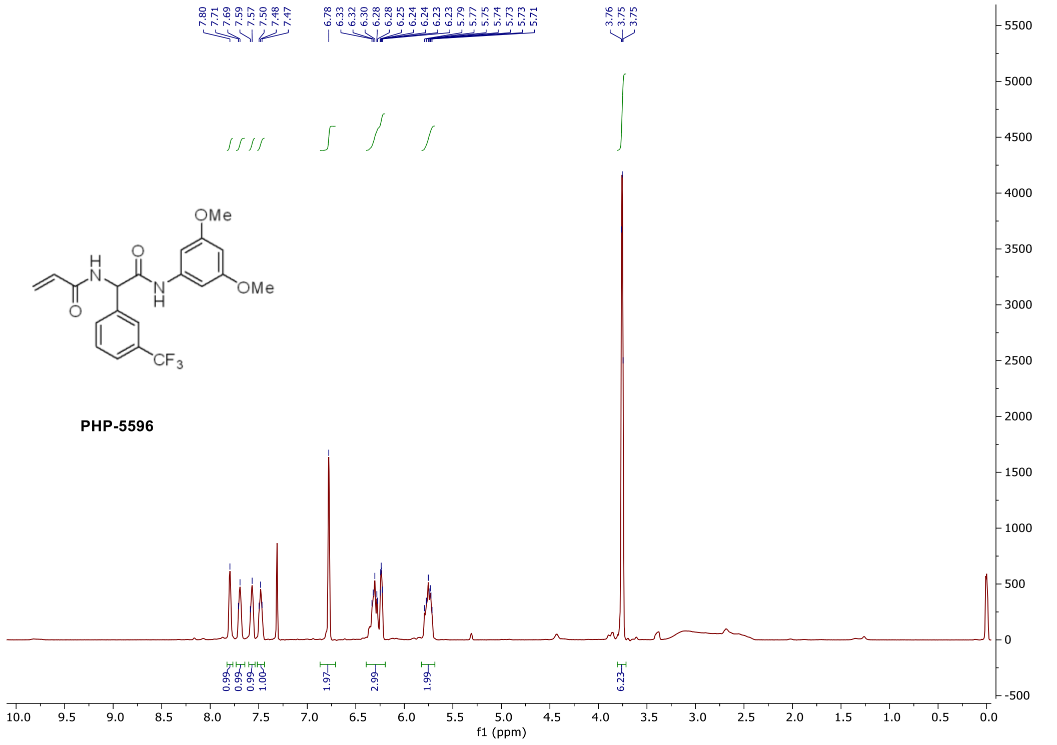

Supplementary Figure 632. ^{1}H NMR (500 MHz, $CDCl_3$) of compound **PHP-5596**.

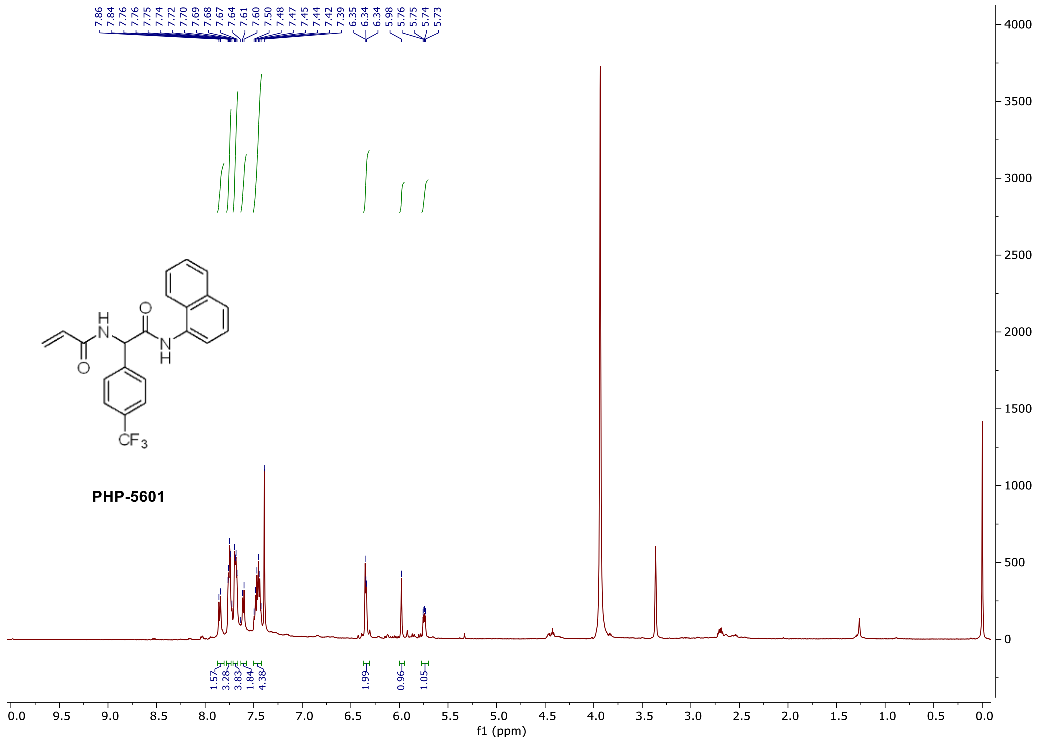

Supplementary Figure 633. ^{1}H NMR (500 MHz, $CDCl_3$) of compound **PHP-5601**.

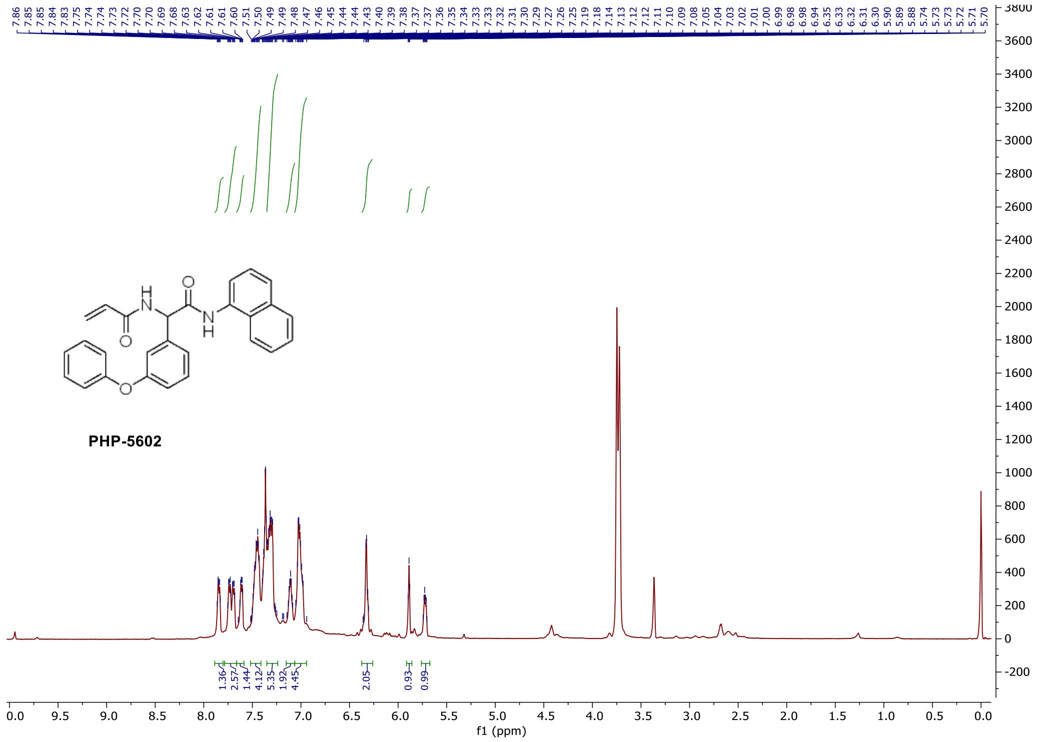

Supplementary Figure 634. ^{1}H NMR (500 MHz, $CDCl_3$) of compound **PHP-5602**.

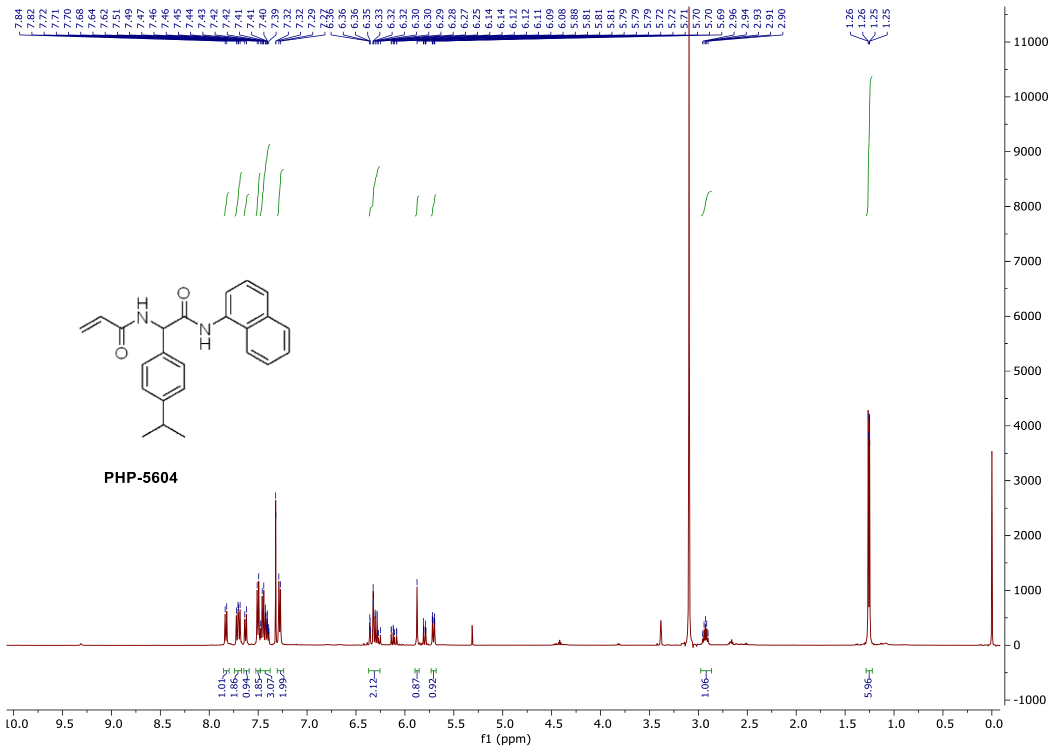

Supplementary Figure 635. ^{1}H NMR (500 MHz, $CDCl_3$ + one drop of CD_3OD) of compound **PHP-5604**.

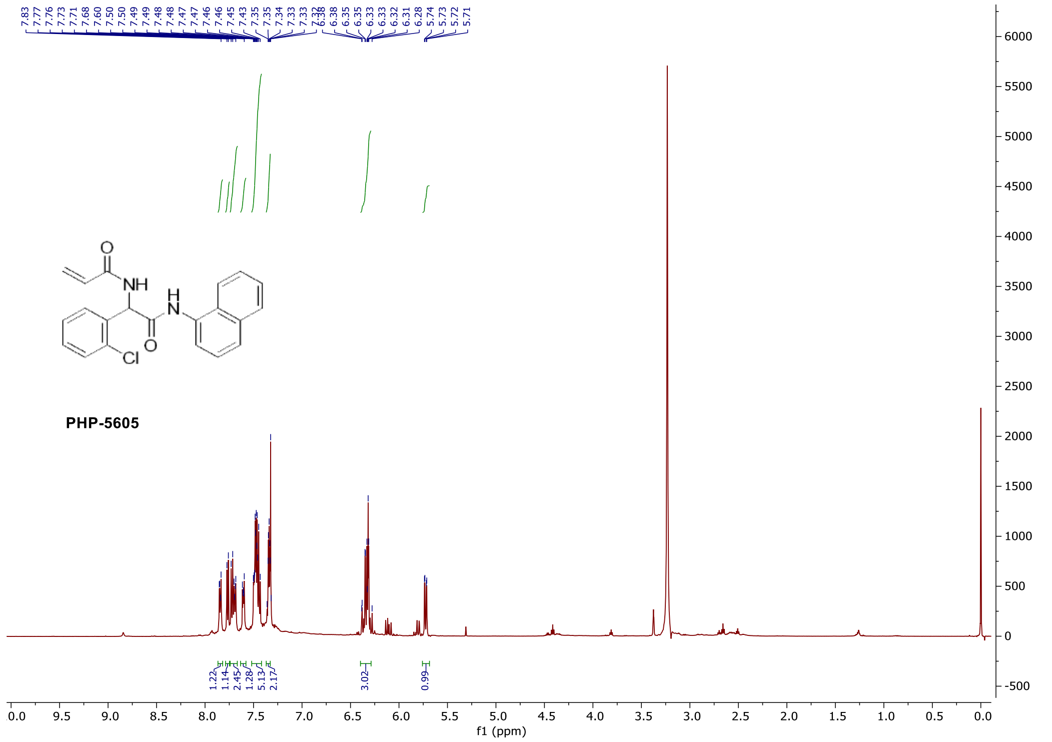

Supplementary Figure 636. ^{1}H NMR (500 MHz, $CDCl_3$ + one drop of CD_3OD) of compound **PHP-5605**.

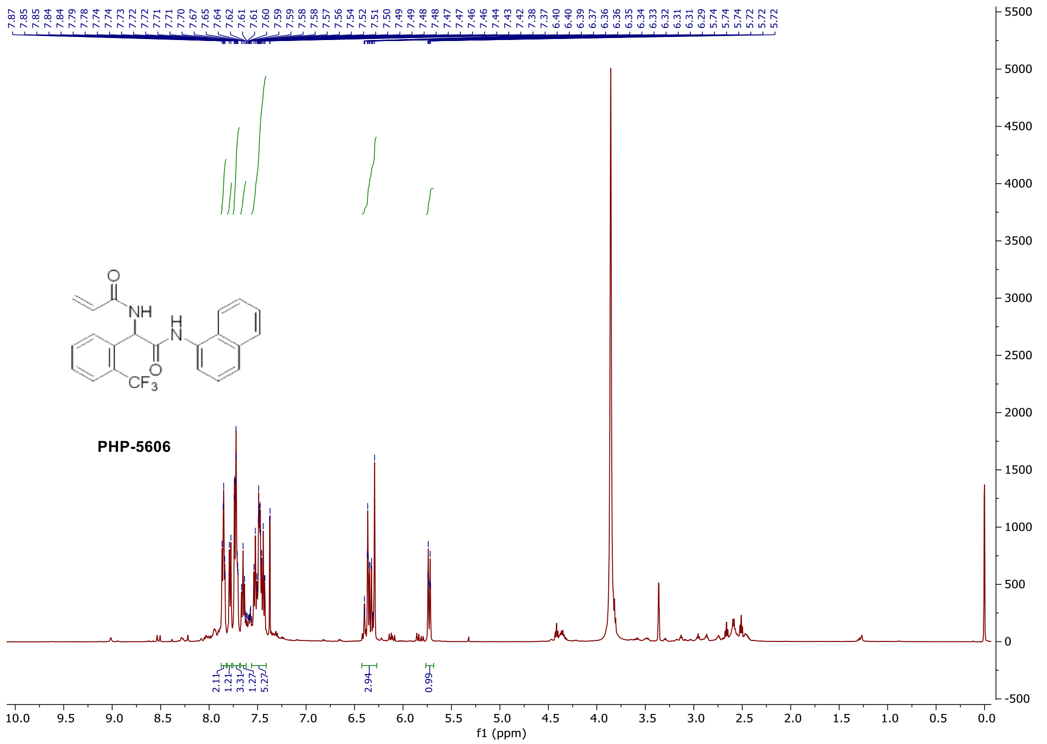

Supplementary Figure 637. ^{1}H NMR (500 MHz, $CDCl_3$ + one drop of CD_3OD) of compound **PHP-5606**.

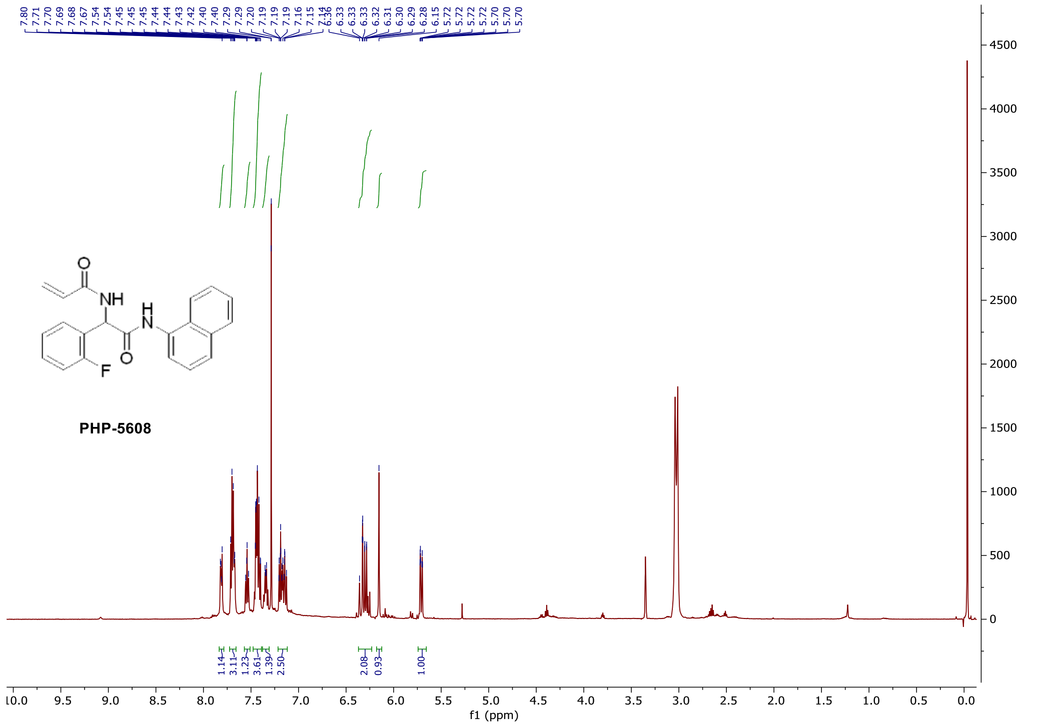

Supplementary Figure 638. ^{1}H NMR (500 MHz, $CDCl_3$ + one drop of CD_3OD) of compound **PHP-5608**.

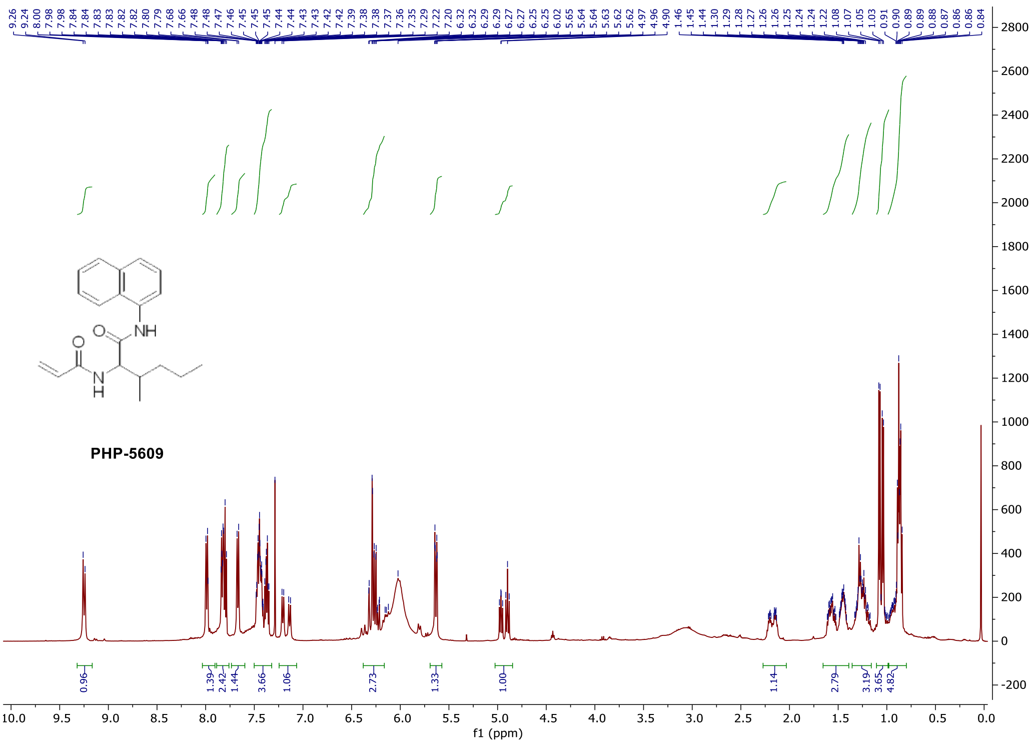

Supplementary Figure 639. ^{1}H NMR (500 MHz, $CDCl_3$ + one drop of CD_3OD) of compound **PHP-5609**.

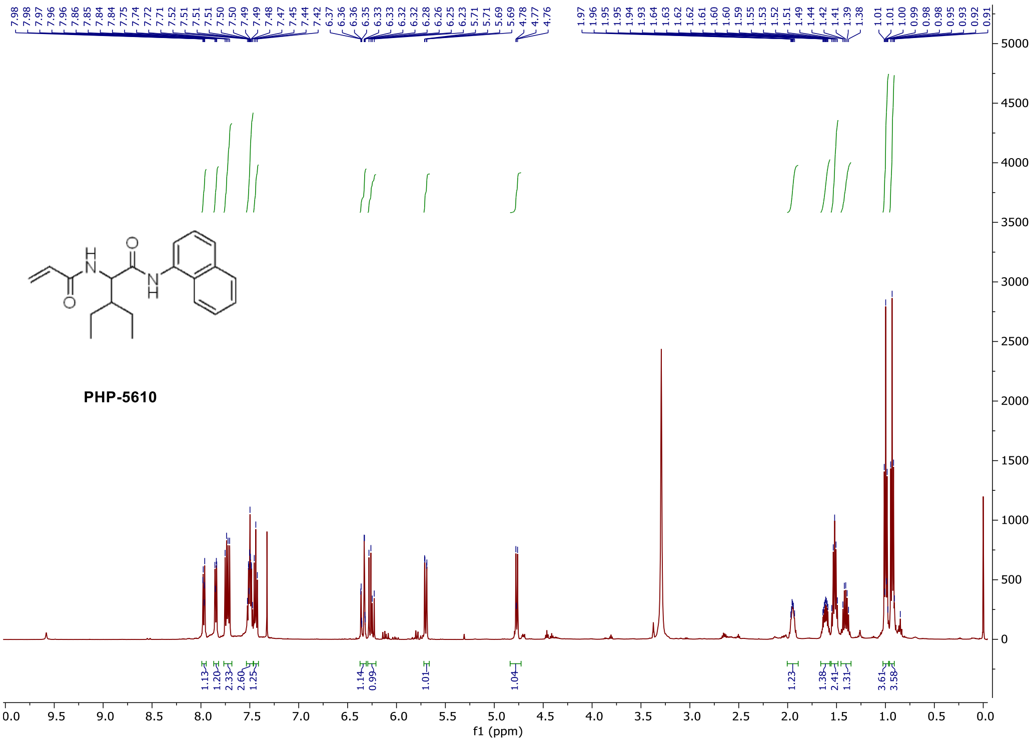

Supplementary Figure 640. ^{1}H NMR (500 MHz, $CDCl_3$ + one drop of CD_3OD) of compound **PHP-5610**.

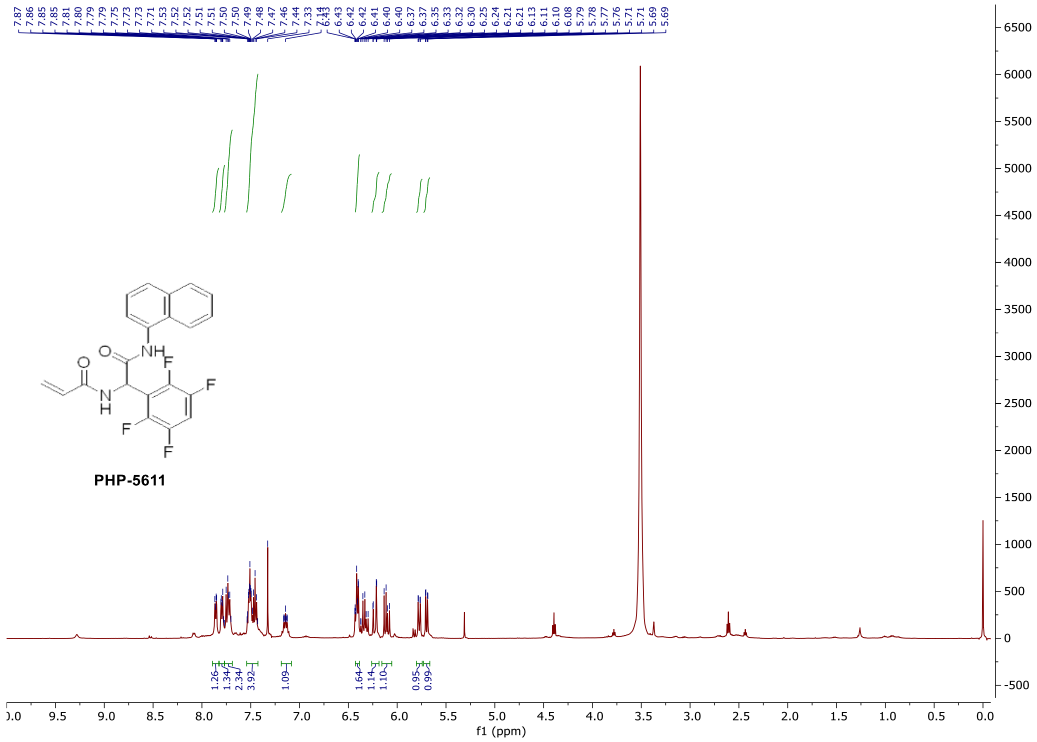

Supplementary Figure 641. ^{1}H NMR (500 MHz, $CDCl_3$) of compound **PHP-5611**.

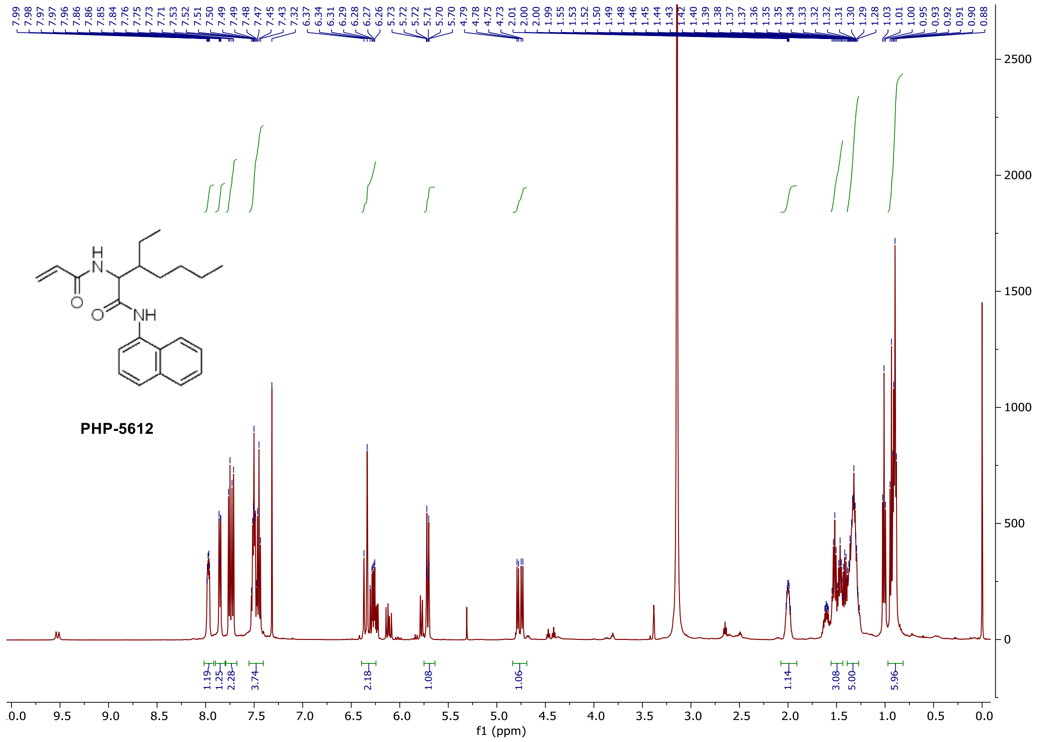

Supplementary Figure 642. ^{1}H NMR (500 MHz, $CDCl_3$ + one drop of CD_3OD) of compound **PHP-5612**.

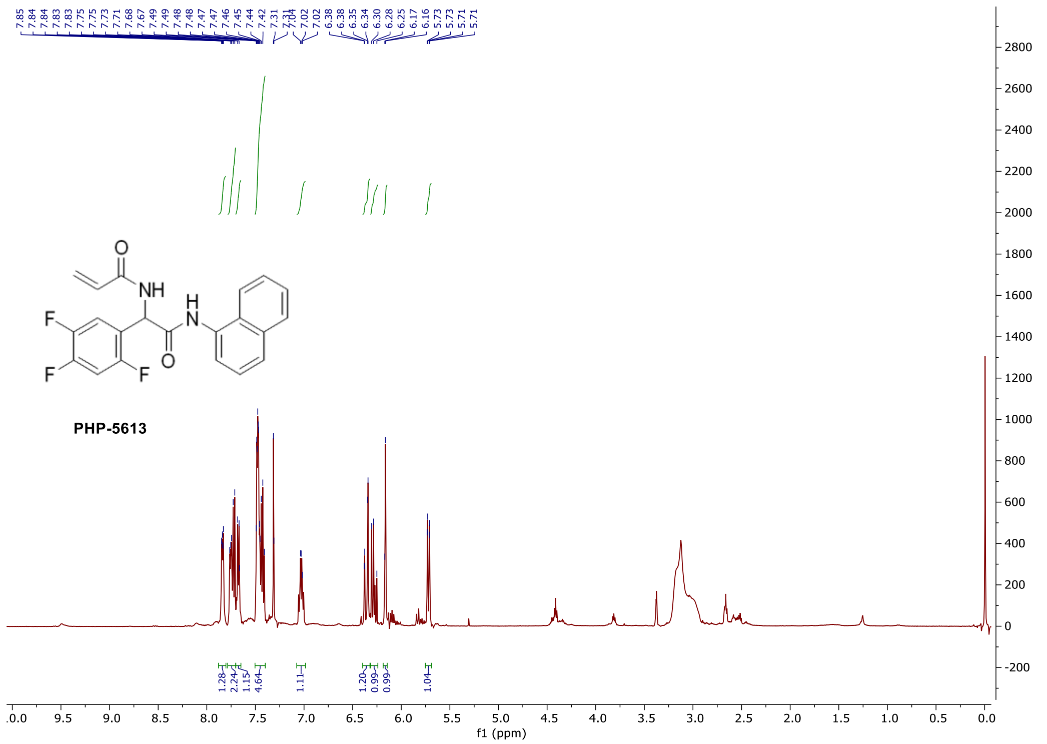

Supplementary Figure 643. ^{1}H NMR (500 MHz, $CDCl_3$ + one drop of CD_3OD) of compound **PHP-5613**.

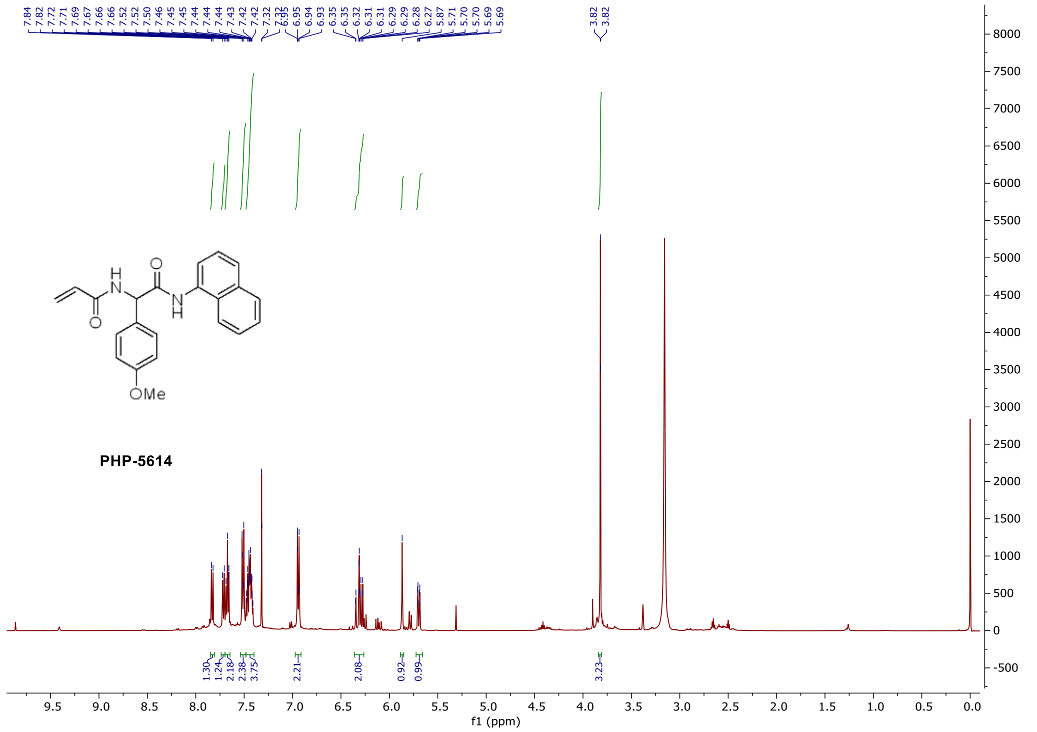

Supplementary Figure 644. ^{1}H NMR (500 MHz, $CDCl_3$ + one drop of CD_3OD) of compound **PHP-5614**.

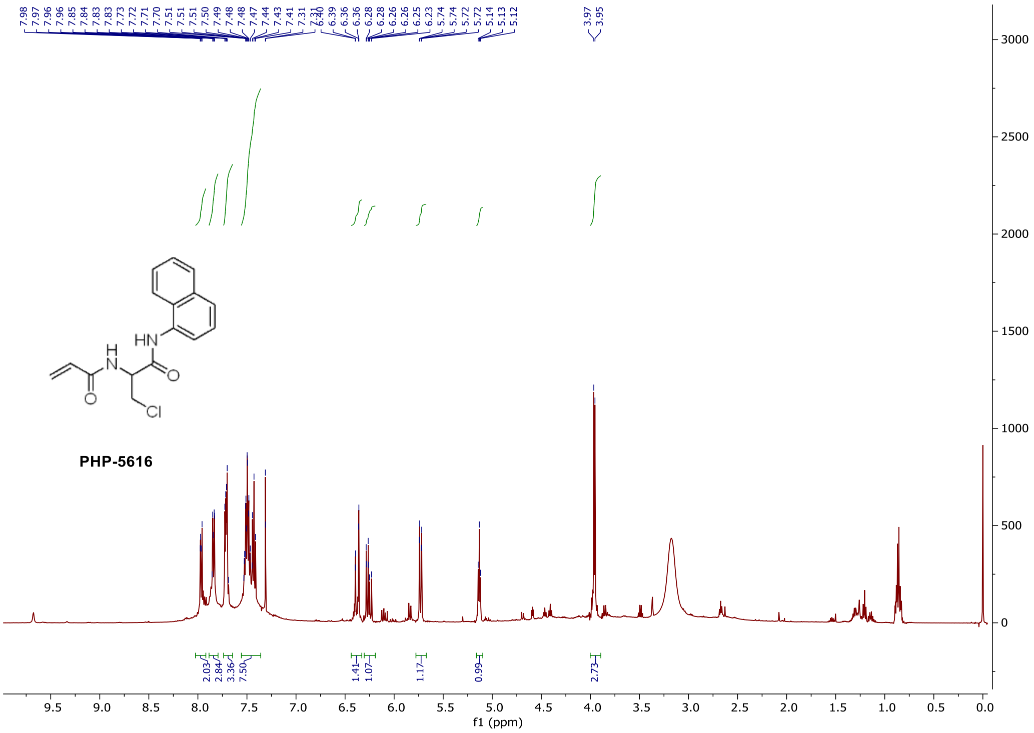

Supplementary Figure 645. ^{1}H NMR (500 MHz, $CDCl_3$ + one drop of CD_3OD) of compound **PHP-5616**.

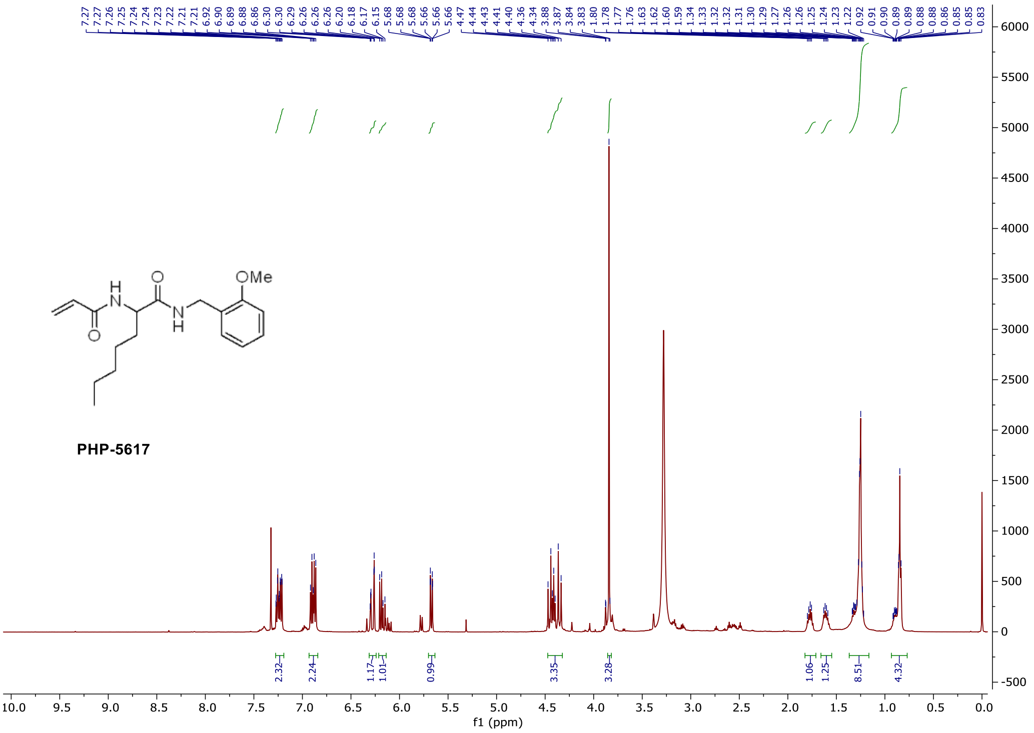

Supplementary Figure 646. ^{1}H NMR (500 MHz, $CDCl_3$ + one drop of CD_3OD) of compound **PHP-5617**.

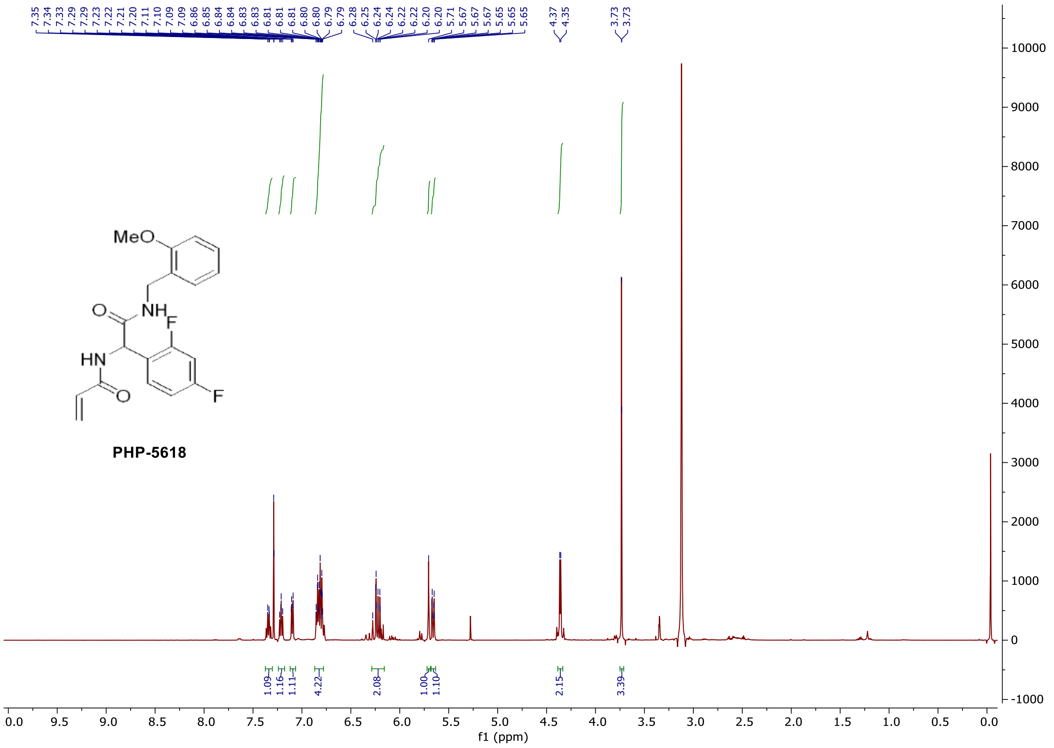

Supplementary Figure 647. ^{1}H NMR (500 MHz, $CDCl_3$ + one drop of CD_3OD) of compound **PHP-5618**.

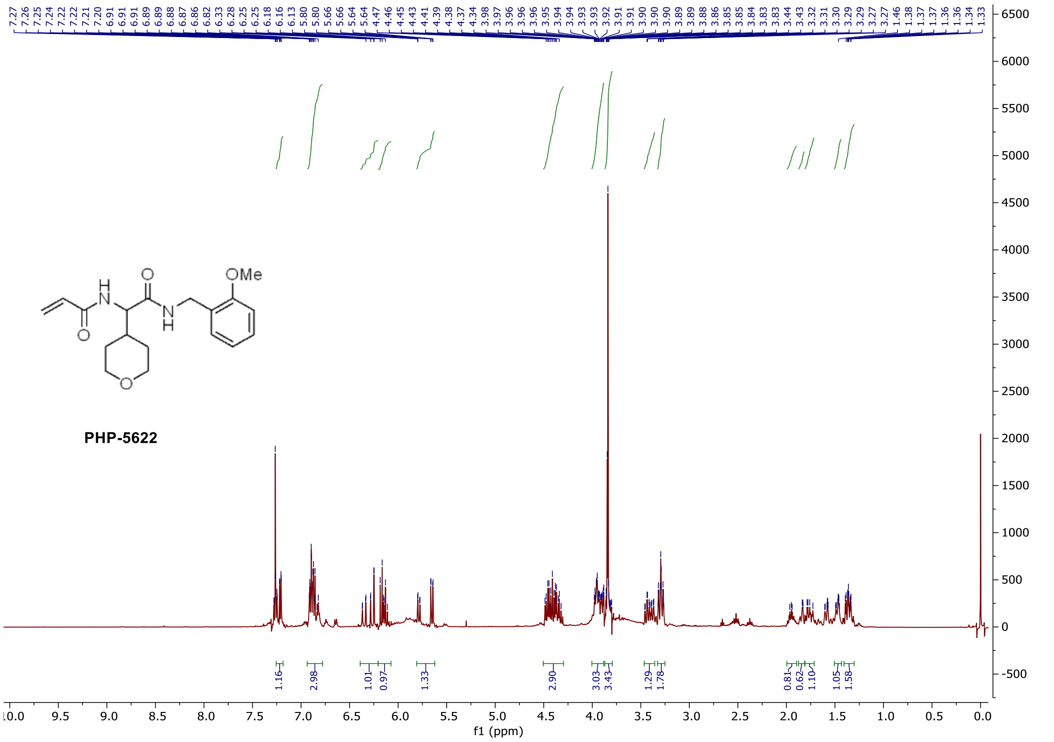

Supplementary Figure 648. ^{1}H NMR (500 MHz, $CDCl_3$) of compound **PHP-5622**.

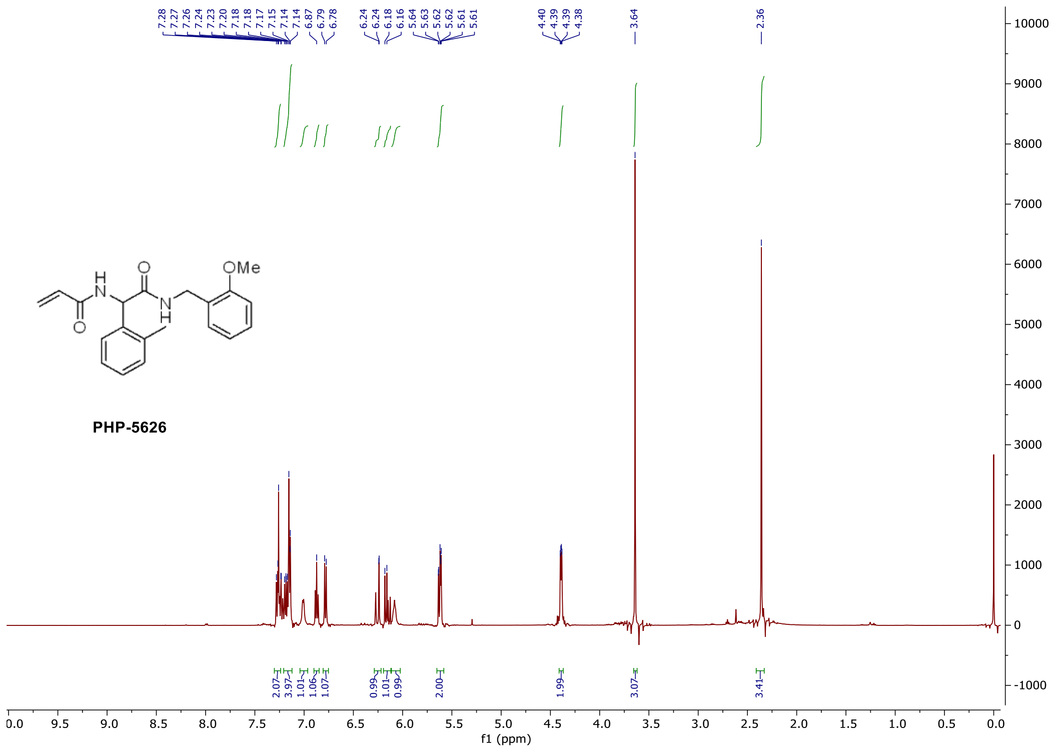

Supplementary Figure 649. ^{1}H NMR (500 MHz, $CDCl_3$) of compound **PHP-5626**.

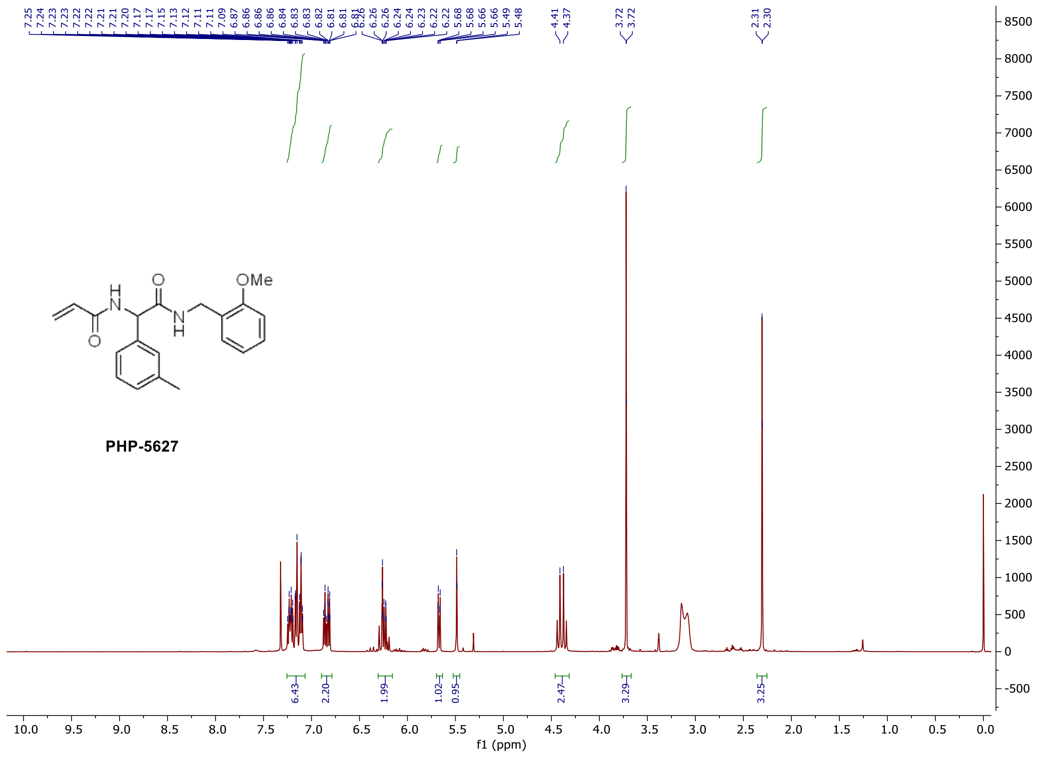

Supplementary Figure 650. ^{1}H NMR (500 MHz, $CDCl_3$ + one drop of CD_3OD) of compound **PHP-5627**.

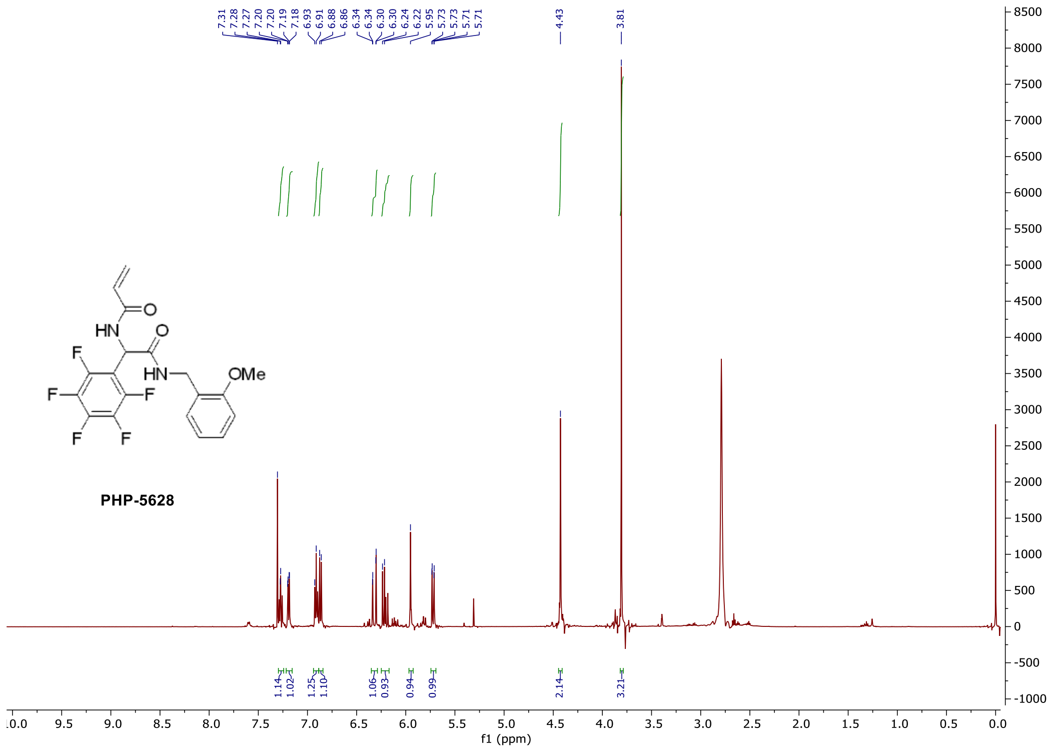

Supplementary Figure 651. ^{1}H NMR (500 MHz, $CDCl_3$) of compound **PHP-5628**.

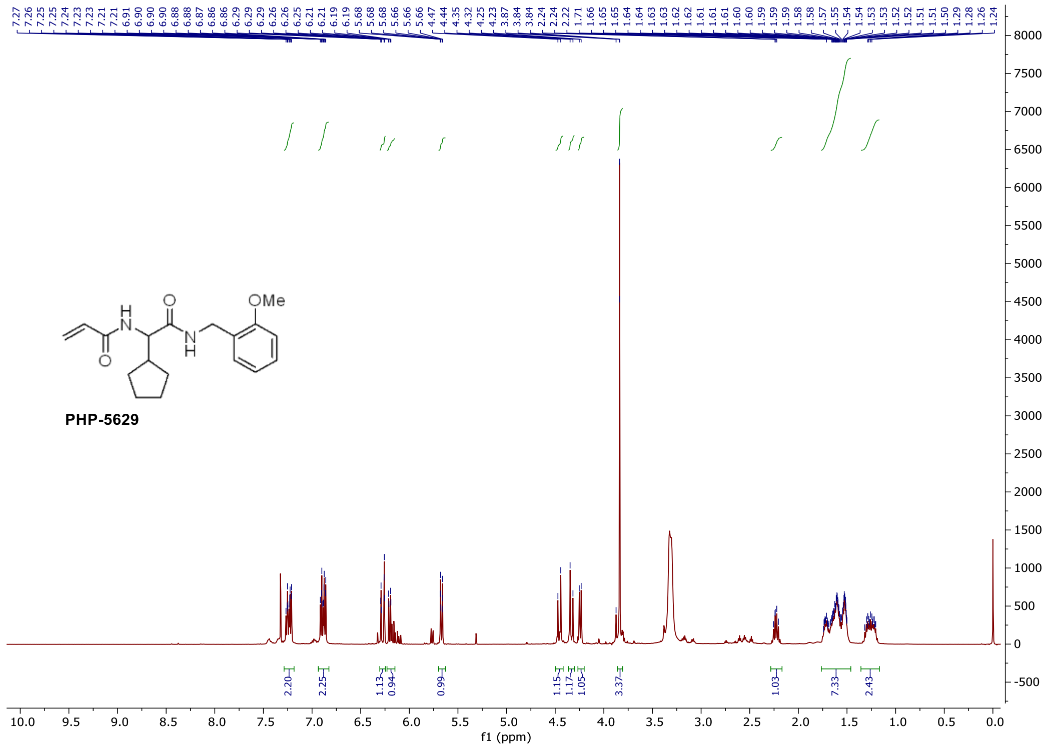

Supplementary Figure 652. ^{1}H NMR (500 MHz, $CDCl_3$ + one drop of CD_3OD) of compound **PHP-5629**.

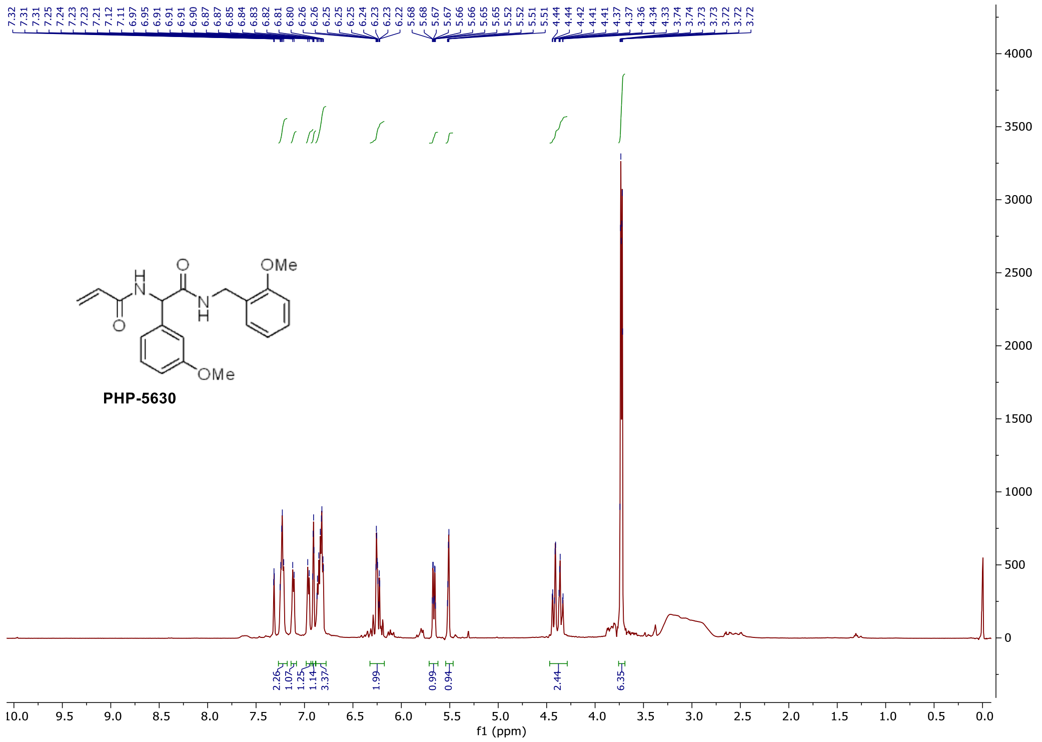

Supplementary Figure 653. ^{1}H NMR (500 MHz, $CDCl_3$ + one drop of CD_3OD) of compound **PHP-5630**.

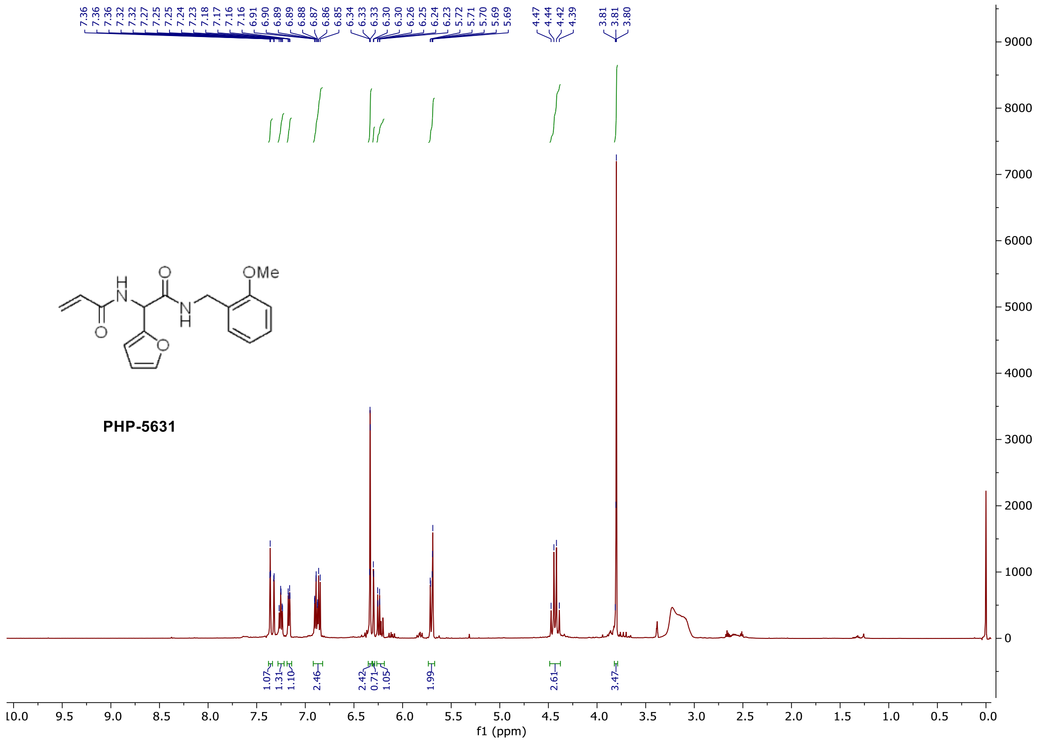

Supplementary Figure 654. ^{1}H NMR (500 MHz, $CDCl_3$ + one drop of CD_3OD) of compound **PHP-5631**.

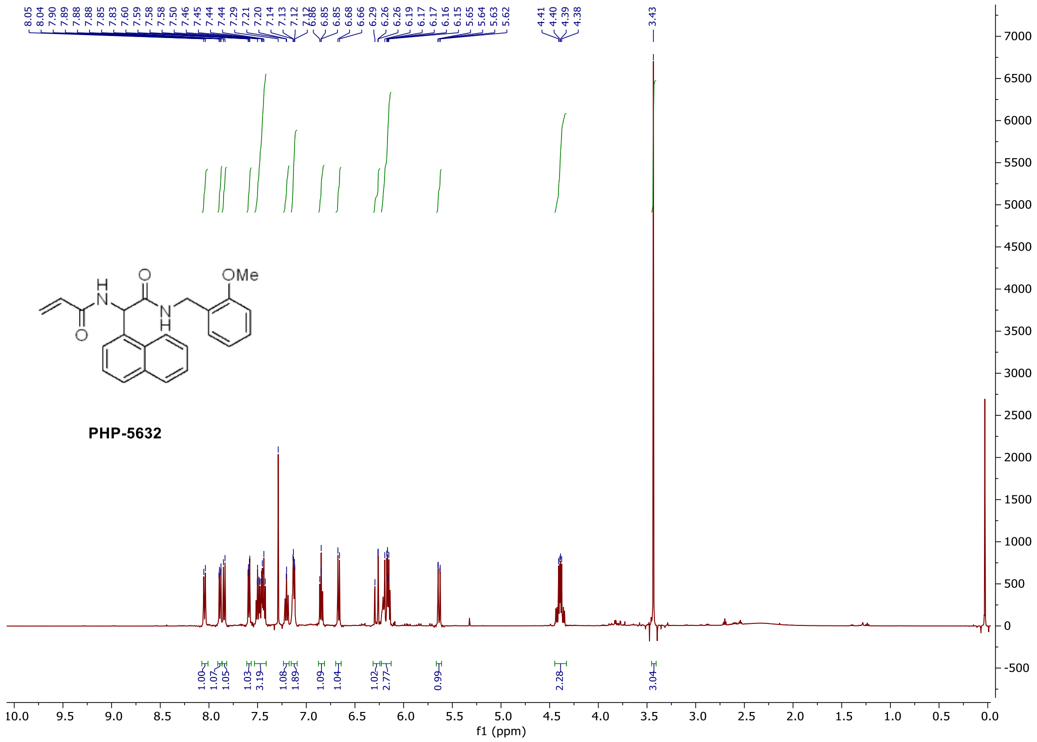

Supplementary Figure 655. ^{1}H NMR (500 MHz, CDCl3) of compound **PHP-5632**.

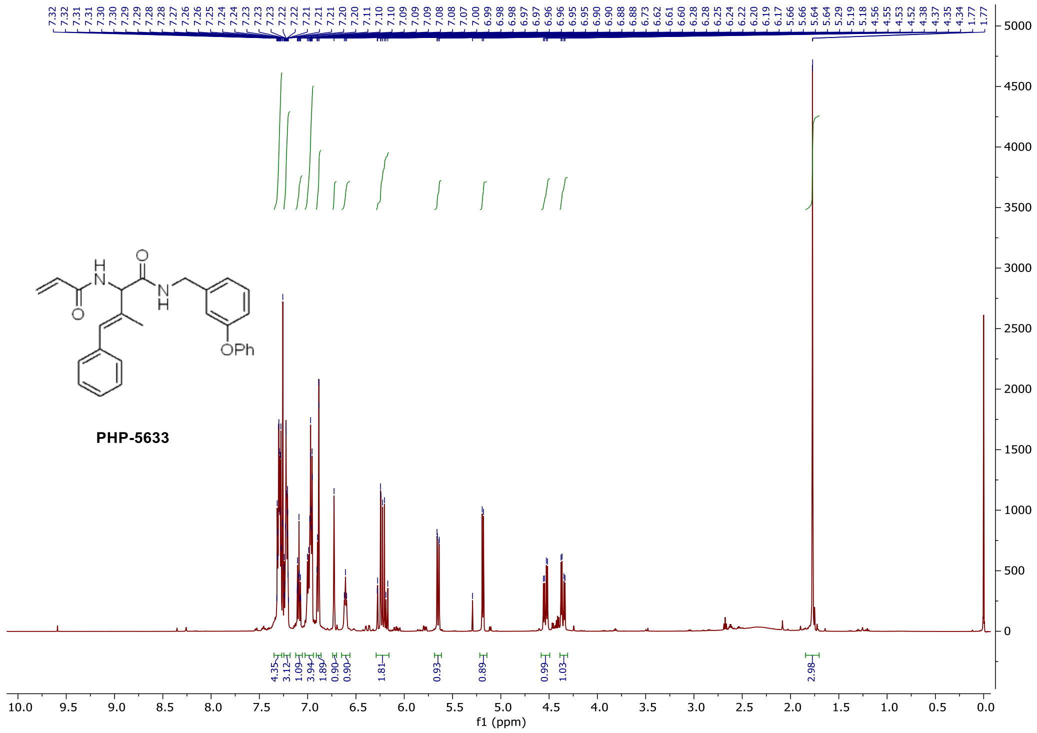

Supplementary Figure 656. ^{1}H NMR (500 MHz, CDCl3) of compound **PHP-5633**.

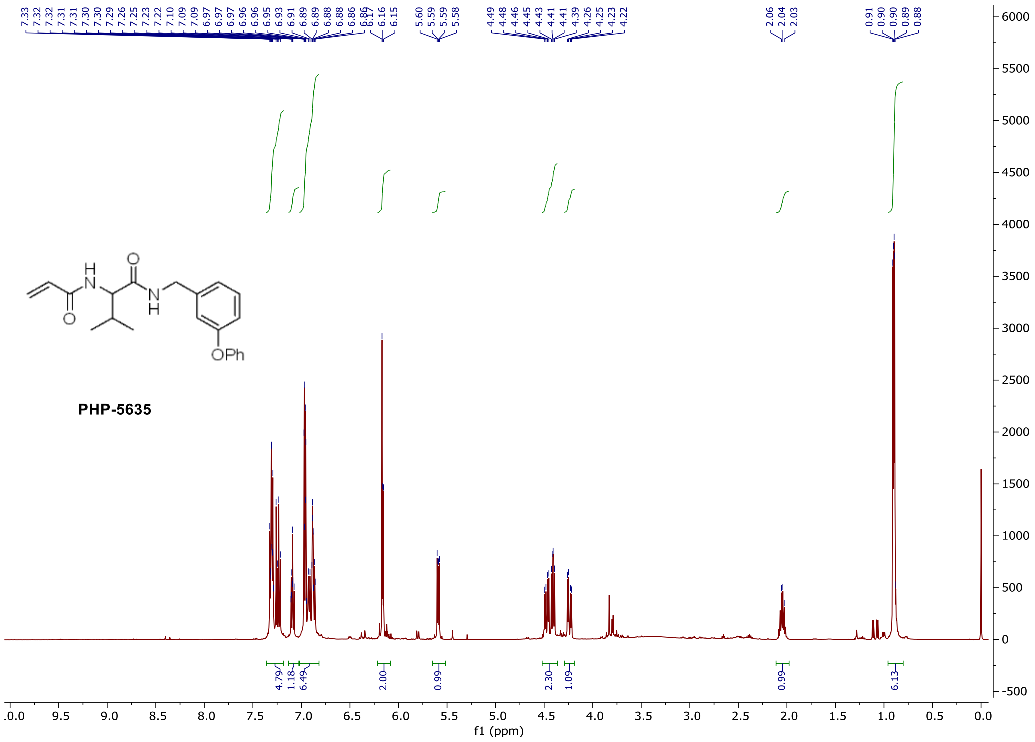

Supplementary Figure 657. ^{1}H NMR (500 MHz, $CDCl_3$) of compound **PHP-5635**.

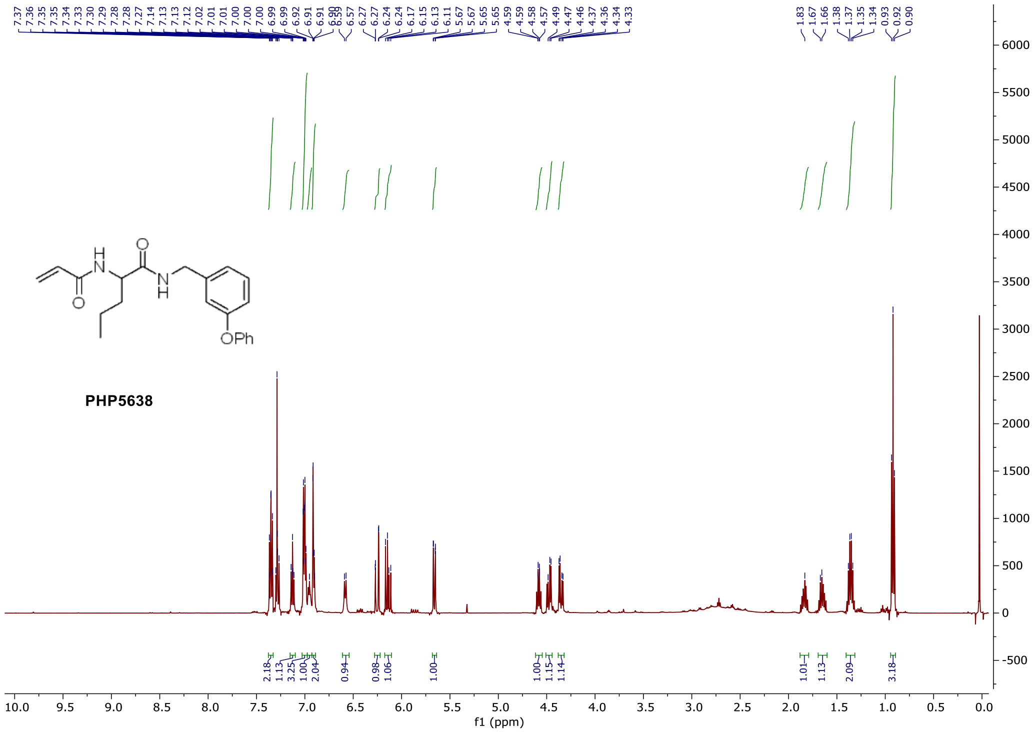

Supplementary Figure 658. ^{1}H NMR (500 MHz, $CDCl_3$) of compound **PHP-5638**.

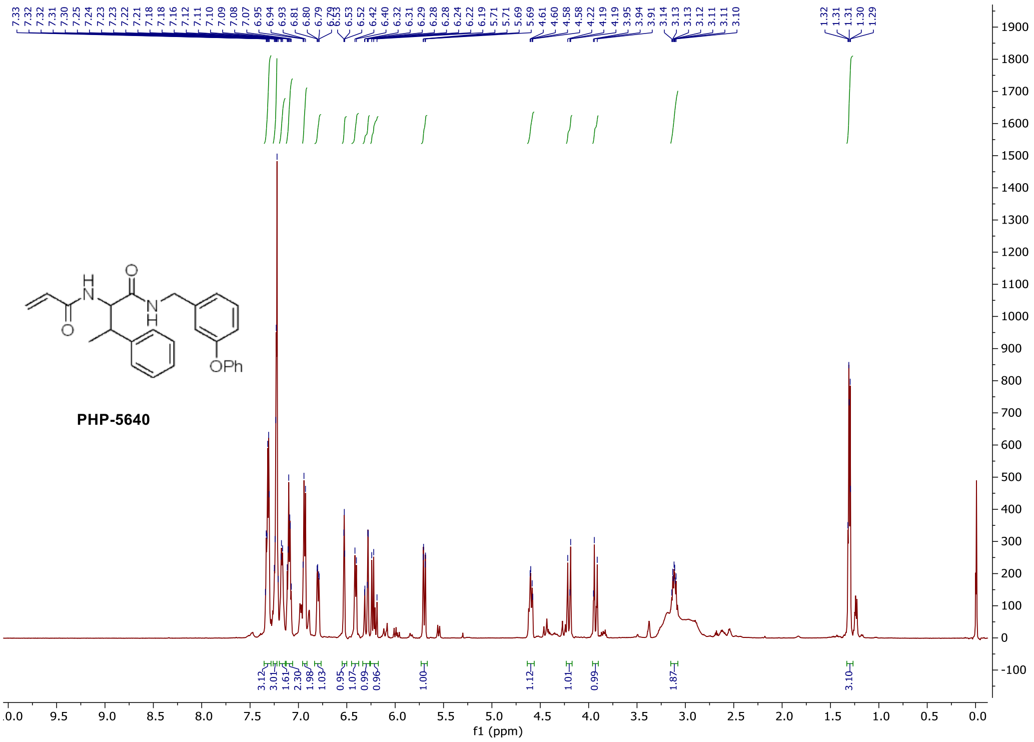

Supplementary Figure 659. ^{1}H NMR (500 MHz, $CDCl_3$ + one drop of CD_3OD) of compound **PHP-5640**.

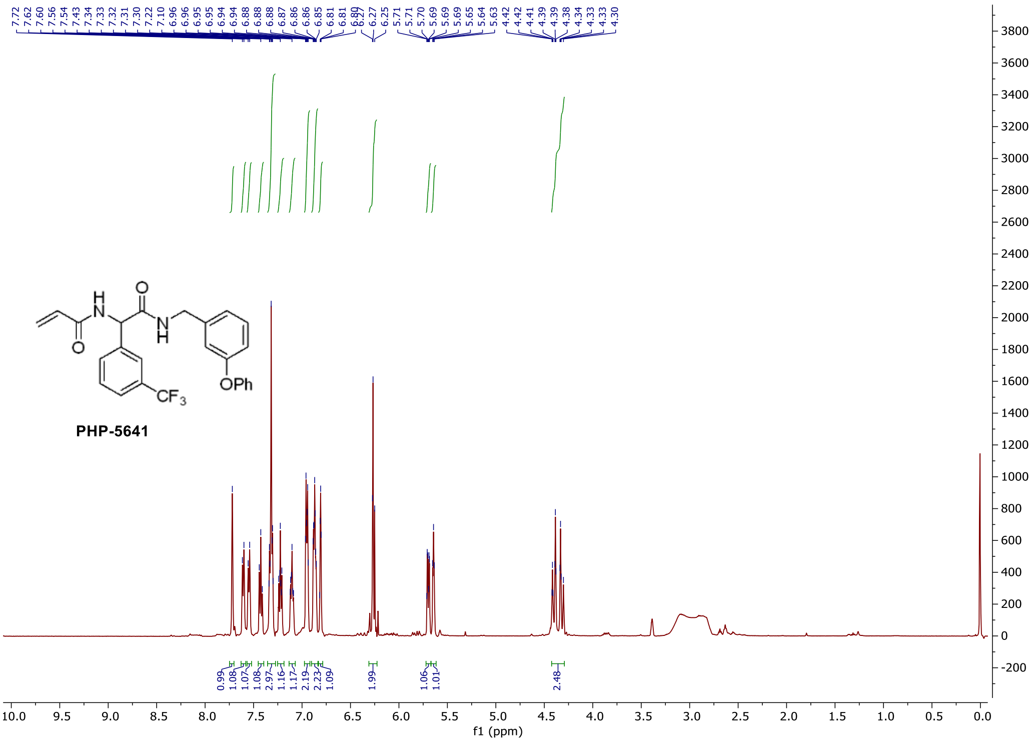

Supplementary Figure 660. ^{1}H NMR (500 MHz, $CDCl_3$ + one drop of CD_3OD) of compound **PHP-5641**.

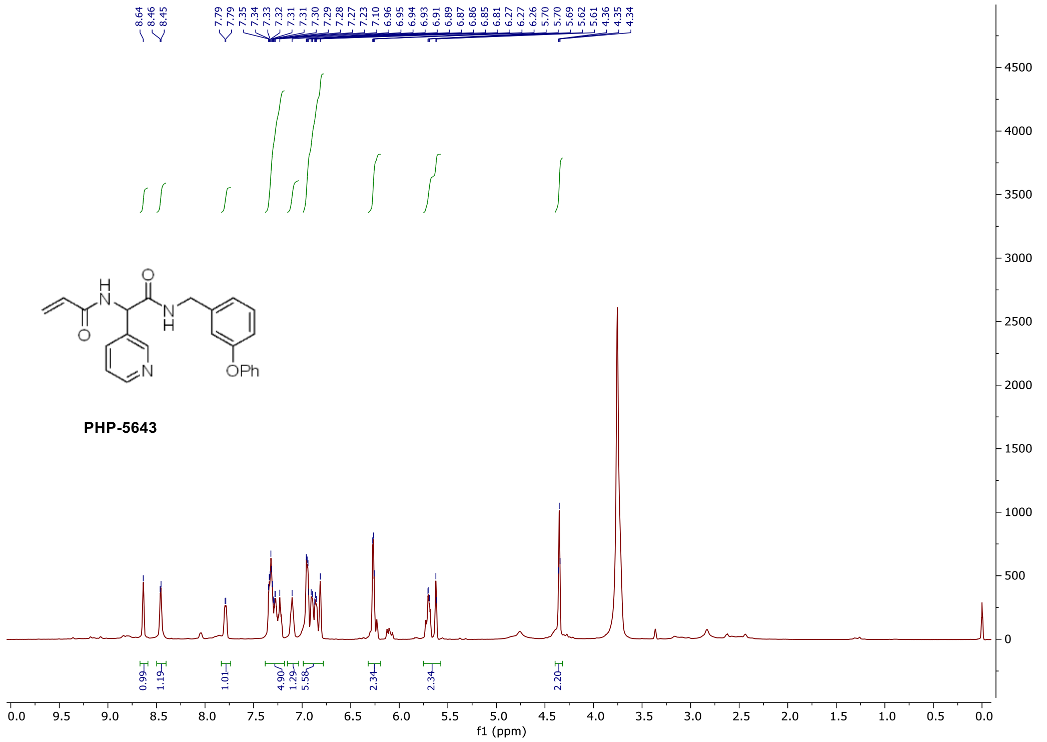

Supplementary Figure 661. ^{1}H NMR (500 MHz, $CDCl_3$ + one drop of CD_3OD) of compound **PHP-5643**.

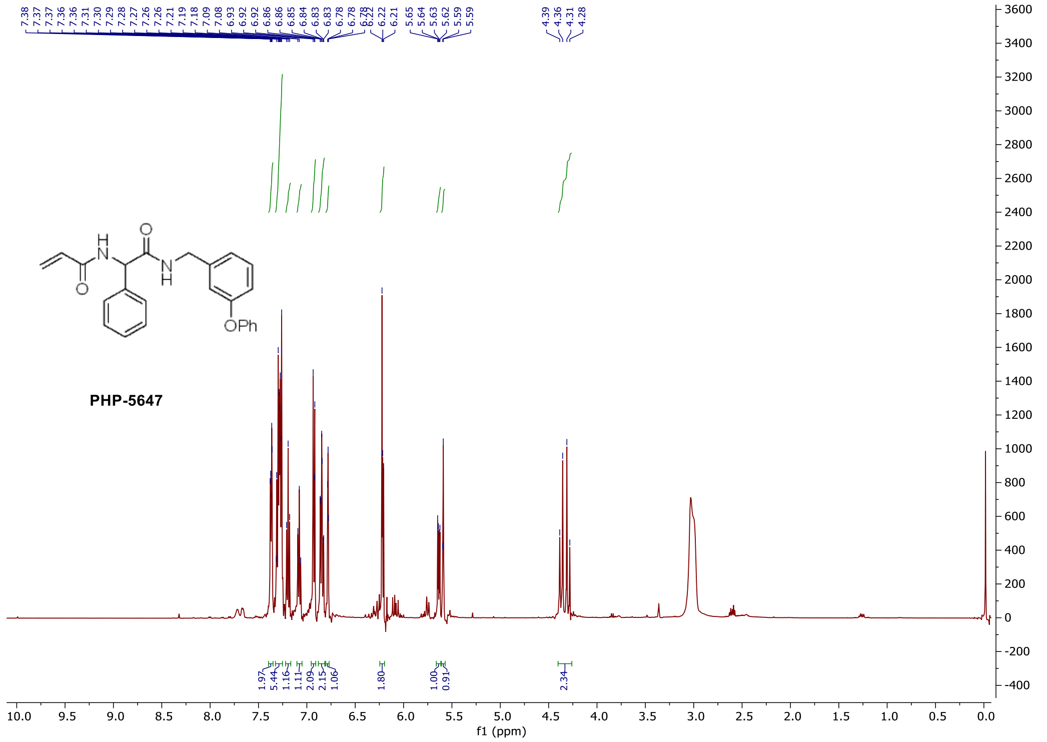

Supplementary Figure 662. ^{1}H NMR (500 MHz, $CDCl_3$ + one drop of CD_3OD) of compound **PHP-5647**.

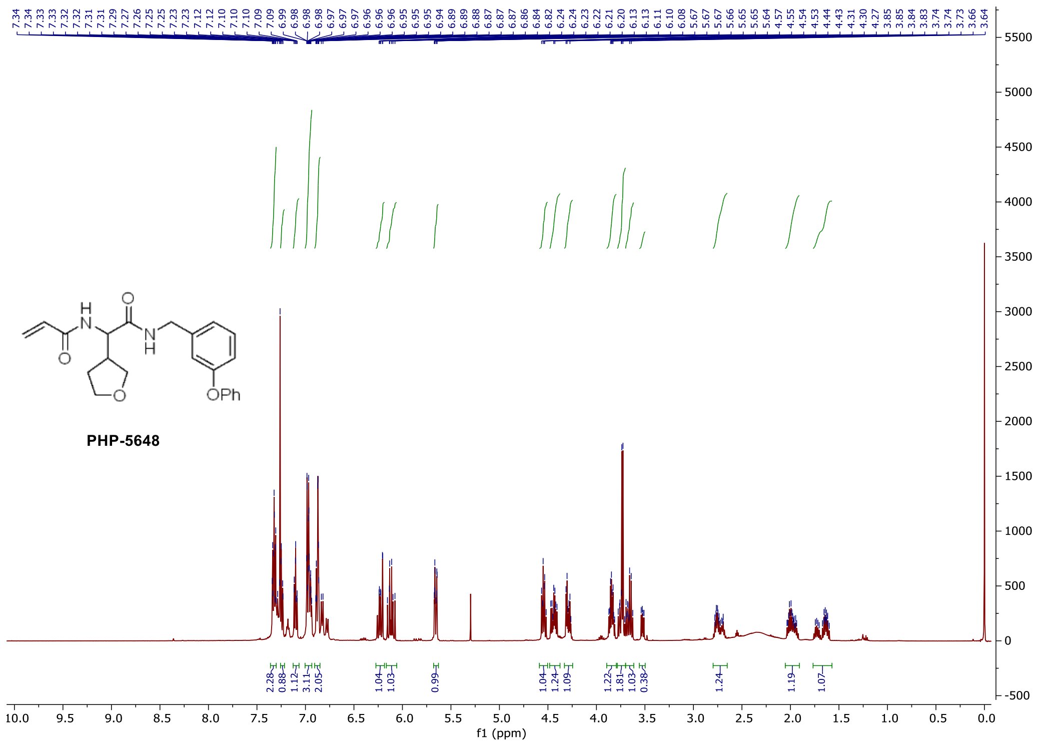

Supplementary Figure 663. ^{1}H NMR (500 MHz, $CDCl_3$) of compound **PHP-5648**.

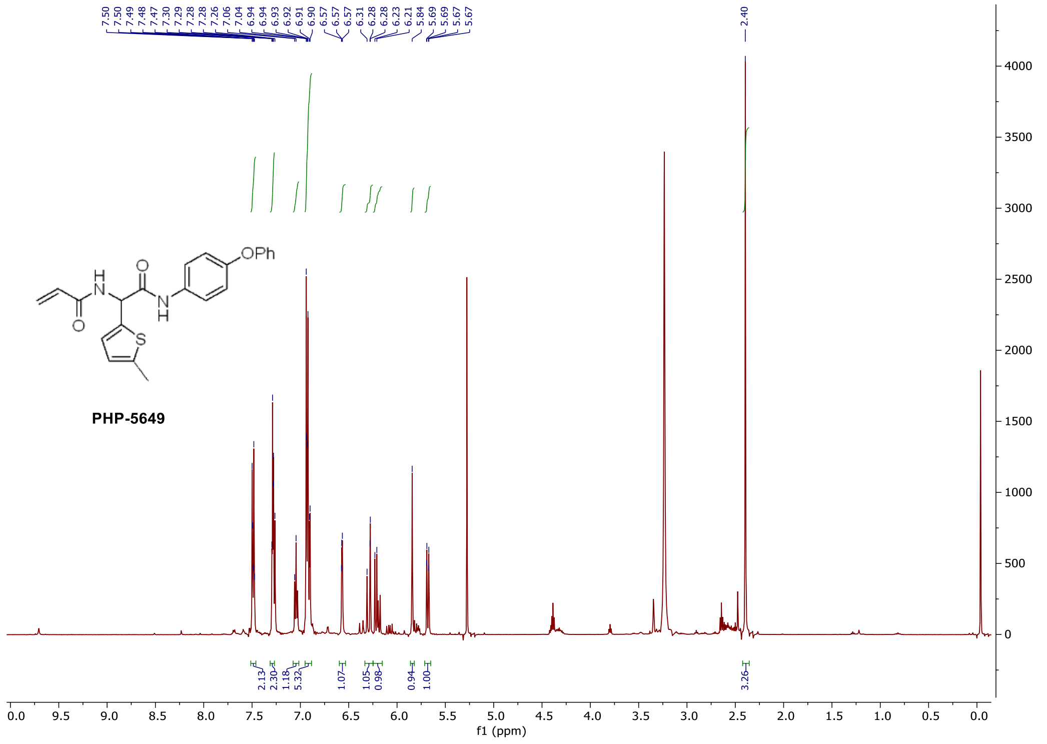

Supplementary Figure 664. ^{1}H NMR (500 MHz, $CDCl_3$ + one drop of CD_3OD) of compound **PHP-5649**.

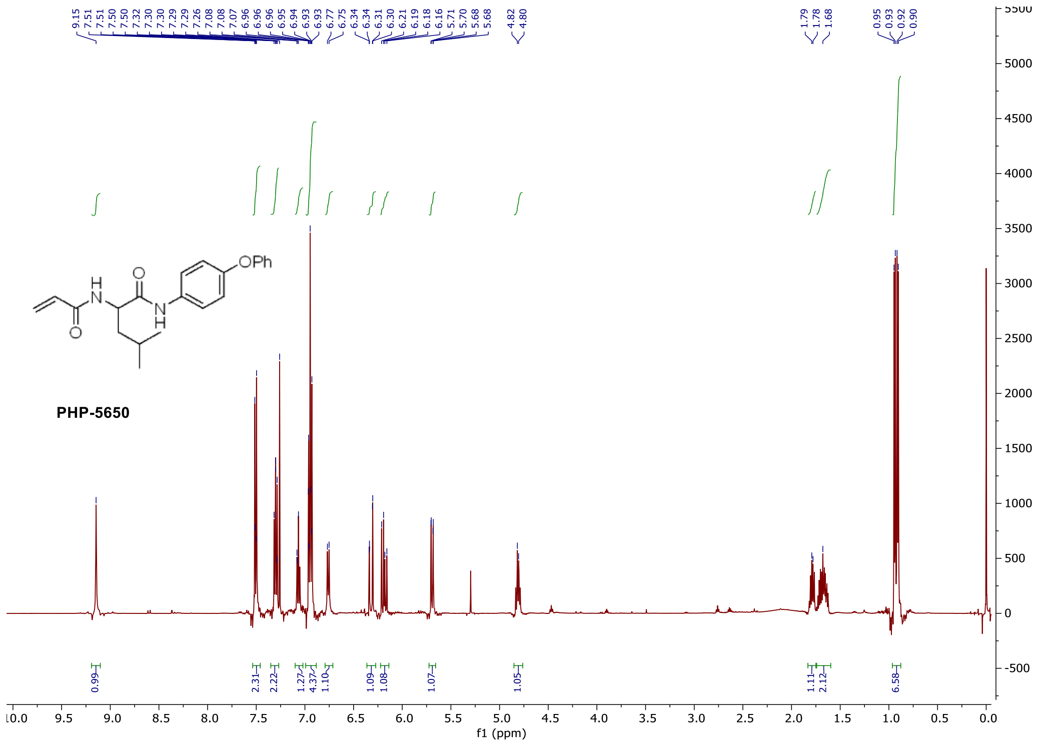

Supplementary Figure 665. ^{1}H NMR (500 MHz, $CDCl_3$) of compound **PHP-5650**.

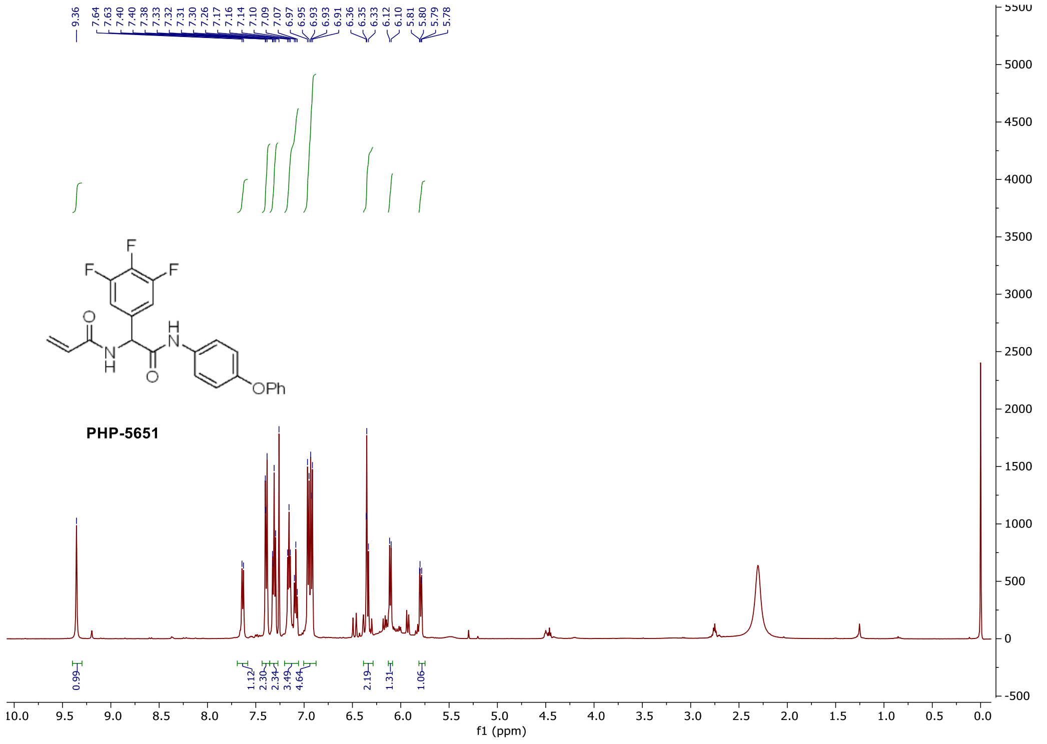

Supplementary Figure 666. ^{1}H NMR (500 MHz, $CDCl_3$) of compound **PHP-5651**.

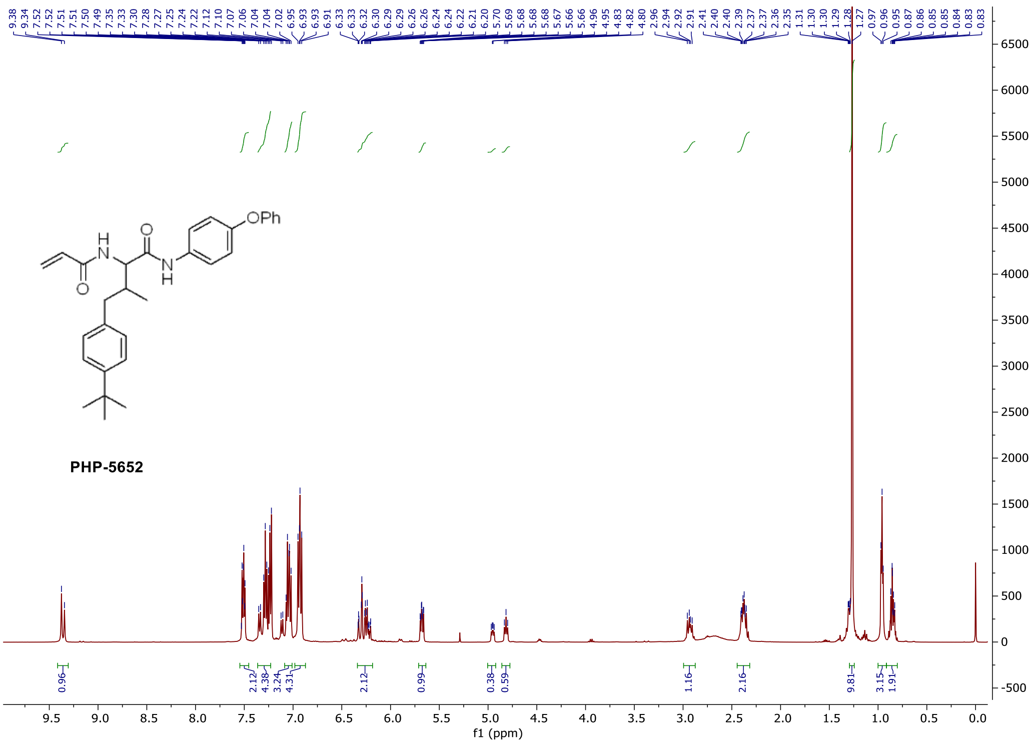

Supplementary Figure 667. ^{1}H NMR (500 MHz, $CDCl_3$) of compound **PHP-5652**.

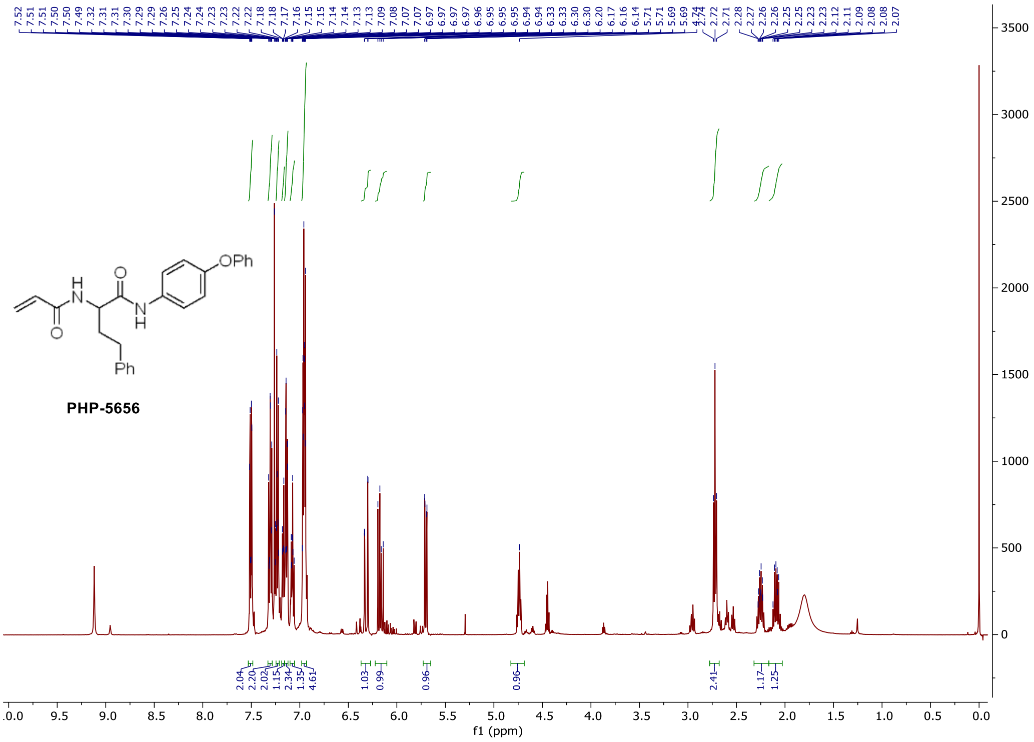

Supplementary Figure 668. ^{1}H NMR (500 MHz, $CDCl_3$) of compound **PHP-5656**.

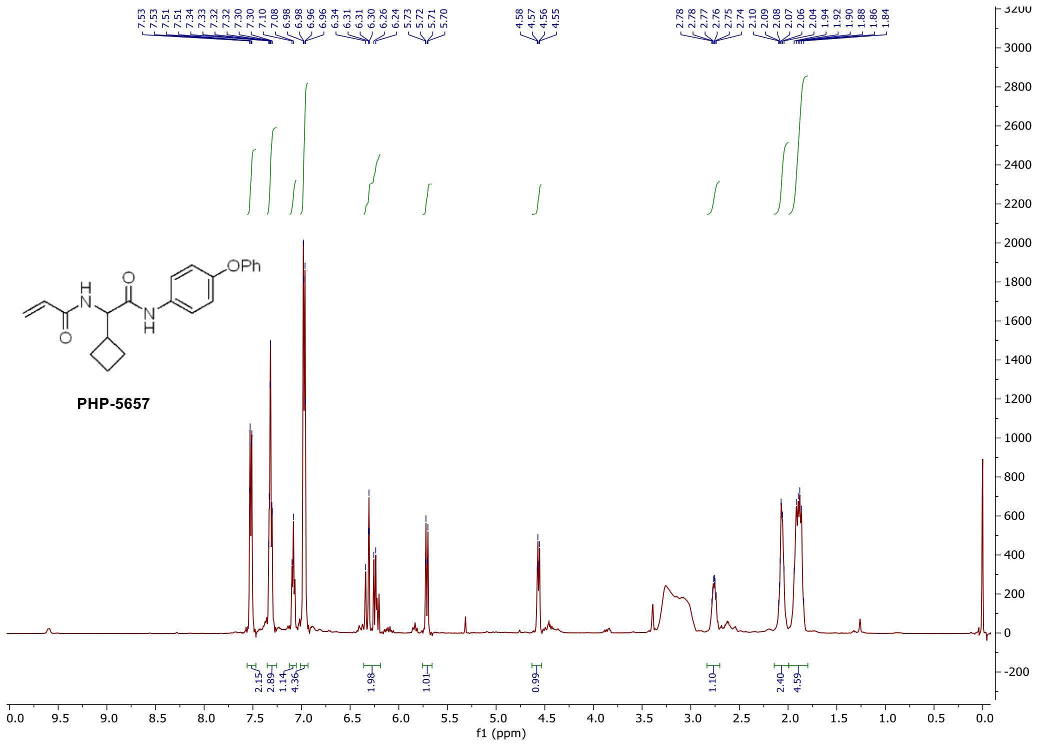

Supplementary Figure 669. ^{1}H NMR (500 MHz, $CDCl_3$ + one drop of CD_3OD) of compound **PHP-5657**.

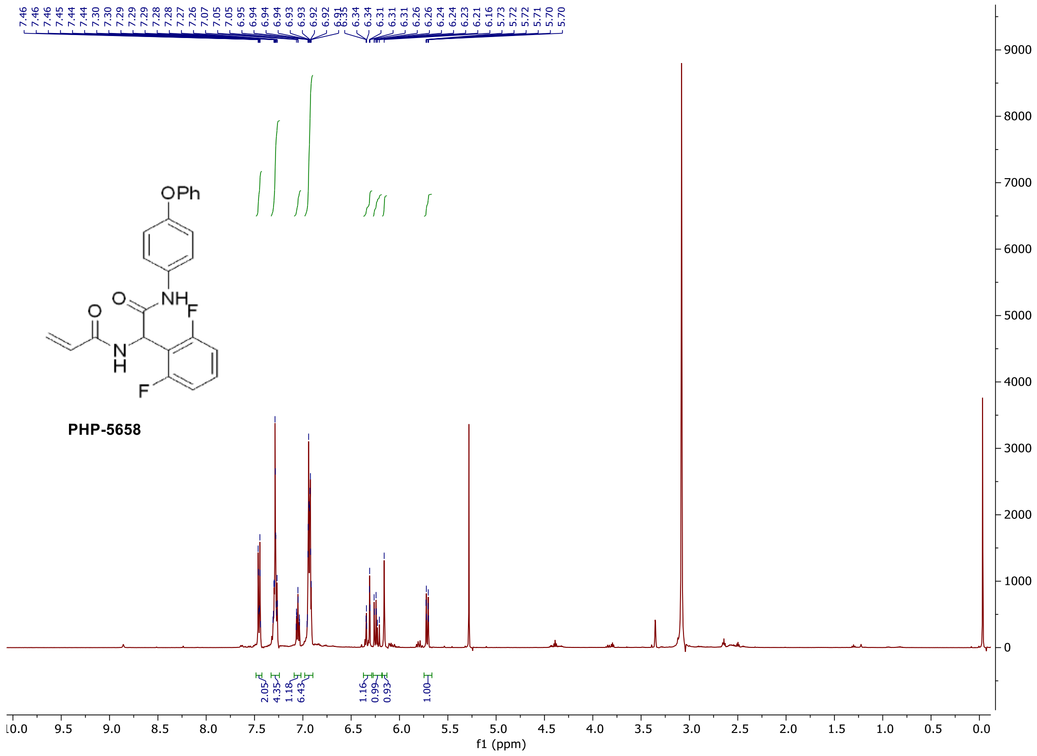

Supplementary Figure 670. ^{1}H NMR (500 MHz, $CDCl_3$ + one drop of CD_3OD) of compound **PHP-5658**.

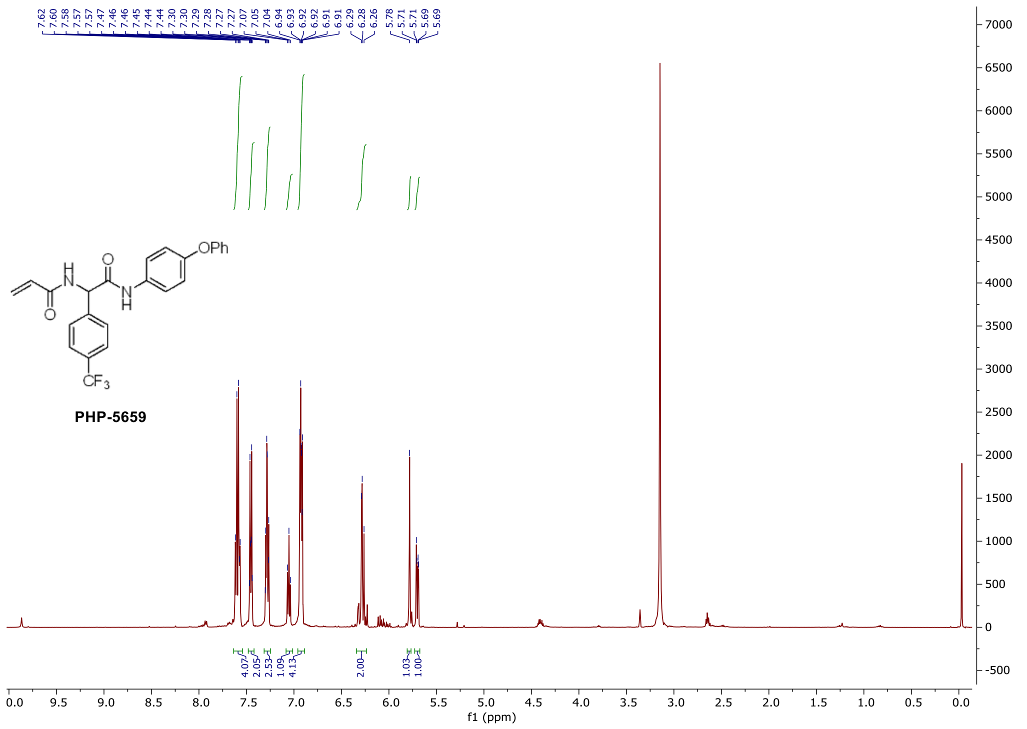

Supplementary Figure 671. ^{1}H NMR (500 MHz, $CDCl_3$ + one drop of CD_3OD) of compound **PHP-5659**.

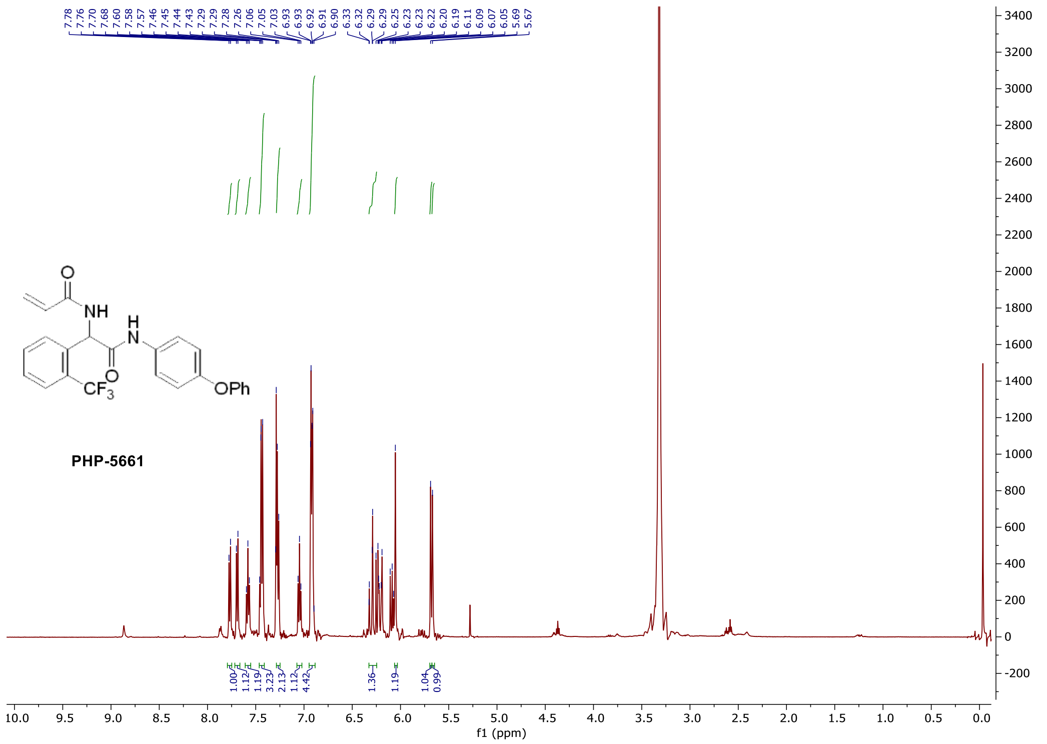

Supplementary Figure 672. ^{1}H NMR (500 MHz, $CDCl_3$ + one drop of CD_3OD) of compound **PHP-5661**.

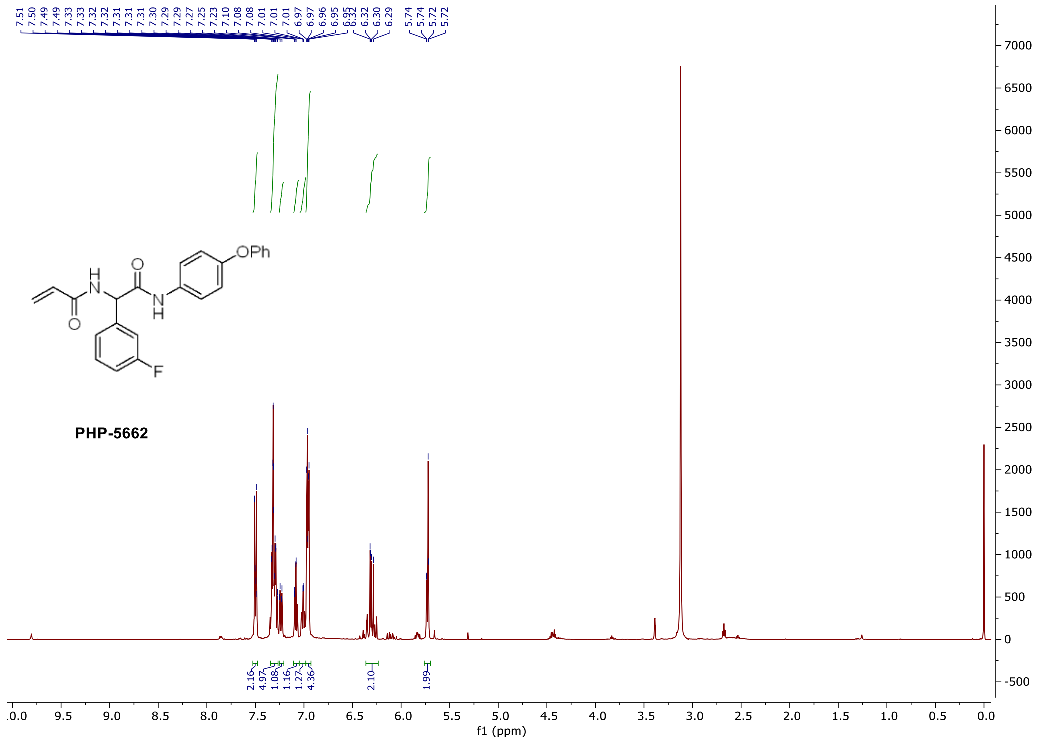

Supplementary Figure 673. ^{1}H NMR (500 MHz, $CDCl_3$ + one drop of CD_3OD) of compound **PHP-5662**.

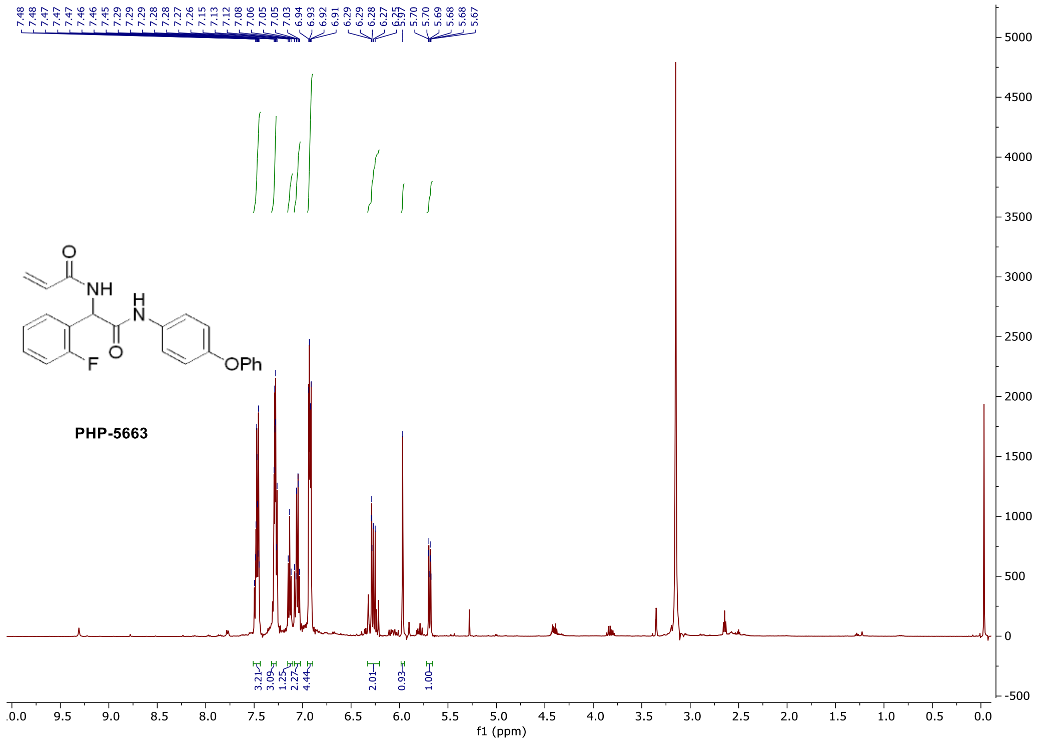

Supplementary Figure 674. ^{1}H NMR (500 MHz, $CDCl_3$ + one drop of CD_3OD) of compound **PHP-5663**.

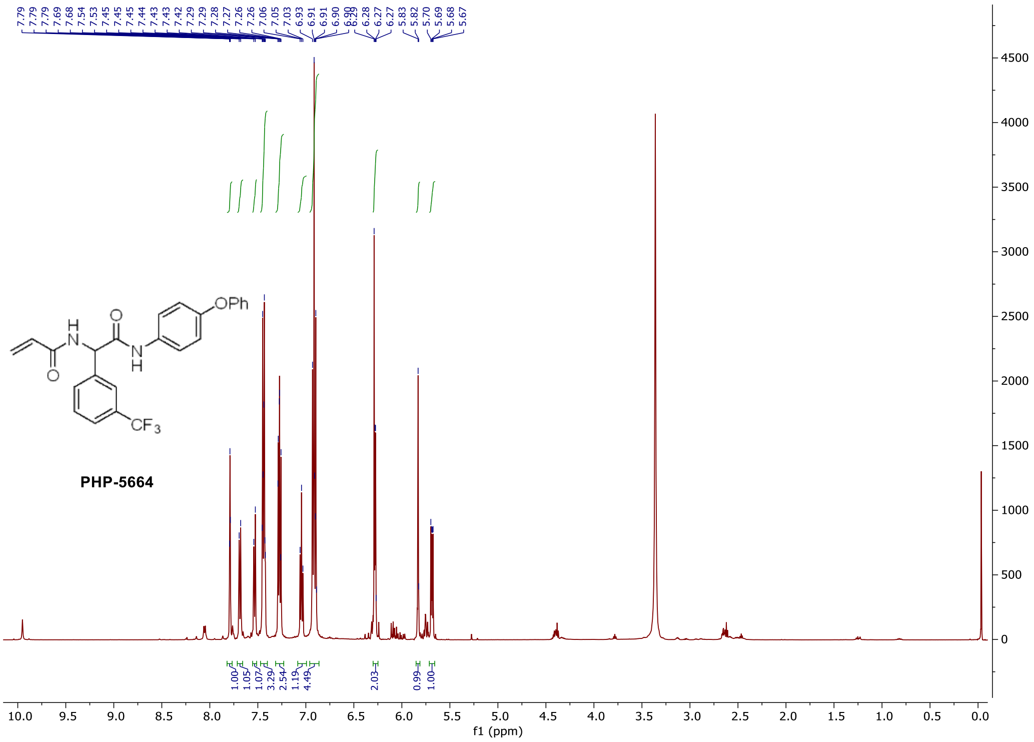

Supplementary Figure 675. ^{1}H NMR (500 MHz, $CDCl_3$ + one drop of CD_3OD) of compound **PHP-5664**.

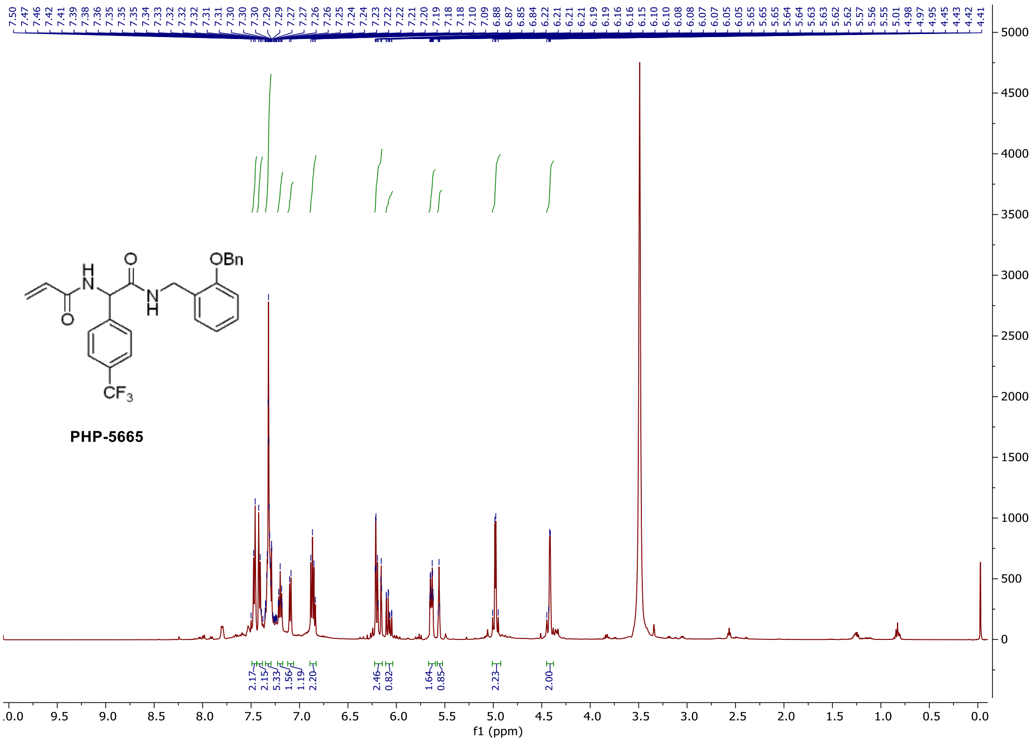

Supplementary Figure 676. ^{1}H NMR (500 MHz, $CDCl_3$) of compound **PHP-5665**.

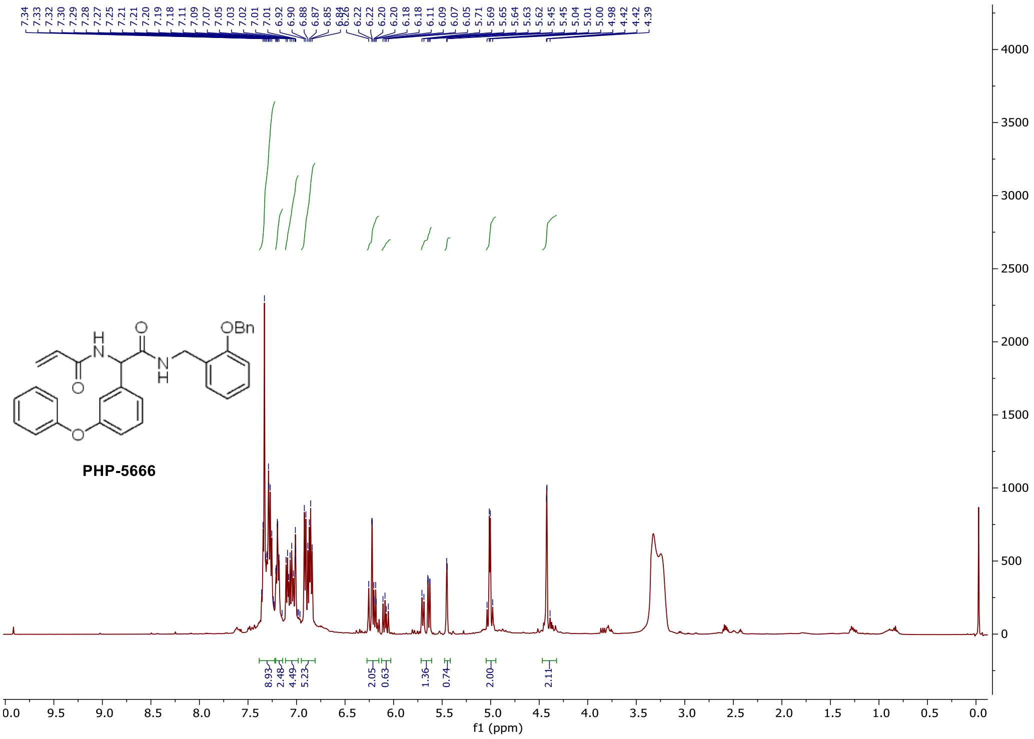

Supplementary Figure 677. ^{1}H NMR (500 MHz, $CDCl_3$ + one drop of CD_3OD) of compound **PHP-5666**.

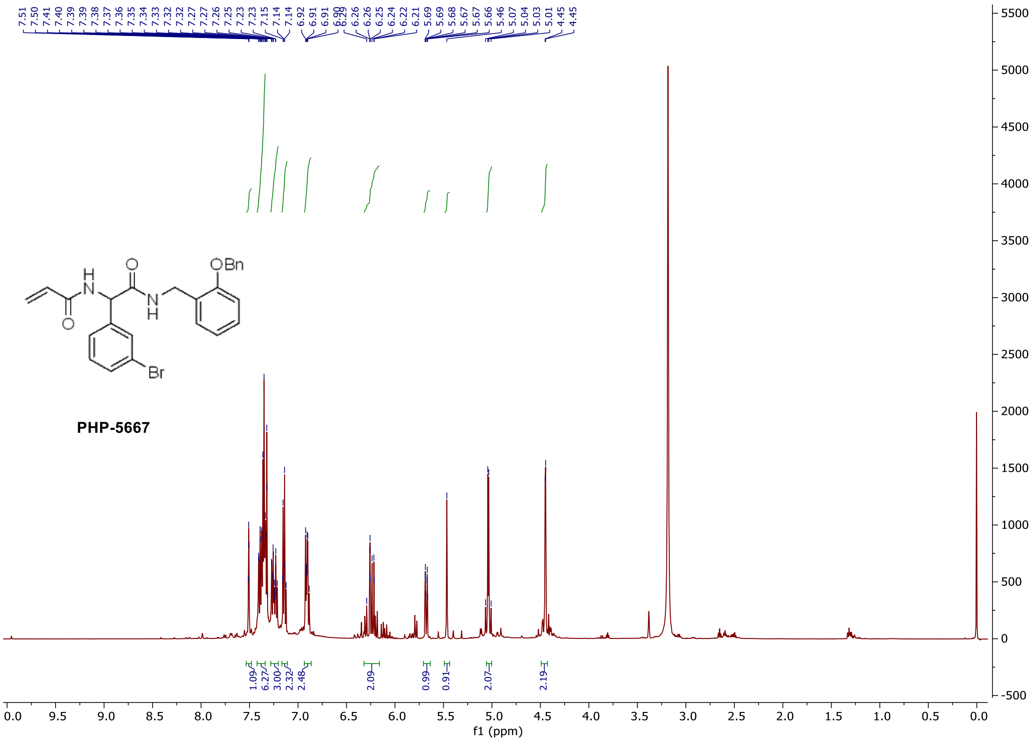

Supplementary Figure 678. ^{1}H NMR (500 MHz, $CDCl_3$ + one drop of CD_3OD) of compound **PHP-5667**.

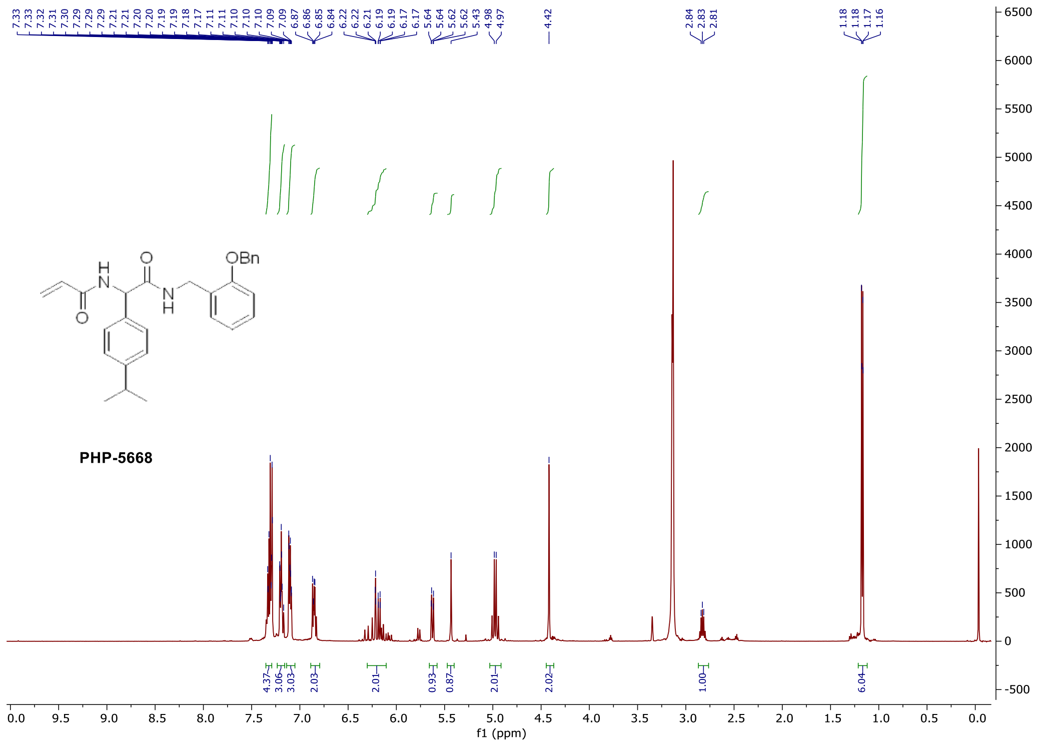

Supplementary Figure 679. ^{1}H NMR (500 MHz, $CDCl_3$ + one drop of CD_3OD) of compound **PHP-5668**.

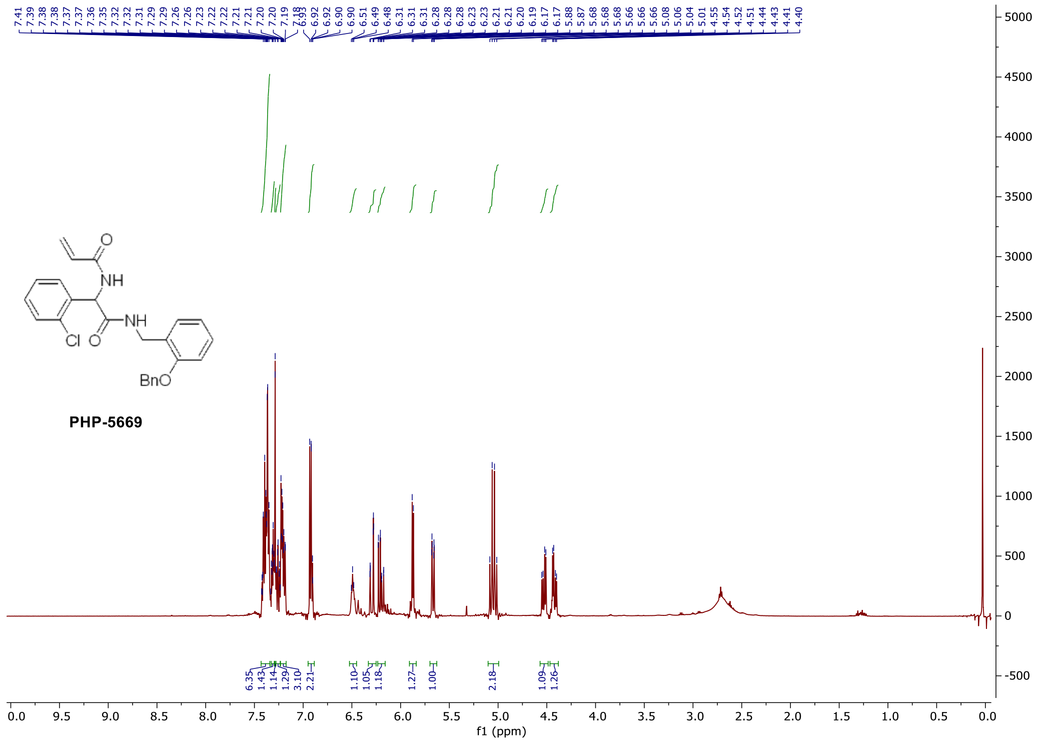

Supplementary Figure 680. ^{1}H NMR (500 MHz, $CDCl_3$) of compound **PHP-5669**.

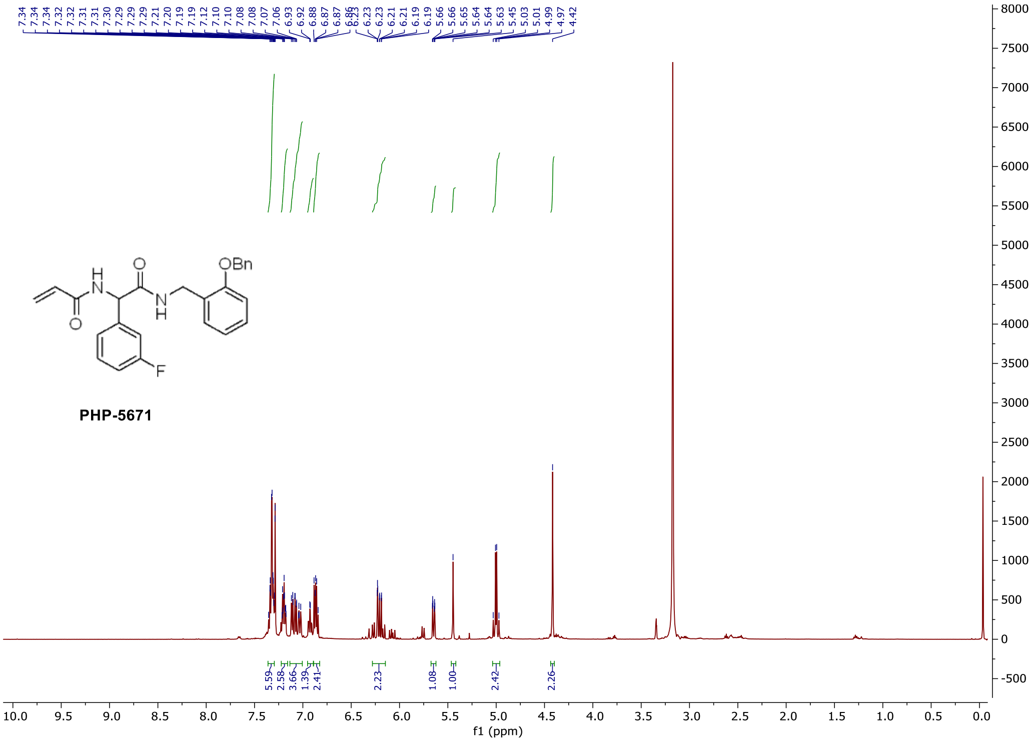

Supplementary Figure 681. ^{1}H NMR (500 MHz, $CDCl_3$ + one drop of CD_3OD) of compound **PHP-5671**.

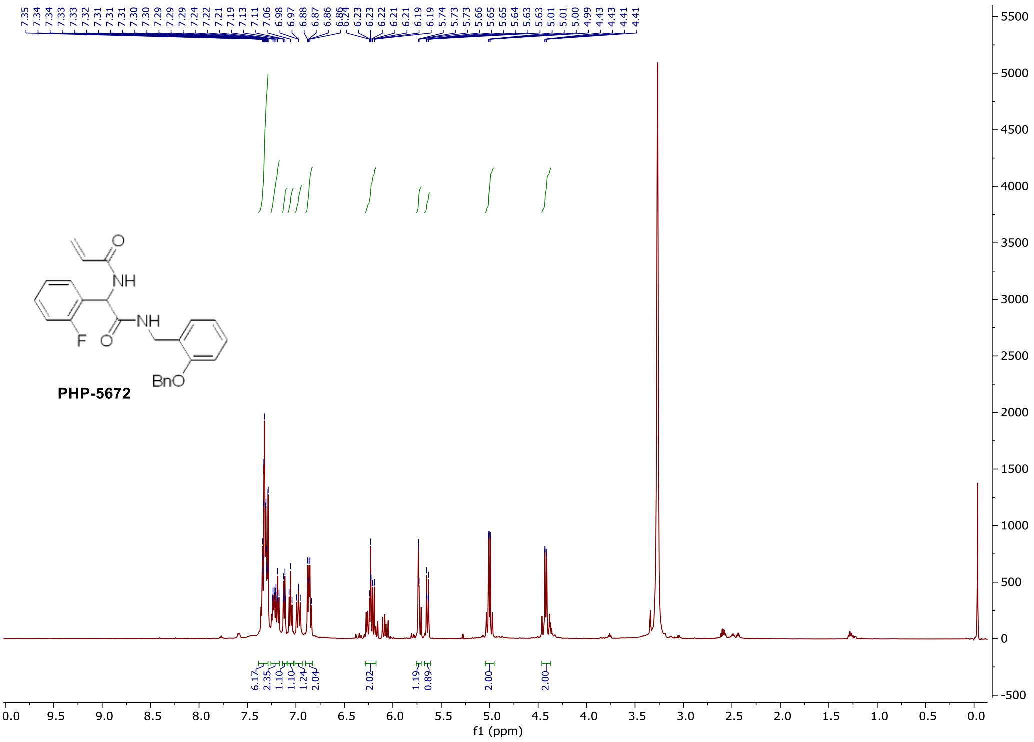

Supplementary Figure 682. ^{1}H NMR (500 MHz, $CDCl_3$ + one drop of CD_3OD) of compound **PHP-5672**.

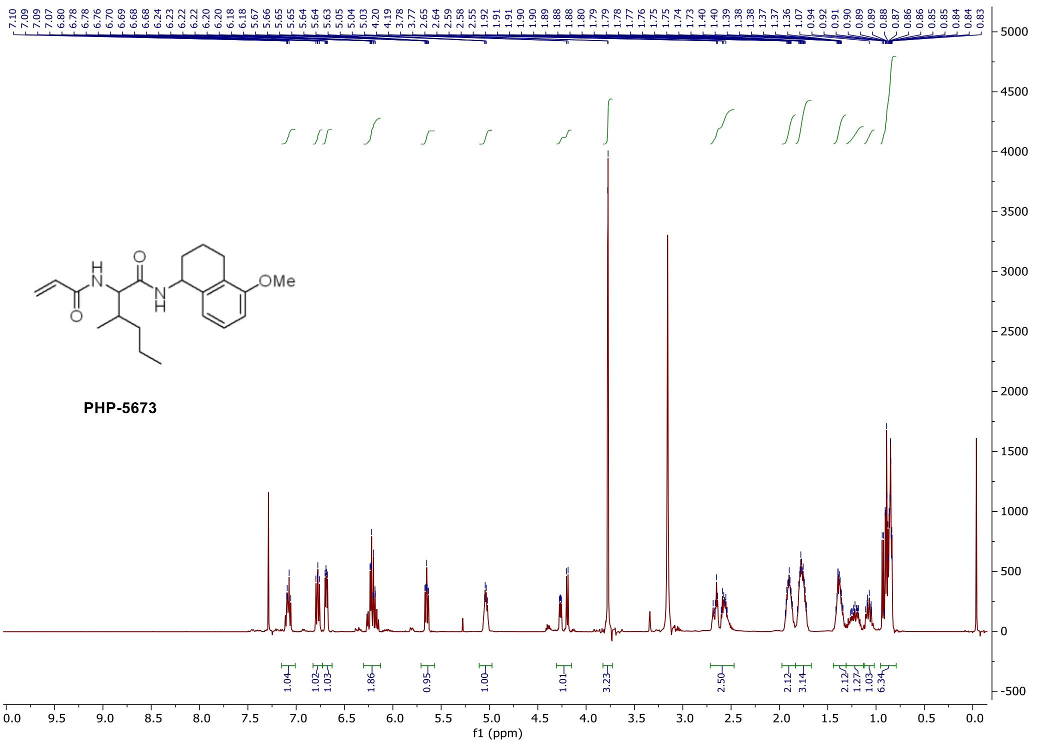

Supplementary Figure 683. ^{1}H NMR (500 MHz, $CDCl_3$ + one drop of CD_3OD) of compound **PHP-5673**.

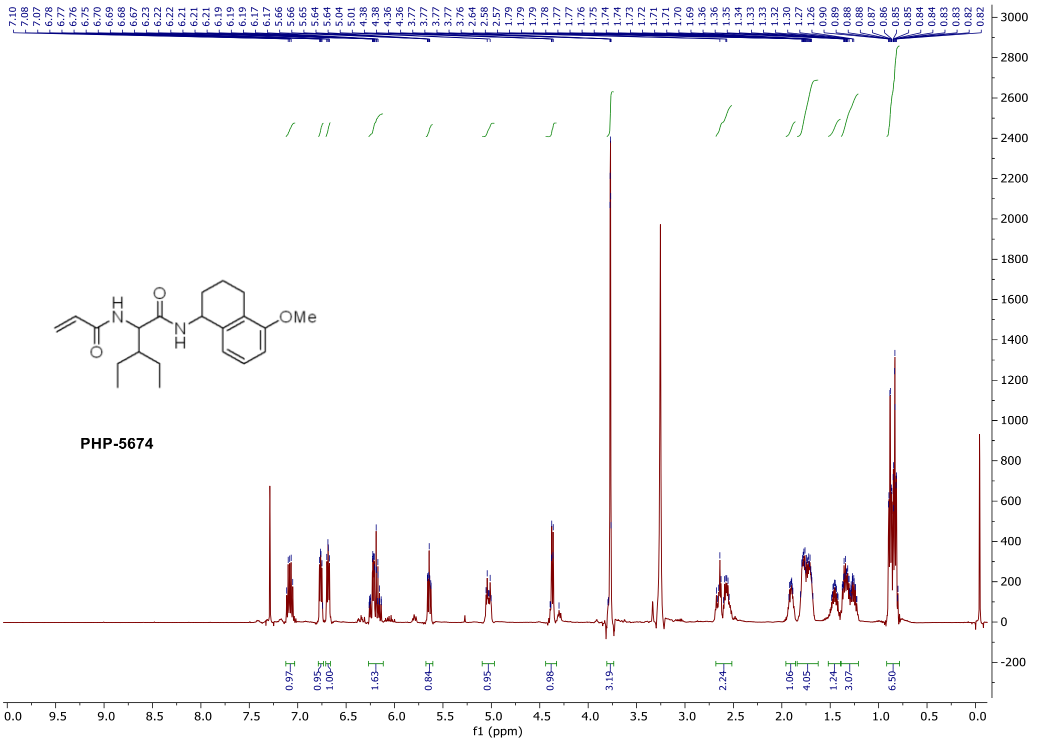

Supplementary Figure 684. ^{1}H NMR (500 MHz, $CDCl_3$ + one drop of CD_3OD) of compound **PHP-5674**.

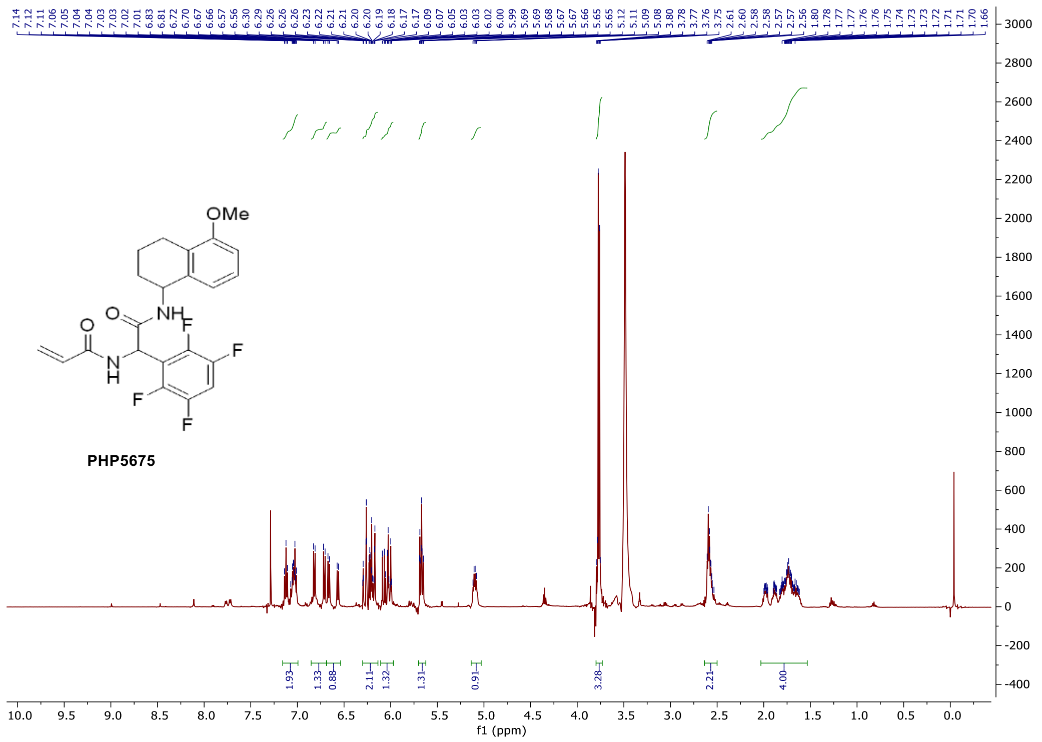

Supplementary Figure 685. ^{1}H NMR (500 MHz, $CDCl_3$) of compound **PHP-5675**.

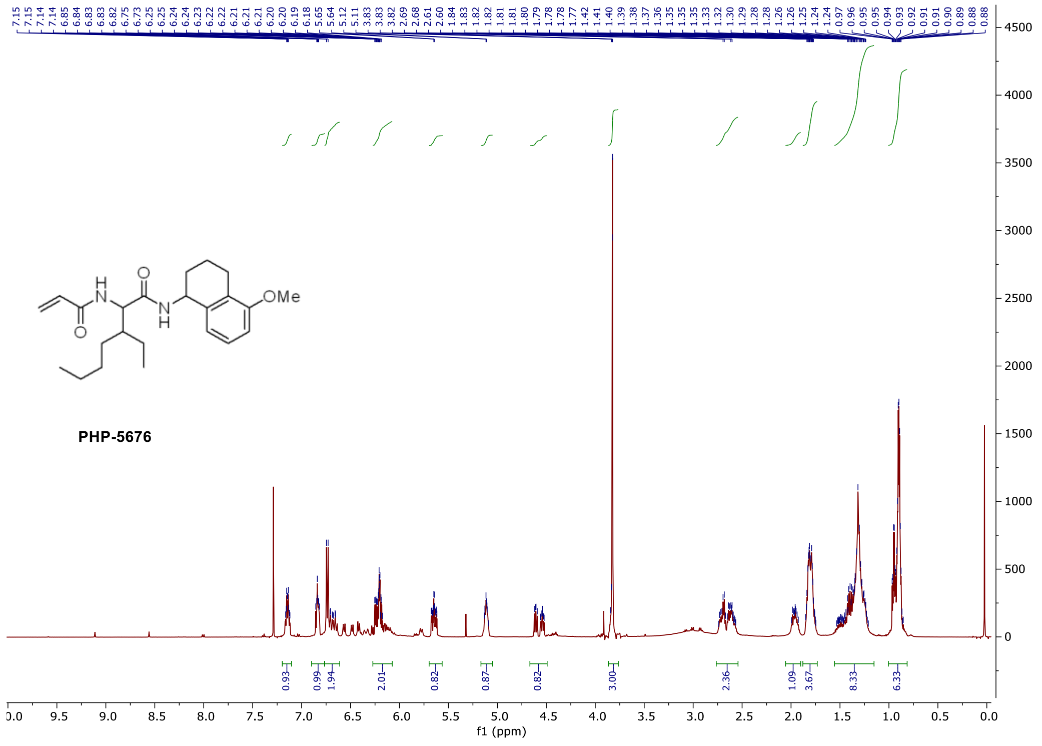

Supplementary Figure 686. ^{1}H NMR (500 MHz, $CDCl_3$) of compound **PHP-5676**.

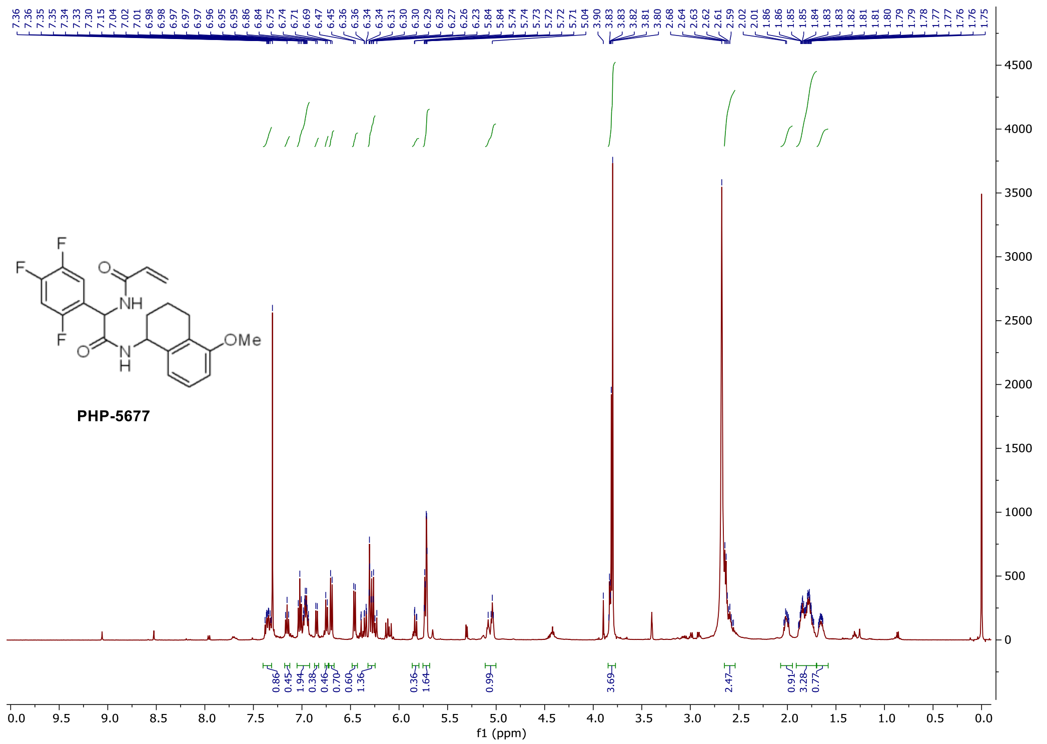

Supplementary Figure 687. ^{1}H NMR (500 MHz, $CDCl_3$) of compound **PHP-5677**.

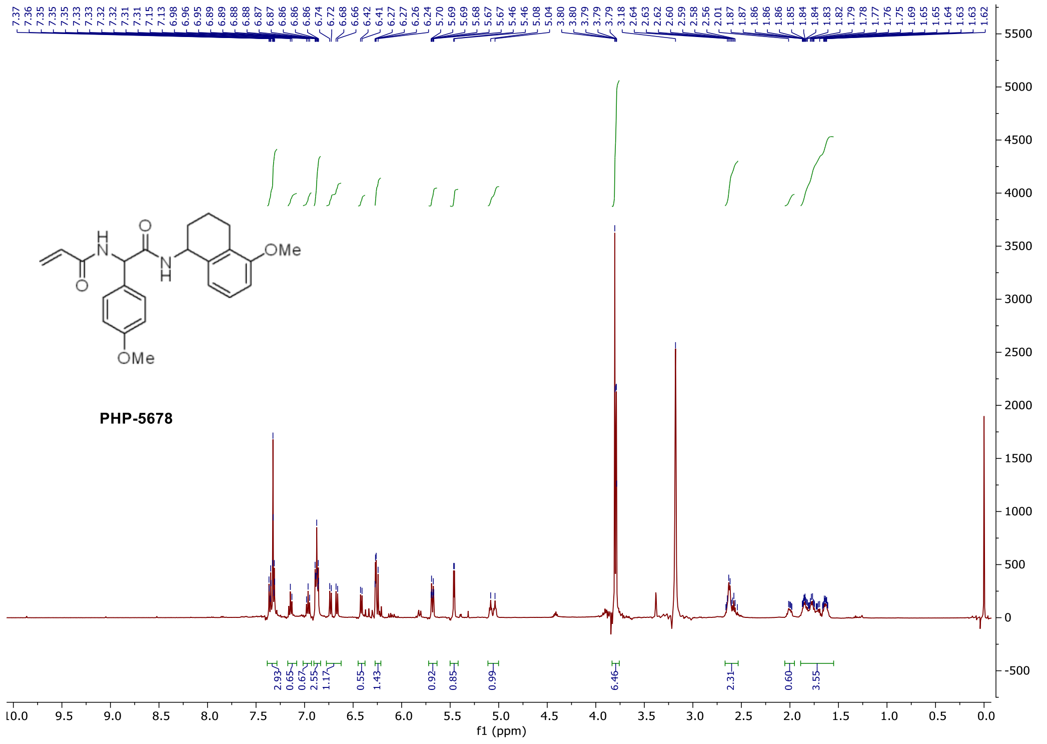

Supplementary Figure 688. ^{1}H NMR (500 MHz, $CDCl_3$ + one drop of CD_3OD) of compound **PHP-5678**.

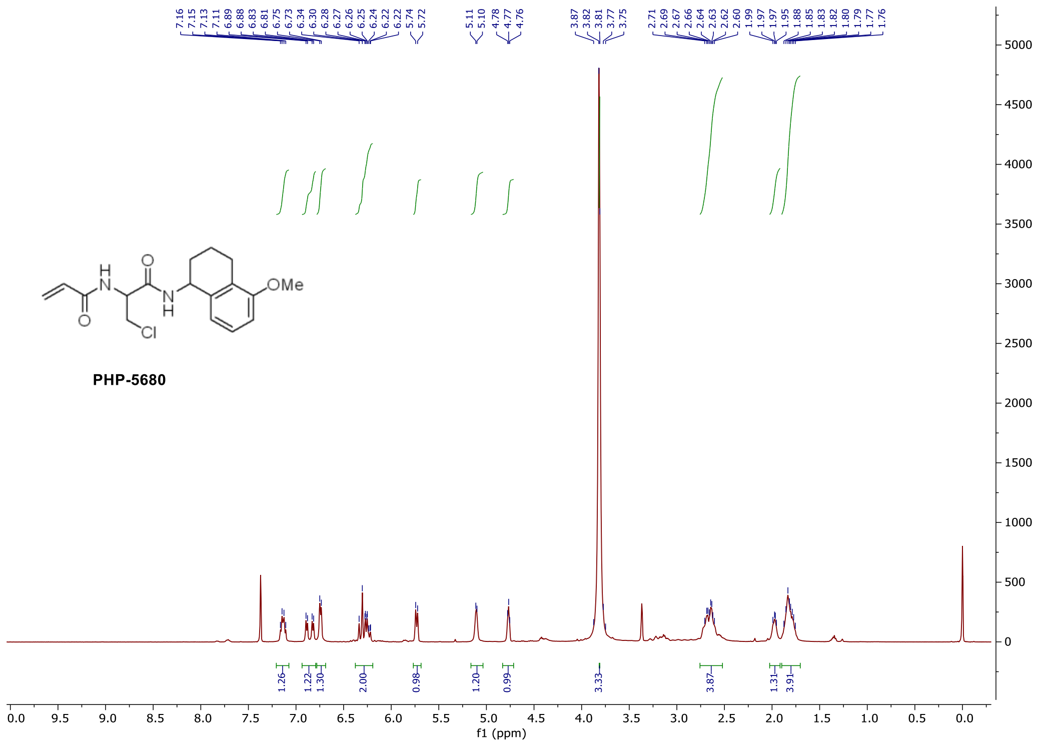

Supplementary Figure 689. ^{1}H NMR (500 MHz, $CDCl_3$) of compound **PHP-5680**.

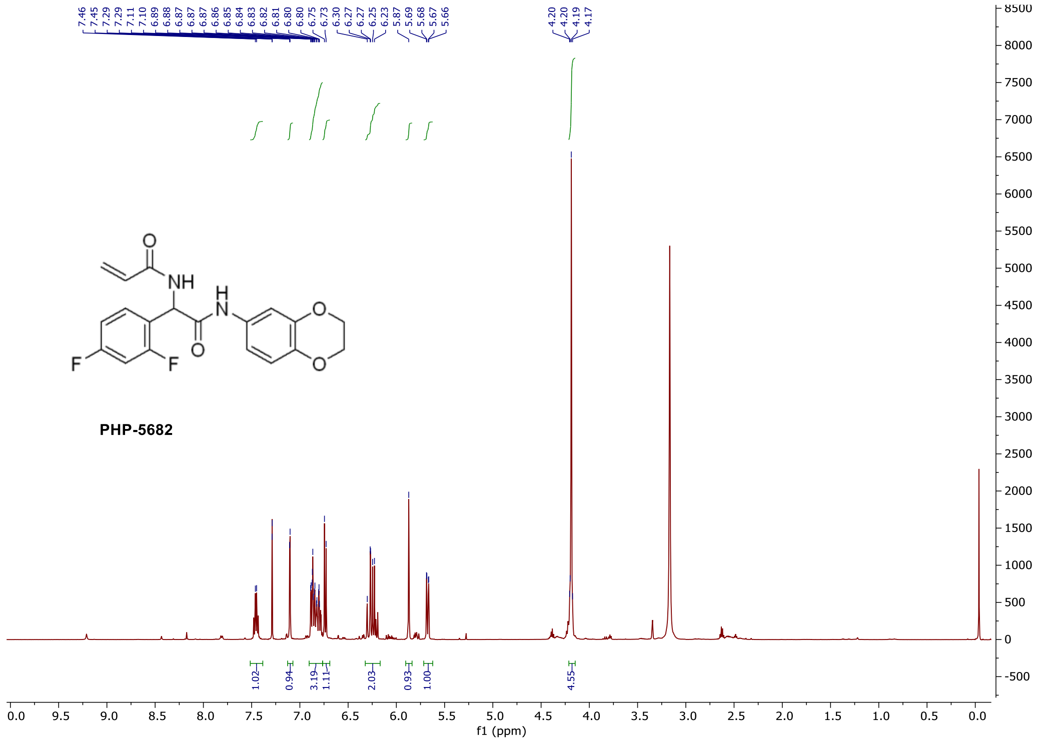

Supplementary Figure 690. ^{1}H NMR (500 MHz, $CDCl_3$ + one drop of CD_3OD) of compound **PHP-5682**.

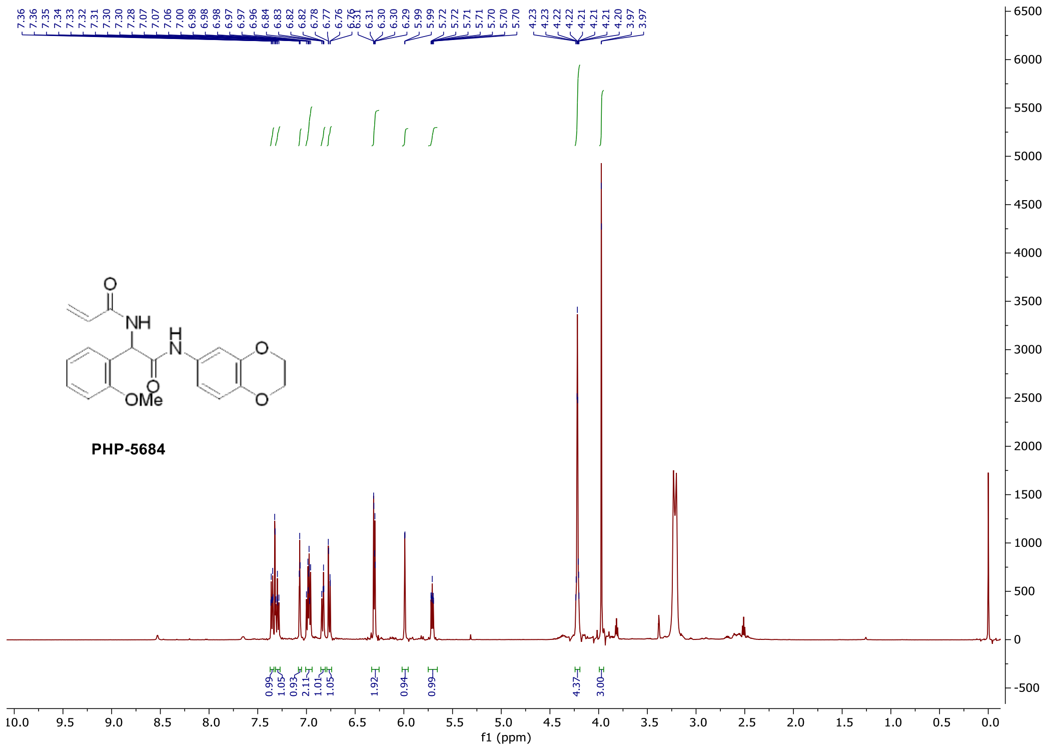

Supplementary Figure 691. ^{1}H NMR (500 MHz, $CDCl_3$ + one drop of CD_3OD) of compound **PHP-5684**.

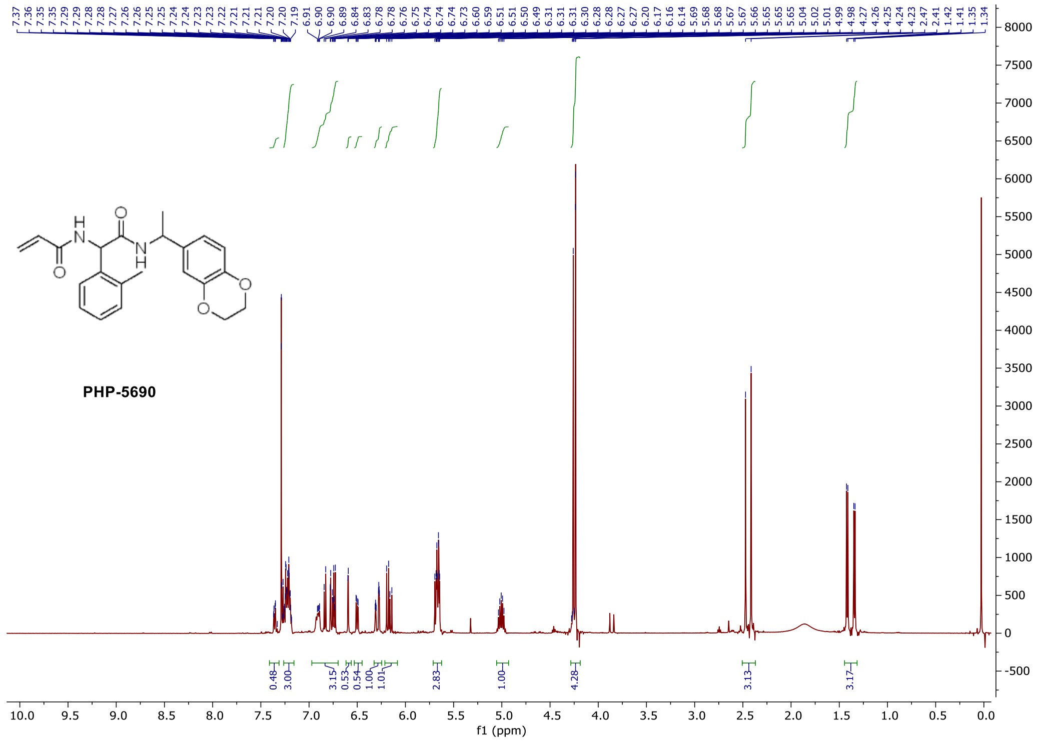

Supplementary Figure 692. ^{1}H NMR (500 MHz, $CDCl_3$) of compound **PHP-5690**.

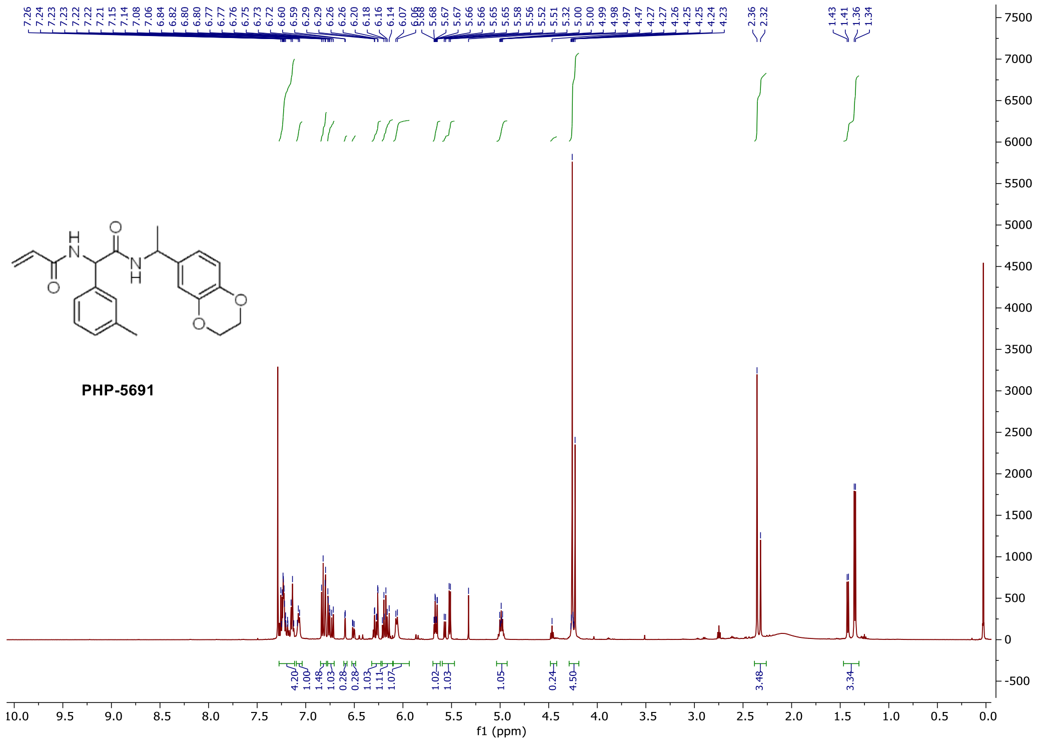

Supplementary Figure 693. ¹H NMR (500 MHz, $CDCl_3$) of compound **PHP-5691**.

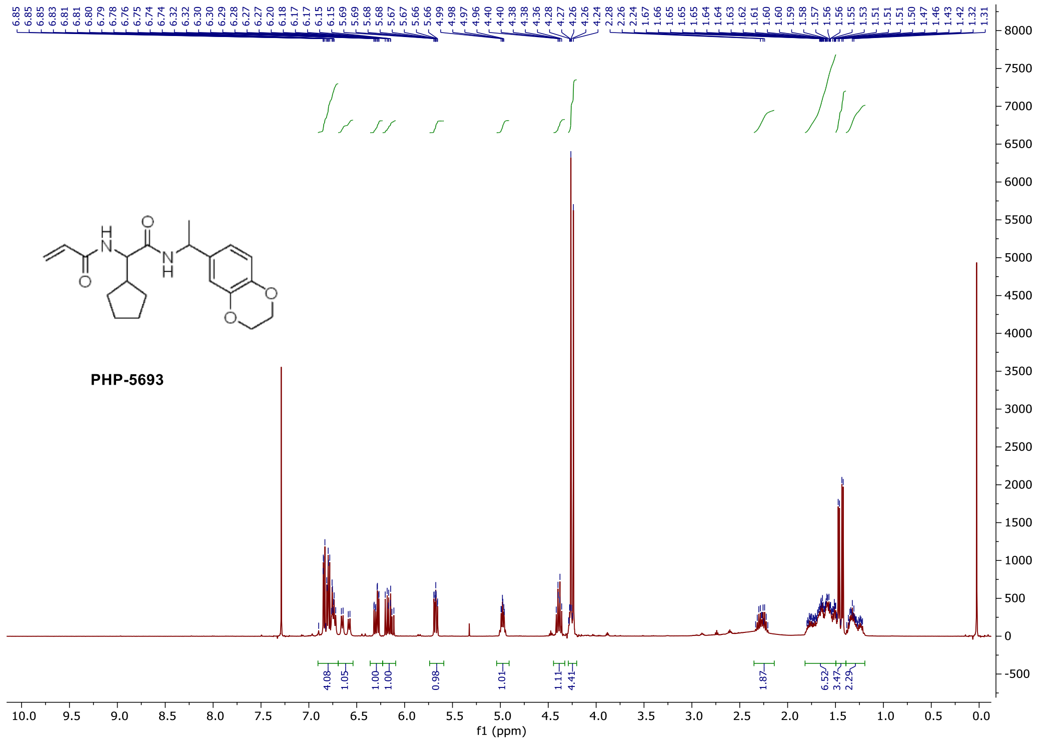

Supplementary Figure 694. ¹H NMR (500 MHz, $CDCl_3$) of compound **PHP-5693**.

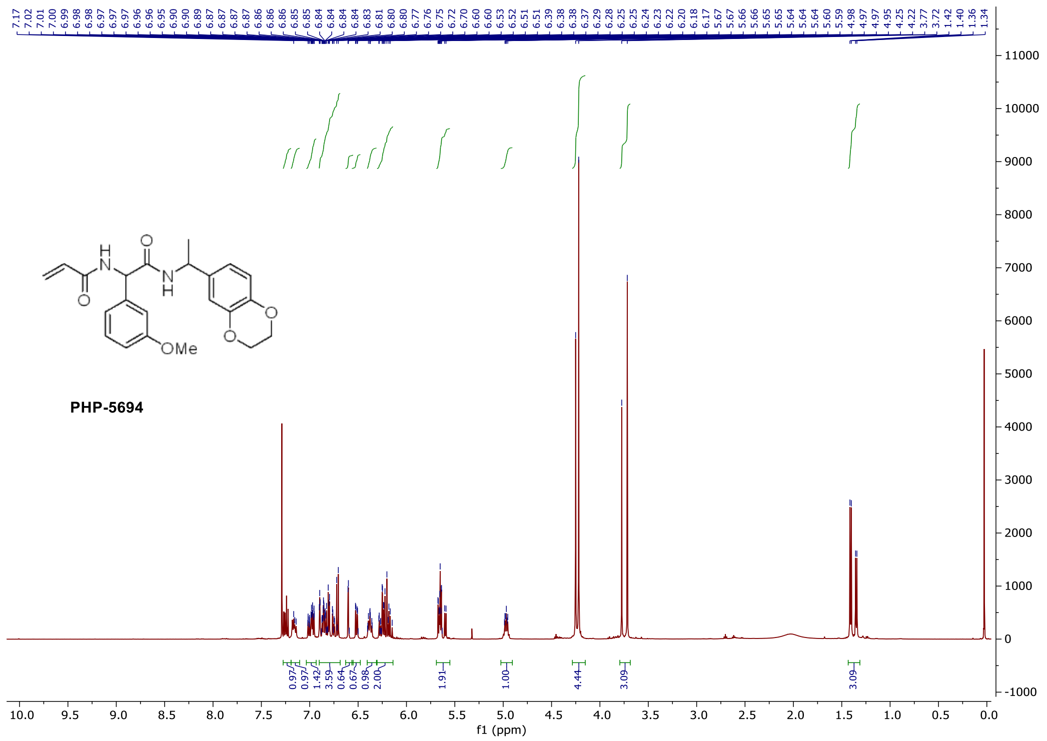

Supplementary Figure 695. ^{1}H NMR (500 MHz, $CDCl_3$) of compound **PHP-5694**.

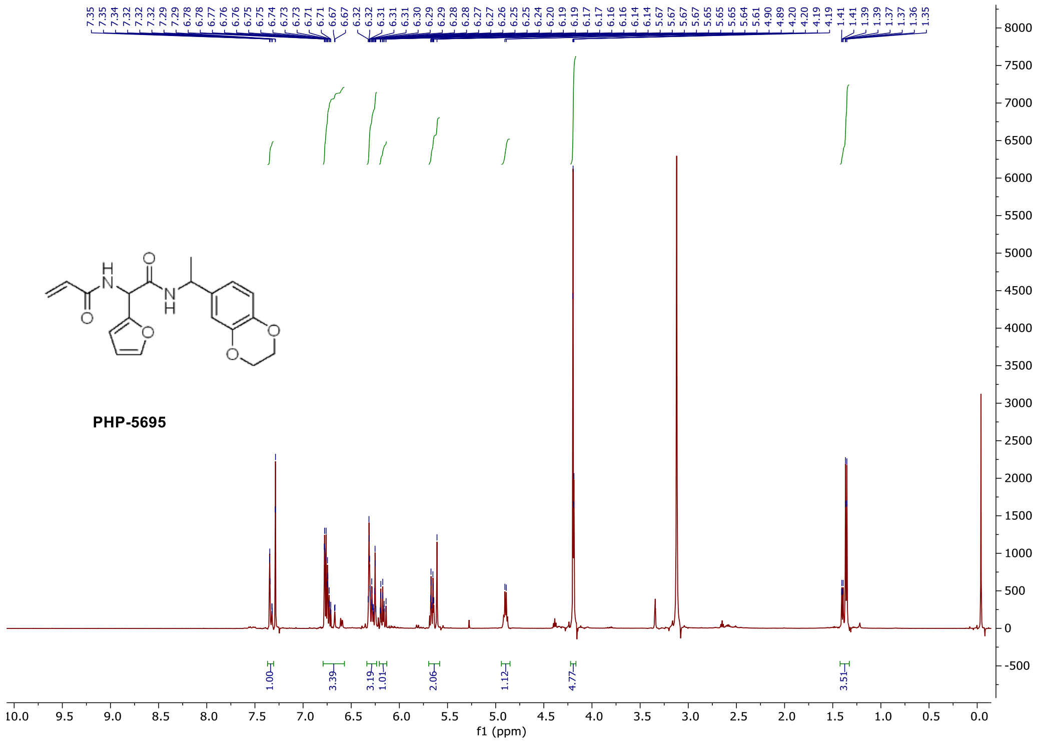

Supplementary Figure 696. ^{1}H NMR (500 MHz, $CDCl_3$ + one drop of CD_3OD) of compound **PHP-5695**.

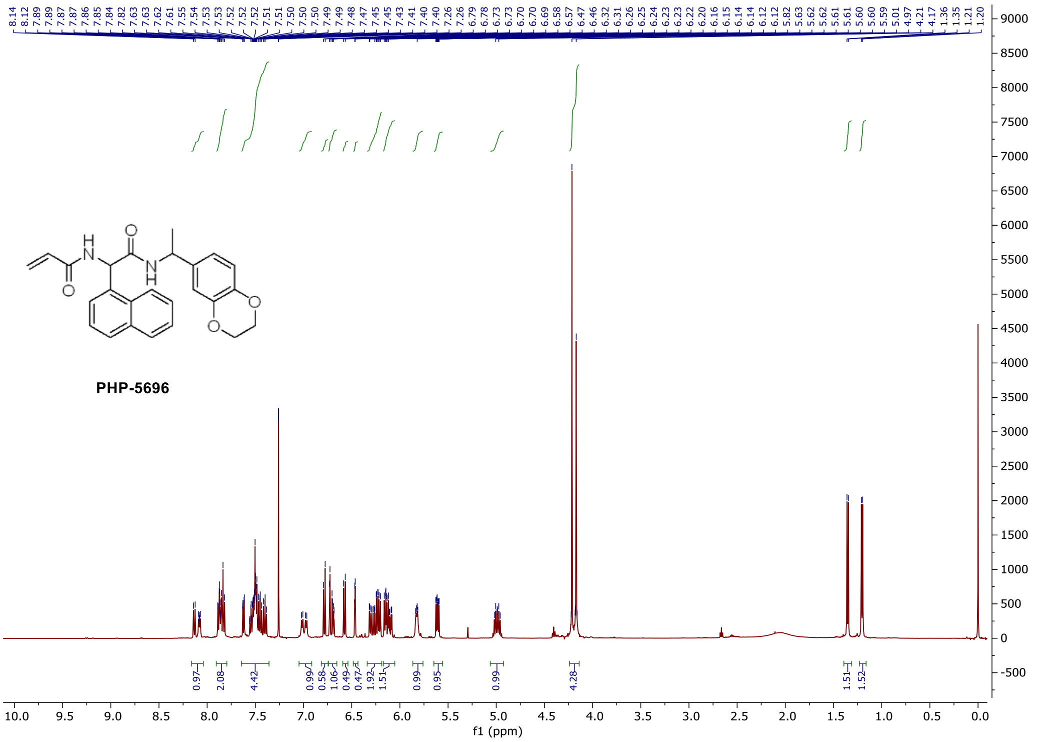

Supplementary Figure 697. ^{1}H NMR (500 MHz, CDCl3) of compound **PHP-5696**.

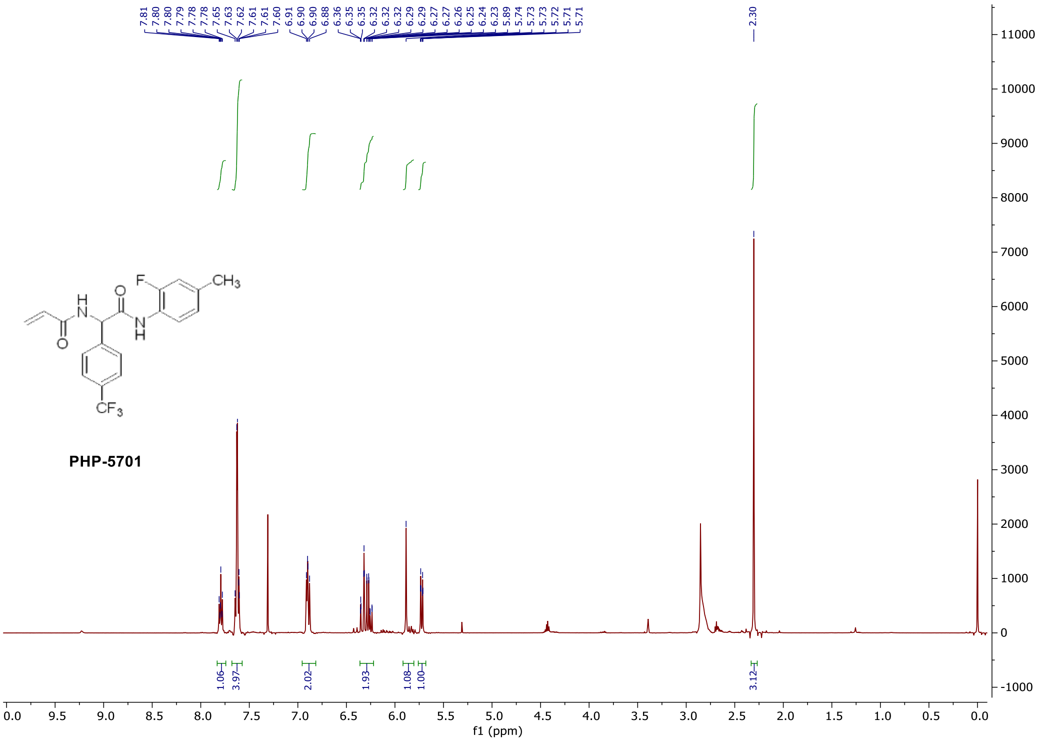

Supplementary Figure 698. ^{1}H NMR (500 MHz, CDCl3) of compound **PHP-5701**.

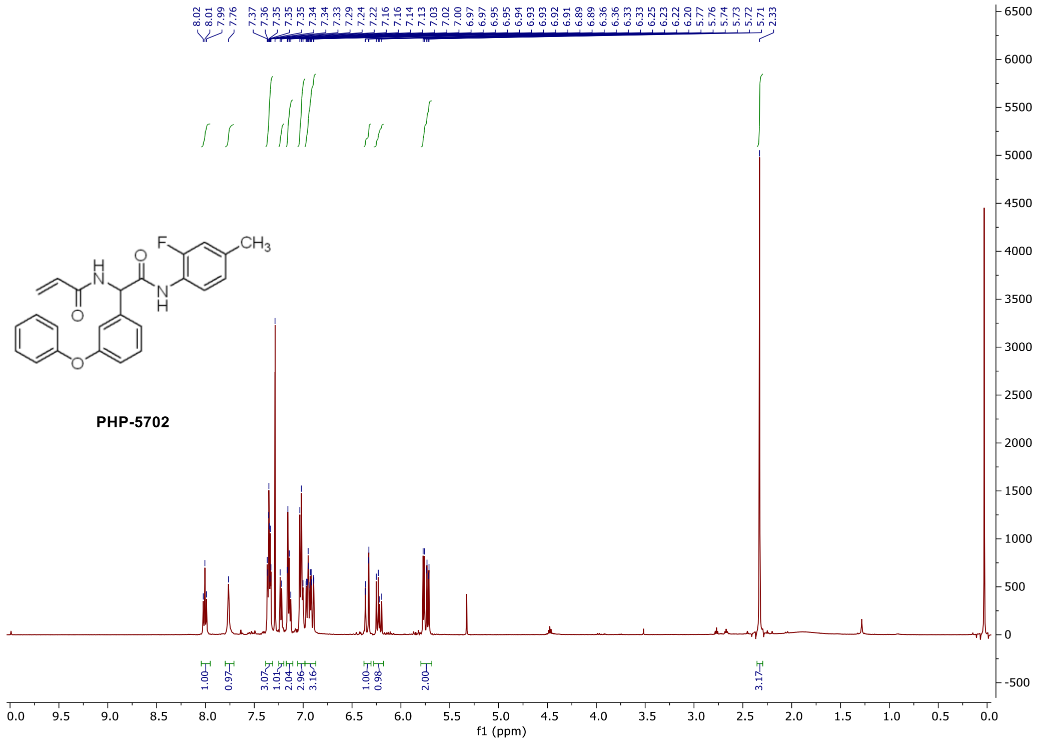

Supplementary Figure 699. ^{1}H NMR (500 MHz, $CDCl_3$) of compound **PHP-5702**.

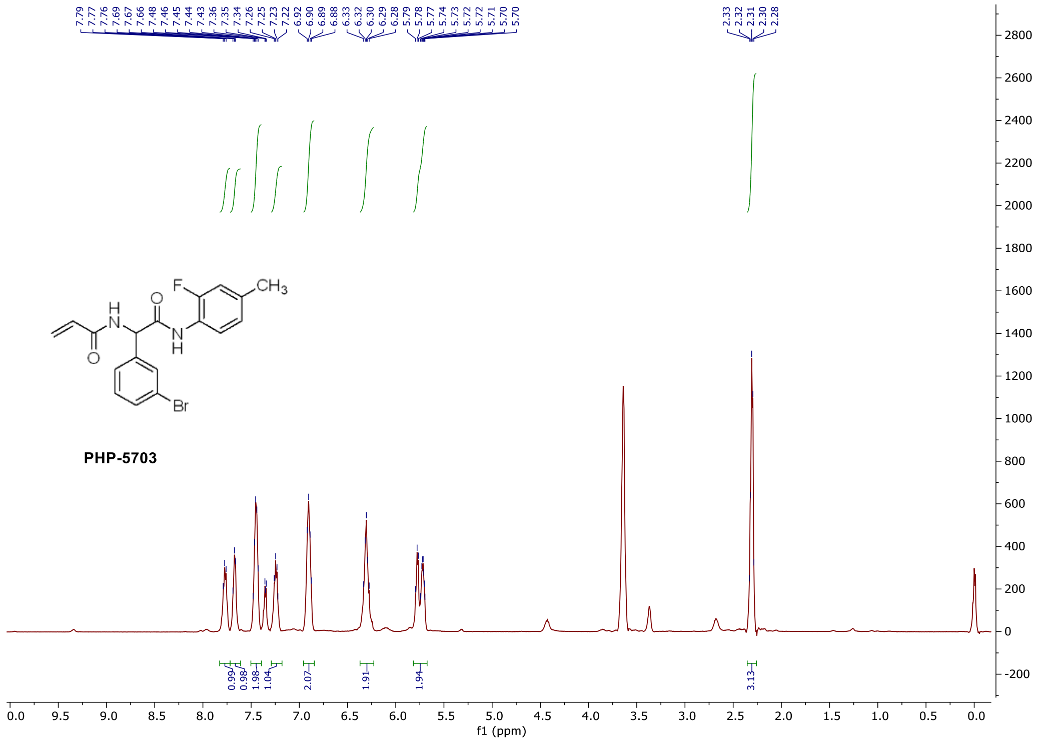

Supplementary Figure 700. ^{1}H NMR (500 MHz, $CDCl_3$ + one drop of CD_3OD) of compound **PHP-5703**.

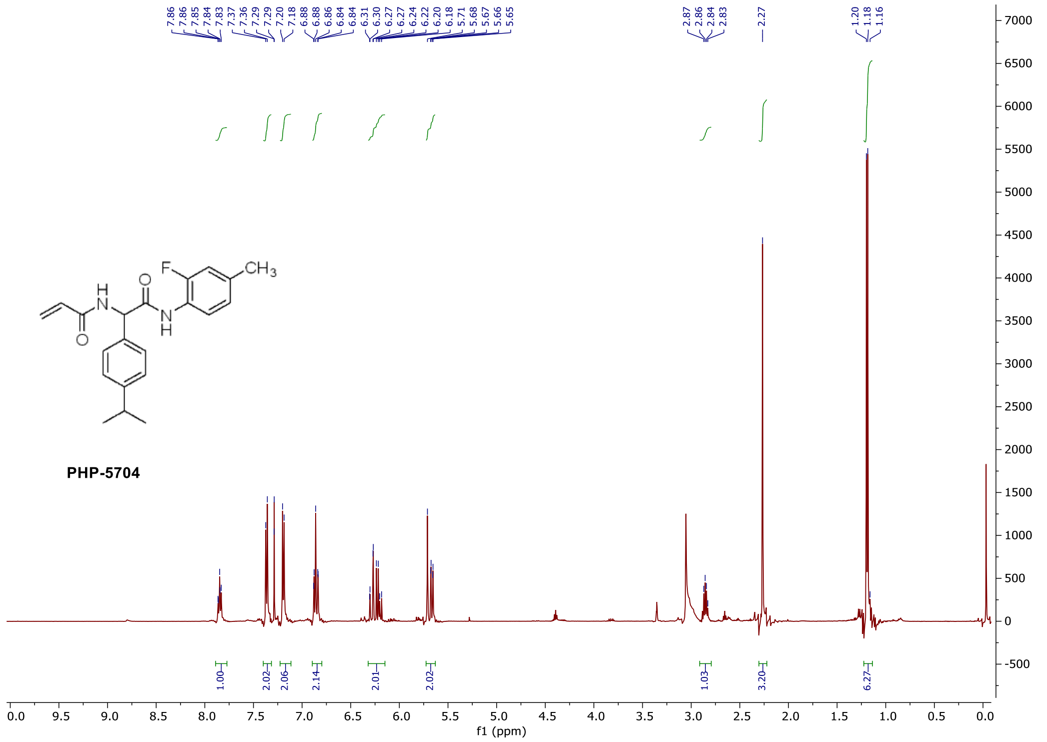

Supplementary Figure 701. ^{1}H NMR (500 MHz, $CDCl_3$ + one drop of CD_3OD) of compound **PHP-5704**.

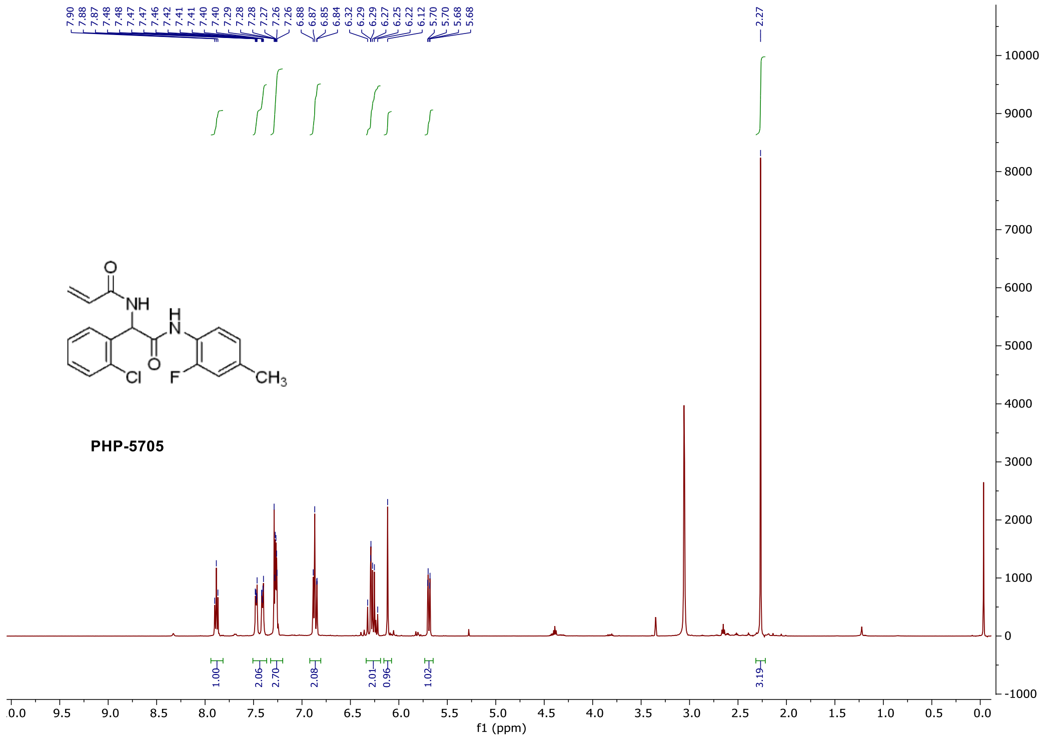

Supplementary Figure 702. ^{1}H NMR (500 MHz, $CDCl_3$ + one drop of CD_3OD) of compound **PHP-5705**.

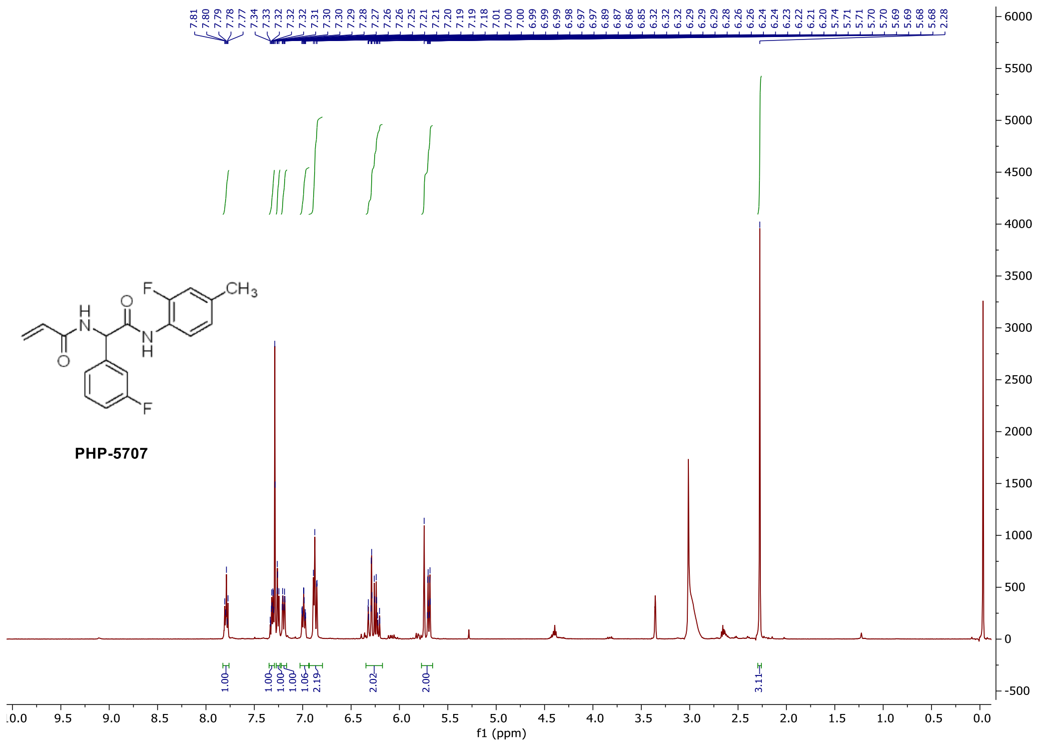

Supplementary Figure 703. ^{1}H NMR (500 MHz, $CDCl_3$ + one drop of CD_3OD) of compound **PHP-5707**.

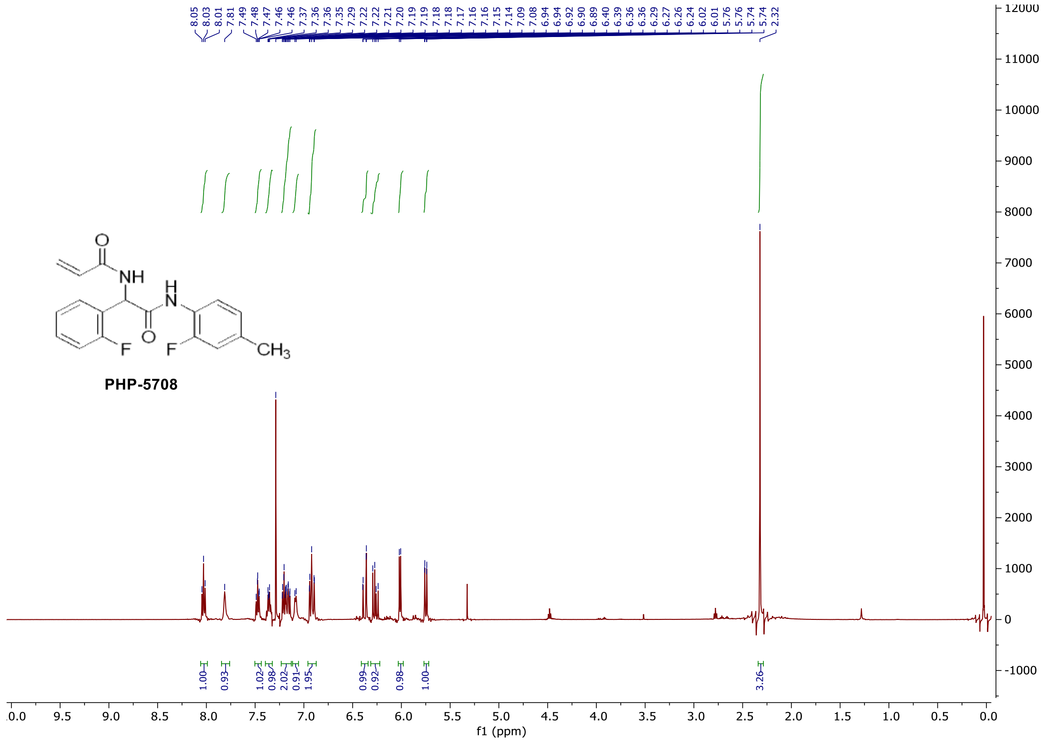

Supplementary Figure 704. ^{1}H NMR (500 MHz, $CDCl_3$) of compound **PHP-5708**.

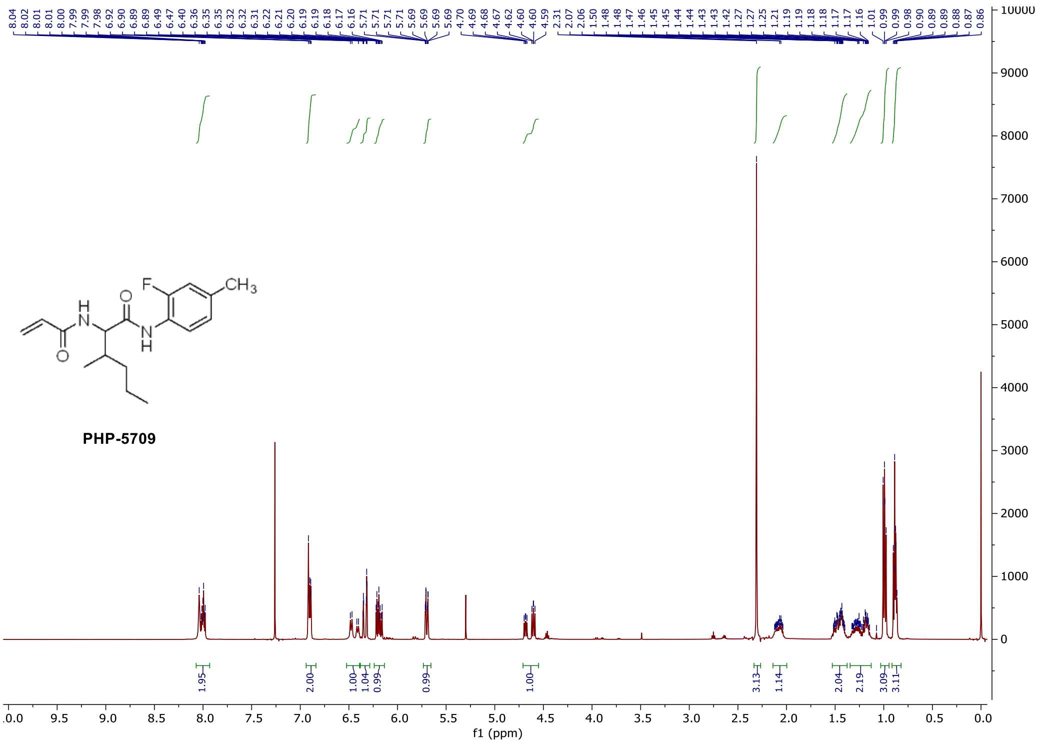

Supplementary Figure 705. ^{1}H NMR (500 MHz, $CDCl_3$) of compound **PHP-5709**.

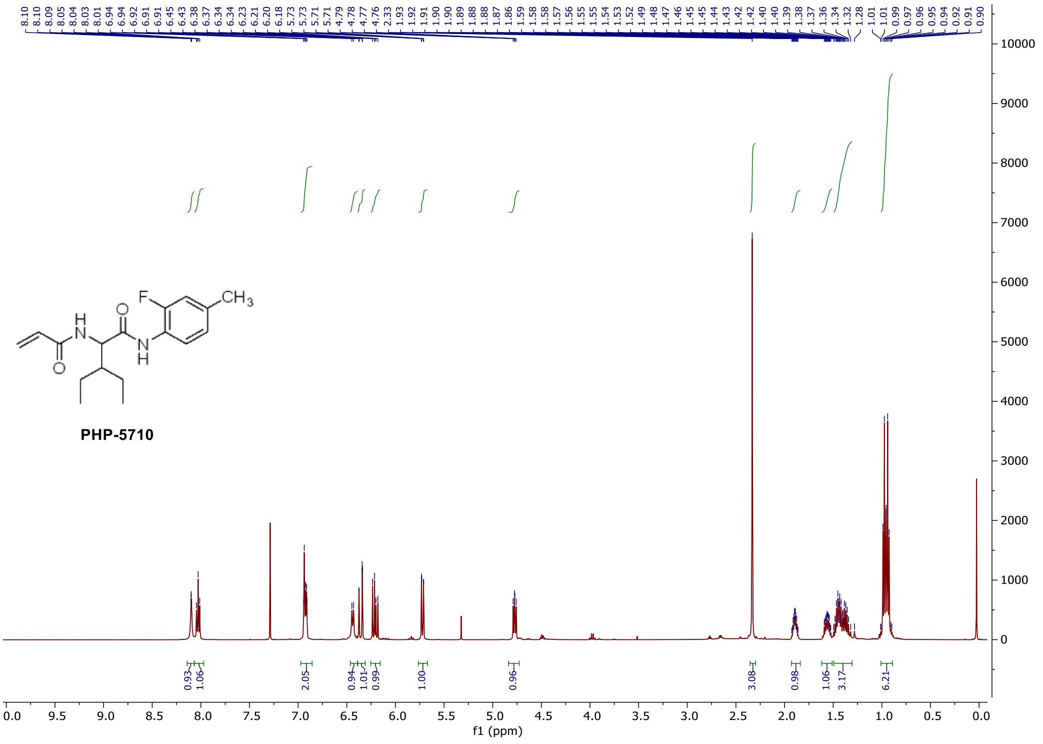

Supplementary Figure 706. ^{1}H NMR (500 MHz, $CDCl_3$) of compound **PHP-5710**.

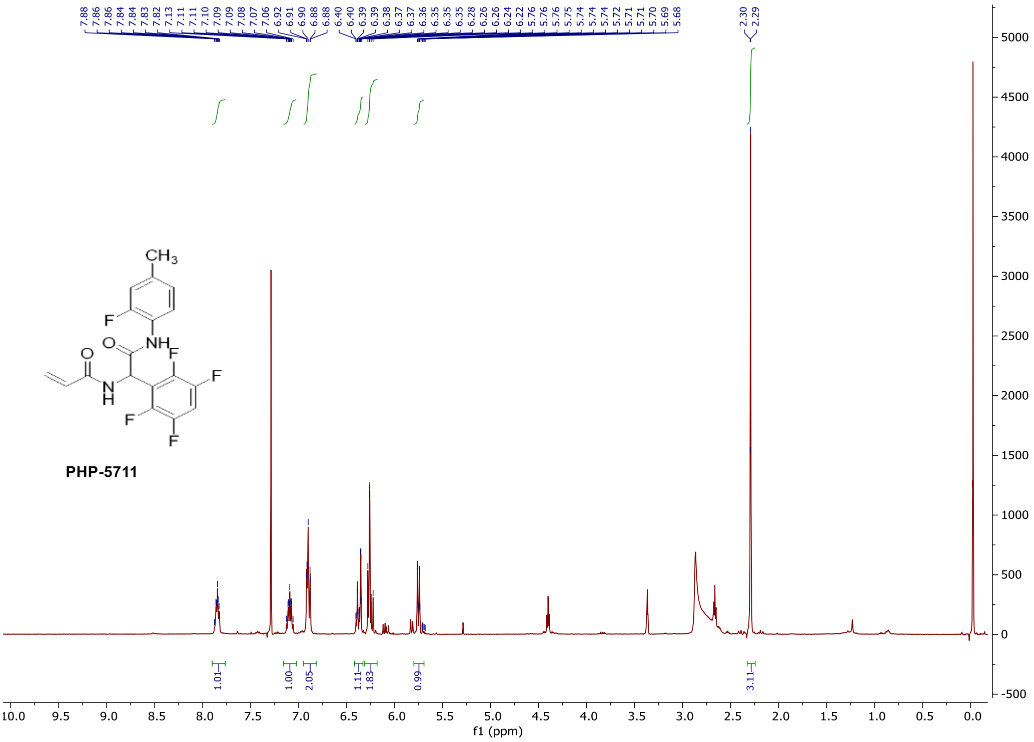

PHP-5711

Supplementary Figure 707. ^{1}H NMR (500 MHz, $CDCl_3$ + one drop of CD_3OD) of compound **PHP-5711**.

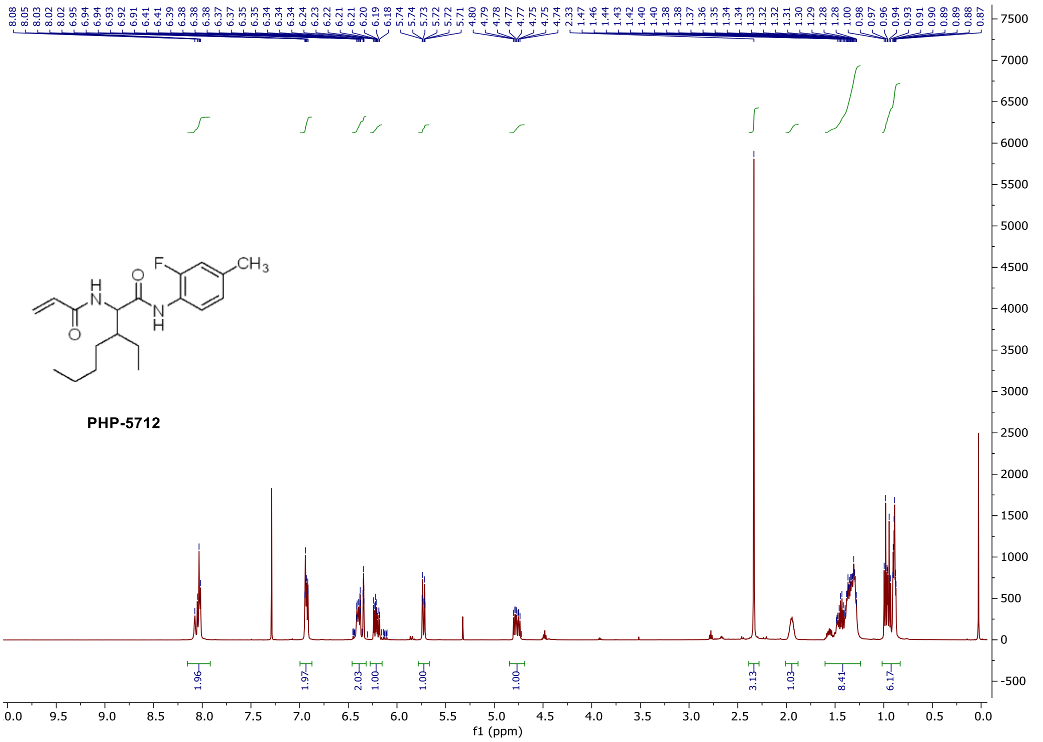

PHP-5712

Supplementary Figure 708. ^{1}H NMR (500 MHz, $CDCl_3$) of compound **PHP-5712**.

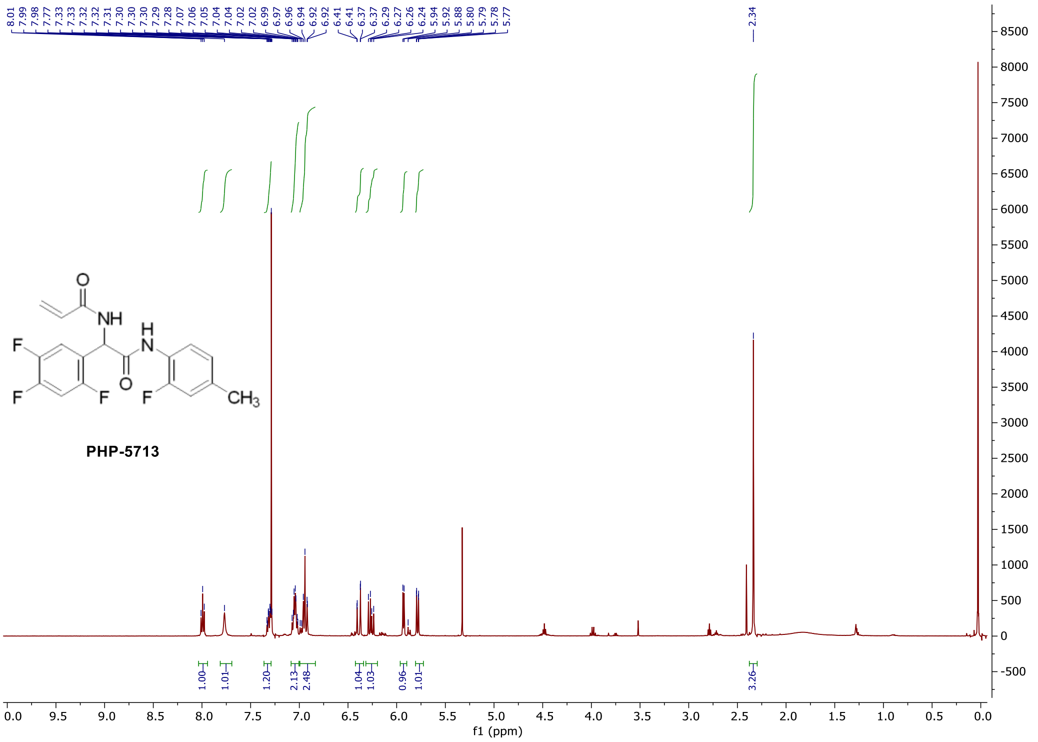

Supplementary Figure 709. ^{1}H NMR (500 MHz, $CDCl_3$) of compound **PHP-5713**.

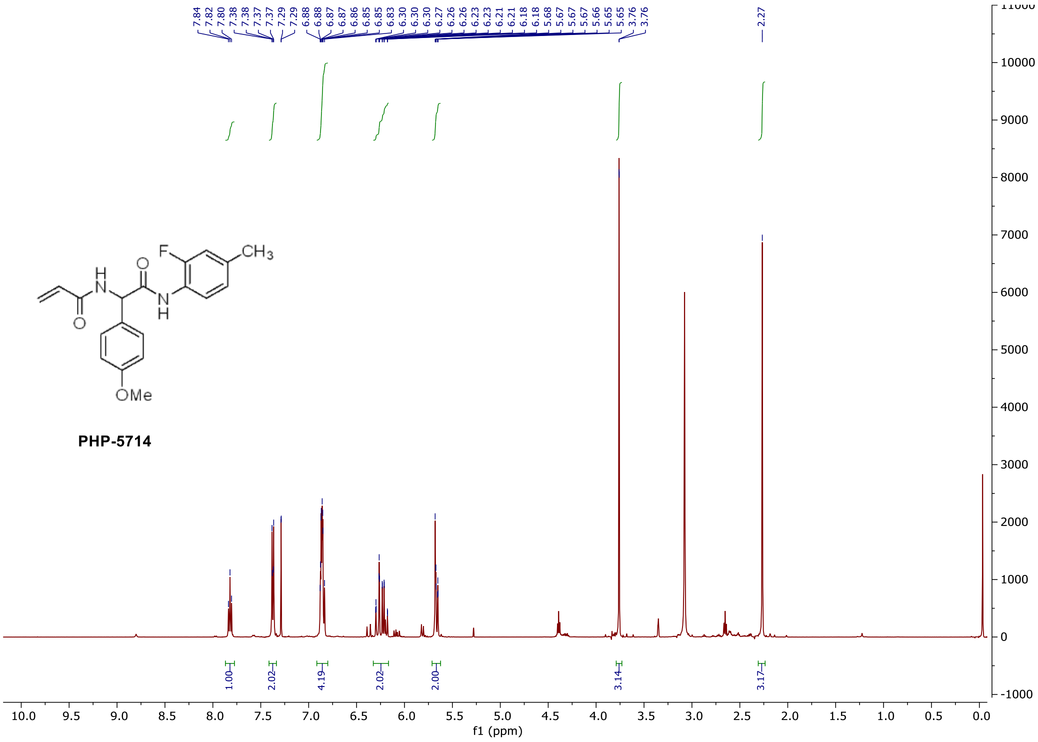

Supplementary Figure 710. ^{1}H NMR (500 MHz, $CDCl_3$ + one drop of CD_3OD) of compound **PHP-5714**.

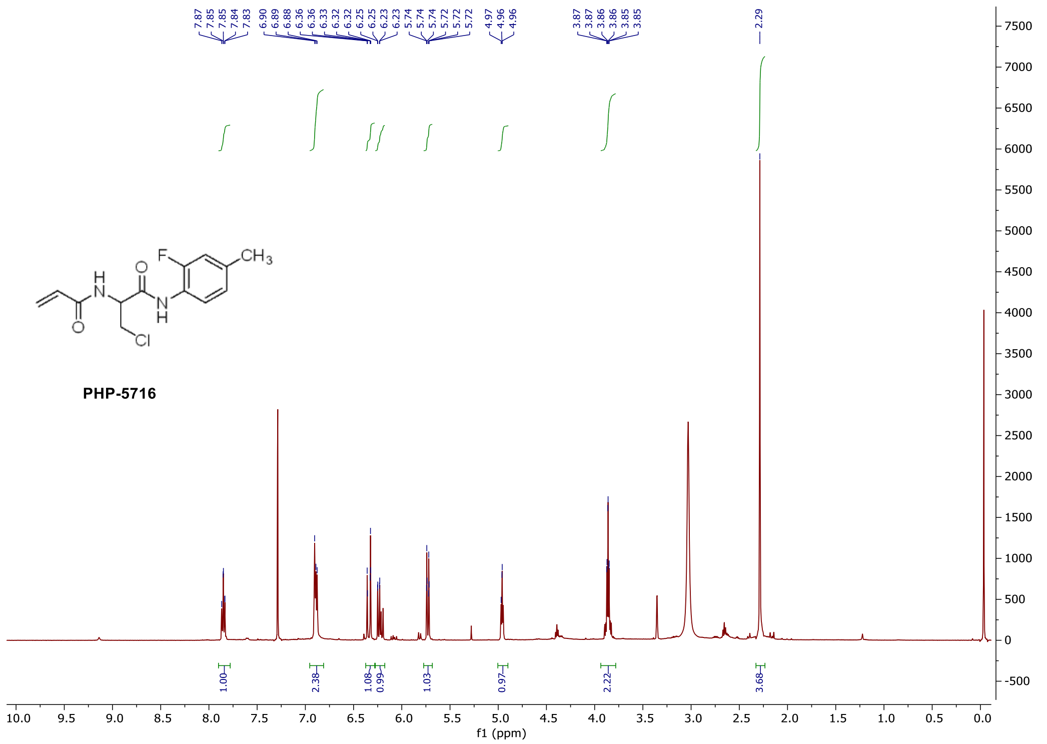

Supplementary Figure 711. ^{1}H NMR (500 MHz, $CDCl_3$ + one drop of CD_3OD) of compound **PHP-5716**.

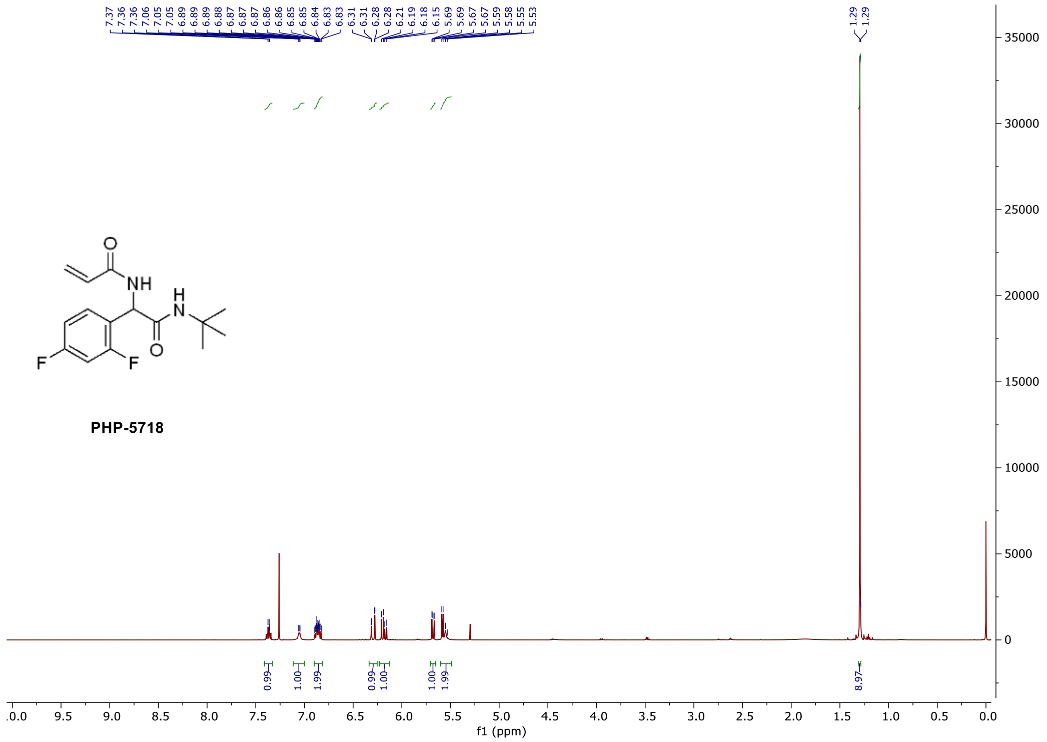

Supplementary Figure 712. ^{1}H NMR (500 MHz, $CDCl_3$) of compound **PHP-5718**.

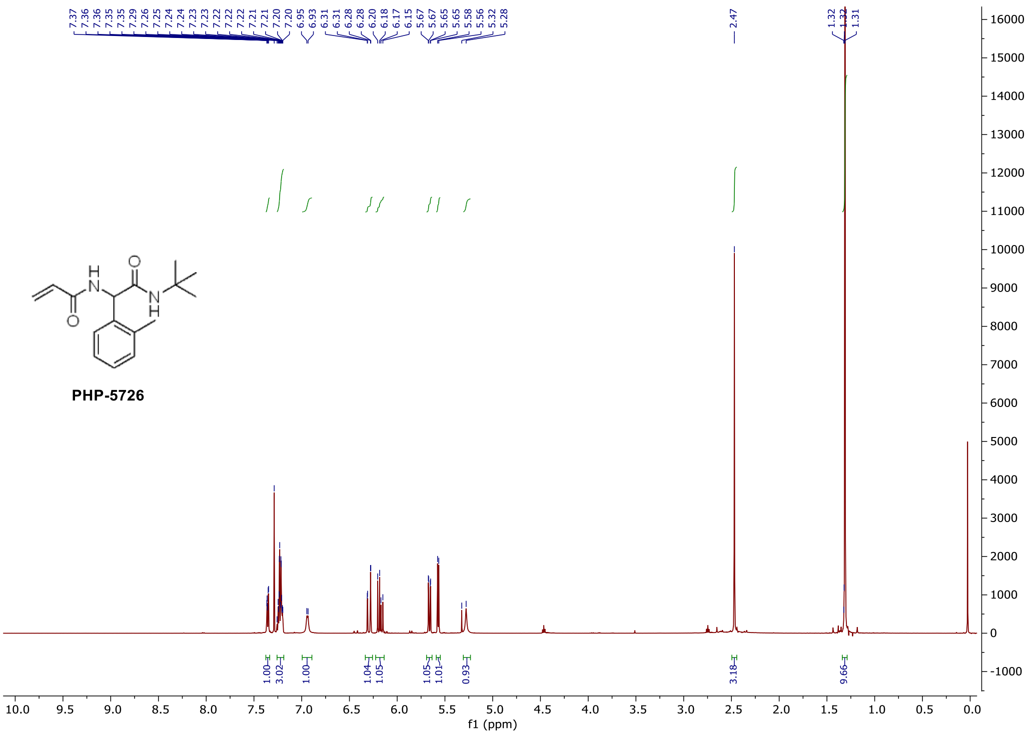

Supplementary Figure 713. ^{1}H NMR (500 MHz, $CDCl_3$) of compound **PHP-5726**.

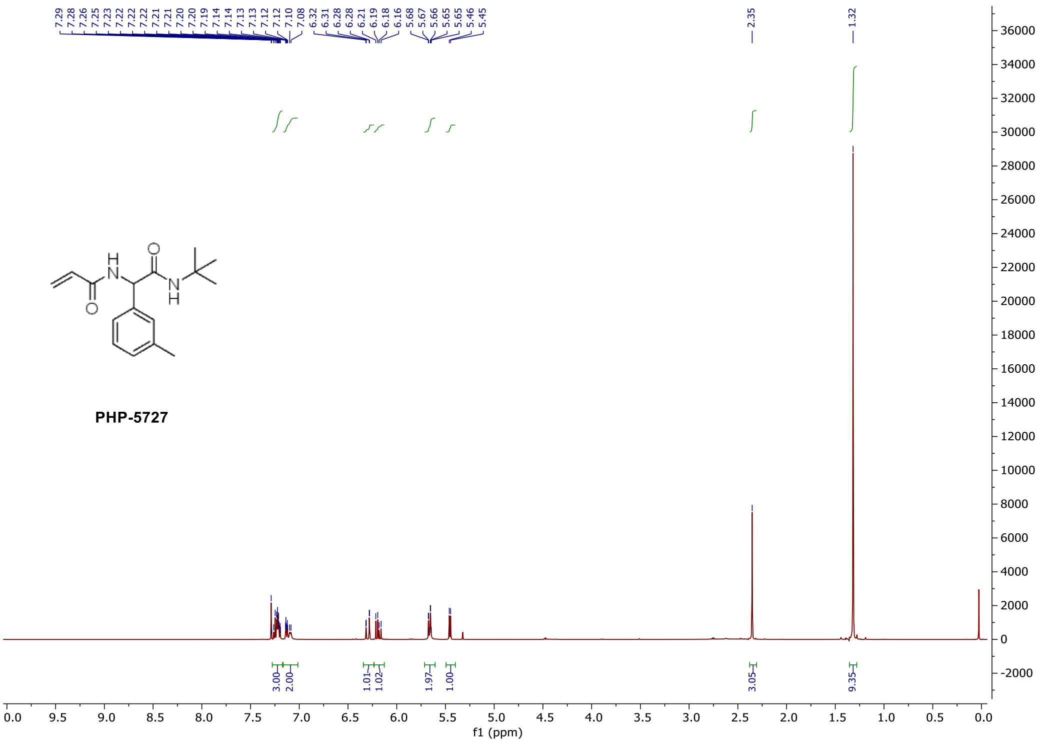

Supplementary Figure 714. ^{1}H NMR (500 MHz, $CDCl_3$) of compound **PHP-5727**.

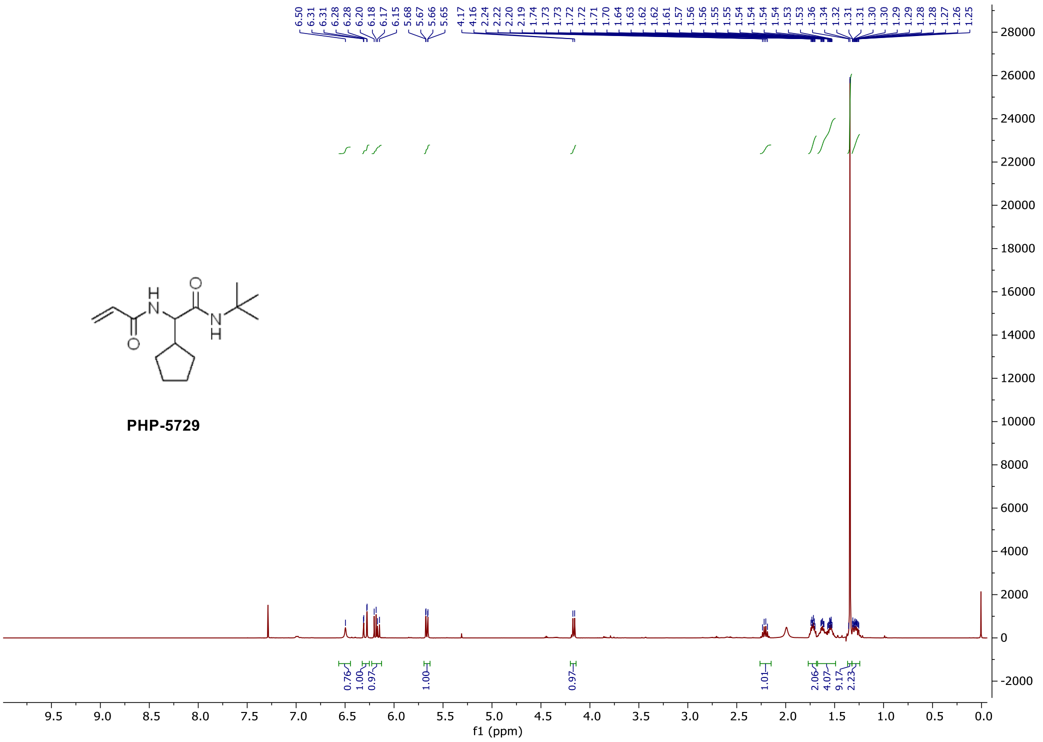

Supplementary Figure 715. ^{1}H NMR (500 MHz, $CDCl_3$) of compound **PHP-5729**.

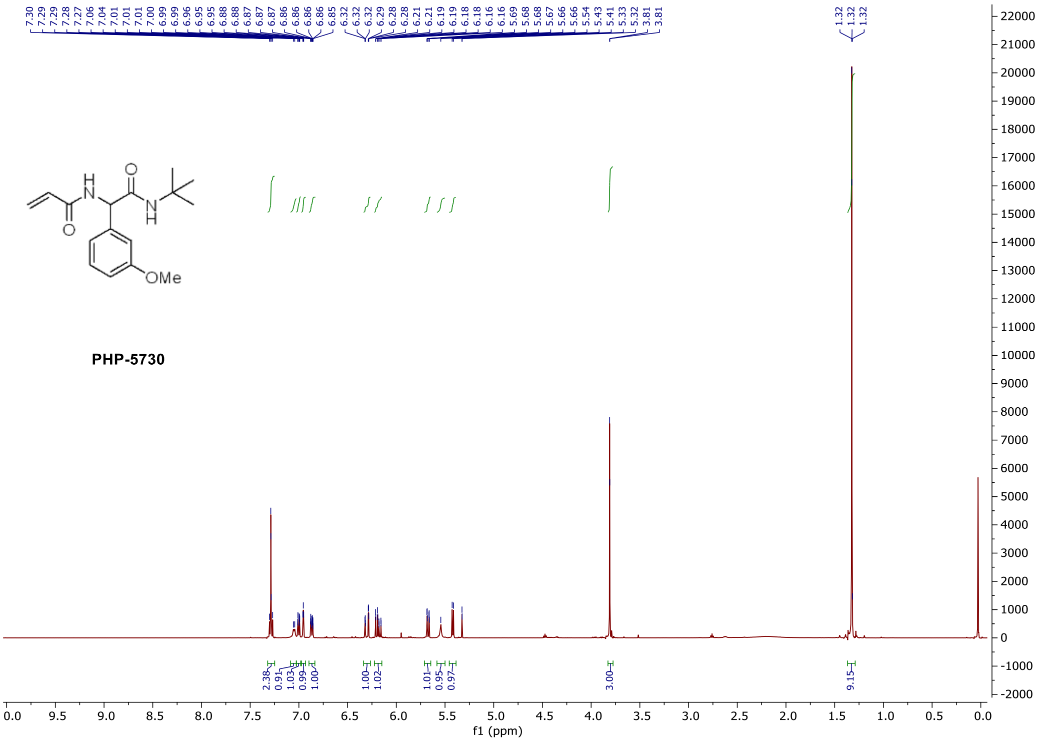

Supplementary Figure 716. ^{1}H NMR (500 MHz, $CDCl_3$) of compound **PHP-5730**.

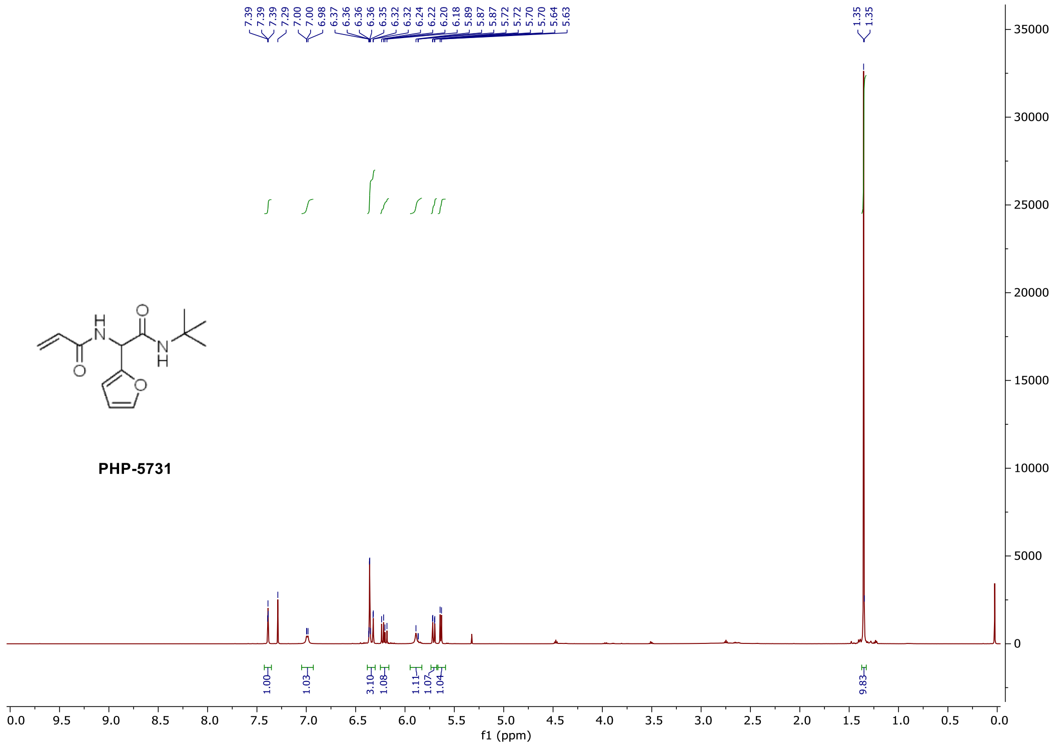

Supplementary Figure 717. ^{1}H NMR (500 MHz, $CDCl_3$) of compound **PHP-5731**.

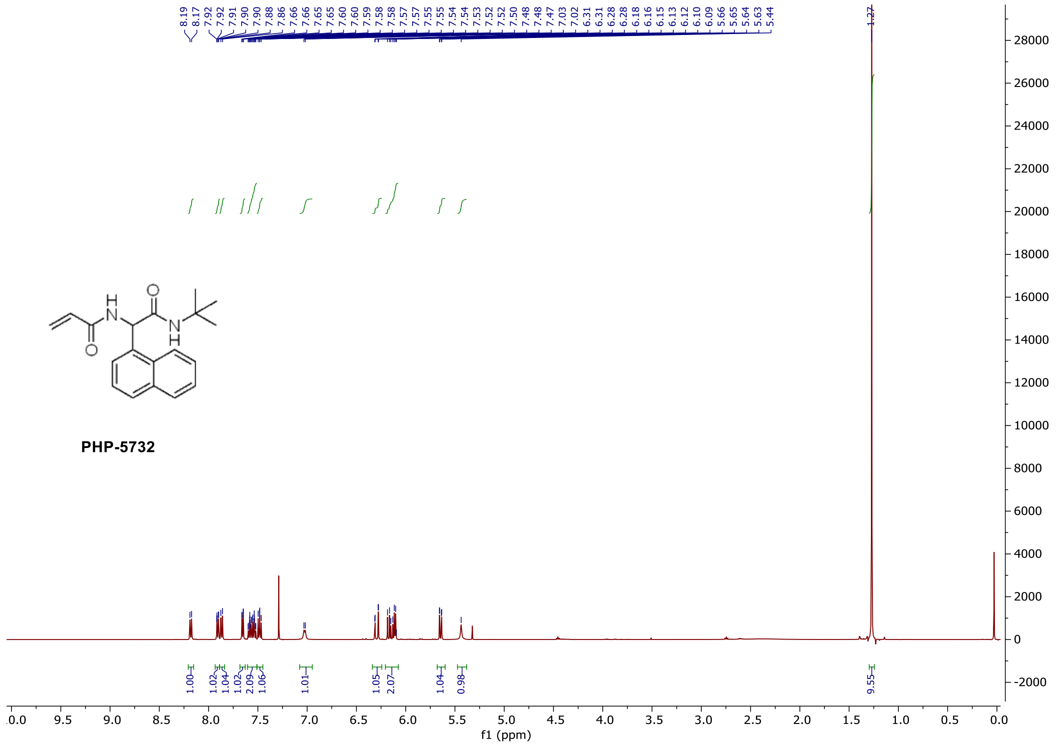

Supplementary Figure 718. ^{1}H NMR (500 MHz, $CDCl_3$) of compound **PHP-5732**.

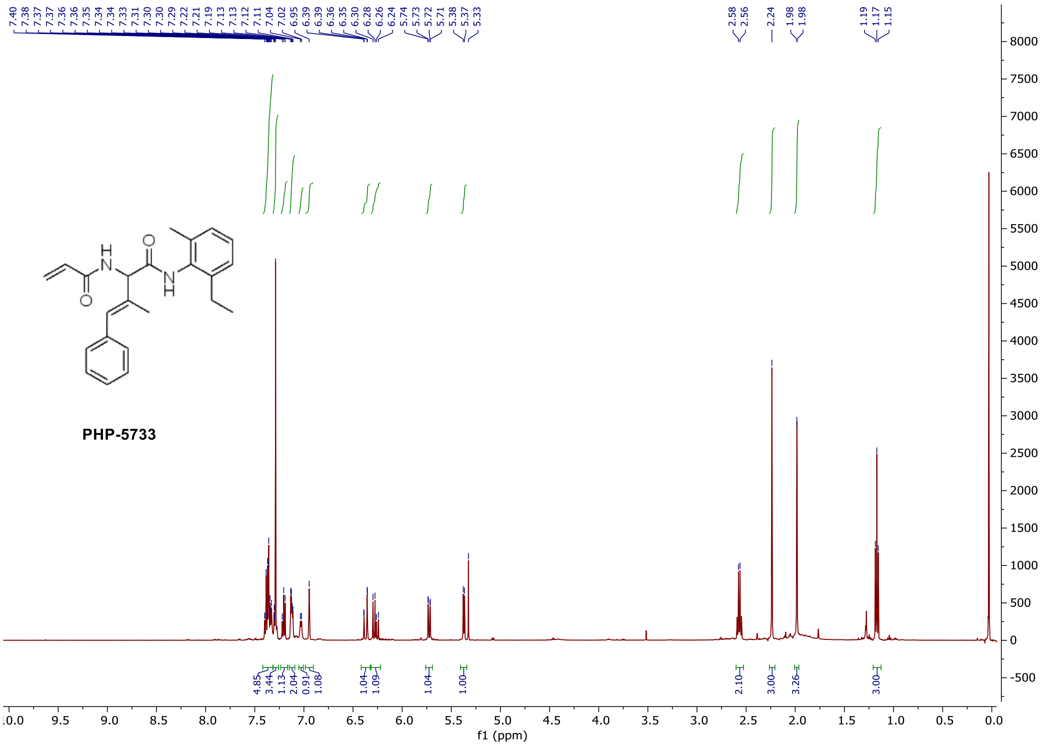

Supplementary Figure 719. ^{1}H NMR (500 MHz, $CDCl_3$) of compound **PHP-5733**.

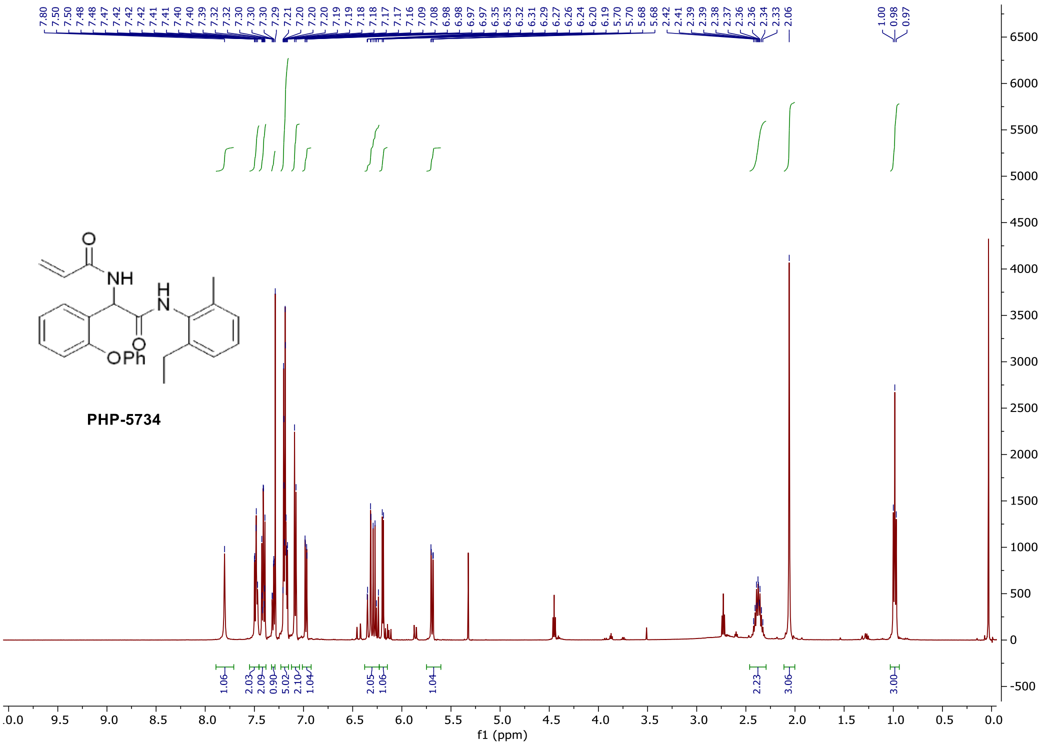

Supplementary Figure 720. ^{1}H NMR (500 MHz, $CDCl_3$) of compound **PHP-5734**.

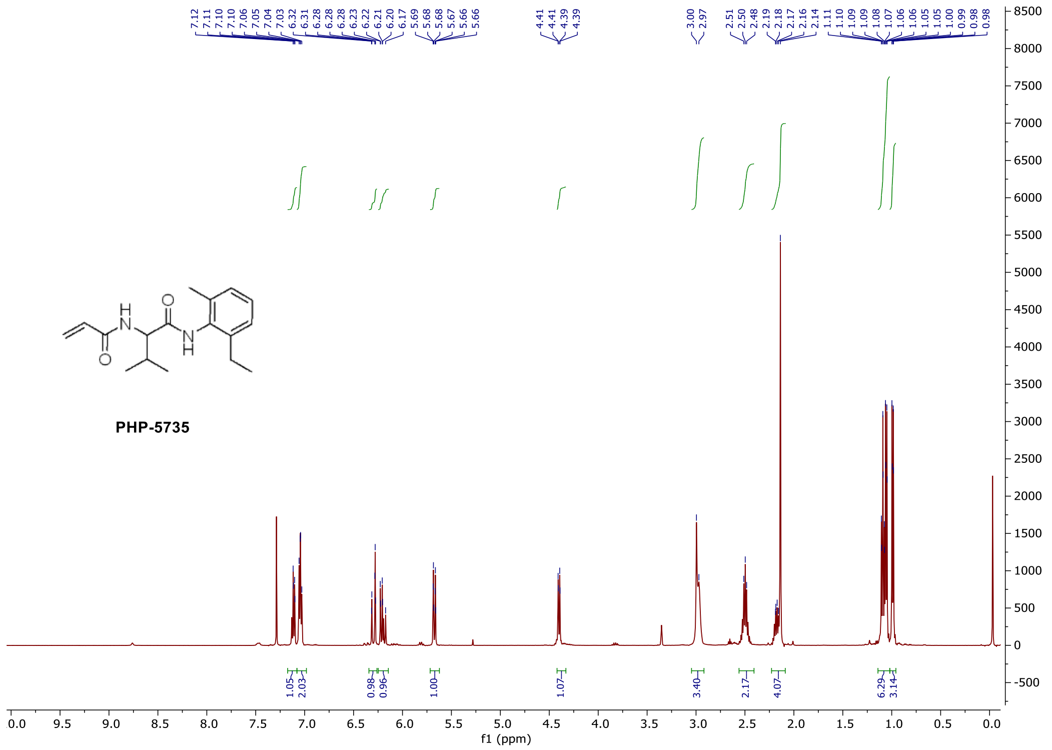

Supplementary Figure 721. ^{1}H NMR (500 MHz, $CDCl_3$ + one drop of CD_3OD) of compound **PHP-5735**.

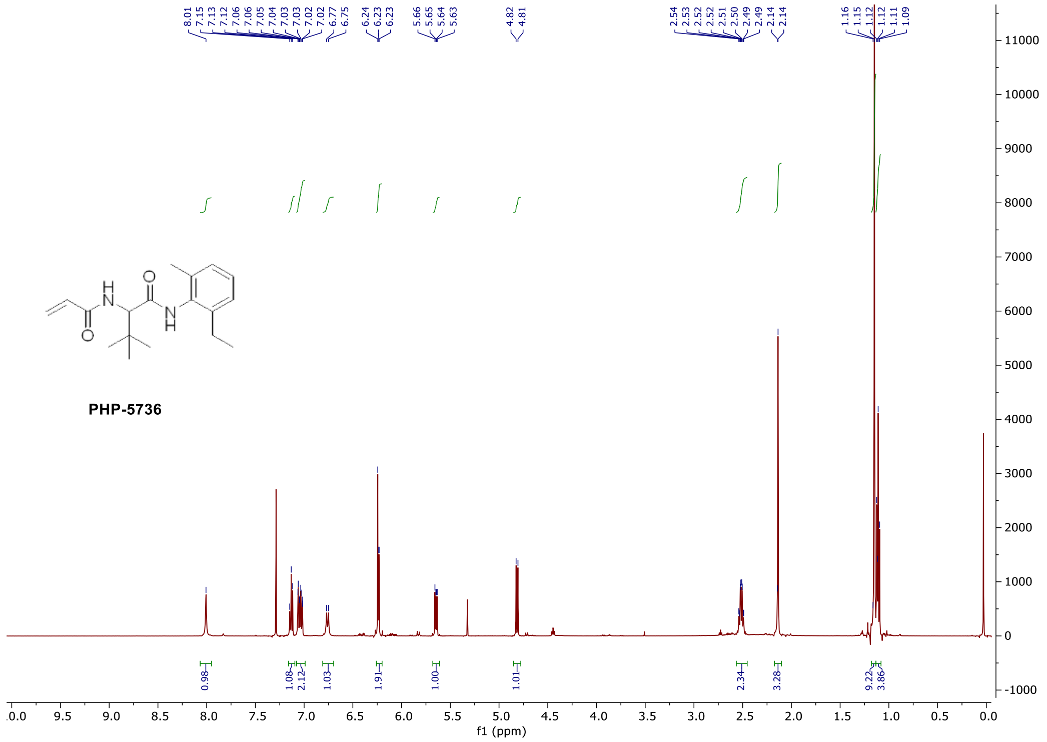

Supplementary Figure 722. ^{1}H NMR (500 MHz, $CDCl_3$) of compound **PHP-5736**.

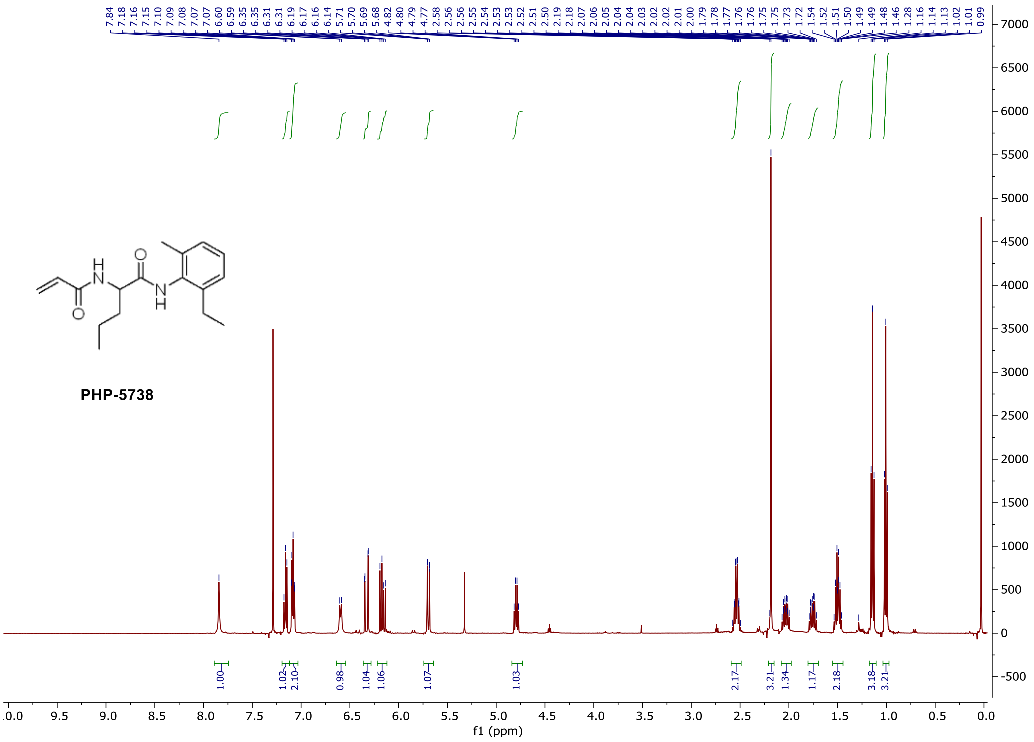

Supplementary Figure 723. ^{1}H NMR (500 MHz, $CDCl_3$) of compound **PHP-5738**.

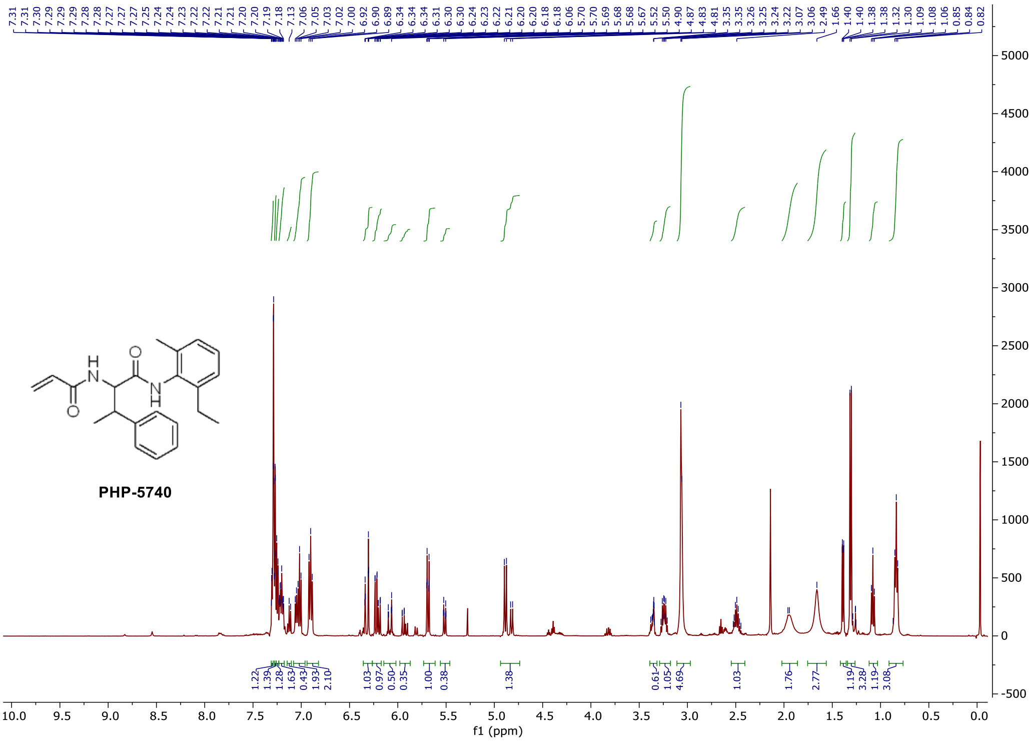

Supplementary Figure 724. ^{1}H NMR (500 MHz, $CDCl_3$) of compound **PHP-5740**.

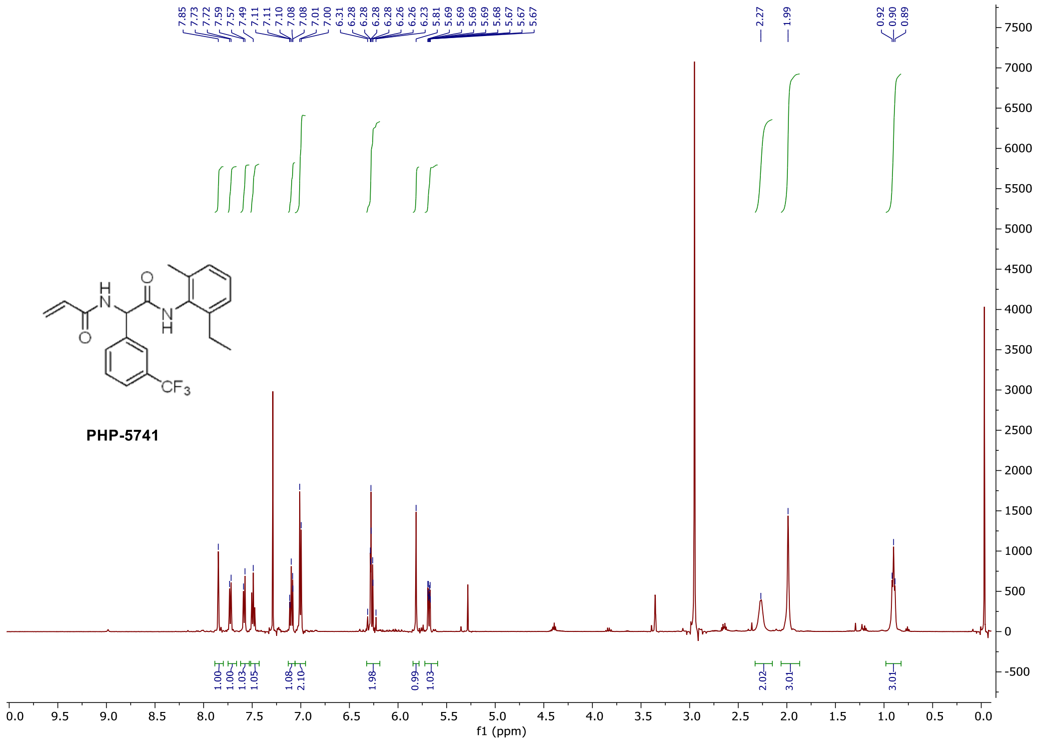

Supplementary Figure 725. ^{1}H NMR (500 MHz, $CDCl_3$) of compound **PHP-5741**.

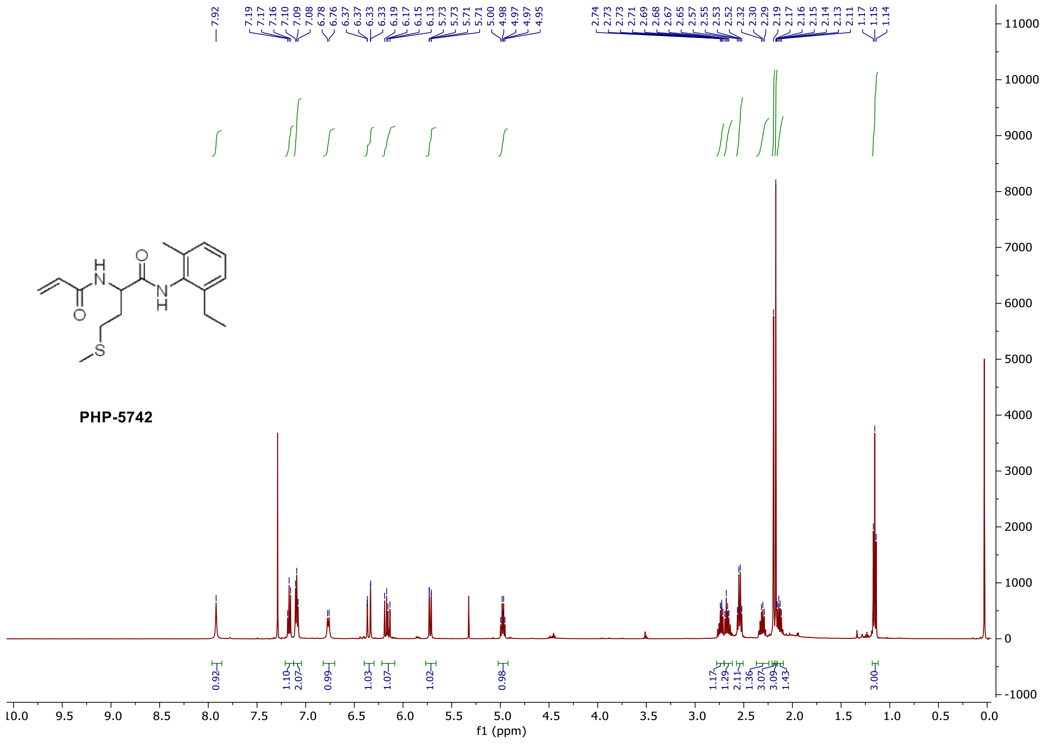

Supplementary Figure 726. ^{1}H NMR (500 MHz, $CDCl_3$) of compound **PHP-5742**.

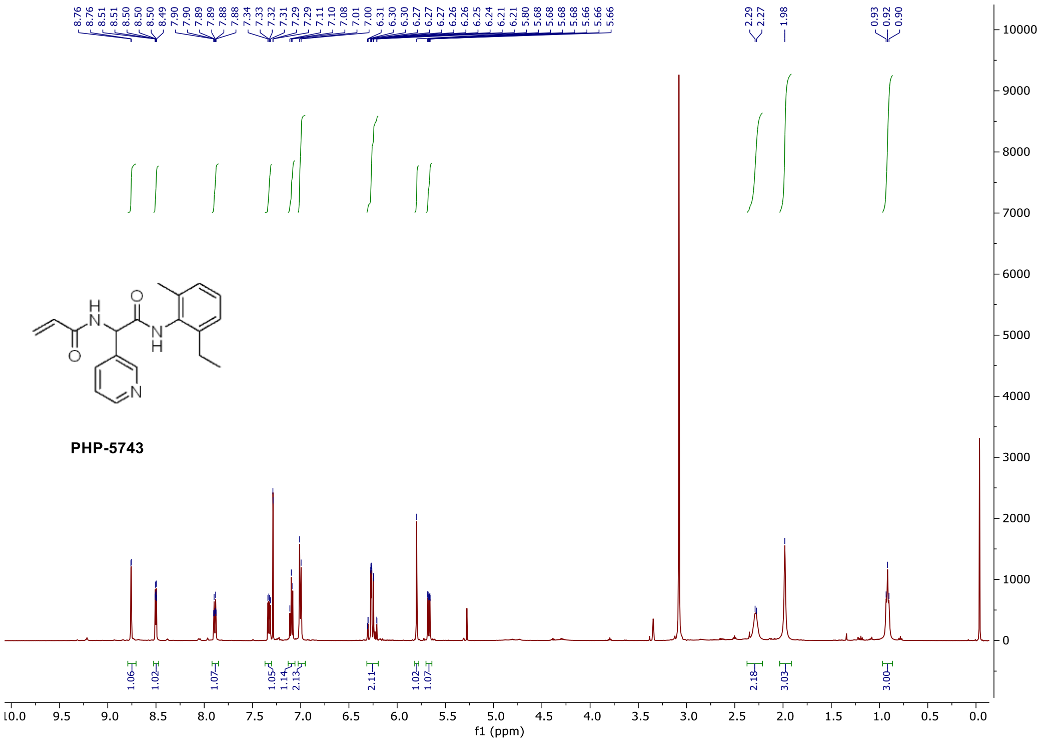

Supplementary Figure 727. ^{1}H NMR (500 MHz, $CDCl_3$ + one drop of CD_3OD) of compound **PHP-5743**.

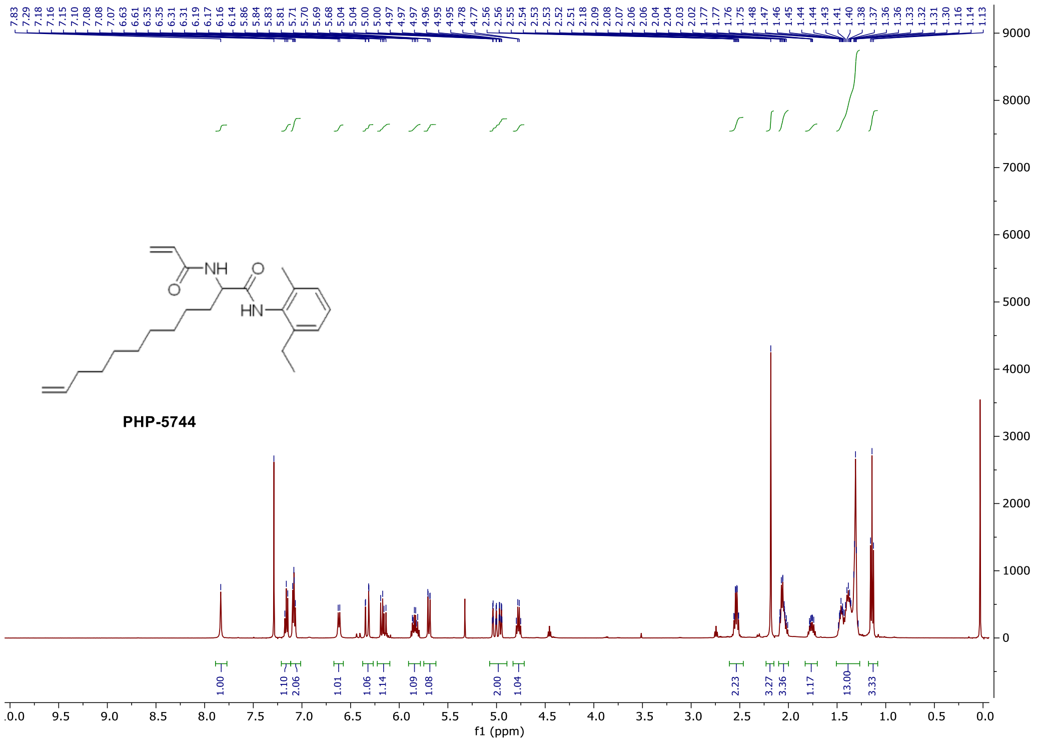

Supplementary Figure 728. ^{1}H NMR (500 MHz, $CDCl_3$) of compound **PHP-5744**.

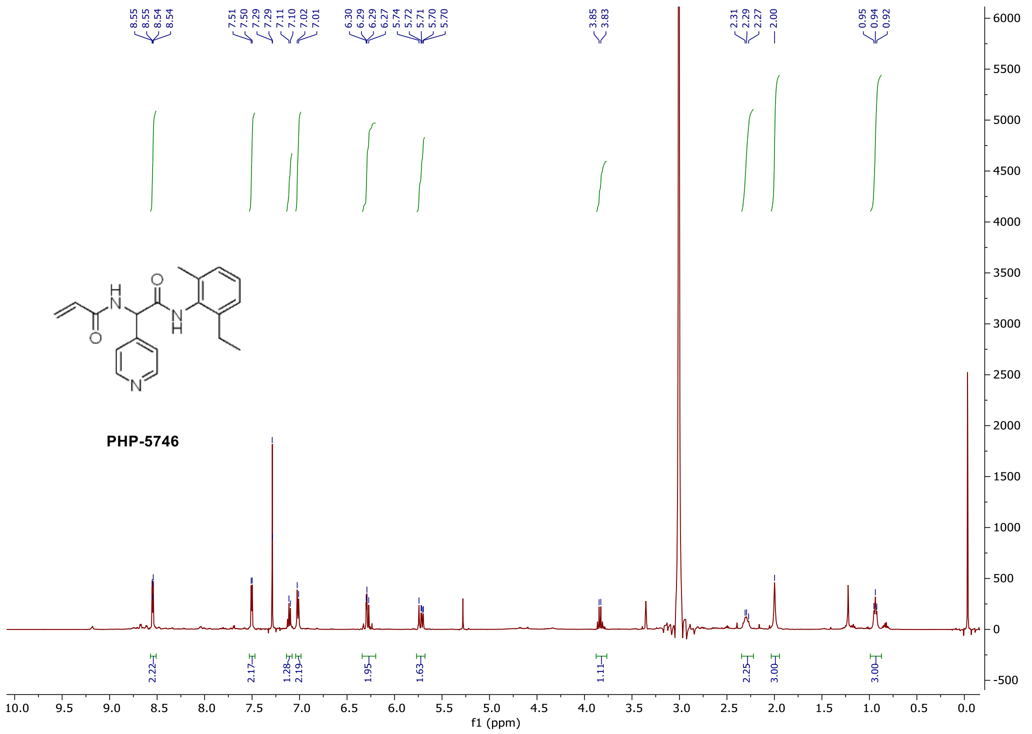

Supplementary Figure 729. ^{1}H NMR (500 MHz, $CDCl_3$ + one drop of CD_3OD) of compound **PHP-5746**.

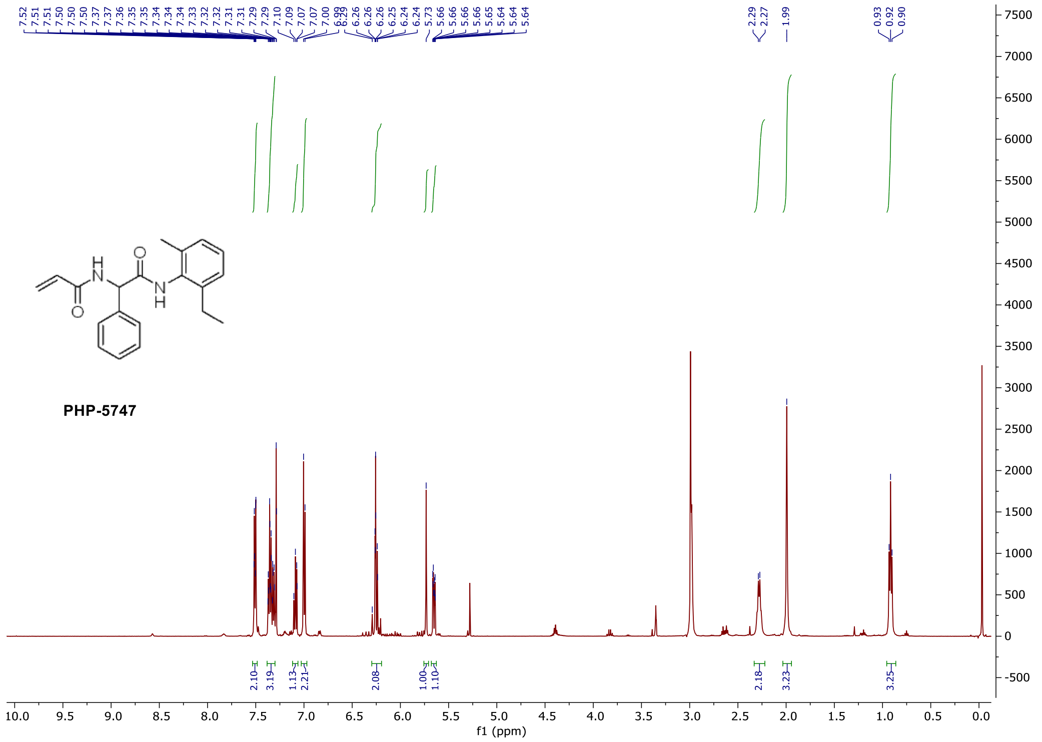

Supplementary Figure 730. ^{1}H NMR (500 MHz, $CDCl_3$ + one drop of CD_3OD) of compound **PHP-5747**.

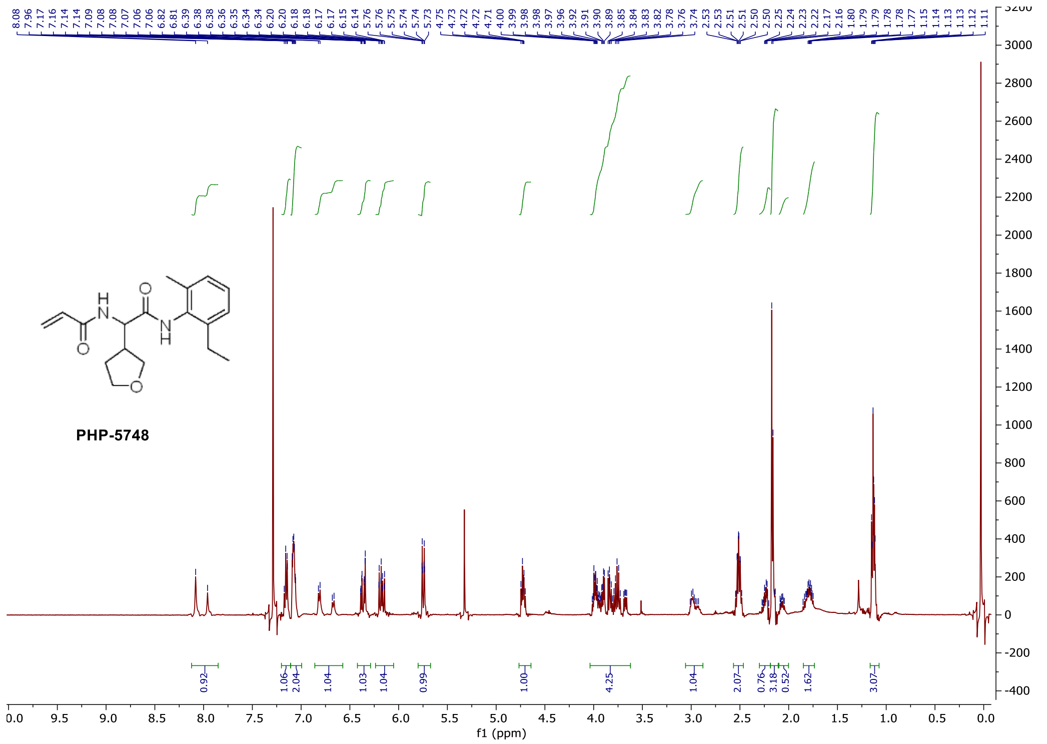

Supplementary Figure 731. ^{1}H NMR (500 MHz, $CDCl_3$) of compound **PHP-5748**.

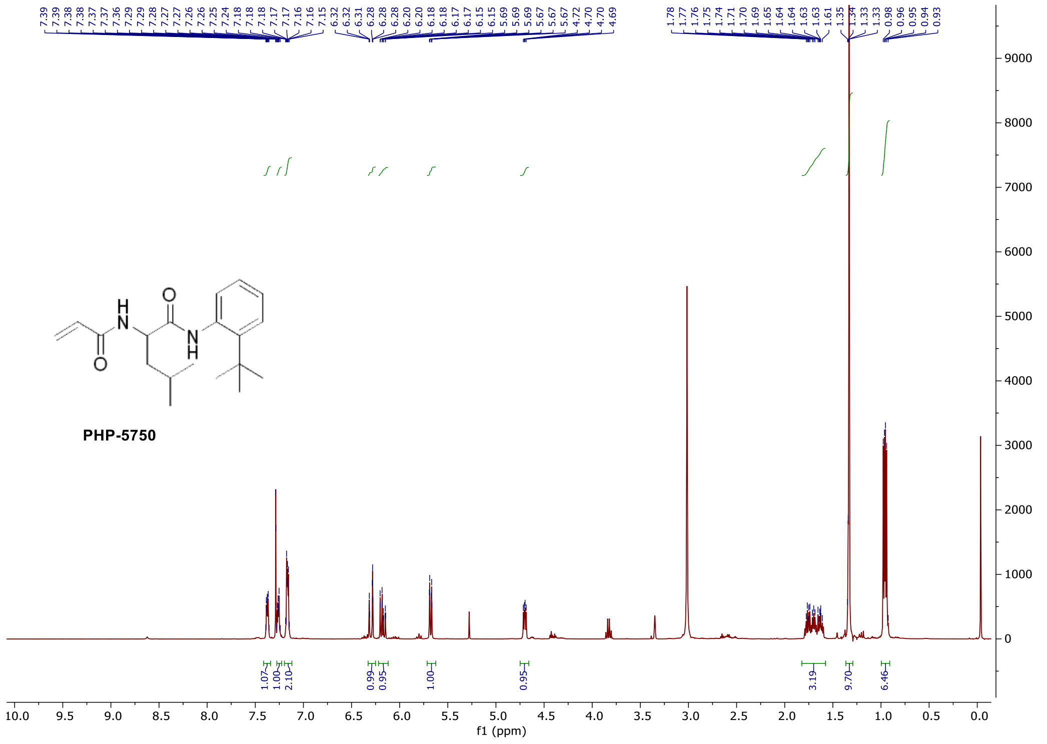

Supplementary Figure 732. ^{1}H NMR (500 MHz, $CDCl_3$ + one drop of CD_3OD) of compound **PHP-5750**.

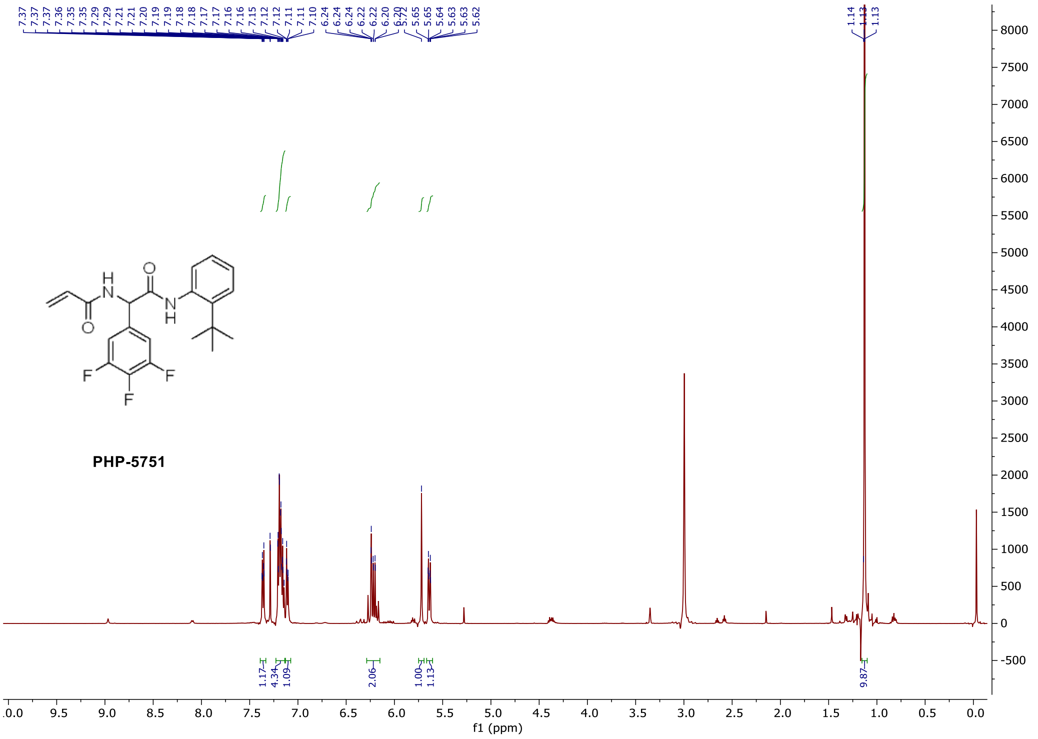

Supplementary Figure 733. ^{1}H NMR (500 MHz, $CDCl_3$ + one drop of CD_3OD) of compound **PHP-5751**.

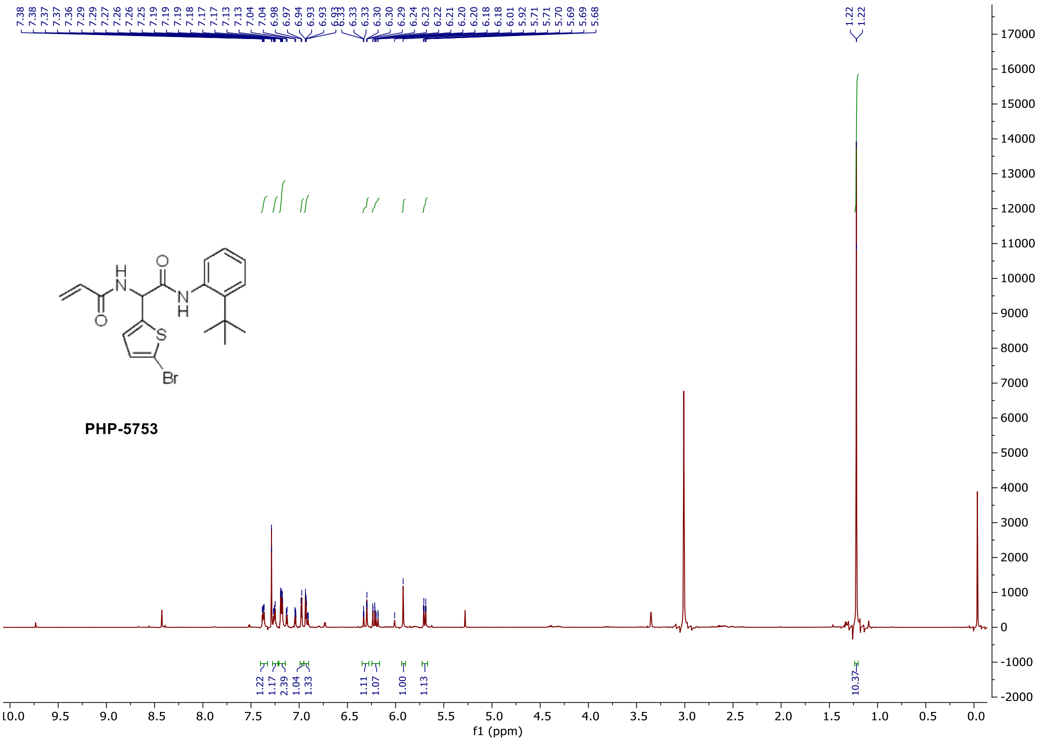

Supplementary Figure 734. ^{1}H NMR (500 MHz, $CDCl_3$) of compound **PHP-5753**.

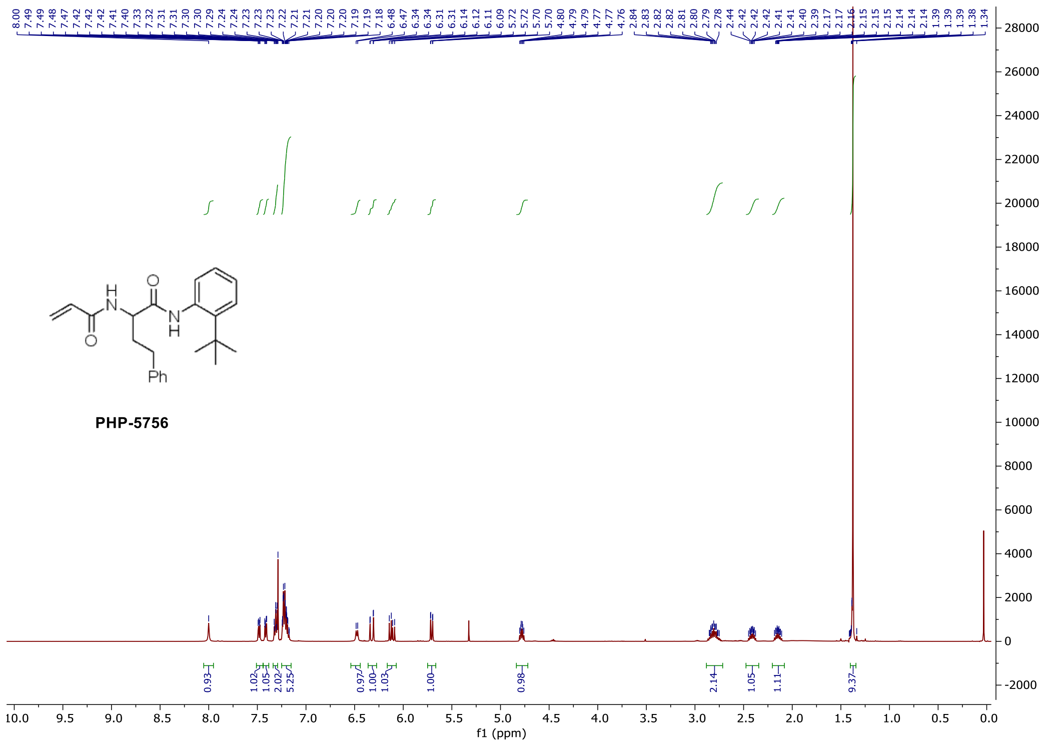

Supplementary Figure 735. ^{1}H NMR (500 MHz, $CDCl_3$) of compound **PHP-5756**.

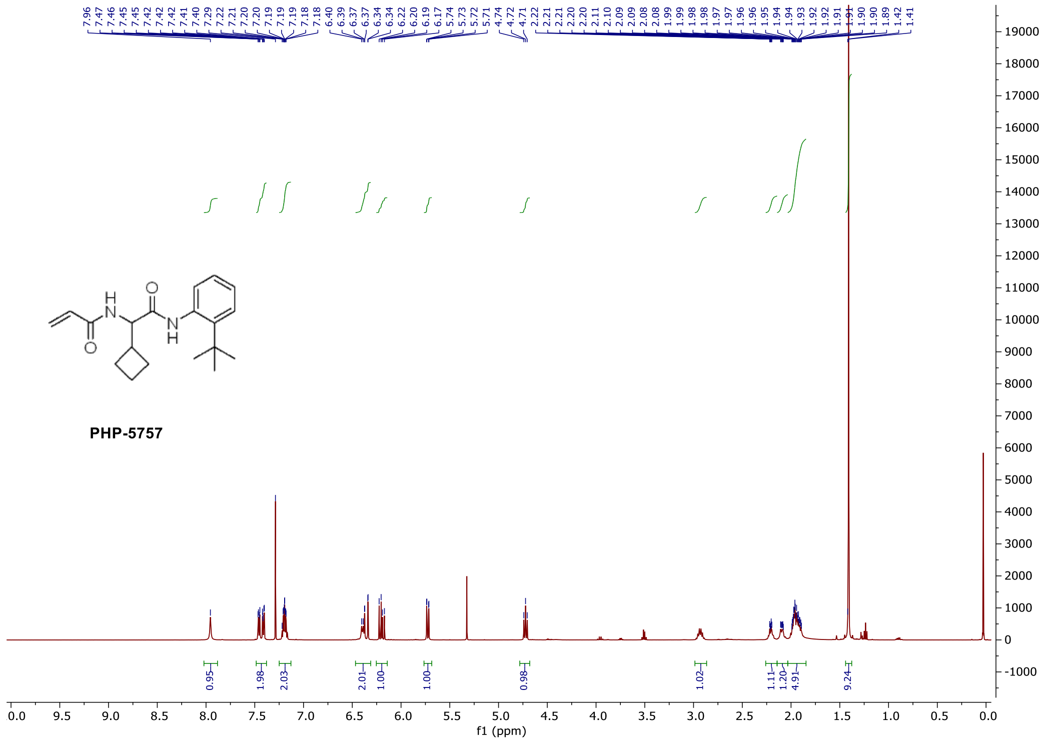

Supplementary Figure 736. ^{1}H NMR (500 MHz, $CDCl_3$) of compound **PHP-5757**.

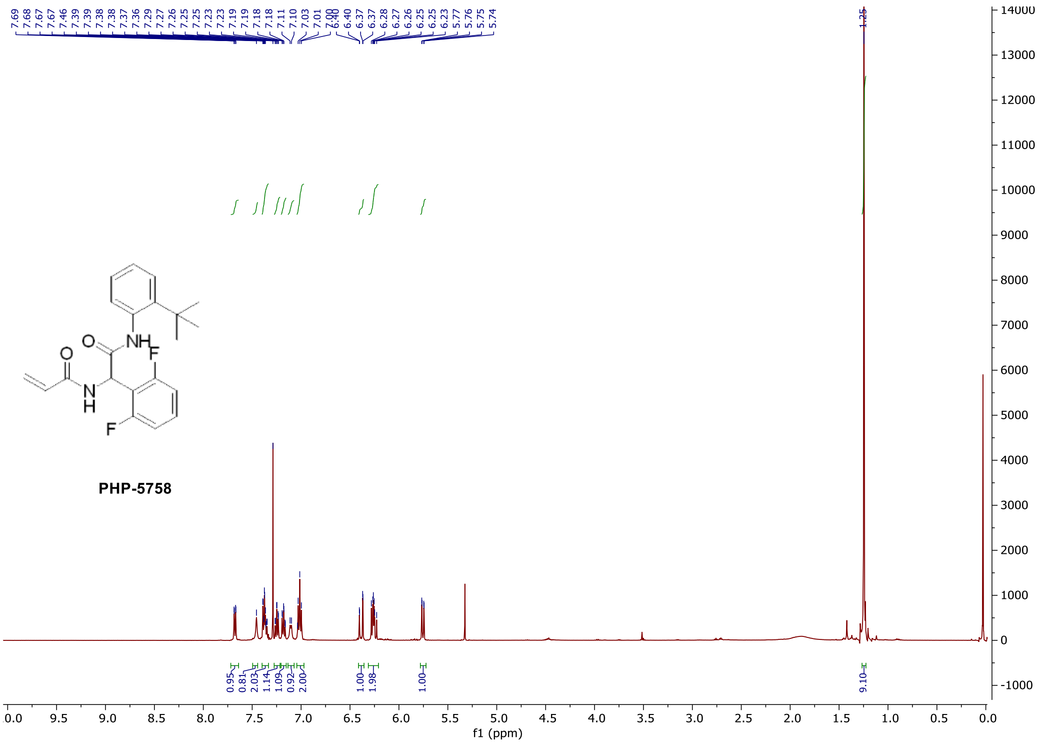

Supplementary Figure 737. ^{1}H NMR (500 MHz, $CDCl_3$) of compound **PHP-5758**.

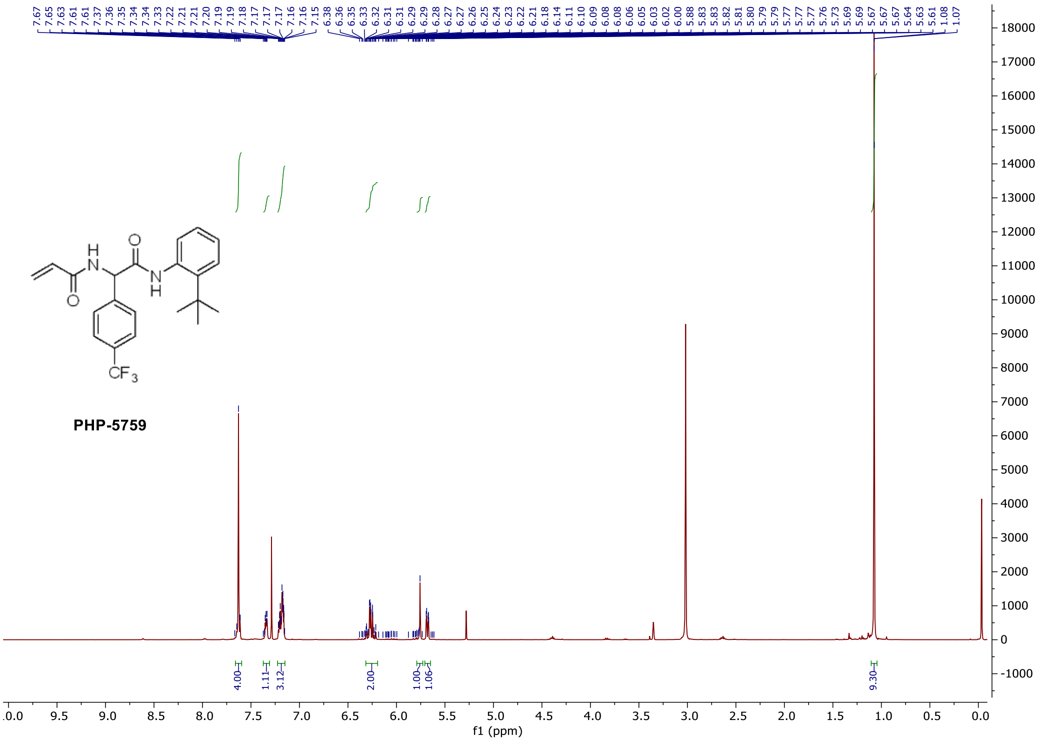

Supplementary Figure 738. ^{1}H NMR (500 MHz, $CDCl_3$ + one drop of CD_3OD) of compound **PHP-5759**.

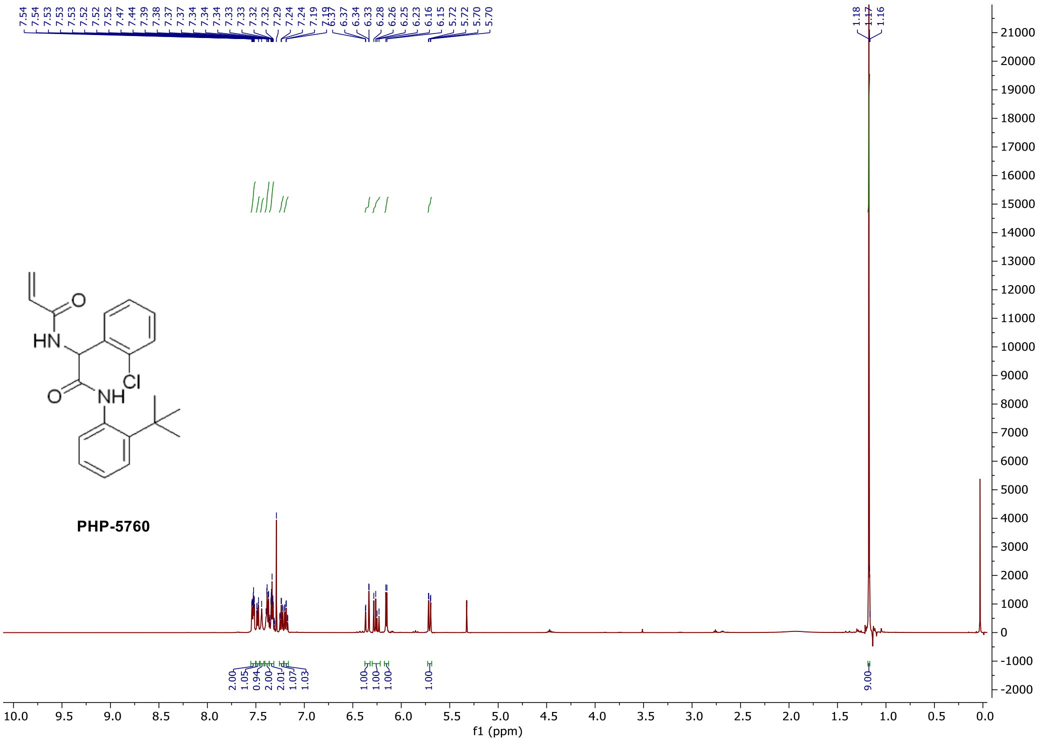

Supplementary Figure 739. ^{1}H NMR (500 MHz, $CDCl_3$) of compound **PHP-5760**.

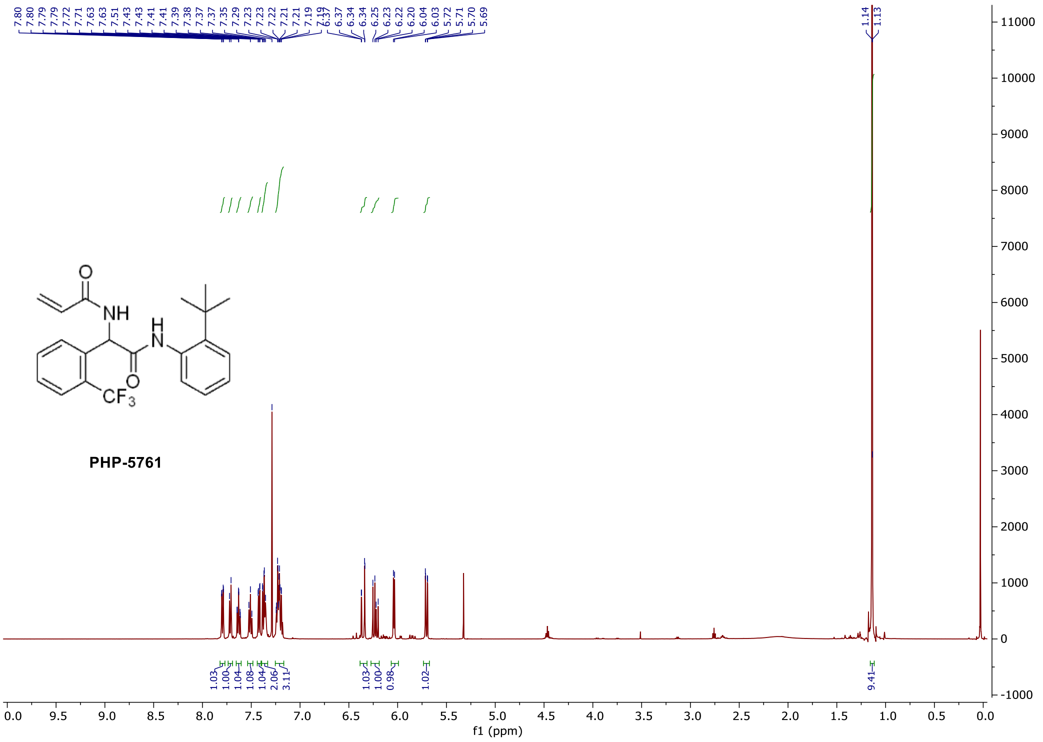

Supplementary Figure 740. ^{1}H NMR (500 MHz, $CDCl_3$) of compound **PHP-5761**.

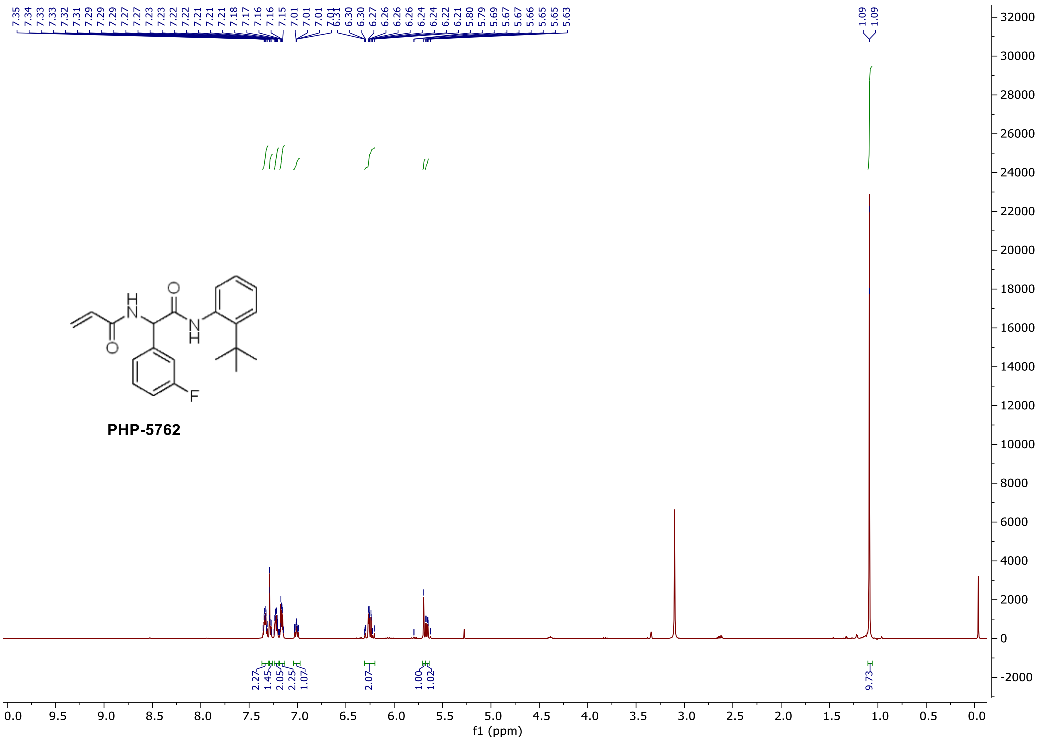

Supplementary Figure 741. ^{1}H NMR (500 MHz, $CDCl_3$ + one drop of CD_3OD) of compound **PHP-5762**.

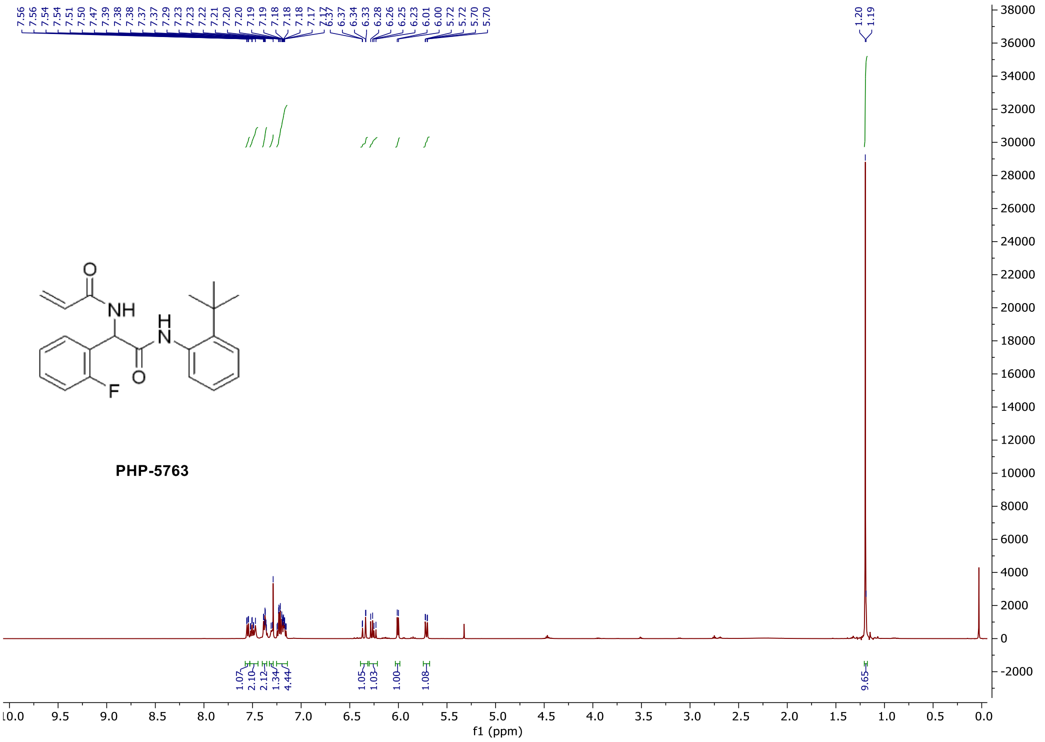

Supplementary Figure 742. ^{1}H NMR (500 MHz, $CDCl_3$) of compound **PHP-5763**.

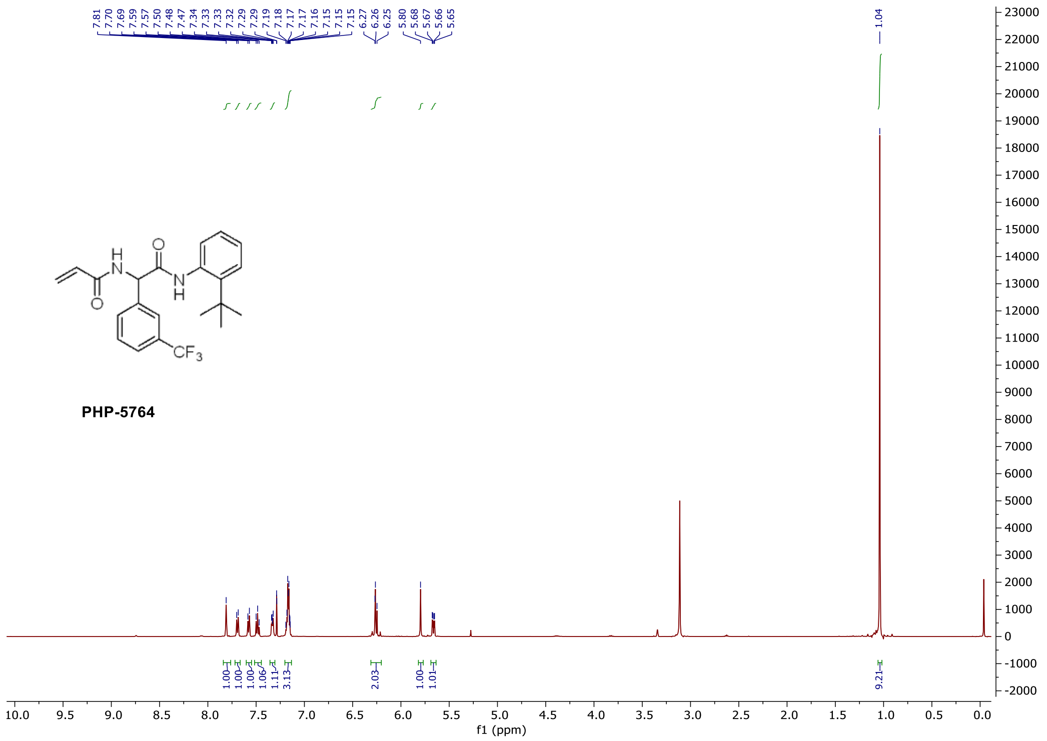

Supplementary Figure 743. ^{1}H NMR (500 MHz, $CDCl_3$ + one drop of CD_3OD) of compound **PHP-5764**.

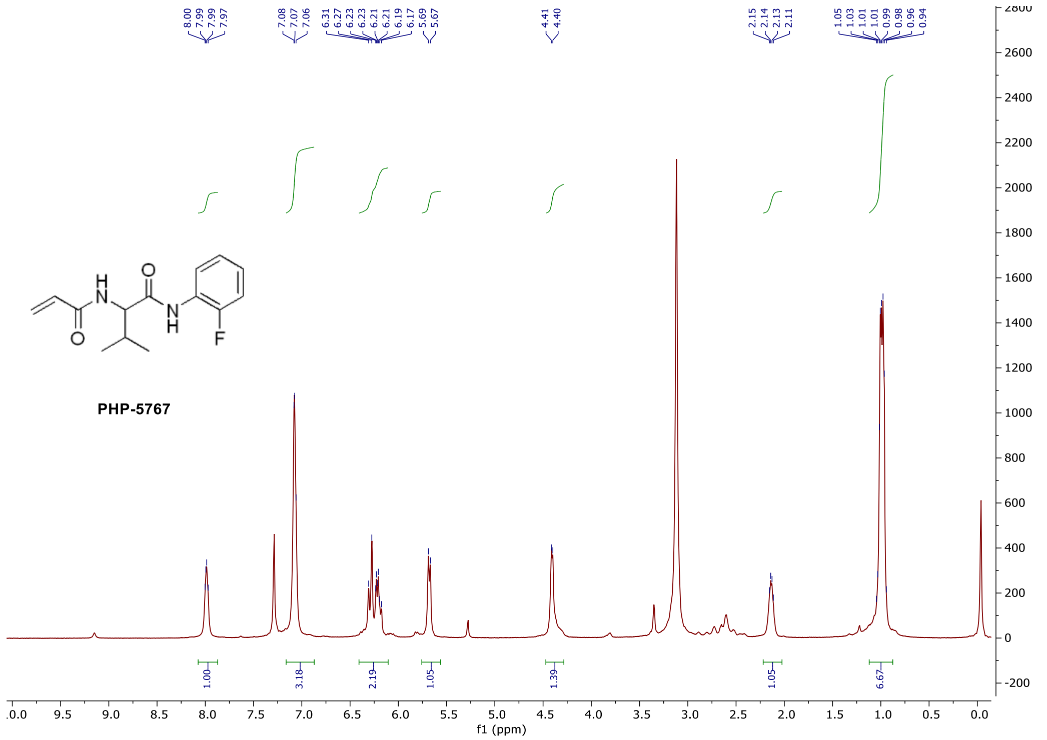

Supplementary Figure 744. ^{1}H NMR (500 MHz, $CDCl_3$ + one drop of CD_3OD) of compound **PHP-5767**.

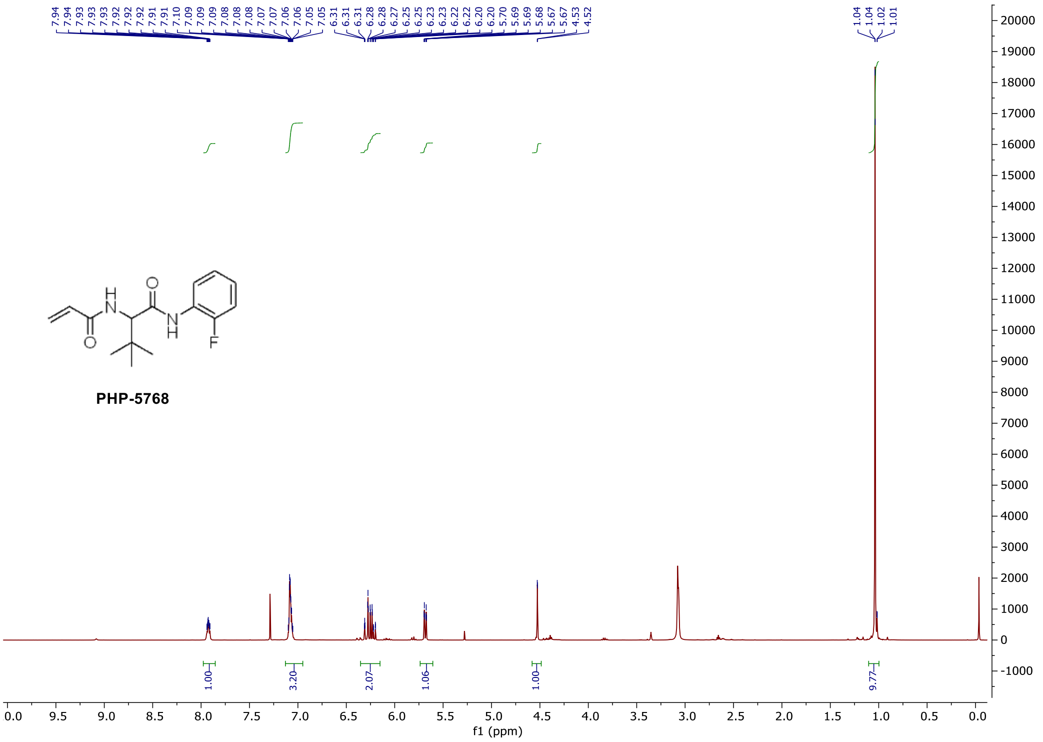

Supplementary Figure 745. ^{1}H NMR (500 MHz, $CDCl_3$ + one drop of CD_3OD) of compound **PHP-5768**.

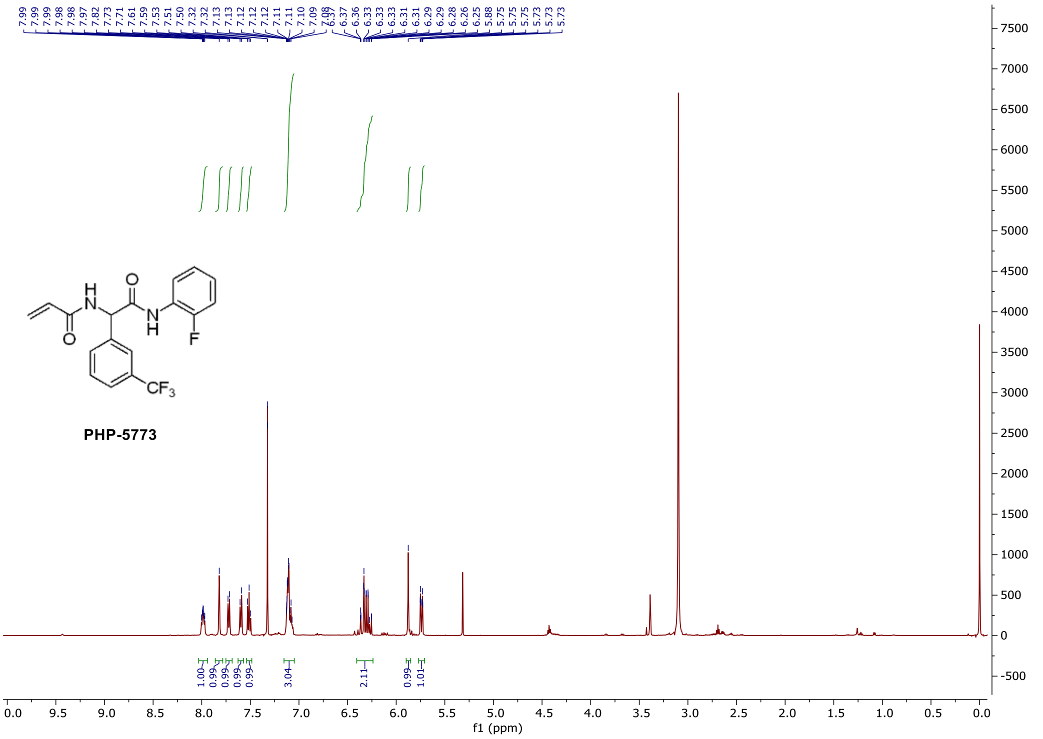

Supplementary Figure 746. ^{1}H NMR (500 MHz, $CDCl_3$ + one drop of CD_3OD) of compound **PHP-5773**.

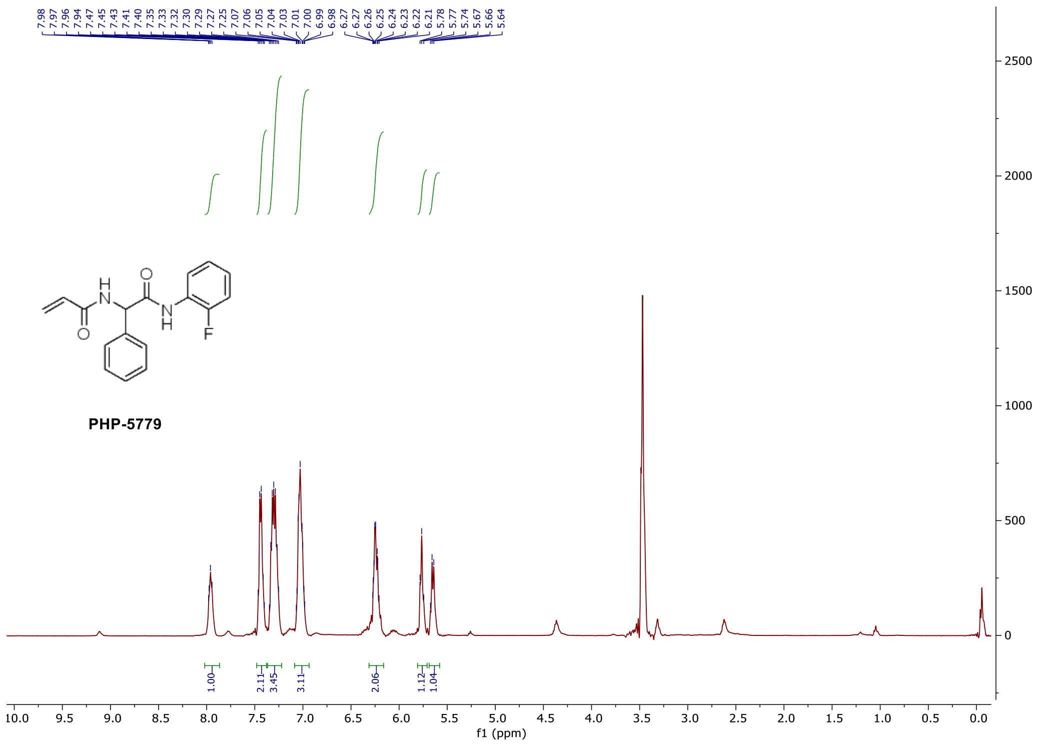

Supplementary Figure 747. ^{1}H NMR (500 MHz, $CDCl_3$ + one drop of CD_3OD) of compound **PHP-5779**.

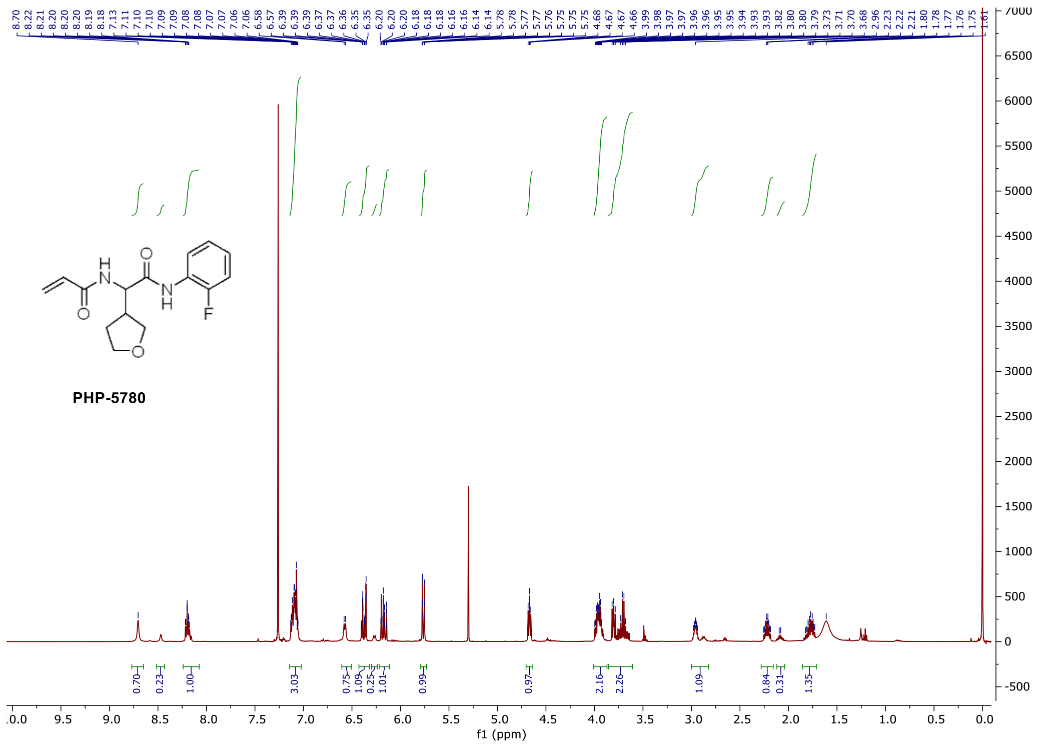

Supplementary Figure 748. ^{1}H NMR (500 MHz, $CDCl_3$) of compound **PHP-5780**.

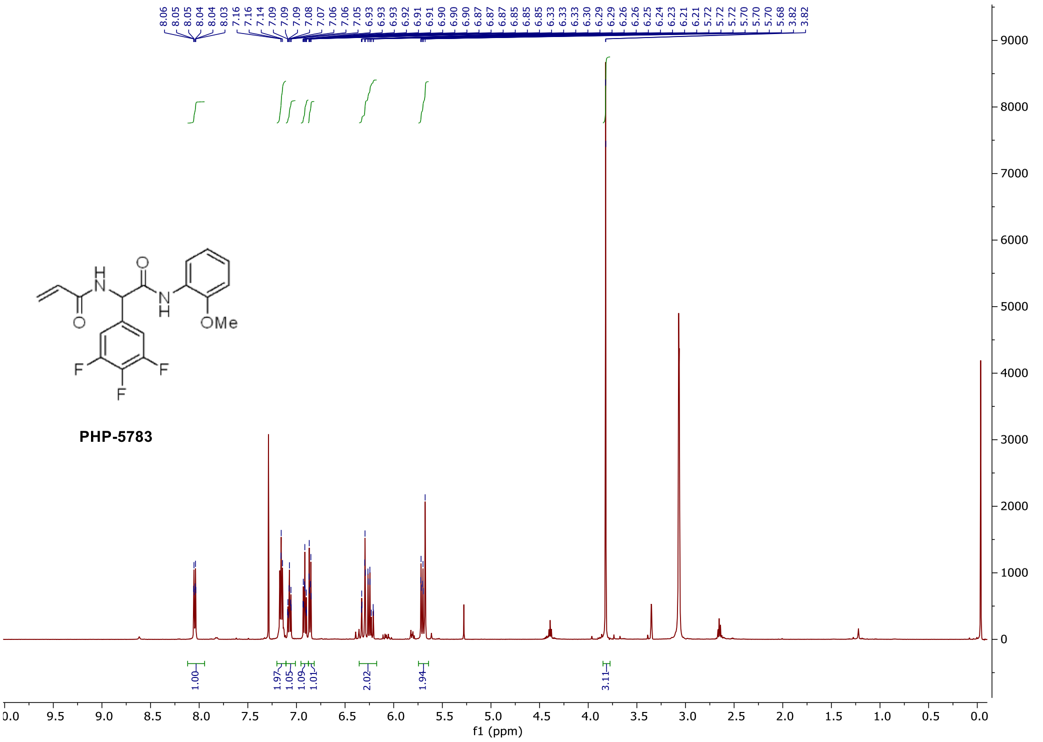

Supplementary Figure 749. ^{1}H NMR (500 MHz, $CDCl_3$ + one drop of CD_3OD) of compound **PHP-5783**.

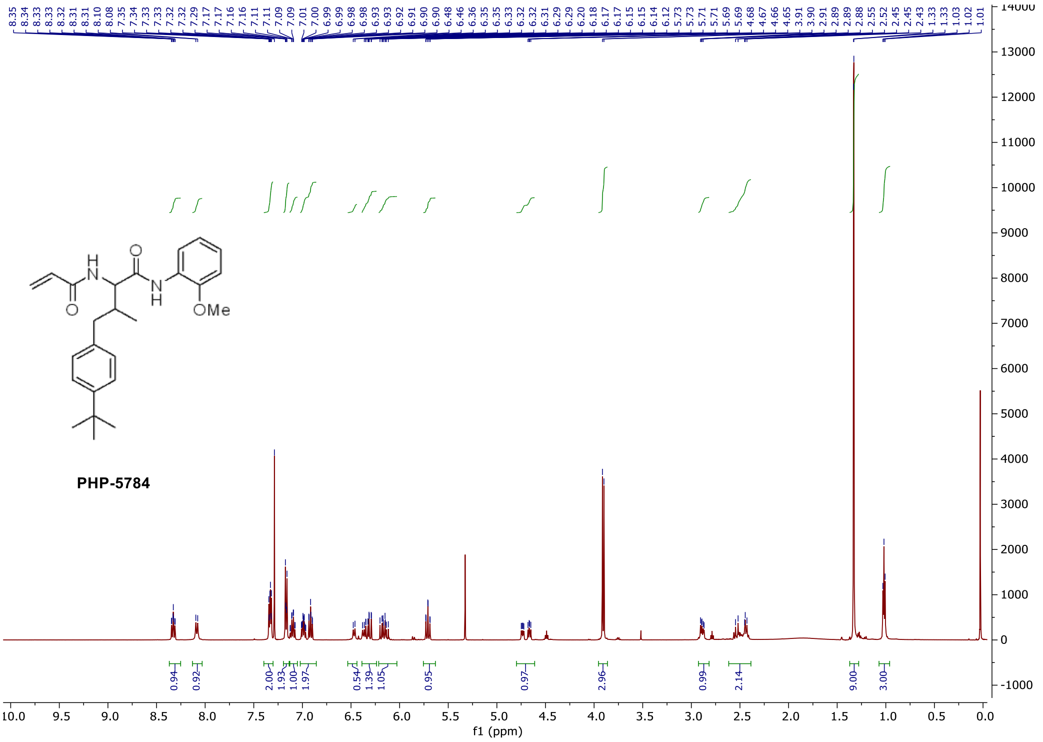

Supplementary Figure 750. ^{1}H NMR (500 MHz, $CDCl_3$) of compound **PHP-5784**.

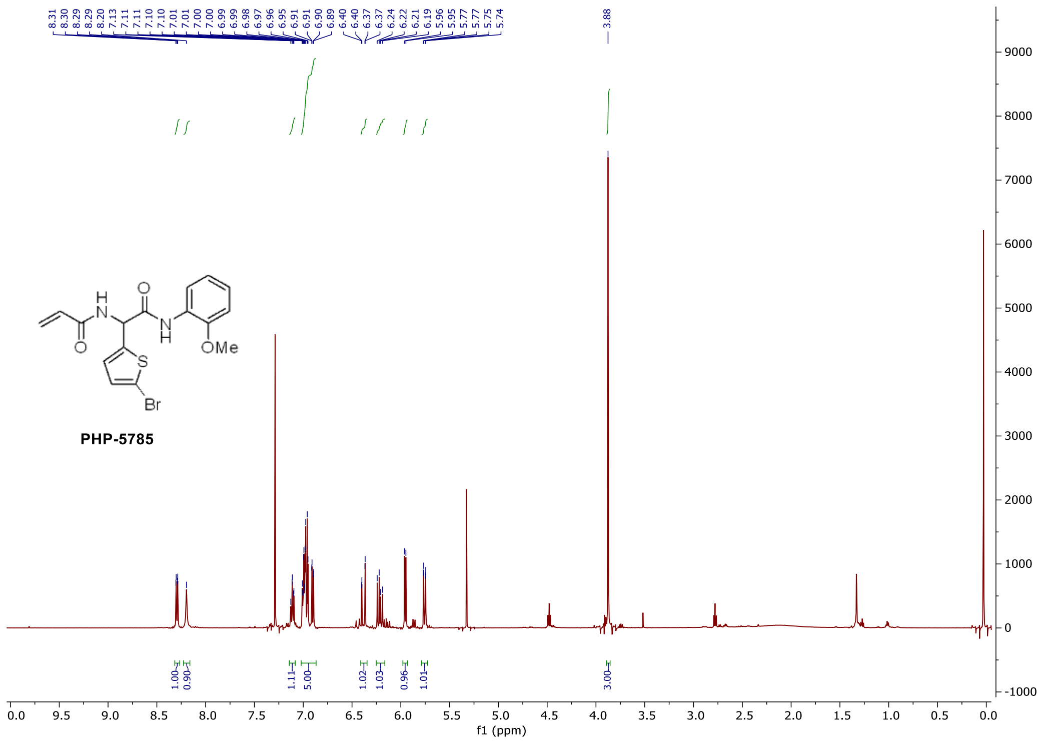

Supplementary Figure 751. ^{1}H NMR (500 MHz, $CDCl_3$) of compound **PHP-5785**.

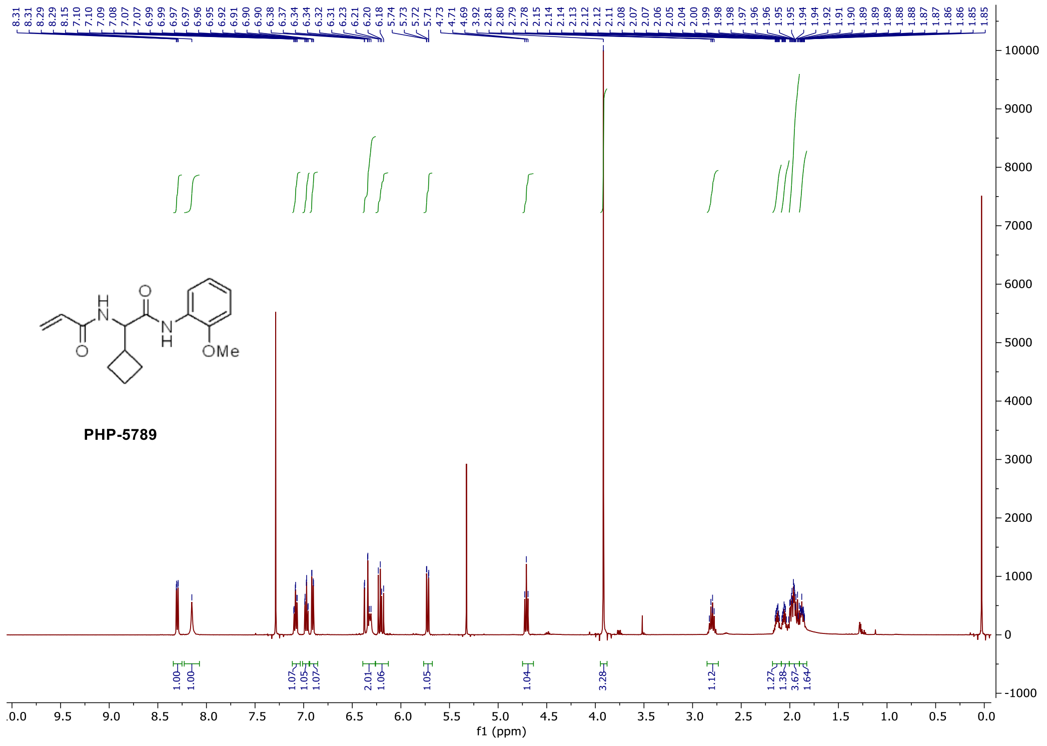

Supplementary Figure 752. ^{1}H NMR (500 MHz, $CDCl_3$) of compound **PHP-5789**.

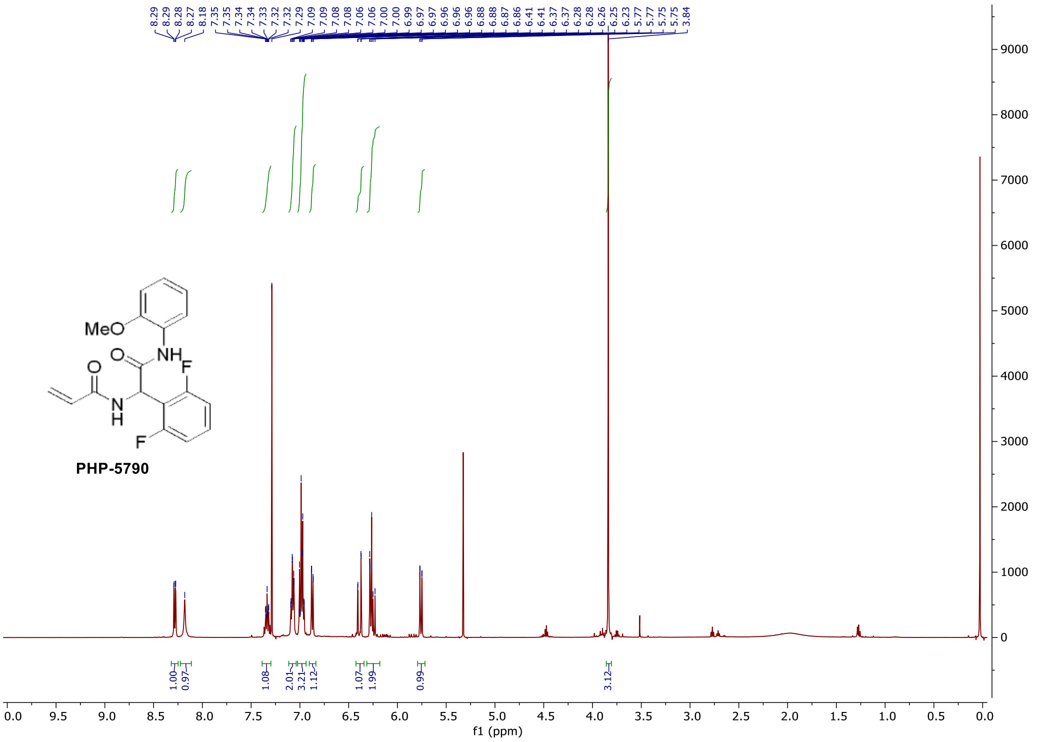

Supplementary Figure 753. ¹H NMR (500 MHz, $CDCl_3$) of compound **PHP-5790**.

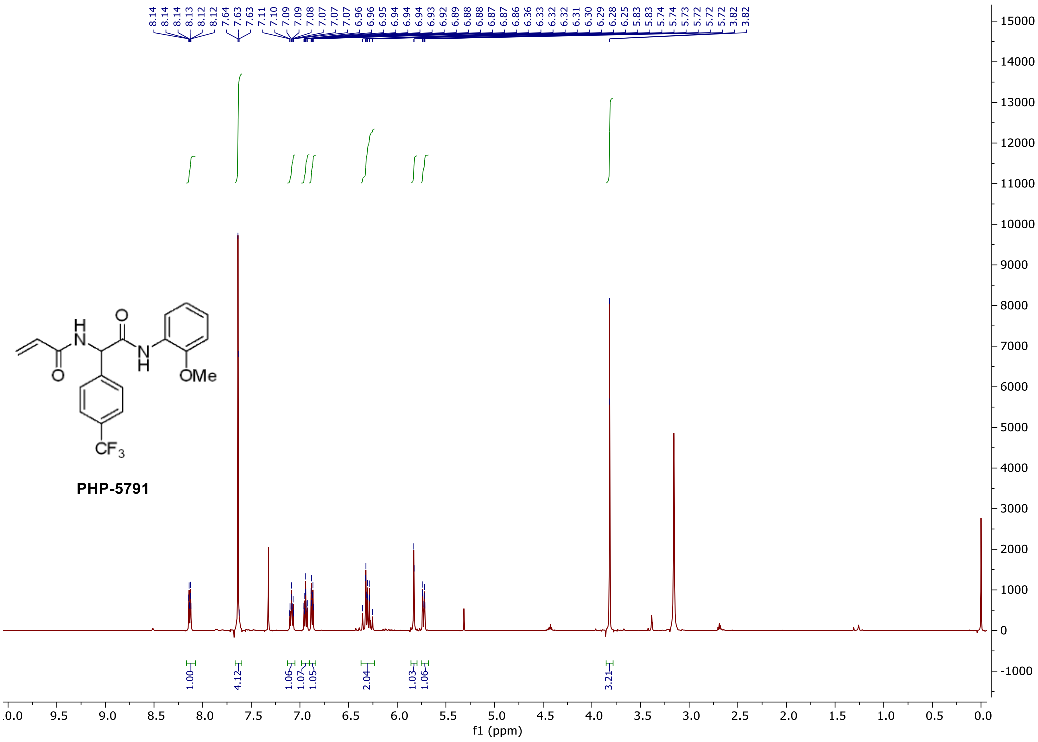

Supplementary Figure 754. ¹H NMR (500 MHz, $CDCl_3$ + one drop of CD_3OD) of compound **PHP-5791**.

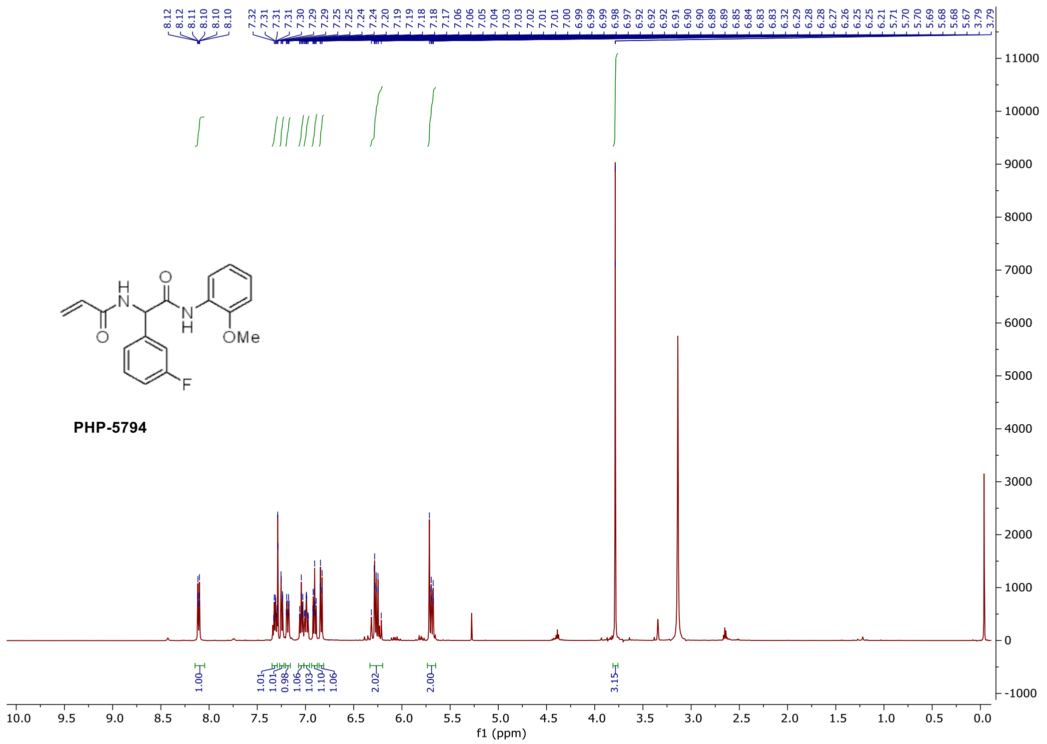

Supplementary Figure 755. ^{1}H NMR (500 MHz, $CDCl_3$ + one drop of CD_3OD) of compound **PHP-5794**.

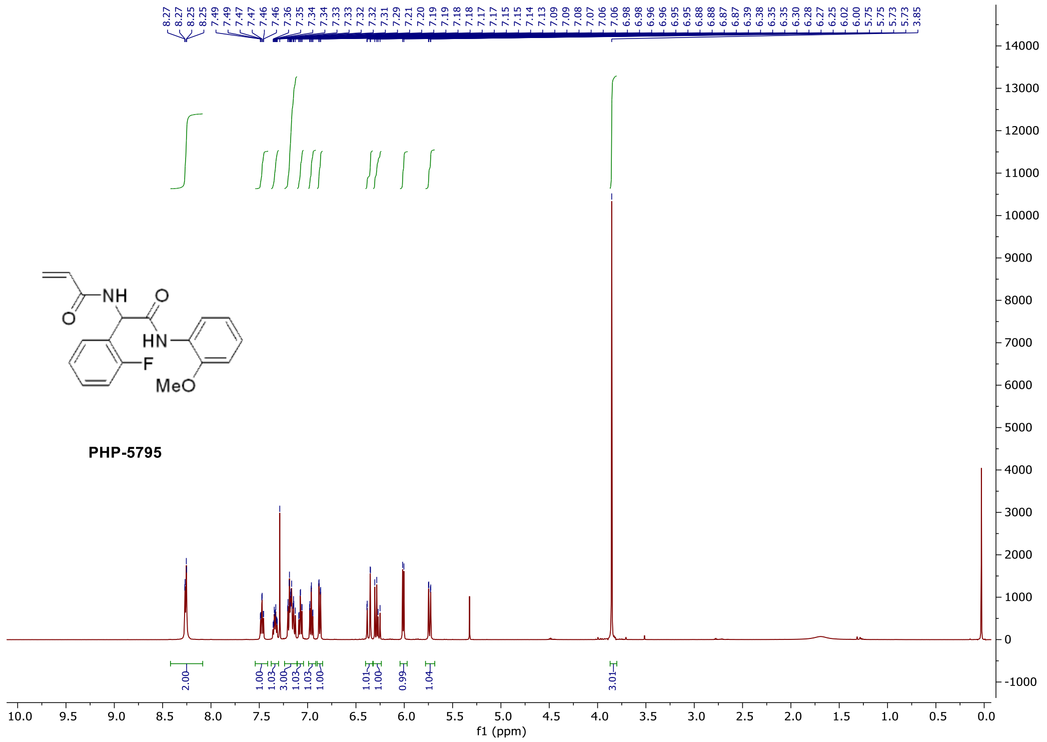

Supplementary Figure 756. ^{1}H NMR (500 MHz, $CDCl_3$) of compound **PHP-5795**.

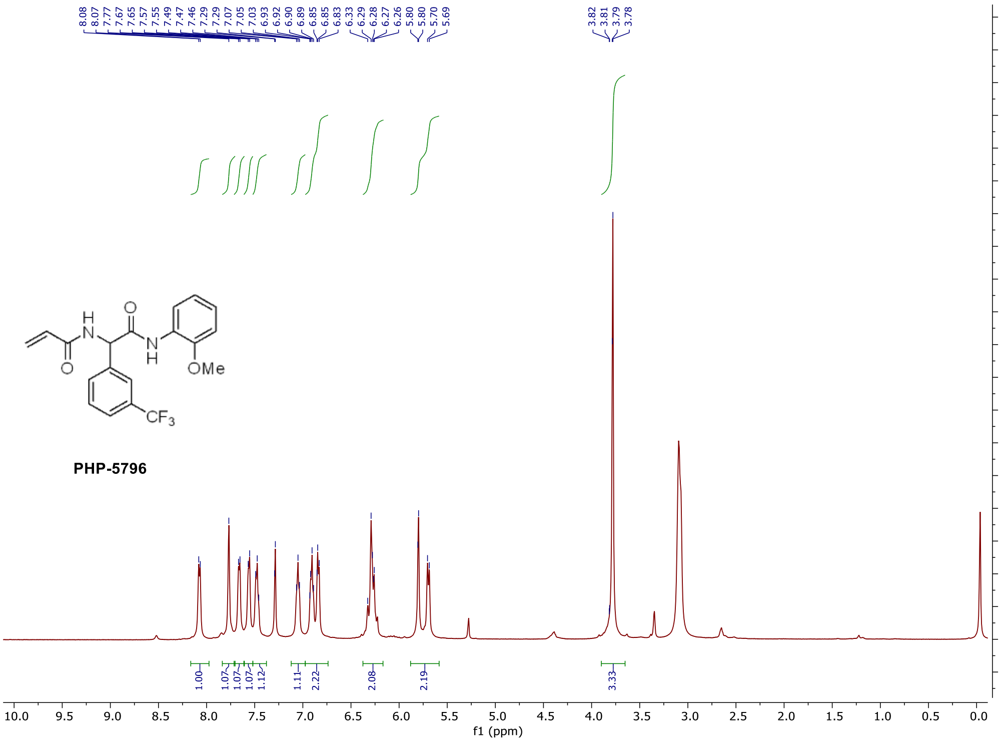

Supplementary Figure 757. ^1H NMR (500 MHz, $CDCl_3$ + one drop of CD_3OD) of compound **PHP-5796**.

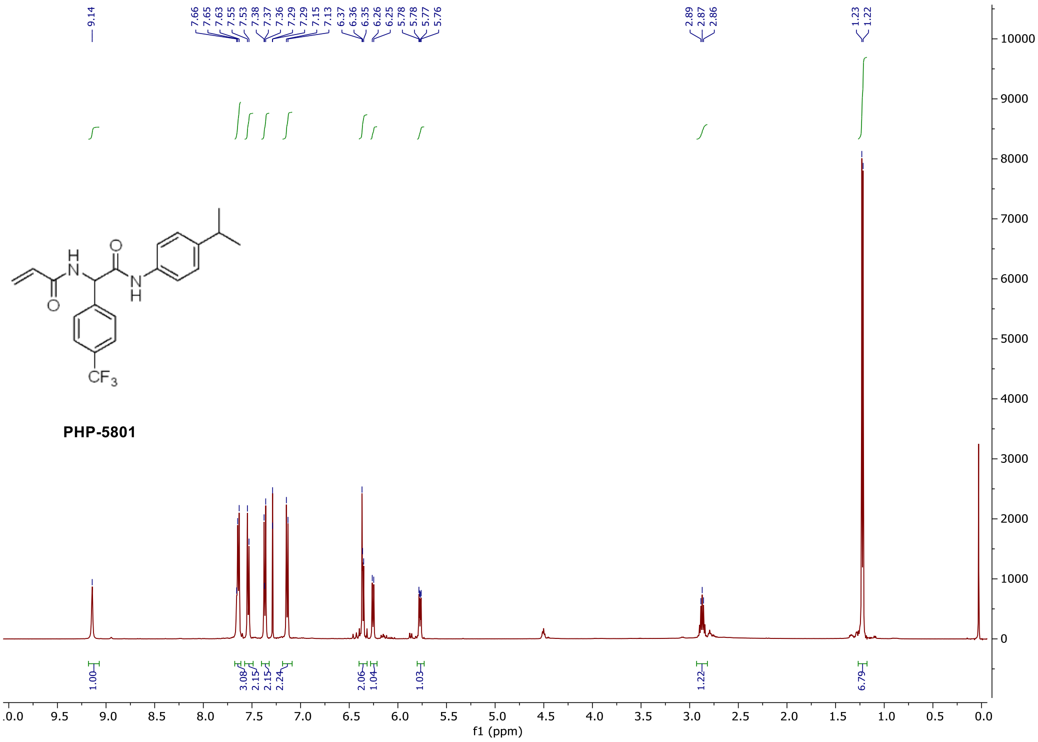

Supplementary Figure 758. ^1H NMR (500 MHz, $CDCl_3$) of compound **PHP-5801**.

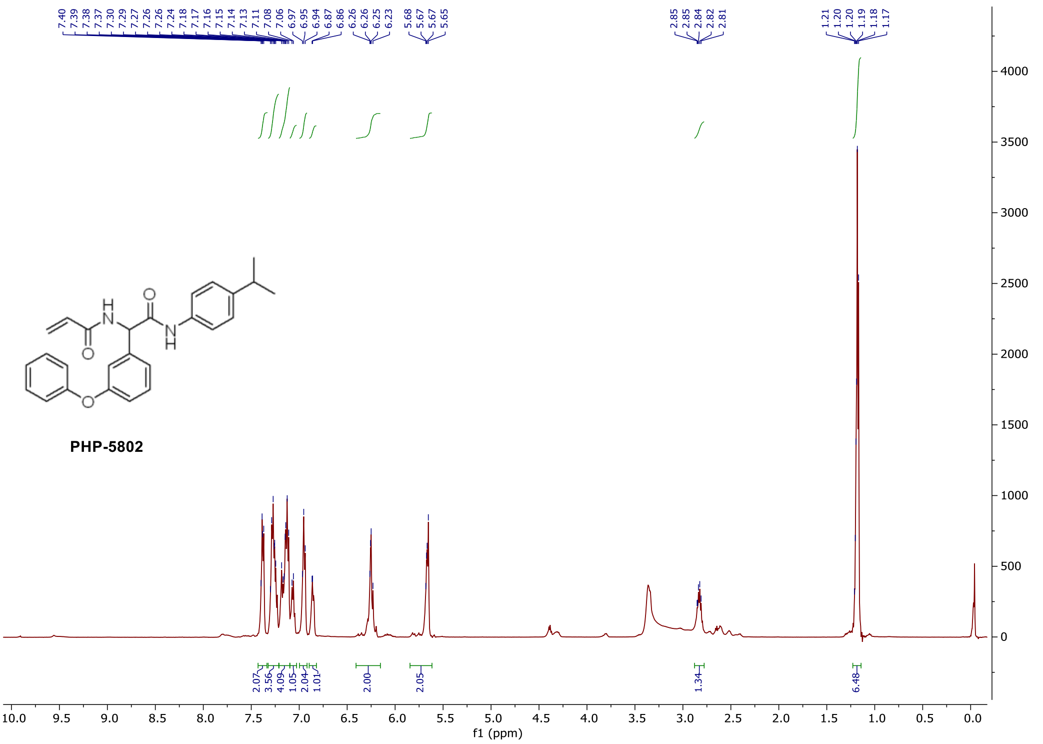

Supplementary Figure 759. ^{1}H NMR (500 MHz, $CDCl_3$ + one drop of CD_3OD) of compound **PHP-5802**.

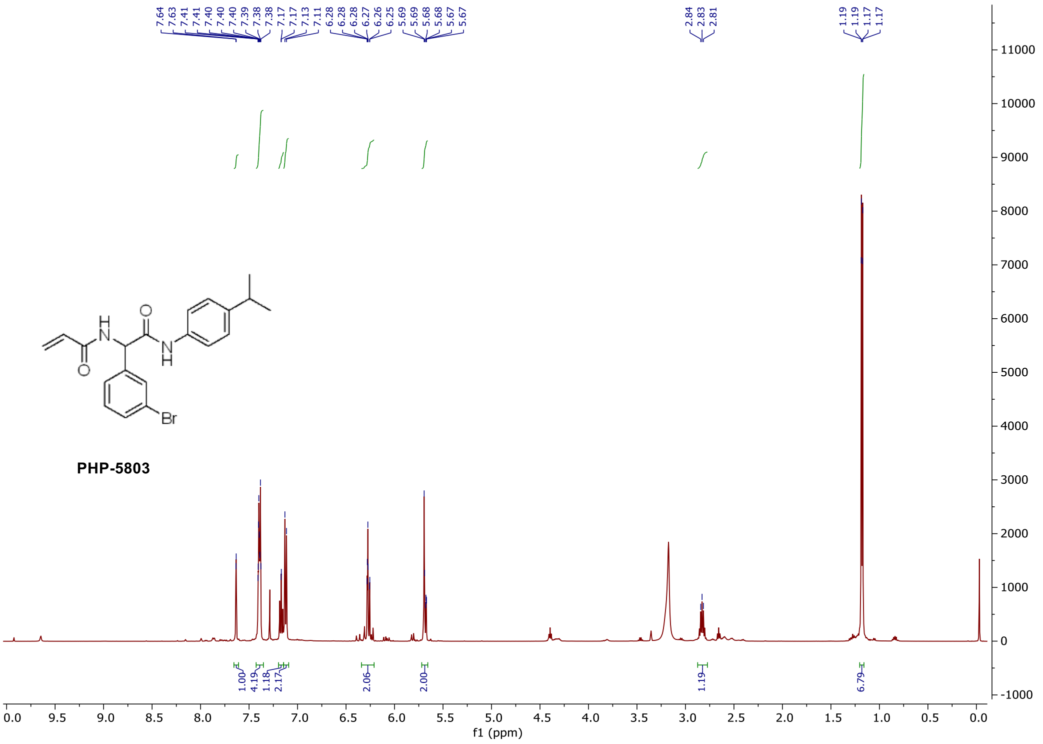

Supplementary Figure 760. ^{1}H NMR (500 MHz, $CDCl_3$ + one drop of CD_3OD) of compound **PHP-5803**.

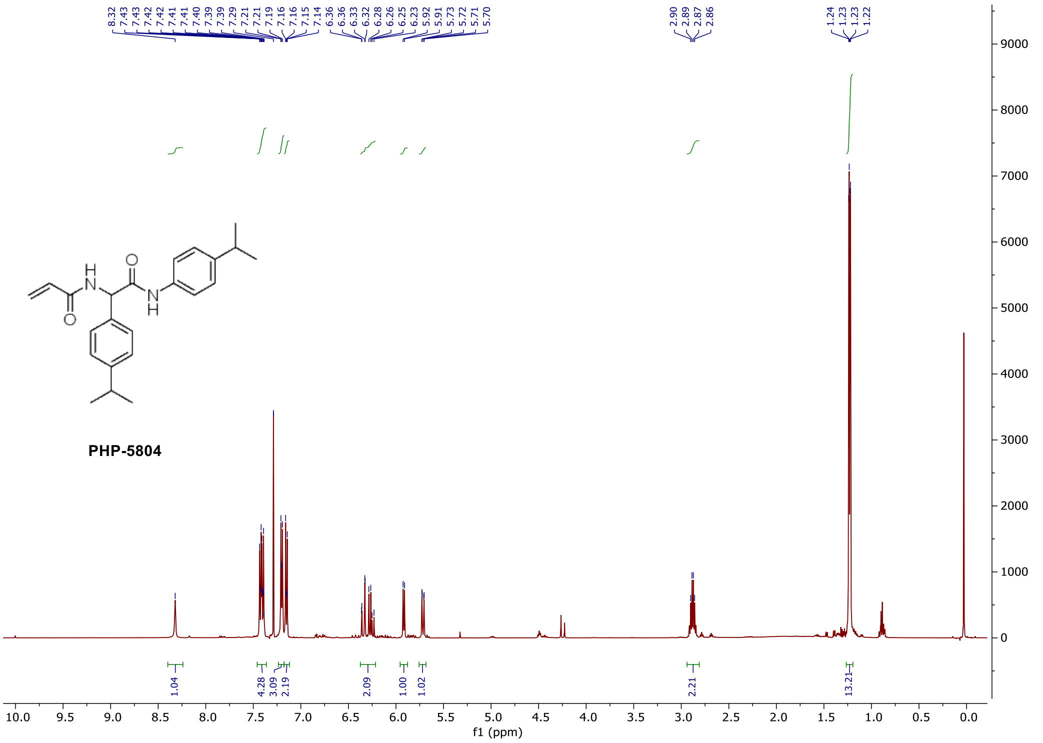

Supplementary Figure 761. ^{1}H NMR (500 MHz, $CDCl_3$) of compound **PHP-5804**.

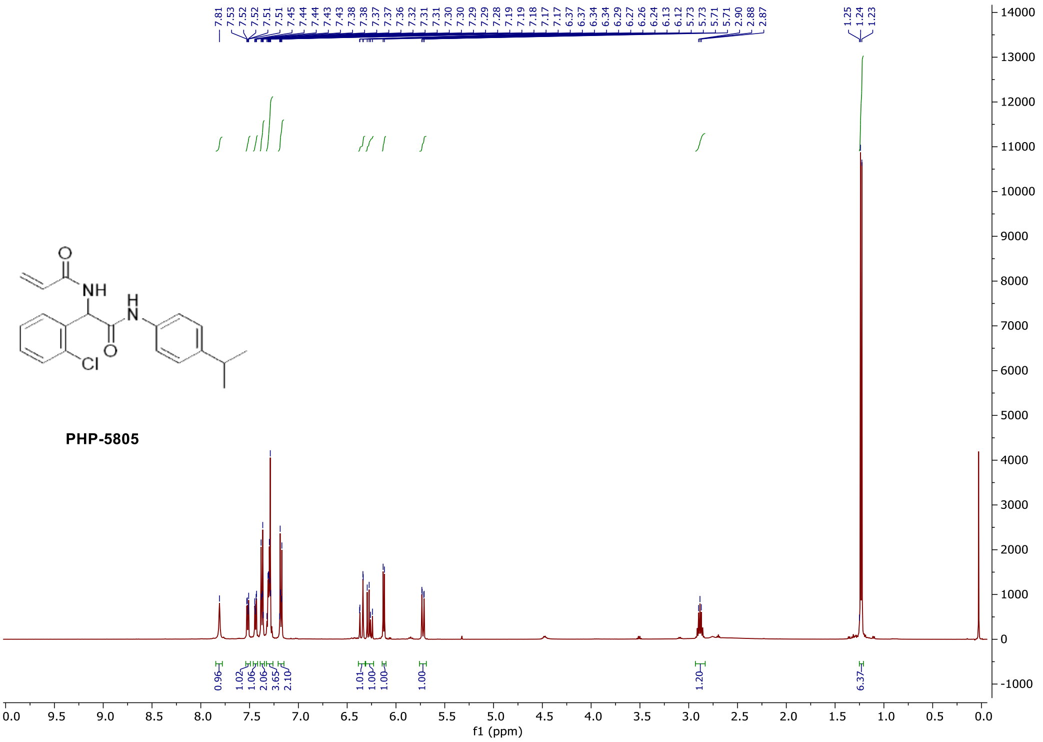

Supplementary Figure 762. ^{1}H NMR (500 MHz, $CDCl_3$) of compound **PHP-5805**.

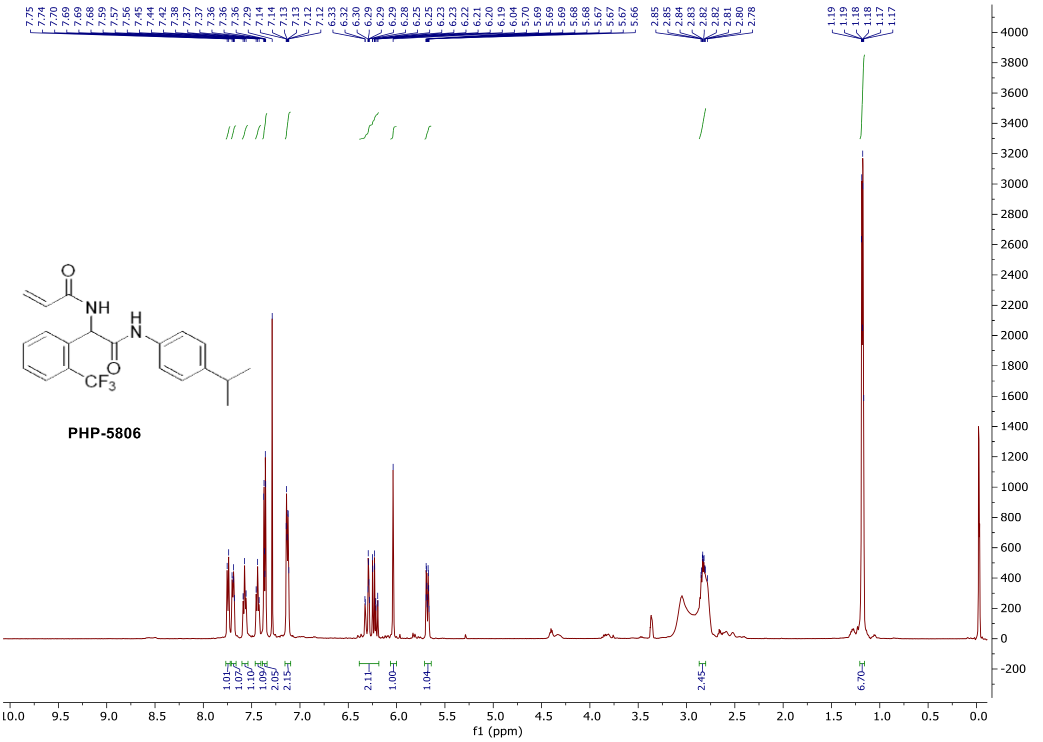

Supplementary Figure 763. ^{1}H NMR (500 MHz, $CDCl_3$ + one drop of CD_3OD) of compound **PHP-5806**.

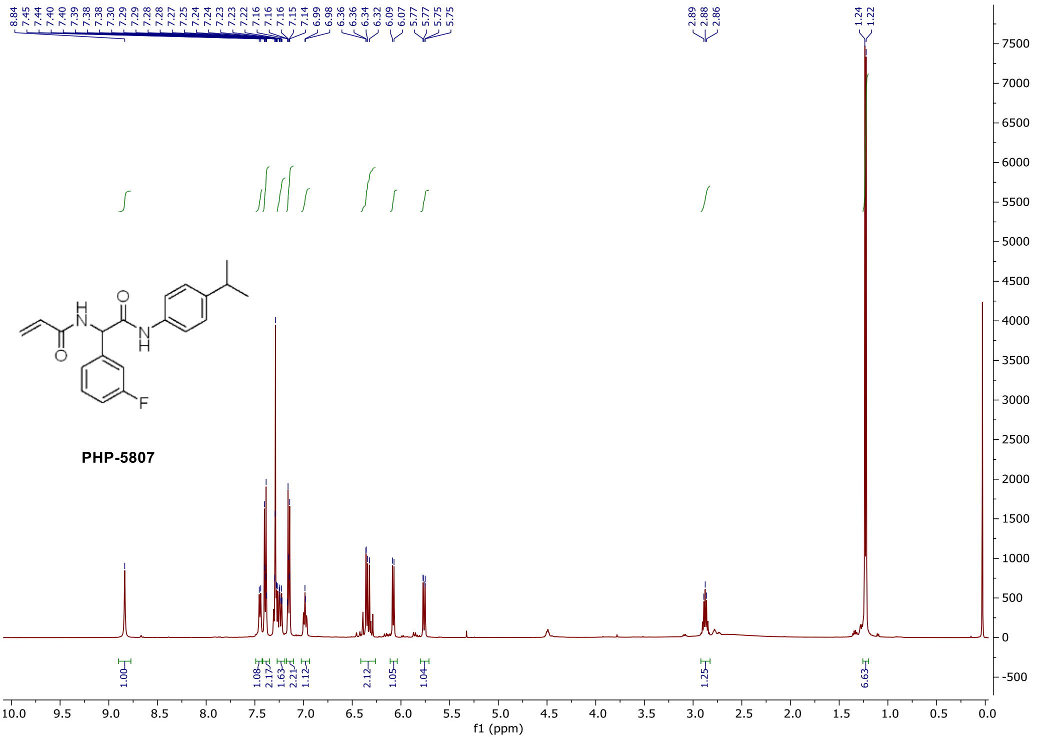

Supplementary Figure 764. ^{1}H NMR (500 MHz, $CDCl_3$) of compound **PHP-5807**.

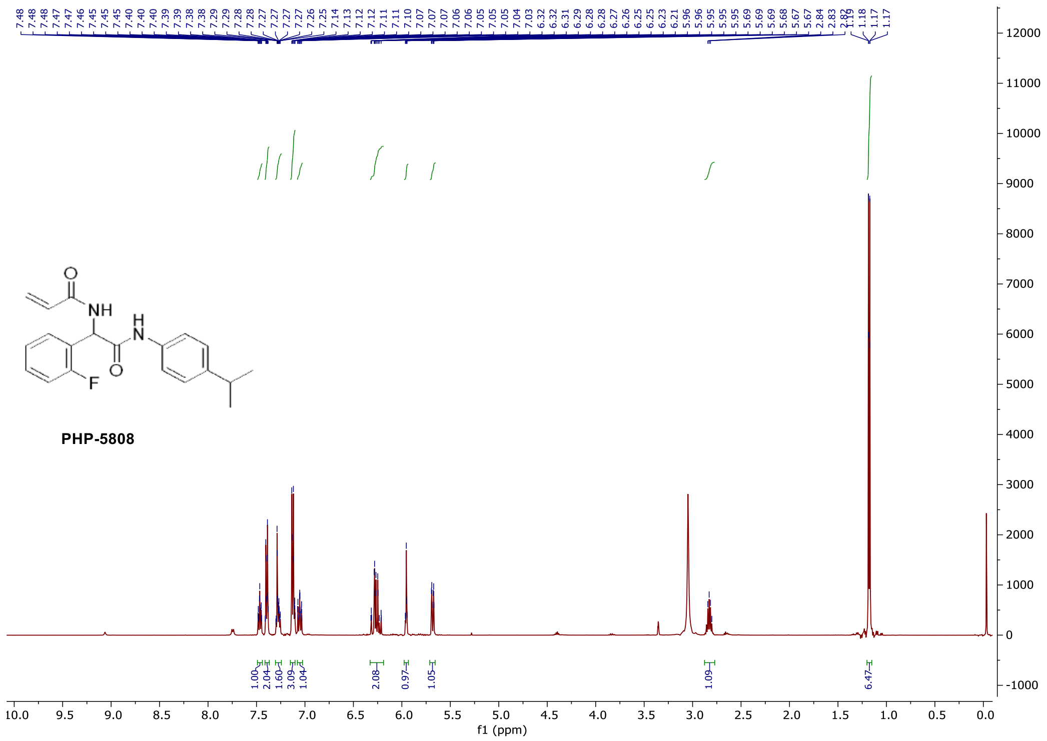

Supplementary Figure 765. ^{1}H NMR (500 MHz, $CDCl_3$ + one drop of CD_3OD) of compound **PHP-5808**.

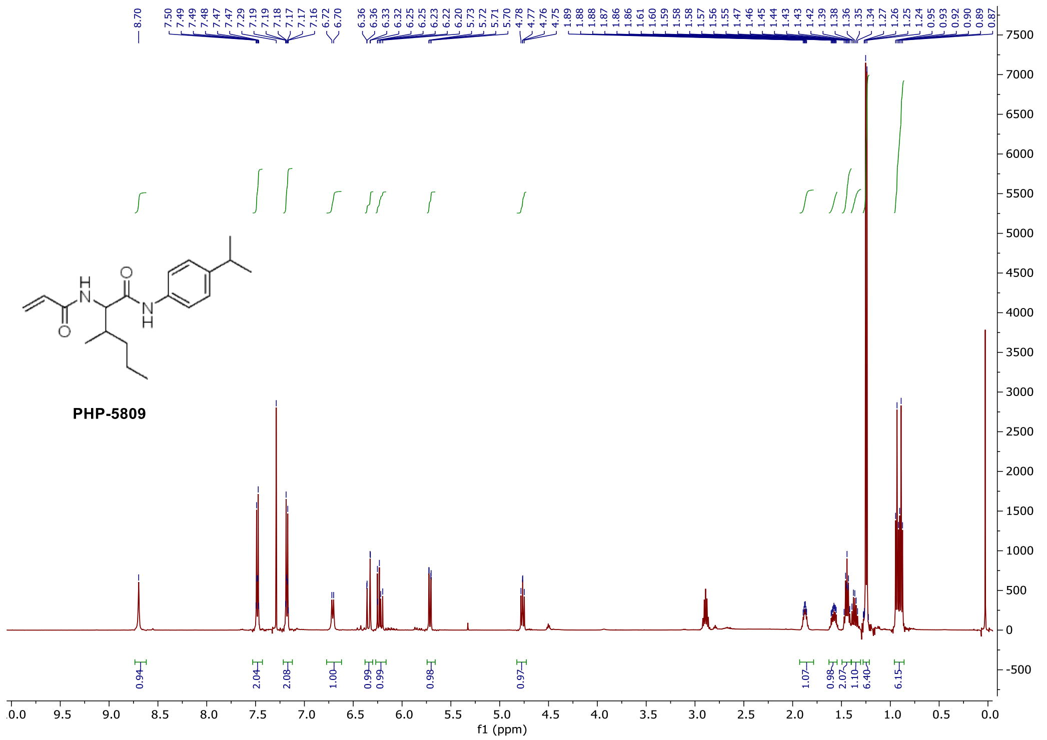

Supplementary Figure 766. ^{1}H NMR (500 MHz, $CDCl_3$) of compound **PHP-5809**.

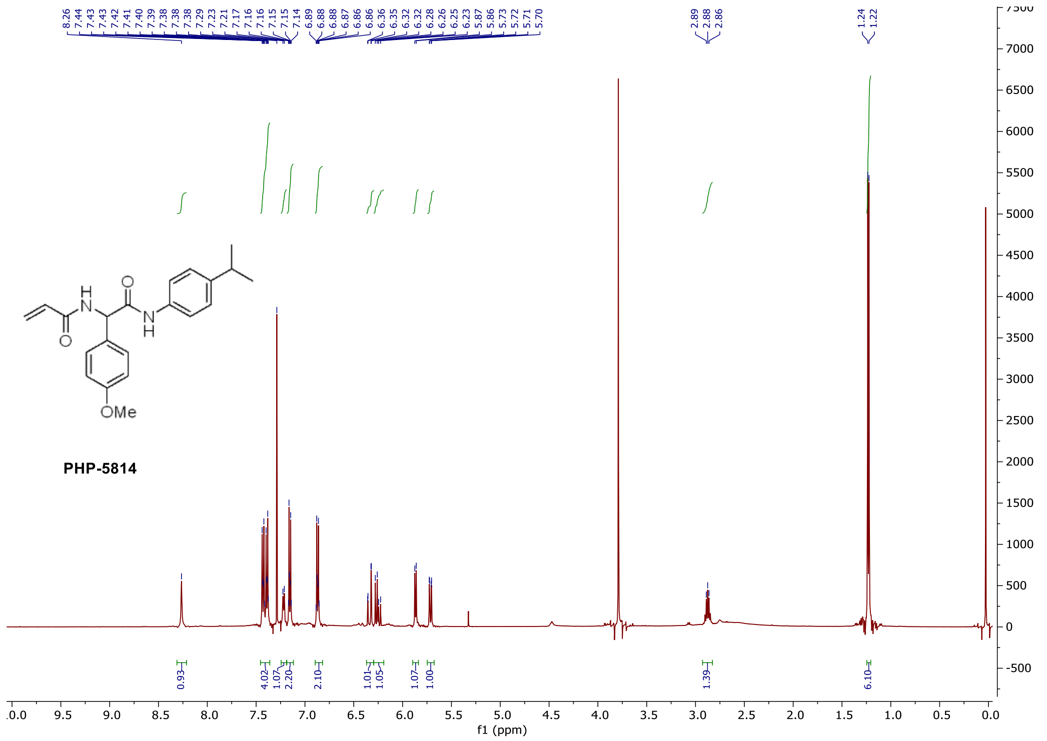

Supplementary Figure 767. ^1H NMR (500 MHz, $CDCl_3$) of compound **PHP-5814**.

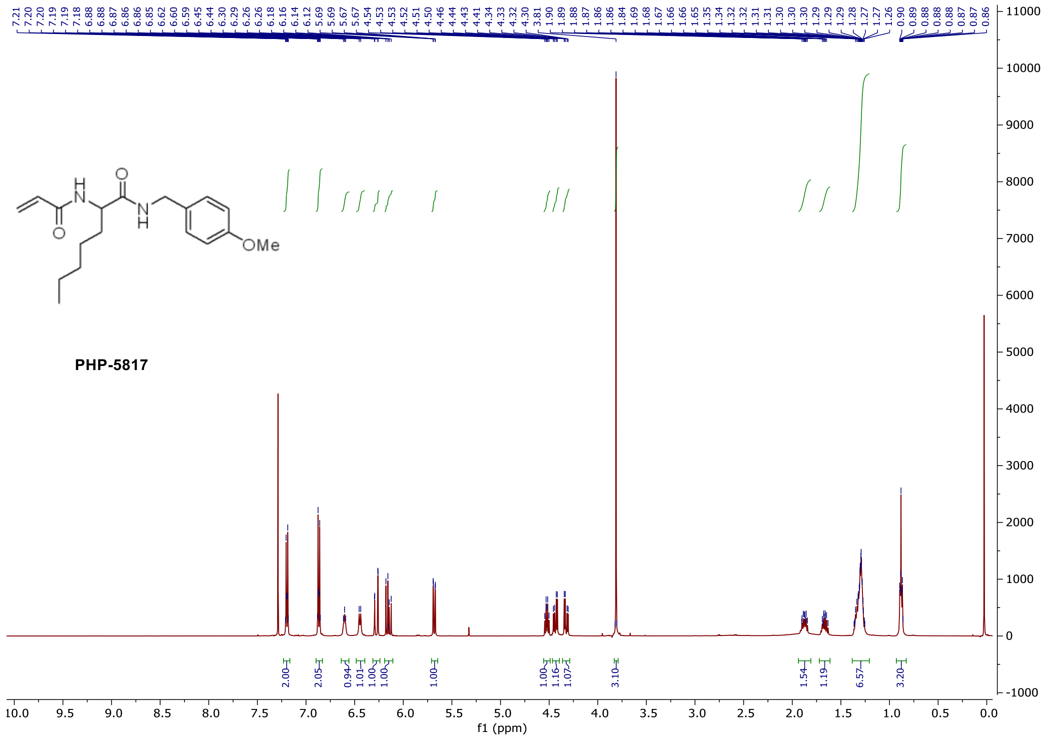

Supplementary Figure 768. ^1H NMR (500 MHz, $CDCl_3$) of compound **PHP-5817**.

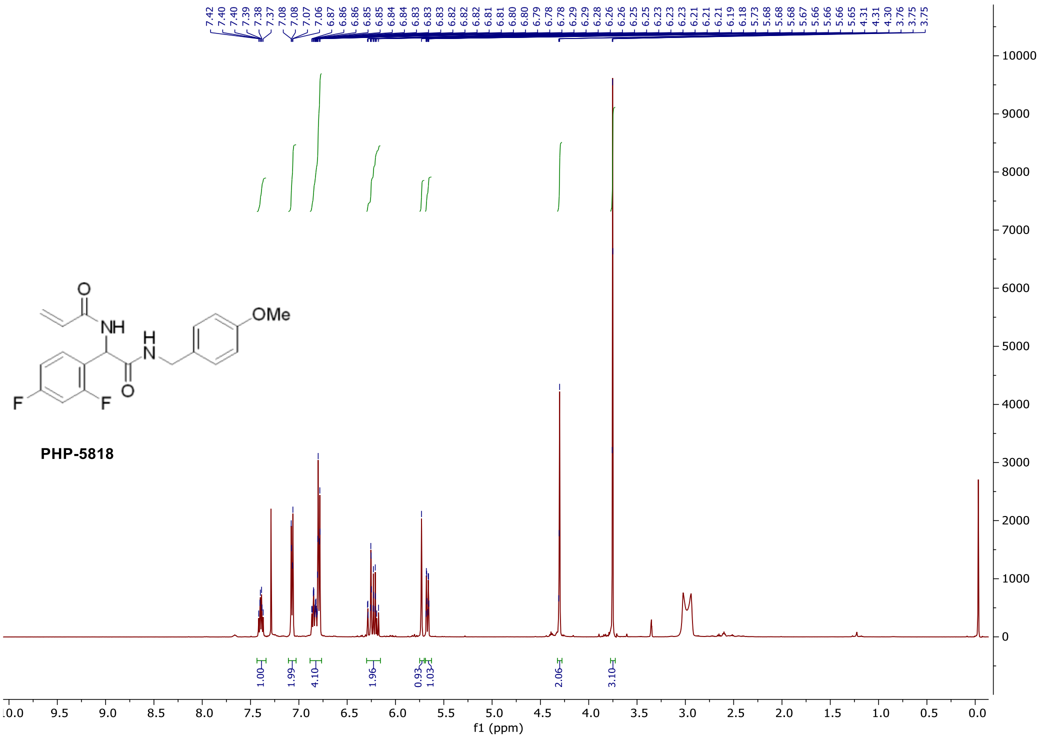

Supplementary Figure 769. ^{1}H NMR (500 MHz, $CDCl_3$ + one drop of CD_3OD) of compound **PHP-5818**.

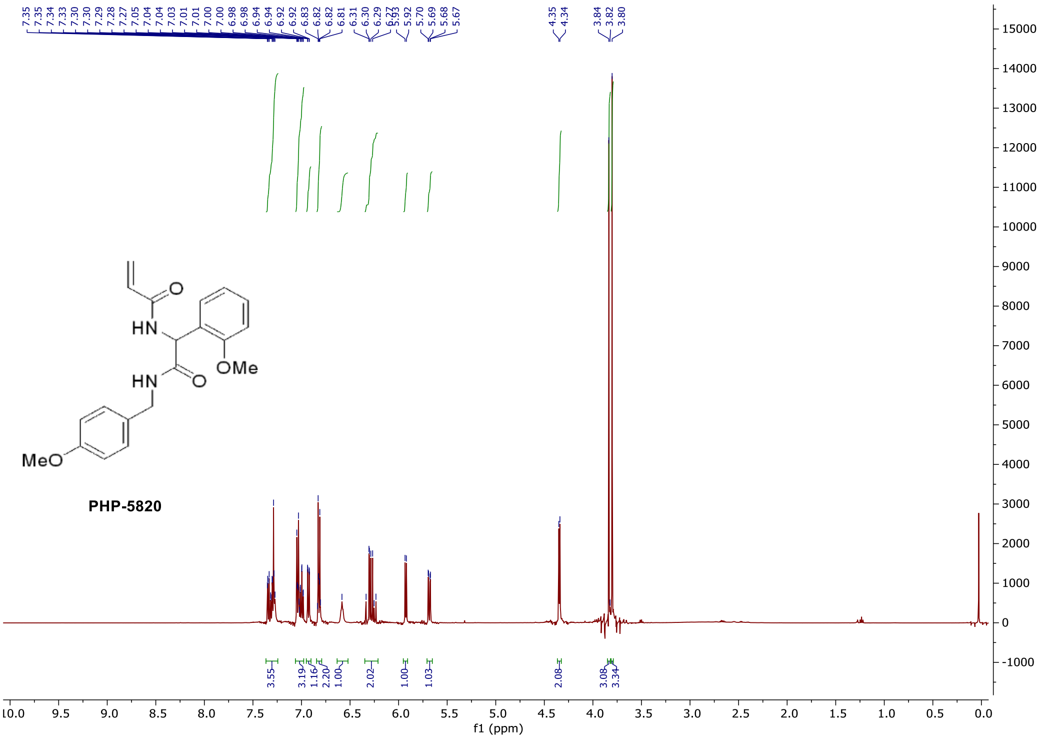

Supplementary Figure 770. ^{1}H NMR (500 MHz, $CDCl_3$) of compound **PHP-5820**.

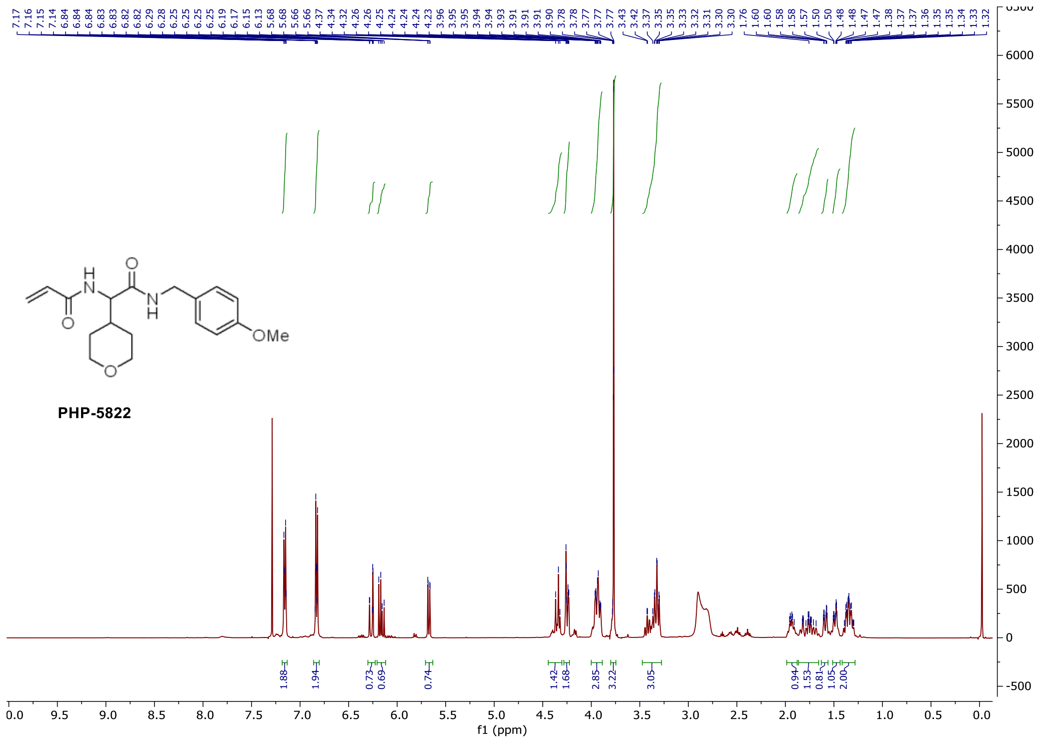

Supplementary Figure 771. ^{1}H NMR (500 MHz, $CDCl_3$ + one drop of CD_3OD) of compound **PHP-5822**.

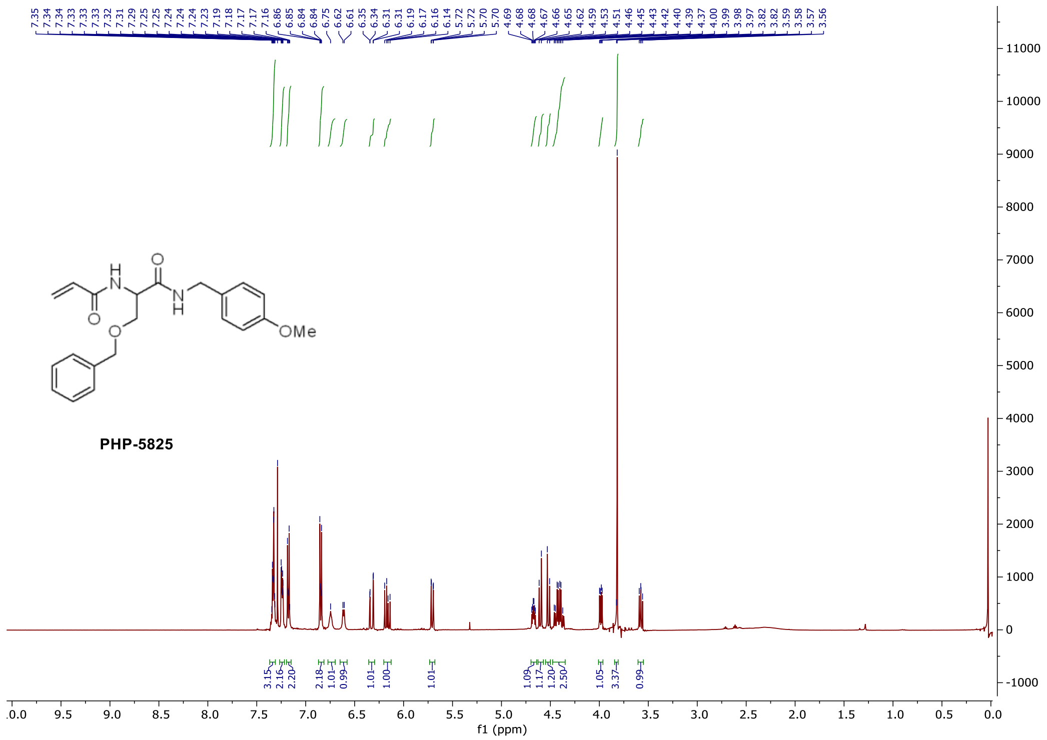

Supplementary Figure 772. ^{1}H NMR (500 MHz, $CDCl_3$) of compound **PHP-5825**.

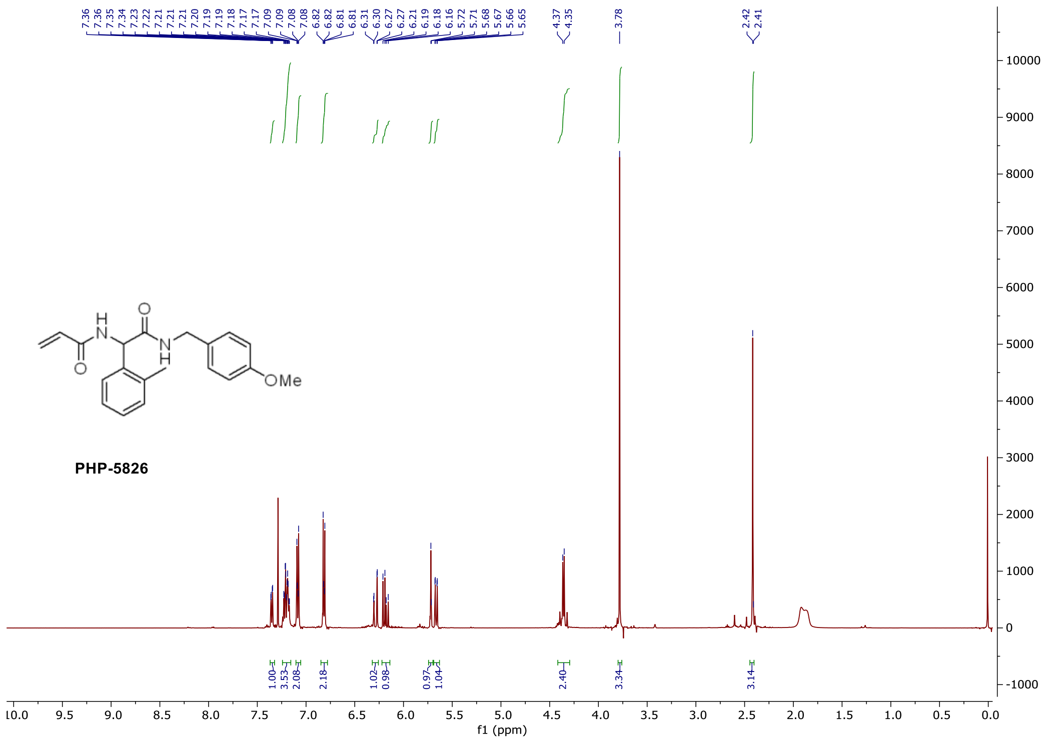

Supplementary Figure 773. ^{1}H NMR (500 MHz, $CDCl_3$) of compound **PHP-5826**.

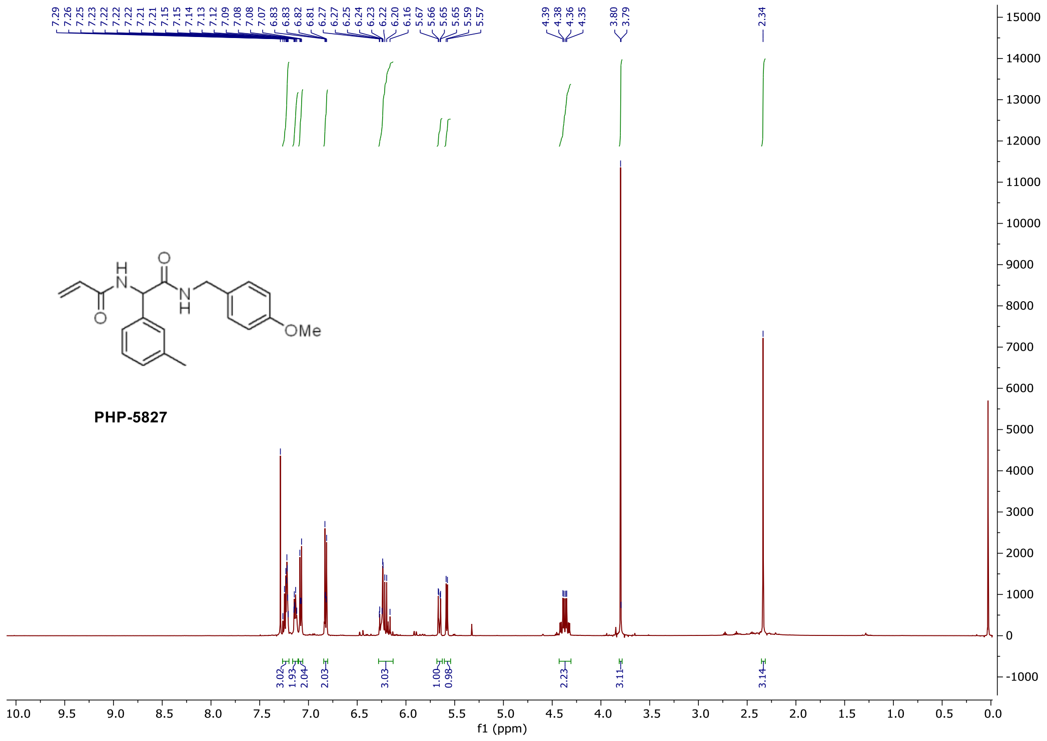

Supplementary Figure 774. ^{1}H NMR (500 MHz, $CDCl_3$) of compound **PHP-5827**.

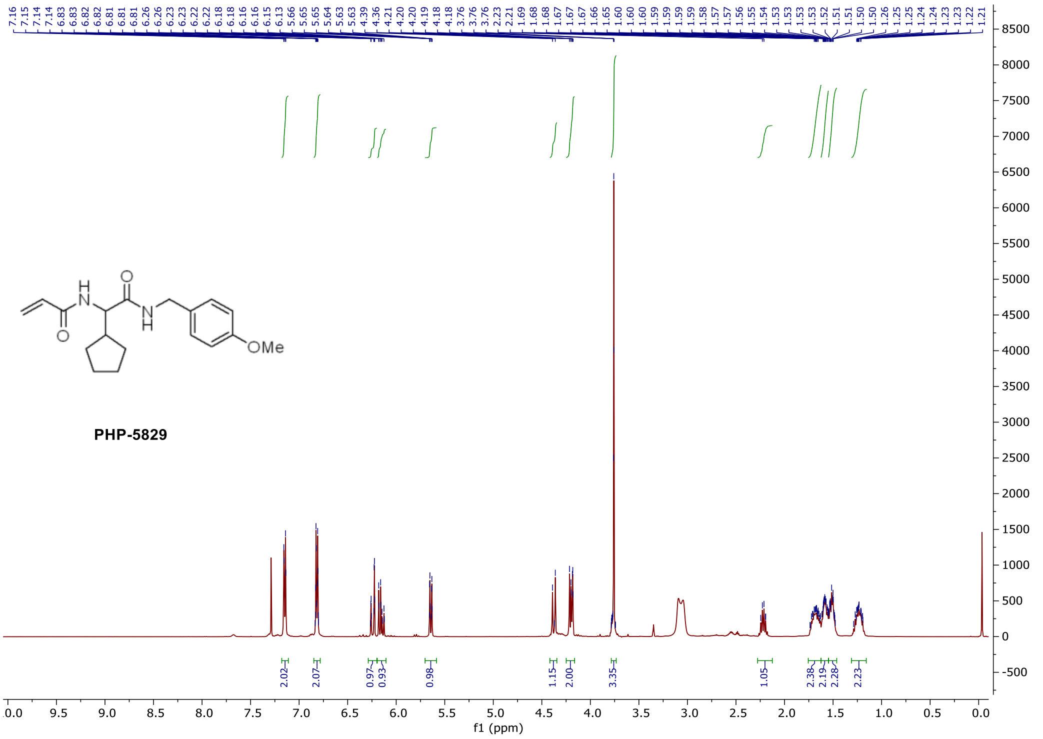

Supplementary Figure 775. ^{1}H NMR (500 MHz, $CDCl_3$ + one drop of CD_3OD) of compound **PHP-5829**.

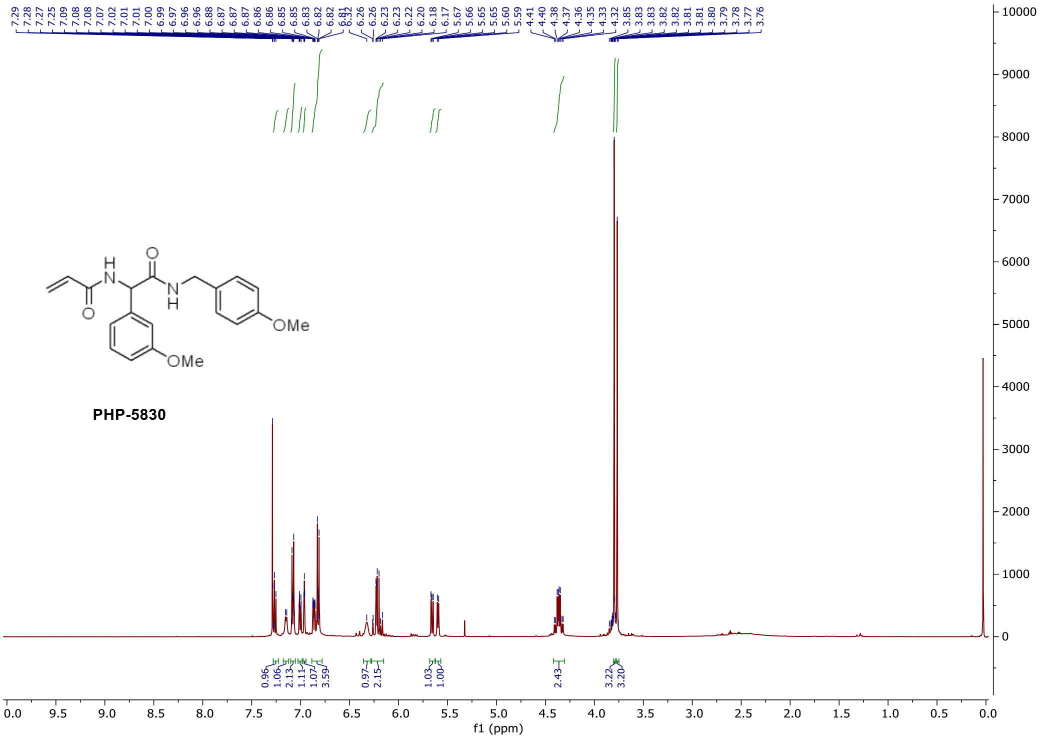

Supplementary Figure 776. ^{1}H NMR (500 MHz, $CDCl_3$) of compound **PHP-5830**.

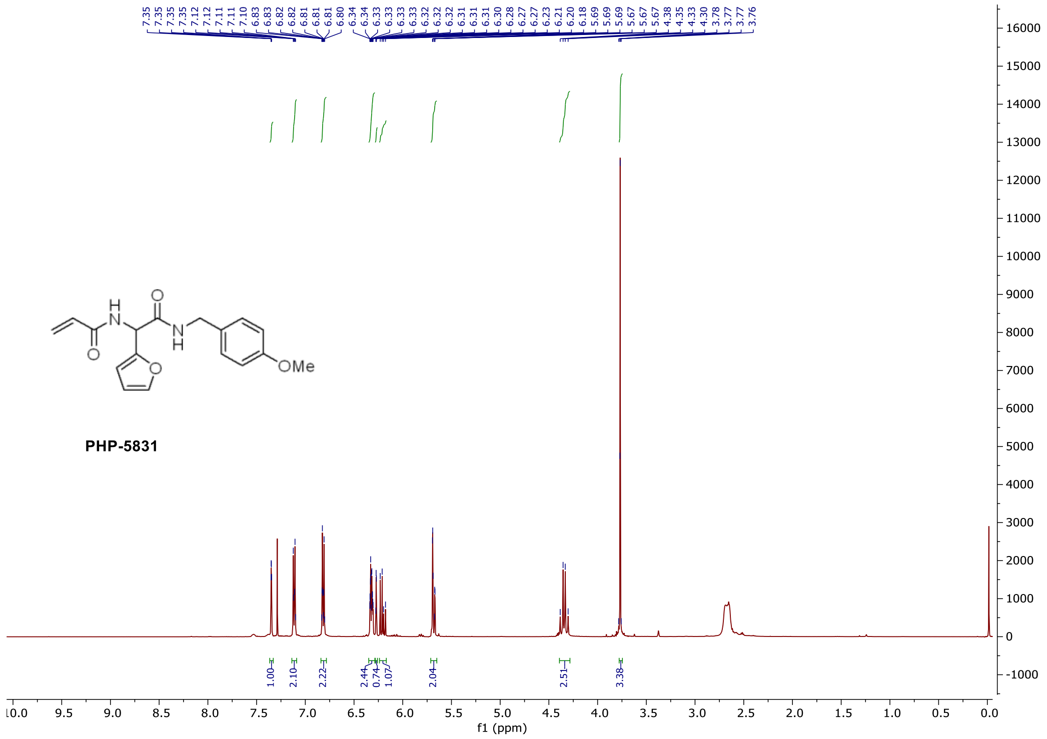

Supplementary Figure 777. ^{1}H NMR (500 MHz, $CDCl_3$) of compound **PHP-5831**.

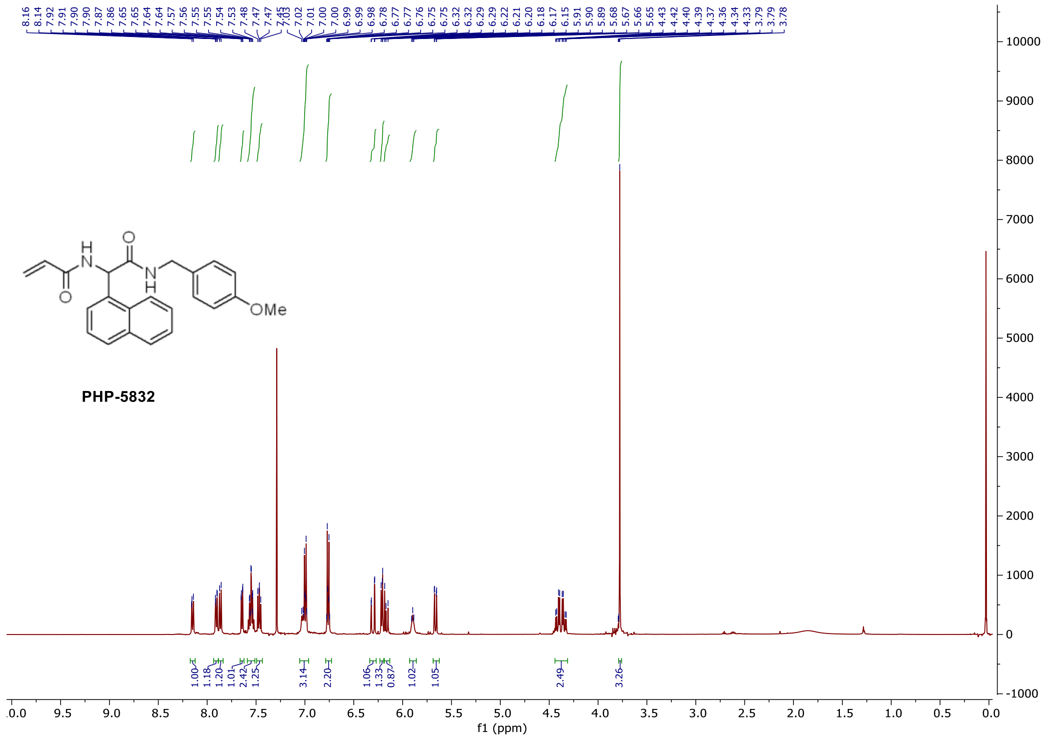

Supplementary Figure 778. ^{1}H NMR (500 MHz, $CDCl_3$) of compound **PHP-5832**.

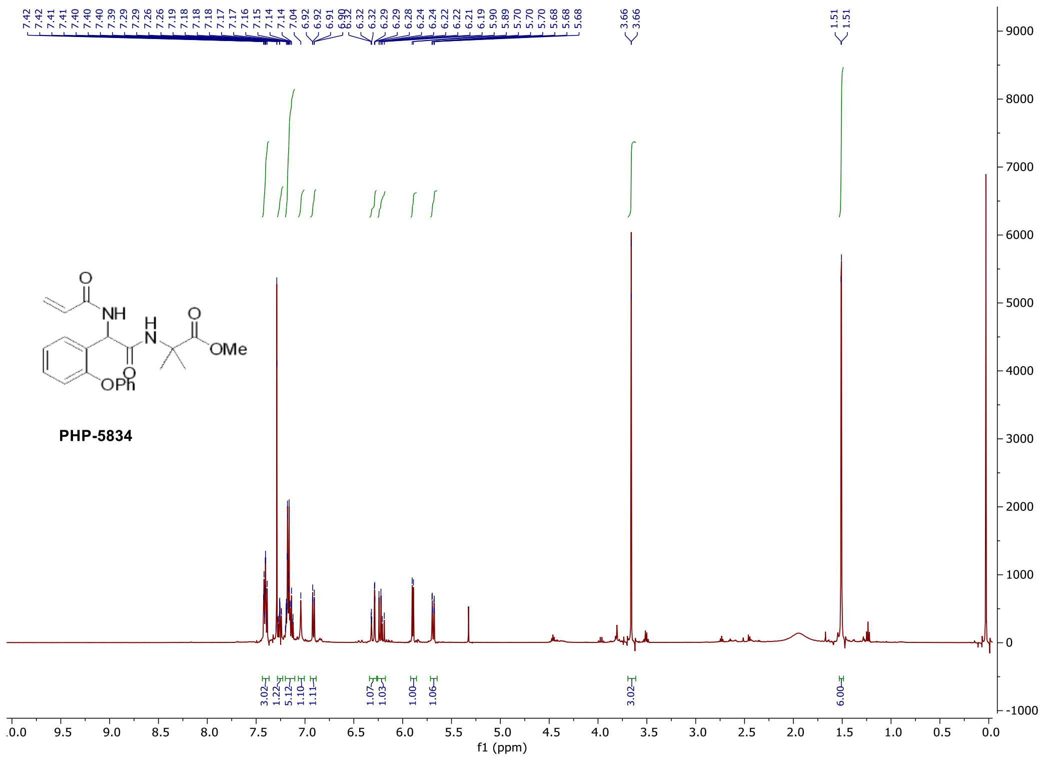

Supplementary Figure 779. ^{1}H NMR (500 MHz, $CDCl_3$) of compound **PHP-5834**.

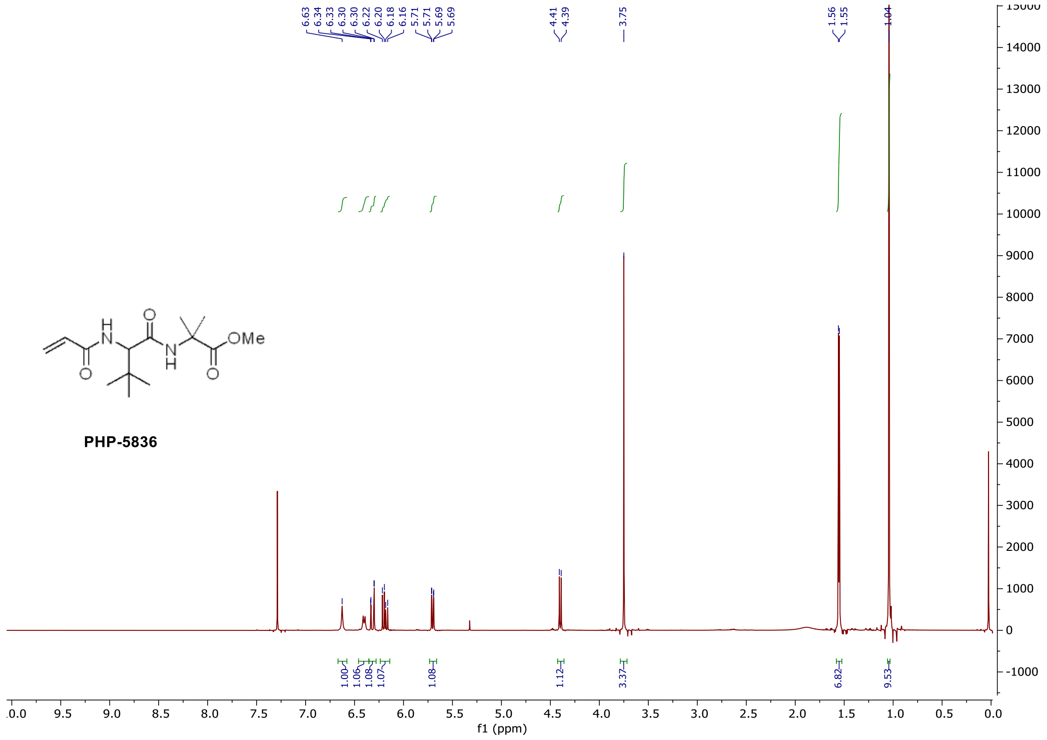

Supplementary Figure 780. ^{1}H NMR (500 MHz, $CDCl_3$) of compound **PHP-5836**.

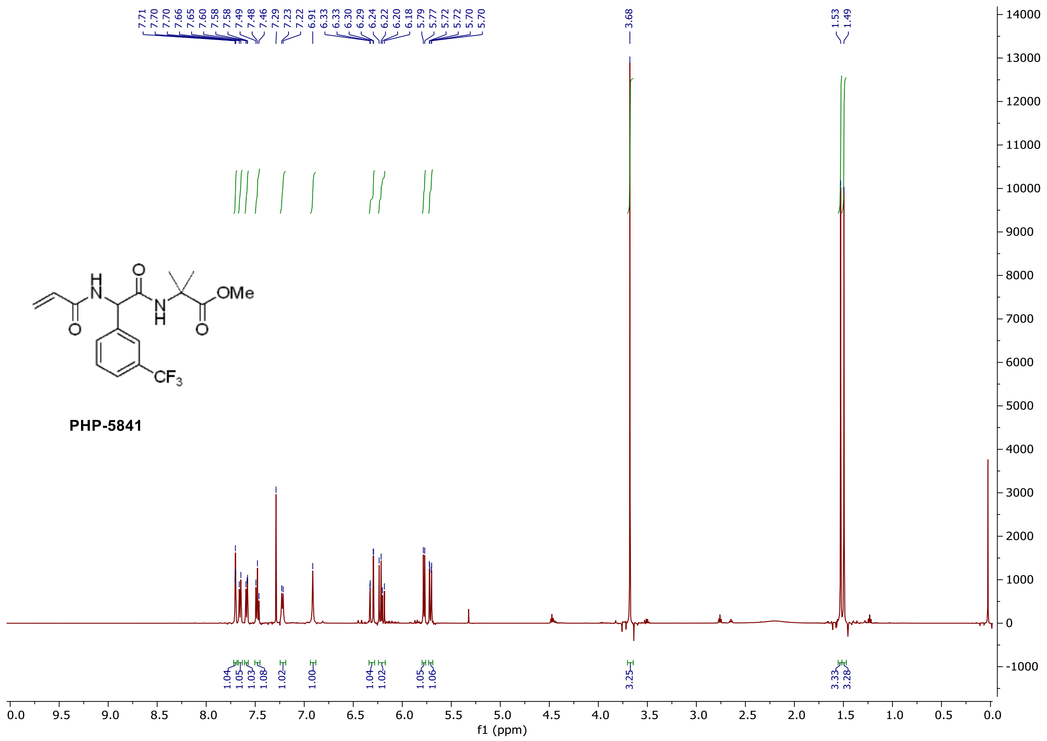

Supplementary Figure 781. ^{1}H NMR (500 MHz, $CDCl_3$) of compound **PHP-5841**.

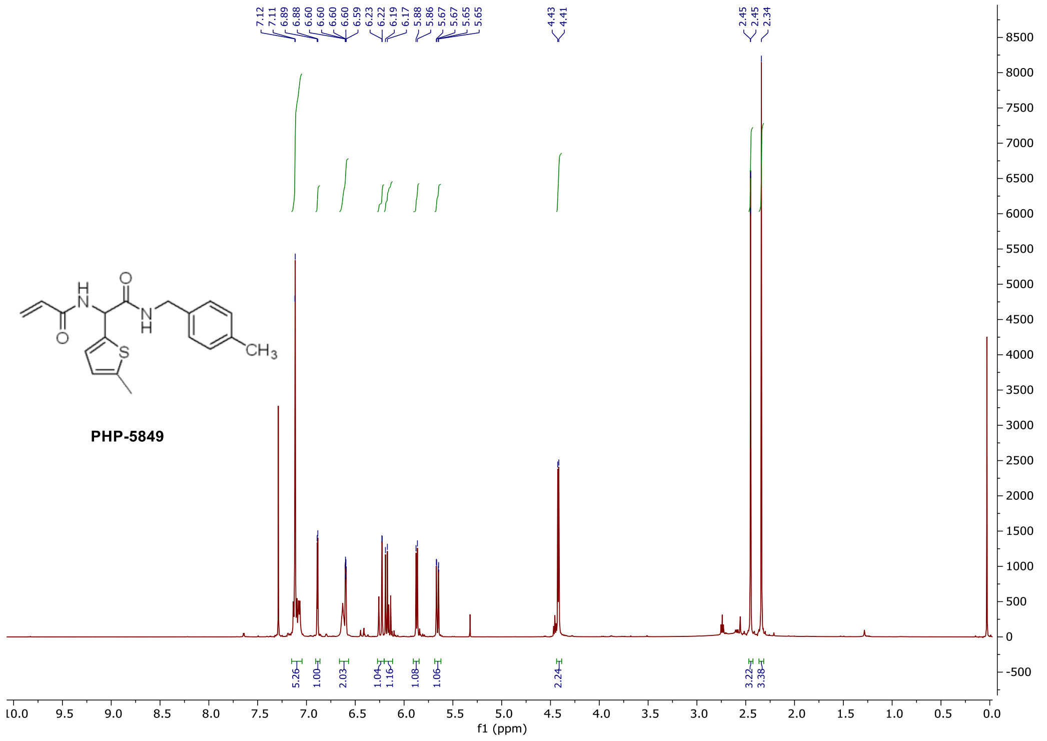

Supplementary Figure 782. ^{1}H NMR (500 MHz, $CDCl_3$) of compound **PHP-5849**.

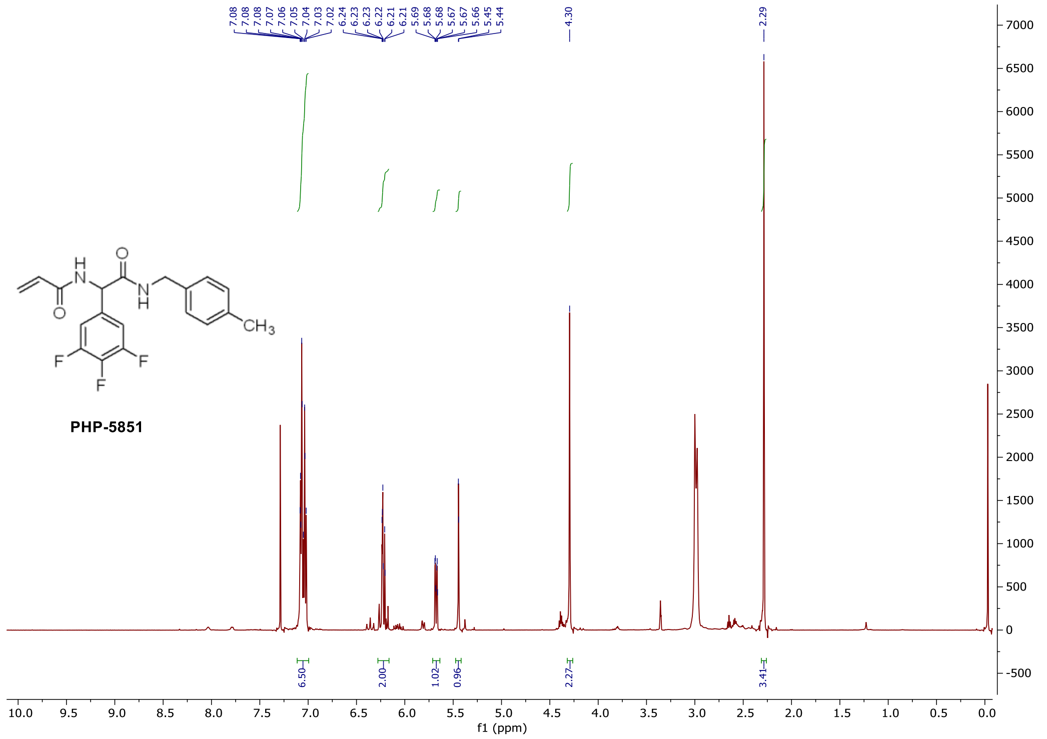

Supplementary Figure 783. ^{1}H NMR (500 MHz, $CDCl_3$ + one drop of CD_3OD) of compound **PHP-5851**.

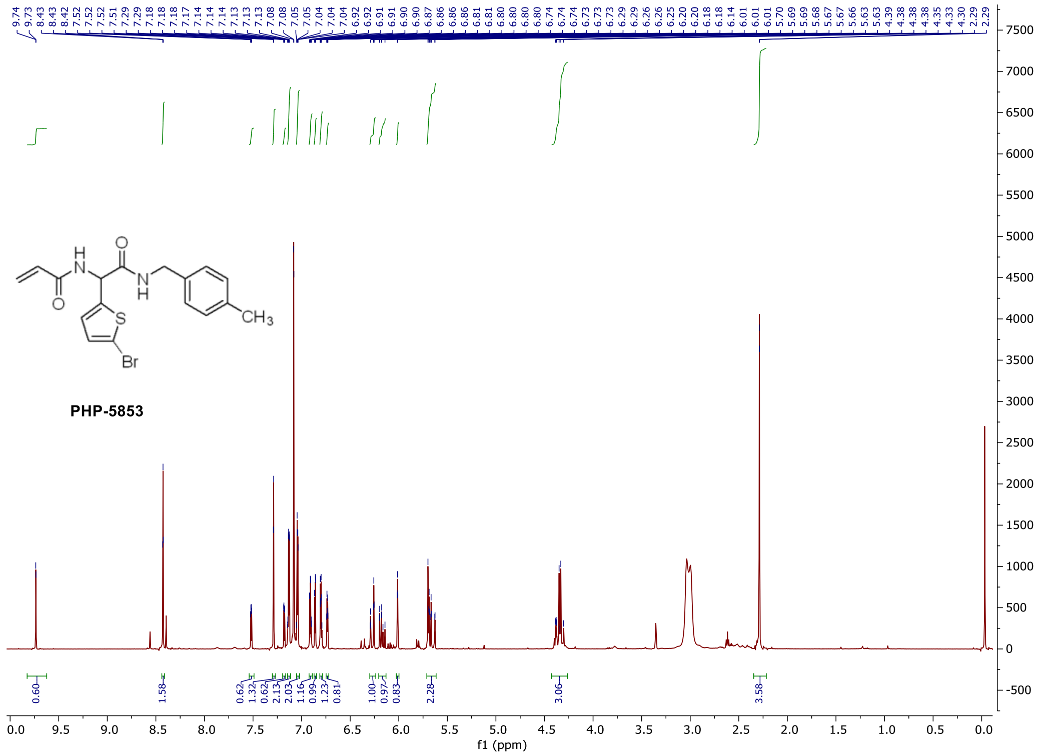

Supplementary Figure 784. ^{1}H NMR (500 MHz, $CDCl_3$ + one drop of CD_3OD) of compound **PHP-5853**.

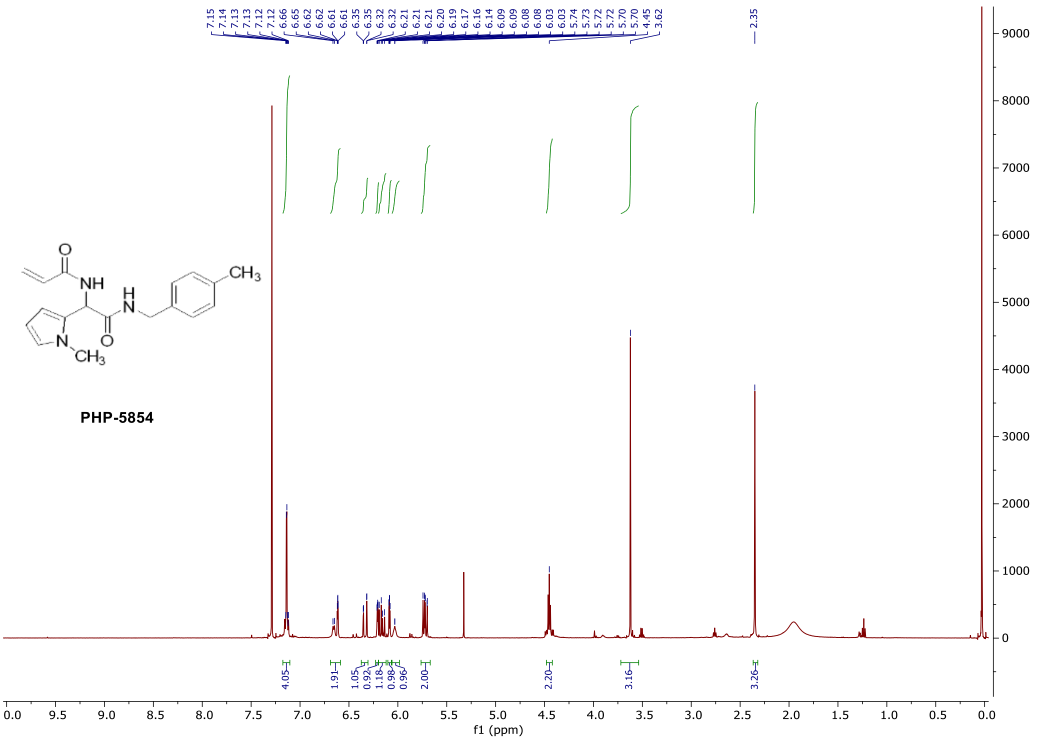

Supplementary Figure 785. ^{1}H NMR (500 MHz, $CDCl_3$) of compound **PHP-5854**.

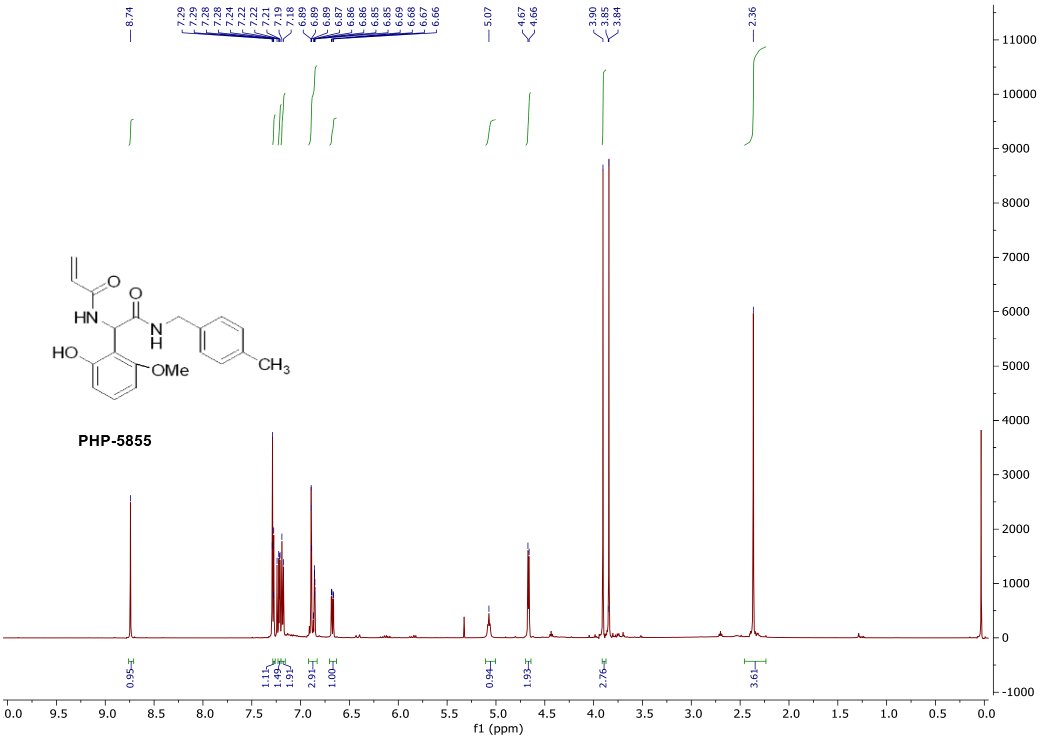

Supplementary Figure 786. ^{1}H NMR (500 MHz, $CDCl_3$) of compound **PHP-5855**.

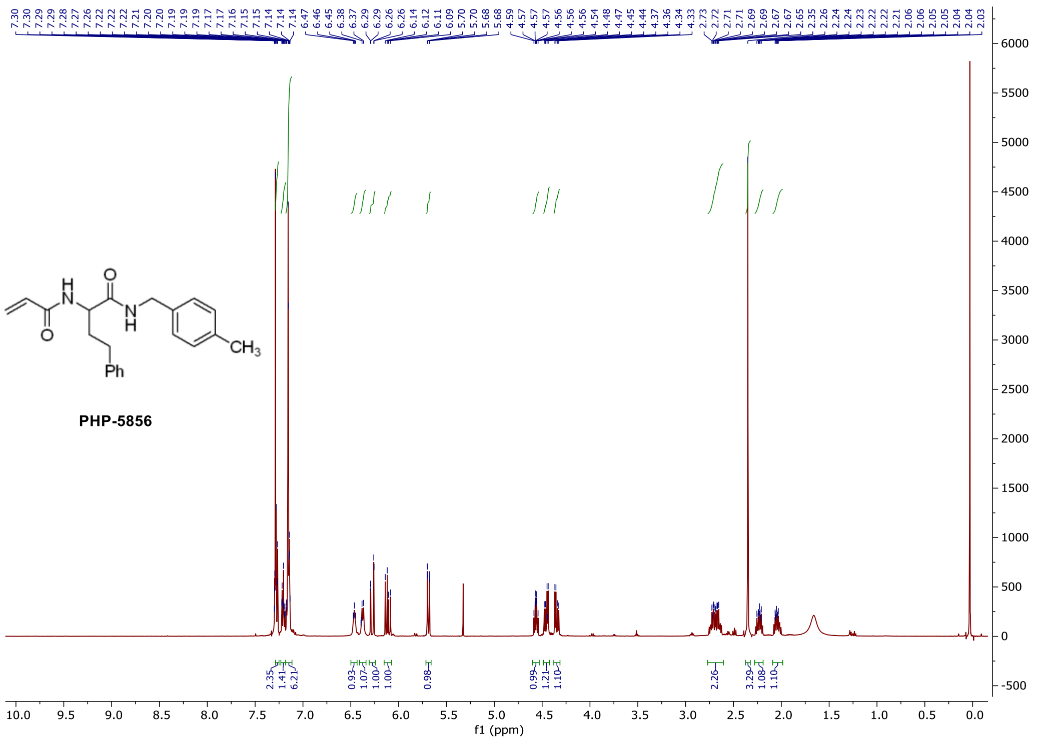

Supplementary Figure 787. ^{1}H NMR (500 MHz, CDCl3) of compound **PHP-5856**.

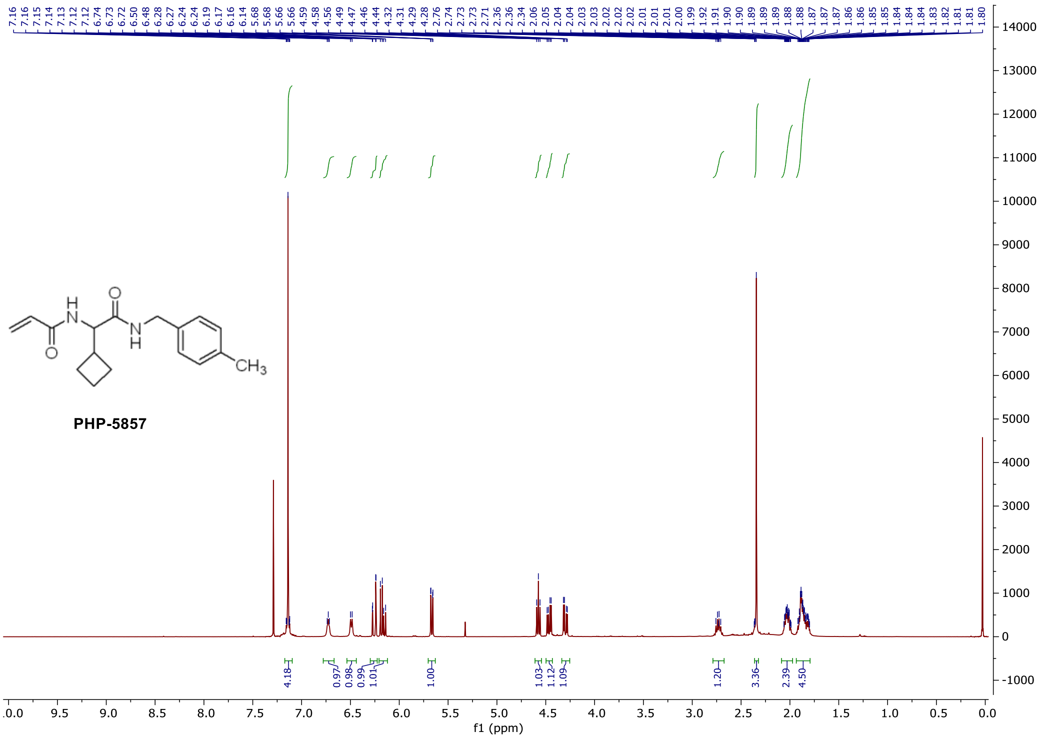

Supplementary Figure 788. ^{1}H NMR (500 MHz, CDCl3) of compound **PHP-5857**.

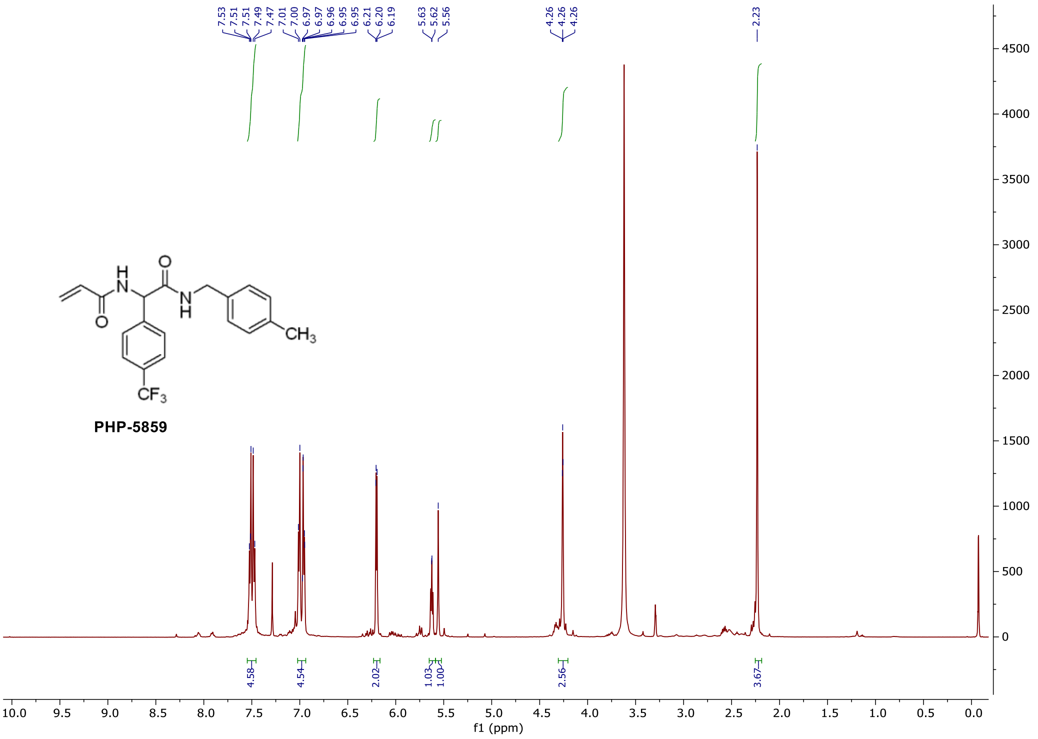

Supplementary Figure 789. ^{1}H NMR (500 MHz, $CDCl_3$) of compound **PHP-5859**.

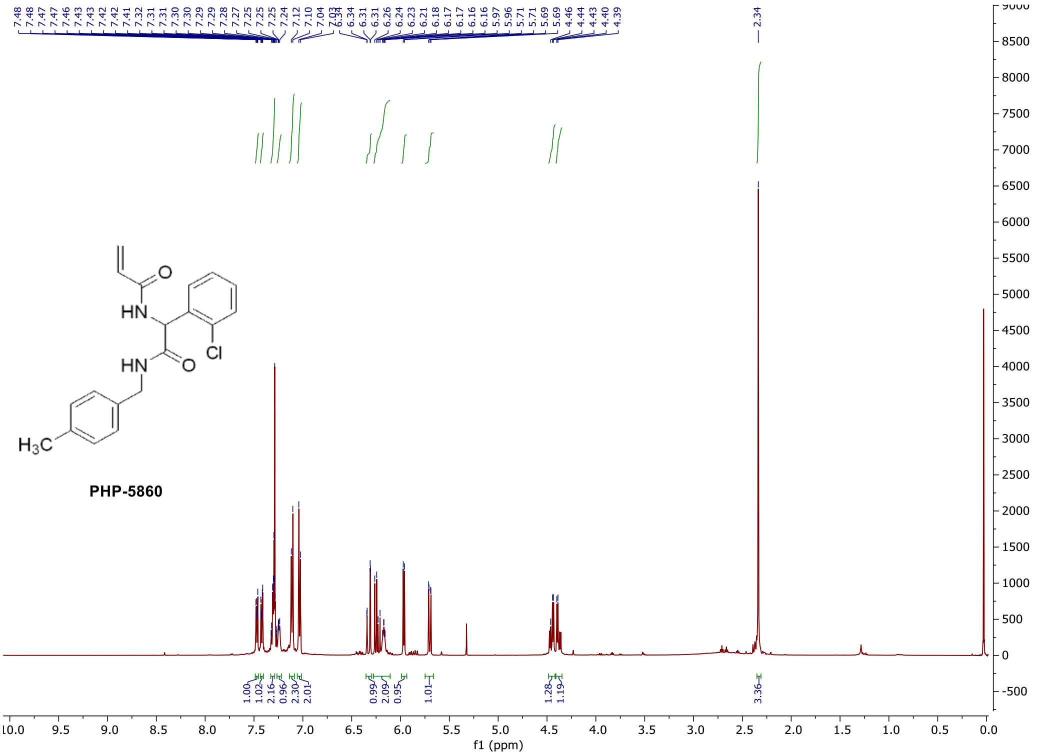

Supplementary Figure 790. ^{1}H NMR (500 MHz, $CDCl_3$) of compound **PHP-5860**.

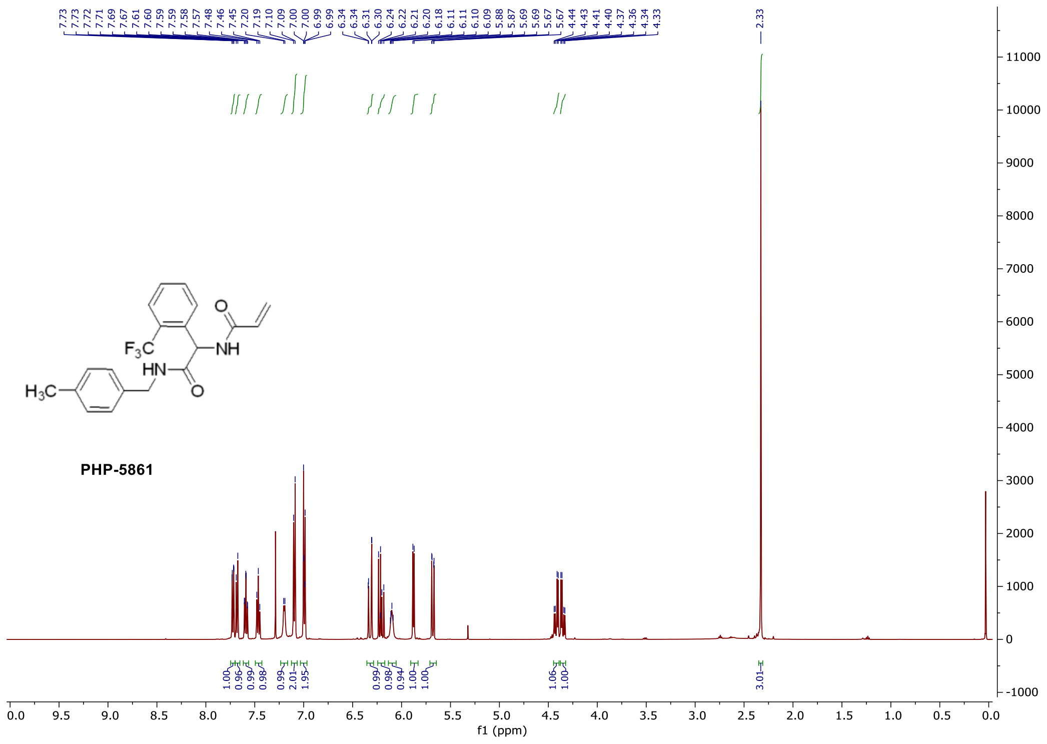

Supplementary Figure 791. ^{1}H NMR (500 MHz, $CDCl_3$) of compound **PHP-5861**.

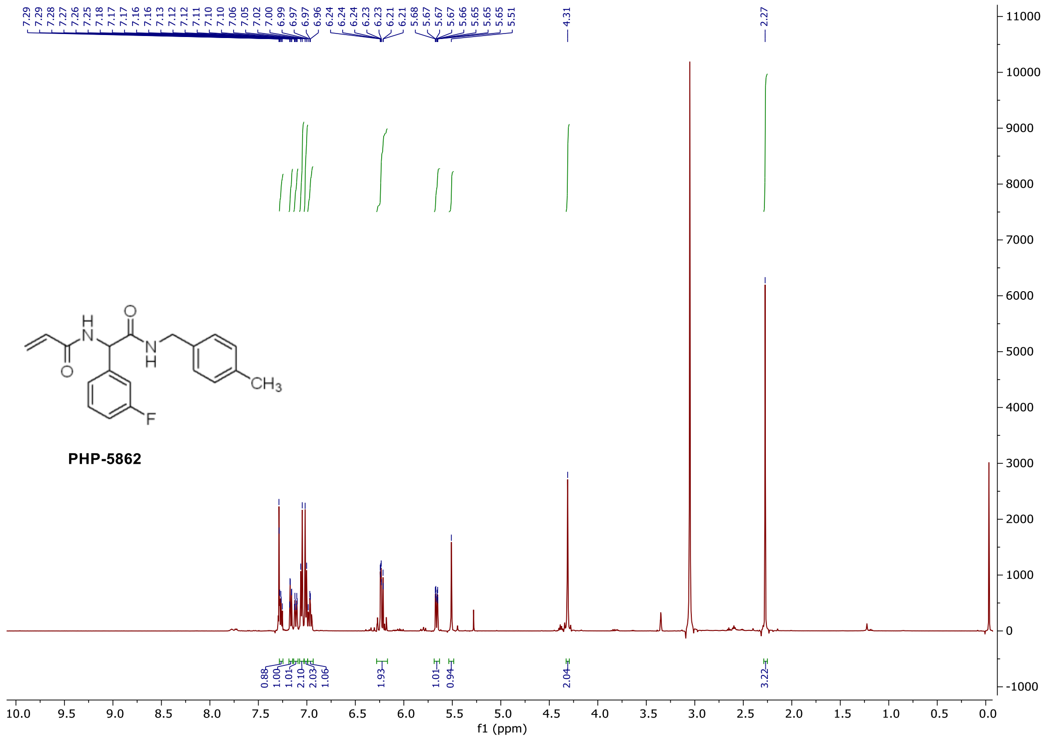

Supplementary Figure 792. ^{1}H NMR (500 MHz, $CDCl_3$ + one drop of CD_3OD) of compound **PHP-5862**.

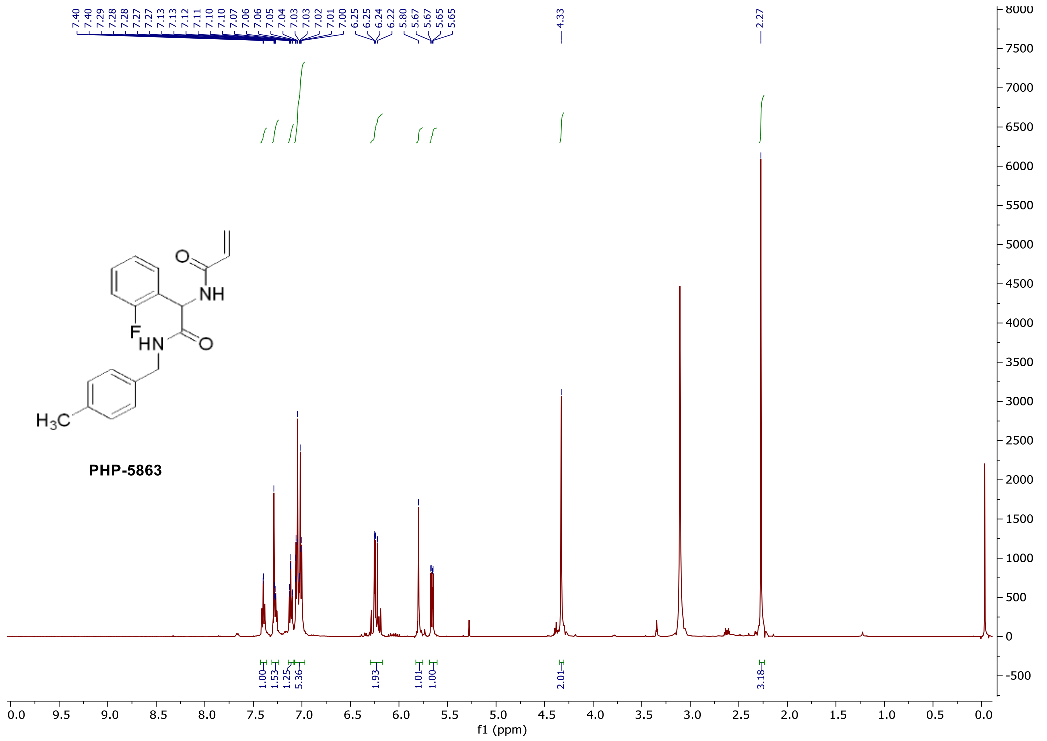

Supplementary Figure 793. ^{1}H NMR (500 MHz, $CDCl_3$ + one drop of CD_3OD) of compound **PHP-5863**.

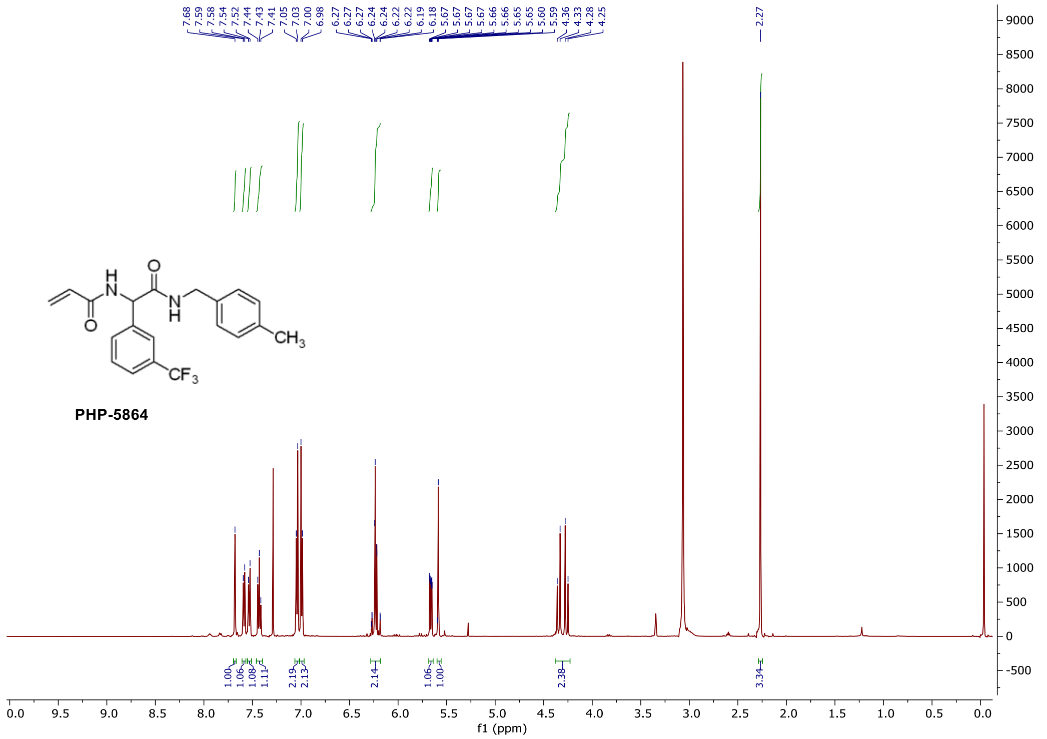

Supplementary Figure 794. ^{1}H NMR (500 MHz, $CDCl_3$ + one drop of CD_3OD) of compound **PHP-5864**.

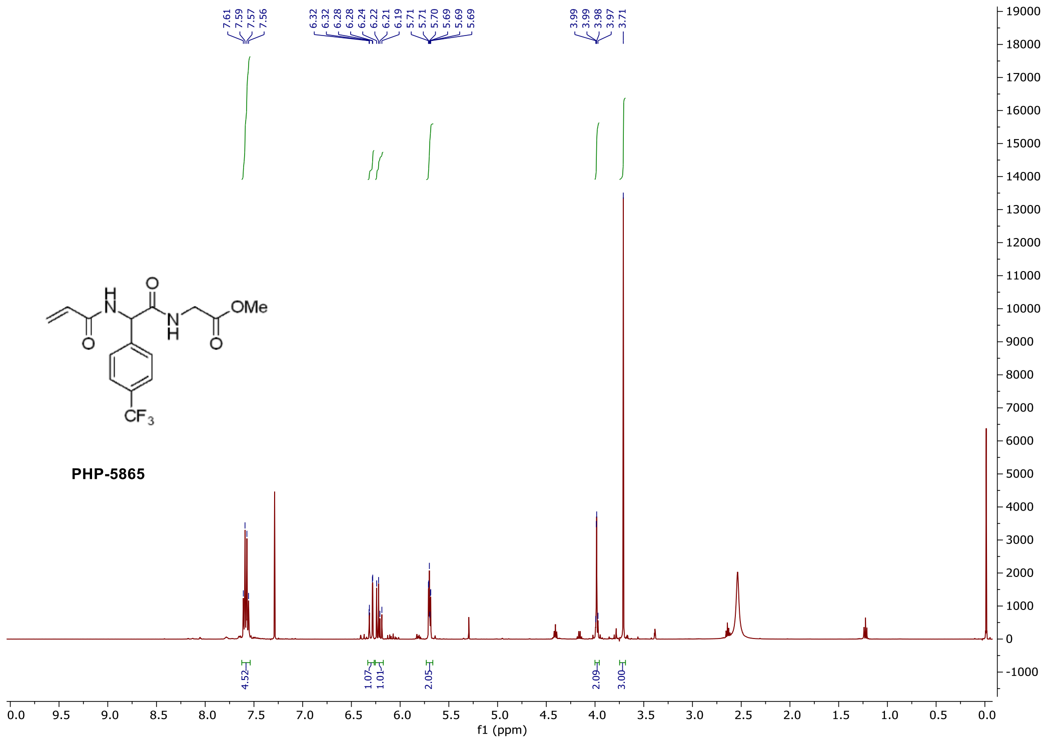

Supplementary Figure 795. ^{1}H NMR (500 MHz, $CDCl_3$) of compound **PHP-5865**.

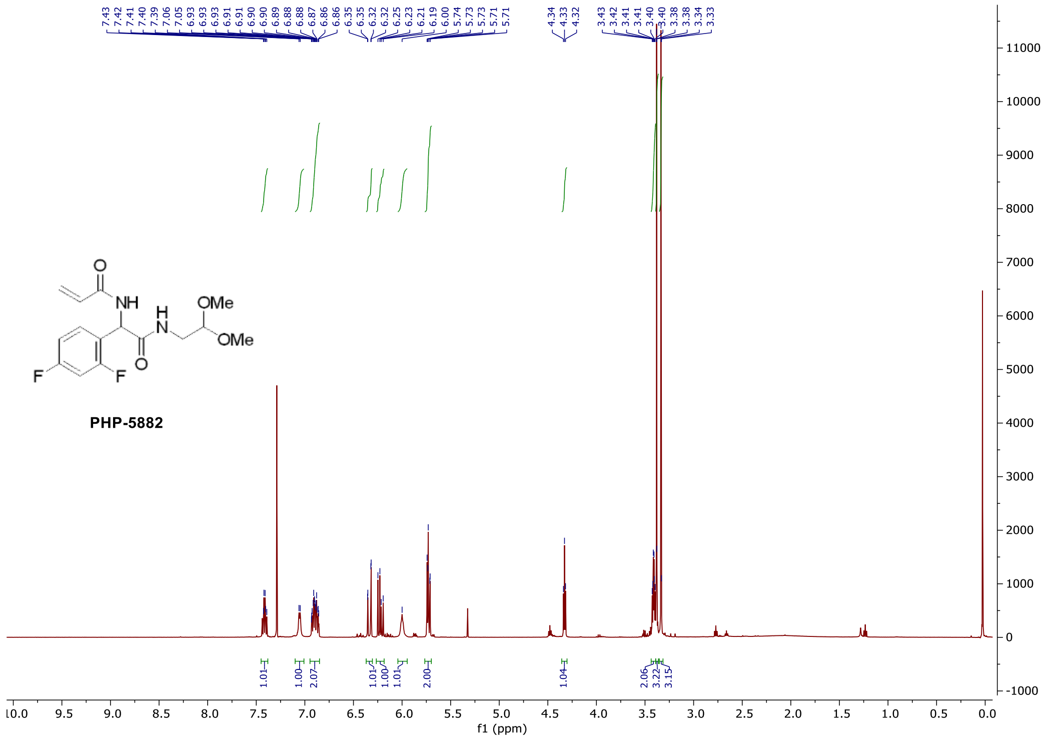

Supplementary Figure 796. ^{1}H NMR (500 MHz, $CDCl_3$) of compound **PHP-5882**.

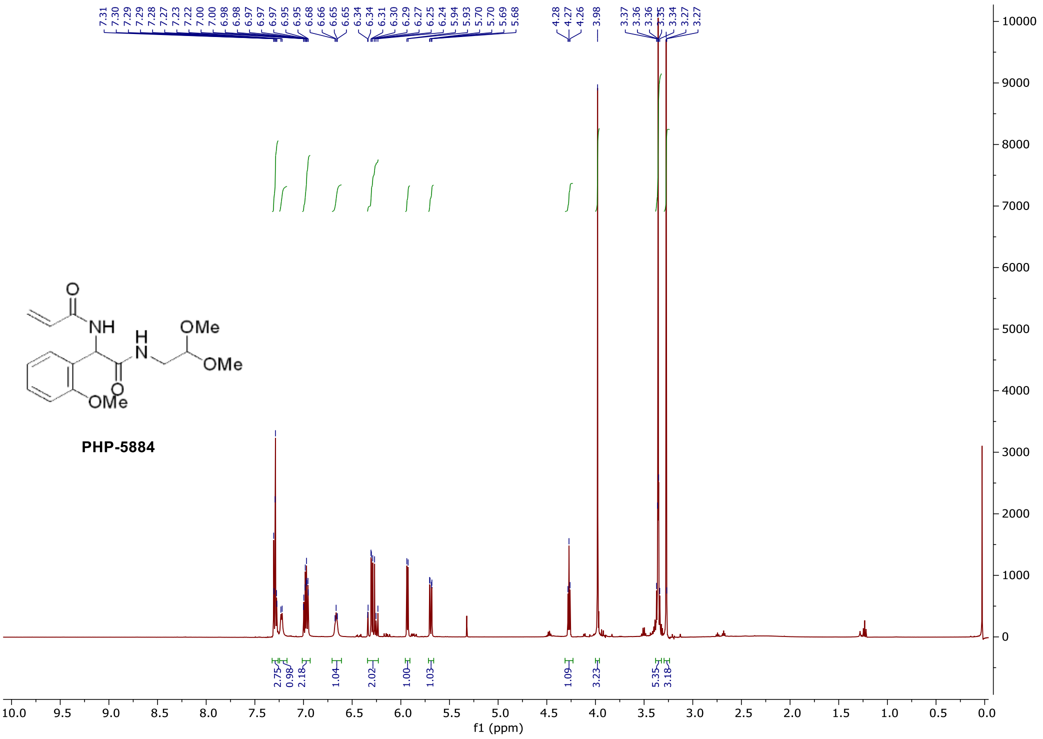

Supplementary Figure 797. ^{1}H NMR (500 MHz, $CDCl_3$) of compound **PHP-5884**.

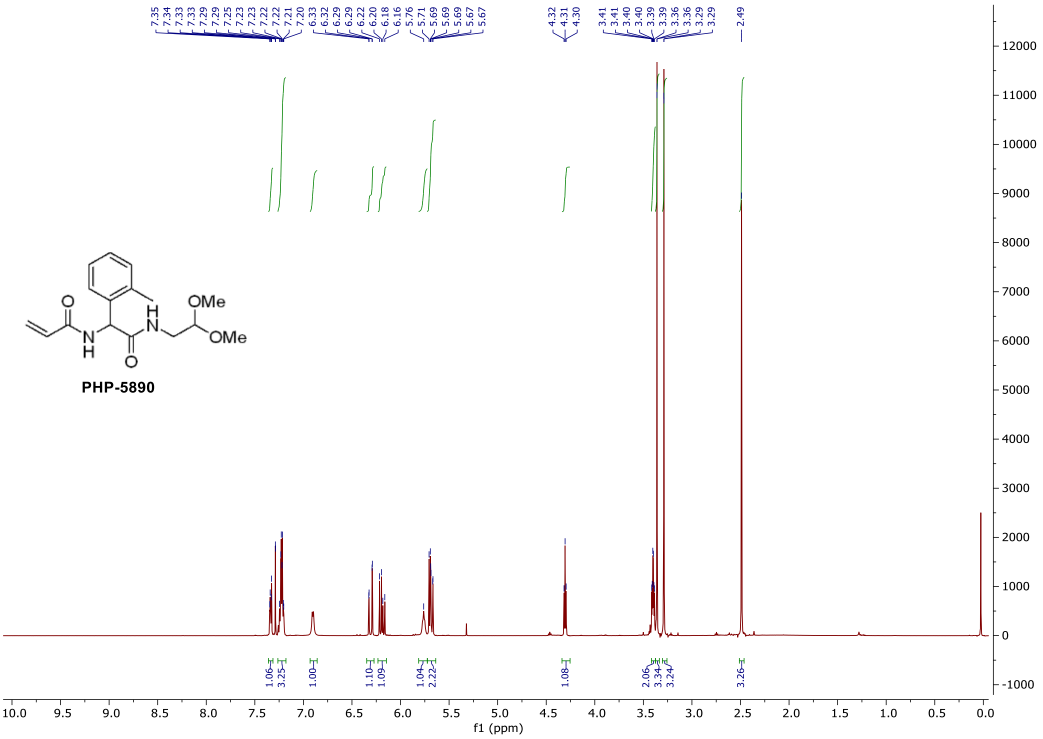

Supplementary Figure 798. ^{1}H NMR (500 MHz, $CDCl_3$) of compound **PHP-5890**.

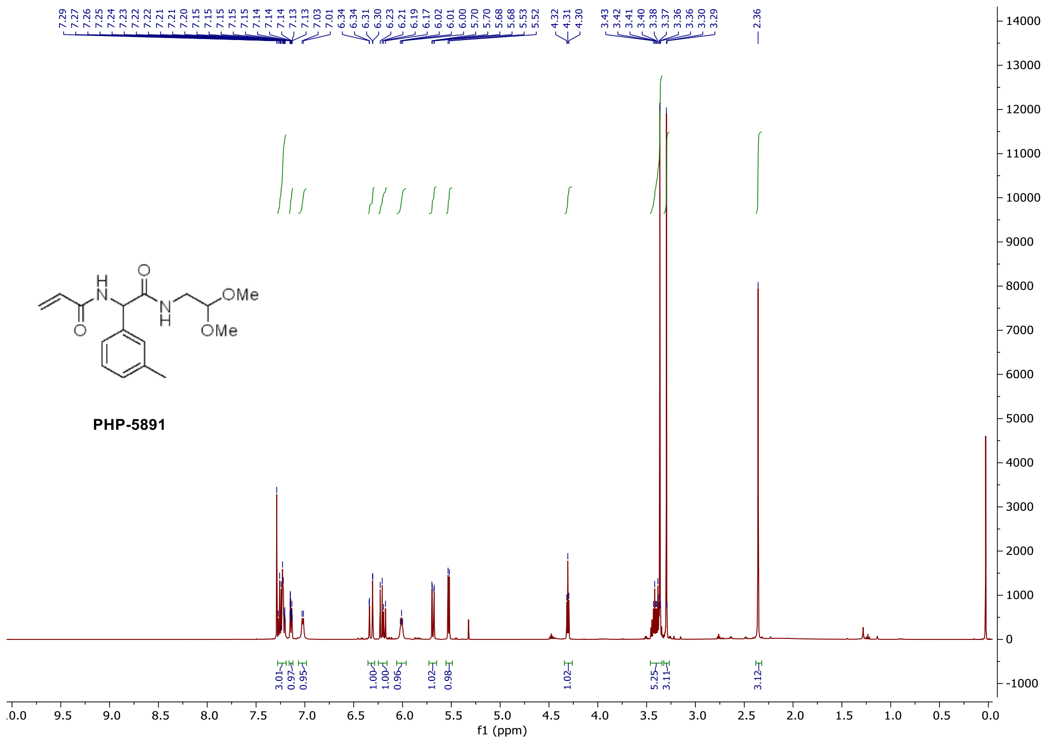

Supplementary Figure 799. ^{1}H NMR (500 MHz, $CDCl_3$) of compound **PHP-5891**.

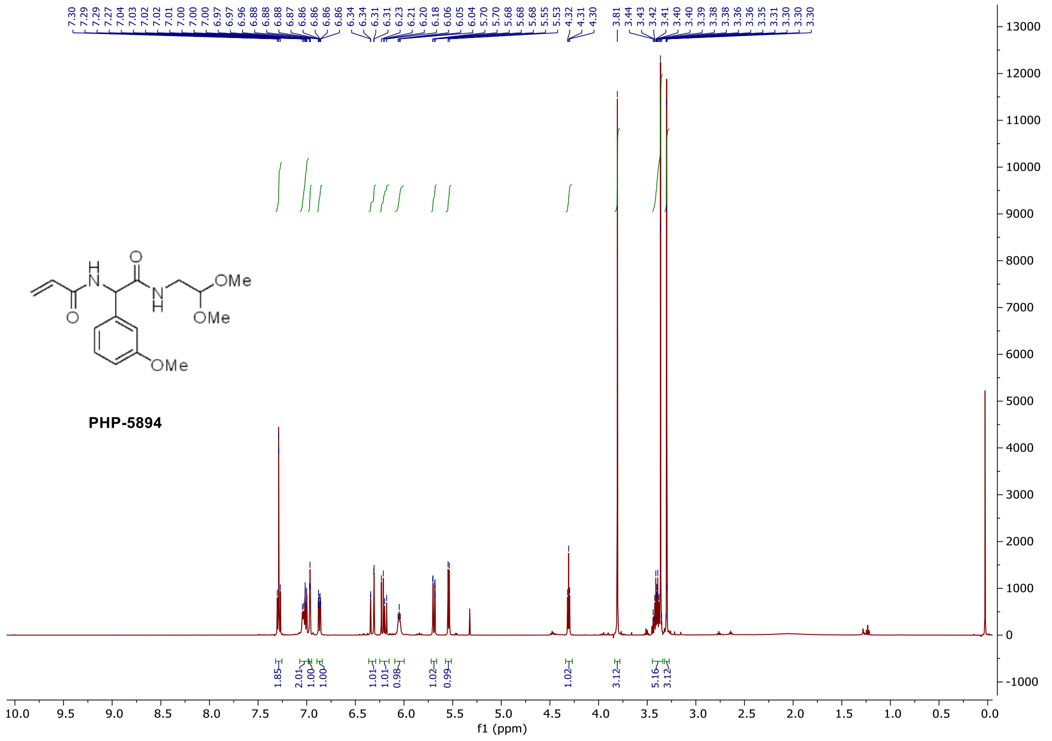

Supplementary Figure 800. ^{1}H NMR (500 MHz, $CDCl_3$) of compound **PHP-5894**.

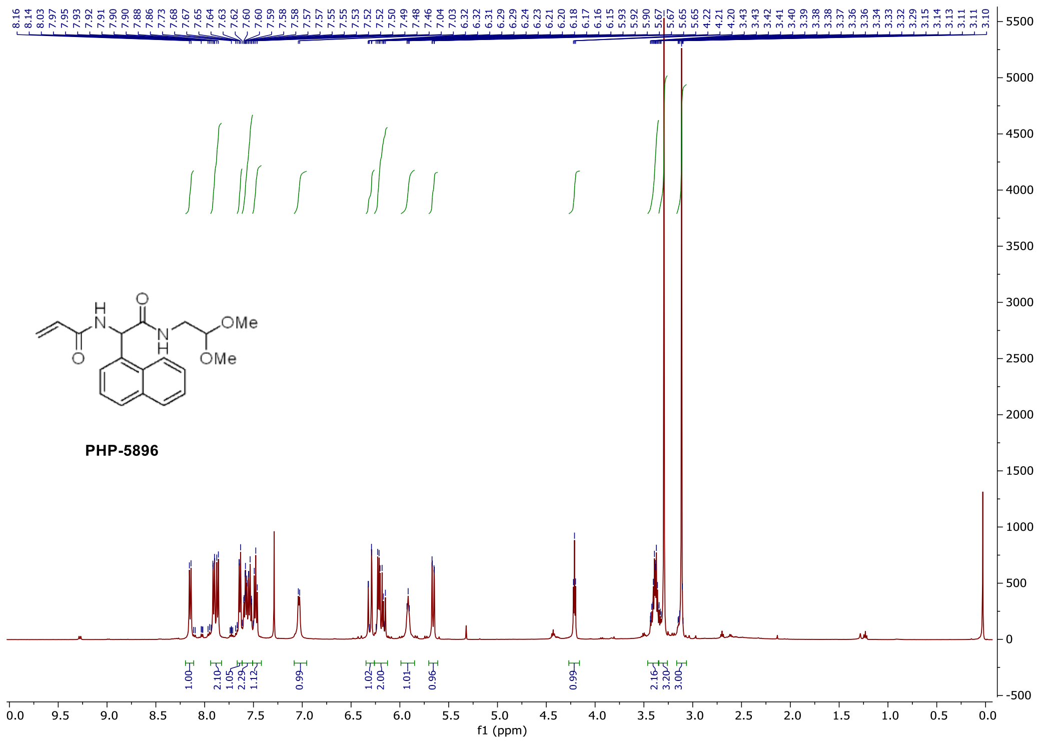

Supplementary Figure 801. ^{1}H NMR (500 MHz, $CDCl_3$) of compound **PHP-5896.**

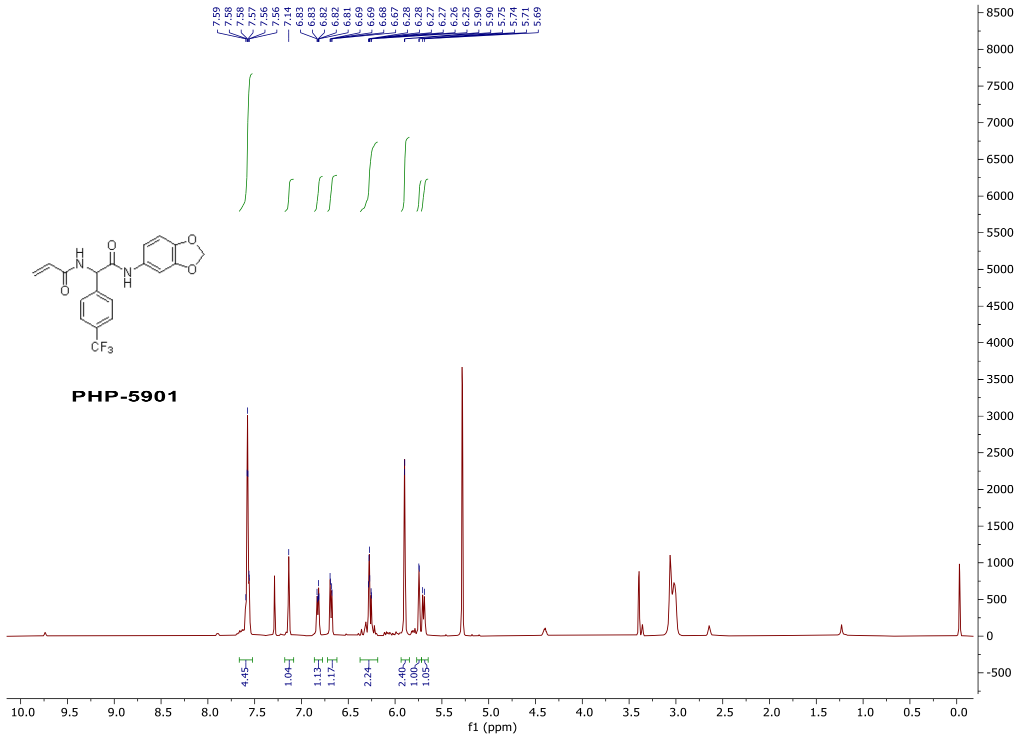

Supplementary Figure 802. ^{1}H NMR (400 MHz, $CDCl_3$ + one drop of CD_3OD) of compound **PHP-5901.**

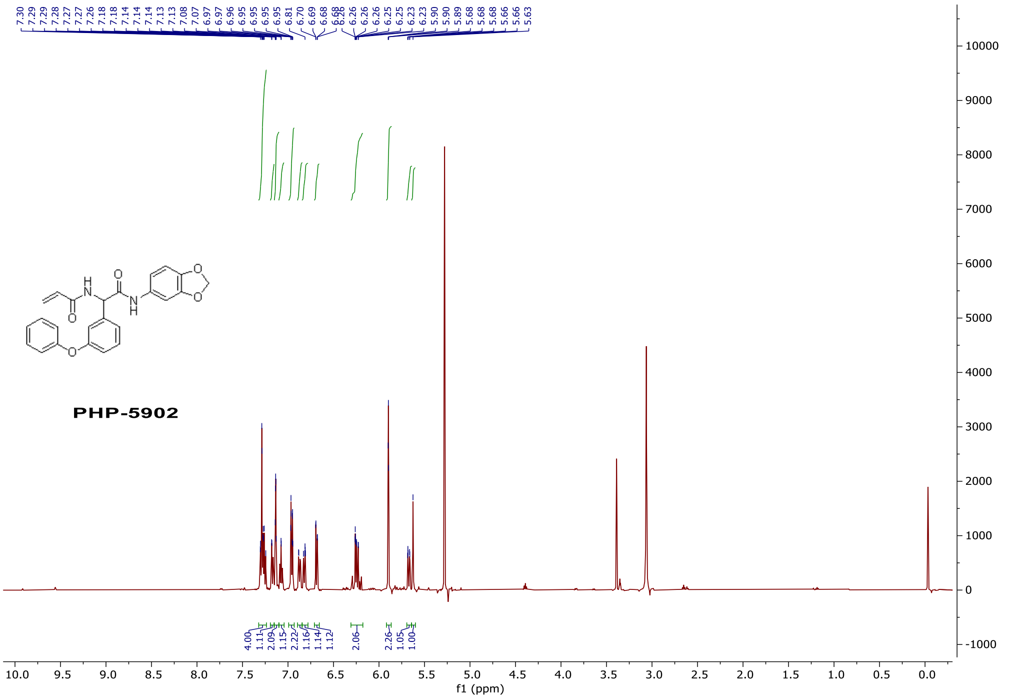

Supplementary Figure 803. ^{1}H NMR (400 MHz, $CDCl_3$ + one drop of CD_3OD) of compound **PHP-5902.**

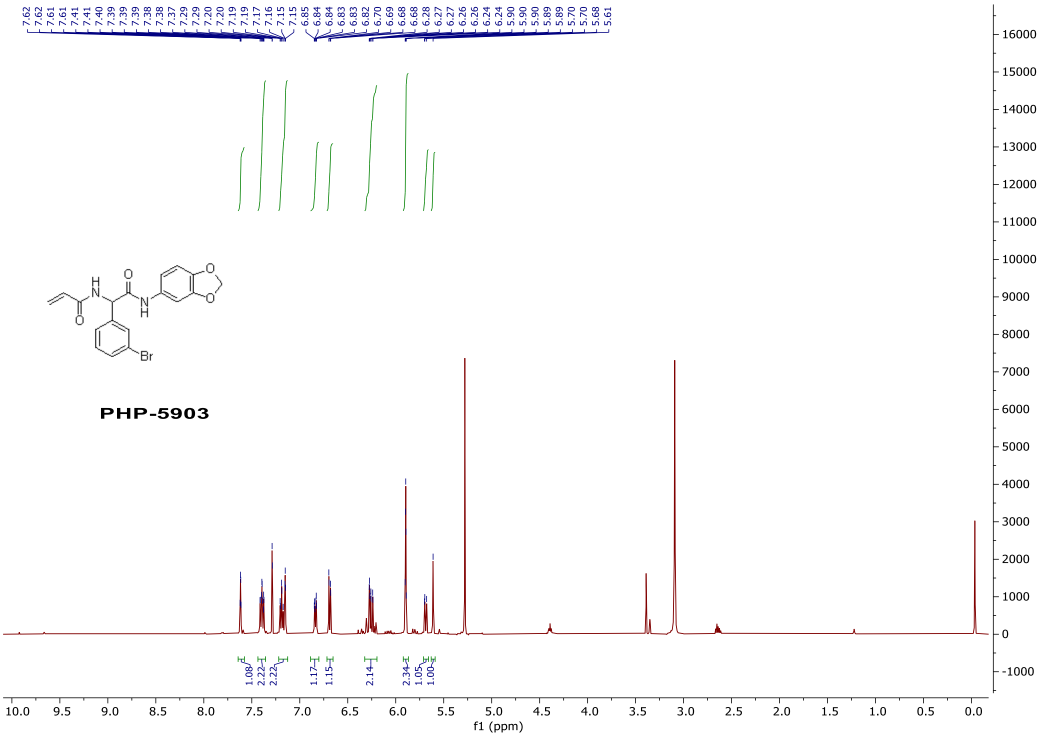

Supplementary Figure 804. ^{1}H NMR (400 MHz, $CDCl_3$ + one drop of CD_3OD) of compound **PHP-5903.**

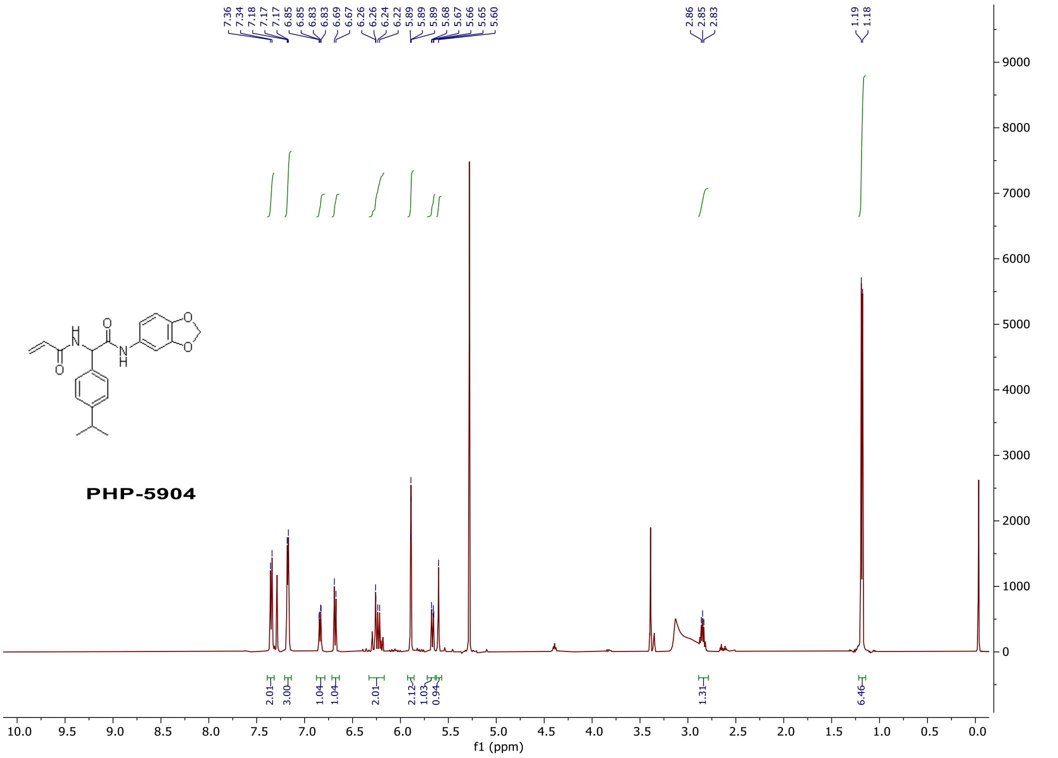

Supplementary Figure 805. ^{1}H NMR (400 MHz, $CDCl_3$ + one drop of CD_3OD) of compound **PHP-5904.**

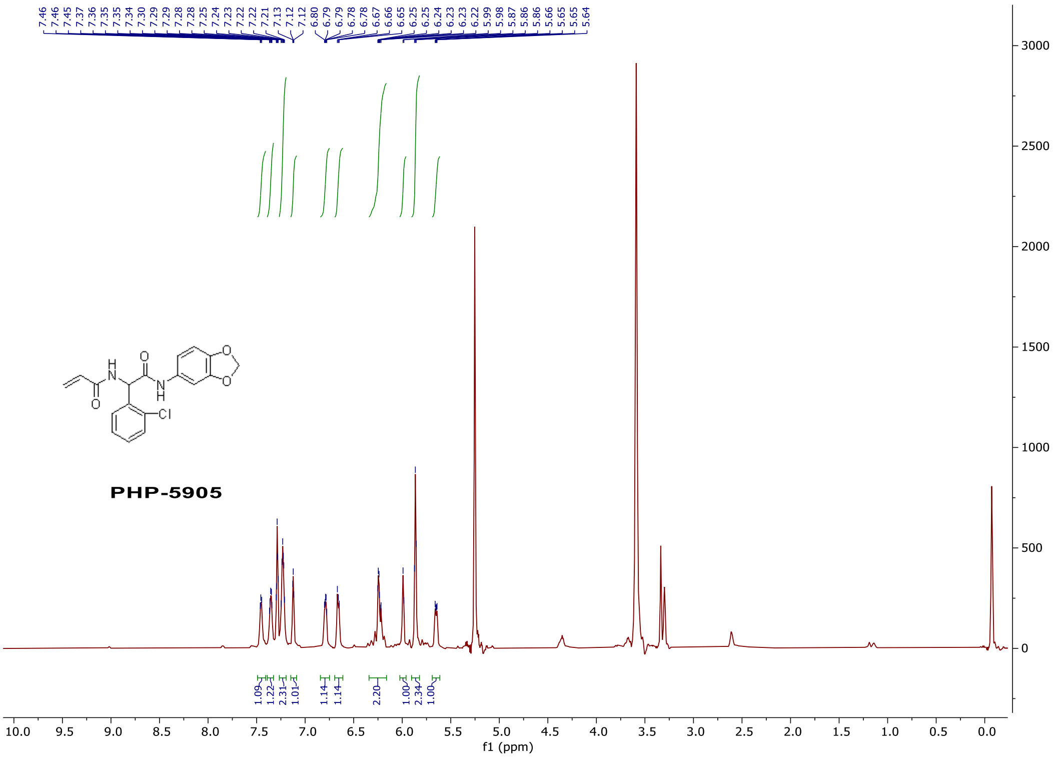

Supplementary Figure 806. ^{1}H NMR (400 MHz, $CDCl_3$ + one drop of CD_3OD) of compound **PHP-5905**.

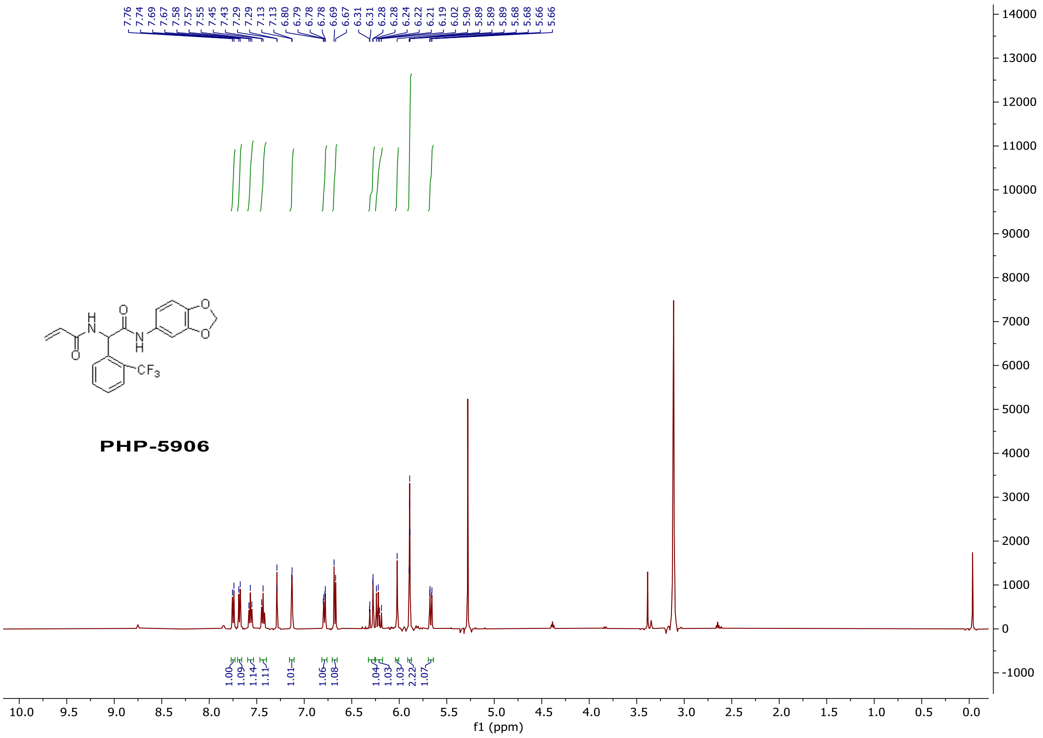

Supplementary Figure 807. ^{1}H NMR (400 MHz, $CDCl_3$ + one drop of CD_3OD) of compound **PHP-5906.**

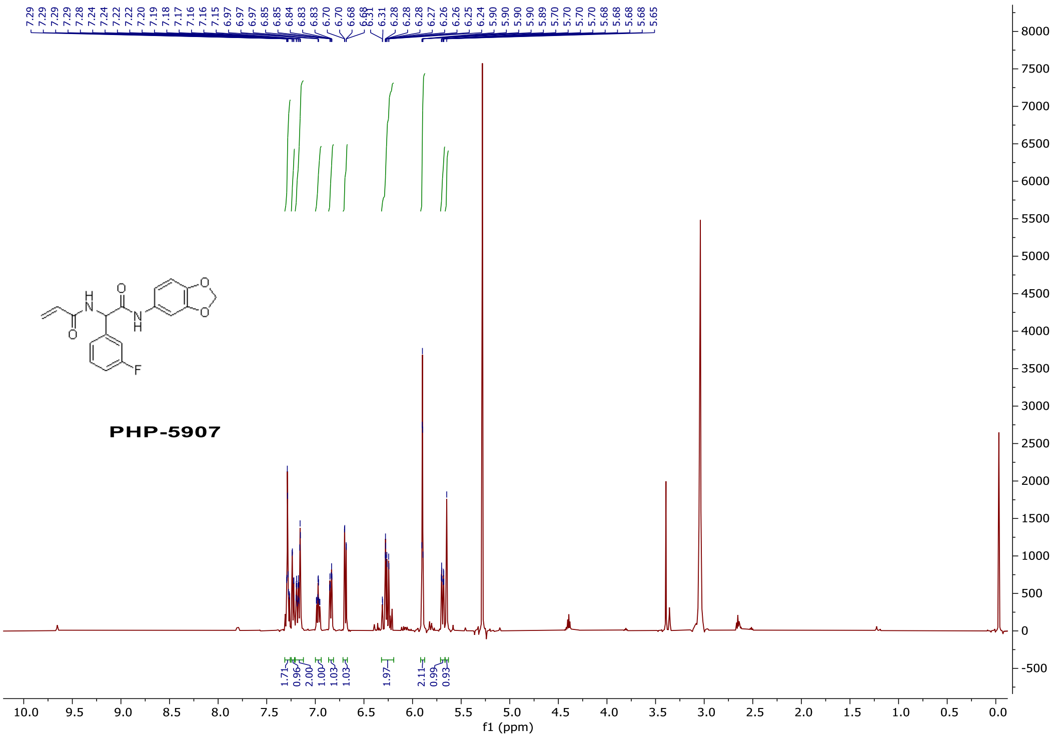

Supplementary Figure 808. ^{1}H NMR (400 MHz, $CDCl_3$ + one drop of CD_3OD) of compound **PHP-5907.**

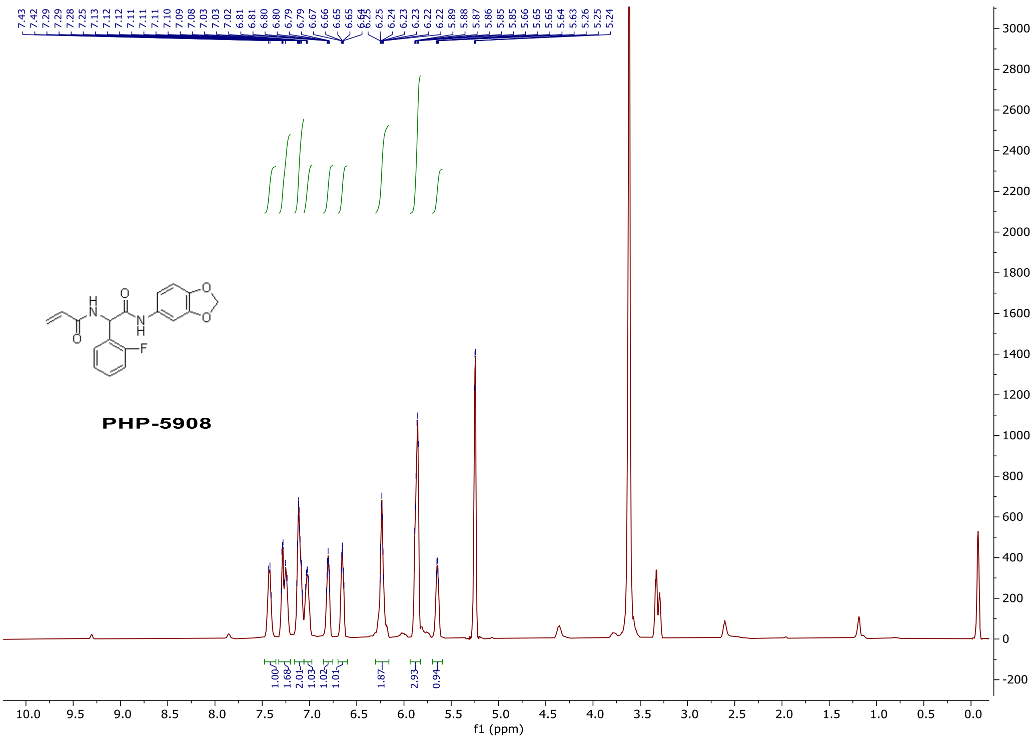

Supplementary Figure 809. ^{1}H NMR (400 MHz, $CDCl_3$ + one drop of CD_3OD) of compound **PHP-5908**.

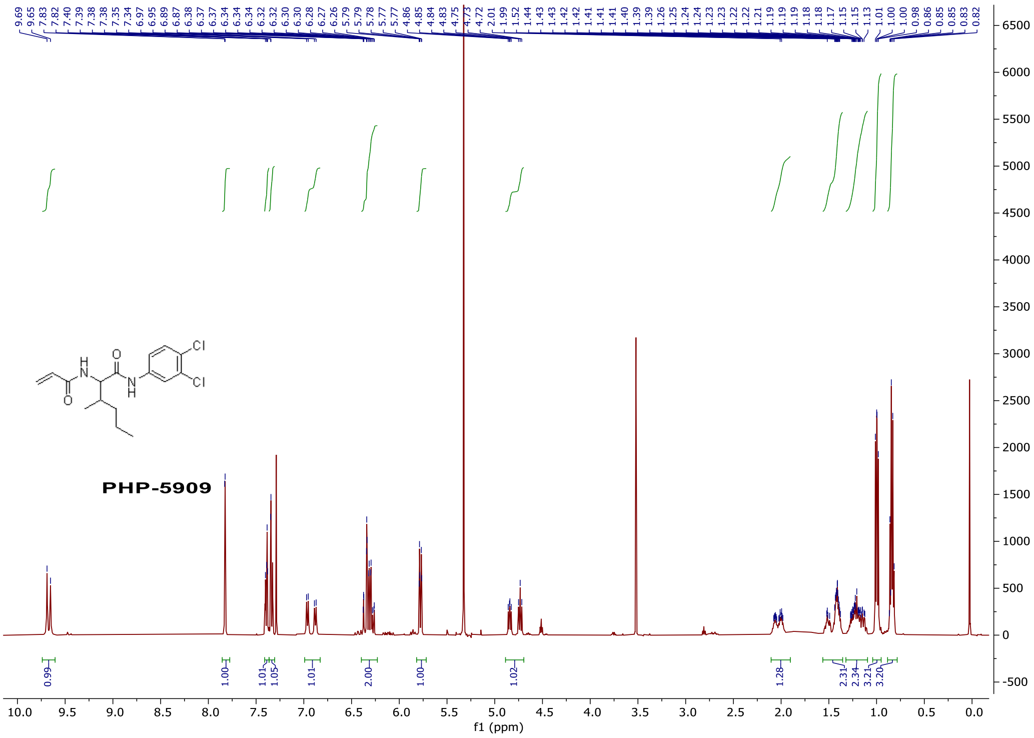

Supplementary Figure 810. ^{1}H NMR (400 MHz, $CDCl_3$ + one drop of CD_3OD) of compound **PHP-5909**.

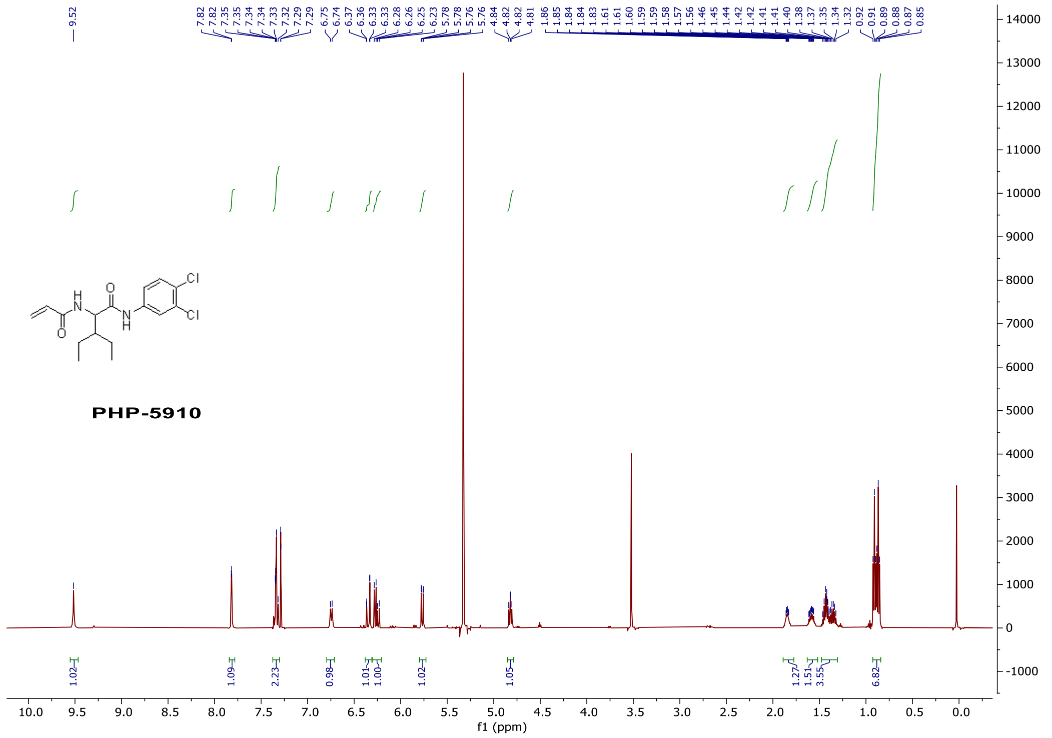

Supplementary Figure 811. ^{1}H NMR (400 MHz, $CDCl_3$ + one drop of CD_3OD) of compound **PHP-5910.**

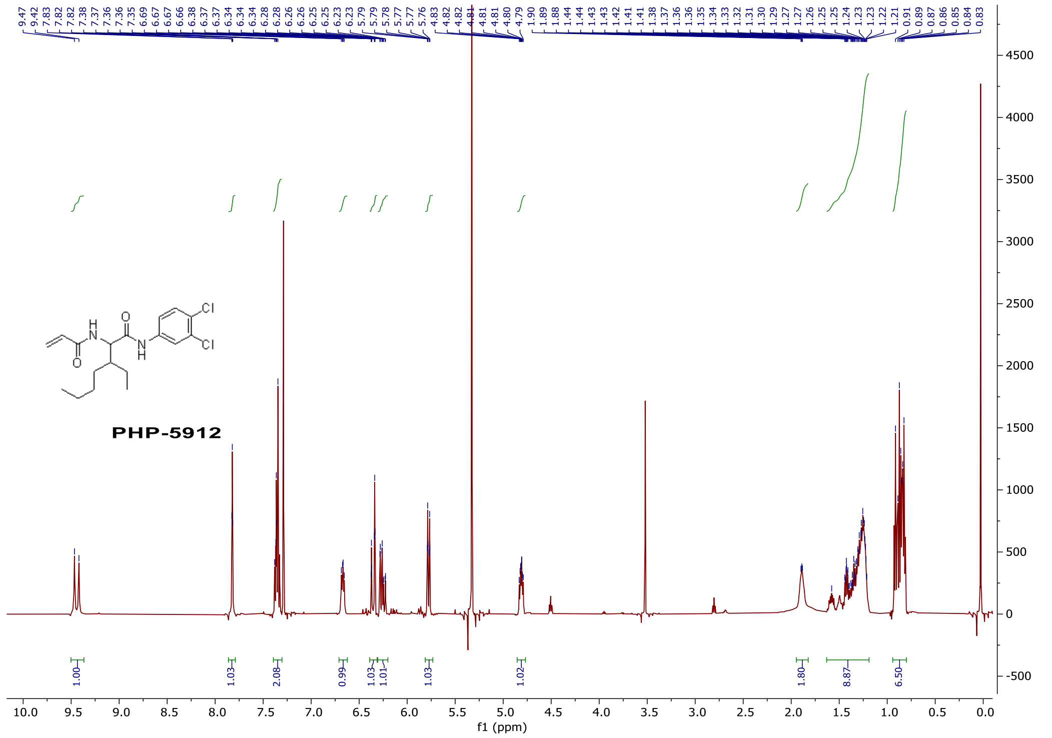

Supplementary Figure 812. ^{1}H NMR (400 MHz, $CDCl_3$ + one drop of CD_3OD) of compound **PHP-5912.**

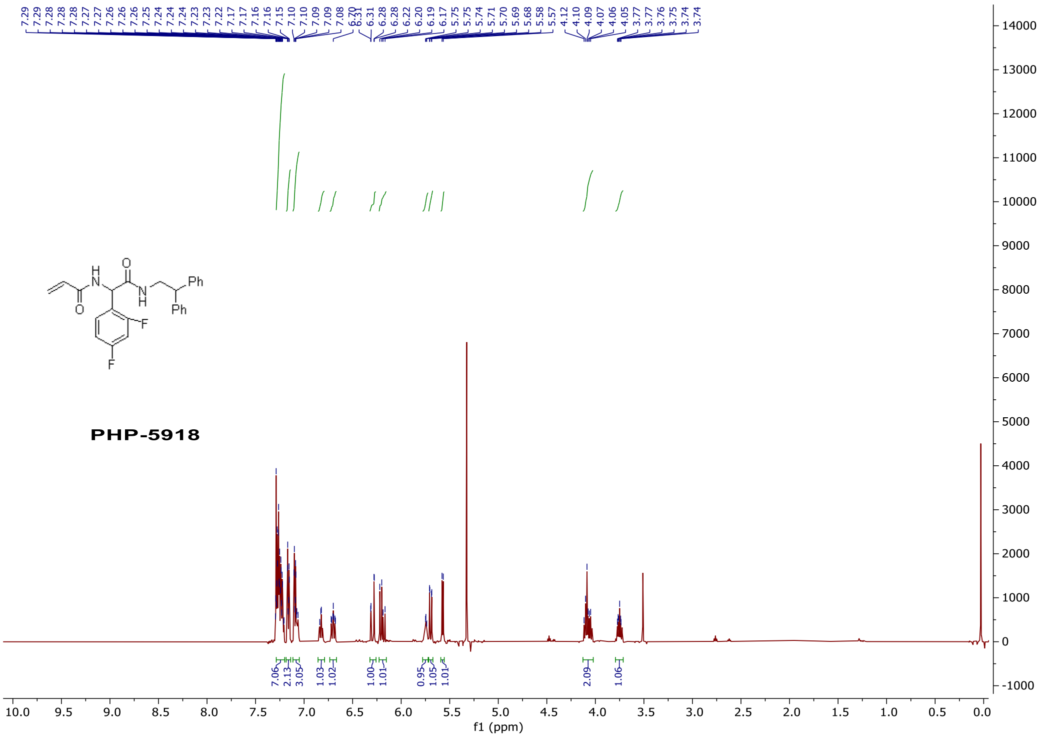

Supplementary Figure 813. ^{1}H NMR (400 MHz, $CDCl_3$ + one drop of CD_3OD) of compound **PHP-5918.**

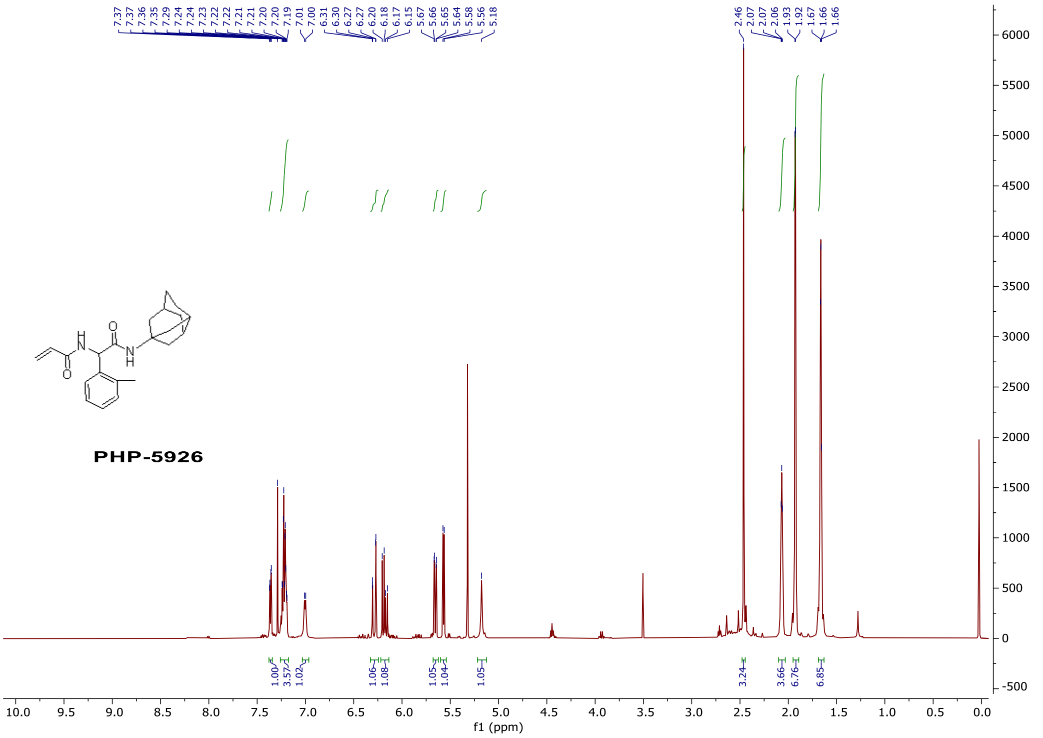

Supplementary Figure 814. ^{1}H NMR (400 MHz, $CDCl_3$ + one drop of CD_3OD) of compound **PHP-5926.**

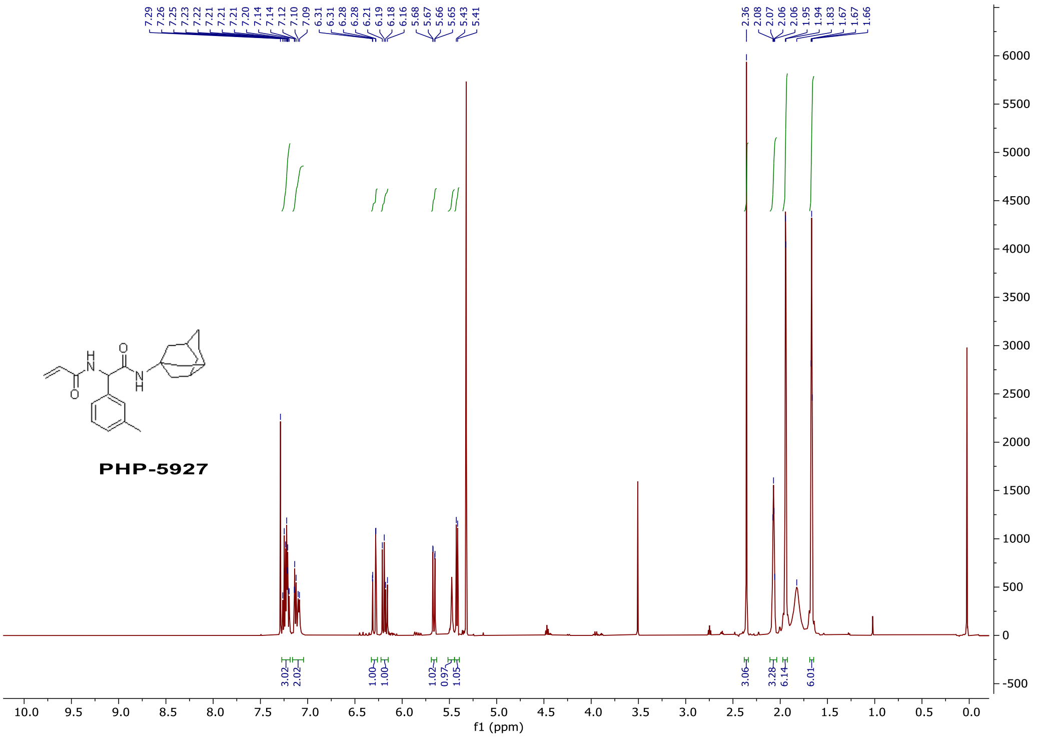

Supplementary Figure 815. ^{1}H NMR (400 MHz, $CDCl_3$ + one drop of CD_3OD) of compound **PHP-5927**.

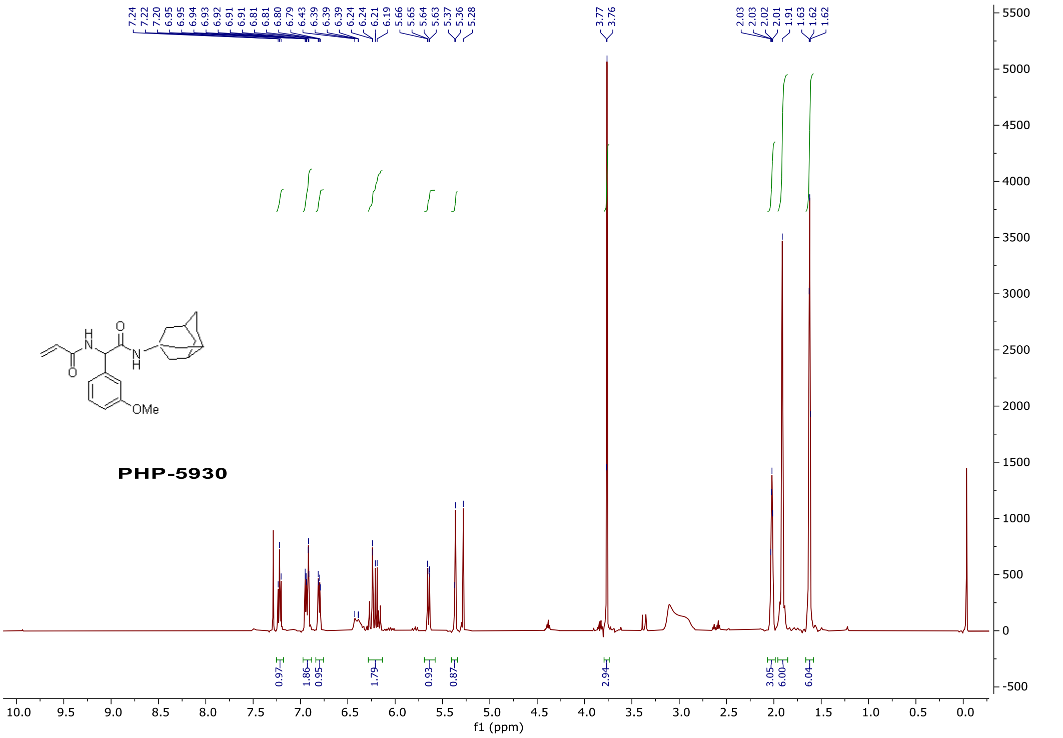

Supplementary Figure 816. ^{1}H NMR (400 MHz, $CDCl_3$ + one drop of CD_3OD) of compound **PHP-5930**.

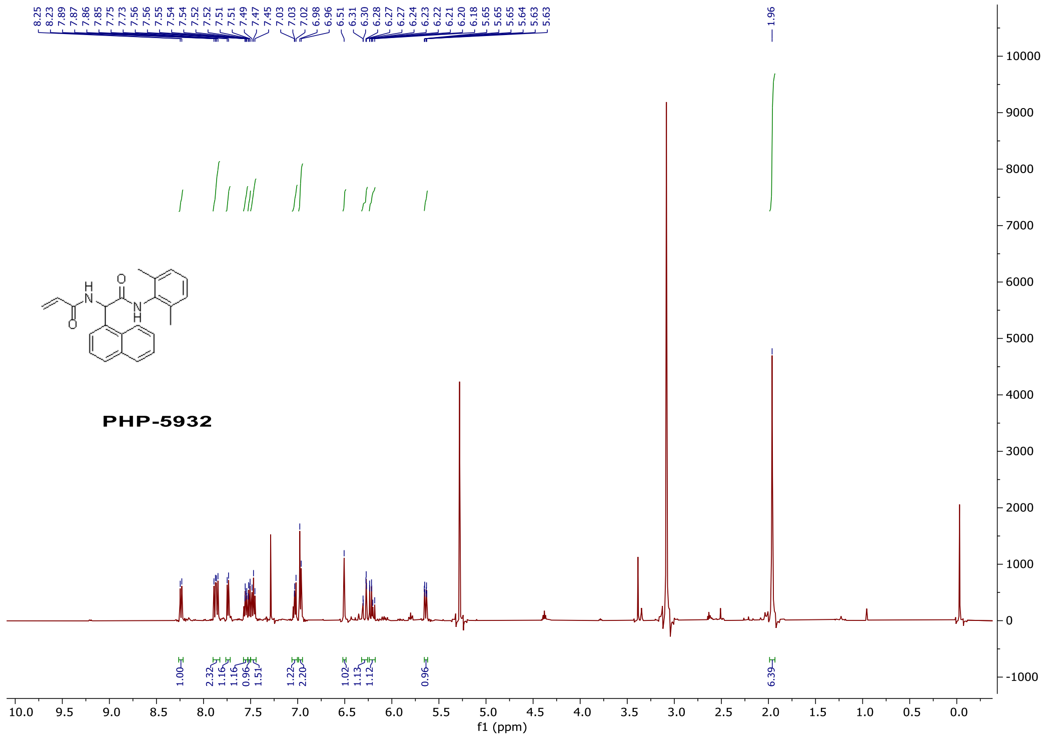

Supplementary Figure 817. ^{1}H NMR (400 MHz, $CDCl_3$ + one drop of CD_3OD) of compound **PHP-5932.**

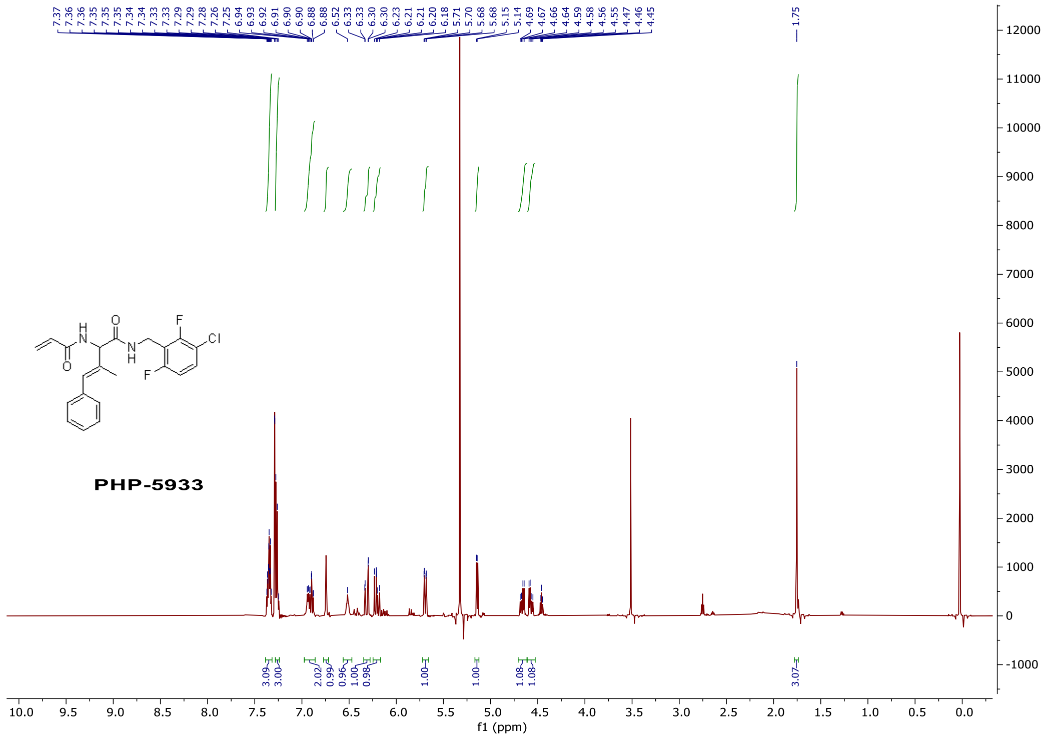

Supplementary Figure 818. ^{1}H NMR (400 MHz, $CDCl_3$ + one drop of CD_3OD) of compound **PHP-5933.**

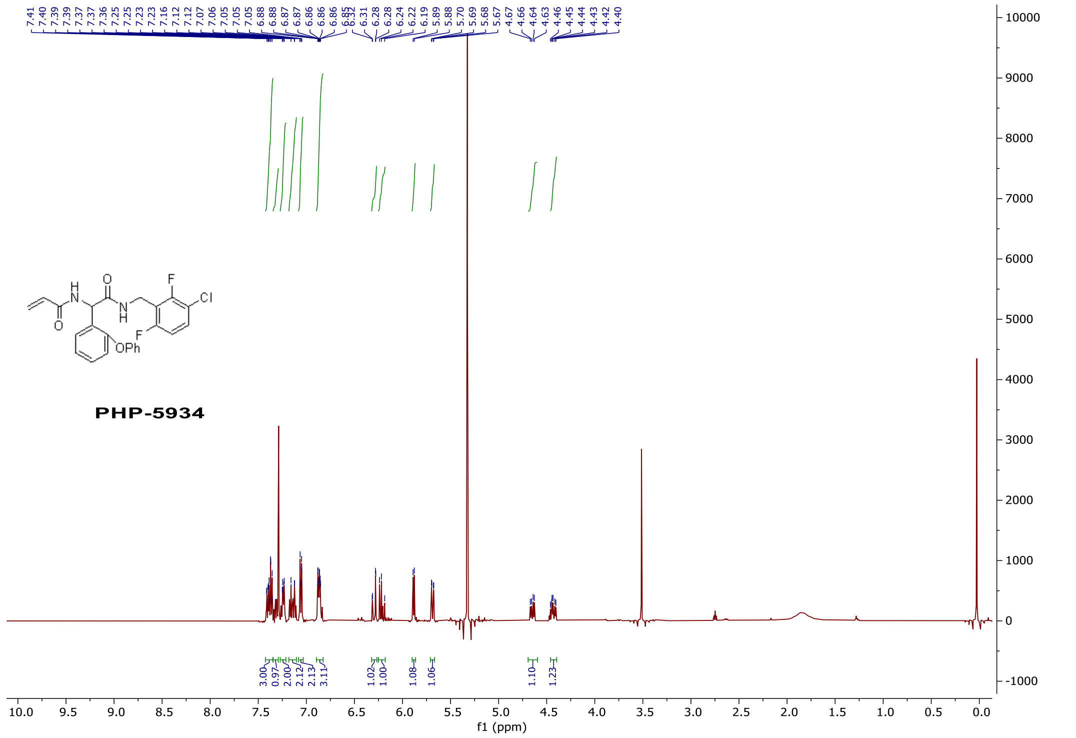

Supplementary Figure 819. ^{1}H NMR (400 MHz, $CDCl_3$ + one drop of CD_3OD) of compound **PHP-5934.**

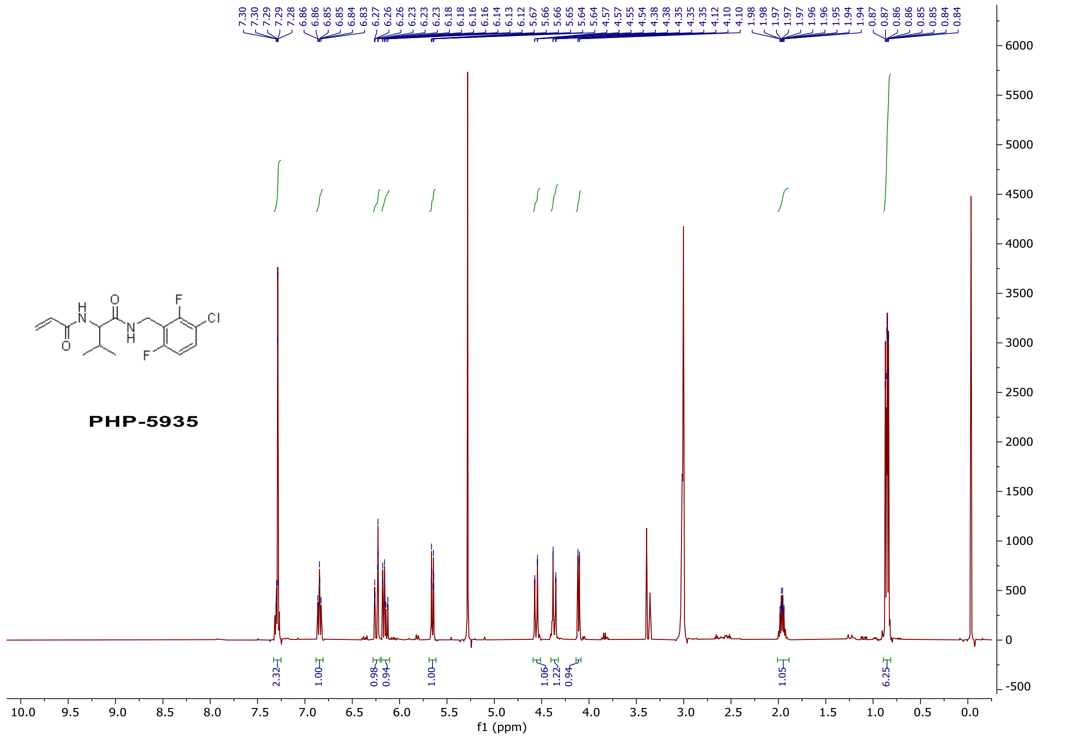

Supplementary Figure 820. ^{1}H NMR (400 MHz, $CDCl_3$ + one drop of CD_3OD) of compound **PHP-5935.**

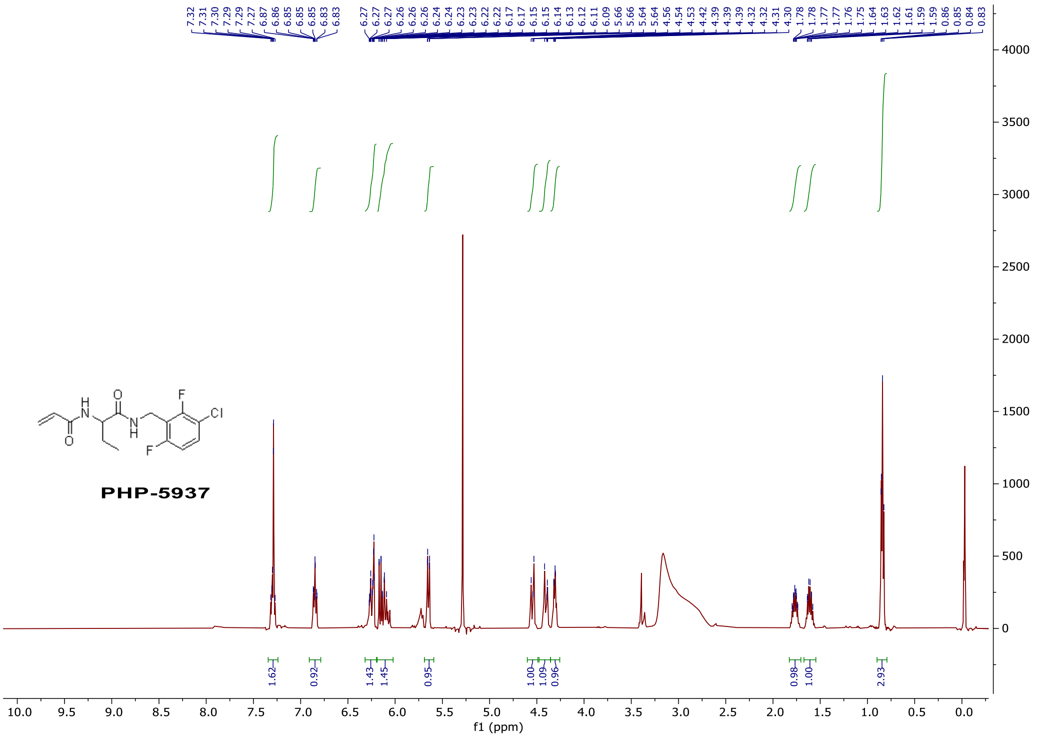

Supplementary Figure 821. ^{1}H NMR (400 MHz, $CDCl_3$ + one drop of CD_3OD) of compound **PHP-5937.**

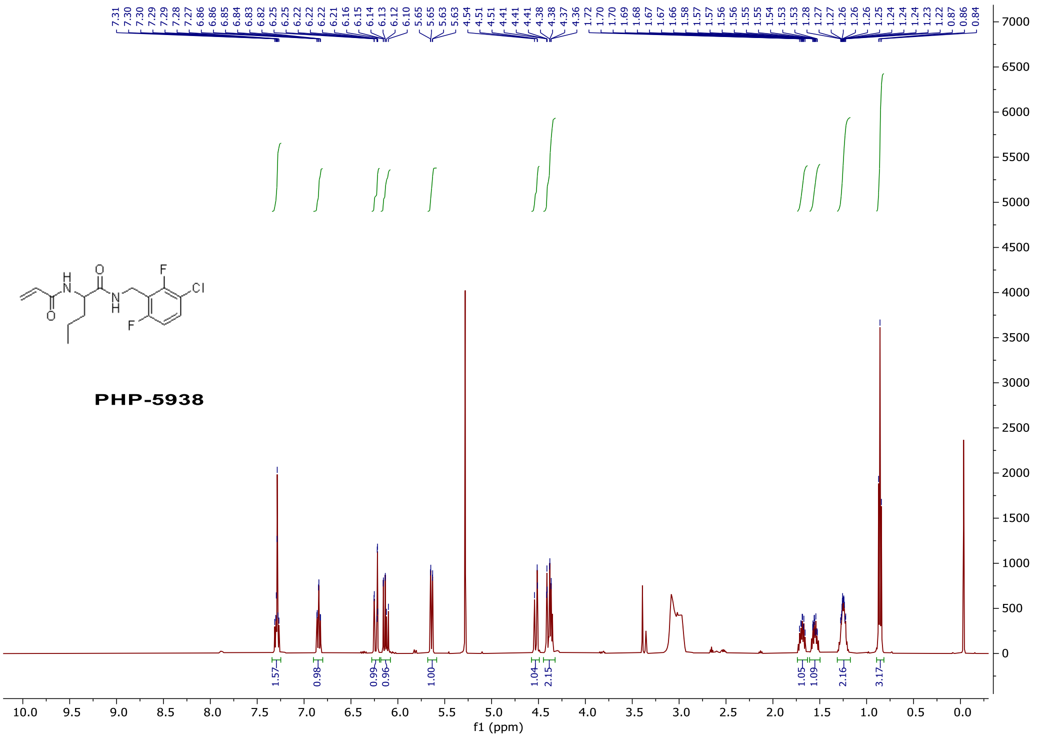

Supplementary Figure 822. ^{1}H NMR (400 MHz, $CDCl_3$ + one drop of CD_3OD) of compound **PHP-5938.**

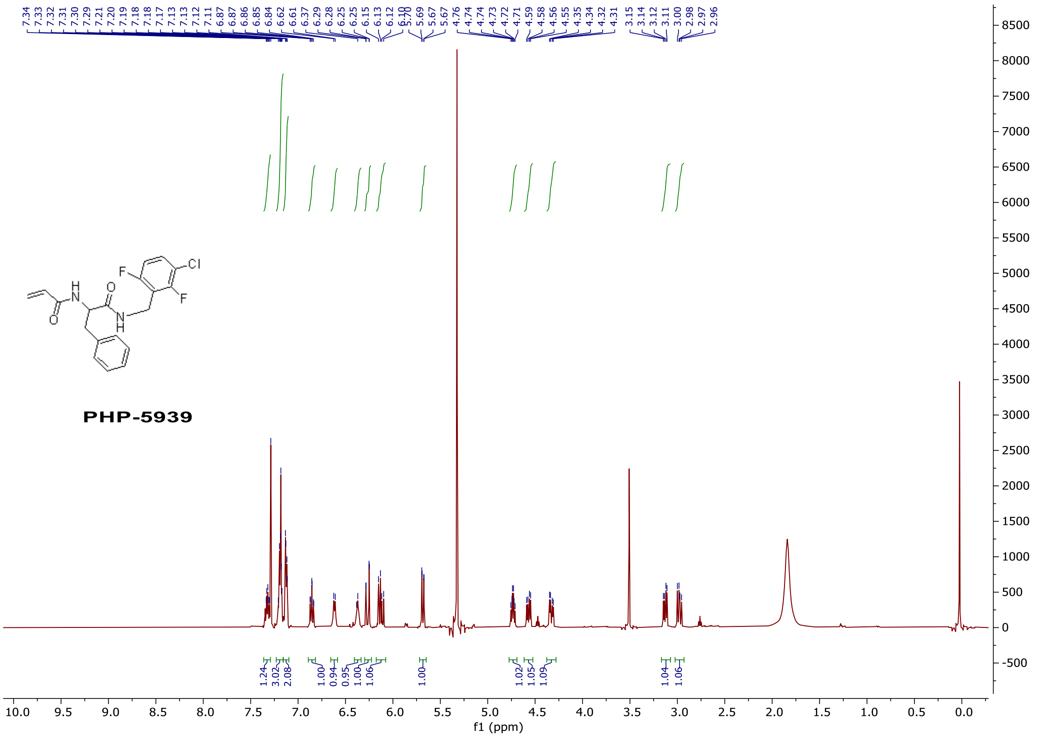

Supplementary Figure 823. ^{1}H NMR (400 MHz, $CDCl_3$ + one drop of CD_3OD) of compound **PHP-5939.**

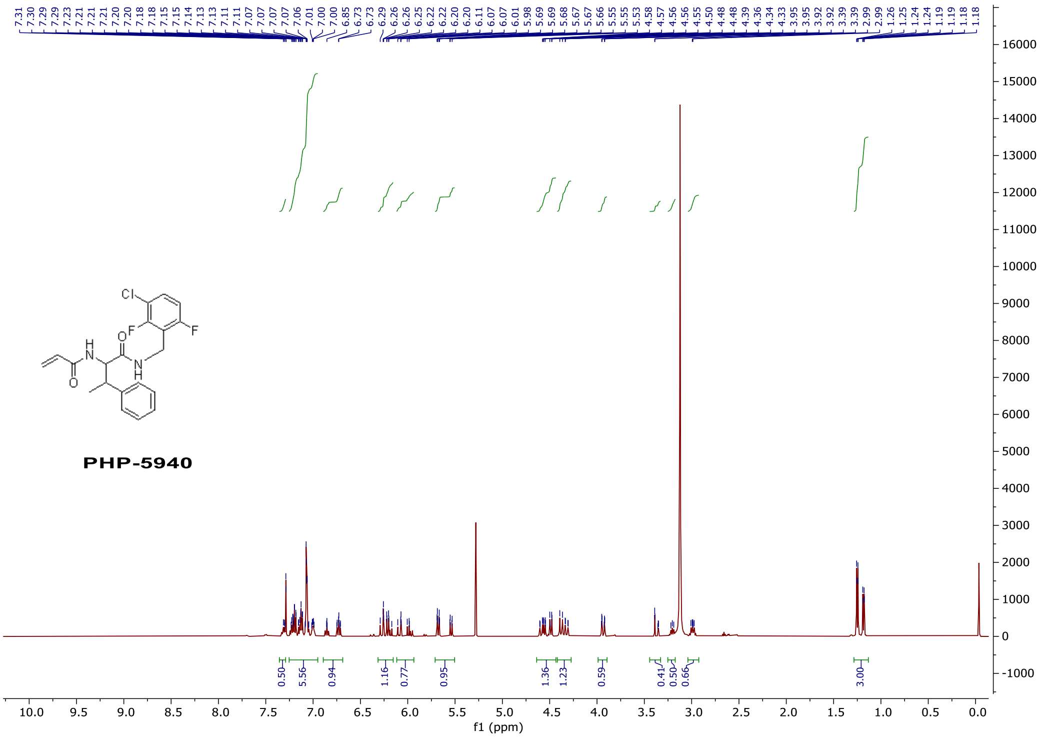

Supplementary Figure 824. ^{1}H NMR (400 MHz, $CDCl_3$ + one drop of CD_3OD) of compound **PHP-5940.**

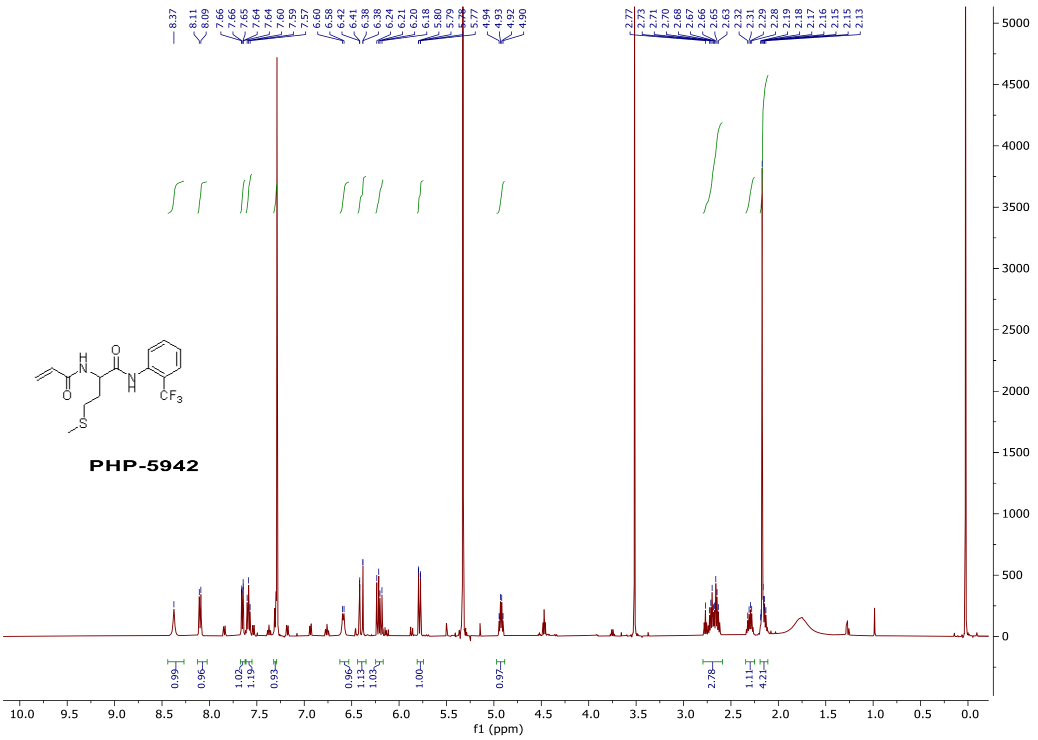

Supplementary Figure 825. ^{1}H NMR (400 MHz, $CDCl_3$ + one drop of CD_3OD) of compound **PHP-5942**.

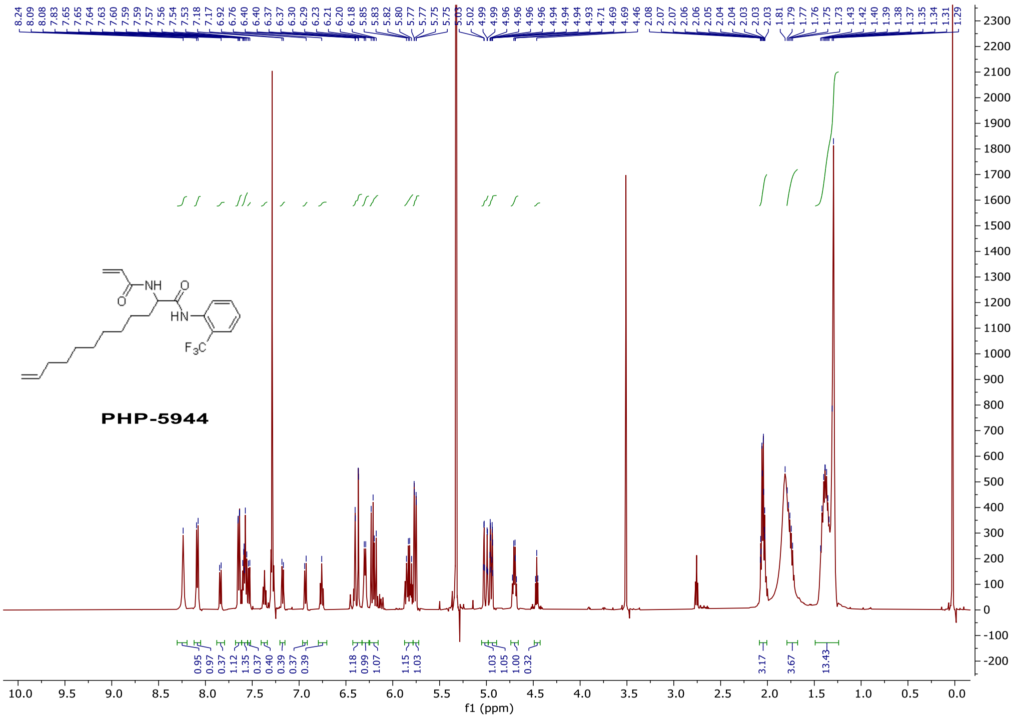

Supplementary Figure 826. ^{1}H NMR (400 MHz, $CDCl_3$ + one drop of CD_3OD) of compound **PHP-5944**.

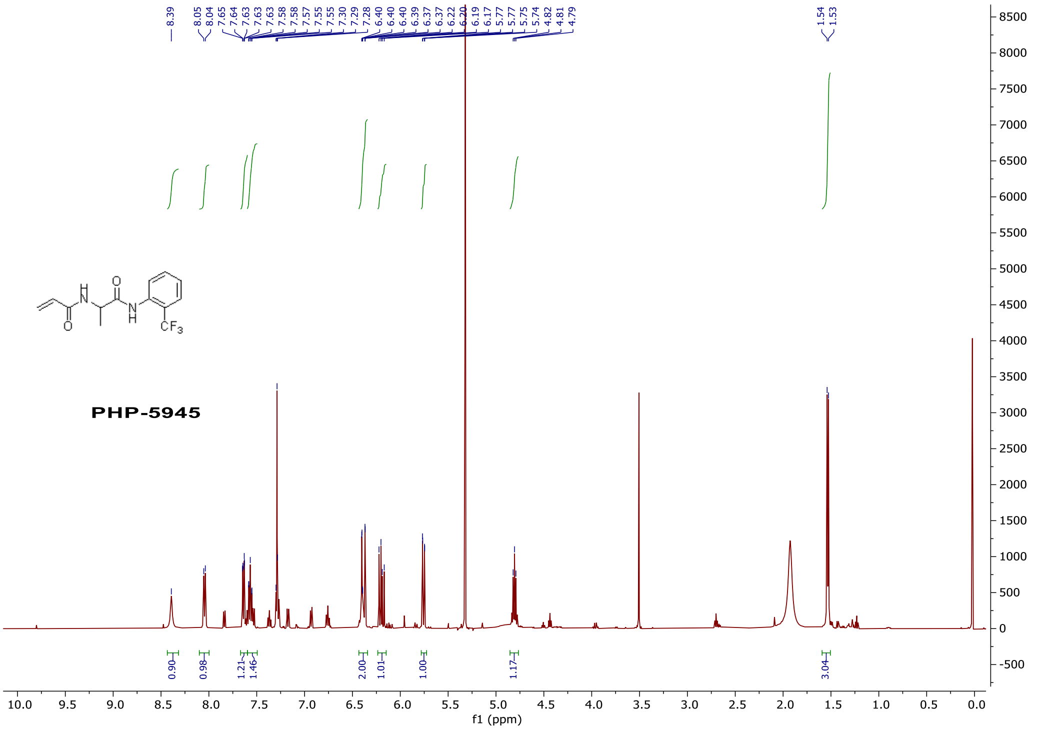

Supplementary Figure 827. ^{1}H NMR (400 MHz, $CDCl_3$ + one drop of CD_3OD) of compound **PHP-5945.**

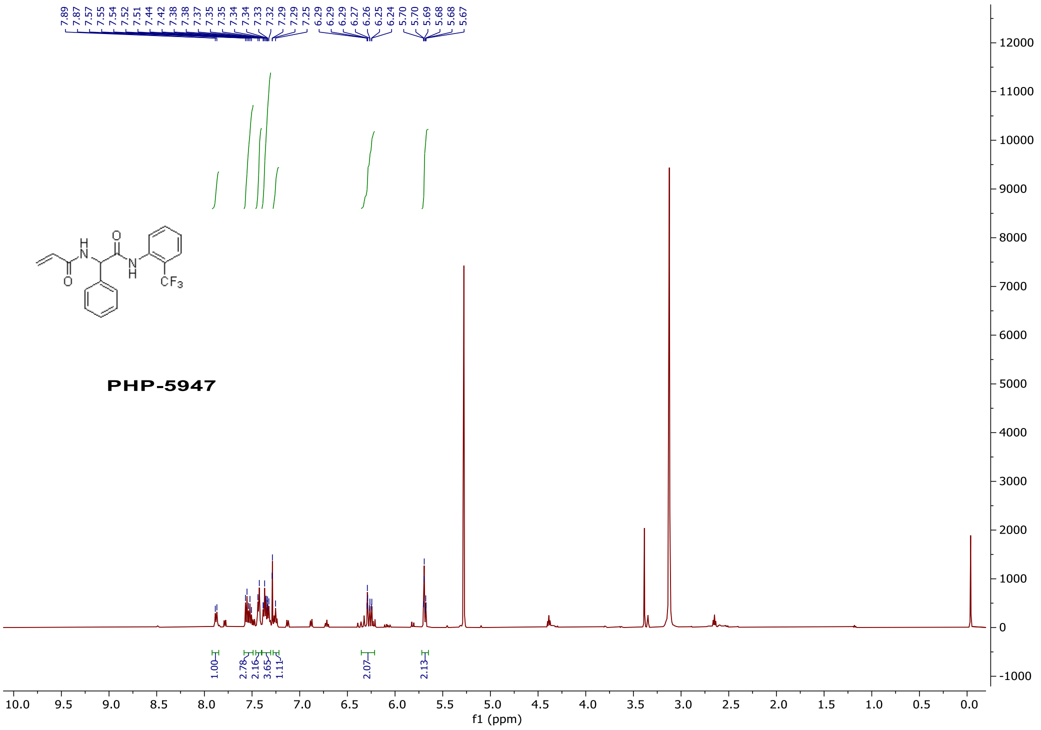

Supplementary Figure 828. ^{1}H NMR (400 MHz, $CDCl_3$ + one drop of CD_3OD) of compound **PHP-5947.**

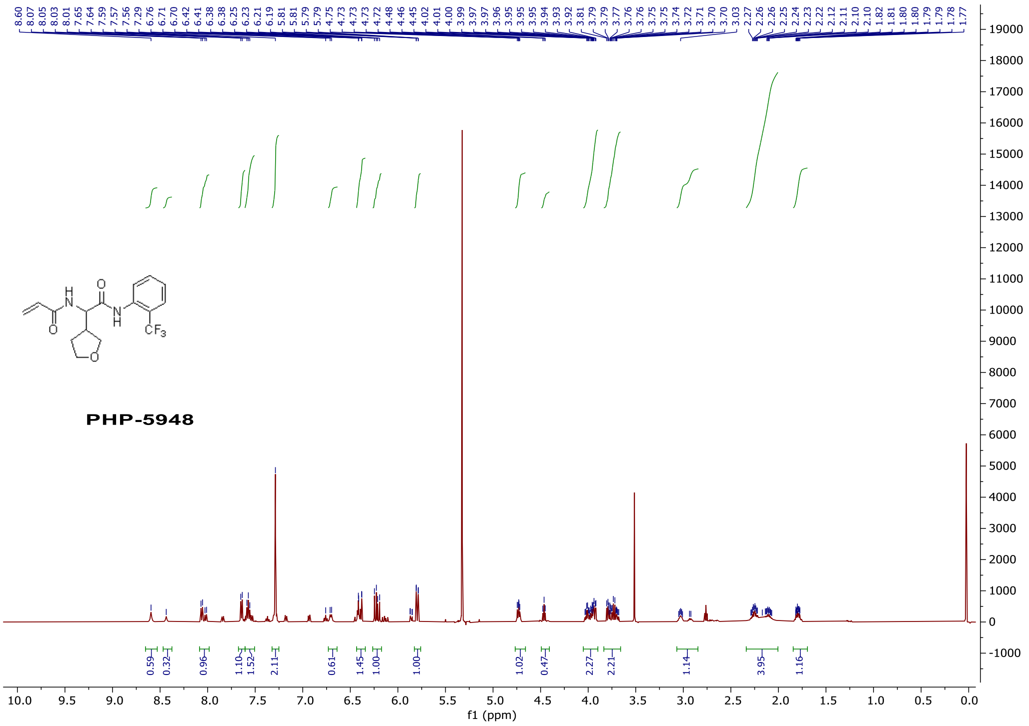

Supplementary Figure 829. ^{1}H NMR (400 MHz, $CDCl_3$ + one drop of CD_3OD) of compound **PHP-5948.**

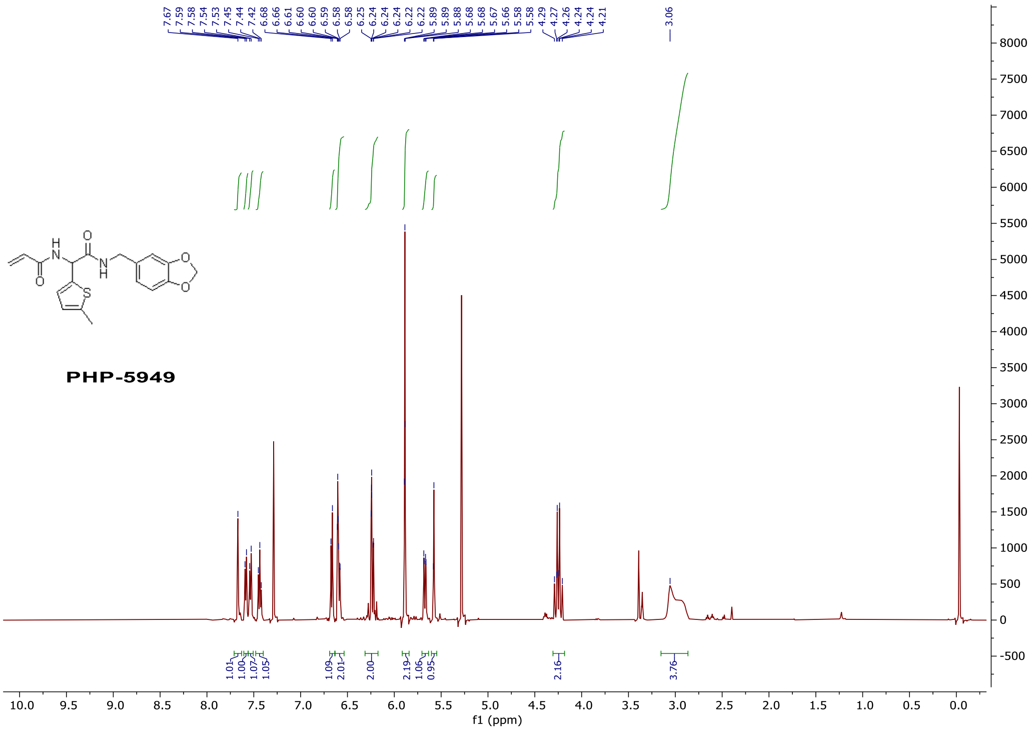

Supplementary Figure 830. ^{1}H NMR (400 MHz, $CDCl_3$ + one drop of CD_3OD) of compound **PHP-5949.**

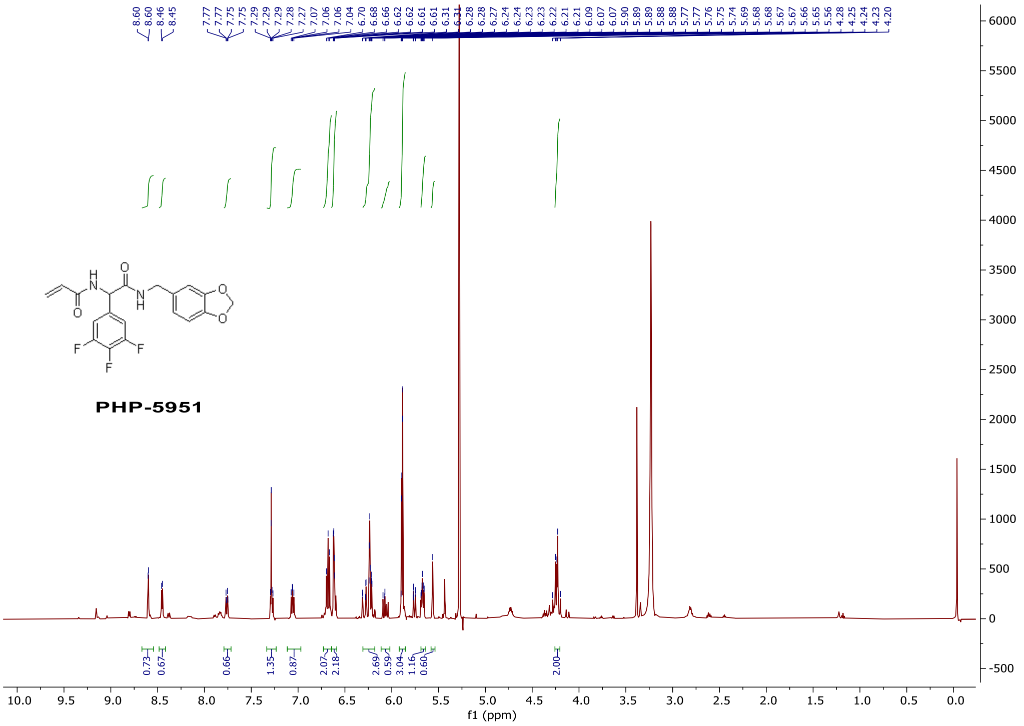

Supplementary Figure 831. ^{1}H NMR (400 MHz, $CDCl_3$ + one drop of CD_3OD) of compound **PHP-5951**.

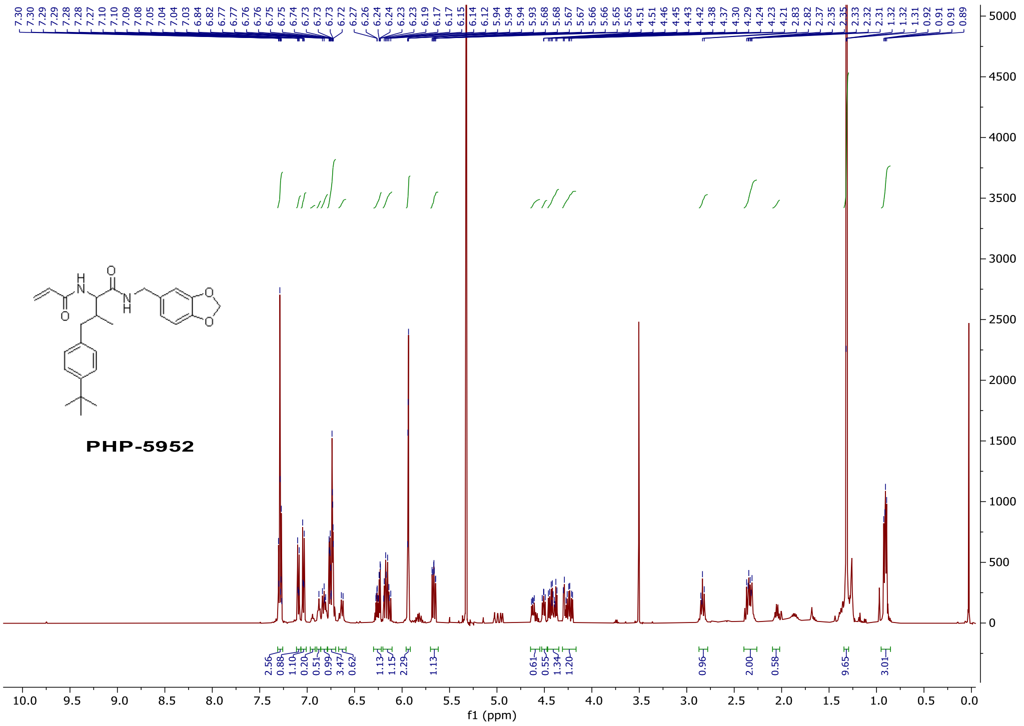

Supplementary Figure 832. ^{1}H NMR (400 MHz, $CDCl_3$ + one drop of CD_3OD) of compound **PHP-5952**.

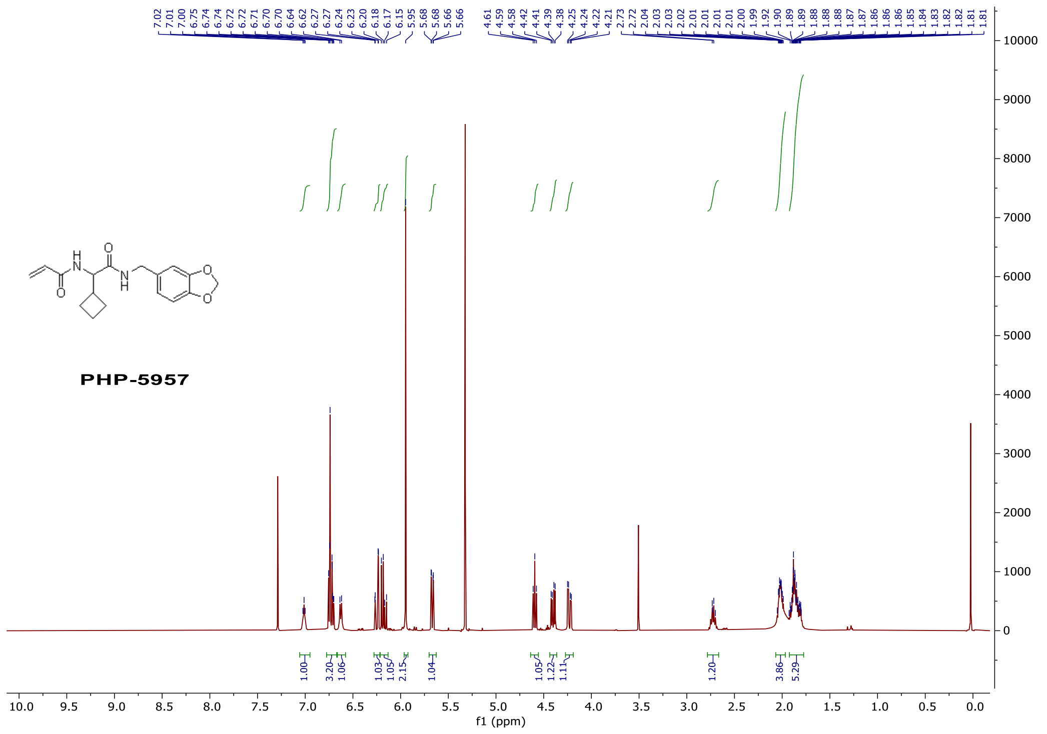

Supplementary Figure 833. ^{1}H NMR (400 MHz, $CDCl_3$ + one drop of CD_3OD) of compound **PHP-5957.**

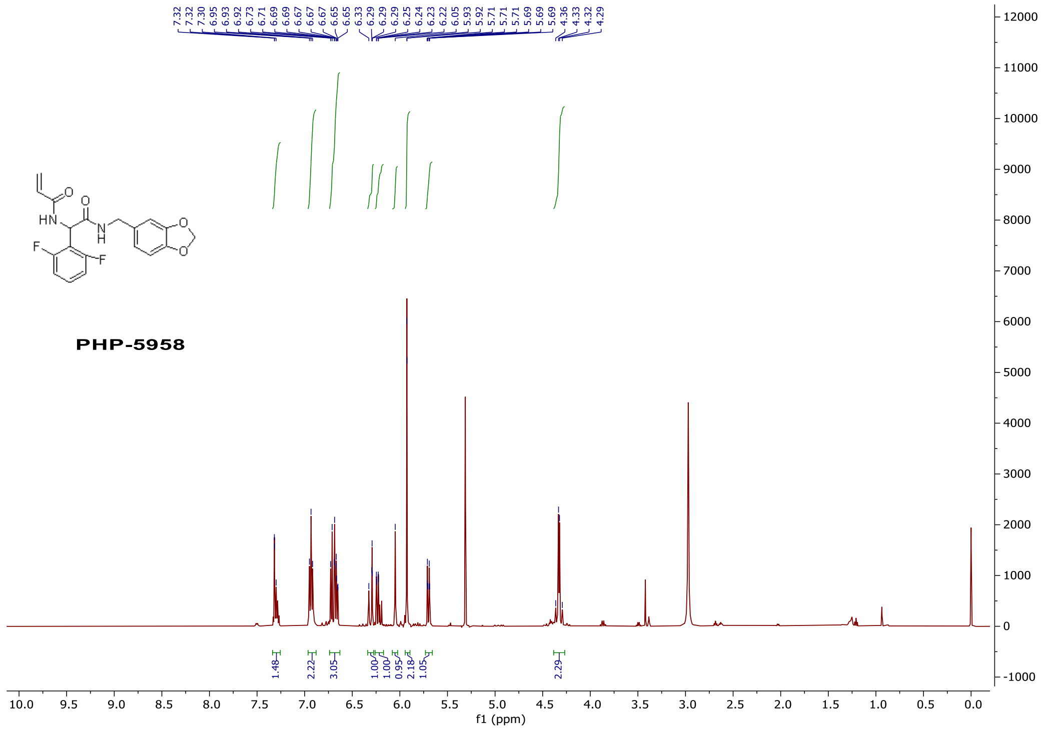

Supplementary Figure 834. ^{1}H NMR (400 MHz, $CDCl_3$ + one drop of CD_3OD) of compound **PHP-5958.**

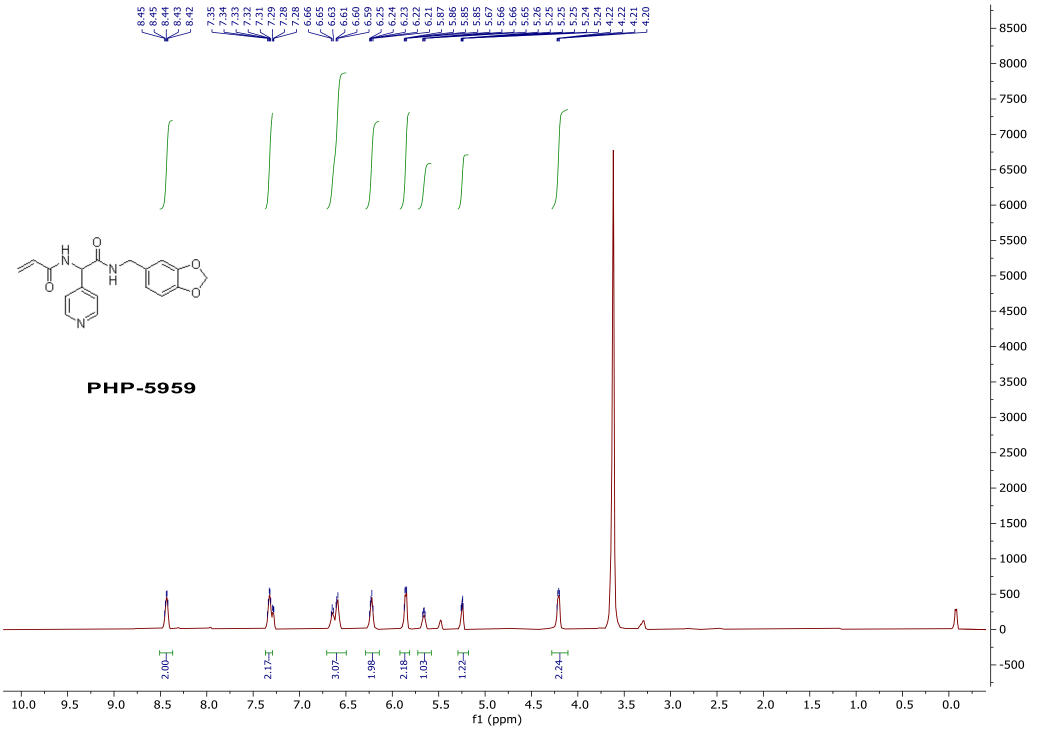

Supplementary Figure 835. ^{1}H NMR (400 MHz, $CDCl_3$ + one drop of CD_3OD) of compound **PHP-5959.**

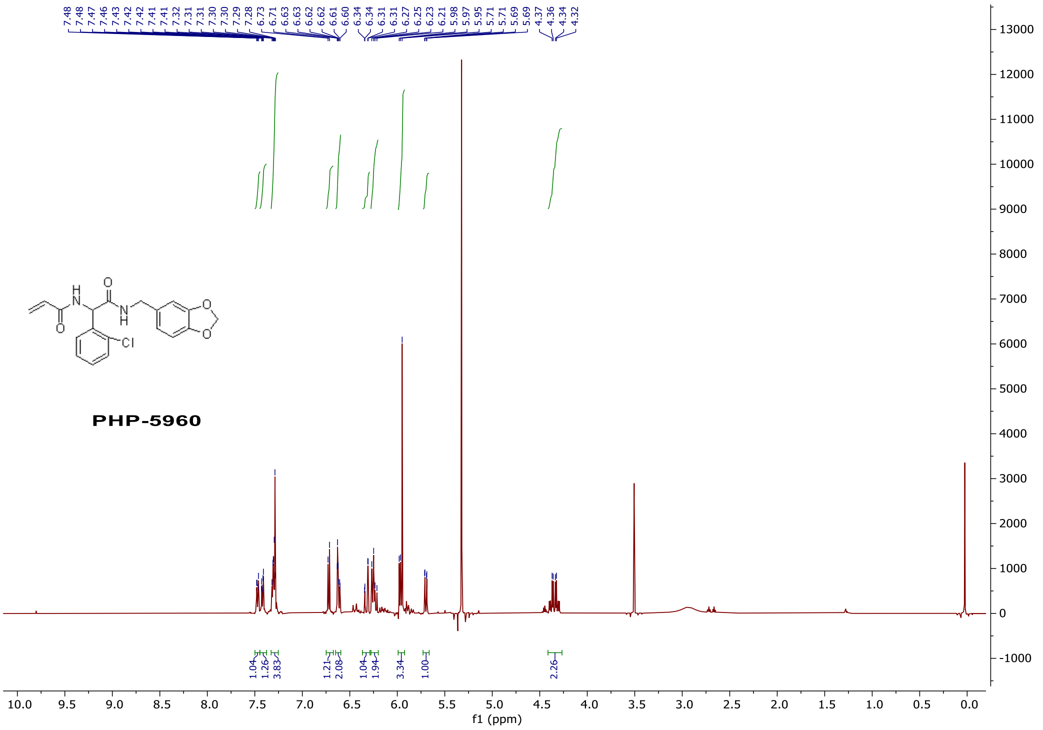

Supplementary Figure 836. ^{1}H NMR (400 MHz, $CDCl_3$ + one drop of CD_3OD) of compound **PHP-5960.**

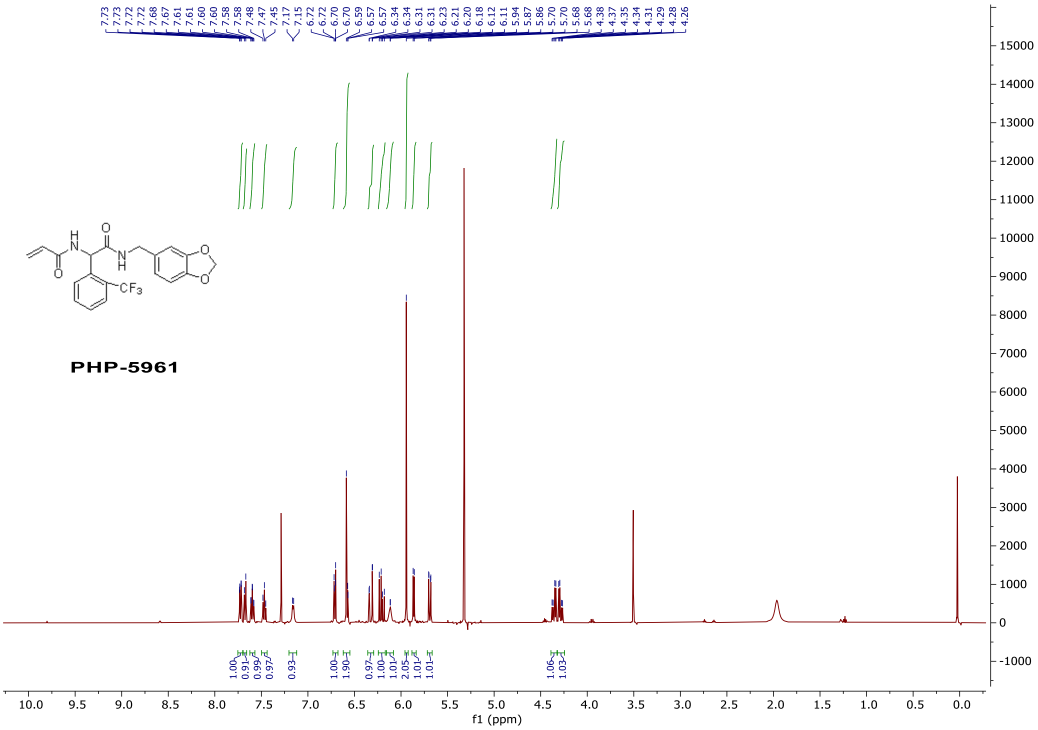

Supplementary Figure 837. ^{1}H NMR (400 MHz, $CDCl_3$ + one drop of CD_3OD) of compound **PHP-5961.**

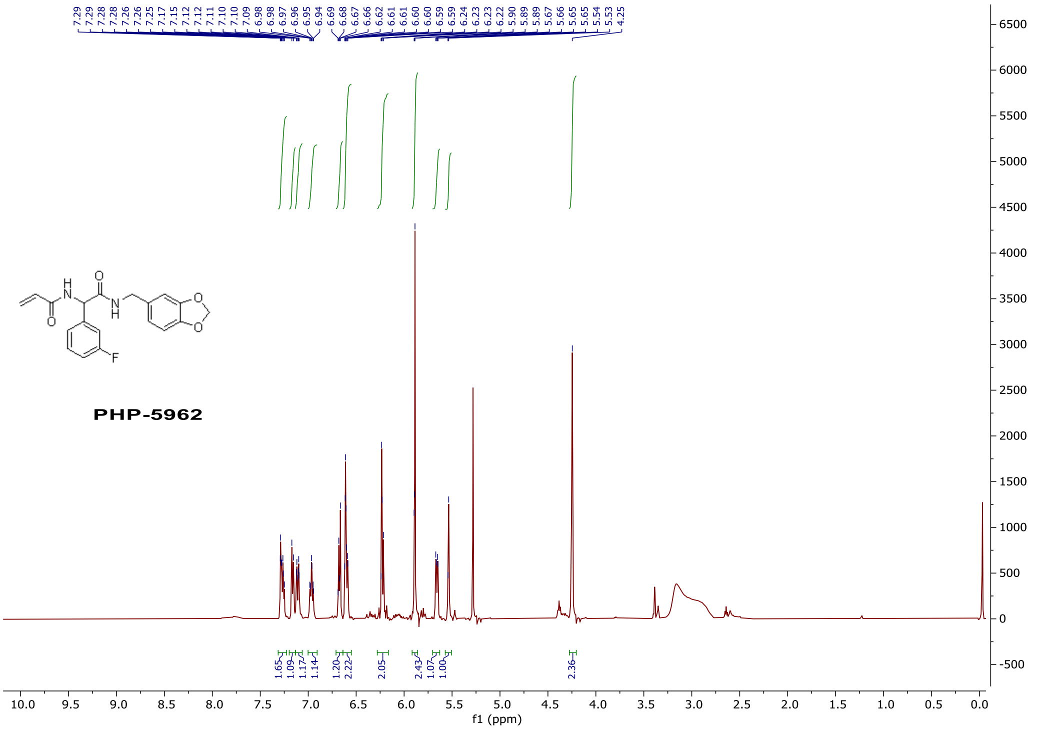

Supplementary Figure 838. ^{1}H NMR (400 MHz, $CDCl_3$ + one drop of CD_3OD) of compound **PHP-5962.**

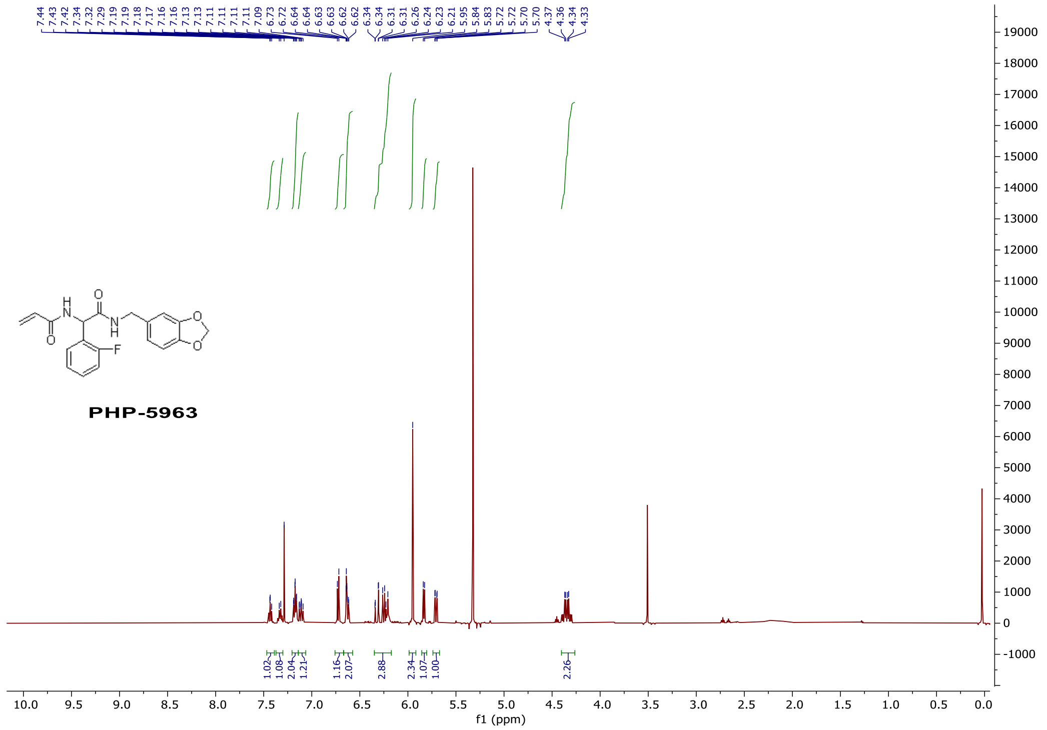

Supplementary Figure 839. ^{1}H NMR (400 MHz, $CDCl_3$ + one drop of CD_3OD) of compound **PHP-5963.**

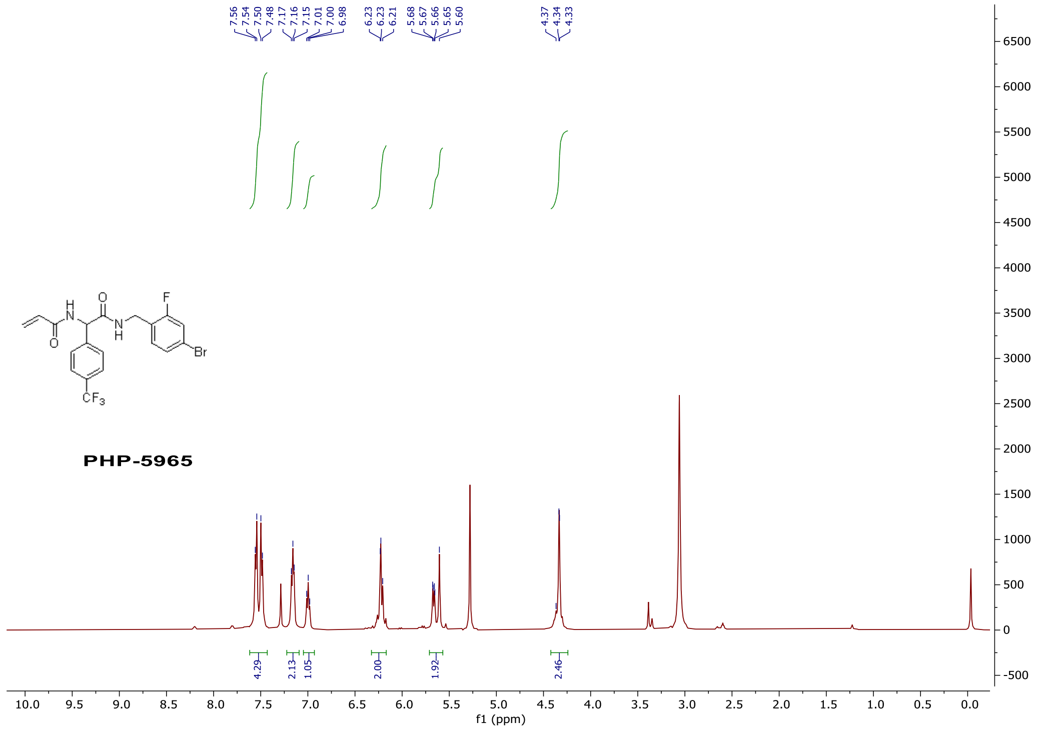

Supplementary Figure 840. ^{1}H NMR (400 MHz, $CDCl_3$ + one drop of CD_3OD) of compound **PHP-5965.**

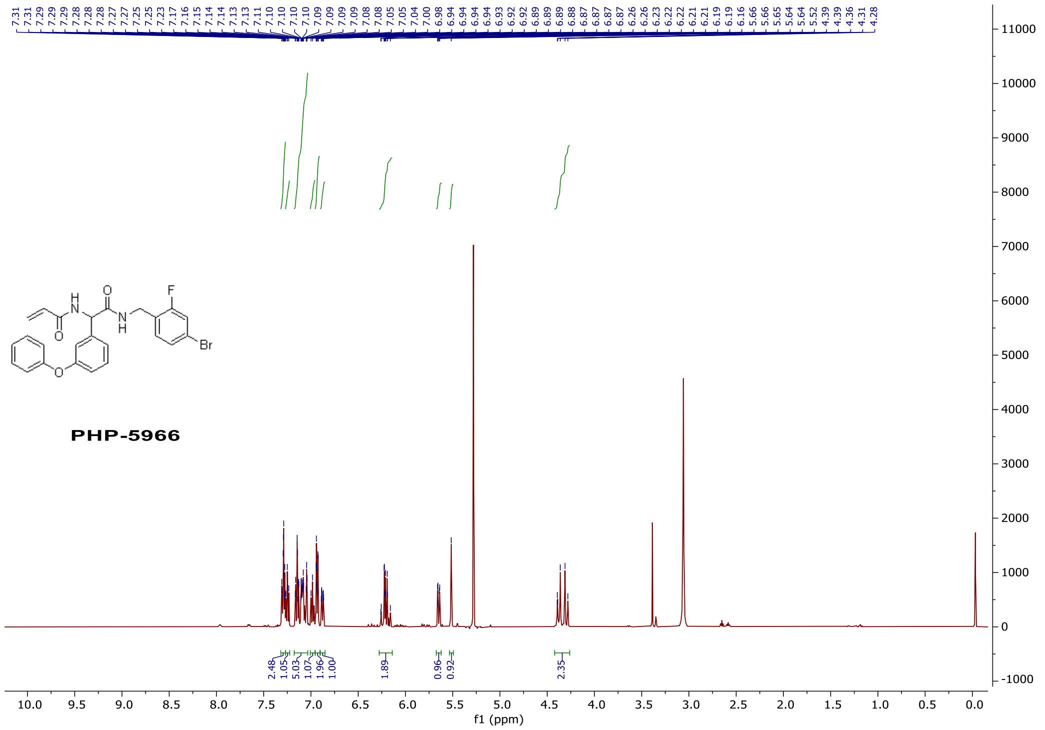

Supplementary Figure 841. ^{1}H NMR (400 MHz, $CDCl_3$ + one drop of CD_3OD) of compound **PHP-5966.**

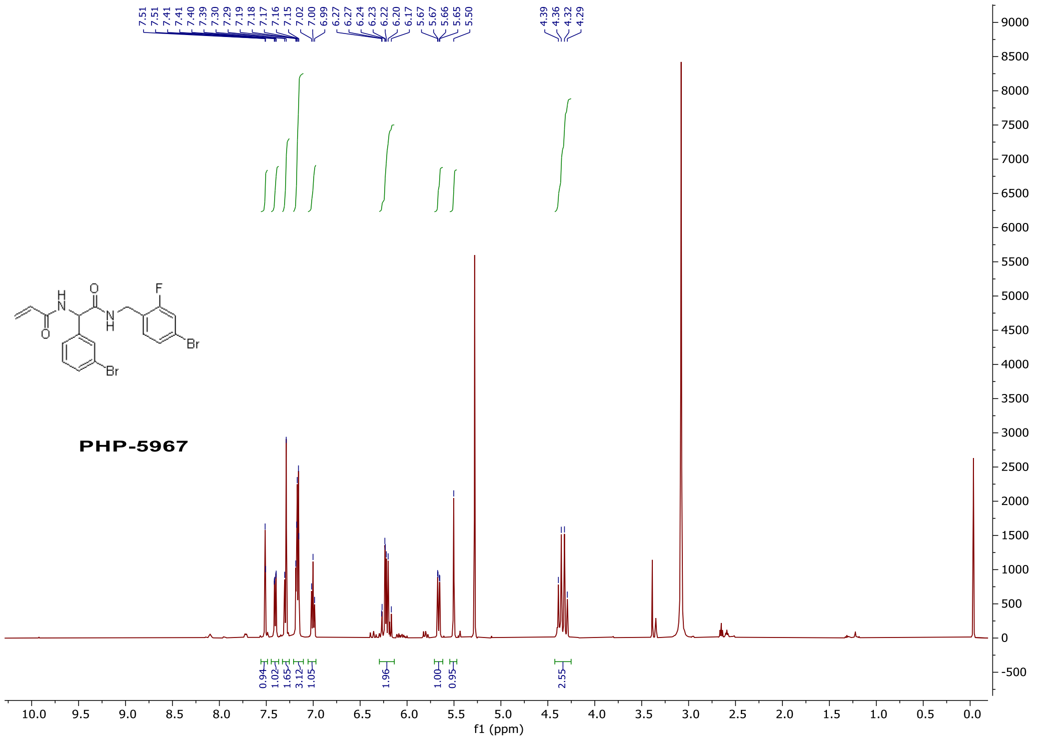

Supplementary Figure 842. ^{1}H NMR (400 MHz, $CDCl_3$ + one drop of CD_3OD) of compound **PHP-5967.**

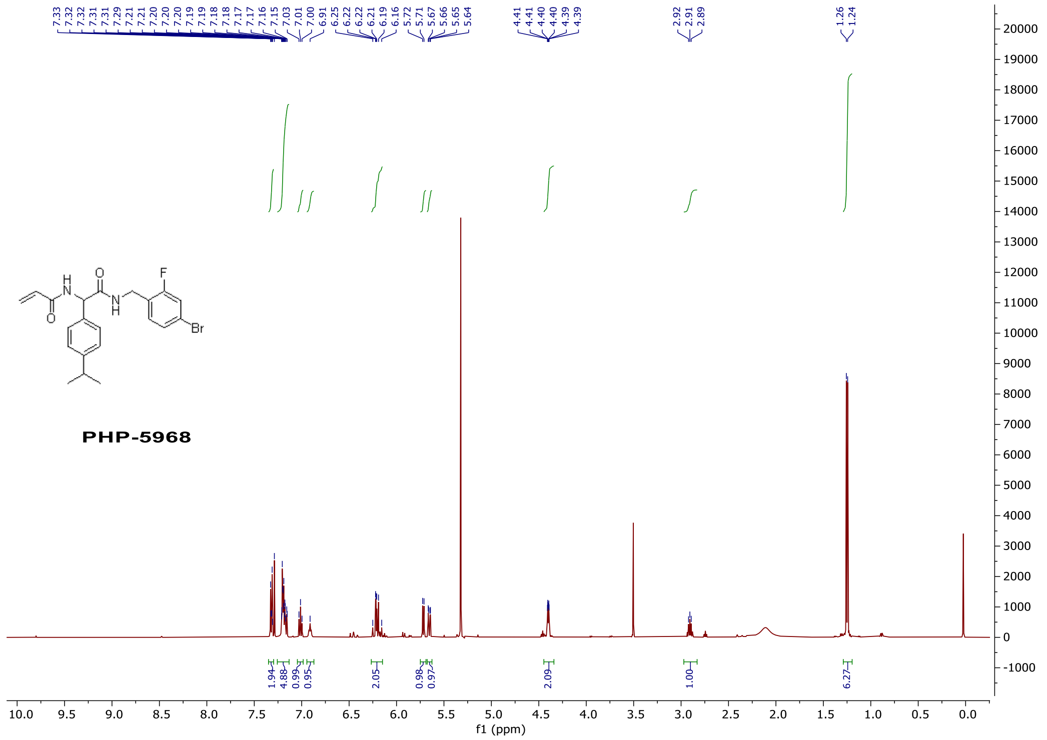

Supplementary Figure 843. ^{1}H NMR (400 MHz, $CDCl_3$ + one drop of CD_3OD) of compound **PHP-5968.**

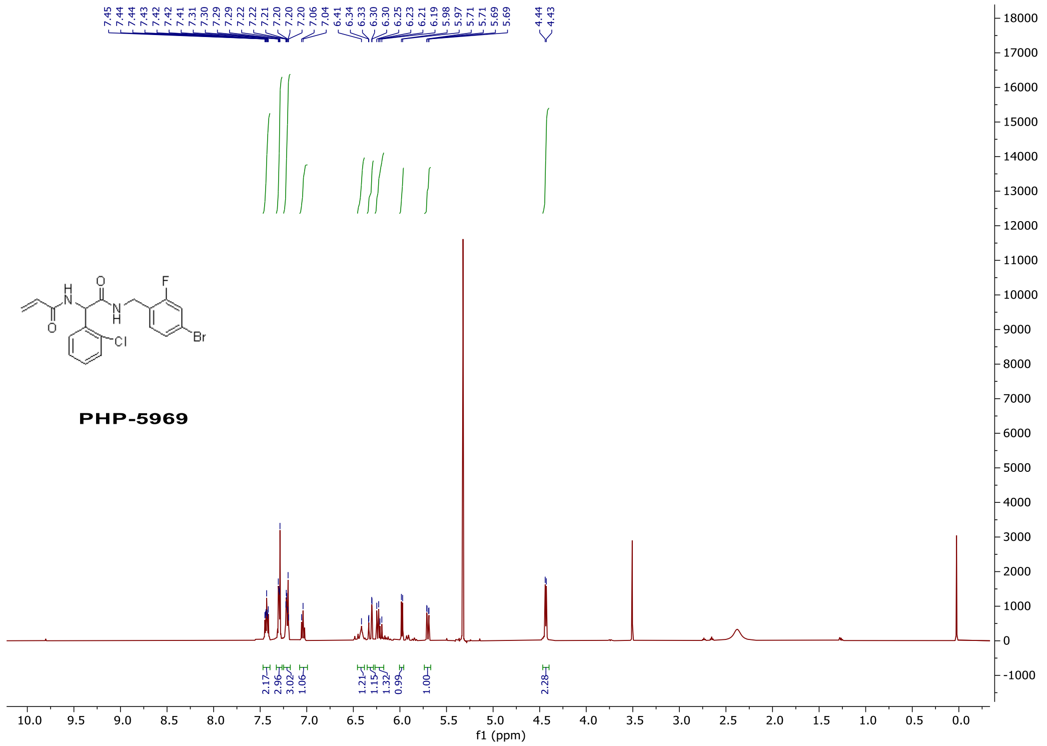

Supplementary Figure 844. ^{1}H NMR (400 MHz, $CDCl_3$ + one drop of CD_3OD) of compound **PHP-5969.**

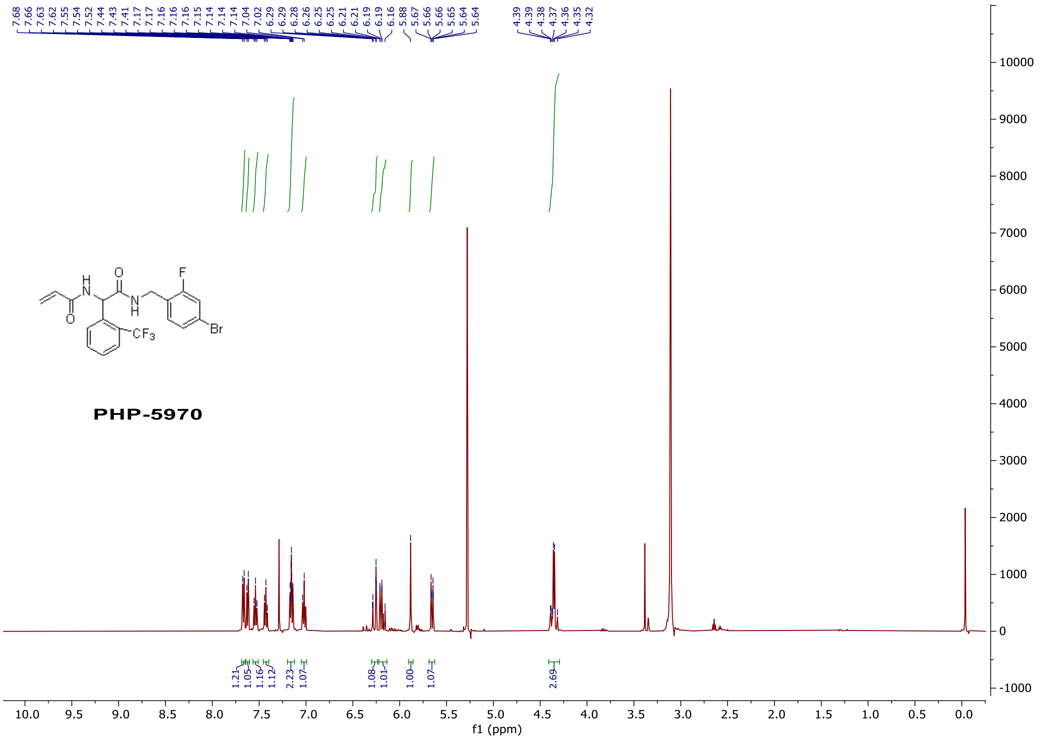

Supplementary Figure 845. ^{1}H NMR (400 MHz, $CDCl_3$ + one drop of CD_3OD) of compound **PHP-5970.**

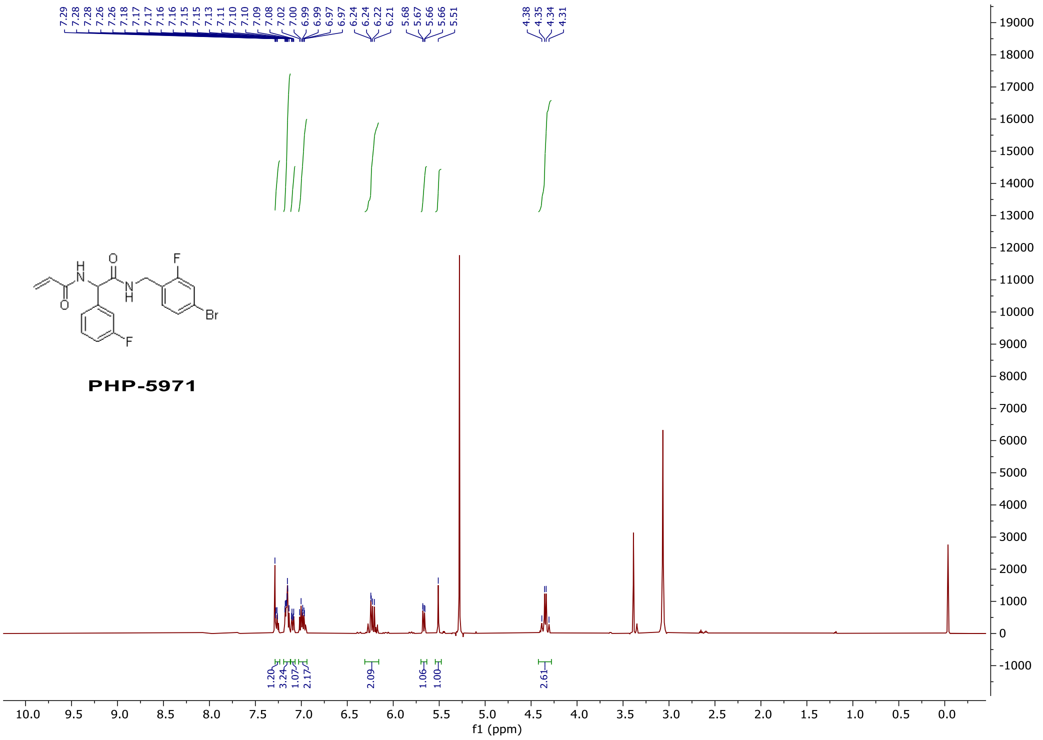

Supplementary Figure 846. ^{1}H NMR (400 MHz, $CDCl_3$ + one drop of CD_3OD) of compound **PHP-5971.**

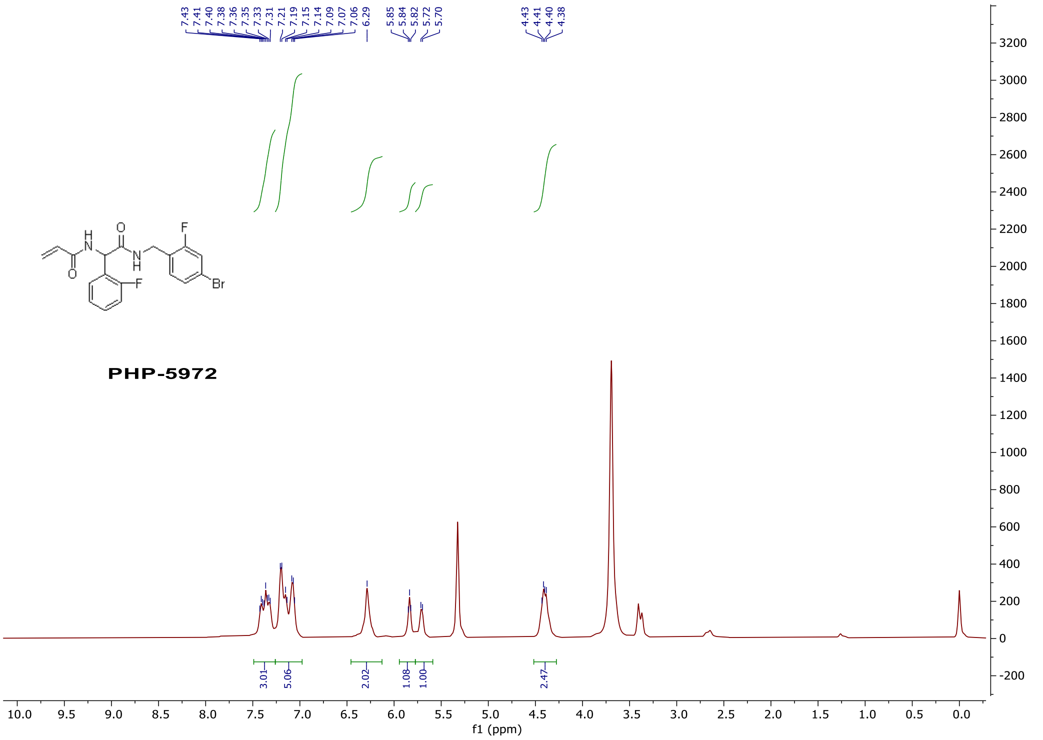

Supplementary Figure 847. ^{1}H NMR (400 MHz, $CDCl_3$ + one drop of CD_3OD) of compound **PHP-5972.**

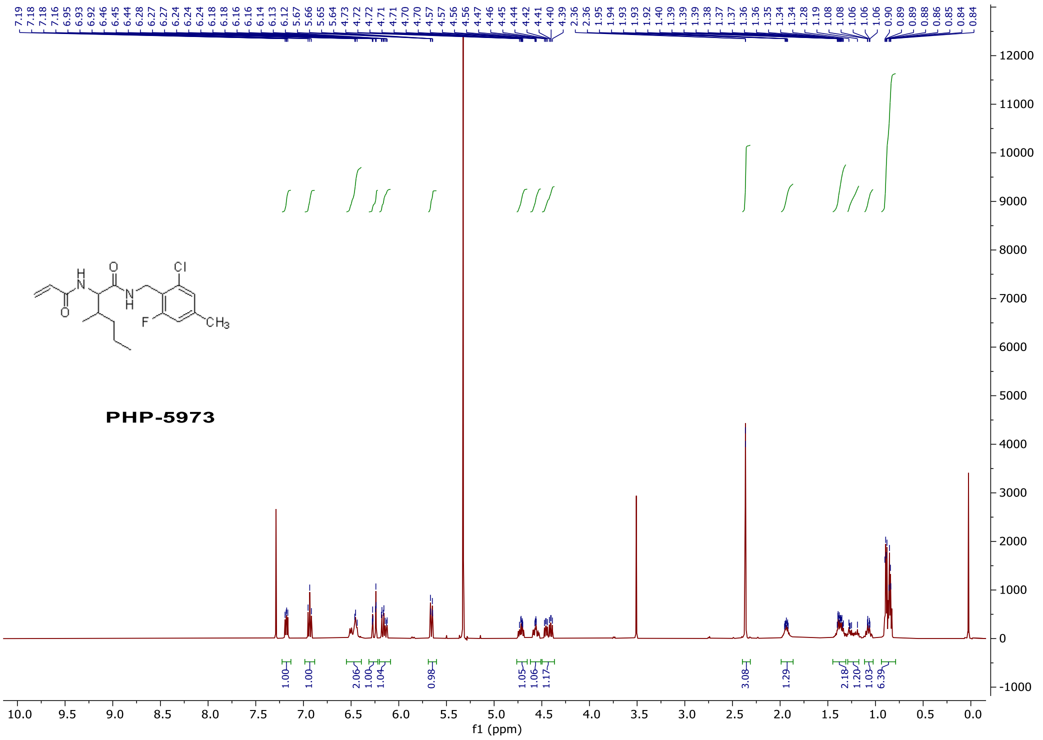

Supplementary Figure 848. ^{1}H NMR (400 MHz, $CDCl_3$ + one drop of CD_3OD) of compound **PHP-5973.**

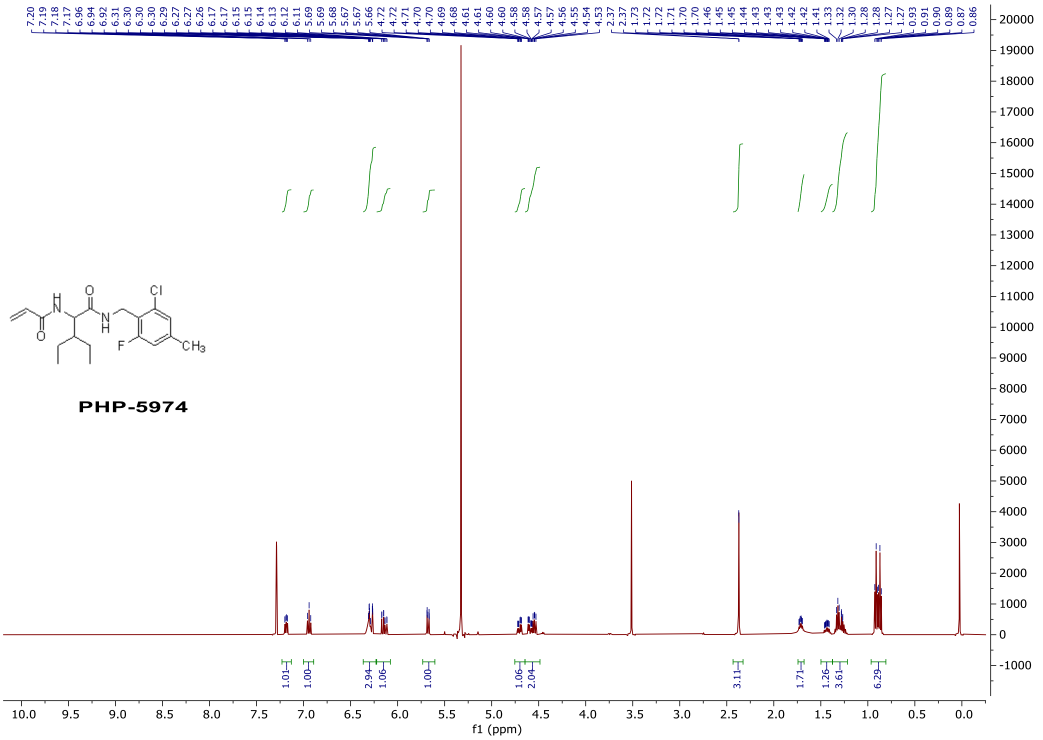

Supplementary Figure 849. ^{1}H NMR (400 MHz, $CDCl_3$ + one drop of CD_3OD) of compound **PHP-5974.**

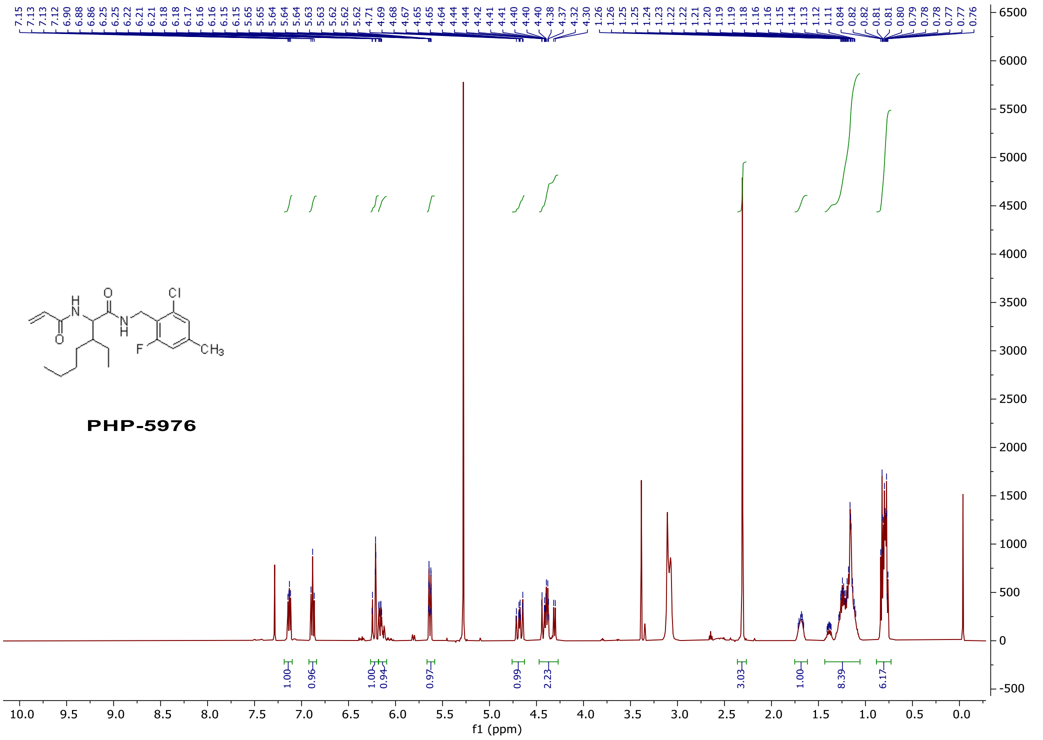

Supplementary Figure 850. ^{1}H NMR (400 MHz, $CDCl_3$ + one drop of CD_3OD) of compound **PHP-5976.**

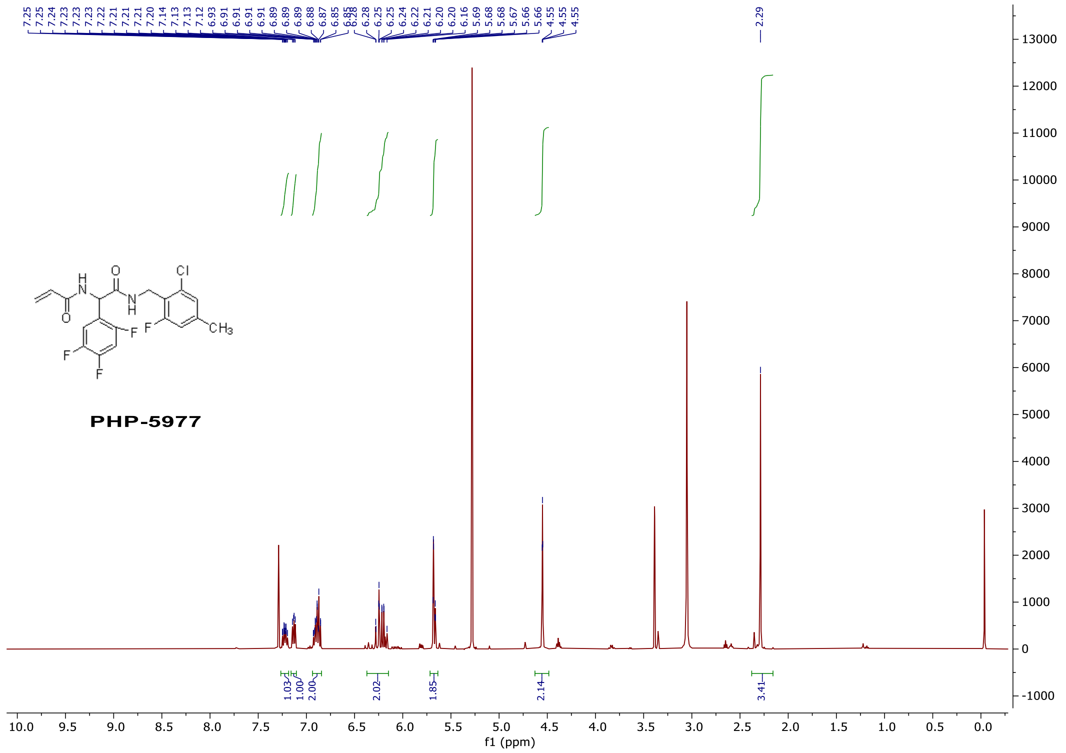

Supplementary Figure 851. ^{1}H NMR (400 MHz, $CDCl_3$ + one drop of CD_3OD) of compound **PHP-5977.**

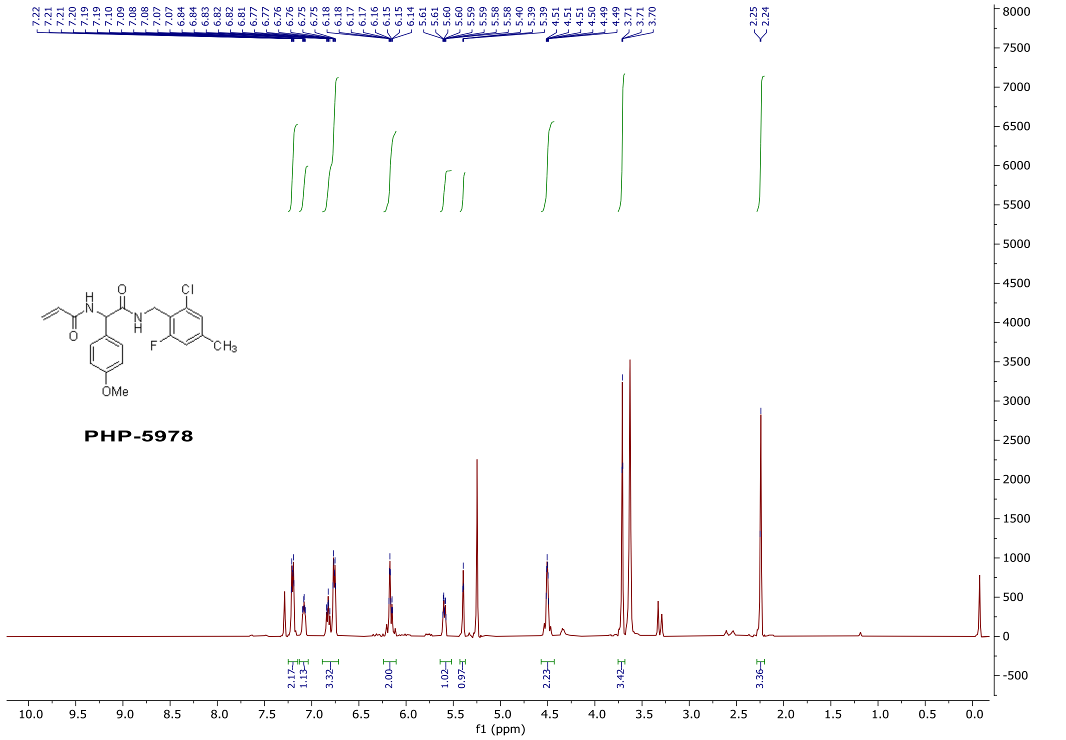

Supplementary Figure 852. ^{1}H NMR (400 MHz, $CDCl_3$ + one drop of CD_3OD) of compound **PHP-5978.**

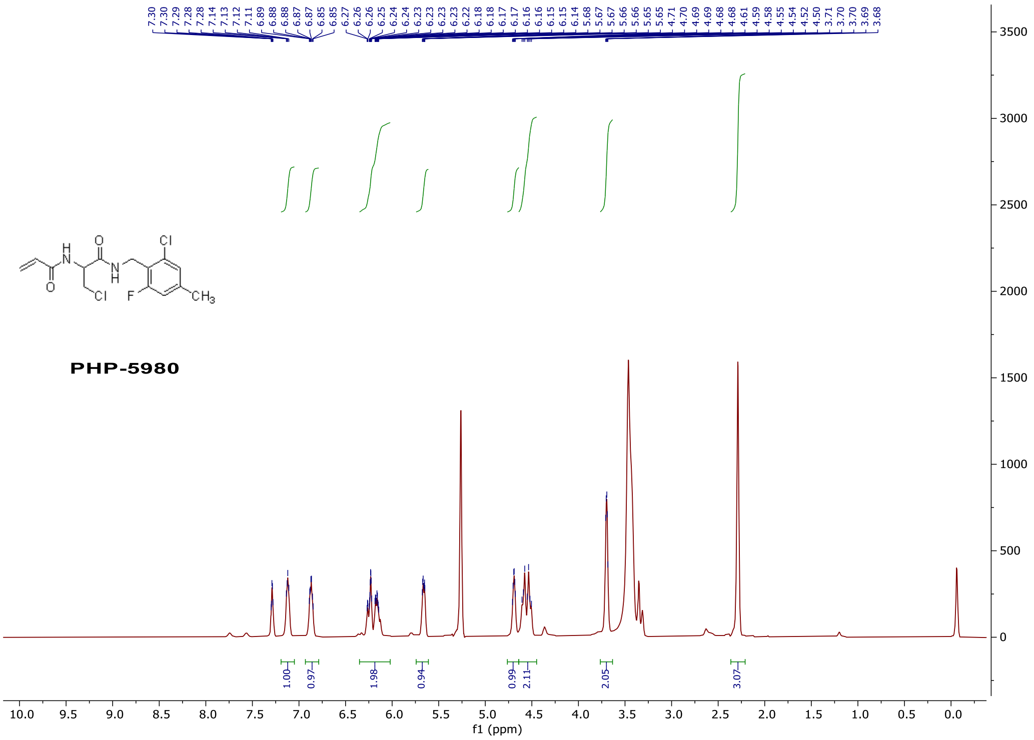

Supplementary Figure 853. ^{1}H NMR (400 MHz, $CDCl_3$ + one drop of CD_3OD) of compound **PHP-5980.**

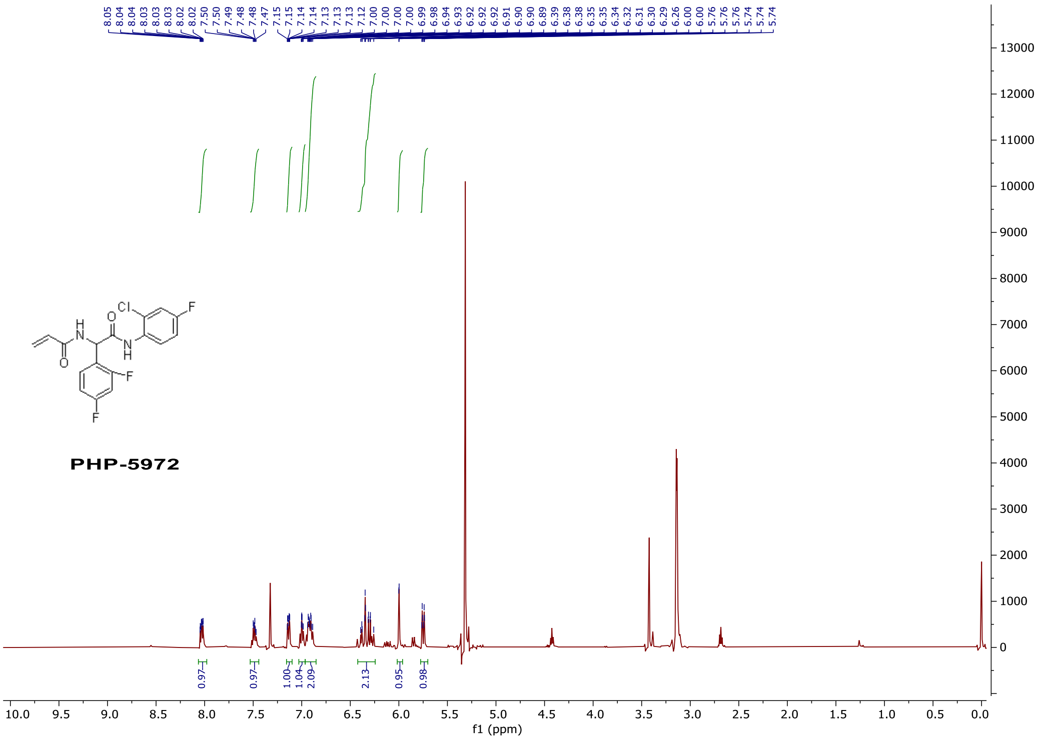

Supplementary Figure 854. ^{1}H NMR (400 MHz, $CDCl_3$ + one drop of CD_3OD) of compound **PHP-5982.**

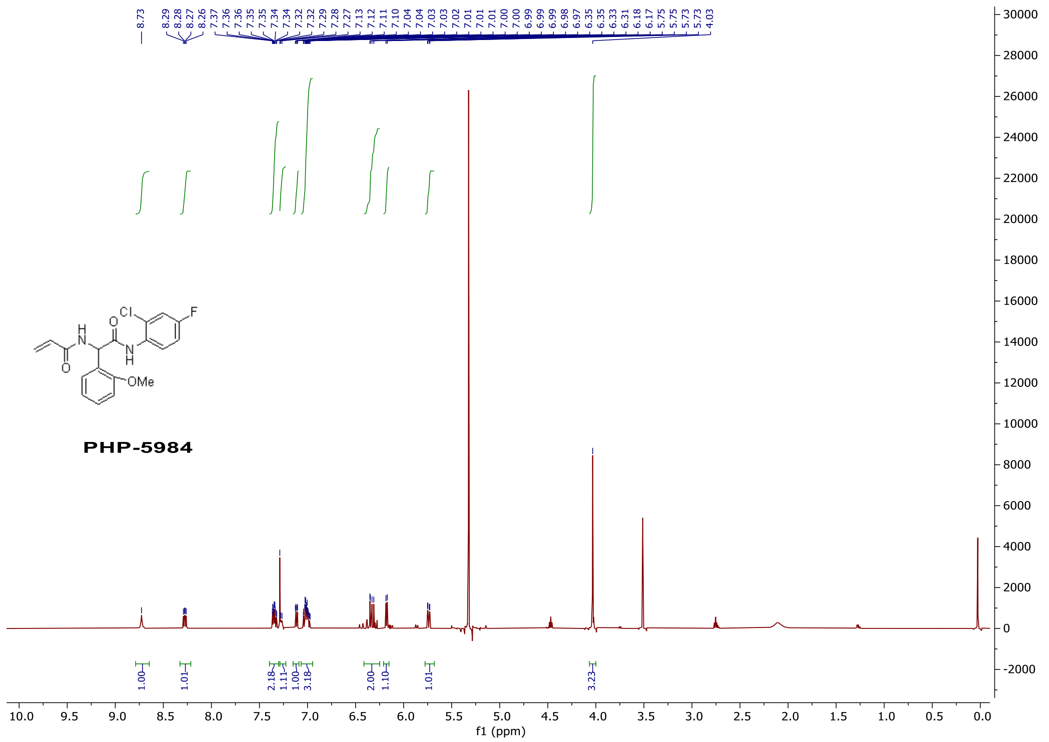

Supplementary Figure 855. ^{1}H NMR (400 MHz, $CDCl_3$ + one drop of CD_3OD) of compound **PHP-5984.**

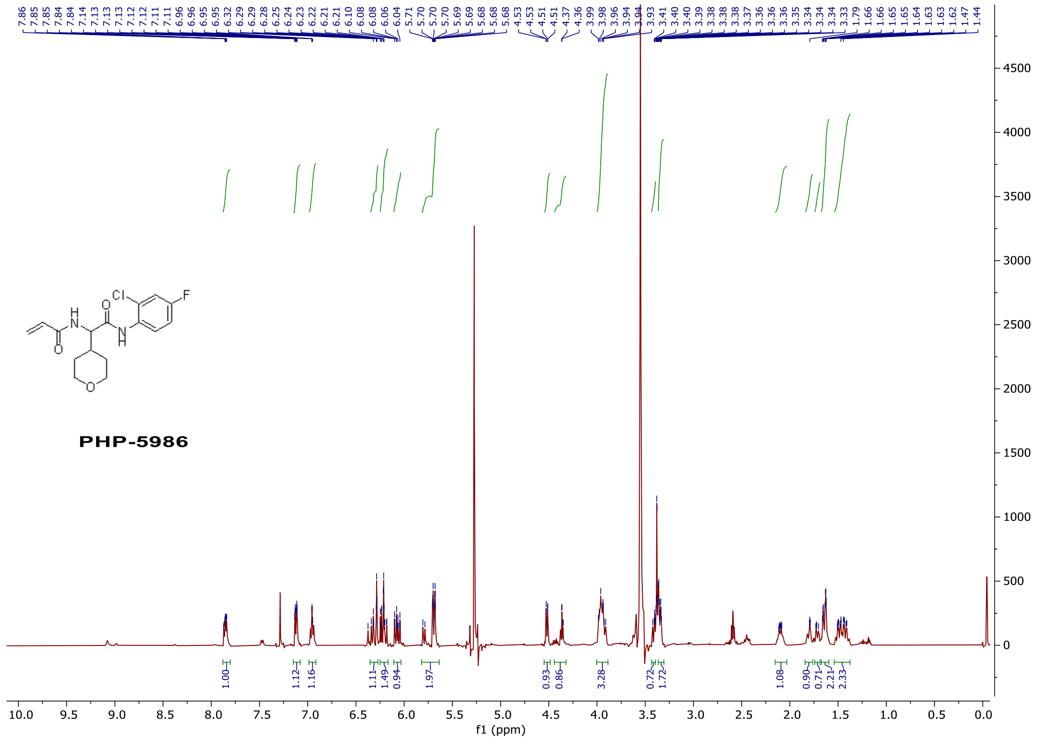

Supplementary Figure 856. ^{1}H NMR (400 MHz, $CDCl_3$ + one drop of CD_3OD) of compound **PHP-5986.**

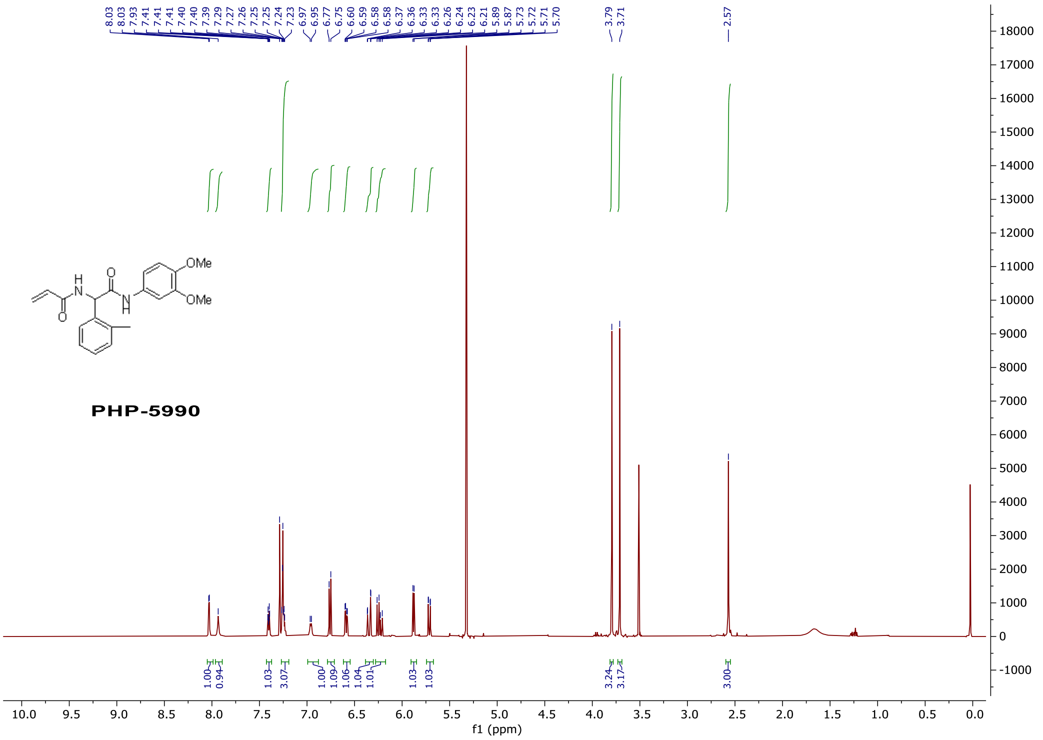

Supplementary Figure 857. ^{1}H NMR (400 MHz, $CDCl_3$ + one drop of CD_3OD) of compound **PHP-5990.**

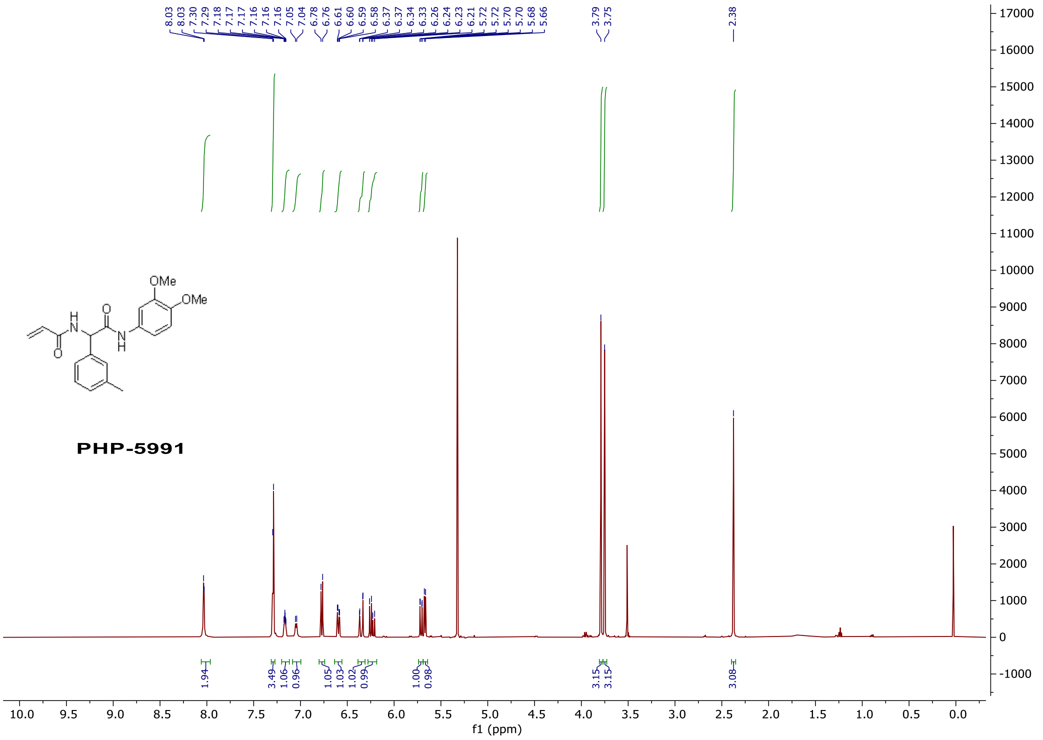

Supplementary Figure 858. ^{1}H NMR (400 MHz, $CDCl_3$ + one drop of CD_3OD) of compound **PHP-5991.**

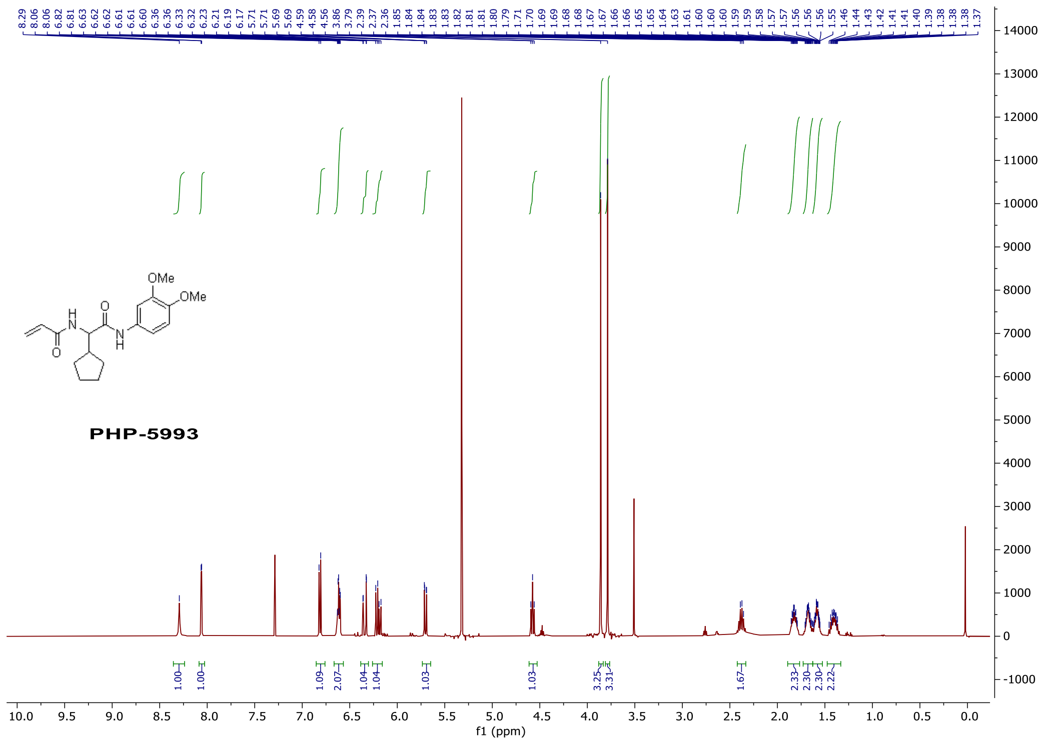

Supplementary Figure 859. ^{1}H NMR (400 MHz, $CDCl_3$ + one drop of CD_3OD) of compound **PHP-5993.**

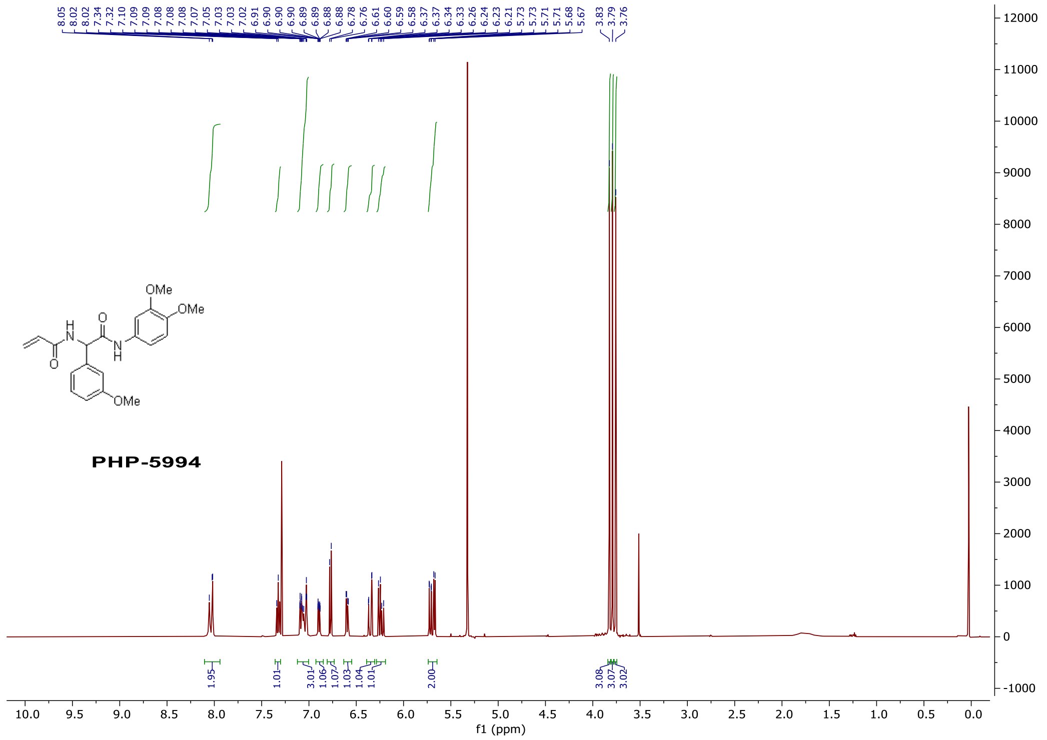

Supplementary Figure 860. ^{1}H NMR (400 MHz, $CDCl_3$ + one drop of CD_3OD) of compound **PHP-5994.**

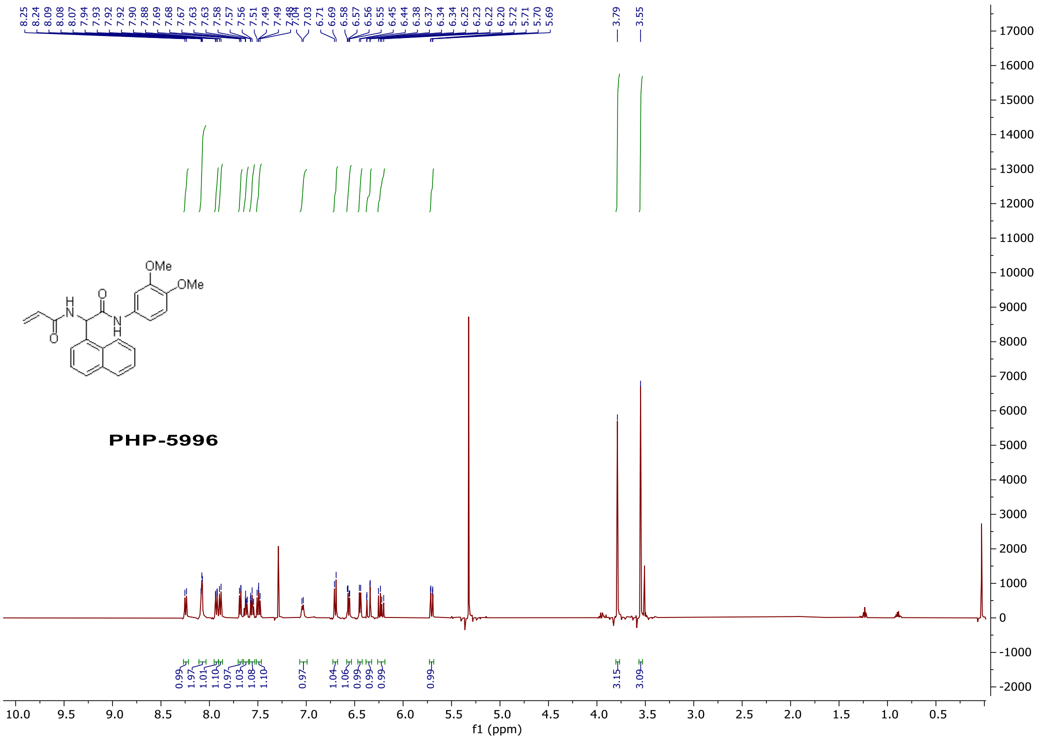

Supplementary Figure 861. ^{1}H NMR (400 MHz, $CDCl_3$ + one drop of CD_3OD) of compound **PHP-5996.**

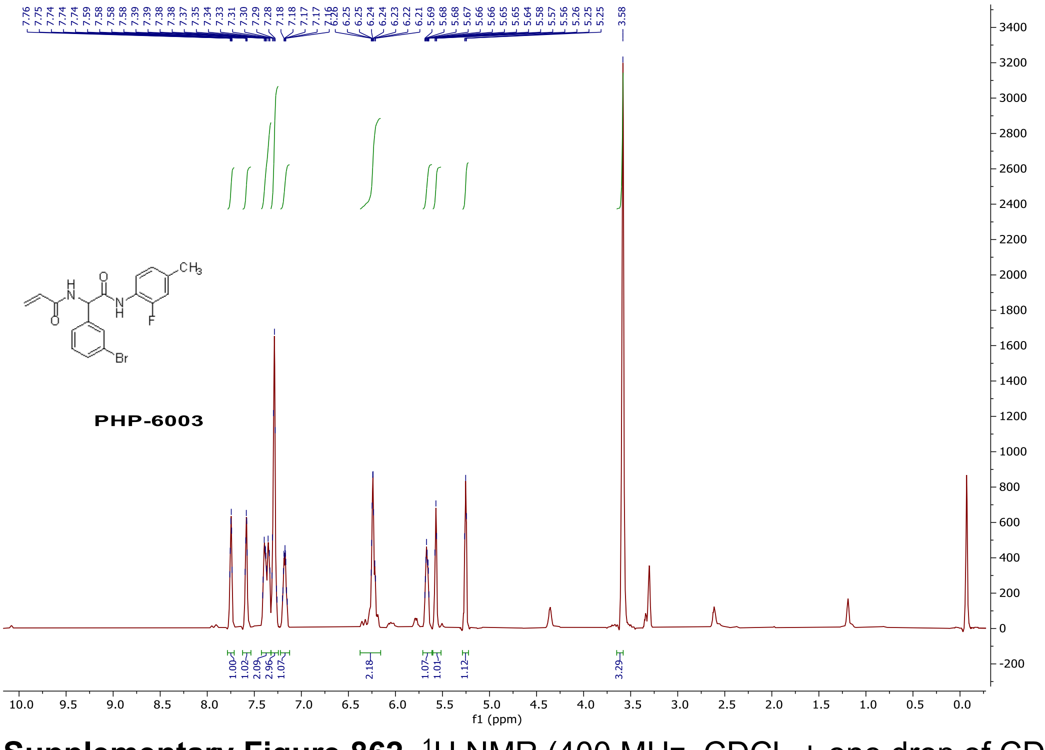

Supplementary Figure 862. ^{1}H NMR (400 MHz, $CDCl_3$ + one drop of CD_3OD) of compound **PHP-6003.**

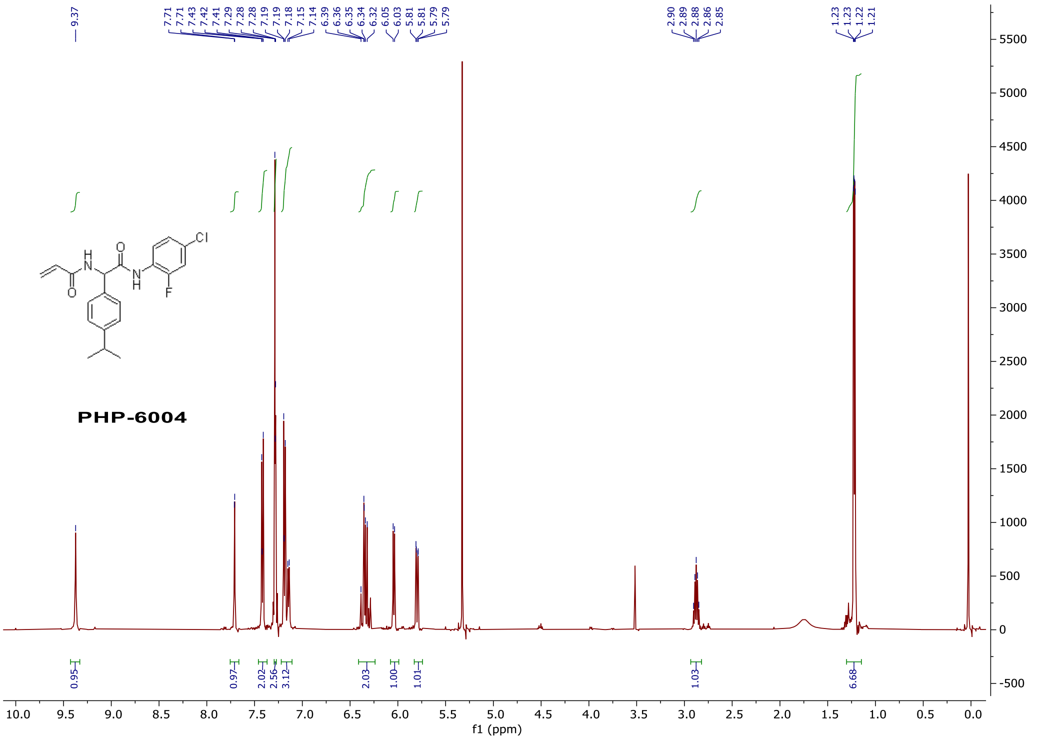

Supplementary Figure 863. ^{1}H NMR (400 MHz, $CDCl_3$ + one drop of CD_3OD) of compound **PHP-6004.**

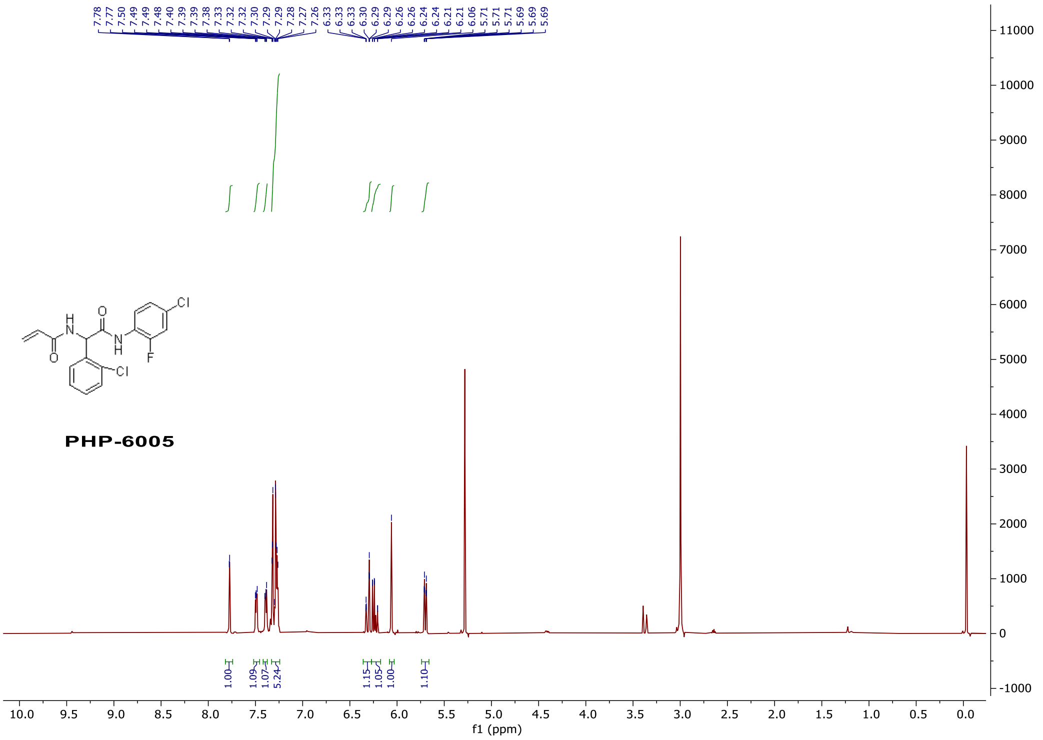

Supplementary Figure 864. ^{1}H NMR (400 MHz, $CDCl_3$ + one drop of CD_3OD) of compound **PHP-6005.**

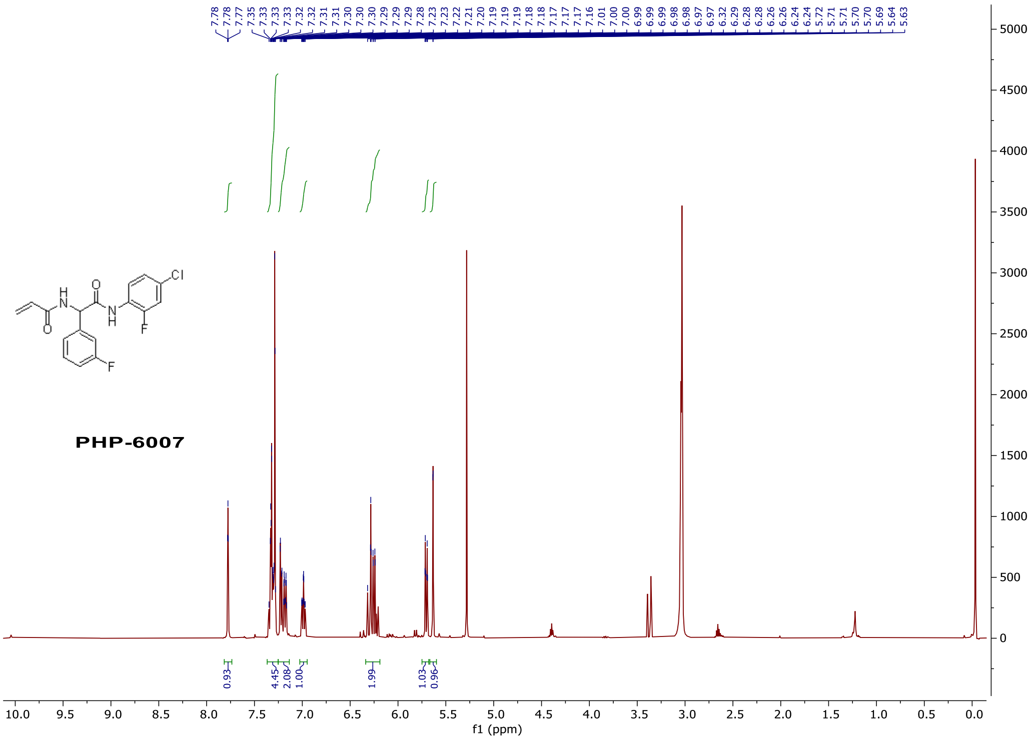

Supplementary Figure 865. ^{1}H NMR (400 MHz, $CDCl_3$ + one drop of CD_3OD) of compound **PHP-6007.**

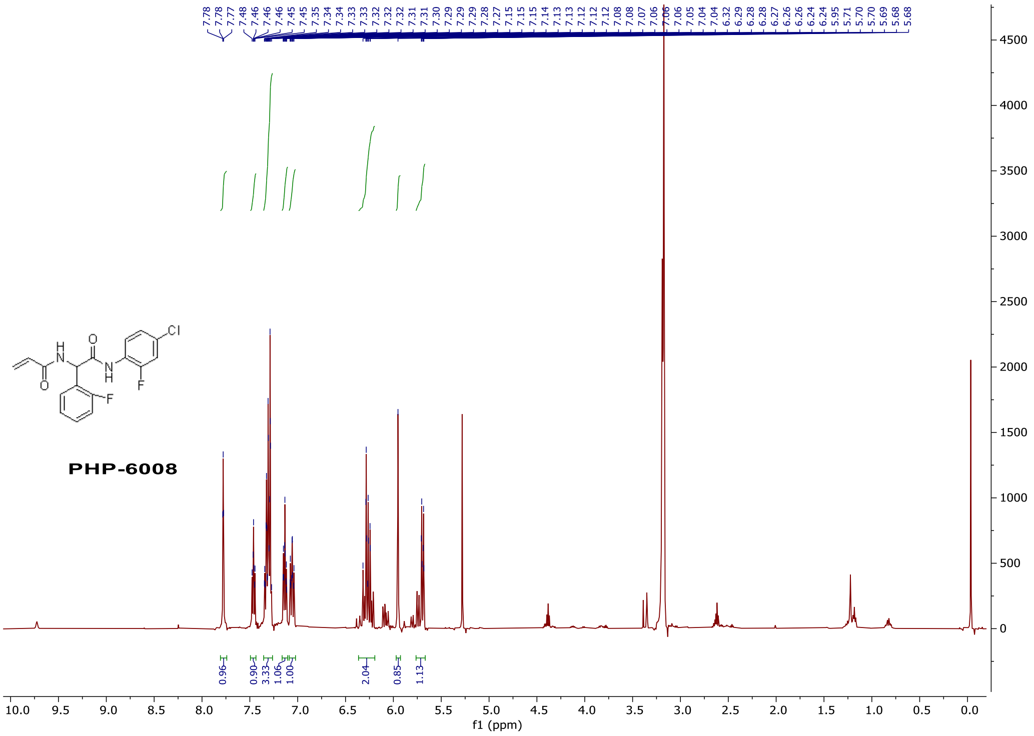

Supplementary Figure 866. ^{1}H NMR (400 MHz, $CDCl_3$ + one drop of CD_3OD) of compound **PHP-6008.**

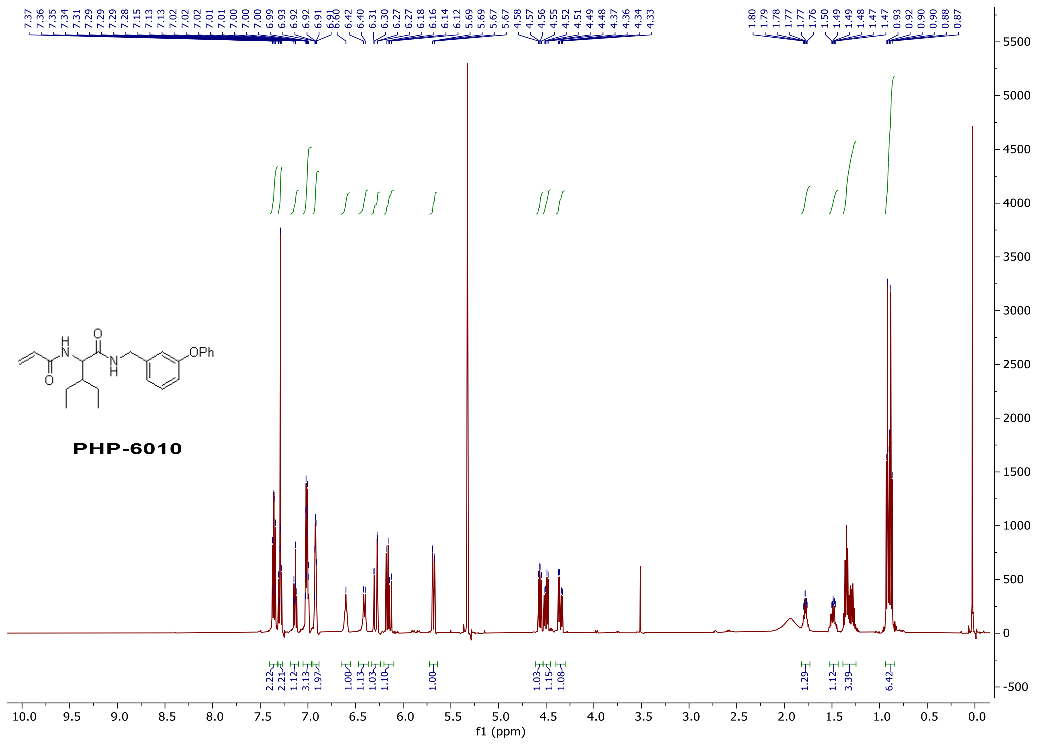

Supplementary Figure 867. ¹H NMR (400 MHz, $CDCl_3$ + one drop of CD_3OD) of compound **PHP-6010.**

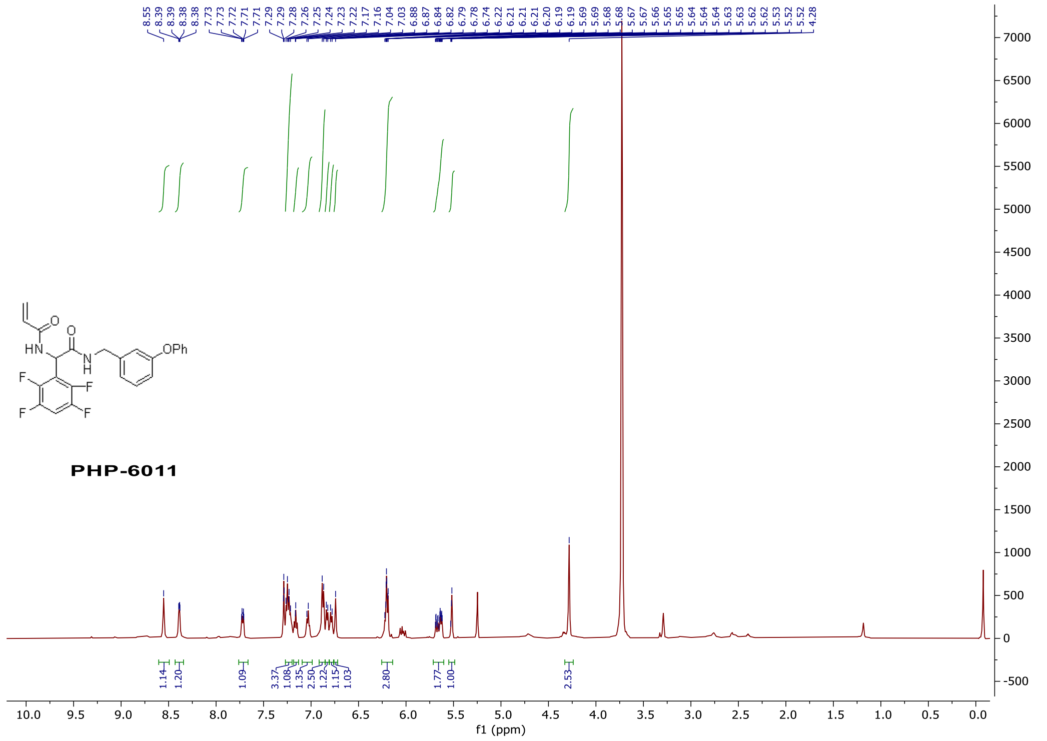

Supplementary Figure 868. ¹H NMR (400 MHz, $CDCl_3$ + one drop of CD_3OD) of compound **PHP-6011.**

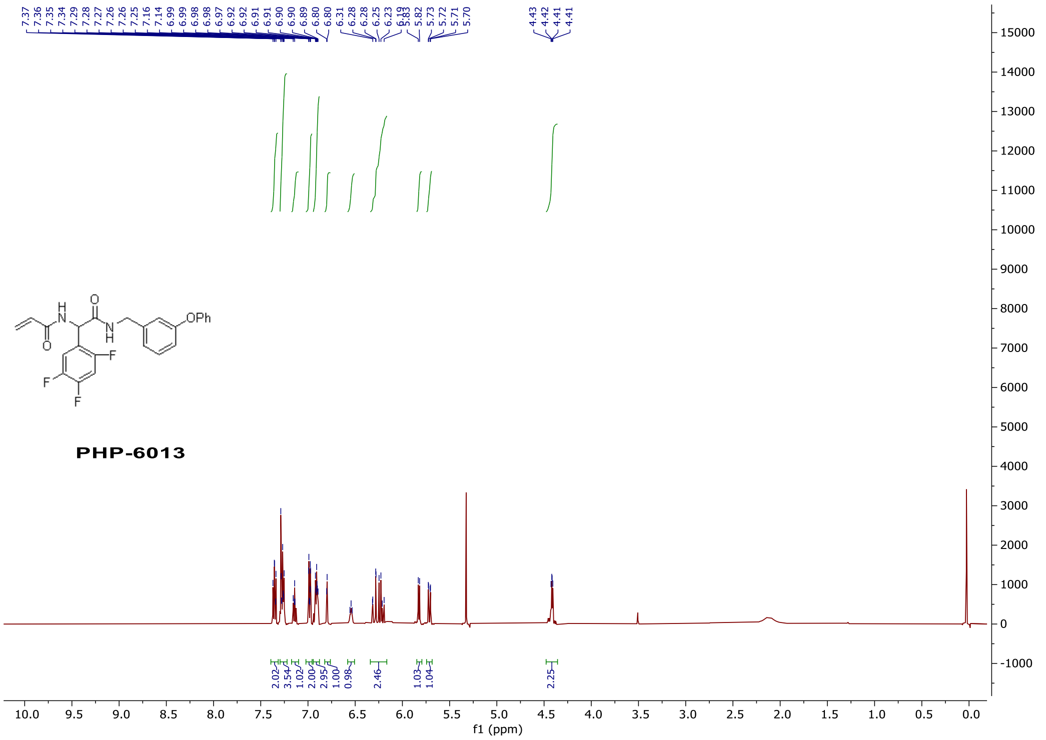

Supplementary Figure 869. ^{1}H NMR (400 MHz, $CDCl_3$ + one drop of CD_3OD) of compound **PHP-6013.**

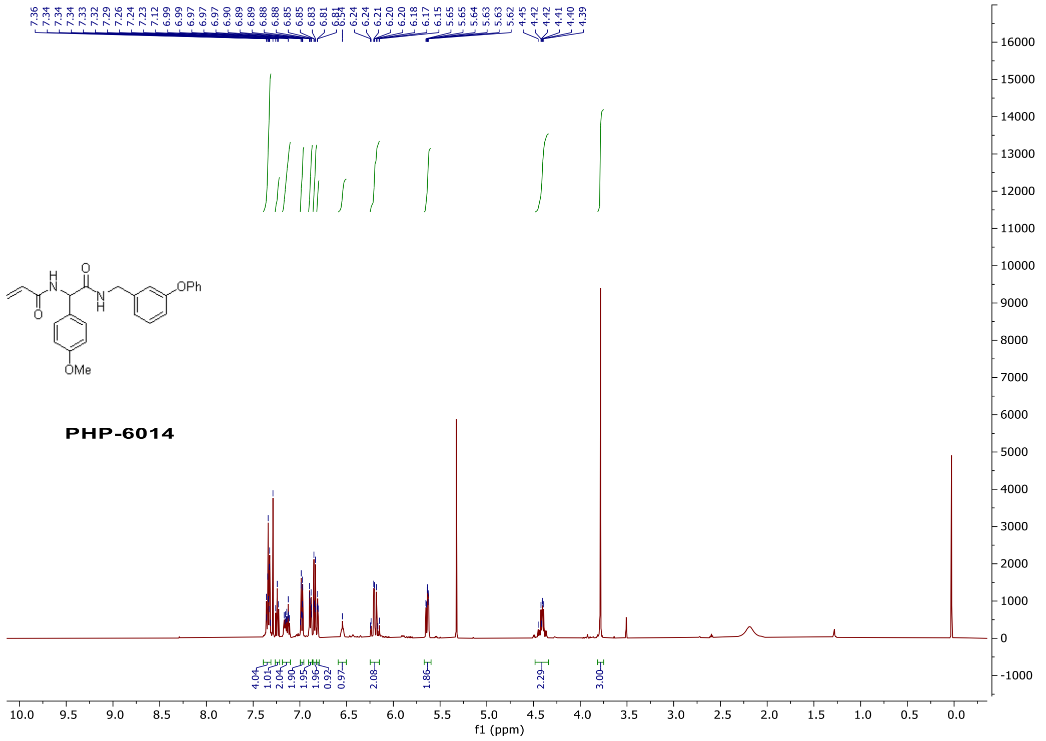

Supplementary Figure 870. ^{1}H NMR (400 MHz, $CDCl_3$ + one drop of CD_3OD) of compound **PHP-6014.**

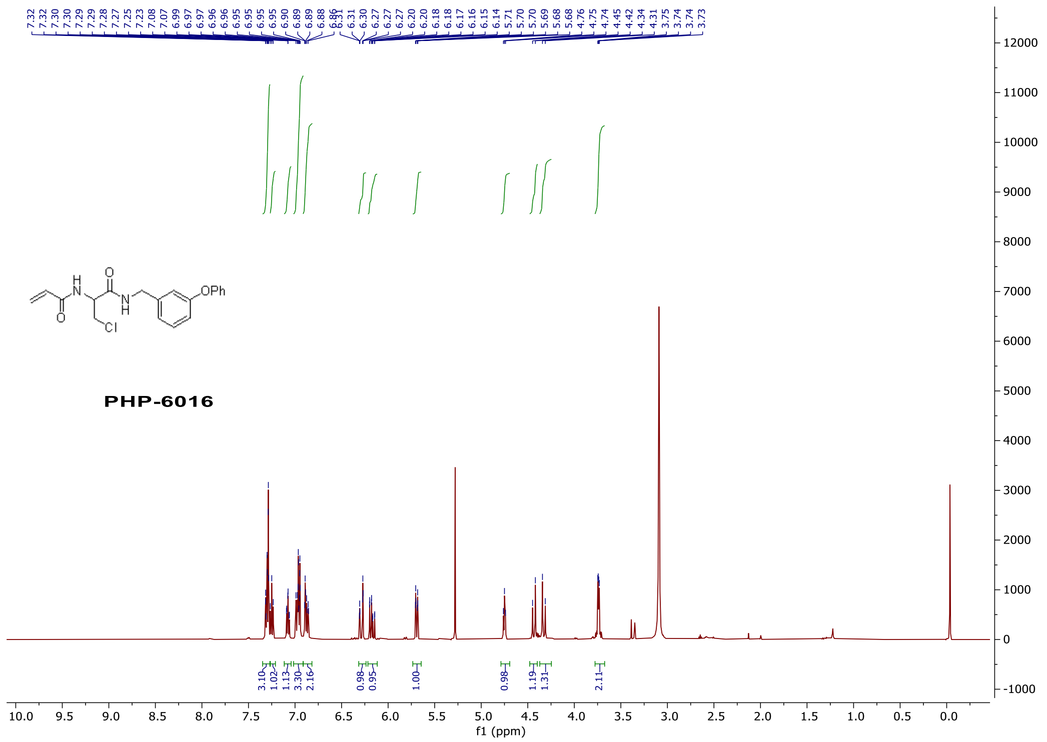

Supplementary Figure 871. ^{1}H NMR (400 MHz, $CDCl_3$ + one drop of CD_3OD) of compound **PHP-6016.**

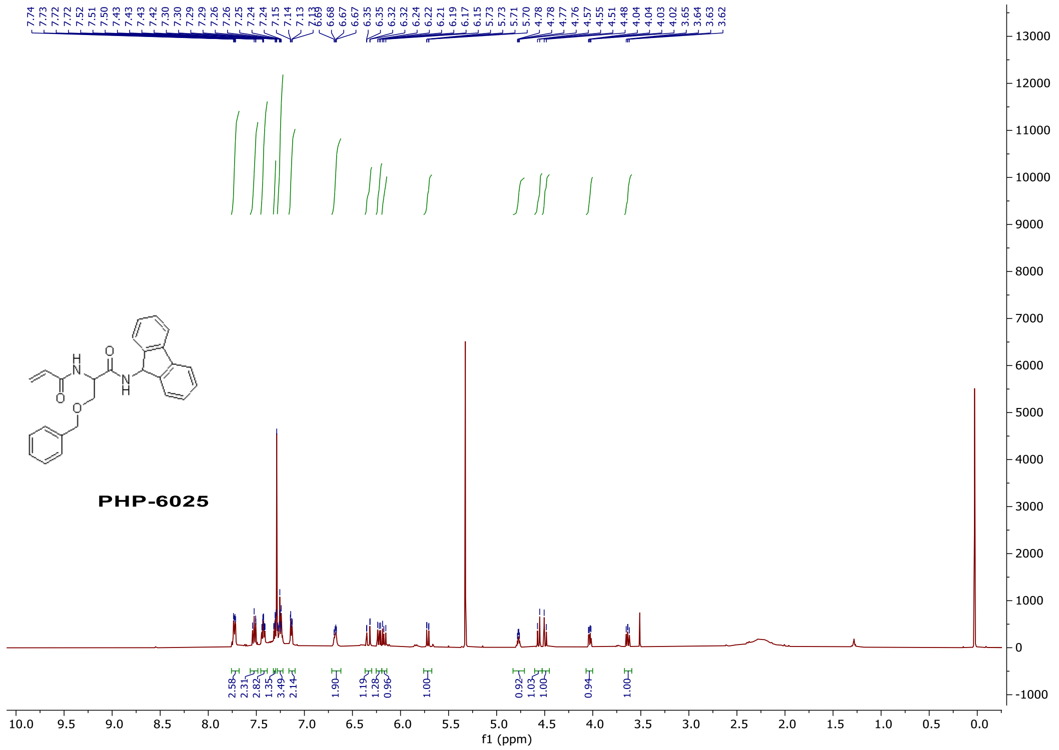

Supplementary Figure 872. ^{1}H NMR (400 MHz, $CDCl_3$ + one drop of CD_3OD) of compound **PHP-6025.**

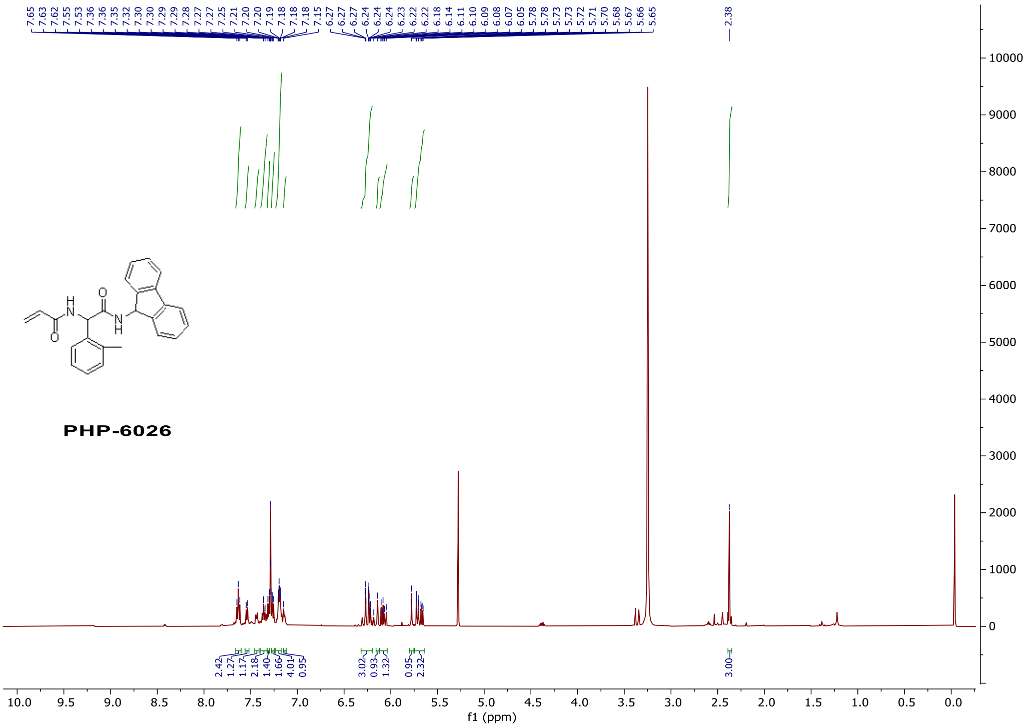

Supplementary Figure 873. ^{1}H NMR (400 MHz, $CDCl_3$ + one drop of CD_3OD) of compound **PHP-6026.**

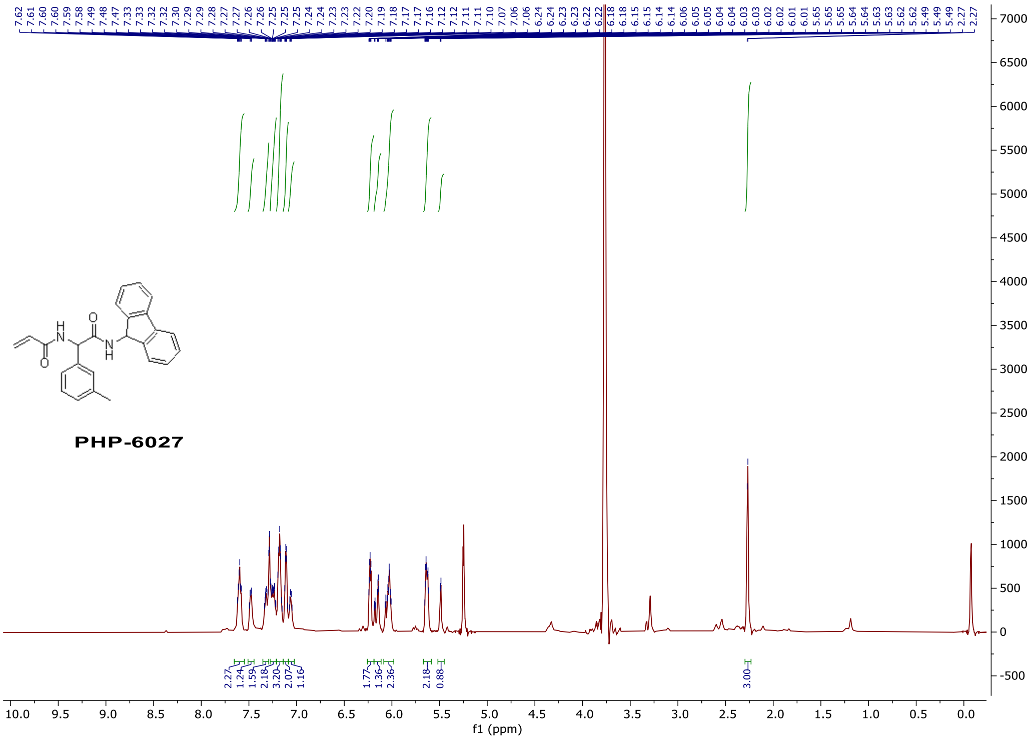

Supplementary Figure 874. ^{1}H NMR (400 MHz, $CDCl_3$ + one drop of CD_3OD) of compound **PHP-6027.**

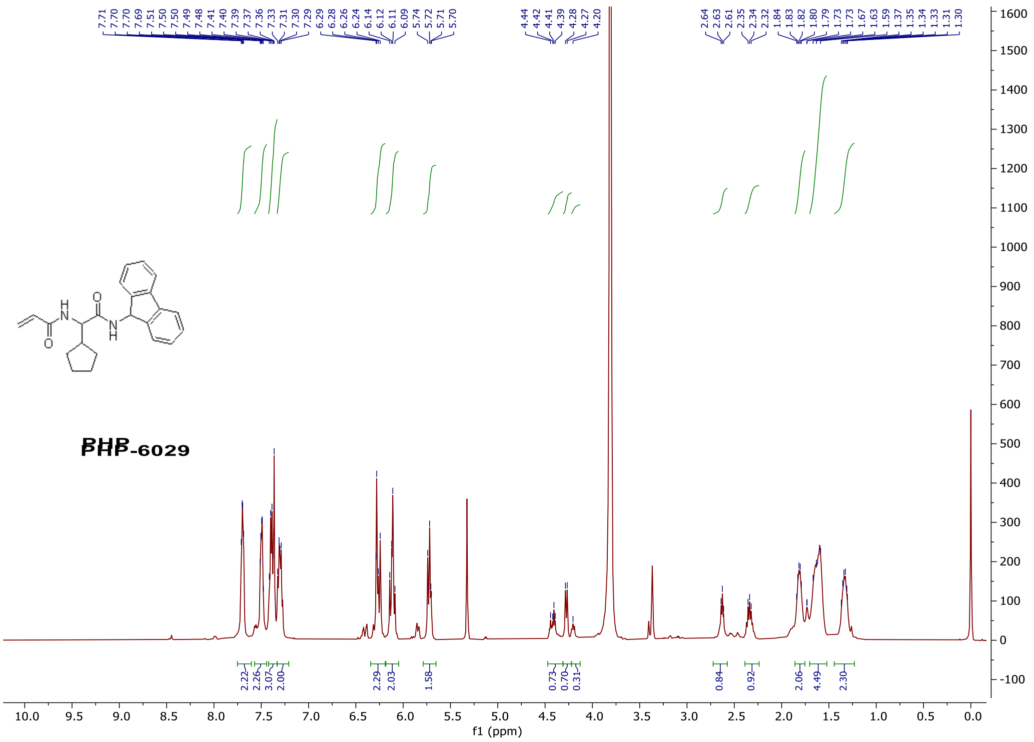

Supplementary Figure 875. ^{1}H NMR (400 MHz, $CDCl_3$ + one drop of CD_3OD) of compound **PHP-6029.**

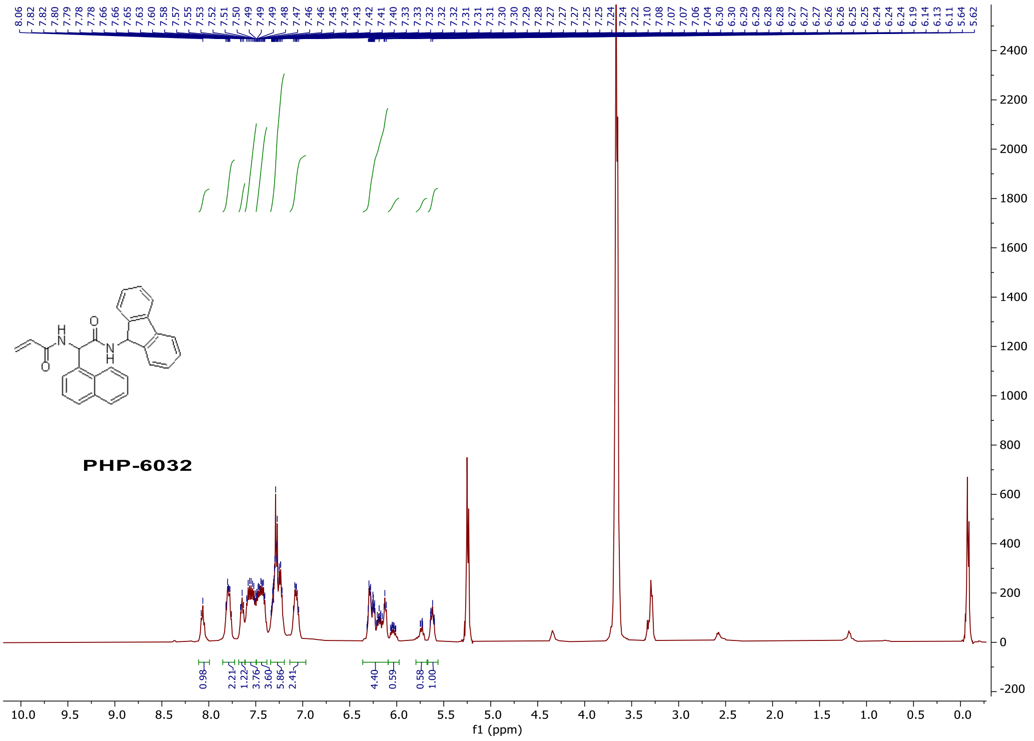

Supplementary Figure 876. ^{1}H NMR (400 MHz, $CDCl_3$ + one drop of CD_3OD) of compound **PHP-6032.**

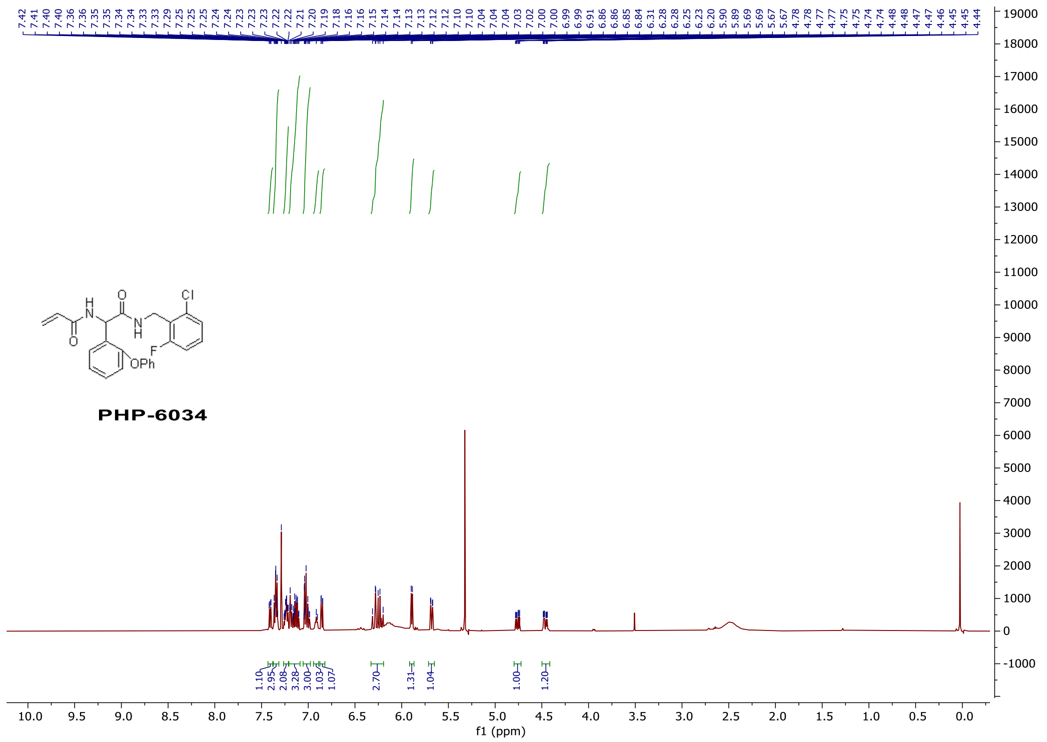

Supplementary Figure 877. ^{1}H NMR (400 MHz, $CDCl_3$ + one drop of CD_3OD) of compound **PHP-6034**.

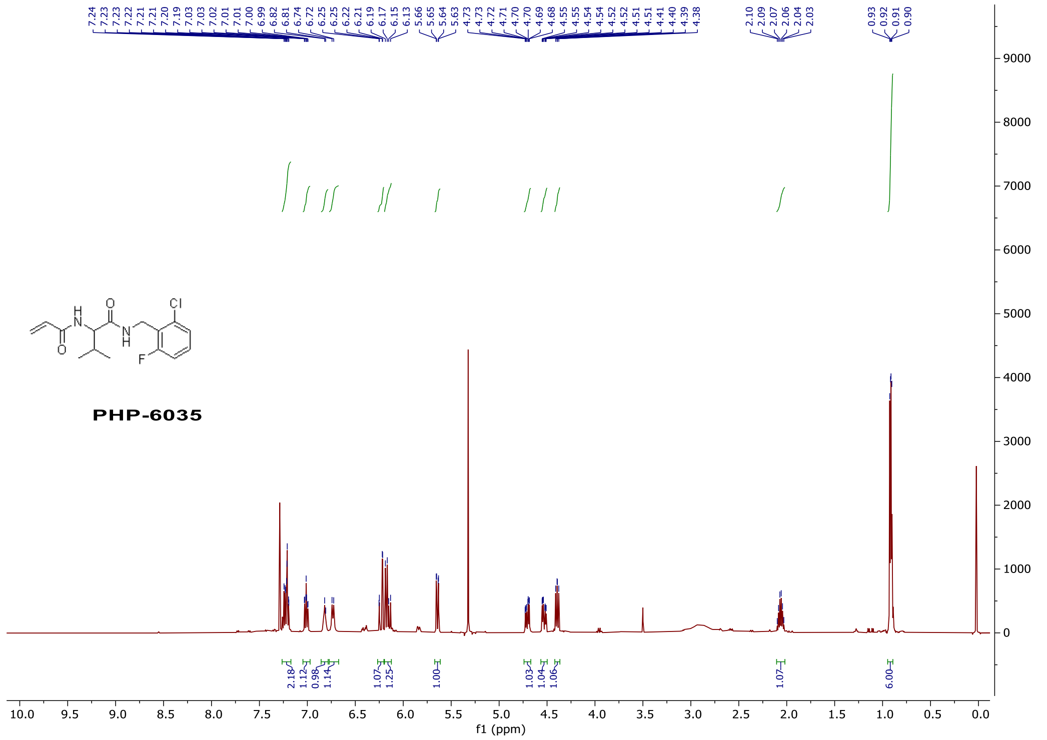

Supplementary Figure 878. ^{1}H NMR (400 MHz, $CDCl_3$ + one drop of CD_3OD) of compound **PHP-6035**.

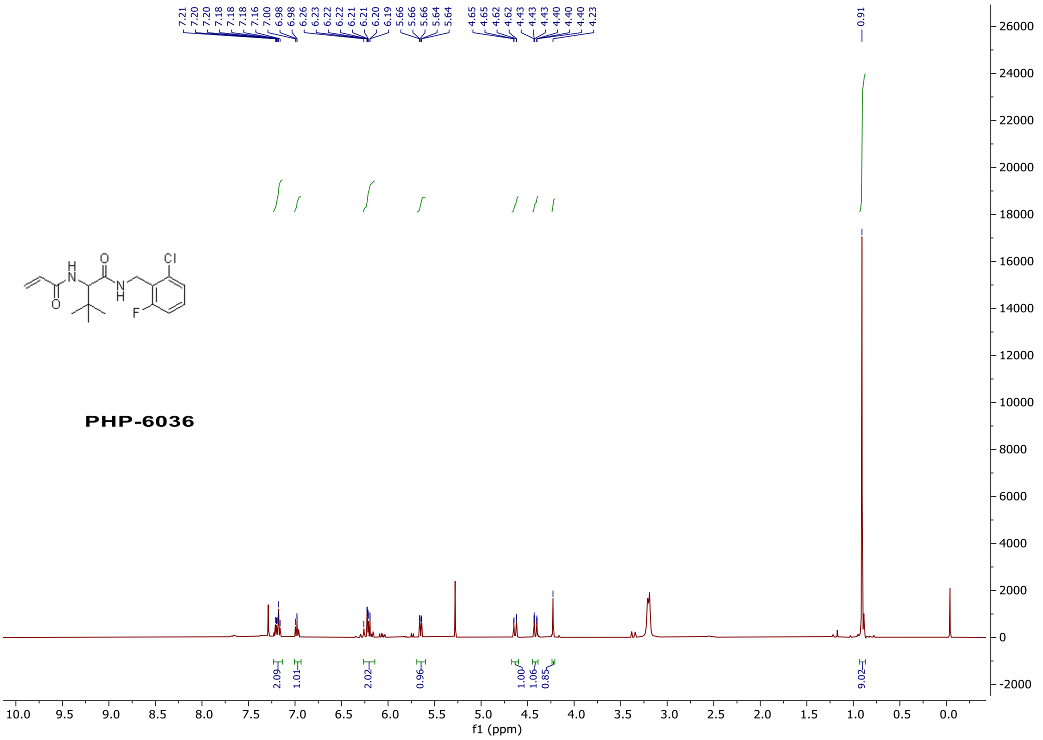

Supplementary Figure 879. ^{1}H NMR (400 MHz, $CDCl_3$ + one drop of CD_3OD) of compound **PHP-6036.**

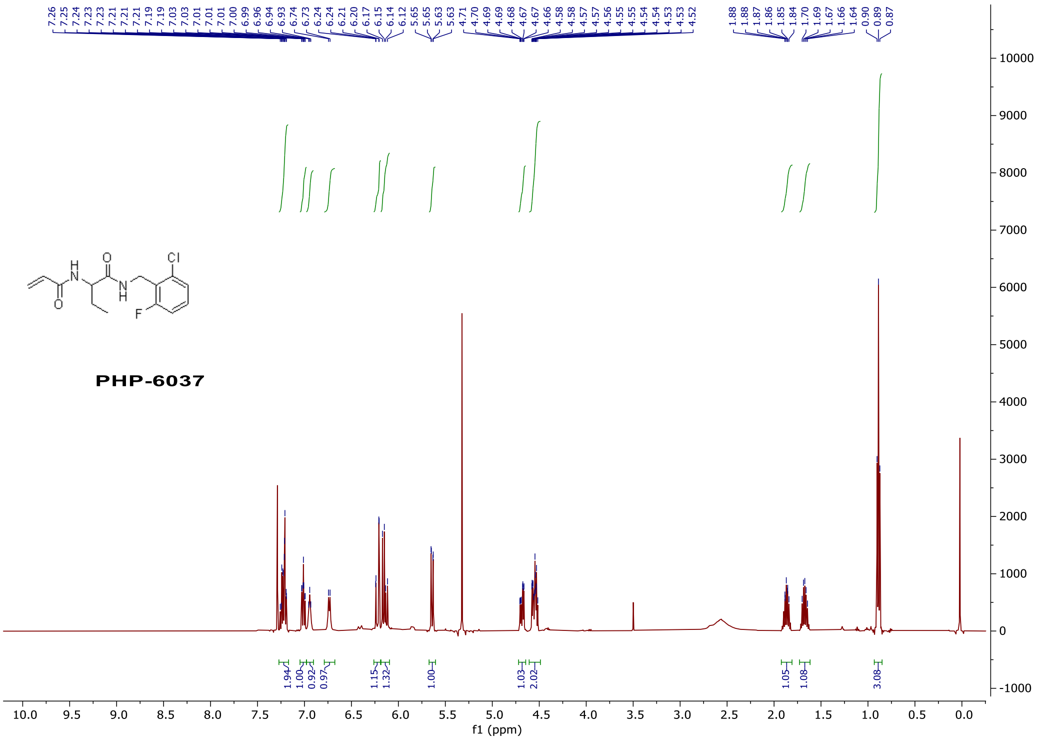

Supplementary Figure 880. ^{1}H NMR (400 MHz, $CDCl_3$ + one drop of CD_3OD) of compound **PHP-6037.**

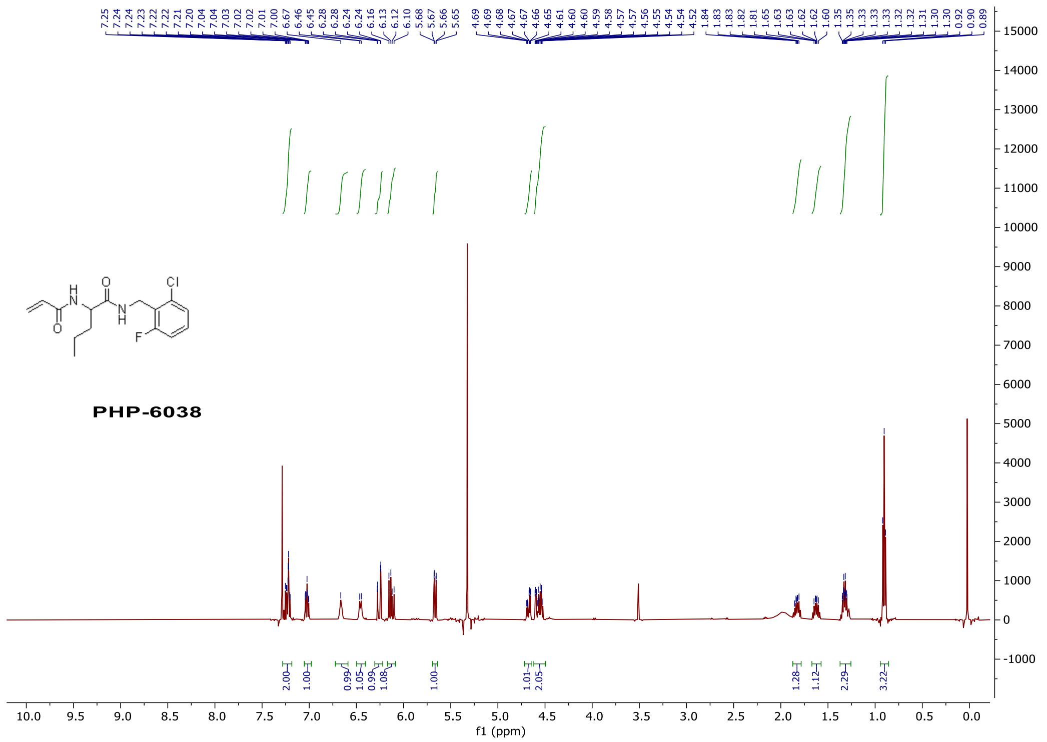

Supplementary Figure 881. ^{1}H NMR (400 MHz, $CDCl_3$ + one drop of CD_3OD) of compound **PHP-6038.**

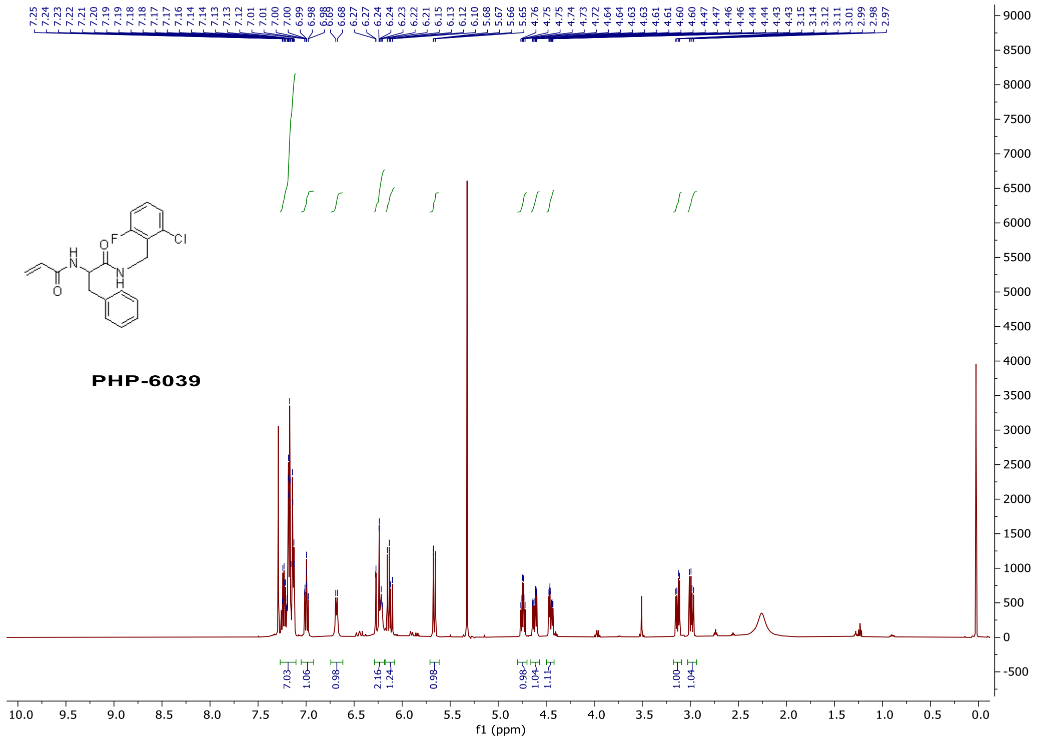

Supplementary Figure 882. ^{1}H NMR (400 MHz, $CDCl_3$ + one drop of CD_3OD) of compound **PHP-6039.**

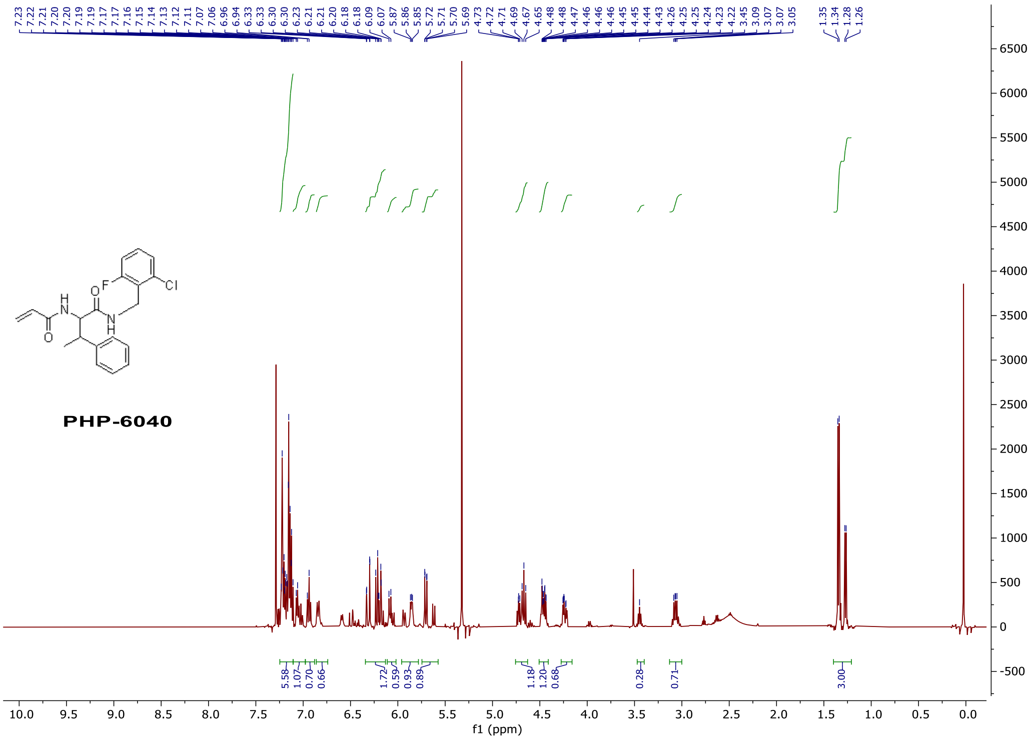

Supplementary Figure 883. ^{1}H NMR (400 MHz, $CDCl_3$ + one drop of CD_3OD) of compound **PHP-6040.**

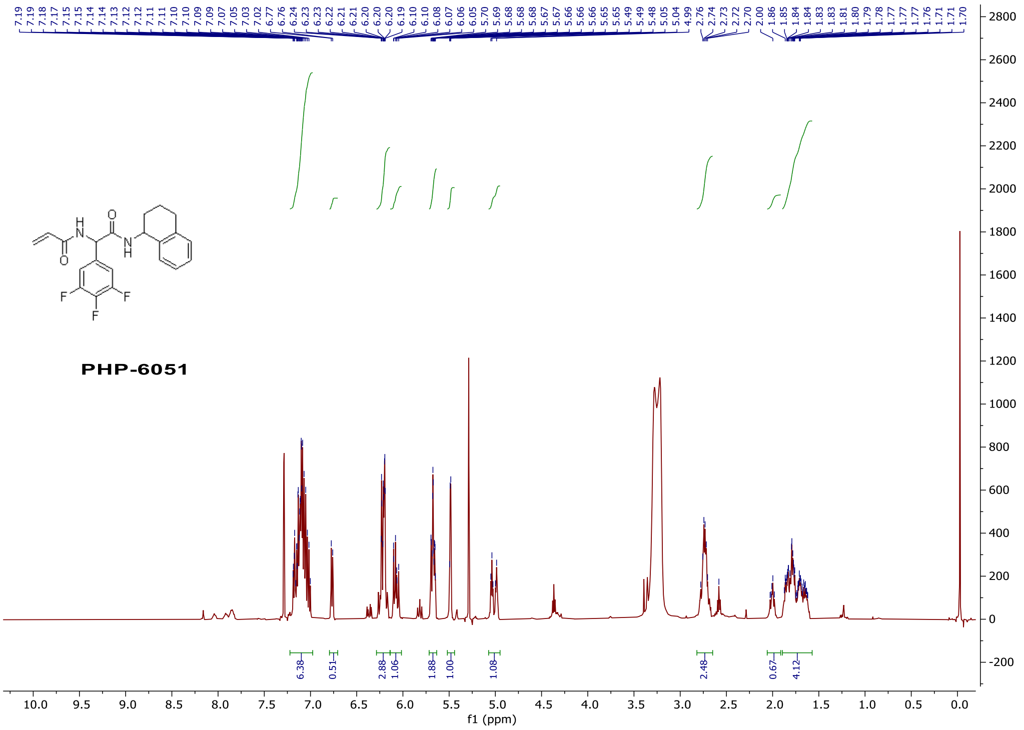

Supplementary Figure 884. ^{1}H NMR (400 MHz, $CDCl_3$ + one drop of CD_3OD) of compound **PHP-6051.**

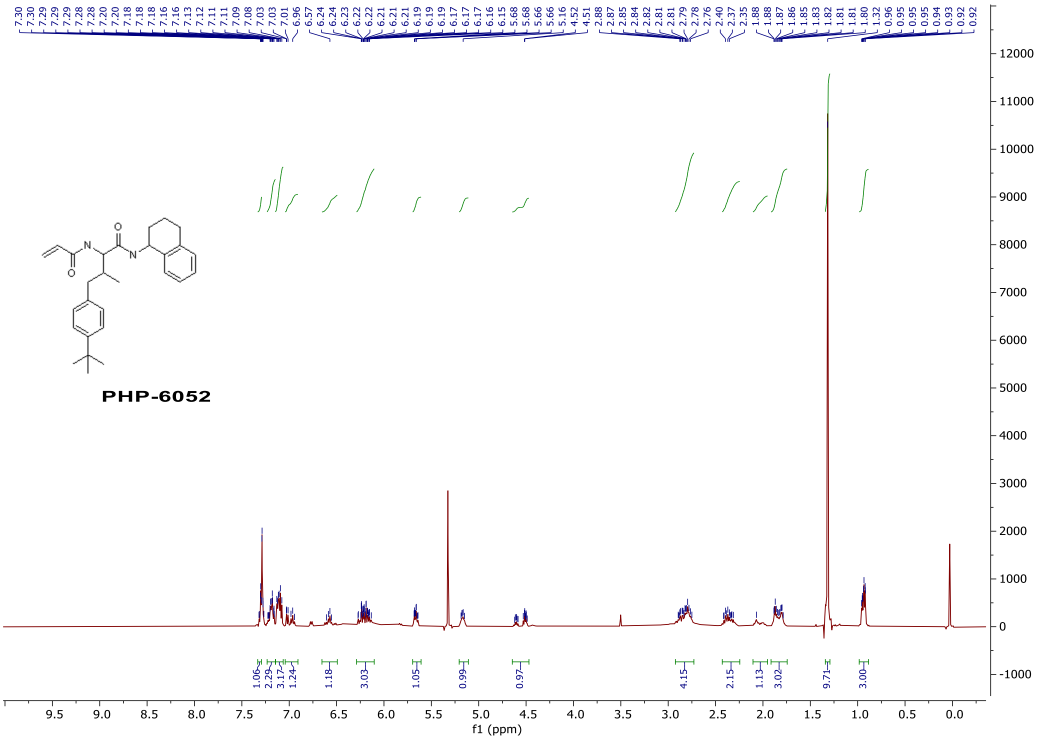

Supplementary Figure 885. ^{1}H NMR (400 MHz, $CDCl_3$ + one drop of CD_3OD) of compound **PHP-6052.**

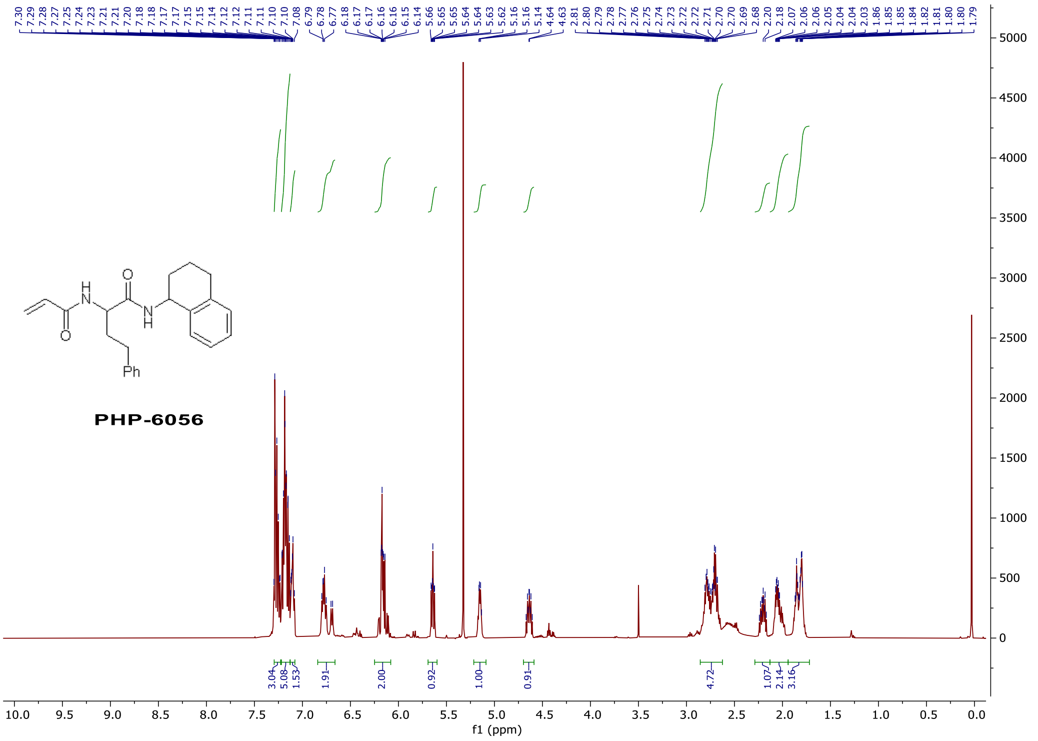

Supplementary Figure 886. ^{1}H NMR (400 MHz, $CDCl_3$ + one drop of CD_3OD) of compound **PHP-6056.**

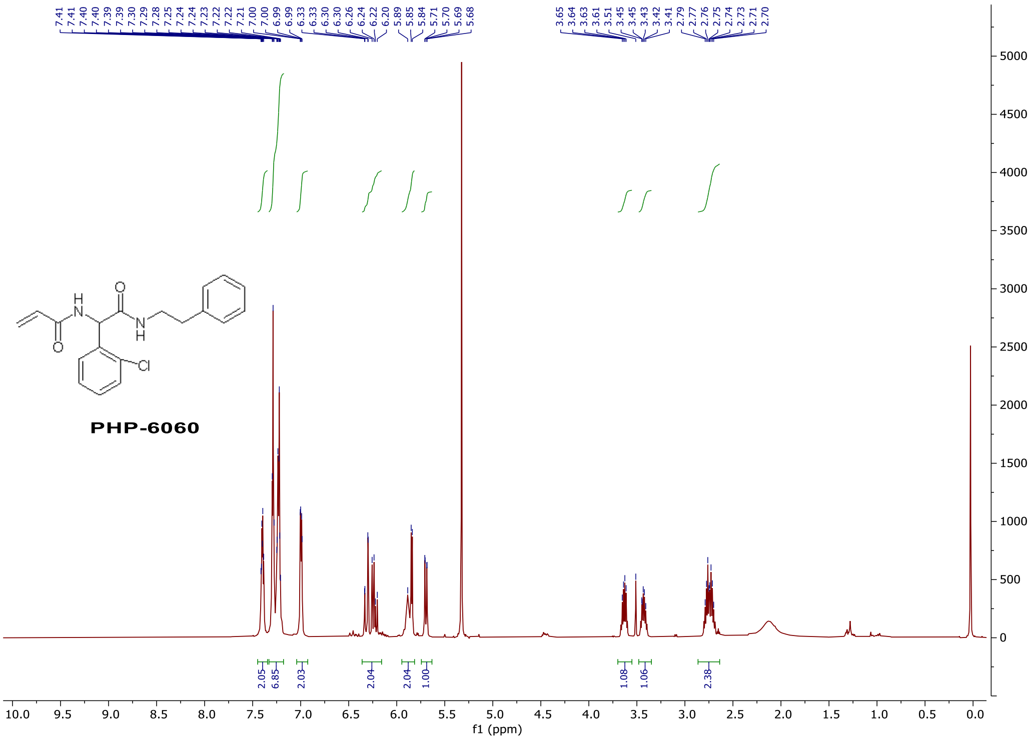

Supplementary Figure 887. ^{1}H NMR (400 MHz, $CDCl_3$ + one drop of CD_3OD) of compound **PHP-6060.**

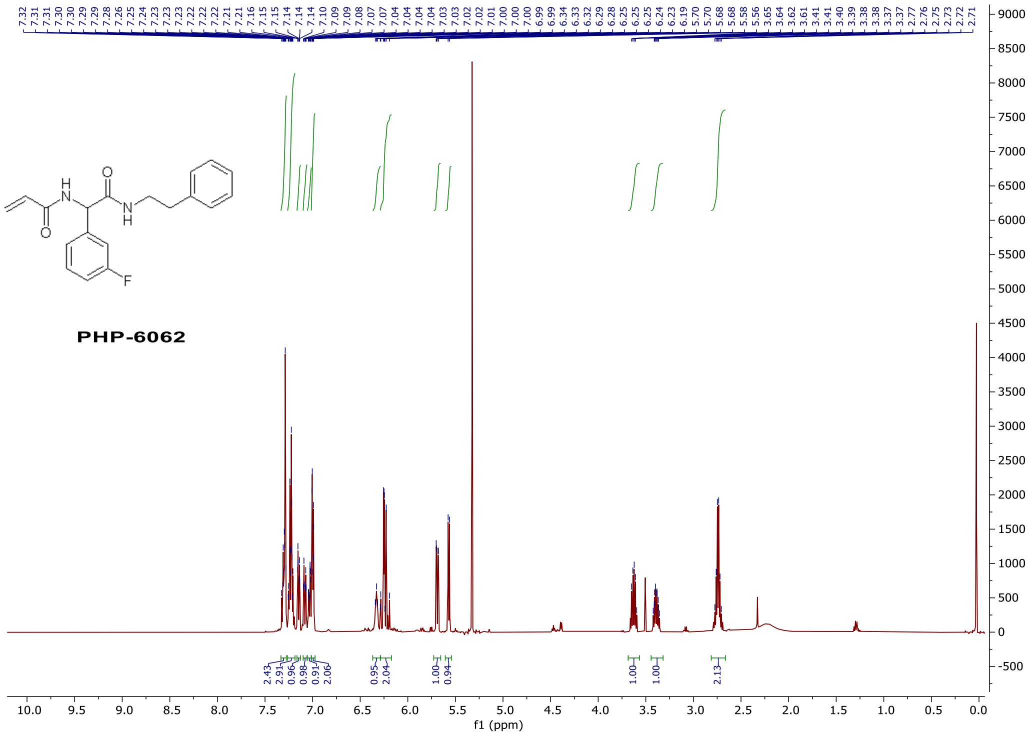

Supplementary Figure 888. ^{1}H NMR (400 MHz, $CDCl_3$ + one drop of CD_3OD) of compound **PHP-6062.**

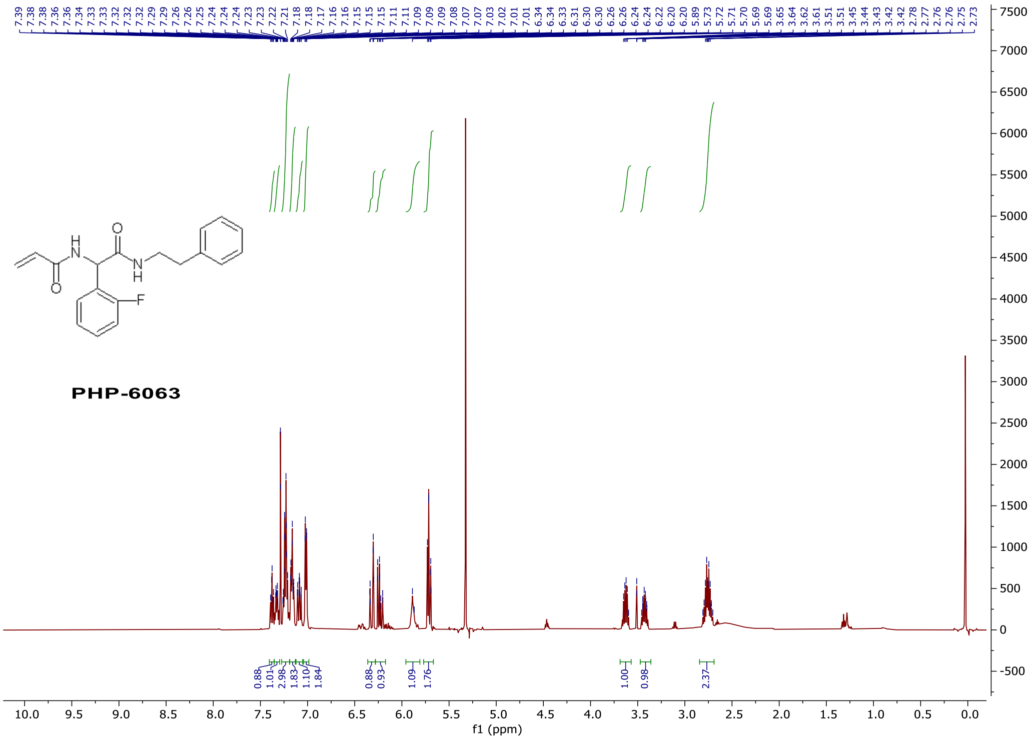

Supplementary Figure 889. ¹H NMR (400 MHz, $CDCl_3$ + one drop of CD_3OD) of compound **PHP-6063.**

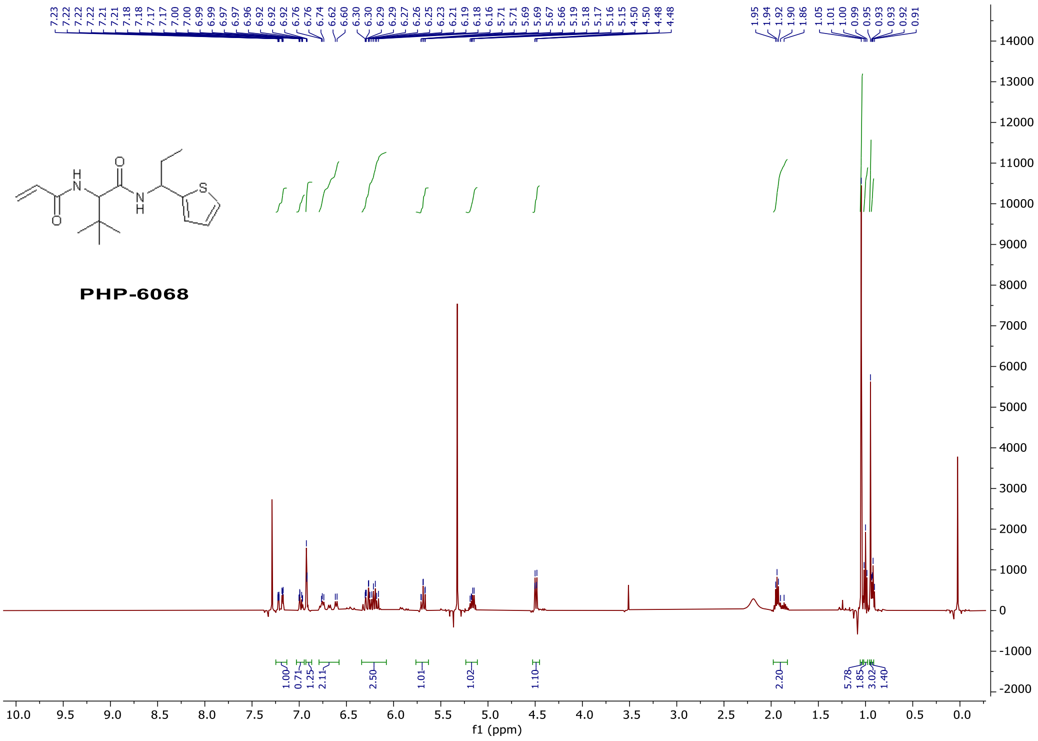

Supplementary Figure 890. ¹H NMR (400 MHz, $CDCl_3$ + one drop of CD_3OD) of compound **PHP-6068.**

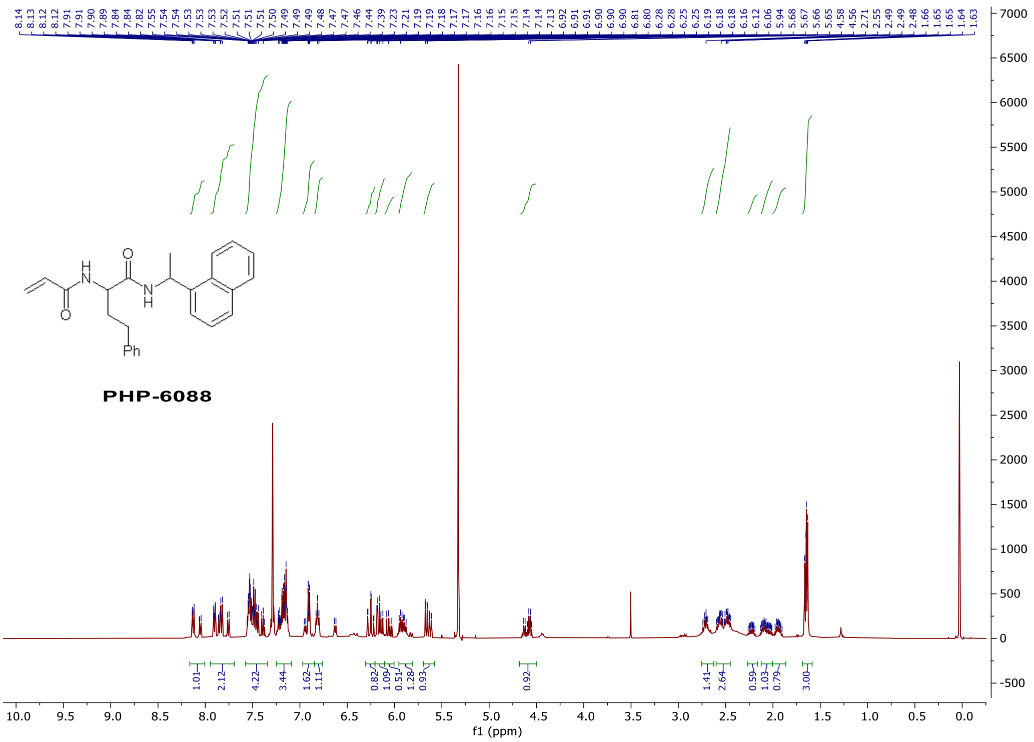

Supplementary Figure 891. ^{1}H NMR (400 MHz, $CDCl_3$ + one drop of CD_3OD) of compound **PHP-6088.**

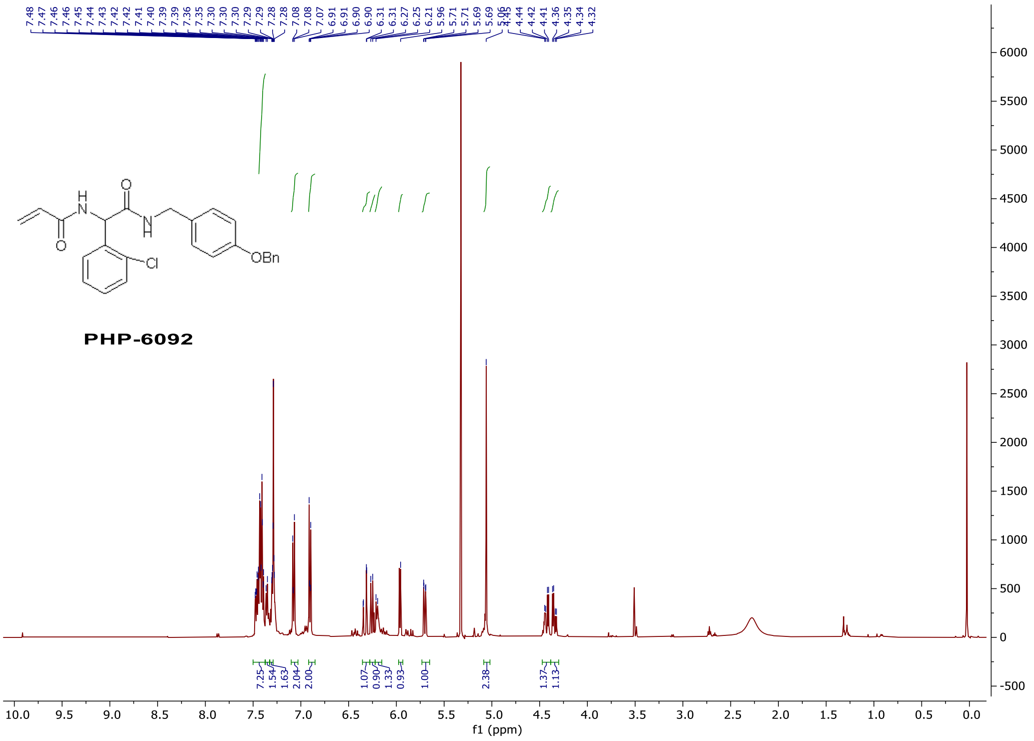

Supplementary Figure 892. ^{1}H NMR (400 MHz, $CDCl_3$ + one drop of CD_3OD) of compound **PHP-6092.**

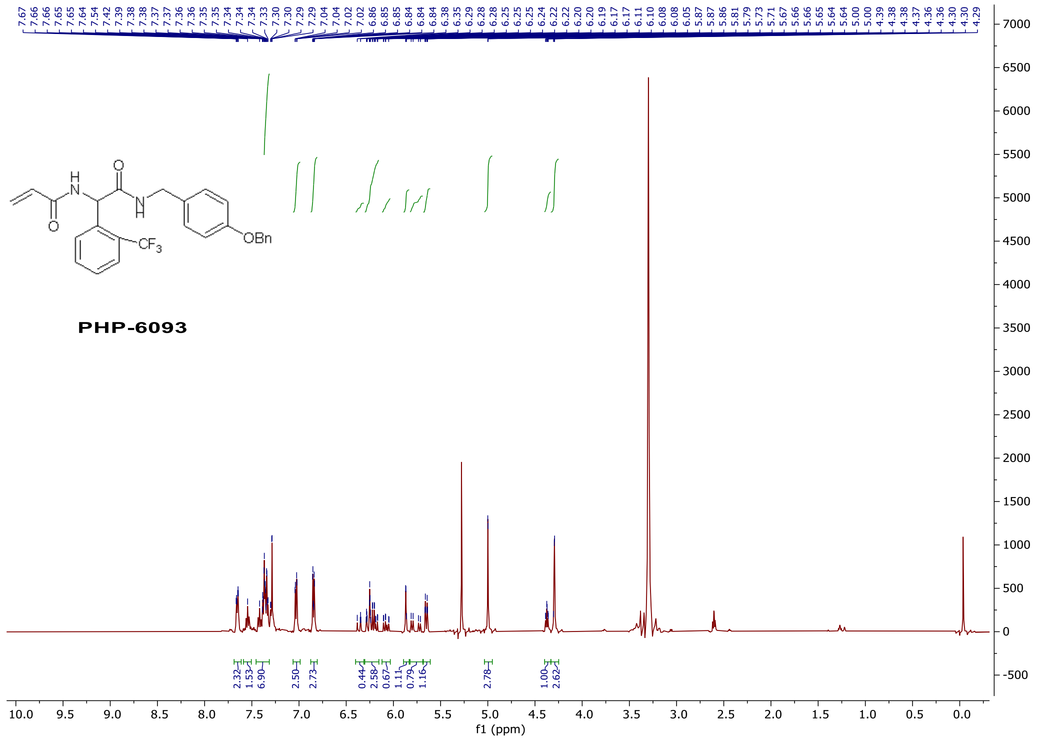

Supplementary Figure 893. ^{1}H NMR (400 MHz, $CDCl_3$ + one drop of CD_3OD) of compound **PHP-6093.**

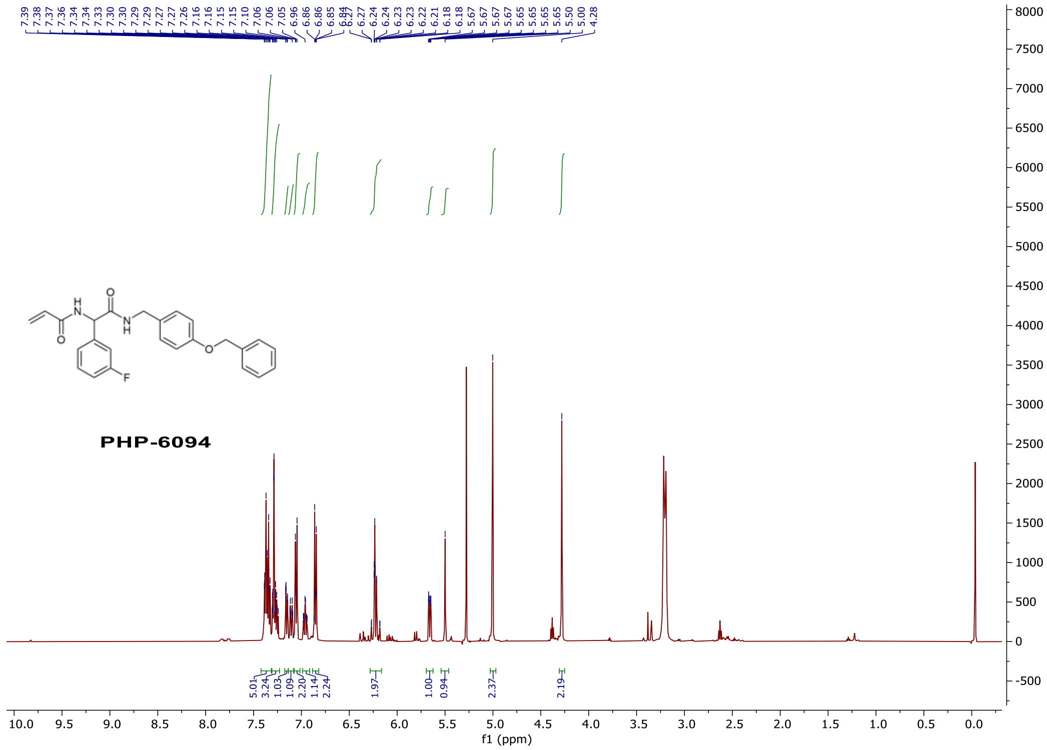

Supplementary Figure 894. ^{1}H NMR (400 MHz, $CDCl_3$ + one drop of CD_3OD) of compound **PHP-6094.**

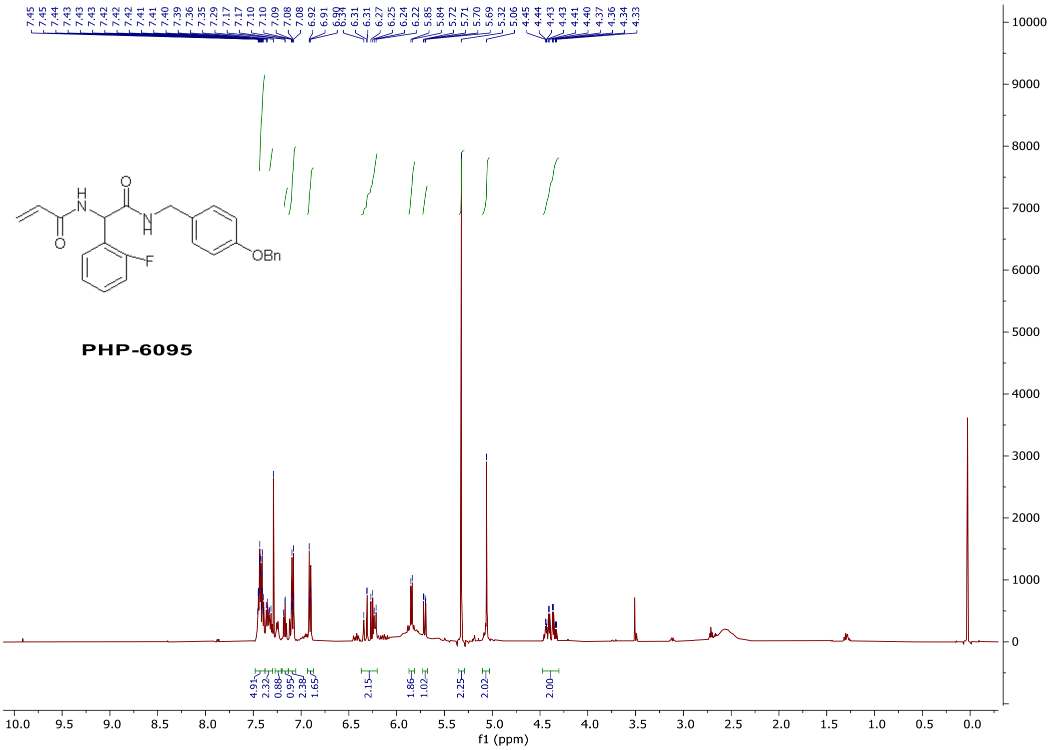

Supplementary Figure 895. ^{1}H NMR (400 MHz, $CDCl_3$ + one drop of CD_3OD) of compound **PHP-6095.**

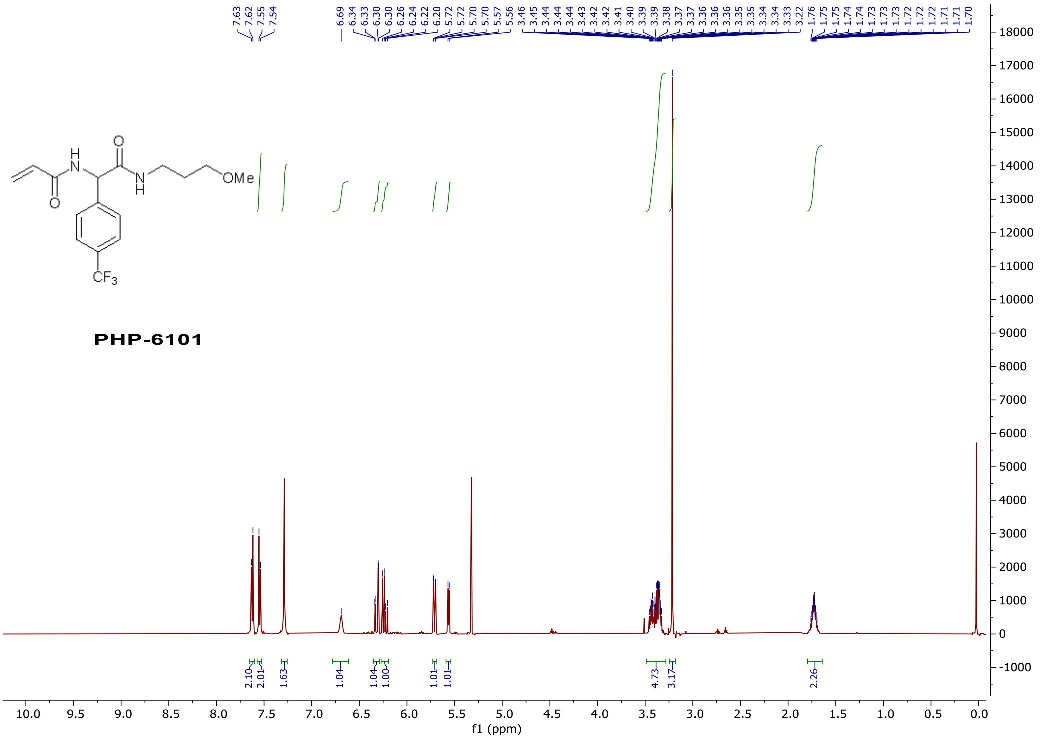

Supplementary Figure 896. ^{1}H NMR (400 MHz, $CDCl_3$ + one drop of CD_3OD) of compound **PHP-6101.**

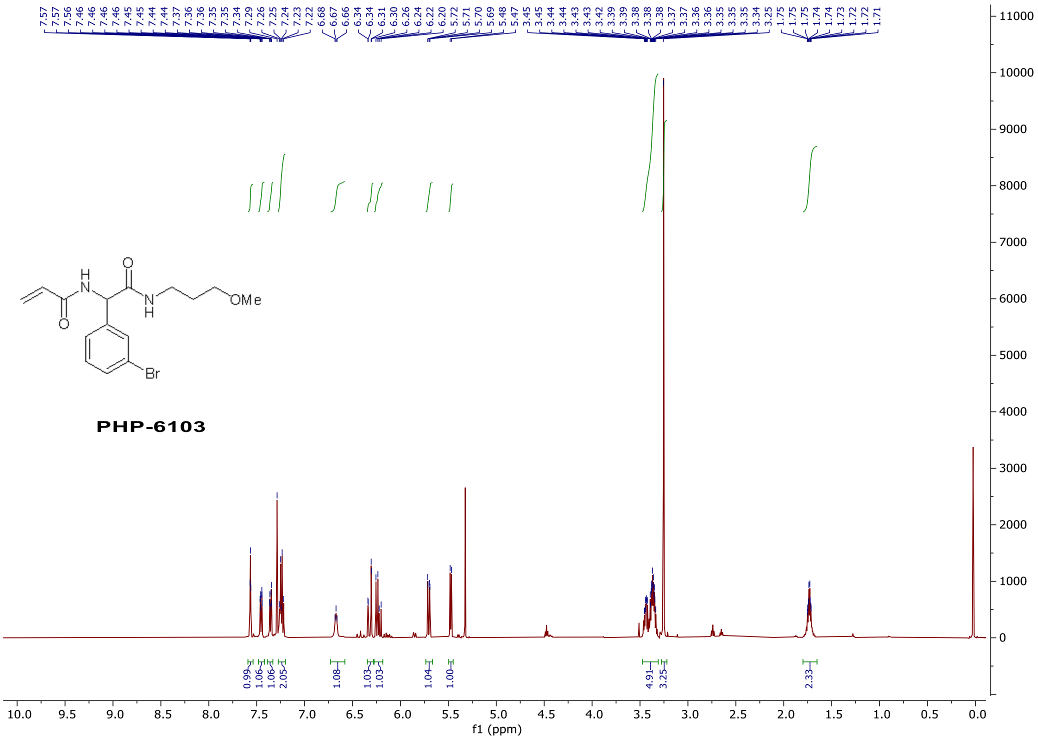

Supplementary Figure 897. ^{1}H NMR (400 MHz, $CDCl_3$ + one drop of CD_3OD) of compound **PHP-6103.**

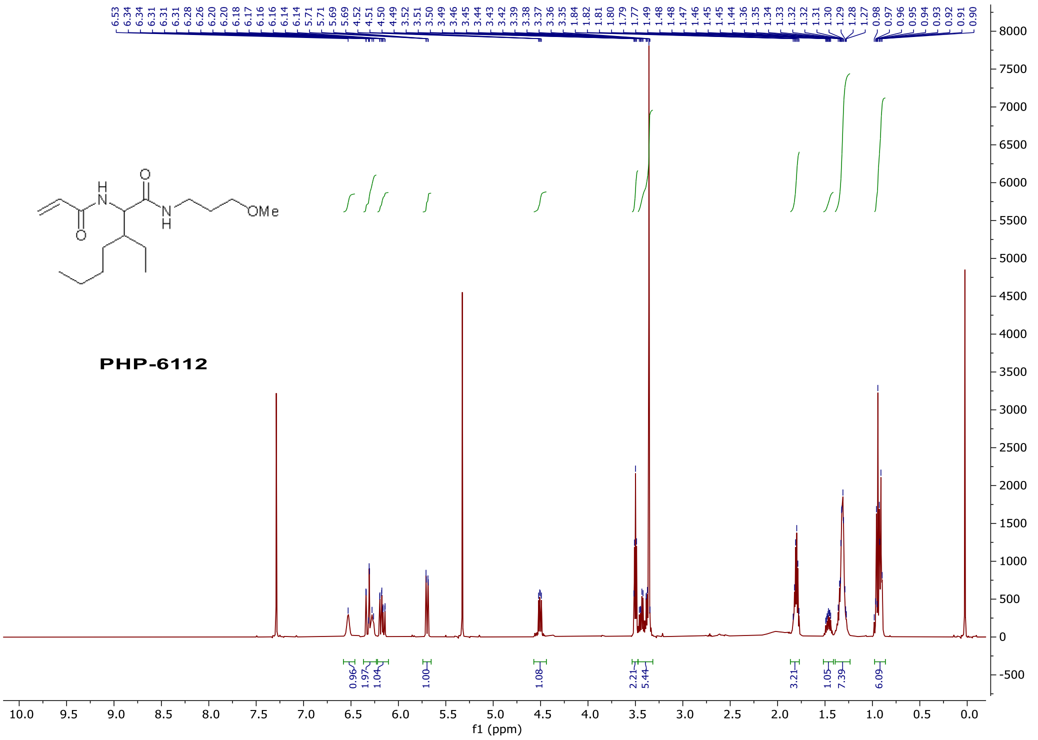

Supplementary Figure 898. ^{1}H NMR (400 MHz, $CDCl_3$ + one drop of CD_3OD) of compound **PHP-6112.**

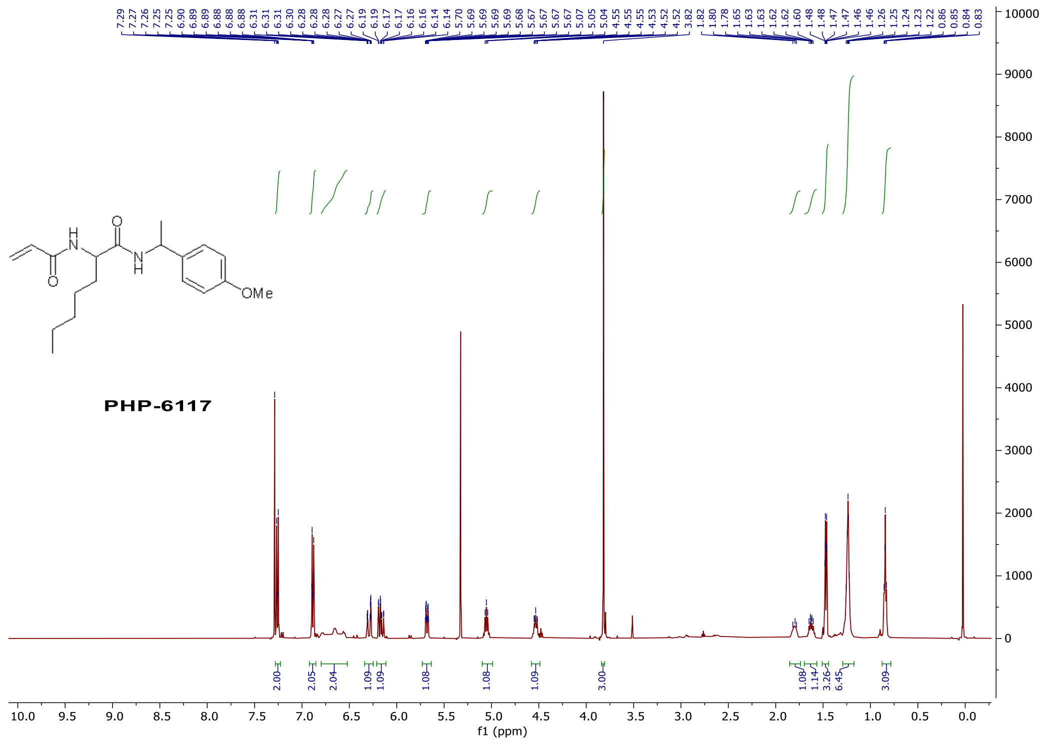

Supplementary Figure 899. ^{1}H NMR (400 MHz, $CDCl_3$ + one drop of CD_3OD) of compound **PHP-6117**.

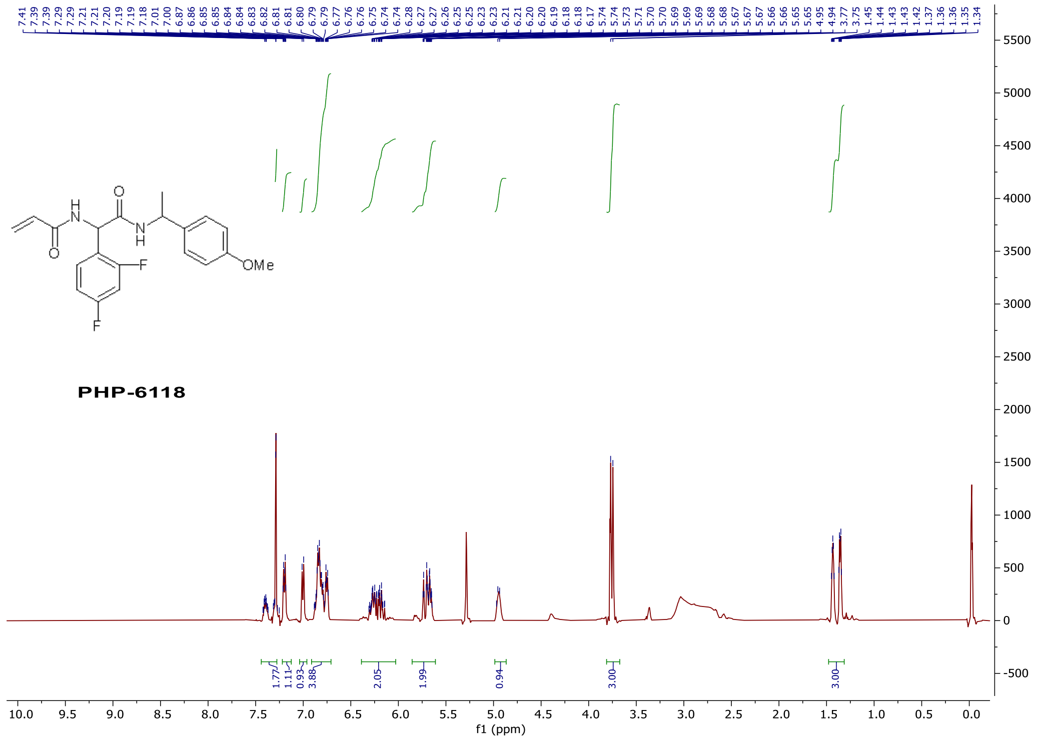

Supplementary Figure 900. ^{1}H NMR (400 MHz, $CDCl_3$ + one drop of CD_3OD) of compound **PHP-6118**.

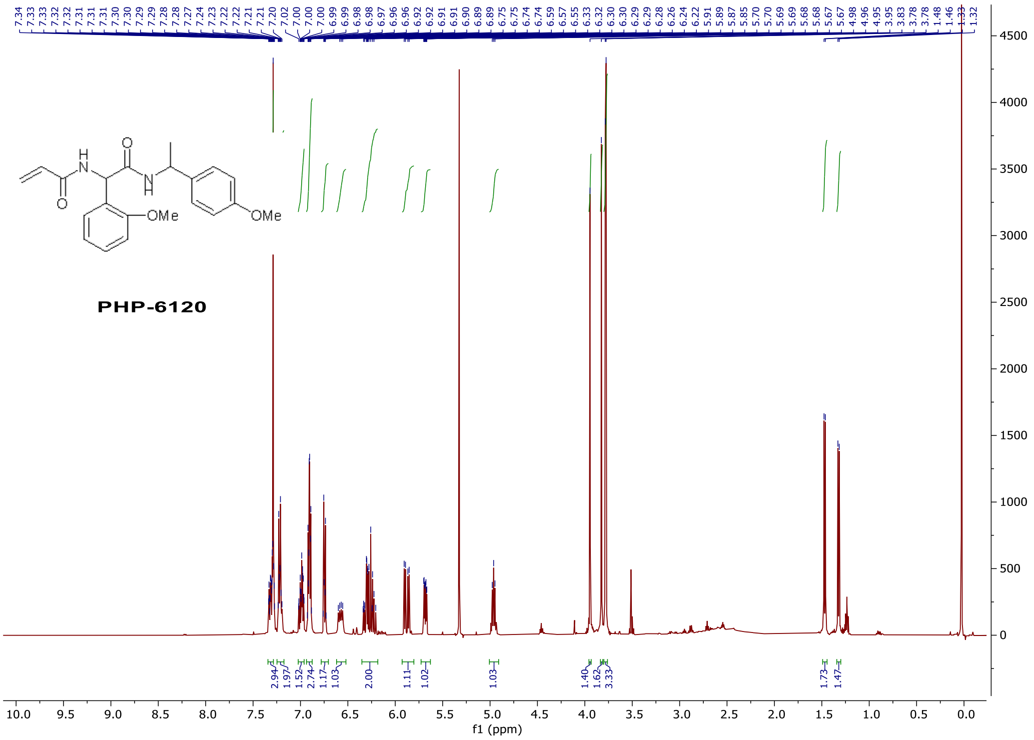

Supplementary Figure 901. ^{1}H NMR (400 MHz, $CDCl_3$ + one drop of CD_3OD) of compound **PHP-6120.**

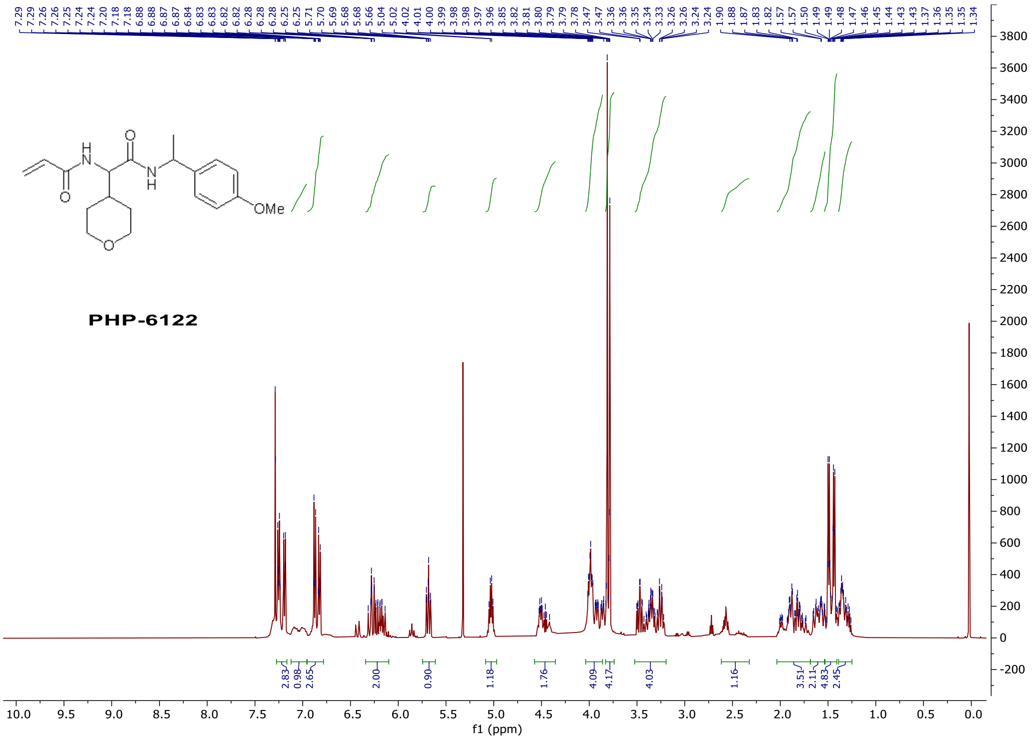

Supplementary Figure 902. ^{1}H NMR (400 MHz, $CDCl_3$ + one drop of CD_3OD) of compound **PHP-6122.**

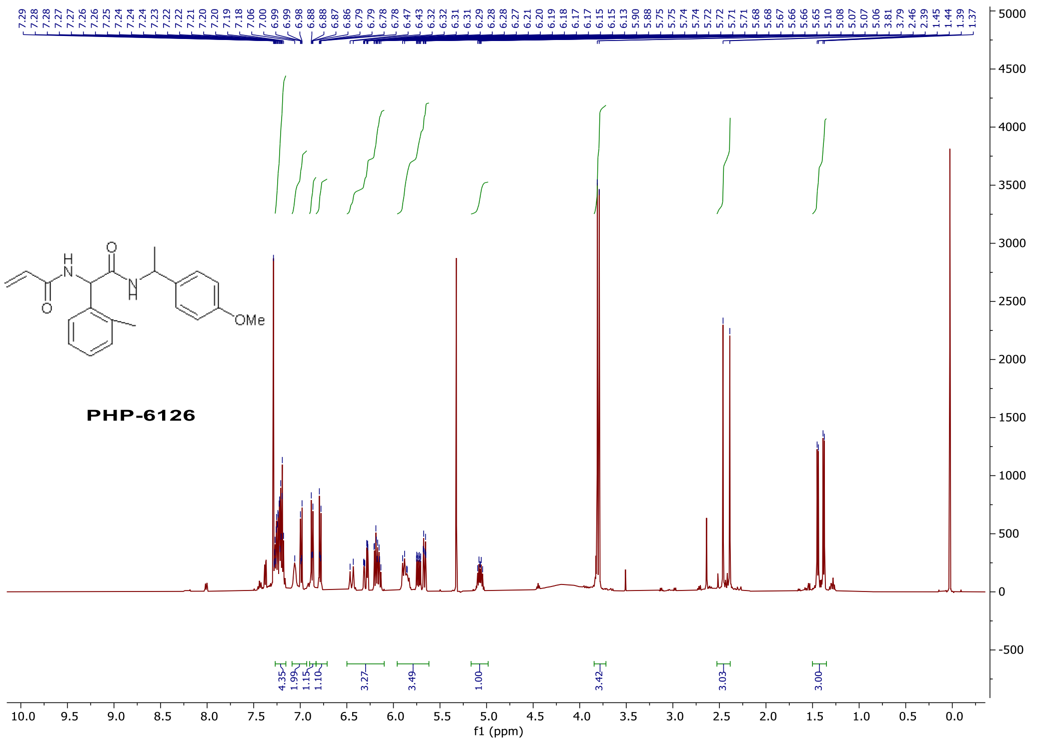

Supplementary Figure 903. ^{1}H NMR (400 MHz, $CDCl_3$ + one drop of CD_3OD) of compound **PHP-6126.**

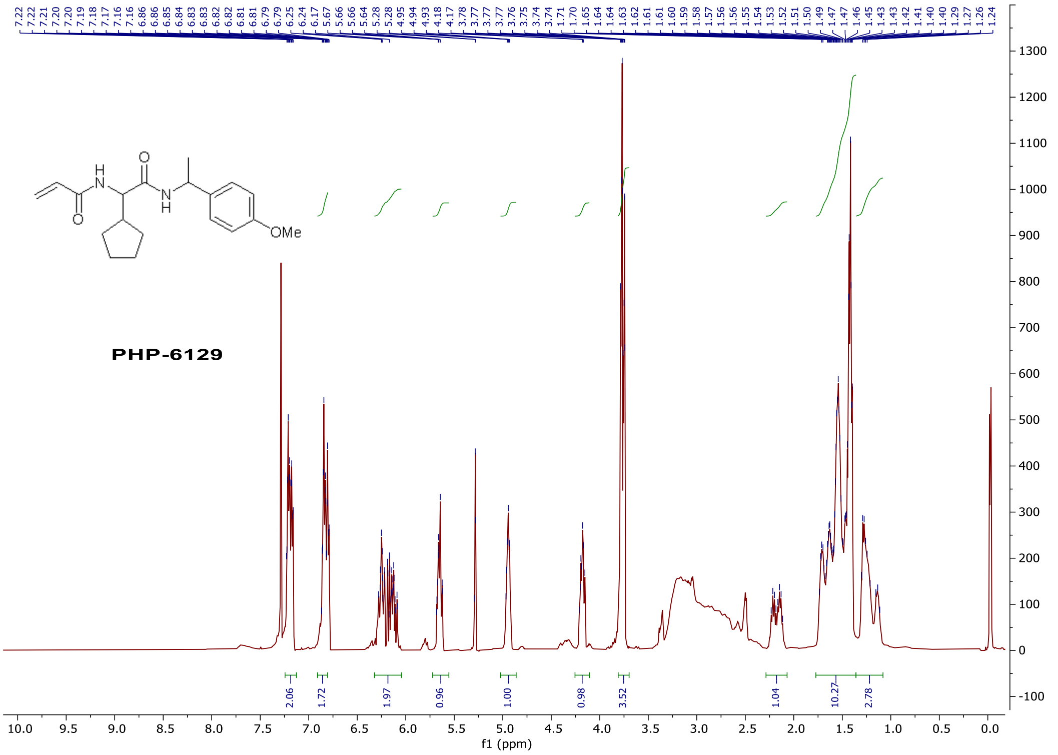

Supplementary Figure 904. ^{1}H NMR (400 MHz, $CDCl_3$ + one drop of CD_3OD) of compound **PHP-6129.**

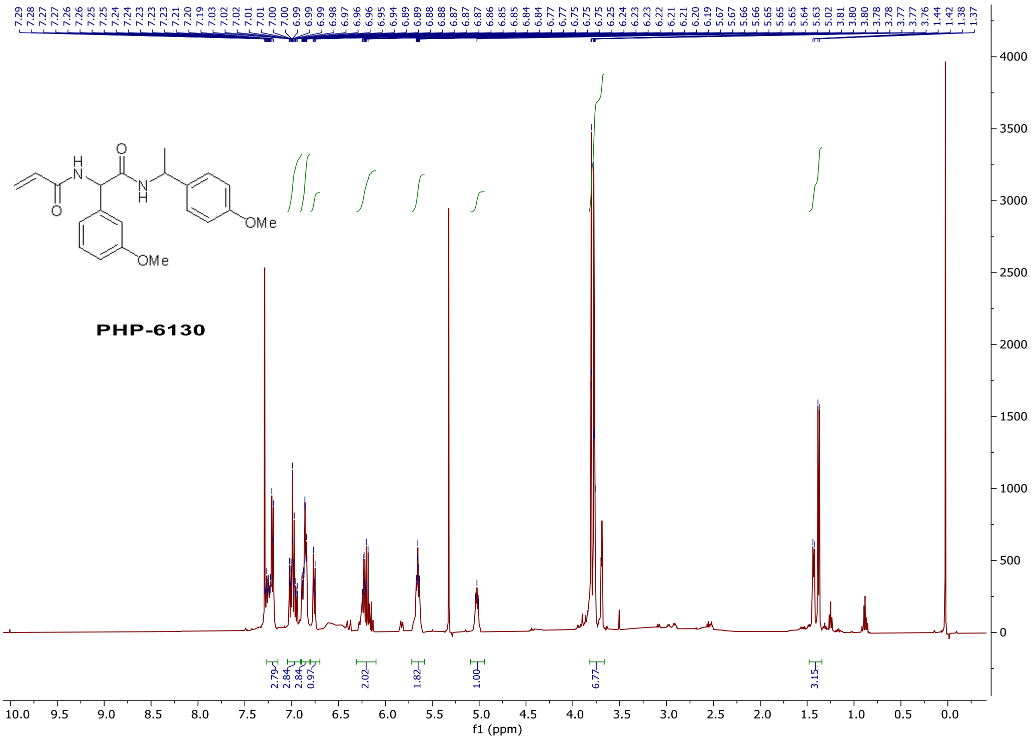

Supplementary Figure 905. ^{1}H NMR (400 MHz, $CDCl_3$ + one drop of CD_3OD) of compound **PHP-6130.**

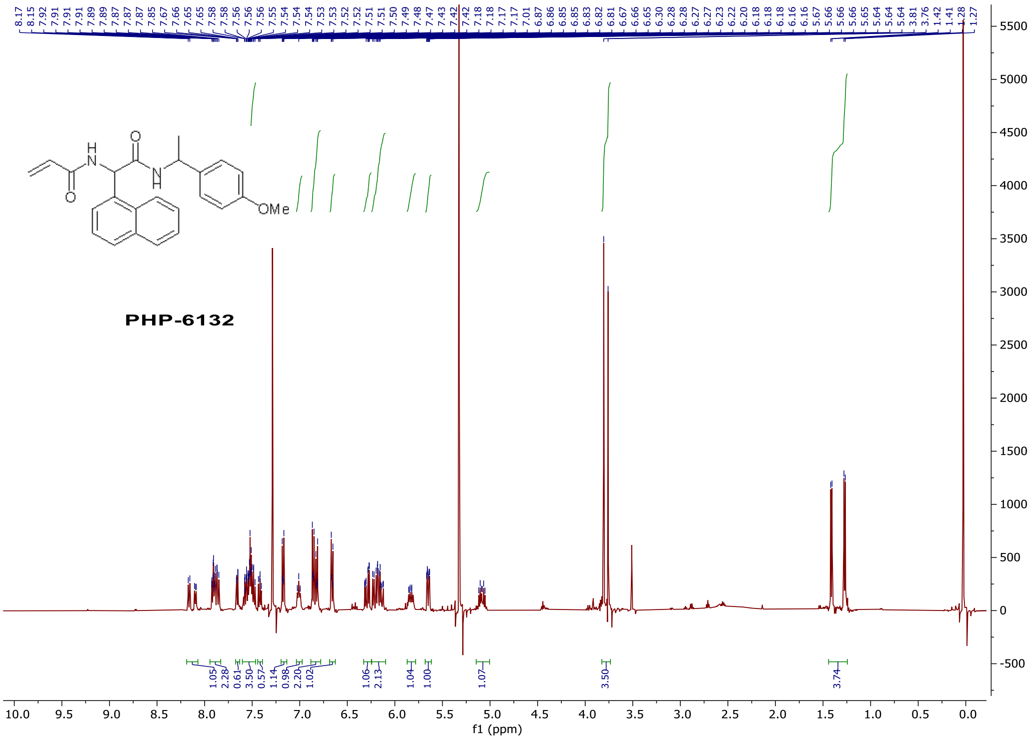

Supplementary Figure 905. ^{1}H NMR (400 MHz, $CDCl_3$ + one drop of CD_3OD) of compound **PHP-6132.**

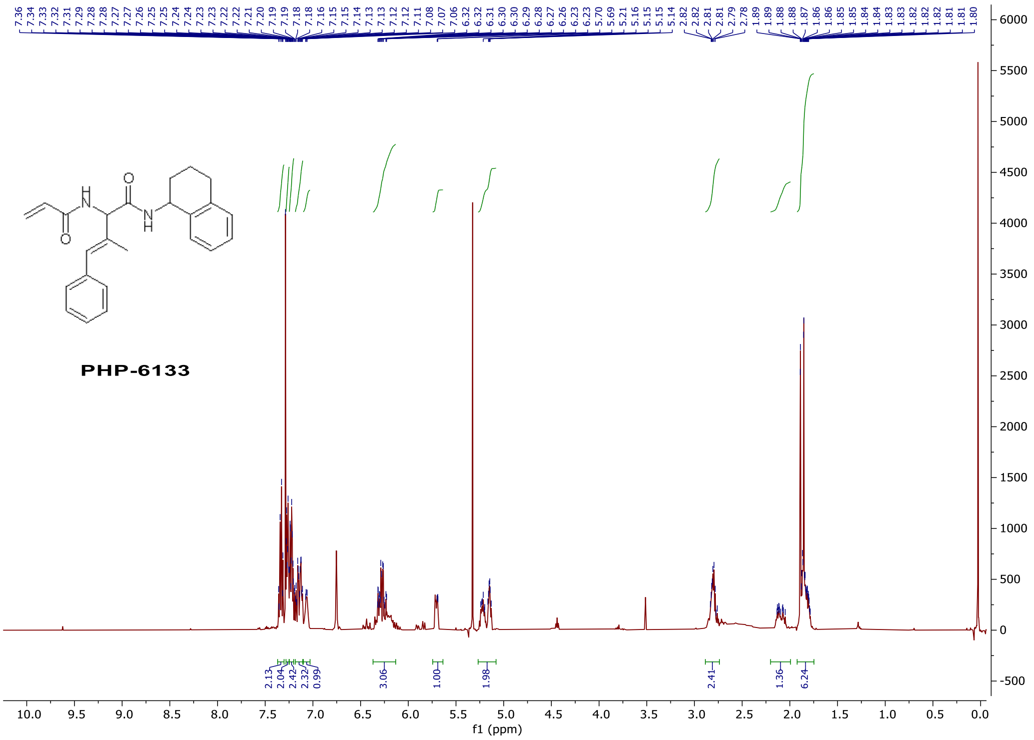

Supplementary Figure 906. ^{1}H NMR (400 MHz, $CDCl_3$ + one drop of CD_3OD) of compound **PHP-6133.**

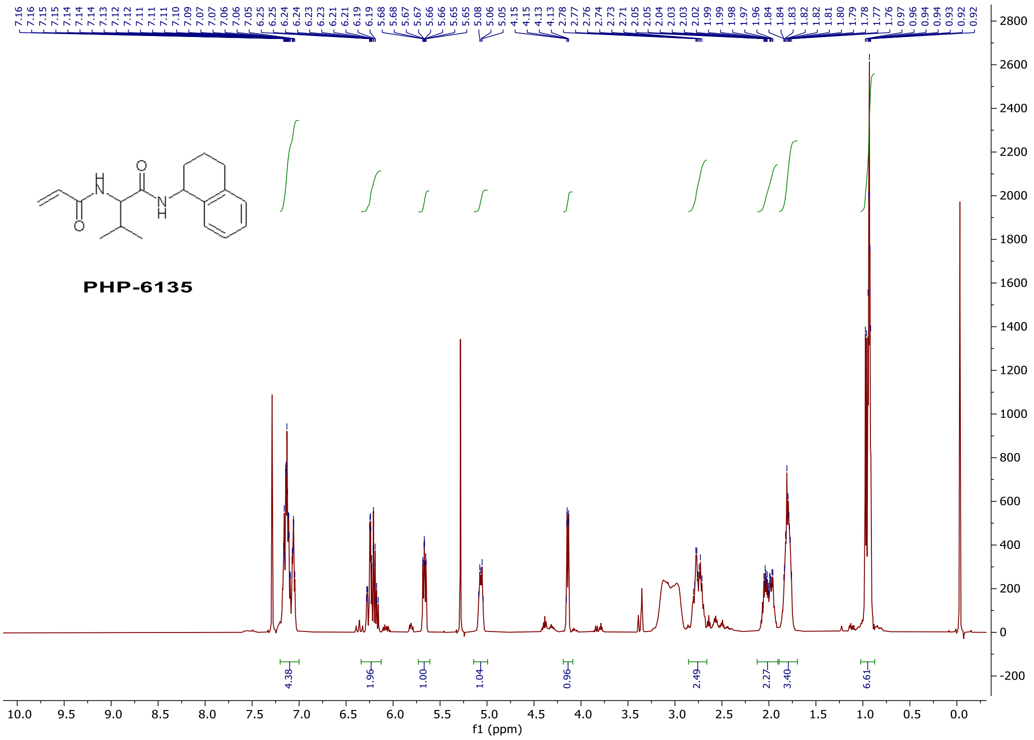

Supplementary Figure 906. ^{1}H NMR (400 MHz, $CDCl_3$ + one drop of CD_3OD) of compound **PHP-6135.**

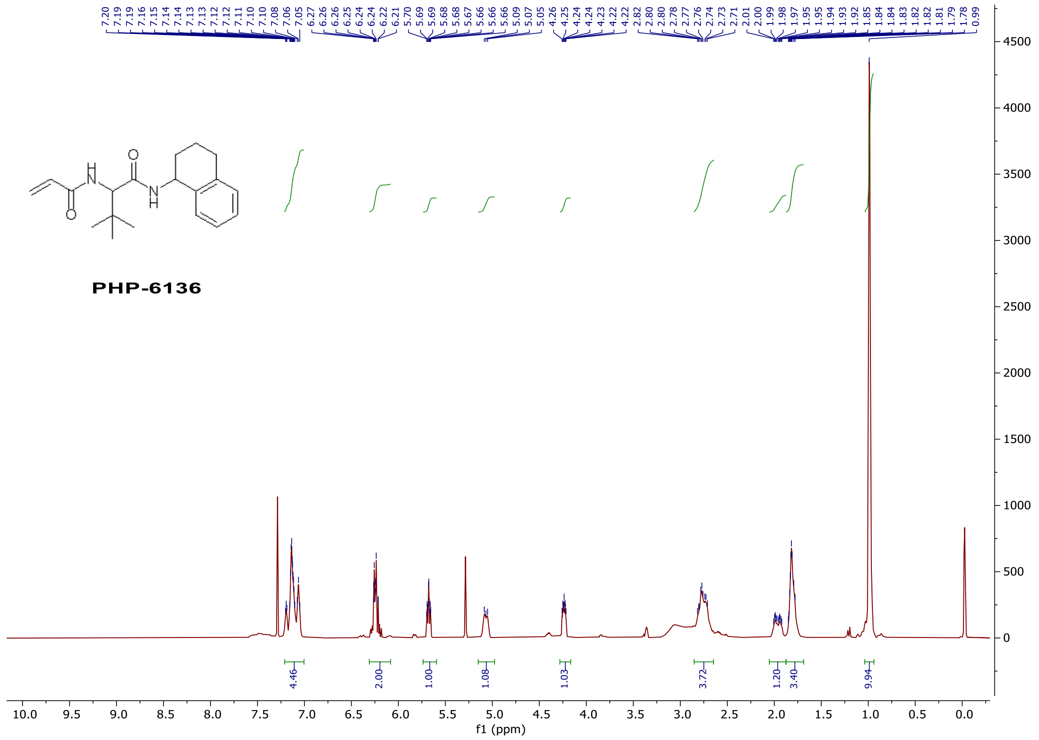

Supplementary Figure 907. ^{1}H NMR (400 MHz, $CDCl_3$ + one drop of CD_3OD) of compound **PHP-6136.**

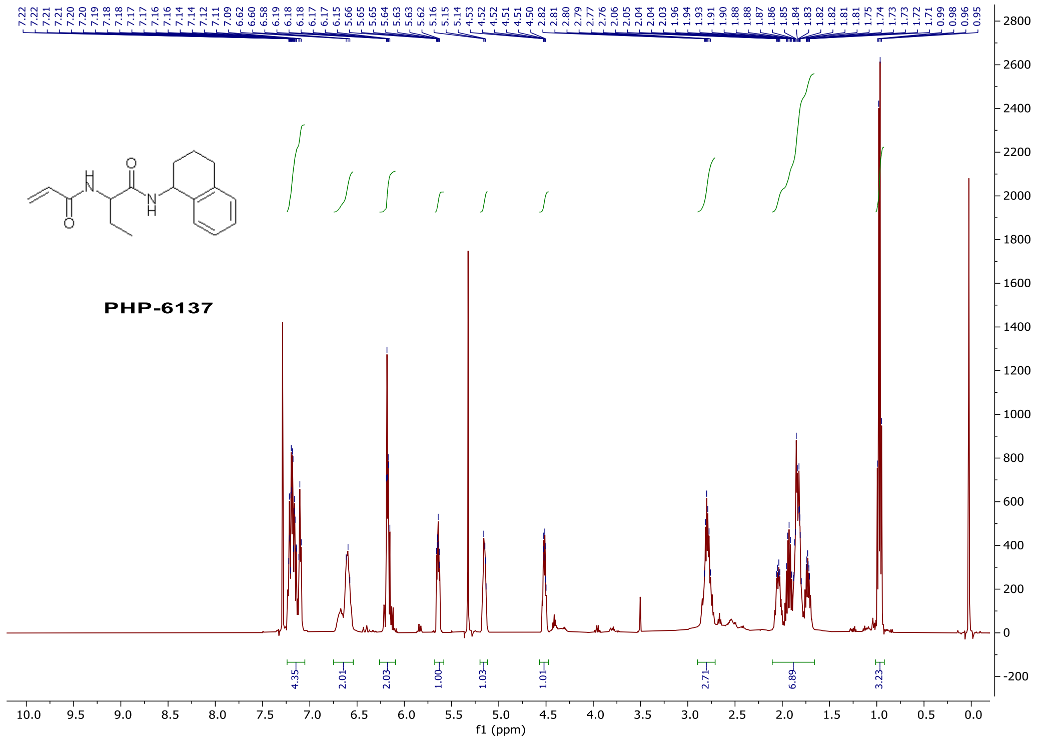

Supplementary Figure 908. ^{1}H NMR (400 MHz, $CDCl_3$ + one drop of CD_3OD) of compound **PHP-6137.**

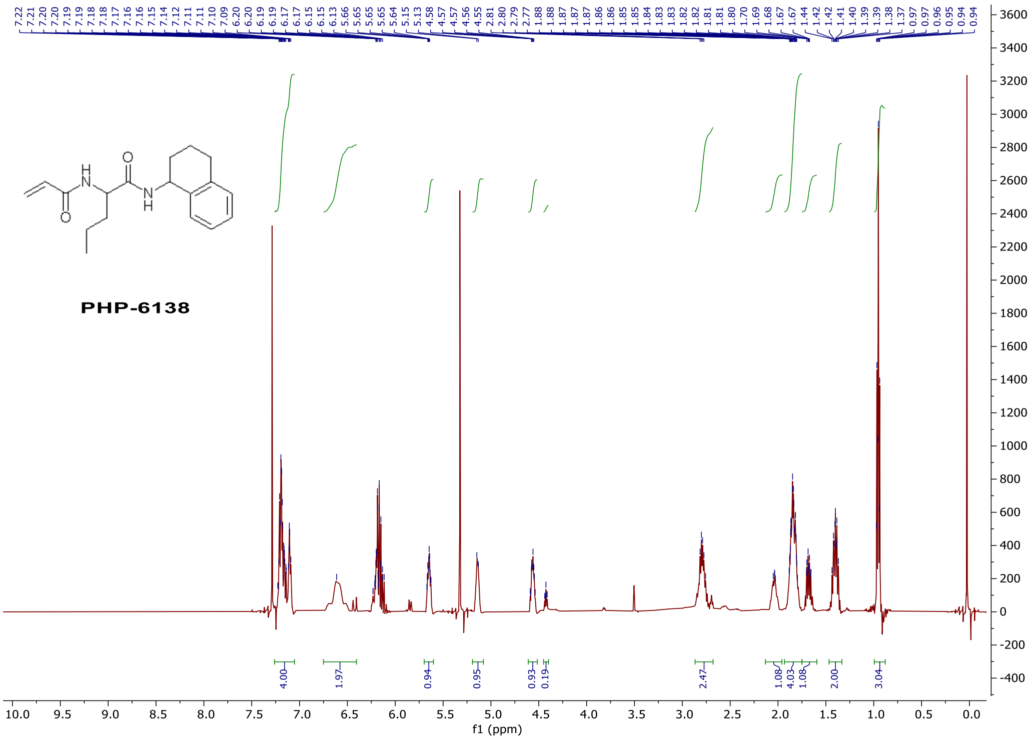

Supplementary Figure 909. ^{1}H NMR (400 MHz, $CDCl_3$ + one drop of CD_3OD) of compound **PHP-6138.**

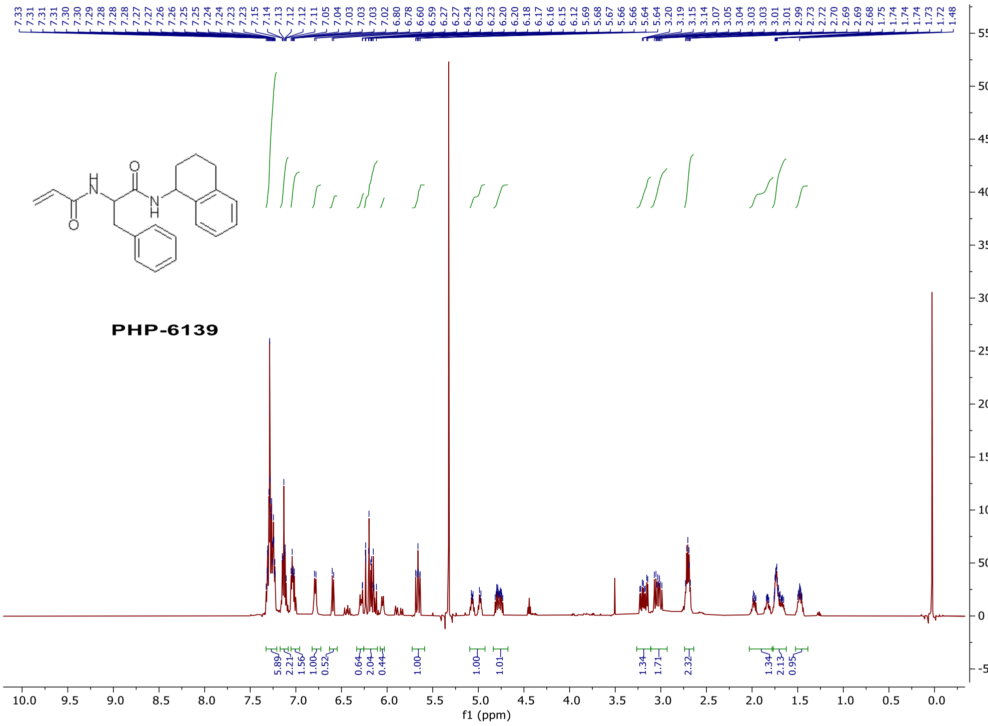

Supplementary Figure 910. ^{1}H NMR (400 MHz, $CDCl_3$ + one drop of CD_3OD) of compound **PHP-6139.**

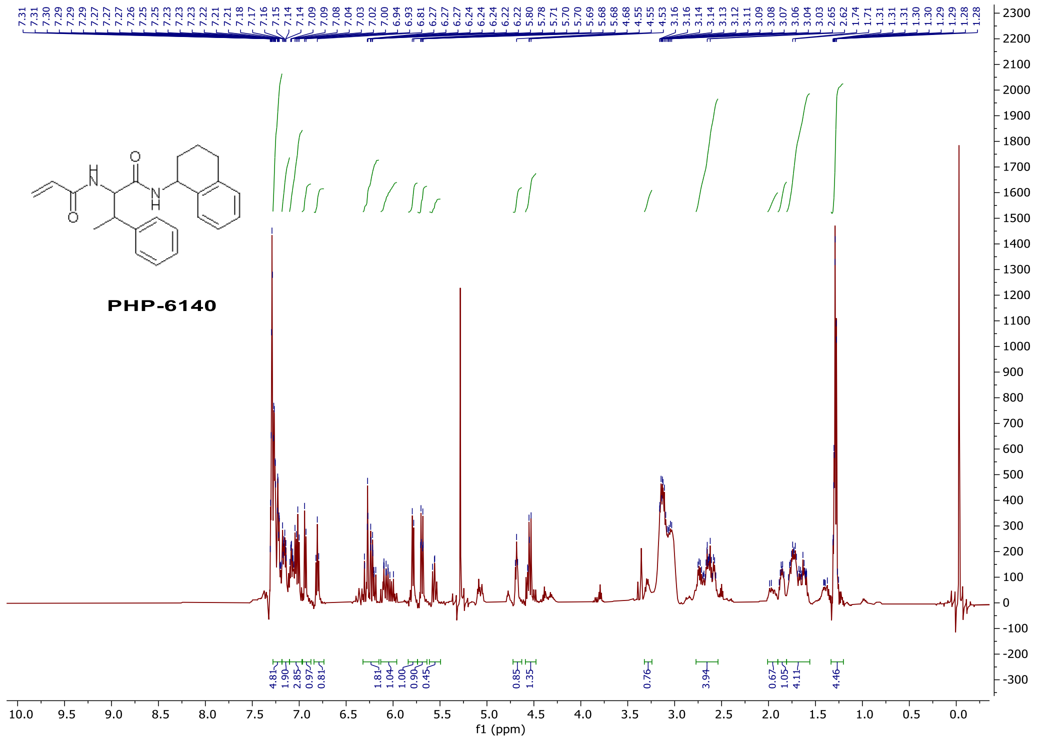

Supplementary Figure 911. ^1H NMR (400 MHz, $CDCl_3$ + one drop of CD_3OD) of compound **PHP-6140.**

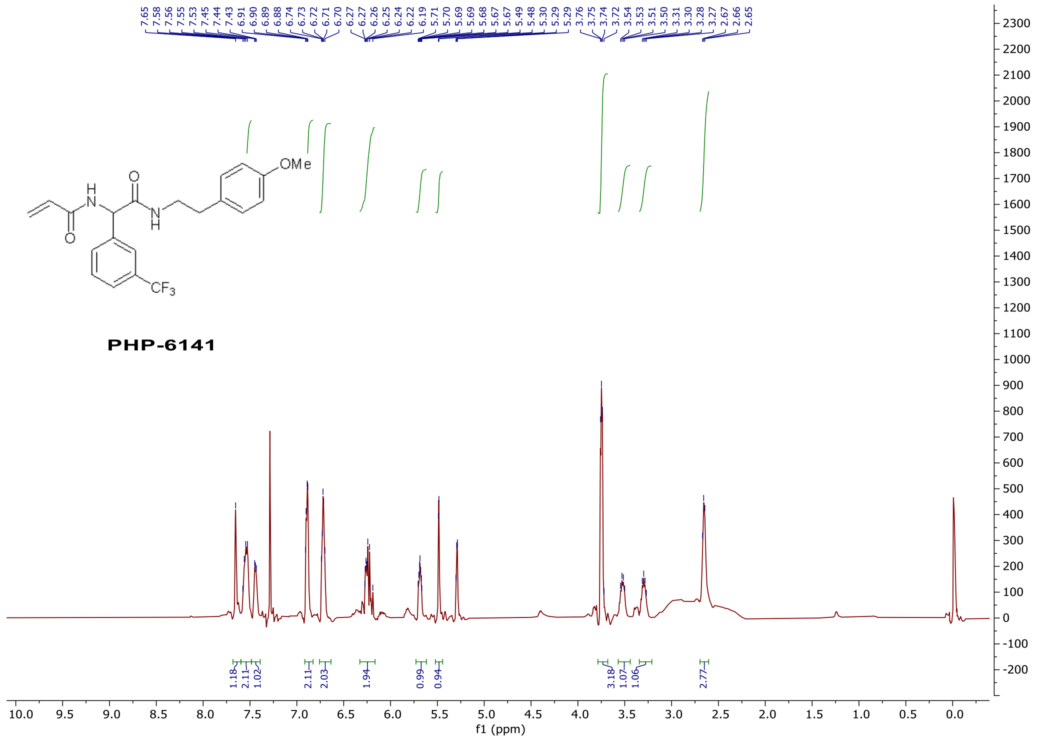

Supplementary Figure 912. ^1H NMR (400 MHz, $CDCl_3$ + one drop of CD_3OD) of compound **PHP-6141.**

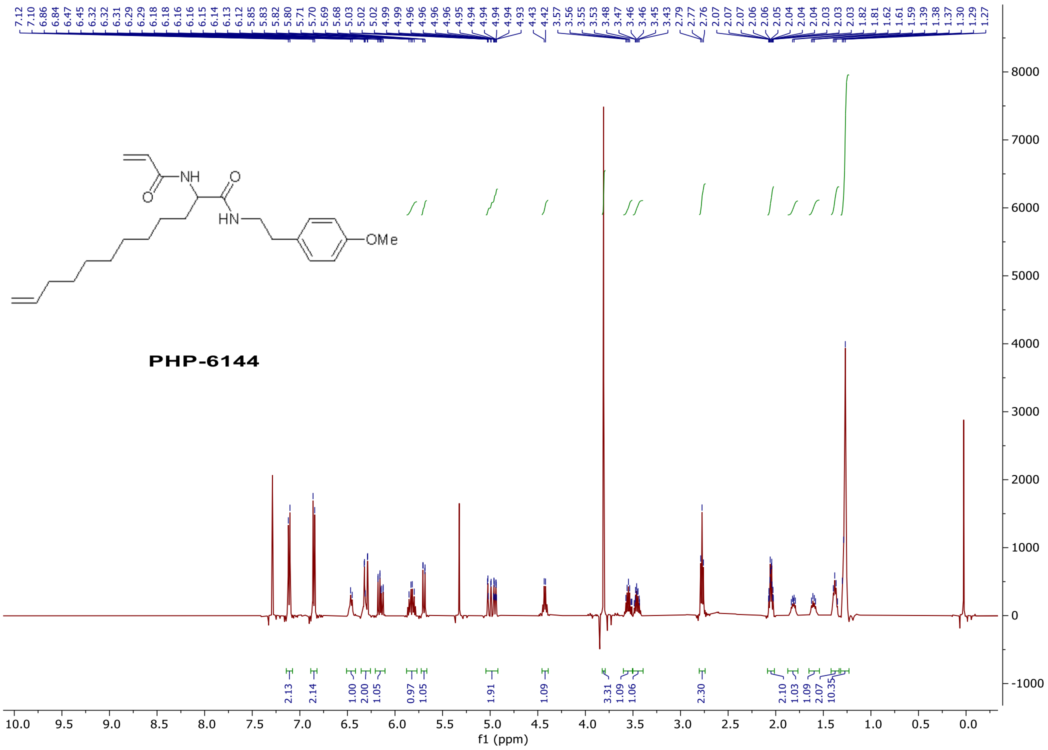

Supplementary Figure 913. ^{1}H NMR (400 MHz, $CDCl_3$ + one drop of CD_3OD) of compound **PHP-6144.**

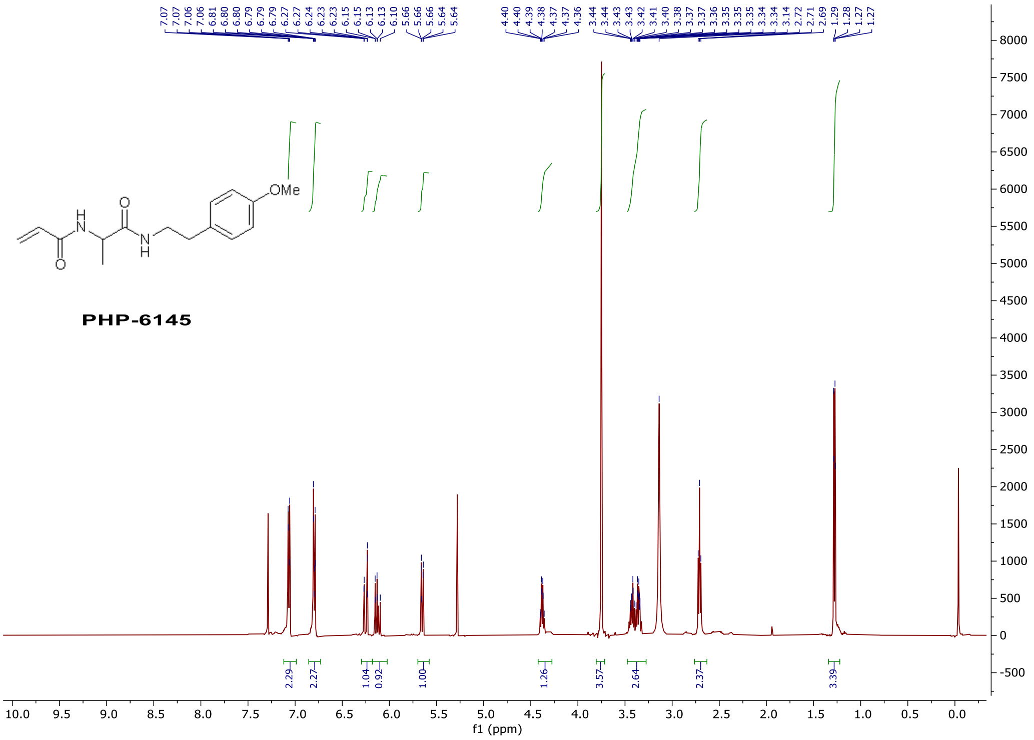

Supplementary Figure 914. ^{1}H NMR (400 MHz, $CDCl_3$ + one drop of CD_3OD) of compound **PHP-6145.**

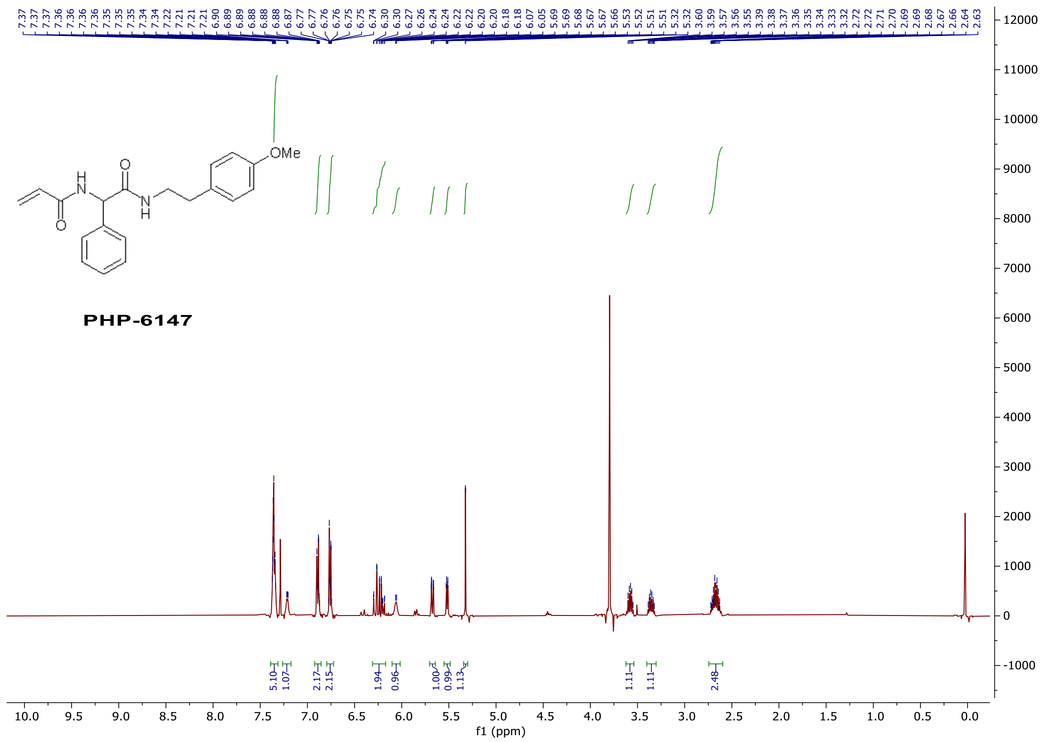

Supplementary Figure 915. ^{1}H NMR (400 MHz, $CDCl_3$ + one drop of CD_3OD) of compound **PHP-6147.**

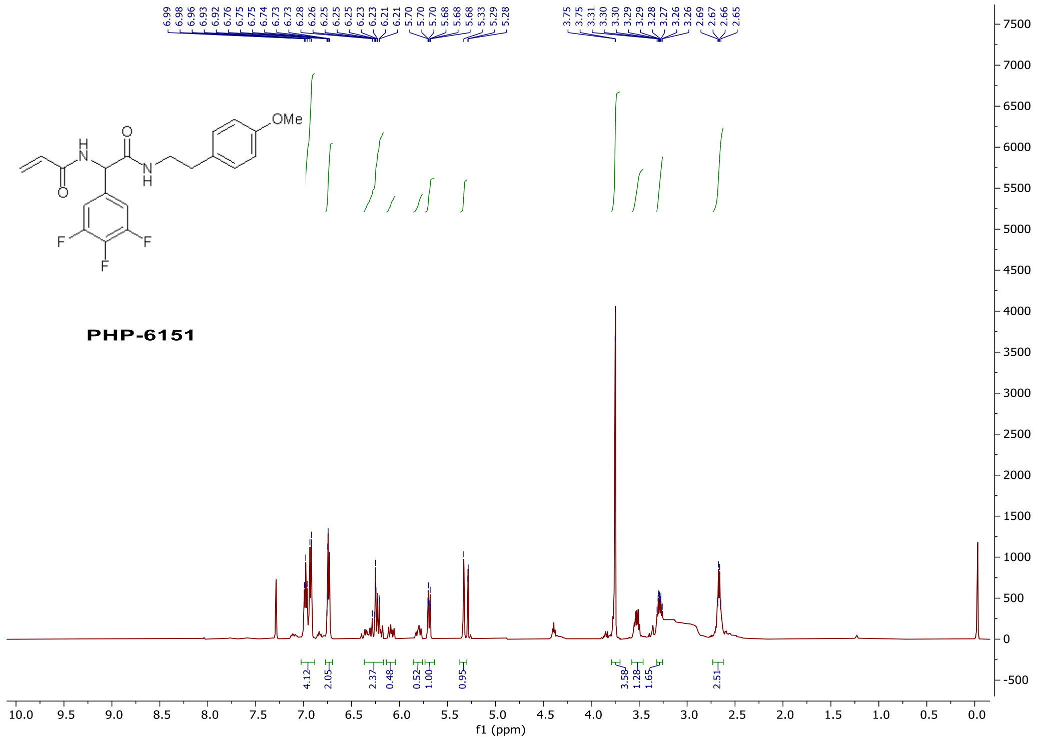

Supplementary Figure 916. ^{1}H NMR (400 MHz, $CDCl_3$ + one drop of CD_3OD) of compound **PHP-6151.**

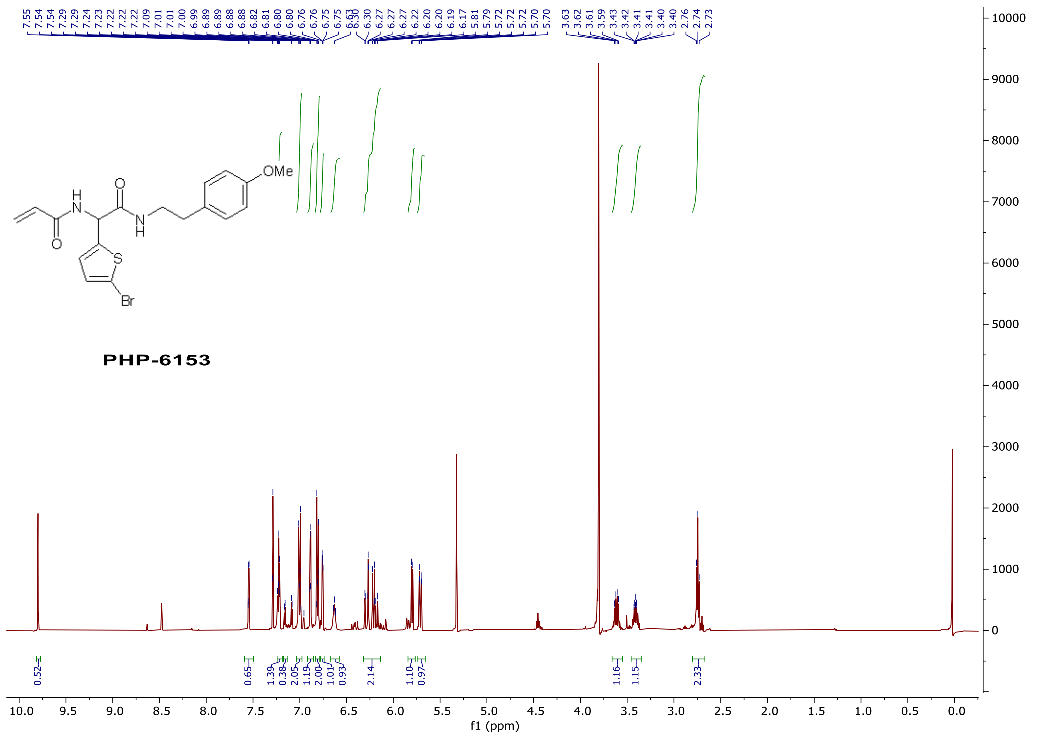

Supplementary Figure 917. ^{1}H NMR (400 MHz, $CDCl_3$ + one drop of CD_3OD) of compound **PHP-6153.**

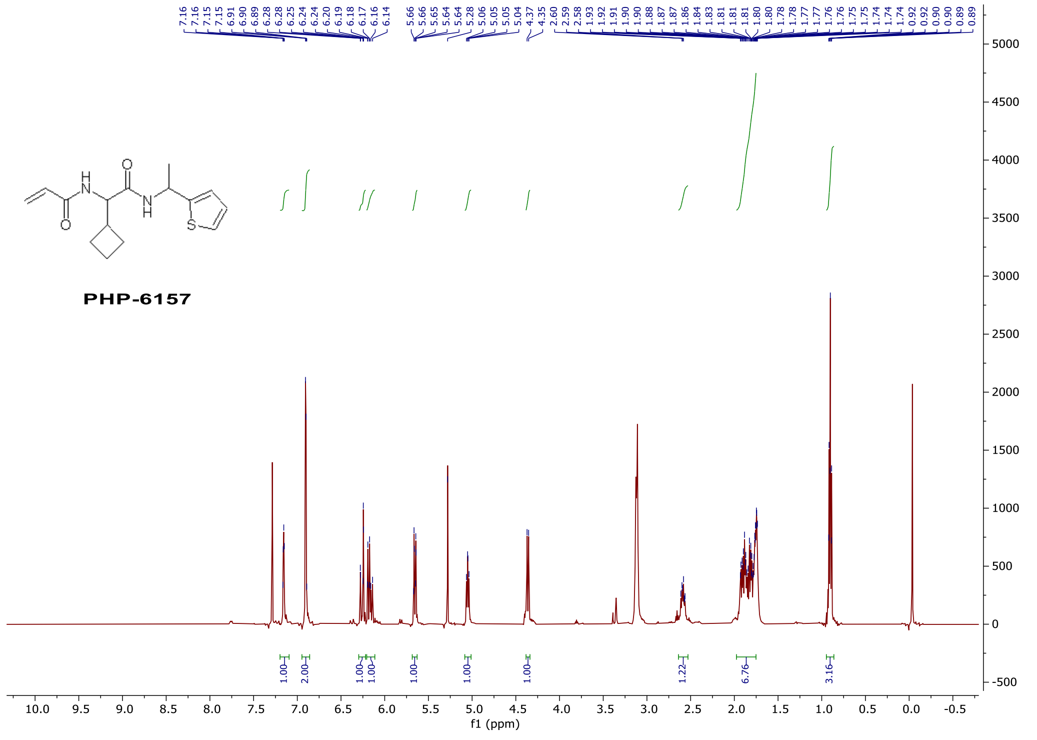

Supplementary Figure 918. ^{1}H NMR (400 MHz, $CDCl_3$ + one drop of CD_3OD) of compound **PHP-6157.**

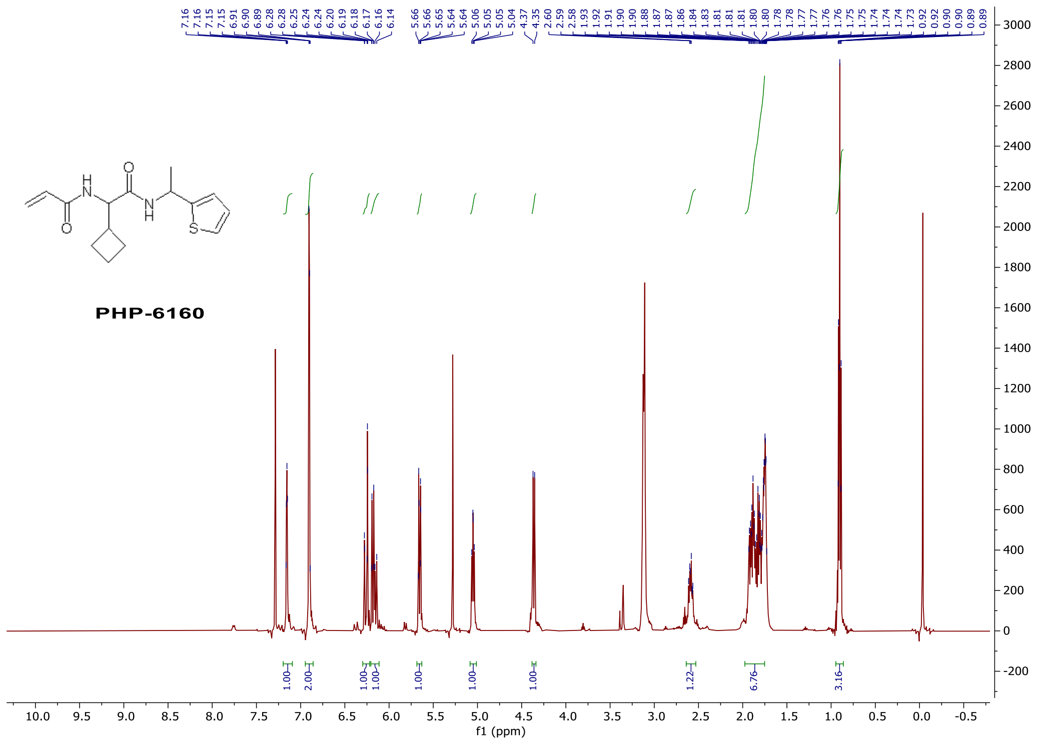

Supplementary Figure 919. ^{1}H NMR (400 MHz, $CDCl_3$ + one drop of CD_3OD) of compound **PHP-6160.**

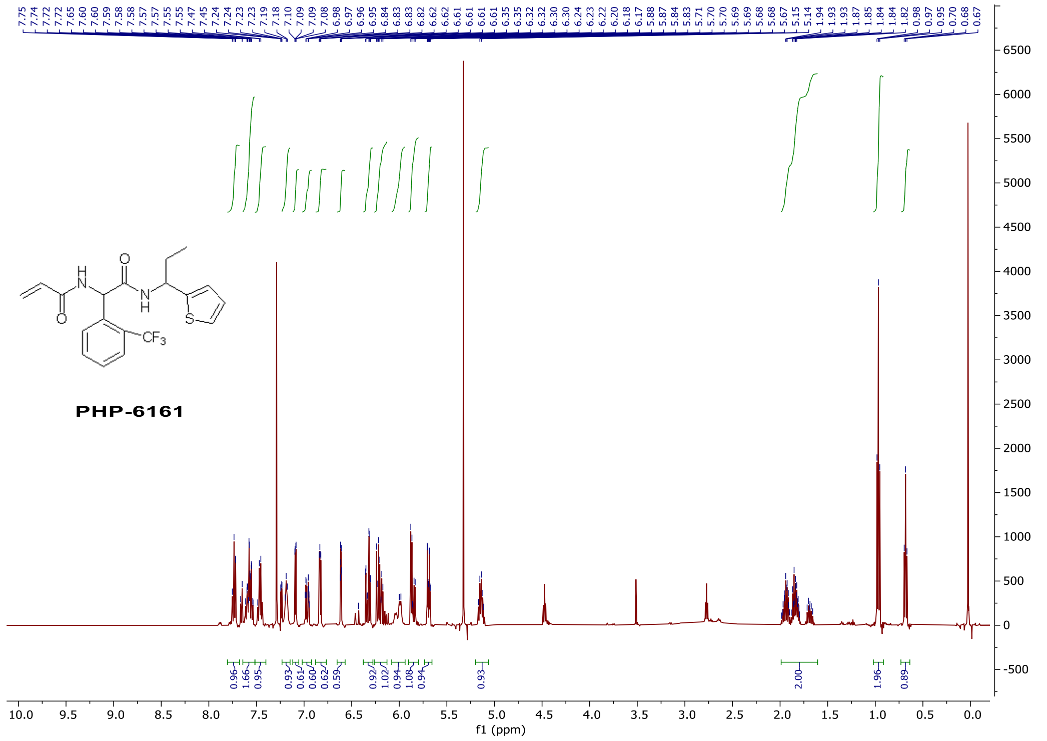

Supplementary Figure 920. ^{1}H NMR (400 MHz, $CDCl_3$ + one drop of CD_3OD) of compound **PHP-6161.**

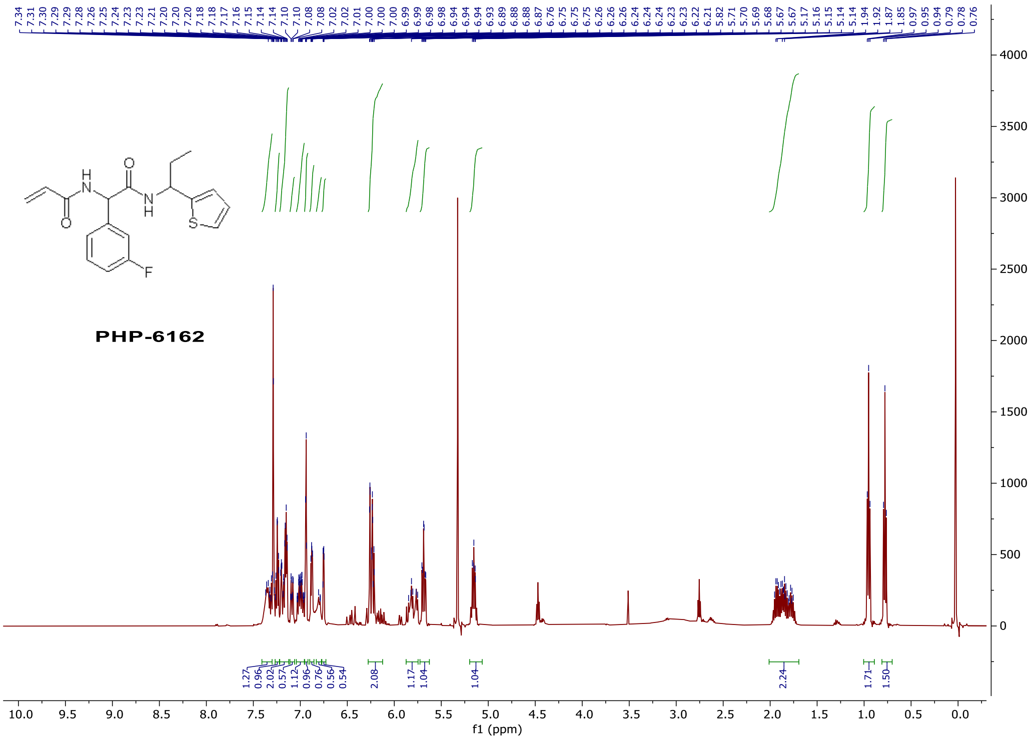

Supplementary Figure 921. ^{1}H NMR (400 MHz, $CDCl_3$ + one drop of CD_3OD) of compound **PHP-6162.**

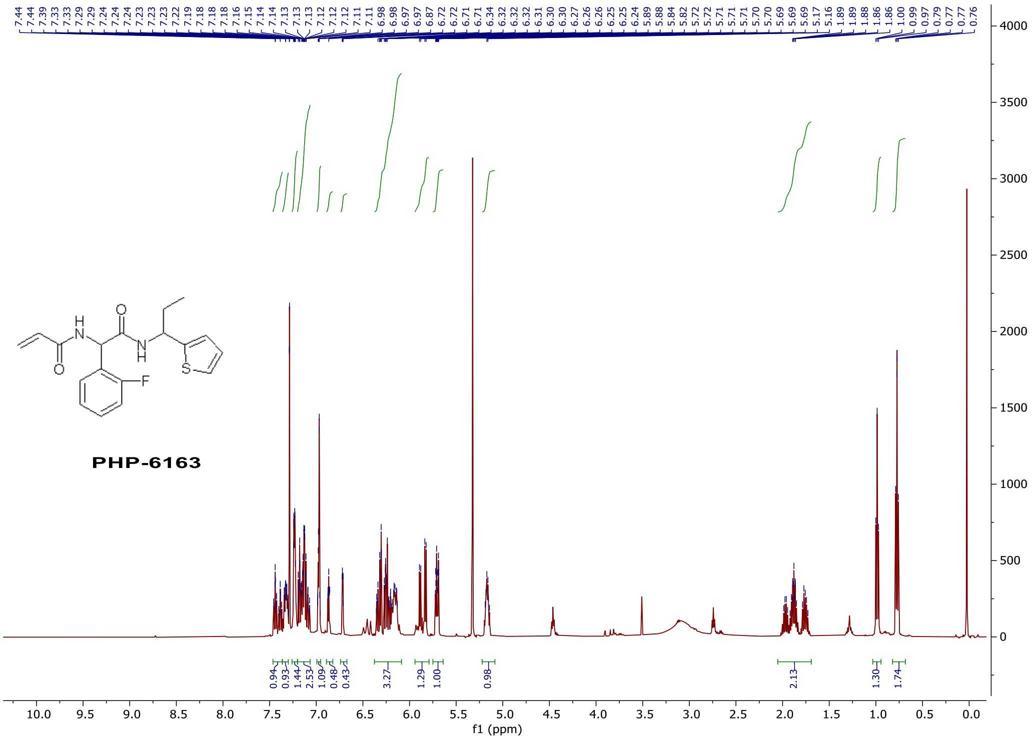

Supplementary Figure 922. ^{1}H NMR (400 MHz, $CDCl_3$ + one drop of CD_3OD) of compound **PHP-6163.**

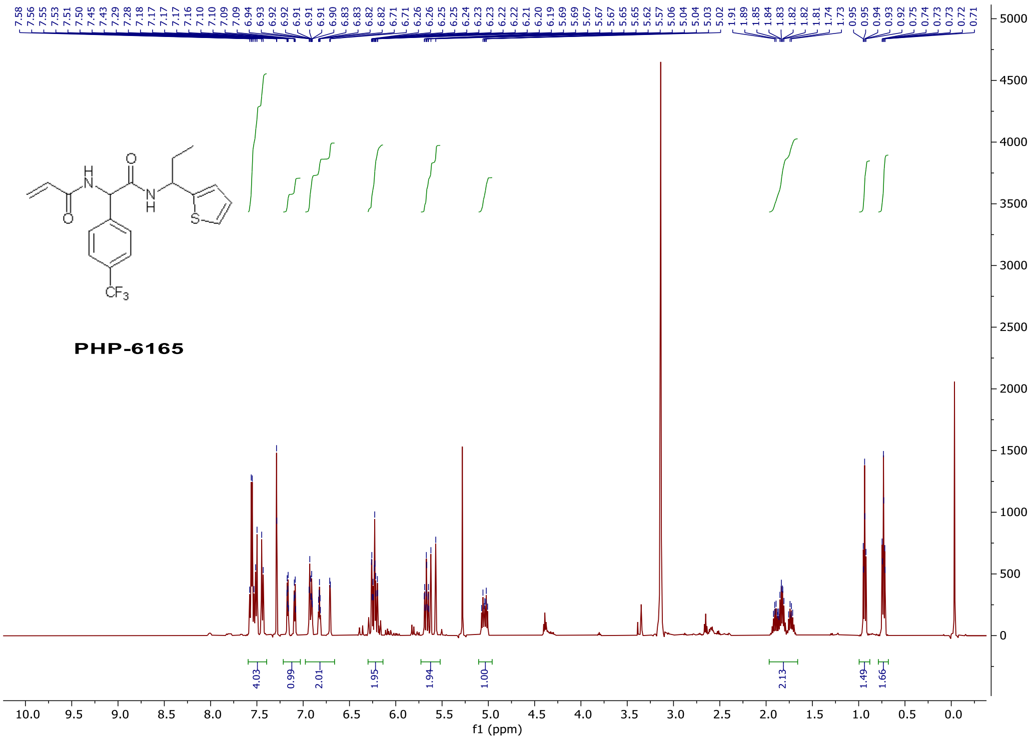

Supplementary Figure 923. ^{1}H NMR (400 MHz, $CDCl_3$ + one drop of CD_3OD) of compound **PHP-6165.**

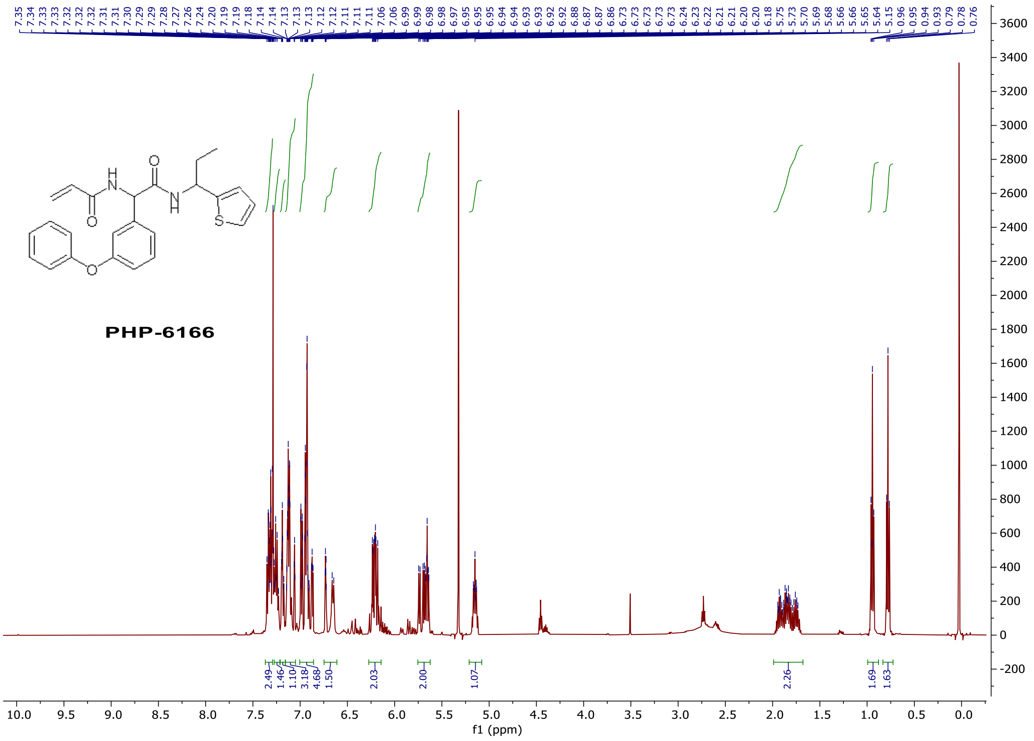

Supplementary Figure 924. ^{1}H NMR (400 MHz, $CDCl_3$ + one drop of CD_3OD) of compound **PHP-6166.**

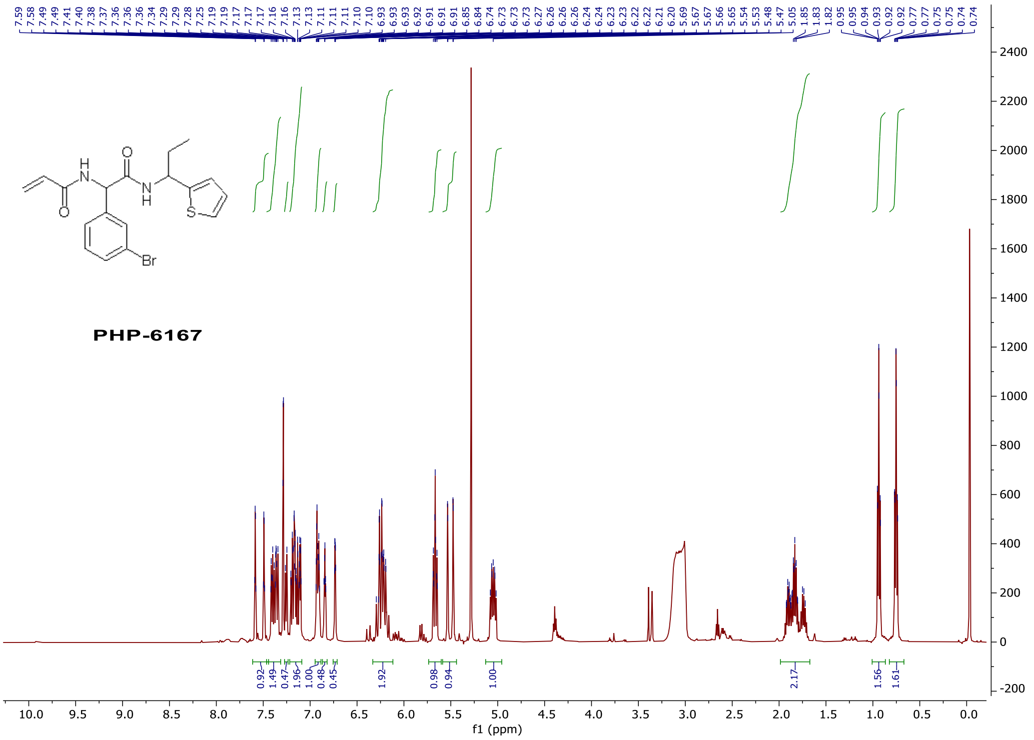

Supplementary Figure 925. ^{1}H NMR (400 MHz, $CDCl_3$ + one drop of CD_3OD) of compound **PHP-6167.**

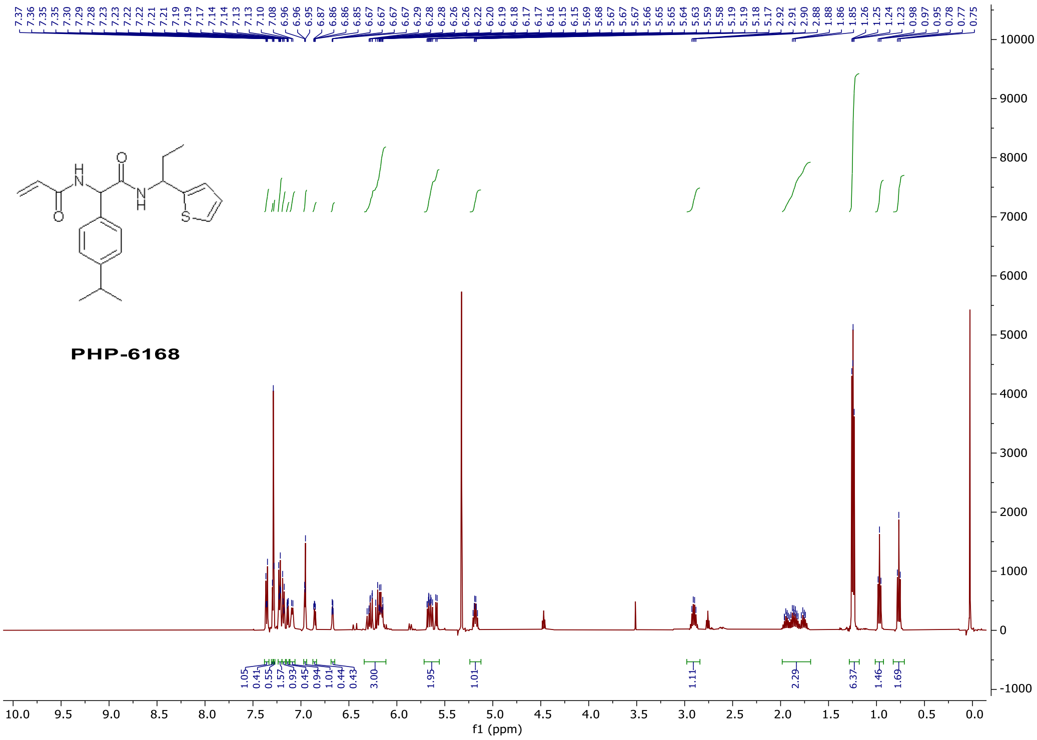

Supplementary Figure 926. ^{1}H NMR (400 MHz, $CDCl_3$ + one drop of CD_3OD) of compound **PHP-6168.**

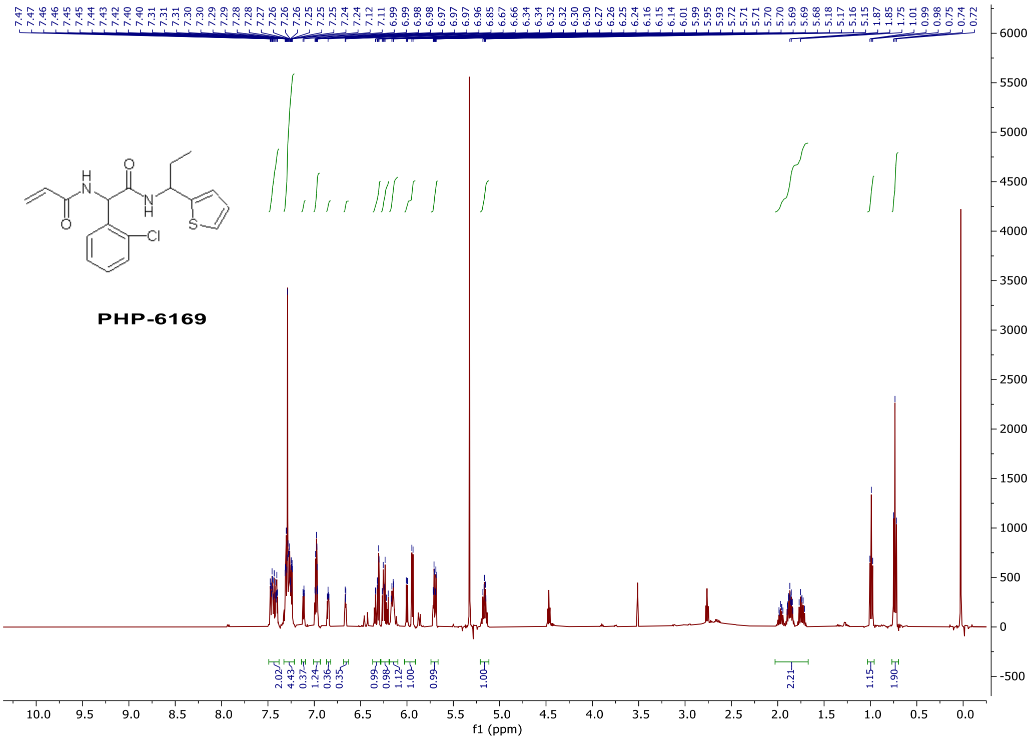

Supplementary Figure 927. ^{1}H NMR (400 MHz, $CDCl_3$ + one drop of CD_3OD) of compound **PHP-6169.**

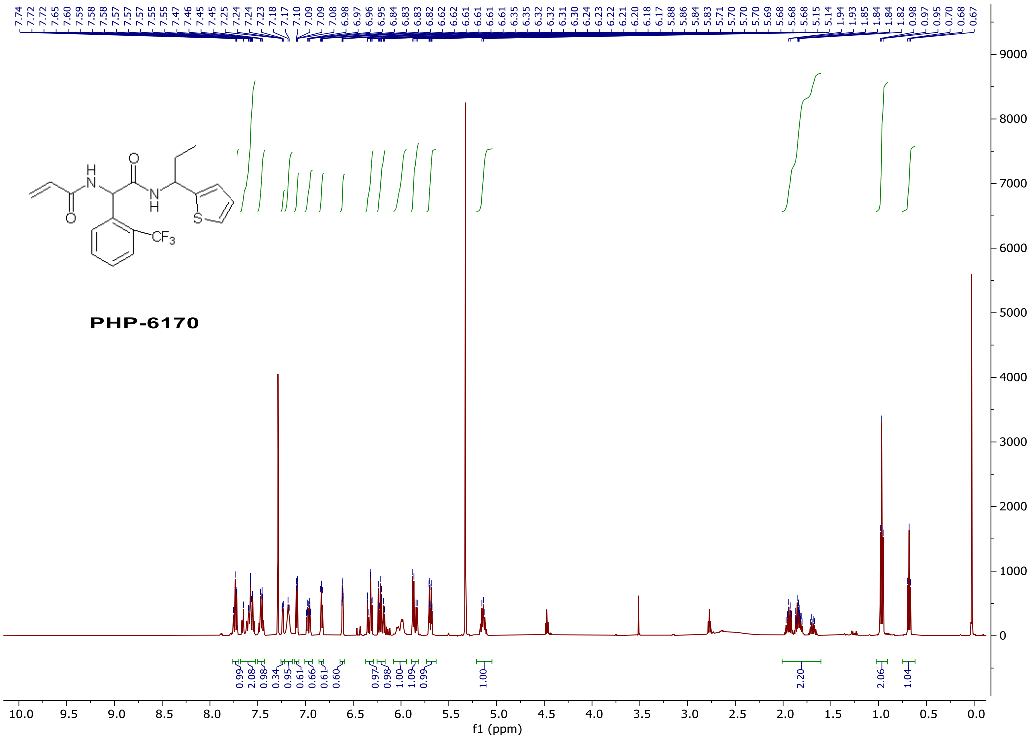

Supplementary Figure 928. ^{1}H NMR (400 MHz, $CDCl_3$ + one drop of CD_3OD) of compound **PHP-6170.**

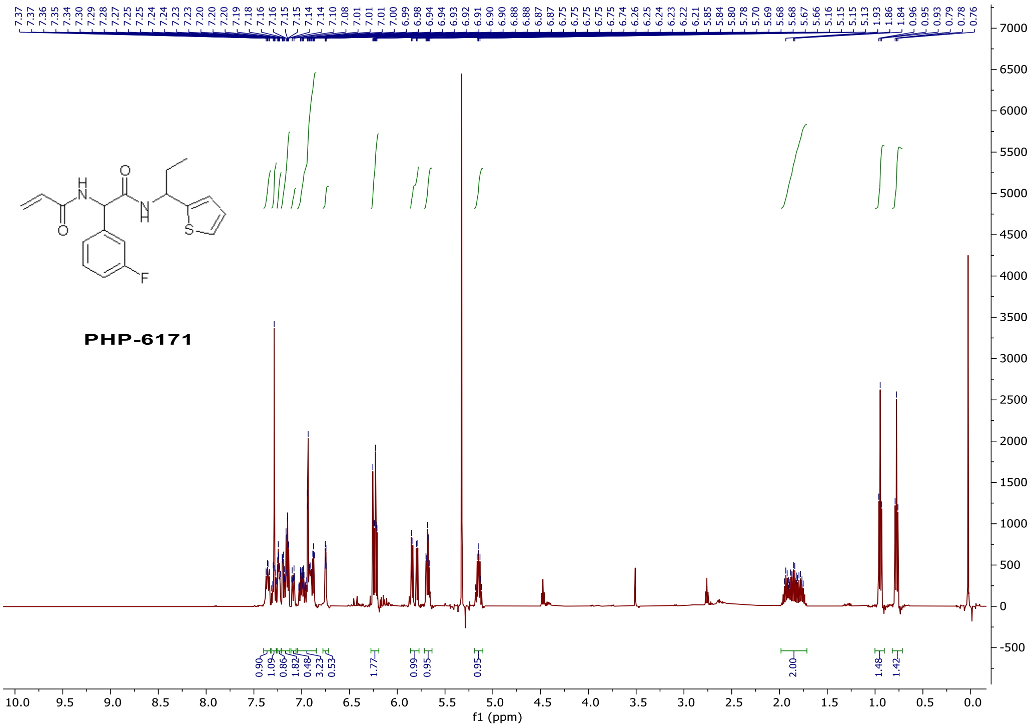

Supplementary Figure 929. ^{1}H NMR (400 MHz, $CDCl_3$ + one drop of CD_3OD) of compound **PHP-6171.**

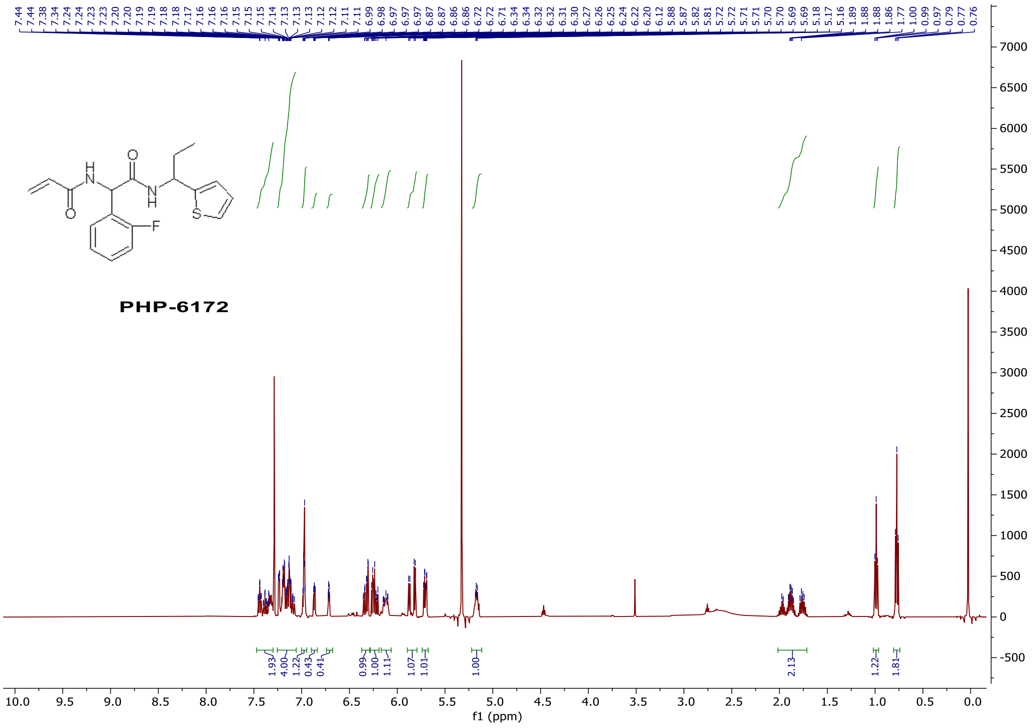

Supplementary Figure 930. ^{1}H NMR (400 MHz, $CDCl_3$ + one drop of CD_3OD) of compound **PHP-6172.**

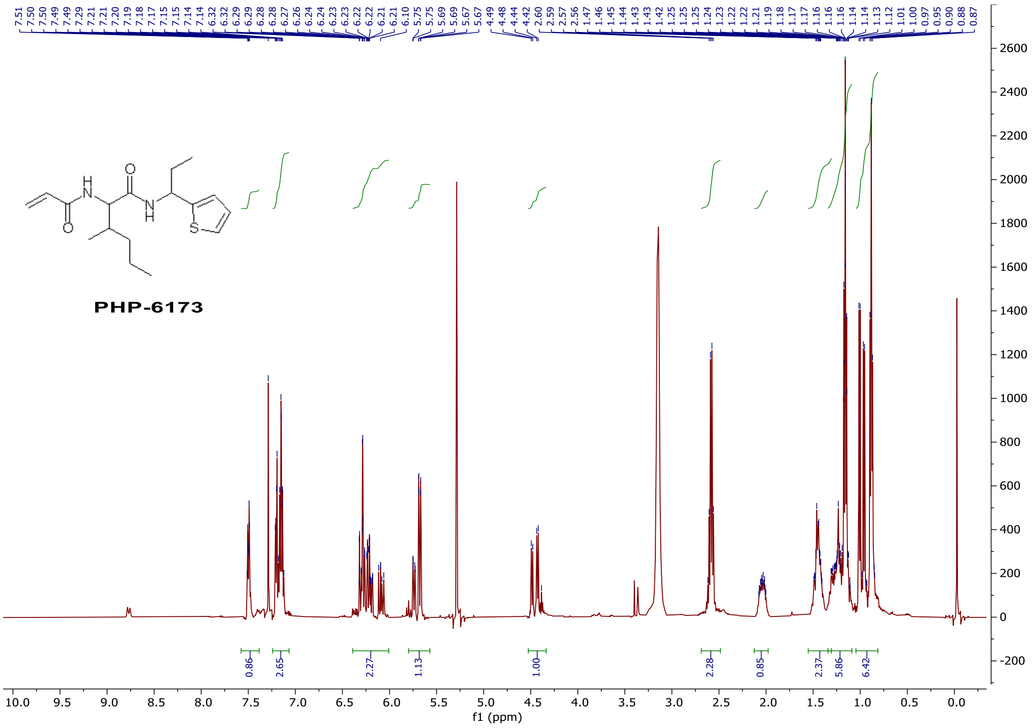

Supplementary Figure 931. ^{1}H NMR (400 MHz, $CDCl_3$ + one drop of CD_3OD) of compound **PHP-6173.**

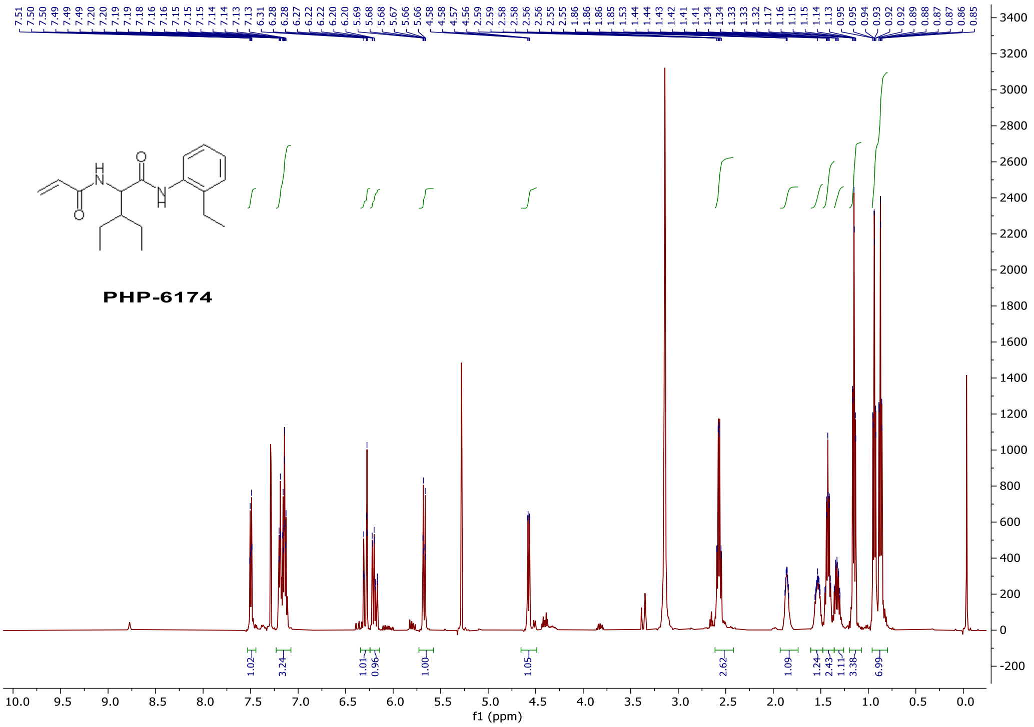

Supplementary Figure 932. ^{1}H NMR (400 MHz, $CDCl_3$ + one drop of CD_3OD) of compound **PHP-6174.**

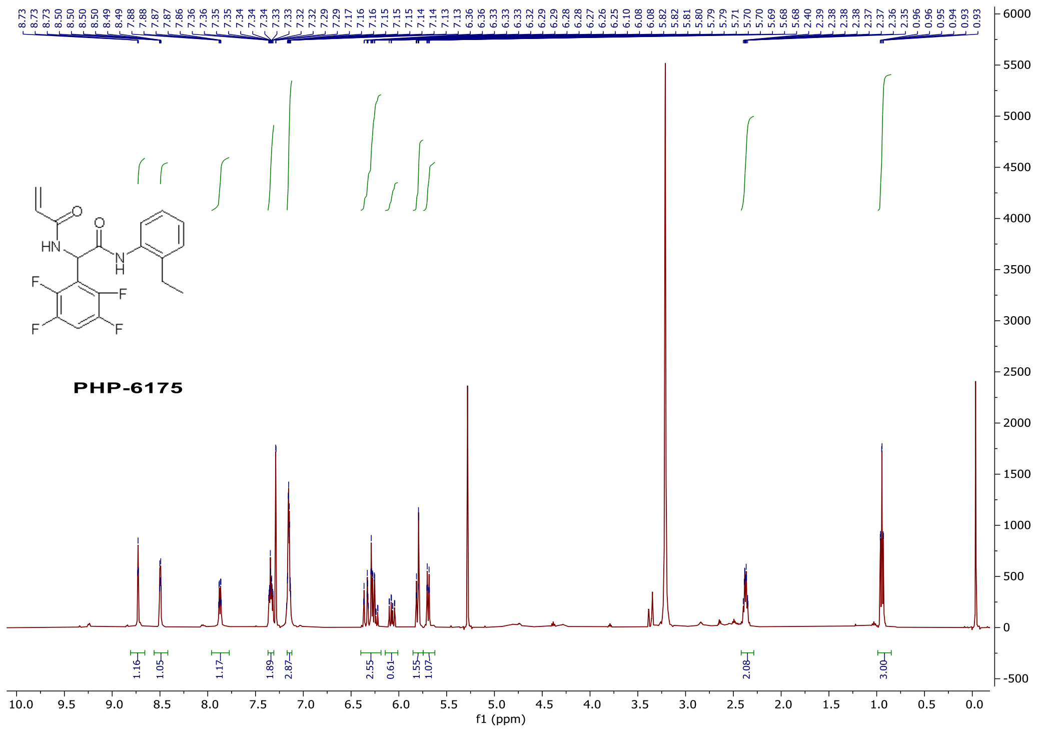

Supplementary Figure 933. ^{1}H NMR (400 MHz, $CDCl_3$ + one drop of CD_3OD) of compound **PHP-6175.**

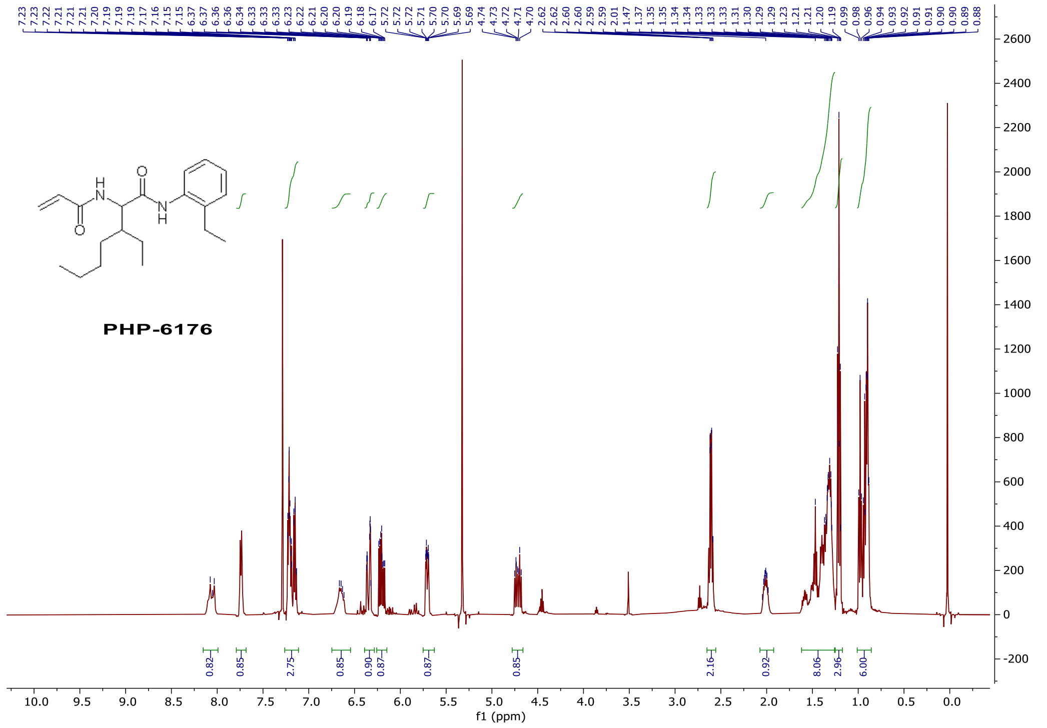

Supplementary Figure 934. ^{1}H NMR (400 MHz, $CDCl_3$ + one drop of CD_3OD) of compound **PHP-6176.**

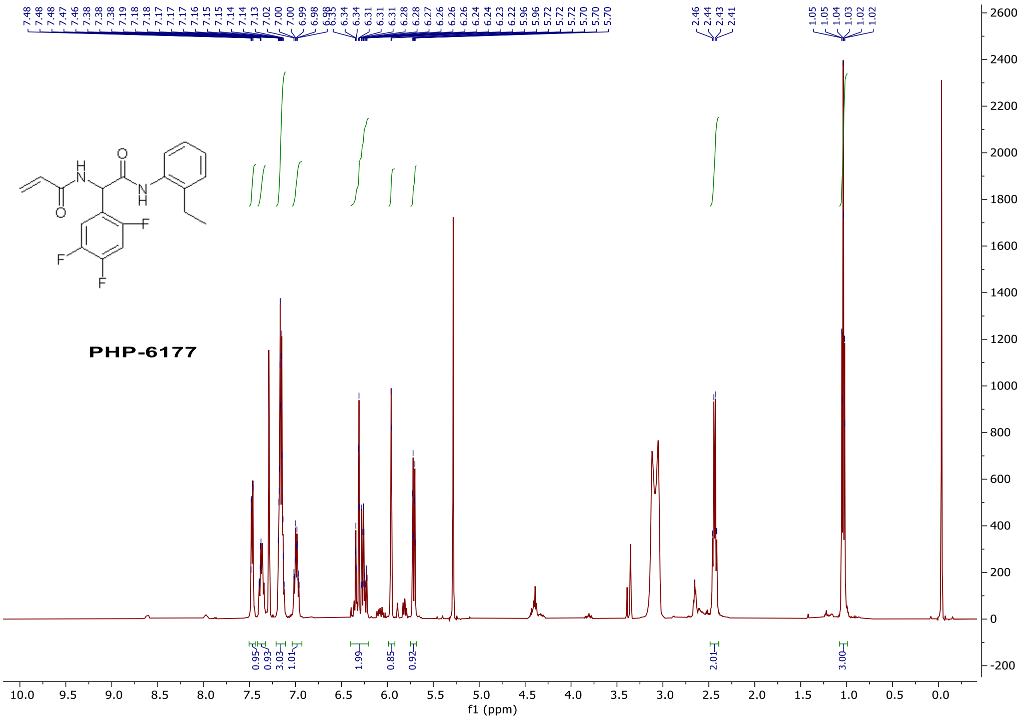

Supplementary Figure 935. ^{1}H NMR (400 MHz, $CDCl_3$ + one drop of CD_3OD) of compound **PHP-6177.**

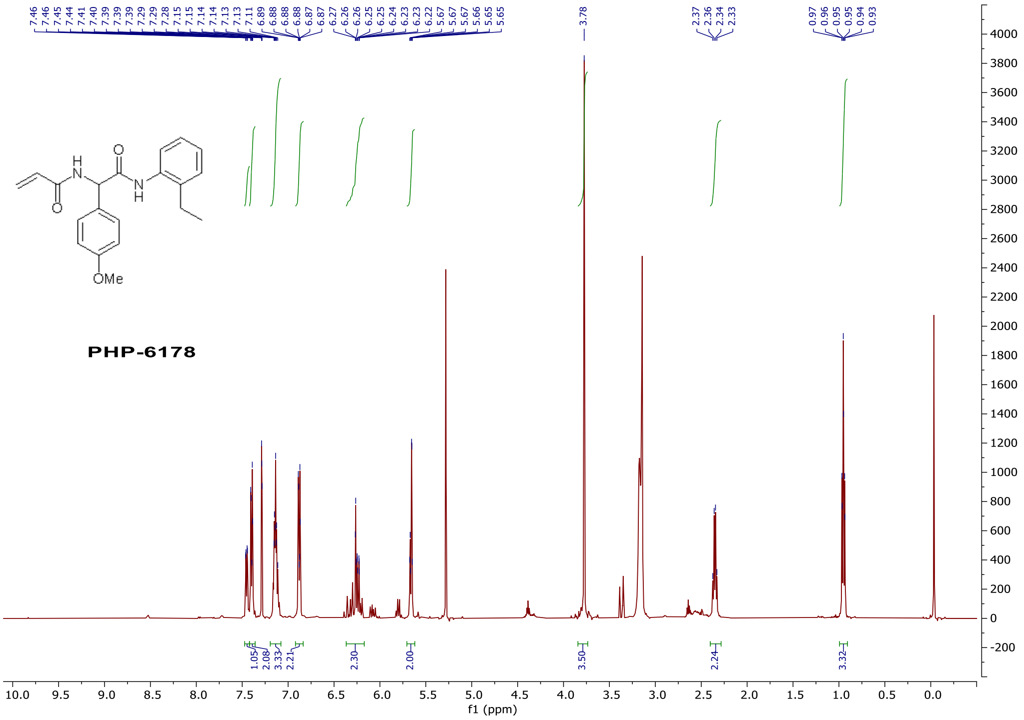

Supplementary Figure 936. ^{1}H NMR (400 MHz, $CDCl_3$ + one drop of CD_3OD) of compound **PHP-6178.**

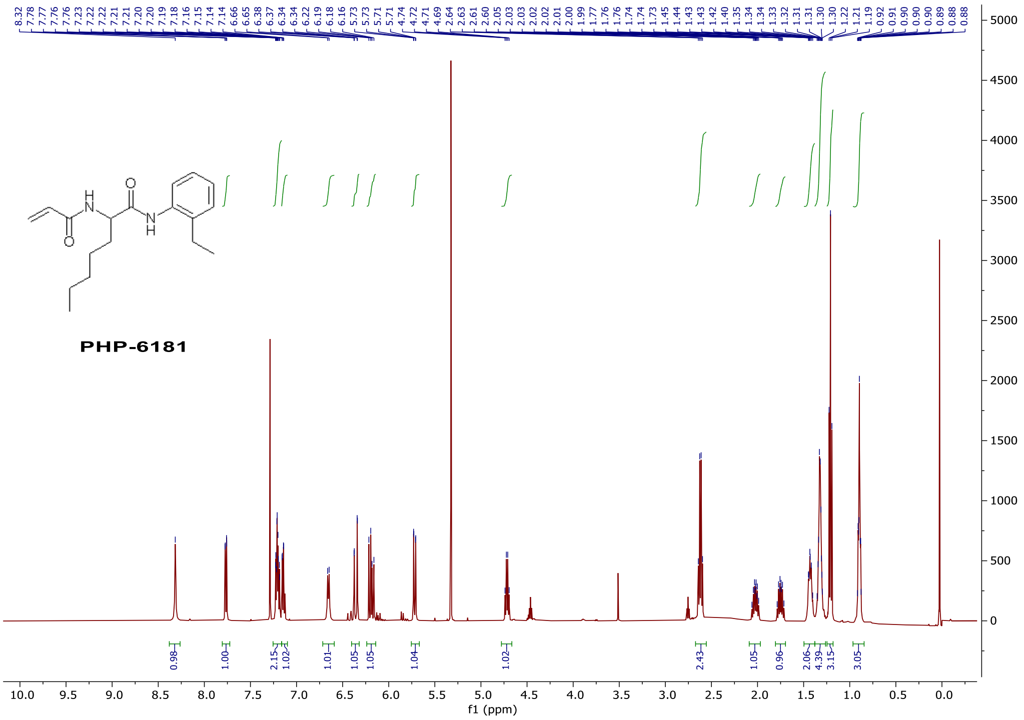

Supplementary Figure 937. ^{1}H NMR (400 MHz, $CDCl_3$ + one drop of CD_3OD) of compound **PHP-6181.**

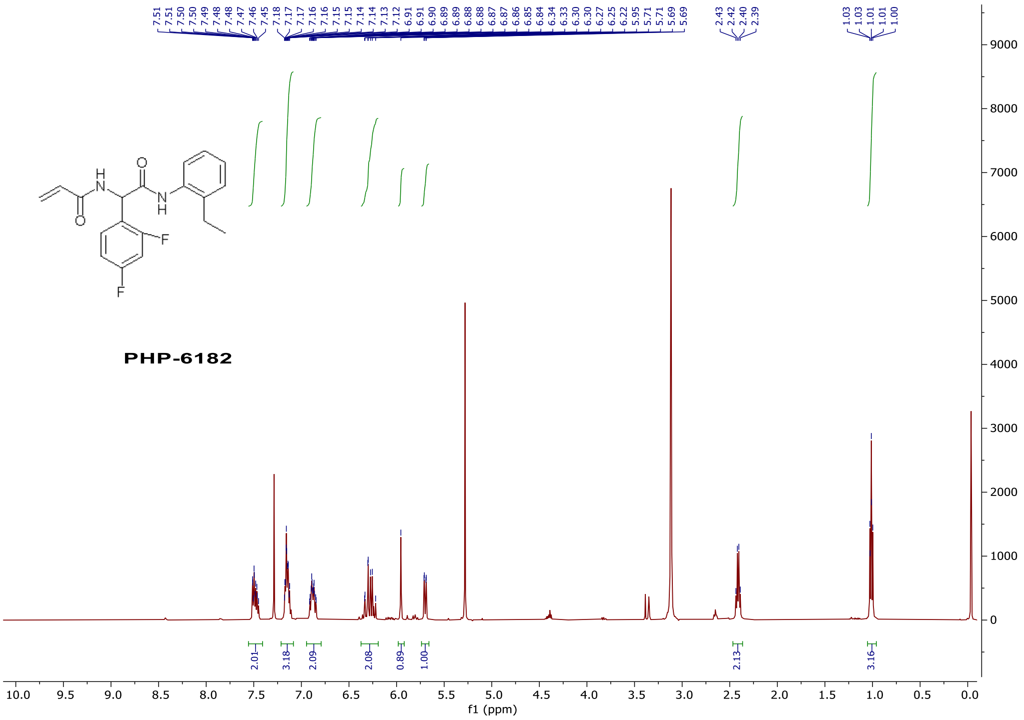

Supplementary Figure 938. ^{1}H NMR (400 MHz, $CDCl_3$ + one drop of CD_3OD) of compound **PHP-6182.**

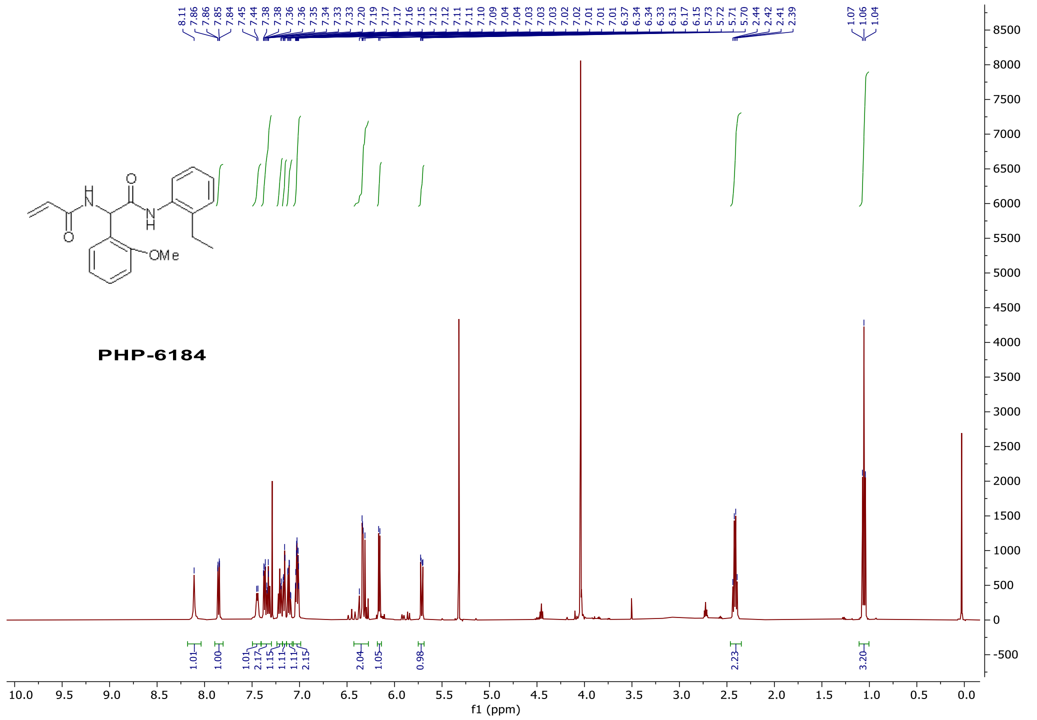

Supplementary Figure 939. ^{1}H NMR (400 MHz, $CDCl_3$ + one drop of CD_3OD) of compound **PHP-6184.**

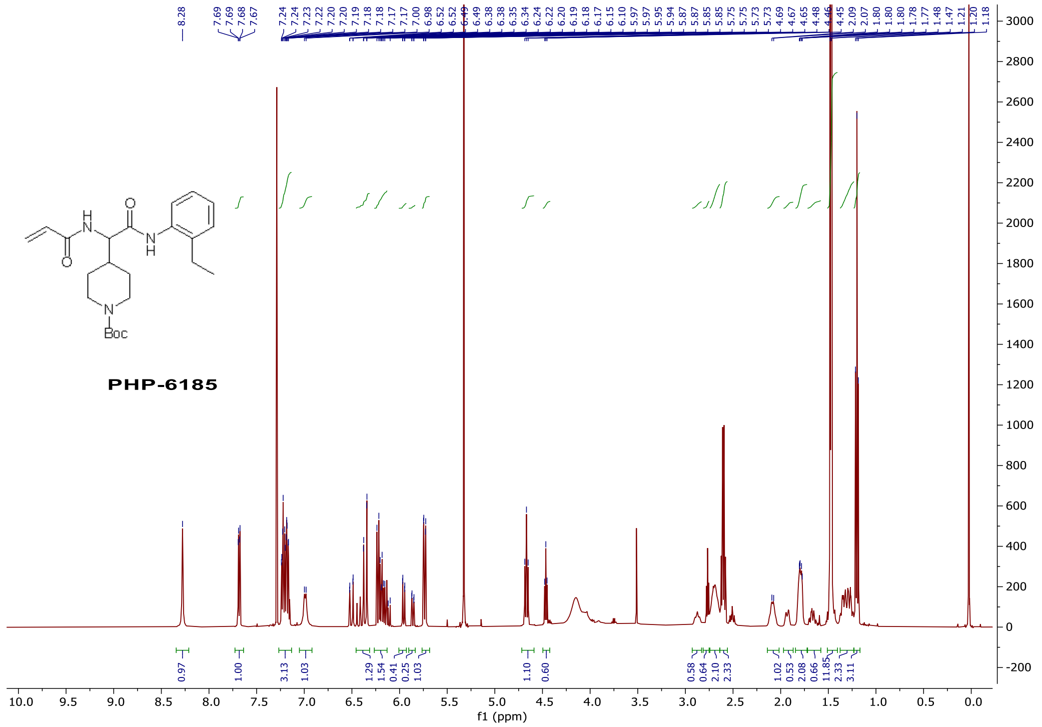

Supplementary Figure 940. ^{1}H NMR (400 MHz, $CDCl_3$ + one drop of CD_3OD) of compound **PHP-6185.**

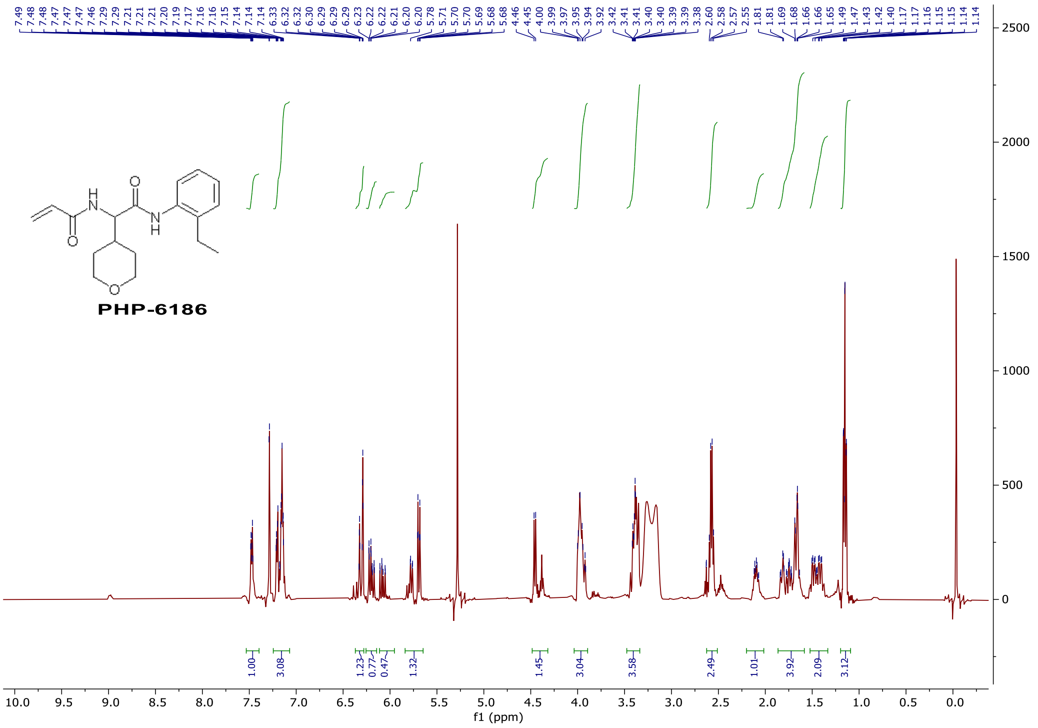

Supplementary Figure 941. ^{1}H NMR (400 MHz, $CDCl_3$ + one drop of CD_3OD) of compound **PHP-6186.**

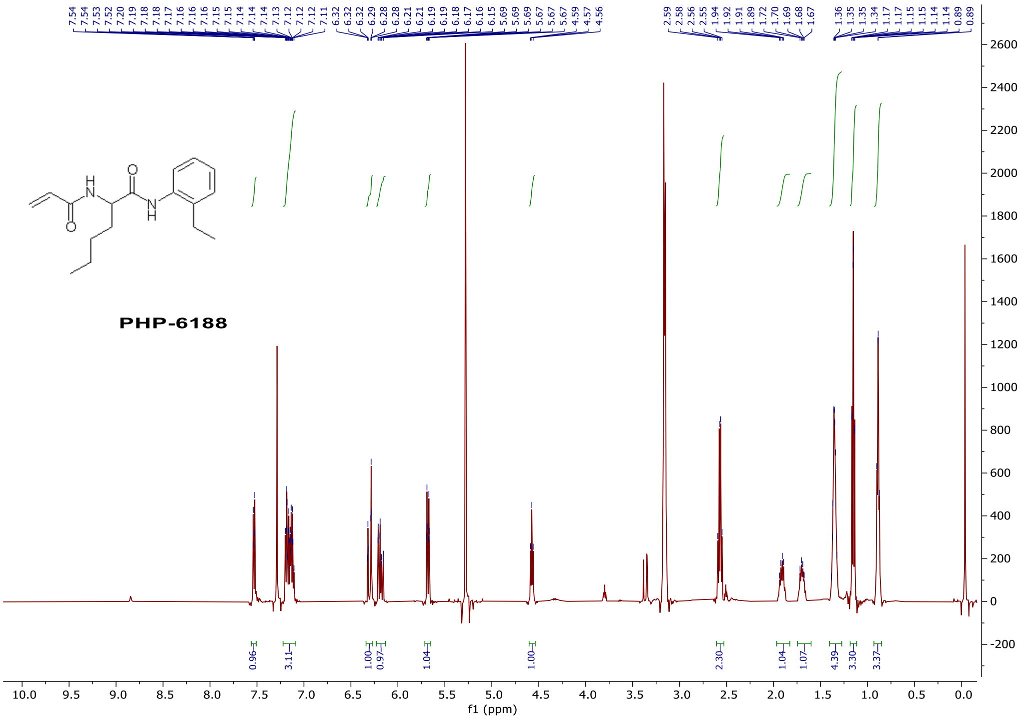

Supplementary Figure 942. ^{1}H NMR (400 MHz, $CDCl_3$ + one drop of CD_3OD) of compound **PHP-6188.**

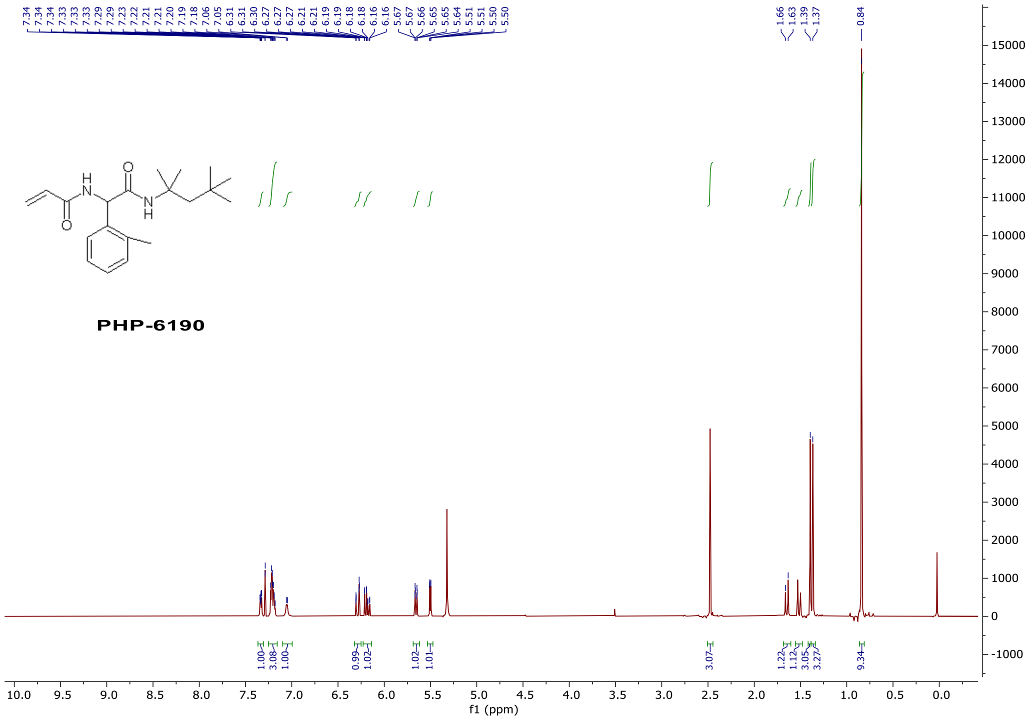

Supplementary Figure 943. ^{1}H NMR (400 MHz, $CDCl_3$ + one drop of CD_3OD) of compound **PHP-6190**.

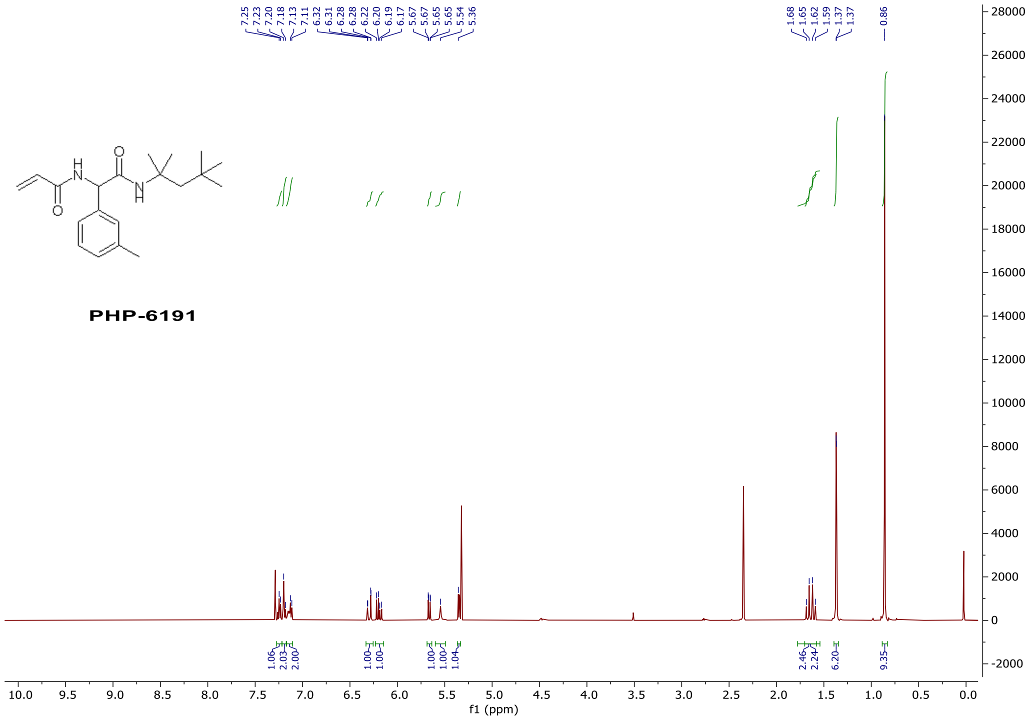

Supplementary Figure 944. ^{1}H NMR (400 MHz, $CDCl_3$ + one drop of CD_3OD) of compound **PHP-6191**.

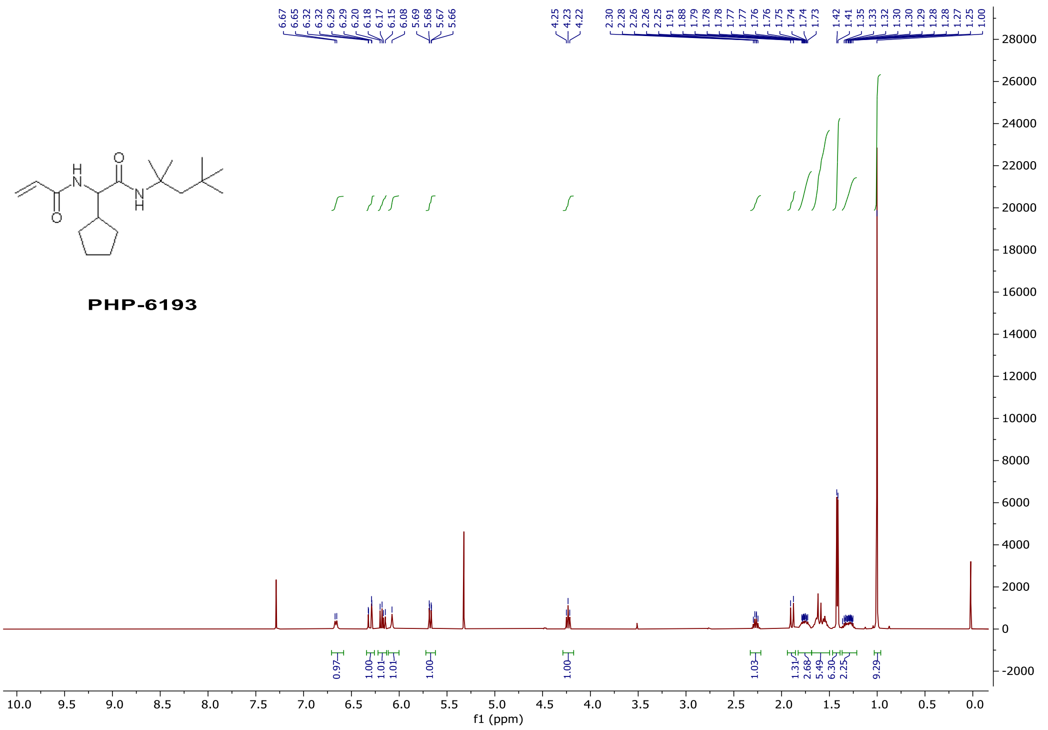

Supplementary Figure 945. ^{1}H NMR (400 MHz, $CDCl_3$ + one drop of CD_3OD) of compound **PHP-6193**.

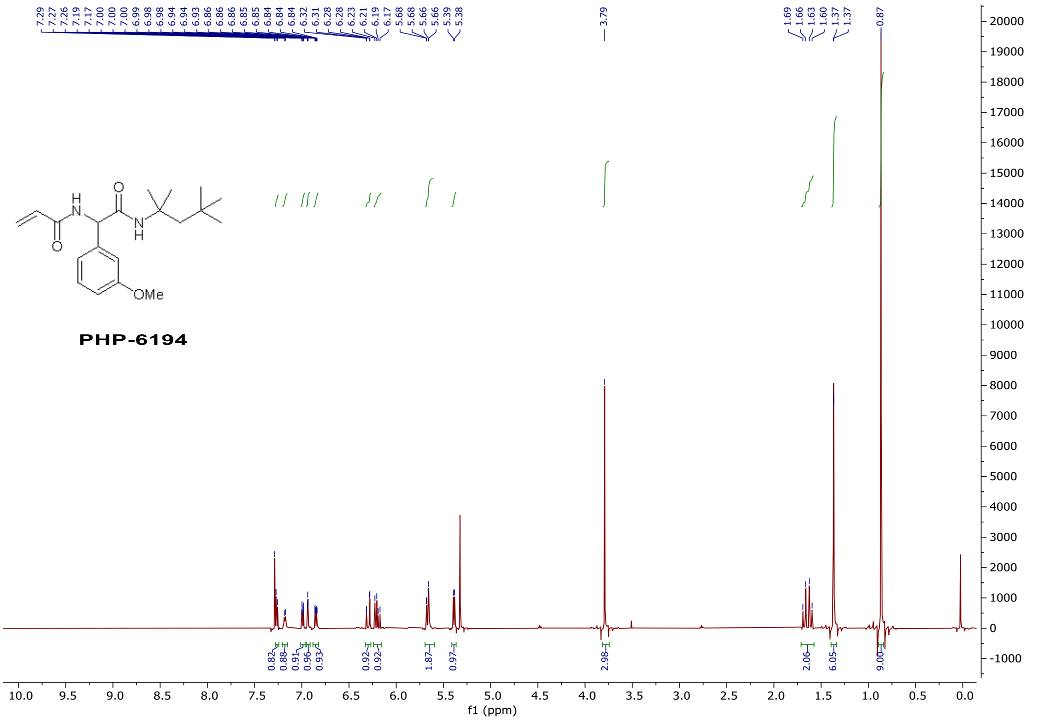

Supplementary Figure 946. ^{1}H NMR (400 MHz, $CDCl_3$ + one drop of CD_3OD) of compound **PHP-6194**.

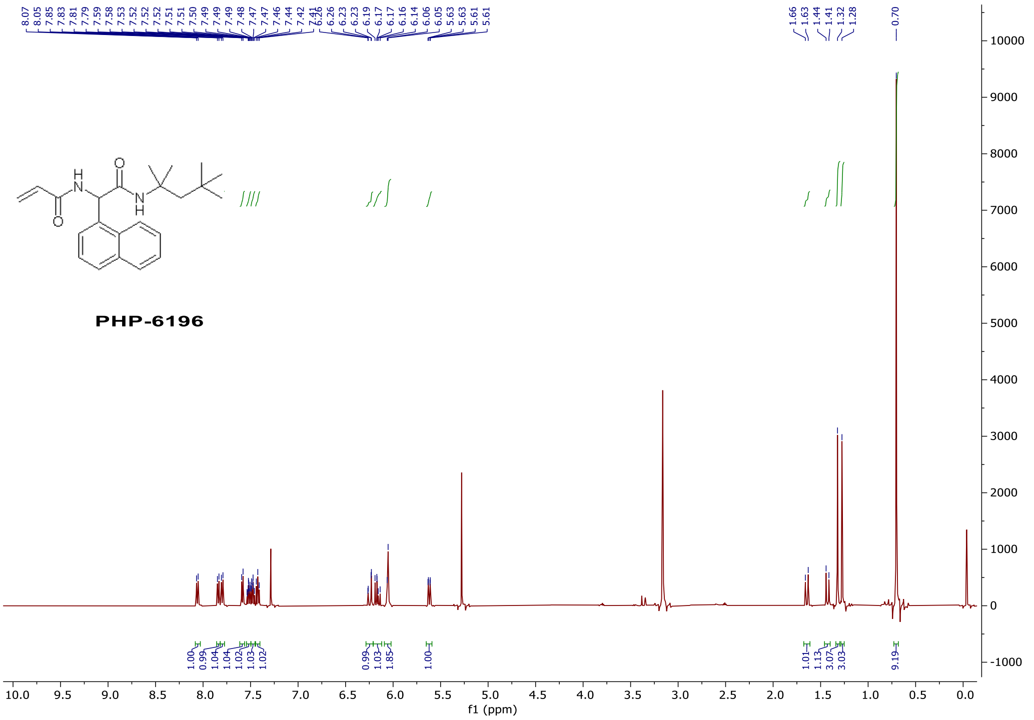

Supplementary Figure 947. ^{1}H NMR (400 MHz, $CDCl_3$ + one drop of CD_3OD) of compound **PHP-6196.**

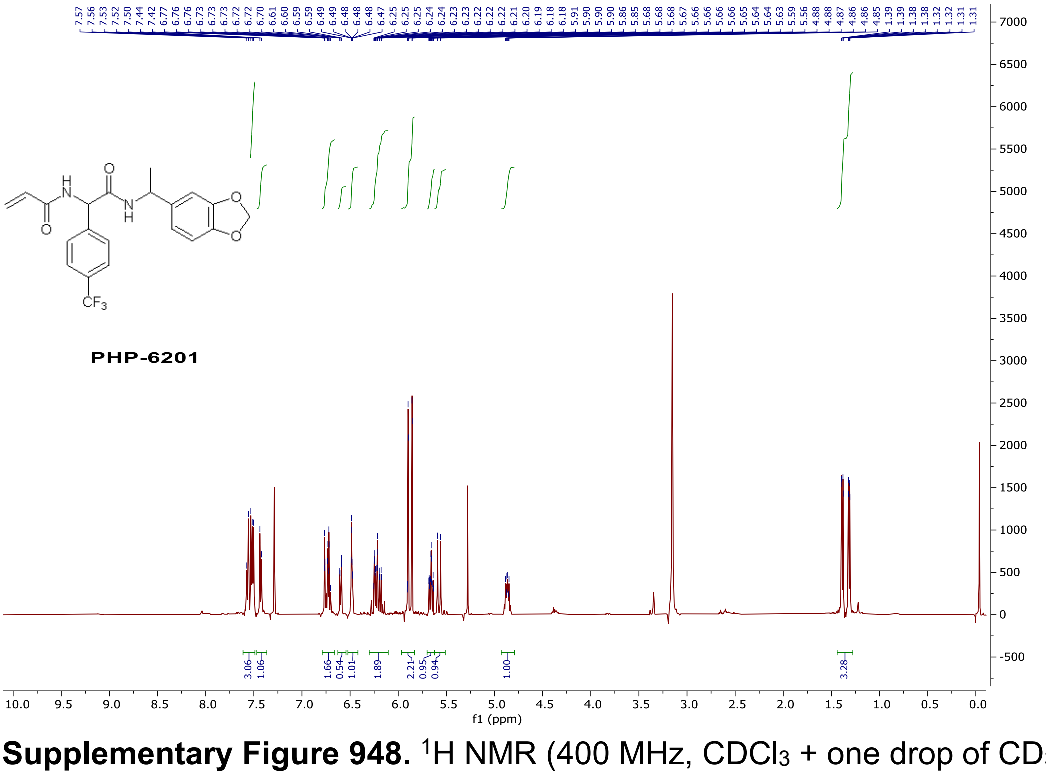

Supplementary Figure 948. ^{1}H NMR (400 MHz, $CDCl_3$ + one drop of CD_3OD) of compound **PHP-6201.**

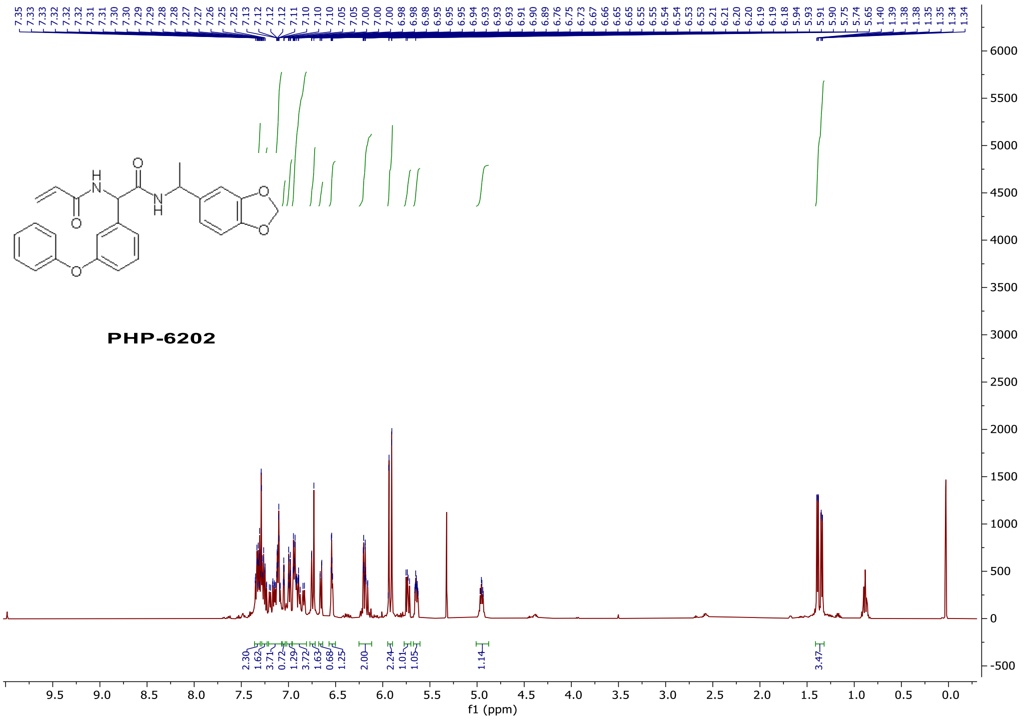

Supplementary Figure 949. ^{1}H NMR (400 MHz, $CDCl_3$ + one drop of CD_3OD) of compound **PHP-6202.**

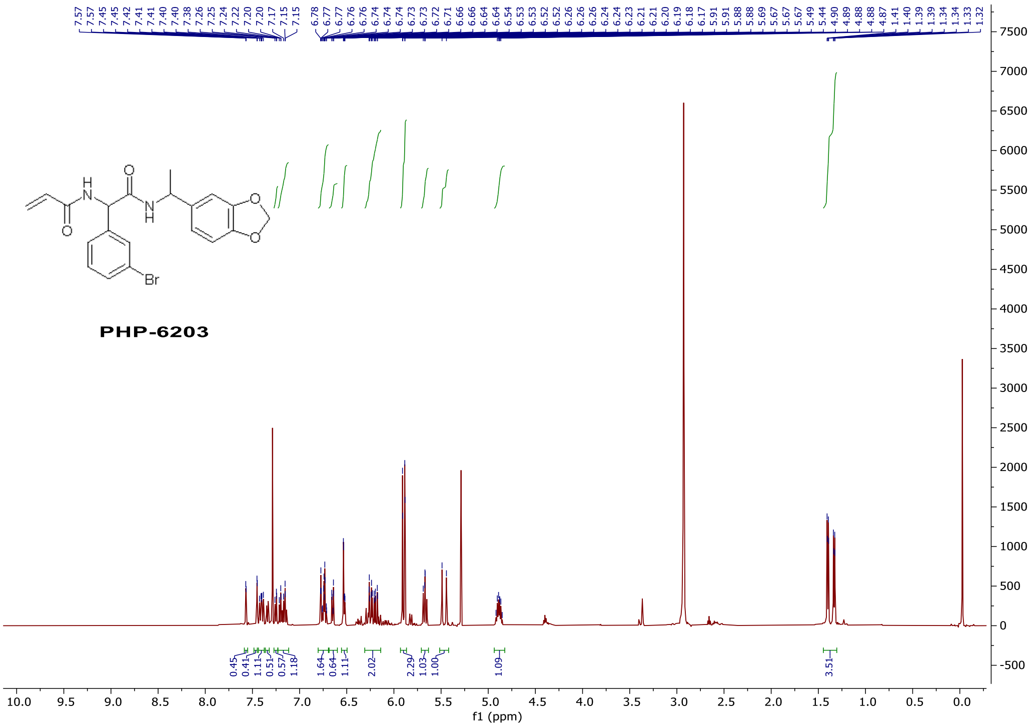

Supplementary Figure 950. ^{1}H NMR (400 MHz, $CDCl_3$ + one drop of CD_3OD) of compound **PHP-6203.**

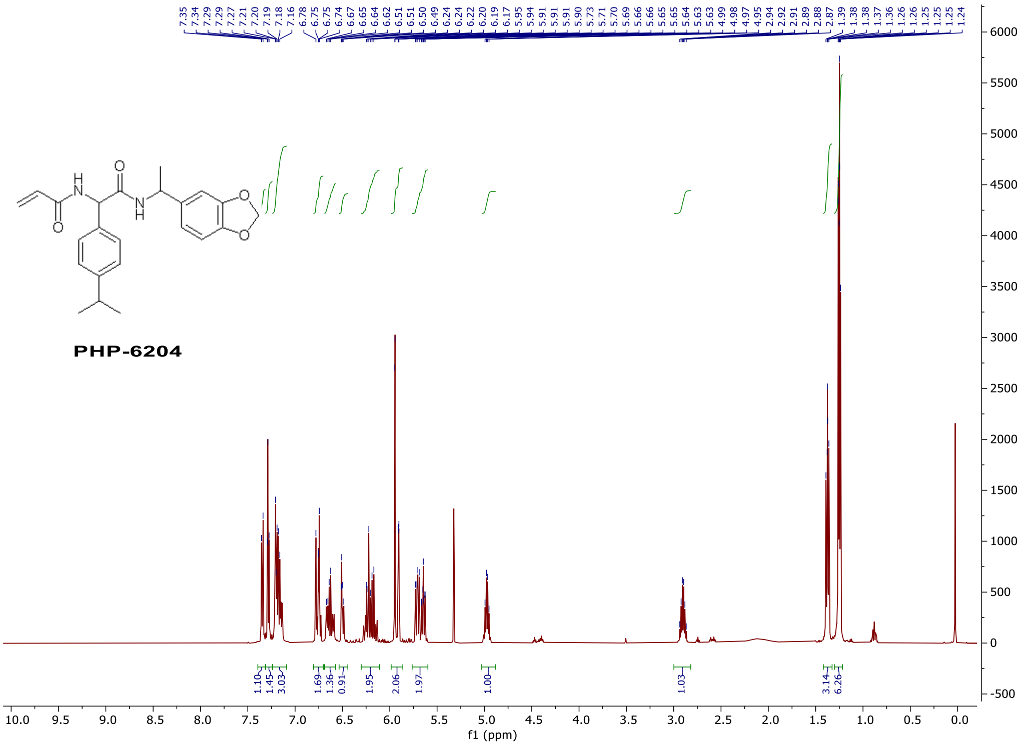

Supplementary Figure 951. ^{1}H NMR (400 MHz, $CDCl_3$ + one drop of CD_3OD) of compound **PHP-6204.**

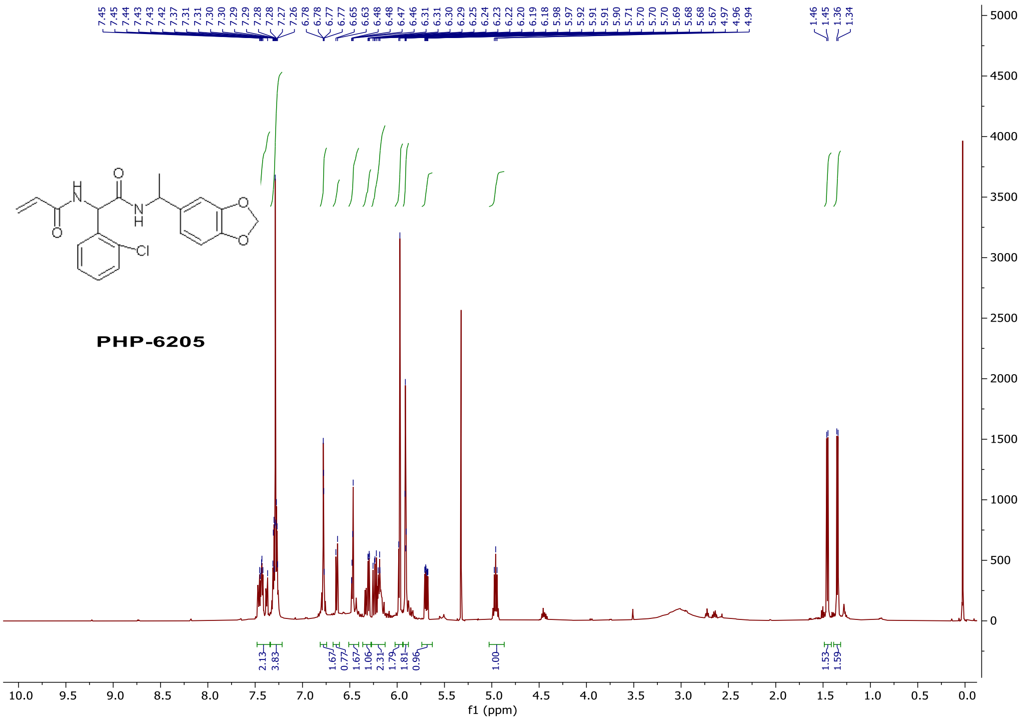

Supplementary Figure 952. ^{1}H NMR (400 MHz, $CDCl_3$ + one drop of CD_3OD) of compound **PHP-6205.**

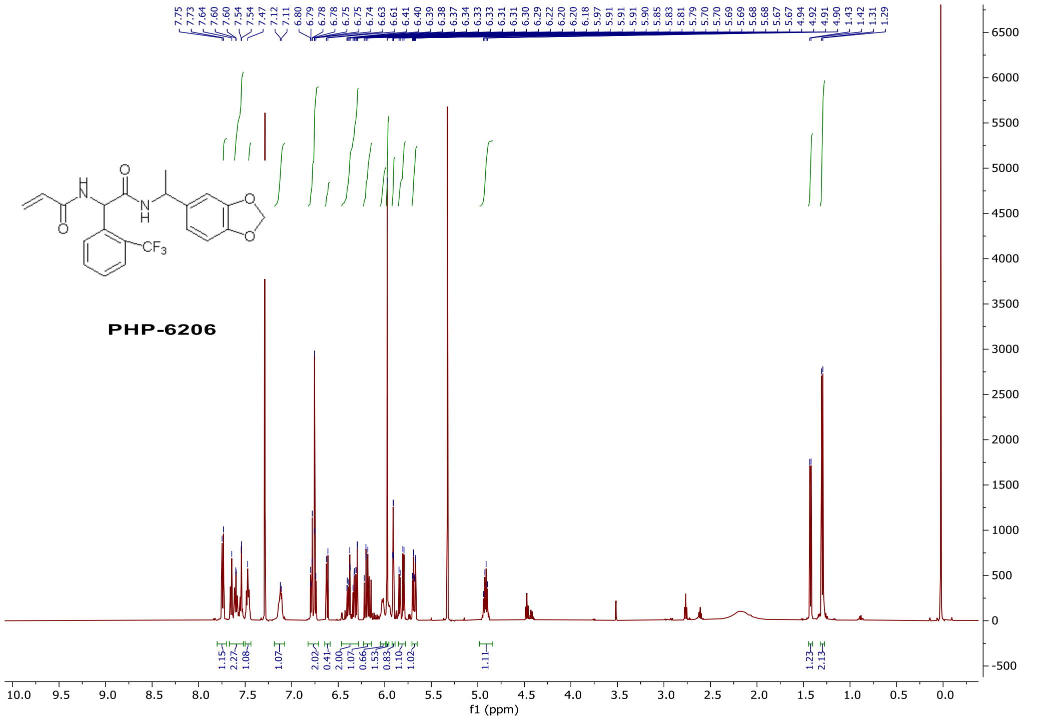

Supplementary Figure 953. ^{1}H NMR (400 MHz, $CDCl_3$ + one drop of CD_3OD) of compound **PHP-6206.**

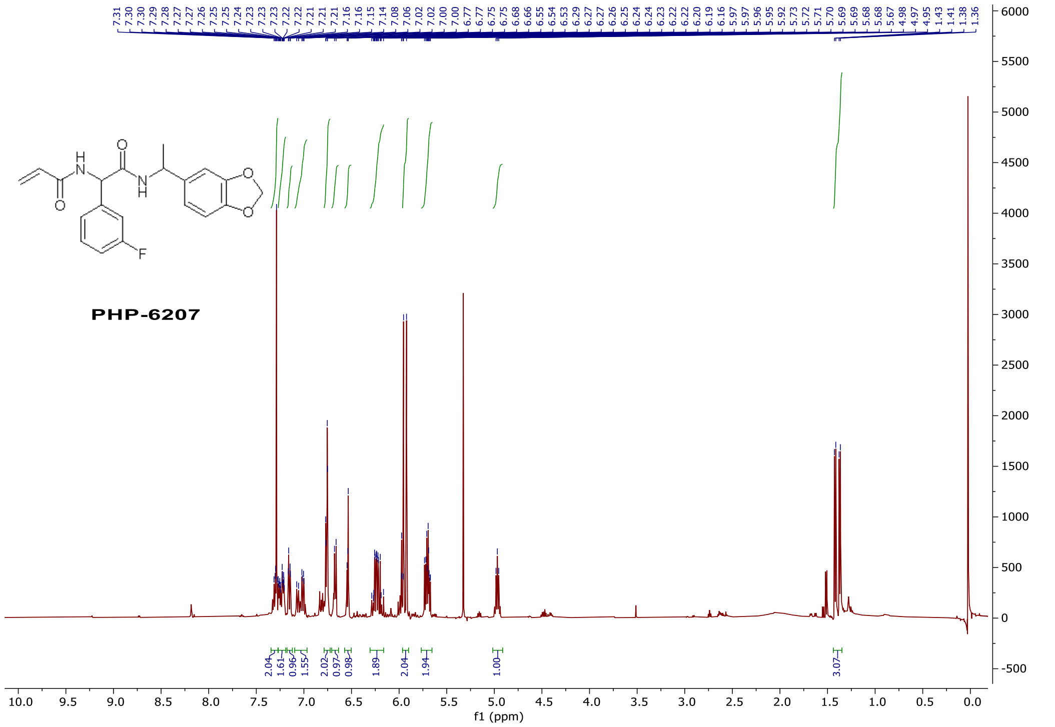

Supplementary Figure 954. ^{1}H NMR (400 MHz, $CDCl_3$ + one drop of CD_3OD) of compound **PHP-6207.**

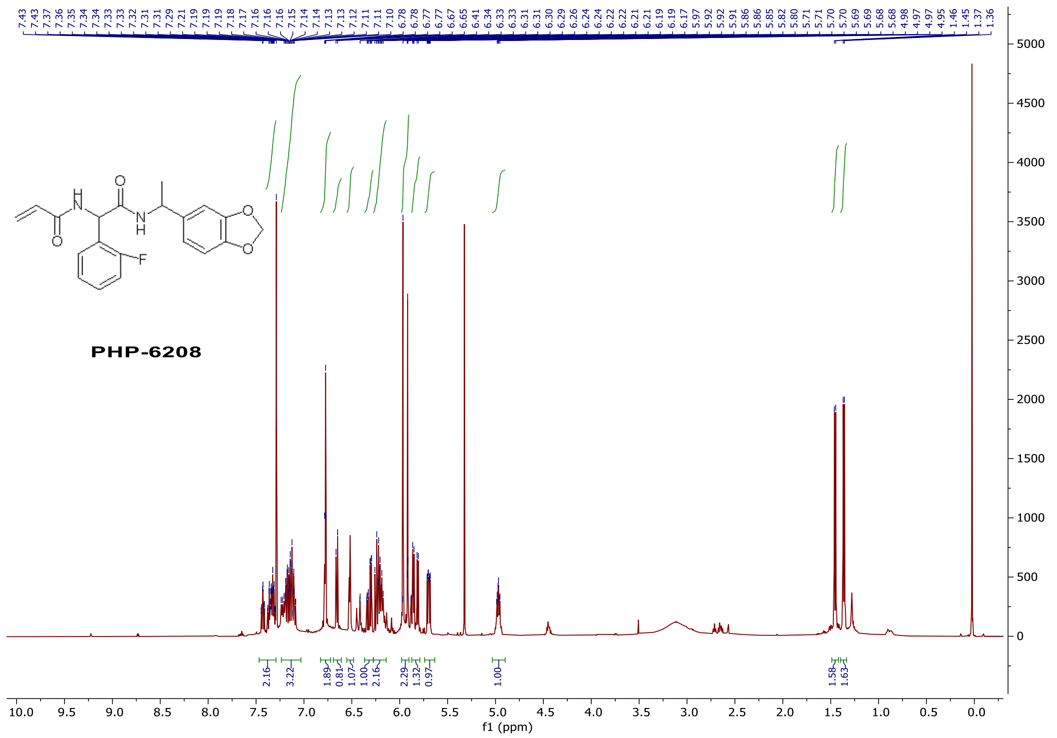

Supplementary Figure 955. ^{1}H NMR (400 MHz, $CDCl_3$ + one drop of CD_3OD) of compound **PHP-6208.**

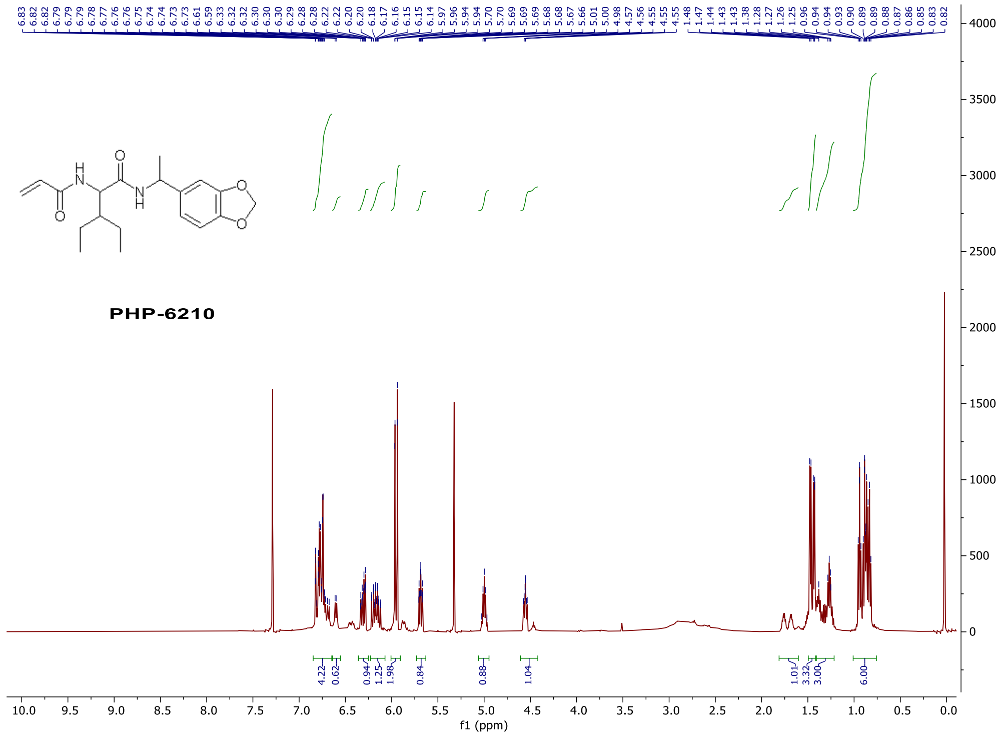

Supplementary Figure 956. ^{1}H NMR (400 MHz, $CDCl_3$ + one drop of CD_3OD) of compound **PHP-6210.**

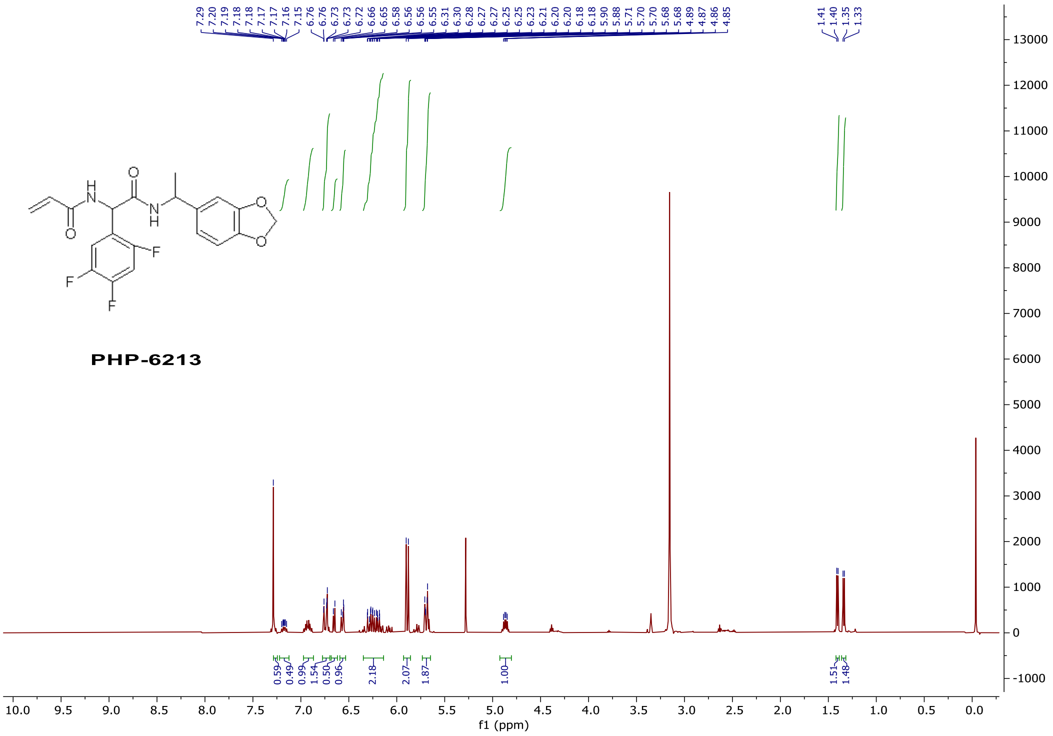

Supplementary Figure 957. ^1H NMR (400 MHz, $CDCl_3$ + one drop of CD_3OD) of compound **PHP-6213.**

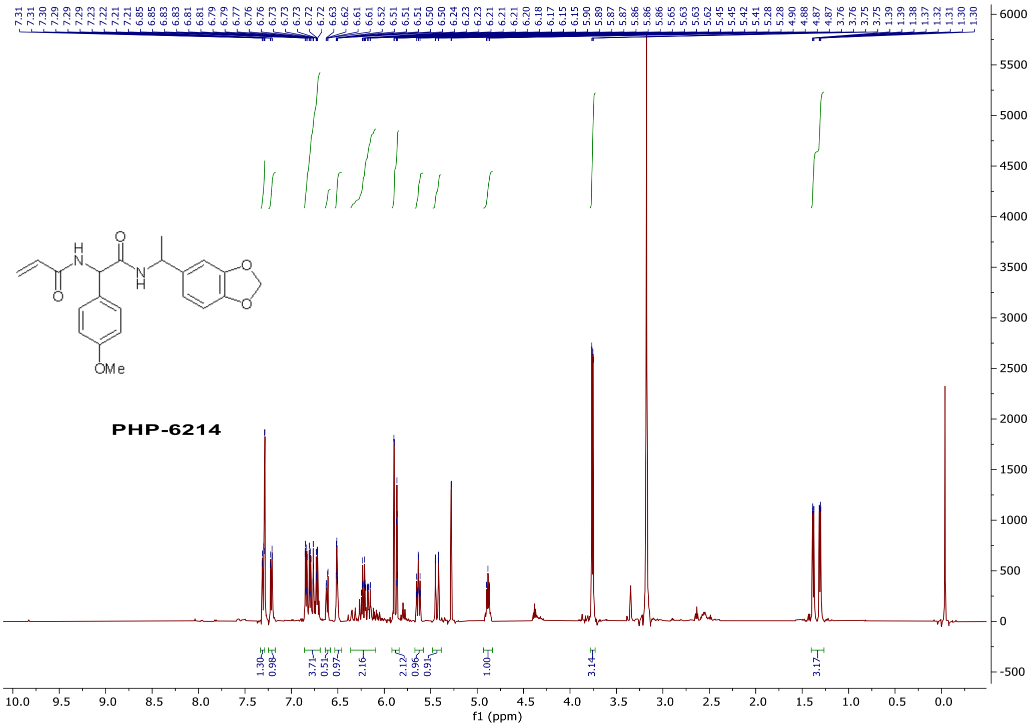

Supplementary Figure 958. ^1H NMR (400 MHz, $CDCl_3$ + one drop of CD_3OD) of compound **PHP-6214.**

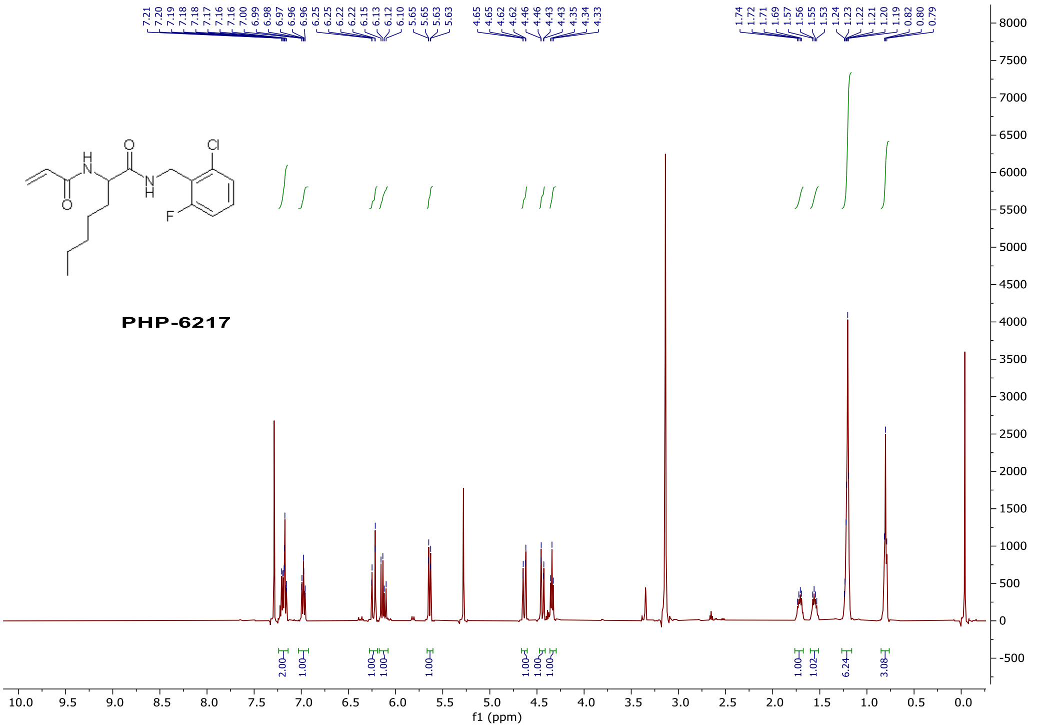

Supplementary Figure 959. ^{1}H NMR (400 MHz, $CDCl_3$ + one drop of CD_3OD) of compound **PHP-6217**.

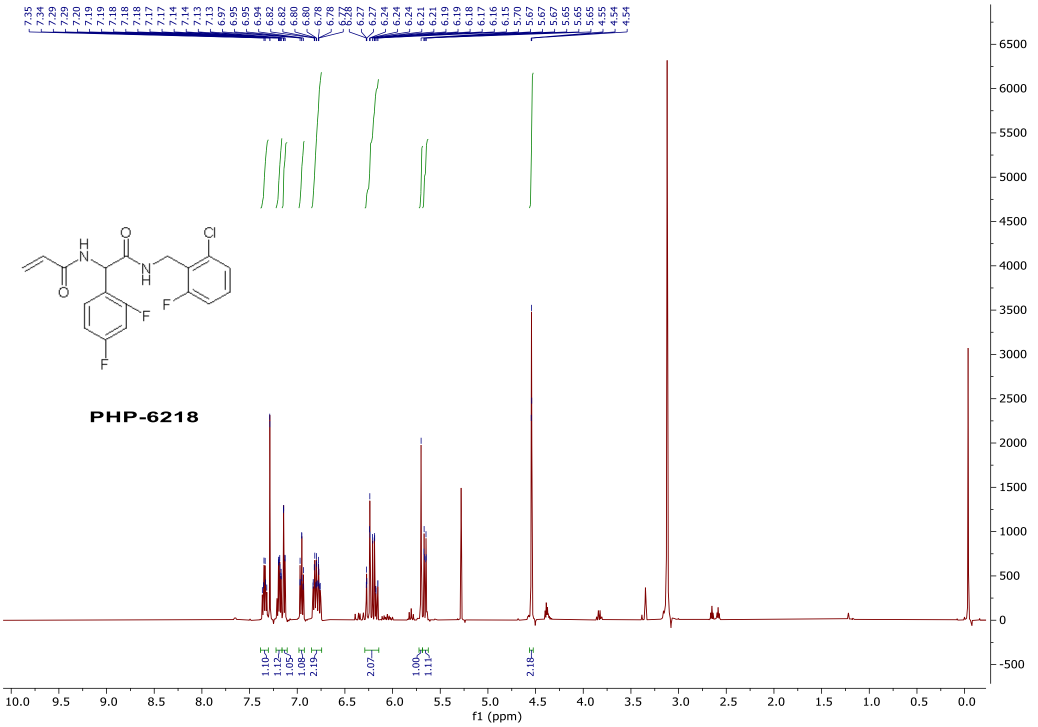

Supplementary Figure 960. ^{1}H NMR (400 MHz, $CDCl_3$ + one drop of CD_3OD) of compound **PHP-6218**.

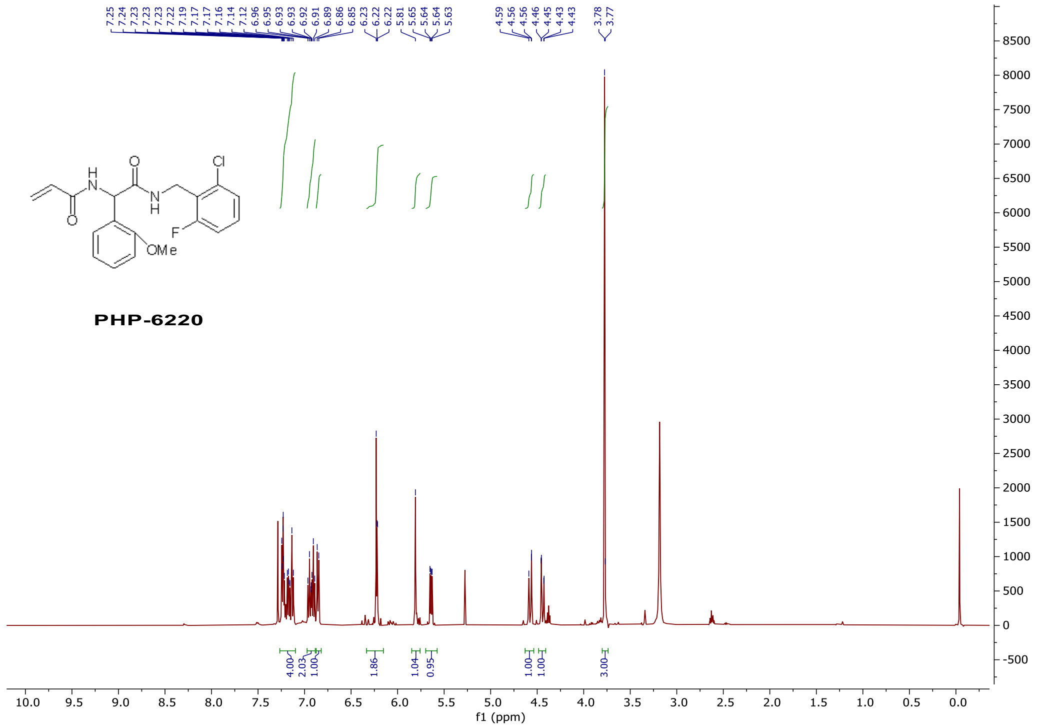

Supplementary Figure 961. ^{1}H NMR (400 MHz, $CDCl_3$ + one drop of CD_3OD) of compound **PHP-6220.**

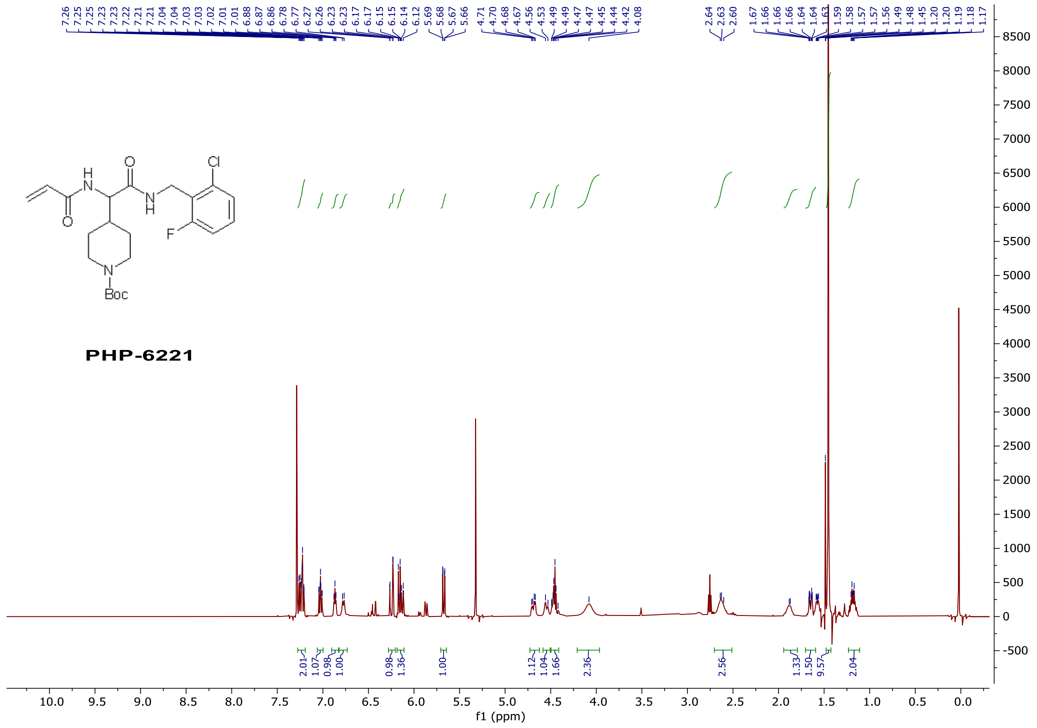

Supplementary Figure 962. ^{1}H NMR (400 MHz, $CDCl_3$ + one drop of CD_3OD) of compound **PHP-6221.**

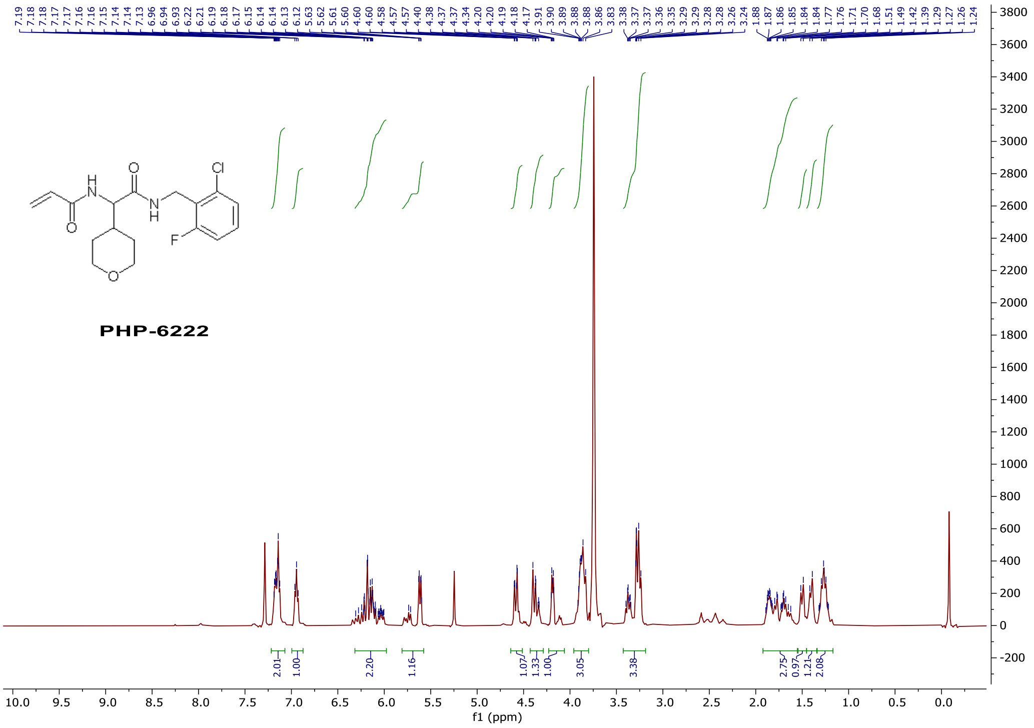

Supplementary Figure 963. ^{1}H NMR (400 MHz, $CDCl_3$ + one drop of CD_3OD) of compound **PHP-6222.**

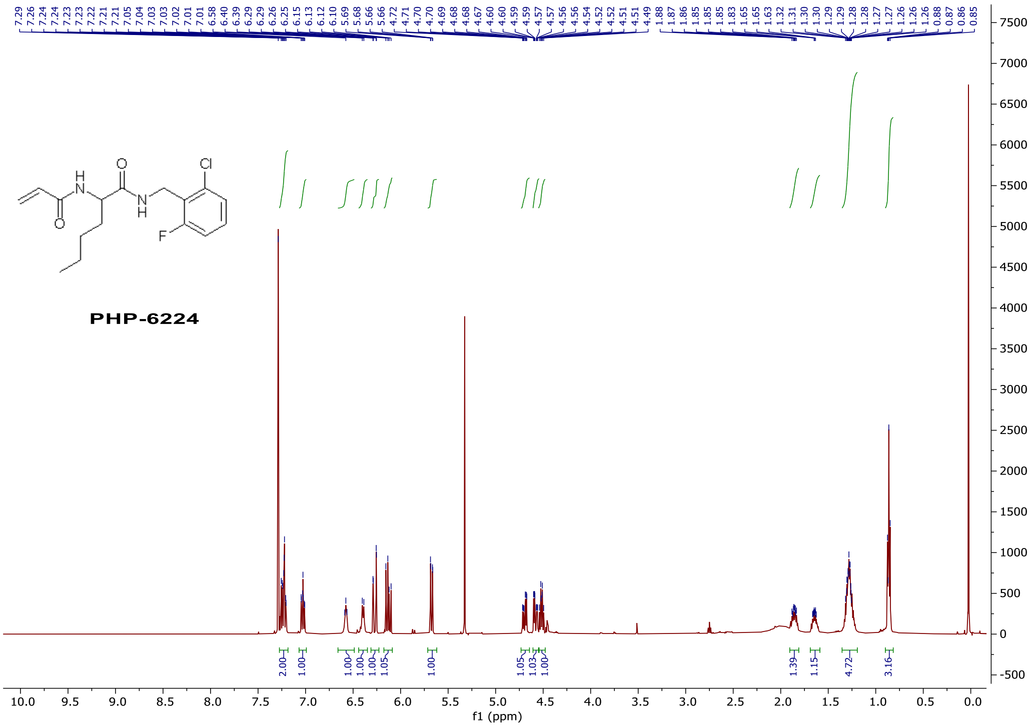

Supplementary Figure 964. ^{1}H NMR (400 MHz, $CDCl_3$ + one drop of CD_3OD) of compound **PHP-6224.**

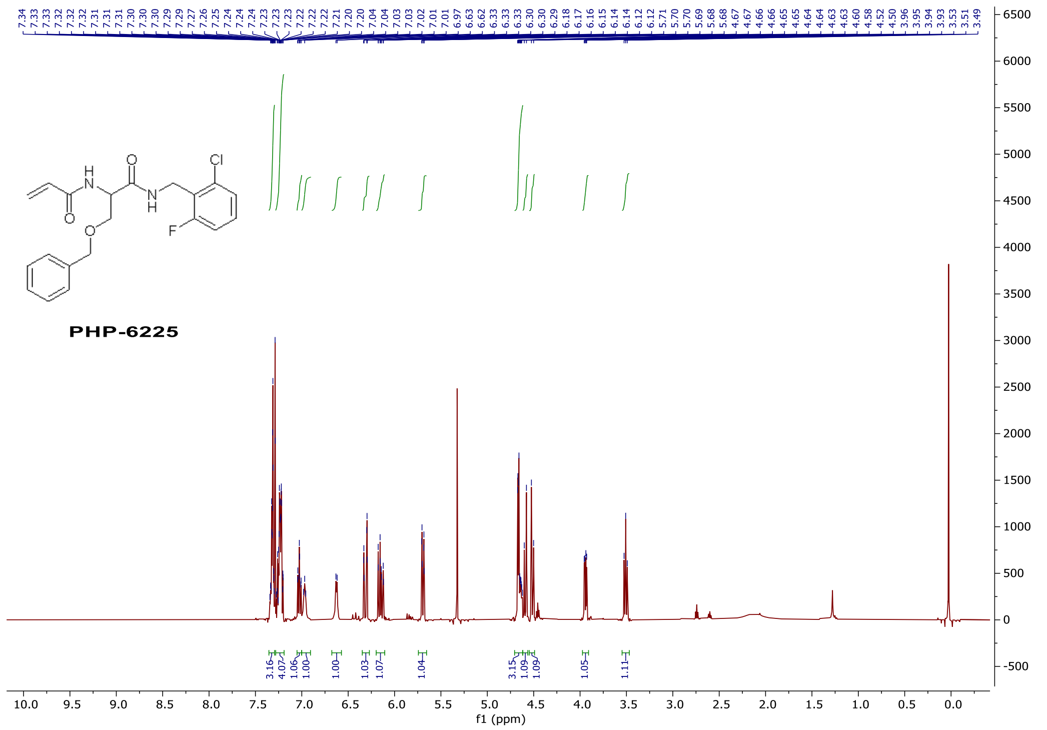

Supplementary Figure 965. ^{1}H NMR (400 MHz, $CDCl_3$ + one drop of CD_3OD) of compound **PHP-6225**.

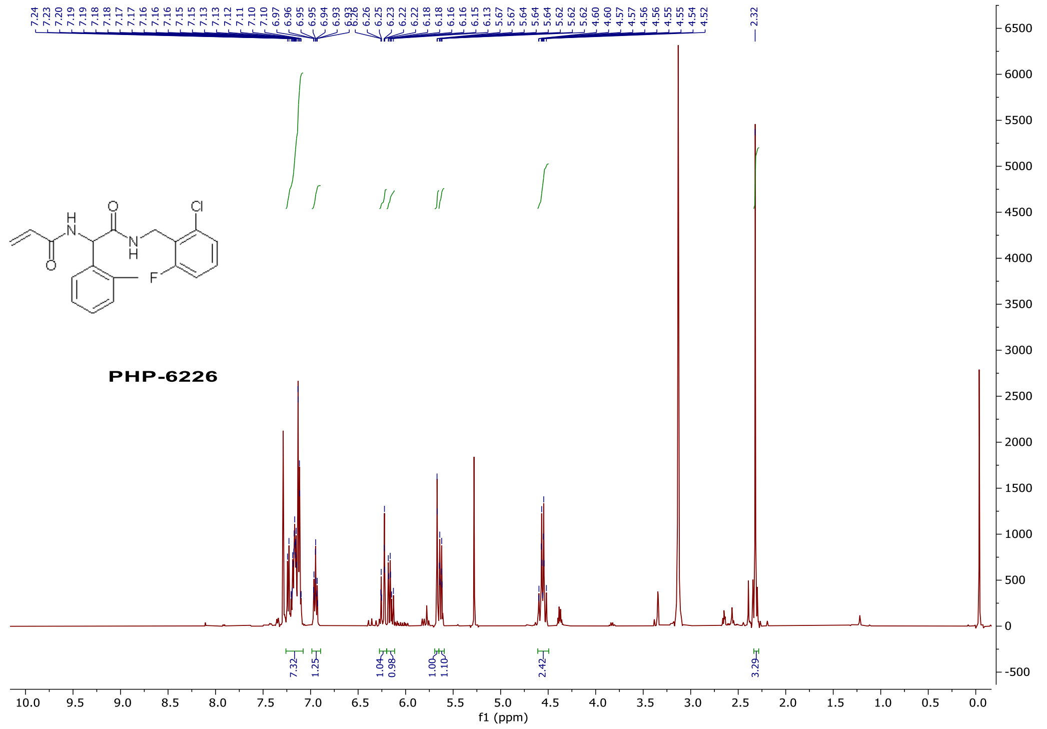

Supplementary Figure 966. ^{1}H NMR (400 MHz, $CDCl_3$ + one drop of CD_3OD) of compound **PHP-6226**.

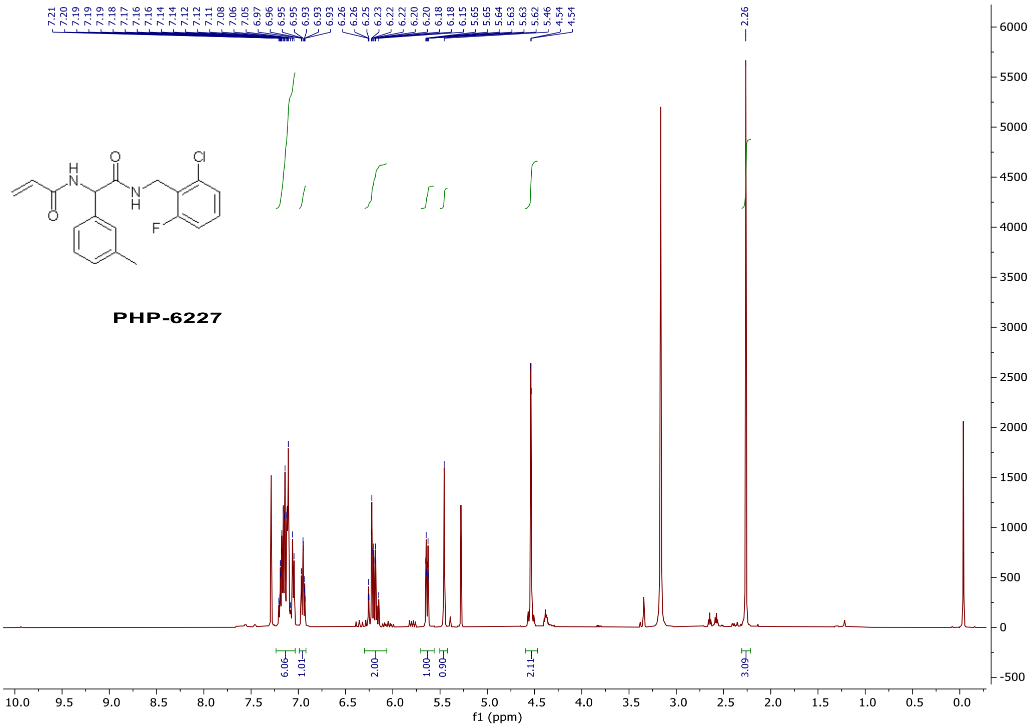

Supplementary Figure 967. ^{1}H NMR (400 MHz, $CDCl_3$ + one drop of CD_3OD) of compound **PHP-622.**

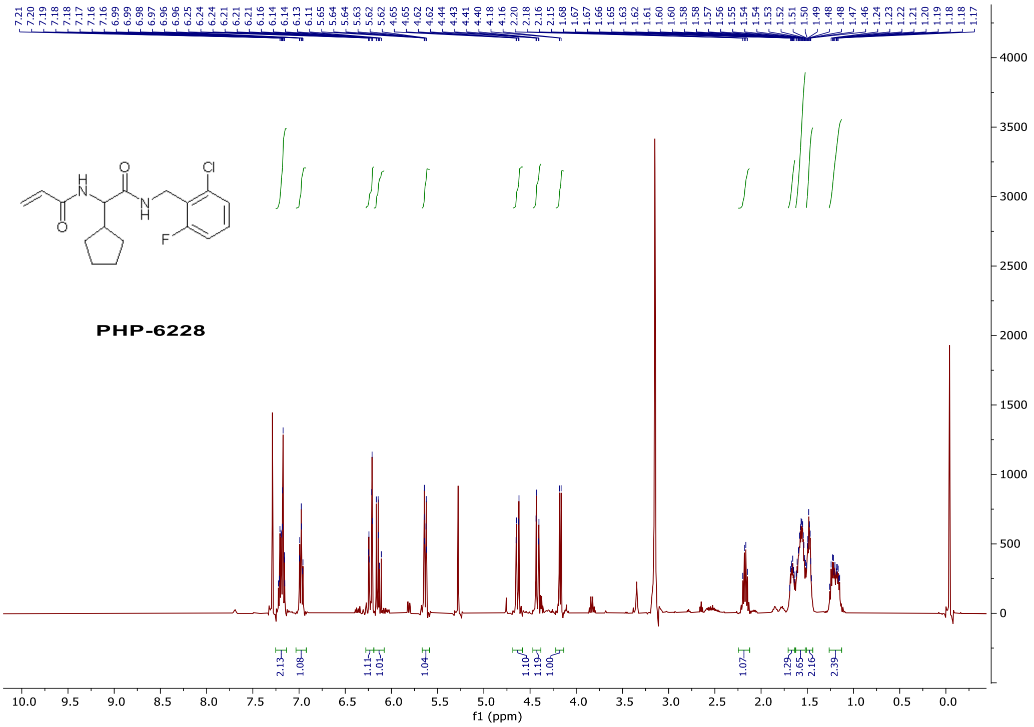

Supplementary Figure 968. ^{1}H NMR (400 MHz, $CDCl_3$ + one drop of CD_3OD) of compound **PHP-6228.**

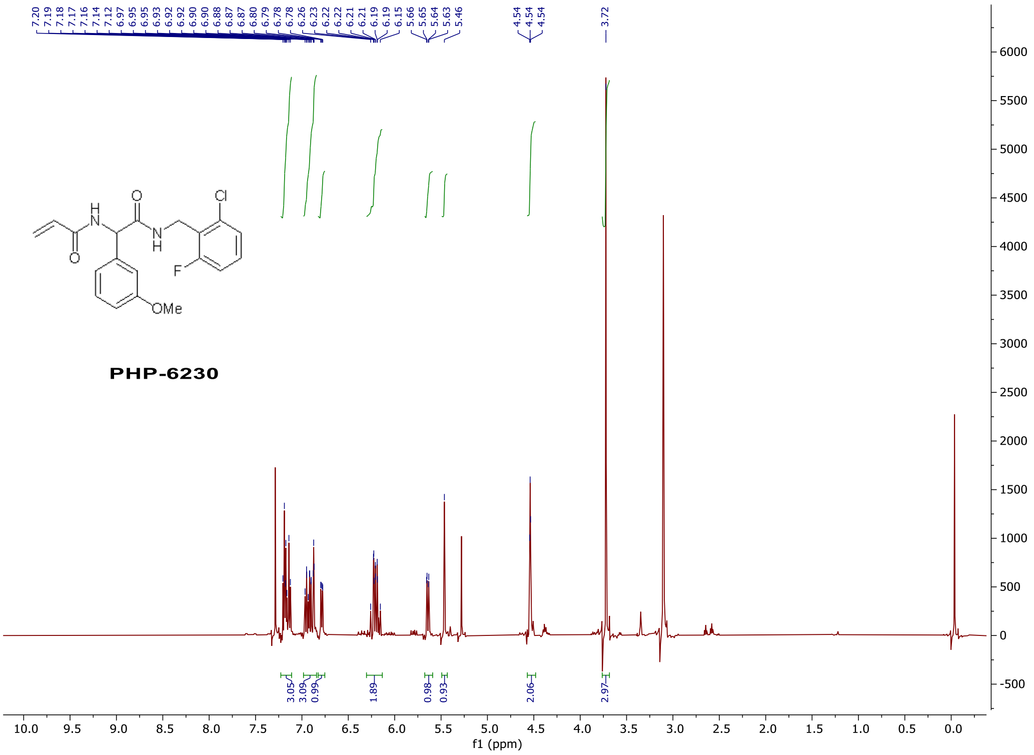

Supplementary Figure 969. ^{1}H NMR (400 MHz, $CDCl_3$ + one drop of CD_3OD) of compound **PHP-6230**.

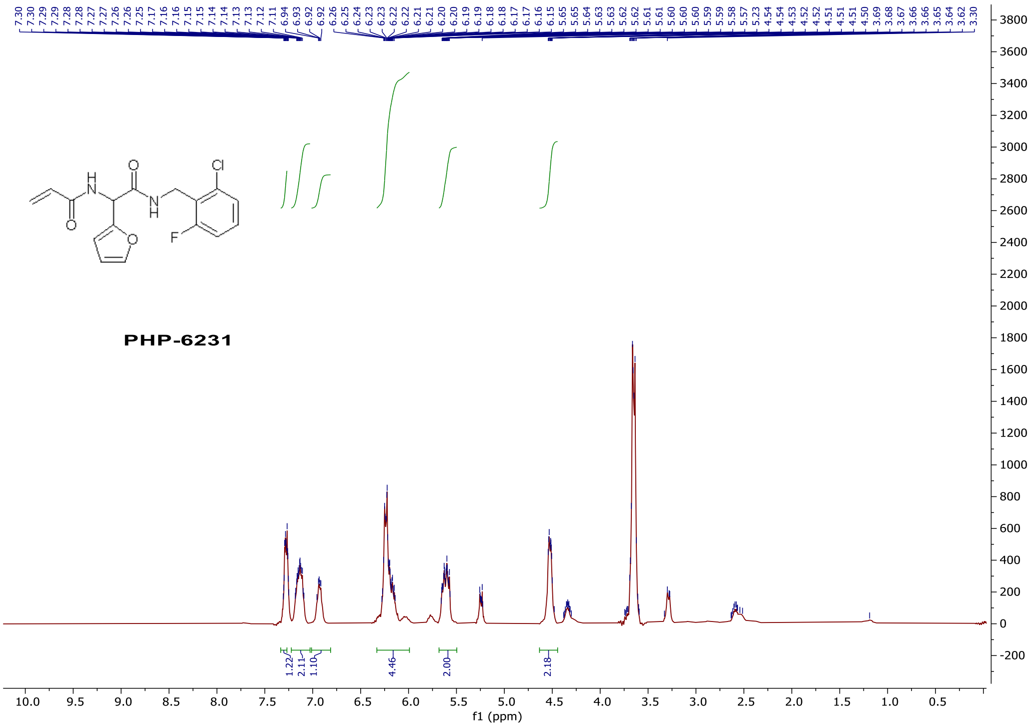

Supplementary Figure 970. ^{1}H NMR (400 MHz, $CDCl_3$ + one drop of CD_3OD) of compound **PHP-6231.**

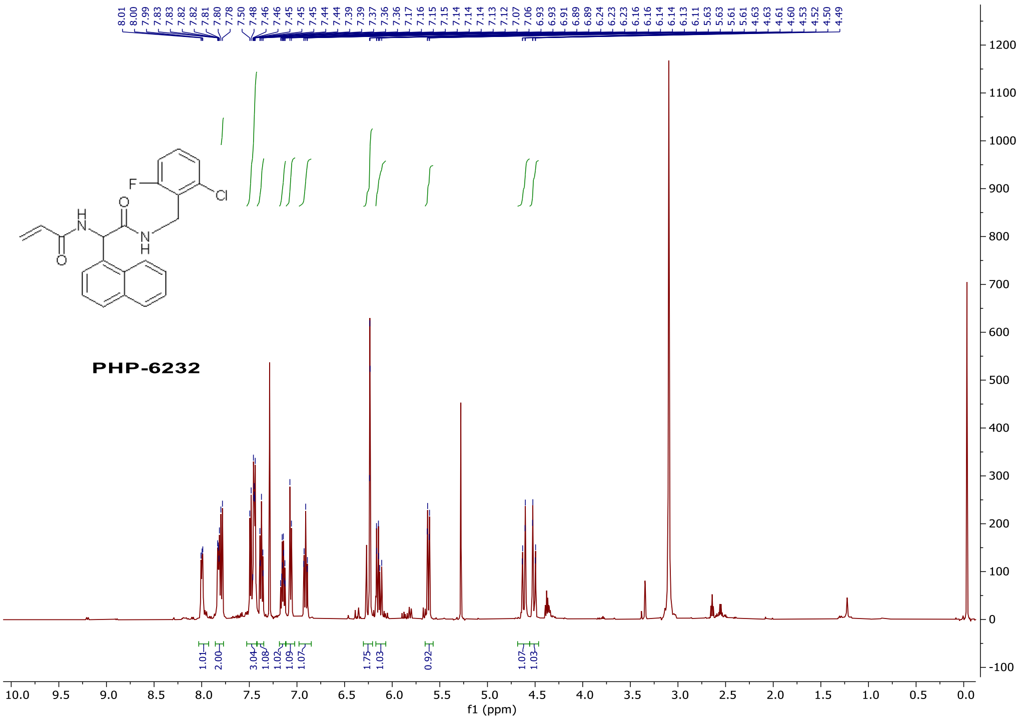

Supplementary Figure 971. ^{1}H NMR (400 MHz, $CDCl_3$ + one drop of CD_3OD) of compound **PHP-6232.**

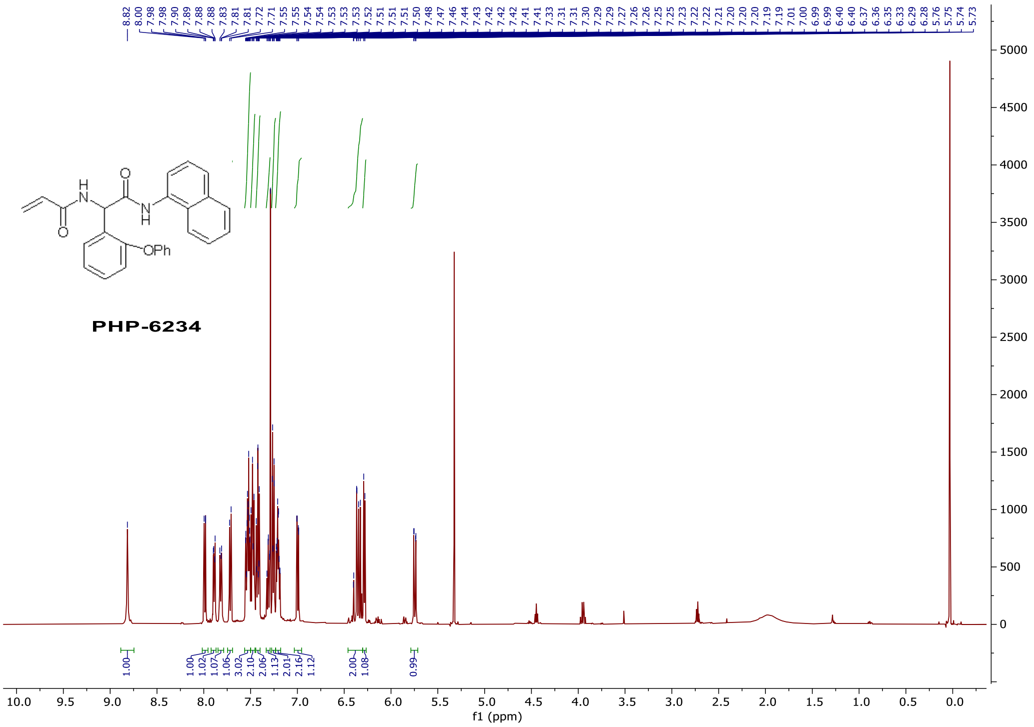

Supplementary Figure 972. ^{1}H NMR (400 MHz, $CDCl_3$ + one drop of CD_3OD) of compound **PHP-6234.**

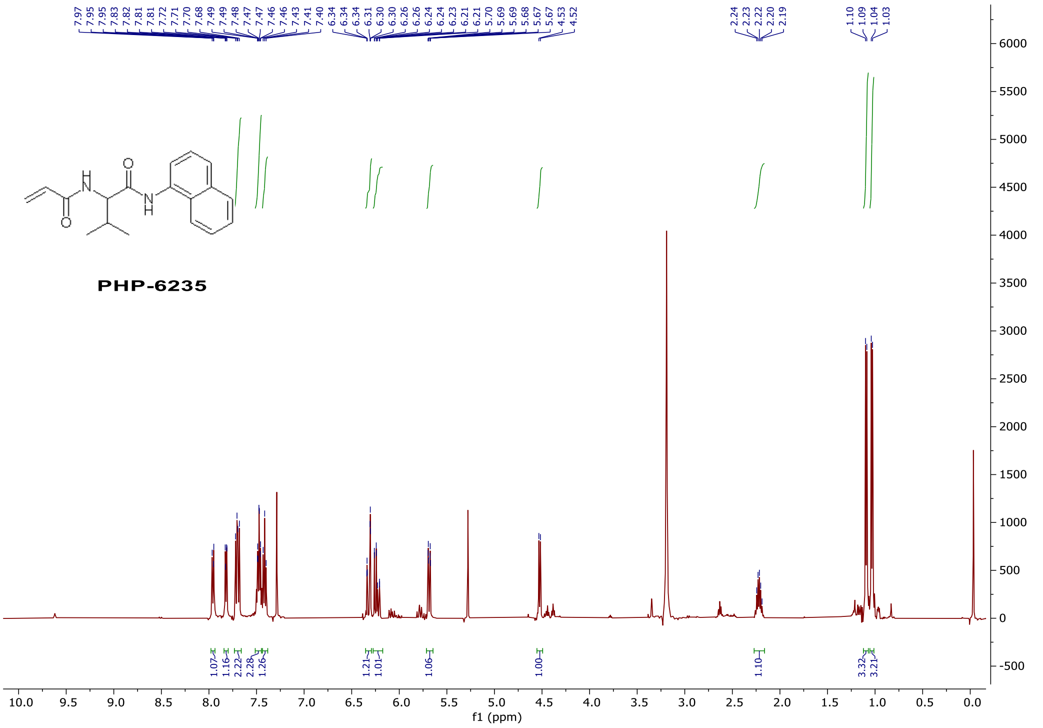

Supplementary Figure 973. ^{1}H NMR (400 MHz, $CDCl_3$ + one drop of CD_3OD) of compound **PHP-6235**.

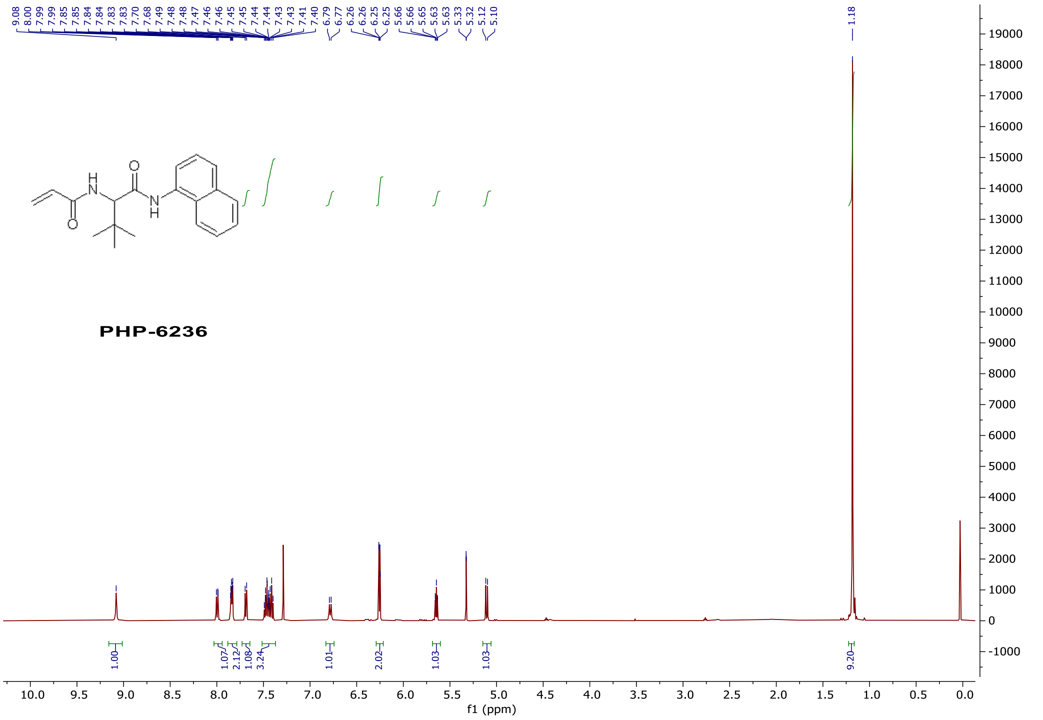

Supplementary Figure 974. ^{1}H NMR (400 MHz, $CDCl_3$ + one drop of CD_3OD) of compound **PHP-6236**.

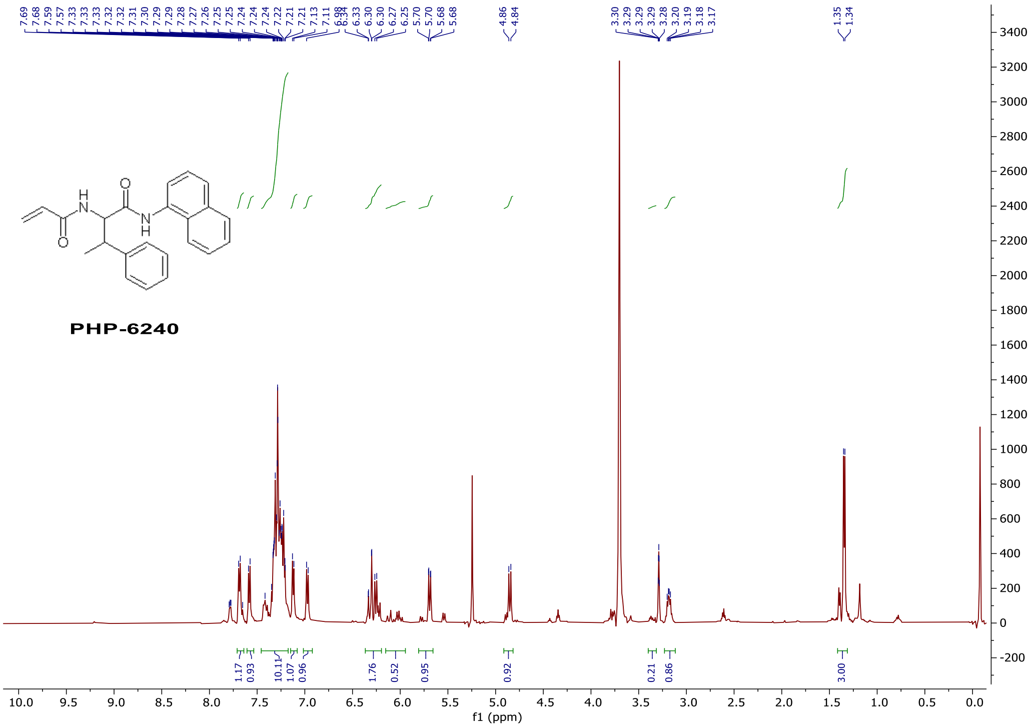

Supplementary Figure 975. ^{1}H NMR (400 MHz, $CDCl_3$ + one drop of CD_3OD) of compound **PHP-6240**.

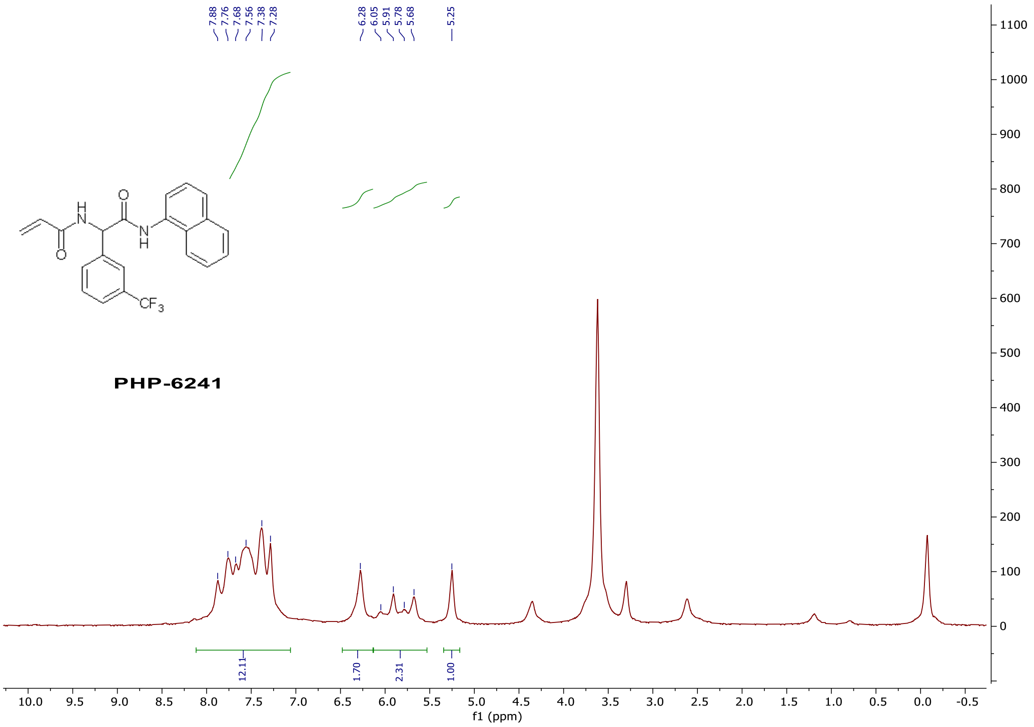

Supplementary Figure 976. ^{1}H NMR (400 MHz, $CDCl_3$ + one drop of CD_3OD) of compound **PHP-6241**.

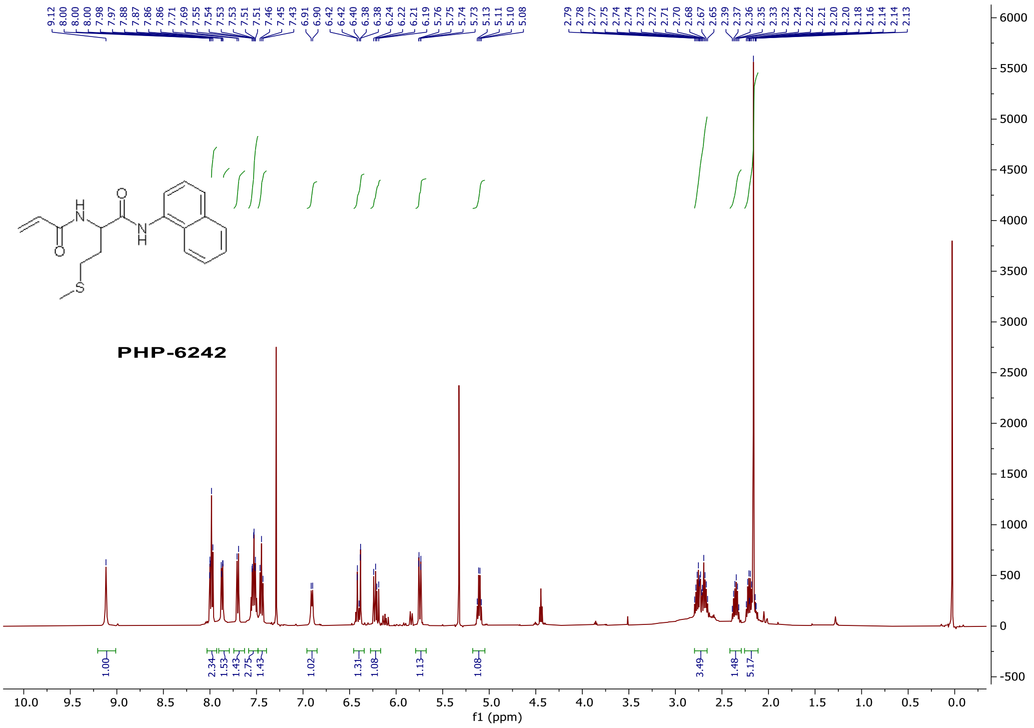

Supplementary Figure 977. ^{1}H NMR (400 MHz, $CDCl_3$ + one drop of CD_3OD) of compound **PHP-6242.**

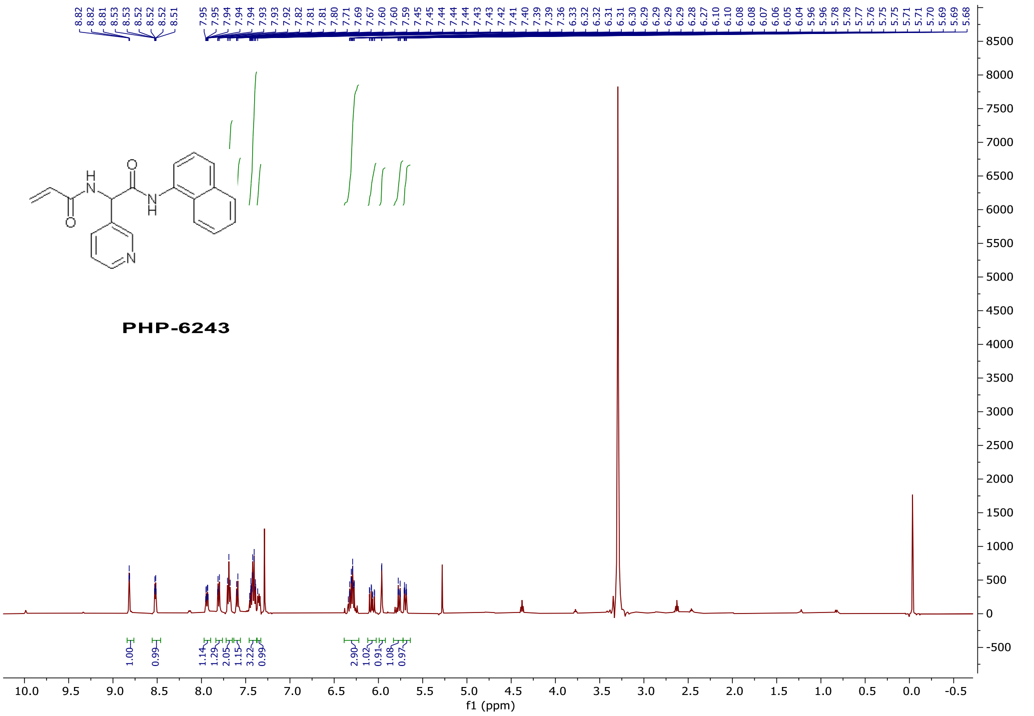

Supplementary Figure 978. ^{1}H NMR (400 MHz, $CDCl_3$ + one drop of CD_3OD) of compound **PHP-6243.**

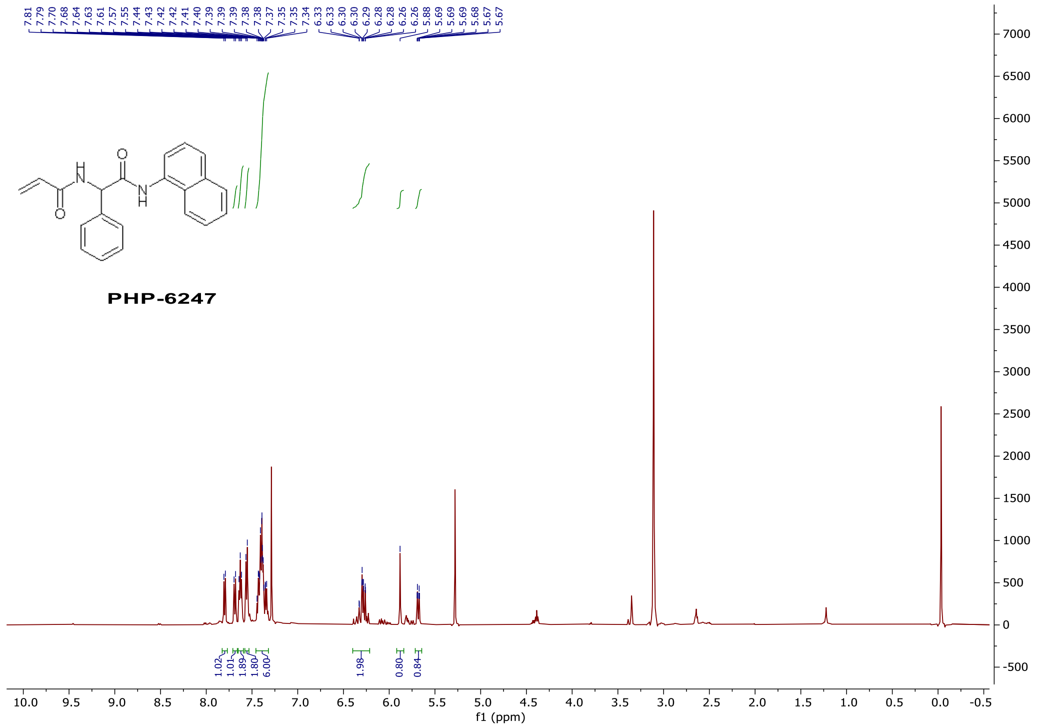

Supplementary Figure 979. ^{1}H NMR (400 MHz, $CDCl_3$ + one drop of CD_3OD) of compound **PHP-6247**.

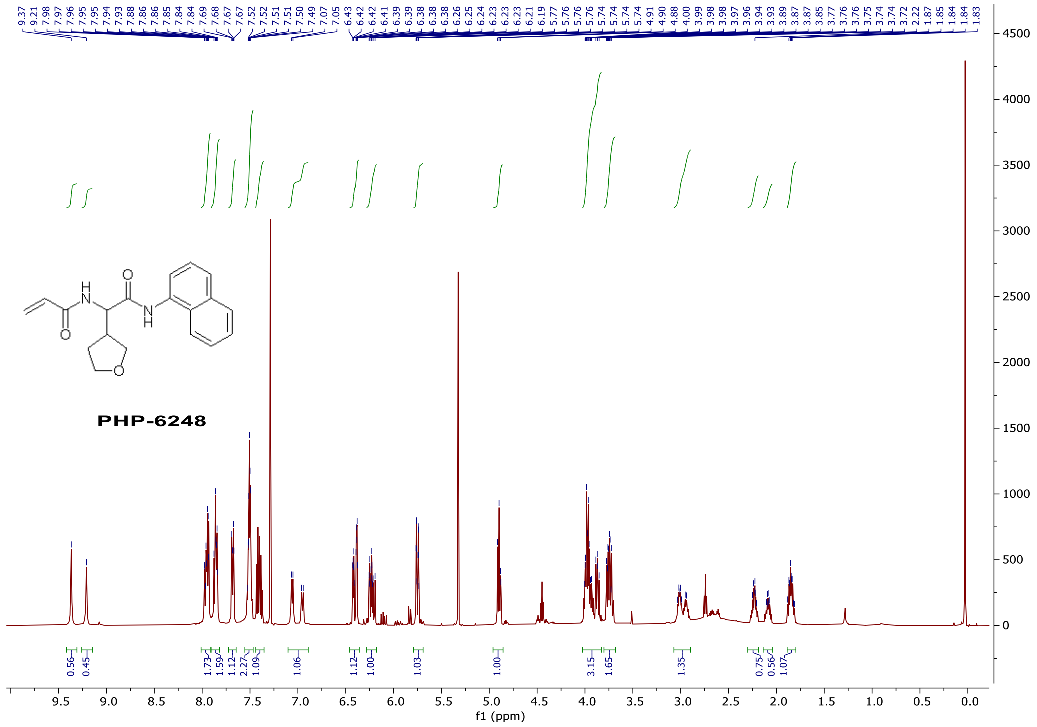

Supplementary Figure 980. ^{1}H NMR (400 MHz, $CDCl_3$ + one drop of CD_3OD) of compound **PHP-6248**.

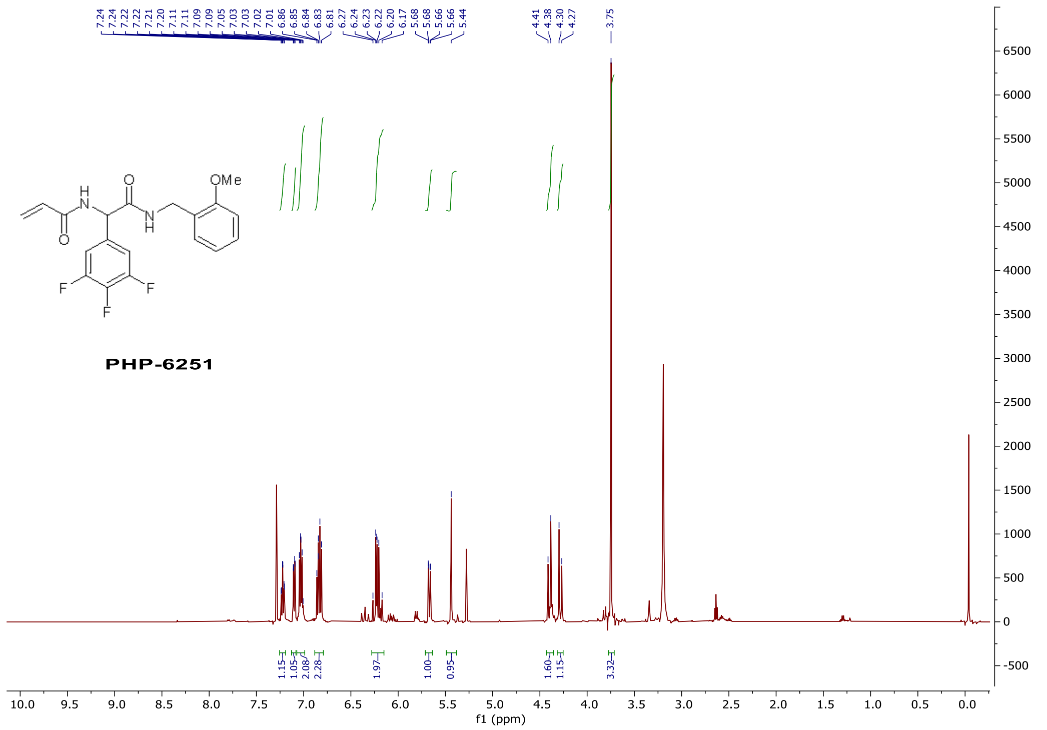

Supplementary Figure 981. ^{1}H NMR (400 MHz, $CDCl_3$ + one drop of CD_3OD) of compound **PHP-6251**.

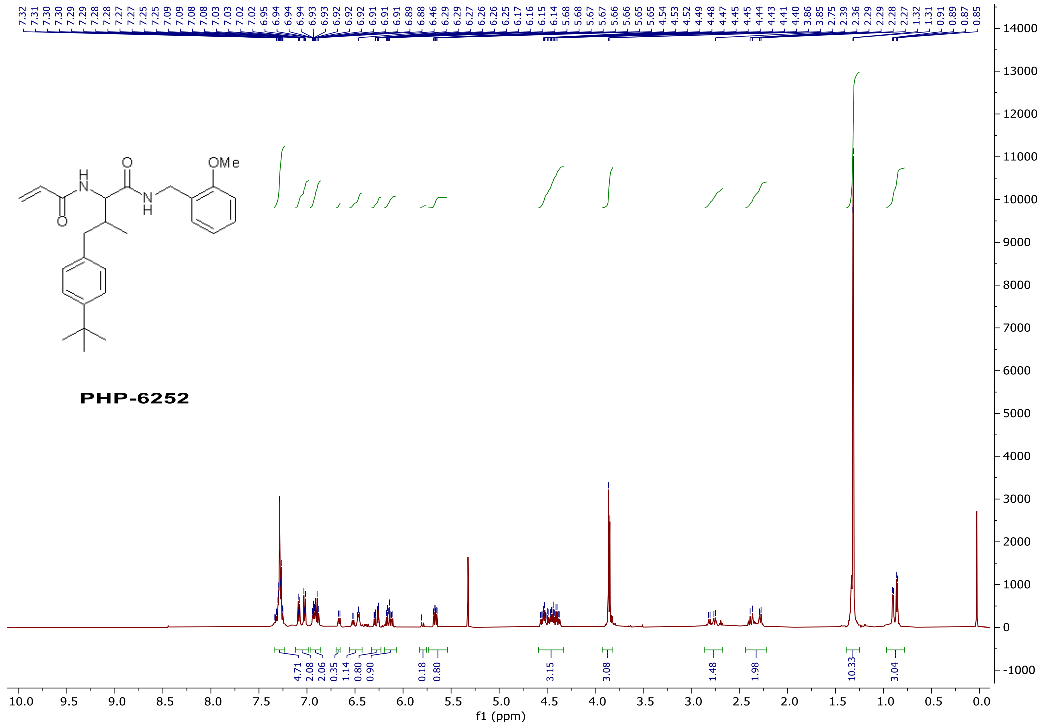

Supplementary Figure 982. ^{1}H NMR (400 MHz, $CDCl_3$ + one drop of CD_3OD) of compound **PHP-6252**.

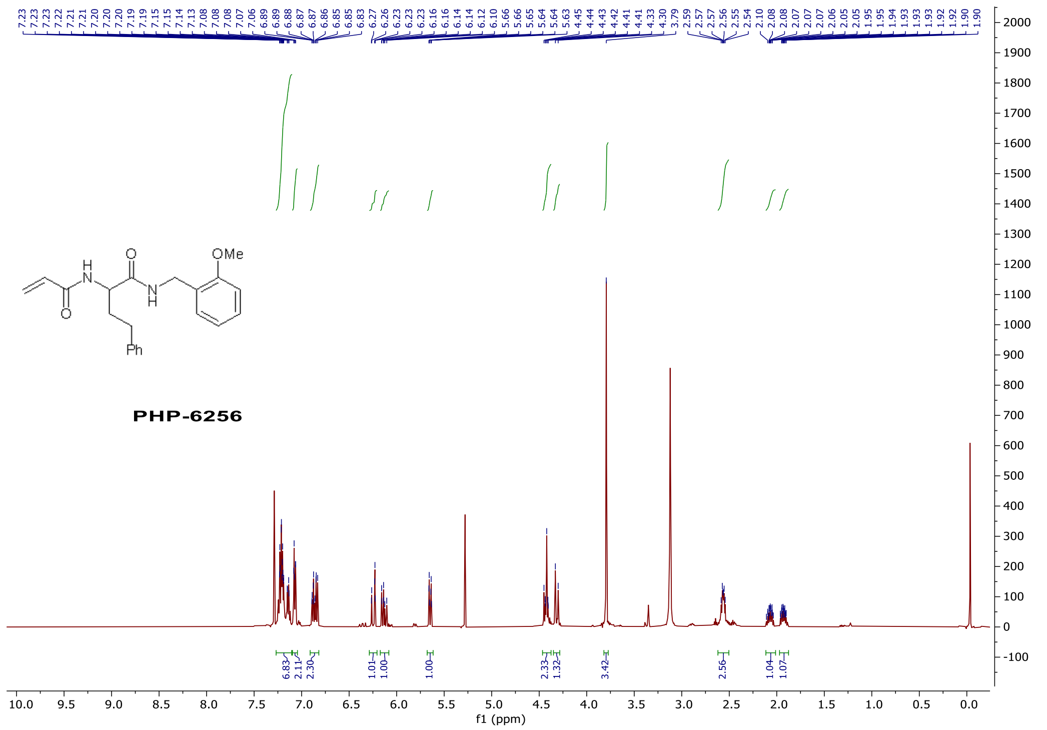

Supplementary Figure 983. ^{1}H NMR (400 MHz, $CDCl_3$ + one drop of CD_3OD) of compound **PHP-6256.**

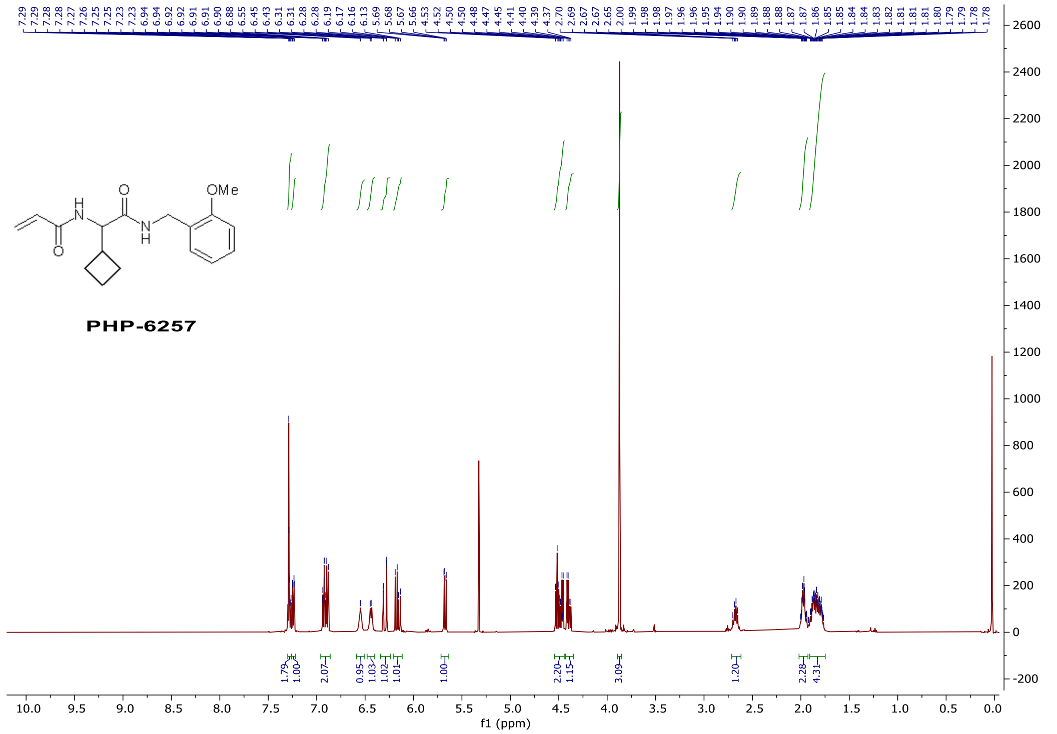

Supplementary Figure 984. ^{1}H NMR (400 MHz, $CDCl_3$ + one drop of CD_3OD) of compound **PHP-6257.**

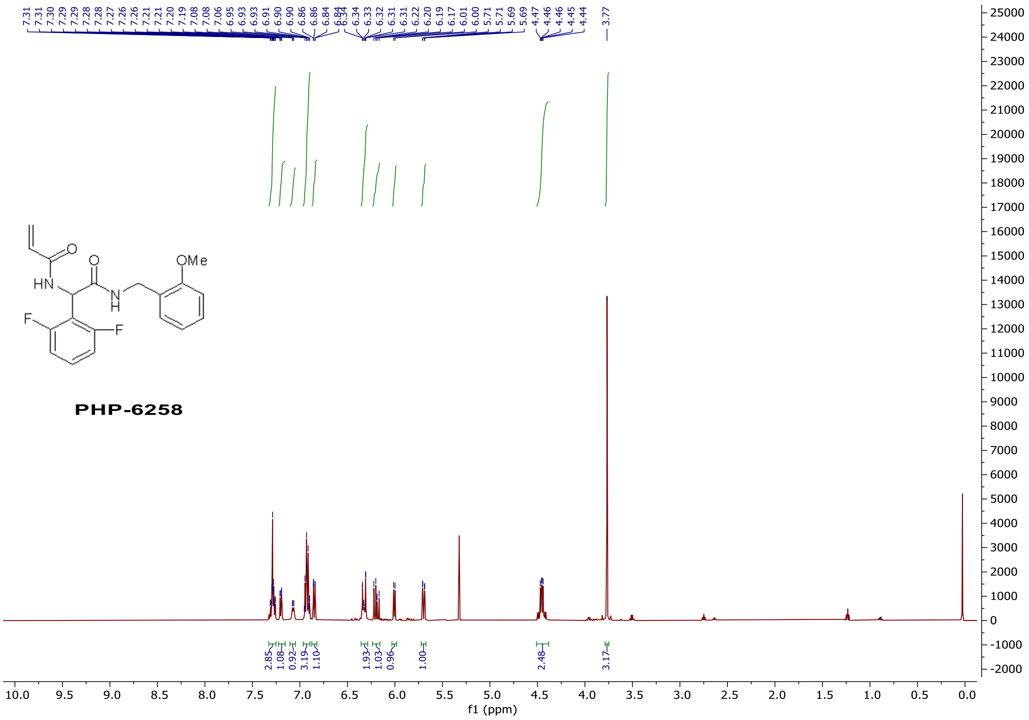

Supplementary Figure 985. ^{1}H NMR (400 MHz, $CDCl_3$ + one drop of CD_3OD) of compound **PHP-6258.**

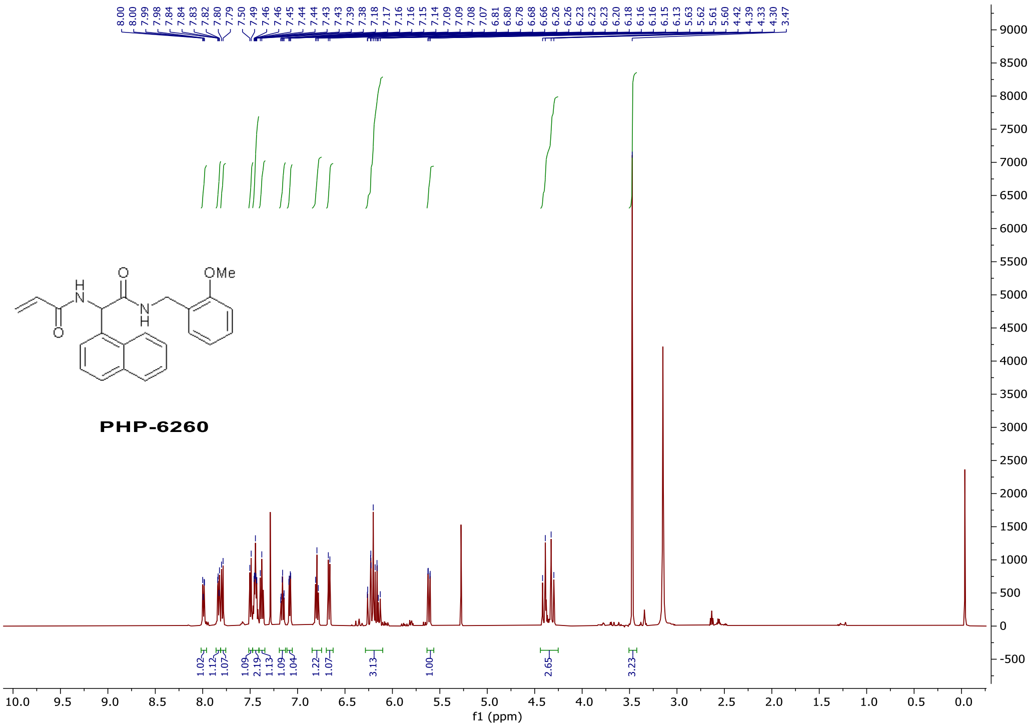

Supplementary Figure 986. ^{1}H NMR (400 MHz, $CDCl_3$ + one drop of CD_3OD) of compound **PHP-6260.**

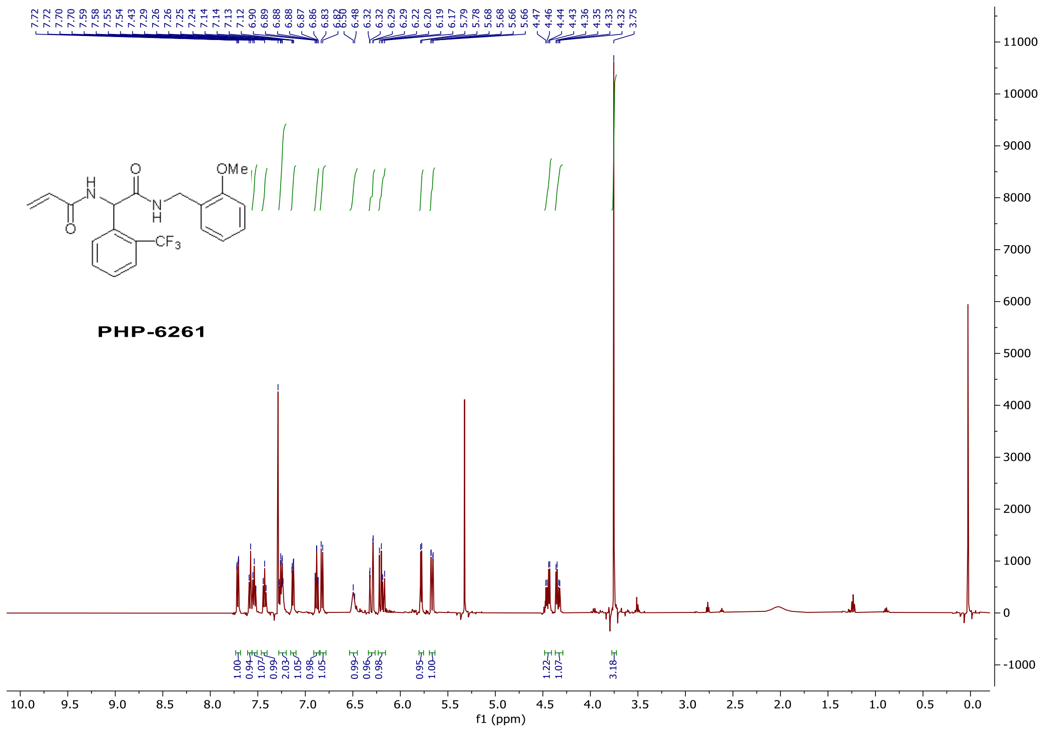

Supplementary Figure 987. ^{1}H NMR (400 MHz, $CDCl_3$ + one drop of CD_3OD) of compound **PHP-6261**.

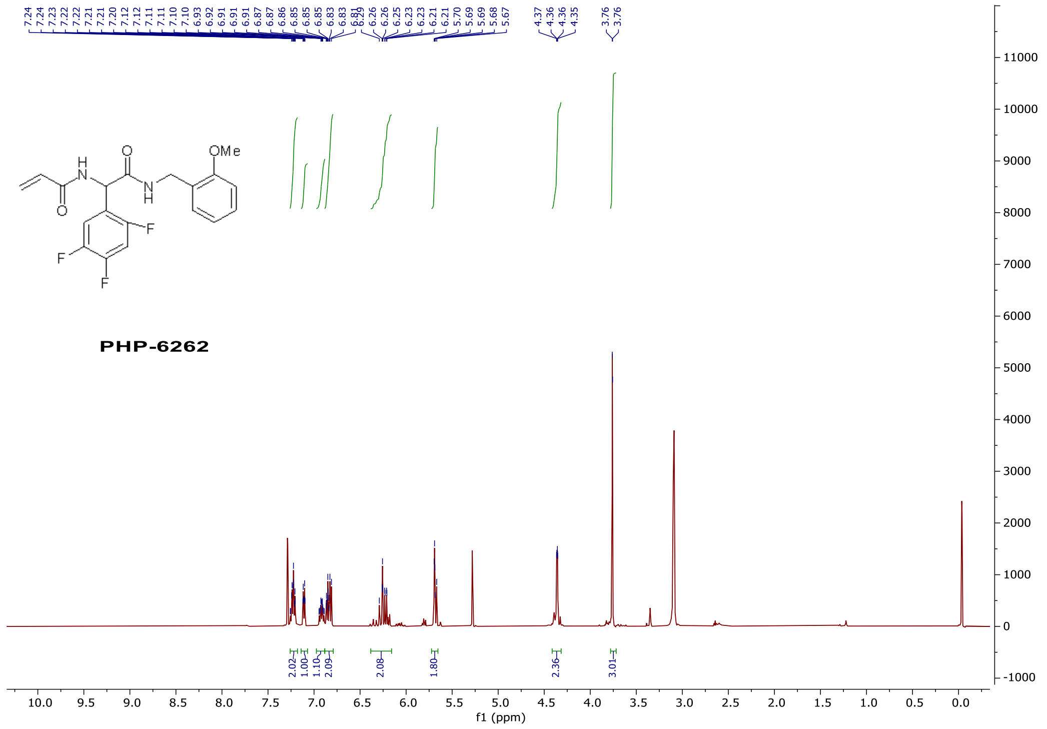

Supplementary Figure 988. ^{1}H NMR (400 MHz, $CDCl_3$ + one drop of CD_3OD) of compound **PHP-6262**.

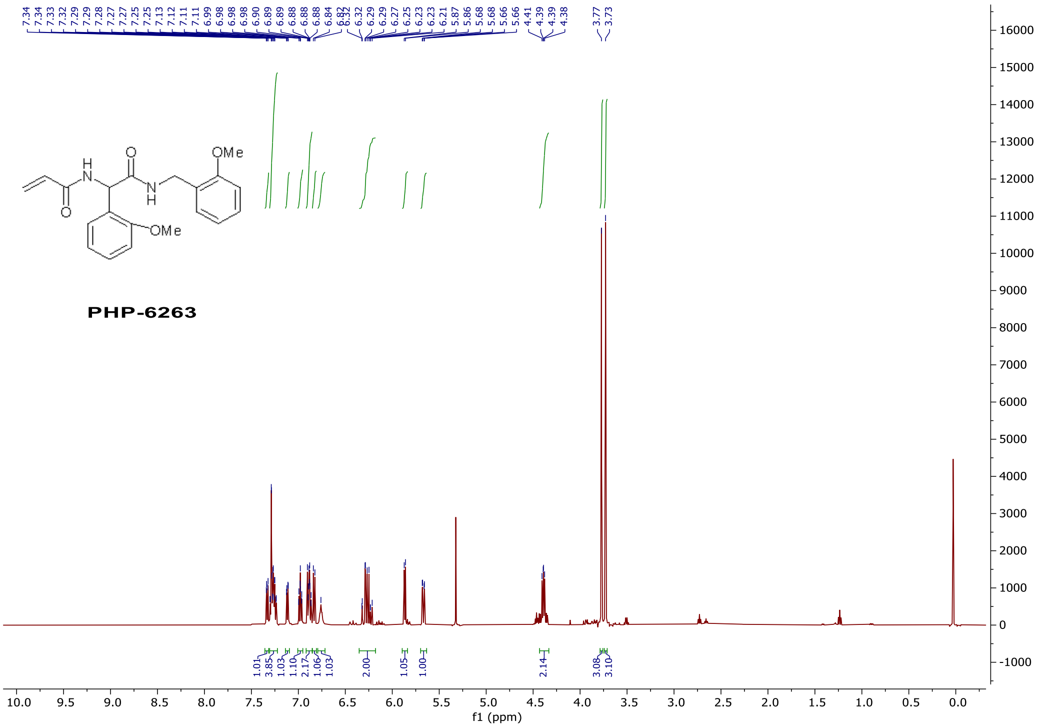

Supplementary Figure 989. ^{1}H NMR (400 MHz, $CDCl_3$ + one drop of CD_3OD) of compound **PHP-6263**.

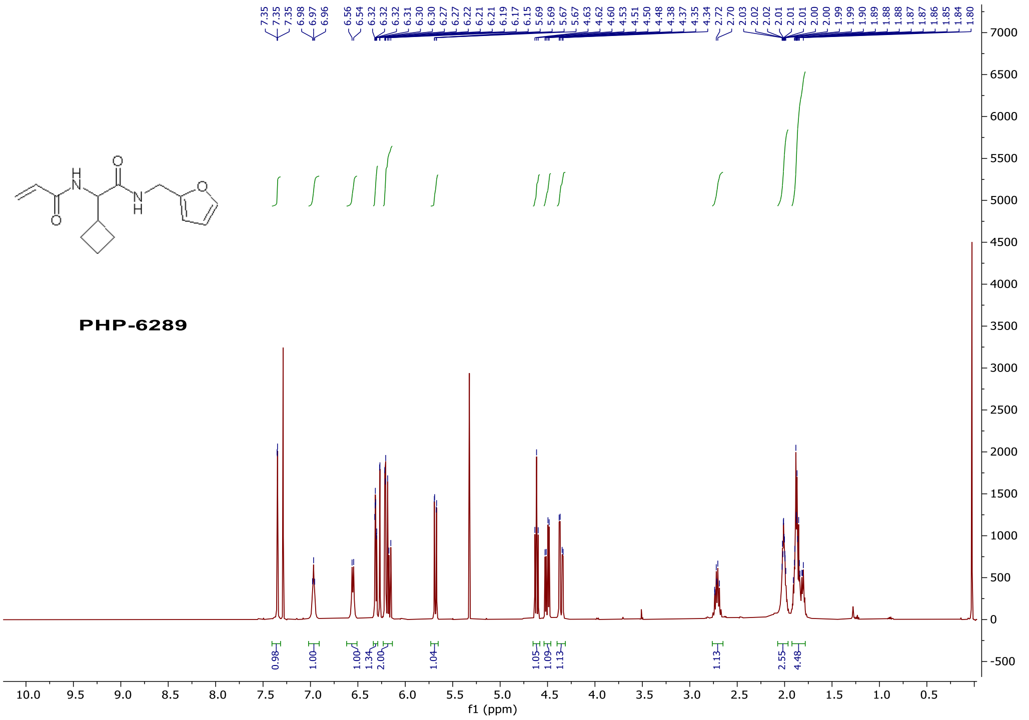

Supplementary Figure 990. ^{1}H NMR (400 MHz, $CDCl_3$ + one drop of CD_3OD) of compound **PHP-6289**.

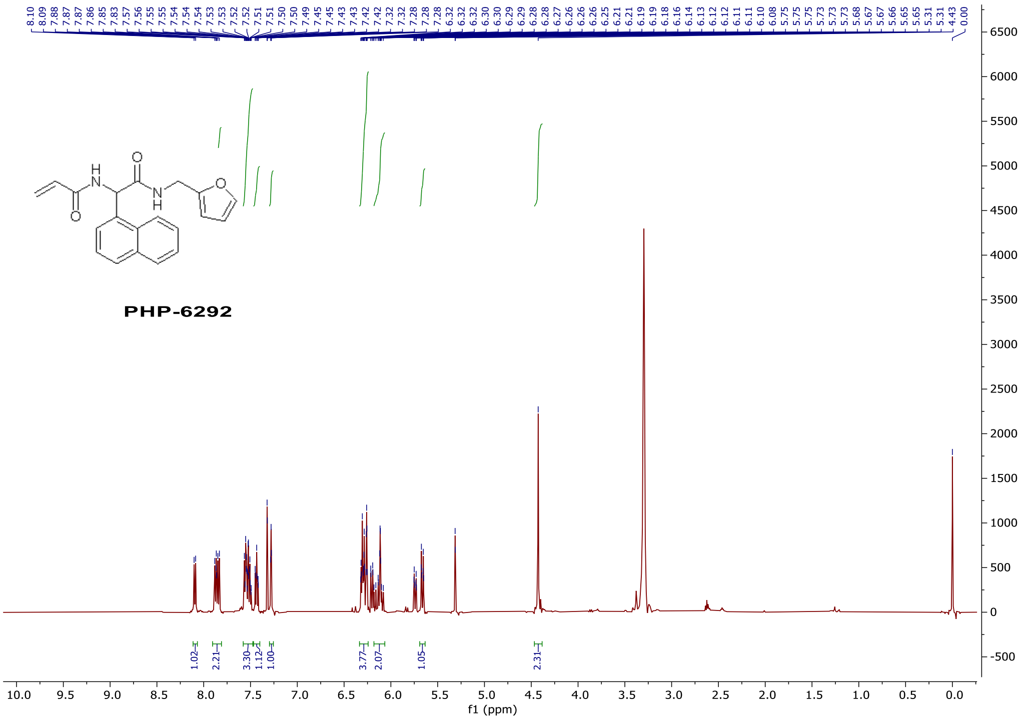

Supplementary Figure 991. ^{1}H NMR (400 MHz, $CDCl_3$ + one drop of CD_3OD) of compound **PHP-6292.**

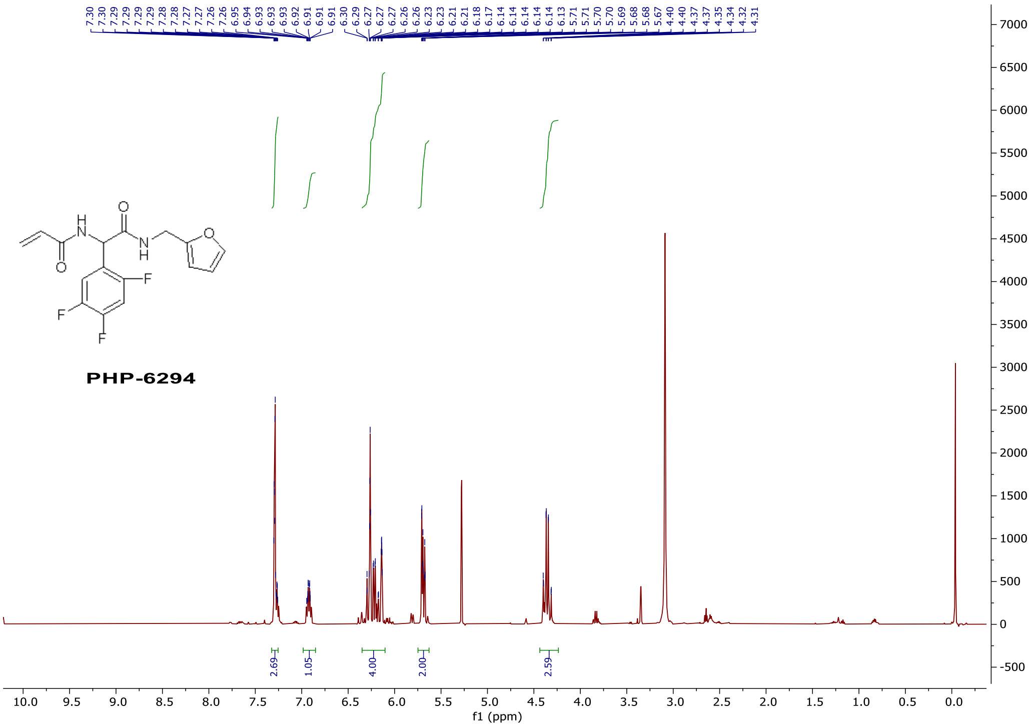

Supplementary Figure 992. ^{1}H NMR (400 MHz, $CDCl_3$ + one drop of CD_3OD) of compound **PHP-6294.**

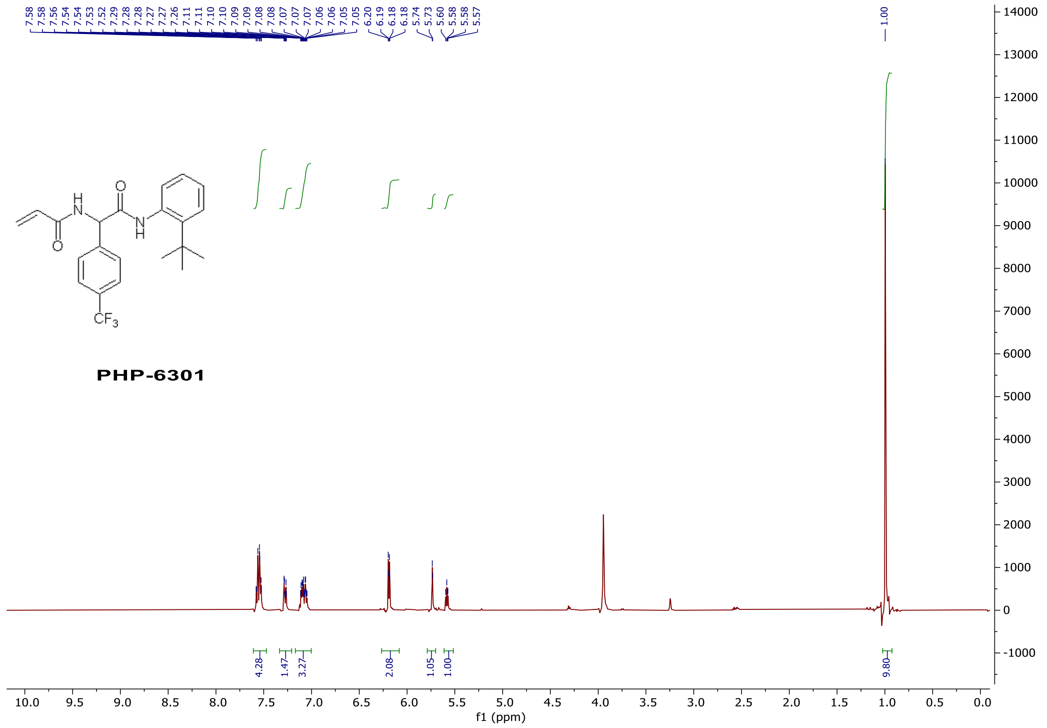

Supplementary Figure 993. ^{1}H NMR (400 MHz, $CDCl_3$ + one drop of CD_3OD) of compound **PHP-6301.**

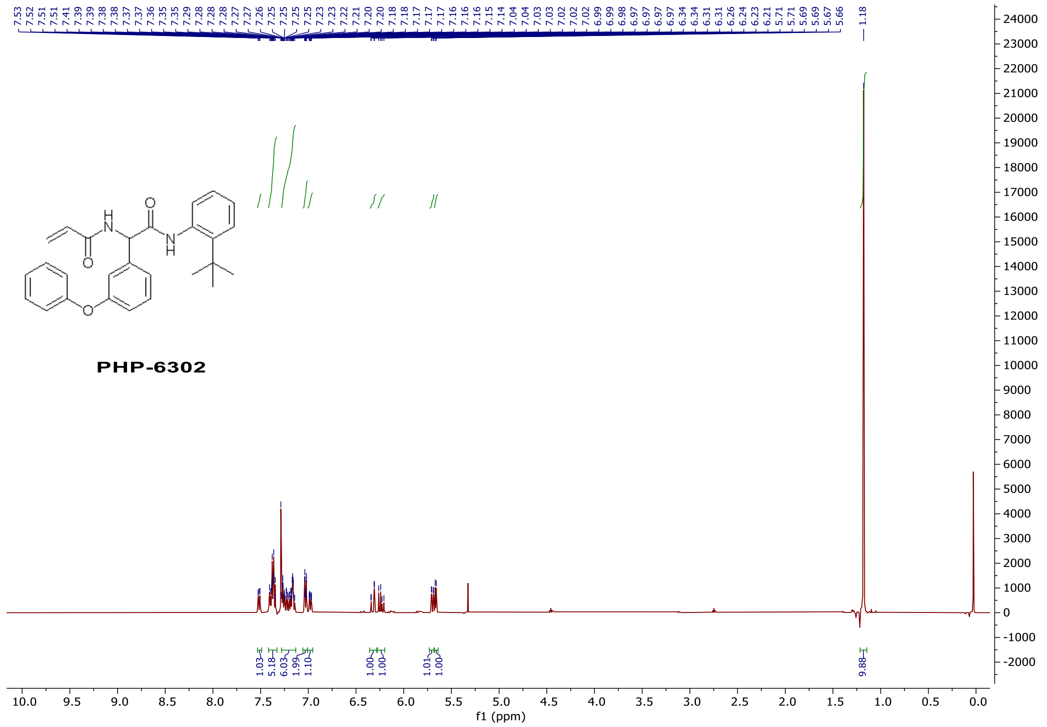

Supplementary Figure 994. ^{1}H NMR (400 MHz, $CDCl_3$ + one drop of CD_3OD) of compound **PHP-6302.**

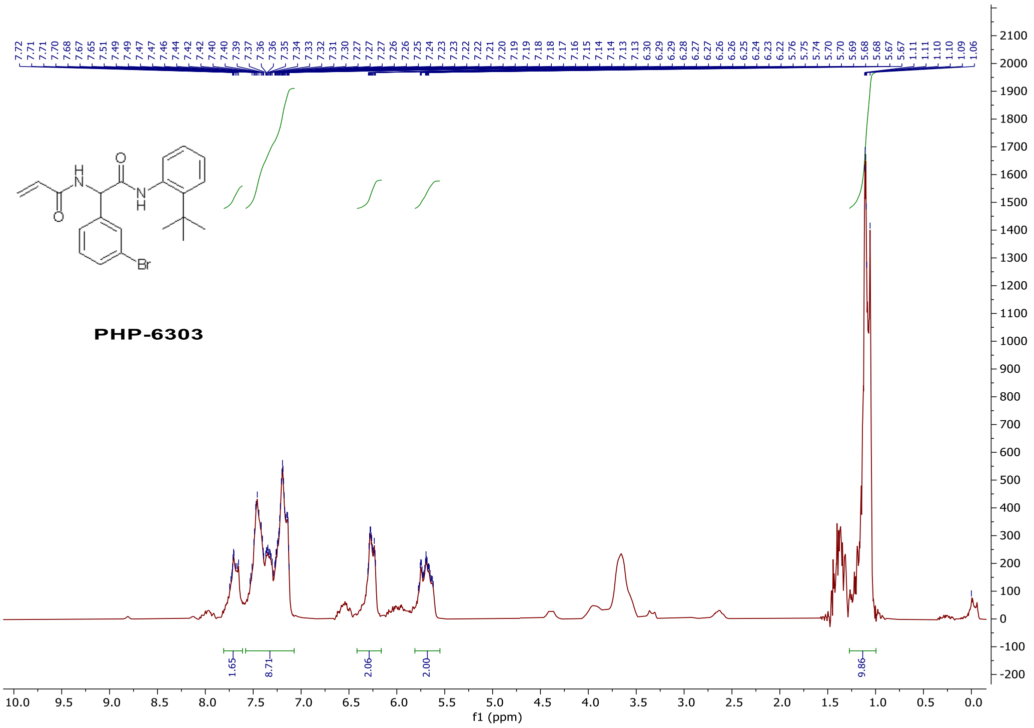

Supplementary Figure 995. ^{1}H NMR (400 MHz, $CDCl_3$ + one drop of CD_3OD) of compound **PHP-6303.**

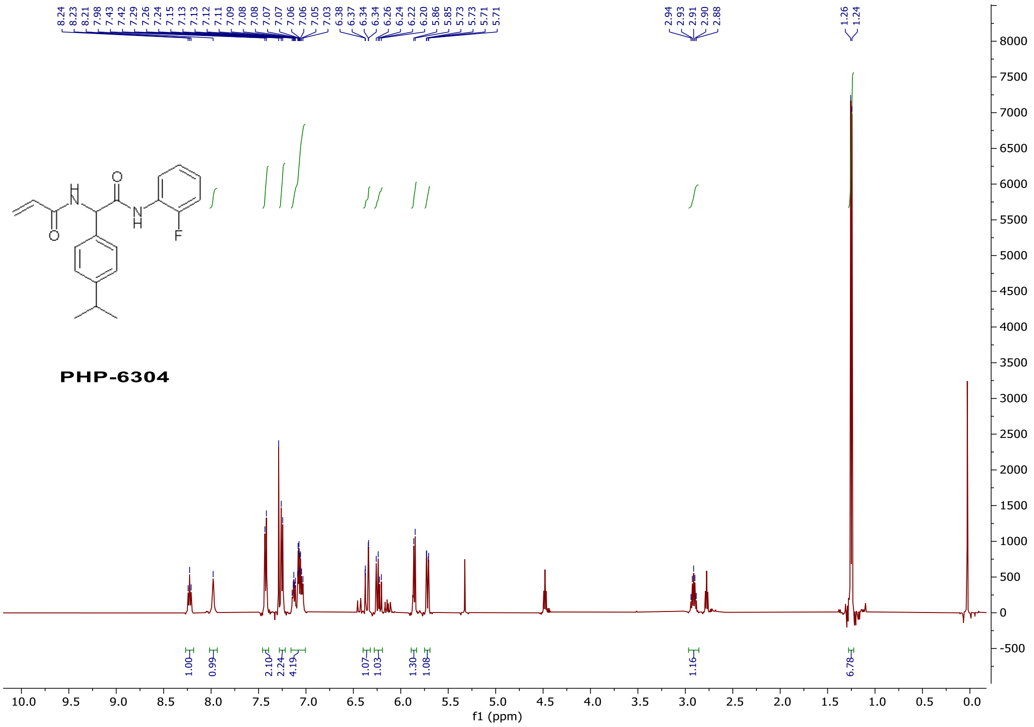

Supplementary Figure 996. ^{1}H NMR (400 MHz, $CDCl_3$ + one drop of CD_3OD) of compound **PHP-6304.**

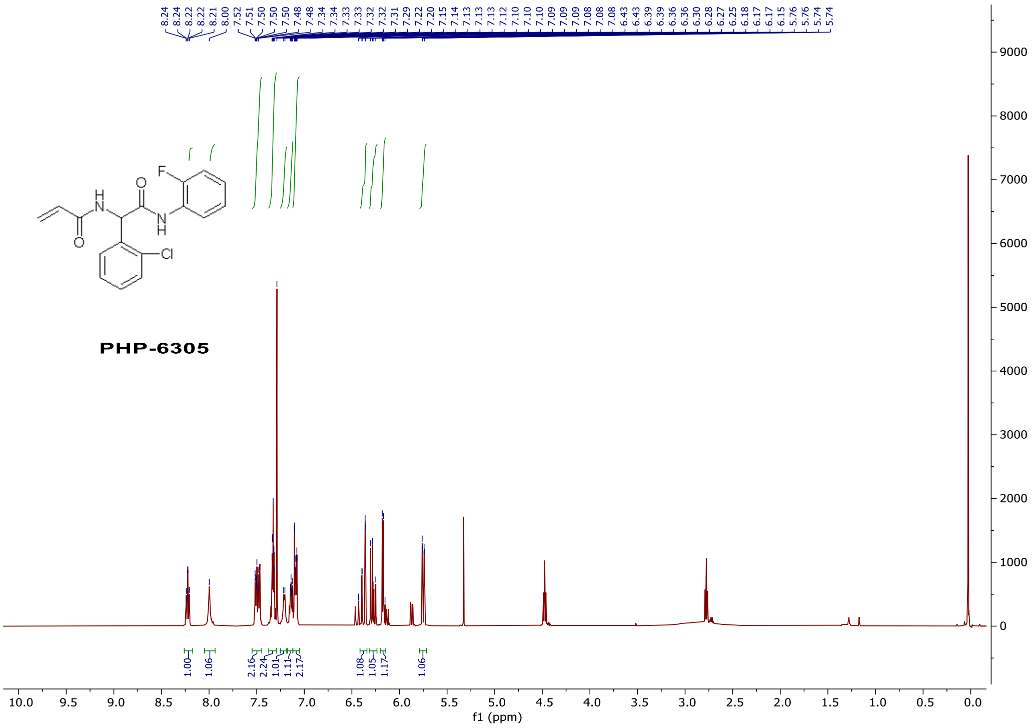

Supplementary Figure 997. ^{1}H NMR (400 MHz, $CDCl_3$ + one drop of CD_3OD) of compound **PHP-6305.**

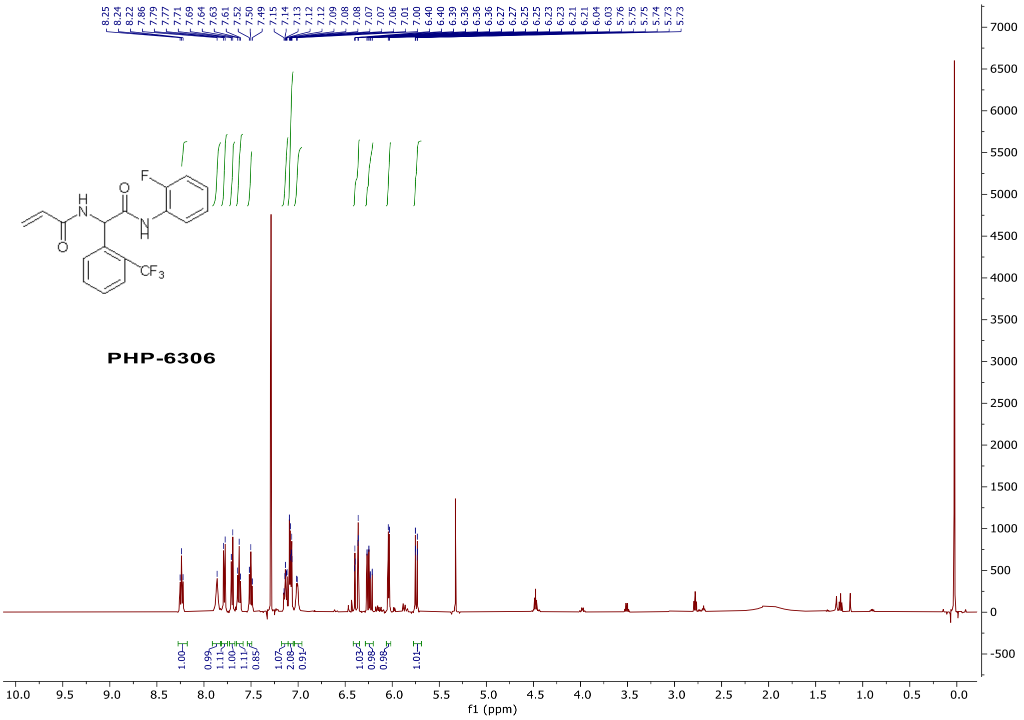

Supplementary Figure 998. ^{1}H NMR (400 MHz, $CDCl_3$ + one drop of CD_3OD) of compound **PHP-6306.**

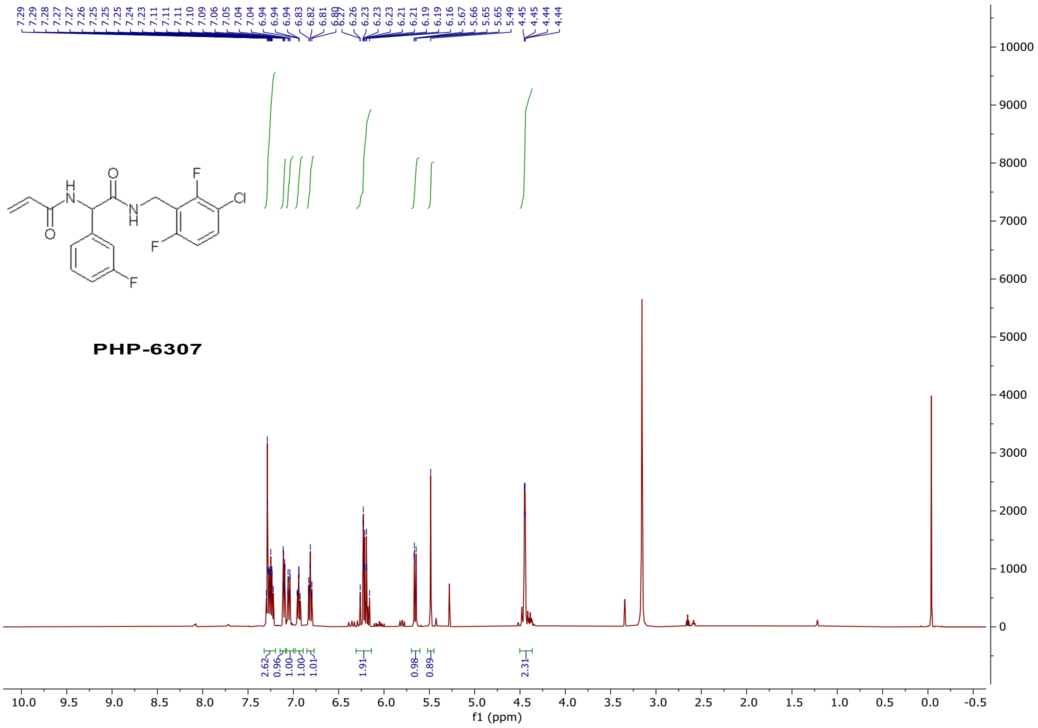

Supplementary Figure 999. ^{1}H NMR (400 MHz, $CDCl_3$ + one drop of CD_3OD) of compound **PHP-6307.**

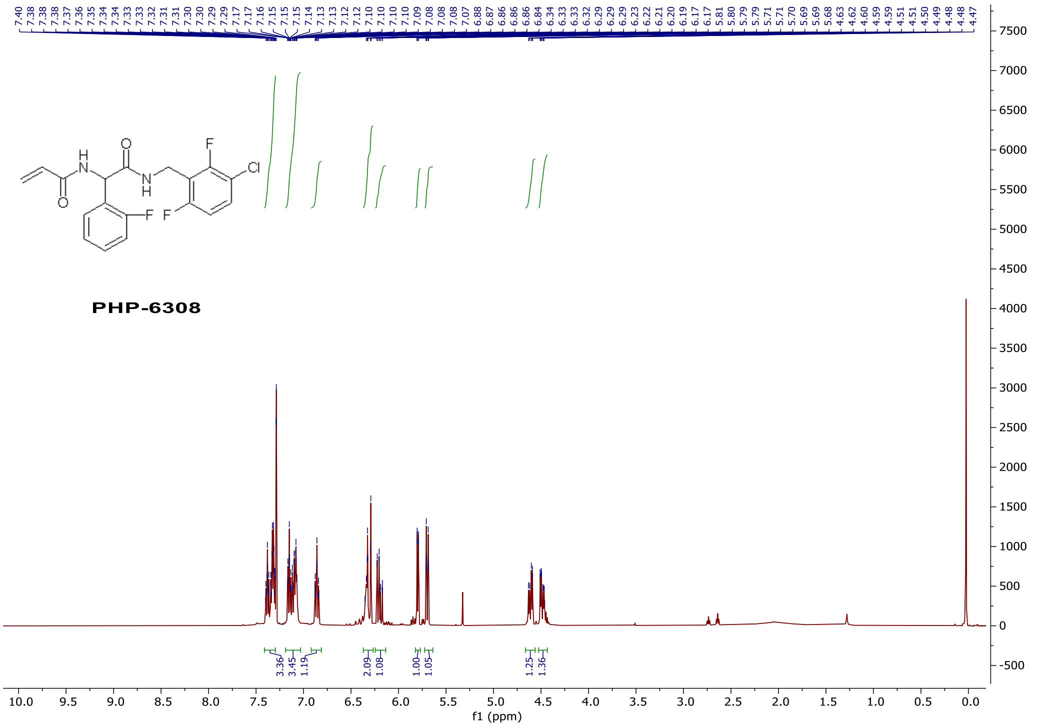

Supplementary Figure 1000. ^{1}H NMR (400 MHz, $CDCl_3$ + one drop of CD_3OD) of compound **PHP-6308.**

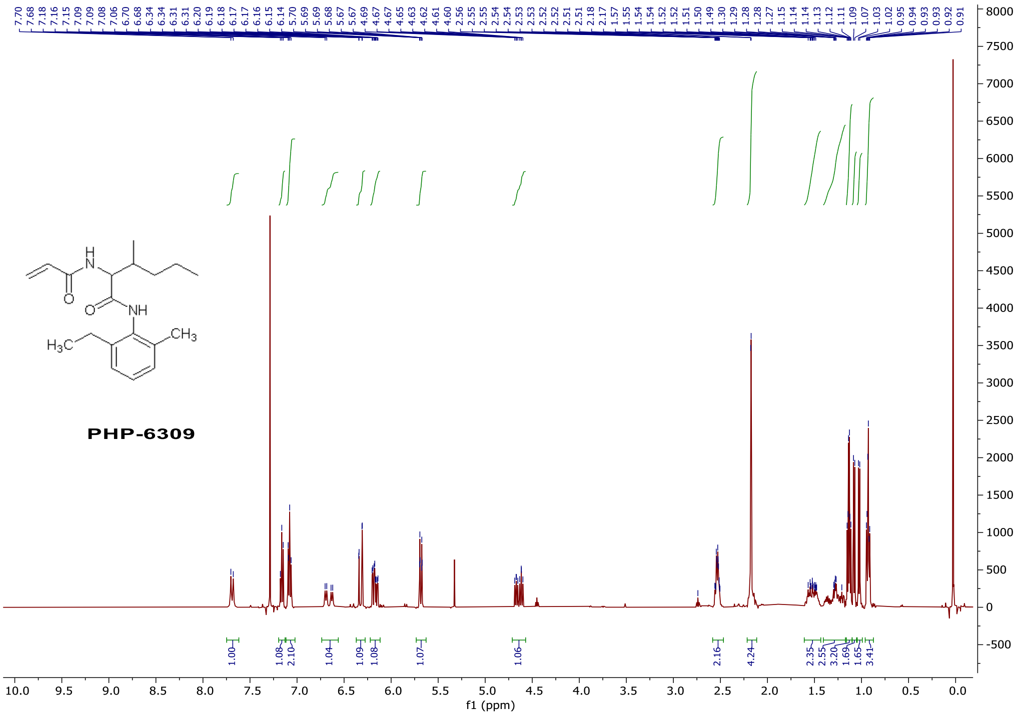

Supplementary Figure 1001. ^{1}H NMR (400 MHz, $CDCl_3$ + one drop of CD_3OD) of compound **PHP-6309.**

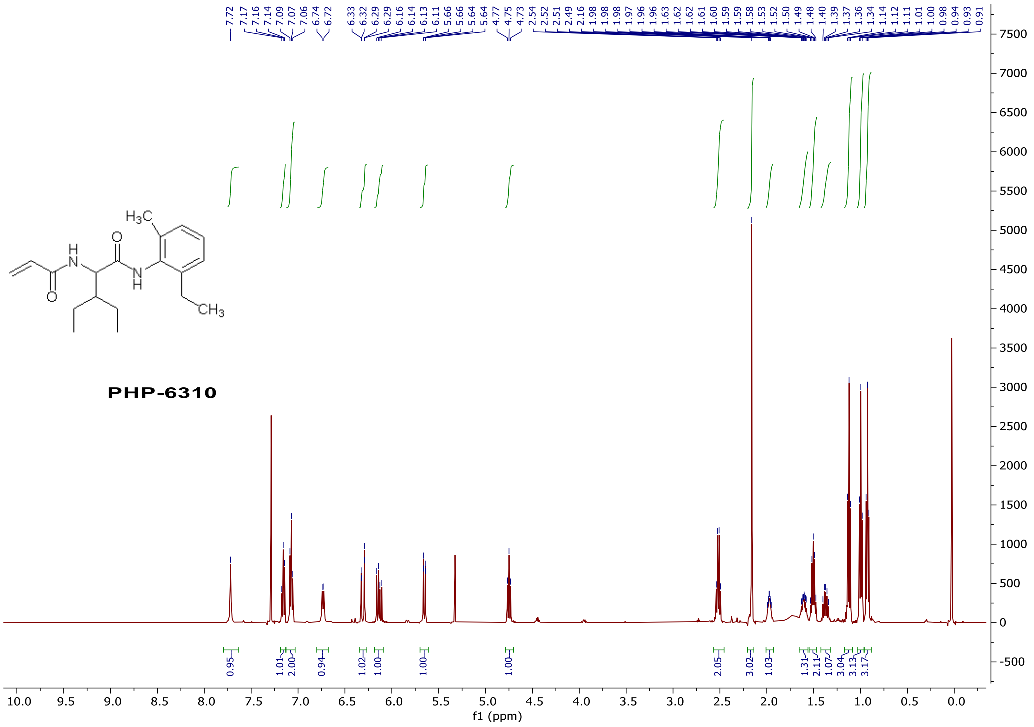

Supplementary Figure 1002. ^{1}H NMR (400 MHz, $CDCl_3$ + one drop of CD_3OD) of compound **PHP-6310.**

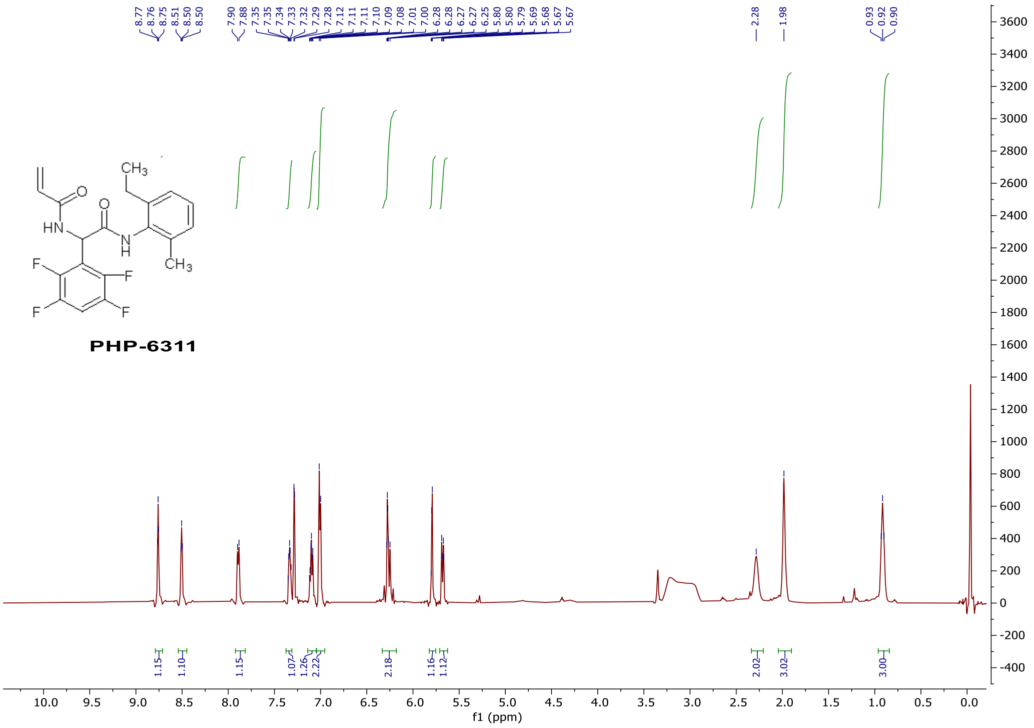

Supplementary Figure 1003. ^{1}H NMR (400 MHz, $CDCl_3$ + one drop of CD_3OD) of compound **PHP-6311.**

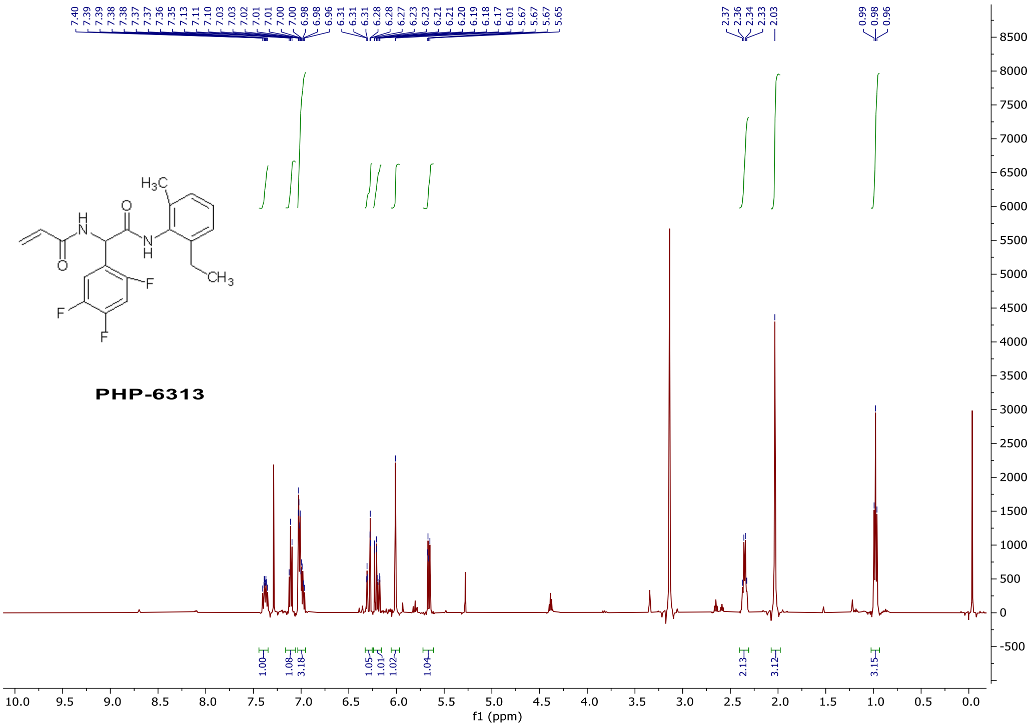

Supplementary Figure 1004. ^{1}H NMR (400 MHz, $CDCl_3$ + one drop of CD_3OD) of compound **PHP-6313.**

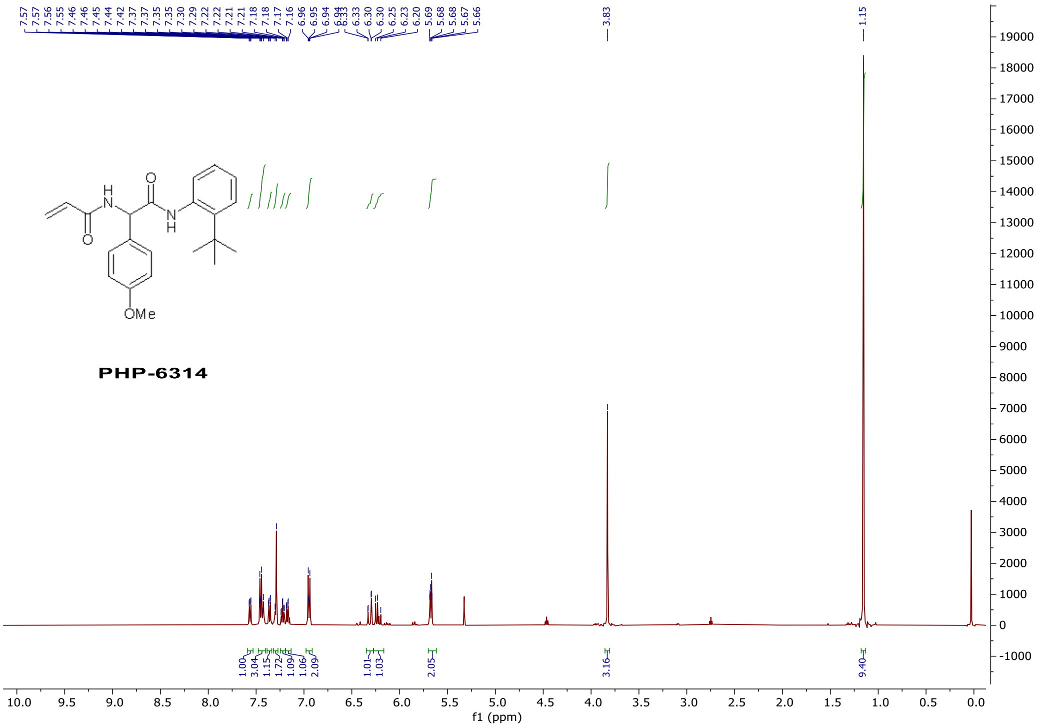

Supplementary Figure 1005. ^{1}H NMR (400 MHz, $CDCl_3$ + one drop of CD_3OD) of compound **PHP-6314.**

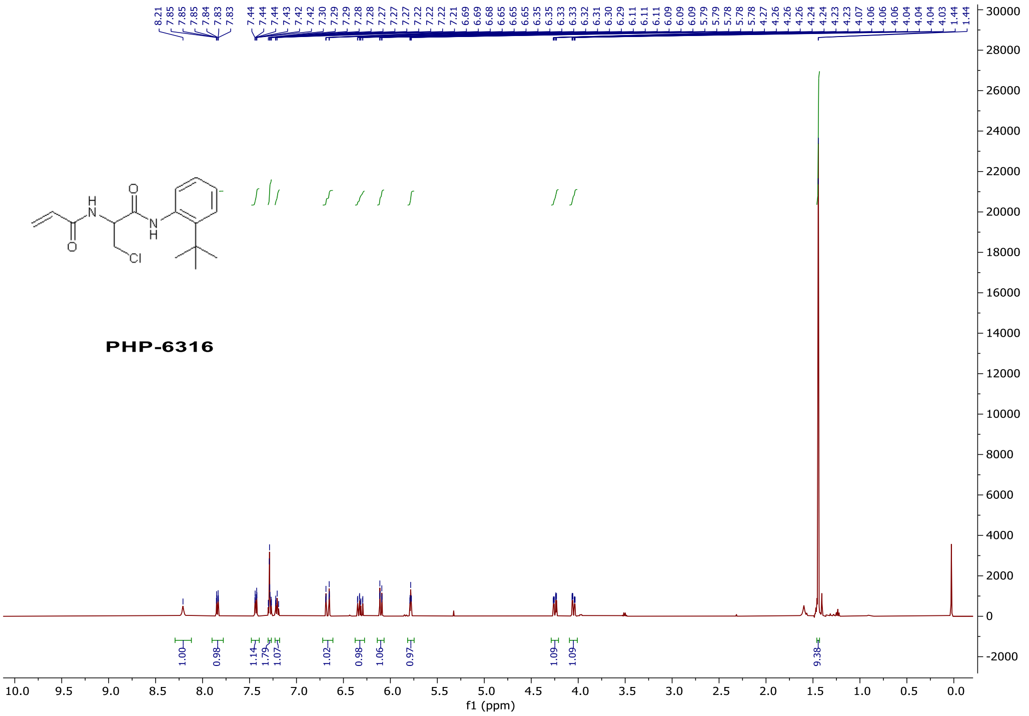

Supplementary Figure 1006. ^{1}H NMR (400 MHz, $CDCl_3$ + one drop of CD_3OD) of compound **PHP-6316.**

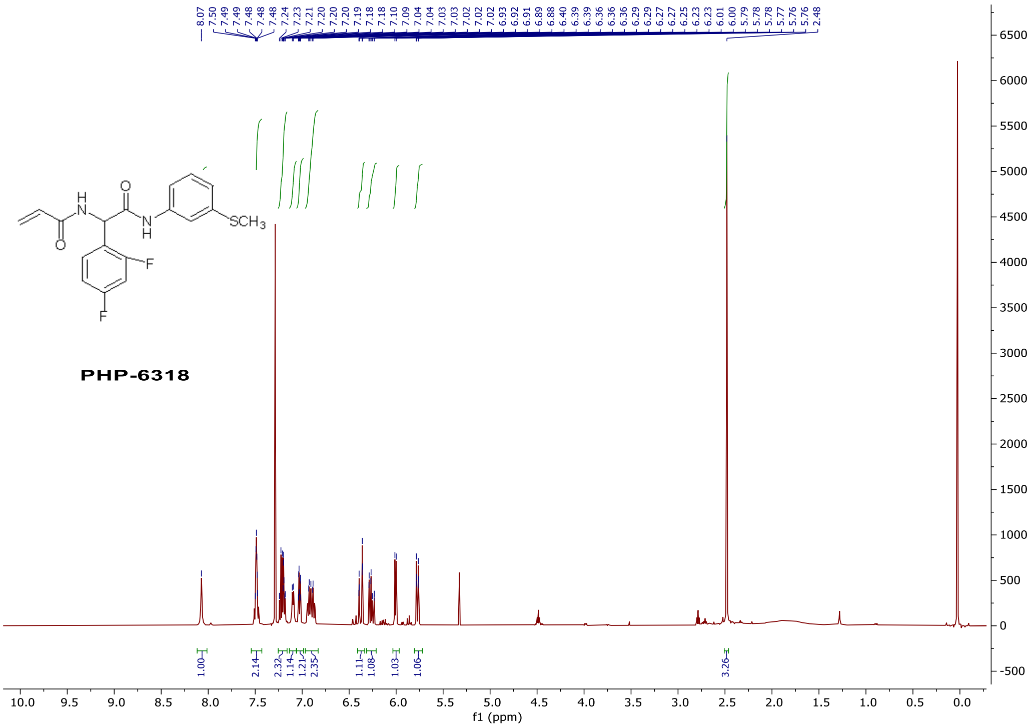

Supplementary Figure 1007. ^{1}H NMR (400 MHz, $CDCl_3$ + one drop of CD_3OD) of compound **PHP-6318**.

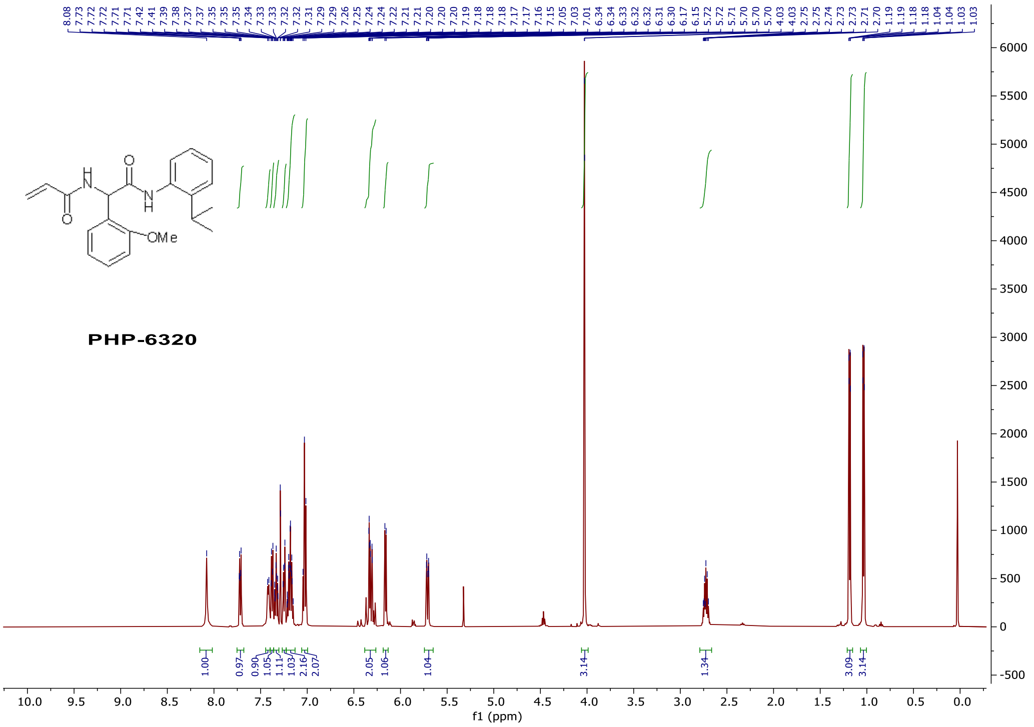

Supplementary Figure 1008. ^{1}H NMR (400 MHz, $CDCl_3$ + one drop of CD_3OD) of compound **PHP-6320**.

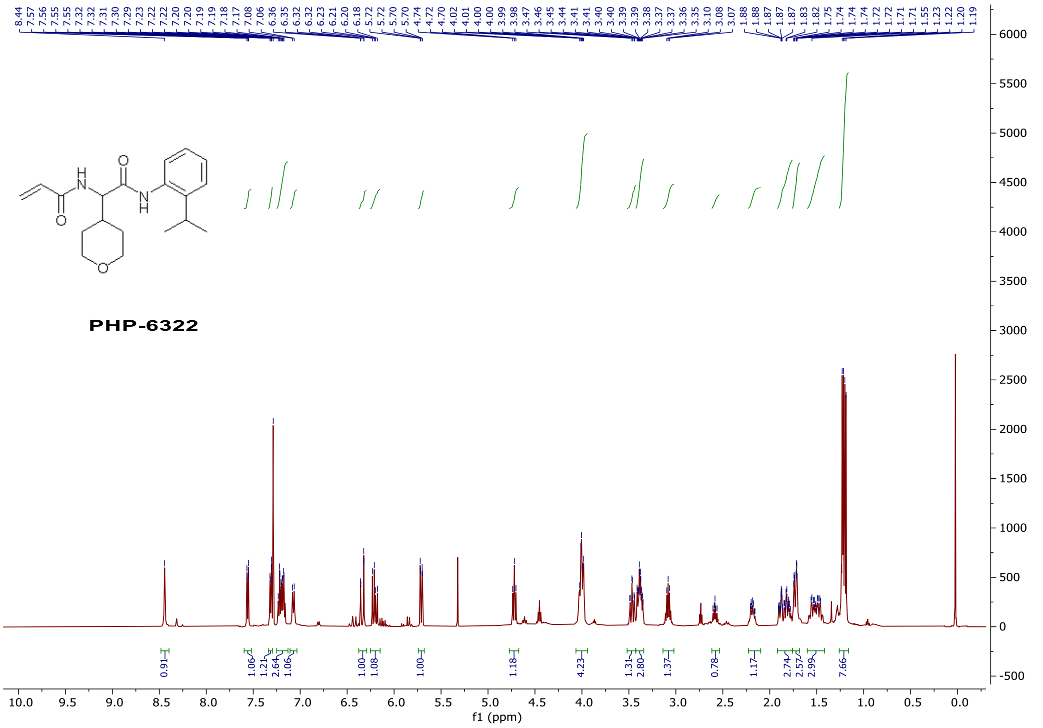

Supplementary Figure 1009. ^{1}H NMR (400 MHz, $CDCl_3$ + one drop of CD_3OD) of compound **PHP-6322.**

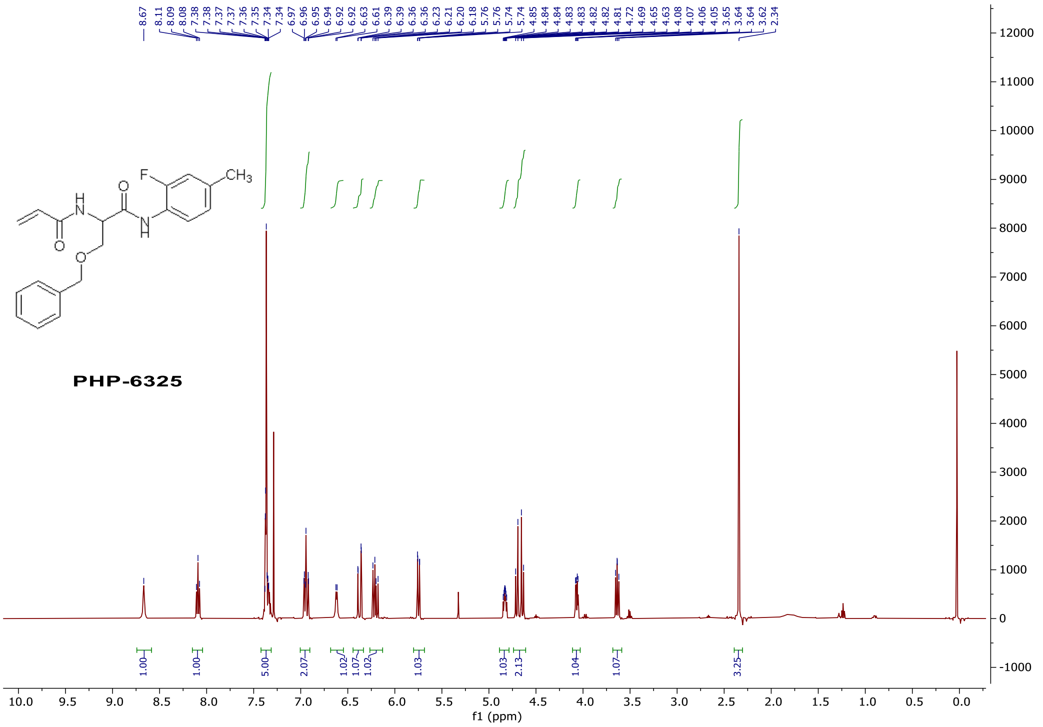

Supplementary Figure 1010. ^{1}H NMR (400 MHz, $CDCl_3$ + one drop of CD_3OD) of compound **PHP-6325.**

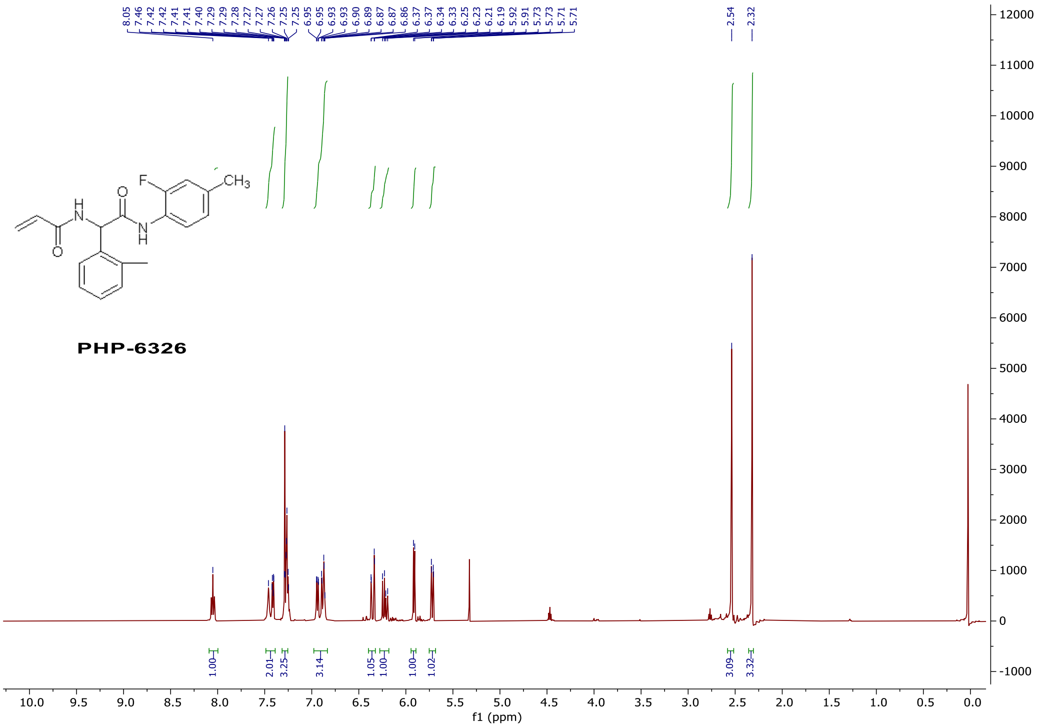

Supplementary Figure 1011. ^{1}H NMR (400 MHz, $CDCl_3$ + one drop of CD_3OD) of compound **PHP-6326.**

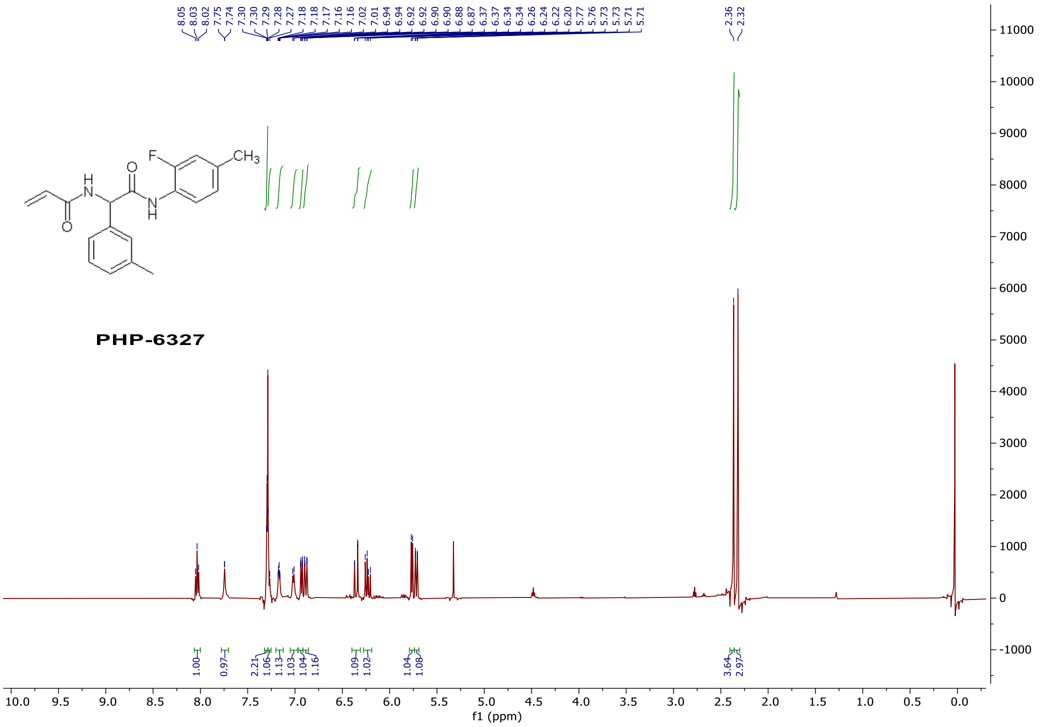

Supplementary Figure 1012. ^{1}H NMR (400 MHz, $CDCl_3$ + one drop of CD_3OD) of compound **PHP-6327.**

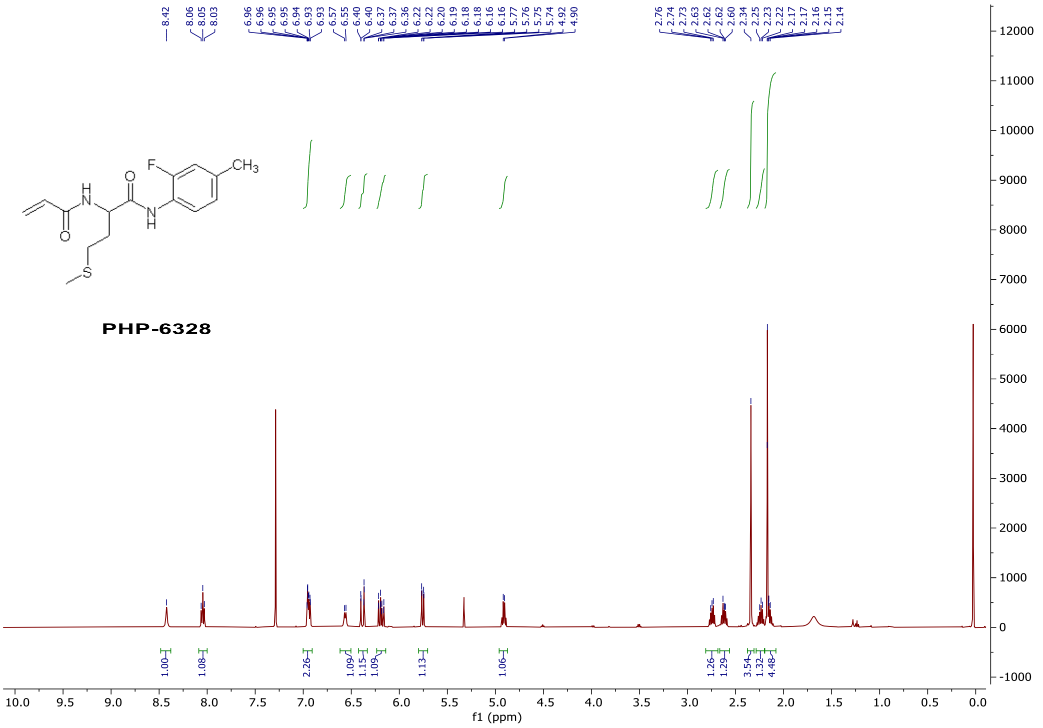

Supplementary Figure 1013. ^{1}H NMR (400 MHz, $CDCl_3$ + one drop of CD_3OD) of compound **PHP-6328**.

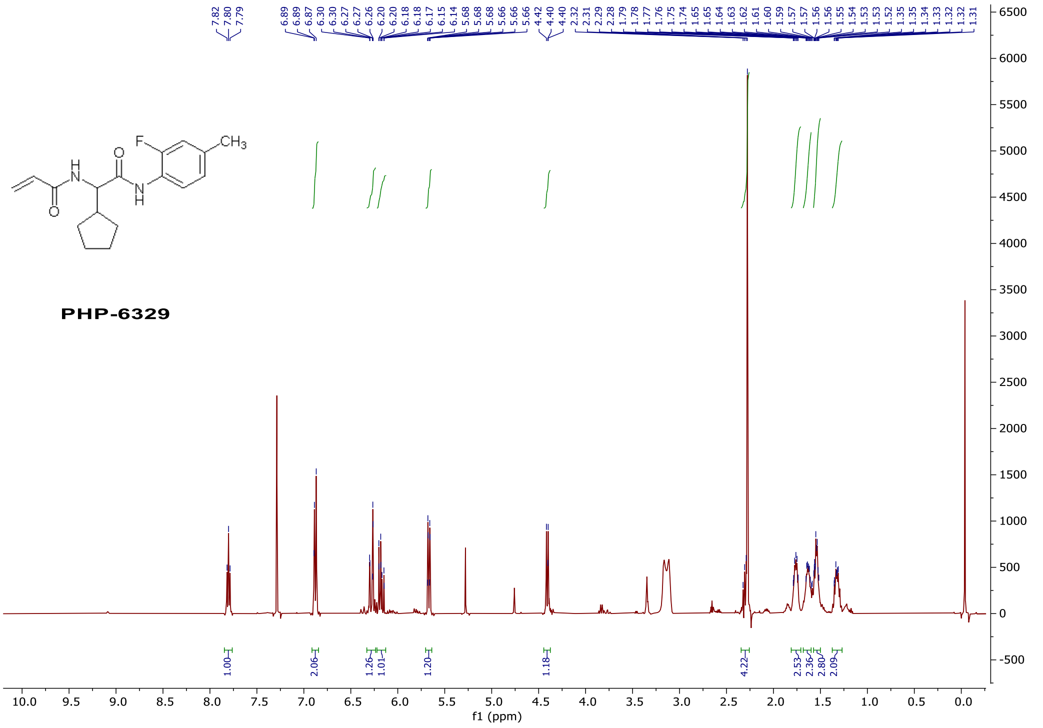

Supplementary Figure 1014. ^{1}H NMR (400 MHz, $CDCl_3$ + one drop of CD_3OD) of compound **PHP-6329**.

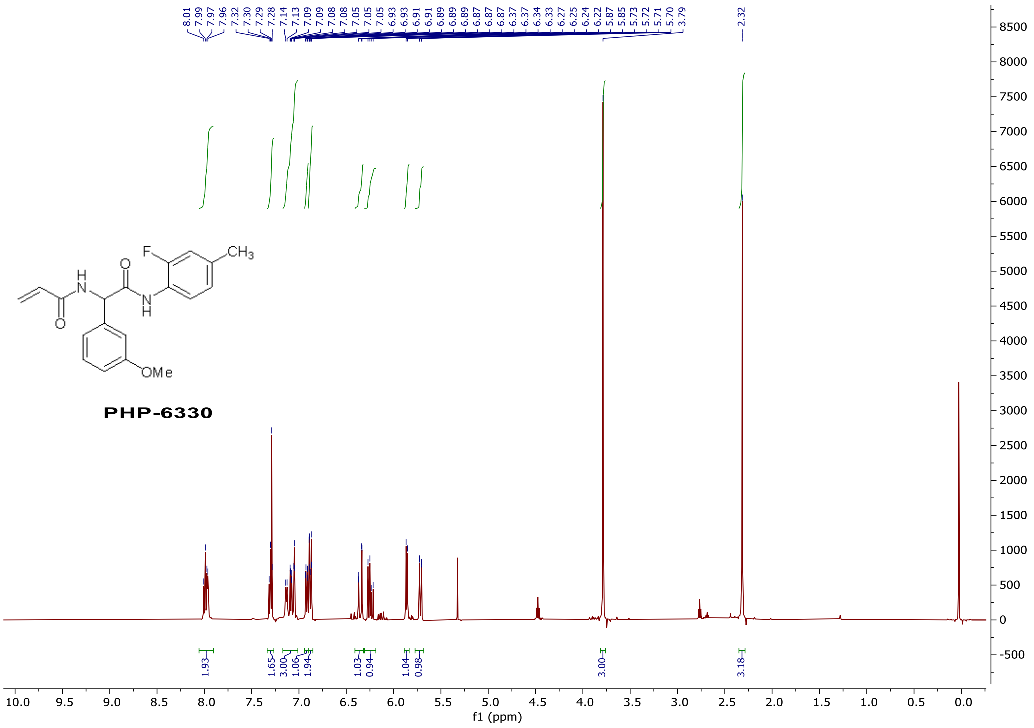

Supplementary Figure 1015. ^{1}H NMR (400 MHz, $CDCl_3$ + one drop of CD_3OD) of compound **PHP-6330.**

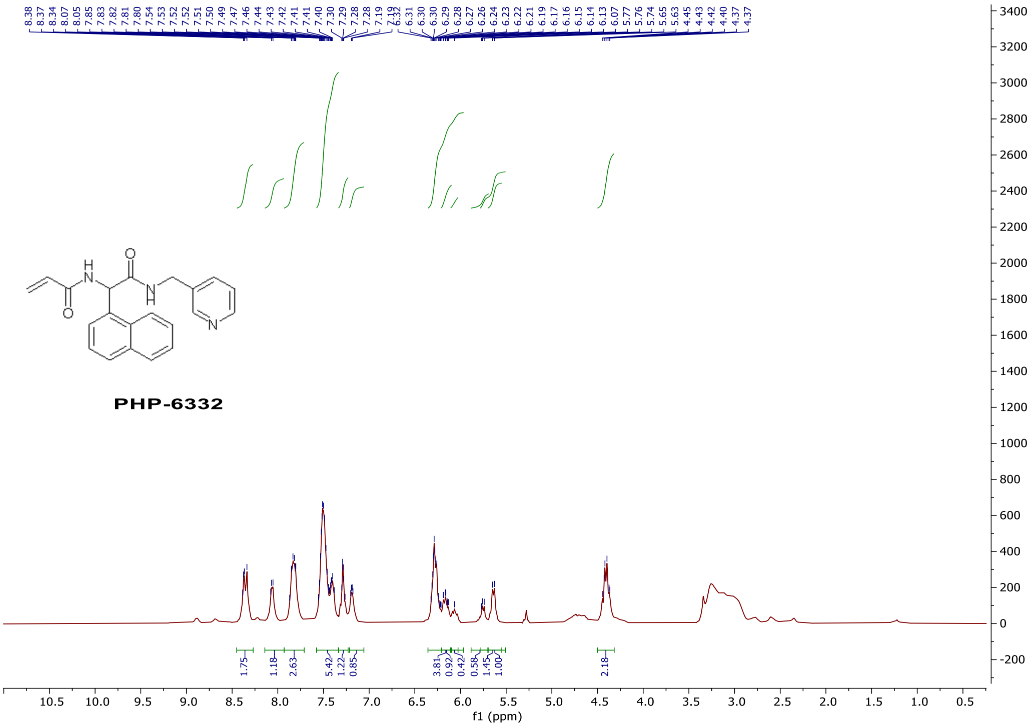

Supplementary Figure 1016. ^{1}H NMR (400 MHz, $CDCl_3$ + one drop of CD_3OD) of compound **PHP-6332.**

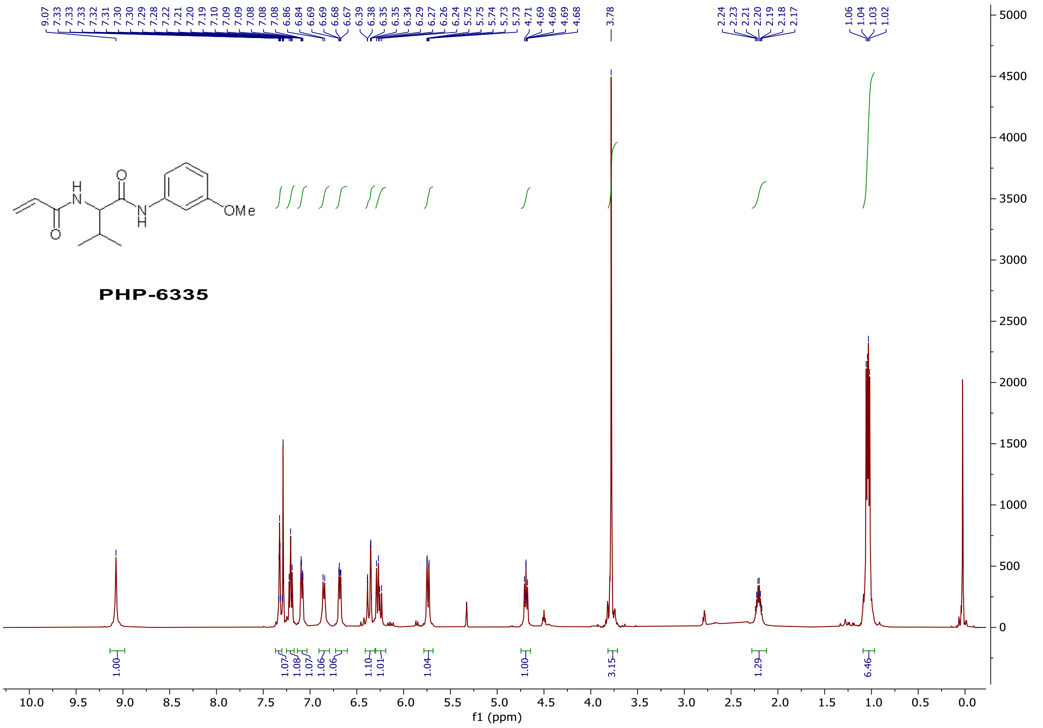

Supplementary Figure 1017. ^{1}H NMR (400 MHz, $CDCl_3$ + one drop of CD_3OD) of compound **PHP-6335.**

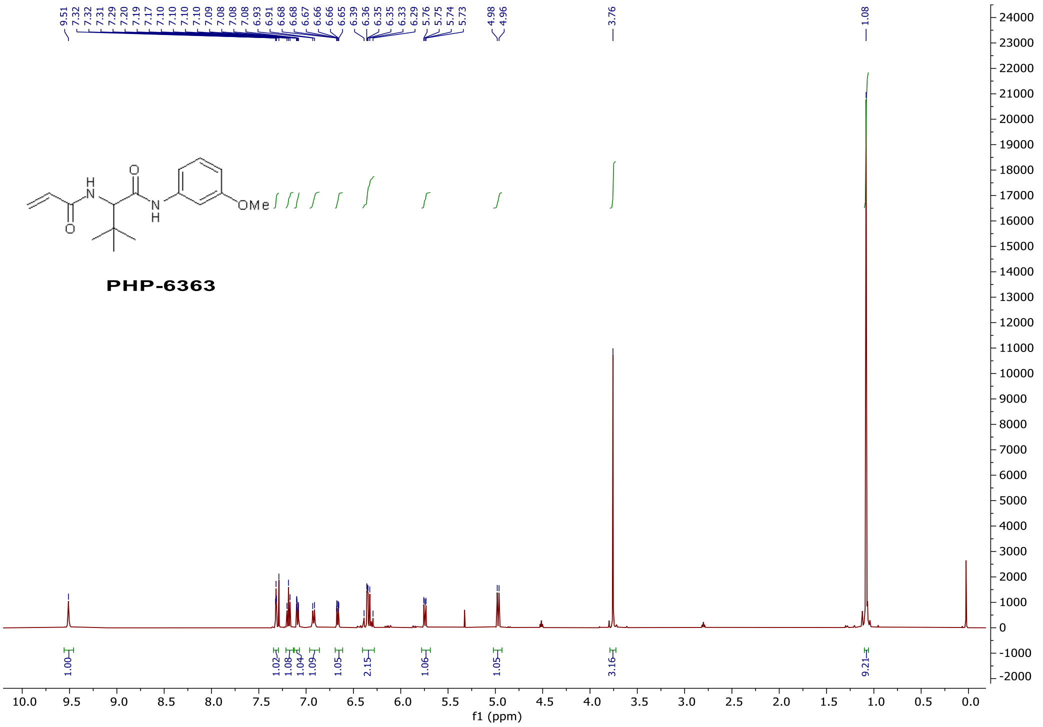

Supplementary Figure 1018. ^{1}H NMR (400 MHz, $CDCl_3$ + one drop of CD_3OD) of compound **PHP-6336.**

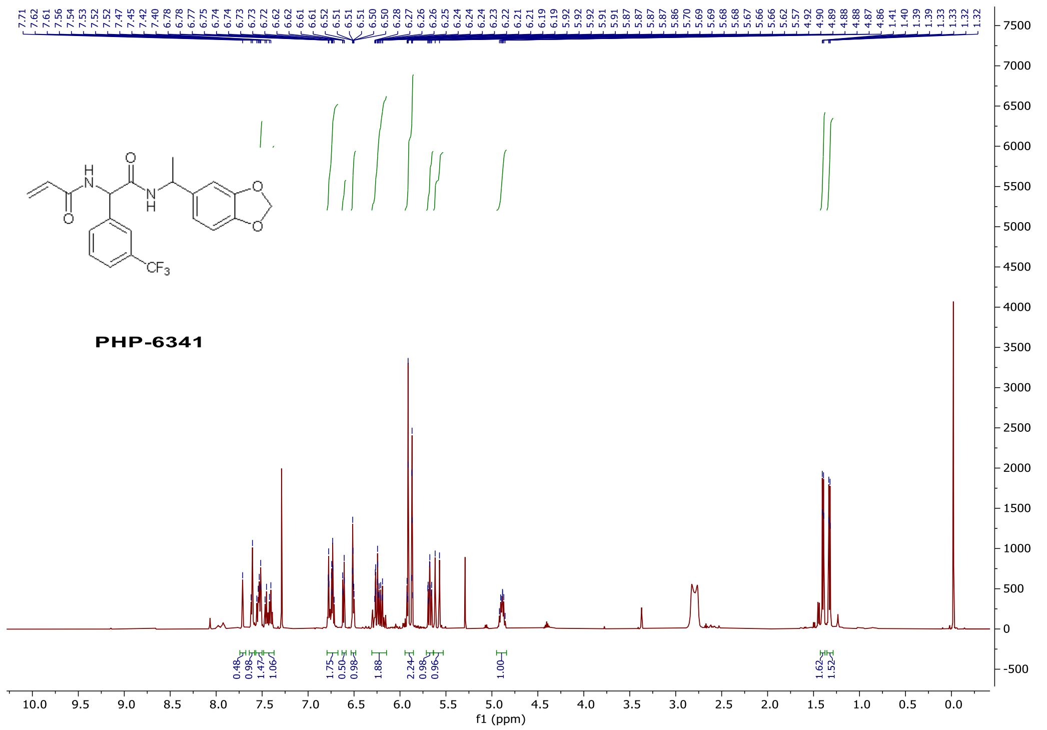

Supplementary Figure 1019. ^{1}H NMR (400 MHz, $CDCl_3$ + one drop of CD_3OD) of compound **PHP-6341.**

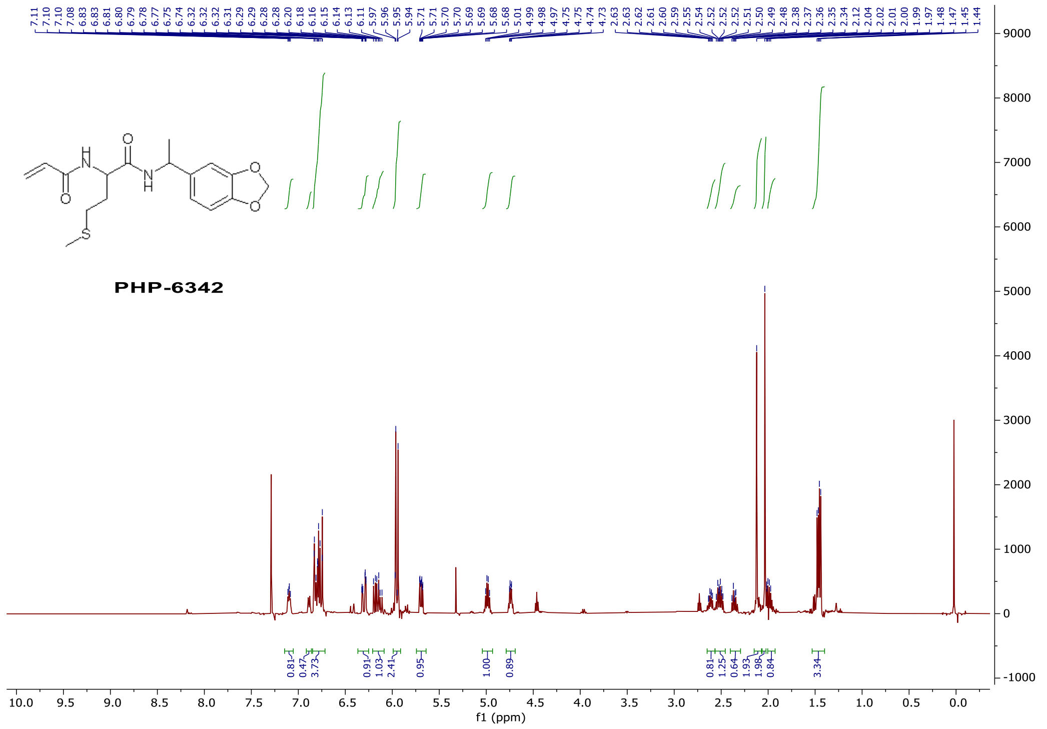

Supplementary Figure 1020. ^{1}H NMR (400 MHz, $CDCl_3$ + one drop of CD_3OD) of compound **PHP-6342.**

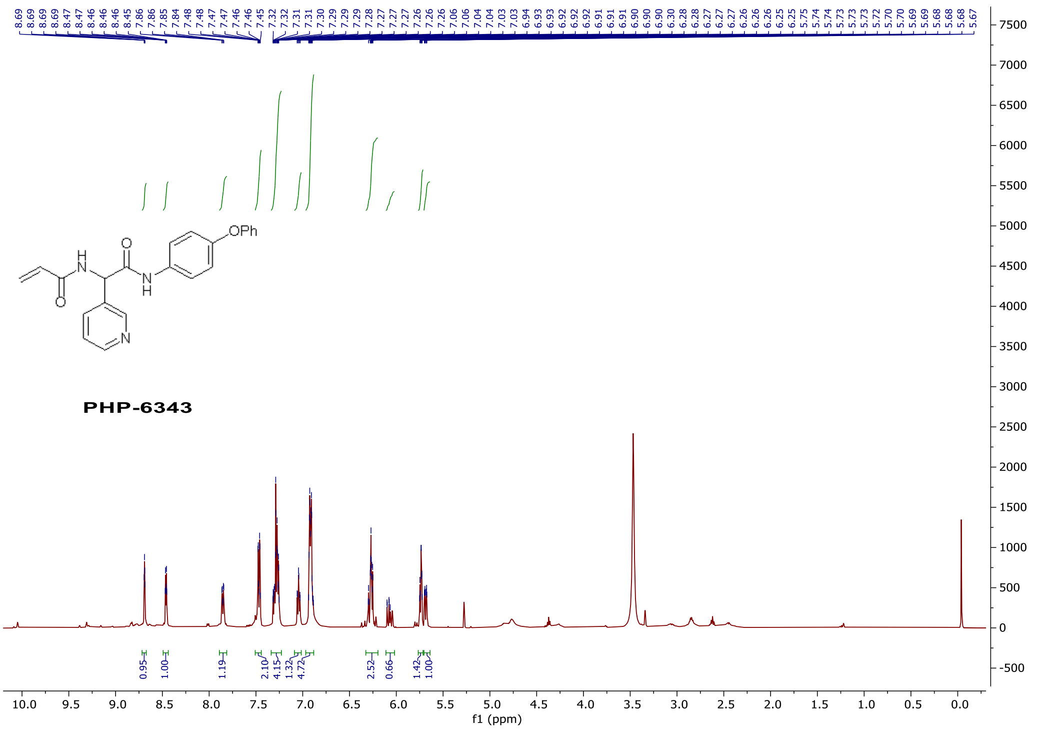

Supplementary Figure 1021. ^{1}H NMR (400 MHz, $CDCl_3$ + one drop of CD_3OD) of compound **PHP-6343.**

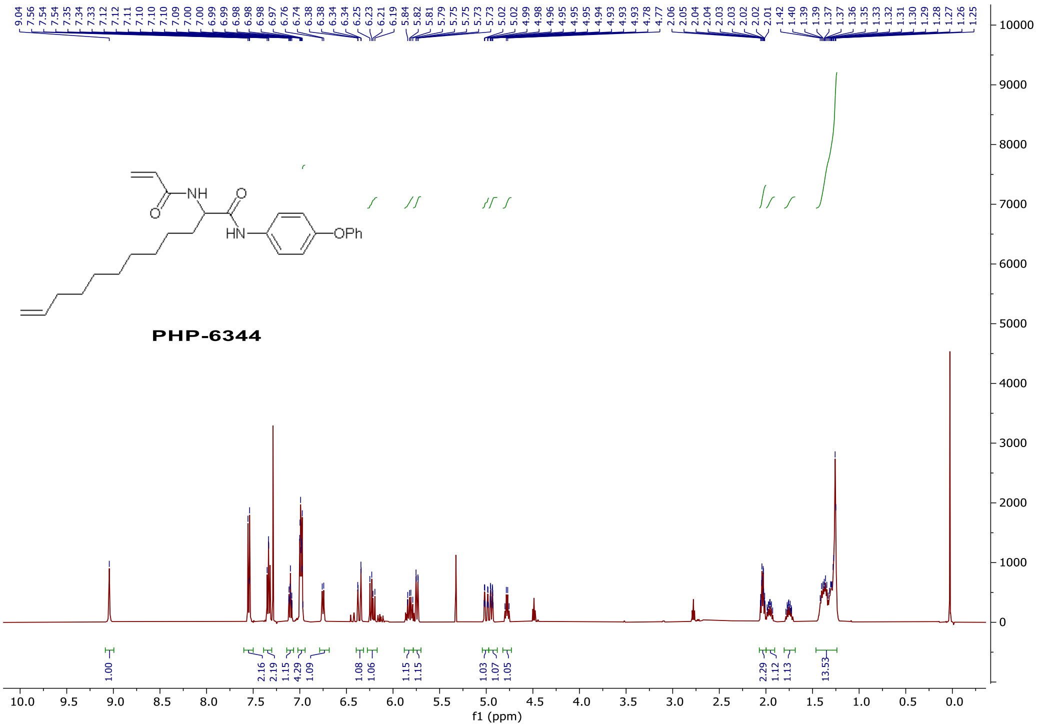

Supplementary Figure 1022. ^{1}H NMR (400 MHz, $CDCl_3$ + one drop of CD_3OD) of compound **PHP-6344.**

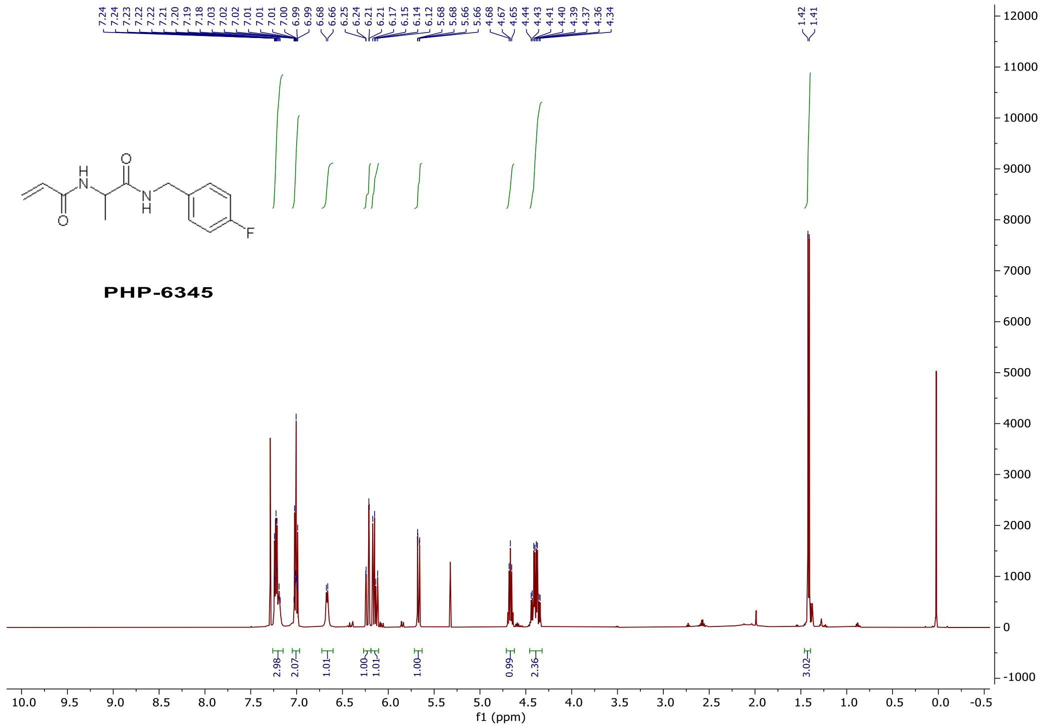

Supplementary Figure 1023. ^{1}H NMR (400 MHz, $CDCl_3$ + one drop of CD_3OD) of compound **PHP-6345.**

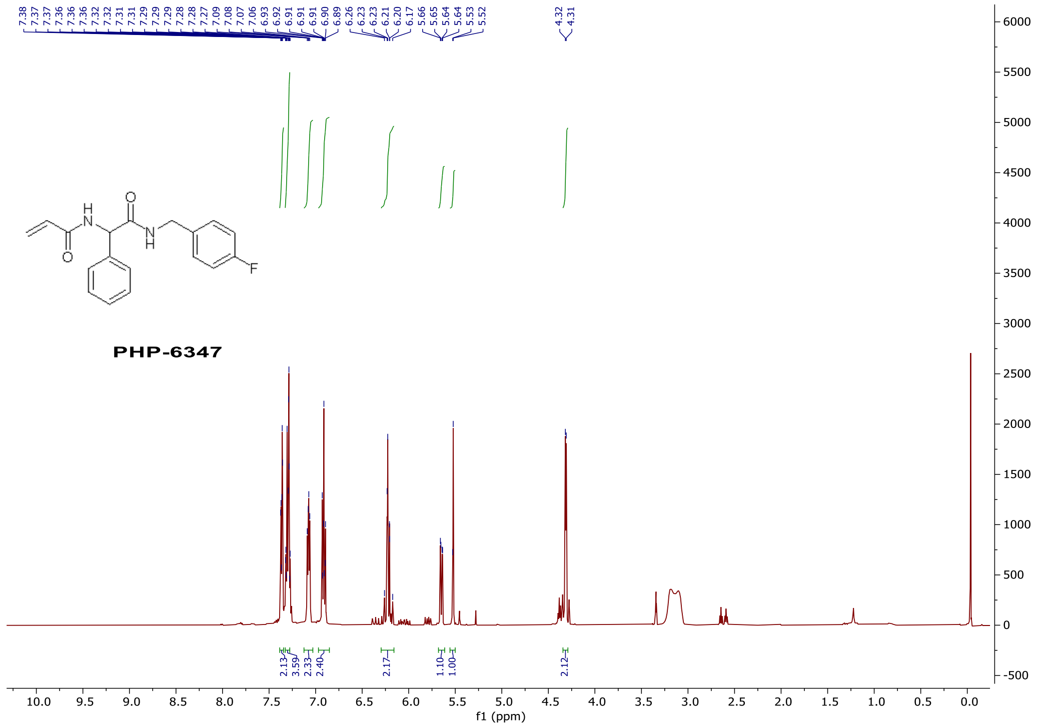

Supplementary Figure 1024. ^{1}H NMR (400 MHz, $CDCl_3$ + one drop of CD_3OD) of compound **PHP-6347.**

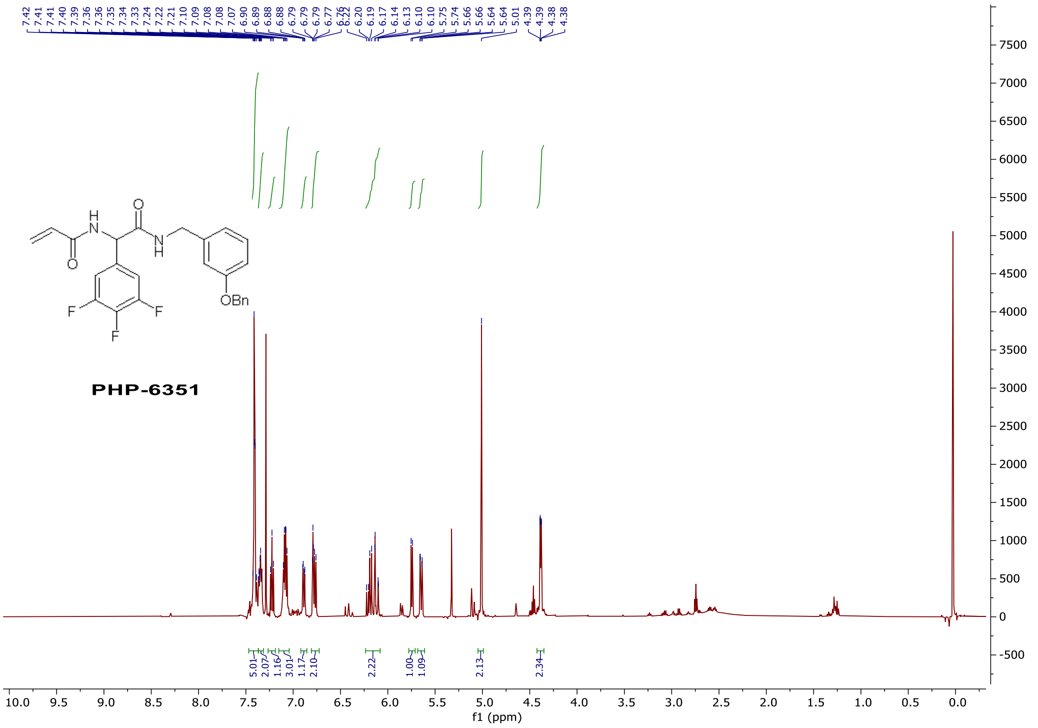

Supplementary Figure 1025. ^1H NMR (400 MHz, $CDCl_3$ + one drop of CD_3OD) of compound **PHP-6351.**

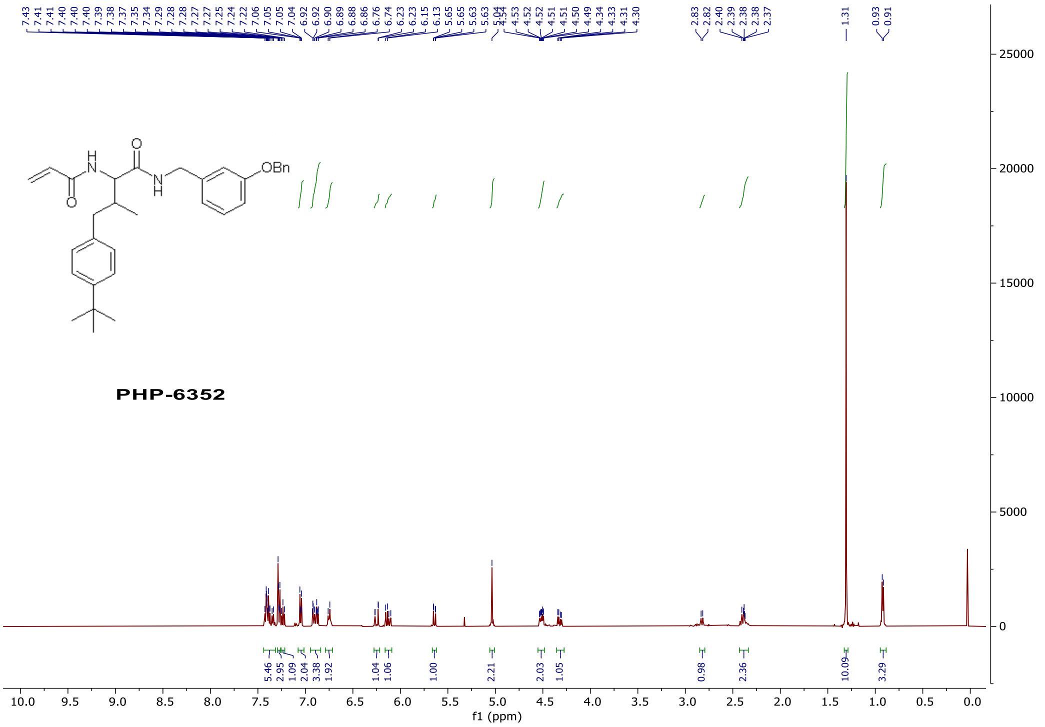

Supplementary Figure 1026. ^1H NMR (400 MHz, $CDCl_3$ + one drop of CD_3OD) of compound **PHP-6352.**

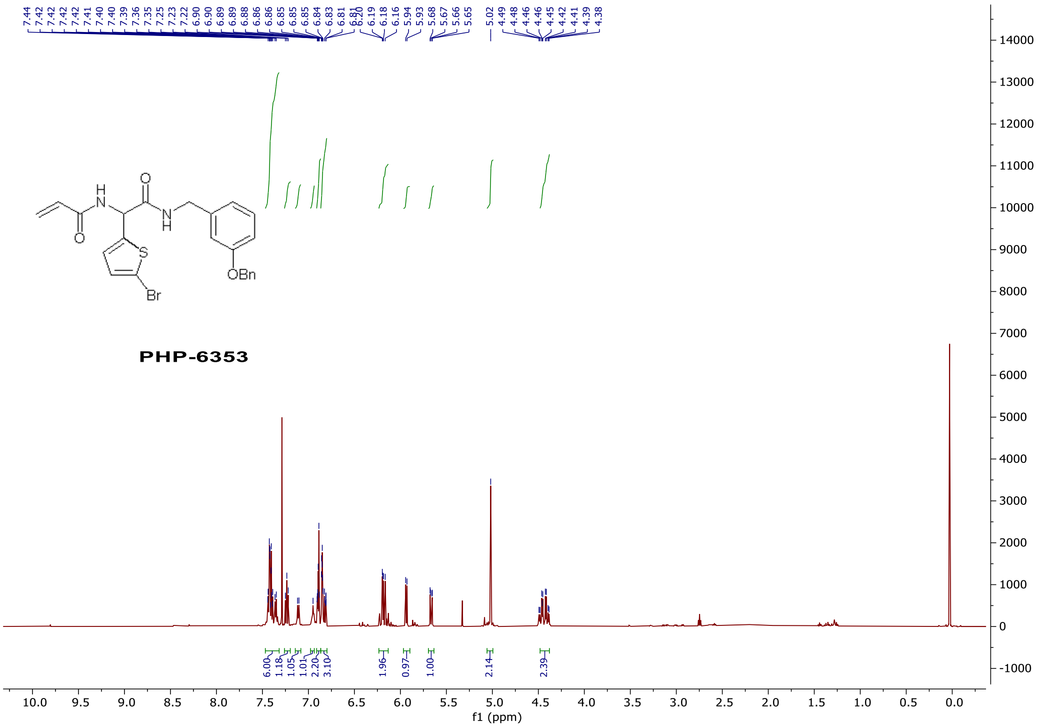

Supplementary Figure 1027. ^1H NMR (400 MHz, $CDCl_3$ + one drop of CD_3OD) of compound **PHP-6353.**

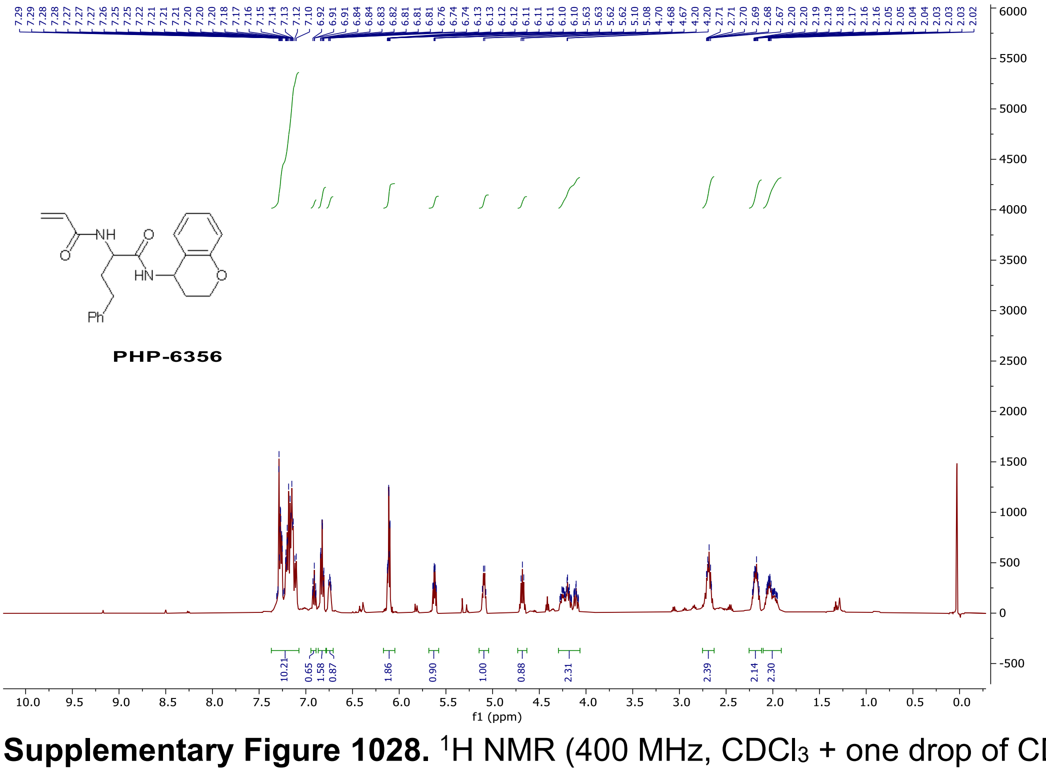

Supplementary Figure 1028. ^1H NMR (400 MHz, $CDCl_3$ + one drop of CD_3OD) of compound **PHP-6356.**

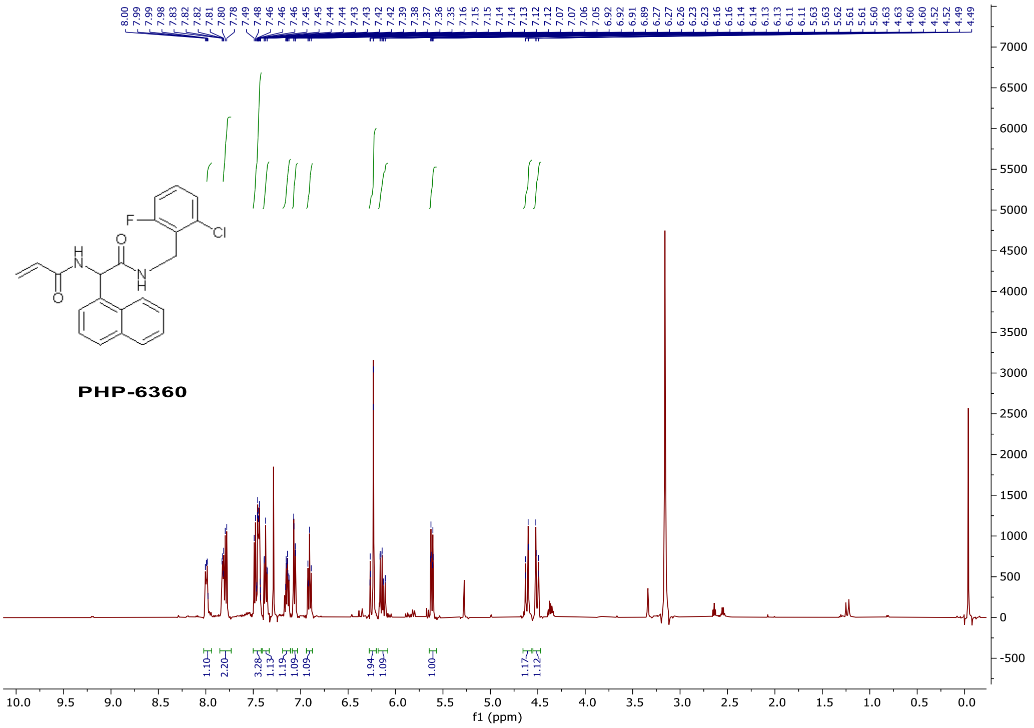

Supplementary Figure 1029. ^{1}H NMR (400 MHz, $CDCl_3$ + one drop of CD_3OD) of compound **PHP-6360.**

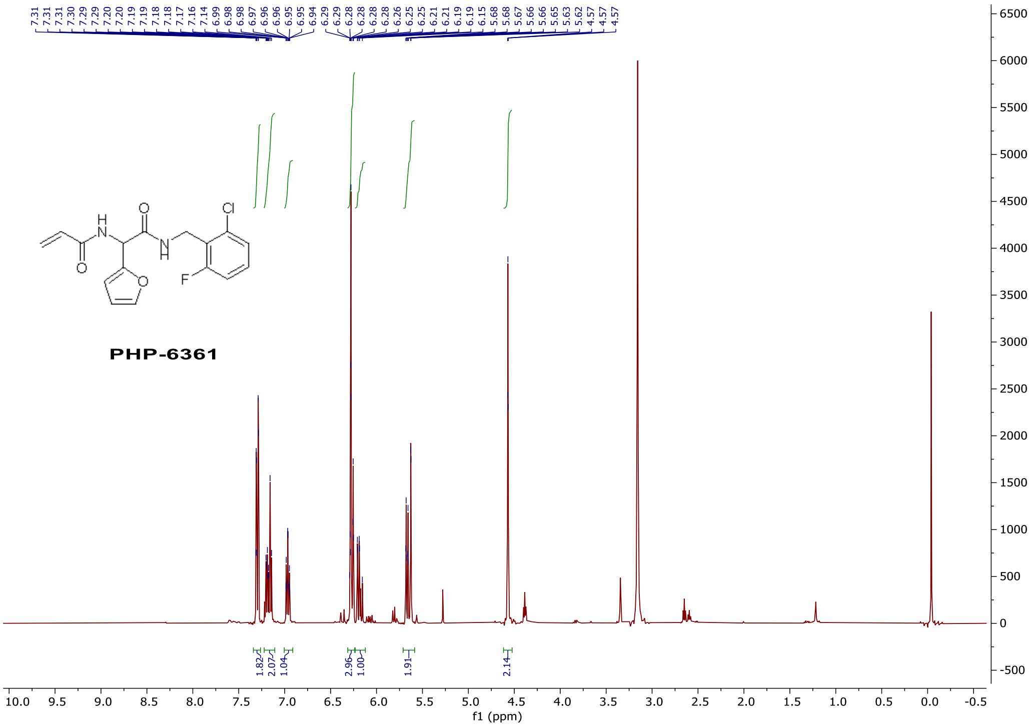

Supplementary Figure 1030. ^{1}H NMR (400 MHz, $CDCl_3$ + one drop of CD_3OD) of compound **PHP-6361.**

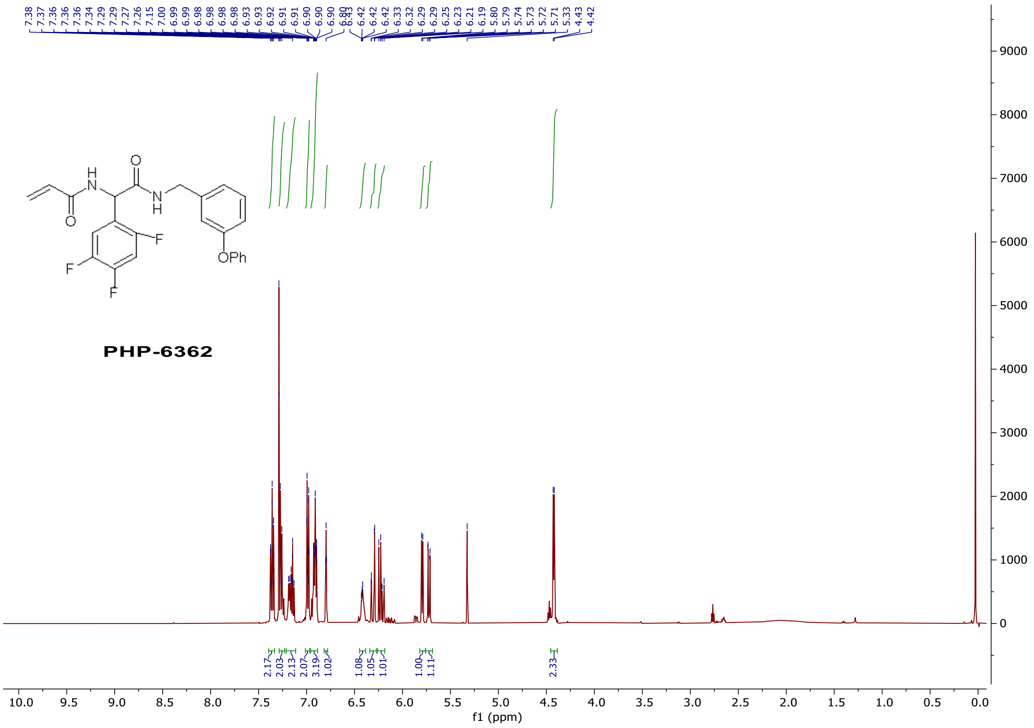

Supplementary Figure 1031. ^{1}H NMR (400 MHz, $CDCl_3$ + one drop of CD_3OD) of compound **PHP-6362.**

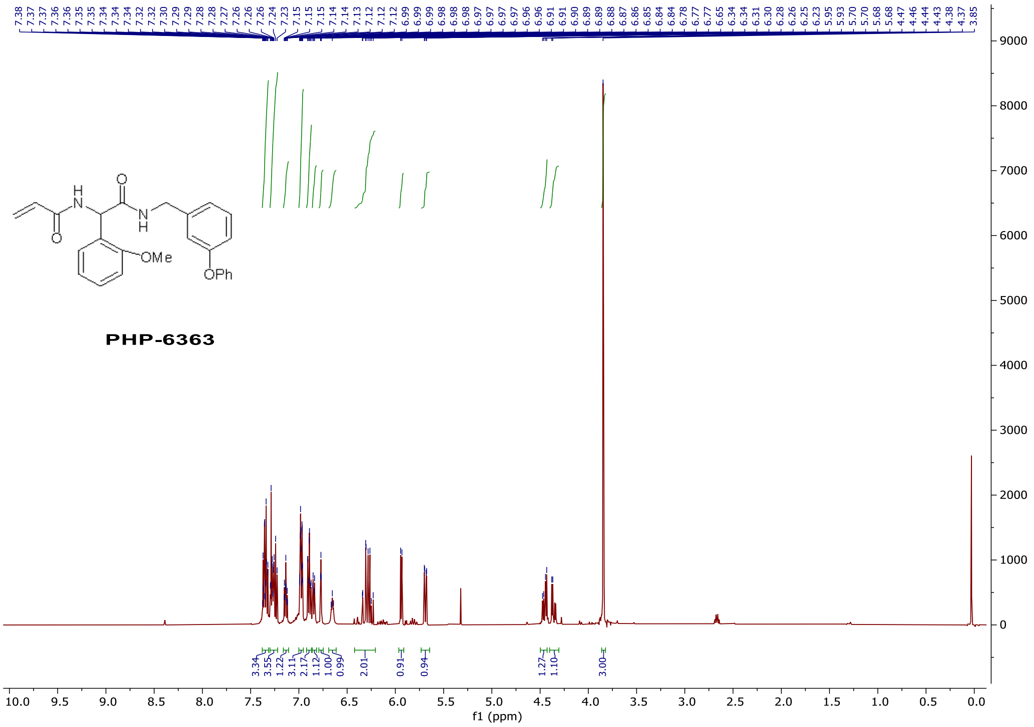

Supplementary Figure 1031. ^{1}H NMR (400 MHz, $CDCl_3$ + one drop of CD_3OD) of compound **PHP-6363.**

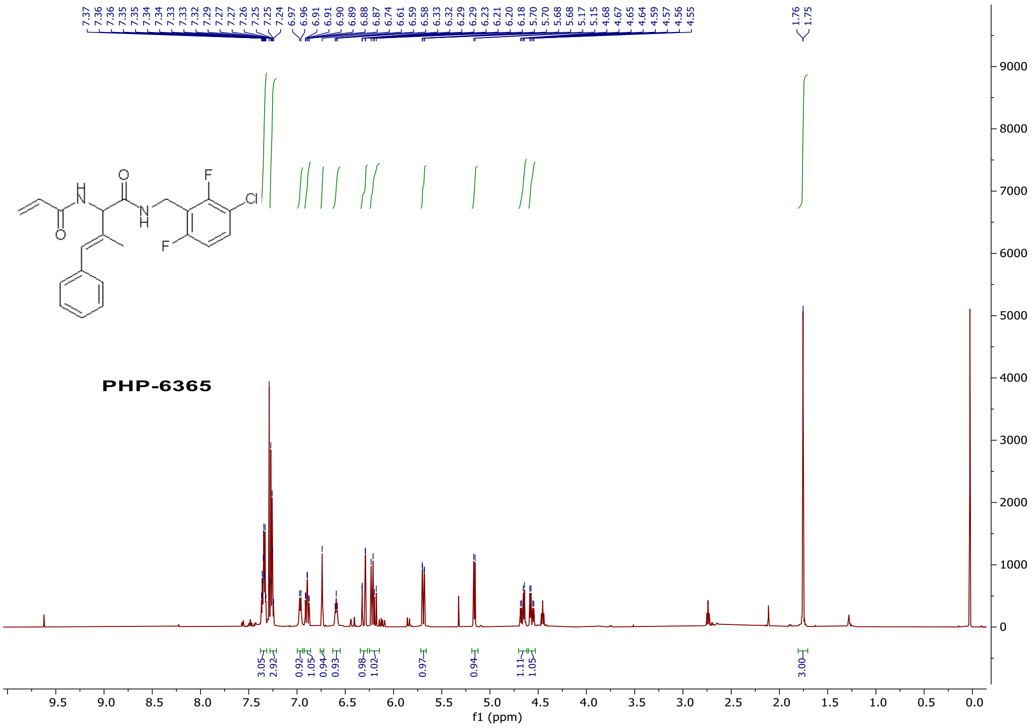

Supplementary Figure 1032. ^{1}H NMR (400 MHz, $CDCl_3$ + one drop of CD_3OD) of compound **PHP-6365.**

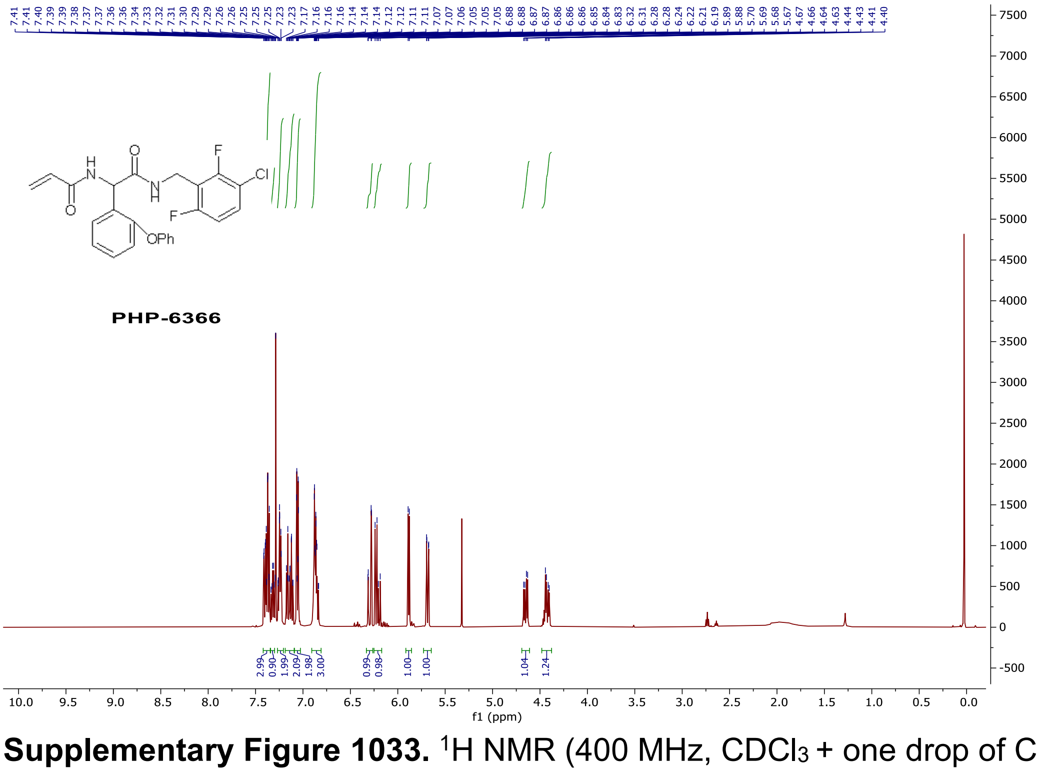

Supplementary Figure 1033. ^{1}H NMR (400 MHz, $CDCl_3$ + one drop of CD_3OD) of compound **PHP-6366.**

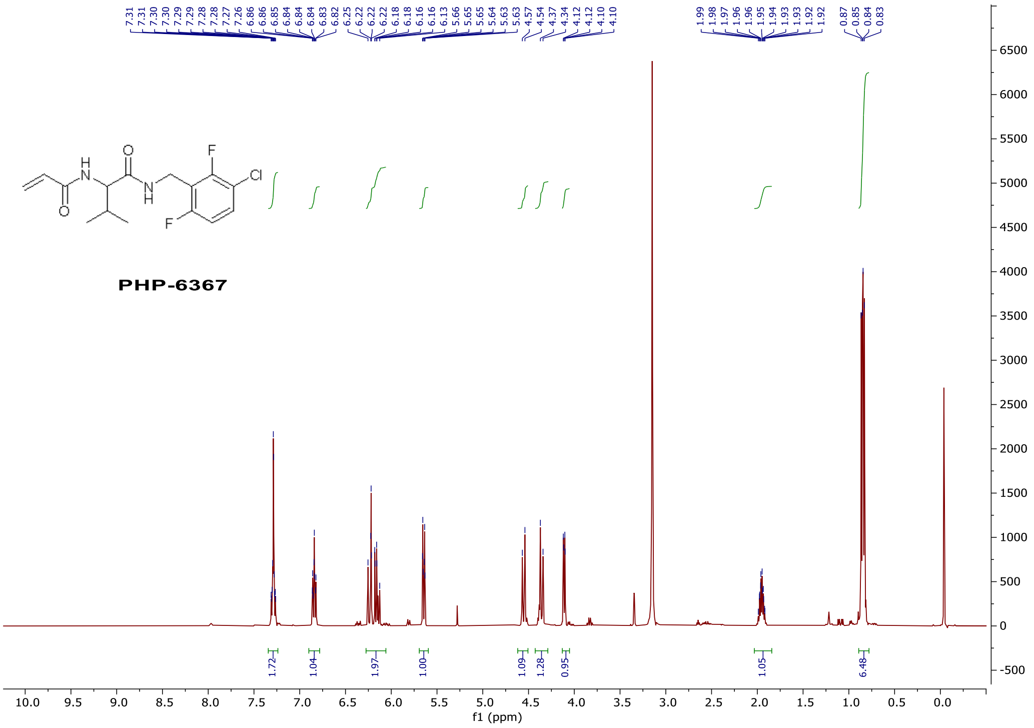

Supplementary Figure 1034. ^{1}H NMR (400 MHz, $CDCl_3$ + one drop of CD_3OD) of compound **PHP-6367.**

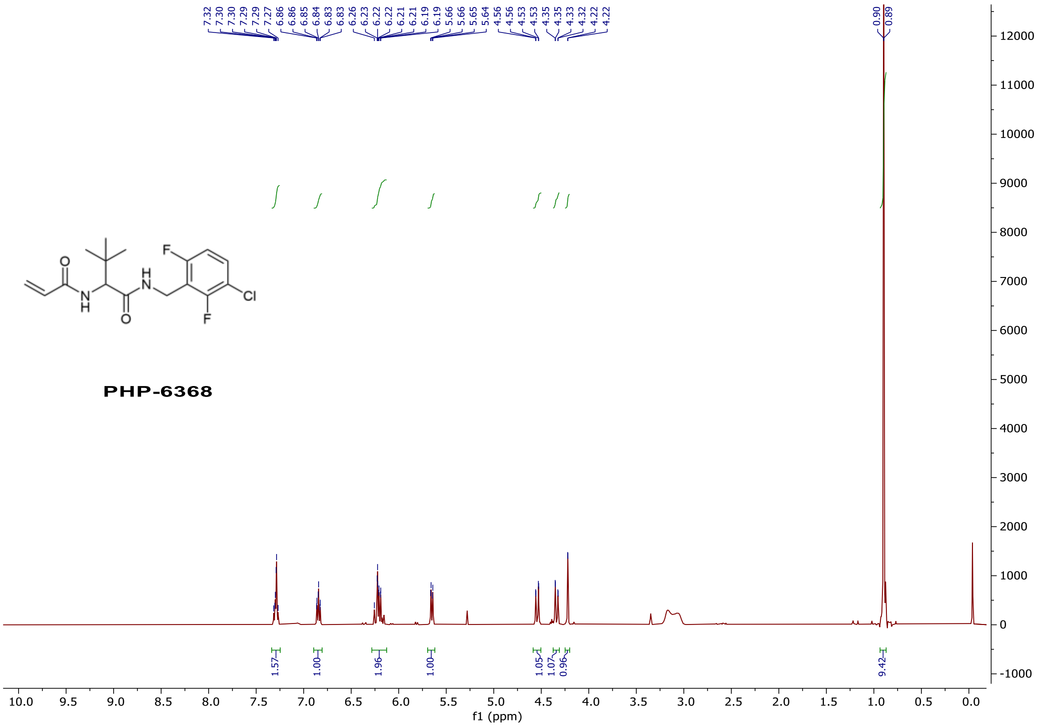

Supplementary Figure 1035. ^{1}H NMR (400 MHz, $CDCl_3$ + one drop of CD_3OD) of compound **PHP-6368.**

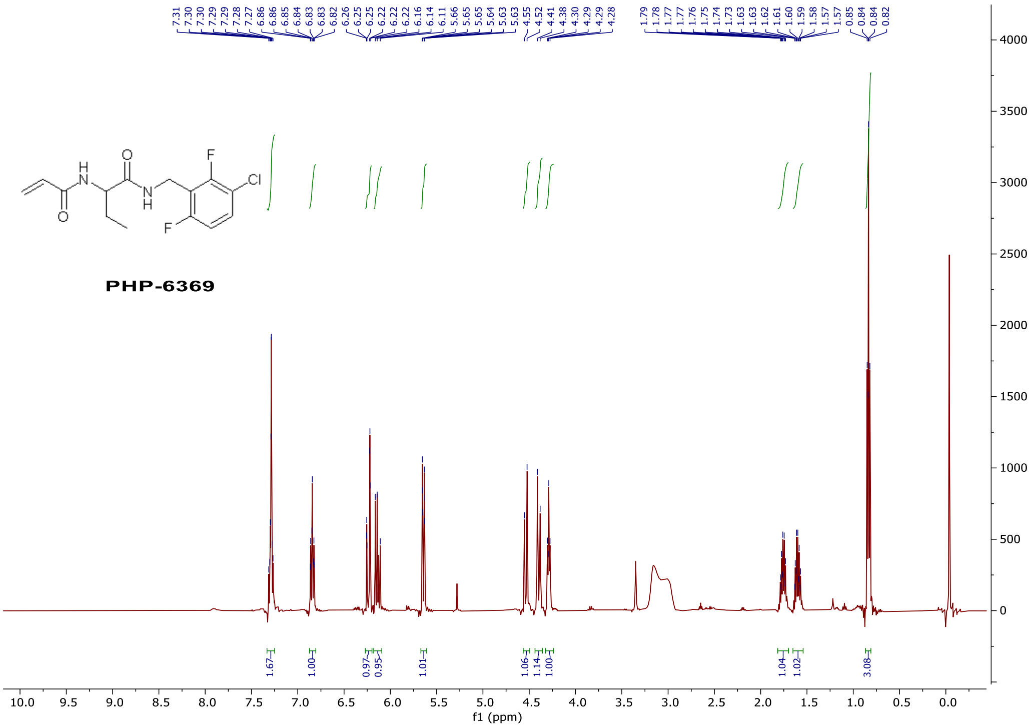

Supplementary Figure 1036. ^{1}H NMR (400 MHz, $CDCl_3$ + one drop of CD_3OD) of compound **PHP-6369.**

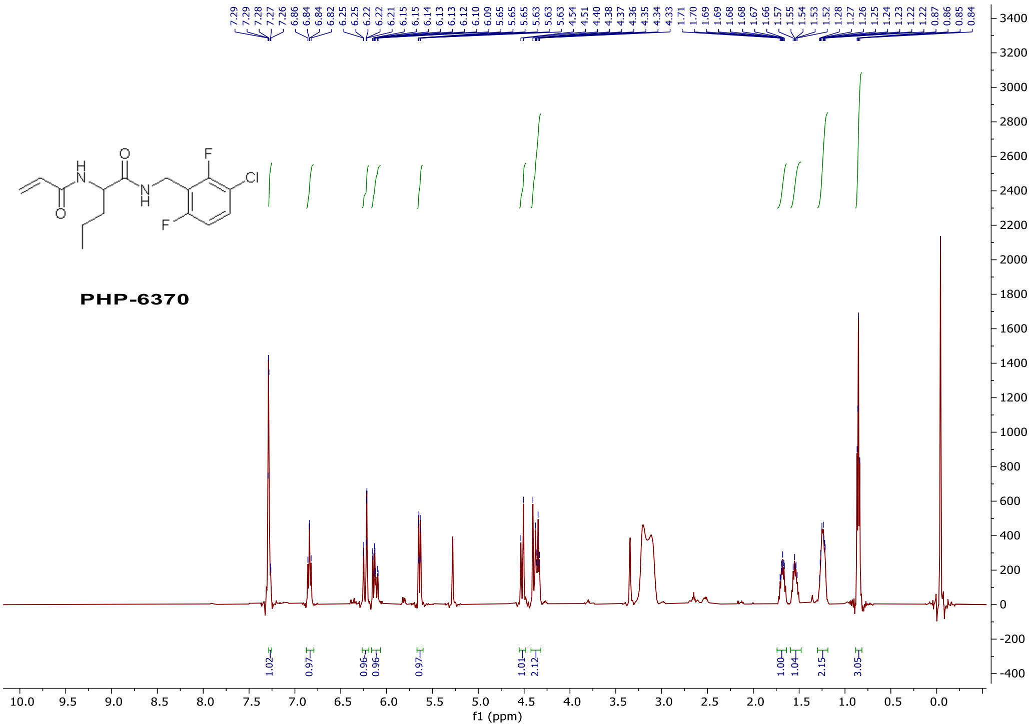

Supplementary Figure 1037. ^{1}H NMR (400 MHz, $CDCl_3$ + one drop of CD_3OD) of compound **PHP-6370.**

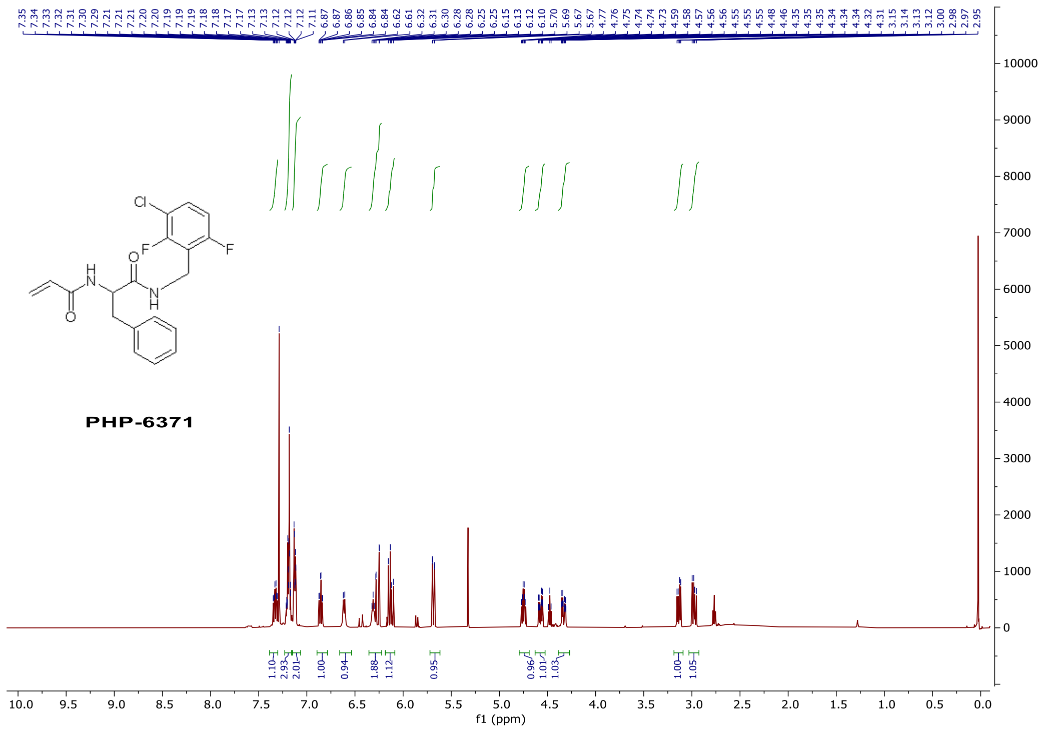

Supplementary Figure 1038. ^{1}H NMR (400 MHz, $CDCl_3$ + one drop of CD_3OD) of compound **PHP-6371.**

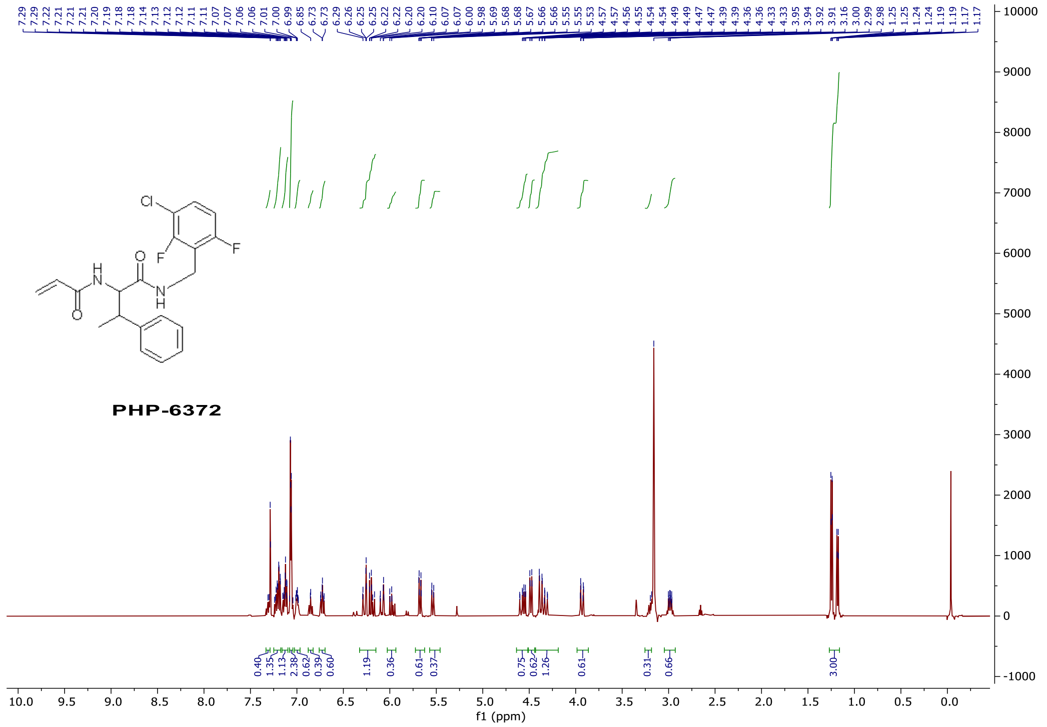

Supplementary Figure 1039. ^{1}H NMR (400 MHz, $CDCl_3$ + one drop of CD_3OD) of compound **PHP-6372.**

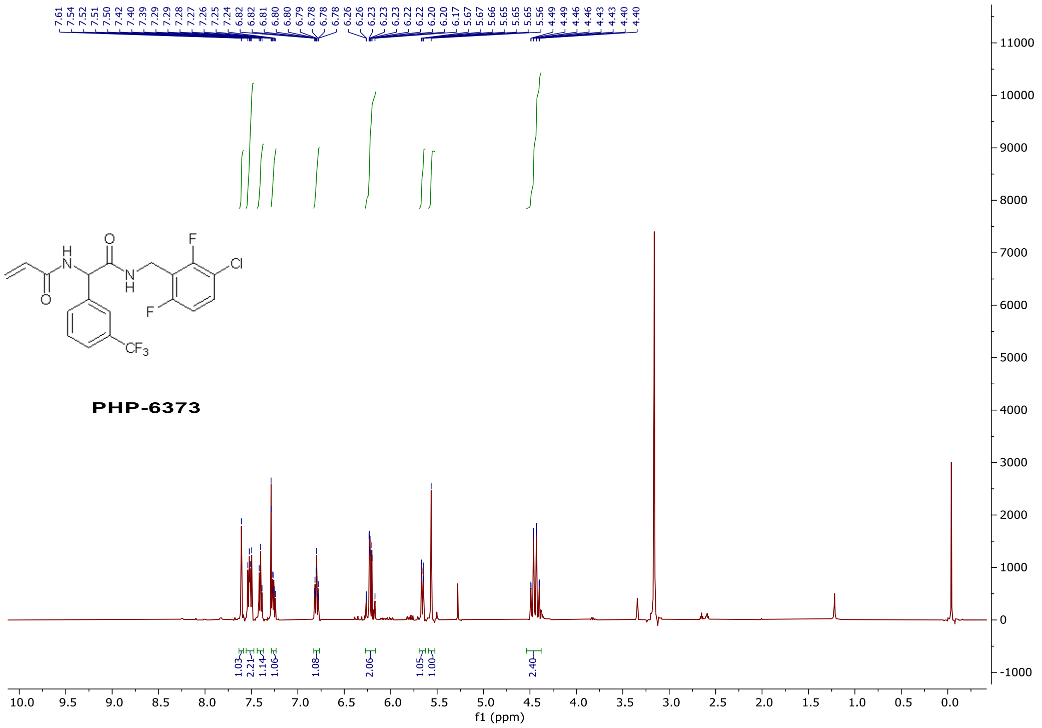

Supplementary Figure 1040. ^{1}H NMR (400 MHz, $CDCl_3$ + one drop of CD_3OD) of compound **PHP-6373.**

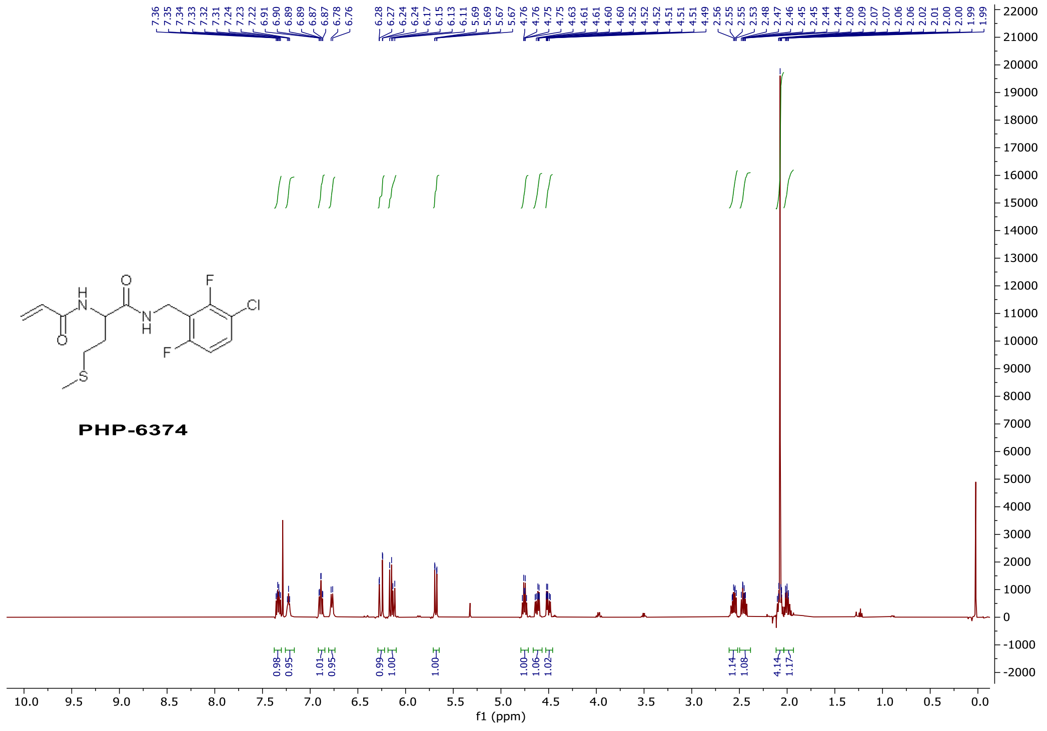

Supplementary Figure 1041. ^{1}H NMR (400 MHz, $CDCl_3$ + one drop of CD_3OD) of compound **PHP-6374.**

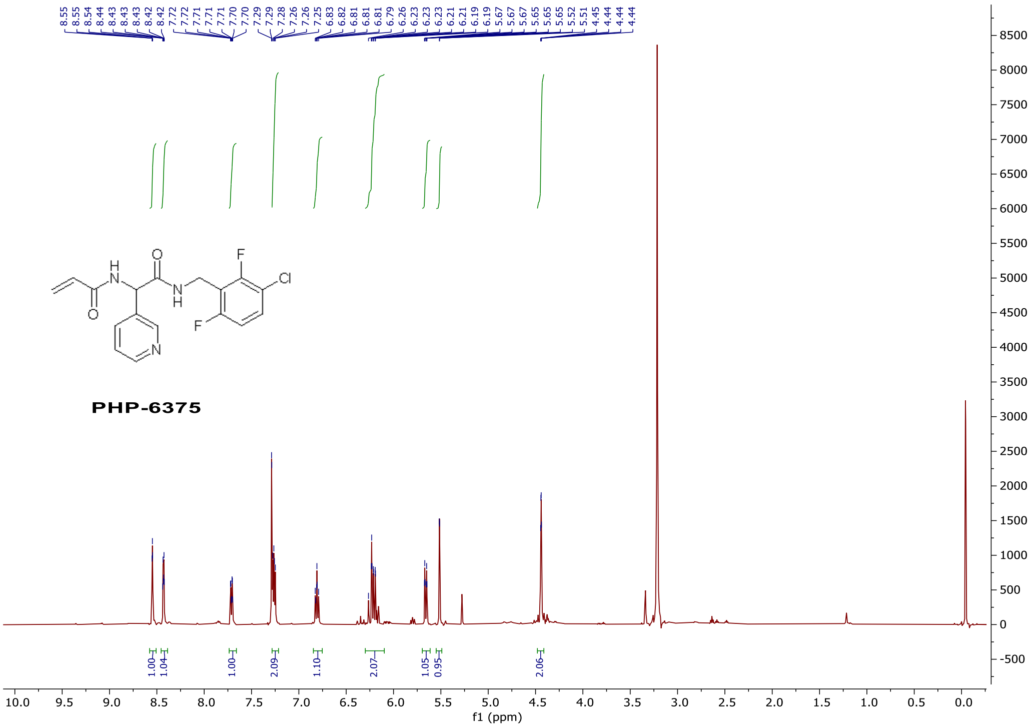

Supplementary Figure 1042. ^{1}H NMR (400 MHz, $CDCl_3$ + one drop of CD_3OD) of compound **PHP-6375.**

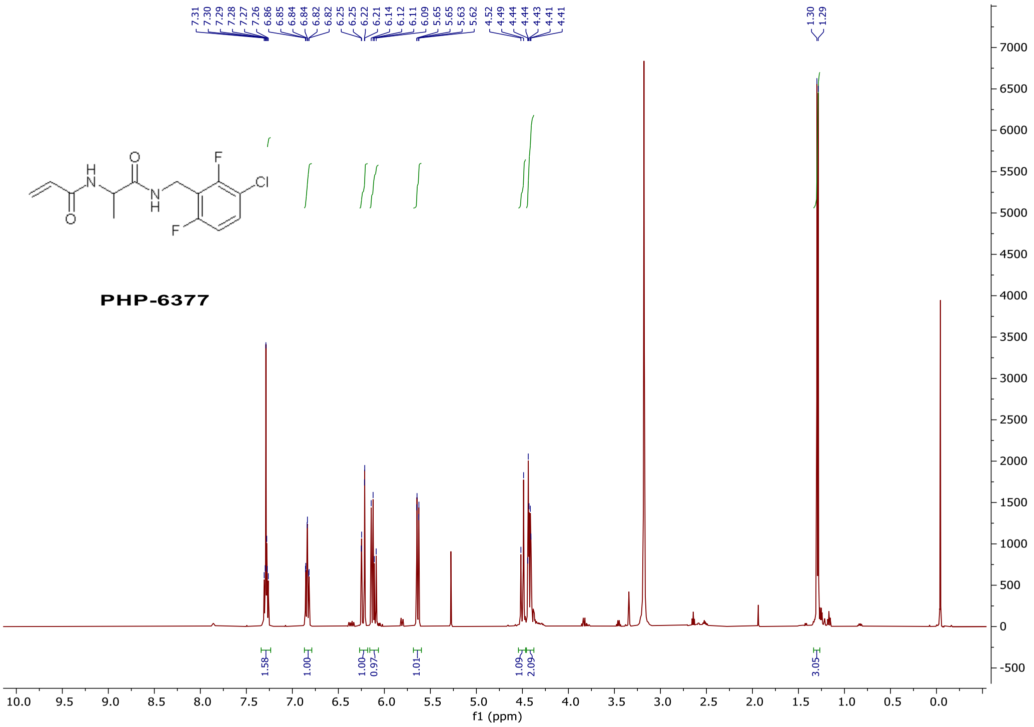

Supplementary Figure 1043. ^{1}H NMR (400 MHz, $CDCl_3$ + one drop of CD_3OD) of compound **PHP-6377.**

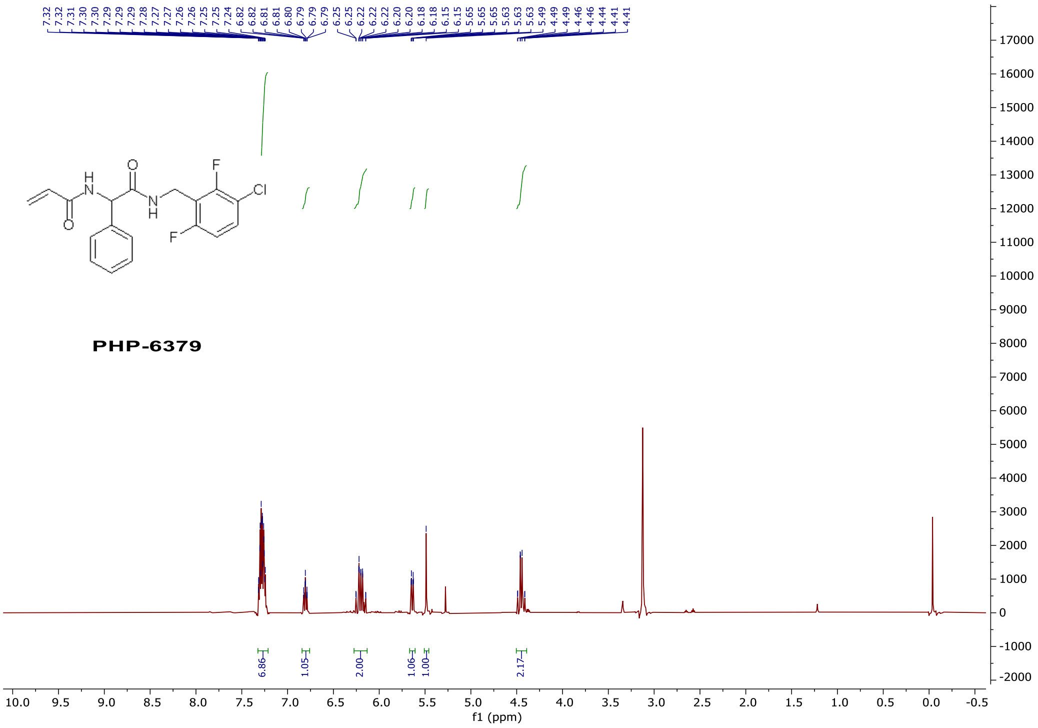

Supplementary Figure 1044. ^{1}H NMR (400 MHz, $CDCl_3$ + one drop of CD_3OD) of compound **PHP-6379**.

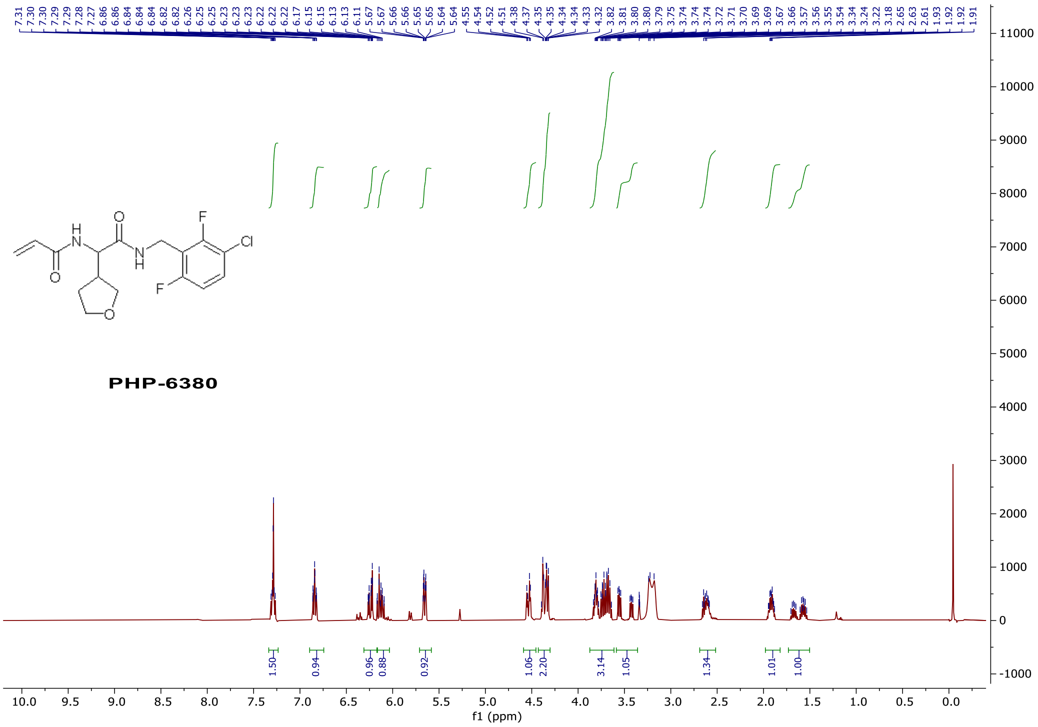

Supplementary Figure 1045. ^{1}H NMR (400 MHz, $CDCl_3$ + one drop of CD_3OD) of compound **PHP-6380**.

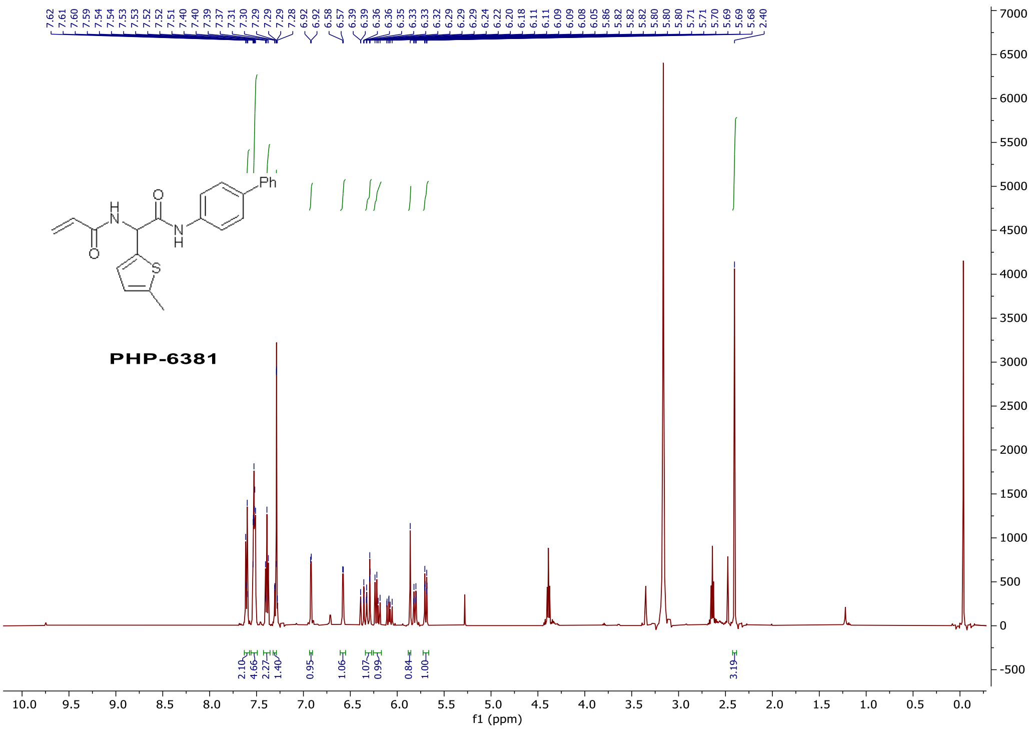

Supplementary Figure 1046. ^{1}H NMR (400 MHz, $CDCl_3$ + one drop of CD_3OD) of compound **PHP-6381.**

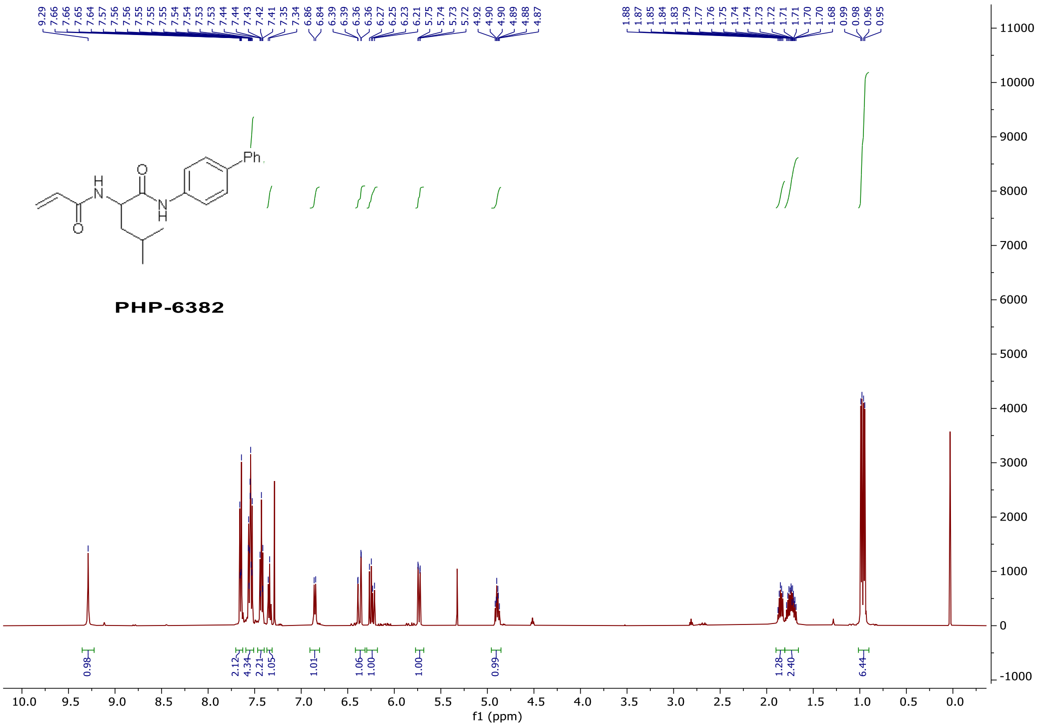

Supplementary Figure 1047. ^{1}H NMR (400 MHz, $CDCl_3$ + one drop of CD_3OD) of compound **PHP-6382.**

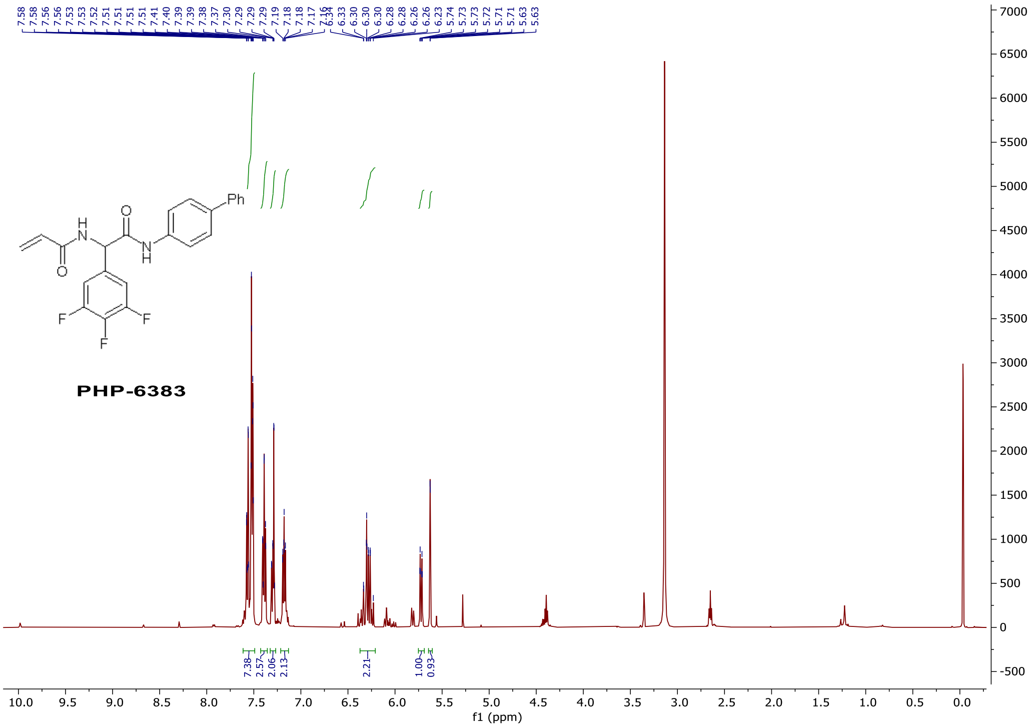

Supplementary Figure 1048. ^{1}H NMR (400 MHz, $CDCl_3$ + one drop of CD_3OD) of compound **PHP-6383.**

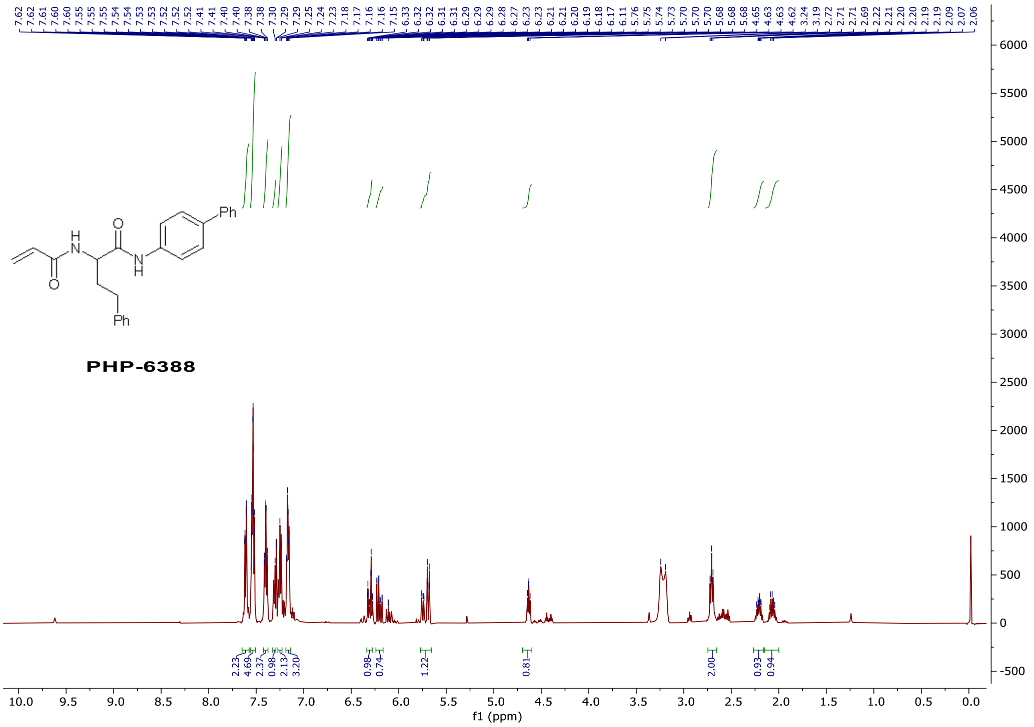

Supplementary Figure 1049. ^{1}H NMR (400 MHz, $CDCl_3$ + one drop of CD_3OD) of compound **PHP-6388.**

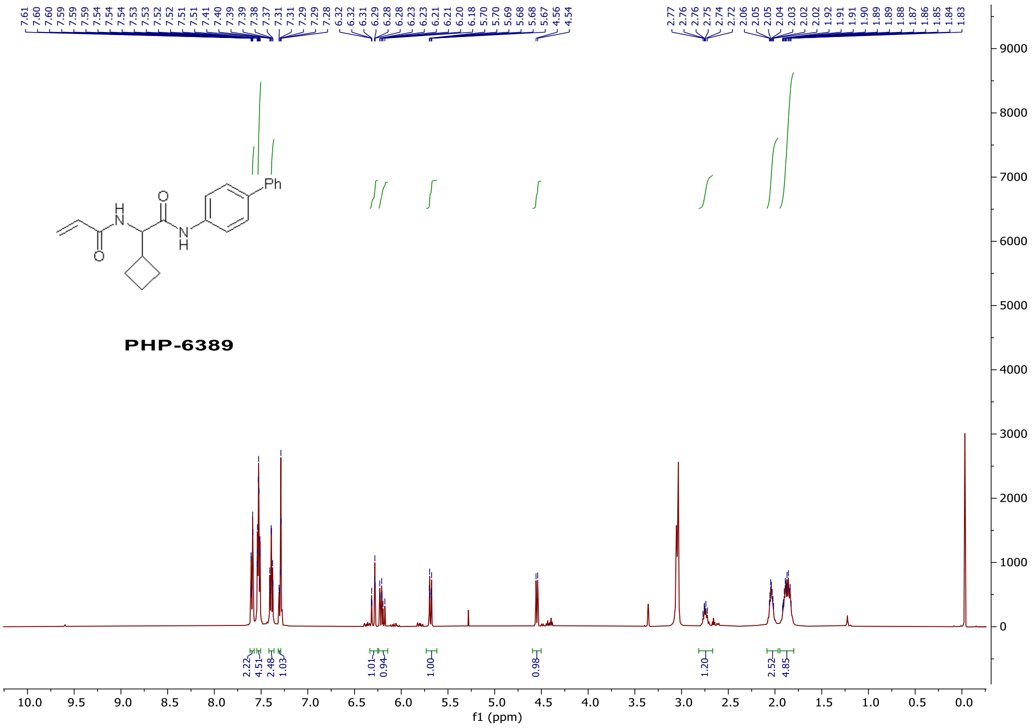

Supplementary Figure 1050. ^{1}H NMR (400 MHz, $CDCl_3$ + one drop of CD_3OD) of compound **PHP-6389.**

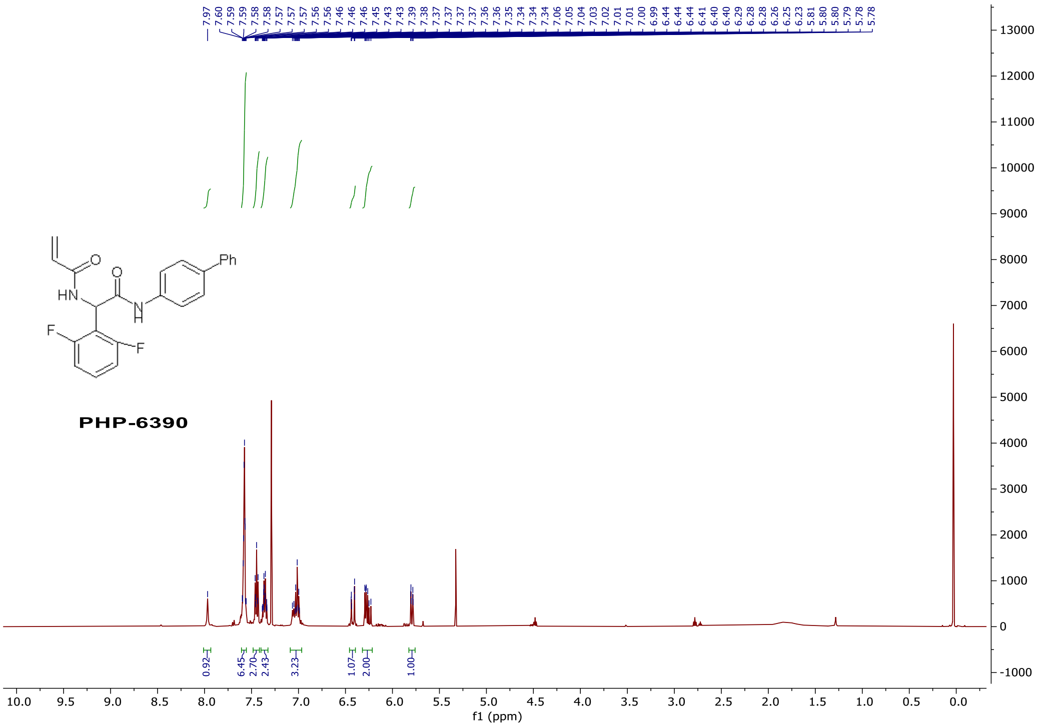

Supplementary Figure 1051. ^{1}H NMR (400 MHz, $CDCl_3$ + one drop of CD_3OD) of compound **PHP-6390.**

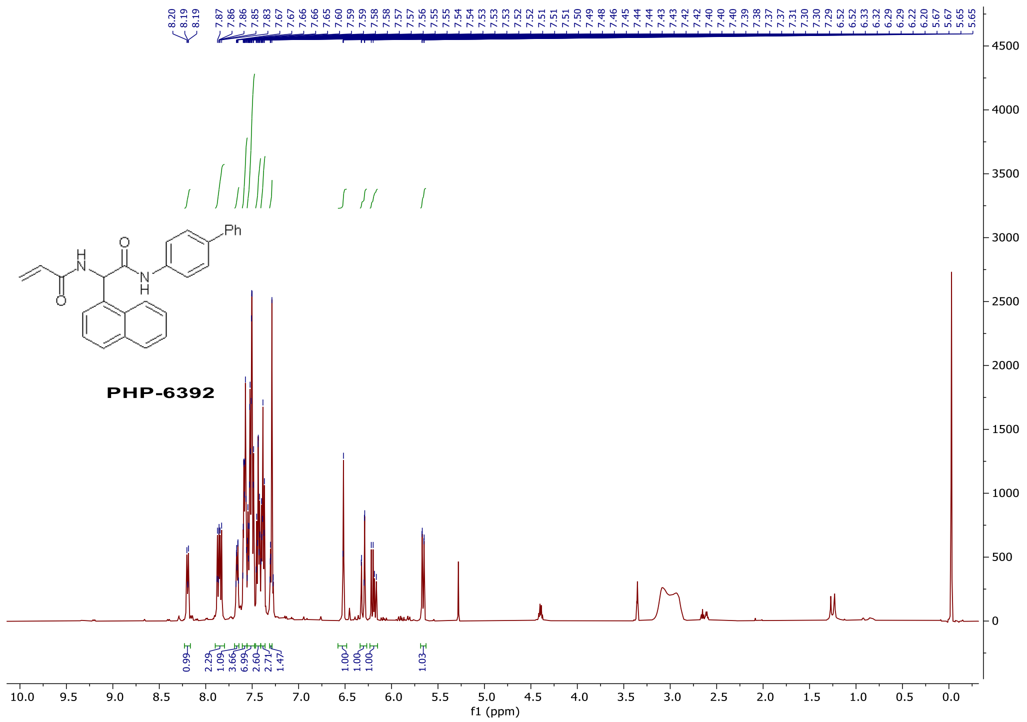

Supplementary Figure 1052. ^{1}H NMR (400 MHz, $CDCl_3$ + one drop of CD_3OD) of compound **PHP-6392.**

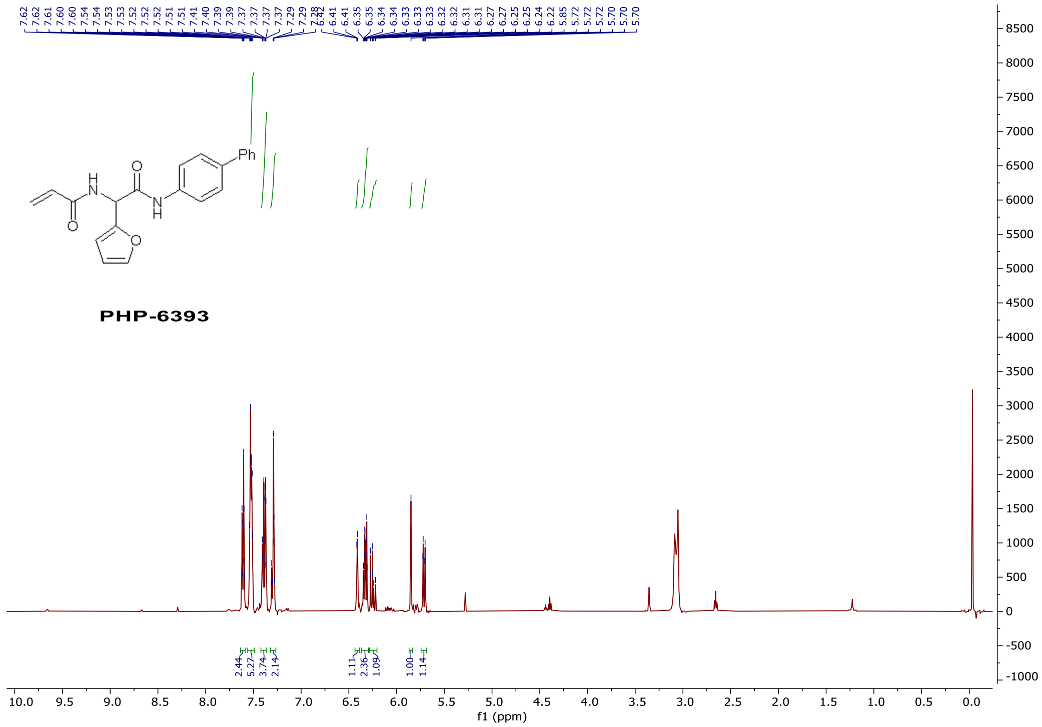

Supplementary Figure 1053. ^{1}H NMR (400 MHz, $CDCl_3$ + one drop of CD_3OD) of compound **PHP-6393.**

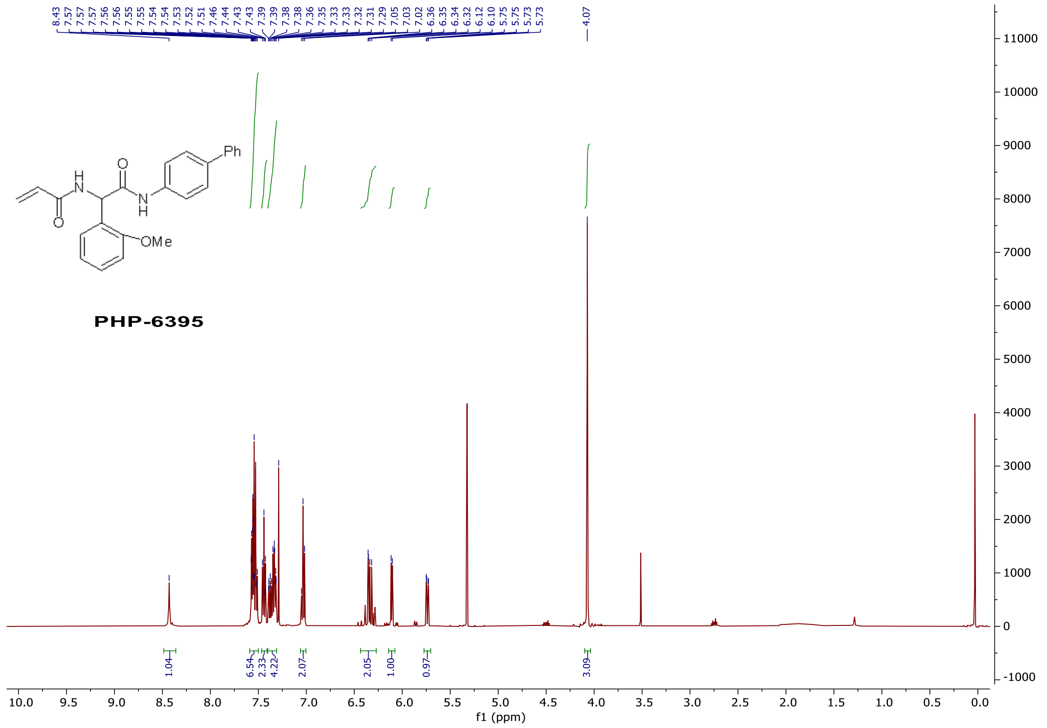

Supplementary Figure 1054. ^{1}H NMR (400 MHz, $CDCl_3$ + one drop of CD_3OD) of compound **PHP-6395.**

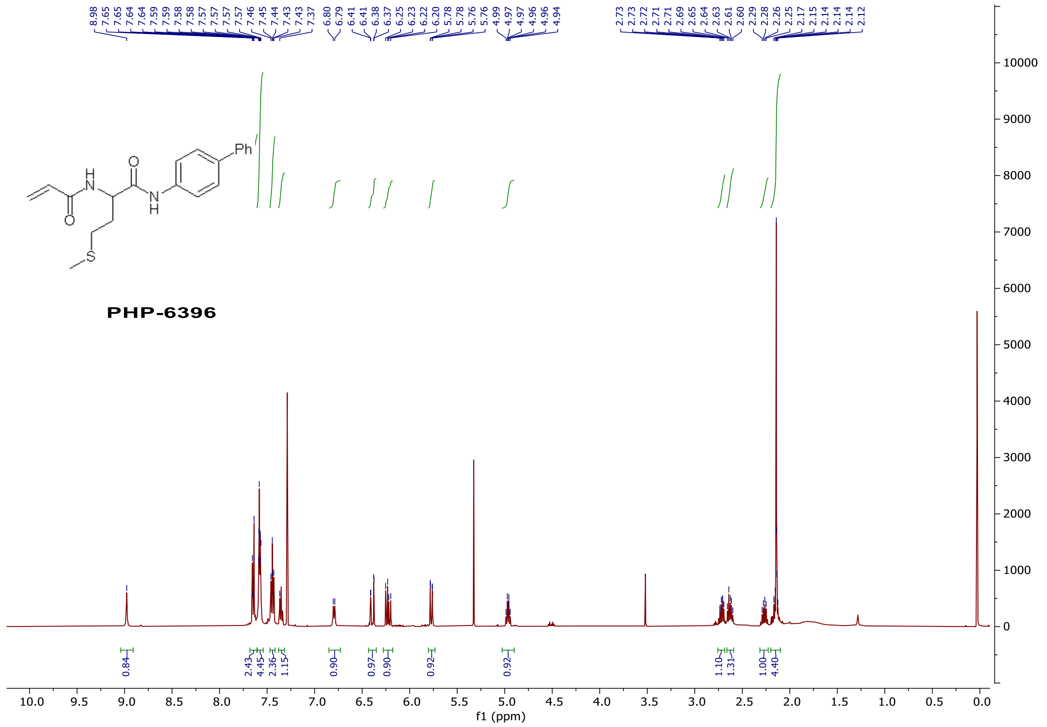

Supplementary Figure 1055. ^{1}H NMR (400 MHz, $CDCl_3$ + one drop of CD_3OD) of compound **PHP-6396.**

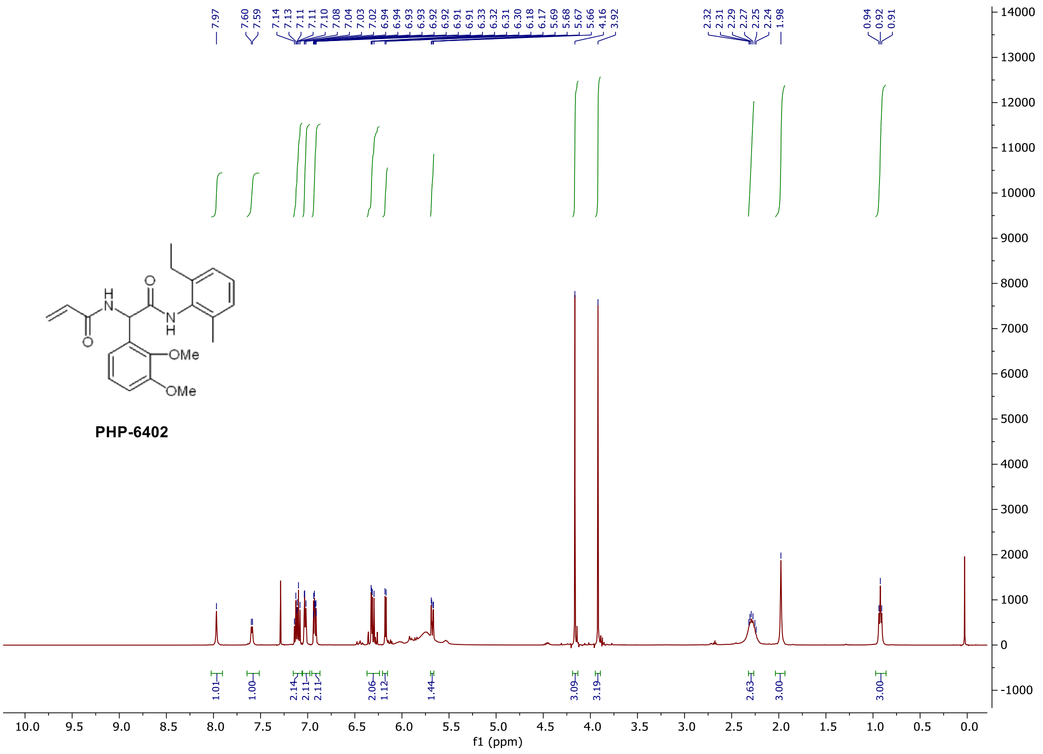

Supplementary Figure 1056. ^{1}H NMR (400 MHz, $CDCl_3$ + one drop of CD_3OD) of compound **PHP-6402.**

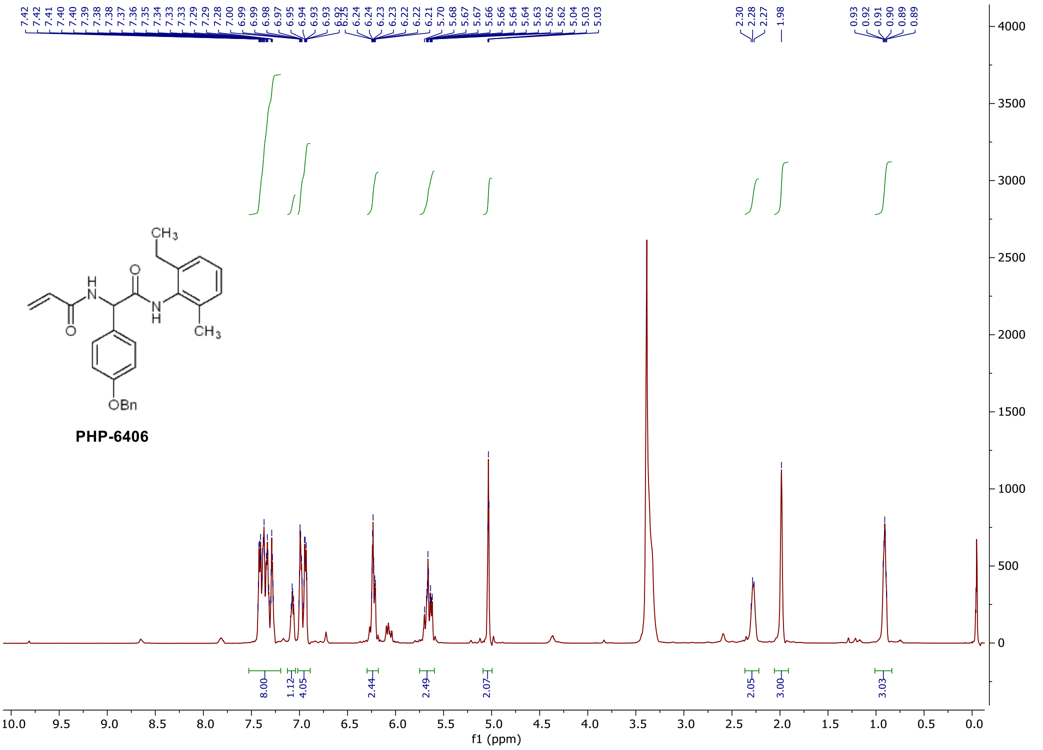

Supplementary Figure 1057. ^{1}H NMR (400 MHz, $CDCl_3$ + one drop of CD_3OD) of compound **PHP-6406.**

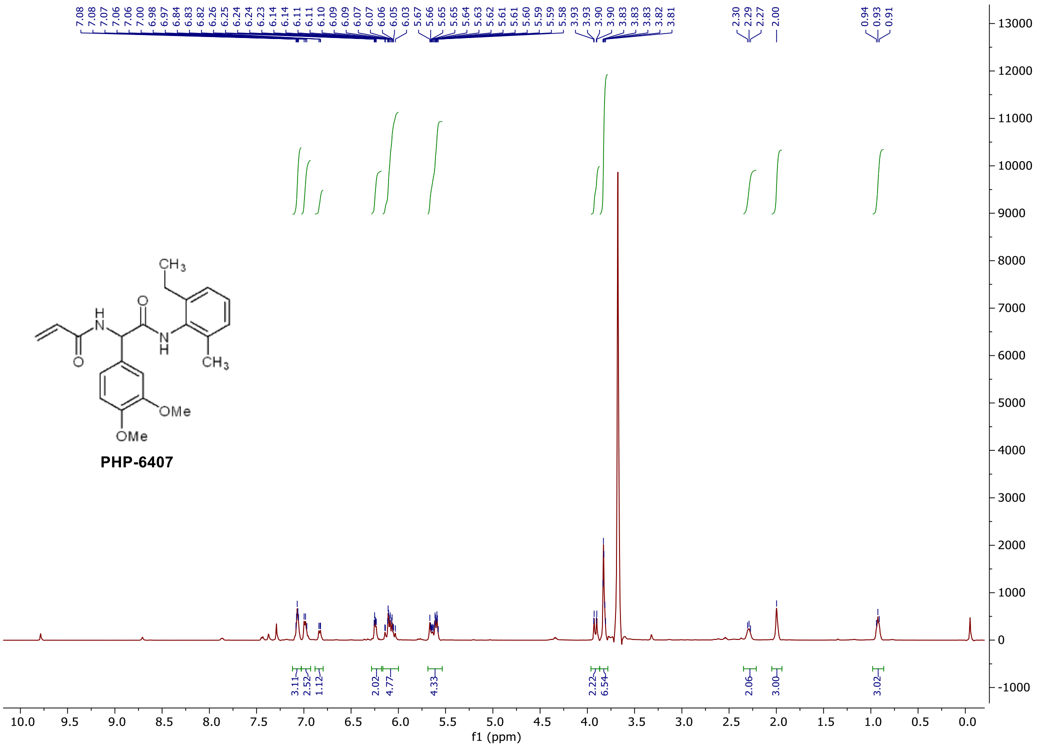

Supplementary Figure 1058. ^{1}H NMR (400 MHz, $CDCl_3$ + one drop of CD_3OD) of compound **PHP-6407**.

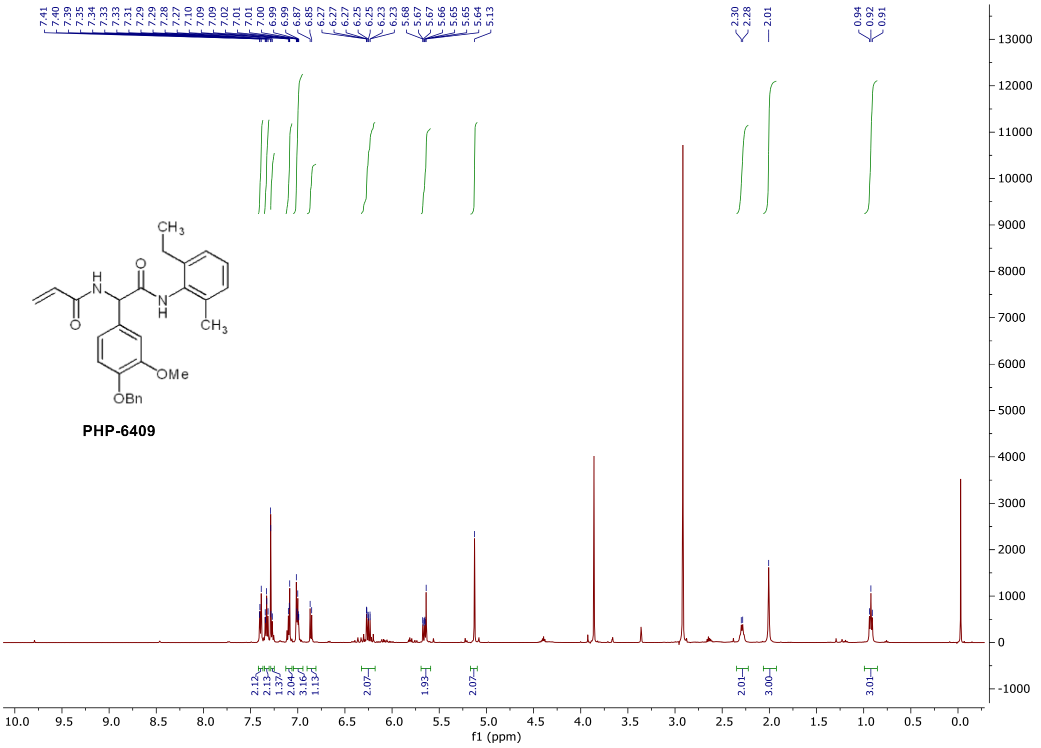

Supplementary Figure 1059. ^{1}H NMR (400 MHz, $CDCl_3$ + one drop of CD_3OD) of compound **PHP-6409**.

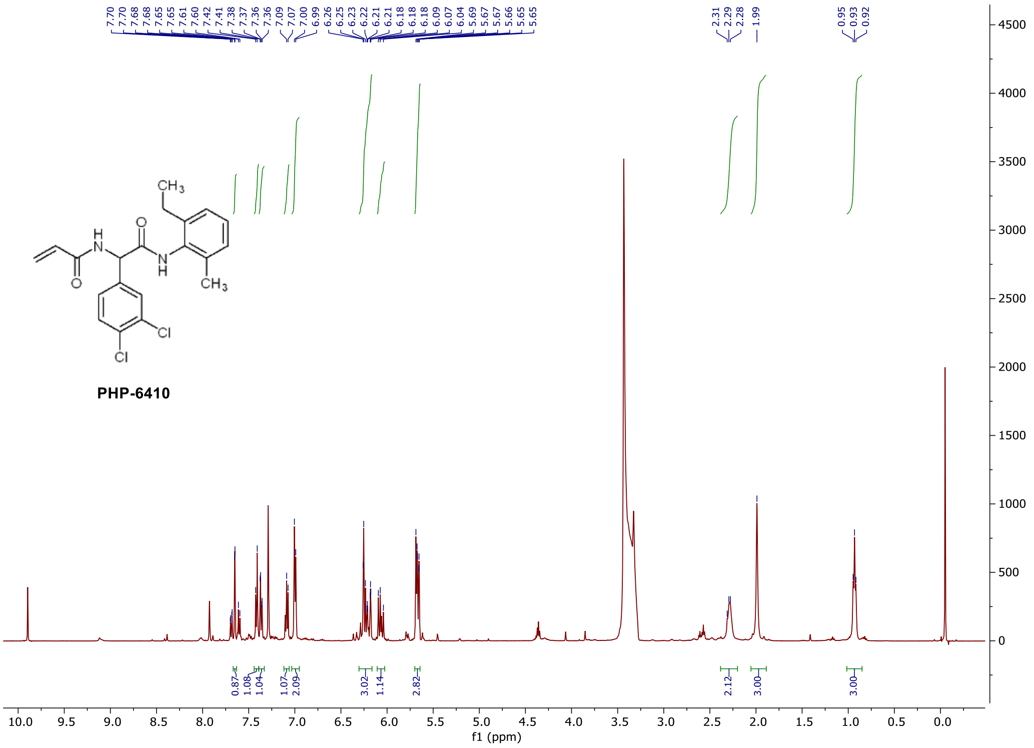

Supplementary Figure 1060. ^{1}H NMR (400 MHz, $CDCl_3$ + one drop of CD_3OD) of compound **PHP-6410.**

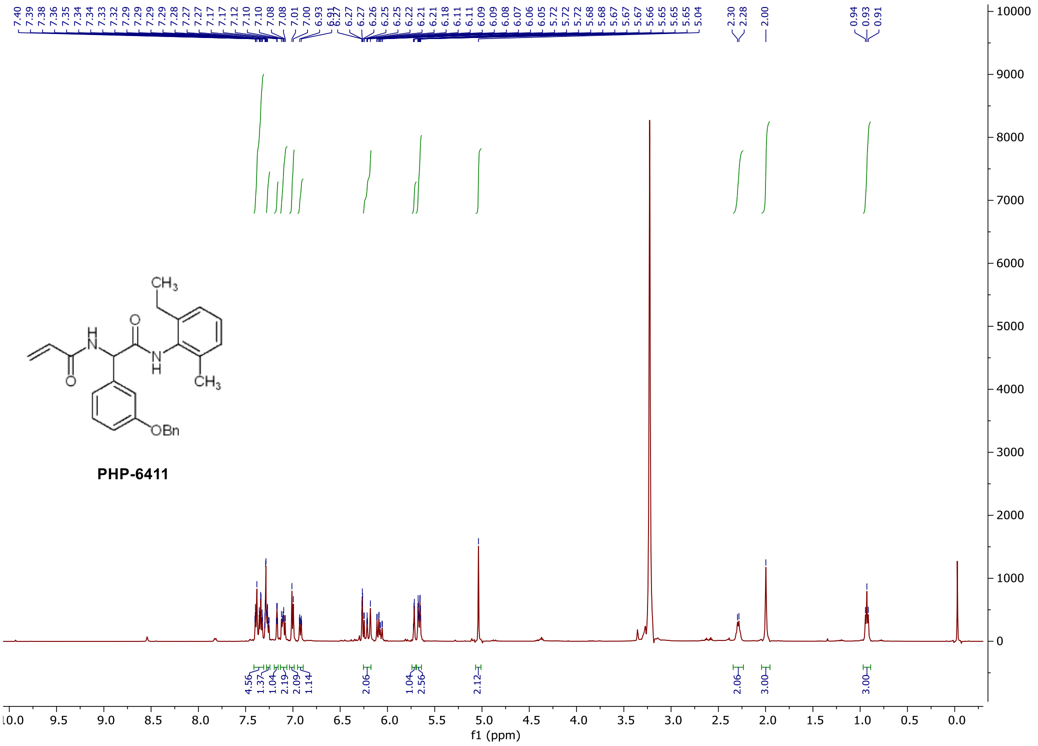

Supplementary Figure 1061. ^{1}H NMR (400 MHz, $CDCl_3$ + one drop of CD_3OD) of compound **PHP-6411.**

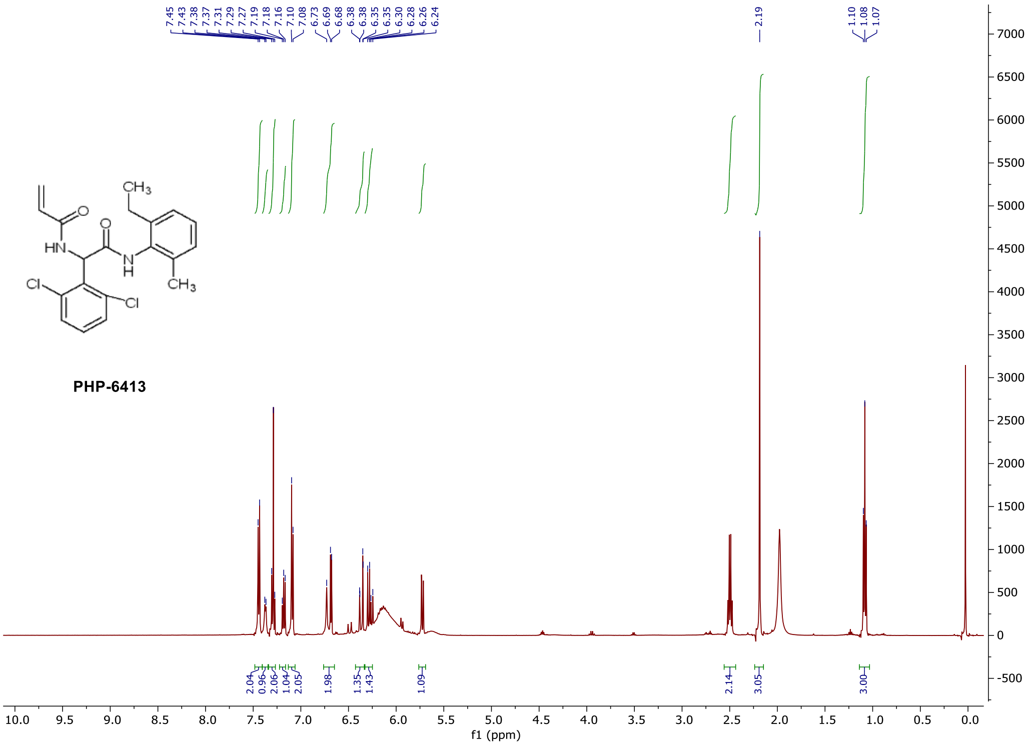

PHP-6413

Supplementary Figure 1062. ^{1}H NMR (400 MHz, $CDCl_3$ + one drop of CD_3OD) of compound **PHP-6413.**

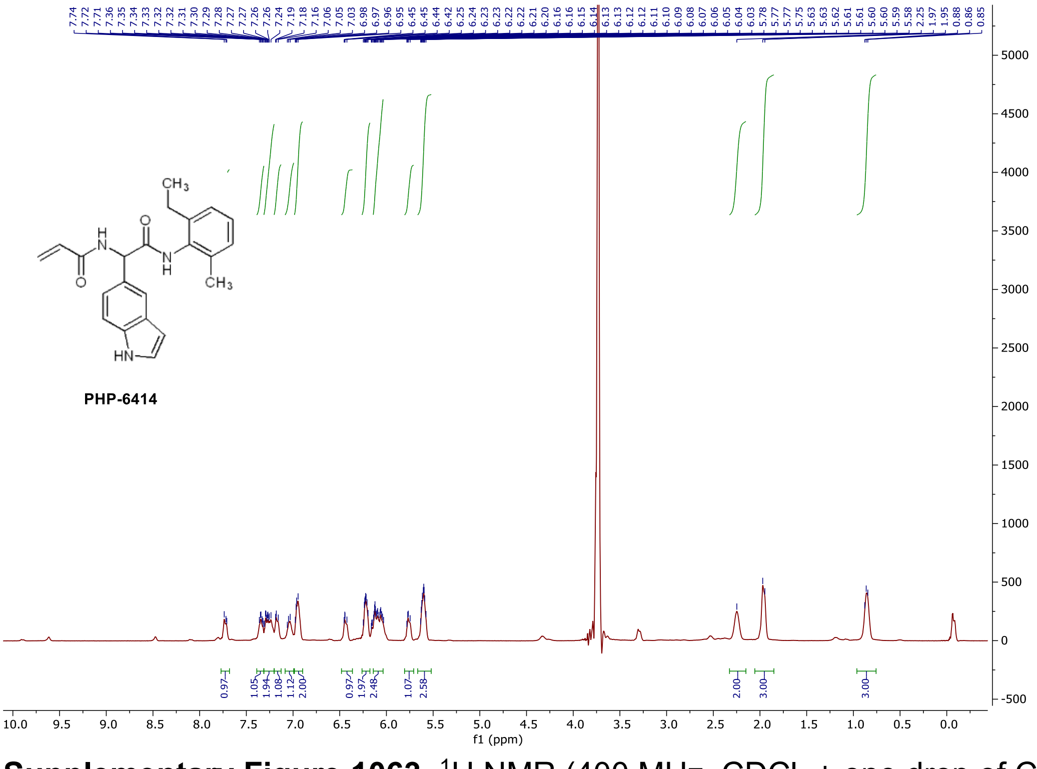

PHP-6414

Supplementary Figure 1063. ^{1}H NMR (400 MHz, $CDCl_3$ + one drop of CD_3OD) of compound **PHP-6414.**

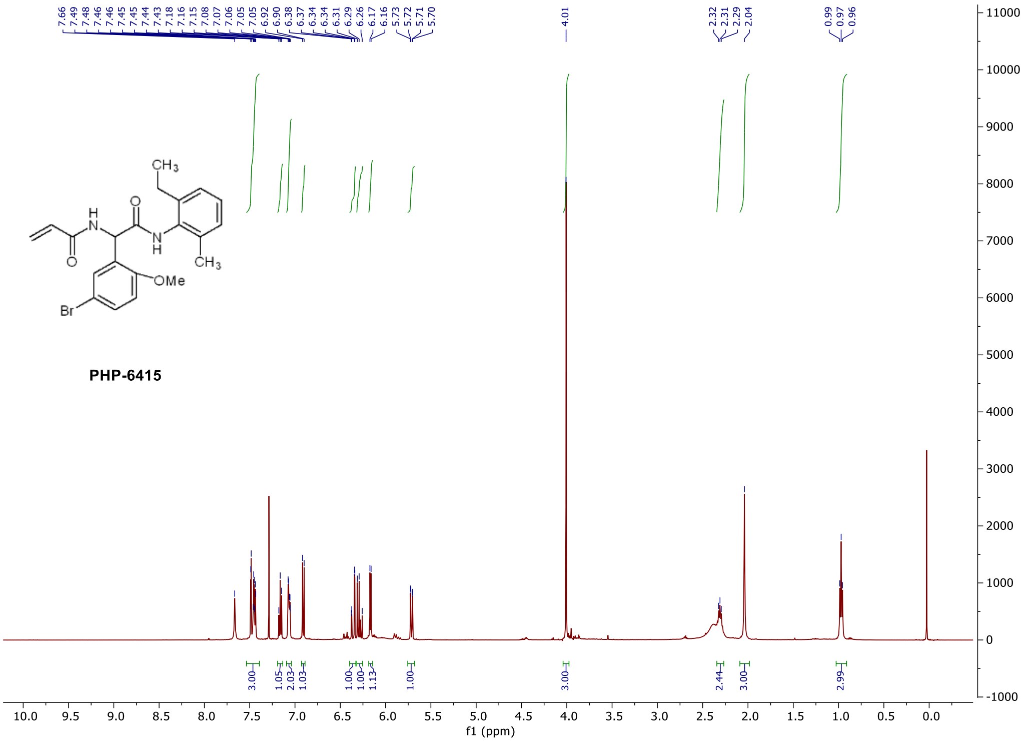

Supplementary Figure 1064. ^{1}H NMR (400 MHz, $CDCl_3$ + one drop of CD_3OD) of compound **PHP-6415.**

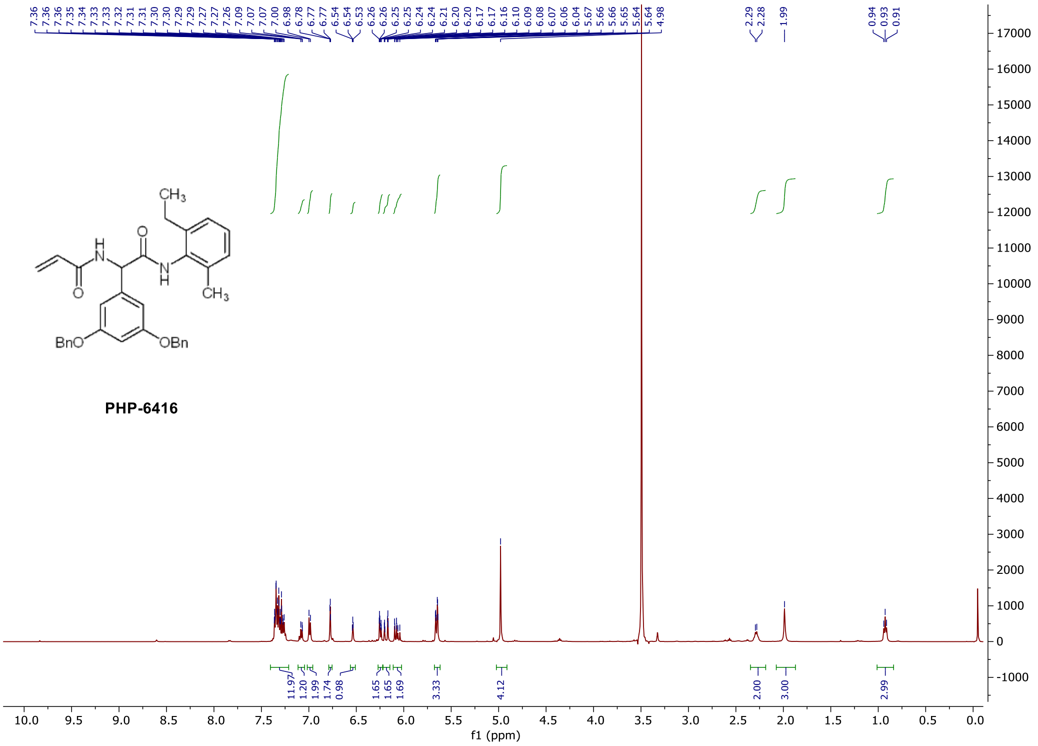

Supplementary Figure 1065. ^{1}H NMR (400 MHz, $CDCl_3$ + one drop of CD_3OD) of compound **PHP-6416.**

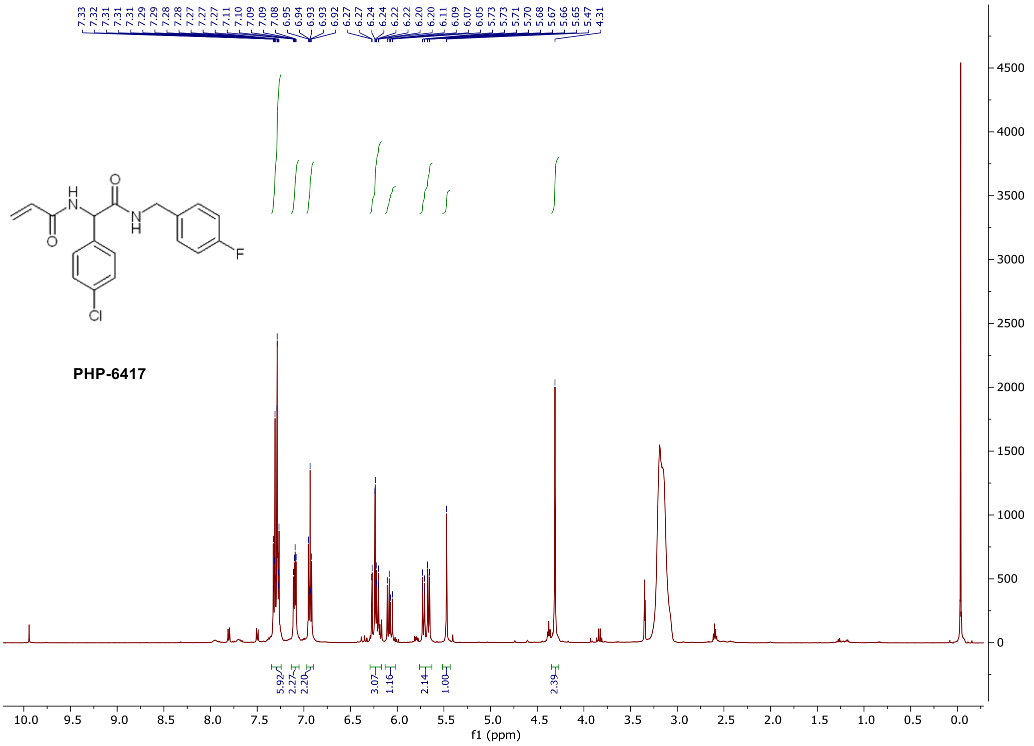

Supplementary Figure 1066. ^{1}H NMR (400 MHz, $CDCl_3$ + one drop of CD_3OD) of compound **PHP-6417.**

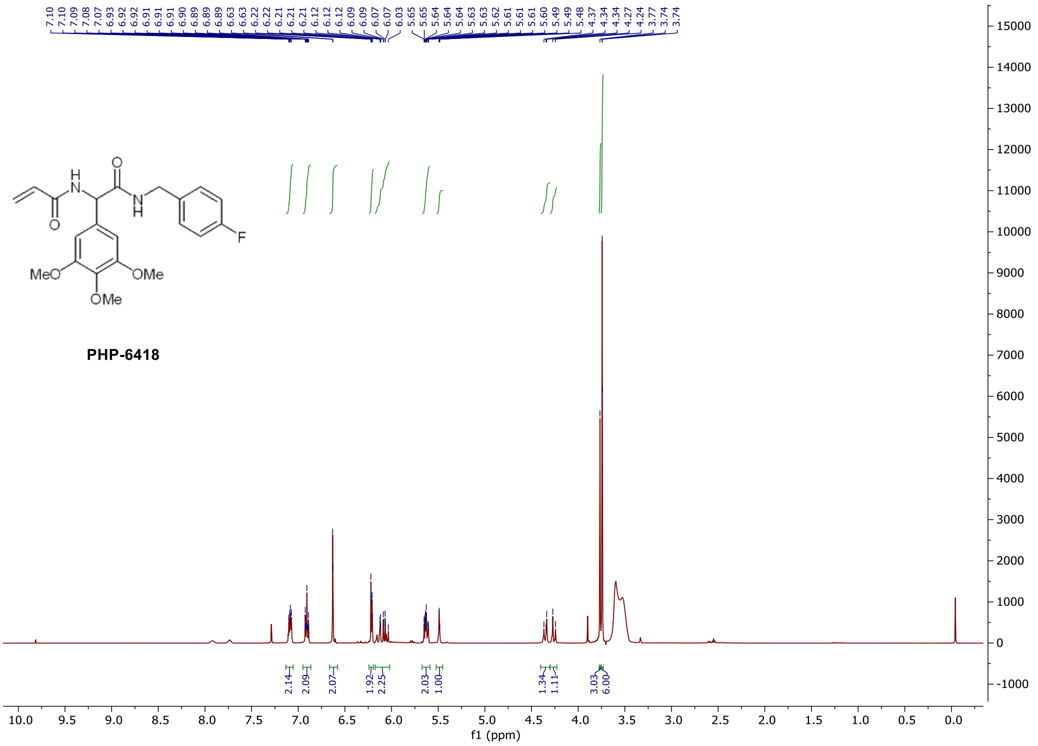

Supplementary Figure 1067. ^{1}H NMR (400 MHz, $CDCl_3$ + one drop of CD_3OD) of compound **PHP-6418.**

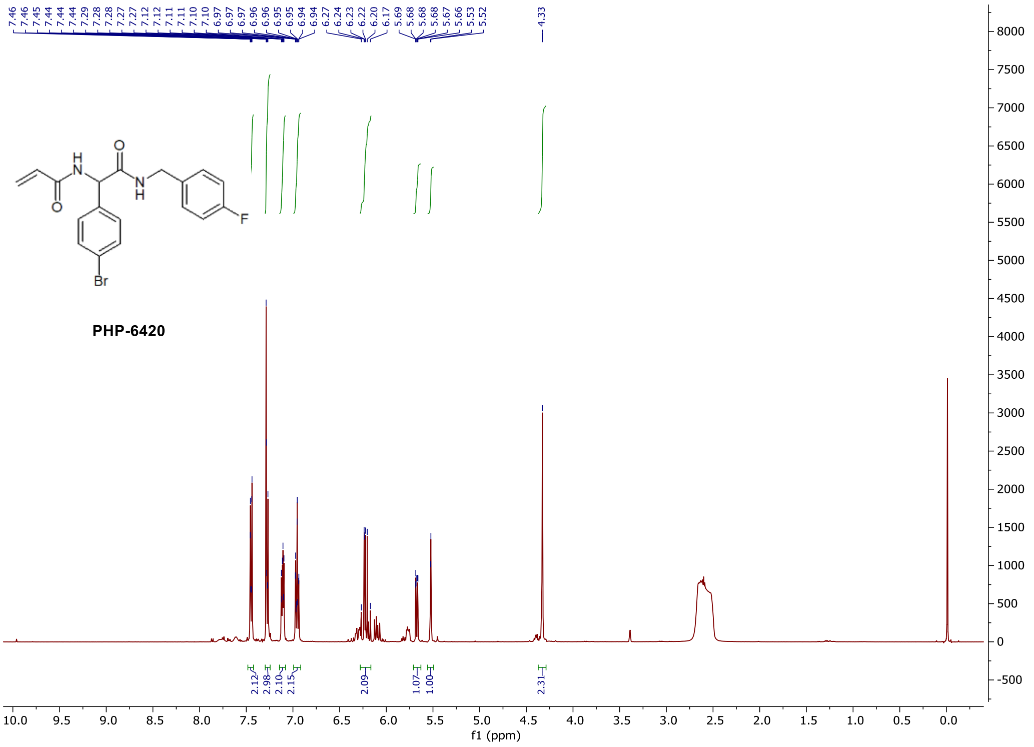

Supplementary Figure 1068. ¹H NMR (400 MHz, $CDCl_3$ + one drop of CD_3OD) of compound **PHP-6420.**

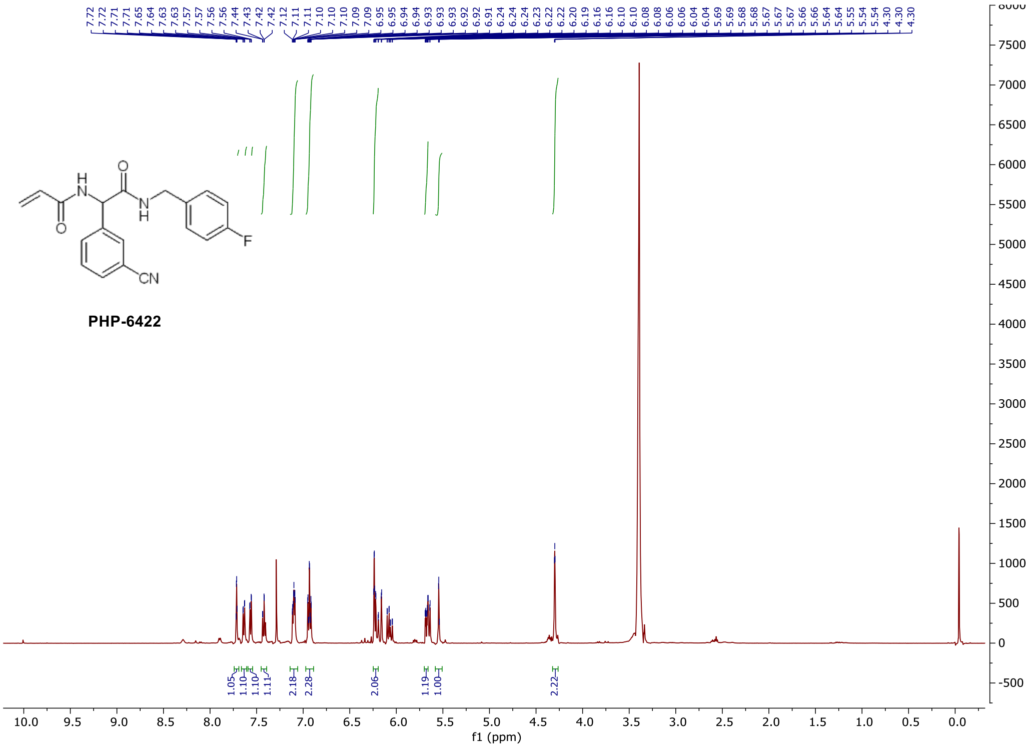

Supplementary Figure 1069. ¹H NMR (400 MHz, $CDCl_3$ + one drop of CD_3OD) of compound **PHP-6422.**

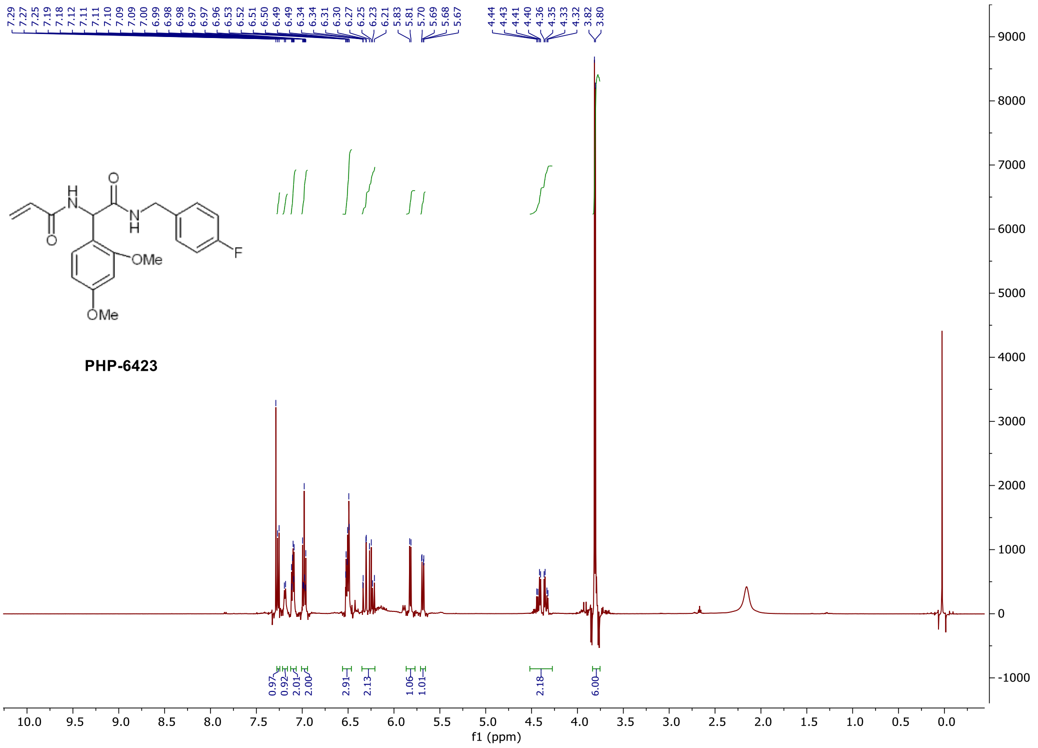

Supplementary Figure 1070. ^{1}H NMR (400 MHz, $CDCl_3$ + one drop of CD_3OD) of compound **PHP-6423.**

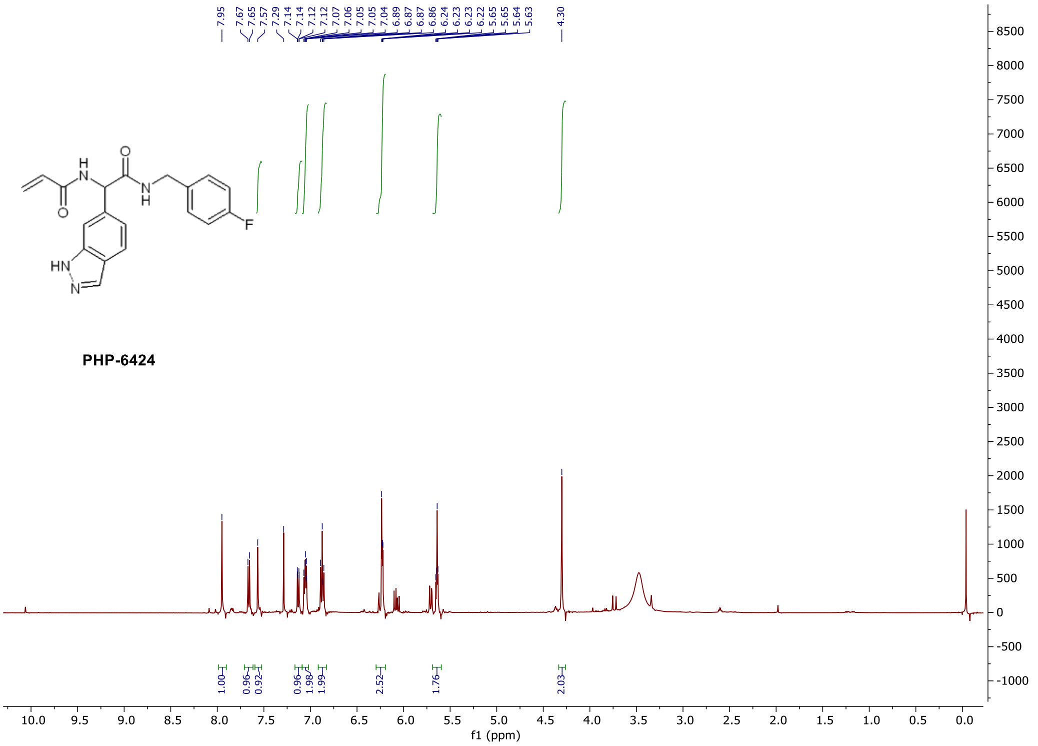

Supplementary Figure 1071. ^{1}H NMR (400 MHz, $CDCl_3$ + one drop of CD_3OD) of compound **PHP-6424.**

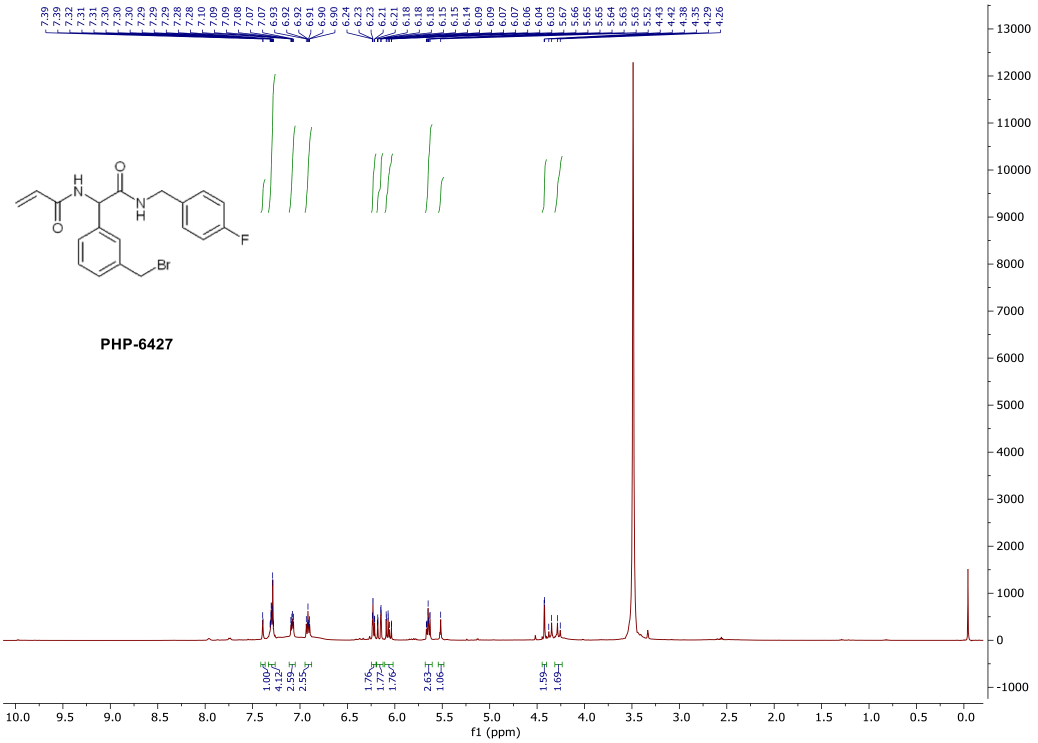

Supplementary Figure 1072. ^{1}H NMR (400 MHz, $CDCl_3$ + one drop of CD_3OD) of compound **PHP-6427**.

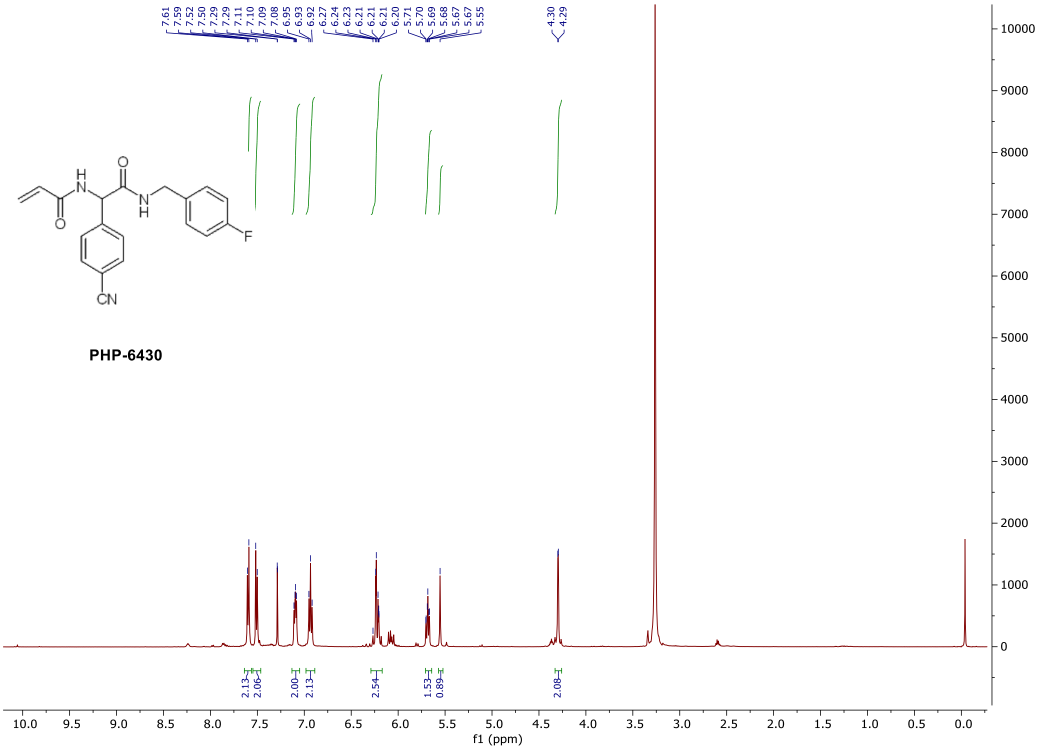

Supplementary Figure 1073. ^{1}H NMR (400 MHz, $CDCl_3$ + one drop of CD_3OD) of compound **PHP-6430**.

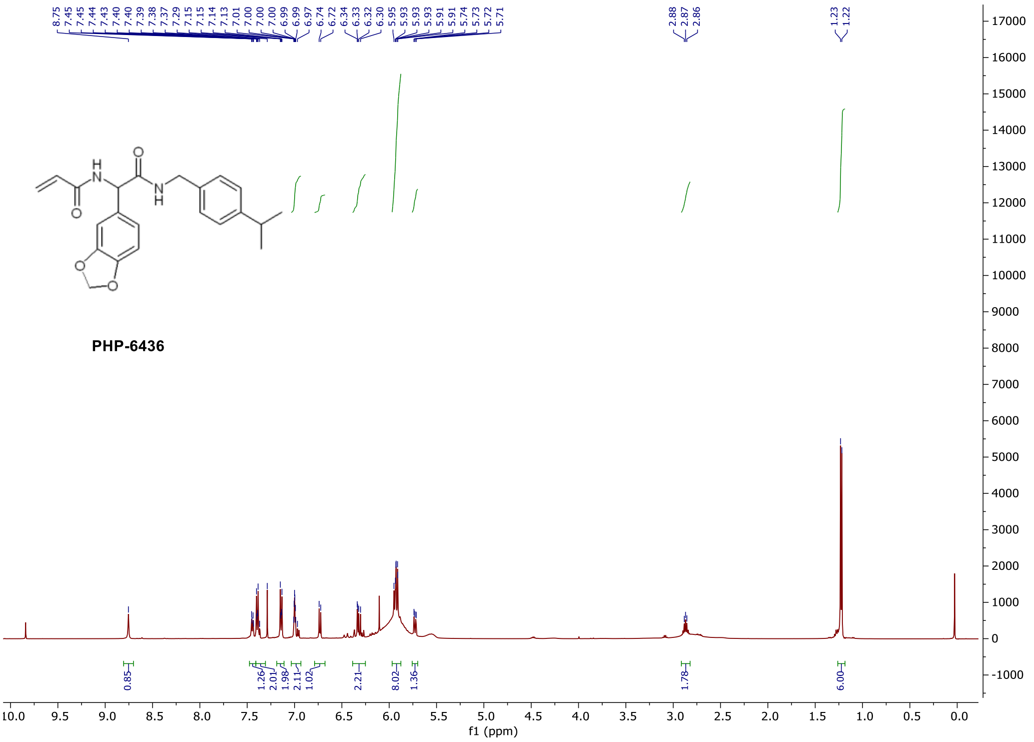

Supplementary Figure 1074. ¹H NMR (400 MHz, CDCl₃ + one drop of CD₃OD) of compound **PHP-6436**.

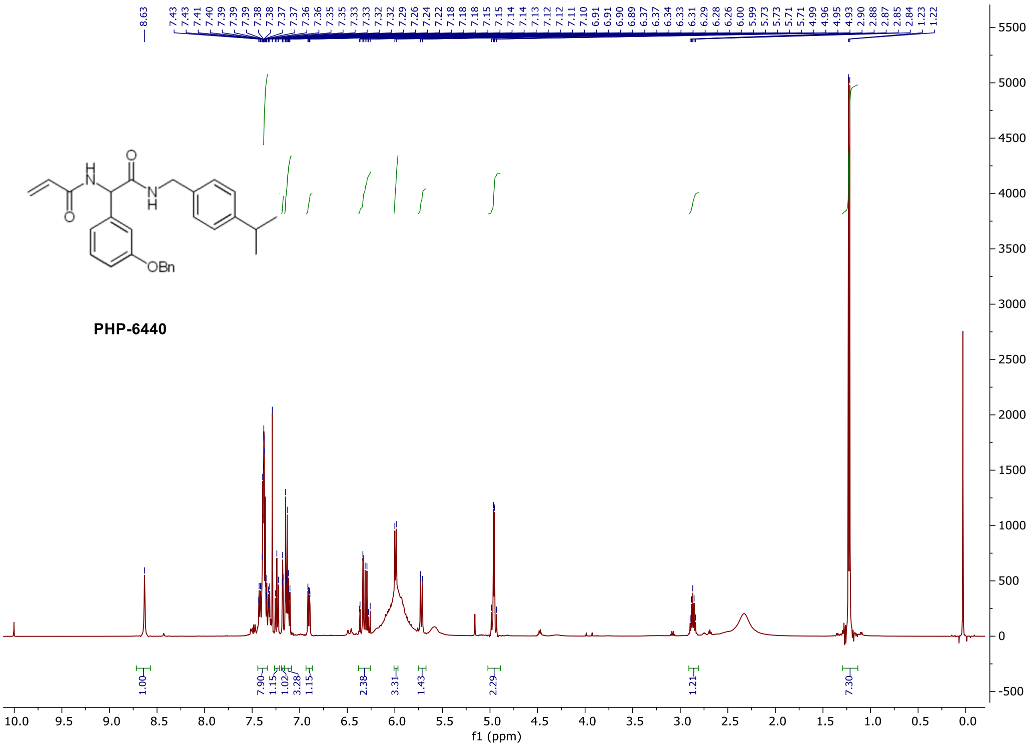

Supplementary Figure 1075. ¹H NMR (400 MHz, CDCl₃ + one drop of CD₃OD) of compound **PHP-6440**.

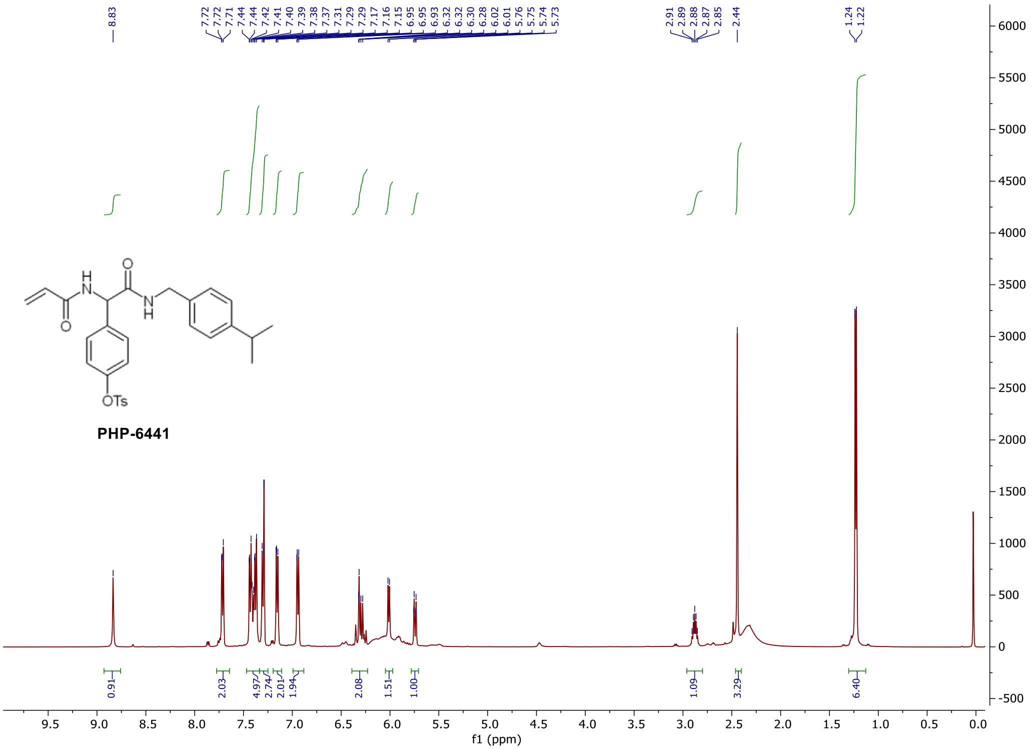

Supplementary Figure 1076. ^{1}H NMR (400 MHz, $CDCl_3$ + one drop of CD_3OD) of compound **PHP-6441**.

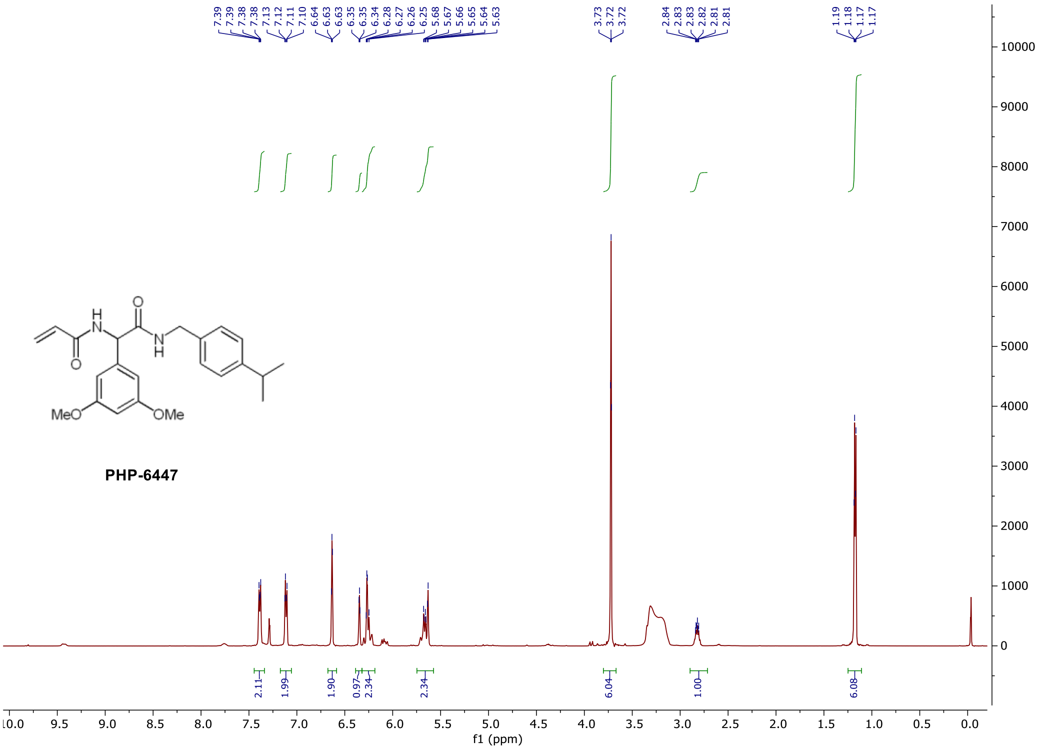

Supplementary Figure 1077. ^{1}H NMR (400 MHz, $CDCl_3$ + one drop of CD_3OD) of compound **PHP-6447**.

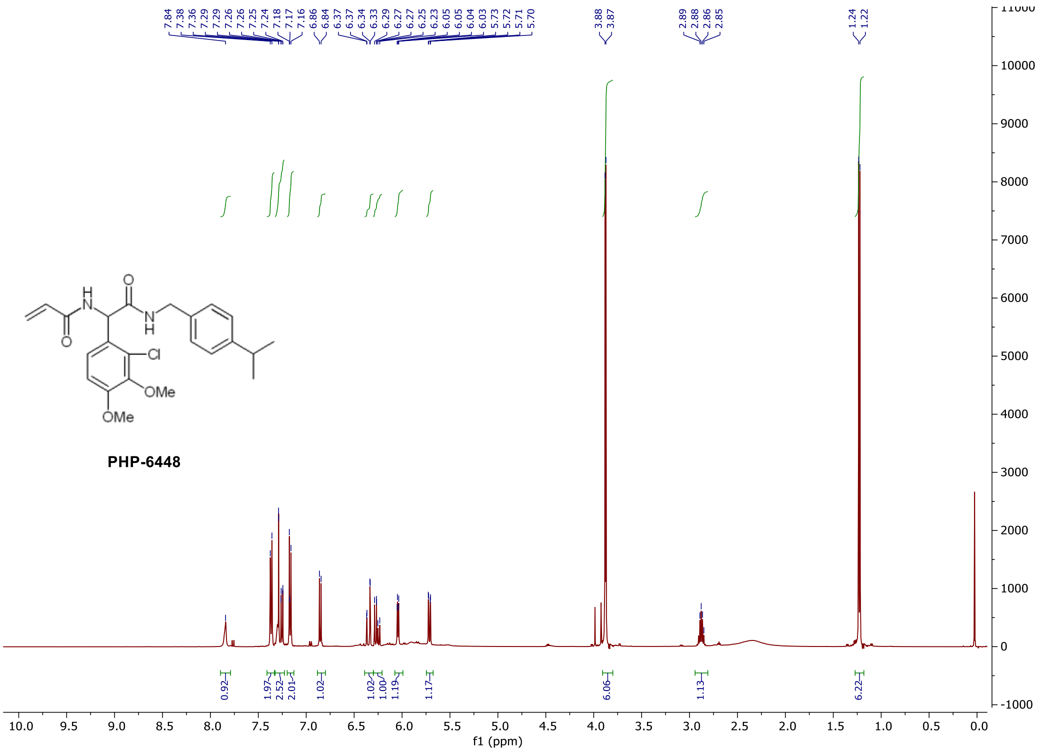

Supplementary Figure 1078. ^{1}H NMR (400 MHz, $CDCl_3$ + one drop of CD_3OD) of compound **PHP-6448.**

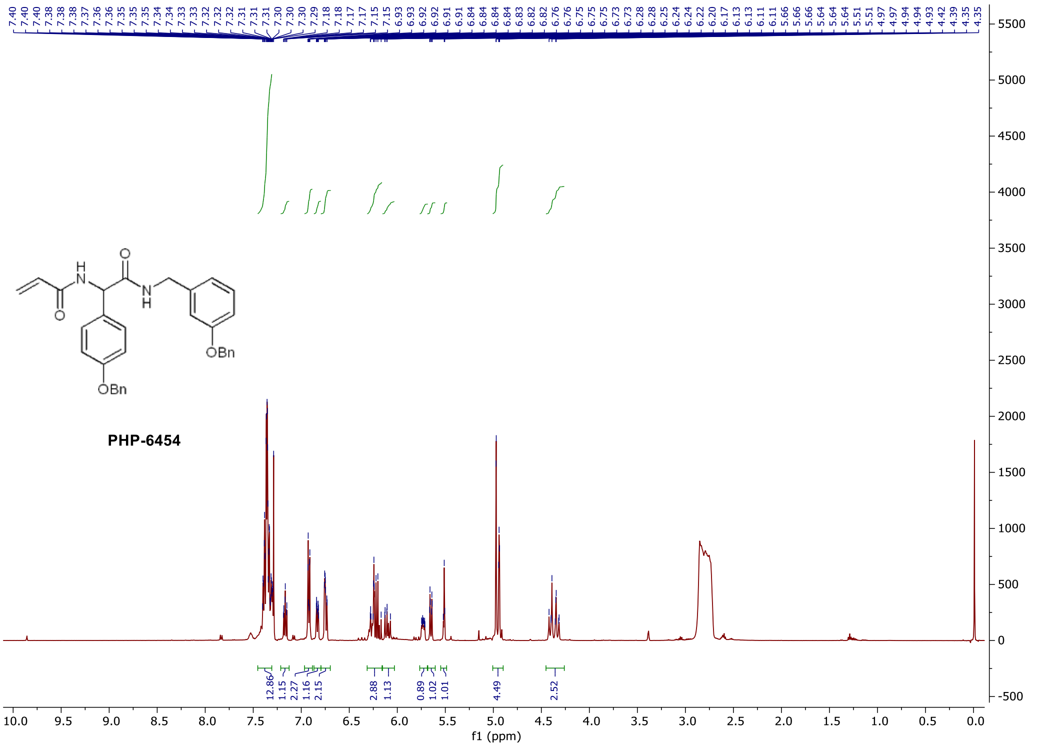

Supplementary Figure 1079. ^{1}H NMR (400 MHz, $CDCl_3$ + one drop of CD_3OD) of compound **PHP-6454.**

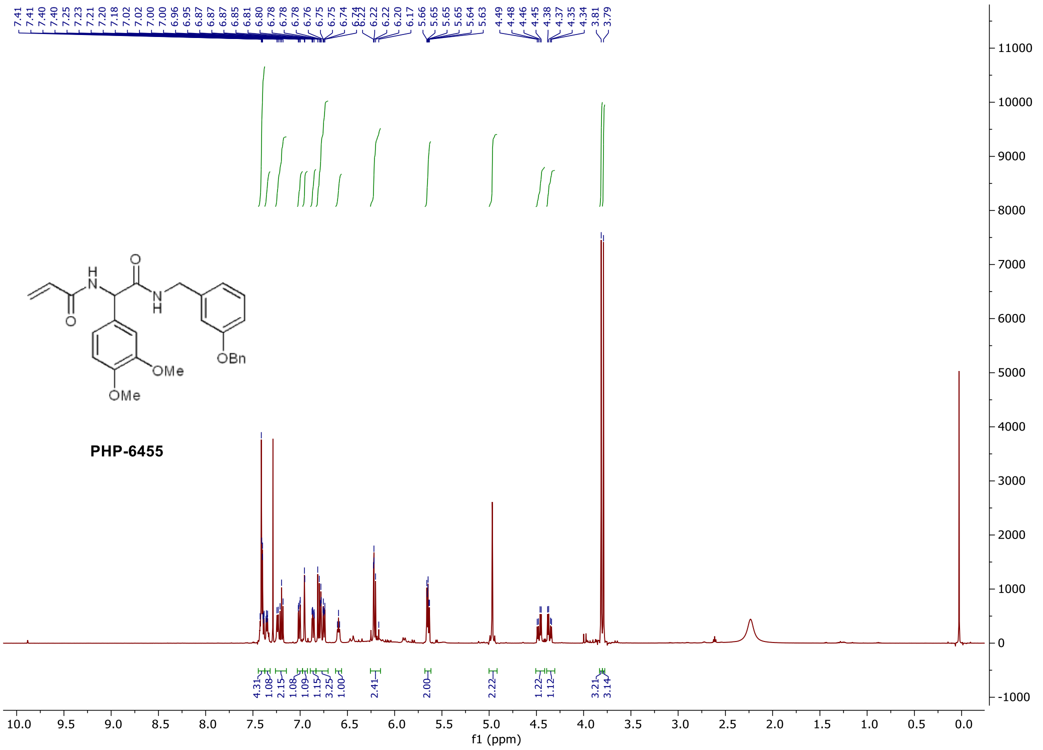

Supplementary Figure 1080. ^{1}H NMR (400 MHz, $CDCl_3$ + one drop of CD_3OD) of compound **PHP-6455.**

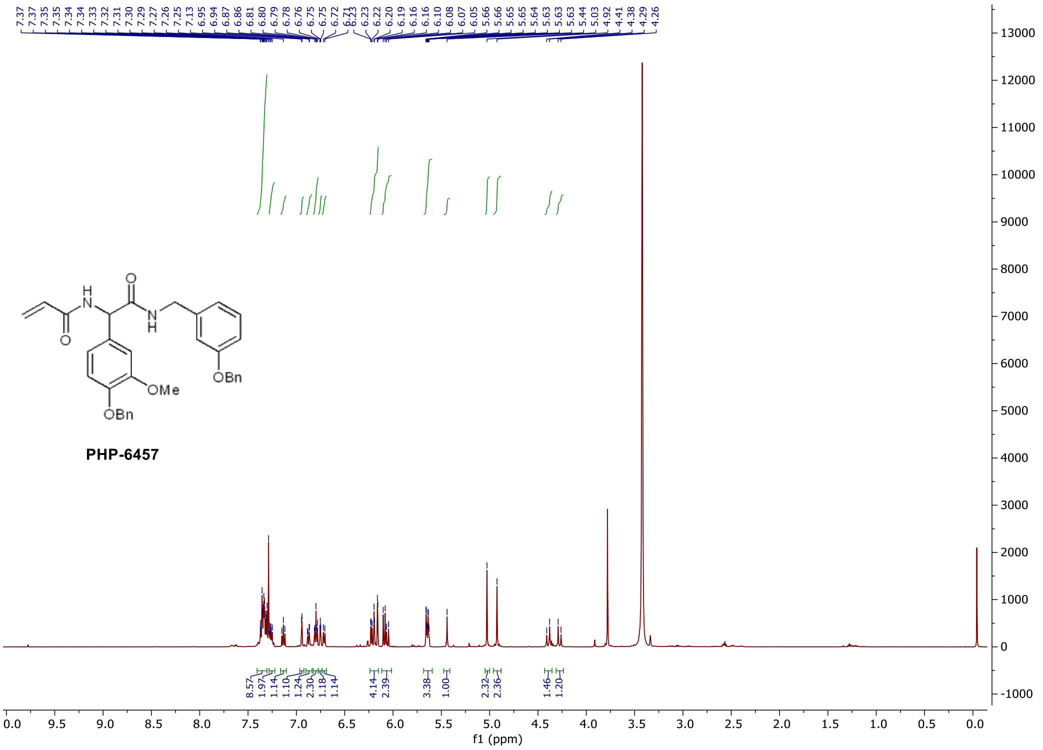

Supplementary Figure 1081. ^{1}H NMR (400 MHz, $CDCl_3$ + one drop of CD_3OD) of compound **PHP-6457.**

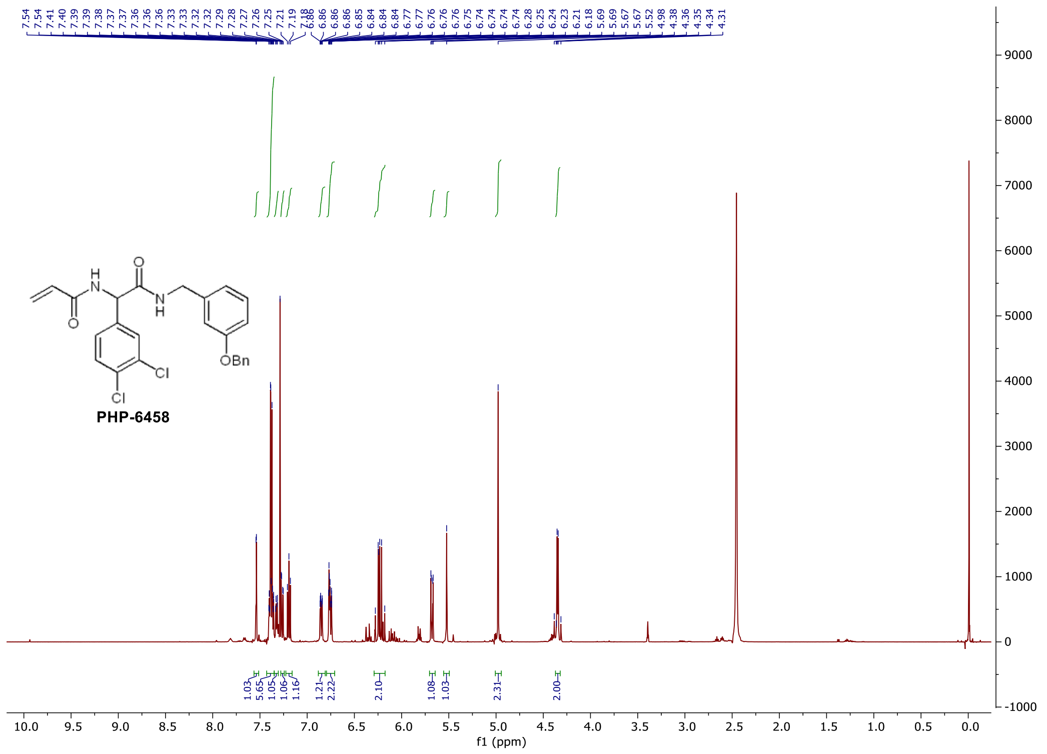

Supplementary Figure 1082. ^{1}H NMR (400 MHz, $CDCl_3$ + one drop of CD_3OD) of compound **PHP-6458**.

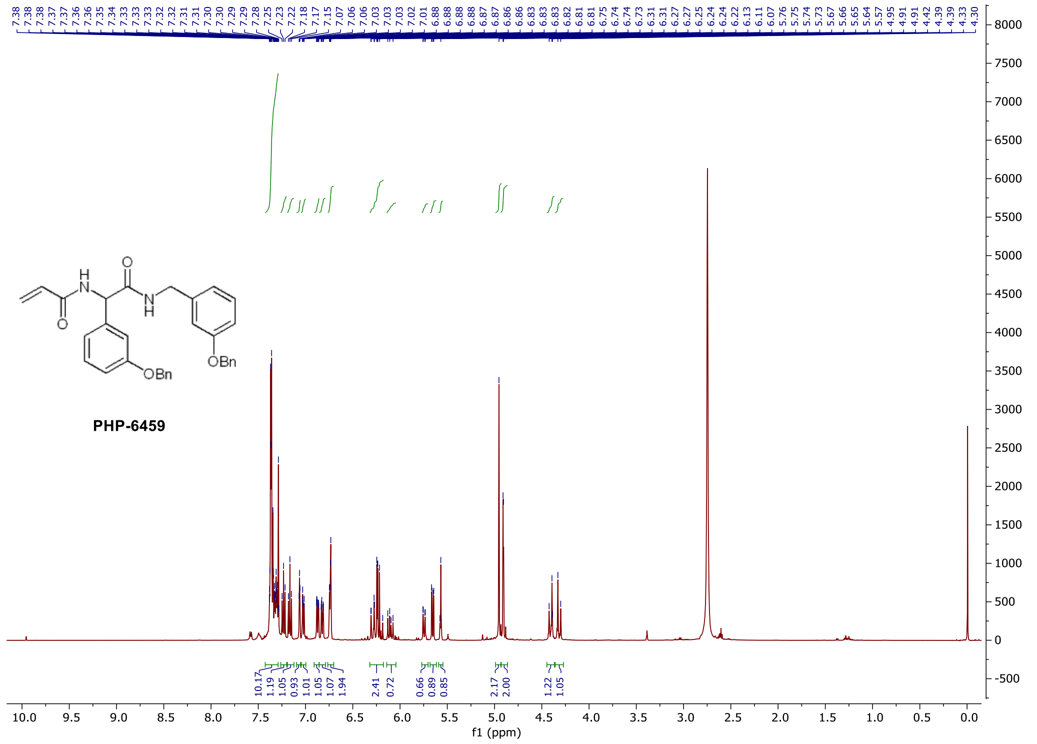

Supplementary Figure 1083. ^{1}H NMR (400 MHz, $CDCl_3$ + one drop of CD_3OD) of compound **PHP-6459**.

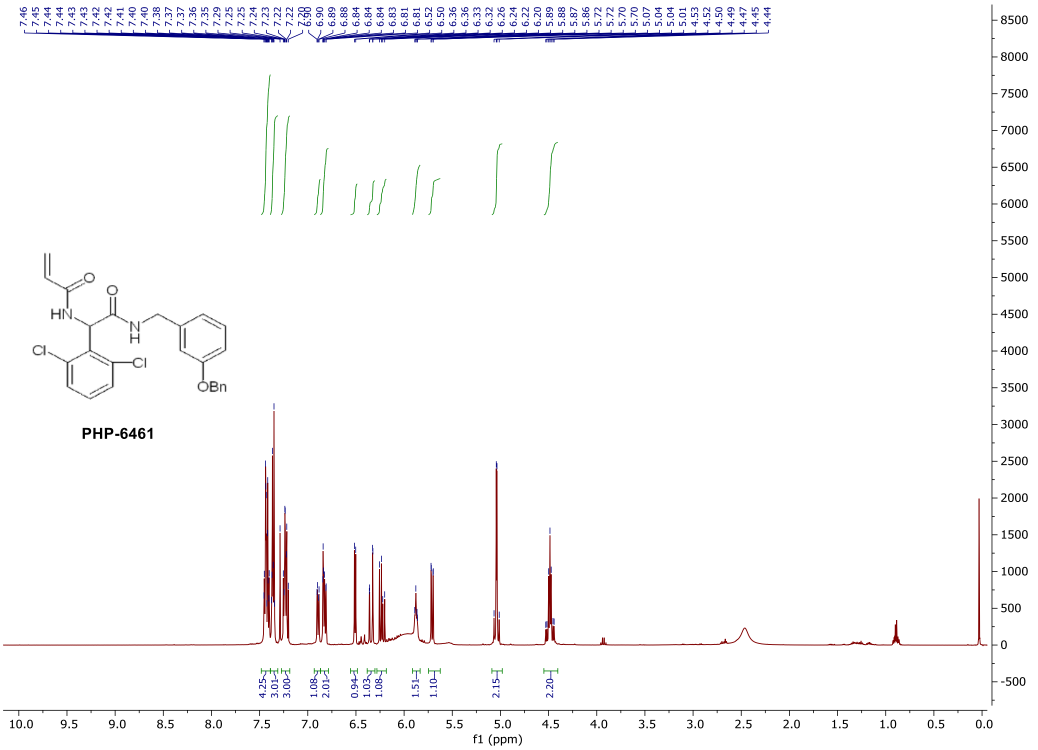

Supplementary Figure 1084. ^{1}H NMR (400 MHz, $CDCl_3$ + one drop of CD_3OD) of compound **PHP-6461**.

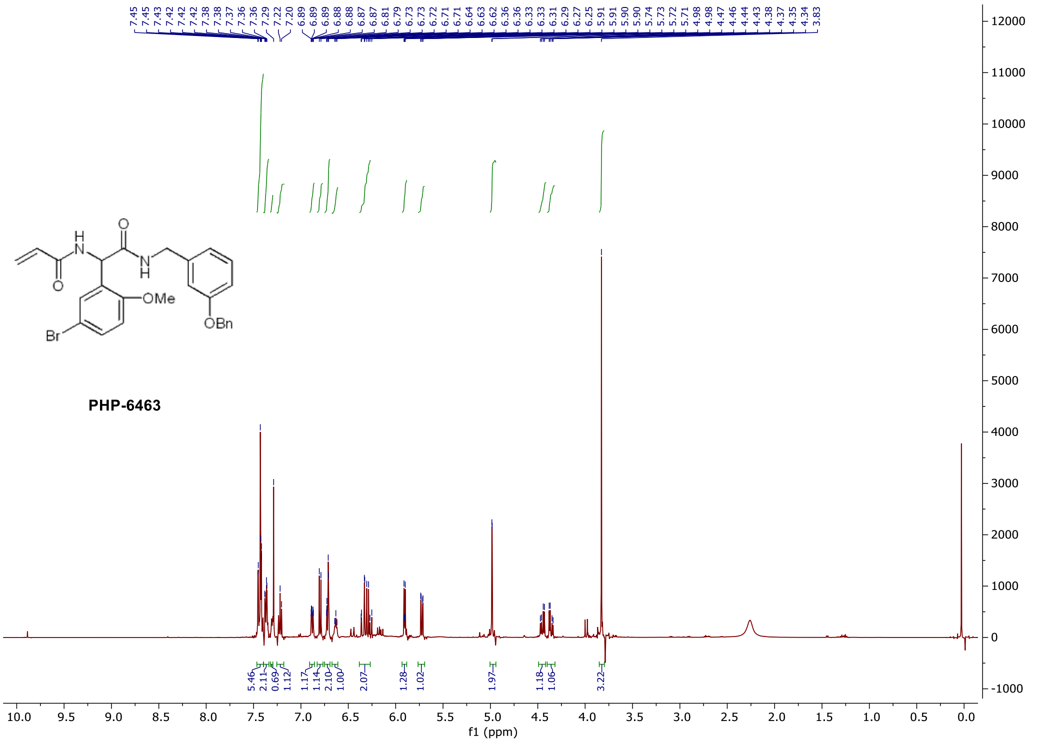

Supplementary Figure 1085. ^{1}H NMR (400 MHz, $CDCl_3$ + one drop of CD_3OD) of compound **PHP-6463**.

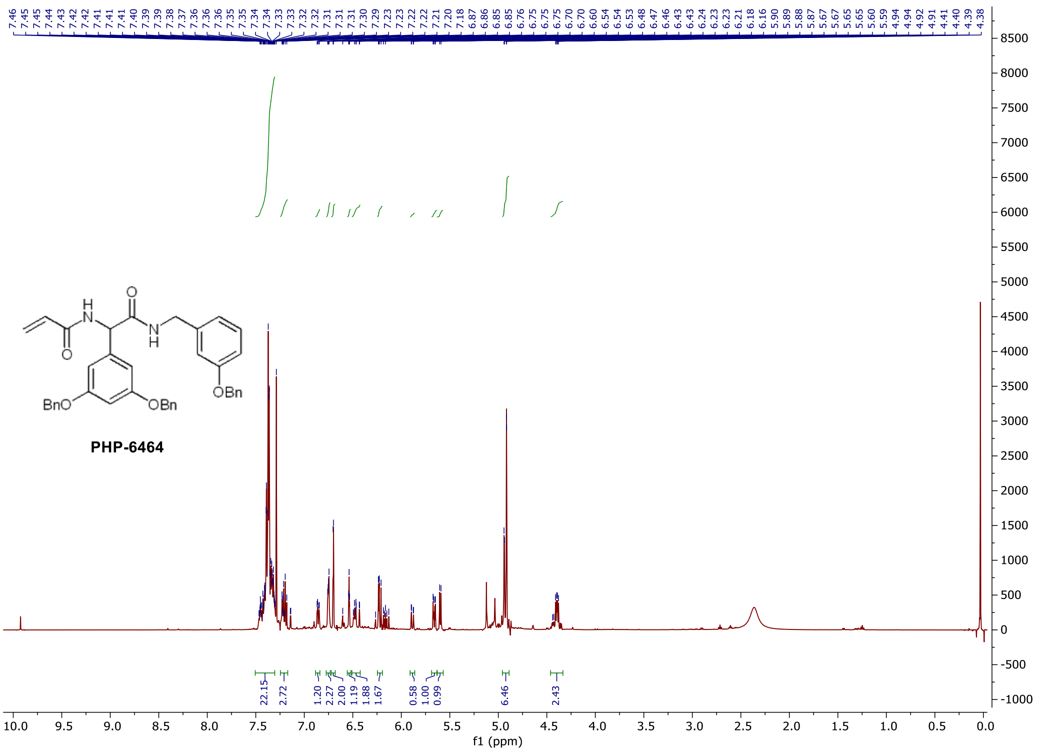

Supplementary Figure 1086. ^{1}H NMR (400 MHz, $CDCl_3$ + one drop of CD_3OD) of compound **PHP-6464.**

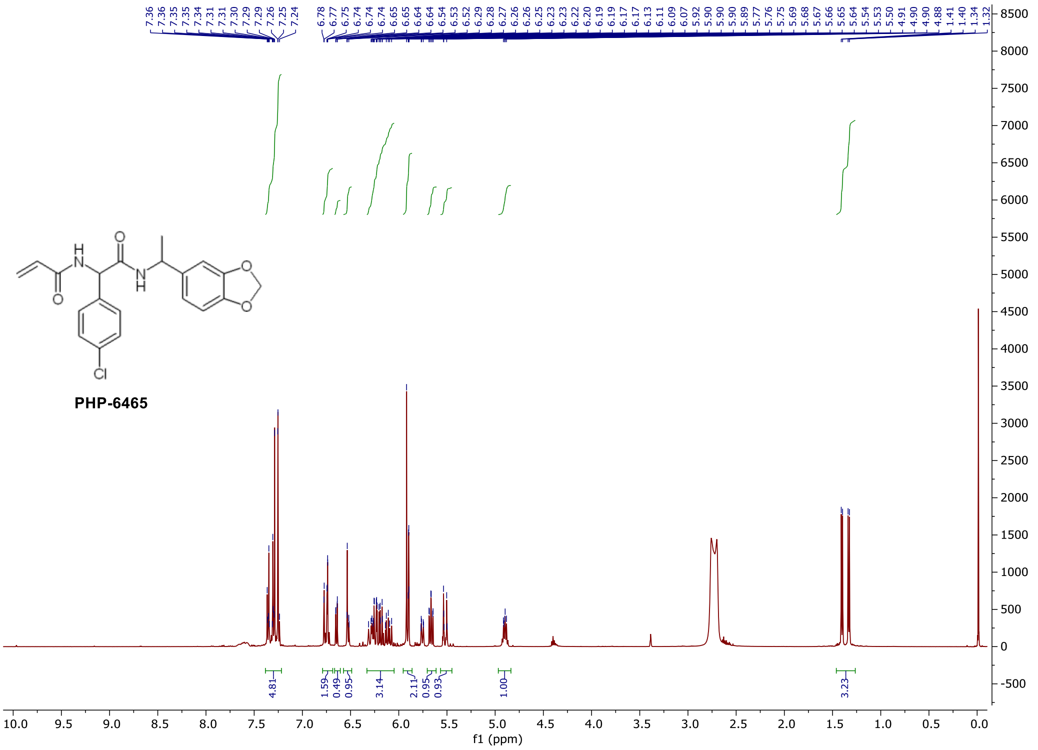

Supplementary Figure 1087. ^{1}H NMR (400 MHz, $CDCl_3$ + one drop of CD_3OD) of compound **PHP-6465.**

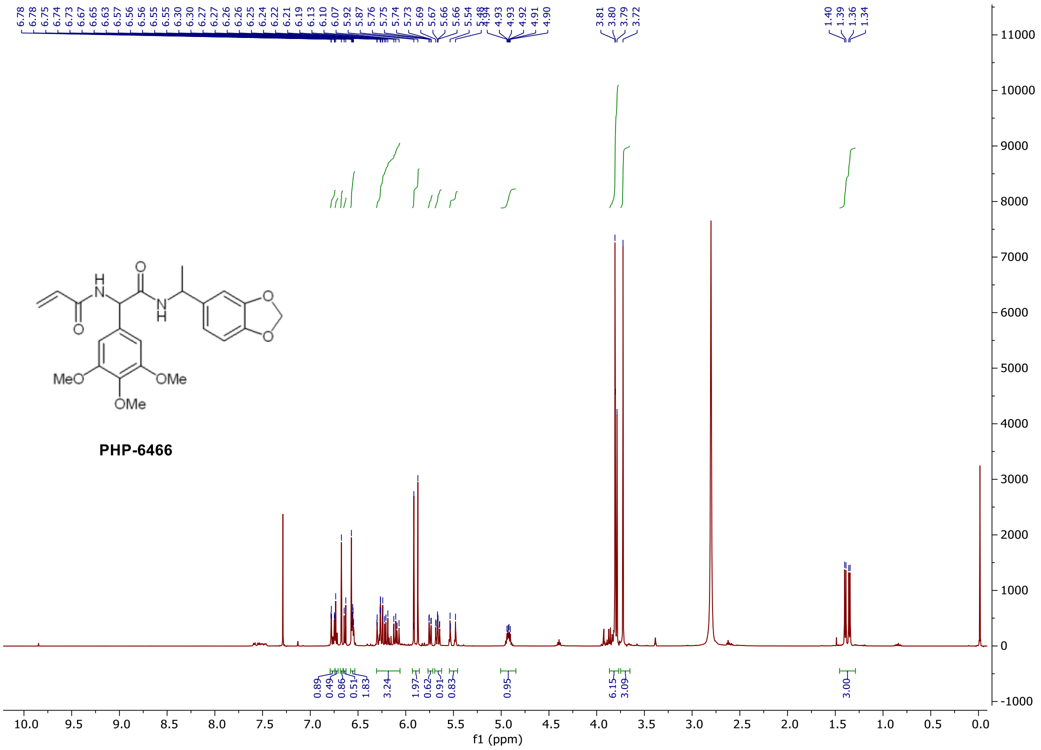

Supplementary Figure 1088. ^{1}H NMR (400 MHz, $CDCl_3$ + one drop of CD_3OD) of compound **PHP-6466.**

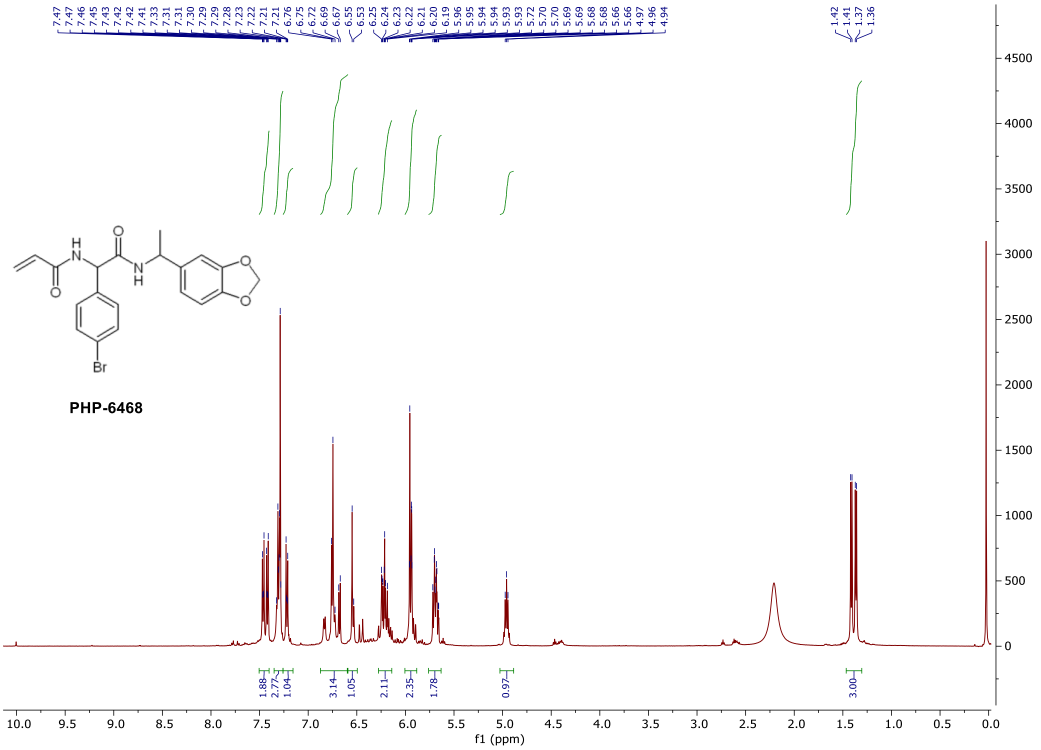

Supplementary Figure 1089. ^{1}H NMR (400 MHz, $CDCl_3$ + one drop of CD_3OD) of compound **PHP-6468.**

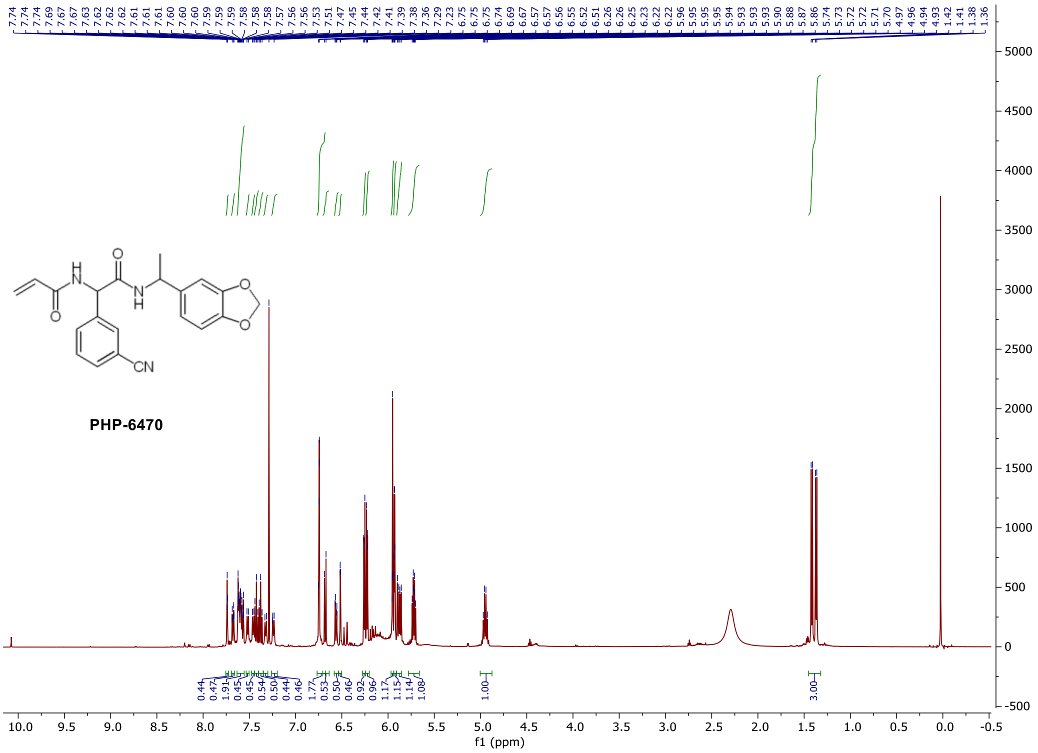

Supplementary Figure 1090. ^{1}H NMR (400 MHz, $CDCl_3$ + one drop of CD_3OD) of compound **PHP-6470.**

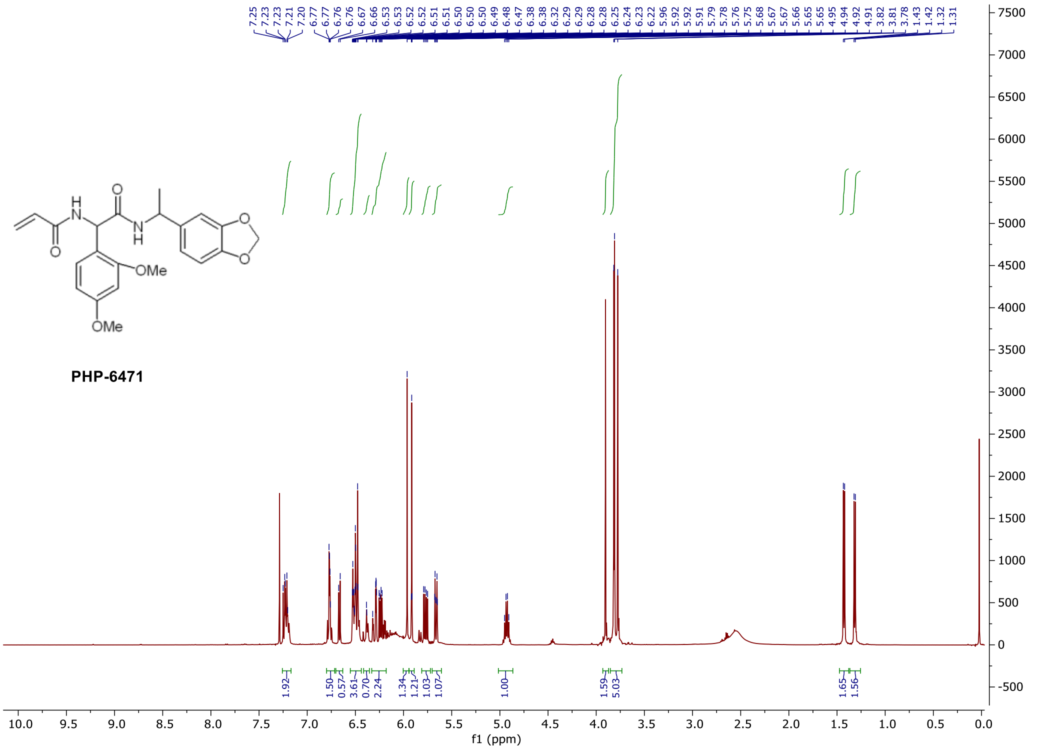

Supplementary Figure 1091. ^{1}H NMR (400 MHz, $CDCl_3$ + one drop of CD_3OD) of compound **PHP-6471.**

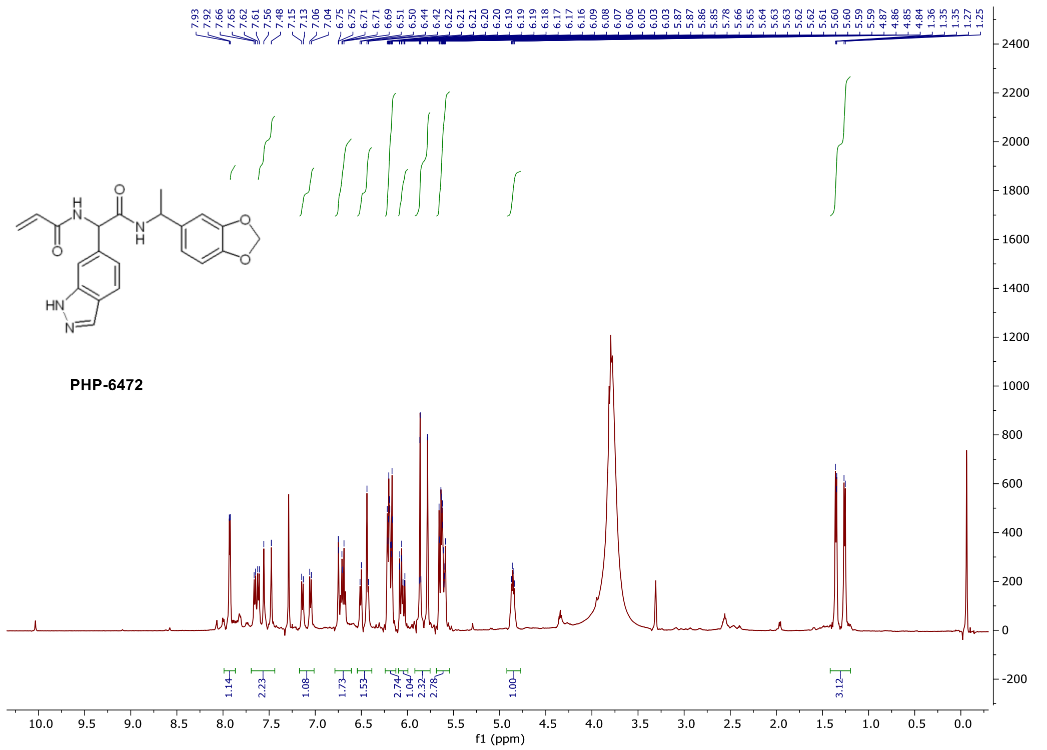

Supplementary Figure 1092. ^{1}H NMR (400 MHz, $CDCl_3$ + one drop of CD_3OD) of compound **PHP-6472.**

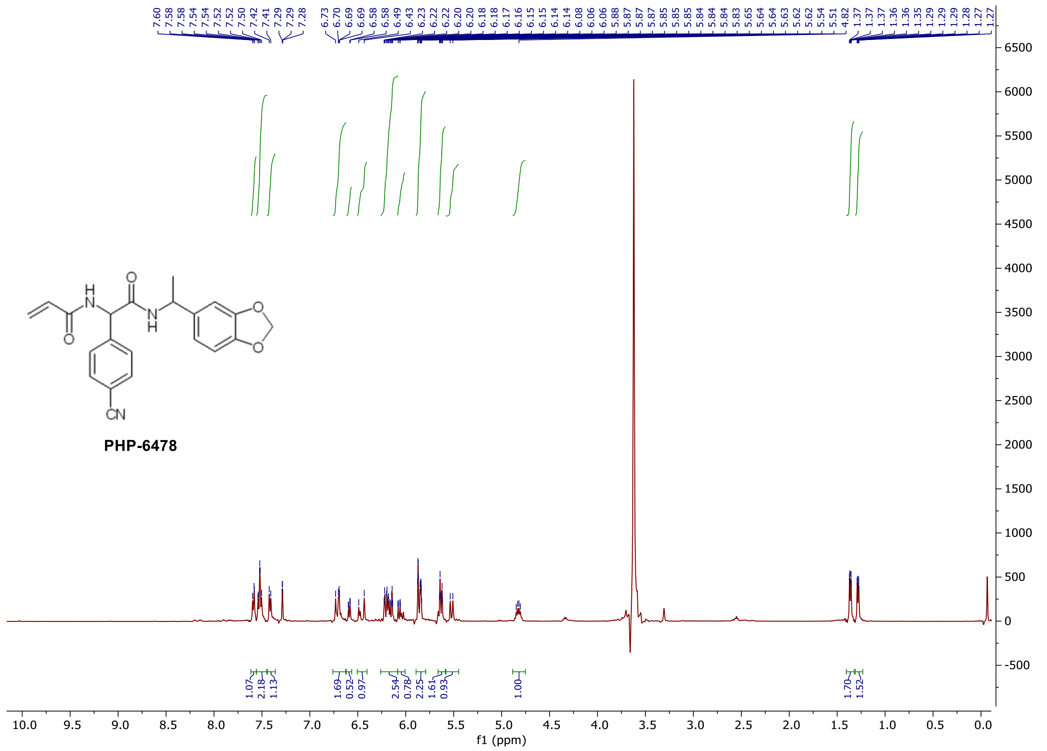

Supplementary Figure 1093. ^{1}H NMR (400 MHz, $CDCl_3$ + one drop of CD_3OD) of compound **PHP-6478.**

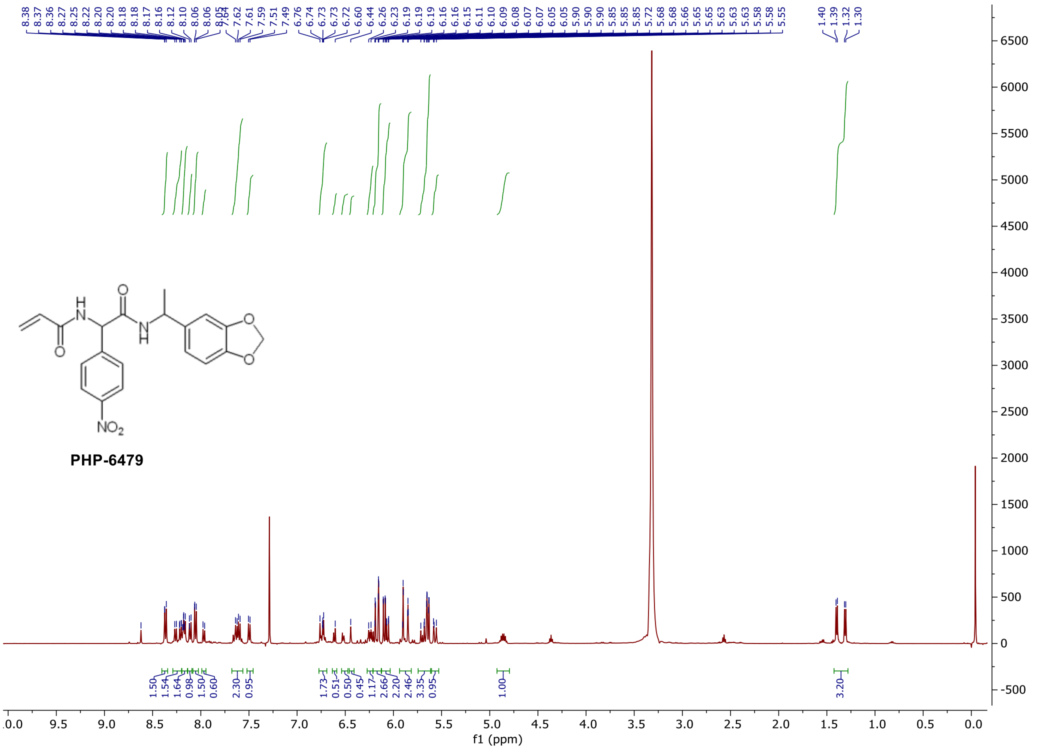

Supplementary Figure 1094. ^{1}H NMR (400 MHz, $CDCl_3$ + one drop of CD_3OD) of compound **PHP-6479.**

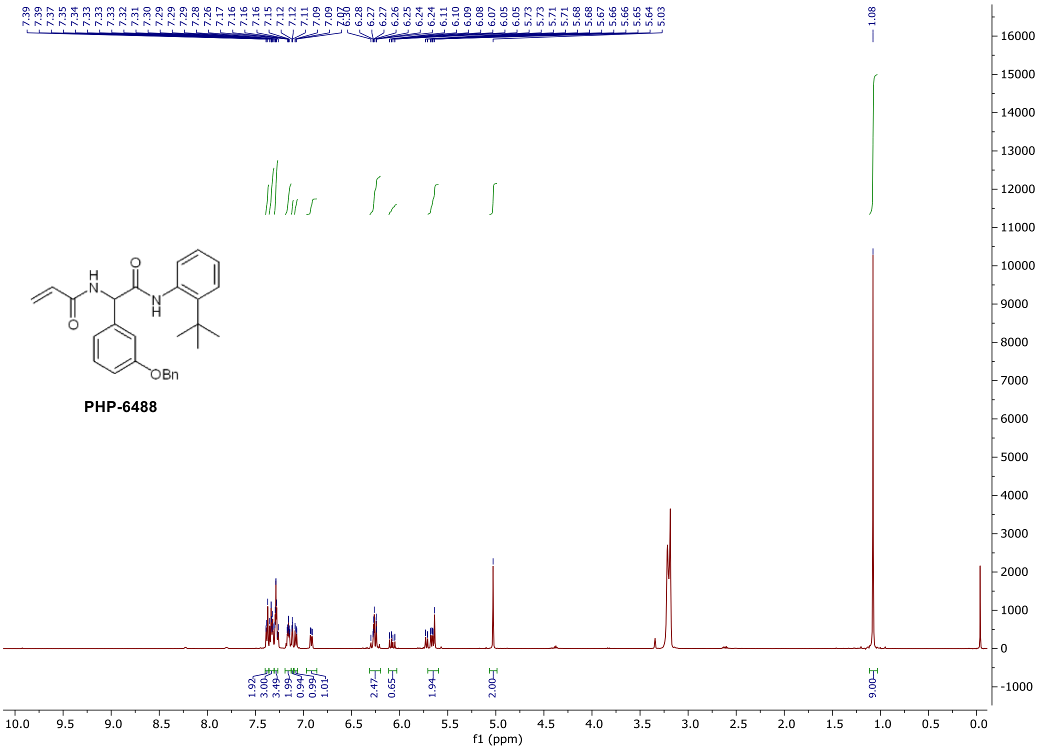

Supplementary Figure 1095. ^{1}H NMR (400 MHz, $CDCl_3$ + one drop of CD_3OD) of compound **PHP-6488.**

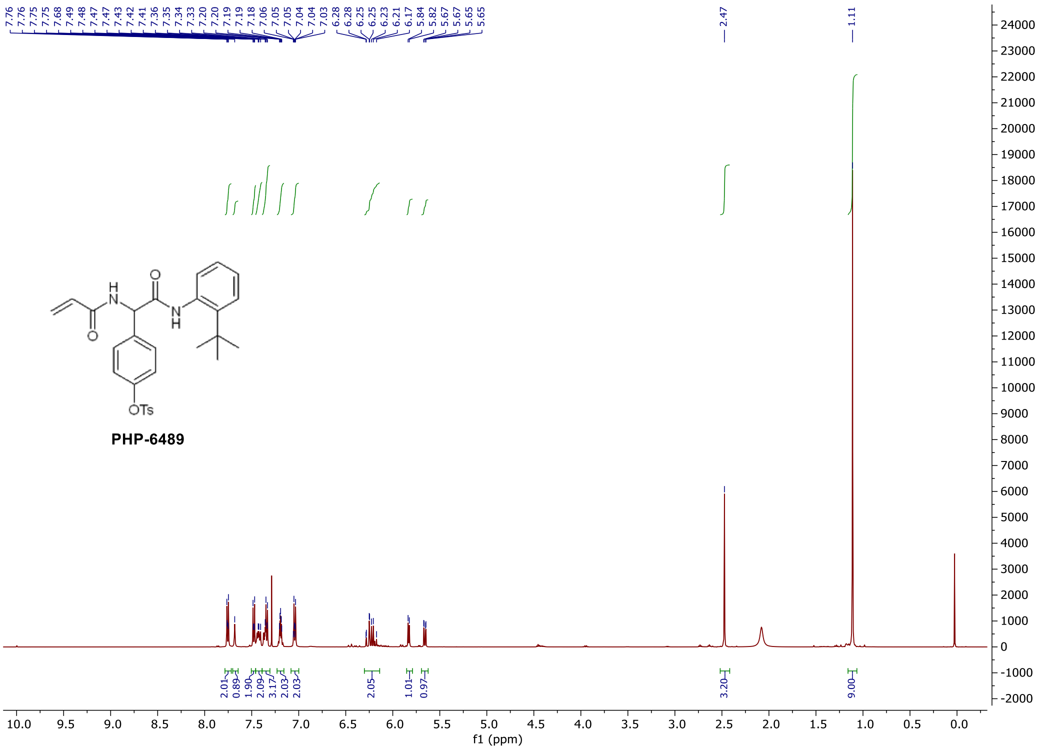

Supplementary Figure 1096. ^{1}H NMR (400 MHz, $CDCl_3$ + one drop of CD_3OD) of compound **PHP-6489.**

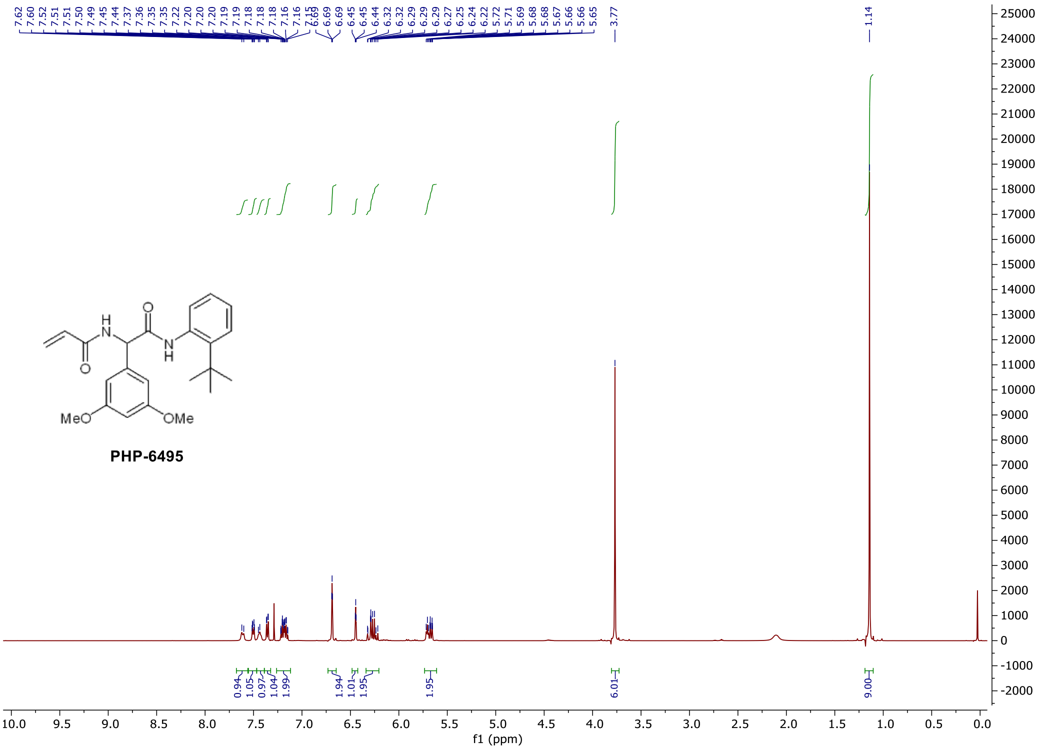

Supplementary Figure 1097. ^{1}H NMR (400 MHz, $CDCl_3$ + one drop of CD_3OD) of compound **PHP-6495.**

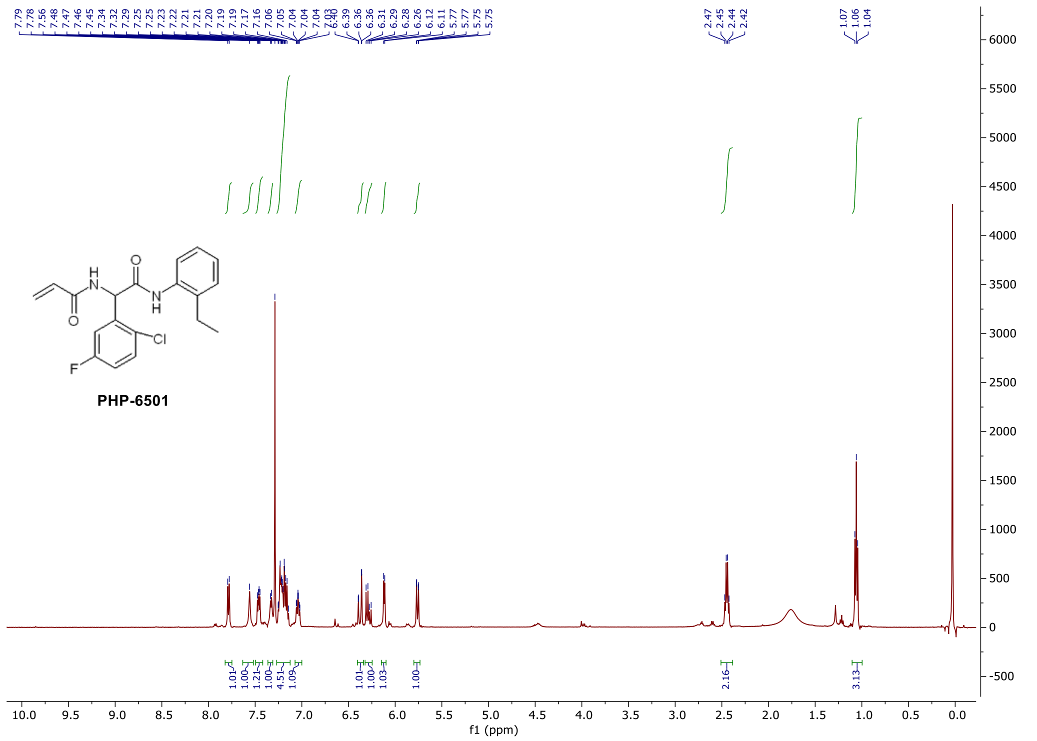

Supplementary Figure 1098. ^{1}H NMR (400 MHz, $CDCl_3$ + one drop of CD_3OD) of compound **PHP-6501.**

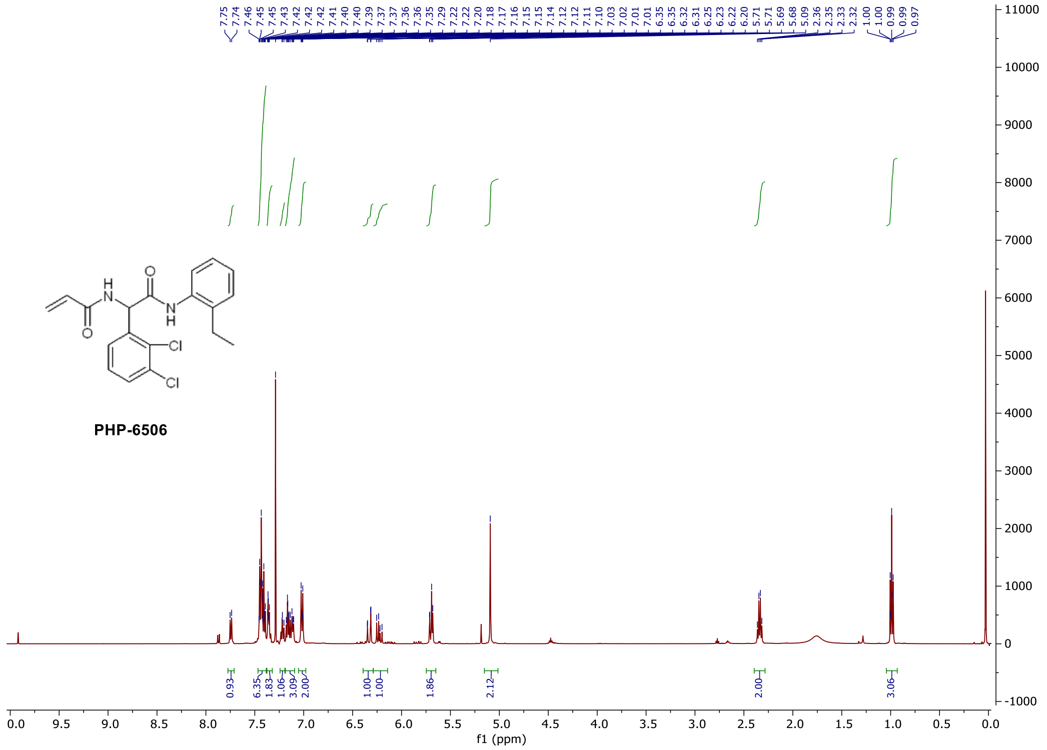

Supplementary Figure 1099. ^{1}H NMR (400 MHz, $CDCl_3$ + one drop of CD_3OD) of compound **PHP-6506.**

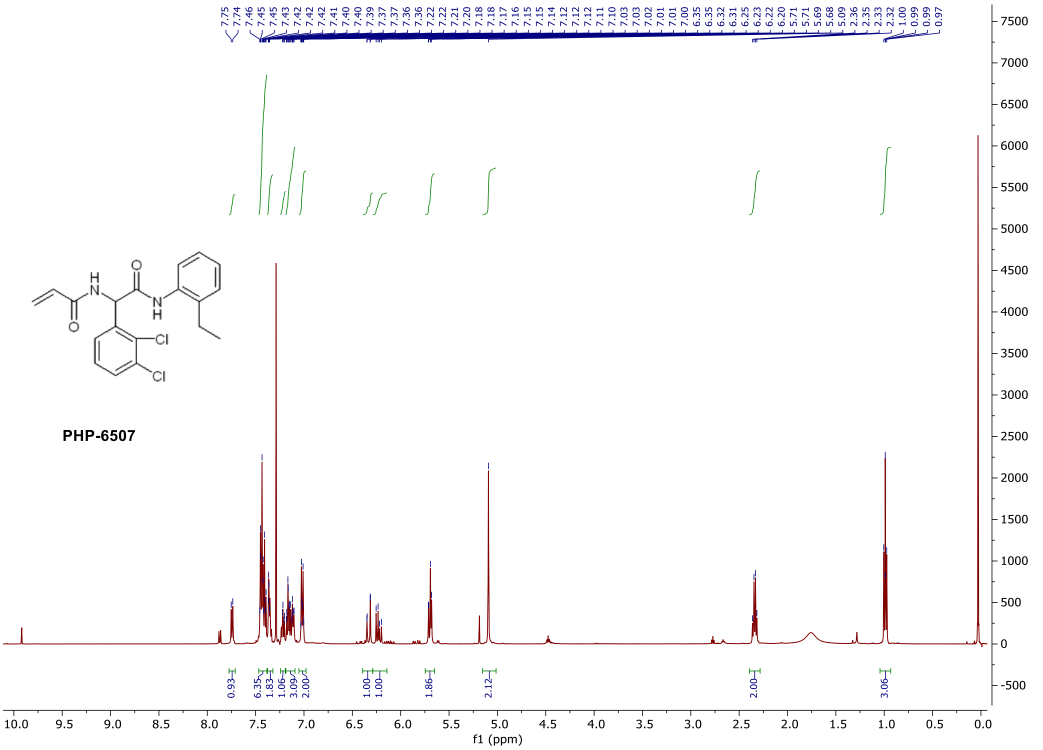

Supplementary Figure 1100. ^{1}H NMR (400 MHz, $CDCl_3$ + one drop of CD_3OD) of compound **PHP-6507.**

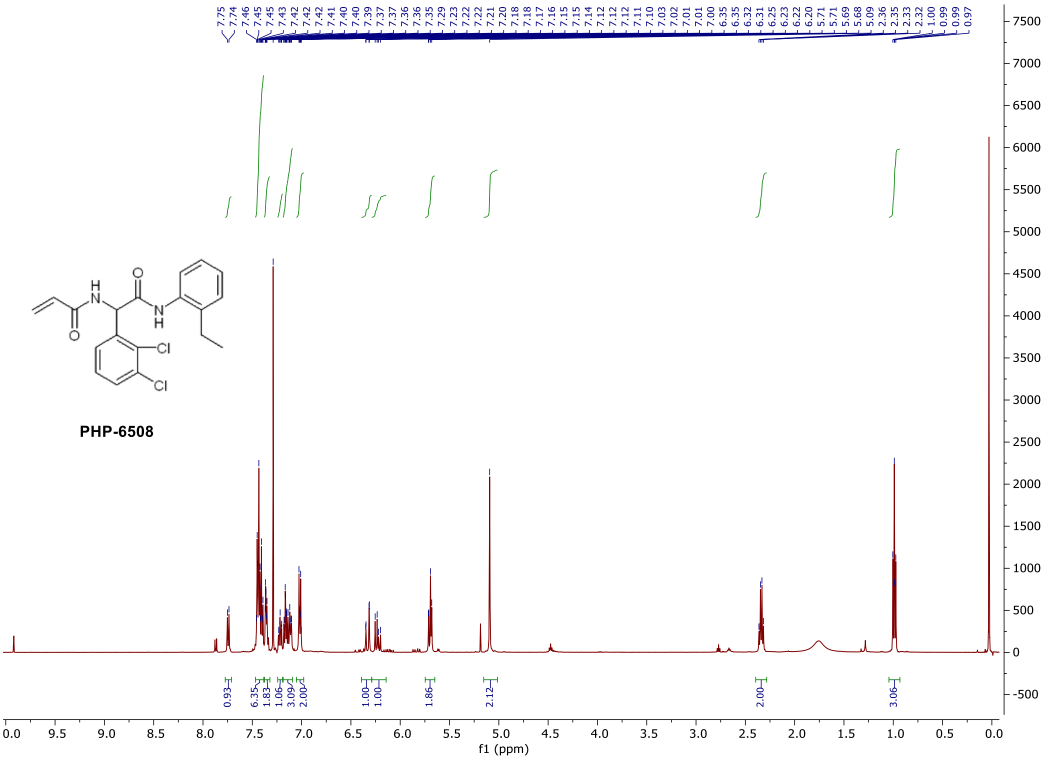

Supplementary Figure 1101. ^{1}H NMR (400 MHz, $CDCl_3$ + one drop of CD_3OD) of compound **PHP-6508.**

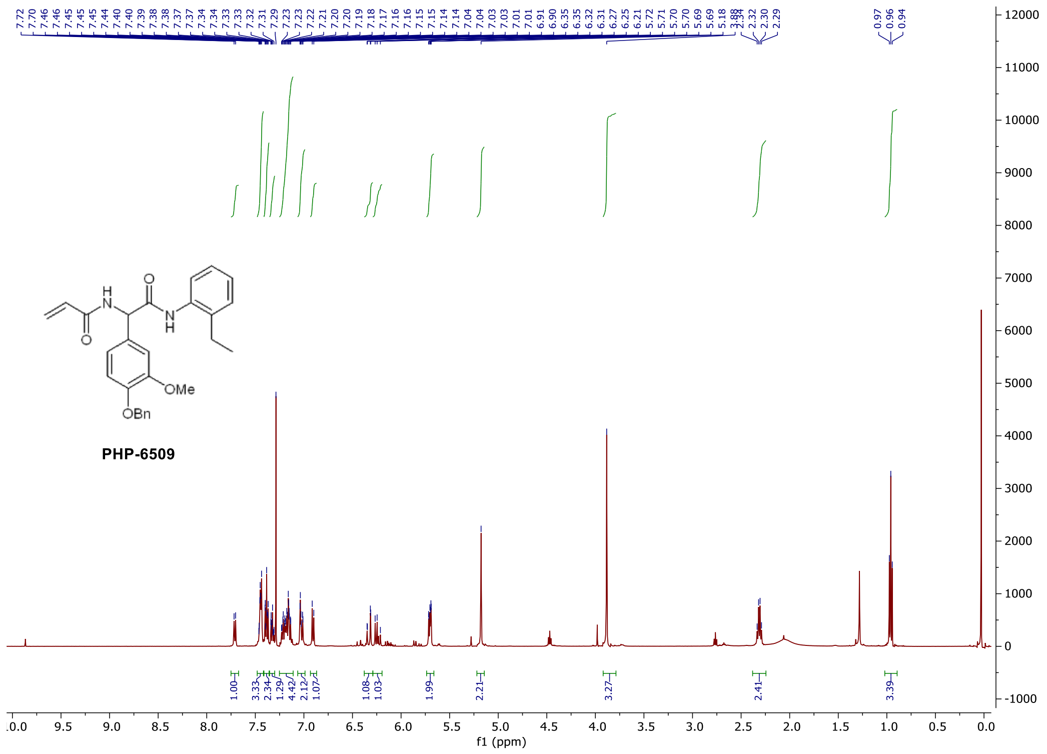

Supplementary Figure 1102. ^{1}H NMR (400 MHz, $CDCl_3$ + one drop of CD_3OD) of compound **PHP-6509.**

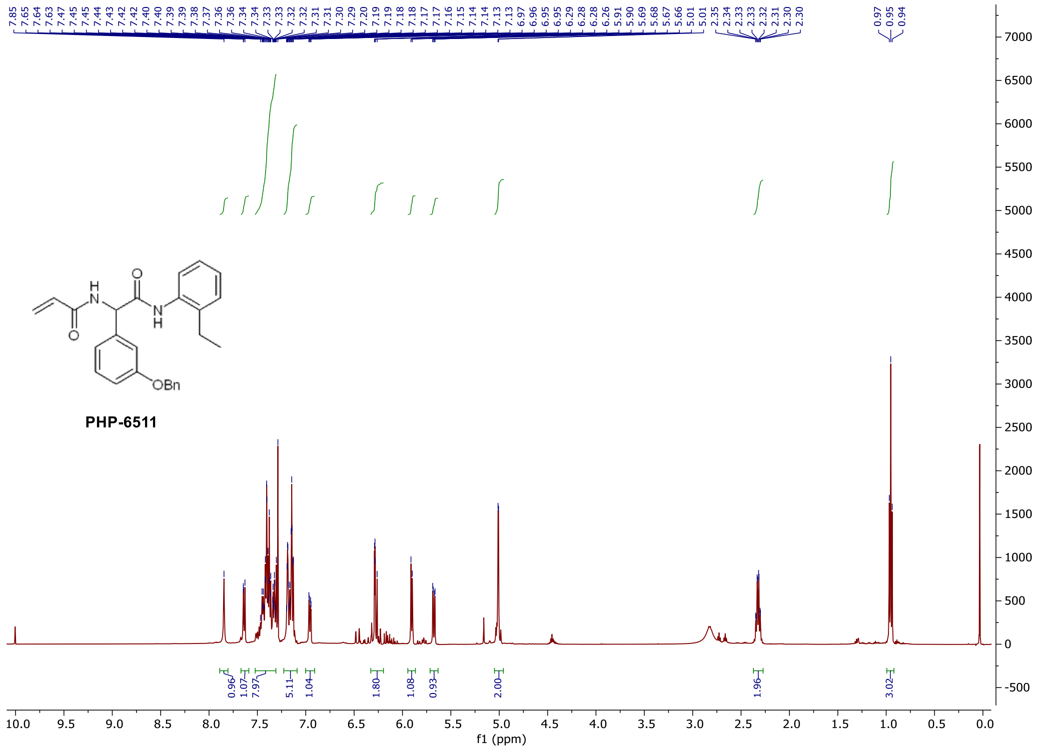

Supplementary Figure 1103. ^{1}H NMR (400 MHz, $CDCl_3$ + one drop of CD_3OD) of compound **PHP-6511.**

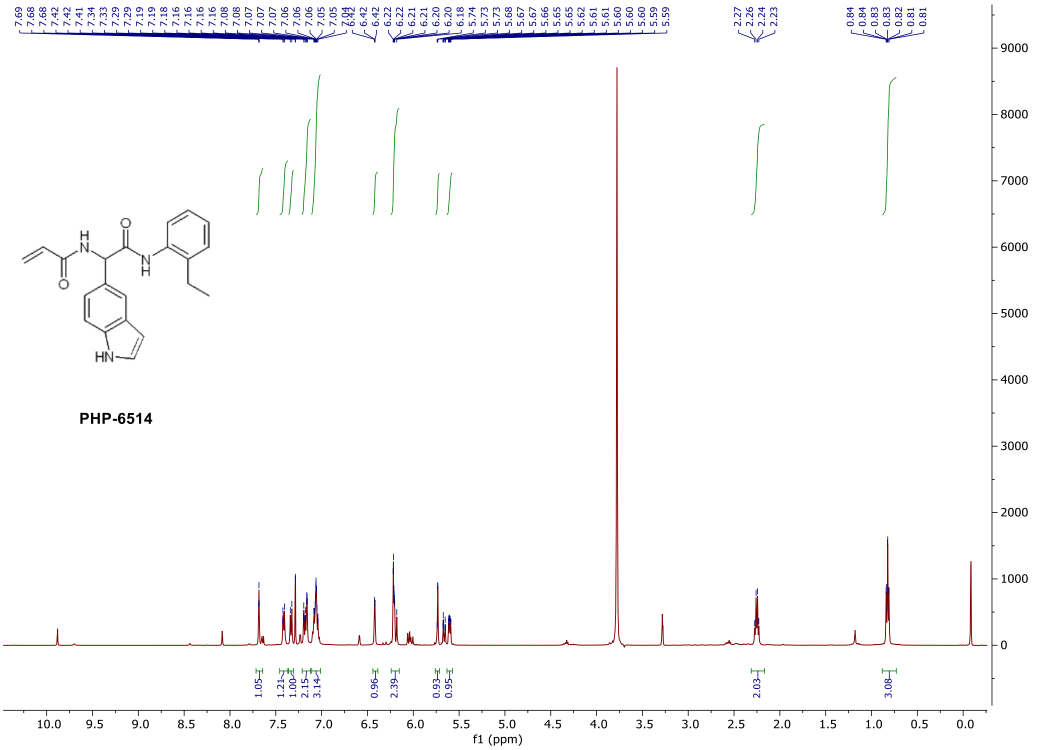

Supplementary Figure 1104. ^{1}H NMR (400 MHz, $CDCl_3$ + one drop of CD_3OD) of compound **PHP-6514.**

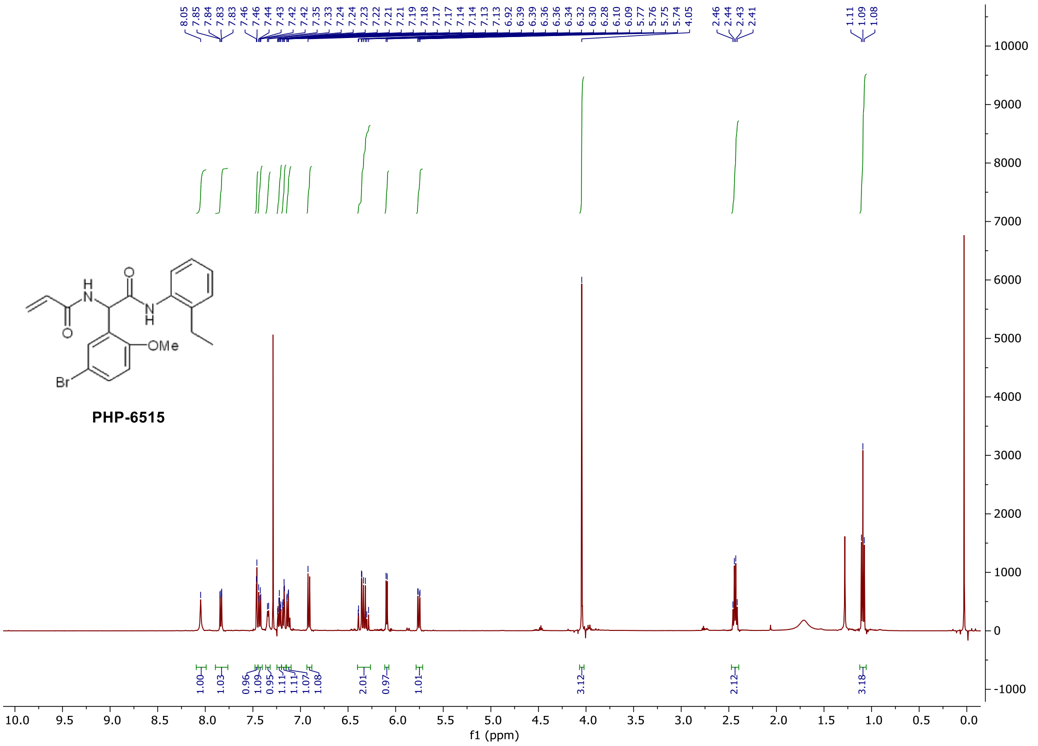

Supplementary Figure 1105. ^{1}H NMR (400 MHz, $CDCl_3$ + one drop of CD_3OD) of compound **PHP-6515.**

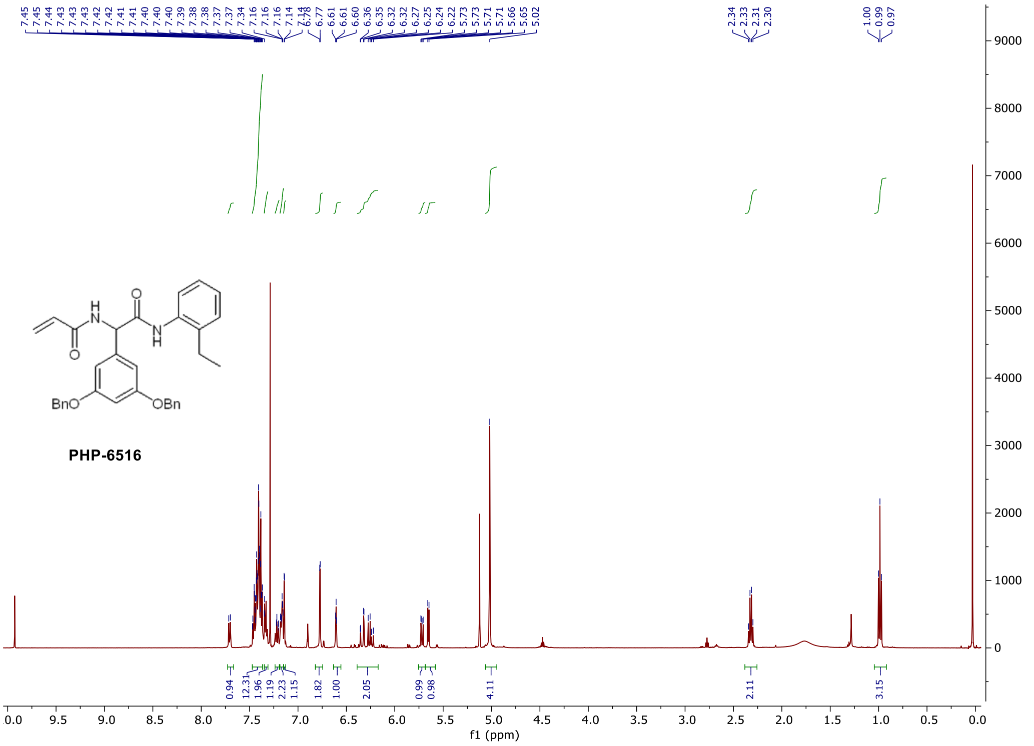

Supplementary Figure 1106. ^{1}H NMR (400 MHz, $CDCl_3$ + one drop of CD_3OD) of compound **PHP-6516.**

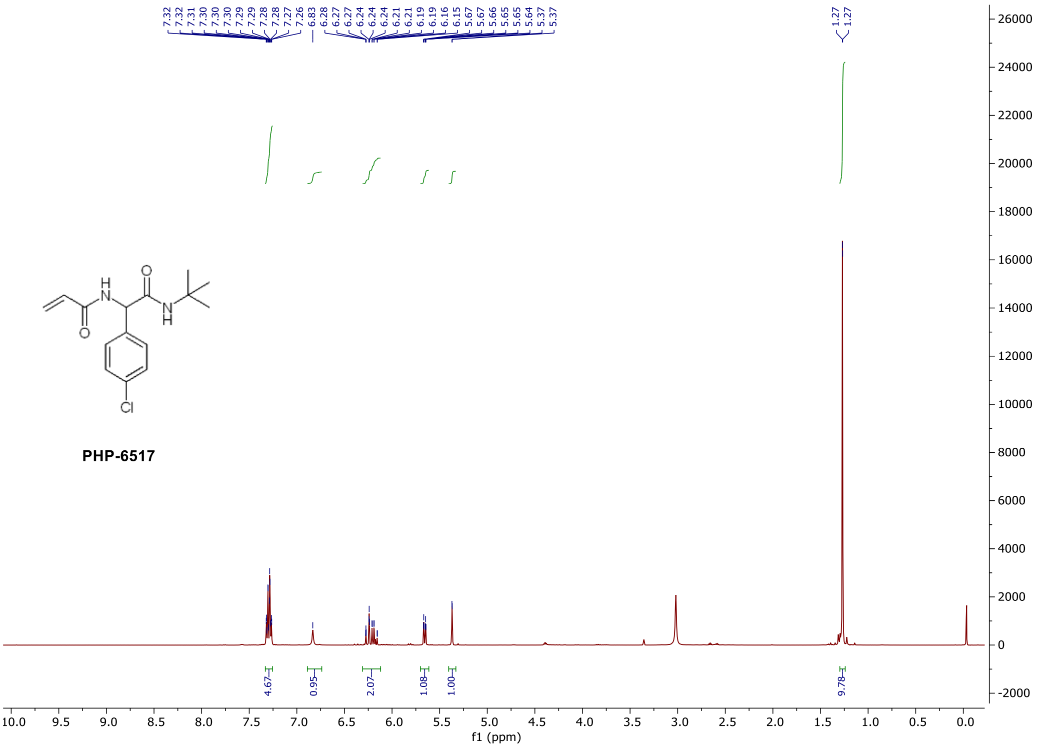

Supplementary Figure 1107. ^{1}H NMR (400 MHz, $CDCl_3$ + one drop of CD_3OD) of compound **PHP-6517.**

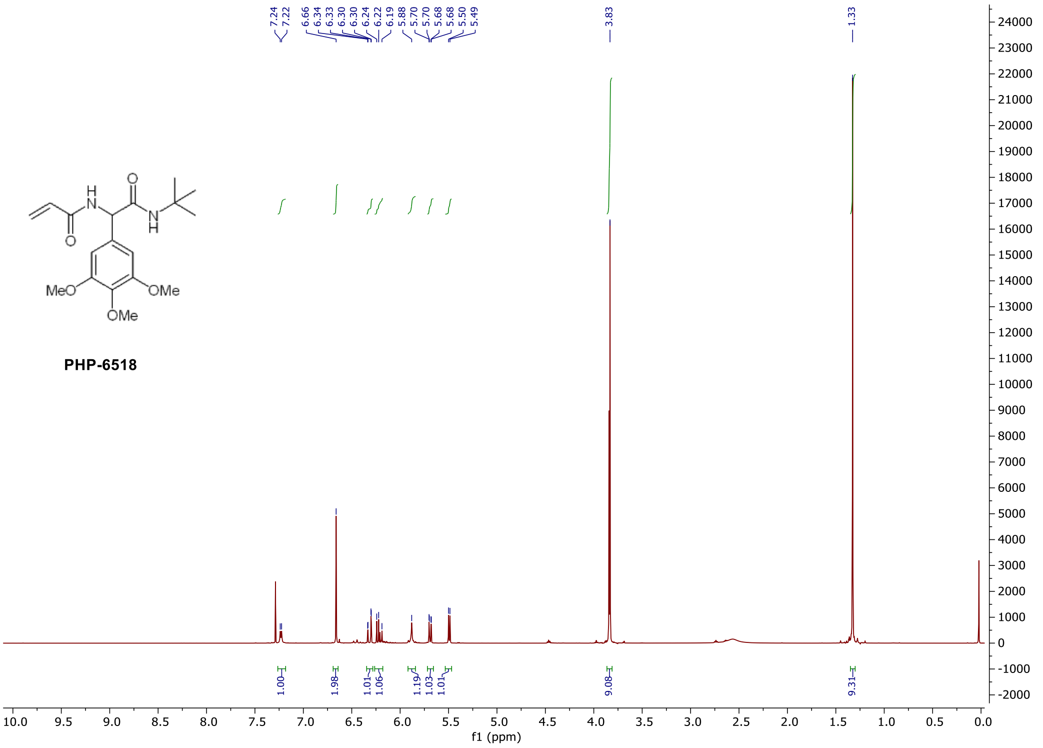

Supplementary Figure 1108. ^{1}H NMR (400 MHz, $CDCl_3$ + one drop of CD_3OD) of compound **PHP-6518.**

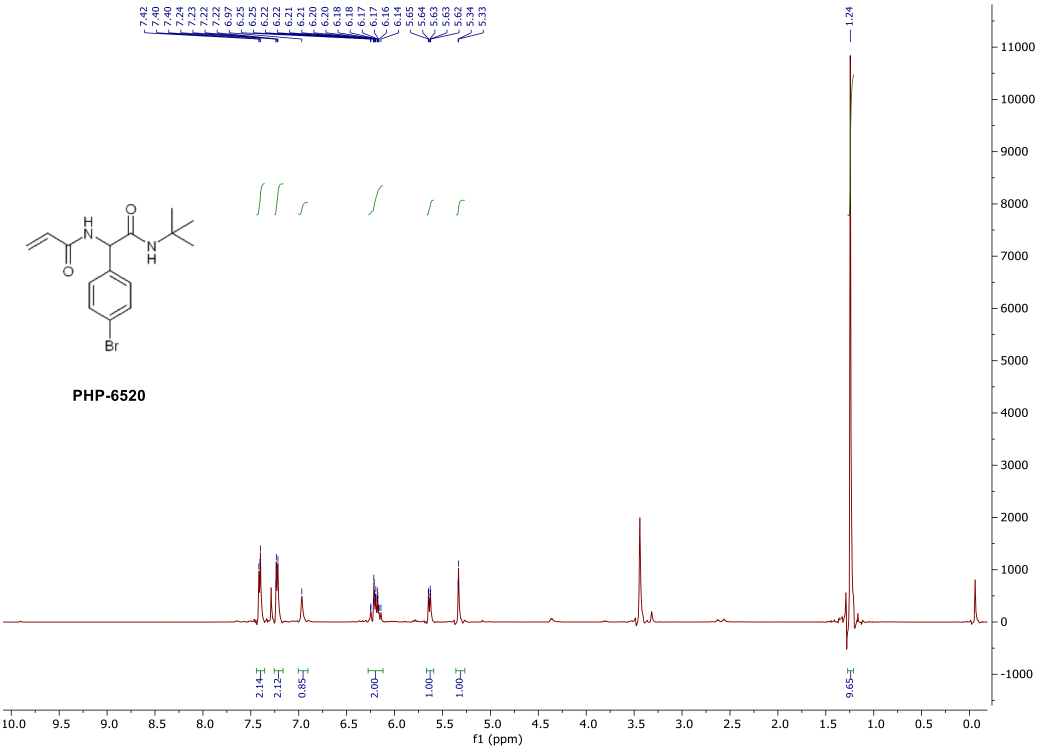

Supplementary Figure 1109. ^{1}H NMR (400 MHz, $CDCl_3$ + one drop of CD_3OD) of compound **PHP-6520.**

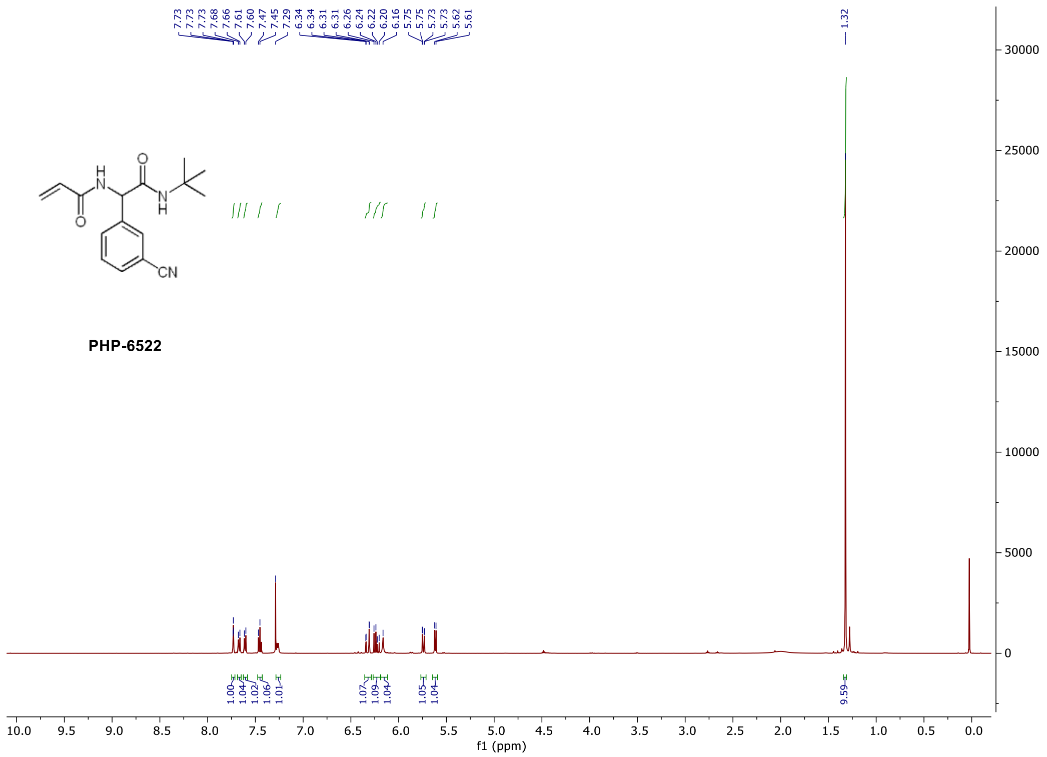

Supplementary Figure 1110. ^{1}H NMR (400 MHz, $CDCl_3$ + one drop of CD_3OD) of compound **PHP-6522.**

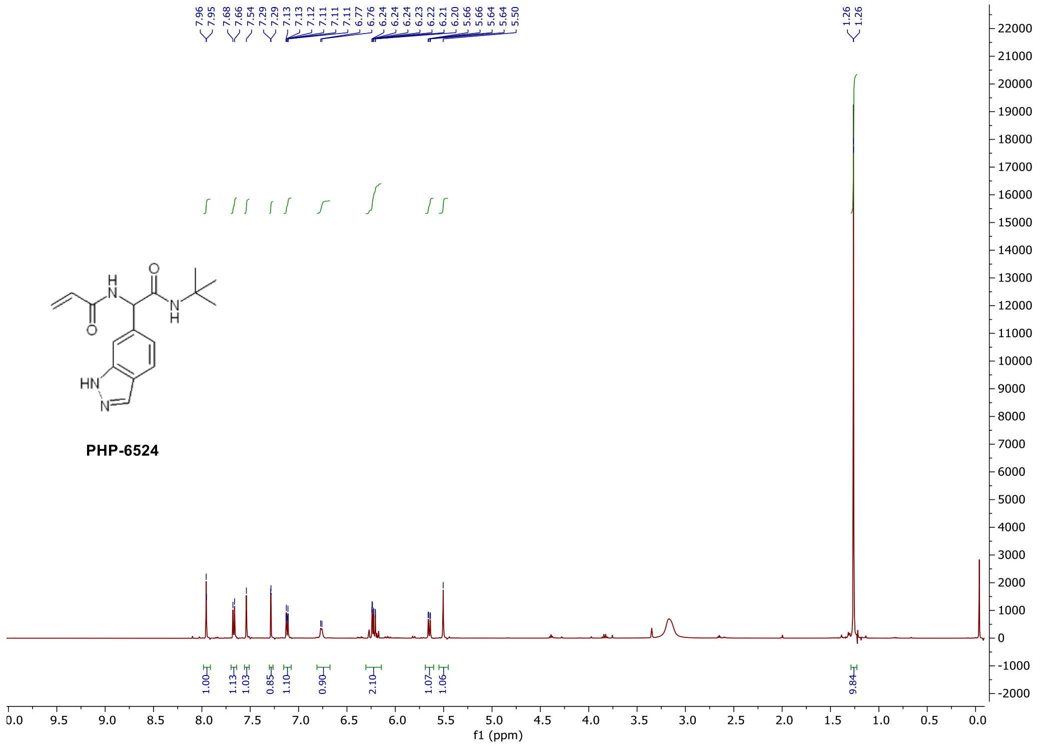

Supplementary Figure 1111. ^{1}H NMR (400 MHz, $CDCl_3$ + one drop of CD_3OD) of compound **PHP-6524.**

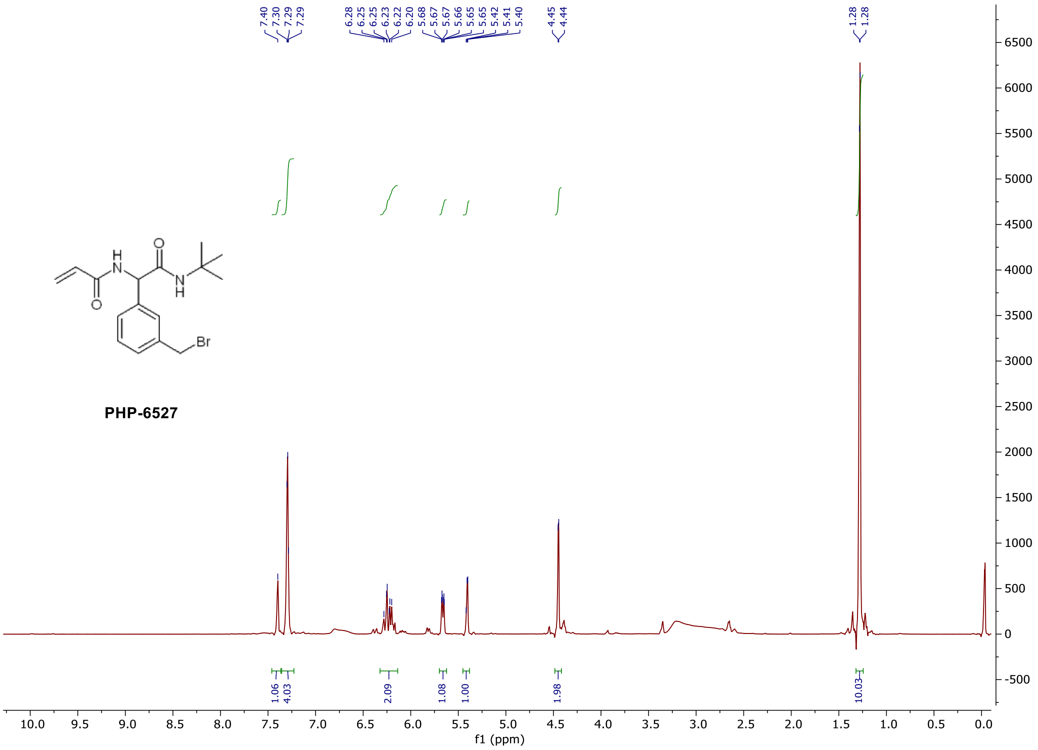

Supplementary Figure 1112. ^{1}H NMR (400 MHz, $CDCl_3$ + one drop of CD_3OD) of compound **PHP-6527.**

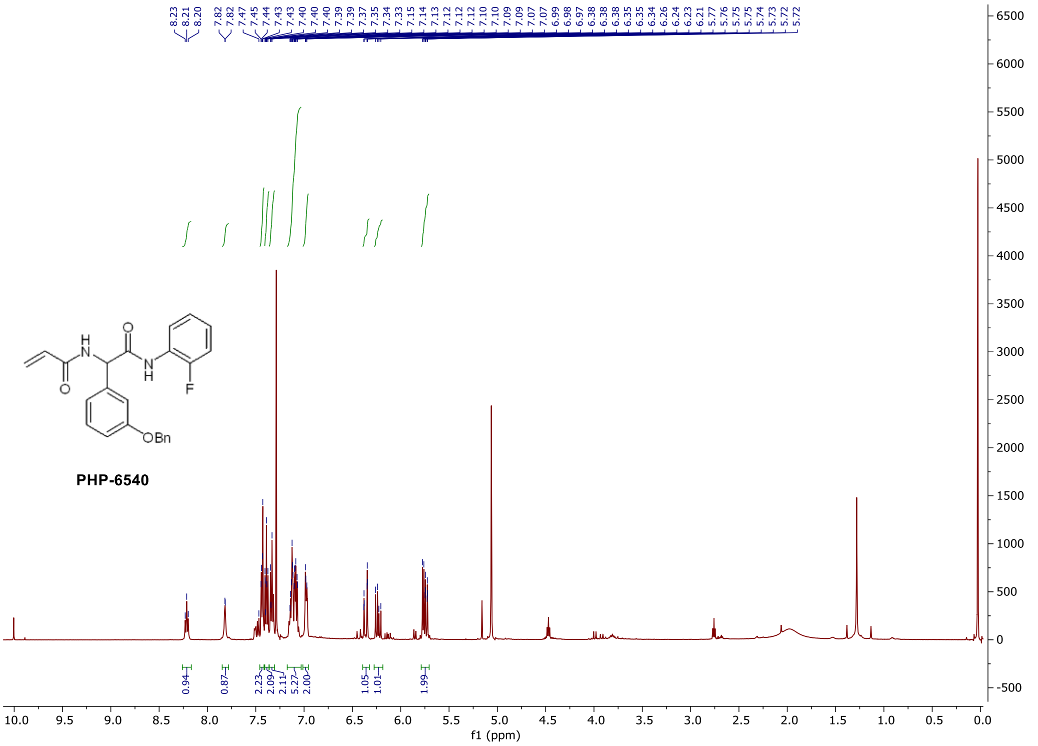

Supplementary Figure 1113. ^{1}H NMR (400 MHz, $CDCl_3$ + one drop of CD_3OD) of compound **PHP-6540.**

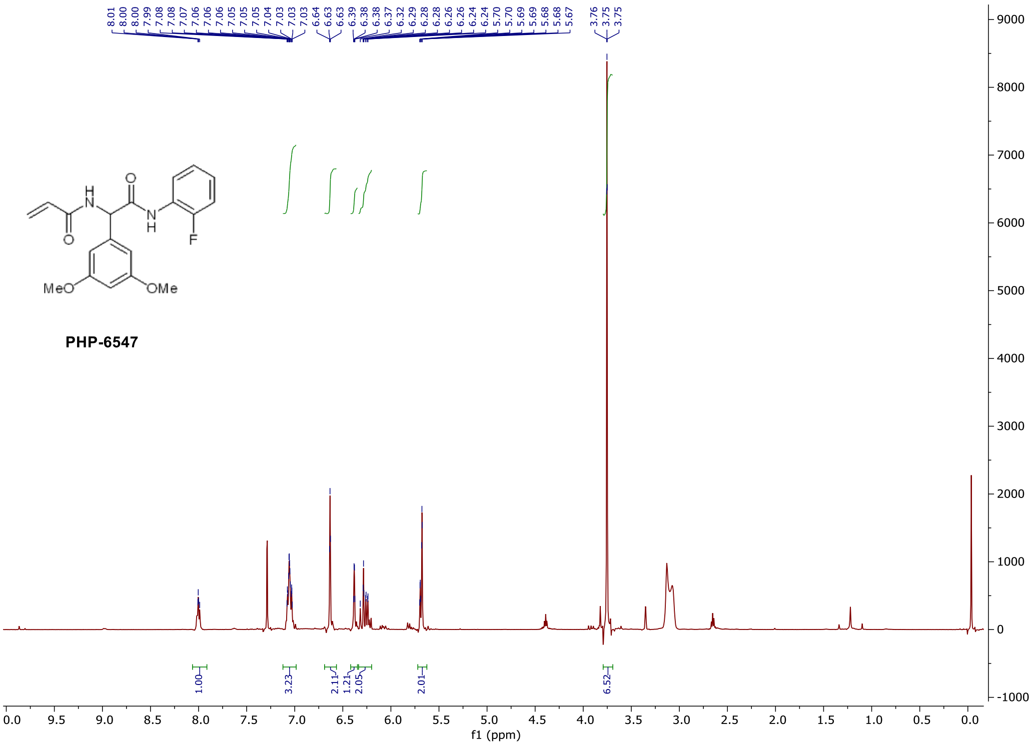

Supplementary Figure 1114. ^{1}H NMR (400 MHz, $CDCl_3$ + one drop of CD_3OD) of compound **PHP-6547.**

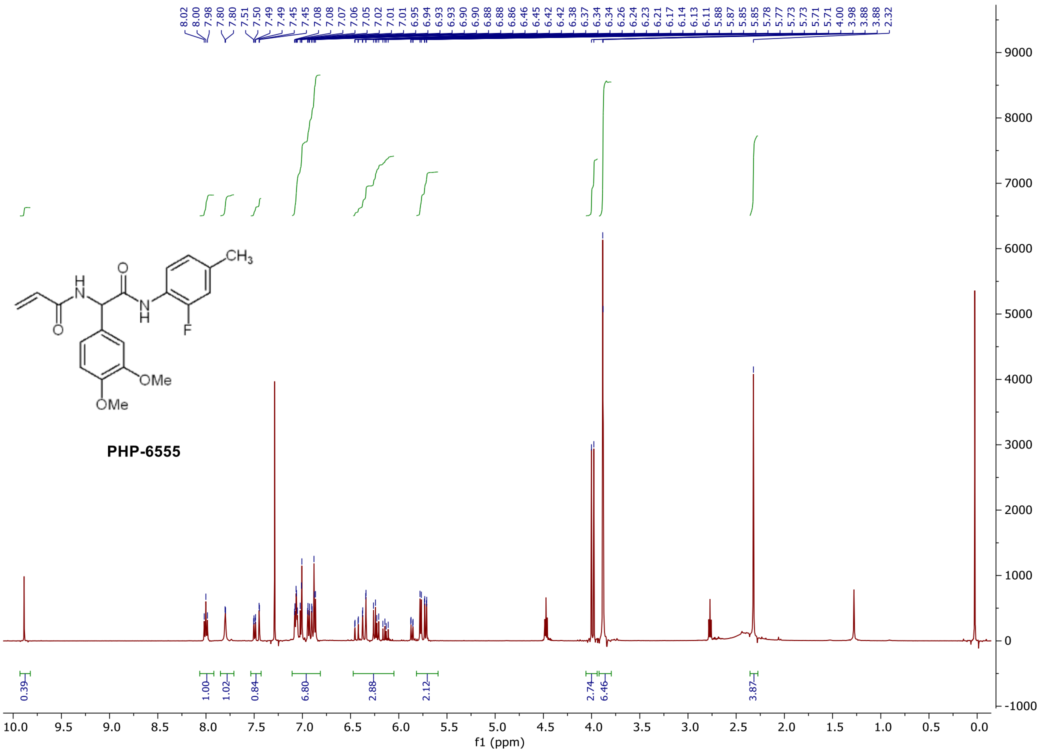

Supplementary Figure 1115. ^{1}H NMR (400 MHz, $CDCl_3$ + one drop of CD_3OD) of compound **PHP-6555.**

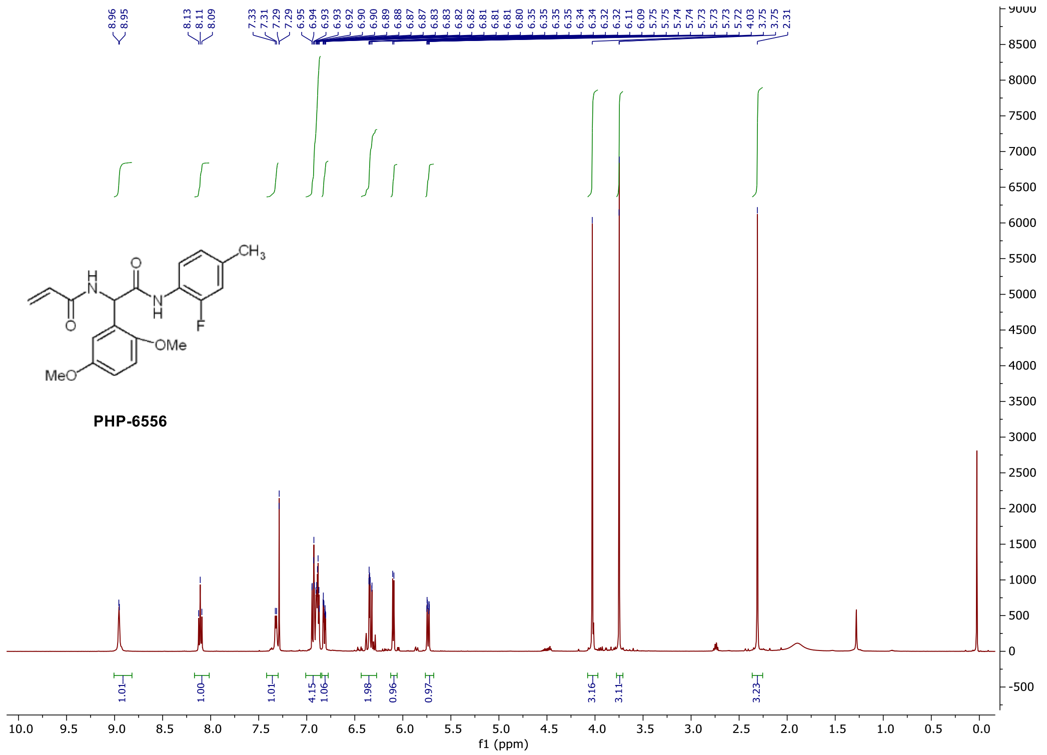

Supplementary Figure 1116. ^{1}H NMR (400 MHz, $CDCl_3$ + one drop of CD_3OD) of compound **PHP-6556.**

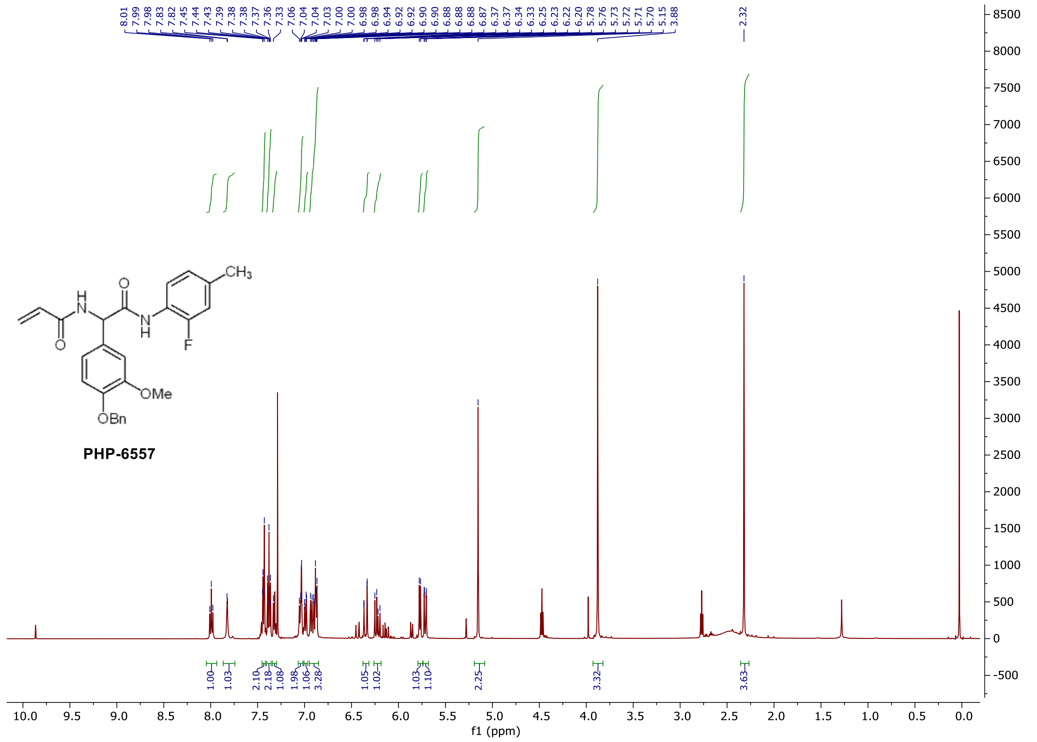

Supplementary Figure 1117. ^{1}H NMR (400 MHz, $CDCl_3$ + one drop of CD_3OD) of compound **PHP-6557.**

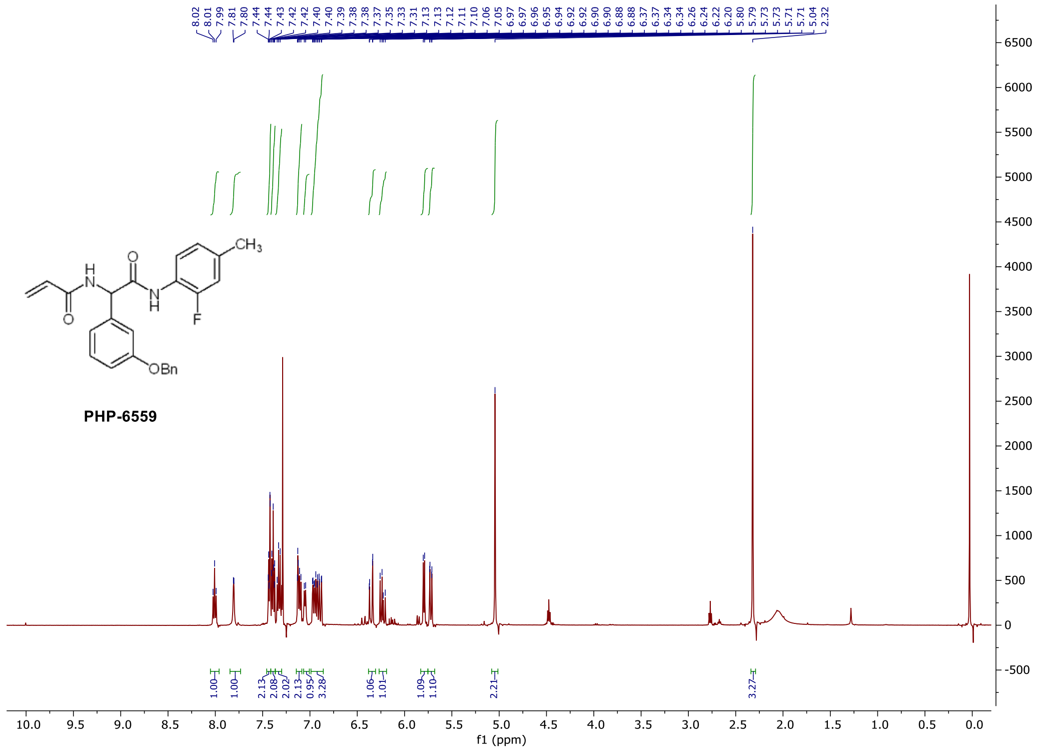

Supplementary Figure 1118. ^{1}H NMR (400 MHz, $CDCl_3$ + one drop of CD_3OD) of compound **PHP-6559.**

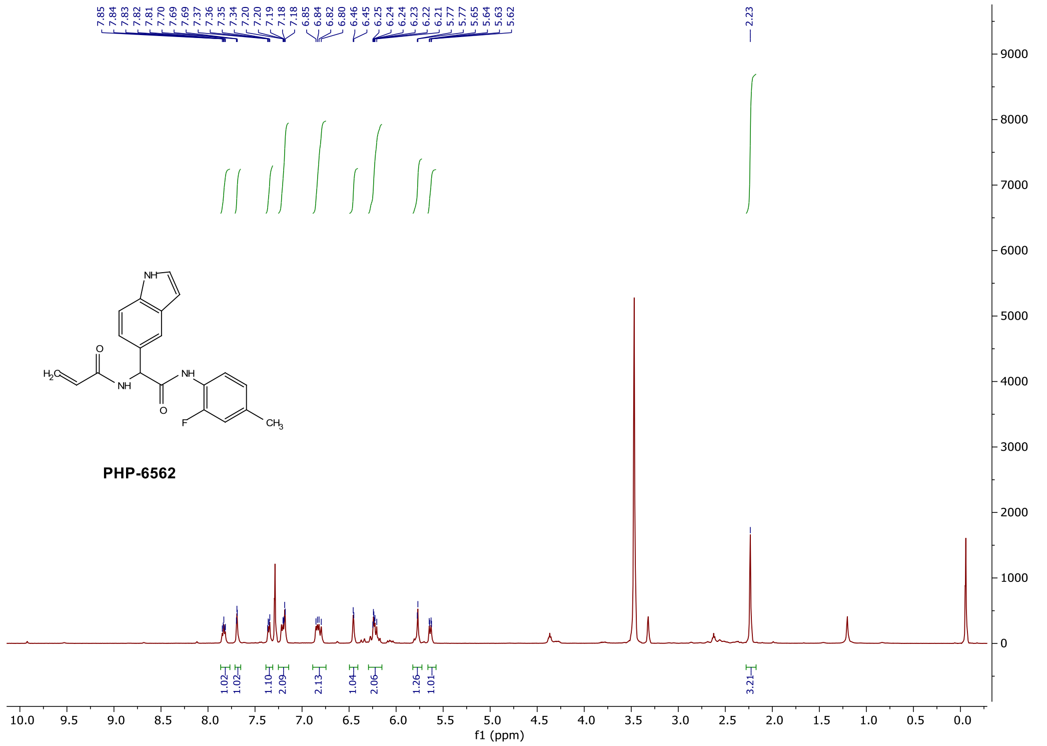

Supplementary Figure 1119. ^{1}H NMR (400 MHz, $CDCl_3$ + one drop of CD_3OD) of compound **PHP-6562.**

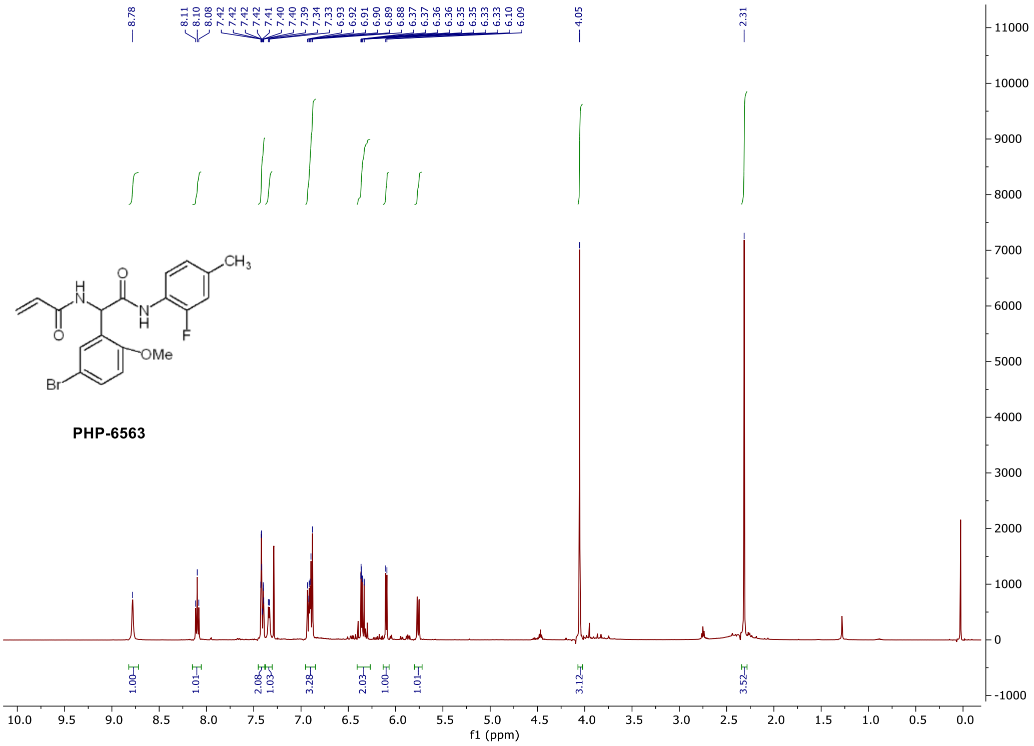

Supplementary Figure 1120. ^{1}H NMR (400 MHz, $CDCl_3$ + one drop of CD_3OD) of compound **PHP-6563**.

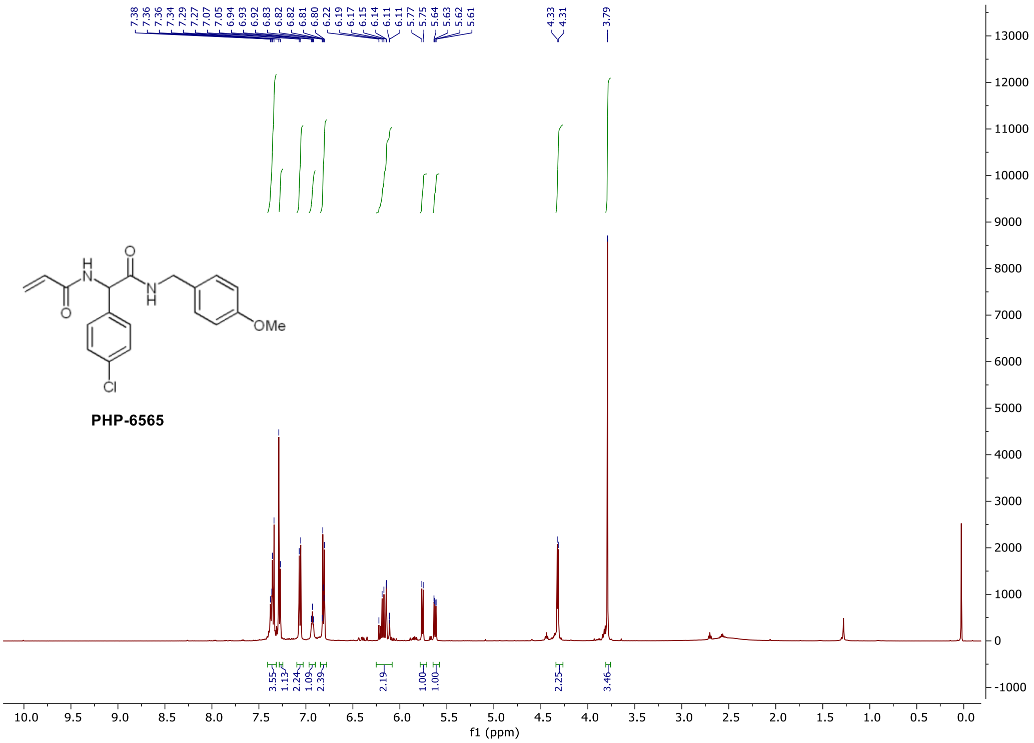

Supplementary Figure 1121. ^{1}H NMR (400 MHz, $CDCl_3$ + one drop of CD_3OD) of compound **PHP-6565**.

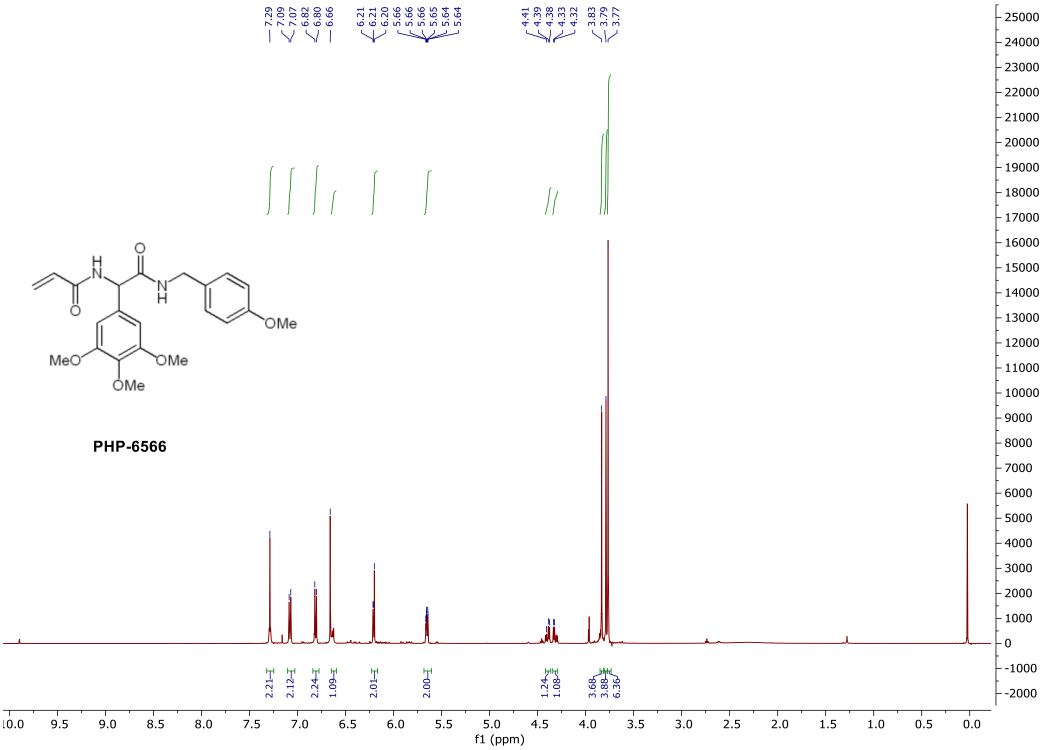

Supplementary Figure 1122. ^{1}H NMR (400 MHz, $CDCl_3$ + one drop of CD_3OD) of compound **PHP-6566.**

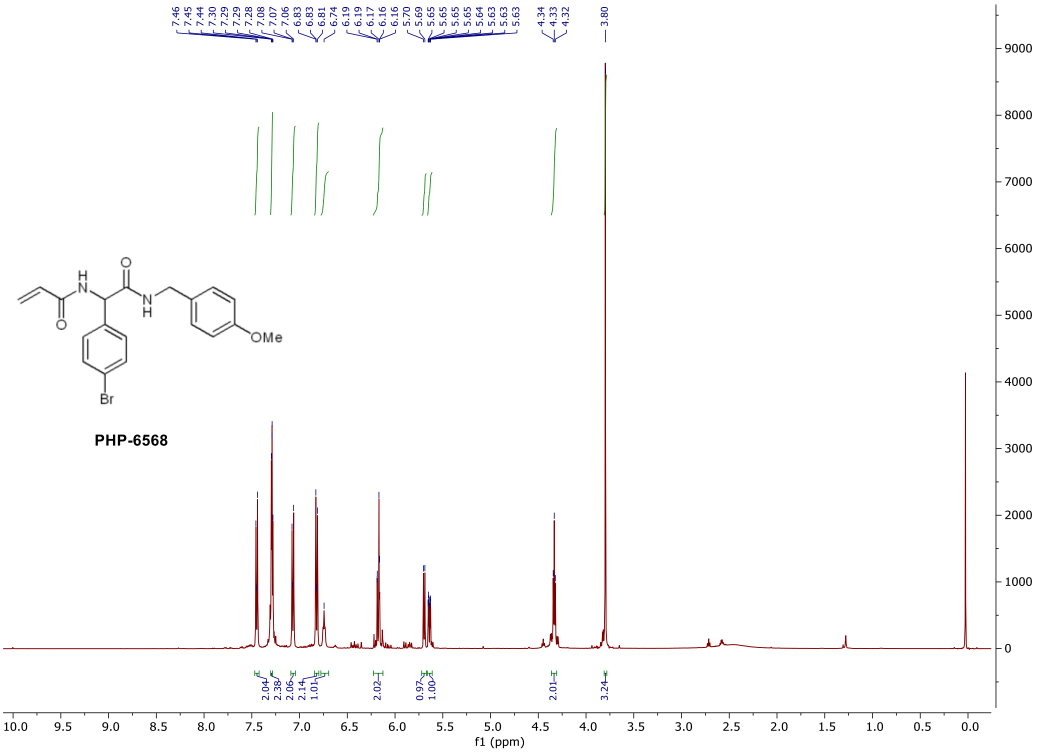

Supplementary Figure 1123. ^{1}H NMR (400 MHz, $CDCl_3$ + one drop of CD_3OD) of compound **PHP-6568.**

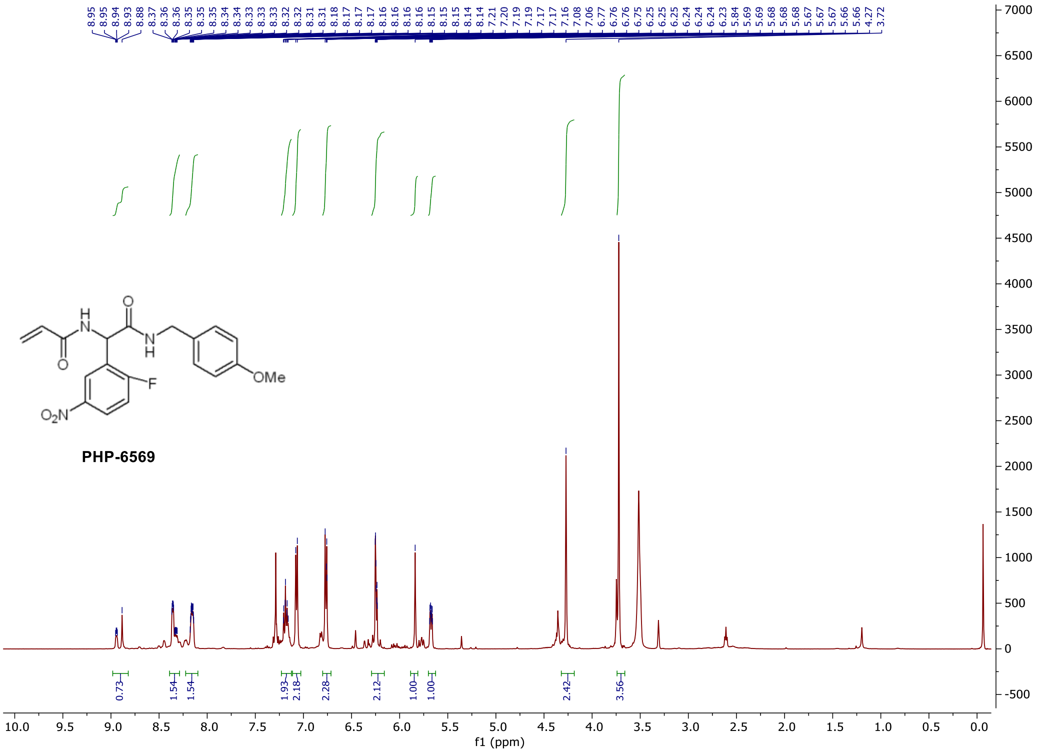

Supplementary Figure 1124. ^{1}H NMR (400 MHz, $CDCl_3$ + one drop of CD_3OD) of compound **PHP-6569.**

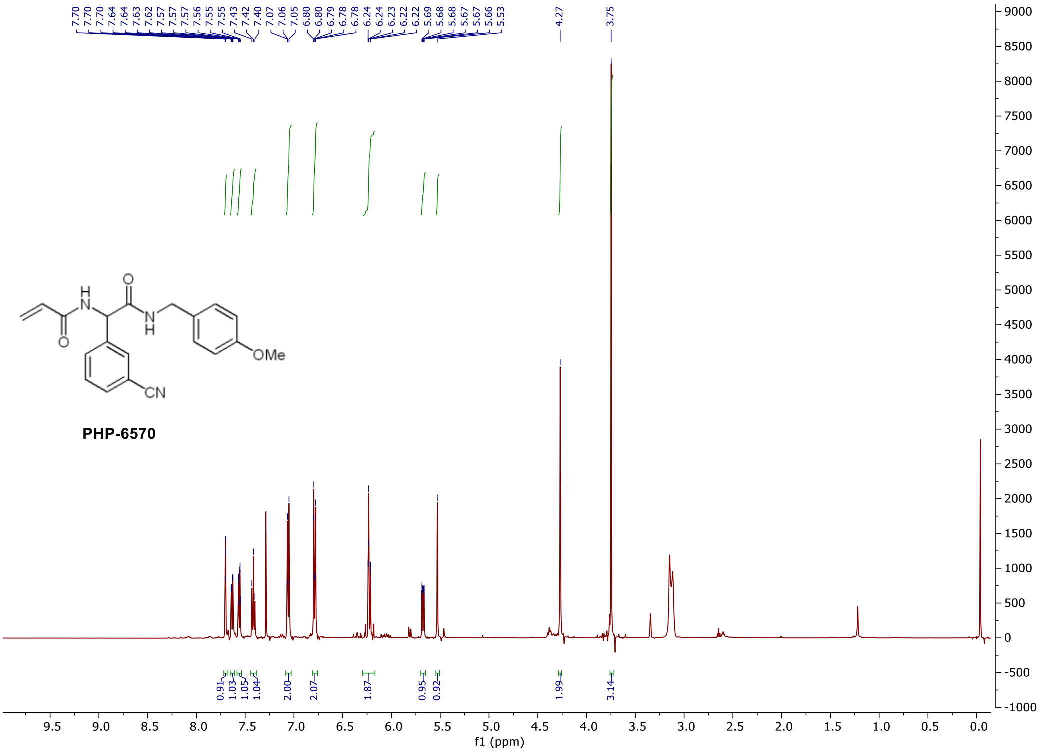

Supplementary Figure 1125. ^{1}H NMR (400 MHz, $CDCl_3$ + one drop of CD_3OD) of compound **PHP-6570.**

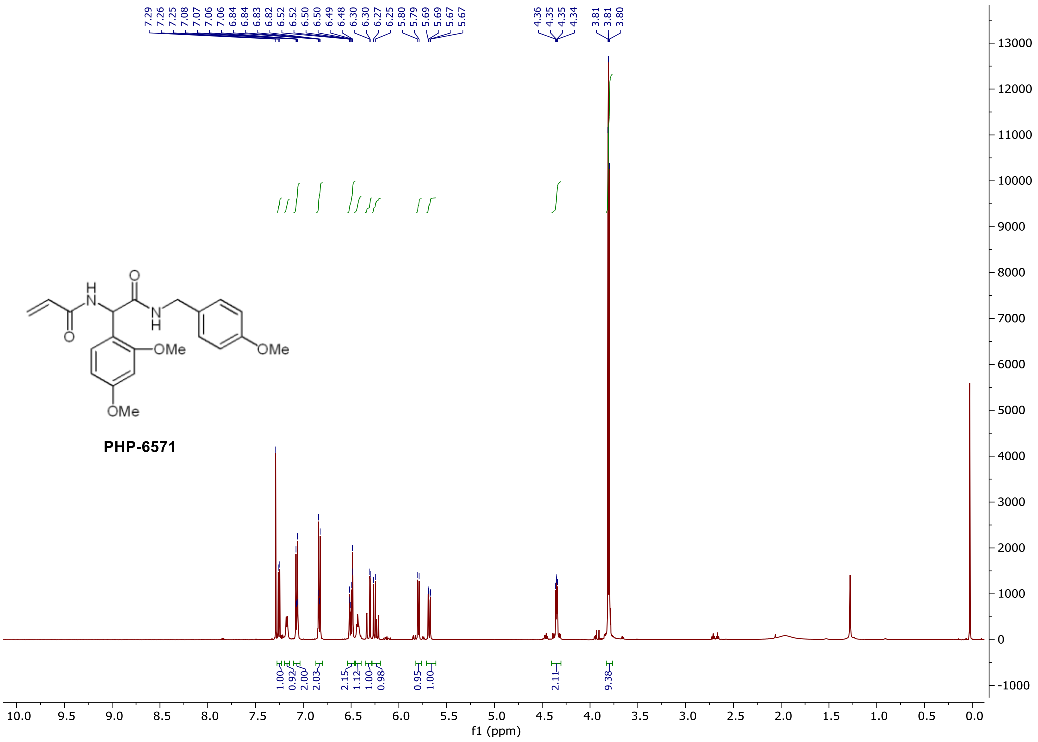

Supplementary Figure 1126. ^{1}H NMR (400 MHz, $CDCl_3$ + one drop of CD_3OD) of compound **PHP-6571.**

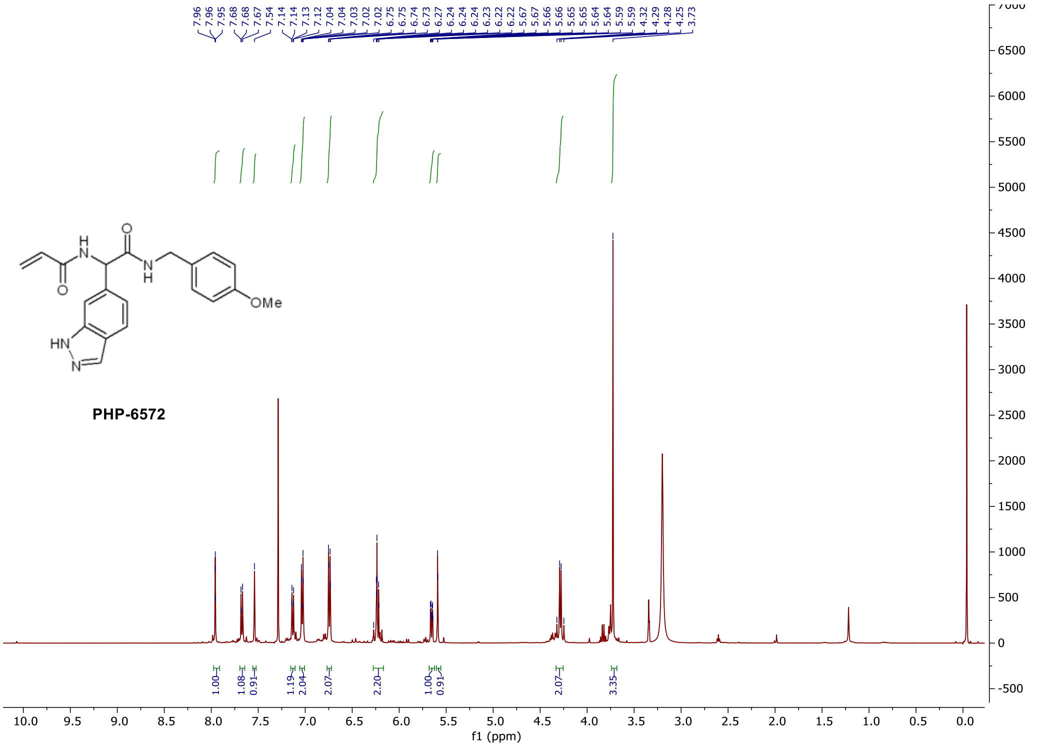

Supplementary Figure 1127. ^{1}H NMR (400 MHz, $CDCl_3$ + one drop of CD_3OD) of compound **PHP-6572.**

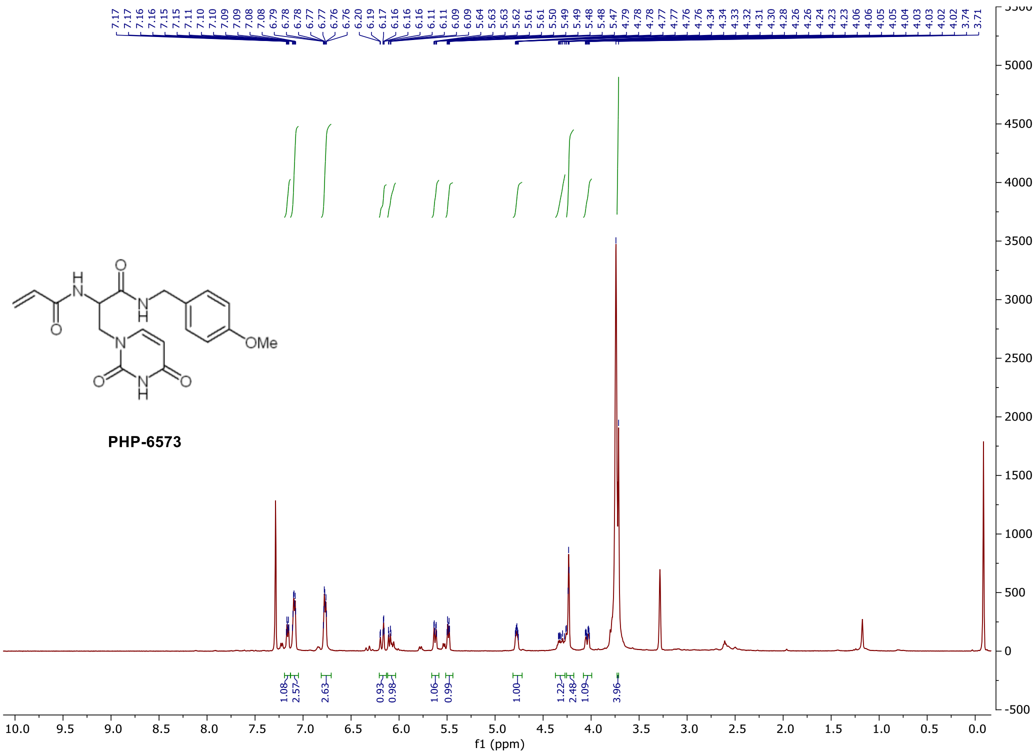

Supplementary Figure 1128. ^{1}H NMR (400 MHz, $CDCl_3$ + one drop of CD_3OD) of compound **PHP-6573.**

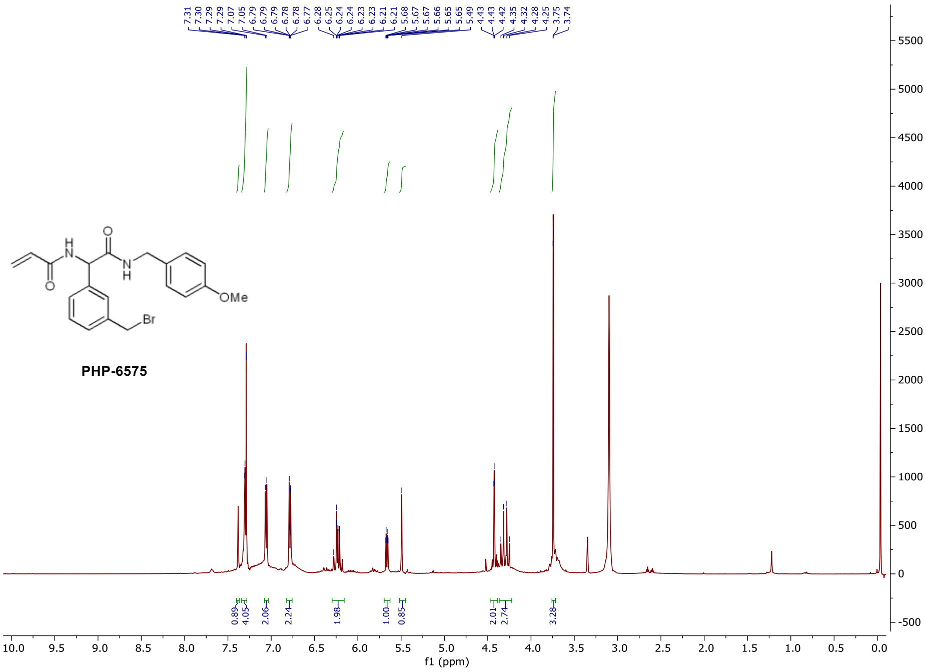

Supplementary Figure 1129. ^{1}H NMR (400 MHz, $CDCl_3$ + one drop of CD_3OD) of compound **PHP-6575.**

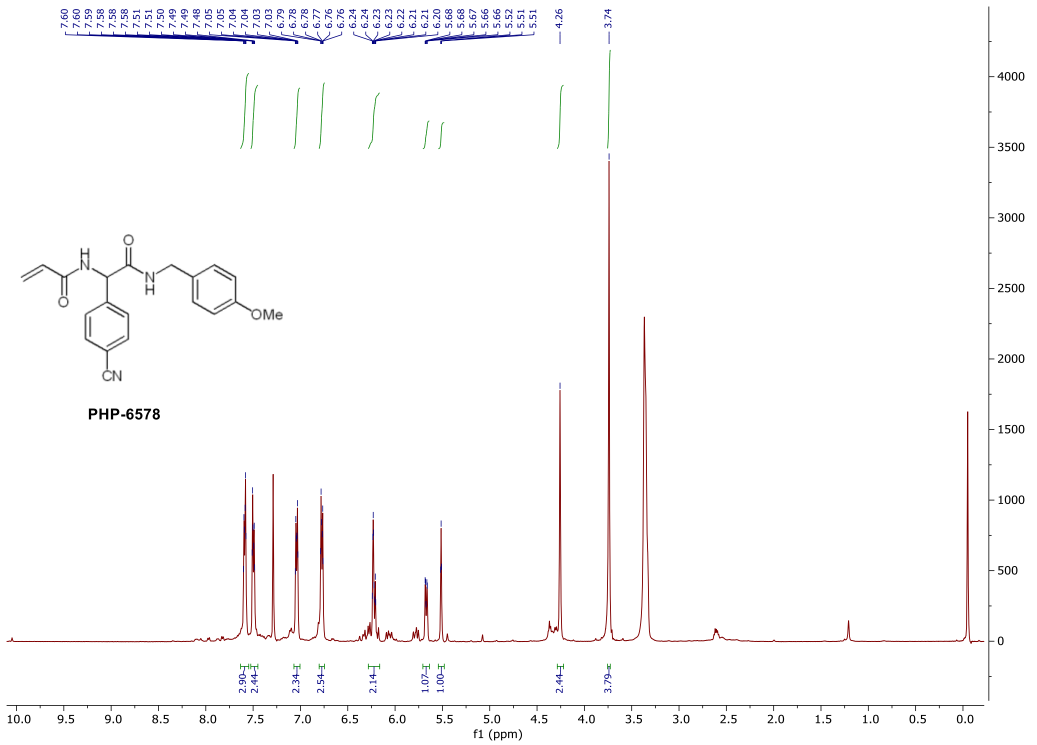

Supplementary Figure 1130. ^{1}H NMR (400 MHz, $CDCl_3$ + one drop of CD_3OD) of compound **PHP-6578.**

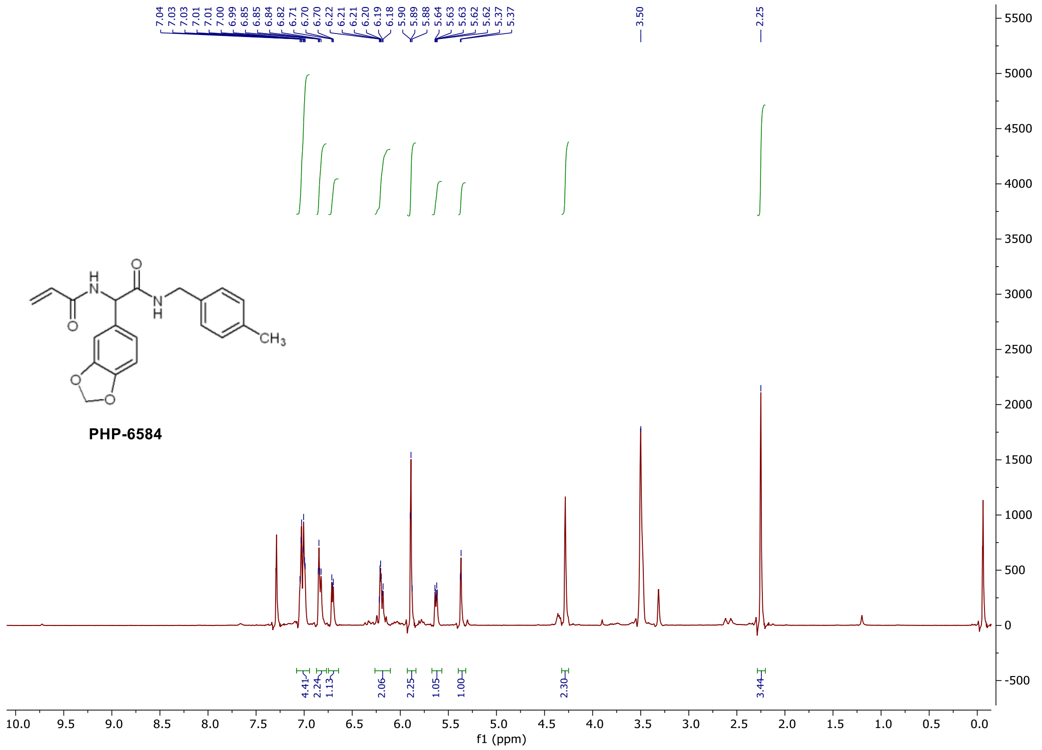

Supplementary Figure 1131. ^{1}H NMR (400 MHz, $CDCl_3$ + one drop of CD_3OD) of compound **PHP-6584.**

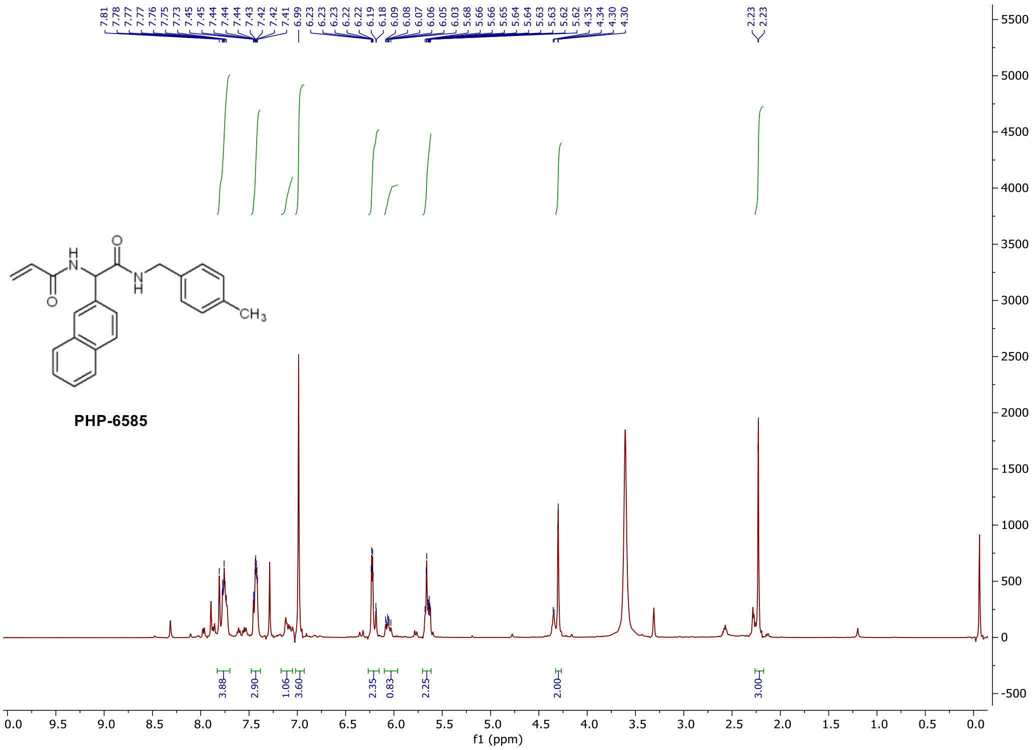

Supplementary Figure 1132. ^{1}H NMR (400 MHz, $CDCl_3$ + one drop of CD_3OD) of compound **PHP-6585.**

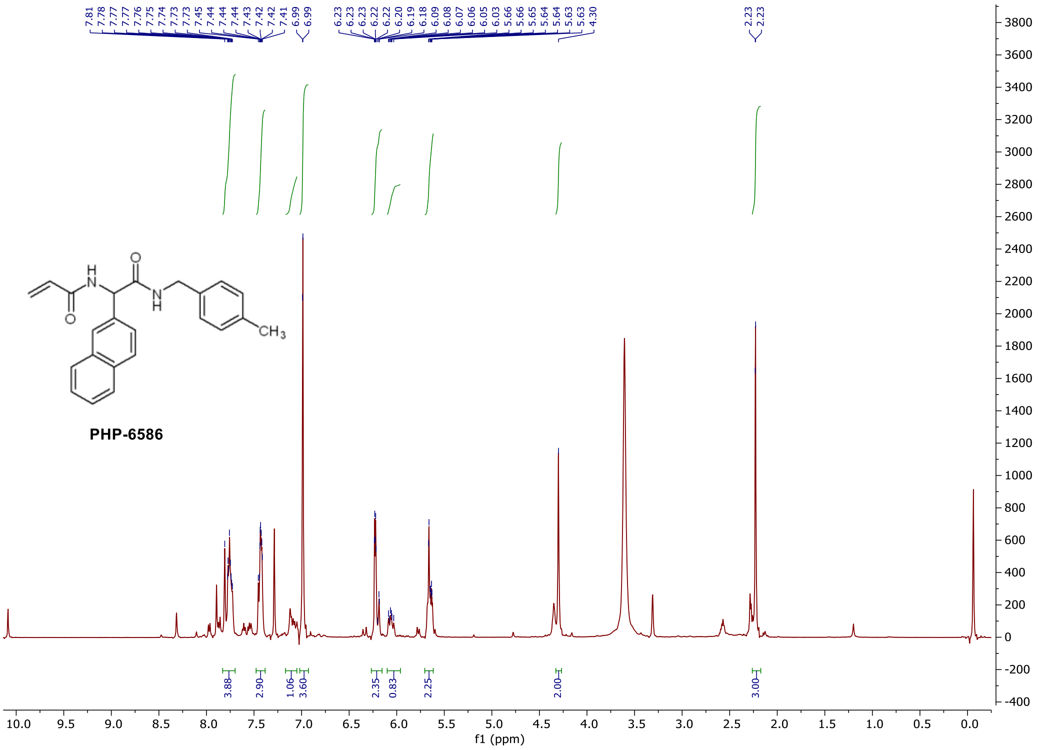

Supplementary Figure 1133. ^{1}H NMR (400 MHz, $CDCl_3$ + one drop of CD_3OD) of compound **PHP-6586.**

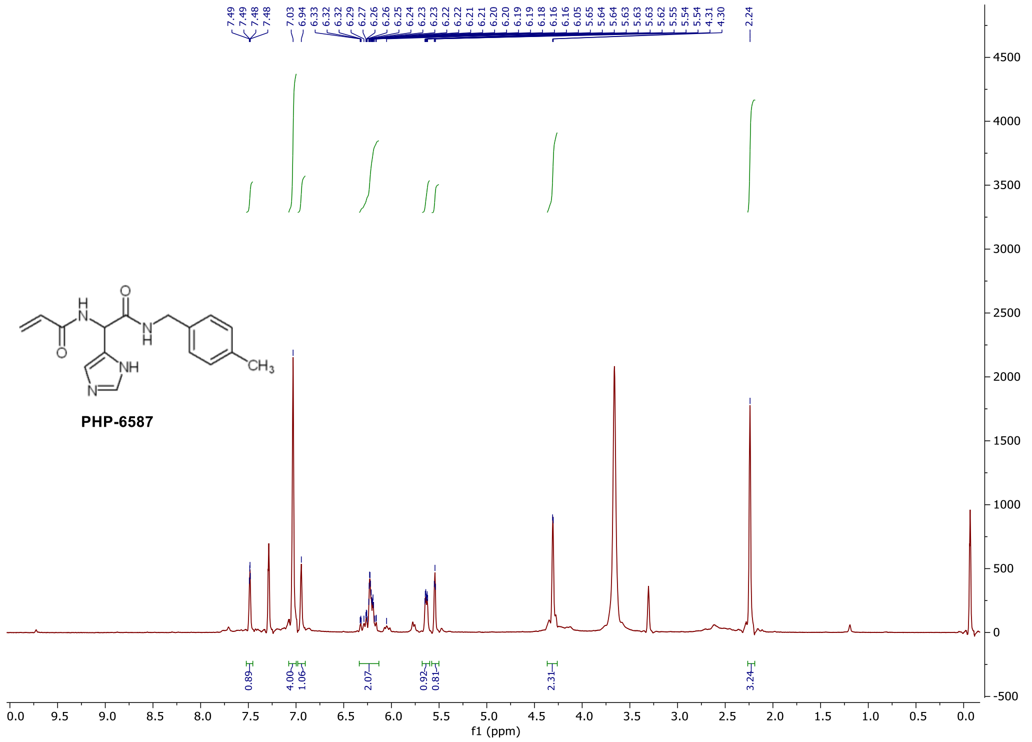

Supplementary Figure 1134. ^{1}H NMR (400 MHz, $CDCl_3$ + one drop of CD_3OD) of compound **PHP-6587.**

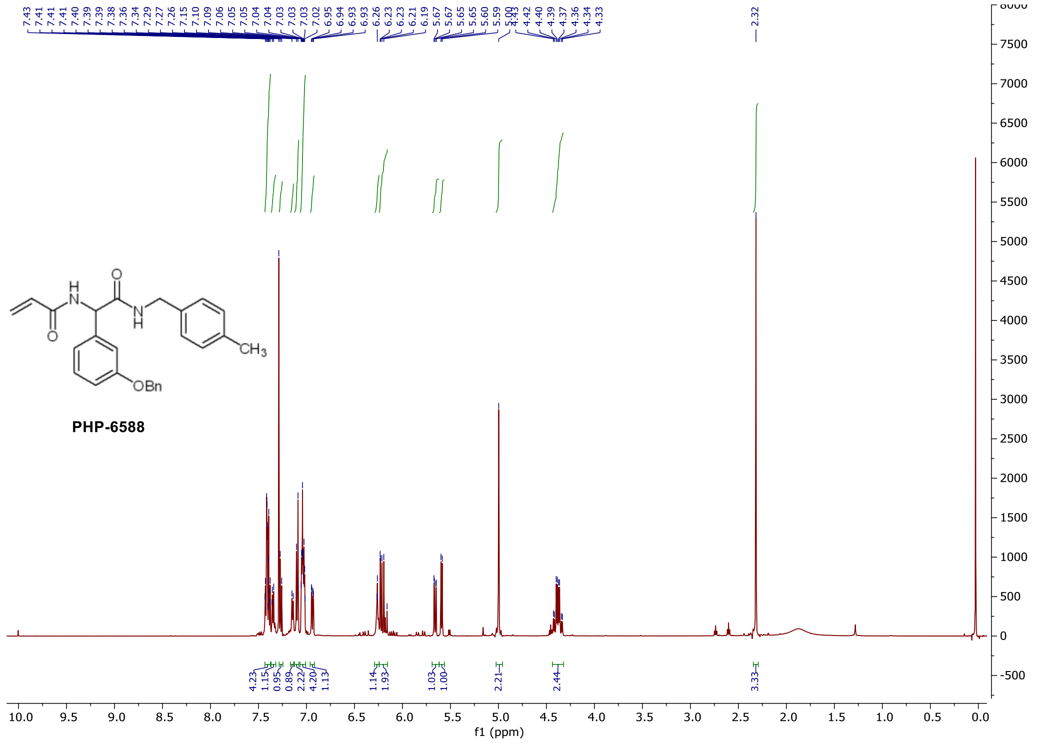

Supplementary Figure 1135. ^{1}H NMR (400 MHz, $CDCl_3$ + one drop of CD_3OD) of compound **PHP-6588.**

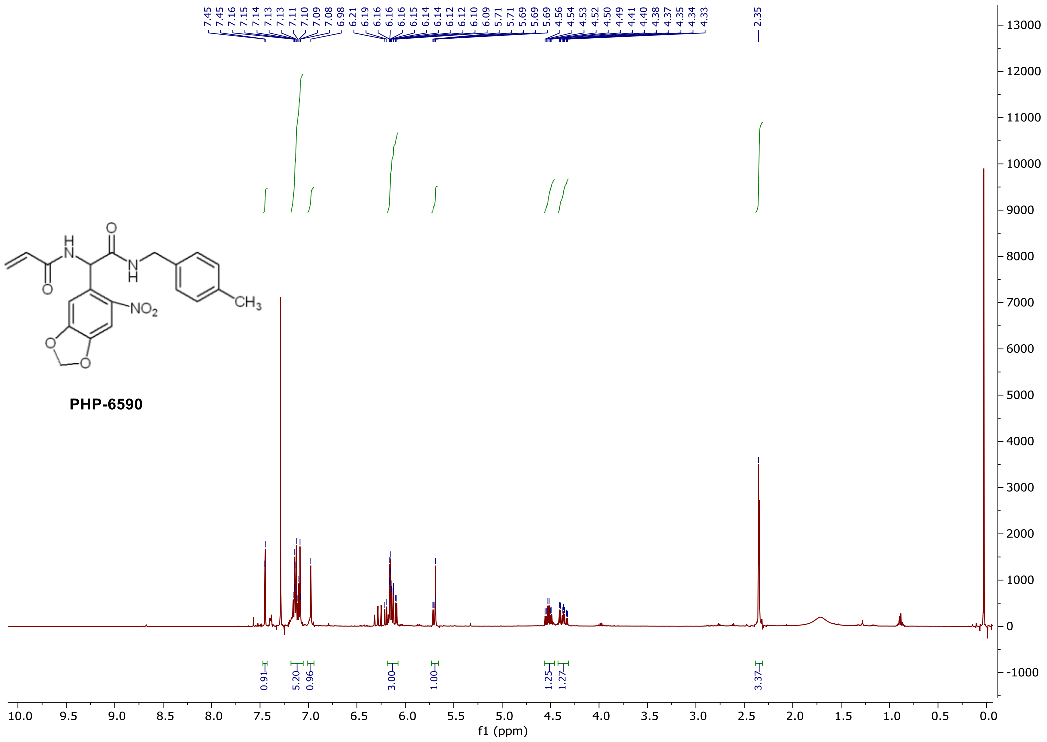

Supplementary Figure 1136. ^{1}H NMR (400 MHz, $CDCl_3$ + one drop of CD_3OD) of compound **PHP-6590.**

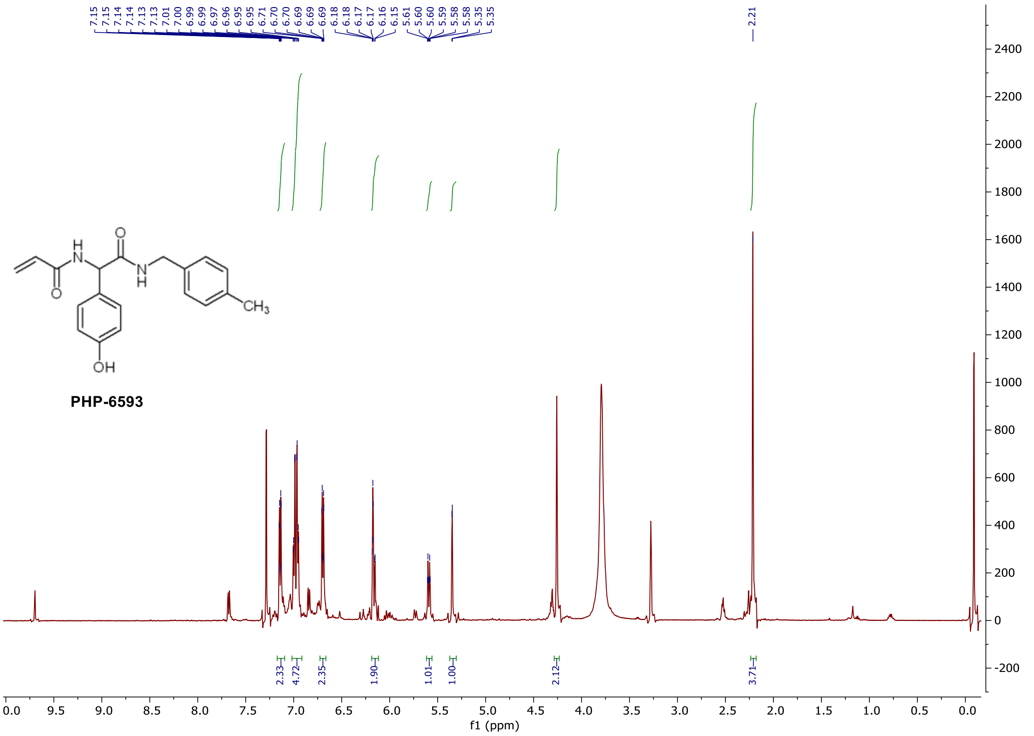

Supplementary Figure 1137. ^{1}H NMR (400 MHz, $CDCl_3$ + one drop of CD_3OD) of compound **PHP-6593.**

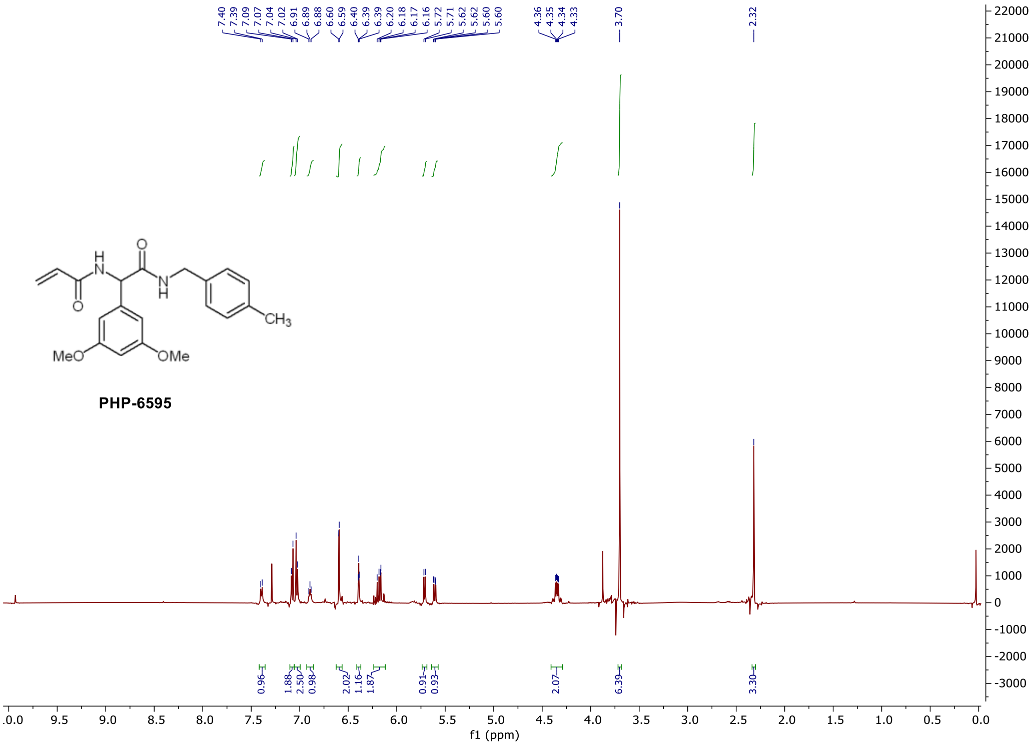

Supplementary Figure 1138. ^{1}H NMR (400 MHz, $CDCl_3$ + one drop of CD_3OD) of compound **PHP-6595.**

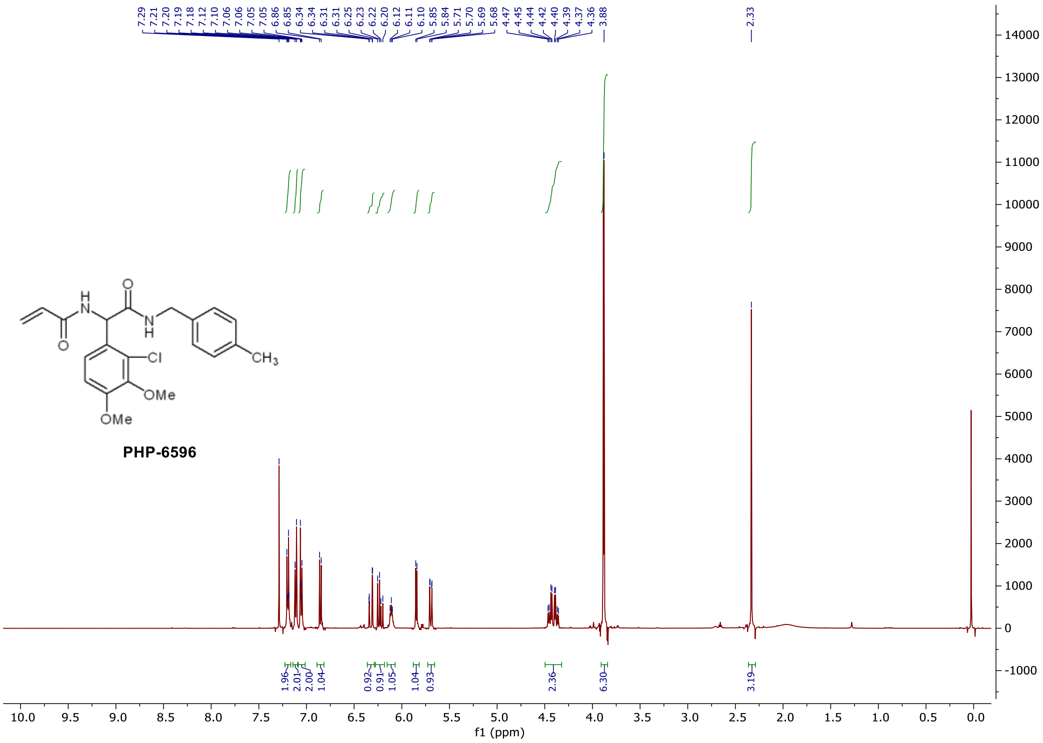

Supplementary Figure 1139. ^{1}H NMR (400 MHz, $CDCl_3$ + one drop of CD_3OD) of compound **PHP-6596.**

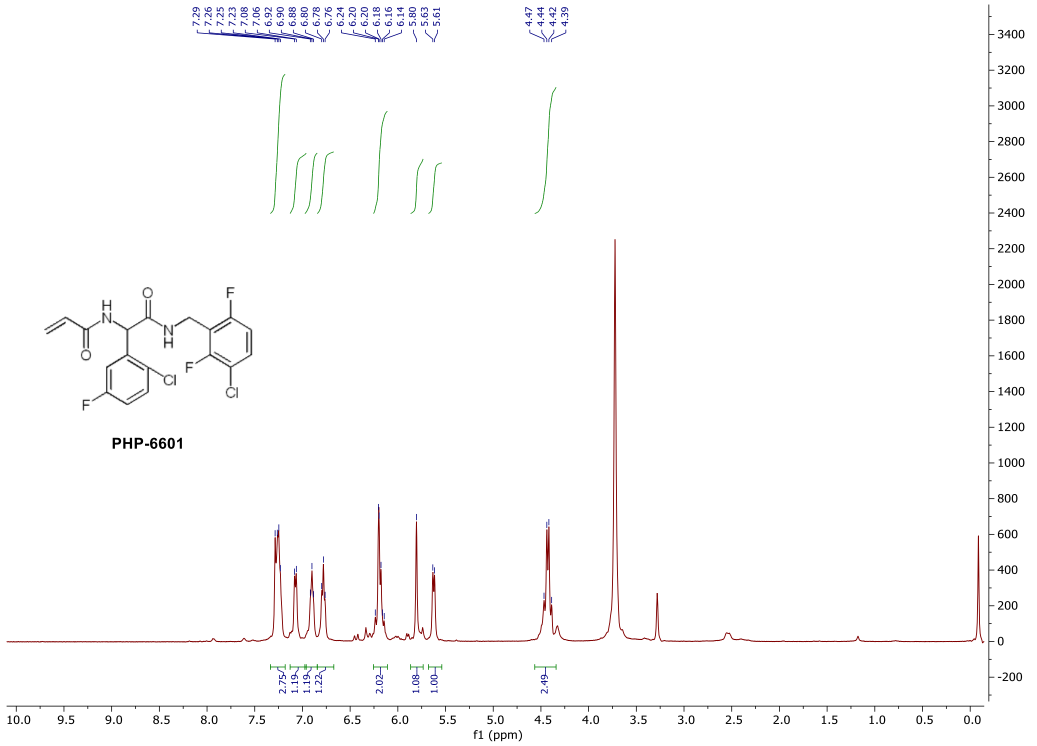

Supplementary Figure 1140. ^{1}H NMR (400 MHz, $CDCl_3$ + one drop of CD_3OD) of compound **PHP-6601.**

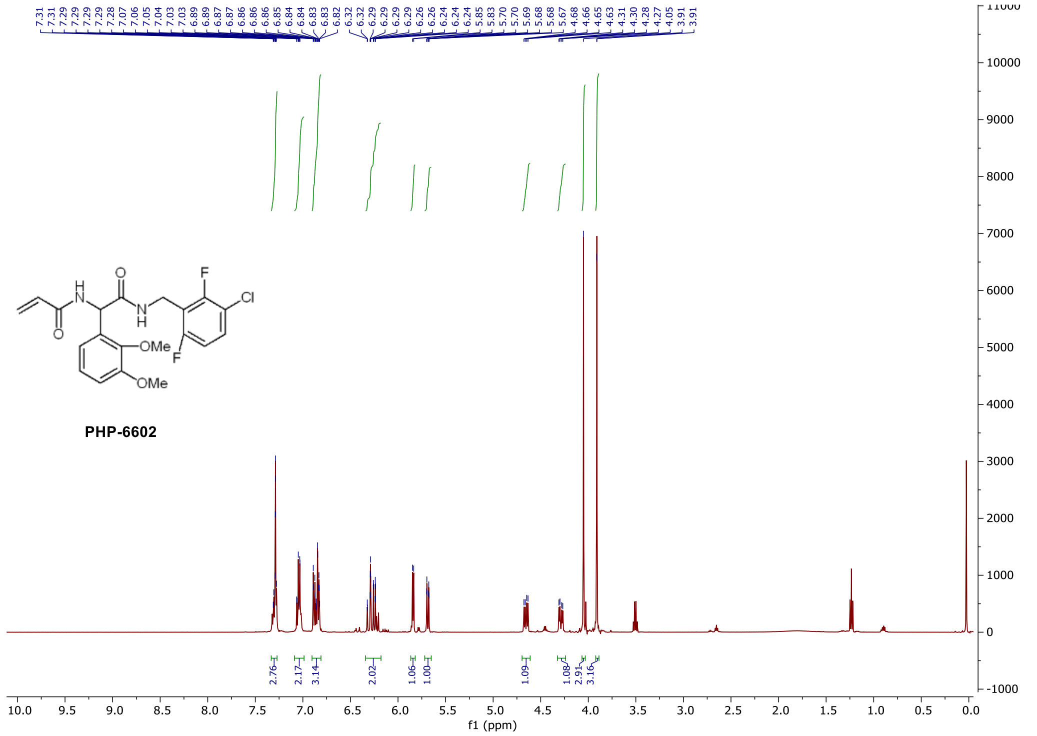

Supplementary Figure 1141. ^{1}H NMR (400 MHz, $CDCl_3$ + one drop of CD_3OD) of compound **PHP-6602.**

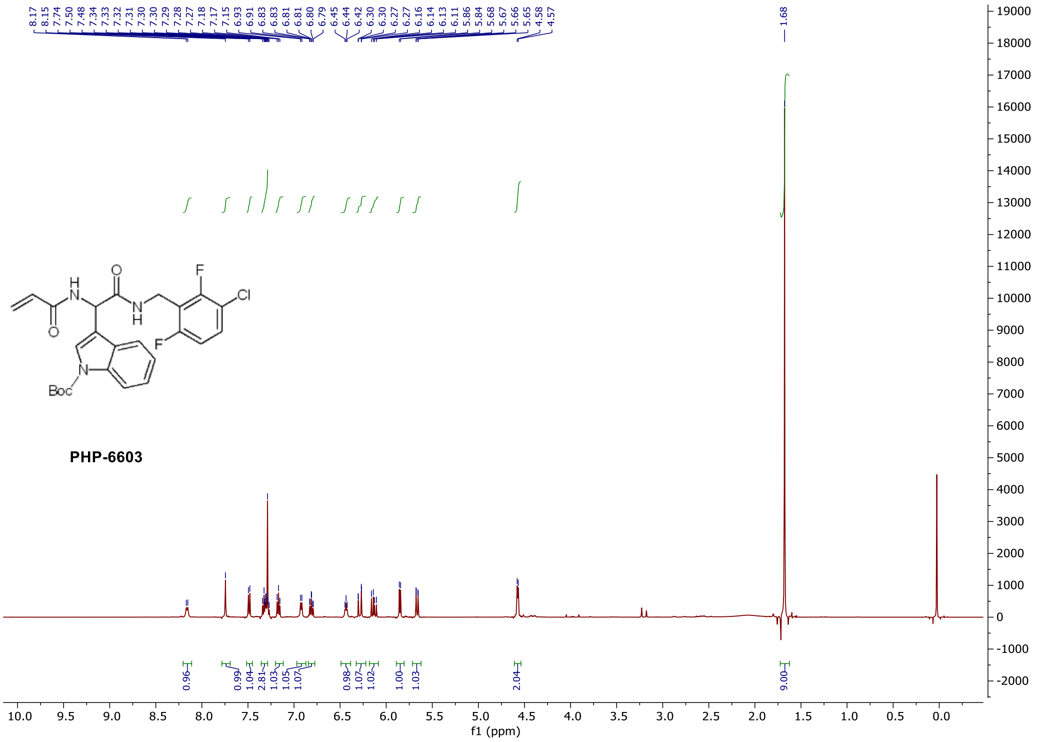

Supplementary Figure 1142. ^{1}H NMR (400 MHz, $CDCl_3$ + one drop of CD_3OD) of compound **PHP-6603**.

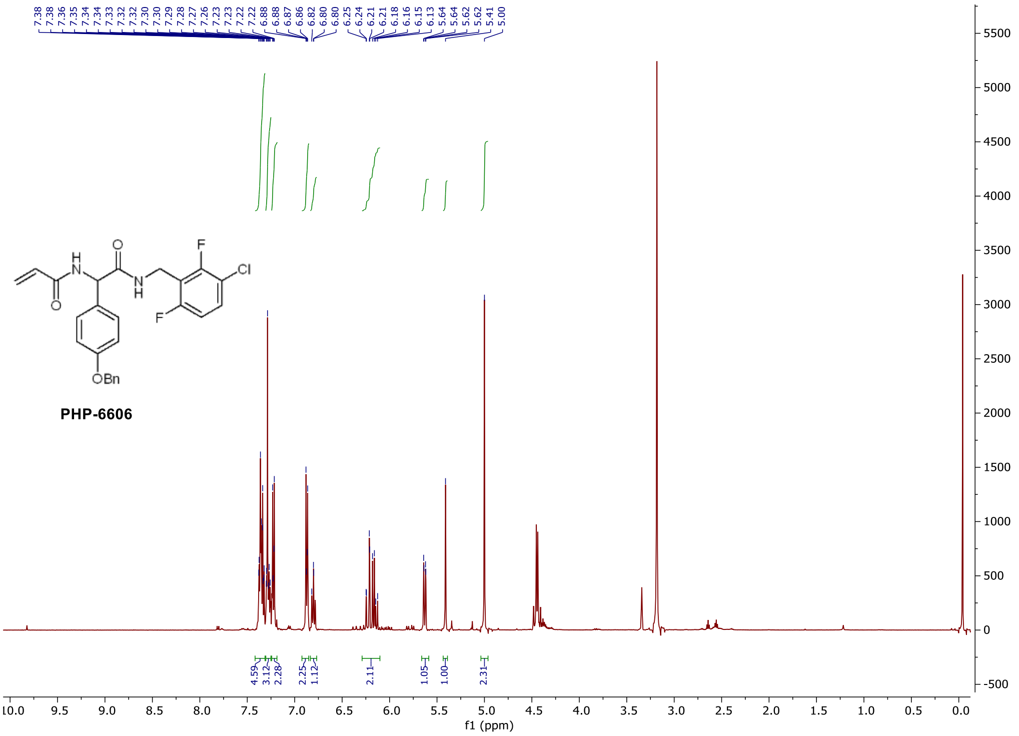

Supplementary Figure 1143. ^{1}H NMR (400 MHz, $CDCl_3$ + one drop of CD_3OD) of compound **PHP-6606**.

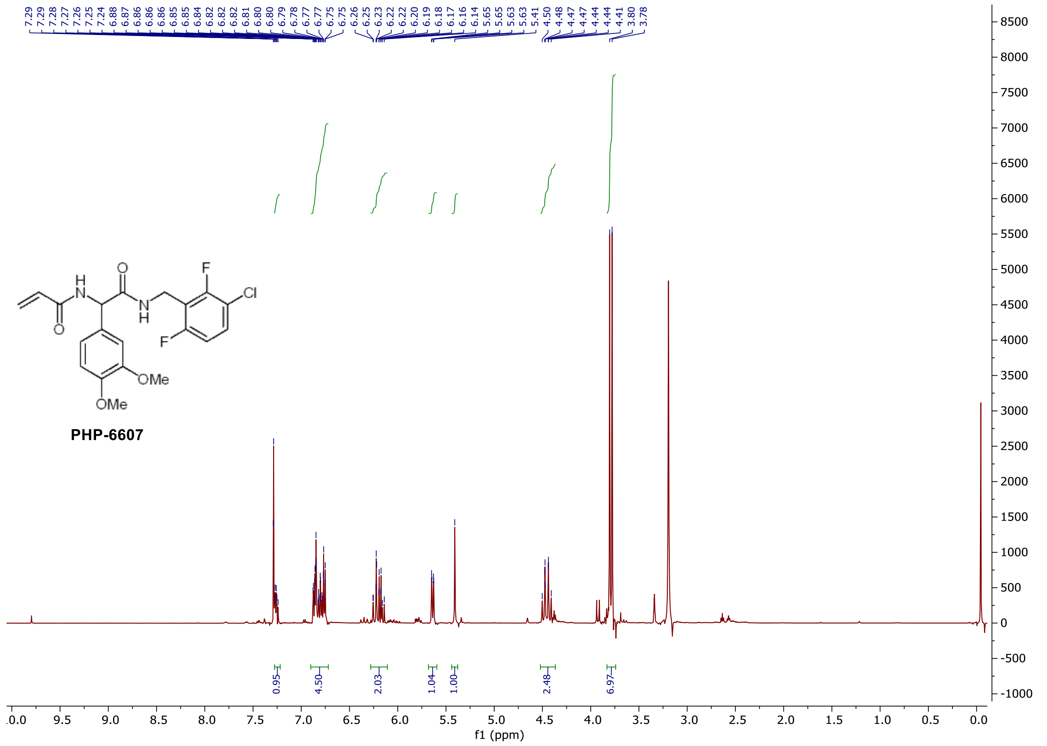

Supplementary Figure 1144. ^{1}H NMR (400 MHz, $CDCl_3$ + one drop of CD_3OD) of compound **PHP-6607**.

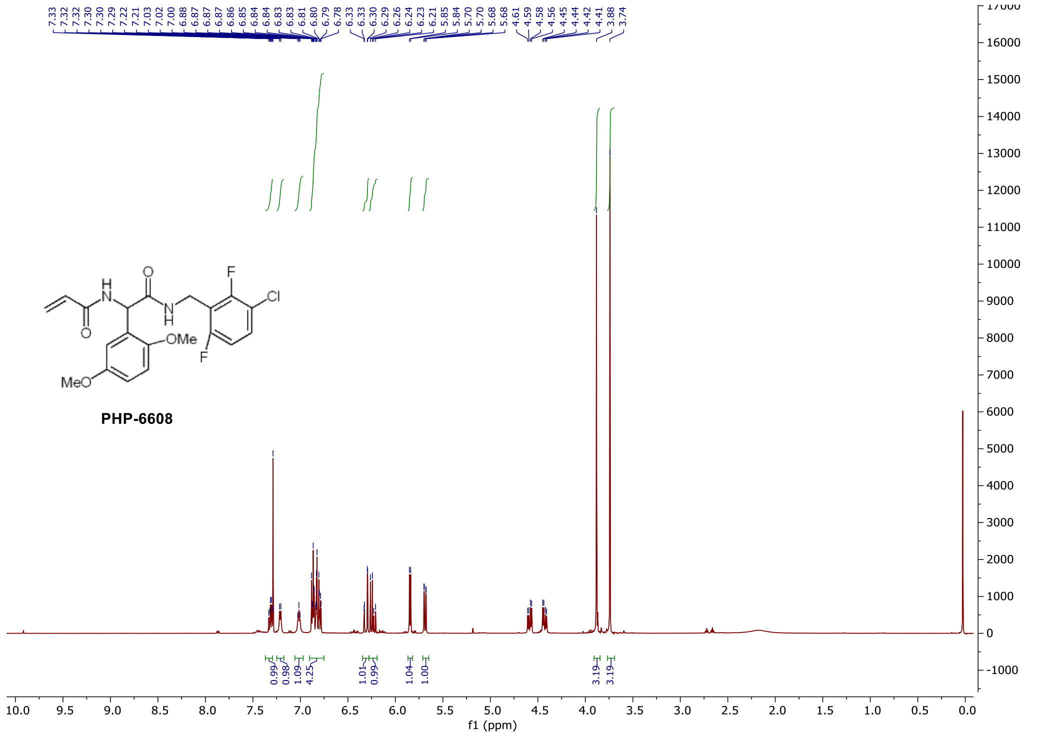

Supplementary Figure 1145. ^{1}H NMR (400 MHz, $CDCl_3$ + one drop of CD_3OD) of compound **PHP-6608**.

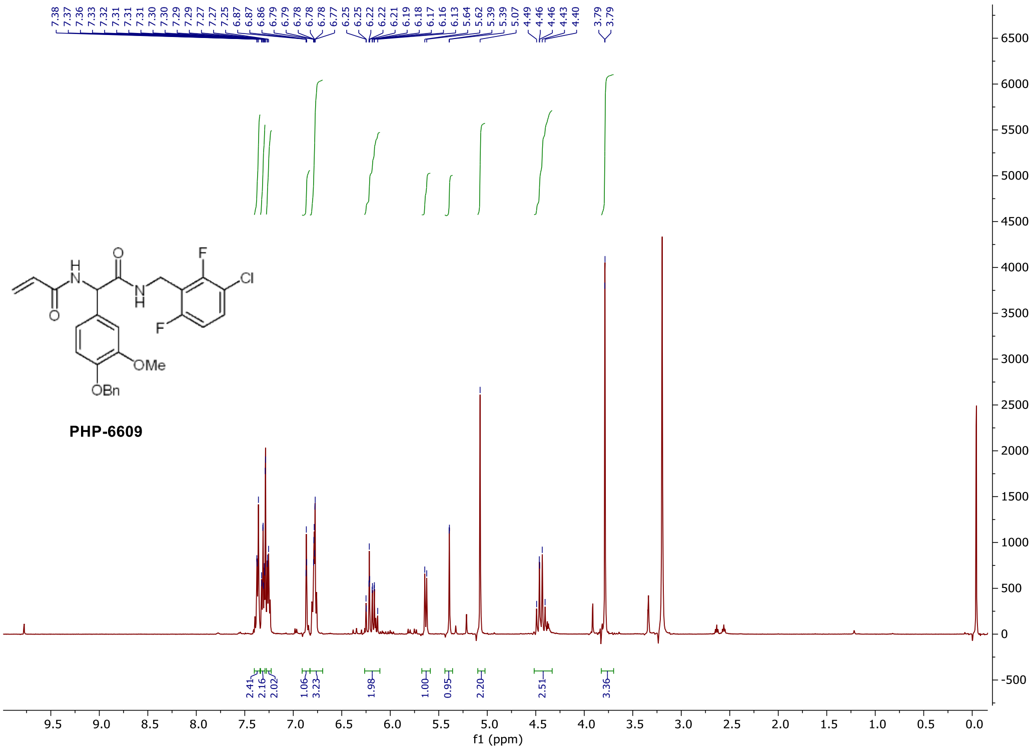

Supplementary Figure 1146. ^{1}H NMR (400 MHz, $CDCl_3$ + one drop of CD_3OD) of compound **PHP-6609.**

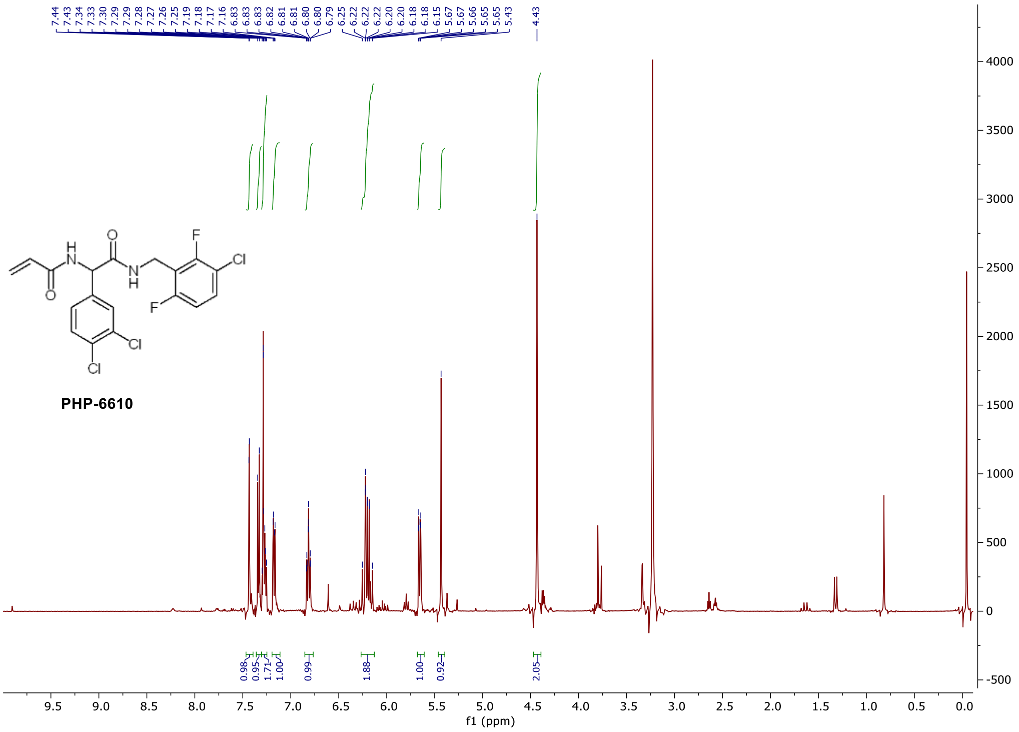

Supplementary Figure 1147. ^{1}H NMR (400 MHz, $CDCl_3$ + one drop of CD_3OD) of compound **PHP-6610.**

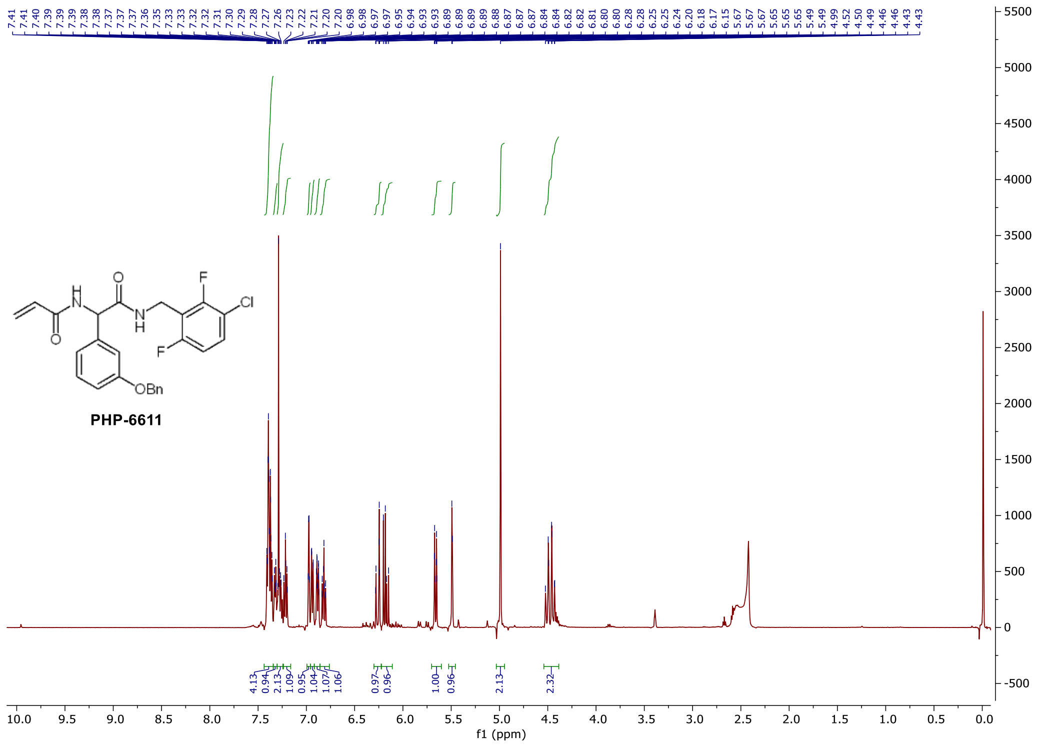

Supplementary Figure 1148. ^{1}H NMR (400 MHz, $CDCl_3$ + one drop of CD_3OD) of compound **PHP-6611.**

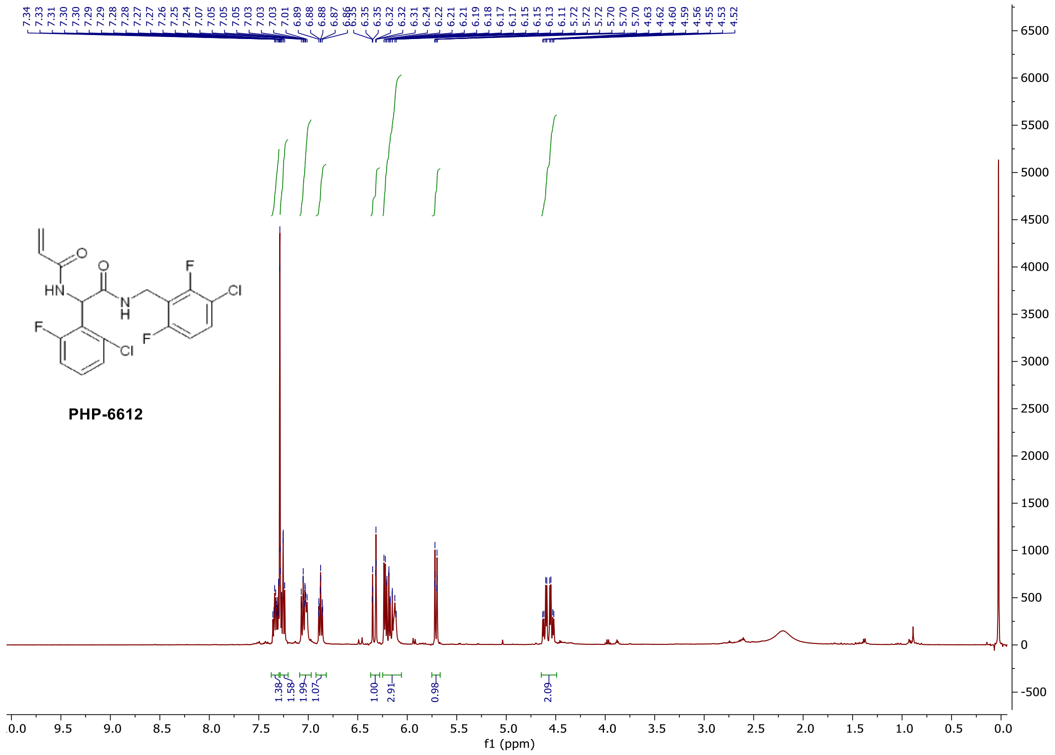

Supplementary Figure 1149. ^{1}H NMR (400 MHz, $CDCl_3$ + one drop of CD_3OD) of compound **PHP-6612.**

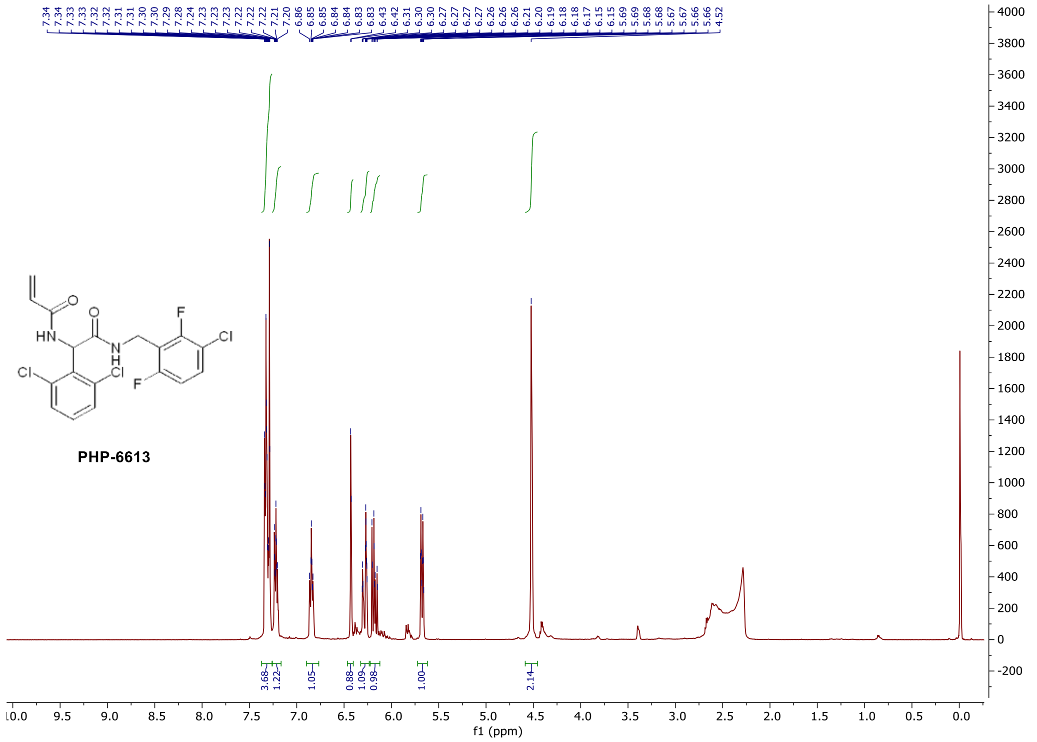

Supplementary Figure 1150. ^{1}H NMR (400 MHz, $CDCl_3$ + one drop of CD_3OD) of compound **PHP-6613**.

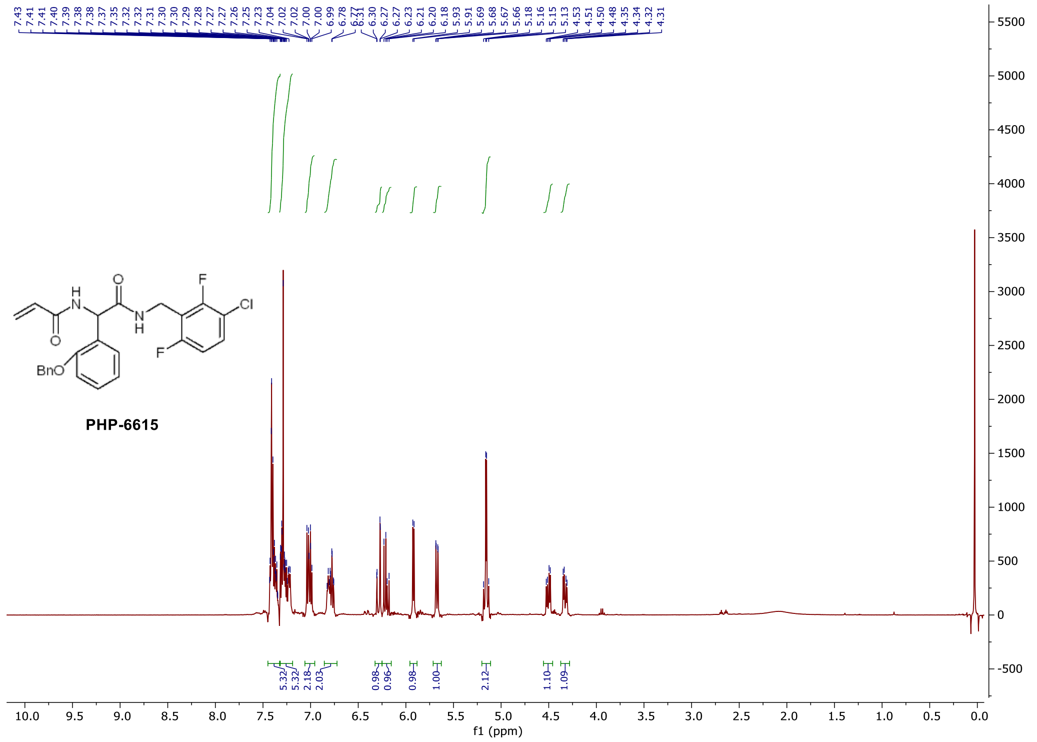

Supplementary Figure 1151. ^{1}H NMR (400 MHz, $CDCl_3$ + one drop of CD_3OD) of compound **PHP-6615**.

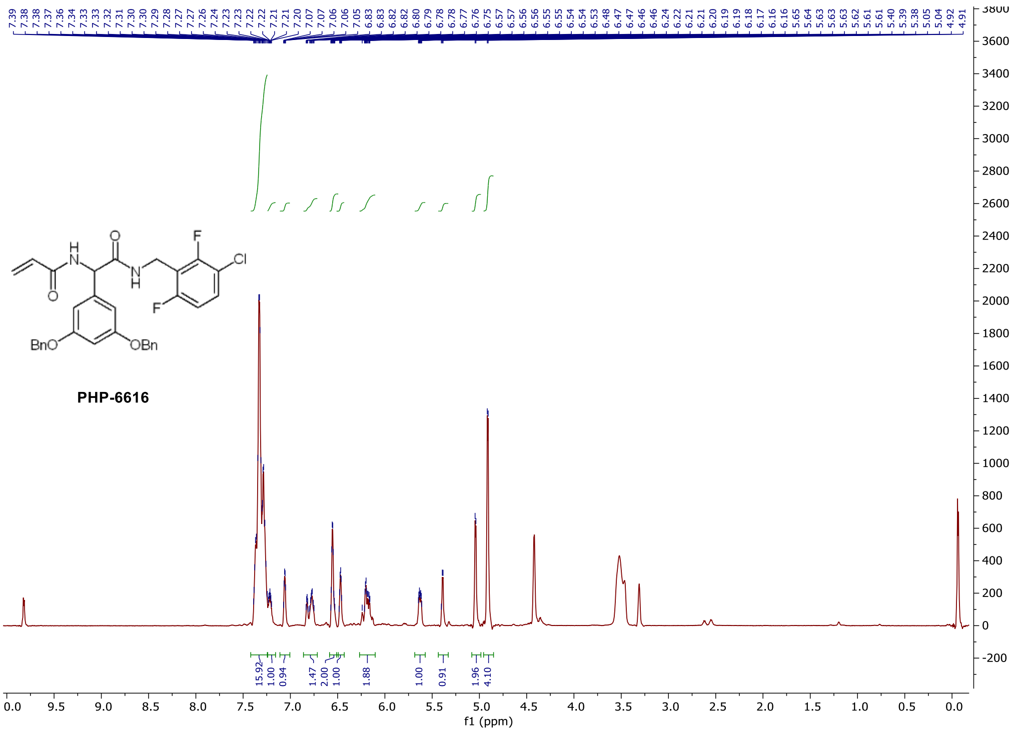

Supplementary Figure 1152. ^{1}H NMR (400 MHz, $CDCl_3$ + one drop of CD_3OD) of compound **PHP-6616.**

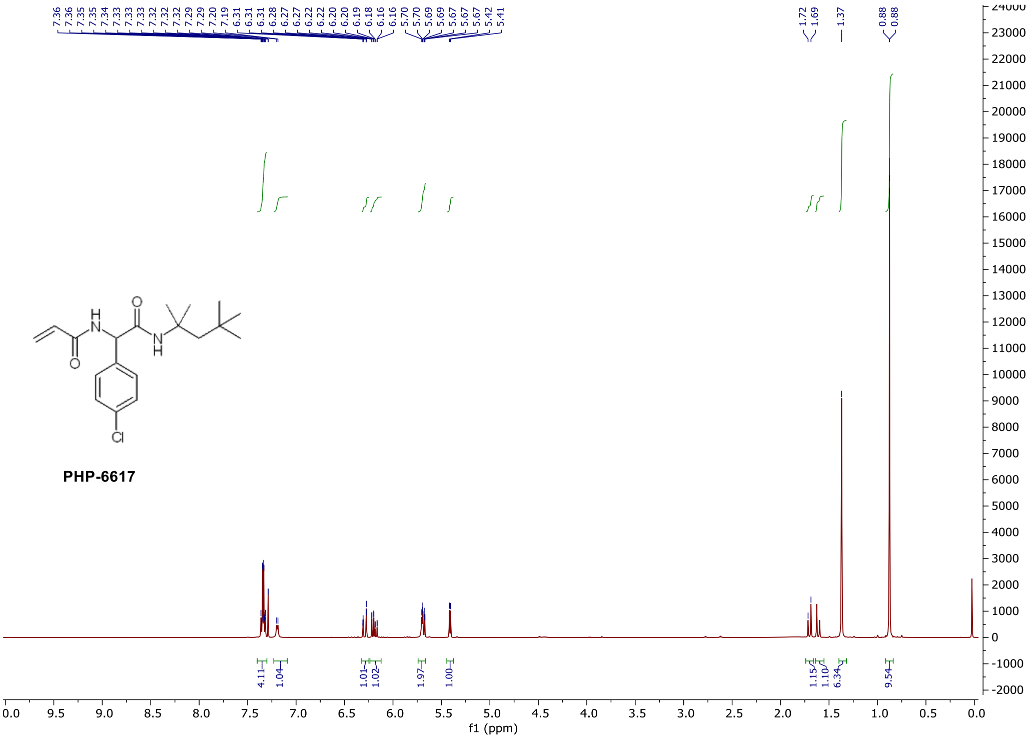

Supplementary Figure 1153. ^{1}H NMR (400 MHz, $CDCl_3$ + one drop of CD_3OD) of compound **PHP-6617.**

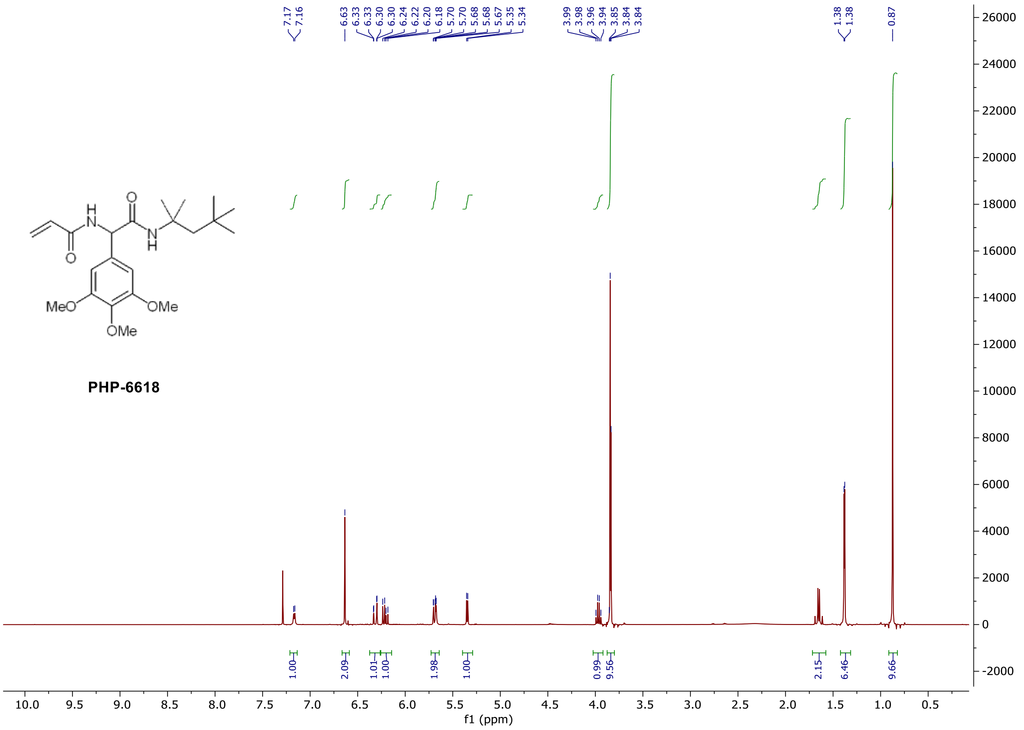

Supplementary Figure 1154. ^{1}H NMR (400 MHz, $CDCl_3$ + one drop of CD_3OD) of compound **PHP-6618.**

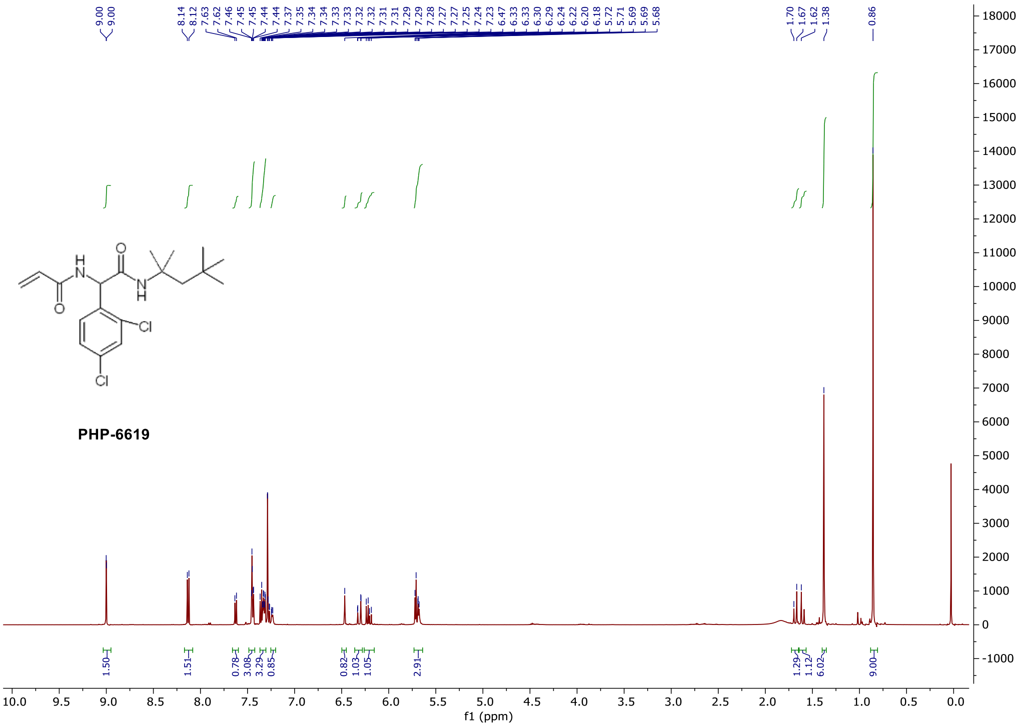

Supplementary Figure 1155. ^{1}H NMR (400 MHz, $CDCl_3$ + one drop of CD_3OD) of compound **PHP-6619.**

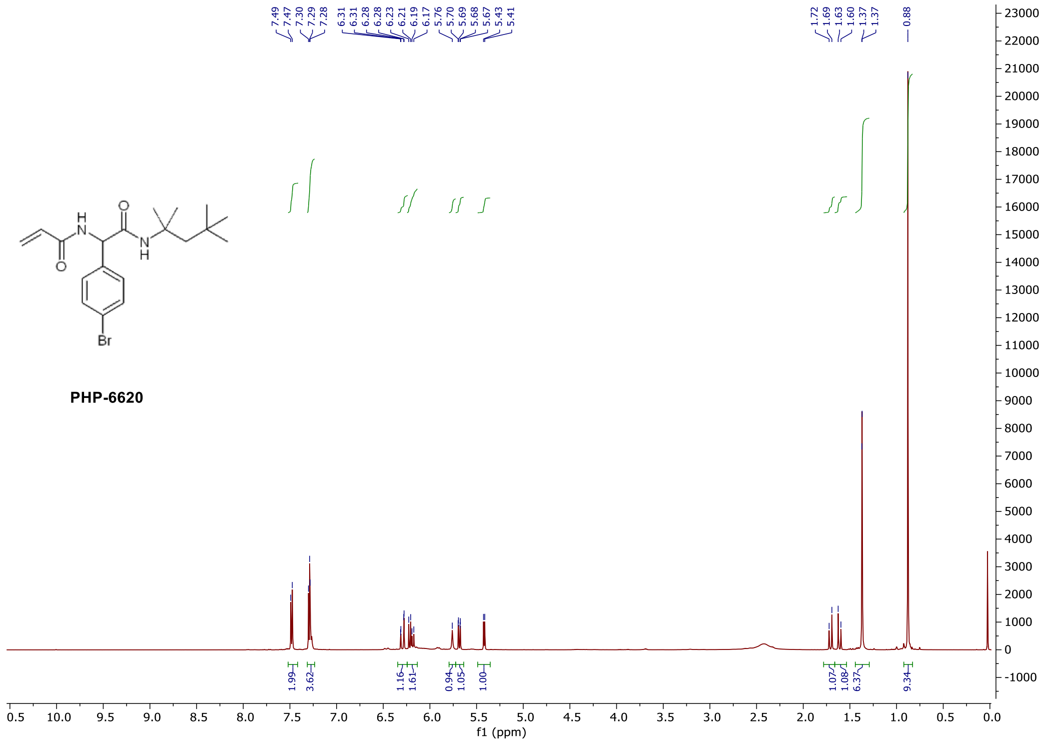

Supplementary Figure 1156. ^{1}H NMR (400 MHz, $CDCl_3$ + one drop of CD_3OD) of compound **PHP-6620.**

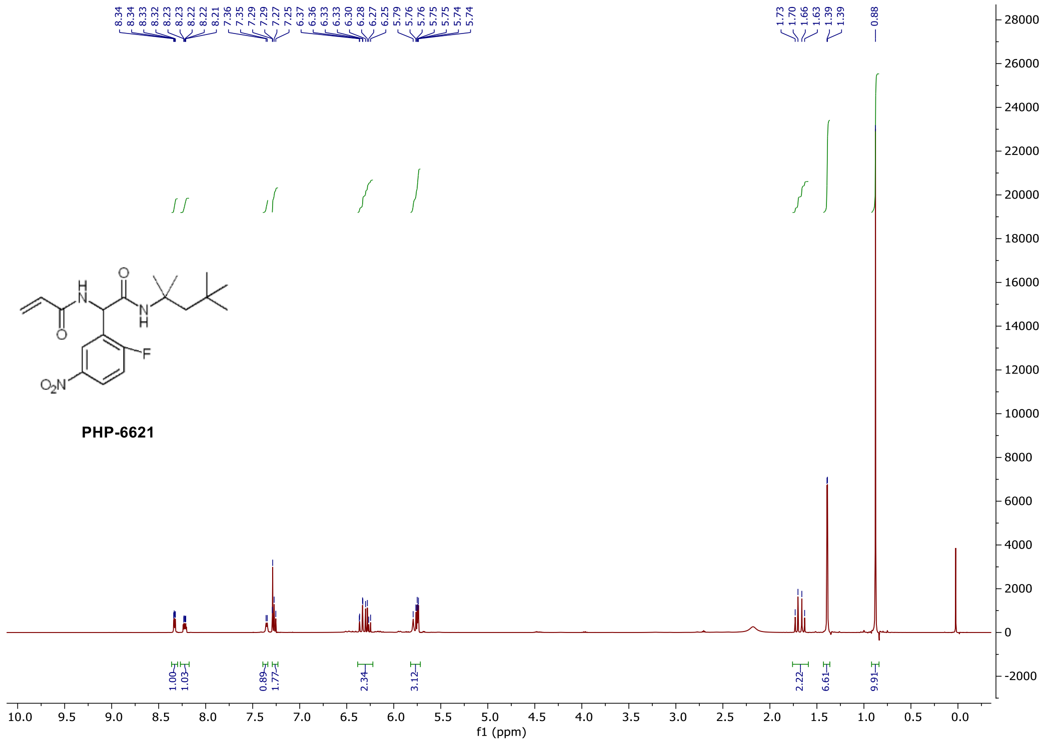

Supplementary Figure 1157. ^{1}H NMR (400 MHz, $CDCl_3$ + one drop of CD_3OD) of compound **PHP-6621.**

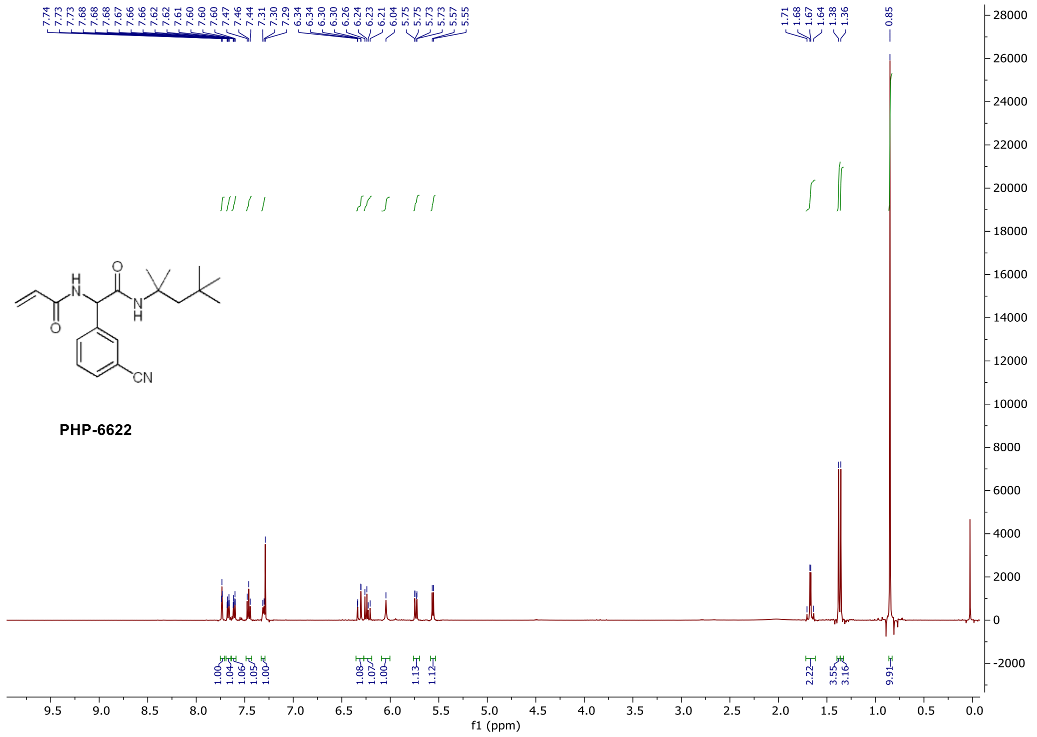

Supplementary Figure 1158. ^{1}H NMR (400 MHz, $CDCl_3$ + one drop of CD_3OD) of compound **PHP-6622**.

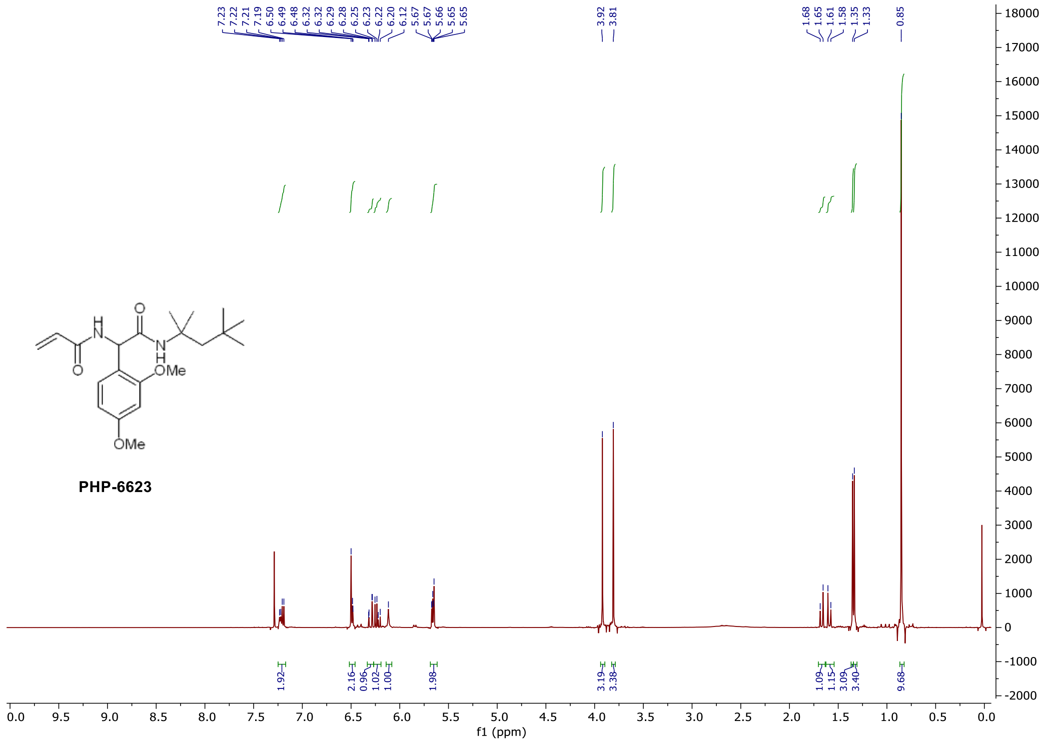

Supplementary Figure 1159. ^{1}H NMR (400 MHz, $CDCl_3$ + one drop of CD_3OD) of compound **PHP-6623**.

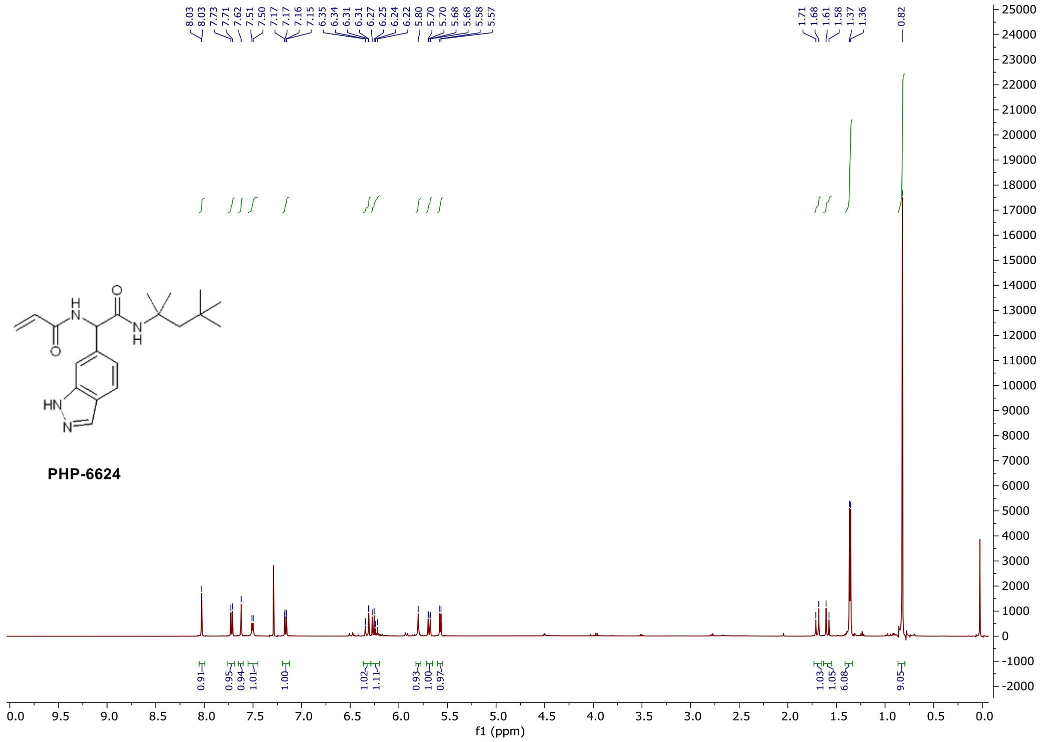

Supplementary Figure 1160. ^{1}H NMR (400 MHz, $CDCl_3$ + one drop of CD_3OD) of compound **PHP-6624.**

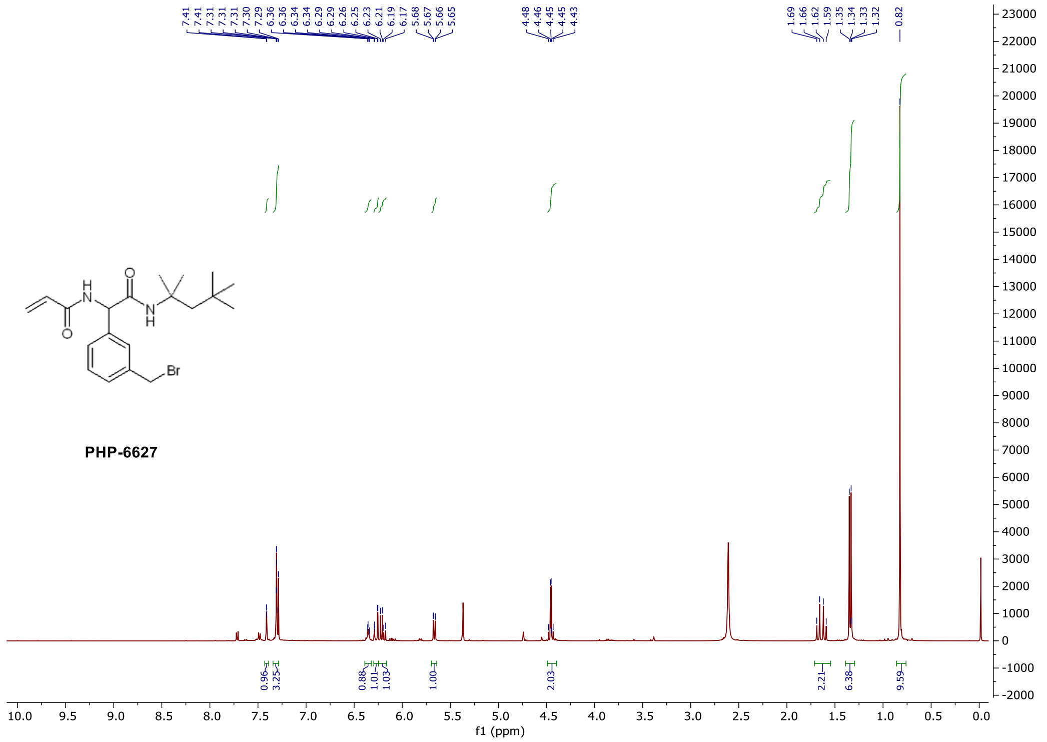

Supplementary Figure 1161. ^{1}H NMR (400 MHz, $CDCl_3$ + one drop of CD_3OD) of compound **PHP-6627.**

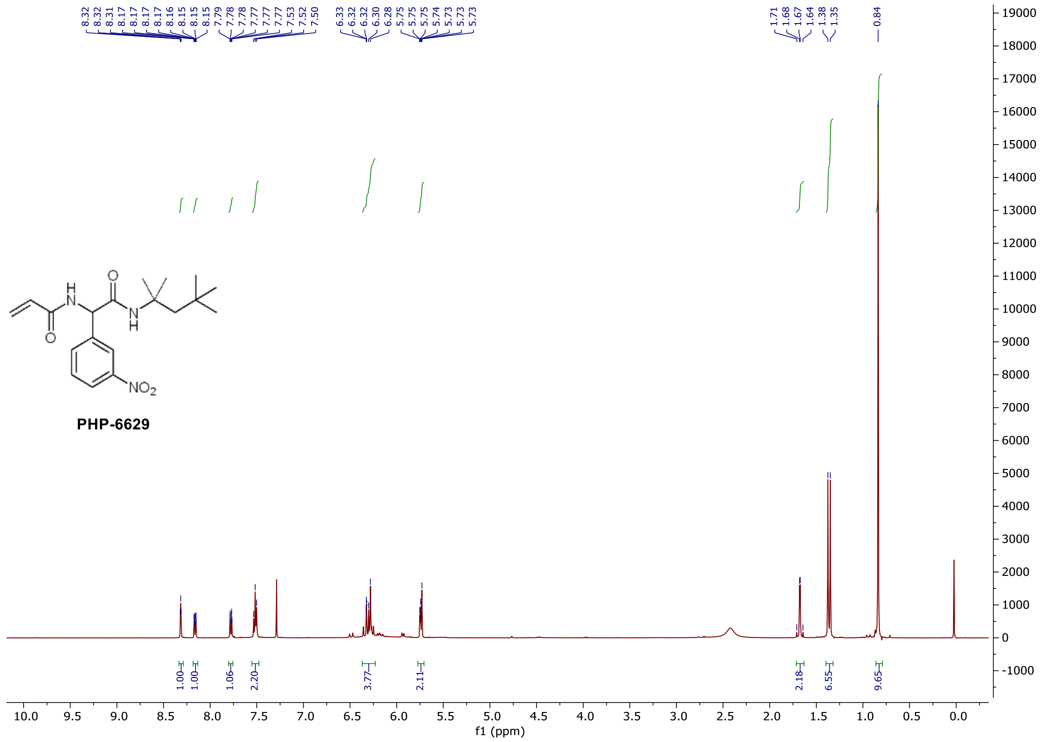

Supplementary Figure 1162. ^{1}H NMR (400 MHz, $CDCl_3$ + one drop of CD_3OD) of compound **PHP-6629**.

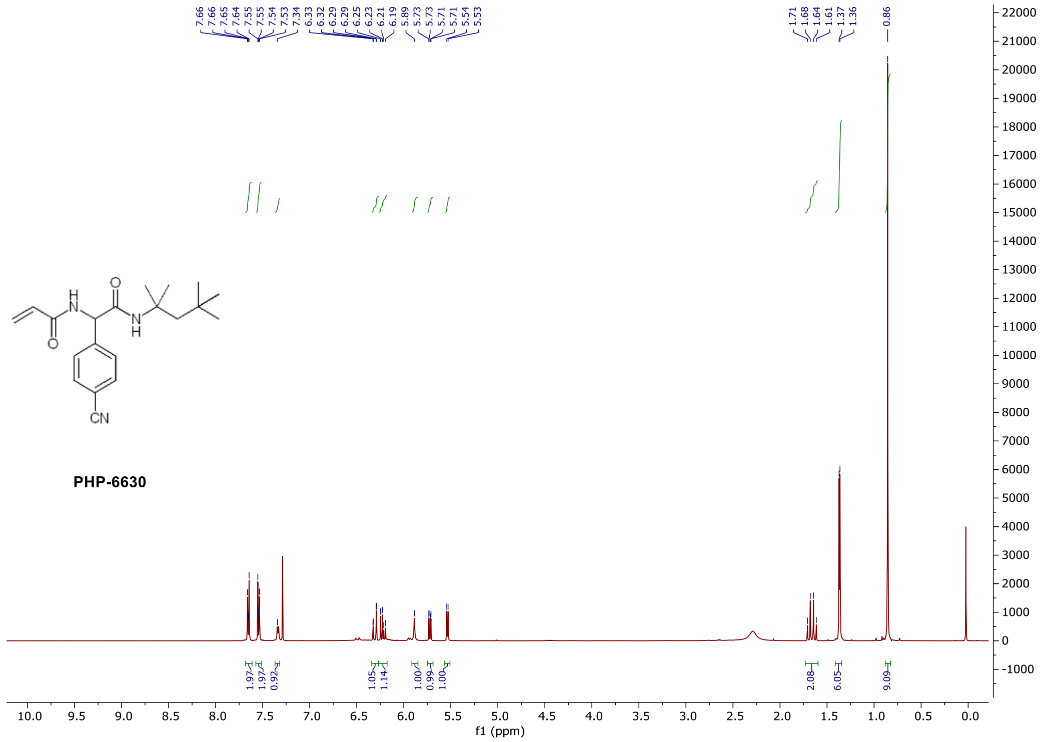

Supplementary Figure 1163. ^{1}H NMR (400 MHz, $CDCl_3$ + one drop of CD_3OD) of compound **PHP-6630**.

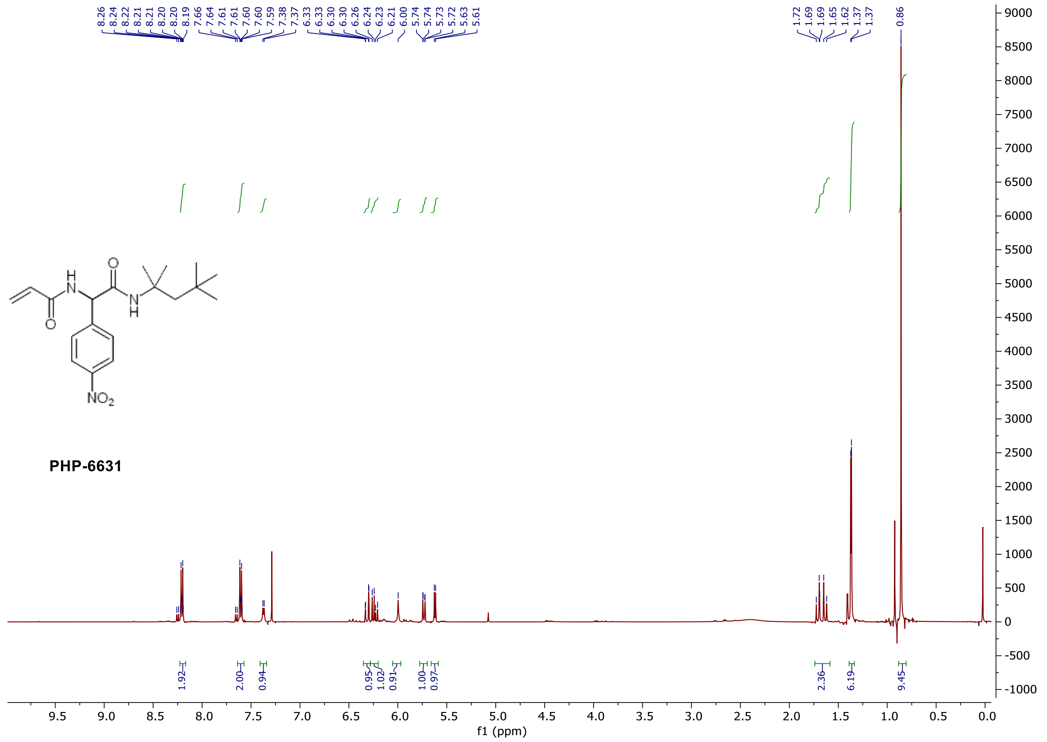

Supplementary Figure 1164. ^{1}H NMR (400 MHz, $CDCl_3$ + one drop of CD_3OD) of compound **PHP-6631.**

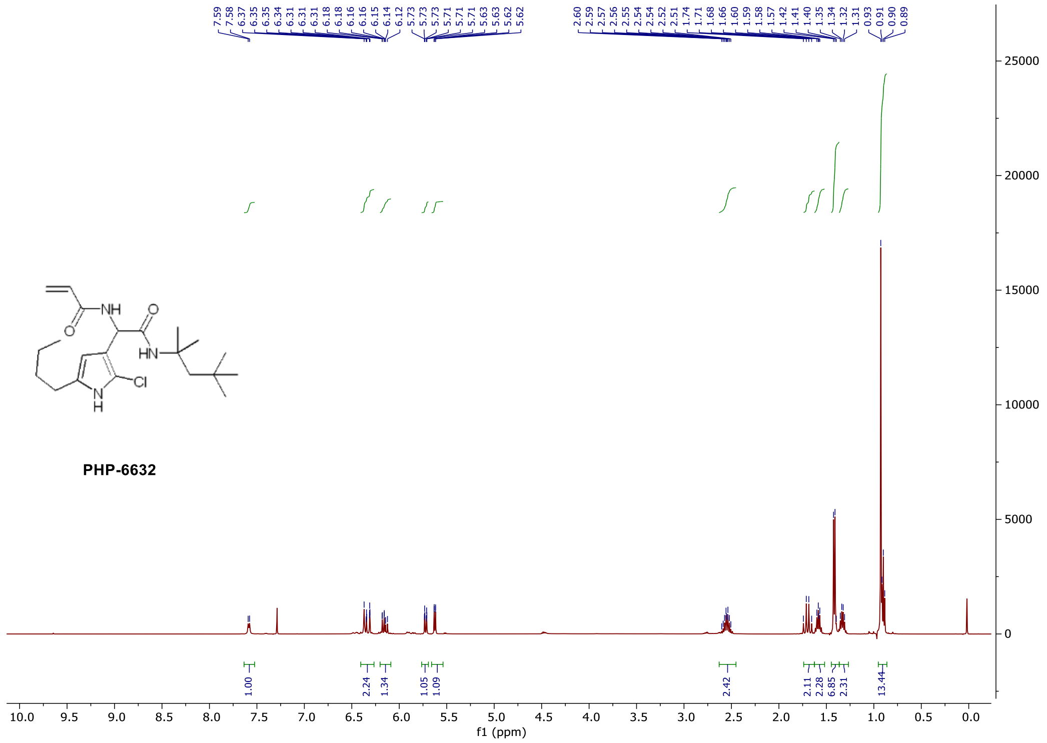

Supplementary Figure 1165. ^{1}H NMR (400 MHz, $CDCl_3$ + one drop of CD_3OD) of compound **PHP-6632.**

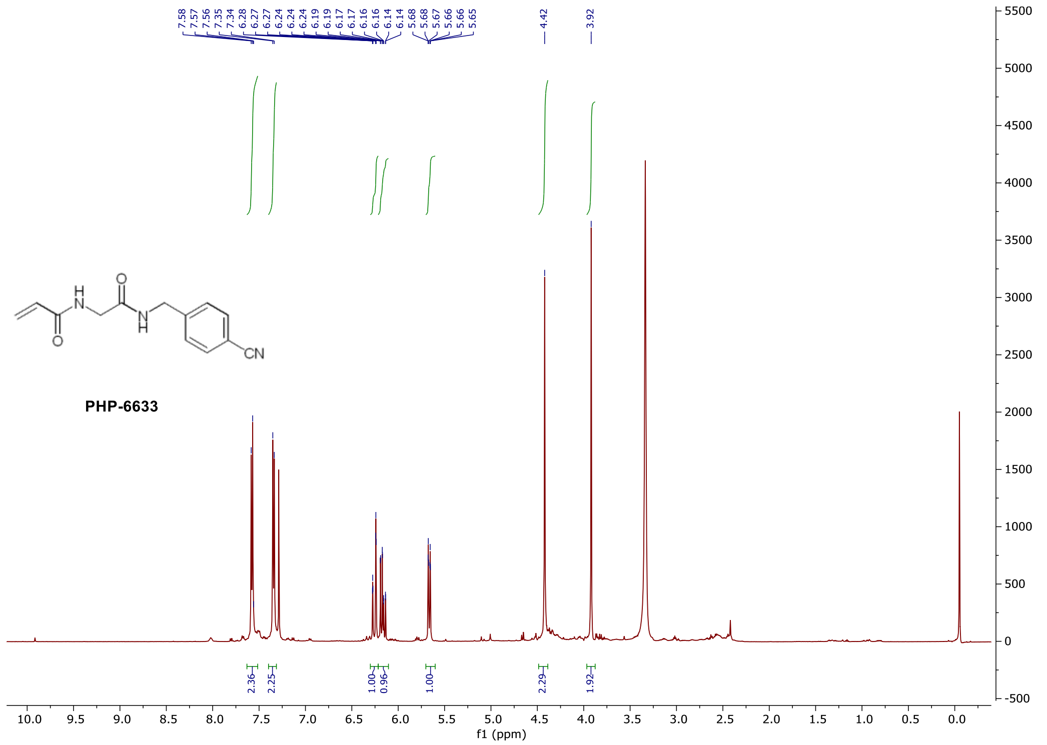

Supplementary Figure 1166. ^{1}H NMR (400 MHz, $CDCl_3$ + one drop of CD_3OD) of compound **PHP-6633.**

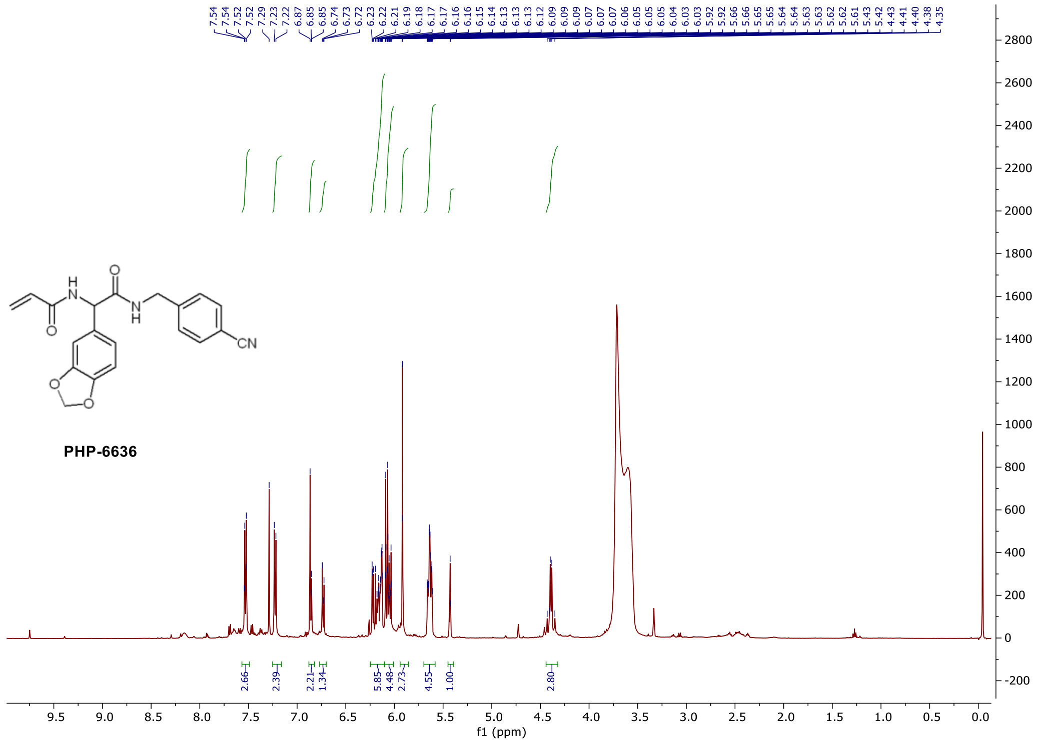

Supplementary Figure 1167. ^{1}H NMR (400 MHz, $CDCl_3$ + one drop of CD_3OD) of compound **PHP-6636.**

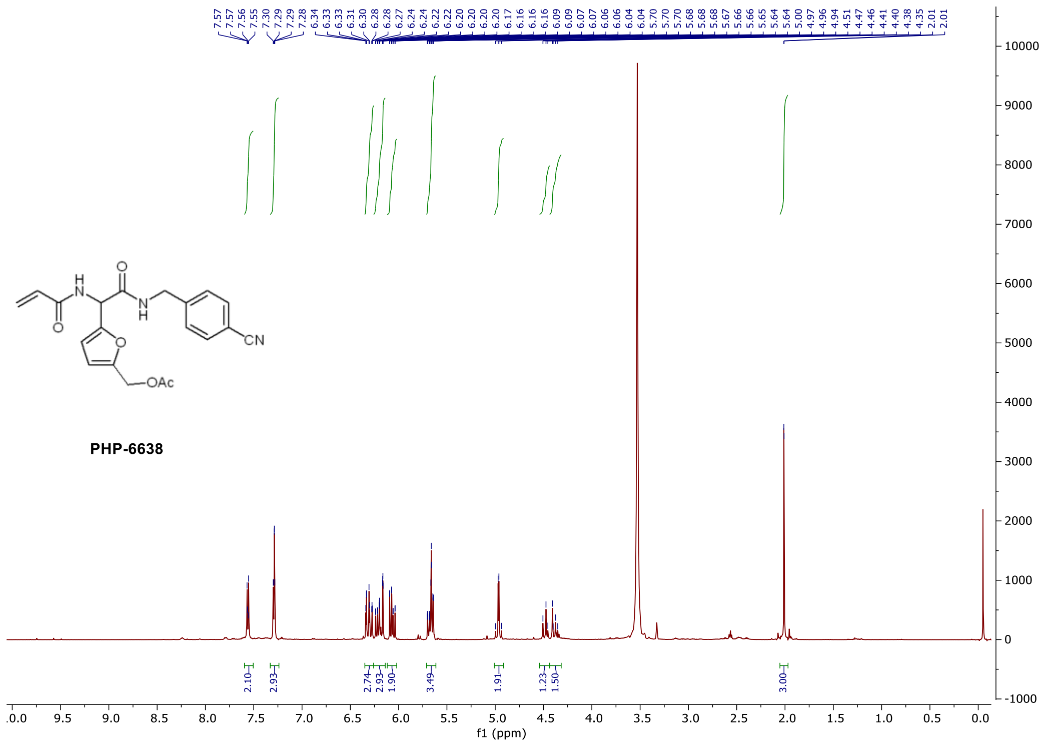

Supplementary Figure 1168. ^{1}H NMR (400 MHz, $CDCl_3$ + one drop of CD_3OD) of compound **PHP-6638.**

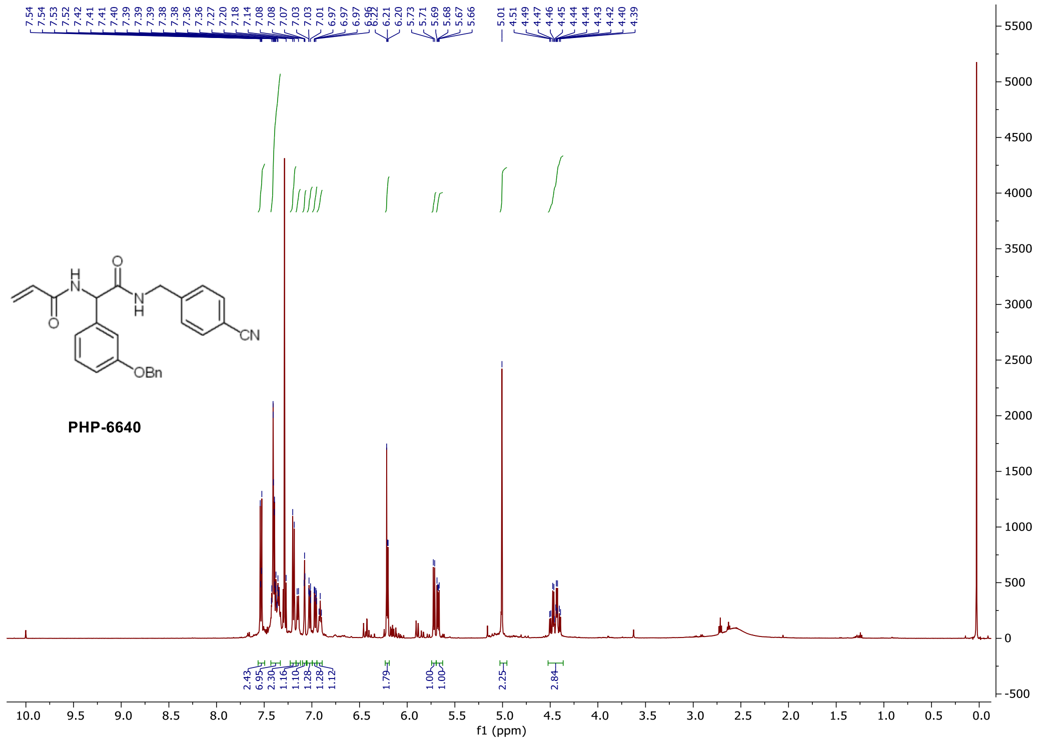

Supplementary Figure 1169. ^{1}H NMR (400 MHz, $CDCl_3$ + one drop of CD_3OD) of compound **PHP-6640.**

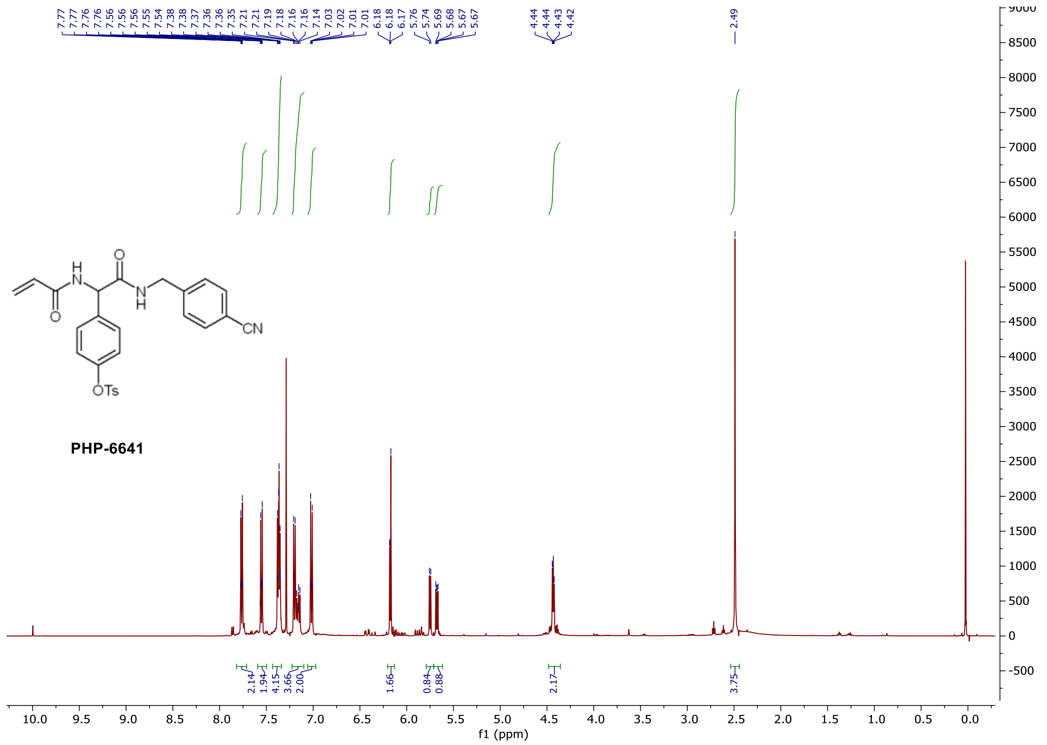

Supplementary Figure 1170. ^{1}H NMR (400 MHz, $CDCl_3$ + one drop of CD_3OD) of compound **PHP-6641.**

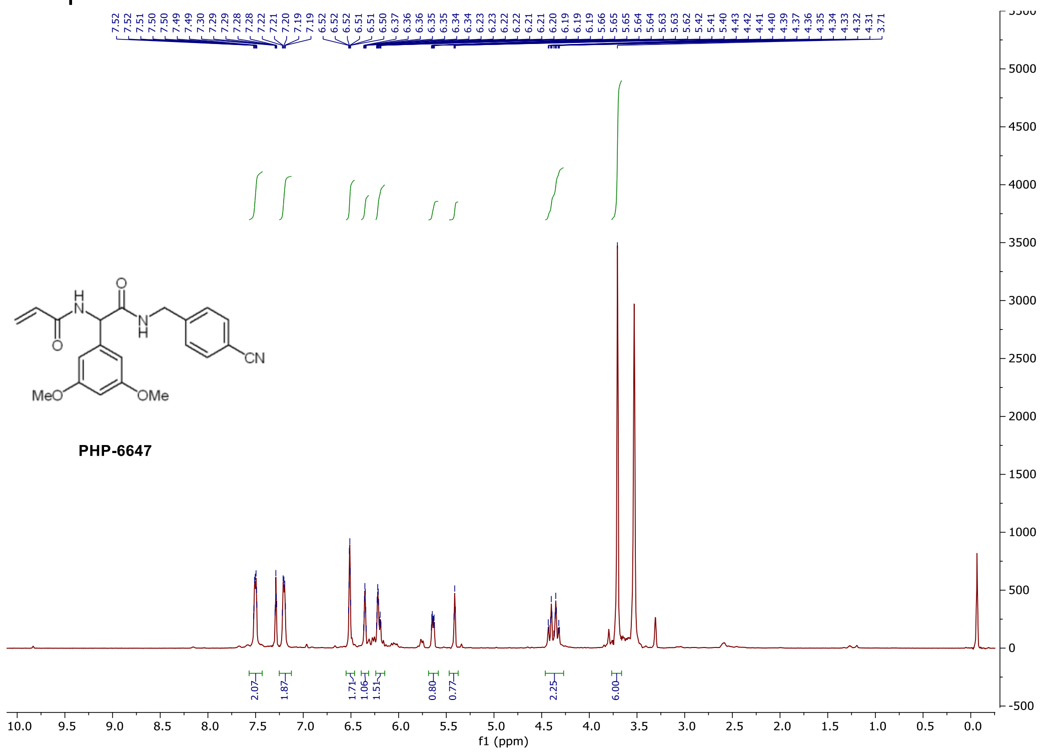

Supplementary Figure 1171. ^{1}H NMR (400 MHz, $CDCl_3$ + one drop of CD_3OD) of compound **PHP-6647.**

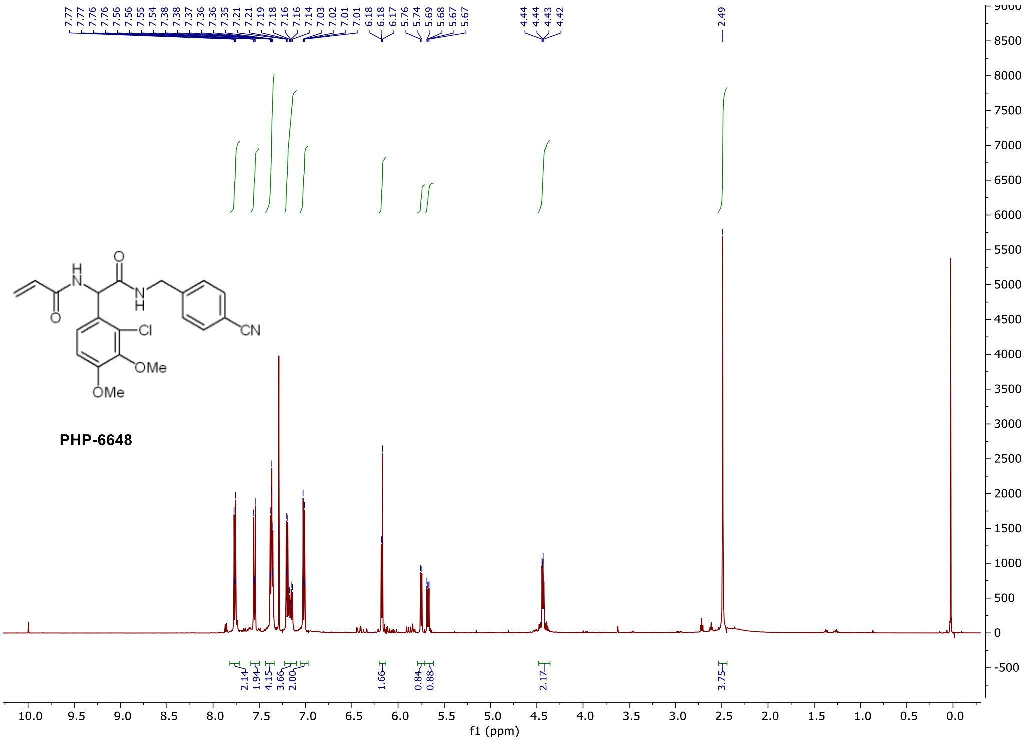

Supplementary Figure 1172. ^{1}H NMR (400 MHz, $CDCl_3$ + one drop of CD_3OD) of compound **PHP-6648.**

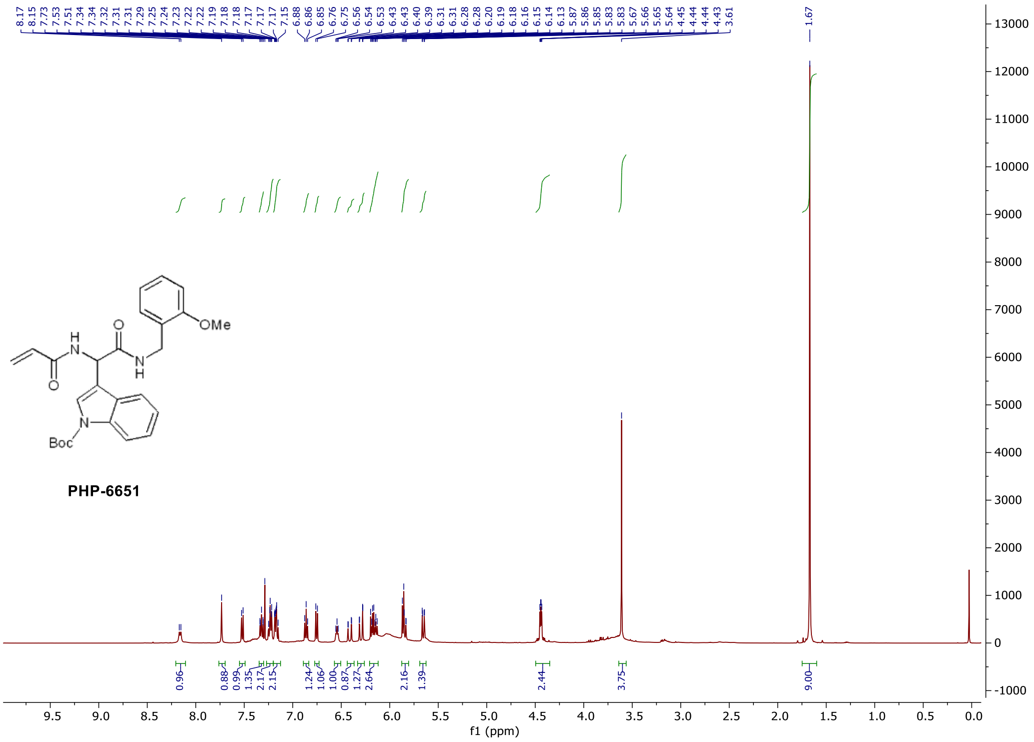

Supplementary Figure 1173. ^{1}H NMR (400 MHz, $CDCl_3$ + one drop of CD_3OD) of compound **PHP-6651.**

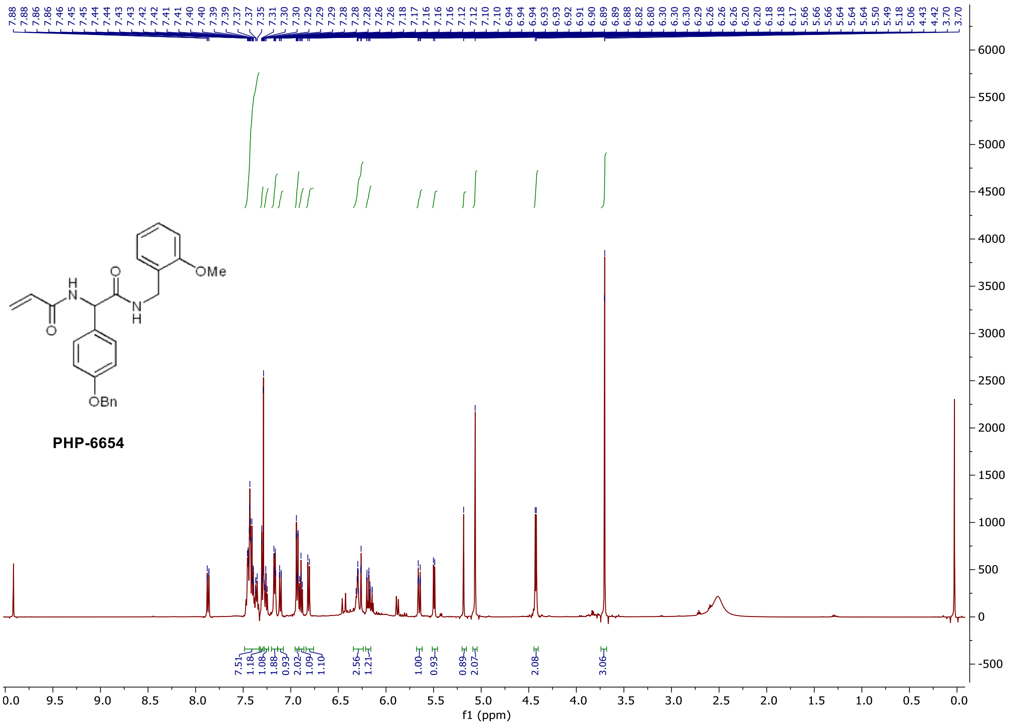

Supplementary Figure 1174. ^{1}H NMR (400 MHz, $CDCl_3$ + one drop of CD_3OD) of compound **PHP-6654.**

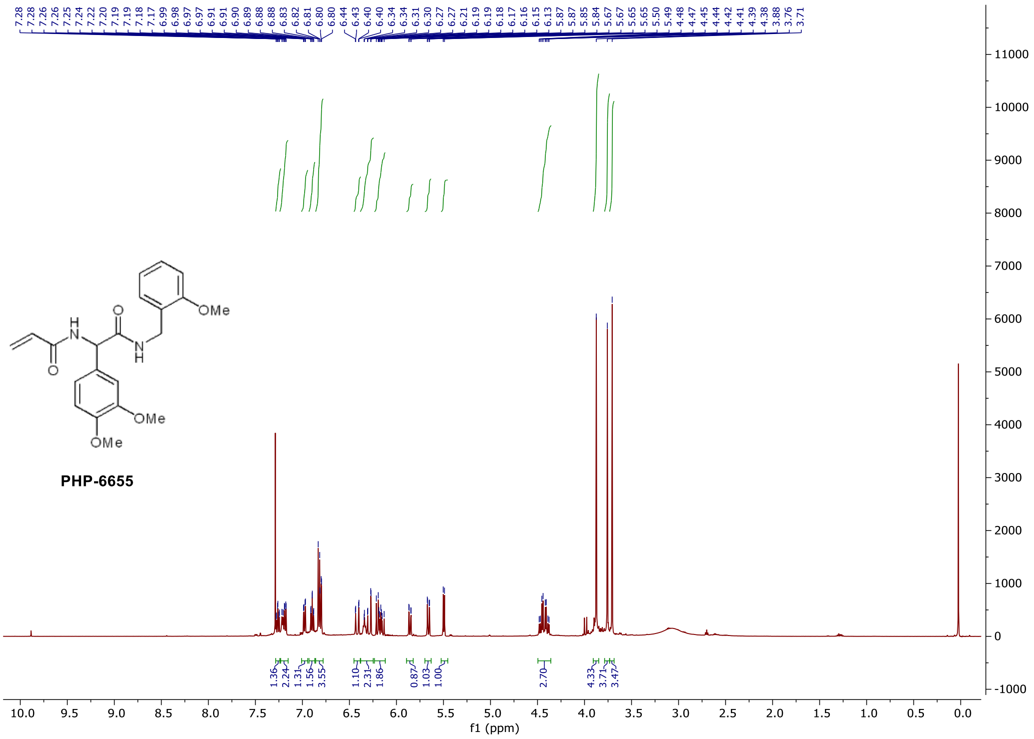

Supplementary Figure 1175. ^{1}H NMR (400 MHz, $CDCl_3$ + one drop of CD_3OD) of compound **PHP-6655.**

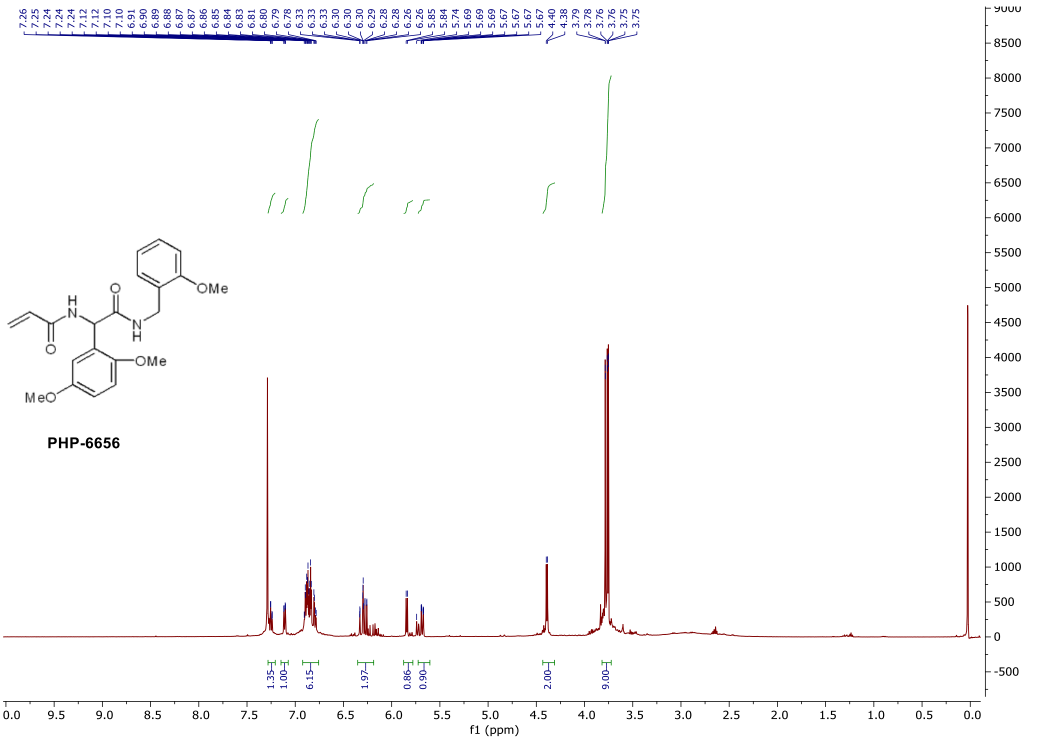

Supplementary Figure 1176. ^{1}H NMR (400 MHz, $CDCl_3$ + one drop of CD_3OD) of compound **PHP-6656.**

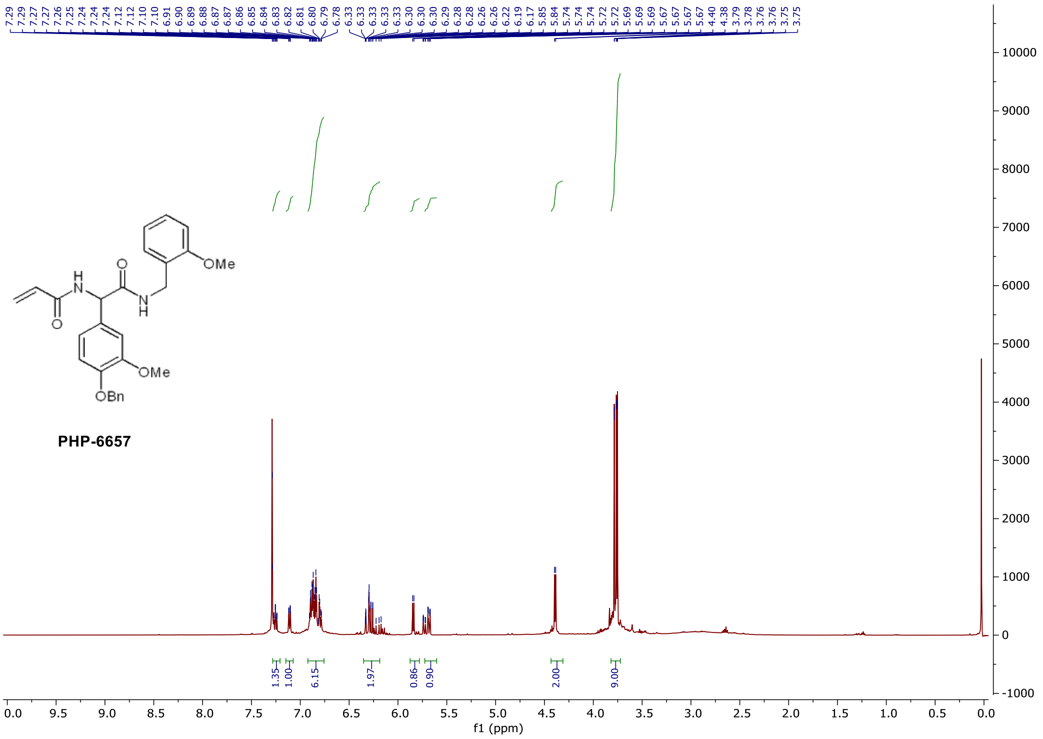

Supplementary Figure 1177. ^{1}H NMR (400 MHz, $CDCl_3$ + one drop of CD_3OD) of compound **PHP-6657.**

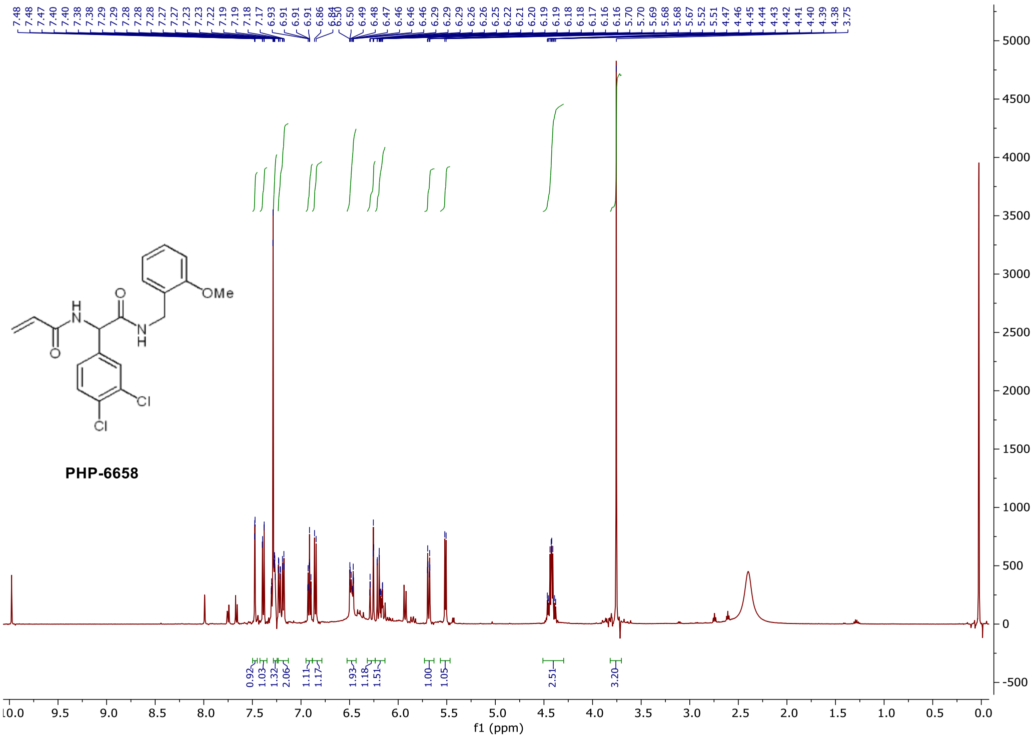

Supplementary Figure 1178. ^{1}H NMR (400 MHz, $CDCl_3$ + one drop of CD_3OD) of compound **PHP-6658.**

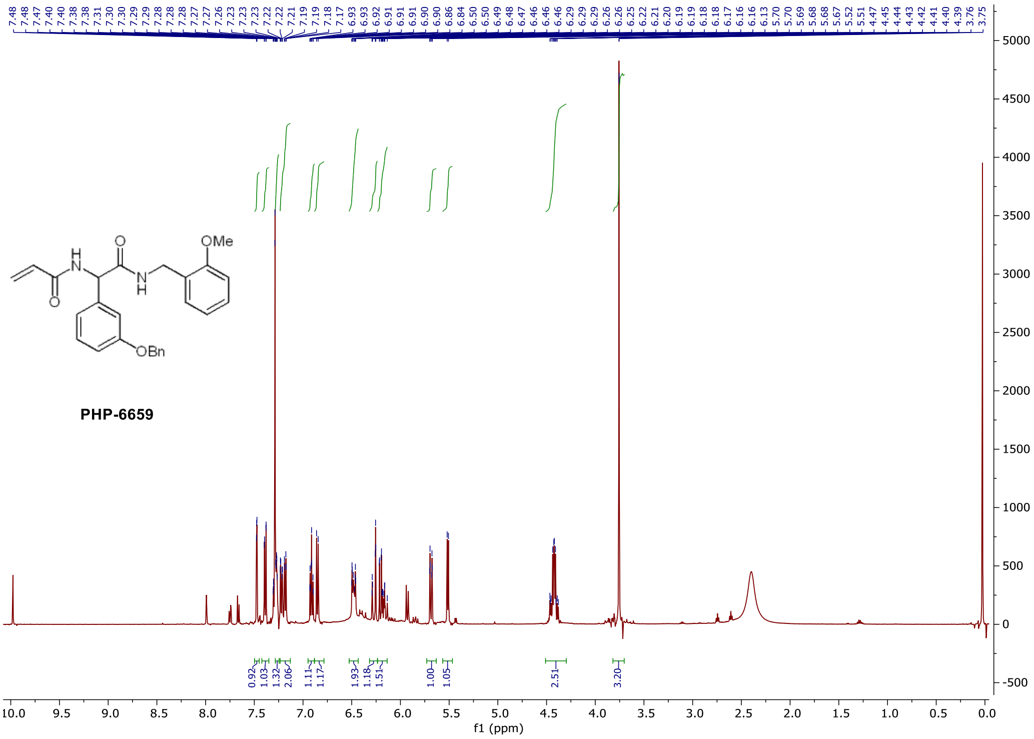

Supplementary Figure 1179. ^{1}H NMR (400 MHz, $CDCl_3$ + one drop of CD_3OD) of compound **PHP-6659.**

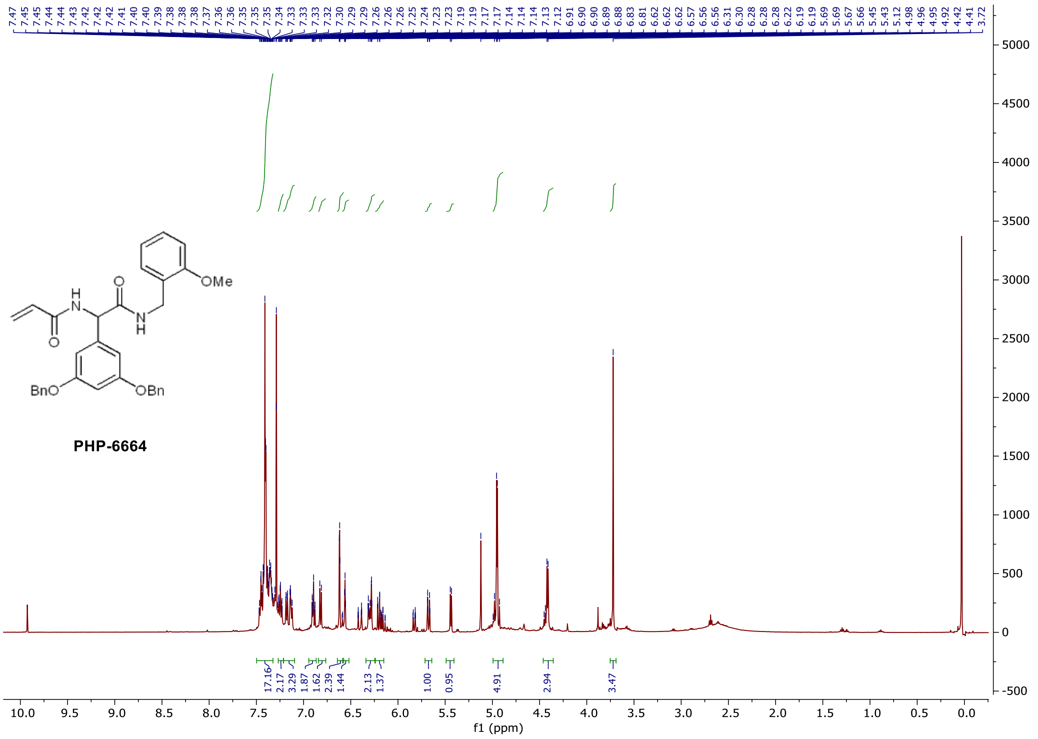

Supplementary Figure 1180. ^{1}H NMR (400 MHz, $CDCl_3$ + one drop of CD_3OD) of compound **PHP-6664.**

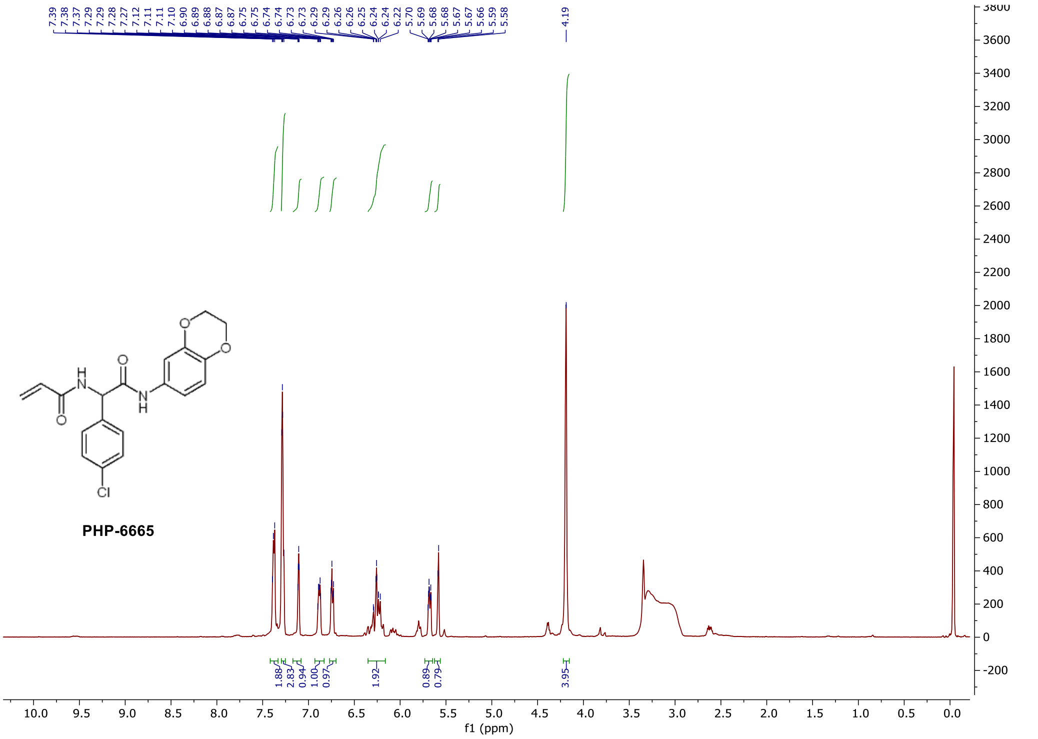

Supplementary Figure 1181. ^{1}H NMR (400 MHz, $CDCl_3$ + one drop of CD_3OD) of compound **PHP-6665.**

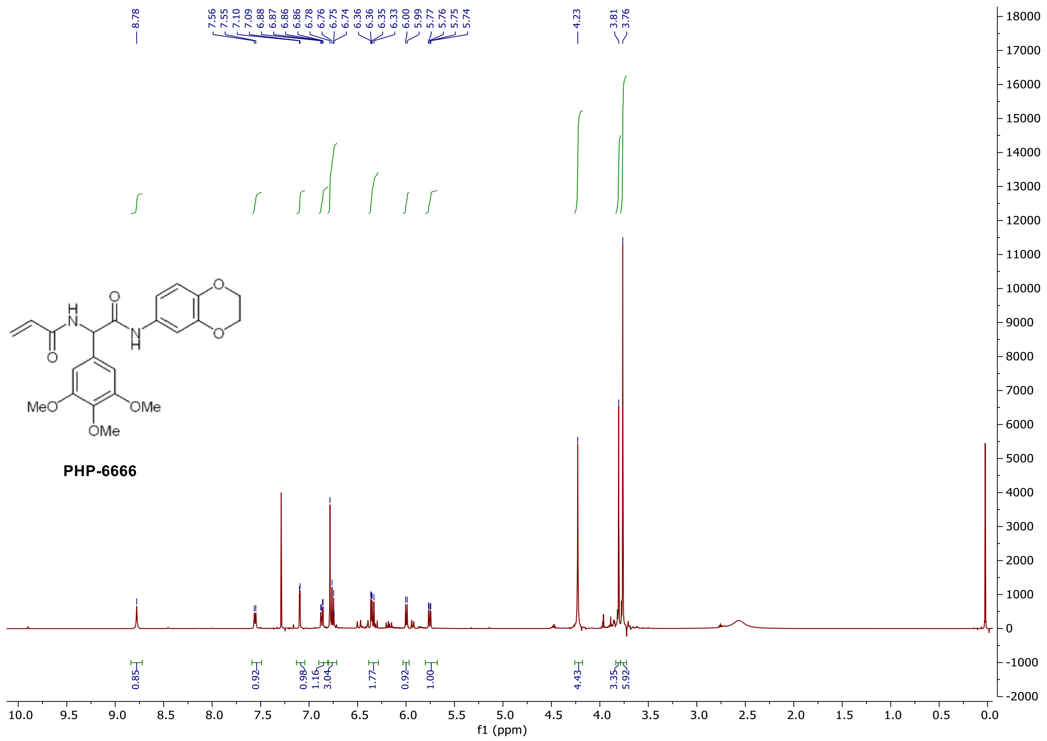

Supplementary Figure 1182. ^1H NMR (400 MHz, $CDCl_3$ + one drop of CD_3OD) of compound **PHP-6666.**

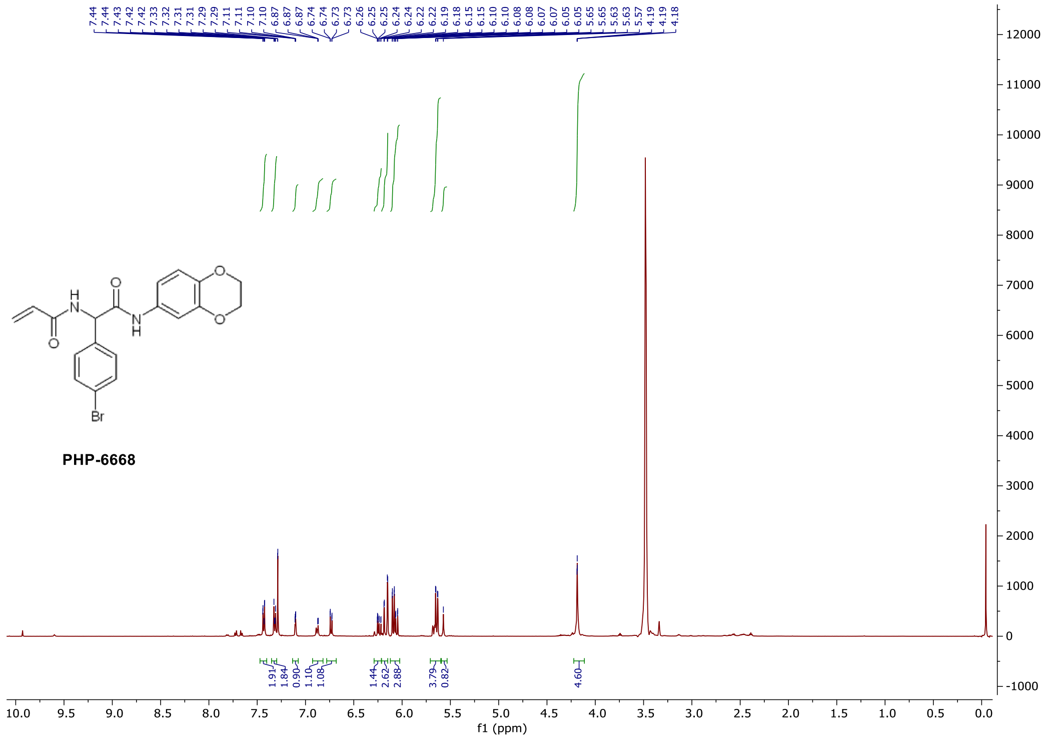

Supplementary Figure 1183. ^1H NMR (400 MHz, $CDCl_3$ + one drop of CD_3OD) of compound **PHP-6668.**

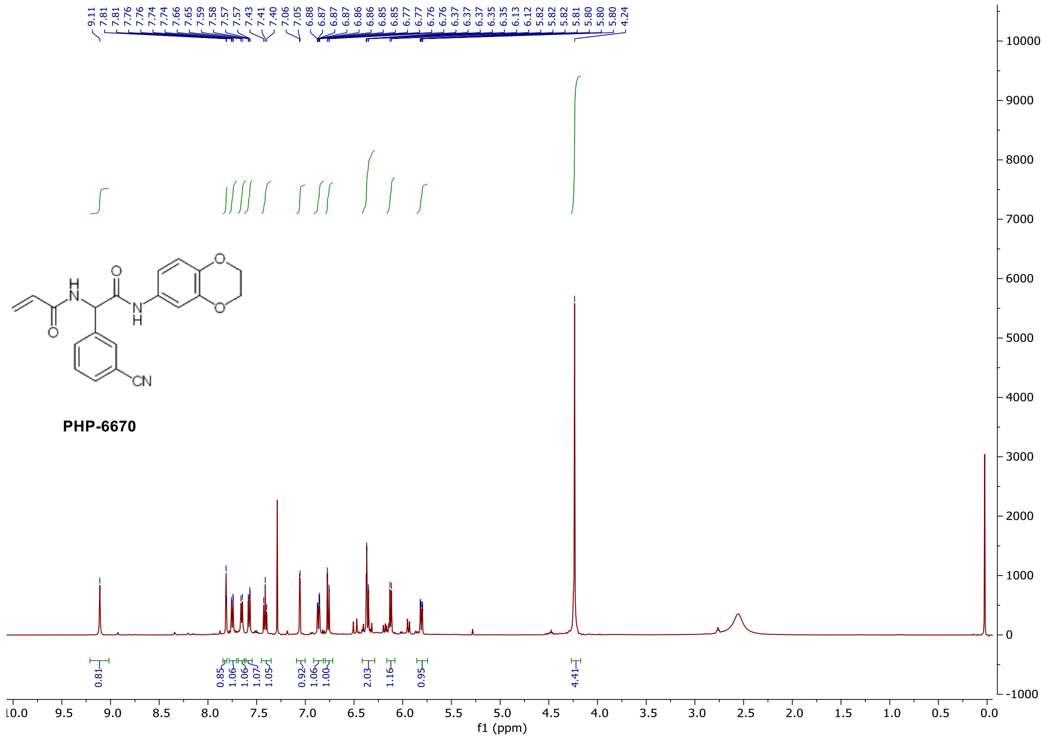

Supplementary Figure 1184. $_1$H NMR (400 MHz, $CDCl_3$ + one drop of CD_3OD) of compound **PHP-6670.**

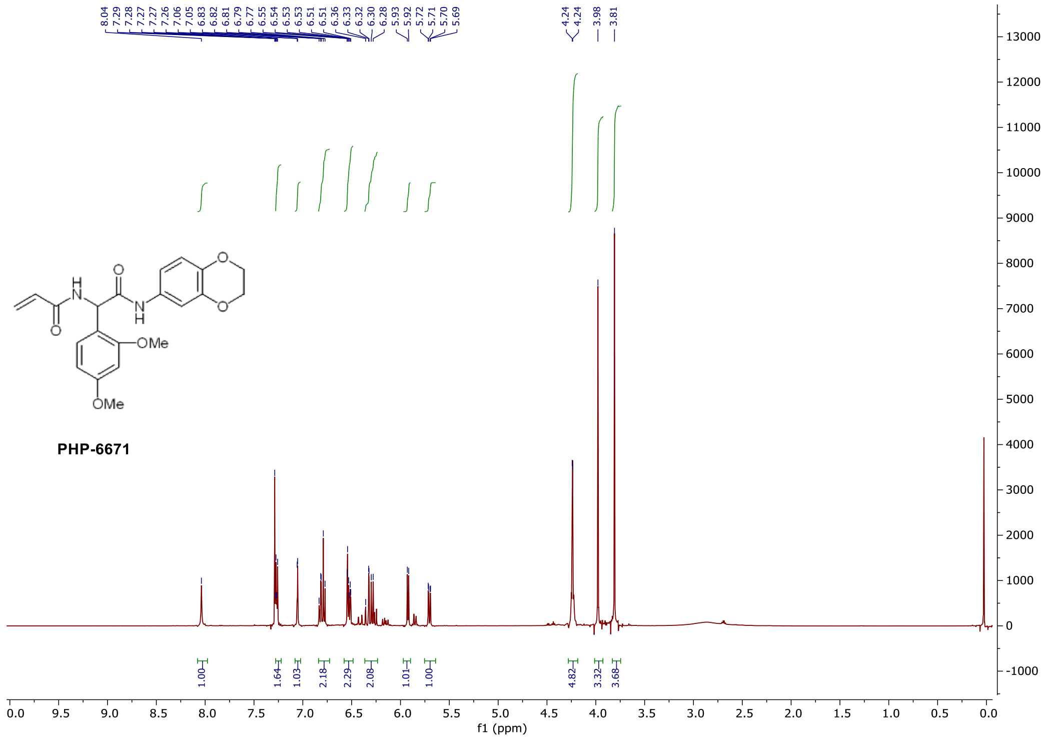

Supplementary Figure 1185. ^{1}H NMR (400 MHz, $CDCl_3$ + one drop of CD_3OD) of compound **PHP-6671.**

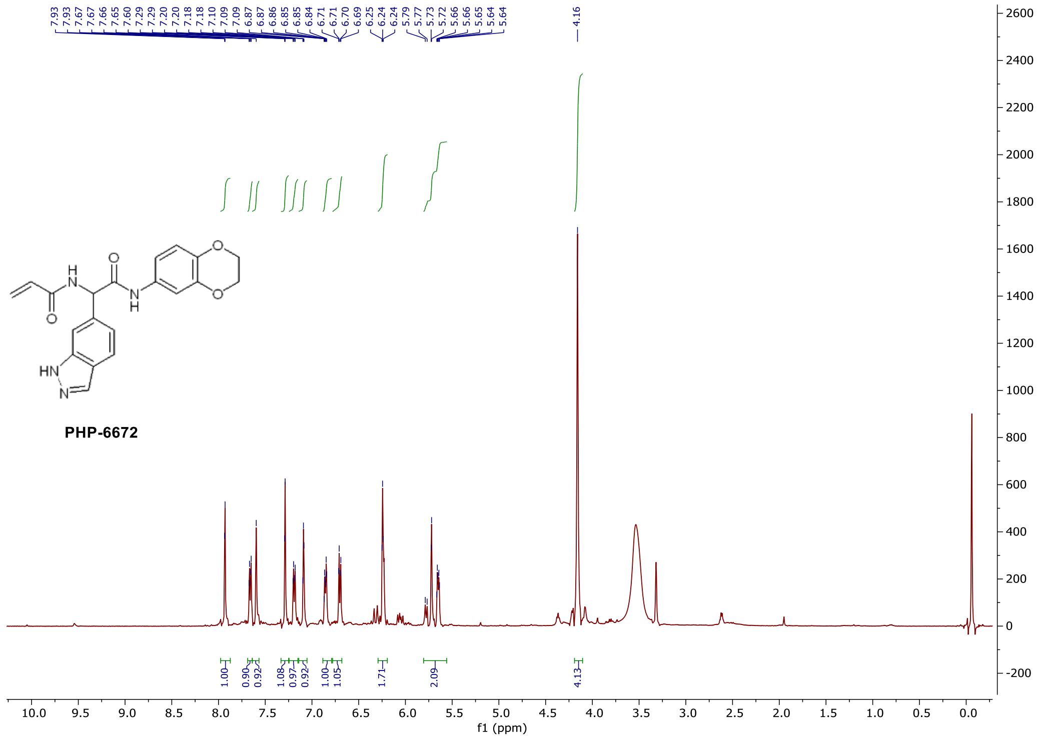

Supplementary Figure 1186. ^{1}H NMR (400 MHz, $CDCl_3$ + one drop of CD_3OD) of compound **PHP-6672.**

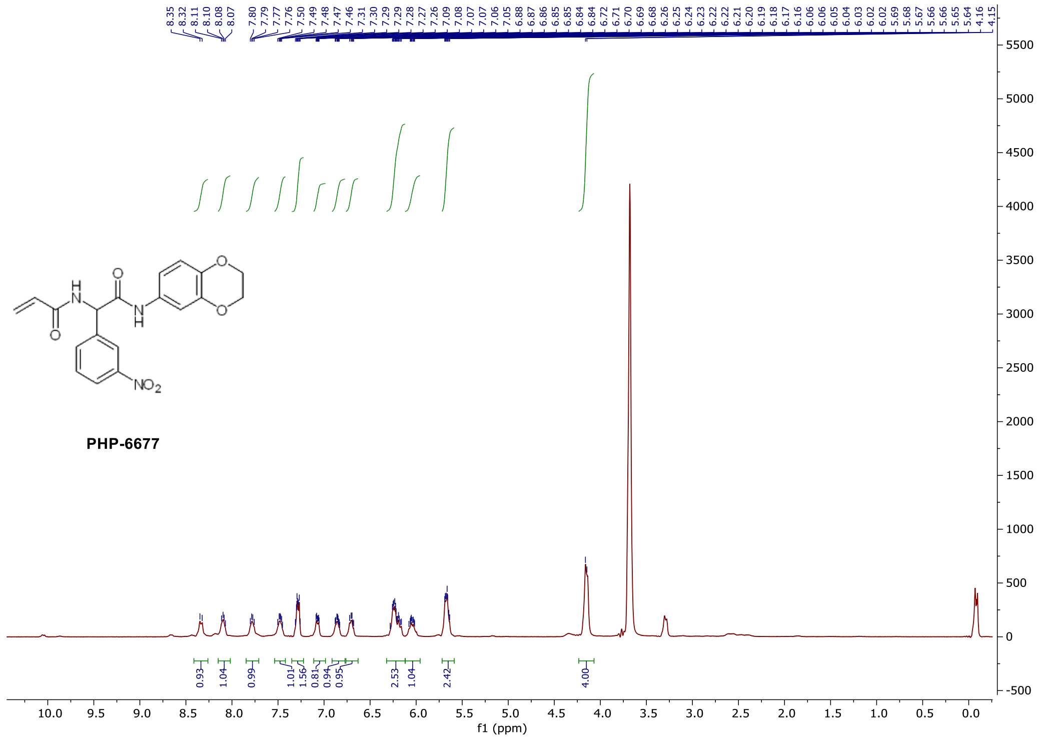

Supplementary Figure 1187. ^{1}H NMR (400 MHz, $CDCl_3$ + one drop of CD_3OD) of compound **PHP-6677.**

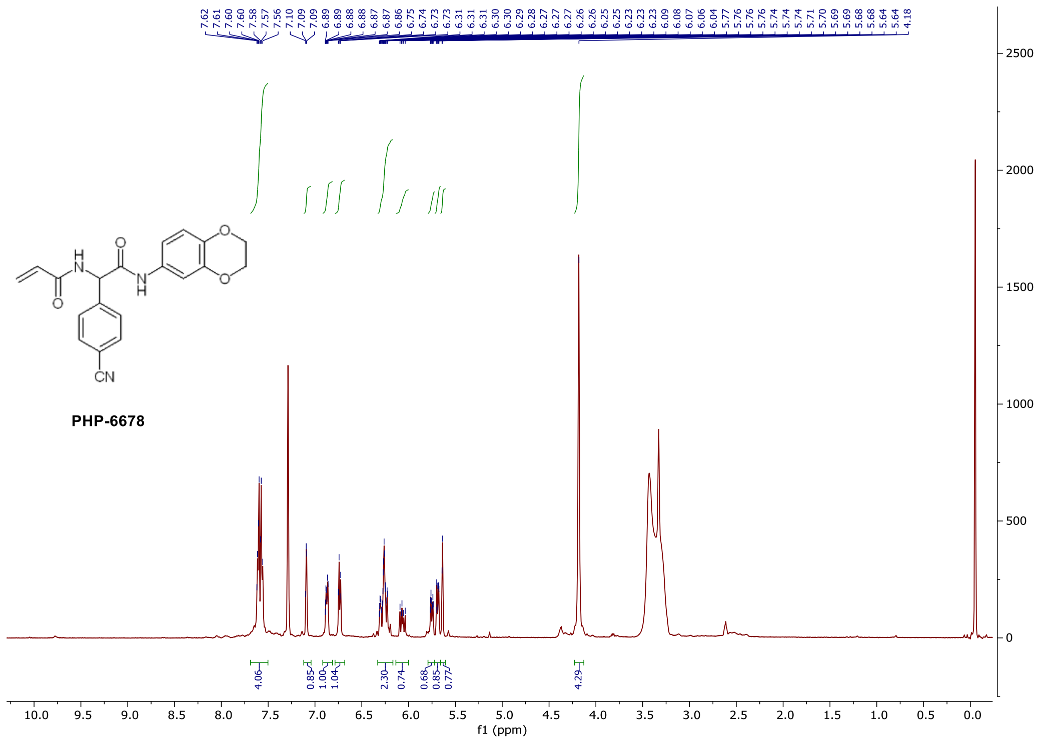

Supplementary Figure 1188. ^{1}H NMR (400 MHz, $CDCl_3$ + one drop of CD_3OD) of compound **PHP-6678.**

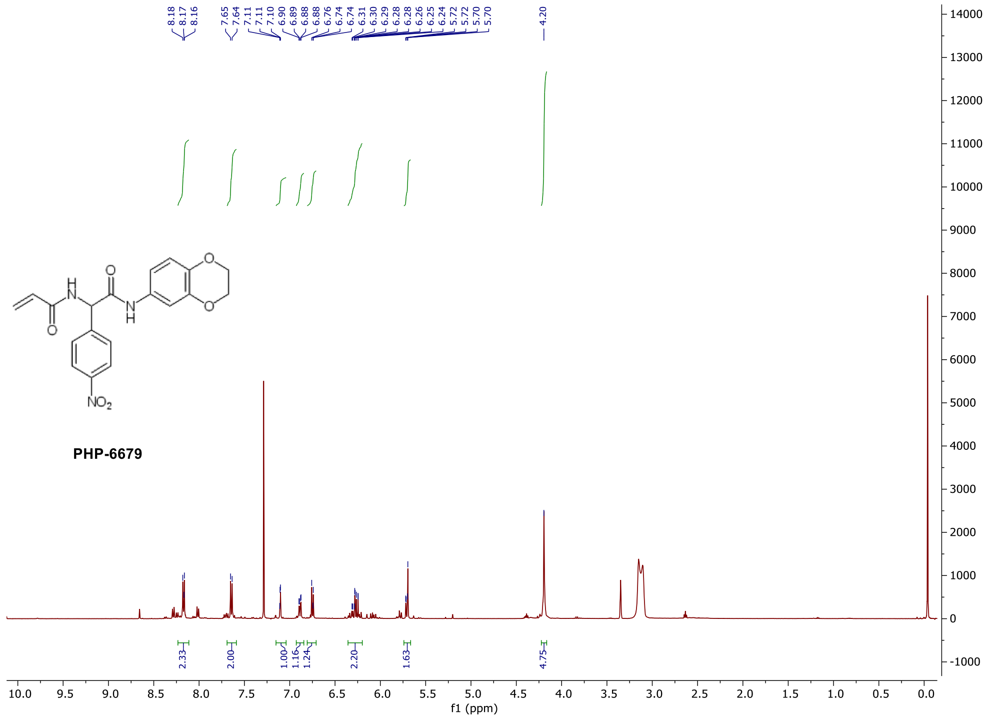

Supplementary Figure 1189. ^{1}H NMR (400 MHz, $CDCl_3$ + one drop of CD_3OD) of compound **PHP-6679.**

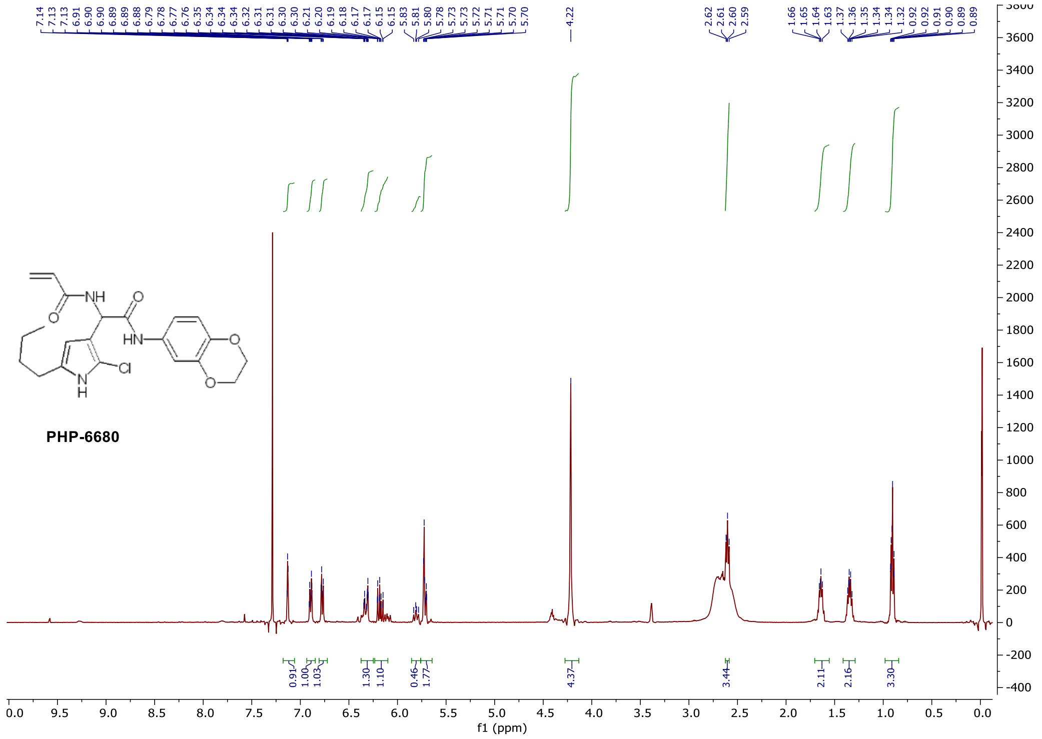

Supplementary Figure 1190. ^{1}H NMR (400 MHz, $CDCl_3$ + one drop of CD_3OD) of compound **PHP-6680.**

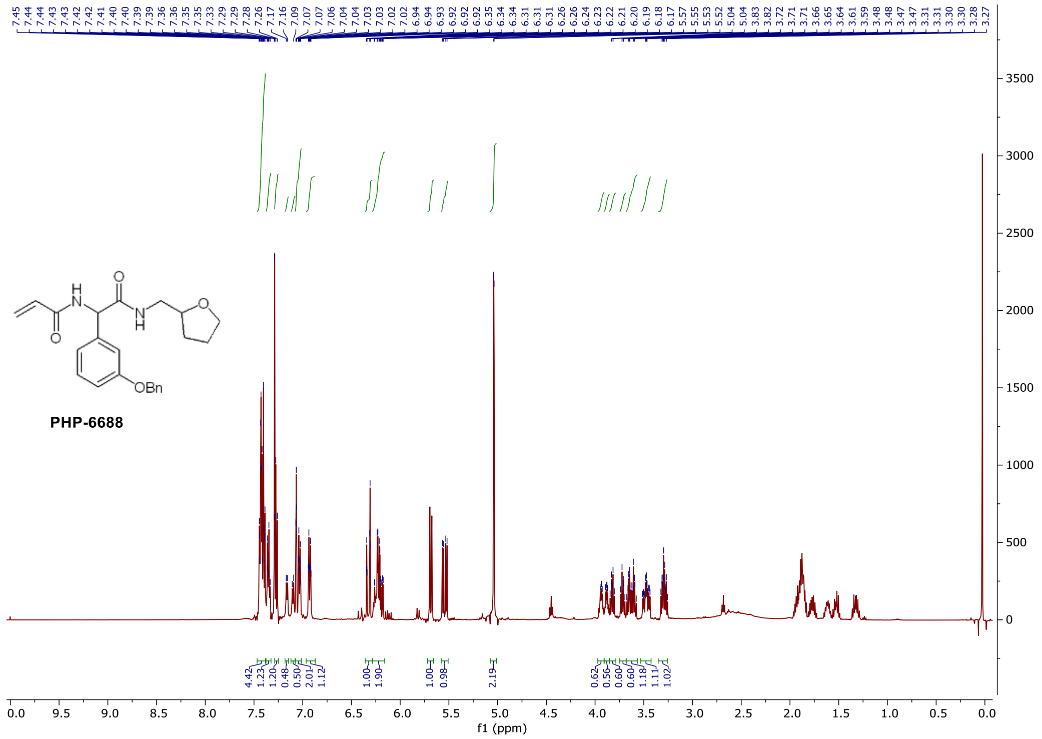

Supplementary Figure 1191. ^{1}H NMR (400 MHz, $CDCl_3$ + one drop of CD_3OD) of compound **PHP-6688.**

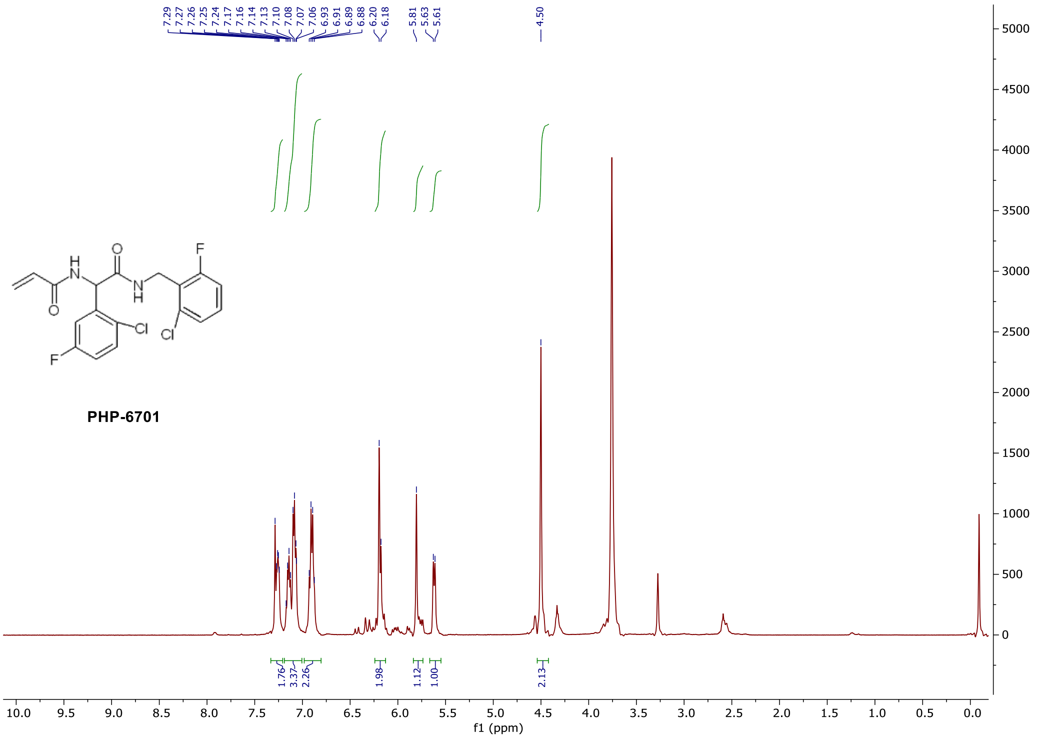

Supplementary Figure 1192. ^{1}H NMR (400 MHz, $CDCl_3$ + one drop of CD_3OD) of compound **PHP-6701.**

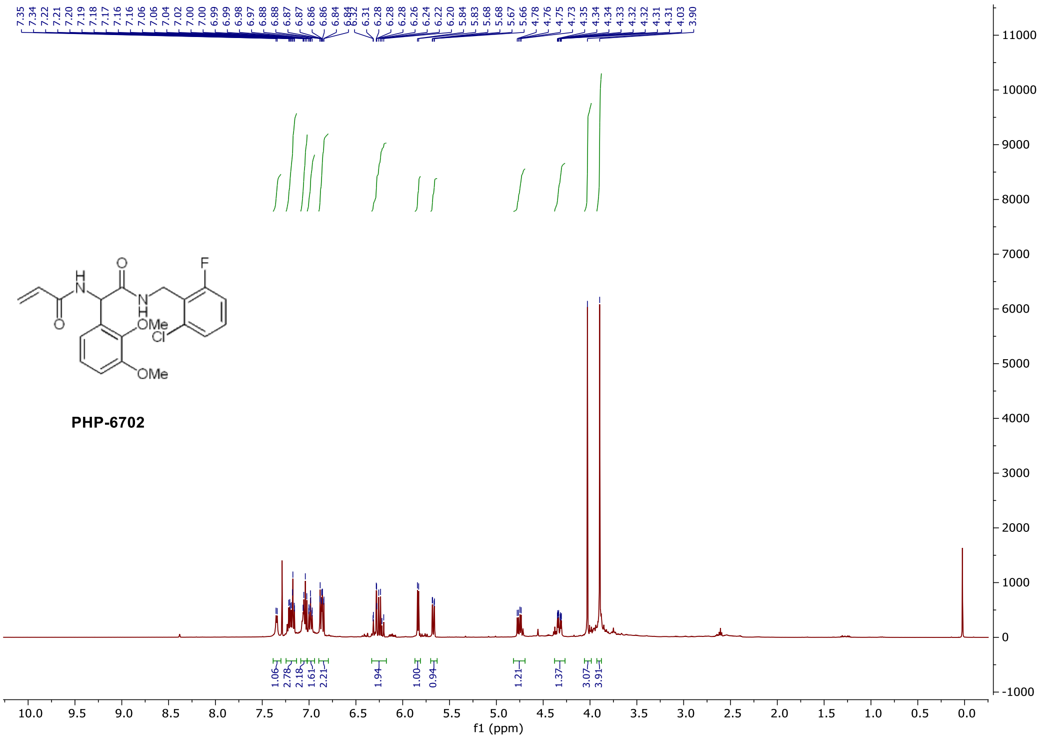

Supplementary Figure 1193. ^{1}H NMR (400 MHz, $CDCl_3$ + one drop of CD_3OD) of compound **PHP-6702.**

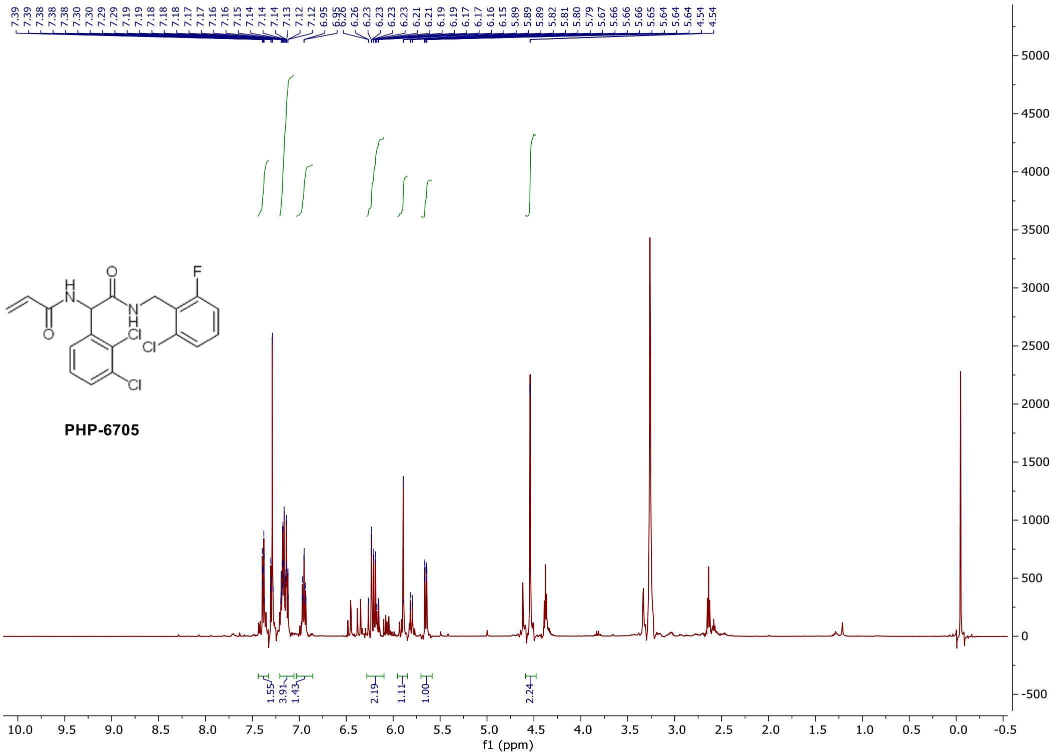

Supplementary Figure 1194. ^{1}H NMR (400 MHz, $CDCl_3$ + one drop of CD_3OD) of compound **PHP-6705**.

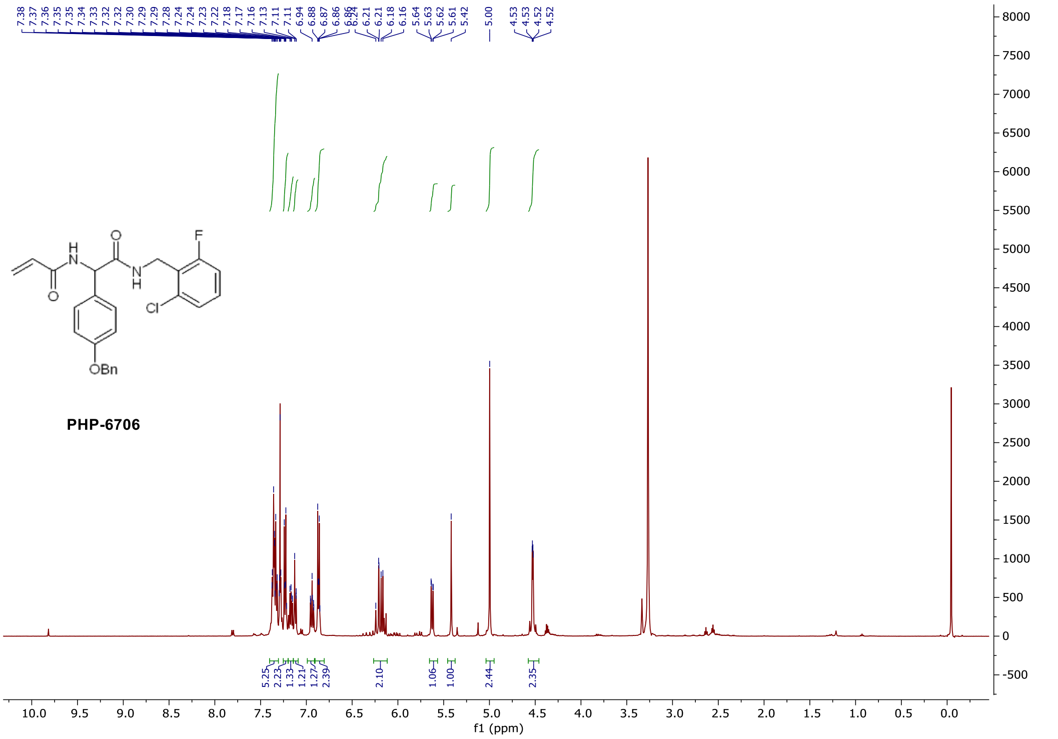

Supplementary Figure 1195. ^{1}H NMR (400 MHz, $CDCl_3$ + one drop of CD_3OD) of compound **PHP-6706**.

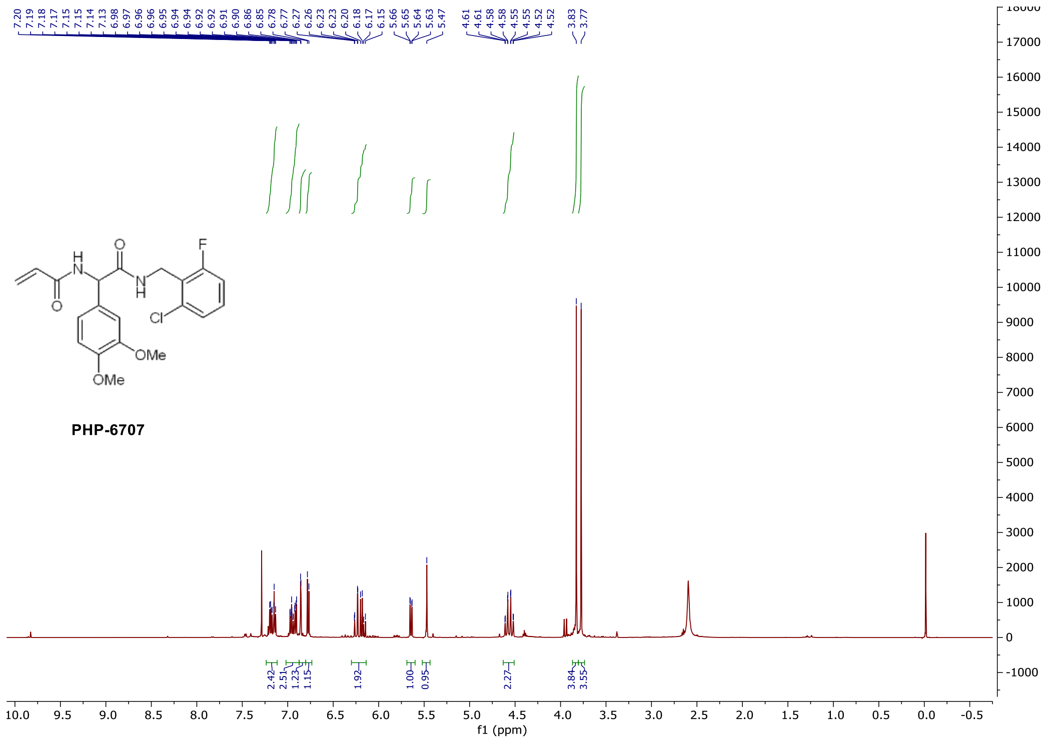

Supplementary Figure 1196. ^1H NMR (400 MHz, $CDCl_3$ + one drop of CD_3OD) of compound **PHP-6707**.

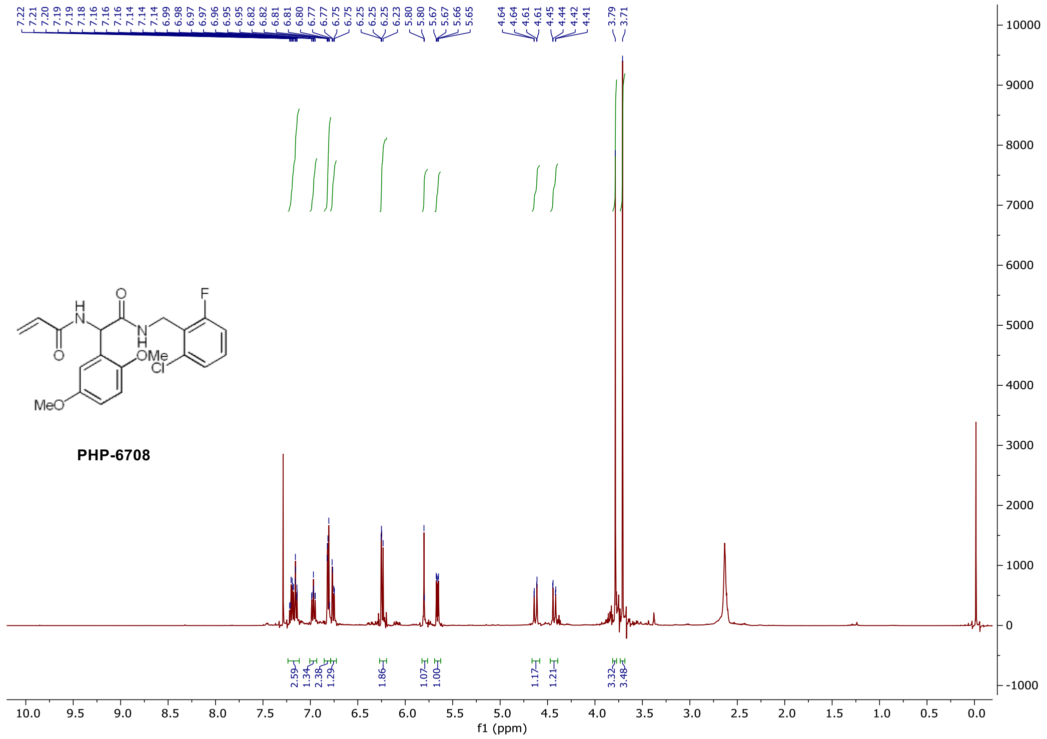

Supplementary Figure 1197. ^1H NMR (400 MHz, $CDCl_3$ + one drop of CD_3OD) of compound **PHP-6708**.

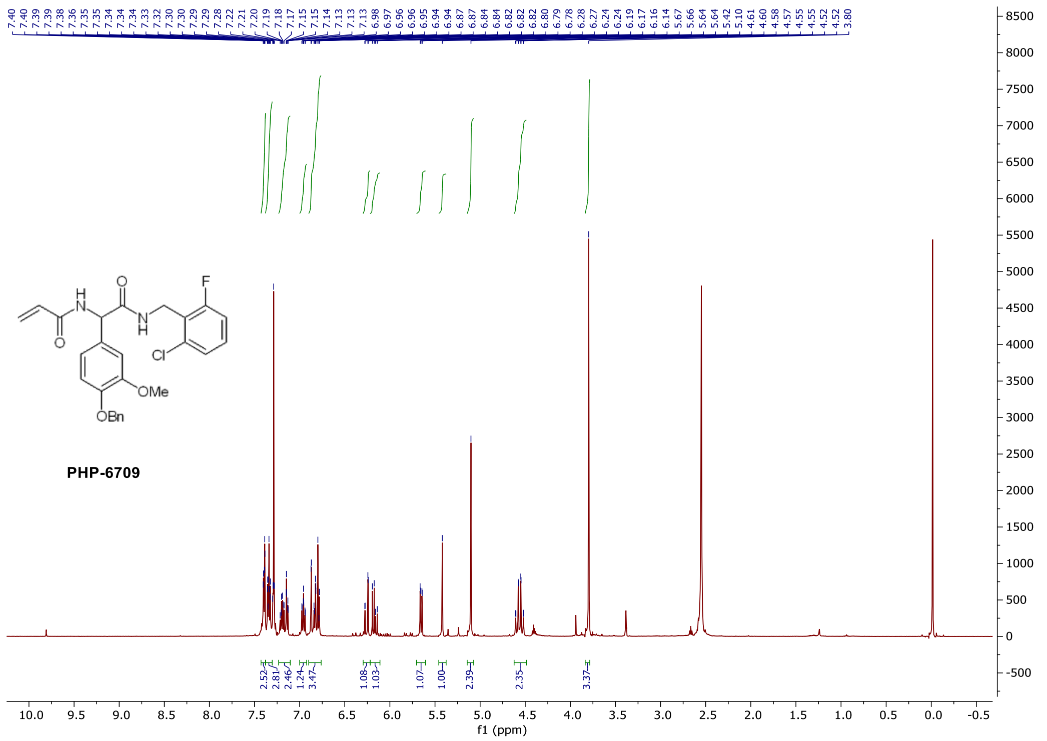

Supplementary Figure 1198. ^{1}H NMR (400 MHz, $CDCl_3$ + one drop of CD_3OD) of compound **PHP-6709.**

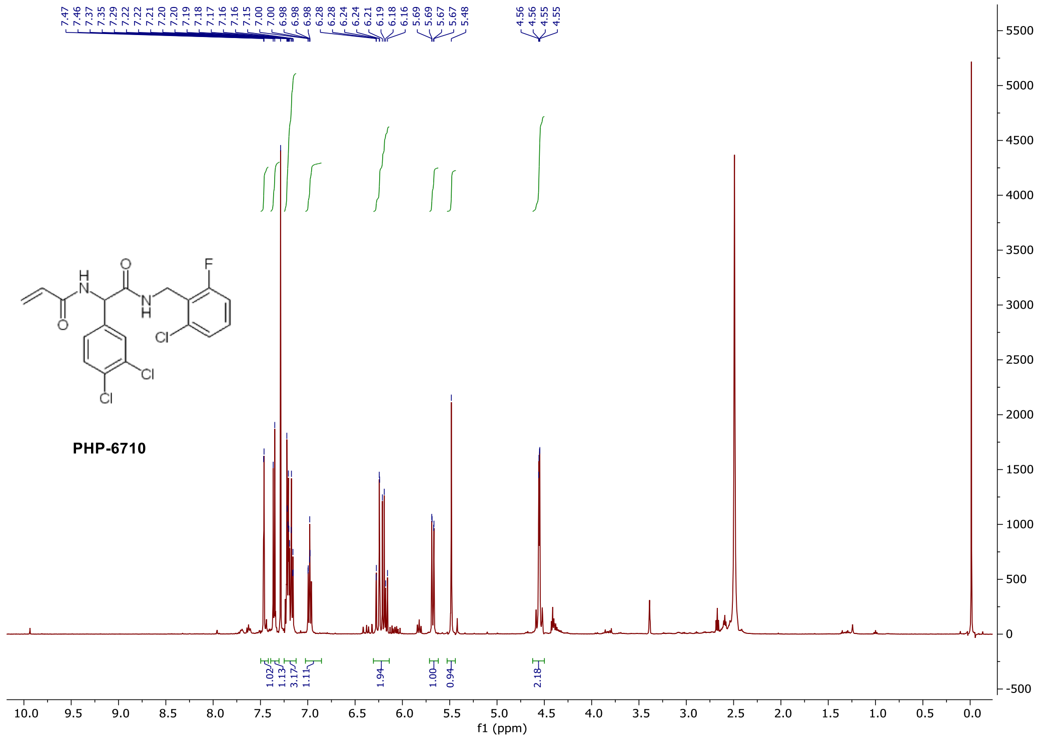

Supplementary Figure 1199. ^{1}H NMR (400 MHz, $CDCl_3$ + one drop of CD_3OD) of compound **PHP-6710.**

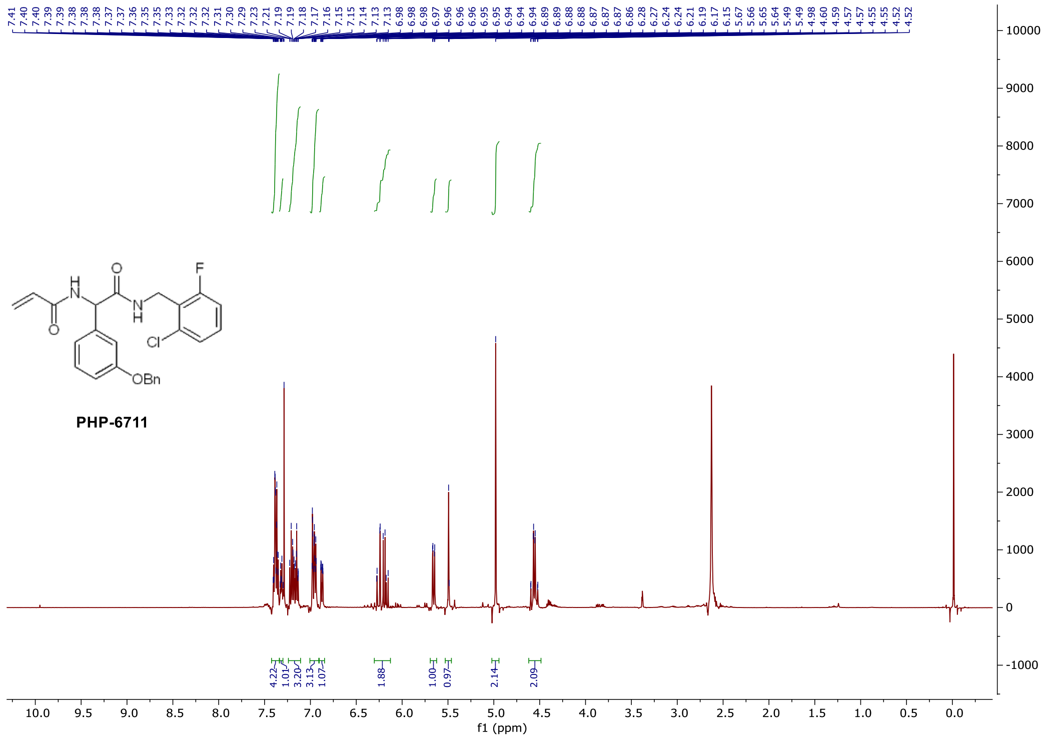

Supplementary Figure 1200. ^{1}H NMR (400 MHz, $CDCl_3$ + one drop of CD_3OD) of compound **PHP-6711.**

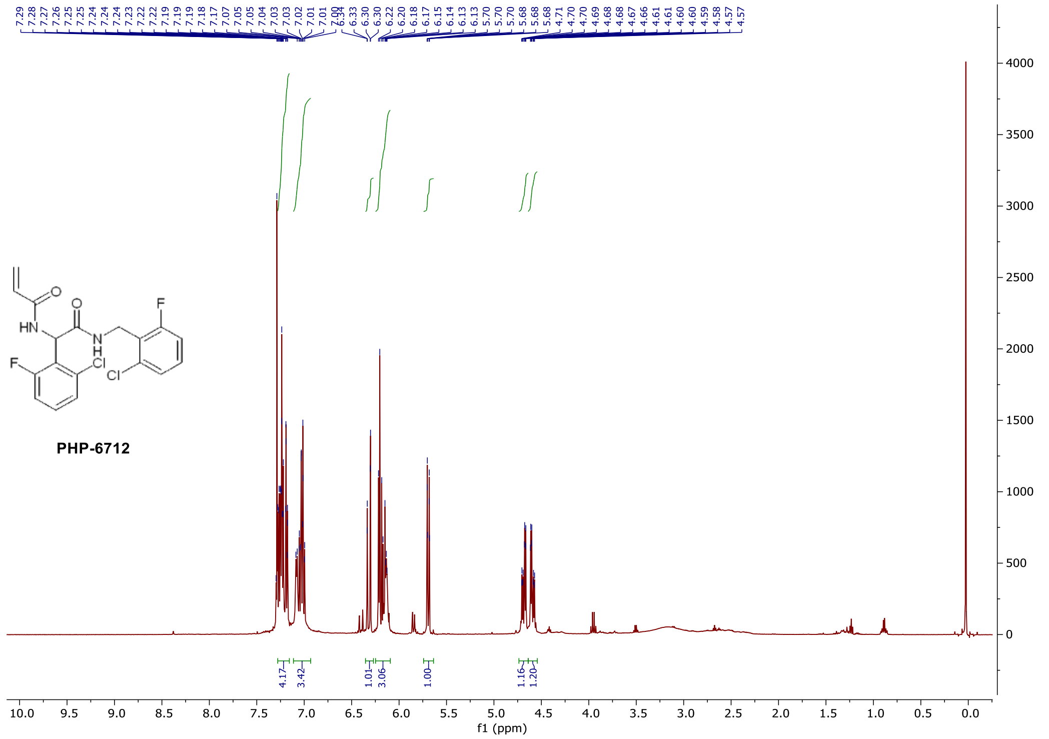

Supplementary Figure 1201. ^{1}H NMR (400 MHz, $CDCl_3$ + one drop of CD_3OD) of compound **PHP-6712.**

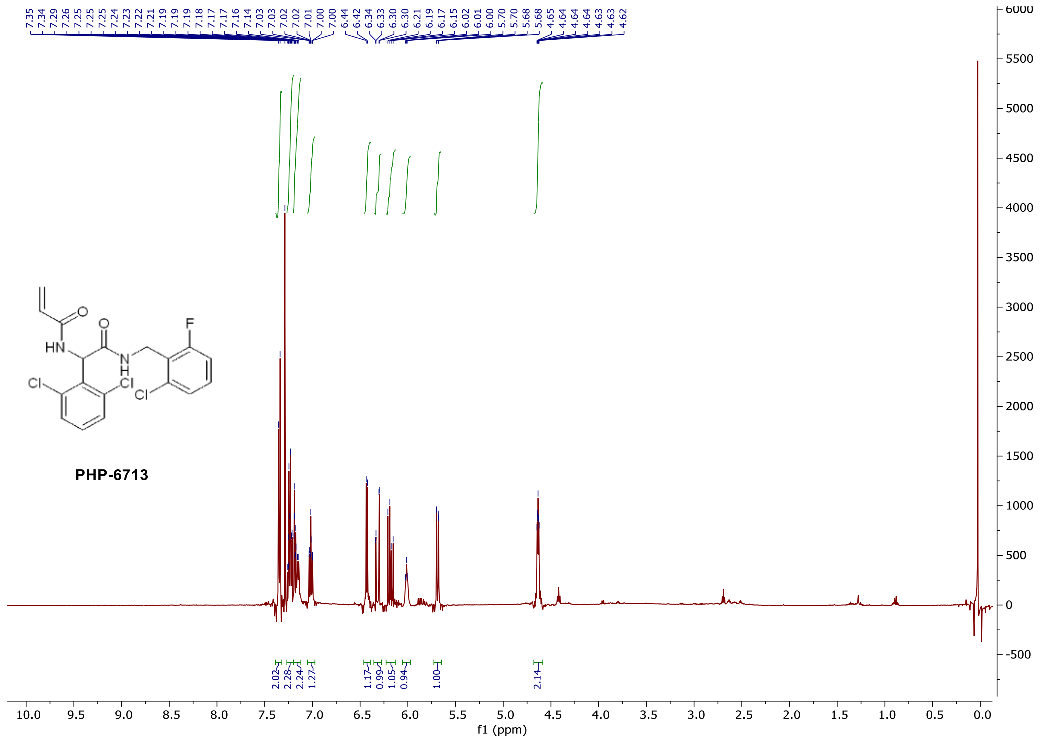

Supplementary Figure 1202. ^{1}H NMR (400 MHz, $CDCl_3$ + one drop of CD_3OD) of compound **PHP-6713.**

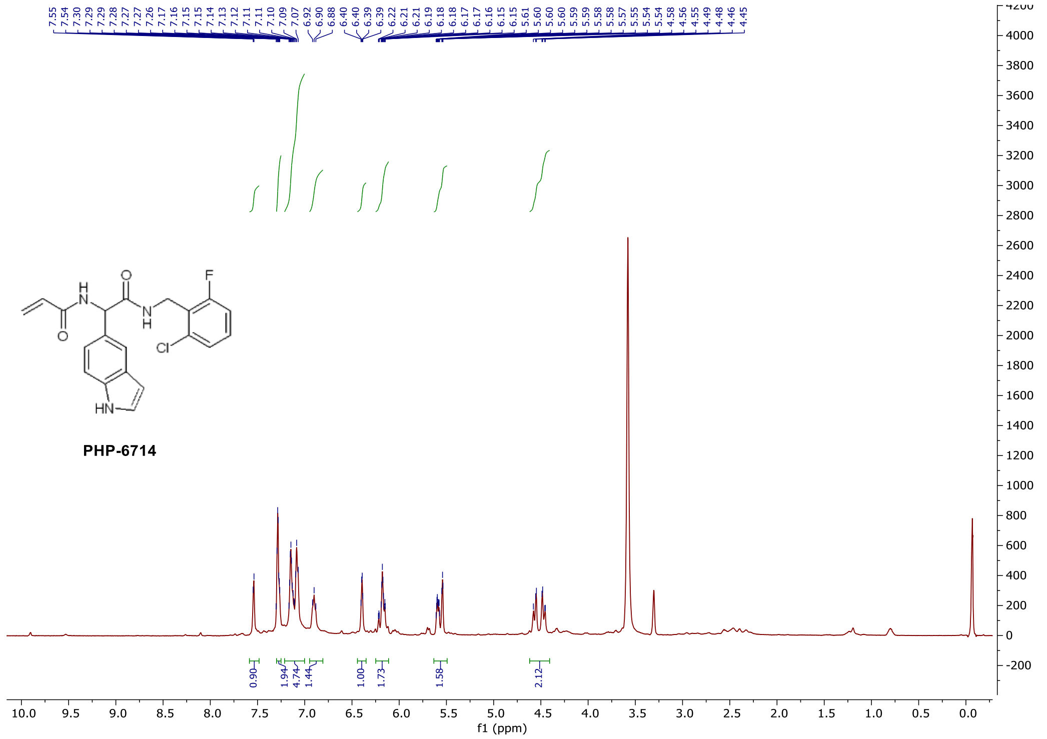

Supplementary Figure 1203. ^{1}H NMR (400 MHz, $CDCl_3$ + one drop of CD_3OD) of compound **PHP-6714.**

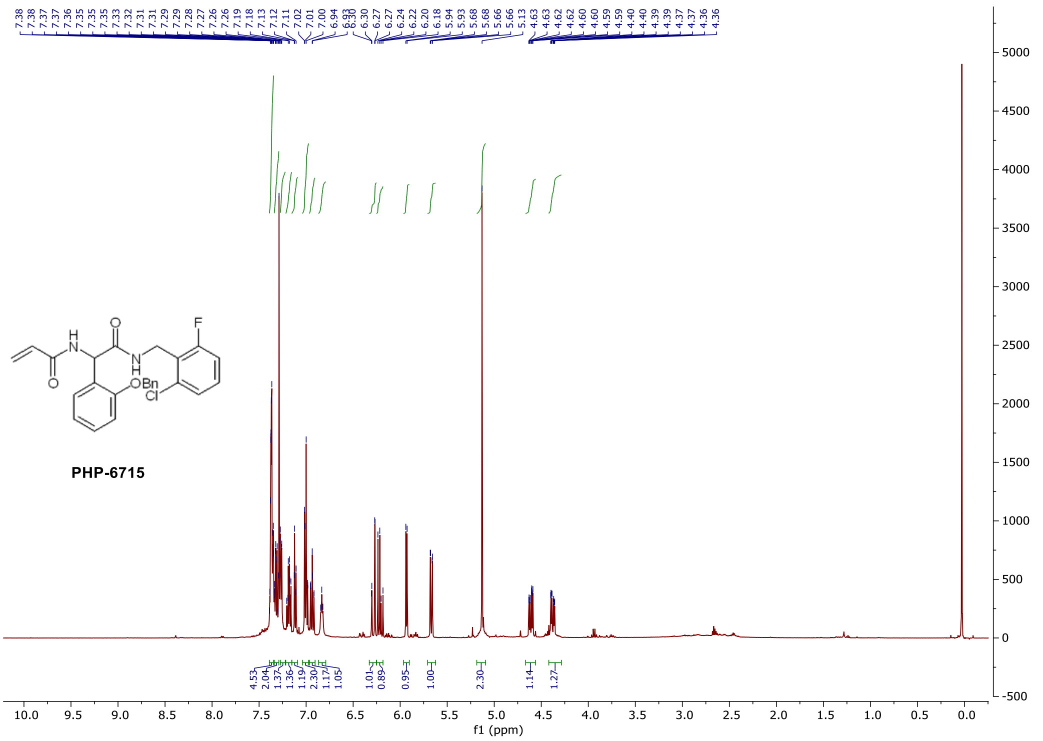

Supplementary Figure 1204. ^{1}H NMR (400 MHz, $CDCl_3$ + one drop of CD_3OD) of compound **PHP-6715.**

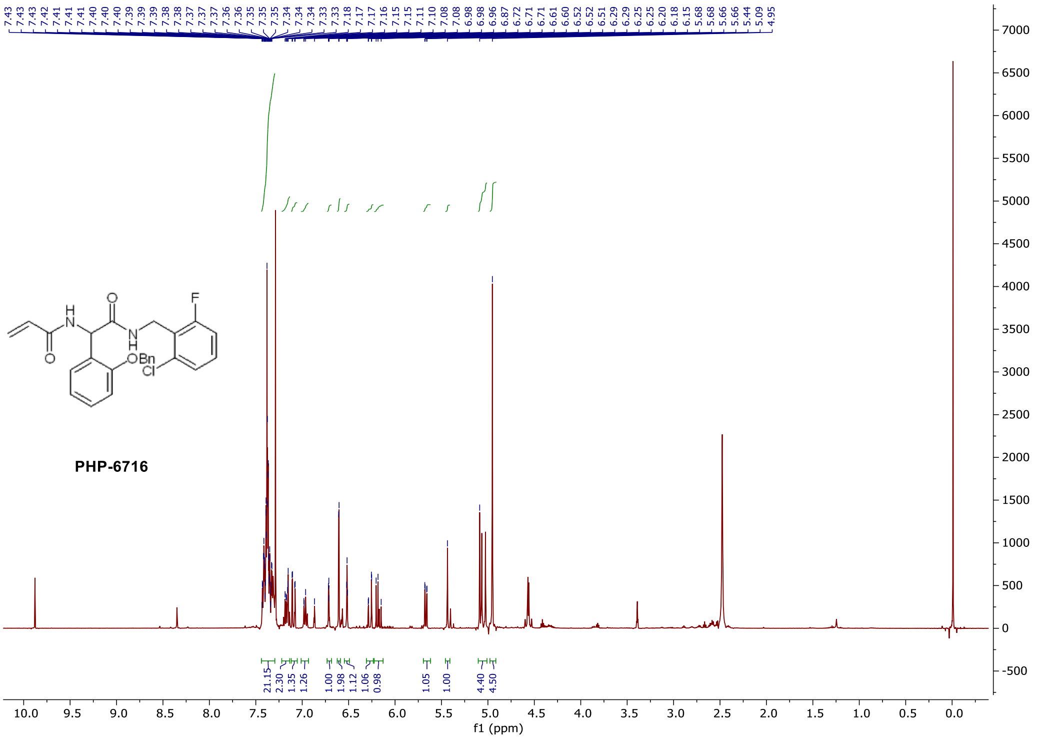

Supplementary Figure 1205. ^{1}H NMR (400 MHz, $CDCl_3$ + one drop of CD_3OD) of compound **PHP-6716.**

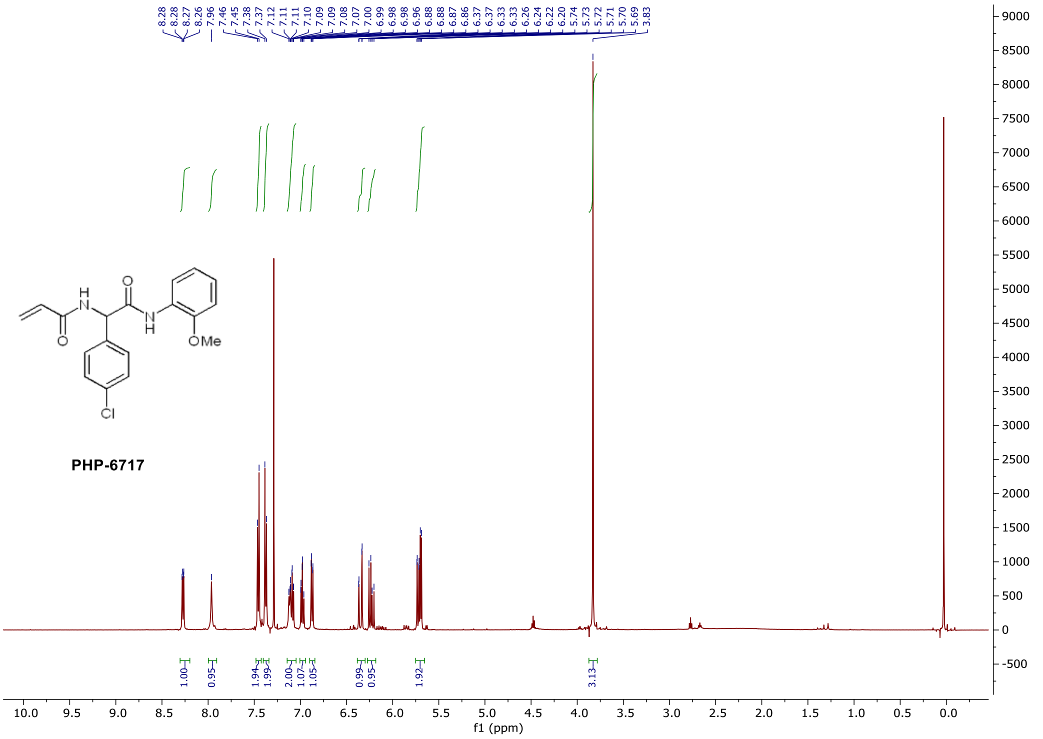

Supplementary Figure 1206. ^{1}H NMR (400 MHz, $CDCl_3$ + one drop of CD_3OD) of compound **PHP-6717.**

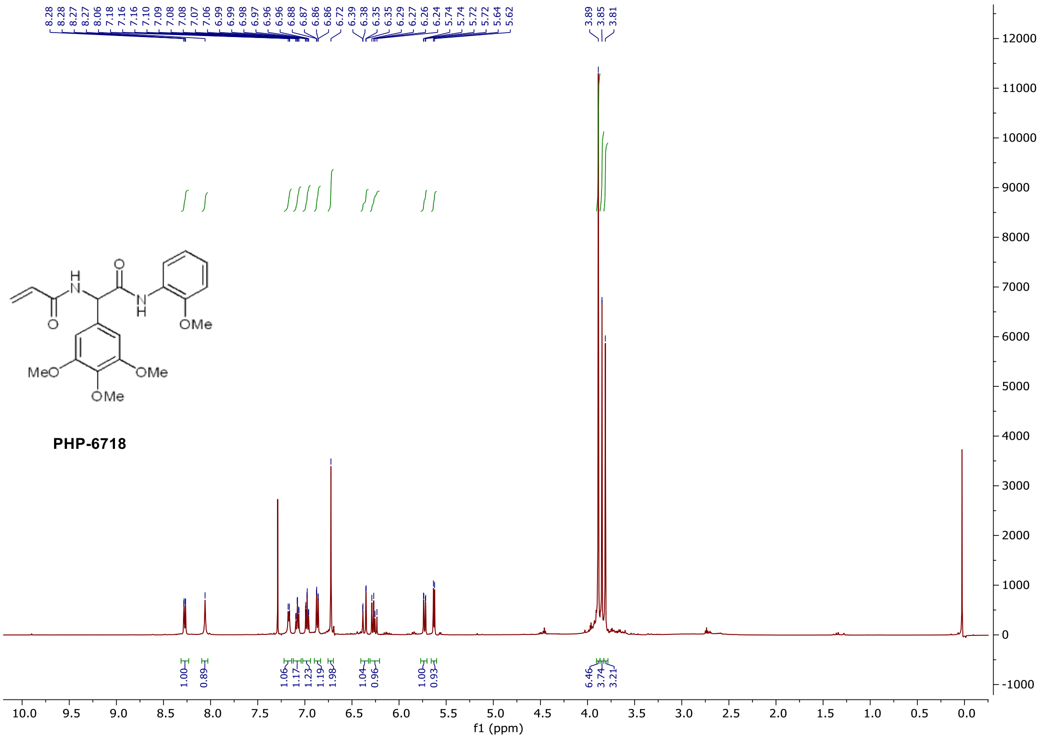

Supplementary Figure 1207. ^{1}H NMR (400 MHz, $CDCl_3$ + one drop of CD_3OD) of compound **PHP-6718.**

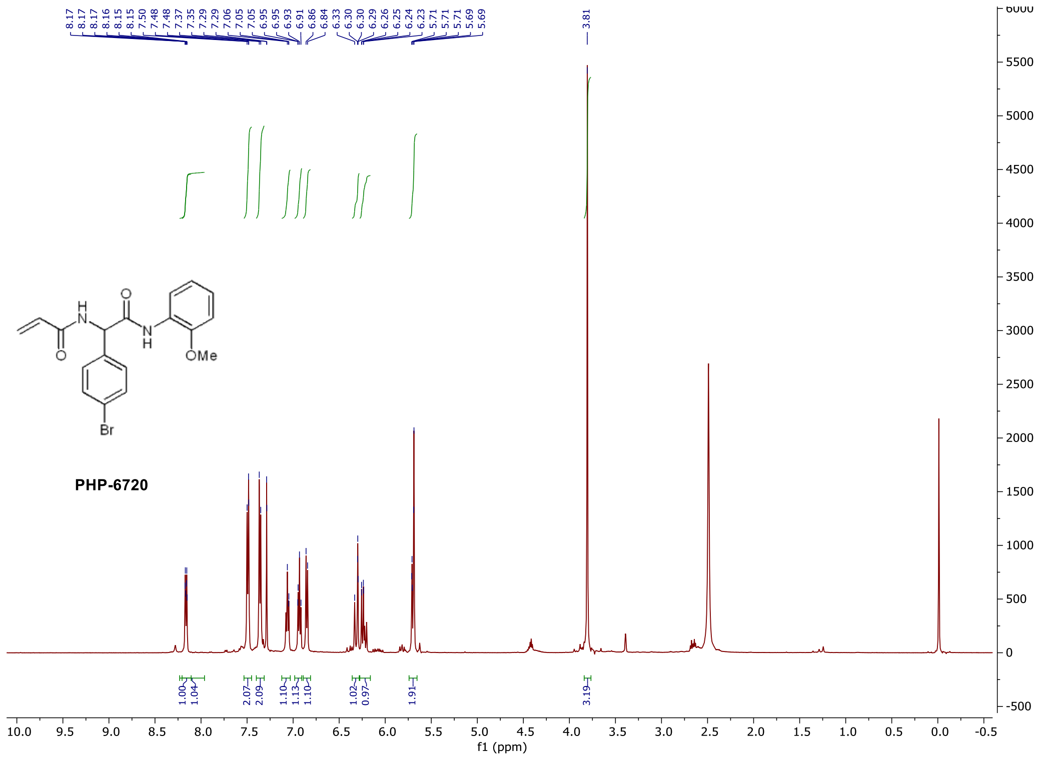

Supplementary Figure 1208. ^{1}H NMR (400 MHz, $CDCl_3$ + one drop of CD_3OD) of compound **PHP-6720.**

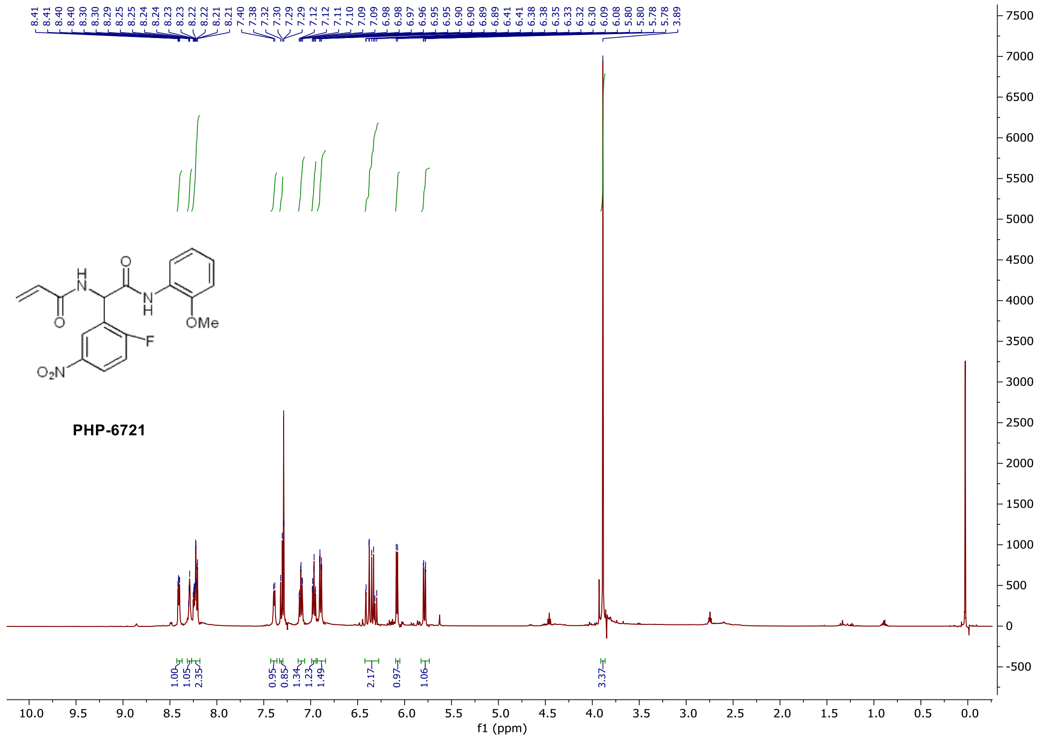

Supplementary Figure 1209. ^{1}H NMR (400 MHz, $CDCl_3$ + one drop of CD_3OD) of compound **PHP-6721.**

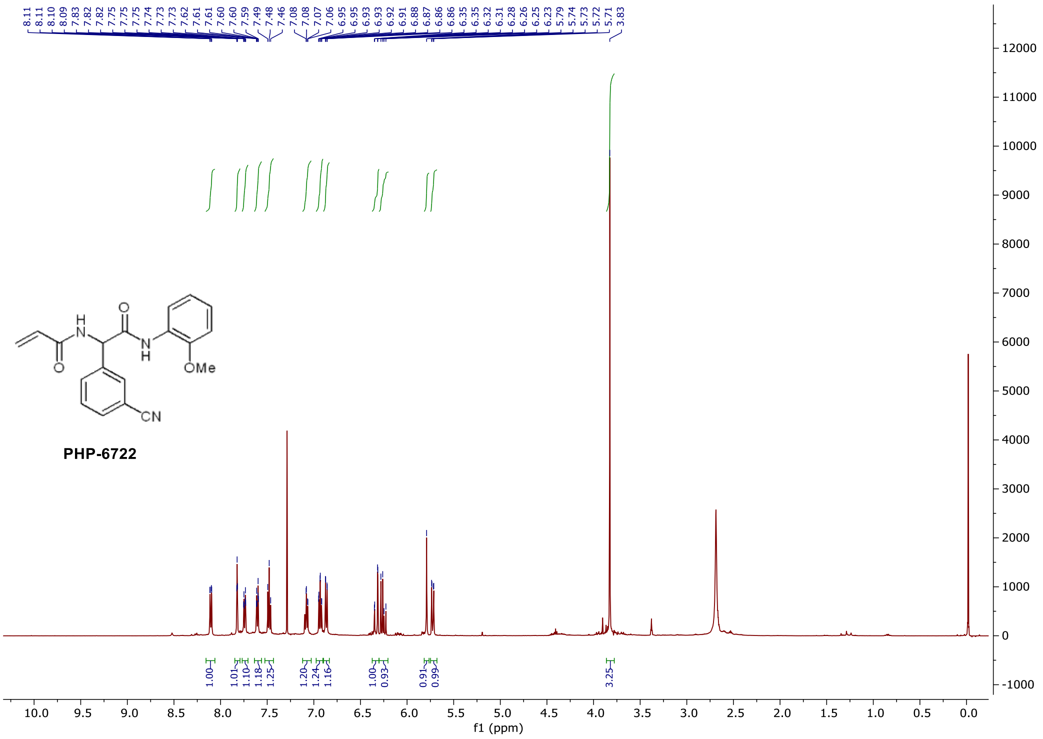

Supplementary Figure 1210. ^{1}H NMR (400 MHz, $CDCl_3$ + one drop of CD_3OD) of compound **PHP-6722.**

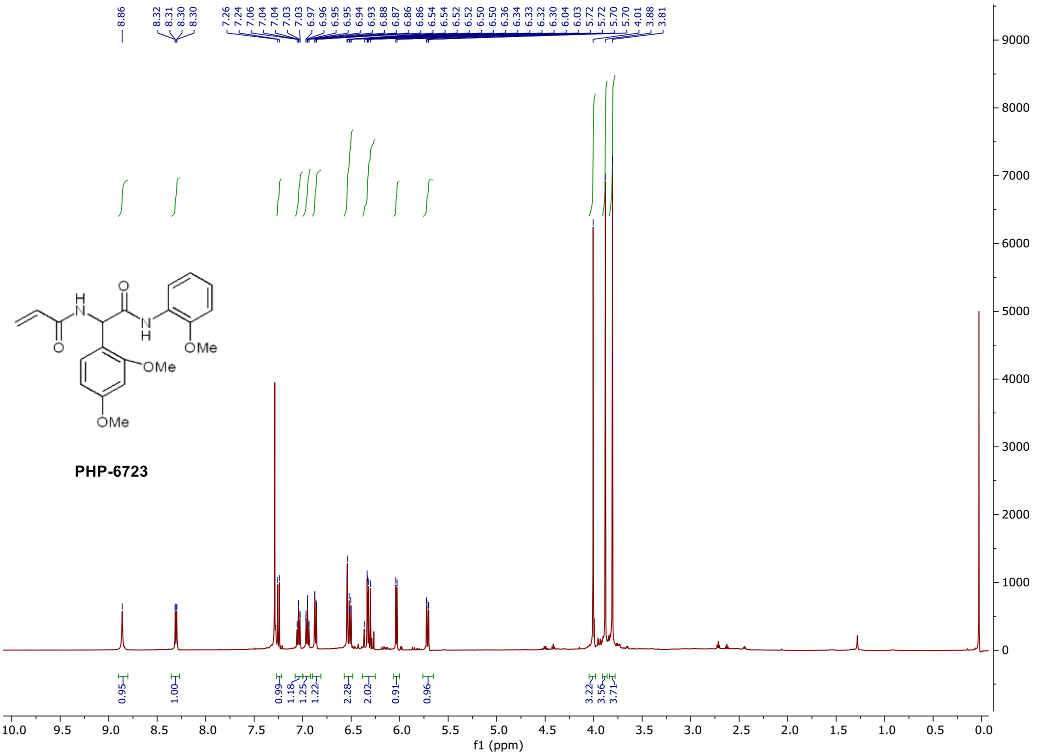

Supplementary Figure 1211. ^{1}H NMR (400 MHz, $CDCl_3$ + one drop of CD_3OD) of compound **PHP-6723.**

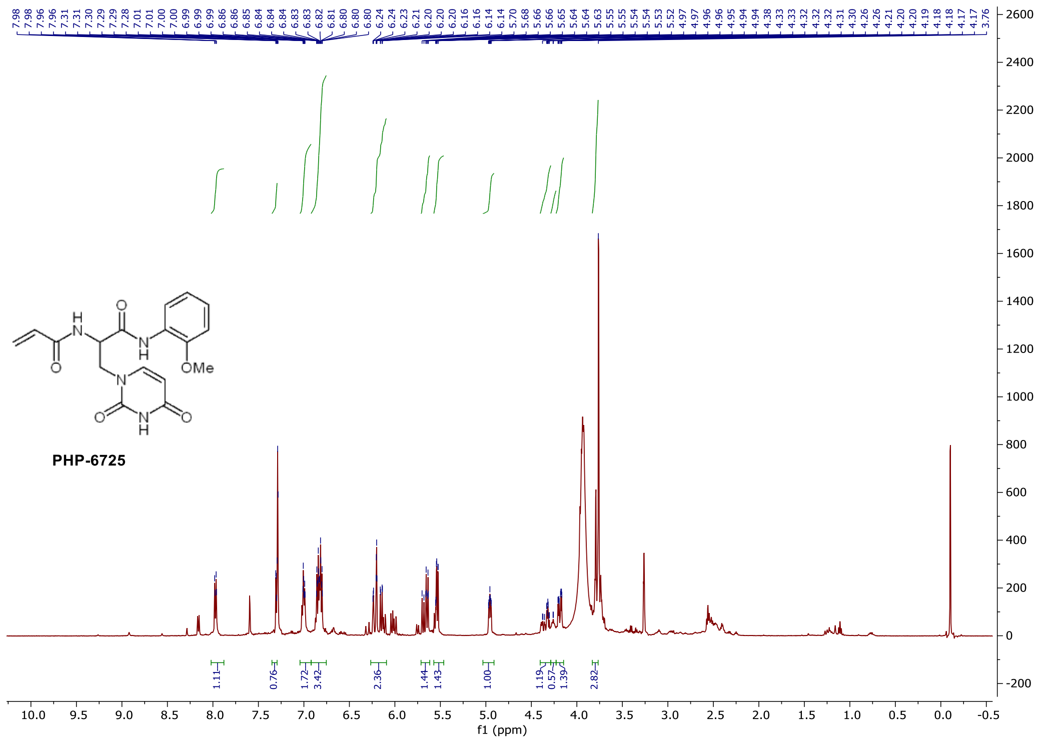

Supplementary Figure 1212. ^{1}H NMR (400 MHz, $CDCl_3$ + one drop of CD_3OD) of compound **PHP-6725.**

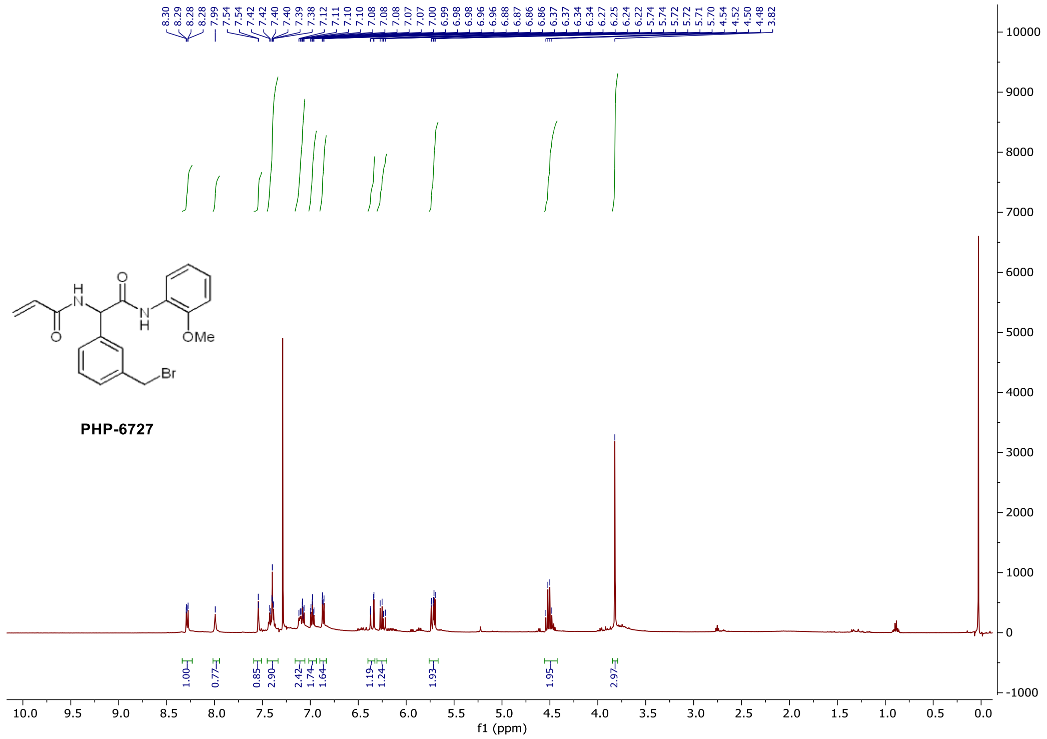

Supplementary Figure 1213. ^{1}H NMR (400 MHz, $CDCl_3$ + one drop of CD_3OD) of compound **PHP-6727.**

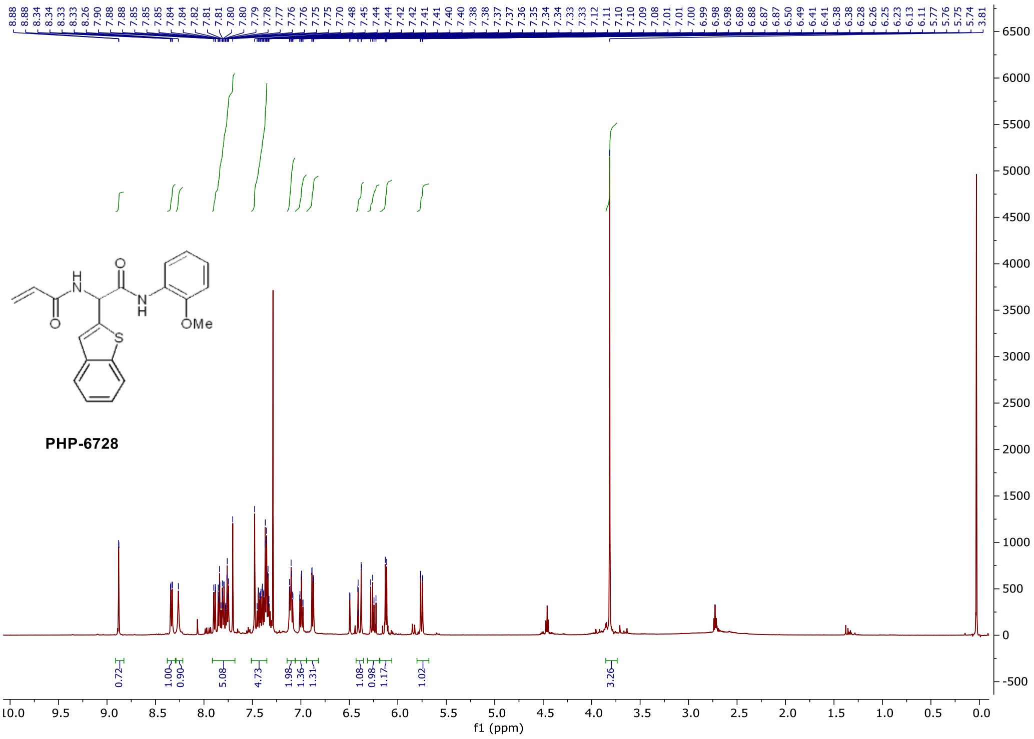

Supplementary Figure 1214. ^{1}H NMR (400 MHz, $CDCl_3$ + one drop of CD_3OD) of compound **PHP-6728.**

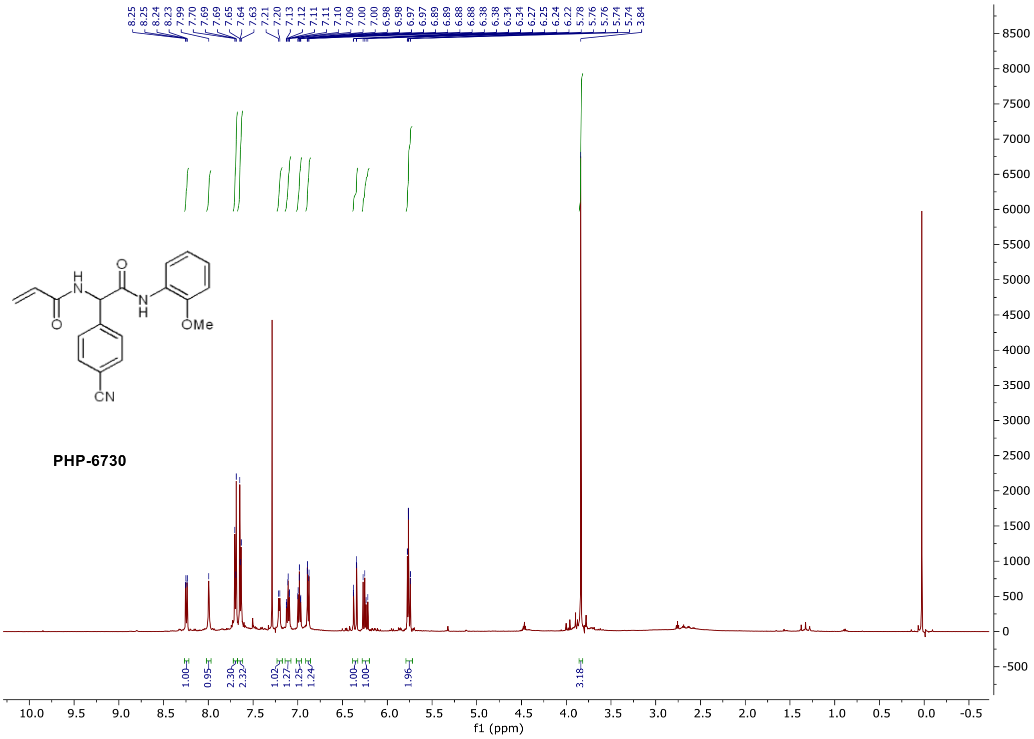

Supplementary Figure 1215. ^{1}H NMR (400 MHz, $CDCl_3$ + one drop of CD_3OD) of compound **PHP-6730.**

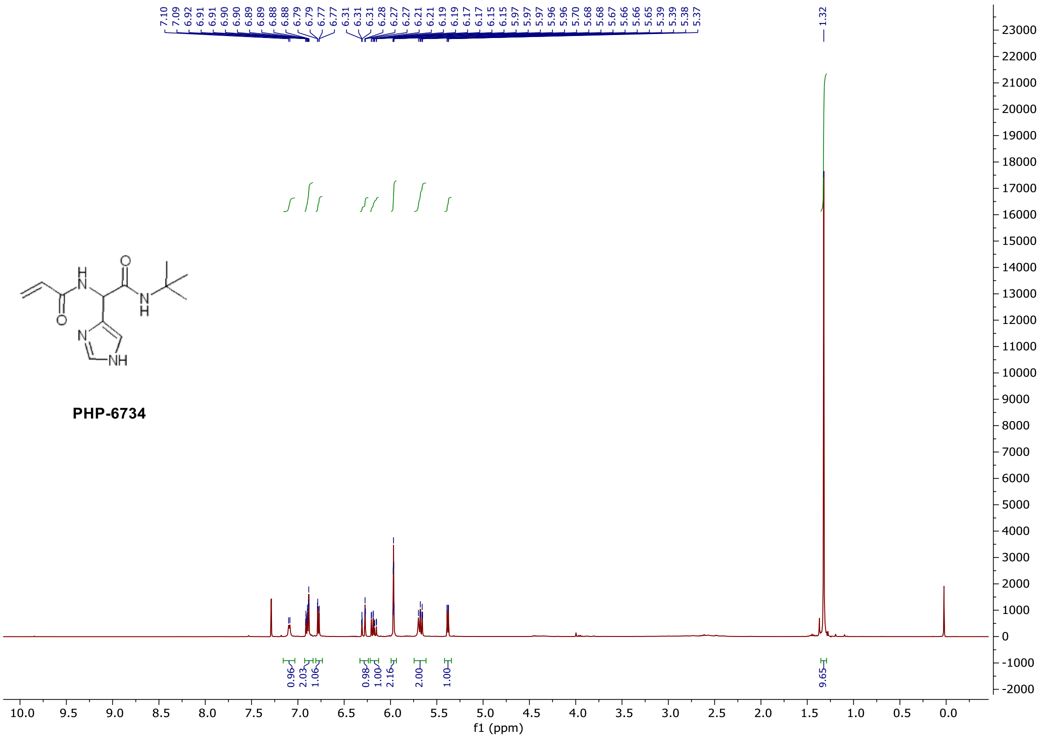

Supplementary Figure 1216. ^{1}H NMR (400 MHz, $CDCl_3$ + one drop of CD_3OD) of compound **PHP-6734.**

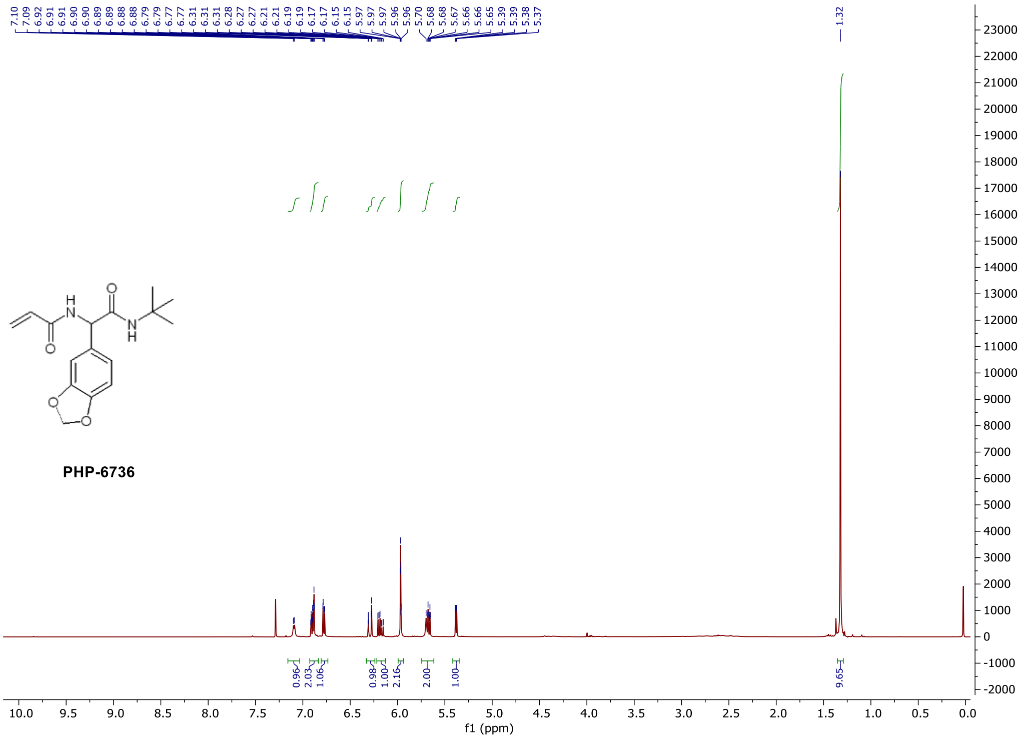

Supplementary Figure 1217. ^{1}H NMR (400 MHz, $CDCl_3$ + one drop of CD_3OD) of compound **PHP-6736.**

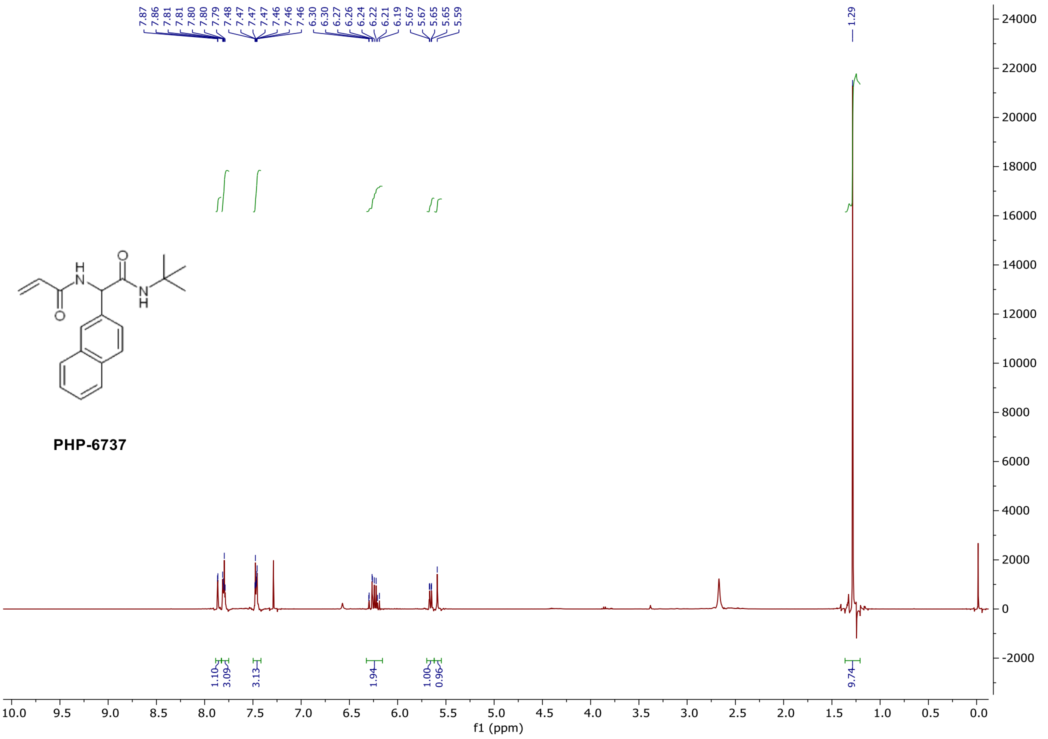

Supplementary Figure 1218. ^{1}H NMR (400 MHz, $CDCl_3$ + one drop of CD_3OD) of compound **PHP-6737.**

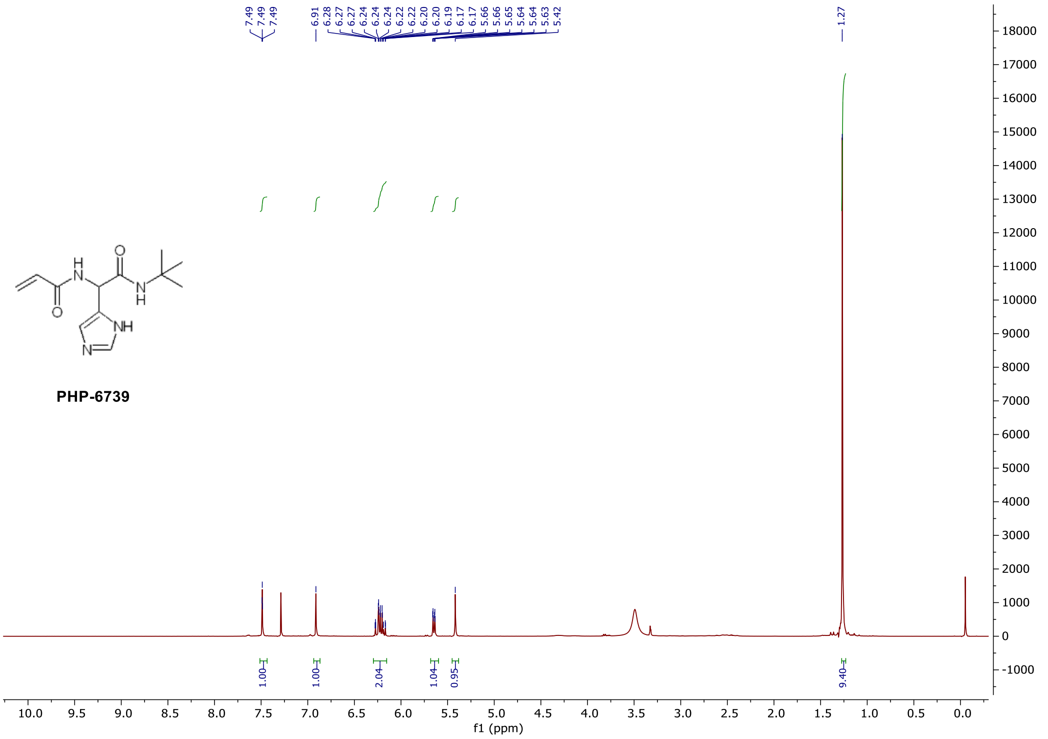

Supplementary Figure 1219. ^{1}H NMR (400 MHz, $CDCl_3$ + one drop of CD_3OD) of compound **PHP-6739.**

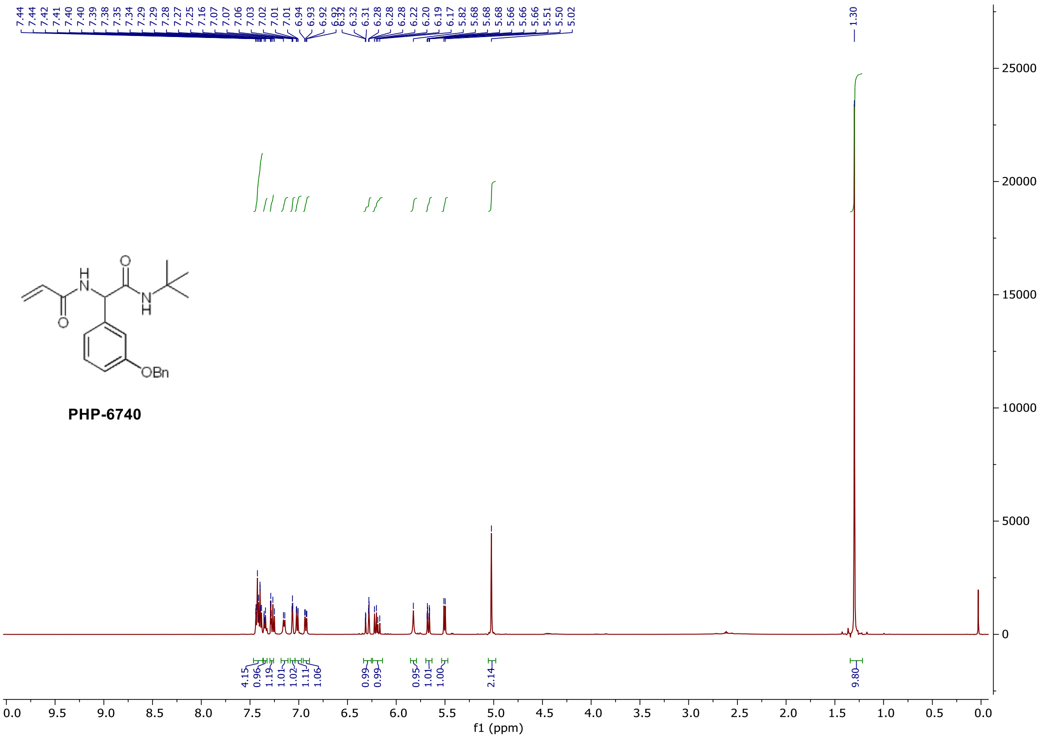

Supplementary Figure 1220. ^{1}H NMR (400 MHz, $CDCl_3$ + one drop of CD_3OD) of compound **PHP-6740.**

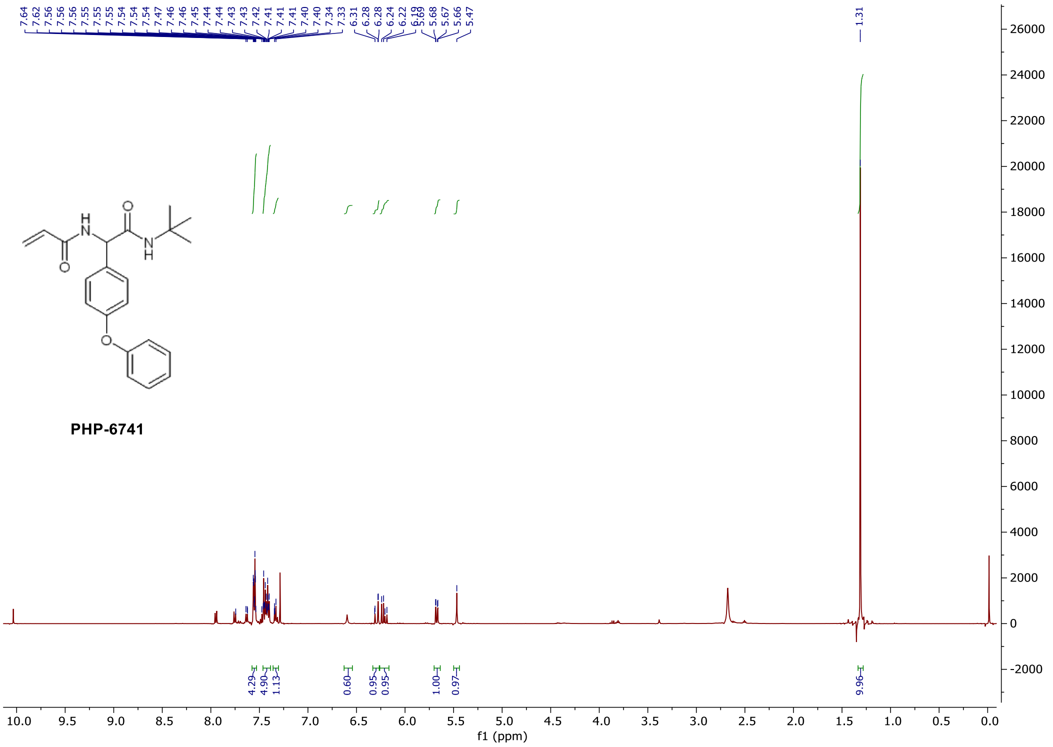

Supplementary Figure 1221. ^{1}H NMR (400 MHz, $CDCl_3$ + one drop of CD_3OD) of compound **PHP-6741.**

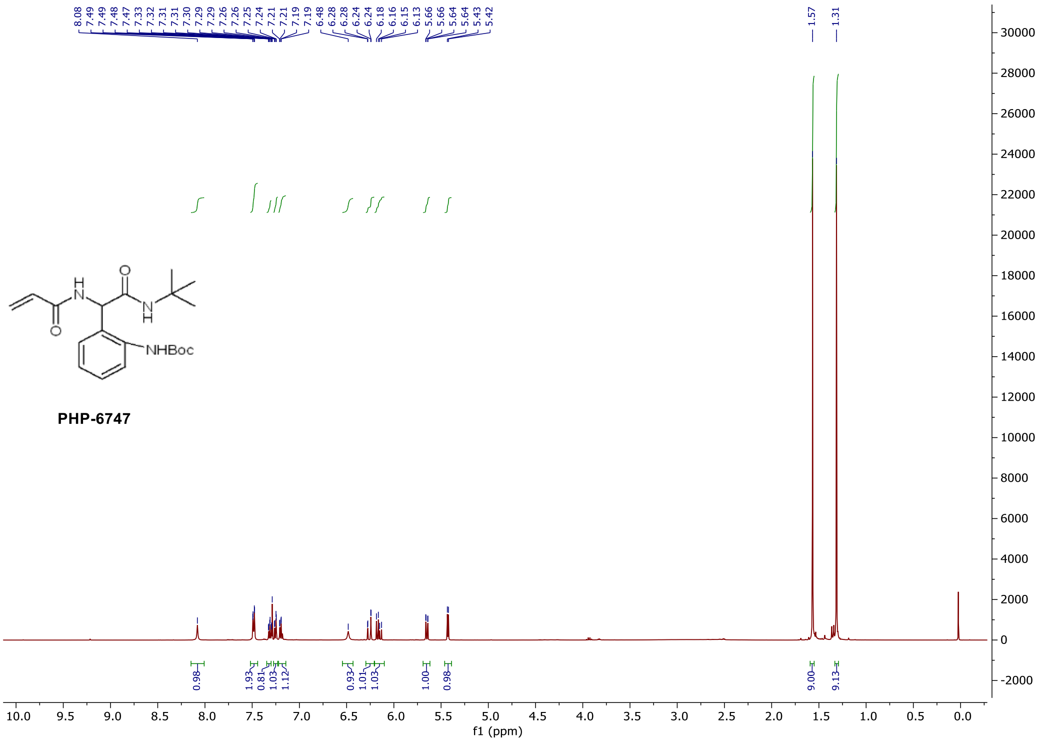

Supplementary Figure 1222. ^{1}H NMR (400 MHz, $CDCl_3$ + one drop of CD_3OD) of compound **PHP-6747.**

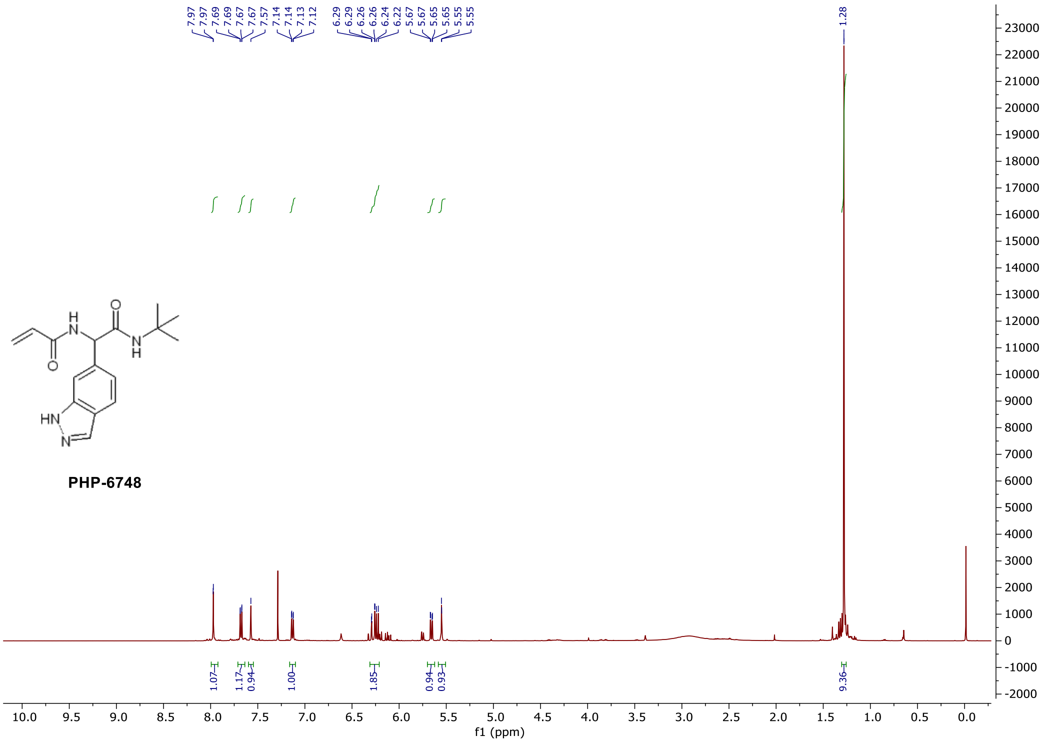

Supplementary Figure 1223. ^{1}H NMR (400 MHz, $CDCl_3$ + one drop of CD_3OD) of compound **PHP-6748.**

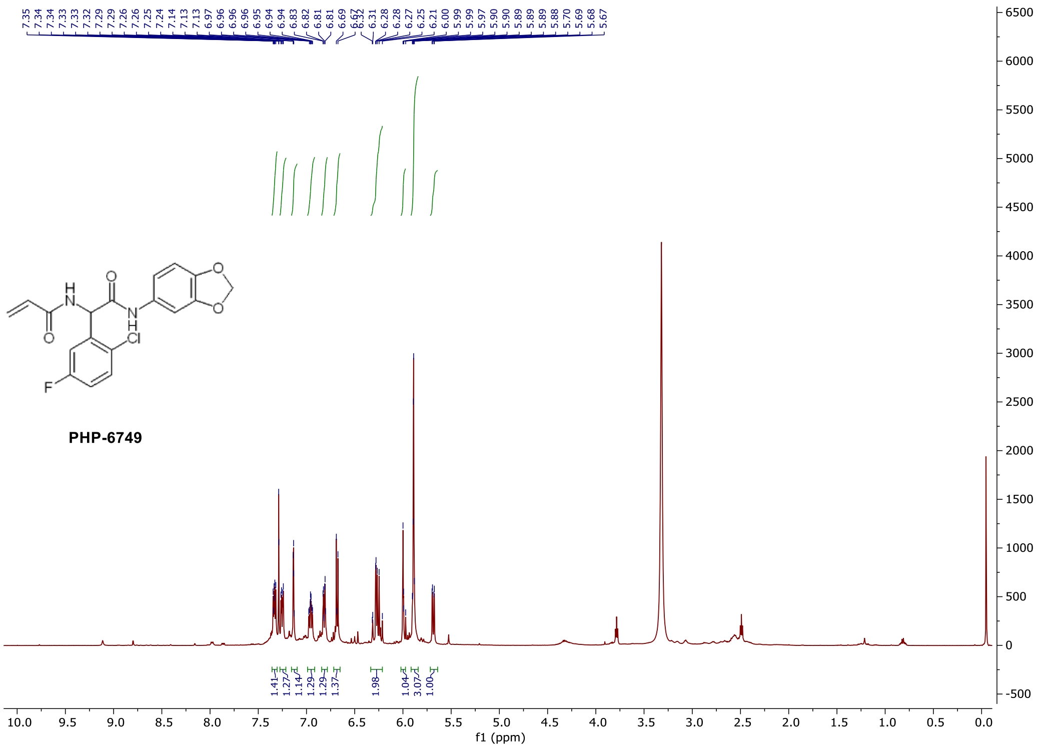

Supplementary Figure 1224. ^{1}H NMR (400 MHz, $CDCl_3$ + one drop of CD_3OD) of compound **PHP-6749.**

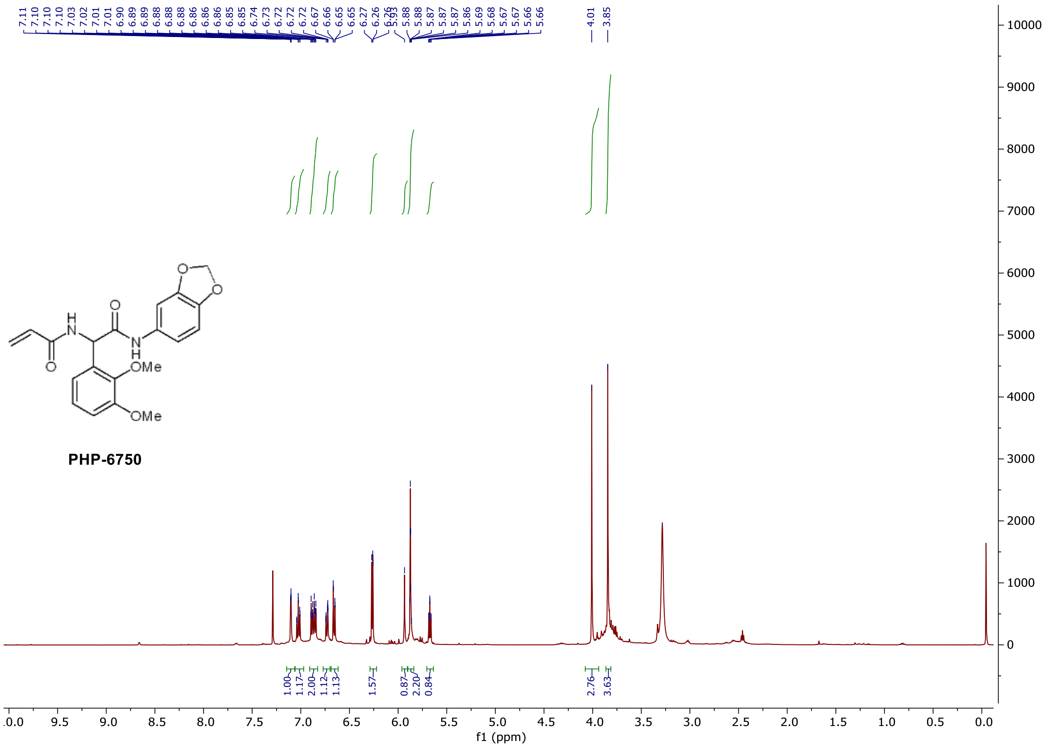

Supplementary Figure 1225. ^{1}H NMR (400 MHz, $CDCl_3$ + one drop of CD_3OD) of compound **PHP-6750.**

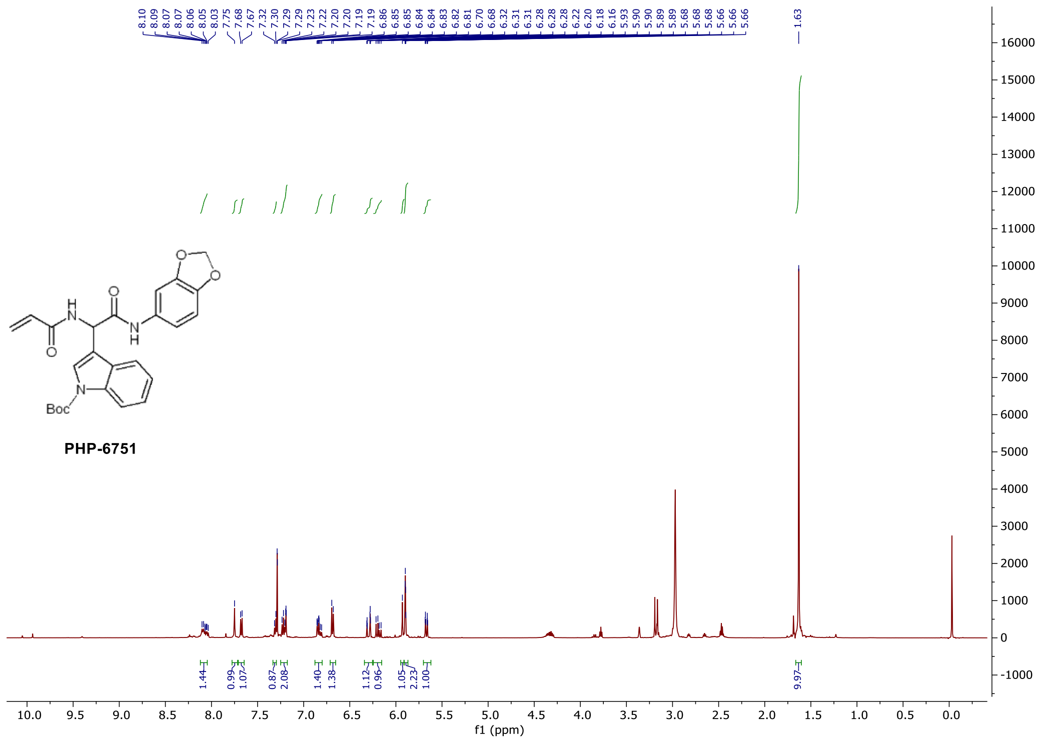

Supplementary Figure 1226. ^{1}H NMR (400 MHz, $CDCl_3$ + one drop of CD_3OD) of compound **PHP-6751.**

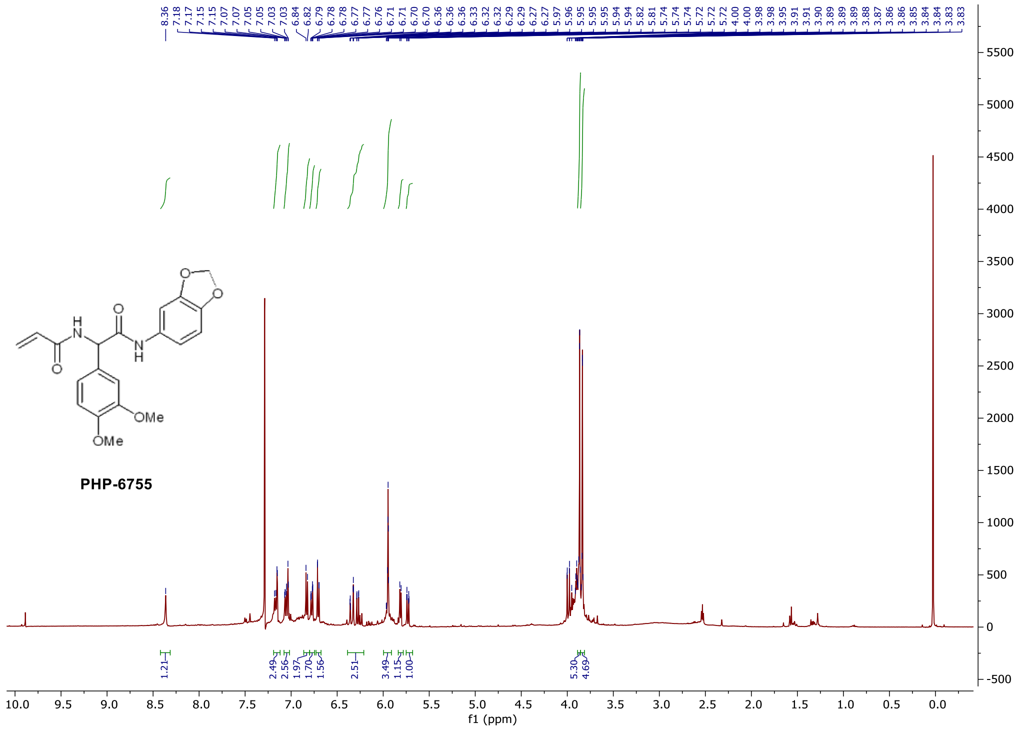

Supplementary Figure 1227. ^{1}H NMR (400 MHz, $CDCl_3$ + one drop of CD_3OD) of compound **PHP-6755.**

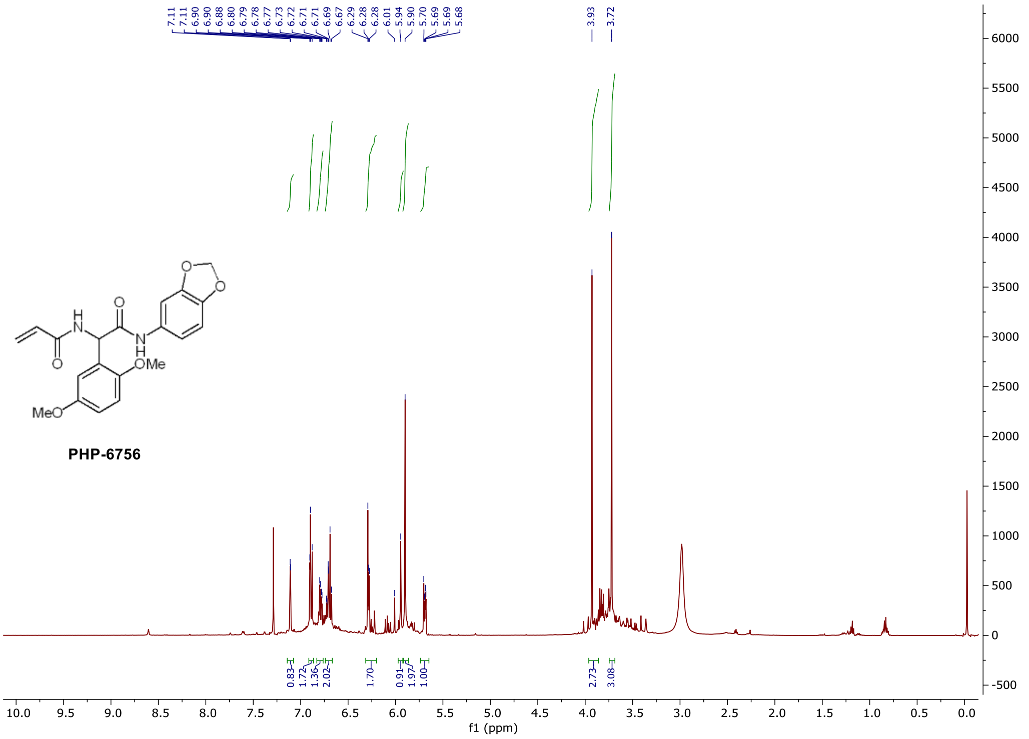

Supplementary Figure 1227. ^{1}H NMR (400 MHz, $CDCl_3$ + one drop of CD_3OD) of compound **PHP-6756.**

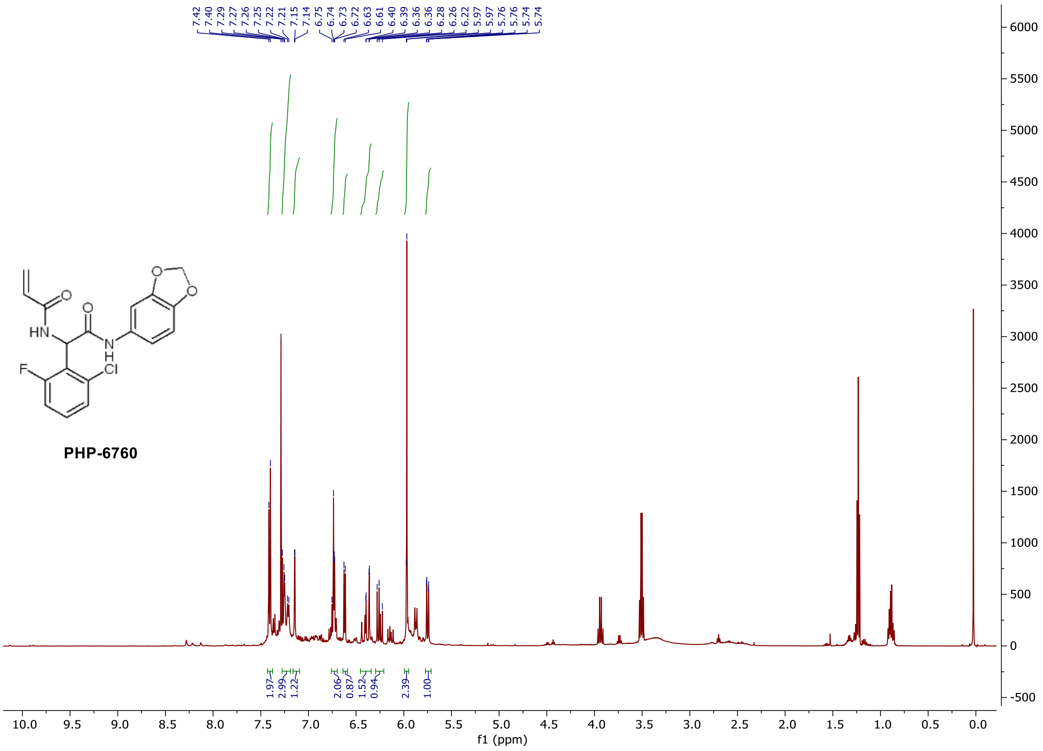

Supplementary Figure 1228. ^{1}H NMR (400 MHz, $CDCl_3$ + one drop of CD_3OD) of compound **PHP-6760.**

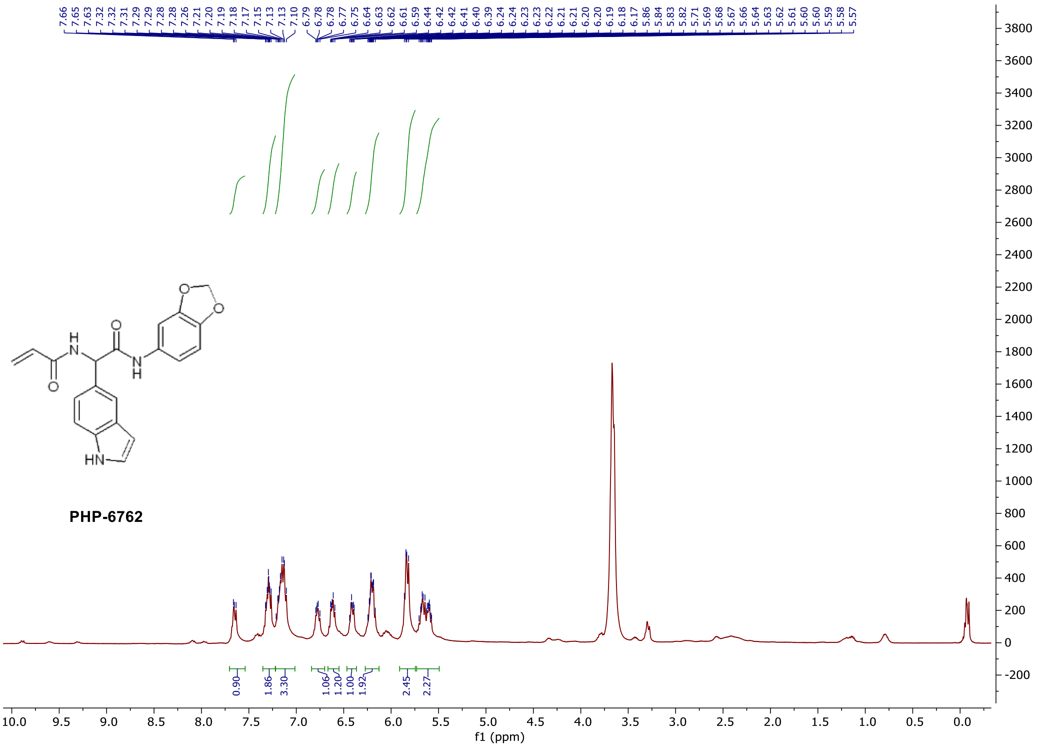

Supplementary Figure 1229. ^1H NMR (400 MHz, $CDCl_3$ + one drop of CD_3OD) of compound **PHP-6762.**

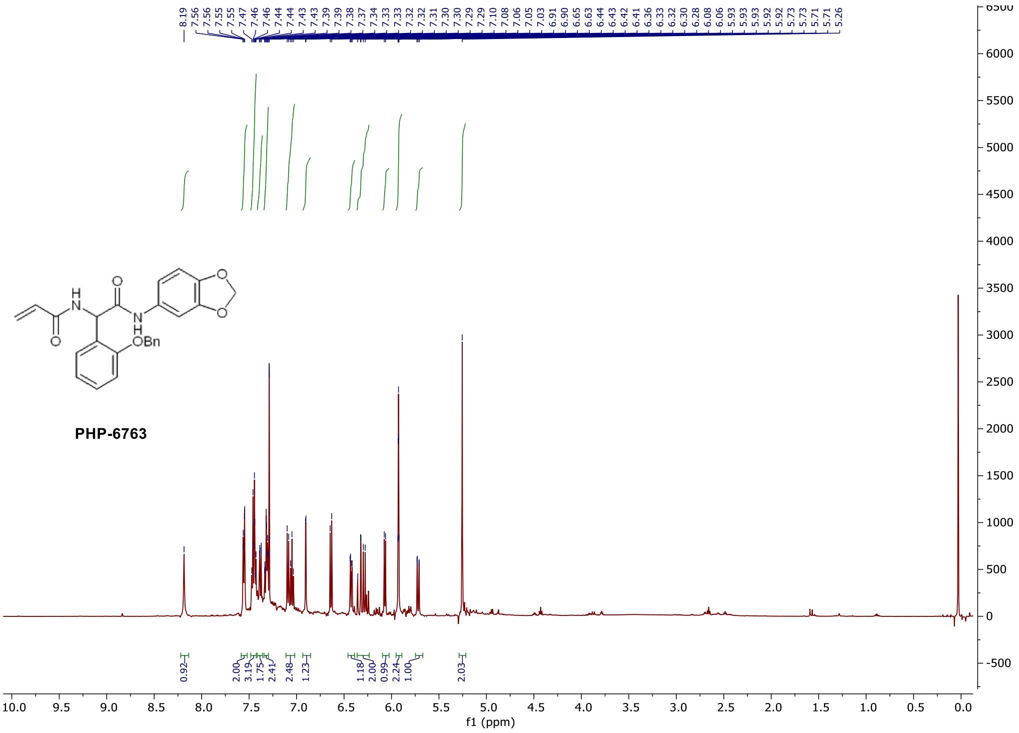

Supplementary Figure 1230. ^1H NMR (400 MHz, $CDCl_3$ + one drop of CD_3OD) of compound **PHP-6763.**

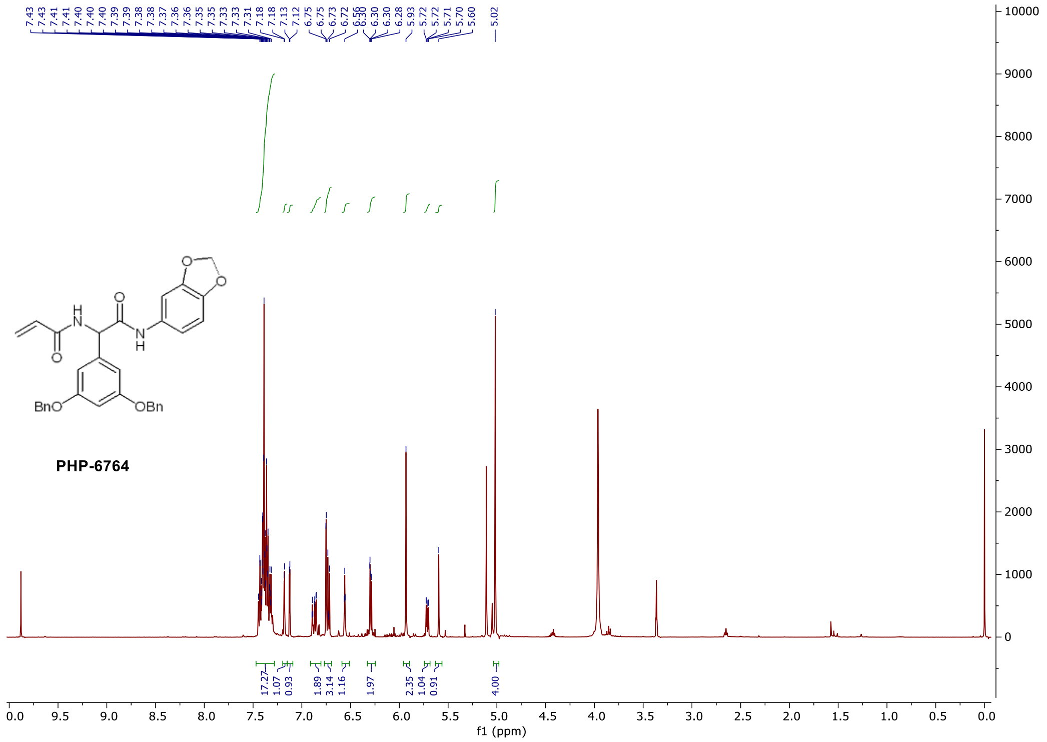

Supplementary Figure 1231. ^{1}H NMR (400 MHz, $CDCl_3$ + one drop of CD_3OD) of compound **PHP-6764.**

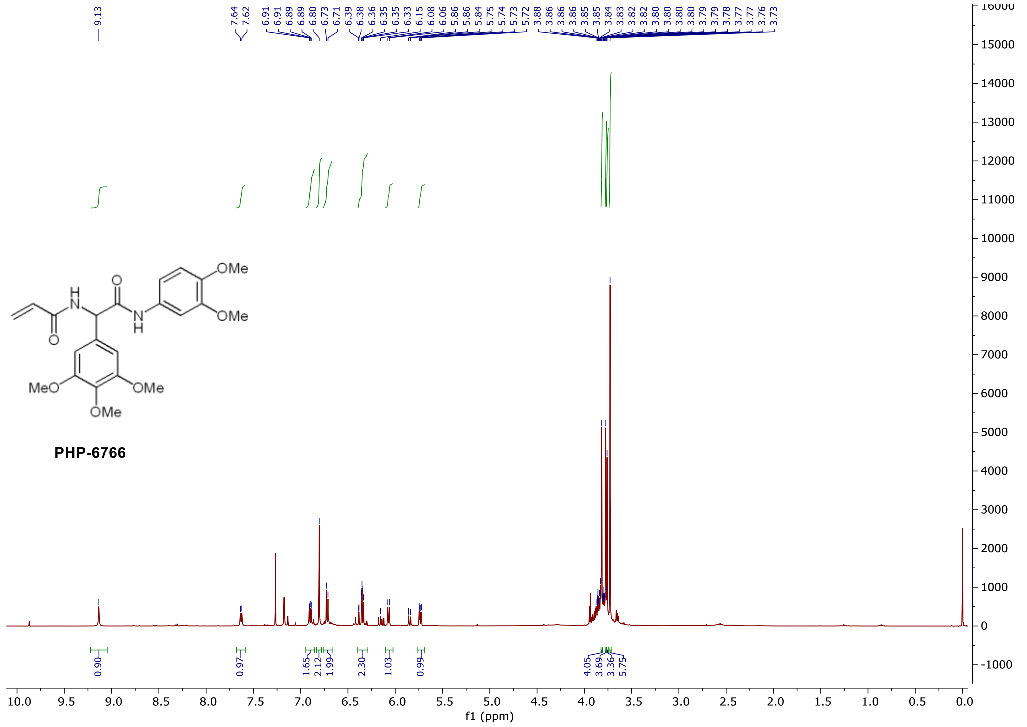

Supplementary Figure 1232. ^{1}H NMR (400 MHz, $CDCl_3$ + one drop of CD_3OD) of compound **PHP-6766.**

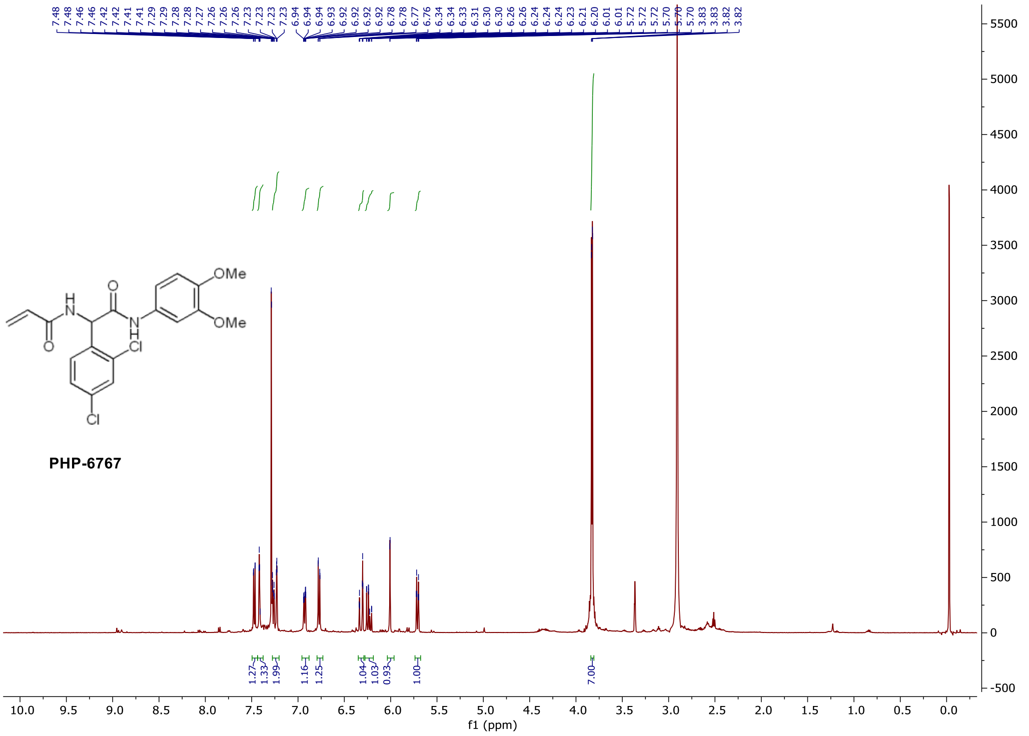

Supplementary Figure 1233. ^{1}H NMR (400 MHz, $CDCl_3$ + one drop of CD_3OD) of compound **PHP-6767.**

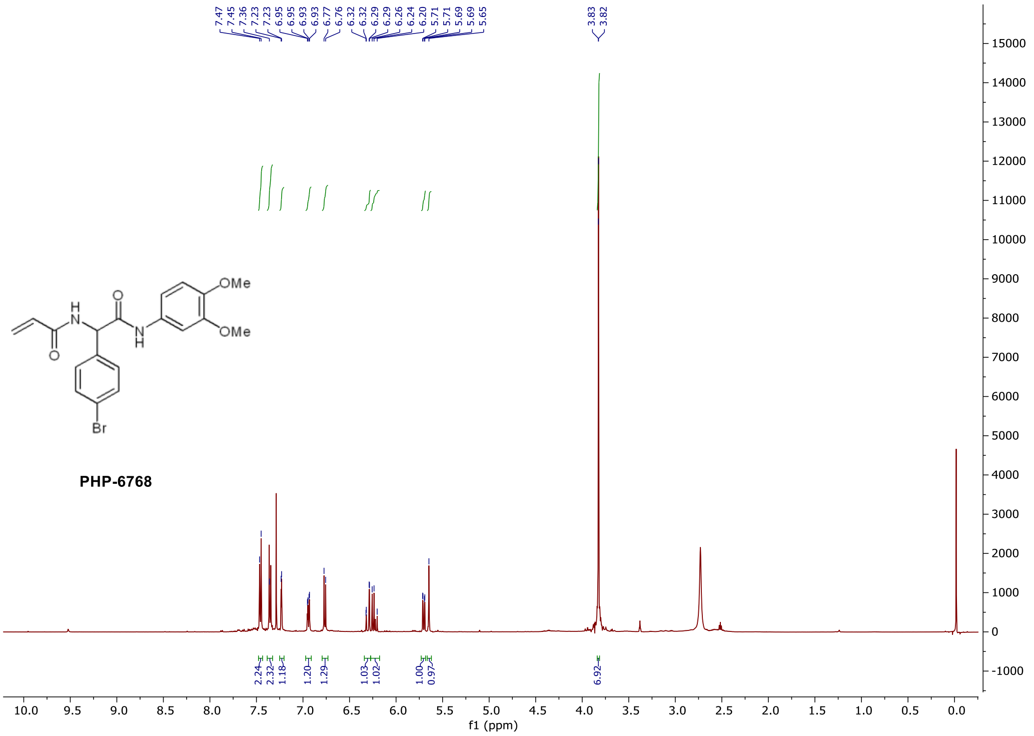

Supplementary Figure 1234. ^{1}H NMR (400 MHz, $CDCl_3$ + one drop of CD_3OD) of compound **PHP-6768.**

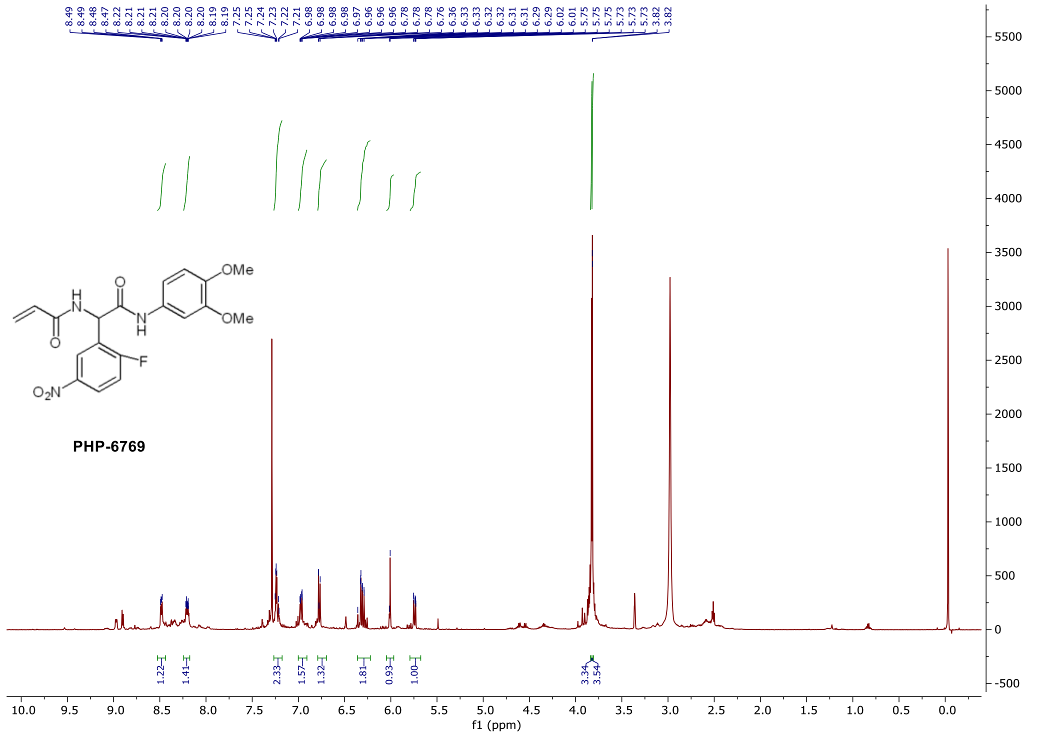

Supplementary Figure 1235. ^{1}H NMR (400 MHz, $CDCl_3$ + one drop of CD_3OD) of compound **PHP-6769**.

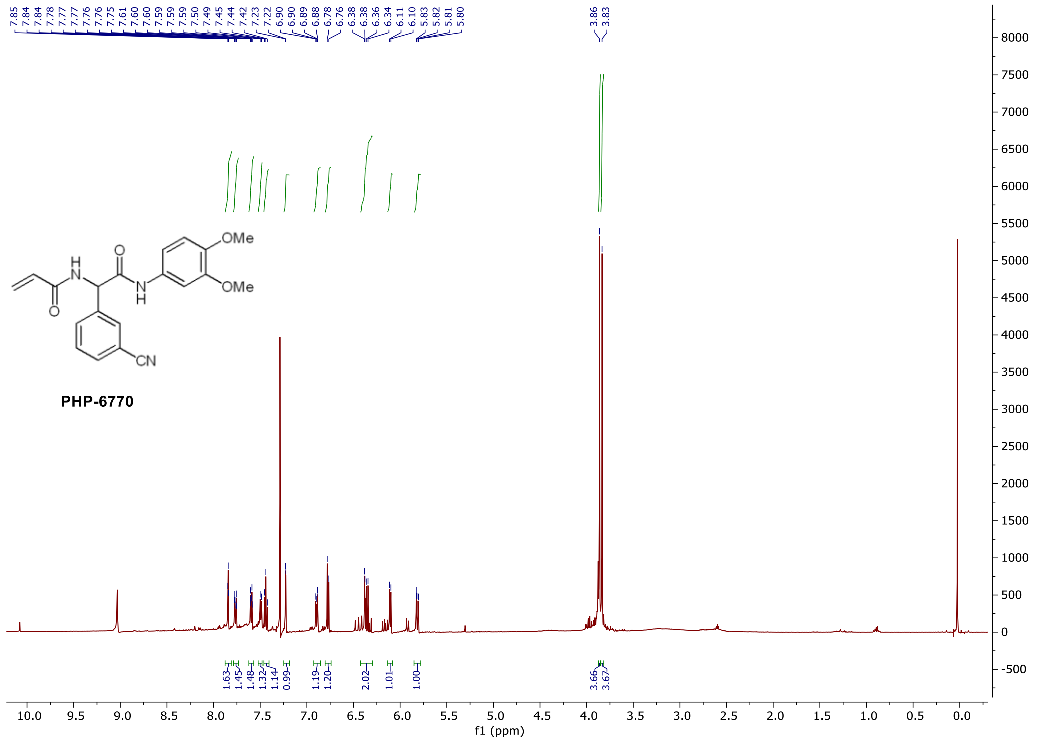

Supplementary Figure 1236. ^{1}H NMR (400 MHz, $CDCl_3$ + one drop of CD_3OD) of compound **PHP-6770**.

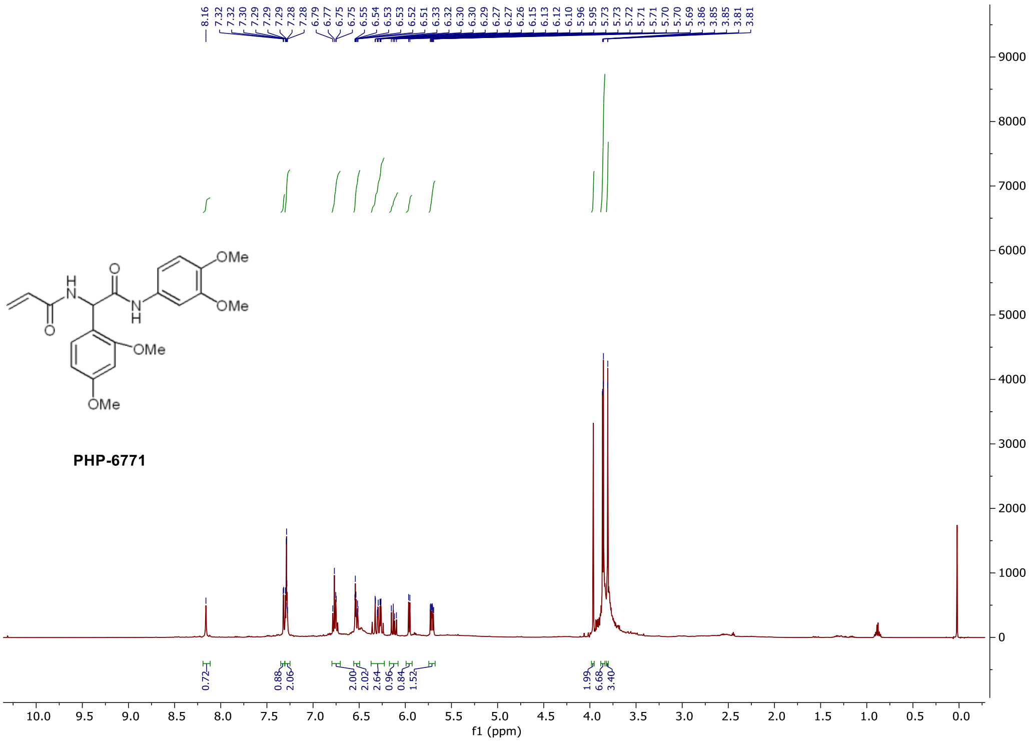

Supplementary Figure 1237. ^{1}H NMR (400 MHz, $CDCl_3$ + one drop of CD_3OD) of compound **PHP-6771.**

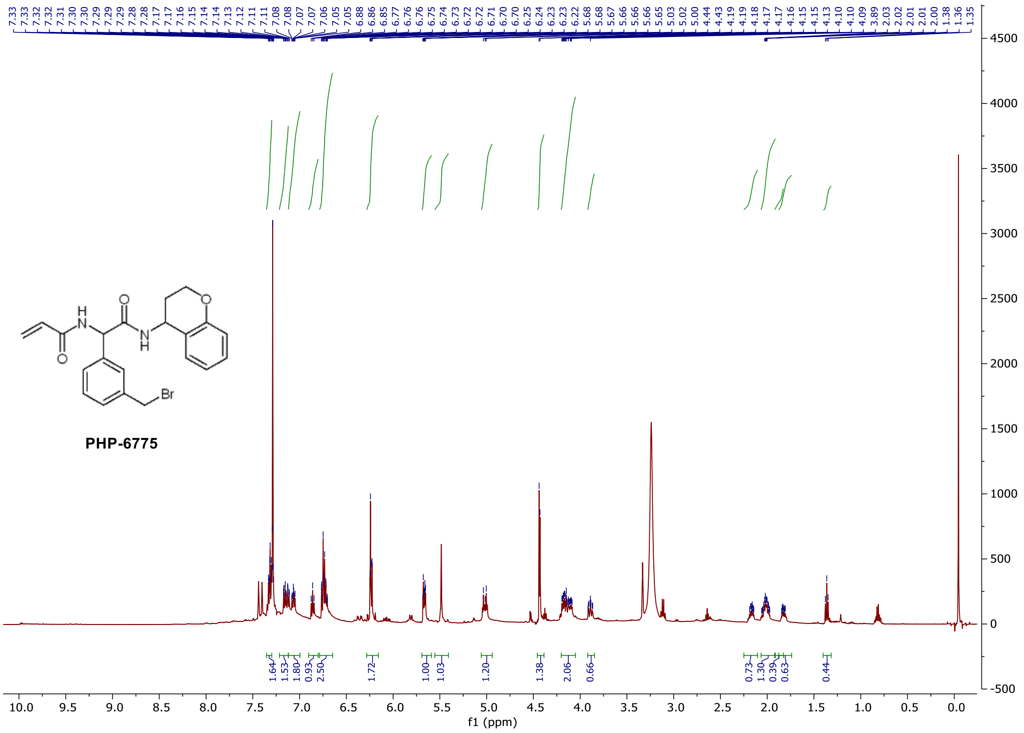

Supplementary Figure 1238. ^{1}H NMR (400 MHz, $CDCl_3$ + one drop of CD_3OD) of compound **PHP-6775.**

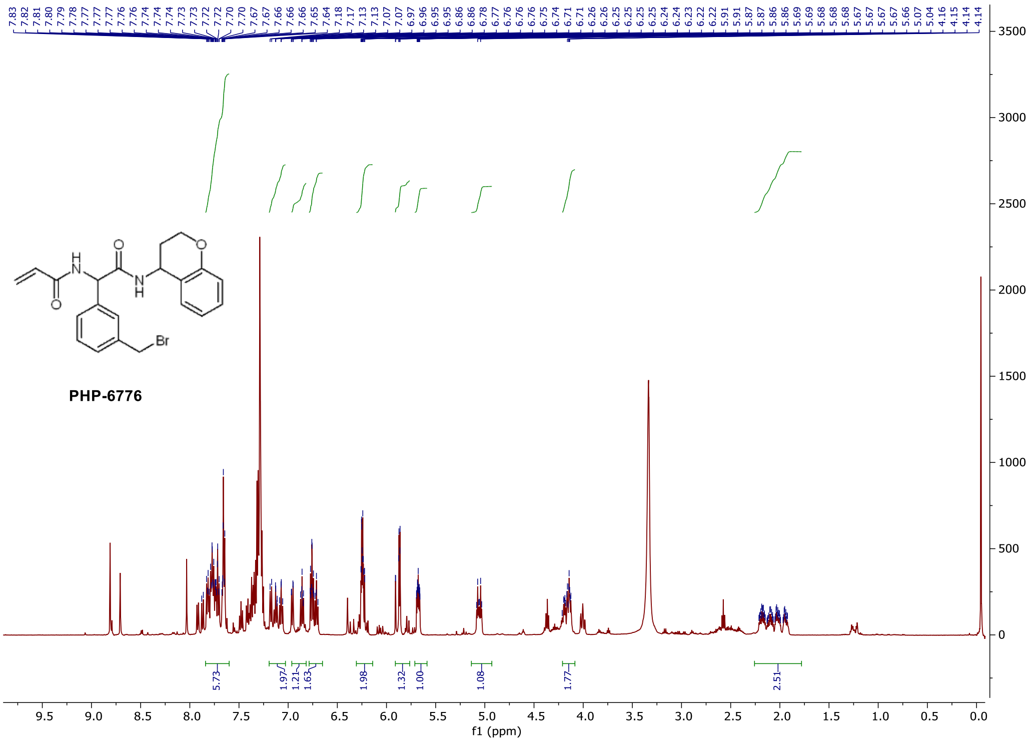

Supplementary Figure 1239. ^{1}H NMR (400 MHz, $CDCl_3$ + one drop of CD_3OD) of compound **PHP-6776.**

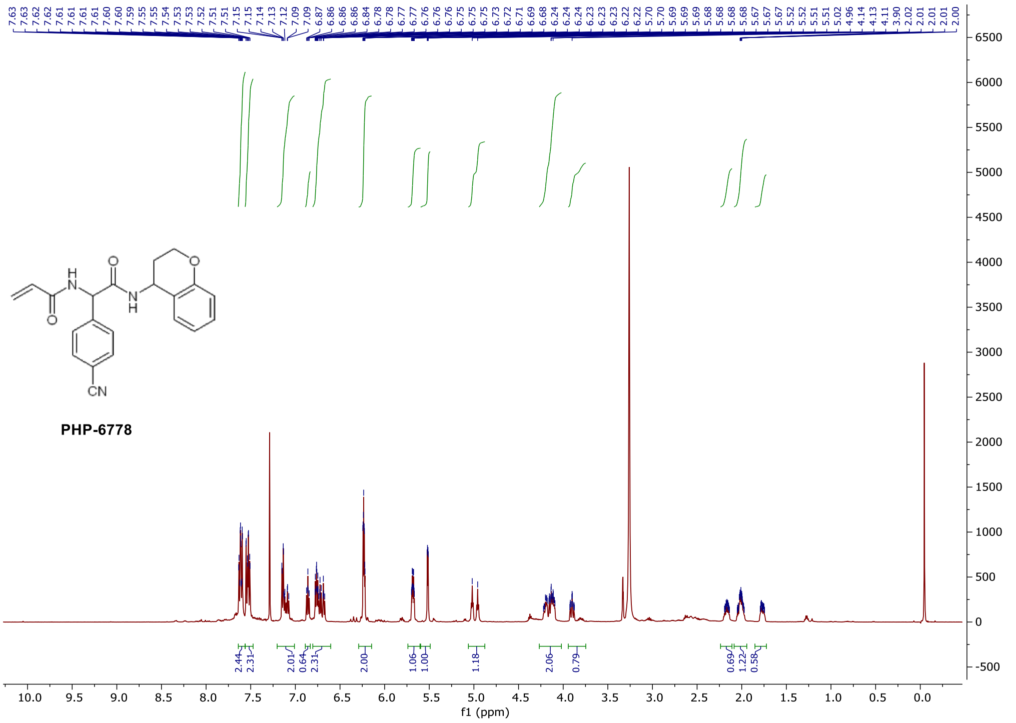

Supplementary Figure 1240. ^{1}H NMR (400 MHz, $CDCl_3$ + one drop of CD_3OD) of compound **PHP-6778.**

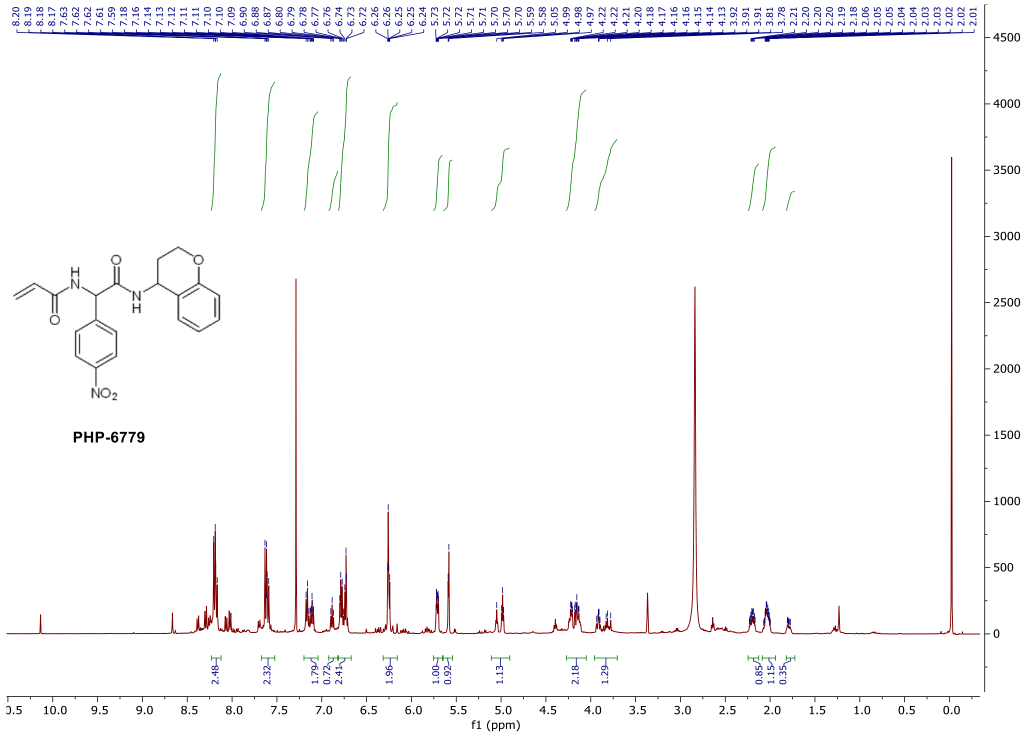

Supplementary Figure 1241. ^{1}H NMR (400 MHz, $CDCl_3$ + one drop of CD_3OD) of compound **PHP-6779.**

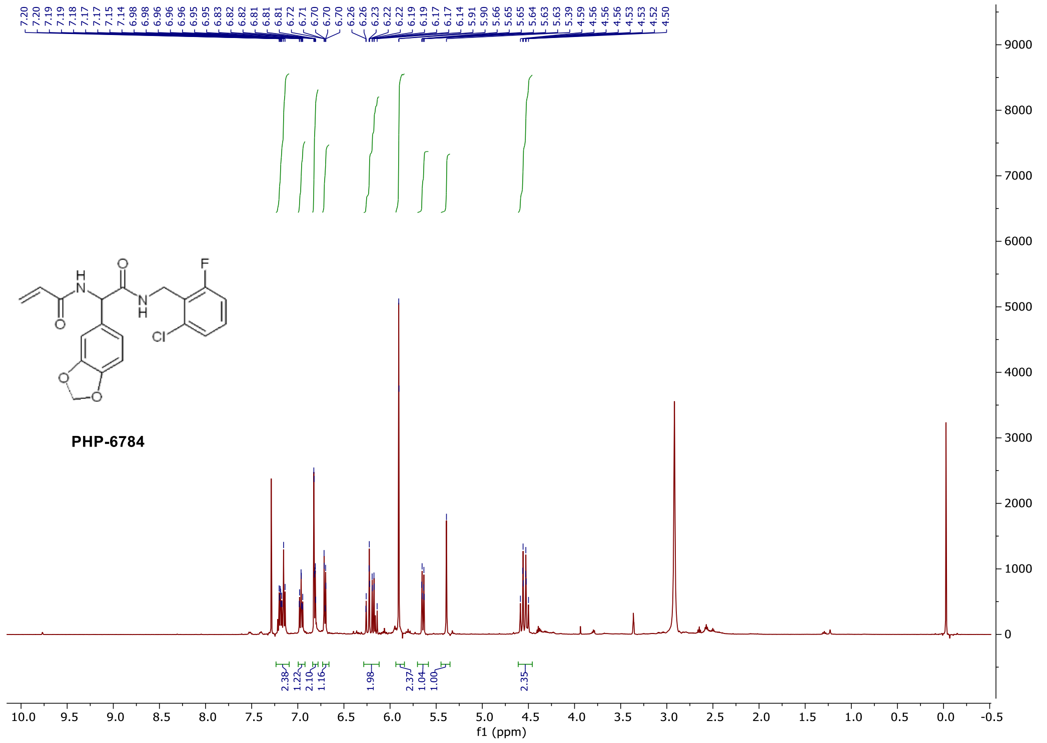

Supplementary Figure 1242. ^{1}H NMR (400 MHz, $CDCl_3$ + one drop of CD_3OD) of compound **PHP-6784.**

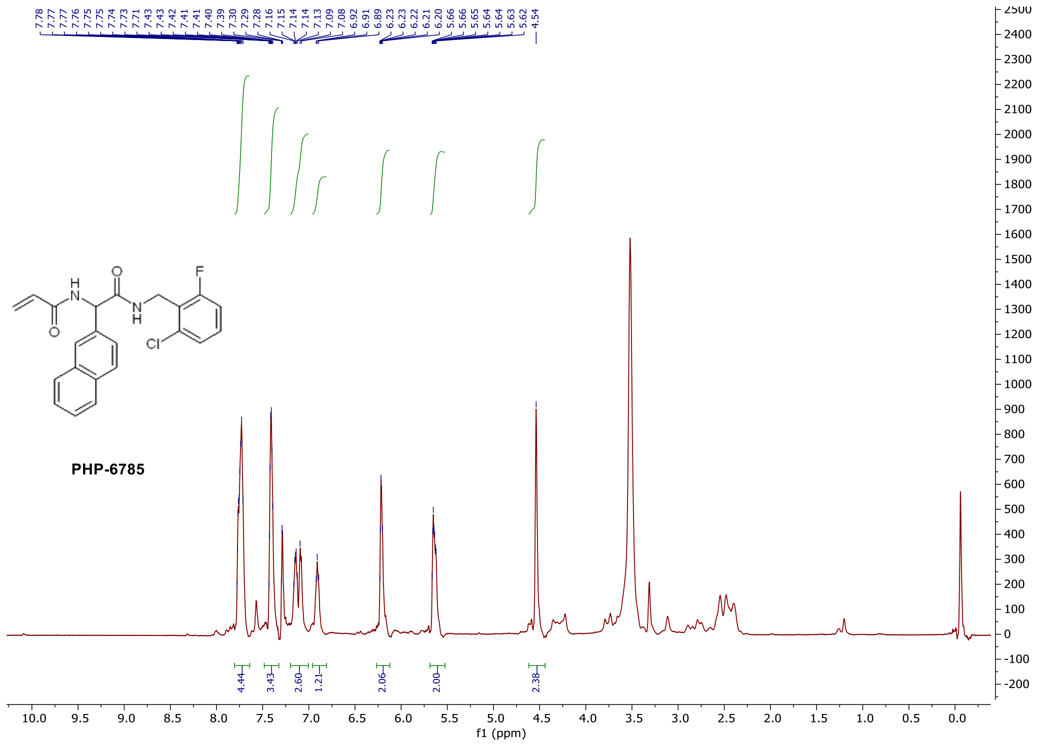

Supplementary Figure 1243. ^{1}H NMR (400 MHz, $CDCl_3$ + one drop of CD_3OD) of compound **PHP-6785**.

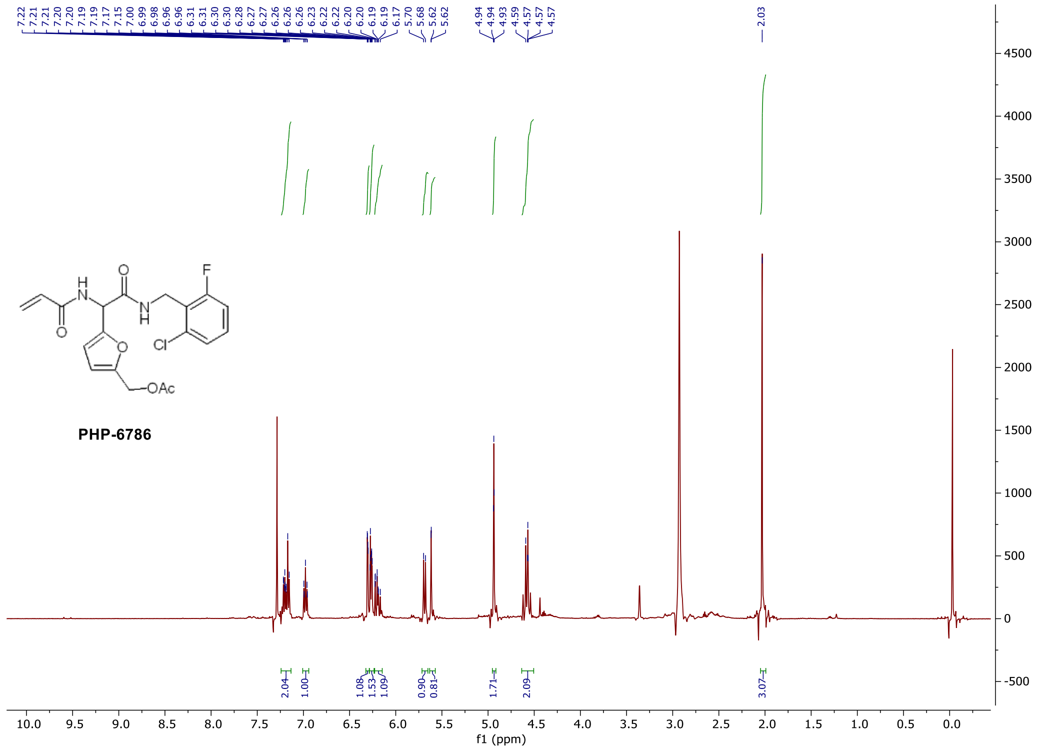

Supplementary Figure 1244. ^{1}H NMR (400 MHz, $CDCl_3$ + one drop of CD_3OD) of compound **PHP-6786**.

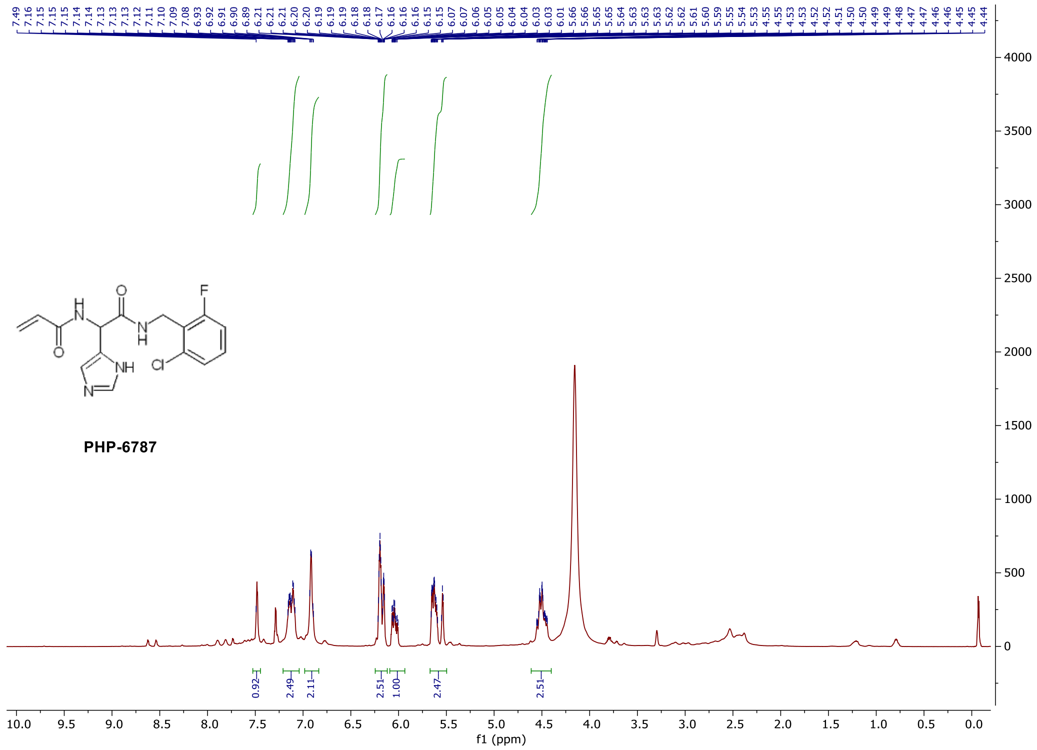

Supplementary Figure 1245. ^{1}H NMR (400 MHz, $CDCl_3$ + one drop of CD_3OD) of compound **PHP-6787.**

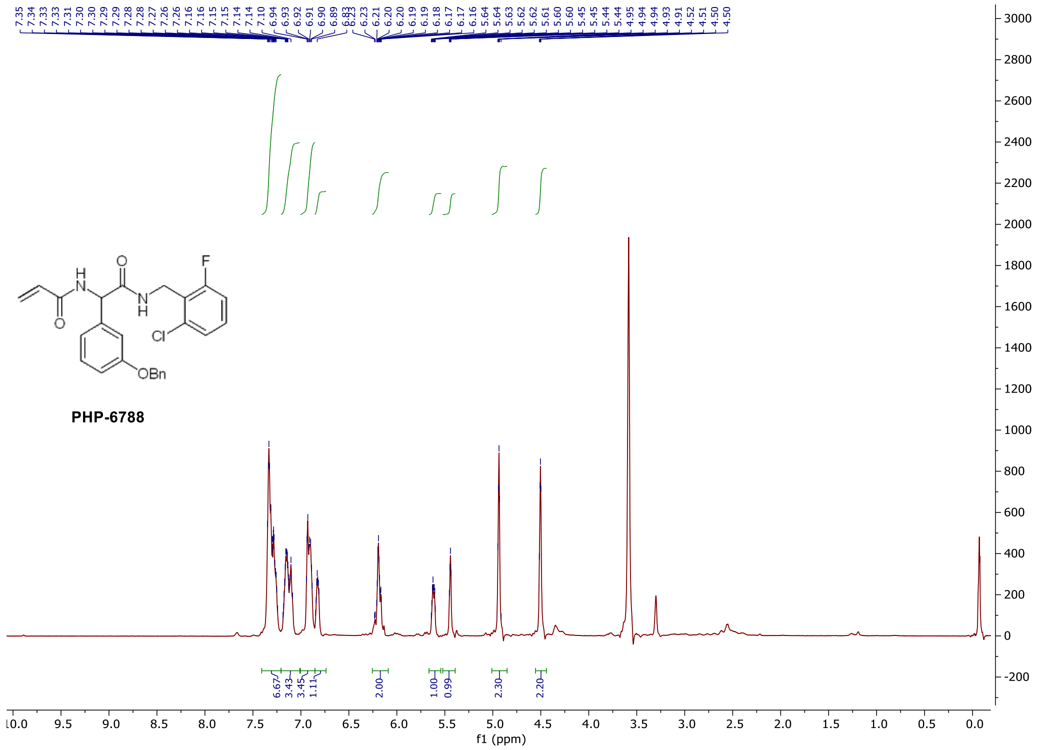

Supplementary Figure 1246. ^{1}H NMR (400 MHz, $CDCl_3$ + one drop of CD_3OD) of compound **PHP-6788.**

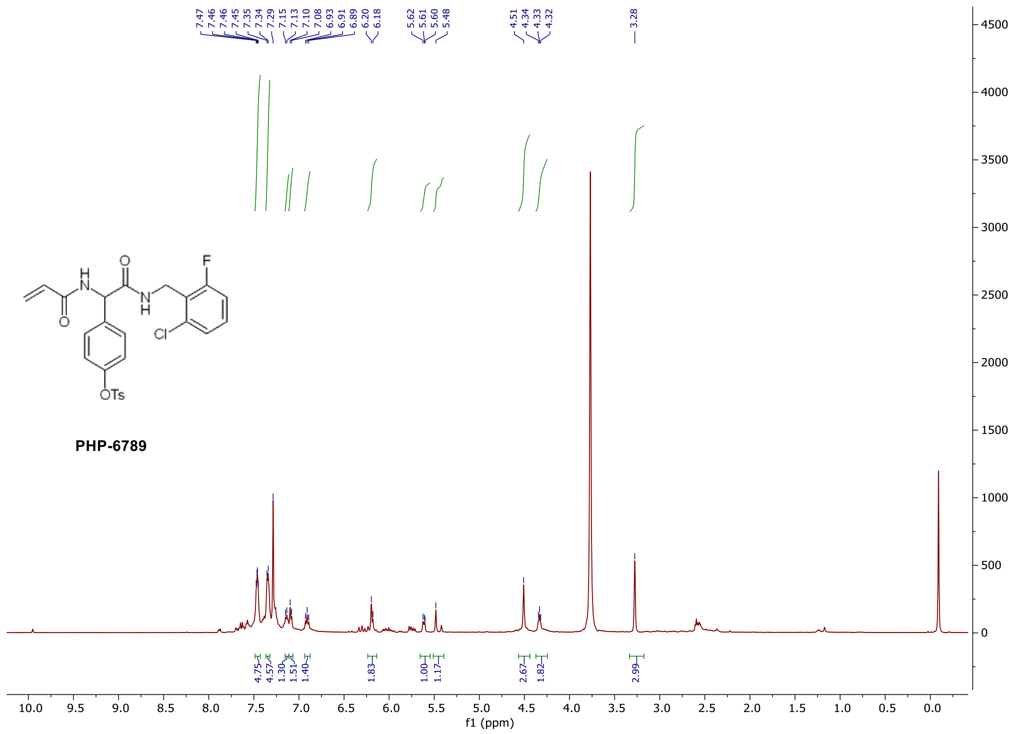

Supplementary Figure 1247. ^{1}H NMR (400 MHz, $CDCl_3$ + one drop of CD_3OD) of compound **PHP-6789.**

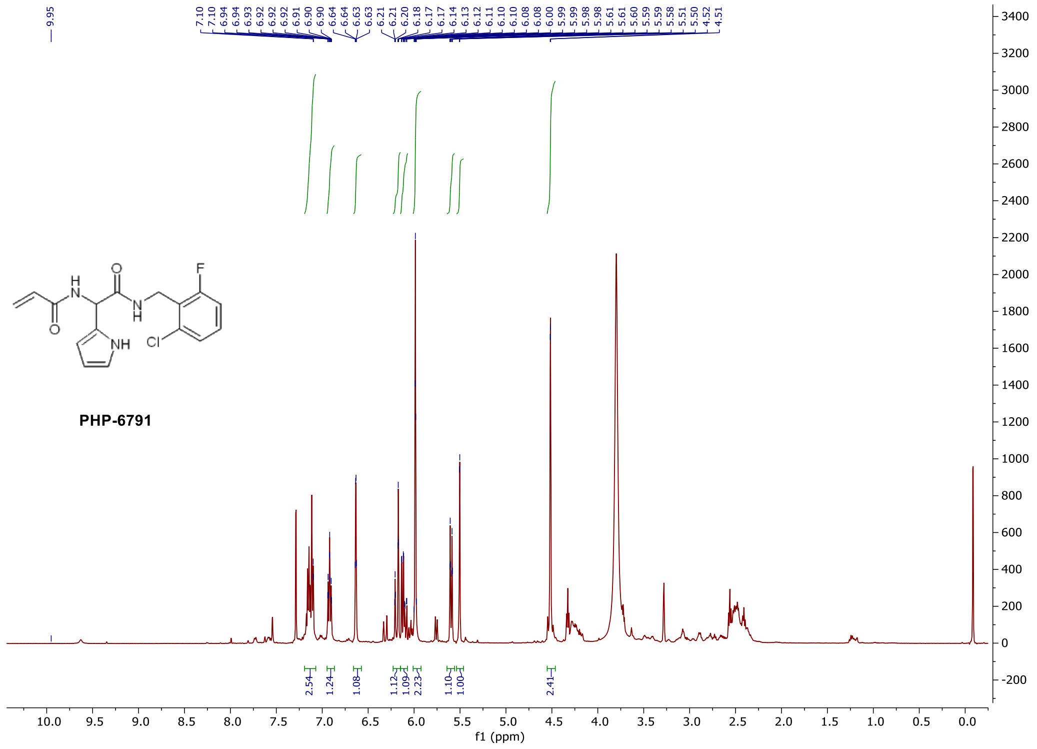

Supplementary Figure 1248. ^{1}H NMR (400 MHz, $CDCl_3$ + one drop of CD_3OD) of compound **PHP-6791.**

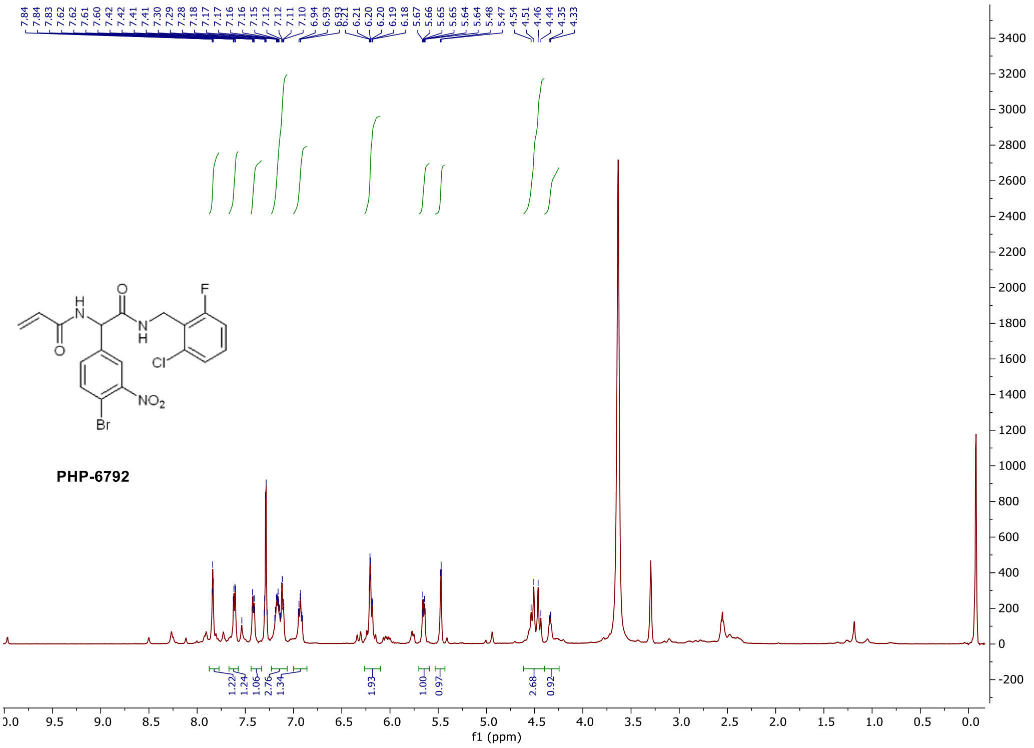

Supplementary Figure 1249. ^{1}H NMR (400 MHz, $CDCl_3$ + one drop of CD_3OD) of compound **PHP-6792**.

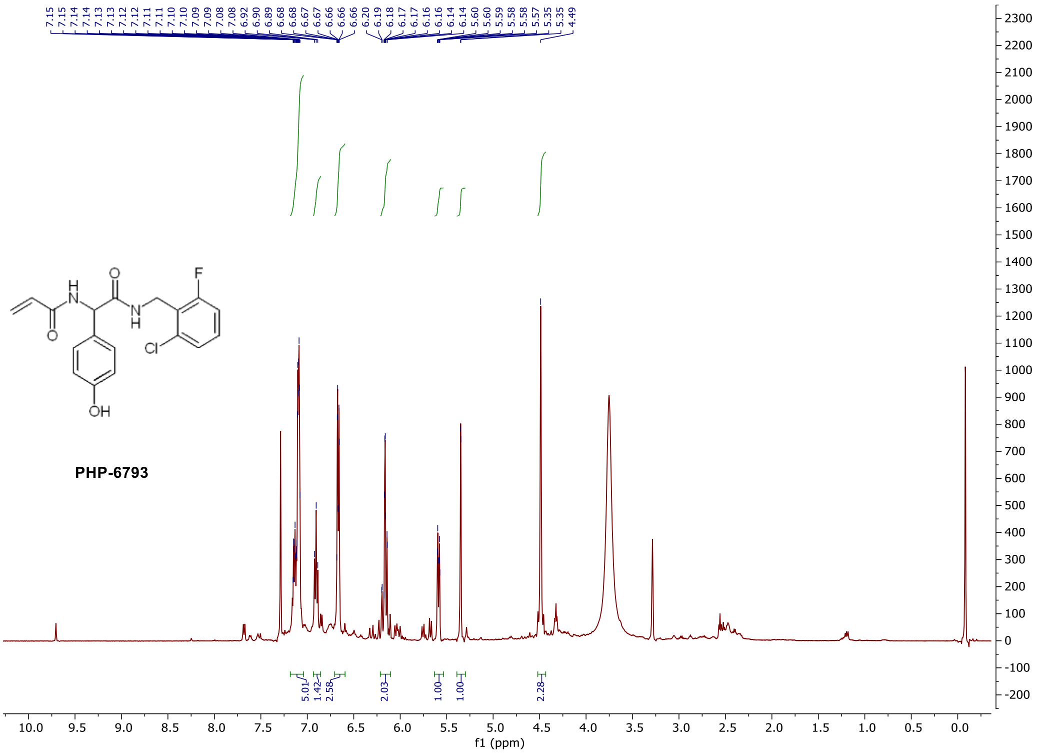

Supplementary Figure 1250. ^{1}H NMR (400 MHz, $CDCl_3$ + one drop of CD_3OD) of compound **PHP-6793**.

9.2 UPLC-MS spectra of the 384-compounds subset

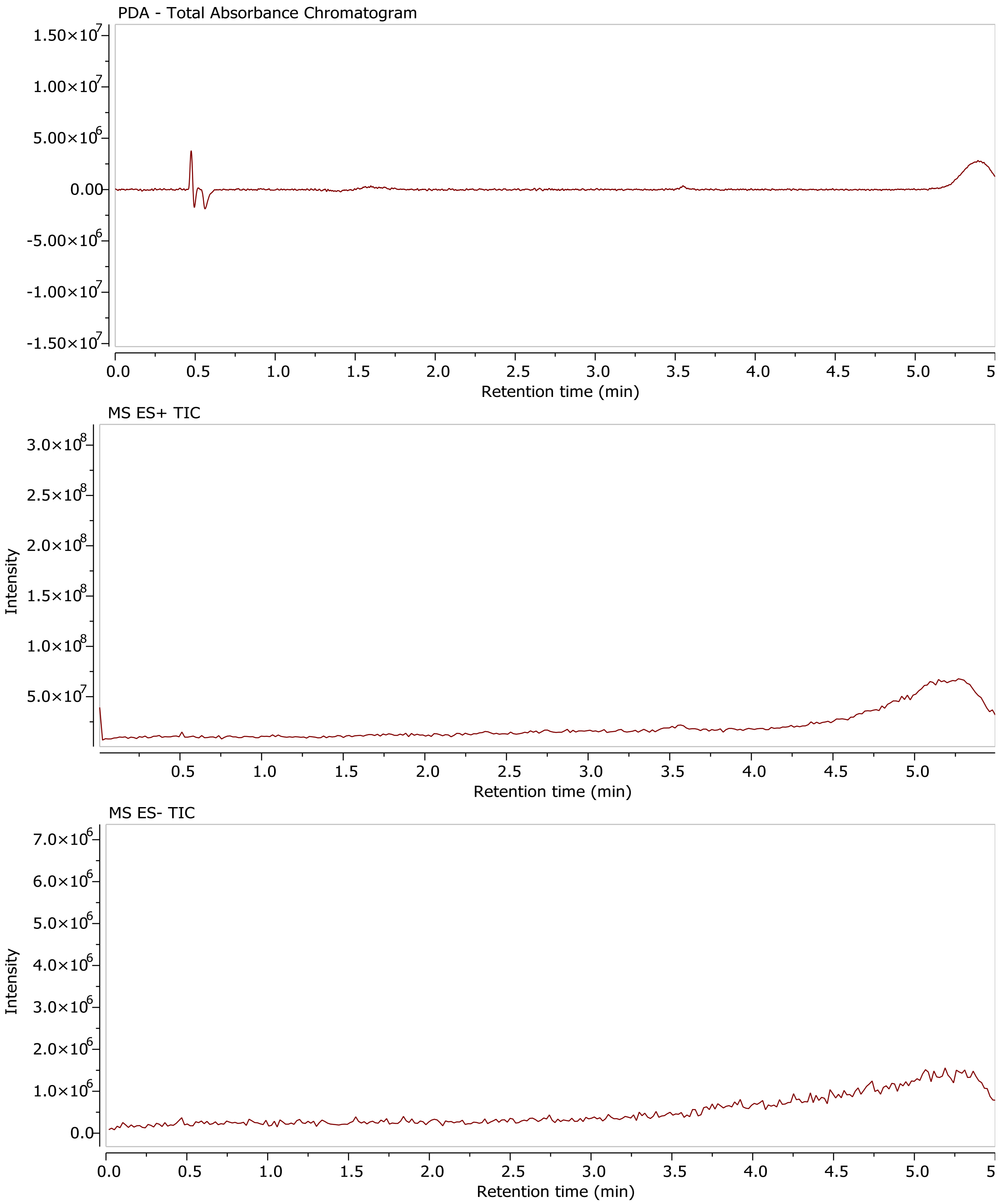

Supplementary figure **1251**. UPLC-MS analysis for blank solvent used for 384-well plate.

Chemical Formula: $C_{16}H_{20}ClFN_2O_2$
Exact Mass: 326.1

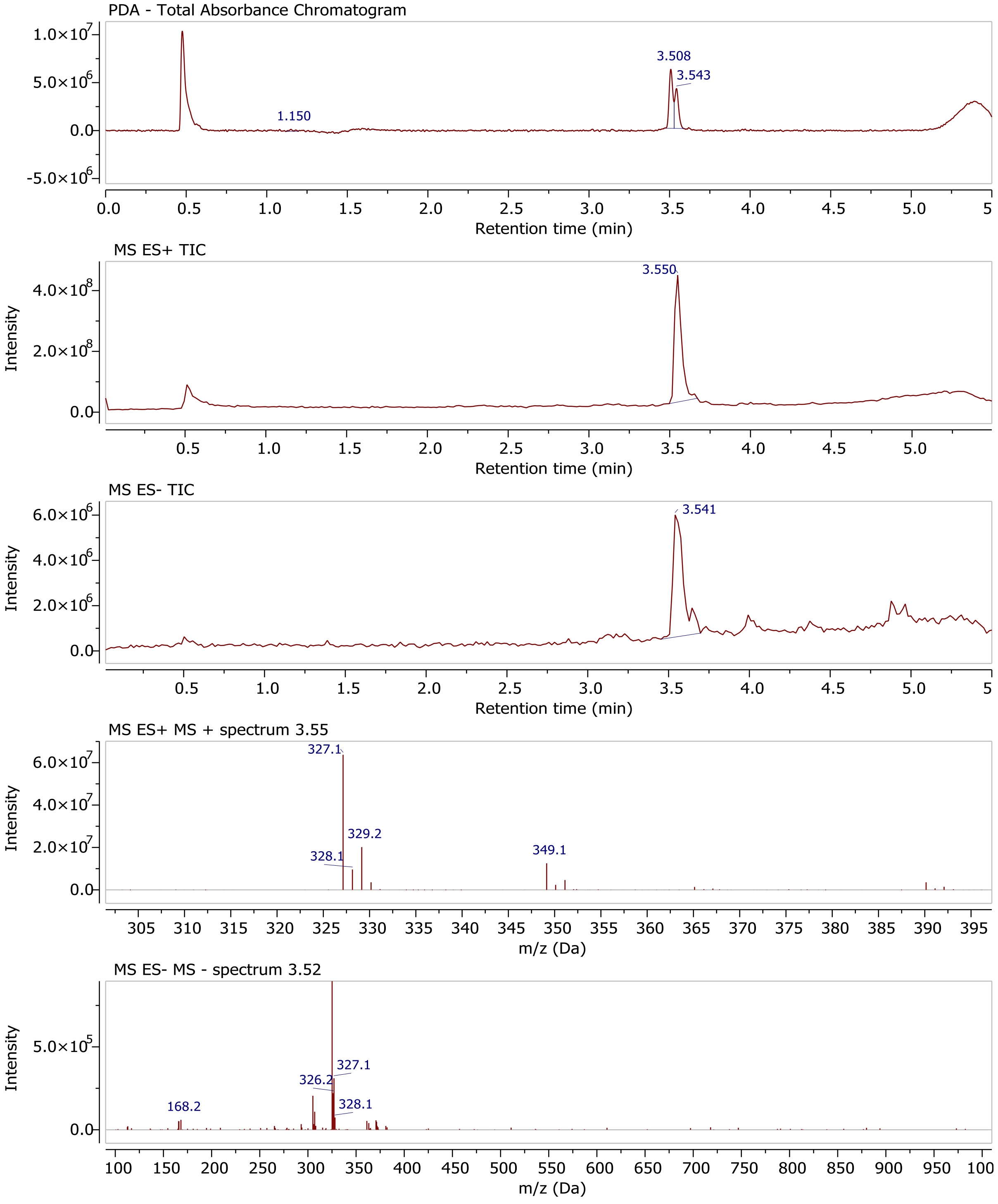

Supplementary figure **1252**. UPLC-MS analysis for compound **PHP-4688**

Chemical Formula: $C_{19}H_{15}ClF_4N_2O_2$
Exact Mass: 414.1

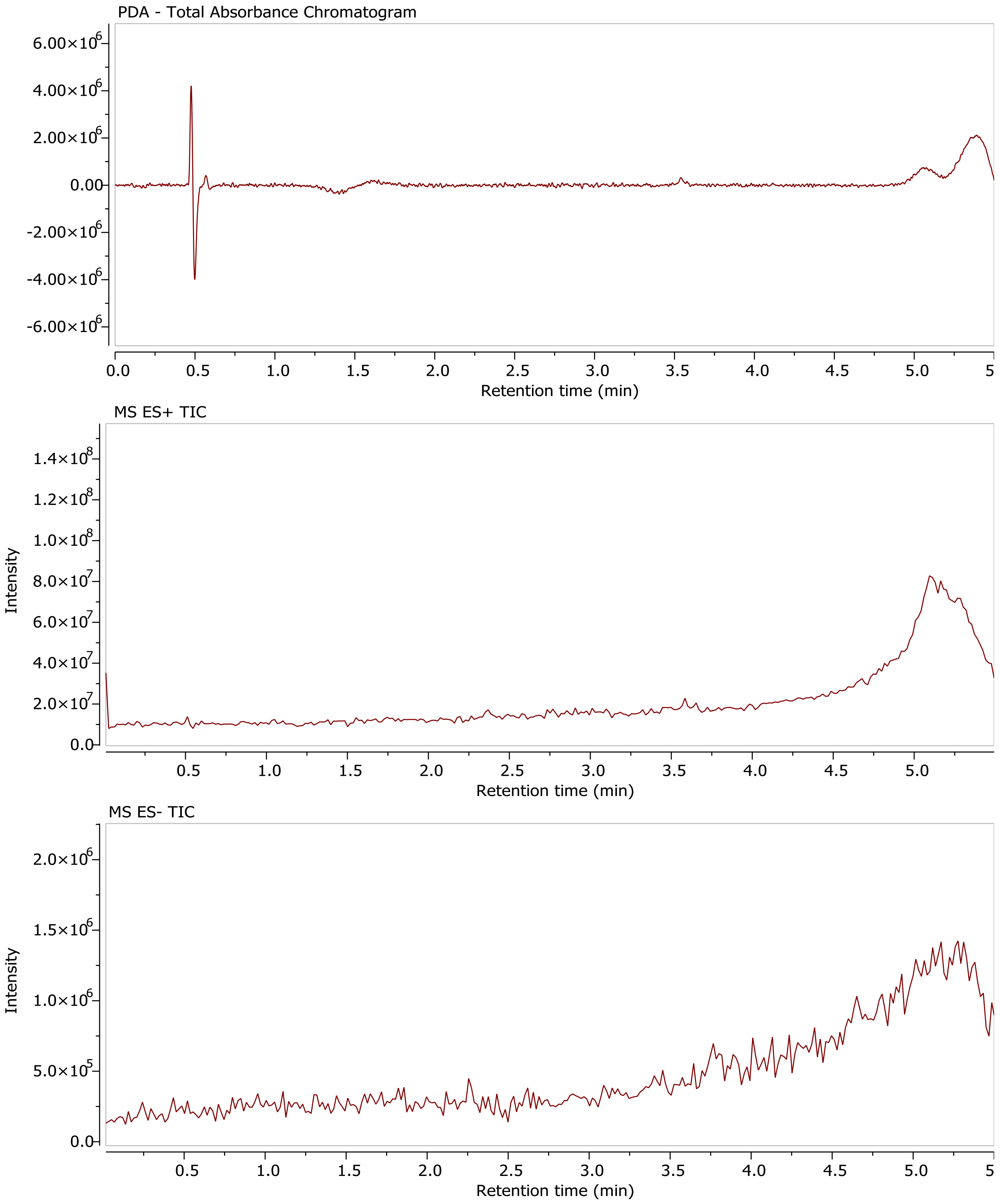

Supplementary figure **1253**. UPLC-MS analysis for compound **PHP-5401**

Chemical Formula: $C_{26}H_{26}N_2O_3$
Exact Mass: 414.2

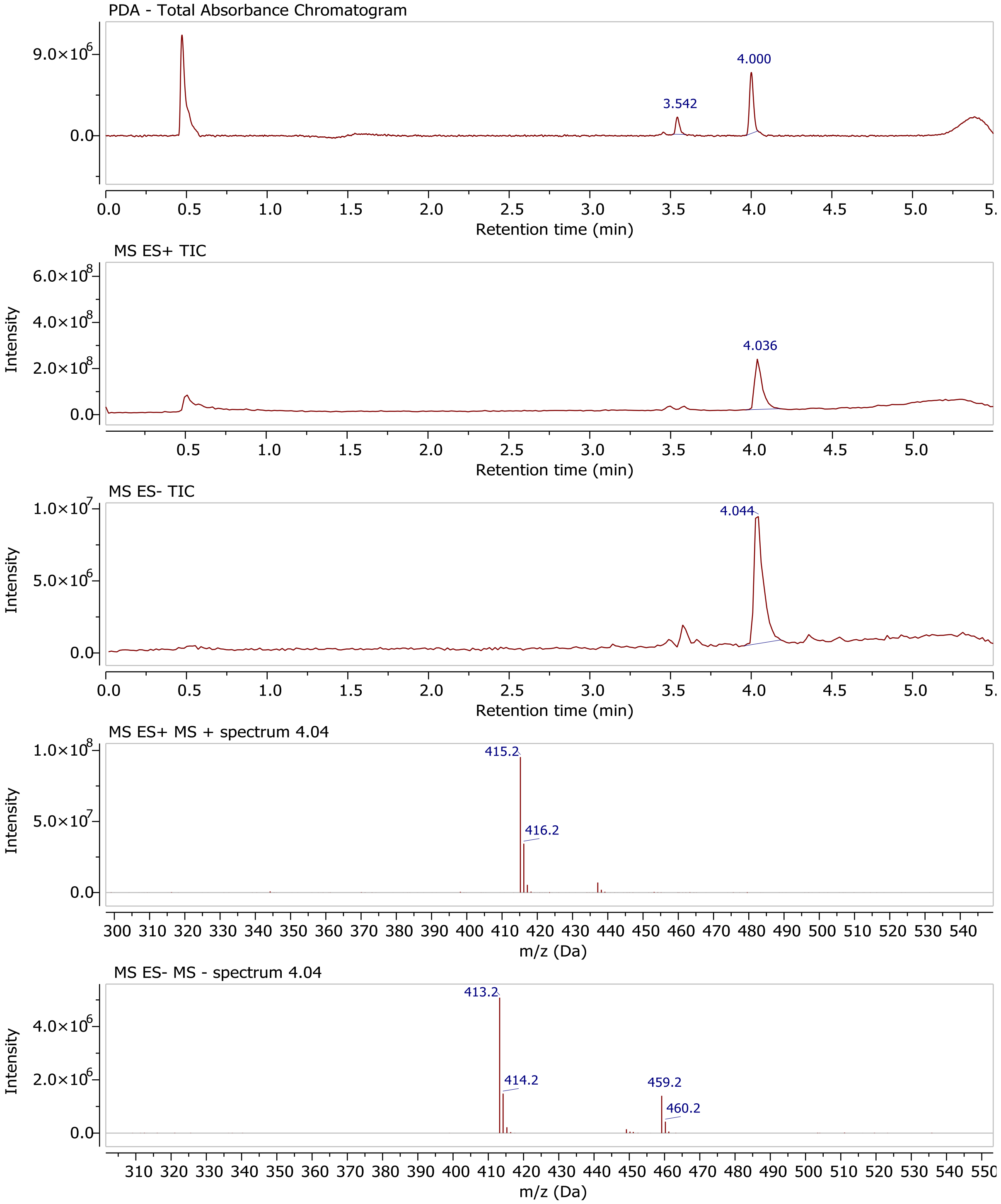

Supplementary figure **1254**. UPLC-MS analysis for compound **PHP-4691**

Chemical Formula: $C_{24}H_{20}ClFN_2O_3$
Exact Mass: 438.1

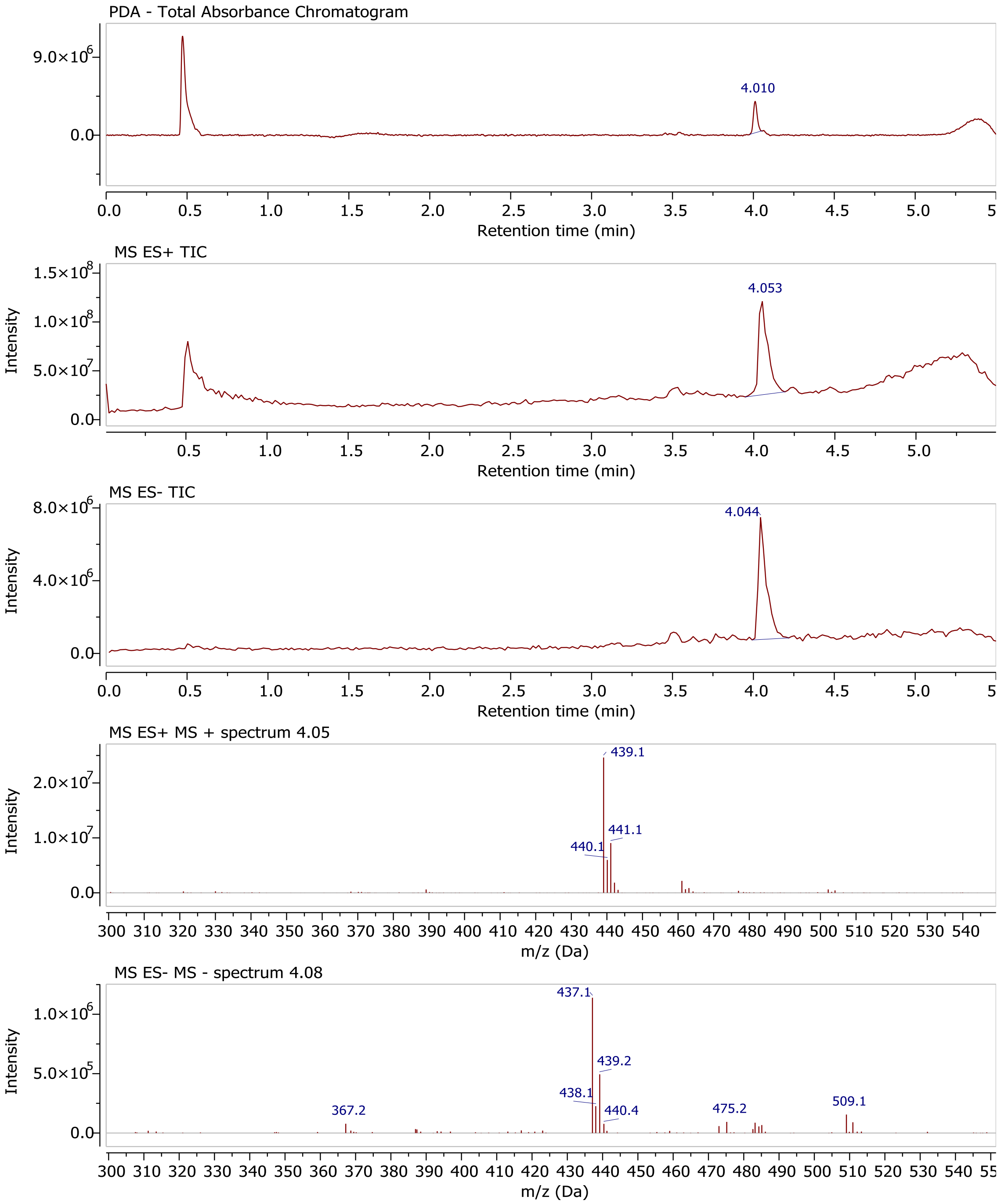

Supplementary figure **1255**. UPLC-MS analysis for compound **PHP-5402**

Chemical Formula: $C_{19}H_{24}N_2O_4$
Exact Mass: 344.2

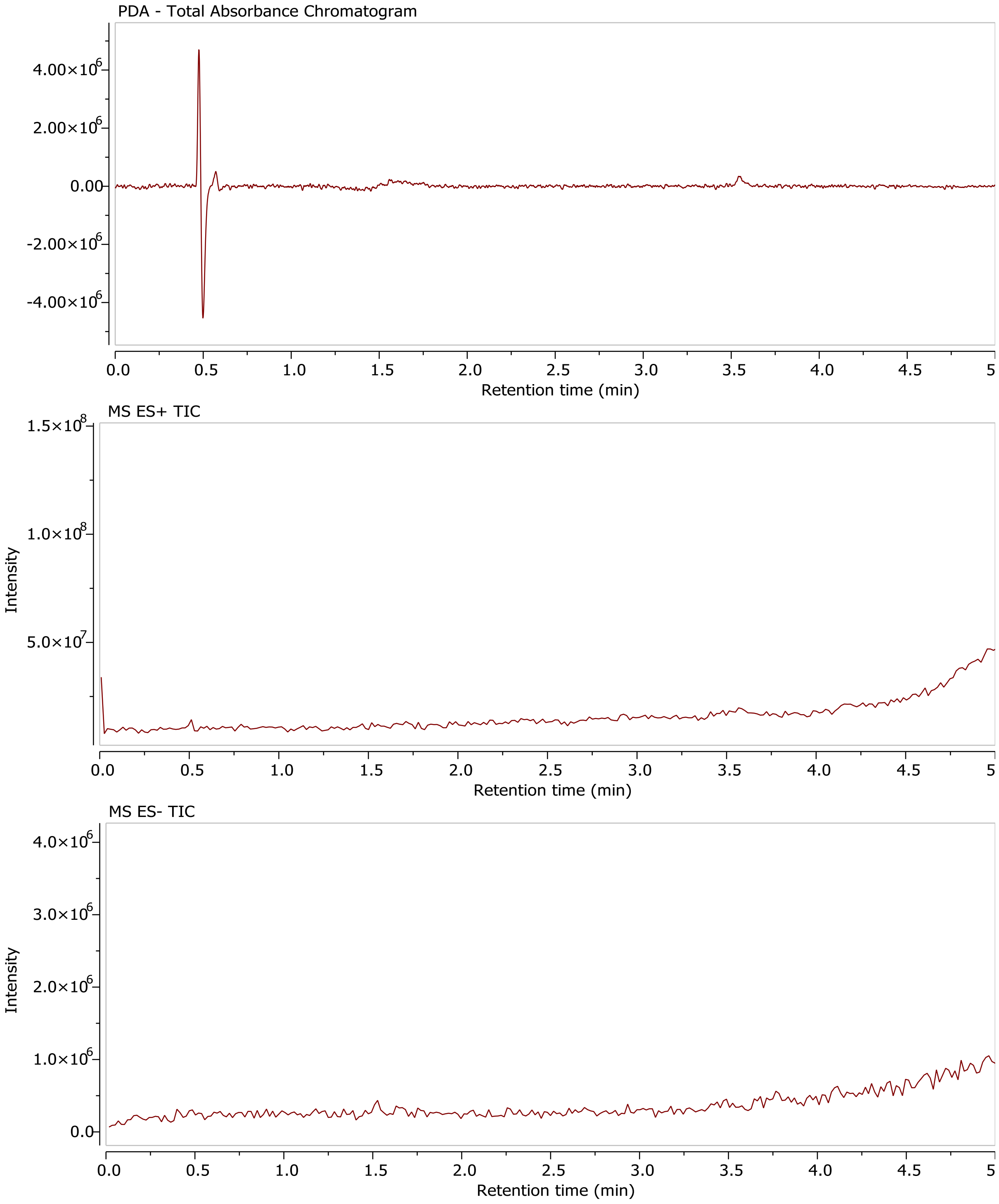

Supplementary figure **1256**. UPLC-MS analysis for compound **PHP-4693**

Chemical Formula: $C_{21}H_{22}ClFN_2O_2$
Exact Mass: 388.1

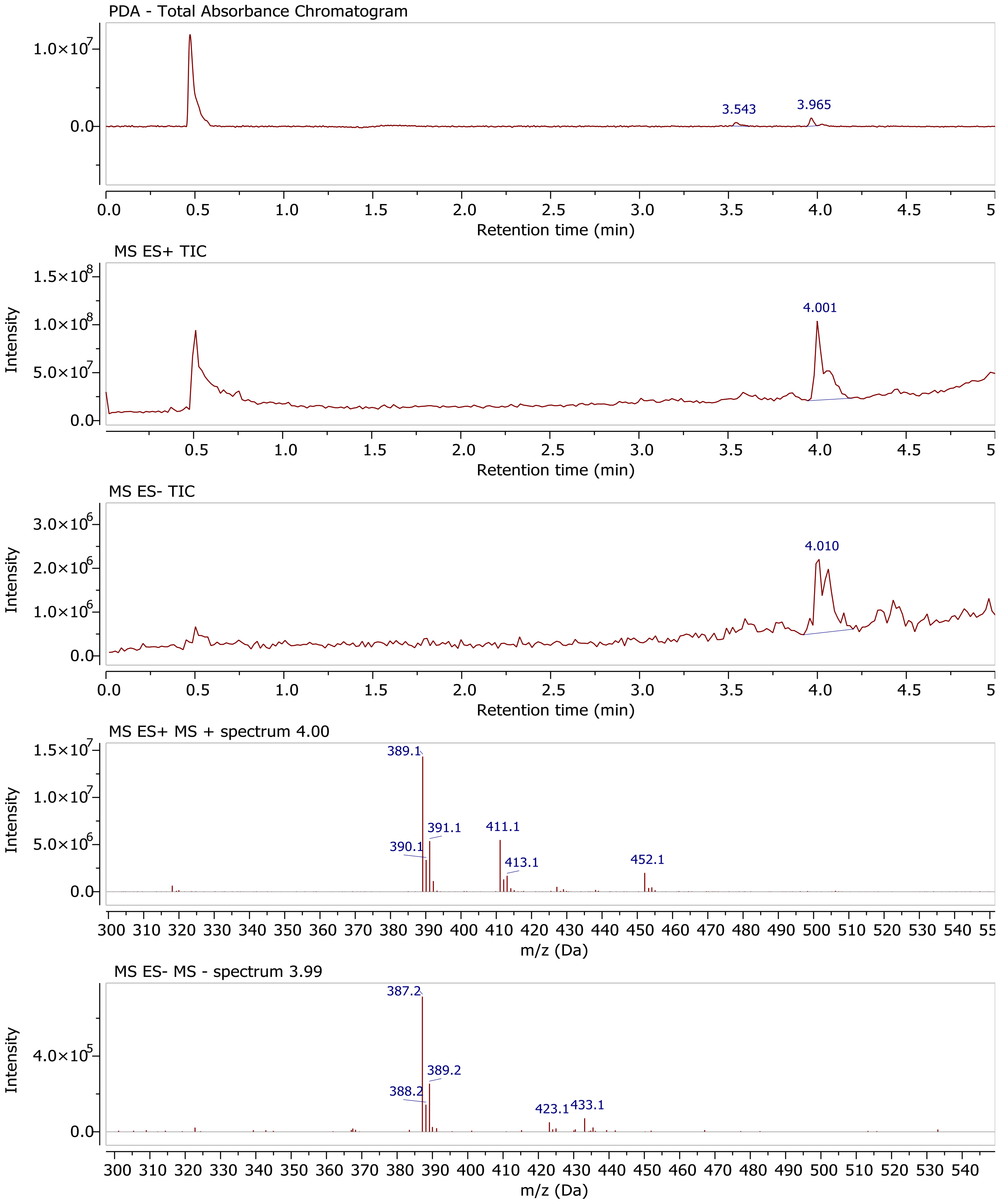

Supplementary figure **1257**. UPLC-MS analysis for compound **PHP-5404**

Chemical Formula: $C_{24}H_{22}N_2O_4$
Exact Mass: 402.2

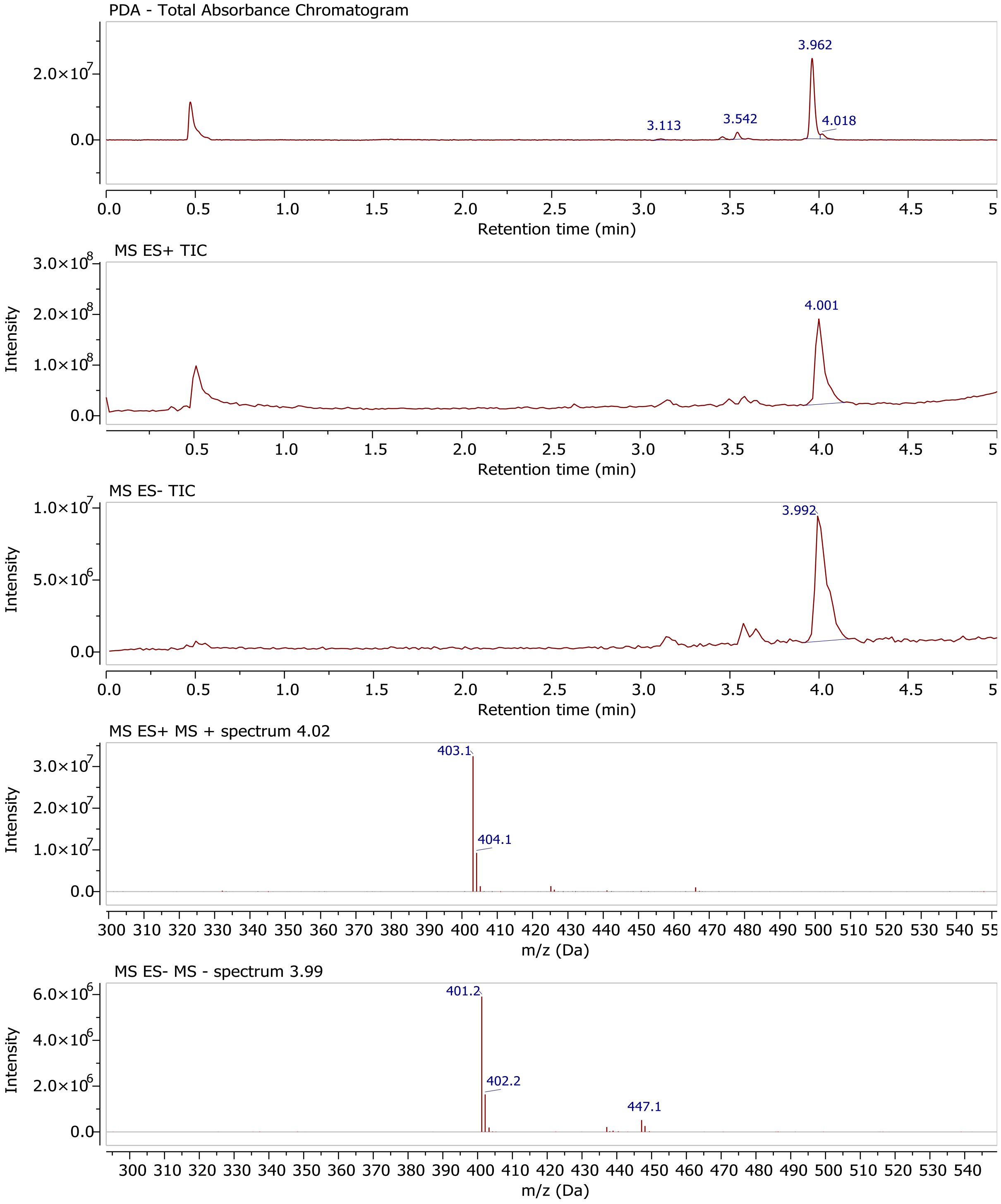

Supplementary figure **1258**. UPLC-MS analysis for compound **PHP-4694**

Chemical Formula: $C_{18}H_{15}Cl_2FN_2O_2$
Exact Mass: 380.0

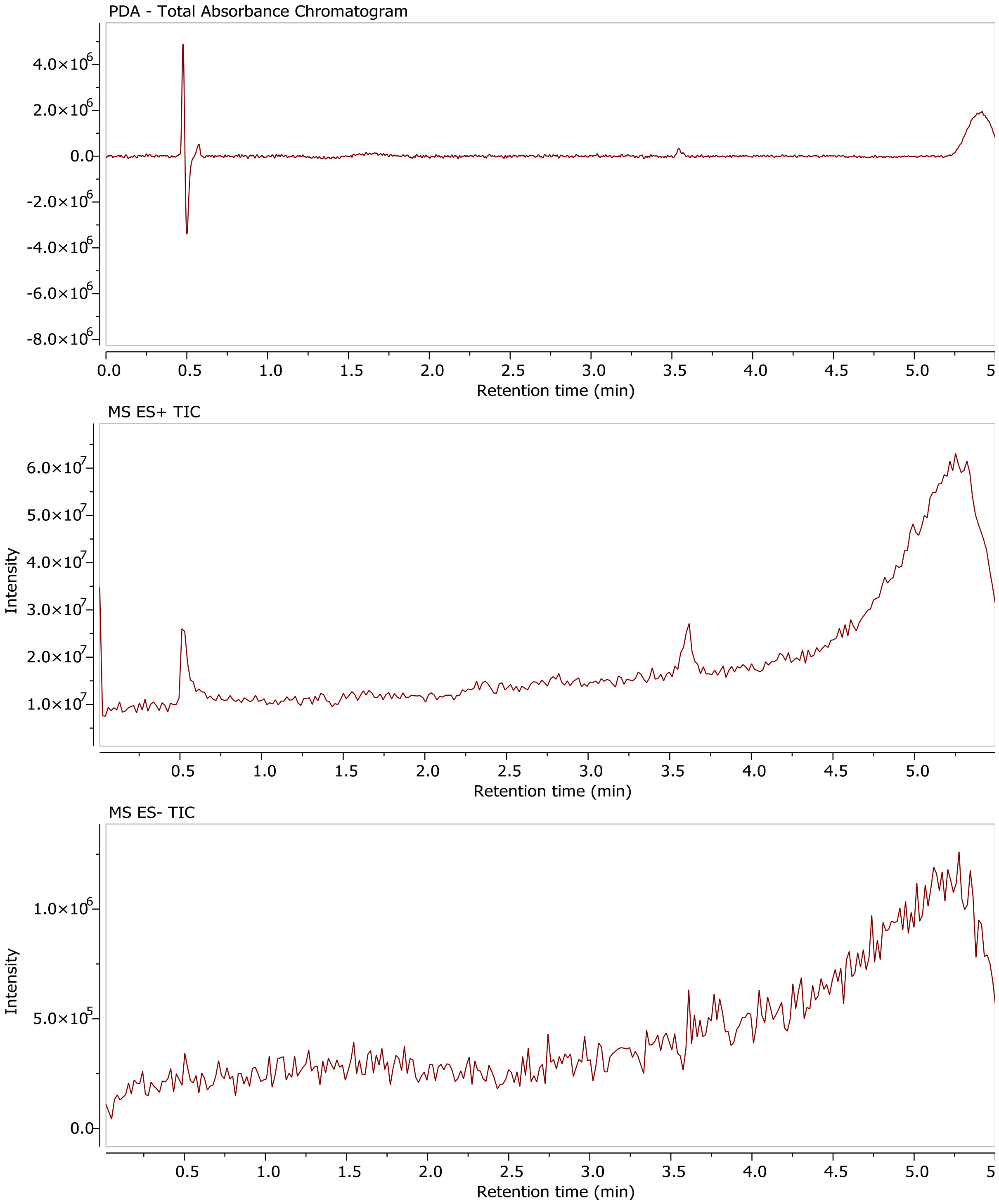

Supplementary figure **1259**. UPLC-MS analysis for compound **PHP-5405**

Chemical Formula: $C_{20}H_{20}ClFN_2O_4$
Exact Mass: 406.1

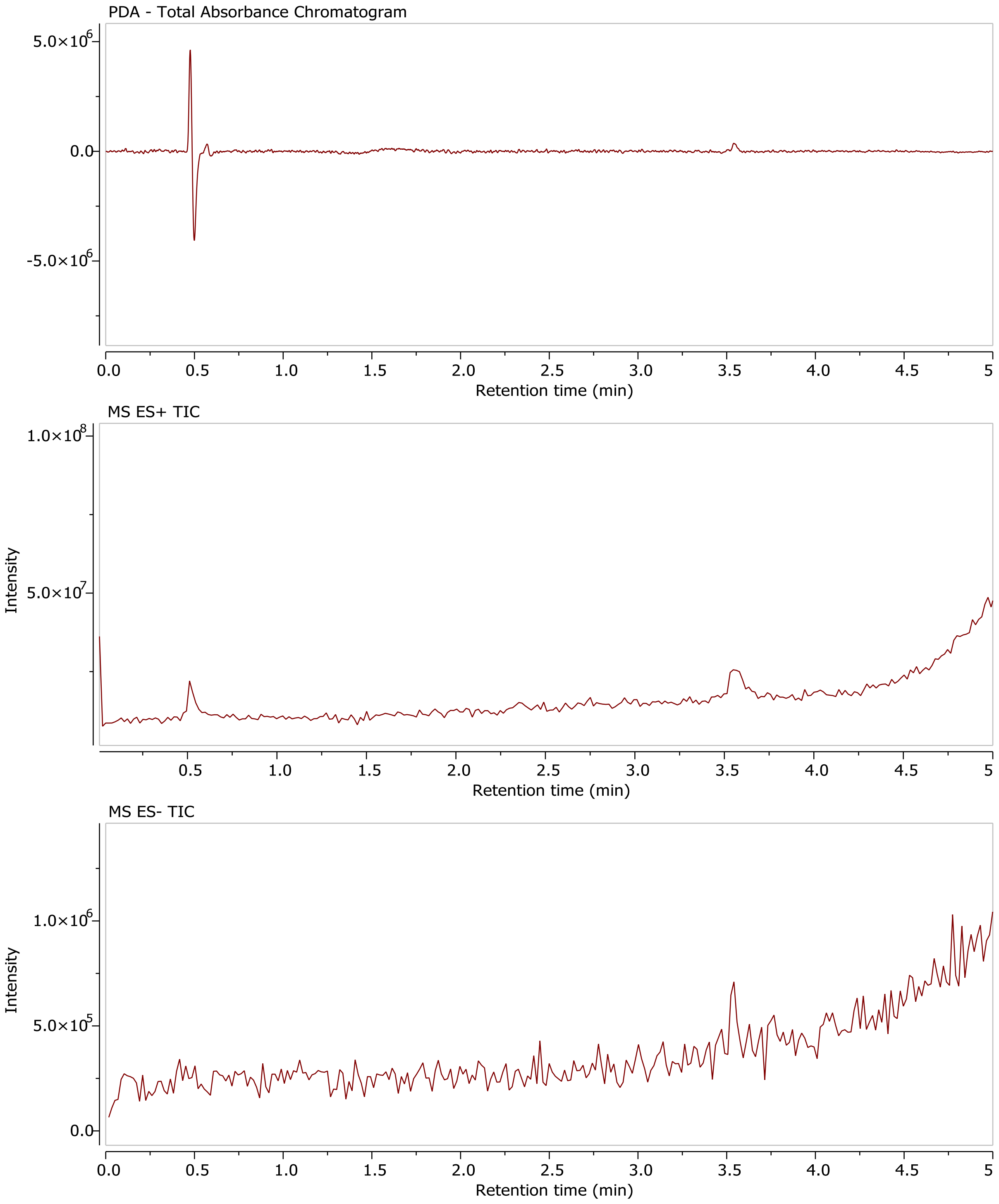

Supplementary figure **1260**. UPLC-MS analysis for compound **PHP-4801**

Chemical Formula: $C_{19}H_{15}ClF_4N_2O_2$
Exact Mass: 414.1

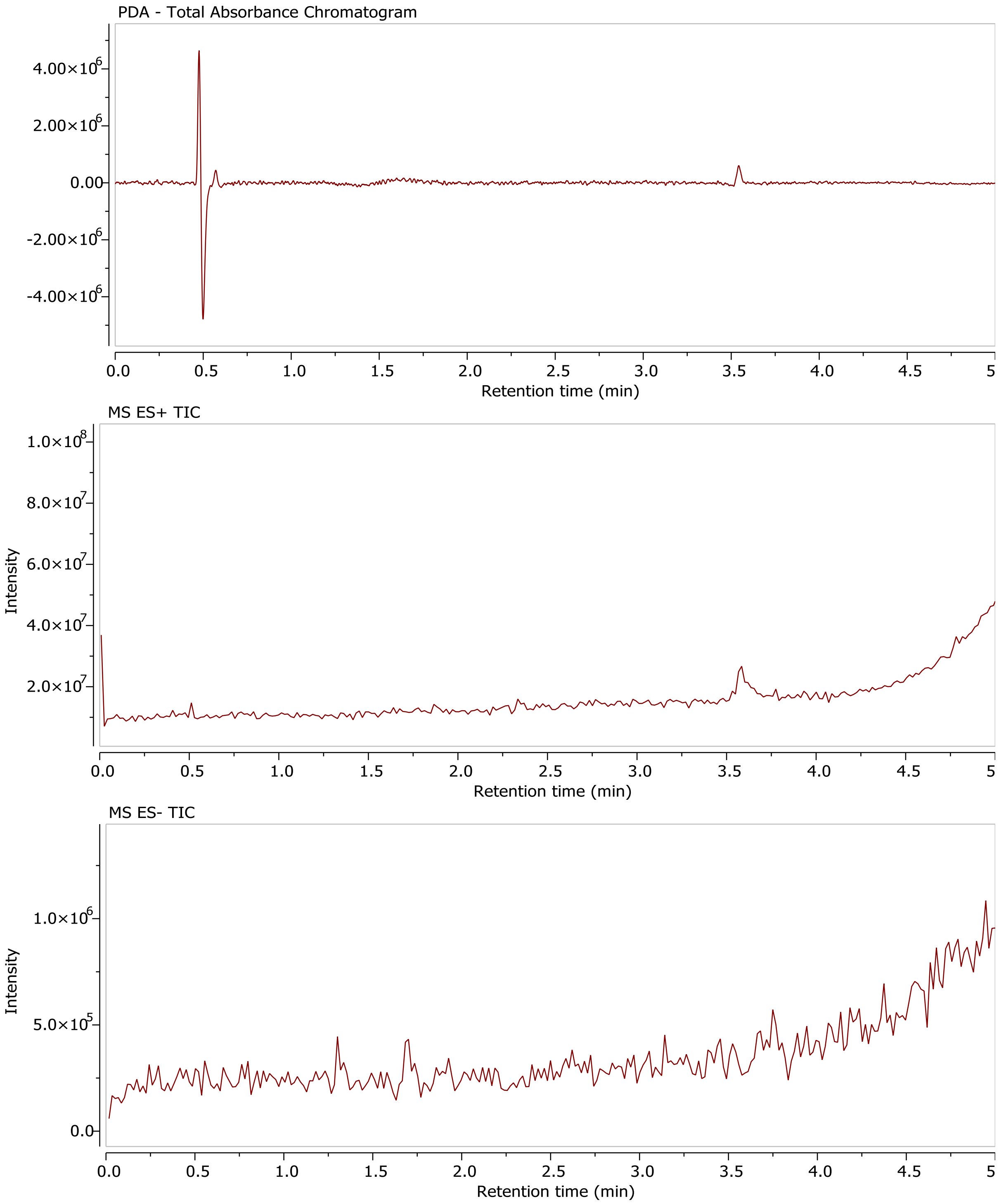

Supplementary figure **1261**. UPLC-MS analysis for compound **PHP-5406**

Chemical Formula: $C_{27}H_{31}N_3O_6$
Exact Mass: 493.2

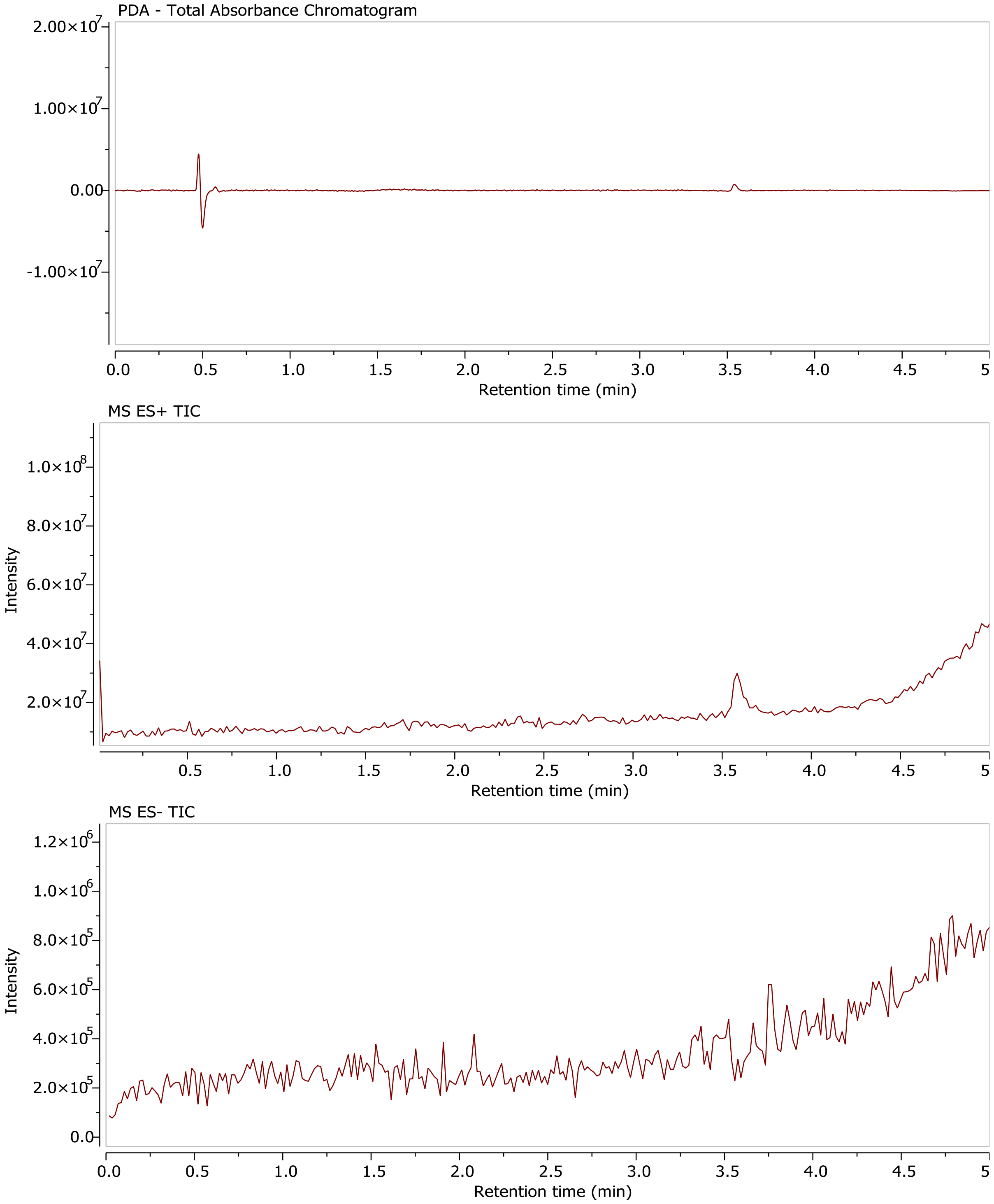

Supplementary figure **1262**. UPLC-MS analysis for compound **PHP-4803**

Chemical Formula: $C_{18}H_{15}ClF_2N_2O_2$
Exact Mass: 364.1

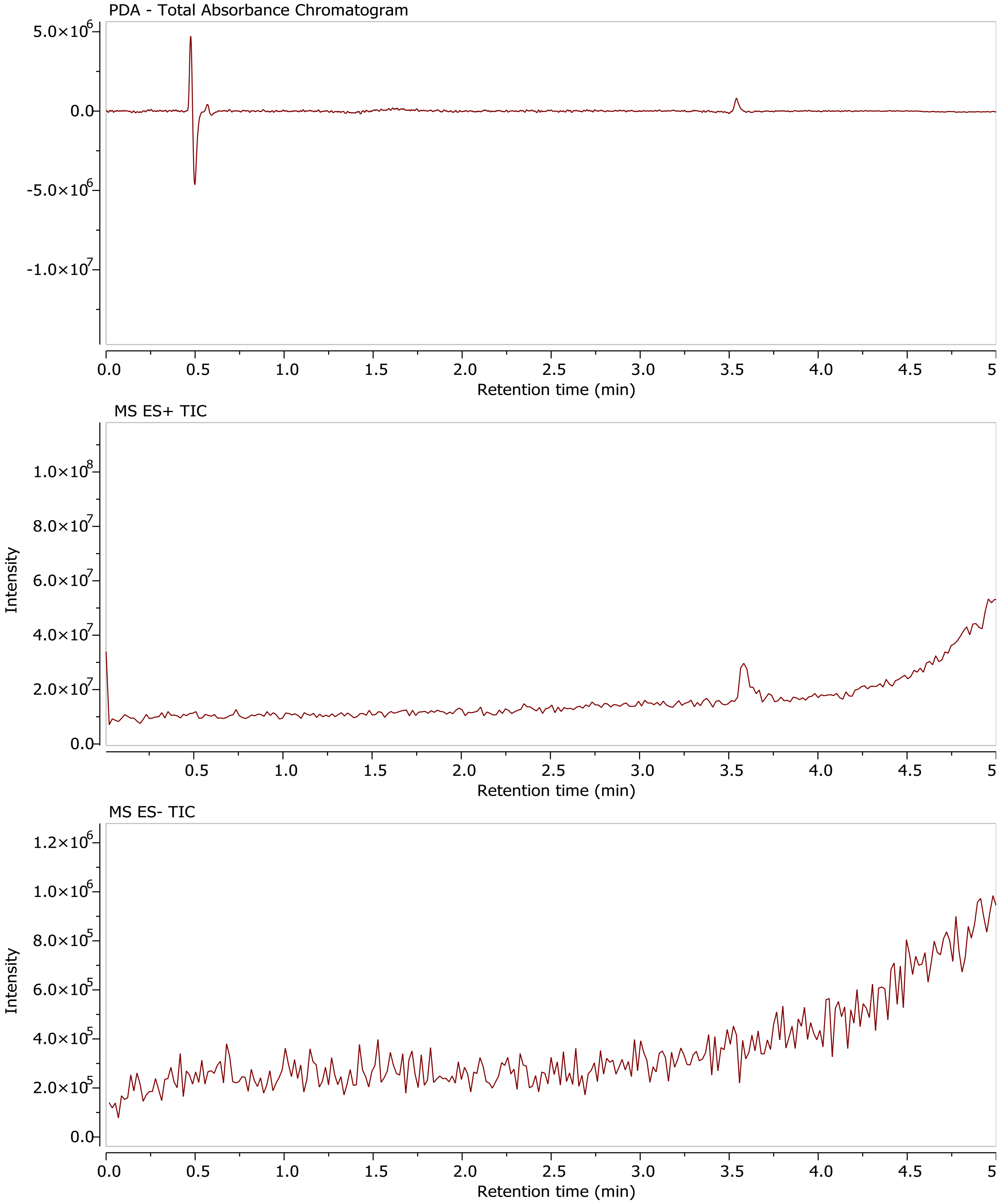

Supplementary figure **1263**. UPLC-MS analysis for compound **PHP-5407**

Chemical Formula: $C_{20}H_{20}Cl_2N_2O_4$
Exact Mass: 422.1

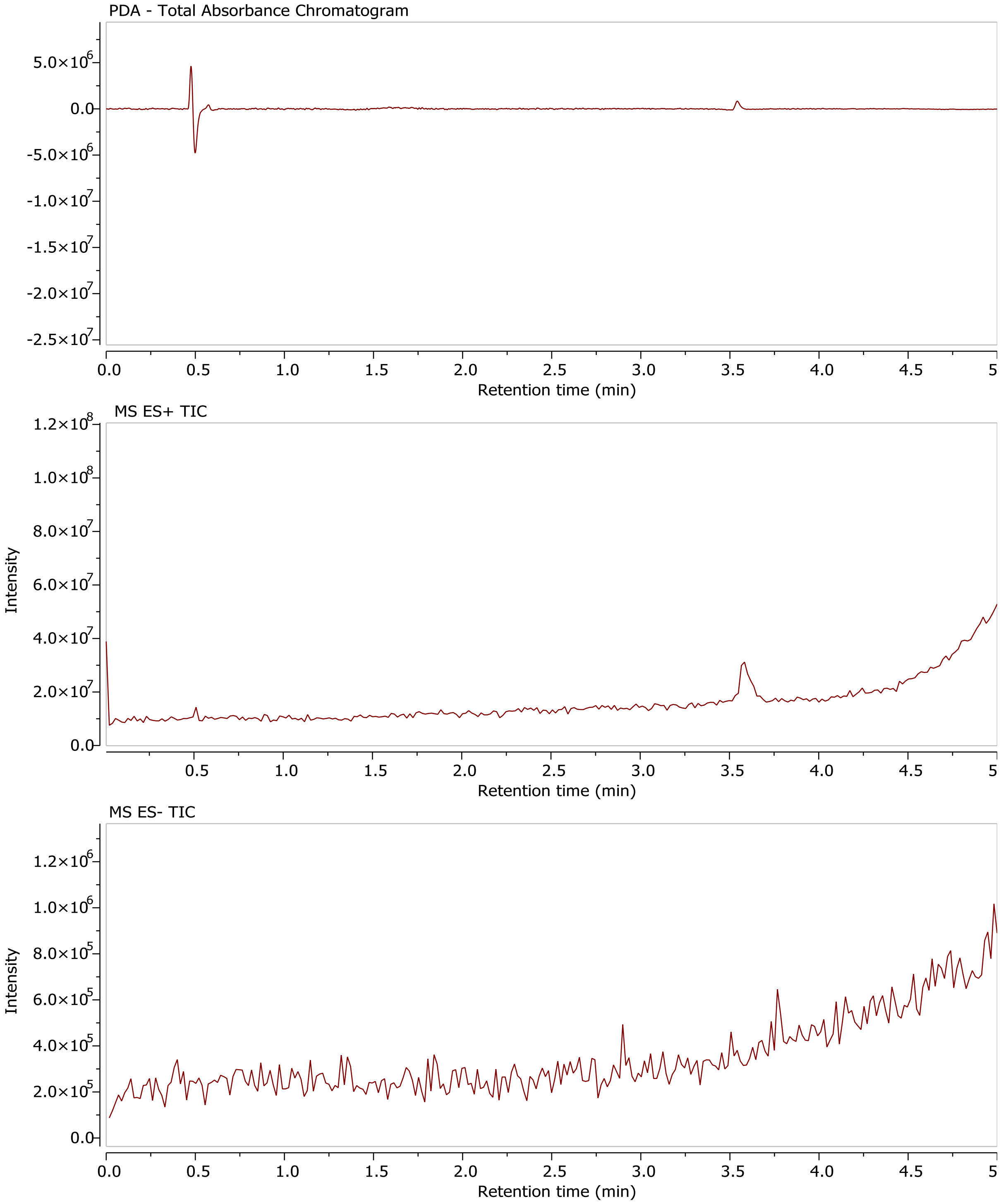

Supplementary figure **1264**. UPLC-MS analysis for compound **PHP-4805**

Chemical Formula: $C_{18}H_{15}ClF_2N_2O_2$
Exact Mass: 364.1

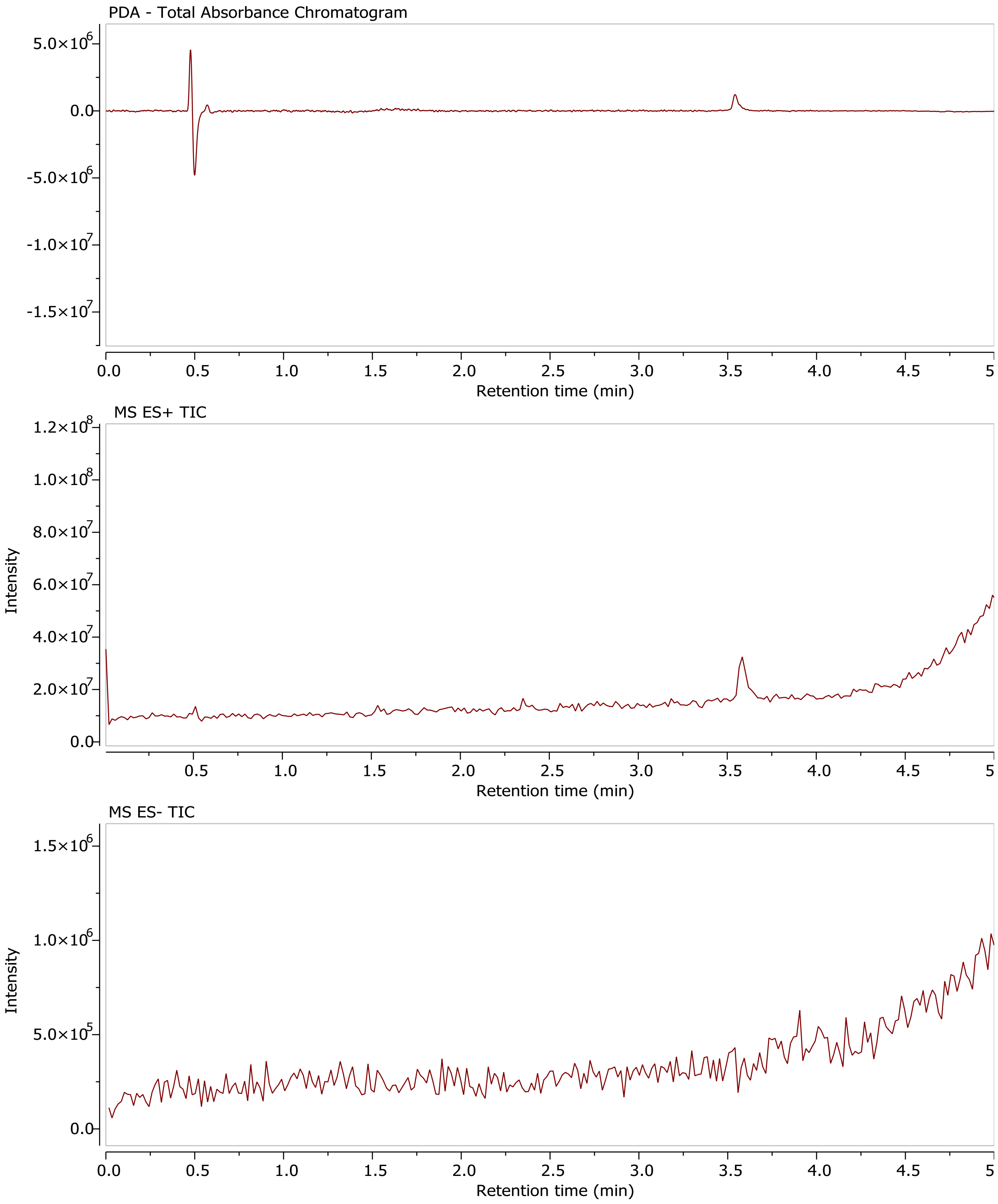

Supplementary figure **1265**. UPLC-MS analysis for compound **PHP-5408**

Chemical Formula: $C_{22}H_{26}N_2O_6$
Exact Mass: 414.2

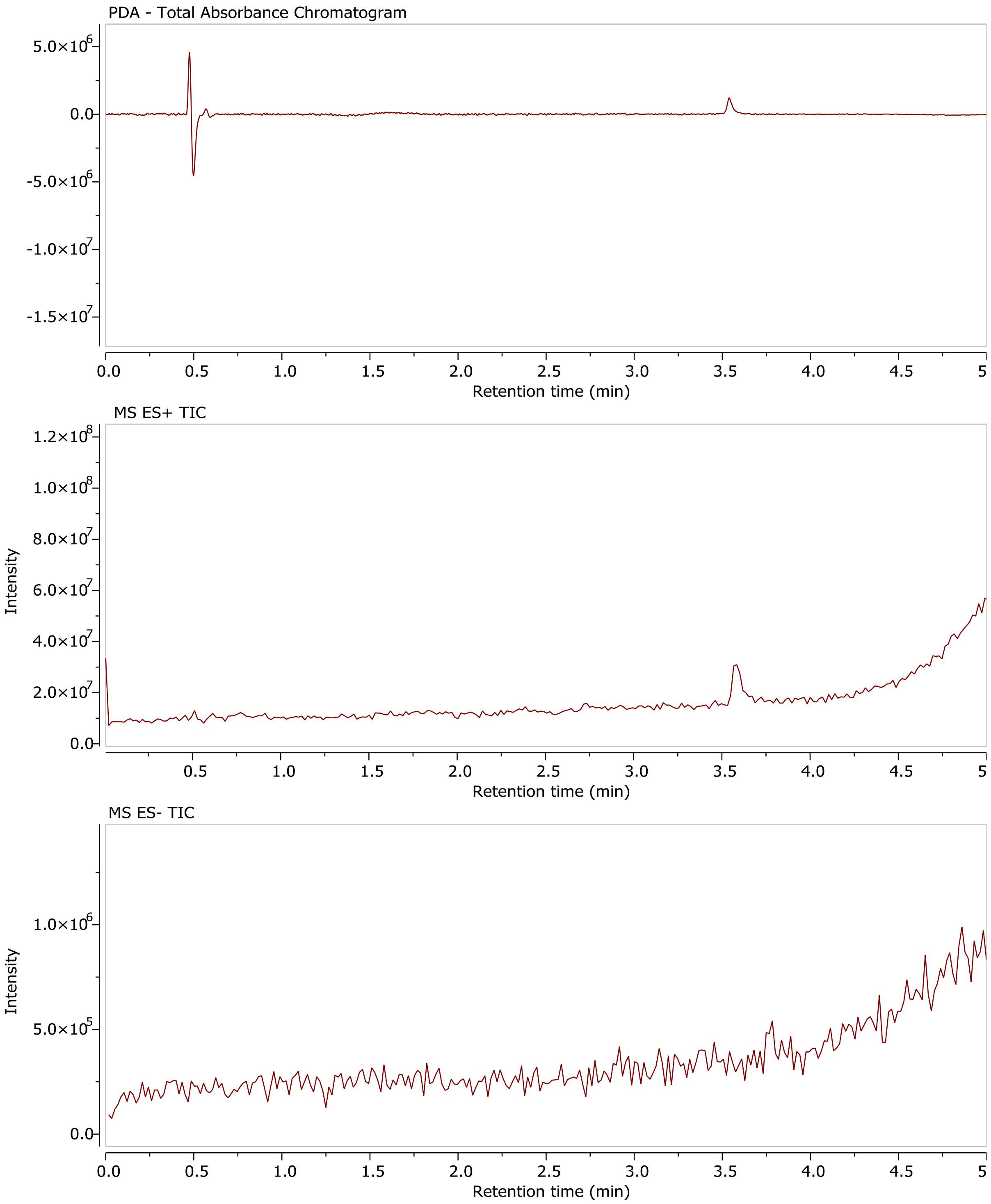

Supplementary figure **1266**. UPLC-MS analysis for compound **PHP-4807**

Chemical Formula: $C_{17}H_{22}ClFN_2O_2$
Exact Mass: 340.1

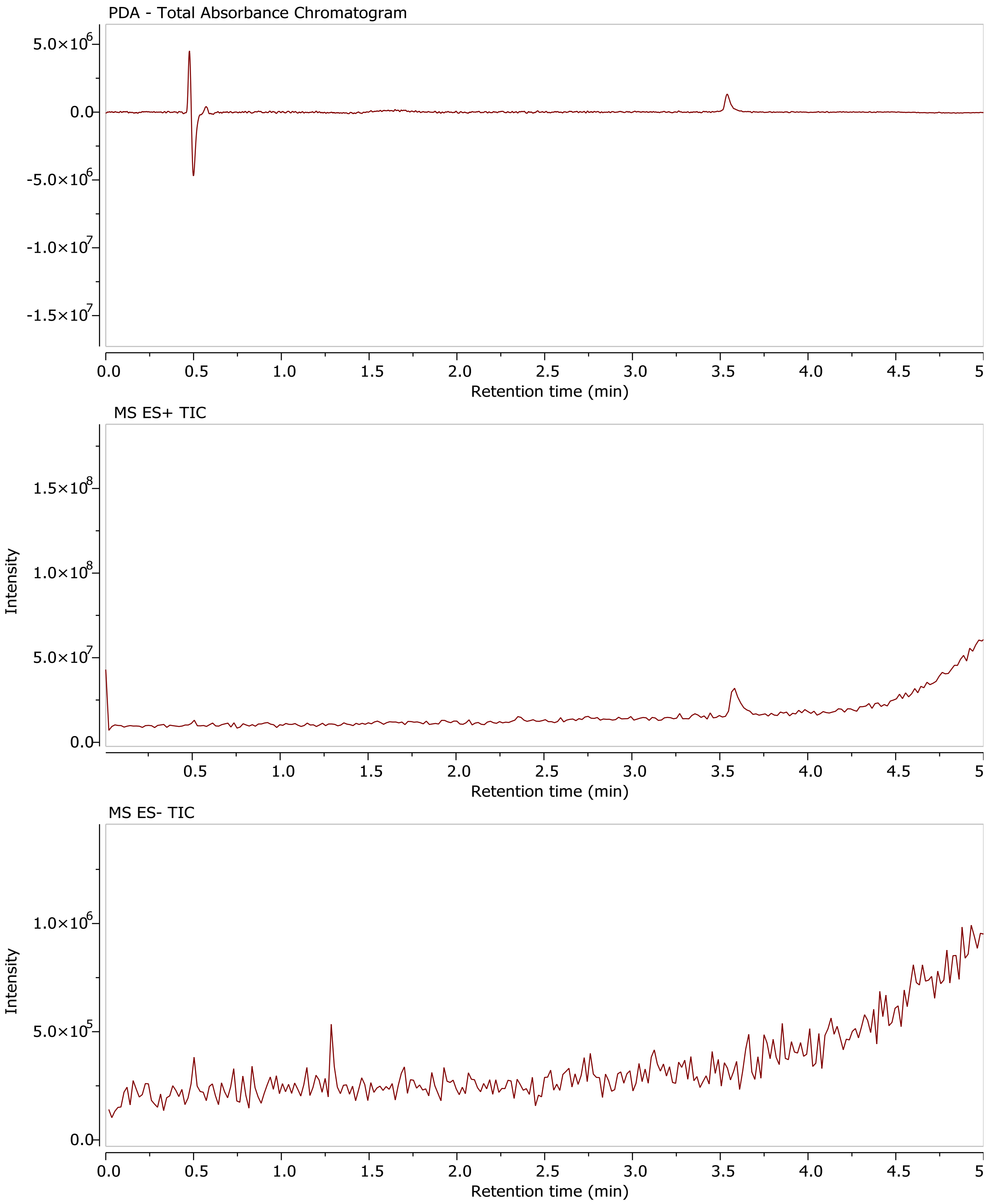

Supplementary figure **1267**. UPLC-MS analysis for compound **PHP-5409**

Chemical Formula: $C_{28}H_{30}N_2O_6$
Exact Mass: 490.2

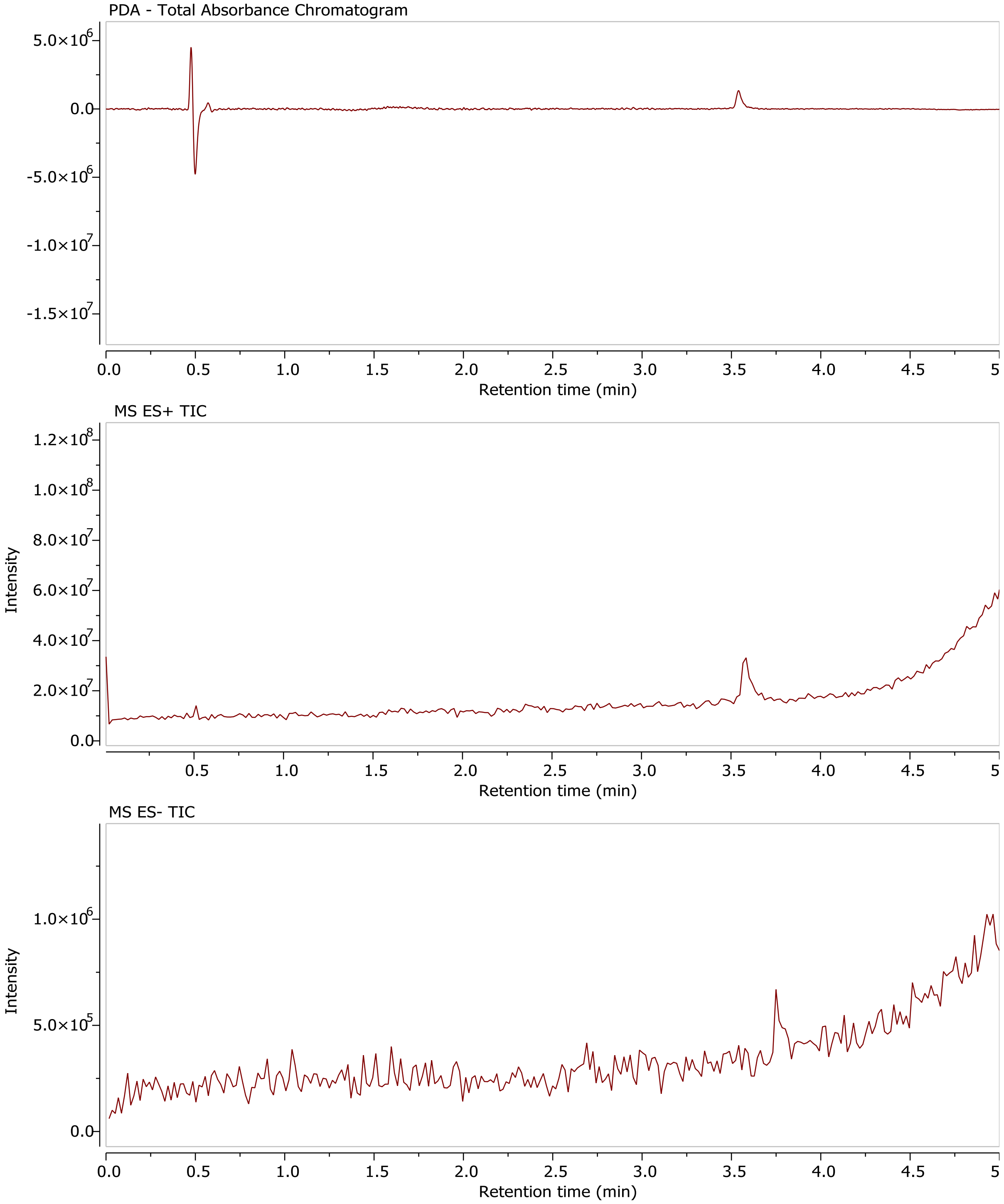

Supplementary figure **1268**. UPLC-MS analysis for compound **PHP-4809**

Chemical Formula: $C_{17}H_{22}ClFN_2O_2$
Exact Mass: 340.1

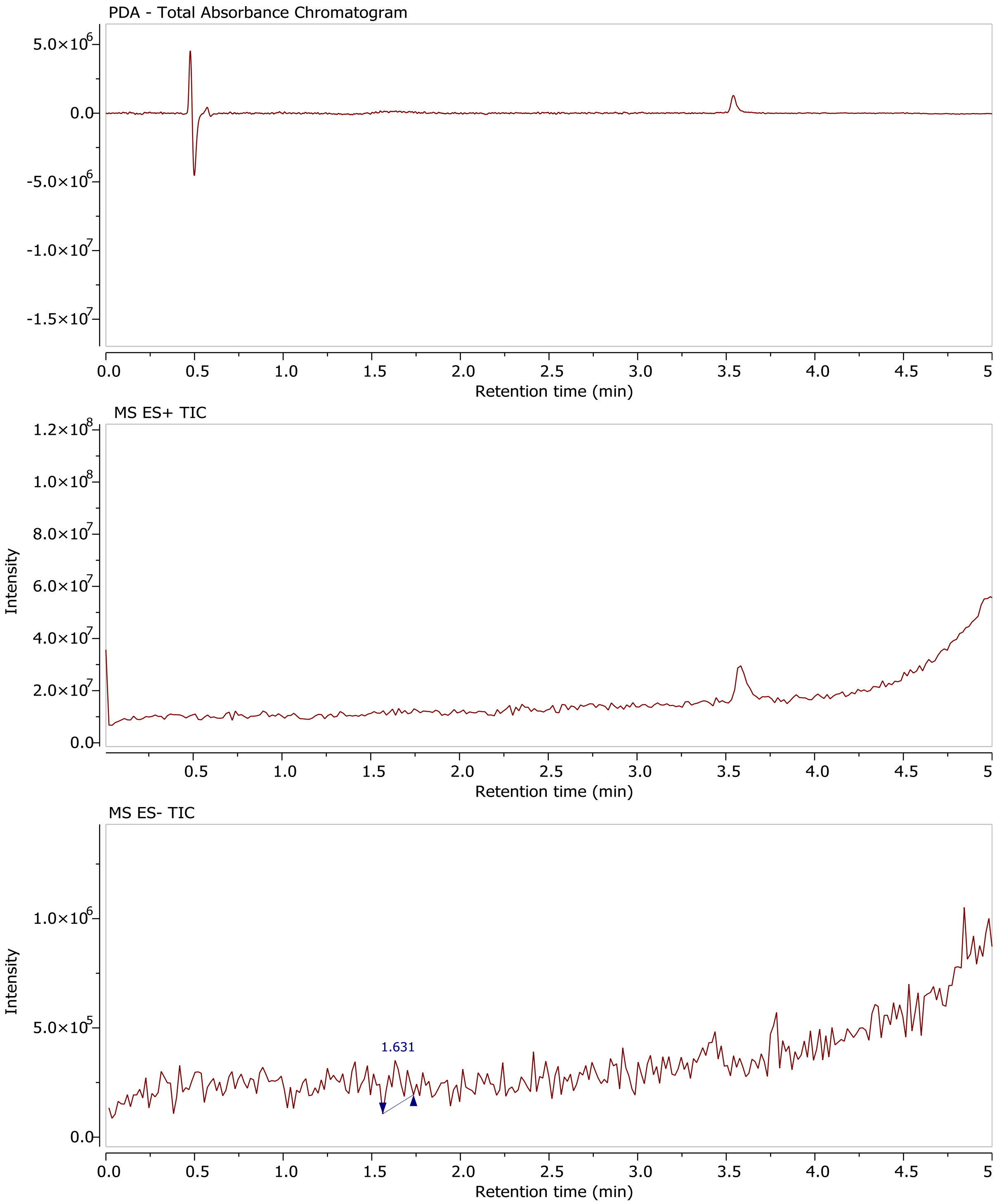

Supplementary figure **1269**. UPLC-MS analysis for compound **PHP-4809**

Chemical Formula: $C_{20}H_{20}Cl_2N_2O_4$
Exact Mass: 422.1

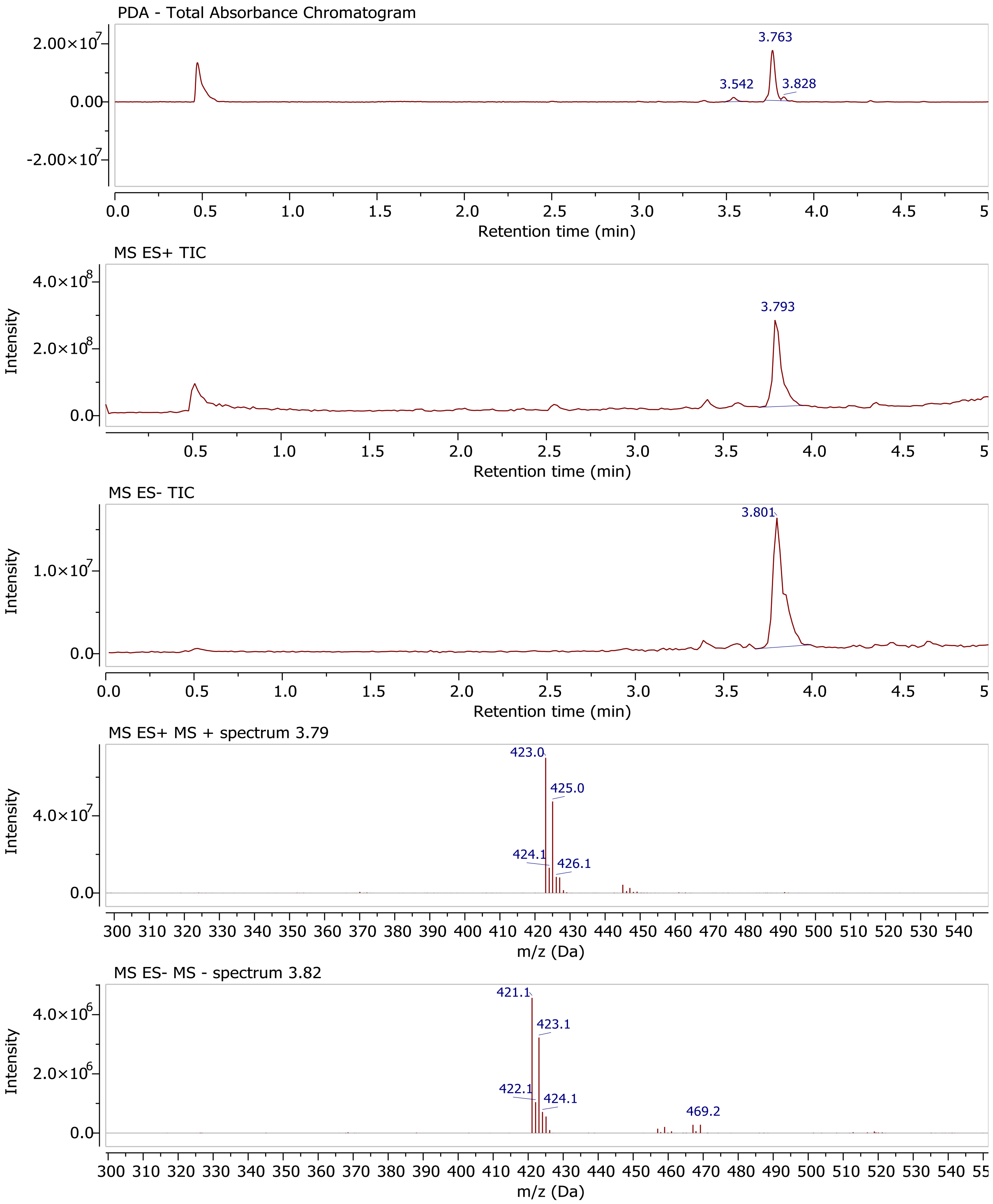

Supplementary figure **1270**. UPLC-MS analysis for compound **PHP-4810**

Chemical Formula: $C_{18}H_{12}ClF_5N_2O_2$
Exact Mass: 418.1

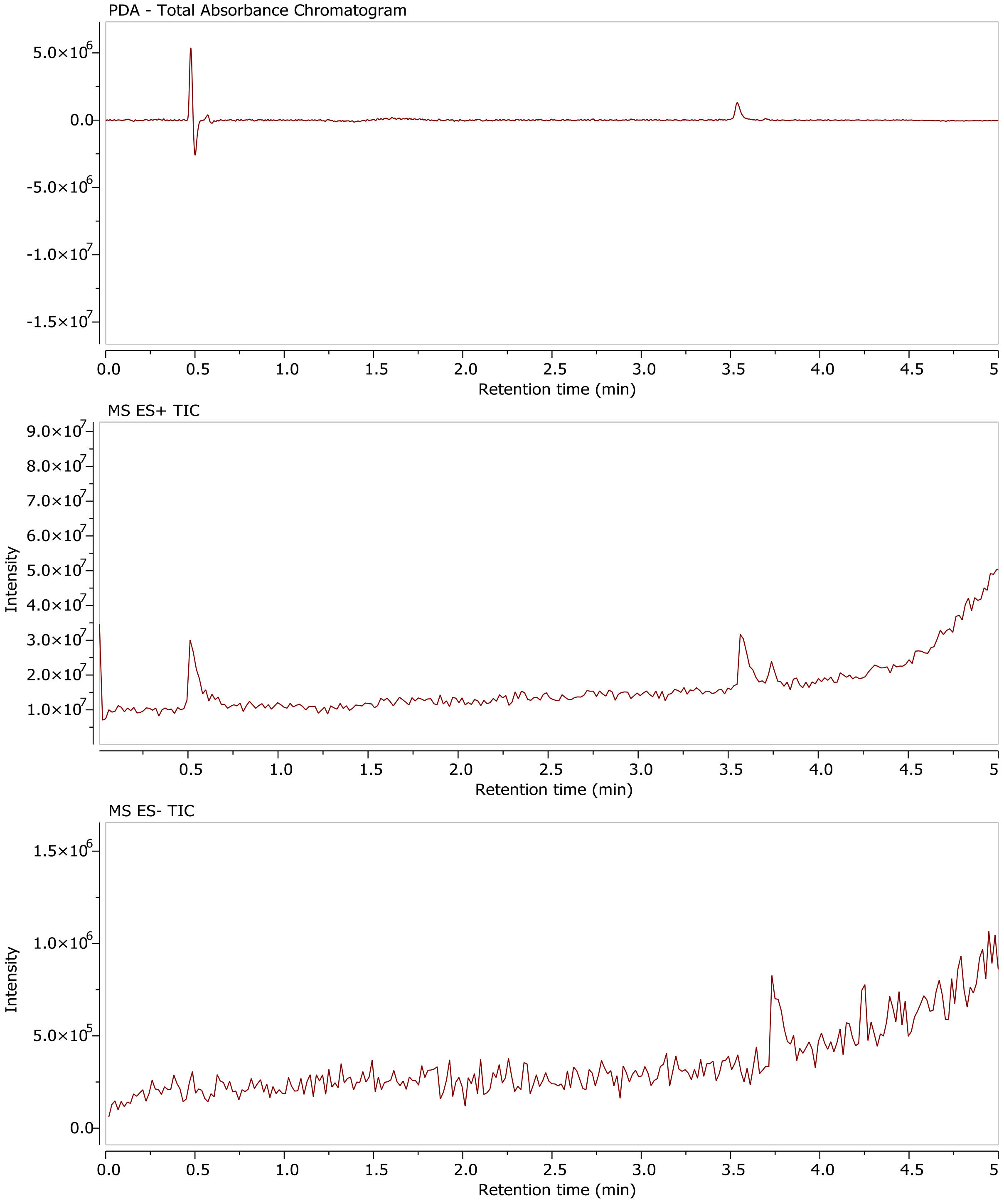

Supplementary figure **1271**. UPLC-MS analysis for compound **PHP-5411**

Chemical Formula: $C_{27}H_{28}N_2O_5$
Exact Mass: 460.2

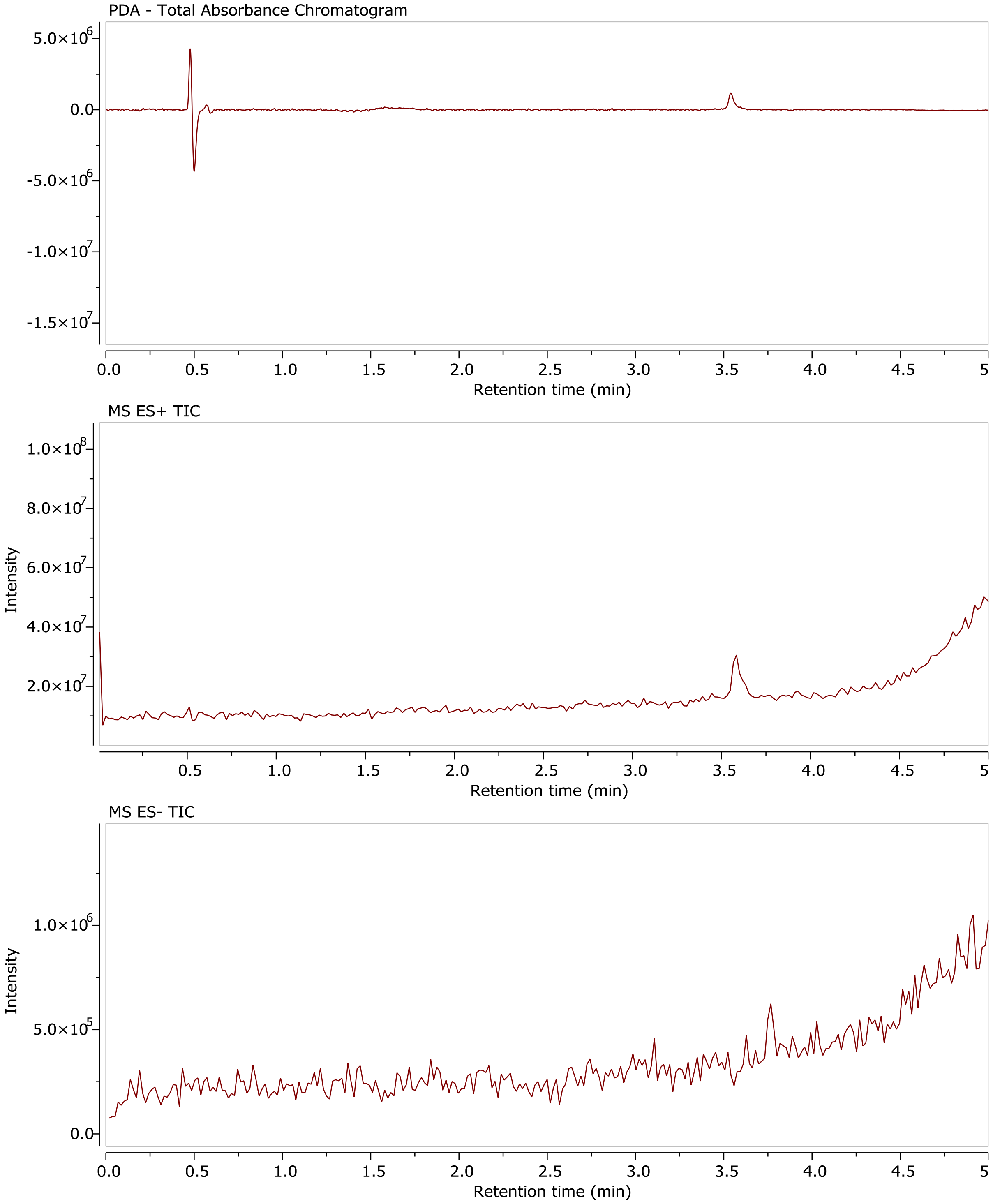

Supplementary figure **1272**. UPLC-MS analysis for compound **PHP-4811**

Chemical Formula: $C_{19}H_{26}ClFN_2O_2$
Exact Mass: 368.2

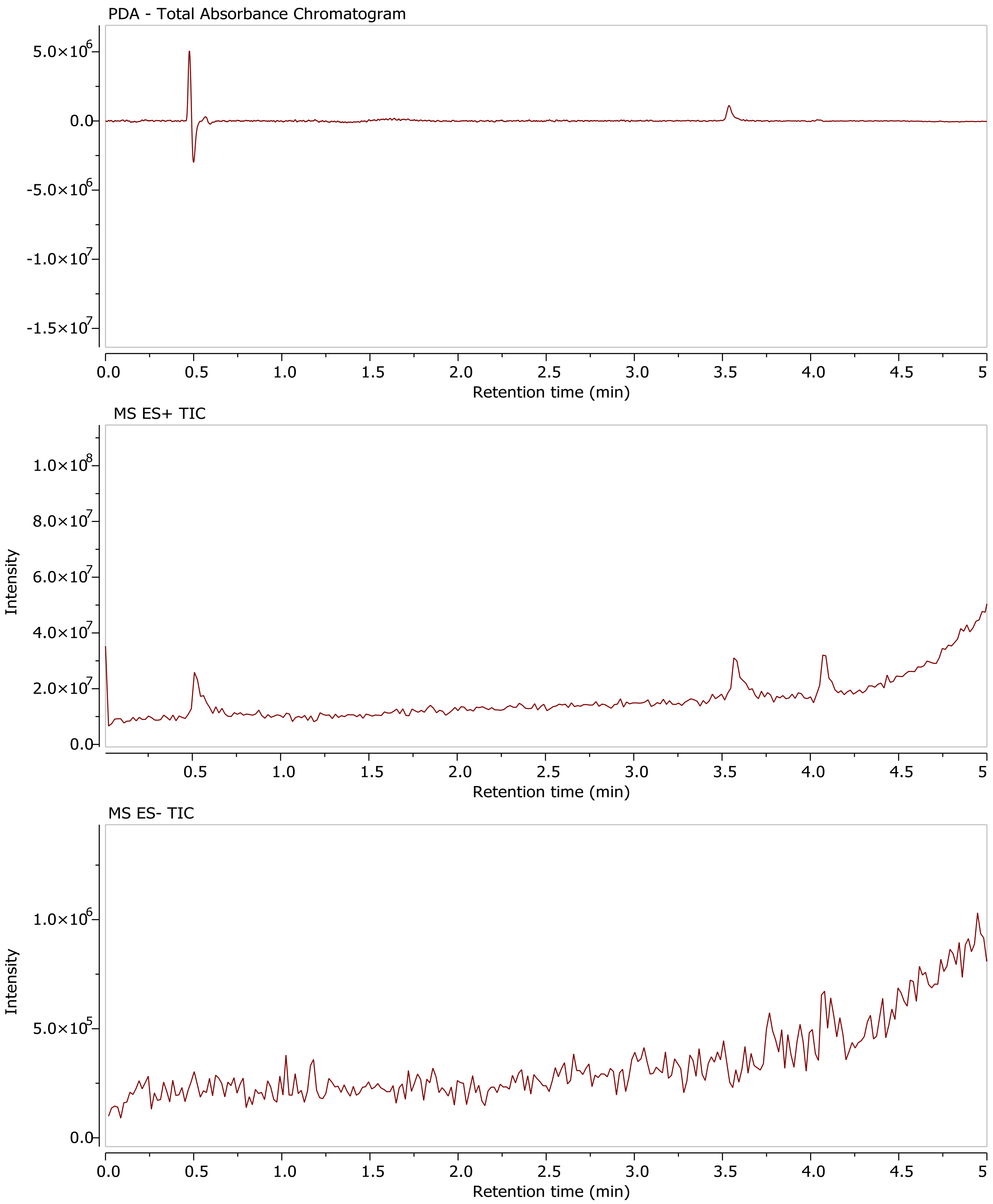

Supplementary figure **1273**. UPLC-MS analysis for compound **PHP-5412**

Chemical Formula: $C_{22}H_{23}N_3O_4$
Exact Mass: 393.2

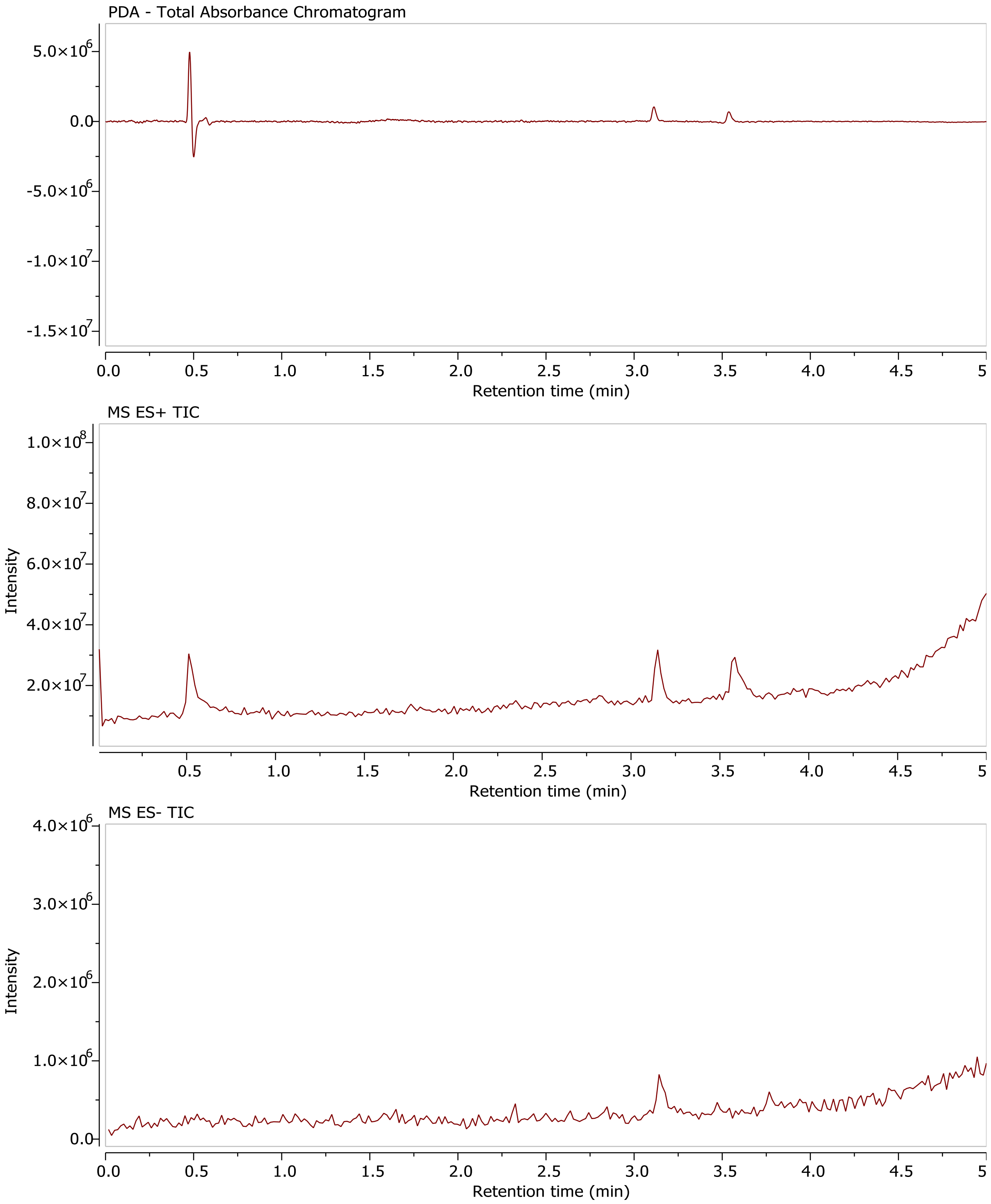

Supplementary figure **1274**. UPLC-MS analysis for compound **PHP-4814**

Chemical Formula: $C_{18}H_{13}ClF_4N_2O_2$
Exact Mass: 400.1

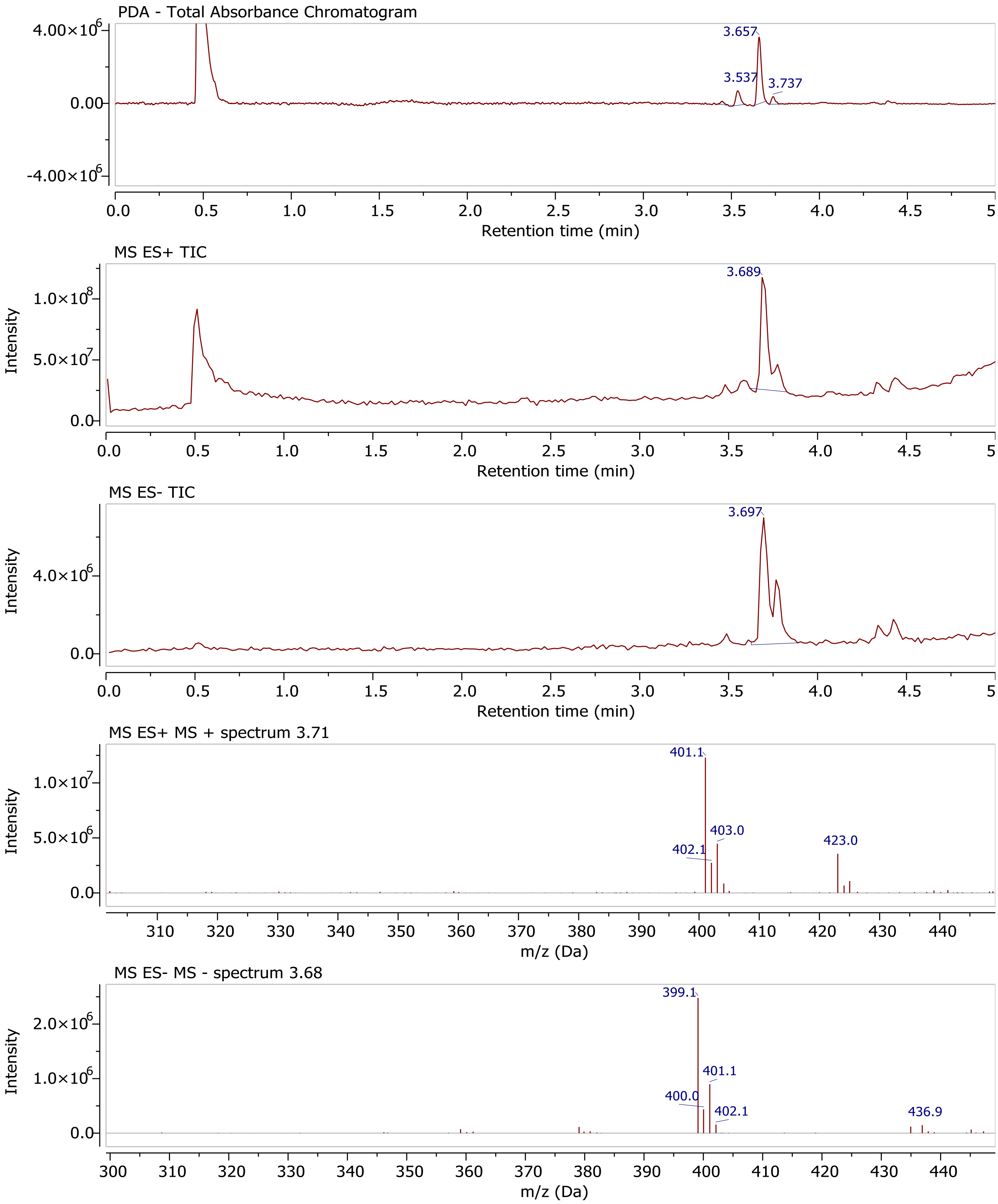

Supplementary figure **1275**. UPLC-MS analysis for compound **PHP-5413**

Chemical Formula: $C_{19}H_{16}BrF_3N_2O_2$
Exact Mass: 440.0

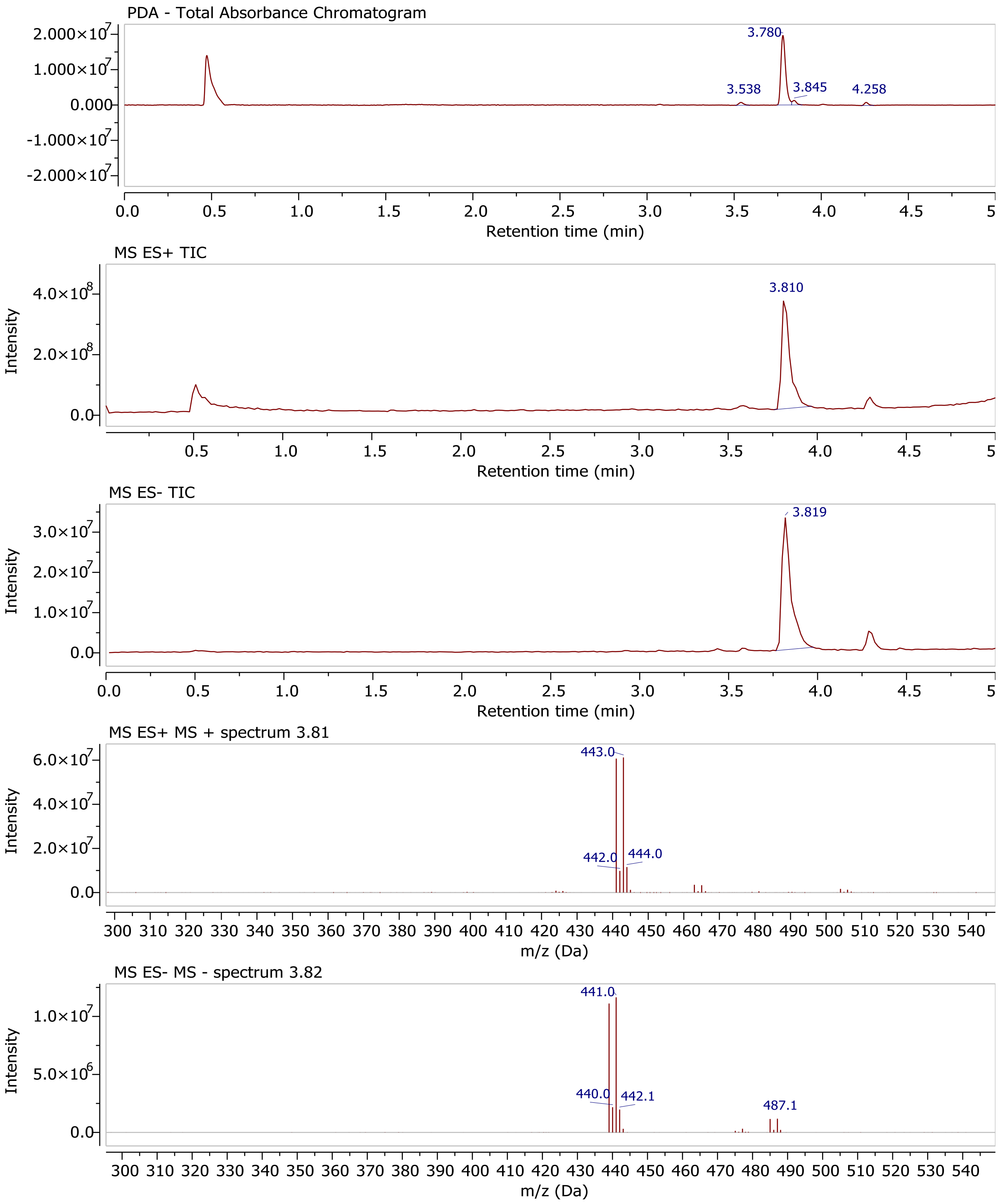

Supplementary figure **1276**. UPLC-MS analysis for compound **PHP-5561**

Chemical Formula: $C_{19}H_{19}FN_2O_3$
Exact Mass: 342.1

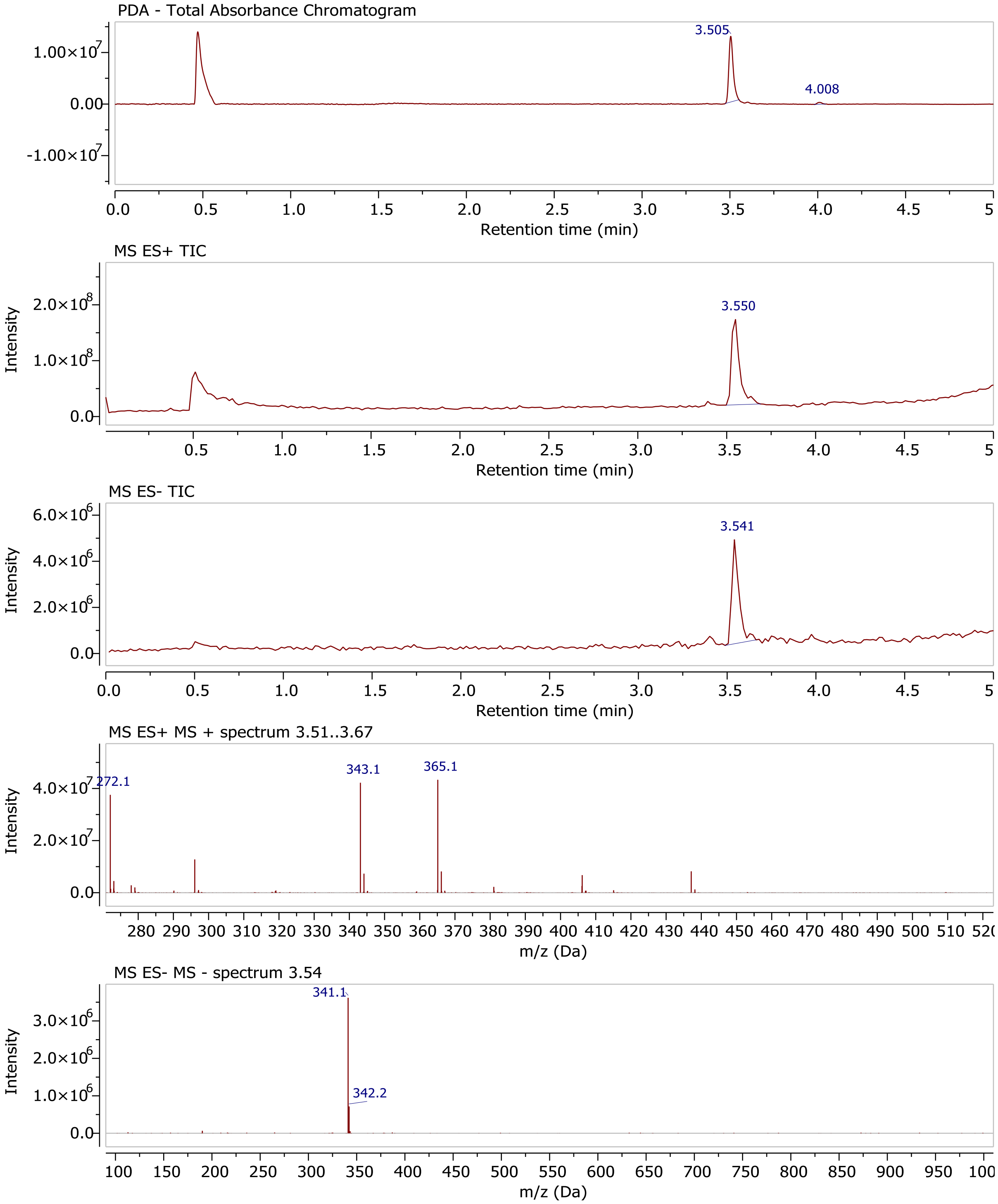

Supplementary figure **1277**. UPLC-MS analysis for compound **PHP-5714**

Chemical Formula: $C_{18}H_{16}BrFN_2O_2$
Exact Mass: 390.0

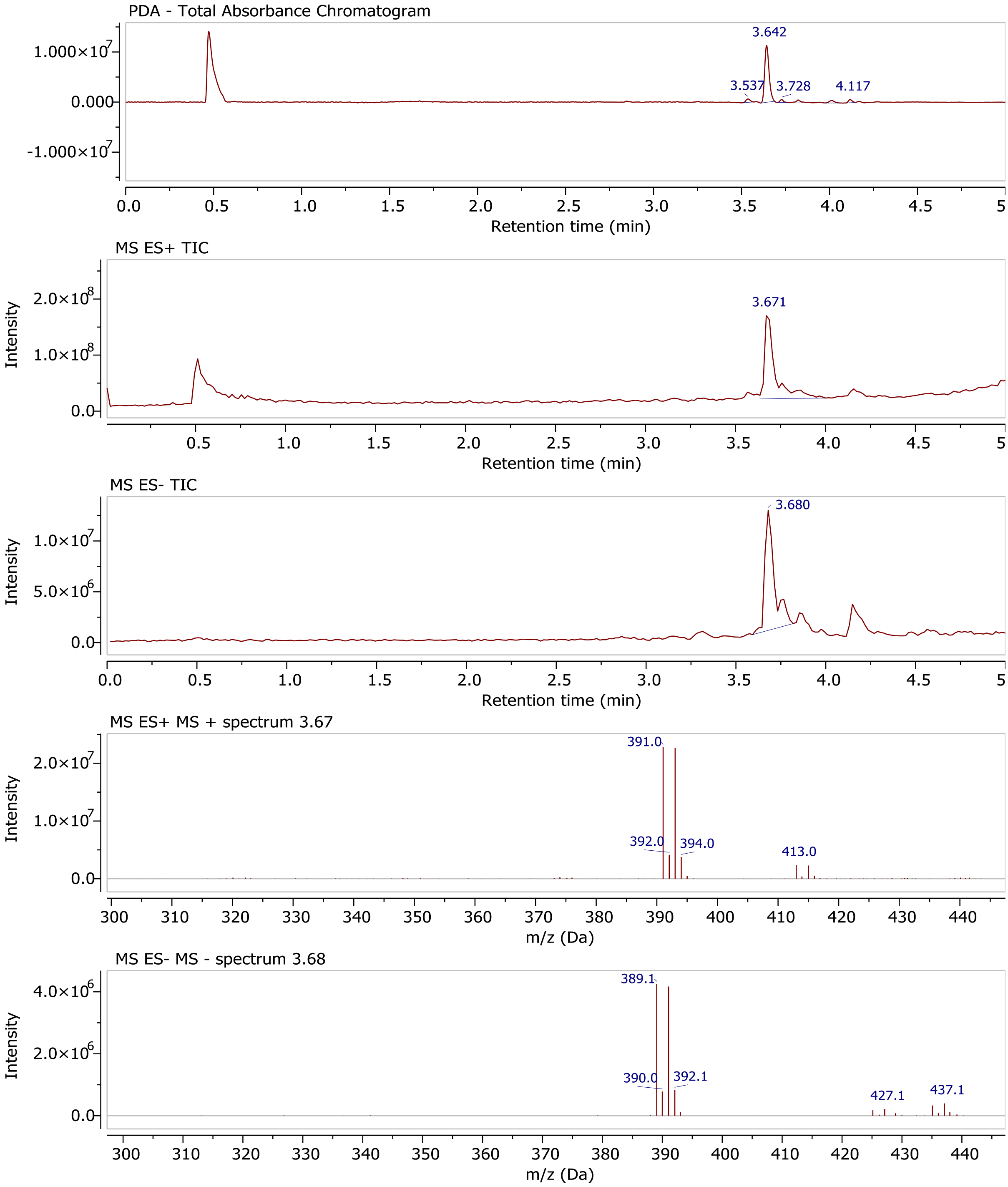

Supplementary figure **1278**. UPLC-MS analysis for compound **PHP-5562**

Chemical Formula: $C_{15}H_{18}F_2N_2O_2$
Exact Mass: 296.1

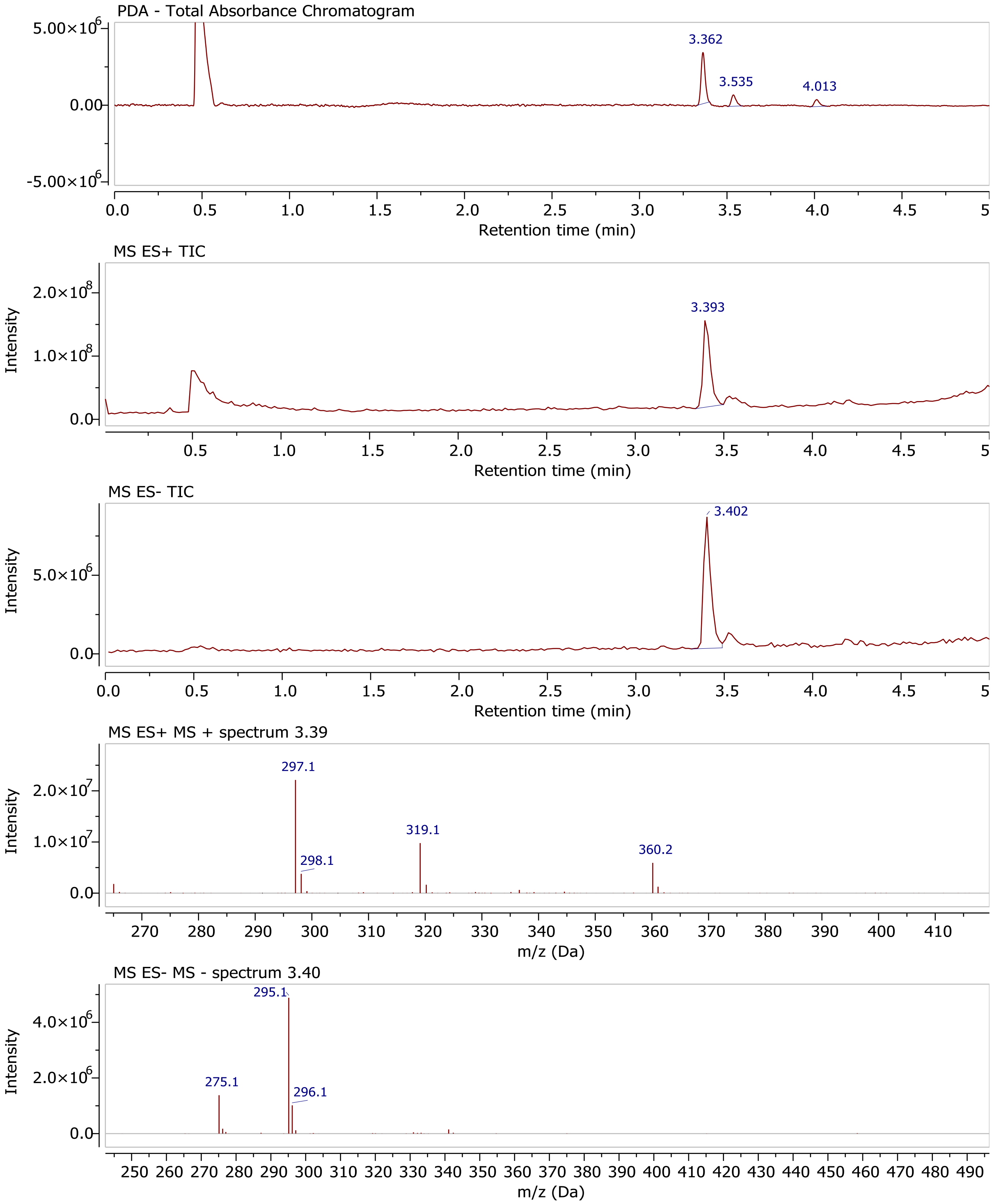

Supplementary figure **1279**. UPLC-MS analysis for compound **PHP-5718**

Chemical Formula: $C_{18}H_{16}BrFN_2O_2$
Exact Mass: 390.0

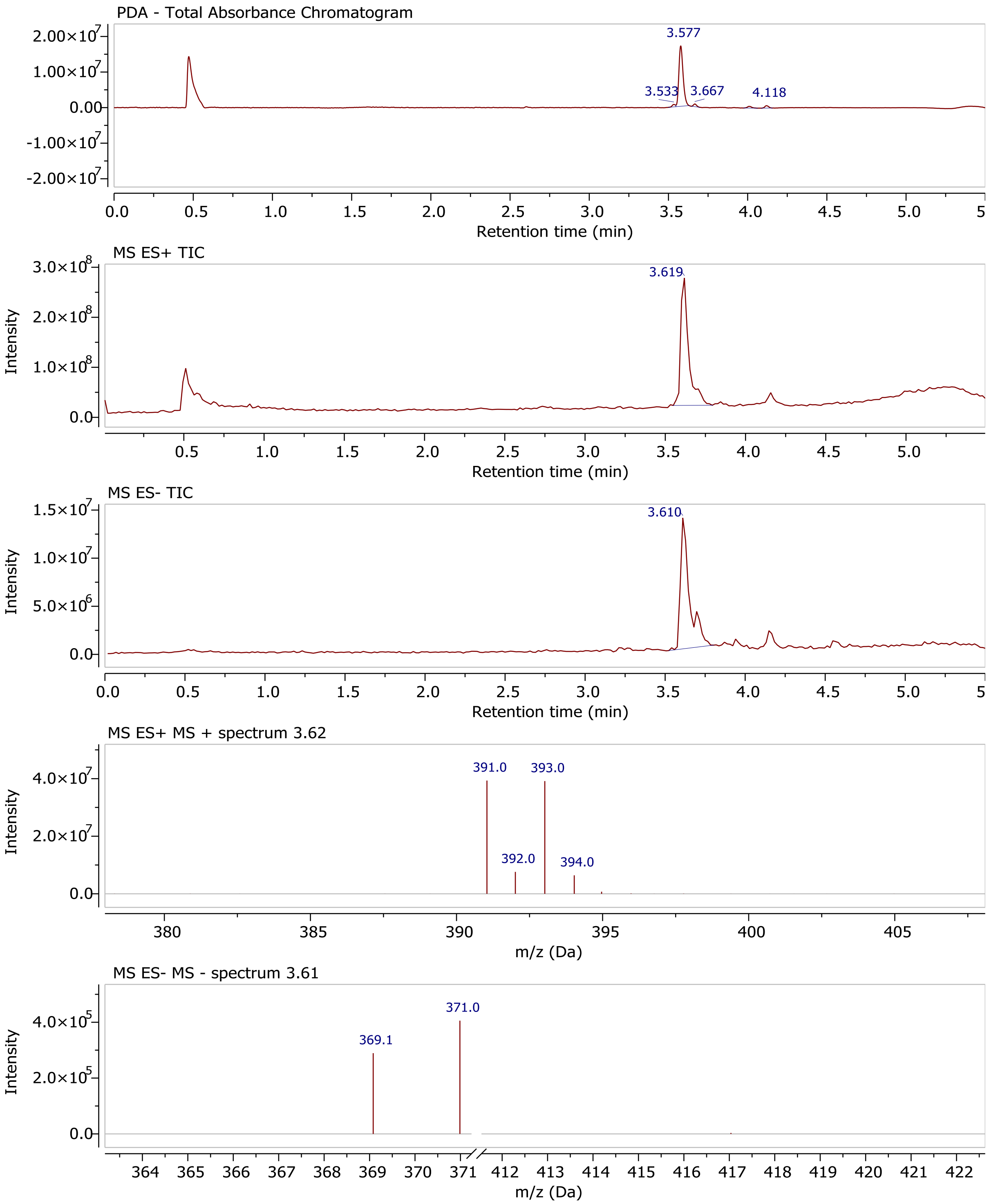

Supplementary figure **1280**. UPLC-MS analysis for compound **PHP-5563**

Chemical Formula: $C_{16}H_{22}N_2O_2$
Exact Mass: 274.2

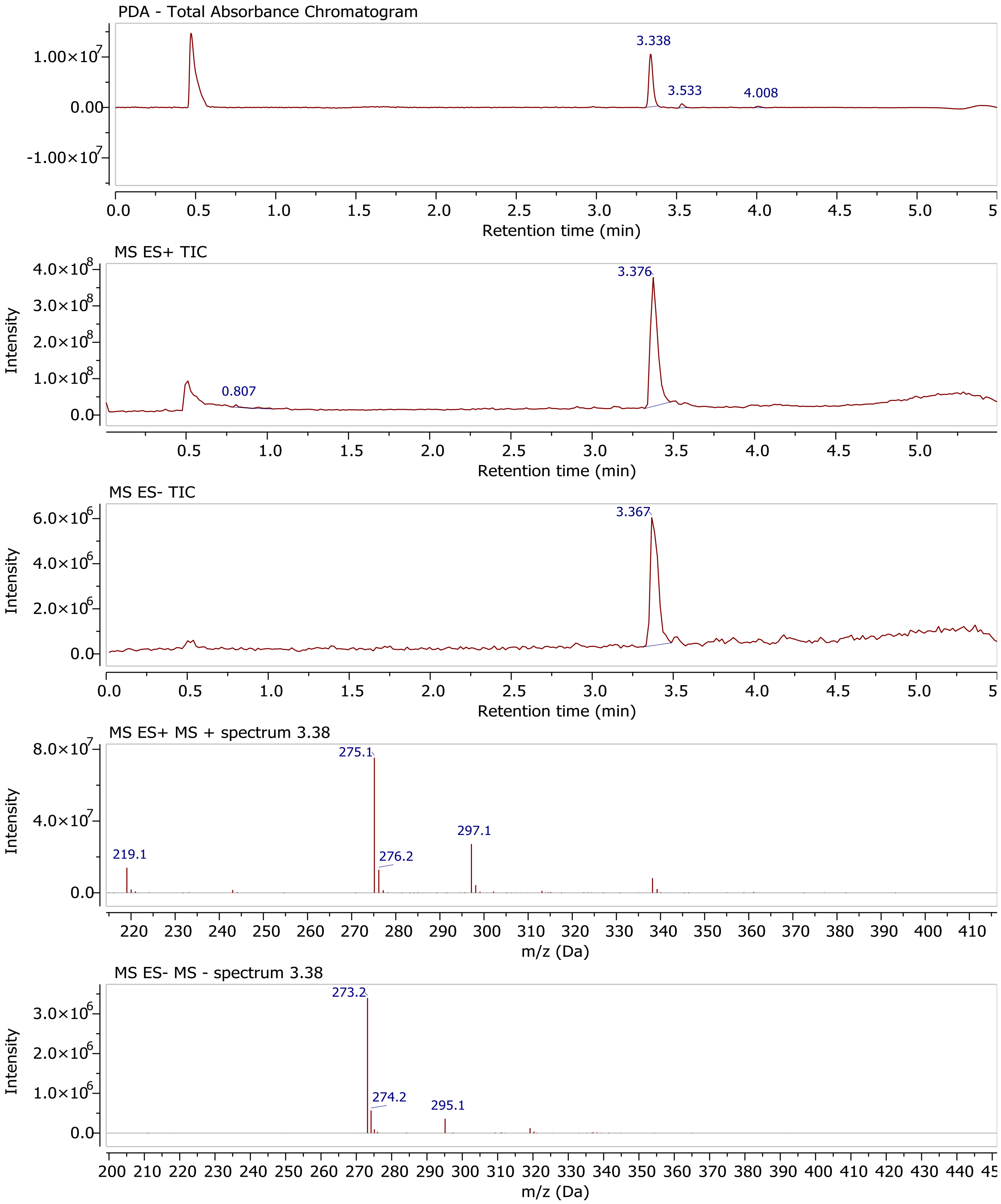

Supplementary figure **1281**. UPLC-MS analysis for compound **PHP-5726**

Chemical Formula: $C_{19}H_{16}BrF_3N_2O_2$
Exact Mass: 440.0

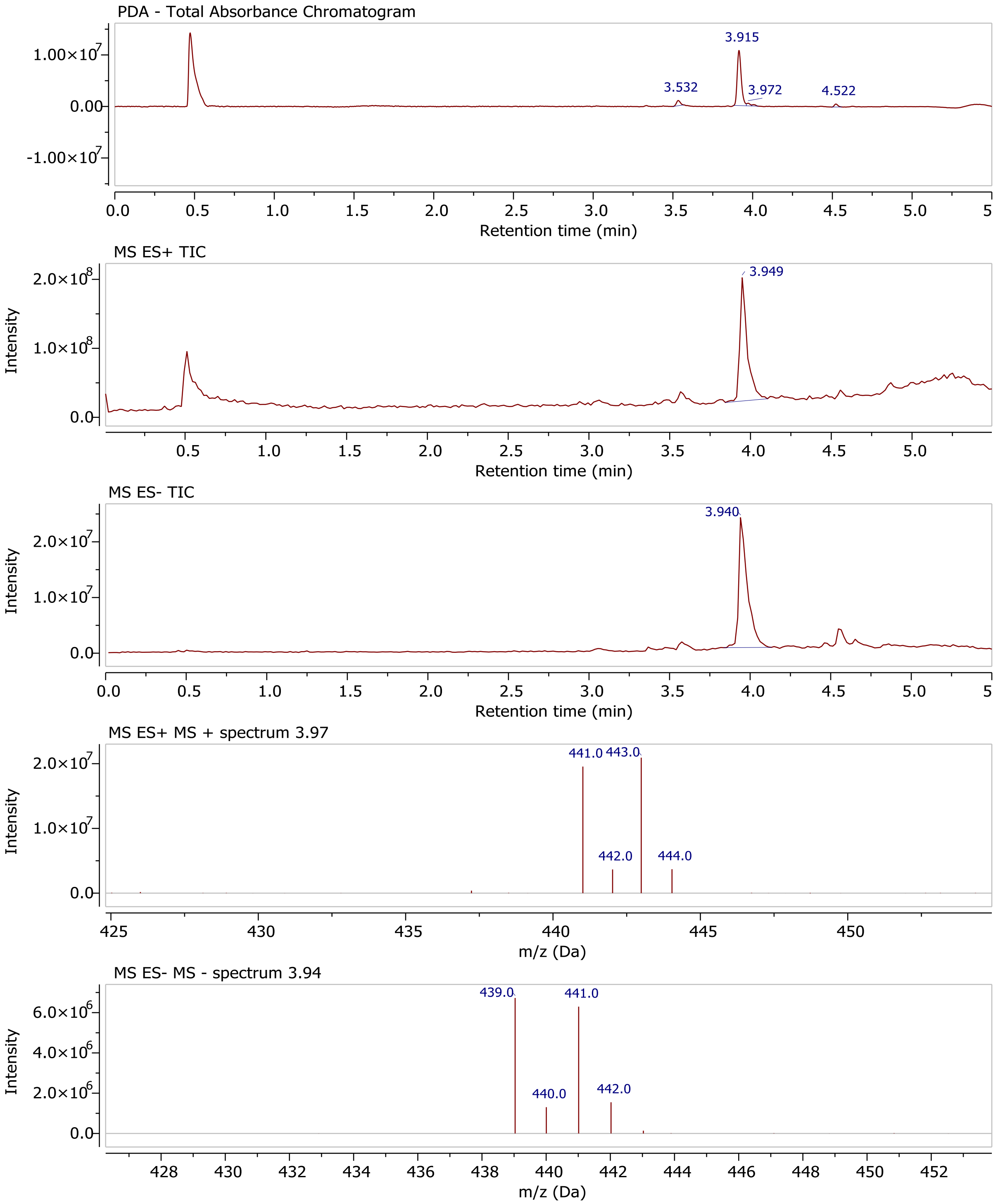

Supplementary figure **1282**. UPLC-MS analysis for compound **PHP-5564**

Chemical Formula: $C_{16}H_{22}N_2O_2$
Exact Mass: 274.2

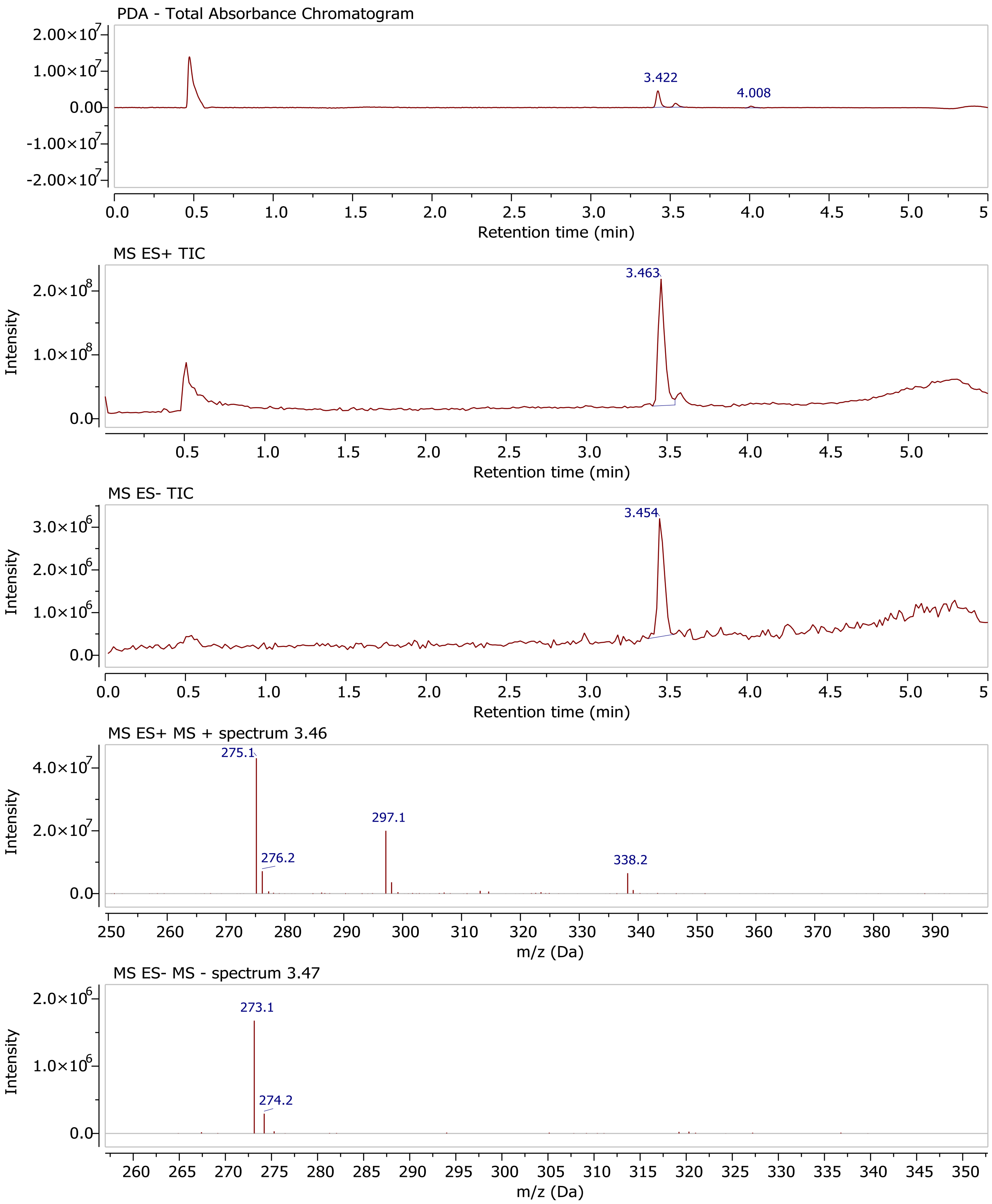

Supplementary figure **1283**. UPLC-MS analysis for compound **PHP-5727**

Chemical Formula: $C_{22}H_{29}N_3O_4$
Exact Mass: 399.2

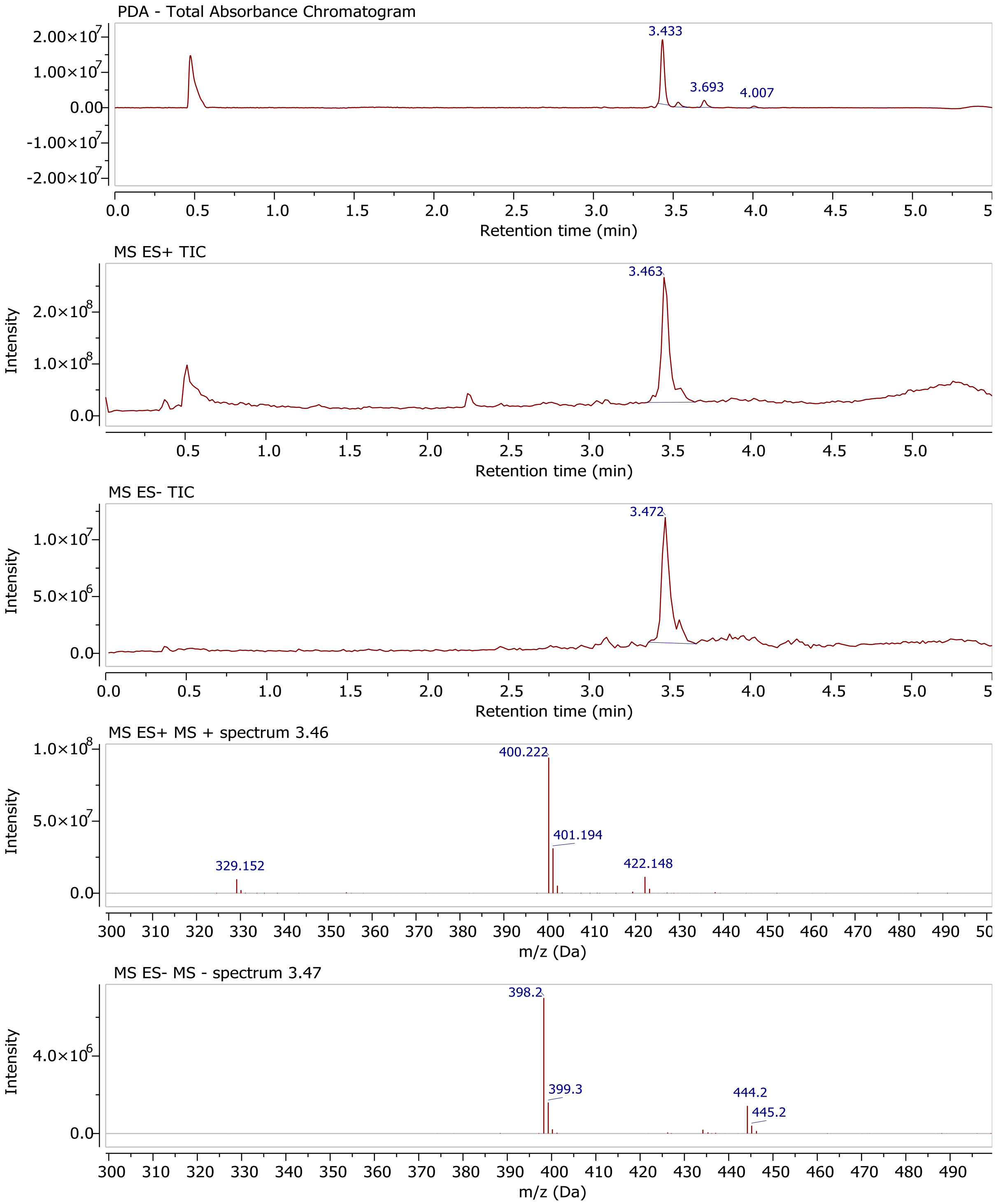

Supplementary figure **1284**. UPLC-MS analysis for compound **PHP-5565**

Chemical Formula: $C_{14}H_{24}N_2O_2$
Exact Mass: 252.2

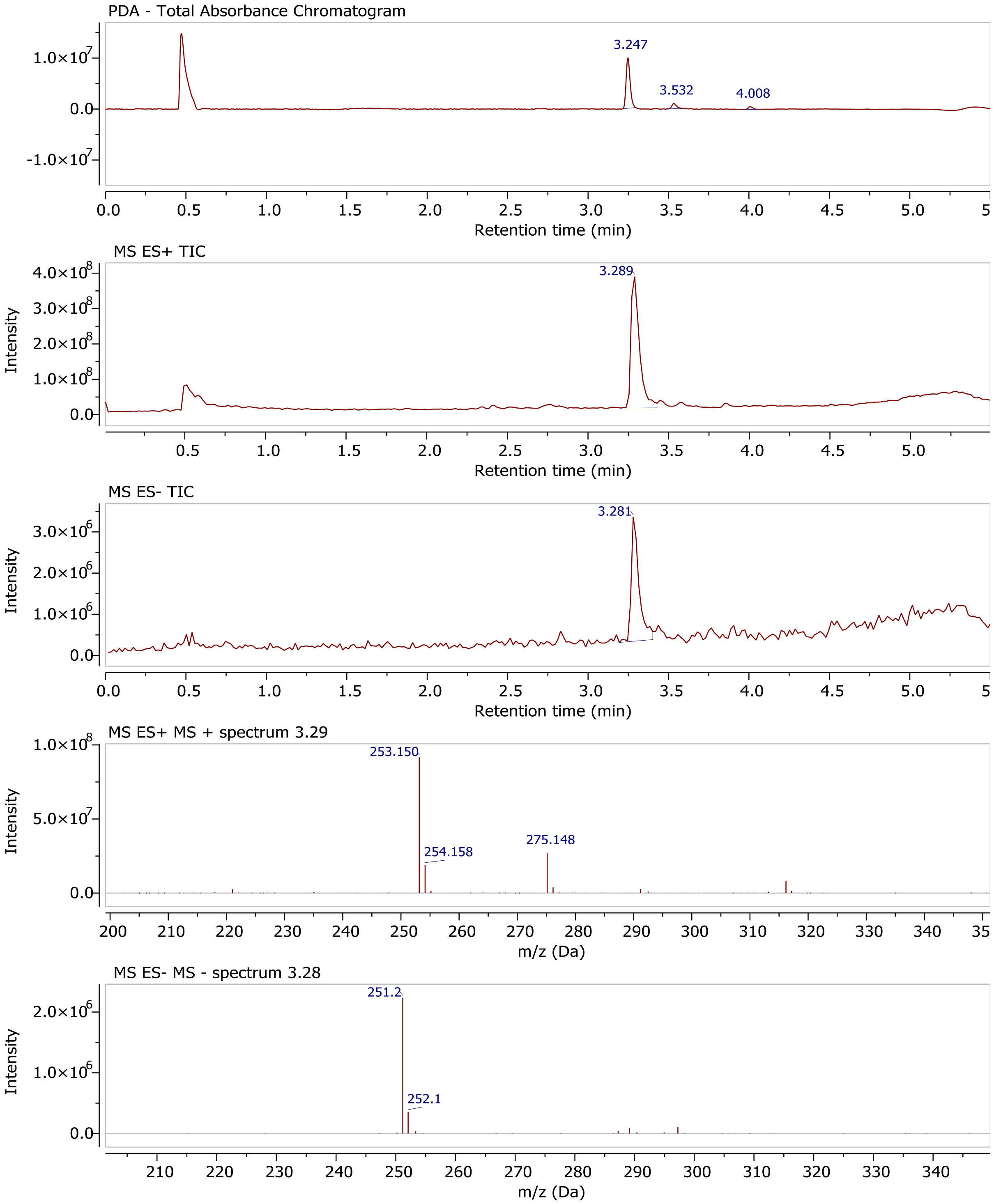

Supplementary figure **1285**. UPLC-MS analysis for compound **PHP-5729**

Chemical Formula: $C_{25}H_{29}N_3O_5$
Exact Mass: 451.2

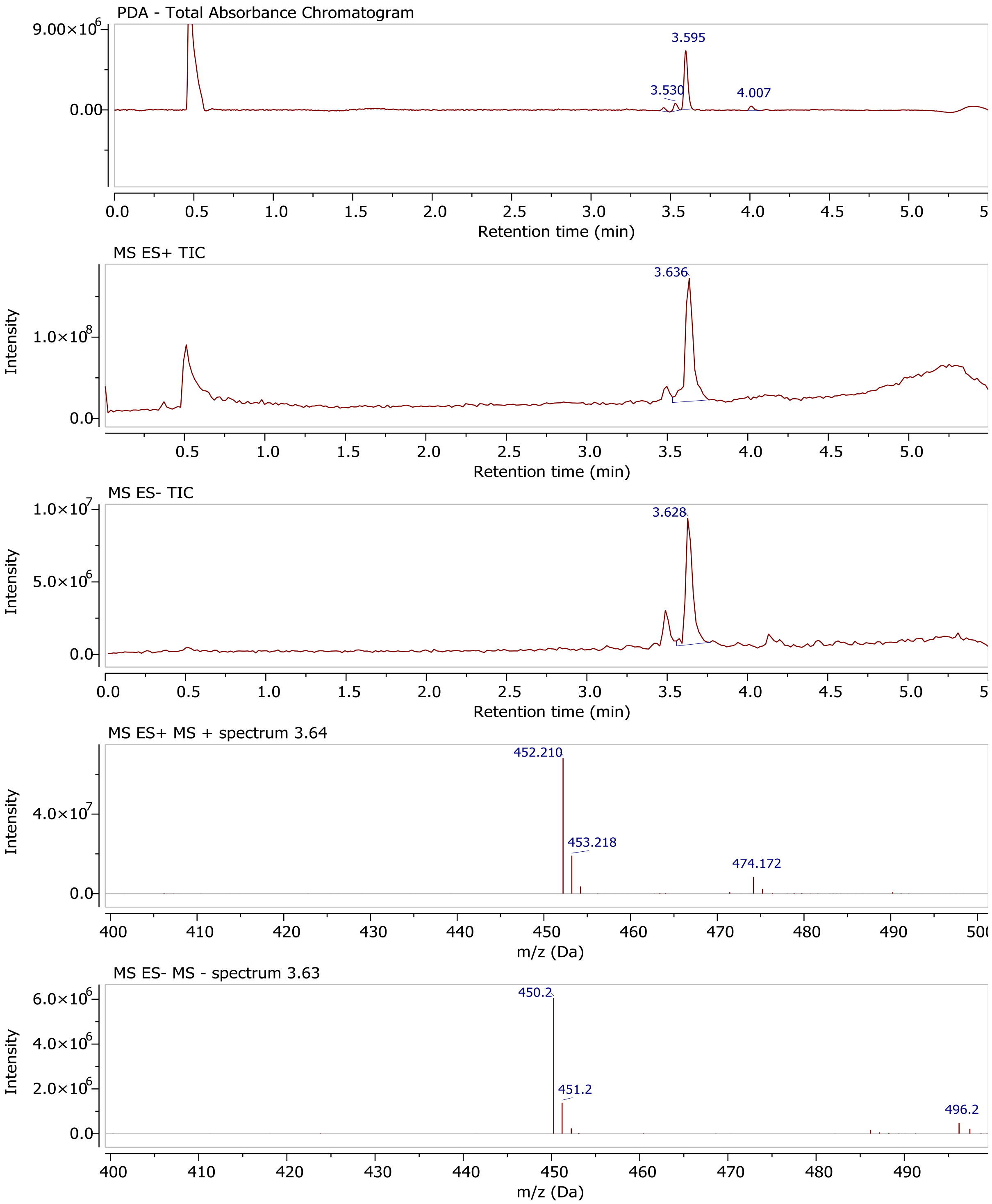

Supplementary figure **1286**. UPLC-MS analysis for compound **PHP-5566**

Chemical Formula: $C_{16}H_{22}N_2O_3$
Exact Mass: 290.2

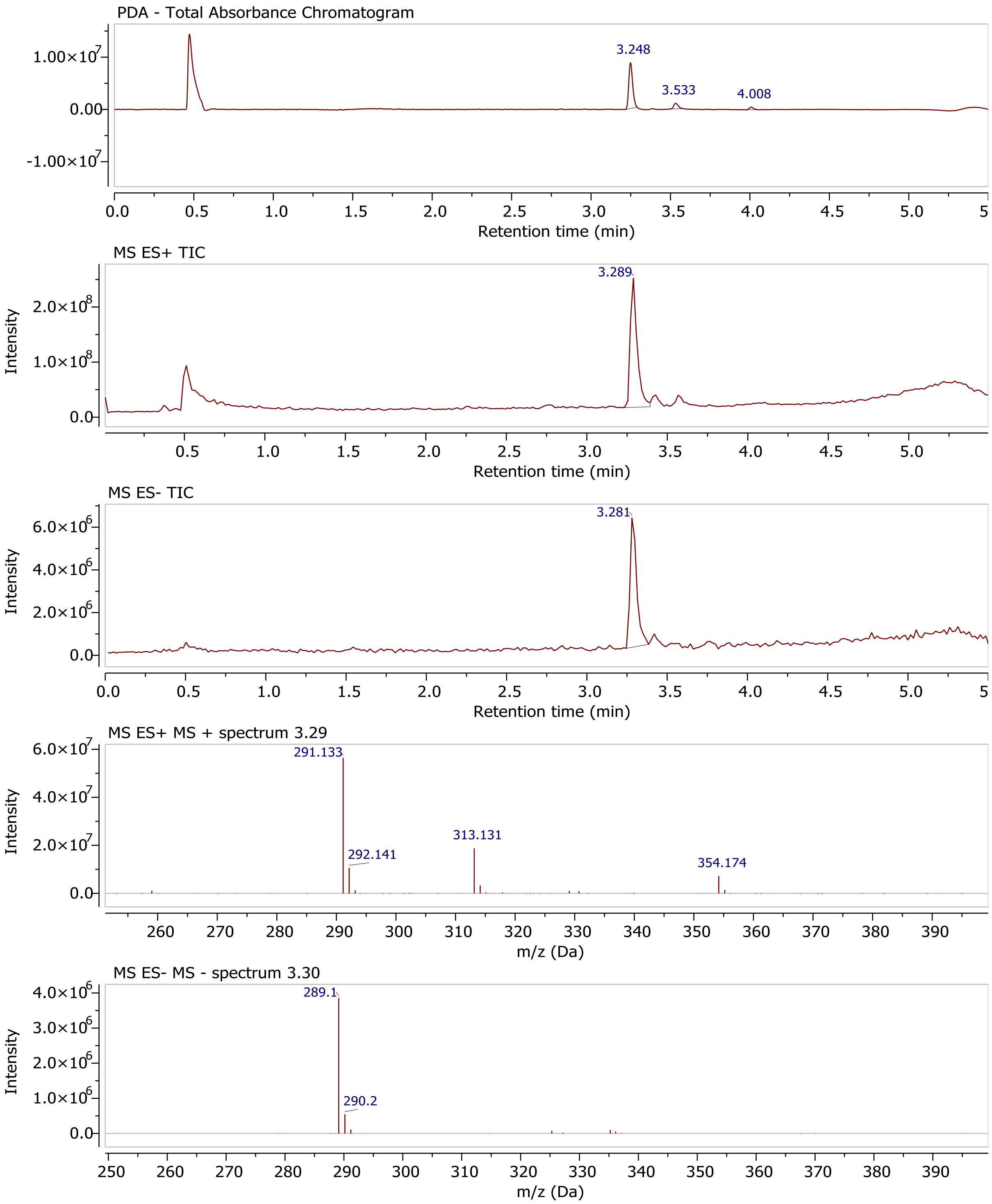

Supplementary figure **1287**. UPLC-MS analysis for compound **PHP-5730**

Chemical Formula: $C_{17}H_{29}N_3O_4$
Exact Mass: 339.2

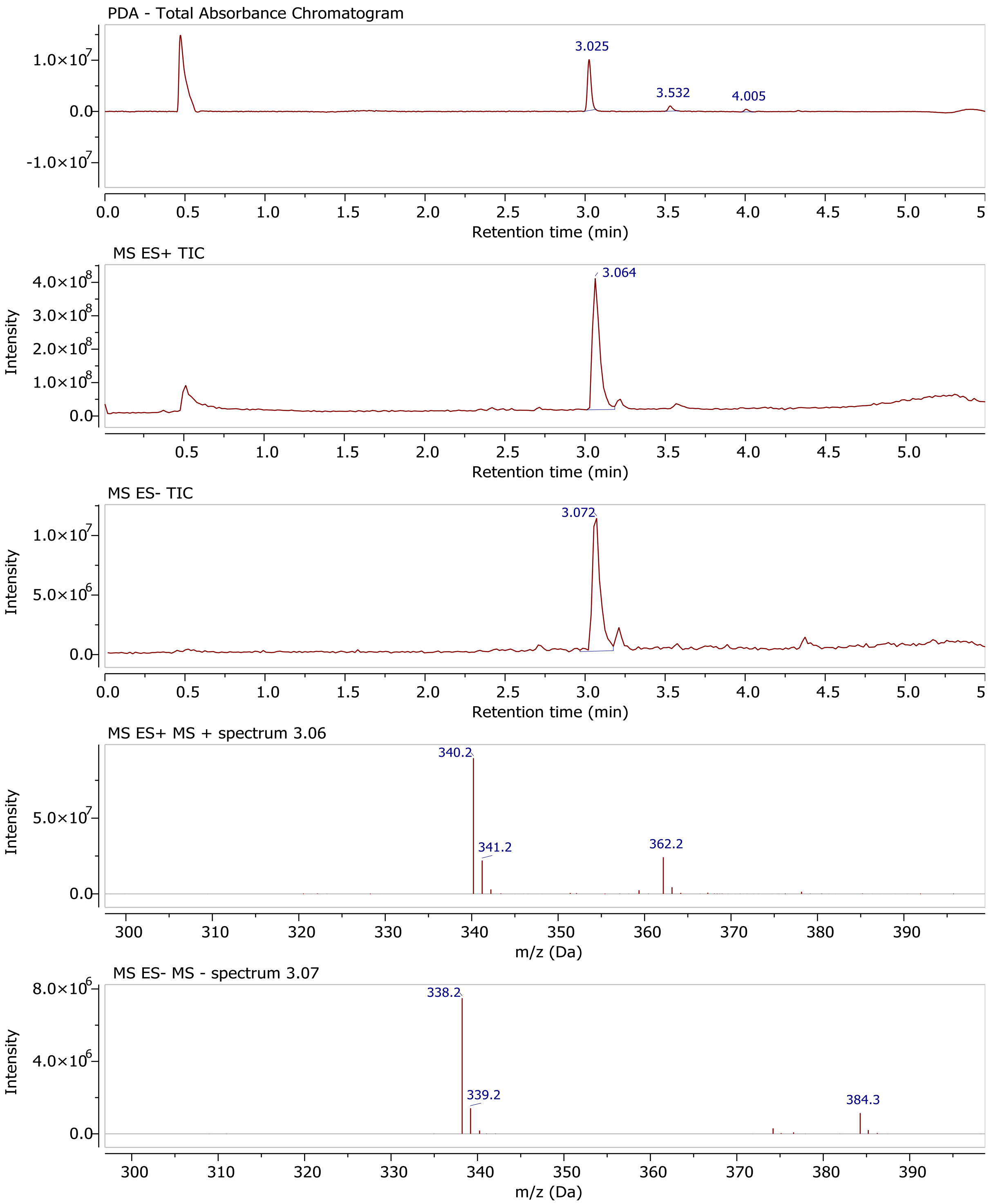

Supplementary figure **1288**. UPLC-MS analysis for compound **PHP-5568**

Chemical Formula: $C_{13}H_{18}N_2O_3$
Exact Mass: 250.1

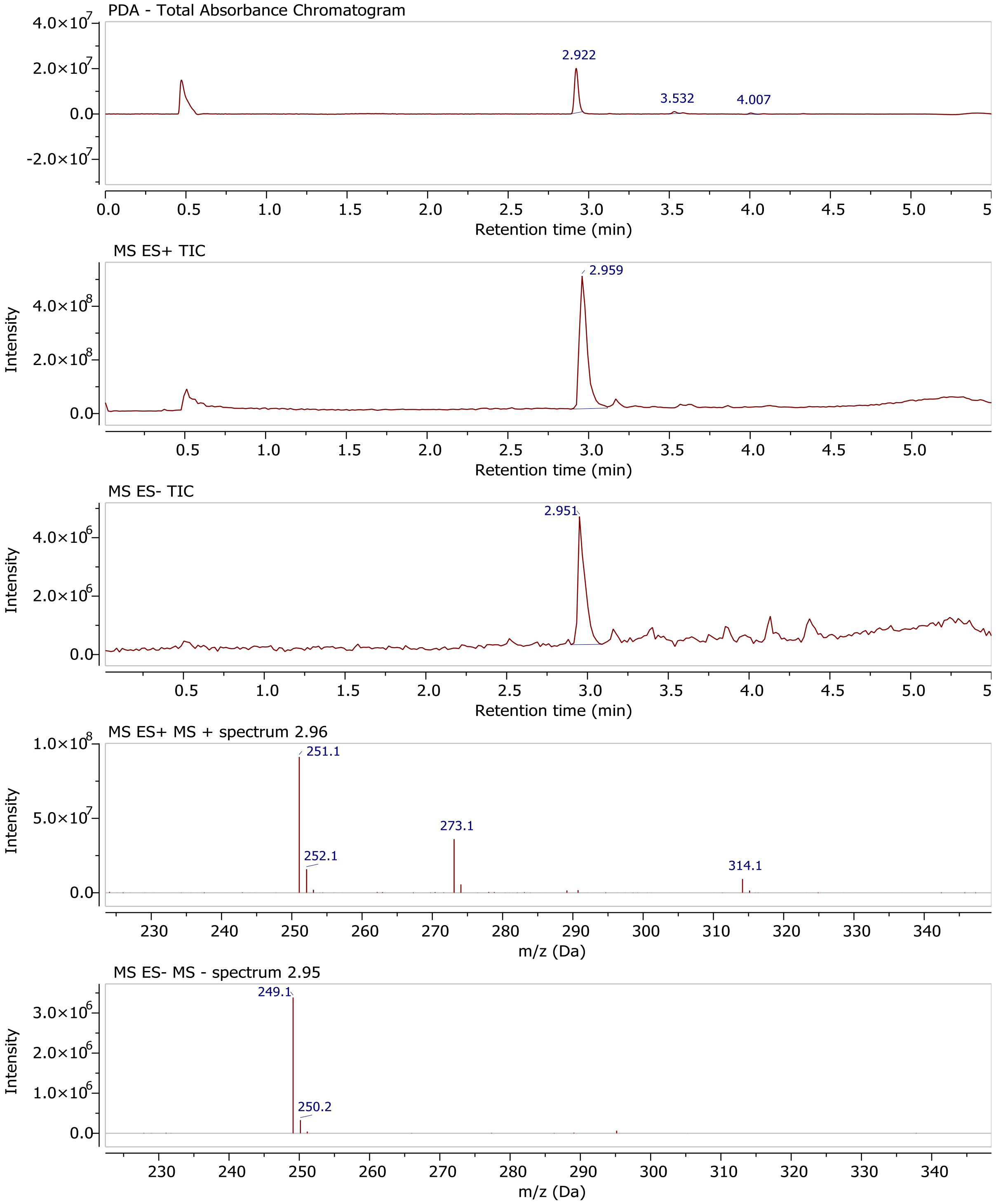

Supplementary figure **1289**. UPLC-MS analysis for compound **PHP-5731**

Chemical Formula: $C_{20}H_{24}F_3N_3O_4$
Exact Mass: 427.2

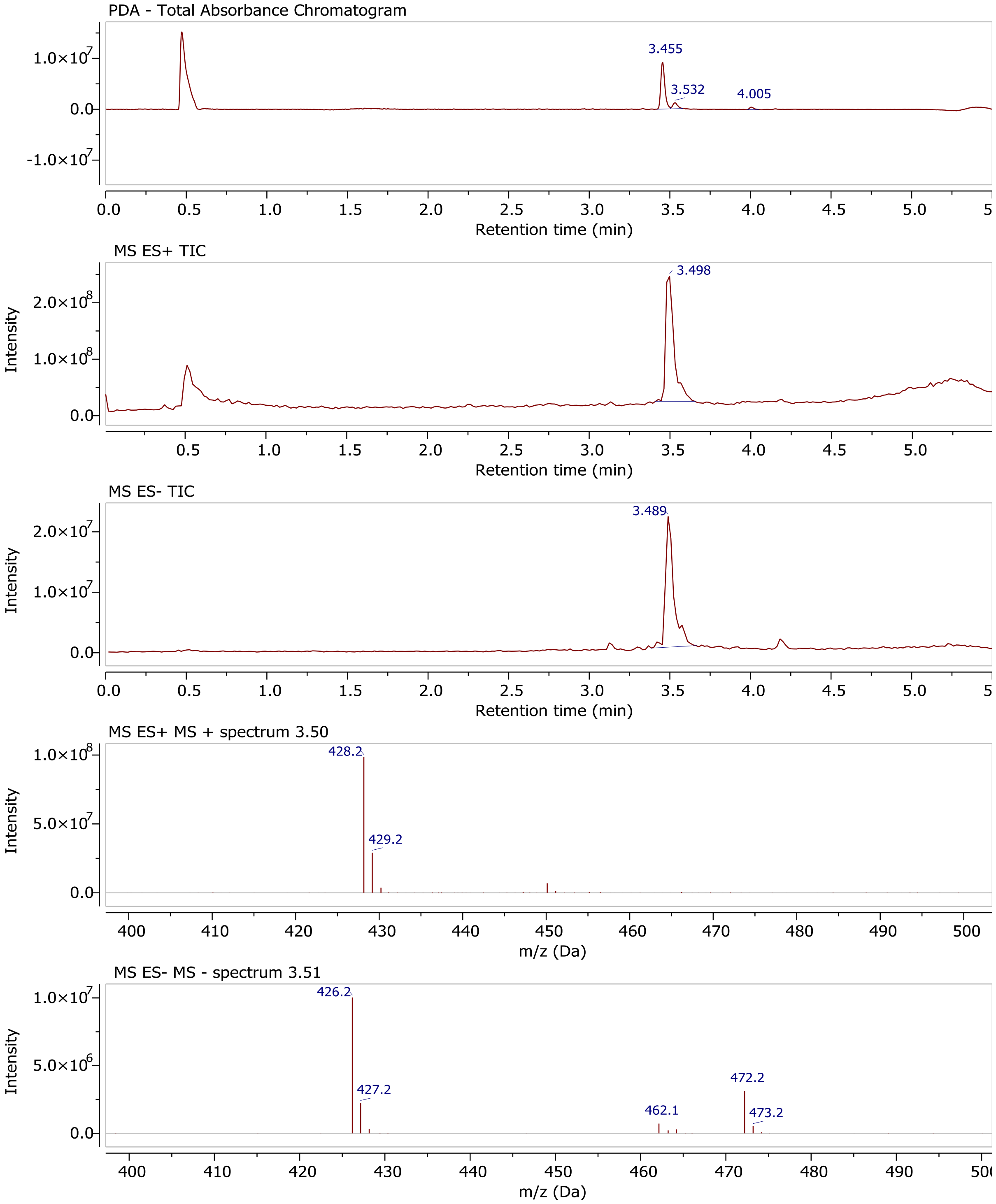

Supplementary figure **1290**. UPLC-MS analysis for compound **PHP-5573**

Chemical Formula: $C_{19}H_{22}N_2O_2$
Exact Mass: 310.2

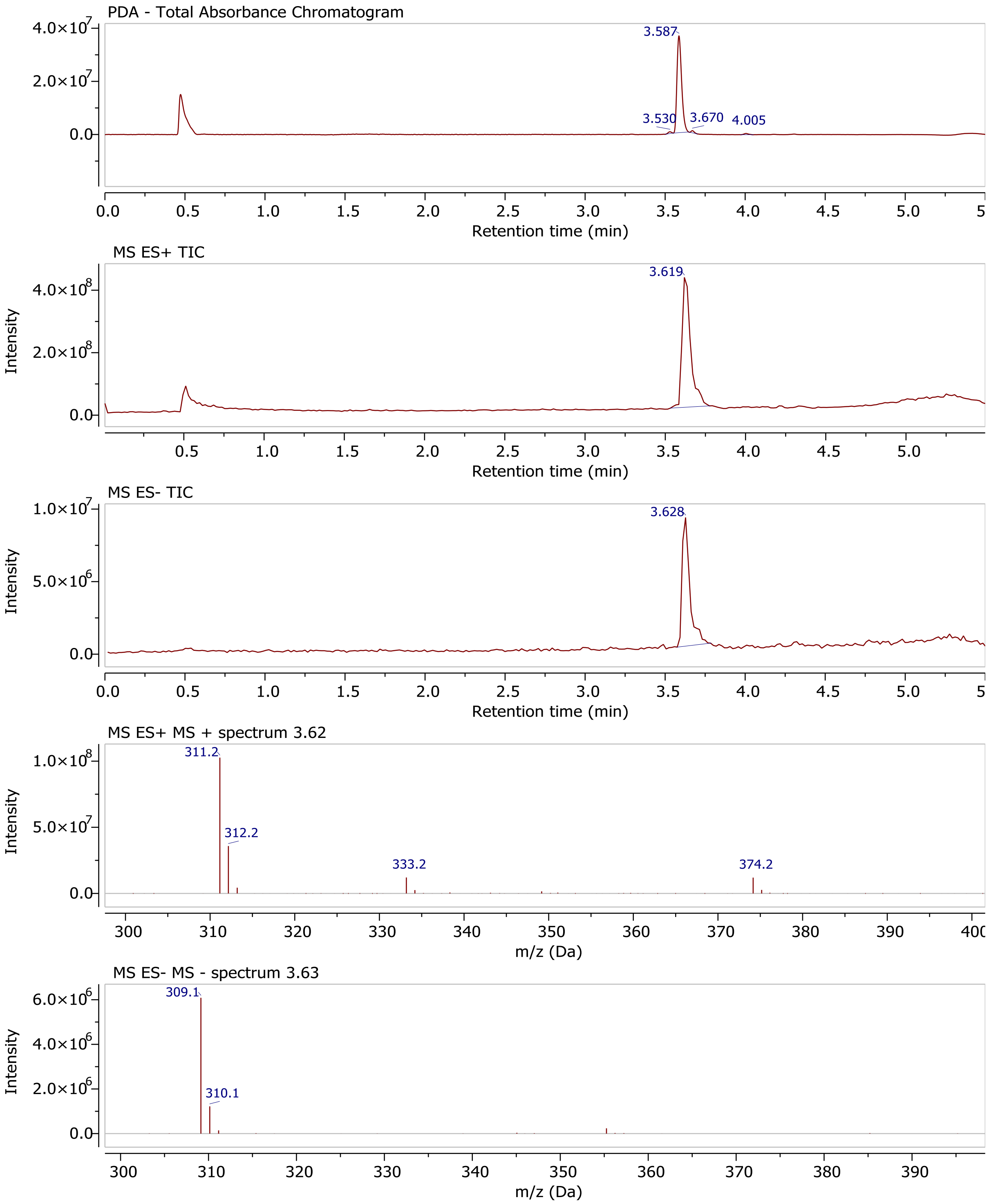

Supplementary figure **1291**. UPLC-MS analysis for compound **PHP-5732**

Chemical Formula: $C_{17}H_{24}N_2O_4$
Exact Mass: 320.2

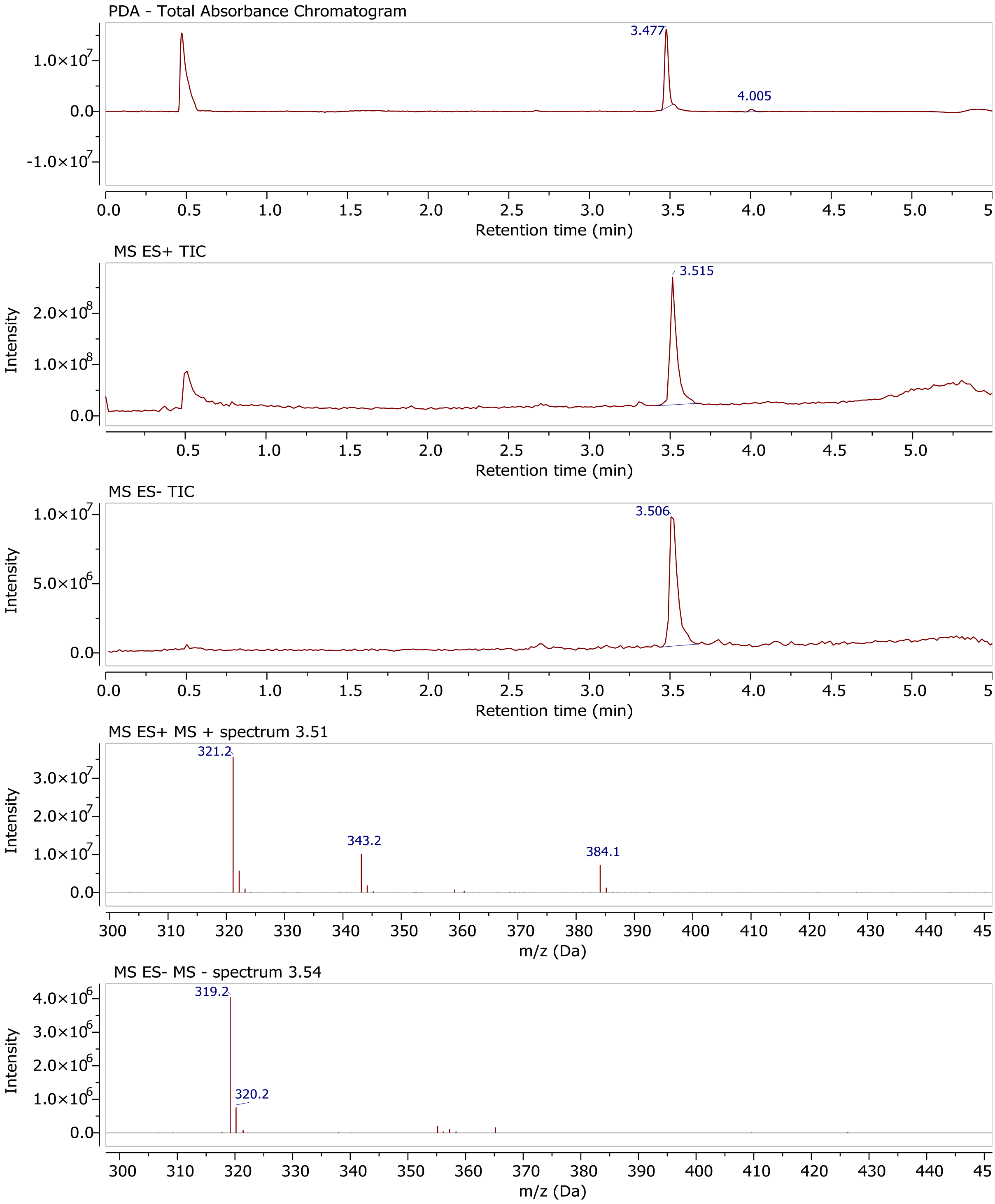

Supplementary figure **1292**. UPLC-MS analysis for compound **PHP-5582**

Chemical Formula: $C_{26}H_{26}N_2O_3$
Exact Mass: 414.2

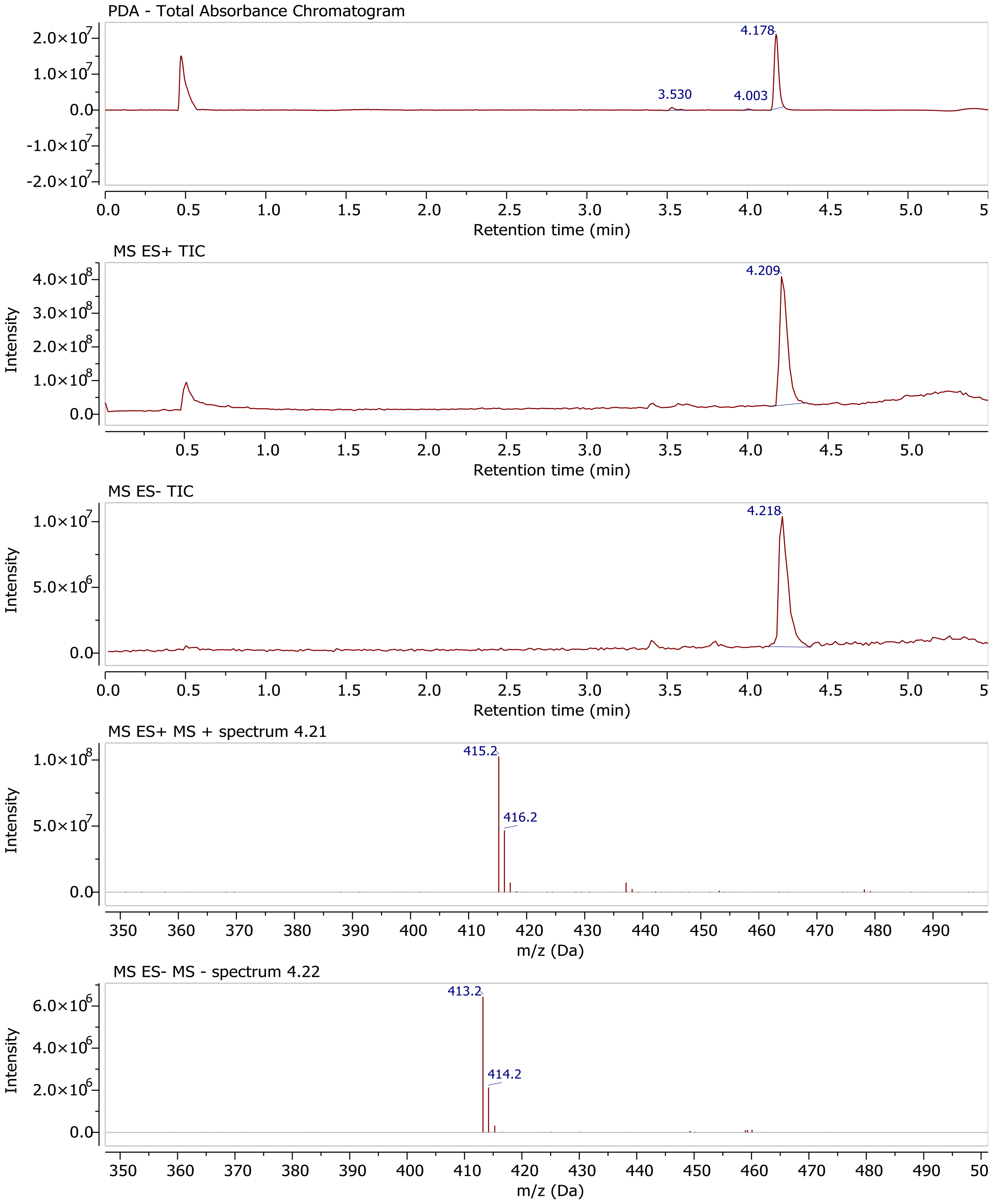

Supplementary figure **1293**. UPLC-MS analysis for compound **PHP-5734**

Chemical Formula: $C_{19}H_{17}F_3N_2O_4$
Exact Mass: 394.1

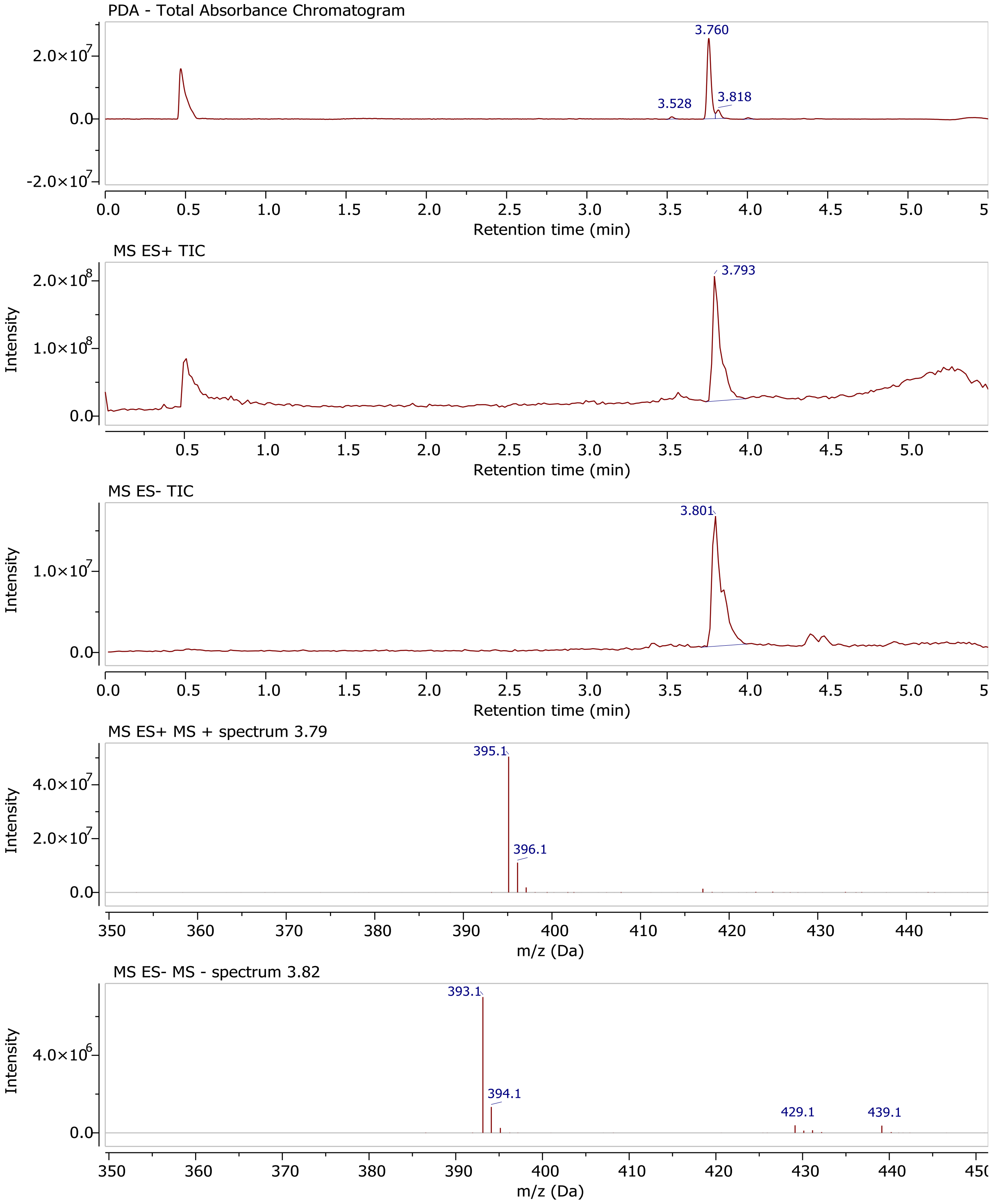

Supplementary figure **1294**. UPLC-MS analysis for compound **PHP-5583**

Chemical Formula: $C_{17}H_{24}N_2O_2$
Exact Mass: 288.2

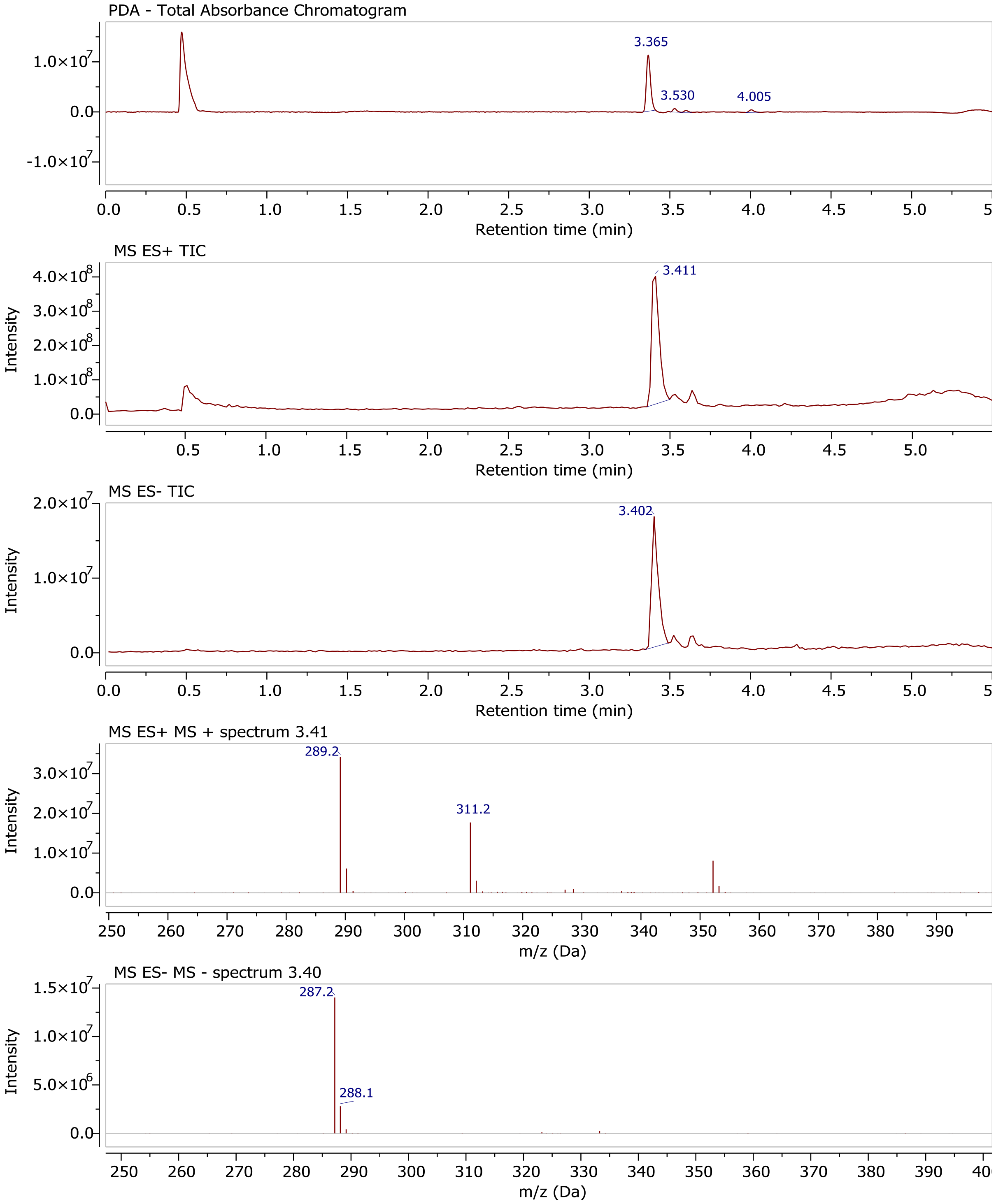

Supplementary figure **1295**. UPLC-MS analysis for compound **PHP-5735**

Chemical Formula: $C_{17}H_{22}N_2O_4$
Exact Mass: 318.2

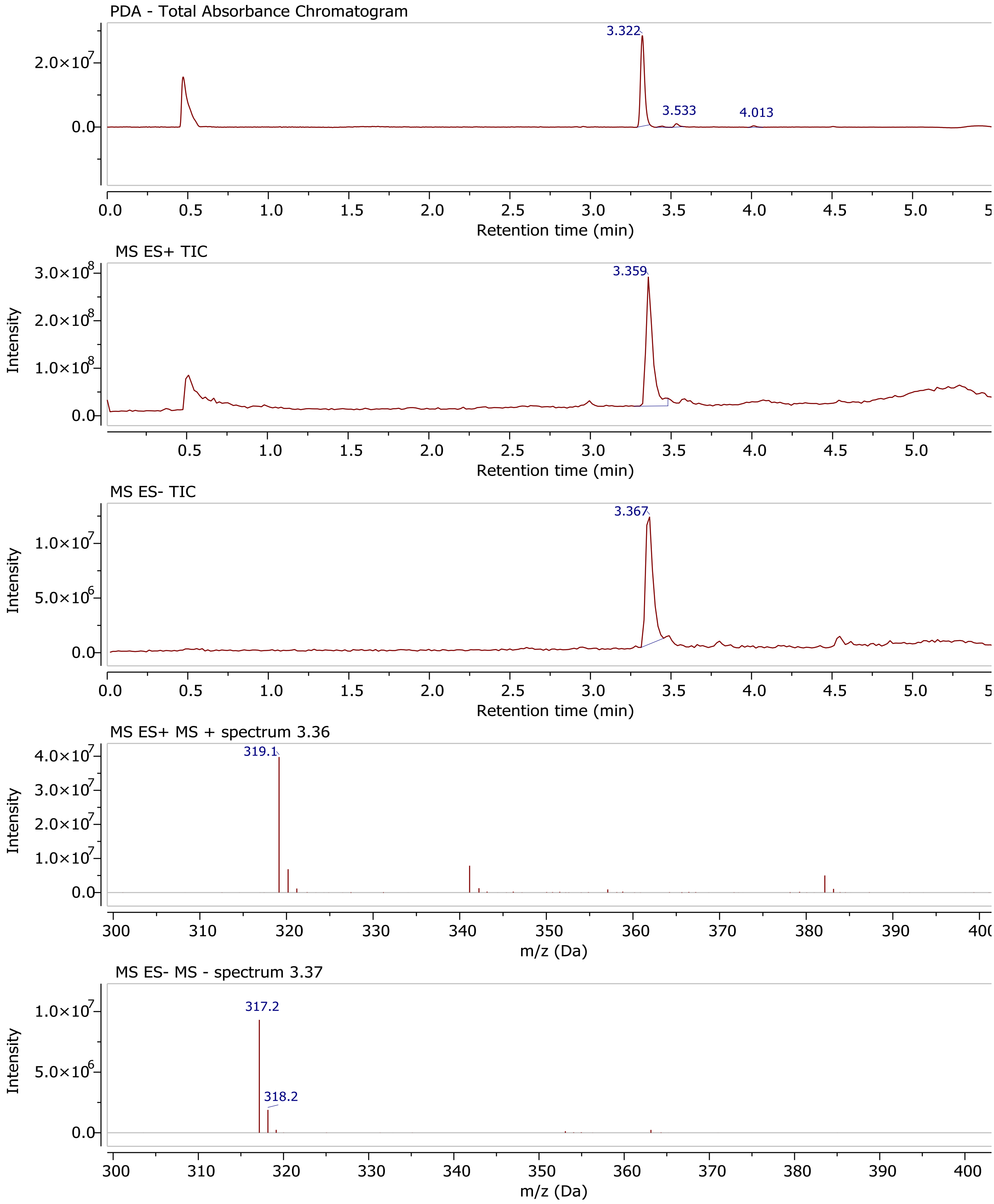

Supplementary figure **1296**. UPLC-MS analysis for compound **PHP-5589**

Chemical Formula: $C_{18}H_{26}N_2O_2$
Exact Mass: 302.2

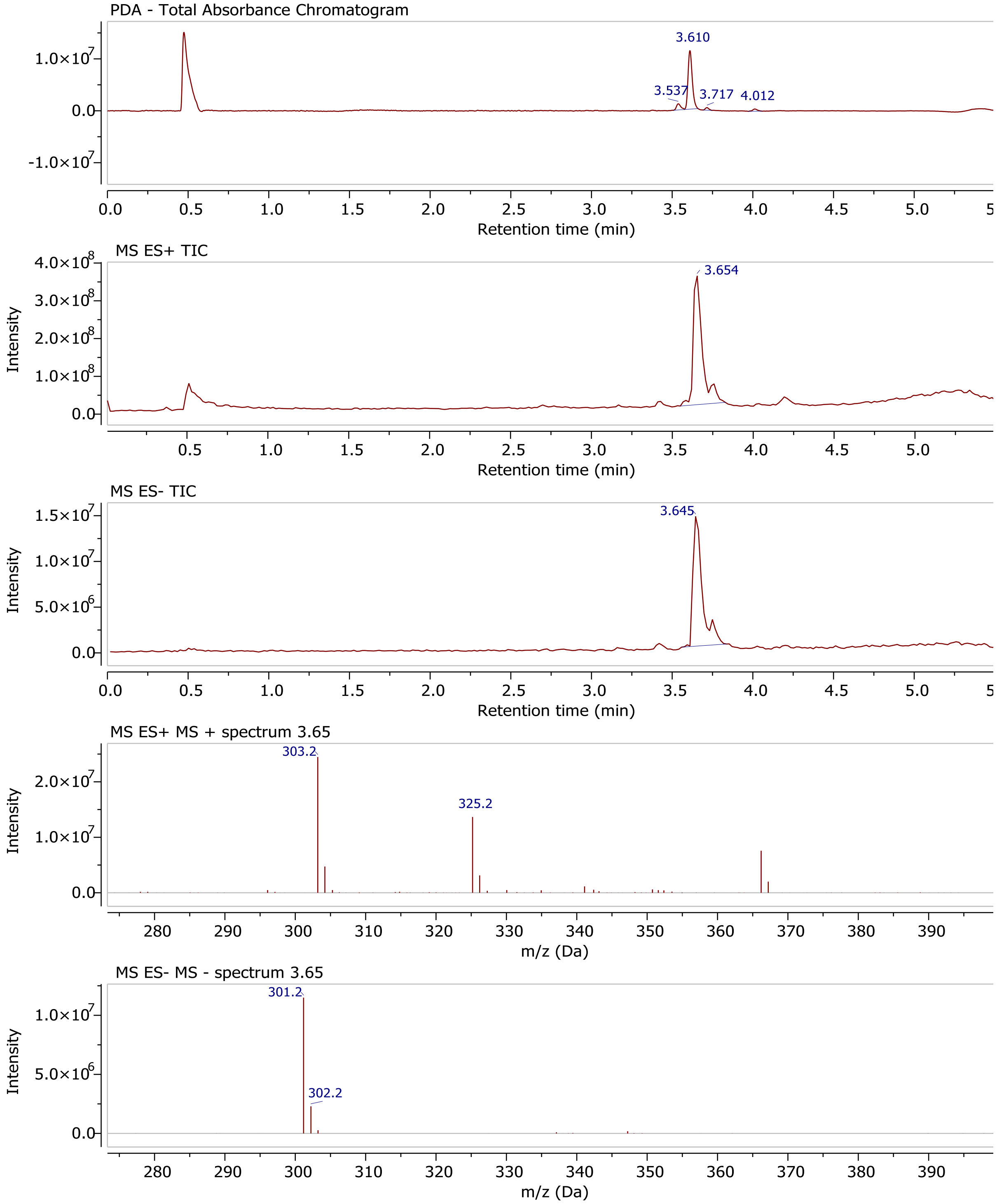

Supplementary figure **1297**. UPLC-MS analysis for compound **PHP-5736**

Chemical Formula: $C_{19}H_{18}F_2N_2O_4$
Exact Mass: 376.1

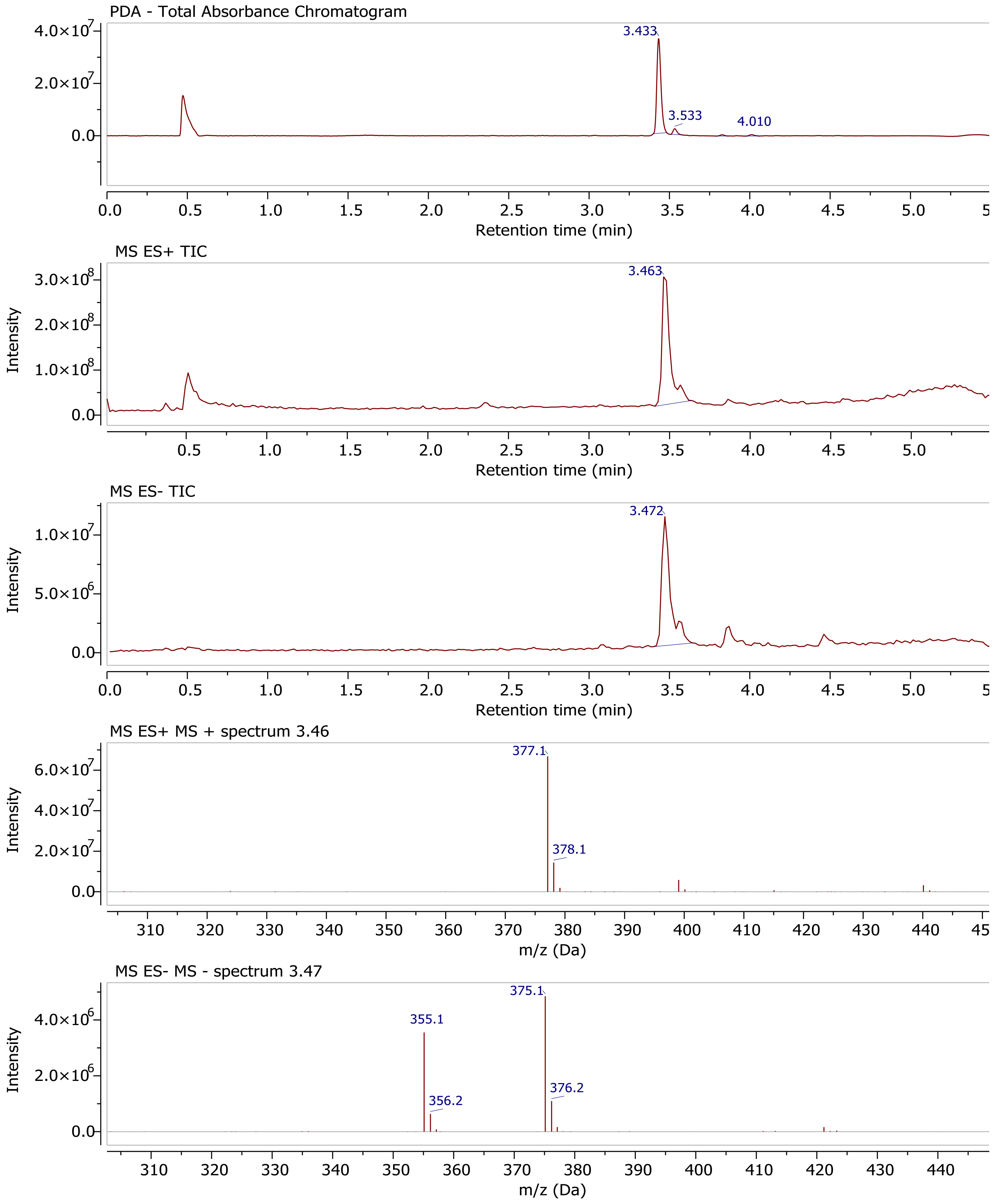

Supplementary figure **1298**. UPLC-MS analysis for compound **PHP-5590**

Chemical Formula: $C_{17}H_{24}N_2O_2$
Exact Mass: 288.2

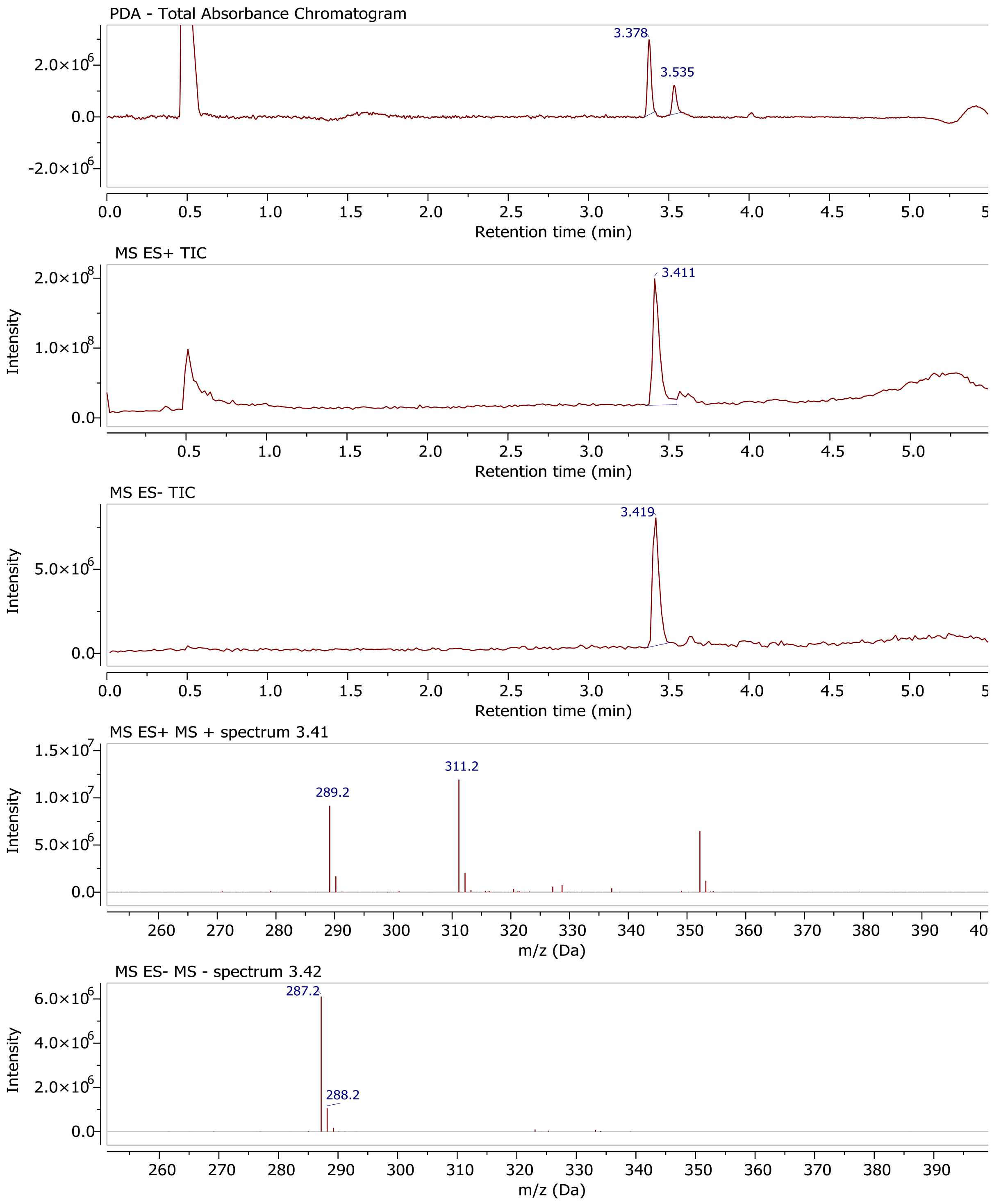

Supplementary figure **1299**. UPLC-MS analysis for compound **PHP-5738**

Chemical Formula: $C_{34}H_{34}N_2O_6$
Exact Mass: 566.2

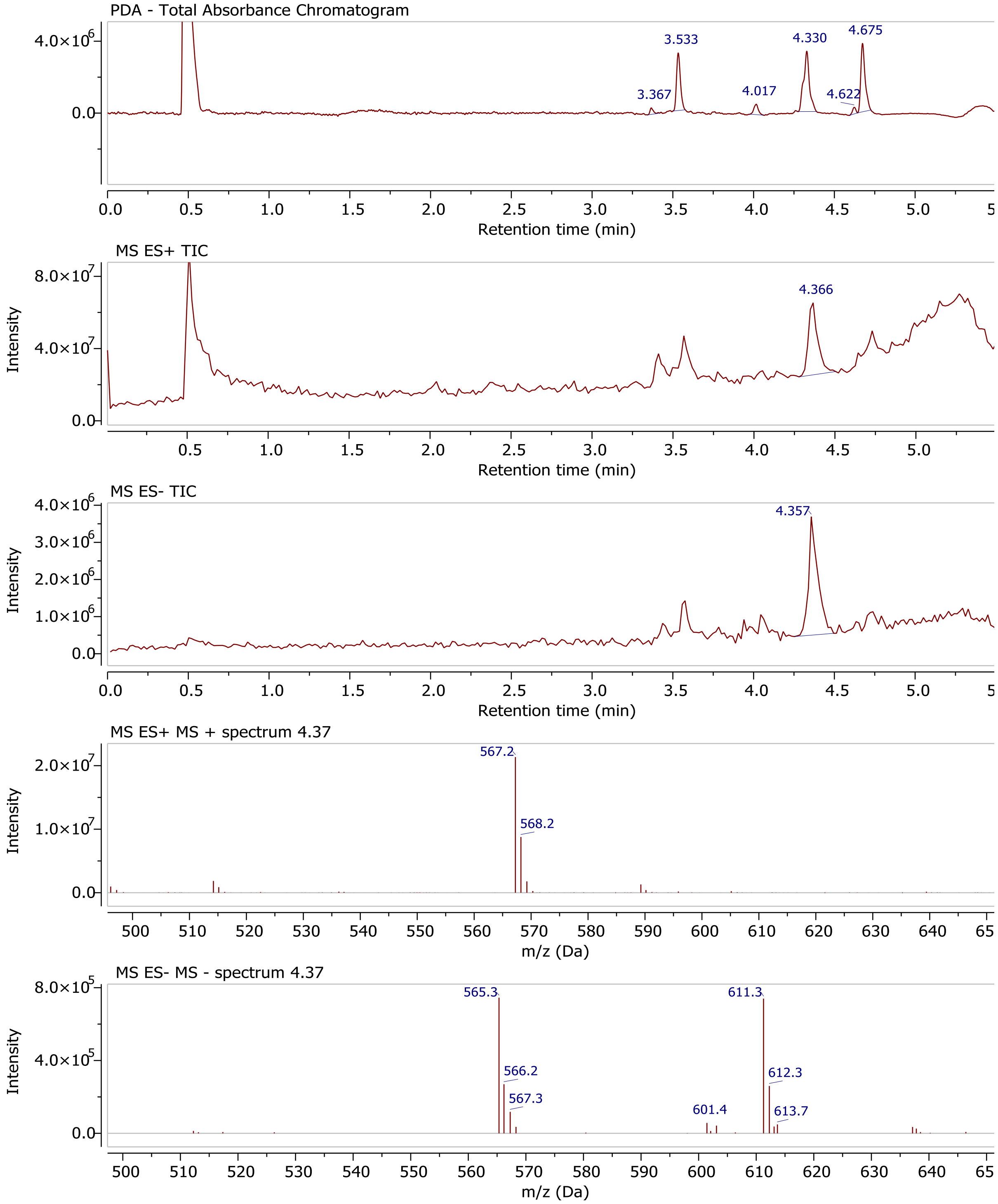

Supplementary figure **1300**. UPLC-MS analysis for compound **PHP-4815**

Chemical Formula: $C_{19}H_{18}F_2N_2O_4$
Exact Mass: 376.1

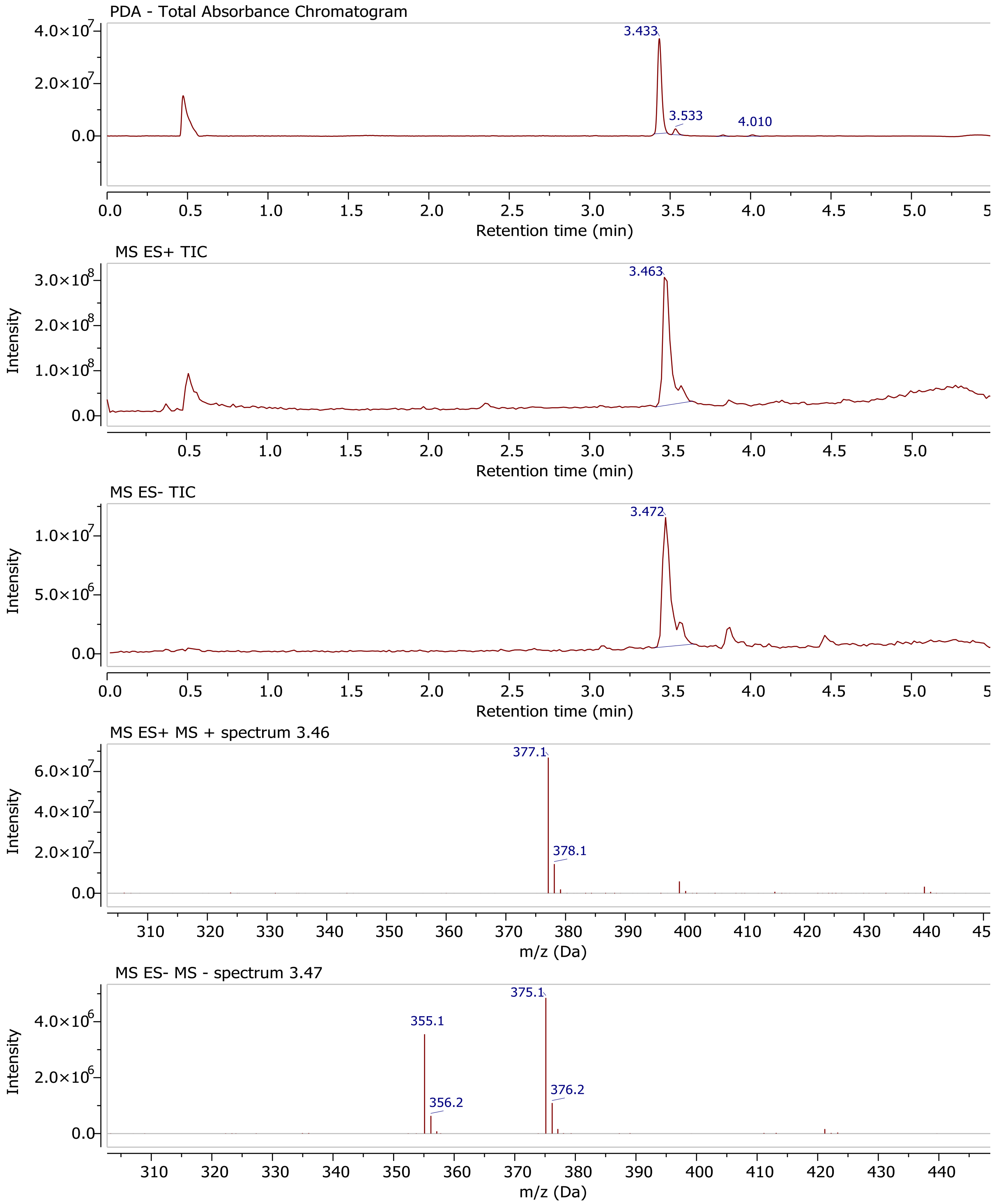

Supplementary figure **1301**. UPLC-MS analysis for compound **PHP-5414**

Chemical Formula: $C_{20}H_{21}ClN_2O_4$
Exact Mass: 388.1

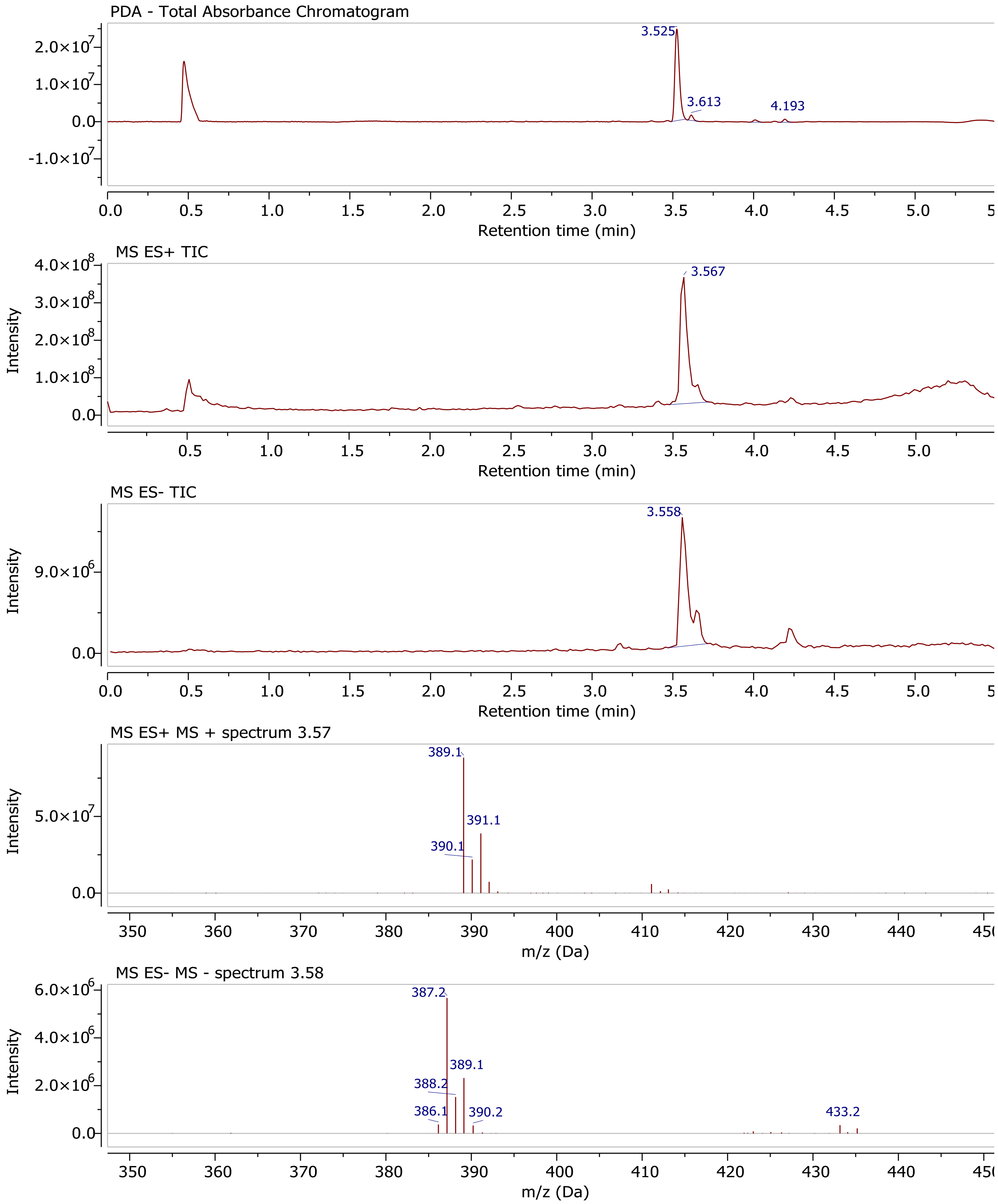

Supplementary figure **1302**. UPLC-MS analysis for compound **PHP-4816**

Chemical Formula: $C_{20}H_{18}ClFN_2O_2$
Exact Mass: 372.1

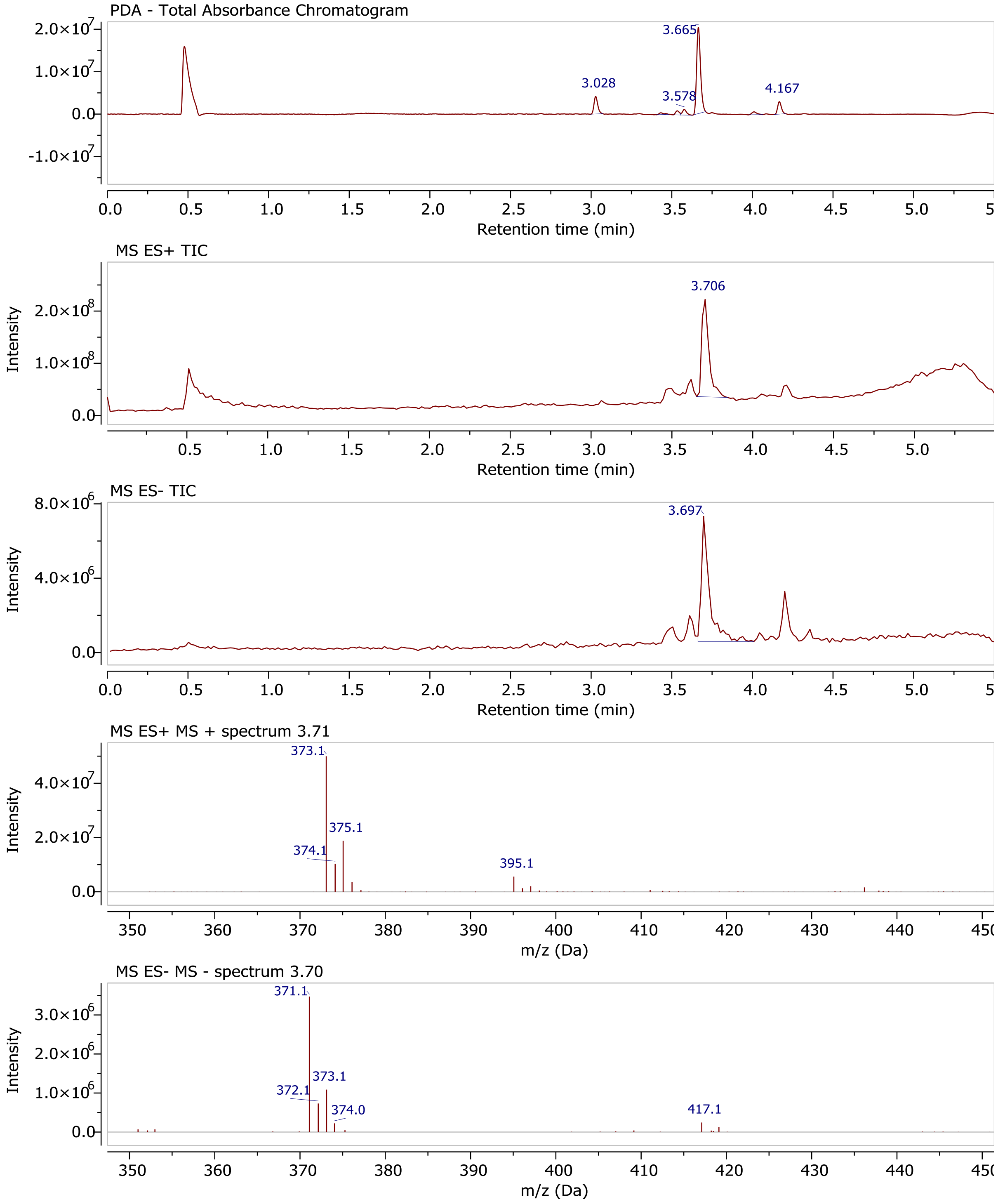

Supplementary figure **1303**. UPLC-MS analysis for compound **PHP-5415**

Chemical Formula: $C_{26}H_{26}N_2O_5$
Exact Mass: 446.2

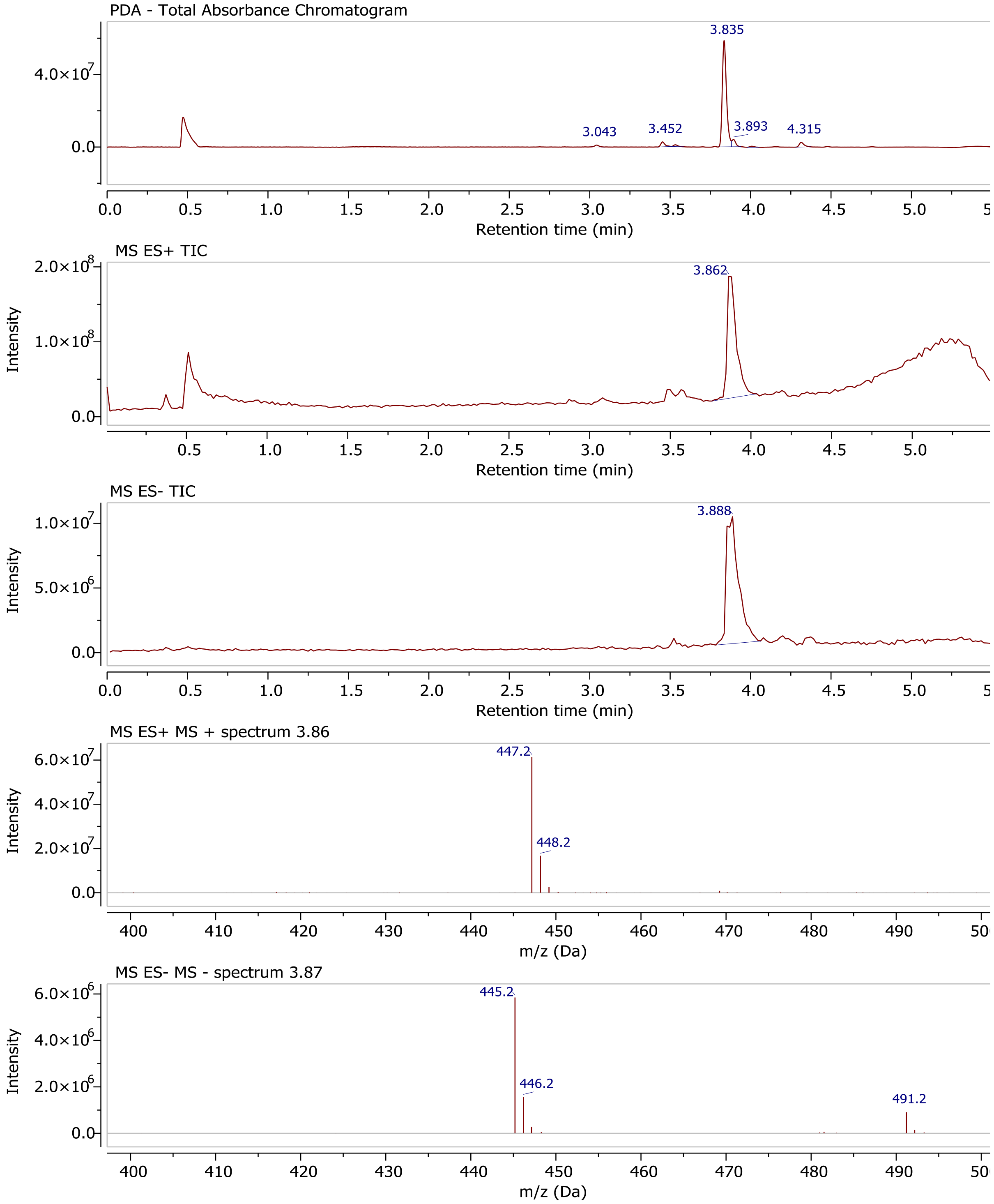

Supplementary figure **1304**. UPLC-MS analysis for compound **PHP-4817**

Chemical Formula: $C_{13}H_{13}Cl_2FN_2O_2$
Exact Mass: 318.0

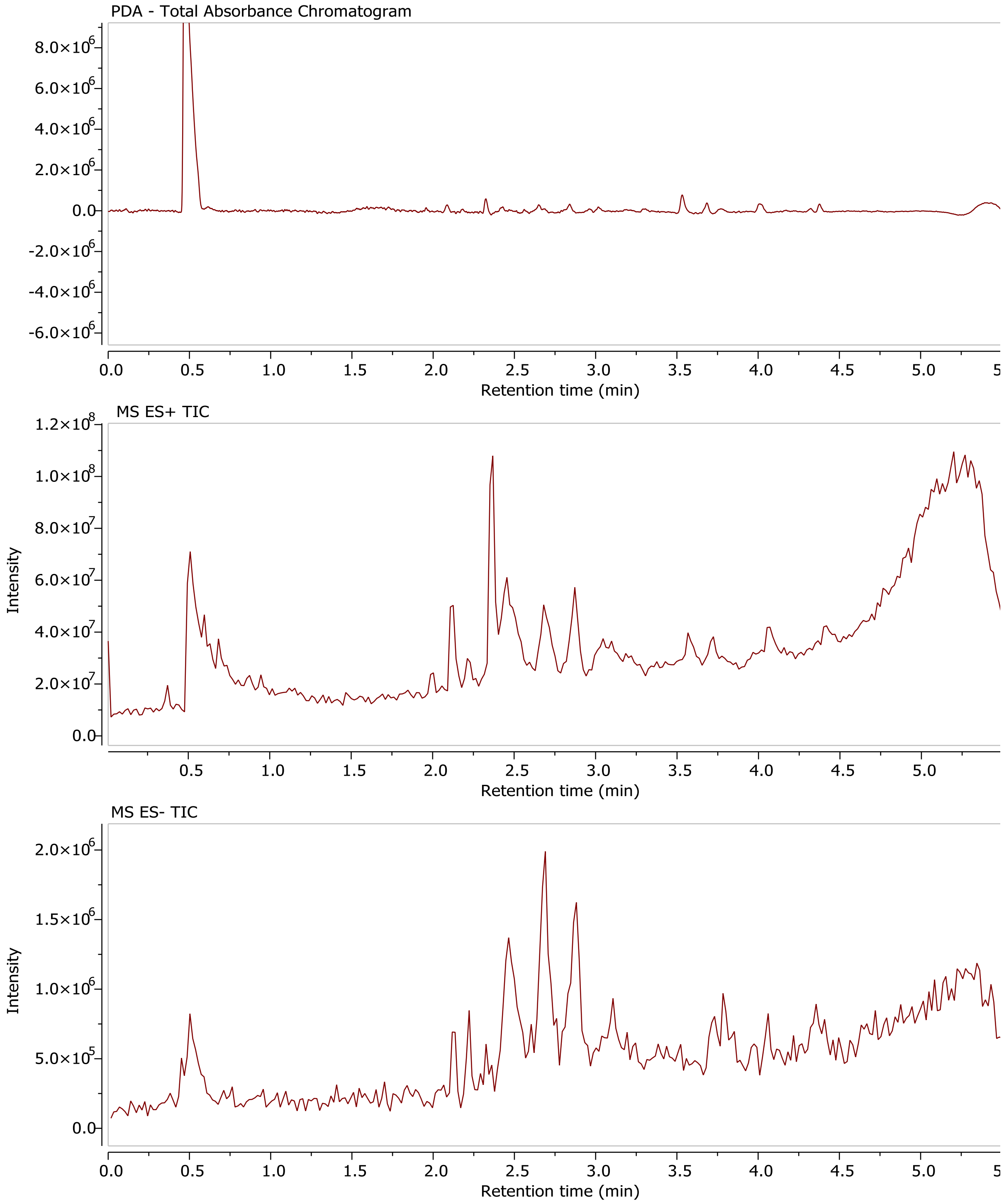

Supplementary figure **1305**. UPLC-MS analysis for compound **PHP-5416**

Chemical Formula: $C_{23}H_{20}N_2O_3$
Exact Mass: 372.1

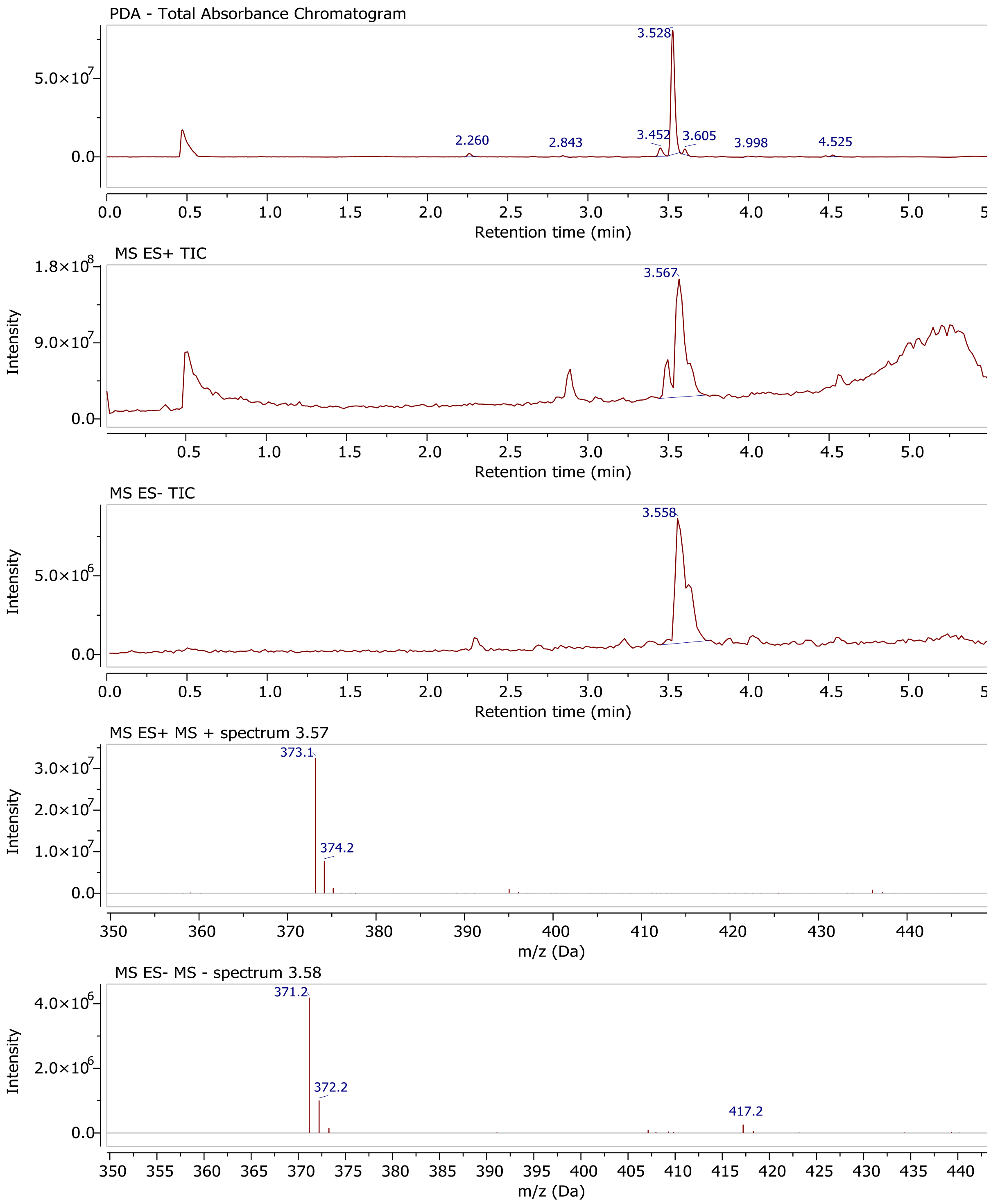

Supplementary figure **1306**. UPLC-MS analysis for compound **PHP-4823**

Chemical Formula: $C_{18}H_{16}F_2N_2O_3$
Exact Mass: 346.1

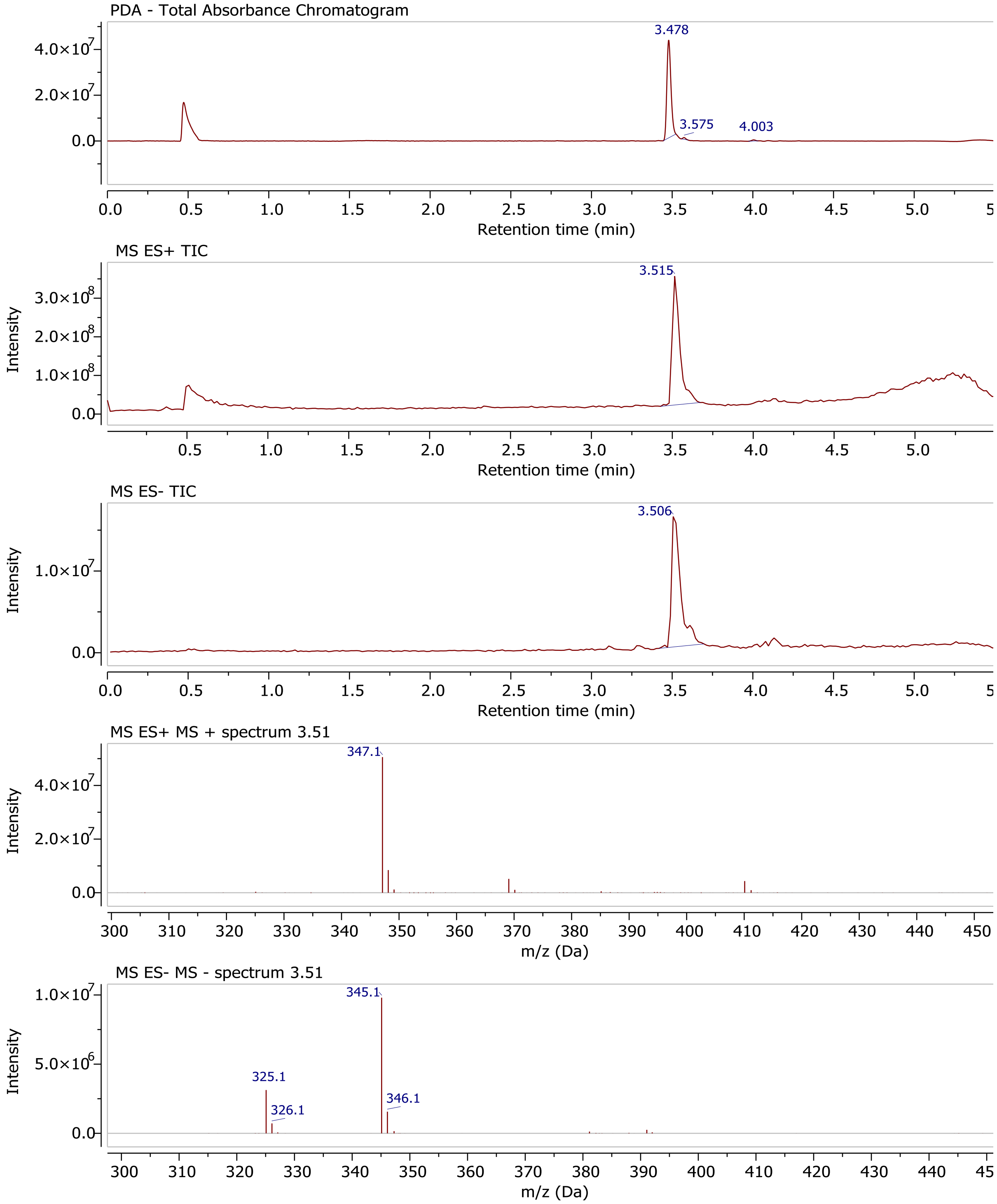

Supplementary figure **1307**. UPLC-MS analysis for compound **PHP-5418**

Chemical Formula: $C_{30}H_{26}N_2O_3$
Exact Mass: 462.2

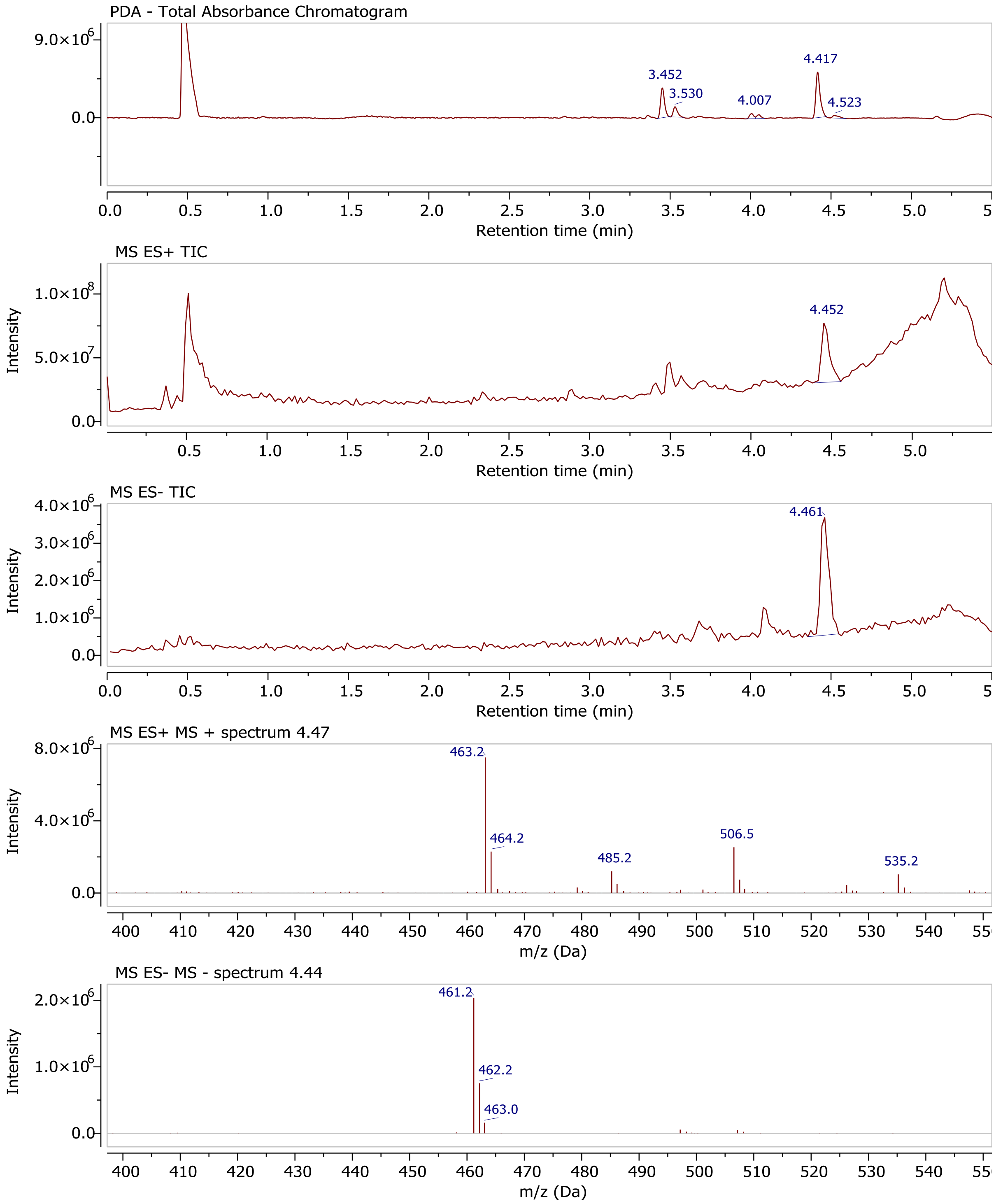

Supplementary figure **1308**. UPLC-MS analysis for compound **PHP-4824**

Chemical Formula: $C_{19}H_{20}N_2O_4$
Exact Mass: 340.1

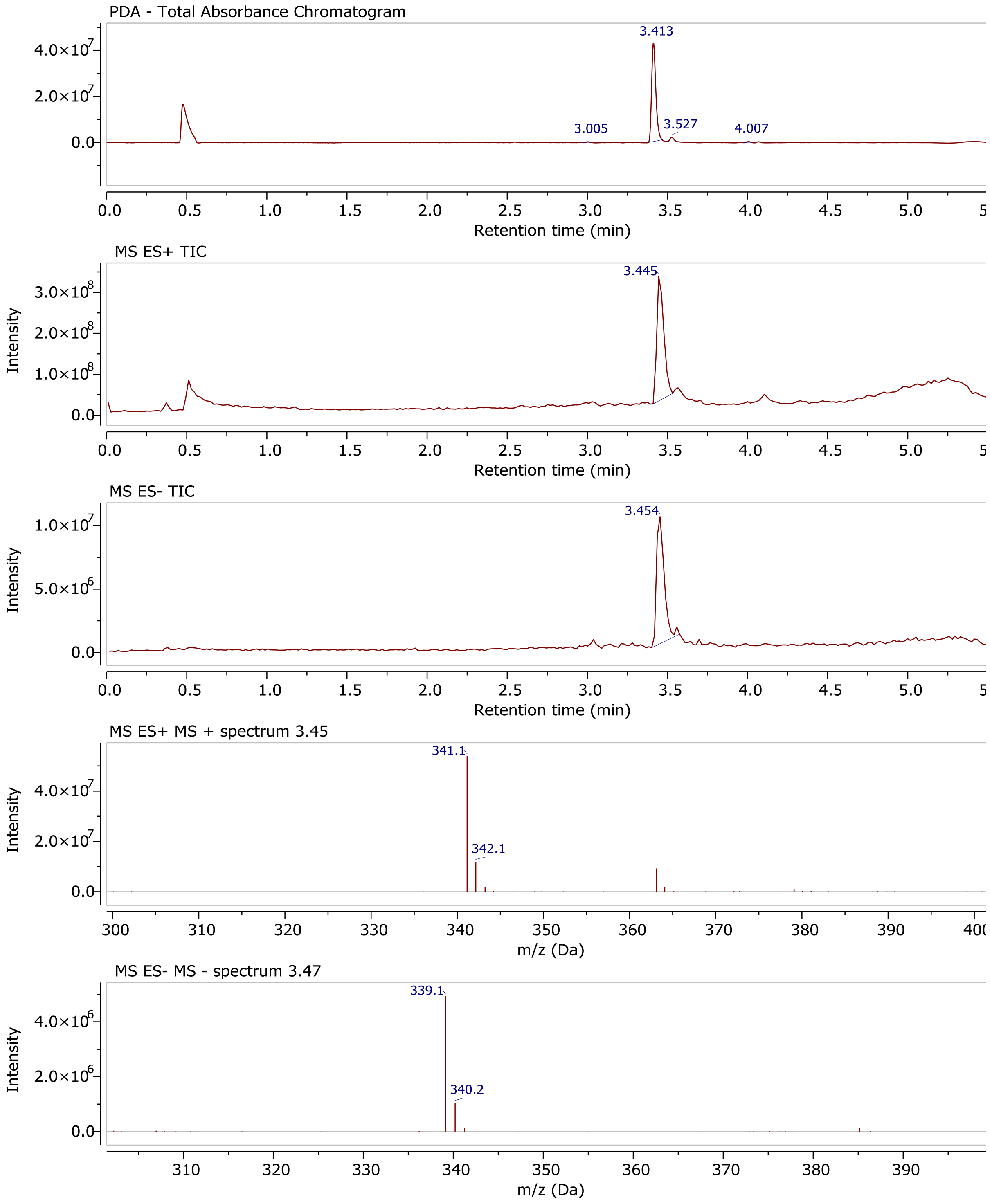

Supplementary figure **1309**. UPLC-MS analysis for compound **PHP-5424**

Chemical Formula: $C_{25}H_{24}N_2O_4$
Exact Mass: 416.2

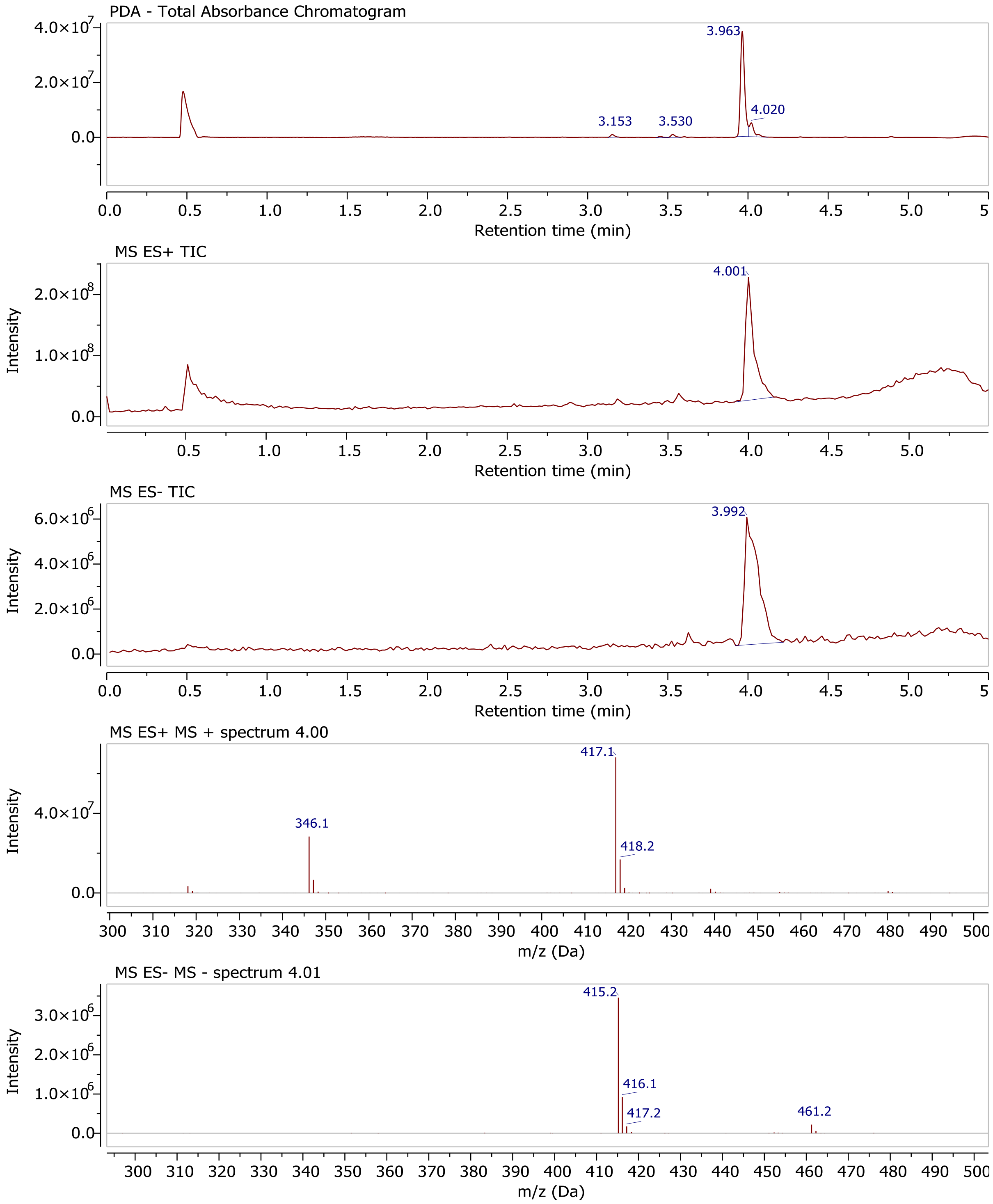

Supplementary figure **1310**. UPLC-MS analysis for compound **PHP-4825**

Chemical Formula: $C_{17}H_{22}N_2O_4$
Exact Mass: 318.2

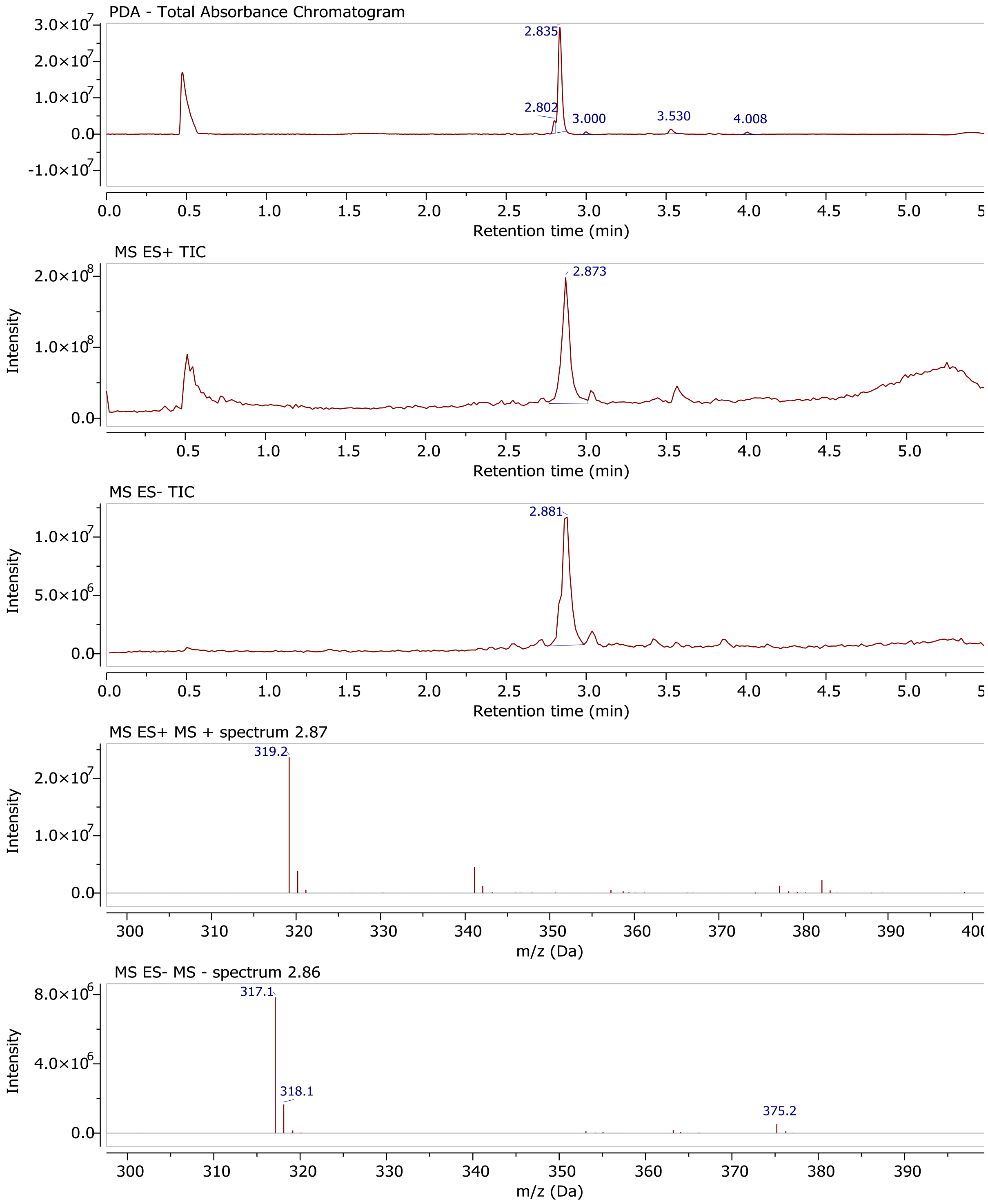

Supplementary figure **1311**. UPLC-MS analysis for compound **PHP-5422**

Chemical Formula: $C_{30}H_{26}N_2O_5S$
Exact Mass: 526.2

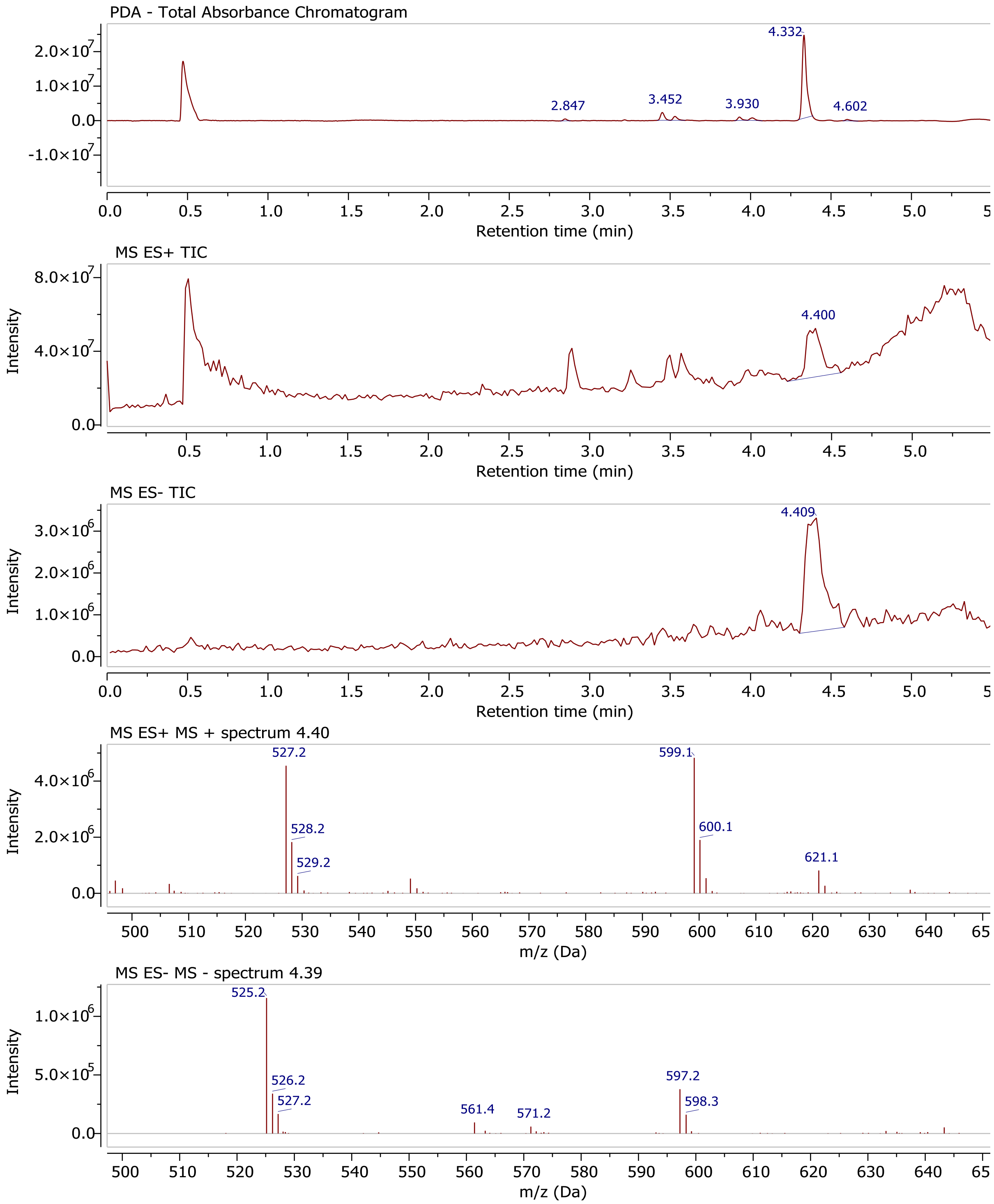

Supplementary figure **1312**. UPLC-MS analysis for compound **PHP-4827**

Chemical Formula: $C_{19}H_{20}N_2O_3$
Exact Mass: 324.1

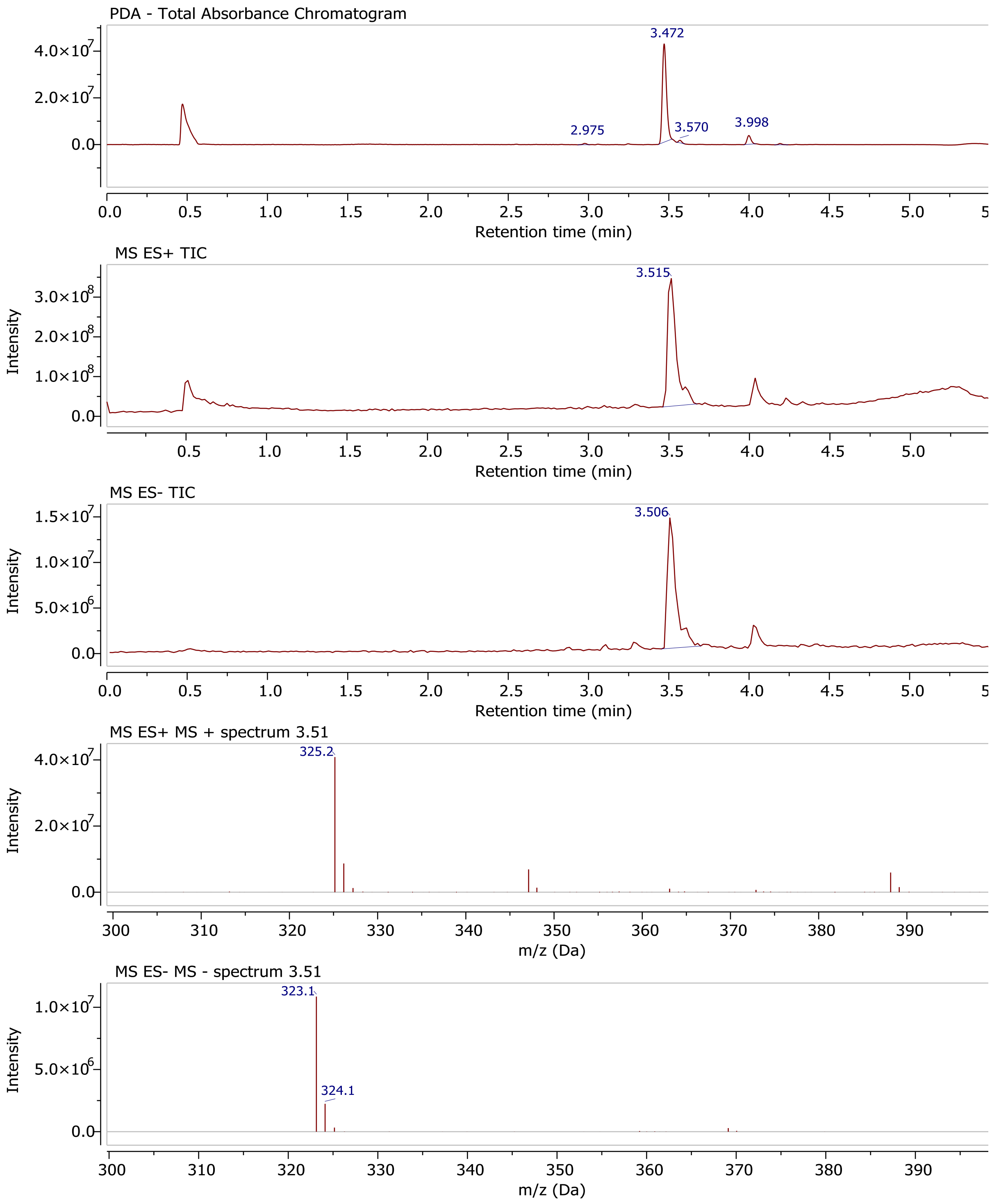

Supplementary figure **1313**. UPLC-MS analysis for compound **PHP-5426**

Chemical Formula: $C_{24}H_{21}BrN_2O_2$
Exact Mass: 448.1

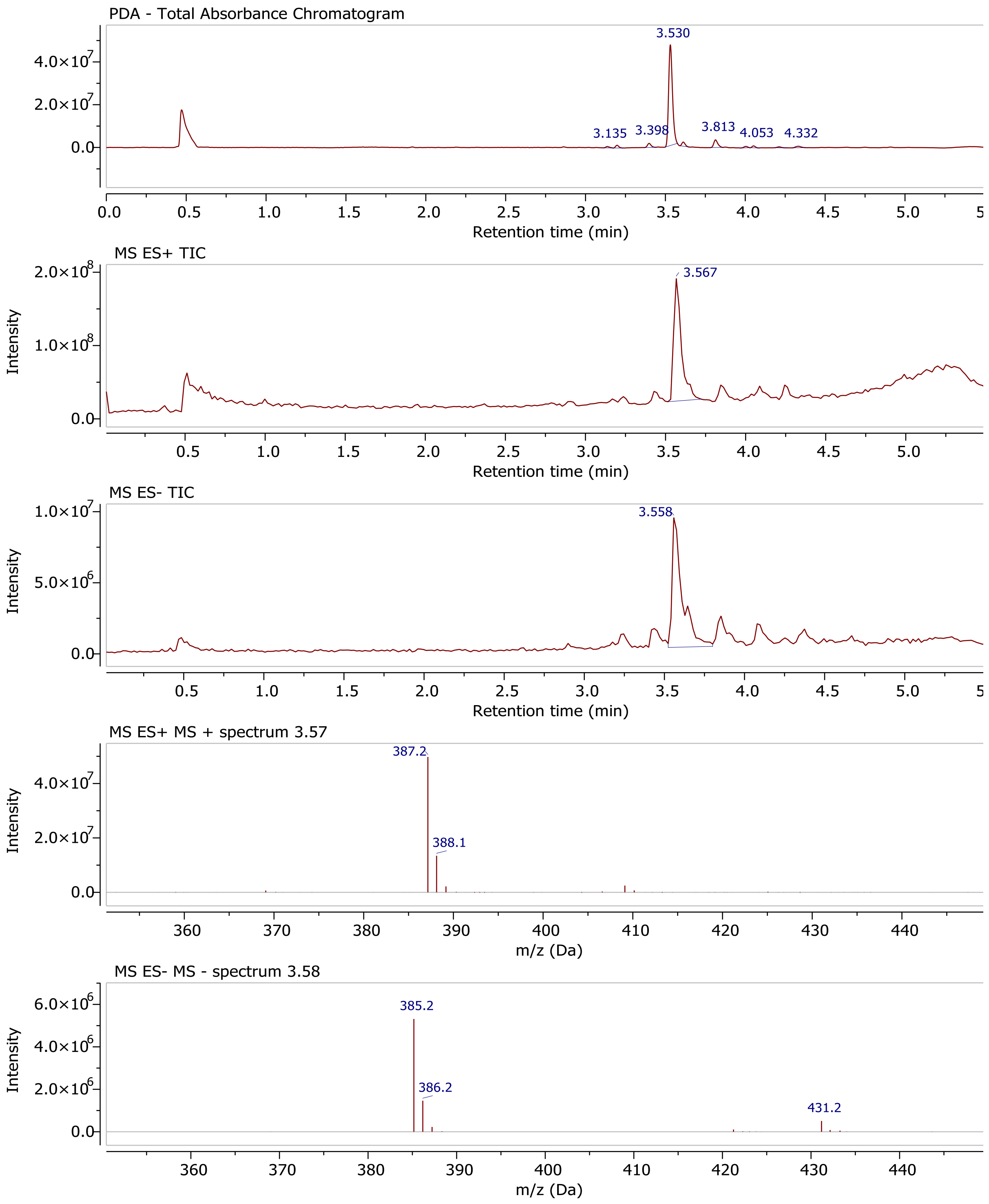

Supplementary figure **1314**. UPLC-MS analysis for compound **PHP-4828**

Chemical Formula: $C_{19}H_{20}N_2O_3$
Exact Mass: 324.1

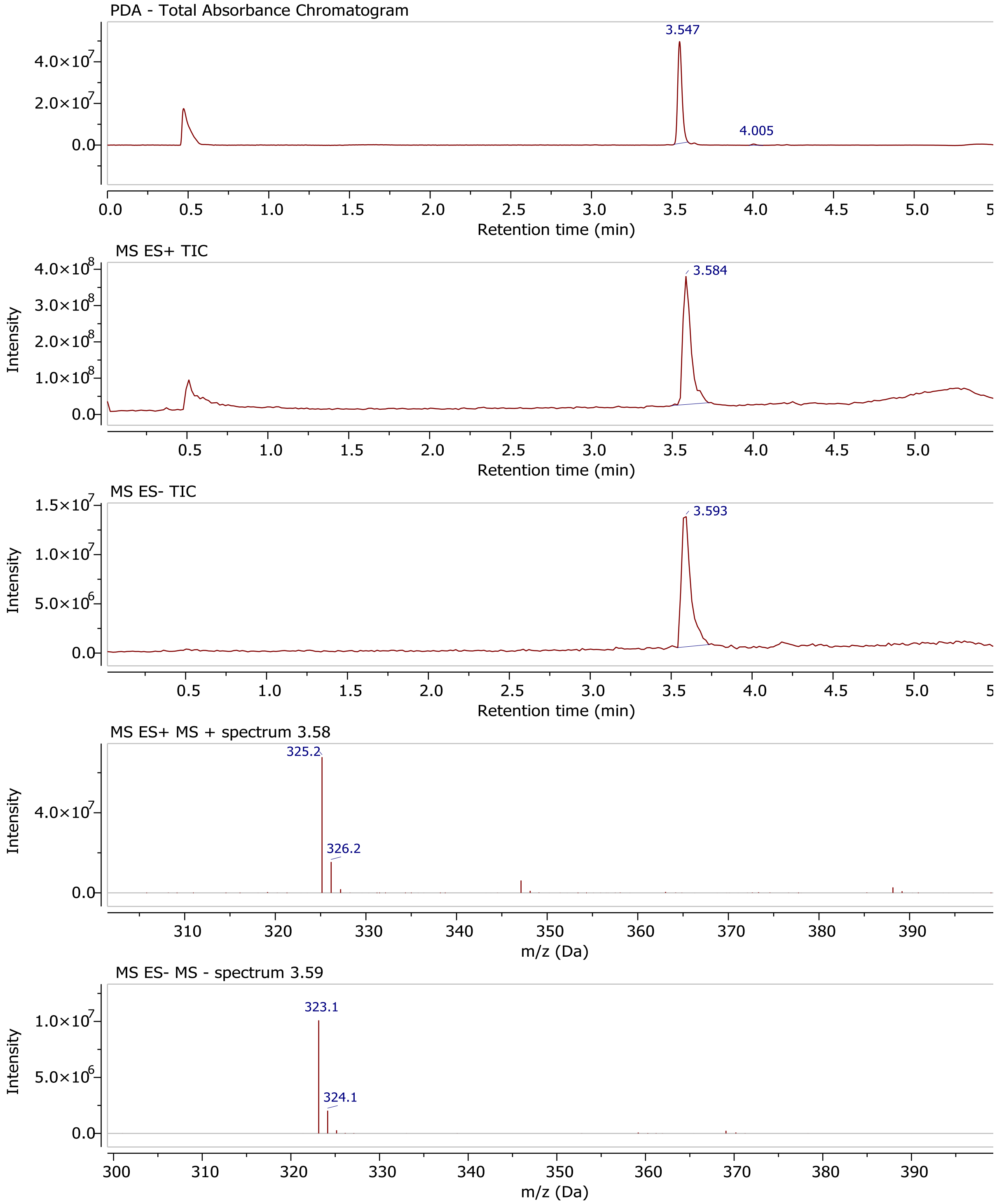

Supplementary figure **1315**. UPLC-MS analysis for compound **PHP-5427**

Chemical Formula: $C_{24}H_{20}N_4O_2$
Exact Mass: 396.2

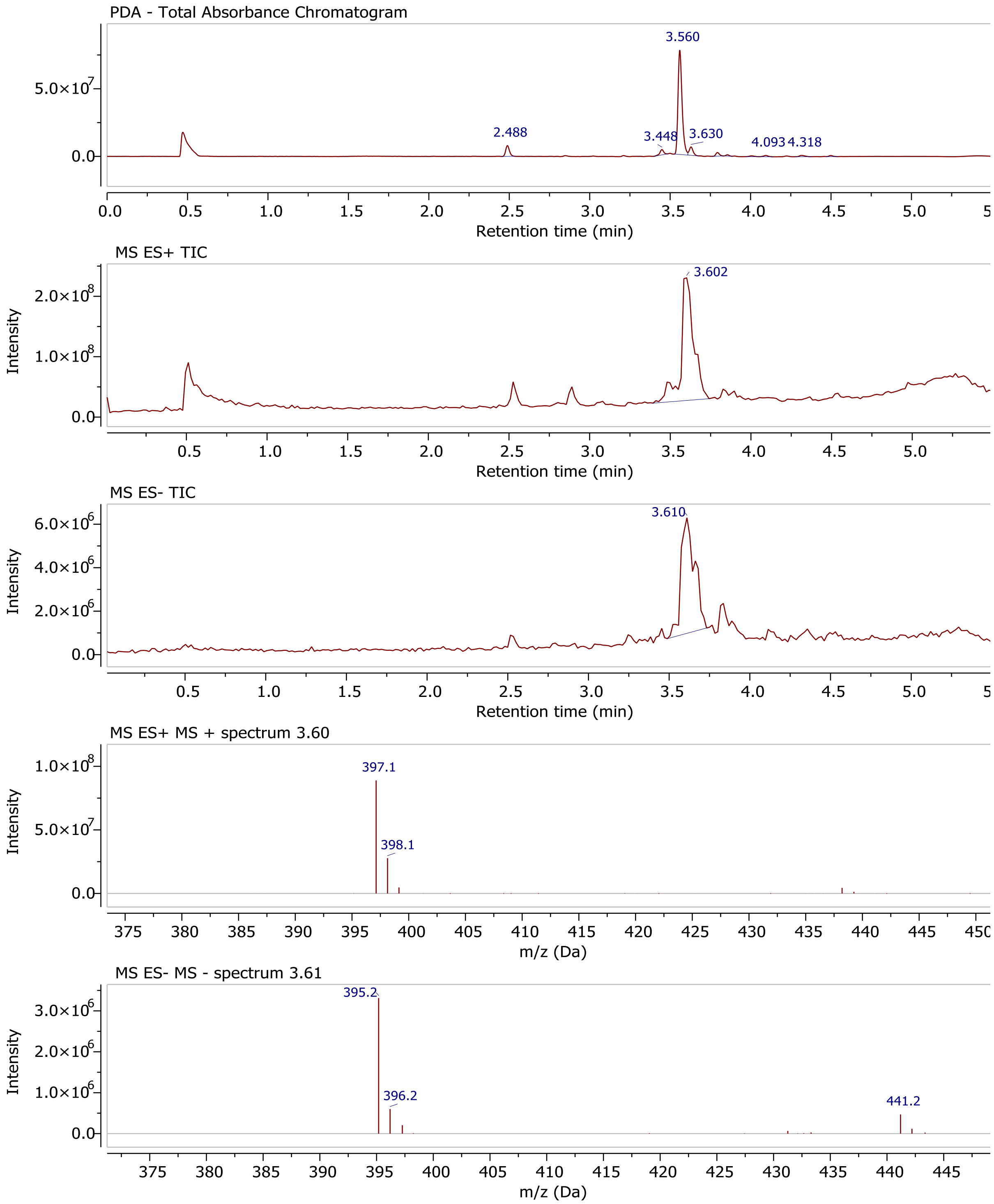

Supplementary figure **1316**. UPLC-MS analysis for compound **PHP-4831**

Chemical Formula: $C_{17}H_{22}N_2O_3$
Exact Mass: 302.2

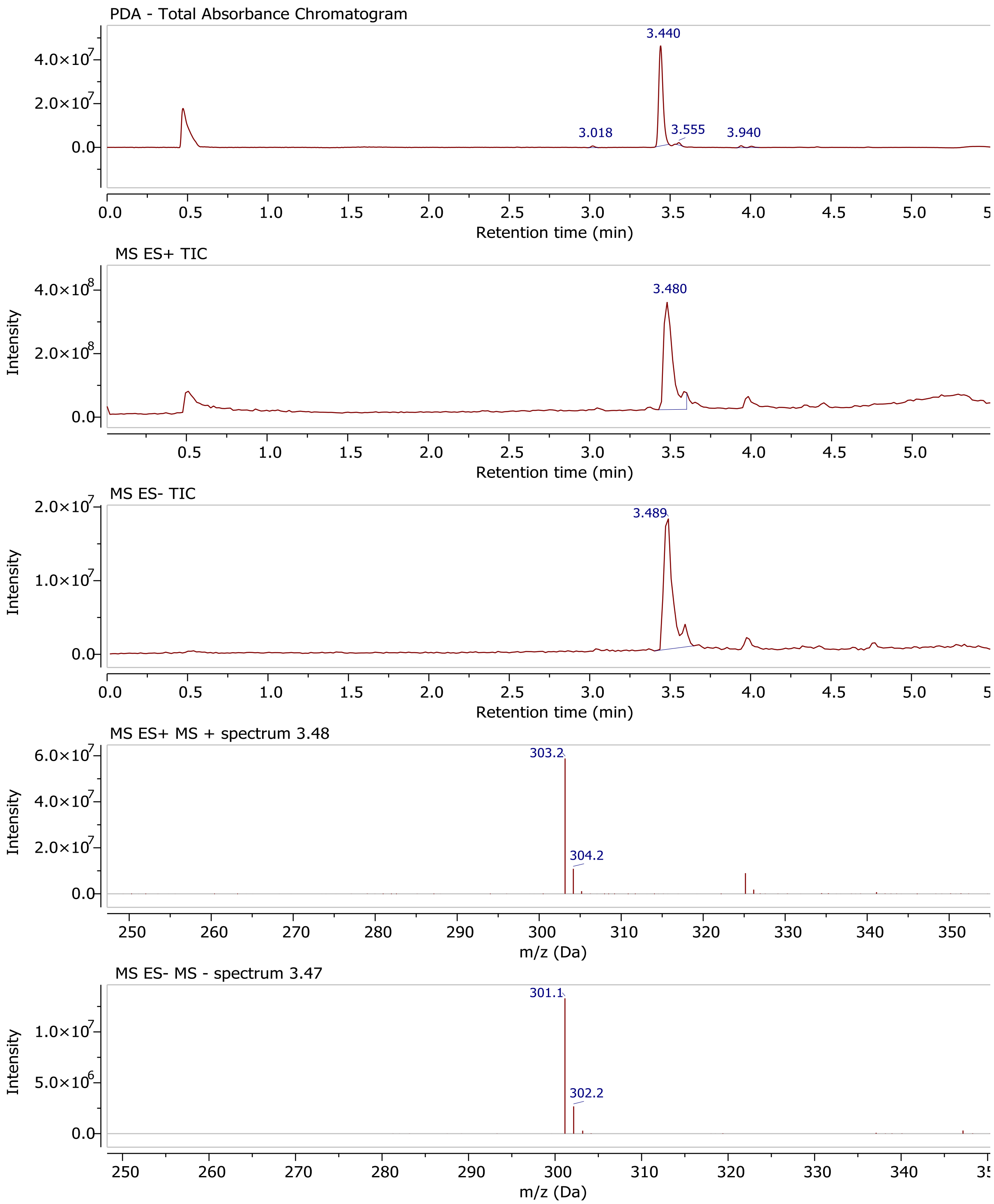

Supplementary figure **1317**. UPLC-MS analysis for compound **PHP-5429**

Chemical Formula: $C_{31}H_{24}N_2O_2$
Exact Mass: 456.2

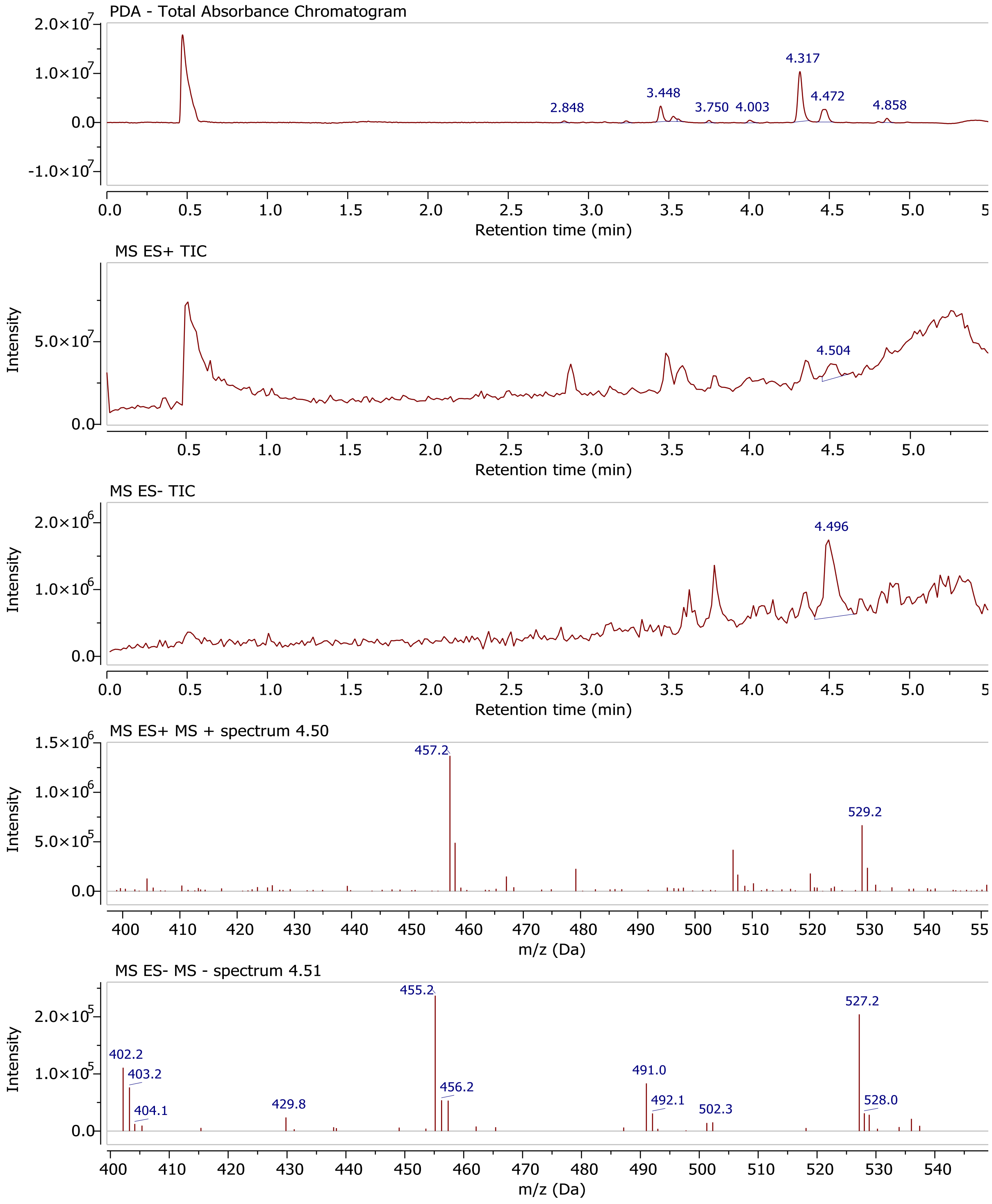

Supplementary figure **1318**. UPLC-MS analysis for compound **PHP-4832**

Chemical Formula: $C_{19}H_{20}N_2O_4$
Exact Mass: 340.1

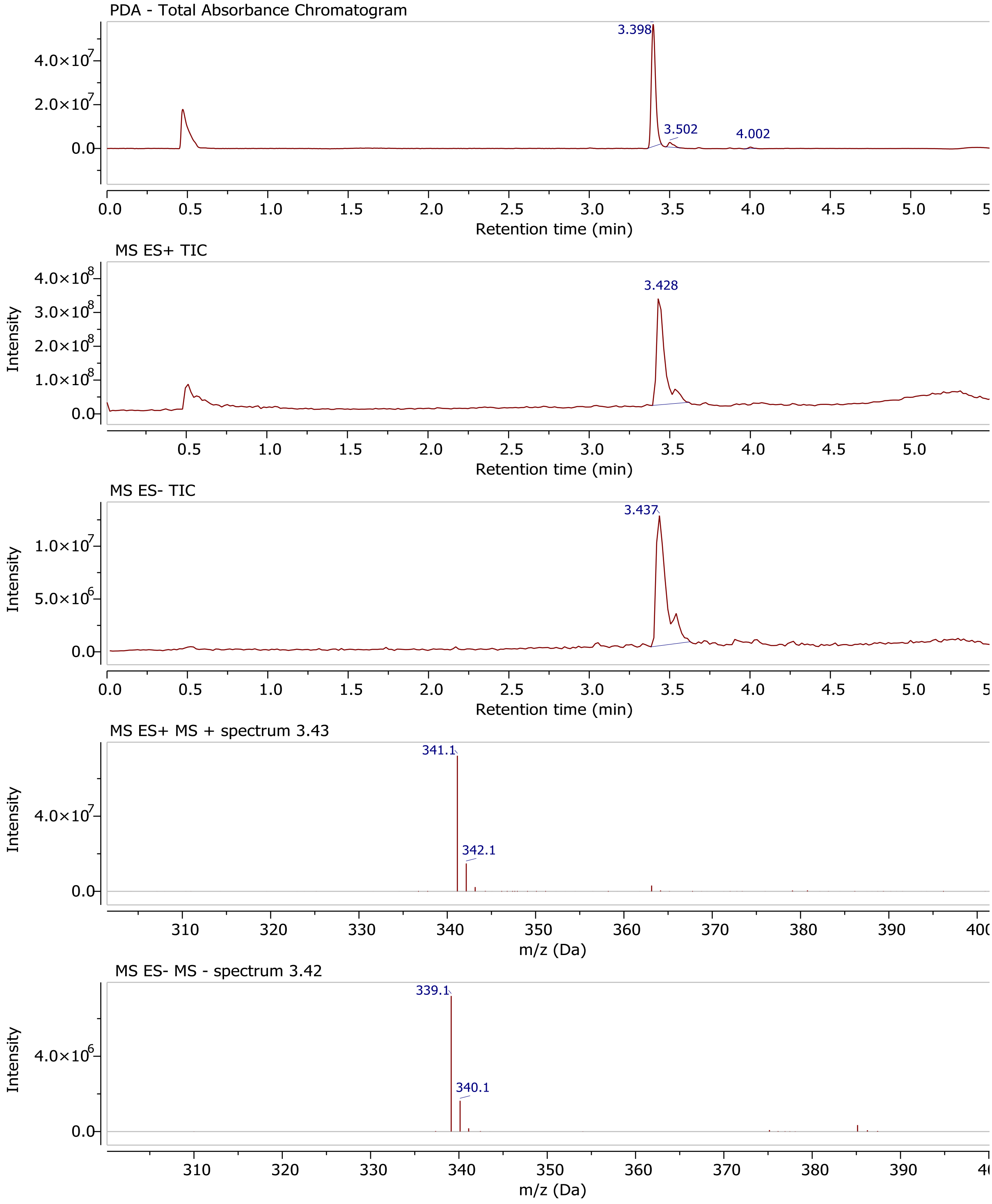

Supplementary figure **1319**. UPLC-MS analysis for compound **PHP-5430**

Chemical Formula: $C_{23}H_{21}N_3O_2$
Exact Mass: 371.2

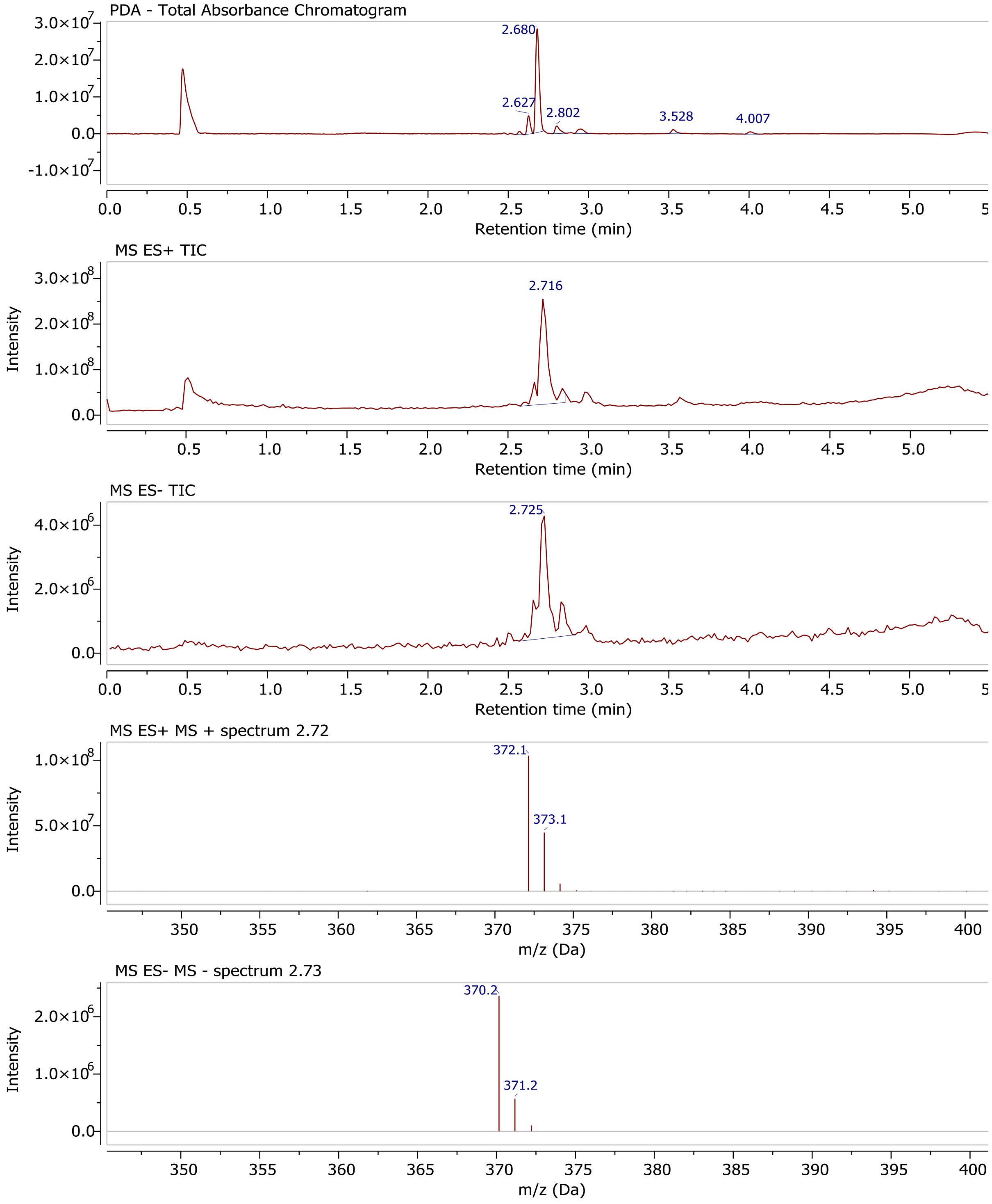

Supplementary figure **1320**. UPLC-MS analysis for compound **PHP-4834**

Chemical Formula: $C_{16}H_{16}N_2O_4$
Exact Mass: 300.1

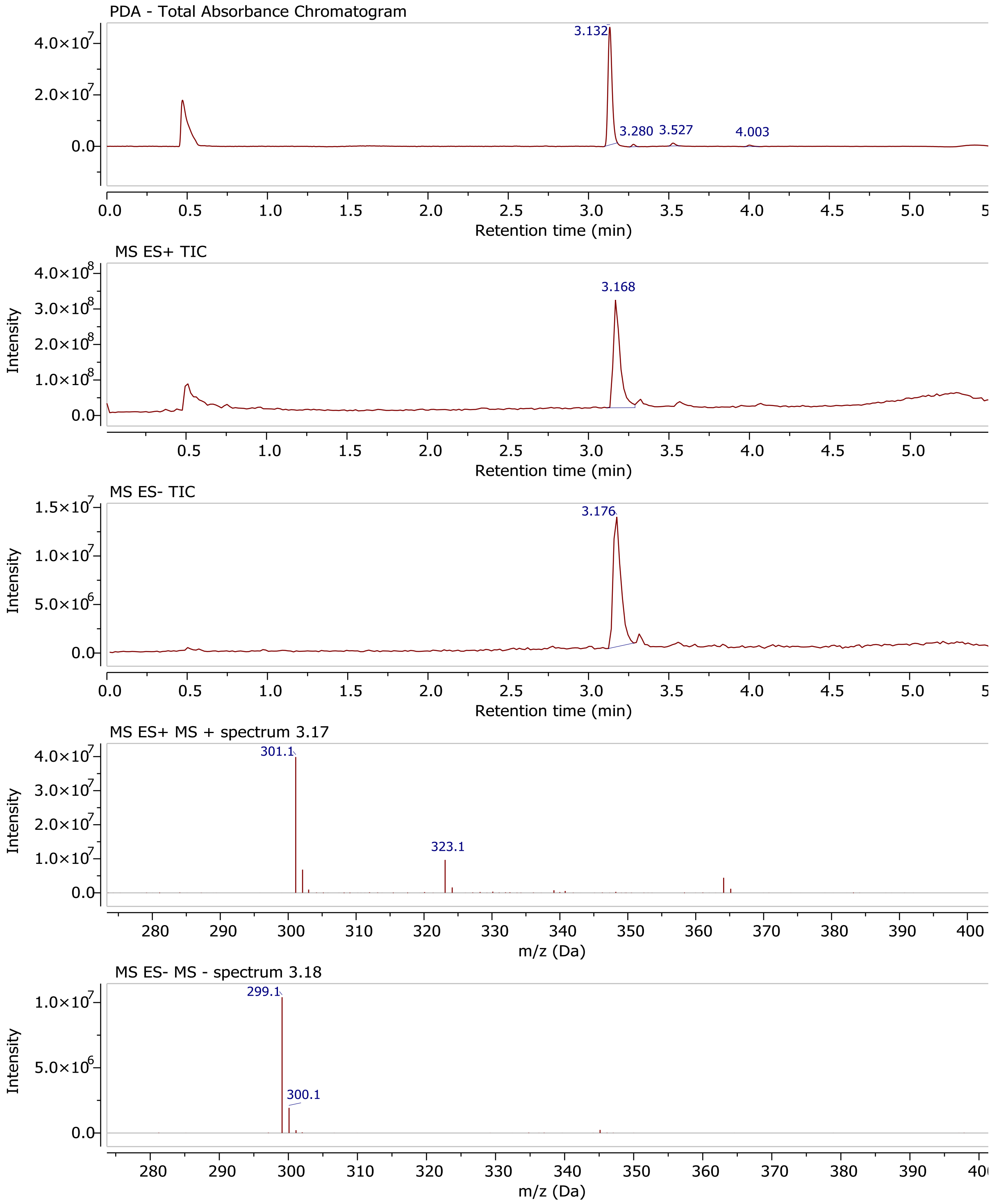

Supplementary figure **1321**. UPLC-MS analysis for compound **PHP-5431**

Chemical Formula: $C_{29}H_{26}N_2O_3$
Exact Mass: 450.2

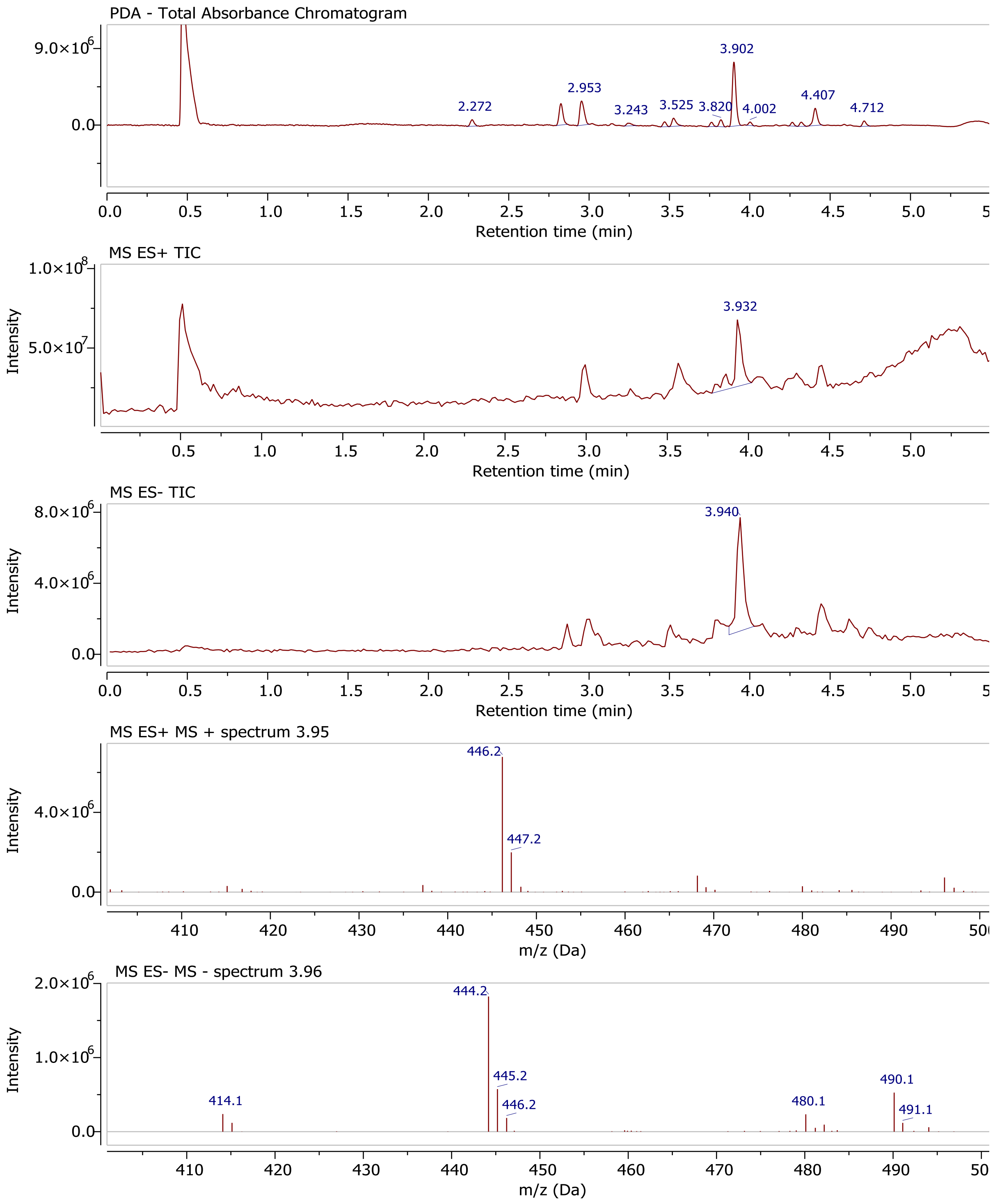

Supplementary figure **1322**. UPLC-MS analysis for compound **PHP-4849**

Chemical Formula: $C_{22}H_{20}N_2O_3$
Exact Mass: 360.1

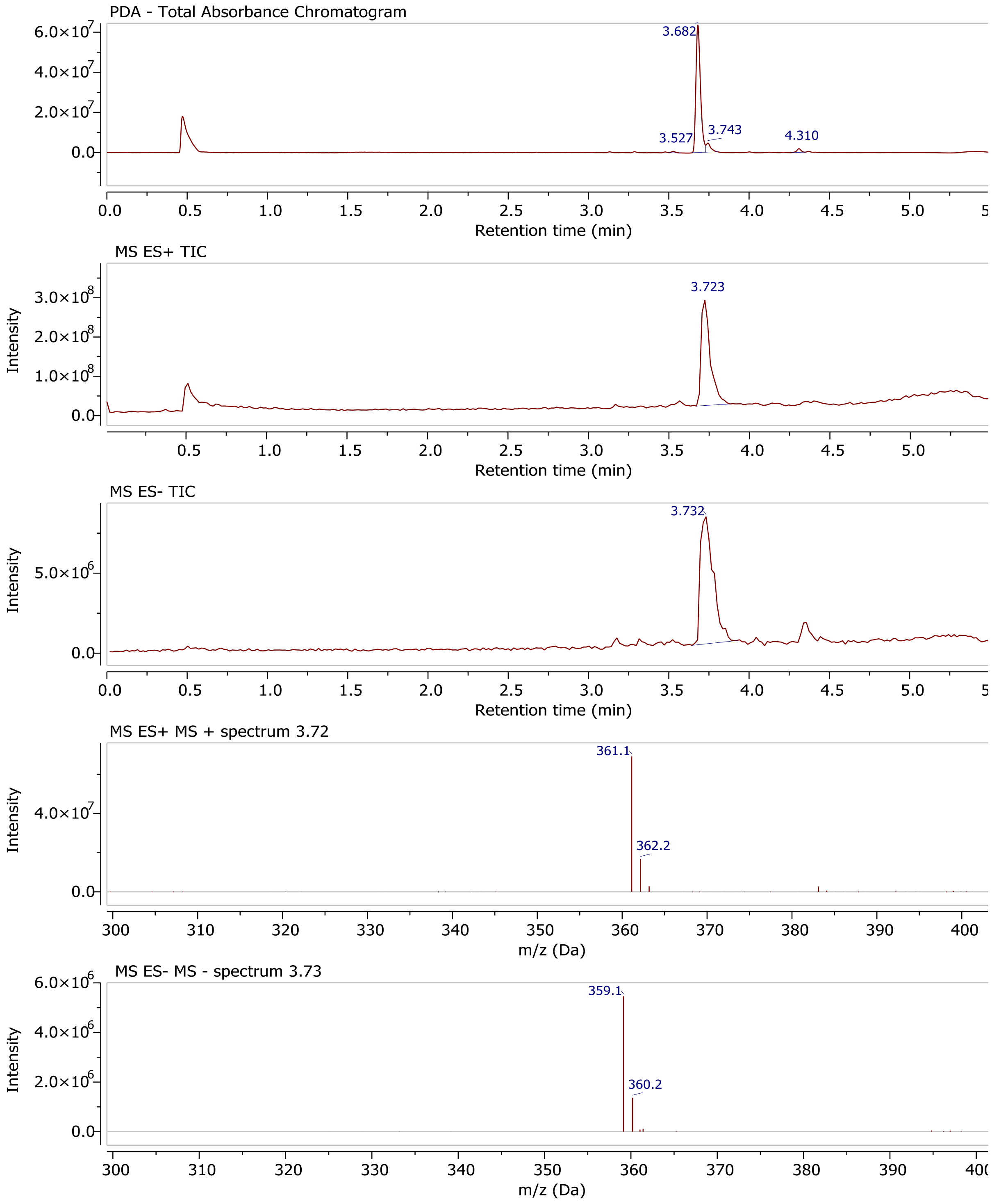

Supplementary figure **1323**. UPLC-MS analysis for compound **PHP-5432**

Chemical Formula: $C_{20}H_{19}F_3N_2O_4$
Exact Mass: 408.1

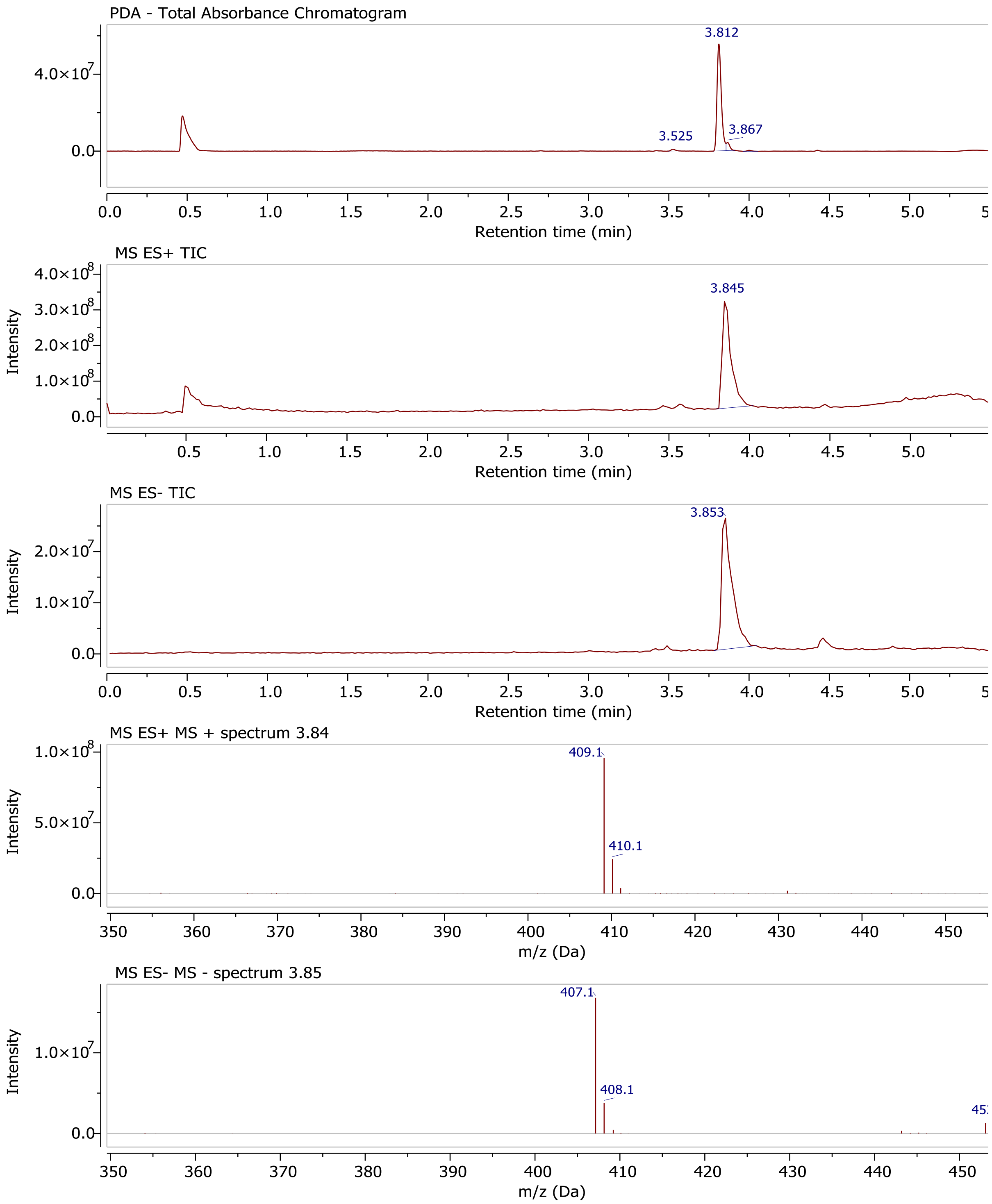

Supplementary figure **1324**. UPLC-MS analysis for compound **PHP-5591**

Chemical Formula: $C_{22}H_{26}N_2O_2$
Exact Mass: 350.2

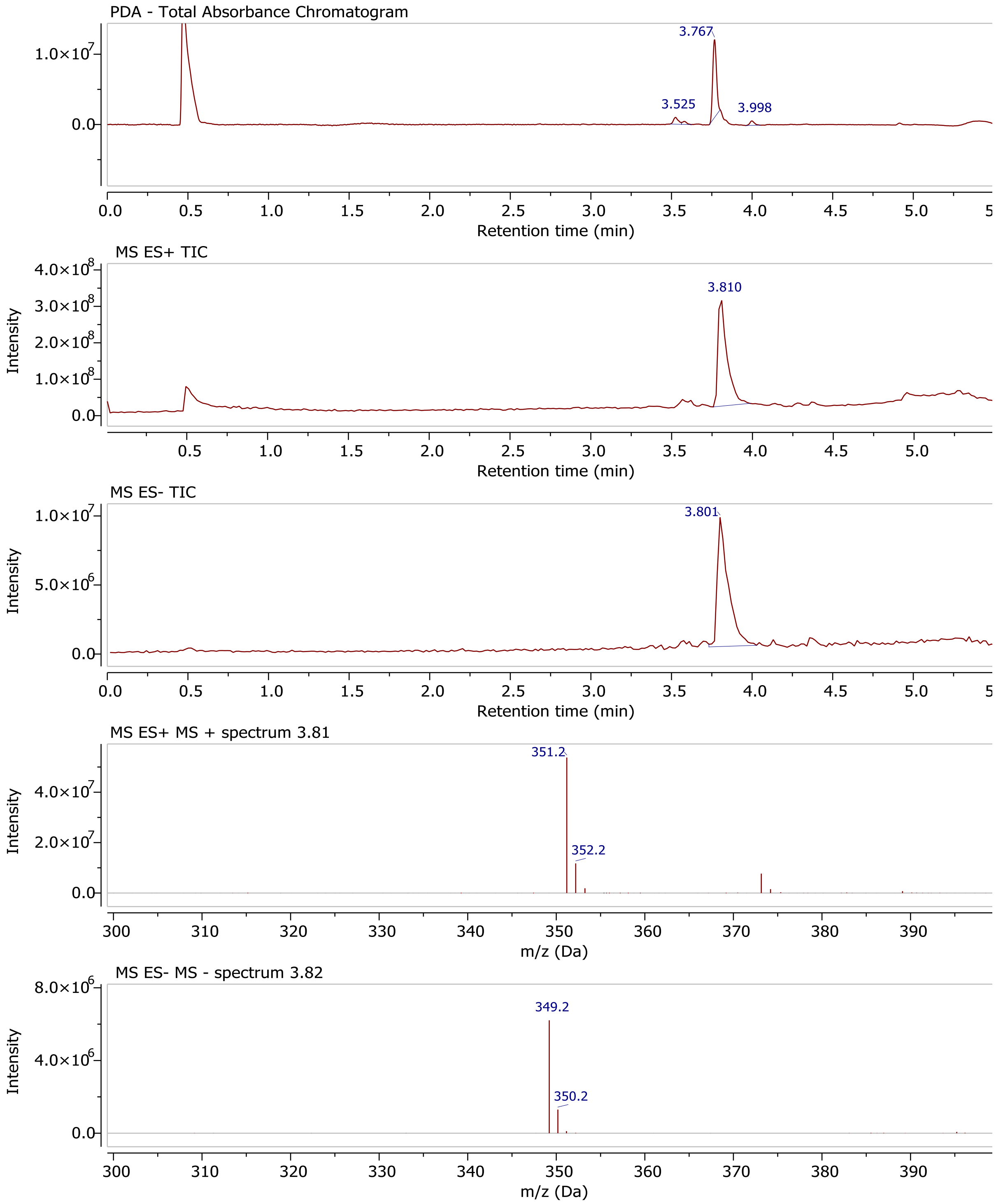

Supplementary figure **1325**. UPLC-MS analysis for compound **PHP-5740**

Chemical Formula: $C_{19}H_{19}ClN_2O_4$
Exact Mass: 374.1

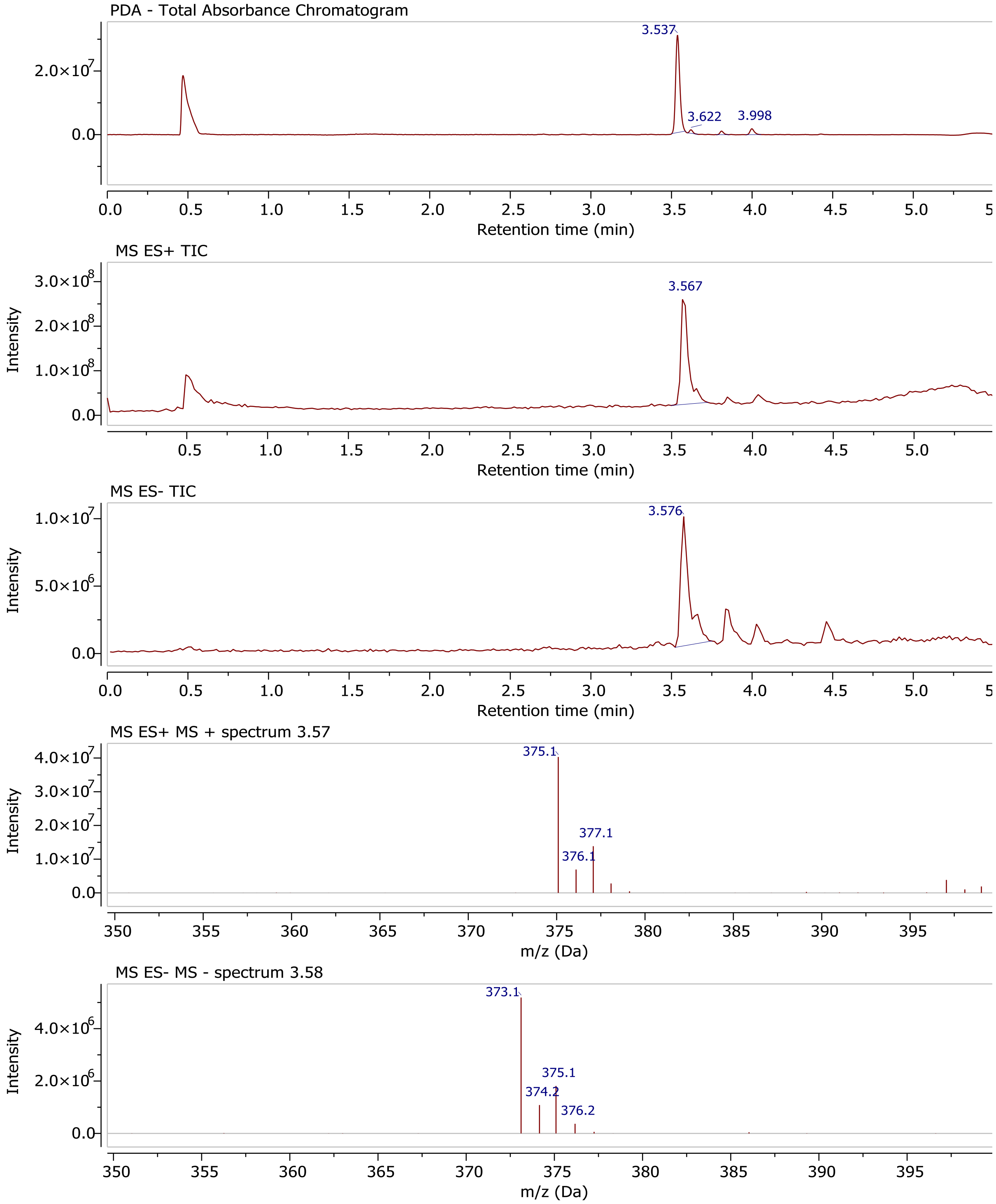

Supplementary figure **1326**. UPLC-MS analysis for compound **PHP-5592**

Chemical Formula: $C_{21}H_{21}F_3N_2O_2$
Exact Mass: 390.2

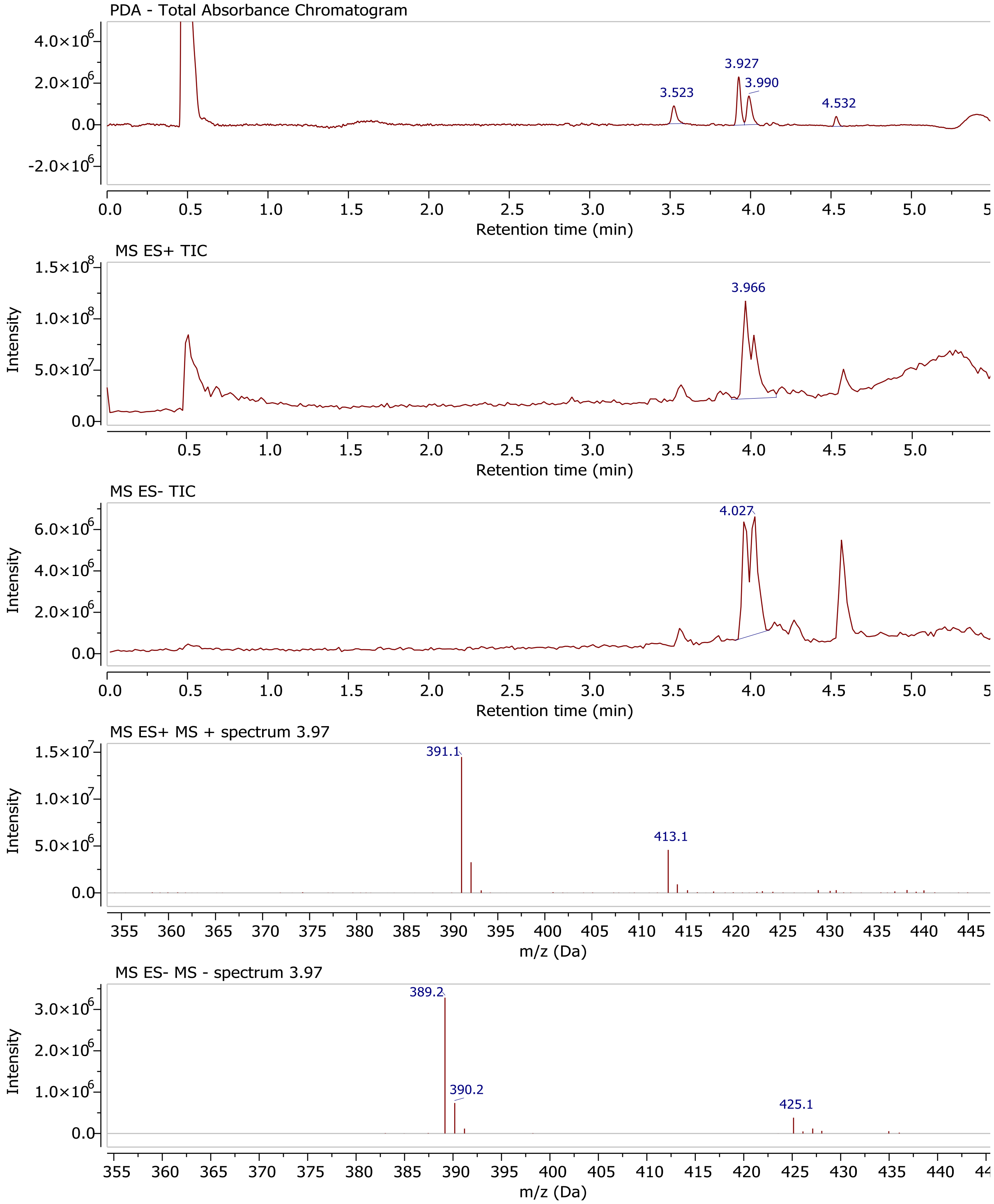

Supplementary figure **1327**. UPLC-MS analysis for compound **PHP-5741**

Chemical Formula: $C_{20}H_{19}F_3N_2O_4$
Exact Mass: 408.1

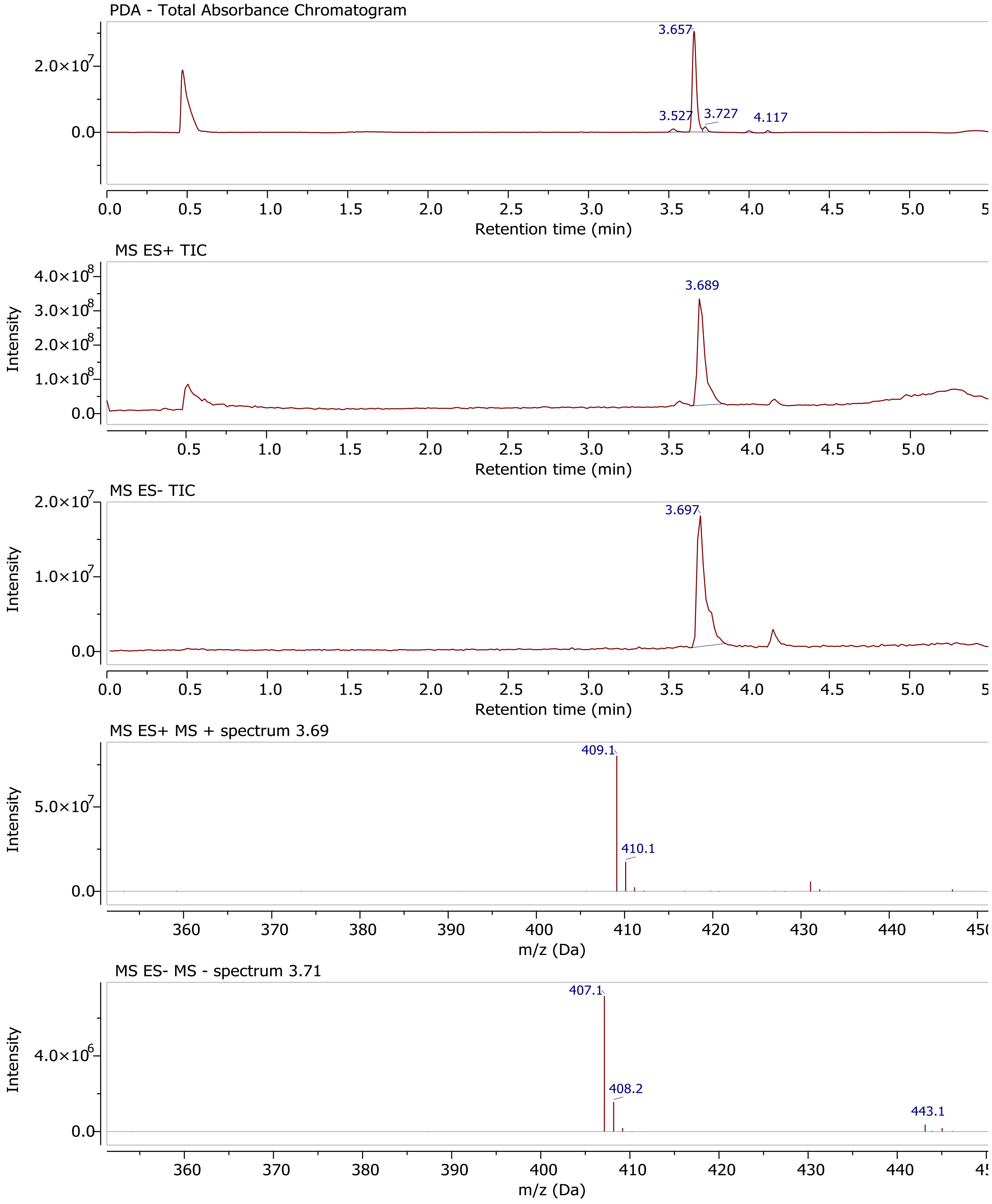

Supplementary figure **1328**. UPLC-MS analysis for compound **PHP-5593**

Chemical Formula: $C_{17}H_{24}N_2O_2S$
Exact Mass: 320.2

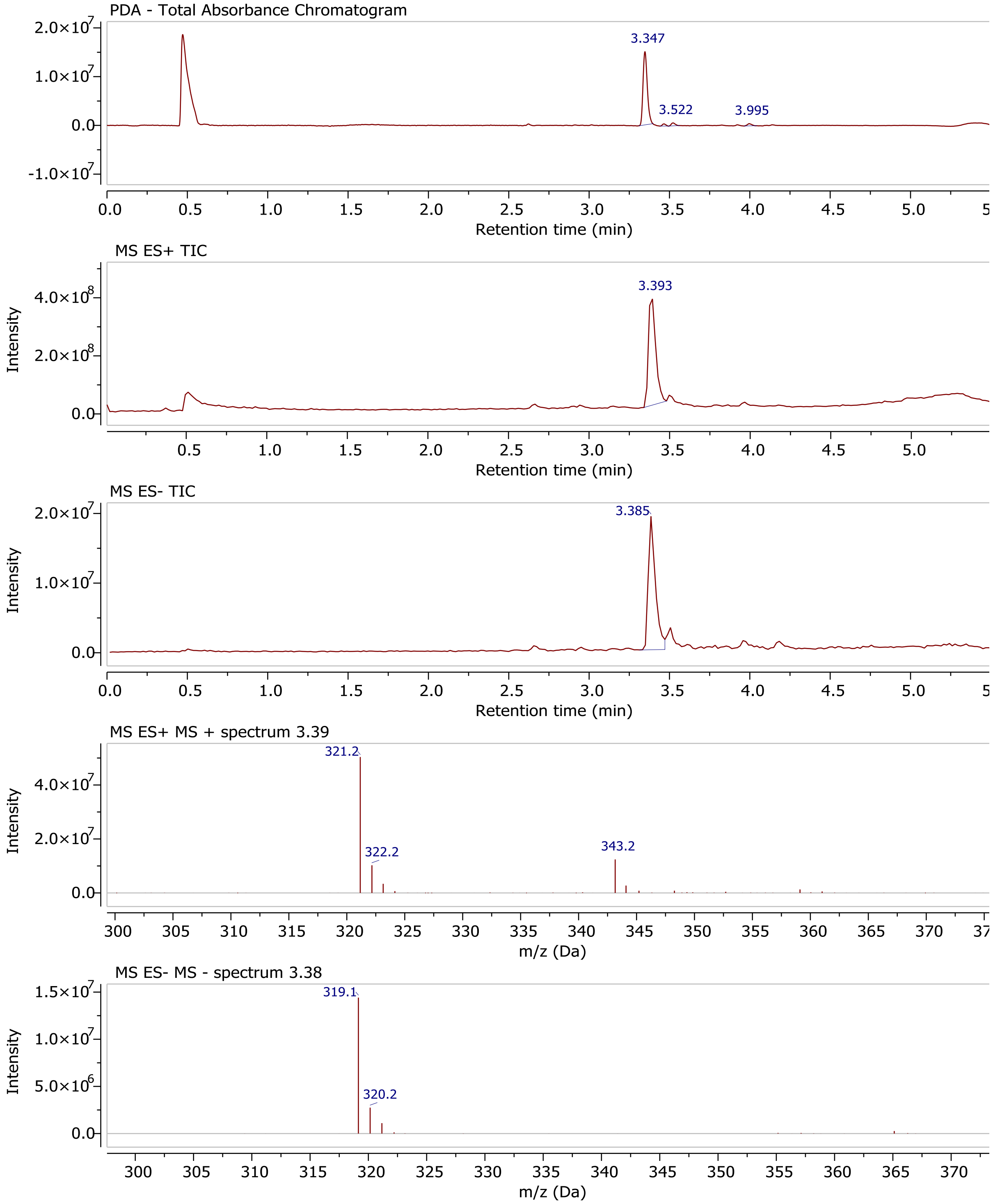

Supplementary figure **1329**. UPLC-MS analysis for compound **PHP-5742**

Chemical Formula: $C_{19}H_{19}FN_2O_4$
Exact Mass: 358.1

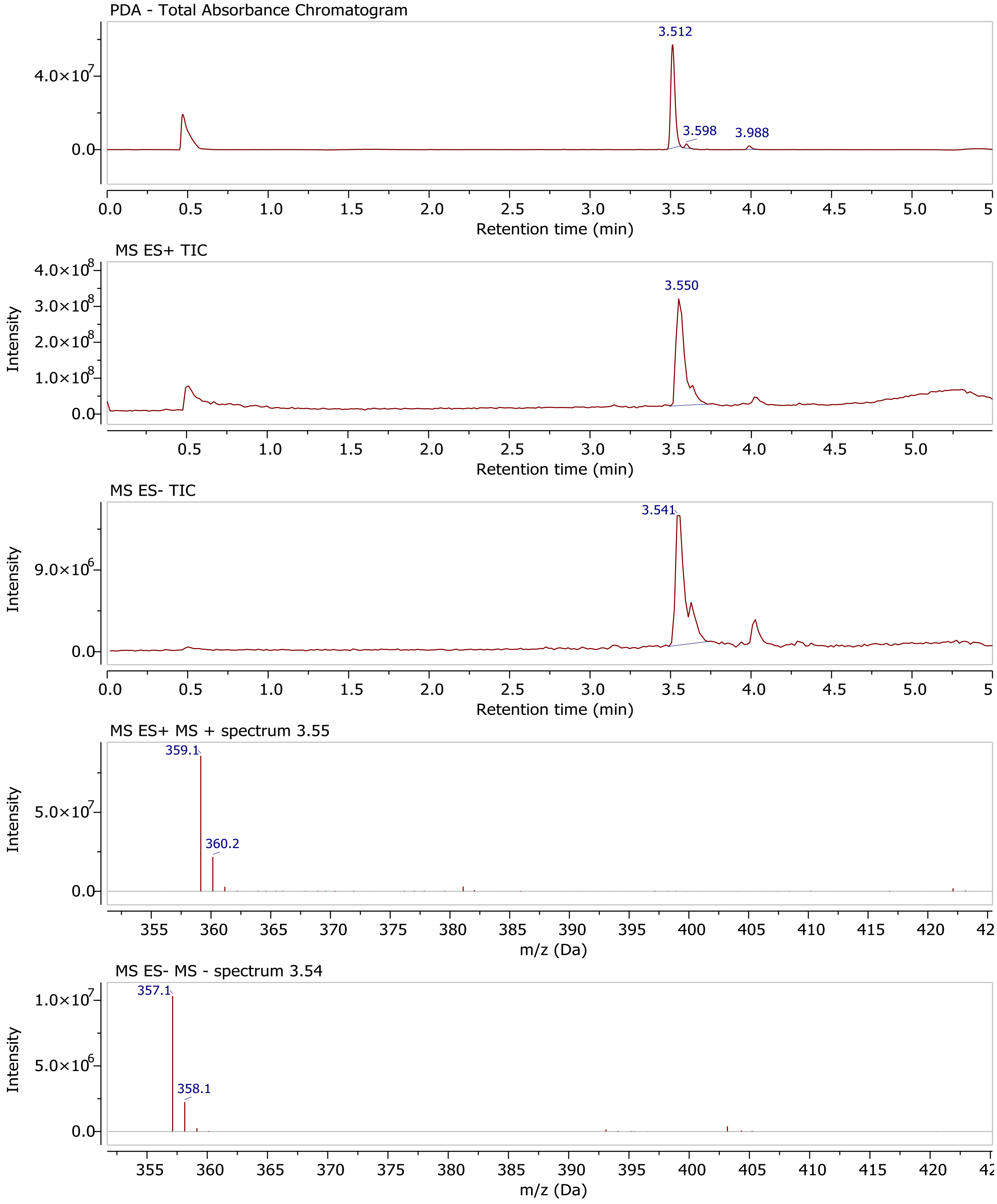

Supplementary figure **1330**. UPLC-MS analysis for compound **PHP-5594**

Chemical Formula: $C_{19}H_{21}N_3O_2$
Exact Mass: 323.2

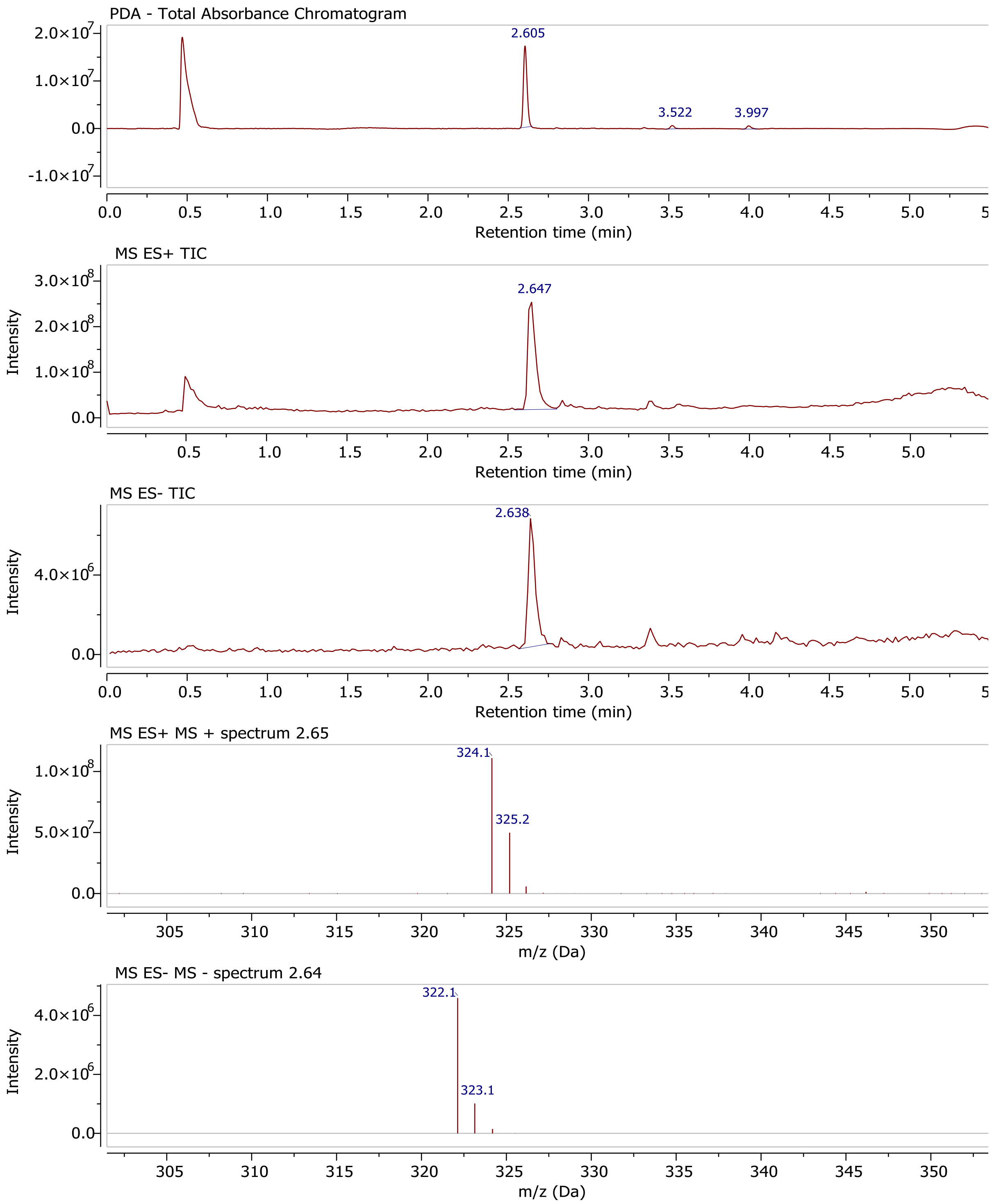

Supplementary figure **1331**. UPLC-MS analysis for compound **PHP-5743**

Chemical Formula: $C_{19}H_{19}FN_2O_4$
Exact Mass: 358.1

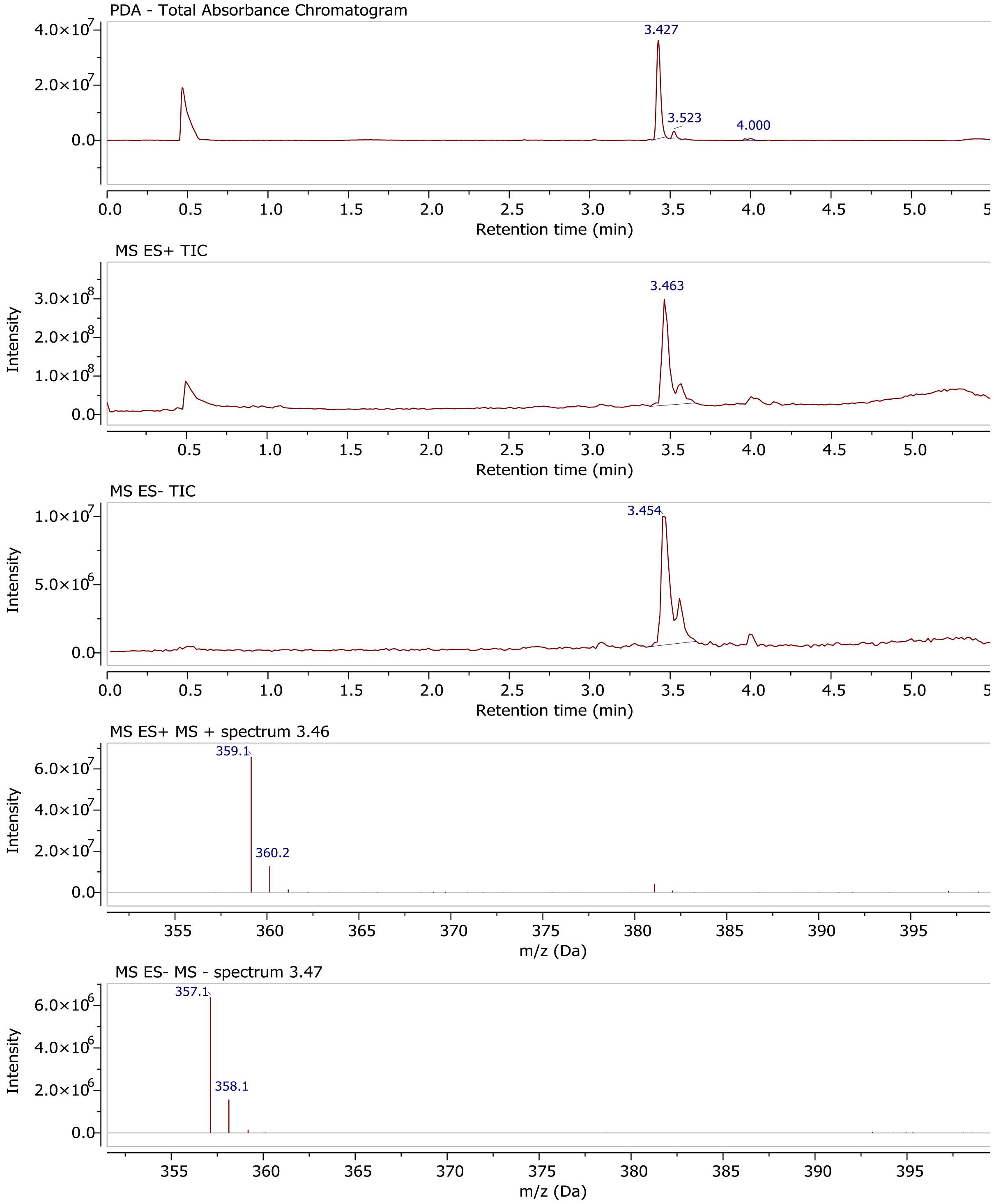

Supplementary figure **1332**. UPLC-MS analysis for compound **PHP-5595**

Chemical Formula: $C_{24}H_{36}N_2O_2$
Exact Mass: 384.3

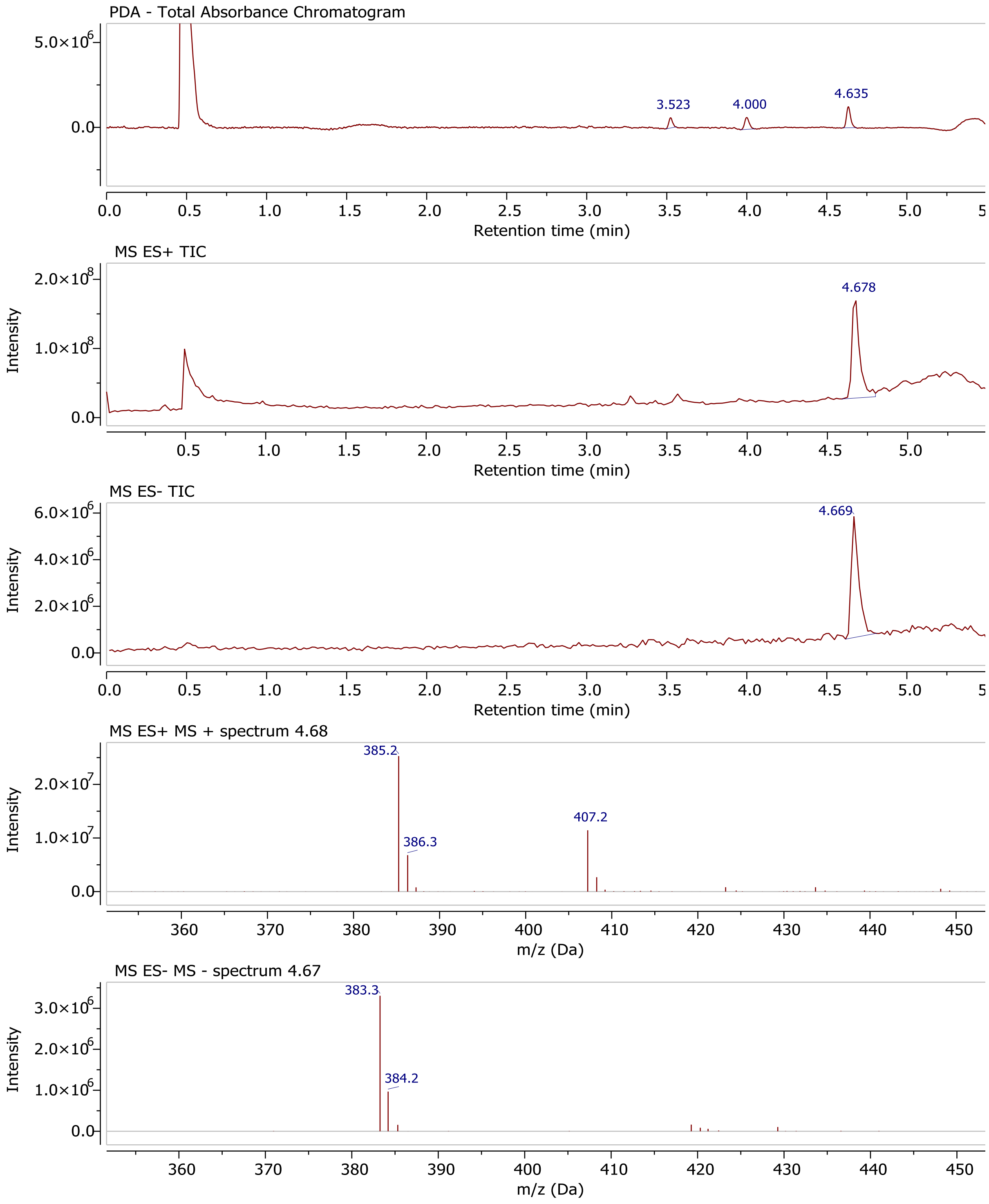

Supplementary figure **1333**. UPLC-MS analysis for compound **PHP-5744**

Chemical Formula: $C_{20}H_{19}F_3N_2O_4$
Exact Mass: 408.1

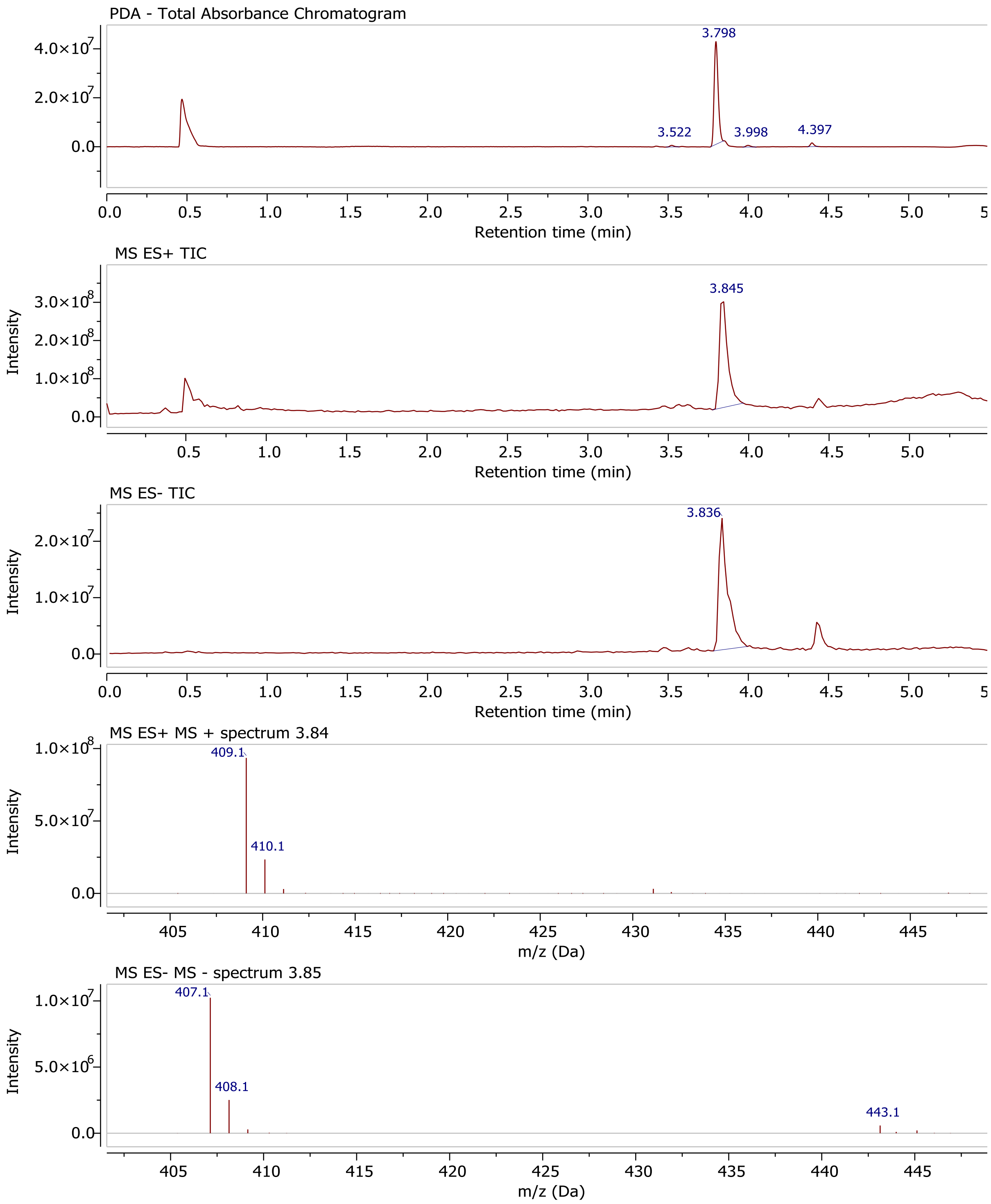

Supplementary figure **1334**. UPLC-MS analysis for compound **PHP-5596**

Chemical Formula: $C_{19}H_{21}N_3O_2$
Exact Mass: 323.2

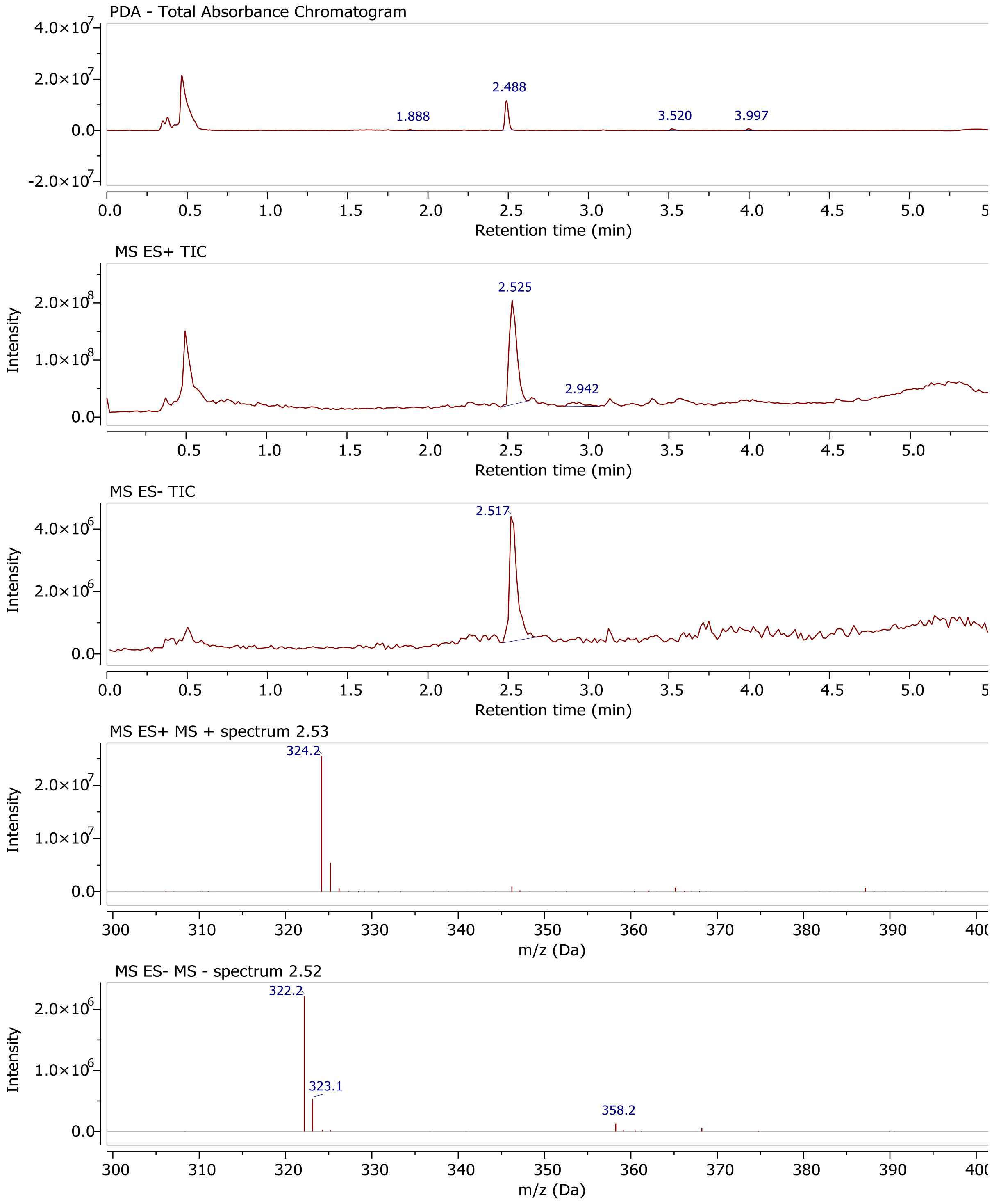

Supplementary figure **1335**. UPLC-MS analysis for compound **PHP-5746**

Chemical Formula: $C_{22}H_{17}F_3N_2O_2$
Exact Mass: 398.1

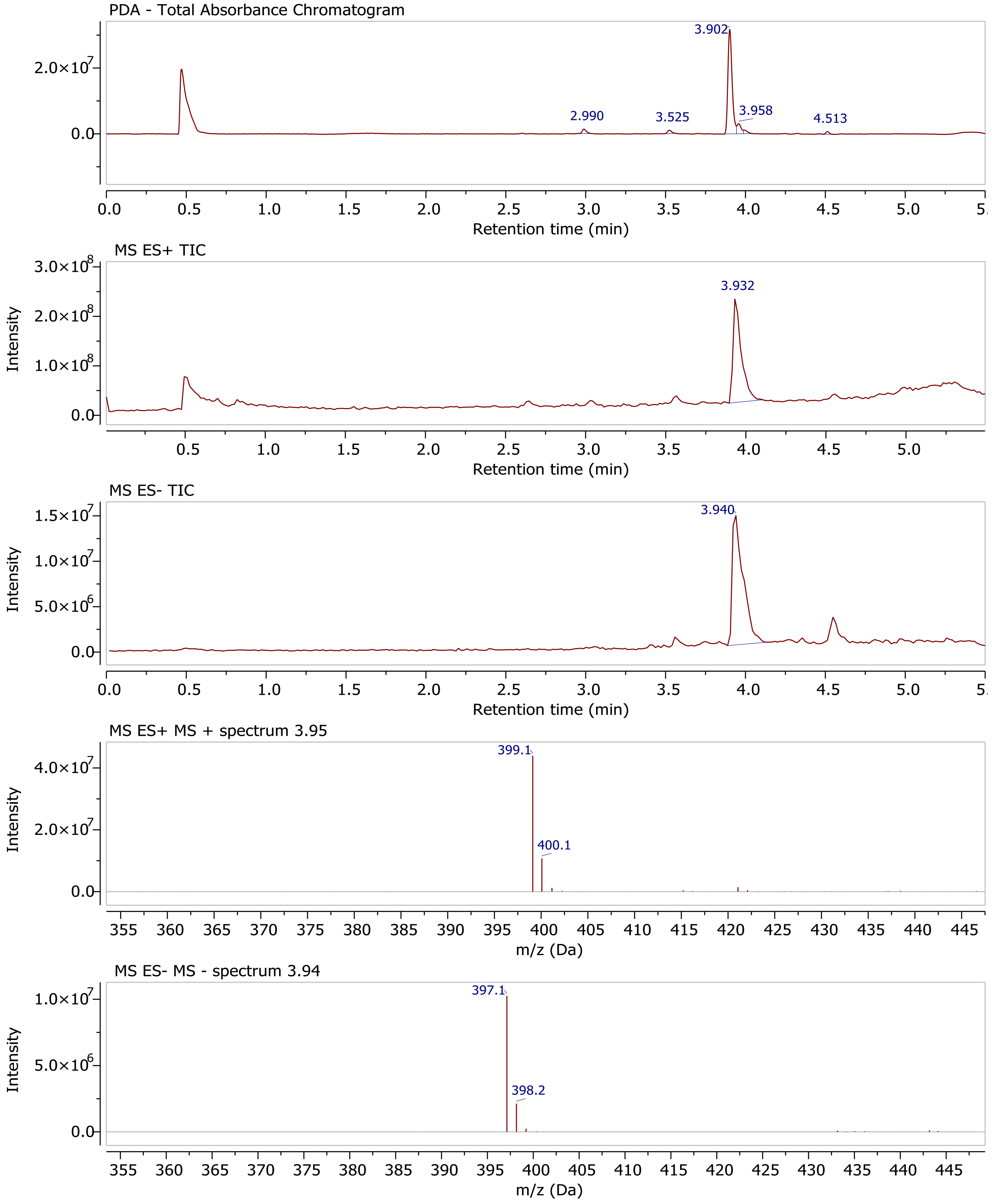

Supplementary figure **1336**. UPLC-MS analysis for compound **PHP-5601**

Chemical Formula: $C_{20}H_{22}N_2O_2$
Exact Mass: 322.2

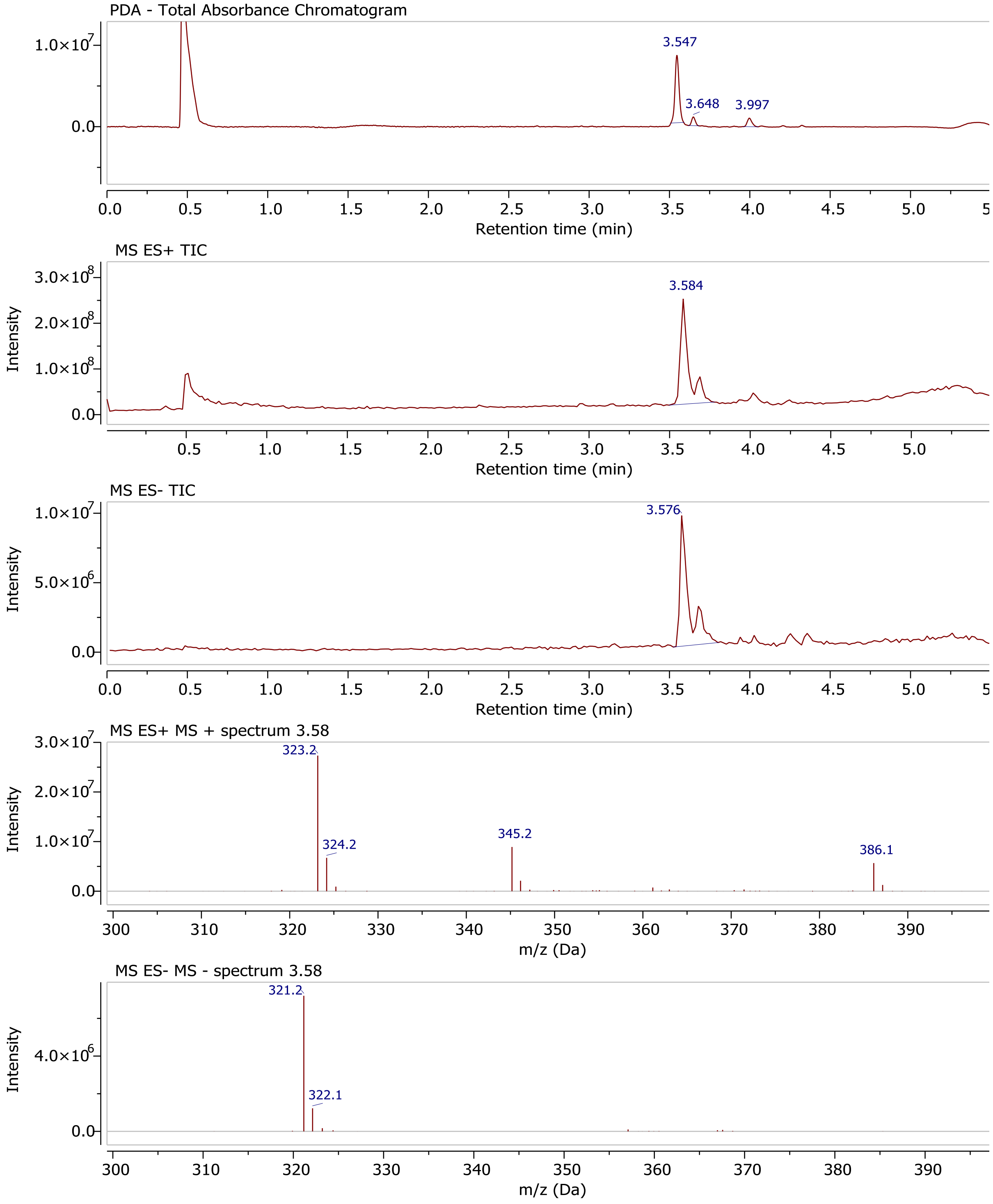

Supplementary figure **1337**. UPLC-MS analysis for compound **PHP-5747**

Chemical Formula: $C_{27}H_{22}N_2O_3$
Exact Mass: 422.2

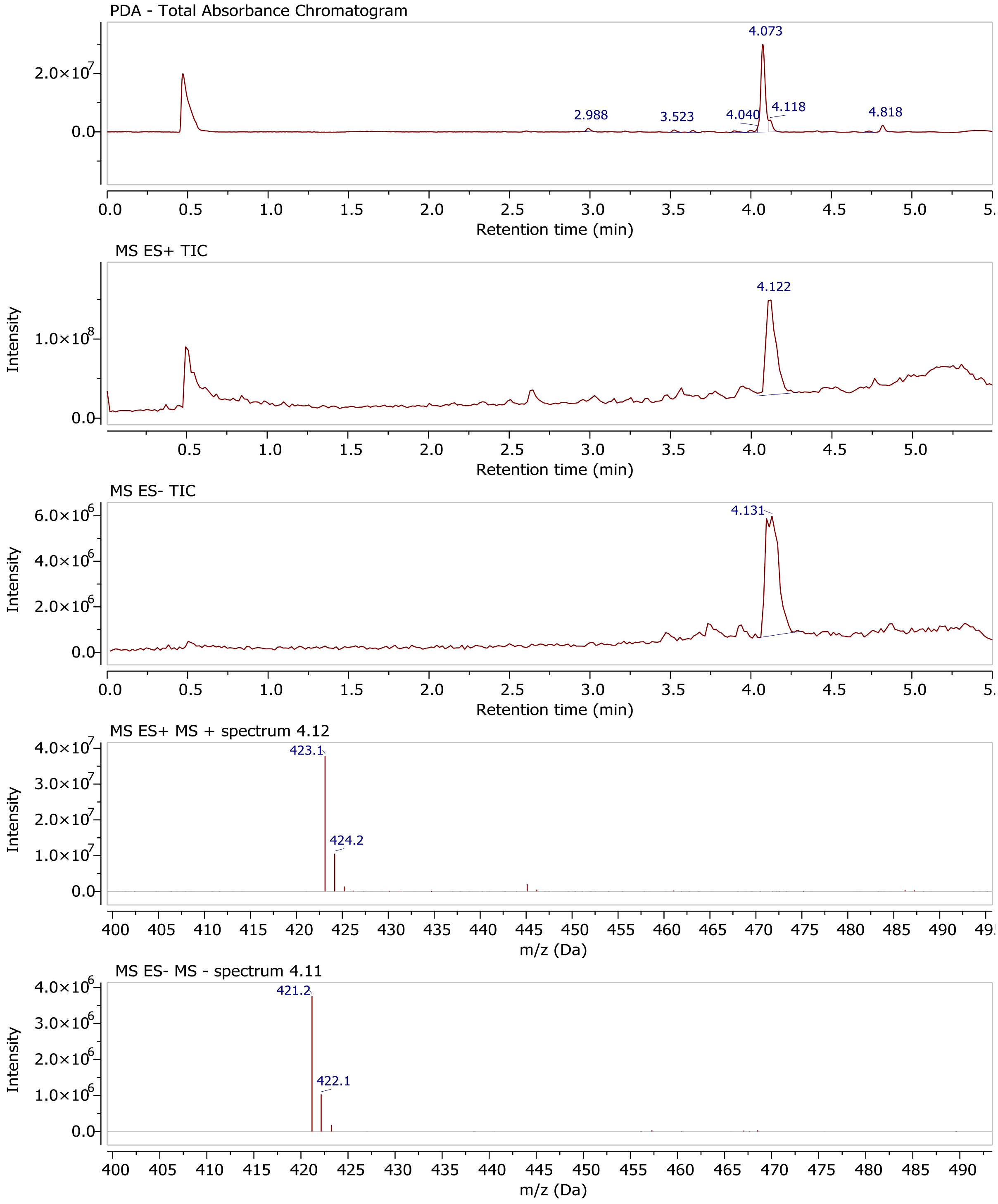

Supplementary figure **1338**. UPLC-MS analysis for compound **PHP-5602**

Chemical Formula: $C_{18}H_{24}N_2O_3$
Exact Mass: 316.2

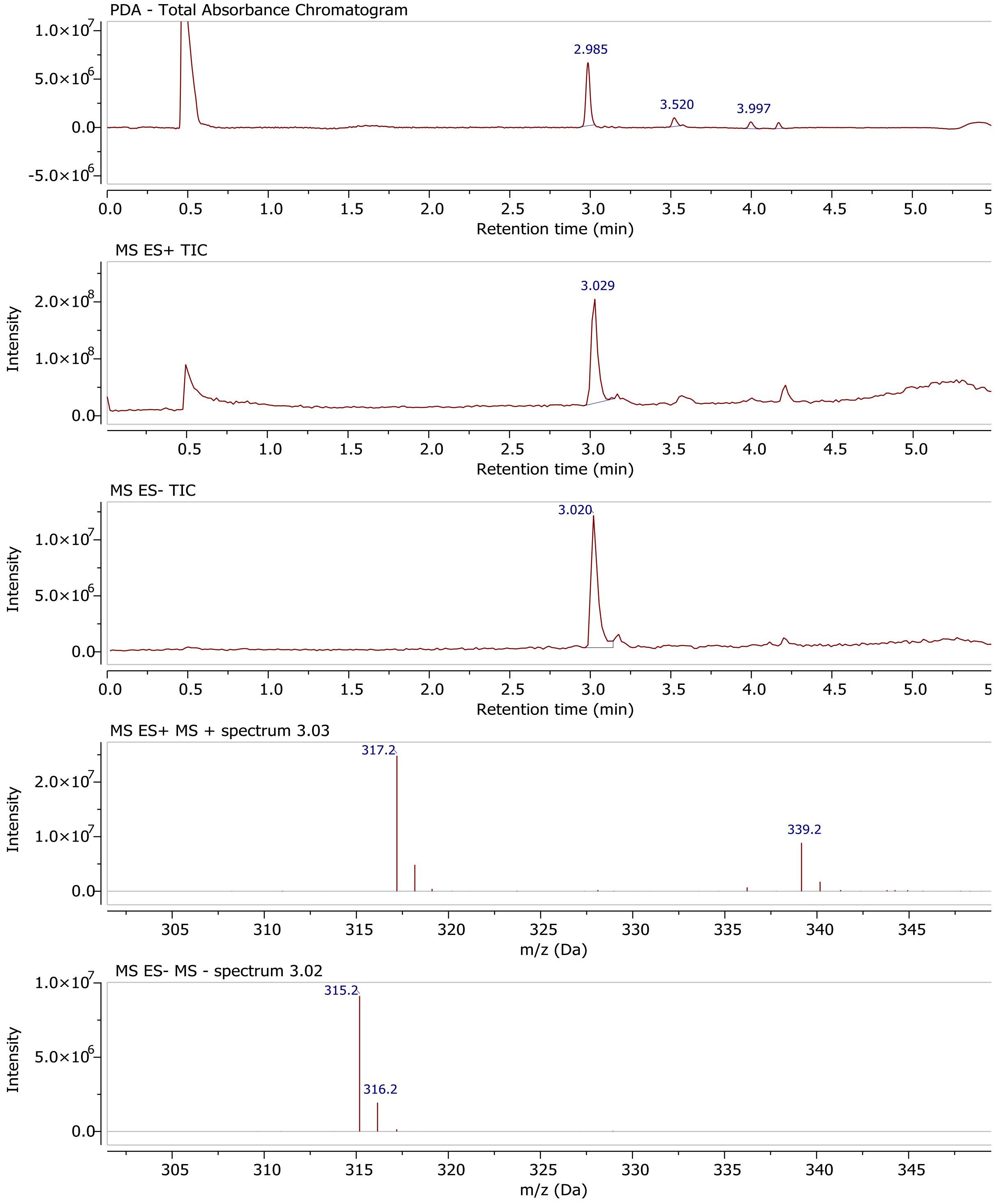

Supplementary figure **1339**. UPLC-MS analysis for compound **PHP-5748**

Chemical Formula: $C_{24}H_{24}N_2O_2$
Exact Mass: 372.2

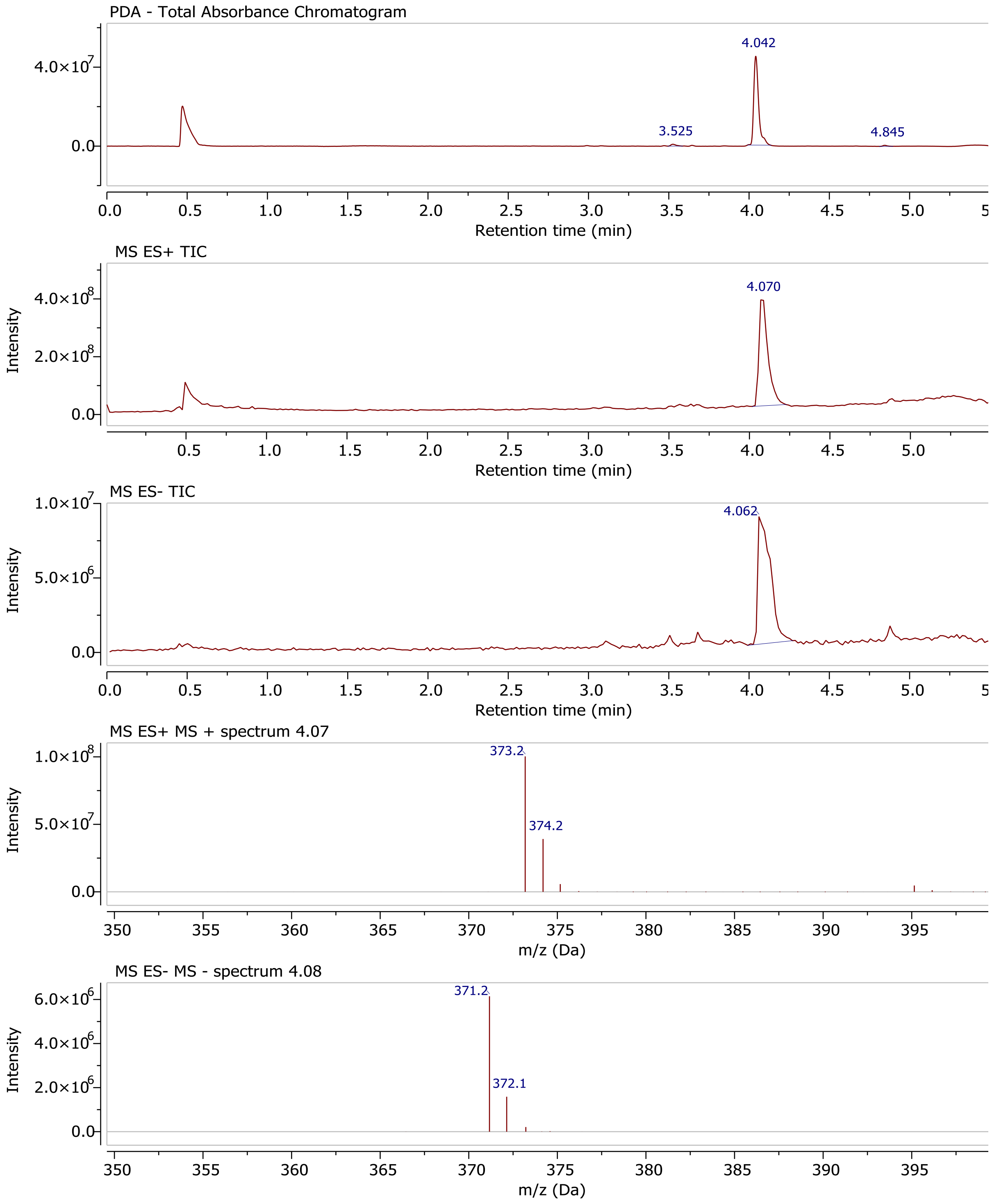

Supplementary figure **1340**. UPLC-MS analysis for compound **PHP-5604**

Chemical Formula: $C_{19}H_{28}N_2O_2$
Exact Mass: 316.2

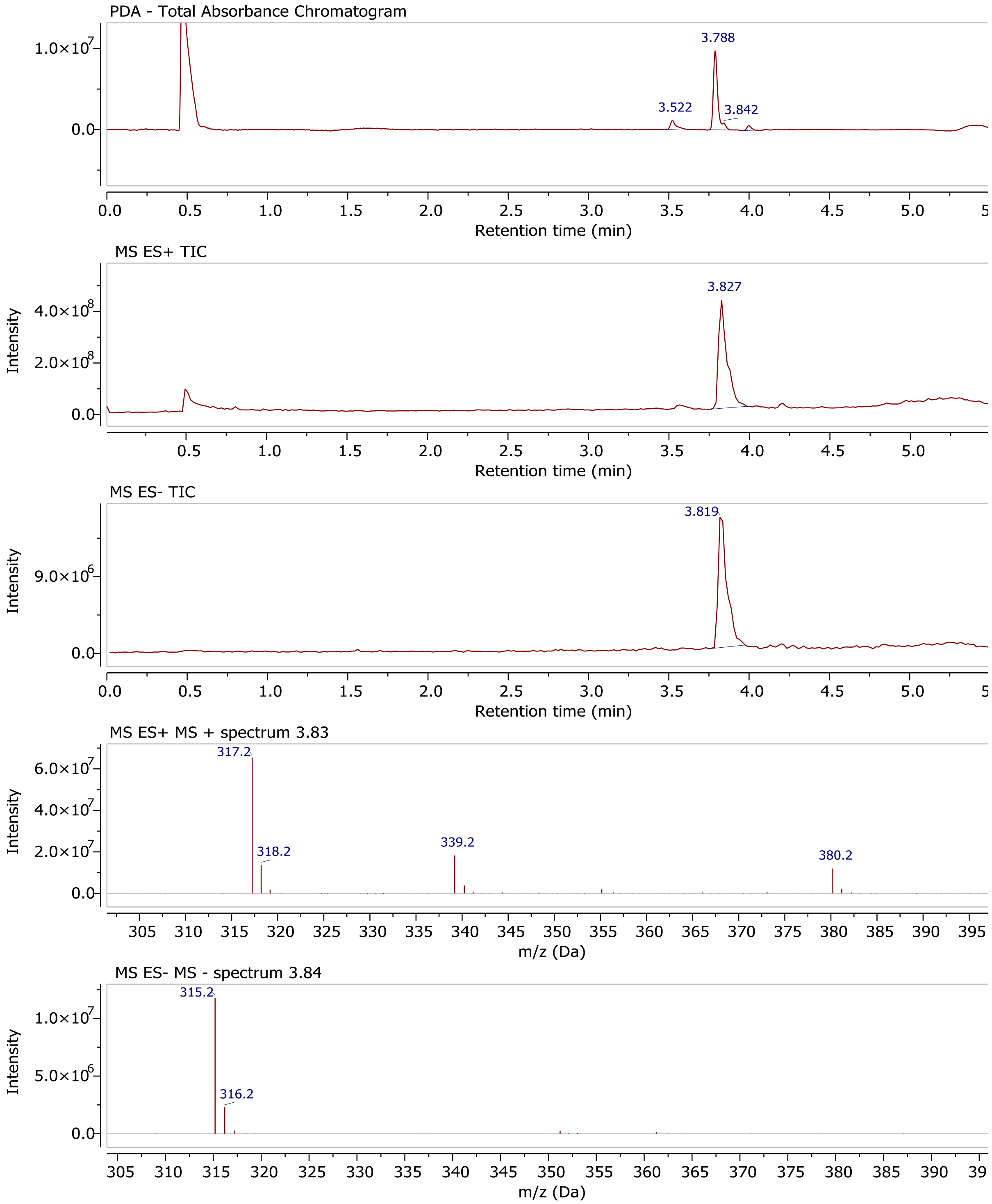

Supplementary figure **1341**. UPLC-MS analysis for compound **PHP-5750**

Chemical Formula: $C_{21}H_{17}ClN_2O_2$
Exact Mass: 364.1

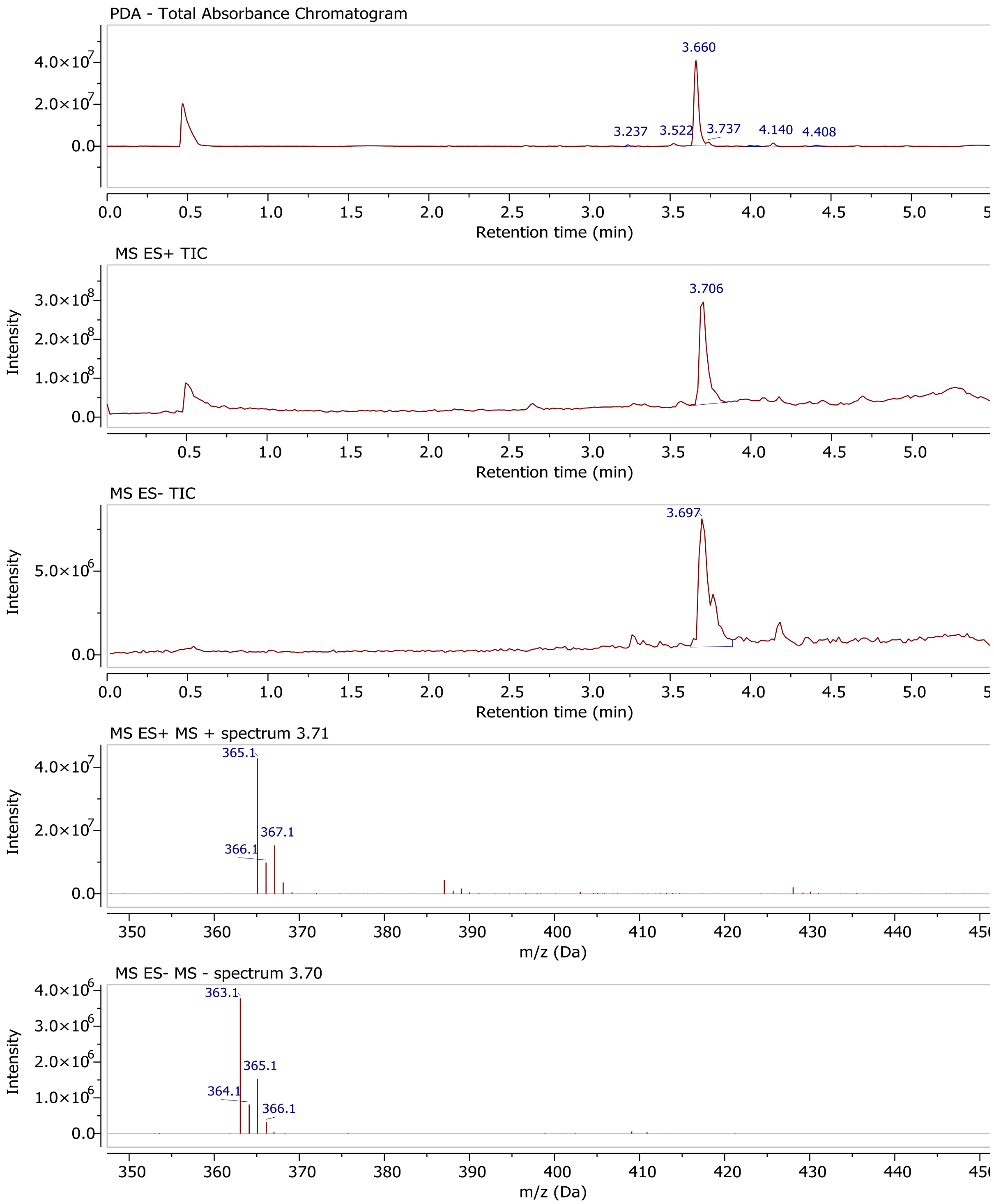

Supplementary figure **1342**. UPLC-MS analysis for compound **PHP-5605**

Chemical Formula: $C_{21}H_{21}F_3N_2O_2$
Exact Mass: 390.2

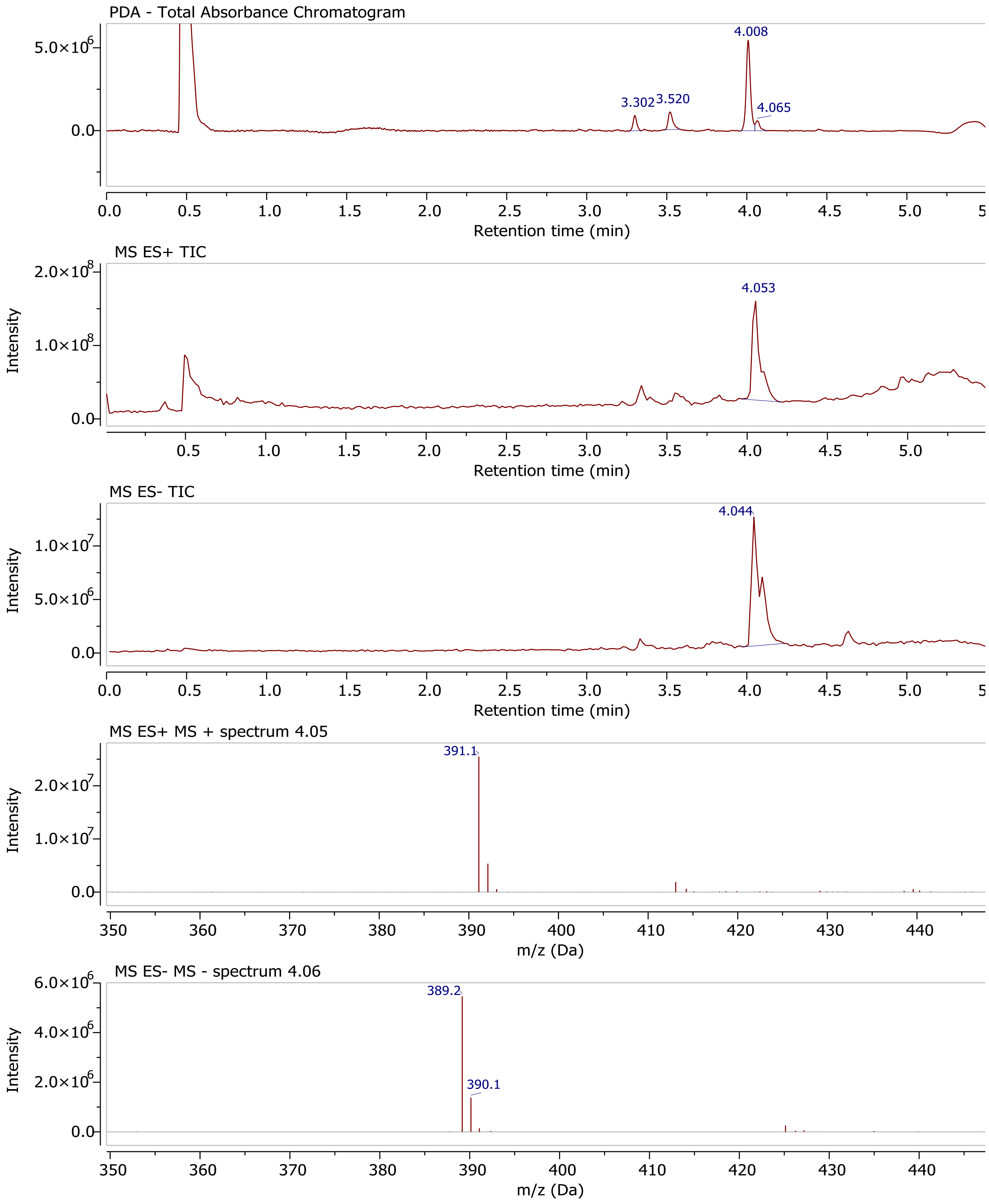

Supplementary figure **1343**. UPLC-MS analysis for compound **PHP-5751**

Chemical Formula: $C_{22}H_{17}F_3N_2O_2$
Exact Mass: 398.1

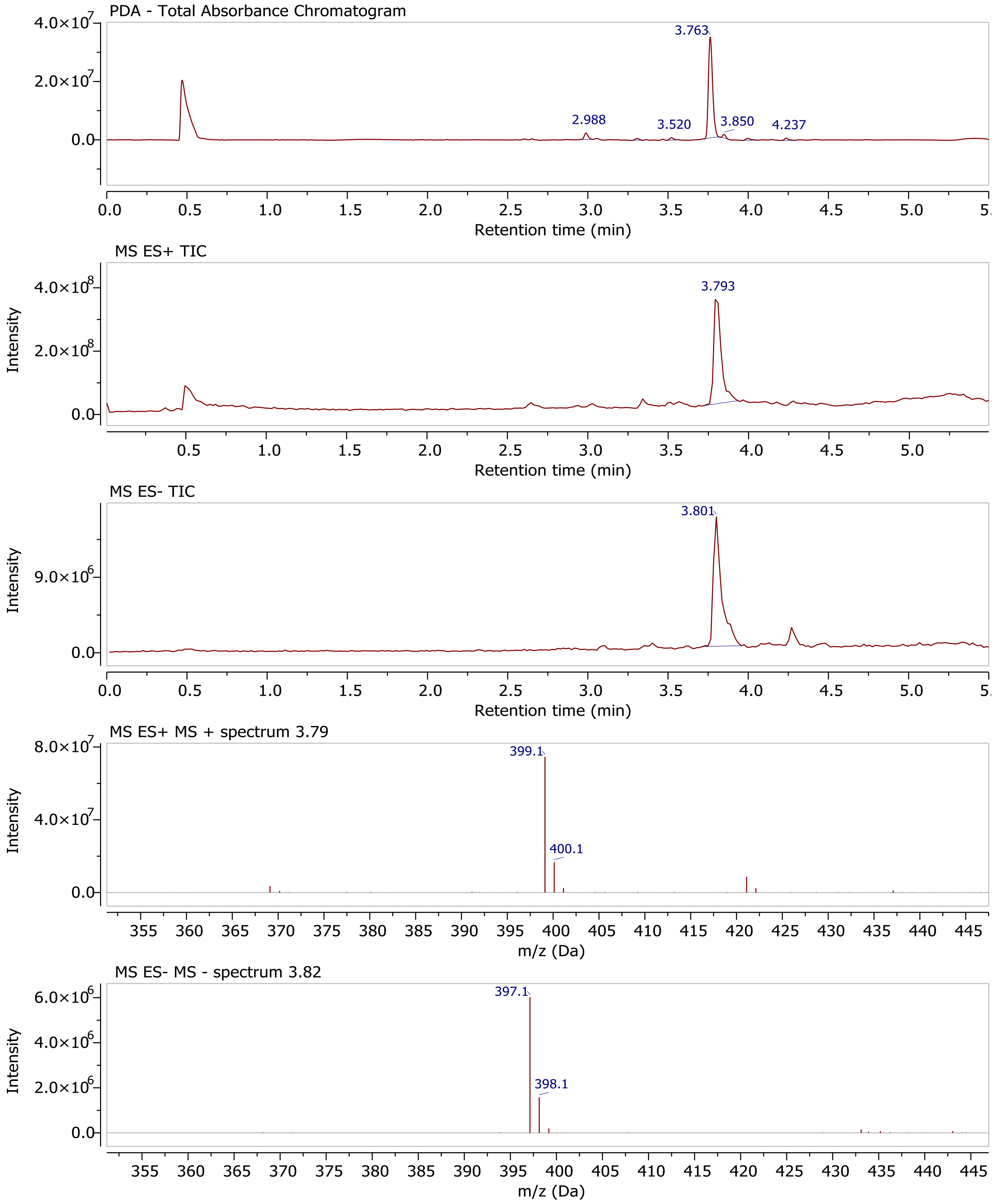

Supplementary figure **1344**. UPLC-MS analysis for compound **PHP-5606**

Chemical Formula: $C_{23}H_{28}N_2O_2$
Exact Mass: 364.2

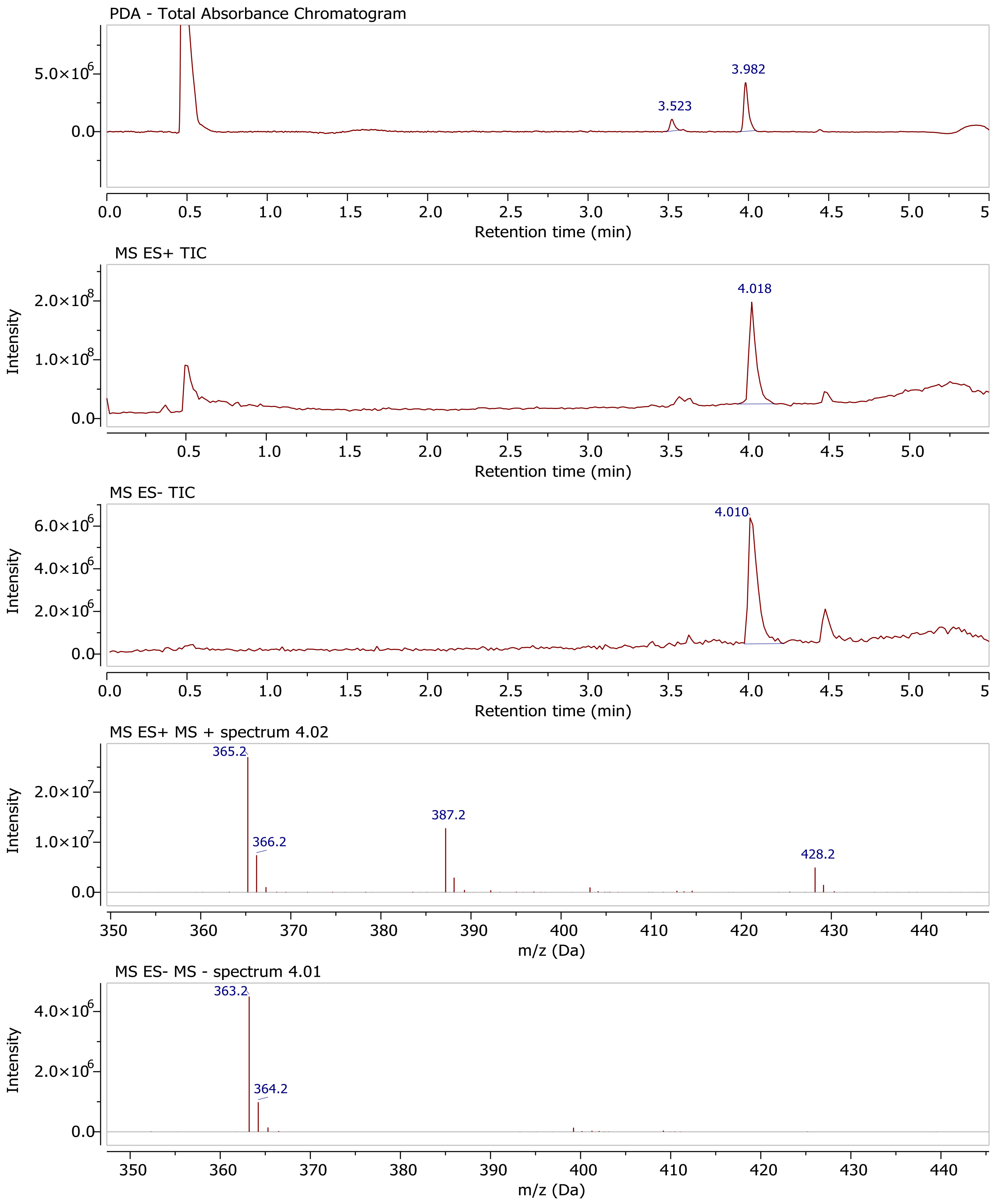

Supplementary figure **1345**. UPLC-MS analysis for compound **PHP-5756**

Chemical Formula: $C_{21}H_{17}FN_2O_2$
Exact Mass: 348.1

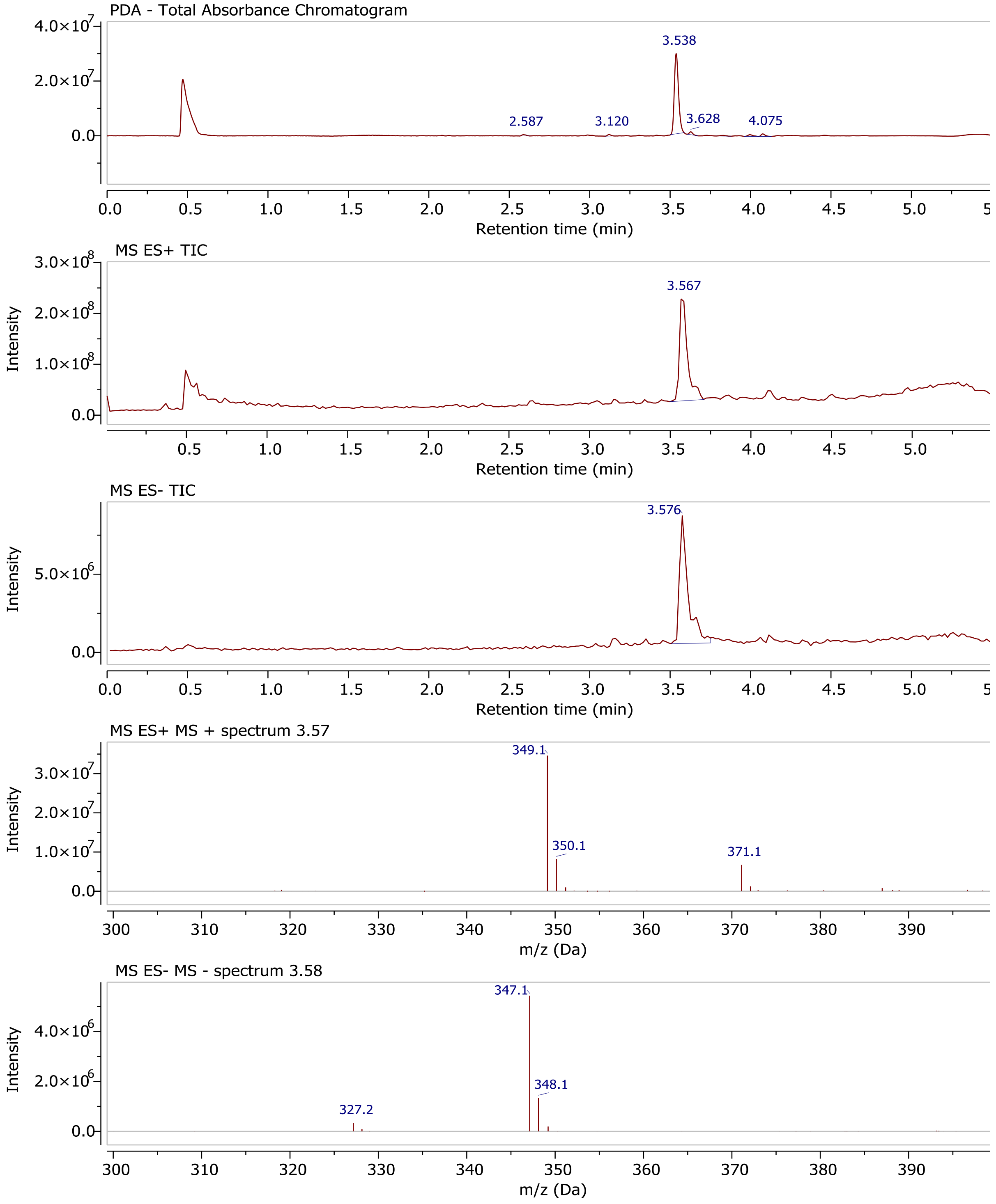

Supplementary figure **1346**. UPLC-MS analysis for compound **PHP-5608**

Chemical Formula: $C_{19}H_{26}N_2O_2$
Exact Mass: 314.2

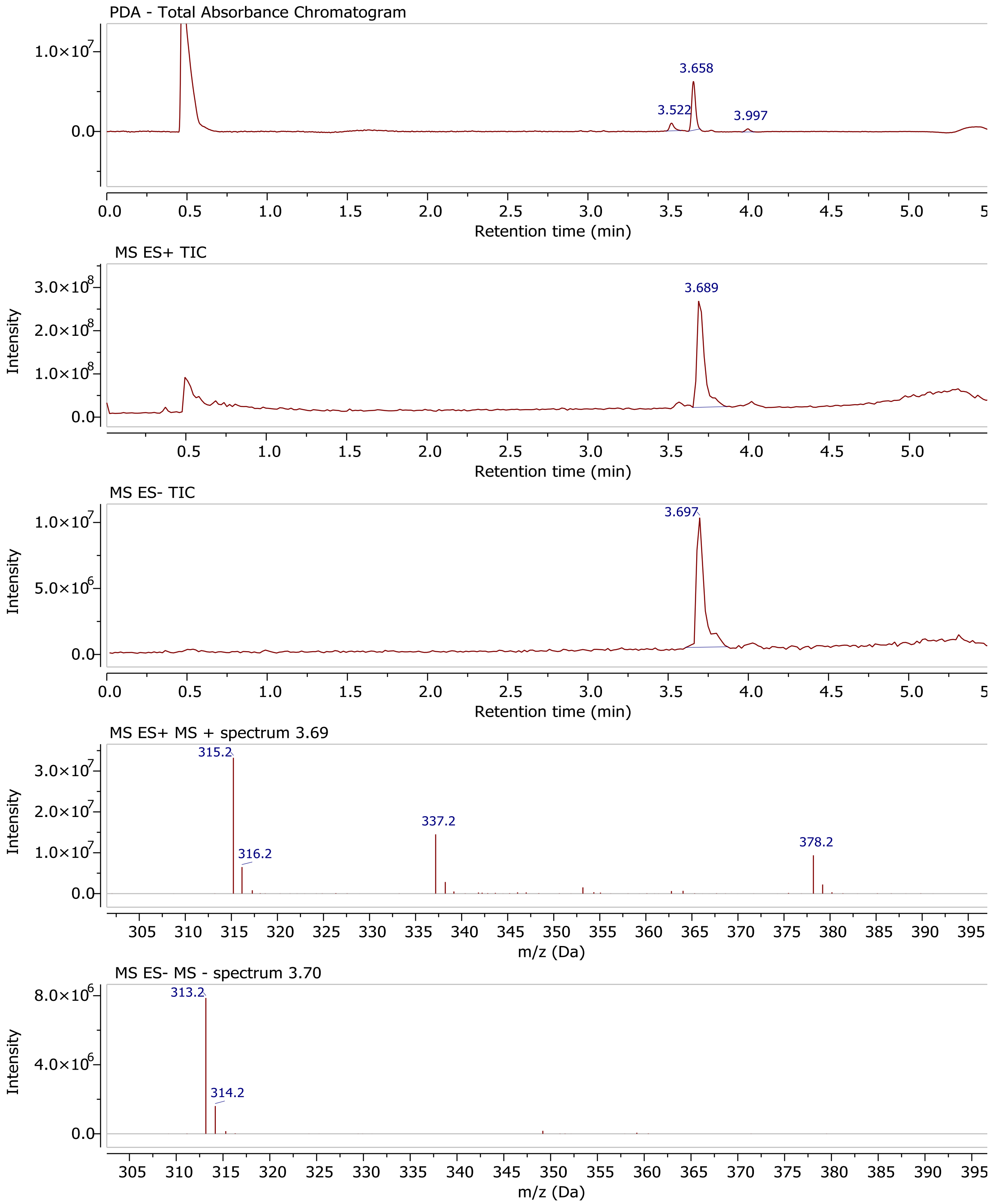

Supplementary figure **1347**. UPLC-MS analysis for compound **PHP-5757**

Chemical Formula: $C_{22}H_{21}N_3O_3S$
Exact Mass: 407.1

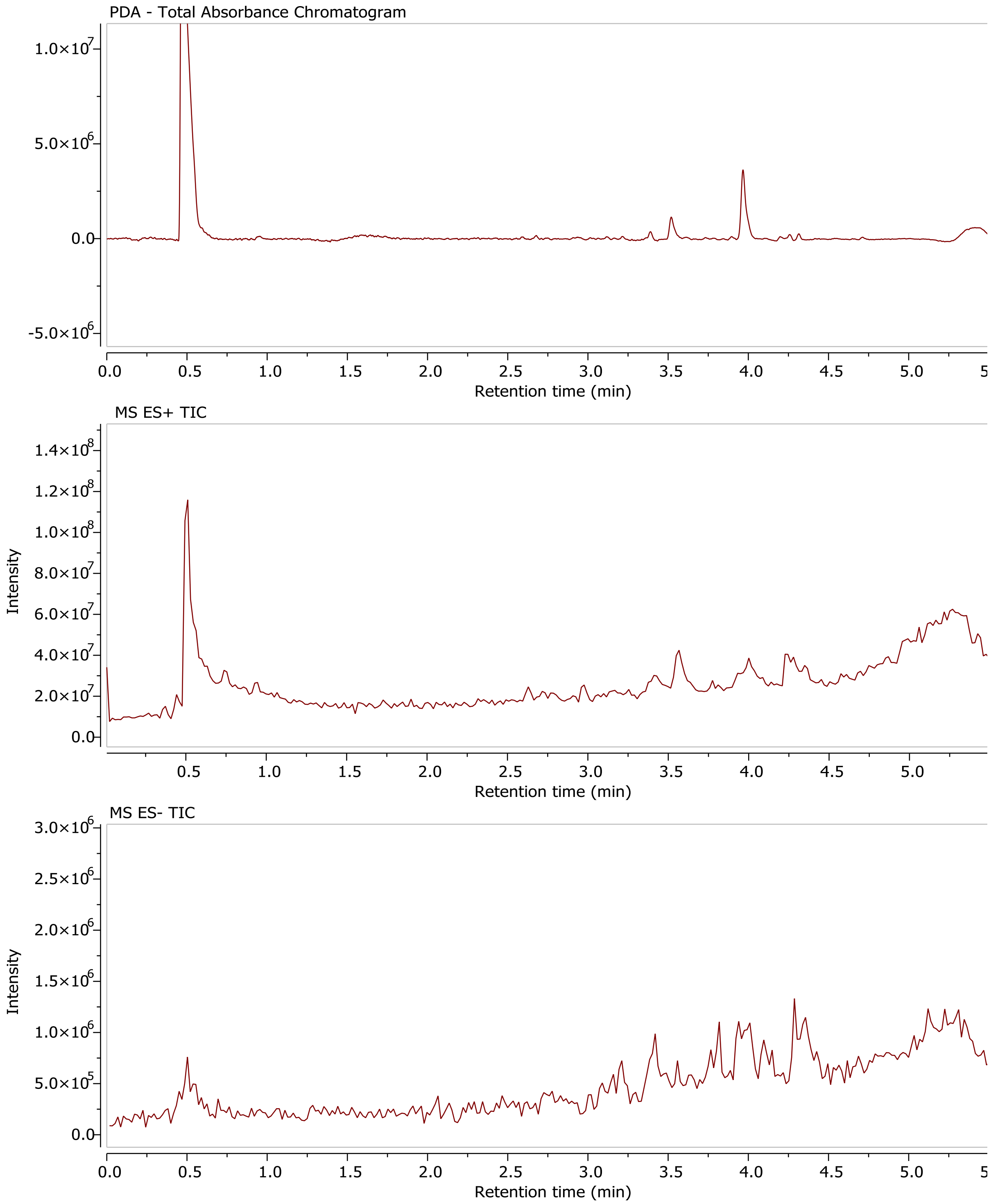

Supplementary figure **1348**. UPLC-MS analysis for compound **PHP-4851**

Chemical Formula: $C_{28}H_{28}N_2O_3$
Exact Mass: 440.2

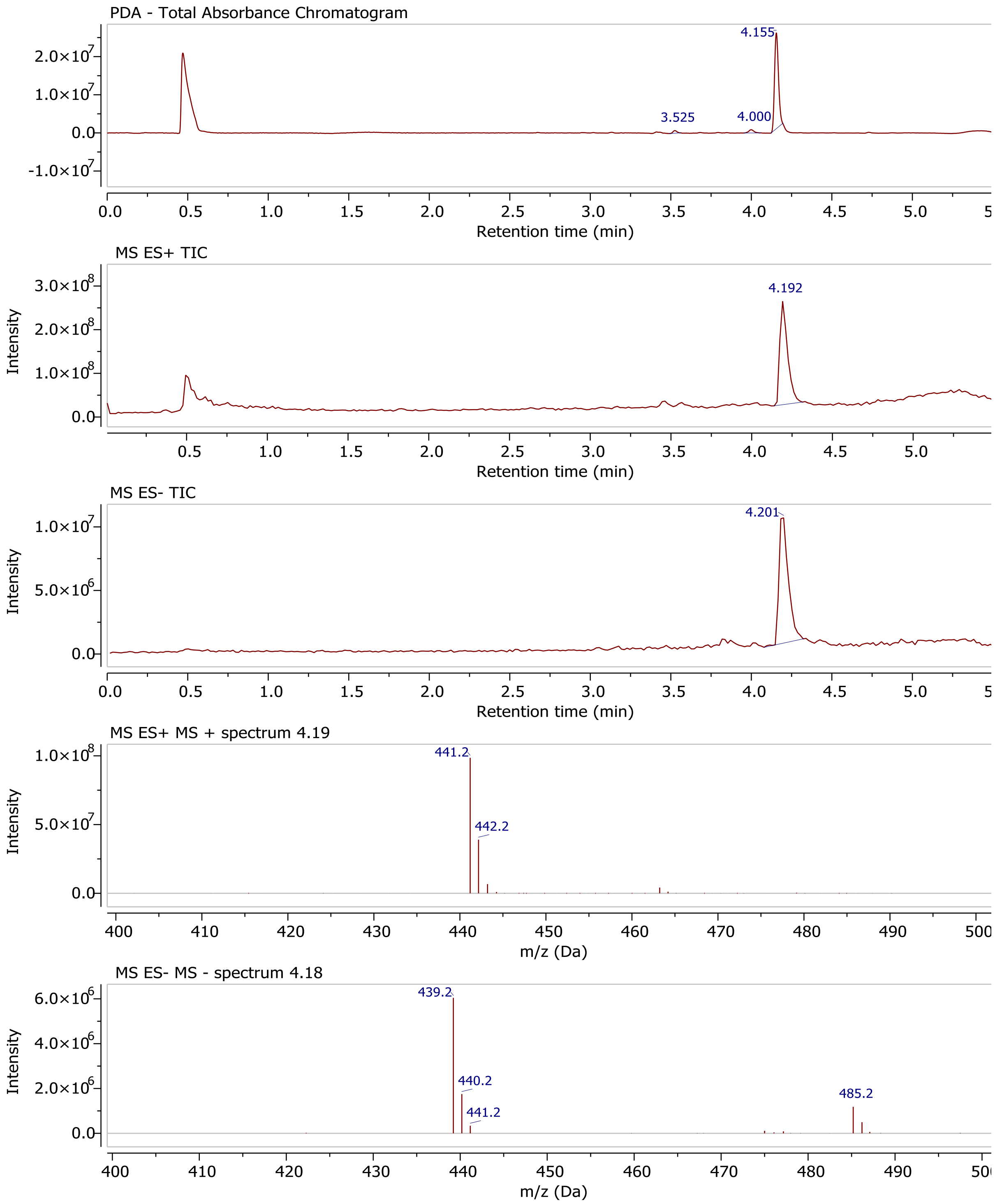

Supplementary figure **1349**. UPLC-MS analysis for compound **PHP-5433**

Chemical Formula: $C_{27}H_{26}N_2O_6$
Exact Mass: 474.2

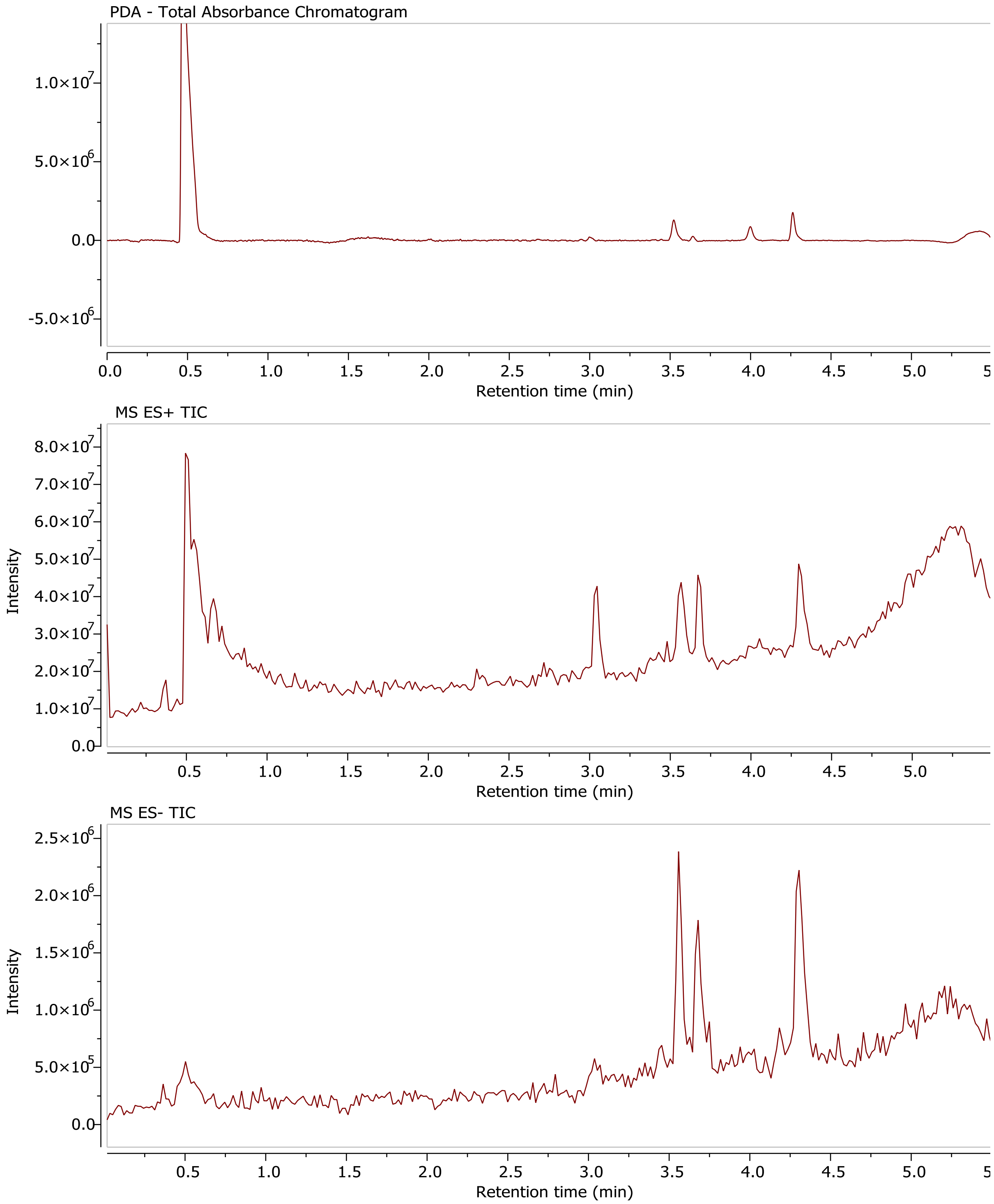

Supplementary figure **1350**. UPLC-MS analysis for compound **PHP-4854**

Chemical Formula: $C_{31}H_{28}N_2O_4$
Exact Mass: 492.2

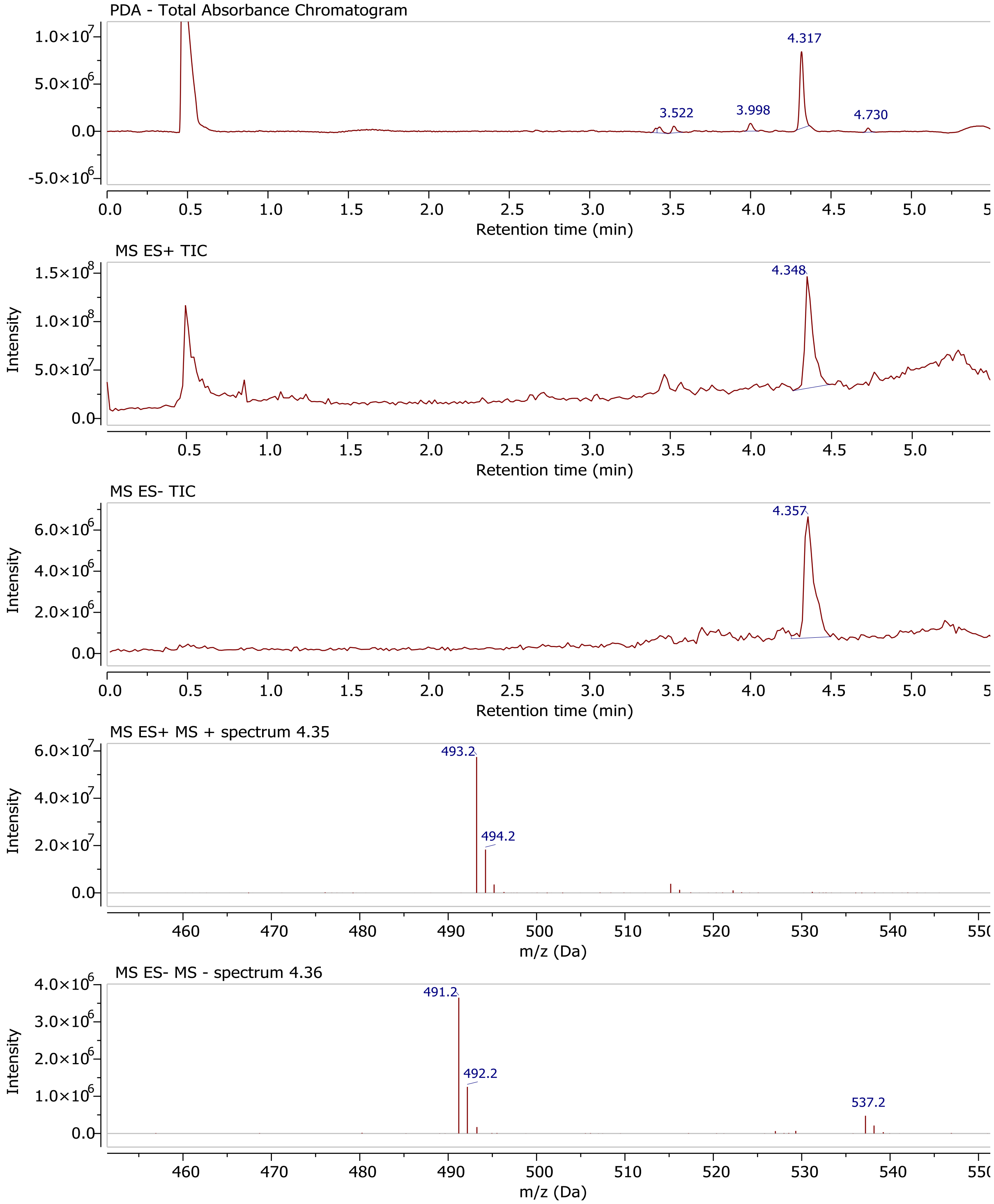

Supplementary figure **1351**. UPLC-MS analysis for compound **PHP-5434**

Chemical Formula: $C_{32}H_{30}N_2O_4$
Exact Mass: 506.2

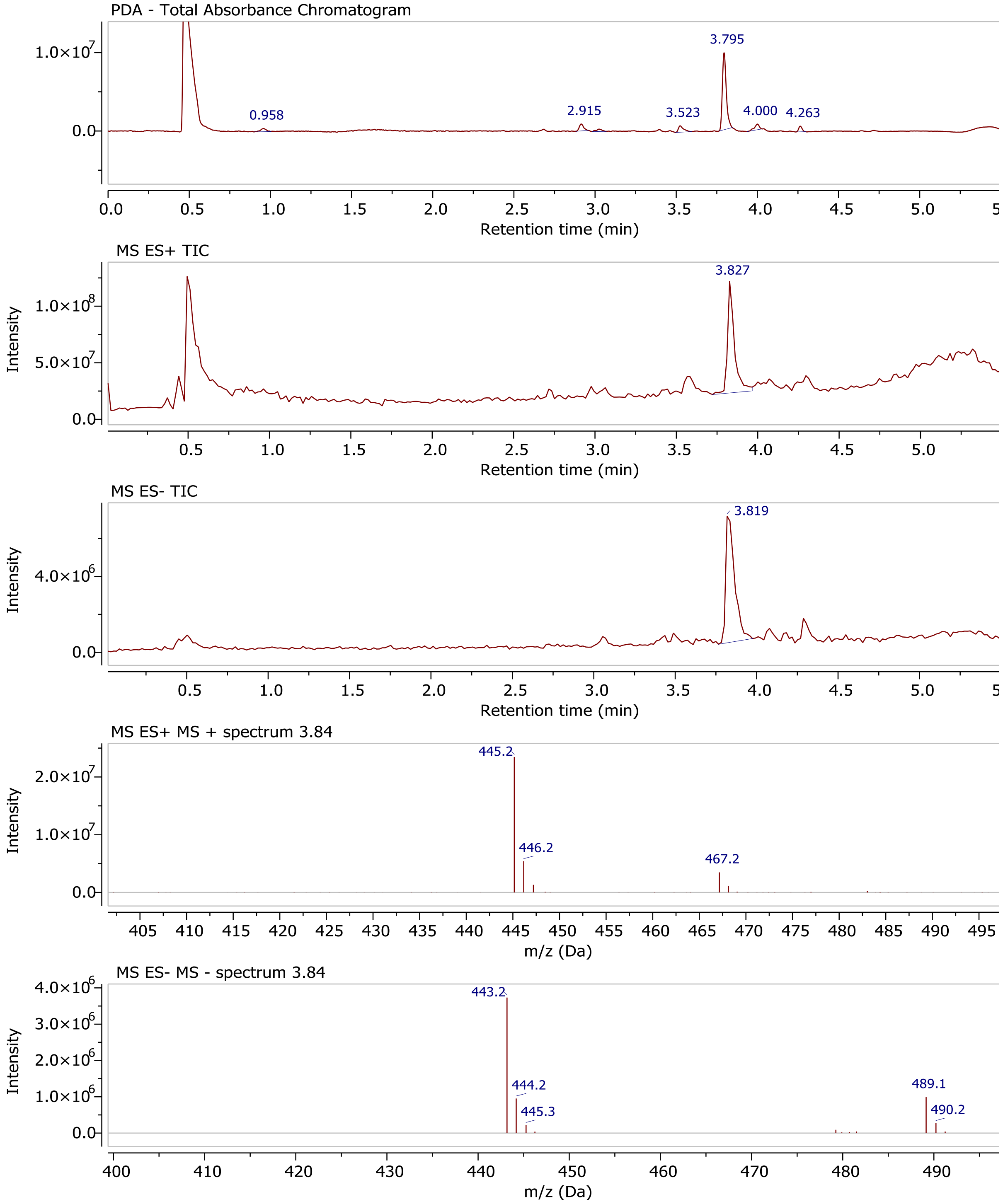

Supplementary figure **1352**. UPLC-MS analysis for compound **PHP-4859**

Chemical Formula: $C_{22}H_{26}N_2O_3$
Exact Mass: 366.2

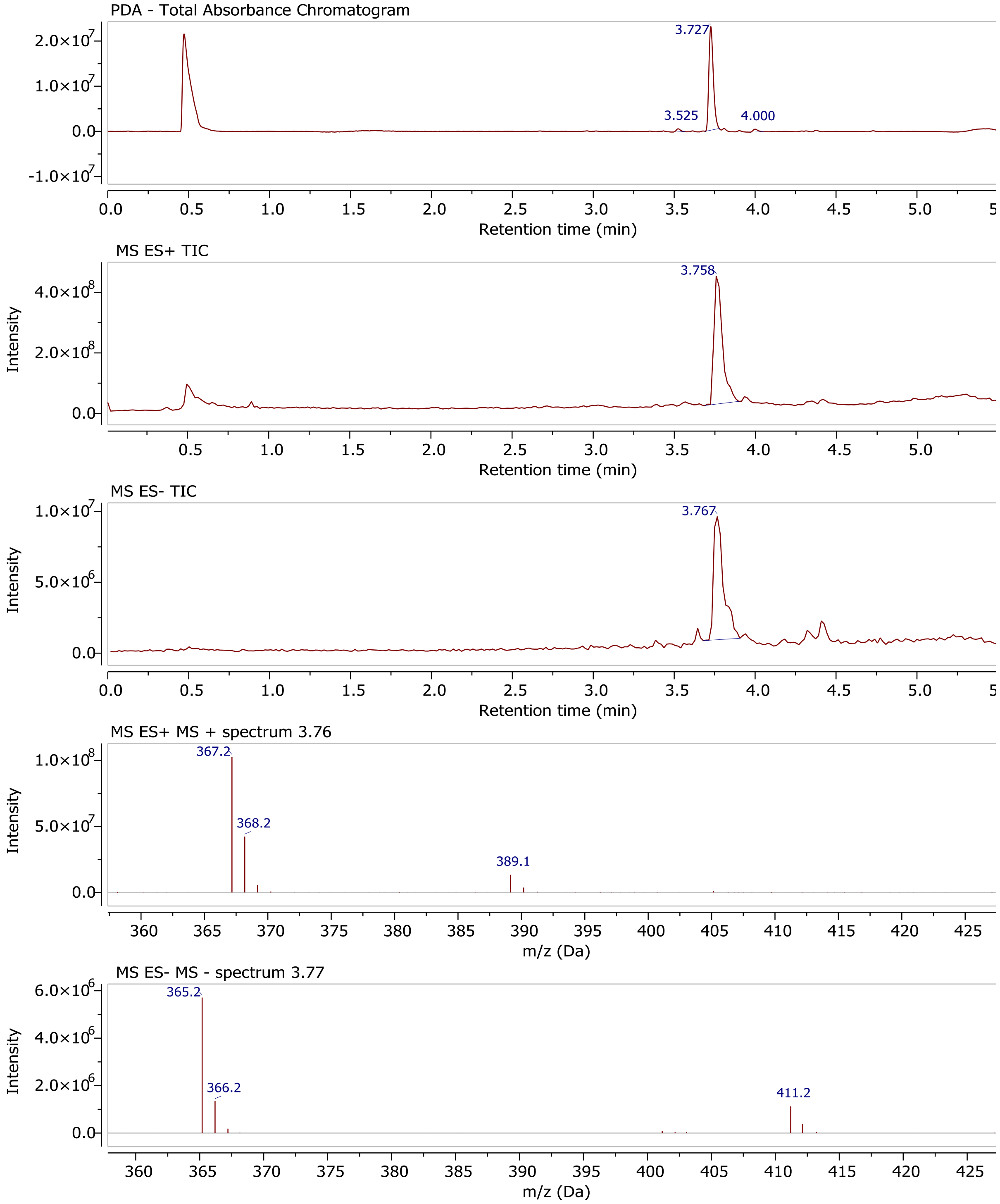

Supplementary figure **1352**. UPLC-MS analysis for compound **PHP-5435**

Chemical Formula: $C_{25}H_{23}ClN_2O_3$
Exact Mass: 434.1

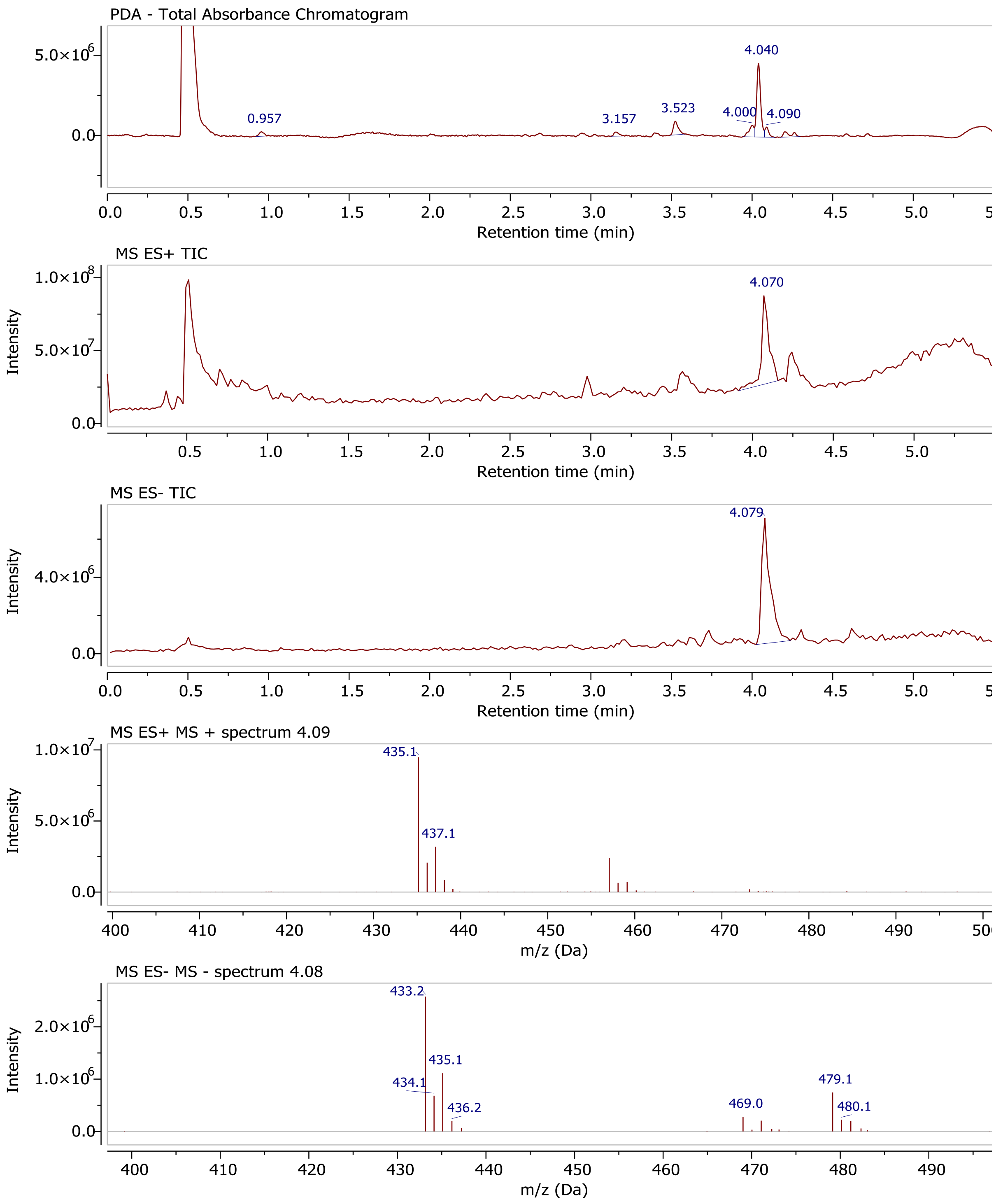

Supplementary figure **1353**. UPLC-MS analysis for compound **PHP-4864**

Chemical Formula: $C_{26}H_{23}F_3N_2O_3$
Exact Mass: 468.2

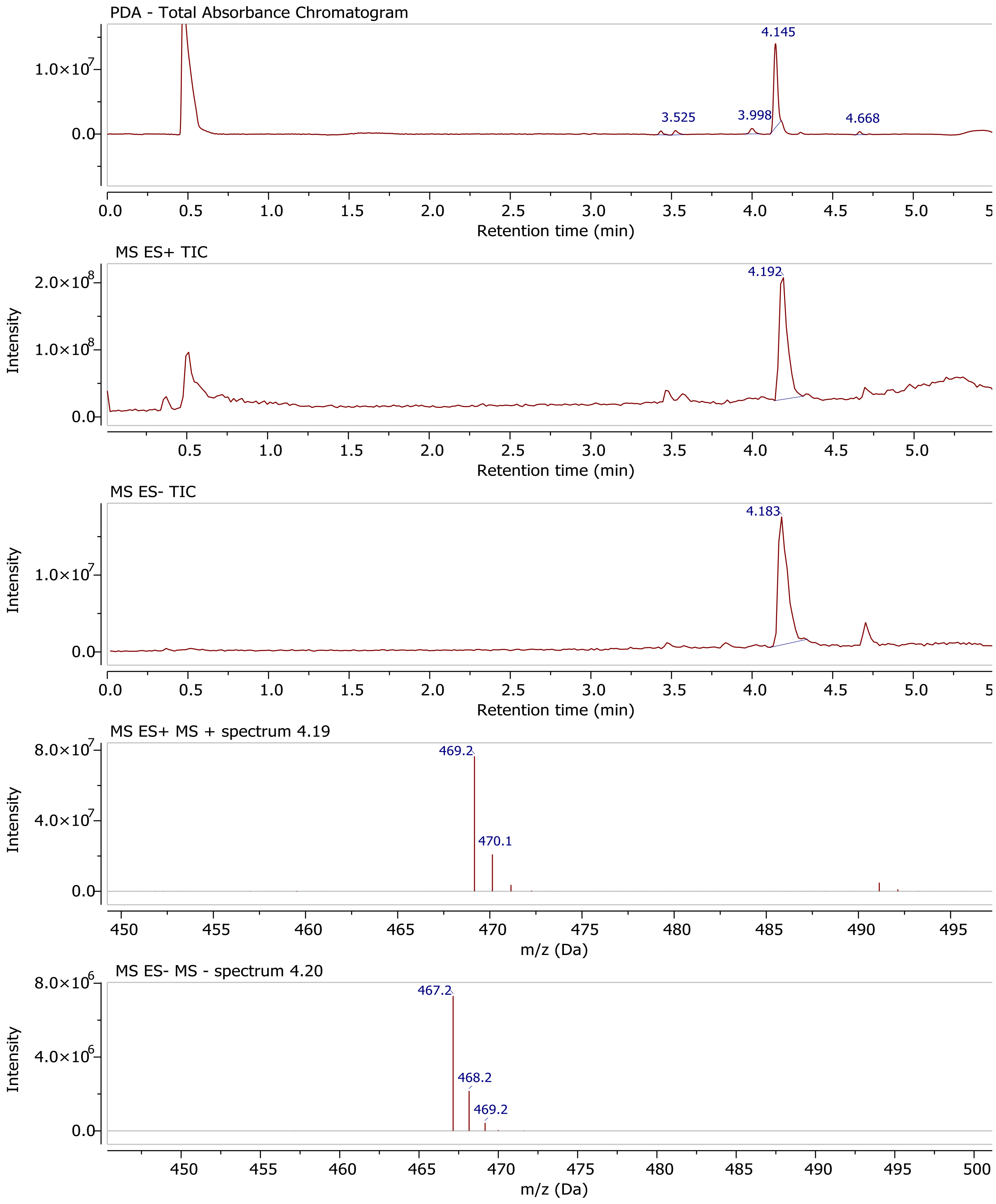

Supplementary figure **1354**. UPLC-MS analysis for compound **PHP-5441**

Chemical Formula: $C_{21}H_{26}N_2O_5S$
Exact Mass: 418.2

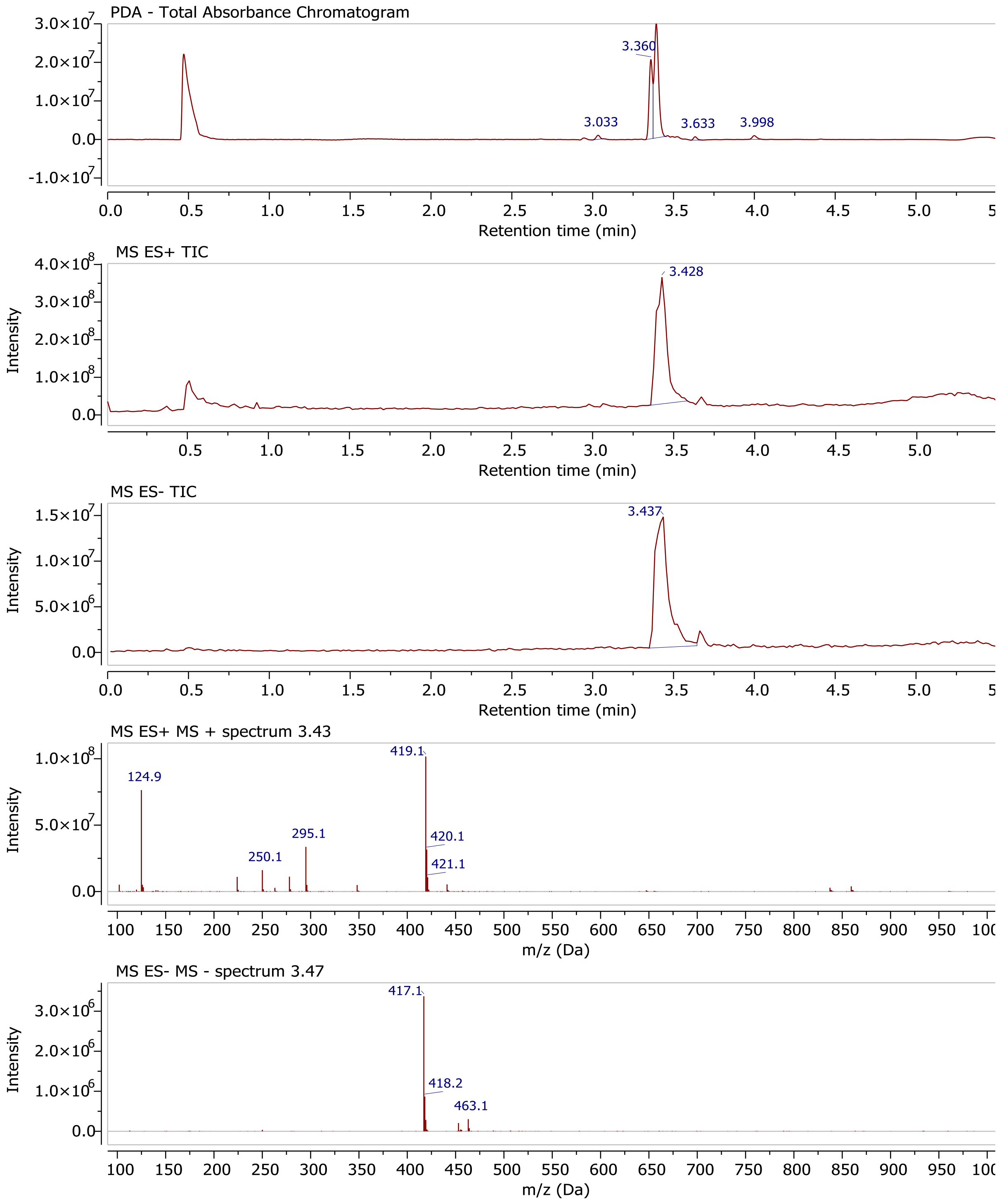

Supplementary figure **1355**. UPLC-MS analysis for compound **PHP-4865**

Chemical Formula: $C_{24}H_{23}N_3O_3$
Exact Mass: 401.2

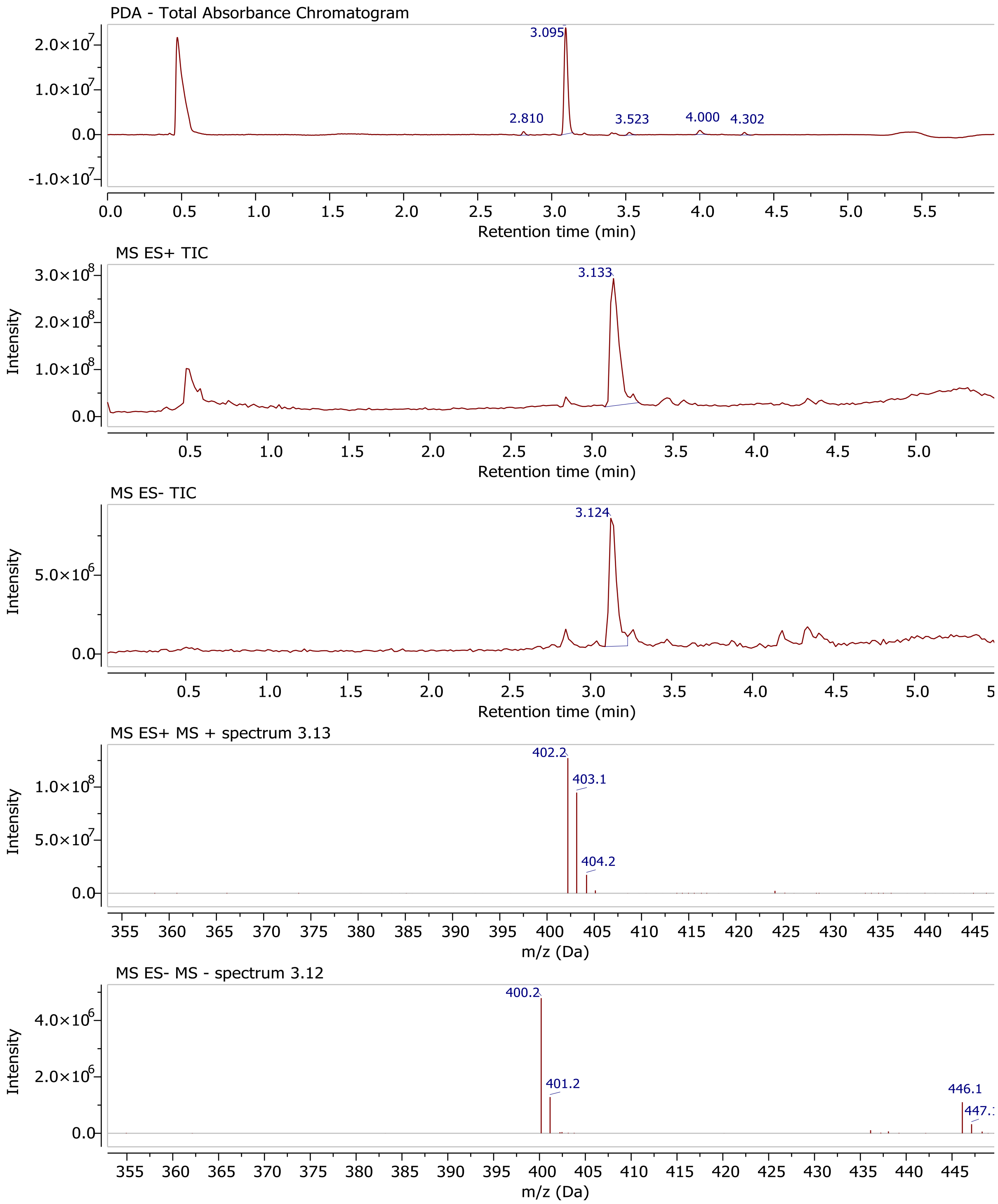

Supplementary figure **1356**. UPLC-MS analysis for compound **PHP-5443**

Chemical Formula: $C_{18}H_{18}Cl_2N_2O_2S$
Exact Mass: 396.0

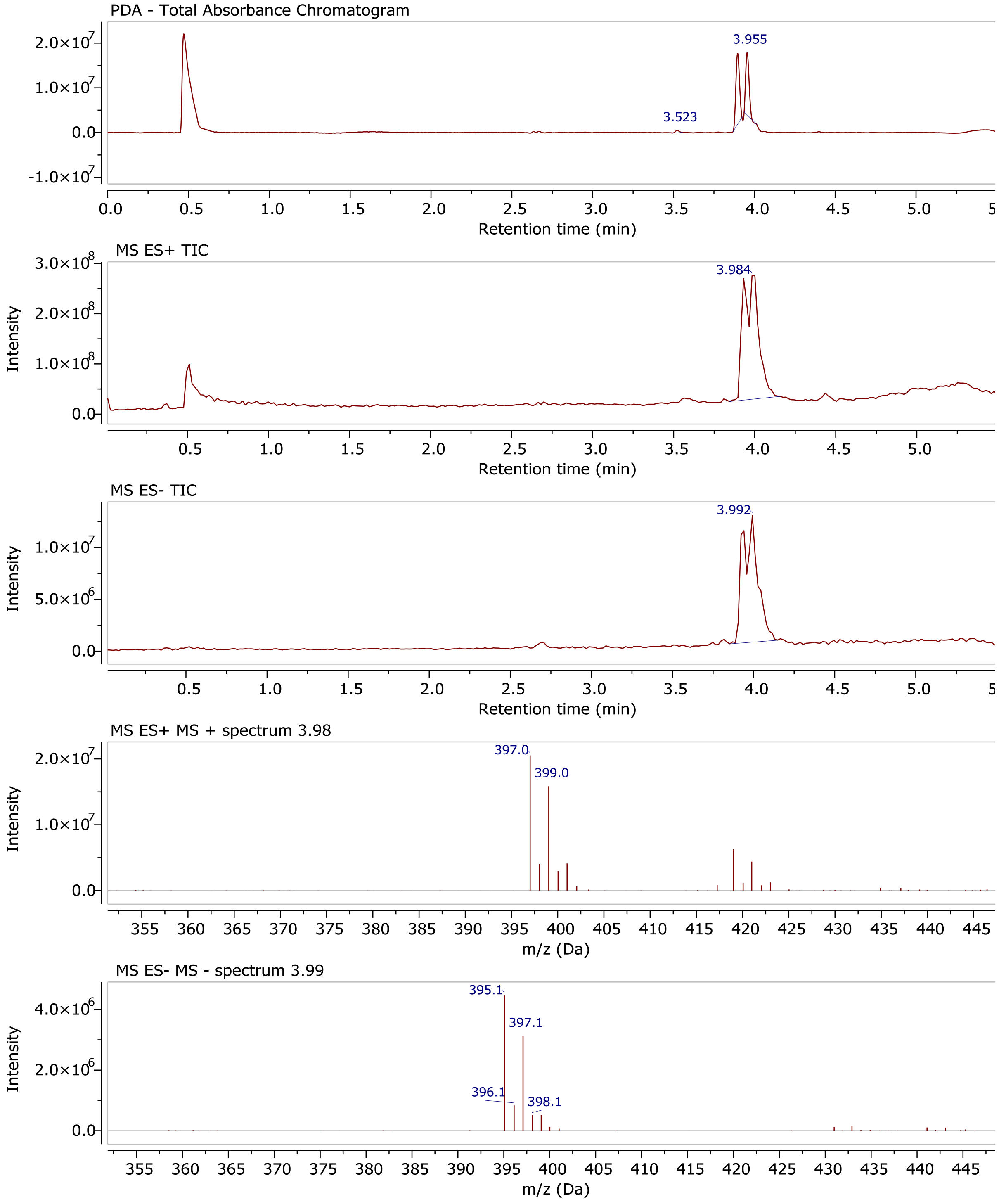

Supplementary figure **1357**. UPLC-MS analysis for compound **PHP-4866**

Chemical Formula: $C_{25}H_{24}N_2O_3$
Exact Mass: 400.2

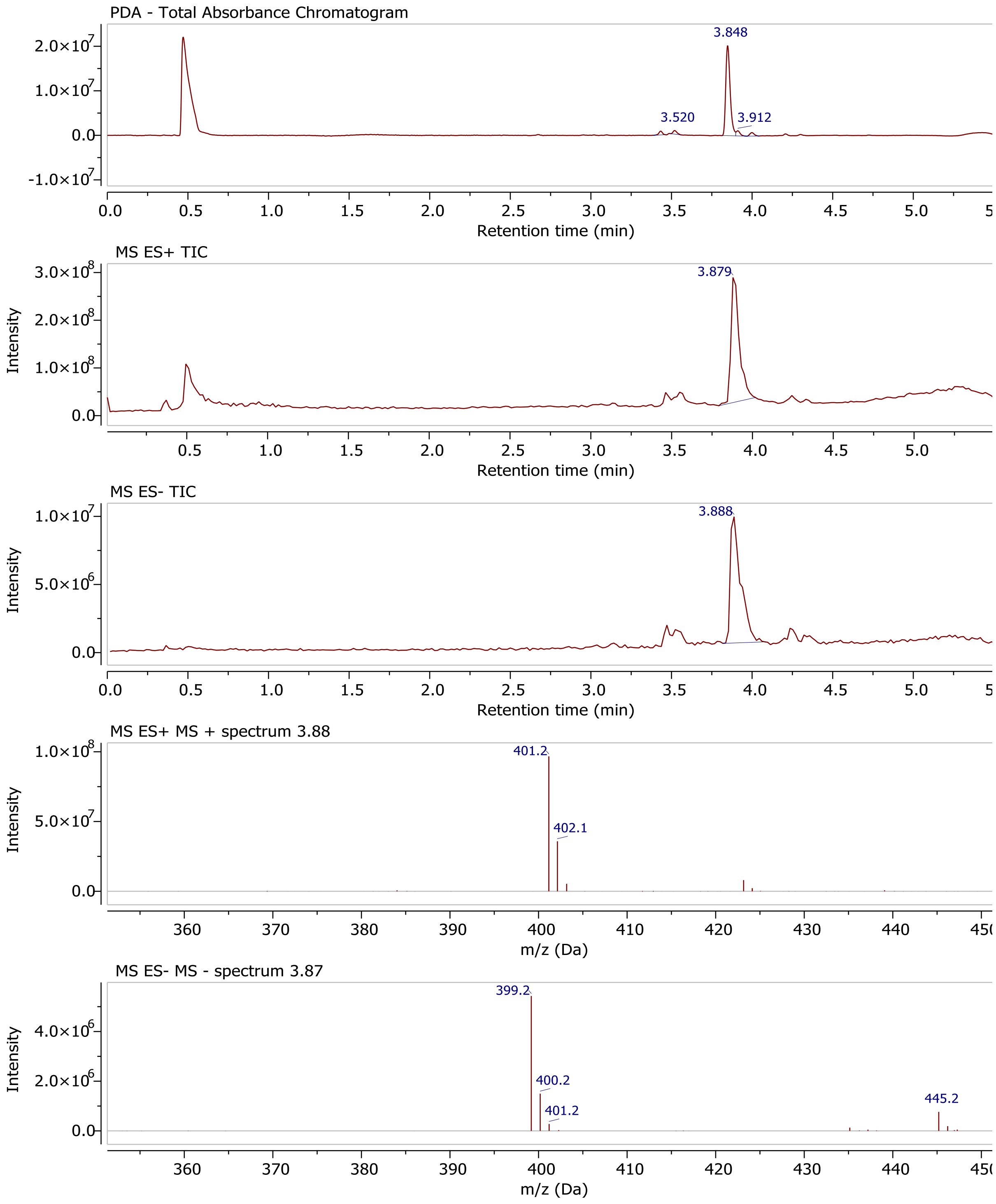

Supplementary figure **1358**. UPLC-MS analysis for compound **PHP-5447**

Chemical Formula: $C_{18}H_{19}BrN_2O_2S$
Exact Mass: 406.0

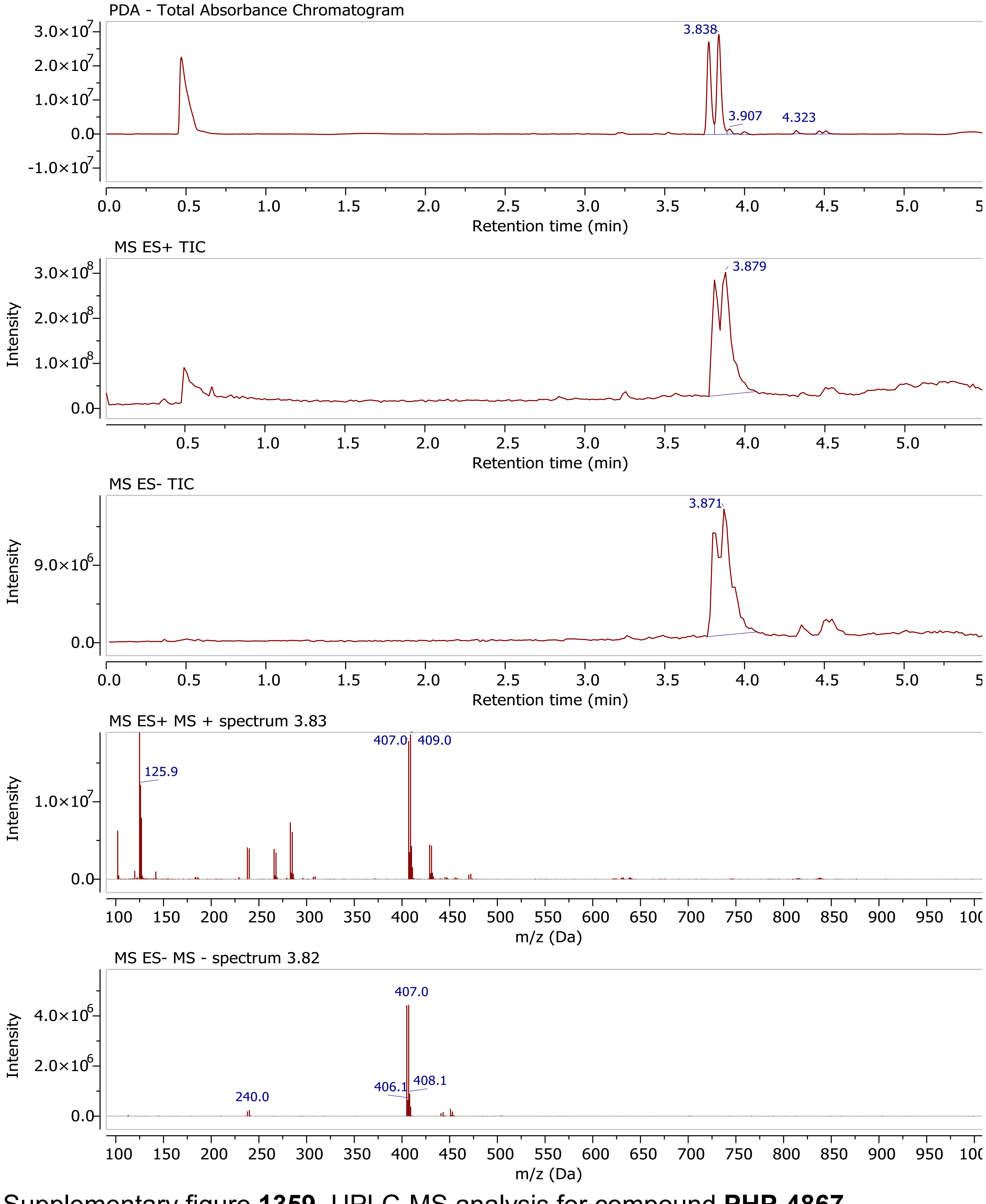

Supplementary figure **1359**. UPLC-MS analysis for compound **PHP-4867**

Chemical Formula: $C_{23}H_{26}N_2O_4$
Exact Mass: 394.2

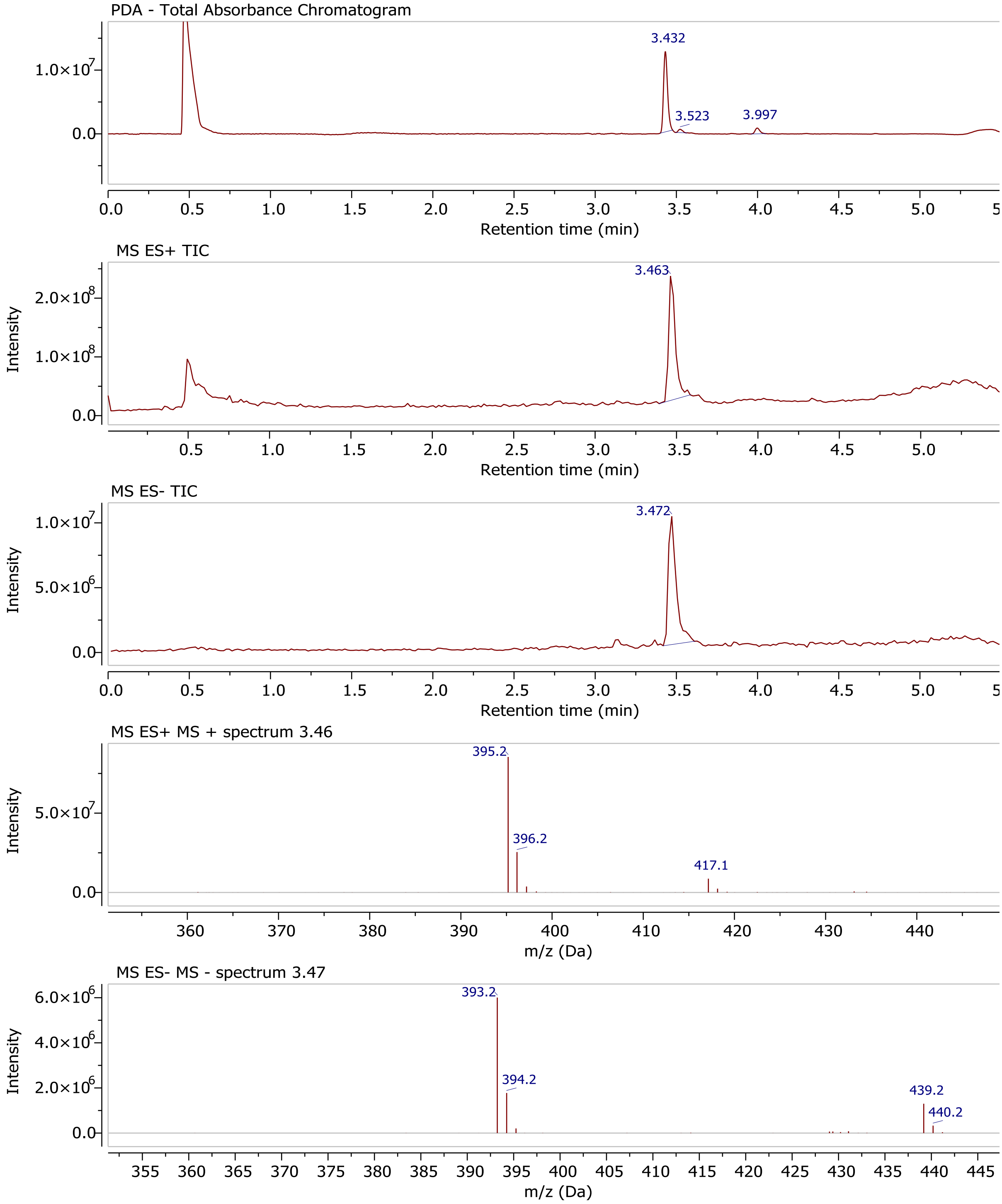

Supplementary figure **1360**. UPLC-MS analysis for compound **PHP-5448**

Chemical Formula: $C_{19}H_{19}N_3O_2S$
Exact Mass: 353.1

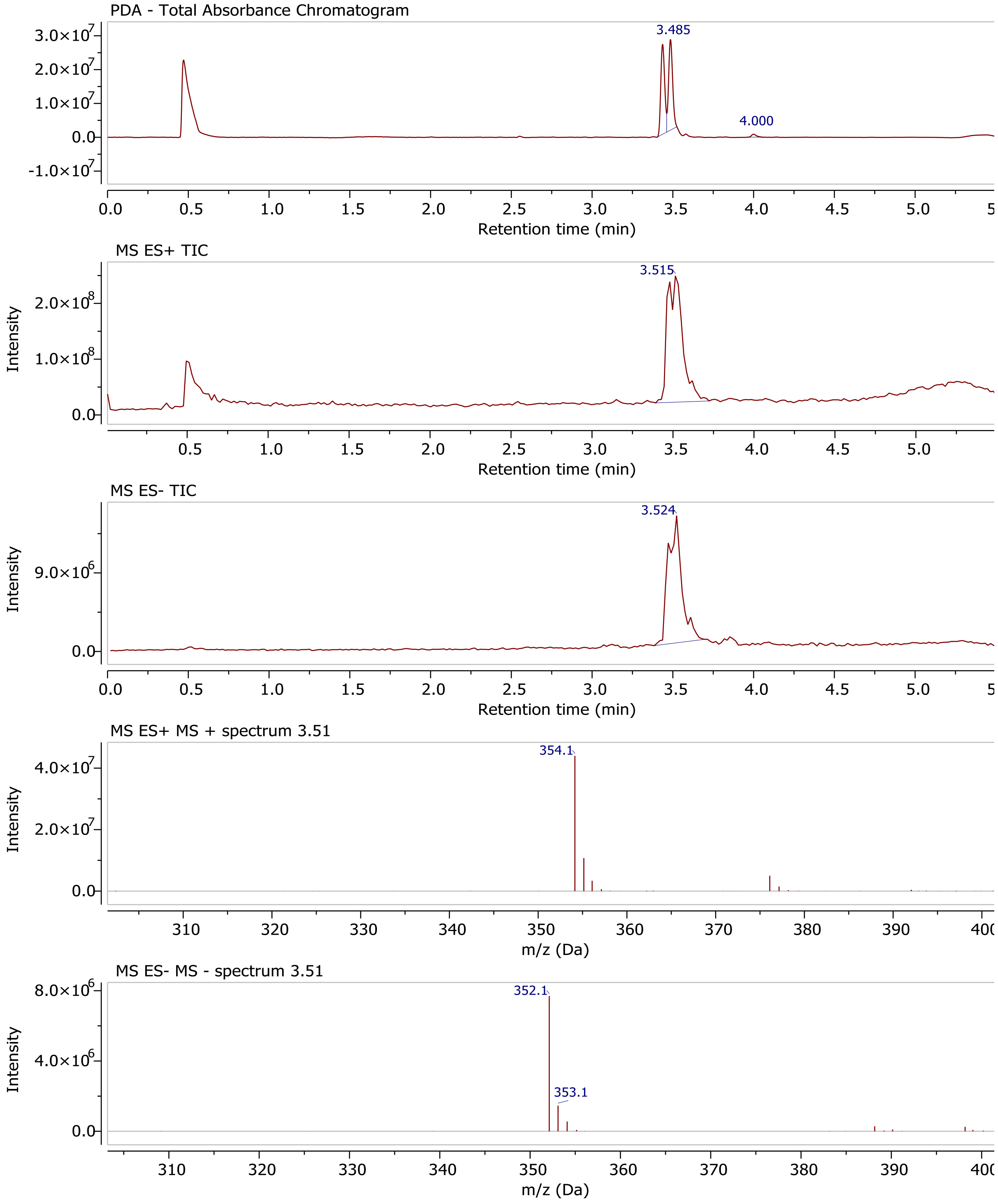

Supplementary figure **1361**. UPLC-MS analysis for compound **PHP-4868**

Chemical Formula: $C_{19}H_{20}N_2O_4S$
Exact Mass: 372.1

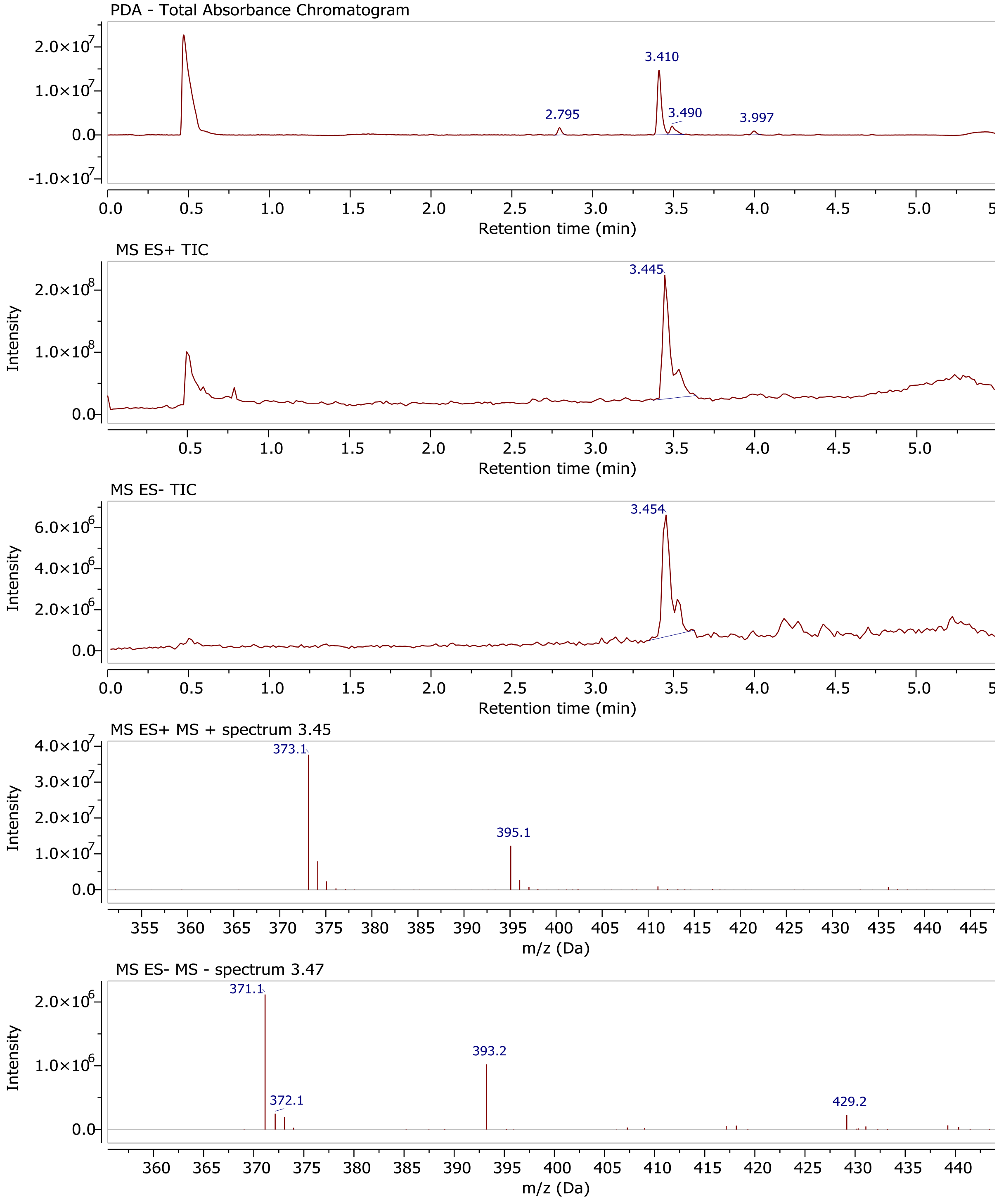

Supplementary figure **1362**. UPLC-MS analysis for compound **PHP-5449**

Chemical Formula: $C_{19}H_{20}FN_3O_3S$
Exact Mass: 389.1

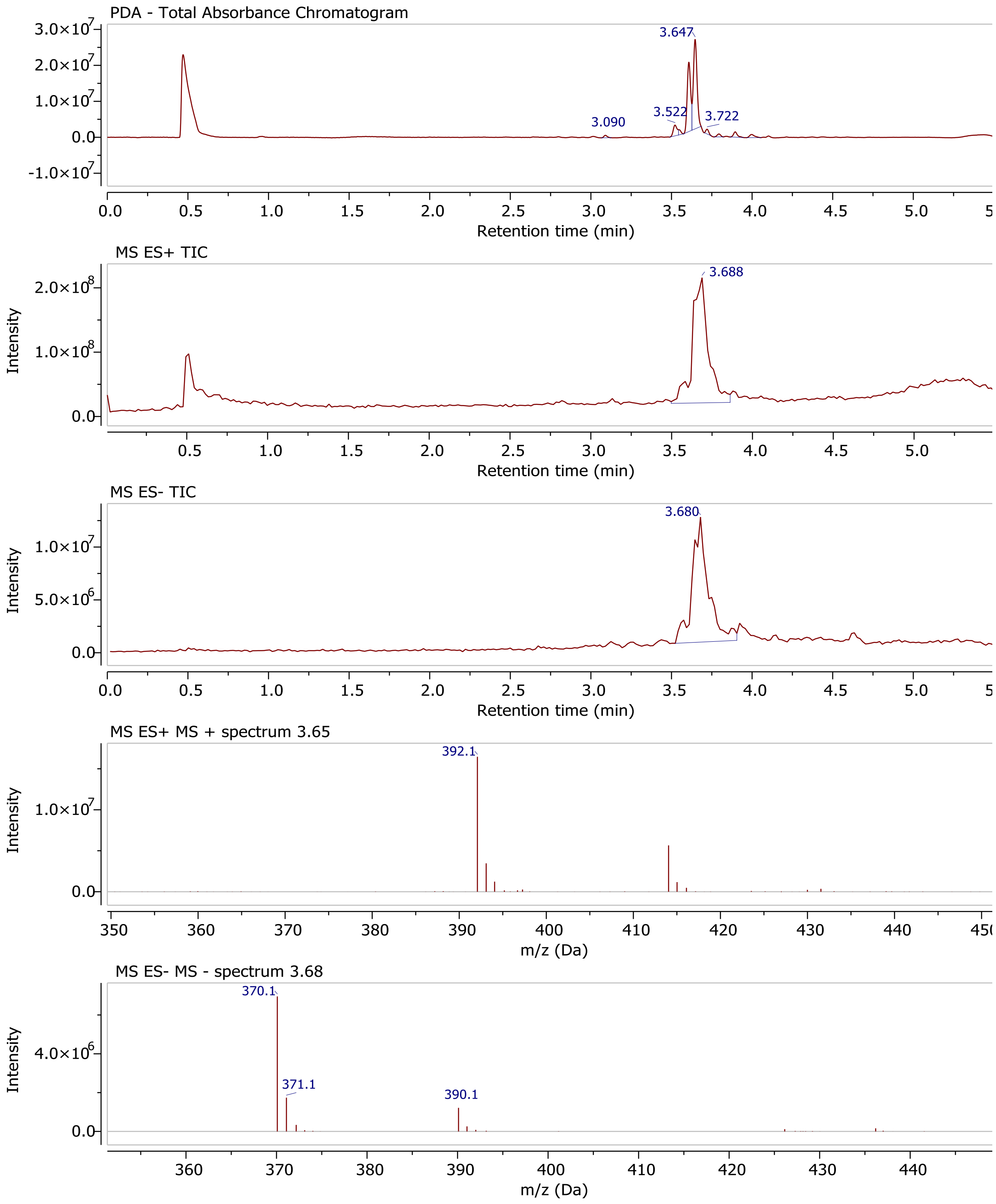

Supplementary figure **1363**. UPLC-MS analysis for compound **PHP-4869**

Chemical Formula: $C_{20}H_{17}F_3N_2O_4$
Exact Mass: 406.1

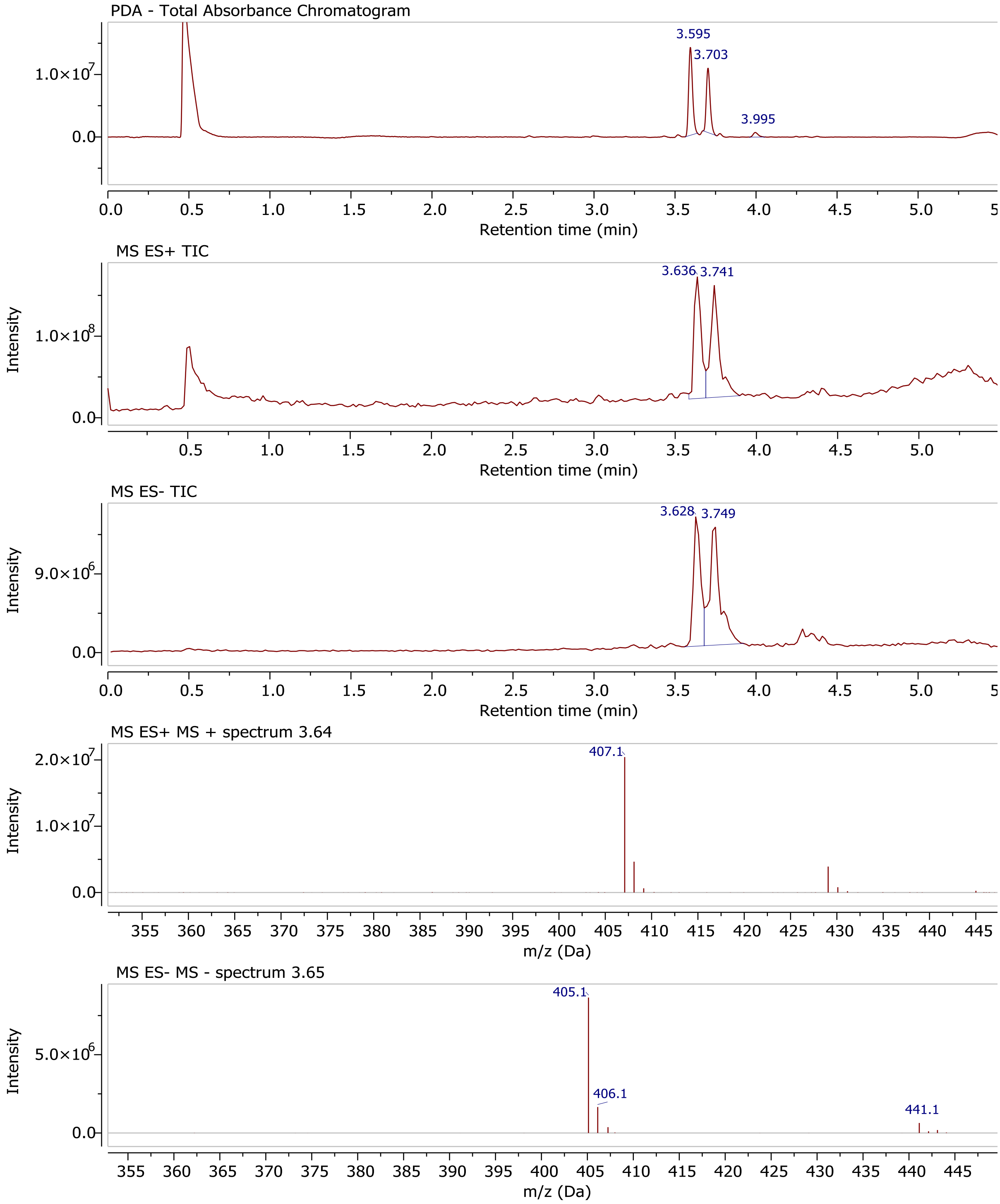

Supplementary figure **1364**. UPLC-MS analysis for compound **PHP-5451**

Chemical Formula: $C_{18}H_{20}N_2O_3S$
Exact Mass: 344.1

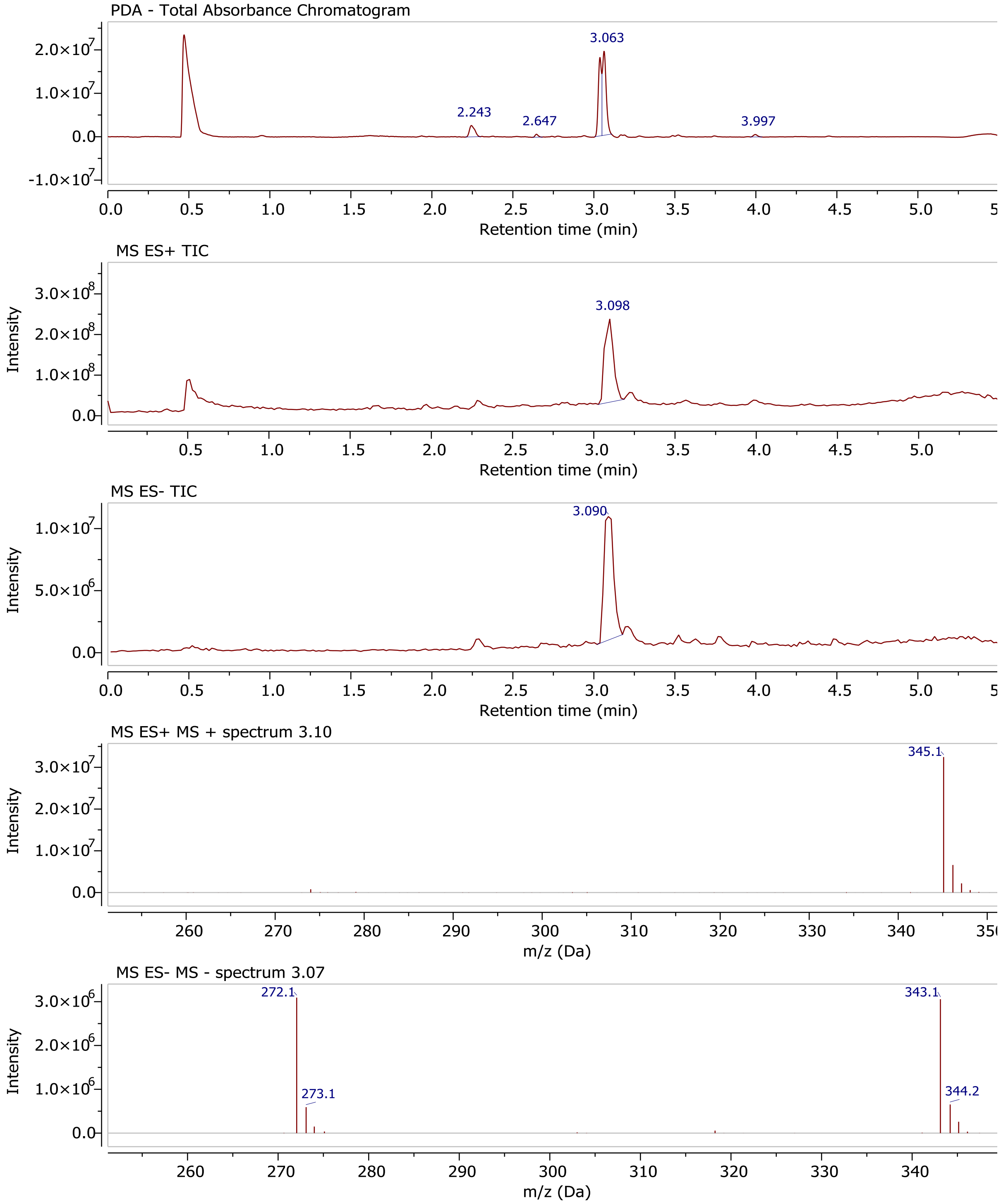

Supplementary figure **1365**. UPLC-MS analysis for compound **PHP-4871**

Chemical Formula: $C_{27}H_{34}N_2O_4$
Exact Mass: 450.3

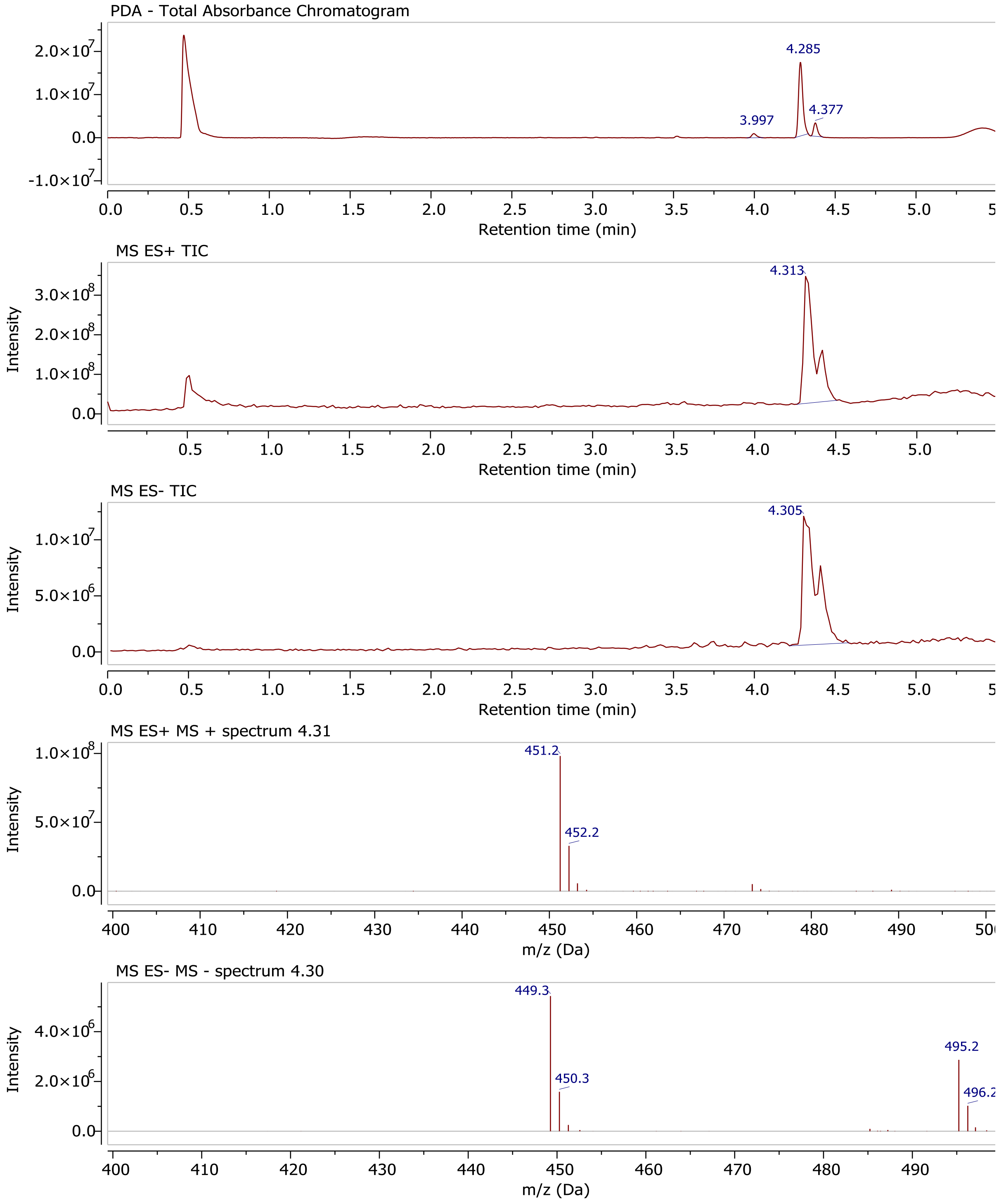

Supplementary figure **1366**. UPLC-MS analysis for compound **PHP-5452**

Chemical Formula: $C_{20}H_{24}N_2O_4S$
Exact Mass: 388.1

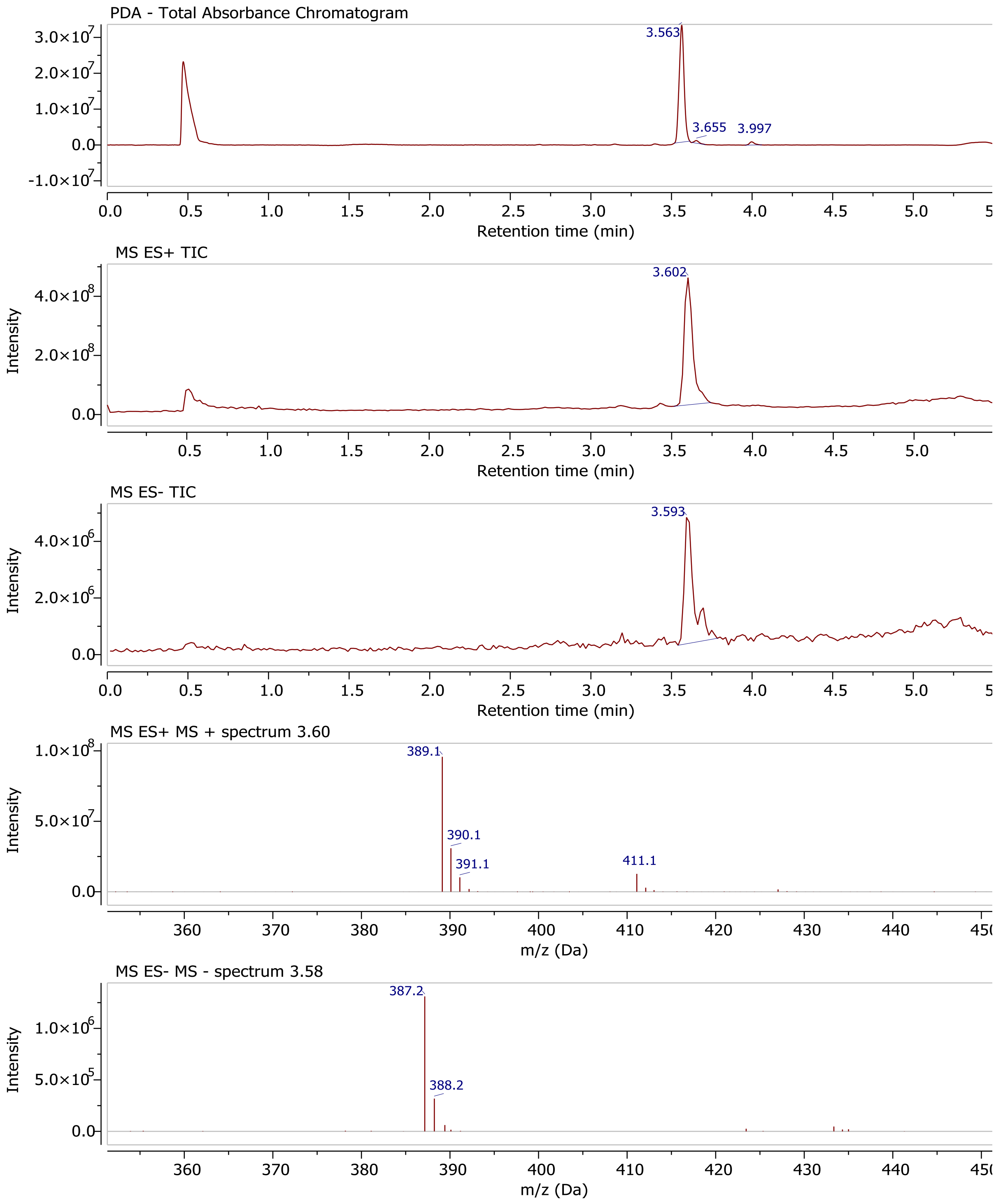

Supplementary figure **1368**. UPLC-MS analysis for compound **PHP-4873**

Chemical Formula: $C_{18}H_{17}BrN_2O_4S$
Exact Mass: 436.0

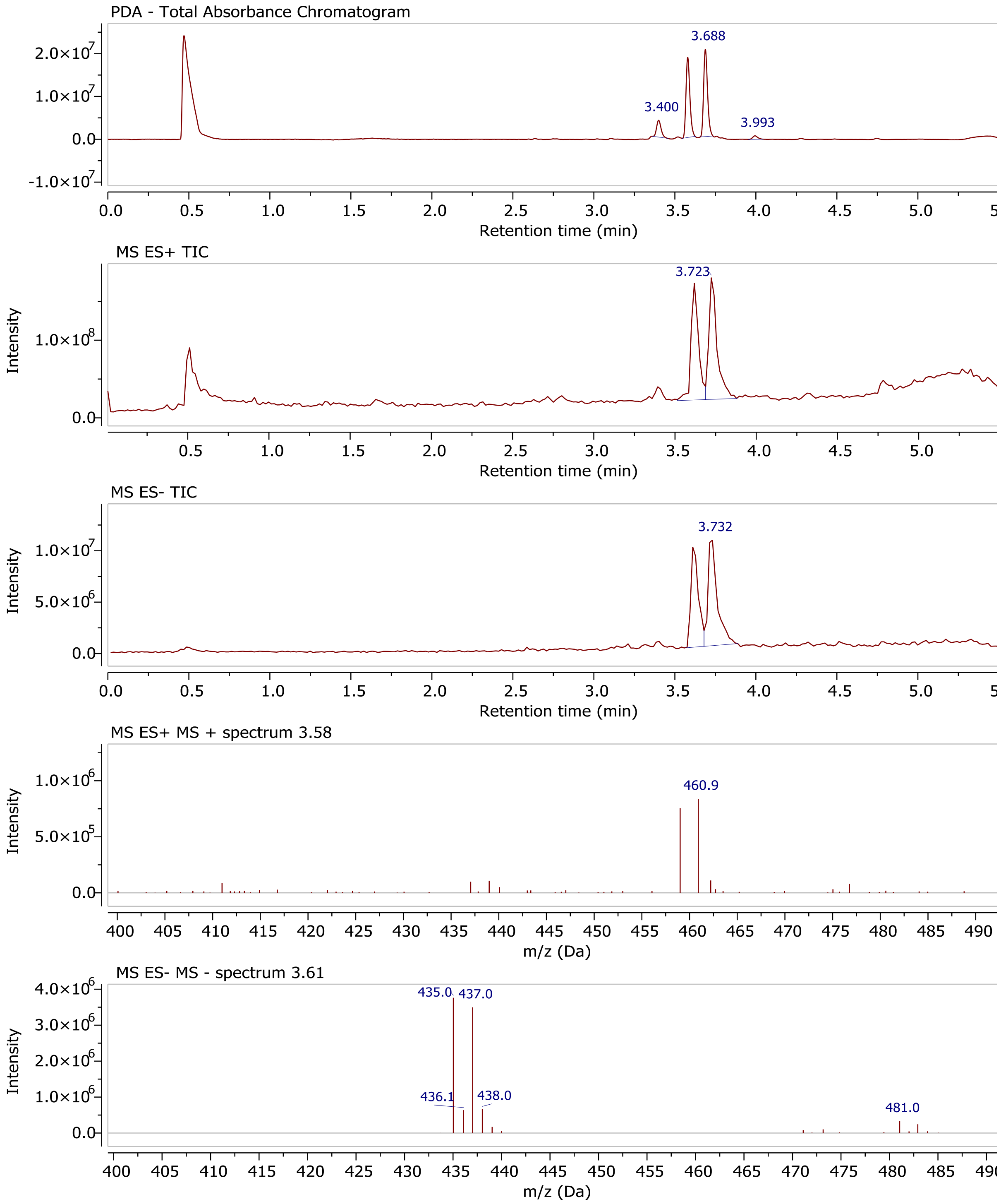

Supplementary figure **1369**. UPLC-MS analysis for compound **PHP-5453**

Chemical Formula: $C_{25}H_{26}N_2O_5S_2$
Exact Mass: 498.1

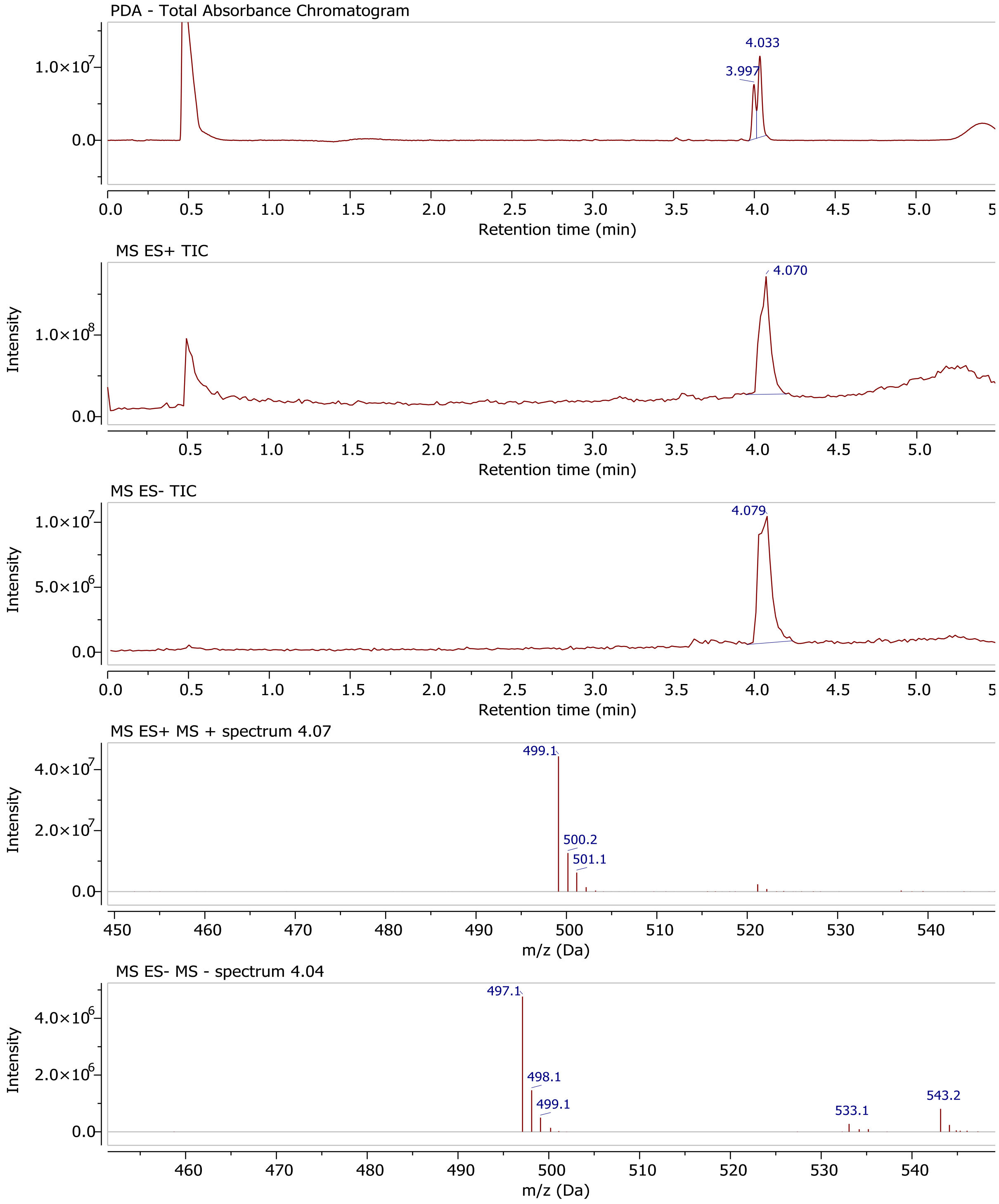

Supplementary figure **1370**. UPLC-MS analysis for compound **PHP-4875**

Chemical Formula: $C_{18}H_{22}N_2O_4$
Exact Mass: 330.2

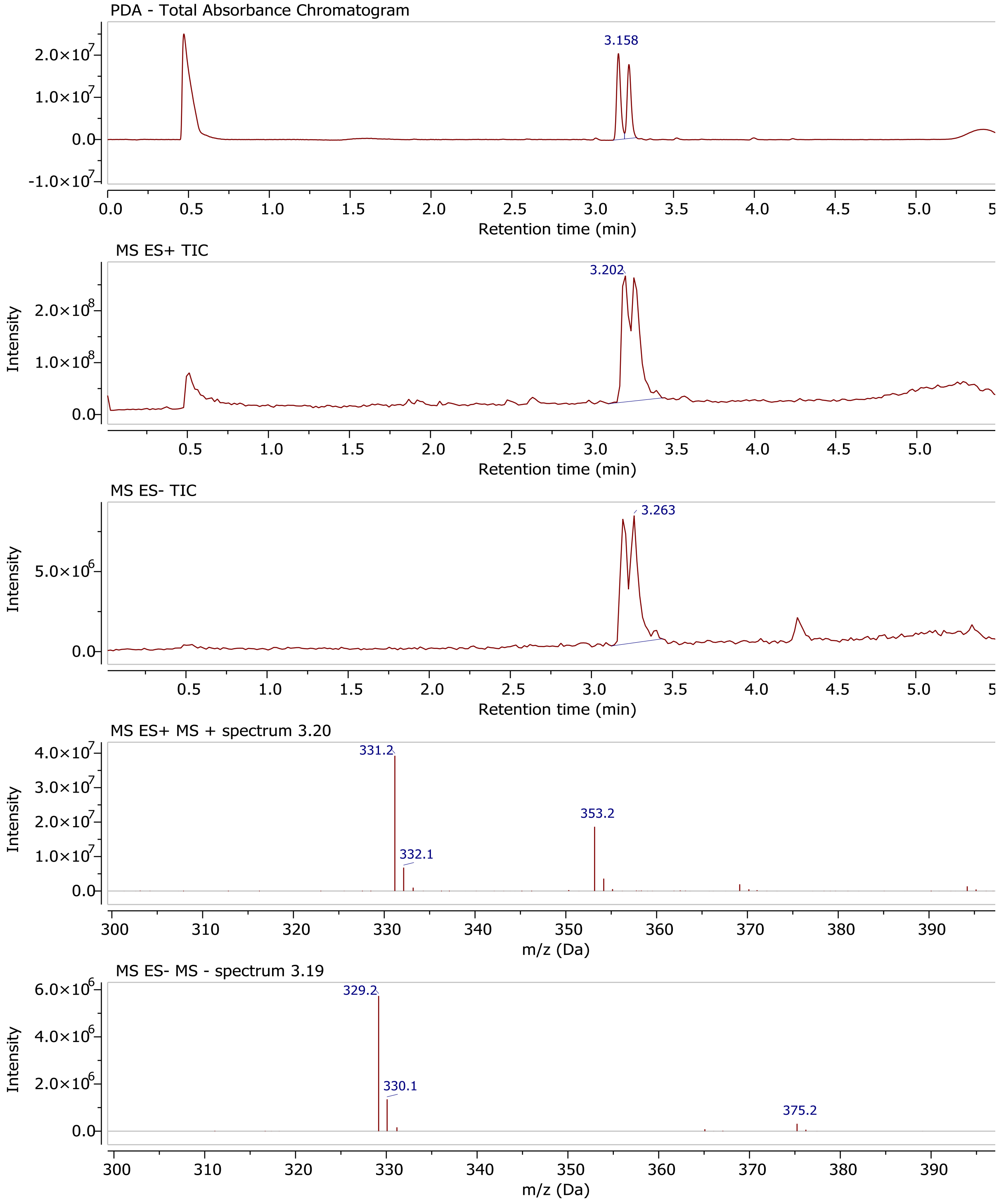

Supplementary figure **1371**. UPLC-MS analysis for compound **PHP-5457**

Chemical Formula: $C_{20}H_{24}N_2O_2$
Exact Mass: 324.2

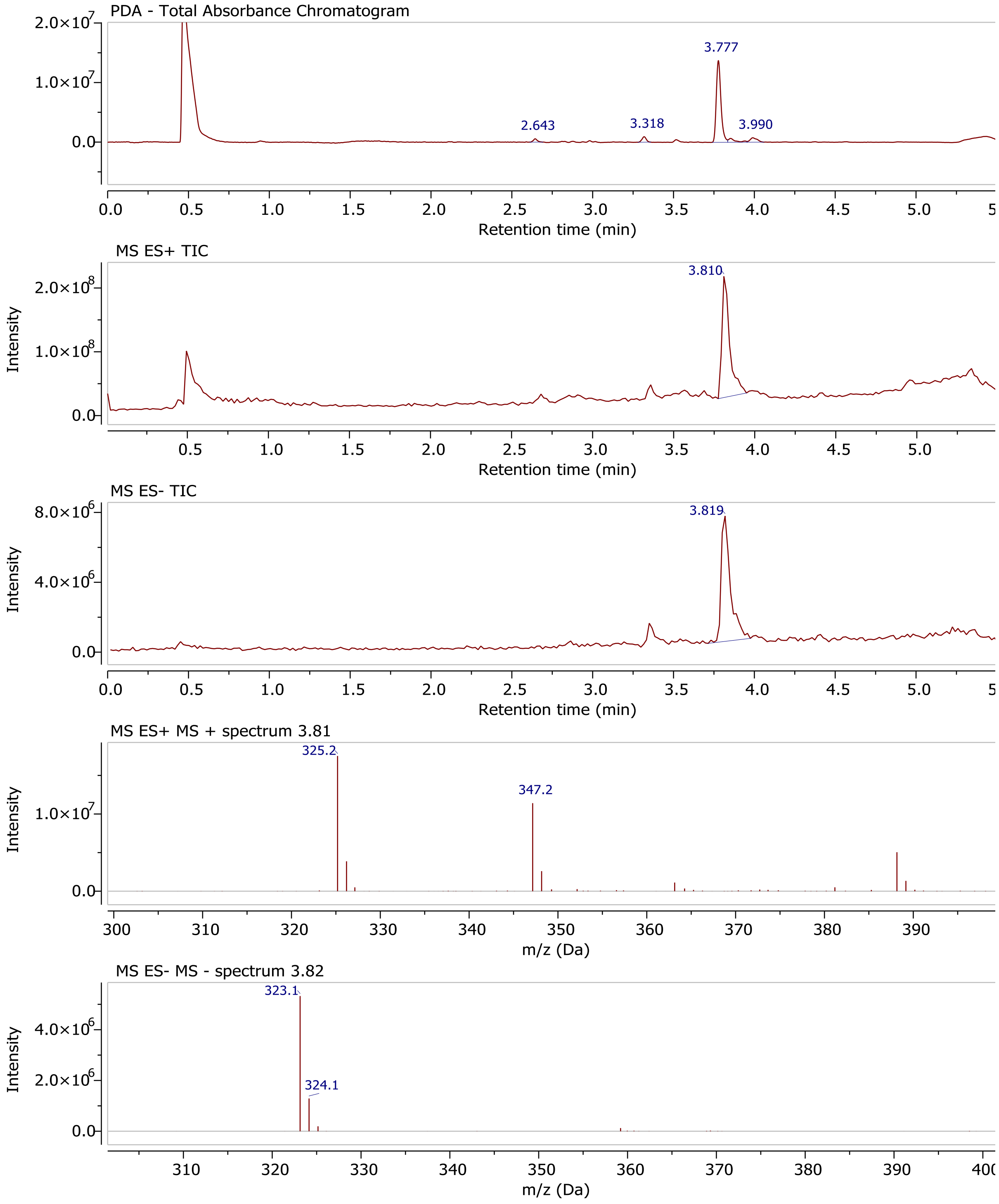

Supplementary figure **1372**. UPLC-MS analysis for compound **PHP-5609**

Chemical Formula: $C_{21}H_{22}F_2N_2O_2$
Exact Mass: 372.2

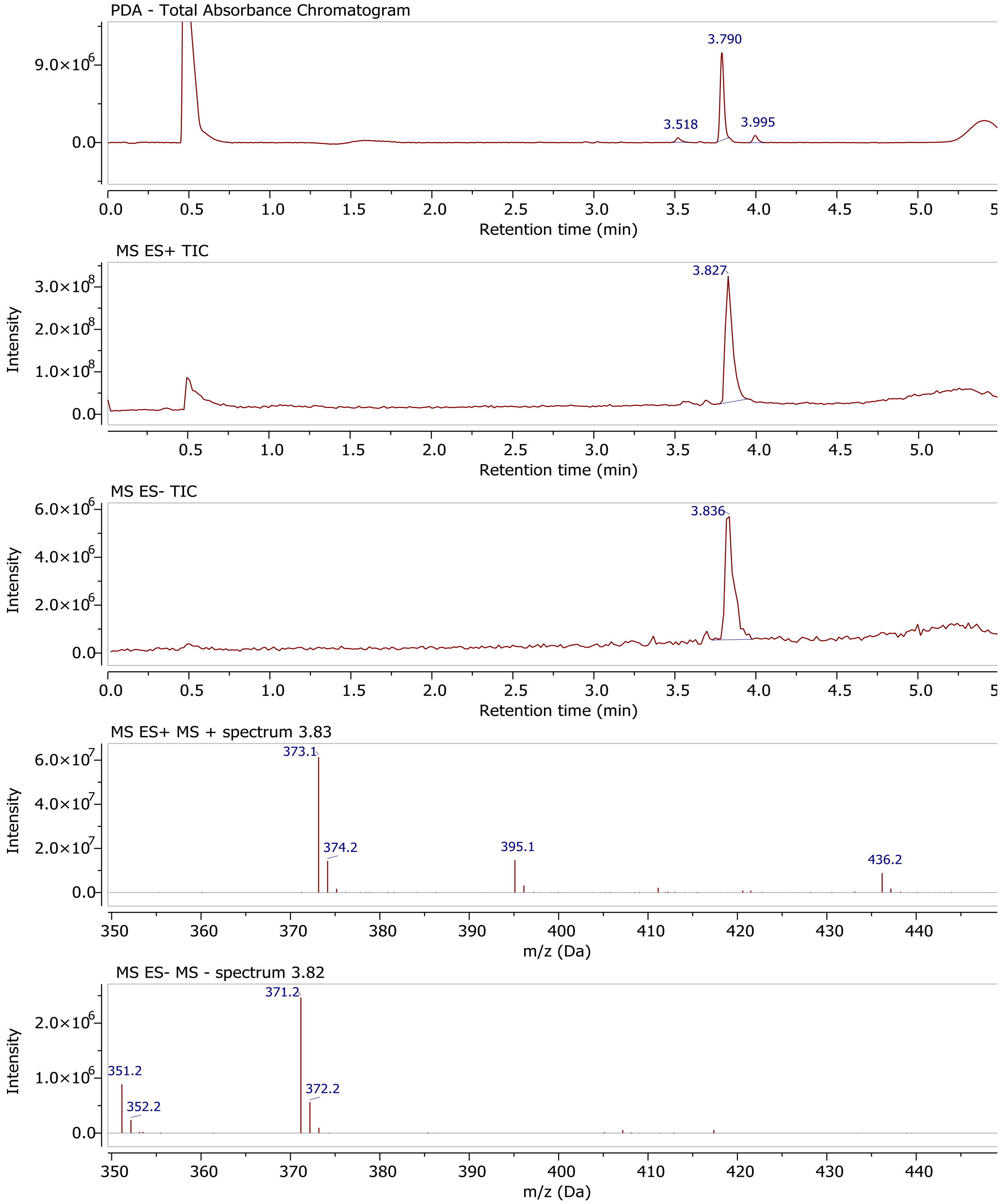

Supplementary figure **1373**. UPLC-MS analysis for compound **PHP-5758**

Chemical Formula: $C_{20}H_{24}N_2O_2$
Exact Mass: 324.2

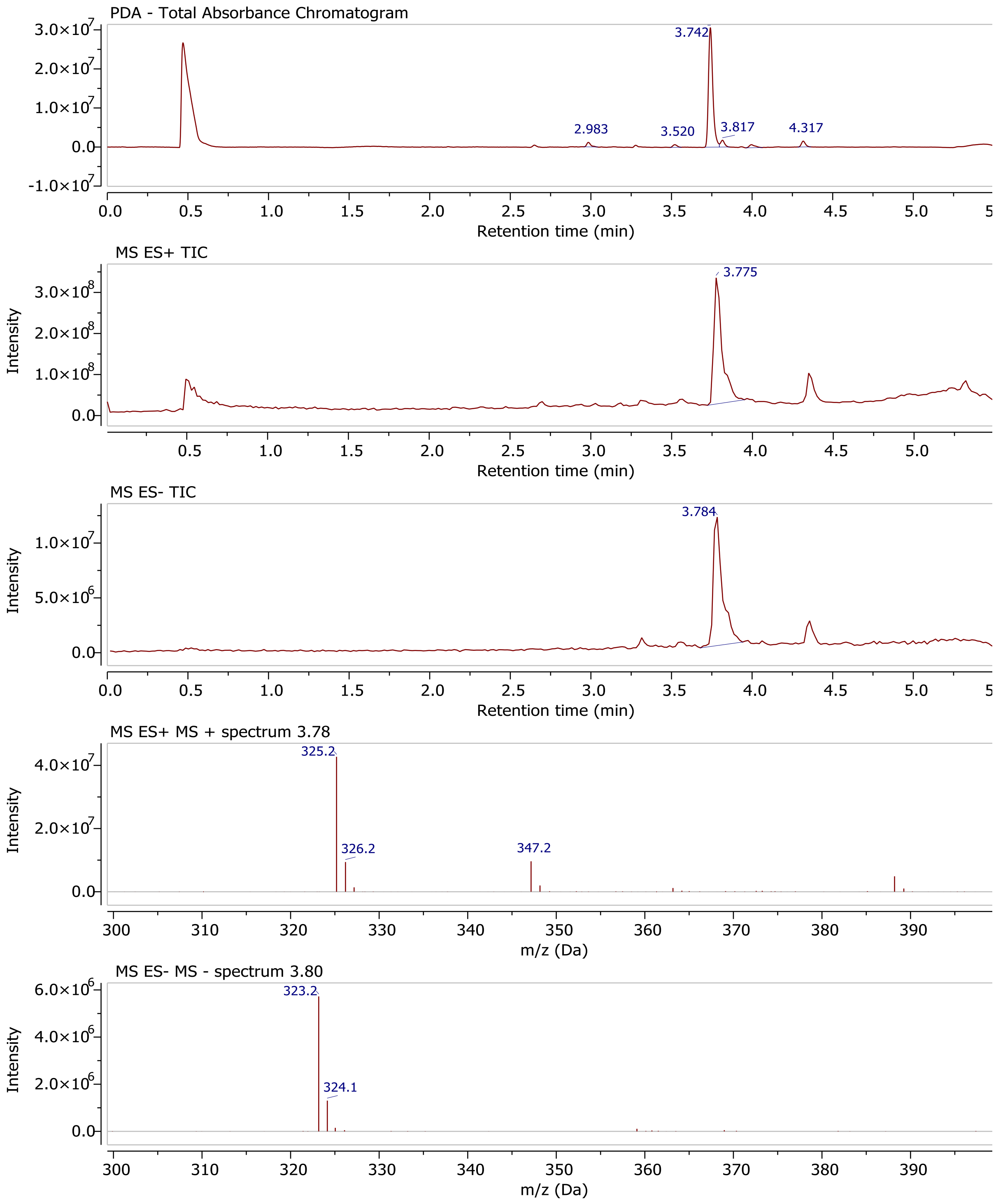

Supplementary figure **1374**. UPLC-MS analysis for compound **PHP-5610**

Chemical Formula: $C_{22}H_{23}F_3N_2O_2$
Exact Mass: 404.2

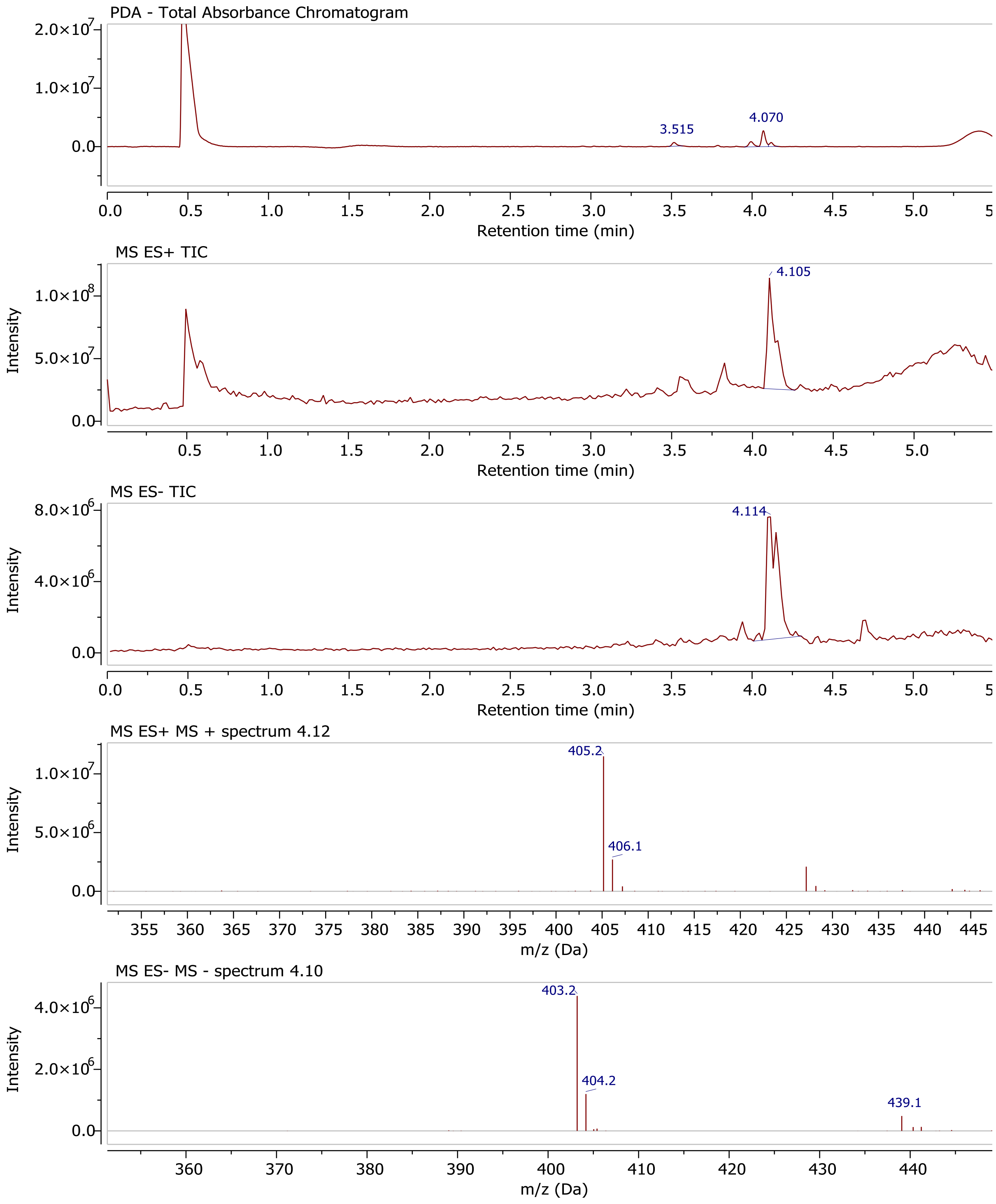

Supplementary figure **1375**. UPLC-MS analysis for compound **PHP-5759**

Chemical Formula: $C_{21}H_{14}F_4N_2O_2$
Exact Mass: 402.1

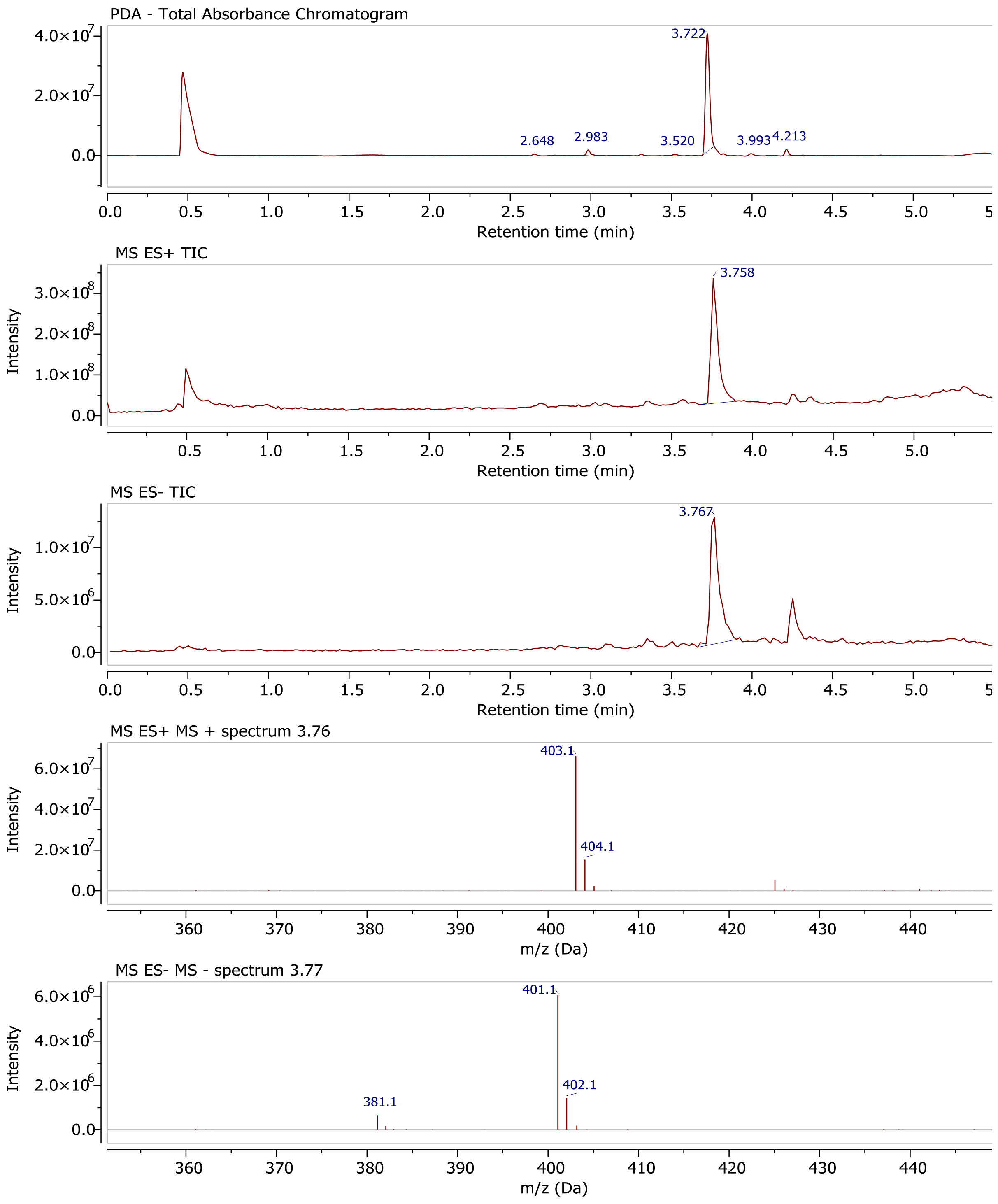

Supplementary figure **1376**. UPLC-MS analysis for compound **PHP-5611**

Chemical Formula: $C_{21}H_{23}ClN_2O_2$
Exact Mass: 370.1

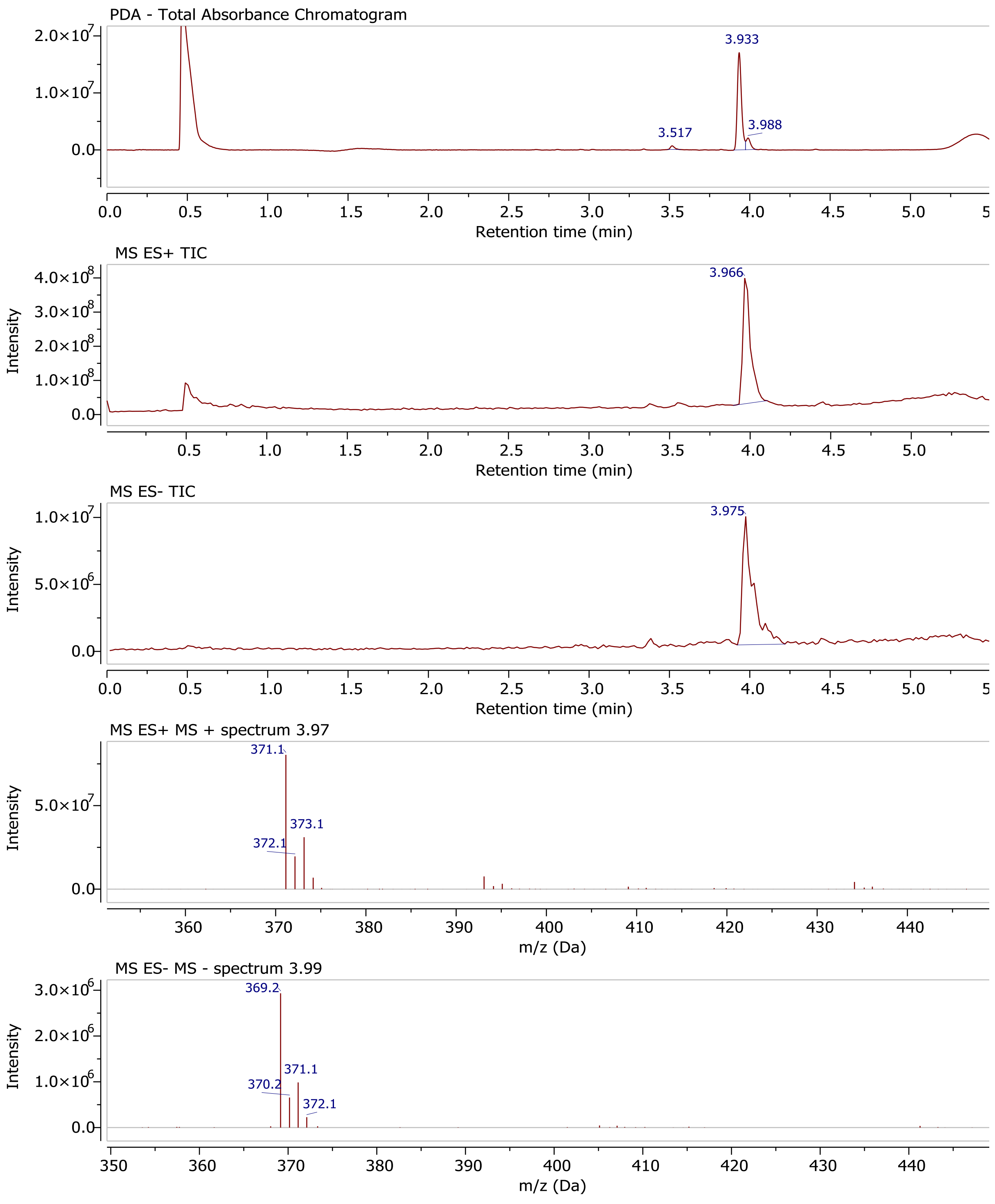

Supplementary figure **1377**. UPLC-MS analysis for compound **PHP-5760**

Chemical Formula: $C_{22}H_{28}N_2O_2$
Exact Mass: 352.2

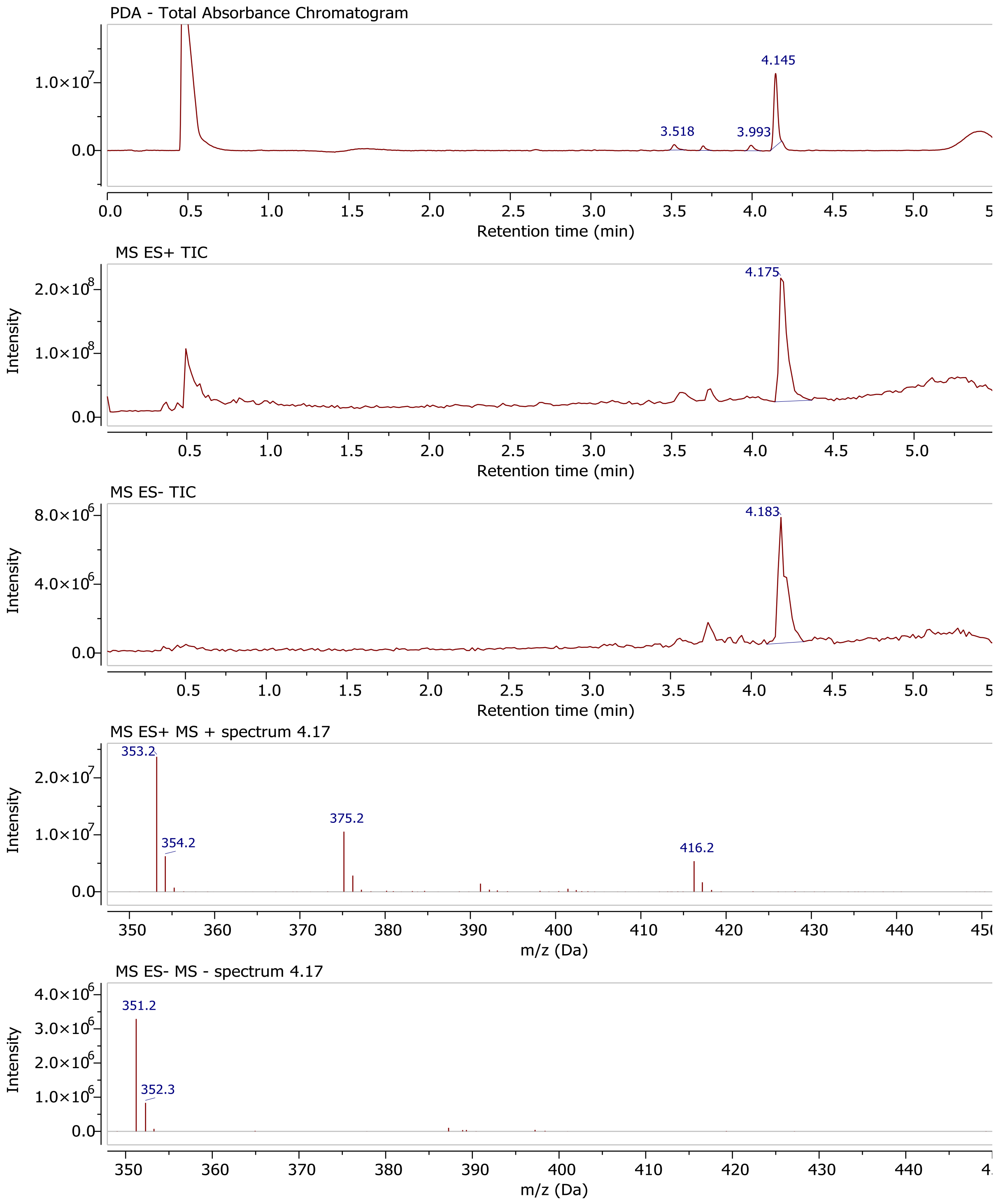

Supplementary figure **1378**. UPLC-MS analysis for compound **PHP-5612**

Chemical Formula: $C_{22}H_{23}F_3N_2O_2$
Exact Mass: 404.2

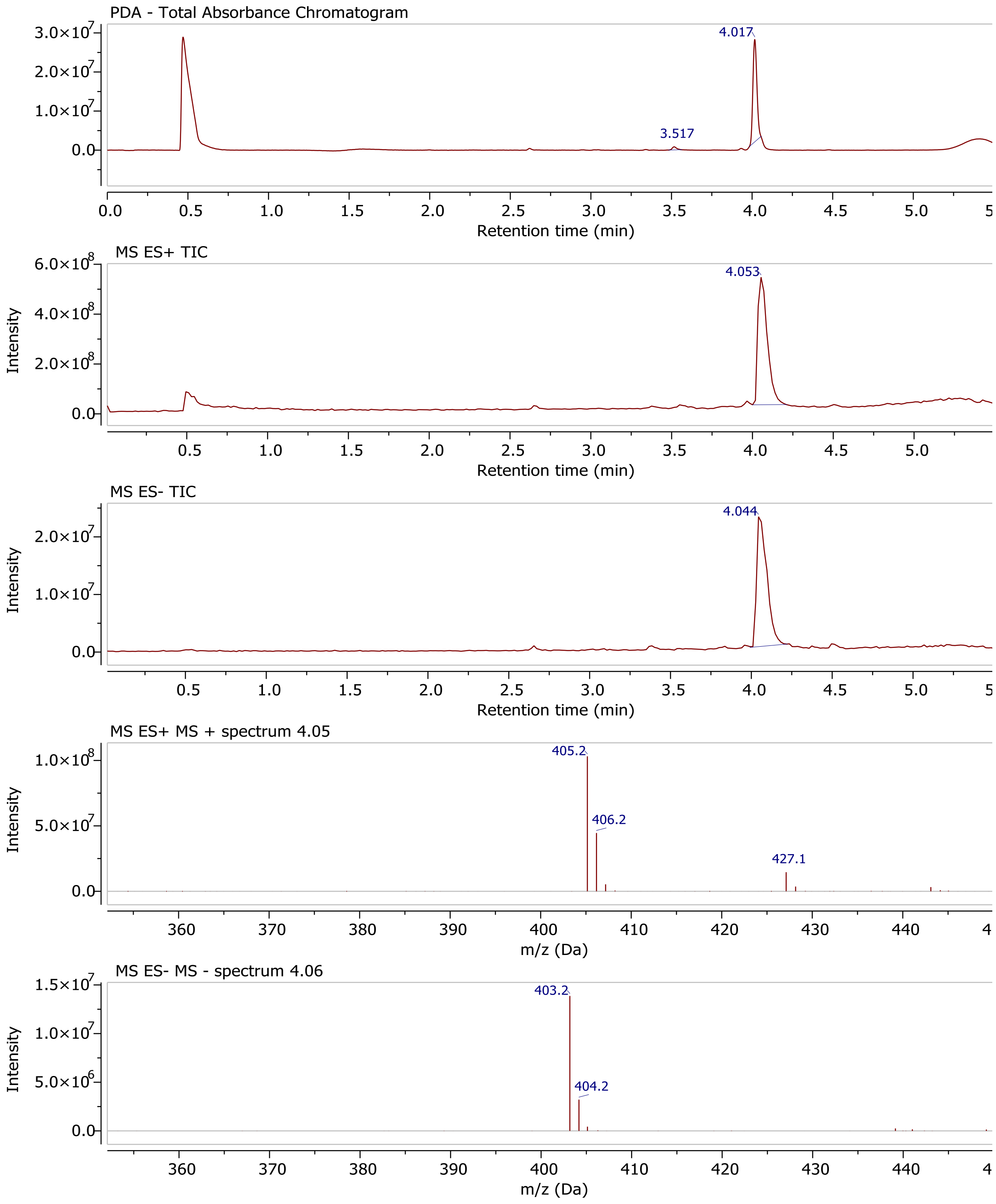

Supplementary figure **1379**. UPLC-MS analysis for compound **PHP-5761**

Chemical Formula: $C_{21}H_{15}F_3N_2O_2$
Exact Mass: 384.1

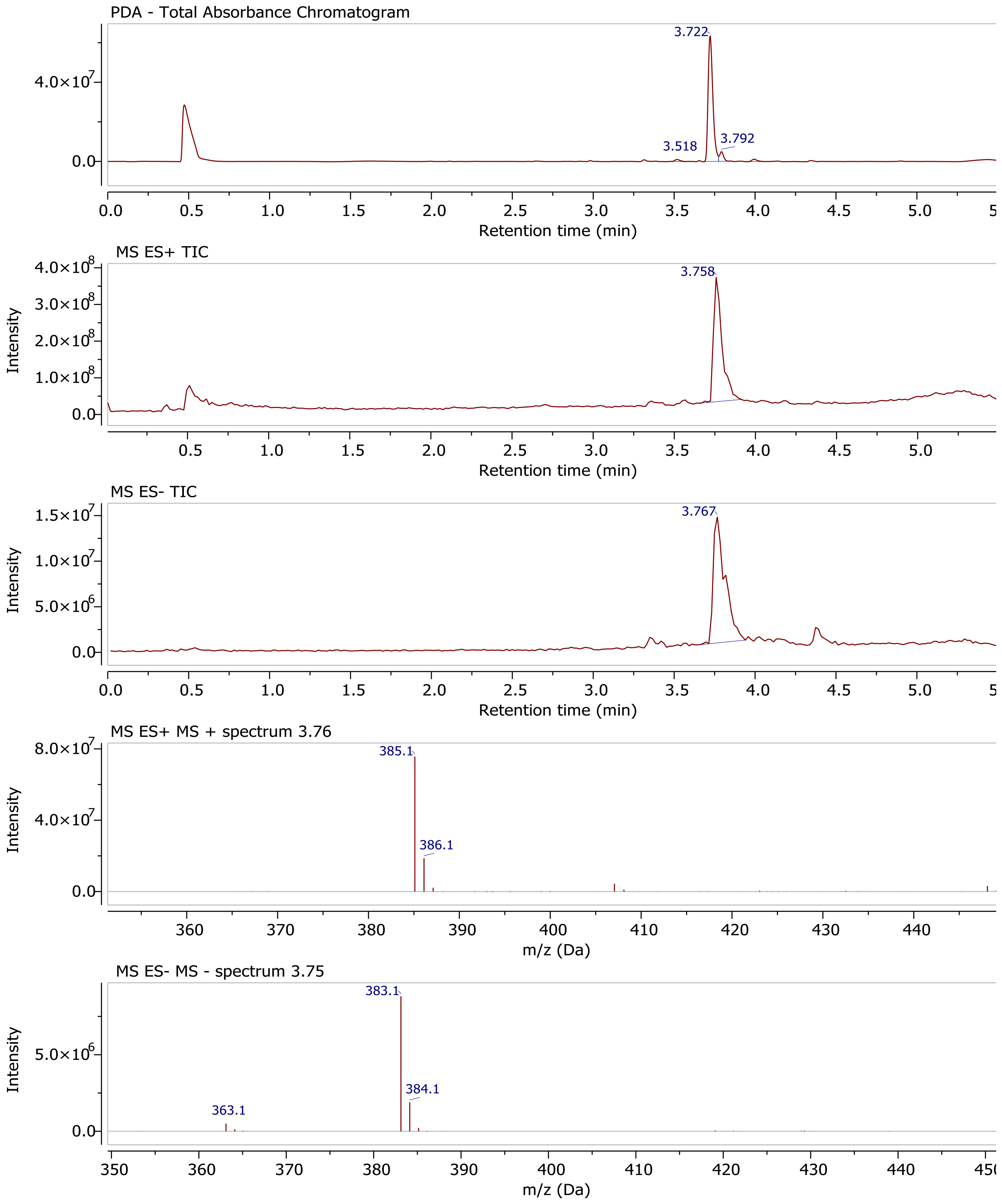

Supplementary figure **1380**. UPLC-MS analysis for compound **PHP-5613**

Chemical Formula: $C_{21}H_{23}FN_2O_2$
Exact Mass: 354.2

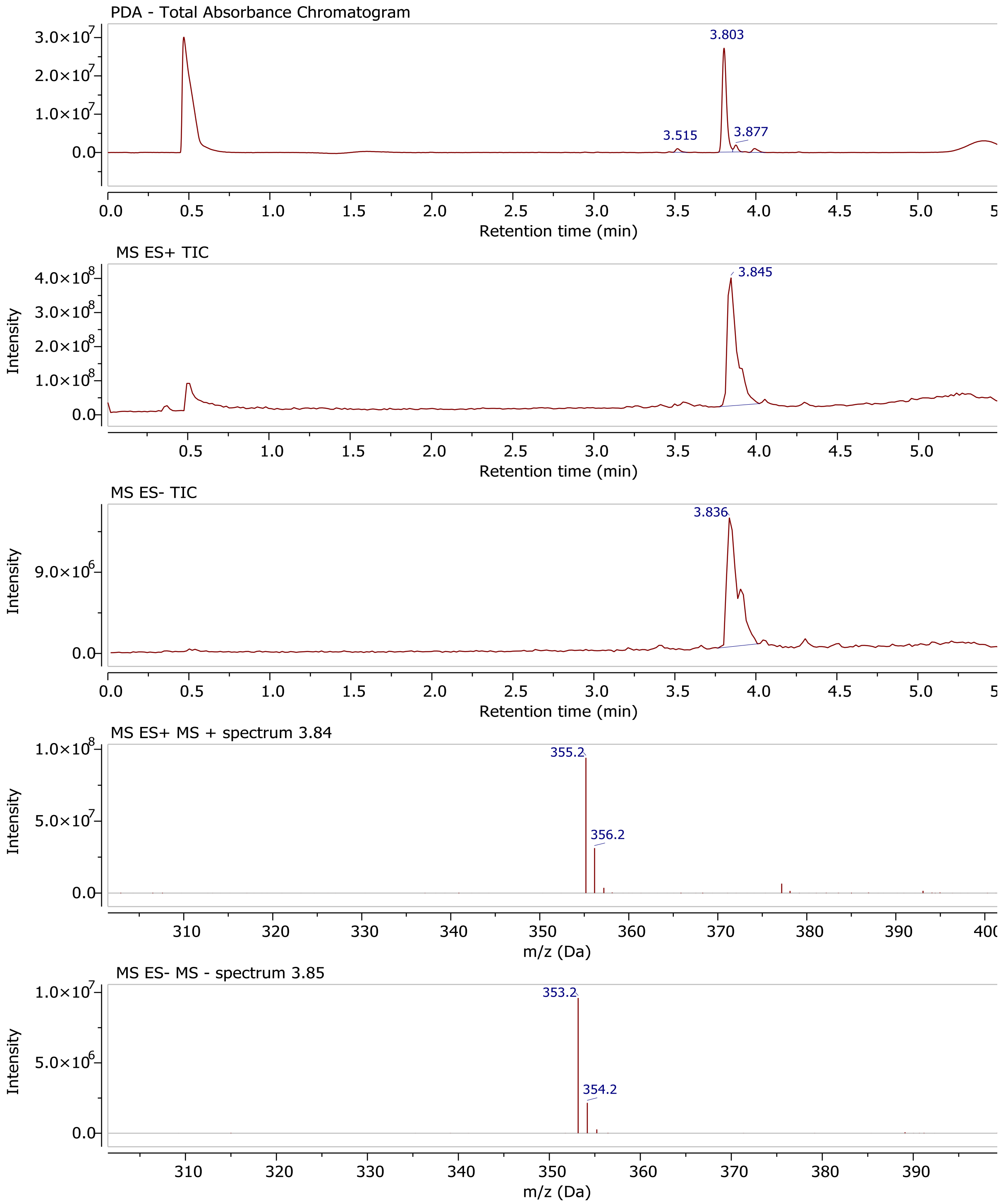

Supplementary figure **1381**. UPLC-MS analysis for compound **PHP-5762**

Chemical Formula: $C_{22}H_{20}N_2O_3$
Exact Mass: 360.1

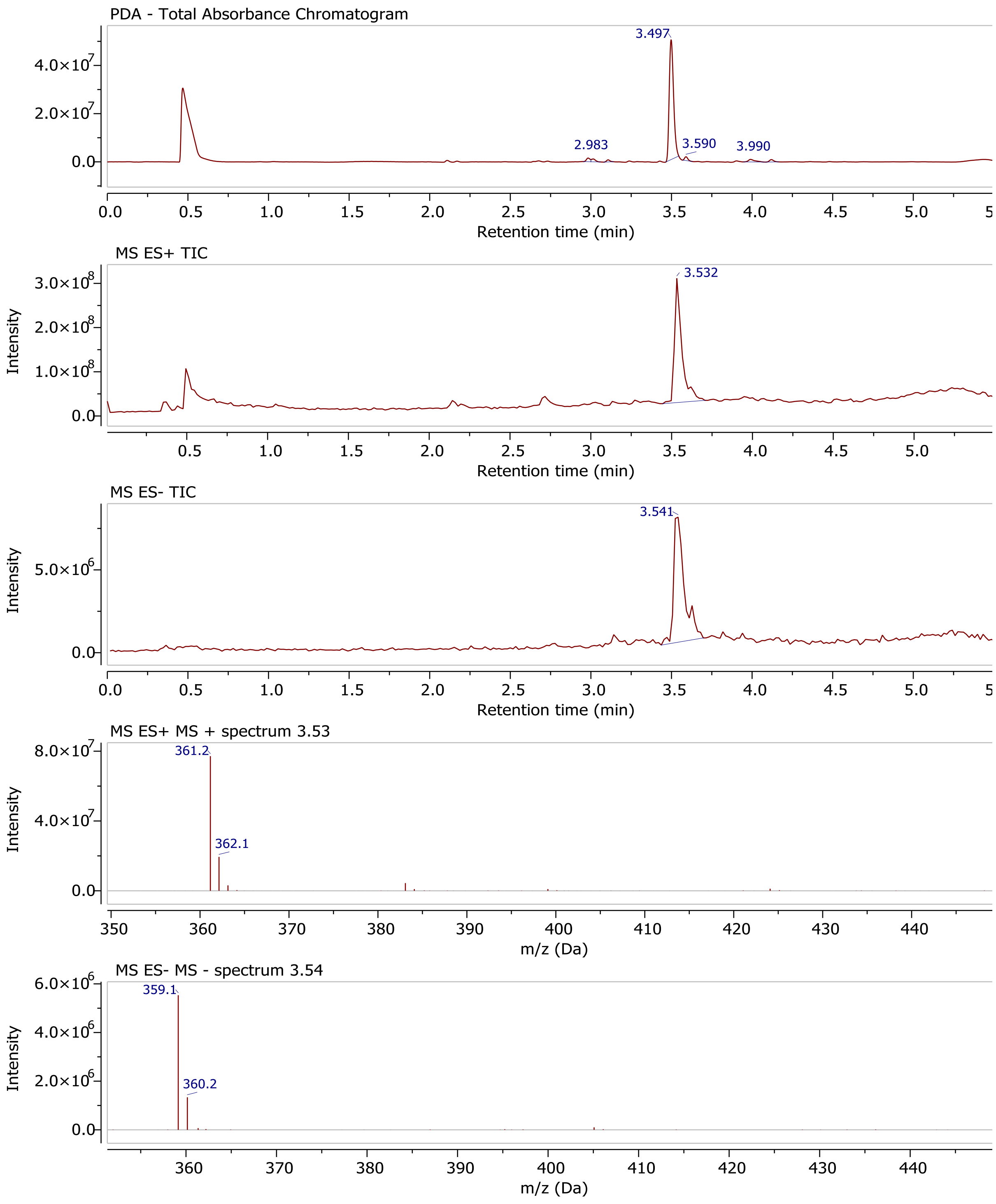

Supplementary figure **1382**. UPLC-MS analysis for compound **PHP-5614**

Chemical Formula: $C_{21}H_{23}FN_2O_2$
Exact Mass: 354.2

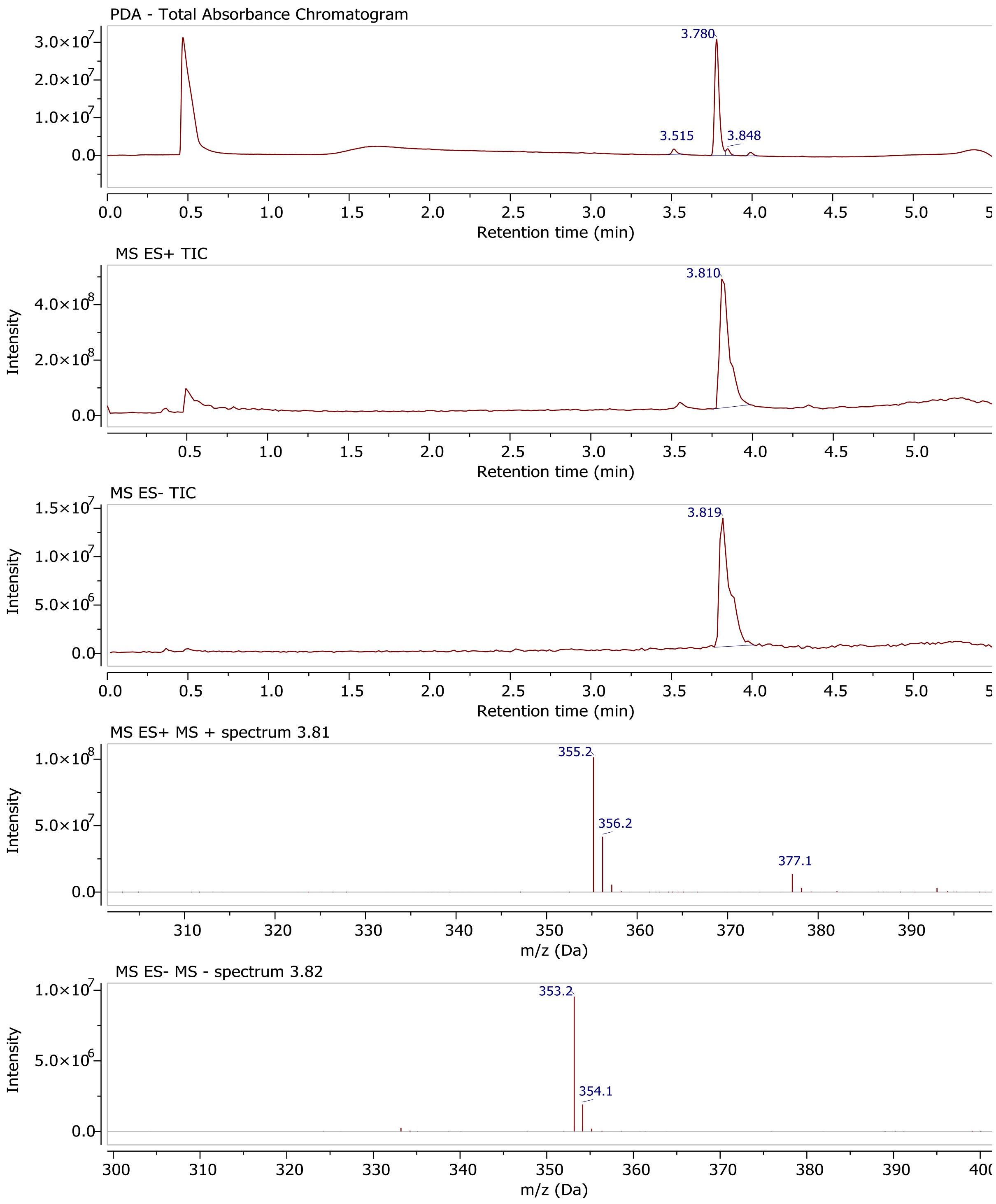

Supplementary figure **1383**. UPLC-MS analysis for compound **PHP-5763**

Chemical Formula: $C_{16}H_{15}ClN_2O_2$
Exact Mass: 302.1

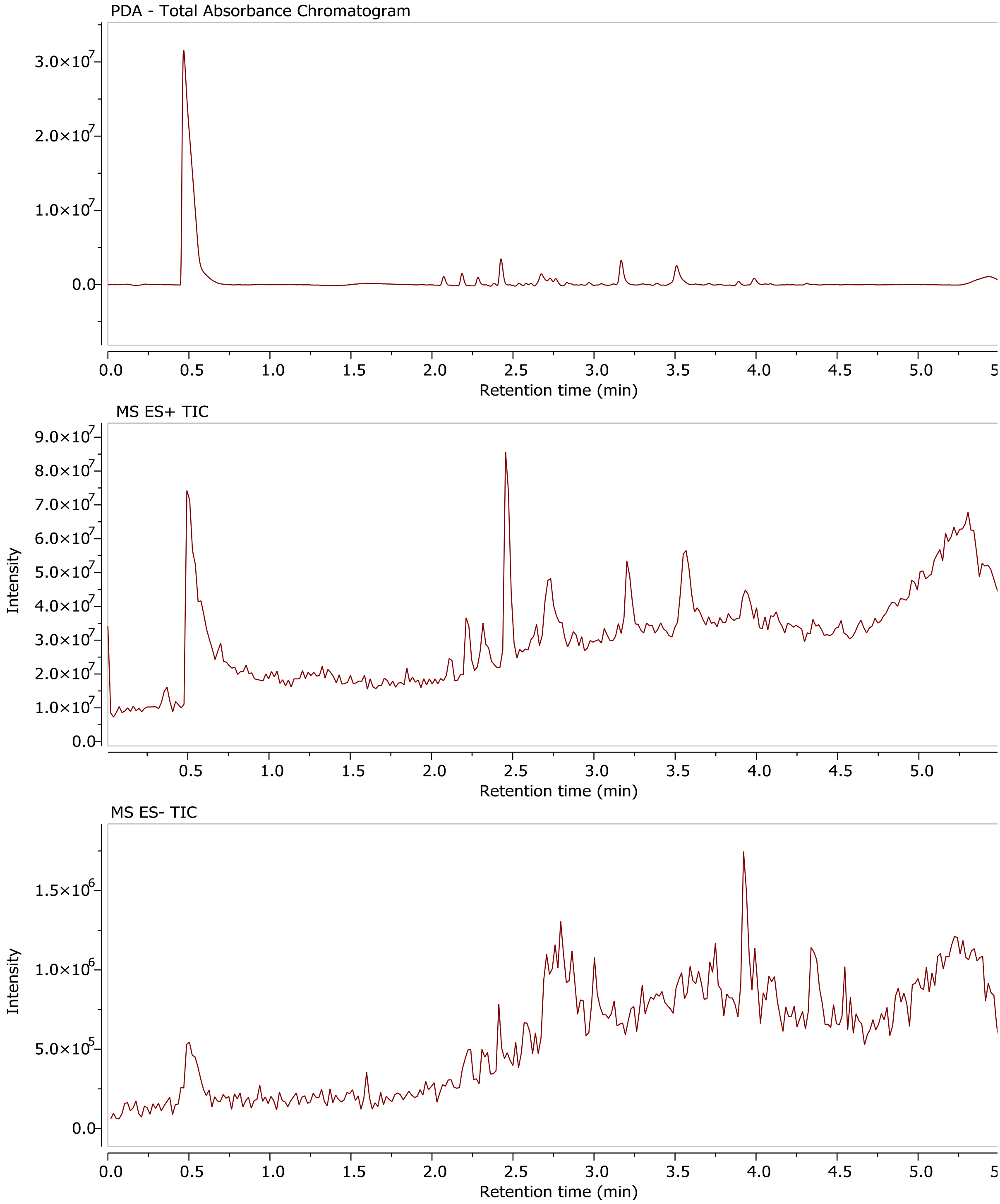

Supplementary figure **1384**. UPLC-MS analysis for compound **PHP-5616**

Chemical Formula: $C_{22}H_{23}F_3N_2O_2$
Exact Mass: 404.2

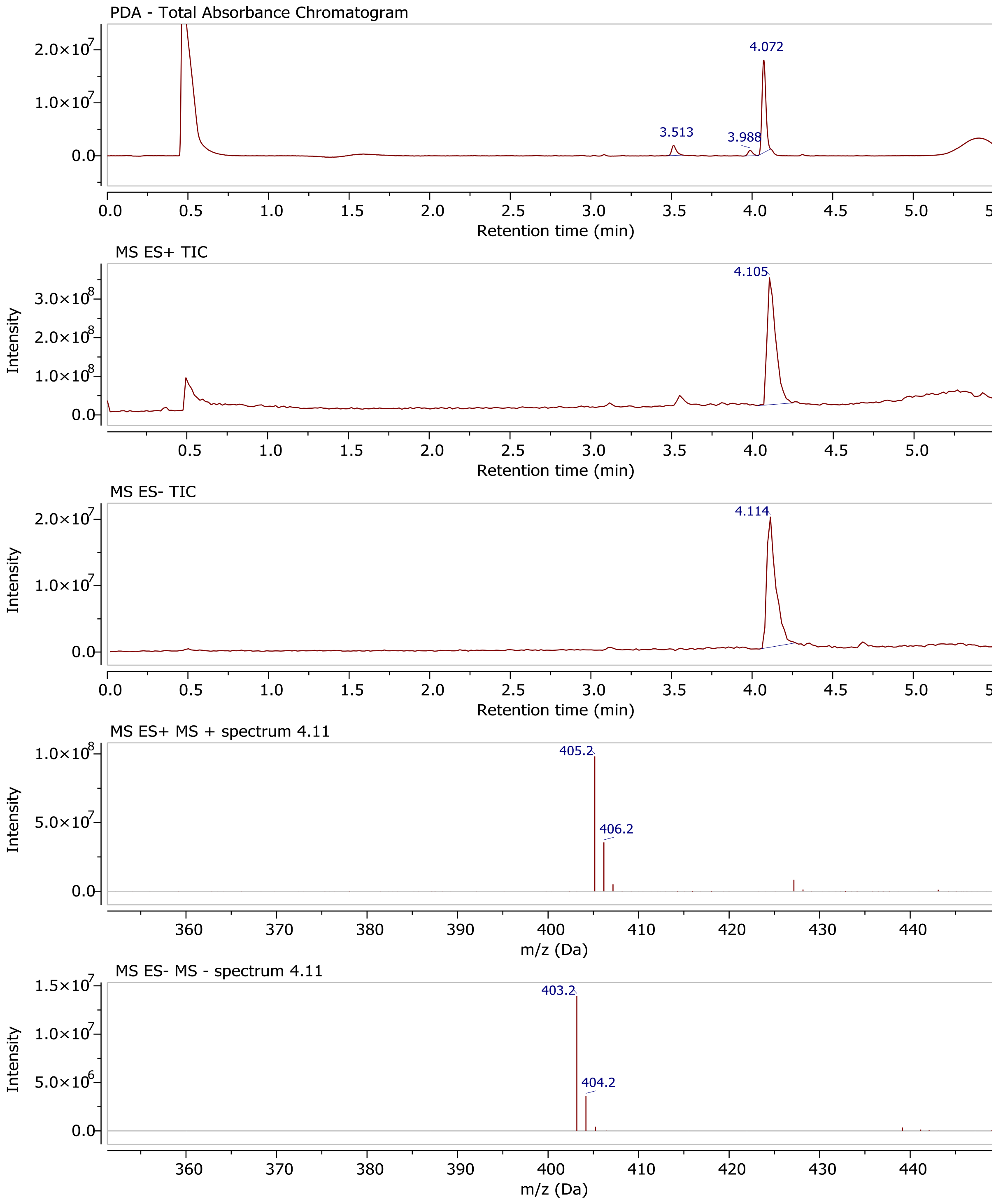

Supplementary figure **1385**. UPLC-MS analysis for compound **PHP-5764**

Chemical Formula: $C_{18}H_{26}N_2O_3$
Exact Mass: 318.2

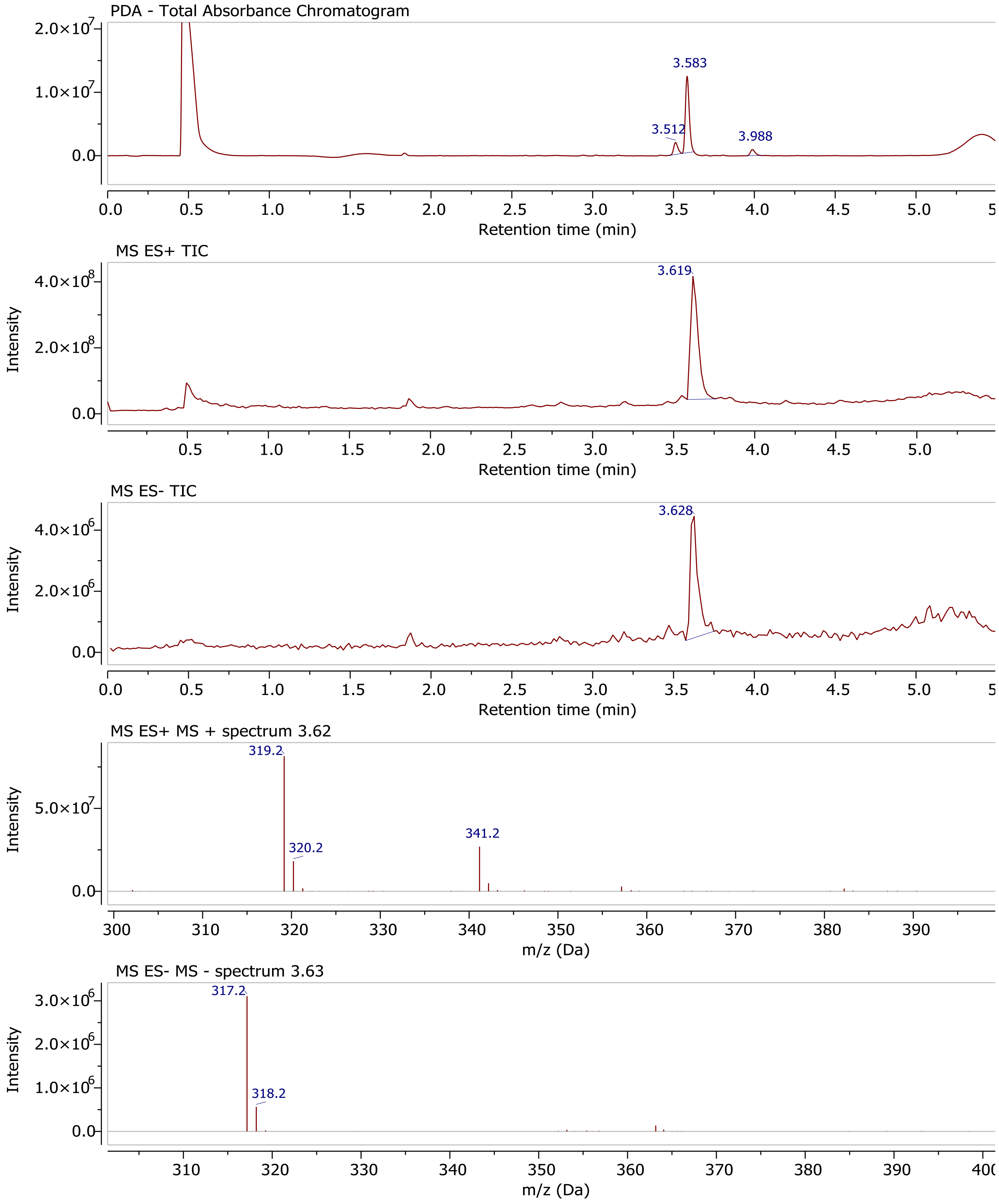

Supplementary figure **1386**. UPLC-MS analysis for compound **PHP-5617**

Chemical Formula: $C_{14}H_{17}FN_2O_2$
Exact Mass: 264.1

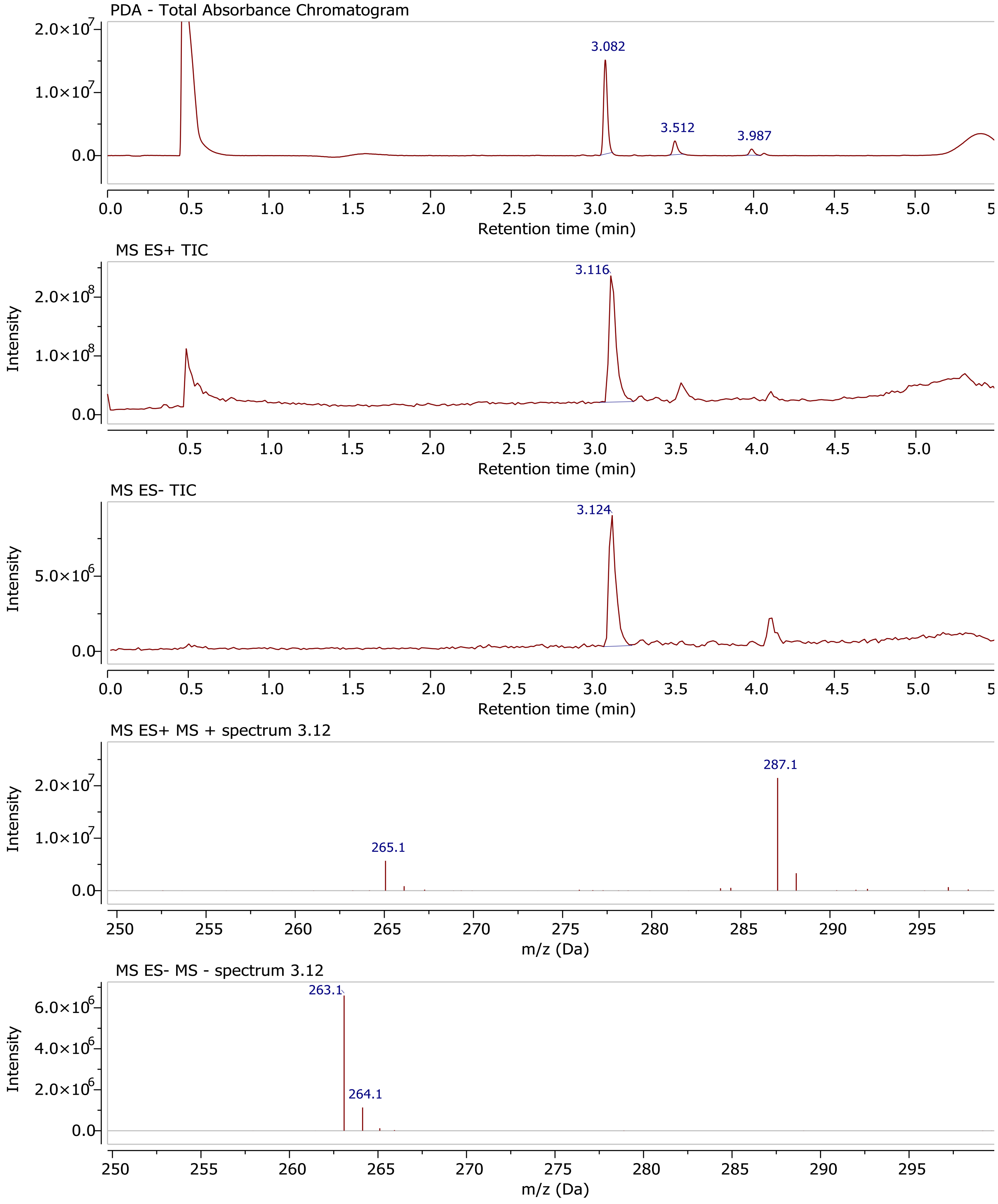

Supplementary figure **1387**. UPLC-MS analysis for compound **PHP-5767**

Chemical Formula: $C_{19}H_{18}F_2N_2O_3$
Exact Mass: 360.1

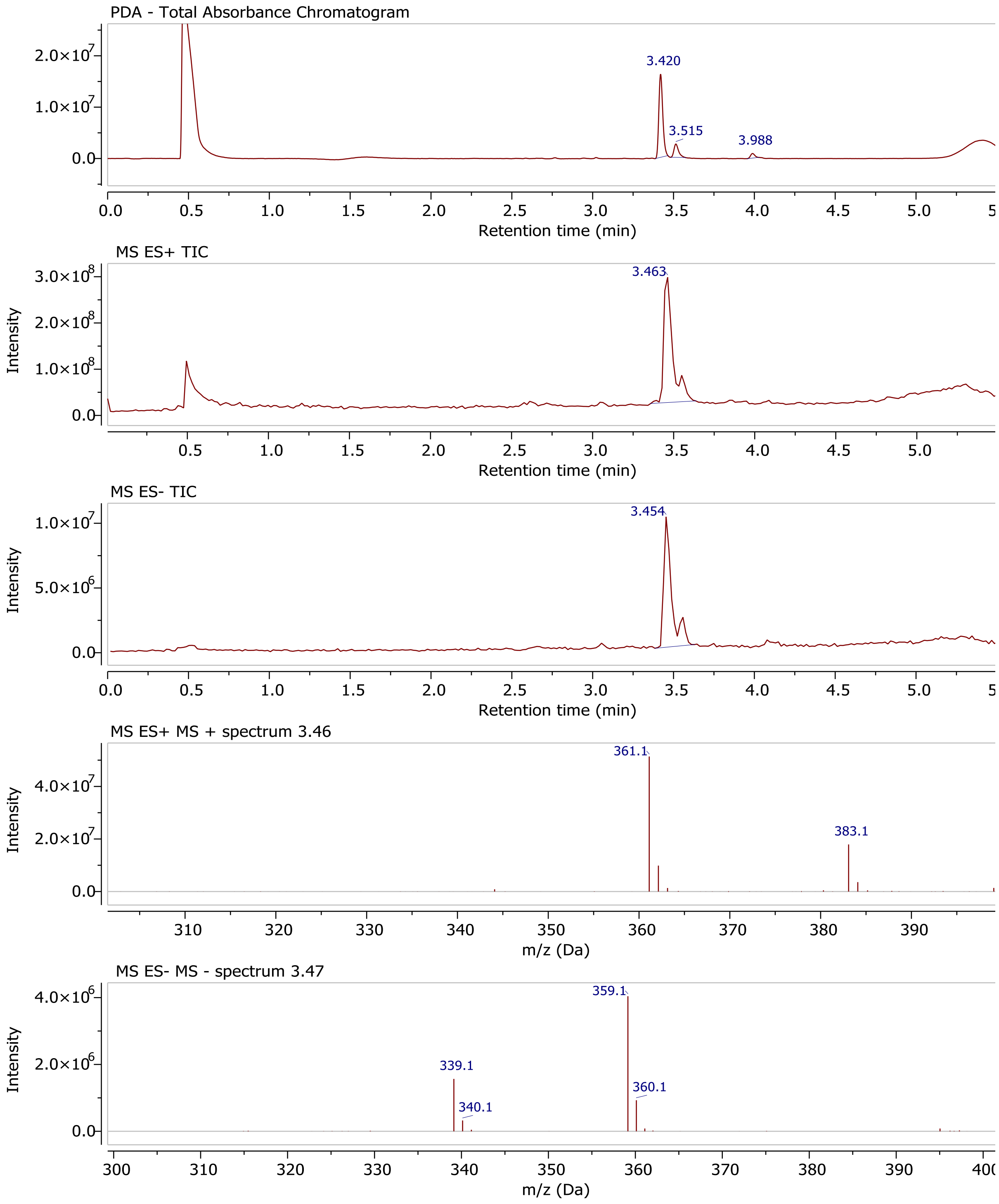

Supplementary figure **1388**. UPLC-MS analysis for compound **PHP-5618**

Chemical Formula: $C_{15}H_{19}FN_2O_2$
Exact Mass: 278.1

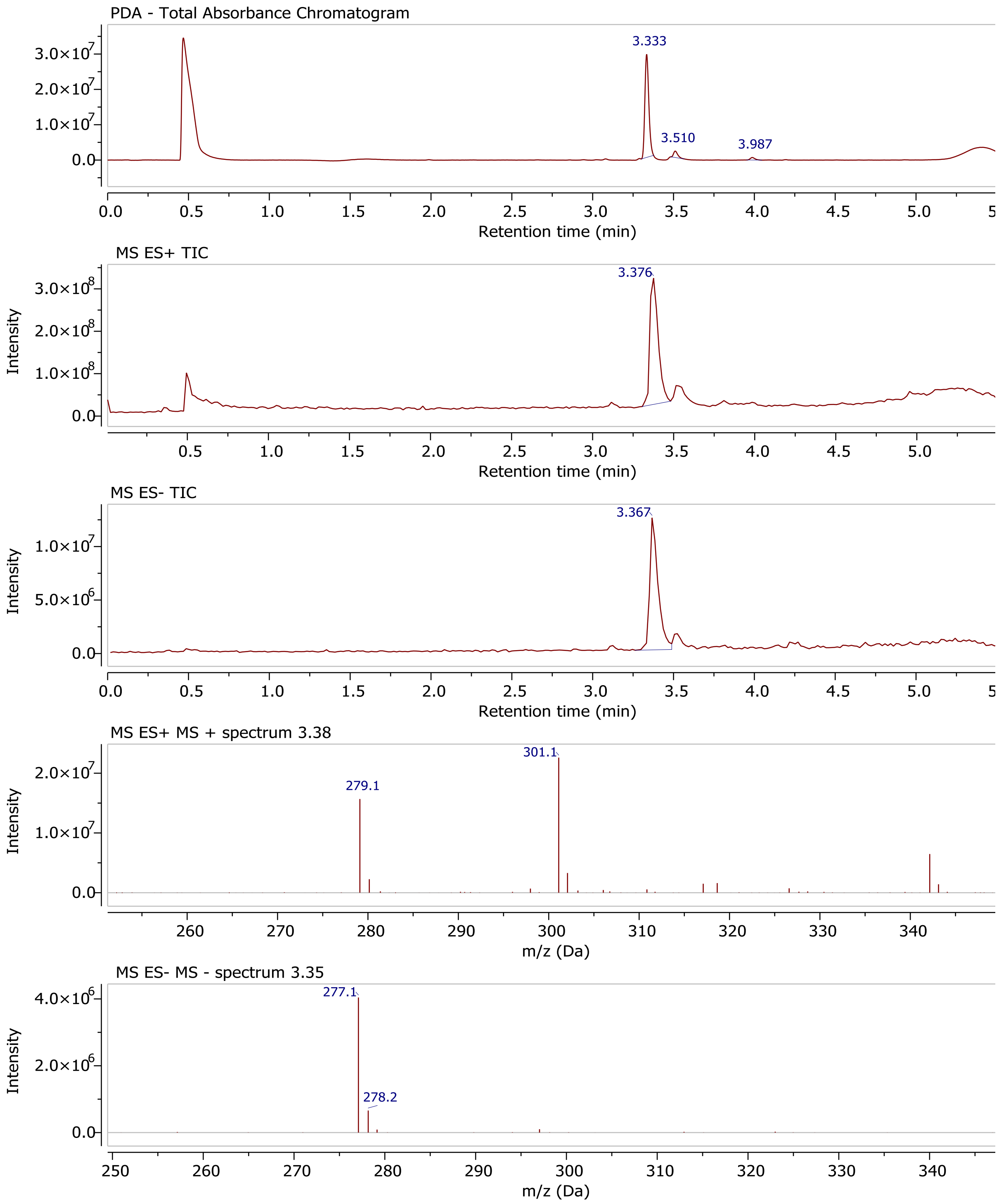

Supplementary figure **1389**. UPLC-MS analysis for compound **PHP-5768**

Chemical Formula: $C_{18}H_{24}N_2O_4$
Exact Mass: 332.2

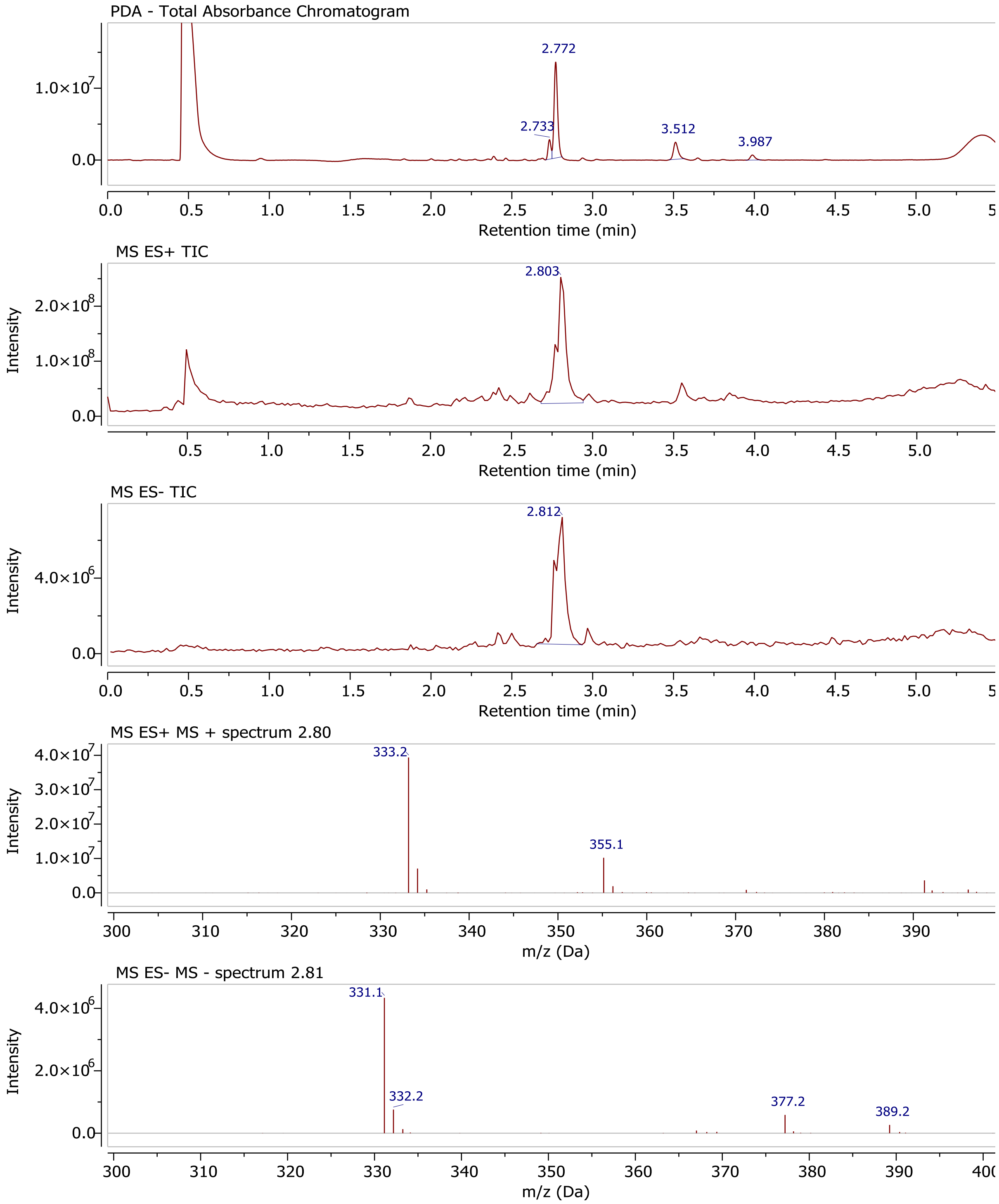

Supplementary figure **1390**. UPLC-MS analysis for compound **PHP-5622**

Chemical Formula: $C_{17}H_{15}FN_2O_2$
Exact Mass: 298.1

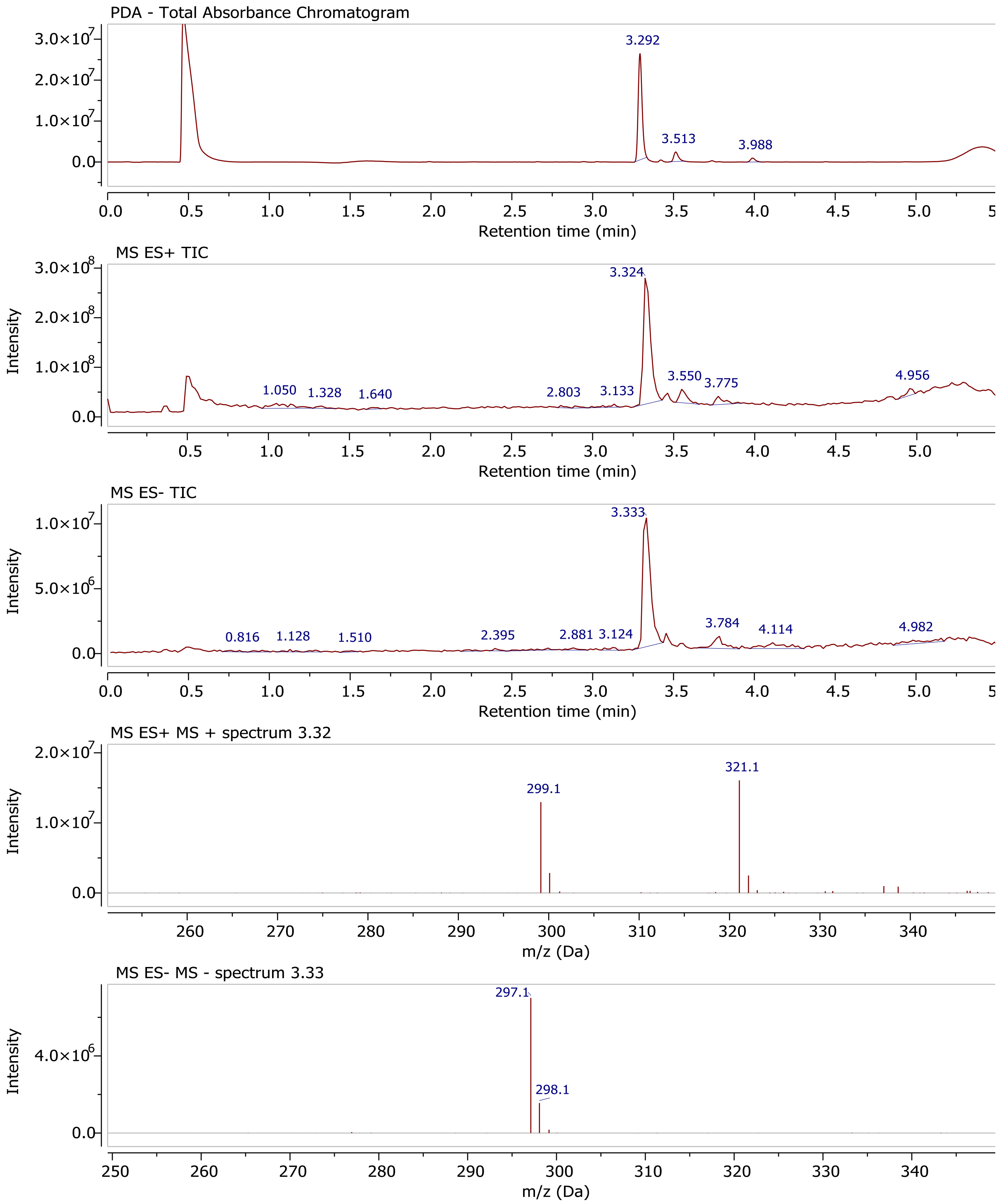

Supplementary figure **1391**. UPLC-MS analysis for compound **PHP-5779**

Chemical Formula: $C_{20}H_{22}N_2O_3$
Exact Mass: 338.2

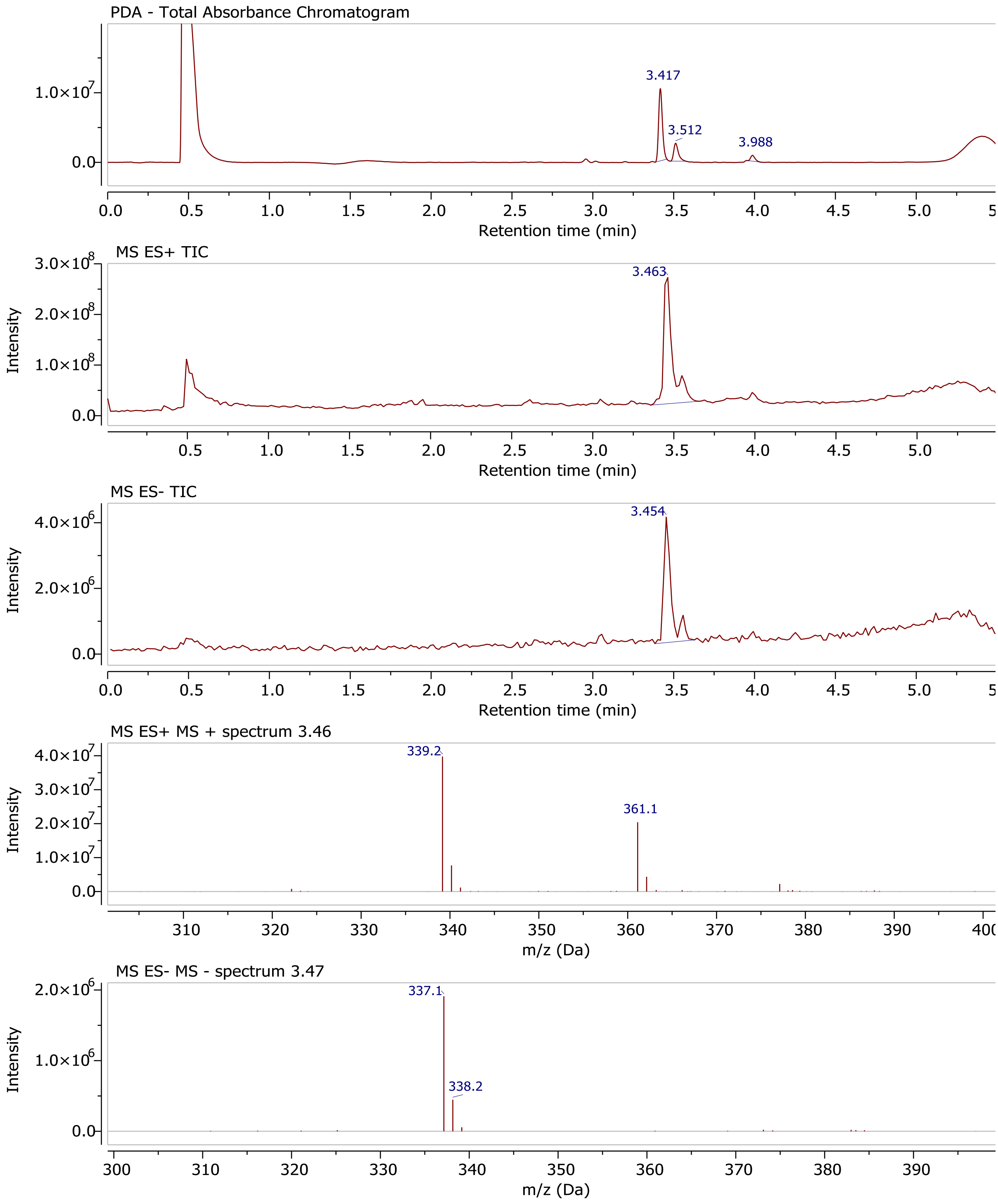

Supplementary figure **1392**. UPLC-MS analysis for compound **PHP-5626**

Chemical Formula: $C_{18}H_{15}F_3N_2O_3$
Exact Mass: 364.1

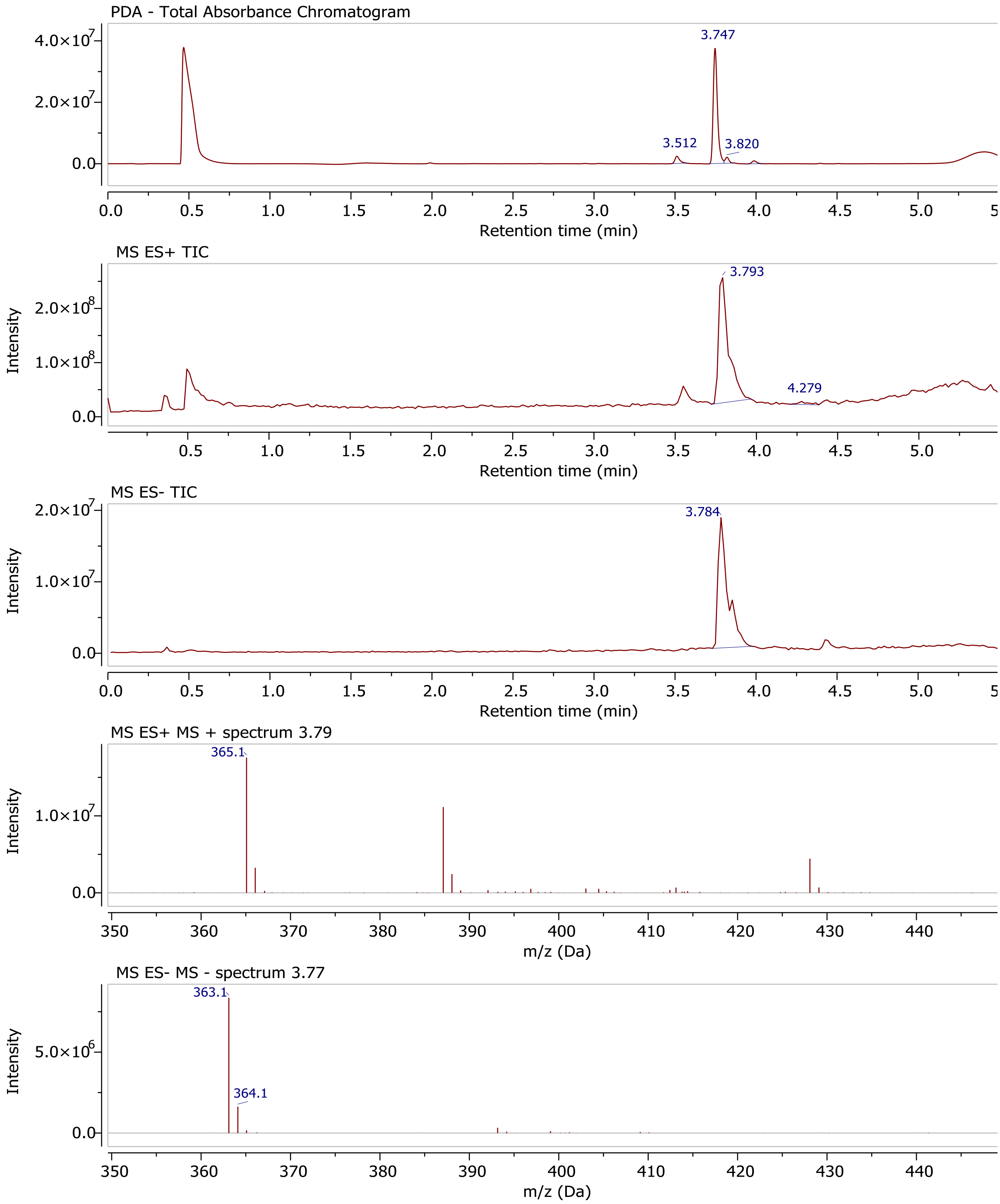

Supplementary figure **1393**. UPLC-MS analysis for compound **PHP-5783**

Chemical Formula: $C_{20}H_{22}N_2O_3$
Exact Mass: 338.2

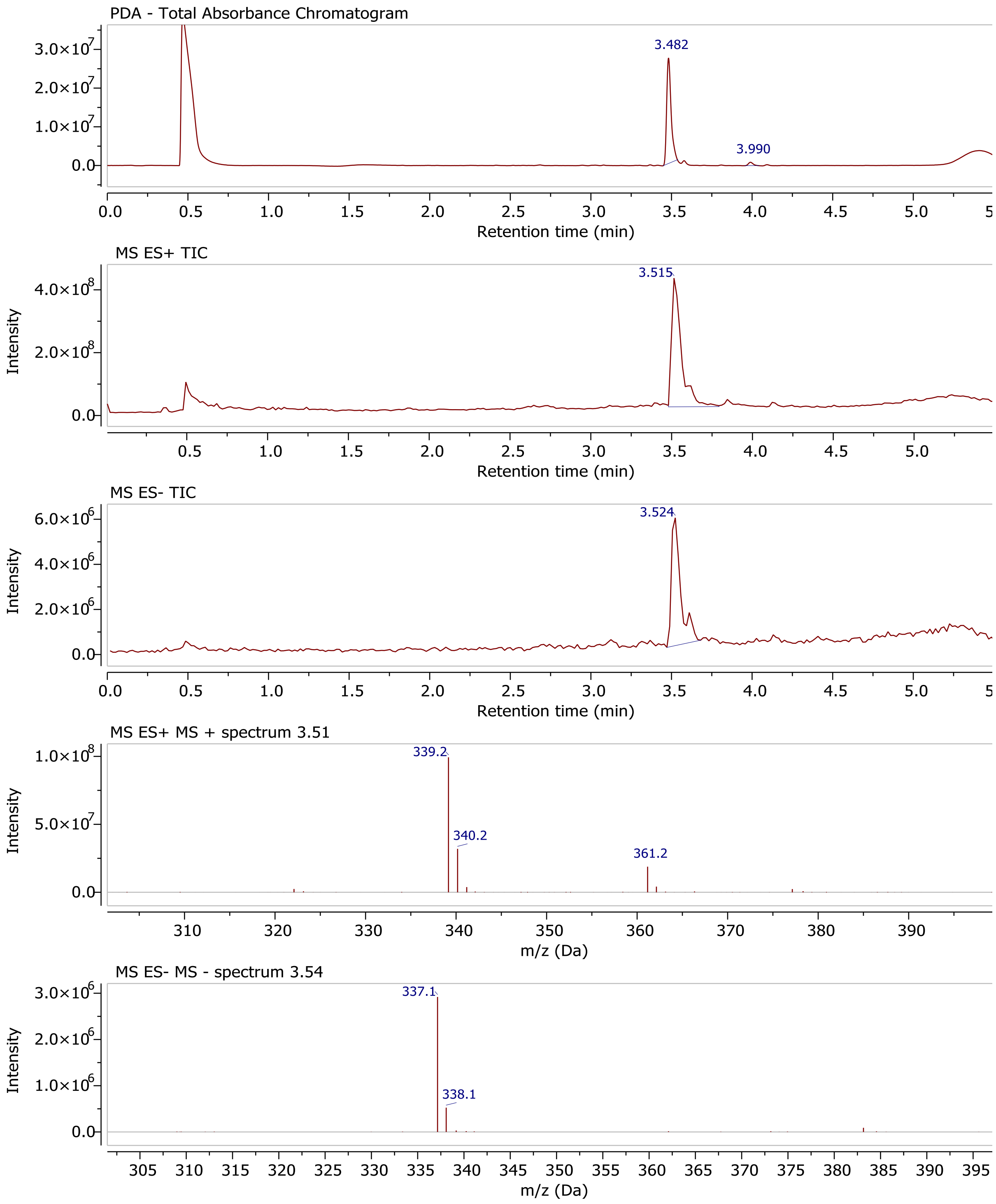

Supplementary figure **1394**. UPLC-MS analysis for compound **PHP-5627**

Chemical Formula: $C_{25}H_{32}N_2O_3$
Exact Mass: 408.2

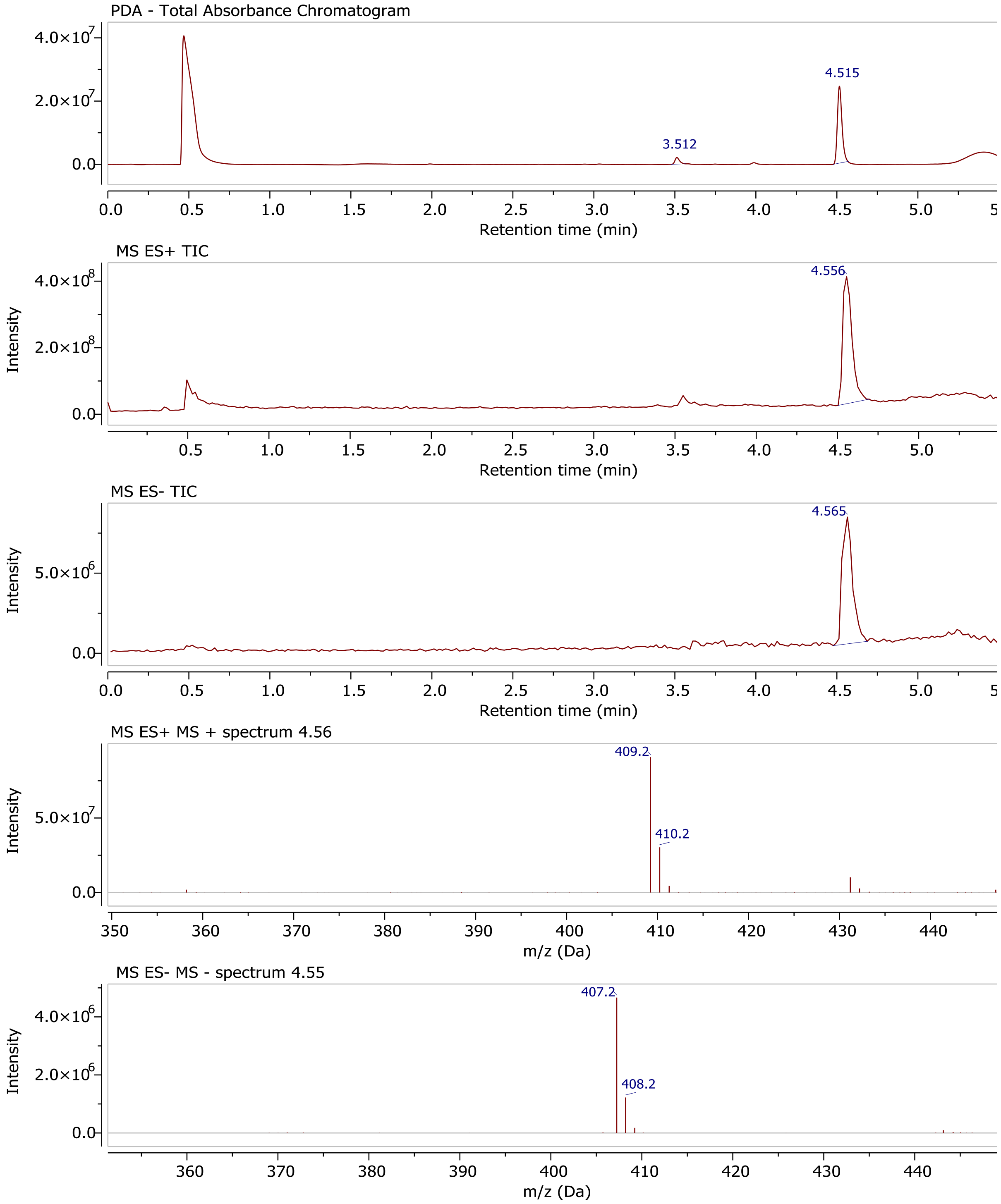

Supplementary figure **1395**. UPLC-MS analysis for compound **PHP-5784**

Chemical Formula: $C_{19}H_{21}BrN_2O_2S$
Exact Mass: 420.1

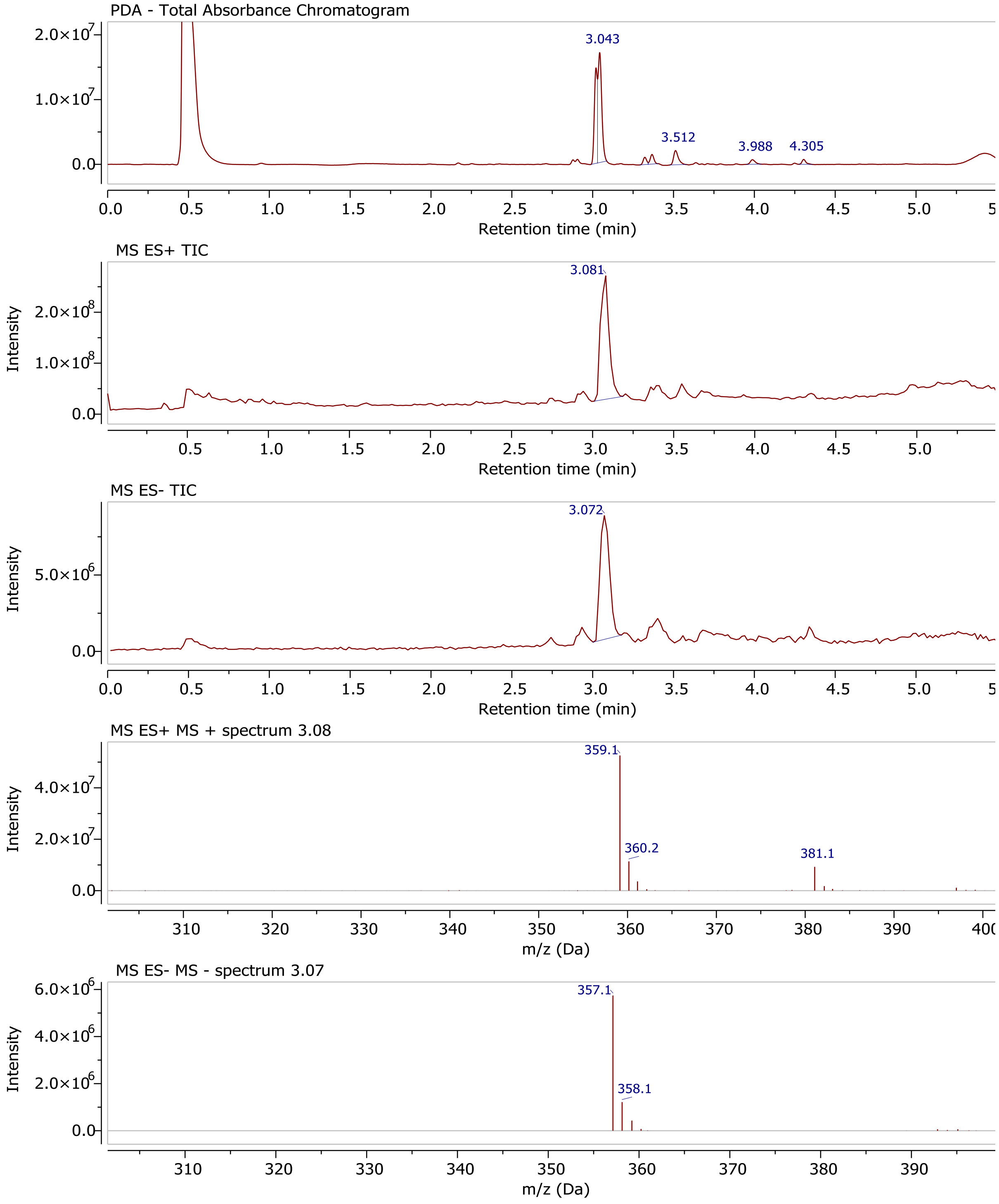

Supplementary figure **1396**. UPLC-MS analysis for compound **PHP-4876**

Chemical Formula: $C_{21}H_{19}F_3N_2O_4$
Exact Mass: 420.1

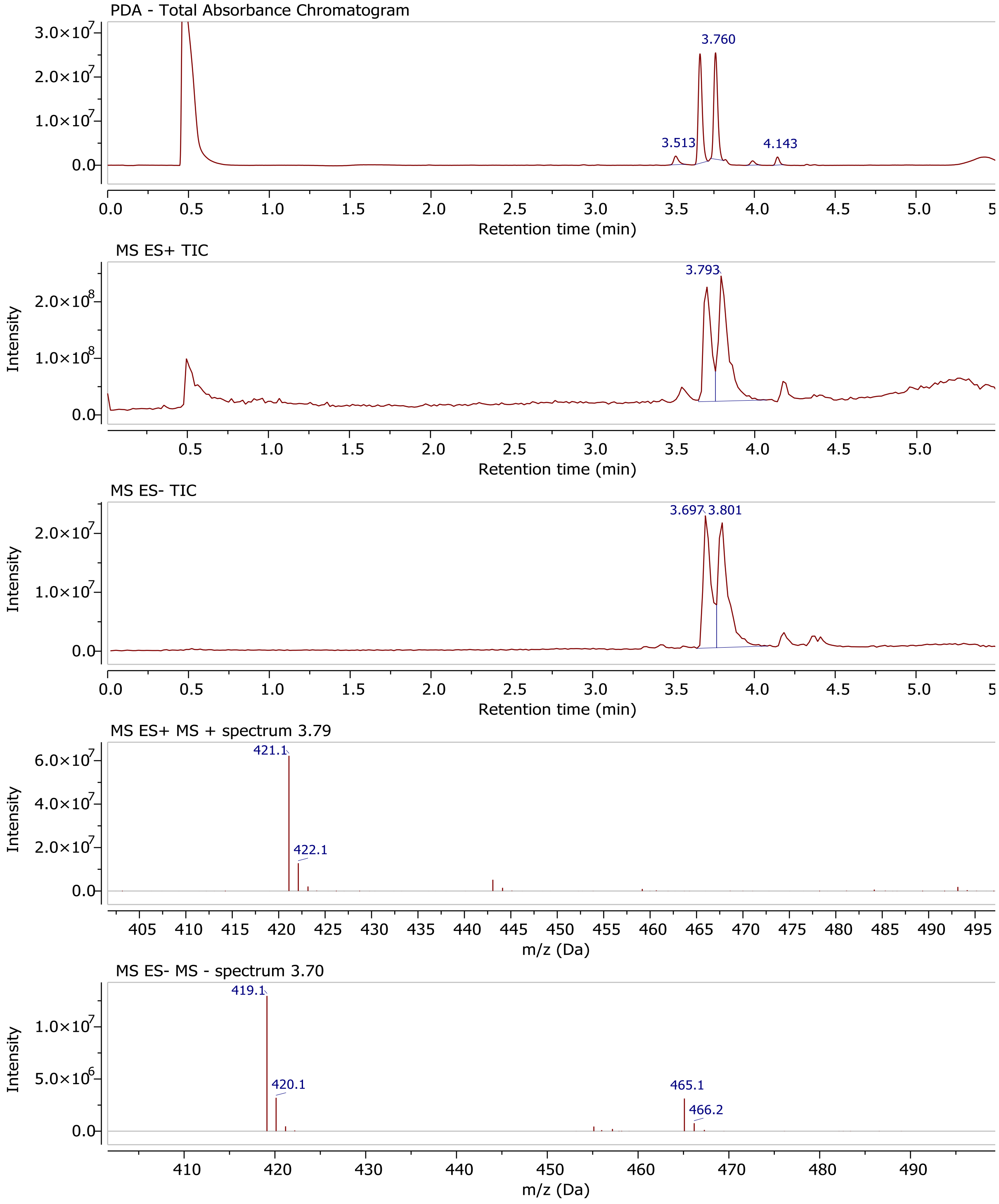

Supplementary figure **1397**. UPLC-MS analysis for compound **PHP-5459**

Chemical Formula: $C_{19}H_{20}N_4O_2S$
Exact Mass: 368.1

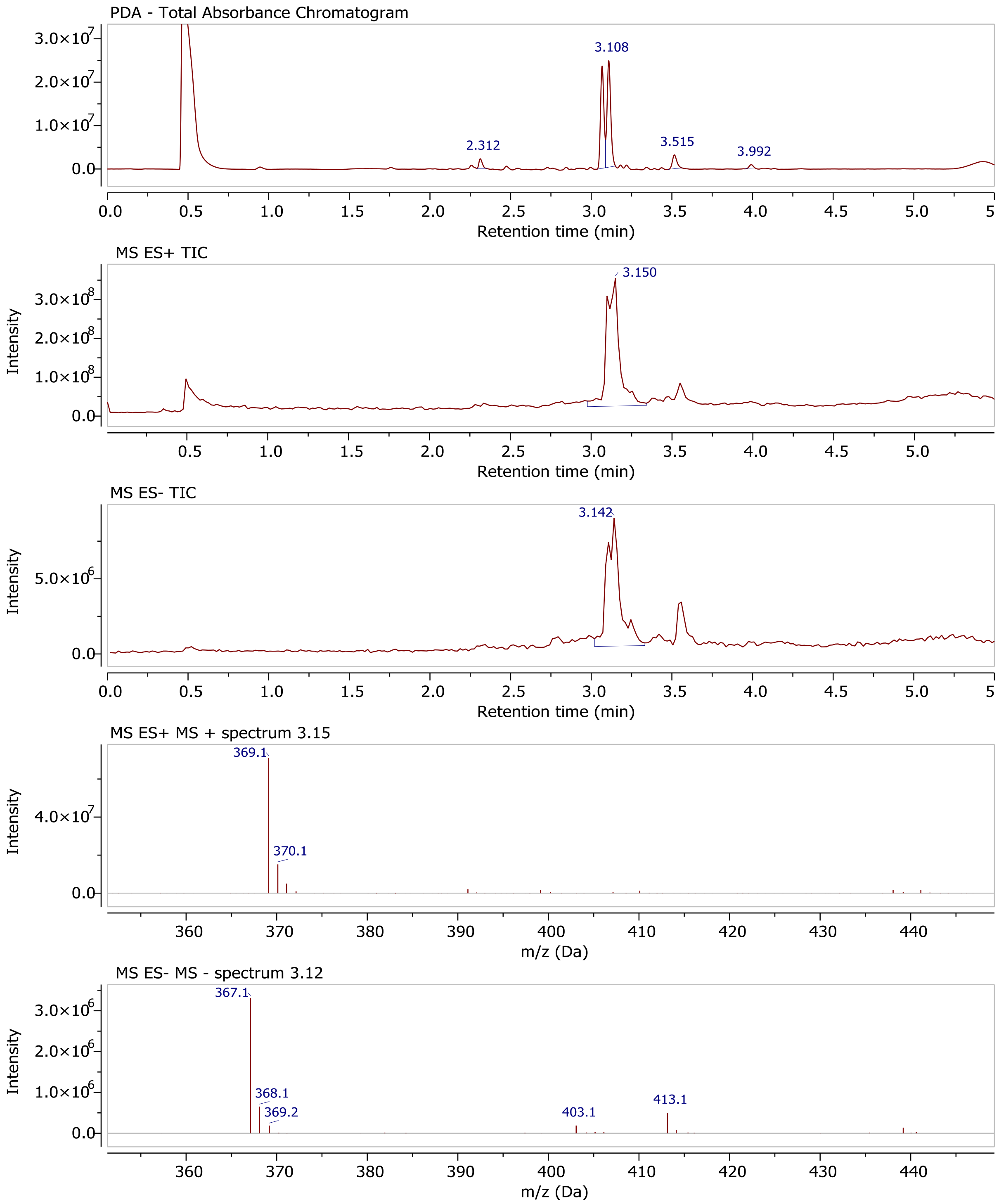

Supplementary figure **1398**. UPLC-MS analysis for compound **PHP-4879**

Chemical Formula: $C_{20}H_{19}ClN_2O_4$
Exact Mass: 386.1

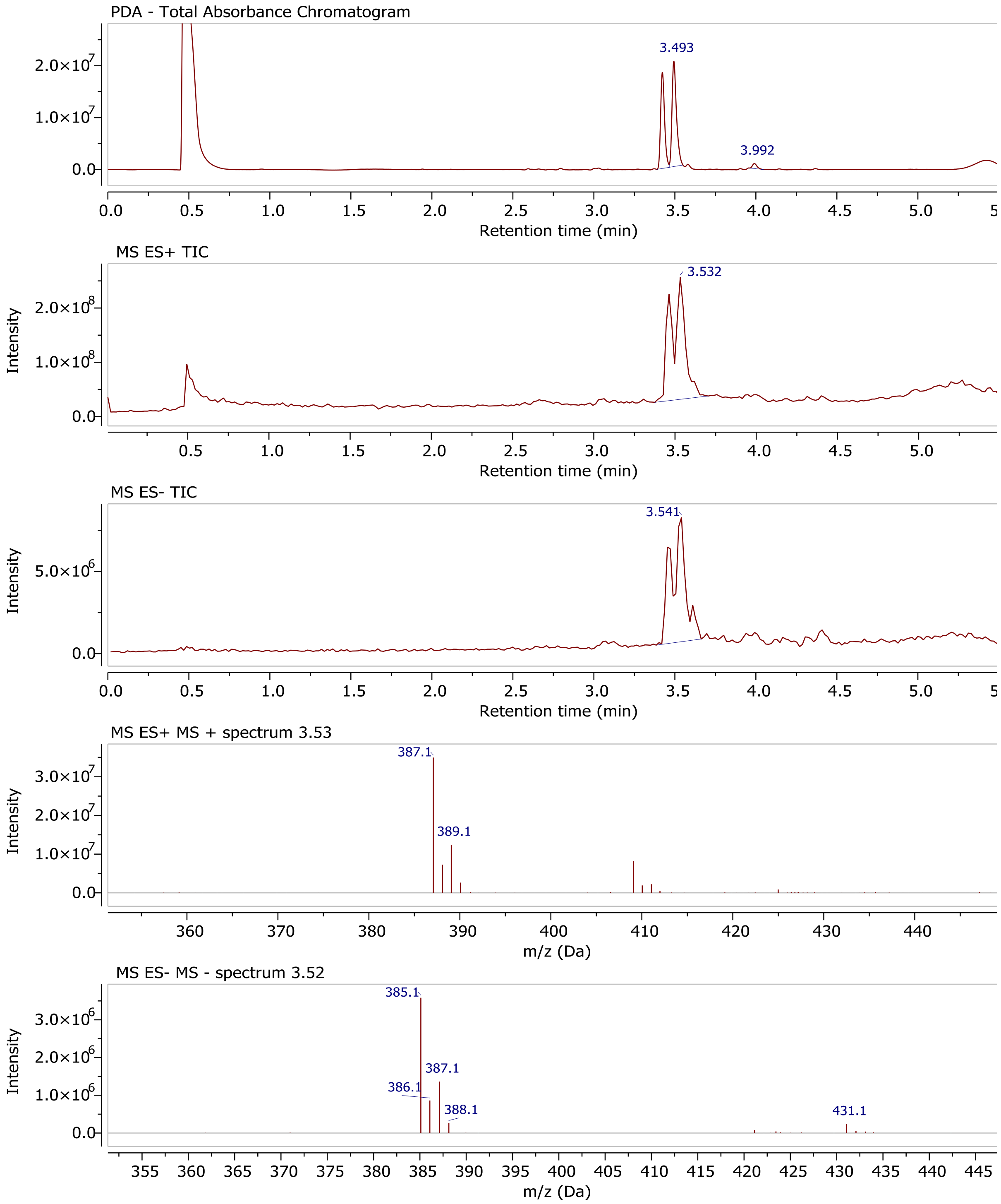

Supplementary figure **1399**. UPLC-MS analysis for compound **PHP-5460**

Chemical Formula: $C_{26}H_{24}N_2O_2S$
Exact Mass: 428.2

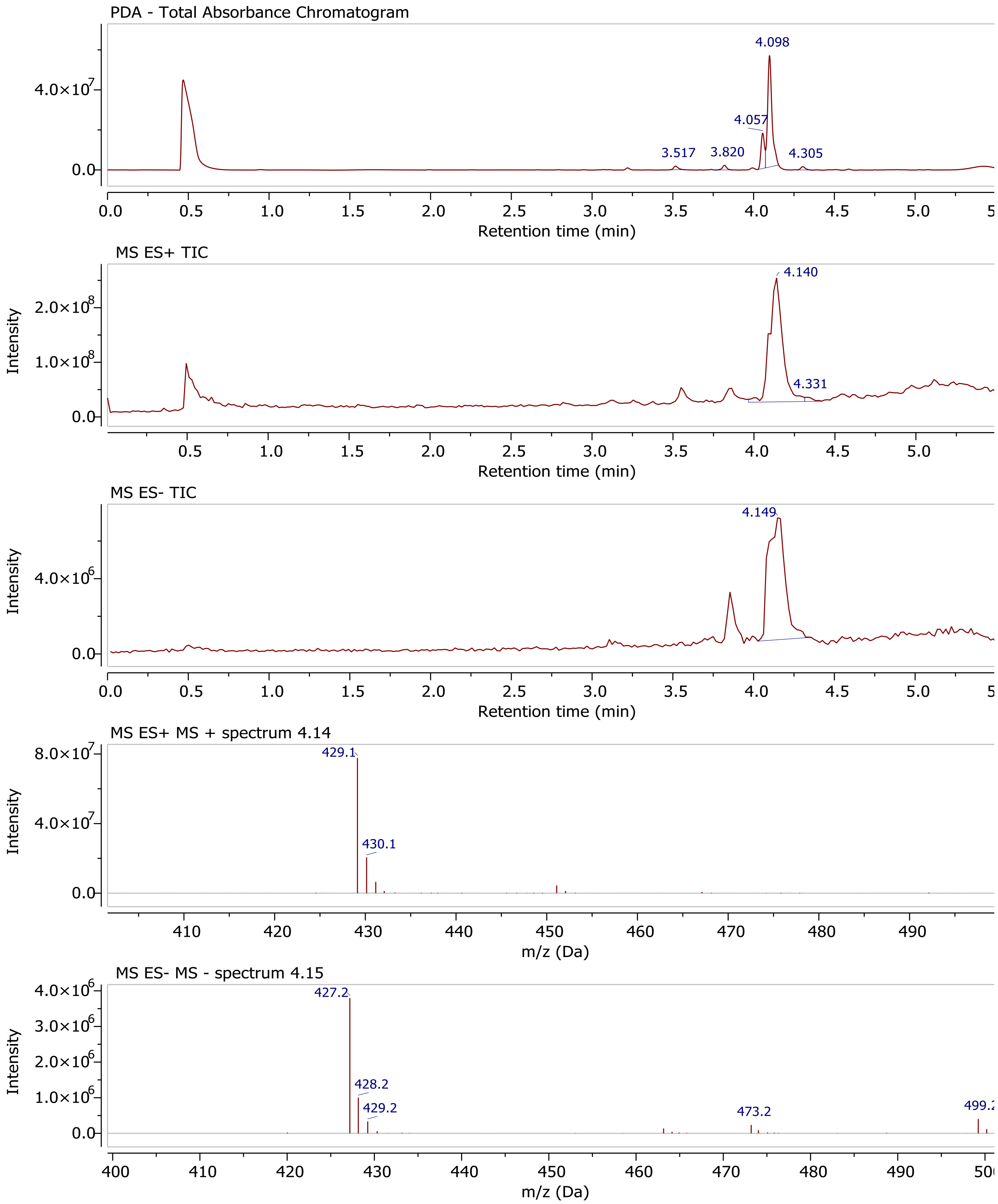

Supplementary figure **1400**. UPLC-MS analysis for compound **PHP-4880**

Chemical Formula: $C_{21}H_{19}F_3N_2O_4$
Exact Mass: 420.1

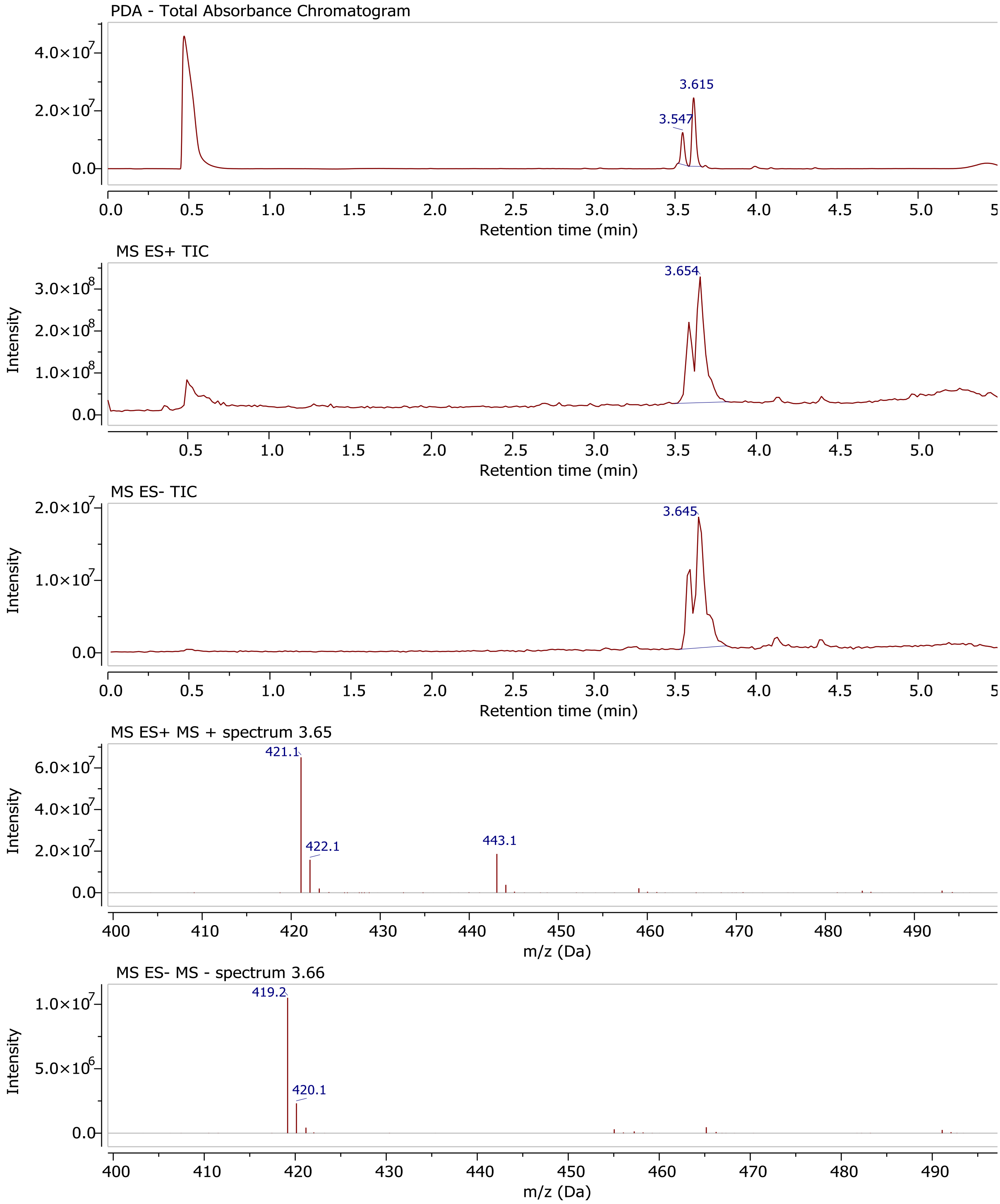

Supplementary figure **1401**. UPLC-MS analysis for compound **PHP-5461**

Chemical Formula: $C_{25}H_{25}ClN_2O_3$
Exact Mass: 436.2

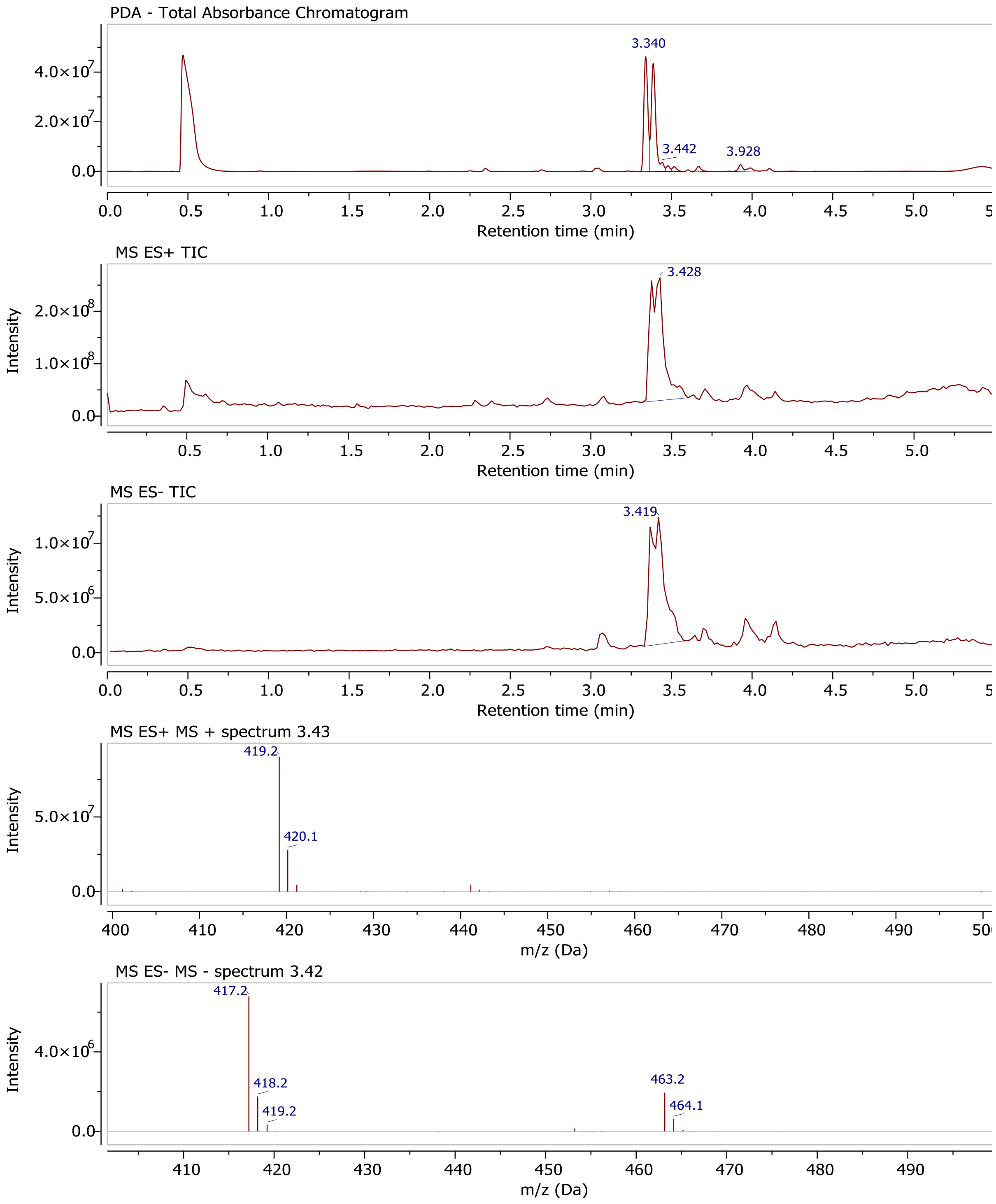

Supplementary figure **1402**. UPLC-MS analysis for compound **PHP-4881**

Chemical Formula: $C_{20}H_{19}FN_2O_4$
Exact Mass: 370.1

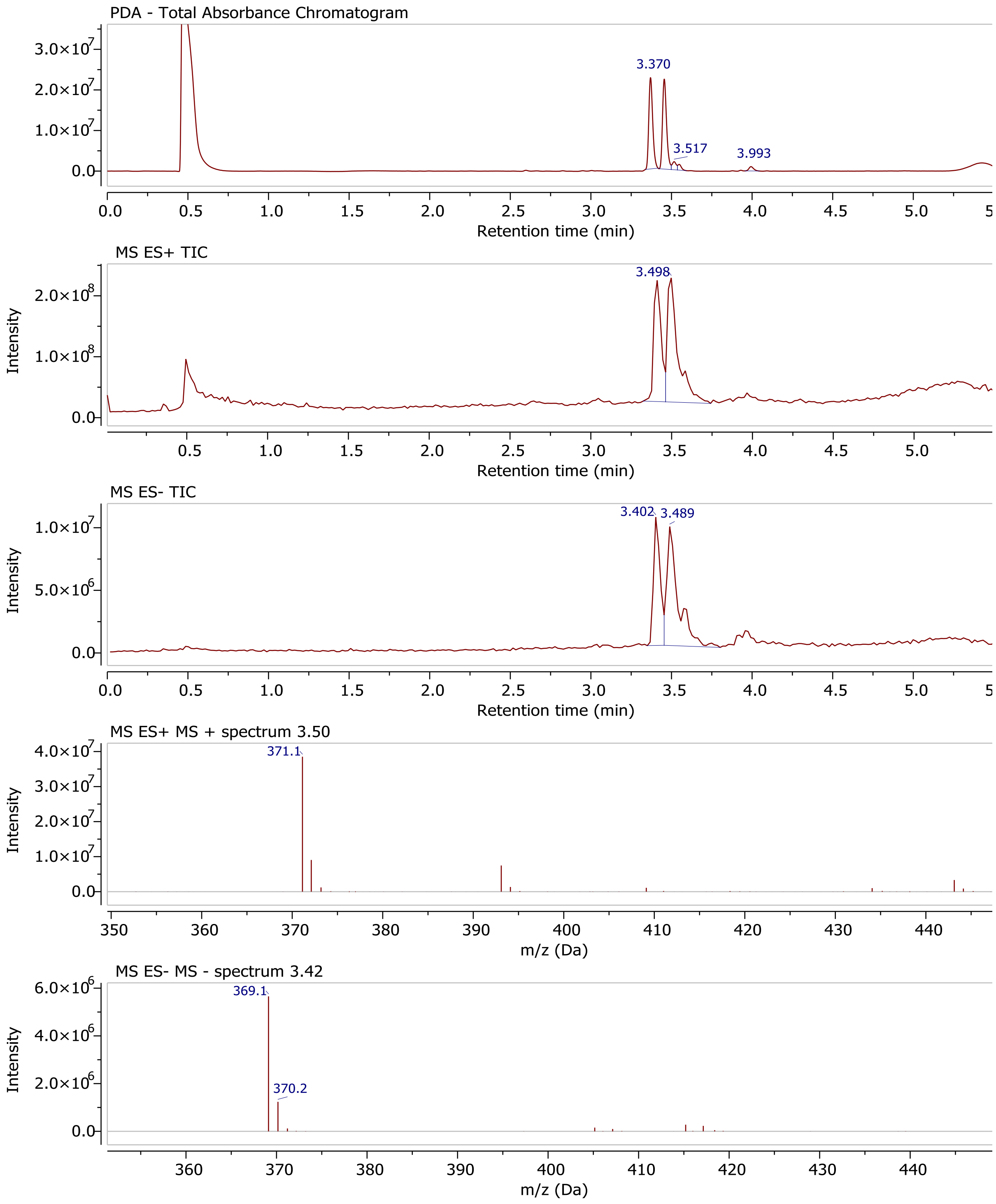

Supplementary figure **1403**. UPLC-MS analysis for compound **PHP-5462**

Chemical Formula: $C_{29}H_{26}N_2O_2$
Exact Mass: 434.2

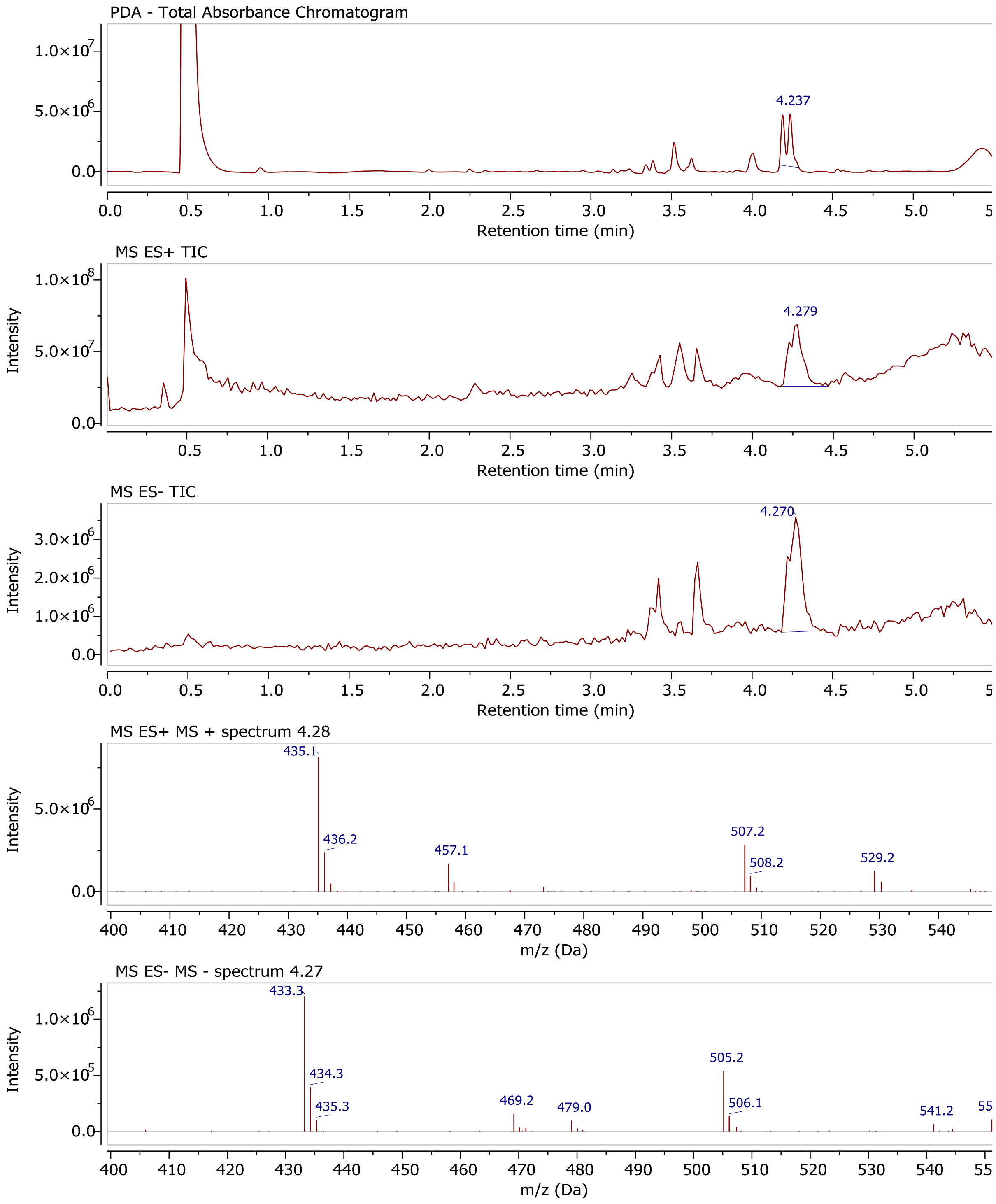

Supplementary figure **1404**. UPLC-MS analysis for compound **PHP-4882**

Chemical Formula: $C_{20}H_{19}FN_2O_4$
Exact Mass: 370.1

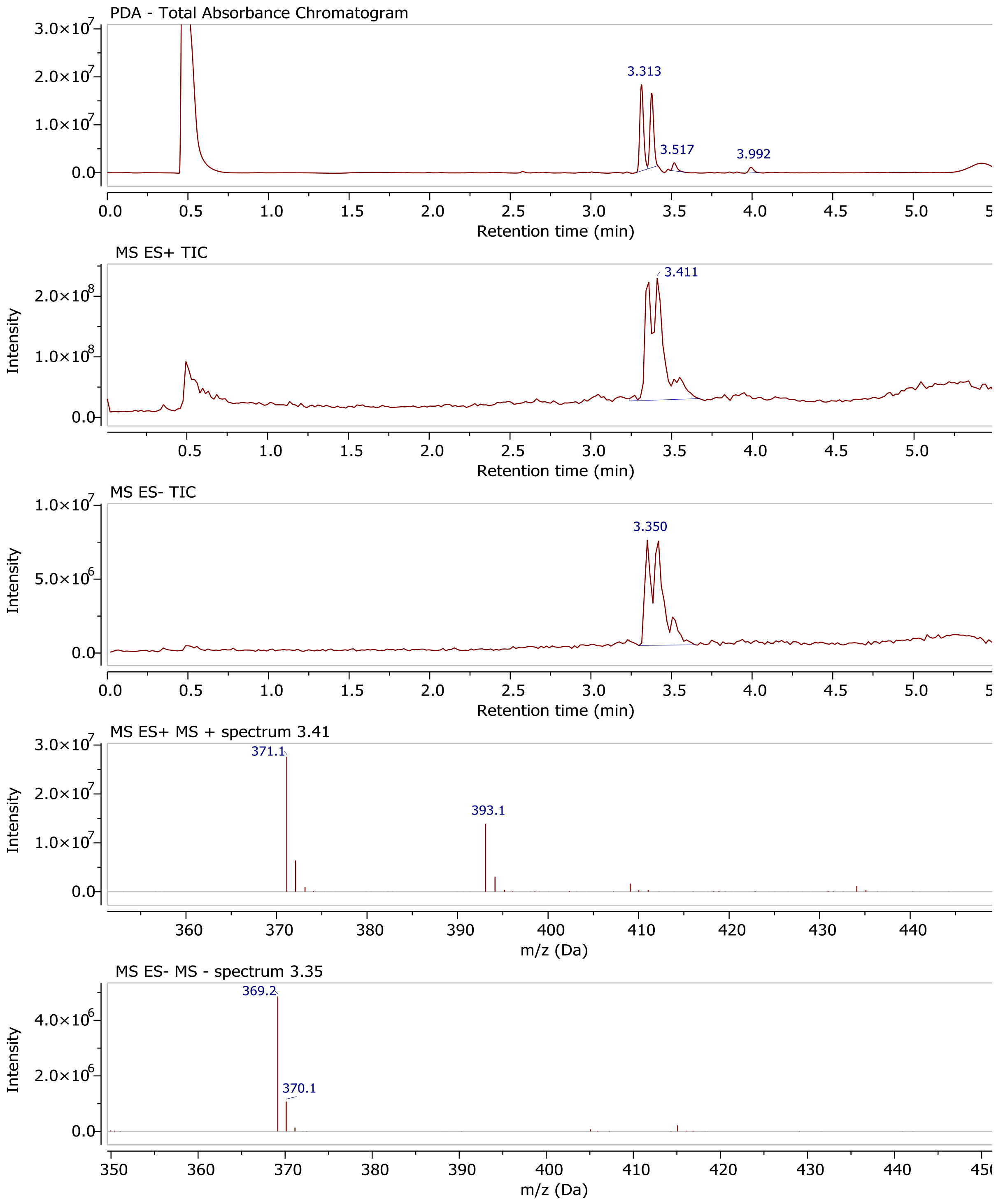

Supplementary figure **1405**. UPLC-MS analysis for compound **PHP-5463**

Chemical Formula: $C_{24}H_{21}N_3O_2$
Exact Mass: 383.2

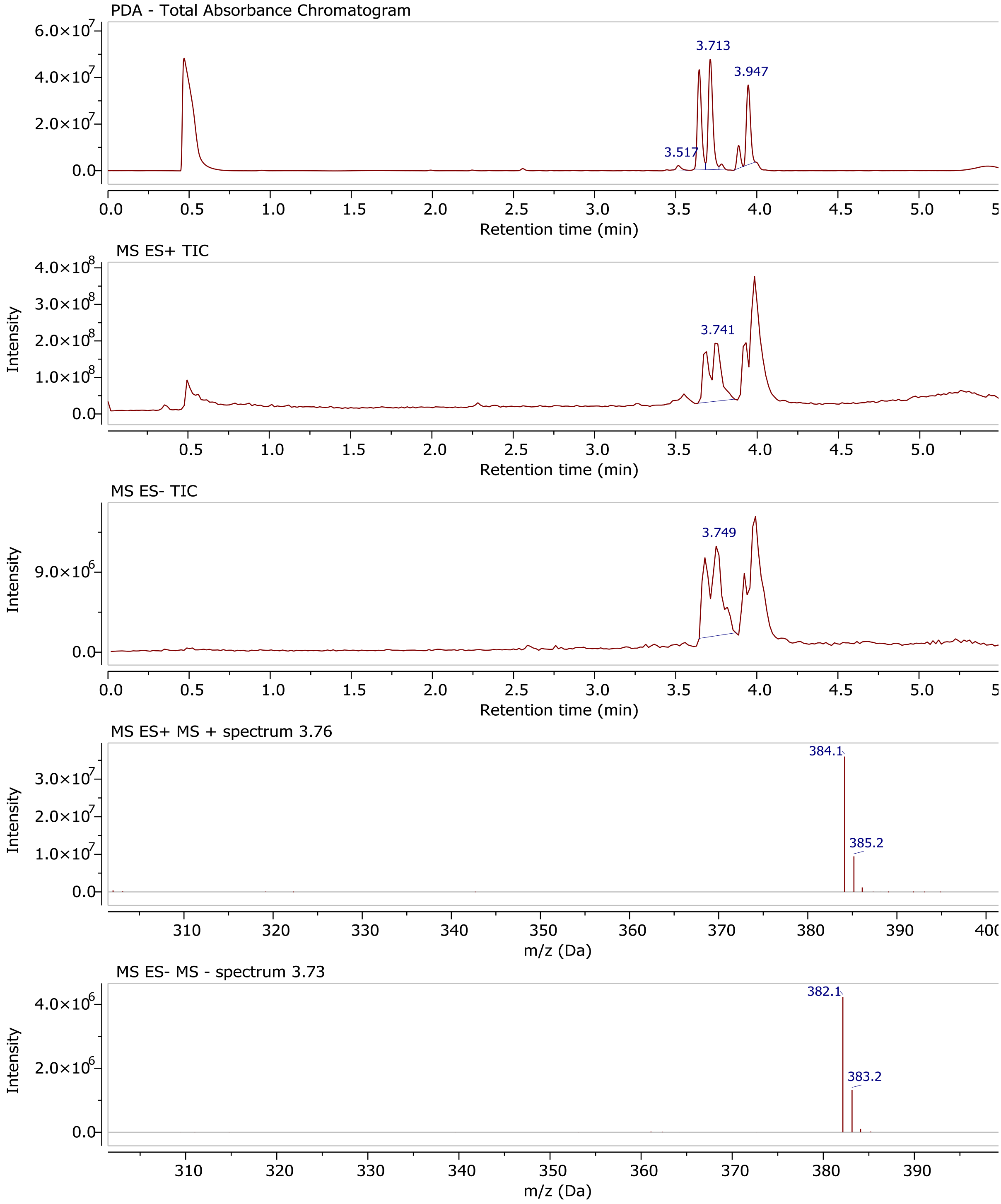

Supplementary figure **1406**. UPLC-MS analysis for compound **PHP-4884**

Chemical Formula: $C_{21}H_{19}F_3N_2O_4$
Exact Mass: 420.1

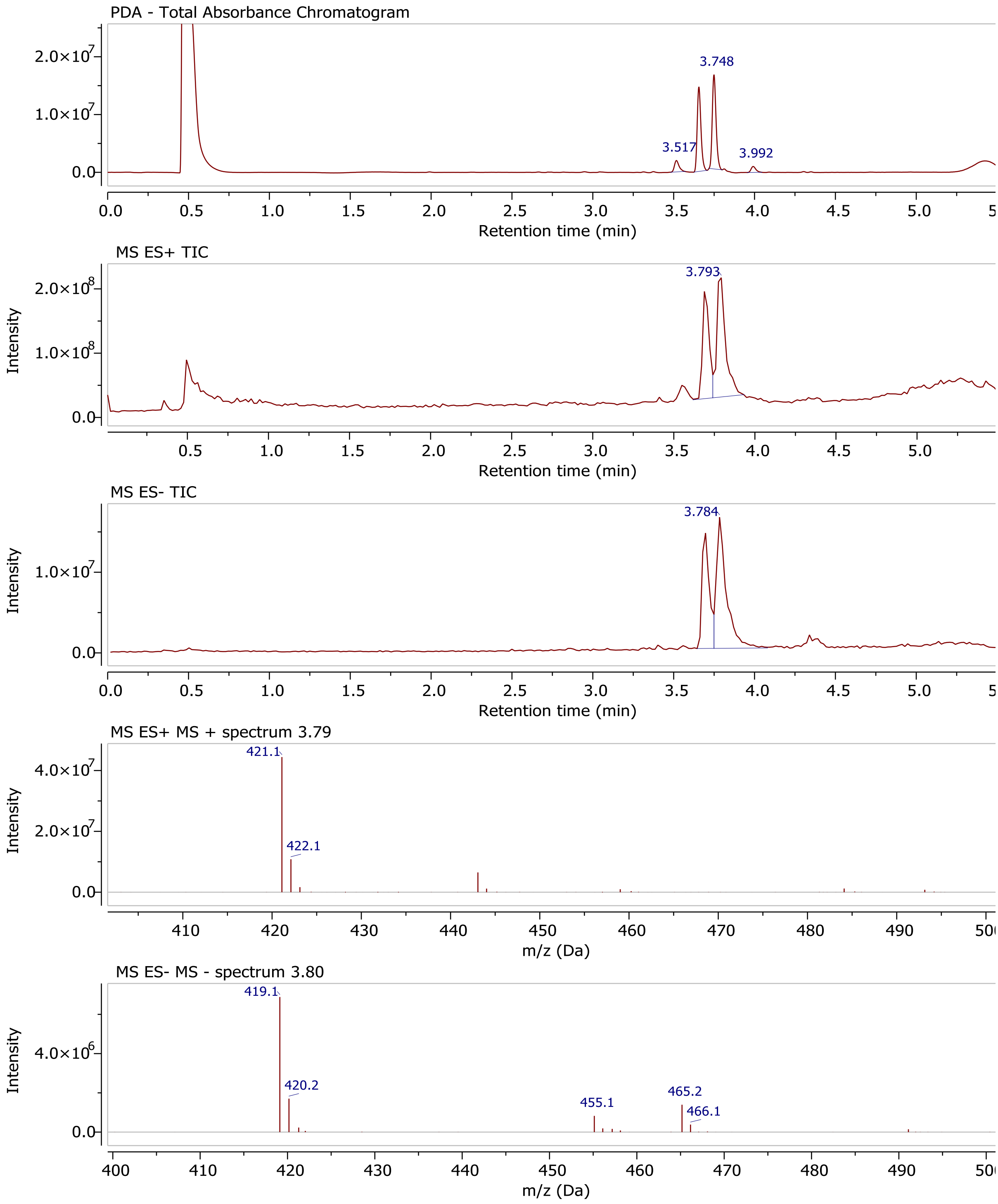

Supplementary figure **1407**. UPLC-MS analysis for compound **PHP-5464**

Chemical Formula: $C_{20}H_{20}N_4O_2$
Exact Mass: 348.2

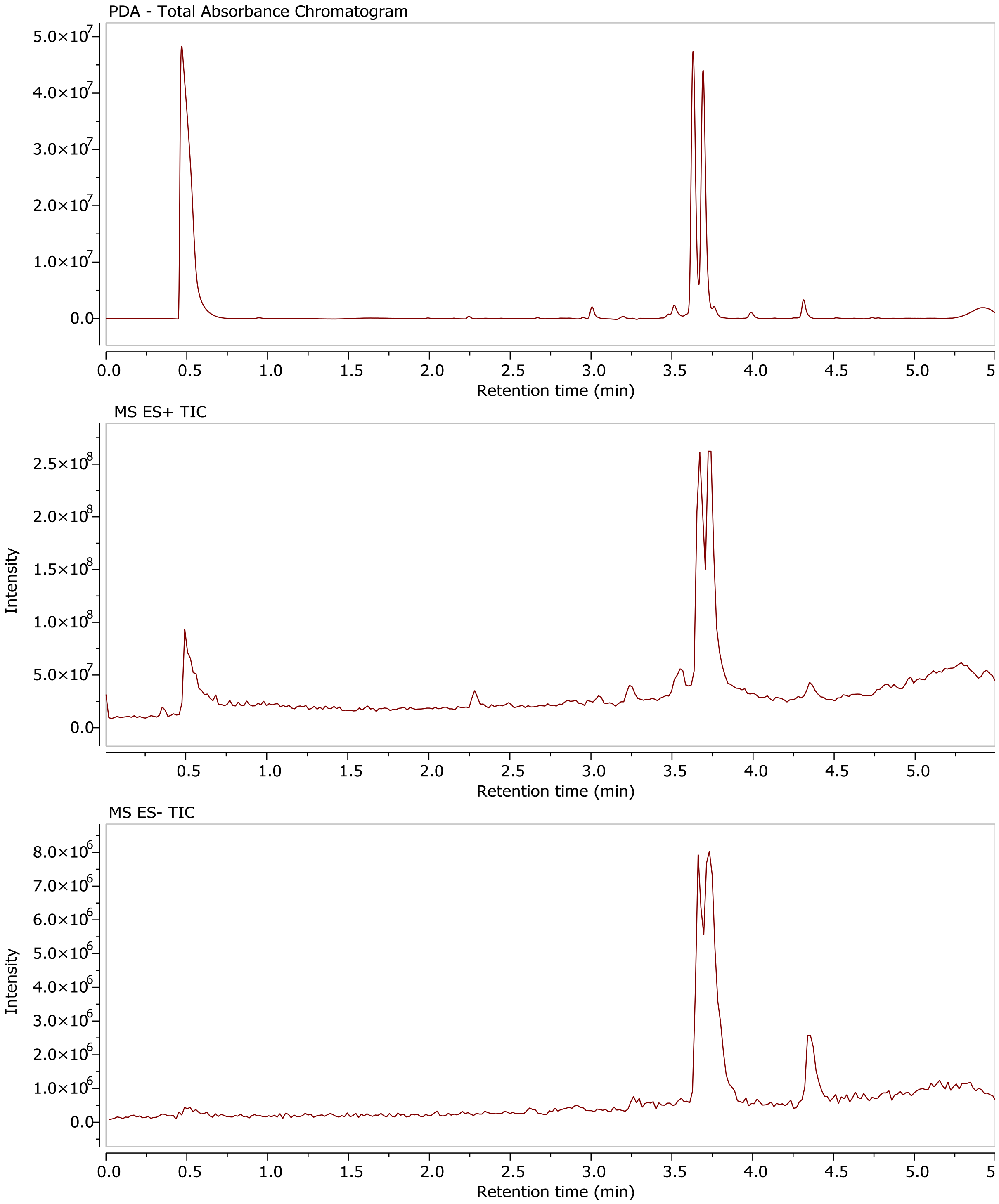

Supplementary figure **1408**. UPLC-MS analysis for compound **PHP-4894**

Chemical Formula: $C_{24}H_{19}F_3N_2O_3$
Exact Mass: 440.1

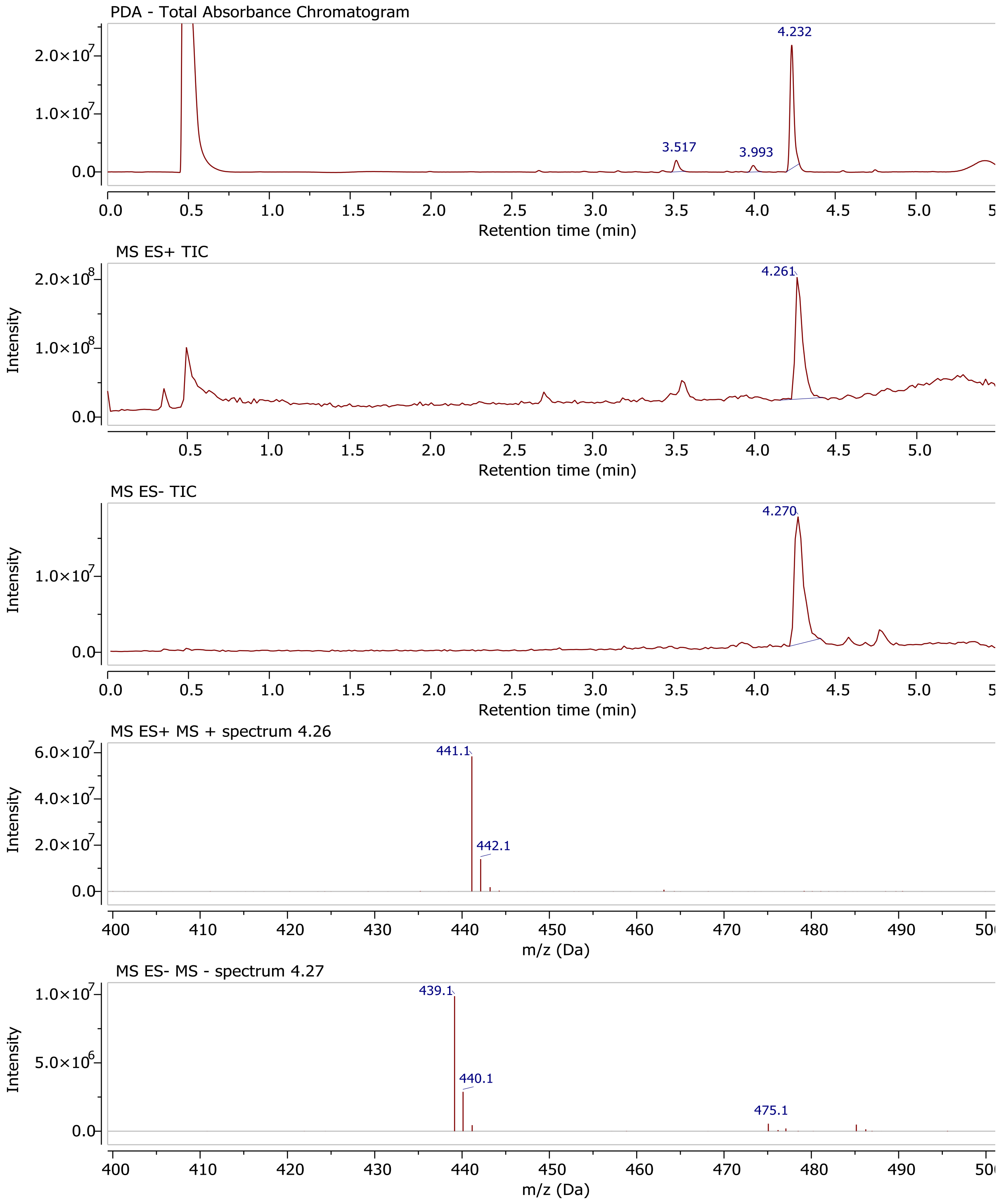

Supplementary figure **1409**. UPLC-MS analysis for compound **PHP-5465**

Chemical Formula: $C_{24}H_{22}N_2O_4$
Exact Mass: 402.2

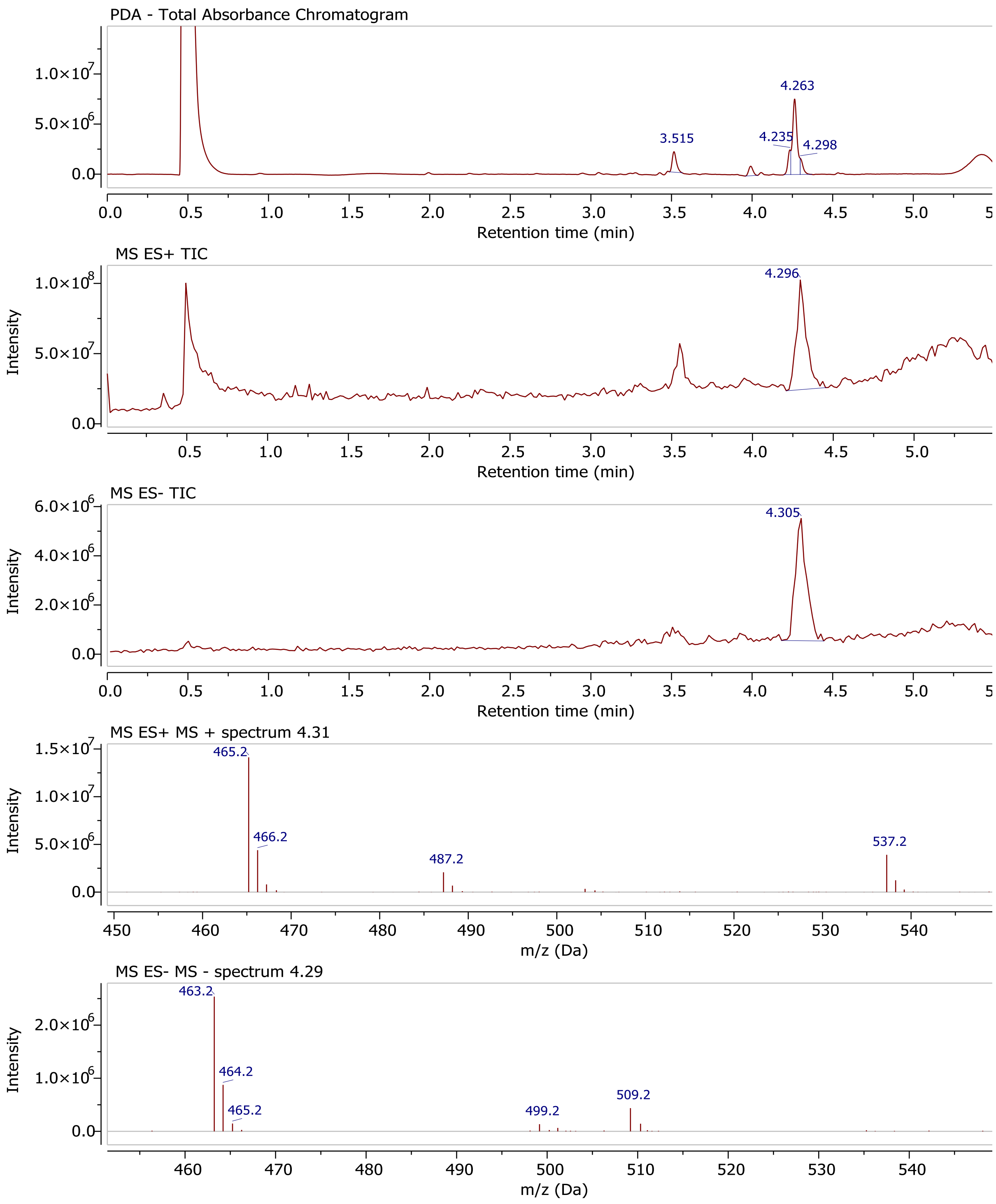

Supplementary figure **1410**. UPLC-MS analysis for compound **PHP-4895**

Chemical Formula: $C_{29}H_{24}N_2O_4$
Exact Mass: 464.2

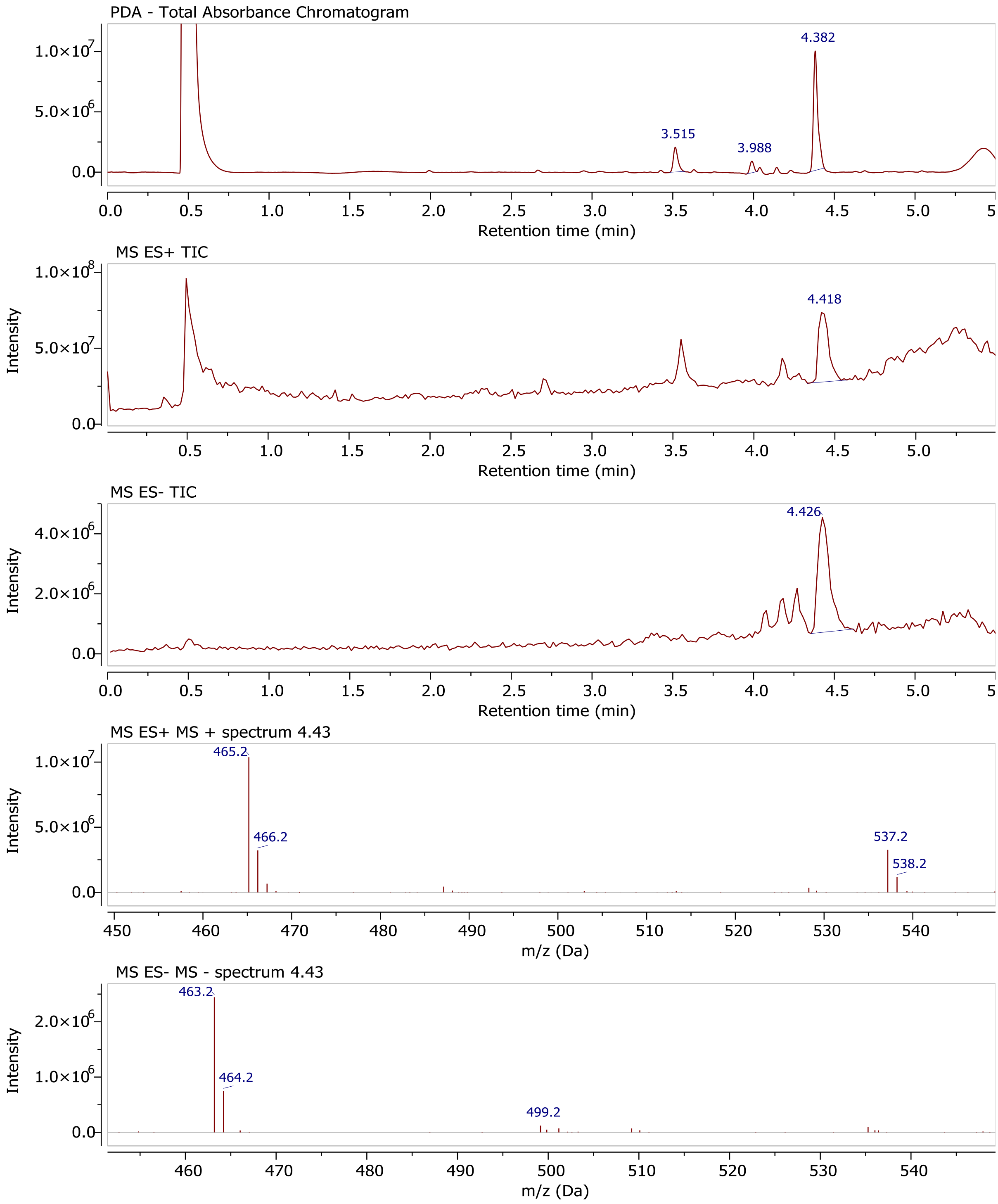

Supplementary figure **1411**. UPLC-MS analysis for compound **PHP-5466**

Chemical Formula: $C_{22}H_{22}N_4O_2$
Exact Mass: 374.2

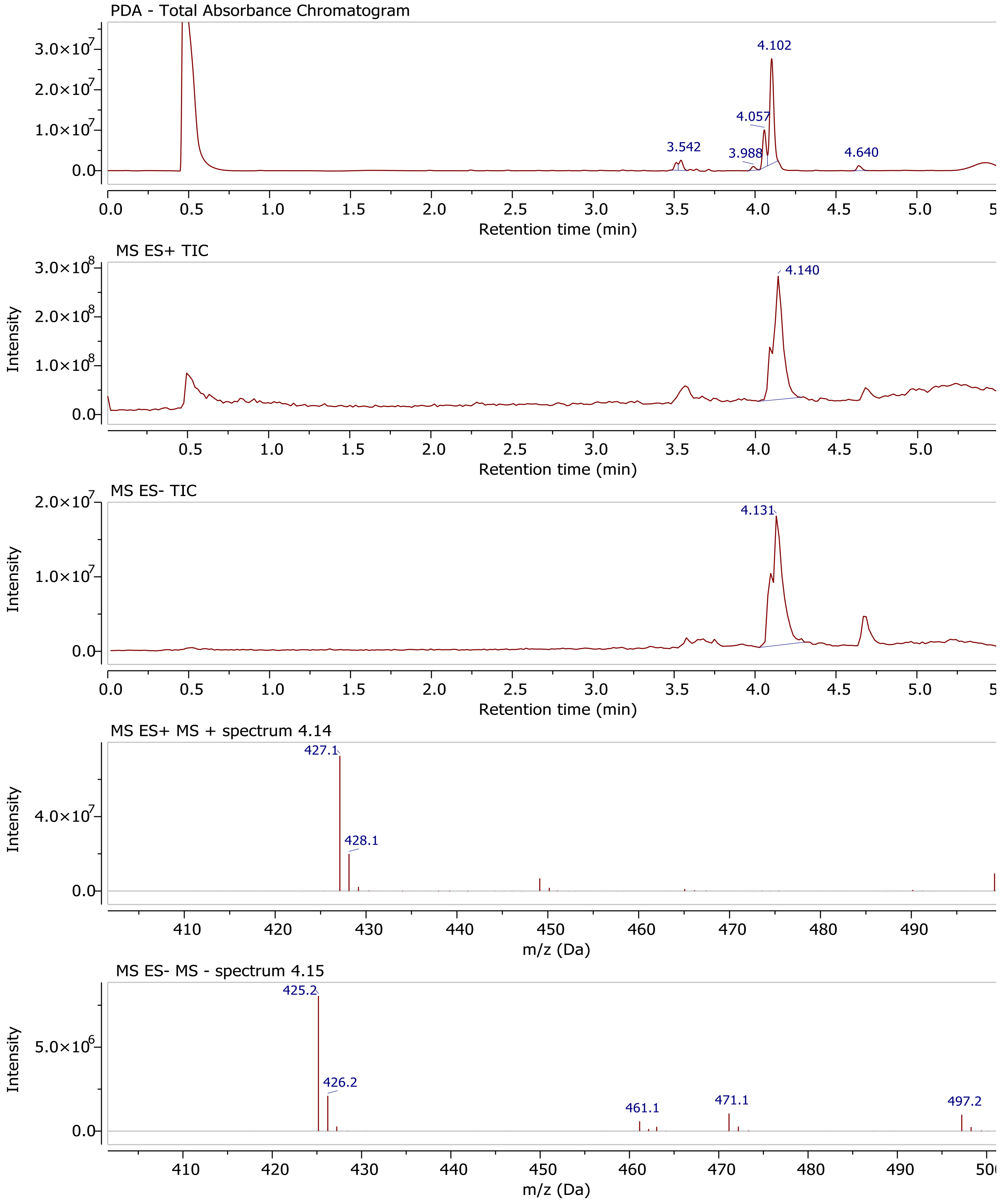

Supplementary figure **1412**. UPLC-MS analysis for compound **PHP-4896**

Chemical Formula: $C_{26}H_{26}N_2O_3$
Exact Mass: 414.2

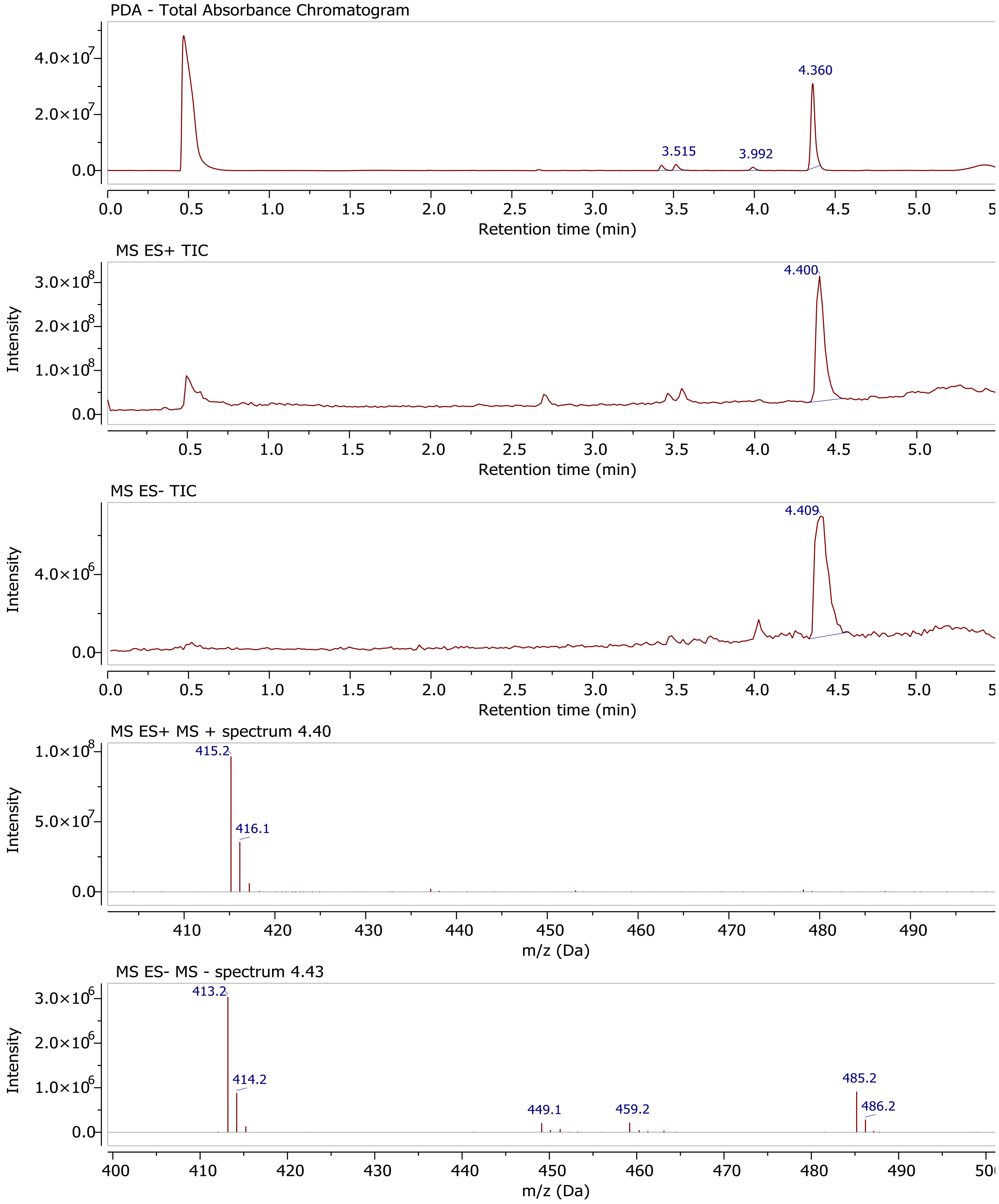

Supplementary figure **1413**. UPLC-MS analysis for compound **PHP-5468**

Chemical Formula: $C_{20}H_{20}ClFN_2O_3$
Exact Mass: 390.1

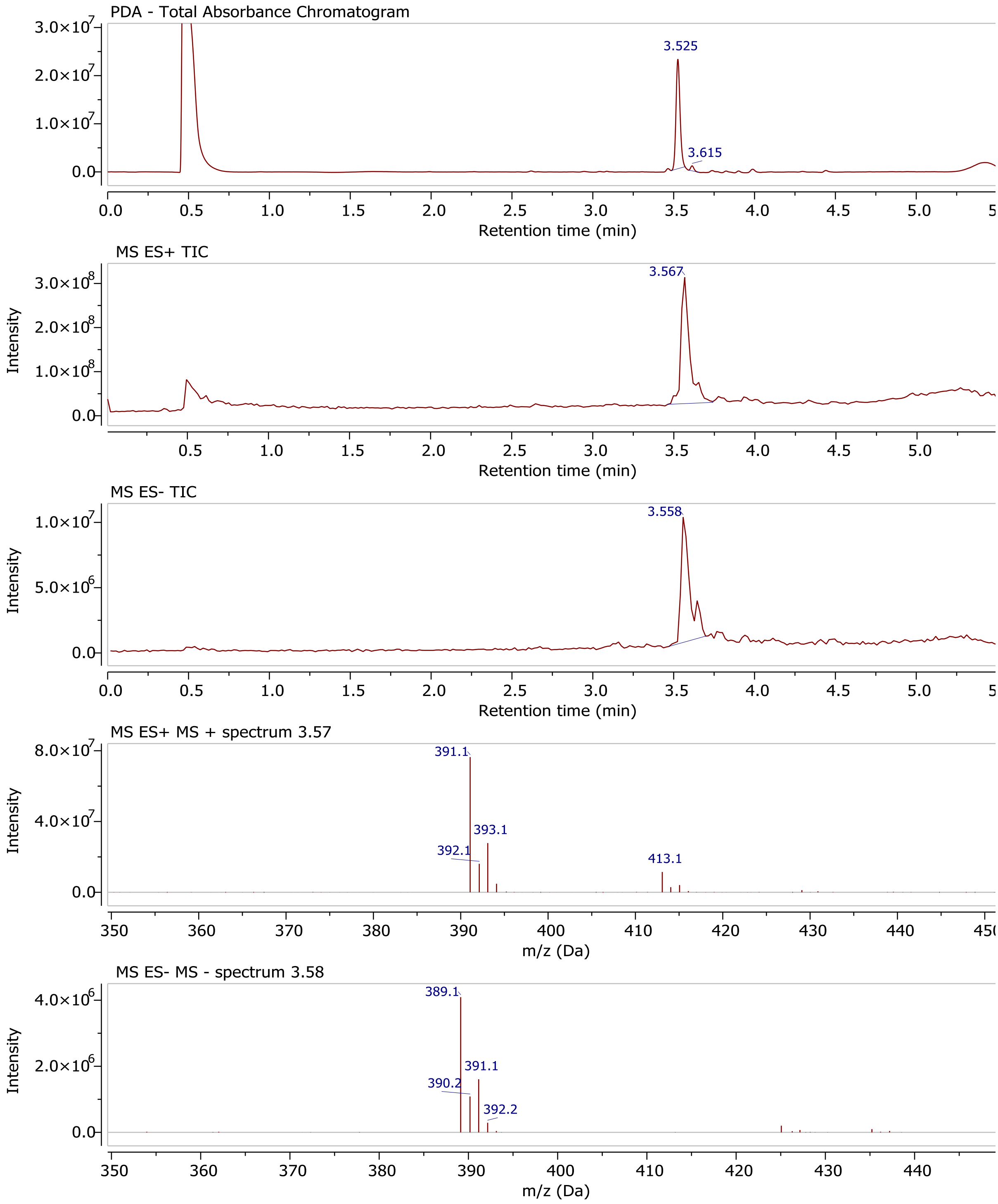

Supplementary figure **1414**. UPLC-MS analysis for compound **PHP-4901**

Chemical Formula: $C_{23}H_{19}ClN_2O_3$
Exact Mass: 406.1

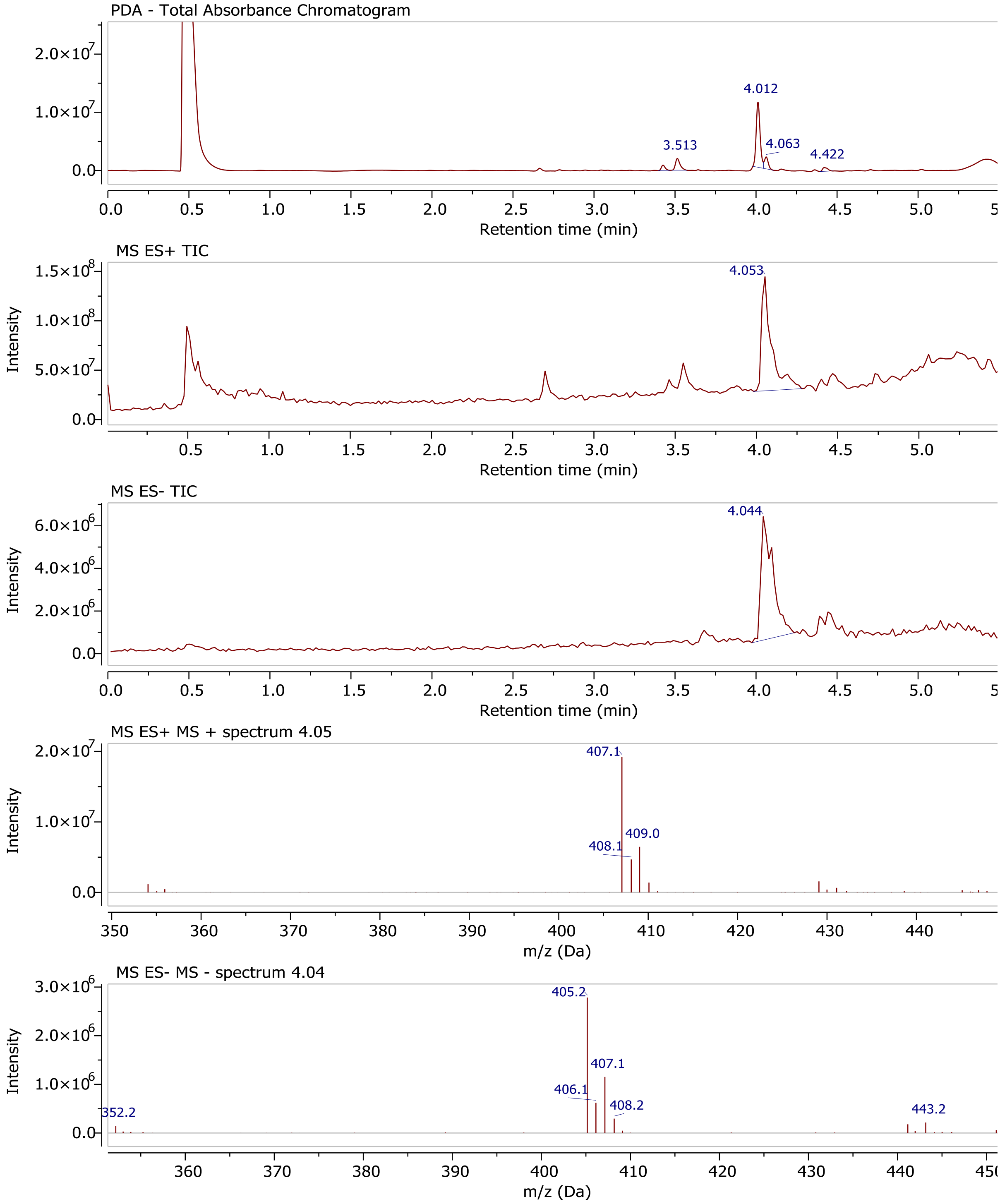

Supplementary figure **1415**. UPLC-MS analysis for compound **PHP-5469**

Chemical Formula: $C_{22}H_{26}N_2O_5$
Exact Mass: 398.2

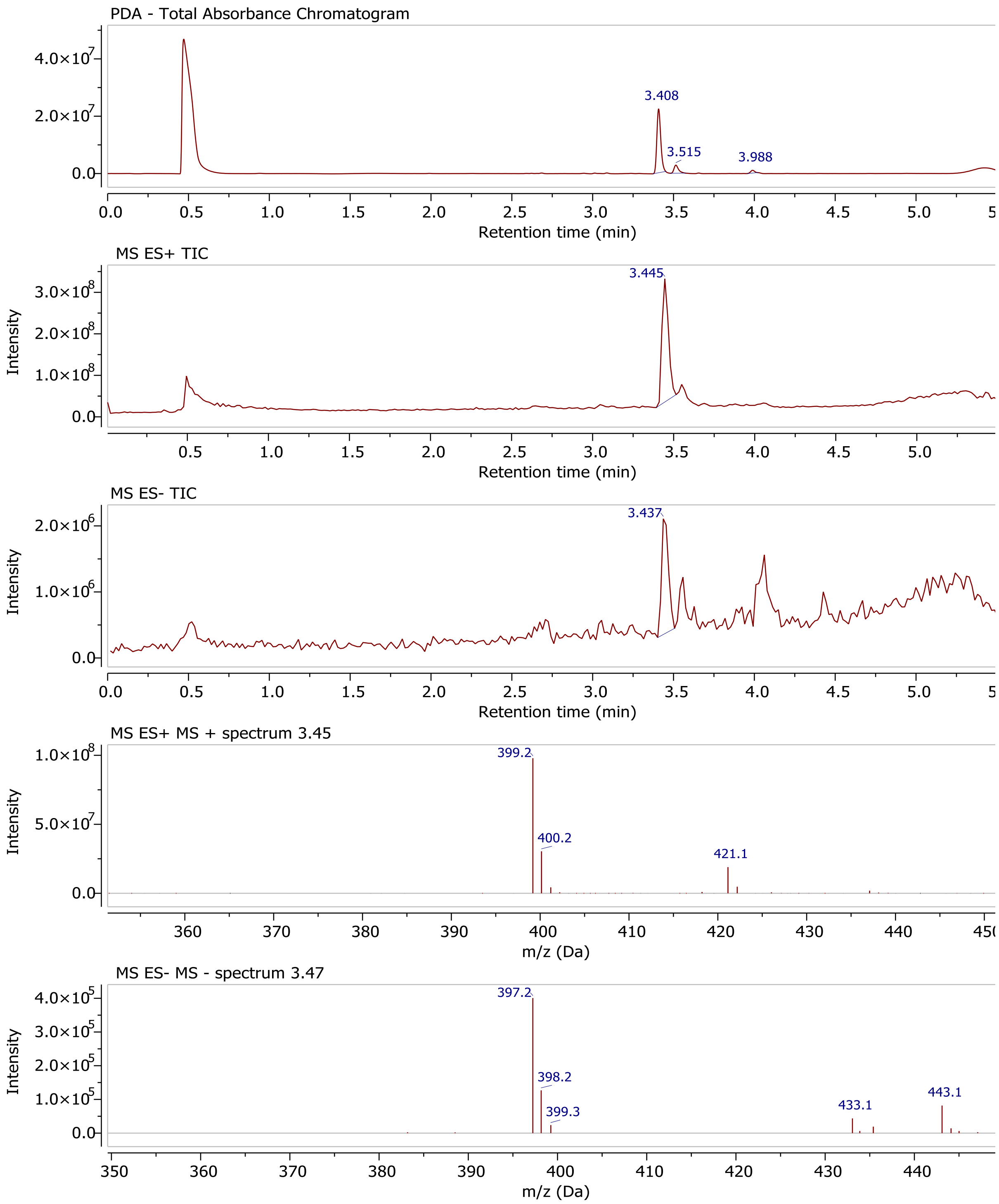

Supplementary figure **1416**. UPLC-MS analysis for compound **PHP-4902**

Chemical Formula: $C_{24}H_{19}F_3N_2O_3$
Exact Mass: 440.1

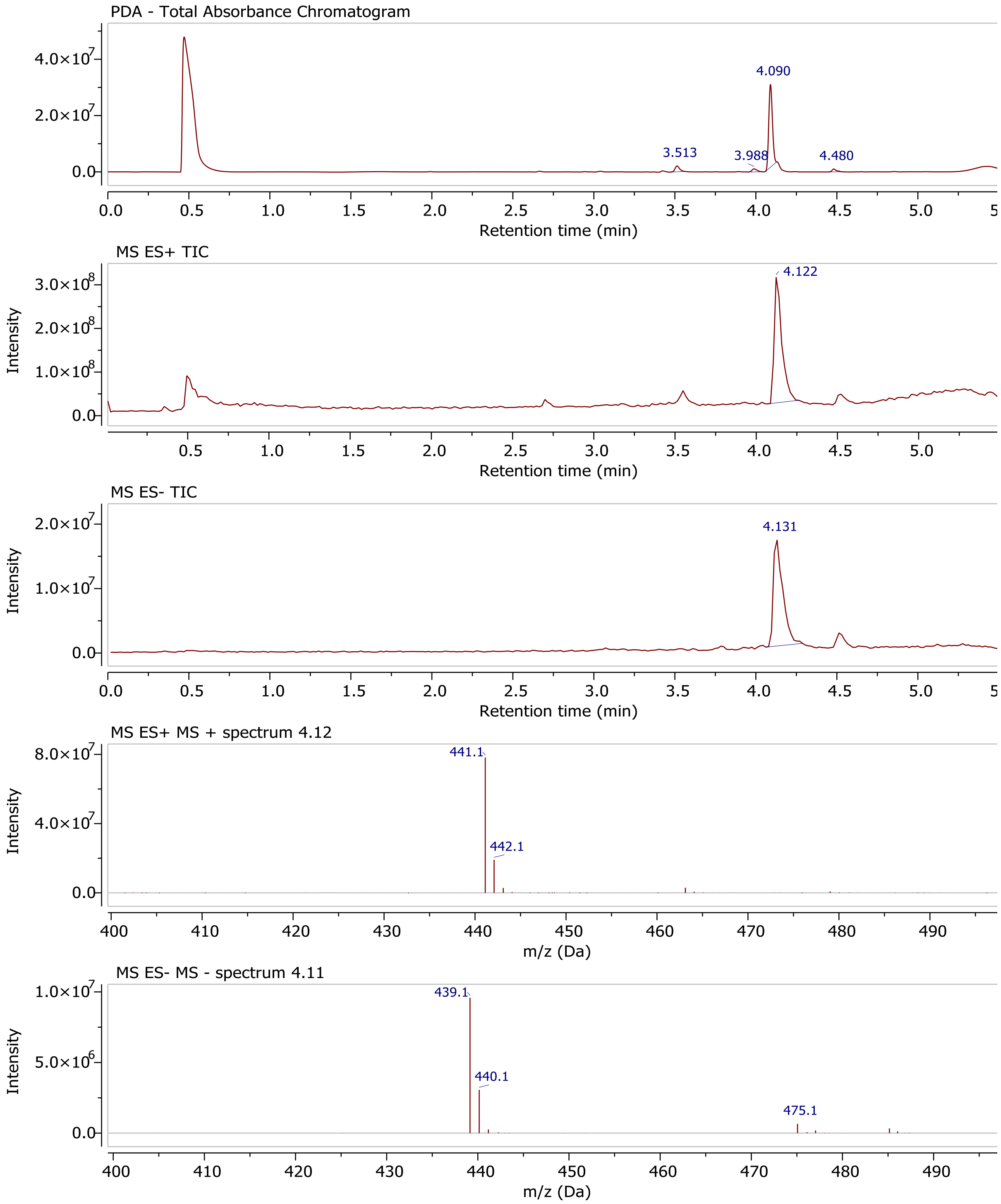

Supplementary figure **1417**. UPLC-MS analysis for compound **PHP-5470**

Chemical Formula: $C_{27}H_{31}N_3O_5$
Exact Mass: 477.2

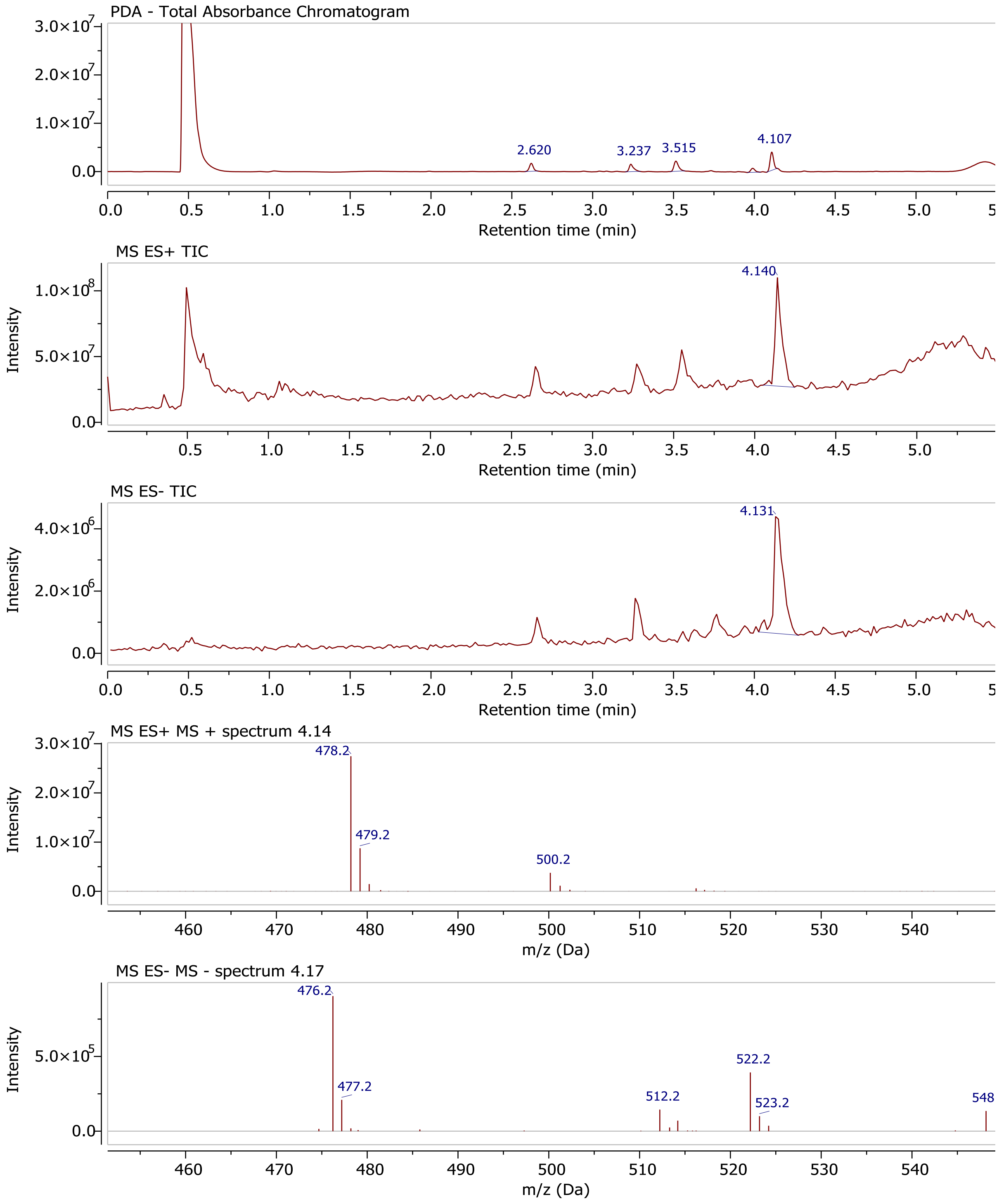

Supplementary figure **1418**. UPLC-MS analysis for compound **PHP-4903**

Chemical Formula: $C_{23}H_{19}FN_2O_3$
Exact Mass: 390.1

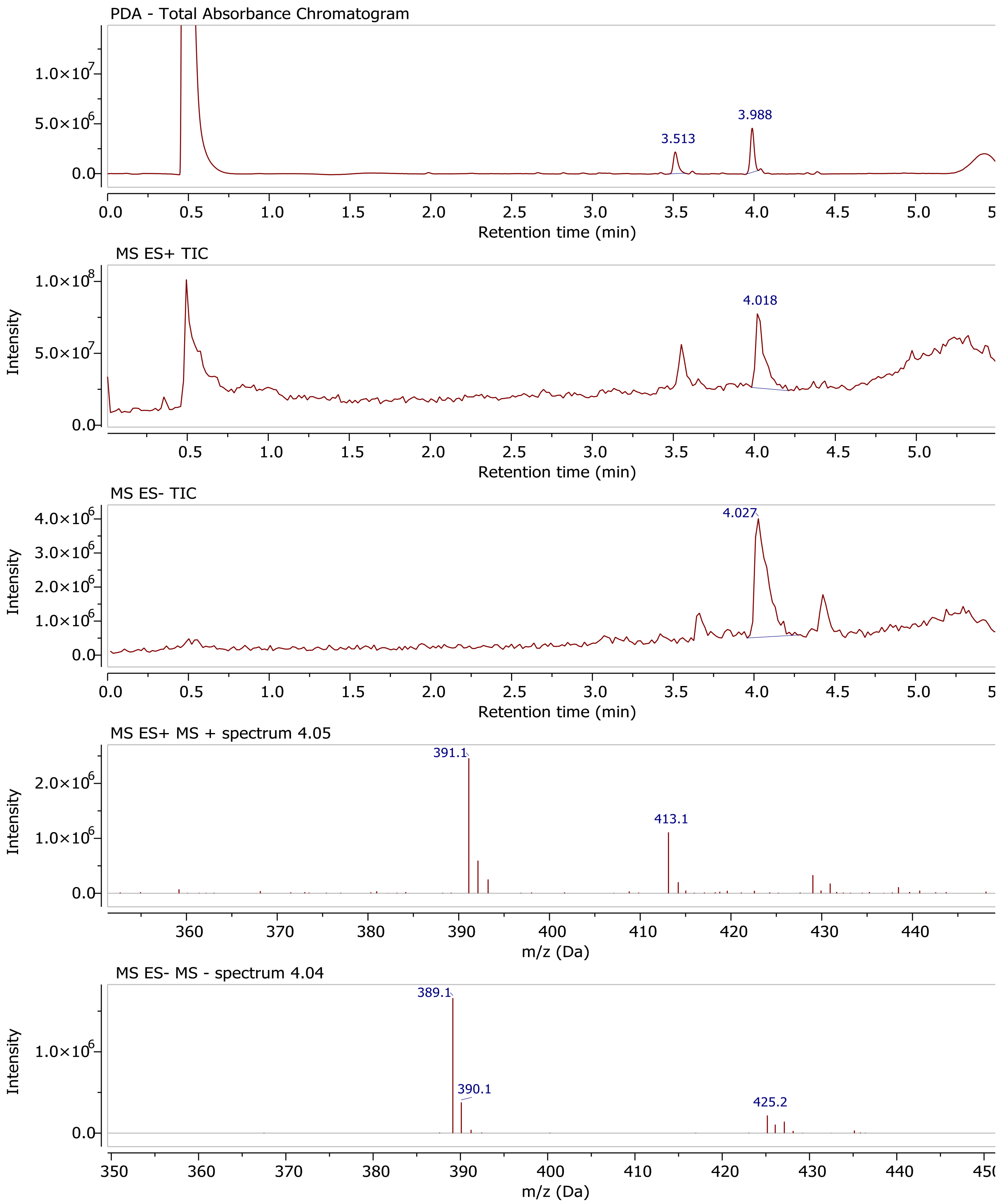

Supplementary figure **1419**. UPLC-MS analysis for compound **PHP-5471**

Chemical Formula: $C_{19}H_{15}F_5N_2O_3$
Exact Mass: 414.1

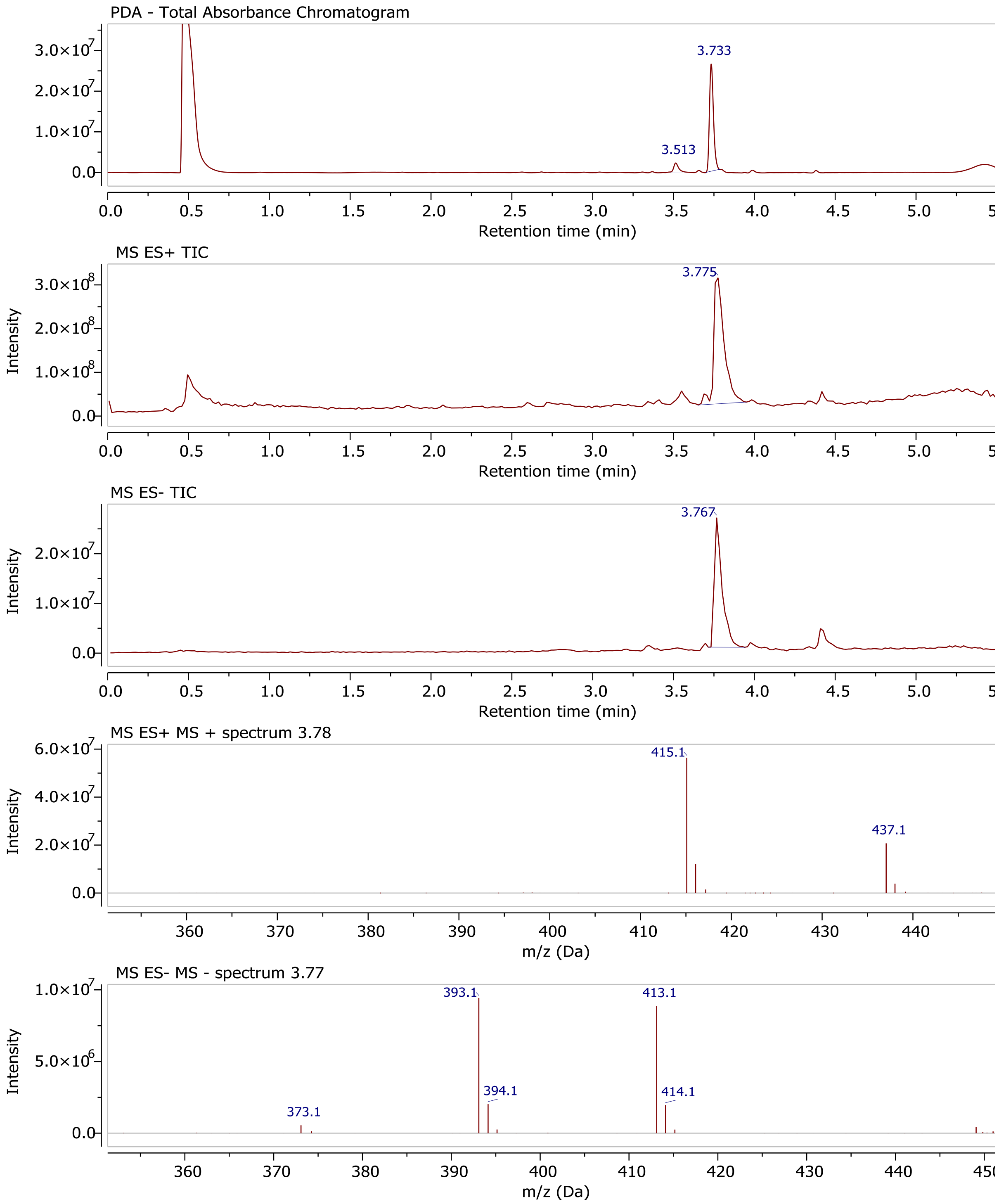

Supplementary figure **1420**. UPLC-MS analysis for compound **PHP-5628**

Chemical Formula: $C_{16}H_{15}BrN_2O_3S$
Exact Mass: 394.0

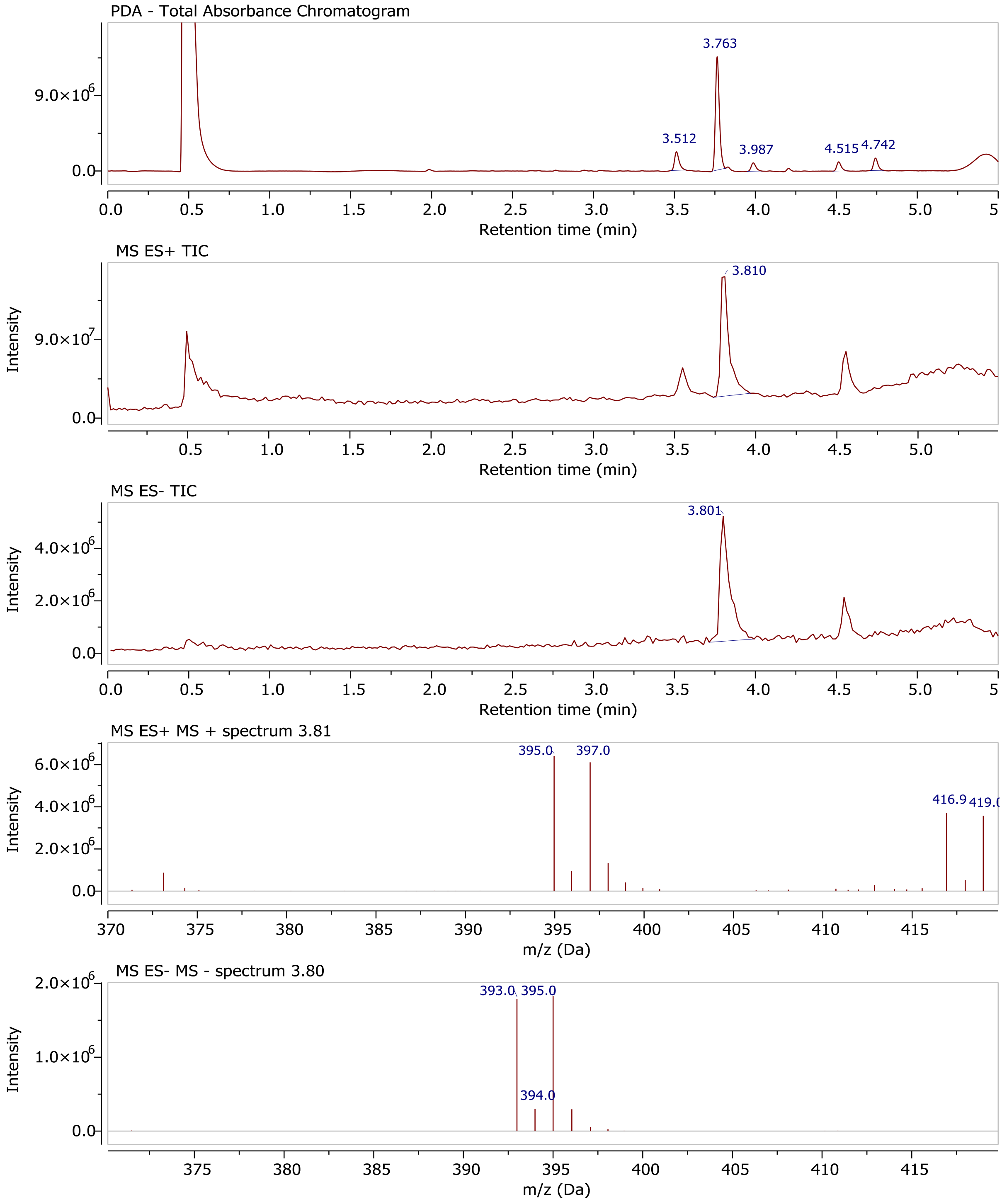

Supplementary figure **1421**. UPLC-MS analysis for compound **PHP-5785**

Chemical Formula: $C_{18}H_{24}N_2O_3$
Exact Mass: 316.2

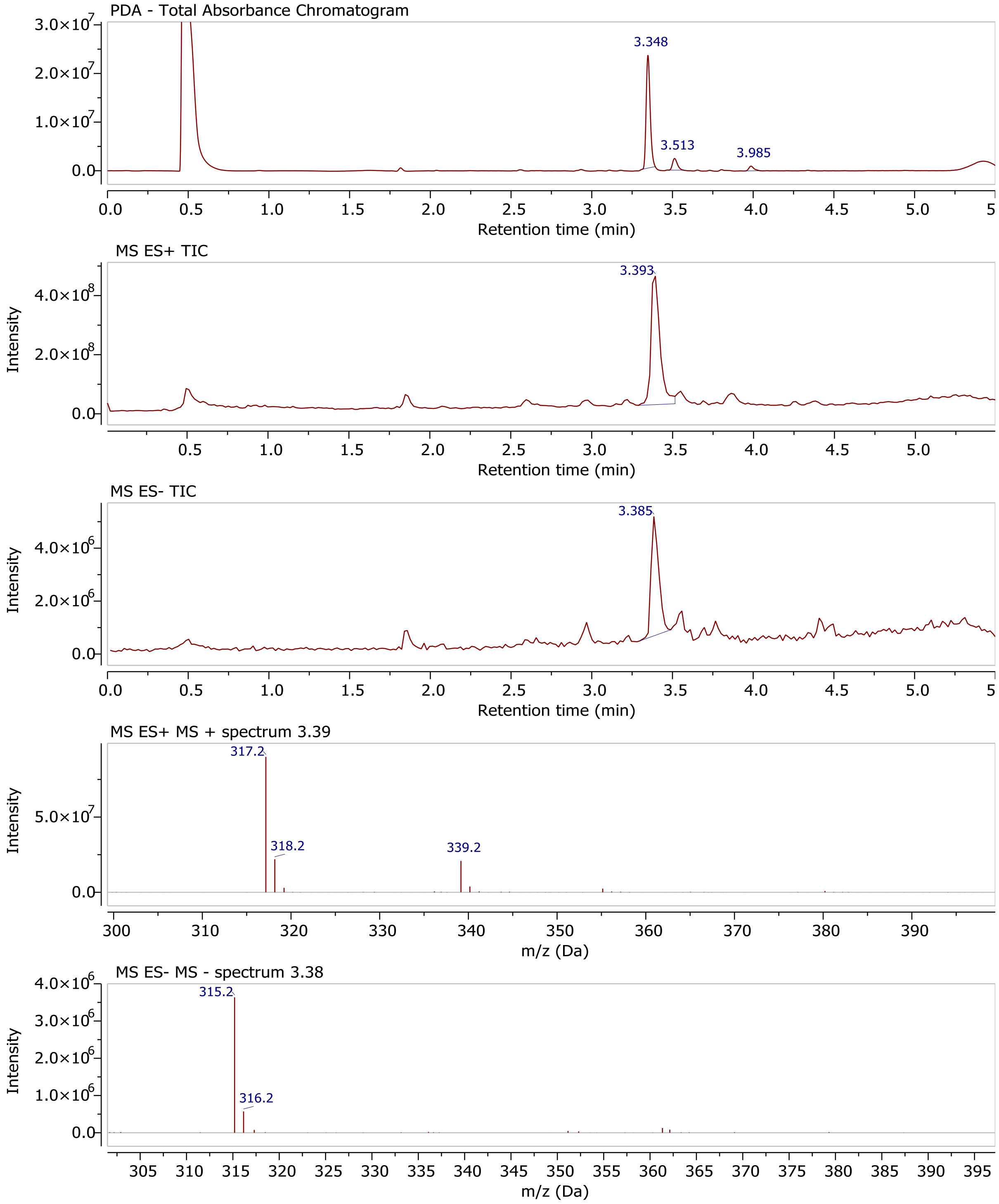

Supplementary figure **1422**. UPLC-MS analysis for compound **PHP-5629**

Chemical Formula: $C_{16}H_{20}N_2O_3$
Exact Mass: 288.1

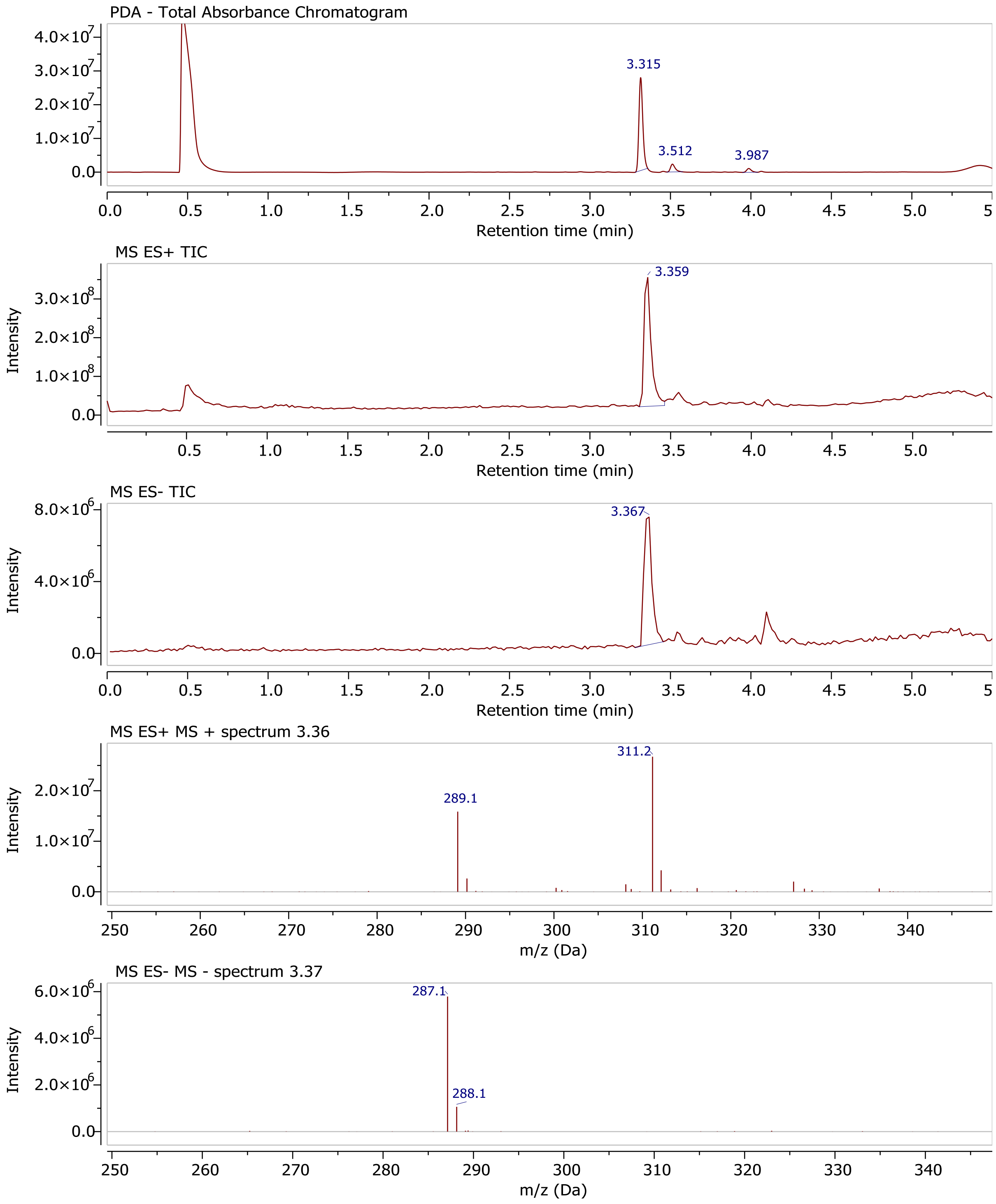

Supplementary figure **1423**. UPLC-MS analysis for compound **PHP-5789**

Chemical Formula: $C_{20}H_{22}N_2O_4$
Exact Mass: 354.2

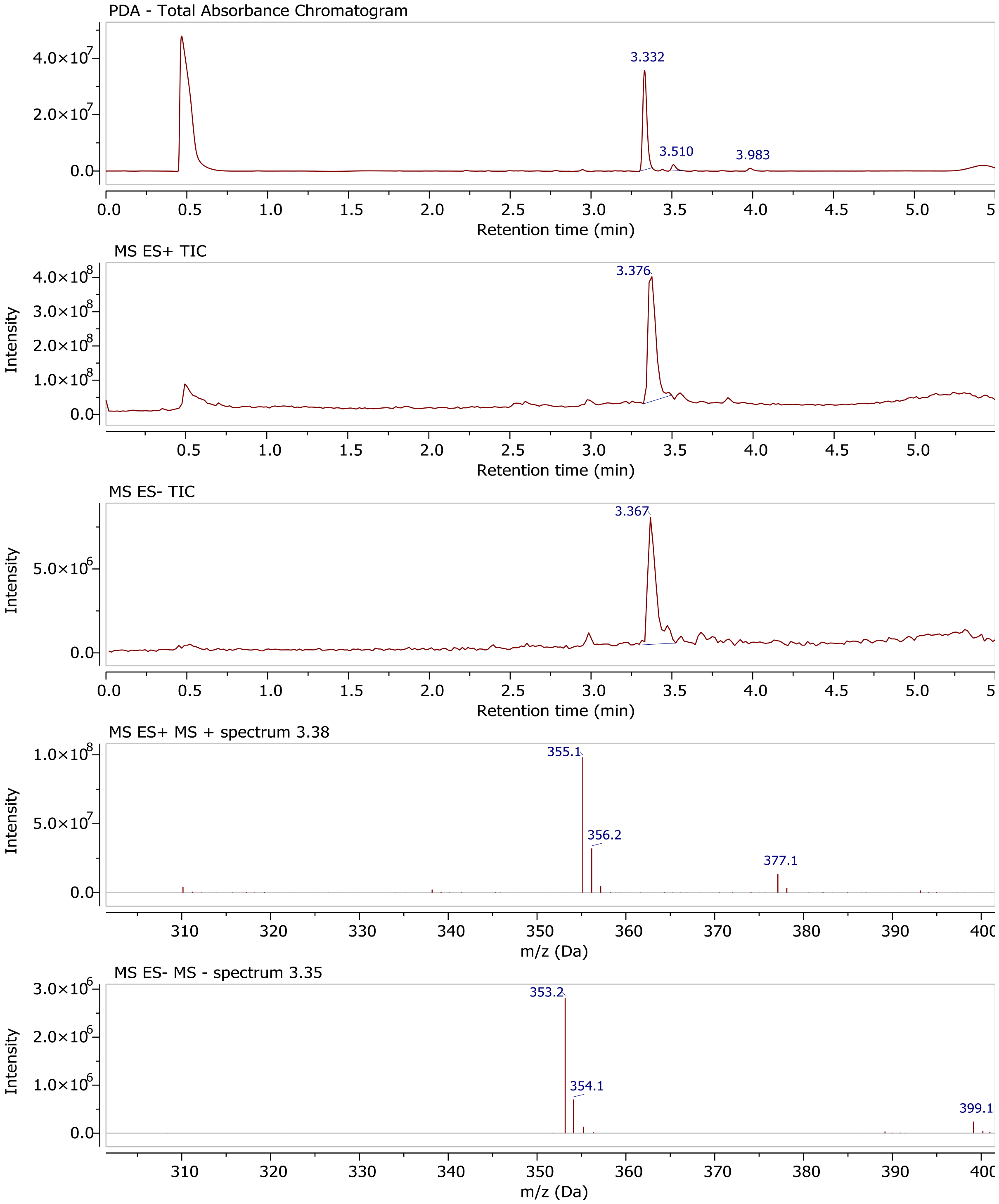

Supplementary figure **1424**. UPLC-MS analysis for compound **PHP-5630**

Chemical Formula: $C_{19}H_{17}F_3N_2O_3$
Exact Mass: 378.1

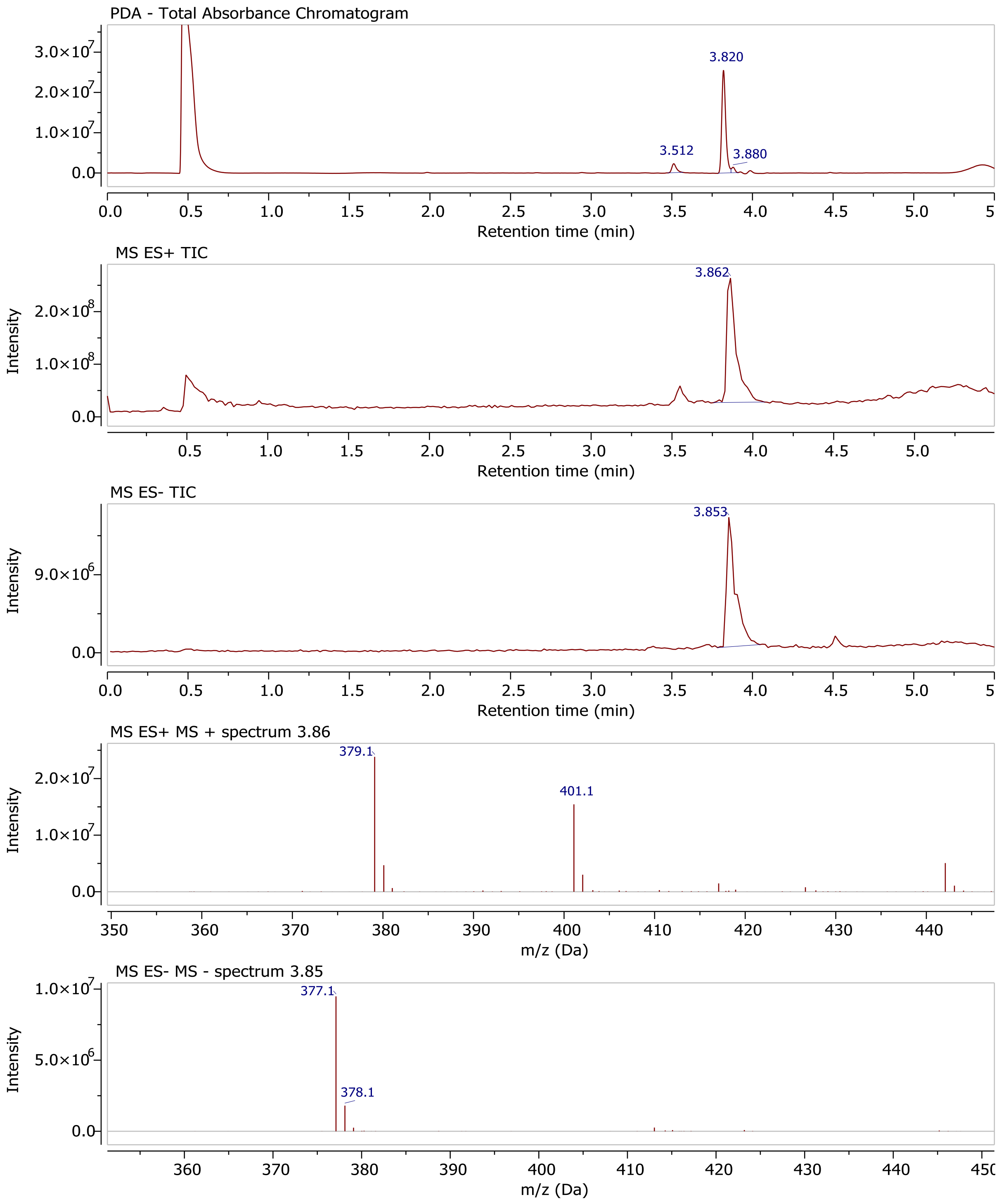

Supplementary figure **1425**. UPLC-MS analysis for compound **PHP-5791**

Chemical Formula: $C_{17}H_{18}N_2O_4$
Exact Mass: 314.1

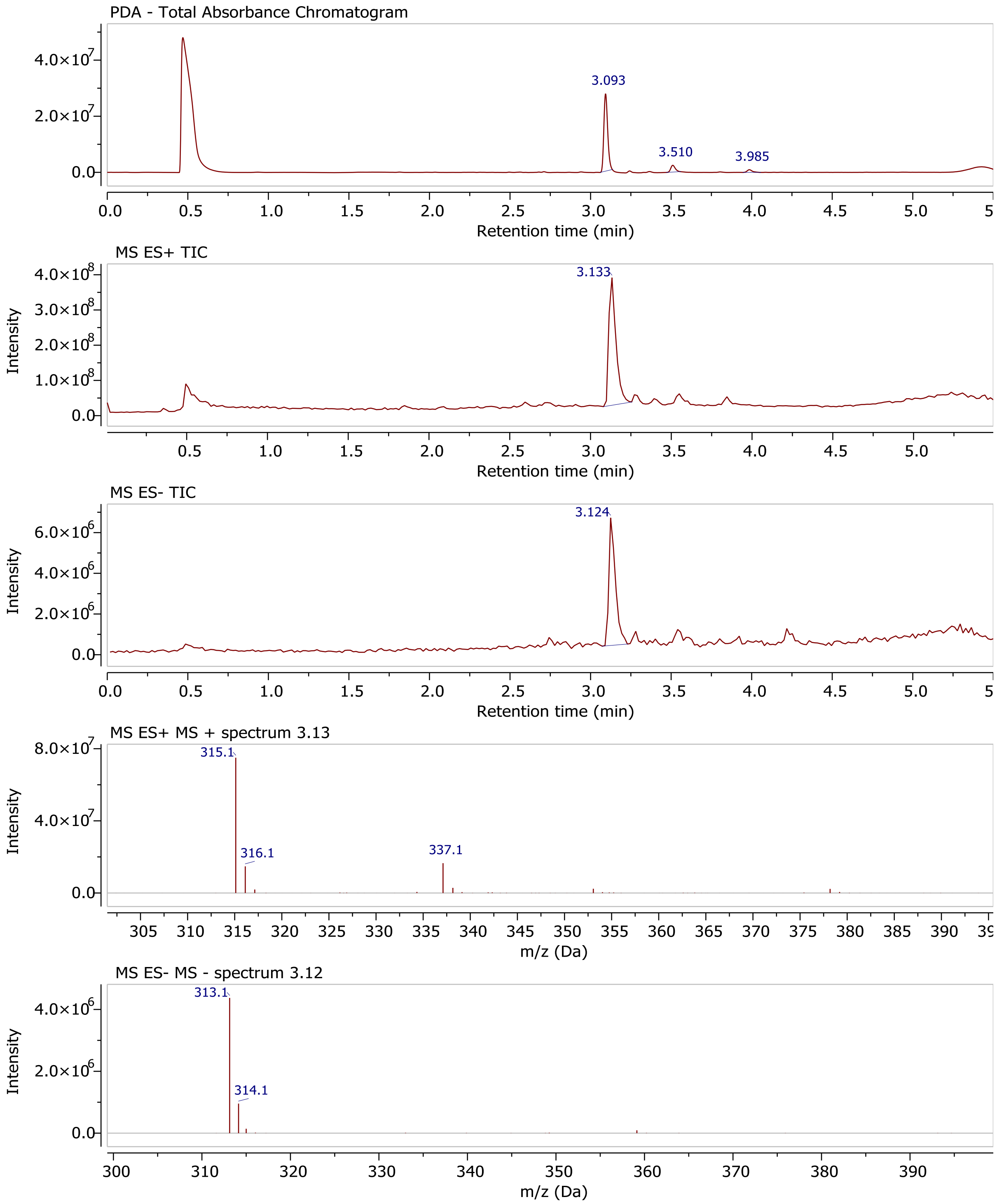

Supplementary figure **1426**. UPLC-MS analysis for compound **PHP-5631**

Chemical Formula: $C_{18}H_{17}FN_2O_3$
Exact Mass: 328.1

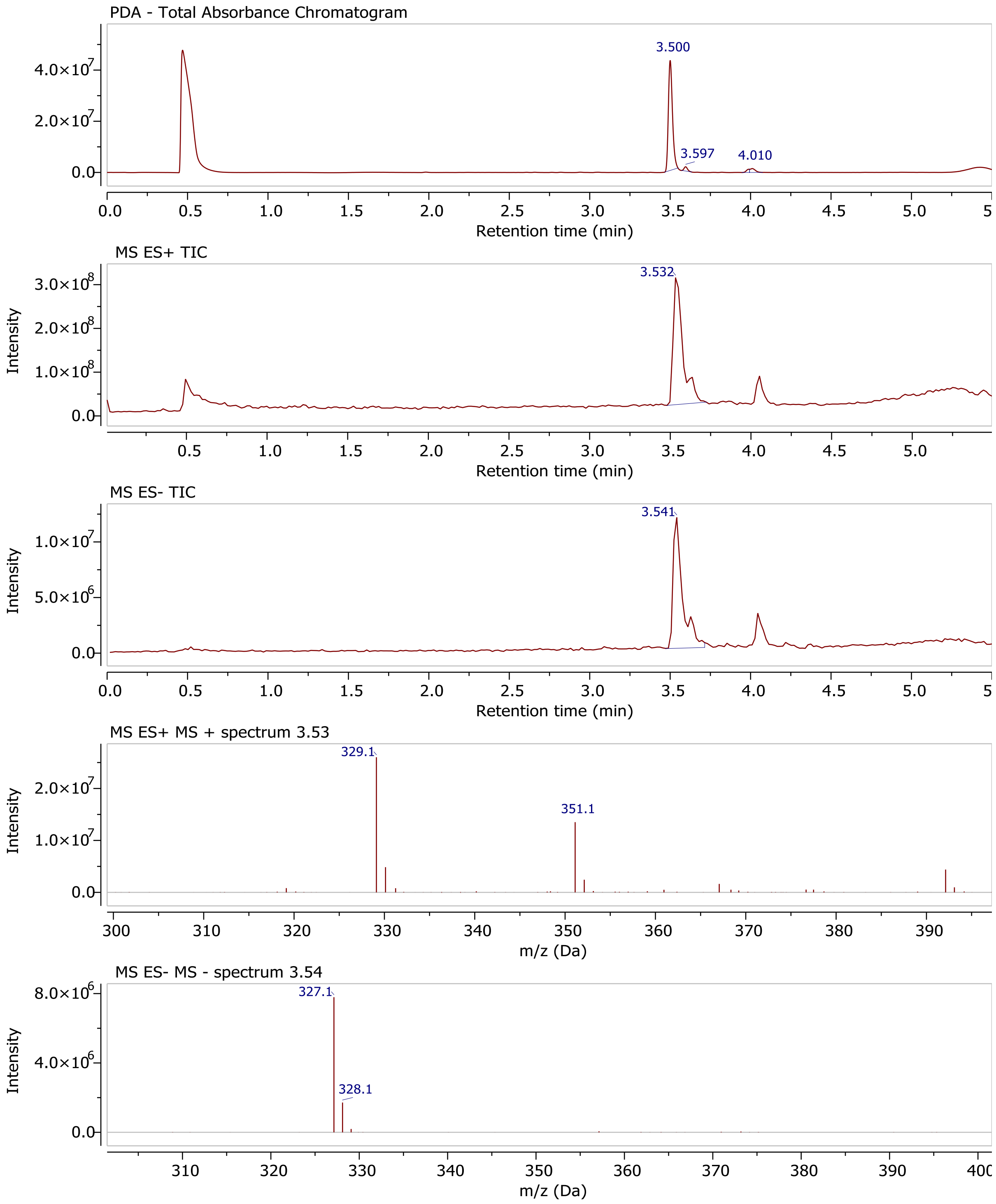

Supplementary figure **1427**. UPLC-MS analysis for compound **PHP-5794**

Chemical Formula: $C_{23}H_{22}N_2O_3$
Exact Mass: 374.2

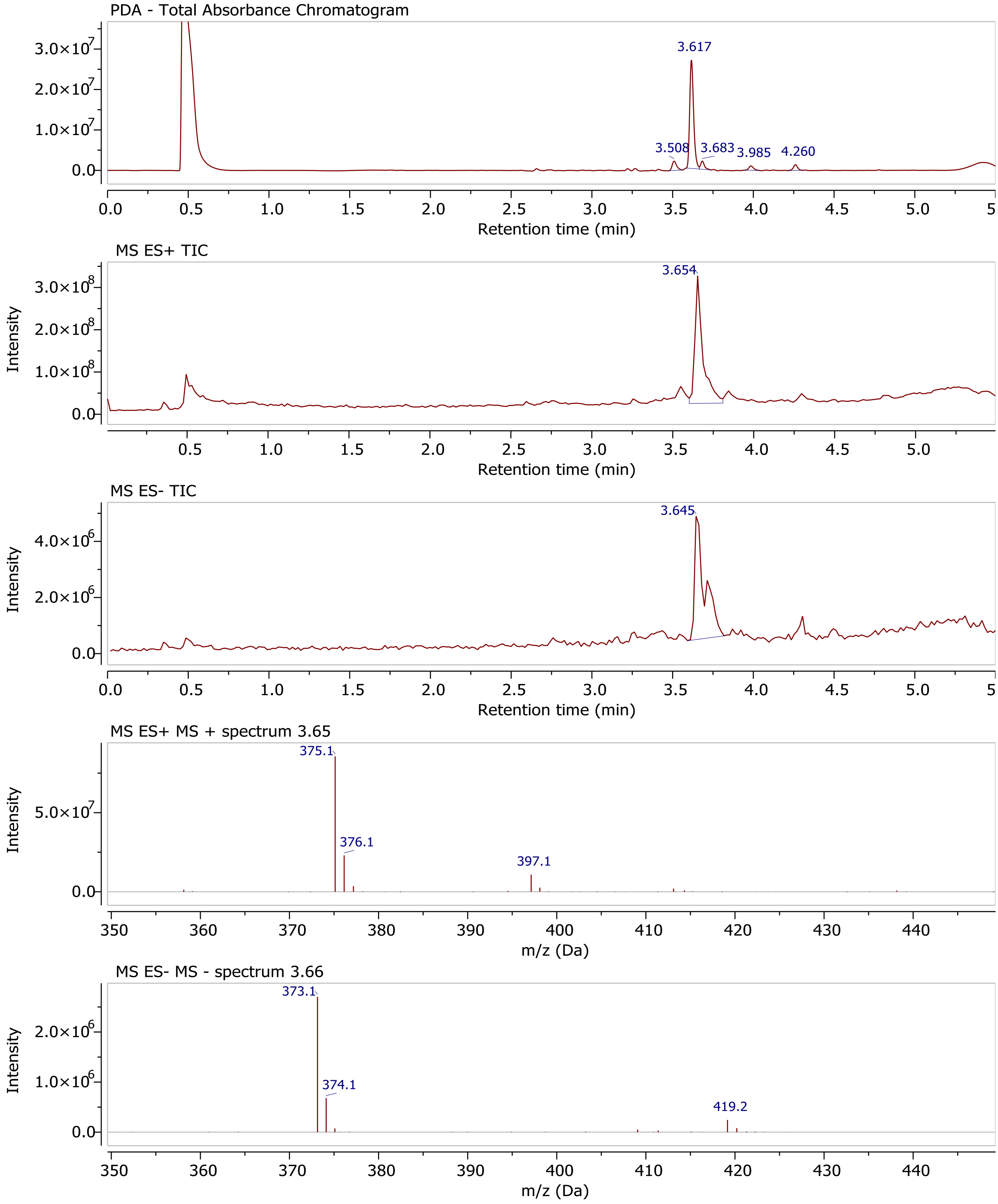

Supplementary figure **1428**. UPLC-MS analysis for compound **PHP-5632**

Chemical Formula: $C_{18}H_{17}FN_2O_3$
Exact Mass: 328.1

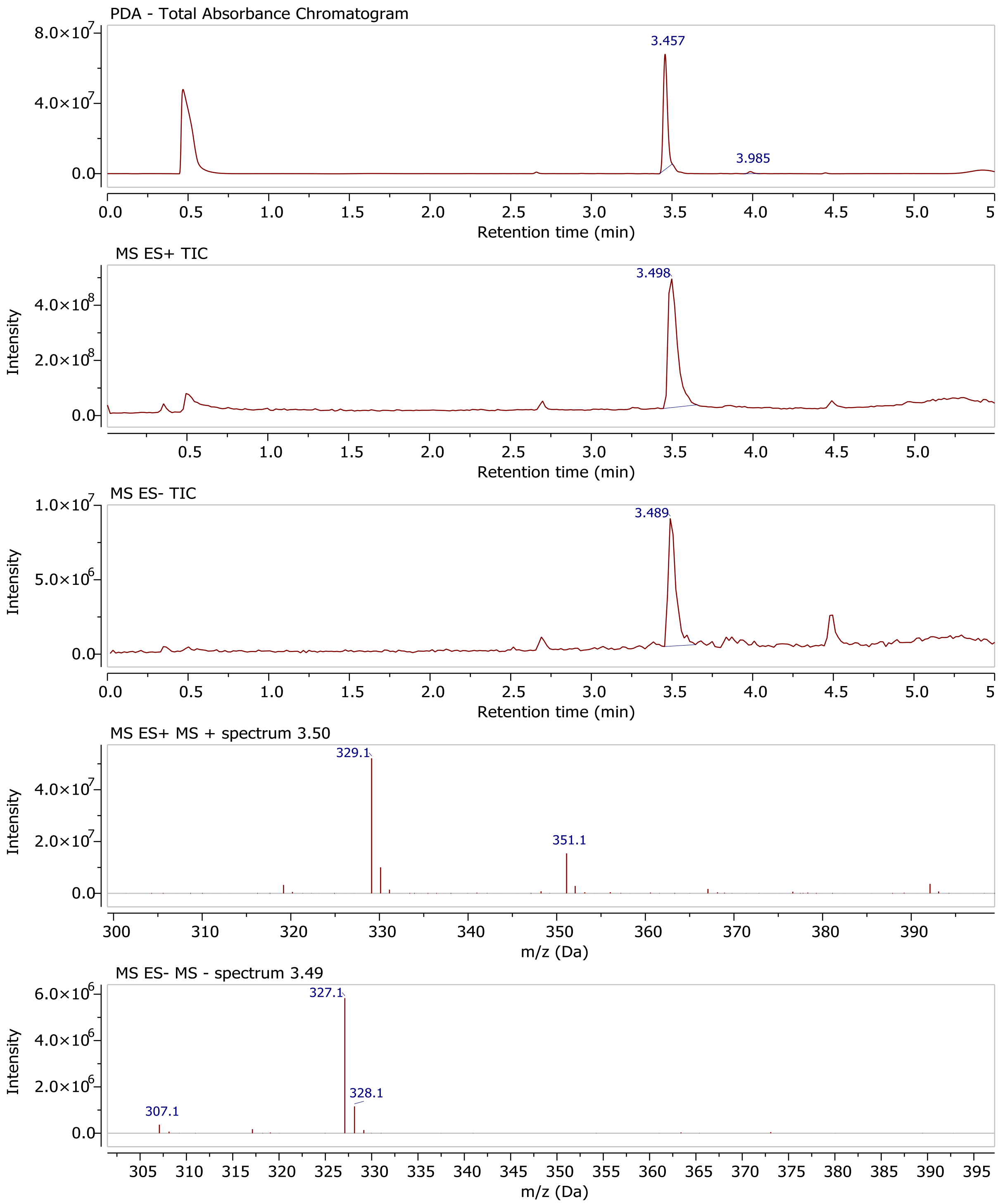

Supplementary figure **1429**. UPLC-MS analysis for compound **PHP-5795**

Chemical Formula: $C_{27}H_{26}N_2O_3$
Exact Mass: 426.2

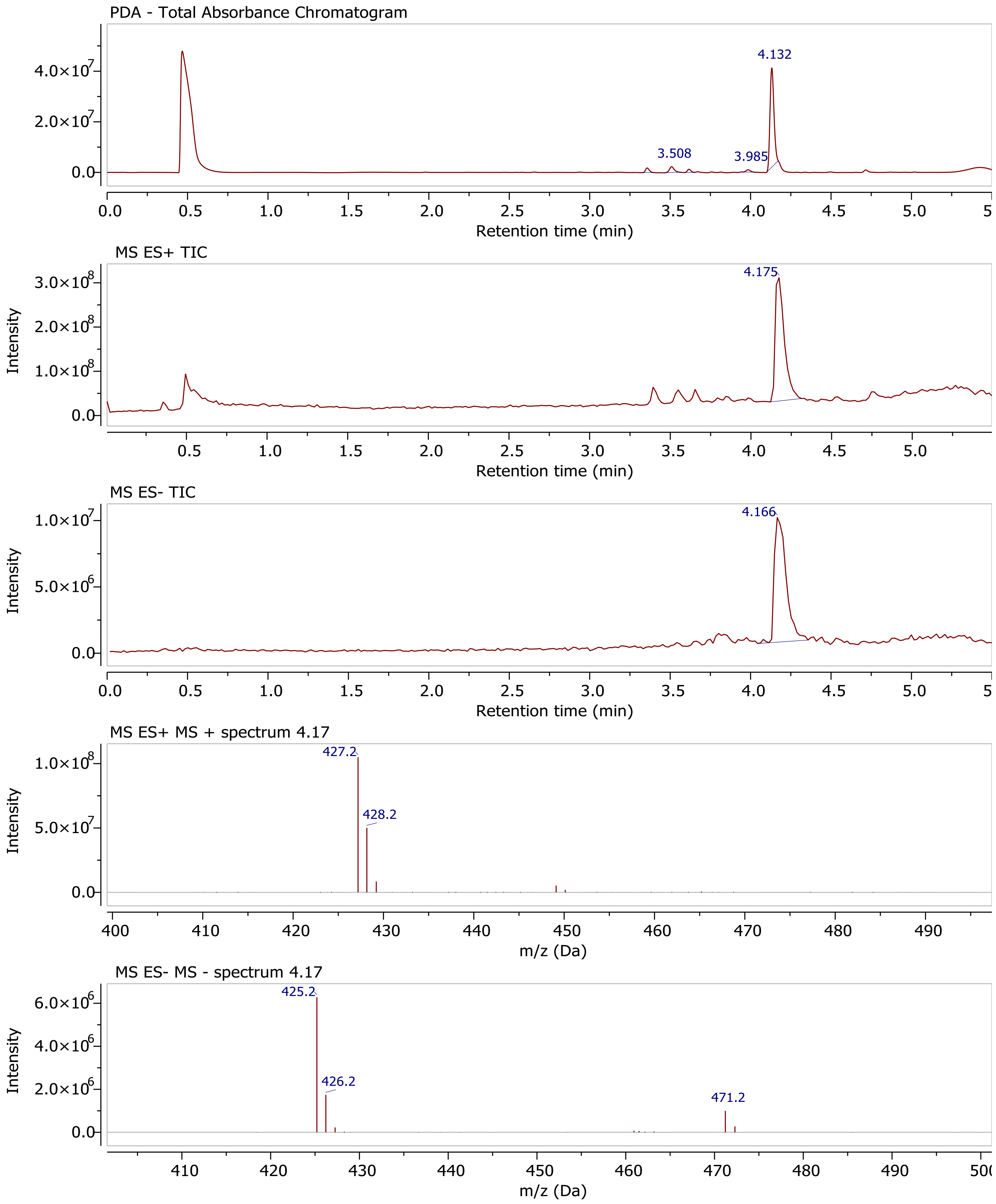

Supplementary figure **1430**. UPLC-MS analysis for compound **PHP-5633**

Chemical Formula: $C_{19}H_{17}F_3N_2O_3$
Exact Mass: 378.1

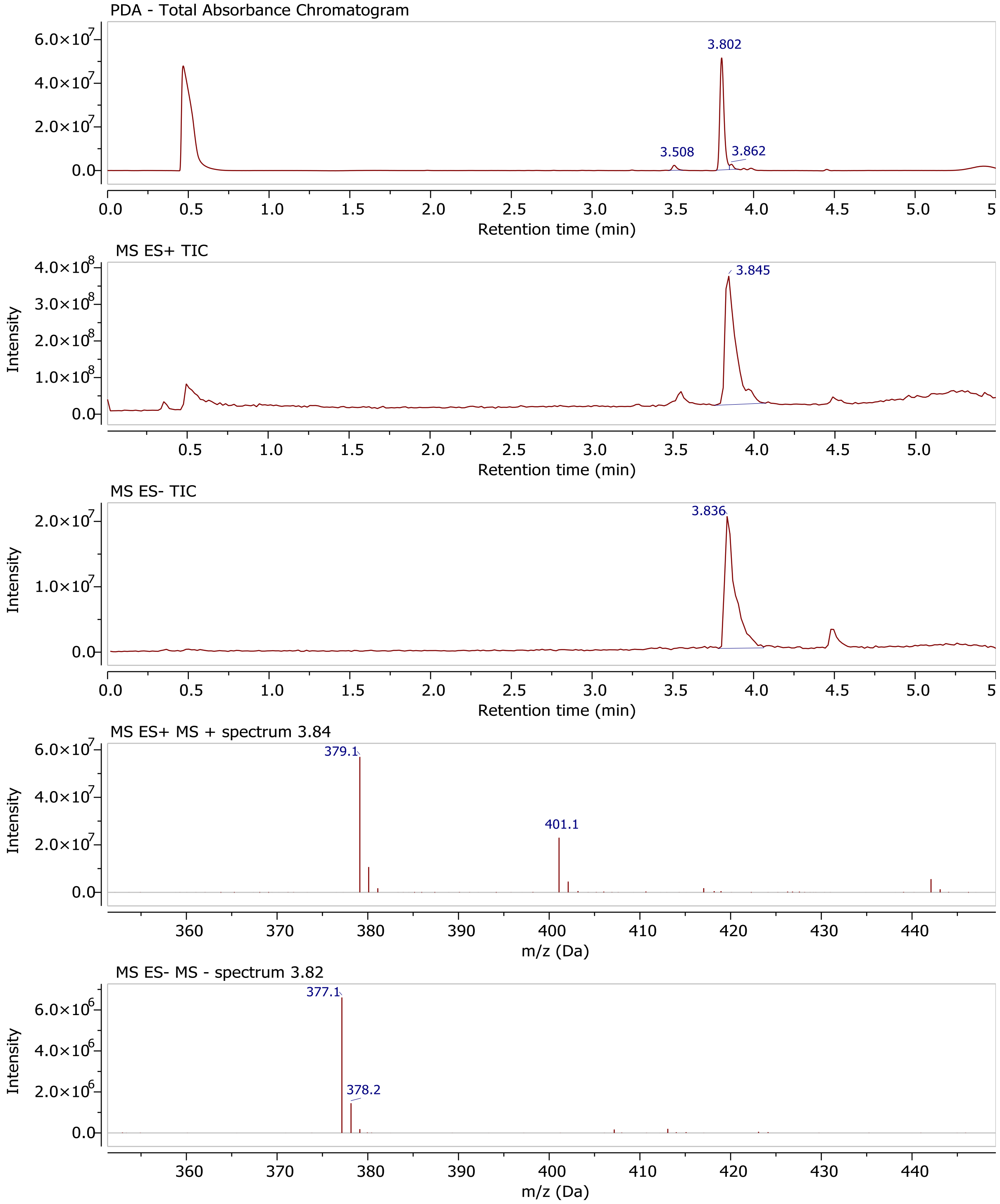

Supplementary figure **1431**. UPLC-MS analysis for compound **PHP-5796**

Chemical Formula: $C_{21}H_{24}N_2O_3$
Exact Mass: 352.2

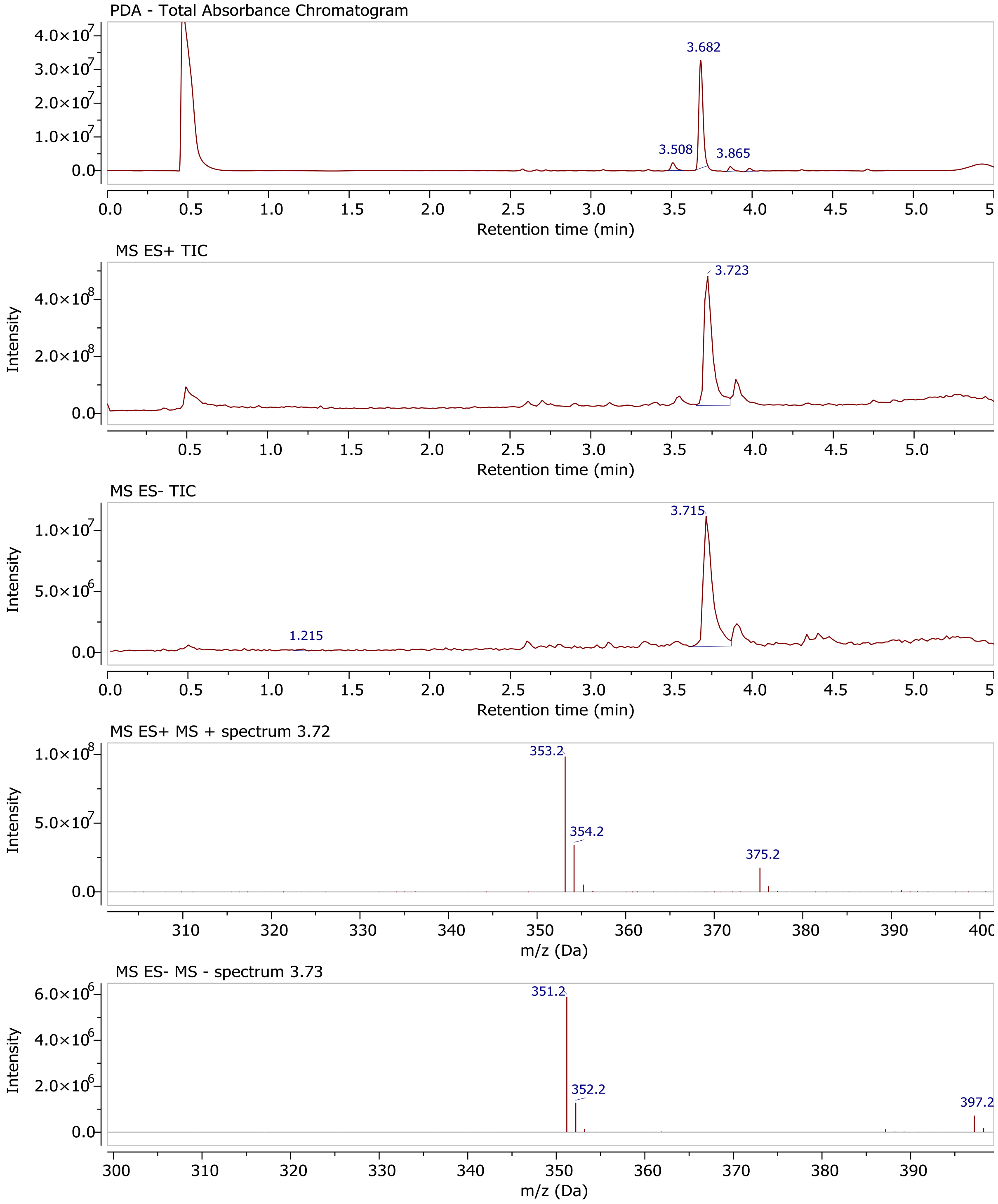

Supplementary figure **1432**. UPLC-MS analysis for compound **PHP-5635**

Chemical Formula: $C_{21}H_{21}F_3N_2O_2$
Exact Mass: 390.2

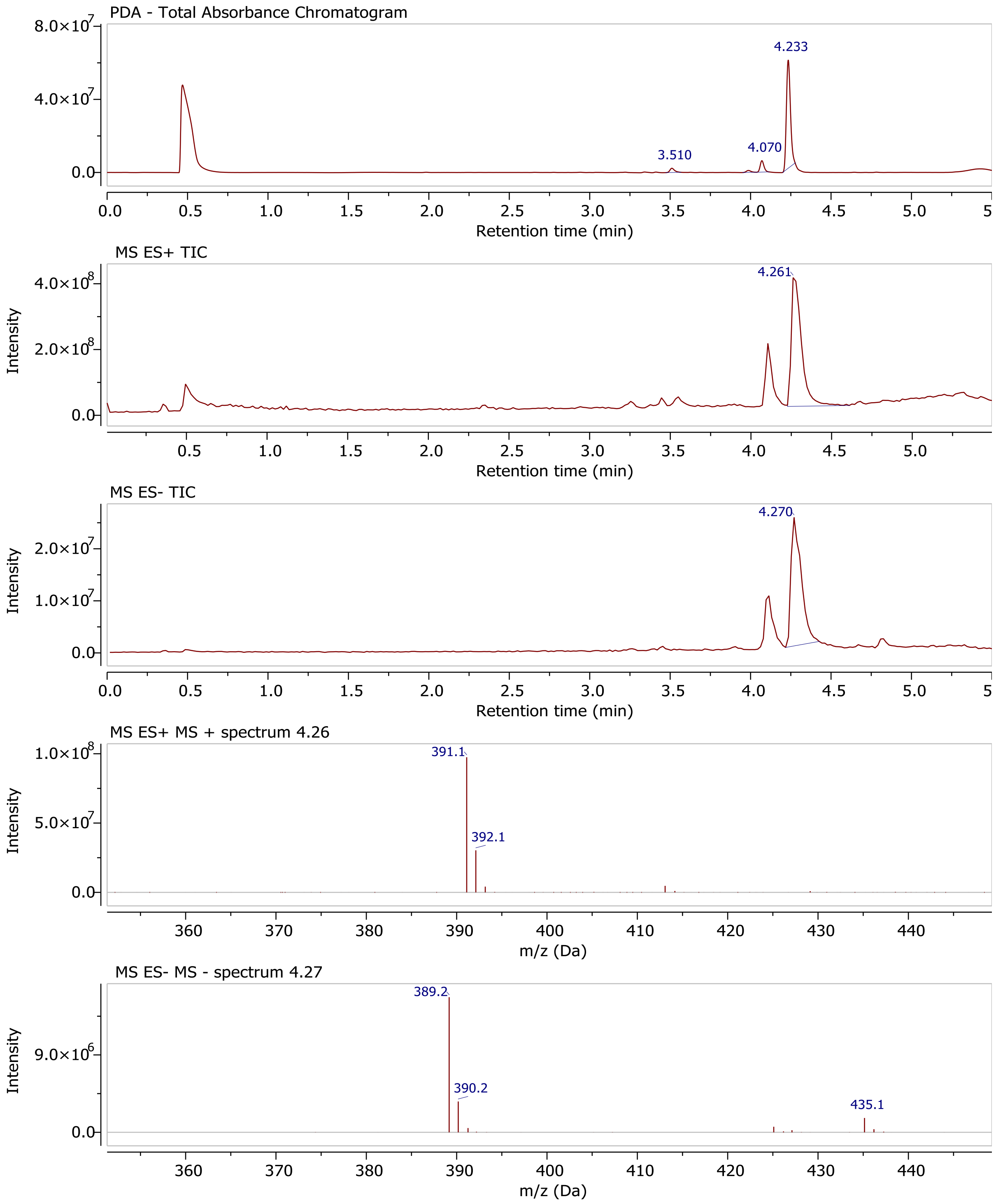

Supplementary figure **1433**. UPLC-MS analysis for compound **PHP-5801**

Chemical Formula: $C_{21}H_{24}N_2O_3$
Exact Mass: 352.2

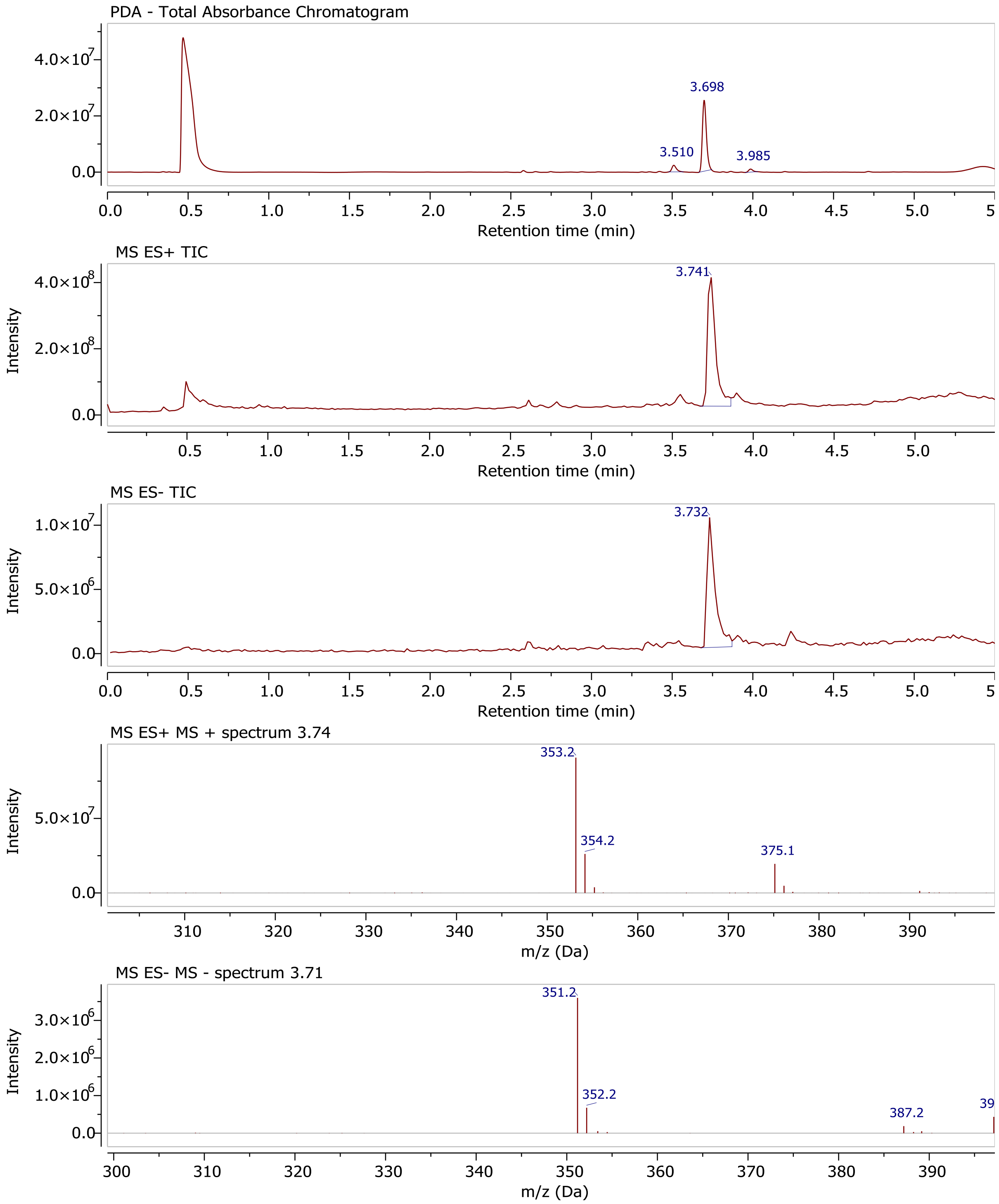

Supplementary figure **1434**. UPLC-MS analysis for compound **PHP-5638**

Chemical Formula: $C_{26}H_{26}N_2O_3$
Exact Mass: 414.2

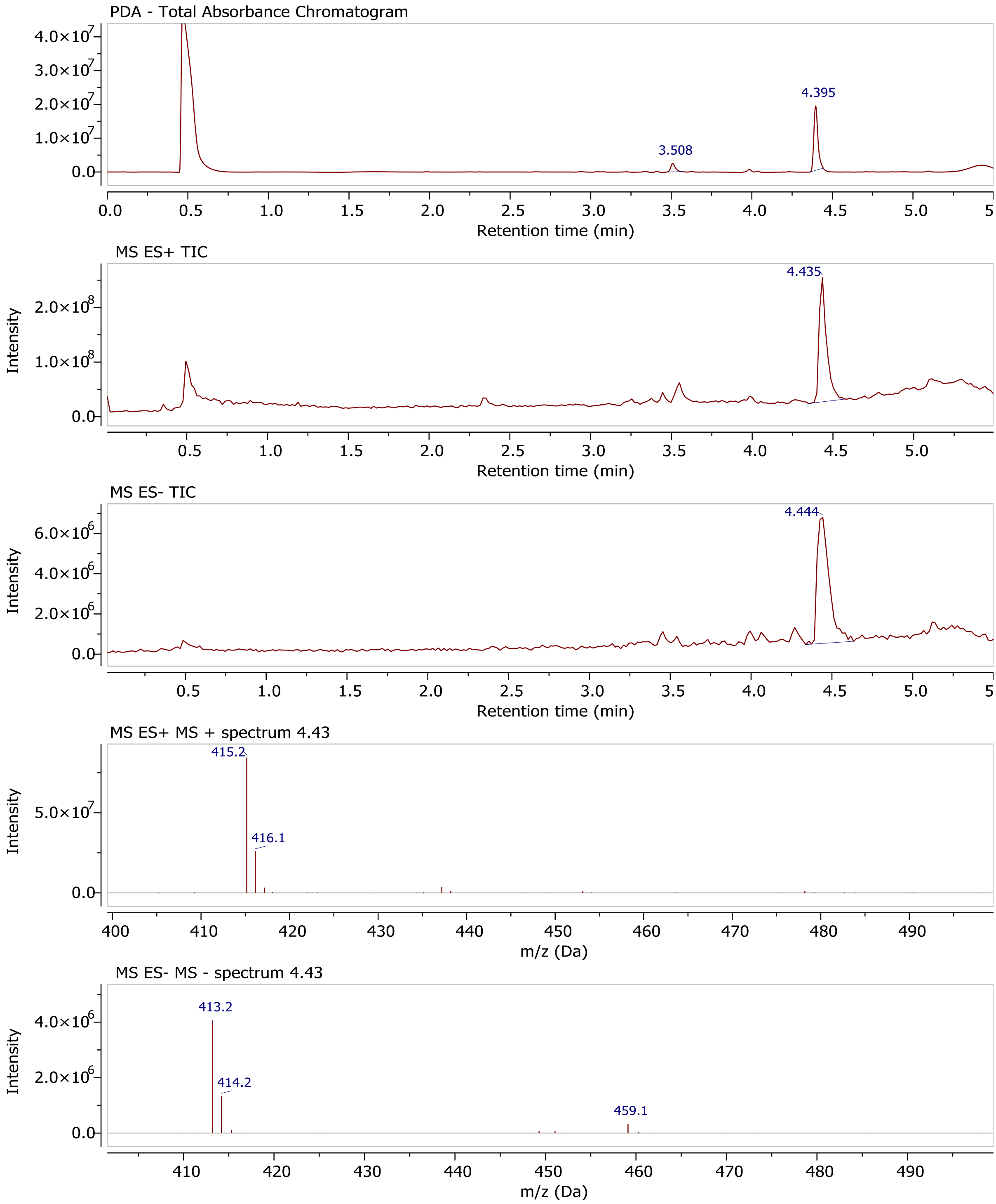

Supplementary figure **1435**. UPLC-MS analysis for compound **PHP-5802**

Chemical Formula: $C_{26}H_{26}N_2O_3$
Exact Mass: 414.2

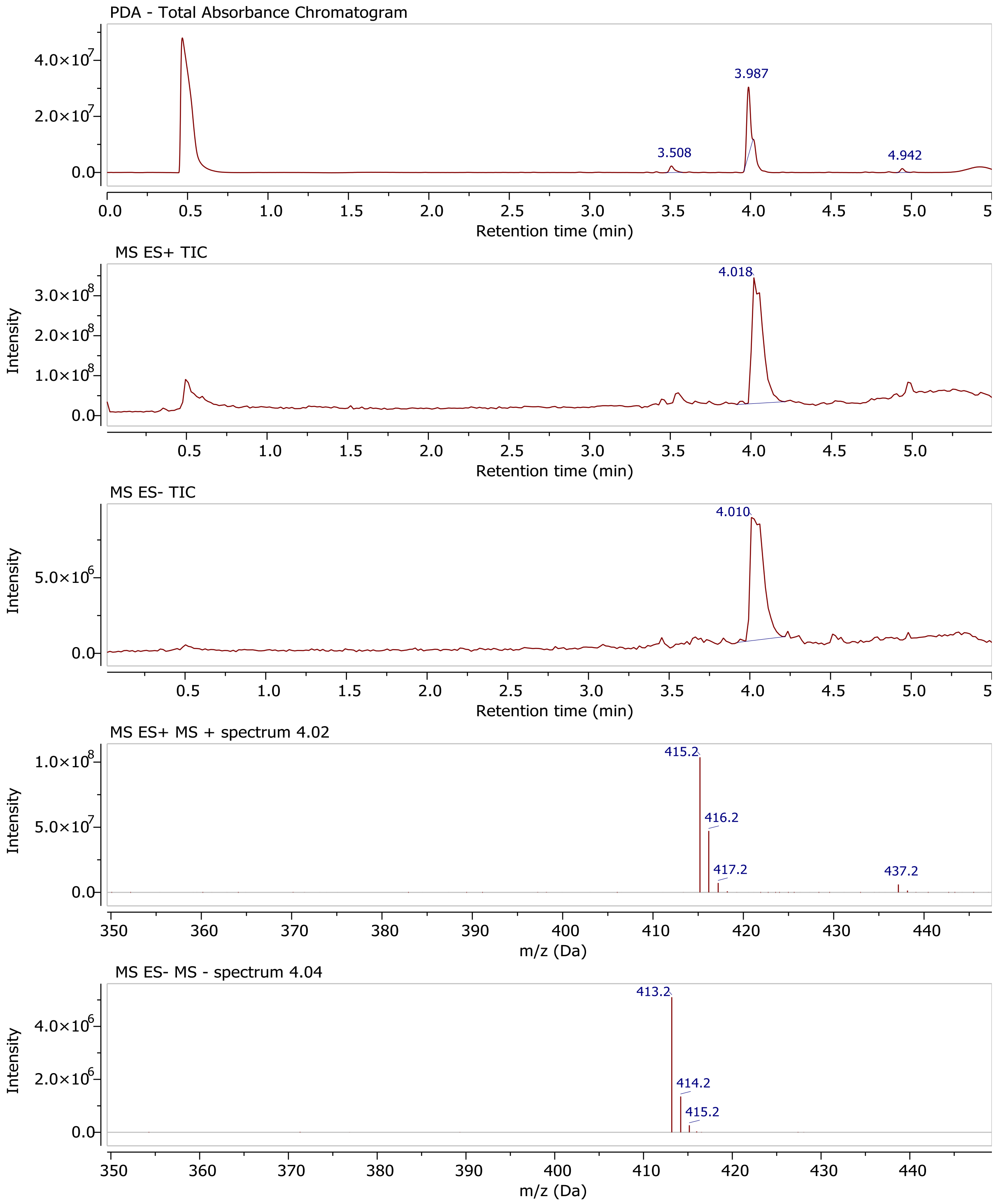

Supplementary figure **1436.** UPLC-MS analysis for compound **PHP-5640**

Chemical Formula: $C_{20}H_{21}BrN_2O_2$
Exact Mass: 400.1

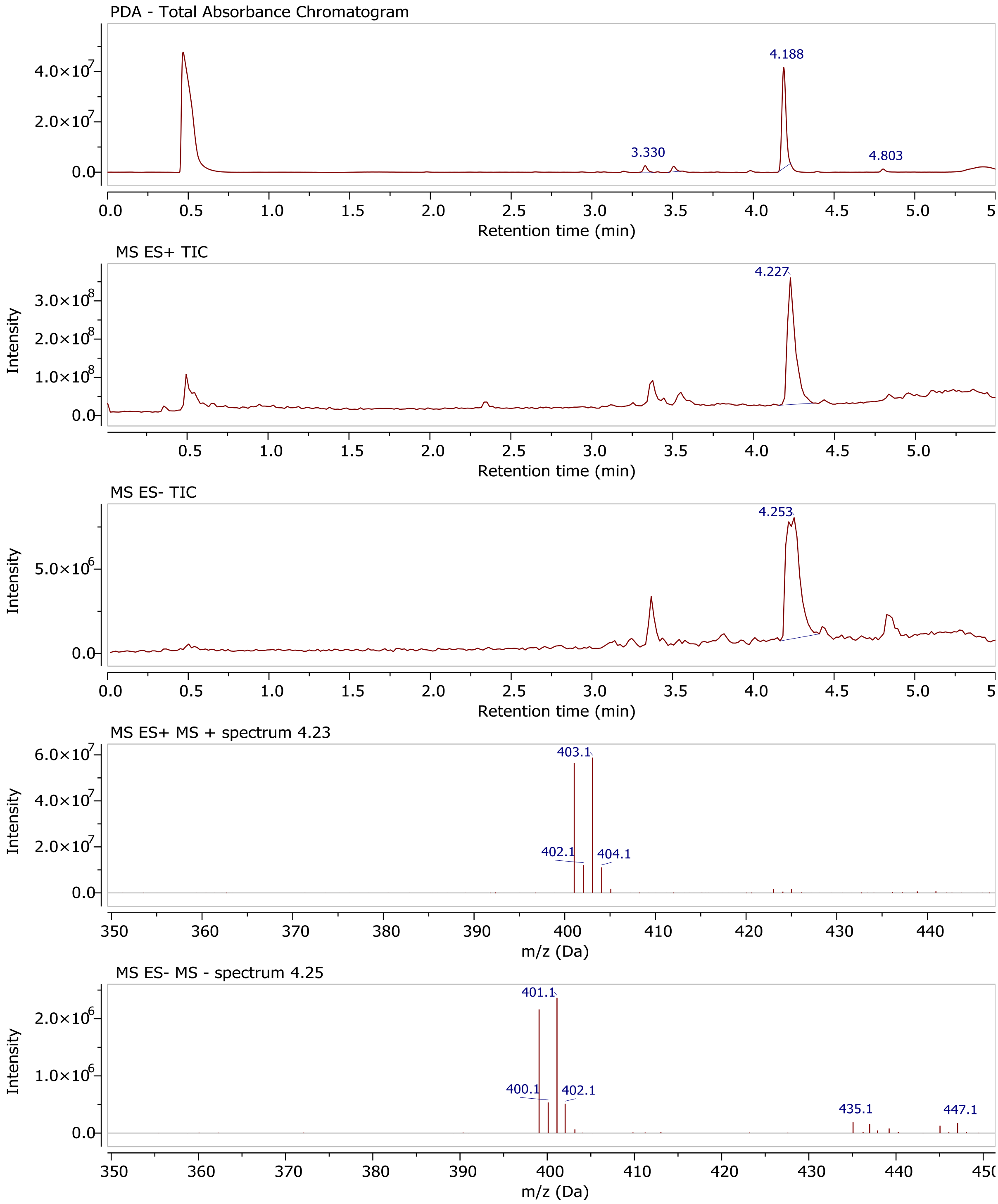

Supplementary figure **1437**. UPLC-MS analysis for compound **PHP-5803**

Chemical Formula: $C_{25}H_{21}F_3N_2O_3$
Exact Mass: 454.2

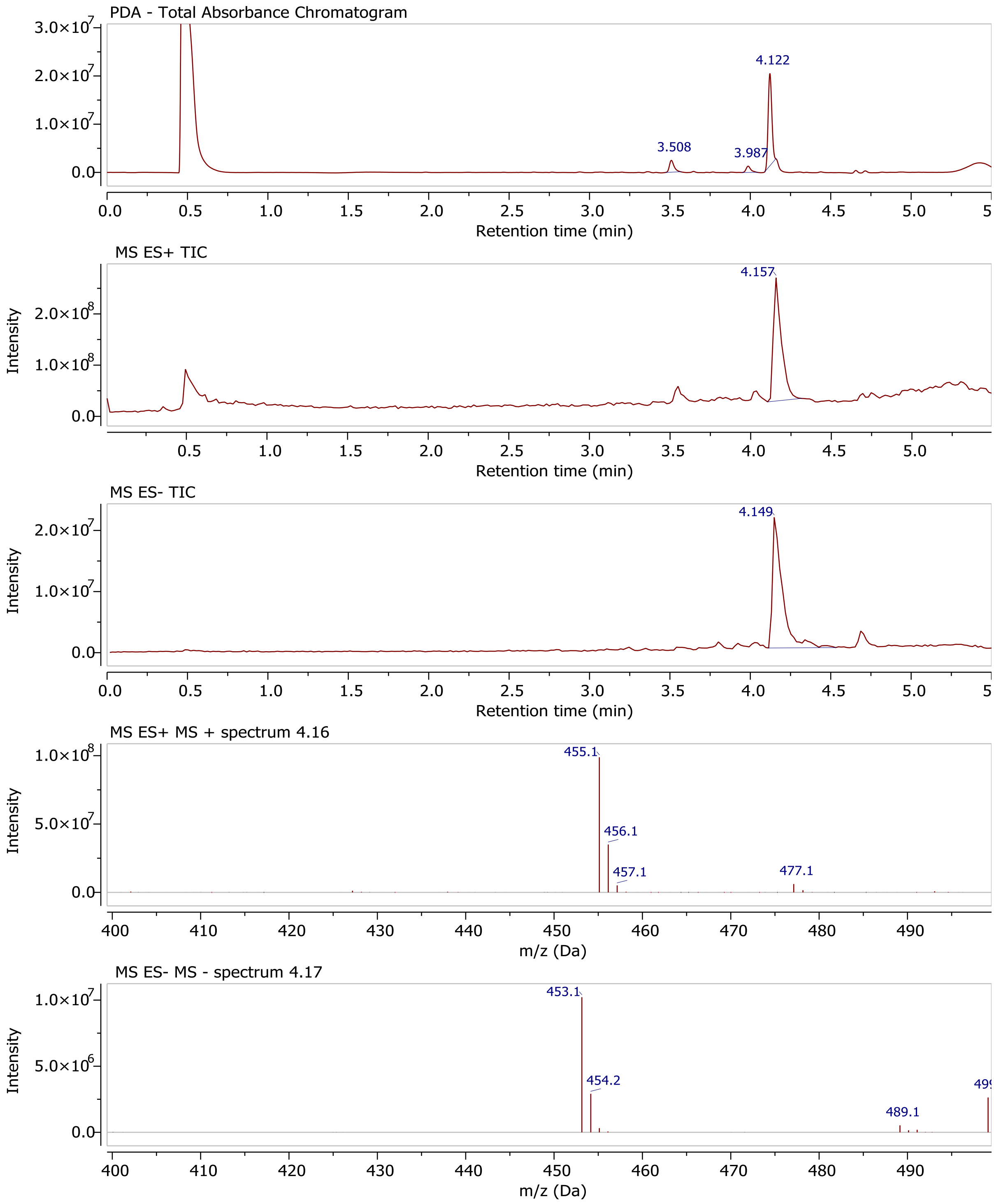

Supplementary figure **1438**. UPLC-MS analysis for compound **PHP-5641**

Chemical Formula: $C_{23}H_{28}N_2O_2$
Exact Mass: 364.2

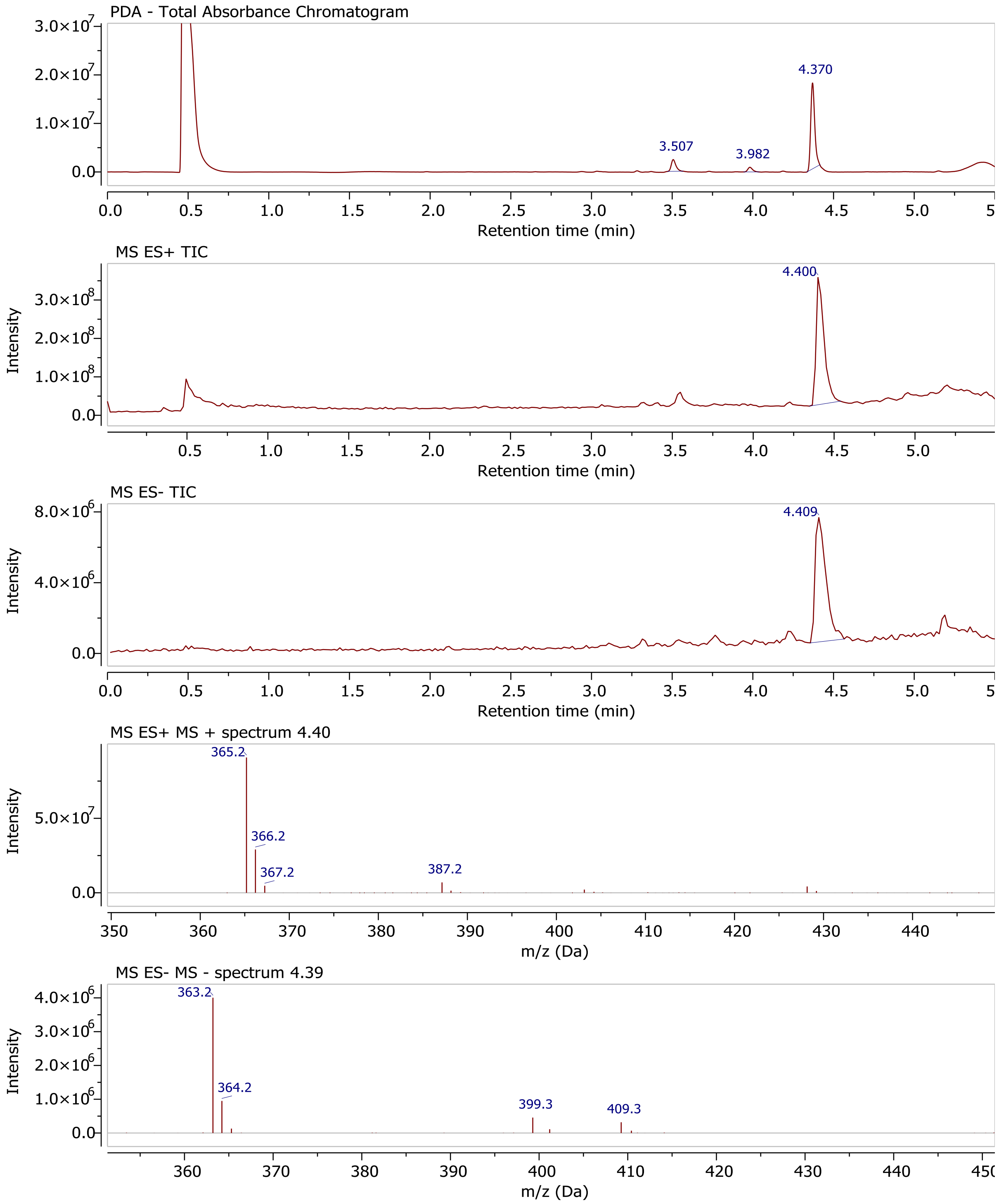

Supplementary figure **1439**. UPLC-MS analysis for compound **PHP-5804**

Chemical Formula: $C_{23}H_{21}N_3O_3$
Exact Mass: 387.2

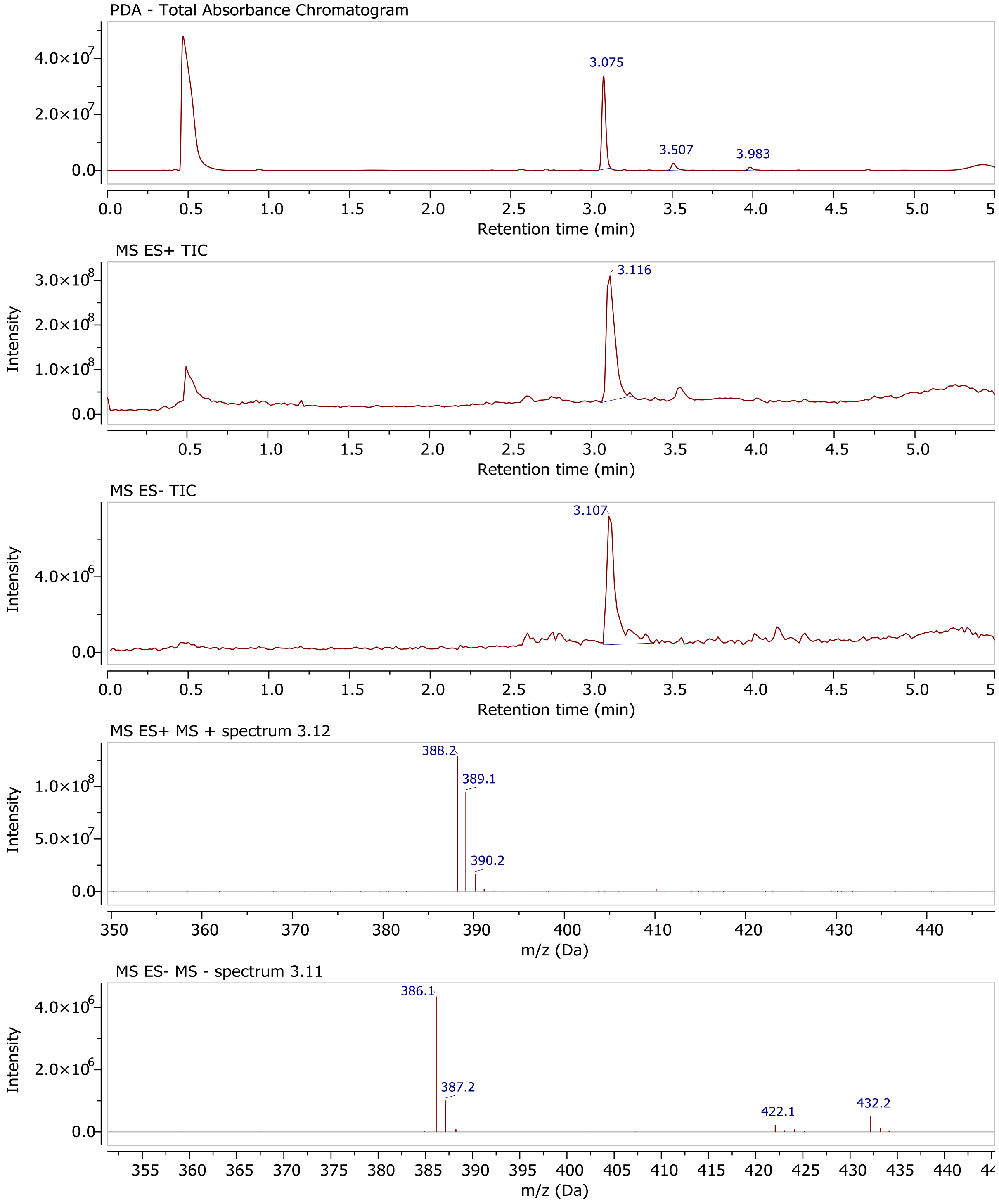

Supplementary figure **1440**. UPLC-MS analysis for compound **PHP-5643**

Chemical Formula: $C_{20}H_{21}ClN_2O_2$
Exact Mass: 356.1

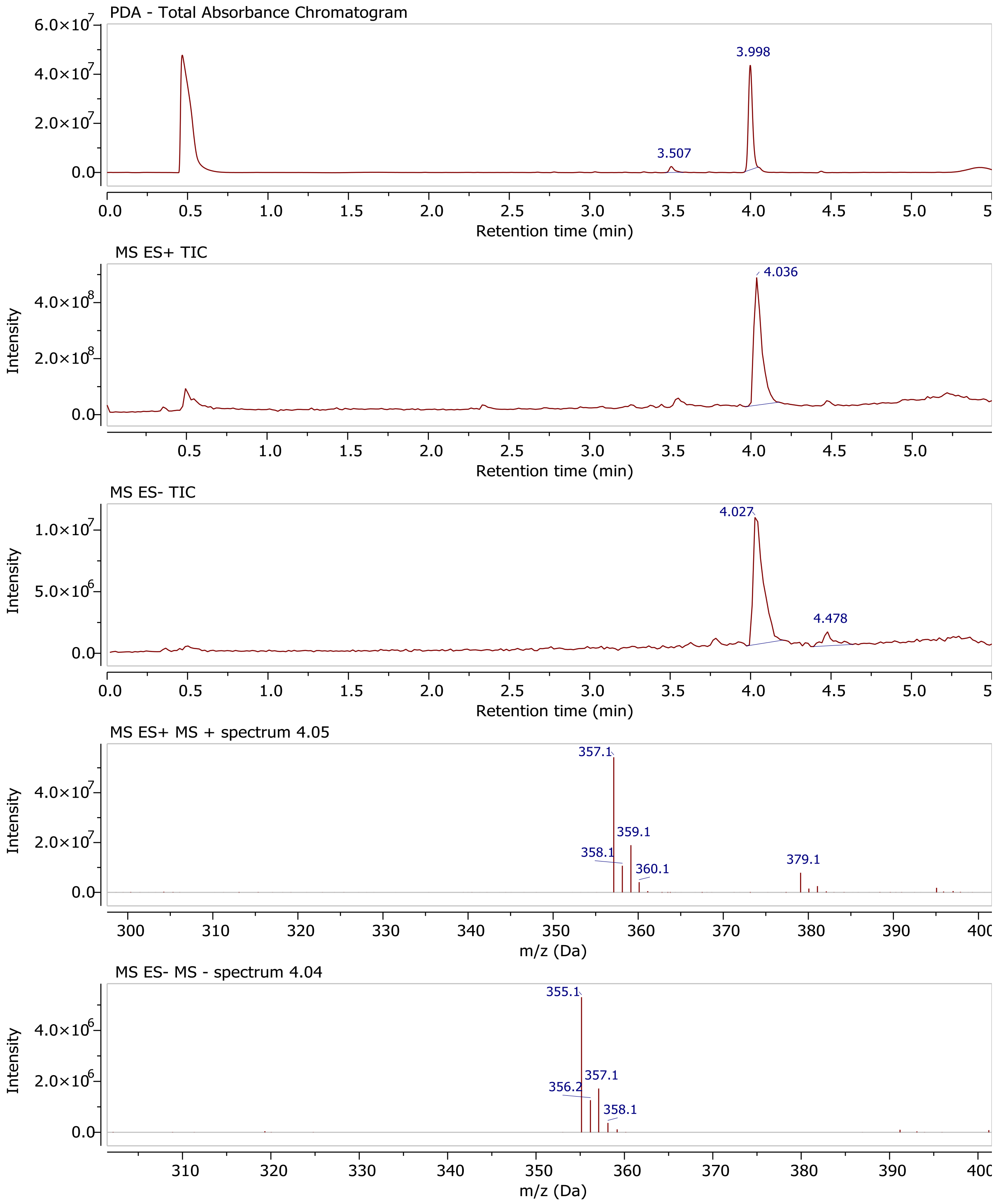

Supplementary figure **1441**. UPLC-MS analysis for compound **PHP-5805**

Chemical Formula: $C_{24}H_{22}N_2O_3$
Exact Mass: 386.2

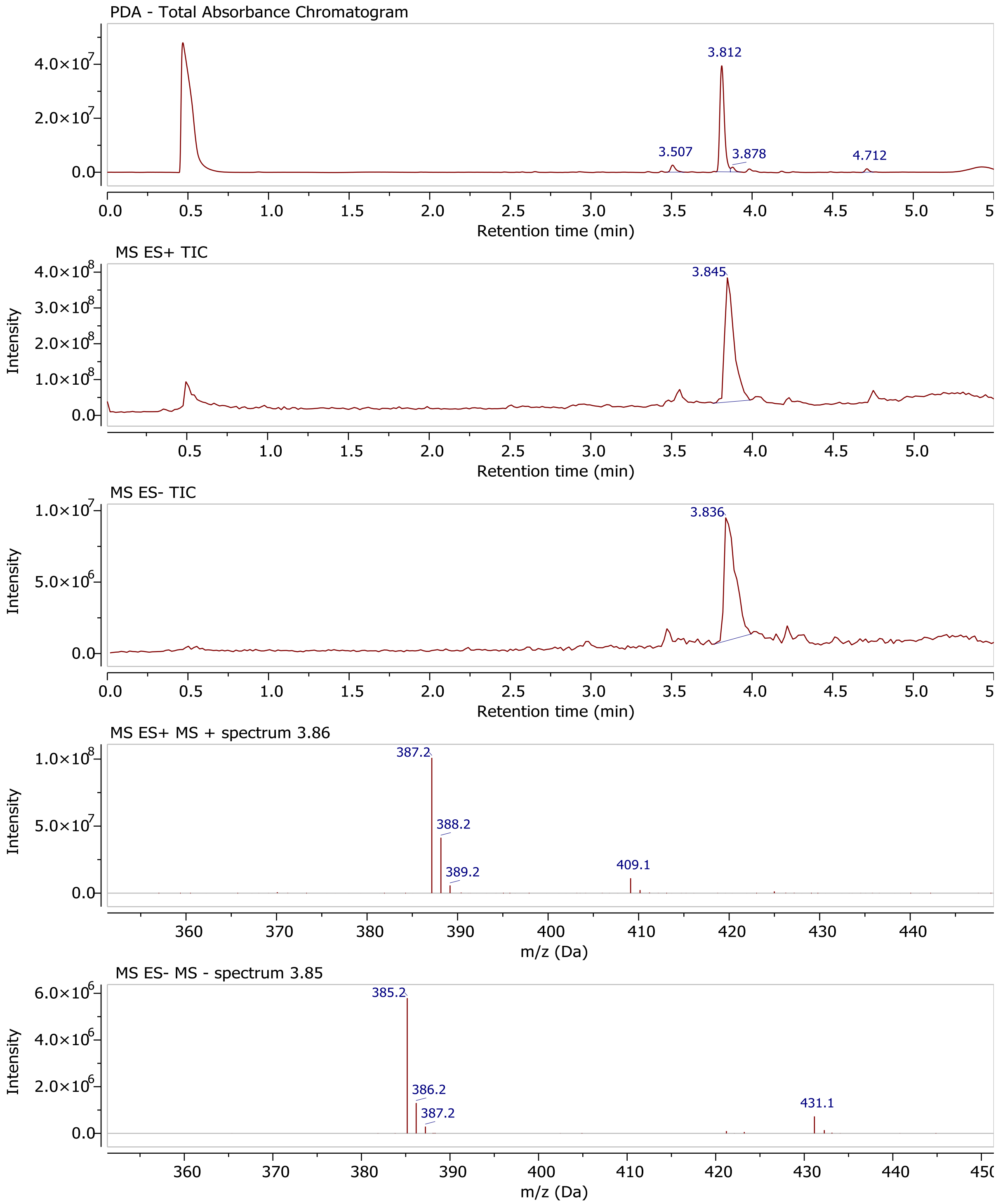

Supplementary figure **1442**. UPLC-MS analysis for compound **PHP-5647**

Chemical Formula: $C_{21}H_{21}F_3N_2O_2$
Exact Mass: 390.2

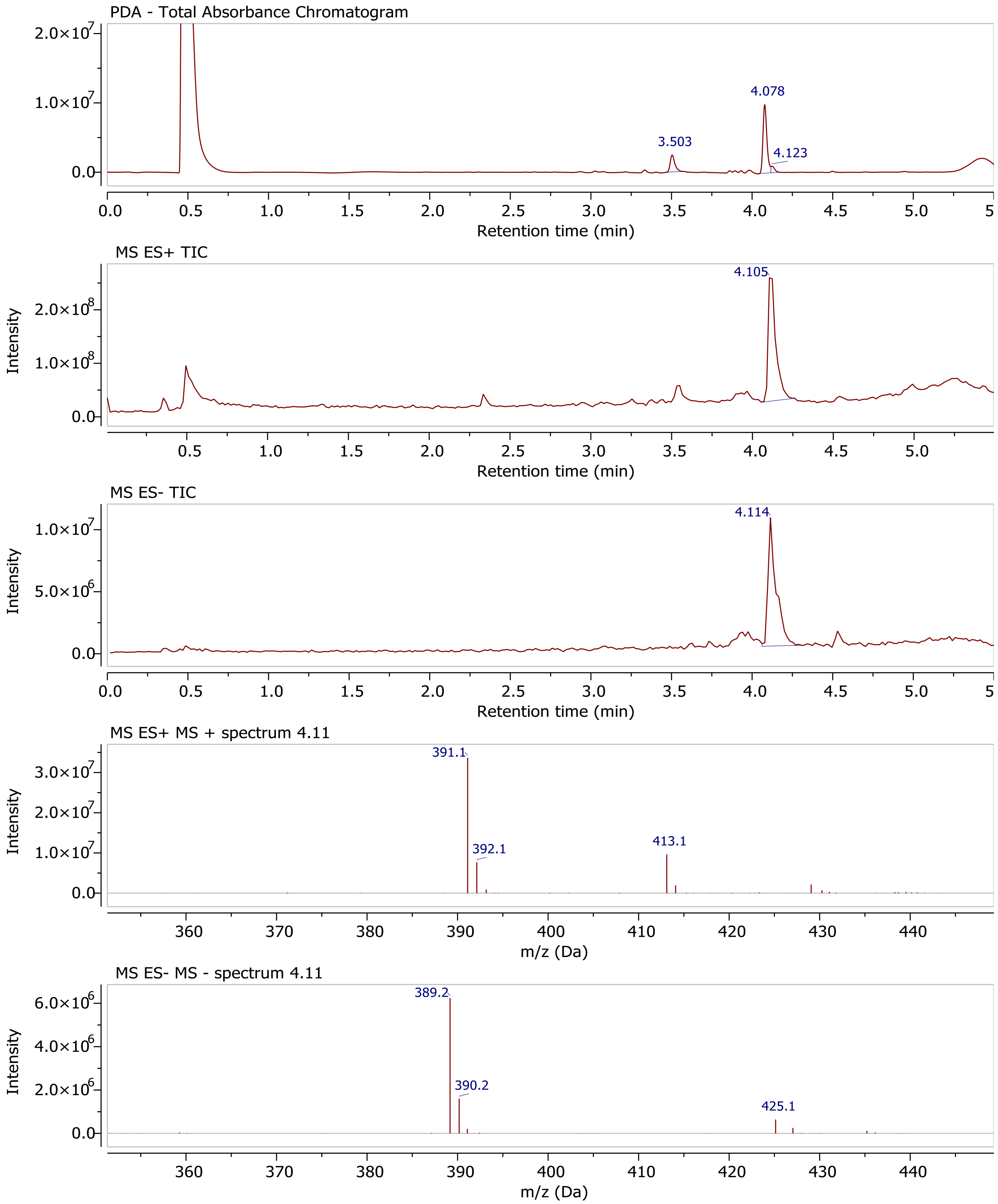

Supplementary figure **1443**. UPLC-MS analysis for compound **PHP-5806**

Chemical Formula: $C_{20}H_{20}Cl_2N_2O_3$
Exact Mass: 406.1

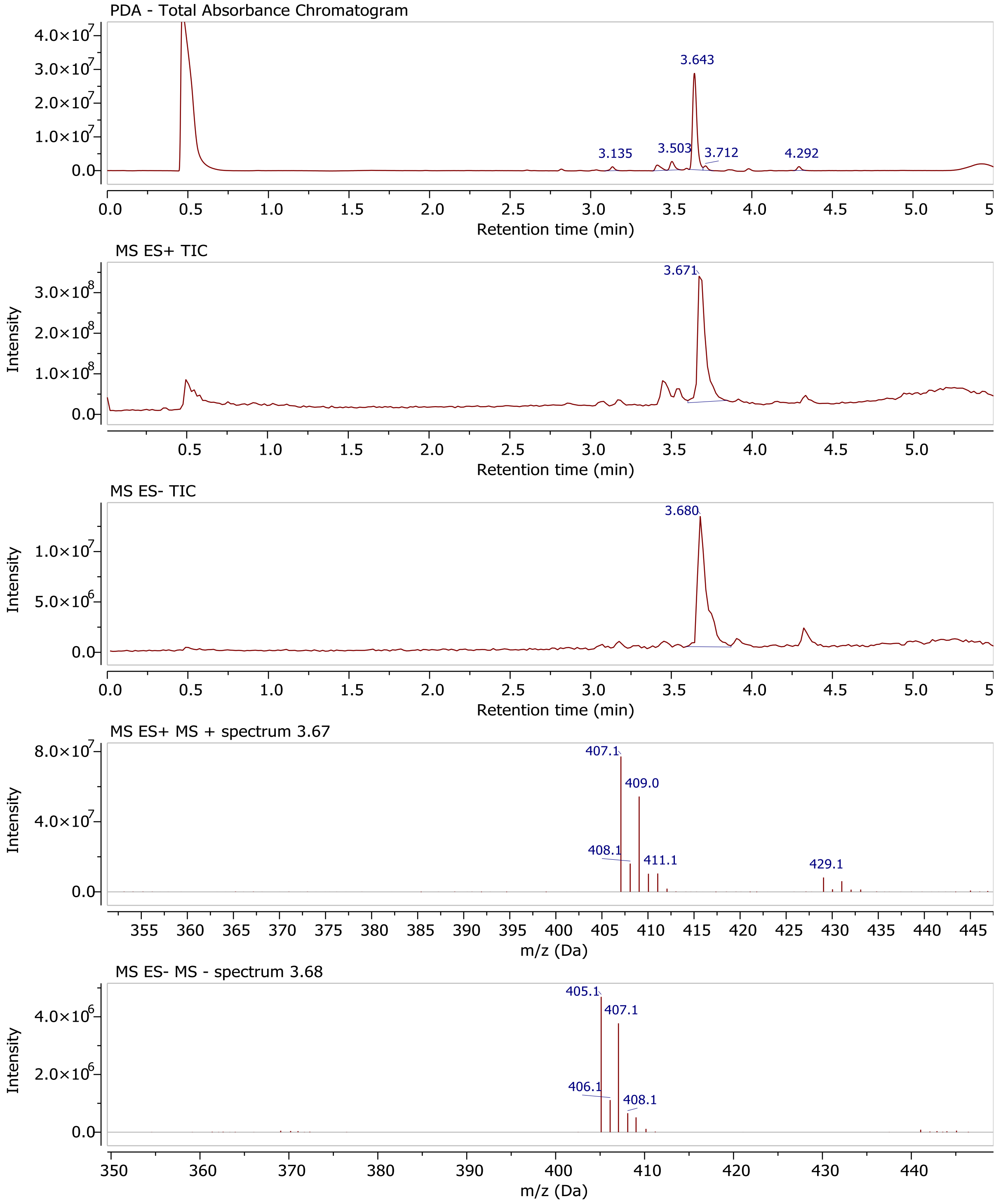

Supplementary figure **1444**. UPLC-MS analysis for compound **PHP-4905**

Chemical Formula: $C_{23}H_{19}FN_2O_3$
Exact Mass: 390.1

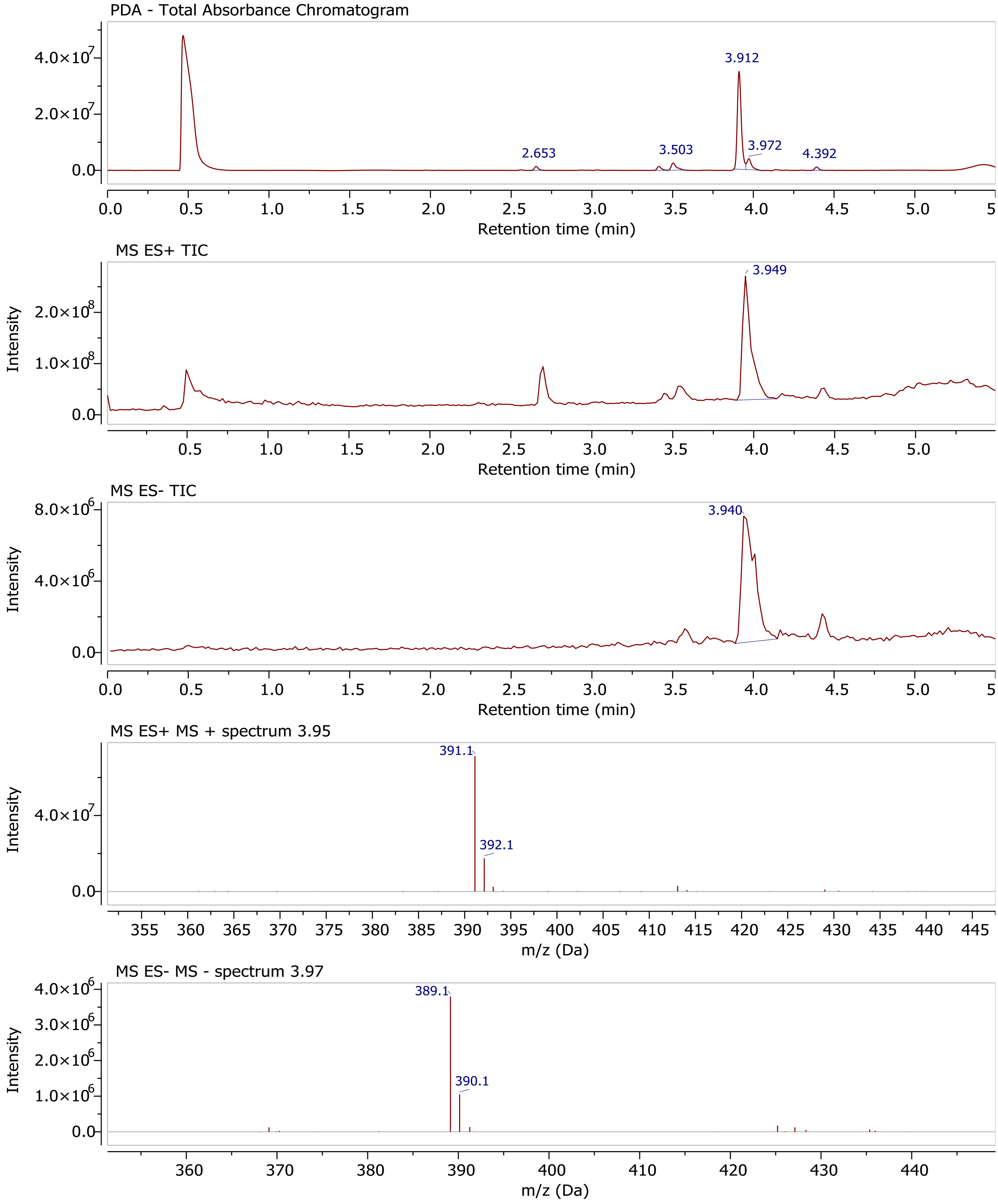

Supplementary figure **1445**. UPLC-MS analysis for compound **PHP-5472**

Chemical Formula: $C_{22}H_{26}N_2O_5$
Exact Mass: 398.2

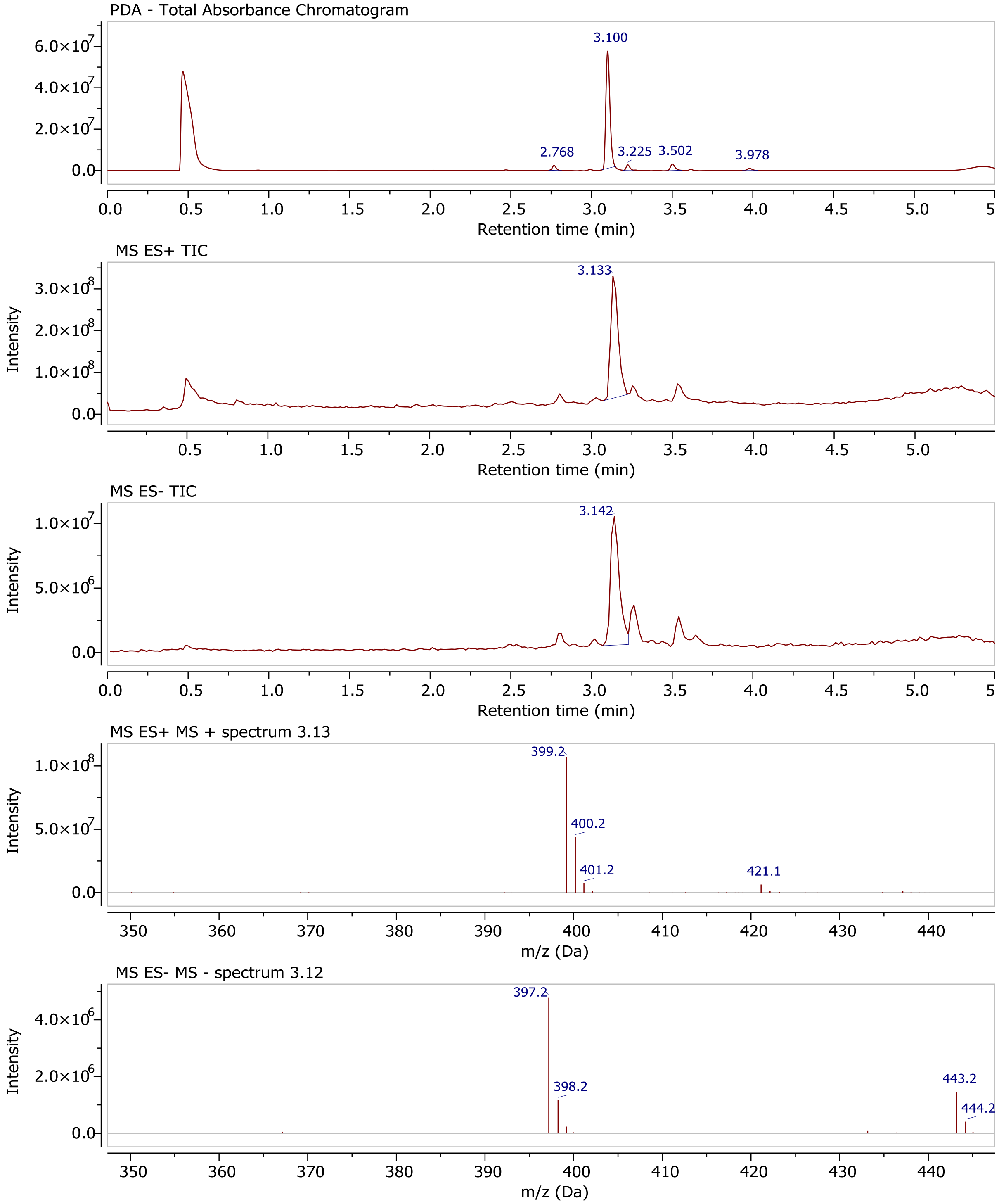

Supplementary figure **1446**. UPLC-MS analysis for compound **PHP-4907**

Chemical Formula: $C_{22}H_{26}N_2O_3$
Exact Mass: 366.2

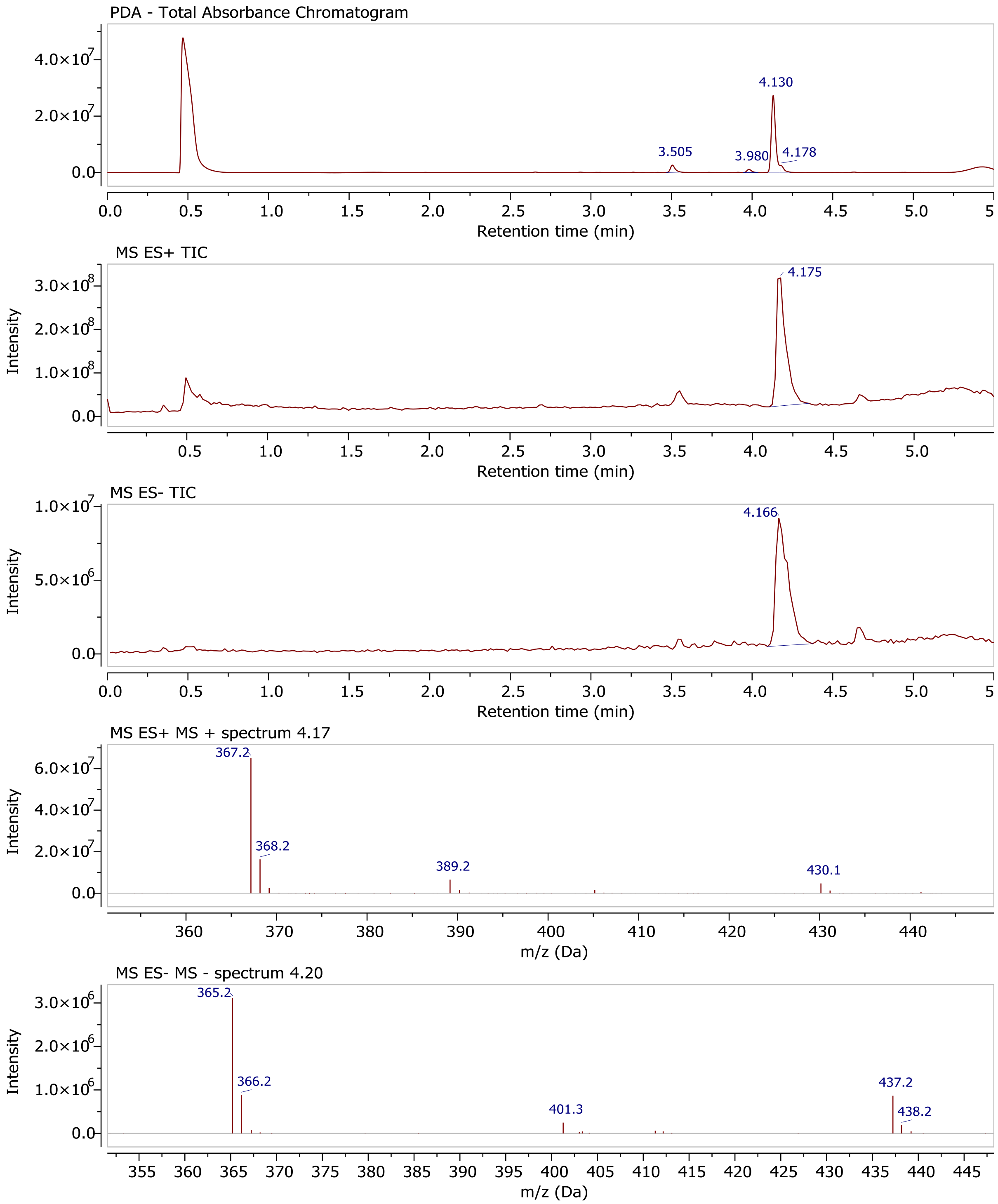

Supplementary figure **1447**. UPLC-MS analysis for compound **PHP-5473**

Chemical Formula: $C_{28}H_{30}N_2O_5$
Exact Mass: 474.2

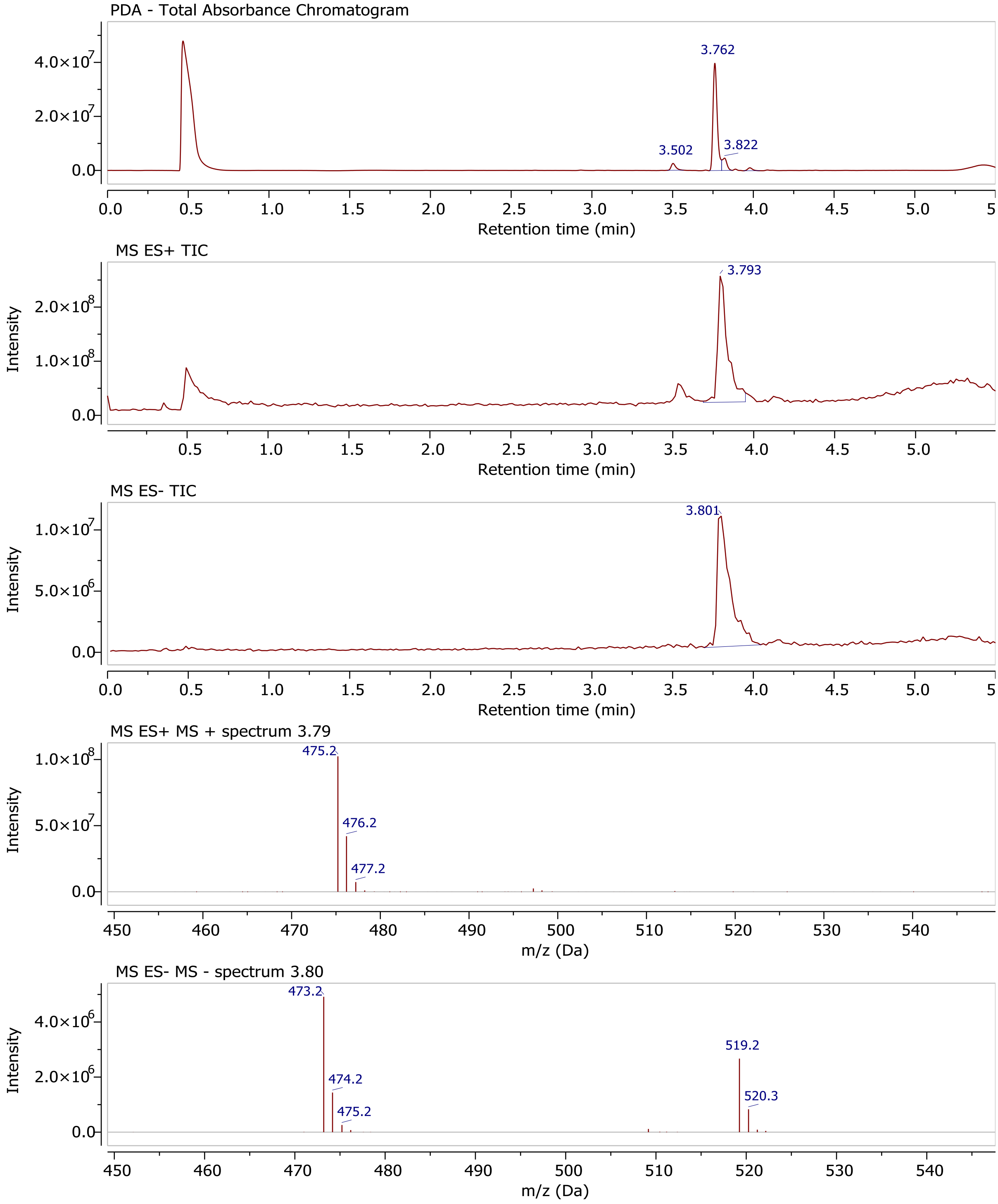

Supplementary figure **1448**. UPLC-MS analysis for compound **PHP-4909**

Chemical Formula: $C_{22}H_{26}N_2O_3$
Exact Mass: 366.2

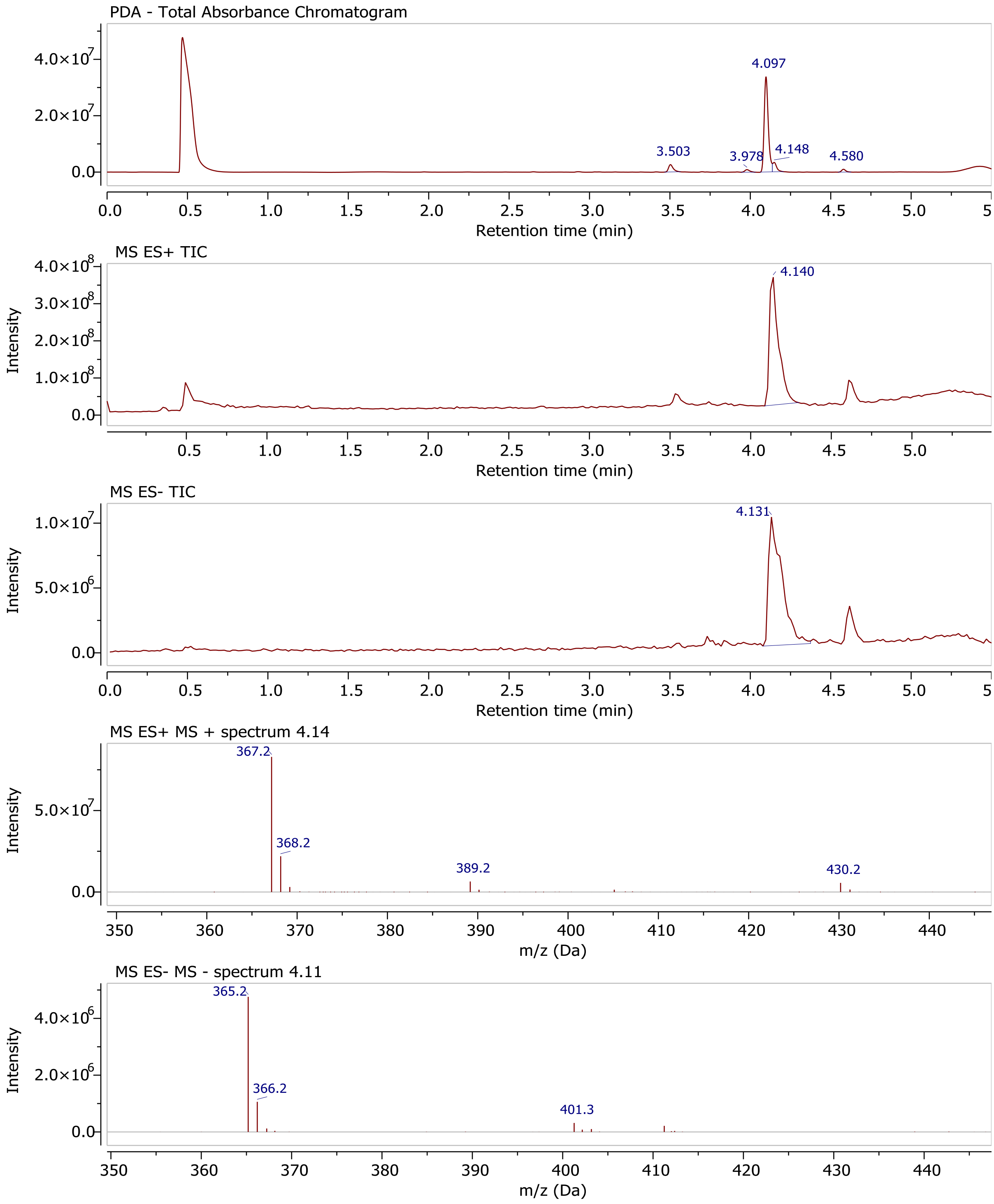

Supplementary figure **1449**. UPLC-MS analysis for compound **PHP-5474**

Chemical Formula: $C_{20}H_{20}Cl_2N_2O_3$
Exact Mass: 406.1

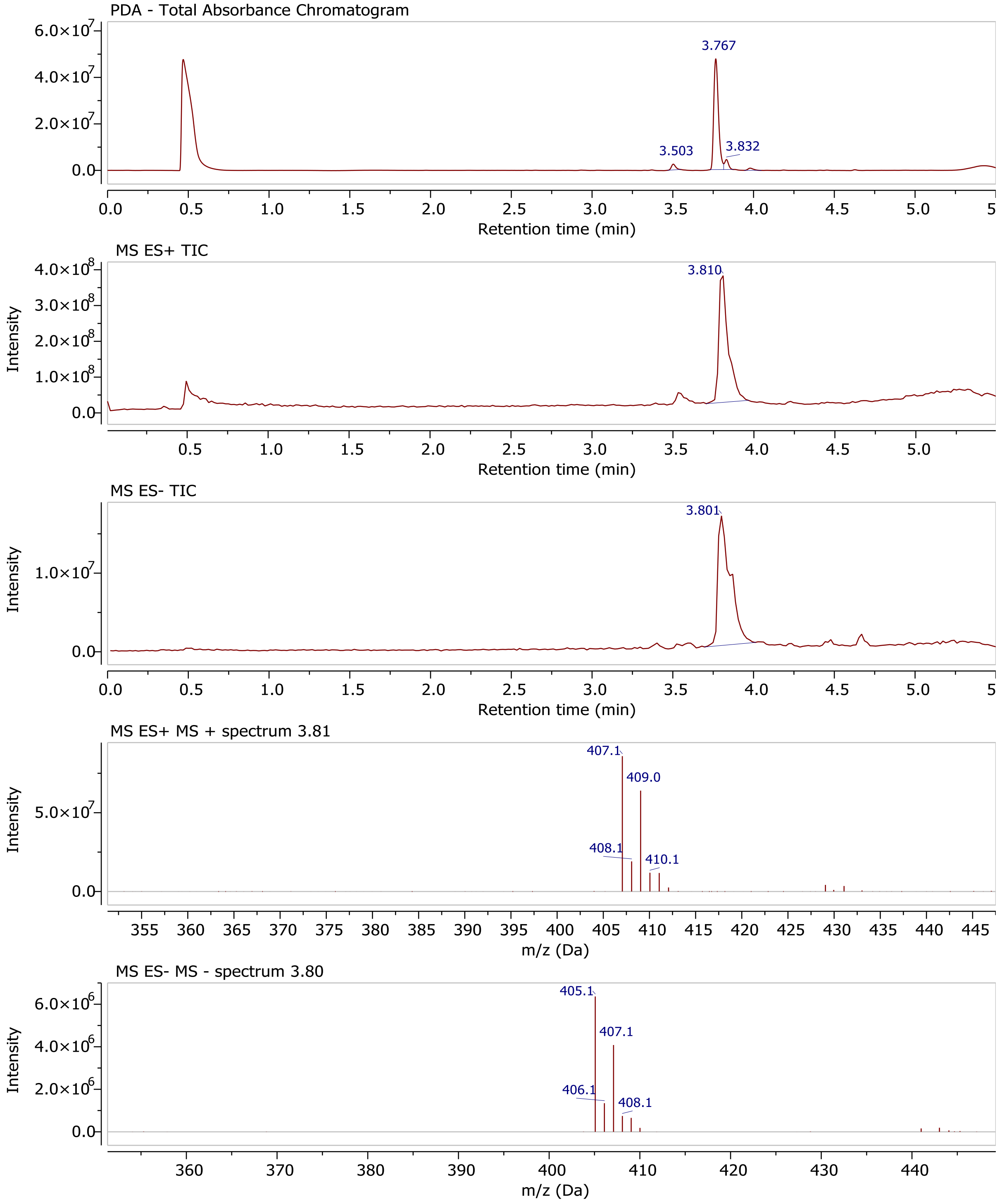

Supplementary figure **1450**. UPLC-MS analysis for compound **PHP-4910**

Chemical Formula: $C_{23}H_{16}F_4N_2O_3$
Exact Mass: 444.1

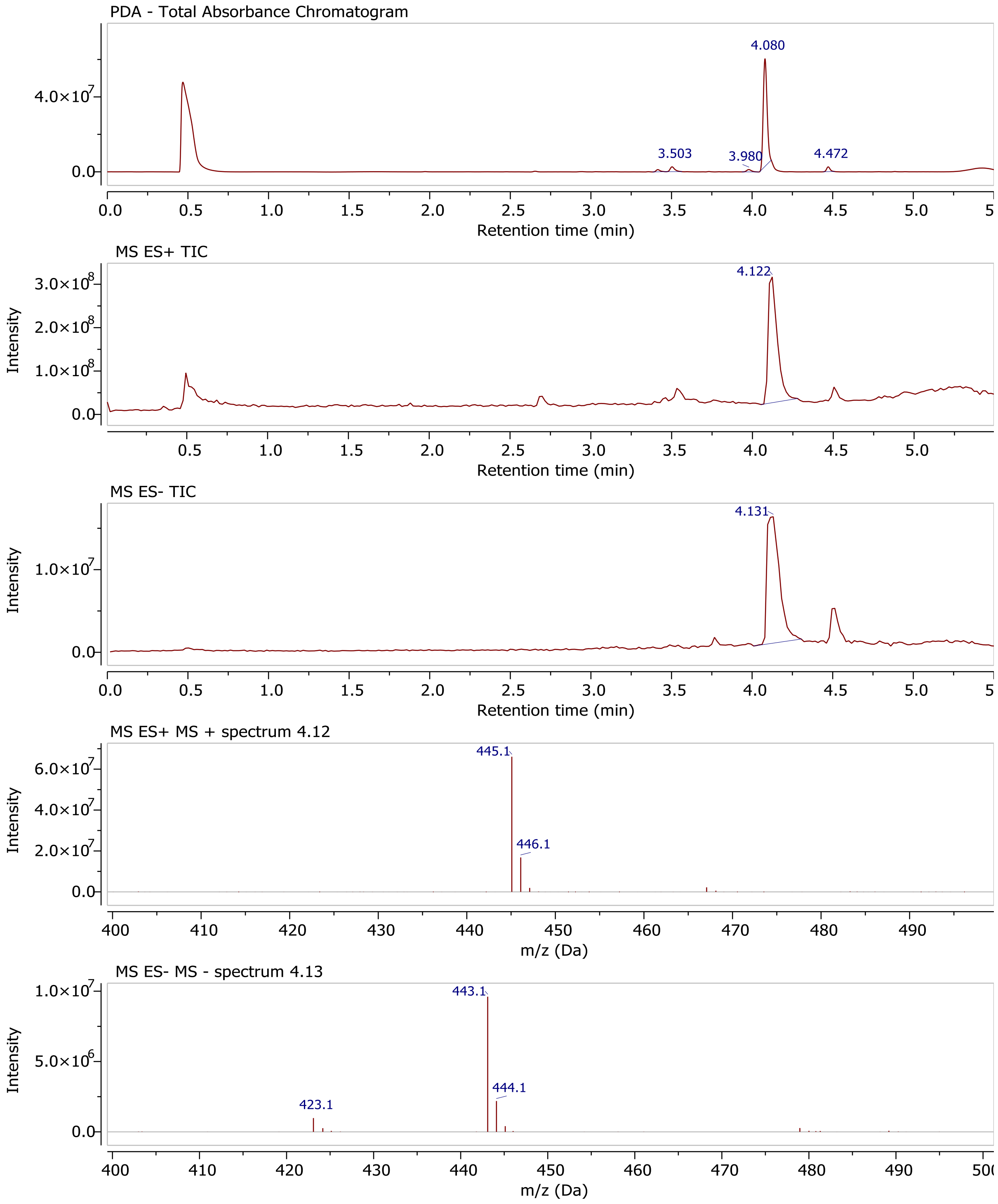

Supplementary figure **1451**. UPLC-MS analysis for compound **PHP-5475**

Chemical Formula: $C_{27}H_{28}N_2O_4$
Exact Mass: 444.2

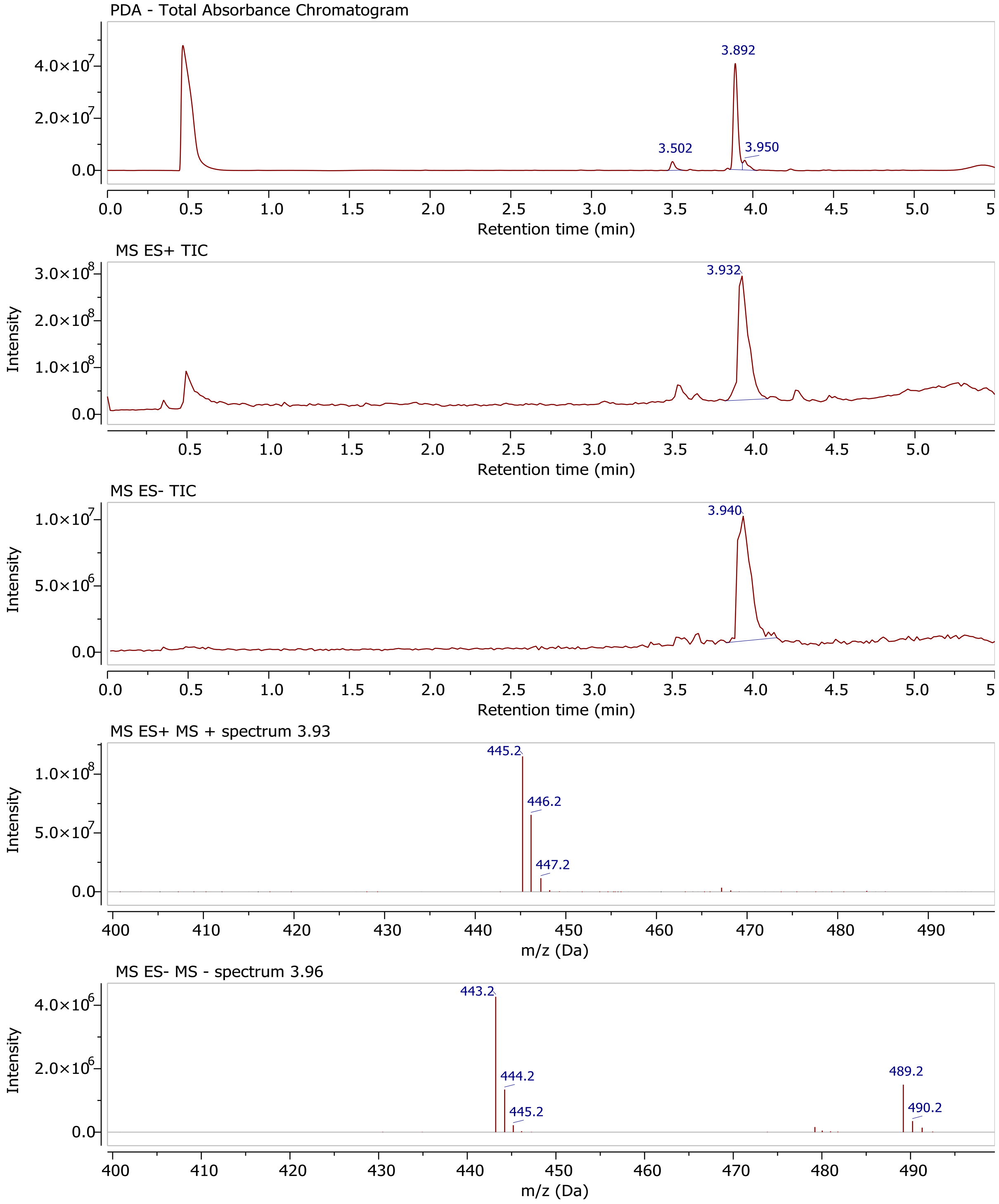

Supplementary figure **1452**. UPLC-MS analysis for compound **PHP-4911**

Chemical Formula: $C_{24}H_{30}N_2O_3$
Exact Mass: 394.2

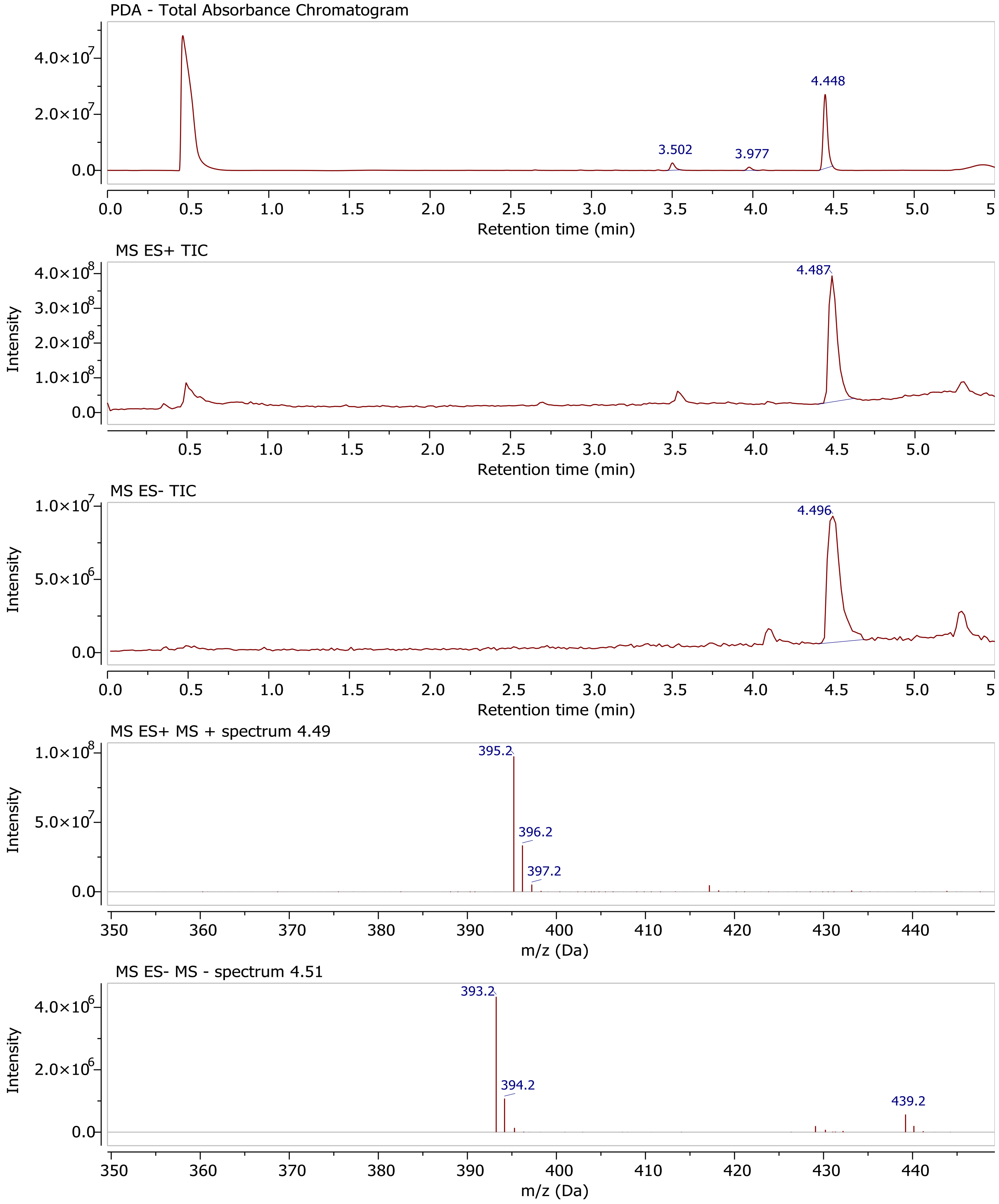

Supplementary figure **1453**. UPLC-MS analysis for compound **PHP-5476**

Chemical Formula: $C_{20}H_{20}ClFN_2O_3$
Exact Mass: 390.1

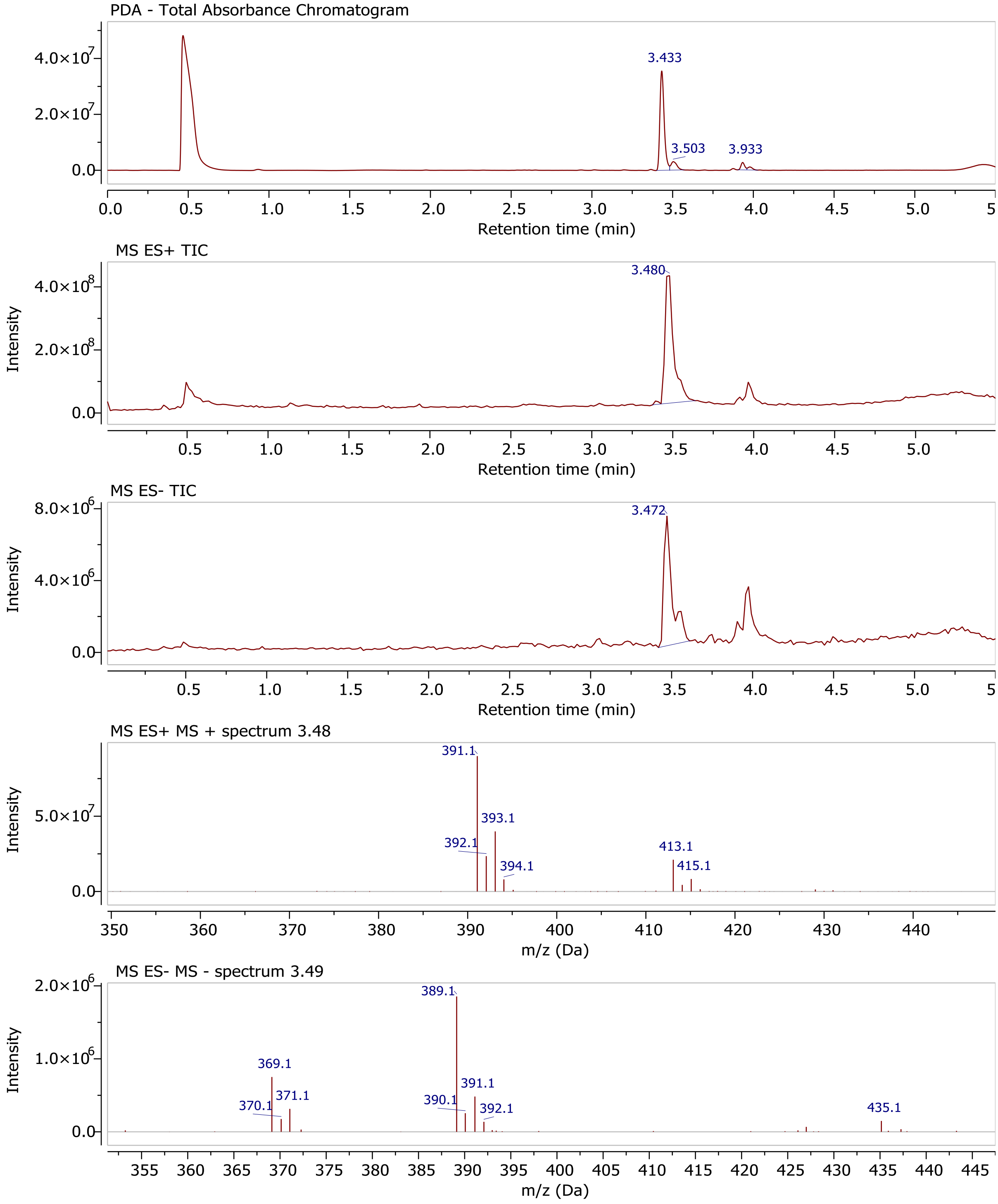

Supplementary figure **1454**. UPLC-MS analysis for compound **PHP-4912**

Chemical Formula: $C_{23}H_{17}F_3N_2O_3$
Exact Mass: 426.1

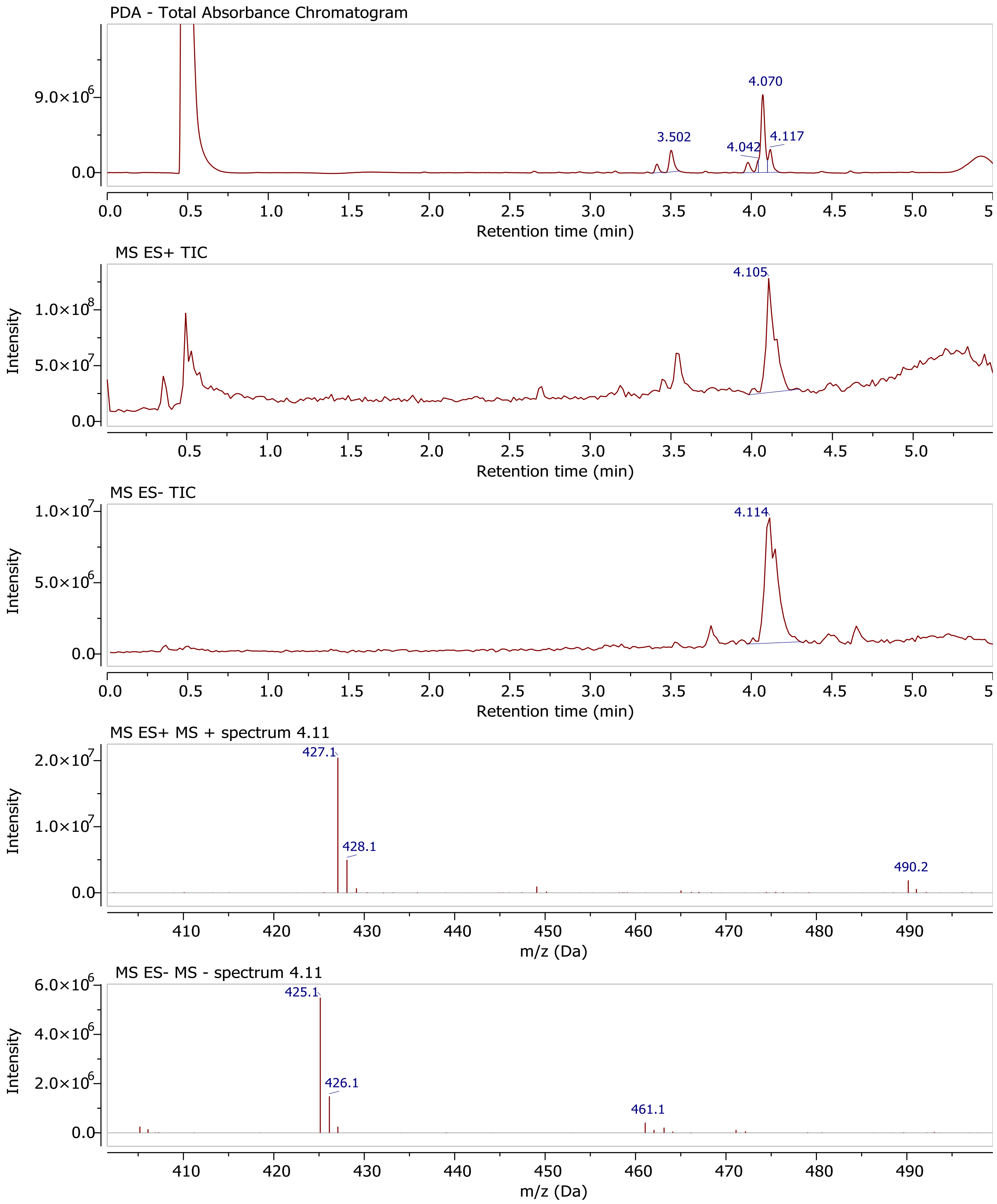

Supplementary figure **1455**. UPLC-MS analysis for compound **PHP-5477**

Chemical Formula: $C_{20}H_{20}Cl_2N_2O_3$
Exact Mass: 406.1

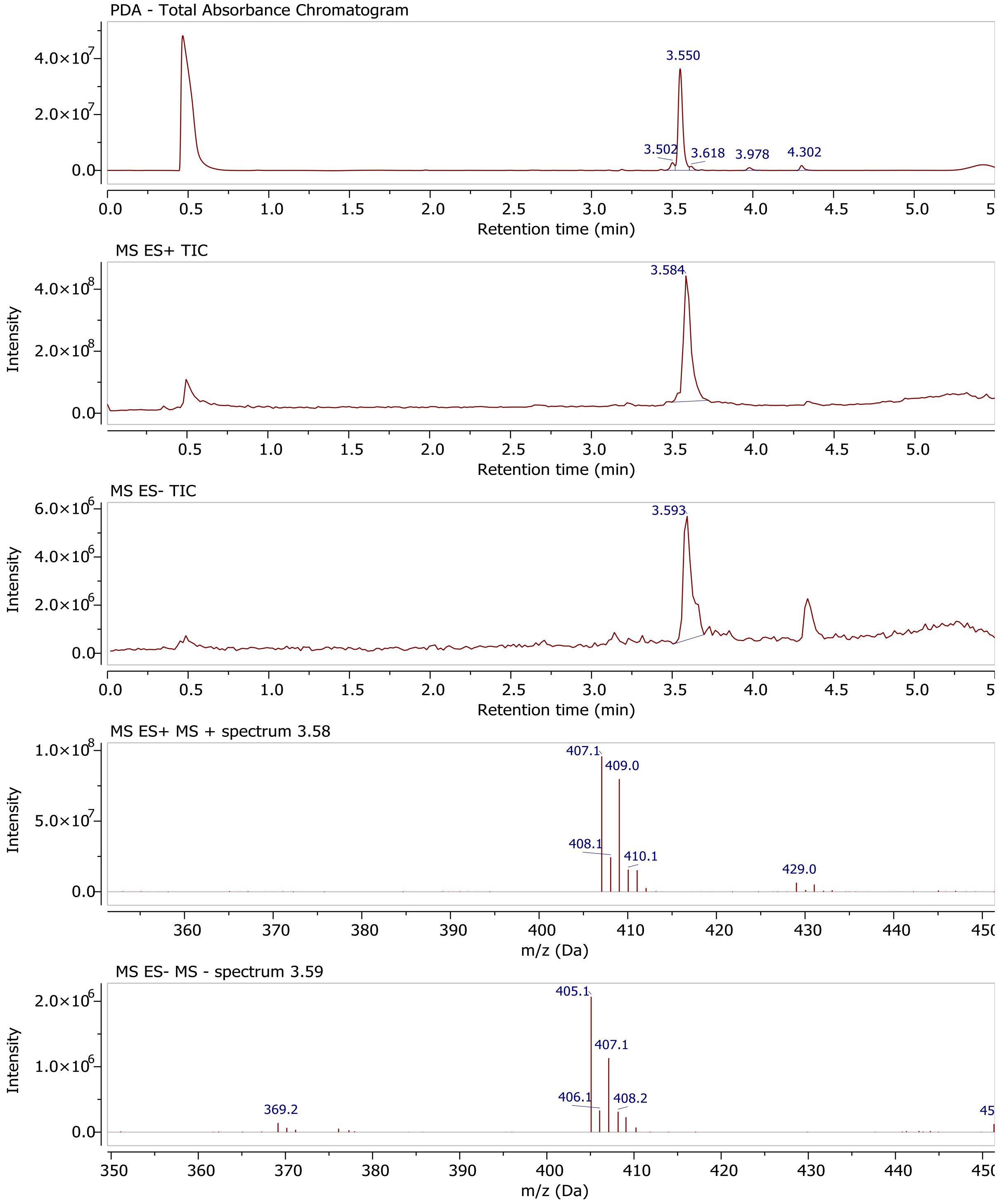

Supplementary figure **1456**. UPLC-MS analysis for compound **PHP-4913**

Chemical Formula: $C_{24}H_{22}N_2O_4$
Exact Mass: 402.2

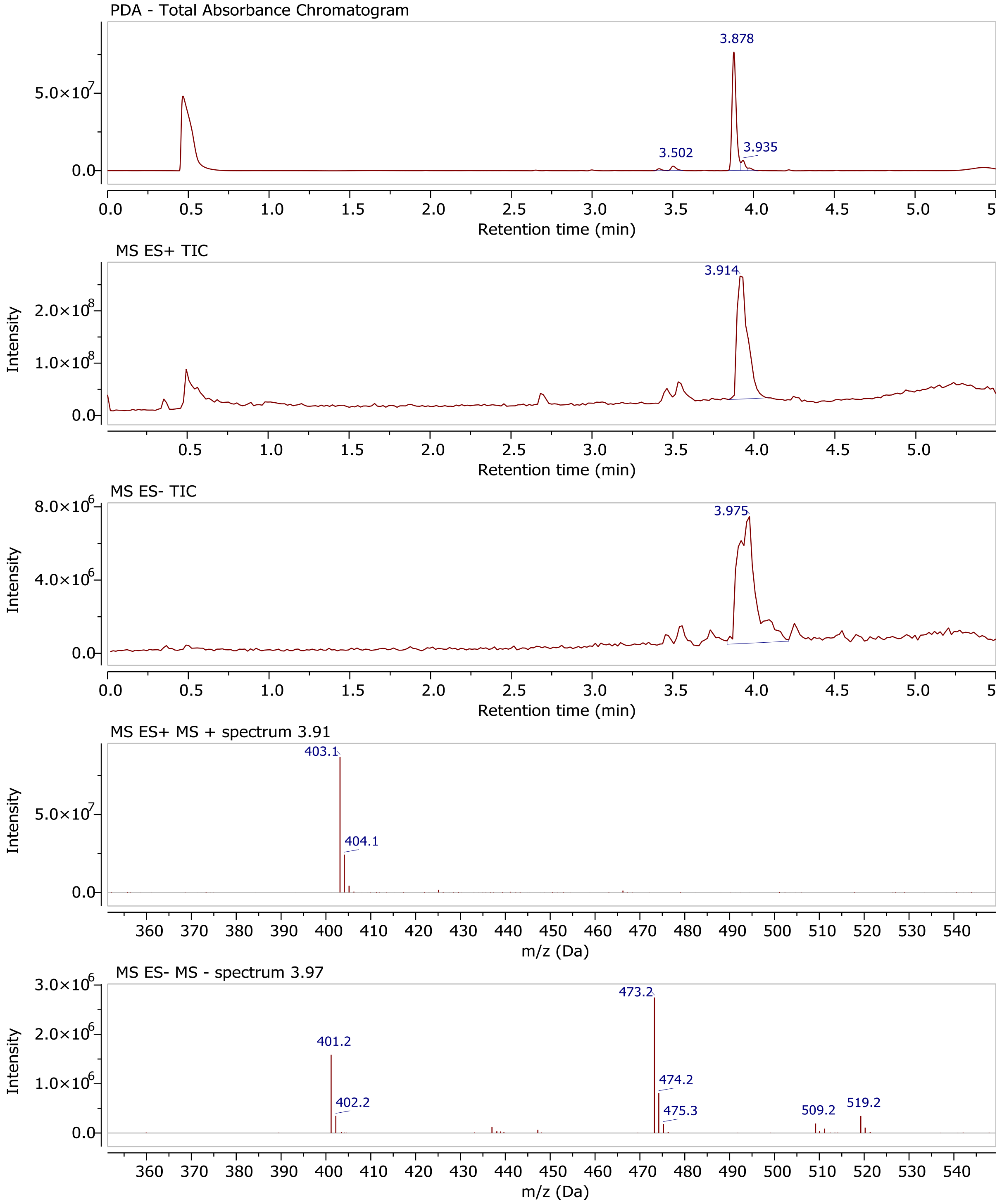

Supplementary figure **1457**. UPLC-MS analysis for compound **PHP-5478**

Chemical Formula: $C_{34}H_{34}N_2O_5$
Exact Mass: 550.2

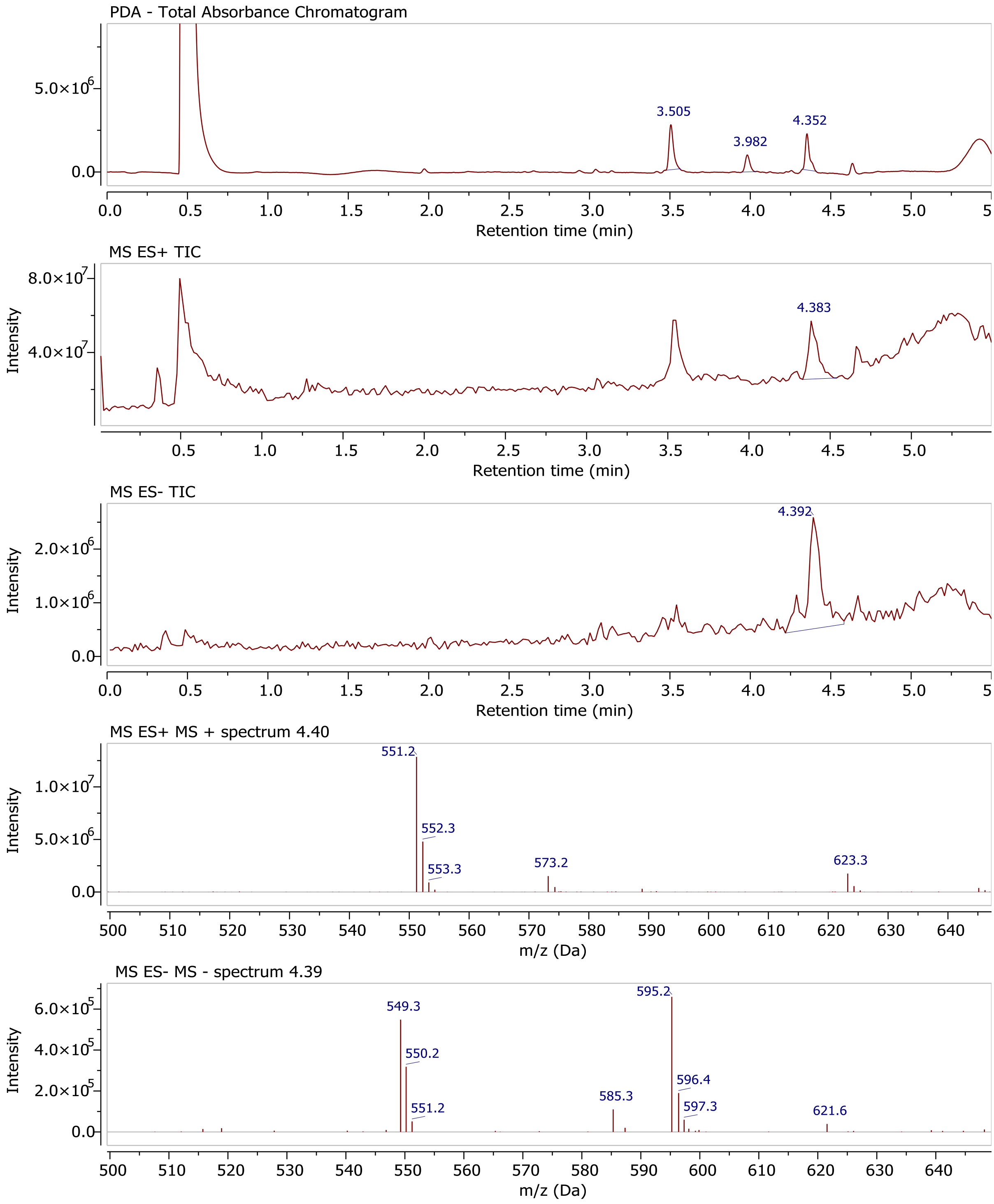

Supplementary figure **1458**. UPLC-MS analysis for compound **PHP-4915**

Chemical Formula: $C_{18}H_{17}ClN_2O_3$
Exact Mass: 344.1

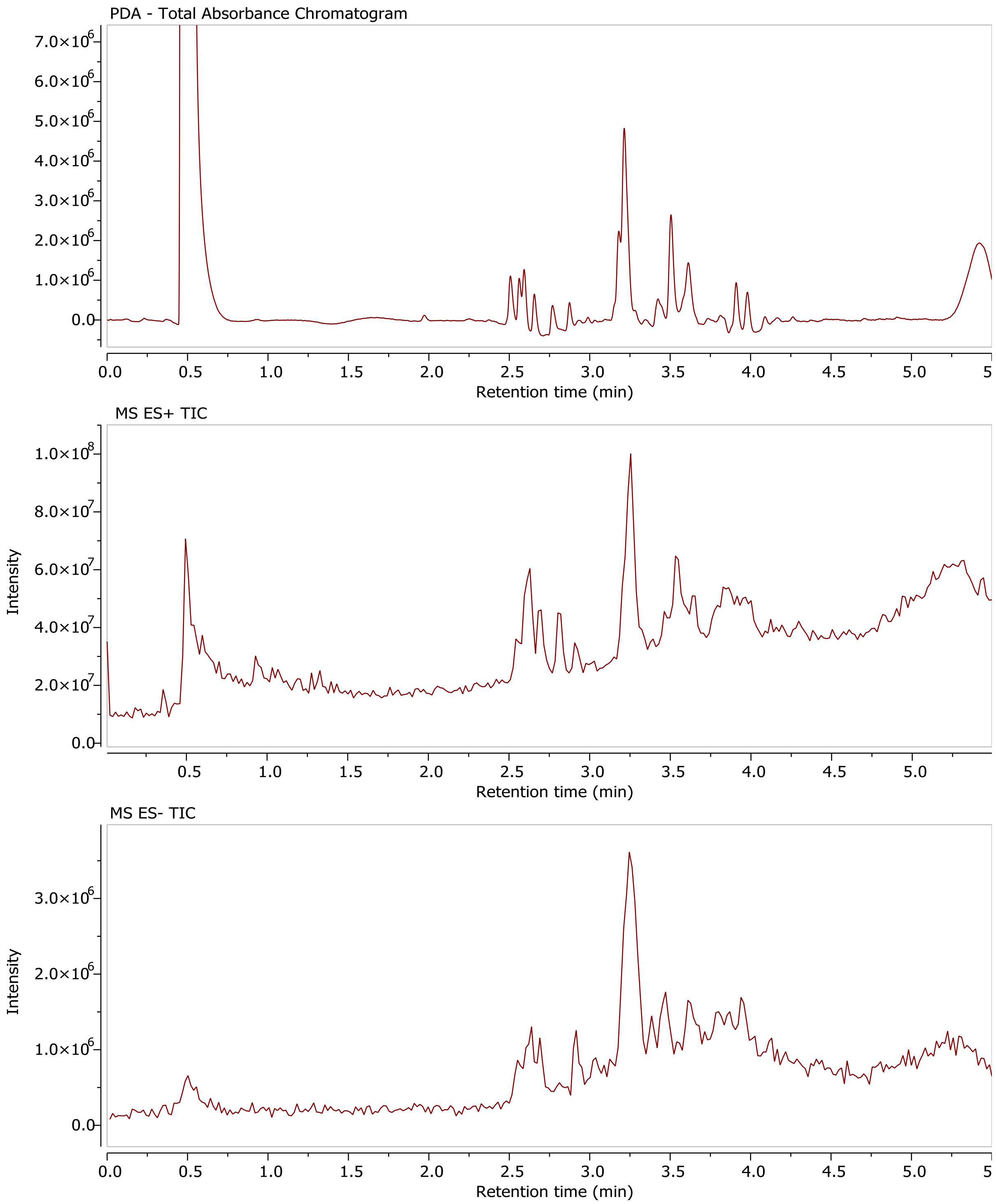

Supplementary figure **1459**. UPLC-MS analysis for compound **PHP-5480**

Chemical Formula: $C_{20}H_{21}ClN_2O_3$
Exact Mass: 372.1

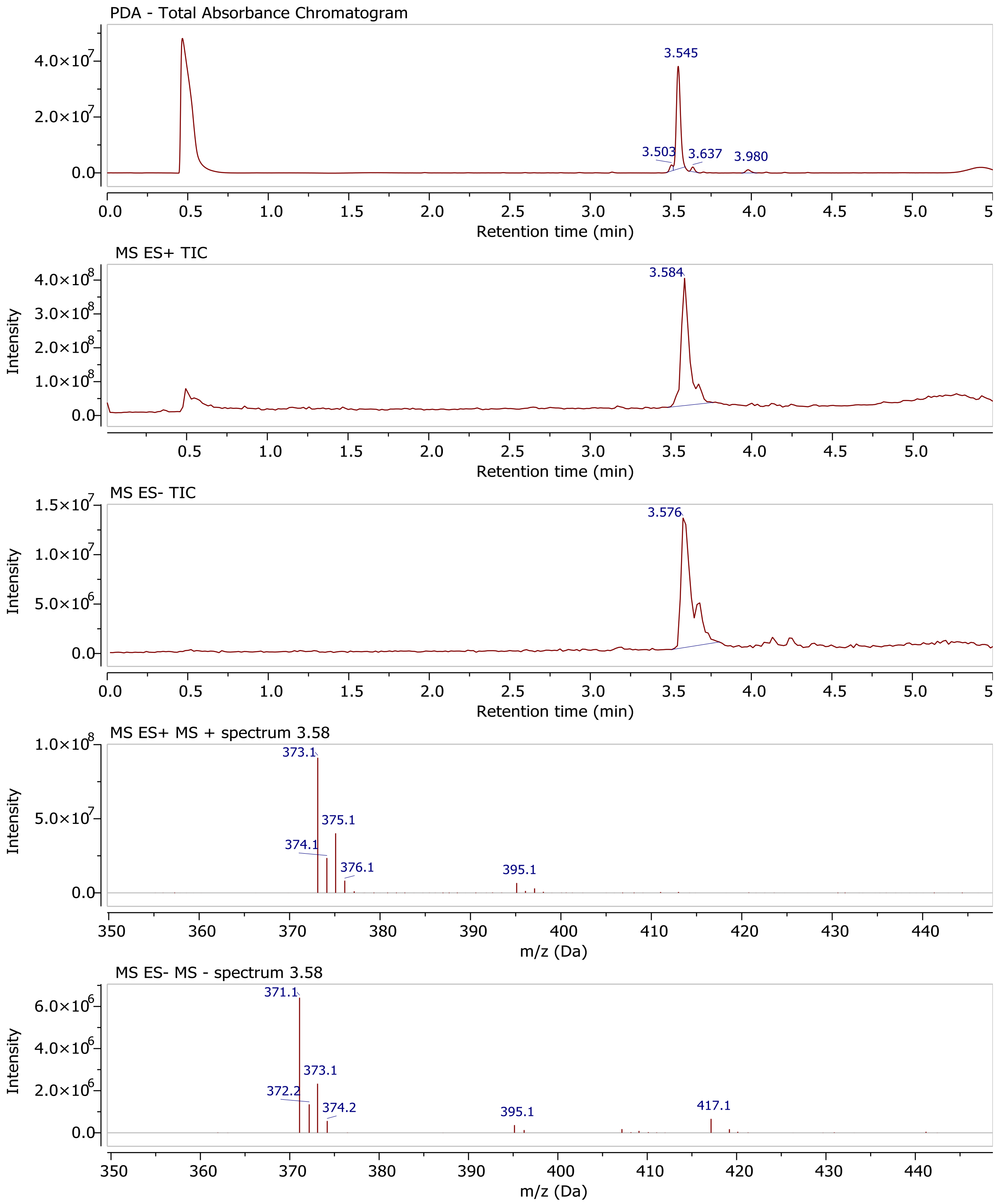

Supplementary figure **1460**. UPLC-MS analysis for compound **PHP-4916**

Chemical Formula: $C_{18}H_{15}F_3N_2O_2$
Exact Mass: 348.1

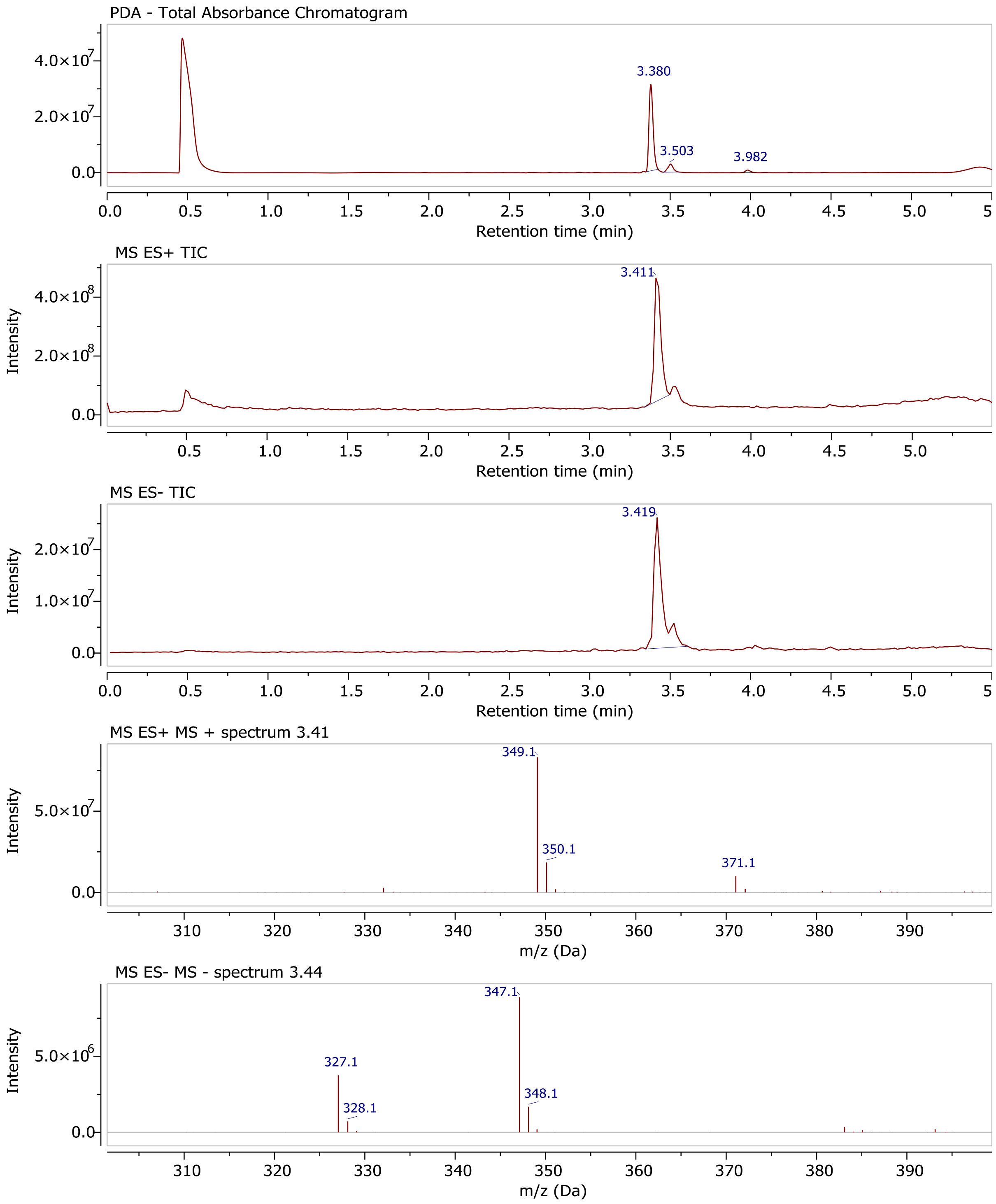

Supplementary figure **1461**. UPLC-MS analysis for compound **PHP-5482**

Chemical Formula: $C_{18}H_{26}N_2O_5$
Exact Mass: 350.2

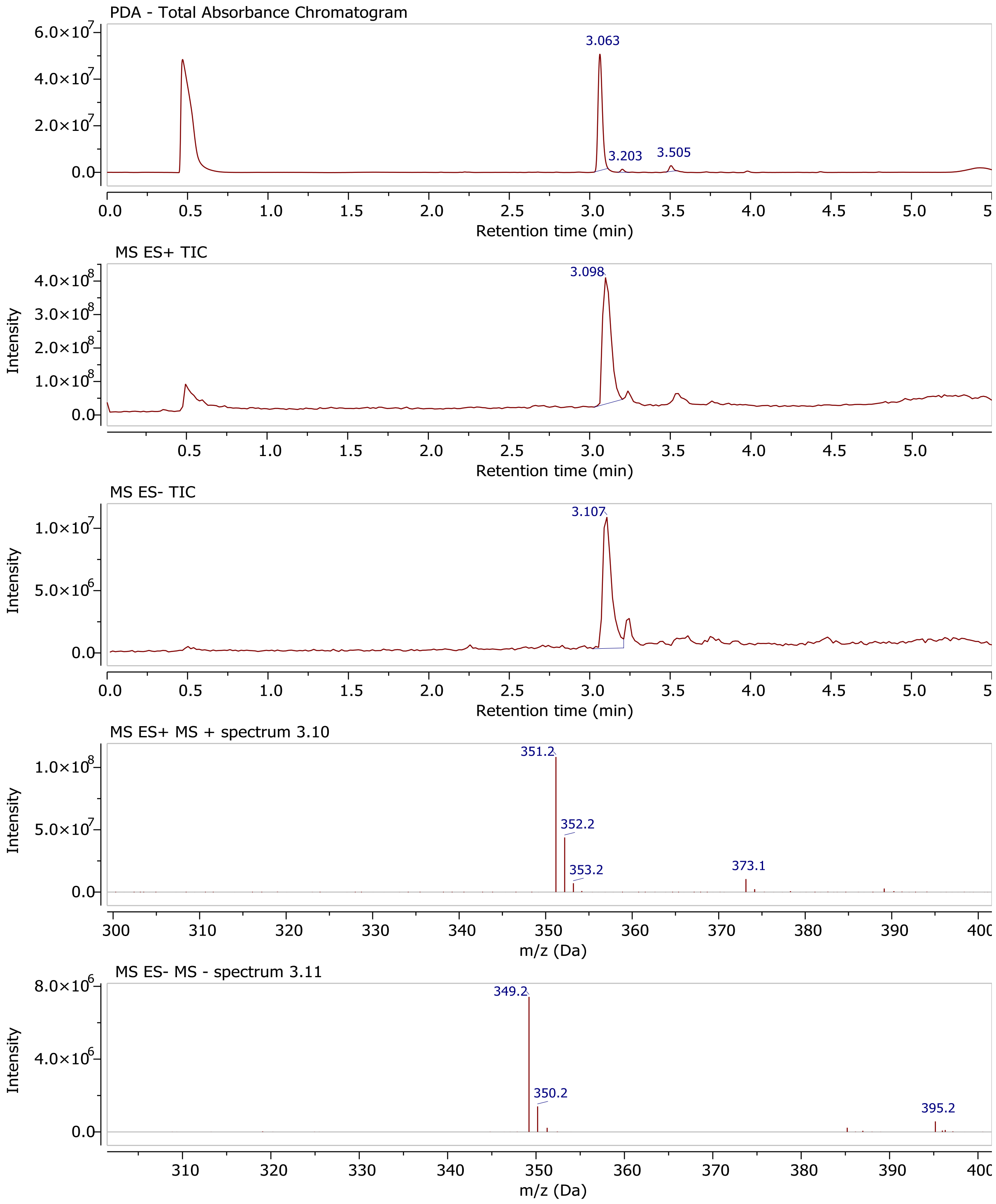

Supplementary figure **1462**. UPLC-MS analysis for compound **PHP-4917**

Chemical Formula: $C_{19}H_{19}FN_2O_3$
Exact Mass: 342.1

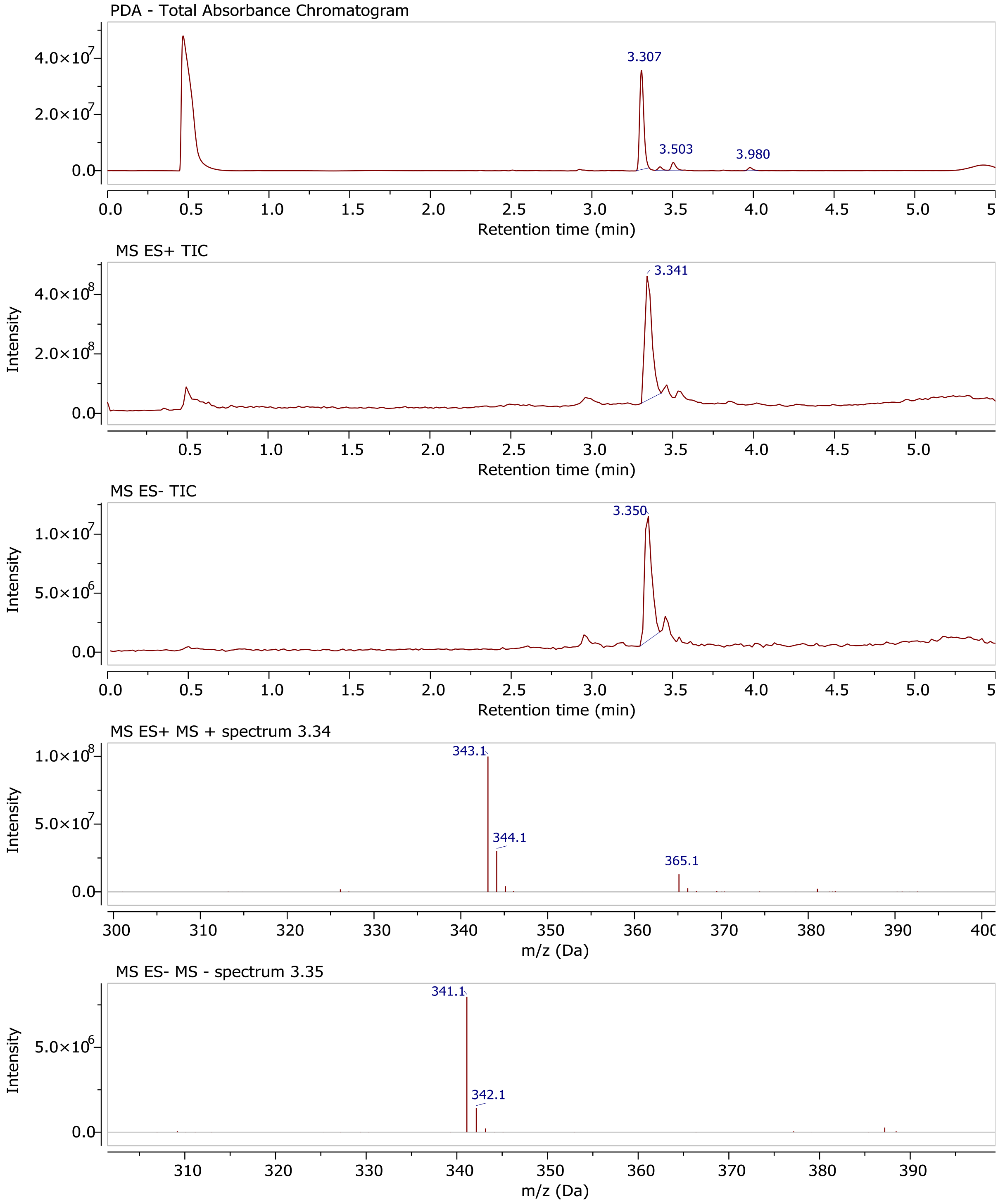

Supplementary figure **1463**. UPLC-MS analysis for compound **PHP-5484**

Chemical Formula: $C_{15}H_{18}Cl_2N_2O_2$
Exact Mass: 328.1

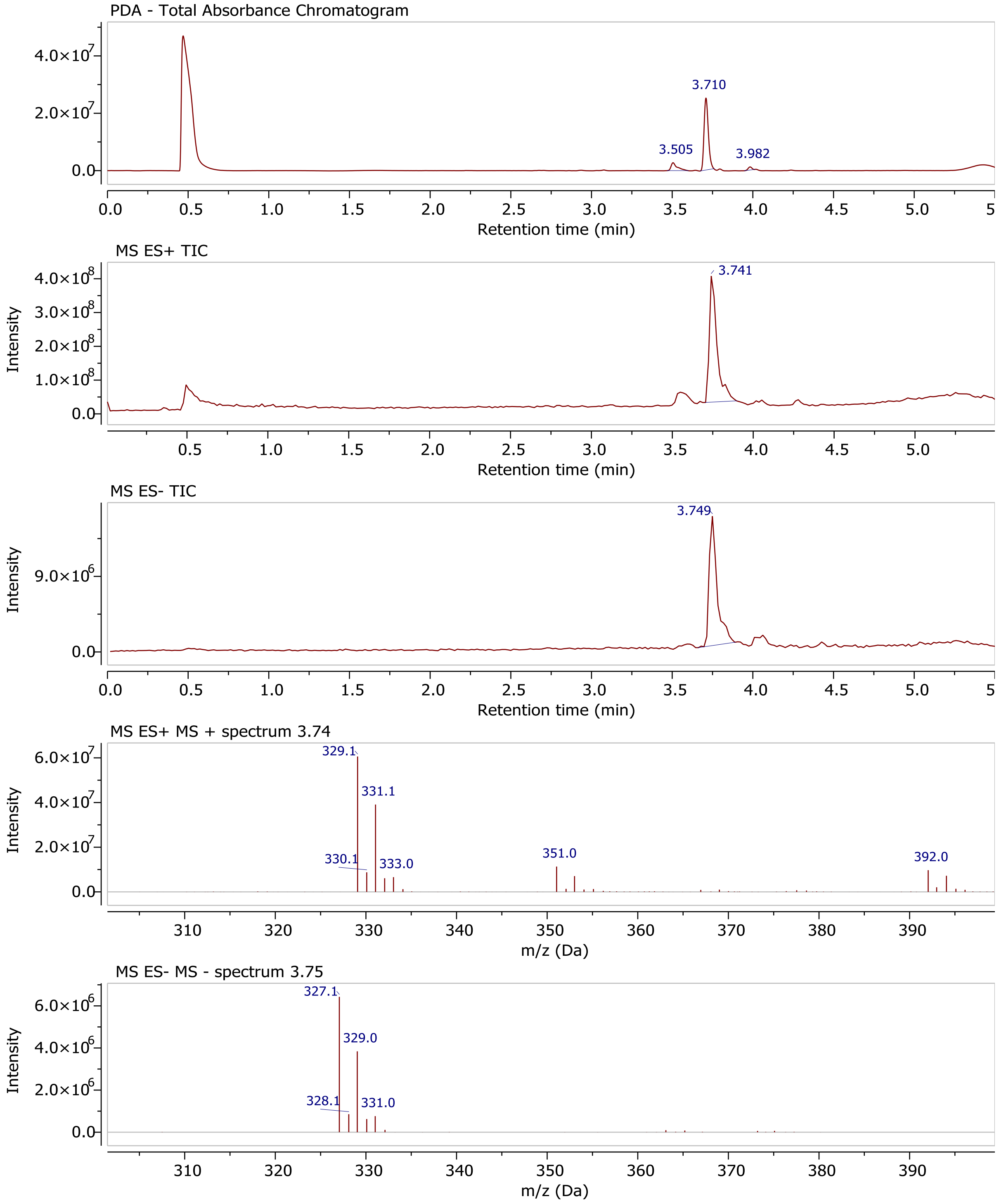

Supplementary figure **1464**. UPLC-MS analysis for compound **PHP-4918**

Chemical Formula: $C_{17}H_{21}FN_2O_3$
Exact Mass: 320.2

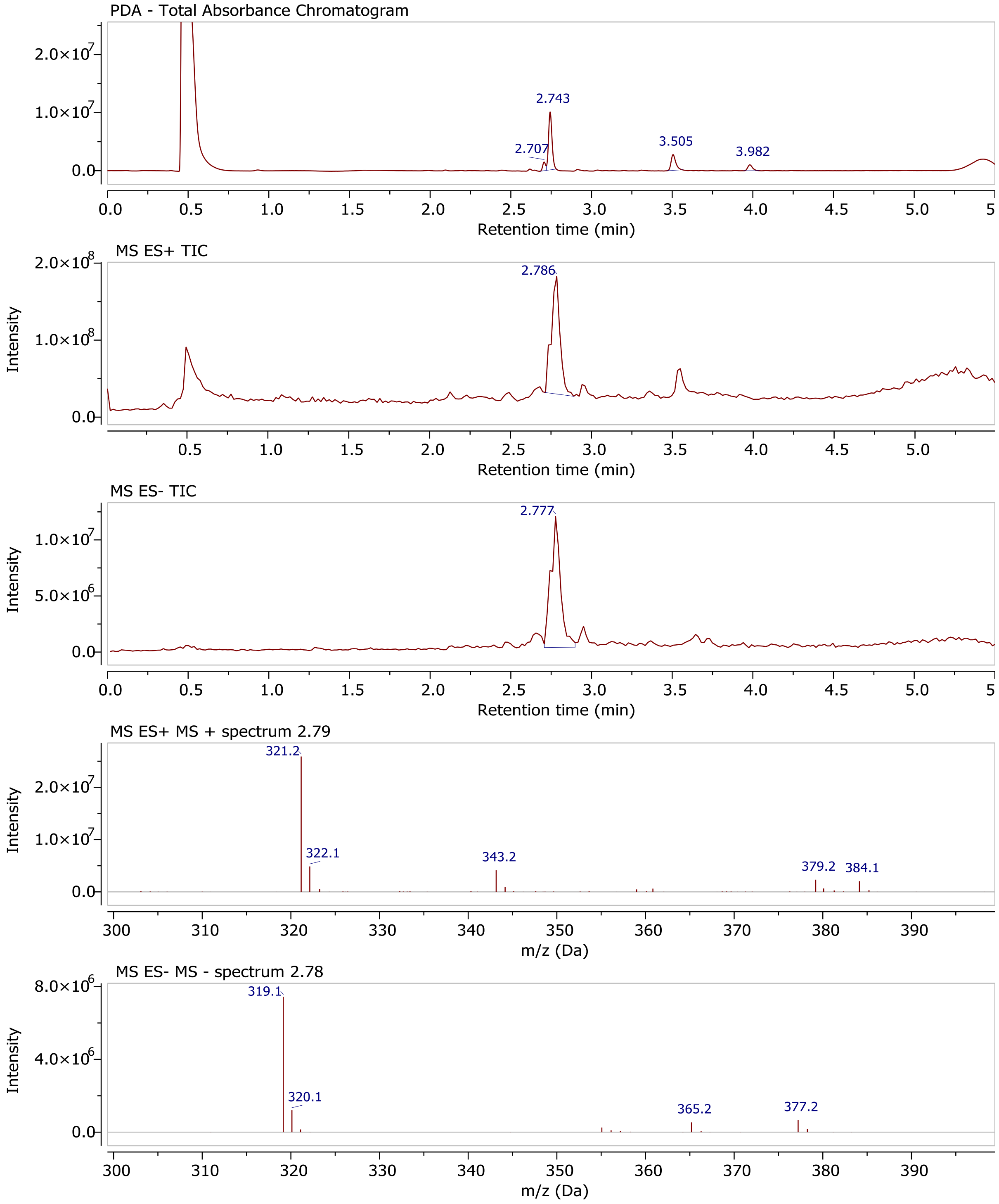

Supplementary figure **1465**. UPLC-MS analysis for compound **PHP-5486**

Chemical Formula: $C_{15}H_{19}BrN_2O_2$
Exact Mass: 338.1

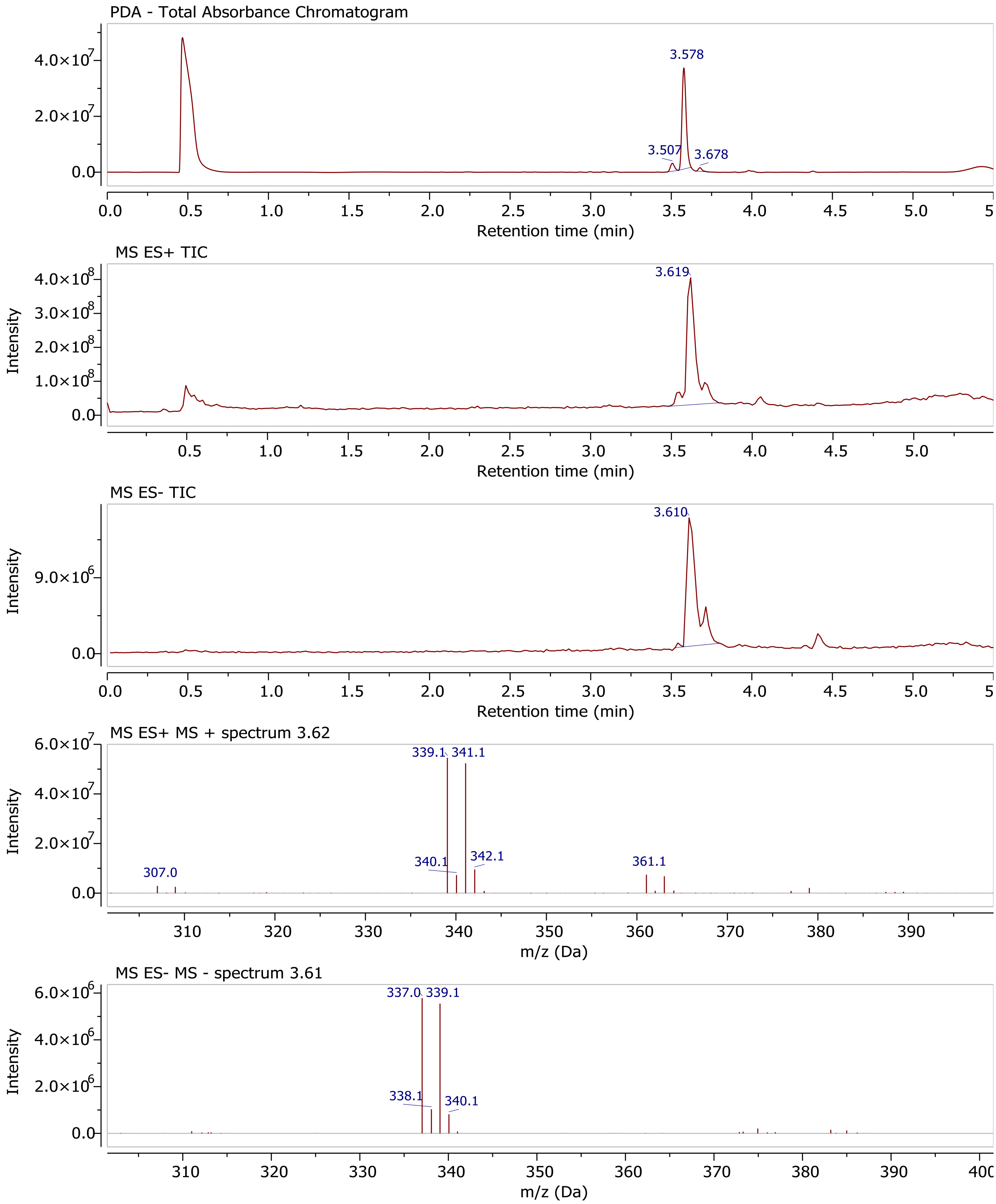

Supplementary figure **1466**. UPLC-MS analysis for compound **PHP-4919**

Chemical Formula: $C_{20}H_{21}FN_2O_3$
Exact Mass: 356.2

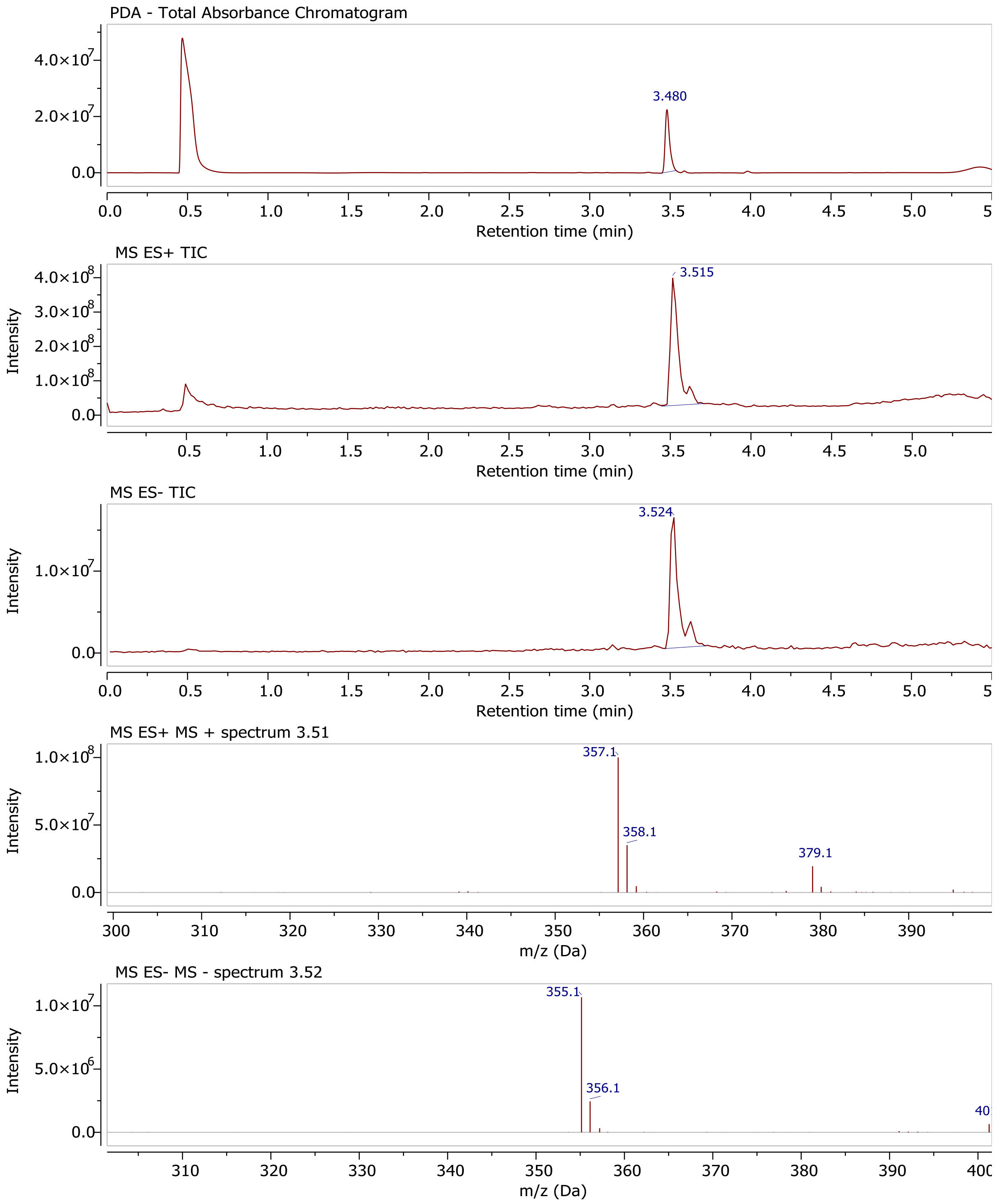

Supplementary figure **1467**. UPLC-MS analysis for compound **PHP-5489**

Chemical Formula: $C_{22}H_{24}N_2O_4$
Exact Mass: 380.2

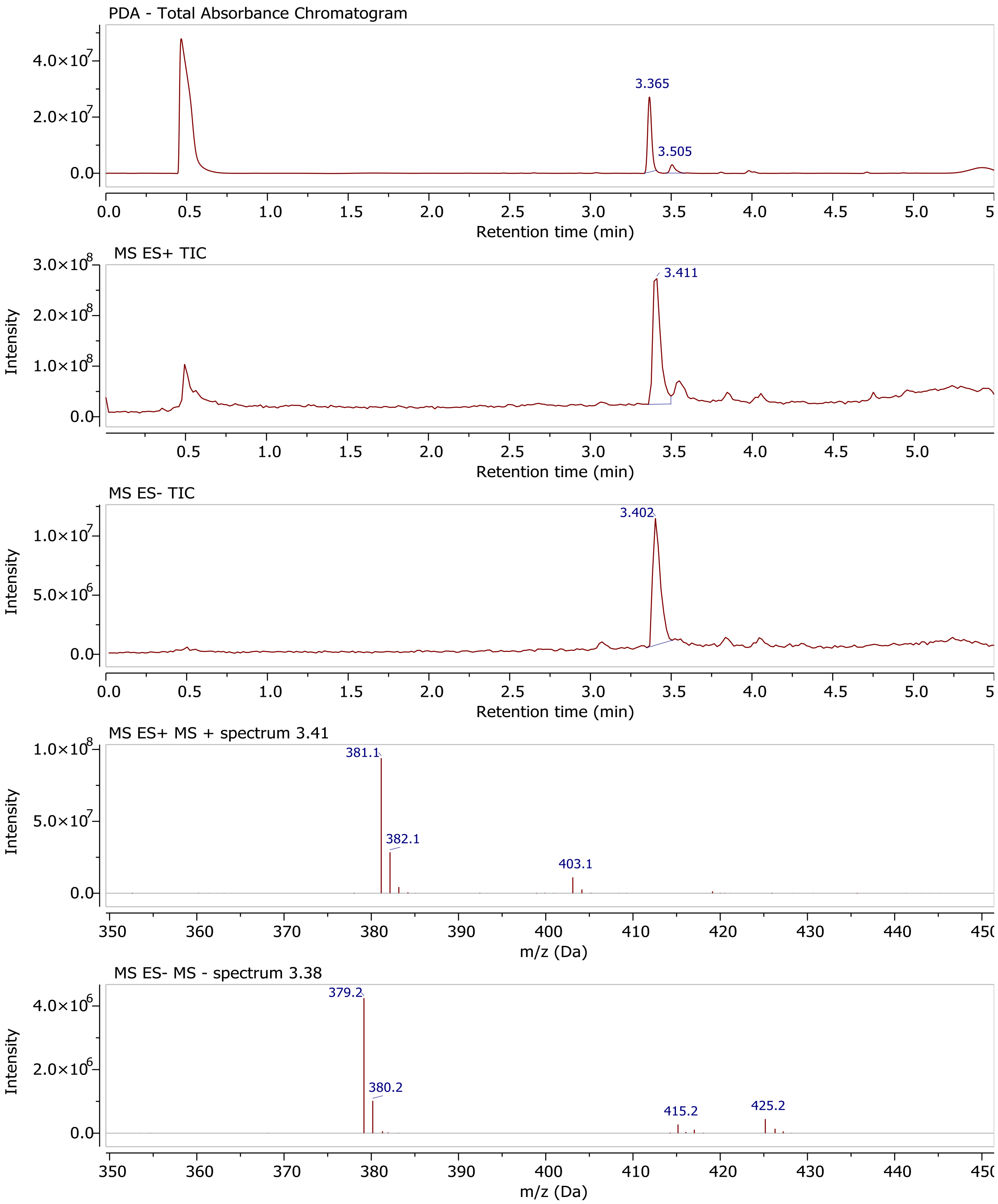

Supplementary figure **1468**. UPLC-MS analysis for compound **PHP-5648**

Chemical Formula: $C_{20}H_{21}FN_2O_2$
Exact Mass: 340.2

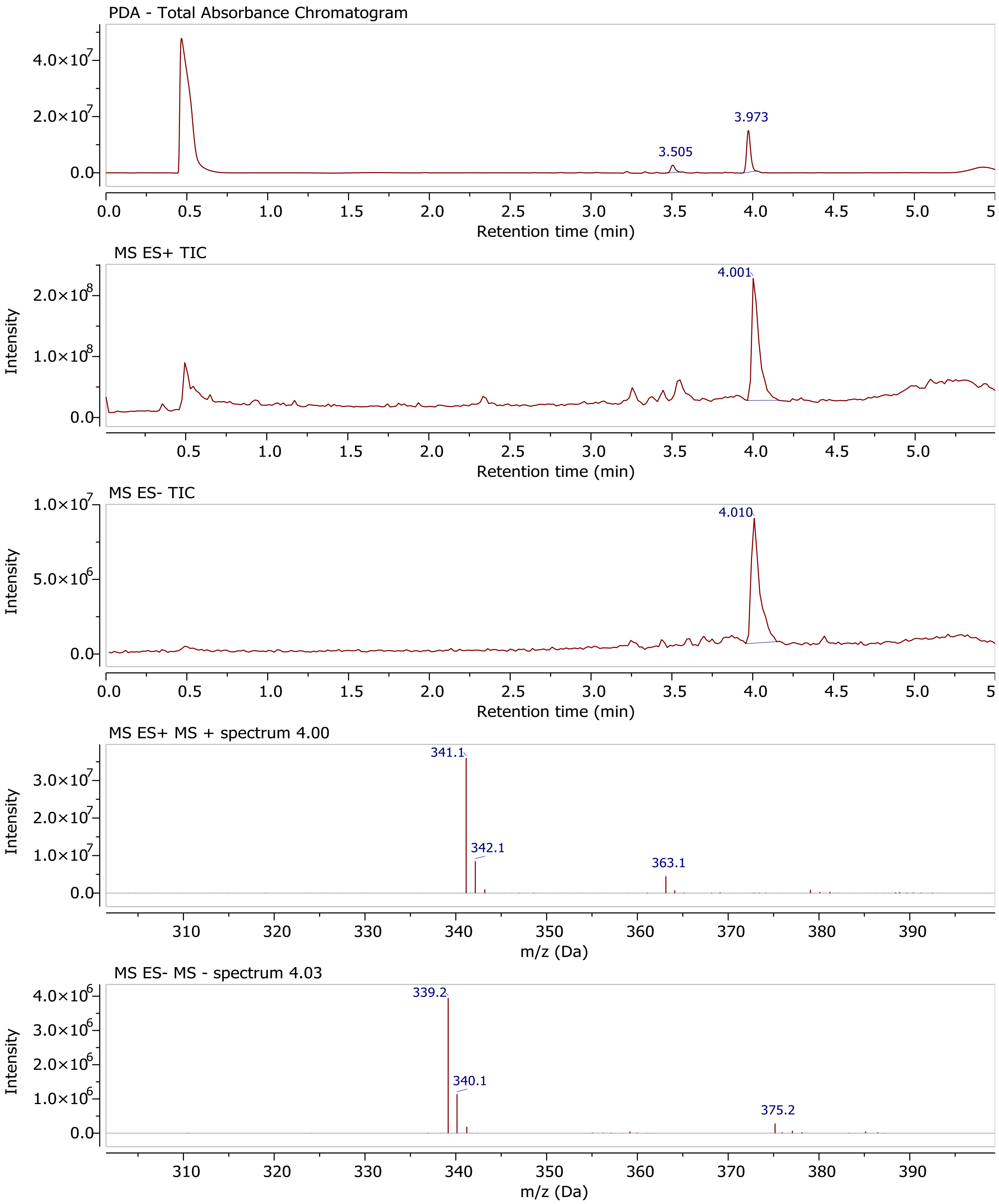

Supplementary figure **1469**. UPLC-MS analysis for compound **PHP-5807**

Chemical Formula: $C_{22}H_{20}N_2O_3S$
Exact Mass: 392.1

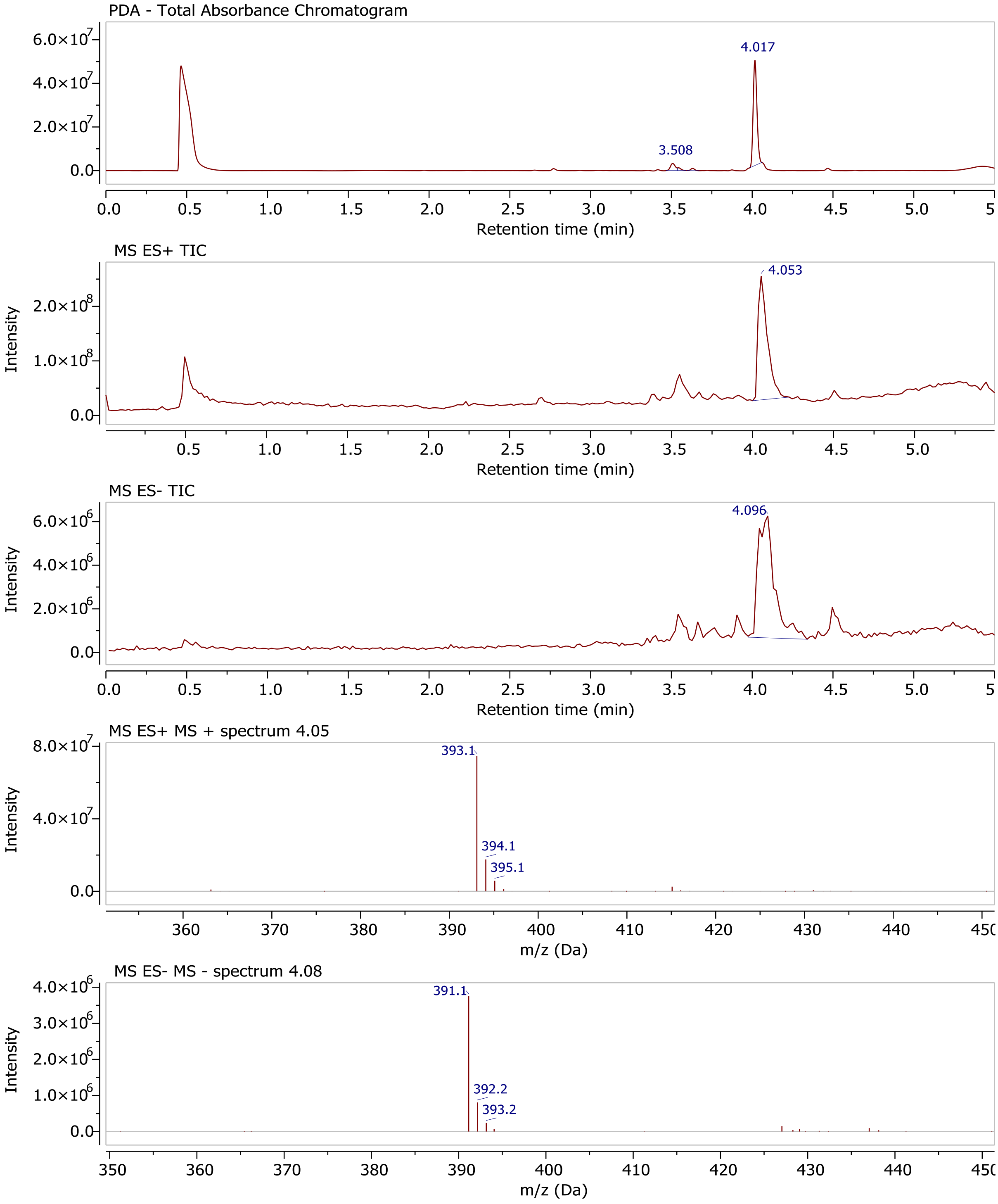

Supplementary figure **1470**. UPLC-MS analysis for compound **PHP-5649**

Chemical Formula: $C_{20}H_{21}FN_2O_2$
Exact Mass: 340.2

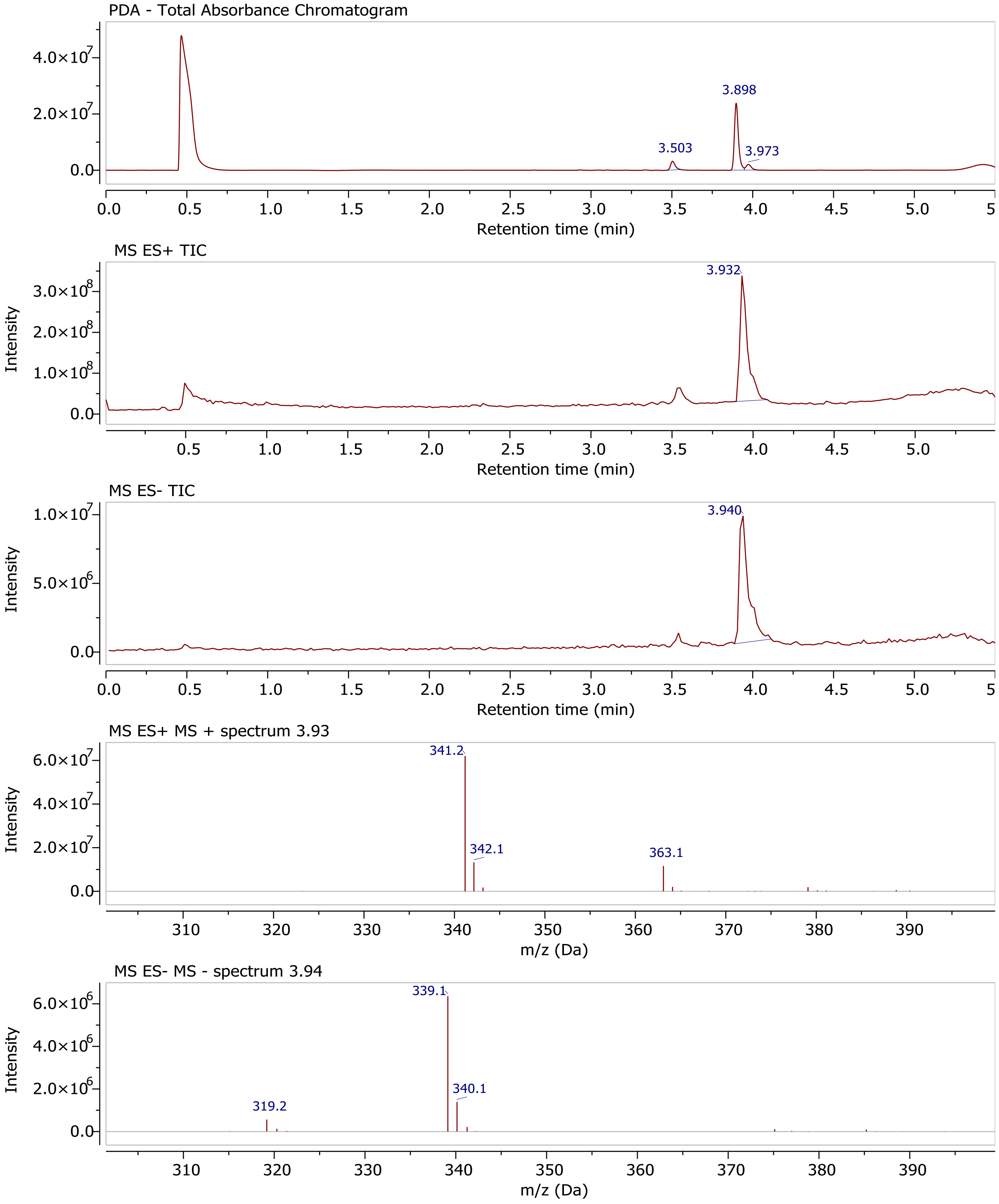

Supplementary figure **1471**. UPLC-MS analysis for compound **PHP-5808**

Chemical Formula: $C_{21}H_{24}N_2O_3$
Exact Mass: 352.2

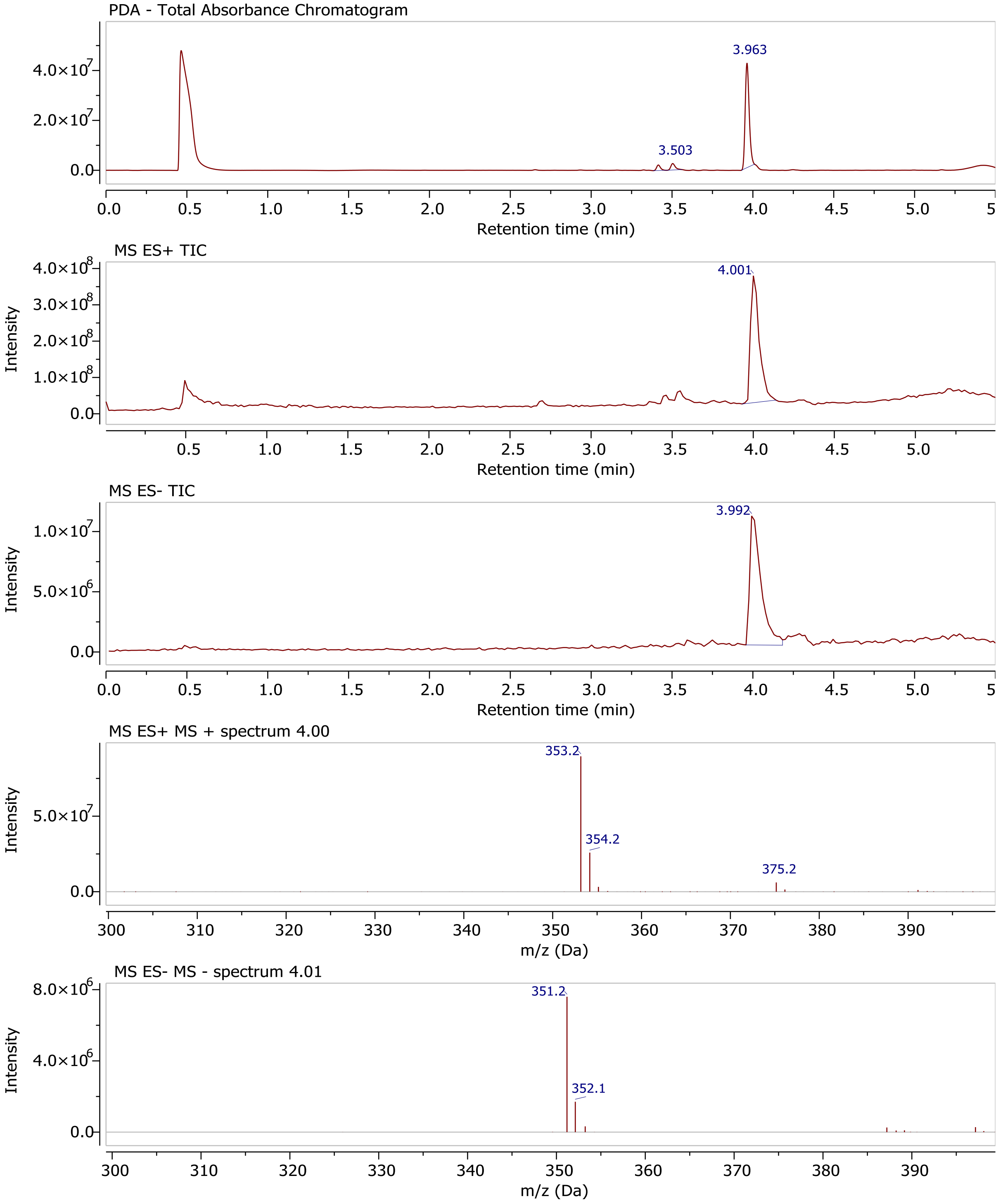

Supplementary figure **1472**. UPLC-MS analysis for compound **PHP-5650**

Chemical Formula: $C_{19}H_{28}N_2O_2$
Exact Mass: 316.2

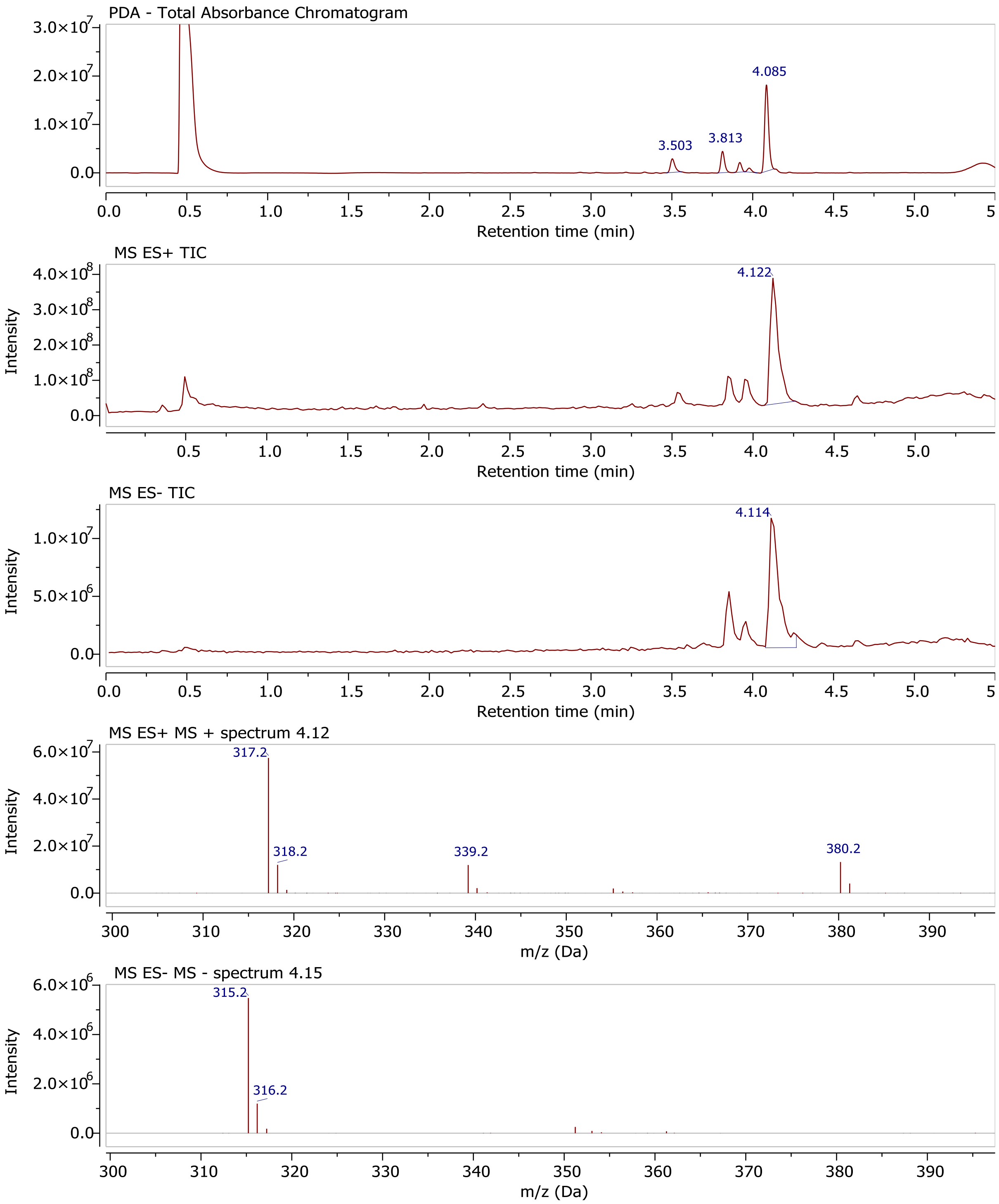

Supplementary figure **1473**. UPLC-MS analysis for compound **PHP-5809**

Chemical Formula: $C_{23}H_{17}F_3N_2O_3$
Exact Mass: 426.1

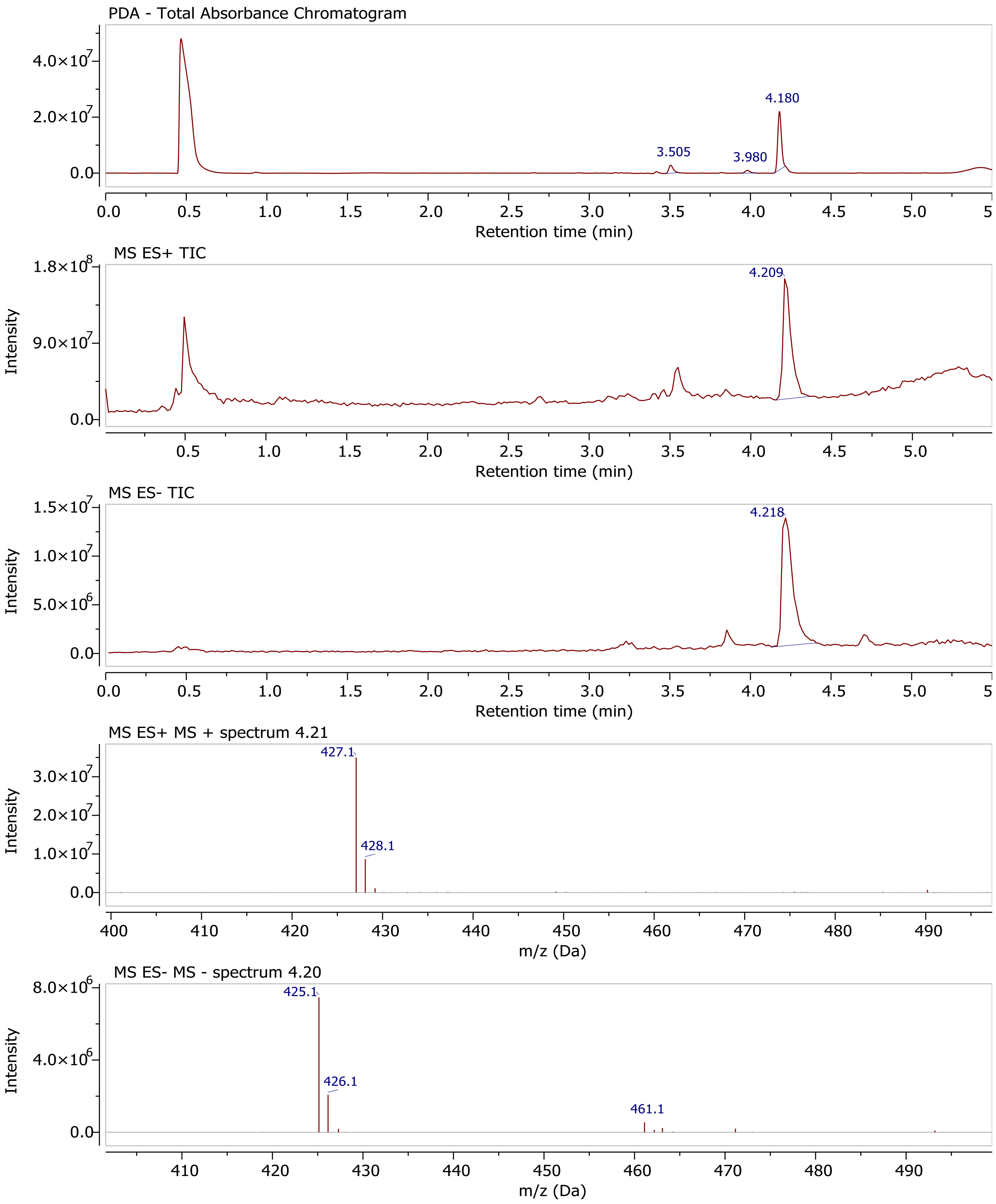

Supplementary figure **1474**. UPLC-MS analysis for compound **PHP-5651**

Chemical Formula: $C_{21}H_{24}N_2O_3$
Exact Mass: 352.2

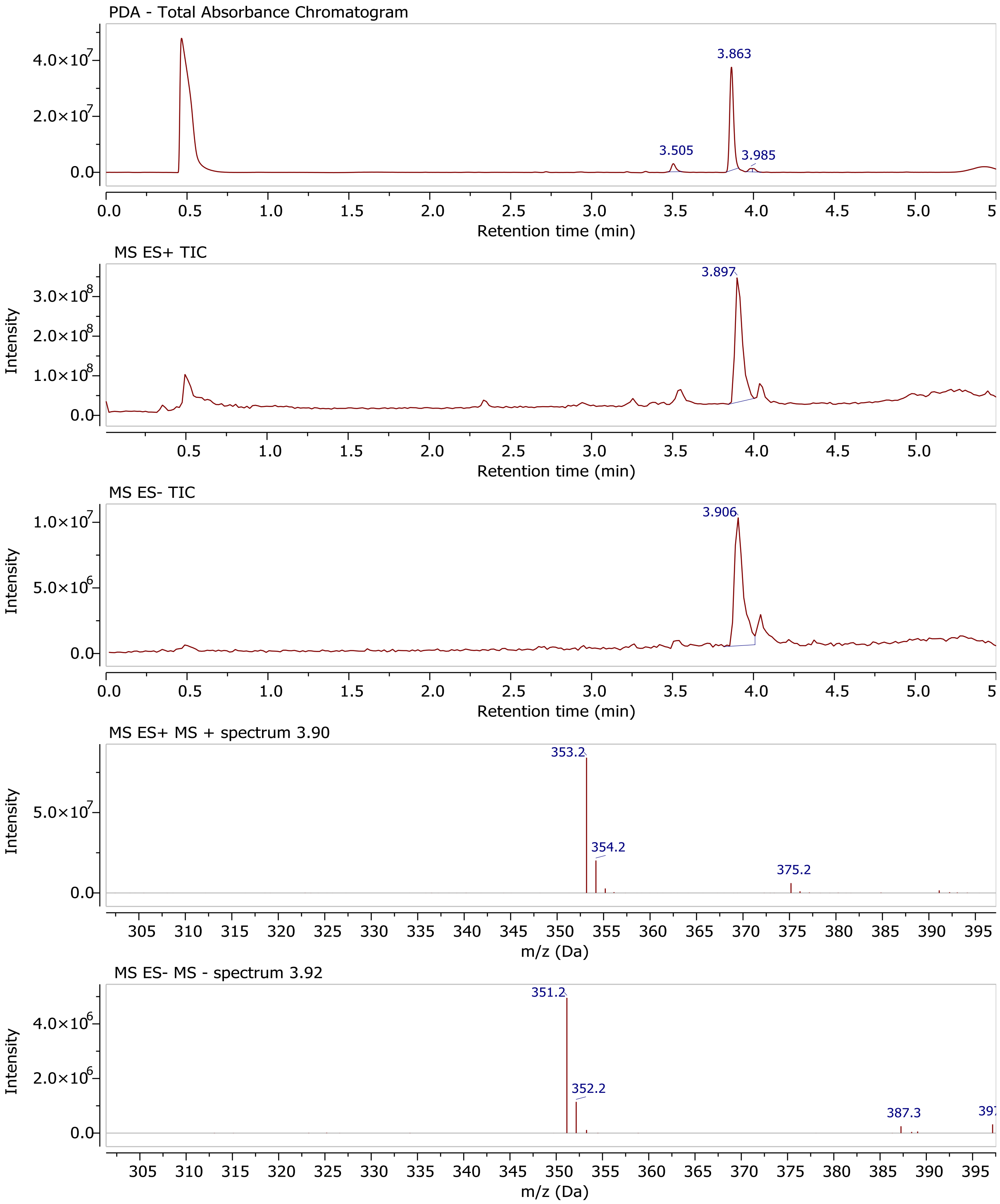

Supplementary figure **1475**. UPLC-MS analysis for compound **PHP-5814**

Chemical Formula: $C_{30}H_{34}N_2O_3$
Exact Mass: 470.3

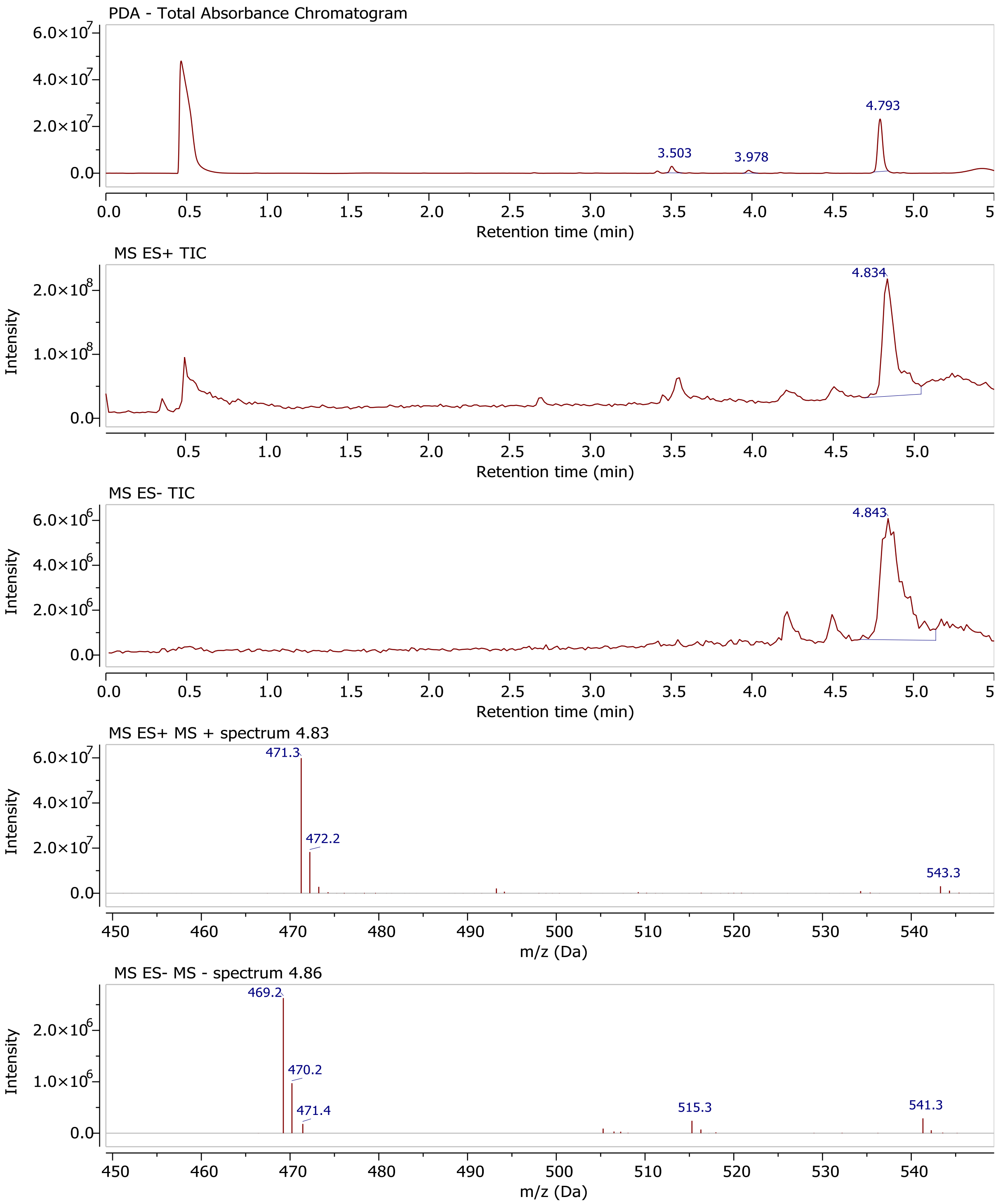

Supplementary figure **1476**. UPLC-MS analysis for compound **PHP-5652**

Chemical Formula: $C_{18}H_{26}N_2O_3$
Exact Mass: 318.2

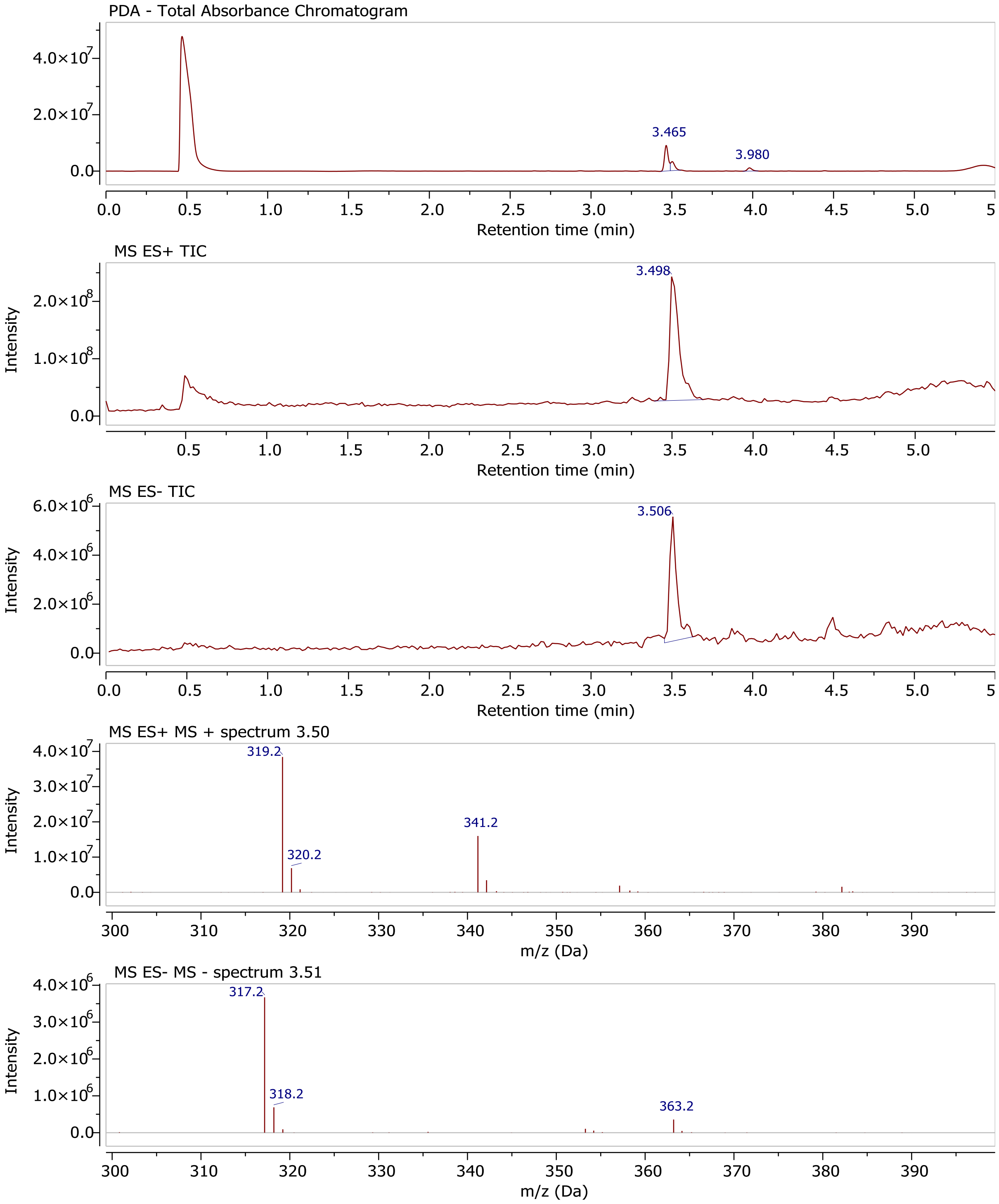

Supplementary figure **1477**. UPLC-MS analysis for compound **PHP-5817**

Chemical Formula: $C_{25}H_{24}N_2O_3$
Exact Mass: 400.2

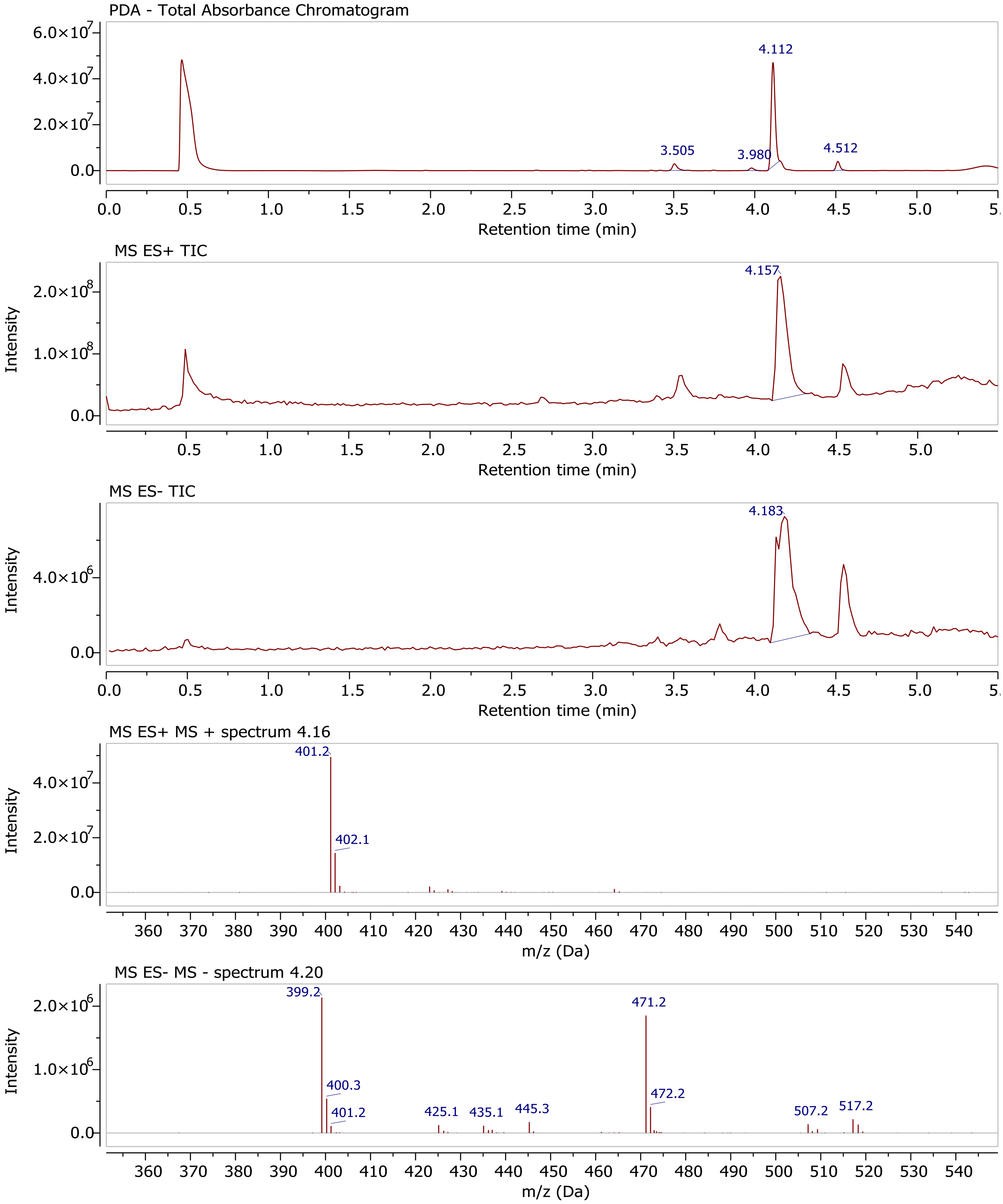

Supplementary figure **1478**. UPLC-MS analysis for compound **PHP-5656**

Chemical Formula: $C_{19}H_{18}F_2N_2O_3$
Exact Mass: 360.1

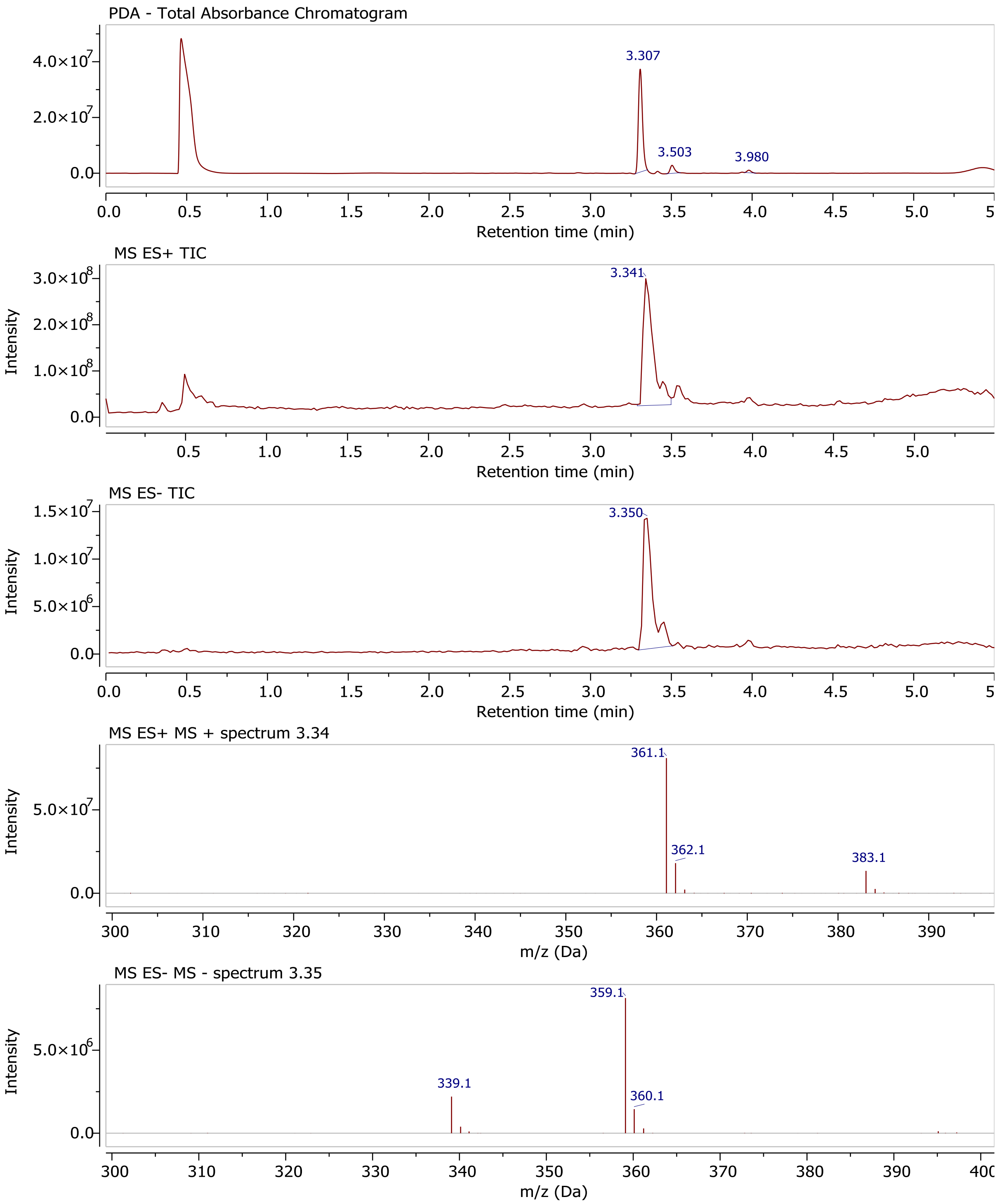

Supplementary figure **1479**. UPLC-MS analysis for compound **PHP-5818**

Chemical Formula: $C_{21}H_{22}N_2O_3$
Exact Mass: 350.2

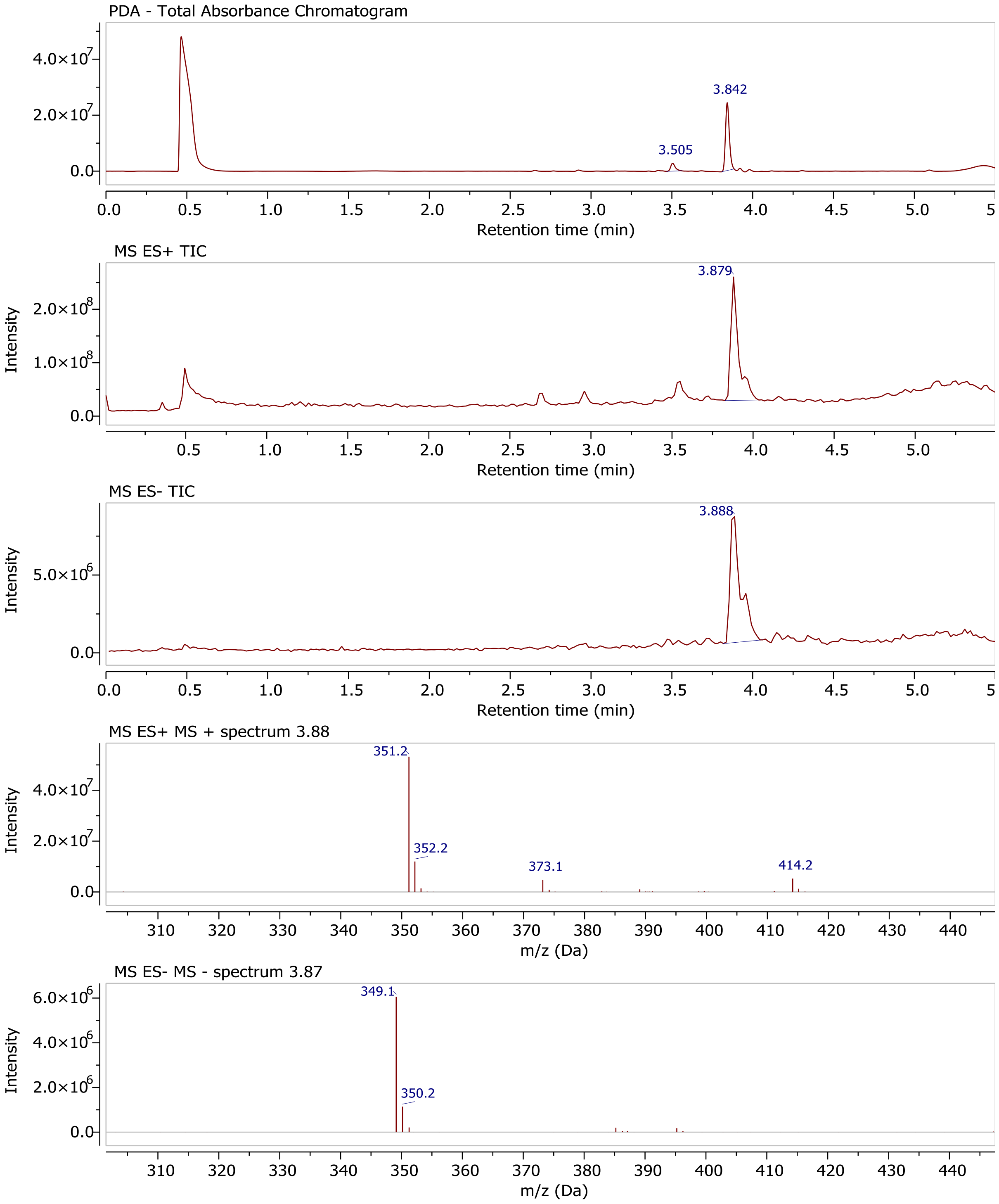

Supplementary figure **1480**. UPLC-MS analysis for compound **PHP-5657**

Chemical Formula: $C_{20}H_{22}N_2O_4$
Exact Mass: 354.2

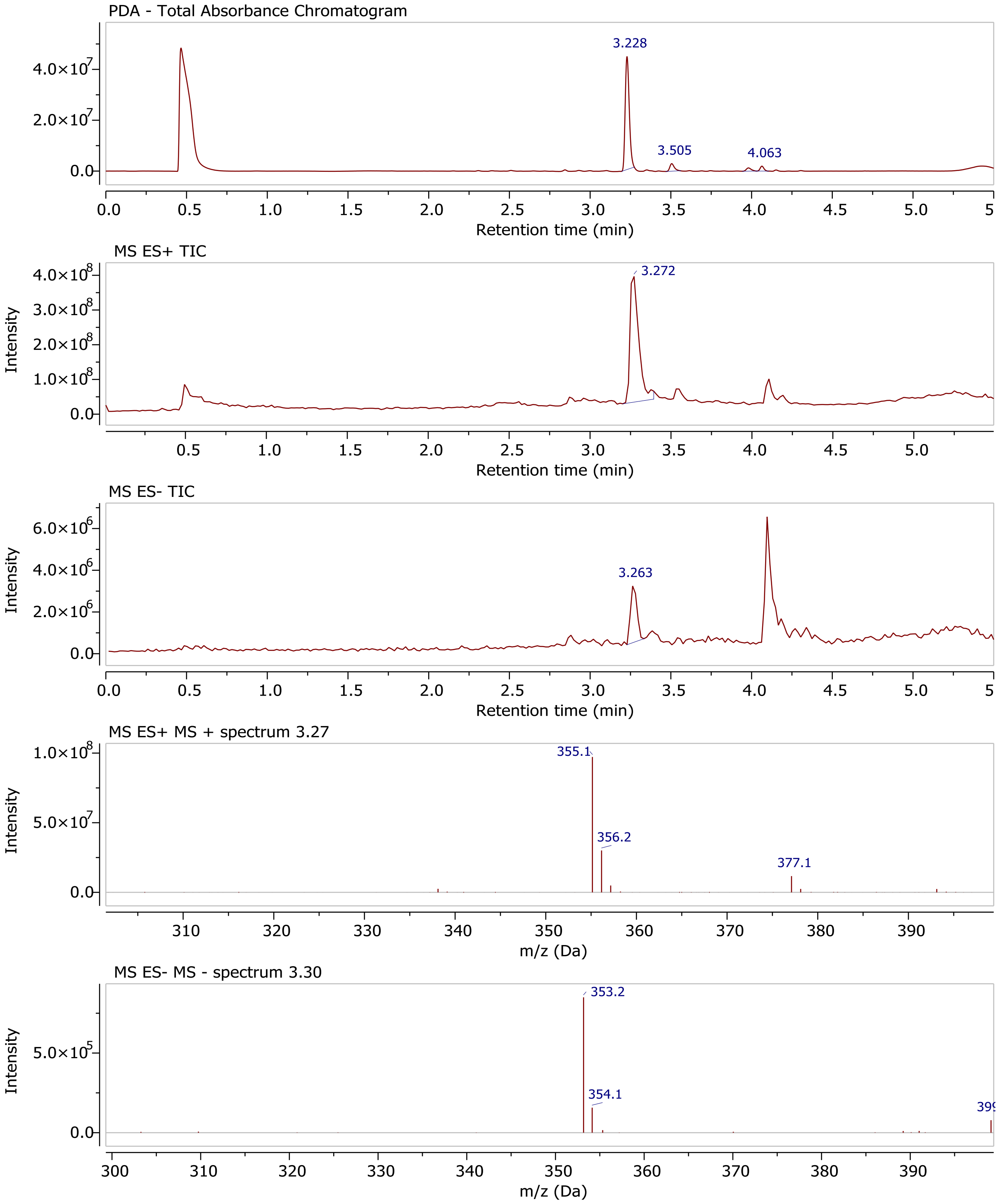

Supplementary figure **1481**. UPLC-MS analysis for compound **PHP-5820**

Chemical Formula: $C_{23}H_{18}F_2N_2O_3$
Exact Mass: 408.1

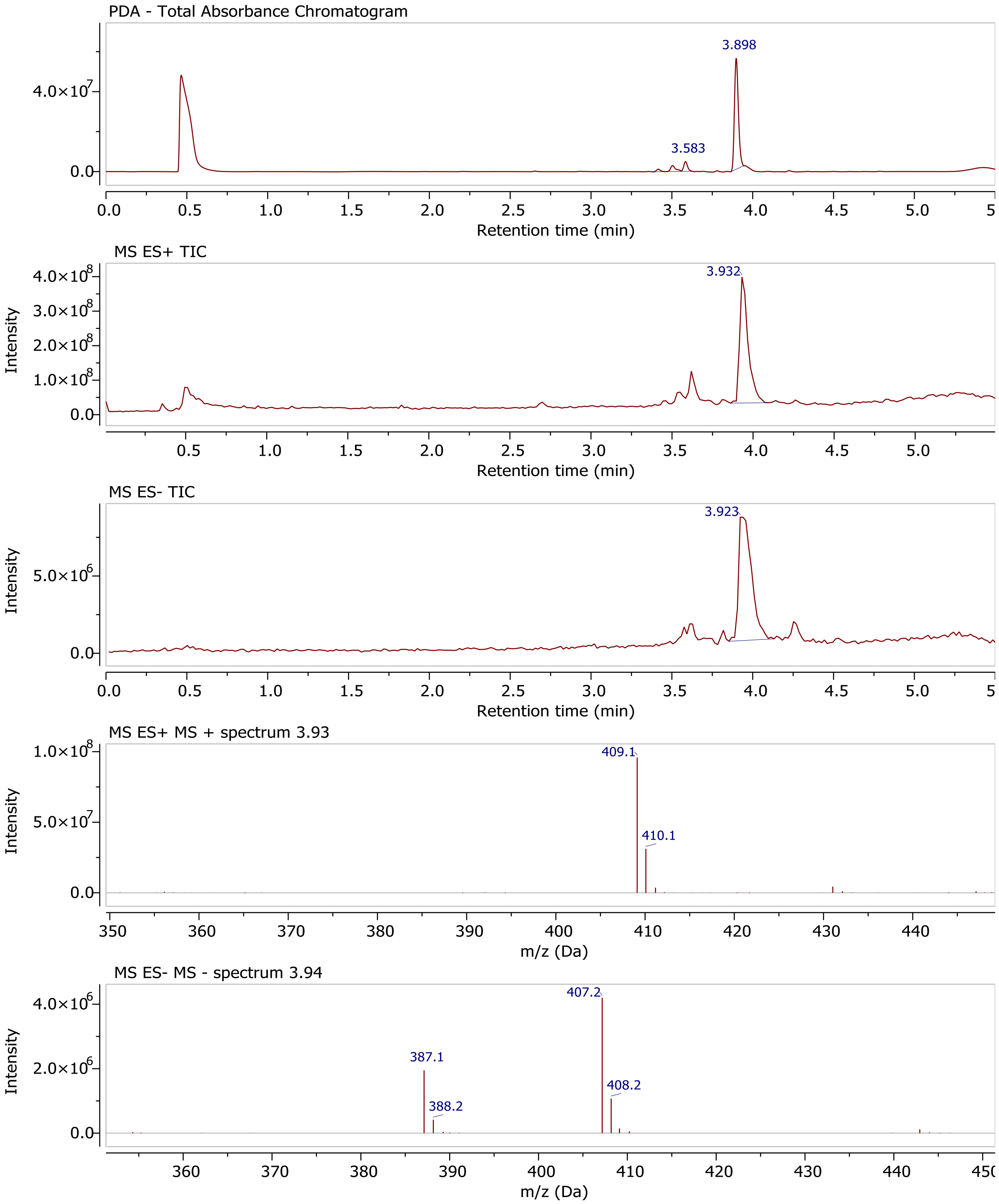

Supplementary figure **1482**. UPLC-MS analysis for compound **PHP-5658**

Chemical Formula: $C_{18}H_{24}N_2O_4$
Exact Mass: 332.2

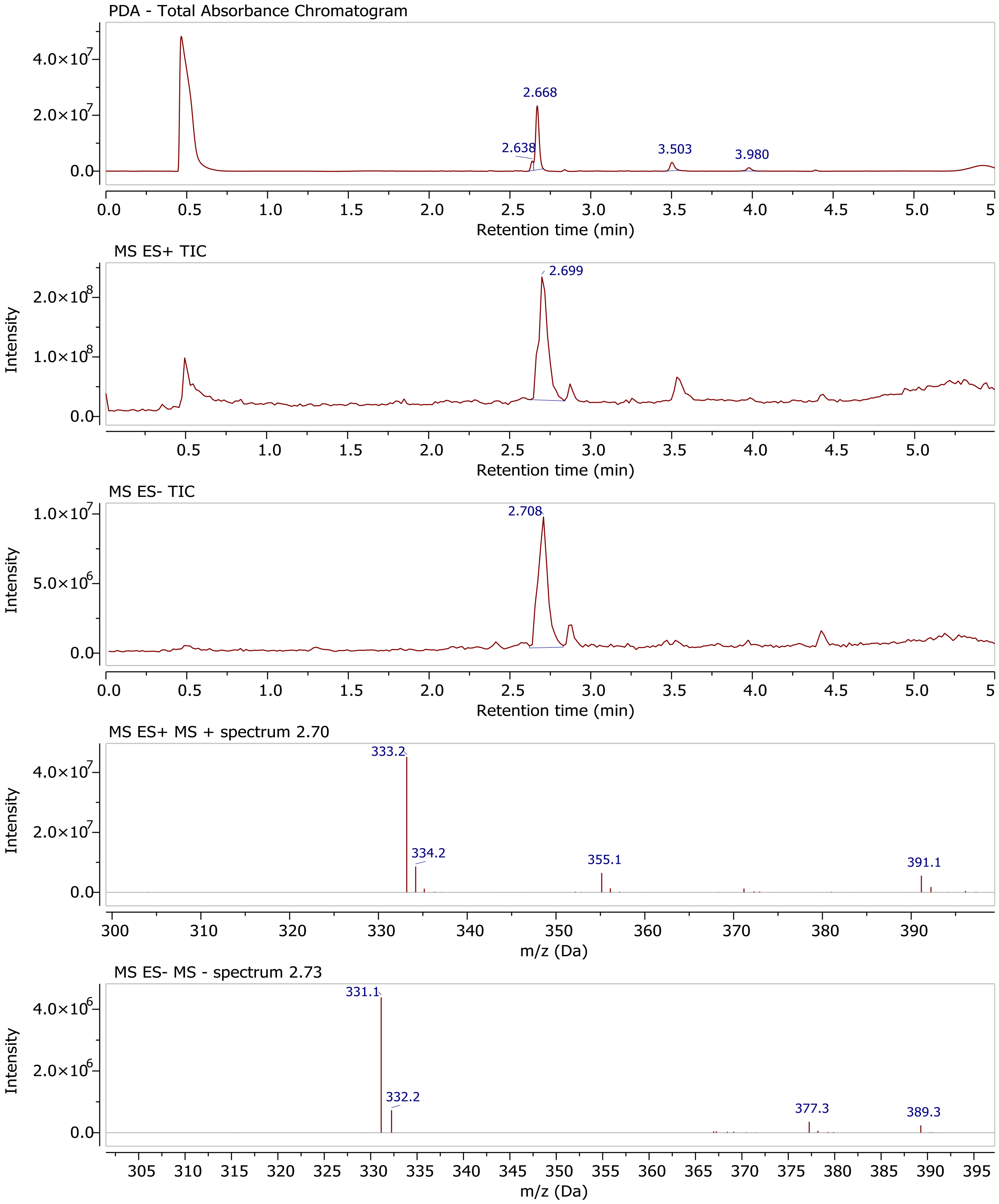

Supplementary figure **1483**. UPLC-MS analysis for compound **PHP-5822**

Chemical Formula: $C_{24}H_{19}F_3N_2O_3$
Exact Mass: 440.1

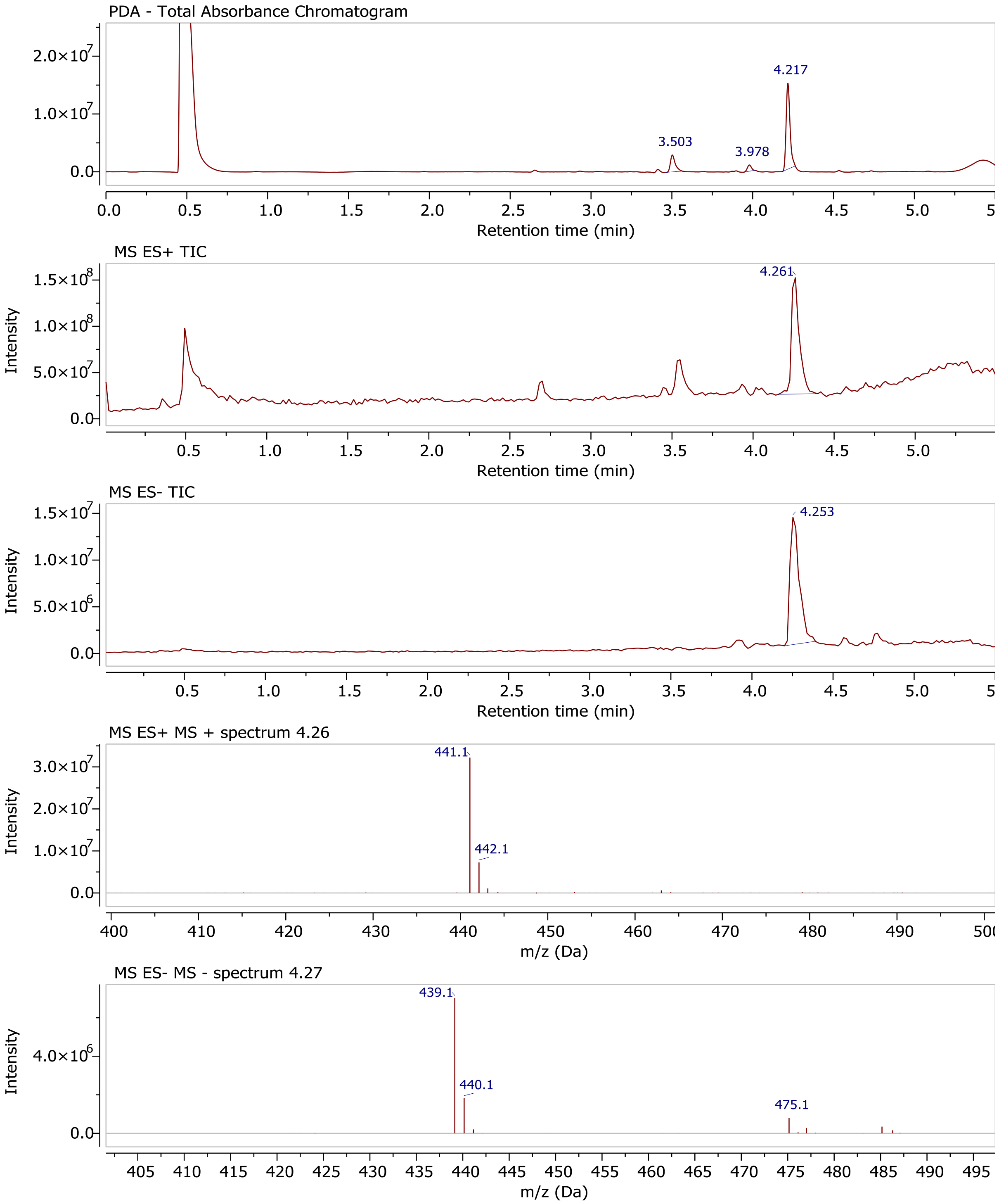

Supplementary figure **1484**. UPLC-MS analysis for compound **PHP-5659**

Chemical Formula: $C_{21}H_{24}N_2O_4$
Exact Mass: 368.2

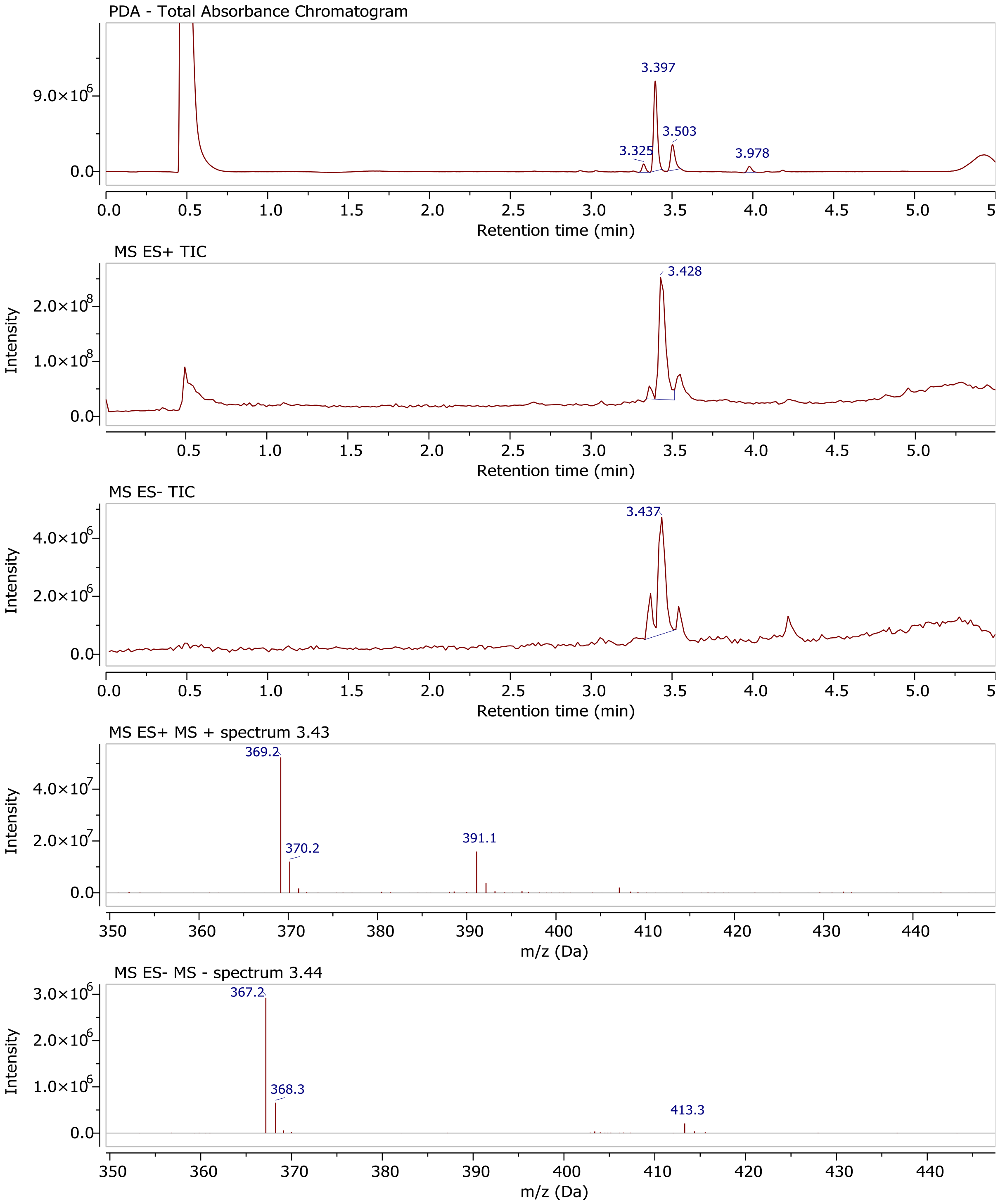

Supplementary figure **1485**. UPLC-MS analysis for compound **PHP-5825**

Chemical Formula: $C_{23}H_{19}ClN_2O_3$
Exact Mass: 406.1

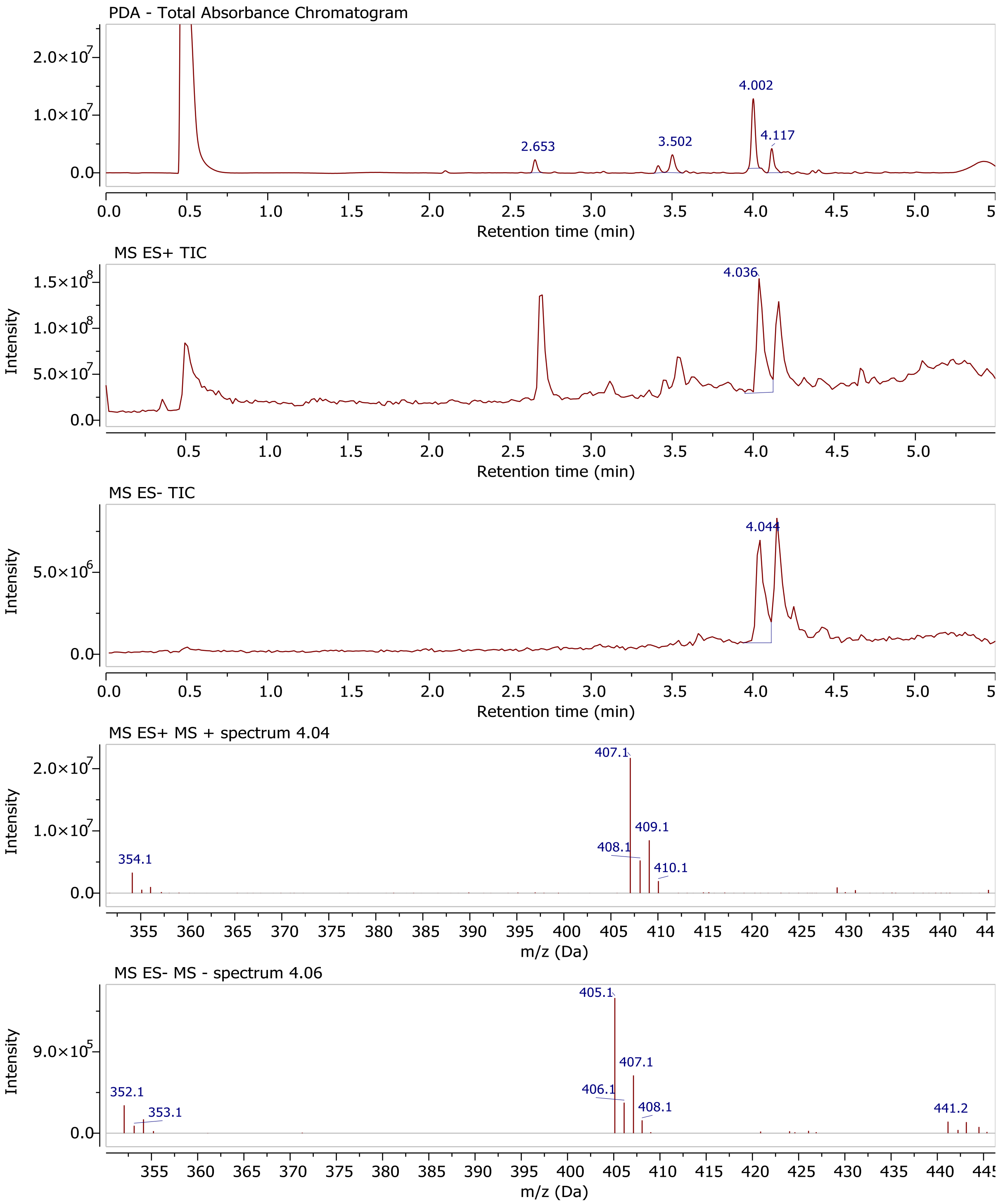

Supplementary figure **1486**. UPLC-MS analysis for compound **PHP-5660**

Chemical Formula: $C_{20}H_{22}N_2O_3$
Exact Mass: 338.2

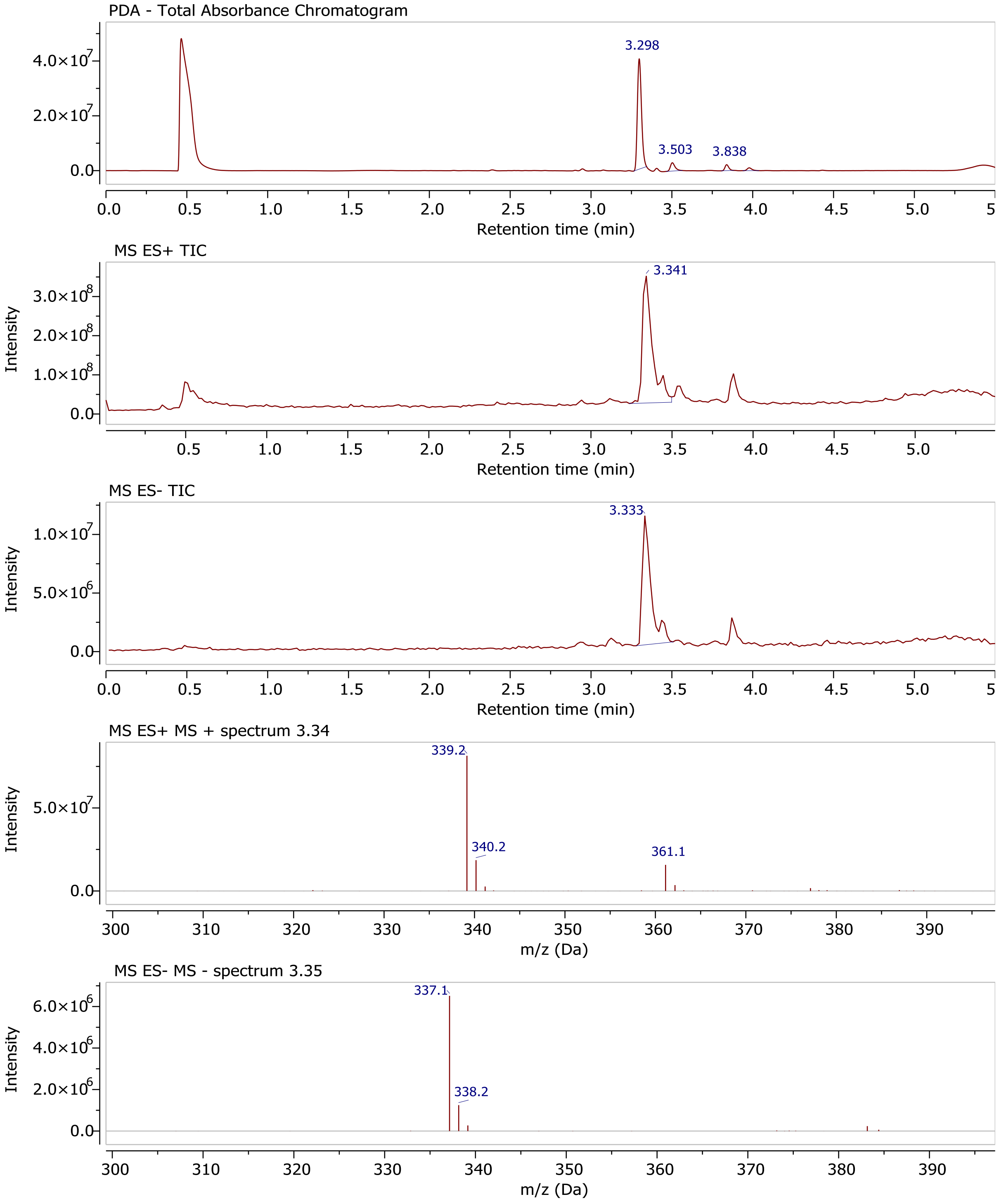

Supplementary figure **1487**. UPLC-MS analysis for compound **PHP-5826**

Chemical Formula: $C_{24}H_{19}F_3N_2O_3$
Exact Mass: 440.1

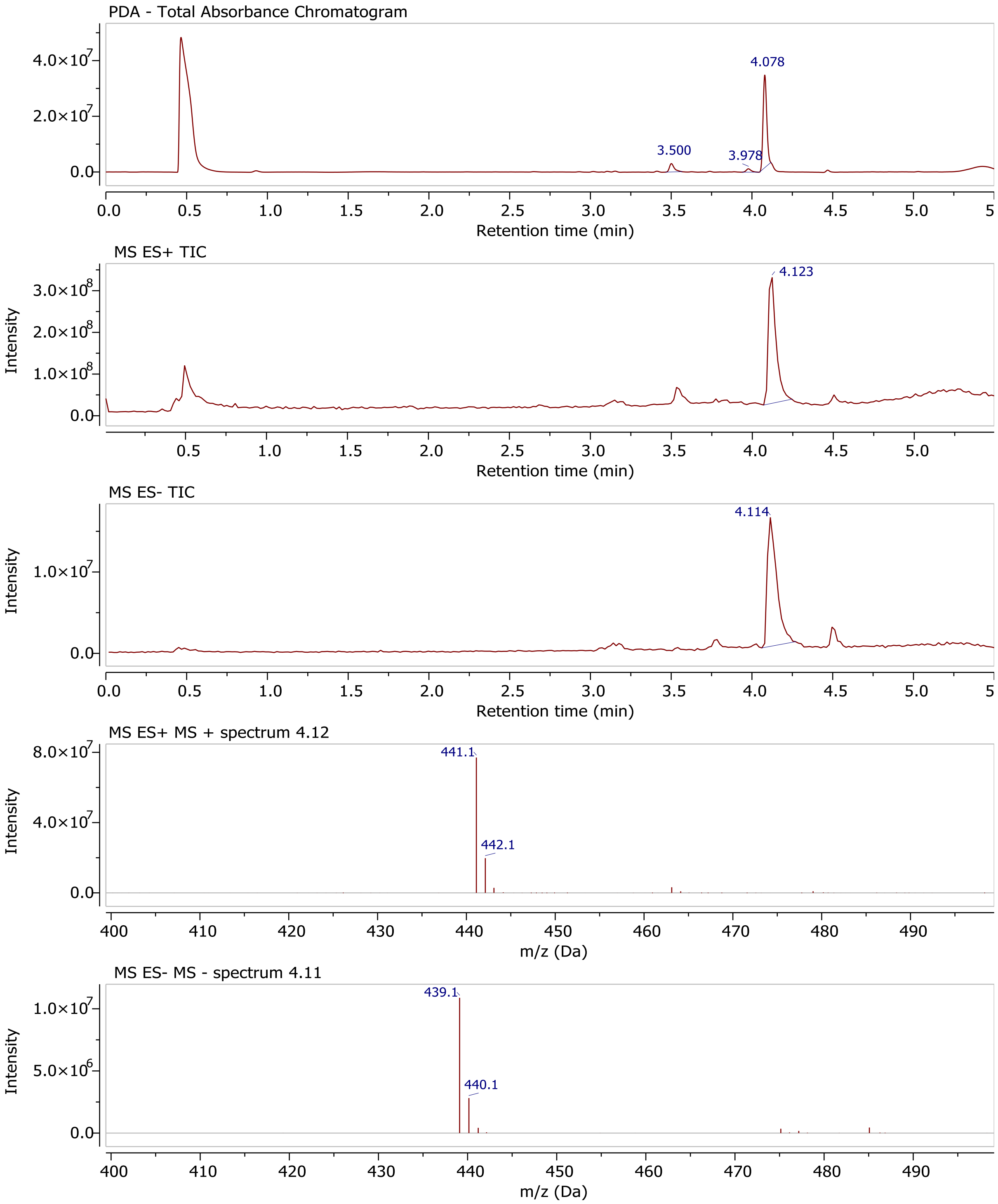

Supplementary figure **1488**. UPLC-MS analysis for compound **PHP-5661**

Chemical Formula: $C_{20}H_{22}N_2O_3$
Exact Mass: 338.2

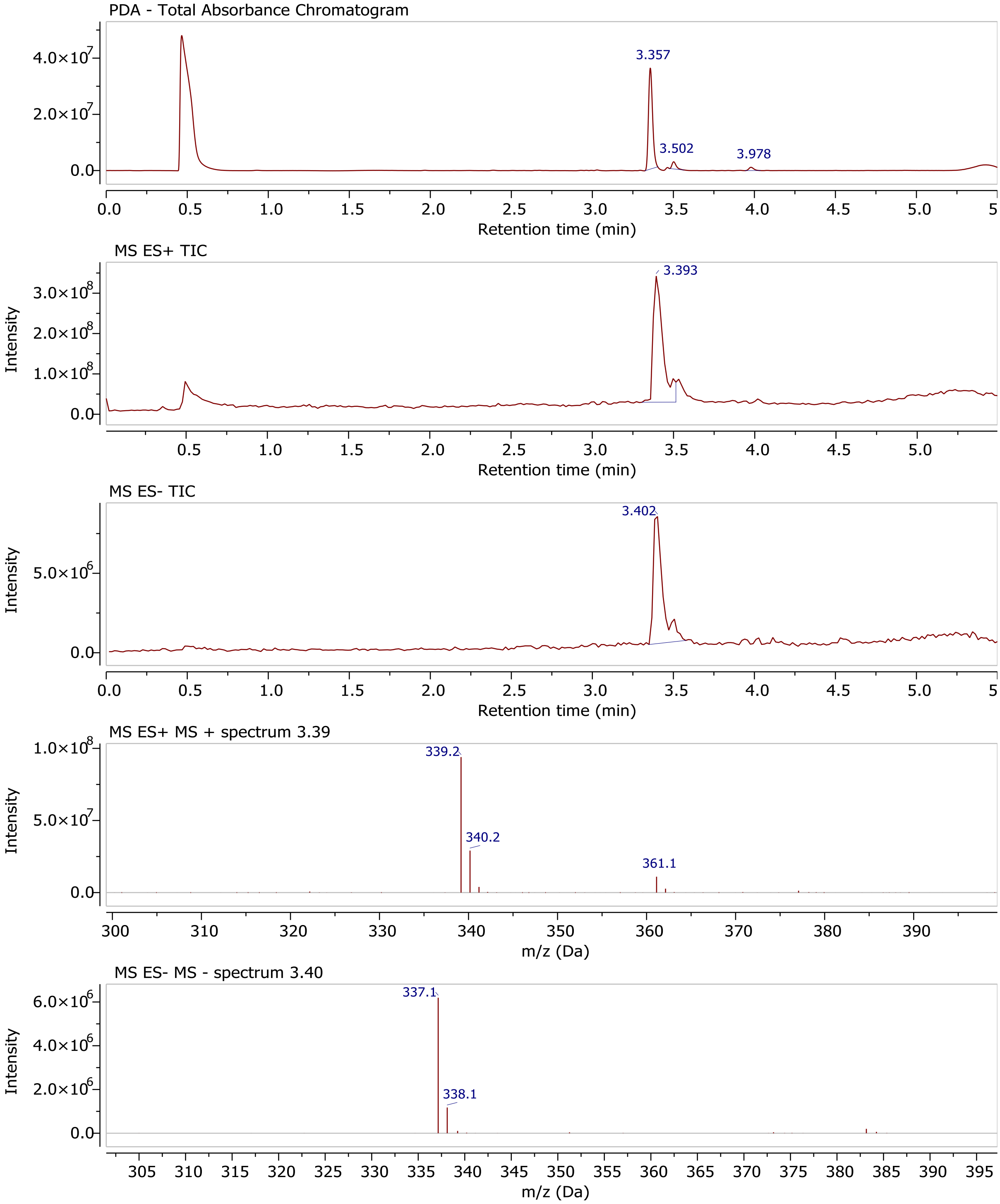

Supplementary figure **1489**. UPLC-MS analysis for compound **PHP-5827**

Chemical Formula: $C_{23}H_{19}FN_2O_3$
Exact Mass: 390.1

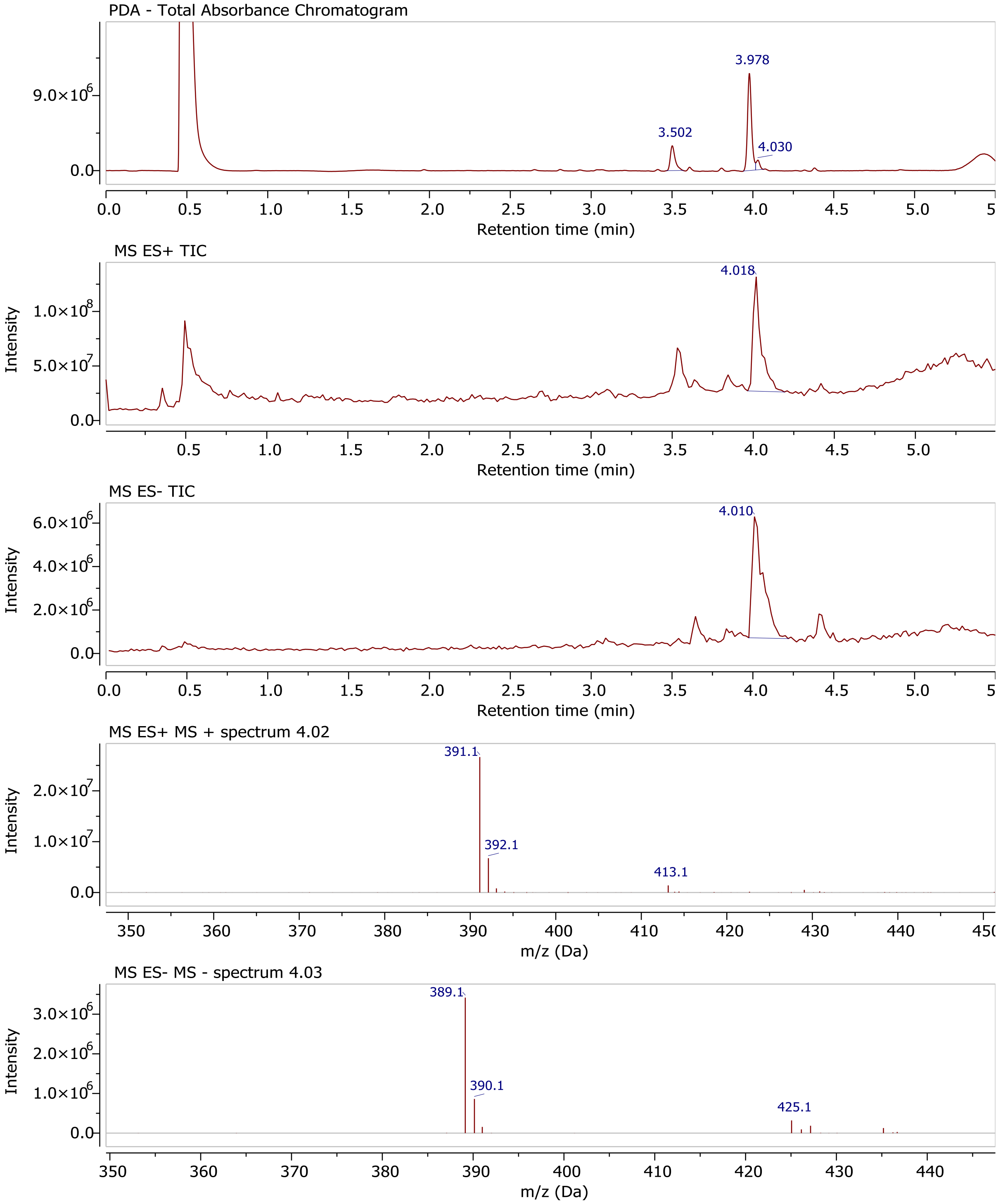

Supplementary figure **1490**. UPLC-MS analysis for compound **PHP-5662**

Chemical Formula: $C_{18}H_{24}N_2O_3$
Exact Mass: 316.2

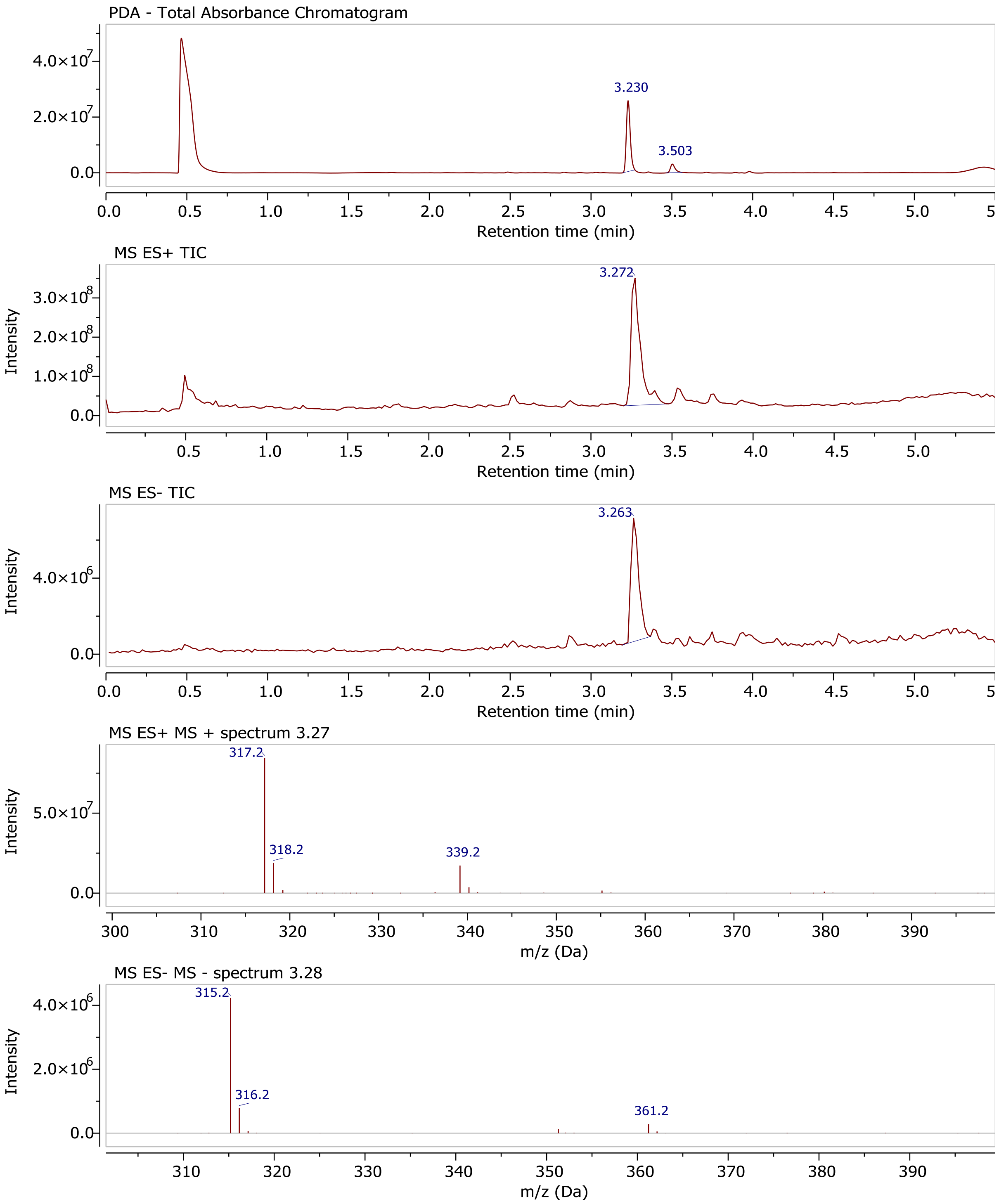

Supplementary figure **1491**. UPLC-MS analysis for compound **PHP-5829**

Chemical Formula: $C_{16}H_{19}N_3O_2$
Exact Mass: 285.1

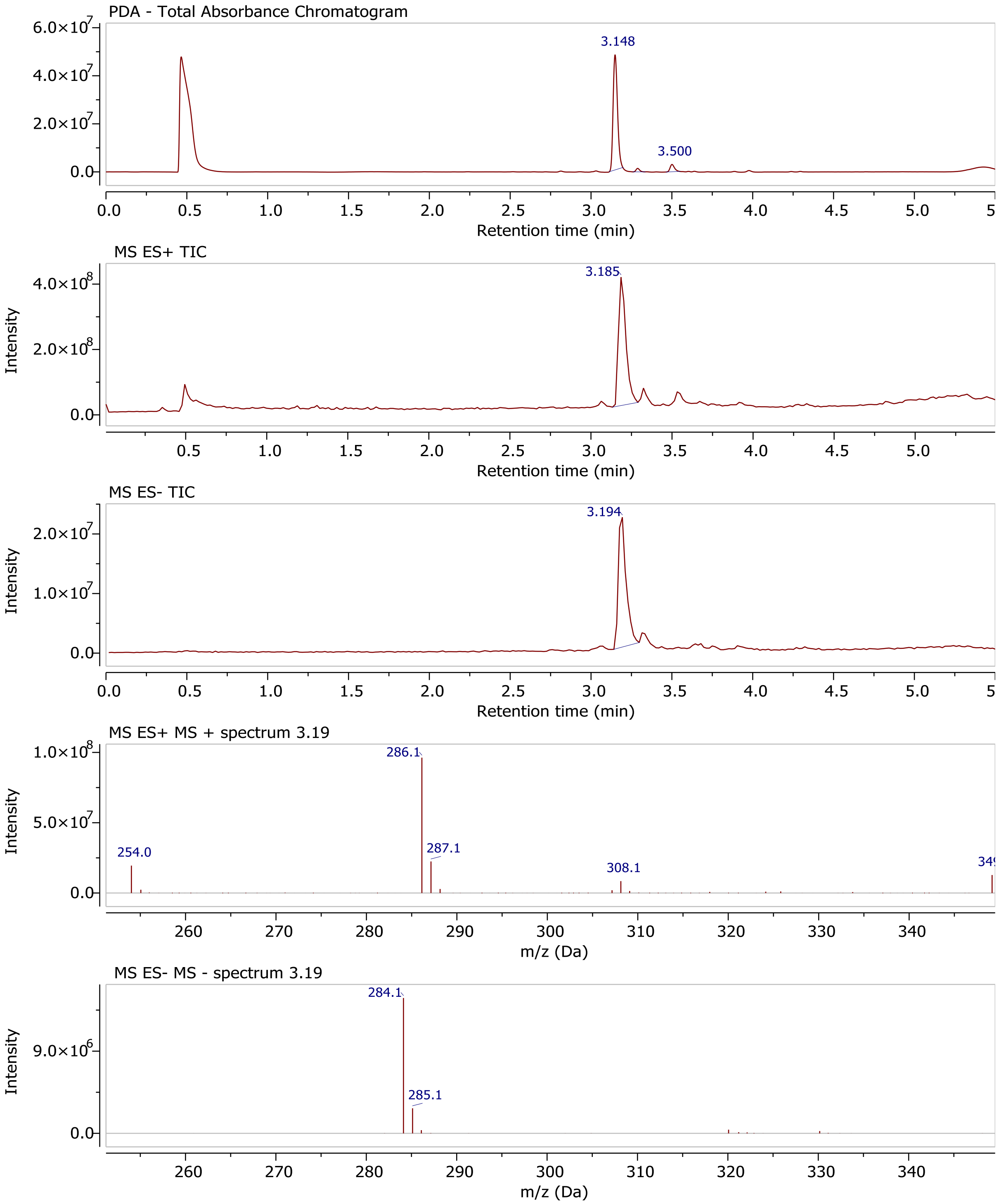

Supplementary figure **1492**. UPLC-MS analysis for compound **PHP-4920**

Chemical Formula: $C_{19}H_{19}FN_2O_2$
Exact Mass: 326.1

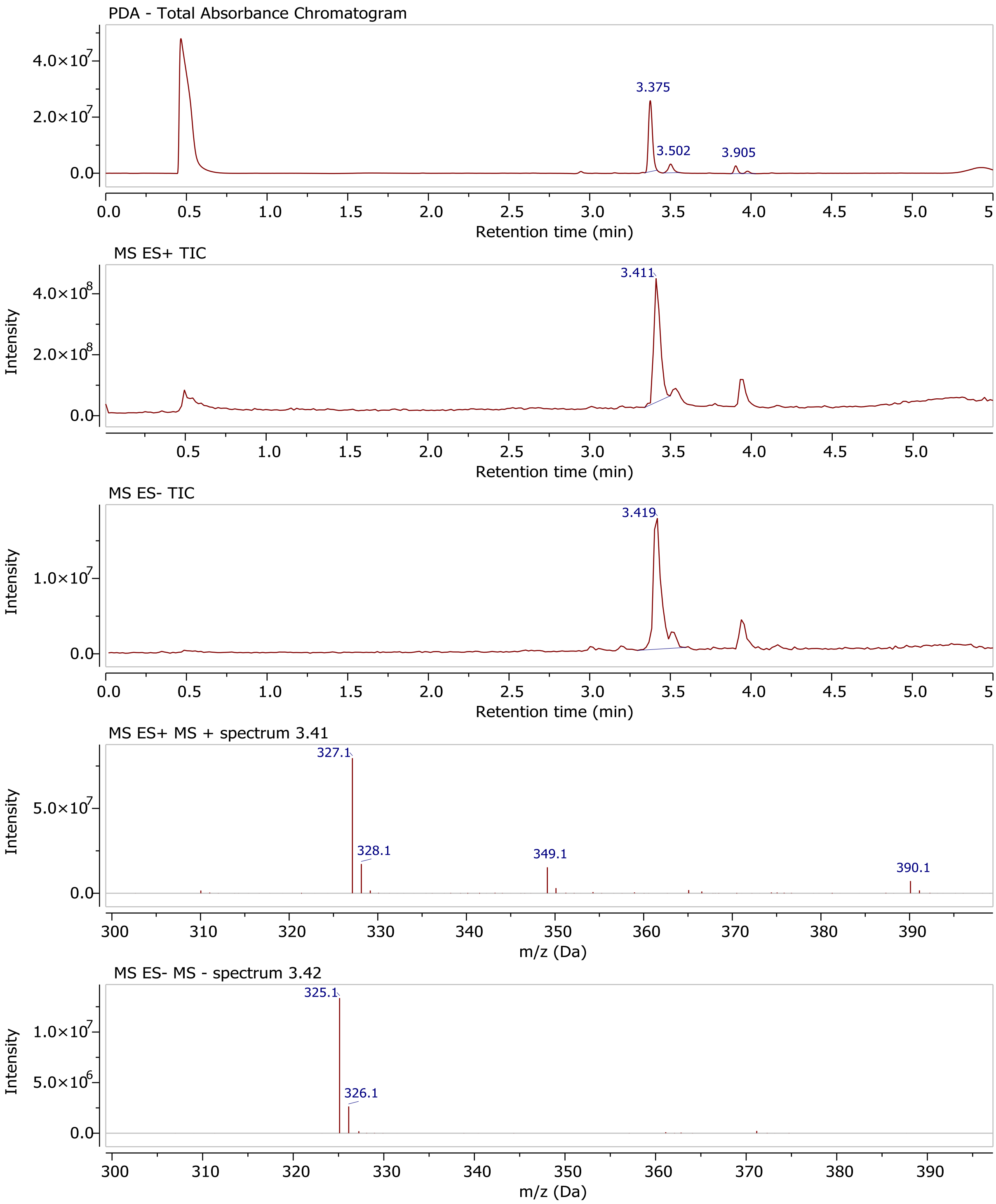

Supplementary figure **1493**. UPLC-MS analysis for compound **PHP-5490**

Chemical Formula: $C_{15}H_{18}FN_3O_4$
Exact Mass: 323.1

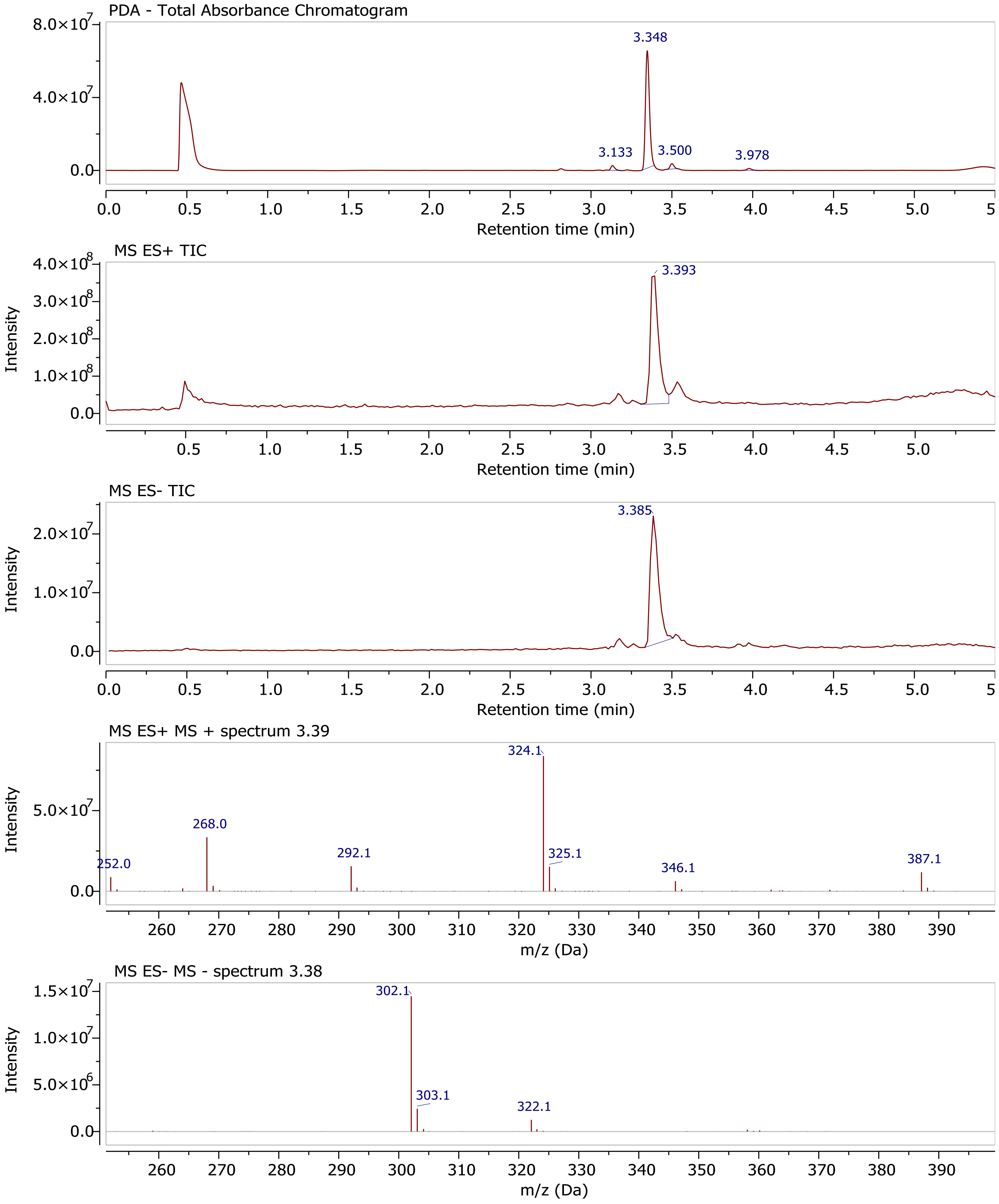

Supplementary figure **1494**. UPLC-MS analysis for compound **PHP-4921**

Chemical Formula: $C_{19}H_{19}FN_2O_2$
Exact Mass: 326.1

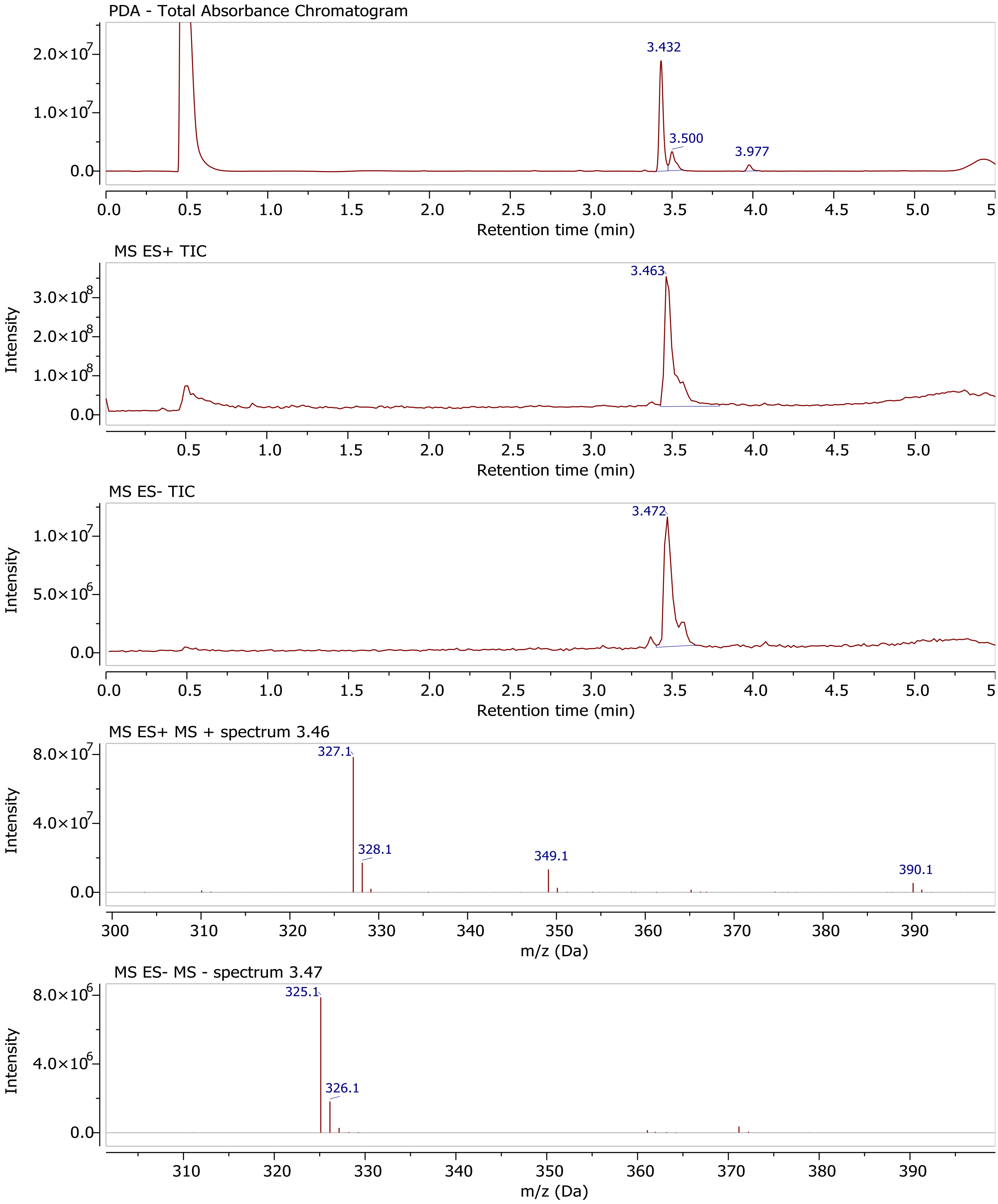

Supplementary figure **1495**. UPLC-MS analysis for compound **PHP-5491**

Chemical Formula: $C_{15}H_{20}N_2O_3$
Exact Mass: 276.1

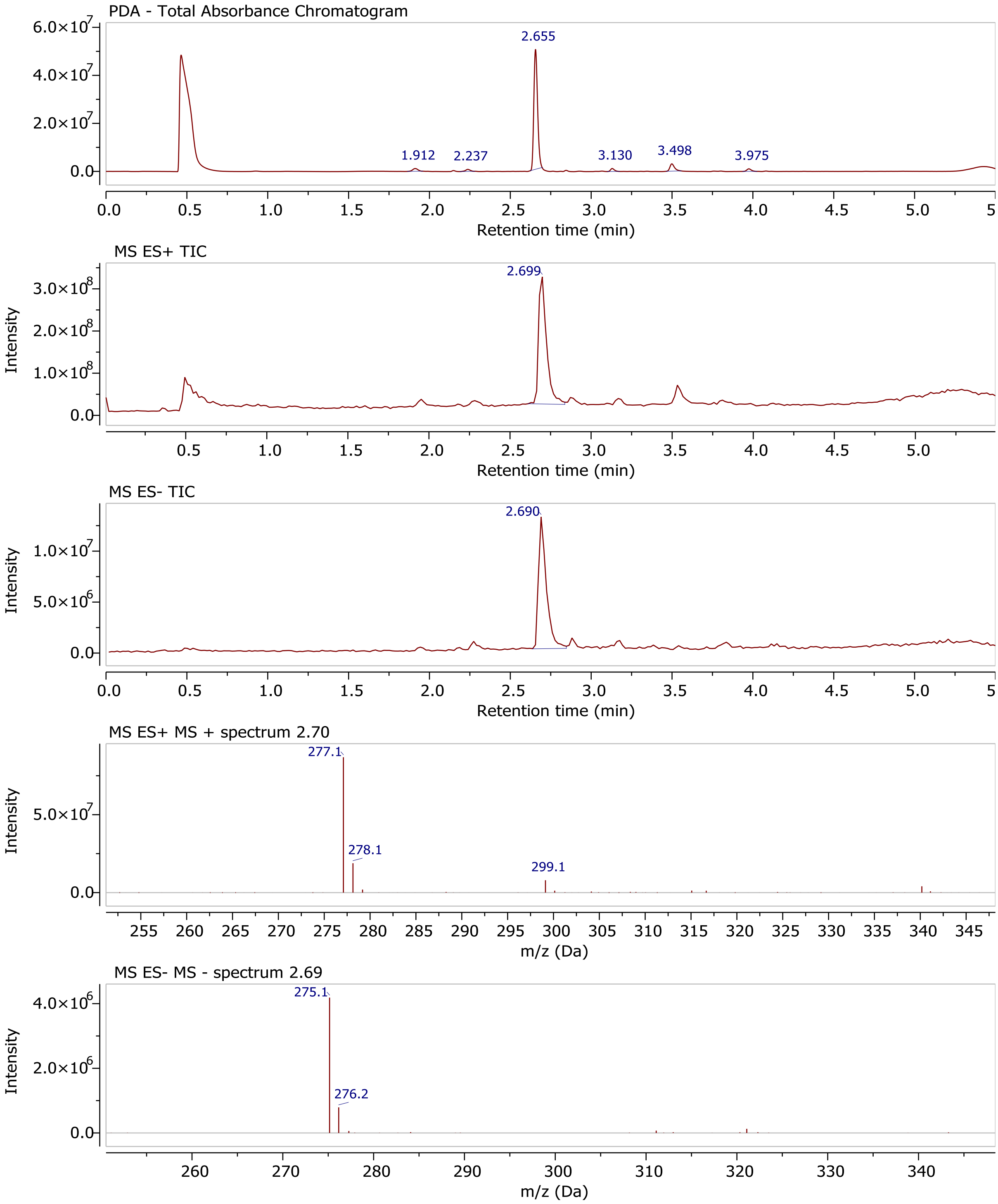

Supplementary figure **1496**. UPLC-MS analysis for compound **PHP-4923**

Chemical Formula: $C_{17}H_{21}FN_2O_2$
Exact Mass: 304.2

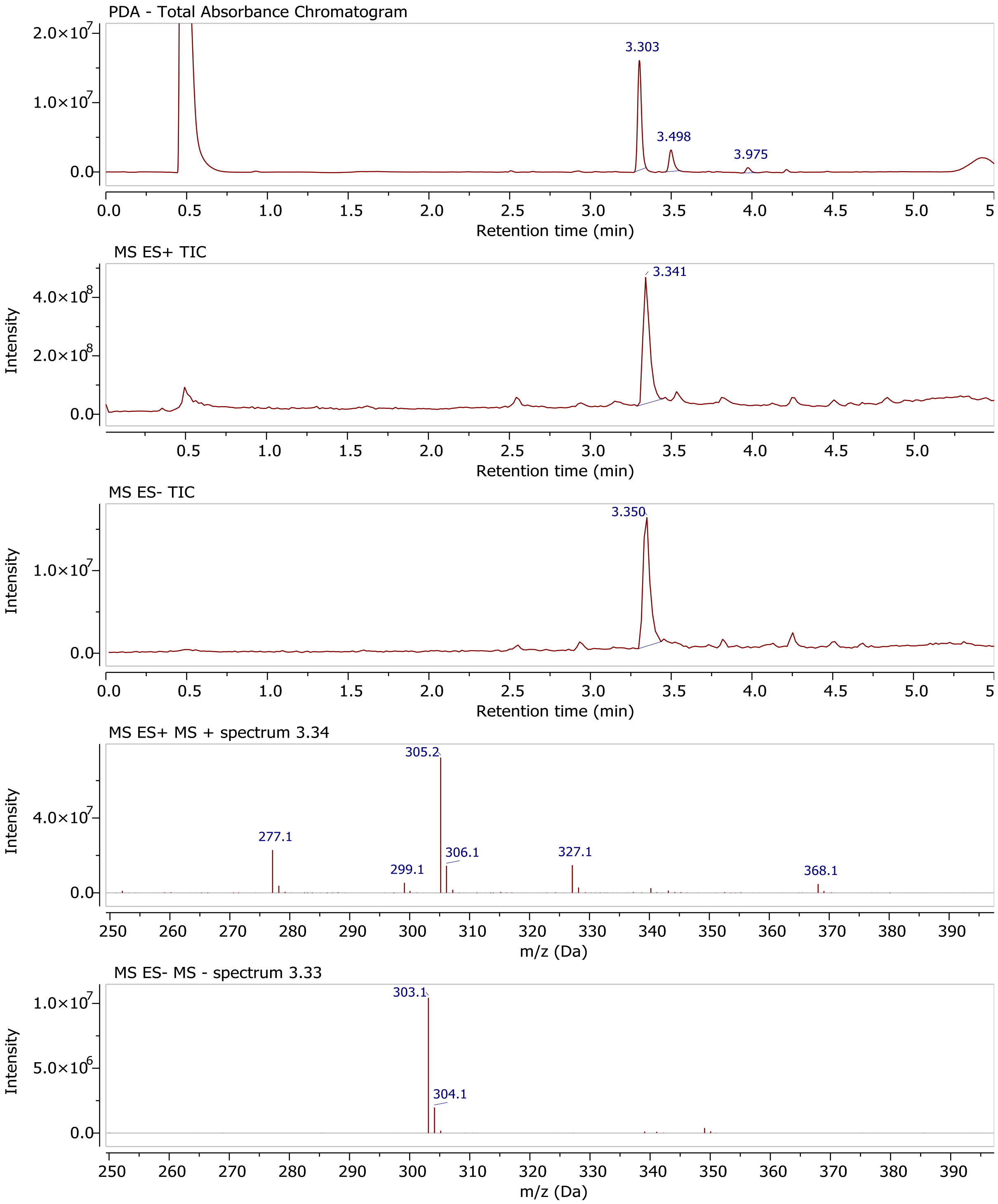

Supplementary figure **1497**. UPLC-MS analysis for compound **PHP-5493**

Chemical Formula: $C_{22}H_{26}N_2O_3$
Exact Mass: 366.2

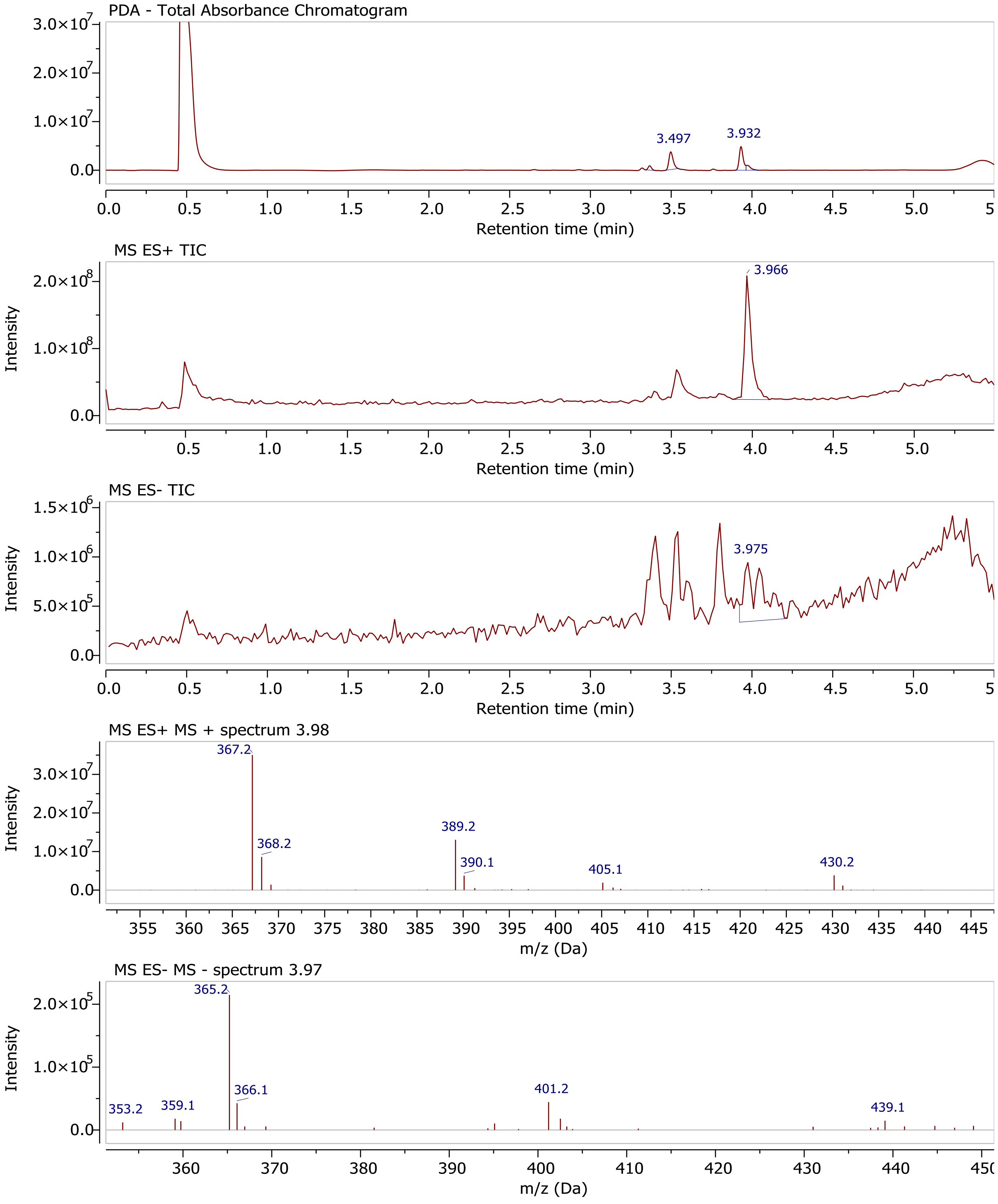

Supplementary figure **1498**. UPLC-MS analysis for compound **PHP-4924**

Chemical Formula: $C_{19}H_{19}FN_2O_3$
Exact Mass: 342.1

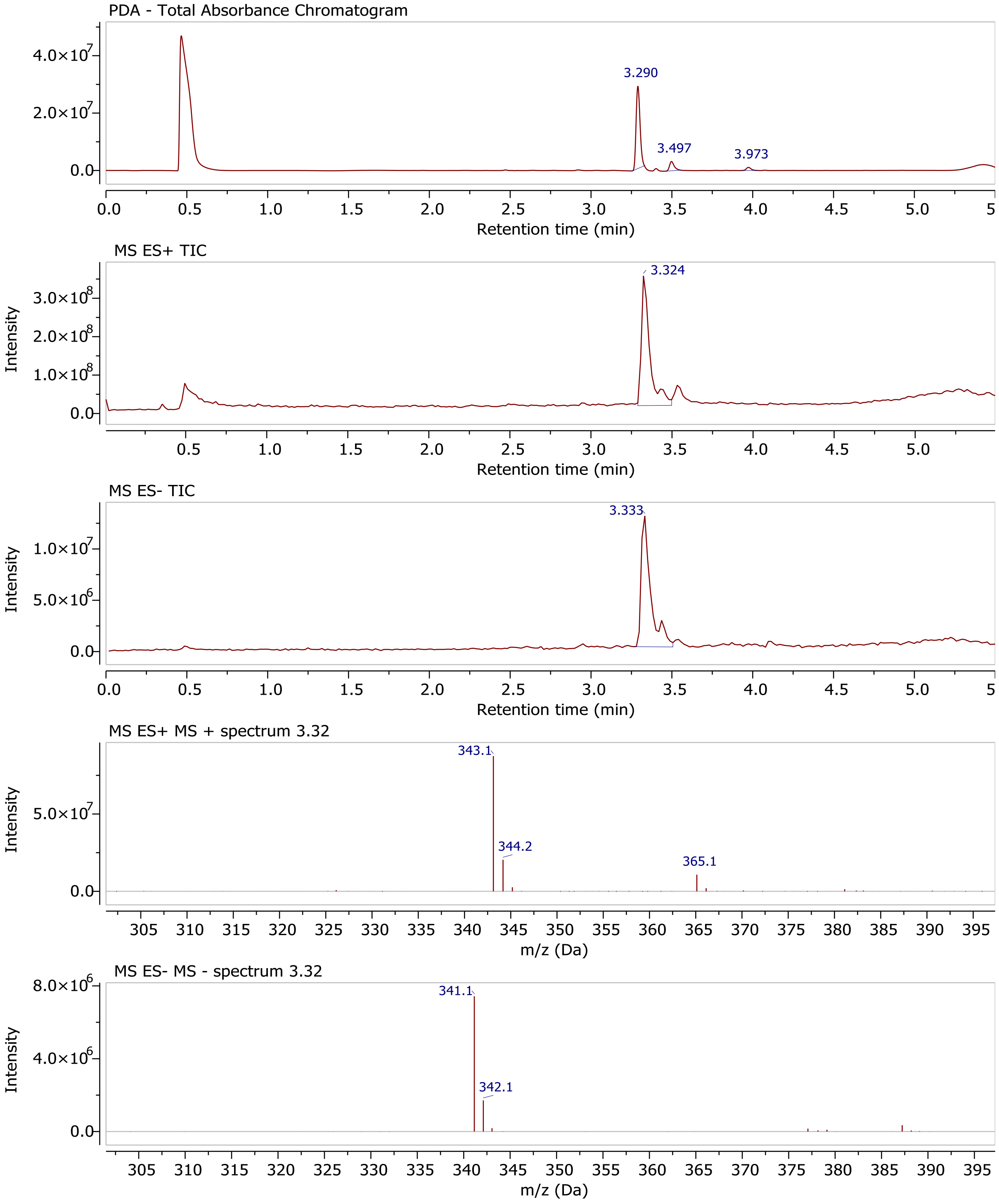

Supplementary figure **1499**. UPLC-MS analysis for compound **PHP-5494**

Chemical Formula: $C_{22}H_{26}N_2O_5S$
Exact Mass: 430.2

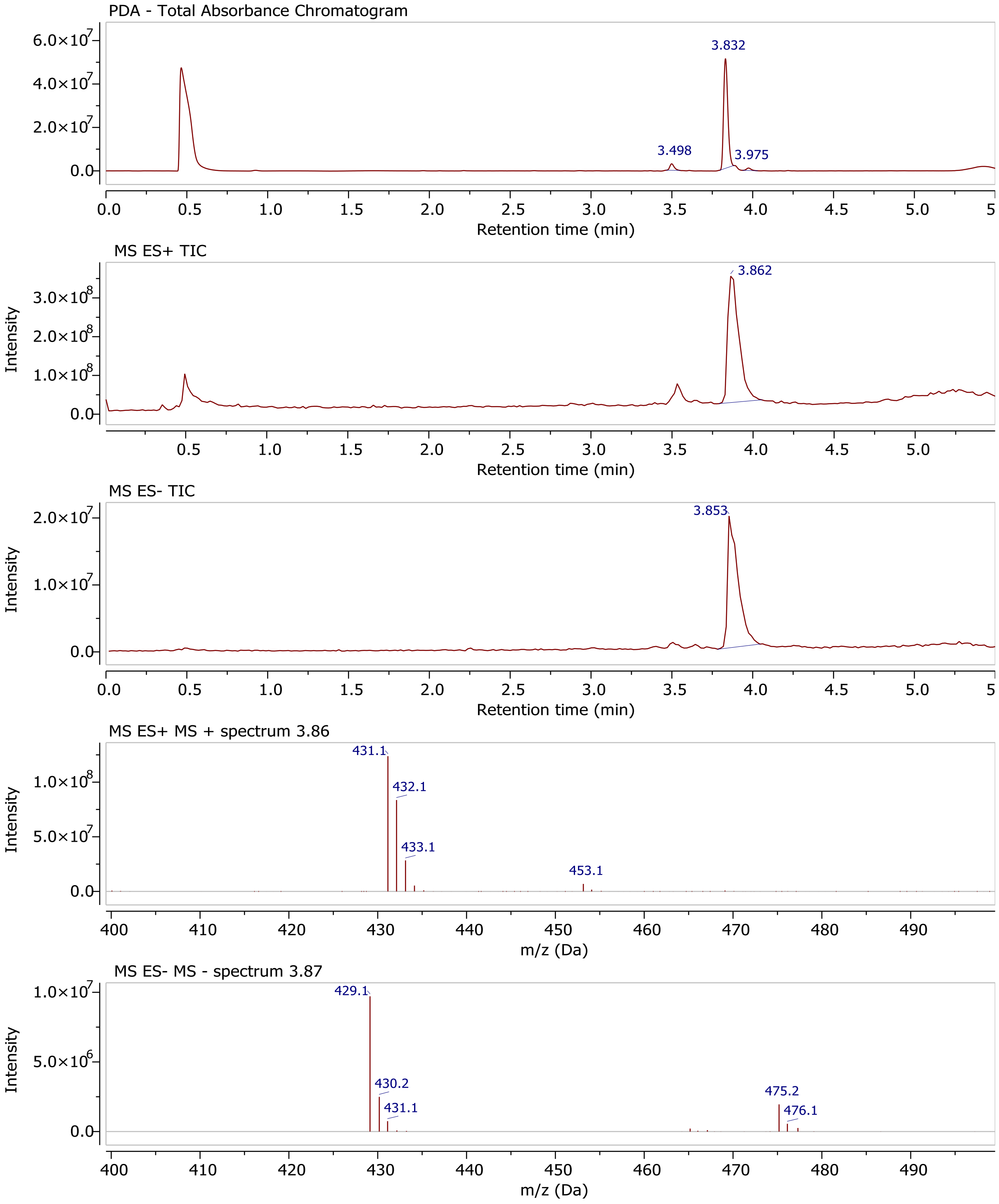

Supplementary figure **1500**. UPLC-MS analysis for compound **PHP-4927**

Chemical Formula: $C_{16}H_{15}FN_2O_3$
Exact Mass: 302.1

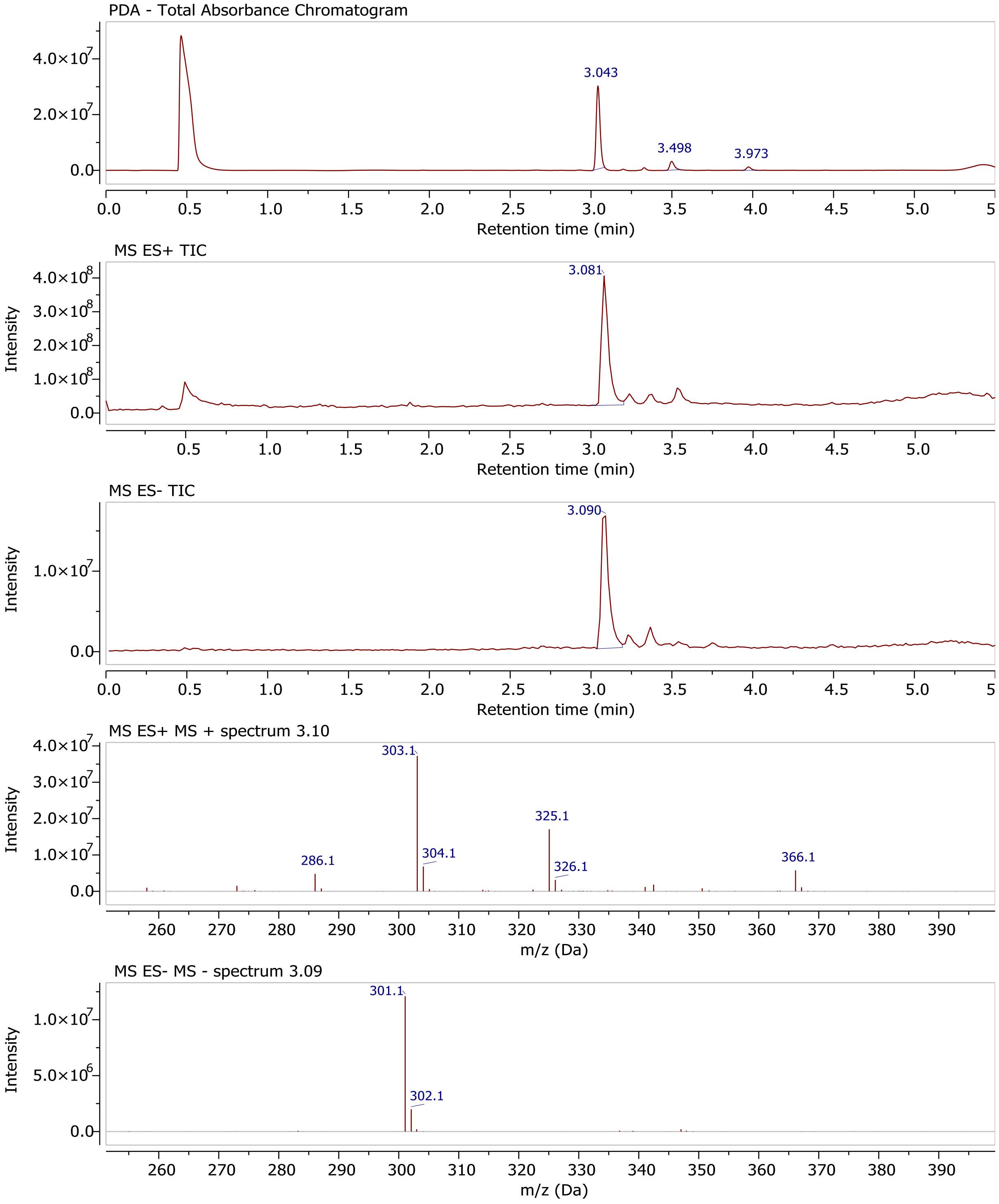

Supplementary figure **1501**. UPLC-MS analysis for compound **PHP-5495**

Chemical Formula: $C_{16}H_{21}BrN_2O_2$
Exact Mass: 352.1

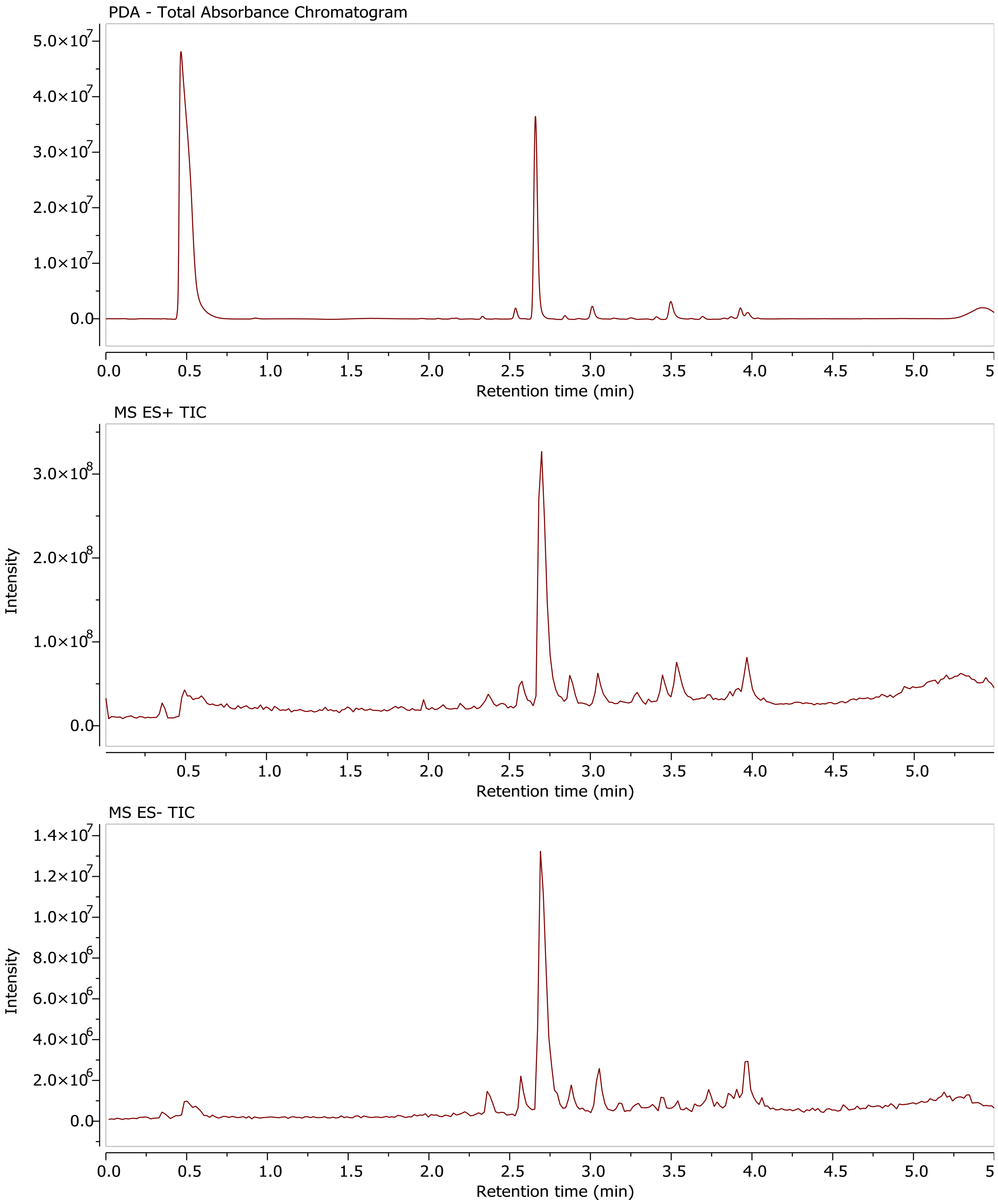

Supplementary figure **1502**. UPLC-MS analysis for compound **PHP-4928**

Chemical Formula: $C_{22}H_{19}FN_2O_2$
Exact Mass: 362.1

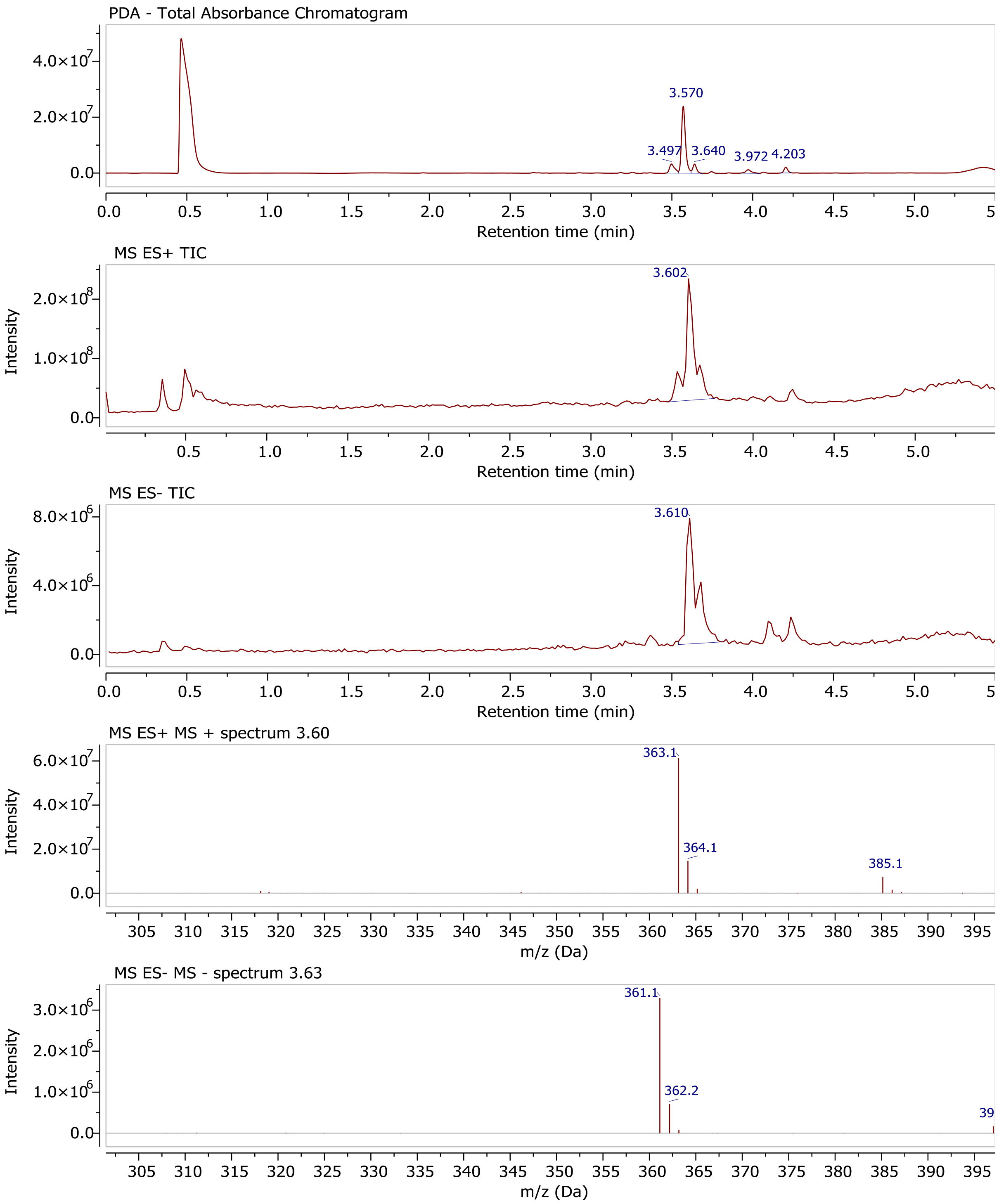

Supplementary figure **1503**. UPLC-MS analysis for compound **PHP-5496**

Chemical Formula: $C_{16}H_{20}N_4O_2$
Exact Mass: 300.2

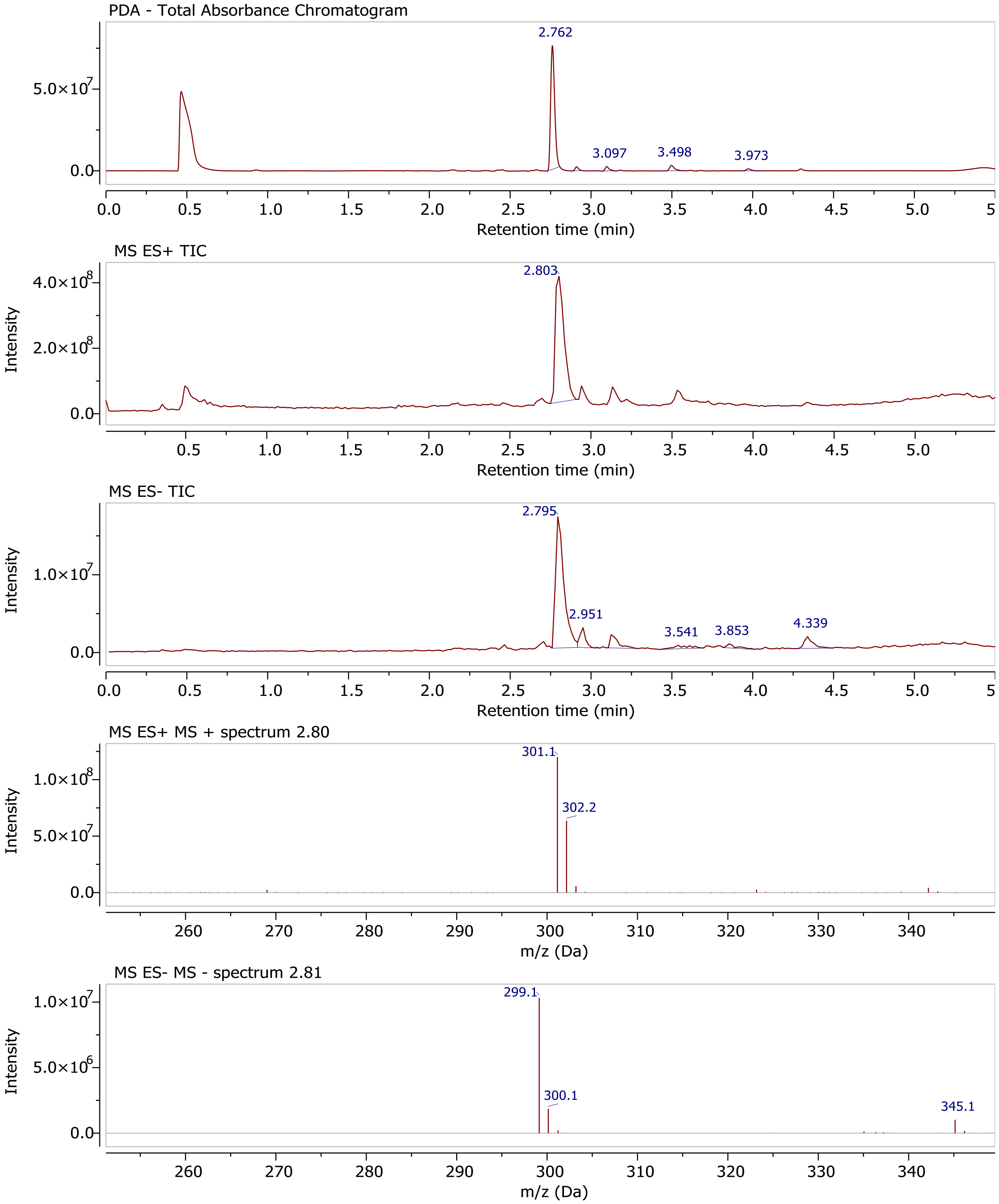

Supplementary figure **1504**. UPLC-MS analysis for compound **PHP-4931**

Chemical Formula: $C_{18}H_{24}N_2O_4$
Exact Mass: 332.2

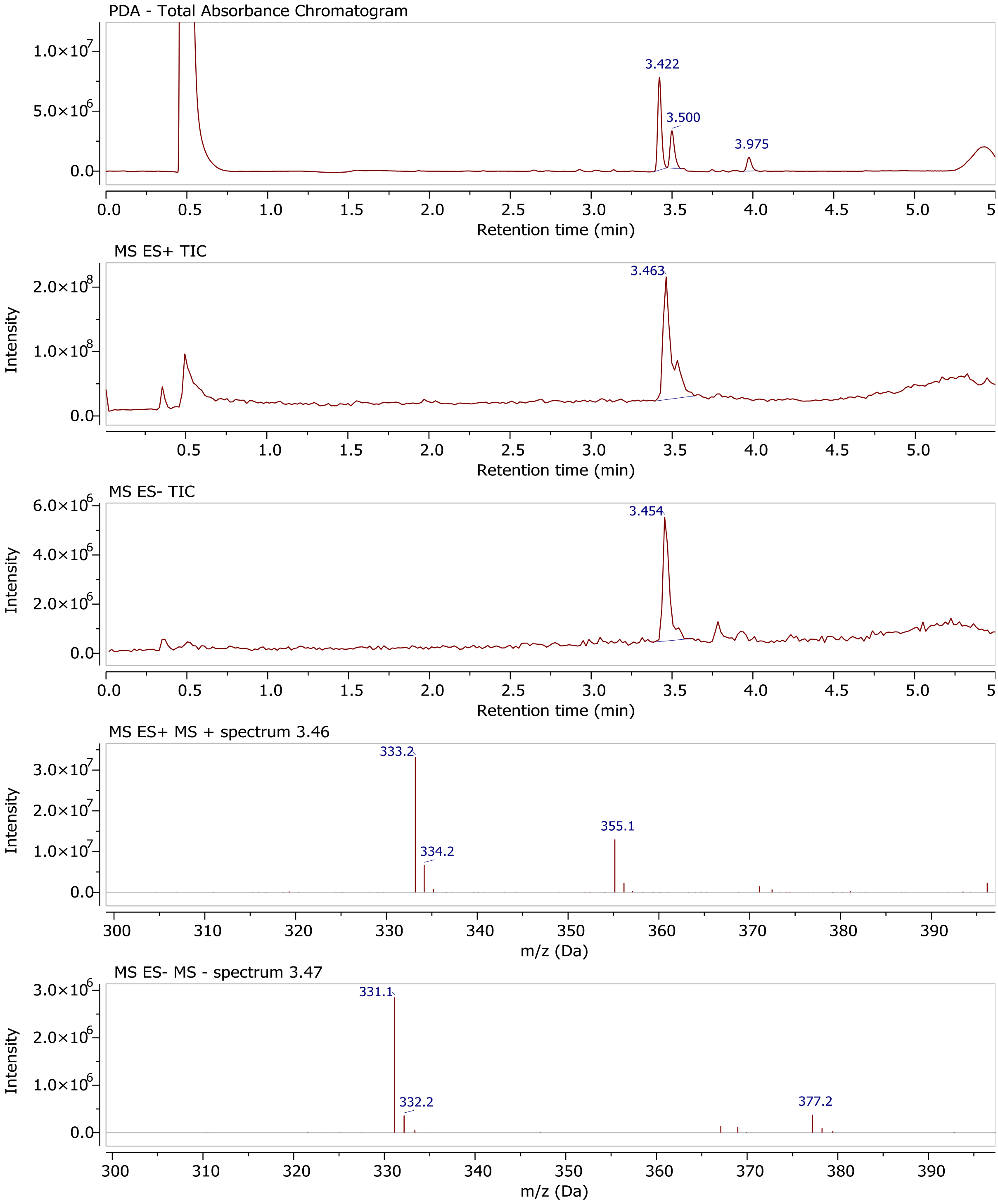

Supplementary figure **1505**. UPLC-MS analysis for compound **PHP-5517**

Chemical Formula: $C_{23}H_{24}N_2O_2$
Exact Mass: 360.2

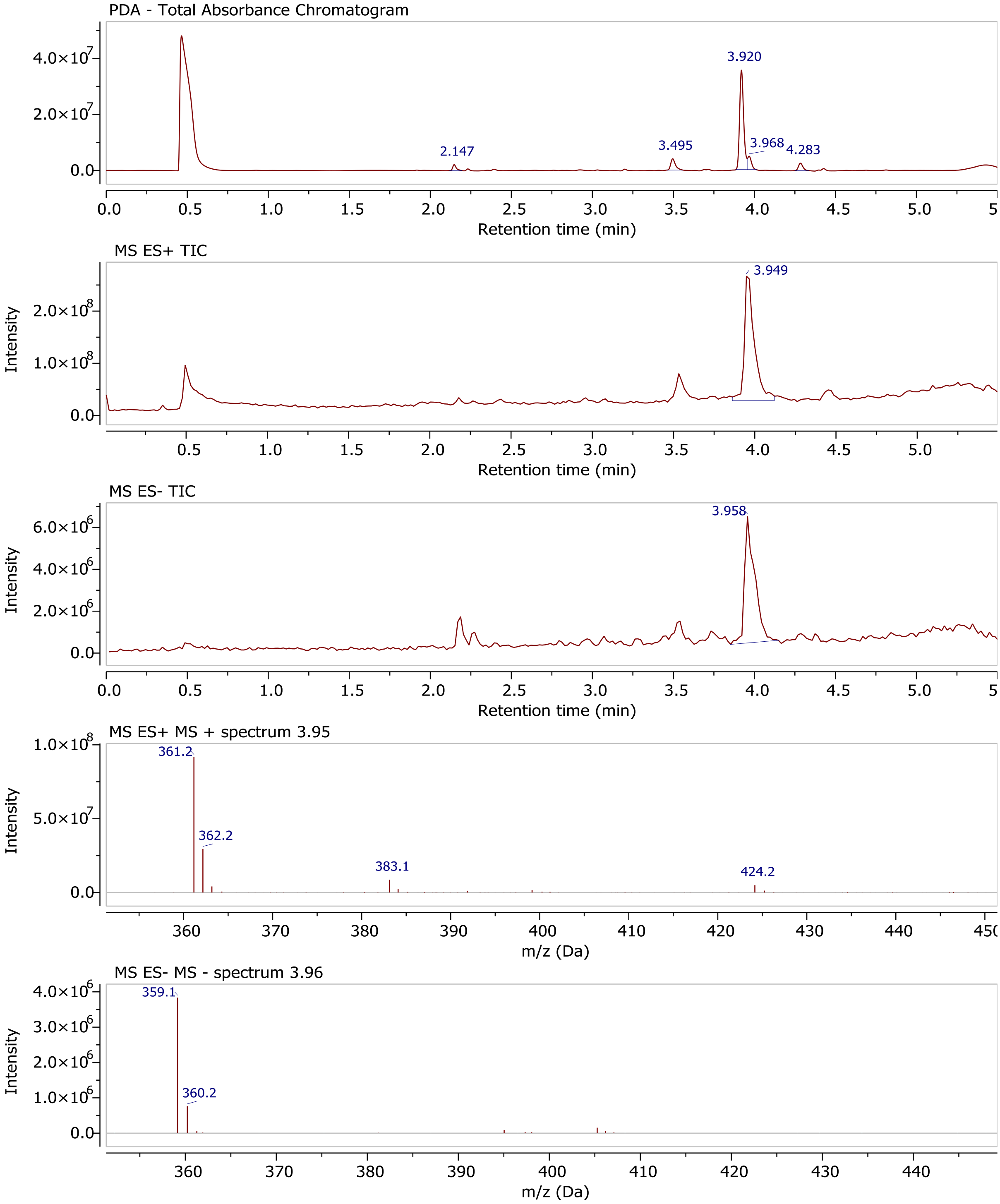

Supplementary figure **1506**. UPLC-MS analysis for compound **PHP-4932**

Chemical Formula: $C_{19}H_{16}F_2N_2O_4$
Exact Mass: 374.1

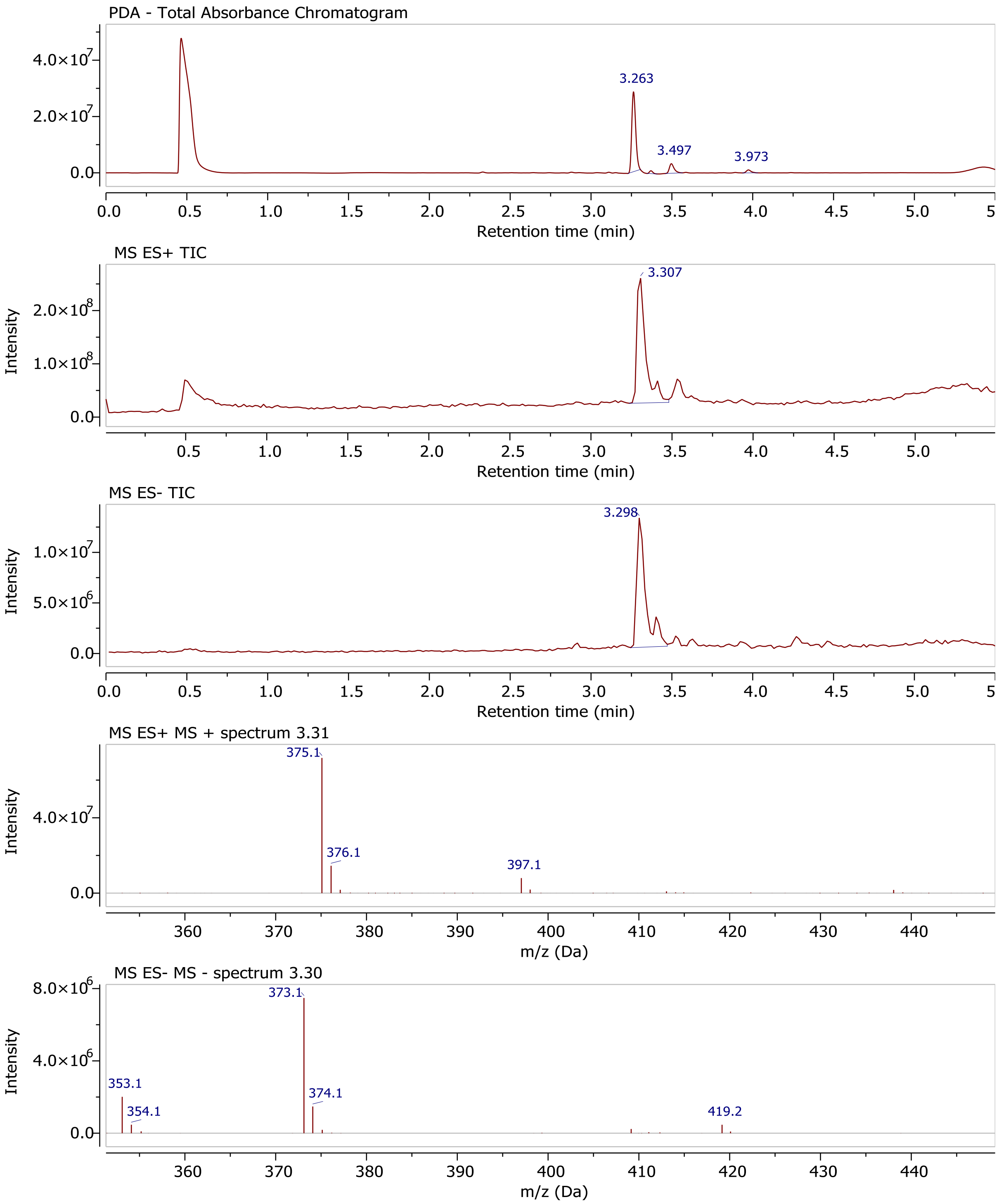

Supplementary figure **1507**. UPLC-MS analysis for compound **PHP-5518**

Chemical Formula: $C_{16}H_{19}N_3O_3$
Exact Mass: 301.1

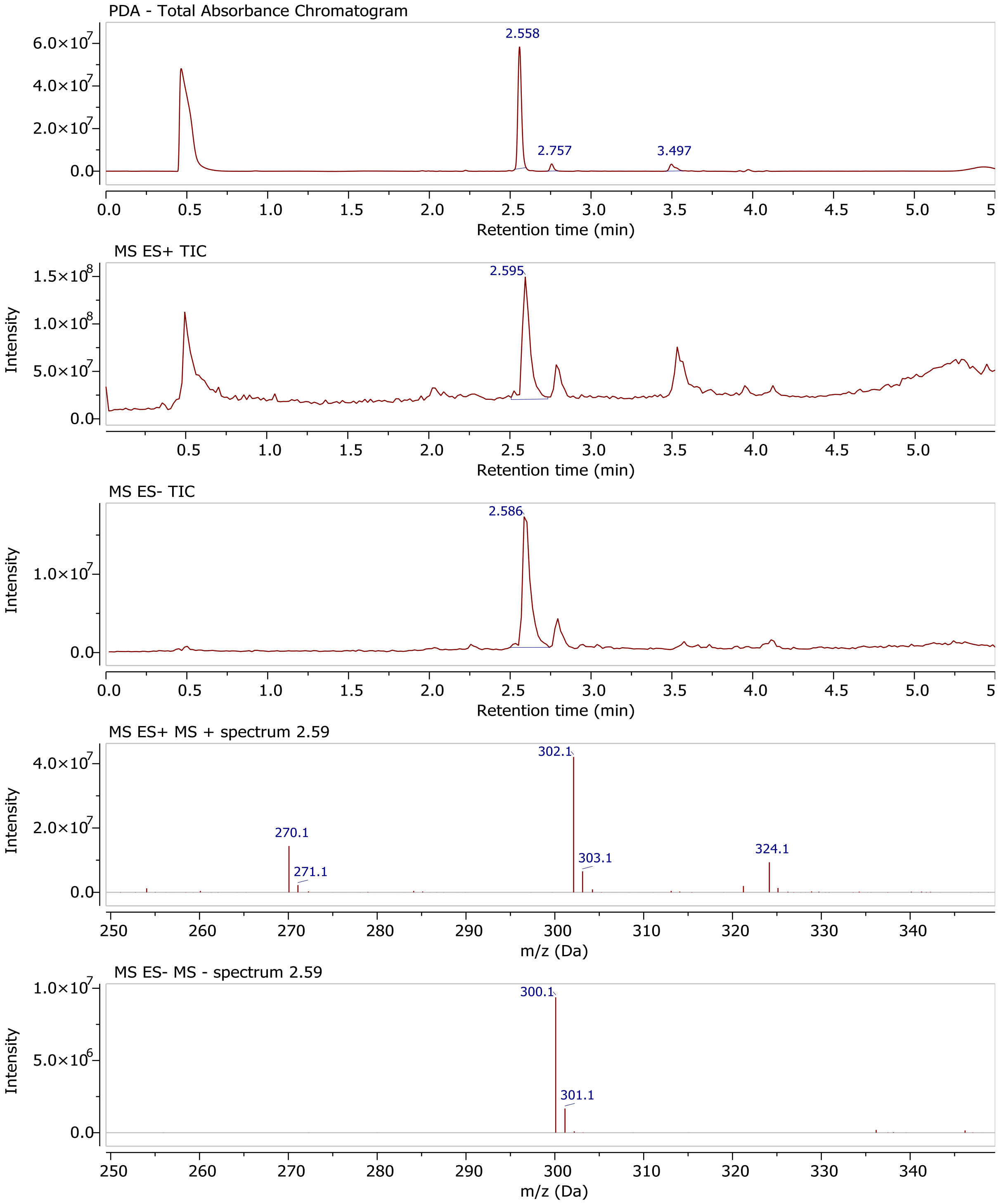

Supplementary figure XXXX. UPLC-MS analysis for compound PHP-4936

Chemical Formula: $C_{20}H_{20}N_2O_5$
Exact Mass: 368.1

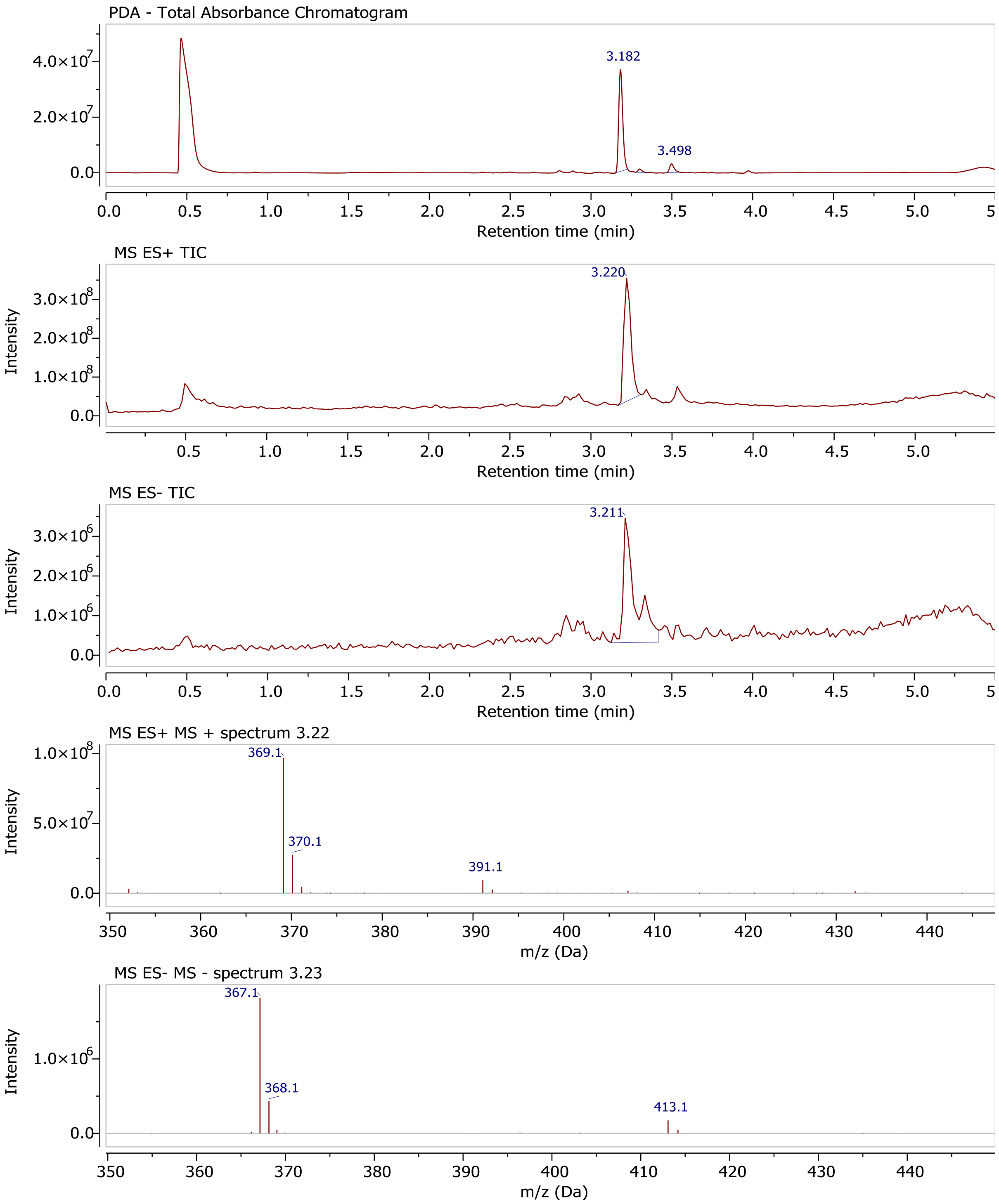

Supplementary figure **1508**. UPLC-MS analysis for compound **PHP-5520**

Chemical Formula: $C_{15}H_{19}N_3O_5$
Exact Mass: 321.1

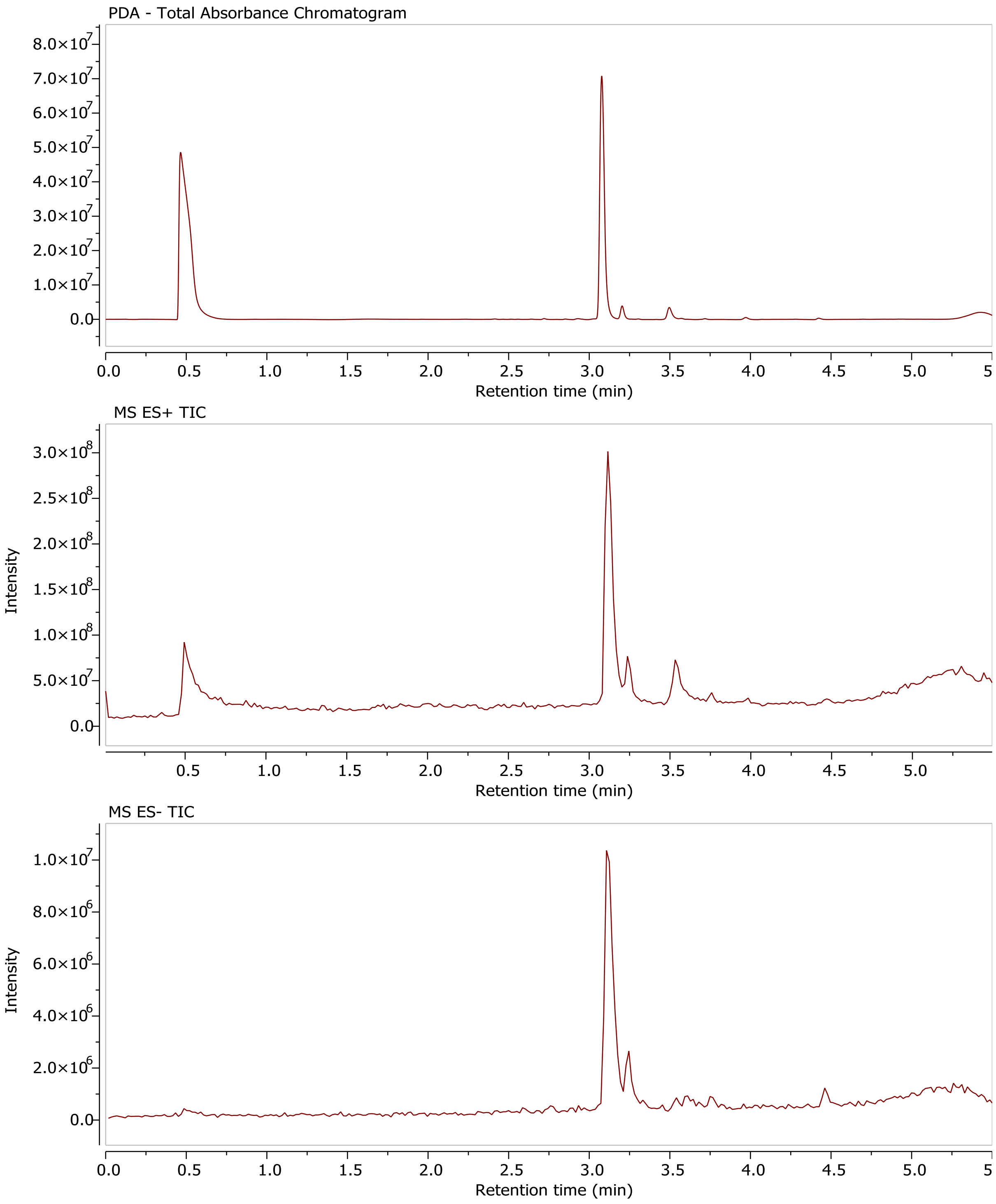

Supplementary figure **1509**. UPLC-MS analysis for compound **PHP-5520**

Chemical Formula: $C_{18}H_{22}N_2O_5$
Exact Mass: 346.2

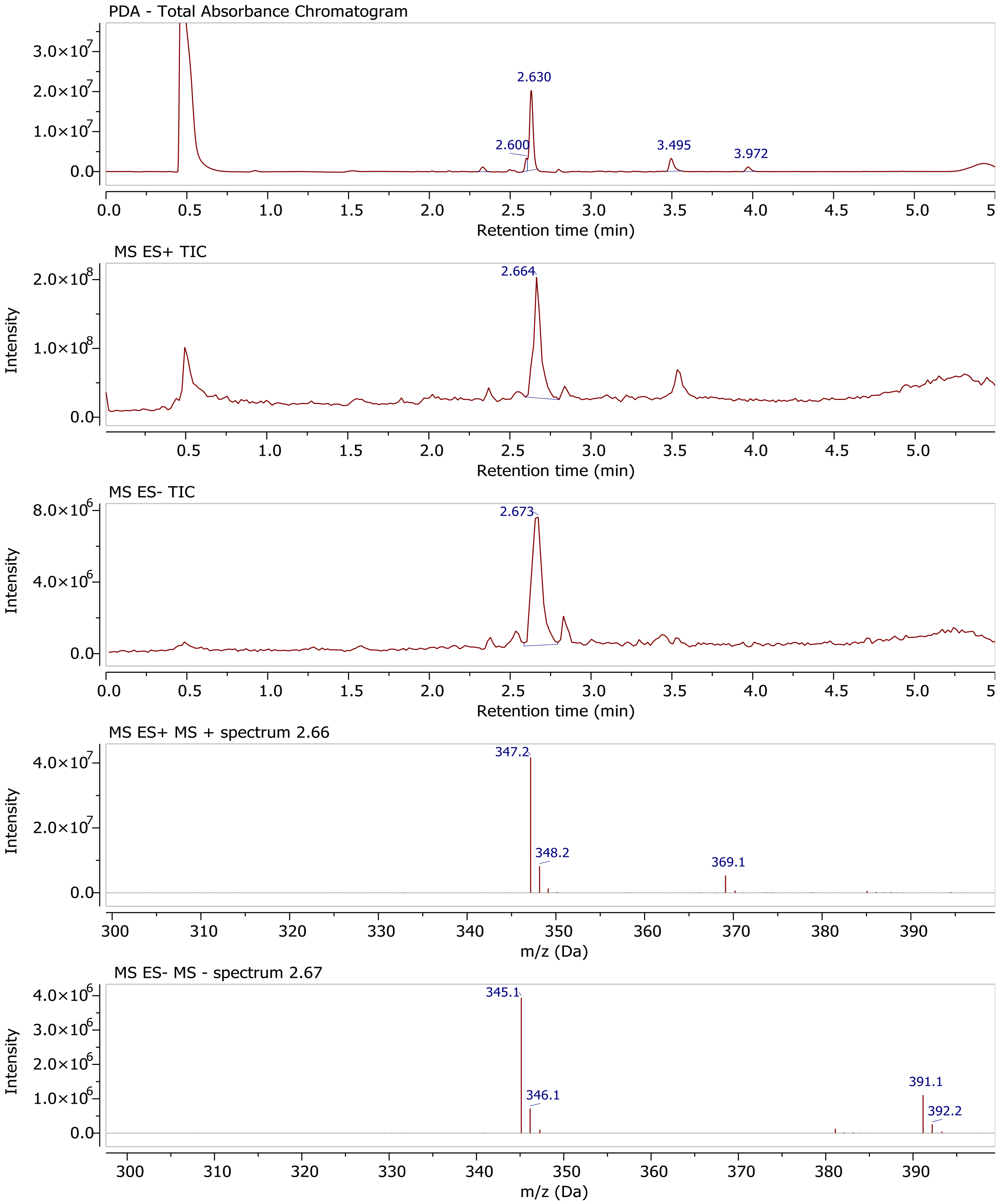

Supplementary figure **1510**. UPLC-MS analysis for compound **PHP-5522**

Chemical Formula: $C_{15}H_{21}N_3O_3$
Exact Mass: 291.2

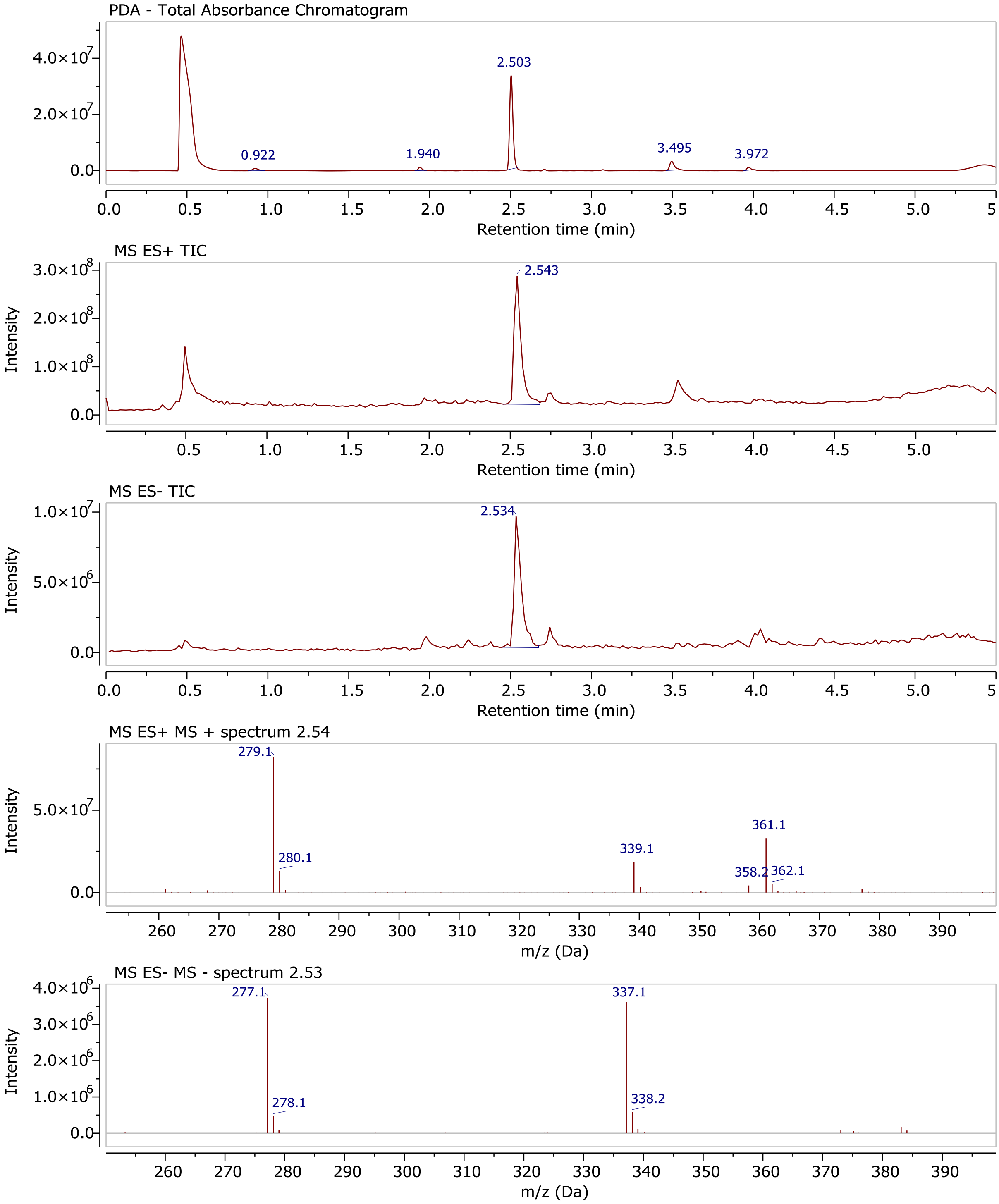

Supplementary figure **1511**. UPLC-MS analysis for compound **PHP-4938**

Chemical Formula: $C_{17}H_{22}N_2O_4$
Exact Mass: 318.2

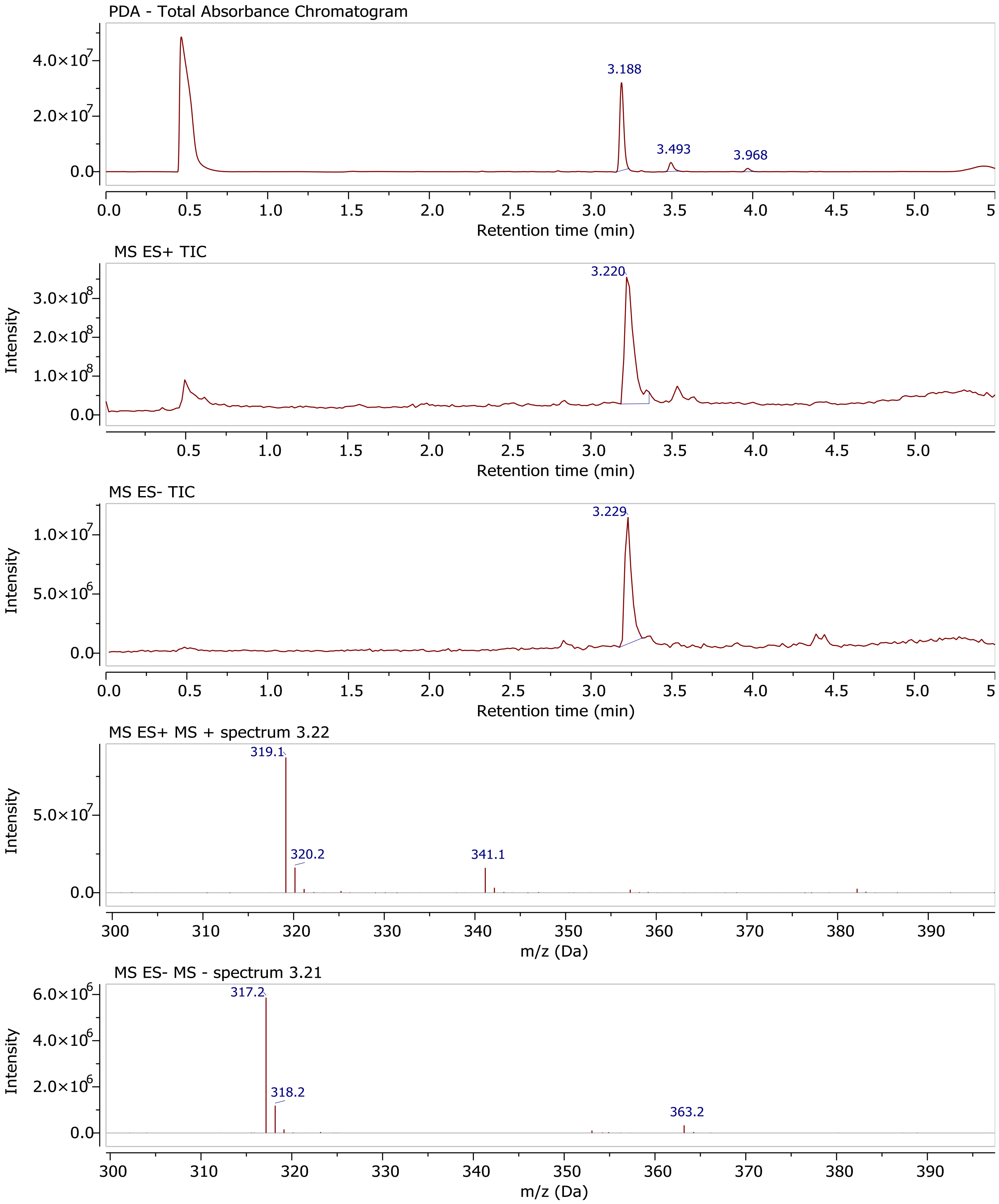

Supplementary figure **1512**. UPLC-MS analysis for compound **PHP-5524**

Chemical Formula: $C_{12}H_{18}N_4O_3$
Exact Mass: 266.1

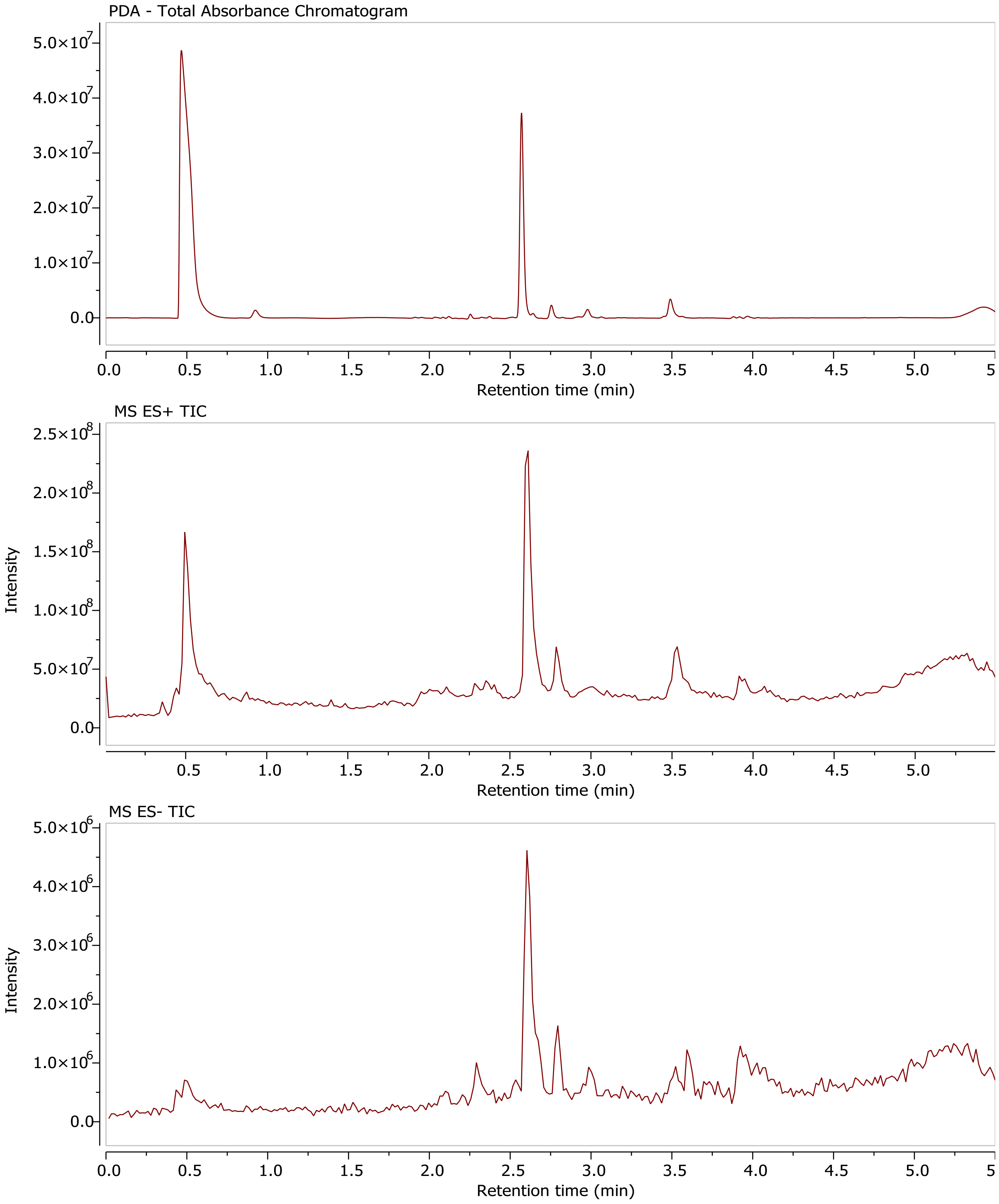

Supplementary figure **1513**. UPLC-MS analysis for compound **PHP-4946**

Chemical Formula: $C_{21}H_{22}N_2O_5$
Exact Mass: 382.2

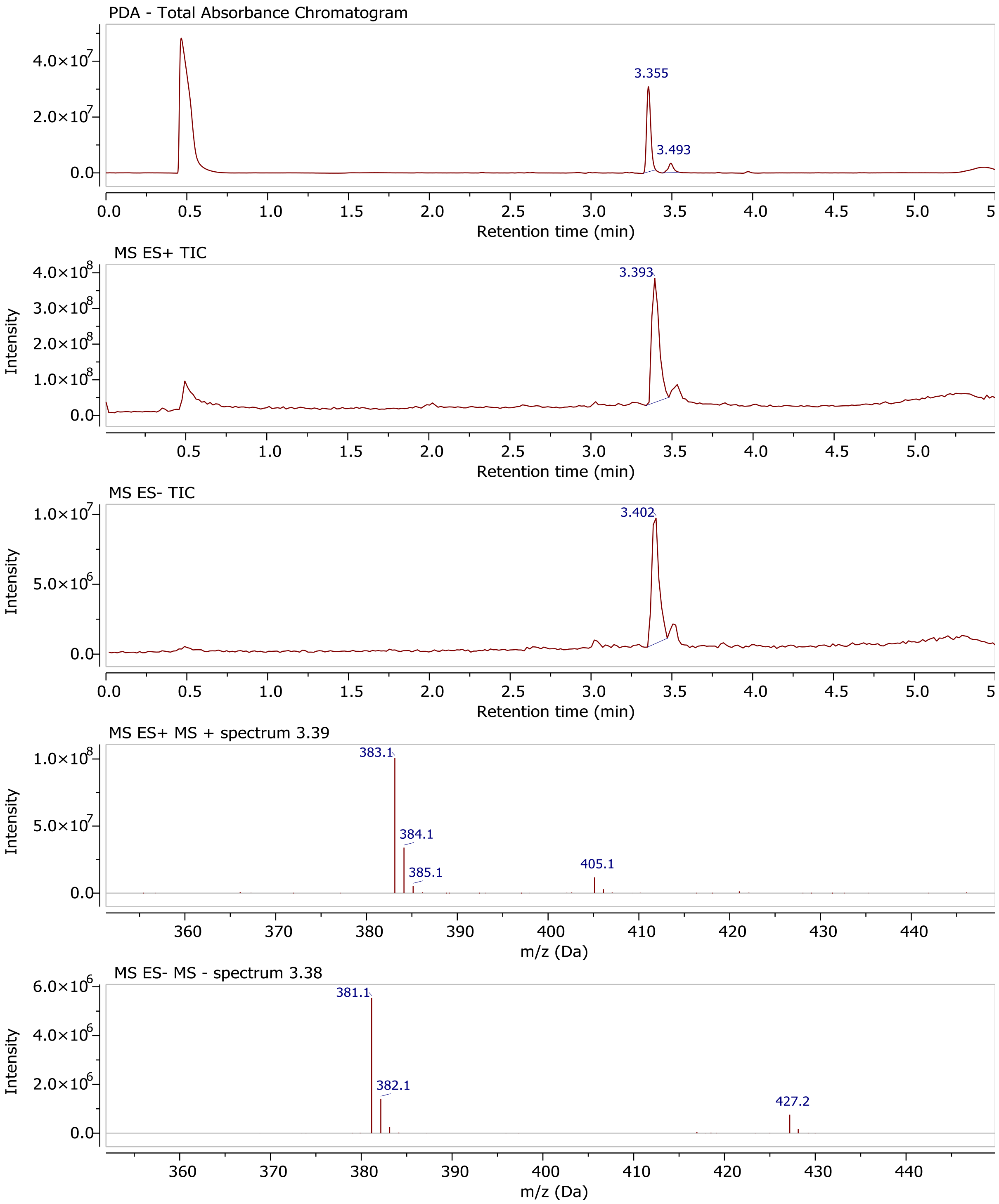

Supplementary figure **1514**. UPLC-MS analysis for compound **PHP-5525**

Chemical Formula: $C_{23}H_{19}FN_2O_3$
Exact Mass: 390.1

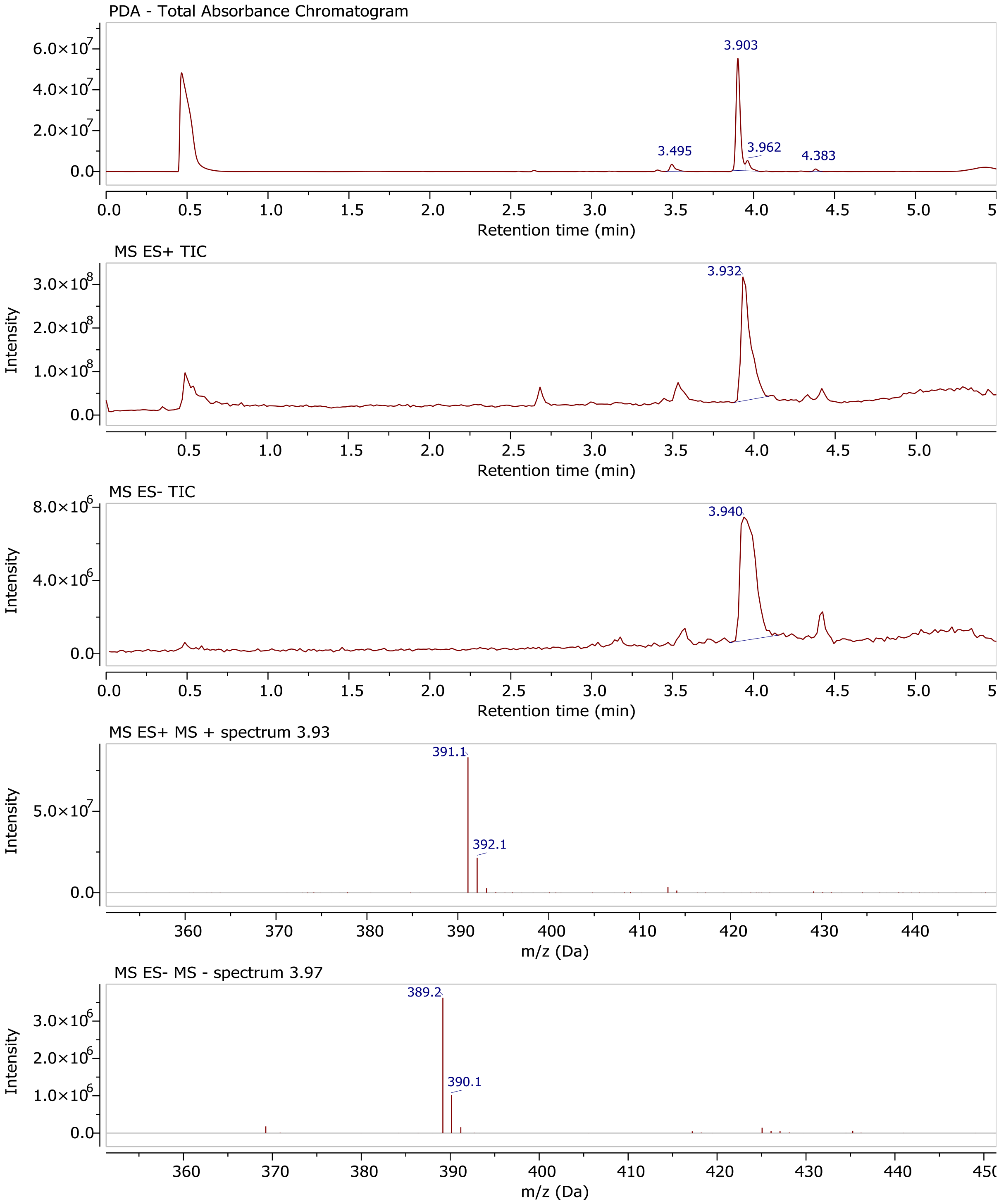

Supplementary figure **1515**. UPLC-MS analysis for compound **PHP-5663**

Chemical Formula: $C_{20}H_{22}N_2O_4$
Exact Mass: 354.2

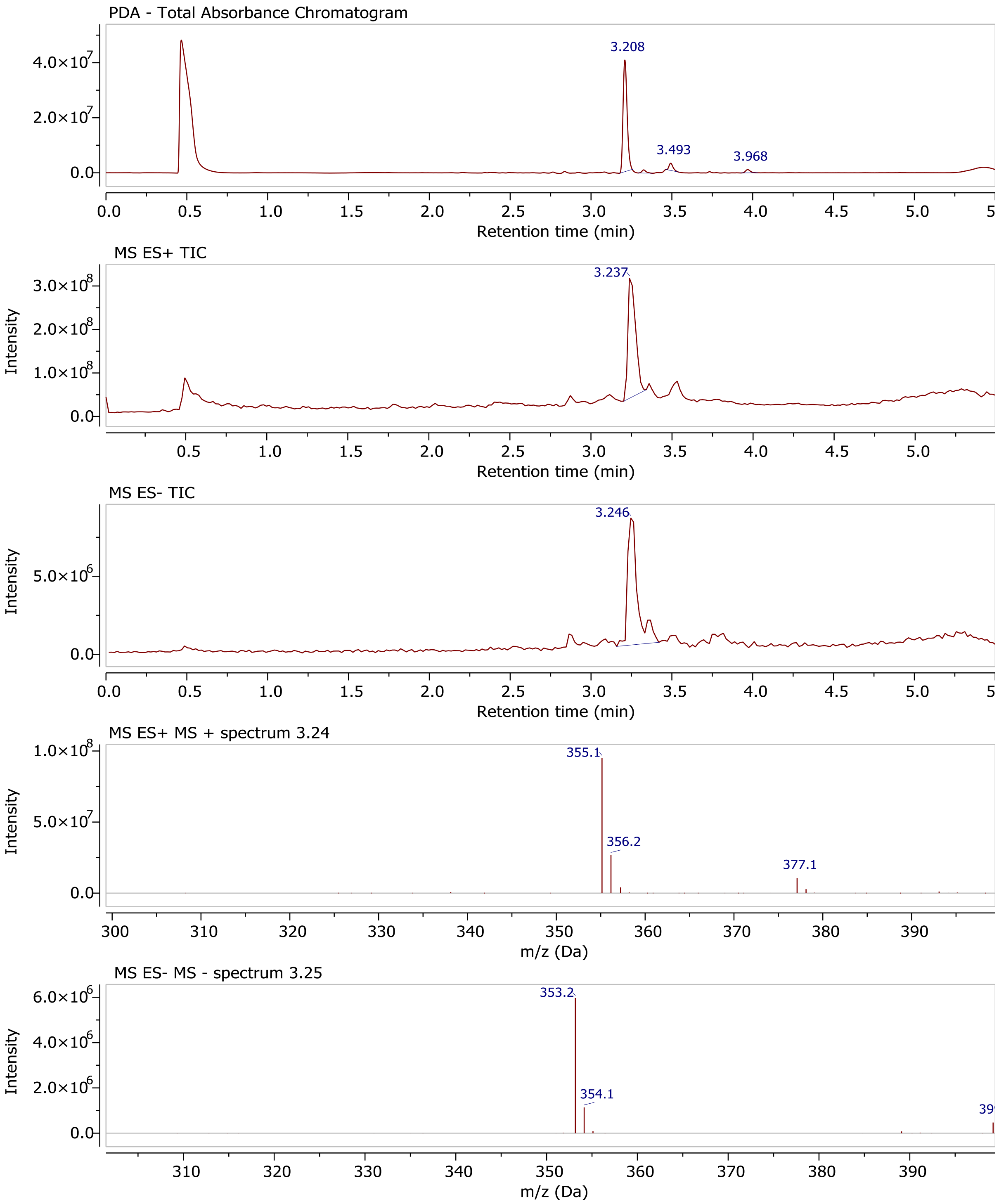

Supplementary figure **1516**. UPLC-MS analysis for compound **PHP-5830**

Chemical Formula: $C_{24}H_{19}F_3N_2O_3$
Exact Mass: 440.1

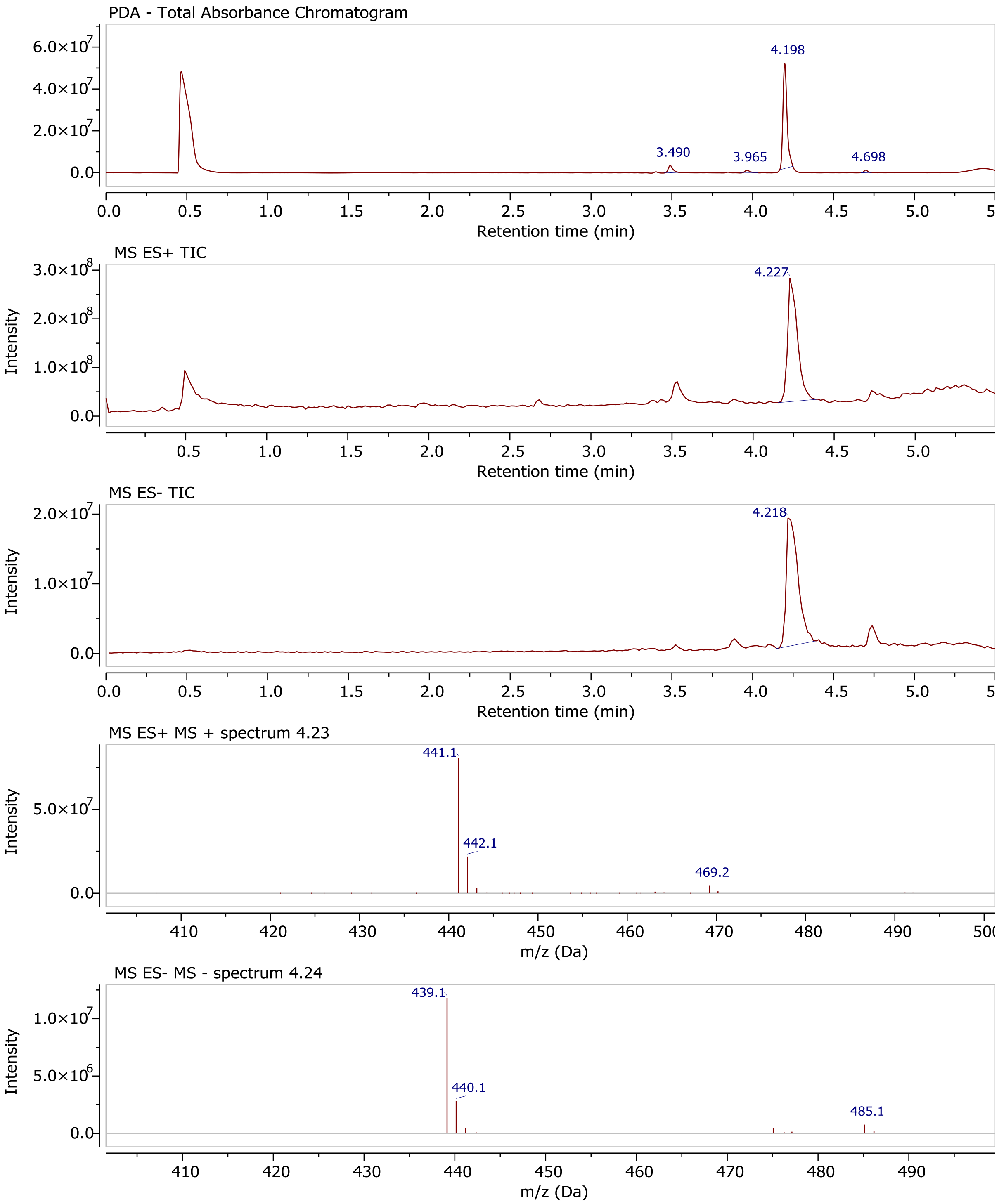

Supplementary figure **1517**. UPLC-MS analysis for compound **PHP-5664**

Chemical Formula: $C_{17}H_{18}N_2O_4$
Exact Mass: 314.1

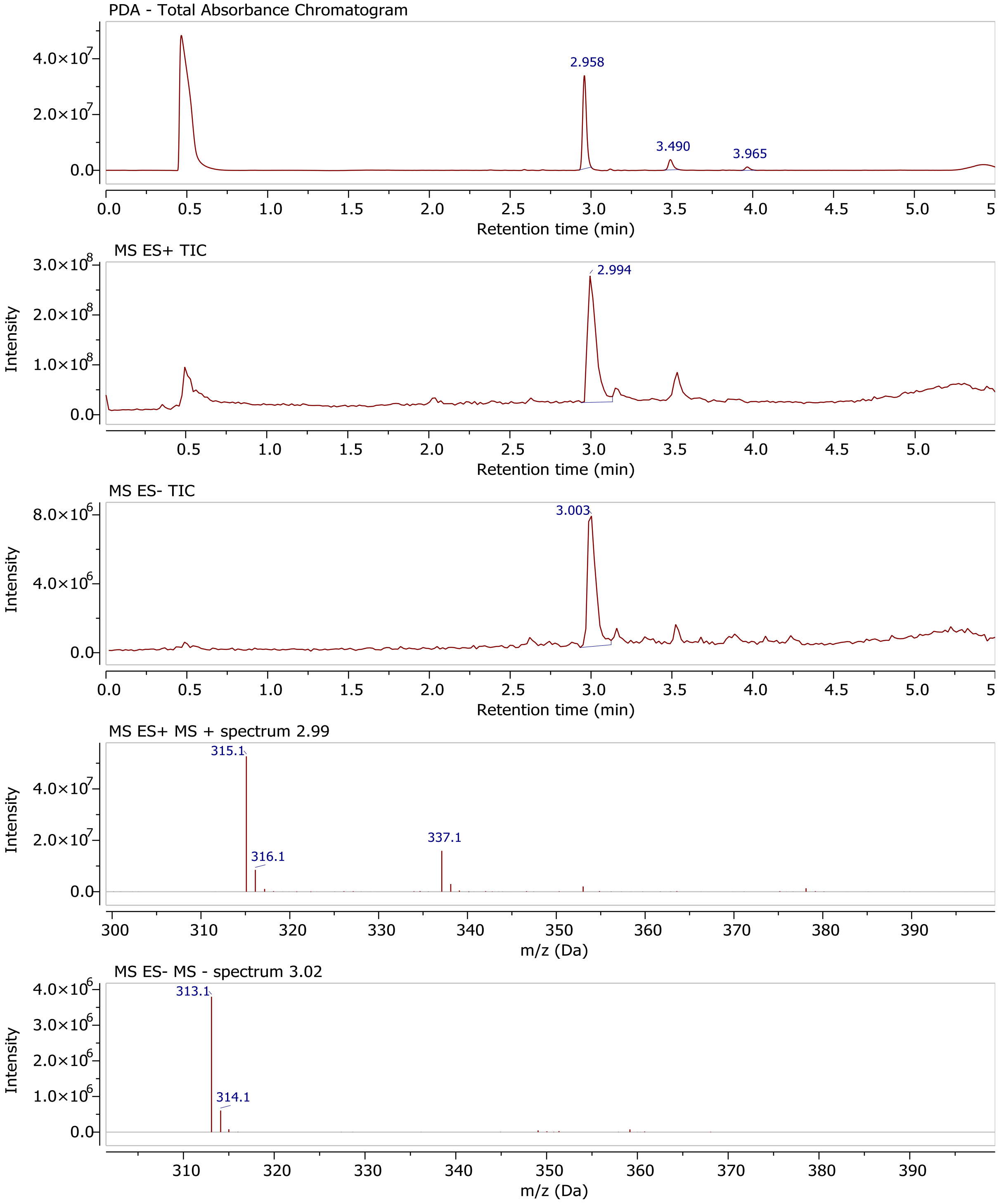

Supplementary figure **1518**. UPLC-MS analysis for compound **PHP-5831**

Chemical Formula: $C_{26}H_{23}F_3N_2O_3$
Exact Mass: 468.2

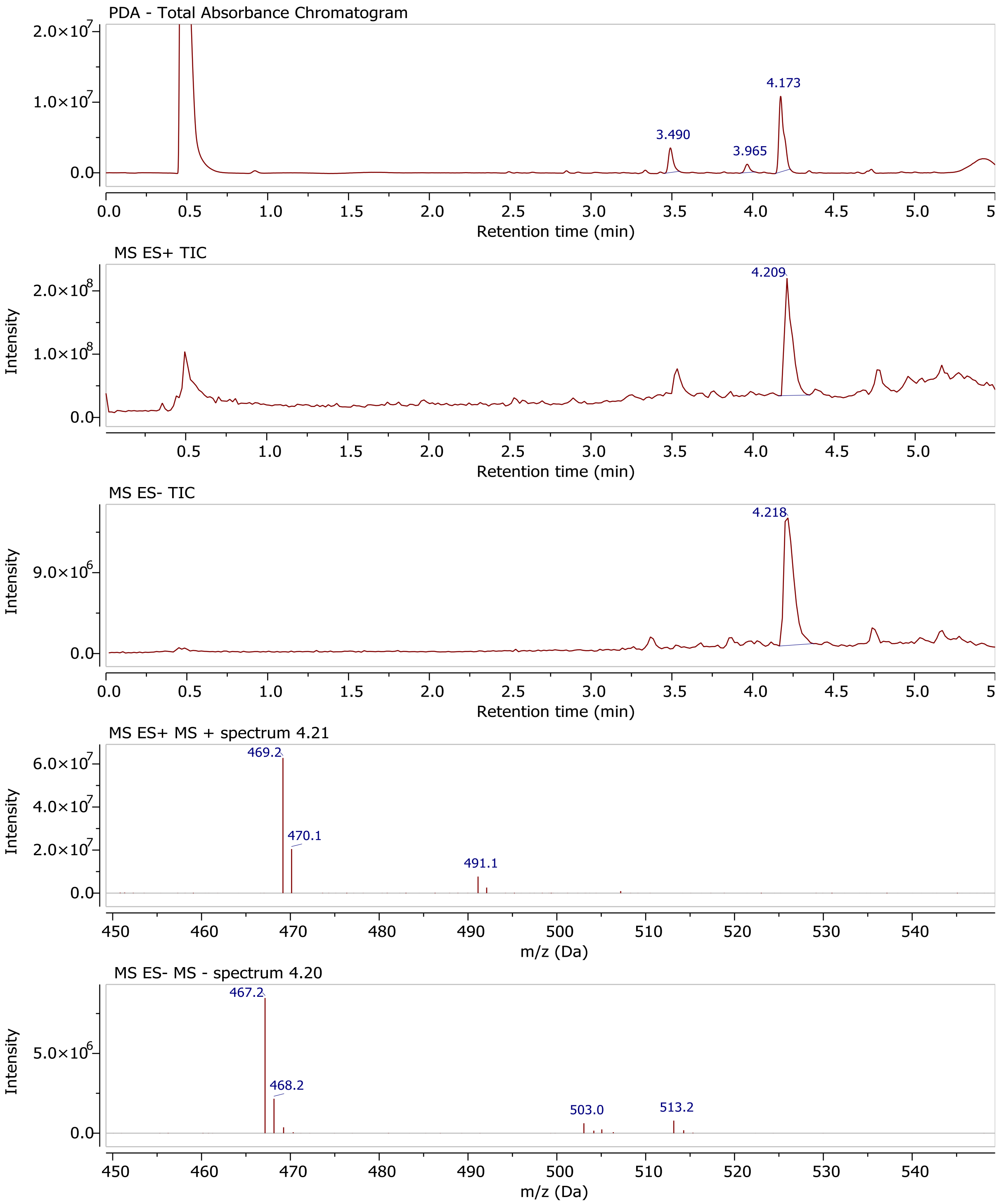

Supplementary figure **1519**. UPLC-MS analysis for compound **PHP-5665**

Chemical Formula: $C_{23}H_{22}N_2O_3$
Exact Mass: 374.2

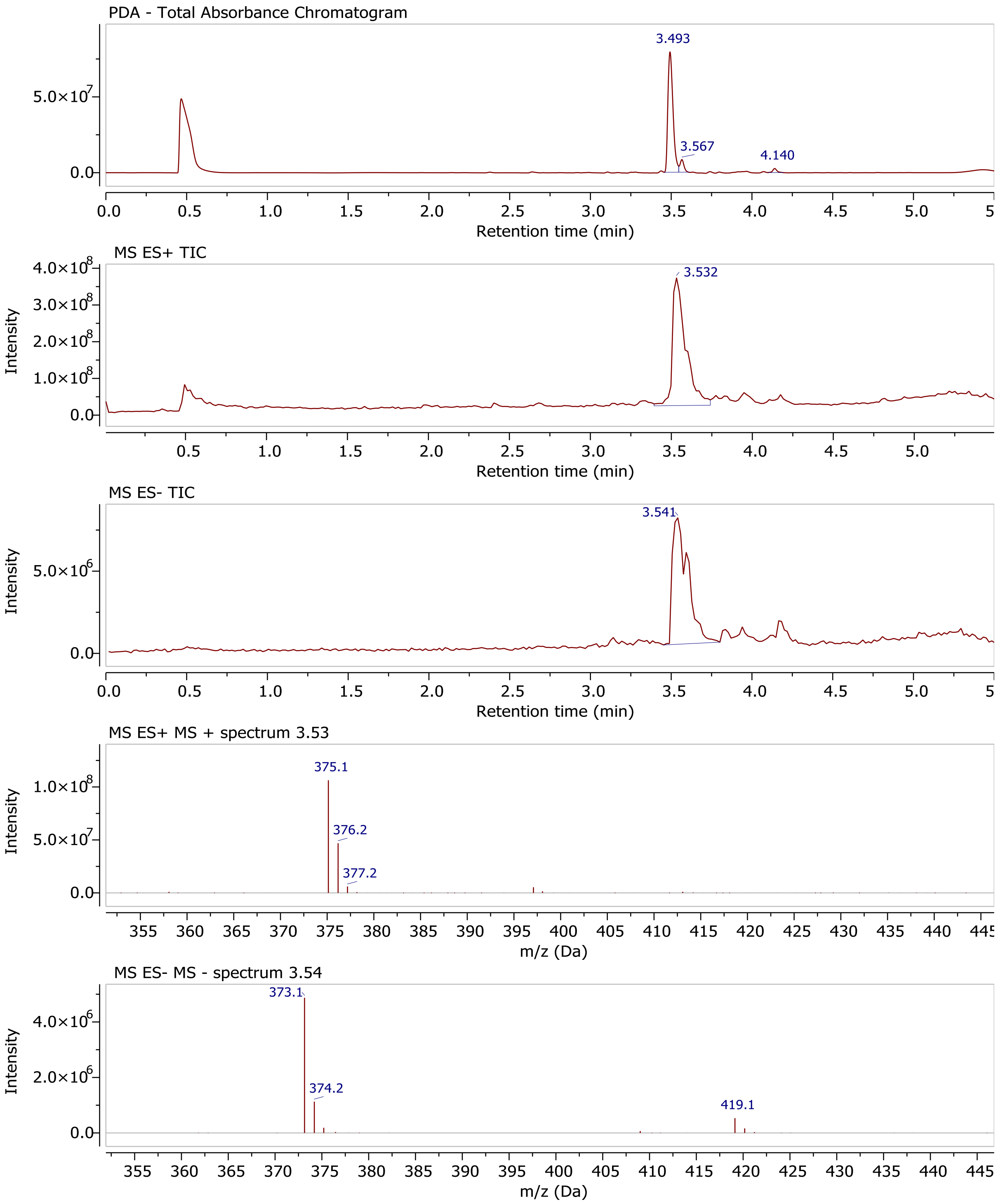

Supplementary figure **1520**. UPLC-MS analysis for compound **PHP-5832**

Chemical Formula: $C_{31}H_{28}N_2O_4$
Exact Mass: 492.2

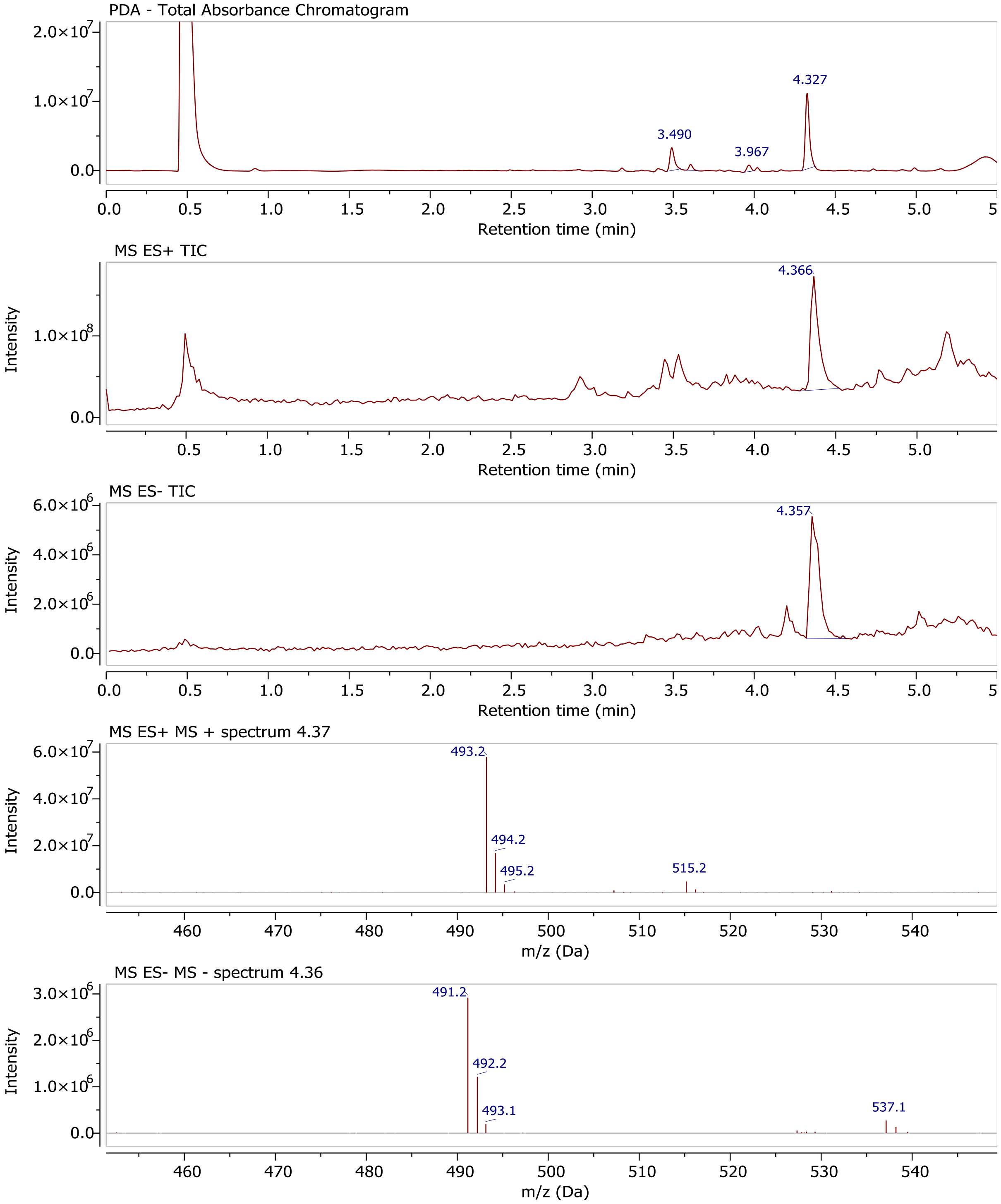

Supplementary figure **1521**. UPLC-MS analysis for compound **PHP-5666**

Chemical Formula: $C_{14}H_{24}N_2O_4$
Exact Mass: 284.2

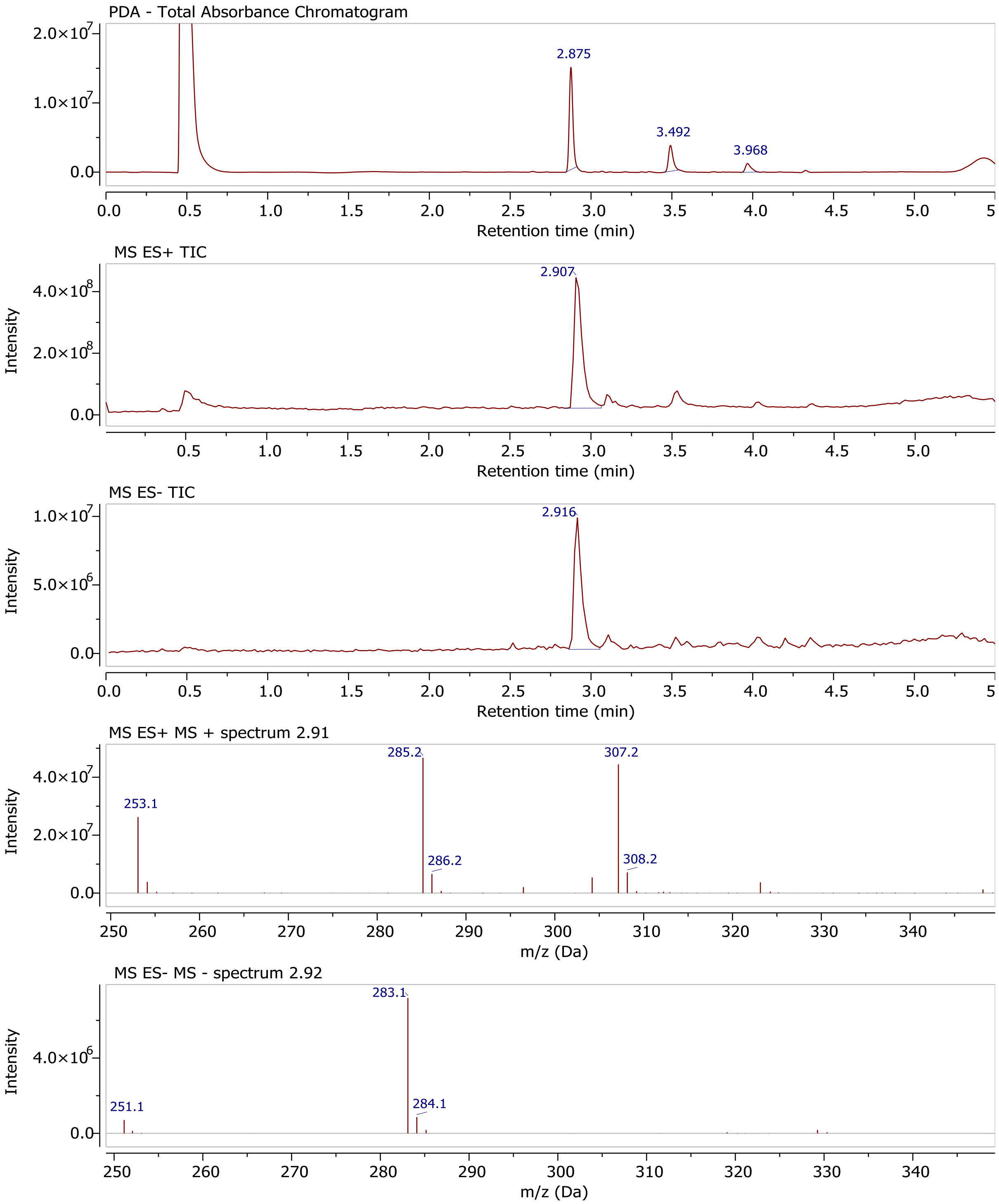

Supplementary figure **1522**. UPLC-MS analysis for compound **PHP-5836**

Chemical Formula: $C_{25}H_{23}BrN_2O_3$
Exact Mass: 478.1

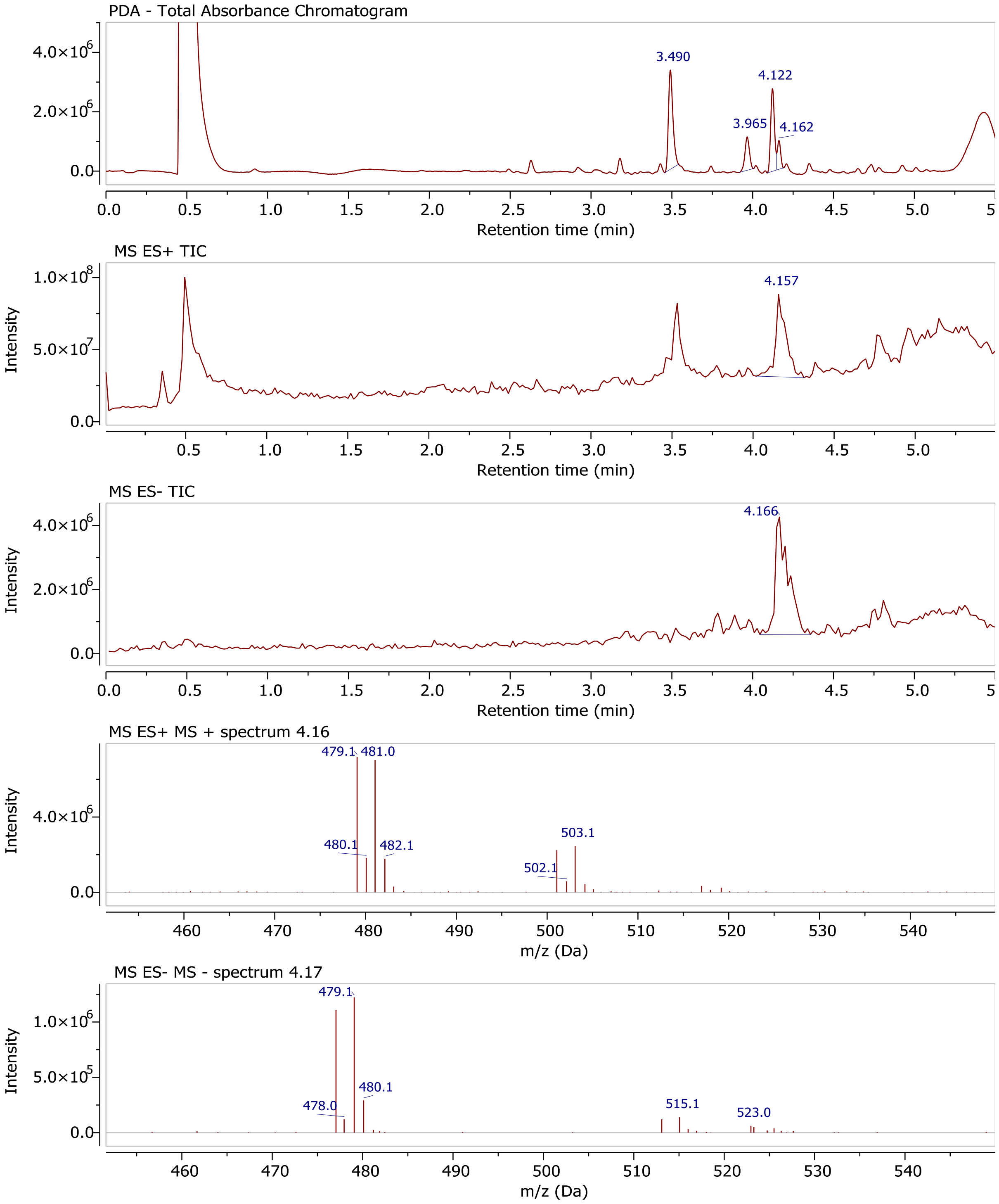

Supplementary figure **1523**. UPLC-MS analysis for compound **PHP-5667**

Chemical Formula: $C_{17}H_{19}F_3N_2O_4$
Exact Mass: 372.1

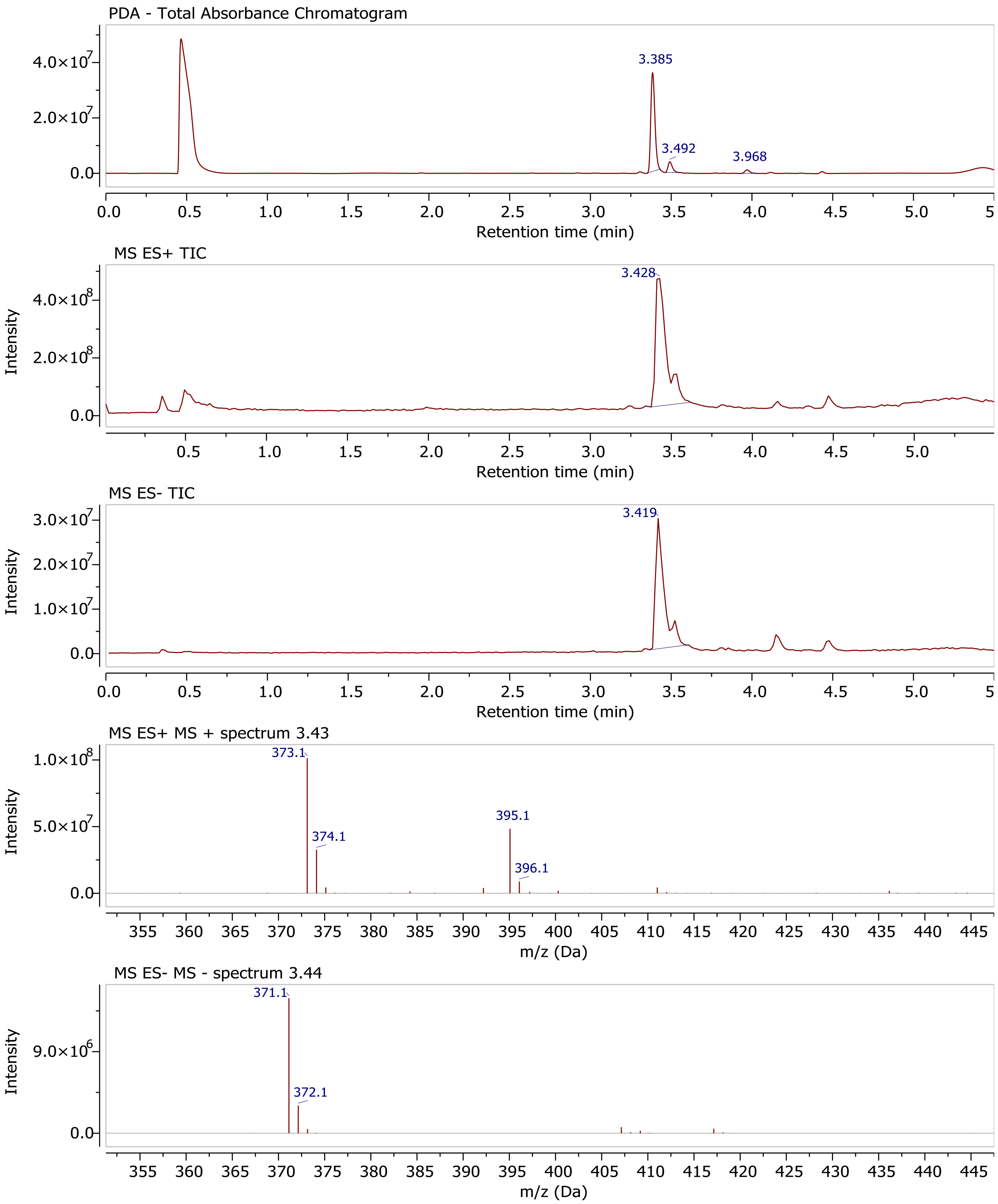

Supplementary figure **1524**. UPLC-MS analysis for compound **PHP-5841**

Chemical Formula: $C_{28}H_{30}N_2O_3$
Exact Mass: 442.2

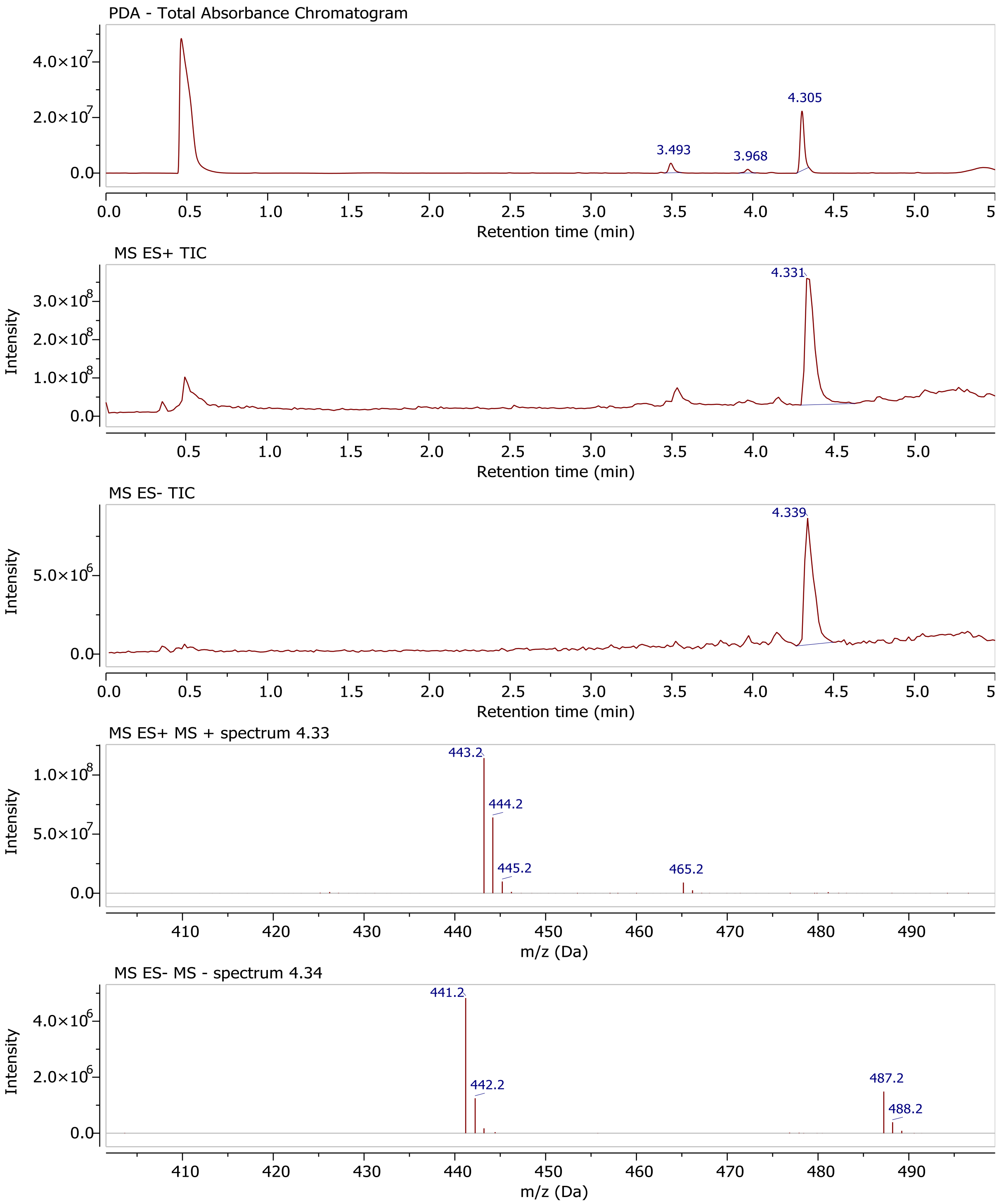

Supplementary figure **1525**. UPLC-MS analysis for compound **PHP-5668**

Chemical Formula: $C_{18}H_{20}N_2O_2S$
Exact Mass: 328.1

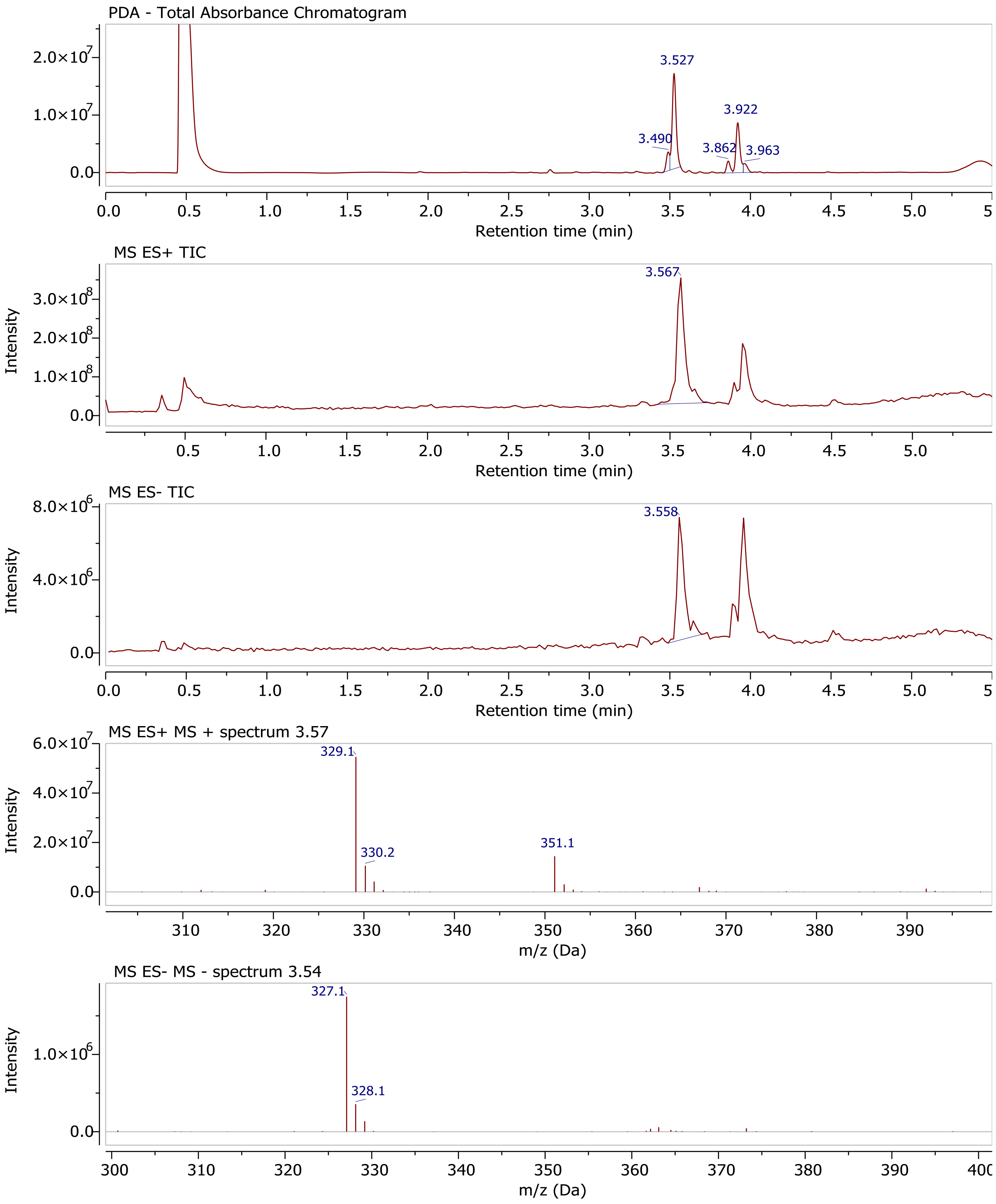

Supplementary figure **1526**. UPLC-MS analysis for compound **PHP-5849**

Chemical Formula: $C_{25}H_{23}ClN_2O_3$
Exact Mass: 434.1

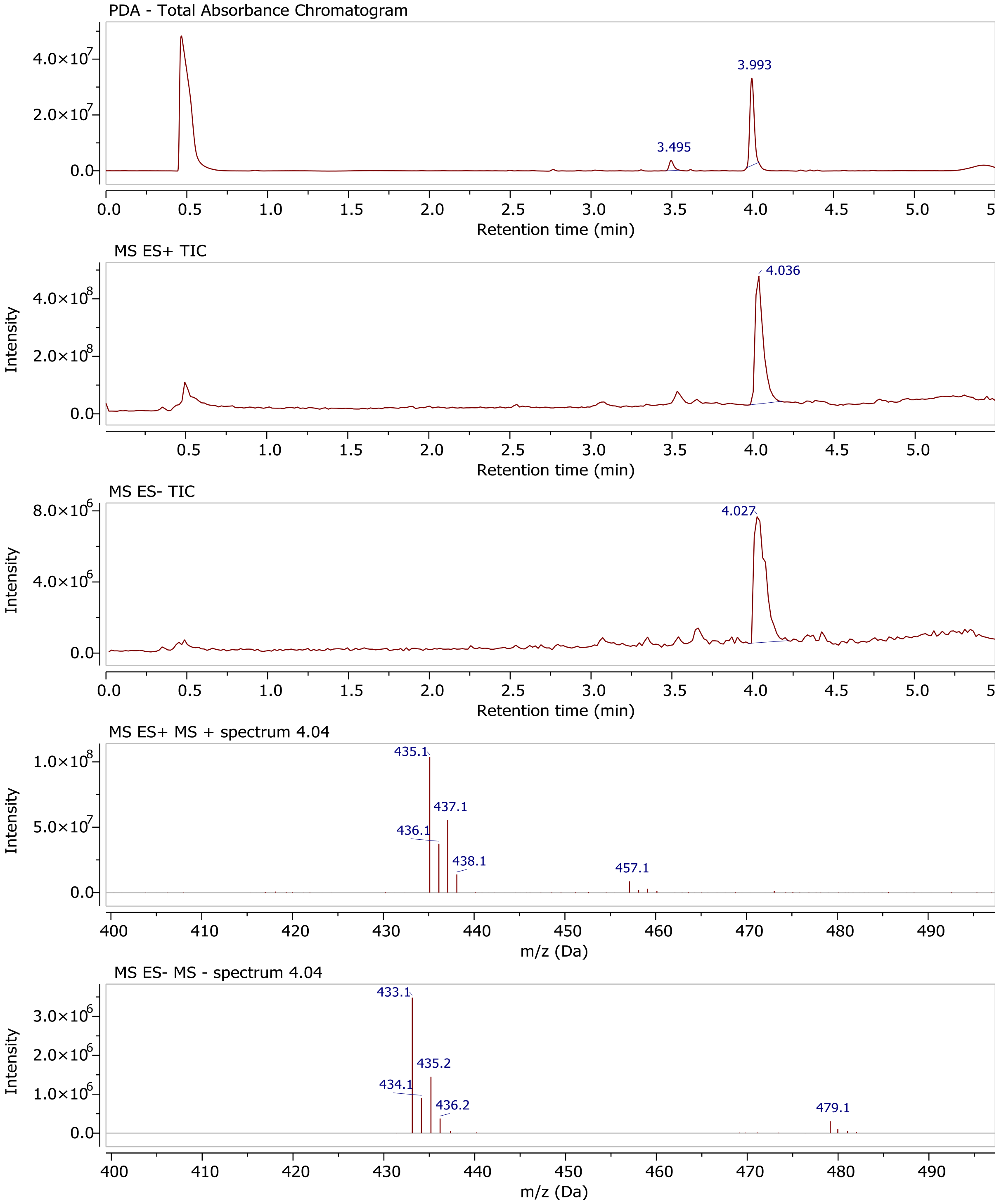

Supplementary figure **1527**. UPLC-MS analysis for compound **PHP-5669**

Chemical Formula: $C_{19}H_{17}F_3N_2O_2$
Exact Mass: 362.1

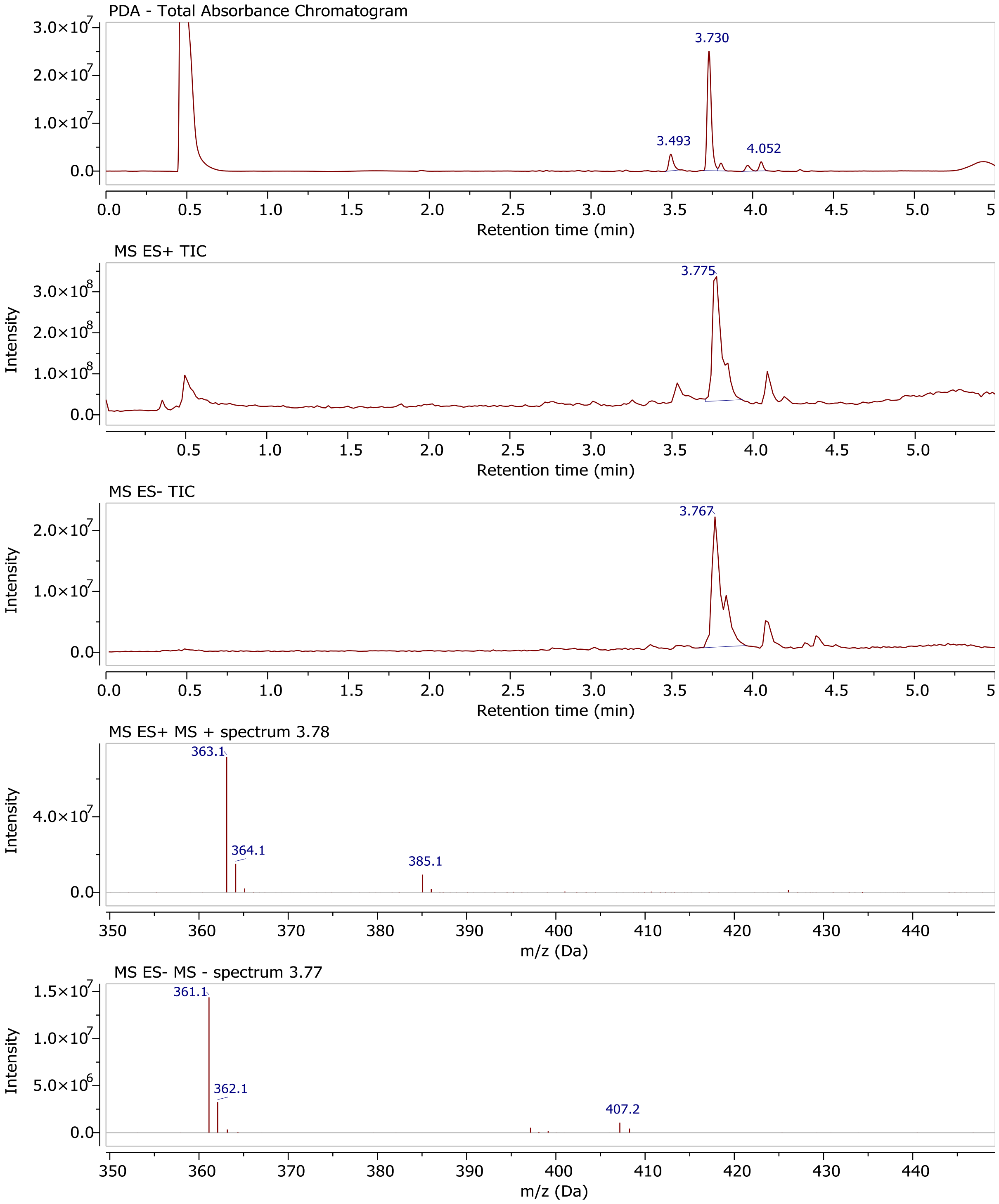

Supplementary figure **1528**. UPLC-MS analysis for compound **PHP-5851**

Chemical Formula: $C_{25}H_{23}FN_2O_3$
Exact Mass: 418.2

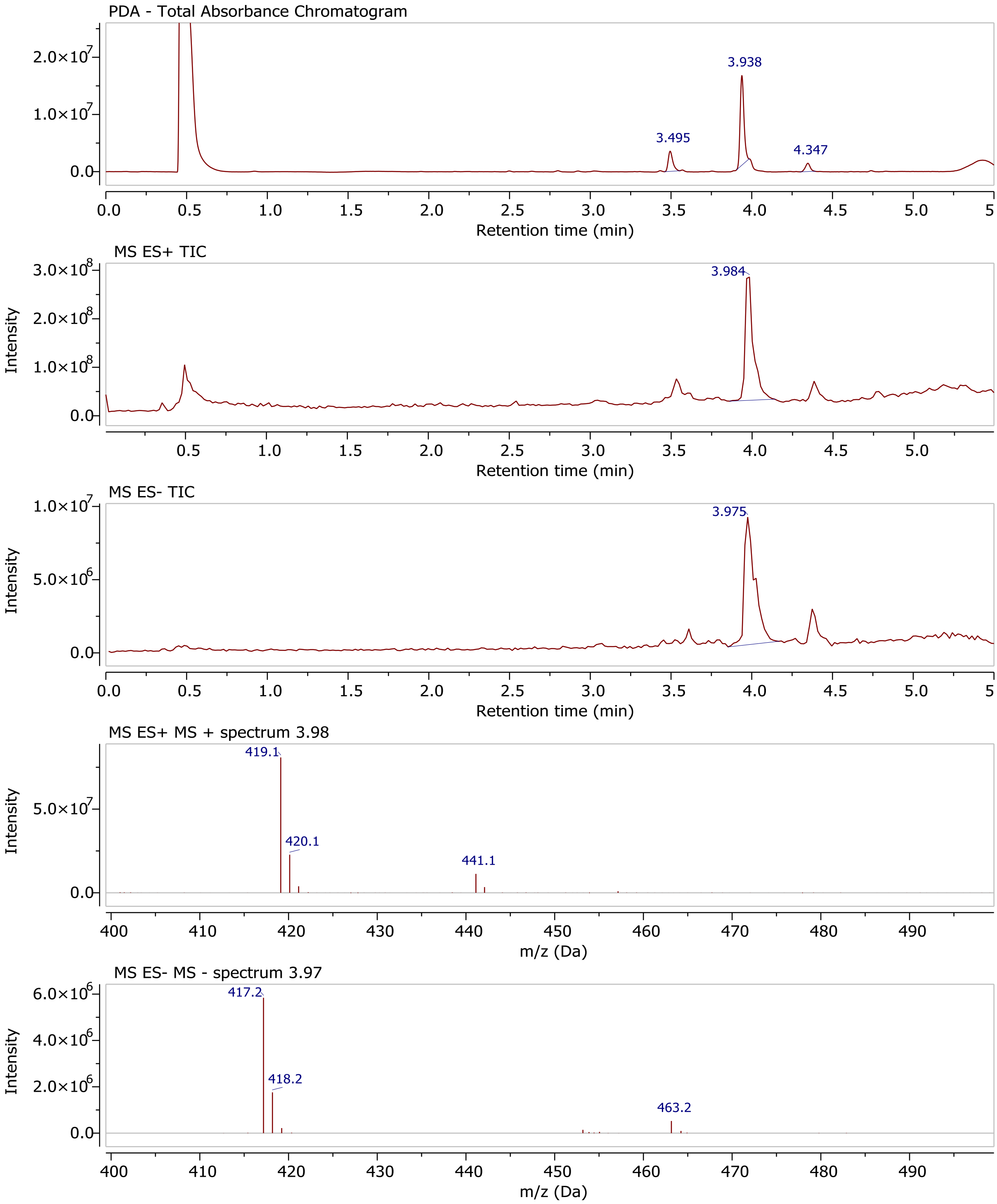

Supplementary figure **1529**. UPLC-MS analysis for compound **PHP-5671**

Chemical Formula: $C_{17}H_{17}BrN_2O_2S$
Exact Mass: 392.0

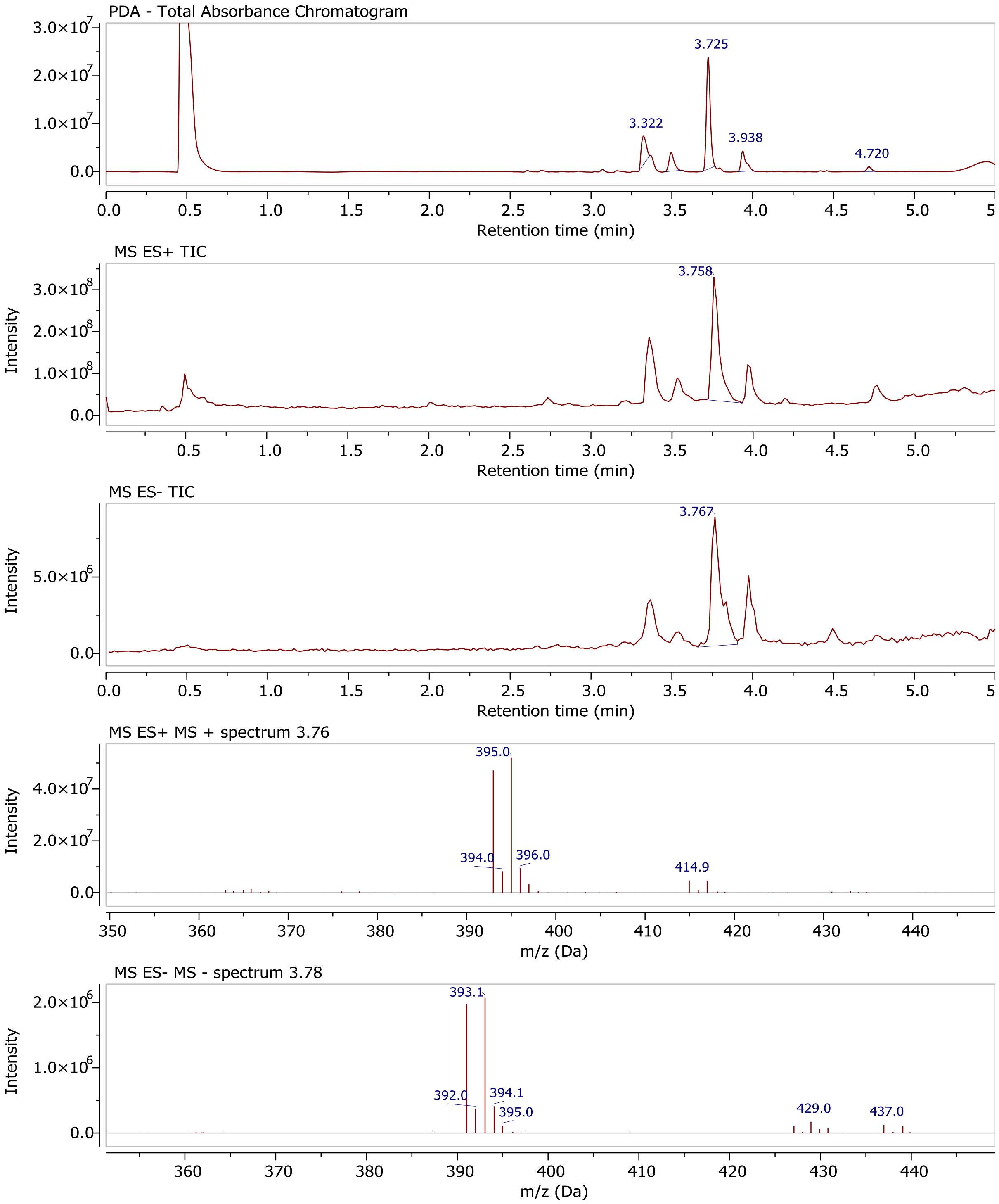

Supplementary figure **1530**. UPLC-MS analysis for compound **PHP-5853**

Chemical Formula: $C_{25}H_{23}FN_2O_3$
Exact Mass: 418.2

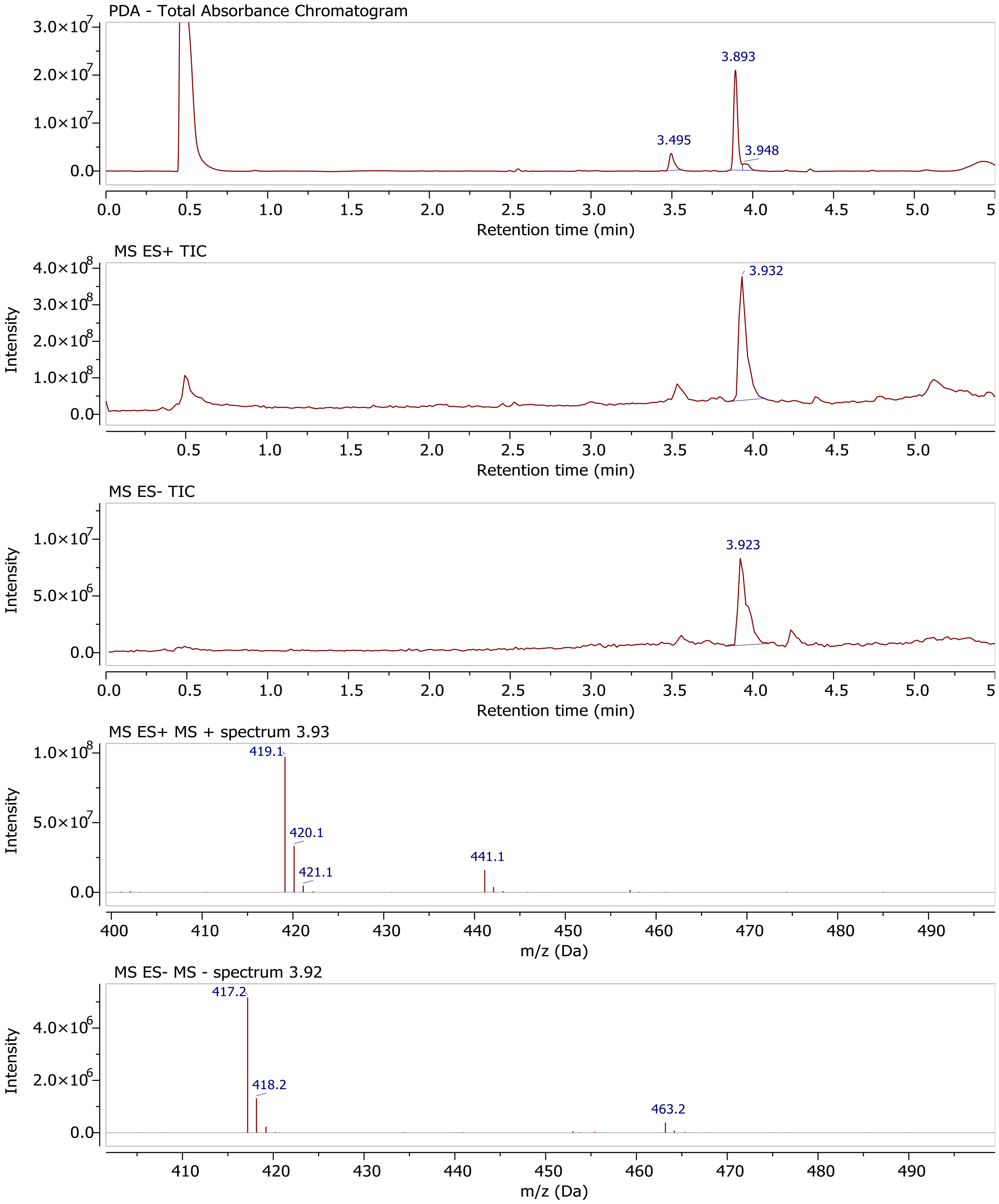

Supplementary figure **1531**. UPLC-MS analysis for compound **PHP-5672**

Chemical Formula: $C_{20}H_{22}N_2O_4$
Exact Mass: 354.2

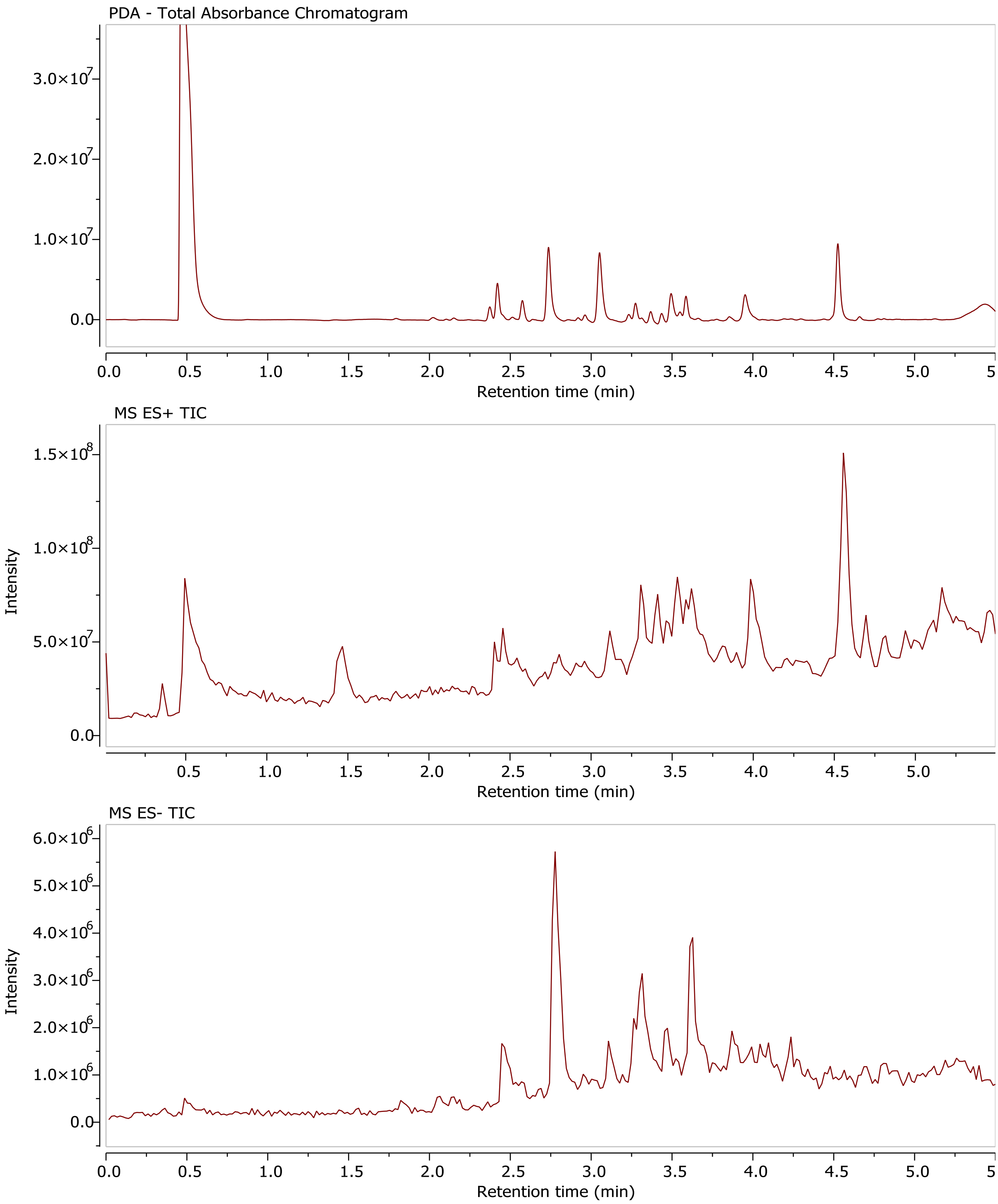

Supplementary figure **1532**. UPLC-MS analysis for compound **PHP-5855**

Chemical Formula: $C_{21}H_{30}N_2O_3$
Exact Mass: 358.2

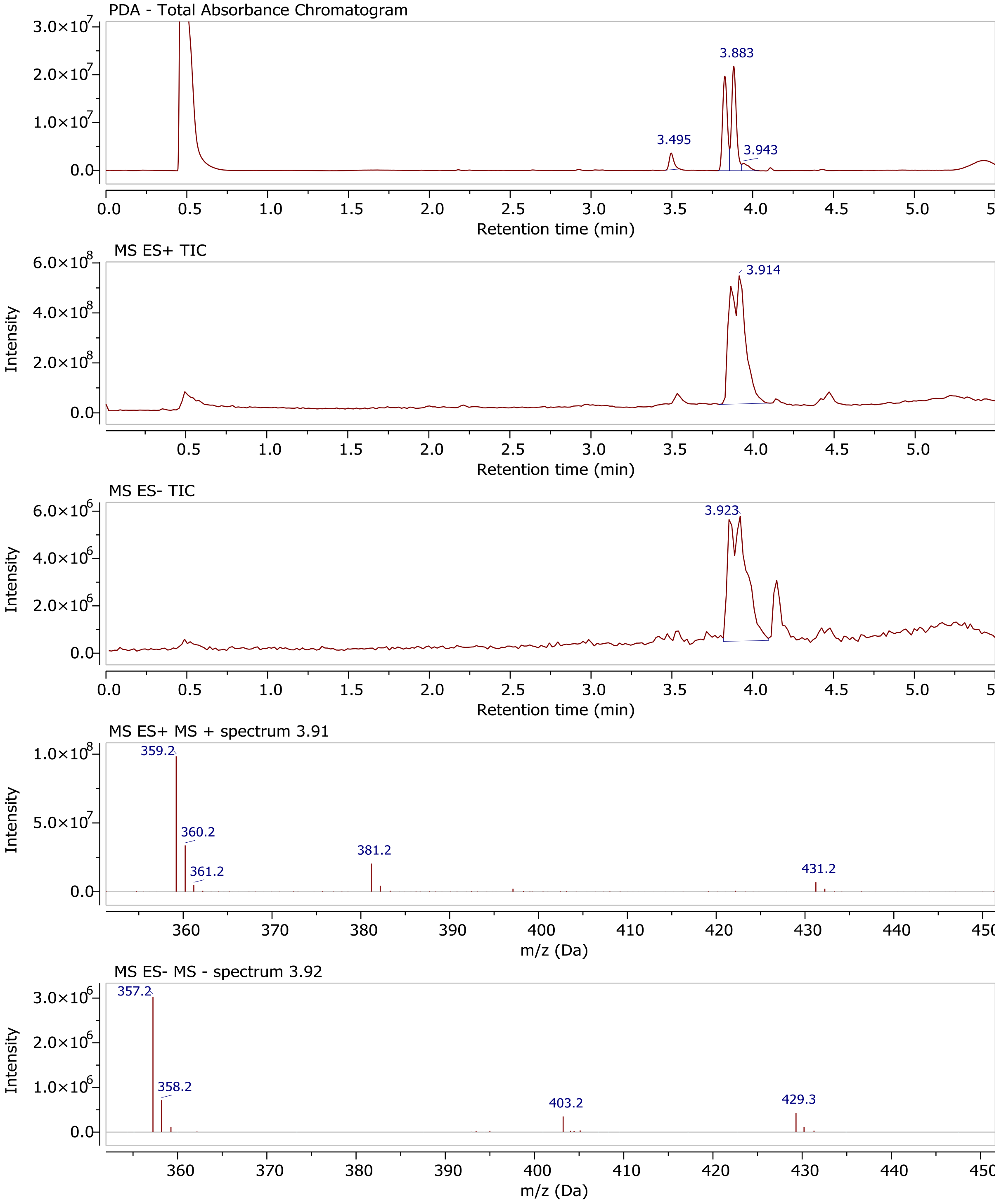

Supplementary figure **1533**. UPLC-MS analysis for compound **PHP-5673**

Chemical Formula: $C_{21}H_{24}N_2O_2$
Exact Mass: 336.2

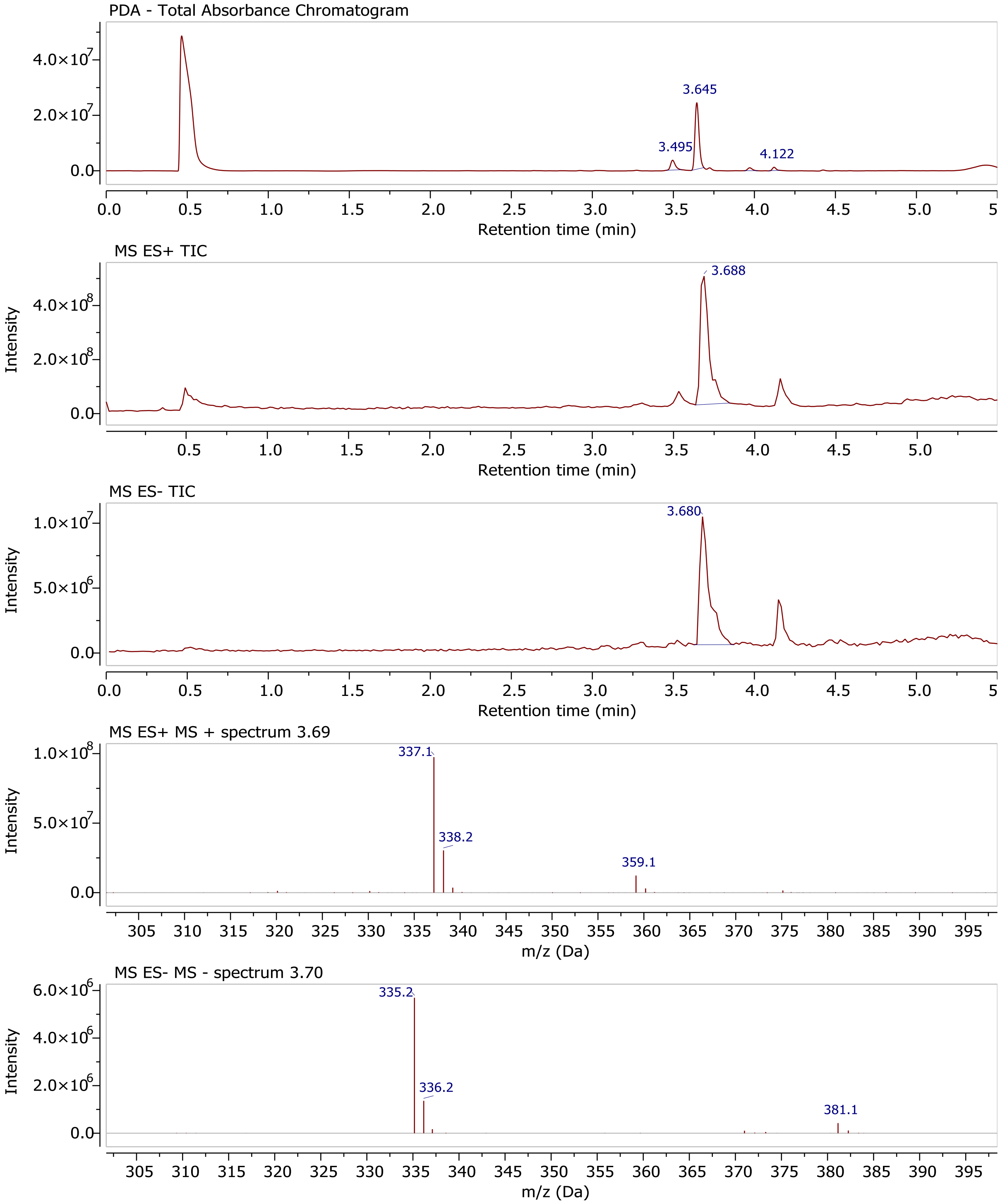

Supplementary figure **1534**. UPLC-MS analysis for compound **PHP-5856**

Chemical Formula: $C_{21}H_{30}N_2O_3$
Exact Mass: 358.2

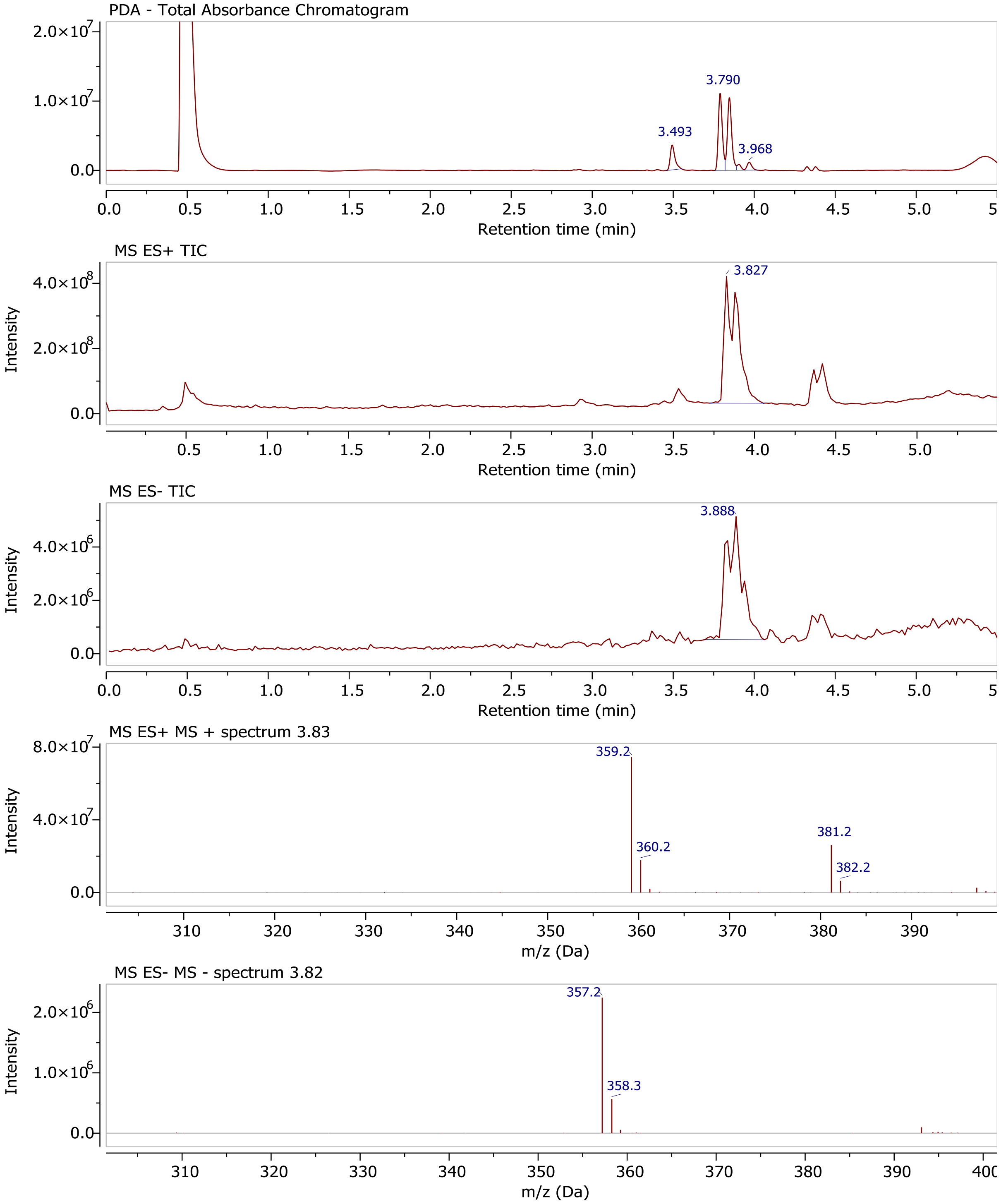

Supplementary figure **1535**. UPLC-MS analysis for compound **PHP-5674**

Chemical Formula: $C_{17}H_{22}N_2O_2$
Exact Mass: 286.2

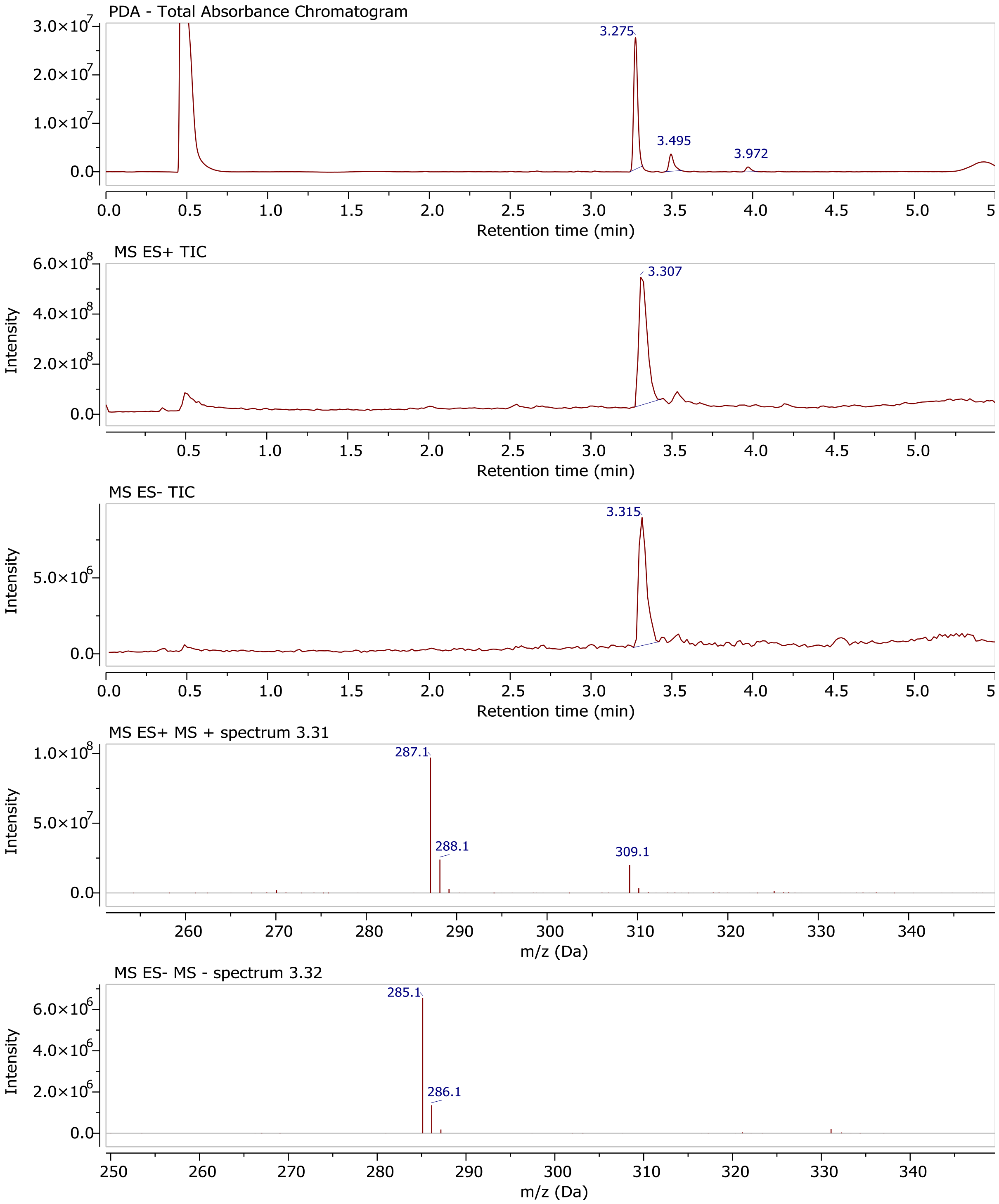

Supplementary figure **1536**. UPLC-MS analysis for compound **PHP-5857**

Chemical Formula: $C_{22}H_{20}F_4N_2O_3$
Exact Mass: 436.1

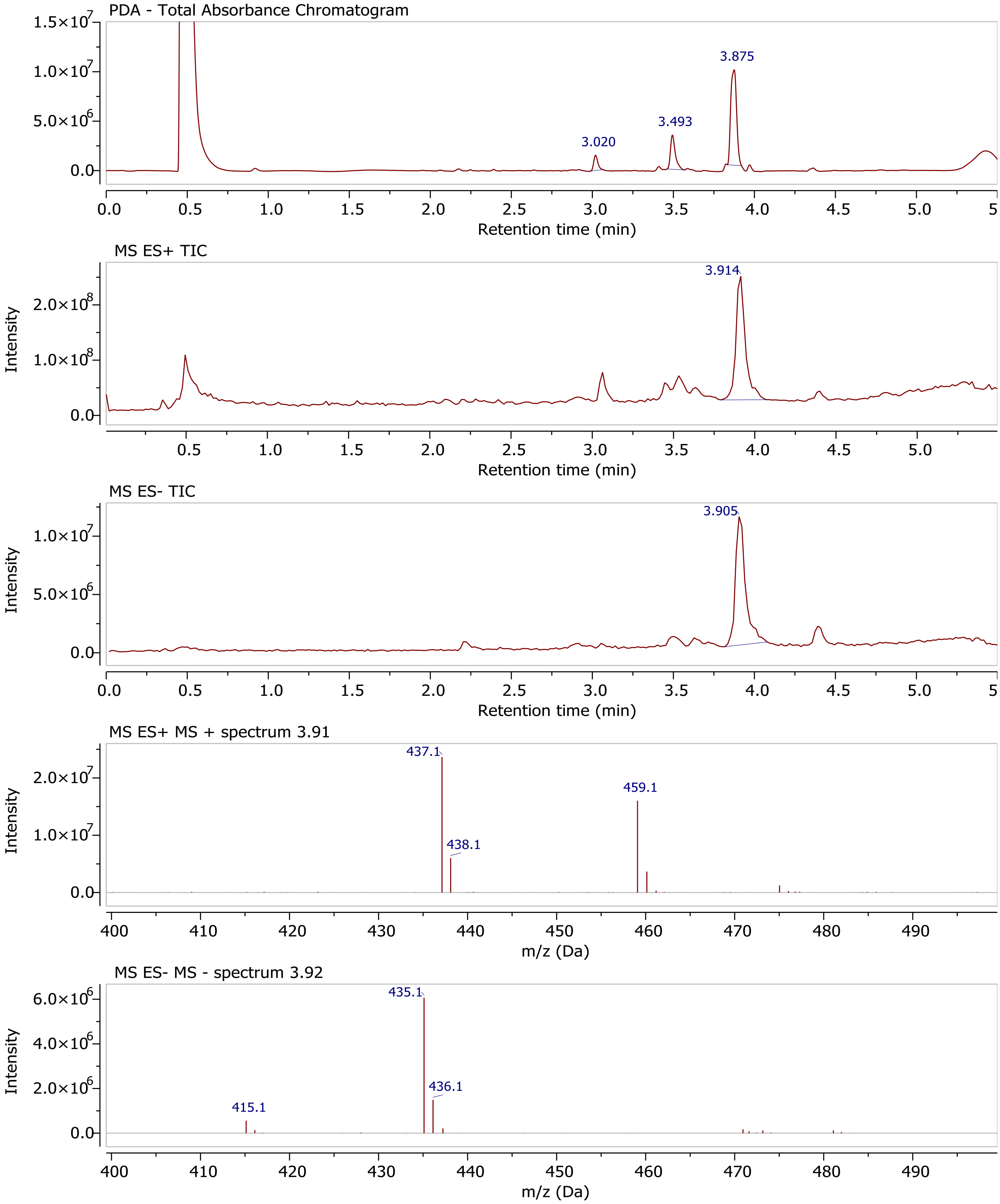

Supplementary figure **1537**. UPLC-MS analysis for compound **PHP-5675**

Chemical Formula: $C_{20}H_{19}F_3N_2O_2$
Exact Mass: 376.1

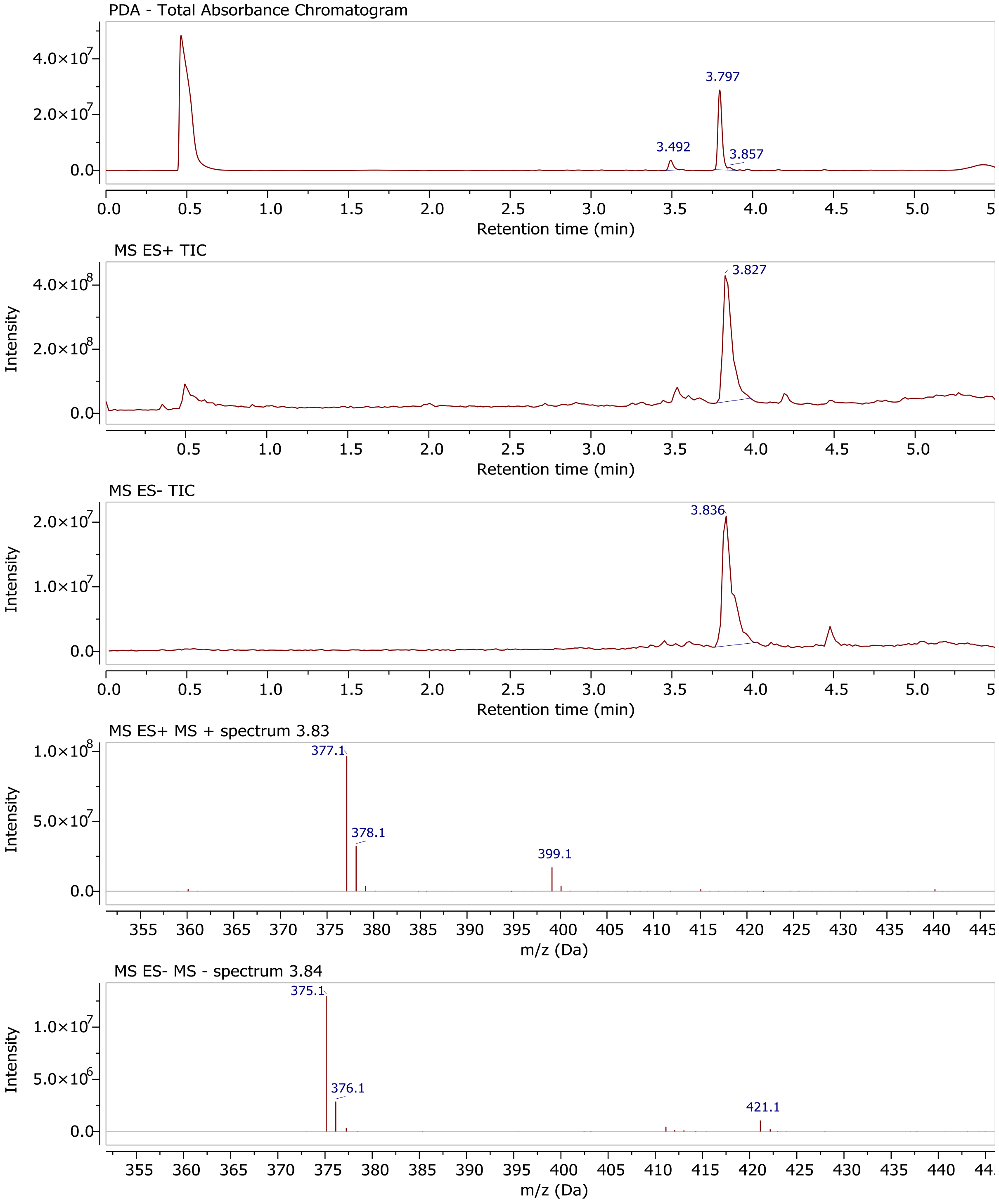

Supplementary figure **1538**. UPLC-MS analysis for compound **PHP-5859**

Chemical Formula: $C_{16}H_{20}N_2O_5$
Exact Mass: 320.1

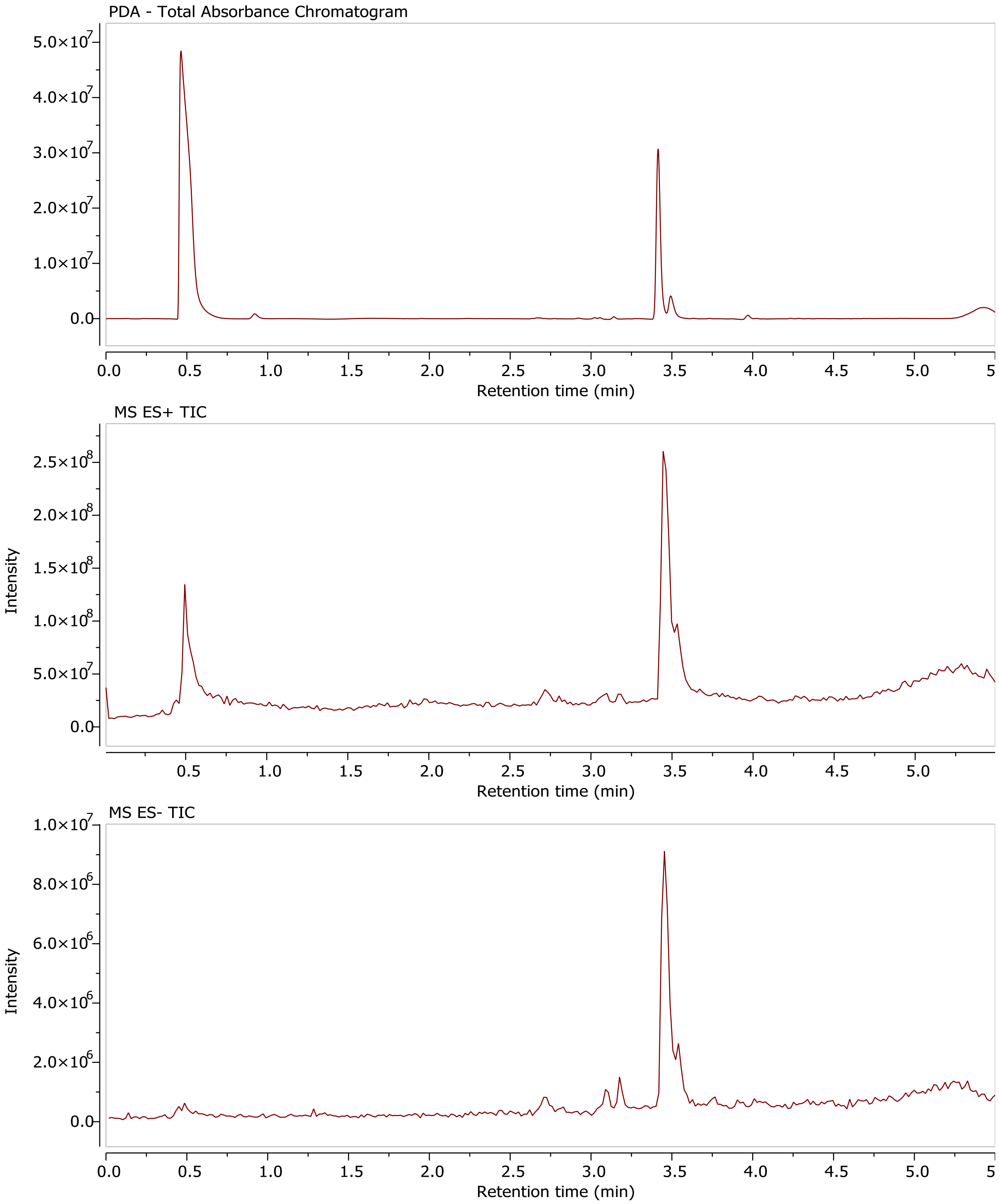

Supplementary figure **1539**. UPLC-MS analysis for compound **PHP-4947**

Chemical Formula: $C_{20}H_{20}N_2O_4$
Exact Mass: 352.1

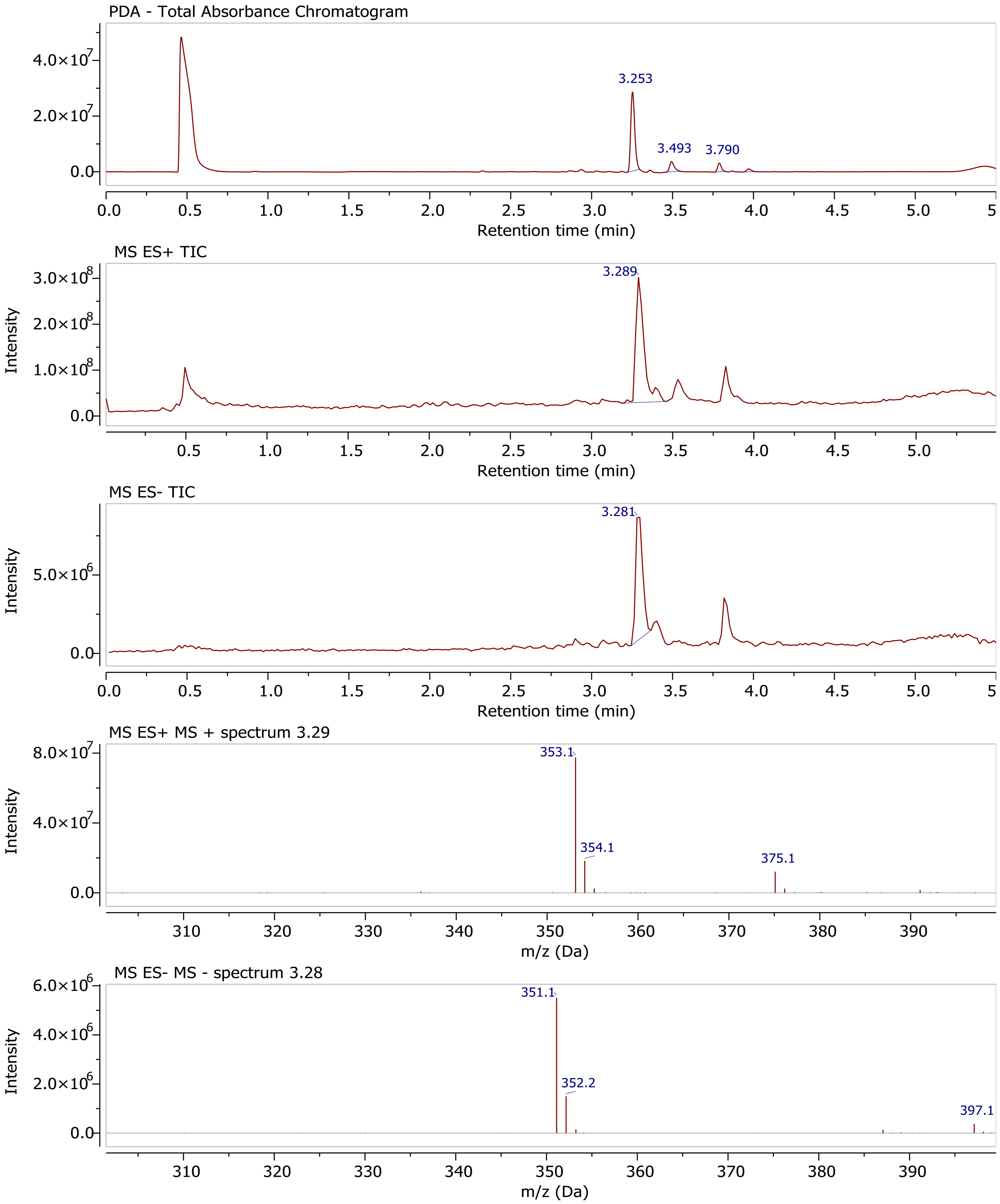

Supplementary figure **1540**. UPLC-MS analysis for compound **PHP-5526**

Chemical Formula: $C_{15}H_{21}N_3O_3$
Exact Mass: 291.2

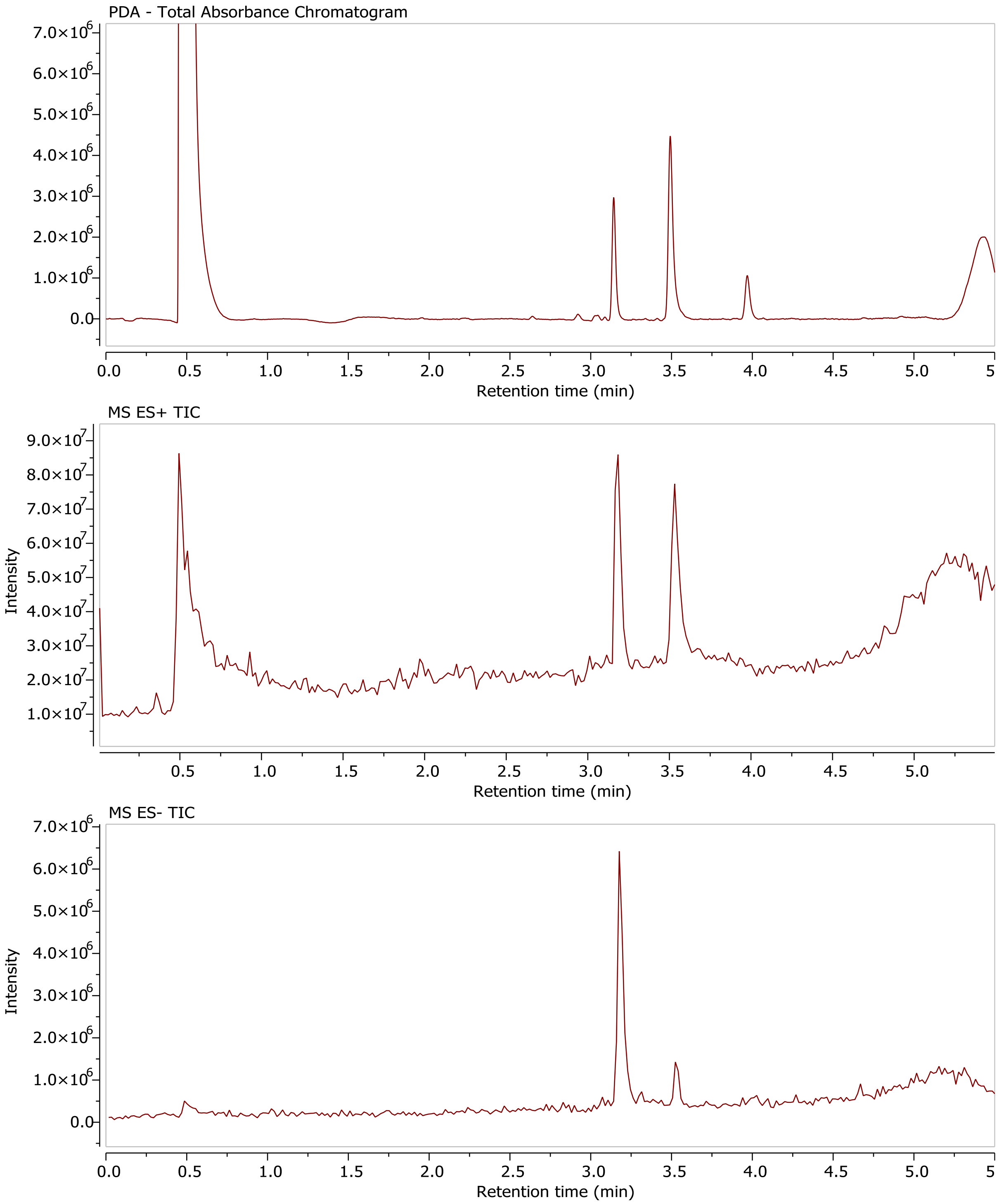

Supplementary figure **1541**. UPLC-MS analysis for compound **PHP-4948**

Chemical Formula: $C_{20}H_{20}N_2O_4$
Exact Mass: 352.1

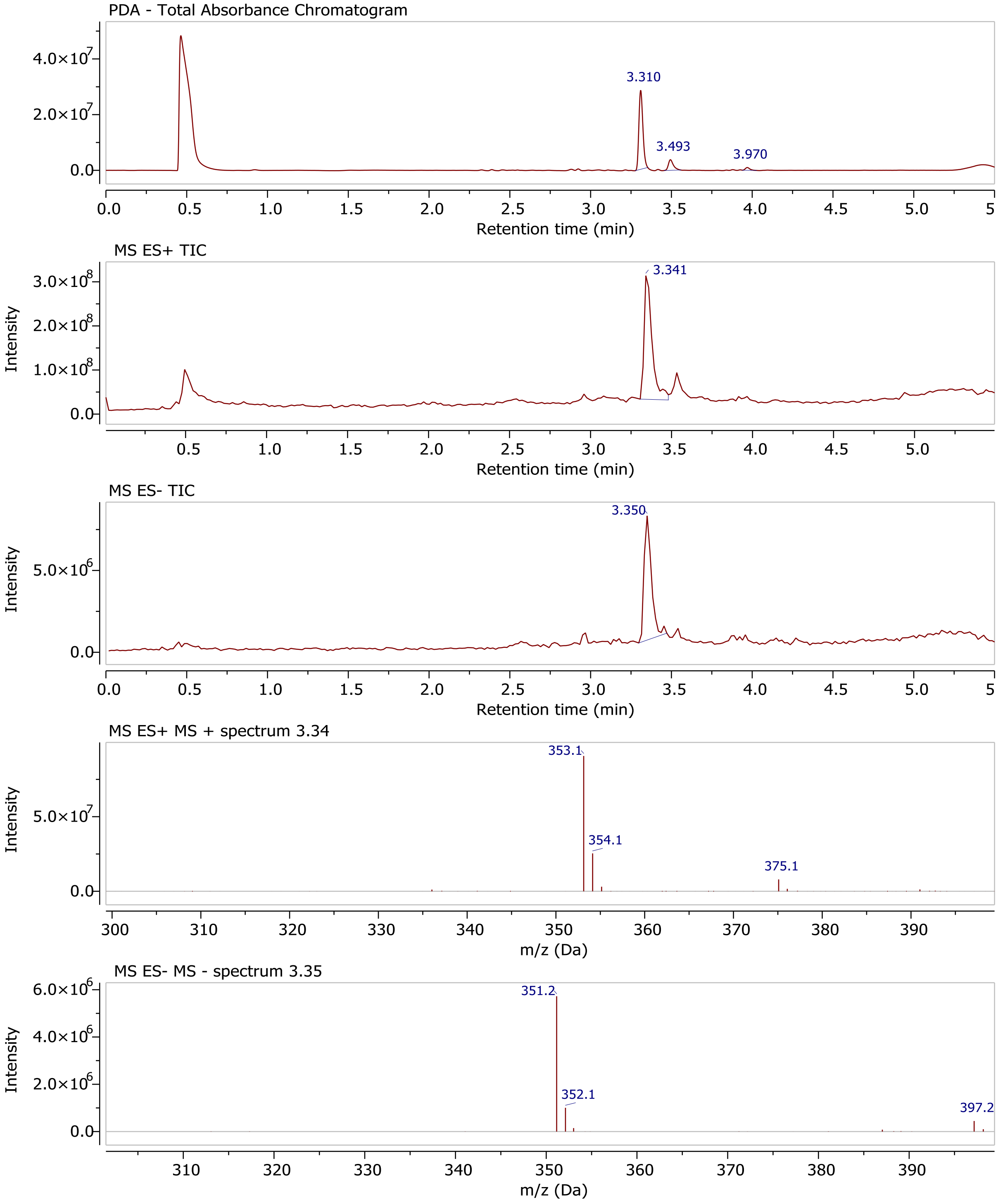

Supplementary figure **1542**. UPLC-MS analysis for compound **PHP-5527**

Chemical Formula: $C_{22}H_{25}ClN_2O_5$
Exact Mass: 432.1

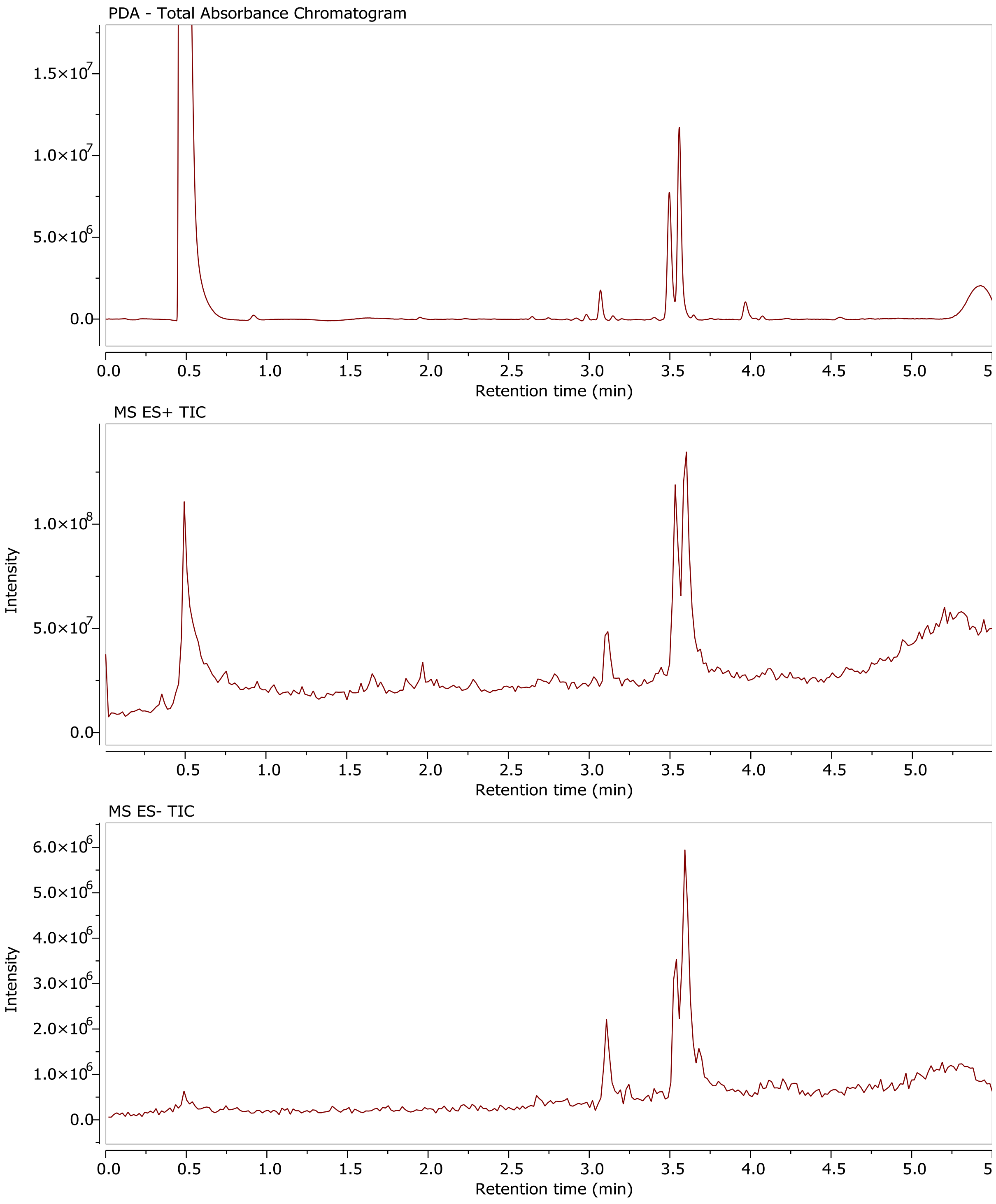

Supplementary figure **1543**. UPLC-MS analysis for compound **PHP-4953**

Chemical Formula: $C_{19}H_{13}F_5N_2O_4$
Exact Mass: 428.1

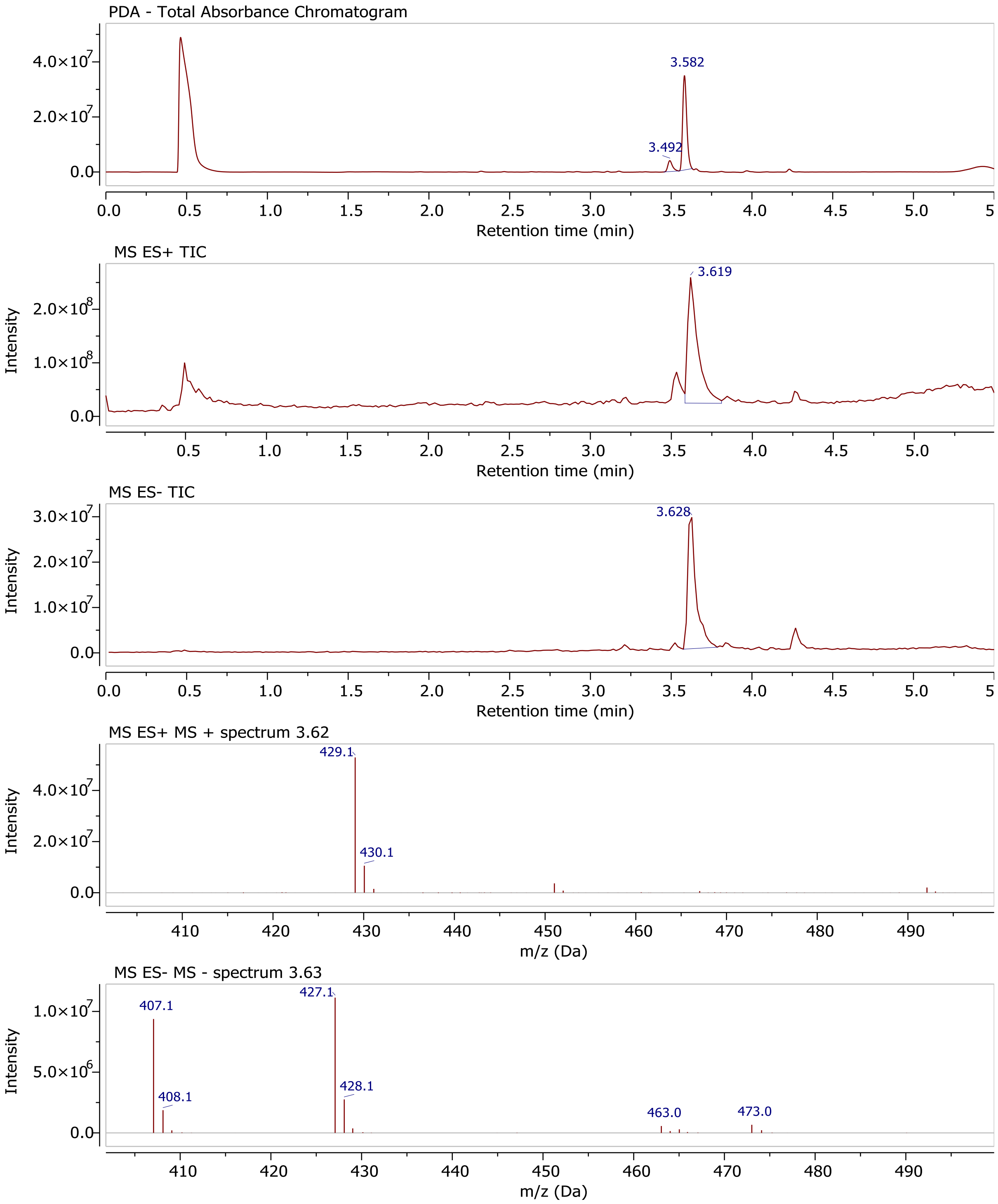

Supplementary figure **1544**. UPLC-MS analysis for compound **PHP-5528**

Chemical Formula: $C_{20}H_{23}N_3O_3$
Exact Mass: 353.2

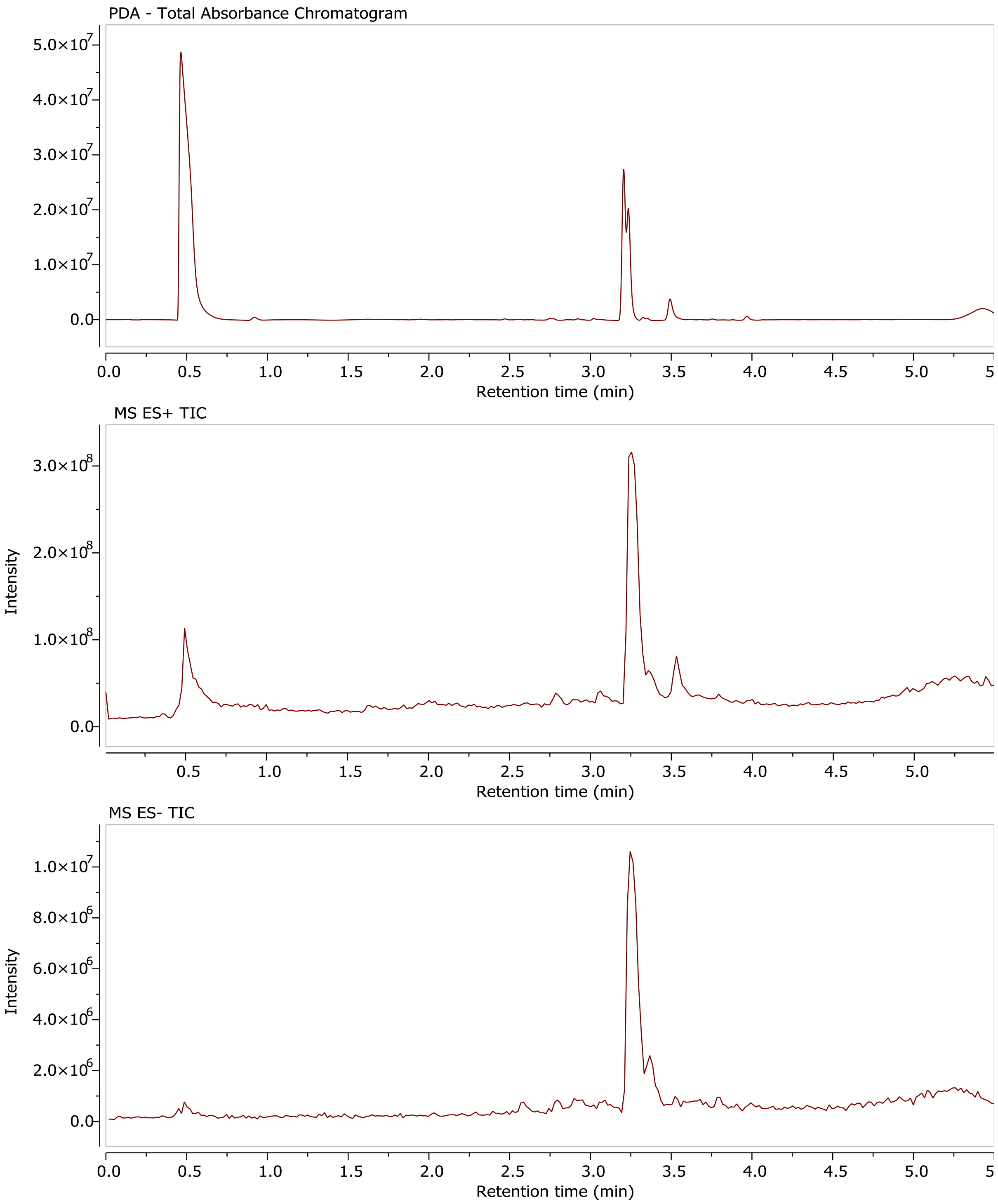

Supplementary figure **1545**. UPLC-MS analysis for compound **PHP-4962**

Chemical Formula: $C_{18}H_{22}N_2O_4$
Exact Mass: 330.2

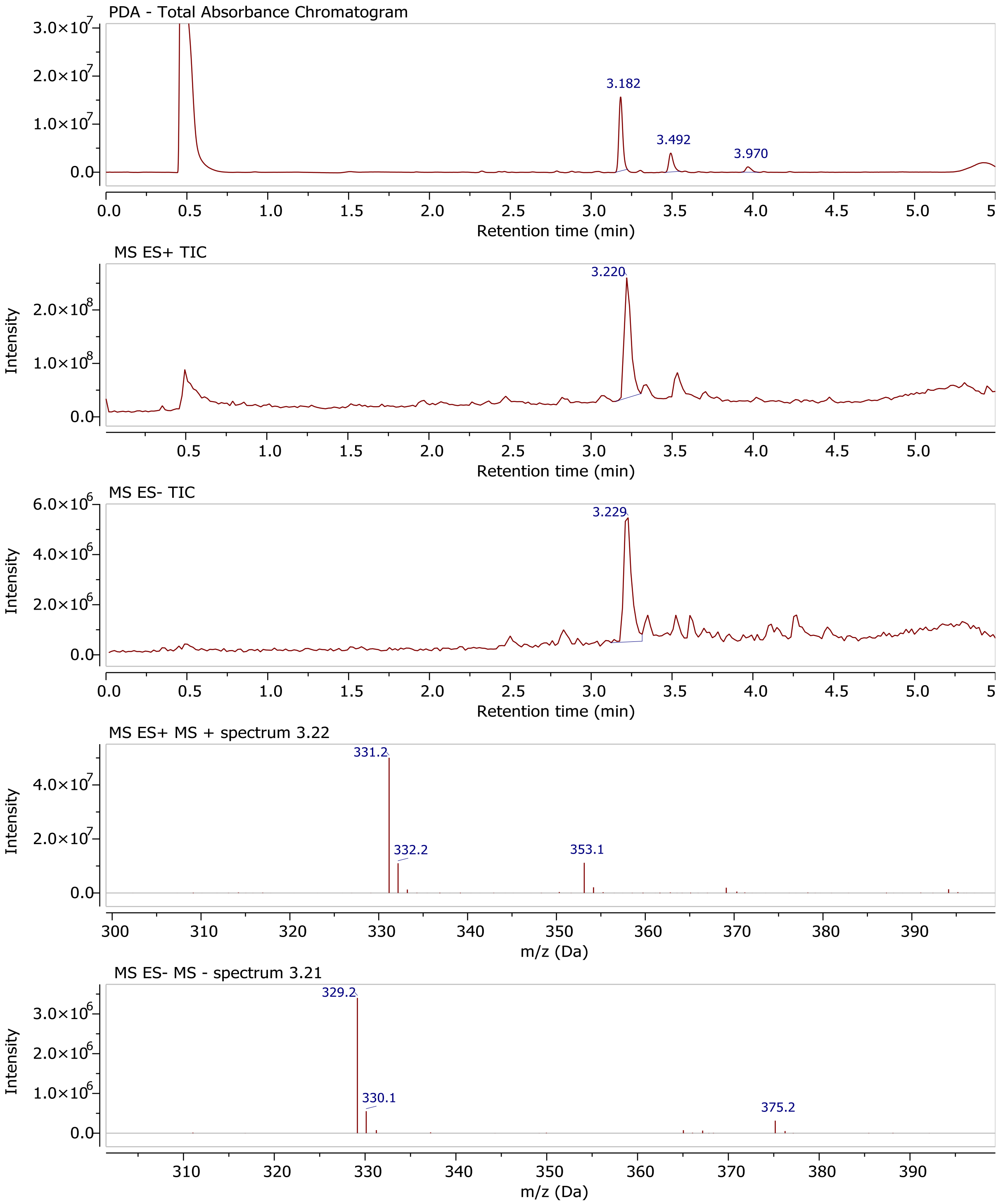

Supplementary figure **1546**. UPLC-MS analysis for compound **PHP-5529**

Chemical Formula: $C_{21}H_{21}N_3O_2$
Exact Mass: 347.2

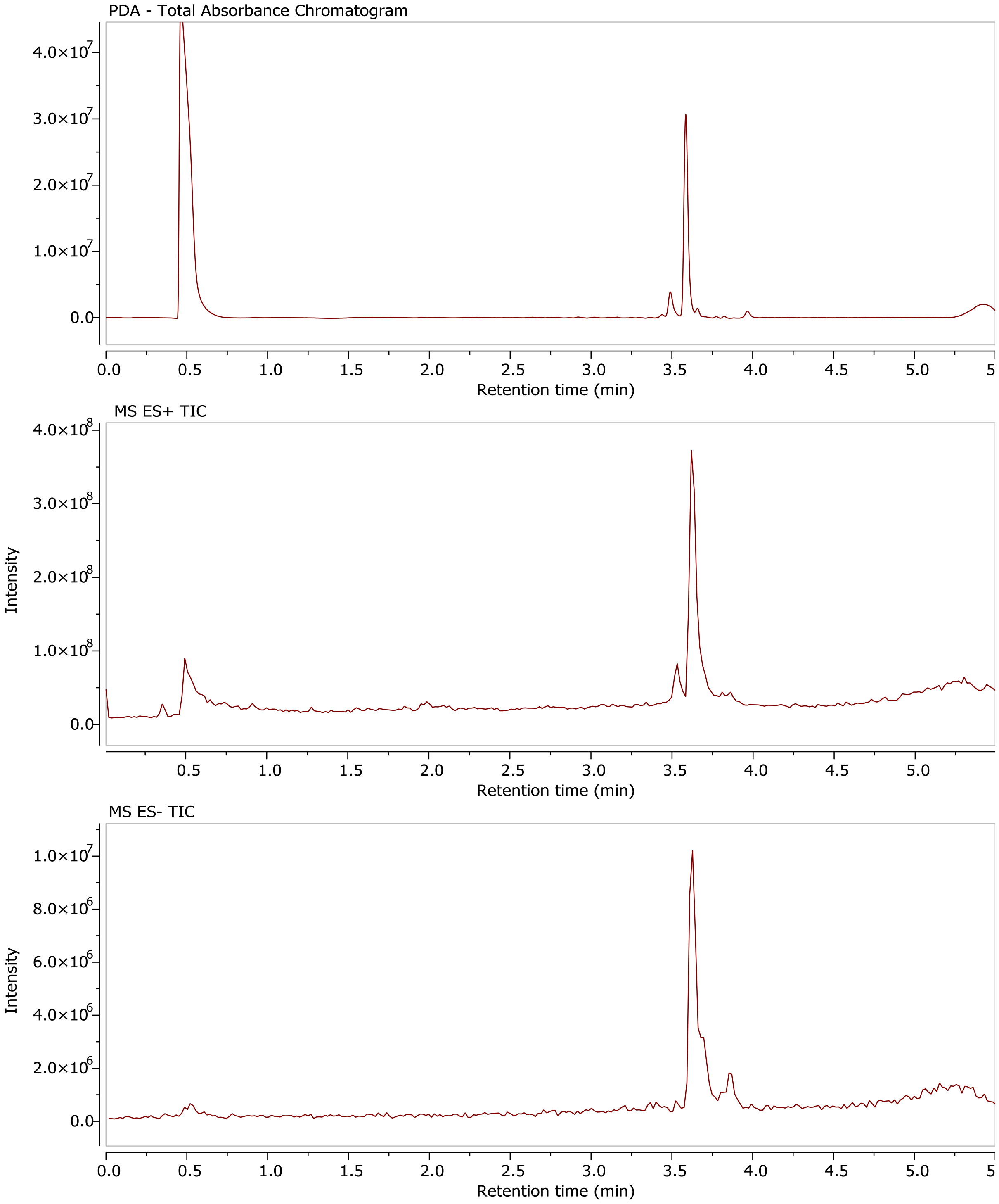

Supplementary figure **1547**. UPLC-MS analysis for compound **PHP-4965**

Chemical Formula: $C_{20}H_{20}N_2O_5$
Exact Mass: 368.1

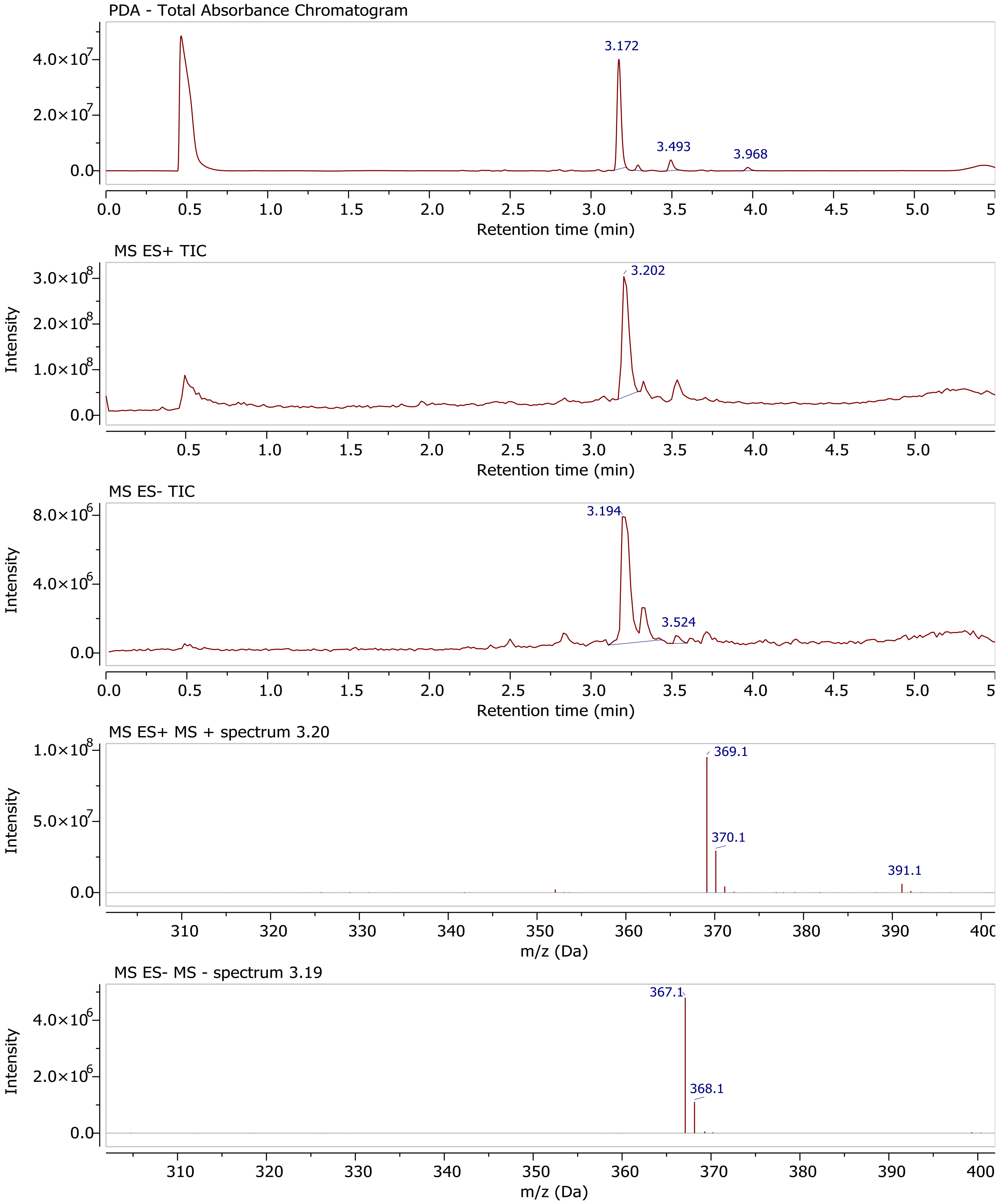

Supplementary figure **1548**. UPLC-MS analysis for compound **PHP-5530**

Chemical Formula: $C_{13}H_{16}N_2O_2$
Exact Mass: 232.1

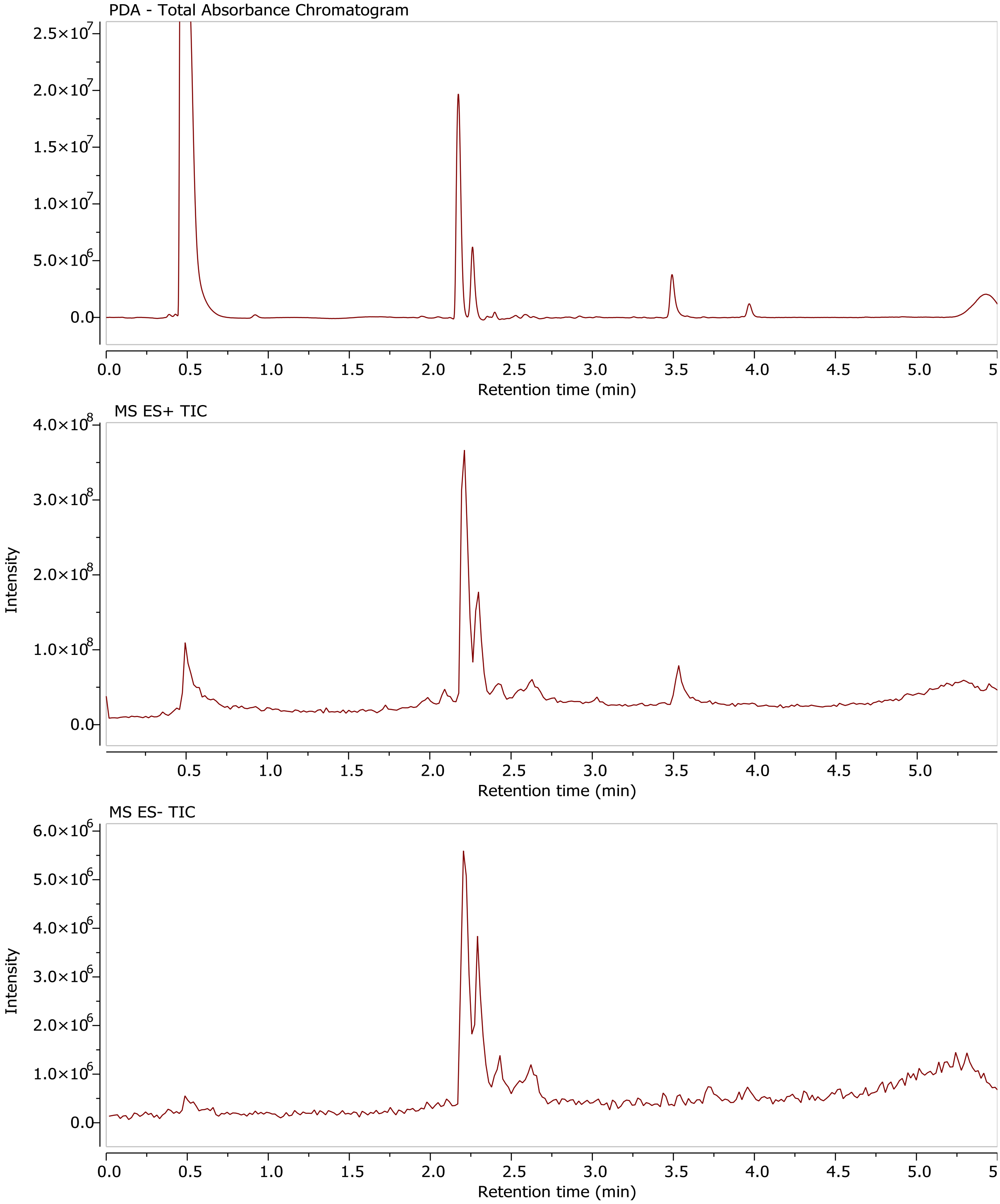

Supplementary figure **1549**. UPLC-MS analysis for compound **PHP-4968**

Chemical Formula: $C_{17}H_{16}N_2O_5$
Exact Mass: 328.1

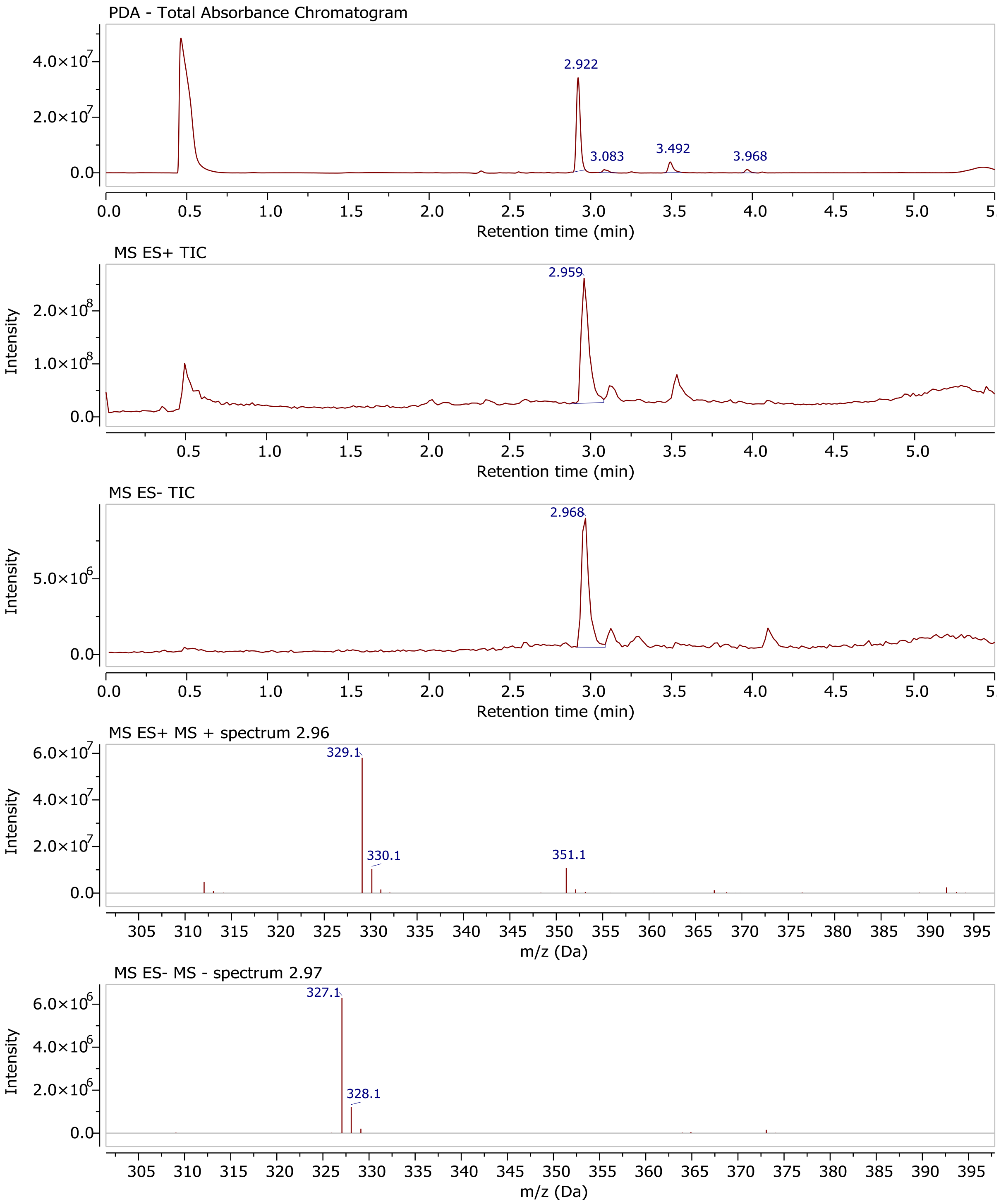

Supplementary figure **1550**. UPLC-MS analysis for compound **PHP-5531**

Chemical Formula: $C_{16}H_{18}N_4O_2$
Exact Mass: 298.1

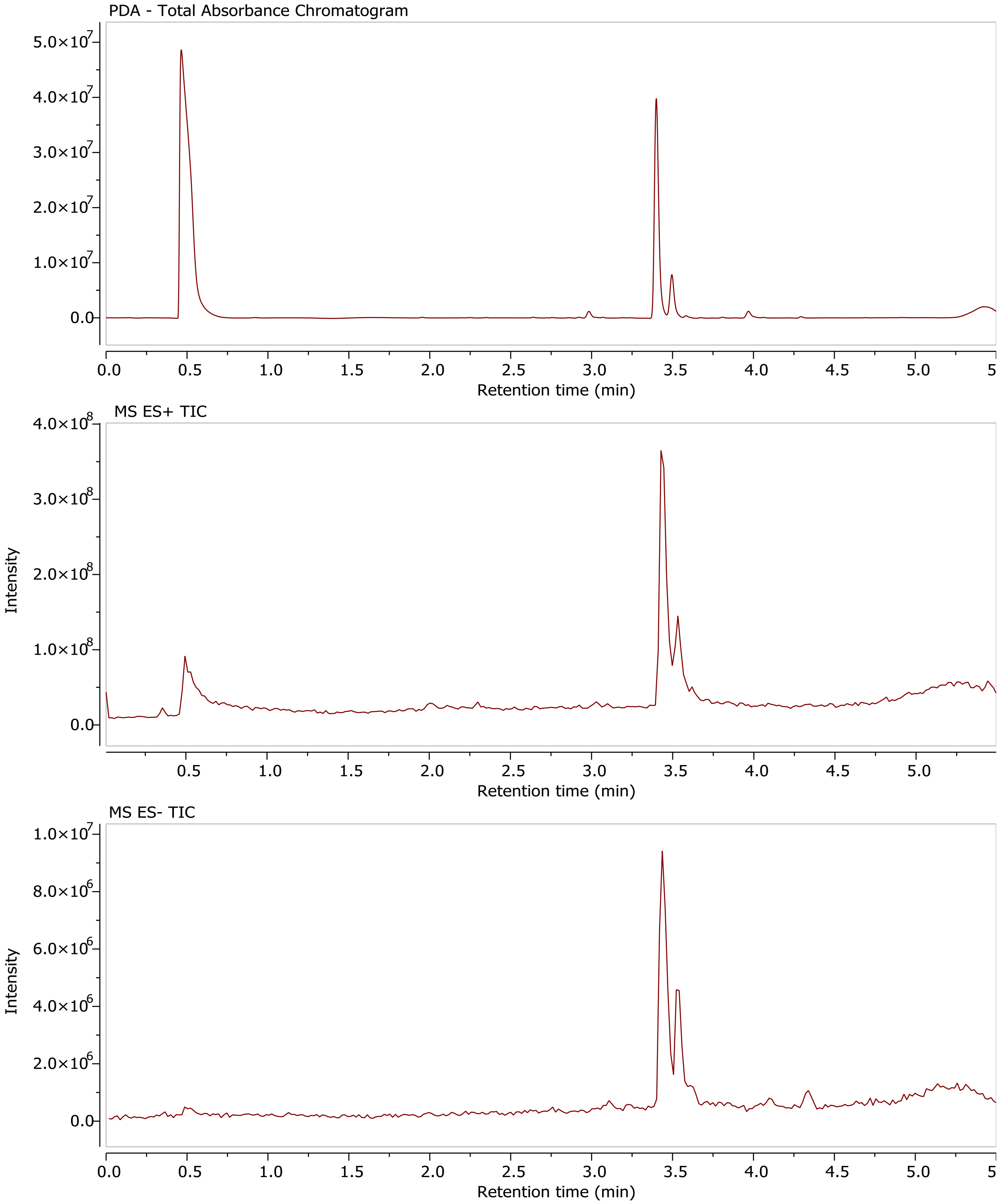

Supplementary figure **1551**. UPLC-MS analysis for compound **PHP-4970**

Chemical Formula: $C_{23}H_{20}N_2O_4$
Exact Mass: 388.1

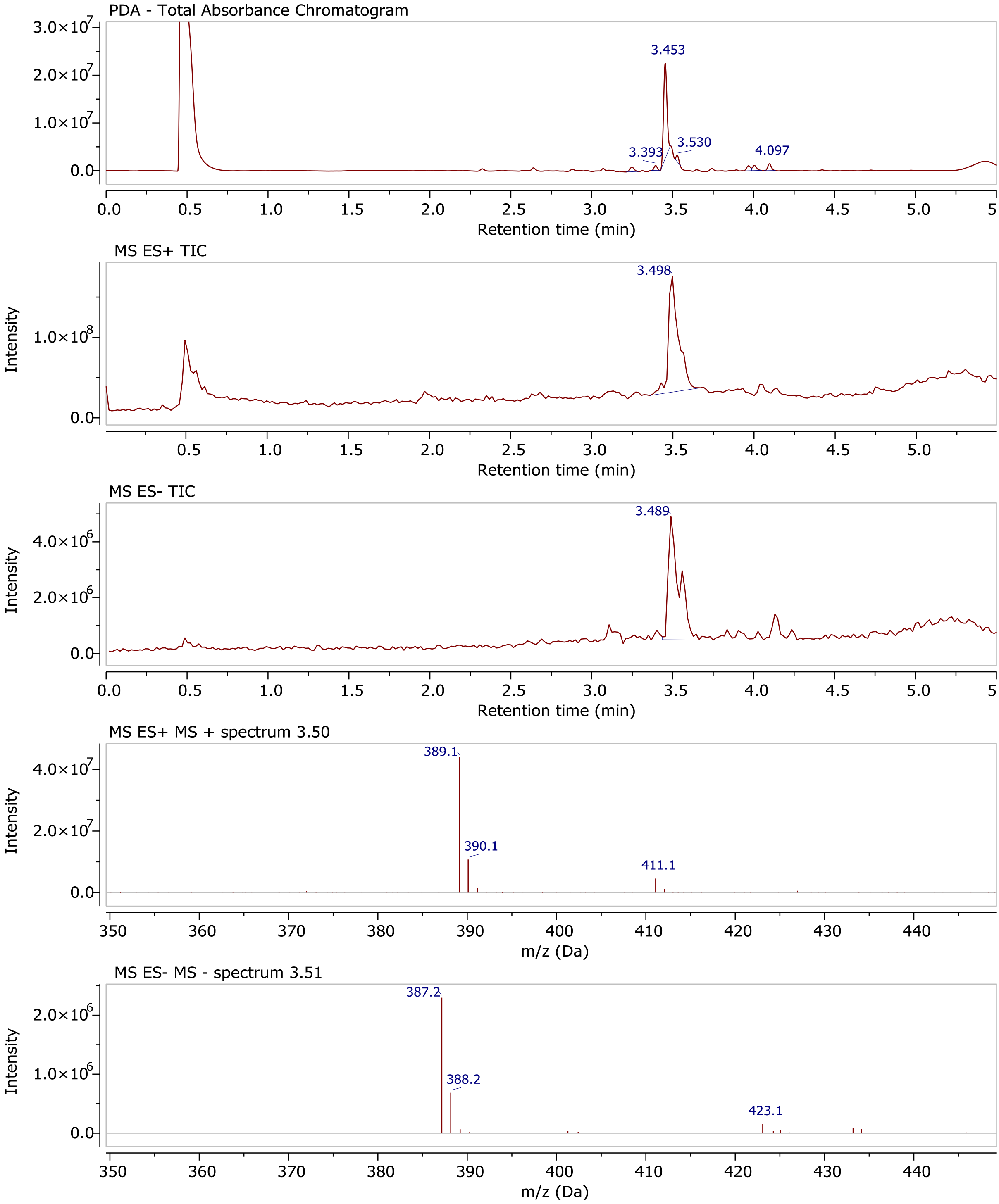

Supplementary figure **1552**. UPLC-MS analysis for compound **PHP-5532**

Chemical Formula: $C_{20}H_{20}N_2O_4$
Exact Mass: 352.1

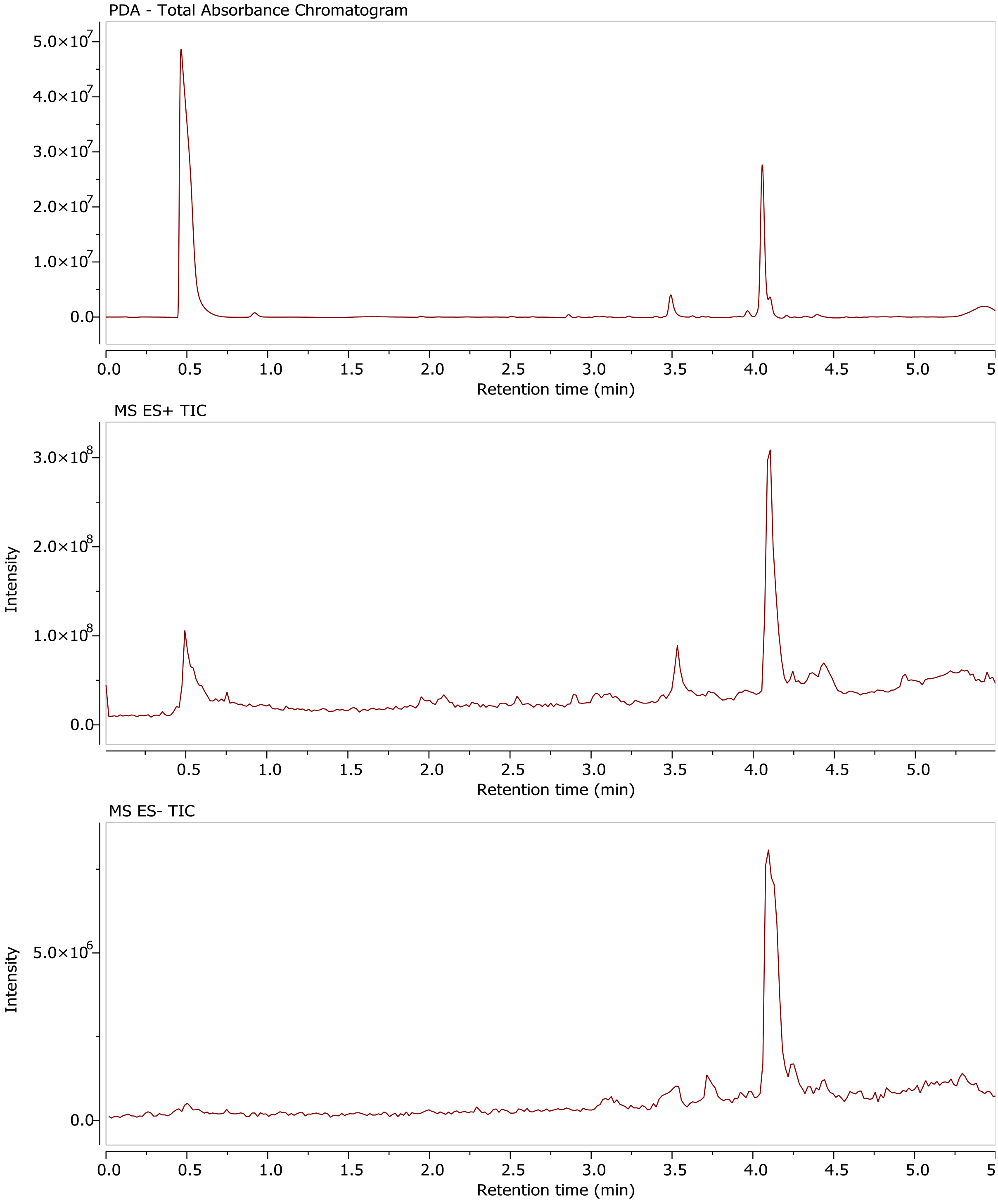

Supplementary figure **1553**. UPLC-MS analysis for compound **PHP-4971**

Chemical Formula: $C_{22}H_{24}N_2O_4$
Exact Mass: 380.2

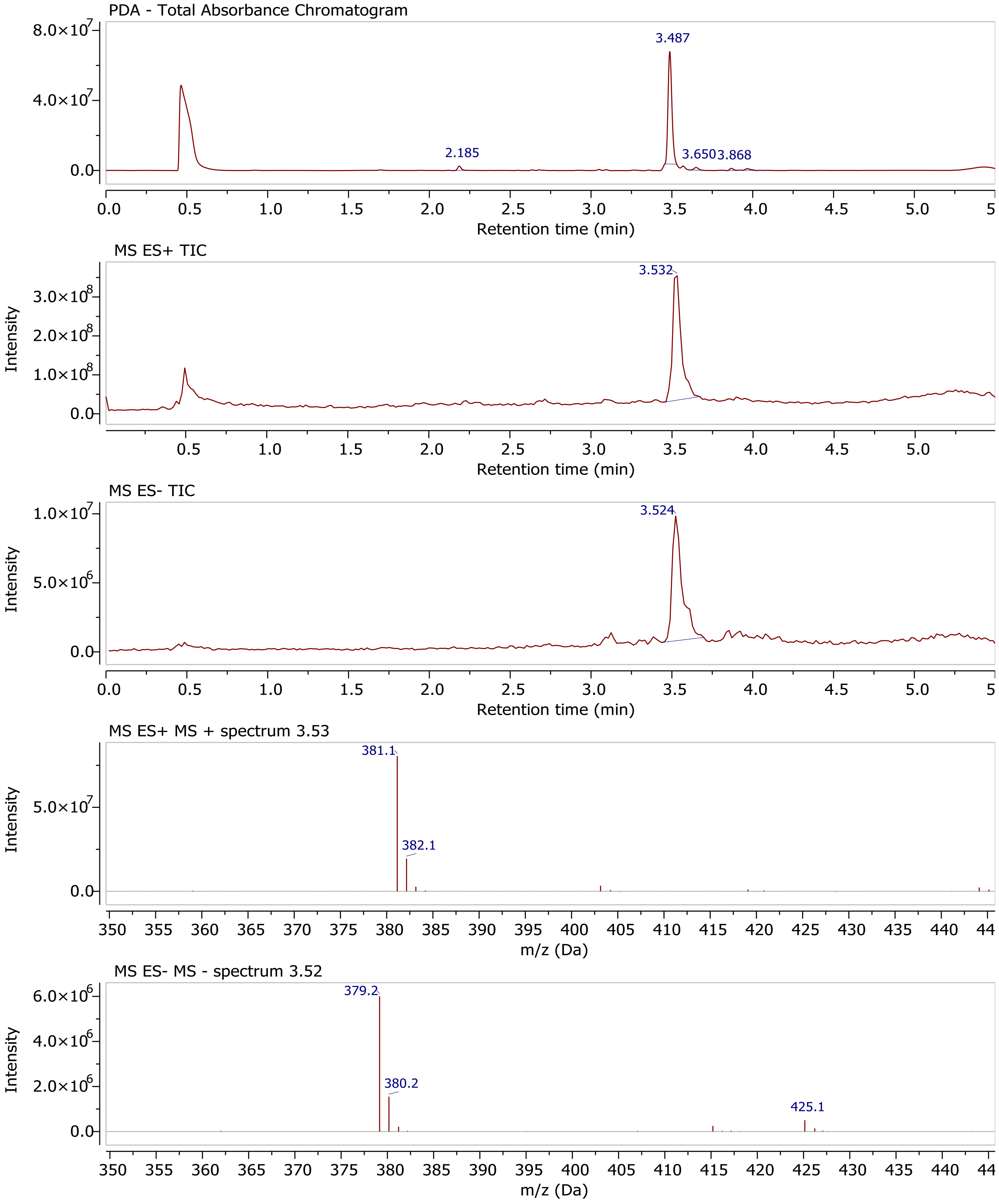

Supplementary figure **1554**. UPLC-MS analysis for compound **PHP-5533**

Chemical Formula: $C_{18}H_{20}N_4O_2$
Exact Mass: 324.2

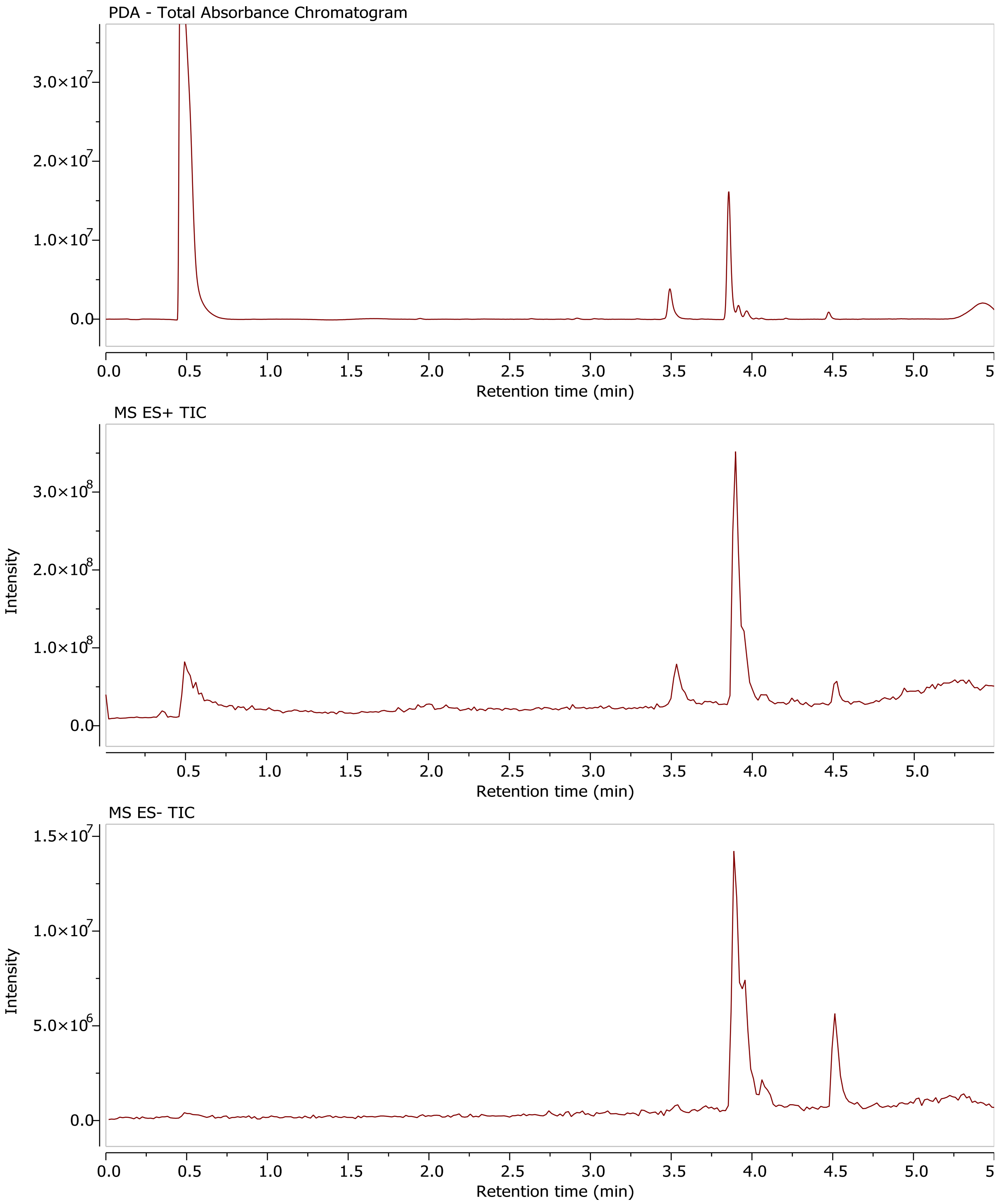

Supplementary figure **1555**. UPLC-MS analysis for compound **PHP-4972**

Chemical Formula: $C_{25}H_{24}N_2O_5$
Exact Mass: 432.2

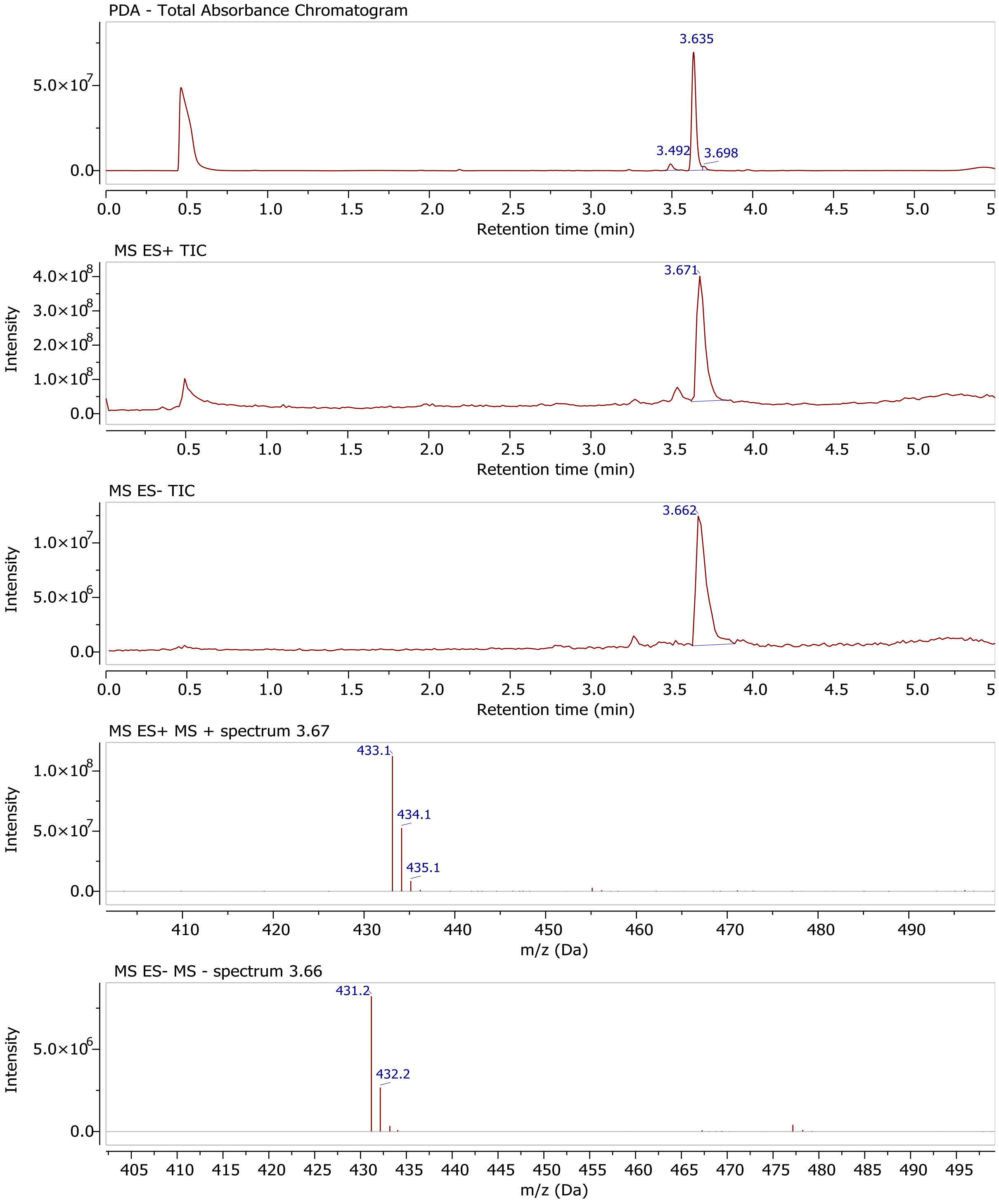

Supplementary figure **1556**. UPLC-MS analysis for compound **PHP-5534**

Chemical Formula: $C_{23}H_{22}N_2O_2$
Exact Mass: 358.2

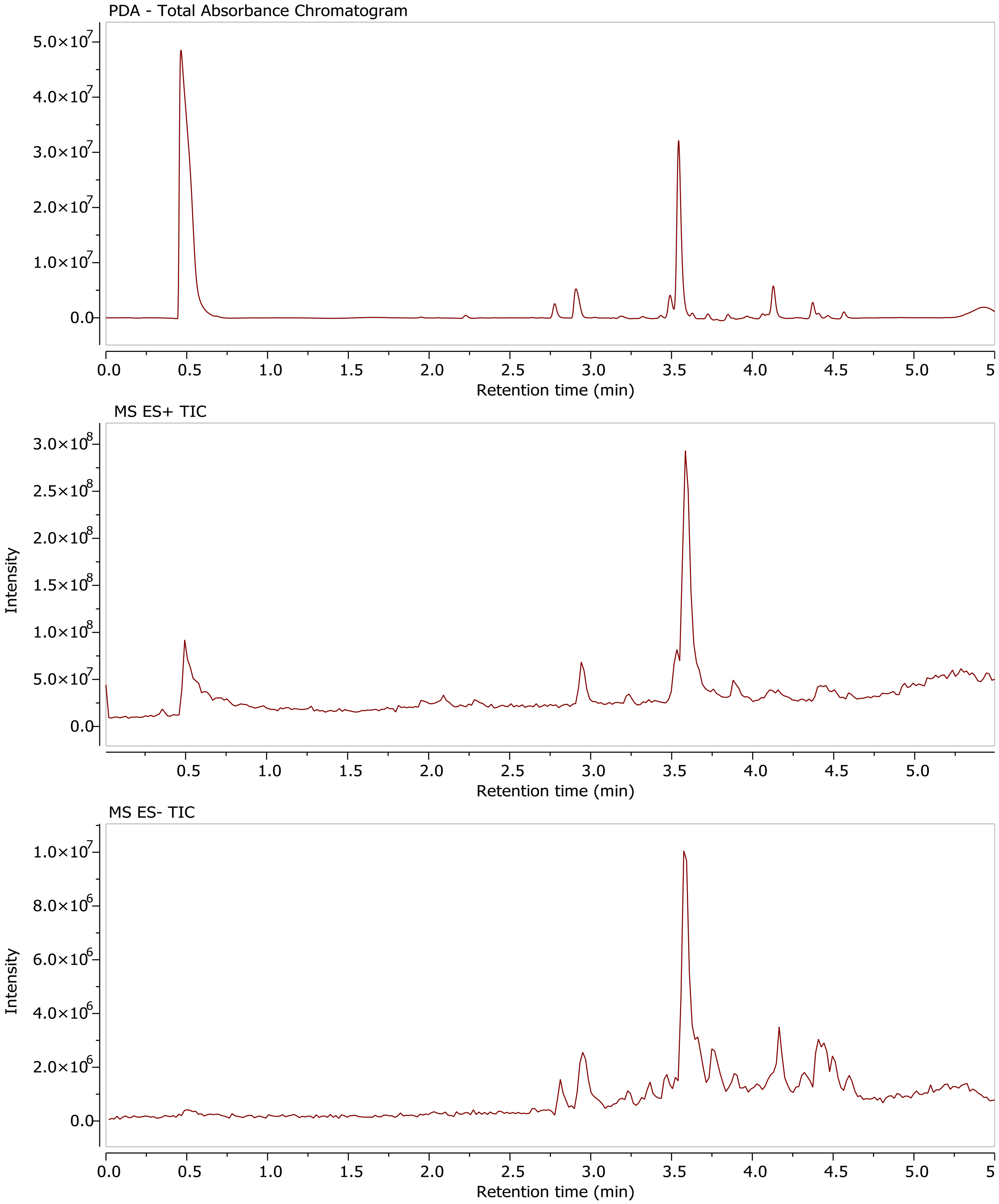

Supplementary figure **1557**. UPLC-MS analysis for compound **PHP-4973**

Chemical Formula: $C_{16}H_{22}N_2O_4$
Exact Mass: 306.2

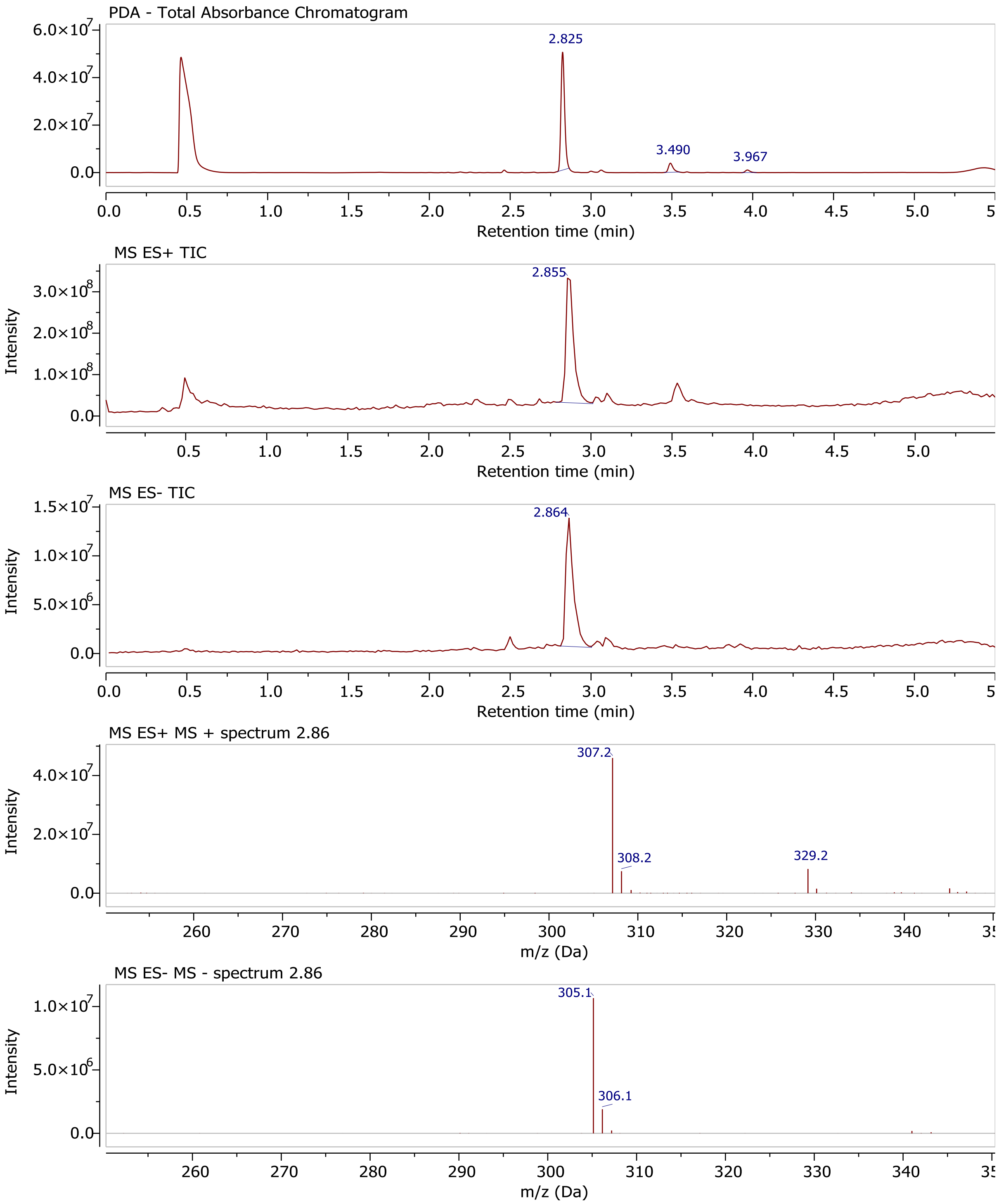

Supplementary figure **1558**. UPLC-MS analysis for compound **PHP-5535**

Chemical Formula: $C_{16}H_{17}N_3O_2S$
Exact Mass: 315.1

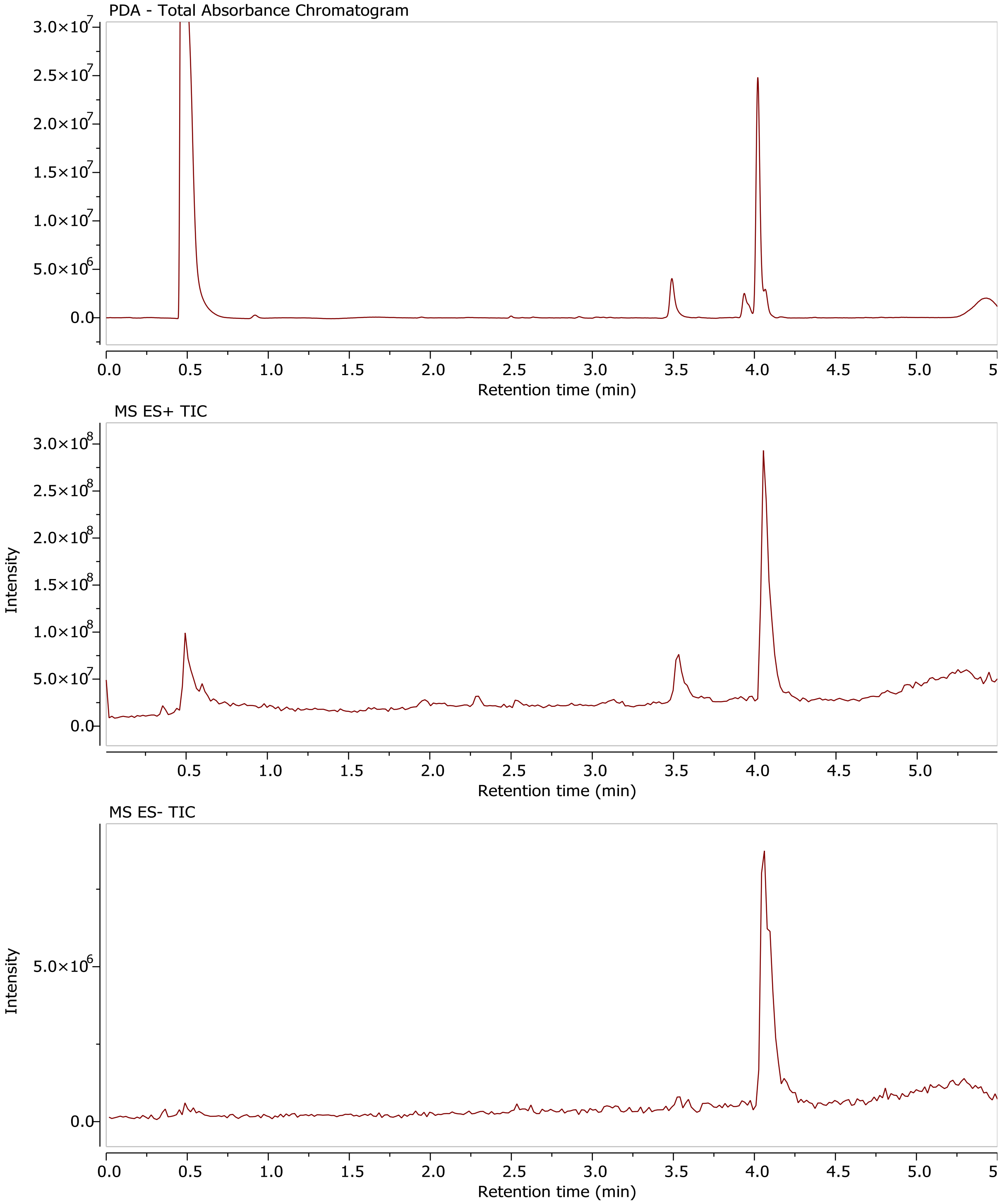

Supplementary figure **1559**. UPLC-MS analysis for compound **PHP-4975**

Chemical Formula: $C_{17}H_{24}N_2O_4$
Exact Mass: 320.2

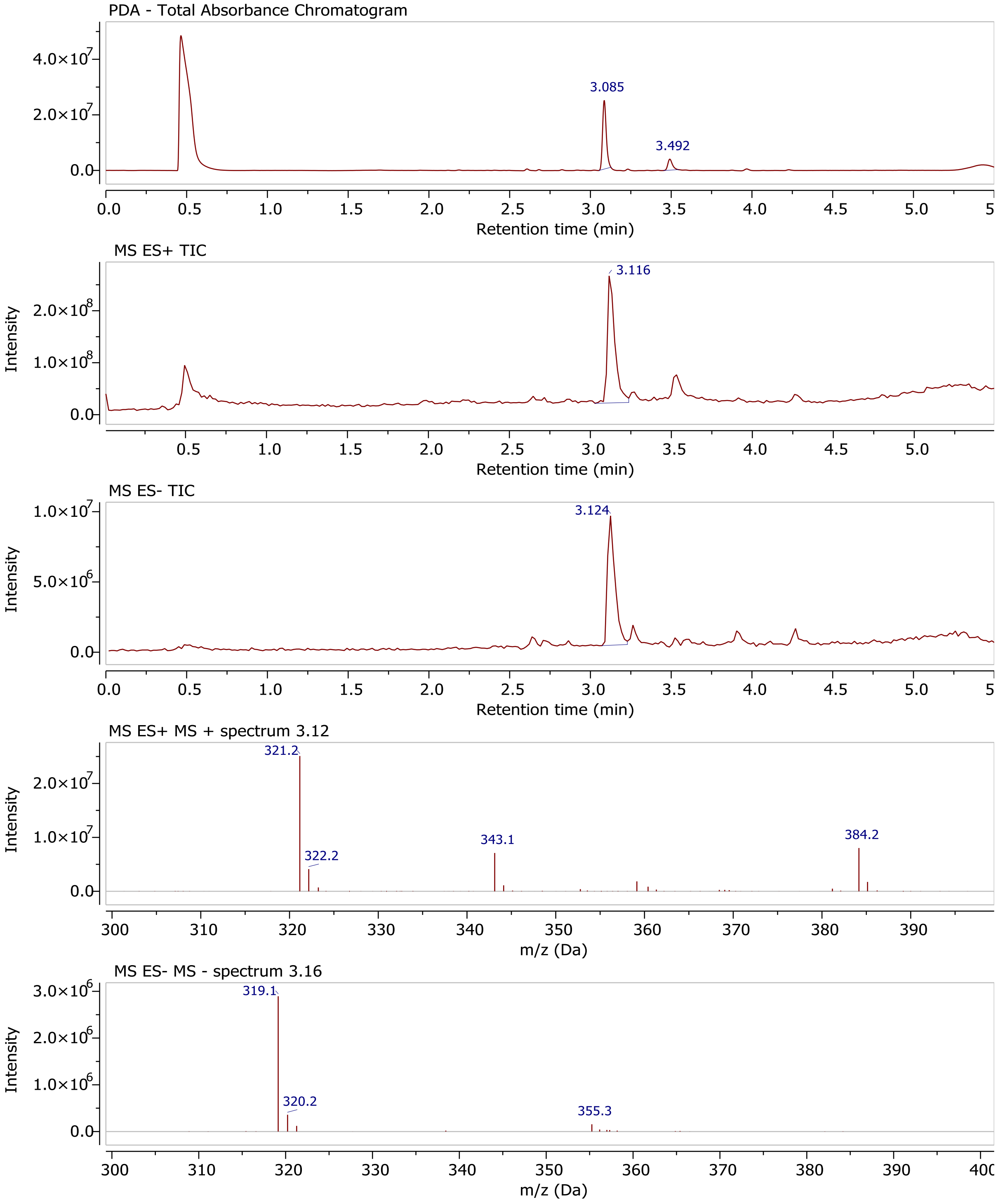

Supplementary figure **1560**. UPLC-MS analysis for compound **PHP-5536**

Chemical Formula: $C_{20}H_{28}N_2O_4$
Exact Mass: 360.2

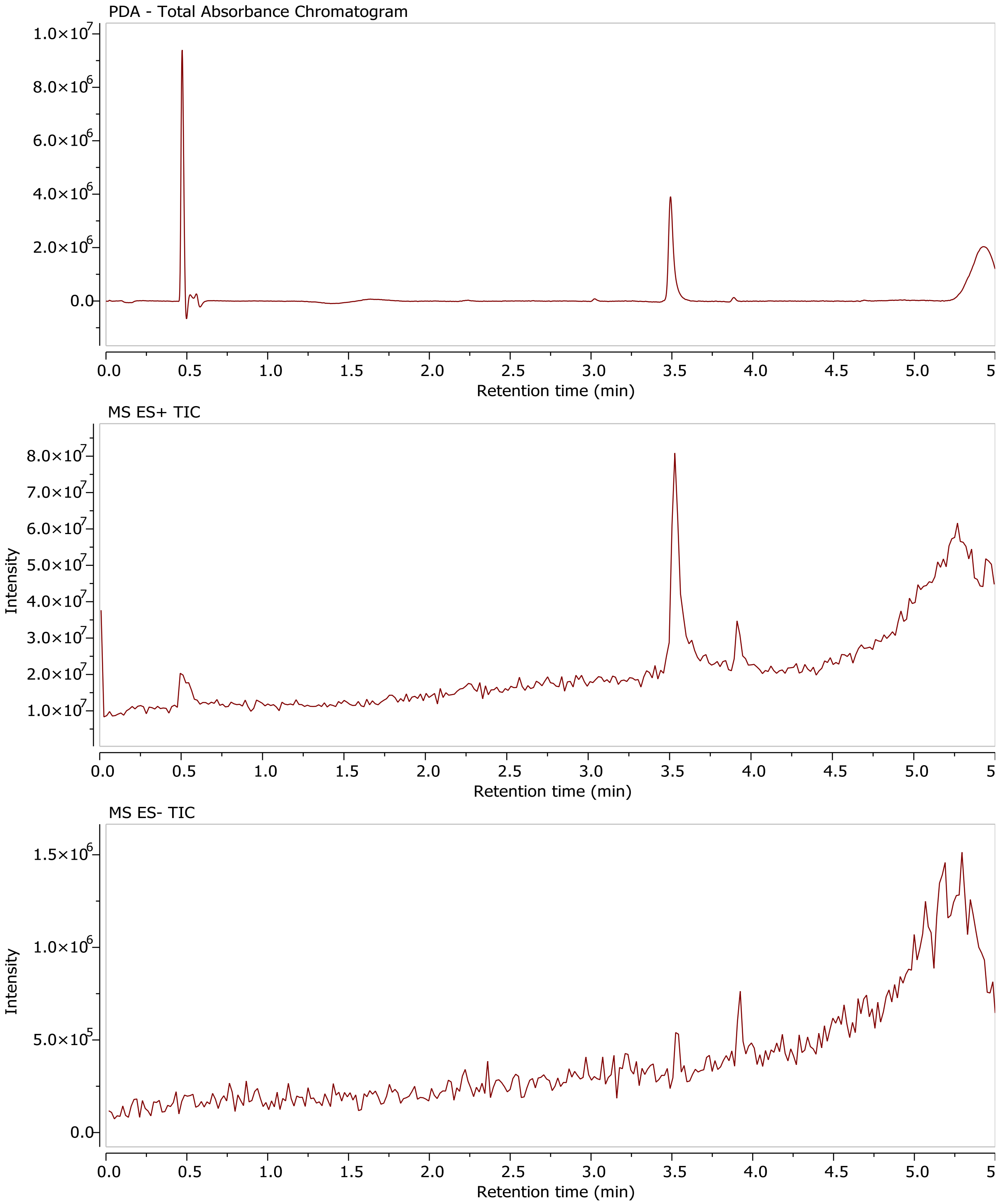

Supplementary figure **1561**. UPLC-MS analysis for compound **PHP-4983**

Chemical Formula: $C_{15}H_{20}N_2O_4$
Exact Mass: 292.1

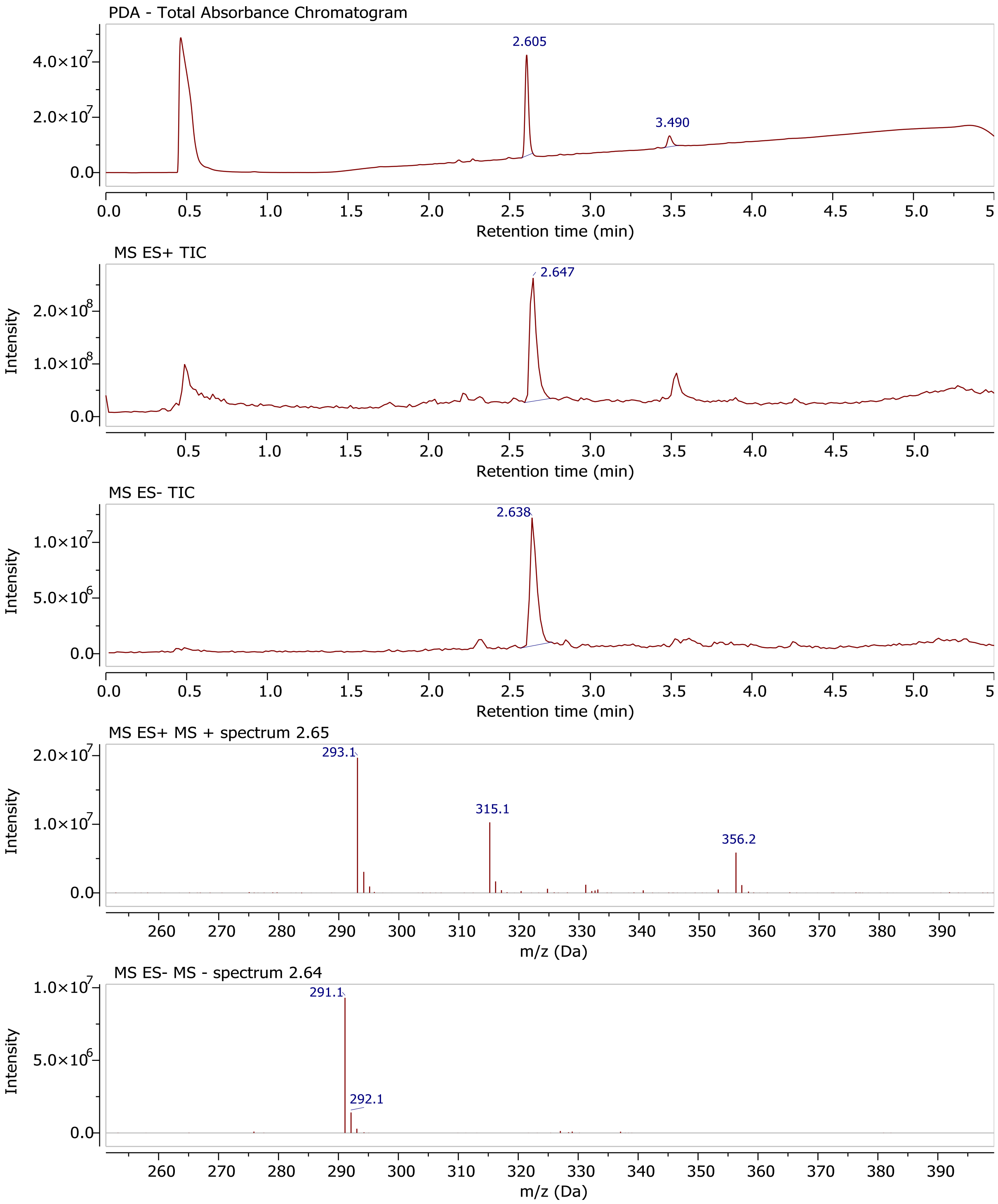

Supplementary figure **1562**. UPLC-MS analysis for compound **PHP-5537**

Chemical Formula: $C_{23}H_{34}N_2O_3$
Exact Mass: 386.3

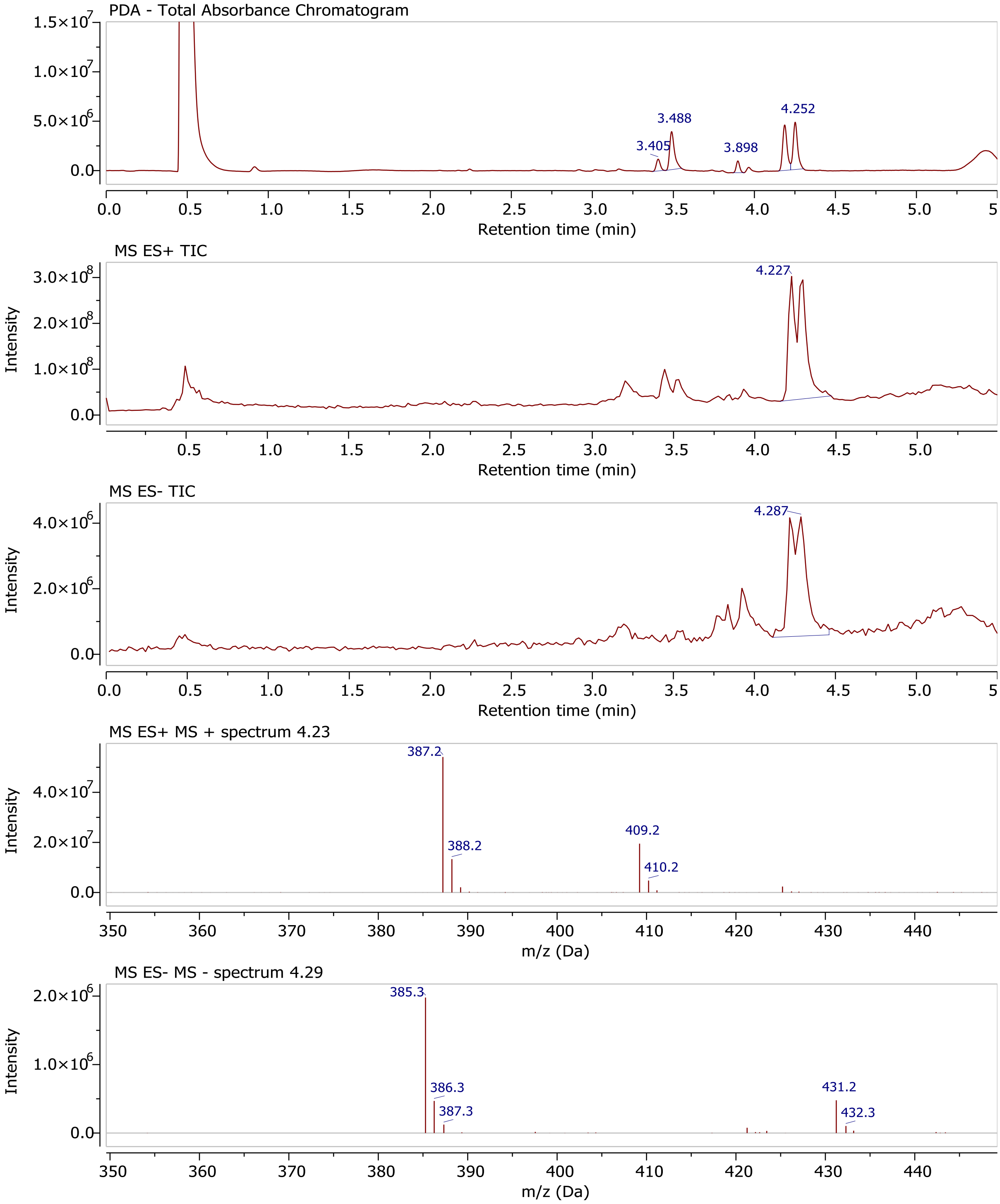

Supplementary figure **1563**. UPLC-MS analysis for compound **PHP-5676**

Chemical Formula: $C_{19}H_{19}ClN_2O_2$
Exact Mass: 342.1

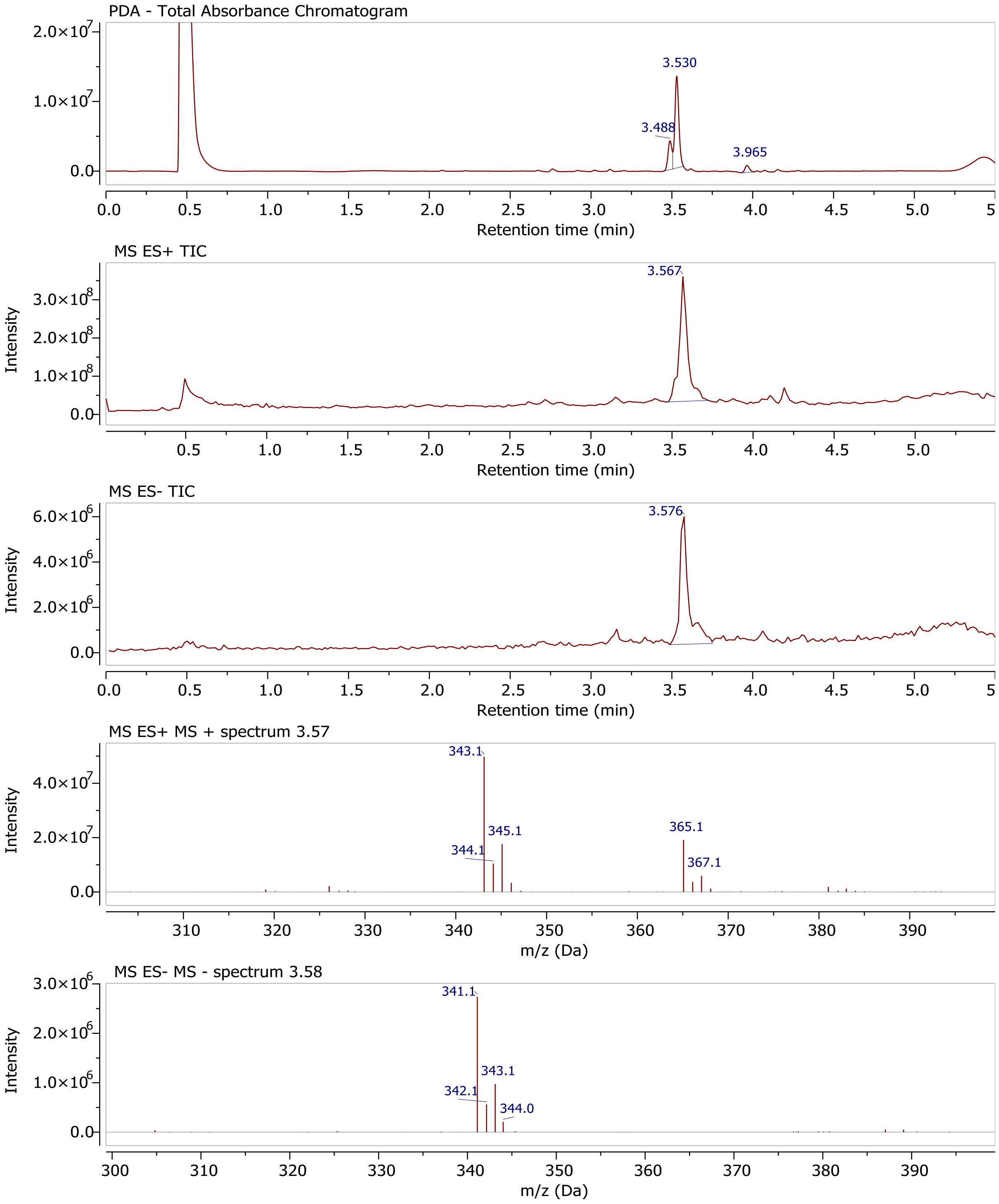

Supplementary figure **1564**. UPLC-MS analysis for compound **PHP-5860**

Chemical Formula: $C_{22}H_{21}F_3N_2O_3$
Exact Mass: 418.2

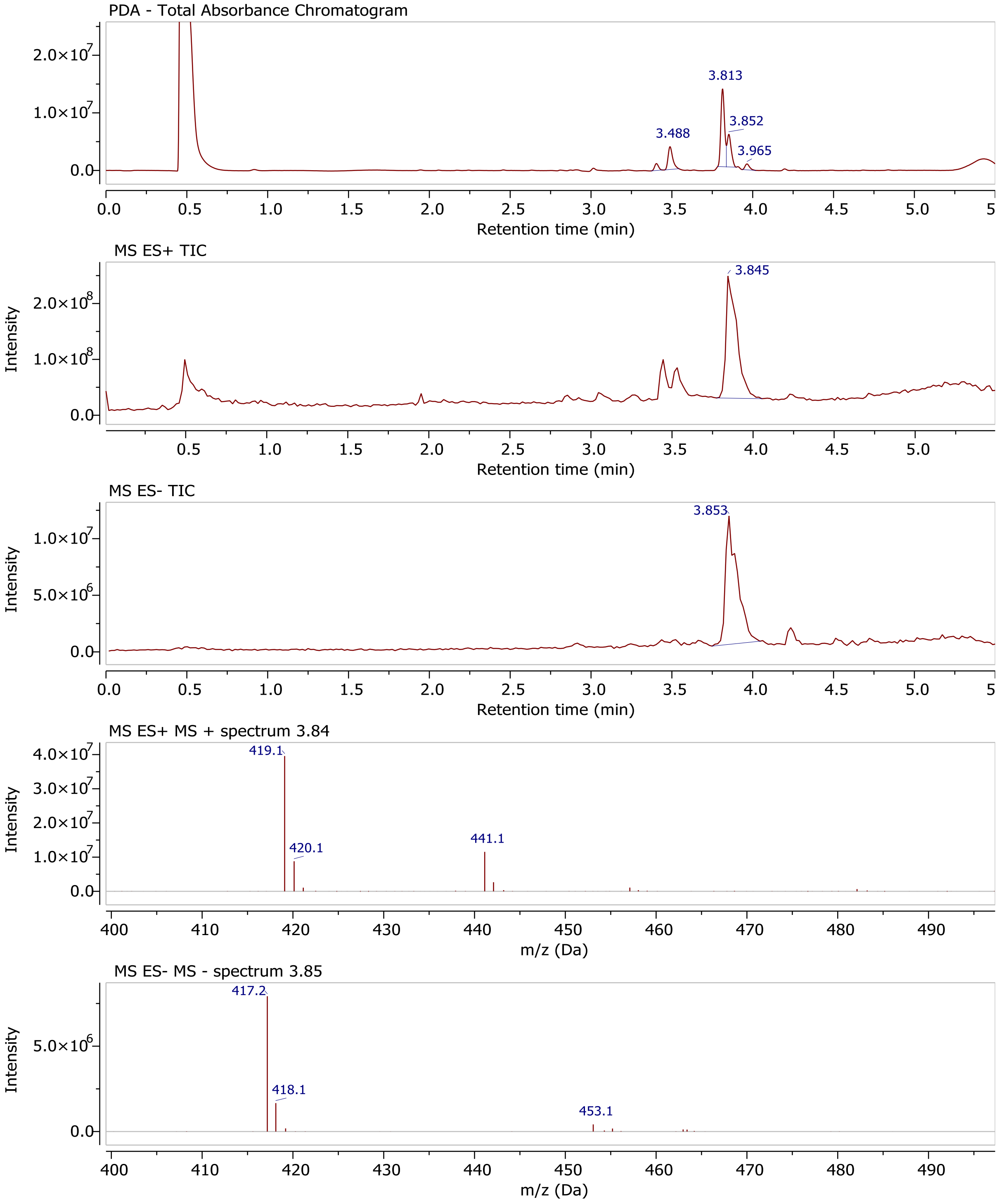

Supplementary figure **1565**. UPLC-MS analysis for compound **PHP-5677**

Chemical Formula: $C_{20}H_{19}F_3N_2O_2$
Exact Mass: 376.1

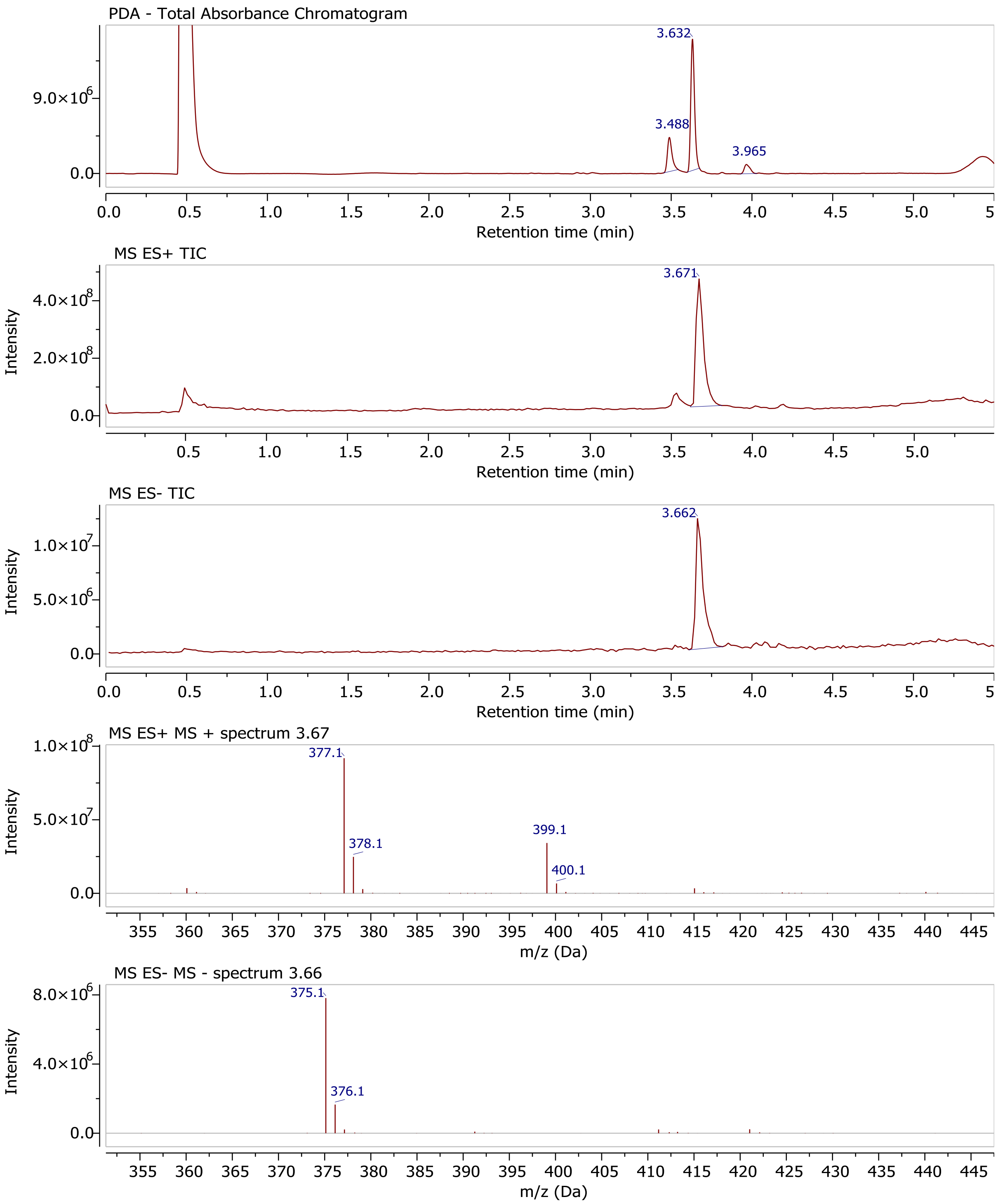

Supplementary figure **1566**. UPLC-MS analysis for compound **PHP-5861**

Chemical Formula: $C_{23}H_{26}N_2O_4$
Exact Mass: 394.2

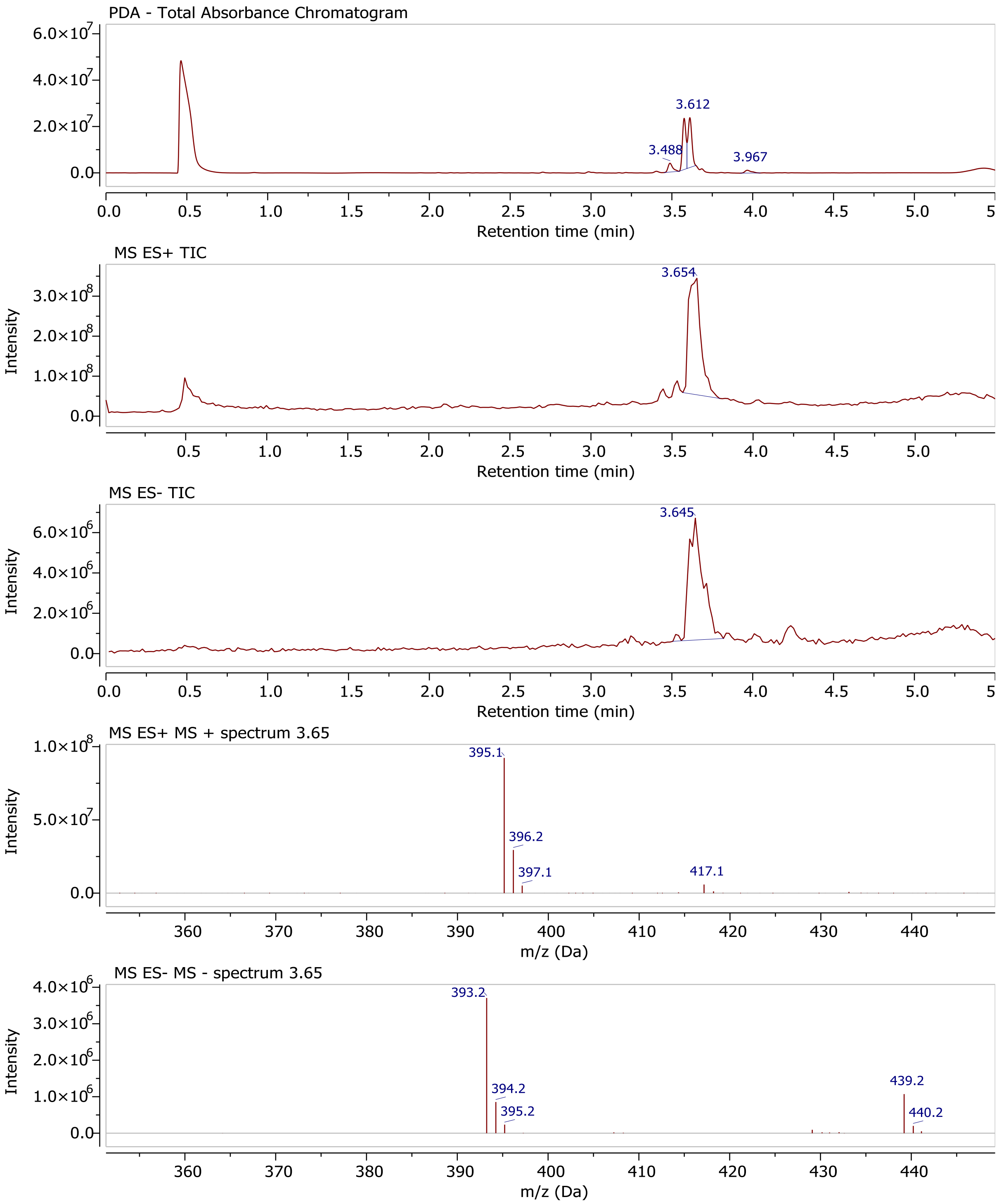

Supplementary figure **1567**. UPLC-MS analysis for compound **PHP-5678**

Chemical Formula: $C_{19}H_{19}FN_2O_2$
Exact Mass: 326.1

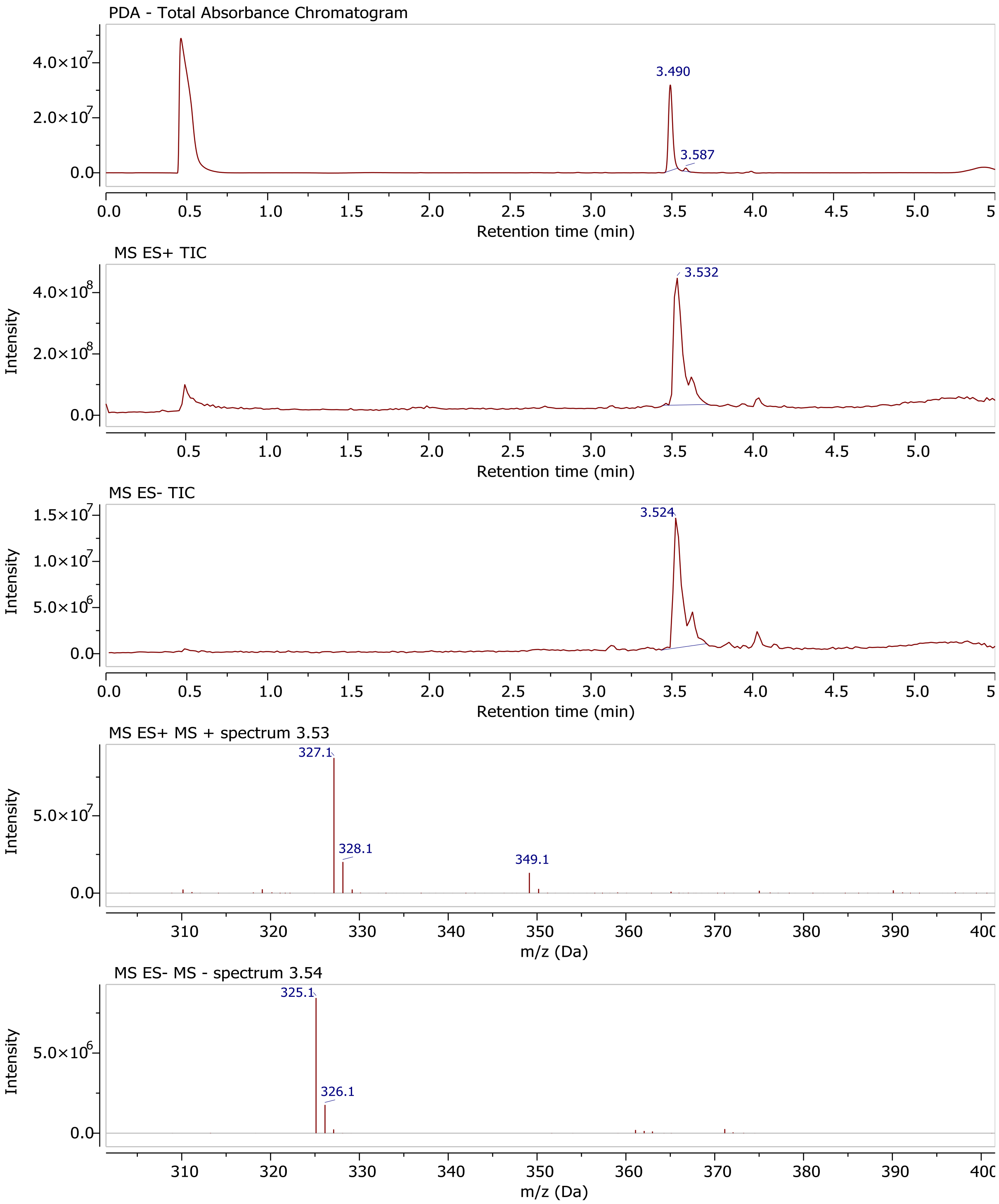

Supplementary figure **1568**. UPLC-MS analysis for compound **PHP-5862**

Chemical Formula: $C_{17}H_{21}ClN_2O_3$
Exact Mass: 336.1

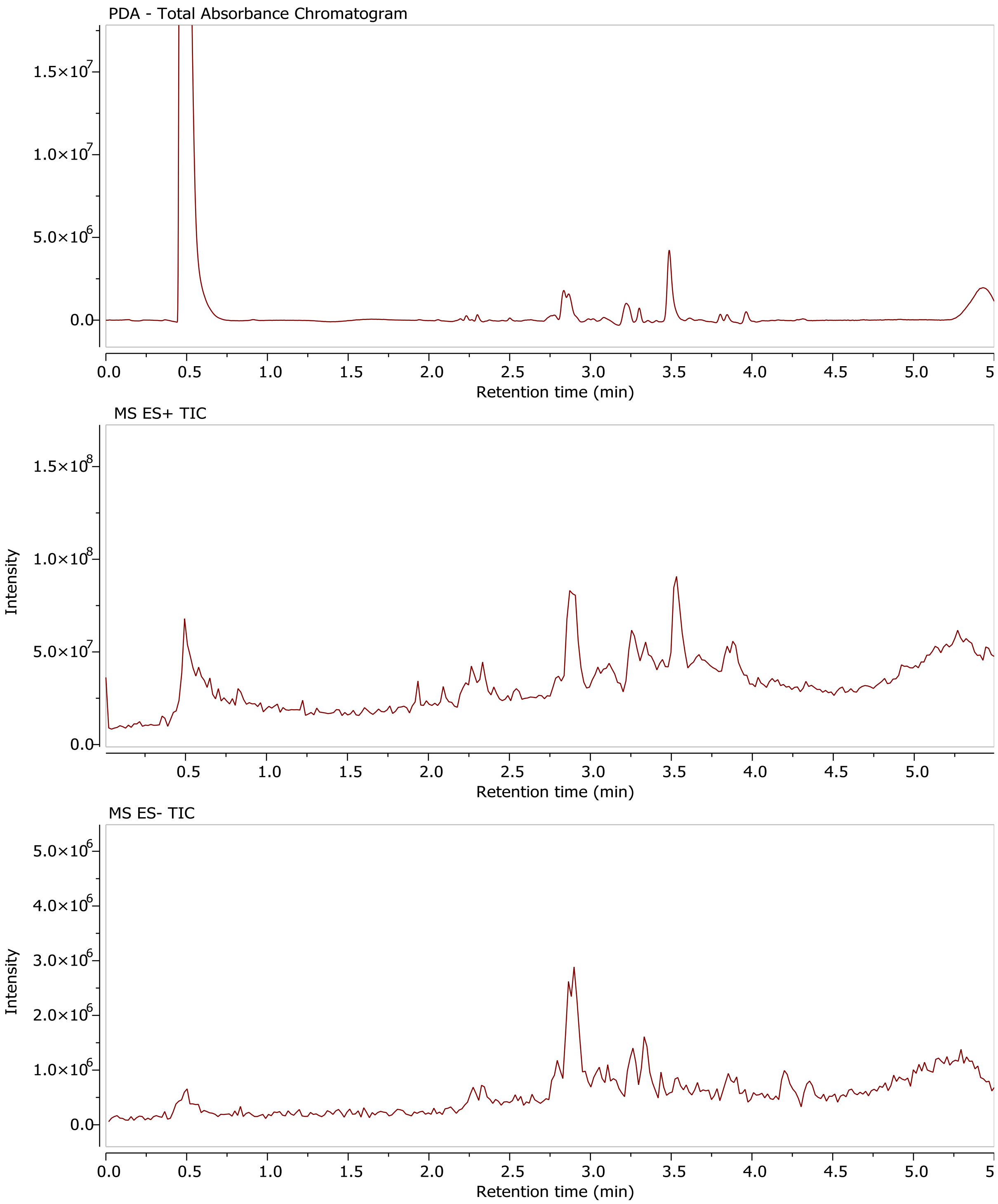

Supplementary figure **1569**. UPLC-MS analysis for compound **PHP-5680**

Chemical Formula: $C_{19}H_{19}FN_2O_2$
Exact Mass: 326.1

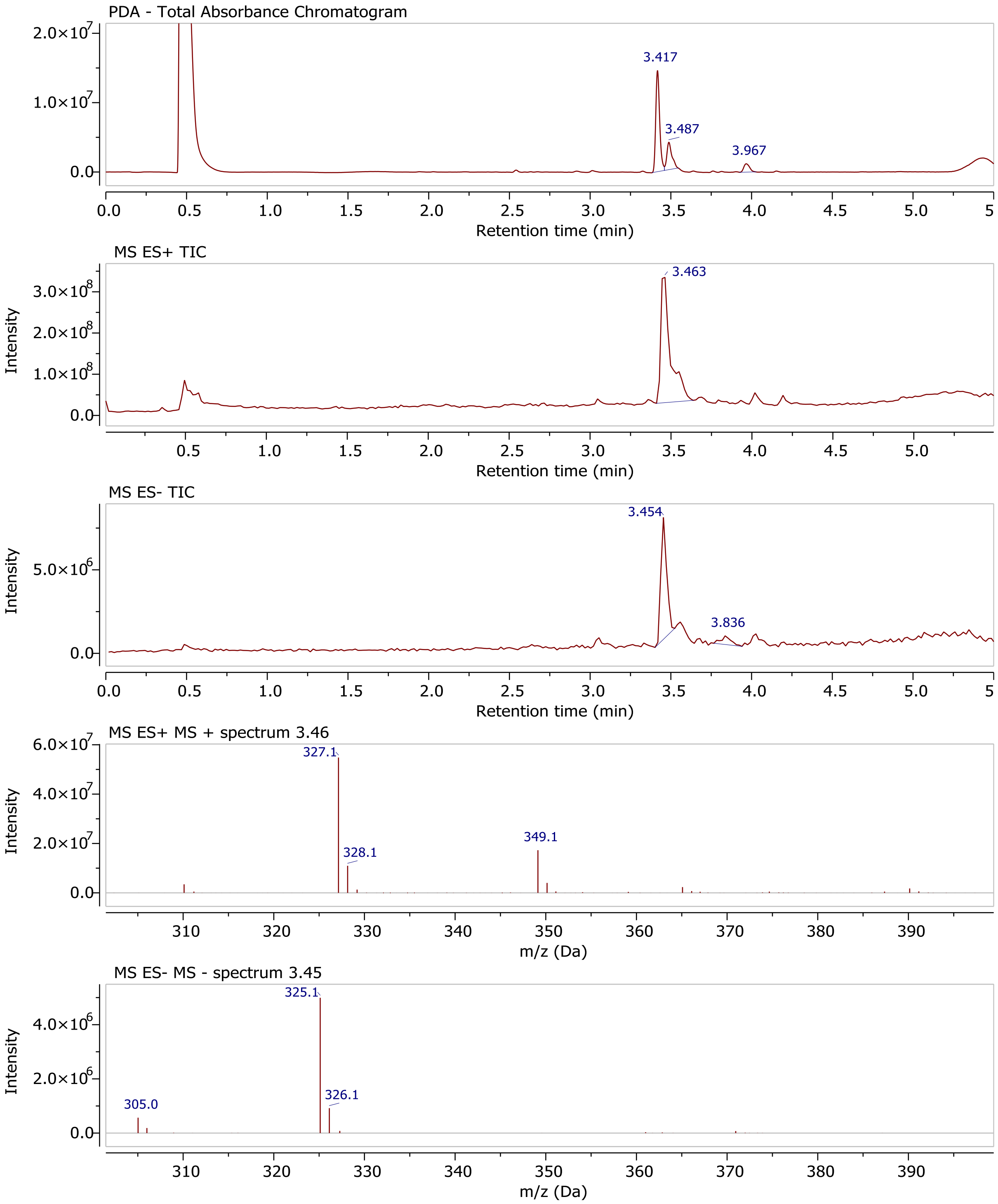

Supplementary figure **1570**. UPLC-MS analysis for compound **PHP-5863**

Chemical Formula: $C_{19}H_{16}F_2N_2O_4$
Exact Mass: 374.1

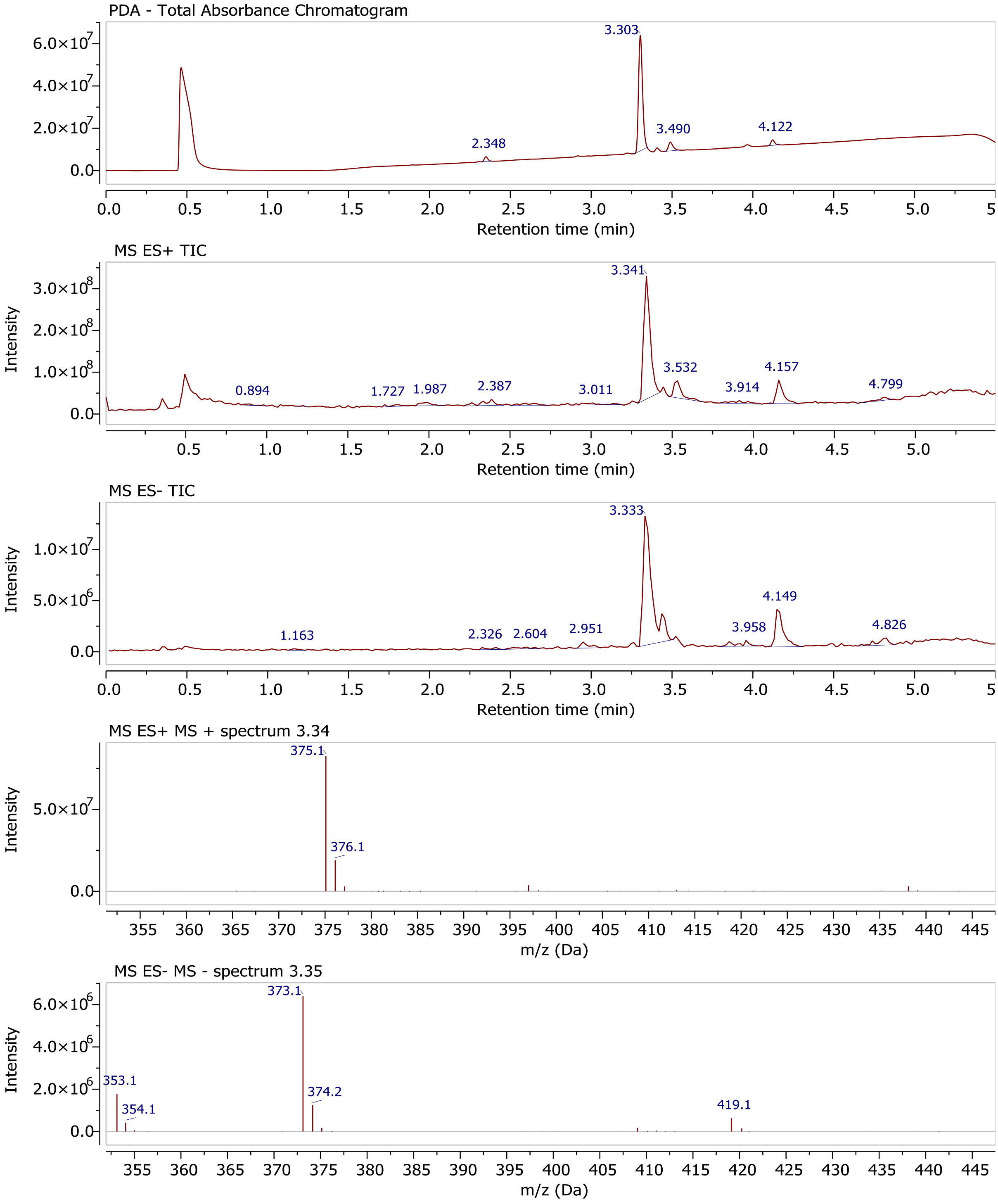

Supplementary figure **1571**. UPLC-MS analysis for compound **PHP-5682**

Chemical Formula: $C_{20}H_{19}F_3N_2O_2$
Exact Mass: 376.1

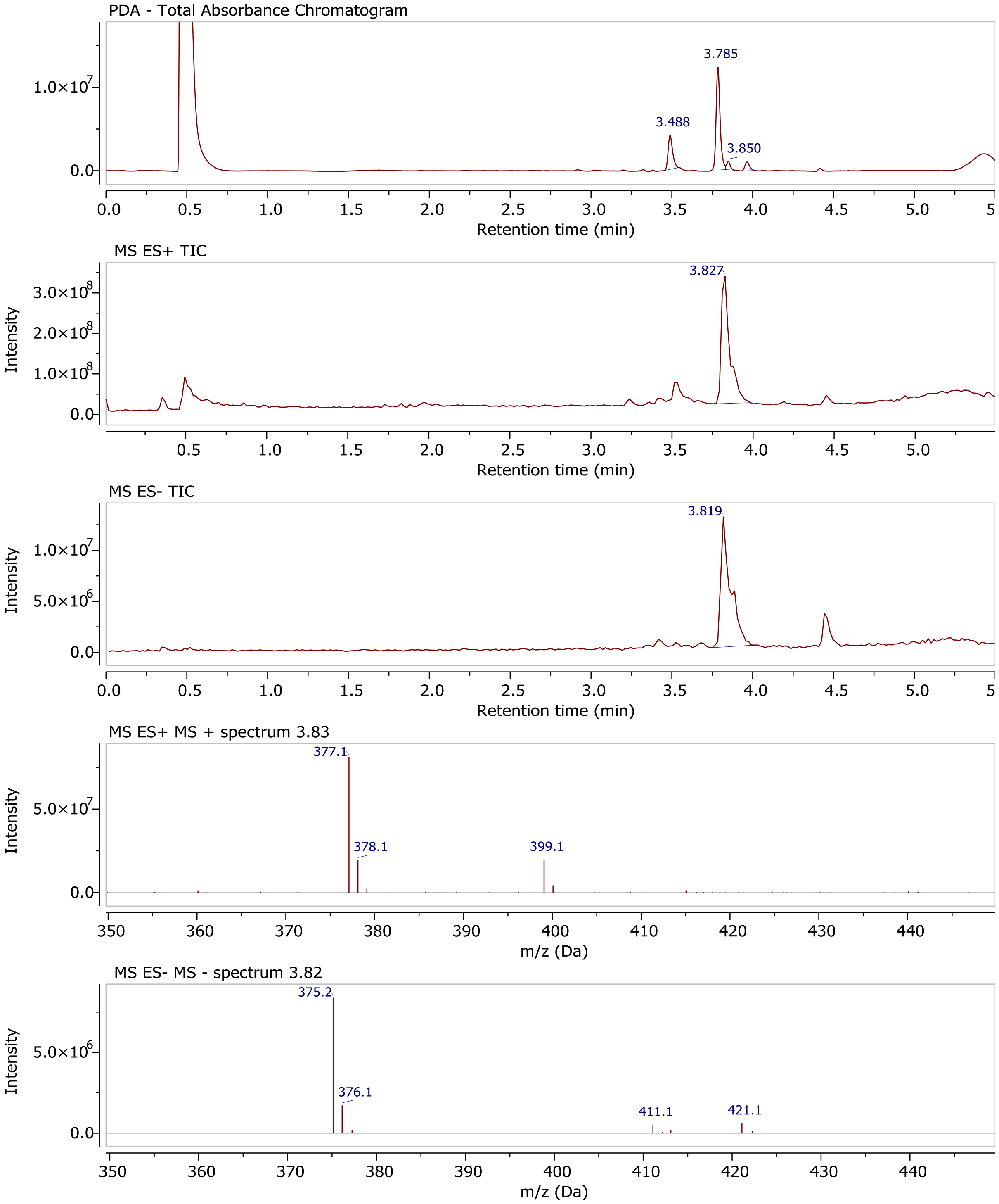

Supplementary figure **1572**. UPLC-MS analysis for compound **PHP-5864**

Chemical Formula: $C_{20}H_{20}N_2O_5$
Exact Mass: 368.1

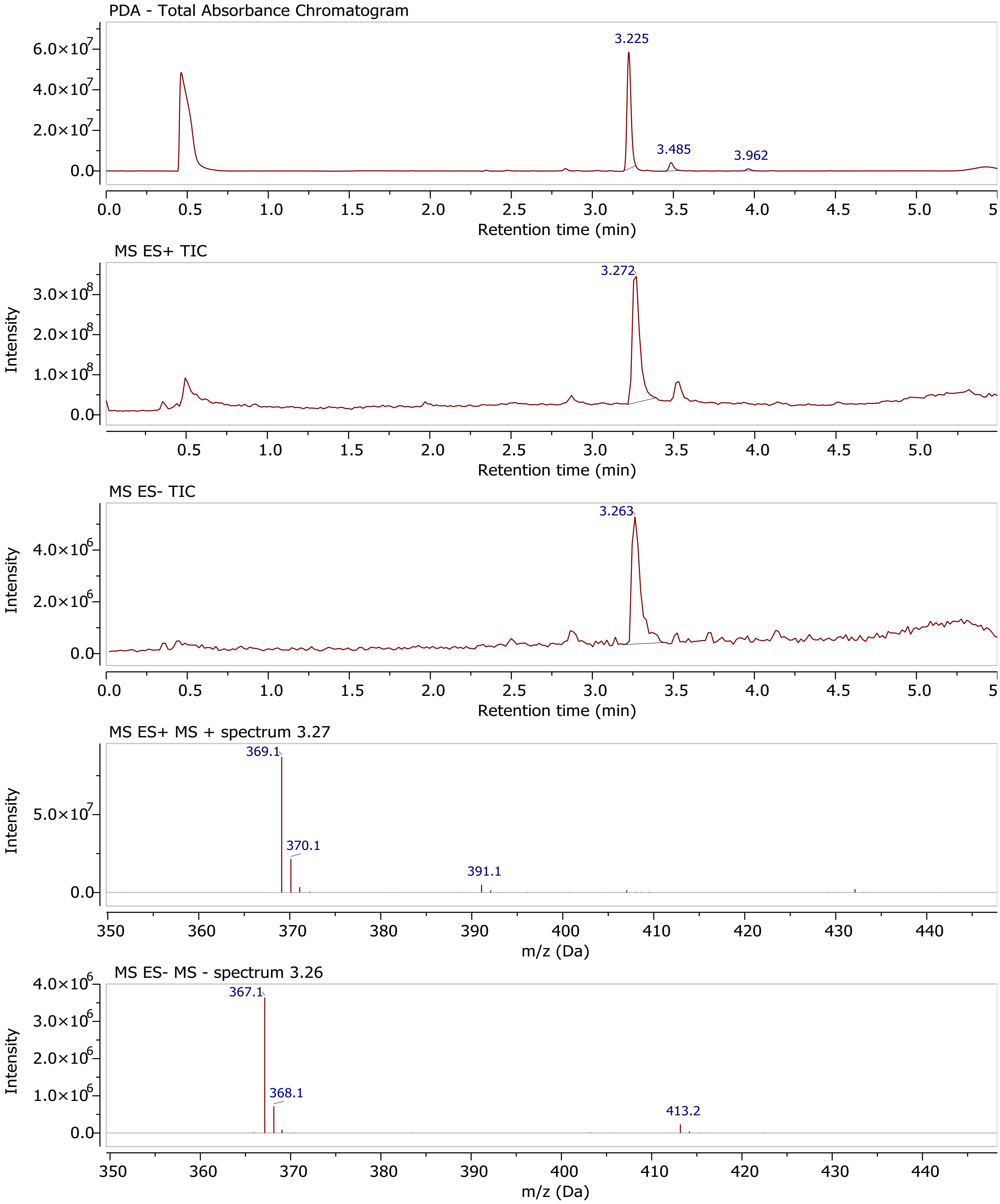

Supplementary figure **1573**. UPLC-MS analysis for compound **PHP-5684**

Chemical Formula: $C_{15}H_{15}F_3N_2O_4$
Exact Mass: 344.1

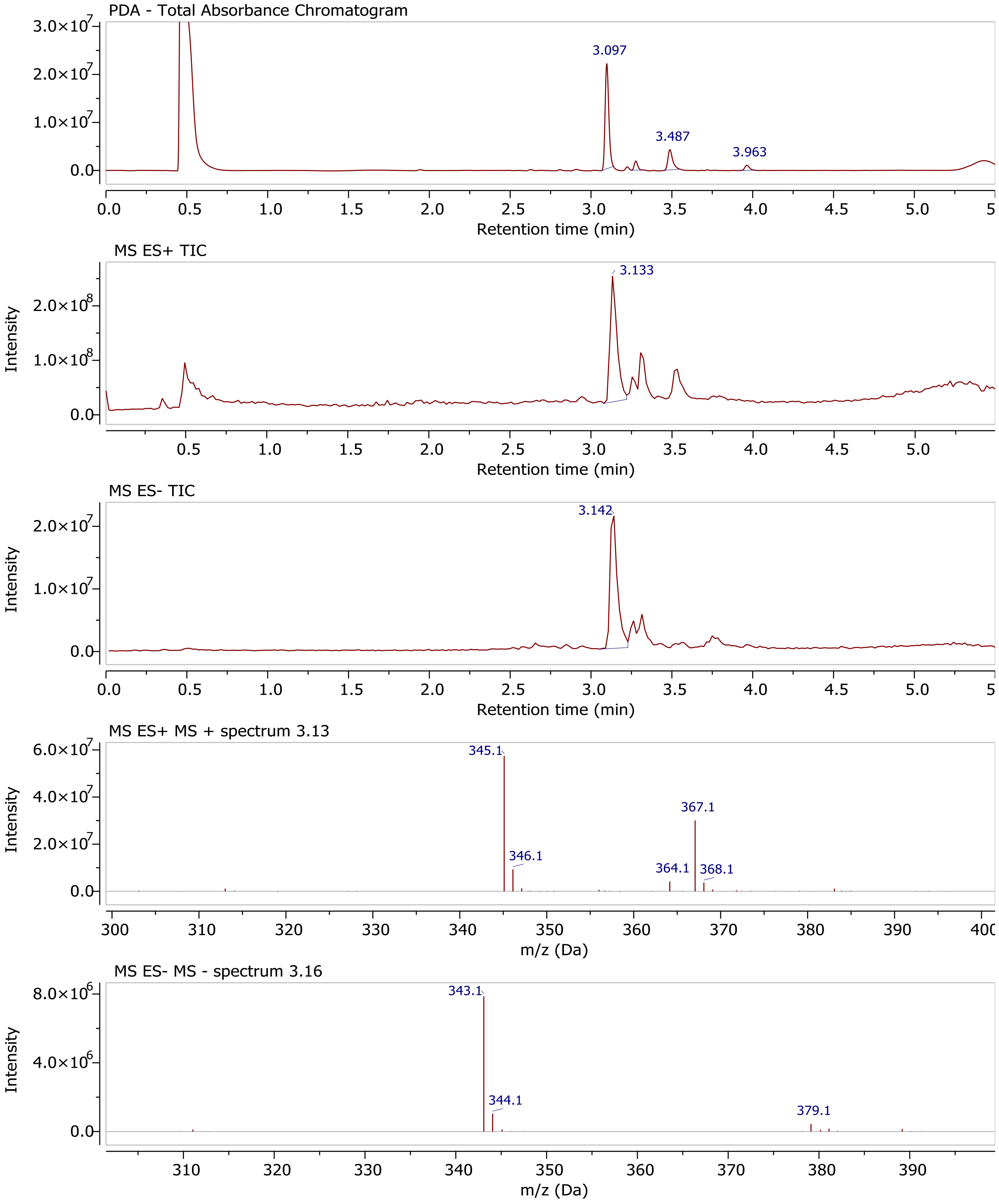

Supplementary figure **1574**. UPLC-MS analysis for compound **PHP-5865**

Chemical Formula: $C_{22}H_{24}N_2O_4$
Exact Mass: 380.2

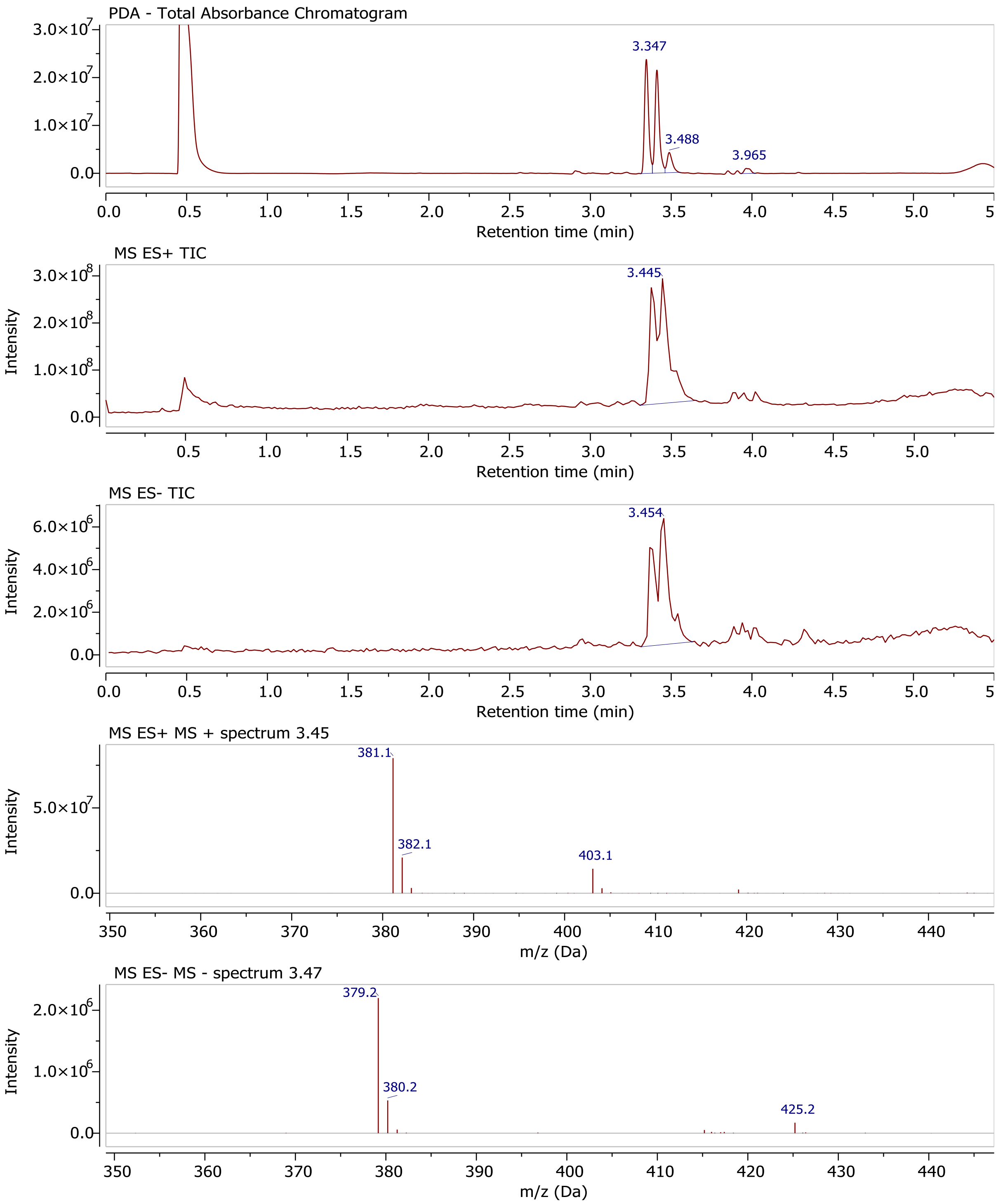

Supplementary figure **1575**. UPLC-MS analysis for compound **PHP-5690**

Chemical Formula: $C_{15}H_{18}F_2N_2O_4$
Exact Mass: 328.1

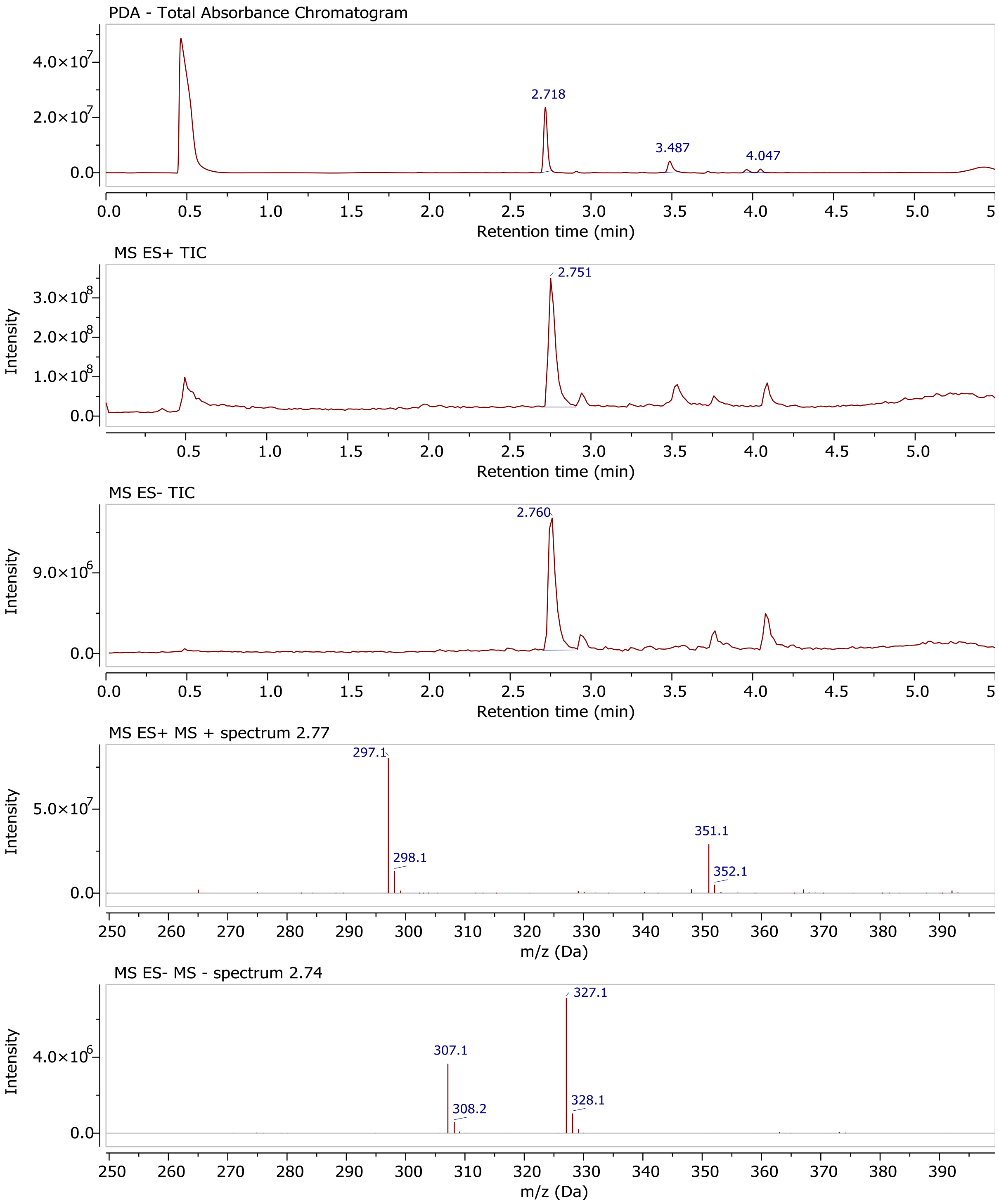

Supplementary figure **1576**. UPLC-MS analysis for compound **PHP-5882**

Chemical Formula: $C_{22}H_{24}N_2O_4$
Exact Mass: 380.2

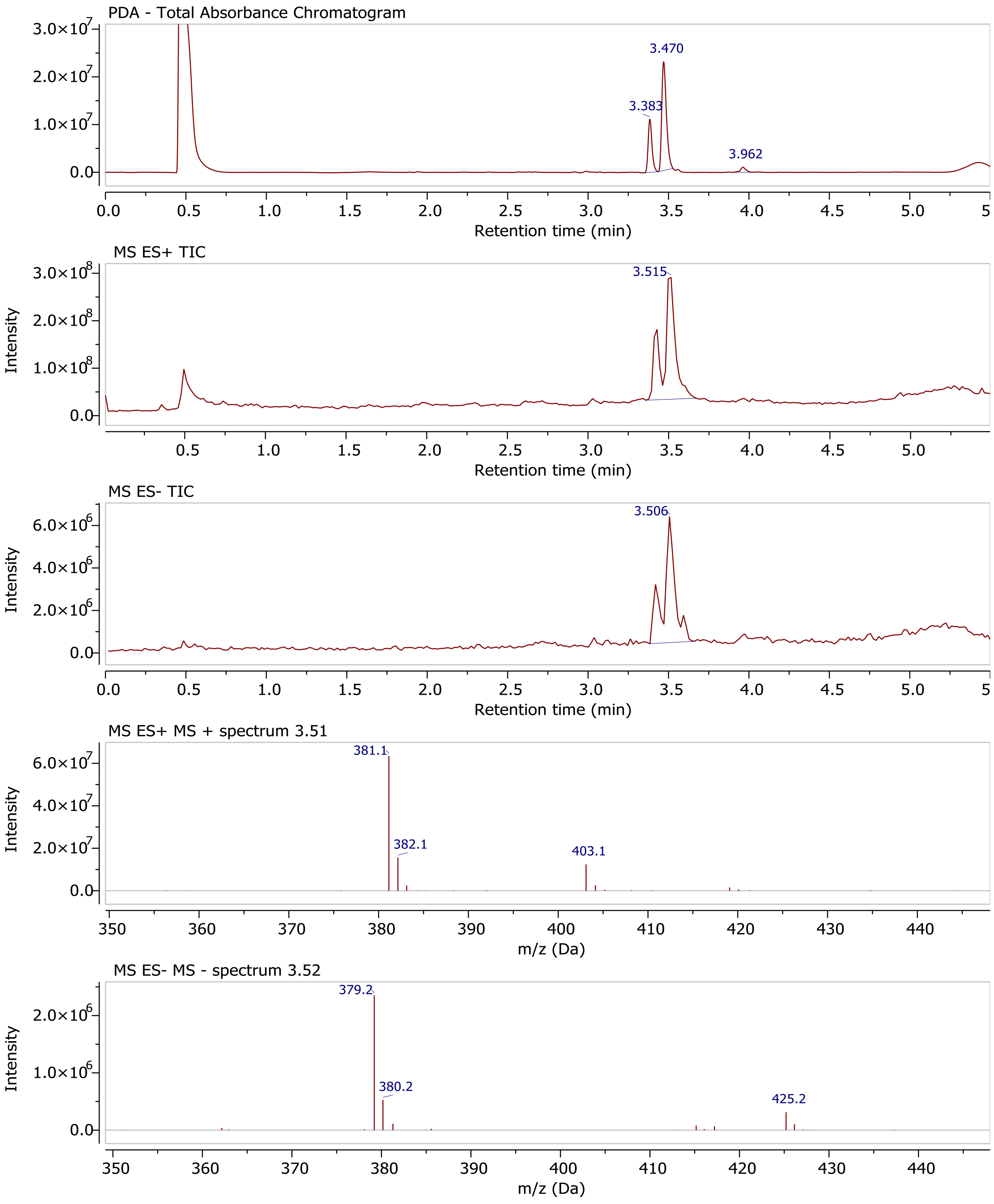

Supplementary figure **1577**. UPLC-MS analysis for compound **PHP-5691**

Chemical Formula: $C_{16}H_{22}N_2O_5$
Exact Mass: 322.2

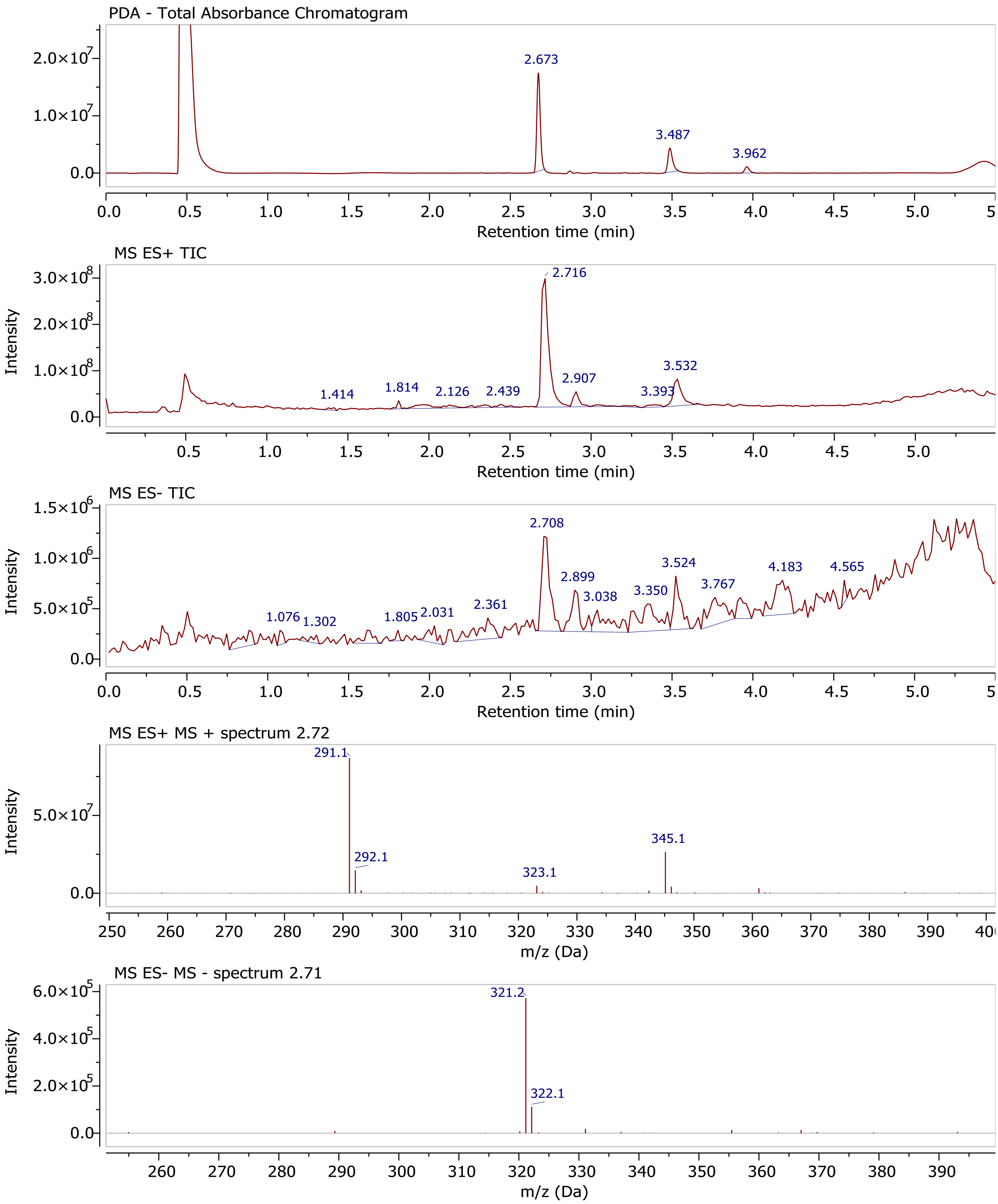

Supplementary figure **1578**. UPLC-MS analysis for compound **PHP-5884**

Chemical Formula: $C_{20}H_{26}N_2O_4$
Exact Mass: 358.2

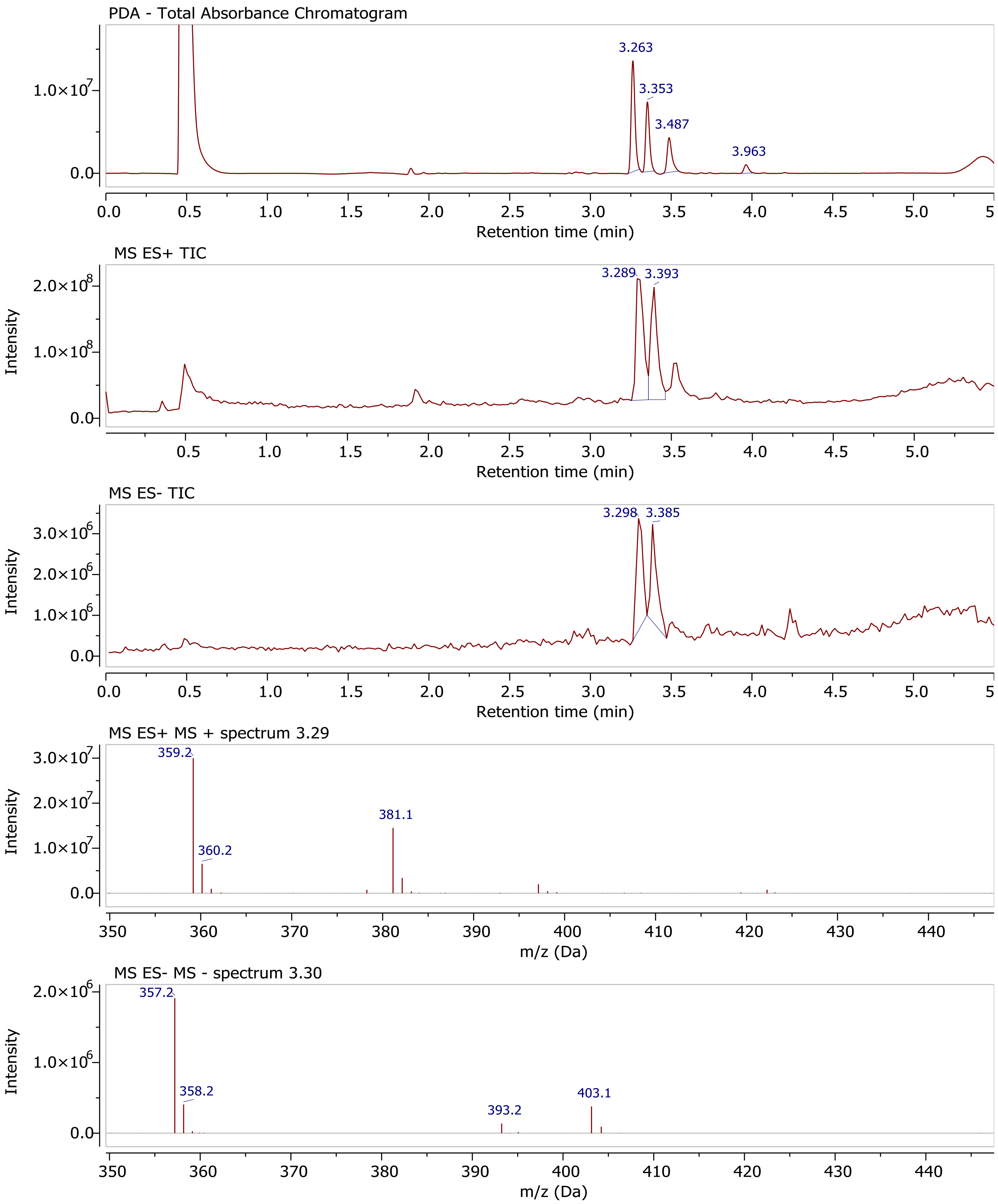

Supplementary figure **1579**. UPLC-MS analysis for compound **PHP-5693**

Chemical Formula: $C_{16}H_{22}N_2O_4$
Exact Mass: 306.2

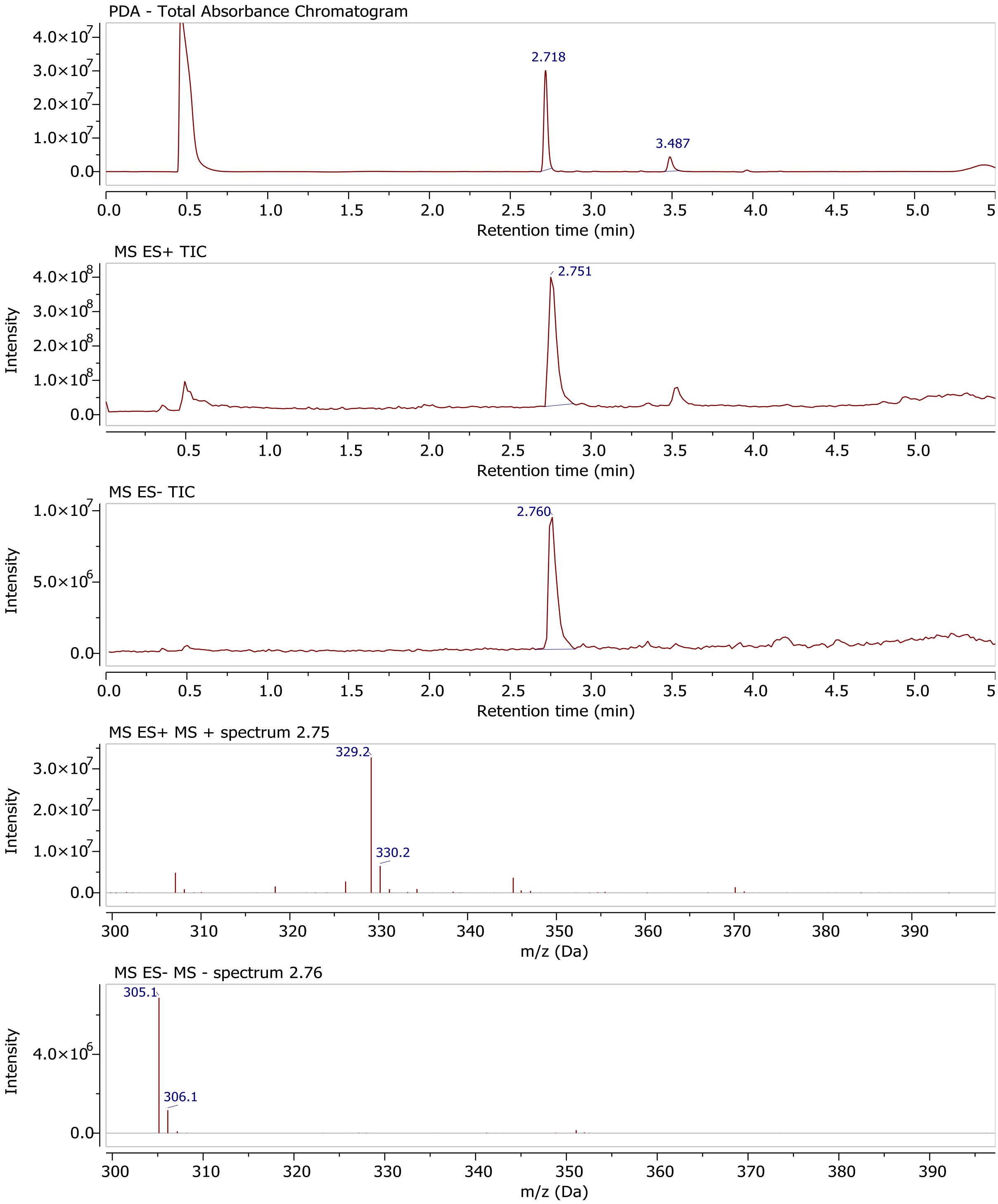

Supplementary figure **1580**. UPLC-MS analysis for compound **PHP-5890**

Chemical Formula: $C_{22}H_{24}N_2O_5$
Exact Mass: 396.2

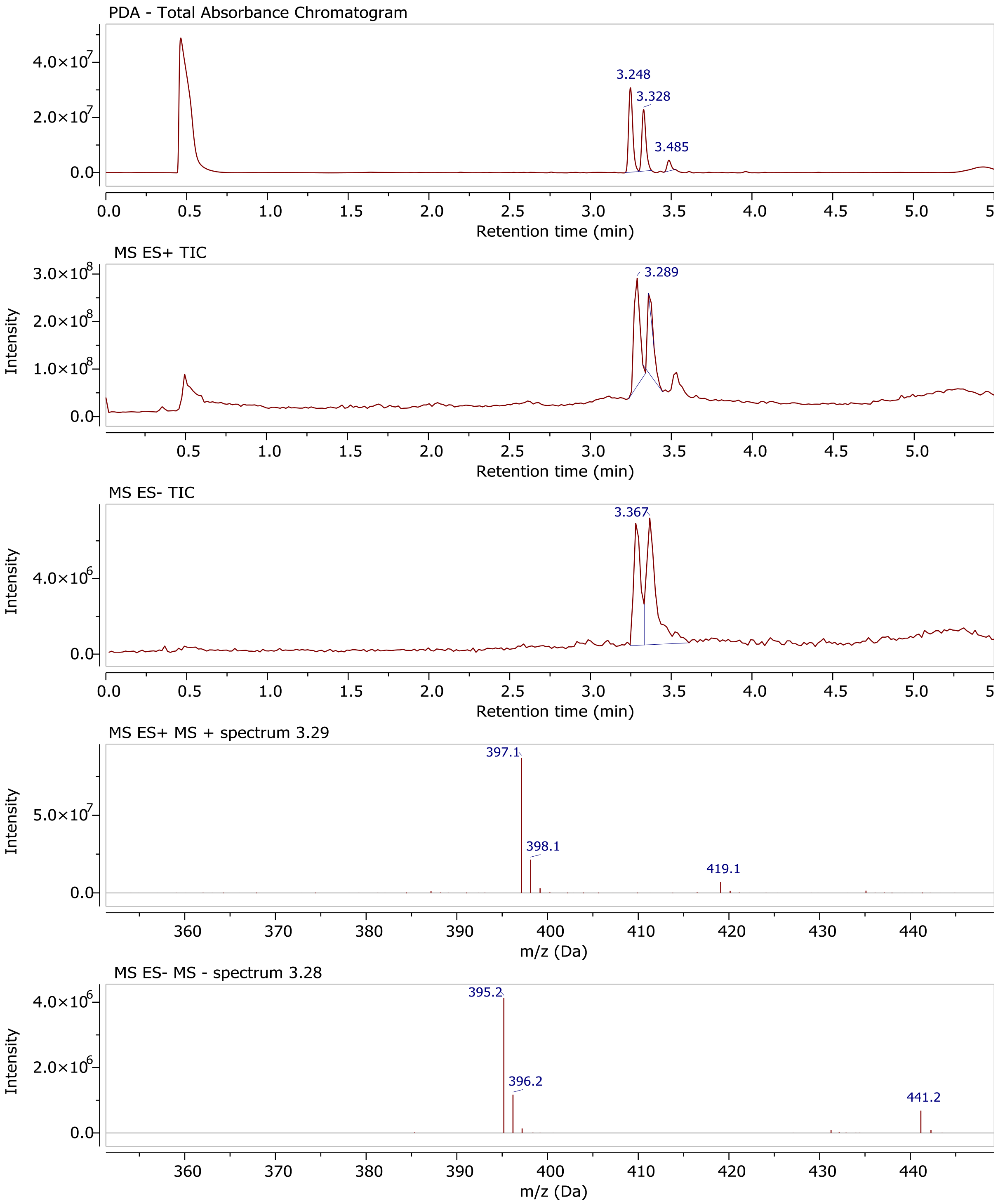

Supplementary figure **1581**. UPLC-MS analysis for compound **PHP-5694**

Chemical Formula: $C_{16}H_{22}N_2O_4$
Exact Mass: 306.2

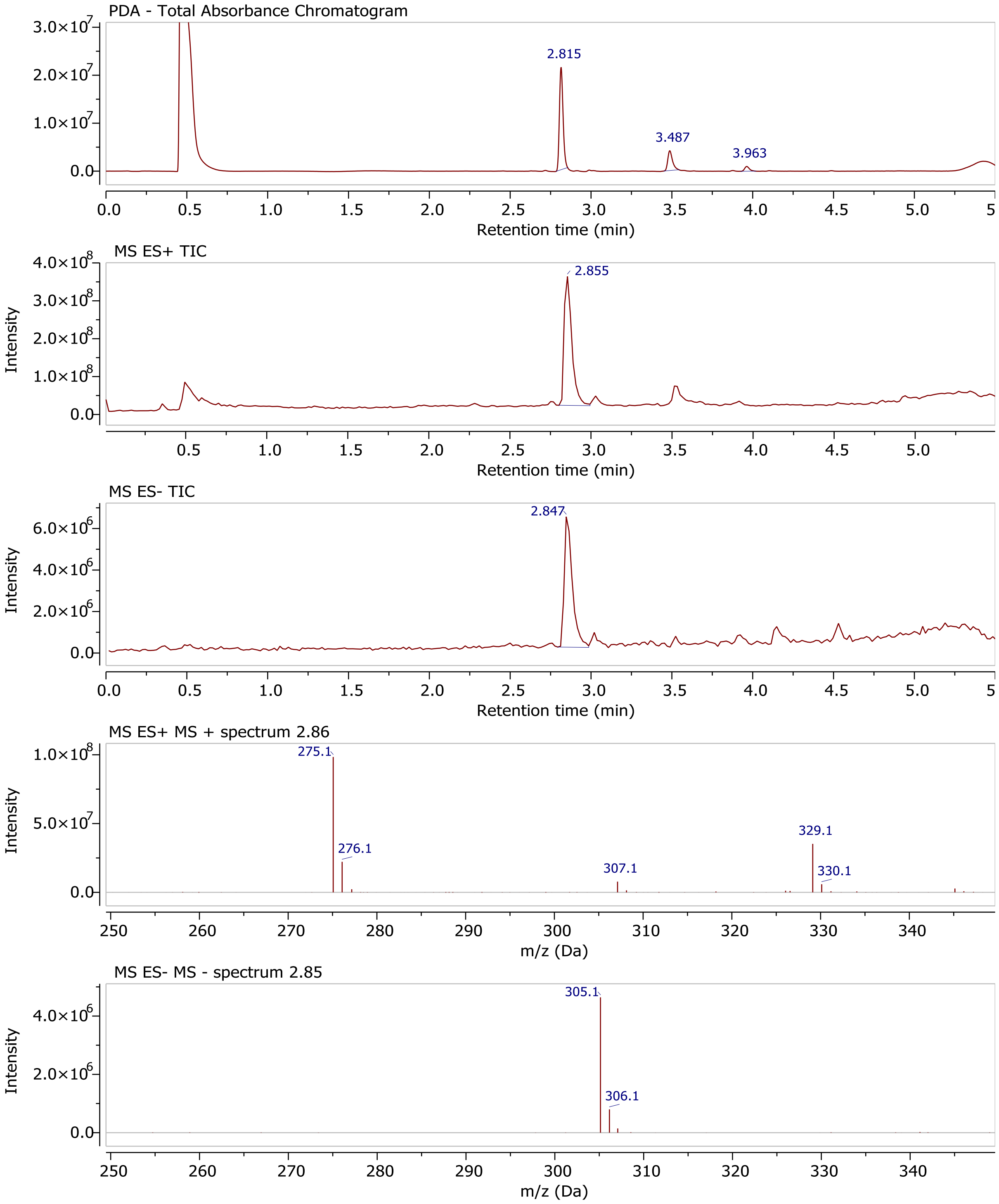

Supplementary figure **1582**. UPLC-MS analysis for compound **PHP-5891**

Chemical Formula: $C_{19}H_{20}N_2O_5$
Exact Mass: 356.1

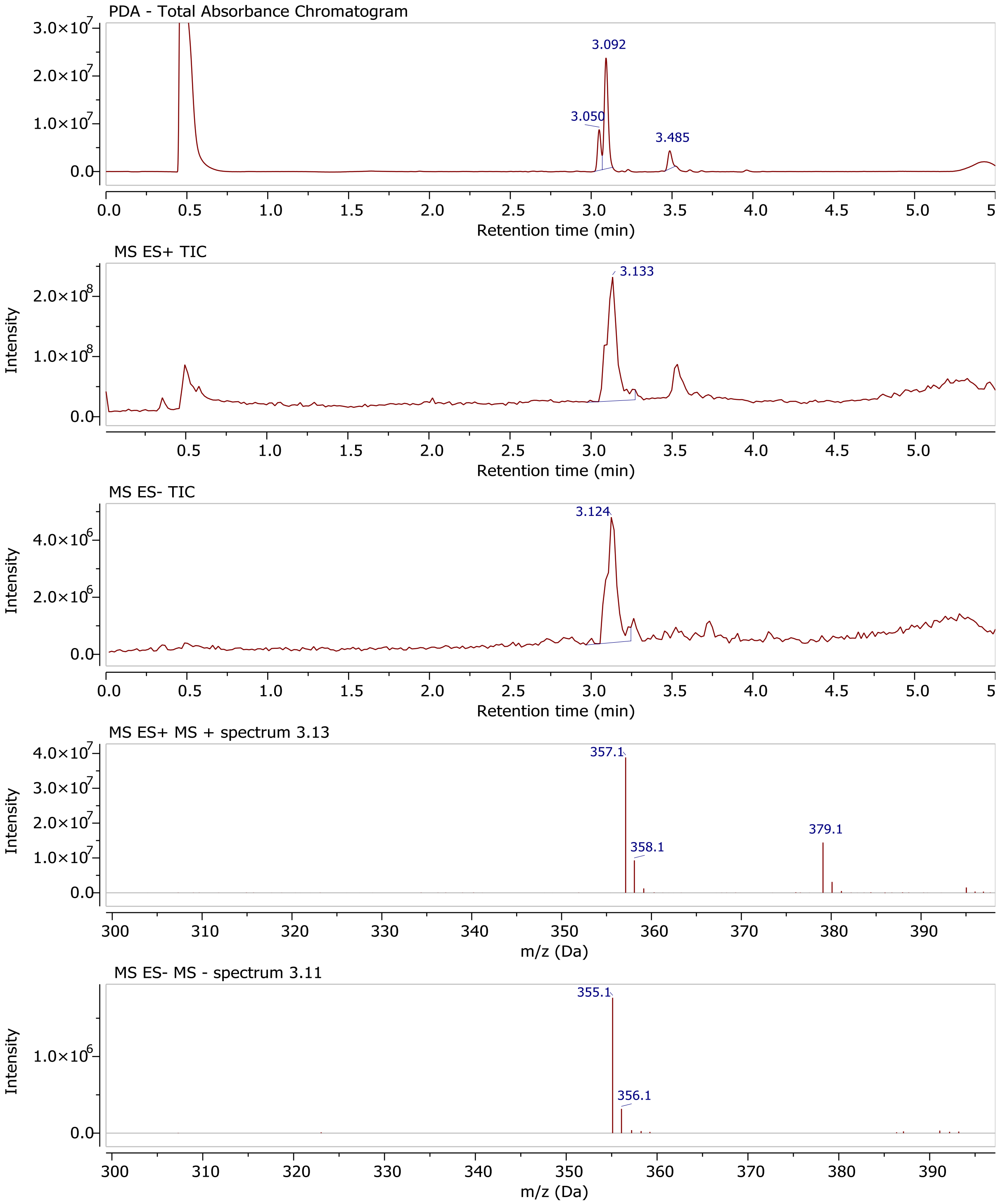

Supplementary figure **1583**. UPLC-MS analysis for compound **PHP-5695**

Chemical Formula: $C_{16}H_{22}N_2O_5$
Exact Mass: 322.2

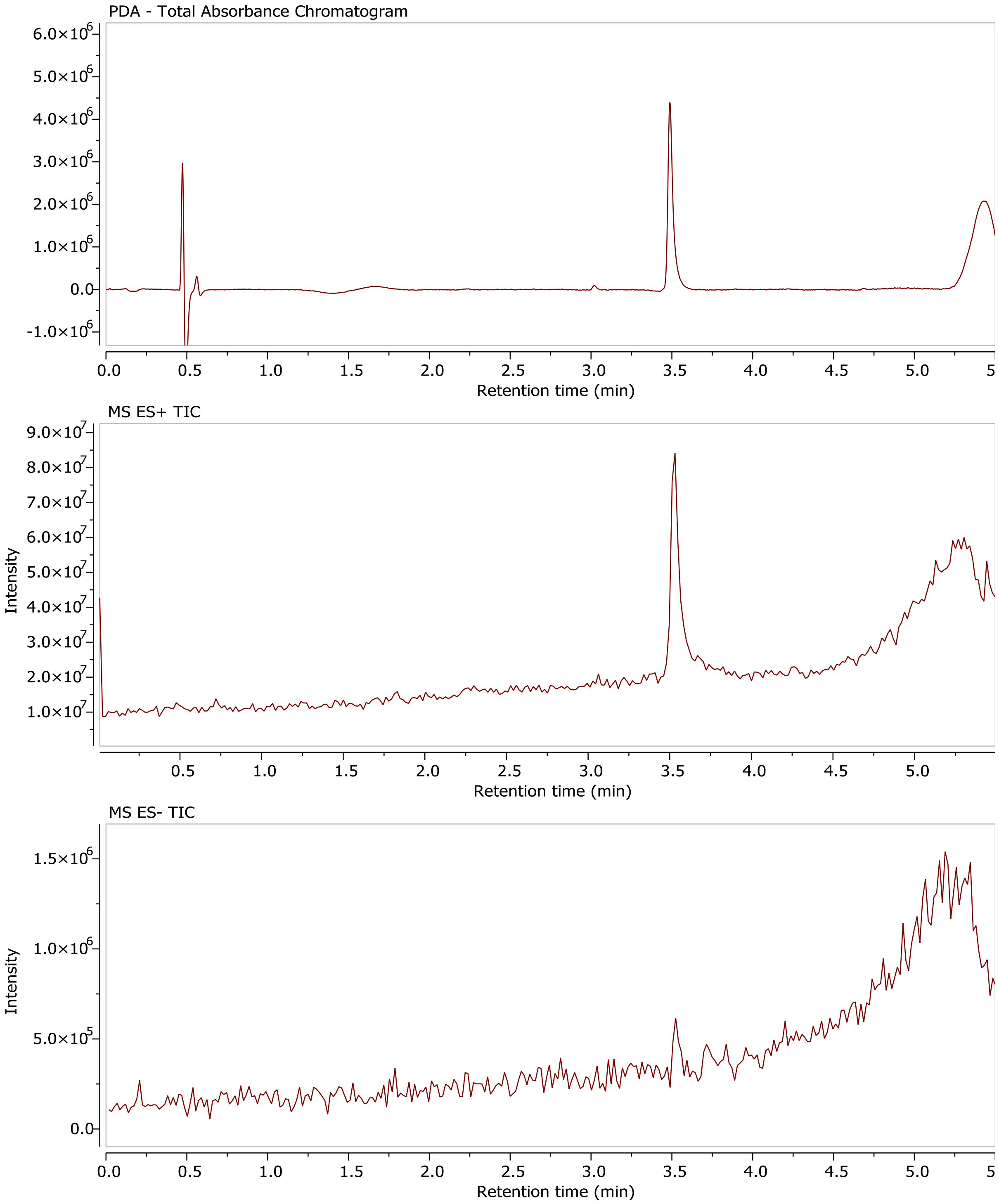

Supplementary figure **1584**. UPLC-MS analysis for compound **PHP-5894**

Chemical Formula: $C_{25}H_{24}N_2O_4$
Exact Mass: 416.2

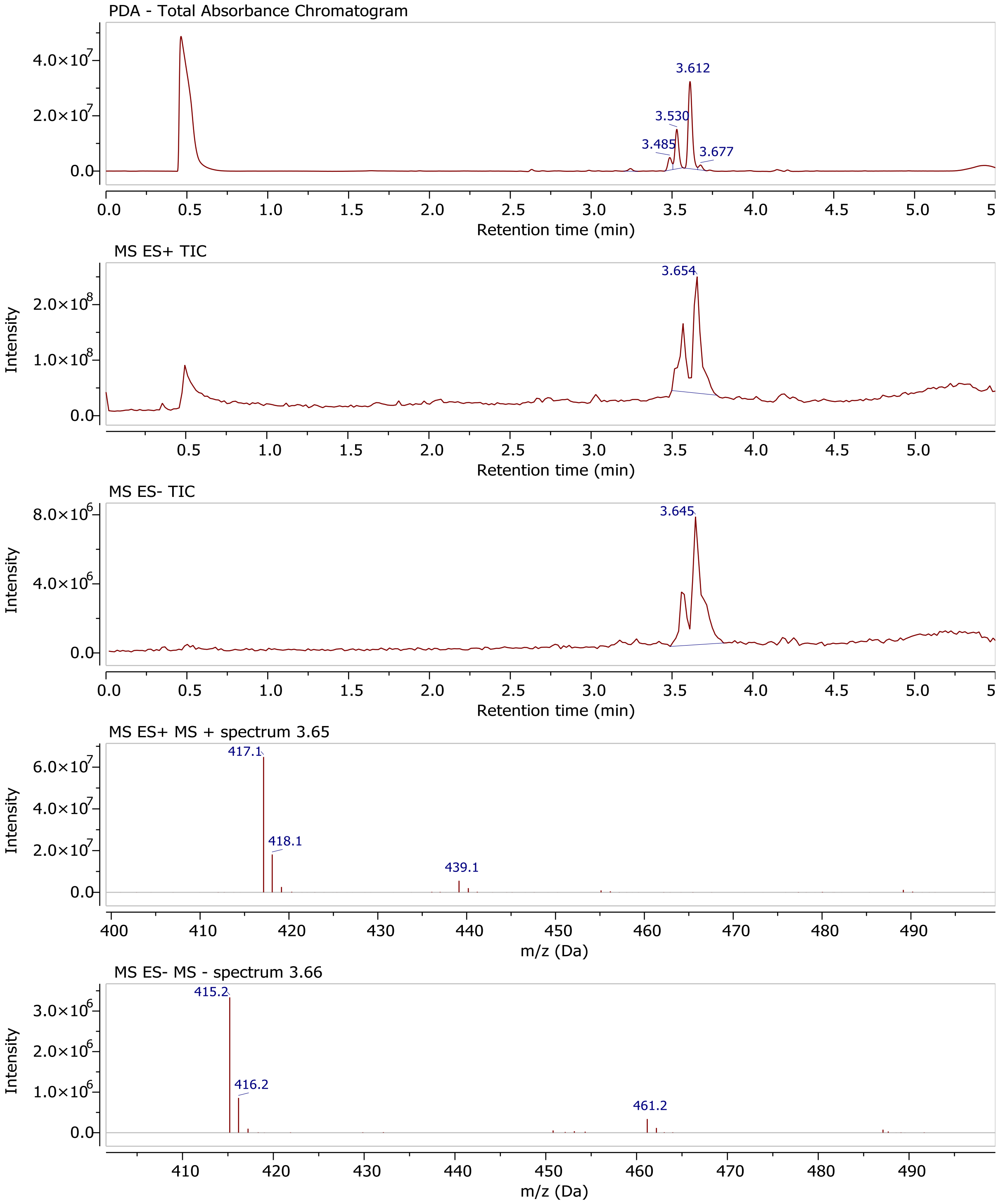

Supplementary figure **1585**. UPLC-MS analysis for compound **PHP-5696**

Chemical Formula: $C_{19}H_{22}N_2O_4$
Exact Mass: 342.2

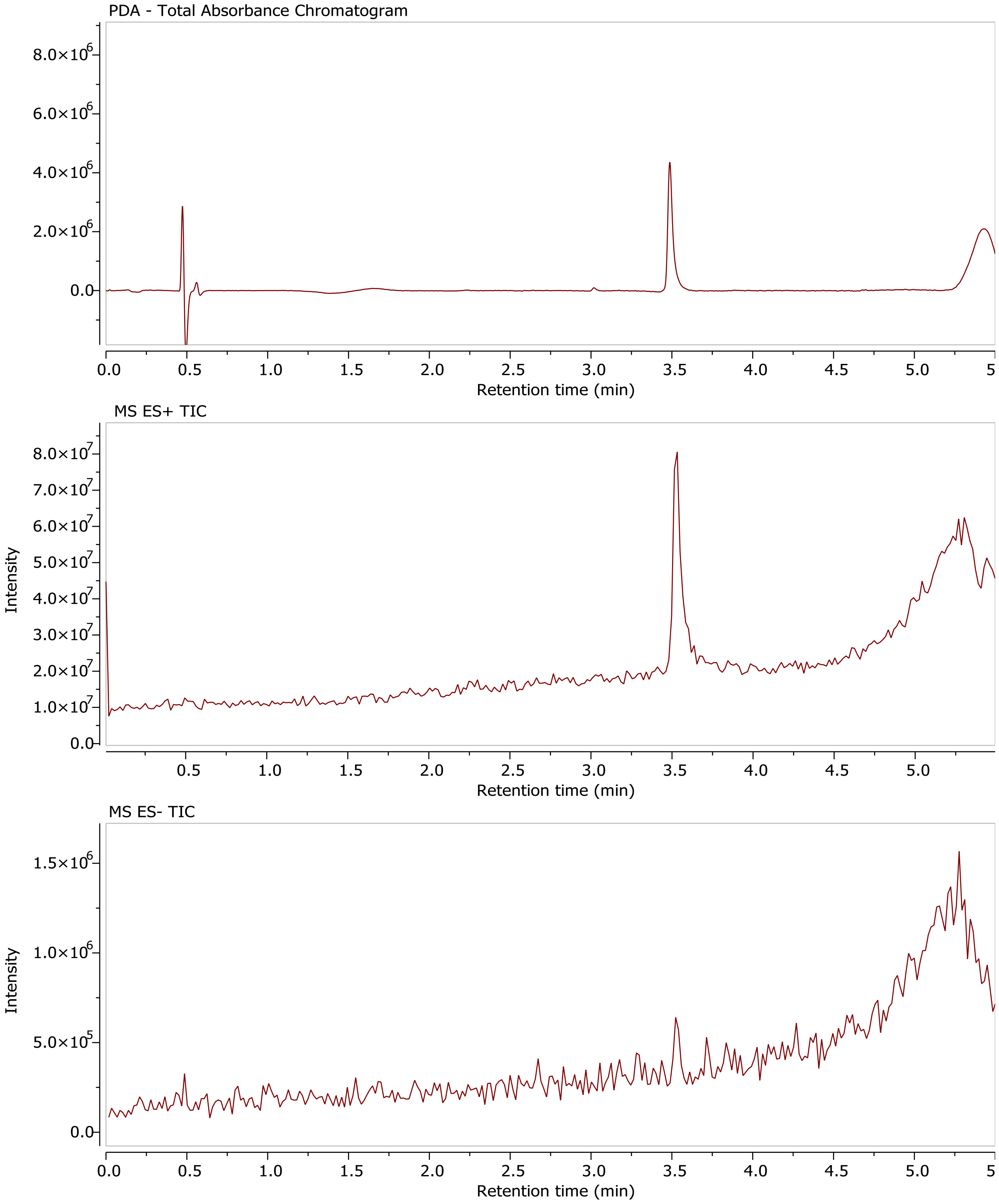

Supplementary figure **1586**. UPLC-MS analysis for compound **PHP-5896**

Chemical Formula: $C_{19}H_{26}ClFN_2O_2$
Exact Mass: 368.2

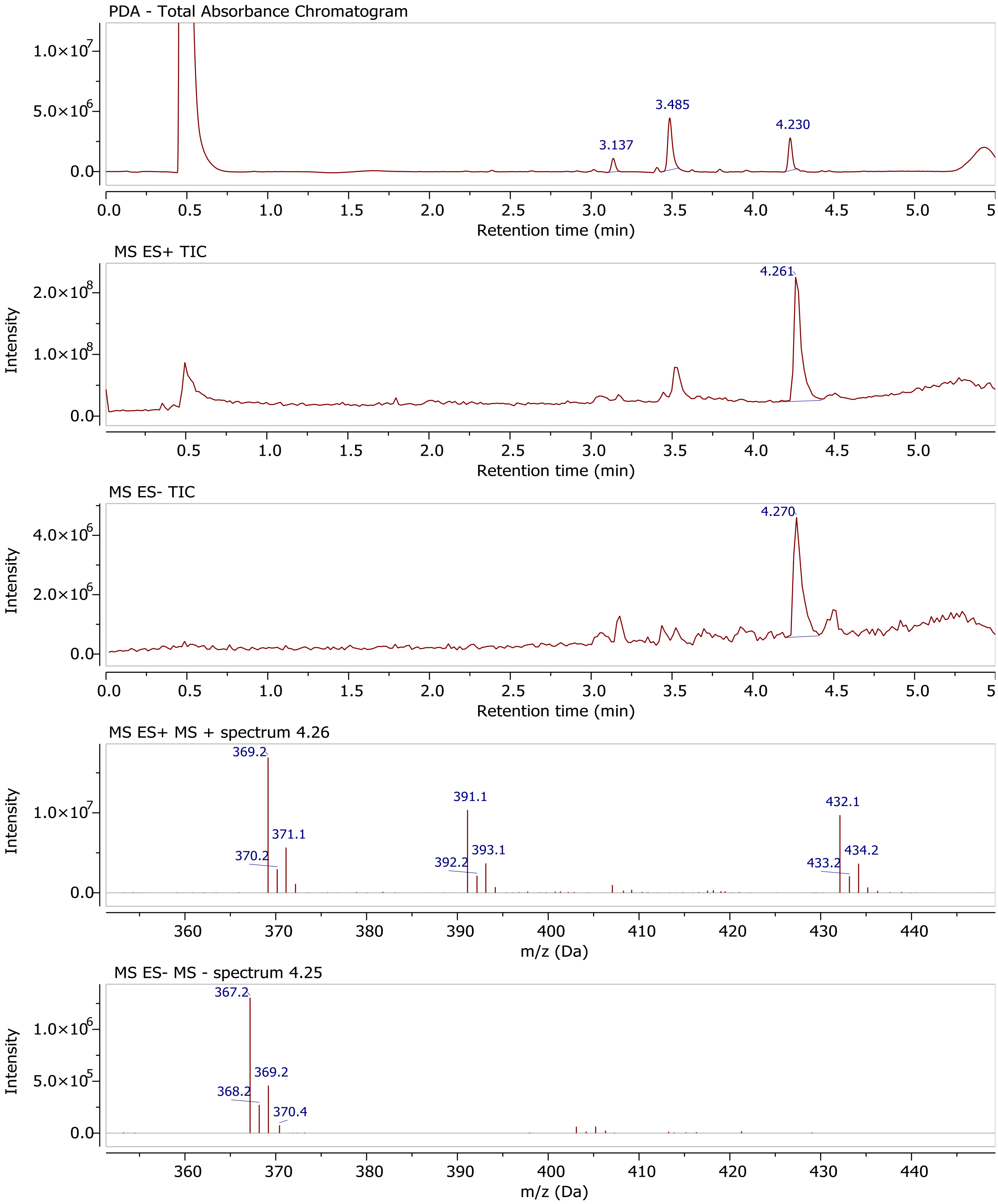

Supplementary figure **1587**. UPLC-MS analysis for compound **PHP-4985**

Chemical Formula: $C_{20}H_{19}F_3N_2O_4$
Exact Mass: 408.1

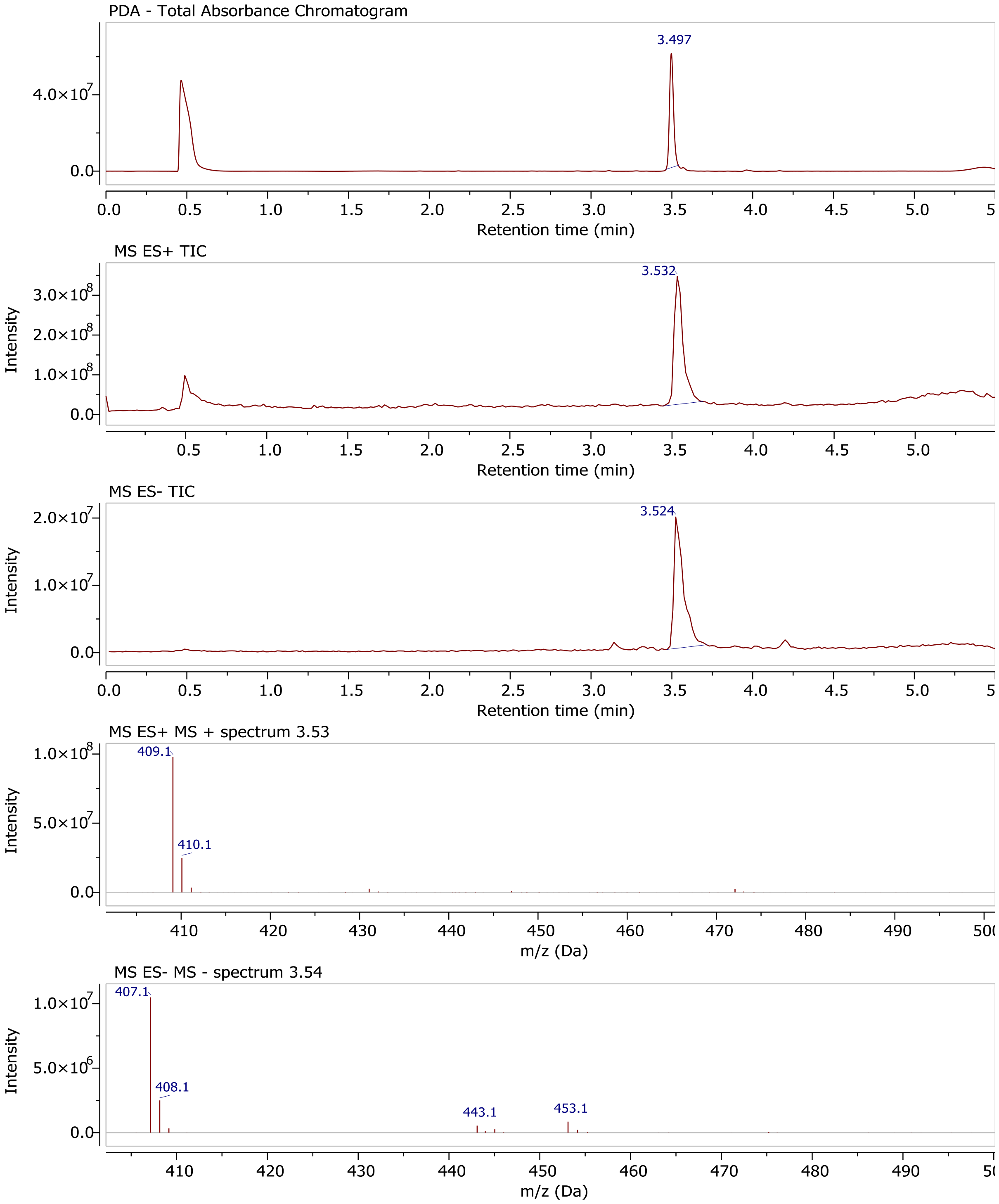

Supplementary figure **1588**. UPLC-MS analysis for compound **PHP-5541**

Chemical Formula: $C_{26}H_{37}N_3O_4$
Exact Mass: 455.3

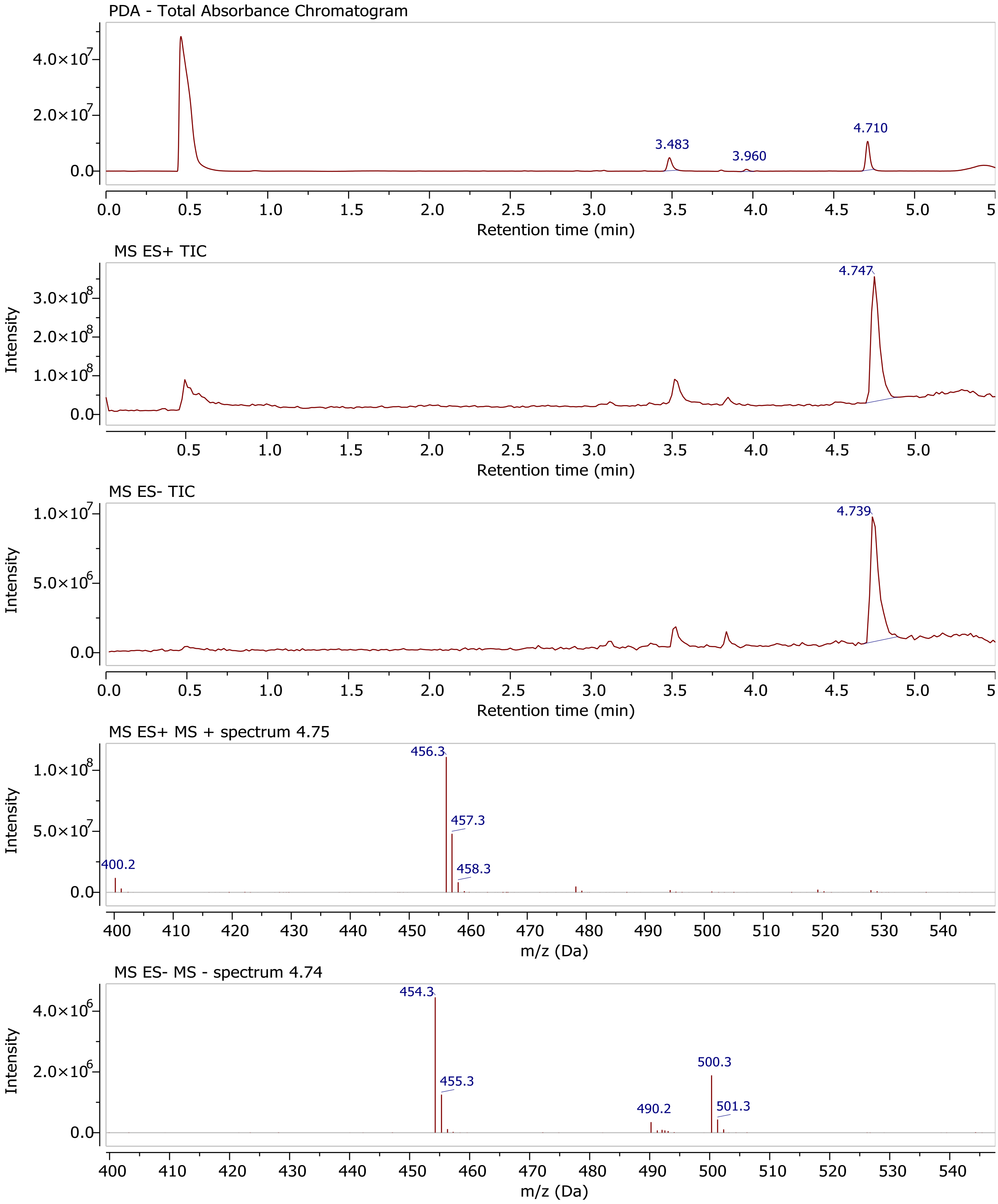

Supplementary figure **1589**. UPLC-MS analysis for compound **PHP-4987**

Chemical Formula: $C_{23}H_{34}N_2O_4$
Exact Mass: 402.3

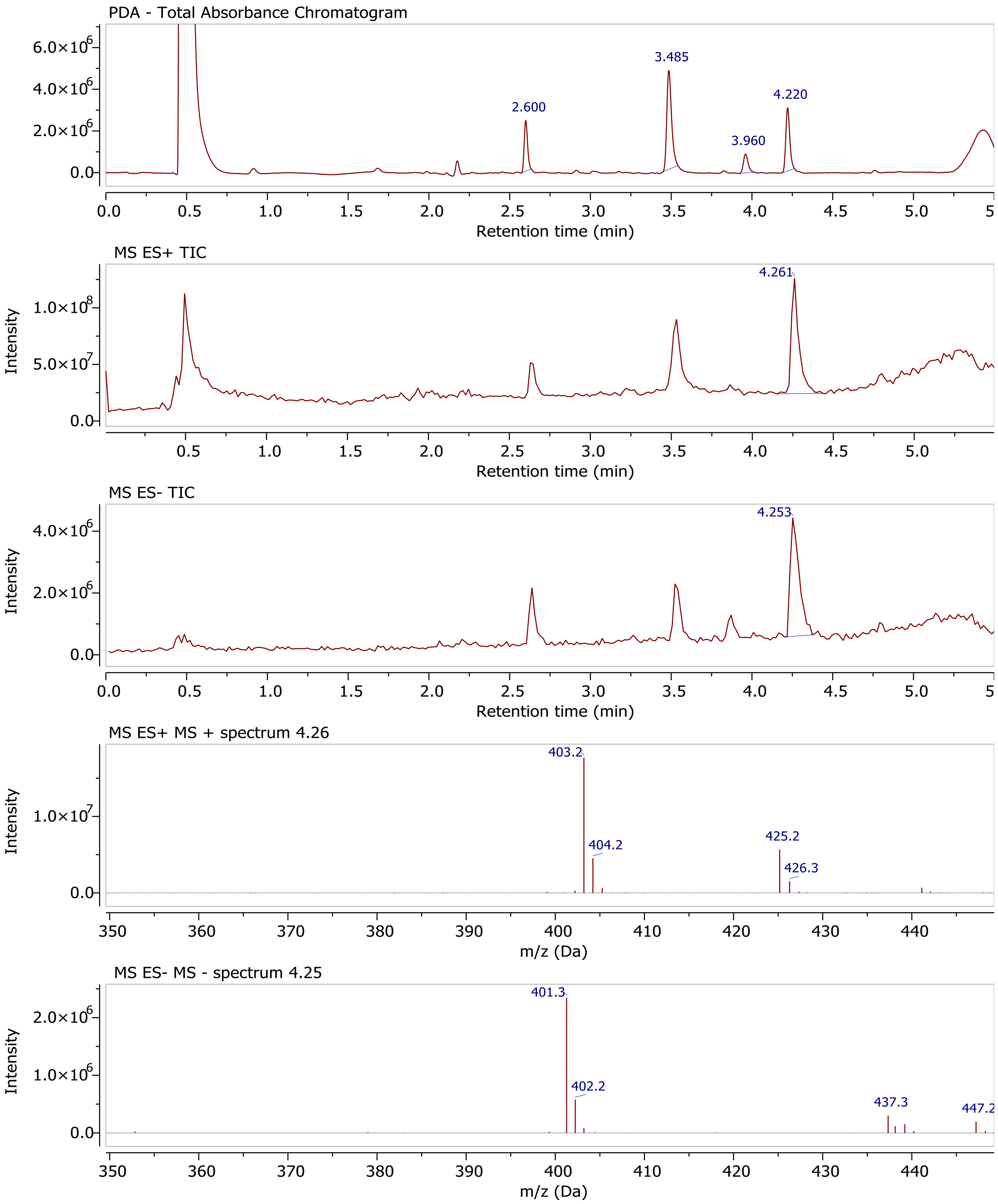

Supplementary figure **1590**. UPLC-MS analysis for compound **PHP-5544**

Chemical Formula: $C_{21}H_{32}N_2O_4$
Exact Mass: 376.2

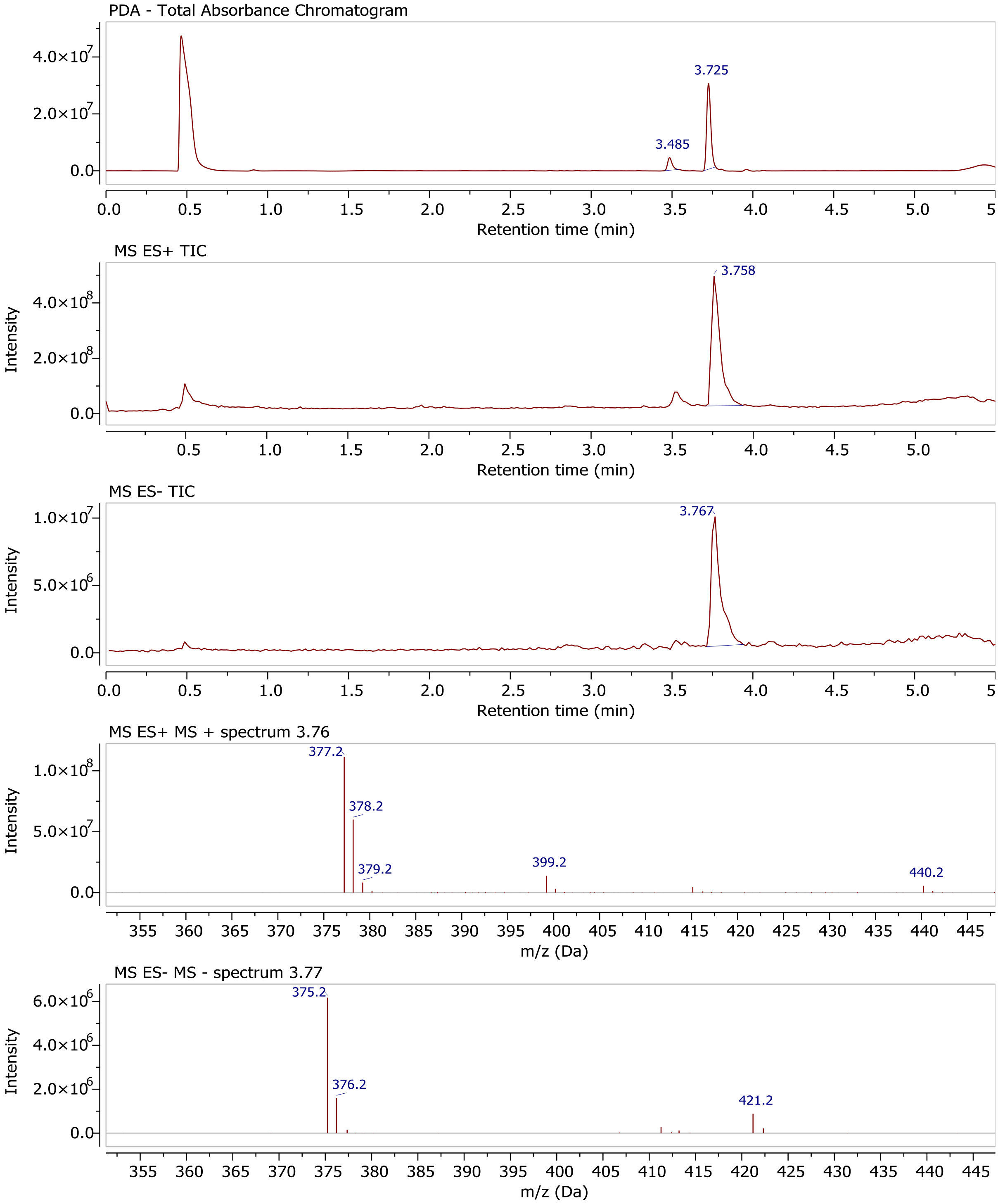

Supplementary figure **1591**. UPLC-MS analysis for compound **PHP-4991**

Chemical Formula: $C_{19}H_{20}N_2O_4$
Exact Mass: 340.1

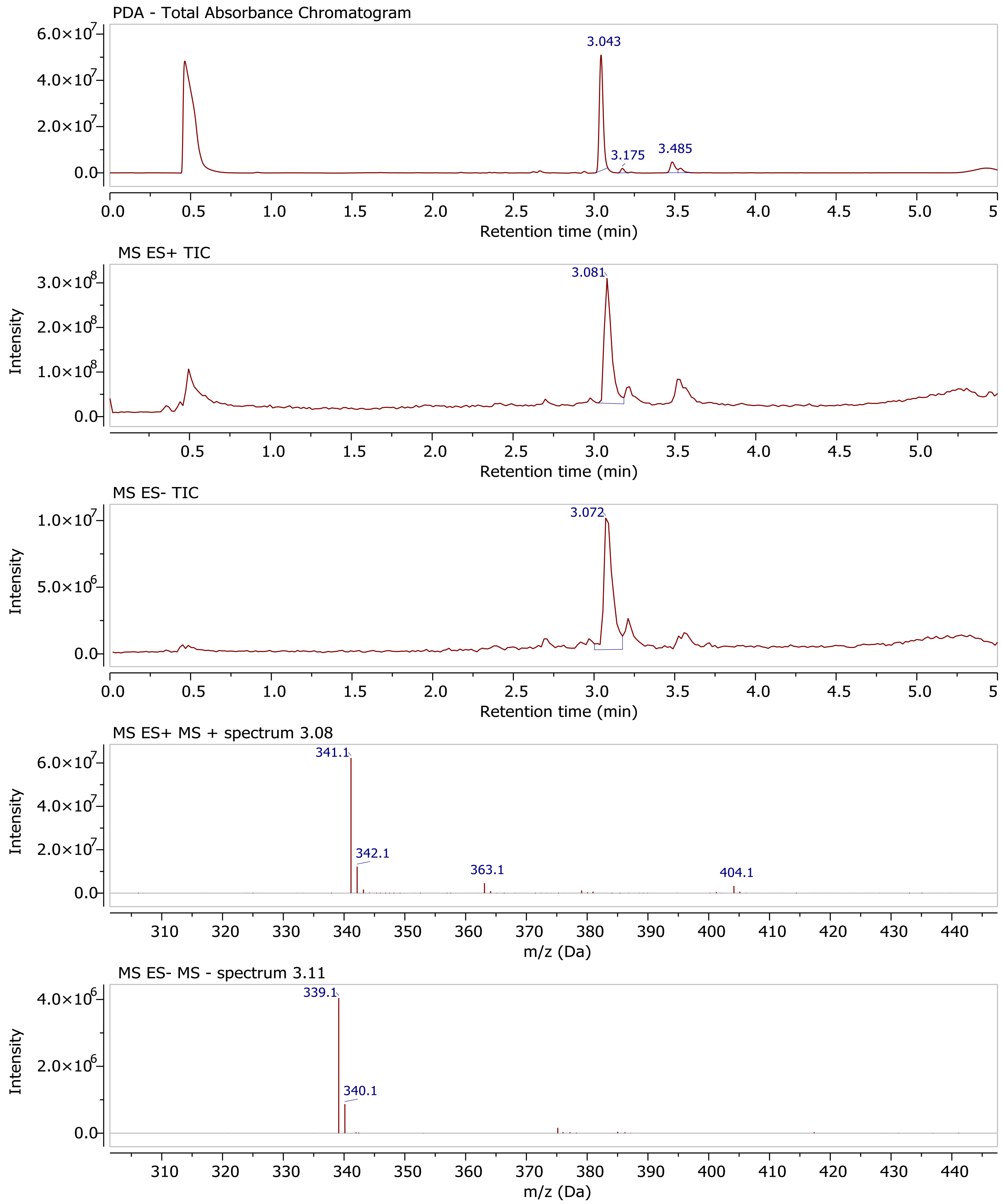

Supplementary figure **1592**. UPLC-MS analysis for compound **PHP-5547**

Chemical Formula: $C_{27}H_{36}N_2O_4$
Exact Mass: 452.3

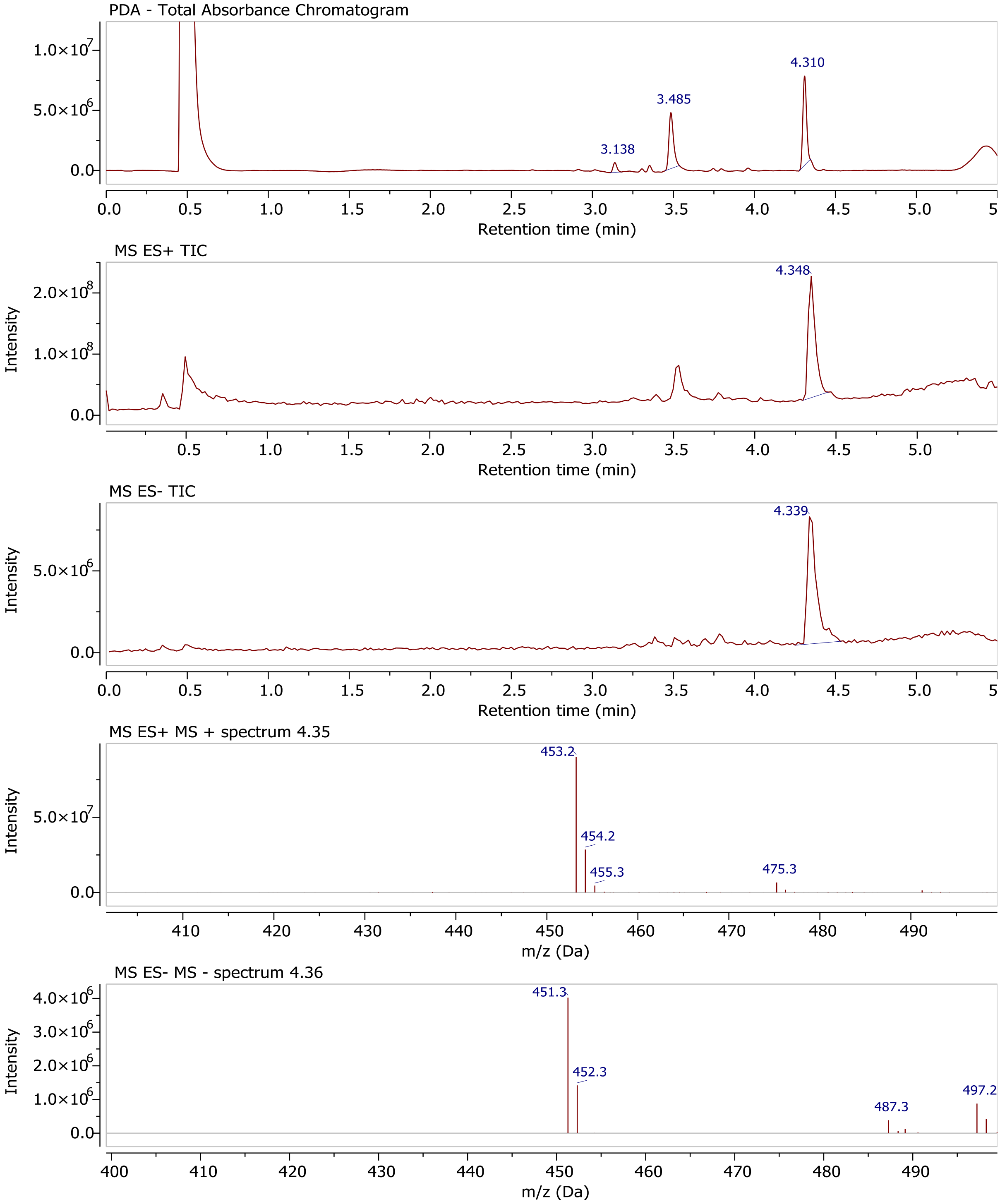

Supplementary figure **1593**. UPLC-MS analysis for compound **PHP-4993**

Chemical Formula: $C_{17}H_{22}N_2O_5$
Exact Mass: 334.2

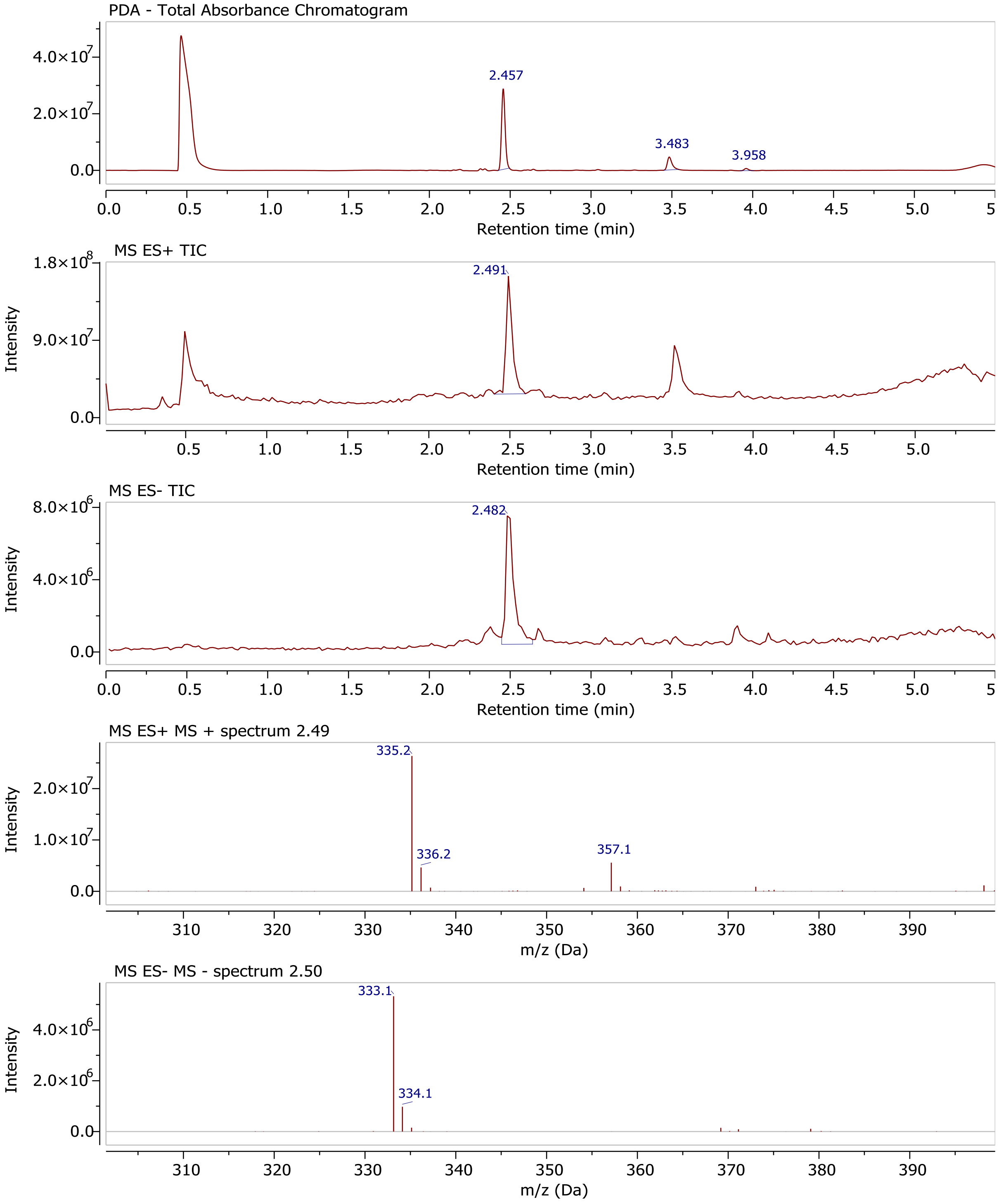

Supplementary figure **1594**. UPLC-MS analysis for compound **PHP-5548**

Chemical Formula: $C_{19}H_{26}Cl_2N_2O_2$
Exact Mass: 384.1

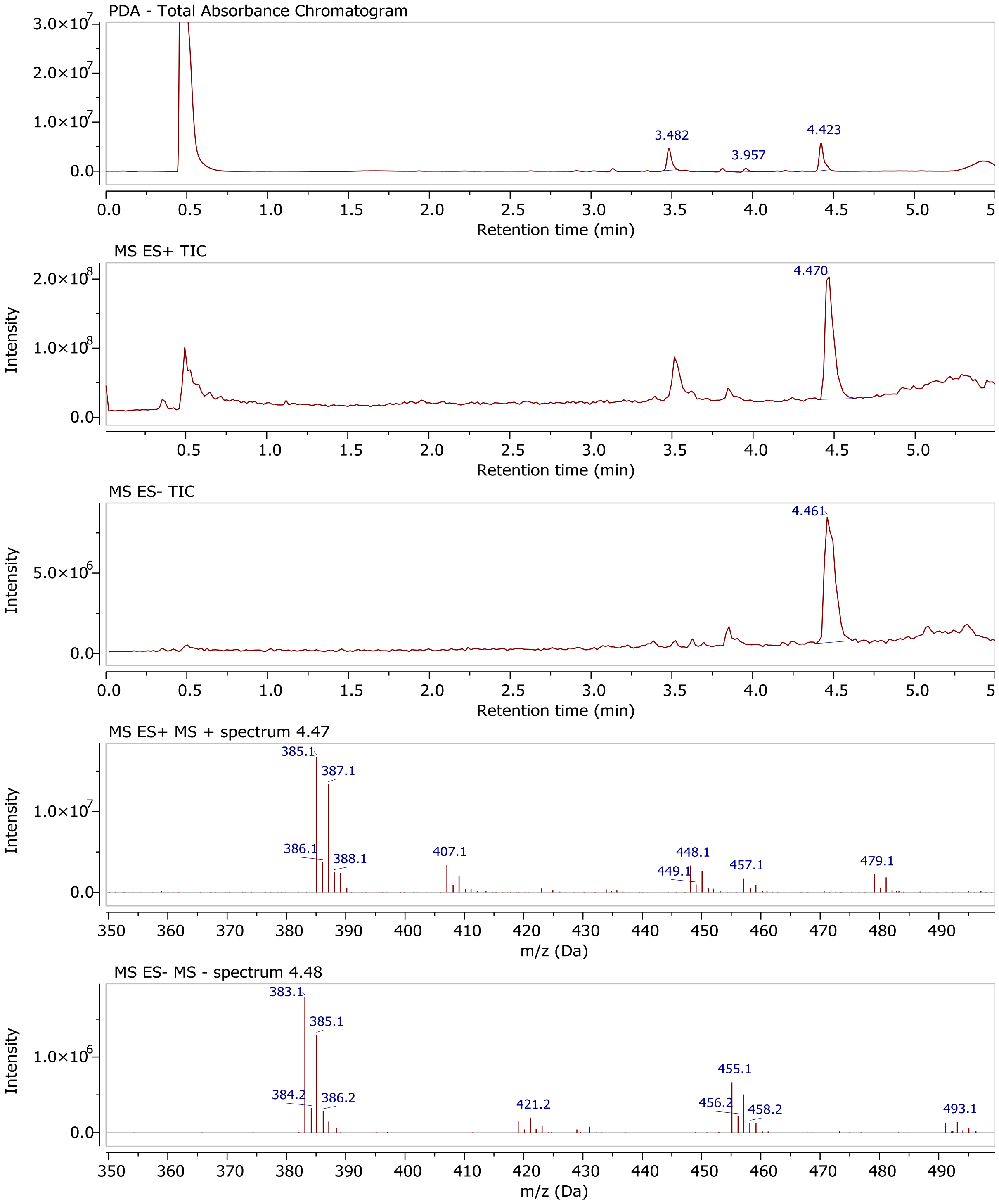

Supplementary figure **1595**. UPLC-MS analysis for compound **PHP-4994**

Chemical Formula: $C_{17}H_{17}BrN_2O_2S$
Exact Mass: 392.0

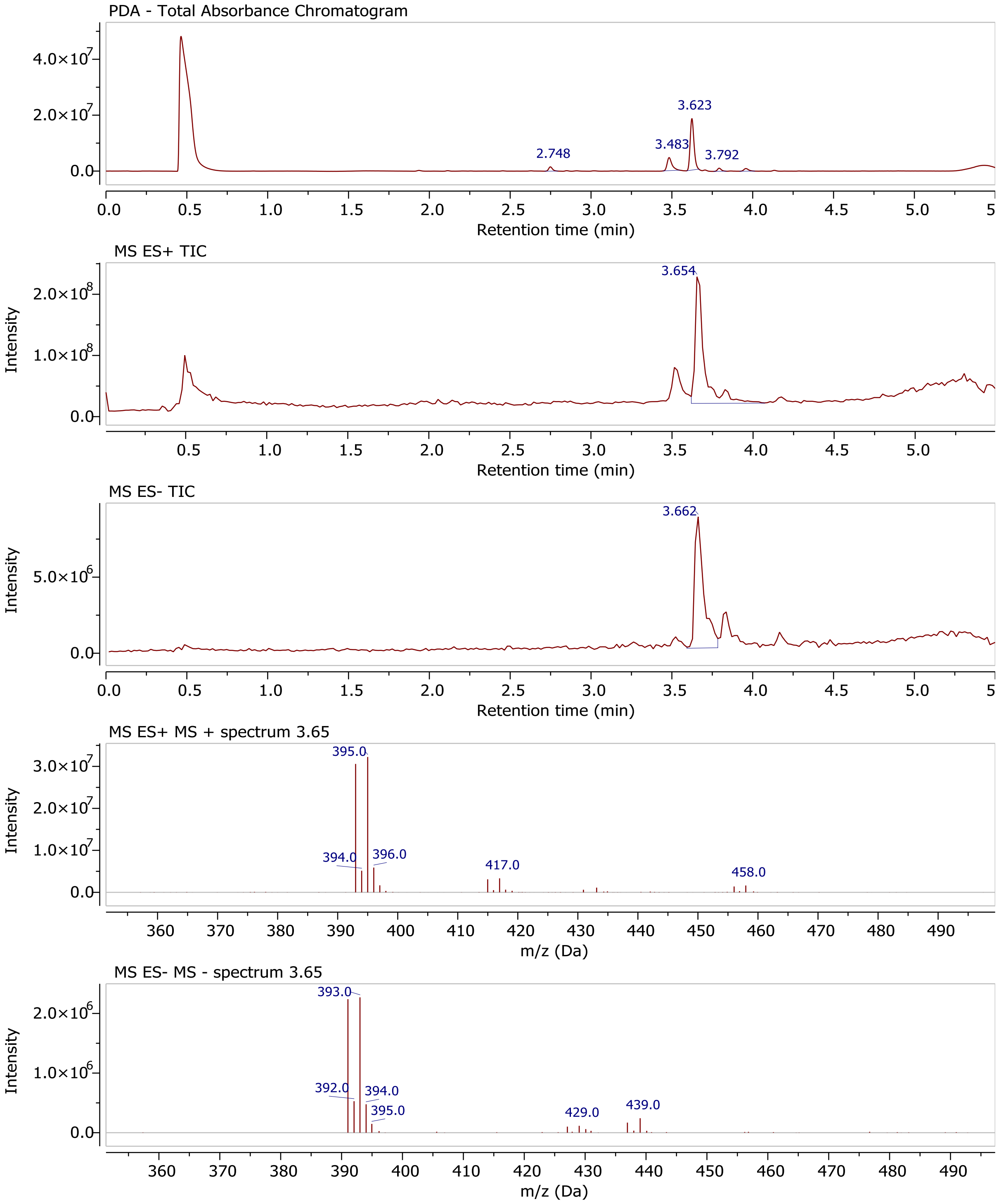

Supplementary figure **1596**. UPLC-MS analysis for compound **PHP-5549**

Chemical Formula: $C_{26}H_{34}N_2O_3$
Exact Mass: 422.3

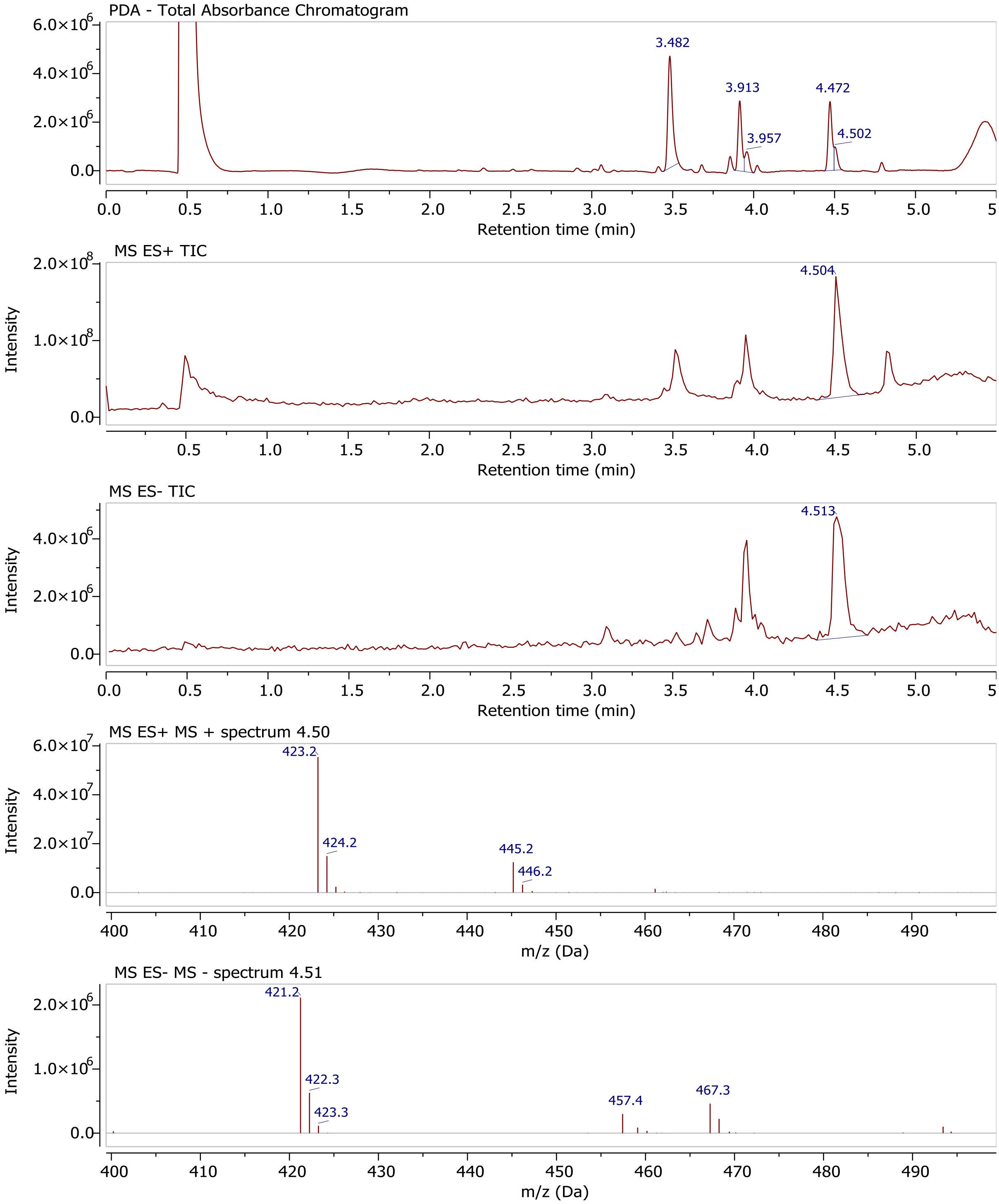

Supplementary figure **1597**. UPLC-MS analysis for compound **PHP-4995**

Chemical Formula: $C_{16}H_{21}BrN_2O_2$
Exact Mass: 352.1

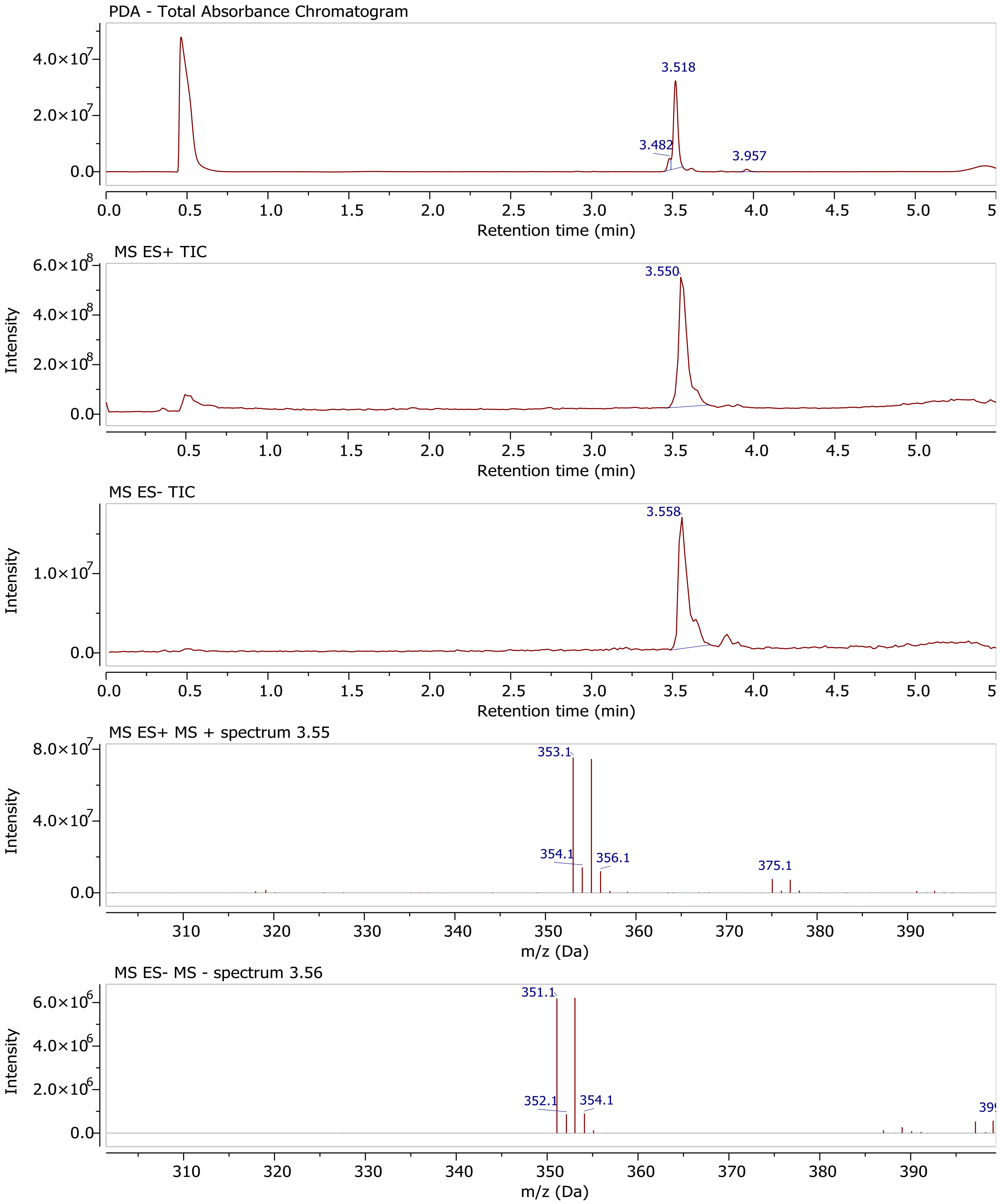

Supplementary figure **1598**. UPLC-MS analysis for compound **PHP-5550**

Chemical Formula: $C_{28}H_{26}N_2O_4$
Exact Mass: 454.2

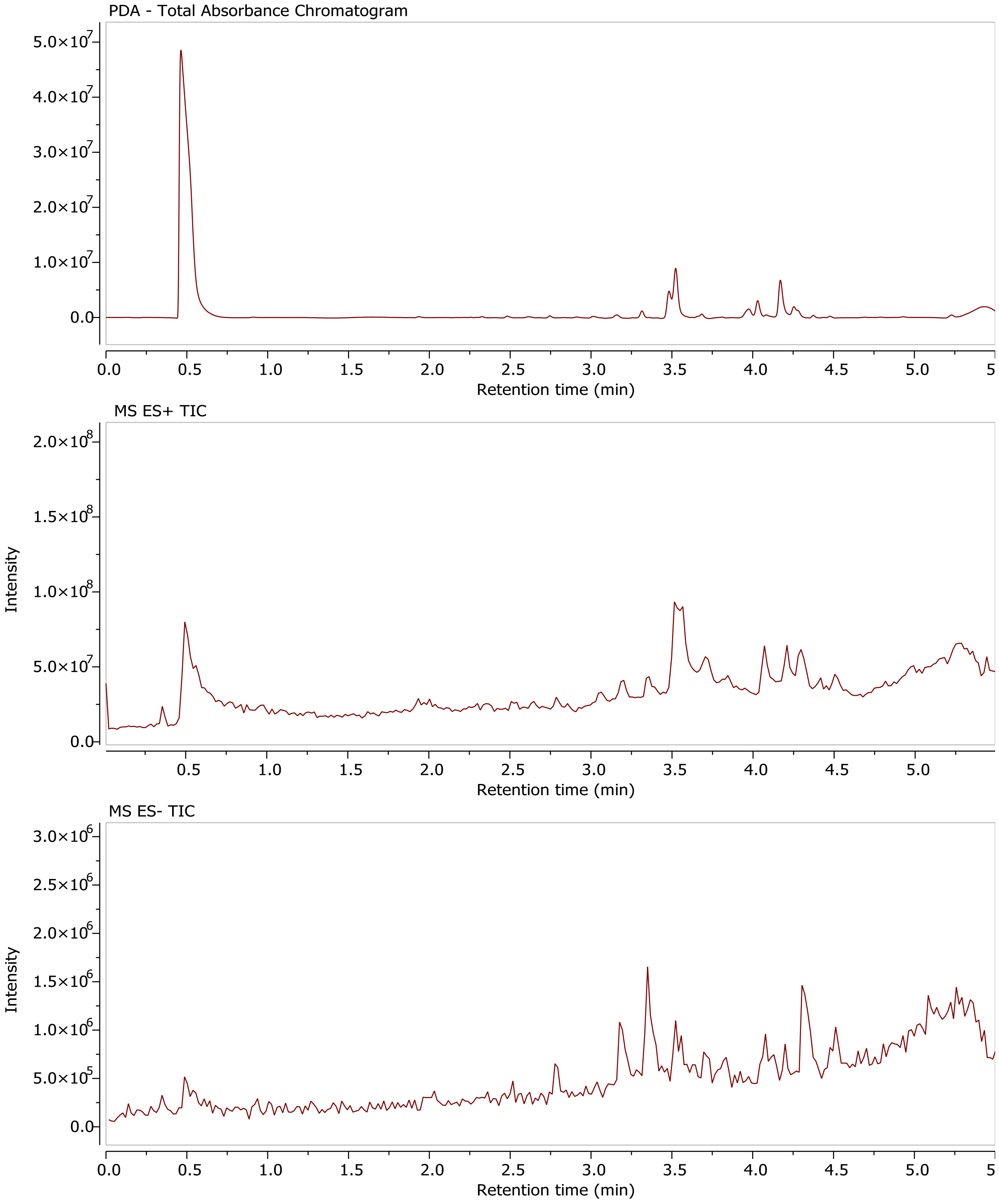

Supplementary figure **1599**. UPLC-MS analysis for compound **PHP-5002**

Chemical Formula: $C_{18}H_{14}BrF_3N_2O_2$
Exact Mass: 426.0

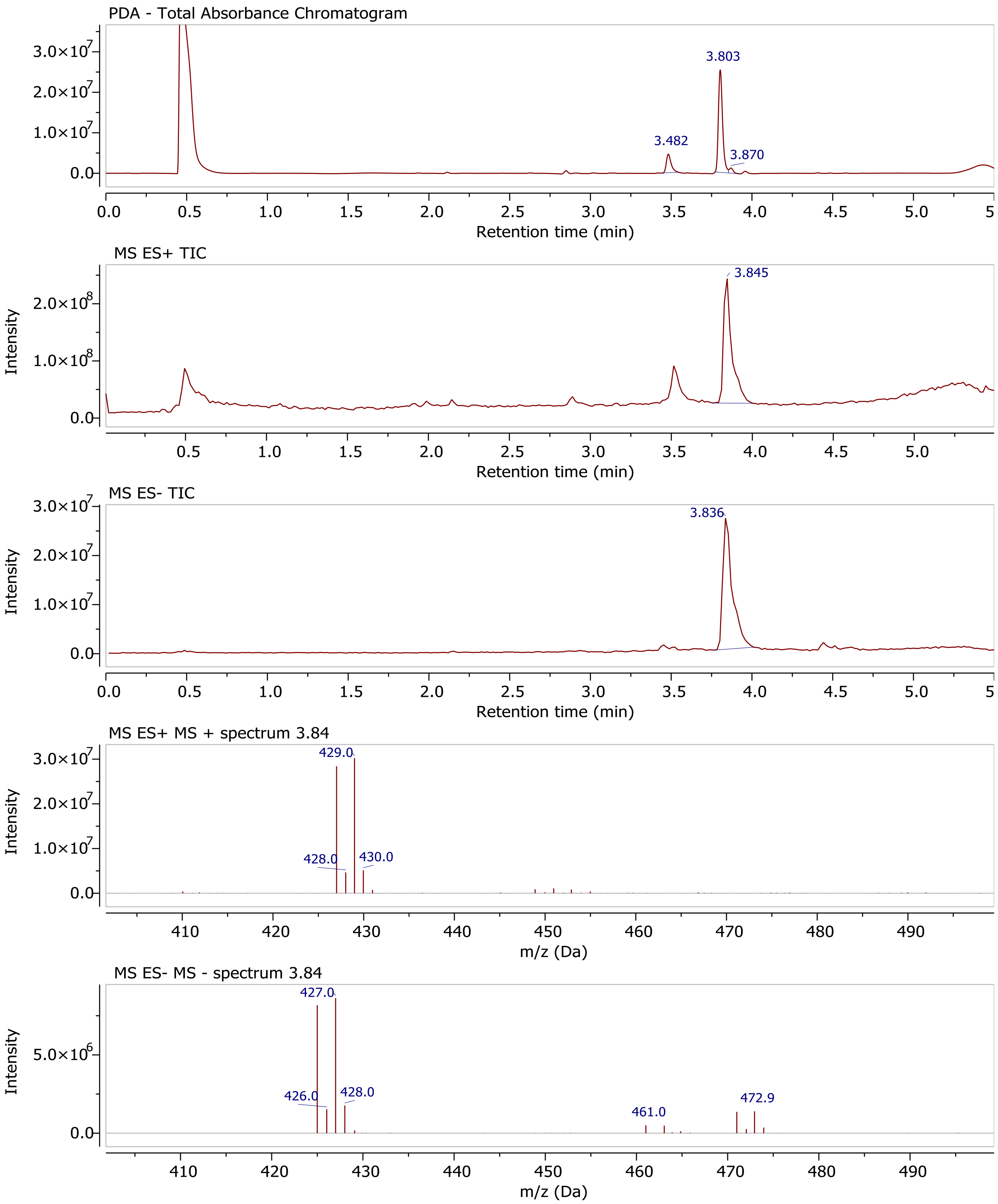

Supplementary figure **1600**. UPLC-MS analysis for compound **PHP-5551**

Chemical Formula: $C_{26}H_{20}ClFN_2O_2$
Exact Mass: 446.1

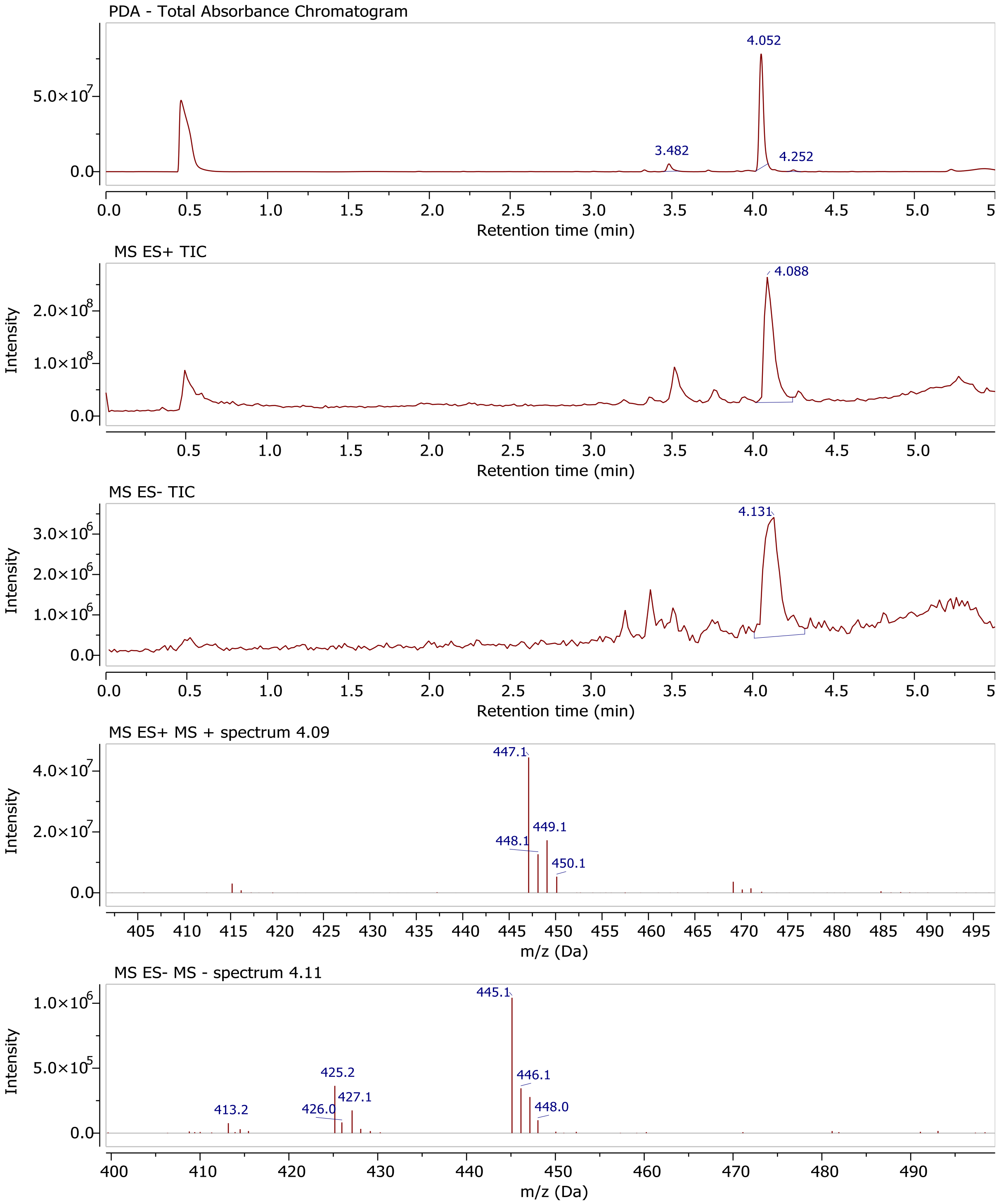

Supplementary figure **1601**. UPLC-MS analysis for compound **PHP-5012**

Chemical Formula: $C_{17}H_{18}BrN_3O_2$
Exact Mass: 375.1

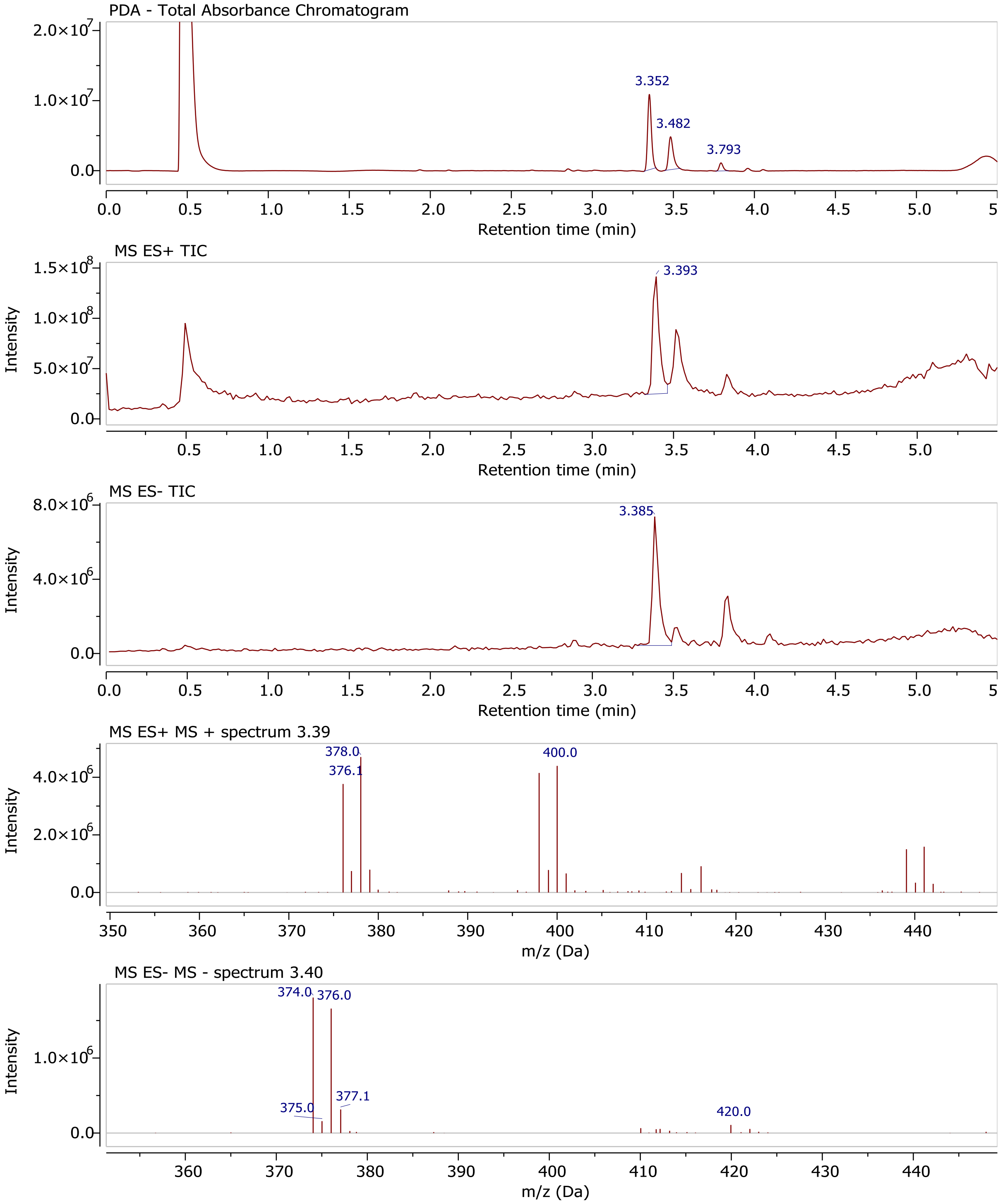

Supplementary figure **1602**. UPLC-MS analysis for compound **PHP-5554**

Chemical Formula: $C_{26}H_{20}Cl_2N_2O_2$
Exact Mass: 462.1

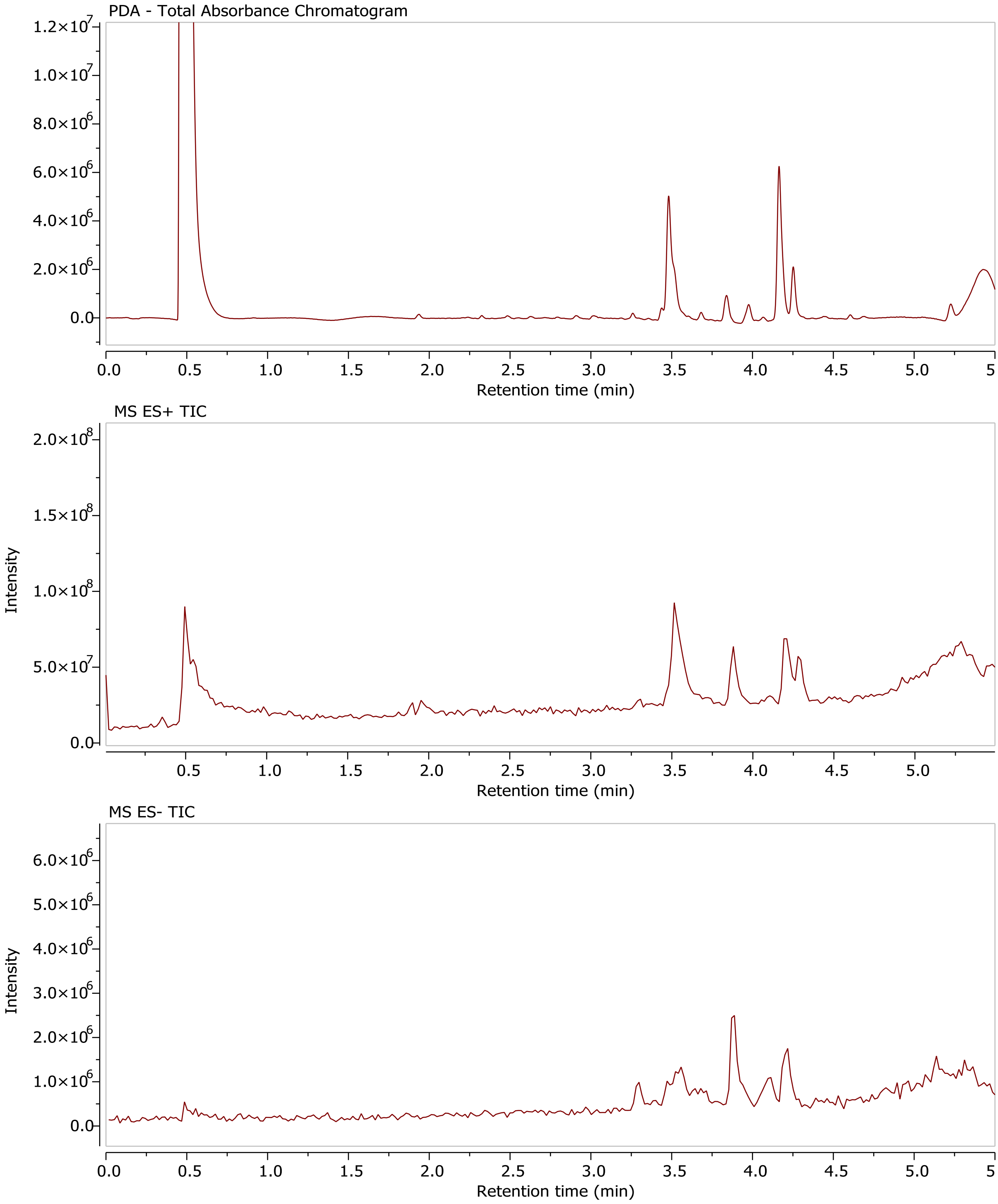

Supplementary figure **1603**. UPLC-MS analysis for compound **PHP-5013**

Chemical Formula: $C_{20}H_{21}BrN_2O_2$
Exact Mass: 400.1

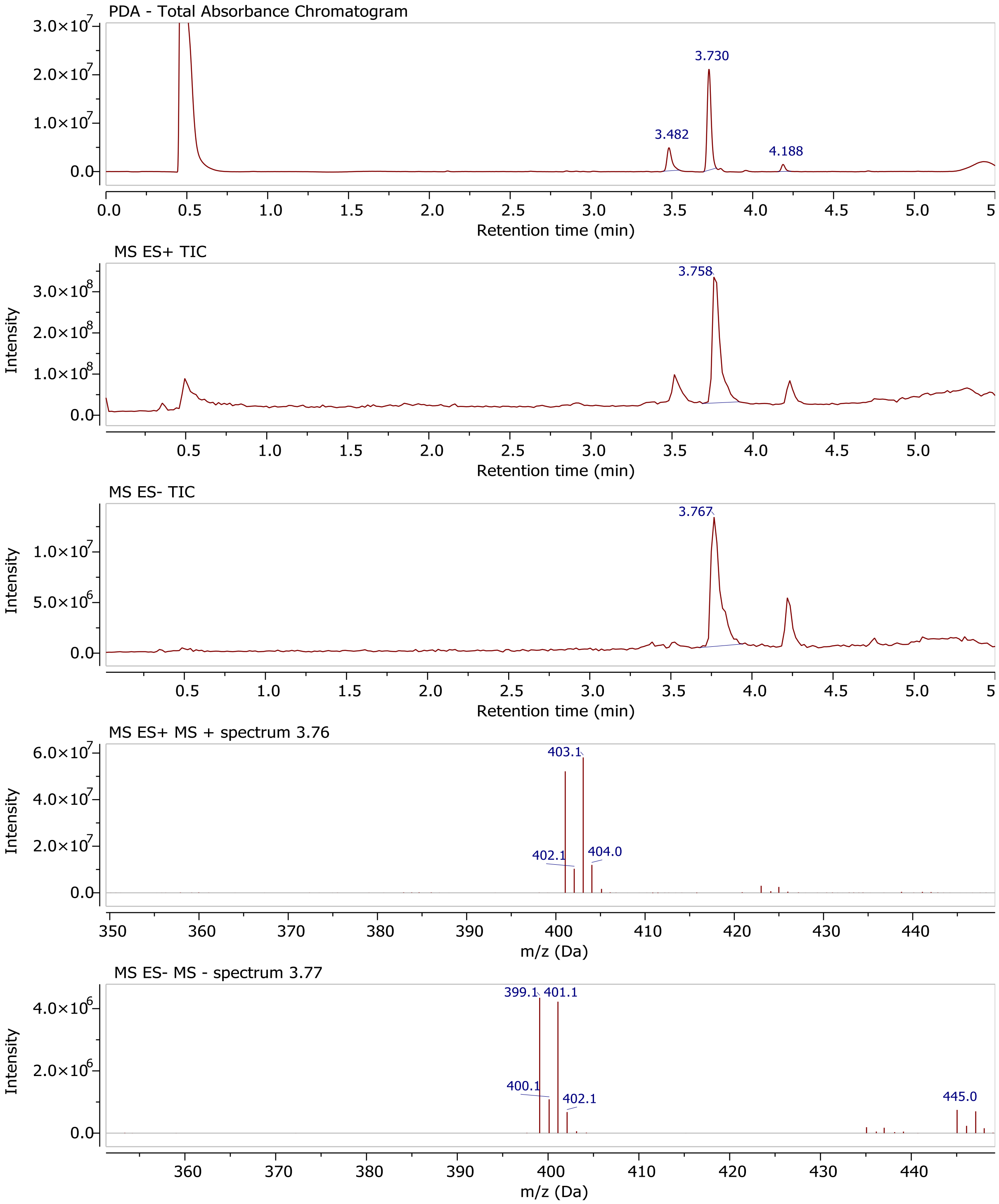

Supplementary figure **1604**. UPLC-MS analysis for compound **PHP-5556**

Chemical Formula: $C_{24}H_{28}N_2O_5$
Exact Mass: 424.2

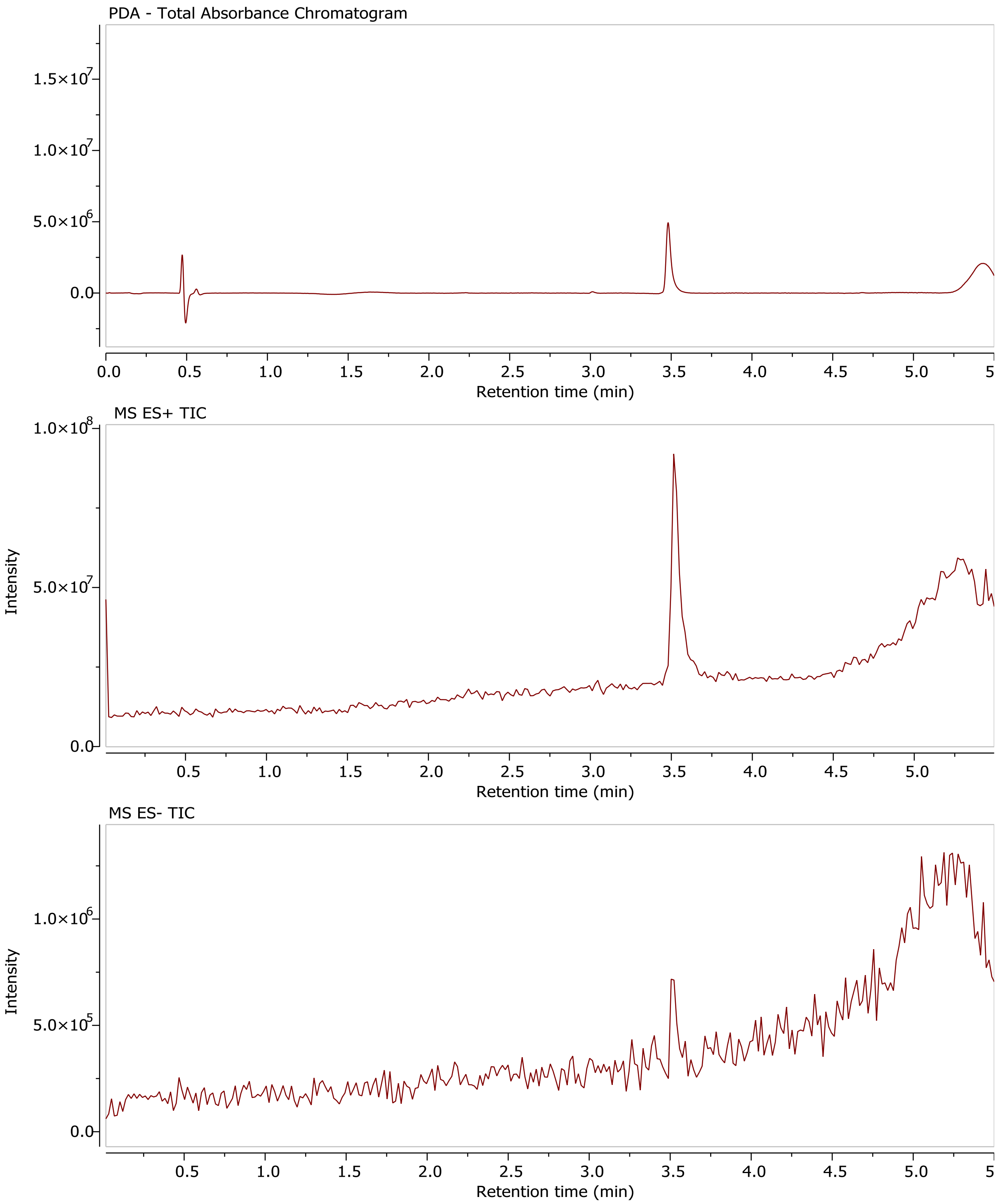

Supplementary figure **1605**. UPLC-MS analysis for compound **PHP-5017**

Chemical Formula: $C_{16}H_{19}BrN_2O_2$
Exact Mass: 350.1

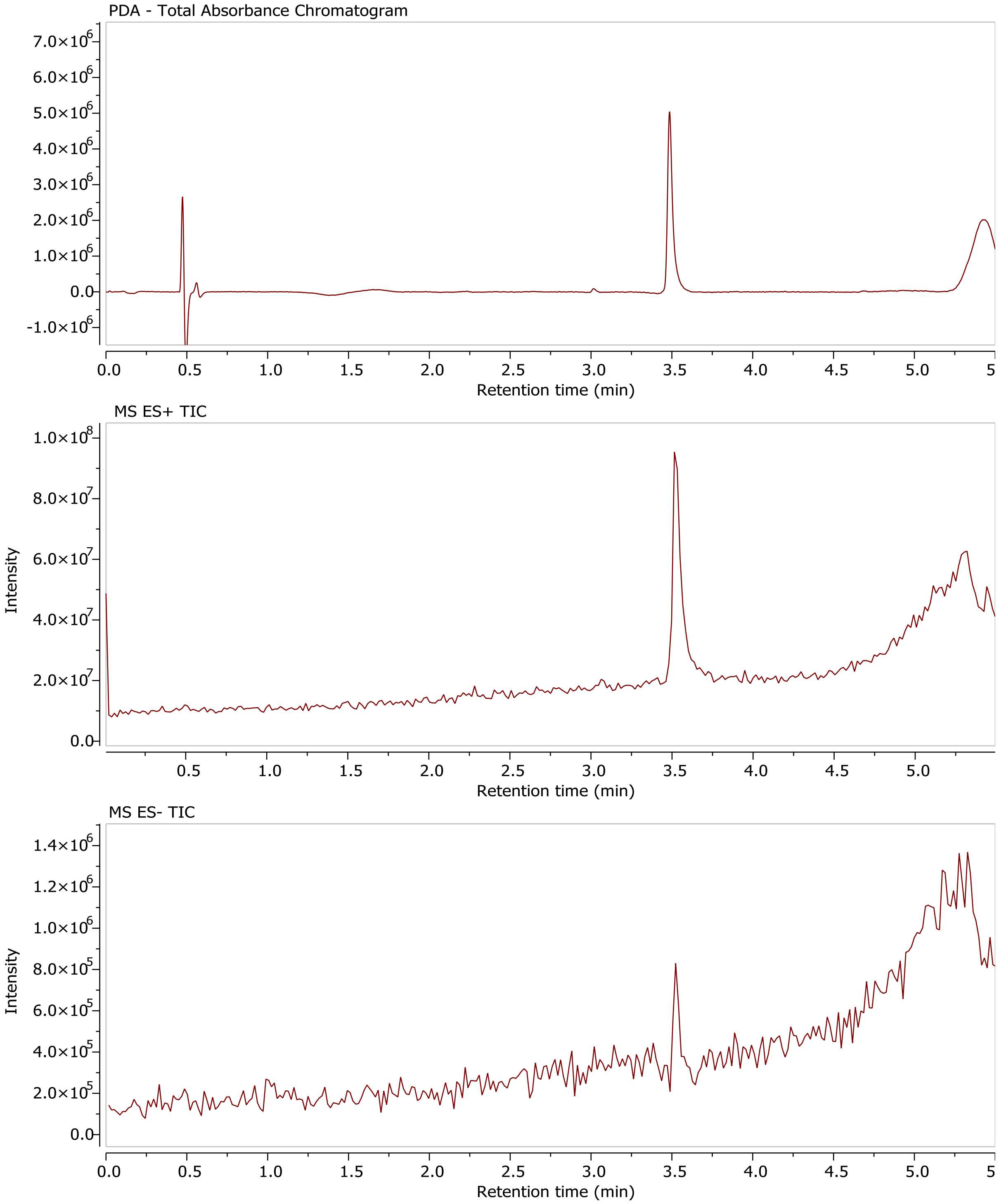

Supplementary figure **1606**. UPLC-MS analysis for compound **PHP-5557**

Chemical Formula: $C_{21}H_{20}Cl_2N_2O_2$
Exact Mass: 402.1

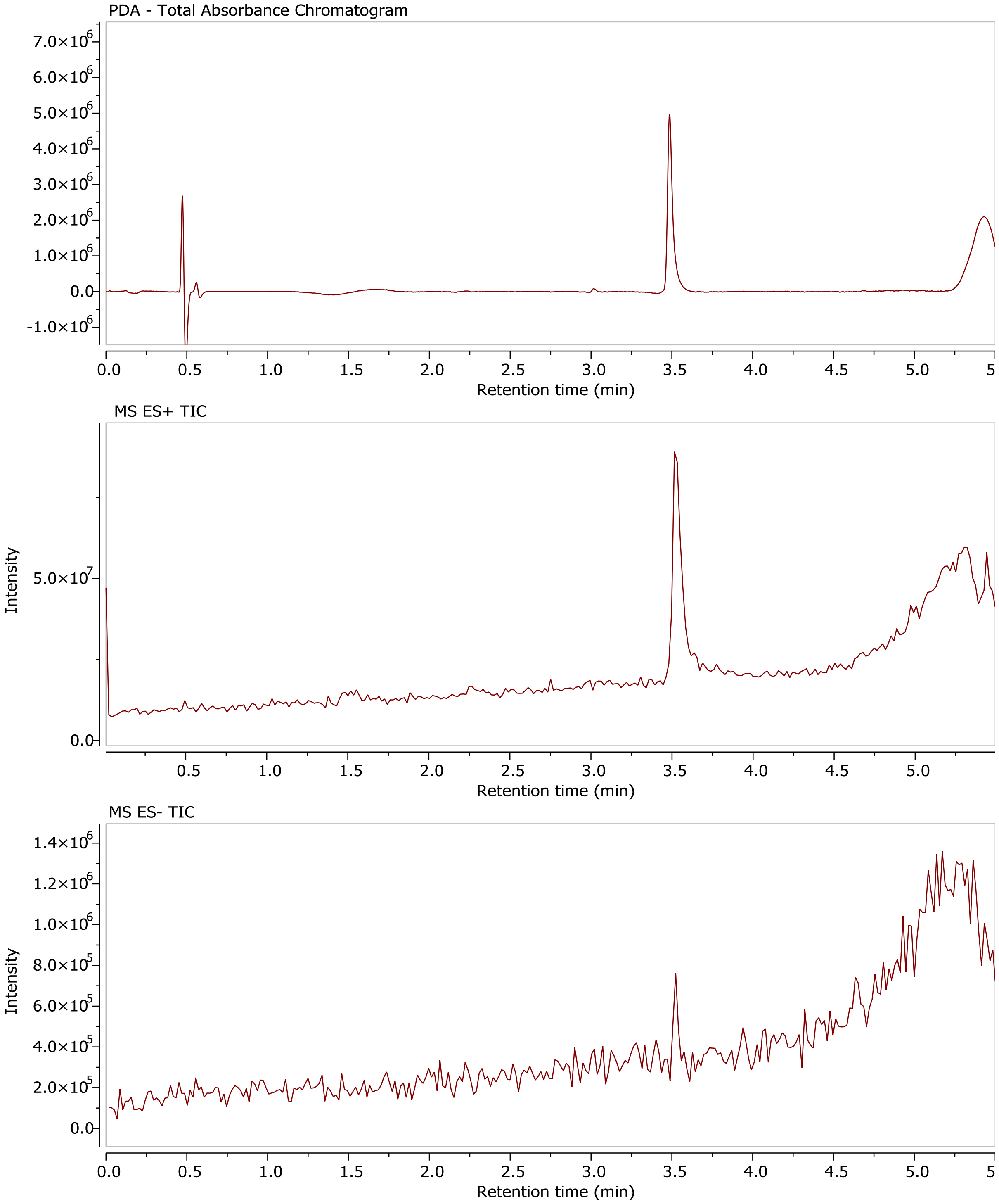

Supplementary figure **1607**. UPLC-MS analysis for compound **PHP-5018**

Chemical Formula: $C_{19}H_{16}BrF_3N_2O_2$
Exact Mass: 440.0

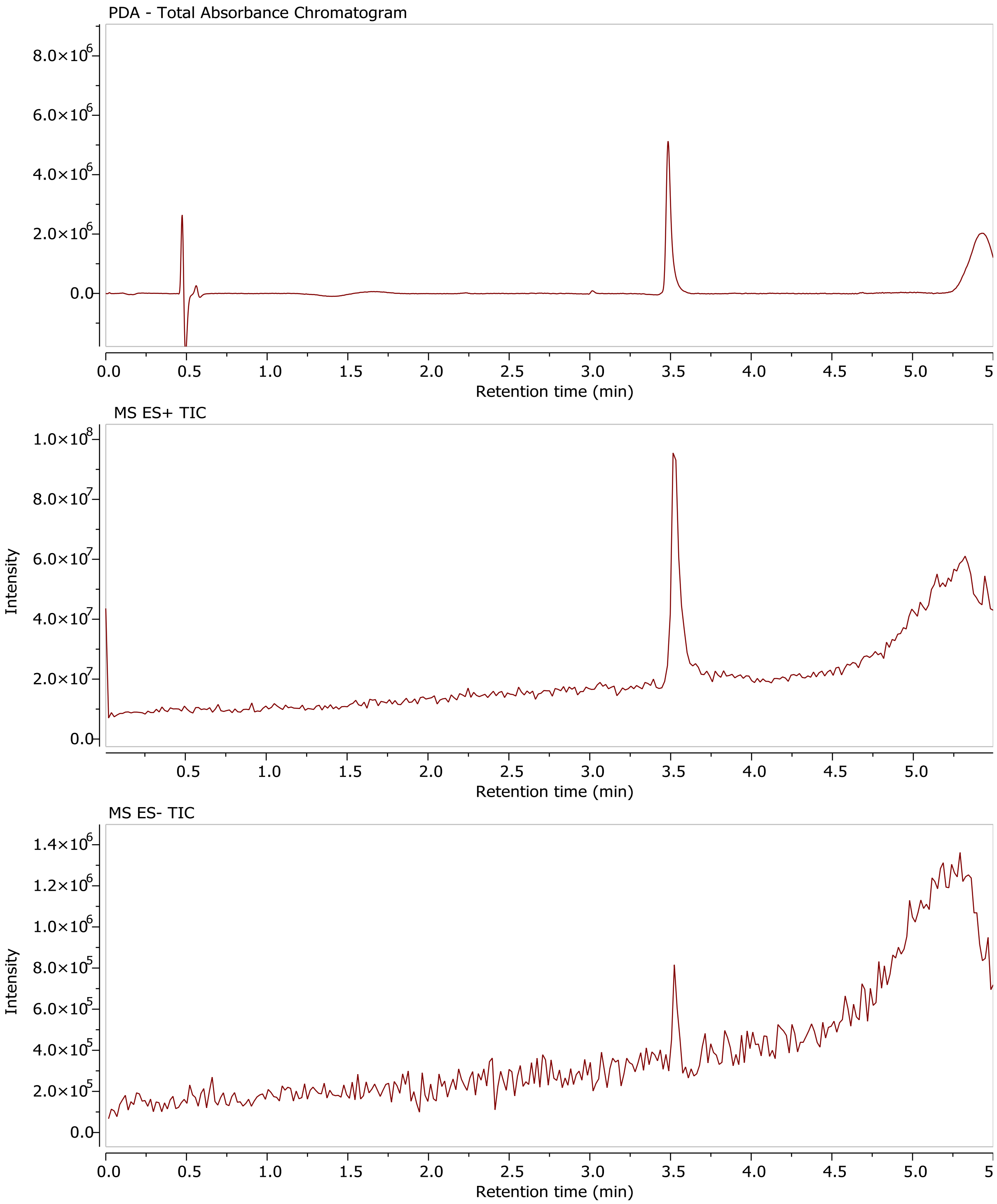

Supplementary figure **1608**. UPLC-MS analysis for compound **PHP-5559**

Chemical Formula: $C_{21}H_{21}BrN_2O_2$
Exact Mass: 412.1

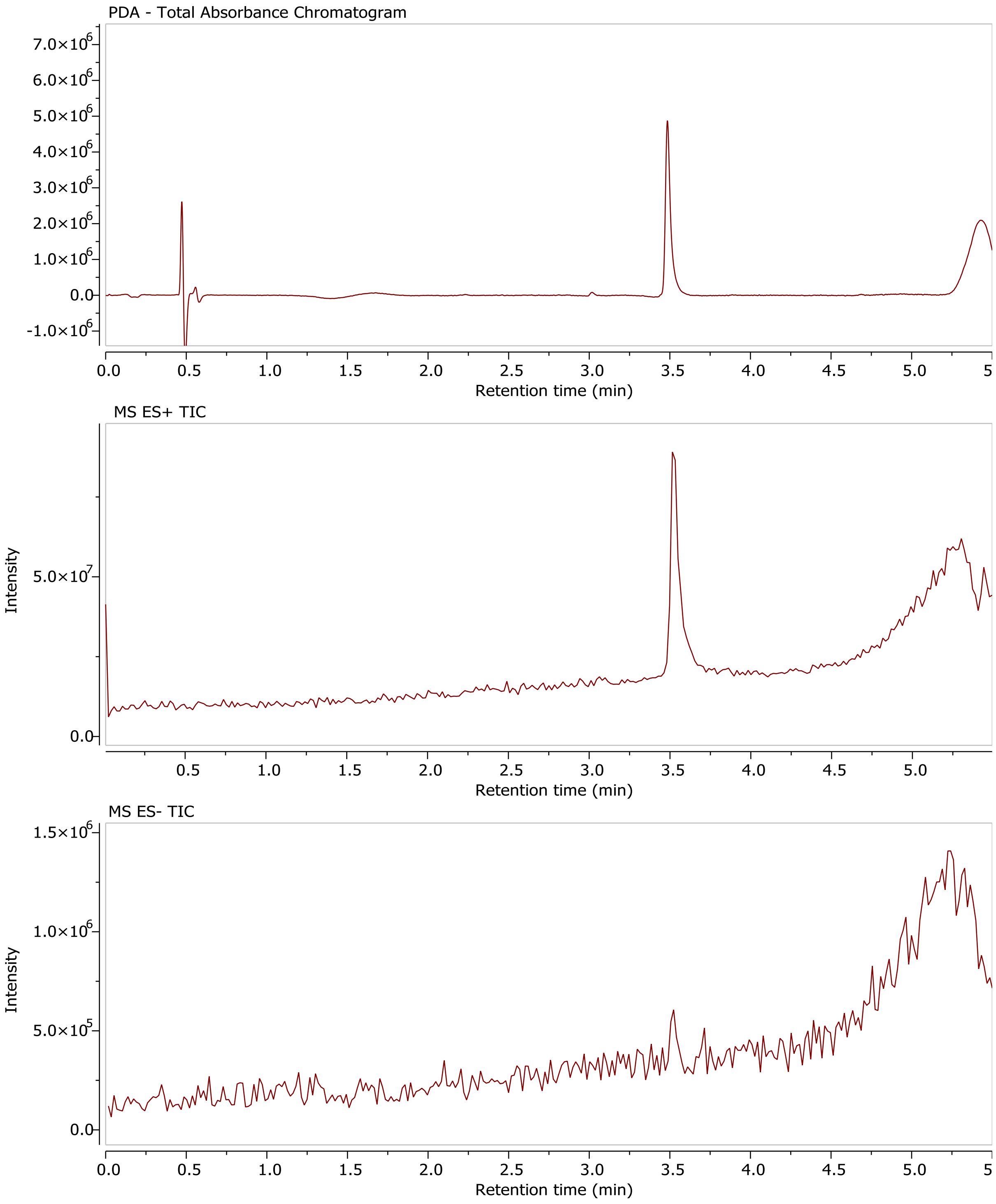

Supplementary figure **1610**. UPLC-MS analysis for compound **PHP-5019**

Chemical Formula: $C_{18}H_{16}BrClN_2O_2$
Exact Mass: 406.0

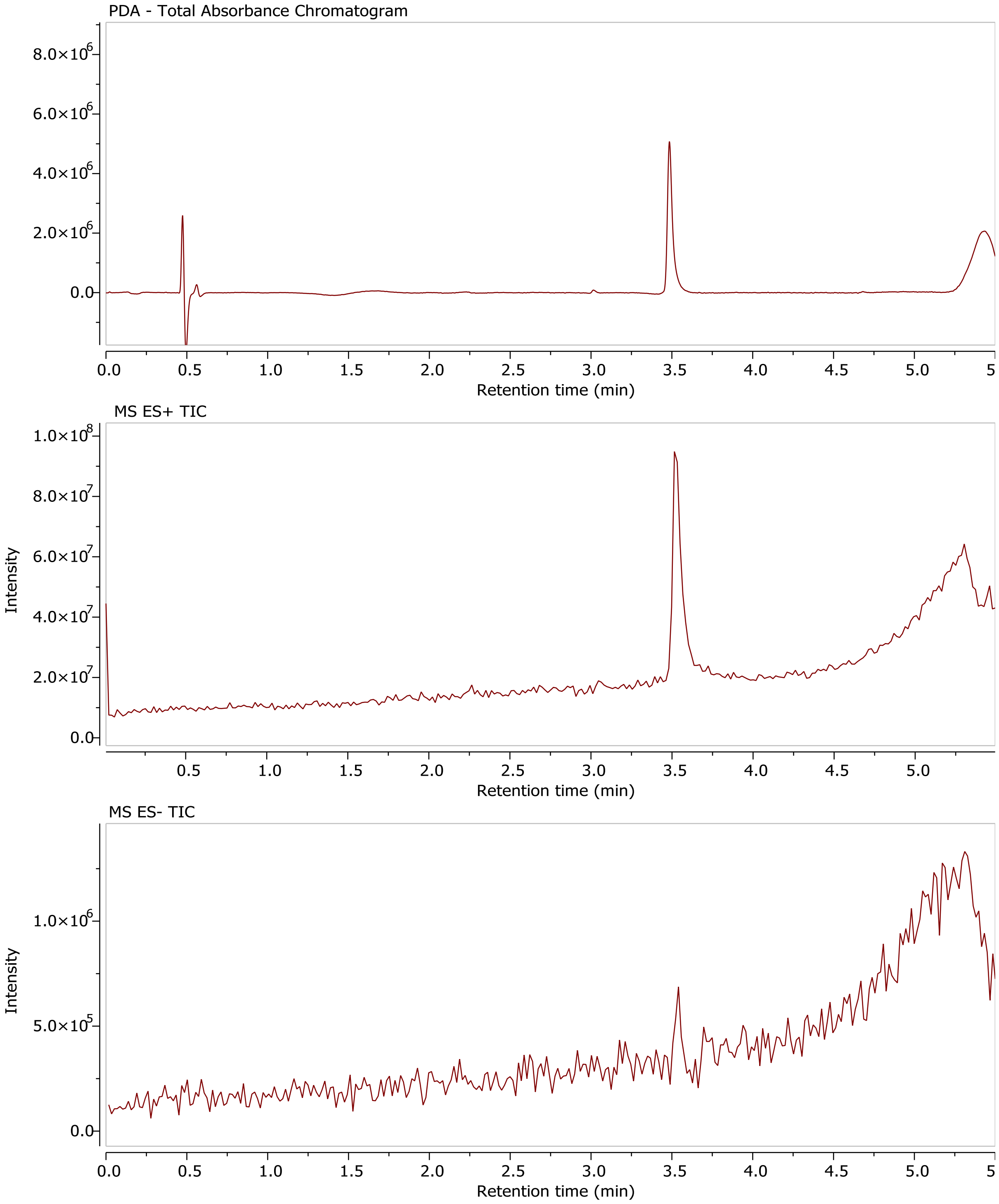

Supplementary figure **1611**. UPLC-MS analysis for compound **PHP-5560**

Chemical Formula: $C_{19}H_{16}F_4N_2O_2$
Exact Mass: 380.1

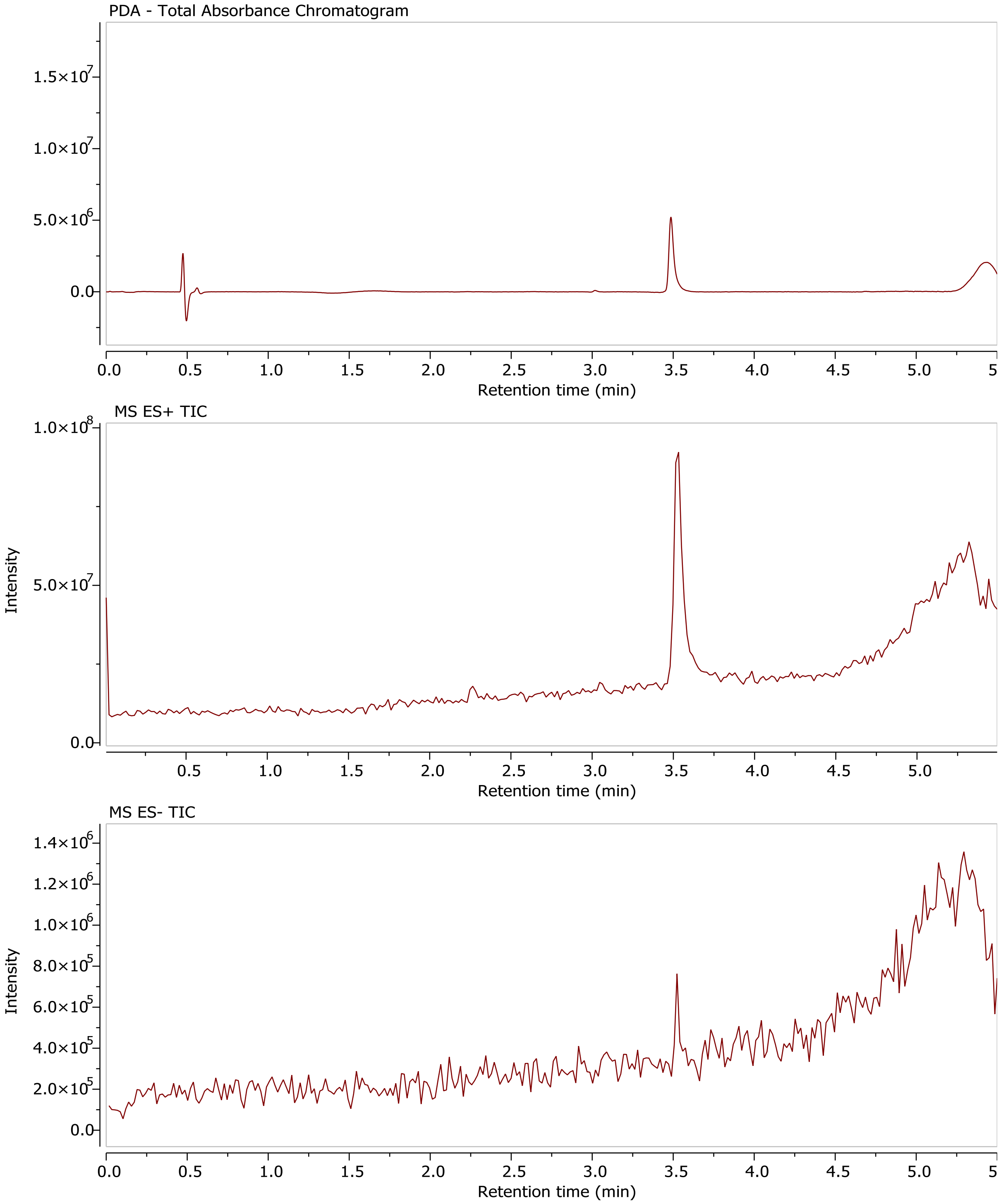

Supplementary figure **1612**. UPLC-MS analysis for compound **PHP-5701**

Chemical Formula: $C_{19}H_{15}F_3N_2O_4$
Exact Mass: 392.1

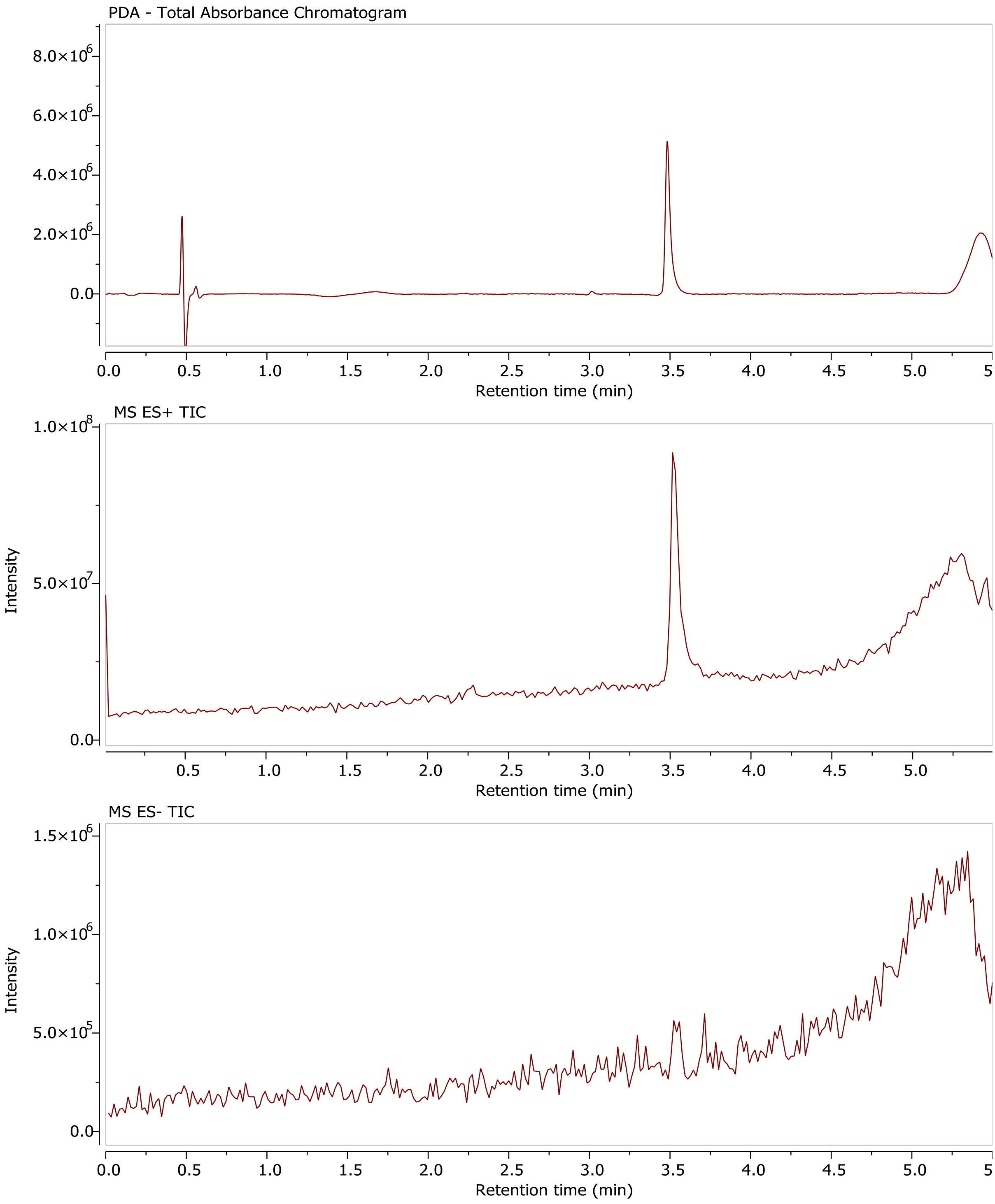

Supplementary figure **1613**. UPLC-MS analysis for compound **PHP-5901**

Chemical Formula: $C_{24}H_{21}FN_2O_3$
Exact Mass: 404.2

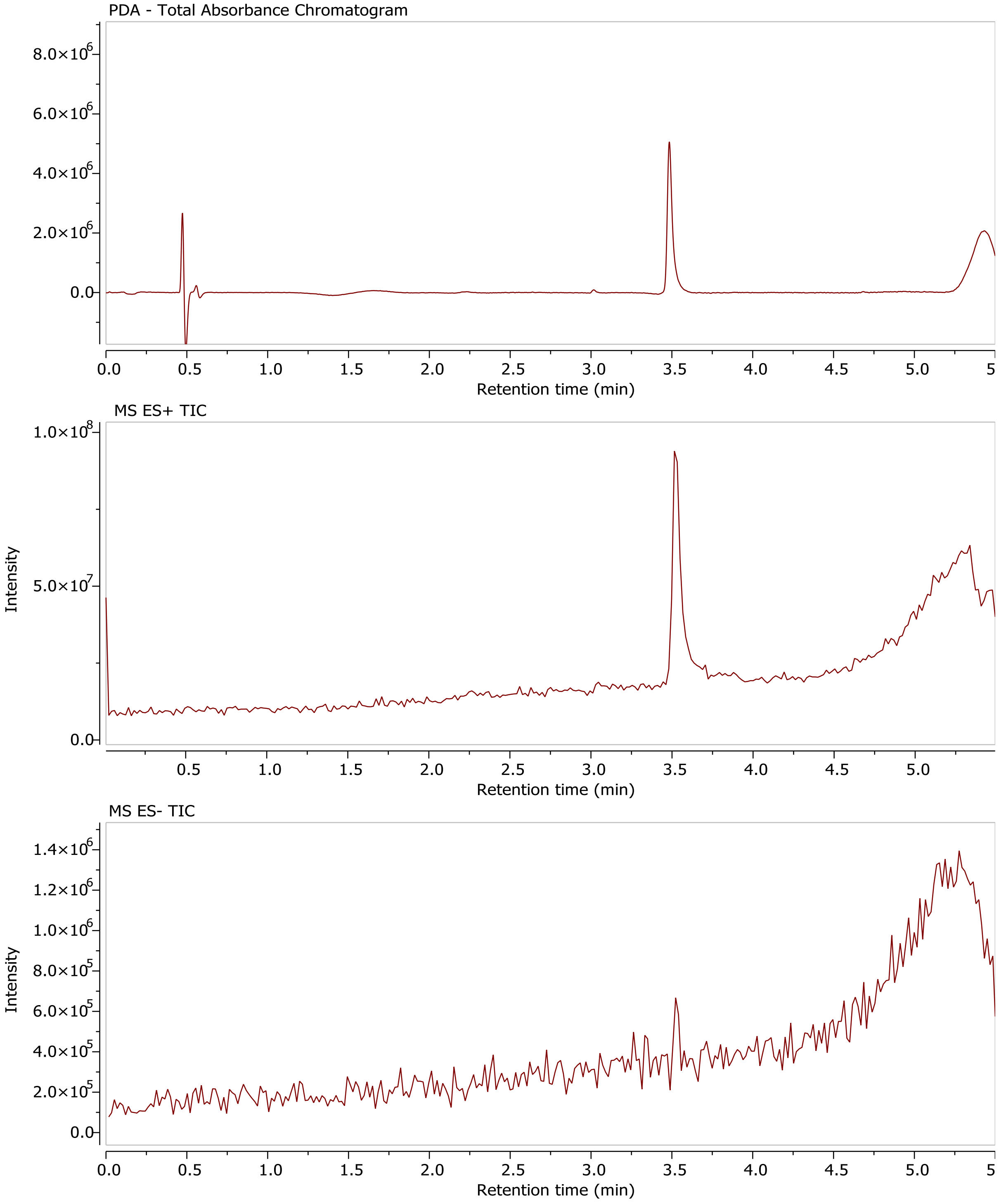

Supplementary figure **1614**. UPLC-MS analysis for compound **PHP-5702**

Chemical Formula: $C_{24}H_{20}N_2O_5$
Exact Mass: 416.1

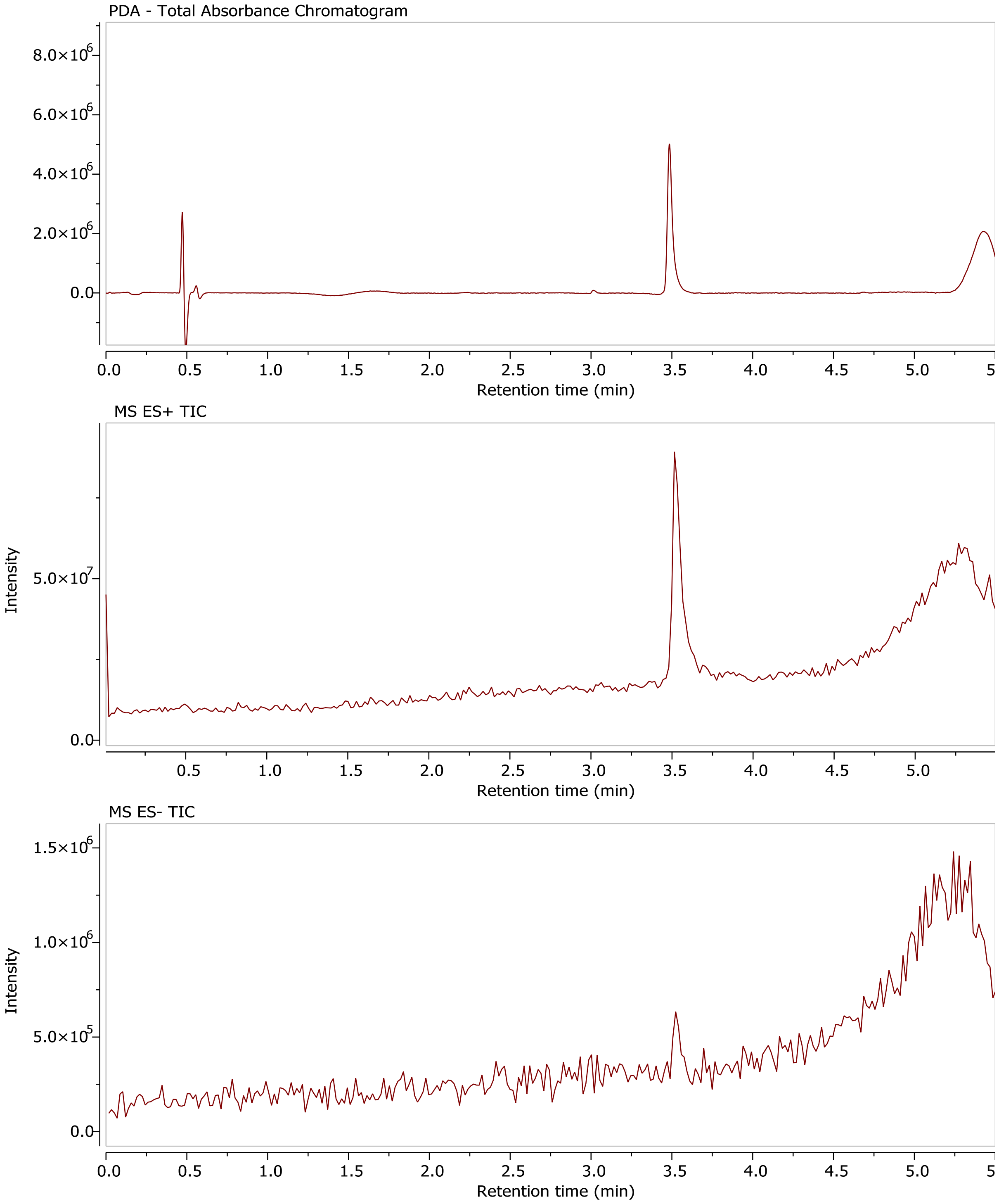

Supplementary figure **1615**. UPLC-MS analysis for compound **PHP-5902**

Chemical Formula: $C_{18}H_{16}BrFN_2O_2$
Exact Mass: 390.0

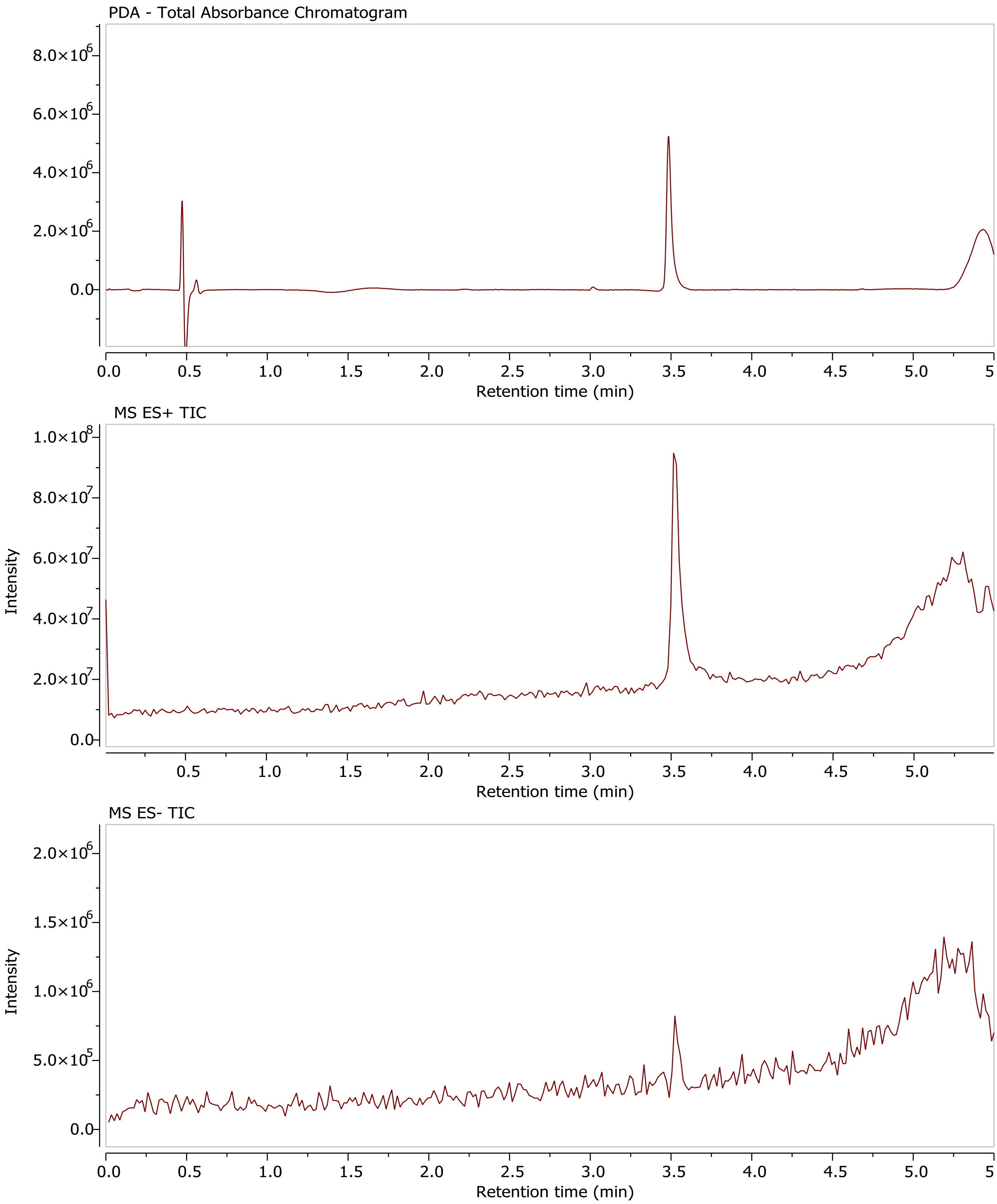

Supplementary figure **1616**. UPLC-MS analysis for compound **PHP-5703**

Chemical Formula: $C_{18}H_{15}BrN_2O_4$
Exact Mass: 402.0

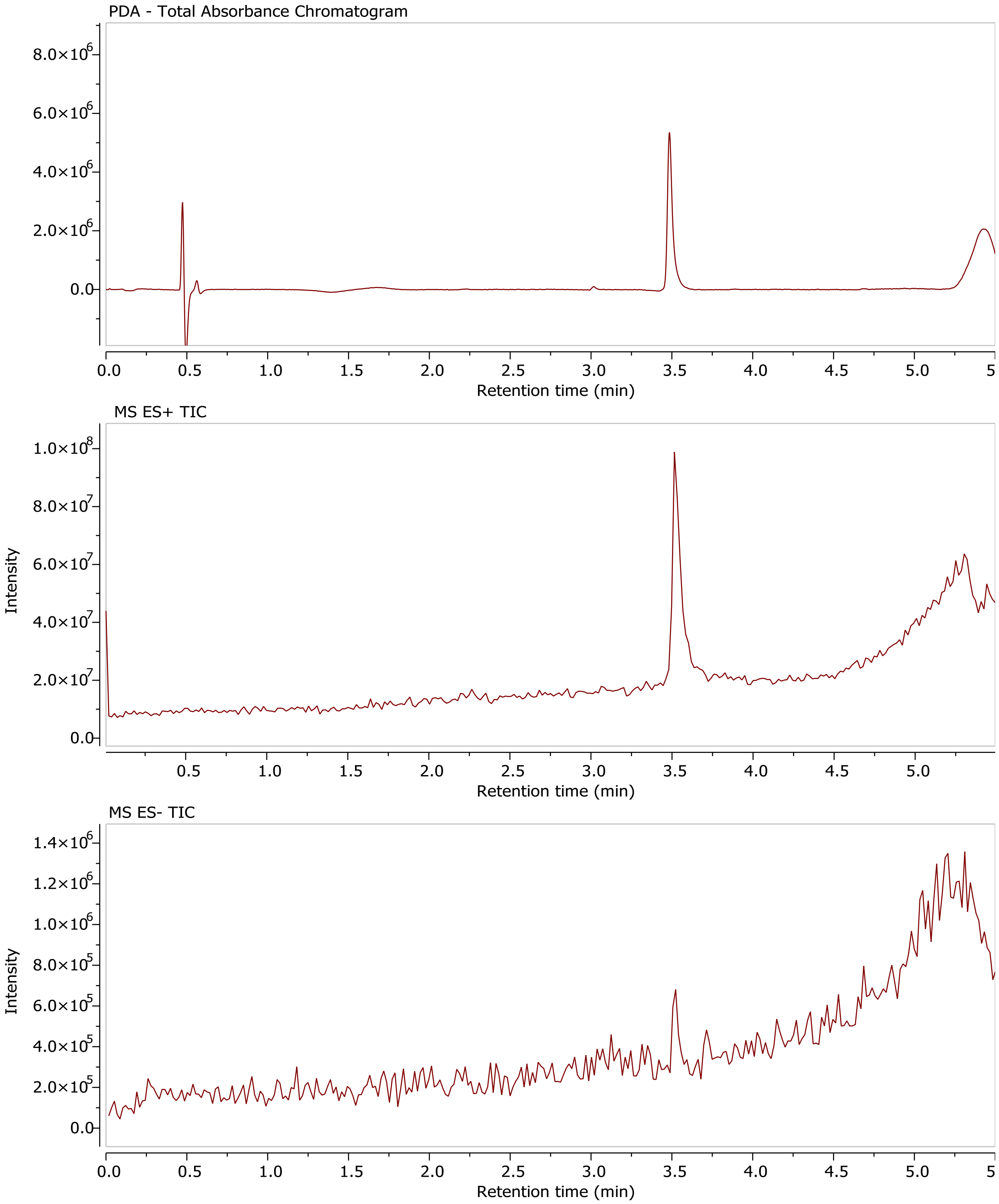

Supplementary figure **1617**. UPLC-MS analysis for compound **PHP-5903**

Chemical Formula: $C_{21}H_{23}FN_2O_2$
Exact Mass: 354.2

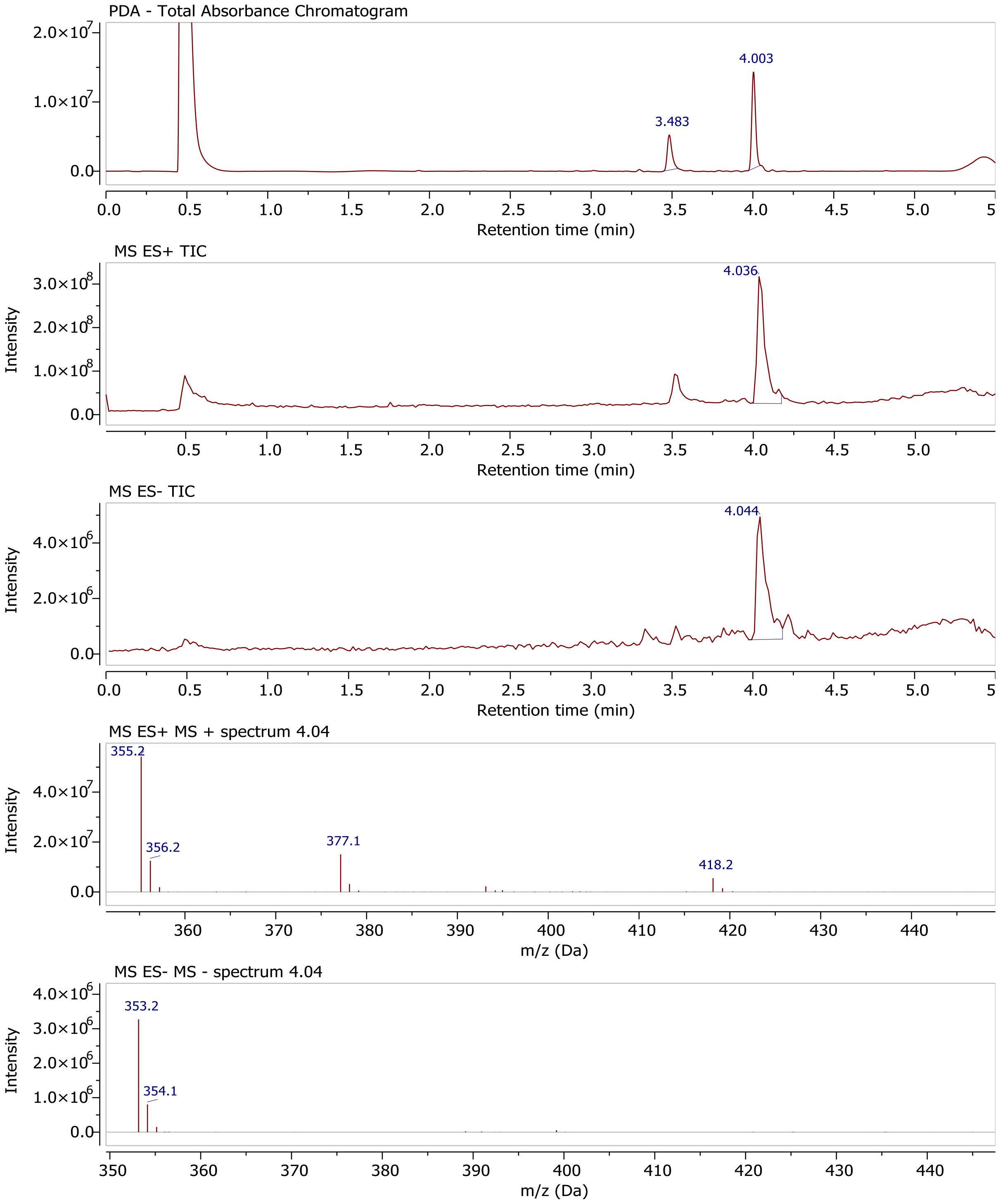

Supplementary figure **1618**. UPLC-MS analysis for compound **PHP-5704**

Chemical Formula: $C_{21}H_{22}N_2O_4$
Exact Mass: 366.2

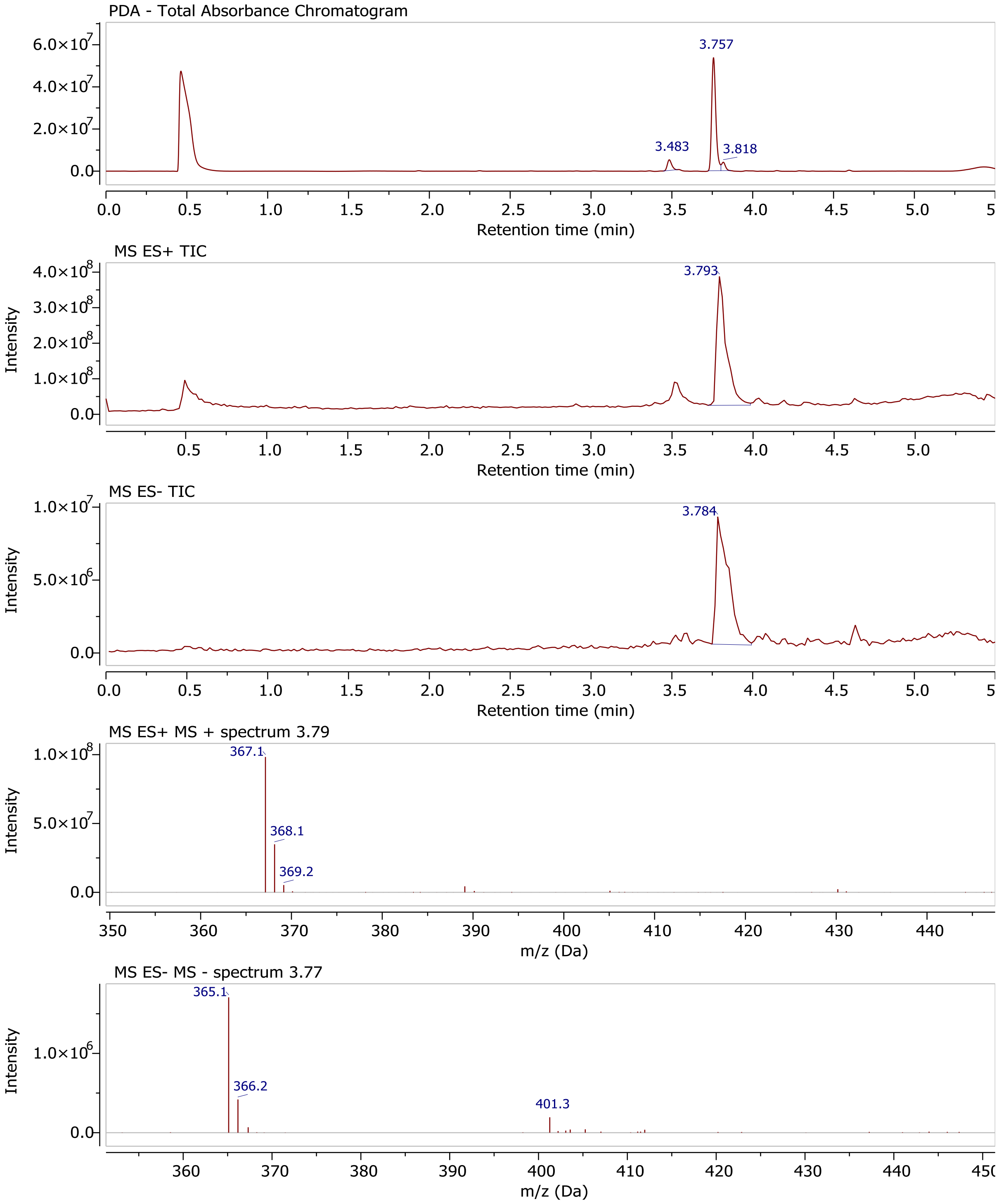

Supplementary figure **1619**. UPLC-MS analysis for compound **PHP-5904**

Chemical Formula: $C_{18}H_{16}ClFN_2O_2$
Exact Mass: 346.1

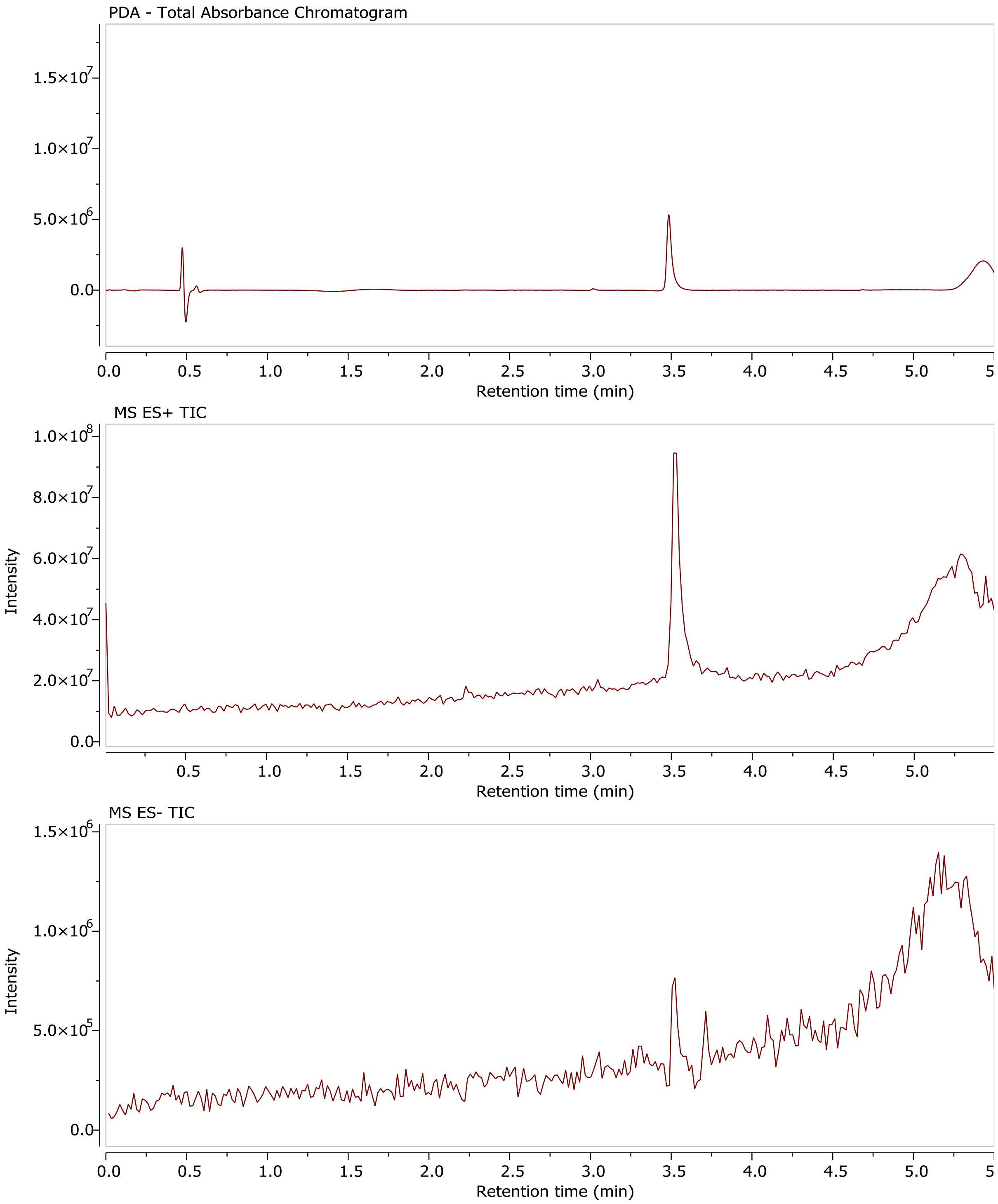

Supplementary figure **1620**. UPLC-MS analysis for compound **PHP-5705**

Chemical Formula: $C_{18}H_{15}ClN_2O_4$
Exact Mass: 358.1

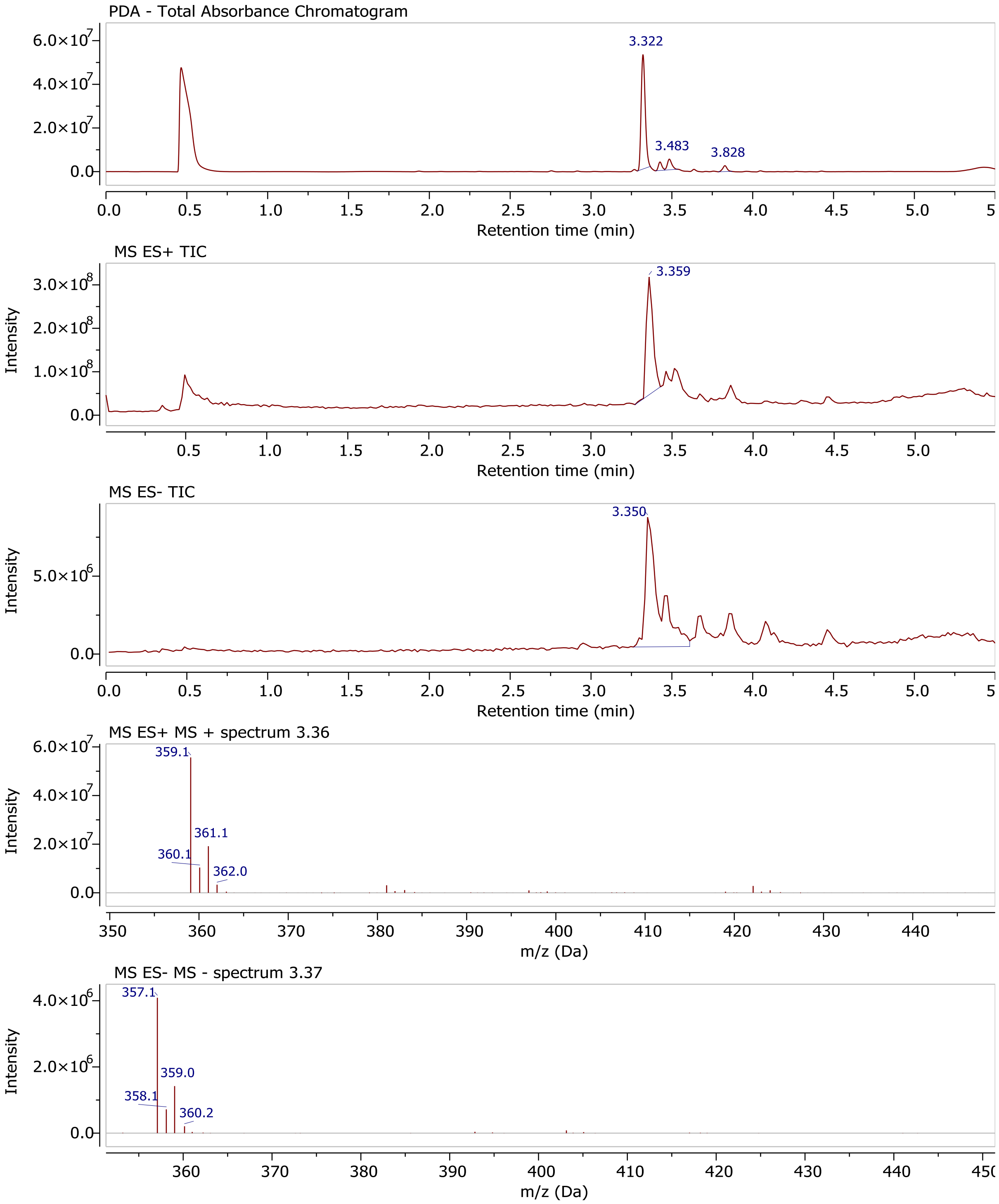

Supplementary figure **1621**. UPLC-MS analysis for compound **PHP-5905**

Chemical Formula: $C_{18}H_{16}F_2N_2O_2$
Exact Mass: 330.1

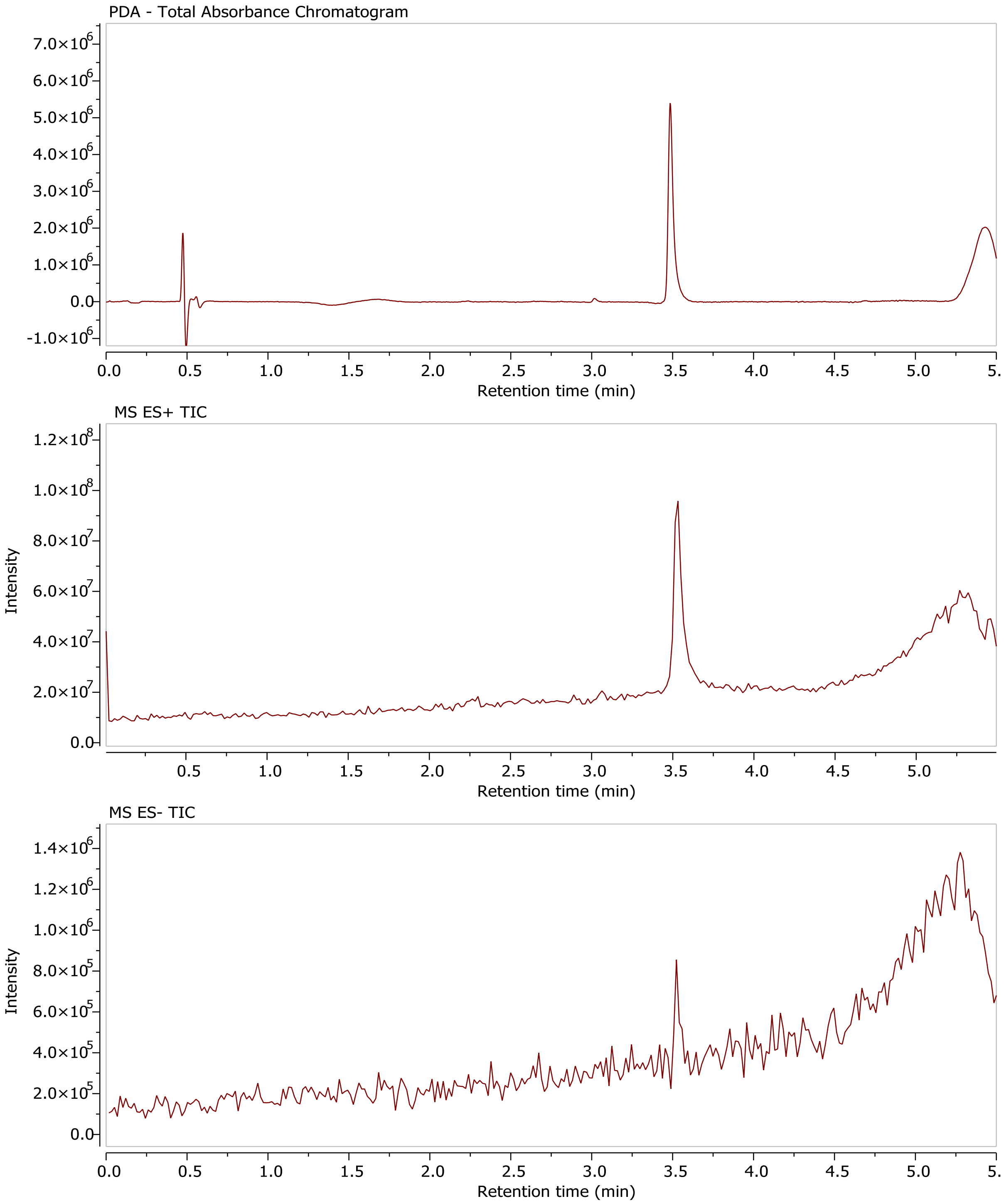

Supplementary figure **1622**. UPLC-MS analysis for compound **PHP-5707**

Chemical Formula: $C_{18}H_{16}F_2N_2O_2$
Exact Mass: 330.1

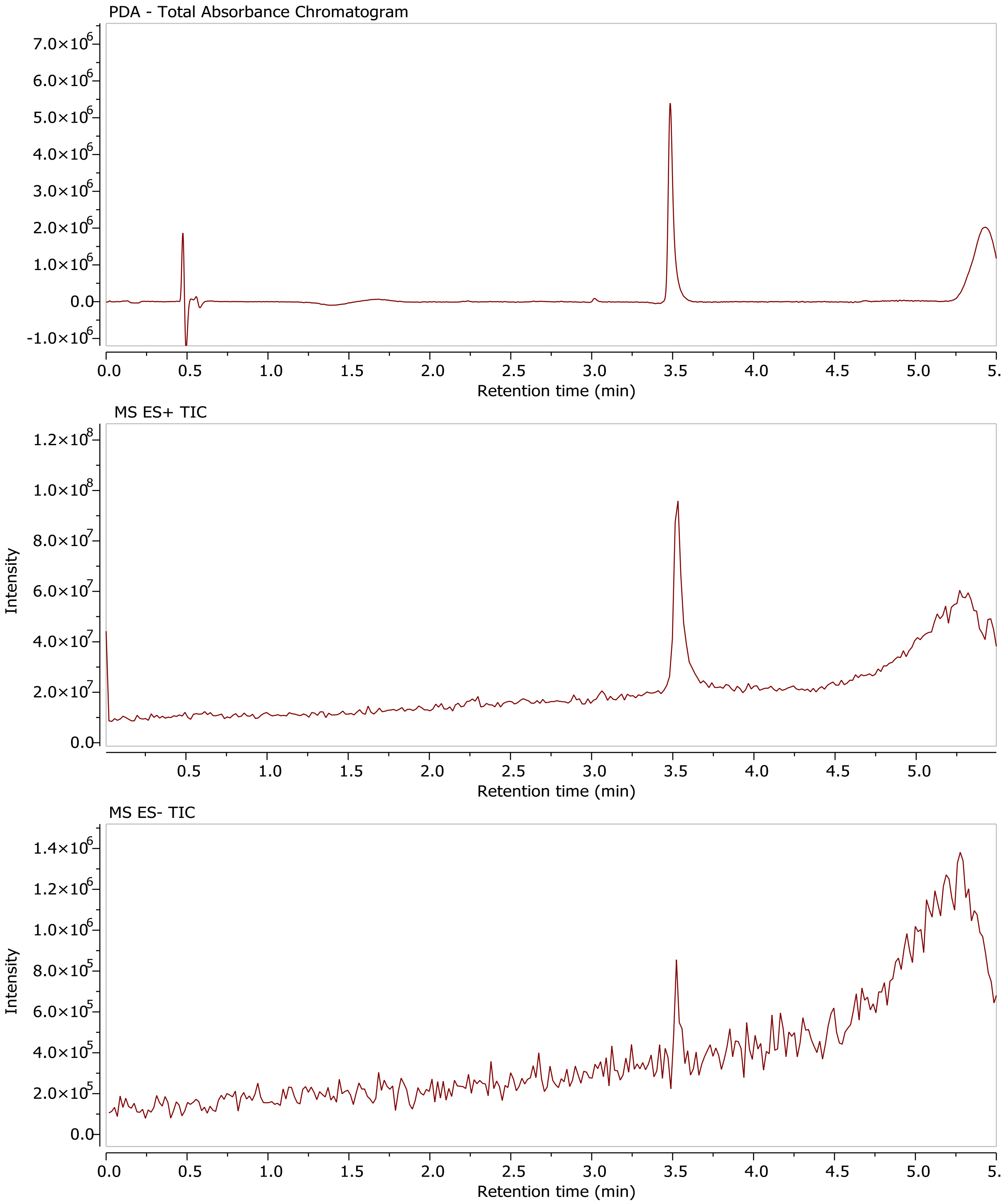

Supplementary figure **1623**. UPLC-MS analysis for compound **PHP-5906**

Chemical Formula: $C_{18}H_{16}F_2N_2O_2$
Exact Mass: 330.1

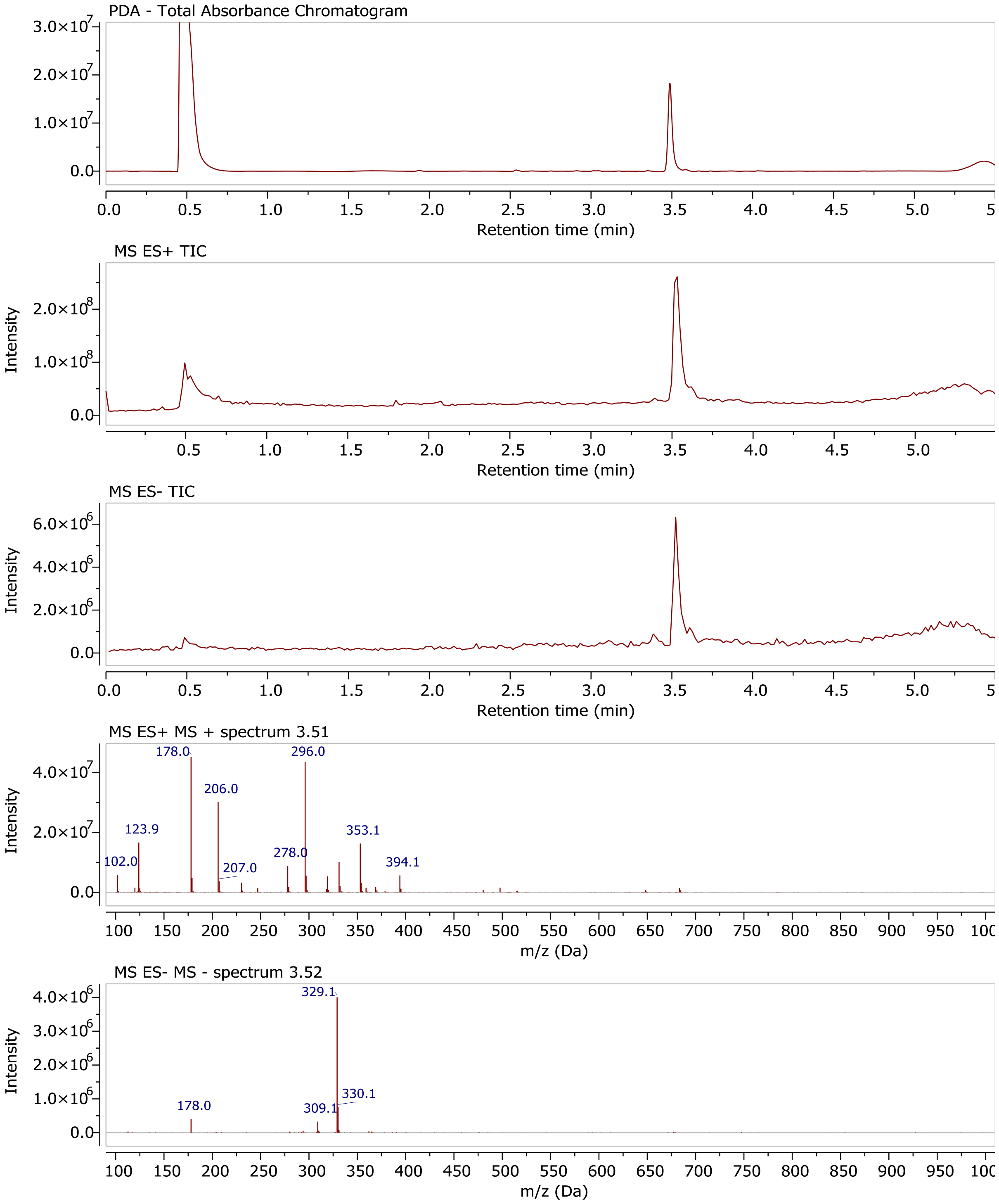

Supplementary figure **1624**. UPLC-MS analysis for compound **PHP-5708**

Chemical Formula: $C_{18}H_{15}FN_2O_4$
Exact Mass: 342.1

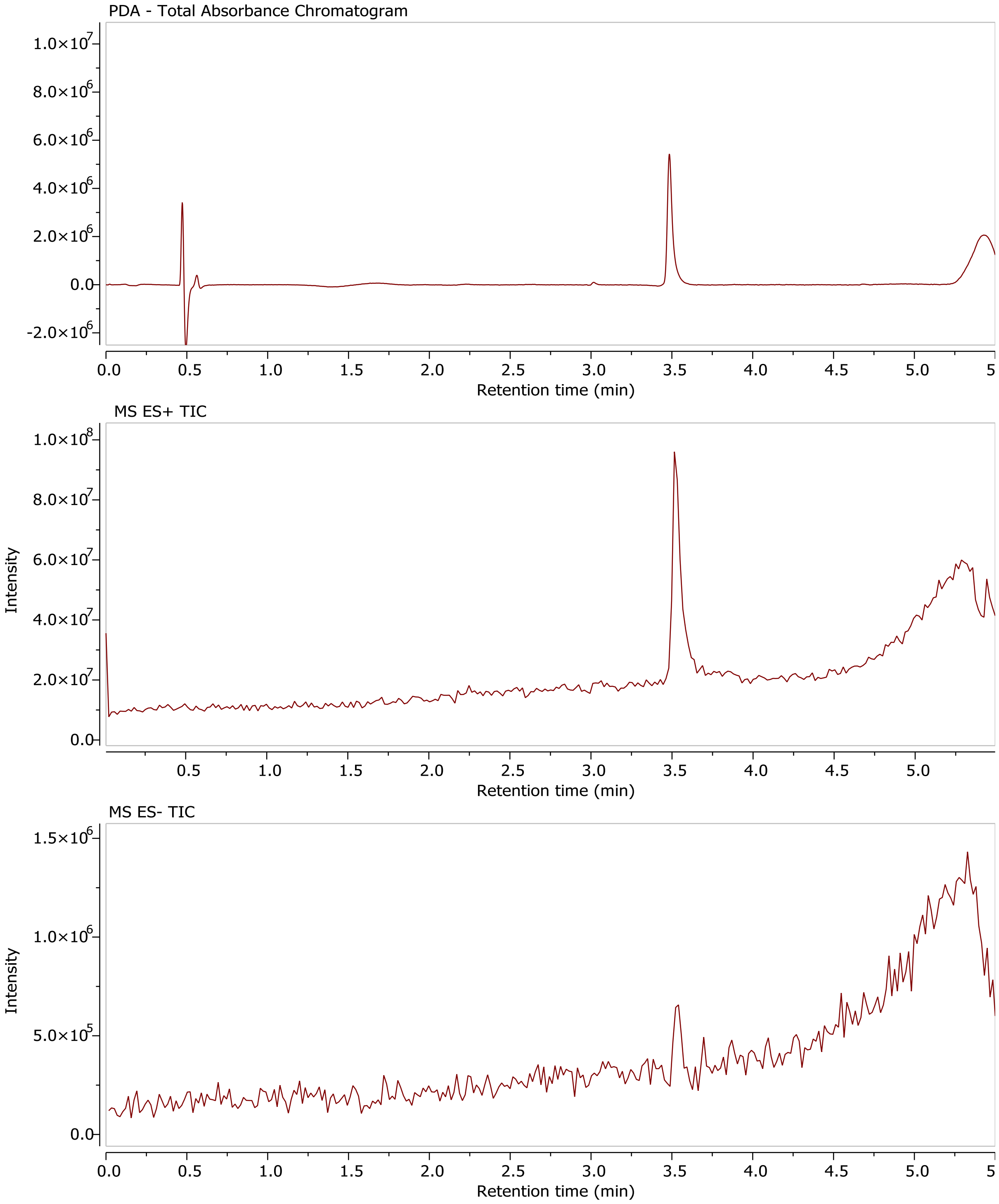

Supplementary figure **1625**. UPLC-MS analysis for compound **PHP-5907**

Chemical Formula: $C_{17}H_{23}FN_2O_2$
Exact Mass: 306.2

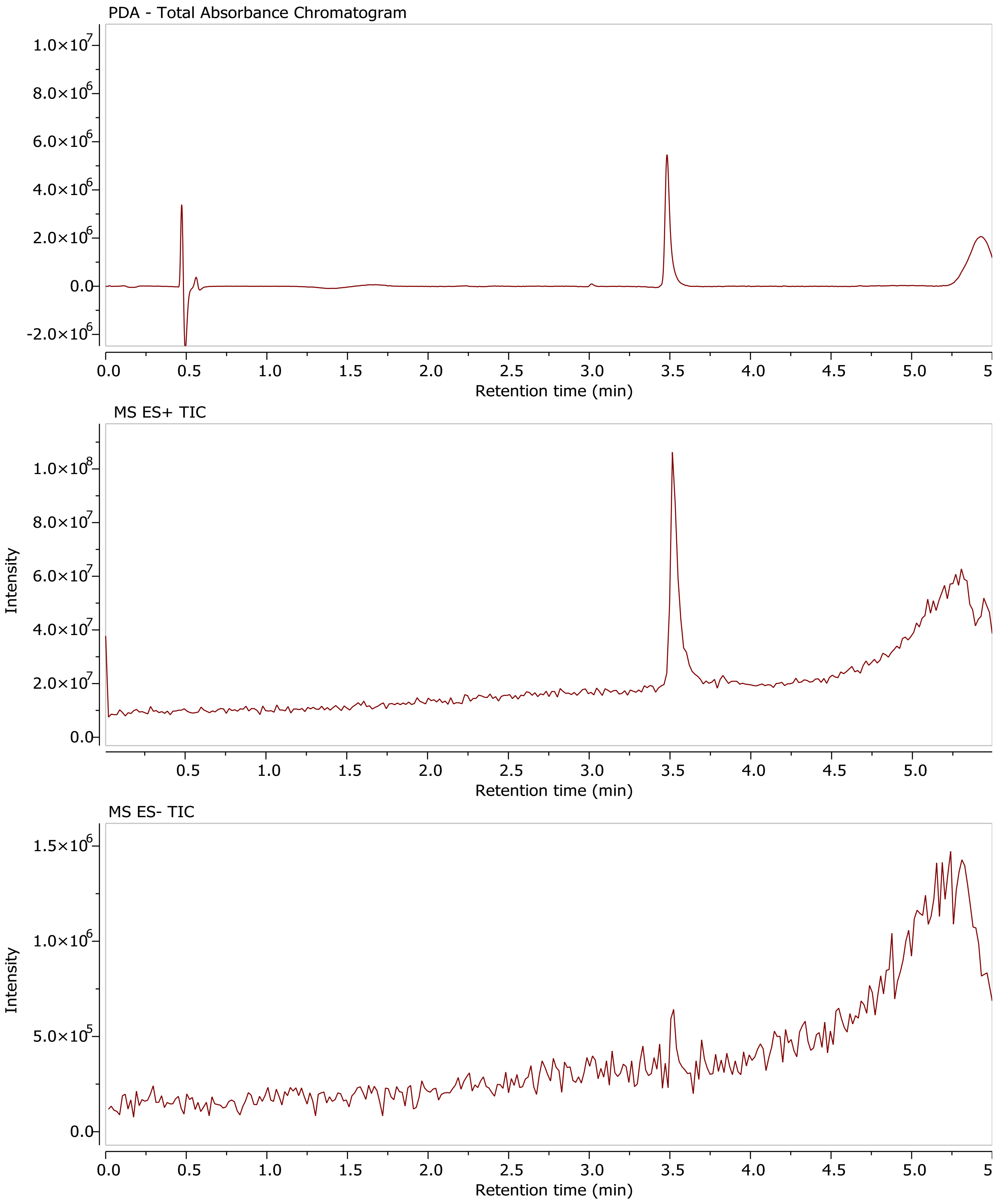

Supplementary figure **1626**. UPLC-MS analysis for compound **PHP-5709**

Chemical Formula: $C_{18}H_{15}FN_2O_4$
Exact Mass: 342.1

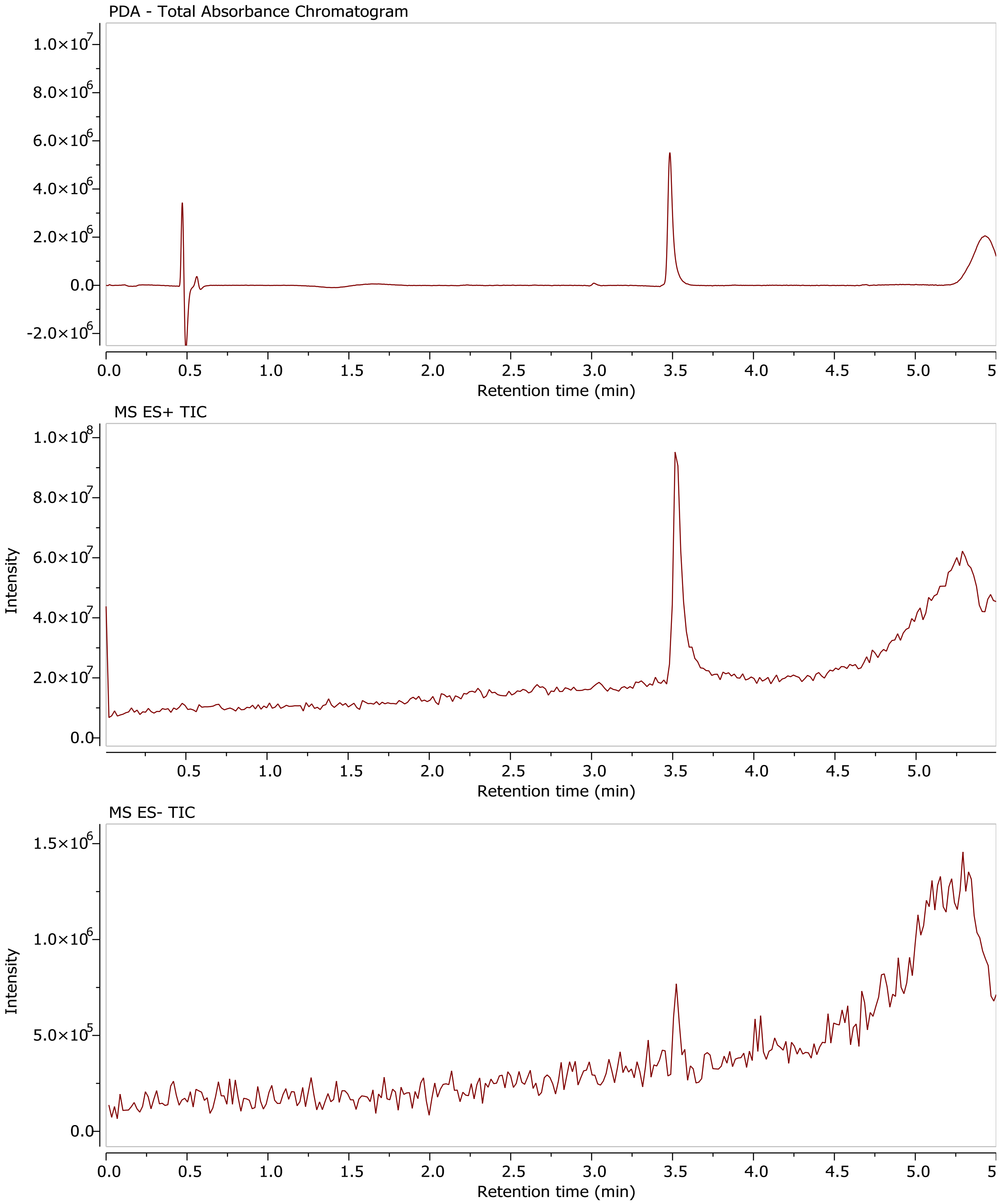

Supplementary figure **1627**. UPLC-MS analysis for compound **PHP-5908**

Chemical Formula: $C_{17}H_{23}FN_2O_2$
Exact Mass: 306.2

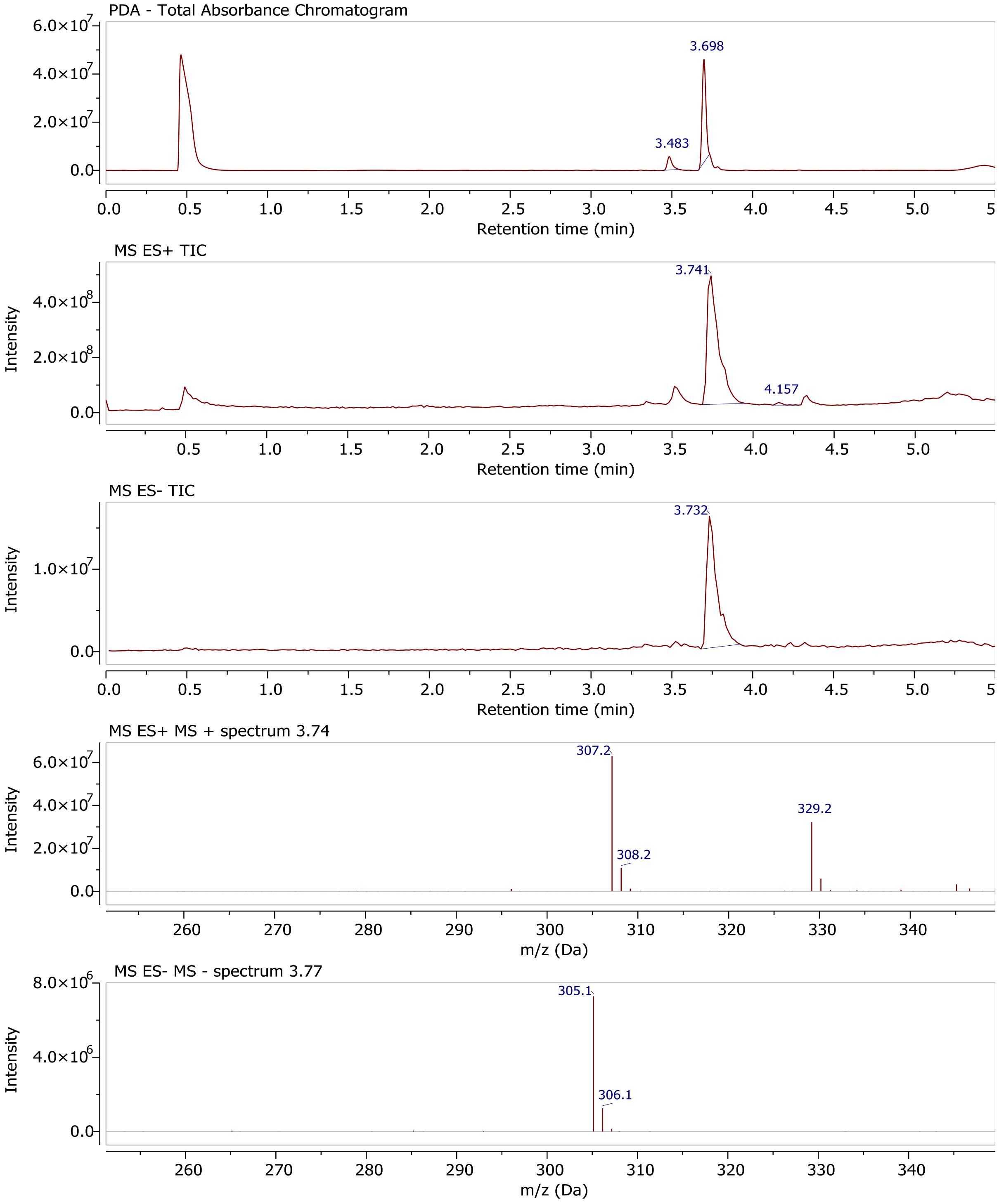

Supplementary figure **1628**. UPLC-MS analysis for compound **PHP-5710**

Chemical Formula: $C_{16}H_{20}Cl_2N_2O_2$
Exact Mass: 342.1

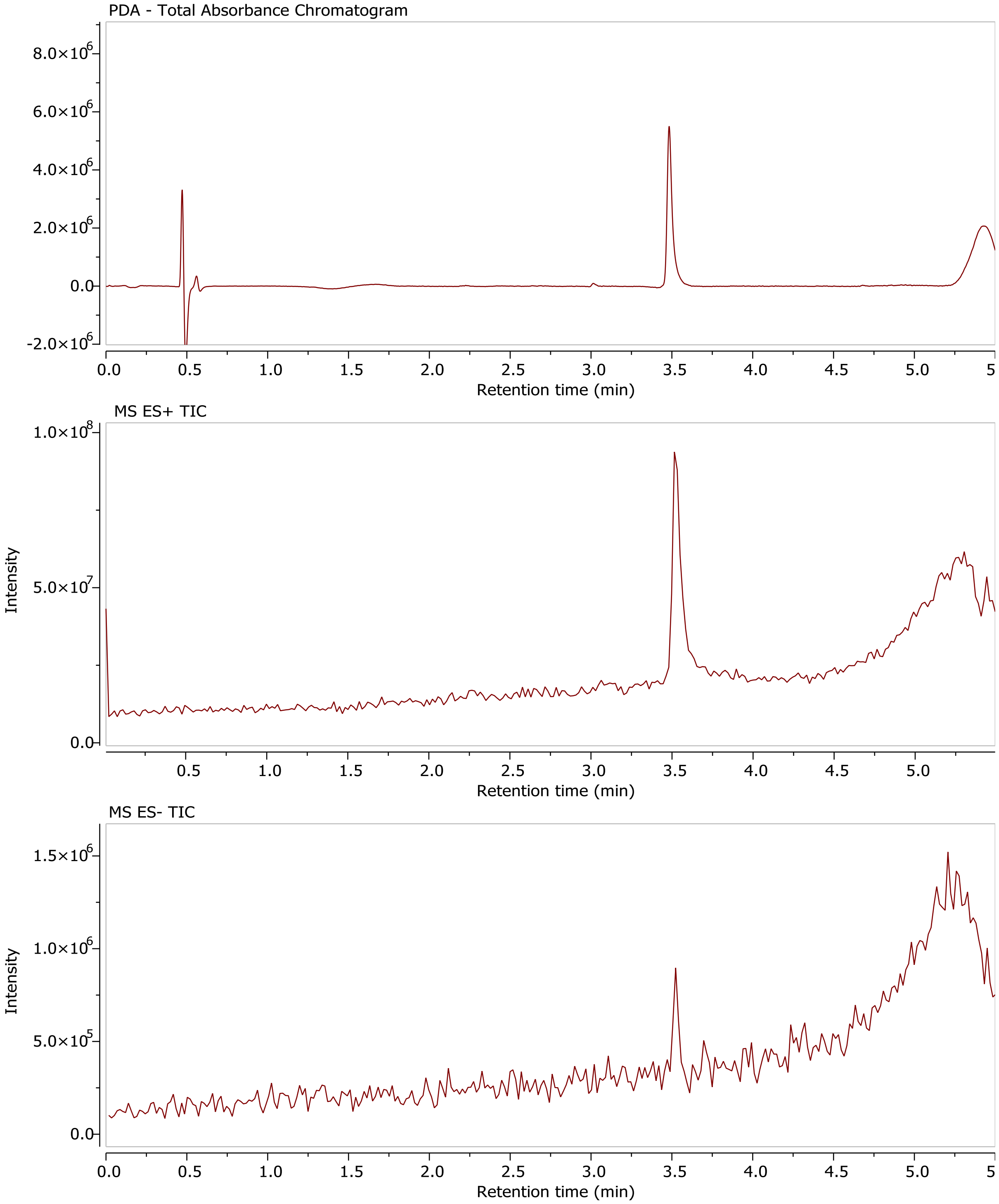

Supplementary figure **1629**. UPLC-MS analysis for compound **PHP-5909**

Chemical Formula: $C_{18}H_{13}F_5N_2O_2$
Exact Mass: 384.1

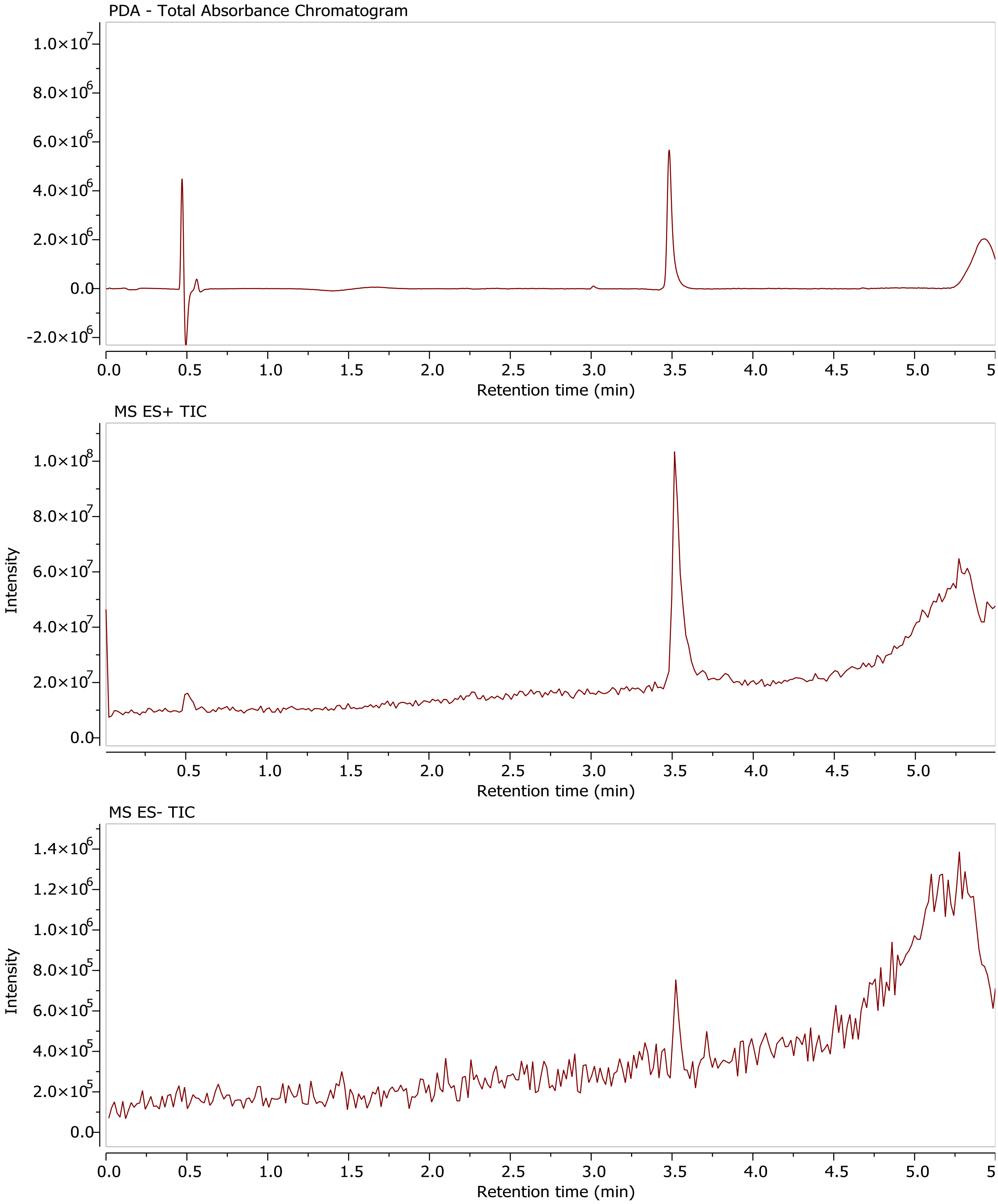

Supplementary figure **1630**. UPLC-MS analysis for compound **PHP-5711**

Chemical Formula: $C_{16}H_{20}Cl_2N_2O_2$
Exact Mass: 342.1

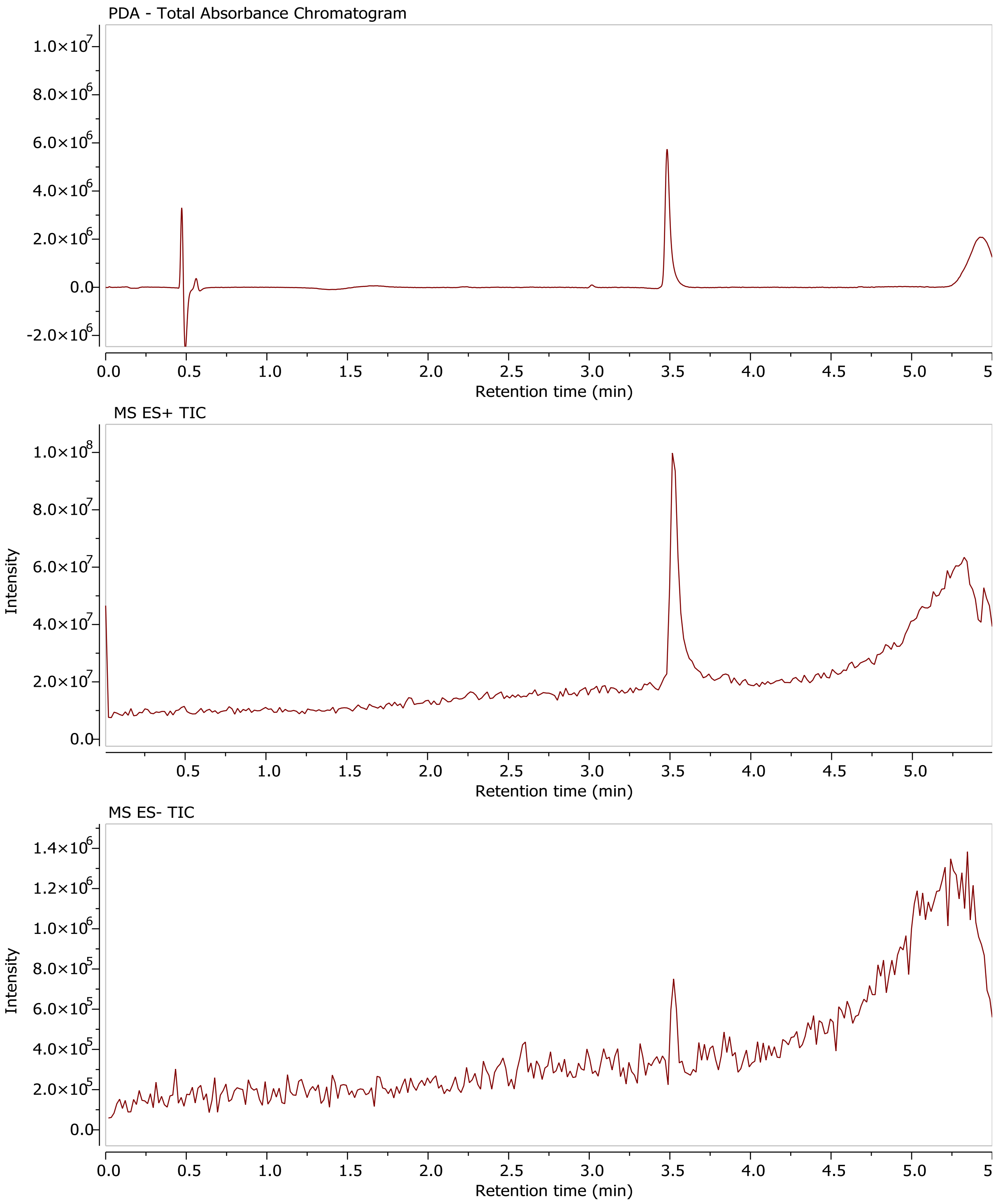

Supplementary figure **1631**. UPLC-MS analysis for compound **PHP-5910**

Chemical Formula: $C_{19}H_{27}FN_2O_2$
Exact Mass: 334.2

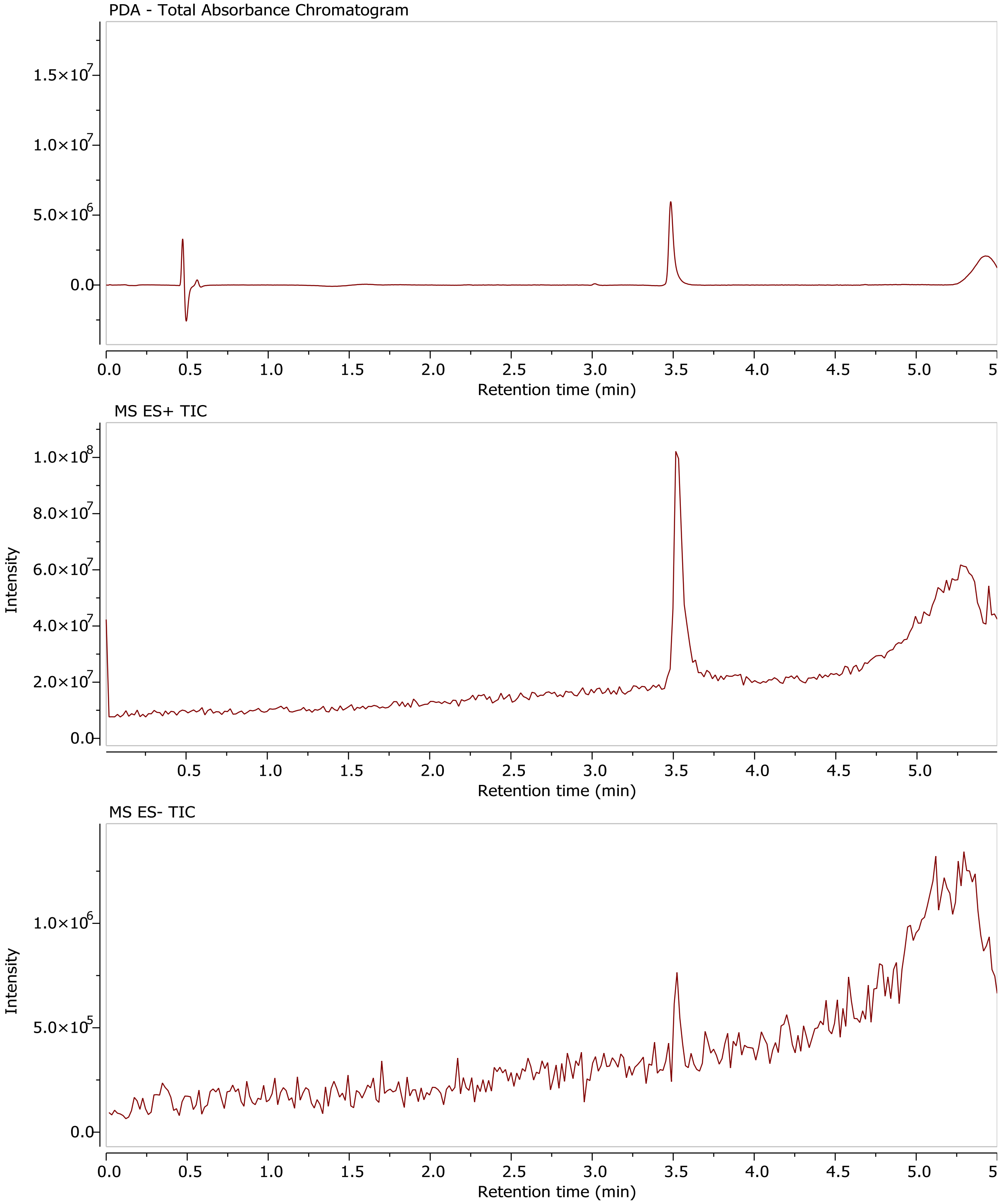

Supplementary figure **1632**. UPLC-MS analysis for compound **PHP-5712**

Chemical Formula: $C_{18}H_{24}Cl_2N_2O_2$
Exact Mass: 370.1

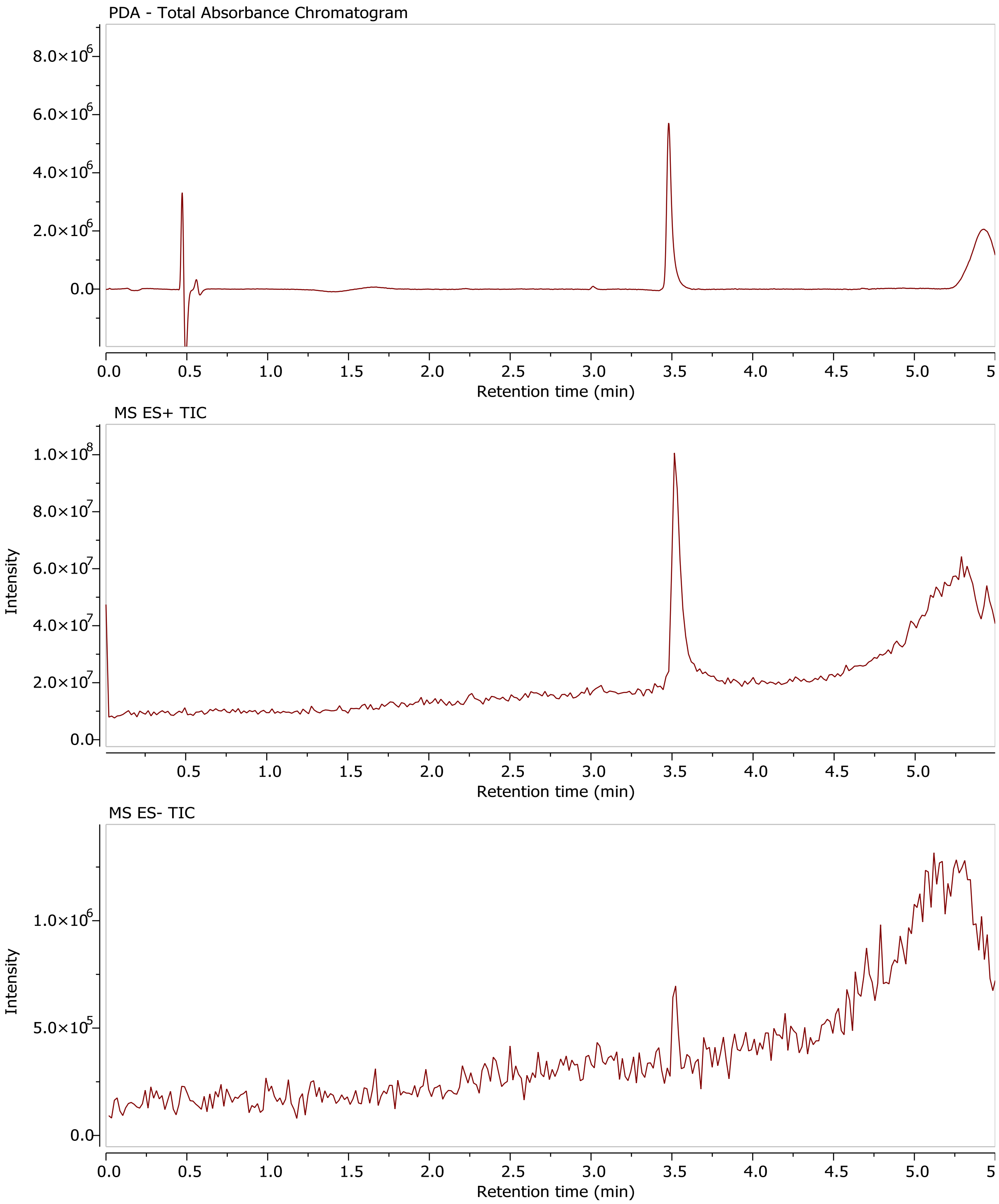

Supplementary figure **1633**. UPLC-MS analysis for compound **PHP-5912**

Chemical Formula: $C_{18}H_{14}F_4N_2O_2$
Exact Mass: 366.1

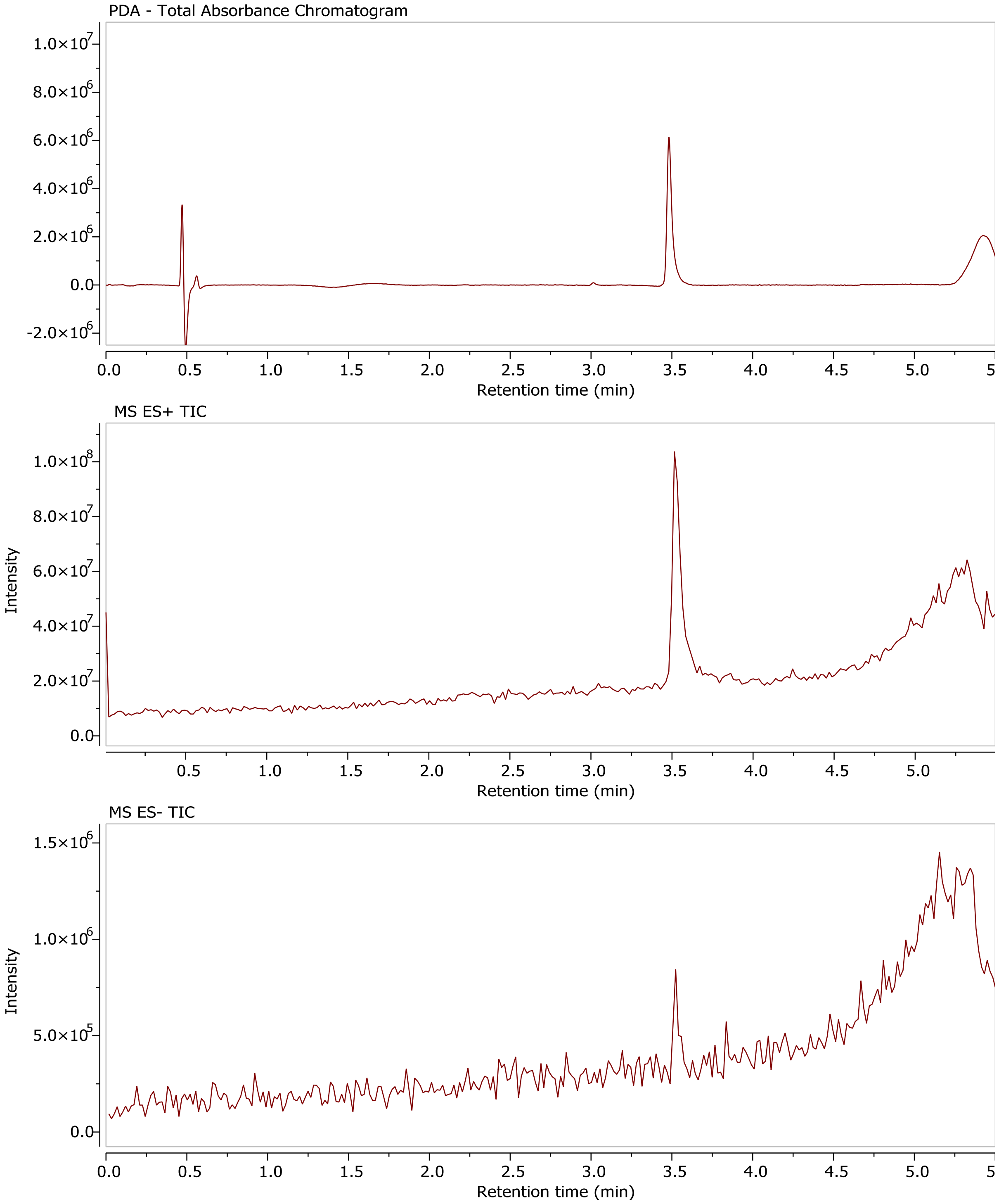

Supplementary figure **1634**. UPLC-MS analysis for compound **PHP-5713**

Chemical Formula: $C_{25}H_{22}F_2N_2O_2$
Exact Mass: 420.2

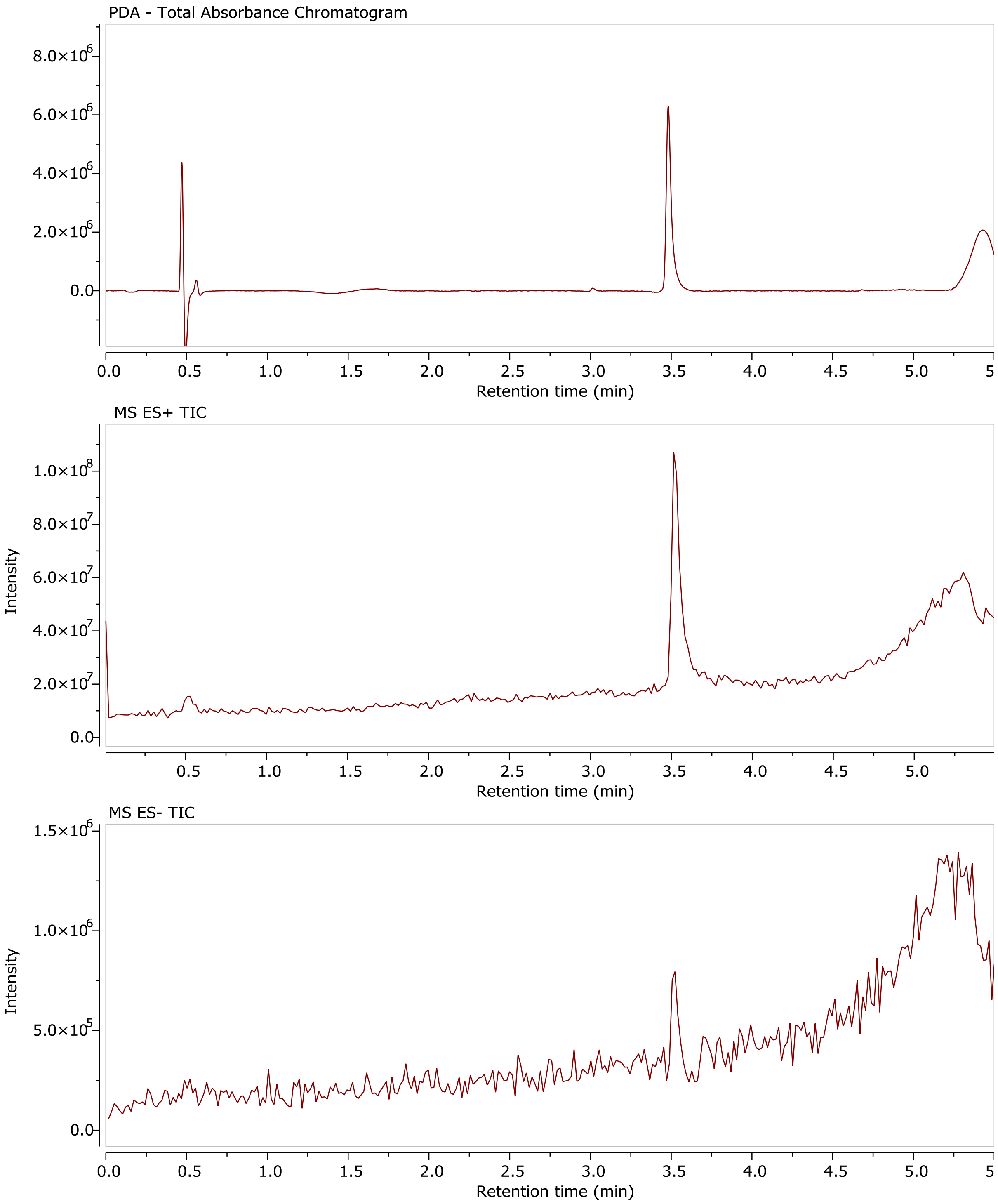

Supplementary figure **1635**. UPLC-MS analysis for compound **PHP-5918**
